# Integration of small RNAs from plasma and cerebrospinal fluid for classification of multiple sclerosis

**DOI:** 10.3389/fgene.2022.1042483

**Published:** 2022-11-17

**Authors:** Maria Needhamsen, Payam Emami Khoonsari, Galina Yurevna Zheleznyakova, Eliane Piket, Michael Hagemann-Jensen, Yanan Han, Jannik Gierlich, Diana Ekman, Maja Jagodic

**Affiliations:** ^1^ Department of Clinical Neuroscience, Karolinska Institutet, Center for Molecular Medicine, Karolinska University Hospital, Stockholm, Sweden; ^2^ Department of Biochemistry and Biophysics, National Bioinformatics Infrastructure Sweden, Science for Life Laboratory, Stockholm University, Stockholm, Sweden; ^3^ Department of Cell and Molecular Biology, Karolinska Institutet, Stockholm, Sweden

**Keywords:** multiple sclerosis (MS), data integration, MCIA, biomarkers, small RNAs, tRNA fragments, DNA methylation

## Abstract

Multiple Sclerosis (MS) is an autoimmune, neurological disease, commonly presenting with a relapsing-remitting form, that later converts to a secondary progressive stage, referred to as RRMS and SPMS, respectively. Early treatment slows disease progression, hence, accurate and early diagnosis is crucial. Recent advances in large-scale data processing and analysis have progressed molecular biomarker development. Here, we focus on small RNA data derived from cell-free cerebrospinal fluid (CSF), cerebrospinal fluid cells, plasma and peripheral blood mononuclear cells as well as CSF cell methylome data, from people with RRMS (*n* = 20), clinically/radiologically isolated syndrome (CIS/RIS, *n* = 2) and neurological disease controls (*n* = 14). We applied multiple co-inertia analysis (MCIA), an unsupervised and thereby unbiased, multivariate method for simultaneous data integration and found that the top latent variable classifies RRMS status with an Area Under the Receiver Operating Characteristics (AUROC) score of 0.82. Variable selection based on Lasso regression reduced features to 44, derived from the small RNAs from plasma (20), CSF cells (8) and cell-free CSF (16), with a marginal reduction in AUROC to 0.79. Samples from SPMS patients (*n* = 6) were subsequently projected on the latent space and differed significantly from RRMS and controls. On contrary, we found no differences between relapse and remission or between inflammatory and non-inflammatory disease controls, suggesting that the latent variable is not prone to inflammatory signals alone, but could be MS-specific. Hence, we here showcase that integration of small RNAs from plasma and CSF can be utilized to distinguish RRMS from SPMS and neurological disease controls.

## 1 Introduction

Multiple Sclerosis (MS) is a complex autoimmune and neurodegenerative disease, which can manifest with clinical symptoms such as impaired vision, fatigue, sensory disturbance, spasticity, pain, and depression among others (McGinley et al., 2021). Due to immune-related attacks on myelin sheaths in the brain and spinal cord, demyelinating lesions can be detected by magnetic resonance imaging (MRI) (Filippi et al., 2019). The majority (85%) of patients presents with the relapsing-remitting course of MS (RRMS) before eventually converting to a more severe, secondary progressive state (SPMS) (McGinley et al., 2021). However, earlier treatment intervention slows disease progression (Harding et al., 2019), thus early and accurate diagnosis is important. Individuals with a single neurological episode are typically classified as clinically isolated syndrome (CIS), and when lesions are discovered “incidentally” in asymptomatic individuals, the diagnosis is referred to as radiologically isolated syndrome (RIS) (Calabrese et al., 2021).

Molecular biomarkers, such as immunoglobulin G (IgG) index, oligoclonal bands and neuro-filament light chain in the cerebrospinal fluid (CSF) are becoming more widely used for supporting MS diagnosis together with patient medical history, clinical examination and magnetic resonance imaging (MRI) emphasizing the importance of moving towards multidisciplinary approaches in disease management (Gallien et al., 2014; Ziemssen et al., 2019). Listed biomarkers are predominantly based on protein and antibody assays, whereas additional molecular biomarkers based on nucleic acids detected by more sensitive methods are also emerging. For example, we previously found evidence that micro (mi)RNA, miR-150, levels are higher in MS patients compared to neurological disease controls and in CIS patients later converting to MS, compared to non-converters, suggesting miR-150 as a potential biomarker for early diagnosis of MS (Bergman et al., 2016; Quintana et al., 2017; Piket et al., 2019). Mature miRNAs are roughly 22 nucleotide long RNAs that regulate gene expression in a post-transcriptional manner (Filipowicz et al., 2008). Other classes of small non-coding RNAs (ncRNAs) include small nucleolar (sno)RNAs, small nuclear (sn)RNAs, transfer (t)RNAs and YRNAs, which have defined roles in RNA modifications, splicing, protein synthesis and DNA replication, respectively (Watson et al., 2019). Emerging evidence suggests that shorter, processed fragments derived from snoRNAs, snRNAs, tRNAs and YRNAs, acquire specific, functional roles. DNA methylation, another putative epigenetic biomarker for MS (Marabita et al., 2017; Ewing et al., 2019; Ringh et al., 2021), typically presented by a methyl group bound to cytosines in a CpG dinucleotide context (Zemach et al., 2010), can affect transcription, differential promoter usage or alternative splicing (Weber et al., 2007; Jones, 2012), and thereby reflect the status of cells of interest.

Previous studies have predominately considered tissue-specific, single omics data, in search of biomarkers. However, recent advances in omics technologies have invited multi-omics and multi-tissue biomarkers to the scene, which allows diagnostic biomarkers and treatment response evaluation to be multi-layered (Olivier et al., 2019). Bioinformatics approaches for integrative analysis are concurrently expanding with sequential and simultaneous method designs (Subramanian et al., 2020). These methods allow the discovery of complex patterns that would otherwise not be detectable using a single data type, potentially leading to higher diagnostic accuracy (Herman et al., 2018) and data-driven molecular subtyping (Jiangzhou et al., 2021).

In this study, we applied a multi-omics, multi-source integration approach to showcase MS biomarker discovery. We profiled small RNAs in CSF (cell-free and cells) and blood (plasma and mononuclear cells) with Small-seq methodology (Faridani et al., 2016; Hagemann-Jensen et al., 2018). Furthermore, CSF cells were profiled with Post-Bisulfite Adaptor Tagging (PBAT), a whole-genome bisulfite sequencing approach (Smallwood et al., 2014; Han et al., 2021). For integration, we applied multiple co-inertia analysis (MCIA), an unsupervised, multivariate method, which allows for simultaneous, unbiased data integration (Meng et al., 2014).

## 2 Materials and methods

### 2.1 Sample collection

Blood and CSF were collected from people with MS (n_RRMS_ = 20, n_SPMS_ = 5 (+1), n_CIS_ = 1, n_RIS_ = 1) and neurological controls (n_NINDC_ = 9, n_INDC_ = 5) as previously described (Zheleznyakova et al., 2021). MS was diagnosed according to the 2017 revised McDonald’s criteria (Thompson et al., 2018) and detailed cohort characteristics are given in supplementary Table S2. The study was approved by the Regional Ethical Board (2009/2107–31/2) and patients signed the informed consent form. In short, blood samples were centrifuged at 1500 *g* for 15 min at room temperature (RT), followed by plasma phase aspiration. CSF was centrifuged immediately after collection at 440 g for 10 min at RT to separate cells from supernatants. All samples were stored at −80°C until further processing.

### 2.2 Small-seq

RNA extraction, Small-seq library preparation, data pre-processing and normalization were conducted as previously described (Zheleznyakova et al., 2021). In short, RNA was extracted from 300 μl of plasma and cell-free CSF using the miRCURY RNA isolation kit (Exiqon, Product #300112, Vedbaek, 2950 Denmark), whereas RNA from CSF cells and PBMCs was extracted using the miRNeasy micro kit (Qiagen, Cat. No. 74004, Germantown, MD 20874, United States). Small-seq libraries were constructed pooled, purified and size selected as previously described (Faridani et al., 2016; Hagemann-Jensen et al., 2018; Zheleznyakova et al., 2021). Libraries were sequenced on HiSeq2500 (Illumina) with read length of 51 nt, single-end. Adapter sequences and CA were removed with Cutadapt v1.8.1 (Martin, 2011). Trimmed reads were subsequently mapped to hg38 using STAR v2.4.0 (Dobin et al., 2013) and annotated using miRbase v21 (Kozomara and Griffiths-Jones, 2014), GtRNAdb v1 (Chan and Lowe, 2009) and Gencode v22 (Frankish et al., 2019) of which the latter was further stratified into biotypes using the getBM function of biomaRt v.2.50.3 (Durinck et al., 2009). Small-seq transcripts with less than 100 Unique Molecular Identifiers (UMI) counts across all samples were filtered out and subsequently normalized using the trimmed mean of M values (TMM)-method from EdgeR (Robinson et al., 2010). TsRNAs were profiled and normalized with MINTmap v1.0 (Loher et al., 2017) and transcripts covered by less than 2/3 of samples in either the RRMS or control groups were filtered out. A standard deviation filter of 0 was also applied to both Small-seq and MINTmap derived transcripts.

### 2.3 PBAT

The post-bisulfite adaptor tagging (PBAT) protocol was conducted as previously described (Smallwood et al., 2014; Han et al., 2021) on lysate from approximately 5000 CSF cells. Library pools were sequenced on a HiSeq2500 in High Output mode (8 lanes/run) at the Babraham Institute Next Generation Sequencing Facility with a sequencing length of 100 nt, single-end reads.

Reads were trimmed 9bp from their 5′end (--clip_rl 9) using Trim Galore version 0.4.1 (http://www.bioinformatics.babraham.ac.uk/projects/trim_galore/). Quality (-q 20) and adapter (-a AGATCGGAAGAGC) trimming were performed using Cutadapt 1.8.1 (Martin, 2011) with a minimum required adapter overlap of 1bp (−O 1) and a maximum trimming error rate of 0.1 (−e 0.1). The minimum required sequencing length after trimming was set at 20bp.

Trimmed and filtered sequencing reads were aligned to GRCh38 with the –pbat option of Bismark 0.14.4 (Krueger and Andrews, 2011) using Bowtie 2 (Langmead and Salzberg, 2012) and the following specified options: -q–phred33 –score-min L, 0,−0.2 –ignore-quals. Alignments with a unique best hit were taken for further processing. Duplicated reads were removed using the deduplicate_bismark function and subsequently top and bottom strands were merged into single CpG dinucleotides using the –merge_CpG option of the Bismark coverage2cytosine module.

The BSmooth function from bsseq v.1.20.0 (Hansen et al., 2012) was applied to the Bismark-derived coverage files with default settings (1 kB window, 70 CpGs) to estimate DNA methylation levels for each sample and each CpG site. Sites with robust coefficient of variation (RCV) (RCV(x_i_) = median (x_i_ - median(x))/median(x)) lower than one and standard deviation of 0 across samples were filtered.

### 2.4 Multi co-inertia analysis

For integration, we performed multi co-inertia analysis (MCIA) (Chessel and Hanafi, 1996) by applying the mbpca function of the mogsa Bioconductor package v.1.22.1 (Meng et al., 2019) with the following parameters: method = "blockLoading”, option = “inertia”, center = TRUE, scale = T, moa = TRUE, svd. solver = “fast”, maxiter = 1000, k = "all”. In brief, each dataset was first centered by mean and standardized by its standard deviation followed by another division by its inertia:

$$X_{k}=\frac{\left(X_{k}\left(n,p\right)-{\bar{Xp}}_{k}\right)/{\sigma p}_{k}}{\sqrt{\overset{n}{\underset{i=1}{\sum }}\overset{p}{\underset{j=1}{\sum }}X_{i,j}^{2}\;}}$$

Where X_k_ refers to expression matrix X from dataset K. Xp_k_ is the mean of the column *p* and σp_k_ is the standard deviation of column *p*. The resulting standardized datasets were then combined followed by singular value decomposition (SVD) to calculate the first eigenvectors:

$$X=\left[X_{1\;}X_{2}\;X_{3}\;...\;X_{k}\right]$$

$$X=U\Sigma V^{T}$$

Where U is the global latent scores and V is the eigenvectors. The deflation of the matrix was performed using partial eigenvectors as:

$$X_{k}=X_{k}-X_{k}{.V}_{k}^{T}$$

Where V_k_^T^ refers to the eigenvectors for variables from dataset k. The collection of all UΣ (after all iterations) is referred to as global latent scores and is referred to as T_*global*_. The partial contributions for each subtype were divided by the contributions of the complete dataset to make fractions and percentages.

$$contrib={\left(\frac{T_{partial}.T_{global}}{T_{global}^{T}.T_{global}}\right)}^{2}\times T_{global}^{T}.T_{global}$$

For projection and estimation eigenvalues for subtypes, we used only the corresponding eigenvectors to estimate latent scores and variance contribution. More specifically, the projections were calculated by:

$$T_{projected}=X_{new}.V^{T}$$

Where *X*_*new*_ is the new unseen standardized dataset (all sub-datasets have been concatenated) and VT is the SVD weights. The variance contribution for each omics was calculated as:

$$T_{k}=X_{k}{.V}_{k}^{T}$$

$$T_{partial}=\left[T_{1\;}T_{2}\;T_{3}\;...\;{\;T}_{k}\right]$$

### 2.5 Clustering and accuracy

Clustering was performed using k-means with k = 2. We calculated the accuracy using the split-join distance (van Dongen, 2000) and the area under the ROC curve. To calculate the area under the Receiver Operating Characteristics (ROC) curve we used an iterative approach (Lee and Fujita, 2007) as follows:

1.Apply k-means clustering with k = 2
2.Compute the fractions RRMS (RRRMS) and Controls (Rcontrol) detected in cluster one and cluster two
3.Determine the true positive cluster by comparing RRRMS and Rcontrol such that cluster one is true positive if RRRMS > Rcontrol otherwise cluster two is the true positive
4.Calculate true positive and false positive fractions
5.Relocate a sample xi from cluster one to cluster two such that the change in the k-means objective function is minimum
6.Calculate true positive and false positive fractions
7.Repeat steps five to six until cluster one becomes an empty cluster
8.Reinstate the k-means clustering results
9.Relocate a sample from cluster two to cluster one such that the change in the k-means objective function is minimum
10.Calculate true positive and false positive fractions
11.Repeat steps 9–10 until cluster two becomes an empty cluster

### 2.6 Variable selection and statistical testing

Variable selection was performed using Lasso regression (Friedman et al., 2010) (glmnet package in R v 4.1–1) with 5-fold cross-validation to estimate *λ*. We used the global scores from MCIA as the dependent and the features as the independent variable. The features with shrunk *ß* = 0 were removed. If no variable from a dataset was left, the entire dataset was removed before performing MCIA on the reduced dataset. The statistical inference was performed using Mann-Whitney test. *p*-values lower than 0.05 were considered statistically significant.

Additional computational details are given in Supplementary Information 1.

## 3 Results

### 3.1 Multi-omics and multi-source approach: Cohort and feature description

**FIGURE 1:**
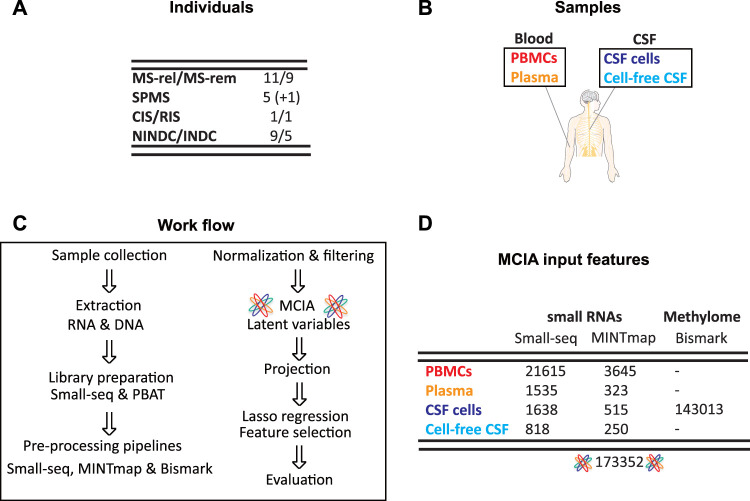
Cohort and sample overview. **(A)** Samples were collected from people with Multiple Sclerosis (MS) in relapse (MS-rel), remission (MS-rem), secondary progressive (SPMS), clinically and radiologically isolated syndrome (CIS/RIS) stages, inflammatory (INDC) and non-inflammatory neurological disease controls (NINDCs). **(B)** Blood and cerebrospinal fluid (CSF) were collected from the same individual and separated into peripheral blood mononuclear cells (PBMCs), plasma, CSF cells and cell-free CSF. **(C)** Work flow. Upon DNA and RNA extraction, sequencing libraries were constructed using Small-seq and PBAT methodology, respectively. Sequencing reads were pre-processed, which includes QC, filtering, mapping, deduplication, and coverage count, using the standard Small-seq and MINTmap pipelines for small RNAs, whereas the methylome data was profiled using Bismark. After normalization and variance filtering, multiple co-inertia analysis (MCIA) was applied, SPMS samples were projected, and latent variables were subsequently extracted. Lasso regression was applied for feature selection followed by evaluation of the reduced feature model. **(D)** Overview of features included in MCIA. The number of features for each source and methodology is given, as well as the total number of features included in downstream integration with MCIA.

Samples from CSF cells, cell-free CSF, plasma and peripheral blood mononuclear cells (PBMCs) were collected from RRMS in relapse (*n* = 11), RRMS during remission (*n* = 9), SPMS (*n* = 5 + 1 from same individual sampled a year later), as well as inflammatory (INDC, *n* = 5) and non-inflammatory neurological disease controls (NINDCs, *n* = 9). Furthermore, samples from two individuals classified as RIS and CIS at the time of collection, were also included (Figures 1A,B). Blood- and CSF-derived biofluid and cellular samples were profiled for small RNAs using Small-seq methodology (Faridani et al., 2016; Hagemann-Jensen et al., 2018) with detailed tRNA-derived small RNAs (tsRNAs) profiled using MINTmap (Loher et al., 2017) (Figure 1C, Supplementary Table S1). For PBMCs, which is the most abundant cellular compartment, 21615 features derived from the Small-seq pipeline and 3645 MINTmap-derived features passed the filtering criteria, whereas for plasma, which is extracellular, 1535 Small-seq and 323 MINTmap-derived features passed (Figure 1D, Supplementary Information 2–10). From the CNS compartment, fewer cells were extracted, which resulted in 1638 and 818 features derived from the Small-seq pipeline and 515 and 250 features derived from MINTmap from CSF cells and cell-free CSF, respectively. CSF cells were furthermore profiled for genome-wide DNA methylation using PBAT technology of which 143013 features with a coefficient of variation (CV) > 1 were identified (Figure 1D, Supplementary Information 2–10).

In total, 173352 features across multi-omics and multi-fluid and cellular sources from 42 overlapping individuals with MS and other neurological diseases were considered for downstream analysis.

### 3.2 Unbiased, simultaneous integration of small RNAs and methylation profiles from cellular and fluid sources to classify RRMS status

**FIGURE 2:**
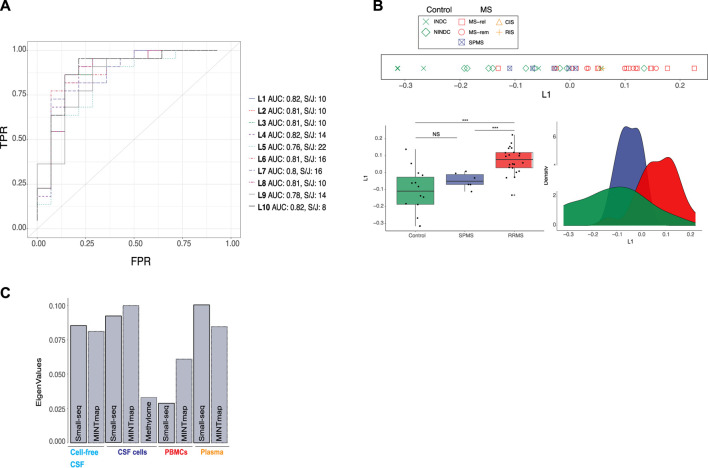
Integration of omics data from cellular and fluid sources separates RRMS from controls and SPMS. **(A)** Multiple co-inertia analysis (MCIA) method was used for simultaneous, unsupervised integration. A total of 10 latent variables (L1-L10), illustrated with different colours and dashed lines, were evaluated using Area Under the Receiver Operating Characteristics (AUROC) analysis and k-means clustering (k = 2) followed by split and join method as indicated by “AUC” and “S/J”, respectively. TPR: true positive rate, FPR: false positive rate **(B)** Separation of controls (green), secondary progressive multiple sclerosis (SPMS, blue) and relapsing–remitting MS (RRMS, red) based on L1, was evaluated with line, density and boxplots as well as Wilcoxon signed-rank test (***: *p*-value < 0.001. NS: not significant). In the line plot MS subtype: relapse (MS-rel), remission (MS-rem) and secondary progressive (SPMS), control subtype: inflammatory (INDC) and non-inflammatory neurological disease controls (NINDCs) and clinically or radiologically isolated syndrome (CIS and RIS) are distinguished by colour and shape. **(C)** Contribution of small RNAs derived by the Small-seq pipeline, Mintmap and methylome derived data to L1.

For simultaneous data integration, we applied multiple co-inertia analysis (MCIA), which is an unsupervised and thereby unbiased method (Meng et al., 2014). MCIA is based on a covariance objective function that attempts to summarize shared patterns among multiple datasets into lower-dimensional latent variables. We initially focused on RRMS, CIS, RIS and control samples due to their diagnostic relevance. Up to ten latent variables (L1-L10), derived from the MCIA analysis applied on RRMS, CIS, RIS, NINDC and INDC samples were considered. Latent variables are ranked according to percentage of variation captured, hence, L1 captures more variance than L10 (Supplementary Figure S1). To evaluate our clinical phenotype of interest, i.e., RRMS and CIS/RIS, across latent variables, Area Under the Receiver Operating Characteristics (AUROC) analysis, was applied. The first MCIA latent variable, L1, distinguished RRMS from all neurological disease controls jointly with an AUROC value of 0.82, with none of the remaining latent variables (L2-L10) having a higher score (Figure 2A). Furthermore, we conducted k-means (k = 2) clustering and subsequently evaluated clusters with a split and join method, which measures the number of moves needed to transform the observed clusters to the ground truth (lower numbers are preferable). Taken together, L1, explaining the majority of variation, was the top choice for prediction of RRMS disease status (Figure 2A, Supplementary Figure S1). Group separation (relapse, remission and CIS/RIS *versus* INDC and NINDC) based on L1 was evaluated with line, box- and density plots (Figure 2B). Wilcoxon signed-rank test confirmed a significant difference (*p* < 0.001) (Figure 2B). Partial eigenvalues (i.e., the relative contribution of variance to the selected latent variable) revealed a relatively even contribution across compartments and datasets, apart from DNA methylation and PBMC transcripts, which contributed less to L1 (Figure 2C).

Subsequently, we projected the six samples from five SPMS individuals onto L1 (Figure 1D). Interestingly, SPMS samples seemed to locate between RRMS and control samples, although a significant difference in L1 was only detected between SPMS and RRMS (*p* < 0.001) based on Wilcoxon signed-rank test (Figure 2B). We further examined whether stratifying the RRMS group into relapse and remission would reveal additional differences between MS subgroups, but this was not the case (Supplementary Figure S2). Neither did we detect significant differences between the NINDC and INDC control groups (Supplementary Figure S2), suggesting that L1 is not solely picking up inflammatory cues, but could be MS-specific.

In summary, the top latent variable, L1, derived from simultaneous, unbiased integration of multi-cellular and fluid sources of small RNA and methylome profiles, distinguished RRMS from other neurological disease controls and SPMS.

### 3.3 Lasso regression reduces the number of features for classification of RRMS

**FIGURE 3:**
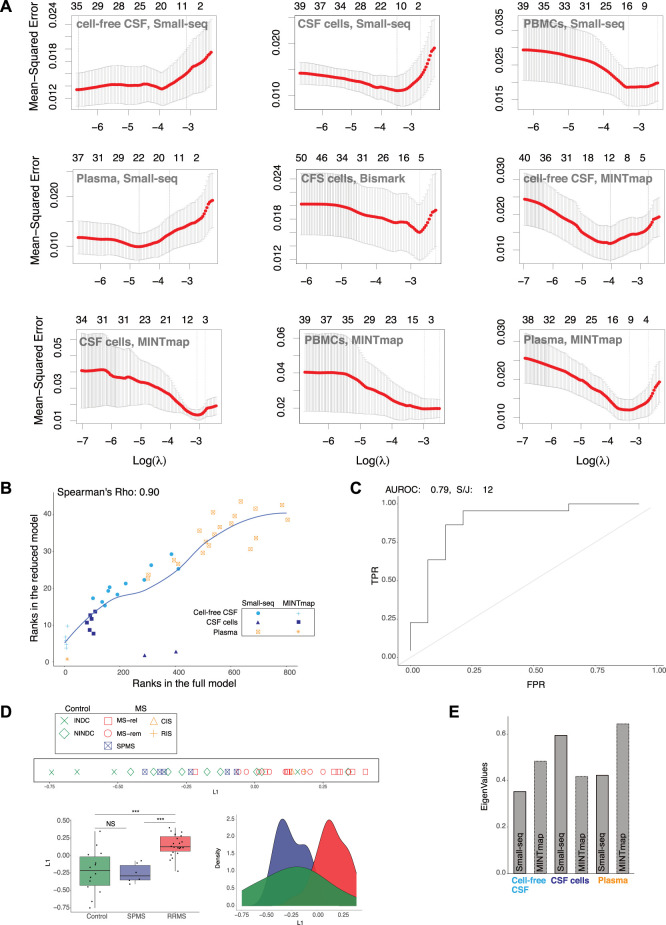
RRMS separation from controls and SPMS remains after feature reduction. **(A)** Lasso regression. Lambda parameter tuning of small RNA data processed through Small-seq and MINTmap pipelines from cell-free cerebrospinal fluid (CSF), CSF cells, plasma and peripheral blood mononuclear cells (PBMCs) as well as methylome data from CSF cells processed through the Bismark pipeline, which were included in the initial, full multiple co-inertia analysis (MCIA) model. **(B)** Scatter plot comparing ranks of the 44 selected features with ranks of the full model (limited to 800). Dots are color-coded and shaped according to cellular and fluid compartment (CSF: cerebral spinal fluid, CSF cells and plasma) and Small-seq and transfer (t)RNA fragments (tRFs)), respectively. **(C)** Area Under the Receiver Operating Characteristics (AUROC) and k-means clustering (k = 2) followed by split and join (S/J) method. **(D)** Separation of relapsing–remitting Multiple Sclerosis (RRMS, blue) and other neurological disease controls (C, red) based on latent variable 1 (L1) of the MCIA analysis derived from reduced variable numbers, was evaluated with line, density and boxplots as well as Wilcoxon signed-rank test (***: *p*-value < 0.001, NS: Not significant). In the line plot MS subtype: relapse (MS-rel), remission (MS-rem) and secondary progressive (SPMS), control subtype: inflammatory (INDC) and non-inflammatory neurological disease controls (NINDCs) and clinically or radiologically isolated syndrome (CIS and RIS) are distinguished by colour and shape. **(E)** Contribution of various features derived from the Small-seq and MINTmap pipelines to MCIA L1 eigenvalues of the reduced model.

To reduce the number of input features for MS classification based on L1 from the full model, we applied Lasso regression. Lambda parameter tuning with 5-fold cross-validation and a filter of 1 standard deviation were applied for each compartment and dataset, which reduced the feature count from 173,352 to 44 (Figure 3A). Comparison of ranks showed that the 44 features in the reduced model were present in the top 800 (of 173,352) ranked features in the full model with a Spearman’s correlation coefficient of 0.90 (Figure 3B). When evaluation methods were re-applied to L1 of the “reduced” model, the AUROC value decreased marginally from 0.82 to 0.79 and the split/join value increased by 2, from 10 to 12 compared to the full model (Figure 3C). Hence, the 44 selected features still provided a robust classification of RRMS compared to controls, which was confirmed by line, density and boxplots of the “reduced” L1 as well as the Wilcoxon signed-rank test (*p* < 0.001) (Figure 3D). Selected features were solely based on small RNAs derived from CSF cells (Quintana et al., 2017), CSF (Weber et al., 2007) and plasma (Herman et al., 2018) (Figure 3E). Hence, the contribution from DNA methylation of CSF cells and Small-seq derived transcripts from PBMCs were no longer considered in L1.

In summary, Lasso regression reduced the number of input features to 44, while consistently allowing for classification of RRMS. Hence, we here demonstrate that data from multi-cellular and fluid sources can be reduced to few features, which is important for future biomarker potential.

### 3.4 Misc- and miRNAs from CSF cells and 3′-tRFs from plasma have major contribution to the RRMS classification

**FIGURE 4:**
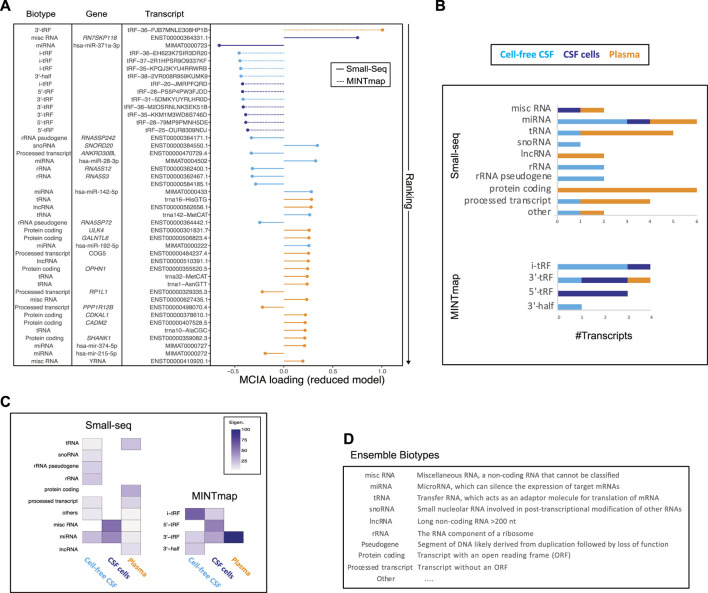
Transcript biotype and source contribution to the reduced model. **(A)** Plotting of ranked multiple co-inertia analysis (MCIA) loadings color-coded according to the source (cell-free CSF: light blue, CSF cells: dark blue and plasma: orange), and line type according to analysis pipeline with full line representing Small-seq and dashed representing MINTmap, respectively. Transcript biotype, gene name and transcript IDs are given. **(B)** Overview of #transcripts from different biotypes derived from plasma (orange), CSF cells (dark blue) and CSF (light blue), which are included in the reduced model. Transcripts were divided into Small-seq and MINTmap pipelines, respectively. **(C)** Heatmap illustrating the contribution of different biotypes and sources, with dark purple illustrating most, and lighter color illustrating less contribution. **(D)** Explanation of various biotypes modified from https://m.ensembl.org/info/genome/genebuild/biotypes.html.

Ranking of individual MCIA loadings from the 44 selected features revealed that among top 15, 12 were tsRNAS derived from MINTmap profiling with the majority derived from the CNS compartment and down-regulated in RRMS compared to controls (Figure 4A, Supplementary Figure S4). However, the top feature, tRF-36-PJB7MNLE308HP1B, derived from plasma was up-regulated in RRMS. The second top-ranked feature was classified as a miscRNA, 7SK small nuclear pseudogene 118 (ENST00000364331.1), which, like the third top-ranked small RNA, hsa-miR-371a-5p, was found to be dysregulated in RRMS in CSF cells. MiRNA was the only biotype represented in all sources, i.e., CSF, CSF cells and plasma, in the reduced model. Other biotypes, such as tRNA, lncRNA, protein coding and processed transcripts were predominantly represented in plasma, whereas snoRNA, rRNA and rRNA pseudogene were represented in cell-free CSF only (Figure 4A). Overall, more than half, 59.4% (19/32), of the features derived from the Small-seq pipeline were derived from plasma, 34.4% (11/32) from CSF and 6.3% (2/32) from CSF cells, in contrast to MINTmap-derived features, where half, 50% (6/12) were derived from CSF cells, 41.7% (5/12) from CSF and only 8.3% (1/12) from plasma (Figure 4A). The majority of MINTmap-derived features were classified as i- (4/12, 33.3%), 5′-(3/12, 25%) and 3′-tRFs (4/12, 33.3%), which are cleaved at the D- and T-loops of mature tRNAs, whereas only 8.3% (1/12) was classified as the longer 3′half, cleaved at the anticodon loop (Figure 4A). The contribution of each biotype to the latent variable of the reduced model, estimated based on the magnitude and number, showed that among CSF cells, miscRNAs and miRNAs were major contributors, whereas, for CSF and plasma, the contribution was more widespread across different biotypes (Figures 4B,C). For MINTmap-derived features, the overall major contributor was 3′-tRNA-derived fragments (tRFs) from plasma, i-tRFs from CSF and 5′-tRFs from CSF cells (Figures 4B,C). Hence, it is interesting to observe that, although plasma only has one MINTmap-derived feature included in the reduced model, it has a major contribution to L1. Boxplots visualizing selected features grouped into RRMS (+ CIS and RIS), SPMS and controls are given in Supplementary Figure S4.

In summary, a mix of transcript biotypes and sources contribute to the latent variable of the reduced model with major contributions coming from misc- and miRNAs from CSF cells and CSF and a 3′tRF from plasma.

## 4 Discussion

In the current study, we utilized an unsupervised approach, based on simultaneous integration of omics data from fluids and cells from the same individuals to distinguish MS from controls. Samples are derived from blood or CSF, which are both of biological relevance to MS due to the immunological component of the disease and proximity of CSF to the target organ. Furthermore, blood and CSF are routinely collected in MS diagnostic evaluation emphasizing their biomarker potential. We focused on small RNAs and DNA methylation and found that small RNAs from cell-free CSF, CSF cells and plasma played a major role in distinguishing MS from controls. Lasso regression selected 44 small RNAs from three sources with just a slight reduction in AUROC (from 0.82 to 0.79) and split/join (from 10 to 12). Hence, we showcase an integrative approach with input of multi-omics data derived from multiple compartments combined with feature reduction for classification of MS.

The majority (12 of 15) of top-ranked features in the reduced model were derived from MINTmap profiled plasma or cell-free CSF small RNAs, predominantly classified as tRFs, which include i-, 5′- and 3′-tRFs, cleaved at the D- and T-loops of mature tRNAs. 3′ and 5′tRFs are 14–30 nucleotides long and have previously been shown to interact with Argonaute complexes, suggesting that tRFs might have post-transcriptional regulatory potential (Kumar et al., 2014; Kuscu et al., 2018) and may also be protected from degradation in extracellular compartments similar to miRNAs (Arroyo et al., 2011; Turchinovich et al., 2011). Bioinformatic tools for prediction of tRF targets genes are starting to emerge, however, we did not manage to predict targets for our 15 candidates using either trftars (Xiao et al., 2021), miRanda (Betel et al., 2008) or TargetScan (Agarwal et al., 2015), of which the latter two, were originally designed for miRNAs, but have previously been utilized for tRF target prediction as well (Wang et al., 2019). However, tRF target prediction is still in its early days, and future tools might include target genes for tRFs identified in our study as well, which could potentially also give insight into molecular mechanisms. Currently, MINTmap (Loher et al., 2017) supports 125,285 tRNAs (based on genomic location), corresponding to 28,824 tRFs. Although, tRFs have previously been linked to diseases including neurological disorders, such as Alzheimer’s (Wu et al., 2021) and Parkinson’s (Magee et al., 2019) disease, as well as immune-related disorders such as Rheumatoid Arthritis (Ormseth et al., 2020) and inflammation (Liu et al., 2018a), specific roles of individual tRFs are still to be elucidated. Noteworthy, tRFs can be excreted through exosomes to recipient cells (Chiou et al., 2018), which could also be the reason, we detect them in plasma and cell-free CSF of MS patients and controls. Other studies have indeed documented enrichment of extracellular tRFs compared to other sncRNA classes in urine, blood serum, saliva or cerebrospinal fluid (Torres and Marti, 2021). Hence, studies on tsRNAs will likely grow in the coming years due to their high prevalence and stability making them excellent biomarker candidates, which might also give insight into molecular mechanisms by target gene prediction.

Another top-ranked small RNA (RN7SKP118) found to be up-regulated in CSF cells of RRMS patients is a pseudogene of 7SK snRNA, which is a master transcriptional regulator of RNA polymerase II (RNAPII), involved in the production of sncRNAs such as enhancer RNAs (eRNAs), sn/snoRNAs and also mRNA (Flynn et al., 2016). Genetic variations in genes coding for proteins in complex with 7SK snRNA, have previously been linked to neurological disorders. For example, biallelic *LARP7* loss-of-function variants have been associated with Alazami syndrome characterized by cognitive disability (Najmabadi et al., 2011; Alazami et al., 2012) and *MEPCE* nonsense variants have been reported in a young boy with developmental delay and seizures accompanied with frontal white matter lesions identified by brain MRI (Schneeberger et al., 2019). Hence, impairment of the 7SK snRNP complex can have neurological implications and has previously been linked to white matter lesions, a well-characterized disease hallmark of MS. Furthermore, altered expression of 7SK snRNA has previously been detected in serum of MS patients (Santoro et al., 2016). Although, RN7SKP118 is classified as a miscRNA due to its pseudogene status, RN7SKP118 has been predicted to also interact with LARP7 (https://rnact.crg.eu/) and could therefore have similar functions as 7SK snRNA.

We also identified several miRNAs in the reduced model. For example, hsa-miR-142–5p, which has previously been found in brain tissue and CSF from MS patients (Mandolesi et al., 2017), serum from SPMS (Regev et al., 2018), and animal model of MS (Talebi et al., 2017). Furthermore, hsa-miR-142–5p has been suggested to be involved in neurogenic differentiation (Yang et al., 2018). Hsa-miR-371a-3p we previously showed to be down-regulated in RRMS compared to NINCs in CFS cells (Zheleznyakova et al., 2021). Hsa-miR-374, which was detected in plasma, has previously been linked to neurodegenerative disorders, such as Amyotrophic lateral sclerosis (Waller et al., 2017), Alzheimer’s (Unterbruner et al., 2018) and Parkinson’s disease (Briggs et al., 2015), as well as immune-related diseases including T‐cell acute lymphoid leukemia (Gimenes-Teixeira et al., 2013; Qian et al., 2015) and inflammatory processes in diabetes (Doumatey et al., 2018). Additional miRNAs, identified in the reduced model, are linked to inflammation, such as hsa-miR-28–3p, which has been detected in germinal center B cells (Schneider et al., 2014) and hsa-miR-192–5p, which has been linked to M1 macrophage activation.

Despite capturing predominantly sncRNAs, we also detected protein coding and processed transcripts, in particular in plasma. Interestingly, Unc-51 Like Kinase 4 (ULK4), one of the selected protein coding transcripts in the reduced model has been proposed as a key regulator of myelination (Liu et al., 2018b) with ULK4 deficiency linked with disrupted white matter integrity (Lang et al., 2014), again a disease hallmark of MS. Furthermore, Oligophrenin 1 (OPHN1), a protein expressed in the brain, which is involved in synaptic maturation and plasticity (Nadif Kasri et al., 2009) was also among the 44 selected transcripts in the reduced model.

Our integrative approach was initially also based on features from CSF cell methylome data. However, plotting of MCIA loadings from each dataset for the full model revealed that the CSF cell methylome had a minor contribution to the latent variable, L1, separating RRMS from controls (NINDC and INDC) and no CSF cell methylome features were included in the final, reduced model. Noticeably, a smoothing algorithm was applied to the PBAT methylome data, due to sparse coverage, which may have compromised the outcome, or perhaps the DNA methylation signal got diluted due to mixed cell populations in the CSF cells. It could also be that extracellular RNAs simply have greater biomarker potential for MS compared to intracellular DNA methylation. Future studies are needed for making further conclusions in this regard.

In summary, we input 378 (42 × 4 × 2 + 42) profiles from cell-free CSF, CSF, plasma and PBMCs, focusing on small RNAs processed through Small-seq and MINTmap pipelines as well as CSF cell methylomes resulting in a total of 173,352 features. All datasets were derived from the same 42 individuals allowing for simultaneous integration. Although we considerably increased the input data using this approach, it is important to note that MS is a heterogeneous disease and that potentially we cover a subgroup of affected individuals. Future studies are needed to uncover whether all, some or none of the selected features separate RRMS from others in independent cohorts and a larger sample size is needed for final AUROC estimates. Here the main purpose was to trial a multi-omics integration approach for future MS biomarker development based on samples commonly collected from MS patients such as blood and CSF.

## Data Availability

Publicly available datasets were analyzed in this study. This data can be found here: Swedish National Data Service (https://doi.org/10.5878/c1mq-9r62).
