# Supplementary material for: Integration of small RNAs from plasma and cerebrospinal fluid for classification of multiple sclerosis
Source: Front Genet. 2022 Nov 17;13:1042483. doi: 10.3389/fgene.2022.1042483 (PMC9713411; doi:10.3389/fgene.2022.1042483)
Supplement: Supplementary file 5 [file DataSheet11.PDF]

3trna1-AsnGTT_1 NA NA
3trna1-HisGTG_1 NA NA
3trna1-PheGAA_1 NA NA
3trna10-AspGTC_1 NA NA
3trna10-MetCAT_1 NA NA
3trna10-SerGCT_1 NA NA
3trna11-ArgACG_1 NA NA
3trna11-GluTTC_1 NA NA
3trna12-ValAAC_1 NA NA
3trna128-GlyGCC_1 NA NA
3trna13-AlaCGC_1 NA NA
3trna13-LysCTT_2 NA NA
3trna14-ThrCGT_1 NA NA
3trna15-ThrCGT_1 NA NA
3trna158-IleAAT_1 NA NA
3trna16-LeuAAG_2 NA NA
3trna16-TyrGTA_2 NA NA
3trna2-SerTGA_1 NA NA
3trna2-TyrGTA_1 NA NA
3trna21-ArgCCT_1 NA NA
3trna26-CysGCA_1 NA NA
3trna26-LeuCAG_1 NA NA
3trna27-CysGCA_1 NA NA
3trna27-LeuTAG_1 NA NA
3trna3-ArgCCT_1 NA NA
3trna32-MetCAT_1 NA NA
3trna39-TrpCCA_1 NA NA
3trna4-ArgTCG_1 NA NA
3trna4-ArgTCG_2 NA NA
3trna4-AspGTC_1 NA NA
3trna4-ThrAGT_1 NA NA
3trna4-TyrGTA_1 NA NA
3trna4-ValTAC_1 NA NA
3trna40-ThrAGT_1 NA NA
3trna47-AsnGTT_1 NA NA
3trna5-GlyGCC_1 NA NA
3trna5-IleTAT_1 NA NA
3trna6-AlaAGC_1 NA NA
3trna6-ProTGG_1 NA NA
3trna6-TrpCCA_1 NA NA
3trna7-ArgACG_1 NA NA
3trna7-AsnGTT_1 NA NA
3trna76-LysTTT_1 NA NA
3trna8-ArgACG_1 NA NA
3trna8-HisGTG_1 NA NA
3trna9-HisGTG_1 NA NA
3trna9-IleAAT_2 NA NA
ENST00000005178.5 PDK4 protein_coding
ENST00000011619.5 RANBP9 protein_coding
ENST00000011653.7 CD4 protein_coding
ENST00000011700.9 VPS13D protein_coding
ENST00000012134.5 HIVEP2 protein_coding
ENST00000016171.5 COX15 protein_coding
ENST00000022615.7 VDAC3 protein_coding

ENST00000025301.3 AKAP11 protein_coding
ENST00000025399.9 STRAP protein_coding
ENST00000033079.6 FAM13B protein_coding
ENST00000040738.8 BOD1L1 protein_coding
ENST00000040877.1 TARBP1 protein_coding
ENST00000046794.8 LCP2 protein_coding
ENST00000053468.3 MRPS10 protein_coding
ENST00000054666.9 VAMP3 protein_coding
ENST00000056233.3 NFE2L3 protein_coding
ENST00000064778.7 FAM168A protein_coding
ENST00000066544.6 CDC27 protein_coding
ENST00000074304.8 INPP4A protein_coding
ENST00000082468.10 BTN3A1 retained_intron
ENST00000162391.6 FOXJ2 protein_coding
ENST00000163416.5 GOLGA5 protein_coding
ENST00000167825.5 ARHGEF10L nonsense_mediated_decay
ENST00000170630.5 IL4R protein_coding
ENST00000173785.4 KLF6 processed_transcript
ENST00000174618.4 MNT protein_coding
ENST00000175091.4 LAPTM4A protein_coding
ENST00000176763.8 STK10 protein_coding
ENST00000177694.1 TBX21 protein_coding
ENST00000179259.5 TIGAR protein_coding
ENST00000181839.7 CDK13 protein_coding
ENST00000182527.3 TRAM2 protein_coding
ENST00000186436.8 TMEM131 protein_coding
ENST00000192788.5 UHRF1BP1 protein_coding
ENST00000193391.7 IMPG2 protein_coding
ENST00000196371.8 OXCT1 protein_coding
ENST00000198765.5 NA NA
ENST00000198939.6 CHERP protein_coding
ENST00000199389.9 EIF2AK1 protein_coding
ENST00000200453.5 PPP1R15A protein_coding
ENST00000200557.9 ADAM11 protein_coding
ENST00000200639.7 LAMP2 protein_coding
ENST00000200652.3 SLC22A4 protein_coding
ENST00000202677.10 RALGAPA2 protein_coding
ENST00000204549.7 PDCD7 protein_coding
ENST00000205061.8 GLG1 protein_coding
ENST00000205636.3 CMTM6 protein_coding
ENST00000209875.7 CBX5 protein_coding
ENST00000209884.4 KLHL20 protein_coding
ENST00000210227.4 PTBP3 protein_coding
ENST00000215730.10 SNAP29 protein_coding
ENST00000215793.11 SF3A1 protein_coding
ENST00000215829.6 SNRPD3 protein_coding
ENST00000215832.9 MAPK1 protein_coding
ENST00000215862.7 MORC2 protein_coding
ENST00000215886.5 LGALS2 protein_coding
ENST00000215956.8 SNU13 protein_coding
ENST00000216038.5 RTCB protein_coding
ENST00000216061.8 PPP6R2 protein_coding
ENST00000216083.6 CBX6 protein_coding
ENST00000216106.5 HMGXB4 protein_coding

ENST00000216133.8 CBX7 protein_coding
ENST00000216181.8 MYH9 protein_coding
ENST00000216223.8 IL2RB protein_coding
ENST00000216264.11 CERK protein_coding
ENST00000216268.5 ZBED4 protein_coding
ENST00000216277.11 PAPOLA protein_coding
ENST00000216281.11 HSP90AA1 protein_coding
ENST00000216294.4 SNAPC1 protein_coding
ENST00000216297.5 SUPT16H protein_coding
ENST00000216373.8 SOS2 protein_coding
ENST00000216468.7 TMED8 protein_coding
ENST00000216484.5 SPTLC2 protein_coding
ENST00000216962.7 PYGB protein_coding
ENST00000217131.5 CTSZ protein_coding
ENST00000217133.1 TUBB1 protein_coding
ENST00000217315.8 TM9SF4 protein_coding
ENST00000217402.2 CHMP4B protein_coding
ENST00000217740.3 RNF125 protein_coding
ENST00000217885.5 NOX1 protein_coding
ENST00000217961.4 STS protein_coding
ENST00000217964.10 NA NA
ENST00000217971.7 PGRMC1 protein_coding
ENST00000218032.6 TLR8 protein_coding
ENST00000218328.11 HUWE1 processed_transcript
ENST00000218340.3 RP2 protein_coding
ENST00000219084.9 CHD9 retained_intron
ENST00000219689.10 USP31 protein_coding
ENST00000220592.8 AGO2 protein_coding
ENST00000220597.4 PAG1 protein_coding
ENST00000220763.8 CPQ protein_coding
ENST00000221930.5 TGFB1 protein_coding
ENST00000222120.6 RAB3D protein_coding
ENST00000222345.9 SIPA1L3 protein_coding
ENST00000222553.6 NAMPT protein_coding
ENST00000222567.5 POLR1F protein_coding
ENST00000222690.9 H2AZ2 protein_coding
ENST00000222725.8 LFNG protein_coding
ENST00000223023.4 WASL protein_coding
ENST00000223073.5 RBM28 protein_coding
ENST00000223122.3 C1GALT1 protein_coding
ENST00000223129.7 RPA3 protein_coding
ENST00000223293.8 GIMAP2 protein_coding
ENST00000223500.8 CHMP5 protein_coding
ENST00000223641.4 SEC61B protein_coding
ENST00000223791.6 AKNA protein_coding
ENST00000223795.2 TNFSF8 protein_coding
ENST00000224140.5 SETX protein_coding
ENST00000224756.11 CCSER2 protein_coding
ENST00000224949.7 PITRM1 protein_coding
ENST00000225235.4 TBC1D12 protein_coding
ENST00000225298.10 UTP18 protein_coding
ENST00000225388.7 NUFIP2 protein_coding
ENST00000225525.3 TAX1BP3 protein_coding
ENST00000225719.7 CPD protein_coding

ENST00000225724.8 GOSR1 protein_coding
ENST00000225737.9 AKAP10 protein_coding
ENST00000225972.7 LRRC59 protein_coding
ENST00000226328.7 RUFY3 protein_coding
ENST00000226524.3 PF4V1 protein_coding
ENST00000228434.6 CD69 protein_coding
ENST00000228463.6 SELPLG protein_coding
ENST00000228862.3 DUSP16 protein_coding
ENST00000228865.2 CREBL2 protein_coding
ENST00000228872.7 CDKN1B protein_coding
ENST00000228928.10 OAS3 protein_coding
ENST00000229214.7 KRR1 protein_coding
ENST00000229402.3 KLRB1 protein_coding
ENST00000229633.6 HINT3 protein_coding
ENST00000229634.12 NCOA7 protein_coding
ENST00000229794.7 MAPK14 protein_coding
ENST00000229812.7 STK38 protein_coding
ENST00000229971.2 FBXL4 protein_coding
ENST00000230122.3 ZBTB24 protein_coding
ENST00000230124.6 FIG4 protein_coding
ENST00000230449.7 EXOC2 protein_coding
ENST00000230568.4 LY86 protein_coding
ENST00000230859.6 TENT4A protein_coding
ENST00000230990.6 HBEGF protein_coding
ENST00000231009.2 GZMK protein_coding
ENST00000231368.8 LNPEP protein_coding
ENST00000231461.8 ST8SIA4 protein_coding
ENST00000232564.6 GNB4 protein_coding
ENST00000233057.7 EIF2AK2 protein_coding
ENST00000233099.5 HEATR5B protein_coding
ENST00000233143.5 TMSB10 protein_coding
ENST00000233202.9 SLC11A1 protein_coding
ENST00000233336.6 TTL protein_coding
ENST00000233468.4 SF3B6 protein_coding
ENST00000233573.6 ITGA4 protein_coding
ENST00000233957.4 IL18R1 protein_coding
ENST00000234115.9 PLEKHB2 protein_coding
ENST00000234179.5 PRKD3 protein_coding
ENST00000234310.6 PPP3R1 protein_coding
ENST00000234313.7 PLEK protein_coding
ENST00000234453.8 PLEKHA3 protein_coding
ENST00000234827.4 TCEANC2 protein_coding
ENST00000235150.4 RNF19B protein_coding
ENST00000235307.6 C1orf21 protein_coding
ENST00000235329.8 MFN2 protein_coding
ENST00000235345.5 SLC35D1 protein_coding
ENST00000235382.6 RGS2 protein_coding
ENST00000235453.7 DENND1B protein_coding
ENST00000235790.7 KDM5B protein_coding
ENST00000236147.4 SELL protein_coding
ENST00000237163.8 DOP1A protein_coding
ENST00000237281.4 FBXO30 protein_coding
ENST00000237380.10 MED28 protein_coding
ENST00000237449.9 NA NA

ENST00000237654.7 CCNI protein_coding
ENST00000237937.6 ZFAND5 protein_coding
ENST00000238256.6 FKBP15 protein_coding
ENST00000238647.4 IRF2BPL protein_coding
ENST00000238738.7 RHOQ protein_coding
ENST00000238789.8 ATAD2B protein_coding
ENST00000238831.7 YIPF4 protein_coding
ENST00000238892.3 CRIPT protein_coding
ENST00000239223.3 DUSP1 protein_coding
ENST00000239231.6 PANK3 protein_coding
ENST00000239374.7 CCDC170 protein_coding
ENST00000239891.3 ALG5 protein_coding
ENST00000239938.4 EGR1 protein_coding
ENST00000239944.5 SERP1 protein_coding
ENST00000240316.4 COIL protein_coding
ENST00000240487.5 TMEM131L protein_coding
ENST00000240617.8 PLBD1 protein_coding
ENST00000240719.6 ZNF549 protein_coding
ENST00000241052.4 CAT protein_coding
ENST00000241393.3 CXCR4 protein_coding
ENST00000242059.8 SCRN1 protein_coding
ENST00000242210.10 NA NA
ENST00000242275.6 SLC25A51 protein_coding
ENST00000242310.4 TAF1L protein_coding
ENST00000242351.8 ZC3HAV1 protein_coding
ENST00000242465.3 SRGN protein_coding
ENST00000242480.3 EGR2 protein_coding
ENST00000242719.3 RNF11 protein_coding
ENST00000242737.5 ITPR2 protein_coding
ENST00000243077.6 LRP1 protein_coding
ENST00000243325.5 RAB9A protein_coding
ENST00000243344.7 TTC21B protein_coding
ENST00000243346.8 NMI protein_coding
ENST00000243903.5 ACTR5 protein_coding
ENST00000244020.4 SRSF6 protein_coding
ENST00000244040.3 RAB22A protein_coding
ENST00000244070.6 PPP4R1L processed_transcript
ENST00000244534.6 H1-3 protein_coding
ENST00000244537.5 H4C6 protein_coding
ENST00000244571.4 AARS2 protein_coding
ENST00000244745.2 SOX4 protein_coding
ENST00000244769.7 ATXN1 protein_coding
ENST00000245138.7 NA NA
ENST00000245414.7 IRF1 protein_coding
ENST00000246006.4 CD93 protein_coding
ENST00000246024.5 TMX4 protein_coding
ENST00000246100.3 FAM110A protein_coding
ENST00000246115.4 S1PR4 protein_coding
ENST00000246149.9 ANKHD1 protein_coding
ENST00000246163.2 MAX protein_coding
ENST00000246229.4 PLAGL2 protein_coding
ENST00000246314.9 AGO3 protein_coding
ENST00000246421.4 SLC35E2A processed_transcript
ENST00000247178.5 ATG14 protein_coding

ENST00000247225.6 SGPP1 protein_coding
ENST00000247226.10 PLEKHG3 protein_coding
ENST00000247295.4 FAM78A processed_transcript
ENST00000247584.6 NA NA
ENST00000247930.4 ZNF777 protein_coding
ENST00000248071.5 KLF2 protein_coding
ENST00000248244.5 TICAM1 protein_coding
ENST00000248248.6 MON1B protein_coding
ENST00000248566.3 SEM1 protein_coding
ENST00000248594.9 PTPN12 protein_coding
ENST00000248598.5 FGL2 protein_coding
ENST00000249071.9 RAC2 protein_coding
ENST00000249700.7 TMOD2 protein_coding
ENST00000249822.7 ARPP19 protein_coding
ENST00000250360.6 SIGLEC9 protein_coding
ENST00000250559.12 RAP1B protein_coding
ENST00000250974.9 ABHD17A protein_coding
ENST00000251047.5 LMAN1 protein_coding
ENST00000251334.6 INTS2 protein_coding
ENST00000251343.8 KHNYN protein_coding
ENST00000251527.8 ESYT2 protein_coding
ENST00000251691.4 ARFGEF3 protein_coding
ENST00000251879.9 RIC1 protein_coding
ENST00000252032.9 DNAAF9 protein_coding
ENST00000252071.7 ACTR3C protein_coding
ENST00000252229.6 MICB protein_coding
ENST00000252322.1 CRACR2A protein_coding
ENST00000252482.6 NA NA
ENST00000252485.7 NECTIN2 protein_coding
ENST00000252655.1 NA NA
ENST00000252674.8 MLLT1 protein_coding
ENST00000252744.5 ZSWIM6 protein_coding
ENST00000252785.3 SCO2 protein_coding
ENST00000252818.4 JUND protein_coding
ENST00000252840.9 ZNF557 protein_coding
ENST00000252936.6 TUBGCP2 protein_coding
ENST00000253039.7 EIF2S3 protein_coding
ENST00000253048.8 ZC3H4 protein_coding
ENST00000253063.3 SESN2 protein_coding
ENST00000253233.4 MTRFR protein_coding
ENST00000253251.11 UBE4B protein_coding
ENST00000253270.10 SLC35D2 protein_coding
ENST00000253329.2 PPIL4 protein_coding
ENST00000253339.8 LATS1 protein_coding
ENST00000253673.5 ADGRE3 protein_coding
ENST00000253692.10 TBC1D5 protein_coding
ENST00000253814.5 NDFIP1 protein_coding
ENST00000253815.3 UBE2D2 protein_coding
ENST00000254101.3 PRKAB2 protein_coding
ENST00000254190.3 CHSY1 protein_coding
ENST00000254235.6 ADCY7 protein_coding
ENST00000254321.8 ZNF700 protein_coding
ENST00000254322.2 DNAJB1 protein_coding
ENST00000254480.8 SMARCC1 protein_coding

ENST00000254508.6 NUP210 protein_coding
ENST00000254528.3 EMILIN2 protein_coding
ENST00000254630.10 PTCD3 protein_coding
ENST00000254691.8 CARD6 protein_coding
ENST00000254803.3 UTP3 protein_coding
ENST00000254810.7 H3-3B protein_coding
ENST00000255304.7 USPL1 protein_coding
ENST00000255882.9 PI4KA protein_coding
ENST00000256015.4 BTG1 protein_coding
ENST00000256078.7 KRAS protein_coding
ENST00000256186.2 NA NA
ENST00000256190.11 SBF2 protein_coding
ENST00000256257.1 RNF122 protein_coding
ENST00000256367.2 TTC9 protein_coding
ENST00000256429.6 MBD2 protein_coding
ENST00000256447.5 CD180 protein_coding
ENST00000256458.4 IRAK2 protein_coding
ENST00000256495.3 BHLHE40 protein_coding
ENST00000256497.7 EDEM1 protein_coding
ENST00000256646.5 NOTCH2 protein_coding
ENST00000256652.7 CD101 protein_coding
ENST00000256854.8 NARS1 protein_coding
ENST00000256858.9 RELCH protein_coding
ENST00000256876.9 IL2RA protein_coding
ENST00000257336.4 BIVM protein_coding
ENST00000257787.5 AKIRIN2 protein_coding
ENST00000257789.4 ORC3 protein_coding
ENST00000258198.5 DYNC1LI2 protein_coding
ENST00000258243.5 URB2 protein_coding
ENST00000258301.5 STX6 protein_coding
ENST00000258349.7 RC3H1 protein_coding
ENST00000258362.6 PNKD protein_coding
ENST00000258418.8 CAB39 protein_coding
ENST00000258439.6 TMEM127 protein_coding
ENST00000258449.2 TGFBRAP1 protein_coding
ENST00000258455.6 MRPS9 protein_coding
ENST00000258484.9 EPC2 protein_coding
ENST00000258526.7 PLXNC1 protein_coding
ENST00000258646.3 RCBTB1 protein_coding
ENST00000258886.11 IREB2 protein_coding
ENST00000258963.6 VEZF1 protein_coding
ENST00000259006.6 LIMD2 protein_coding
ENST00000259021.7 KAT7 protein_coding
ENST00000259235.6 SAP130 protein_coding
ENST00000259253.9 UGGT1 protein_coding
ENST00000259324.5 LRRC8A protein_coding
ENST00000259339.5 TOR1B protein_coding
ENST00000259455.2 GABBR2 protein_coding
ENST00000259512.7 DERL1 protein_coding
ENST00000259605.9 RNF38 protein_coding
ENST00000259698.7 RIPOR2 protein_coding
ENST00000259939.3 RNF144B protein_coding
ENST00000259963.3 FAM8A1 protein_coding
ENST00000259997.8 NA NA

ENST00000260010.6 TLR2 protein_coding
ENST00000260184.10 NA NA
ENST00000260197.10 SORL1 protein_coding
ENST00000260270.2 FDX1 protein_coding
ENST00000260323.14 UNC13C protein_coding
ENST00000260356.5 THBS1 protein_coding
ENST00000260408.6 ADAM10 protein_coding
ENST00000260563.4 RTCA protein_coding
ENST00000260569.4 CEP68 protein_coding
ENST00000260585.10 SELENOI protein_coding
ENST00000260641.8 ACTR2 protein_coding
ENST00000260665.10 LRPPRC protein_coding
ENST00000260743.8 CALHM2 protein_coding
ENST00000260746.5 ARL3 protein_coding
ENST00000260762.9 EXOC6 protein_coding
ENST00000260810.8 TOPBP1 protein_coding
ENST00000260818.9 DNAJC13 protein_coding
ENST00000260908.10 SLFN12L protein_coding
ENST00000261024.5 SLC40A1 protein_coding
ENST00000261173.5 ATP2B1 protein_coding
ENST00000261187.7 SLC16A7 protein_coding
ENST00000261203.6 LIN7A nonsense_mediated_decay
ENST00000261211.6 CDK17 protein_coding
ENST00000261226.7 TMCC3 protein_coding
ENST00000261233.7 IRAK3 protein_coding
ENST00000261254.6 CCND2 protein_coding
ENST00000261263.4 RAB21 protein_coding
ENST00000261267.5 LYZ protein_coding
ENST00000261318.4 SPRING1 protein_coding
ENST00000261381.6 XYLT1 protein_coding
ENST00000261396.5 NUP133 protein_coding
ENST00000261415.10 CERT1 protein_coding
ENST00000261427.8 UBE2K protein_coding
ENST00000261438.8 KLF3 protein_coding
ENST00000261461.5 PPP2R5A protein_coding
ENST00000261482.7 REEP5 protein_coding
ENST00000261483.4 MAN2A1 protein_coding
ENST00000261489.5 TSC22D1 protein_coding
ENST00000261497.7 USP22 protein_coding
ENST00000261537.6 MIB1 protein_coding
ENST00000261596.7 LPIN2 protein_coding
ENST00000261609.10 HERC2 protein_coding
ENST00000261622.4 SLC7A5 protein_coding
ENST00000261637.4 UTP20 protein_coding
ENST00000261657.5 CLEC16A protein_coding
ENST00000261667.6 KPNA3 protein_coding
ENST00000261716.6 TAOK1 protein_coding
ENST00000261721.7 BTBD1 protein_coding
ENST00000261733.5 ALDH2 protein_coding
ENST00000261758.5 MESD protein_coding
ENST00000261772.11 AARS1 protein_coding
ENST00000261778.1 TANGO6 protein_coding
ENST00000261797.6 NDST1 protein_coding
ENST00000261811.5 CYSTM1 protein_coding

ENST00000261845.6 MAPK6 protein_coding
ENST00000261858.5 GLCE protein_coding
ENST00000261900.6 CCNT1 protein_coding
ENST00000261942.6 FAF2 protein_coding
ENST00000262031.8 RBMS2 protein_coding
ENST00000262056.12 EIF4B protein_coding
ENST00000262065.6 MMD miRNA
ENST00000262077.2 NUP153 miRNA
ENST00000262096.11 ZDHHC2 miRNA
ENST00000262160.9 SMAD2 miRNA
ENST00000262193.6 PSMB1 miRNA
ENST00000262215.6 ARFGEF1 miRNA
ENST00000262225.6 TMED2 miRNA
ENST00000262238.7 YY1 miRNA
ENST00000262241.6 RCOR1 miRNA
ENST00000262244.5 MOB3B miRNA
ENST00000262318.11 CLCN7 miRNA
ENST00000262346.6 ANKRD13C miRNA
ENST00000262367.8 CREBBP miRNA
ENST00000262384.3 N4BP1 miRNA
ENST00000262430.5 MLYCD miRNA
ENST00000262455.6 ERP44 miRNA
ENST00000262456.5 INVS miRNA
ENST00000262487.4 ISM1 miRNA
ENST00000262519.11 SETD1A miRNA
ENST00000262554.5 SPTLC1 miRNA
ENST00000262577.5 ZC3H3 miRNA
ENST00000262607.3 ADA2 miRNA
ENST00000262644.7 BPNT2 miRNA
ENST00000262719.8 PHLPP1 miRNA
ENST00000262768.10 TIMP2 miRNA
ENST00000262809.7 ELL miRNA
ENST00000262811.9 MAST3 miRNA
ENST00000262825.8 NA miRNA
ENST00000262839.2 TRPC5 miRNA
ENST00000262848.5 PRKX miRNA
ENST00000262861.7 NA miRNA
ENST00000262878.4 SAMHD1 miRNA
ENST00000262919.8 ATRN miRNA
ENST00000262929.8 SIRPB1 miRNA
ENST00000262940.10 RASA4 miRNA
ENST00000262941.7 ZSCAN25 miRNA
ENST00000262962.10 YJU2 miRNA
ENST00000262971.2 PIAS4 miRNA
ENST00000262982.2 CSE1L miRNA
ENST00000262992.7 INPP4B miRNA
ENST00000263026.8 EEF2K miRNA
ENST00000263050.3 AKAP7 miRNA
ENST00000263063.7 MTPAP miRNA
ENST00000263073.9 SMG6 miRNA
ENST00000263095.9 ZNF264 miRNA
ENST00000263102.6 CCDC6 miRNA
ENST00000263150.7 WDR37 miRNA
ENST00000263168.3 CAPZA1 miRNA

ENST00000263228.3 UBE2R2 miRNA
ENST00000263238.5 ACTR3 miRNA
ENST00000263239.5 DDX18 miRNA
ENST00000263253.7 EP300 miRNA
ENST00000263256.6 DESI1 miRNA
ENST00000263268.9 MREG miRNA
ENST00000263275.4 OPA3 miRNA
ENST00000263372.4 KCNK6 miRNA
ENST00000263377.5 BRD4 miRNA
ENST00000263379.3 IL27RA miRNA
ENST00000263384.10 FAM32A miRNA
ENST00000263398.9 CD44 miRNA
ENST00000263464.6 BIRC3 miRNA
ENST00000263579.4 DCPS miRNA
ENST00000263620.6 ARID3A miRNA
ENST00000263635.7 TANC1 miRNA
ENST00000263636.4 LY75 miRNA
ENST00000263642.2 NA miRNA
ENST00000263708.5 PTPN4 miRNA
ENST00000263733.4 FAM20B miRNA
ENST00000263754.4 KAT2B miRNA
ENST00000263805.7 ZNF106 miRNA
ENST00000263812.7 SLC25A12 miRNA
ENST00000263826.8 AKT3 miRNA
ENST00000263831.10 DESI2 miRNA
ENST00000263847.4 OSBP miRNA
ENST00000263904.4 STAM2 miRNA
ENST00000263918.7 STRN miRNA
ENST00000263955.7 STK17B miRNA
ENST00000263967.3 PIK3CA miRNA
ENST00000263979.6 MAN1C1 miRNA
ENST00000264028.4 ARCN1 miRNA
ENST00000264033.4 CBL miRNA
ENST00000264156.2 MCM6 miRNA
ENST00000264170.7 KYNU miRNA
ENST00000264192.6 CYTIP miRNA
ENST00000264193.2 NA miRNA
ENST00000264211.11 EIF4G3 miRNA
ENST00000264235.11 GSK3B miRNA
ENST00000264245.7 ARHGAP31 miRNA
ENST00000264313.9 SLAIN2 miRNA
ENST00000264316.7 TXK miRNA
ENST00000264343.4 ARHGAP24 miRNA
ENST00000264345.6 HERC3 miRNA
ENST00000264346.10 HERC6 miRNA
ENST00000264360.6 PCDH10 miRNA
ENST00000264380.7 PIKFYVE miRNA
ENST00000264409.4 GPAT3 miRNA
ENST00000264414.7 CUL3 miRNA
ENST00000264431.7 RAPGEF2 miRNA
ENST00000264433.9 FNIP2 miRNA
ENST00000264449.13 ATP8A1 miRNA
ENST00000264474.3 CSTA miRNA
ENST00000264501.7 KIAA1109 miRNA

ENST00000264572.10 RAP1GDS1 miRNA
ENST00000264657.8 STAT3 miRNA
ENST00000264658.9 FBXL20 miRNA
ENST00000264709.6 DNMT3A miRNA
ENST00000264710.4 RAB10 miRNA
ENST00000264716.7 FOSL2 miRNA
ENST00000264779.6 GPBP1 miRNA
ENST00000264790.5 MMRN1 miRNA
ENST00000264824.4 LYL1 miRNA
ENST00000264864.7 PI4K2B miRNA
ENST00000264870.6 F13A1 miRNA
ENST00000264899.9 NA miRNA
ENST00000264914.7 ARSB miRNA
ENST00000264930.8 SLC12A7 miRNA
ENST00000264934.5 MTMR12 miRNA
ENST00000264951.7 XRN1 miRNA
ENST00000265012.4 GCNT2 miRNA
ENST00000265018.3 FAM184B miRNA
ENST00000265044.5 SSR3 miRNA
ENST00000265069.11 ZFR protein_coding
ENST00000265070.6 GOLPH3 protein_coding
ENST00000265081.6 MSH3 trna
ENST00000265085.8 CPEB4 trna
ENST00000265093.4 ATP6V0E1 trna
ENST00000265138.3 ARRDC3 trna
ENST00000265271.6 RBM27 trna
ENST00000265343.8 AFF4 trna
ENST00000265344.7 KCTD20 trna
ENST00000265354.5 SRF trna
ENST00000265361.6 SEMA3C trna
ENST00000265381.5 APBA1 trna
ENST00000265404.5 STAP1 trna
ENST00000265447.7 ANXA11 trna
ENST00000265450.5 TSPAN14 trna
ENST00000265453.7 NA trna
ENST00000265562.4 PTPN23 trna
ENST00000265631.8 SLC25A13 trna
ENST00000265717.4 PRKAR2B trna
ENST00000265734.7 CDK6 trna
ENST00000265742.6 ANKIB1 trna
ENST00000265870.6 SLC25A16 trna
ENST00000265970.10 PIK3C2A trna
ENST00000265990.9 BTAF1 trna
ENST00000265997.4 CPEB3 trna
ENST00000266037.9 DOCK3 trna
ENST00000266069.4 GID8 trna
ENST00000266079.4 PRPF6 trna
ENST00000266397.5 ERP27 trna
ENST00000266427.3 ETV6 trna
ENST00000266517.7 ETNK1 trna
ENST00000266589.9 SCAF11 trna
ENST00000266604.5 LLPH trna
ENST00000266659.6 GLIPR1 trna
ENST00000267068.5 N4BP2L2 trna

ENST00000267113.4 ESYT1 trna
ENST00000267116.7 ANKRD52 trna
ENST00000267163.4 RB1 trna
ENST00000267202.5 VPS37B trna
ENST00000267229.10 RBM26 trna
ENST00000267328.4 RAB20 trna
ENST00000267339.5 ANKRD10 trna
ENST00000267460.7 PELI2 trna
ENST00000267549.4 GPR65 trna
ENST00000267569.5 JDP2 trna
ENST00000267812.3 MFAP1 trna
ENST00000267890.9 TTBK2 trna
ENST00000267984.3 TLNRD1 trna
ENST00000268042.6 ARRDC4 trna
ENST00000268154.7 ZNF710 trna
ENST00000268184.9 CRTC3 trna
ENST00000268349.7 FTO trna
ENST00000268389.5 IGSF6 trna
ENST00000268489.8 ZFHX3 trna
ENST00000268893.9 TSPOAP1 trna
ENST00000269033.6 SSH2 trna
ENST00000269143.6 AFG3L2 trna
ENST00000269209.6 GAREM1 trna
ENST00000269214.8 ESCO1 trna
ENST00000269300.7 PIK3R5 trna
ENST00000269518.9 PMAIP1 trna
ENST00000269829.5 ZNF544 trna
ENST00000269856.4 FEM1A trna
ENST00000269967.3 CCDC97 trna
ENST00000270001.10 ZFP14 trna
ENST00000270139.6 IFNAR1 trna
ENST00000270162.6 SIK1 trna
ENST00000270190.7 NA trna
ENST00000271139.11 MOB3C trna
ENST00000271375.5 SFT2D2 trna
ENST00000271583.6 TOR1AIP1 trna
ENST00000271588.7 HMCN1 trna
ENST00000271628.8 SF3B4 trna
ENST00000271638.2 S100A11 trna
ENST00000272091.7 SDE2 trna
ENST00000272117.6 ITPKB trna
ENST00000272163.7 LBR trna
ENST00000272217.5 ARL8A trna
ENST00000272233.5 RHOB trna
ENST00000272249.6 HNRNPLL trna
ENST00000272322.7 VPS54 trna
ENST00000272427.9 EXOC6B trna
ENST00000272542.6 SLC20A1 trna
ENST00000272716.8 SLC4A10 trna
ENST00000273062.5 CTDSP1 trna
ENST00000273153.8 CSRNP1 trna
ENST00000273221.7 IQSEC1 trna
ENST00000273317.4 LIMD1 trna
ENST00000273398.6 ATP6V1A trna

ENST00000273432.7 MED12L trna
ENST00000273550.10 FTH1 trna
ENST00000273908.4 SCD5 trna
ENST00000274008.4 SPATA5 trna
ENST00000274056.10 MARCHF1 trna
ENST00000274217.3 OTULINL trna
ENST00000274306.6 GZMA trna
ENST00000274457.4 FEM1C trna
ENST00000274498.7 ARHGAP26 trna
ENST00000274609.5 ADAMTS2 trna
ENST00000274680.7 FARS2 trna
ENST00000274787.2 HIGD2A trna
ENST00000274793.10 PLA2G7 trna
ENST00000274811.7 RNF44 trna
ENST00000274813.3 MMUT trna
ENST00000274849.2 ABT1 trna
ENST00000275034.4 PHIP trna
ENST00000275072.4 PM20D2 trna
ENST00000275233.10 SHPRH trna
ENST00000275364.6 GNA12 trna
ENST00000275732.5 GIGYF1 trna
ENST00000275767.3 TMEM140 trna
ENST00000276055.3 CHST7 trna
ENST00000276062.8 NDUFB11 trna
ENST00000276072.6 TAF1 trna
ENST00000276110.5 IL2RG trna
ENST00000276202.7 DOCK11 trna
ENST00000276282.6 MFHAS1 trna
ENST00000276590.4 LACTB2 trna
ENST00000276898.3 RIC1 trna
ENST00000277165.9 FAM120A trna
ENST00000277198.5 AOPEP trna
ENST00000277537.9 SEC16A trna
ENST00000277541.6 NA trna
ENST00000277554.3 NACC2 trna
ENST00000277575.5 USP6NL trna
ENST00000277817.9 HERC4 trna
ENST00000277865.4 GLUD1 trna
ENST00000277900.11 ADD3 trna
ENST00000278499.5 SESN3 trna
ENST00000278505.4 ENDOD1 trna
ENST00000278590.6 ZC3H12C trna
ENST00000278612.8 NPAT trna
ENST00000278742.5 ST14 trna
ENST00000279034.9 SOGA1 trna
ENST00000279101.8 CABLES2 trna
ENST00000279451.7 CNKSR2 trna
ENST00000279488.7 DUSP6 trna
ENST00000279873.10 ARID5B trna
ENST00000280377.8 USP15 trna
ENST00000280527.5 CRIM1 trna
ENST00000280571.9 RILPL2 trna
ENST00000280591.9 TRNT1 trna
ENST00000280699.12 NGLY1 trna

ENST00000280772.5 ANK3 trna
ENST00000280780.6 FAM53B trna
ENST00000280871.7 SLC2A13 trna
ENST00000280876.6 GXYLT1 trna
ENST00000281092.7 FER trna
ENST00000281156.4 KHDRBS2 trna
ENST00000281419.6 ASAP2 trna
ENST00000281474.8 NA trna
ENST00000281496.6 VWA8 trna
ENST00000281513.8 NBAS trna
ENST00000281589.4 PABPC3 trna
ENST00000281631.8 PARP8 trna
ENST00000281828.6 FARSB trna
ENST00000281923.3 MGAT5 trna
ENST00000281924.6 TMEM163 trna
ENST00000281928.6 MED13L trna
ENST00000282003.6 OBI1 trna
ENST00000282007.6 ZC3H13 trna
ENST00000282030.5 NA trna
ENST00000282032.3 ARL14EP trna
ENST00000282077.6 PDK1 trna
ENST00000282096.7 PDE3B trna
ENST00000282146.4 KCNK13 trna
ENST00000282169.8 UHMK1 trna
ENST00000282251.8 CWF19L2 trna
ENST00000282344.9 USP12 trna
ENST00000282388.3 ZFP36L2 trna
ENST00000282570.3 GMCL1 trna
ENST00000282574.7 TIA1 trna
ENST00000282588.6 ITGA1 trna
ENST00000283131.3 SMARCA5 trna
ENST00000283147.6 BMP6 trna
ENST00000283195.9 RANBP2 trna
ENST00000283206.7 TMEM87B trna
ENST00000283285.8 CD96 trna
ENST00000283415.3 LPCAT1 trna
ENST00000283632.4 RMND5A trna
ENST00000283646.4 RPIA trna
ENST00000283684.7 NA trna
ENST00000283882.3 CFDP1 trna
ENST00000283943.8 TRIP12 trna
ENST00000284049.6 CHD1 trna
ENST00000284268.6 ANKH trna
ENST00000284273.5 UBASH3B trna
ENST00000284274.4 OTULIN trna
ENST00000284320.5 TOMM70 trna
ENST00000284384.6 PRKCA trna
ENST00000284476.7 DISP1 trna
ENST00000284548.14 OBSCN trna
ENST00000284898.9 NA trna
ENST00000285018.4 WNT7A trna
ENST00000285667.3 HSPA13 trna
ENST00000285873.7 GEMIN5 trna
ENST00000285968.9 NUP205 trna

ENST00000286067.3 NA trna
ENST00000286091.7 PDIA4 trna
ENST00000286181.6 FAM126B trna
ENST00000286364.6 RASA2 trna
ENST00000286548.7 GNAQ trna
ENST00000286713.5 STOM trna
ENST00000286760.5 WHAMM trna
ENST00000287156.7 UBE2L6 trna
ENST00000287474.8 FRRS1 trna
ENST00000287497.11 NA trna
ENST00000287814.4 TIMP4 trna
ENST00000287912.6 RBM33 trna
ENST00000287934.3 FZD1 trna
ENST00000288266.6 APPL1 trna
ENST00000288422.3 TAB3 trna
ENST00000288502.7 TMEM268 trna
ENST00000288520.8 ASTN2 trna
ENST00000288670.12 FMNL2 trna
ENST00000288943.4 DUSP2 trna
ENST00000289153.5 PIK3CB trna
ENST00000289316.2 H2BC5 trna
ENST00000289382.6 CNOT11 trna
ENST00000289495.7 PPP1R9A trna
ENST00000289703.7 ELK4 trna
ENST00000289893.7 MACF1 trna
ENST00000289902.1 FCER1G trna
ENST00000290039.5 CACHD1 trna
ENST00000290209.8 SLC12A6 trna
ENST00000290551.4 BTG2 trna
ENST00000290650.7 UBR1 trna
ENST00000290795.6 GPBP1L1 trna
ENST00000290855.9 CLEC2D protein_coding
ENST00000291041.5 PSKH1 protein_coding
ENST00000291232.4 TNFRSF13C protein_coding
ENST00000291688.4 MCM3AP protein_coding
ENST00000291900.5 ZER1 protein_coding
ENST00000292176.2 ZBTB7B protein_coding
ENST00000292432.8 HK3 protein_coding
ENST00000292562.10 ZNF251 protein_coding
ENST00000292599.3 MAML1 protein_coding
ENST00000292782.7 DCUN1D1 protein_coding
ENST00000293303.4 KLHL10 protein_coding
ENST00000293328.6 STAT5B protein_coding
ENST00000293373.9 NCKAP1L protein_coding
ENST00000293648.7 ZNF562 protein_coding
ENST00000293662.7 TAMALIN protein_coding
ENST00000294353.6 ZYG11B protein_coding
ENST00000294383.6 USP24 protein_coding
ENST00000294507.3 LAPTM5 protein_coding
ENST00000294599.7 MEGF6 protein_coding
ENST00000294738.5 DENND1B processed_transcript
ENST00000294981.7 MAPKAPK2 protein_coding
ENST00000295025.11 REL protein_coding
ENST00000295030.5 PEX13 protein_coding

ENST00000295087.11 ARL5A protein_coding
ENST00000295133.8 FBXO41 protein_coding
ENST00000295211.5 SERPINB8 protein_coding
ENST00000295268.3 STPG2 protein_coding
ENST00000295317.3 RNF149 protein_coding
ENST00000295321.7 IWS1 protein_coding
ENST00000295324.3 CDC42EP3 protein_coding
ENST00000295470.8 HNRNPDL protein_coding
ENST00000295500.7 GPR155 protein_coding
ENST00000295628.3 LRRC58 protein_coding
ENST00000295770.3 STT3B protein_coding
ENST00000295797.4 PRKCI protein_coding
ENST00000295839.9 PPM1L protein_coding
ENST00000295888.7 WDFY3 protein_coding
ENST00000295920.7 GMPS protein_coding
ENST00000295925.5 CCNL1 protein_coding
ENST00000296028.3 PPBP protein_coding
ENST00000296029.3 PF4 protein_coding
ENST00000296088.10 SNRK protein_coding
ENST00000296121.5 KIAA1143 protein_coding
ENST00000296122.9 PPP1CB protein_coding
ENST00000296126.6 WDR43 protein_coding
ENST00000296137.5 FYCO1 protein_coding
ENST00000296140.3 CCR1 protein_coding
ENST00000296161.7 DTX3L protein_coding
ENST00000296220.5 OSBPL11 protein_coding
ENST00000296255.6 RPN1 protein_coding
ENST00000296328.7 UBXN7 protein_coding
ENST00000296490.6 WDR82 protein_coding
ENST00000296499.5 NDST3 protein_coding
ENST00000296519.5 CEP44 protein_coding
ENST00000296530.11 GASK1B protein_coding
ENST00000296555.8 FBXW7 protein_coding
ENST00000296564.8 ICE1 protein_coding
ENST00000296582.6 TMEM184C protein_coding
ENST00000296603.4 LMBRD2 protein_coding
ENST00000296642.4 PGGT1B processed_transcript
ENST00000296657.6 ANKRD33B protein_coding
ENST00000296754.6 ERAP1 protein_coding
ENST00000296800.4 PRKAA1 protein_coding
ENST00000296824.3 CCDC127 protein_coding
ENST00000296861.2 TNFRSF21 protein_coding
ENST00000296953.5 CREBRF protein_coding
ENST00000297109.9 SAP30L protein_coding
ENST00000297135.6 COG5 protein_coding
ENST00000297239.10 NA NA
ENST00000297273.7 CASD1 protein_coding
ENST00000297293.5 LMTK2 protein_coding
ENST00000297303.4 XKR6 protein_coding
ENST00000297338.5 RAD21 protein_coding
ENST00000297534.6 FMC1 protein_coding
ENST00000297632.7 TMEM65 protein_coding
ENST00000297689.3 NFIL3 protein_coding
ENST00000297785.6 ALDH1A1 protein_coding

ENST00000297814.5 KIF27 protein_coding
ENST00000298125.6 WDFY2 protein_coding
ENST00000298130.4 SPTSSA protein_coding
ENST00000298139.5 WRN protein_coding
ENST00000298173.6 GTF2A1 nonsense_mediated_decay
ENST00000298198.4 PGM2L1 protein_coding
ENST00000298281.7 PCF11 protein_coding
ENST00000298289.6 RPL36AL protein_coding
ENST00000298316.6 ARF6 protein_coding
ENST00000298492.5 ABRAXAS2 protein_coding
ENST00000298510.3 PRDX3 protein_coding
ENST00000298532.2 SNAPC4 protein_coding
ENST00000298566.2 BCL2L14 nonsense_mediated_decay
ENST00000298571.6 BORCS5 protein_coding
ENST00000298784.4 SHLD2 protein_coding
ENST00000298875.7 CPSF2 protein_coding
ENST00000298912.7 CLMN protein_coding
ENST00000298942.4 PTER protein_coding
ENST00000298999.6 R3HCC1L protein_coding
ENST00000299045.6 TCP11L2 protein_coding
ENST00000299138.10 VPS35 protein_coding
ENST00000299163.6 HIF1AN protein_coding
ENST00000299192.7 HEATR3 protein_coding
ENST00000299297.7 SGPL1 protein_coding
ENST00000299314.10 GNPTAB protein_coding
ENST00000299413.6 TRIM44 protein_coding
ENST00000299543.8 CTDP1 protein_coding
ENST00000299563.4 RNF169 protein_coding
ENST00000299608.5 TMX3 protein_coding
ENST00000299633.6 HDGFL3 protein_coding
ENST00000299641.7 NDST2 protein_coding
ENST00000299665.2 CLEC4D protein_coding
ENST00000299687.8 ZNF407 protein_coding
ENST00000299927.4 ZNF592 protein_coding
ENST00000299977.7 SLFN5 protein_coding
ENST00000299980.7 AP1G1 protein_coding
ENST00000300026.3 PPIB protein_coding
ENST00000300060.6 ANPEP protein_coding
ENST00000300086.4 TERF2IP protein_coding
ENST00000300146.9 PATL1 protein_coding
ENST00000300291.8 NUDT21 protein_coding
ENST00000300584.6 TBC1D2B protein_coding
ENST00000300589.5 NOD2 protein_coding
ENST00000300648.6 GCN1 protein_coding
ENST00000300933.7 TPM4 nonsense_mediated_decay
ENST00000300935.6 RAB8A protein_coding
ENST00000301015.12 PIEZO1 protein_coding
ENST00000301030.7 ANKRD11 protein_coding
ENST00000301042.6 ZNF641 protein_coding
ENST00000301067.10 KMT2D protein_coding
ENST00000301180.8 DIP2B protein_coding
ENST00000301335.8 SLC43A2 protein_coding
ENST00000301785.5 HNRNPUL2 protein_coding
ENST00000301831.7 ULK4 protein_coding

ENST00000301838.4 FADD protein_coding
ENST00000301998.4 B3GNT2 protein_coding
ENST00000302071.5 SESN1 protein_coding
ENST00000302188.6 RBKS protein_coding
ENST00000302277.6 ZNF804A protein_coding
ENST00000302342.3 ZNF217 protein_coding
ENST00000302418.4 KIF5B protein_coding
ENST00000302424.10 TRIM8 protein_coding
ENST00000302472.3 PTGER4 protein_coding
ENST00000302516.8 SF3B3 protein_coding
ENST00000302558.6 RGPD8 protein_coding
ENST00000302692.6 SLC25A33 protein_coding
ENST00000302754.5 JUNB protein_coding
ENST00000302764.7 NUDCD2 protein_coding
ENST00000302806.5 FRYL retained_intron
ENST00000302850.8 INSR protein_coding
ENST00000303004.4 CEBPB protein_coding
ENST00000303025.9 ARHGAP6 protein_coding
ENST00000303115.6 IL7R protein_coding
ENST00000303221.8 EMB protein_coding
ENST00000303230.4 HCN1 protein_coding
ENST00000303236.6 EIF2AK3 protein_coding
ENST00000303459.9 METTL15 protein_coding
ENST00000303531.10 NA NA
ENST00000303538.11 PDS5A protein_coding
ENST00000303545.3 RNF139 protein_coding
ENST00000303562.7 FOS protein_coding
ENST00000303575.7 TMED10 protein_coding
ENST00000303577.6 PCBP1 protein_coding
ENST00000303596.2 THAP11 protein_coding
ENST00000303635.10 CAMTA1 protein_coding
ENST00000303694.5 CHST11 protein_coding
ENST00000303843.7 REPS2 protein_coding
ENST00000303910.3 H2AC8 protein_coding
ENST00000304056.7 KBTBD2 protein_coding
ENST00000304061.7 RIOX1 protein_coding
ENST00000304084.11 CLEC7A protein_coding
ENST00000304141.4 CAVIN2 protein_coding
ENST00000304195.6 FRMD3 protein_coding
ENST00000304218.4 H1-4 protein_coding
ENST00000304385.6 TMEM154 protein_coding
ENST00000304414.10 ARL6IP1 protein_coding
ENST00000304434.9 ELOVL5 protein_coding
ENST00000304465.7 SRGAP2C protein_coding
ENST00000304621.9 TTC39C protein_coding
ENST00000304677.2 RNASE6 protein_coding
ENST00000304685.7 RGL1 protein_coding
ENST00000304743.5 PCNX1 protein_coding
ENST00000304808.9 PAFAH1B2 protein_coding
ENST00000304863.5 UQCRFS1 protein_coding
ENST00000304932.4 SUGT1P3 retained_intron
ENST00000304987.3 SIK2 protein_coding
ENST00000305097.5 P2RY1 protein_coding
ENST00000305135.8 YEATS2 protein_coding

ENST00000305352.6 S1PR1 protein_coding
ENST00000305386.3 MGAT2 protein_coding
ENST00000305447.4 GRM5 protein_coding
ENST00000305510.3 CNNM3 protein_coding
ENST00000305626.5 RAB33B protein_coding
ENST00000305766.9 PHACTR2 protein_coding
ENST00000305799.8 TET3 protein_coding
ENST00000305963.2 MMGT1 protein_coding
ENST00000305978.5 SCAND1 protein_coding
ENST00000305988.5 ADRB2 protein_coding
ENST00000306010.7 MGMT protein_coding
ENST00000306024.3 LSM3 protein_coding
ENST00000306031.5 DPYD protein_coding
ENST00000306065.7 ANKRD27 protein_coding
ENST00000306085.9 TRIM56 protein_coding
ENST00000306103.2 HSPBAP1 protein_coding
ENST00000306156.6 PRKCE protein_coding
ENST00000306167.10 MTM1 processed_transcript
ENST00000306193.6 CBR4 protein_coding
ENST00000306320.9 RETREG1 protein_coding
ENST00000306434.6 MAT2A protein_coding
ENST00000306682.6 RASA4B protein_coding
ENST00000306726.5 NA NA
ENST00000306730.6 AVEN protein_coding
ENST00000306749.2 FASN protein_coding
ENST00000306802.7 KIAA1109 protein_coding
ENST00000306867.8 NA NA
ENST00000306917.4 FEM1B protein_coding
ENST00000306984.6 MPLKIP protein_coding
ENST00000307050.5 OTUD7A protein_coding
ENST00000307102.8 MAP2K1 protein_coding
ENST00000307106.3 SMIM4 protein_coding
ENST00000307145.3 KLF13 protein_coding
ENST00000307149.8 COG7 protein_coding
ENST00000307194.5 GIMAP1 protein_coding
ENST00000307201.4 ABHD15 protein_coding
ENST00000307216.9 TMEM87A protein_coding
ENST00000307271.3 GIMAP8 protein_coding
ENST00000307297.7 PATJ protein_coding
ENST00000307300.7 NDUFA10 protein_coding
ENST00000307333.7 ANTXR2 protein_coding
ENST00000307340.6 USH2A protein_coding
ENST00000307367.2 CLCN5 protein_coding
ENST00000307564.7 AKNA protein_coding
ENST00000307630.4 YWHAG protein_coding
ENST00000307637.4 C3AR1 protein_coding
ENST00000307659.5 KIAA0232 protein_coding
ENST00000307714.11 KHDRBS1 processed_transcript
ENST00000307750.4 ZBTB5 protein_coding
ENST00000307771.7 ZRSR2 protein_coding
ENST00000307808.9 AFF1 protein_coding
ENST00000307826.4 MGAT1 protein_coding
ENST00000307940.3 MCL1 protein_coding
ENST00000307968.10 FNIP1 protein_coding

ENST00000308025.6 DDX23 protein_coding
ENST00000308080.8 EMILIN2 processed_transcript
ENST00000308086.2 THAP2 protein_coding
ENST00000308191.9 PDE7B protein_coding
ENST00000308249.3 PPM1E protein_coding
ENST00000308330.2 LEMD3 protein_coding
ENST00000308377.7 SLFN11 protein_coding
ENST00000308385.6 DUSP6 protein_coding
ENST00000308488.9 RSF1 protein_coding
ENST00000308666.3 ABCD2 protein_coding
ENST00000308675.5 LINGO2 protein_coding
ENST00000308683.2 ZNF622 protein_coding
ENST00000308783.8 KDM2A protein_coding
ENST00000308873.9 RUNX3 protein_coding
ENST00000308942.4 SIPA1L2 protein_coding
ENST00000309027.4 GOLIM4 protein_coding
ENST00000309096.4 GPBP1 retained_intron
ENST00000309134.8 TMEM9B protein_coding
ENST00000309137.11 PSME3IP1 protein_coding
ENST00000309241.8 PPARGC1B protein_coding
ENST00000309279.10 NA NA
ENST00000309311.6 EEF2 protein_coding
ENST00000309334.5 ARID5B protein_coding
ENST00000309395.5 MOB1B protein_coding
ENST00000309415.7 SH3RF3 protein_coding
ENST00000309434.8 NA NA
ENST00000309495.5 NA NA
ENST00000309575.4 CHST2 protein_coding
ENST00000309733.5 FAM241A protein_coding
ENST00000309822.5 UTP23 protein_coding
ENST00000309902.9 ZNF407 protein_coding
ENST00000309909.8 FBXW8 protein_coding
ENST00000309934.4 SEC22A protein_coding
ENST00000309955.6 CFLAR protein_coding
ENST00000309957.2 NAXD protein_coding
ENST00000309964.7 CLOCK protein_coding
ENST00000309983.4 TPMT protein_coding
ENST00000309993.2 MSL2 protein_coding
ENST00000310002.4 CLEC7A protein_coding
ENST00000310015.9 SP3 protein_coding
ENST00000310078.11 AGFG1 protein_coding
ENST00000310128.7 KCNE3 protein_coding
ENST00000310389.5 ARL10 protein_coding
ENST00000310421.4 VCPIP1 protein_coding
ENST00000310430.9 TNKS protein_coding
ENST00000310512.3 MEPCE protein_coding
ENST00000310528.8 SUGT1 protein_coding
ENST00000310806.7 MIS18BP1 protein_coding
ENST00000310823.6 ADAM17 protein_coding
ENST00000310924.5 TBC1D16 protein_coding
ENST00000310954.6 SLCO4C1 protein_coding
ENST00000310981.5 SFT2D3 protein_coding
ENST00000311085.8 DMXL1 protein_coding
ENST00000311122.5 ARMH3 protein_coding

ENST00000311127.7 HEG1 protein_coding
ENST00000311128.8 DENND6A protein_coding
ENST00000311151.8 DPYSL2 protein_coding
ENST00000311160.12 TNS3 protein_coding
ENST00000311317.7 WASHC4 retained_intron
ENST00000311380.7 ARHGAP12 protein_coding
ENST00000311403.4 ZBTB4 protein_coding
ENST00000311413.4 RNF26 protein_coding
ENST00000311417.5 ZMAT3 protein_coding
ENST00000311601.5 SH3PXD2B protein_coding
ENST00000311604.6 LSP1 protein_coding
ENST00000311637.8 ATP9A protein_coding
ENST00000311694.6 HECTD4 retained_intron
ENST00000311806.6 OXSR1 protein_coding
ENST00000311912.5 TLR8 protein_coding
ENST00000311922.3 TRIB1 protein_coding
ENST00000311936.6 KRAS protein_coding
ENST00000311956.7 ARHGAP1 protein_coding
ENST00000312033.3 AKNA protein_coding
ENST00000312349.4 FNDC9 protein_coding
ENST00000312350.8 KCNK10 protein_coding
ENST00000312397.8 SART1 protein_coding
ENST00000312405.9 CAMSAP1 protein_coding
ENST00000312493.5 PEAK1 protein_coding
ENST00000312584.3 TNFRSF10D protein_coding
ENST00000312655.7 YPEL2 protein_coding
ENST00000312916.10 AGGF1 protein_coding
ENST00000312938.4 ZBTB11 protein_coding
ENST00000313064.2 LINC00471 lncRNA
ENST00000313116.10 ZNF41 protein_coding
ENST00000313132.7 VDAC2 protein_coding
ENST00000313156.3 CHST12 processed_transcript
ENST00000313342.8 transcribed_unprocessed_pseudogene
ENST00000313349.3 SERTAD2 protein_coding
ENST00000313368.7 TAF7 protein_coding
ENST00000313543.4 GIMAP7 protein_coding
ENST00000313766.5 FAM20C protein_coding
ENST00000314045.10 DDX54 protein_coding
ENST00000314073.8 BICRAL protein_coding
ENST00000314100.9 BTBD9 protein_coding
ENST00000314117.7 RELL1 protein_coding
ENST00000314133.3 COX8A protein_coding
ENST00000314138.9 RPL27A protein_coding
ENST00000314146.10 ASAH1 protein_coding
ENST00000314328.12 SPECC1L protein_coding
ENST00000314358.8 KDM3B protein_coding
ENST00000314393.5 ZHX2 protein_coding
ENST00000314401.3 CD300LB protein_coding
ENST00000314442.7 P2RX4 retained_intron
ENST00000314565.7 BORCS5 protein_coding
ENST00000314622.7 NSUN3 protein_coding
ENST00000314845.6 AMBRA1 protein_coding
ENST00000314888.9 TLN1 protein_coding
ENST00000314940.6 HNRNPA0 protein_coding

ENST00000315141.5 LEO1 protein_coding
ENST00000315147.8 NAIP retained_intron
ENST00000315150.8 RAB43 protein_coding
ENST00000315285.6 SPAST protein_coding
ENST00000315289.5 RTKN2 protein_coding
ENST00000315357.9 DAZ3 protein_coding
ENST00000315377.4 RPS6 protein_coding
ENST00000315475.6 ZNF354C protein_coding
ENST00000315563.9 RRP12 protein_coding
ENST00000315576.6 ADGRE2 protein_coding
ENST00000315684.11 NA NA
ENST00000315707.3 LINC00324 lncRNA
ENST00000315765.9 HEPHL1 protein_coding
ENST00000315872.9 ROCK2 protein_coding
ENST00000315927.7 YOD1 protein_coding
ENST00000315994.6 SH3PXD2A processed_transcript
ENST00000316059.6 ZFP91 protein_coding
ENST00000316157.6 LARP4B protein_coding
ENST00000316292.12 EEF1A1 protein_coding
ENST00000316334.4 LNX2 protein_coding
ENST00000316355.8 CCNG2 protein_coding
ENST00000316485.9 NA NA
ENST00000316509.9 VAMP2 protein_coding
ENST00000316549.9 SLC9A9 protein_coding
ENST00000316594.5 HNRNPH2 protein_coding
ENST00000316626.5 GSK3B protein_coding
ENST00000316637.6 DEF6 protein_coding
ENST00000316836.4 PNMA1 protein_coding
ENST00000316881.7 TRIM25 protein_coding
ENST00000316985.6 NSD3 protein_coding
ENST00000317025.11 NSD3 protein_coding
ENST00000317118.11 PDE4D protein_coding
ENST00000317122.1 FLJ37453 lncRNA
ENST00000317271.2 PVRIG protein_coding
ENST00000317571.6 TTC39C protein_coding
ENST00000317586.7 DISC1 protein_coding
ENST00000317623.7 PCNX4 protein_coding
ENST00000317673.8 NA NA
ENST00000317677.8 CPNE1 protein_coding
ENST00000318037.3 RNF168 protein_coding
ENST00000318160.4 GREM2 protein_coding
ENST00000318469.2 GAPT protein_coding
ENST00000318471.4 PEX7 protein_coding
ENST00000318524.6 NFX1 protein_coding
ENST00000318560.5 ABL1 protein_coding
ENST00000318636.6 CA5B protein_coding
ENST00000318779.6 FOXP1 protein_coding
ENST00000318803.6 ST6GALNAC5 nonsense_mediated_decay
ENST00000318948.6 NRIP1 protein_coding
ENST00000319041.6 SH3BGRL3 protein_coding
ENST00000319080.10 MLXIP protein_coding
ENST00000319119.4 ZBTB34 protein_coding
ENST00000319121.3 KLHL11 protein_coding
ENST00000319211.4 F2R protein_coding

ENST00000319363.9 IL17RA protein_coding
ENST00000319397.6 ETS1 protein_coding
ENST00000319406.7 LCLAT1 protein_coding
ENST00000319555.6 ZFPM1 protein_coding
ENST00000319562.9 FARP1 protein_coding
ENST00000319584.9 ARID1B protein_coding
ENST00000319685.11 TMTC1 processed_transcript
ENST00000319688.5 KIDINS220 protein_coding
ENST00000319725.9 FUBP3 protein_coding
ENST00000319921.4 RNF213 protein_coding
ENST00000319945.9 RNF157 protein_coding
ENST00000319974.5 C2orf69 protein_coding
ENST00000320054.4 PRPF18 protein_coding
ENST00000320095.10 METRNL protein_coding
ENST00000320216.9 ITGB2 protein_coding
ENST00000320248.3 KBTBD11 protein_coding
ENST00000320254.5 LRRC37A protein_coding
ENST00000320280.8 SCAMP1 protein_coding
ENST00000320285.7 AGPAT4 protein_coding
ENST00000320451.6 ZNF121 protein_coding
ENST00000320486.6 NA NA
ENST00000320578.2 RAB39A protein_coding
ENST00000320580.7 PACS1 protein_coding
ENST00000320876.9 SMCHD1 protein_coding
ENST00000320954.9 CRTAP protein_coding
ENST00000321037.4 ENPP4 protein_coding
ENST00000321063.7 PLXNA4 protein_coding
ENST00000321117.8 DNMT3A protein_coding
ENST00000321250.11 LEPROTL1 protein_coding
ENST00000321276.7 SGPP2 protein_coding
ENST00000321407.2 GCC1 protein_coding
ENST00000321437.7 RAB8B protein_coding
ENST00000321464.6 ZBTB38 protein_coding
ENST00000321725.9 STAB1 protein_coding
ENST00000321792.5 RBMXL1 protein_coding
ENST00000321867.5 ULK1 protein_coding
ENST00000321949.11 CRTC1 protein_coding
ENST00000321990.4 ATAD5 protein_coding
ENST00000322028.4 POLR2L protein_coding
ENST00000322030.11 SET protein_coding
ENST00000322153.10 IL12RB1 protein_coding
ENST00000322244.8 UBA6 protein_coding
ENST00000322282.10 NA NA
ENST00000322313.7 WDR33 protein_coding
ENST00000322348.5 GCNT4 protein_coding
ENST00000322349.11 EEA1 protein_coding
ENST00000322357.7 ZBTB7A protein_coding
ENST00000322611.3 BASP1 protein_coding
ENST00000322716.7 EPM2AIP1 protein_coding
ENST00000322723.7 NCL protein_coding
ENST00000322831.6 ZCCHC7 protein_coding
ENST00000322927.2 ZNF335 protein_coding
ENST00000322941.3 HIC1 protein_coding
ENST00000323013.6 RRP7A protein_coding

ENST00000323037.4 ZNRF2 protein_coding
ENST00000323076.5 LCP1 protein_coding
ENST00000323387.5 SHLD2P3 unprocessed_pseudogene
ENST00000323482.7 ACSS1 protein_coding
ENST00000323496.5 TMSB4XP4 processed_pseudogene
ENST00000323534.3 RAP2B protein_coding
ENST00000323570.6 ELMOD2 protein_coding
ENST00000323571.7 WIPF2 protein_coding
ENST00000323599.9 KMT5B nonsense_mediated_decay
ENST00000323816.7 GAS7 protein_coding
ENST00000323853.8 SNRNP200 protein_coding
ENST00000323883.10 PIP4K2A protein_coding
ENST00000323929.6 MRE11 protein_coding
ENST00000323959.7 CSTF3 protein_coding
ENST00000324093.7 PLXND1 miRNA
ENST00000324106.8 CD28 miRNA
ENST00000324109.7 DNMBP miRNA
ENST00000324142.4 PCNX2 miRNA
ENST00000324198.9 GRK3 miRNA
ENST00000324288.11 CIITA miRNA
ENST00000324309.7 CCDC149 miRNA
ENST00000324344.7 DCUN1D3 miRNA
ENST00000324460.6 HSPA5 miRNA
ENST00000324489.5 MTURN miRNA
ENST00000324501.8 PSEN1 miRNA
ENST00000324559.8 ANO5 miRNA
ENST00000324607.7 MBOAT1 miRNA
ENST00000324677.10 SSH2 miRNA
ENST00000324696.7 ASCC3 miRNA
ENST00000324731.7 GLULP4 miRNA
ENST00000324817.4 MED14 miRNA
ENST00000324856.10 ARID1A miRNA
ENST00000325000.4 RPL10P16 miRNA
ENST00000325094.8 TMEM33 miRNA
ENST00000325144.4 ZBTB2 miRNA
ENST00000325212.9 CEP295 miRNA
ENST00000325222.7 CUL1 miRNA
ENST00000325233.3 C8orf34 miRNA
ENST00000325234.5 PLCB3 miRNA
ENST00000325239.8 WDFY4 miRNA
ENST00000325279.4 KLHL36 miRNA
ENST00000325349.6 EEF1A1P16 miRNA
ENST00000325602.5 P2RY13 miRNA
ENST00000325612.5 miRNA
ENST00000325658.3 PPM1A miRNA
ENST00000326071.7 NOL4L miRNA
ENST00000326094.4 ISCA1 miRNA
ENST00000326134.8 MROH1 miRNA
ENST00000326151.8 NFKBIZ miRNA
ENST00000326172.8 NFKBIZ miRNA
ENST00000326277.4 SNX18 miRNA
ENST00000326294.3 PTPRCAP miRNA
ENST00000326351.10 WASHC3 miRNA
ENST00000326495.8 SSH1 miRNA

ENST00000326648.4 ZNF609 miRNA
ENST00000326654.5 ERGIC1 miRNA
ENST00000326756.3 ZNF518B miRNA
ENST00000326765.9 APOLD1 miRNA
ENST00000326793.9 ACAP2 miRNA
ENST00000326912.7 NDUFAF3 miRNA
ENST00000326956.10 SSBP3 miRNA
ENST00000326965.6 TAGAP miRNA
ENST00000327026.6 CCDC57 miRNA
ENST00000327086.7 SEPTIN7P13 miRNA
ENST00000327134.6 PAK2 miRNA
ENST00000327423.9 PRR14L miRNA
ENST00000327443.7 SP1 miRNA
ENST00000327490.7 UBALD2 miRNA
ENST00000327492.3 TOB2 miRNA
ENST00000327505.5 AGPAT3 miRNA
ENST00000327520.7 JMJD1C miRNA
ENST00000327761.6 RASSF1 miRNA
ENST00000327827.10 C16orf72 miRNA
ENST00000327892.11 TUBB miRNA
ENST00000328046.8 KLHL15 miRNA
ENST00000328078.2 GLUD2 miRNA
ENST00000328090.8 TASOR2 miRNA
ENST00000328118.6 NA miRNA
ENST00000328142.3 ATAD1 miRNA
ENST00000328235.4 FAM120C miRNA
ENST00000328249.3 ERMAP miRNA
ENST00000328252.3 PAPPA miRNA
ENST00000328299.3 ST6GALNAC3 miRNA
ENST00000328306.4 SLC9A7 miRNA
ENST00000328554.7 RTL10 miRNA
ENST00000328557.4 NRROS miRNA
ENST00000328654.8 ZNF26 miRNA
ENST00000328737.5 ANKRD44 miRNA
ENST00000328767.8 TMTC4 miRNA
ENST00000328771.7 DHX34 miRNA
ENST00000328902.8 GIMAP6 miRNA
ENST00000328914.4 FOXK1 miRNA
ENST00000328963.8 P2RX7 miRNA
ENST00000329016.10 SORCS2 miRNA
ENST00000329021.8 NFAM1 miRNA
ENST00000329099.4 RFLNB miRNA
ENST00000329134.8 UTY miRNA
ENST00000329235.5 AP1S2 miRNA
ENST00000329236.8 RBM10 miRNA
ENST00000329240.7 ARL17A miRNA
ENST00000329286.6 NA miRNA
ENST00000329335.3 RP1L1 miRNA
ENST00000329366.7 HNMT miRNA
ENST00000329378.7 KIAA0825 miRNA
ENST00000329410.3 C16orf54 miRNA
ENST00000329421.7 MARCKSL1 miRNA
ENST00000329474.6 CARMIL1 miRNA
ENST00000329563.7 TSPO miRNA

ENST00000329565.5 SNN miRNA
ENST00000329613.7 TSHZ2 miRNA
ENST00000329627.10 PEX26 miRNA
ENST00000330232.7 ADA2 miRNA
ENST00000330236.6 ZKSCAN8 miRNA
ENST00000330317.5 WDR4 miRNA
ENST00000330452.6 PRKCD miRNA
ENST00000330494.10 CHD3 miRNA
ENST00000330501.10 ZNF397 miRNA
ENST00000330588.4 NA miRNA
ENST00000330714.6 MX2 miRNA
ENST00000330793.1 CD300C miRNA
ENST00000330843.7 RAB11FIP1 miRNA
ENST00000330871.3 SOCS3 miRNA
ENST00000330943.7 SNX20 miRNA
ENST00000330953.5 CCR4 miRNA
ENST00000330965.4 RPL9P7 miRNA
ENST00000330992.8 COPG2 miRNA
ENST00000331113.7 KCND2 miRNA
ENST00000331129.3 ID2 miRNA
ENST00000331173.5 CSTF2T miRNA
ENST00000331194.8 NA miRNA
ENST00000331222.4 CLN8 miRNA
ENST00000331244.8 GLRX3 miRNA
ENST00000331327.4 PURA miRNA
ENST00000331351.5 HS3ST4 miRNA
ENST00000331380.3 H2AC20 miRNA
ENST00000331469.5 CD8B protein_coding
ENST00000331491.1 H3C13 protein_coding
ENST00000331497.3 NGRN trna
ENST00000331569.5 ZNF703 trna
ENST00000331710.8 TBK1 trna
ENST00000331825.9 FTL trna
ENST00000332129.5 KIF21B trna
ENST00000332220.9 YBX1 trna
ENST00000332245.9 NA trna
ENST00000332290.3 C6orf120 trna
ENST00000332303.4 NSMCE3 trna
ENST00000332345.9 LIMS1 trna
ENST00000332408.7 SHC4 trna
ENST00000332482.7 EP400 trna
ENST00000332499.3 HEXIM1 trna
ENST00000332687.9 RLIM trna
ENST00000332707.8 XPOT trna
ENST00000332935.6 trna
ENST00000333010.6 JAKMIP2 trna
ENST00000333027.6 MTMR3 trna
ENST00000333059.5 NA trna
ENST00000333070.4 LCK trna
ENST00000333190.4 ZNF804B trna
ENST00000333209.4 GPRIN3 trna
ENST00000333395.9 EWSR1 trna
ENST00000333412.3 NA trna
ENST00000333421.6 ABHD17B trna

ENST00000333432.7 POLR2J2 trna
ENST00000333483.13 METTL2A trna
ENST00000333617.7 LSAMP trna
ENST00000333681.4 BCL2 trna
ENST00000333750.8 CRACR2A trna
ENST00000333762.5 H1-10 trna
ENST00000333896.5 SPTBN1 trna
ENST00000334003.10 GSAP trna
ENST00000334035.8 CPEB4 trna
ENST00000334062.7 RASA3 trna
ENST00000334095.4 ZNF761 trna
ENST00000334103.10 NA trna
ENST00000334220.7 DLST trna
ENST00000334232.7 EDARADD trna
ENST00000334256.7 KPNA4 trna
ENST00000334270.2 TTF1 trna
ENST00000334351.7 PNRC2 trna
ENST00000334376.4 NA trna
ENST00000334425.1 GPR141 trna
ENST00000334448.5 CCDC88C trna
ENST00000334464.6 PDZD8 trna
ENST00000334512.8 ZMIZ1 trna
ENST00000334528.12 FMN1 trna
ENST00000334534.8 SLX4IP trna
ENST00000334546.5 PPP2R3A trna
ENST00000334660.8 CHP1 trna
ENST00000334801.6 BCL9L trna
ENST00000334828.5 PGAM1 trna
ENST00000334841.9 CMC1 trna
ENST00000334914.10 NA trna
ENST00000334937.7 ZNF81 trna
ENST00000334961.10 NF2 trna
ENST00000334976.9 EMC10 trna
ENST00000335090.6 ZNF480 trna
ENST00000335148.6 ETNK1 trna
ENST00000335154.8 FMNL3 trna
ENST00000335179.5 TMSB4XP2 trna
ENST00000335221.8 NA trna
ENST00000335258.8 ZFAND4 trna
ENST00000335271.8 MAP4 trna
ENST00000335295.4 HBB trna
ENST00000335327.5 WASF3 trna
ENST00000335387.8 RC3H2 trna
ENST00000335420.8 KLHDC10 trna
ENST00000335475.5 KCNQ1 trna
ENST00000335500.7 GPATCH8 trna
ENST00000335508.9 SF3B1 trna
ENST00000335626.7 TCF20 trna
ENST00000335661.6 BCL2A1 trna
ENST00000335670.9 RORA trna
ENST00000335727.12 TNRC6B trna
ENST00000335730.5 LOXHD1 trna
ENST00000335766.2 CDKN2D trna
ENST00000335891.5 DCAF1 trna

ENST00000336032.3 PNRC1 trna
ENST00000336061.2 RASSF3 trna
ENST00000336095.9 RNF24 trna
ENST00000336112.7 ASXL2 trna
ENST00000336125.5 ARL17A trna
ENST00000336126.5 PCGF5 trna
ENST00000336156.8 KIAA0930 trna
ENST00000336273.6 MTDH trna
ENST00000336314.7 LARP1 trna
ENST00000336332.5 ZXDC trna
ENST00000336415.7 CEP170 trna
ENST00000336454.3 CDC14A trna
ENST00000336577.7 MMP25 trna
ENST00000336592.9 CUL4B trna
ENST00000336615.7 PNPLA2 trna
ENST00000336733.9 SLC25A26 trna
ENST00000336735.7 SEL1L trna
ENST00000336812.4 DHX15 trna
ENST00000336949.6 MID1IP1 trna
ENST00000337049.7 OPRM1 trna
ENST00000337273.8 TNPO1 trna
ENST00000337304.2 ATF4 trna
ENST00000337318.6 FAM53B trna
ENST00000337343.7 DGKH trna
ENST00000337387.4 WTAP trna
ENST00000337400.5 PPARD trna
ENST00000337478.2 ITPRIP trna
ENST00000337659.9 PRPF4B trna
ENST00000337752.5 IRF1-AS1 trna
ENST00000337787.3 SMG1P1 trna
ENST00000337815.7 ZNF664 trna
ENST00000337970.6 HNRNPF trna
ENST00000338045.6 LIMS1 trna
ENST00000338051.4 C5orf24 trna
ENST00000338086.8 PUM2 trna
ENST00000338087.8 SLA trna
ENST00000338205.8 ECPAS trna
ENST00000338222.6 UBQLN2 trna
ENST00000338230.3 ZNF600 trna
ENST00000338244.4 SLC23A2 trna
ENST00000338305.6 LRFN2 trna
ENST00000338313.5 TAGAP trna
ENST00000338366.5 TAF13 trna
ENST00000338368.6 PRKDC trna
ENST00000338427.9 NUDCD3 trna
ENST00000338435.7 GLS trna
ENST00000338450.7 ING1 trna
ENST00000338488.7 NR4A3 trna
ENST00000338560.10 TRPV2 trna
ENST00000338565.3 ZFX trna
ENST00000338597.8 SRSF10 trna
ENST00000338732.6 LFNG trna
ENST00000338783.6 GNAS trna
ENST00000338797.9 CRTC1 trna

ENST00000338850.5 RTN3 trna
ENST00000338962.7 LRP1 trna
ENST00000339063.6 NA trna
ENST00000339145.7 IFI6 trna
ENST00000339209.7 PHF10 trna
ENST00000339275.8 OASL trna
ENST00000339282.10 ZNF564 trna
ENST00000339364.8 PIK3AP1 trna
ENST00000339365.2 SEC24C trna
ENST00000339397.4 SRFBP1 trna
ENST00000339399.4 ANP32B trna
ENST00000339430.8 LITAF trna
ENST00000339642.9 ZNF254 trna
ENST00000339659.7 NA trna
ENST00000339697.4 SERINC1 trna
ENST00000339701.6 CSMD3 trna
ENST00000339728.5 ARL4C trna
ENST00000339766.7 CLEC2A trna
ENST00000339875.8 MARCHF1 trna
ENST00000339950.4 USP1 trna
ENST00000340149.5 GAB2 trna
ENST00000340160.6 TSPAN18 trna
ENST00000340170.7 HIRA trna
ENST00000340301.5 PKD1P6-NPIPP1 trna
ENST00000340398.4 H3-5 trna
ENST00000340434.4 PTAR1 trna
ENST00000340450.6 CAP1 trna
ENST00000340457.8 UPK3BL1 trna
ENST00000340473.7 GTF2F2 trna
ENST00000340510.7 LINC00265 trna
ENST00000340513.4 TPX2 trna
ENST00000340535.10 MIA3 trna
ENST00000340553.6 FOXN2 trna
ENST00000340648.5 RRP1B trna
ENST00000340697.3 trna
ENST00000340833.4 CDC25B trna
ENST00000340855.9 IDS trna
ENST00000340857.3 H1-0 trna
ENST00000340881.2 PPIL4 trna
ENST00000340913.9 HNRNPA1 trna
ENST00000340972.7 MAP2K5 trna
ENST00000341028.8 CDK11B trna
ENST00000341041.7 SYCP2L trna
ENST00000341119.8 SLC25A40 trna
ENST00000341148.6 RBM33 trna
ENST00000341217.6 FAM222B trna
ENST00000341259.5 SH2B3 trna
ENST00000341322.4 BRWD1 trna
ENST00000341360.5 ATP2B4 trna
ENST00000341376.9 NFYA trna
ENST00000341421.6 TRAK1 trna
ENST00000341446.8 BRI3BP trna
ENST00000341472.8 SYNE2 trna
ENST00000341502.8 MIA2 trna

ENST00000341517.7 PDE4B trna
ENST00000341567.7 TMEM248 trna
ENST00000341653.5 MAX trna
ENST00000341700.6 GNA14 trna
ENST00000341772.4 DNER trna
ENST00000341776.5 JARID2 trna
ENST00000341861.8 DPP8 trna
ENST00000341901.4 SBK1 trna
ENST00000342016.6 CIR1 trna
ENST00000342101.6 IFNAR2 trna
ENST00000342294.4 NA trna
ENST00000342320.7 RBPJ trna
ENST00000342374.4 SERINC3 trna
ENST00000342462.4 TMPPE trna
ENST00000342505.4 JAK1 trna
ENST00000342558.3 PPP3CB trna
ENST00000342609.6 RAB1AP1 trna
ENST00000342645.7 HIPK2 trna
ENST00000342652.6 INPP5A trna
ENST00000342745.4 SUSD6 trna
ENST00000342988.6 SMAD4 trna
ENST00000342992.9 TTN trna
ENST00000343003.9 SPATA13 trna
ENST00000343053.4 NELFB trna
ENST00000343122.6 BACH2 trna
ENST00000343123.4 trna
ENST00000343137.7 PRDM2 trna
ENST00000343200.8 VCAN trna
ENST00000343242.7 MAFK trna
ENST00000343258.7 VAV3 trna
ENST00000343276.4 ASB7 trna
ENST00000343307.4 B3GLCT trna
ENST00000343325.7 ZNF791 trna
ENST00000343327.5 PTBP3 trna
ENST00000343420.6 CD55 trna
ENST00000343439.8 NA trna
ENST00000343638.8 UBTF trna
ENST00000343677.3 H1-2 trna
ENST00000343706.7 SEZ6L trna
ENST00000343736.7 TSPOAP1 protein_coding
ENST00000343738.9 PKD1P6 transcribed_unprocessed_pseudogene
ENST00000343742.5 LRRK2 protein_coding
ENST00000343762.5 CDYL protein_coding
ENST00000343788.9 SUGT1 protein_coding
ENST00000343805.9 SP140 protein_coding
ENST00000343933.8 CORO2A protein_coding
ENST00000343936.4 ZC3H6 protein_coding
ENST00000343984.5 SEPTIN6 protein_coding
ENST00000343999.1 MIR3667HG lncRNA
ENST00000344086.7 IL6R protein_coding
ENST00000344096.3 DYRK2 protein_coding
ENST00000344099.3 ZNF14 protein_coding
ENST00000344102.8 WARS1 protein_coding
ENST00000344111.3 COX19 protein_coding

ENST00000344138.7 GRAP2 protein_coding
ENST00000344201.6 CBFA2T2 protein_coding
ENST00000344271.5 STX2 nonsense_mediated_decay
ENST00000344293.5 TAF3 protein_coding
ENST00000344327.6 TRPC6 protein_coding
ENST00000344359.6 RBL1 protein_coding
ENST00000344517.4 ABCB10 protein_coding
ENST00000344523.7 NR6A1 protein_coding
ENST00000344532.6 RCBTB2 protein_coding
ENST00000344548.6 CDC42 protein_coding
ENST00000344579.5 TPRG1L protein_coding
ENST00000344651.4 UNC119B protein_coding
ENST00000344691.7 RUNX1 protein_coding
ENST00000344698.5 PCNX2 protein_coding
ENST00000344700.6 RPS10 protein_coding
ENST00000344726.8 HMGN3 protein_coding
ENST00000344736.7 NA NA
ENST00000344754.4 SIGLEC1 protein_coding
ENST00000344756.7 INSIG1 protein_coding
ENST00000344861.8 ZDHHC7 nonsense_mediated_decay
ENST00000344862.8 PUS7L protein_coding
ENST00000344893.4 lncRNA
ENST00000344908.8 MAP3K2 protein_coding
ENST00000344920.7 NNT protein_coding
ENST00000345015.4 KCNQ1 processed_transcript
ENST00000345136.6 PLEC protein_coding
ENST00000345317.5 SKAP2 protein_coding
ENST00000345392.2 VHL protein_coding
ENST00000345541.6 WAC retained_intron
ENST00000345714.7 SGK3 protein_coding
ENST00000345724.6 MALT1 protein_coding
ENST00000346183.6 NFATC3 protein_coding
ENST00000346198.5 TCF7L2 protein_coding
ENST00000346234.6 OSTF1 protein_coding
ENST00000346248.6 CHST15 protein_coding
ENST00000346617.7 ZFP64 protein_coding
ENST00000346786.2 MYL9 protein_coding
ENST00000346817.8 CASP10 protein_coding
ENST00000346911.4 IL6STP1 processed_pseudogene
ENST00000347037.8 SYNE1 processed_transcript
ENST00000347055.3 KRCC1 protein_coding
ENST00000347255.5 RABGAP1L protein_coding
ENST00000347264.2 RNU1-75P snRNA
ENST00000347359.3 STOM protein_coding
ENST00000347486.7 SUPT6H protein_coding
ENST00000347596.2 GUCY1A2 protein_coding
ENST00000347635.7 NUP50 protein_coding
ENST00000347662.7 TRAF3 protein_coding
ENST00000347785.6 CD5 protein_coding
ENST00000347982.7 NSD1 protein_coding
ENST00000348031.5 UBOX5 protein_coding
ENST00000348035.7 RAC1 protein_coding
ENST00000348120.5 RGS7 protein_coding
ENST00000348124.5 TRAF6 protein_coding

ENST00000348165.8 UBE3C protein_coding
ENST00000348264.5 CLCC1 protein_coding
ENST00000348428.6 NA NA
ENST00000348520.9 KLC1 protein_coding
ENST00000348564.9 PTPRC protein_coding
ENST00000348610.3 SENP7 protein_coding
ENST00000348811.5 WSB1 protein_coding
ENST00000348911.9 SBF1 protein_coding
ENST00000349014.6 ADNP protein_coding
ENST00000349033.8 TEX14 protein_coding
ENST00000349139.5 WDR3 protein_coding
ENST00000349225.2 RLIM protein_coding
ENST00000349379.5 SNX3 protein_coding
ENST00000349556.4 CEP43 protein_coding
ENST00000349598.6 EXOC1 protein_coding
ENST00000349606.4 MYLIP protein_coding
ENST00000349663.6 KDM5C processed_transcript
ENST00000349820.9 IQCB1 protein_coding
ENST00000349984.6 CCR6 protein_coding
ENST00000349995.8 COG3 protein_coding
ENST00000350021.2 NDUFB6 protein_coding
ENST00000350082.8 CILK1 protein_coding
ENST00000350092.7 PTBP1 protein_coding
ENST00000350221.3 FSIP1 protein_coding
ENST00000350249.6 PPP2R5C protein_coding
ENST00000350339.3 FCN2 protein_coding
ENST00000350356.6 PRKACA processed_transcript
ENST00000350425.5 NA NA
ENST00000350721.7 ATR protein_coding
ENST00000351097.8 USP25 protein_coding
ENST00000351298.7 PLD1 protein_coding
ENST00000351578.9 FYB1 protein_coding
ENST00000351593.8 NA NA
ENST00000351677.5 PTPN11 protein_coding
ENST00000351691.8 TRAF3 protein_coding
ENST00000351773.6 ZNF37A protein_coding
ENST00000351808.8 IFT88 protein_coding
ENST00000351839.6 HNRNPK protein_coding
ENST00000352133.2 SLC37A1 protein_coding
ENST00000352435.7 PRDX5 protein_coding
ENST00000352580.6 CD8A protein_coding
ENST00000352645.4 ZC3H7B protein_coding
ENST00000352689.9 MKLN1 protein_coding
ENST00000352966.8 PKIA protein_coding
ENST00000353205.5 NFYA protein_coding
ENST00000353231.8 CLEC7A protein_coding
ENST00000353267.6 CREB1 protein_coding
ENST00000353411.9 SKP1 protein_coding
ENST00000353796.6 RBAK protein_coding
ENST00000353801.6 HSP90AB1 protein_coding
ENST00000353868.5 NRF1 protein_coding
ENST00000353999.6 EIF4H protein_coding
ENST00000354069.6 PID1 protein_coding
ENST00000354241.5 TRIM4 protein_coding

ENST00000354258.4 TAP1 protein_coding
ENST00000354289.7 NAMPT protein_coding
ENST00000354300.4 PPTC7 protein_coding
ENST00000354325.2 TRERF1 protein_coding
ENST00000354352.8 SLC11A1 nonsense_mediated_decay
ENST00000354366.6 NRDE2 protein_coding
ENST00000354396.5 ARNT protein_coding
ENST00000354445.2 PLAAT4 protein_coding
ENST00000354451.5 CRYBB2P1 processed_transcript
ENST00000354480.2 RBM3 protein_coding
ENST00000354485.5 KANK1 retained_intron
ENST00000354504.6 VPS54 protein_coding
ENST00000354558.6 CKAP5 protein_coding
ENST00000354619.8 ERO1B protein_coding
ENST00000354656.3 ZNF44 nonsense_mediated_decay
ENST00000354665.7 DIDO1 protein_coding
ENST00000354674.4 SYNE1 protein_coding
ENST00000354725.6 SND1 protein_coding
ENST00000354811.5 RPL15 protein_coding
ENST00000354817.6 FAR1 protein_coding
ENST00000354883.9 SLC25A26 protein_coding
ENST00000354897.3 CELF2 protein_coding
ENST00000354906.6 MIA3 protein_coding
ENST00000354922.3 PNRC1 protein_coding
ENST00000355057.2 H4C11 protein_coding
ENST00000355072.8 HTT protein_coding
ENST00000355081.3 KLHL28 protein_coding
ENST00000355085.3 C5AR1 protein_coding
ENST00000355095.7 ZNF736 protein_coding
ENST00000355100.3 NA NA
ENST00000355125.3 BTBD7 nonsense_mediated_decay
ENST00000355155.4 VPS13B nonsense_mediated_decay
ENST00000355163.6 NA NA
ENST00000355193.5 NA NA
ENST00000355238.9 SYNCRIP protein_coding
ENST00000355295.4 TDRD7 protein_coding
ENST00000355312.6 GIT2 protein_coding
ENST00000355327.6 THSD4 protein_coding
ENST00000355439.5 NA NA
ENST00000355469.4 NFAM1 nonsense_mediated_decay
ENST00000355512.9 RGS6 protein_coding
ENST00000355520.5 OPHN1 protein_coding
ENST00000355621.6 UBE2H protein_coding
ENST00000355622.6 TLR4 protein_coding
ENST00000355628.8 TASOR protein_coding
ENST00000355640.3 XIAP protein_coding
ENST00000355681.3 FNBP1 protein_coding
ENST00000355703.3 PCNX3 protein_coding
ENST00000355754.6 GBP4 protein_coding
ENST00000355755.6 KIF16B protein_coding
ENST00000355773.5 SLC17A5 protein_coding
ENST00000355815.7 SREBF1 protein_coding
ENST00000355867.7 SVIL protein_coding
ENST00000355898.5 ZNF507 protein_coding

ENST00000355928.6 LCK retained_intron
ENST00000355946.5 SH3PXD2A protein_coding
ENST00000355947.5 TRIM4 protein_coding
ENST00000355999.4 STK39 protein_coding
ENST00000356016.6 LCOR protein_coding
ENST00000356064.3 ARFIP1 protein_coding
ENST00000356068.2 NA NA
ENST00000356136.6 UVRAG protein_coding
ENST00000356147.3 PTPRA protein_coding
ENST00000356156.6 NPIPB5 protein_coding
ENST00000356162.5 NA NA
ENST00000356166.9 FBRS protein_coding
ENST00000356183.7 GTF3C1 protein_coding
ENST00000356290.7 NA NA
ENST00000356297.4 PXDNL protein_coding
ENST00000356303.5 EEF1A1 protein_coding
ENST00000356321.4 ZNF770 protein_coding
ENST00000356331.8 SH3BP2 protein_coding
ENST00000356338.9 MYO5A protein_coding
ENST00000356352.5 UPF2 protein_coding
ENST00000356384.4 RXRA processed_transcript
ENST00000356386.5 BTN3A2 protein_coding
ENST00000356408.3 CARD11 protein_coding
ENST00000356413.4 IGBP1 protein_coding
ENST00000356427.2 ATF7IP2 protein_coding
ENST00000356458.6 EML6 protein_coding
ENST00000356489.8 BCKDHB protein_coding
ENST00000356554.6 MAN1A2 protein_coding
ENST00000356576.7 COG6 nonsense_mediated_decay
ENST00000356674.7 HNRNPA2B1 protein_coding
ENST00000356689.6 S100PBP processed_transcript
ENST00000356692.8 PPP4R2 protein_coding
ENST00000356740.5 XXYLT1 protein_coding
ENST00000356763.6 PIK3R4 protein_coding
ENST00000356785.4 APBB1IP protein_coding
ENST00000356798.9 ITGAL protein_coding
ENST00000356825.7 SMAD2 protein_coding
ENST00000356840.6 MYLIP protein_coding
ENST00000356865.9 ATP10A protein_coding
ENST00000356892.3 SASH3 protein_coding
ENST00000356906.6 NBR2 lncRNA
ENST00000356916.6 EIF4G3 protein_coding
ENST00000356920.8 POTEE protein_coding
ENST00000356936.5 NCL protein_coding
ENST00000356937.6 CDK11A retained_intron
ENST00000356950.1 H2BC12 protein_coding
ENST00000356956.4 IGF2R protein_coding
ENST00000356978.7 CALM1 protein_coding
ENST00000357028.5 MED27 protein_coding
ENST00000357065.7 HNRNPF protein_coding
ENST00000357066.6 MOB3A protein_coding
ENST00000357071.7 ECE1 protein_coding
ENST00000357085.4 TRIM38 protein_coding
ENST00000357103.4 ADIPOR2 protein_coding

ENST00000357137.7 XPNPEP3 protein_coding
ENST00000357164.3 GM2A protein_coding
ENST00000357167.7 BTBD11 protein_coding
ENST00000357182.7 ZMYM6 protein_coding
ENST00000357195.6 BCL11B protein_coding
ENST00000357214.5 SFPQ protein_coding
ENST00000357304.7 NA NA
ENST00000357311.6 SRPK2 protein_coding
ENST00000357344.7 TCAF2 protein_coding
ENST00000357393.5 AKNAD1 protein_coding
ENST00000357402.8 MVP protein_coding
ENST00000357457.7 CERT1 nonsense_mediated_decay
ENST00000357462.8 NA NA
ENST00000357464.4 TENT4B nonsense_mediated_decay
ENST00000357503.2 TOR4A protein_coding
ENST00000357537.4 GPALPP1 protein_coding
ENST00000357563.4 SETD3 nonsense_mediated_decay
ENST00000357568.6 CAPN3 protein_coding
ENST00000357640.7 DENND2D protein_coding
ENST00000357650.7 SLC30A7 protein_coding
ENST00000357727.5 CREB5 protein_coding
ENST00000357731.8 NEGR1 protein_coding
ENST00000357732.7 RTN4 protein_coding
ENST00000357742.7 MCTP2 protein_coding
ENST00000357778.6 NA NA
ENST00000357802.5 RRP7BP processed_transcript
ENST00000357854.6 G3BP2 protein_coding
ENST00000357869.6 ADCY6 protein_coding
ENST00000357878.5 HMX3 protein_coding
ENST00000357949.4 SERTAD1 protein_coding
ENST00000357992.7 ELK4 protein_coding
ENST00000358028.7 NIPAL3 protein_coding
ENST00000358053.3 OSBPL2 protein_coding
ENST00000358073.2 lncRNA
ENST00000358100.5 NA NA
ENST00000358156.6 SRSF2 protein_coding
ENST00000358164.8 ITGAL protein_coding
ENST00000358187.2 ITPRIP protein_coding
ENST00000358200.5 NA NA
ENST00000358232.9 ELP2 protein_coding
ENST00000358246.4 MS4A7 protein_coding
ENST00000358307.5 FUT8 protein_coding
ENST00000358357.6 NA NA
ENST00000358465.5 TRIM33 protein_coding
ENST00000358544.5 VPS13B protein_coding
ENST00000358546.3 RAB21 retained_intron
ENST00000358550.5 SIPA1L1 protein_coding
ENST00000358600.6 ADGRE5 protein_coding
ENST00000358609.4 RPL27AP processed_pseudogene
ENST00000358625.3 WDR5 protein_coding
ENST00000358637.4 ASTN2 protein_coding
ENST00000358662.7 SP110 protein_coding
ENST00000358689.7 ENTPD4 protein_coding
ENST00000358691.8 HELZ protein_coding

ENST00000358704.4 ZBTB18 protein_coding
ENST00000358730.5 RFX3 protein_coding
ENST00000358738.3 ZBTB1 protein_coding
ENST00000358739.4 H2AC13 protein_coding
ENST00000358746.5 TTC37 protein_coding
ENST00000358752.3 FUT4 protein_coding
ENST00000358763.5 SYN3 protein_coding
ENST00000358771.4 SIRPA protein_coding
ENST00000358912.4 PELI1 protein_coding
ENST00000358935.2 MARCHF5 protein_coding
ENST00000358951.5 RAB3GAP2 protein_coding
ENST00000359013.4 TGFBR2 protein_coding
ENST00000359015.4 MAP3K5 protein_coding
ENST00000359039.4 KLHL9 protein_coding
ENST00000359060.5 SSU72 protein_coding
ENST00000359165.5 EEF1A1P4 processed_pseudogene
ENST00000359193.3 H2AC11 protein_coding
ENST00000359204.4 FRA10AC1 protein_coding
ENST00000359263.7 MYOF protein_coding
ENST00000359297.5 STK40 protein_coding
ENST00000359303.3 H3C12 protein_coding
ENST00000359314.5 CD2AP protein_coding
ENST00000359315.5 TPGS1 protein_coding
ENST00000359359.5 C6orf89 protein_coding
ENST00000359376.6 NA NA
ENST00000359426.6 HK1 protein_coding
ENST00000359445.6 EFL1 protein_coding
ENST00000359446.8 LDLRAD4 protein_coding
ENST00000359520.10 TECPR2 protein_coding
ENST00000359524.6 RUNX2 protein_coding
ENST00000359544.2 UBAP1 protein_coding
ENST00000359564.2 DSE protein_coding
ENST00000359600.5 ZSWIM5 protein_coding
ENST00000359606.3 CBFA2T2 protein_coding
ENST00000359653.4 ZRANB1 protein_coding
ENST00000359676.8 NOL4L protein_coding
ENST00000359720.3 MGST1 processed_transcript
ENST00000359727.7 NIPA2 protein_coding
ENST00000359798.7 ZNF808 protein_coding
ENST00000359801.6 SIRPB2 protein_coding
ENST00000359872.6 ASIC2 protein_coding
ENST00000359890.6 RBM23 protein_coding
ENST00000359933.5 ATG2B protein_coding
ENST00000359943.6 FAM3C protein_coding
ENST00000359979.7 CCSER2 protein_coding
ENST00000359980.5 ZFYVE27 protein_coding
ENST00000359983.6 ATP2A3 protein_coding
ENST00000360004.5 HLA-DRB1 protein_coding
ENST00000360064.7 NA NA
ENST00000360104.6 KMT2C protein_coding
ENST00000360117.6 ZNF107 nonsense_mediated_decay
ENST00000360121.4 SPN protein_coding
ENST00000360184.7 DYNC1H1 protein_coding
ENST00000360254.3 DISP1 processed_transcript

ENST00000360256.7 F8 protein_coding
ENST00000360270.6 MSN protein_coding
ENST00000360338.3 ZNF460 protein_coding
ENST00000360359.4 ANKS1A protein_coding
ENST00000360540.8 PCMTD1 protein_coding
ENST00000360565.6 SCAF1 protein_coding
ENST00000360612.4 PIM3 protein_coding
ENST00000360629.3 ARGLU1 processed_transcript
ENST00000360647.7 ZNF148 protein_coding
ENST00000360655.7 NAV2 protein_coding
ENST00000360672.2 SLA2 protein_coding
ENST00000360697.4 ADARB1 protein_coding
ENST00000360718.5 SQSTM1 protein_coding
ENST00000360726.3 PAQR8 protein_coding
ENST00000360768.3 VKORC1L1 protein_coding
ENST00000360844.6 SIGLEC14 protein_coding
ENST00000360845.2 GNL3L protein_coding
ENST00000360851.3 RGL1 protein_coding
ENST00000360890.5 WDFY4 protein_coding
ENST00000360902.1 TRRAP nonsense_mediated_decay
ENST00000360938.6 ETS2 protein_coding
ENST00000360982.2 NCKAP1 protein_coding
ENST00000360995.7 AEBP2 protein_coding
ENST00000361024.5 DENND4C protein_coding
ENST00000361034.6 GRAMD4 protein_coding
ENST00000361050.3 MPEG1 protein_coding
ENST00000361060.5 GFPT1 protein_coding
ENST00000361112.5 NA NA
ENST00000361127.5 LRIG2 protein_coding
ENST00000361138.6 SMC5 protein_coding
ENST00000361166.7 NF2 protein_coding
ENST00000361171.6 MVB12B protein_coding
ENST00000361183.6 CAPZA2 protein_coding
ENST00000361198.8 LDB1 protein_coding
ENST00000361226.6 RUSC2 protein_coding
ENST00000361227.2 MT-ND3 protein_coding
ENST00000361236.6 TMEM123 protein_coding
ENST00000361246.5 CDC42BPB protein_coding
ENST00000361264.7 DCAF12 protein_coding
ENST00000361283.2 CHAMP1 protein_coding
ENST00000361309.5 CD47 protein_coding
ENST00000361310.6 ZNF438 protein_coding
ENST00000361311.4 CLSTN1 protein_coding
ENST00000361335.1 MT-ND4L protein_coding
ENST00000361337.2 TOP1 protein_coding
ENST00000361357.6 MAP3K3 protein_coding
ENST00000361367.5 CTR9 protein_coding
ENST00000361368.5 SMURF1 protein_coding
ENST00000361371.8 LTN1 protein_coding
ENST00000361381.2 MT-ND4 protein_coding
ENST00000361390.2 MT-ND1 protein_coding
ENST00000361409.2 ARHGEF11 protein_coding
ENST00000361421.1 TOX protein_coding
ENST00000361428.2 ZNF318 protein_coding

ENST00000361434.6 ADGRL1 protein_coding
ENST00000361441.4 APOBEC3C protein_coding
ENST00000361445.7 MTOR protein_coding
ENST00000361453.3 MT-ND2 protein_coding
ENST00000361462.5 TOGARAM1 protein_coding
ENST00000361464.6 OGA protein_coding
ENST00000361479.8 MPHOSPH8 protein_coding
ENST00000361496.3 RCSD1 protein_coding
ENST00000361522.4 NA NA
ENST00000361544.9 CDC14A protein_coding
ENST00000361557.4 PJA2 protein_coding
ENST00000361565.7 IPO9 protein_coding
ENST00000361567.2 MT-ND5 protein_coding
ENST00000361573.2 SLC9A8 protein_coding
ENST00000361587.3 HIPK1 protein_coding
ENST00000361624.2 MT-CO1 protein_coding
ENST00000361648.5 ATP11C protein_coding
ENST00000361660.7 TYW1 nonsense_mediated_decay
ENST00000361671.8 NAA35 protein_coding
ENST00000361681.2 MT-ND6 protein_coding
ENST00000361690.6 SRPK1 protein_coding
ENST00000361717.3 TIFA protein_coding
ENST00000361727.6 CNTNAP2 protein_coding
ENST00000361739.1 MT-CO2 protein_coding
ENST00000361752.6 QKI protein_coding
ENST00000361758.7 QKI nonsense_mediated_decay
ENST00000361771.6 WDTC1 protein_coding
ENST00000361781.5 SGMS1 protein_coding
ENST00000361789.2 MT-CYB protein_coding
ENST00000361799.5 RNF220 protein_coding
ENST00000361804.4 SMC3 protein_coding
ENST00000361813.5 SMG5 protein_coding
ENST00000361828.5 OPA1 protein_coding
ENST00000361832.2 BLOC1S2 processed_transcript
ENST00000361842.6 XAF1 protein_coding
ENST00000361851.1 MT-ATP8 protein_coding
ENST00000361875.6 TSC22D2 protein_coding
ENST00000361899.2 MT-ATP6 protein_coding
ENST00000361904.6 CELF1 protein_coding
ENST00000361911.8 ZNF326 protein_coding
ENST00000361951.4 NA NA
ENST00000361971.8 PLXNB3 protein_coding
ENST00000361972.7 SFMBT2 protein_coding
ENST00000361976.5 NA NA
ENST00000362018.2 MTCP1 protein_coding
ENST00000362058.2 CROCCP2 processed_transcript
ENST00000362063.5 ZNF652 protein_coding
ENST00000362074.7 NOTCH2NLA protein_coding
ENST00000362079.2 MT-CO3 protein_coding
ENST00000362104.2 MIR30E miRNA
ENST00000362154.1 MIR103A2 miRNA
ENST00000362165.1 MIR103A1 miRNA
ENST00000362181.2 MIR361 miRNA
ENST00000362212.1 MIR342 miRNA

ENST00000362220.1 MIR326 miRNA
ENST00000362263.2 MIRLET7D miRNA
ENST00000362295.2 MIRLET7A1 miRNA
ENST00000362302.2 MIR331 miRNA
ENST00000362309.2 MIRLET7I miRNA
ENST00000362330.2 Y_RNA misc_RNA
ENST00000362352.1 Y_RNA misc_RNA
ENST00000362353.1 Y_RNA misc_RNA
ENST00000362354.1 Y_RNA misc_RNA
ENST00000362371.1 Y_RNA misc_RNA
ENST00000362375.1 Y_RNA misc_RNA
ENST00000362378.1 RNU6-28P snRNA
ENST00000362396.1 SNORA60 snoRNA
ENST00000362400.1 RNA5S12 rRNA
ENST00000362412.1 snoRNA
ENST00000362415.1 Y_RNA misc_RNA
ENST00000362416.1 RNU6-1094P snRNA
ENST00000362420.1 Y_RNA misc_RNA
ENST00000362421.1 Y_RNA misc_RNA
ENST00000362423.1 SNORA21 snoRNA
ENST00000362433.1 Y_RNA misc_RNA
ENST00000362443.1 RNU4-82P snRNA
ENST00000362452.1 RNA5SP183 miRNA
ENST00000362455.1 RNU4-39P miRNA
ENST00000362464.1 RNA5S11 miRNA
ENST00000362467.1 RNA5S3 miRNA
ENST00000362477.1 RNU5E-1 miRNA
ENST00000362482.1 RNA5S1 miRNA
ENST00000362484.1 RNA5SP273 miRNA
ENST00000362487.1 Y_RNA miRNA
ENST00000362496.1 Y_RNA miRNA
ENST00000362507.1 RNU5F-1 miRNA
ENST00000362512.1 RNU12 miRNA
ENST00000362526.1 RNA5S5 miRNA
ENST00000362530.1 RNY4P19 miRNA
ENST00000362532.1 RNA5SP308 miRNA
ENST00000362535.1 SNORA1B miRNA
ENST00000362539.1 Y_RNA miRNA
ENST00000362540.1 Y_RNA miRNA
ENST00000362541.1 SNORD62 miRNA
ENST00000362545.1 RNA5SP370 miRNA
ENST00000362552.1 VTRNA3-1P miRNA
ENST00000362554.1 Y_RNA miRNA
ENST00000362566.1 SNORD9 miRNA
ENST00000362572.1 Y_RNA miRNA
ENST00000362574.1 Y_RNA miRNA
ENST00000362591.1 Y_RNA miRNA
ENST00000362599.1 RNU1-62P miRNA
ENST00000362606.1 Y_RNA miRNA
ENST00000362607.1 SNORA31 miRNA
ENST00000362610.1 RNA5SP389 miRNA
ENST00000362627.1 RNU1-94P miRNA
ENST00000362645.1 Y_RNA miRNA
ENST00000362646.1 Y_RNA miRNA

ENST00000362653.1 RNA5SP226 miRNA
ENST00000362680.1 Y_RNA miRNA
ENST00000362692.1 RNU6-37P miRNA
ENST00000362694.1 RNA5SP502 miRNA
ENST00000362697.1 Y_RNA miRNA
ENST00000362698.1 RNU5A-1 miRNA
ENST00000362704.1 SNORD18C miRNA
ENST00000362710.1 Y_RNA miRNA
ENST00000362721.1 Y_RNA miRNA
ENST00000362725.1 Y_RNA miRNA
ENST00000362735.1 RNY4P24 miRNA
ENST00000362739.1 RNA5SP124 miRNA
ENST00000362750.1 RNA5SP258 miRNA
ENST00000362759.1 RNU1-14P miRNA
ENST00000362761.1 SNORD33 miRNA
ENST00000362765.1 Y_RNA miRNA
ENST00000362782.2 RNU1-35P miRNA
ENST00000362797.1 Y_RNA miRNA
ENST00000362803.1 SNORD16 miRNA
ENST00000362805.1 miRNA
ENST00000362813.1 RN7SKP185 miRNA
ENST00000362817.1 RNU1-38P miRNA
ENST00000362821.1 RN7SKP173 miRNA
ENST00000362839.1 RNU4-23P miRNA
ENST00000362862.1 Y_RNA miRNA
ENST00000362874.1 SNORD36A miRNA
ENST00000362881.1 Y_RNA miRNA
ENST00000362883.1 SNORD104 miRNA
ENST00000362886.1 Y_RNA miRNA
ENST00000362901.1 RNA5SP426 miRNA
ENST00000362911.1 Y_RNA miRNA
ENST00000362915.1 SNORA52 miRNA
ENST00000362918.1 RNY3P2 miRNA
ENST00000362931.1 Y_RNA miRNA
ENST00000362935.1 RNU1-134P miRNA
ENST00000362967.1 NA miRNA
ENST00000362969.1 RNA5SP150 miRNA
ENST00000362973.1 RNA5SP358 miRNA
ENST00000363004.1 RNA5SP452 miRNA
ENST00000363005.1 Y_RNA miRNA
ENST00000363009.1 RNVU1-22 miRNA
ENST00000363010.1 RNU6-888P miRNA
ENST00000363013.1 RN7SKP90 miRNA
ENST00000363014.1 Y_RNA miRNA
ENST00000363030.1 NA miRNA
ENST00000363040.1 RNA5S10 miRNA
ENST00000363046.1 RMRP miRNA
ENST00000363062.1 RNU1-18P miRNA
ENST00000363064.1 SNORD81 miRNA
ENST00000363066.1 Y_RNA miRNA
ENST00000363068.1 Y_RNA miRNA
ENST00000363079.2 Y_RNA miRNA
ENST00000363091.1 SNORD1B miRNA
ENST00000363094.1 Y_RNA miRNA

ENST00000363120.1 VTRNA1-1 miRNA
ENST00000363124.1 RNA5SP145 miRNA
ENST00000363156.1 U8 miRNA
ENST00000363166.1 RNA5SP65 miRNA
ENST00000363171.1 Y_RNA miRNA
ENST00000363179.1 Y_RNA miRNA
ENST00000363187.1 RN7SKP63 miRNA
ENST00000363188.1 RNA5SP218 miRNA
ENST00000363194.1 RNY4P9 miRNA
ENST00000363214.1 SNORD68 miRNA
ENST00000363217.1 SNORA73B miRNA
ENST00000363220.1 Y_RNA miRNA
ENST00000363248.1 Y_RNA miRNA
ENST00000363251.2 Y_RNA miRNA
ENST00000363282.1 RNU6-216P miRNA
ENST00000363286.1 RNU5B-1 miRNA
ENST00000363294.1 Y_RNA miRNA
ENST00000363298.1 RNA5SP350 miRNA
ENST00000363299.1 RNU5D-1 miRNA
ENST00000363301.1 Y_RNA miRNA
ENST00000363306.1 RNU1-19P miRNA
ENST00000363312.1 Telomerase-vert miRNA
ENST00000363315.1 SNORD1C miRNA
ENST00000363331.1 Y_RNA miRNA
ENST00000363334.1 RNU1-22P miRNA
ENST00000363338.1 Y_RNA miRNA
ENST00000363341.1 RNY4P27 miRNA
ENST00000363367.1 SNORA70 miRNA
ENST00000363373.1 RN7SKP79 miRNA
ENST00000363376.1 RNA5SP263 miRNA
ENST00000363389.1 SNORD35A miRNA
ENST00000363391.1 Y_RNA miRNA
ENST00000363405.1 RNA5SP199 miRNA
ENST00000363408.1 RNA5SP352 miRNA
ENST00000363421.1 Y_RNA miRNA
ENST00000363426.1 RNU1-83P snRNA
ENST00000363435.1 Y_RNA misc_RNA
ENST00000363439.1 Y_RNA trna
ENST00000363442.1 RN7SKP255 trna
ENST00000363444.1 Y_RNA trna
ENST00000363450.1 SNORA63 trna
ENST00000363473.1 RNA5S8 trna
ENST00000363484.1 SNORA71D trna
ENST00000363485.1 SNORA72 trna
ENST00000363500.1 RNA5S17 trna
ENST00000363509.1 RN7SKP45 trna
ENST00000363511.1 RNA5S4 trna
ENST00000363524.1 SNORA38B trna
ENST00000363538.1 RNA5SP74 trna
ENST00000363549.1 Y_RNA trna
ENST00000363552.1 trna
ENST00000363558.1 Y_RNA trna
ENST00000363564.1 RNA5-8SP2 trna
ENST00000363566.1 Y_RNA trna

ENST00000363593.1 SNORD118 trna
ENST00000363617.1 RNA5SP322 trna
ENST00000363618.1 RN7SKP203 trna
ENST00000363624.1 Y_RNA trna
ENST00000363626.1 U8 trna
ENST00000363632.1 Y_RNA trna
ENST00000363636.1 Y_RNA trna
ENST00000363651.1 Y_RNA trna
ENST00000363656.1 RNY4P14 trna
ENST00000363660.1 SNORD35B trna
ENST00000363664.1 SNORA33 trna
ENST00000363667.1 RNY4P6 trna
ENST00000363673.1 RN7SKP176 trna
ENST00000363674.1 Y_RNA trna
ENST00000363688.1 RNA5SP429 trna
ENST00000363743.1 RNA5SP349 trna
ENST00000363745.1 Y_RNA trna
ENST00000363753.1 SNORD18A trna
ENST00000363754.1 RNA5S6 trna
ENST00000363781.1 Y_RNA trna
ENST00000363804.1 RN7SKP160 trna
ENST00000363811.1 RNU6-263P trna
ENST00000363836.1 trna
ENST00000363858.1 RNU6-271P trna
ENST00000363867.1 Y_RNA trna
ENST00000363871.1 RNA5SP161 trna
ENST00000363872.1 Y_RNA trna
ENST00000363880.1 Y_RNA trna
ENST00000363891.1 RN7SKP113 trna
ENST00000363894.1 Y_RNA trna
ENST00000363899.1 Y_RNA trna
ENST00000363913.1 RN7SKP180 trna
ENST00000363915.1 SNORD8 trna
ENST00000363922.1 SNORA80A trna
ENST00000363925.1 RNU4-1 trna
ENST00000363946.1 SNORA38 trna
ENST00000363959.1 Y_RNA trna
ENST00000363961.1 SNORD36B trna
ENST00000363963.2 NA trna
ENST00000363964.1 Y_RNA trna
ENST00000363969.1 RNA5SP48 trna
ENST00000363977.1 Y_RNA trna
ENST00000363979.1 Y_RNA trna
ENST00000363981.1 SNORD27 trna
ENST00000363985.1 Y_RNA trna
ENST00000364009.1 SNORD14E trna
ENST00000364018.1 Y_RNA trna
ENST00000364019.1 RNU4-13P trna
ENST00000364033.1 RNU1-42P trna
ENST00000364043.1 SNORD46 trna
ENST00000364089.1 SNORA74A trna
ENST00000364102.1 RNU5A-8P trna
ENST00000364112.1 Y_RNA trna
ENST00000364113.1 SNORA3A trna

ENST00000364128.1 Y_RNA trna
ENST00000364139.1 trna
ENST00000364161.1 Y_RNA trna
ENST00000364165.1 RNA5SP469 trna
ENST00000364171.1 RNA5SP242 trna
ENST00000364189.1 RNA5SP336 trna
ENST00000364201.1 Y_RNA trna
ENST00000364228.1 RNY1 trna
ENST00000364234.1 RNU6-11P trna
ENST00000364243.1 RNU6-647P trna
ENST00000364251.1 RNY1P12 trna
ENST00000364259.1 SNORA58B trna
ENST00000364275.1 RNA5SP175 trna
ENST00000364294.1 RNU4-36P trna
ENST00000364298.1 RNA5SP368 trna
ENST00000364300.1 RNU1-132P trna
ENST00000364308.1 Y_RNA trna
ENST00000364315.1 RNA5SP202 trna
ENST00000364326.1 Y_RNA trna
ENST00000364329.1 trna
ENST00000364331.1 RN7SKP118 trna
ENST00000364337.1 Y_RNA trna
ENST00000364338.1 Y_RNA trna
ENST00000364346.1 Y_RNA trna
ENST00000364348.1 Y_RNA trna
ENST00000364357.1 NA trna
ENST00000364359.1 SNORA63D trna
ENST00000364369.1 Y_RNA trna
ENST00000364407.1 Y_RNA trna
ENST00000364409.1 Y_RNA trna
ENST00000364419.1 RN7SKP76 trna
ENST00000364432.1 SNORA65 trna
ENST00000364442.1 RNA5SP72 trna
ENST00000364445.1 RN7SKP227 trna
ENST00000364447.1 RNU4-59P trna
ENST00000364451.1 RNA5S9 trna
ENST00000364469.1 Y_RNA trna
ENST00000364473.1 Y_RNA trna
ENST00000364478.1 RNU105B trna
ENST00000364481.1 Y_RNA trna
ENST00000364485.1 RNA5S14 trna
ENST00000364486.1 RNA5SP500 trna
ENST00000364488.1 RN7SKP193 trna
ENST00000364502.1 RNU6-1251P trna
ENST00000364507.1 RNY4P23 trna
ENST00000364520.1 RNU6-1141P trna
ENST00000364533.1 SNORD14B trna
ENST00000364535.1 Y_RNA trna
ENST00000364542.1 Y_RNA trna
ENST00000364543.1 RNA5SP141 trna
ENST00000364545.1 RNA5SP204 trna
ENST00000364558.1 RN7SKP71 trna
ENST00000364569.1 RNU4-67P trna
ENST00000364577.1 RNA5SP509 trna

ENST00000364578.1 SNORA63C trna
ENST00000364581.1 Y_RNA trna
ENST00000364587.1 SNORA55 trna
ENST00000364588.1 RNU4-4P trna
ENST00000364596.1 Y_RNA trna
ENST00000364600.1 RNY4P7 trna
ENST00000364604.1 RNU6-164P trna
ENST00000364613.1 Y_RNA trna
ENST00000364617.1 SNORD45B trna
ENST00000364622.1 RNA5SP78 trna
ENST00000364639.2 Y_RNA trna
ENST00000364642.1 SNORA71C trna
ENST00000364647.1 RNU6-707P trna
ENST00000364659.1 Y_RNA trna
ENST00000364663.1 RN7SKP237 trna
ENST00000364678.1 Y_RNA trna
ENST00000364685.1 Y_RNA trna
ENST00000364688.1 RNVU1-6 trna
ENST00000364696.1 Y_RNA trna
ENST00000364718.1 RNA5S2 trna
ENST00000364722.1 trna
ENST00000364725.1 RNA5SP132 trna
ENST00000364729.1 SNORA71A trna
ENST00000364730.1 RN7SKP124 trna
ENST00000364740.1 RNA5SP84 trna
ENST00000364768.2 RNY4P16 trna
ENST00000364773.1 SNORA14A trna
ENST00000364774.2 Y_RNA trna
ENST00000364778.1 RNU4-91P trna
ENST00000364784.1 RNU6-7 trna
ENST00000364805.1 SNORD32A trna
ENST00000364811.2 Y_RNA trna
ENST00000364814.1 RN7SKP239 trna
ENST00000364838.1 RNA5SP359 trna
ENST00000364849.1 SNORD87 trna
ENST00000364853.2 Y_RNA trna
ENST00000364874.1 RNU6-34P trna
ENST00000364879.1 Y_RNA trna
ENST00000364884.1 SNORD52 trna
ENST00000364888.1 RN7SKP55 trna
ENST00000364893.1 RNA5SP267 trna
ENST00000364902.1 SNORA5C trna
ENST00000364908.1 Y_RNA trna
ENST00000364915.1 SNORD117 trna
ENST00000364916.1 Y_RNA trna
ENST00000364923.1 RN7SKP9 trna
ENST00000364930.1 Y_RNA trna
ENST00000364931.1 RNU5E-4P trna
ENST00000364938.1 SNORA73A trna
ENST00000364939.1 U8 trna
ENST00000364948.1 RNY4P17 trna
ENST00000364950.1 Y_RNA trna
ENST00000364952.1 RNA5SP149 trna
ENST00000364953.1 SNORD48 trna

ENST00000364968.1 SNORD1A trna
ENST00000364977.1 SNORD31B trna
ENST00000364991.1 RNA5SP298 trna
ENST00000364995.1 SNORD50B trna
ENST00000365005.1 RN7SKP178 trna
ENST00000365012.1 trna
ENST00000365028.1 SNORA72 trna
ENST00000365030.1 RNY1P13 trna
ENST00000365031.1 RN7SKP48 trna
ENST00000365050.1 RNA5SP442 trna
ENST00000365055.1 RNA5S15 trna
ENST00000365063.1 Y_RNA trna
ENST00000365068.1 Y_RNA trna
ENST00000365075.1 SNORA70 trna
ENST00000365085.1 RNY3P1 trna
ENST00000365096.1 RNA5-8SP4 trna
ENST00000365128.1 SNORA23 trna
ENST00000365138.1 Y_RNA trna
ENST00000365157.1 Y_RNA trna
ENST00000365161.1 SNORD38A trna
ENST00000365172.1 SNORD49B trna
ENST00000365184.1 RNA5SP152 trna
ENST00000365186.1 RNA5SP19 trna
ENST00000365188.1 RN7SKP80 trna
ENST00000365208.1 Y_RNA trna
ENST00000365223.1 SNORD58C trna
ENST00000365241.1 VTRNA1-2 trna
ENST00000365254.1 RNY4P3 trna
ENST00000365267.1 Y_RNA trna
ENST00000365274.1 Y_RNA trna
ENST00000365281.1 RNY4P28 trna
ENST00000365304.1 RNA5-8SP5 trna
ENST00000365312.1 Y_RNA trna
ENST00000365317.1 RNA5SP355 trna
ENST00000365328.1 7SK trna
ENST00000365370.1 RNU6-737P trna
ENST00000365382.1 SNORD14C trna
ENST00000365387.1 RNA5S16 trna
ENST00000365390.1 RN7SKP69 trna
ENST00000365393.1 RNA5SP22 trna
ENST00000365394.1 RNA5SP77 trna
ENST00000365399.1 U8 trna
ENST00000365403.1 Y_RNA trna
ENST00000365430.1 RNU6-487P trna
ENST00000365436.1 Y_RNA trna
ENST00000365444.1 SNORD6 trna
ENST00000365459.1 RNU6-609P trna
ENST00000365462.1 Y_RNA misc_RNA
ENST00000365465.1 snoRNA
ENST00000365467.1 RNU6-8 snRNA
ENST00000365474.1 RN7SKP208 misc_RNA
ENST00000365477.1 RNU1-16P snRNA
ENST00000365481.1 RN7SKP204 misc_RNA
ENST00000365484.1 RNY3 misc_RNA

ENST00000365487.1 Y_RNA misc_RNA
ENST00000365493.1 SNORA62 snoRNA
ENST00000365498.1 Y_RNA misc_RNA
ENST00000365510.1 RNU1-55P snRNA
ENST00000365519.1 SNORA70 snoRNA
ENST00000365522.1 RN7SKP292 misc_RNA
ENST00000365528.1 RNU6-61P snRNA
ENST00000365529.1 Y_RNA misc_RNA
ENST00000365530.1 SNORD82 snoRNA
ENST00000365536.1 RN7SKP187 misc_RNA
ENST00000365537.1 Y_RNA misc_RNA
ENST00000365538.2 RNVU1-21 snRNA
ENST00000365541.1 RNA5SP259 rRNA_pseudogene
ENST00000365571.2 RNY4P10 misc_RNA
ENST00000365574.1 RNU5E-6P snRNA
ENST00000365599.1 Y_RNA misc_RNA
ENST00000365602.1 RNA5SP248 rRNA_pseudogene
ENST00000365604.1 RNA5SP283 rRNA_pseudogene
ENST00000365607.2 SNORD25 snoRNA
ENST00000365633.1 SNORD34 snoRNA
ENST00000365642.1 RN7SKP230 misc_RNA
ENST00000365645.1 VTRNA1-3 misc_RNA
ENST00000365651.1 RNA5S7 rRNA
ENST00000365652.1 Y_RNA misc_RNA
ENST00000365656.1 RNA5S13 rRNA
ENST00000365663.1 Y_RNA misc_RNA
ENST00000365664.1 RNU6-1329P snRNA
ENST00000365668.1 RNU4-2 snRNA
ENST00000365696.3 MIR421 miRNA
ENST00000366206.2 STRN3 processed_transcript
ENST00000366210.2 SEPTIN2 processed_transcript
ENST00000366221.3 retained_intron
ENST00000366272.5 MLXIP protein_coding
ENST00000366413.5 NA NA
ENST00000366425.3 GP1BB protein_coding
ENST00000366438.3 ATAD2B processed_transcript
ENST00000366442.2 SHC1 protein_coding
ENST00000366478.2 NA NA
ENST00000366481.3 TRIM58 protein_coding
ENST00000366497.5 NLRP3 protein_coding
ENST00000366508.4 AHCTF1 protein_coding
ENST00000366510.3 SCCPDH protein_coding
ENST00000366511.1 CNST protein_coding
ENST00000366512.6 CNST protein_coding
ENST00000366513.7 CNST protein_coding
ENST00000366514.4 TFB2M protein_coding
ENST00000366525.6 HNRNPU retained_intron
ENST00000366527.3 HNRNPU retained_intron
ENST00000366535.3 ADSS2 protein_coding
ENST00000366553.1 CHML protein_coding
ENST00000366560.3 FH protein_coding
ENST00000366576.3 MTR protein_coding
ENST00000366577.8 MTR protein_coding
ENST00000366581.5 HEATR1 protein_coding

ENST00000366589.1 ERO1B processed_transcript
ENST00000366591.4 GPR137B retained_intron
ENST00000366595.6 NID1 protein_coding
ENST00000366607.4 TOMM20 protein_coding
ENST00000366610.6 IRF2BP2 protein_coding
ENST00000366617.3 SLC35F3 protein_coding
ENST00000366618.6 SLC35F3 protein_coding
ENST00000366637.6 DISC1 protein_coding
ENST00000366641.3 EGLN1 protein_coding
ENST00000366645.1 EXOC8 protein_coding
ENST00000366672.4 GALNT2 protein_coding
ENST00000366676.4 NA NA
ENST00000366683.3 ACTA1 protein_coding
ENST00000366686.1 CCSAP protein_coding
ENST00000366691.3 RHOU protein_coding
ENST00000366711.3 IBA57 protein_coding
ENST00000366780.7 PHF10 protein_coding
ENST00000366784.1 ITPKB protein_coding
ENST00000366794.8 PARP1 protein_coding
ENST00000366812.5 ACBD3 protein_coding
ENST00000366813.1 H3-3A protein_coding
ENST00000366814.3 H3-3A protein_coding
ENST00000366835.6 TMEM63A protein_coding
ENST00000366839.7 SRP9 protein_coding
ENST00000366892.4 PRKN protein_coding
ENST00000366905.3 AGPAT4 protein_coding
ENST00000366922.2 IARS2 protein_coding
ENST00000366932.3 RRP15 protein_coding
ENST00000366934.3 GPATCH2 protein_coding
ENST00000366935.6 GPATCH2 protein_coding
ENST00000366957.8 SMYD2 protein_coding
ENST00000366978.4 NSL1 protein_coding
ENST00000366996.1 LPGAT1 protein_coding
ENST00000366997.7 LPGAT1 protein_coding
ENST00000367001.4 SLC30A1 protein_coding
ENST00000367004.3 TRAF5 protein_coding
ENST00000367007.4 KCNH1 protein_coding
ENST00000367009.2 HHAT protein_coding
ENST00000367033.3 PLXNA2 protein_coding
ENST00000367050.7 CR1 processed_transcript
ENST00000367054.5 SOD2 protein_coding
ENST00000367063.5 CD55 protein_coding
ENST00000367085.3 DYNLT1 retained_intron
ENST00000367088.1 DYNLT1 retained_intron
ENST00000367090.3 TMEM181 protein_coding
ENST00000367103.3 MAPKAPK2 protein_coding
ENST00000367123.6 SRGAP2C protein_coding
ENST00000367142.4 NUCKS1 protein_coding
ENST00000367157.3 NUAK2 protein_coding
ENST00000367162.6 DSTYK protein_coding
ENST00000367164.1 RBBP5 protein_coding
ENST00000367178.6 SCAF8 protein_coding
ENST00000367180.4 MDM4 protein_coding
ENST00000367182.6 MDM4 protein_coding

ENST00000367188.4 PPP1R15B protein_coding
ENST00000367196.3 CTSD protein_coding
ENST00000367210.1 ZC3H11A protein_coding
ENST00000367218.6 ATP2B4 protein_coding
ENST00000367257.7 SYNE1 protein_coding
ENST00000367258.1 KLHL12 protein_coding
ENST00000367259.1 KLHL12 protein_coding
ENST00000367262.3 RABIF protein_coding
ENST00000367272.4 PPP1R12BP1 unprocessed_pseudogene
ENST00000367326.1 PLEKHG1 protein_coding
ENST00000367352.3 ZNF281 protein_coding
ENST00000367364.4 PTPRC protein_coding
ENST00000367379.4 PTPRC protein_coding
ENST00000367385.7 NEK7 protein_coding
ENST00000367404.7 NUP43 protein_coding
ENST00000367419.8 GINM1 protein_coding
ENST00000367435.3 CDC73 protein_coding
ENST00000367445.6 RO60 protein_coding
ENST00000367455.7 UCHL5 protein_coding
ENST00000367460.3 RGS18 protein_coding
ENST00000367463.4 UST protein_coding
ENST00000367466.3 PLA2G4A protein_coding
ENST00000367467.6 SASH1 protein_coding
ENST00000367469.4 SASH1 retained_intron
ENST00000367475.5 STXBP5 protein_coding
ENST00000367478.7 TPR protein_coding
ENST00000367480.6 STXBP5 protein_coding
ENST00000367495.3 RAB32 protein_coding
ENST00000367497.1 IVNS1ABP protein_coding
ENST00000367501.6 SWT1 protein_coding
ENST00000367503.6 NA NA
ENST00000367506.8 TRMT1L protein_coding
ENST00000367511.3 NIBAN1 protein_coding
ENST00000367514.6 C1orf21 processed_transcript
ENST00000367518.3 TSEN15 retained_intron
ENST00000367524.6 UTRN protein_coding
ENST00000367525.3 UTRN protein_coding
ENST00000367526.7 UTRN protein_coding
ENST00000367535.6 NCF2 protein_coding
ENST00000367545.6 UTRN protein_coding
ENST00000367549.3 DHX9 protein_coding
ENST00000367568.4 STX11 protein_coding
ENST00000367571.2 PLAGL1 protein_coding
ENST00000367572.2 PLAGL1 protein_coding
ENST00000367576.5 LTV1 protein_coding
ENST00000367577.6 IER5 protein_coding
ENST00000367580.5 MR1 protein_coding
ENST00000367584.7 PHACTR2 protein_coding
ENST00000367589.3 XPR1 protein_coding
ENST00000367591.4 PEX3 protein_coding
ENST00000367595.3 ACBD6 protein_coding
ENST00000367600.5 QSOX1 protein_coding
ENST00000367603.5 HIVEP2 protein_coding
ENST00000367607.6 CEP350 protein_coding

ENST00000367619.6 SOAT1 protein_coding
ENST00000367621.1 VTA1 protein_coding
ENST00000367658.2 HECA protein_coding
ENST00000367660.3 ABRACL protein_coding
ENST00000367688.3 RABGAP1L protein_coding
ENST00000367690.4 RABGAP1L protein_coding
ENST00000367694.2 RC3H1 protein_coding
ENST00000367696.5 RC3H1 protein_coding
ENST00000367701.8 ZBTB37 protein_coding
ENST00000367716.3 PRDX6-AS1 lncRNA
ENST00000367733.5 DNM3 protein_coding
ENST00000367765.4 KIFAP3 protein_coding
ENST00000367797.6 F5 protein_coding
ENST00000367804.4 SLC19A2 protein_coding
ENST00000367815.7 ATP1B1 protein_coding
ENST00000367833.5 TIPRL protein_coding
ENST00000367840.3 DCAF6 protein_coding
ENST00000367848.1 ADCY10 protein_coding
ENST00000367875.1 POGK protein_coding
ENST00000367879.7 UCK2 protein_coding
ENST00000367925.4 UAP1 protein_coding
ENST00000367928.4 VNN1 protein_coding
ENST00000367937.4 STX7 protein_coding
ENST00000367940.2 OLFML2B protein_coding
ENST00000367941.5 STX7 protein_coding
ENST00000367942.3 ATF6 protein_coding
ENST00000368016.6 ARHGAP30 protein_coding
ENST00000368035.1 LY9 protein_coding
ENST00000368045.3 CD48 protein_coding
ENST00000368046.6 CD48 protein_coding
ENST00000368047.3 CD84 processed_transcript
ENST00000368054.6 CD84 protein_coding
ENST00000368055.1 SLAMF6 protein_coding
ENST00000368057.6 SLAMF6 protein_coding
ENST00000368069.6 COPA protein_coding
ENST00000368096.4 TAGLN2 protein_coding
ENST00000368114.1 FCER1A protein_coding
ENST00000368141.4 MNDA protein_coding
ENST00000368149.2 ARHGAP18 protein_coding
ENST00000368171.3 CD1D protein_coding
ENST00000368192.7 ETV3 protein_coding
ENST00000368198.6 SH2D2A protein_coding
ENST00000368261.6 CCT3 processed_transcript
ENST00000368282.1 SEMA4A protein_coding
ENST00000368287.1 ECHDC1 retained_intron
ENST00000368324.4 SYT11 protein_coding
ENST00000368440.4 SMPDL3A protein_coding
ENST00000368455.7 HSF2 protein_coding
ENST00000368463.6 PBXIP1 protein_coding
ENST00000368468.3 MAN1A1 protein_coding
ENST00000368474.7 ADAR protein_coding
ENST00000368478.2 MCM9 processed_transcript
ENST00000368485.6 IL6R protein_coding
ENST00000368489.3 ATP8B2 protein_coding

ENST00000368491.6 CEP85L protein_coding
ENST00000368494.3 NUS1 protein_coding
ENST00000368498.5 GOPC protein_coding
ENST00000368516.1 C1orf43 protein_coding
ENST00000368545.6 TPM3 retained_intron
ENST00000368553.4 NUP210L protein_coding
ENST00000368562.4 TUBGCP2 retained_intron
ENST00000368564.4 KPNA5 protein_coding
ENST00000368608.3 TSPYL1 protein_coding
ENST00000368622.4 STK32C protein_coding
ENST00000368646.5 DENND4B protein_coding
ENST00000368655.4 GATAD2B protein_coding
ENST00000368686.1 CHTOP protein_coding
ENST00000368715.4 S100A4 protein_coding
ENST00000368732.4 S100A8 protein_coding
ENST00000368737.4 S100A12 protein_coding
ENST00000368738.3 S100A9 protein_coding
ENST00000368809.1 S100A10 protein_coding
ENST00000368838.1 SNX27 protein_coding
ENST00000368839.1 LHPP protein_coding
ENST00000368845.5 OAT protein_coding
ENST00000368847.4 MFSD4B protein_coding
ENST00000368850.4 SLC16A10 protein_coding
ENST00000368859.5 BUB3 protein_coding
ENST00000368869.7 ACADSB protein_coding
ENST00000368873.4 PI4KB protein_coding
ENST00000368877.8 AMD1 protein_coding
ENST00000368910.3 TNFAIP8L2 protein_coding
ENST00000368918.6 GABPB2 protein_coding
ENST00000368921.4 MLLT11 protein_coding
ENST00000368930.4 CDC40 protein_coding
ENST00000368941.1 FIG4 protein_coding
ENST00000368985.6 CTSS protein_coding
ENST00000369002.7 SEC63 protein_coding
ENST00000369016.7 ENSA protein_coding
ENST00000369025.5 SCML4 protein_coding
ENST00000369026.2 MCL1 protein_coding
ENST00000369031.4 PDSS2 protein_coding
ENST00000369037.7 PDSS2 protein_coding
ENST00000369046.7 QRSL1 protein_coding
ENST00000369066.6 NA NA
ENST00000369067.6 RPRD2 protein_coding
ENST00000369068.4 RPRD2 protein_coding
ENST00000369073.3 PLPP4 processed_transcript
ENST00000369075.6 SEC23IP protein_coding
ENST00000369077.3 MCMBP protein_coding
ENST00000369086.1 TIAL1 protein_coding
ENST00000369089.3 PRDM1 protein_coding
ENST00000369110.6 PREP protein_coding
ENST00000369115.3 ANP32E protein_coding
ENST00000369143.2 ASCC3 protein_coding
ENST00000369153.2 BOLA1 protein_coding
ENST00000369155.3 H2BC21 protein_coding
ENST00000369158.1 H3C14 protein_coding

ENST00000369159.2 H2AC18 protein_coding
ENST00000369160.3 NA NA
ENST00000369162.5 ASCC3 protein_coding
ENST00000369163.3 H3C10 protein_coding
ENST00000369167.2 H2BC18 protein_coding
ENST00000369183.7 FAM204A protein_coding
ENST00000369199.4 RAB11FIP2 protein_coding
ENST00000369207.2 ENO4 protein_coding
ENST00000369217.7 CCNC protein_coding
ENST00000369227.7 NBPF19 protein_coding
ENST00000369239.8 PNISR protein_coding
ENST00000369246.1 FHIP2A protein_coding
ENST00000369248.7 FHIP2A protein_coding
ENST00000369251.5 MMS22L protein_coding
ENST00000369266.6 NA NA
ENST00000369278.4 UFL1 protein_coding
ENST00000369285.6 CCDC186 protein_coding
ENST00000369287.6 CCDC186 protein_coding
ENST00000369301.3 NHLRC2 protein_coding
ENST00000369320.1 MAP3K7 protein_coding
ENST00000369325.6 MAP3K7 protein_coding
ENST00000369387.4 H3P6 processed_pseudogene
ENST00000369404.3 ZDHHC6 protein_coding
ENST00000369439.4 TMLHE protein_coding
ENST00000369443.8 GDAP2 protein_coding
ENST00000369448.3 TENT5C protein_coding
ENST00000369466.7 TTF2 protein_coding
ENST00000369472.1 PNRC1 protein_coding
ENST00000369475.6 RNGTT protein_coding
ENST00000369477.1 CD2 protein_coding
ENST00000369478.3 CD2 protein_coding
ENST00000369487.3 CD58 protein_coding
ENST00000369494.4 ATP1A1 protein_coding
ENST00000369496.7 ATP1A1 protein_coding
ENST00000369502.1 SLC22A15 protein_coding
ENST00000369503.7 SLC22A15 protein_coding
ENST00000369535.4 NRAS protein_coding
ENST00000369536.8 RARS2 protein_coding
ENST00000369553.4 HIPK1 protein_coding
ENST00000369559.7 HIPK1 protein_coding
ENST00000369568.7 GAB3 protein_coding
ENST00000369572.3 SMIM8 processed_transcript
ENST00000369578.5 ZNF292 processed_transcript
ENST00000369583.3 DUSP5 protein_coding
ENST00000369622.6 SYNCRIP protein_coding
ENST00000369679.4 CYB5R4 protein_coding
ENST00000369681.8 CYB5R4 protein_coding
ENST00000369699.7 NA NA
ENST00000369705.3 ME1 protein_coding
ENST00000369709.3 RAP1A protein_coding
ENST00000369739.6 DOP1A protein_coding
ENST00000369751.2 IBTK retained_intron
ENST00000369754.6 TENT5A protein_coding
ENST00000369755.3 SLK protein_coding

ENST00000369760.7 BCKDHB protein_coding
ENST00000369764.1 STN1 protein_coding
ENST00000369769.3 KCNA3 protein_coding
ENST00000369780.7 NEURL1 protein_coding
ENST00000369797.6 PDCD11 protein_coding
ENST00000369838.5 SH3BGRL2 protein_coding
ENST00000369851.5 GNAI3 protein_coding
ENST00000369878.7 CNNM2 protein_coding
ENST00000369889.5 WBP1L protein_coding
ENST00000369902.6 SUFU protein_coding
ENST00000369927.7 TPD52L2 protein_coding
ENST00000369963.5 IMPG1 protein_coding
ENST00000369976.4 CLCC1 protein_coding
ENST00000369983.3 GBF1 protein_coding
ENST00000369984.4 HCFC1 protein_coding
ENST00000370021.1 PRPF38B protein_coding
ENST00000370033.7 ARMH3 protein_coding
ENST00000370035.6 FAM102B protein_coding
ENST00000370041.4 SLC25A24 protein_coding
ENST00000370050.8 TMEM30A protein_coding
ENST00000370069.4 GMEB2 protein_coding
ENST00000370077.1 GMEB2 protein_coding
ENST00000370082.1 HELZ2 retained_intron
ENST00000370094.6 OGA protein_coding
ENST00000370111.4 SLC30A7 protein_coding
ENST00000370112.7 SLC30A7 protein_coding
ENST00000370124.6 CDC14A protein_coding
ENST00000370131.3 DBT protein_coding
ENST00000370132.7 DBT protein_coding
ENST00000370141.5 TRMT13 protein_coding
ENST00000370152.6 MFSD14A protein_coding
ENST00000370156.3 NA NA
ENST00000370179.6 PPDPF protein_coding
ENST00000370192.6 DPYD protein_coding
ENST00000370251.3 ZNF275 protein_coding
ENST00000370267.1 DR1 protein_coding
ENST00000370269.3 SLF2 protein_coding
ENST00000370271.6 SLF2 protein_coding
ENST00000370272.7 DR1 protein_coding
ENST00000370280.1 TMED5 protein_coding
ENST00000370290.6 TMED5 nonsense_mediated_decay
ENST00000370310.4 DIPK1A protein_coding
ENST00000370315.3 CGAS protein_coding
ENST00000370334.4 YTHDF1 protein_coding
ENST00000370366.1 DIDO1 protein_coding
ENST00000370371.7 DIDO1 protein_coding
ENST00000370379.1 NA NA
ENST00000370382.6 BTBD8 retained_intron
ENST00000370387.2 MTMR1 retained_intron
ENST00000370392.4 KCNQ5 protein_coding
ENST00000370393.4 MTM1 retained_intron
ENST00000370397.7 CHUK protein_coding
ENST00000370408.2 ERLIN1 protein_coding
ENST00000370435.4 OGFRL1 protein_coding

ENST00000370438.2 lncRNA
ENST00000370452.6 SMAP1 protein_coding
ENST00000370454.7 LRRC8C protein_coding
ENST00000370461.4 OGFR protein_coding
ENST00000370466.3 GBP2 protein_coding
ENST00000370473.4 GBP1 protein_coding
ENST00000370485.5 NA NA
ENST00000370505.6 NA NA
ENST00000370509.4 CREG1 protein_coding
ENST00000370513.8 PKN2 protein_coding
ENST00000370546.4 HPSE2 protein_coding
ENST00000370548.3 protein_coding
ENST00000370551.7 HS2ST1 protein_coding
ENST00000370570.4 LMBRD1 protein_coding
ENST00000370574.3 ZNHIT6 protein_coding
ENST00000370586.5 R3HCC1L protein_coding
ENST00000370618.6 NA NA
ENST00000370620.4 ARHGEF6 protein_coding
ENST00000370625.1 CTBS nonsense_mediated_decay
ENST00000370628.2 CD40LG protein_coding
ENST00000370631.3 PI4K2A protein_coding
ENST00000370641.3 GNG5 protein_coding
ENST00000370651.4 protein_coding
ENST00000370654.5 RPF1 protein_coding
ENST00000370659.1 FKBP1C protein_coding
ENST00000370664.3 UBTD1 protein_coding
ENST00000370688.6 PRKACB protein_coding
ENST00000370701.4 SLC9A6 protein_coding
ENST00000370708.7 ZNF451 protein_coding
ENST00000370751.8 IFI44L protein_coding
ENST00000370766.6 ZNF75D protein_coding
ENST00000370768.5 FUBP1 protein_coding
ENST00000370791.6 MIGA1 protein_coding
ENST00000370800.4 PHF6 protein_coding
ENST00000370828.3 GPC4 protein_coding
ENST00000370913.5 ELOVL5 protein_coding
ENST00000370927.4 ENOX2 protein_coding
ENST00000370952.3 LRRC40 protein_coding
ENST00000370958.4 LRRC7 protein_coding
ENST00000370990.5 SERBP1 protein_coding
ENST00000371000.4 IL12RB2 protein_coding
ENST00000371012.5 MIER1 protein_coding
ENST00000371019.3 FRAT2 protein_coding
ENST00000371021.4 FRAT1 protein_coding
ENST00000371030.2 ZNF831 protein_coding
ENST00000371059.6 LEPR protein_coding
ENST00000371083.4 PGM1 protein_coding
ENST00000371109.3 PIK3AP1 protein_coding
ENST00000371118.1 ATG4C protein_coding
ENST00000371120.6 ATG4C protein_coding
ENST00000371142.7 TM9SF3 protein_coding
ENST00000371144.6 STAG2 protein_coding
ENST00000371177.2 TM2D1 protein_coding
ENST00000371219.2 RBM38 protein_coding

ENST00000371222.3 JUN protein_coding
ENST00000371230.1 DAB1 protein_coding
ENST00000371248.3 VPS37B protein_coding
ENST00000371331.1 TCEANC2 protein_coding
ENST00000371332.7 HELLS protein_coding
ENST00000371335.4 LAMP2 protein_coding
ENST00000371337.3 TMEM59 protein_coding
ENST00000371344.4 TMEM59 protein_coding
ENST00000371429.3 NDC1 protein_coding
ENST00000371436.9 RUNX2 protein_coding
ENST00000371437.4 NDUFA1 protein_coding
ENST00000371458.1 SUPT3H protein_coding
ENST00000371459.4 SUPT3H protein_coding
ENST00000371477.3 CDC5L protein_coding
ENST00000371489.4 MYOF protein_coding
ENST00000371497.8 TSHZ2 protein_coding
ENST00000371541.4 TUT4 processed_transcript
ENST00000371543.4 EXOC6 protein_coding
ENST00000371554.2 HSP90AB1 protein_coding
ENST00000371602.7 ADNP protein_coding
ENST00000371627.4 TNKS2 protein_coding
ENST00000371642.1 IL13RA1 protein_coding
ENST00000371648.4 EDF1 protein_coding
ENST00000371656.3 PEDS1 protein_coding
ENST00000371658.3 PEDS1 nonsense_mediated_decay
ENST00000371666.6 IL13RA1 protein_coding
ENST00000371711.4 B4GALT5 protein_coding
ENST00000371730.5 EPS15 protein_coding
ENST00000371744.4 ZNFX1 protein_coding
ENST00000371752.4 ZNFX1 protein_coding
ENST00000371753.4 NACC2 miRNA
ENST00000371795.4 IFIT5 miRNA
ENST00000371804.3 IFIT1 miRNA
ENST00000371806.3 FCN1 miRNA
ENST00000371811.4 IFIT3 miRNA
ENST00000371834.5 BRD3 miRNA
ENST00000371835.7 BRD4 miRNA
ENST00000371836.1 AGBL4 miRNA
ENST00000371837.4 LIPA miRNA
ENST00000371841.1 SPATA6 miRNA
ENST00000371848.3 WDR44 miRNA
ENST00000371873.8 CMPK1 miRNA
ENST00000371917.4 ARFGEF2 miRNA
ENST00000371927.6 STAMBPL1 miRNA
ENST00000371933.6 EFCAB14 miRNA
ENST00000371941.3 PREX1 miRNA
ENST00000371953.6 PTEN miRNA
ENST00000371957.3 STKLD1 miRNA
ENST00000371982.3 SURF4 miRNA
ENST00000371989.6 SURF4 miRNA
ENST00000371997.3 NCOA3 miRNA
ENST00000371998.6 NCOA3 miRNA
ENST00000372008.5 MAST2 miRNA
ENST00000372009.3 NA miRNA

ENST00000372025.4 TMEM69 miRNA
ENST00000372037.6 BMPR1A miRNA
ENST00000372059.5 AMMECR1 miRNA
ENST00000372075.1 WAPL miRNA
ENST00000372084.4 TESK2 miRNA
ENST00000372102.3 TP53RK miRNA
ENST00000372123.4 GFI1B miRNA
ENST00000372134.4 GHITM miRNA
ENST00000372146.4 GTF3C4 miRNA
ENST00000372164.6 TSPAN14 miRNA
ENST00000372179.6 NA miRNA
ENST00000372190.6 RAPGEF1 miRNA
ENST00000372271.3 FAM78A miRNA
ENST00000372291.3 NCOA5 miRNA
ENST00000372317.6 CALCOCO2 miRNA
ENST00000372333.3 ZCCHC24 miRNA
ENST00000372390.7 TSC22D3 miRNA
ENST00000372396.3 KDM4A miRNA
ENST00000372409.6 PCIF1 miRNA
ENST00000372410.6 GPR107 miRNA
ENST00000372450.7 SZT2 miRNA
ENST00000372454.5 TJAP1 miRNA
ENST00000372489.2 TMEM156 miRNA
ENST00000372491.3 IER5L miRNA
ENST00000372499.4 LRMDA miRNA
ENST00000372525.5 C1orf50 miRNA
ENST00000372571.4 FOXJ3 miRNA
ENST00000372577.2 NUP188 miRNA
ENST00000372582.4 IL1RAPL2 miRNA
ENST00000372583.4 HIVEP3 miRNA
ENST00000372687.4 SAMD8 miRNA
ENST00000372690.6 NA miRNA
ENST00000372708.4 SMAP2 miRNA
ENST00000372711.1 KAT6B miRNA
ENST00000372718.6 SMAP2 miRNA
ENST00000372734.3 ADK miRNA
ENST00000372745.1 AP3M1 miRNA
ENST00000372759.3 ZMPSTE24 miRNA
ENST00000372770.4 GLE1 miRNA
ENST00000372771.4 RLF miRNA
ENST00000372775.2 PPT1 miRNA
ENST00000372801.4 STK4 miRNA
ENST00000372805.6 CAP1 miRNA
ENST00000372836.4 CNPY3 miRNA
ENST00000372837.3 CHCHD1 miRNA
ENST00000372839.6 YWHAB miRNA
ENST00000372870.4 SLC27A4 miRNA
ENST00000372876.1 TBCC miRNA
ENST00000372901.1 UBR2 miRNA
ENST00000372903.5 UBR2 miRNA
ENST00000372969.6 NDUFS5 miRNA
ENST00000372970.5 OSER1 miRNA
ENST00000372987.7 CCND3 miRNA
ENST00000373008.5 P4HA1 miRNA

ENST00000373009.6 NA miRNA
ENST00000373027.4 INPP5B miRNA
ENST00000373036.4 MTF1 miRNA
ENST00000373054.5 NA miRNA
ENST00000373061.6 NA miRNA
ENST00000373062.6 GNL2 miRNA
ENST00000373069.8 SLC25A25 miRNA
ENST00000373087.6 ZC3H12A miRNA
ENST00000373109.5 SPOCK2 miRNA
ENST00000373173.5 APOOL miRNA
ENST00000373177.2 HDX miRNA
ENST00000373178.4 ADPRS miRNA
ENST00000373181.7 MOCS1 miRNA
ENST00000373189.5 SLC29A3 miRNA
ENST00000373191.7 AGO3 miRNA
ENST00000373202.6 SGPL1 miRNA
ENST00000373204.4 AGO1 miRNA
ENST00000373209.2 PRF1 miRNA
ENST00000373210.3 AGO4 miRNA
ENST00000373212.5 SH3BGRL miRNA
ENST00000373218.4 EIF4EBP2 miRNA
ENST00000373233.6 CHD6 miRNA
ENST00000373236.1 SAR1A miRNA
ENST00000373239.2 SAR1A miRNA
ENST00000373275.4 BRWD3 miRNA
ENST00000373304.3 CYSLTR1 miRNA
ENST00000373313.2 MAFB miRNA
ENST00000373316.4 PGK1 miRNA
ENST00000373322.1 LRSAM1 miRNA
ENST00000373323.7 DHX35 miRNA
ENST00000373331.2 PPP1R16B miRNA
ENST00000373336.3 MAGT1 miRNA
ENST00000373365.4 GLO1 miRNA
ENST00000373373.7 UPRT miRNA
ENST00000373381.7 CSMD2 miRNA
ENST00000373389.5 ZFAND3 miRNA
ENST00000373428.5 ZNF362 miRNA
ENST00000373434.4 RALGPS1 miRNA
ENST00000373449.5 AK2 miRNA
ENST00000373451.7 CMTR1 miRNA
ENST00000373452.2 ZBTB34 miRNA
ENST00000373457.1 ZBTB43 miRNA
ENST00000373469.1 CTNNBL1 miRNA
ENST00000373491.3 TBC1D22B protein_coding
ENST00000373493.8 RBBP4 protein_coding
ENST00000373509.5 PIM1 trna
ENST00000373547.7 PPP6C trna
ENST00000373559.7 HDAC8 trna
ENST00000373596.4 NEK6 trna
ENST00000373609.1 TXLNA trna
ENST00000373618.1 DENND1A trna
ENST00000373622.8 RPN2 trna
ENST00000373626.3 RPS4X trna
ENST00000373644.4 TET1 trna

ENST00000373659.3 ZBTB6 trna
ENST00000373670.4 RC3H2 trna
ENST00000373685.1 C6orf89 trna
ENST00000373695.1 GCNA trna
ENST00000373698.6 PTGS1 trna
ENST00000373701.6 OGT trna
ENST00000373715.9 SRSF3 trna
ENST00000373719.6 OGT trna
ENST00000373758.4 REEP3 trna
ENST00000373773.6 NDRG3 trna
ENST00000373795.5 SRSF4 trna
ENST00000373797.1 EPB41 trna
ENST00000373807.4 GSN trna
ENST00000373821.5 SRPK1 trna
ENST00000373836.3 PHACTR4 trna
ENST00000373840.7 RAB14 trna
ENST00000373847.4 CNTRL trna
ENST00000373857.6 PTAFR trna
ENST00000373864.4 EYA3 trna
ENST00000373865.5 CNTRL trna
ENST00000373886.6 BICC1 trna
ENST00000373887.6 TRAF1 trna
ENST00000373903.1 PSMD5 trna
ENST00000373930.3 MEGF9 trna
ENST00000373932.3 AAR2 trna
ENST00000373935.3 IPMK trna
ENST00000373943.7 STX12 trna
ENST00000373955.4 PCDH15 trna
ENST00000373976.6 PRKG1 trna
ENST00000373986.6 ASTN2 trna
ENST00000374004.4 FGR trna
ENST00000374012.6 PHF20 trna
ENST00000374021.1 ILRUN trna
ENST00000374023.6 ILRUN trna
ENST00000374050.3 ATP6V1G1 trna
ENST00000374080.6 MED12 trna
ENST00000374084.2 SLC9A1 trna
ENST00000374086.3 SLC9A1 trna
ENST00000374104.6 RBM12 trna
ENST00000374136.4 RGS3 trna
ENST00000374161.6 WDFY4 trna
ENST00000374174.1 MAPK8 trna
ENST00000374198.4 PRPF4 trna
ENST00000374206.3 CDC26 trna
ENST00000374212.4 SLC31A1 trna
ENST00000374220.3 SLC31A2 trna
ENST00000374232.6 SNX30 trna
ENST00000374244.3 KIAA1958 trna
ENST00000374257.4 PTBP3 trna
ENST00000374264.5 SUSD1 trna
ENST00000374281.2 ADD1 trna
ENST00000374284.4 PAFAH2 trna
ENST00000374293.4 GNG10 trna
ENST00000374315.1 SELENON trna

ENST00000374329.1 MAN1C1 trna
ENST00000374343.4 MACO1 trna
ENST00000374359.1 WASHC2C trna
ENST00000374370.1 ZFAND4 trna
ENST00000374383.1 ECPAS trna
ENST00000374393.4 RCAN3 trna
ENST00000374395.7 RCAN3 trna
ENST00000374430.5 LPAR1 trna
ENST00000374466.3 CSGALNACT2 trna
ENST00000374467.3 BAK1 trna
ENST00000374468.1 PNRC2 trna
ENST00000374472.4 CNR2 trna
ENST00000374479.3 FUCA1 trna
ENST00000374491.3 EDEM2 trna
ENST00000374518.5 BMS1 trna
ENST00000374557.4 EPB41L4B trna
ENST00000374574.2 BMPR2 trna
ENST00000374586.6 TMEM245 trna
ENST00000374597.3 STARD8 trna
ENST00000374643.6 YIPF6 trna
ENST00000374650.6 CASP10 trna
ENST00000374704.6 CCNY trna
ENST00000374707.1 FSD1L trna
ENST00000374730.2 USP48 trna
ENST00000374732.6 USP48 trna
ENST00000374733.1 ABCA1 trna
ENST00000374736.6 ABCA1 trna
ENST00000374746.4 CUL2 trna
ENST00000374767.4 NIPSNAP3A trna
ENST00000374773.4 PARD3 trna
ENST00000374778.4 OPCML trna
ENST00000374807.8 LAS1L trna
ENST00000374846.3 DYNLRB1 trna
ENST00000374852.3 MTMR8 trna
ENST00000374865.4 MRPL50 trna
ENST00000374900.4 FAAH2 trna
ENST00000374930.6 NUP35 trna
ENST00000374933.3 EIF4G3 trna
ENST00000374940.3 HLA-DQA2 trna
ENST00000374975.3 HLA-DRB5 trna
ENST00000374980.2 EIF2S2 trna
ENST00000374982.5 HLA-DRA trna
ENST00000374998.3 MGAT5B trna
ENST00000375003.5 HP1BP3 trna
ENST00000375030.5 NA trna
ENST00000375063.4 TBC1D2 trna
ENST00000375064.4 TBC1D2 trna
ENST00000375110.5 EPC1 trna
ENST00000375118.1 TRMO trna
ENST00000375120.3 OTUD3 trna
ENST00000375144.4 CAPZB trna
ENST00000375147.6 NCBP1 trna
ENST00000375167.1 H2AX trna
ENST00000375218.3 UBR4 trna

ENST00000375223.4 NA trna
ENST00000375224.1 UBR4 trna
ENST00000375249.4 HABP4 trna
ENST00000375254.6 UBR4 trna
ENST00000375311.1 ZNF438 trna
ENST00000375321.1 MAP3K8 trna
ENST00000375322.2 MAP3K8 trna
ENST00000375340.9 SMC1A trna
ENST00000375344.6 MFSD14B trna
ENST00000375350.3 NSD1 trna
ENST00000375365.2 IQSEC2 trna
ENST00000375383.6 KDM5C trna
ENST00000375389.6 FAM120A trna
ENST00000375400.6 SVIL trna
ENST00000375433.3 RCC2 trna
ENST00000375436.7 RCC2 trna
ENST00000375440.7 CUL4A trna
ENST00000375448.4 PADI4 trna
ENST00000375453.4 PADI4 trna
ENST00000375469.4 SUSD3 trna
ENST00000375481.1 PADI2 trna
ENST00000375495.6 ZNF484 trna
ENST00000375512.3 BICD2 trna
ENST00000375522.1 IPPK trna
ENST00000375571.5 MAPRE1 trna
ENST00000375587.6 CENPP trna
ENST00000375592.6 FBXO42 trna
ENST00000375647.4 ZBTB40 trna
ENST00000375650.4 HSPA1B trna
ENST00000375669.6 TUBGCP3 trna
ENST00000375677.4 NOL4L trna
ENST00000375702.5 AXIN2 trna
ENST00000375704.6 NA trna
ENST00000375712.3 KIF3B trna
ENST00000375723.4 ARHGEF7 trna
ENST00000375731.7 AUH trna
ENST00000375746.1 SYK trna
ENST00000375759.6 SPEN trna
ENST00000375772.6 MAGED1 trna
ENST00000375773.5 KYNU trna
ENST00000375793.2 PLEKHM2 trna
ENST00000375838.4 DNAJC16 trna
ENST00000375846.3 S1PR3 trna
ENST00000375856.4 IRS2 trna
ENST00000375887.7 TNFSF13B trna
ENST00000375898.3 ABHD13 trna
ENST00000375915.3 NALF1 trna
ENST00000375947.1 TUT7 trna
ENST00000375948.2 TUT7 trna
ENST00000375954.1 ERCC5 trna
ENST00000375957.4 TUT7 trna
ENST00000375958.3 ERCC5 trna
ENST00000375963.6 TUT7 trna
ENST00000375972.6 NA trna

ENST00000375980.7 EFHD2 trna
ENST00000376033.2 PRRC2A trna
ENST00000376040.2 NAA35 trna
ENST00000376049.4 AIF1 trna
ENST00000376062.5 BCL2L1 trna
ENST00000376065.7 TPP2 trna
ENST00000376080.2 AGTPBP1 trna
ENST00000376081.7 AGTPBP1 trna
ENST00000376083.6 AGTPBP1 trna
ENST00000376087.4 ANKRD26 trna
ENST00000376091.6 CLCN5 trna
ENST00000376104.5 DLG2 trna
ENST00000376113.5 BIN1 trna
ENST00000376131.7 FGF14 trna
ENST00000376140.3 ABI1 trna
ENST00000376236.7 APBB1IP trna
ENST00000376256.2 HNRNPK trna
ENST00000376285.4 PCCA trna
ENST00000376300.5 CCDC93 trna
ENST00000376322.6 PLP2 trna
ENST00000376334.3 FRG1JP trna
ENST00000376351.3 GPR158 trna
ENST00000376354.4 CLYBL trna
ENST00000376377.2 IER3 trna
ENST00000376381.3 MBTD1 trna
ENST00000376387.4 TM9SF2 trna
ENST00000376414.4 GPR183 trna
ENST00000376436.4 ZNF337 trna
ENST00000376462.4 KIAA1217 trna
ENST00000376463.2 TLE1 trna
ENST00000376491.6 CLCN6 trna
ENST00000376495.4 OTUD1 trna
ENST00000376497.6 CLCN6 trna
ENST00000376509.4 PIM2 trna
ENST00000376511.5 PPP1R10 trna
ENST00000376552.5 TLE4 trna
ENST00000376554.7 STK24 trna
ENST00000376573.7 PIP4K2A trna
ENST00000376585.4 MTHFR trna
ENST00000376621.6 GNL1 trna
ENST00000376630.4 HLA-E trna
ENST00000376634.7 NA trna
ENST00000376705.3 HS6ST3 trna
ENST00000376723.6 UGGT1 trna
ENST00000376726.3 ACSS1 trna
ENST00000376758.1 PPP1R11 trna
ENST00000376767.6 PCSK5 trna
ENST00000376795.6 DNAJC3 trna
ENST00000376810.5 UBIAD1 trna
ENST00000376834.6 CARNMT1 trna
ENST00000376838.4 MTOR trna
ENST00000376840.7 TBC1D8 trna
ENST00000376887.7 NA trna
ENST00000376896.6 RORB trna

ENST00000376925.6 CST3 trna
ENST00000376926.7 MAP3K14 protein_coding
ENST00000376935.4 SMIM13 nonsense_mediated_decay
ENST00000376943.6 ZNF182 protein_coding
ENST00000376946.2 DNAJC1 retained_intron
ENST00000376950.4 ZNF81 protein_coding
ENST00000376954.4 ZNF81 protein_coding
ENST00000376958.4 GPR180 protein_coding
ENST00000376962.8 ZFAND5 protein_coding
ENST00000376980.6 DNAJC1 protein_coding
ENST00000377026.4 NAPB protein_coding
ENST00000377045.7 ARAF protein_coding
ENST00000377047.7 GPC6 protein_coding
ENST00000377057.4 CEMIP2 retained_intron
ENST00000377059.6 MLLT10 protein_coding
ENST00000377067.6 GPC5 protein_coding
ENST00000377075.2 CNNM4 protein_coding
ENST00000377083.4 KIF1B protein_coding
ENST00000377093.7 KIF1B protein_coding
ENST00000377111.5 TRPM3 protein_coding
ENST00000377126.3 KLF9 protein_coding
ENST00000377153.4 UBE4B protein_coding
ENST00000377154.1 OR5V1 protein_coding
ENST00000377191.4 XRN2 protein_coding
ENST00000377200.8 PTAR1 protein_coding
ENST00000377238.2 PLXDC2 processed_transcript
ENST00000377242.6 PLXDC2 protein_coding
ENST00000377252.4 PLXDC2 protein_coding
ENST00000377256.1 CTNNBIP1 protein_coding
ENST00000377259.4 TJP2 retained_intron
ENST00000377269.3 UBA1 protein_coding
ENST00000377275.3 ARL5B protein_coding
ENST00000377284.4 PIP5K1B protein_coding
ENST00000377291.2 HRH2 protein_coding
ENST00000377304.5 NSUN6 protein_coding
ENST00000377346.7 PIK3CD protein_coding
ENST00000377365.3 INSYN2B protein_coding
ENST00000377387.4 SF1 protein_coding
ENST00000377401.3 H2BC13 protein_coding
ENST00000377452.3 DTD1 protein_coding
ENST00000377459.2 H2AC12 protein_coding
ENST00000377465.4 SEC23B protein_coding
ENST00000377474.3 KCTD12 protein_coding
ENST00000377524.6 STAM protein_coding
ENST00000377560.8 HECTD4 protein_coding
ENST00000377575.2 HMGN4 protein_coding
ENST00000377602.4 ST8SIA6 protein_coding
ENST00000377636.6 TBC1D4 protein_coding
ENST00000377666.5 NA NA
ENST00000377669.5 KLF12 protein_coding
ENST00000377705.5 NOL9 protein_coding
ENST00000377707.3 SHB protein_coding
ENST00000377724.6 DCAF10 protein_coding
ENST00000377727.1 H4C8 protein_coding

ENST00000377745.3 H4C2 protein_coding
ENST00000377777.5 H2BC5 protein_coding
ENST00000377791.3 H2AC6 protein_coding
ENST00000377801.6 PLCXD3 protein_coding
ENST00000377803.3 H4C3 protein_coding
ENST00000377833.7 CUBN protein_coding
ENST00000377844.7 KLHL1 protein_coding
ENST00000377877.4 RNF38 protein_coding
ENST00000377882.6 TDRD3 protein_coding
ENST00000377911.1 RSU1 processed_transcript
ENST00000377959.4 GLIPR2 protein_coding
ENST00000377966.3 RECK protein_coding
ENST00000377973.4 CIR1 protein_coding
ENST00000377982.7 ACTR2 protein_coding
ENST00000378004.6 NA NA
ENST00000378013.2 ARHGAP26 processed_transcript
ENST00000378024.7 AHNAK protein_coding
ENST00000378026.4 CKAP4 protein_coding
ENST00000378036.4 MINDY3 protein_coding
ENST00000378078.4 RGP1 protein_coding
ENST00000378083.6 KCNAB2 protein_coding
ENST00000378102.3 C6orf62 protein_coding
ENST00000378119.7 C6orf62 protein_coding
ENST00000378150.1 NMT2 protein_coding
ENST00000378154.1 CASK protein_coding
ENST00000378164.5 TAF2 protein_coding
ENST00000378168.5 CASK protein_coding
ENST00000378180.7 NA NA
ENST00000378192.2 TLN1 retained_intron
ENST00000378242.1 DCLRE1C protein_coding
ENST00000378251.2 LRRC47 protein_coding
ENST00000378289.7 DCLRE1C protein_coding
ENST00000378308.5 USP9X protein_coding
ENST00000378383.4 FNDC3A protein_coding
ENST00000378402.8 PKHD1L1 protein_coding
ENST00000378421.1 CXorf38 protein_coding
ENST00000378438.7 ATP6AP2 protein_coding
ENST00000378470.4 FAM107B protein_coding
ENST00000378494.6 CAMKMT protein_coding
ENST00000378496.7 NA NA
ENST00000378505.5 NA NA
ENST00000378511.6 PREPL protein_coding
ENST00000378512.4 RER1 protein_coding
ENST00000378536.4 SKI protein_coding
ENST00000378539.5 PLXNA4 protein_coding
ENST00000378549.5 ITM2B protein_coding
ENST00000378557.1 FAM214B protein_coding
ENST00000378588.4 CYBB protein_coding
ENST00000378595.6 AFF4 protein_coding
ENST00000378609.7 GNB1 protein_coding
ENST00000378610.1 CDKAL1 protein_coding
ENST00000378614.7 SEPHS1 protein_coding
ENST00000378616.3 XK protein_coding
ENST00000378718.2 DGKZP1 processed_pseudogene

ENST00000378725.3 SSU72 retained_intron
ENST00000378726.1 SSU72 retained_intron
ENST00000378770.1 HSP90AA4P processed_transcript
ENST00000378789.3 HAO1 protein_coding
ENST00000378818.2 TP53I13 protein_coding
ENST00000378843.5 KIF13A protein_coding
ENST00000378845.4 CAMK1D protein_coding
ENST00000378930.6 TAB3 protein_coding
ENST00000378941.4 GK protein_coding
ENST00000378945.6 GK protein_coding
ENST00000378962.3 TASL protein_coding
ENST00000378993.4 IL1RAPL1 protein_coding
ENST00000379070.3 CDS2 processed_transcript
ENST00000379078.1 FAM219A protein_coding
ENST00000379091.7 ZBTB10 protein_coding
ENST00000379143.8 PCNA protein_coding
ENST00000379153.3 CD83 protein_coding
ENST00000379161.4 NUFIP1 protein_coding
ENST00000379162.7 PDK3 protein_coding
ENST00000379165.7 NFATC3 retained_intron
ENST00000379177.4 ZFX protein_coding
ENST00000379213.3 STRN protein_coding
ENST00000379221.3 DNAJC15 protein_coding
ENST00000379224.8 ISG20 protein_coding
ENST00000379237.5 USP6NL protein_coding
ENST00000379251.6 SAT1 retained_intron
ENST00000379277.5 TMEM230 protein_coding
ENST00000379278.3 GFOD1 processed_transcript
ENST00000379284.1 GFOD1 protein_coding
ENST00000379310.6 VWA8 protein_coding
ENST00000379343.5 SLC30A6 protein_coding
ENST00000379357.8 NA NA
ENST00000379359.3 RGCC protein_coding
ENST00000379374.4 PHEX protein_coding
ENST00000379376.2 RASSF2 protein_coding
ENST00000379400.6 RASSF2 protein_coding
ENST00000379403.2 BTBD1 protein_coding
ENST00000379404.4 SMS protein_coding
ENST00000379419.7 PDE7A protein_coding
ENST00000379426.1 TMEM170B protein_coding
ENST00000379433.5 NEDD9 protein_coding
ENST00000379448.7 ZNF827 protein_coding
ENST00000379449.9 ASPH protein_coding
ENST00000379487.4 WBP4 protein_coding
ENST00000379515.3 TPTE2P5 processed_transcript
ENST00000379521.7 NFX1 retained_intron
ENST00000379550.4 ELF2 protein_coding
ENST00000379561.5 FOXO1 protein_coding
ENST00000379565.6 RPS6KA3 protein_coding
ENST00000379593.1 EIF1AX protein_coding
ENST00000379607.8 EIF1AX protein_coding
ENST00000379629.6 BABAM2 protein_coding
ENST00000379632.5 BABAM2 protein_coding
ENST00000379656.6 ARRDC2 protein_coding

ENST00000379660.4 SLC35B3 protein_coding
ENST00000379697.6 SH3KBP1 protein_coding
ENST00000379698.7 SH3KBP1 protein_coding
ENST00000379711.2 SFMBT2 protein_coding
ENST00000379713.6 SFMBT2 protein_coding
ENST00000379715.8 EEF1E1 protein_coding
ENST00000379716.4 SH3KBP1 protein_coding
ENST00000379719.6 IPO7 protein_coding
ENST00000379731.4 B4GALT1 protein_coding
ENST00000379751.4 CENPB protein_coding
ENST00000379775.7 PFKFB3 protein_coding
ENST00000379799.6 PAM protein_coding
ENST00000379800.4 CSNK1A1L protein_coding
ENST00000379858.1 TOPORS protein_coding
ENST00000379868.4 DDX58 protein_coding
ENST00000379869.6 ADGRG2 protein_coding
ENST00000379887.7 ARIH1 protein_coding
ENST00000379915.4 nonsense_mediated_decay
ENST00000379923.4 ACO1 protein_coding
ENST00000379928.4 lncRNA
ENST00000379939.5 NBEA protein_coding
ENST00000379942.4 PHKA2 protein_coding
ENST00000379958.2 SAMD9 protein_coding
ENST00000379995.1 C9orf72 protein_coding
ENST00000380013.7 TET2 protein_coding
ENST00000380027.4 TRIM5 protein_coding
ENST00000380038.6 KLC1 protein_coding
ENST00000380060.6 NHS protein_coding
ENST00000380071.6 RFC3 protein_coding
ENST00000380121.6 N4BP2L2 processed_transcript
ENST00000380155.3 SYAP1 protein_coding
ENST00000380217.1 FRY processed_transcript
ENST00000380221.6 TRAPPC10 protein_coding
ENST00000380250.6 NA NA
ENST00000380259.5 nonsense_mediated_decay
ENST00000380266.3 FASTKD5 protein_coding
ENST00000380298.2 SLC22A23 protein_coding
ENST00000380308.6 ZRSR2 protein_coding
ENST00000380313.1 CA5B processed_transcript
ENST00000380333.4 CA5BP1 processed_transcript
ENST00000380342.3 CLTRN protein_coding
ENST00000380381.3 RPS6 protein_coding
ENST00000380409.2 RIPK1 protein_coding
ENST00000380490.4 ALOX5AP protein_coding
ENST00000380496.4 HAUS6 protein_coding
ENST00000380498.9 CD68 protein_coding
ENST00000380502.6 HAUS6 protein_coding
ENST00000380520.4 SERPINB6 protein_coding
ENST00000380527.2 RRAGA protein_coding
ENST00000380590.3 NA NA
ENST00000380598.4 SMG1P4 processed_transcript
ENST00000380599.7 ZBTB4 protein_coding
ENST00000380629.5 BNIP3L protein_coding
ENST00000380633.1 TMSB4X protein_coding

ENST00000380635.4 TMSB4X protein_coding
ENST00000380636.1 TMSB4X protein_coding
ENST00000380641.4 CNTLN protein_coding
ENST00000380647.6 CNTLN protein_coding
ENST00000380649.6 HADHA protein_coding
ENST00000380650.4 RPAP3 protein_coding
ENST00000380659.3 TLR7 protein_coding
ENST00000380663.6 PRPS2 protein_coding
ENST00000380675.3 NA NA
ENST00000380680.4 UBL3 protein_coding
ENST00000380698.4 SERPINB9 protein_coding
ENST00000380701.6 CCDC171 protein_coding
ENST00000380702.5 PDE4A protein_coding
ENST00000380736.4 ARHGAP6 protein_coding
ENST00000380738.7 PSIP1 protein_coding
ENST00000380739.5 SERPINB1 protein_coding
ENST00000380752.8 SLC7A1 protein_coding
ENST00000380764.1 WRNIP1 protein_coding
ENST00000380776.4 KCNQ1 protein_coding
ENST00000380800.6 BRWD1 protein_coding
ENST00000380805.5 GMDS retained_intron
ENST00000380814.4 SLC46A3 protein_coding
ENST00000380815.4 GMDS protein_coding
ENST00000380821.6 SNAPC3 protein_coding
ENST00000380829.4 CLCN4 protein_coding
ENST00000380853.1 TTC39B retained_intron
ENST00000380858.1 SLC2A13 protein_coding
ENST00000380861.7 WWC3 protein_coding
ENST00000380916.7 ZDHHC21 protein_coding
ENST00000380927.5 SECISBP2L protein_coding
ENST00000380943.5 ERBIN protein_coding
ENST00000380946.3 KLF6 processed_transcript
ENST00000380956.7 IRF4 protein_coding
ENST00000380958.6 PAN3 protein_coding
ENST00000380987.2 FLT3 nonsense_mediated_decay
ENST00000381007.7 SGTB protein_coding
ENST00000381018.6 TRIM23 protein_coding
ENST00000381024.4 ATAD2B protein_coding
ENST00000381051.5 MIRLET7BHG lncRNA
ENST00000381054.3 RESF1 protein_coding
ENST00000381070.6 CWC27 protein_coding
ENST00000381103.5 KIF2A protein_coding
ENST00000381151.3 SLC5A3 protein_coding
ENST00000381177.4 CD99 nonsense_mediated_decay
ENST00000381196.7 PTPRD protein_coding
ENST00000381231.4 SMG1P1 processed_transcript
ENST00000381232.6 SIPA1L1 protein_coding
ENST00000381283.6 ITSN1 protein_coding
ENST00000381287.7 IL6ST protein_coding
ENST00000381293.5 IL6ST protein_coding
ENST00000381297.7 P2RY8 protein_coding
ENST00000381312.4 ADARB2 protein_coding
ENST00000381317.6 ASMTL protein_coding
ENST00000381323.6 CYRIA protein_coding

ENST00000381340.6 ITPR2 protein_coding
ENST00000381373.3 UHRF2 protein_coding
ENST00000381401.8 SLC25A6 protein_coding
ENST00000381410.4 SNX18 protein_coding
ENST00000381440.4 ITPRIPL2 protein_coding
ENST00000381448.7 SMG1P4 processed_transcript
ENST00000381465.2 TRIB2 protein_coding
ENST00000381482.6 STK35 protein_coding
ENST00000381496.6 DIP2C protein_coding
ENST00000381527.6 CDK8 protein_coding
ENST00000381570.6 RNF6 protein_coding
ENST00000381580.4 SIRPG protein_coding
ENST00000381596.1 SIRPB1 processed_transcript
ENST00000381621.4 SIRPD protein_coding
ENST00000381625.7 PPP2R3B protein_coding
ENST00000381630.2 SIRPB2 retained_intron
ENST00000381638.5 ZZEF1 protein_coding
ENST00000381646.5 TPTEP2 transcribed_unprocessed_pseudogene
ENST00000381652.3 JAK2 protein_coding
ENST00000381679.7 SON protein_coding
ENST00000381685.8 NOL10 protein_coding
ENST00000381732.3 RCL1 protein_coding
ENST00000381774.7 CPPED1 protein_coding
ENST00000381781.2 ARRDC5 protein_coding
ENST00000381793.5 RBM47 protein_coding
ENST00000381801.5 MTMR6 protein_coding
ENST00000381810.6 NA NA
ENST00000381815.7 GART protein_coding
ENST00000381835.8 CIITA protein_coding
ENST00000381843.5 NUP155 protein_coding
ENST00000381844.7 YWHAQ protein_coding
ENST00000381858.4 CDC37L1 protein_coding
ENST00000381867.4 SNPH protein_coding
ENST00000381903.2 KLRC3 protein_coding
ENST00000381908.6 KLRD1 protein_coding
ENST00000381930.6 KLHL5 protein_coding
ENST00000381938.3 TMEM156 protein_coding
ENST00000381984.2 RFX3 protein_coding
ENST00000381989.3 PARP4 protein_coding
ENST00000382004.6 RFX3 protein_coding
ENST00000382040.3 RSAD2 protein_coding
ENST00000382070.6 CREBBP protein_coding
ENST00000382073.3 CLEC6A protein_coding
ENST00000382079.3 C1QTNF3-AMACR nonsense_mediated_decay
ENST00000382080.4 SGCZ protein_coding
ENST00000382093.5 ADI1 protein_coding
ENST00000382159.6 GNG7 protein_coding
ENST00000382172.3 MIPEP protein_coding
ENST00000382194.4 SMARCA2 protein_coding
ENST00000382247.4 DCAF16 protein_coding
ENST00000382252.4 ZNF316 protein_coding
ENST00000382296.4 DAZ4 protein_coding
ENST00000382329.1 DOCK8 protein_coding
ENST00000382331.4 DOCK8 retained_intron

ENST00000382341.4 DOCK8 retained_intron
ENST00000382422.5 BAZ1A protein_coding
ENST00000382433.4 DAZ2 protein_coding
ENST00000382490.8 MSRA protein_coding
ENST00000382496.8 SEMA5A protein_coding
ENST00000382584.7 TTC14 protein_coding
ENST00000382592.4 LATS2 protein_coding
ENST00000382713.8 DOK6 protein_coding
ENST00000382751.3 URB1 protein_coding
ENST00000382753.4 TMEM128 protein_coding
ENST00000382812.4 CRYL1 protein_coding
ENST00000382815.7 NA NA
ENST00000382870.6 ZMYM2 retained_intron
ENST00000382871.3 ZMYM2 protein_coding
ENST00000382881.6 ZMYM2 retained_intron
ENST00000382893.2 UTY protein_coding
ENST00000382907.7 ZMYM5 protein_coding
ENST00000382927.3 ME2 protein_coding
ENST00000382938.3 APBA2 retained_intron
ENST00000383032.4 TBL1Y protein_coding
ENST00000383432.6 RALBP1 protein_coding
ENST00000383440.3 NA NA
ENST00000383620.4 HLA-H unprocessed_pseudogene
ENST00000383661.3 DTX3L protein_coding
ENST00000383736.3 MAP4 protein_coding
ENST00000383850.1 Y_RNA misc_RNA
ENST00000383858.1 RNVU1-7 snRNA
ENST00000383860.1 RNU6-46P snRNA
ENST00000383861.1 RNU1-28P snRNA
ENST00000383866.1 RNU6-47P snRNA
ENST00000383869.1 RNU1-27P snRNA
ENST00000383870.1 SNORA57 snoRNA
ENST00000383873.1 RNU6-25P snRNA
ENST00000383874.1 RNU6-431P snRNA
ENST00000383875.1 SNORD58A snoRNA
ENST00000383884.1 SNORD24 snoRNA
ENST00000383885.1 SNORA2A snoRNA
ENST00000383886.1 RNU6-1336P snRNA
ENST00000383890.1 RNY1P5 misc_RNA
ENST00000383893.1 SNORD45C snoRNA
ENST00000383894.1 SNORD116-14 snoRNA
ENST00000383895.1 SNORA69 snoRNA
ENST00000383897.1 RNU1-39P snRNA
ENST00000383898.1 RNU6-1 snRNA
ENST00000383903.1 SNORD60 snoRNA
ENST00000383906.1 SNORA80B snoRNA
ENST00000383924.1 Y_RNA misc_RNA
ENST00000383925.1 RNU1-1 snRNA
ENST00000383929.1 SNORD116-17 snoRNA
ENST00000383932.1 Y_RNA misc_RNA
ENST00000383934.1 SNORA70 snoRNA
ENST00000383948.1 RNU6-32P snRNA
ENST00000383953.1 SNORD21 snoRNA
ENST00000383961.1 SNORD116-18 snoRNA

ENST00000383966.1 SNORA56 snoRNA
ENST00000383972.1 Y_RNA misc_RNA
ENST00000383975.1 RNU1-11P snRNA
ENST00000383978.1 Y_RNA misc_RNA
ENST00000383990.1 Y_RNA misc_RNA
ENST00000383994.1 Y_RNA misc_RNA
ENST00000384000.1 SNORD116-9 snoRNA
ENST00000384001.1 Y_RNA misc_RNA
ENST00000384010.1 RNVU1-18 snRNA
ENST00000384027.1 SNORD101 snoRNA
ENST00000384028.1 SNORA30 snoRNA
ENST00000384033.1 SNORA6 snoRNA
ENST00000384036.1 RNU6-10P snRNA
ENST00000384045.1 RNU6-44P snRNA
ENST00000384048.1 SNORD37 snoRNA
ENST00000384050.1 RNU6-637P snRNA
ENST00000384063.1 Y_RNA misc_RNA
ENST00000384068.1 Y_RNA misc_RNA
ENST00000384070.1 Y_RNA misc_RNA
ENST00000384072.1 SNORA32 snoRNA
ENST00000384076.1 RNU6-968P snRNA
ENST00000384081.1 Y_RNA misc_RNA
ENST00000384084.1 SNORA10 snoRNA
ENST00000384087.1 Y_RNA misc_RNA
ENST00000384090.1 Y_RNA misc_RNA
ENST00000384093.1 RNU1-138P snRNA
ENST00000384095.1 Y_RNA misc_RNA
ENST00000384096.1 SNORA24 snoRNA
ENST00000384097.1 Y_RNA misc_RNA
ENST00000384101.1 RNVU1-30 snRNA
ENST00000384105.1 RNU6V snRNA
ENST00000384106.1 RNU6-20P snRNA
ENST00000384107.1 SNORA1 snoRNA
ENST00000384111.1 SNORA5A snoRNA
ENST00000384113.1 Y_RNA misc_RNA
ENST00000384119.1 Y_RNA misc_RNA
ENST00000384123.1 Y_RNA misc_RNA
ENST00000384138.1 Y_RNA misc_RNA
ENST00000384143.1 RNU6-398P snRNA
ENST00000384147.1 SNORD26 snoRNA
ENST00000384148.1 RNU6-761P snRNA
ENST00000384155.1 RNA5SP87 rRNA_pseudogene
ENST00000384158.1 SNORA75 snoRNA
ENST00000384161.1 RNU6-48P snRNA
ENST00000384165.1 RNU6-42P snRNA
ENST00000384170.1 SNORA9B snoRNA
ENST00000384172.1 RNU6-36P snRNA
ENST00000384173.1 RNU6-98P snRNA
ENST00000384174.1 SNORA72 snoRNA
ENST00000384176.1 SNORA24B snoRNA
ENST00000384178.1 Y_RNA misc_RNA
ENST00000384181.1 RNU1-136P snRNA
ENST00000384187.1 Y_RNA misc_RNA
ENST00000384198.1 Y_RNA misc_RNA

ENST00000384202.1 Y_RNA misc_RNA
ENST00000384205.1 RNU6-4P snRNA
ENST00000384209.1 RNU4-52P snRNA
ENST00000384210.1 SNORA70B snoRNA
ENST00000384214.1 SNORD15A snoRNA
ENST00000384215.1 SNORA9 snoRNA
ENST00000384220.1 SNORA20B snoRNA
ENST00000384221.1 SNORA36A snoRNA
ENST00000384223.1 Y_RNA misc_RNA
ENST00000384227.1 RNU6-1201P snRNA
ENST00000384229.1 SNORD49A snoRNA
ENST00000384238.1 RNU6-5P snRNA
ENST00000384245.1 RNU6-17P snRNA
ENST00000384248.1 RNU6-13P snRNA
ENST00000384251.1 Y_RNA misc_RNA
ENST00000384252.1 SNORD61 snoRNA
ENST00000384254.1 RNU6-40P snRNA
ENST00000384262.1 SNORD63 snoRNA
ENST00000384265.1 RNU6-574P snRNA
ENST00000384268.1 Y_RNA misc_RNA
ENST00000384269.1 RNU6-842P snRNA
ENST00000384273.1 RNU6-820P snRNA
ENST00000384274.1 SNORD116-2 snoRNA
ENST00000384278.1 RNU1-2 snRNA
ENST00000384280.1 RNU6-891P snRNA
ENST00000384281.1 SNORA54 snoRNA
ENST00000384282.1 Y_RNA misc_RNA
ENST00000384284.1 RNY4P13 misc_RNA
ENST00000384287.1 SNORD116-3 snoRNA
ENST00000384289.1 SNORA36C snoRNA
ENST00000384290.1 Y_RNA misc_RNA
ENST00000384297.1 Y_RNA misc_RNA
ENST00000384304.1 SNORD59A snoRNA
ENST00000384312.1 Y_RNA misc_RNA
ENST00000384314.1 RNU6-3P snRNA
ENST00000384320.2 SNORD51 snoRNA
ENST00000384322.1 Y_RNA misc_RNA
ENST00000384323.1 SNORA27 snoRNA
ENST00000384324.1 RNU6-378P miRNA
ENST00000384333.1 Y_RNA miRNA
ENST00000384335.1 SNORD116-1 miRNA
ENST00000384339.1 SNORA72 miRNA
ENST00000384340.1 RNU6-463P miRNA
ENST00000384341.1 Y_RNA miRNA
ENST00000384342.2 SNORA16A miRNA
ENST00000384344.1 RNU6-39P miRNA
ENST00000384355.1 RNU6-22P miRNA
ENST00000384356.1 SNORA72 miRNA
ENST00000384358.1 Y_RNA miRNA
ENST00000384360.1 SNORA7B miRNA
ENST00000384365.1 SNORD116-8 miRNA
ENST00000384370.1 SNORA70 miRNA
ENST00000384373.1 Y_RNA miRNA
ENST00000384376.1 RNU6-434P miRNA

ENST00000384377.1 Y_RNA miRNA
ENST00000384382.1 RNVU1-32 miRNA
ENST00000384384.1 SNORA25 miRNA
ENST00000384385.1 RNU6-16P miRNA
ENST00000384388.1 RNU6-31P miRNA
ENST00000384390.1 SNORD14D miRNA
ENST00000384395.1 Y_RNA miRNA
ENST00000384401.1 miRNA
ENST00000384404.1 SNORD116-7 miRNA
ENST00000384413.1 Y_RNA miRNA
ENST00000384416.1 SNORA18 miRNA
ENST00000384419.1 Y_RNA miRNA
ENST00000384423.1 SNORA67 miRNA
ENST00000384425.1 RNU1-46P miRNA
ENST00000384430.1 SNORD116-22 miRNA
ENST00000384432.1 Y_RNA miRNA
ENST00000384436.1 SNORA70 miRNA
ENST00000384437.1 SNORA68 miRNA
ENST00000384445.1 SNORD116-15 miRNA
ENST00000384446.1 RNU1-67P miRNA
ENST00000384452.1 SNORA14B miRNA
ENST00000384462.1 SNORD116-5 miRNA
ENST00000384471.1 RNU6-45P miRNA
ENST00000384472.1 RNU1-148P miRNA
ENST00000384476.1 RNVU1-15 miRNA
ENST00000384478.1 Y_RNA miRNA
ENST00000384488.1 SNORA80D miRNA
ENST00000384499.1 RNVU1-2 miRNA
ENST00000384504.1 SNORA37 miRNA
ENST00000384507.1 SNORD116-21 miRNA
ENST00000384511.1 Y_RNA miRNA
ENST00000384512.1 SNORD45A miRNA
ENST00000384516.1 SNORD116-29 miRNA
ENST00000384519.1 RNU6-1005P miRNA
ENST00000384527.1 RNU6-18P miRNA
ENST00000384529.1 SNORD116-20 miRNA
ENST00000384530.1 RNU6-35P miRNA
ENST00000384533.1 SNORD116-16 miRNA
ENST00000384534.1 RNU6-15P miRNA
ENST00000384541.1 Y_RNA miRNA
ENST00000384549.1 SNORD116-24 miRNA
ENST00000384550.1 SNORD20 miRNA
ENST00000384552.1 Y_RNA miRNA
ENST00000384561.1 RNU6-30P miRNA
ENST00000384567.1 SNORD7 miRNA
ENST00000384574.1 SNORA8 miRNA
ENST00000384577.1 RNU6-145P miRNA
ENST00000384581.1 SNORA61 miRNA
ENST00000384582.1 RNU6-429P miRNA
ENST00000384583.1 SNORA2B miRNA
ENST00000384584.1 SNORA44 miRNA
ENST00000384587.1 Y_RNA miRNA
ENST00000384592.1 RNU1-89P miRNA
ENST00000384599.1 Y_RNA miRNA

ENST00000384600.1 RNU6-73P miRNA
ENST00000384604.1 RNU6-12P miRNA
ENST00000384606.1 RNU6-658P miRNA
ENST00000384609.1 NA miRNA
ENST00000384610.1 RNVU1-1 miRNA
ENST00000384613.1 RNU6-225P miRNA
ENST00000384619.1 RNVU1-17 miRNA
ENST00000384626.1 Y_RNA miRNA
ENST00000384627.1 RNU6-2 miRNA
ENST00000384630.1 RNU6-14P miRNA
ENST00000384637.1 RNU6-29P miRNA
ENST00000384645.1 SNORD116-23 miRNA
ENST00000384653.1 Y_RNA miRNA
ENST00000384656.1 Y_RNA miRNA
ENST00000384657.1 Y_RNA miRNA
ENST00000384659.1 RNU1-4 miRNA
ENST00000384662.1 SNORA20 miRNA
ENST00000384665.1 Y_RNA miRNA
ENST00000384673.1 Y_RNA miRNA
ENST00000384674.1 SNORA64 miRNA
ENST00000384675.1 SNORA41 miRNA
ENST00000384676.1 miRNA
ENST00000384677.1 Y_RNA miRNA
ENST00000384693.1 SNORD30 miRNA
ENST00000384706.1 SNORD28 miRNA
ENST00000384710.1 RNU6-21P miRNA
ENST00000384711.1 SNORD116-6 miRNA
ENST00000384714.1 SNORD15B miRNA
ENST00000384718.1 RNU6-19P miRNA
ENST00000384729.1 SNORD116-19 miRNA
ENST00000384737.1 SNORA19 miRNA
ENST00000384741.1 RNU6-41P miRNA
ENST00000384743.1 RNY1P7 miRNA
ENST00000384744.1 SNORA80E miRNA
ENST00000384750.1 Y_RNA miRNA
ENST00000384753.1 Y_RNA miRNA
ENST00000384756.1 SNORD22 miRNA
ENST00000384762.1 SNORA46 miRNA
ENST00000384763.1 Y_RNA miRNA
ENST00000384765.1 SNORA7A miRNA
ENST00000384766.1 Y_RNA miRNA
ENST00000384768.1 Y_RNA miRNA
ENST00000384769.1 SNORD102 miRNA
ENST00000384770.1 RNVU1-14 miRNA
ENST00000384776.1 RNU6-9 miRNA
ENST00000384782.1 RNU1-3 miRNA
ENST00000384792.1 SNORA66 miRNA
ENST00000384793.1 RNU6-33P miRNA
ENST00000384835.2 MIR142 miRNA
ENST00000384914.1 MIR153-1 miRNA
ENST00000384994.1 MIR612 miRNA
ENST00000385054.1 MIR26A2 miRNA
ENST00000385069.1 MIR599 miRNA
ENST00000385197.1 MIR33A trna

ENST00000385227.1 MIR30C1 trna
ENST00000385271.1 MIR16-1 trna
ENST00000385277.1 MIRLET7F2 trna
ENST00000385573.1 SNORA40B trna
ENST00000386037.1 SNORD94 trna
ENST00000386062.1 SNORD73A trna
ENST00000386157.1 SNORA49 trna
ENST00000386307.1 SNORD12C trna
ENST00000386347.1 MT-TL1 trna
ENST00000386683.1 SNORD64 trna
ENST00000386745.1 SNORD83B trna
ENST00000386747.1 SNORD83A trna
ENST00000386847.1 SNORA48 trna
ENST00000386910.1 SNORD105 trna
ENST00000386967.1 SNORD41 trna
ENST00000386972.1 MIR24-2 trna
ENST00000387069.1 RNU11 trna
ENST00000387314.1 MT-TF trna
ENST00000387342.1 MT-TV trna
ENST00000387347.2 MT-RNR2 trna
ENST00000387365.1 MT-TI trna
ENST00000387372.1 MT-TQ trna
ENST00000387377.1 MT-TM trna
ENST00000387382.1 MT-TW trna
ENST00000387392.1 MT-TA trna
ENST00000387400.1 MT-TN trna
ENST00000387405.1 MT-TC trna
ENST00000387409.1 MT-TY trna
ENST00000387416.2 MT-TS1 trna
ENST00000387419.1 MT-TD trna
ENST00000387421.1 MT-TK trna
ENST00000387429.1 MT-TG trna
ENST00000387439.1 MT-TR trna
ENST00000387441.1 MT-TH trna
ENST00000387449.1 MT-TS2 trna
ENST00000387456.1 MT-TL2 trna
ENST00000387459.1 MT-TE trna
ENST00000387460.2 MT-TT trna
ENST00000387461.2 MT-TP trna
ENST00000387943.1 RNU6ATAC2P trna
ENST00000388090.1 SNORA40 trna
ENST00000388738.6 KIAA1109 trna
ENST00000388882.5 TNFAIP8 trna
ENST00000388940.7 SCFD2 trna
ENST00000388962.4 SELPLG trna
ENST00000389003.6 KIAA0100 trna
ENST00000389005.5 NCBP3 trna
ENST00000389010.6 BMP2K trna
ENST00000389045.6 TRIP12 trna
ENST00000389103.4 UBE3C trna
ENST00000389120.6 RNF20 trna
ENST00000389171.4 FLCN trna
ENST00000389195.5 LTN1 trna
ENST00000389224.6 PDPK1 trna

ENST00000389262.7 DGCR2 trna
ENST00000389341.8 POU2F2 trna
ENST00000389395.6 MFSD12 trna
ENST00000389400.4 RPS3AP5 trna
ENST00000389418.7 PTPRN2 trna
ENST00000389506.8 KMT2A trna
ENST00000389520.7 TANC2 trna
ENST00000389531.6 SDK1 trna
ENST00000389562.5 NA trna
ENST00000389568.6 ALKBH8 trna
ENST00000389622.5 CRYBG3 trna
ENST00000389629.7 RTF1 trna
ENST00000389680.2 MT-RNR1 trna
ENST00000389740.2 GPR149 trna
ENST00000389794.6 NA trna
ENST00000389797.6 LRCH1 trna
ENST00000389826.6 EIF4E3 trna
ENST00000389834.7 TMEM87A trna
ENST00000389856.8 CCDC88C trna
ENST00000389857.9 CCDC88C trna
ENST00000389900.7 RNF216 trna
ENST00000389915.3 FAM168B trna
ENST00000389993.6 PSME4 trna
ENST00000390013.3 NA trna
ENST00000390136.2 NA trna
ENST00000390237.2 IGKC trna
ENST00000390243.2 IGKV4-1 trna
ENST00000390321.2 IGLC1 trna
ENST00000390323.2 IGLC2 trna
ENST00000390325.2 IGLC3 trna
ENST00000390382.3 TRBV7-5 trna
ENST00000390539.2 IGHA2 trna
ENST00000390542.5 IGHG1 trna
ENST00000390545.2 IGHG2 trna
ENST00000390547.3 IGHA1 trna
ENST00000390551.5 IGHG3 trna
ENST00000390555.3 IGHGP trna
ENST00000390559.5 IGHM trna
ENST00000390609.3 IGHV3-23 trna
ENST00000390654.6 COL23A1 trna
ENST00000390833.1 SNORD67 trna
ENST00000390842.1 U8 trna
ENST00000390846.1 trna
ENST00000390851.1 RNU1-82P trna
ENST00000390856.1 SNORD66 trna
ENST00000390874.1 RNA5SP207 trna
ENST00000390880.2 U3 trna
ENST00000390893.2 U3 trna
ENST00000390904.1 trna
ENST00000390930.1 SNORD17 trna
ENST00000390947.2 U8 trna
ENST00000390962.1 SNORD65C trna
ENST00000390981.1 SNORD89 trna
ENST00000390994.1 SNORD72 trna

ENST00000391002.1 SNORD12 trna
ENST00000391004.1 Y_RNA trna
ENST00000391007.1 SNORD70B trna
ENST00000391017.1 Y_RNA trna
ENST00000391023.1 Y_RNA trna
ENST00000391033.1 Y_RNA trna
ENST00000391040.1 trna
ENST00000391061.1 trna
ENST00000391079.1 SNORD65 trna
ENST00000391094.1 RNA5SP323 trna
ENST00000391100.1 SNORA74B trna
ENST00000391107.1 RNY4P18 trna
ENST00000391112.1 SNORD77B trna
ENST00000391131.1 RNA5SP252 trna
ENST00000391141.1 SNORA53 trna
ENST00000391145.1 SNORD90 trna
ENST00000391146.1 Y_RNA trna
ENST00000391150.1 SNORD69 trna
ENST00000391152.1 RNA5SP286 trna
ENST00000391162.1 SNORA12 trna
ENST00000391185.2 SNORA17A trna
ENST00000391196.1 SNORD86 trna
ENST00000391232.1 SNORD70 trna
ENST00000391269.1 RNA5SP482 trna
ENST00000391286.1 SNORA26 trna
ENST00000391303.1 RNU1-56P trna
ENST00000391305.1 SNORA3B trna
ENST00000391313.1 SNORD58 trna
ENST00000391491.2 RPL15P3 trna
ENST00000391611.5 PCNX4 trna
ENST00000391614.6 JCHAIN trna
ENST00000391746.4 LILRB2 trna
ENST00000391760.1 OSCAR trna
ENST00000391768.2 MYADM trna
ENST00000391769.2 MYADM trna
ENST00000391775.6 NLRP12 trna
ENST00000391791.4 PPP2R1A trna
ENST00000391796.6 CD33 trna
ENST00000391827.2 NLRP3 trna
ENST00000391836.2 SMYD3 trna
ENST00000391959.4 PPP1R12B trna
ENST00000391974.3 NEK7 trna
ENST00000391979.5 NA trna
ENST00000392000.4 LRRFIP1 trna
ENST00000392028.7 SERTAD3 trna
ENST00000392029.5 QSOX1 trna
ENST00000392043.3 ABL2 trna
ENST00000392048.6 SP110 trna
ENST00000392062.5 RHBDD1 trna
ENST00000392078.6 NA trna
ENST00000392128.5 SMARCAL1 trna
ENST00000392132.5 XRCC5 trna
ENST00000392177.7 NA trna
ENST00000392185.6 SNX9 trna

ENST00000392217.3 FXYD5 trna
ENST00000392218.5 FXYD5 trna
ENST00000392238.2 FAM117B trna
ENST00000392254.5 PYHIN1 trna
ENST00000392301.3 trna
ENST00000392323.5 STAT1 trna
ENST00000392348.5 BCLAF1 trna
ENST00000392391.6 PIGX trna
ENST00000392403.6 ASH1L trna
ENST00000392452.2 MB21D2 trna
ENST00000392455.6 CCDC50 trna
ENST00000392456.3 CCDC50 trna
ENST00000392464.3 RNF34 trna
ENST00000392500.6 CEP85L trna
ENST00000392517.2 NA trna
ENST00000392546.5 WIPF1 trna
ENST00000392547.5 WIPF1 trna
ENST00000392558.4 RPS27 trna
ENST00000392561.6 RBM19 trna
ENST00000392584.2 HAT1 trna
ENST00000392597.4 PTPN11 trna
ENST00000392619.1 CD300E trna
ENST00000392621.4 CD300LB trna
ENST00000392625.6 CD300A trna
ENST00000392632.5 UBR3 trna
ENST00000392644.7 ARMC2 trna
ENST00000392650.6 SDK2 trna
ENST00000392687.4 CERS6 trna
ENST00000392710.7 PRKAR1A trna
ENST00000392723.4 POGZ trna
ENST00000392730.2 RPL13P12 trna
ENST00000392753.6 NA trna
ENST00000392754.6 FAM53B trna
ENST00000392755.2 RBM33 trna
ENST00000392757.7 LHPP trna
ENST00000392761.3 RBM33 trna
ENST00000392776.3 SAE1 trna
ENST00000392782.4 BAZ2B trna
ENST00000392796.6 WDSUB1 trna
ENST00000392857.8 ORC4 trna
ENST00000392859.6 ARCN1 trna
ENST00000392861.5 ZEB2 trna
ENST00000392870.2 GRK5 trna
ENST00000392873.3 KMT2A trna
ENST00000392901.7 SHTN1 trna
ENST00000392910.5 ZYX trna
ENST00000392948.2 H3-2 trna
ENST00000392952.6 ABLIM1 trna
ENST00000392962.3 ADGRE2 trna
ENST00000392963.5 NBPF1 trna
ENST00000392985.3 ZNF292 trna
ENST00000393001.1 AMMECR1L trna
ENST00000393003.6 USP32 trna
ENST00000393006.4 WDR33 trna

ENST00000393043.4 CLTC trna
ENST00000393063.4 DICER1 trna
ENST00000393065.5 TRIM37 trna
ENST00000393077.2 VTI1A trna
ENST00000393085.3 MTPN trna
ENST00000393100.3 ALKBH8 trna
ENST00000393132.2 BPGM trna
ENST00000393158.5 PDGFD trna
ENST00000393160.6 RALGDS trna
ENST00000393164.5 ATP2B1 protein_coding
ENST00000393241.7 MRE11 protein_coding
ENST00000393249.5 OSBPL8 protein_coding
ENST00000393250.7 OSBPL8 protein_coding
ENST00000393256.6 BCL2L11 protein_coding
ENST00000393262.3 NA NA
ENST00000393266.2 processed_pseudogene
ENST00000393293.4 ABL1 protein_coding
ENST00000393299.5 FAM135A retained_intron
ENST00000393301.4 CTSC protein_coding
ENST00000393348.5 NCK2 protein_coding
ENST00000393349.2 NCK2 protein_coding
ENST00000393350.1 MAF protein_coding
ENST00000393354.5 B4GALNT2 protein_coding
ENST00000393387.4 PHF3 protein_coding
ENST00000393416.5 MDM2 protein_coding
ENST00000393471.2 LIPT1 protein_coding
ENST00000393481.5 TES protein_coding
ENST00000393485.4 TFEC protein_coding
ENST00000393486.4 MDFIC protein_coding
ENST00000393488.2 PHYKPL nonsense_mediated_decay
ENST00000393555.3 DYRK2 protein_coding
ENST00000393593.6 IRF2 protein_coding
ENST00000393596.2 P2RY2 protein_coding
ENST00000393603.5 COG5 protein_coding
ENST00000393605.6 ARAP1 protein_coding
ENST00000393609.6 ARAP1 protein_coding
ENST00000393630.6 MON2 protein_coding
ENST00000393640.7 ST3GAL2 protein_coding
ENST00000393704.3 MFAP3L protein_coding
ENST00000393711.6 RNF121 nonsense_mediated_decay
ENST00000393726.6 PALLD protein_coding
ENST00000393742.5 NFAT5 nonsense_mediated_decay
ENST00000393743.6 DDX60 protein_coding
ENST00000393792.2 UBTD2 protein_coding
ENST00000393802.5 FBXW11 protein_coding
ENST00000393808.6 ST3GAL5 protein_coding
ENST00000393896.5 ACLY protein_coding
ENST00000393906.2 TBC1D10A protein_coding
ENST00000393956.6 FBXW7 protein_coding
ENST00000393979.3 RBM14 protein_coding
ENST00000393991.4 TSC22D4 protein_coding
ENST00000394000.5 PILRA protein_coding
ENST00000394030.5 CBLB protein_coding
ENST00000394085.6 SENP7 protein_coding

ENST00000394089.5 RARA protein_coding
ENST00000394114.2 CASC3 retained_intron
ENST00000394123.6 G3BP1 protein_coding
ENST00000394142.6 NA NA
ENST00000394168.1 BICRAL protein_coding
ENST00000394183.2 lncRNA
ENST00000394199.5 NEK6 protein_coding
ENST00000394219.6 NA NA
ENST00000394224.3 SIPA1 protein_coding
ENST00000394264.4 PABIR1 protein_coding
ENST00000394290.3 FTH1P3 processed_pseudogene
ENST00000394294.6 FBXL20 protein_coding
ENST00000394323.2 ERV3-1 protein_coding
ENST00000394455.5 ZFYVE26 retained_intron
ENST00000394457.6 AHSA2P processed_transcript
ENST00000394477.4 STAG2 protein_coding
ENST00000394478.1 STAG2 protein_coding
ENST00000394479.3 REL protein_coding
ENST00000394510.5 AKAP13 protein_coding
ENST00000394534.5 AKAP9 protein_coding
ENST00000394564.4 AKAP9 retained_intron
ENST00000394583.6 ZNF326 nonsense_mediated_decay
ENST00000394597.5 RFFL protein_coding
ENST00000394600.6 MTIF2 protein_coding
ENST00000394609.5 RTN4 protein_coding
ENST00000394652.5 IL16 protein_coding
ENST00000394660.5 IL16 protein_coding
ENST00000394665.4 RPL34 protein_coding
ENST00000394670.7 ZNF207 protein_coding
ENST00000394686.3 SGMS2 protein_coding
ENST00000394689.2 BRD7 protein_coding
ENST00000394714.5 ELAPOR2 nonsense_mediated_decay
ENST00000394715.1 ZBTB25 protein_coding
ENST00000394722.6 ANKHD1 protein_coding
ENST00000394725.2 SIAH1 protein_coding
ENST00000394752.6 SFMBT1 protein_coding
ENST00000394764.2 NA NA
ENST00000394768.5 SYNE2 retained_intron
ENST00000394779.6 SHOC1 protein_coding
ENST00000394790.2 FUT11 protein_coding
ENST00000394829.5 PPP3CB protein_coding
ENST00000394847.3 ANXA7 protein_coding
ENST00000394848.4 SSH2 retained_intron
ENST00000394861.2 SOCS5 protein_coding
ENST00000394878.2 RPLP0P6 processed_pseudogene
ENST00000394903.5 DNAJB12 protein_coding
ENST00000394949.7 SIN3A protein_coding
ENST00000394957.6 VSIR protein_coding
ENST00000394979.2 ZNF646 protein_coding
ENST00000395002.5 FAM13A protein_coding
ENST00000395003.4 JADE2 protein_coding
ENST00000395038.5 SOS1 protein_coding
ENST00000395042.1 RGS19 protein_coding
ENST00000395059.5 SRCAP protein_coding

ENST00000395060.4 NA NA
ENST00000395076.7 PPM1A protein_coding
ENST00000395125.1 DAAM1 protein_coding
ENST00000395183.5 ARHGAP24 protein_coding
ENST00000395251.4 lncRNA
ENST00000395270.4 POM121 protein_coding
ENST00000395290.5 CELF1 protein_coding
ENST00000395323.6 LBH protein_coding
ENST00000395339.6 PSMB8 protein_coding
ENST00000395340.4 DIDO1 protein_coding
ENST00000395343.4 DIDO1 protein_coding
ENST00000395344.6 MADD protein_coding
ENST00000395374.1 PHF20L1 retained_intron
ENST00000395389.2 SPN protein_coding
ENST00000395391.1 ZNF107 nonsense_mediated_decay
ENST00000395443.5 QRICH1 protein_coding
ENST00000395454.5 SCAF11 protein_coding
ENST00000395521.6 GCH1 processed_transcript
ENST00000395635.4 GRAP protein_coding
ENST00000395659.1 ATP5F1E protein_coding
ENST00000395686.6 ERO1A protein_coding
ENST00000395699.3 PURB protein_coding
ENST00000395713.5 TRPS1 protein_coding
ENST00000395715.6 TRPS1 protein_coding
ENST00000395719.6 G3BP2 protein_coding
ENST00000395720.2 ANKDD1A protein_coding
ENST00000395744.6 PPP6R2 protein_coding
ENST00000395754.4 IL21R protein_coding
ENST00000395769.5 MARCHF8 protein_coding
ENST00000395770.3 LNPEP protein_coding
ENST00000395782.4 PEMT protein_coding
ENST00000395784.1 LNPEP processed_transcript
ENST00000395788.3 SNHG32 lncRNA
ENST00000395789.4 SNHG32 lncRNA
ENST00000395807.2 MPRIP processed_transcript
ENST00000395827.4 TRABD protein_coding
ENST00000395848.4 NCOR1 protein_coding
ENST00000395856.3 NCOA1 protein_coding
ENST00000395857.6 NA NA
ENST00000395859.2 VCPKMT protein_coding
ENST00000395862.6 ATP6V1C1 protein_coding
ENST00000395884.3 KLF10 protein_coding
ENST00000395896.7 NA NA
ENST00000395898.3 VPS13C protein_coding
ENST00000395934.2 ELP4 protein_coding
ENST00000395957.5 YWHAZ protein_coding
ENST00000395969.5 VPS41 protein_coding
ENST00000396009.6 NFATC2 protein_coding
ENST00000396020.6 RPS29 protein_coding
ENST00000396027.7 XRCC4 protein_coding
ENST00000396040.5 ELMO1 protein_coding
ENST00000396049.4 MOB1A protein_coding
ENST00000396051.2 MOB1B protein_coding
ENST00000396053.7 RBM4 protein_coding

ENST00000396078.6 TCAIM protein_coding
ENST00000396136.5 ADAM10 nonsense_mediated_decay
ENST00000396137.4 JMY protein_coding
ENST00000396150.4 ZNF586 protein_coding
ENST00000396152.5 NA NA
ENST00000396175.4 TRAK1 protein_coding
ENST00000396200.3 PDP1 protein_coding
ENST00000396296.6 YME1L1 protein_coding
ENST00000396298.5 CREB5 protein_coding
ENST00000396307.5 RAB27A protein_coding
ENST00000396364.6 NA NA
ENST00000396373.7 ETV6 protein_coding
ENST00000396384.1 TUBB protein_coding
ENST00000396444.6 USP8 protein_coding
ENST00000396478.3 IPP protein_coding
ENST00000396507.6 CLEC12A protein_coding
ENST00000396512.3 ACO2 protein_coding
ENST00000396578.6 COL4A3 protein_coding
ENST00000396610.5 CMC1 retained_intron
ENST00000396625.3 COL4A4 protein_coding
ENST00000396667.6 TMEM106B protein_coding
ENST00000396696.1 RAB2A retained_intron
ENST00000396816.1 IL6ST protein_coding
ENST00000396821.6 DDX17 protein_coding
ENST00000396827.3 ZSCAN12 protein_coding
ENST00000396830.2 IFFO1 retained_intron
ENST00000396946.7 CARD11 protein_coding
ENST00000396979.1 RHOG protein_coding
ENST00000396984.1 H2BC4 protein_coding
ENST00000397006.2 PRKAA1 retained_intron
ENST00000397007.7 NUP98 protein_coding
ENST00000397023.4 PPARG nonsense_mediated_decay
ENST00000397033.5 ITGA4 protein_coding
ENST00000397040.4 RALGAPB protein_coding
ENST00000397041.6 ATP2A3 protein_coding
ENST00000397061.3 NUDT19 protein_coding
ENST00000397077.4 ATP2B2 protein_coding
ENST00000397128.5 PRKAA1 protein_coding
ENST00000397131.1 BLCAP protein_coding
ENST00000397149.3 SMU1 protein_coding
ENST00000397150.4 MANBAL protein_coding
ENST00000397151.1 MANBAL protein_coding
ENST00000397174.5 SCMH1 protein_coding
ENST00000397195.8 PAFAH1B1 protein_coding
ENST00000397261.6 ARPC4 protein_coding
ENST00000397265.6 NINJ2 protein_coding
ENST00000397282.2 ACTR3B protein_coding
ENST00000397305.3 RBFOX2 protein_coding
ENST00000397311.4 JARID2 protein_coding
ENST00000397390.3 processed_pseudogene
ENST00000397497.7 PTK2B retained_intron
ENST00000397519.5 PSIP1 protein_coding
ENST00000397527.4 CEP250 protein_coding
ENST00000397532.6 SLC7A7 protein_coding

ENST00000397553.5 UQCC1 nonsense_mediated_decay
ENST00000397560.5 KDM7A protein_coding
ENST00000397573.4 BMF protein_coding
ENST00000397591.2 THBS1 protein_coding
ENST00000397628.4 PRMT2 protein_coding
ENST00000397643.3 CUX2 protein_coding
ENST00000397713.4 AMZ2P1 processed_transcript
ENST00000397717.5 NQO2 protein_coding
ENST00000397753.4 ZBTB44 protein_coding
ENST00000397766.3 PGBD4 protein_coding
ENST00000397775.6 PPP3CC protein_coding
ENST00000397786.5 MED13 protein_coding
ENST00000397797.1 HBA1 protein_coding
ENST00000397807.5 NA NA
ENST00000397820.4 C19orf38 protein_coding
ENST00000397828.3 AGO3 protein_coding
ENST00000397852.4 ITGB2 protein_coding
ENST00000397885.2 PUM3 protein_coding
ENST00000397886.3 PTTG1IP protein_coding
ENST00000397887.6 PTTG1IP protein_coding
ENST00000397898.6 SUMO3 protein_coding
ENST00000397906.5 TTC28 protein_coding
ENST00000397912.3 PRKRIP1 protein_coding
ENST00000397992.5 ZFHX3 protein_coding
ENST00000397997.5 FRS2 protein_coding
ENST00000398004.3 SLC35E3 protein_coding
ENST00000398040.7 CMIP protein_coding
ENST00000398058.4 AGPAT3 protein_coding
ENST00000398071.4 NOP14 protein_coding
ENST00000398073.5 CTDSP2 protein_coding
ENST00000398093.6 NA NA
ENST00000398110.5 TPST2 protein_coding
ENST00000398117.1 BCL2 protein_coding
ENST00000398146.3 RGPD2 protein_coding
ENST00000398155.4 TXLNG protein_coding
ENST00000398197.5 TBC1D32 processed_transcript
ENST00000398212.5 TBC1D32 protein_coding
ENST00000398219.2 MRPL37 protein_coding
ENST00000398249.7 GSK3A protein_coding
ENST00000398258.6 CD47 protein_coding
ENST00000398259.2 RPS3AP26 processed_pseudogene
ENST00000398263.5 TGOLN2 protein_coding
ENST00000398316.6 FNDC3A protein_coding
ENST00000398319.5 CABIN1 protein_coding
ENST00000398326.2 FILIP1L protein_coding
ENST00000398337.7 INF2 protein_coding
ENST00000398367.1 UBASH3A protein_coding
ENST00000398379.2 POM121C protein_coding
ENST00000398392.2 CGGBP1 protein_coding
ENST00000398395.6 OFD1 nonsense_mediated_decay
ENST00000398529.6 RAB1A protein_coding
ENST00000398571.5 USP34 protein_coding
ENST00000398580.3 EYS protein_coding
ENST00000398602.2 NAA20 protein_coding

ENST00000398632.3 MX2 retained_intron
ENST00000398637.8 SLC38A1 protein_coding
ENST00000398665.6 DOT1L protein_coding
ENST00000398671.2 RC3H2 protein_coding
ENST00000398675.6 GXYLT1 protein_coding
ENST00000398676.3 CCAR1 retained_intron
ENST00000398684.5 CRCP protein_coding
ENST00000398731.3 AEBP2 protein_coding
ENST00000398763.7 MICU1 protein_coding
ENST00000398782.2 RRBP1 protein_coding
ENST00000398792.3 CDH23 retained_intron
ENST00000398810.5 GRB10 protein_coding
ENST00000398844.5 SEC24A protein_coding
ENST00000398868.6 ATL3 protein_coding
ENST00000398870.6 PIK3C3 protein_coding
ENST00000398873.4 YDJC protein_coding
ENST00000398881.3 TOMM6 protein_coding
ENST00000398944.6 EIF3CL protein_coding
ENST00000398947.1 NAA15 protein_coding
ENST00000398956.2 DYRK1A protein_coding
ENST00000398985.1 KSR1 retained_intron
ENST00000398986.5 NA NA
ENST00000399010.4 TTC3 protein_coding
ENST00000399082.6 HLA-DQB1 protein_coding
ENST00000399100.5 SEC24B protein_coding
ENST00000399120.4 HLCS protein_coding
ENST00000399138.4 ALKBH5 protein_coding
ENST00000399151.3 DOP1B protein_coding
ENST00000399196.1 LINC00243 lncRNA
ENST00000399220.2 CX3CR1 protein_coding
ENST00000399229.5 MYO5A protein_coding
ENST00000399237.5 RUNX1 protein_coding
ENST00000399240.4 RUNX1 protein_coding
ENST00000399262.5 JMJD1C protein_coding
ENST00000399284.1 KCNE1 protein_coding
ENST00000399287.6 HPS5 retained_intron
ENST00000399310.3 CRPPA protein_coding
ENST00000399339.5 WASHC2A protein_coding
ENST00000399402.6 GLB1 protein_coding
ENST00000399410.6 ABCC1 protein_coding
ENST00000399434.2 TNRC18 protein_coding
ENST00000399469.2 HIVEP1 nonsense_mediated_decay
ENST00000399503.3 MAP3K1 protein_coding
ENST00000399541.5 ALOX12-AS1 lncRNA
ENST00000399598.5 UBXN2B protein_coding
ENST00000399600.7 SCIMP protein_coding
ENST00000399613.1 PAN3 protein_coding
ENST00000399627.3 SBF1 retained_intron
ENST00000399702.4 processed_transcript
ENST00000399764.3 YWHAZP6 processed_pseudogene
ENST00000399777.1 BCL2L13 nonsense_mediated_decay
ENST00000399788.5 KDM5A protein_coding
ENST00000399799.2 ROCK1 protein_coding
ENST00000399850.6 CELF2 protein_coding

ENST00000399855.2 HIBCH protein_coding
ENST00000399868.2 LINC02649 lncRNA
ENST00000399921.4 BACH1 protein_coding
ENST00000399971.7 transcribed_unprocessed_pseudogene
ENST00000399973.1 USP16 protein_coding
ENST00000399974.4 MTRNR2L4 protein_coding
ENST00000400000.5 VAPA protein_coding
ENST00000400020.6 ANKRD12 protein_coding
ENST00000400075.3 GABPA protein_coding
ENST00000400135.4 CHODL protein_coding
ENST00000400145.5 DLGAP1 protein_coding
ENST00000400181.7 KDM1A protein_coding
ENST00000400198.6 ARGLU1 protein_coding
ENST00000400239.5 ZBTB40 protein_coding
ENST00000400393.3 DLEU7 protein_coding
ENST00000400454.4 DSCAM protein_coding
ENST00000400457.3 PCDH11Y protein_coding
ENST00000400470.3 RAB6A processed_transcript
ENST00000400522.7 EFCAB8 protein_coding
ENST00000400540.4 LINC00544 lncRNA
ENST00000400564.4 SAMSN1 protein_coding
ENST00000400569.6 NA NA
ENST00000400602.2 NA NA
ENST00000400706.3 WASH8P unprocessed_pseudogene
ENST00000400758.5 PRKAR1B protein_coding
ENST00000400788.7 TMEM242 protein_coding
ENST00000400794.6 WDR47 protein_coding
ENST00000400797.3 KAZN protein_coding
ENST00000400864.3 lncRNA
ENST00000400888.2 CCR2 protein_coding
ENST00000400904.6 CTNNBIP1 protein_coding
ENST00000400908.5 RERE protein_coding
ENST00000401004.2 H3P4 unprocessed_pseudogene
ENST00000401030.3 SELENOF protein_coding
ENST00000401042.6 MIER1 protein_coding
ENST00000401087.3 NBPF17P unprocessed_pseudogene
ENST00000401376.3 MIR941-4 miRNA
ENST00000401394.4 CTCF protein_coding
ENST00000401395.1 KIAA1671 protein_coding
ENST00000401406.3 UQCR10 protein_coding
ENST00000401529.3 RAC2 protein_coding
ENST00000401578.2 processed_pseudogene
ENST00000401701.1 MYH9 protein_coding
ENST00000401723.4 TOGARAM2 protein_coding
ENST00000401743.5 CD14 protein_coding
ENST00000401753.4 ROCK2 protein_coding
ENST00000401787.6 KDM4C protein_coding
ENST00000401830.3 FTH1P10 processed_transcript
ENST00000401882.2 NA NA
ENST00000401884.4 SNED1 protein_coding
ENST00000401970.2 LHFPL3 protein_coding
ENST00000402003.6 YPEL5 protein_coding
ENST00000402010.5 MARK2 protein_coding
ENST00000402038.6 PARG protein_coding

ENST00000402061.6 CCDC134 protein_coding
ENST00000402089.4 TMSB4XP8 processed_pseudogene
ENST00000402092.5 SEPTIN2 protein_coding
ENST00000402203.4 TNRC6B protein_coding
ENST00000402219.5 SOS1 protein_coding
ENST00000402255.4 APOBEC3A protein_coding
ENST00000402280.4 NLRC4 protein_coding
ENST00000402311.4 RABGAP1 protein_coding
ENST00000402380.3 ATXN10 protein_coding
ENST00000402437.2 MVB12B protein_coding
ENST00000402449.7 RAB37 protein_coding
ENST00000402468.3 C1GALT1 protein_coding
ENST00000402522.1 TBC1D1 protein_coding
ENST00000402604.5 AGAP1 protein_coding
ENST00000402630.4 MRTFA protein_coding
ENST00000402685.5 PDZRN4 protein_coding
ENST00000402711.5 EML4 protein_coding
ENST00000402739.7 CTNNA2 protein_coding
ENST00000402742.1 unprocessed_pseudogene
ENST00000402849.4 SNRPD3 protein_coding
ENST00000402863.3 PHACTR2 retained_intron
ENST00000402876.3 PARVB processed_transcript
ENST00000402914.4 MACROD2 protein_coding
ENST00000402924.4 BLVRA protein_coding
ENST00000403018.2 AUTS2 protein_coding
ENST00000403056.2 BTBD9 protein_coding
ENST00000403078.6 NA NA
ENST00000403094.3 FASTKD2 protein_coding
ENST00000403150.4 MAFK nonsense_mediated_decay
ENST00000403167.4 TTYH3 protein_coding
ENST00000403258.1 ACTBP8 processed_pseudogene
ENST00000403263.4 INO80D protein_coding
ENST00000403363.4 NAGA protein_coding
ENST00000403463.1 OSM protein_coding
ENST00000403490.2 IMP3 protein_coding
ENST00000403503.1 YPEL1 protein_coding
ENST00000403538.1 TPT1P4 processed_pseudogene
ENST00000403579.1 SIRT1 protein_coding
ENST00000403609.1 TANK protein_coding
ENST00000403663.5 TRIOBP protein_coding
ENST00000403683.1 H3C15 protein_coding
ENST00000403696.4 PRR5 protein_coding
ENST00000403703.4 UNKL nonsense_mediated_decay
ENST00000403732.2 RPS6KA1 protein_coding
ENST00000403744.6 PACSIN2 protein_coding
ENST00000403750.4 PPARGC1B protein_coding
ENST00000403821.5 R3HDM2 protein_coding
ENST00000403853.6 CAMKMT protein_coding
ENST00000403856.1 THADA protein_coding
ENST00000403997.2 SDF4 protein_coding
ENST00000404039.4 DPP6 protein_coding
ENST00000404121.5 NCOR2 protein_coding
ENST00000404136.2 CWC22 protein_coding
ENST00000404146.1 MTCYBP36 processed_pseudogene

ENST00000404251.1 BAZ1B protein_coding
ENST00000404291.3 STAG3L1 processed_transcript
ENST00000404360.4 ZNF12 protein_coding
ENST00000404395.3 STAT3 protein_coding
ENST00000404459.1 LIPN protein_coding
ENST00000404460.4 PLEKHB2 protein_coding
ENST00000404582.2 MORF4L1P1 processed_pseudogene
ENST00000404590.1 ACVR2A protein_coding
ENST00000404627.2 DOCK7 protein_coding
ENST00000404739.6 NT5C2 protein_coding
ENST00000404742.4 ESR1 protein_coding
ENST00000404767.6 INTS1 protein_coding
ENST00000404796.2 nonsense_mediated_decay
ENST00000404816.5 LTBP1 protein_coding
ENST00000404843.4 ASXL2 protein_coding
ENST00000404876.1 TCF20 protein_coding
ENST00000404989.1 PARVB protein_coding
ENST00000405123.6 ERLEC1 protein_coding
ENST00000405151.2 RPS4XP9 processed_pseudogene
ENST00000405240.4 RTN4 protein_coding
ENST00000405276.2 YWHAZP10 processed_pseudogene
ENST00000405289.4 EHBP1 protein_coding
ENST00000405333.4 ODC1 protein_coding
ENST00000405334.4 EIF2AK2 protein_coding
ENST00000405359.2 processed_pseudogene
ENST00000405420.2 SUMF1 protein_coding
ENST00000405482.4 EHBP1 protein_coding
ENST00000405506.2 XRCC6 protein_coding
ENST00000405510.4 SUN2 protein_coding
ENST00000405573.2 MADD protein_coding
ENST00000405615.2 CADM2 protein_coding
ENST00000405737.2 ELF1 protein_coding
ENST00000405755.5 PDE4D protein_coding
ENST00000405767.1 B3GNT2 protein_coding
ENST00000405772.4 CBLB protein_coding
ENST00000405785.4 MIOS protein_coding
ENST00000405805.4 HMGB1 protein_coding
ENST00000405808.4 FBXO11 protein_coding
ENST00000405810.2 RPL23P8 processed_pseudogene
ENST00000405854.4 MIR3667HG lncRNA
ENST00000405953.4 BCL2L11 protein_coding
ENST00000405995.4 NA NA
ENST00000406022.5 RPL10 protein_coding
ENST00000406041.4 ACYP2 protein_coding
ENST00000406048.2 NR4A2 protein_coding
ENST00000406175.3 EML4 retained_intron
ENST00000406200.2 BCOR miRNA
ENST00000406220.1 FEZ2 miRNA
ENST00000406226.1 STON1 miRNA
ENST00000406230.4 CSF2RB miRNA
ENST00000406272.5 NR2C2 miRNA
ENST00000406274.1 RPS4XP7 miRNA
ENST00000406297.6 AAK1 miRNA
ENST00000406326.4 DPP6 miRNA

ENST00000406334.3 miRNA
ENST00000406337.4 KAT6A miRNA
ENST00000406354.1 SETD7 miRNA
ENST00000406360.1 FOXO3 miRNA
ENST00000406434.4 CYRIA miRNA
ENST00000406438.4 SMCR8 miRNA
ENST00000406453.3 SLC29A4 miRNA
ENST00000406486.7 KIAA1671 miRNA
ENST00000406520.6 COMT miRNA
ENST00000406533.6 KCNMA1 miRNA
ENST00000406606.6 VAV2 miRNA
ENST00000406629.1 MTMR3 miRNA
ENST00000406659.3 DNMT3A miRNA
ENST00000406664.4 TBC1D1 miRNA
ENST00000406696.4 HS6ST2 miRNA
ENST00000406697.4 C1S miRNA
ENST00000406733.1 TBC1D22A miRNA
ENST00000406757.2 GPC3 miRNA
ENST00000406768.1 PSEN1 miRNA
ENST00000406775.5 AUTS2 miRNA
ENST00000406785.5 SLC8A1 miRNA
ENST00000406829.2 UMAD1 miRNA
ENST00000406875.6 HIPK2 miRNA
ENST00000407071.5 KIF26B miRNA
ENST00000407075.3 GRAP2 miRNA
ENST00000407082.3 SMARCB1 miRNA
ENST00000407131.4 CAMKMT miRNA
ENST00000407142.4 PPM1F miRNA
ENST00000407236.4 PPARA miRNA
ENST00000407275.2 ANKMY1 miRNA
ENST00000407343.3 PTPN12 miRNA
ENST00000407418.6 CBX6 miRNA
ENST00000407449.4 PIKFYVE miRNA
ENST00000407528.5 CADM2 miRNA
ENST00000407598.2 HIC2 miRNA
ENST00000407637.4 HERC3 miRNA
ENST00000407643.4 TTYH3 miRNA
ENST00000407673.4 CACNA1I miRNA
ENST00000407674.4 NA miRNA
ENST00000407704.1 ITSN2 miRNA
ENST00000407712.1 CBLB miRNA
ENST00000407787.4 PUS10 miRNA
ENST00000407797.4 IRF1-AS1 miRNA
ENST00000407893.3 MEMO1 miRNA
ENST00000407925.4 LTBP1 miRNA
ENST00000407940.1 miRNA
ENST00000407982.5 STRBP miRNA
ENST00000408028.5 SLC8A1 miRNA
ENST00000408031.1 GRAMD4 miRNA
ENST00000408038.5 BTRC miRNA
ENST00000408042.4 KIF16B miRNA
ENST00000408061.1 SNORD88C miRNA
ENST00000408139.1 SNORD111 miRNA
ENST00000408175.1 SNORA11B miRNA

ENST00000408189.1 SNORD110 miRNA
ENST00000408314.1 SNORD88A miRNA
ENST00000408376.1 SNORA79 miRNA
ENST00000408472.1 RN7SKP175 miRNA
ENST00000408493.2 SNORA81 miRNA
ENST00000408534.2 U3 miRNA
ENST00000408564.2 SNORA2C miRNA
ENST00000408573.1 SNORD100 miRNA
ENST00000408587.1 SNORD111B miRNA
ENST00000408612.1 SNORD99 miRNA
ENST00000408632.2 NA miRNA
ENST00000408684.1 miRNA
ENST00000408712.1 miRNA
ENST00000408716.1 SNORA77 miRNA
ENST00000408749.1 RNU6ATAC miRNA
ENST00000408789.1 SNORA11 miRNA
ENST00000408792.1 SNORA3C miRNA
ENST00000408813.1 SNORD93 miRNA
ENST00000408876.1 SNORD23 miRNA
ENST00000408881.1 MIR1256 miRNA
ENST00000408938.2 FAM200A miRNA
ENST00000408958.4 BTBD9 miRNA
ENST00000408965.3 CEBPD miRNA
ENST00000408968.3 IFITM1 miRNA
ENST00000408984.6 WWOX miRNA
ENST00000408998.1 WDR33 miRNA
ENST00000408999.3 RGPD4 miRNA
ENST00000409000.4 RPL31 miRNA
ENST00000409010.1 COQ10B miRNA
ENST00000409024.3 POLR1A miRNA
ENST00000409032.4 UXS1 miRNA
ENST00000409040.1 EML4 miRNA
ENST00000409053.1 RHBDD1 miRNA
ENST00000409069.4 OSBPL3 miRNA
ENST00000409080.4 ITGA6 miRNA
ENST00000409096.4 CUL3 miRNA
ENST00000409108.5 NR4A2 miRNA
ENST00000409111.2 NA miRNA
ENST00000409112.4 SP100 miRNA
ENST00000409126.4 SPATA13 miRNA
ENST00000409133.1 TRABD2A miRNA
ENST00000409136.4 ITPRID2 miRNA
ENST00000409171.4 AGFG1 miRNA
ENST00000409174.1 HK2 miRNA
ENST00000409176.5 MAP3K20 miRNA
ENST00000409179.2 NA miRNA
ENST00000409185.4 FAM168B miRNA
ENST00000409213.4 NCKAP5 miRNA
ENST00000409232.6 TGOLN2 miRNA
ENST00000409262.6 TET3 miRNA
ENST00000409263.4 FCHSD2 miRNA
ENST00000409271.4 EDAR miRNA
ENST00000409279.1 PLEKHB2 miRNA
ENST00000409283.5 ACVR1 miRNA

ENST00000409284.1 CCDC141 miRNA
ENST00000409307.4 NA miRNA
ENST00000409330.4 NBN miRNA
ENST00000409347.4 UNC50 miRNA
ENST00000409354.5 WDPCP miRNA
ENST00000409367.4 SNX10 protein_coding
ENST00000409369.1 IL18RAP protein_coding
ENST00000409375.1 AP1S3 trna
ENST00000409377.1 PPP3R1 trna
ENST00000409398.4 COQ10B trna
ENST00000409415.6 WIPF1 trna
ENST00000409442.2 MXD1 trna
ENST00000409473.2 PPM1B trna
ENST00000409486.5 PPM1B trna
ENST00000409487.6 ZEB2 trna
ENST00000409497.4 SCRN1 trna
ENST00000409500.6 GSTK1 trna
ENST00000409512.4 KYNU trna
ENST00000409540.6 INPP4A trna
ENST00000409544.4 ZNF638 trna
ENST00000409552.4 CLEC16A trna
ENST00000409563.4 CALM2 trna
ENST00000409572.4 NR4A2 trna
ENST00000409579.4 AFF3 trna
ENST00000409592.6 DOCK10 trna
ENST00000409593.4 PMS1 trna
ENST00000409600.4 BZW1 trna
ENST00000409602.1 POTEJ trna
ENST00000409614.1 SERF2 trna
ENST00000409627.5 SUMO1 trna
ENST00000409652.4 APOL6 trna
ENST00000409654.4 EPC2 trna
ENST00000409656.4 PSD4 trna
ENST00000409658.6 WDR33 trna
ENST00000409660.4 LNPK trna
ENST00000409674.4 GPD2 trna
ENST00000409677.4 TRIP12 trna
ENST00000409681.1 POLR1A trna
ENST00000409694.5 SYNE1 trna
ENST00000409738.4 RBM14 trna
ENST00000409757.7 TBC1D14 trna
ENST00000409760.1 VAMP8 trna
ENST00000409769.5 CLK1 trna
ENST00000409781.1 CD8A trna
ENST00000409785.7 KCMF1 trna
ENST00000409806.6 ZC3H12D trna
ENST00000409810.5 CHMP3 trna
ENST00000409815.5 SP110 trna
ENST00000409817.1 CXCR4 trna
ENST00000409833.4 ATF2 trna
ENST00000409838.1 SNX10 trna
ENST00000409845.1 trna
ENST00000409848.3 TACR1 trna
ENST00000409853.4 FCHSD2 trna

ENST00000409869.4 ARHGAP15 trna
ENST00000409884.4 LRRTM4 trna
ENST00000409886.3 RGPD3 trna
ENST00000409887.3 LRRC75A trna
ENST00000409888.1 AGPS trna
ENST00000409922.4 CHN2 trna
ENST00000409923.4 FAM126A trna
ENST00000409926.2 trna
ENST00000409947.4 MAP3K2 trna
ENST00000409964.5 CHN2 trna
ENST00000409995.4 STAT4 trna
ENST00000410000.2 MXD1 trna
ENST00000410015.5 KYNU trna
ENST00000410026.5 NABP1 trna
ENST00000410031.4 TBC1D14 trna
ENST00000410041.1 DYSF trna
ENST00000410069.1 CCDC126 trna
ENST00000410074.4 DOCK5 trna
ENST00000410077.2 JAKMIP1 trna
ENST00000410080.4 PRPF40A trna
ENST00000410084.6 CAB39 trna
ENST00000410093.4 LIMS1 trna
ENST00000410108.4 BET1L trna
ENST00000410112.5 EXOC6B trna
ENST00000410136.1 RN7SKP154 trna
ENST00000410144.1 RNU2-3P trna
ENST00000410216.1 RNY4P30 trna
ENST00000410290.1 RNU2-17P trna
ENST00000410292.1 Y_RNA trna
ENST00000410344.1 RNU2-33P trna
ENST00000410361.1 RNU2-36P trna
ENST00000410396.1 RNU2-2P trna
ENST00000410411.1 RN7SKP139 trna
ENST00000410413.1 SNORD19C trna
ENST00000410420.1 RN7SKP221 trna
ENST00000410423.1 RNU2-29P trna
ENST00000410433.1 SNORD12B trna
ENST00000410438.1 SNORA36B trna
ENST00000410451.1 RNA5SP203 trna
ENST00000410457.1 RNU2-28P trna
ENST00000410482.1 RNU2-59P trna
ENST00000410508.1 RNU2-26P trna
ENST00000410519.1 RN7SKP143 trna
ENST00000410533.1 RNU2-5P trna
ENST00000410535.1 Y_RNA trna
ENST00000410545.1 RNU2-23P trna
ENST00000410557.1 SNORA79B trna
ENST00000410569.1 RNU4-25P trna
ENST00000410577.1 Y_RNA trna
ENST00000410597.1 Y_RNA trna
ENST00000410635.1 RN7SKP236 trna
ENST00000410647.1 Y_RNA trna
ENST00000410669.1 Y_RNA trna
ENST00000410680.1 RNU2-52P trna

ENST00000410694.1 RNU2-48P trna
ENST00000410695.1 RNU2-37P trna
ENST00000410698.2 RN7SKP131 trna
ENST00000410712.1 RNU2-16P trna
ENST00000410717.1 Y_RNA trna
ENST00000410718.1 RNU2-70P trna
ENST00000410766.1 Y_RNA trna
ENST00000410769.1 Y_RNA trna
ENST00000410792.1 RNU2-63P trna
ENST00000410794.1 RNU2-7P trna
ENST00000410801.1 RNY4P29 trna
ENST00000410804.1 RNU4-6P trna
ENST00000410809.1 RNA5SP229 trna
ENST00000410818.1 RNU4-46P trna
ENST00000410856.1 RNU2-38P trna
ENST00000410874.1 RNA5SP225 trna
ENST00000410878.1 RNU2-68P trna
ENST00000410894.1 RN7SKP286 trna
ENST00000410920.1 Y_RNA trna
ENST00000410940.1 RNU4-78P trna
ENST00000410948.1 RN7SKP261 trna
ENST00000410949.1 Y_RNA trna
ENST00000410980.2 Y_RNA trna
ENST00000411013.2 NA trna
ENST00000411042.1 RN7SKP228 trna
ENST00000411044.1 RN7SKP94 trna
ENST00000411053.1 RNU2-14P trna
ENST00000411065.1 Y_RNA trna
ENST00000411069.1 RNU2-61P trna
ENST00000411204.1 RN7SKP207 trna
ENST00000411222.1 Y_RNA trna
ENST00000411288.1 Y_RNA trna
ENST00000411292.1 SNORD71 trna
ENST00000411315.1 RNU2-64P trna
ENST00000411317.1 NA trna
ENST00000411366.1 RNY3P8 trna
ENST00000411367.1 RN7SKP108 trna
ENST00000411404.1 RNU2-6P trna
ENST00000411416.5 PLAC8 trna
ENST00000411423.1 SF3A1 trna
ENST00000411466.5 SRCAP trna
ENST00000411512.2 trna
ENST00000411529.5 HNRNPA3 trna
ENST00000411542.1 trna
ENST00000411544.4 SERINC3 trna
ENST00000411546.3 SMG1P5 trna
ENST00000411553.2 HCG11 trna
ENST00000411579.1 MHENCR trna
ENST00000411612.4 GSE1 trna
ENST00000411670.4 CCDC18-AS1 trna
ENST00000411677.1 GNAQ trna
ENST00000411680.1 PLXNB2 trna
ENST00000411683.1 EHD1 trna
ENST00000411688.1 CYP27A1 trna

ENST00000411698.5 MDM1 trna
ENST00000411702.6 RNF213 trna
ENST00000411769.1 CRKL trna
ENST00000411780.1 BLCAP trna
ENST00000411790.1 TTLL11 trna
ENST00000411812.1 RNF216 trna
ENST00000411827.1 CDC42 trna
ENST00000411888.4 EIF4G3 trna
ENST00000411900.4 PKP4 trna
ENST00000411912.4 USP34 trna
ENST00000411915.1 TXN2 trna
ENST00000411932.1 NRIP1 trna
ENST00000411938.1 LSM8 trna
ENST00000411973.3 TRAPPC12 trna
ENST00000411975.4 ABCA13 trna
ENST00000411985.4 BRCC3 trna
ENST00000411987.4 MKRN2 trna
ENST00000411993.1 HDAC9 trna
ENST00000411998.1 IL10RB-DT trna
ENST00000412001.1 JMY trna
ENST00000412011.4 ITSN2 trna
ENST00000412025.4 ACVR1 trna
ENST00000412051.4 C7orf50 trna
ENST00000412091.1 LINC00174 trna
ENST00000412095.1 PSMB8-AS1 trna
ENST00000412097.4 RPL15 trna
ENST00000412116.4 ANKHD1 trna
ENST00000412128.1 PTMA trna
ENST00000412141.1 LINC00963 trna
ENST00000412217.1 PAPOLG trna
ENST00000412242.1 KDM5C-IT1 trna
ENST00000412247.1 GLS trna
ENST00000412261.4 LDAH trna
ENST00000412268.1 CD300E trna
ENST00000412306.1 TENT5A trna
ENST00000412393.4 LINC00342 trna
ENST00000412409.3 trna
ENST00000412411.1 LARP4B trna
ENST00000412414.5 KLF7 trna
ENST00000412416.4 SNX10 trna
ENST00000412431.2 NA trna
ENST00000412441.4 ADAM22 trna
ENST00000412450.1 SETD2 trna
ENST00000412480.5 PDE4B trna
ENST00000412482.1 MFSD6 trna
ENST00000412504.5 IL1RAP trna
ENST00000412507.1 TRIM56 trna
ENST00000412517.3 SLC43A2 trna
ENST00000412545.1 RPS20P14 trna
ENST00000412585.5 HLA-B trna
ENST00000412681.2 NRGN trna
ENST00000412692.4 GSE1 trna
ENST00000412743.1 PRR14L trna
ENST00000412784.2 NA trna

ENST00000412793.4 PTK2B trna
ENST00000412805.4 HADHB trna
ENST00000412806.1 SH3BP5 trna
ENST00000412810.5 NA trna
ENST00000412832.1 JOSD1 trna
ENST00000412835.1 trna
ENST00000412865.1 DUSP18 trna
ENST00000412869.4 PCYT1A trna
ENST00000412887.4 PDCD6IP trna
ENST00000412899.4 ITGA6 trna
ENST00000412910.1 C3orf20 trna
ENST00000412911.4 STX16 trna
ENST00000412918.1 ITPKB-IT1 trna
ENST00000412946.5 TPT1-AS1 trna
ENST00000412952.1 BCOR trna
ENST00000412962.1 CROCCP2 trna
ENST00000412972.1 BCL2L1-AS1 trna
ENST00000412979.1 IWS1 nonsense_mediated_decay
ENST00000413009.5 ACOX3 protein_coding
ENST00000413027.1 RPL3P2 processed_pseudogene
ENST00000413034.2 FAM185A protein_coding
ENST00000413035.4 IPO9-AS1 lncRNA
ENST00000413054.4 SLC25A26 protein_coding
ENST00000413079.1 PFKP protein_coding
ENST00000413081.1 TNRC18 protein_coding
ENST00000413082.1 DPM1 protein_coding
ENST00000413123.4 ATF2 processed_transcript
ENST00000413141.4 VPS41 protein_coding
ENST00000413152.3 NA NA
ENST00000413170.1 PCCA protein_coding
ENST00000413194.1 NR2C2 protein_coding
ENST00000413202.1 CYTOR lncRNA
ENST00000413269.3 TMEM252-DT lncRNA
ENST00000413284.4 SP100 protein_coding
ENST00000413297.4 RALY protein_coding
ENST00000413307.5 CAMSAP2 protein_coding
ENST00000413316.1 ACSL3 protein_coding
ENST00000413340.4 KPNA5 protein_coding
ENST00000413366.6 PRKCA protein_coding
ENST00000413409.5 PTPRC protein_coding
ENST00000413433.4 NOD1 protein_coding
ENST00000413447.1 CYCS protein_coding
ENST00000413479.4 SLC8A1-AS1 lncRNA
ENST00000413510.3 DLEU1 lncRNA
ENST00000413522.1 SNORD56 snoRNA
ENST00000413531.4 WARS2-AS1 lncRNA
ENST00000413543.5 JADE1 protein_coding
ENST00000413546.1 TP53BP1 protein_coding
ENST00000413551.1 TNS3 protein_coding
ENST00000413553.1 SIK3 protein_coding
ENST00000413559.1 STX16-NPEPL1 retained_intron
ENST00000413569.4 FOXN2 protein_coding
ENST00000413582.5 RFFL protein_coding
ENST00000413584.1 UBXN7 protein_coding

ENST00000413588.1 ANKIB1 nonsense_mediated_decay
ENST00000413605.5 CDK19 protein_coding
ENST00000413628.4 GNG12-AS1 lncRNA
ENST00000413697.4 REV1 nonsense_mediated_decay
ENST00000413712.5 TMEM245 protein_coding
ENST00000413722.4 NUTM2A-AS1 lncRNA
ENST00000413728.5 ARHGEF3 protein_coding
ENST00000413751.1 ACVR1 protein_coding
ENST00000413759.1 TPT1P9 processed_pseudogene
ENST00000413761.5 ZBED5 protein_coding
ENST00000413769.1 RBMXL1 protein_coding
ENST00000413828.3 CYP1B1-AS1 lncRNA
ENST00000413831.1 REV3L nonsense_mediated_decay
ENST00000413848.1 lncRNA
ENST00000413887.1 lncRNA
ENST00000413888.1 RPL4P5 processed_pseudogene
ENST00000413917.4 EIF3B protein_coding
ENST00000413971.4 TOP2B nonsense_mediated_decay
ENST00000413995.1 MRPL32 nonsense_mediated_decay
ENST00000414006.1 ENTPD1-AS1 lncRNA
ENST00000414008.2 HCG27 lncRNA
ENST00000414023.5 RRAS2 protein_coding
ENST00000414029.1 SLC35F6 nonsense_mediated_decay
ENST00000414030.4 CYTOR lncRNA
ENST00000414041.1 TBXAS1 nonsense_mediated_decay
ENST00000414070.1 processed_pseudogene
ENST00000414075.4 GAS5 lncRNA
ENST00000414080.1 BLCAP protein_coding
ENST00000414084.1 ZMAT3 protein_coding
ENST00000414108.4 WAC nonsense_mediated_decay
ENST00000414113.4 VOPP1 protein_coding
ENST00000414122.1 FBP1 protein_coding
ENST00000414157.1 ITGB1-DT lncRNA
ENST00000414174.1 SF3B1 nonsense_mediated_decay
ENST00000414218.1 LMBR1 nonsense_mediated_decay
ENST00000414250.1 FKBP15 protein_coding
ENST00000414273.1 MTCO1P12 unprocessed_pseudogene
ENST00000414296.2 DPY19L2P3 transcribed_unprocessed_pseudogene
ENST00000414303.5 AMY2A protein_coding
ENST00000414308.1 IATPR lncRNA
ENST00000414316.4 EIF3L protein_coding
ENST00000414318.2 TBC1D5 processed_transcript
ENST00000414320.1 INO80D protein_coding
ENST00000414369.2 ASB3 processed_transcript
ENST00000414380.1 SH2D3C protein_coding
ENST00000414419.5 RFFL protein_coding
ENST00000414489.1 MYADM protein_coding
ENST00000414498.1 STEAP4 protein_coding
ENST00000414513.5 CD99P1 retained_intron
ENST00000414521.5 MGAT4A protein_coding
ENST00000414539.1 lncRNA
ENST00000414558.2 ATG4C protein_coding
ENST00000414563.5 ZDHHC14 protein_coding
ENST00000414568.4 PRKAR1B protein_coding

ENST00000414637.1 AOAH nonsense_mediated_decay
ENST00000414641.4 TRIM22 protein_coding
ENST00000414644.4 RMDN2 protein_coding
ENST00000414670.2 ITGB1 protein_coding
ENST00000414678.5 ARID1B protein_coding
ENST00000414681.1 CREB1 protein_coding
ENST00000414717.4 ATG7 protein_coding
ENST00000414723.1 GCA protein_coding
ENST00000414739.3 CASTOR3 processed_transcript
ENST00000414750.1 LINC01934 lncRNA
ENST00000414770.4 IFNGR1 protein_coding
ENST00000414781.1 RAPGEF1 protein_coding
ENST00000414802.4 CNOT4 protein_coding
ENST00000414804.4 HERC2P3 processed_transcript
ENST00000414861.5 RIF1 protein_coding
ENST00000414862.4 TRA2B protein_coding
ENST00000414897.4 TRIM22 nonsense_mediated_decay
ENST00000414911.1 lncRNA
ENST00000414912.2 BTN3A1 protein_coding
ENST00000414977.4 GALNT3 protein_coding
ENST00000414984.4 LARS2 protein_coding
ENST00000414996.1 MTMR14 nonsense_mediated_decay
ENST00000415009.4 FBXL18 nonsense_mediated_decay
ENST00000415035.1 STAT1 nonsense_mediated_decay
ENST00000415045.1 NLGN1 protein_coding
ENST00000415139.4 MAP3K8 protein_coding
ENST00000415187.1 TMSB4XP6 processed_pseudogene
ENST00000415188.1 USP4 protein_coding
ENST00000415224.4 PARVG protein_coding
ENST00000415248.1 ZNF749 protein_coding
ENST00000415251.5 PHTF2 protein_coding
ENST00000415264.4 HS1BP3 protein_coding
ENST00000415277.2 transcribed_unprocessed_pseudogene
ENST00000415278.1 EEF1A1P11 processed_pseudogene
ENST00000415325.4 DLD nonsense_mediated_decay
ENST00000415357.1 LINC01090 lncRNA
ENST00000415384.1 AFF3 protein_coding
ENST00000415387.1 lncRNA
ENST00000415434.4 LINC01237 lncRNA
ENST00000415452.4 RUBCN protein_coding
ENST00000415454.1 GLB1 protein_coding
ENST00000415471.2 unprocessed_pseudogene
ENST00000415482.5 PTPN12 protein_coding
ENST00000415488.1 ESR1 protein_coding
ENST00000415513.1 KMT2E processed_transcript
ENST00000415527.1 SRSF7 nonsense_mediated_decay
ENST00000415537.1 HLA-C protein_coding
ENST00000415553.1 KANSL1L protein_coding
ENST00000415555.4 DDR2 protein_coding
ENST00000415565.1 CAPN7 protein_coding
ENST00000415570.1 EIF2AK3 processed_transcript
ENST00000415574.5 SMPD4BP transcribed_processed_pseudogene
ENST00000415589.1 lncRNA
ENST00000415617.4 INPP5D protein_coding

ENST00000415659.2 PPIC retained_intron
ENST00000415684.4 TSEN2 protein_coding
ENST00000415690.5 NR3C1 protein_coding
ENST00000415724.2 ZDHHC20 protein_coding
ENST00000415732.1 DHX32 protein_coding
ENST00000415750.4 CCDC146 protein_coding
ENST00000415780.1 ZRANB2-AS2 lncRNA
ENST00000415845.1 STAT5B protein_coding
ENST00000415850.5 ACVR1B protein_coding
ENST00000415852.1 AZI2 protein_coding
ENST00000415861.2 CD164 retained_intron
ENST00000415868.4 MBTD1 protein_coding
ENST00000415896.1 POT1-AS1 lncRNA
ENST00000415906.1 LPP protein_coding
ENST00000415912.5 ECE1 protein_coding
ENST00000415929.4 TNS3 protein_coding
ENST00000415943.5 BTBD11 protein_coding
ENST00000415947.1 PARD3B nonsense_mediated_decay
ENST00000415952.1 OSBPL3 protein_coding
ENST00000415967.2 NIPSNAP2 nonsense_mediated_decay
ENST00000415980.1 FIG4 protein_coding
ENST00000415997.1 APP protein_coding
ENST00000416006.6 LONP2 nonsense_mediated_decay
ENST00000416045.4 NAA35 processed_transcript
ENST00000416052.1 DHDDS protein_coding
ENST00000416061.1 lncRNA
ENST00000416067.4 TEKT4P2 processed_transcript
ENST00000416076.1 lncRNA
ENST00000416082.1 EEF1A1P29 processed_pseudogene
ENST00000416093.1 RAF1 nonsense_mediated_decay
ENST00000416108.4 RBM39 nonsense_mediated_decay
ENST00000416121.4 AGBL4 protein_coding
ENST00000416135.4 RAD50 protein_coding
ENST00000416140.4 TFEB protein_coding
ENST00000416160.1 ITSN2 protein_coding
ENST00000416166.1 IFFO2 protein_coding
ENST00000416172.1 ZNF609 protein_coding
ENST00000416199.4 MED14 protein_coding
ENST00000416215.5 PTPN6 retained_intron
ENST00000416222.1 ATL2 nonsense_mediated_decay
ENST00000416263.3 TOB1-AS1 lncRNA
ENST00000416301.4 ENTPD1-AS1 lncRNA
ENST00000416305.1 ARHGAP21 protein_coding
ENST00000416320.1 processed_pseudogene
ENST00000416385.1 NA NA
ENST00000416400.1 VPS54 nonsense_mediated_decay
ENST00000416411.1 MIR3667HG lncRNA
ENST00000416425.4 LRRFIP2 protein_coding
ENST00000416438.5 LINC00475 processed_transcript
ENST00000416447.1 MX2 protein_coding
ENST00000416449.4 CDK5RAP2 protein_coding
ENST00000416482.1 AUTS2 processed_transcript
ENST00000416494.5 FAM234B nonsense_mediated_decay
ENST00000416495.5 SESN3 protein_coding

ENST00000416500.4 LCP1 protein_coding
ENST00000416510.1 lncRNA
ENST00000416569.2 XKR6 protein_coding
ENST00000416585.1 SNX30 protein_coding
ENST00000416587.4 RNF144A protein_coding
ENST00000416683.4 NBEAL2 protein_coding
ENST00000416718.2 MTCO3P12 unprocessed_pseudogene
ENST00000416732.4 HIBCH protein_coding
ENST00000416735.4 SPECC1L processed_transcript
ENST00000416739.4 PARP11 nonsense_mediated_decay
ENST00000416754.1 RUNX1 protein_coding
ENST00000416801.5 TAX1BP1 nonsense_mediated_decay
ENST00000416804.1 HLA-DPB1 protein_coding
ENST00000416824.1 LINC00957 lncRNA
ENST00000416836.4 PLCB4 protein_coding
ENST00000416888.4 GPD2 nonsense_mediated_decay
ENST00000416904.4 ZNF740 protein_coding
ENST00000416918.4 TRAPPC12 protein_coding
ENST00000416931.1 MTND1P23 unprocessed_pseudogene
ENST00000416957.4 FNDC3B protein_coding
ENST00000416992.5 MKLN1 protein_coding
ENST00000416999.1 LINC-PINT lncRNA
ENST00000417007.1 IFNAR2 nonsense_mediated_decay
ENST00000417017.4 ATG16L1 protein_coding
ENST00000417023.4 DDX39B protein_coding
ENST00000417046.1 CEP350 protein_coding
ENST00000417056.4 NIBAN1 protein_coding
ENST00000417088.2 RPS28 retained_intron
ENST00000417101.1 ACTB protein_coding
ENST00000417137.4 SMARCB1 protein_coding
ENST00000417167.2 MED4 protein_coding
ENST00000417175.1 CCNT2 nonsense_mediated_decay
ENST00000417194.4 processed_transcript
ENST00000417209.2 RGS21 protein_coding
ENST00000417216.3 RBMS1P1 processed_pseudogene
ENST00000417271.1 SLC8A1 protein_coding
ENST00000417273.1 lncRNA
ENST00000417332.4 SUN2 protein_coding
ENST00000417344.2 CCNT1 nonsense_mediated_decay
ENST00000417361.1 FANCL protein_coding
ENST00000417387.1 CASD1 nonsense_mediated_decay
ENST00000417389.1 TM9SF4 nonsense_mediated_decay
ENST00000417392.4 ST6GAL1 protein_coding
ENST00000417396.2 ADAMTS6 processed_transcript
ENST00000417403.4 ABCA13 nonsense_mediated_decay
ENST00000417405.1 RUBCNL protein_coding
ENST00000417448.1 VCP protein_coding
ENST00000417458.4 MROH8 protein_coding
ENST00000417461.4 NBAS protein_coding
ENST00000417477.2 ARID4A protein_coding
ENST00000417497.4 TTC28-AS1 lncRNA
ENST00000417508.1 VPS16 protein_coding
ENST00000417523.4 TRRAP protein_coding
ENST00000417537.1 NAMPT protein_coding

ENST00000417549.1 EEF1A1P3 processed_pseudogene
ENST00000417551.4 DLD nonsense_mediated_decay
ENST00000417555.2 PARGP1 transcribed_unprocessed_pseudogene
ENST00000417564.2 DIP2A protein_coding
ENST00000417615.1 RPL3P4 processed_pseudogene
ENST00000417657.1 processed_pseudogene
ENST00000417661.1 INPP5D protein_coding
ENST00000417662.1 IFRD1 protein_coding
ENST00000417682.5 SFI1 protein_coding
ENST00000417717.5 SATB1 protein_coding
ENST00000417723.1 TAMM41 nonsense_mediated_decay
ENST00000417724.1 HLA-DPA1 protein_coding
ENST00000417748.1 CFLAR protein_coding
ENST00000417751.4 LINC00276 lncRNA
ENST00000417762.4 SHPRH protein_coding
ENST00000417765.1 lncRNA
ENST00000417768.1 TCAIM nonsense_mediated_decay
ENST00000417811.2 DPY19L1P1 unprocessed_pseudogene
ENST00000417816.2 NEBL protein_coding
ENST00000417833.2 TGFBR3 protein_coding
ENST00000417836.1 TLE4 protein_coding
ENST00000417843.2 lncRNA
ENST00000417860.4 NUTM2A-AS1 lncRNA
ENST00000417878.1 RPL3P7 processed_pseudogene
ENST00000417895.1 NEK7 processed_transcript
ENST00000417905.1 RBM5 protein_coding
ENST00000417936.4 SH3BP5 protein_coding
ENST00000417938.2 PPIG nonsense_mediated_decay
ENST00000417942.4 XIST lncRNA
ENST00000417954.4 SLC19A1 protein_coding
ENST00000417956.5 CELF2 protein_coding
ENST00000417958.4 MFSD6 protein_coding
ENST00000417975.1 LINC01725 lncRNA
ENST00000418005.1 SLC39A10 protein_coding
ENST00000418025.1 lncRNA
ENST00000418032.3 GATAD2A nonsense_mediated_decay
ENST00000418038.4 STK24 protein_coding
ENST00000418039.2 TBC1D2B retained_intron
ENST00000418061.1 KMT2C protein_coding
ENST00000418066.2 ANXA4 processed_transcript
ENST00000418101.1 MAP4K4 protein_coding
ENST00000418114.4 RAPH1 protein_coding
ENST00000418115.4 RHOA protein_coding
ENST00000418123.1 TRIP12 protein_coding
ENST00000418133.4 PACSIN2 protein_coding
ENST00000418162.1 DESI2 protein_coding
ENST00000418165.4 LINC01376 lncRNA
ENST00000418192.2 LINC01145 processed_transcript
ENST00000418194.5 SP3 protein_coding
ENST00000418218.4 UICLM lncRNA
ENST00000418236.4 ARHGAP26 protein_coding
ENST00000418239.1 NRCAM protein_coding
ENST00000418246.1 PSMF1 processed_transcript
ENST00000418259.4 ATG2A protein_coding

ENST00000418292.1 lncRNA
ENST00000418331.5 PTPRJ protein_coding
ENST00000418336.5 PKNOX1 retained_intron
ENST00000418338.1 ZNF136 protein_coding
ENST00000418351.1 ACTBP7 processed_pseudogene
ENST00000418375.1 TMEM248 protein_coding
ENST00000418381.4 NA NA
ENST00000418450.4 PARL protein_coding
ENST00000418500.1 COL10A1 protein_coding
ENST00000418505.2 ANKMY1 processed_transcript
ENST00000418510.1 YES1P1 processed_pseudogene
ENST00000418526.5 CNTN5 protein_coding
ENST00000418541.5 MTCH1 protein_coding
ENST00000418546.1 lncRNA
ENST00000418547.1 CD99L2 protein_coding
ENST00000418561.2 TTC17 protein_coding
ENST00000418592.1 SMG1P1 processed_transcript
ENST00000418596.6 FAM126B protein_coding
ENST00000418611.4 SACM1L protein_coding
ENST00000418620.1 SP3 processed_transcript
ENST00000418627.1 TBC1D25 protein_coding
ENST00000418629.5 TMEM131 nonsense_mediated_decay
ENST00000418646.4 GPCPD1 protein_coding
ENST00000418673.1 KMT2C protein_coding
ENST00000418674.1 PTPRC protein_coding
ENST00000418678.4 ATP11A protein_coding
ENST00000418681.5 NUBPL processed_transcript
ENST00000418721.4 KAT6A retained_intron
ENST00000418783.1 processed_pseudogene
ENST00000418785.4 TMEM168 protein_coding
ENST00000418791.4 KANSL1L protein_coding
ENST00000418794.1 NCAPH2 nonsense_mediated_decay
ENST00000418841.4 WDR81 protein_coding
ENST00000418849.1 CMC1 processed_transcript
ENST00000418850.1 lncRNA
ENST00000418888.4 NA NA
ENST00000418919.5 GNS protein_coding
ENST00000418920.4 CYTIP protein_coding
ENST00000418924.5 RIN3 processed_transcript
ENST00000418939.1 PPP1R2 nonsense_mediated_decay
ENST00000418945.1 lncRNA
ENST00000418975.1 LSP1 protein_coding
ENST00000418984.6 EEA1 nonsense_mediated_decay
ENST00000418987.2 ACTG1P19 processed_pseudogene
ENST00000418988.2 PSMA1 protein_coding
ENST00000418994.1 CAPN7 nonsense_mediated_decay
ENST00000419002.5 COLEC11 processed_transcript
ENST00000419020.1 DDX39B protein_coding
ENST00000419025.1 EEF1A1P8 processed_pseudogene
ENST00000419026.4 SENP5 protein_coding
ENST00000419035.1 EPCAM-DT lncRNA
ENST00000419036.4 SLC4A7 protein_coding
ENST00000419040.5 CASP8AP2 protein_coding
ENST00000419065.5 PSMG4 protein_coding

ENST00000419070.5 IMMT protein_coding
ENST00000419073.1 LST1 processed_transcript
ENST00000419169.4 SMG7 protein_coding
ENST00000419177.3 GOLGA4 processed_transcript
ENST00000419187.5 SRGAP2 protein_coding
ENST00000419211.1 lncRNA
ENST00000419215.2 ZNF655 processed_transcript
ENST00000419216.1 PRKAR2A protein_coding
ENST00000419276.1 RPL31 nonsense_mediated_decay
ENST00000419286.2 ATM processed_transcript
ENST00000419325.4 KIAA1109 protein_coding
ENST00000419333.4 PCYT1A protein_coding
ENST00000419338.4 DDX39B protein_coding
ENST00000419349.1 GPX1 protein_coding
ENST00000419358.4 TEX264 protein_coding
ENST00000419390.2 ANKRD36B nonsense_mediated_decay
ENST00000419402.1 NCF2 protein_coding
ENST00000419408.4 NDUFA10 protein_coding
ENST00000419425.1 lncRNA
ENST00000419435.4 OPA1 protein_coding
ENST00000419442.1 RAPGEF1 protein_coding
ENST00000419445.4 PGGT1B protein_coding
ENST00000419448.1 ATP11A processed_transcript
ENST00000419455.1 ARHGAP15 nonsense_mediated_decay
ENST00000419472.1 VPS13A protein_coding
ENST00000419475.1 PHETA2 protein_coding
ENST00000419499.1 LINC00402 lncRNA
ENST00000419517.2 CEP85L protein_coding
ENST00000419531.2 LINC01135 lncRNA
ENST00000419533.1 UBR4 retained_intron
ENST00000419535.1 ELMO1-AS1 lncRNA
ENST00000419541.3 VGLL4 protein_coding
ENST00000419548.4 SIGLEC1 protein_coding
ENST00000419556.2 THEM7P transcribed_unitary_pseudogene
ENST00000419562.5 DERL1 protein_coding
ENST00000419579.1 RPS12P23 processed_pseudogene
ENST00000419580.5 ABI3 protein_coding
ENST00000419608.2 ZNF317 retained_intron
ENST00000419612.1 NOL4L protein_coding
ENST00000419616.4 RAD23B protein_coding
ENST00000419629.1 SDCBPP3 processed_pseudogene
ENST00000419636.1 NPRL3 protein_coding
ENST00000419660.1 NCOA7 protein_coding
ENST00000419661.4 OGDH protein_coding
ENST00000419699.1 GATD3A protein_coding
ENST00000419706.5 BTBD9 protein_coding
ENST00000419735.6 ATXN7L1 protein_coding
ENST00000419744.1 ATRAID protein_coding
ENST00000419748.4 NA NA
ENST00000419791.3 NT5DC1 protein_coding
ENST00000419810.5 HPCAL1 nonsense_mediated_decay
ENST00000419825.2 LDAH protein_coding
ENST00000419827.1 HSPA8P8 processed_pseudogene
ENST00000419842.1 PLCL2 protein_coding

ENST00000419843.1 RASGRP2 protein_coding
ENST00000419854.1 ZNF587 retained_intron
ENST00000419890.3 TVP23C protein_coding
ENST00000419902.1 NIFK-AS1 lncRNA
ENST00000419932.1 NA NA
ENST00000419940.4 TGFA protein_coding
ENST00000419948.4 HP1BP3 protein_coding
ENST00000419958.4 RPA2 protein_coding
ENST00000419985.4 PROC nonsense_mediated_decay
ENST00000420000.5 lncRNA
ENST00000420027.3 NA NA
ENST00000420042.1 RARA processed_transcript
ENST00000420068.1 IFNAR2 processed_transcript
ENST00000420095.1 LMCD1-AS1 lncRNA
ENST00000420104.4 ARPC2 protein_coding
ENST00000420110.1 HLA-L transcribed_unprocessed_pseudogene
ENST00000420115.5 CDV3 protein_coding
ENST00000420121.4 SSBP3 retained_intron
ENST00000420124.2 KMT2B protein_coding
ENST00000420141.2 NUP210 retained_intron
ENST00000420164.4 PIGF nonsense_mediated_decay
ENST00000420195.1 SH3BP5-AS1 lncRNA
ENST00000420223.4 ZCWPW2 protein_coding
ENST00000420292.1 LYN processed_transcript
ENST00000420329.5 CRTC3 protein_coding
ENST00000420397.1 MARCHF7 protein_coding
ENST00000420424.1 NUTM2A-AS1 lncRNA
ENST00000420475.1 KLF4 protein_coding
ENST00000420482.4 PTPN4 protein_coding
ENST00000420525.1 MLLT10 protein_coding
ENST00000420535.1 LOXL3 protein_coding
ENST00000420541.5 TNFSF10 protein_coding
ENST00000420584.3 TAF11 protein_coding
ENST00000420622.5 KCNH5 protein_coding
ENST00000420658.4 AFAP1 protein_coding
ENST00000420665.4 LRRFIP1 protein_coding
ENST00000420670.4 SEMA4D protein_coding
ENST00000420679.1 SPOPL nonsense_mediated_decay
ENST00000420683.1 STK17B protein_coding
ENST00000420699.5 ANKRD36 protein_coding
ENST00000420716.2 NA NA
ENST00000420717.4 PTPN18 processed_transcript
ENST00000420722.2 SBF2 protein_coding
ENST00000420771.2 PHACTR2 retained_intron
ENST00000420807.1 LINC01031 lncRNA
ENST00000420822.1 CCNYL1 protein_coding
ENST00000420835.4 JAZF1 processed_transcript
ENST00000420843.5 ARRB1 protein_coding
ENST00000420847.2 KDM4C protein_coding
ENST00000420848.3 WASHC2C protein_coding
ENST00000420849.4 MED15 nonsense_mediated_decay
ENST00000420850.1 ENTPD3-AS1 lncRNA
ENST00000420886.5 SBNO1 protein_coding
ENST00000420893.5 FAM13B protein_coding

ENST00000420922.5 FTCDNL1 miRNA
ENST00000420927.4 ULK4 miRNA
ENST00000420959.5 CORO1C miRNA
ENST00000420969.1 AHCTF1P1 miRNA
ENST00000420981.2 SERPINB9P1 miRNA
ENST00000420999.1 EFHC2 miRNA
ENST00000421035.2 UTRN miRNA
ENST00000421037.1 MARCHF7 miRNA
ENST00000421043.4 ZNF277 miRNA
ENST00000421059.1 POR miRNA
ENST00000421069.4 VPS8 miRNA
ENST00000421101.1 LRRN3 miRNA
ENST00000421109.5 NR2F2 miRNA
ENST00000421113.4 MAD1L1 miRNA
ENST00000421126.4 AP1B1 miRNA
ENST00000421138.5 RECQL miRNA
ENST00000421147.4 LINC02474 miRNA
ENST00000421162.1 BARD1 miRNA
ENST00000421188.1 SETD5 miRNA
ENST00000421195.1 EEF1A1P14 miRNA
ENST00000421200.1 LENG8 miRNA
ENST00000421203.5 TBC1D7 miRNA
ENST00000421246.1 CDC42P6 miRNA
ENST00000421249.2 VIRMA miRNA
ENST00000421257.1 MIR646HG miRNA
ENST00000421278.2 UBAP2 miRNA
ENST00000421281.1 NT5C2 miRNA
ENST00000421296.1 IFRD1 miRNA
ENST00000421304.4 BUD23 miRNA
ENST00000421323.1 miRNA
ENST00000421325.3 NA miRNA
ENST00000421339.3 TBC1D1 miRNA
ENST00000421350.1 MICA miRNA
ENST00000421365.2 IFIH1 miRNA
ENST00000421406.1 ZNF731P miRNA
ENST00000421433.4 GIGYF2 miRNA
ENST00000421459.2 VIM miRNA
ENST00000421460.1 FGF13 miRNA
ENST00000421480.2 miRNA
ENST00000421483.5 RAP2C-AS1 miRNA
ENST00000421486.1 PPP1R21 miRNA
ENST00000421514.1 GOLGA1 miRNA
ENST00000421515.5 NKIRAS1 miRNA
ENST00000421521.4 ARHGAP26 miRNA
ENST00000421523.4 HDAC8 miRNA
ENST00000421524.5 SGCZ miRNA
ENST00000421535.4 MAN1A2 miRNA
ENST00000421539.1 CSF2RB miRNA
ENST00000421541.1 SON miRNA
ENST00000421573.4 SPATS2L miRNA
ENST00000421641.1 FUBP1 miRNA
ENST00000421682.1 RBM6 miRNA
ENST00000421686.5 miRNA
ENST00000421707.1 MBNL3 miRNA

ENST00000421708.1 MIDEAS miRNA
ENST00000421709.1 NR4A2 miRNA
ENST00000421710.4 SNAPC3 miRNA
ENST00000421723.1 NCEH1 miRNA
ENST00000421730.1 FAM126A miRNA
ENST00000421745.5 BIRC6 miRNA
ENST00000421751.1 PFKP miRNA
ENST00000421758.4 DLEU2 miRNA
ENST00000421768.1 LARGE1 miRNA
ENST00000421802.1 IFNGR2 miRNA
ENST00000421806.1 LCOR miRNA
ENST00000421812.2 ZNF697 miRNA
ENST00000421816.5 GCDH miRNA
ENST00000421827.2 UBXN11 miRNA
ENST00000421851.1 RC3H1-IT1 miRNA
ENST00000421865.2 LAMA2 miRNA
ENST00000421882.4 MAP4K4 miRNA
ENST00000421890.4 ZNF815P miRNA
ENST00000421915.4 AGBL5 miRNA
ENST00000421946.2 ANKRD36 miRNA
ENST00000422050.1 BBIP1 miRNA
ENST00000422078.1 SLC35A3 miRNA
ENST00000422087.4 PNPLA8 miRNA
ENST00000422091.1 ARHGAP4 miRNA
ENST00000422095.1 ANKIB1 miRNA
ENST00000422098.4 XIAP miRNA
ENST00000422138.1 MROH8 miRNA
ENST00000422149.1 MAP3K20 miRNA
ENST00000422239.4 FKBP14-AS1 miRNA
ENST00000422278.1 FOXJ3 miRNA
ENST00000422297.4 NEK6 miRNA
ENST00000422304.1 POLR2J4 miRNA
ENST00000422321.4 PIP4K2A miRNA
ENST00000422329.1 MAN1A2 miRNA
ENST00000422336.4 PACSIN2 miRNA
ENST00000422375.1 FCHSD2 miRNA
ENST00000422435.2 KIF21B miRNA
ENST00000422452.2 TENM1 miRNA
ENST00000422459.1 PELATON miRNA
ENST00000422467.1 NA miRNA
ENST00000422486.1 RPL4P4 miRNA
ENST00000422495.4 PIKFYVE miRNA
ENST00000422521.2 ACYP2 miRNA
ENST00000422544.2 PDGFC miRNA
ENST00000422579.1 MADD miRNA
ENST00000422591.4 HACL1 miRNA
ENST00000422633.1 EIF4A1P2 miRNA
ENST00000422679.1 MIR1302-9HG miRNA
ENST00000422684.1 DOCK10 miRNA
ENST00000422737.4 ARID2 miRNA
ENST00000422754.1 IVNS1ABP miRNA
ENST00000422769.5 PPP2R5E miRNA
ENST00000422781.4 RHOA miRNA
ENST00000422786.1 EIF2AK1 miRNA

ENST00000422803.2 NA miRNA
ENST00000422835.2 SPATA5 miRNA
ENST00000422851.1 MRTFA miRNA
ENST00000422875.4 TRAPPC10 miRNA
ENST00000422877.1 SEC23B miRNA
ENST00000422886.4 ANKRD44 miRNA
ENST00000422910.2 ZDHHC14 miRNA
ENST00000422918.4 ARHGAP4 miRNA
ENST00000422927.1 KCNMB2 miRNA
ENST00000422936.4 MEMO1 miRNA
ENST00000422974.2 ZFP91-CNTF miRNA
ENST00000422980.1 DPYD-AS1 miRNA
ENST00000422993.2 CELF1 miRNA
ENST00000422997.5 GALNT11 protein_coding
ENST00000423022.1 KBTBD2 protein_coding
ENST00000423048.4 ALMS1 trna
ENST00000423068.1 EIF2S3 trna
ENST00000423075.1 trna
ENST00000423084.1 trna
ENST00000423088.4 MAP4 trna
ENST00000423112.2 RFX3-AS1 trna
ENST00000423113.4 ITGB1 trna
ENST00000423118.4 PKD1 trna
ENST00000423123.1 DIRC3 trna
ENST00000423156.1 SACS trna
ENST00000423183.1 ACTG1P12 trna
ENST00000423206.4 TIAM1 trna
ENST00000423239.5 RC3H2 trna
ENST00000423243.4 ATE1 trna
ENST00000423282.1 STAT1 trna
ENST00000423293.1 ACTBP15 trna
ENST00000423294.4 ELAPOR2 trna
ENST00000423296.5 WDR48 trna
ENST00000423345.4 PRAM1 trna
ENST00000423351.4 PBRM1 trna
ENST00000423414.4 LINC-PINT trna
ENST00000423424.4 FNDC3B trna
ENST00000423430.1 SLC23A2 trna
ENST00000423461.5 MTX2 trna
ENST00000423463.5 VPS13A trna
ENST00000423465.1 RAB18 trna
ENST00000423484.2 ZNF736 trna
ENST00000423490.4 INPP5A trna
ENST00000423495.1 LPIN1 trna
ENST00000423507.5 PLXNA4 trna
ENST00000423517.5 PCLO trna
ENST00000423531.2 ACAP2 trna
ENST00000423555.4 SMARCA2 trna
ENST00000423559.2 SUFU trna
ENST00000423596.4 SH3BGR trna
ENST00000423610.1 trna
ENST00000423627.4 ZNF107 trna
ENST00000423633.4 SLC20A1 trna
ENST00000423656.4 DCAF1 trna

ENST00000423674.4 HPCAL1 trna
ENST00000423686.1 MRPS31P5 trna
ENST00000423698.5 ERCC1 trna
ENST00000423785.4 NEK6 trna
ENST00000423841.1 EIF1P3 trna
ENST00000423846.4 CYTOR trna
ENST00000423899.1 GUSBP4 trna
ENST00000423954.3 IL6ST trna
ENST00000423998.1 GTF3C2 trna
ENST00000424002.1 MTND4P24 trna
ENST00000424007.1 ARHGAP26 trna
ENST00000424022.2 LBR trna
ENST00000424046.1 MICAL3 trna
ENST00000424049.1 NA trna
ENST00000424071.4 ATG7 trna
ENST00000424110.1 PUM2 trna
ENST00000424117.4 INO80D trna
ENST00000424127.2 GAB3 trna
ENST00000424163.2 FBXO11 trna
ENST00000424170.4 LINC01934 trna
ENST00000424212.1 ELMO1 trna
ENST00000424225.1 TOP2B trna
ENST00000424252.2 trna
ENST00000424254.5 FBXO28 trna
ENST00000424287.4 MED15 trna
ENST00000424293.4 NPIPB10P trna
ENST00000424300.4 QRICH1 trna
ENST00000424306.4 trna
ENST00000424317.4 ANKRD44 trna
ENST00000424332.1 trna
ENST00000424354.4 SREBF2 trna
ENST00000424425.4 KIAA1109 trna
ENST00000424441.1 BRWD1 trna
ENST00000424461.4 CASP8 trna
ENST00000424491.4 trna
ENST00000424496.1 trna
ENST00000424519.1 NA trna
ENST00000424527.4 PCCA trna
ENST00000424533.4 IPO11 trna
ENST00000424567.1 TMSB4XP1 trna
ENST00000424573.1 RPL39P3 trna
ENST00000424583.5 MEF2B trna
ENST00000424632.4 RNF149 trna
ENST00000424643.3 SDCBPP2 trna
ENST00000424646.5 NR3C1 trna
ENST00000424712.5 PIK3C2B trna
ENST00000424722.4 STAT1 trna
ENST00000424723.4 MTA1 trna
ENST00000424754.4 NCSTN trna
ENST00000424846.3 TPM3P9 trna
ENST00000424848.2 CDK6 trna
ENST00000424854.5 PCSK5 trna
ENST00000424861.5 STK3 trna
ENST00000424877.4 KMT2C trna

ENST00000424878.3 APOL3 trna
ENST00000424945.4 MAP4K2 trna
ENST00000424947.5 SENP6 trna
ENST00000424977.1 CAP1 trna
ENST00000424982.1 DPYD-IT1 trna
ENST00000424990.4 CSAD trna
ENST00000424999.1 PIP5K1A trna
ENST00000425012.5 DAZAP2 trna
ENST00000425030.1 CFLAR trna
ENST00000425032.6 USP8 trna
ENST00000425039.3 PMS2P2 trna
ENST00000425121.1 NA trna
ENST00000425123.1 ACTG1P4 trna
ENST00000425132.4 METTL21A trna
ENST00000425134.2 TXNIP trna
ENST00000425147.1 RPL13AP7 trna
ENST00000425169.1 TYMP trna
ENST00000425198.1 SEPTIN7 trna
ENST00000425217.4 KSR2 trna
ENST00000425234.5 BTN3A1 trna
ENST00000425240.1 PRKX trna
ENST00000425247.5 RAP1B trna
ENST00000425290.1 trna
ENST00000425320.1 MEIKIN trna
ENST00000425393.1 RAB3GAP1 trna
ENST00000425413.4 UBR4 trna
ENST00000425470.1 TANK-AS1 trna
ENST00000425508.5 BBS9 trna
ENST00000425518.4 SMARCC1 trna
ENST00000425521.1 ZBTB21 trna
ENST00000425534.6 EIF4E3 trna
ENST00000425586.4 DLEU2 trna
ENST00000425604.4 ZNRD1ASP trna
ENST00000425608.4 RBM6 trna
ENST00000425647.1 CDK5RAP2 trna
ENST00000425648.1 trna
ENST00000425669.1 trna
ENST00000425682.1 HNRNPLL trna
ENST00000425700.3 C4B trna
ENST00000425705.2 DMTF1 trna
ENST00000425708.5 ZNF445 trna
ENST00000425709.1 LINC01505 trna
ENST00000425715.1 HIBADH trna
ENST00000425756.1 NCK2 trna
ENST00000425771.4 GAS5 trna
ENST00000425792.4 UBE2E2 trna
ENST00000425797.2 DTX2P1 trna
ENST00000425799.5 SATB1-AS1 trna
ENST00000425818.2 MAP2K1 trna
ENST00000425843.1 HSPA8P1 trna
ENST00000425867.2 ADGRV1 trna
ENST00000425868.4 BBX trna
ENST00000425871.1 RPF2 trna
ENST00000425884.4 WARS2-AS1 trna

ENST00000425897.2 MARK2 trna
ENST00000425932.4 C3orf35 trna
ENST00000425954.1 PLXNB2 trna
ENST00000426031.4 FYTTD1 trna
ENST00000426073.5 ARHGEF7 trna
ENST00000426079.4 ACBD5 trna
ENST00000426106.1 NME8 trna
ENST00000426121.1 IKZF1 trna
ENST00000426126.5 ZDHHC17 trna
ENST00000426150.4 SSBP3 trna
ENST00000426156.1 FAM3C trna
ENST00000426180.1 HERPUD2 trna
ENST00000426221.4 SCML4 trna
ENST00000426228.1 KAT2B trna
ENST00000426229.1 ADIPOR1 trna
ENST00000426246.2 USP42 trna
ENST00000426296.1 SIGLEC11 trna
ENST00000426304.4 NHEJ1 trna
ENST00000426367.1 trna
ENST00000426414.5 WDFY3 trna
ENST00000426431.2 SP1 trna
ENST00000426450.1 PALS2 trna
ENST00000426477.1 GSAP trna
ENST00000426482.3 NBPF19 trna
ENST00000426496.5 PRRC2C trna
ENST00000426501.4 HERC2P3 trna
ENST00000426517.1 DNAJB4 trna
ENST00000426518.4 OGG1 trna
ENST00000426524.4 KAT6A trna
ENST00000426545.1 RPL5P34 trna
ENST00000426564.5 USP9Y trna
ENST00000426608.4 PTGS1 trna
ENST00000426620.4 OXSR1 trna
ENST00000426639.4 COL24A1 trna
ENST00000426646.1 TRAT1 trna
ENST00000426648.1 BAZ2B trna
ENST00000426668.1 PAK2 trna
ENST00000426683.4 HERC3 trna
ENST00000426727.5 UBE2Q2 trna
ENST00000426740.4 FCGR3A trna
ENST00000426751.5 DMAC2L trna
ENST00000426755.4 PIGX trna
ENST00000426767.1 ZNRF2P2 trna
ENST00000426768.2 ARHGEF7 trna
ENST00000426776.4 ARID2 trna
ENST00000426789.4 TFRC trna
ENST00000426828.4 CCT6P3 trna
ENST00000426838.7 SETBP1 trna
ENST00000426851.5 ZNF398 trna
ENST00000426867.1 SNORD62B trna
ENST00000426886.1 SMIM12 trna
ENST00000426907.4 HUWE1 trna
ENST00000426923.2 PLAC8 trna
ENST00000426956.1 SOAT1 trna

ENST00000426968.2 HHAT trna
ENST00000426978.4 CD36 trna
ENST00000426988.1 SESTD1 trna
ENST00000426991.2 ANKRD10-IT1 trna
ENST00000427003.6 RNF213 trna
ENST00000427008.1 PNPLA8 trna
ENST00000427022.1 trna
ENST00000427025.5 NHSL1 trna
ENST00000427039.1 trna
ENST00000427041.1 NGLY1 trna
ENST00000427060.5 SLA trna
ENST00000427105.1 ZNF564 trna
ENST00000427110.5 PTPRC trna
ENST00000427112.5 USP40 trna
ENST00000427154.1 trna
ENST00000427175.2 RALGAPA2 trna
ENST00000427180.4 SEPTIN9 trna
ENST00000427207.4 MDFIC trna
ENST00000427222.2 PPP6R2 trna
ENST00000427233.4 GIGYF2 trna
ENST00000427242.1 FTLP3 trna
ENST00000427274.5 GOSR1 trna
ENST00000427290.1 LINC02884 trna
ENST00000427312.1 MRPS18A trna
ENST00000427329.4 RAD18 trna
ENST00000427334.4 BTN3A1 protein_coding
ENST00000427391.1 FIRRE lncRNA
ENST00000427426.1 MTCO2P12 unprocessed_pseudogene
ENST00000427434.1 ANAPC1P4 transcribed_unprocessed_pseudogene
ENST00000427444.1 HFM1 protein_coding
ENST00000427449.1 HLA-DQB2 protein_coding
ENST00000427455.1 HERPUD2 protein_coding
ENST00000427457.1 HECW2 protein_coding
ENST00000427458.1 lncRNA
ENST00000427466.1 THEMIS2 protein_coding
ENST00000427522.5 HELZ2 protein_coding
ENST00000427560.1 DISC1 processed_transcript
ENST00000427574.5 EMSY retained_intron
ENST00000427603.4 MAP4K4 protein_coding
ENST00000427606.2 ZNF252P processed_transcript
ENST00000427628.4 ARID2 processed_transcript
ENST00000427649.4 NA NA
ENST00000427660.5 NUDCD1 protein_coding
ENST00000427665.1 GOLPH3L protein_coding
ENST00000427691.4 MIR646HG lncRNA
ENST00000427693.1 HLX protein_coding
ENST00000427718.5 NA NA
ENST00000427759.4 ATG7 protein_coding
ENST00000427765.1 FAM120A protein_coding
ENST00000427771.1 TRAK1 protein_coding
ENST00000427791.1 BCR protein_coding
ENST00000427792.1 NBAS nonsense_mediated_decay
ENST00000427802.2 FSTL5 protein_coding
ENST00000427819.4 lncRNA

ENST00000427820.1 MIR646HG lncRNA
ENST00000427846.1 ENTPD1-AS1 lncRNA
ENST00000427898.4 IDI1 protein_coding
ENST00000427902.4 ZEB2 protein_coding
ENST00000427943.1 PSPC1 protein_coding
ENST00000427960.4 WDR75 nonsense_mediated_decay
ENST00000427965.5 RRN3P2 lncRNA
ENST00000427980.5 ATXN1L protein_coding
ENST00000427991.5 NA NA
ENST00000427997.4 ANAPC1 protein_coding
ENST00000428005.1 SLC4A7 retained_intron
ENST00000428008.4 ZFAS1 lncRNA
ENST00000428021.1 VRK2 protein_coding
ENST00000428056.5 IDS protein_coding
ENST00000428064.4 LIMS1 protein_coding
ENST00000428068.4 ADGRA2 protein_coding
ENST00000428085.1 MFSD9 nonsense_mediated_decay
ENST00000428092.1 PBX3 protein_coding
ENST00000428095.1 UBXN7 protein_coding
ENST00000428097.4 VOPP1 protein_coding
ENST00000428128.1 RGPD1 nonsense_mediated_decay
ENST00000428191.1 ANKRD44-IT1 lncRNA
ENST00000428216.3 MAVS protein_coding
ENST00000428241.1 OSBPL10 protein_coding
ENST00000428265.4 CYP20A1 nonsense_mediated_decay
ENST00000428308.5 TENT2 protein_coding
ENST00000428311.1 protein_coding
ENST00000428360.2 NAGK processed_transcript
ENST00000428370.1 transcribed_unprocessed_pseudogene
ENST00000428382.2 UIMC1 protein_coding
ENST00000428393.3 NA NA
ENST00000428430.1 ABLIM1 protein_coding
ENST00000428443.6 SESTD1 protein_coding
ENST00000428457.1 TNS3 protein_coding
ENST00000428463.4 ZNF630 nonsense_mediated_decay
ENST00000428466.1 RASSF4 protein_coding
ENST00000428492.4 THRB protein_coding
ENST00000428504.1 transcribed_unprocessed_pseudogene
ENST00000428512.1 CALM2P2 processed_pseudogene
ENST00000428514.1 SNORD62A snoRNA
ENST00000428519.1 RBMS1 protein_coding
ENST00000428527.1 TTC27 nonsense_mediated_decay
ENST00000428541.1 lncRNA
ENST00000428553.1 processed_pseudogene
ENST00000428617.1 MAP3K13 protein_coding
ENST00000428655.4 KANSL1L protein_coding
ENST00000428762.4 RELN protein_coding
ENST00000428783.1 FRY processed_transcript
ENST00000428799.1 XPNPEP3 nonsense_mediated_decay
ENST00000428800.4 LIPA protein_coding
ENST00000428807.4 MADD protein_coding
ENST00000428832.2 EIF4A1P10 processed_pseudogene
ENST00000428857.1 CCNT2-AS1 lncRNA
ENST00000428870.5 KDM4C protein_coding

ENST00000428878.5 HIPK2 protein_coding
ENST00000428879.4 MMADHC protein_coding
ENST00000428881.1 RPL4P3 processed_pseudogene
ENST00000428892.1 APOBEC3C nonsense_mediated_decay
ENST00000428921.1 ZC3H13 retained_intron
ENST00000428953.1 CCDC186 protein_coding
ENST00000428959.4 TRIP12 protein_coding
ENST00000428982.4 SYNGAP1 protein_coding
ENST00000428992.2 ARL5A protein_coding
ENST00000428993.1 CAMKMT nonsense_mediated_decay
ENST00000429018.4 GOLGA4 protein_coding
ENST00000429045.5 IFT74 protein_coding
ENST00000429074.1 lncRNA
ENST00000429104.1 TIMM23B-AGAP6 processed_transcript
ENST00000429124.4 FTX lncRNA
ENST00000429136.4 ATP13A3 retained_intron
ENST00000429139.1 MIR181A2HG lncRNA
ENST00000429175.6 FLI1 nonsense_mediated_decay
ENST00000429214.1 lncRNA
ENST00000429235.1 TEX10 protein_coding
ENST00000429236.4 SYNJ1 protein_coding
ENST00000429238.2 protein_coding
ENST00000429242.6 GATAD2A protein_coding
ENST00000429246.4 ARPC1B protein_coding
ENST00000429258.5 CDC123 protein_coding
ENST00000429270.1 BBX protein_coding
ENST00000429289.4 EPHA1-AS1 lncRNA
ENST00000429309.1 RPL30P4 processed_pseudogene
ENST00000429315.3 KIF9-AS1 lncRNA
ENST00000429326.4 POT1 nonsense_mediated_decay
ENST00000429327.1 MTCO1P3 processed_pseudogene
ENST00000429339.4 RAE1 protein_coding
ENST00000429369.4 AZI2 protein_coding
ENST00000429378.4 LUC7L protein_coding
ENST00000429387.4 SIRPD protein_coding
ENST00000429434.3 ZNF140 protein_coding
ENST00000429492.5 OSBPL10 protein_coding
ENST00000429575.1 OLA1 protein_coding
ENST00000429636.1 lncRNA
ENST00000429642.1 IDI1 protein_coding
ENST00000429644.5 GBE1 protein_coding
ENST00000429656.1 unprocessed_pseudogene
ENST00000429659.6 UBA6 retained_intron
ENST00000429674.4 SLC25A40 nonsense_mediated_decay
ENST00000429686.4 ARAP1 protein_coding
ENST00000429703.5 R3HDM1 protein_coding
ENST00000429715.1 MYCBP2 protein_coding
ENST00000429775.2 RPS23P8 processed_pseudogene
ENST00000429781.1 LINC00476 lncRNA
ENST00000429798.1 LIMD1-AS1 lncRNA
ENST00000429810.2 PDPR2P retained_intron
ENST00000429821.4 lncRNA
ENST00000429829.4 XIST lncRNA
ENST00000429842.1 NBAS protein_coding

ENST00000429843.1 BACH1-IT3 lncRNA
ENST00000429860.1 OGA protein_coding
ENST00000429881.1 CASP8 protein_coding
ENST00000429899.1 ATG4B retained_intron
ENST00000429901.1 LINC-PINT retained_intron
ENST00000429904.5 TMEM176B protein_coding
ENST00000429924.5 TBC1D5 protein_coding
ENST00000429947.1 LINC01237 lncRNA
ENST00000429976.4 TRANK1 protein_coding
ENST00000429978.4 GTDC1 nonsense_mediated_decay
ENST00000429985.1 OST4 protein_coding
ENST00000429988.2 PCNX2 retained_intron
ENST00000429989.6 TSPAN14 protein_coding
ENST00000429990.1 NPIPB15 protein_coding
ENST00000430075.4 UGGT1 nonsense_mediated_decay
ENST00000430079.4 ANOS2P processed_transcript
ENST00000430086.1 CCDC88A processed_transcript
ENST00000430120.3 NOS1AP nonsense_mediated_decay
ENST00000430151.1 HLA-K unprocessed_pseudogene
ENST00000430185.4 SUN2 processed_transcript
ENST00000430192.4 LINC00299 lncRNA
ENST00000430200.1 HDAC4 protein_coding
ENST00000430220.5 LARGE1 protein_coding
ENST00000430234.4 CLASP1 processed_transcript
ENST00000430247.1 lncRNA
ENST00000430254.1 TRAK2 protein_coding
ENST00000430262.2 ZNF652 protein_coding
ENST00000430295.4 SVIL-AS1 processed_transcript
ENST00000430321.4 UBR3 nonsense_mediated_decay
ENST00000430328.5 RIF1 protein_coding
ENST00000430339.4 BCL6 protein_coding
ENST00000430340.4 LPP protein_coding
ENST00000430350.2 PRNP protein_coding
ENST00000430368.5 TCTN3 protein_coding
ENST00000430408.1 CNOT10 protein_coding
ENST00000430409.4 MAPKAPK3 protein_coding
ENST00000430412.4 SLC39A10 nonsense_mediated_decay
ENST00000430421.4 LILRA6 nonsense_mediated_decay
ENST00000430429.1 lncRNA
ENST00000430432.4 JAZF1 protein_coding
ENST00000430436.4 RALGAPA2 protein_coding
ENST00000430455.1 MLLT10 nonsense_mediated_decay
ENST00000430457.1 lncRNA
ENST00000430458.1 AFG1L processed_transcript
ENST00000430461.4 ARMCX4 processed_transcript
ENST00000430530.4 ZNF815P processed_transcript
ENST00000430536.5 DHRSX processed_transcript
ENST00000430575.1 RPS4Y1 protein_coding
ENST00000430584.1 GSAP processed_transcript
ENST00000430603.1 MAP3K8 retained_intron
ENST00000430705.4 PTPRA protein_coding
ENST00000430718.4 TTLL3 protein_coding
ENST00000430750.1 UBE3C nonsense_mediated_decay
ENST00000430762.5 PPP3CB protein_coding

ENST00000430772.4 FTX lncRNA
ENST00000430776.2 LINC02770 lncRNA
ENST00000430789.1 AFF3 nonsense_mediated_decay
ENST00000430796.1 UFL1-AS1 lncRNA
ENST00000430804.4 LRRK2 nonsense_mediated_decay
ENST00000430825.3 LMBR1 processed_transcript
ENST00000430834.1 DGKD nonsense_mediated_decay
ENST00000430907.2 FTH1P10 transcribed_processed_pseudogene
ENST00000430918.4 HDLBP protein_coding
ENST00000430929.1 NA NA
ENST00000430968.4 SPOPL nonsense_mediated_decay
ENST00000430969.4 TNRC18 protein_coding
ENST00000430976.4 PTPN4 nonsense_mediated_decay
ENST00000430979.1 QRICH1 protein_coding
ENST00000430989.1 SUCLG1 processed_transcript
ENST00000430996.2 DHRS3 protein_coding
ENST00000431023.4 LARS2 processed_transcript
ENST00000431037.1 PMS2P1 transcribed_unprocessed_pseudogene
ENST00000431043.1 SNHG5 retained_intron
ENST00000431048.4 PTPRA protein_coding
ENST00000431051.4 PSMD1 nonsense_mediated_decay
ENST00000431065.1 WDPCP protein_coding
ENST00000431078.1 CNTNAP5 protein_coding
ENST00000431087.1 ROCK2 protein_coding
ENST00000431098.1 RPL12P16 transcribed_processed_pseudogene
ENST00000431105.1 GOLGA4 protein_coding
ENST00000431143.4 STXBP5-AS1 lncRNA
ENST00000431155.1 GRAMD4 protein_coding
ENST00000431156.5 TTI2 protein_coding
ENST00000431164.1 SH3KBP1 protein_coding
ENST00000431196.2 KDM6A protein_coding
ENST00000431201.4 PISD protein_coding
ENST00000431210.2 XRRA1 retained_intron
ENST00000431222.5 F13A1 protein_coding
ENST00000431245.2 KMO processed_transcript
ENST00000431250.1 MTMR14 protein_coding
ENST00000431283.1 PTPN4 protein_coding
ENST00000431308.4 lncRNA
ENST00000431312.2 DDX17 retained_intron
ENST00000431319.1 NOL10 protein_coding
ENST00000431357.1 USP4 protein_coding
ENST00000431431.5 SEC14L1 protein_coding
ENST00000431432.1 lncRNA
ENST00000431454.2 BIRC6 nonsense_mediated_decay
ENST00000431458.1 FTLP2 processed_pseudogene
ENST00000431467.1 ING3 protein_coding
ENST00000431508.4 ANKHD1 protein_coding
ENST00000431510.1 SLC25A38 protein_coding
ENST00000431519.5 CDV3 protein_coding
ENST00000431520.4 IKZF2 nonsense_mediated_decay
ENST00000431533.5 RPL28 protein_coding
ENST00000431544.2 RNF215 protein_coding
ENST00000431571.4 CNTRL nonsense_mediated_decay
ENST00000431574.1 MYL12BP3 processed_pseudogene

ENST00000431614.4 PPP1R21 nonsense_mediated_decay
ENST00000431661.1 LINC00189 lncRNA
ENST00000431664.5 SAR1A protein_coding
ENST00000431668.1 GALNT11 protein_coding
ENST00000431681.4 SMG1P1 processed_transcript
ENST00000431687.4 ZNF217 protein_coding
ENST00000431736.5 UBE4A protein_coding
ENST00000431803.2 CDC42-IT1 lncRNA
ENST00000431824.2 IGF2BP1 protein_coding
ENST00000431832.1 PER2 protein_coding
ENST00000431840.2 lncRNA
ENST00000431842.5 TASOR protein_coding
ENST00000431865.1 AFG1L nonsense_mediated_decay
ENST00000431893.3 SEC16A protein_coding
ENST00000431914.4 CTNNB1 protein_coding
ENST00000431916.4 KIAA0319L protein_coding
ENST00000431917.4 ATG16L1 protein_coding
ENST00000431929.2 RHOA processed_transcript
ENST00000431932.5 DENND4A protein_coding
ENST00000431936.1 EIF4BP6 processed_pseudogene
ENST00000431946.1 MICAL1 protein_coding
ENST00000432018.4 IL1B protein_coding
ENST00000432021.3 NA NA
ENST00000432042.4 RNF123 protein_coding
ENST00000432043.2 FCHSD2 nonsense_mediated_decay
ENST00000432045.5 lncRNA
ENST00000432050.4 lncRNA
ENST00000432054.5 TMCC1 protein_coding
ENST00000432058.1 STAT1 protein_coding
ENST00000432066.2 SP4 protein_coding
ENST00000432067.1 FAM242A lncRNA
ENST00000432093.1 LSP1 protein_coding
ENST00000432102.4 NCF1B processed_transcript
ENST00000432103.5 RTN1 retained_intron
ENST00000432105.4 PUM2 nonsense_mediated_decay
ENST00000432131.4 UPP1 protein_coding
ENST00000432132.2 EIF4BP3 processed_pseudogene
ENST00000432179.1 BCL2L11 protein_coding
ENST00000432223.2 FAM168A retained_intron
ENST00000432228.1 HSPA9P1 processed_pseudogene
ENST00000432231.1 nonsense_mediated_decay
ENST00000432234.4 SH3KBP1 protein_coding
ENST00000432248.1 DAGLB protein_coding
ENST00000432261.5 RNF24 protein_coding
ENST00000432266.4 UBE2V1 processed_transcript
ENST00000432270.1 PIGT protein_coding
ENST00000432278.4 ETS2 protein_coding
ENST00000432282.4 ZC3H11A protein_coding
ENST00000432293.2 NA NA
ENST00000432296.1 MIR181A1HG lncRNA
ENST00000432297.5 PILRA protein_coding
ENST00000432301.4 ANKHD1 protein_coding
ENST00000432305.5 THOC3 protein_coding
ENST00000432306.4 VTI1A protein_coding

ENST00000432307.1 EIF4BP7 processed_pseudogene
ENST00000432334.1 RIN2 processed_transcript
ENST00000432336.1 CHST12 protein_coding
ENST00000432358.1 TULP4 processed_transcript
ENST00000432372.5 NRP1 protein_coding
ENST00000432373.1 AP1B1P1 processed_transcript
ENST00000432376.4 PLCL2 protein_coding
ENST00000432404.4 SULT4A1 nonsense_mediated_decay
ENST00000432444.1 TMEM43 nonsense_mediated_decay
ENST00000432455.4 PLXNB2 protein_coding
ENST00000432457.2 HNRNPA3 protein_coding
ENST00000432467.1 NSDHL protein_coding
ENST00000432485.1 PRR14L nonsense_mediated_decay
ENST00000432502.1 NUP50-DT lncRNA
ENST00000432508.3 MTG1 protein_coding
ENST00000432511.1 LINC01136 lncRNA
ENST00000432517.1 transcribed_processed_pseudogene
ENST00000432525.4 DDX17 processed_transcript
ENST00000432534.4 CPVL protein_coding
ENST00000432566.5 GOLGA8A protein_coding
ENST00000432608.4 TEX41 lncRNA
ENST00000432610.1 PIP4K2A processed_transcript
ENST00000432686.4 UTRN protein_coding
ENST00000432704.4 GATAD2A protein_coding
ENST00000432725.1 SF1 protein_coding
ENST00000432729.4 ZMAT3 protein_coding
ENST00000432741.4 CCDC18-AS1 lncRNA
ENST00000432757.4 IL3RA protein_coding
ENST00000432775.5 CCSER1 protein_coding
ENST00000432781.1 YEATS2 protein_coding
ENST00000432786.4 ARPC1A nonsense_mediated_decay
ENST00000432798.1 STAT4 protein_coding
ENST00000432820.1 NAP1L4P3 processed_pseudogene
ENST00000432842.5 NA NA
ENST00000432850.1 RNF144A protein_coding
ENST00000432854.4 DBNL protein_coding
ENST00000432887.4 TCP11L1 protein_coding
ENST00000432931.4 RBM17 protein_coding
ENST00000432959.1 TMEM127 protein_coding
ENST00000432979.4 SP100 protein_coding
ENST00000432992.5 GAS7 protein_coding
ENST00000433011.4 ARMCX4 nonsense_mediated_decay
ENST00000433031.1 unprocessed_pseudogene
ENST00000433033.2 HNF1A-AS1 lncRNA
ENST00000433061.1 LINC01278 lncRNA
ENST00000433097.4 NA NA
ENST00000433113.1 lncRNA
ENST00000433131.1 lncRNA
ENST00000433147.1 DEPDC5 protein_coding
ENST00000433197.3 ERN1 protein_coding
ENST00000433211.5 CTNNA3 protein_coding
ENST00000433228.1 lncRNA
ENST00000433246.4 ELMO1 protein_coding
ENST00000433296.4 LANCL1-AS1 lncRNA

ENST00000433310.5 lncRNA
ENST00000433336.1 SACM1L protein_coding
ENST00000433355.5 SHPRH nonsense_mediated_decay
ENST00000433416.4 TTC27 nonsense_mediated_decay
ENST00000433419.2 CCDC144CP transcribed_processed_pseudogene
ENST00000433439.1 CNOT9 nonsense_mediated_decay
ENST00000433442.1 UST-AS2 lncRNA
ENST00000433445.1 CFLAR protein_coding
ENST00000433448.2 DOCK2 nonsense_mediated_decay
ENST00000433449.4 KLHL18 nonsense_mediated_decay
ENST00000433477.3 CTSO protein_coding
ENST00000433483.1 MAPKAP1 protein_coding
ENST00000433499.1 SYP-AS1 lncRNA
ENST00000433514.4 LHFPL3-AS1 lncRNA
ENST00000433543.2 NOP58 nonsense_mediated_decay
ENST00000433557.1 UTRN protein_coding
ENST00000433566.7 XPO7 protein_coding
ENST00000433568.4 UBE2F nonsense_mediated_decay
ENST00000433571.2 NCOA7 processed_transcript
ENST00000433628.2 CNNM2 protein_coding
ENST00000433632.1 SULF2 processed_transcript
ENST00000433650.4 SEMA4D protein_coding
ENST00000433655.4 CTSD nonsense_mediated_decay
ENST00000433675.1 RPS15P4 processed_pseudogene
ENST00000433677.5 CPPED1 protein_coding
ENST00000433680.1 TNFAIP3 protein_coding
ENST00000433732.1 XIST lncRNA
ENST00000433736.5 ITGAV protein_coding
ENST00000433749.4 ZZZ3 protein_coding
ENST00000433797.4 NA NA
ENST00000433814.4 PCGF3 protein_coding
ENST00000433829.1 FANCC protein_coding
ENST00000433853.1 processed_pseudogene
ENST00000433868.4 ATP11C protein_coding
ENST00000433878.4 FYCO1 protein_coding
ENST00000433883.1 THOC2 nonsense_mediated_decay
ENST00000433888.4 PTPN4 nonsense_mediated_decay
ENST00000433921.4 ZNF142 nonsense_mediated_decay
ENST00000433959.4 VOPP1 protein_coding
ENST00000433971.4 TPRG1 protein_coding
ENST00000434005.1 EEF1A1P6 processed_pseudogene
ENST00000434009.4 REV3L nonsense_mediated_decay
ENST00000434033.4 CR1 retained_intron
ENST00000434060.1 MTURN protein_coding
ENST00000434084.1 GSAP processed_transcript
ENST00000434137.2 UBE2F protein_coding
ENST00000434185.1 SLC25A17 protein_coding
ENST00000434200.4 NA NA
ENST00000434238.1 WDR43 protein_coding
ENST00000434243.4 EDEM1 nonsense_mediated_decay
ENST00000434260.1 CBX7 protein_coding
ENST00000434267.4 MAP4 protein_coding
ENST00000434319.5 CLEC12A protein_coding
ENST00000434333.1 HLA-B protein_coding

ENST00000434358.2 THEMIS protein_coding
ENST00000434361.4 TNRC18 protein_coding
ENST00000434363.1 lncRNA
ENST00000434372.2 FAU protein_coding
ENST00000434373.2 BBS9 protein_coding
ENST00000434382.2 VKORC1L1 protein_coding
ENST00000434385.2 STK38L retained_intron
ENST00000434408.1 TOB2 protein_coding
ENST00000434415.4 ZNF783 protein_coding
ENST00000434418.1 LINC01090 lncRNA
ENST00000434420.1 TBC1D5 protein_coding
ENST00000434423.4 POM121 protein_coding
ENST00000434433.1 SENP5 protein_coding
ENST00000434438.5 HIP1 protein_coding
ENST00000434451.1 TNS3 protein_coding
ENST00000434457.5 DENND4C protein_coding
ENST00000434469.1 NBEAL1 protein_coding
ENST00000434500.4 CCT6P1 transcribed_unprocessed_pseudogene
ENST00000434517.1 TOP3B protein_coding
ENST00000434520.4 ARMH1 processed_transcript
ENST00000434578.5 TMEM51 protein_coding
ENST00000434585.4 RAB11FIP3 protein_coding
ENST00000434602.4 ZNF800 protein_coding
ENST00000434609.1 ELF4 protein_coding
ENST00000434618.5 TAPBP protein_coding
ENST00000434648.4 CAPN2 protein_coding
ENST00000434663.4 GSN processed_transcript
ENST00000434667.3 SCAF4 protein_coding
ENST00000434724.5 GSPT1 protein_coding
ENST00000434748.2 FBRSL1 protein_coding
ENST00000434749.4 LRRFIP2 protein_coding
ENST00000434786.2 lncRNA
ENST00000434794.1 LRP1B protein_coding
ENST00000434796.4 GAS5 retained_intron
ENST00000434834.4 NDUFA6-DT lncRNA
ENST00000434835.2 MSL2 protein_coding
ENST00000434851.1 EIF3FP3 processed_pseudogene
ENST00000434854.1 RBM41 protein_coding
ENST00000434858.4 TAF1B nonsense_mediated_decay
ENST00000434893.1 GUSBP11 lncRNA
ENST00000434910.1 processed_pseudogene
ENST00000434911.5 TLK1 protein_coding
ENST00000434913.1 FTH1P20 processed_pseudogene
ENST00000434934.1 GALM protein_coding
ENST00000434963.1 RPL32 protein_coding
ENST00000434977.1 PHACTR1 protein_coding
ENST00000434978.5 STXBP4 protein_coding
ENST00000434990.1 lncRNA
ENST00000435030.4 KIF5C protein_coding
ENST00000435034.4 TMEM38B protein_coding
ENST00000435041.2 UBE2J1 protein_coding
ENST00000435047.4 SESTD1 protein_coding
ENST00000435070.5 CPSF6 protein_coding
ENST00000435072.1 BACH1 protein_coding

ENST00000435096.1 LUC7L2 retained_intron
ENST00000435128.2 ANXA2P2 processed_pseudogene
ENST00000435159.2 TMEM132C protein_coding
ENST00000435168.5 SMAP2 protein_coding
ENST00000435193.1 ICOS protein_coding
ENST00000435197.2 SMG1P3 processed_transcript
ENST00000435212.2 MTATP8P2 processed_pseudogene
ENST00000435224.2 ABTB2 protein_coding
ENST00000435237.1 miRNA
ENST00000435241.1 FTH1P16 miRNA
ENST00000435254.1 miRNA
ENST00000435268.1 TMEM127 miRNA
ENST00000435290.1 CX3CR1 miRNA
ENST00000435322.1 miRNA
ENST00000435347.6 LASP1 miRNA
ENST00000435371.1 NA miRNA
ENST00000435386.1 AOAH miRNA
ENST00000435402.1 NA miRNA
ENST00000435409.5 INTS3 miRNA
ENST00000435411.4 LINC01934 miRNA
ENST00000435439.4 MIRLET7BHG miRNA
ENST00000435454.4 NR2C2 miRNA
ENST00000435468.1 TBC1D2B miRNA
ENST00000435485.4 LINC01505 miRNA
ENST00000435504.7 ASXL2 miRNA
ENST00000435514.3 ESYT2 miRNA
ENST00000435520.4 ENTPD6 miRNA
ENST00000435523.4 LINC-PINT miRNA
ENST00000435524.2 miRNA
ENST00000435525.1 CTDSPL miRNA
ENST00000435544.5 PPP1R12C miRNA
ENST00000435549.1 SDCCAG8 miRNA
ENST00000435568.1 KRT18P46 miRNA
ENST00000435576.2 TRABD2B miRNA
ENST00000435581.5 CD99P1 miRNA
ENST00000435602.1 KLF7 miRNA
ENST00000435606.1 LDLRAD4 miRNA
ENST00000435634.2 EXOSC6 miRNA
ENST00000435683.5 ABCA7 miRNA
ENST00000435711.4 HNRNPA3 miRNA
ENST00000435720.4 PSMF1 miRNA
ENST00000435722.6 DIP2A miRNA
ENST00000435723.1 MTPN miRNA
ENST00000435731.4 SDHAP1 miRNA
ENST00000435762.2 RAB31 miRNA
ENST00000435773.2 MAIP1 miRNA
ENST00000435829.5 OXNAD1 miRNA
ENST00000435836.4 ZMYND8 miRNA
ENST00000435859.1 BTG3P1 miRNA
ENST00000435868.1 miRNA
ENST00000435891.4 CLSTN1 miRNA
ENST00000435916.1 DYNC1I2P1 miRNA
ENST00000435937.4 KCNAB2 miRNA
ENST00000435943.1 ADAP1 miRNA

ENST00000435946.1 miRNA
ENST00000435962.5 TC2N miRNA
ENST00000435966.1 LINC00630 miRNA
ENST00000435988.1 NCF1B miRNA
ENST00000436015.5 KIAA2026 miRNA
ENST00000436018.1 NA miRNA
ENST00000436022.2 EFCC1 miRNA
ENST00000436046.1 FRY miRNA
ENST00000436054.1 LINC01504 miRNA
ENST00000436111.1 PKN2 miRNA
ENST00000436129.2 TAF4 miRNA
ENST00000436133.4 PRKACB miRNA
ENST00000436146.1 RPL15 miRNA
ENST00000436154.1 WDR37 miRNA
ENST00000436171.2 PANX1 miRNA
ENST00000436178.1 IDE miRNA
ENST00000436179.1 RALA miRNA
ENST00000436213.4 GLRA4 miRNA
ENST00000436216.1 EXT1 miRNA
ENST00000436233.7 SEC14L1 miRNA
ENST00000436236.1 STT3B miRNA
ENST00000436239.4 GNPAT miRNA
ENST00000436241.1 UXS1 miRNA
ENST00000436269.1 USP34 miRNA
ENST00000436277.4 MAST4 miRNA
ENST00000436279.1 AGPAT4 miRNA
ENST00000436287.1 ACTBP11 miRNA
ENST00000436322.1 TRABD2A miRNA
ENST00000436339.4 ARMC9 miRNA
ENST00000436396.1 NA miRNA
ENST00000436399.5 PTGES3 miRNA
ENST00000436427.1 YBX1 miRNA
ENST00000436441.4 SETX miRNA
ENST00000436444.4 NA miRNA
ENST00000436448.1 ZYX miRNA
ENST00000436459.2 EEF1A1P5 miRNA
ENST00000436481.1 RHBDD1 miRNA
ENST00000436527.4 SPN miRNA
ENST00000436577.3 CDK14 miRNA
ENST00000436596.1 SNHG7 miRNA
ENST00000436639.5 SESN1 miRNA
ENST00000436647.1 FOSL2 miRNA
ENST00000436673.4 UPP1 miRNA
ENST00000436712.2 GAPVD1 miRNA
ENST00000436716.1 SLC44A1 miRNA
ENST00000436732.4 DPP10 miRNA
ENST00000436783.4 ATP6AP2 miRNA
ENST00000436787.4 WDR33 miRNA
ENST00000436817.4 HK1 miRNA
ENST00000436829.1 MINDY3 miRNA
ENST00000436857.4 STAG3L3 miRNA
ENST00000436880.2 MIR181A1HG miRNA
ENST00000436894.1 NCL miRNA
ENST00000436901.2 KLF3-AS1 miRNA

ENST00000436911.5 TRGC2 miRNA
ENST00000436924.4 BABAM2 miRNA
ENST00000436941.1 TLDC2 miRNA
ENST00000436947.1 THADA miRNA
ENST00000436949.4 SEMA3A miRNA
ENST00000436950.4 miRNA
ENST00000436970.4 BANP miRNA
ENST00000436992.4 ZNF800 miRNA
ENST00000437008.4 miRNA
ENST00000437030.4 DNAJB6 miRNA
ENST00000437035.4 MIR646HG miRNA
ENST00000437048.5 VPS53 miRNA
ENST00000437070.1 RNF168 miRNA
ENST00000437072.4 H2AZ2 miRNA
ENST00000437084.1 GCK miRNA
ENST00000437099.5 GAS7 miRNA
ENST00000437101.4 XXYLT1 miRNA
ENST00000437115.2 FGF14-IT1 miRNA
ENST00000437116.2 TAPBP miRNA
ENST00000437131.1 GOLGA4 miRNA
ENST00000437146.1 GNB1 miRNA
ENST00000437190.1 PRDX6-AS1 lncRNA
ENST00000437196.1 EXT1 nonsense_mediated_decay
ENST00000437201.4 SBDSP1 trna
ENST00000437205.4 ABCC5 trna
ENST00000437222.1 ZNF217 trna
ENST00000437232.4 VIM-AS1 trna
ENST00000437258.4 AATBC trna
ENST00000437313.1 TSPAN32 trna
ENST00000437404.2 STAU1 trna
ENST00000437413.2 SMG1P3 trna
ENST00000437485.4 CASTOR3 trna
ENST00000437495.1 ANKHD1-EIF4EBP3 trna
ENST00000437500.4 RBM5 trna
ENST00000437515.1 SRGAP2-AS1 trna
ENST00000437545.4 MAP4K3 trna
ENST00000437550.2 HCST trna
ENST00000437561.1 CYTOR trna
ENST00000437623.1 TANK trna
ENST00000437629.5 SHLD2 trna
ENST00000437630.1 PSMD14 trna
ENST00000437650.2 HLA-DRB6 trna
ENST00000437653.1 ACTG1P23 trna
ENST00000437704.3 PLCL1 trna
ENST00000437722.1 VGLL4 trna
ENST00000437765.1 YIPF4 trna
ENST00000437799.1 ANXA11 trna
ENST00000437800.5 CD4 trna
ENST00000437811.1 HLA-DPA1 trna
ENST00000437813.6 TNFRSF1A trna
ENST00000437814.1 METTL15 trna
ENST00000437821.2 PRKAR2A trna
ENST00000437827.1 SNRK trna
ENST00000437857.2 CCDC181 trna

ENST00000437877.1 MAD1L1 trna
ENST00000437883.1 trna
ENST00000437890.1 RPS13P2 trna
ENST00000437898.1 EMC1-AS1 trna
ENST00000437955.1 SULF2 trna
ENST00000437973.2 MAPKAP1 trna
ENST00000437992.2 RPL9 trna
ENST00000437997.1 MTMR14 trna
ENST00000438005.1 MTND5P28 trna
ENST00000438018.4 MTMR1 trna
ENST00000438031.2 TMED2 trna
ENST00000438054.1 BCL2L11 trna
ENST00000438066.1 SPEN trna
ENST00000438077.1 BCL6 trna
ENST00000438097.4 UBE3A trna
ENST00000438114.1 NA trna
ENST00000438181.4 trna
ENST00000438184.2 NFU1 trna
ENST00000438191.1 RPE trna
ENST00000438215.1 PIGU trna
ENST00000438229.5 OXR1 trna
ENST00000438243.2 PI4KB trna
ENST00000438253.1 STX16 trna
ENST00000438277.4 UGGT1 trna
ENST00000438331.4 ANXA11 trna
ENST00000438356.1 RBM33 trna
ENST00000438357.1 RPS6KA3 trna
ENST00000438362.5 CSDE1 trna
ENST00000438374.4 LRRFIP2 trna
ENST00000438382.2 NCF1C trna
ENST00000438393.4 BRD1 trna
ENST00000438394.1 MNDA trna
ENST00000438395.4 VPS50 trna
ENST00000438444.4 COA1 trna
ENST00000438446.1 FYCO1 trna
ENST00000438473.4 MLLT10 trna
ENST00000438486.1 IFI44 trna
ENST00000438490.1 RALGAPB trna
ENST00000438510.4 HLA-DMB trna
ENST00000438527.6 NA trna
ENST00000438571.4 GTF3A trna
ENST00000438590.4 ST6GAL1 trna
ENST00000438647.3 RAPGEF1 trna
ENST00000438704.5 HMGB2 trna
ENST00000438709.5 ANKRD36B trna
ENST00000438715.1 TMEM131 trna
ENST00000438732.2 FLNA trna
ENST00000438817.5 CD96 trna
ENST00000438819.1 CLIP4 trna
ENST00000438858.1 trna
ENST00000438912.1 RBM6 trna
ENST00000438952.4 SYNJ1 trna
ENST00000438959.4 MAD1L1 trna
ENST00000438961.1 DNAAF5 trna

ENST00000438962.1 MED15 trna
ENST00000438975.4 EIF4G3 trna
ENST00000439036.4 MAP2K5 trna
ENST00000439136.2 trna
ENST00000439151.5 NSD1 trna
ENST00000439174.5 GNA13 trna
ENST00000439189.1 RPL13AP5 trna
ENST00000439213.4 IFNGR2 trna
ENST00000439232.2 SNORA71B trna
ENST00000439247.2 PCNX3 trna
ENST00000439301.4 NA trna
ENST00000439325.1 trna
ENST00000439363.4 EIF2B3 trna
ENST00000439369.5 SCLT1 trna
ENST00000439375.5 DNAH14 trna
ENST00000439384.4 CHN2 trna
ENST00000439392.1 C21orf91-OT1 trna
ENST00000439402.1 DNAJB6 trna
ENST00000439432.1 SMAP1 trna
ENST00000439456.4 ZMYND11 trna
ENST00000439467.4 CACNB4 trna
ENST00000439494.1 MIR4435-2HG trna
ENST00000439515.2 TBC1D14 trna
ENST00000439559.2 LINC00863 trna
ENST00000439561.5 COPB1 trna
ENST00000439601.1 LINC01237 trna
ENST00000439638.1 MDN1 trna
ENST00000439642.1 RUBCNL trna
ENST00000439666.1 ACAP2 trna
ENST00000439677.4 BANP trna
ENST00000439681.1 UBR3 trna
ENST00000439696.2 ZFP36L1 trna
ENST00000439701.1 IKZF1 trna
ENST00000439708.1 STEAP1B trna
ENST00000439719.4 LINC00869 trna
ENST00000439758.3 ACAP2 trna
ENST00000439759.5 DIAPH2-AS1 trna
ENST00000439798.1 LINC01891 trna
ENST00000439819.4 DICER1-AS1 trna
ENST00000439830.4 ANKRD28 trna
ENST00000439853.4 LRRC8B trna
ENST00000439871.1 CXCR2P1 trna
ENST00000439883.1 ANKIB1 trna
ENST00000439915.1 ATAD2B trna
ENST00000439962.1 EDEM3 trna
ENST00000439983.4 DBNL trna
ENST00000439987.5 IFT140 trna
ENST00000440001.2 TRAF3IP2-AS1 trna
ENST00000440004.1 trna
ENST00000440051.1 TTC7A trna
ENST00000440079.1 FGD5-AS1 trna
ENST00000440081.2 TNRC18 trna
ENST00000440097.4 LIMD1 trna
ENST00000440108.4 RBM33 trna

ENST00000440126.6 APP trna
ENST00000440150.4 WARS2-AS1 trna
ENST00000440186.4 PTPN12 trna
ENST00000440195.2 SMG1P3 trna
ENST00000440200.4 trna
ENST00000440289.5 PTPRJ trna
ENST00000440317.1 YWHAZP2 trna
ENST00000440335.1 trna
ENST00000440395.1 TRAF3IP2-AS1 trna
ENST00000440400.1 ACTN4 trna
ENST00000440456.4 PIK3IP1-DT trna
ENST00000440465.1 NAMPTP1 trna
ENST00000440467.4 ABLIM1 trna
ENST00000440469.1 FBXO45 trna
ENST00000440482.2 ZFAND3 trna
ENST00000440577.4 PUM2 trna
ENST00000440600.5 INTS7 trna
ENST00000440613.1 CDC123 trna
ENST00000440628.4 GNAI2 trna
ENST00000440647.1 CHD6 trna
ENST00000440650.5 PIK3CG trna
ENST00000440698.1 trna
ENST00000440702.4 FAM20B trna
ENST00000440705.5 VRK2 trna
ENST00000440706.2 PLEKHA8 trna
ENST00000440732.4 CASP8 trna
ENST00000440742.2 LRRFIP2 trna
ENST00000440744.2 trna
ENST00000440765.2 SIL1 trna
ENST00000440803.6 PARGP1 trna
ENST00000440804.6 SIGLEC9 trna
ENST00000440806.2 STX12 trna
ENST00000440807.1 trna
ENST00000440816.4 MIATNB trna
ENST00000440838.4 PSMD1 trna
ENST00000440859.6 CBLL1 trna
ENST00000440865.1 HNRNPU trna
ENST00000440869.5 PHACTR2 trna
ENST00000440875.4 ZEB2 trna
ENST00000440898.1 SECISBP2 trna
ENST00000440906.5 HELB trna
ENST00000440927.1 SCML4 trna
ENST00000440943.4 NRDC trna
ENST00000440951.1 ATP1A1 trna
ENST00000440964.1 SLC25A20 trna
ENST00000440983.1 WDR43 trna
ENST00000441027.4 NBEAL2 trna
ENST00000441037.5 AGBL1 trna
ENST00000441040.2 ZNF84 trna
ENST00000441045.4 NAMPT trna
ENST00000441059.4 OSBPL3 trna
ENST00000441075.1 MIR4435-2HG trna
ENST00000441084.1 YIPF4 trna
ENST00000441088.4 NA trna

ENST00000441089.1 PTPN4 trna
ENST00000441093.1 trna
ENST00000441097.1 PSMB7 trna
ENST00000441099.4 TRAPPC12 trna
ENST00000441128.4 TMEM50B trna
ENST00000441143.1 BAZ2B trna
ENST00000441145.1 OOEP trna
ENST00000441156.4 GNAI2 trna
ENST00000441188.2 AP2B1P1 trna
ENST00000441240.1 LPAR1 trna
ENST00000441246.5 POLR2B trna
ENST00000441255.5 LINC02384 trna
ENST00000441269.2 LRRC8D trna
ENST00000441279.4 DIS3L2 trna
ENST00000441323.4 DARS1 trna
ENST00000441340.1 PLEKHO1 trna
ENST00000441345.2 NUDT16-DT trna
ENST00000441350.2 VPS13B trna
ENST00000441379.4 ITGB2-AS1 trna
ENST00000441382.1 CPN1 trna
ENST00000441386.2 BHLHE40-AS1 trna
ENST00000441429.1 RPS9 trna
ENST00000441447.1 ZMYM4 trna
ENST00000441474.1 TMEM168 trna
ENST00000441488.5 KMT5B trna
ENST00000441564.6 PSD4 trna
ENST00000441595.2 MPP7 trna
ENST00000441606.2 ANO6 trna
ENST00000441624.1 trna
ENST00000441633.2 MTCO1P53 trna
ENST00000441707.4 MRRF trna
ENST00000441733.4 RBCK1 trna
ENST00000441738.1 TIMP1 trna
ENST00000441746.1 lncRNA
ENST00000441751.4 TNRC6B protein_coding
ENST00000441755.4 NBAS protein_coding
ENST00000441760.2 lncRNA
ENST00000441890.4 UBAP2L protein_coding
ENST00000441977.1 ZMYND8 protein_coding
ENST00000441982.4 NCAPG2 protein_coding
ENST00000442018.4 BCOR protein_coding
ENST00000442021.5 NCF1 protein_coding
ENST00000442023.1 ST6GAL1 protein_coding
ENST00000442024.4 HUS1 nonsense_mediated_decay
ENST00000442026.1 LINC01320 lncRNA
ENST00000442034.4 RAB3GAP1 protein_coding
ENST00000442035.4 UBA1 protein_coding
ENST00000442071.1 IFNAR1 protein_coding
ENST00000442081.5 HIVEP1 protein_coding
ENST00000442087.2 PFN1P1 processed_pseudogene
ENST00000442138.5 PRRC1 protein_coding
ENST00000442141.4 SELENOI protein_coding
ENST00000442146.1 lncRNA
ENST00000442183.1 ANKIB1 protein_coding

ENST00000442252.1 STEAP1B processed_transcript
ENST00000442263.4 INTS6 protein_coding
ENST00000442267.2 TBC1D9 protein_coding
ENST00000442293.4 MIR4435-2HG lncRNA
ENST00000442296.4 MXI1 protein_coding
ENST00000442336.1 FCGR3A protein_coding
ENST00000442352.1 PSPC1P1 unprocessed_pseudogene
ENST00000442383.1 OSER1-DT lncRNA
ENST00000442417.4 lncRNA
ENST00000442430.1 PIM2 protein_coding
ENST00000442434.1 DIP2A-IT1 lncRNA
ENST00000442435.3 ADAMTSL4-AS2 lncRNA
ENST00000442489.1 CDKN1B protein_coding
ENST00000442504.4 ELMO1 protein_coding
ENST00000442506.4 NBAS protein_coding
ENST00000442516.1 DTX2 protein_coding
ENST00000442524.3 DGKD protein_coding
ENST00000442541.1 METTL8 protein_coding
ENST00000442544.5 DCC protein_coding
ENST00000442560.4 RFX3 protein_coding
ENST00000442563.4 RABGEF1 protein_coding
ENST00000442578.4 LINC00174 lncRNA
ENST00000442587.1 RAD23B protein_coding
ENST00000442588.4 NEK7 protein_coding
ENST00000442625.1 MCF2L nonsense_mediated_decay
ENST00000442635.2 ZNF518A processed_transcript
ENST00000442637.1 NRSN2-AS1 lncRNA
ENST00000442677.3 CDH23 protein_coding
ENST00000442692.2 TPCN2 processed_transcript
ENST00000442707.1 KIAA1109 protein_coding
ENST00000442713.5 DYM protein_coding
ENST00000442760.2 TPT1 retained_intron
ENST00000442767.1 PRPSAP1 protein_coding
ENST00000442794.1 lncRNA
ENST00000442800.1 nonsense_mediated_decay
ENST00000442805.1 RALY protein_coding
ENST00000442809.1 LINC01266 lncRNA
ENST00000442830.4 PTPRE protein_coding
ENST00000442834.5 YY1AP1 retained_intron
ENST00000442858.1 BBS9 processed_transcript
ENST00000442860.4 CCDC18-AS1 lncRNA
ENST00000442898.4 WASHC1 protein_coding
ENST00000442940.1 PNPLA4 protein_coding
ENST00000442956.1 LINC00299 lncRNA
ENST00000442977.5 OXR1 protein_coding
ENST00000442985.1 SPEN protein_coding
ENST00000442996.1 lncRNA
ENST00000443024.5 IARS1 protein_coding
ENST00000443083.4 EXOC2 protein_coding
ENST00000443112.5 WDR26 retained_intron
ENST00000443149.2 KIAA2026 processed_transcript
ENST00000443175.1 PIK3IP1 protein_coding
ENST00000443183.4 DLG1 protein_coding
ENST00000443185.5 ZNF516 protein_coding

ENST00000443187.1 PRKD3 protein_coding
ENST00000443206.2 TOM1 protein_coding
ENST00000443213.4 SRSF7 nonsense_mediated_decay
ENST00000443217.4 LPP protein_coding
ENST00000443253.1 BBX protein_coding
ENST00000443259.1 GRM7 nonsense_mediated_decay
ENST00000443275.2 POU2F1 protein_coding
ENST00000443280.4 DNAJB6 protein_coding
ENST00000443297.4 ACTR3 protein_coding
ENST00000443302.4 PHF8 protein_coding
ENST00000443361.1 RALGAPA1P1 processed_pseudogene
ENST00000443387.2 ZNF146 protein_coding
ENST00000443402.5 TRGC1 TR_C_gene
ENST00000443439.5 ERAP1 protein_coding
ENST00000443490.4 MIRLET7BHG lncRNA
ENST00000443501.1 FNDC3B protein_coding
ENST00000443515.1 lncRNA
ENST00000443562.1 LRRC8C-DT lncRNA
ENST00000443567.4 CAPN7 nonsense_mediated_decay
ENST00000443574.1 HLA-DQB1 processed_transcript
ENST00000443587.4 DLEU2 lncRNA
ENST00000443590.1 EEF1A1P12 processed_pseudogene
ENST00000443595.1 FAM185BP transcribed_unprocessed_pseudogene
ENST00000443615.1 HP1BP3 protein_coding
ENST00000443617.5 HERC1 protein_coding
ENST00000443621.1 LINC00393 lncRNA
ENST00000443623.4 LINC-PINT lncRNA
ENST00000443624.4 RPL11 retained_intron
ENST00000443629.4 CERK nonsense_mediated_decay
ENST00000443694.2 ITPR1 protein_coding
ENST00000443726.2 BCL11B protein_coding
ENST00000443727.1 LRCH3 nonsense_mediated_decay
ENST00000443790.1 EDEM1 nonsense_mediated_decay
ENST00000443796.4 EPHA4 protein_coding
ENST00000443807.1 GBP5 protein_coding
ENST00000443808.1 MAST4 protein_coding
ENST00000443817.1 CPED1 protein_coding
ENST00000443824.4 CTPS2 protein_coding
ENST00000443829.4 NBEAL2 protein_coding
ENST00000443837.1 RNF216-IT1 lncRNA
ENST00000443879.2 ZDHHC3 protein_coding
ENST00000443896.4 PIKFYVE nonsense_mediated_decay
ENST00000443945.5 LRCH1 retained_intron
ENST00000443950.5 HNRNPF protein_coding
ENST00000443977.1 PRKD3 protein_coding
ENST00000443981.4 PTPRN protein_coding
ENST00000444016.4 PEAR1 nonsense_mediated_decay
ENST00000444045.1 RPA2 protein_coding
ENST00000444051.1 ZNRD1ASP processed_transcript
ENST00000444082.1 unprocessed_pseudogene
ENST00000444102.1 lncRNA
ENST00000444128.2 NCOA7 protein_coding
ENST00000444166.1 COMMD1 protein_coding
ENST00000444179.4 SHC1 protein_coding

ENST00000444191.4 HCFC1 protein_coding
ENST00000444207.1 FBXO7 protein_coding
ENST00000444226.1 MAPKAP1 protein_coding
ENST00000444232.2 nonsense_mediated_decay
ENST00000444244.1 lncRNA
ENST00000444248.1 WASF4P processed_pseudogene
ENST00000444261.1 MDM4 protein_coding
ENST00000444263.4 LINC00278 lncRNA
ENST00000444265.5 CASC15 lncRNA
ENST00000444272.1 SYNCRIP protein_coding
ENST00000444273.5 ZNF451 retained_intron
ENST00000444278.2 DEF6 protein_coding
ENST00000444316.2 BANK1 protein_coding
ENST00000444317.1 MFSD6 protein_coding
ENST00000444332.1 ARL8B retained_intron
ENST00000444341.1 SATB1 protein_coding
ENST00000444342.2 TGOLN2 protein_coding
ENST00000444430.2 CASP8 protein_coding
ENST00000444458.4 CCDC88A protein_coding
ENST00000444487.1 BCL3 protein_coding
ENST00000444489.4 LINC00894 lncRNA
ENST00000444495.1 NA NA
ENST00000444545.4 NEMP2 nonsense_mediated_decay
ENST00000444569.4 DOCK5 protein_coding
ENST00000444574.1 STK24 protein_coding
ENST00000444620.2 ARID4B protein_coding
ENST00000444621.1 HLA-F protein_coding
ENST00000444636.4 SP140L protein_coding
ENST00000444670.4 ARID2 protein_coding
ENST00000444676.4 OGDH protein_coding
ENST00000444688.1 LINC00299 lncRNA
ENST00000444712.4 SNX13 nonsense_mediated_decay
ENST00000444757.4 SLC39A7 protein_coding
ENST00000444770.1 lncRNA
ENST00000444774.3 OGT protein_coding
ENST00000444776.1 EIPR1 protein_coding
ENST00000444815.2 GNAQP1 processed_pseudogene
ENST00000444839.1 GATAD2A protein_coding
ENST00000444844.1 TRIM22 protein_coding
ENST00000444861.4 EIF4G1 protein_coding
ENST00000444872.3 MED27 processed_transcript
ENST00000444933.4 CEP250-AS1 lncRNA
ENST00000444990.4 LINC01881 retained_intron
ENST00000444995.6 LRP5L processed_transcript
ENST00000445038.4 BCAS4 protein_coding
ENST00000445046.1 SMAP1 protein_coding
ENST00000445063.4 WDR35 nonsense_mediated_decay
ENST00000445069.3 CLPB processed_transcript
ENST00000445072.1 LINC01035 lncRNA
ENST00000445108.4 ARID4A protein_coding
ENST00000445125.2 processed_pseudogene
ENST00000445184.1 LINC01004 lncRNA
ENST00000445214.1 TFEB protein_coding
ENST00000445220.4 SHANK3 protein_coding

ENST00000445223.1 F13A1 protein_coding
ENST00000445237.2 HDAC7 protein_coding
ENST00000445239.1 WDR26 protein_coding
ENST00000445288.4 RSBN1L protein_coding
ENST00000445293.5 lncRNA
ENST00000445308.1 D2HGDH processed_transcript
ENST00000445310.1 KCNQ5 processed_transcript
ENST00000445322.1 ELMO1 protein_coding
ENST00000445335.1 IFRD1 protein_coding
ENST00000445387.4 SETD2 nonsense_mediated_decay
ENST00000445418.1 TMEM18-DT lncRNA
ENST00000445422.4 CABIN1 protein_coding
ENST00000445425.4 RHOA protein_coding
ENST00000445445.4 RASGRP2 nonsense_mediated_decay
ENST00000445452.1 lncRNA
ENST00000445485.1 RCL1 processed_transcript
ENST00000445501.4 KCNAB2 protein_coding
ENST00000445516.1 KRIT1 nonsense_mediated_decay
ENST00000445533.1 FARS2 protein_coding
ENST00000445566.1 EFHD2 protein_coding
ENST00000445572.4 TPTE2P6 transcribed_unprocessed_pseudogene
ENST00000445582.4 AGPAT3 protein_coding
ENST00000445603.1 SHLD1 protein_coding
ENST00000445605.4 CYRIA protein_coding
ENST00000445633.4 PFKFB4 nonsense_mediated_decay
ENST00000445695.1 CBWD4P unprocessed_pseudogene
ENST00000445699.4 ING3 protein_coding
ENST00000445700.4 NA NA
ENST00000445706.4 PACSIN2 processed_transcript
ENST00000445708.1 lncRNA
ENST00000445728.6 ZNF473 protein_coding
ENST00000445737.2 UBAC2-AS1 lncRNA
ENST00000445741.4 SAMD12 nonsense_mediated_decay
ENST00000445830.1 YWHAB protein_coding
ENST00000445842.1 NKTR protein_coding
ENST00000445846.1 STAM nonsense_mediated_decay
ENST00000445855.1 R3HDM1 protein_coding
ENST00000445877.4 RMI1 protein_coding
ENST00000445878.1 FLJ31356 lncRNA
ENST00000445886.4 FOXJ3 protein_coding
ENST00000445889.4 ERCC3 nonsense_mediated_decay
ENST00000445922.2 BAG5 protein_coding
ENST00000445943.4 DOCK4 protein_coding
ENST00000445960.4 TMEM131L nonsense_mediated_decay
ENST00000445964.4 INPP5D protein_coding
ENST00000445971.1 CYP27A1 nonsense_mediated_decay
ENST00000445976.1 lncRNA
ENST00000446031.1 CAP1 protein_coding
ENST00000446041.5 SLC25A36 protein_coding
ENST00000446103.4 PBRM1 protein_coding
ENST00000446110.1 ATG7 protein_coding
ENST00000446128.1 CALN1 protein_coding
ENST00000446170.1 ST6GAL1 protein_coding
ENST00000446176.5 FNBP1 protein_coding

ENST00000446180.4 KIAA1109 protein_coding
ENST00000446231.5 SMG1 protein_coding
ENST00000446258.4 RANGAP1 protein_coding
ENST00000446263.1 MTND4LP1 processed_pseudogene
ENST00000446273.1 TNRC6B protein_coding
ENST00000446284.4 FKBP15 protein_coding
ENST00000446317.1 SF3A3P1 processed_pseudogene
ENST00000446336.1 MIATNB lncRNA
ENST00000446375.1 DPY19L1 protein_coding
ENST00000446377.3 NA NA
ENST00000446382.2 ACTG1P14 processed_pseudogene
ENST00000446386.2 MPZL3 nonsense_mediated_decay
ENST00000446446.4 CHN2 protein_coding
ENST00000446450.5 ATG7 protein_coding
ENST00000446471.1 RBM6 protein_coding
ENST00000446496.1 SEC63 nonsense_mediated_decay
ENST00000446506.1 CYTH4 protein_coding
ENST00000446515.1 KPNA6 protein_coding
ENST00000446523.4 PDIA3 nonsense_mediated_decay
ENST00000446531.1 H2AZ2 protein_coding
ENST00000446537.4 UBOX5-AS1 lncRNA
ENST00000446544.5 AHCYL2 protein_coding
ENST00000446564.4 COA1 nonsense_mediated_decay
ENST00000446609.5 PLEKHM1 nonsense_mediated_decay
ENST00000446617.1 SAMD9 protein_coding
ENST00000446619.1 YWHAQ protein_coding
ENST00000446662.4 SMG1P1 processed_transcript
ENST00000446672.2 PARP4P2 unprocessed_pseudogene
ENST00000446712.4 LILRA5 retained_intron
ENST00000446718.1 AVL9 protein_coding
ENST00000446732.4 GLB1 nonsense_mediated_decay
ENST00000446749.1 ZNF385D nonsense_mediated_decay
ENST00000446750.1 HUWE1 protein_coding
ENST00000446758.4 SESTD1 nonsense_mediated_decay
ENST00000446781.3 KIF5C-AS1 lncRNA
ENST00000446803.5 TBC1D1 protein_coding
ENST00000446809.2 CLEC4E retained_intron
ENST00000446815.4 MKLN1 protein_coding
ENST00000446816.1 STARD7-AS1 lncRNA
ENST00000446818.5 TBC1D5 protein_coding
ENST00000446821.4 ACTR3 nonsense_mediated_decay
ENST00000446853.1 FAM106A lncRNA
ENST00000446876.1 HDAC4 protein_coding
ENST00000446896.6 ARL5A nonsense_mediated_decay
ENST00000446897.1 RPS4XP11 processed_pseudogene
ENST00000446916.2 ATRN protein_coding
ENST00000446922.5 HMGB2 protein_coding
ENST00000446924.4 BRWD1 nonsense_mediated_decay
ENST00000446946.1 ALDH1A1 protein_coding
ENST00000446947.5 TBC1D14 protein_coding
ENST00000446951.1 PPIL2 processed_transcript
ENST00000446958.1 URGCP protein_coding
ENST00000446959.4 SAMD9L protein_coding
ENST00000447012.6 NAIP nonsense_mediated_decay

ENST00000447054.4 DGKG processed_transcript
ENST00000447060.4 NA NA
ENST00000447062.2 WDTC1 nonsense_mediated_decay
ENST00000447071.4 NCF4 protein_coding
ENST00000447117.1 PTENP1 transcribed_processed_pseudogene
ENST00000447121.2 CASP3 protein_coding
ENST00000447170.1 SELENOI protein_coding
ENST00000447186.1 ZNF143 protein_coding
ENST00000447215.4 IMMP2L protein_coding
ENST00000447221.1 SNHG7 lncRNA
ENST00000447246.1 LRPPRC protein_coding
ENST00000447259.2 RPL4P2 processed_pseudogene
ENST00000447267.3 SDCBP protein_coding
ENST00000447287.4 WASF1 protein_coding
ENST00000447299.1 MRPS25 nonsense_mediated_decay
ENST00000447355.1 HNRNPKP2 processed_pseudogene
ENST00000447379.1 NEK6 protein_coding
ENST00000447404.5 STRBP protein_coding
ENST00000447413.1 lncRNA
ENST00000447426.1 CPVL protein_coding
ENST00000447430.1 LINC00513 lncRNA
ENST00000447441.4 CBLB protein_coding
ENST00000447466.1 DLG1 protein_coding
ENST00000447473.5 IFI16 protein_coding
ENST00000447484.4 DGKD protein_coding
ENST00000447489.1 ANXA11 protein_coding
ENST00000447507.1 SNHG3 retained_intron
ENST00000447558.1 GCC2 processed_transcript
ENST00000447585.1 OFD1P5Y unprocessed_pseudogene
ENST00000447596.4 AK3 protein_coding
ENST00000447620.4 JAZF1 protein_coding
ENST00000447633.1 PSMD1 retained_intron
ENST00000447643.1 lncRNA
ENST00000447645.4 PLEKHM3 protein_coding
ENST00000447667.2 WNK1 protein_coding
ENST00000447673.1 SLC35F5 protein_coding
ENST00000447683.2 OTUB1 retained_intron
ENST00000447713.1 ANKRD44 nonsense_mediated_decay
ENST00000447718.1 EEF1B2P3 processed_pseudogene
ENST00000447745.4 CTDSPL protein_coding
ENST00000447751.4 GDI2 protein_coding
ENST00000447759.4 KANSL3 nonsense_mediated_decay
ENST00000447833.1 PDS5B protein_coding
ENST00000447845.1 NA NA
ENST00000447858.1 LY86-AS1 lncRNA
ENST00000447906.5 OTUD4 protein_coding
ENST00000447935.2 CTNNBL1 protein_coding
ENST00000447964.1 TOR1AIP1 protein_coding
ENST00000447995.2 PTPN12 nonsense_mediated_decay
ENST00000448008.1 NCOR2 protein_coding
ENST00000448011.3 NPIPP1 processed_transcript
ENST00000448020.1 CHRM3 protein_coding
ENST00000448023.5 NA NA
ENST00000448025.4 AGAP1 protein_coding

ENST00000448034.4 TRIM33 protein_coding
ENST00000448038.2 SYNE1 retained_intron
ENST00000448045.1 ANO10 protein_coding
ENST00000448096.1 lncRNA
ENST00000448097.5 KDM6B protein_coding
ENST00000448156.3 OSBPL2 protein_coding
ENST00000448188.1 SNORD57 snoRNA
ENST00000448193.4 WAC protein_coding
ENST00000448217.2 SCAP protein_coding
ENST00000448238.2 NIPBL protein_coding
ENST00000448246.1 SP4 nonsense_mediated_decay
ENST00000448281.5 TATDN2 protein_coding
ENST00000448287.4 AGPAT3 protein_coding
ENST00000448301.5 CTSS protein_coding
ENST00000448331.6 nonsense_mediated_decay
ENST00000448348.3 STX7 processed_transcript
ENST00000448368.4 LARP4B protein_coding
ENST00000448387.5 GOLGA8N protein_coding
ENST00000448389.1 lncRNA
ENST00000448393.5 IFI16 protein_coding
ENST00000448408.4 ST6GAL1 protein_coding
ENST00000448413.4 SUMF1 nonsense_mediated_decay
ENST00000448420.4 MYADM protein_coding
ENST00000448427.1 MRPL33 nonsense_mediated_decay
ENST00000448463.1 lncRNA
ENST00000448464.5 TP53INP1 protein_coding
ENST00000448504.5 ARSG protein_coding
ENST00000448530.4 VCP protein_coding
ENST00000448549.1 TRA2A nonsense_mediated_decay
ENST00000448576.5 GATAD2A processed_transcript
ENST00000448614.1 NCOR2 protein_coding
ENST00000448692.4 CA5BP1 processed_transcript
ENST00000448723.1 ADAM12 protein_coding
ENST00000448732.1 R3HDM2 protein_coding
ENST00000448790.5 TOX4 protein_coding
ENST00000448801.4 NA NA
ENST00000448833.4 VPS41 nonsense_mediated_decay
ENST00000448841.4 WBP1L protein_coding
ENST00000448850.4 APP protein_coding
ENST00000448879.4 SEMA3A protein_coding
ENST00000448880.4 NDUFA10 protein_coding
ENST00000448912.1 RPL27AP3 processed_pseudogene
ENST00000448926.4 LMBR1 nonsense_mediated_decay
ENST00000448947.2 C6orf223 lncRNA
ENST00000448959.4 CPVL protein_coding
ENST00000448962.4 RPS9 nonsense_mediated_decay
ENST00000448993.1 SNORC protein_coding
ENST00000449017.4 MIATNB lncRNA
ENST00000449061.2 GUSBP1 transcribed_unprocessed_pseudogene
ENST00000449062.1 RTF2 protein_coding
ENST00000449064.4 KCTD7 protein_coding
ENST00000449068.1 SLC1A2 protein_coding
ENST00000449081.5 KCTD20 protein_coding
ENST00000449089.5 FNBP1 protein_coding

ENST00000449098.4 NA NA
ENST00000449111.4 LINC00894 lncRNA
ENST00000449120.1 PI4KA protein_coding
ENST00000449131.5 BEST1 protein_coding
ENST00000449141.2 DSTN nonsense_mediated_decay
ENST00000449143.2 lncRNA
ENST00000449152.1 STK17B protein_coding
ENST00000449165.4 C2CD2 protein_coding
ENST00000449169.1 RBM15-AS1 lncRNA
ENST00000449186.2 RPRD1B protein_coding
ENST00000449189.1 PKN2 protein_coding
ENST00000449190.4 RFX3 protein_coding
ENST00000449191.1 UBE2F-SCLY nonsense_mediated_decay
ENST00000449197.1 ENTPD1-AS1 lncRNA
ENST00000449224.1 CRTAP protein_coding
ENST00000449251.1 KIAA1109 protein_coding
ENST00000449264.2 TNF protein_coding
ENST00000449284.1 MBNL2 protein_coding
ENST00000449305.4 CCDC18-AS1 lncRNA
ENST00000449306.1 PARL protein_coding
ENST00000449307.3 processed_transcript
ENST00000449311.4 CAP1 protein_coding
ENST00000449337.5 RORA protein_coding
ENST00000449370.5 MAN1A2 protein_coding
ENST00000449413.1 HLA-DRB9 unprocessed_pseudogene
ENST00000449440.4 LRRC8B protein_coding
ENST00000449469.4 SNHG17 retained_intron
ENST00000449491.1 USP25 protein_coding
ENST00000449543.4 MTMR14 nonsense_mediated_decay
ENST00000449545.2 TIAM2 processed_transcript
ENST00000449565.1 EAF1 nonsense_mediated_decay
ENST00000449611.4 CD99 processed_transcript
ENST00000449612.2 ACOT9 retained_intron
ENST00000449648.1 PRKCQ-AS1 lncRNA
ENST00000449649.1 RALB protein_coding
ENST00000449651.4 ANTXR2 nonsense_mediated_decay
ENST00000449684.5 LIMS1 processed_transcript
ENST00000449699.4 NDUFS1 protein_coding
ENST00000449714.1 MMADHC-DT lncRNA
ENST00000449732.5 CDYL protein_coding
ENST00000449768.2 OAS2 protein_coding
ENST00000449779.4 GSAP retained_intron
ENST00000449801.4 CPVL protein_coding
ENST00000449802.4 NBEAL1 protein_coding
ENST00000449812.2 LINC01358 lncRNA
ENST00000449821.1 TTI1 protein_coding
ENST00000449842.1 LINC01344 lncRNA
ENST00000449850.1 KDM1B protein_coding
ENST00000449869.1 lncRNA
ENST00000449909.6 DMXL2 protein_coding
ENST00000449920.4 POR protein_coding
ENST00000449930.4 POGK protein_coding
ENST00000449949.4 ZBTB21 protein_coding
ENST00000449959.5 CLEC12A retained_intron

ENST00000449975.1 CLASP1 protein_coding
ENST00000449985.5 TMEM107 protein_coding
ENST00000449987.5 RBM26 protein_coding
ENST00000449997.1 DBNL retained_intron
ENST00000450019.4 TANGO2 nonsense_mediated_decay
ENST00000450022.1 TPST2 protein_coding
ENST00000450053.6 NBEAL2 protein_coding
ENST00000450074.4 SEC23B protein_coding
ENST00000450116.5 RPS21 protein_coding
ENST00000450121.4 ABCG1 protein_coding
ENST00000450123.5 BCL6 protein_coding
ENST00000450176.1 KIF16B protein_coding
ENST00000450180.4 GPR141 protein_coding
ENST00000450198.4 SMARCA2 protein_coding
ENST00000450208.1 transcribed_processed_pseudogene
ENST00000450235.4 MAD1L1 processed_transcript
ENST00000450253.5 EIF4E miRNA
ENST00000450273.1 BCL2L1 miRNA
ENST00000450287.2 TMEM72-AS1 miRNA
ENST00000450288.3 PDXDC1 miRNA
ENST00000450295.4 SEMA4D miRNA
ENST00000450315.6 ADGRE1 miRNA
ENST00000450349.3 NA miRNA
ENST00000450368.1 BTBD3 miRNA
ENST00000450374.1 PTBP3 miRNA
ENST00000450393.4 ABHD12 miRNA
ENST00000450402.1 SPSB1 miRNA
ENST00000450411.1 ILKAP miRNA
ENST00000450436.1 SEPTIN7P2 miRNA
ENST00000450439.4 CR1 miRNA
ENST00000450443.1 miRNA
ENST00000450446.5 FAM168A miRNA
ENST00000450460.4 PDS5B miRNA
ENST00000450482.4 SS18L1 miRNA
ENST00000450485.5 KNTC1 miRNA
ENST00000450494.1 SLC7A1 miRNA
ENST00000450498.1 IFI44L miRNA
ENST00000450508.1 UHRF2 miRNA
ENST00000450548.4 DCAF6 miRNA
ENST00000450551.1 miRNA
ENST00000450574.4 PHTF2 miRNA
ENST00000450599.5 DDX5 miRNA
ENST00000450625.1 SH3BP5 miRNA
ENST00000450644.1 AP1S2 miRNA
ENST00000450657.1 ZNF277 miRNA
ENST00000450670.4 TRIM22 miRNA
ENST00000450673.3 ARL17B miRNA
ENST00000450676.1 FTH1P7 miRNA
ENST00000450686.1 LINC01004 miRNA
ENST00000450737.2 PARP11 miRNA
ENST00000450787.1 SFI1 miRNA
ENST00000450791.5 GOLGA3 miRNA
ENST00000450820.2 RSRP1 miRNA
ENST00000450835.1 GLB1 miRNA

ENST00000450858.1 ZBTB43 miRNA
ENST00000450863.5 GOLGA4 miRNA
ENST00000450898.1 SATB1 miRNA
ENST00000450932.2 SLF1 miRNA
ENST00000450941.1 RPL26P19 miRNA
ENST00000450957.1 SAP130 miRNA
ENST00000450969.4 CTNNB1 miRNA
ENST00000450980.1 miRNA
ENST00000451018.6 MCTP2 miRNA
ENST00000451053.2 VPS33A miRNA
ENST00000451058.4 MED15 miRNA
ENST00000451061.4 OFD1P6Y miRNA
ENST00000451065.1 IL10RB miRNA
ENST00000451078.4 MIDEAS miRNA
ENST00000451127.2 TEX22 miRNA
ENST00000451155.1 RARS2 miRNA
ENST00000451156.1 TRPS1 miRNA
ENST00000451158.4 ZKSCAN5 miRNA
ENST00000451167.1 ZNF487 miRNA
ENST00000451241.2 ATXN10 miRNA
ENST00000451244.1 KLF7 miRNA
ENST00000451259.1 ARHGAP26 miRNA
ENST00000451310.1 SEPTIN2 miRNA
ENST00000451331.1 SOS1 miRNA
ENST00000451391.4 IDH1 miRNA
ENST00000451407.3 INPP5D miRNA
ENST00000451419.1 ACAA1 miRNA
ENST00000451442.4 APMAP miRNA
ENST00000451443.1 PLCB1-IT1 miRNA
ENST00000451469.1 EEF1A1P24 miRNA
ENST00000451486.4 miRNA
ENST00000451498.2 ZAP70 miRNA
ENST00000451528.2 ST8SIA4 miRNA
ENST00000451531.5 POTEI miRNA
ENST00000451560.1 TMEM38B miRNA
ENST00000451561.1 SPAG16 miRNA
ENST00000451579.1 LINC02248 miRNA
ENST00000451583.1 NBDY miRNA
ENST00000451599.5 ITPR2 miRNA
ENST00000451608.2 miRNA
ENST00000451619.1 F13A1 miRNA
ENST00000451653.4 SEC22C miRNA
ENST00000451674.5 VGLL4 miRNA
ENST00000451680.1 MAPKAPK3 miRNA
ENST00000451692.4 NA miRNA
ENST00000451695.4 XRCC5 miRNA
ENST00000451702.2 UBA1 miRNA
ENST00000451703.4 TRAK2 miRNA
ENST00000451708.4 MITF miRNA
ENST00000451724.5 LRP1 miRNA
ENST00000451759.4 RGS5 miRNA
ENST00000451813.5 TRPC4AP miRNA
ENST00000451819.4 KANSL3 miRNA
ENST00000451836.1 ITPRID2 miRNA

ENST00000451838.1 SHOC2 miRNA
ENST00000451855.1 NA miRNA
ENST00000451859.4 RFX3 miRNA
ENST00000451884.4 MIR4435-2HG miRNA
ENST00000451893.4 AOPEP miRNA
ENST00000451940.5 NUTM2A-AS1 miRNA
ENST00000451973.1 miRNA
ENST00000452057.1 KANSL1L-AS1 miRNA
ENST00000452075.6 ZNF37BP miRNA
ENST00000452080.4 TBRG1 miRNA
ENST00000452107.5 LANCL2 miRNA
ENST00000452138.2 FBXO7 miRNA
ENST00000452140.4 VPS8 miRNA
ENST00000452151.1 SLC6A6 miRNA
ENST00000452187.5 ZRANB3 miRNA
ENST00000452196.5 HIP1R miRNA
ENST00000452202.4 C2 miRNA
ENST00000452231.4 BCL2L11 miRNA
ENST00000452260.5 SATB1 miRNA
ENST00000452270.4 PHF20 miRNA
ENST00000452274.5 CLASP1 miRNA
ENST00000452313.4 NPIPB7 miRNA
ENST00000452345.1 SP100 miRNA
ENST00000452376.4 AP1S2 miRNA
ENST00000452383.2 ARAP1 miRNA
ENST00000452398.4 KCNH8 miRNA
ENST00000452400.5 NCOA2 miRNA
ENST00000452463.4 CREB3L2 miRNA
ENST00000452464.5 SCARB2 miRNA
ENST00000452474.4 CREB1 protein_coding
ENST00000452476.4 LYAR protein_coding
ENST00000452479.5 ARSG trna
ENST00000452506.1 TMLHE-AS1 trna
ENST00000452508.5 ATM trna
ENST00000452521.1 CCNT2 trna
ENST00000452532.1 trna
ENST00000452544.1 ARID1B trna
ENST00000452554.2 ANAPC1P2 trna
ENST00000452564.1 PIKFYVE trna
ENST00000452581.4 OXNAD1 trna
ENST00000452584.4 TTTY14 trna
ENST00000452614.4 TMEM87B trna
ENST00000452655.5 RBBP6 trna
ENST00000452661.1 DBNL trna
ENST00000452673.5 CANX trna
ENST00000452684.2 SOD2 trna
ENST00000452725.4 STAMBP trna
ENST00000452741.1 trna
ENST00000452749.1 KMT2C trna
ENST00000452767.1 SAR1A trna
ENST00000452775.1 SLC6A6 trna
ENST00000452786.2 IKZF2 trna
ENST00000452796.5 DCAKD trna
ENST00000452799.4 FAM126B trna

ENST00000452818.1 HIRA trna
ENST00000452824.4 TBL1X trna
ENST00000452826.2 FGF7P6 trna
ENST00000452835.1 IFI44L trna
ENST00000452844.4 MUC20-OT1 trna
ENST00000452894.4 UBE2E2 trna
ENST00000452898.2 NA trna
ENST00000452901.4 LINC01362 trna
ENST00000452906.3 HIGD1A trna
ENST00000452915.3 MCUB trna
ENST00000452941.1 CALM1P2 trna
ENST00000452978.4 DHX57 trna
ENST00000452991.1 SESTD1 trna
ENST00000453011.4 trna
ENST00000453024.4 CTNNB1 trna
ENST00000453046.4 SQSTM1 trna
ENST00000453068.1 CYP51A1-AS1 trna
ENST00000453079.1 PACSIN2 trna
ENST00000453089.2 ZC3H12C trna
ENST00000453092.3 GTF2IP4 trna
ENST00000453108.1 GNB1L trna
ENST00000453112.4 VOPP1 trna
ENST00000453116.4 MXI1 trna
ENST00000453117.1 TPST2 trna
ENST00000453133.1 USP34 trna
ENST00000453142.2 MIS18BP1 trna
ENST00000453194.1 MYH16 trna
ENST00000453225.4 STARD3NL trna
ENST00000453256.4 VOPP1 trna
ENST00000453258.5 ENTPD1 trna
ENST00000453259.2 LYSMD3 trna
ENST00000453357.5 UNC5D trna
ENST00000453359.4 IL1RAP trna
ENST00000453371.2 AGAP1 trna
ENST00000453395.4 PSLNR trna
ENST00000453401.5 NIN trna
ENST00000453412.1 RBKS trna
ENST00000453448.1 KCMF1 trna
ENST00000453452.1 HEBP2 trna
ENST00000453454.4 trna
ENST00000453463.1 DSE trna
ENST00000453478.1 MIR4435-2HG trna
ENST00000453485.1 TRIP12 trna
ENST00000453515.1 JADE2 trna
ENST00000453516.4 ARFGAP3 trna
ENST00000453517.4 trna
ENST00000453536.4 RFTN1 trna
ENST00000453553.1 USP25 trna
ENST00000453558.1 HCG17 trna
ENST00000453572.1 trna
ENST00000453628.1 TLK1 trna
ENST00000453662.5 WASH2P trna
ENST00000453665.1 trna
ENST00000453666.2 LHFPL3-AS2 trna

ENST00000453676.4 SMYD3 trna
ENST00000453677.1 KMT2E-AS1 trna
ENST00000453678.4 trna
ENST00000453734.1 USP34 trna
ENST00000453770.1 trna
ENST00000453839.4 PAPOLG trna
ENST00000453877.4 BACH2 trna
ENST00000453888.6 PARVG trna
ENST00000453892.1 MMP24OS trna
ENST00000453915.1 trna
ENST00000453922.1 IL15RA trna
ENST00000453929.5 FASTKD1 trna
ENST00000453938.1 SETD3 trna
ENST00000453980.3 MARCHF8 trna
ENST00000453989.2 TFDP1 trna
ENST00000454011.5 RHOA trna
ENST00000454041.1 JAZF1 trna
ENST00000454043.1 LINC00299 trna
ENST00000454083.1 SPATA13 trna
ENST00000454092.1 MTND4LP30 trna
ENST00000454127.2 CA5B trna
ENST00000454158.5 RELL1 trna
ENST00000454165.1 BAG6 trna
ENST00000454195.1 NDUFS1 trna
ENST00000454222.4 MTERF1 trna
ENST00000454240.1 SMARCC1 trna
ENST00000454304.5 CMTM7 trna
ENST00000454323.1 CUL3 trna
ENST00000454337.1 trna
ENST00000454358.5 NDUFAF6 trna
ENST00000454368.2 KDELR2 trna
ENST00000454383.4 ADAP1 trna
ENST00000454429.2 UBE2R2-AS1 trna
ENST00000454433.4 CAMKMT trna
ENST00000454465.1 trna
ENST00000454523.4 YY1AP1 trna
ENST00000454540.4 BBX trna
ENST00000454542.4 HDAC4 trna
ENST00000454575.4 DOCK7 trna
ENST00000454577.1 TPTEP2 trna
ENST00000454583.5 RIF1 trna
ENST00000454600.1 LINC01524 trna
ENST00000454610.2 PSMG4 trna
ENST00000454618.4 STYXL1 trna
ENST00000454622.2 trna
ENST00000454638.4 ENTPD1-AS1 trna
ENST00000454652.5 KLHL24 trna
ENST00000454666.2 NA trna
ENST00000454681.2 trna
ENST00000454688.4 NRF1 trna
ENST00000454690.1 TTC27 trna
ENST00000454752.3 trna
ENST00000454778.4 TPST2 trna
ENST00000454806.1 WASHC2A trna

ENST00000454832.1 NA trna
ENST00000454842.2 RNF217 trna
ENST00000454869.1 trna
ENST00000454919.1 ARHGAP12 trna
ENST00000454922.5 GARS1-DT trna
ENST00000454925.1 ZNF185 trna
ENST00000454977.1 EIPR1-IT1 trna
ENST00000455022.1 UTRN trna
ENST00000455053.4 THOC2 trna
ENST00000455062.2 NCF1 trna
ENST00000455070.1 ZNFX1 trna
ENST00000455076.1 TNRC18 trna
ENST00000455083.4 CNTN4 trna
ENST00000455098.2 PDE3B trna
ENST00000455108.4 LILRB2 trna
ENST00000455119.4 ELMO1 trna
ENST00000455125.1 SUN2 trna
ENST00000455145.4 PILRB trna
ENST00000455148.1 EHD1 trna
ENST00000455177.3 PRMT2 trna
ENST00000455207.4 trna
ENST00000455208.4 DNAJB14 trna
ENST00000455219.6 LINC01122 trna
ENST00000455222.1 QSOX2 trna
ENST00000455250.1 PDIA3 trna
ENST00000455277.1 SMYD3 trna
ENST00000455281.1 XXYLT1 trna
ENST00000455301.2 CNTNAP2 trna
ENST00000455322.5 CLASP1 trna
ENST00000455338.5 NAP1L4 trna
ENST00000455353.4 TBXAS1 trna
ENST00000455357.4 trna
ENST00000455395.4 FTX trna
ENST00000455399.1 NA trna
ENST00000455411.1 JADE3 trna
ENST00000455416.1 trna
ENST00000455428.4 WIPF1 trna
ENST00000455467.4 CDC14A trna
ENST00000455500.4 NME8 trna
ENST00000455506.1 TRMO trna
ENST00000455508.1 TIAM1 trna
ENST00000455532.4 TASP1 trna
ENST00000455558.2 GRK3 trna
ENST00000455575.1 TMEM243 trna
ENST00000455576.1 RARB trna
ENST00000455580.4 PPP1CB trna
ENST00000455587.2 OGT trna
ENST00000455628.1 NA trna
ENST00000455659.1 COMMD1 trna
ENST00000455661.4 PTPRE trna
ENST00000455677.1 RAD50 trna
ENST00000455703.1 trna
ENST00000455707.1 trna
ENST00000455726.4 EWSR1 trna

ENST00000455804.1 PKMP1 trna
ENST00000455833.5 IFFO2 trna
ENST00000455835.2 MYADM-AS1 trna
ENST00000455867.4 BRWD1 trna
ENST00000455879.4 ELMO1 trna
ENST00000455891.4 CMTR1 trna
ENST00000455978.1 PPP1R21 trna
ENST00000455979.1 SAMD11 trna
ENST00000456040.1 R3HDM1 trna
ENST00000456046.1 trna
ENST00000456084.1 SYNJ1 trna
ENST00000456104.4 NUTM2A-AS1 trna
ENST00000456125.1 trna
ENST00000456149.1 ARMH3 trna
ENST00000456170.4 ERI3 trna
ENST00000456174.5 HDAC9 trna
ENST00000456178.4 DEPDC5 trna
ENST00000456197.1 TRRAP trna
ENST00000456256.5 PLCB2 trna
ENST00000456350.4 MMP24OS trna
ENST00000456357.5 BRI3 trna
ENST00000456367.4 PCDH9 trna
ENST00000456374.2 STAG3L1 trna
ENST00000456385.4 CLIP4 trna
ENST00000456390.4 CREB3L2 trna
ENST00000456424.2 trna
ENST00000456471.2 ADPGK trna
ENST00000456481.1 trna
ENST00000456483.3 ESR1 trna
ENST00000456512.1 CCDC138 trna
ENST00000456517.2 HIPK3 trna
ENST00000456575.1 ARPC2 trna
ENST00000456581.1 C10orf143 trna
ENST00000456584.4 RABGAP1 trna
ENST00000456586.4 RPL37A trna
ENST00000456594.1 AGFG1 trna
ENST00000456600.1 EIF6 trna
ENST00000456601.1 trna
ENST00000456638.2 ZSWIM8-AS1 trna
ENST00000456642.1 PPP6C trna
ENST00000456650.6 GLIPR1 trna
ENST00000456682.1 KCMF1 protein_coding
ENST00000456723.1 SELENOTP1 processed_pseudogene
ENST00000456729.1 MYH9 protein_coding
ENST00000456782.1 DISC1-IT1 lncRNA
ENST00000456790.1 MMP24OS protein_coding
ENST00000456818.4 TUBA4A protein_coding
ENST00000456820.2 IST1 protein_coding
ENST00000456827.4 LIPA protein_coding
ENST00000456866.2 STAU1 protein_coding
ENST00000456903.7 ATF7 protein_coding
ENST00000456928.2 HSPA8P5 processed_pseudogene
ENST00000456966.1 ETS2 protein_coding
ENST00000456990.1 THEMIS2 protein_coding

ENST00000456993.4 ERCC6L2 nonsense_mediated_decay
ENST00000457021.1 GALNTL6 processed_transcript
ENST00000457054.5 PDCD6IP protein_coding
ENST00000457055.1 VPS41 protein_coding
ENST00000457064.1 MAPKAPK3 protein_coding
ENST00000457077.1 RAB43 protein_coding
ENST00000457080.4 NA NA
ENST00000457085.1 SMS protein_coding
ENST00000457105.2 FBXW4 processed_transcript
ENST00000457116.1 lncRNA
ENST00000457131.1 RPL32 protein_coding
ENST00000457135.1 ARID2 protein_coding
ENST00000457145.4 RPS6KA3 protein_coding
ENST00000457152.3 ARID3A nonsense_mediated_decay
ENST00000457184.4 EPC2 protein_coding
ENST00000457186.2 TAX1BP1 protein_coding
ENST00000457197.2 IRAK3 protein_coding
ENST00000457206.1 PLEKHM3 protein_coding
ENST00000457226.1 SMARCA2 protein_coding
ENST00000457227.2 JAKMIP1 processed_transcript
ENST00000457242.1 LPP protein_coding
ENST00000457254.4 COX19 nonsense_mediated_decay
ENST00000457268.4 TFEC protein_coding
ENST00000457296.4 FGR protein_coding
ENST00000457312.1 SF3B4 protein_coding
ENST00000457316.4 GLS protein_coding
ENST00000457331.1 ZKSCAN7-AS1 lncRNA
ENST00000457332.1 EIF2S3 processed_transcript
ENST00000457352.3 NSUN5P2 transcribed_unprocessed_pseudogene
ENST00000457361.4 IKZF2 protein_coding
ENST00000457368.2 NEDD1 protein_coding
ENST00000457389.5 ZKSCAN8 protein_coding
ENST00000457407.1 lncRNA
ENST00000457423.1 RPS15AP1 processed_pseudogene
ENST00000457437.1 CRYBG1 protein_coding
ENST00000457444.4 PSMA2 nonsense_mediated_decay
ENST00000457496.4 BBX protein_coding
ENST00000457540.1 MTND2P28 unprocessed_pseudogene
ENST00000457554.1 processed_pseudogene
ENST00000457571.1 ERI3 protein_coding
ENST00000457586.1 PRNP protein_coding
ENST00000457599.5 ARID1A protein_coding
ENST00000457607.1 DHX30 protein_coding
ENST00000457622.1 ACTG1P9 processed_pseudogene
ENST00000457653.6 ADCY10P1 transcribed_unprocessed_pseudogene
ENST00000457662.2 TRIM13 protein_coding
ENST00000457666.1 UBAC2 protein_coding
ENST00000457696.1 RABGAP1L protein_coding
ENST00000457715.1 TBPL1 protein_coding
ENST00000457718.4 TNS3 protein_coding
ENST00000457722.5 NECAP2 protein_coding
ENST00000457753.4 UBA1 protein_coding
ENST00000457761.5 HAT1 nonsense_mediated_decay
ENST00000457768.4 CAPZB processed_transcript

ENST00000457782.4 ZNF589 nonsense_mediated_decay
ENST00000457811.1 RAD23B protein_coding
ENST00000457830.1 GRM8 protein_coding
ENST00000457831.1 XRCC6P2 processed_pseudogene
ENST00000457843.1 NALCN-AS1 lncRNA
ENST00000457855.1 BRPF1 protein_coding
ENST00000457889.1 CDC42EP3 protein_coding
ENST00000457898.1 LINC01772 lncRNA
ENST00000457905.6 DIP2A protein_coding
ENST00000457914.4 PRKAR2A protein_coding
ENST00000457942.1 CHCHD3 processed_transcript
ENST00000457946.1 ZMYM4 protein_coding
ENST00000457962.4 KLF7 protein_coding
ENST00000457976.1 lncRNA
ENST00000457986.4 NA NA
ENST00000458001.2 GAS6-AS1 lncRNA
ENST00000458007.2 lncRNA
ENST00000458013.5 SNX27 protein_coding
ENST00000458015.1 SLX9 protein_coding
ENST00000458069.4 IRF1 protein_coding
ENST00000458097.4 PKN2-AS1 lncRNA
ENST00000458113.5 NCOR1 retained_intron
ENST00000458143.5 ZNF746 protein_coding
ENST00000458166.5 KCNAB2 protein_coding
ENST00000458173.3 TACC3 protein_coding
ENST00000458187.4 SYS1-DBNDD2 nonsense_mediated_decay
ENST00000458207.4 PPIEL processed_transcript
ENST00000458212.1 AGFG1 protein_coding
ENST00000458219.1 AIG1 protein_coding
ENST00000458220.1 GAS5 retained_intron
ENST00000458222.4 PYHIN1 protein_coding
ENST00000458269.5 STRADB protein_coding
ENST00000458270.2 ZNF623 protein_coding
ENST00000458283.4 PCCA protein_coding
ENST00000458317.5 TNS3 protein_coding
ENST00000458332.1 RPS2P46 processed_pseudogene
ENST00000458337.4 COMMD1 protein_coding
ENST00000458341.1 SP140L protein_coding
ENST00000458358.4 ITGA6 protein_coding
ENST00000458377.1 SNED1-AS1 lncRNA
ENST00000458405.4 CPVL protein_coding
ENST00000458455.1 RPL11 protein_coding
ENST00000458510.1 ATAD2B protein_coding
ENST00000458535.5 CMTM8 protein_coding
ENST00000458550.4 NA NA
ENST00000458551.1 SENP2 nonsense_mediated_decay
ENST00000458552.1 EXOC6 protein_coding
ENST00000458603.1 NFE2L2 protein_coding
ENST00000458608.1 DOCK10 nonsense_mediated_decay
ENST00000458610.5 CD28 protein_coding
ENST00000458659.2 TSC22D1 protein_coding
ENST00000458669.1 PKD1P1 unprocessed_pseudogene
ENST00000458683.1 LINC01036 lncRNA
ENST00000458691.1 EIF4A2P4 processed_pseudogene

ENST00000458700.4 STAG2 protein_coding
ENST00000458707.1 VPS50 protein_coding
ENST00000458721.4 VPS8 nonsense_mediated_decay
ENST00000458722.4 TBXAS1 protein_coding
ENST00000458723.1 ITPRIP protein_coding
ENST00000458748.1 SCARNA2 scaRNA
ENST00000458762.1 snoRNA
ENST00000458763.2 ZZZ3 processed_transcript
ENST00000458770.1 SNORD105B snoRNA
ENST00000458790.1 SNORA13 snoRNA
ENST00000458797.1 SCARNA7 scaRNA
ENST00000458811.1 RNU7-1 snRNA
ENST00000458838.1 SNORD121B snoRNA
ENST00000458862.1 SNORA47 snoRNA
ENST00000458892.1 SNORD127 snoRNA
ENST00000458893.1 SNORD42B snoRNA
ENST00000458902.1 Y_RNA misc_RNA
ENST00000458909.1 RNA5SP246 rRNA_pseudogene
ENST00000458961.1 SNORD109B snoRNA
ENST00000458981.1 Y_RNA misc_RNA
ENST00000459004.1 SCARNA18 snoRNA
ENST00000459006.1 Y_RNA misc_RNA
ENST00000459083.1 SNORD4B snoRNA
ENST00000459091.1 Y_RNA misc_RNA
ENST00000459124.2 SNORD11 snoRNA
ENST00000459155.1 SCARNA12 snoRNA
ENST00000459157.1 snoRNA
ENST00000459159.1 SNORD54 snoRNA
ENST00000459163.1 SNORD2 snoRNA
ENST00000459174.1 SNORD4A snoRNA
ENST00000459187.1 SNORD97 snoRNA
ENST00000459189.1 Y_RNA misc_RNA
ENST00000459229.1 SNORA84 snoRNA
ENST00000459249.1 RNU6ATAC28P snRNA
ENST00000459254.1 RNY4P25 misc_RNA
ENST00000459255.1 SCARNA10 snoRNA
ENST00000459274.1 RNU1-88P snRNA
ENST00000459286.1 RN7SKP198 misc_RNA
ENST00000459299.1 SNORD13 snoRNA
ENST00000459326.1 SNORA59A snoRNA
ENST00000459342.1 SNORD5 snoRNA
ENST00000459373.1 SNORA78 snoRNA
ENST00000459386.1 SNORD121A snoRNA
ENST00000459421.1 MIR2110 miRNA
ENST00000459424.1 Y_RNA misc_RNA
ENST00000459433.1 SNORD107 snoRNA
ENST00000459475.1 SNORD126 snoRNA
ENST00000459492.1 SNORD13P3 snoRNA
ENST00000459503.1 RNA5SP103 rRNA_pseudogene
ENST00000459538.1 SNORD125 snoRNA
ENST00000459548.1 MIR1976 miRNA
ENST00000459577.1 SNORD124 snoRNA
ENST00000459579.1 SNORD10 snoRNA
ENST00000459584.1 SNORD42A snoRNA

ENST00000459623.1 SNORD19B snoRNA
ENST00000459626.1 EXOC4 processed_transcript
ENST00000459627.1 SH3BP5 processed_transcript
ENST00000459629.1 AVL9 processed_transcript
ENST00000459689.1 SUPT3H processed_transcript
ENST00000459690.4 PPM1B processed_transcript
ENST00000459723.1 CLEC16A protein_coding
ENST00000459725.1 RPL13AP20 processed_pseudogene
ENST00000459731.1 MAD1L1 processed_transcript
ENST00000459735.4 PTBP2 retained_intron
ENST00000459738.4 ADD3 processed_transcript
ENST00000459742.1 lncRNA
ENST00000459744.4 COP1 protein_coding
ENST00000459747.1 MBNL1 protein_coding
ENST00000459760.1 OGT processed_transcript
ENST00000459773.1 SETDB1 processed_transcript
ENST00000459787.1 GYPC processed_transcript
ENST00000459813.1 KIDINS220 protein_coding
ENST00000459824.1 CABIN1 retained_intron
ENST00000459857.4 PRR5 processed_transcript
ENST00000459859.1 RPL3 processed_transcript
ENST00000459864.1 PLD5 protein_coding
ENST00000459872.1 GCNT2 processed_transcript
ENST00000459880.1 NDUFA5 retained_intron
ENST00000459897.1 LPP processed_transcript
ENST00000459901.4 ANKMY1 retained_intron
ENST00000459916.1 ASB3 processed_transcript
ENST00000459929.4 IVNS1ABP processed_transcript
ENST00000459937.4 CYREN processed_transcript
ENST00000459960.1 MYH9 retained_intron
ENST00000459971.1 IL17RA retained_intron
ENST00000459988.1 UBAP2 retained_intron
ENST00000459993.4 TASOR retained_intron
ENST00000459994.2 SDF4 processed_transcript
ENST00000460006.4 CDS2 protein_coding
ENST00000460017.1 STK40 processed_transcript
ENST00000460019.1 DPYD processed_transcript
ENST00000460027.1 PDE4DIP processed_transcript
ENST00000460035.1 CCDC12 retained_intron
ENST00000460053.1 SPOCK2 processed_transcript
ENST00000460056.5 RXFP1 protein_coding
ENST00000460059.1 TAX1BP1 processed_transcript
ENST00000460072.4 THUMPD2 retained_intron
ENST00000460085.1 ATP6V1E1 processed_transcript
ENST00000460090.4 CCDC125 processed_transcript
ENST00000460109.4 AHCYL2 protein_coding
ENST00000460138.4 TXNDC5 retained_intron
ENST00000460140.4 CASP10 retained_intron
ENST00000460143.1 KYNU retained_intron
ENST00000460145.2 WDFY2 processed_transcript
ENST00000460162.1 PTPN4 processed_transcript
ENST00000460166.1 MBNL1 processed_transcript
ENST00000460168.1 HERC4 retained_intron
ENST00000460179.1 NPL processed_transcript

ENST00000460184.2 STYXL1 processed_transcript
ENST00000460190.1 LCP1 processed_transcript
ENST00000460191.1 DNTTIP2 processed_transcript
ENST00000460218.4 CALM2 retained_intron
ENST00000460219.2 MTCH1 protein_coding
ENST00000460231.4 PCNP nonsense_mediated_decay
ENST00000460236.4 PHF20L1 nonsense_mediated_decay
ENST00000460256.1 KANSL1L processed_transcript
ENST00000460260.1 SERPINB1 processed_transcript
ENST00000460276.4 NCOR1 nonsense_mediated_decay
ENST00000460278.4 ANKRD28 processed_transcript
ENST00000460284.4 XRCC5 retained_intron
ENST00000460295.1 MYD88 retained_intron
ENST00000460309.1 CLEC2D protein_coding
ENST00000460333.4 VPS13D retained_intron
ENST00000460346.4 EXOC4 processed_transcript
ENST00000460355.1 NBEAL1 retained_intron
ENST00000460369.2 SLC2A1 protein_coding
ENST00000460378.1 EPB41 retained_intron
ENST00000460403.1 POMP protein_coding
ENST00000460424.4 ACAA1 retained_intron
ENST00000460428.4 SFPQ nonsense_mediated_decay
ENST00000460439.4 ANXA4 processed_transcript
ENST00000460443.4 CEPT1 processed_transcript
ENST00000460450.1 TTL processed_transcript
ENST00000460454.1 TRIM22 retained_intron
ENST00000460462.1 RAP2C processed_transcript
ENST00000460468.4 ANK3 protein_coding
ENST00000460469.1 NMD3 protein_coding
ENST00000460470.1 DDX50 processed_transcript
ENST00000460488.4 MICU2 processed_transcript
ENST00000460490.1 FKBP1A processed_transcript
ENST00000460495.4 ARMC8 retained_intron
ENST00000460510.4 FAS processed_transcript
ENST00000460536.1 SZT2 retained_intron
ENST00000460537.3 RN7SL151P misc_RNA
ENST00000460540.1 IP6K1 protein_coding
ENST00000460562.4 UBAC2 processed_transcript
ENST00000460566.4 PITPNB retained_intron
ENST00000460567.4 SUCLG2 protein_coding
ENST00000460589.4 RPL3 retained_intron
ENST00000460604.1 HIVEP3 processed_transcript
ENST00000460610.1 RAF1 retained_intron
ENST00000460613.4 TPRG1 processed_transcript
ENST00000460618.1 UTRN processed_transcript
ENST00000460639.2 ATRX retained_intron
ENST00000460646.4 LRRFIP2 retained_intron
ENST00000460650.4 SELL processed_transcript
ENST00000460659.4 RERE processed_transcript
ENST00000460660.1 BZW1 retained_intron
ENST00000460671.1 OMA1 processed_transcript
ENST00000460672.4 P2RY8 protein_coding
ENST00000460682.1 RPL37P6 transcribed_processed_pseudogene
ENST00000460683.1 PARP14 nonsense_mediated_decay

ENST00000460690.4 NPL processed_transcript
ENST00000460692.2 TIAM2 processed_transcript
ENST00000460706.4 PTBP2 retained_intron
ENST00000460724.1 PRPSAP2 protein_coding
ENST00000460736.1 FOSL2 processed_transcript
ENST00000460740.1 NDUFV3 processed_transcript
ENST00000460744.1 CD96 protein_coding
ENST00000460749.1 NT5DC1 nonsense_mediated_decay
ENST00000460761.1 RPS9 retained_intron
ENST00000460768.1 MGAT4A processed_transcript
ENST00000460781.1 CCDC93 processed_transcript
ENST00000460786.1 MBOAT2 processed_transcript
ENST00000460796.1 retained_intron
ENST00000460805.4 FOXP1 retained_intron
ENST00000460818.1 TBC1D15 processed_transcript
ENST00000460822.1 HPS3 nonsense_mediated_decay
ENST00000460828.4 PCMT1 nonsense_mediated_decay
ENST00000460842.4 TSC22D1 processed_transcript
ENST00000460845.4 ZC3HAV1 protein_coding
ENST00000460846.1 ANKRD10 processed_transcript
ENST00000460874.5 MTAP protein_coding
ENST00000460889.4 HCP5 lncRNA
ENST00000460897.1 GLCCI1 retained_intron
ENST00000460910.4 NKTR processed_transcript
ENST00000460913.1 CDK19 processed_transcript
ENST00000460933.4 RYK protein_coding
ENST00000460950.1 PRDX6 processed_transcript
ENST00000460961.1 CFLAR retained_intron
ENST00000460976.4 CTBP2 processed_transcript
ENST00000460977.1 lncRNA
ENST00000460979.2 FOXK1 protein_coding
ENST00000460983.1 KCTD20 protein_coding
ENST00000461000.1 STK39 processed_transcript
ENST00000461010.4 PPP2R5D protein_coding
ENST00000461021.1 EIF4A2 retained_intron
ENST00000461022.1 RPS3AP21 processed_pseudogene
ENST00000461048.4 SEPTIN2 processed_transcript
ENST00000461055.2 NEDD9 processed_transcript
ENST00000461082.1 GRAP2 retained_intron
ENST00000461102.1 COQ4 processed_transcript
ENST00000461113.4 HDAC4 retained_intron
ENST00000461124.1 KLF6 processed_transcript
ENST00000461133.6 CLASP2 protein_coding
ENST00000461139.4 TKT processed_transcript
ENST00000461147.1 RALGAPB retained_intron
ENST00000461153.5 ANKRD36 protein_coding
ENST00000461161.4 AHCYL2 processed_transcript
ENST00000461167.1 NIBAN1 processed_transcript
ENST00000461203.4 IRF1-AS1 retained_intron
ENST00000461204.1 ABHD12 nonsense_mediated_decay
ENST00000461206.1 ST3GAL5 processed_transcript
ENST00000461211.4 NSFL1C processed_transcript
ENST00000461212.4 INTS7 retained_intron
ENST00000461236.1 UHRF2 processed_transcript

ENST00000461237.4 EVI2A protein_coding
ENST00000461278.1 ATG7 retained_intron
ENST00000461298.1 FCGR2A processed_transcript
ENST00000461312.4 KIAA0319L processed_transcript
ENST00000461314.4 ARHGAP26 processed_transcript
ENST00000461325.1 STAB1 retained_intron
ENST00000461354.4 SLMAP retained_intron
ENST00000461364.1 SMIM7 processed_transcript
ENST00000461371.4 CYP20A1 processed_transcript
ENST00000461375.1 ST3GAL3 processed_transcript
ENST00000461383.1 VCL processed_transcript
ENST00000461400.1 GCNT2 retained_intron
ENST00000461405.1 PHF20 retained_intron
ENST00000461409.1 HDAC9 processed_transcript
ENST00000461423.1 RALGAPB retained_intron
ENST00000461436.1 MBNL1 processed_transcript
ENST00000461440.4 TES retained_intron
ENST00000461447.1 GLUL processed_transcript
ENST00000461449.4 DRAM2 processed_transcript
ENST00000461472.1 GATA3 processed_transcript
ENST00000461481.4 TAB2 processed_transcript
ENST00000461491.4 NGLY1 processed_transcript
ENST00000461502.1 lncRNA
ENST00000461526.1 LYST processed_transcript
ENST00000461529.1 PRKAG2 processed_transcript
ENST00000461540.2 CCT4 processed_transcript
ENST00000461541.4 P2RY10 processed_transcript
ENST00000461545.1 SOS1 processed_transcript
ENST00000461549.1 DCAF10 processed_transcript
ENST00000461582.1 MARCHF7 processed_transcript
ENST00000461601.4 TTC7A processed_transcript
ENST00000461610.4 GPR141 processed_transcript
ENST00000461612.1 PRPF4B processed_transcript
ENST00000461613.1 RABGAP1L retained_intron
ENST00000461622.1 GUSB retained_intron
ENST00000461627.1 ARRDC1 nonsense_mediated_decay
ENST00000461629.1 IL1RAP retained_intron
ENST00000461645.1 OGA processed_transcript
ENST00000461650.1 STAMBPL1 processed_transcript
ENST00000461651.1 TBCE processed_transcript
ENST00000461654.1 TMEM39A protein_coding
ENST00000461660.1 ATP13A3 retained_intron
ENST00000461661.1 ITCH nonsense_mediated_decay
ENST00000461669.4 ALG13 processed_transcript
ENST00000461673.1 UFL1 processed_transcript
ENST00000461680.1 RAD54L2 retained_intron
ENST00000461689.4 NEK4 protein_coding
ENST00000461696.1 ANKRD28 processed_transcript
ENST00000461705.1 ZNF512 retained_intron
ENST00000461715.1 TMEM164 processed_transcript
ENST00000461724.4 NFKBIZ protein_coding
ENST00000461739.1 ARHGAP4 retained_intron
ENST00000461758.5 TLE4 nonsense_mediated_decay
ENST00000461777.1 MOSPD2 retained_intron

ENST00000461780.1 PPARGC1B processed_transcript
ENST00000461790.1 RCSD1 processed_transcript
ENST00000461791.1 NRROS retained_intron
ENST00000461813.4 KLHL24 processed_transcript
ENST00000461814.1 processed_pseudogene
ENST00000461821.1 ZBTB11 protein_coding
ENST00000461830.5 COP1 retained_intron
ENST00000461832.1 SNHG12 retained_intron
ENST00000461849.1 RBM39 retained_intron
ENST00000461872.5 SYNE1 retained_intron
ENST00000461878.4 CAPZA2 retained_intron
ENST00000461893.4 GNB1 retained_intron
ENST00000461904.1 GTF2F2 processed_transcript
ENST00000461909.1 RAB2B processed_transcript
ENST00000461918.2 APLP2 retained_intron
ENST00000461919.4 DNAJB12 protein_coding
ENST00000461933.1 SEC62 processed_transcript
ENST00000461940.4 GIMAP4 protein_coding
ENST00000461945.1 CARMIL1 protein_coding
ENST00000461948.2 DHRS12 protein_coding
ENST00000461952.1 RPL5 retained_intron
ENST00000461955.1 ATG2A retained_intron
ENST00000461958.2 ZNF746 protein_coding
ENST00000461963.1 HBP1 processed_transcript
ENST00000461965.4 GLS retained_intron
ENST00000461972.4 FAM228B processed_transcript
ENST00000461981.4 CLK1 retained_intron
ENST00000461985.1 CDA processed_transcript
ENST00000461987.1 DAG1 processed_transcript
ENST00000461993.1 CAP1 processed_transcript
ENST00000462000.1 OLA1 retained_intron
ENST00000462003.4 RAB10 processed_transcript
ENST00000462025.1 RBM5 retained_intron
ENST00000462032.1 EPB41 retained_intron
ENST00000462034.1 ARPC2 processed_transcript
ENST00000462042.4 RABGGTB processed_transcript
ENST00000462052.1 KDELR2 retained_intron
ENST00000462055.4 EXOC4 retained_intron
ENST00000462067.1 MRPS15 processed_transcript
ENST00000462074.1 PPP1R12B processed_transcript
ENST00000462075.1 ZNF33B protein_coding
ENST00000462101.1 CEP85L processed_transcript
ENST00000462103.1 MACF1 processed_transcript
ENST00000462122.1 TRAF3IP1 retained_intron
ENST00000462139.1 ZNF496 processed_transcript
ENST00000462143.4 RGS3 protein_coding
ENST00000462159.1 SASS6 processed_transcript
ENST00000462160.4 STK32C processed_transcript
ENST00000462163.1 RPN2 nonsense_mediated_decay
ENST00000462171.1 TMCO4 processed_transcript
ENST00000462185.1 THADA processed_transcript
ENST00000462201.1 IKZF1 retained_intron
ENST00000462202.2 TRIM8 protein_coding
ENST00000462207.1 PBRM1 retained_intron

ENST00000462214.4 ADARB1 processed_transcript
ENST00000462226.1 SPIRE1 protein_coding
ENST00000462235.4 STT3B processed_transcript
ENST00000462245.1 DENND1A processed_transcript
ENST00000462262.1 BACH1 processed_transcript
ENST00000462266.1 GRB2 processed_transcript
ENST00000462275.4 TBXAS1 processed_transcript
ENST00000462276.1 RTF1 processed_transcript
ENST00000462294.1 PIK3CB protein_coding
ENST00000462296.4 RPL22 protein_coding
ENST00000462315.4 PARP9 protein_coding
ENST00000462321.1 PTPN18 retained_intron
ENST00000462326.4 VOPP1 processed_transcript
ENST00000462328.2 DDX27 nonsense_mediated_decay
ENST00000462342.1 COG5 retained_intron
ENST00000462355.2 ZEB2 protein_coding
ENST00000462358.1 ADSS2 processed_transcript
ENST00000462363.5 PTPRC retained_intron
ENST00000462366.1 ROCK2 processed_transcript
ENST00000462376.1 LYST retained_intron
ENST00000462406.1 CHST15 protein_coding
ENST00000462421.1 DDX39B processed_transcript
ENST00000462444.1 GLUL processed_transcript
ENST00000462445.1 SRGN processed_transcript
ENST00000462448.1 STK17A miRNA
ENST00000462450.4 MEI1 miRNA
ENST00000462455.1 PLEKHM2 miRNA
ENST00000462507.1 FBH1 miRNA
ENST00000462513.4 CBWD1 miRNA
ENST00000462515.1 EIPR1 miRNA
ENST00000462527.2 EIF3A miRNA
ENST00000462537.3 CDKN1A miRNA
ENST00000462540.1 TMEM131L miRNA
ENST00000462544.1 ST7 miRNA
ENST00000462568.1 A2M miRNA
ENST00000462580.1 TUBGCP3 miRNA
ENST00000462590.1 FLNA miRNA
ENST00000462592.4 ERMP1 miRNA
ENST00000462598.6 FYN miRNA
ENST00000462618.1 DOCK8 miRNA
ENST00000462625.1 LIMK2 miRNA
ENST00000462628.4 LILRB1 miRNA
ENST00000462639.1 SAT1 miRNA
ENST00000462646.4 MICOS10 miRNA
ENST00000462655.1 ZC3H12D miRNA
ENST00000462668.4 SMC4 miRNA
ENST00000462694.1 PTEN miRNA
ENST00000462696.1 MBOAT2 miRNA
ENST00000462702.1 WDFY1 miRNA
ENST00000462717.1 PSMD6-AS1 miRNA
ENST00000462720.5 PRKCE miRNA
ENST00000462736.1 PRKX miRNA
ENST00000462753.4 TMEM209 miRNA
ENST00000462758.1 LPP miRNA

ENST00000462760.1 ATP5F1C miRNA
ENST00000462763.4 CFLAR miRNA
ENST00000462766.2 FNBP1 miRNA
ENST00000462776.2 S100A6 miRNA
ENST00000462777.1 HLA-F miRNA
ENST00000462810.1 SNRK miRNA
ENST00000462818.1 STAG1 miRNA
ENST00000462828.1 TFEC miRNA
ENST00000462844.4 SPIN1 miRNA
ENST00000462855.1 ATP6V1A miRNA
ENST00000462875.1 BAG6 miRNA
ENST00000462885.1 RPL18AP3 miRNA
ENST00000462906.1 PPP1R2 miRNA
ENST00000462927.4 CYTH4 miRNA
ENST00000462942.2 EHMT1 miRNA
ENST00000462951.2 S100A6 miRNA
ENST00000462957.1 PSMB1 miRNA
ENST00000462958.4 BIN1 miRNA
ENST00000462965.1 ARPC5 miRNA
ENST00000462968.2 CD46 miRNA
ENST00000462987.2 NA miRNA
ENST00000462989.5 DTNBP1 miRNA
ENST00000462994.1 OGA miRNA
ENST00000463008.1 DARS1 miRNA
ENST00000463012.1 SDCCAG8 miRNA
ENST00000463013.1 KDM3A miRNA
ENST00000463016.1 CDK19 miRNA
ENST00000463020.1 SND1 miRNA
ENST00000463021.1 PNISR miRNA
ENST00000463027.1 MYH9 miRNA
ENST00000463032.4 LYRM4 miRNA
ENST00000463033.1 HDHD5 miRNA
ENST00000463042.1 SDCCAG8 miRNA
ENST00000463044.1 STT3B miRNA
ENST00000463055.1 COX7A2L miRNA
ENST00000463056.4 ZMIZ2 miRNA
ENST00000463066.1 HLA-DPA1 miRNA
ENST00000463070.1 APP miRNA
ENST00000463074.1 EIF4E2 miRNA
ENST00000463078.1 ARPC1B miRNA
ENST00000463089.5 TIAL1 miRNA
ENST00000463091.2 PRSS46P miRNA
ENST00000463098.4 RBM39 miRNA
ENST00000463102.4 PCID2 miRNA
ENST00000463108.4 SELL miRNA
ENST00000463109.2 TDRD3 miRNA
ENST00000463133.1 POLDIP3 miRNA
ENST00000463139.4 PSMD6 miRNA
ENST00000463140.1 MAP3K5 miRNA
ENST00000463153.1 ABHD5 miRNA
ENST00000463156.1 ANKRD10 miRNA
ENST00000463163.4 TAF1 miRNA
ENST00000463172.4 HDAC1 miRNA
ENST00000463180.4 SETD5 miRNA

ENST00000463204.4 ARIH2 miRNA
ENST00000463210.5 RUFY2 miRNA
ENST00000463216.4 TTC3 miRNA
ENST00000463222.1 VRK2 miRNA
ENST00000463234.1 STXBP4 miRNA
ENST00000463236.4 NA miRNA
ENST00000463253.2 SAMD3 miRNA
ENST00000463255.1 miRNA
ENST00000463262.1 SLC25A30 miRNA
ENST00000463270.1 YME1L1 miRNA
ENST00000463276.1 RCAN1 miRNA
ENST00000463277.4 SFXN5 miRNA
ENST00000463278.1 miRNA
ENST00000463286.1 DCAF12 miRNA
ENST00000463306.1 DZIP3 miRNA
ENST00000463317.4 VAMP7 miRNA
ENST00000463328.4 TRA2B miRNA
ENST00000463334.2 ECE1 miRNA
ENST00000463340.1 ANXA11 miRNA
ENST00000463343.1 SF1 miRNA
ENST00000463347.4 SACM1L miRNA
ENST00000463358.4 ADAP1 miRNA
ENST00000463369.4 SHQ1 miRNA
ENST00000463378.4 RBBP4 miRNA
ENST00000463397.3 RN7SL674P miRNA
ENST00000463408.1 MYLK-AS1 miRNA
ENST00000463413.1 KLHDC10 miRNA
ENST00000463415.1 LCOR miRNA
ENST00000463417.4 CAPN7 miRNA
ENST00000463419.1 NIN miRNA
ENST00000463427.4 TMEM87B miRNA
ENST00000463428.4 PHACTR4 miRNA
ENST00000463431.4 TLE4 miRNA
ENST00000463434.4 GUSBP2 miRNA
ENST00000463438.4 CCDC174 miRNA
ENST00000463472.1 ODF3B miRNA
ENST00000463477.4 PTPRD miRNA
ENST00000463478.1 HERC4 retained_intron
ENST00000463496.1 AHR nonsense_mediated_decay
ENST00000463497.4 ZBTB20 trna
ENST00000463501.4 STIM2 trna
ENST00000463502.1 TANK trna
ENST00000463504.4 OSBPL10 trna
ENST00000463508.2 NA trna
ENST00000463526.1 CMSS1 trna
ENST00000463546.4 SH3BGRL trna
ENST00000463560.1 GLMN trna
ENST00000463566.1 FRY trna
ENST00000463574.1 HLA-B trna
ENST00000463586.1 PRRC2C trna
ENST00000463589.4 DNAJC25 trna
ENST00000463599.4 U2AF1 trna
ENST00000463621.3 CLASP1 trna
ENST00000463623.1 LIPA trna

ENST00000463625.5 ZCCHC7 trna
ENST00000463643.4 ZAP70 trna
ENST00000463657.1 ANXA11 trna
ENST00000463659.1 SACM1L trna
ENST00000463660.1 GBP2 trna
ENST00000463663.5 PLEKHA1 trna
ENST00000463668.1 DOP1B trna
ENST00000463681.1 AHSA2P trna
ENST00000463682.1 DRAM2 trna
ENST00000463690.1 ARL6IP6 trna
ENST00000463705.4 CHRNA4 trna
ENST00000463708.1 UQCRC1 trna
ENST00000463709.1 TM9SF2 trna
ENST00000463725.4 UMAD1 trna
ENST00000463727.1 PTPRE trna
ENST00000463743.4 MYOF trna
ENST00000463749.1 PPP1R16B trna
ENST00000463764.2 TRANK1 trna
ENST00000463775.1 EIF4G3 trna
ENST00000463779.3 RN7SL752P trna
ENST00000463786.2 DNAJB12 trna
ENST00000463828.1 RIPOR2 trna
ENST00000463836.1 RPL22L1 trna
ENST00000463839.2 ITM2B trna
ENST00000463847.1 ZFAND3 trna
ENST00000463854.4 STAM2 trna
ENST00000463857.1 TEX264 trna
ENST00000463877.1 SRSF11 trna
ENST00000463879.1 ANKRD44 trna
ENST00000463912.4 LUC7L2 trna
ENST00000463926.3 RN7SL43P trna
ENST00000463956.1 MYD88 trna
ENST00000463959.1 MTR trna
ENST00000463963.1 HS6ST1 trna
ENST00000463972.1 SLC39A7 trna
ENST00000463975.1 MOB1A trna
ENST00000463991.4 AKT3 trna
ENST00000463997.1 CAPN2 trna
ENST00000463999.3 RN7SL515P trna
ENST00000464011.1 TFRC trna
ENST00000464013.4 RBM6 trna
ENST00000464024.1 NA trna
ENST00000464025.4 LMO2 trna
ENST00000464026.1 GDAP2 trna
ENST00000464044.1 LST1 trna
ENST00000464051.1 SEMA4D trna
ENST00000464053.1 NA trna
ENST00000464063.1 SLC25A12 trna
ENST00000464070.1 CYREN trna
ENST00000464071.1 FTO trna
ENST00000464072.1 STAT1 trna
ENST00000464099.4 EEF1AKMT2 trna
ENST00000464101.2 UBA5 trna
ENST00000464102.4 SACM1L trna

ENST00000464116.2 XRCC6 trna
ENST00000464124.2 trna
ENST00000464132.1 MCL1 trna
ENST00000464133.1 SETX trna
ENST00000464138.1 SSR3 trna
ENST00000464140.1 ST6GALNAC3 trna
ENST00000464147.1 ORC2 trna
ENST00000464154.1 ATP5PB trna
ENST00000464168.4 USP4 trna
ENST00000464170.1 CATSPERE trna
ENST00000464177.1 TMEM164 trna
ENST00000464181.2 ARMC8 trna
ENST00000464189.4 HIRA trna
ENST00000464194.2 MIPEP trna
ENST00000464202.1 SUN2 trna
ENST00000464210.1 TRIM24 trna
ENST00000464213.1 CD36 trna
ENST00000464215.1 ADARB1 trna
ENST00000464233.4 ROBO1 trna
ENST00000464236.1 ANKRD13C trna
ENST00000464262.5 ELMO1 trna
ENST00000464271.1 LINC00877 trna
ENST00000464273.1 PPP1CB trna
ENST00000464300.5 PARP15 trna
ENST00000464301.1 SLC39A10 trna
ENST00000464302.1 RGS2 trna
ENST00000464313.1 PTPN12 trna
ENST00000464329.1 CD53 trna
ENST00000464347.2 EXOC6B trna
ENST00000464348.4 IPO9 trna
ENST00000464356.5 MEF2D trna
ENST00000464373.1 NADK trna
ENST00000464374.4 TTC21B trna
ENST00000464379.4 TLN1 trna
ENST00000464380.4 NA trna
ENST00000464390.1 BNIP2 trna
ENST00000464398.1 SMYD3 trna
ENST00000464399.2 ACSS2 trna
ENST00000464407.1 CREB1 trna
ENST00000464412.4 LBH trna
ENST00000464423.1 UFM1 trna
ENST00000464435.1 USP25 trna
ENST00000464444.1 RPS6P25 trna
ENST00000464451.4 SEC61A1 trna
ENST00000464452.1 PANK2 trna
ENST00000464461.1 GIMAP1 trna
ENST00000464465.5 CSF3R trna
ENST00000464470.1 N4BP2L1 trna
ENST00000464472.3 RN7SL508P trna
ENST00000464478.4 EPS15 trna
ENST00000464482.1 ATXN10 trna
ENST00000464497.4 CLTA trna
ENST00000464542.4 COG5 trna
ENST00000464552.1 PRKD3 trna

ENST00000464554.4 DIS3L2 trna
ENST00000464559.1 PSD4 trna
ENST00000464560.4 ZBTB20 trna
ENST00000464569.1 LAPTM5 trna
ENST00000464575.1 CCNL1 trna
ENST00000464577.1 USP48 trna
ENST00000464588.1 ZSWIM5 trna
ENST00000464591.1 C1orf162 trna
ENST00000464606.4 ZC3HAV1 trna
ENST00000464608.4 LRRFIP1 trna
ENST00000464611.1 ACTB trna
ENST00000464628.1 SCAF8 trna
ENST00000464636.1 NA trna
ENST00000464649.1 BCL2L13 trna
ENST00000464651.1 NDUFB8 trna
ENST00000464654.1 TM9SF3 trna
ENST00000464661.4 HM13 trna
ENST00000464680.5 ADAMTS6 trna
ENST00000464689.1 CMTM7 trna
ENST00000464717.4 PAXIP1 trna
ENST00000464741.2 GRID1 trna
ENST00000464753.1 MTIF3 trna
ENST00000464766.1 DNAJC13 trna
ENST00000464787.1 UBP1 trna
ENST00000464789.2 SUPT7L trna
ENST00000464790.1 EIF3L trna
ENST00000464812.1 NUDCD3 trna
ENST00000464818.1 PRKCD trna
ENST00000464827.1 ST6GAL1 trna
ENST00000464831.1 FAM78A trna
ENST00000464838.1 ARHGAP26 trna
ENST00000464839.4 GBP2 trna
ENST00000464875.1 DENND6A trna
ENST00000464920.1 ENO1 trna
ENST00000464932.1 CCNT2 trna
ENST00000464940.1 KPNA1 trna
ENST00000464954.1 MPZL1 trna
ENST00000464960.4 GNAS trna
ENST00000464967.4 trna
ENST00000464972.4 RERE trna
ENST00000464976.1 RAPGEF4 trna
ENST00000464984.1 SVIL trna
ENST00000465002.1 TLN1 trna
ENST00000465018.1 XPO4 trna
ENST00000465039.4 LIMD1 trna
ENST00000465074.1 XRN1 trna
ENST00000465079.1 ARHGEF2 trna
ENST00000465082.4 TMBIM1 trna
ENST00000465089.2 PBX1 trna
ENST00000465096.4 NDUFV2 trna
ENST00000465112.4 SRPK2 trna
ENST00000465122.4 PDCD6IP trna
ENST00000465127.1 trna
ENST00000465128.3 PABIR2 trna

ENST00000465130.1 BIRC6 trna
ENST00000465144.1 FLNA trna
ENST00000465148.2 CCNJ trna
ENST00000465151.4 MAT2A trna
ENST00000465155.4 ATF3 trna
ENST00000465160.2 EXD3 trna
ENST00000465185.2 GPR89A trna
ENST00000465187.1 EDEM1 trna
ENST00000465204.4 FBXO11 trna
ENST00000465223.1 NCSTN trna
ENST00000465229.4 EXOSC3 trna
ENST00000465235.1 MFSD1 trna
ENST00000465240.1 CARS1 trna
ENST00000465249.1 ZBTB20-AS5 trna
ENST00000465266.1 ZNF678 trna
ENST00000465304.4 PARP15 trna
ENST00000465311.1 ZNF41 trna
ENST00000465331.1 CARS1 trna
ENST00000465349.4 LYST trna
ENST00000465370.1 NSF trna
ENST00000465375.4 DAP3 trna
ENST00000465376.4 JAK1 trna
ENST00000465379.1 SP3 trna
ENST00000465381.4 TASP1 trna
ENST00000465386.1 USP9X trna
ENST00000465387.4 RPL22 trna
ENST00000465393.1 TNIK trna
ENST00000465412.4 RABGAP1L trna
ENST00000465421.4 SIK3 trna
ENST00000465422.1 NA trna
ENST00000465436.4 trna
ENST00000465443.4 PLK3 trna
ENST00000465446.1 MXD1 trna
ENST00000465454.1 JPT1 trna
ENST00000465459.2 HLA-F trna
ENST00000465461.5 CUX1 trna
ENST00000465467.1 EPS15 trna
ENST00000465476.1 NFKBIZ trna
ENST00000465484.1 AFF4 trna
ENST00000465487.4 SLC4A7 trna
ENST00000465491.4 CLEC16A trna
ENST00000465505.2 SNX4 trna
ENST00000465522.4 ITGA4 trna
ENST00000465529.1 TOM1 trna
ENST00000465534.4 CD55 trna
ENST00000465535.1 EIF2A trna
ENST00000465539.1 ZNF271P trna
ENST00000465556.1 TMCO3 trna
ENST00000465566.2 EHMT1 trna
ENST00000465567.1 TTC19 retained_intron
ENST00000465579.1 MOV10 processed_transcript
ENST00000465582.4 SSBP1 protein_coding
ENST00000465584.4 NKTR retained_intron
ENST00000465614.1 LMO2 retained_intron

ENST00000465625.1 TTC14 processed_transcript
ENST00000465627.4 UBA3 nonsense_mediated_decay
ENST00000465629.4 RNF103 retained_intron
ENST00000465630.1 UBR3 processed_transcript
ENST00000465645.1 ATP5IF1 protein_coding
ENST00000465646.4 SFI1 processed_transcript
ENST00000465661.1 FAM126A processed_transcript
ENST00000465671.1 SOGA1 nonsense_mediated_decay
ENST00000465676.1 EPHB2 processed_transcript
ENST00000465690.5 BTN3A1 retained_intron
ENST00000465709.4 SUSD3 protein_coding
ENST00000465721.1 CNOT4 processed_transcript
ENST00000465723.1 RASAL2 processed_transcript
ENST00000465742.2 FOXP1-AS1 lncRNA
ENST00000465754.5 CD72 processed_transcript
ENST00000465769.1 SULF2 retained_intron
ENST00000465821.4 FAM102A retained_intron
ENST00000465848.1 DEGS1 processed_transcript
ENST00000465851.1 SLC39A10 retained_intron
ENST00000465870.4 LRRFIP1 retained_intron
ENST00000465881.1 HNRNPU processed_transcript
ENST00000465884.1 TBC1D5 processed_transcript
ENST00000465896.4 PHC3 processed_transcript
ENST00000465908.4 DNAJB6 processed_transcript
ENST00000465910.4 WDFY4 retained_intron
ENST00000465911.1 DNAJC11 processed_transcript
ENST00000465926.4 TAF8 protein_coding
ENST00000465931.1 NA NA
ENST00000465937.1 LIMK2 retained_intron
ENST00000465942.1 COG3 processed_transcript
ENST00000465949.1 FUBP3 processed_transcript
ENST00000465950.4 SCAF11 protein_coding
ENST00000465952.4 USPL1 processed_transcript
ENST00000465980.1 ATG3 retained_intron
ENST00000465982.4 WAS processed_transcript
ENST00000465984.4 SLC11A1 retained_intron
ENST00000465985.1 SAMHD1 protein_coding
ENST00000465990.4 SPATA6 processed_transcript
ENST00000466003.1 POLR3C processed_transcript
ENST00000466017.1 VPS41 processed_transcript
ENST00000466023.1 CAPZA2 processed_transcript
ENST00000466032.1 CHMP3 processed_transcript
ENST00000466055.1 CPED1 processed_transcript
ENST00000466057.1 PNISR retained_intron
ENST00000466058.1 KDM3A processed_transcript
ENST00000466062.2 ZNF292 protein_coding
ENST00000466066.1 CAPZA1 processed_transcript
ENST00000466067.1 WDR43 retained_intron
ENST00000466078.1 PDS5B retained_intron
ENST00000466080.1 VDAC1 processed_transcript
ENST00000466101.1 CCNL1 retained_intron
ENST00000466105.4 CCSER2 nonsense_mediated_decay
ENST00000466117.1 TMEM184B retained_intron
ENST00000466124.1 PRKCB protein_coding

ENST00000466126.1 PARP9 protein_coding
ENST00000466137.1 NMRK1 processed_transcript
ENST00000466161.1 LRIG2 processed_transcript
ENST00000466162.4 PI4KA processed_transcript
ENST00000466171.1 CCDC93 retained_intron
ENST00000466173.1 UBAP2L processed_transcript
ENST00000466177.5 PELI1 processed_transcript
ENST00000466182.4 WDR91 retained_intron
ENST00000466185.1 PRPF4B processed_transcript
ENST00000466220.1 TLK1 processed_transcript
ENST00000466224.1 GON4L processed_transcript
ENST00000466227.5 YWHAE nonsense_mediated_decay
ENST00000466234.1 SELENOT retained_intron
ENST00000466253.1 DCAF8 retained_intron
ENST00000466254.1 TRBC2 TR_C_gene
ENST00000466256.5 CROCC processed_transcript
ENST00000466273.4 PPP1R12B nonsense_mediated_decay
ENST00000466274.1 NCL retained_intron
ENST00000466278.1 MRTFA processed_transcript
ENST00000466288.1 CXCR4 processed_transcript
ENST00000466295.1 KMT5B retained_intron
ENST00000466297.1 processed_pseudogene
ENST00000466307.1 TMEM140 processed_transcript
ENST00000466316.1 TRMT11 nonsense_mediated_decay
ENST00000466319.1 FLNA retained_intron
ENST00000466325.1 FLNA retained_intron
ENST00000466340.1 SELL retained_intron
ENST00000466342.1 SQSTM1 retained_intron
ENST00000466347.4 GIMAP5 processed_transcript
ENST00000466359.1 NFX1 retained_intron
ENST00000466361.1 ZBTB8OS retained_intron
ENST00000466362.1 EIF4A2 retained_intron
ENST00000466366.1 YY1AP1 processed_transcript
ENST00000466373.1 UFD1 retained_intron
ENST00000466377.1 processed_pseudogene
ENST00000466379.1 UBE4B retained_intron
ENST00000466382.1 ARID1A nonsense_mediated_decay
ENST00000466392.1 EDEM3 retained_intron
ENST00000466396.4 CLTA protein_coding
ENST00000466424.4 SHISA5 processed_transcript
ENST00000466428.4 TLE4 retained_intron
ENST00000466437.1 PDK1 retained_intron
ENST00000466453.1 APP processed_transcript
ENST00000466470.4 ZC3H11A processed_transcript
ENST00000466476.1 IGF2BP2 processed_transcript
ENST00000466478.4 RNF13 protein_coding
ENST00000466484.1 ARHGAP19 protein_coding
ENST00000466489.1 PMF1 nonsense_mediated_decay
ENST00000466501.1 transcribed_processed_pseudogene
ENST00000466507.2 TTC13 nonsense_mediated_decay
ENST00000466513.1 CLTC retained_intron
ENST00000466516.1 JAZF1 processed_transcript
ENST00000466527.1 BIRC6 retained_intron
ENST00000466531.4 BCL2L14 processed_transcript

ENST00000466539.1 PSME4 processed_transcript
ENST00000466553.1 NKTR retained_intron
ENST00000466555.1 TM9SF2 processed_transcript
ENST00000466557.5 lncRNA
ENST00000466565.1 MBNL1 processed_transcript
ENST00000466596.4 HS3ST3B1 nonsense_mediated_decay
ENST00000466597.1 PARP12 retained_intron
ENST00000466600.1 PLA2G4A processed_transcript
ENST00000466604.4 OSBPL10 processed_transcript
ENST00000466609.1 WWP1P1 processed_pseudogene
ENST00000466613.2 SDHB retained_intron
ENST00000466644.4 NA NA
ENST00000466647.4 INVS processed_transcript
ENST00000466653.1 ARID4B retained_intron
ENST00000466673.1 KDM4C processed_transcript
ENST00000466674.4 RPL22L1 protein_coding
ENST00000466702.1 BCLAF3 processed_transcript
ENST00000466745.4 SFPQ processed_transcript
ENST00000466749.4 ARMC8 protein_coding
ENST00000466755.1 THBS1 retained_intron
ENST00000466758.4 ATP11B nonsense_mediated_decay
ENST00000466759.4 TMEM222 protein_coding
ENST00000466760.4 WDR49 protein_coding
ENST00000466762.1 ARMC8 retained_intron
ENST00000466765.1 TKT retained_intron
ENST00000466766.1 HM13 protein_coding
ENST00000466777.1 TNFSF10 retained_intron
ENST00000466778.2 EVI5 processed_transcript
ENST00000466782.1 ARHGEF10L processed_transcript
ENST00000466784.5 INTS6 processed_transcript
ENST00000466788.1 FHIT processed_transcript
ENST00000466794.4 MAN2B1 retained_intron
ENST00000466800.1 processed_pseudogene
ENST00000466832.4 TRA2B retained_intron
ENST00000466833.4 ZNF782 processed_transcript
ENST00000466840.4 ERCC6L2 processed_transcript
ENST00000466845.1 LRCH3 retained_intron
ENST00000466850.1 ARIH2 retained_intron
ENST00000466876.1 ACAP2 protein_coding
ENST00000466892.4 HLA-C retained_intron
ENST00000466899.4 GNB4 protein_coding
ENST00000466910.4 SRRM1 processed_transcript
ENST00000466916.1 TLN1 retained_intron
ENST00000466924.1 AHCYL2 protein_coding
ENST00000466929.4 CARD19 processed_transcript
ENST00000466946.4 ATP11A processed_transcript
ENST00000466971.1 NFX1 retained_intron
ENST00000466977.1 MTO1 retained_intron
ENST00000466982.1 SFXN3 processed_transcript
ENST00000466992.1 AK9 retained_intron
ENST00000466996.4 LNP1 nonsense_mediated_decay
ENST00000467009.1 GON4L processed_transcript
ENST00000467022.1 NAA50 processed_transcript
ENST00000467025.1 TAPBP protein_coding

ENST00000467026.4 MCM3AP processed_transcript
ENST00000467053.4 ARHGEF7 protein_coding
ENST00000467056.4 ITPR1 protein_coding
ENST00000467058.1 LRPPRC retained_intron
ENST00000467065.1 UBXN4 processed_transcript
ENST00000467075.1 RPL11 nonsense_mediated_decay
ENST00000467077.1 XRN1 retained_intron
ENST00000467080.1 RBM17 retained_intron
ENST00000467114.2 PRPF40A retained_intron
ENST00000467115.1 MTATP8P1 unprocessed_pseudogene
ENST00000467128.4 USP34 processed_transcript
ENST00000467141.1 MSL3 protein_coding
ENST00000467149.1 WIPF1 processed_transcript
ENST00000467174.5 MFN1 protein_coding
ENST00000467184.4 BAZ2B retained_intron
ENST00000467200.5 ZMYND8 protein_coding
ENST00000467202.1 GPR141 processed_transcript
ENST00000467220.1 DYNC2I1 retained_intron
ENST00000467231.4 ZNF644 processed_transcript
ENST00000467238.1 SND1 processed_transcript
ENST00000467249.1 HNRNPH3 processed_transcript
ENST00000467267.1 CAMTA1 processed_transcript
ENST00000467270.1 processed_pseudogene
ENST00000467279.1 DDX17 processed_transcript
ENST00000467290.1 SLC25A53 processed_transcript
ENST00000467302.4 PRPF38B processed_transcript
ENST00000467309.4 TAF1 nonsense_mediated_decay
ENST00000467310.1 SEPTIN6 processed_transcript
ENST00000467311.4 SNTB2 nonsense_mediated_decay
ENST00000467322.1 ZBTB41 nonsense_mediated_decay
ENST00000467326.4 VPS50 processed_transcript
ENST00000467329.4 PRPF3 processed_transcript
ENST00000467332.1 CGGBP1 protein_coding
ENST00000467348.1 U2SURP protein_coding
ENST00000467350.1 RERE processed_transcript
ENST00000467352.1 GTDC1 processed_transcript
ENST00000467356.4 JMJD1C retained_intron
ENST00000467362.1 CEPT1 processed_transcript
ENST00000467366.1 PTPRE protein_coding
ENST00000467369.1 HCP5 lncRNA
ENST00000467371.1 SPTBN1 retained_intron
ENST00000467380.1 NT5C2 processed_transcript
ENST00000467391.1 MGME1 processed_transcript
ENST00000467393.4 INPP5D processed_transcript
ENST00000467395.2 CTBP2 processed_transcript
ENST00000467404.5 CAMTA1 protein_coding
ENST00000467446.5 NSRP1 retained_intron
ENST00000467479.1 NEGR1 processed_transcript
ENST00000467480.1 PIM3 retained_intron
ENST00000467509.4 OMA1 processed_transcript
ENST00000467549.4 ROBO1 protein_coding
ENST00000467567.1 RSF1 retained_intron
ENST00000467570.4 PHC3 protein_coding
ENST00000467577.1 RUNX1 processed_transcript

ENST00000467586.4 EXOC7 retained_intron
ENST00000467593.1 PSMB9 retained_intron
ENST00000467601.1 PRRC2C processed_transcript
ENST00000467603.1 BLCAP processed_transcript
ENST00000467606.4 ITGB1BP1 protein_coding
ENST00000467610.1 BHLHE40 retained_intron
ENST00000467613.4 UBP1 processed_transcript
ENST00000467616.4 SON processed_transcript
ENST00000467622.2 ARPC3 retained_intron
ENST00000467625.4 PIK3AP1 processed_transcript
ENST00000467628.1 SATB1 retained_intron
ENST00000467644.4 CMC1 processed_transcript
ENST00000467652.4 ZBTB8OS retained_intron
ENST00000467655.1 CEP97 nonsense_mediated_decay
ENST00000467659.1 DGCR2 retained_intron
ENST00000467662.4 PPP1R18 processed_transcript
ENST00000467668.1 THADA processed_transcript
ENST00000467673.4 MACF1 retained_intron
ENST00000467687.1 WDPCP processed_transcript
ENST00000467709.5 DOCK5 nonsense_mediated_decay
ENST00000467716.1 C1GALT1 processed_transcript
ENST00000467730.1 NAMPT retained_intron
ENST00000467731.1 SCAF4 retained_intron
ENST00000467744.2 CHM processed_transcript
ENST00000467757.4 IKZF3 protein_coding
ENST00000467764.1 NA NA
ENST00000467766.4 UBE2E1 protein_coding
ENST00000467782.4 RREB1 protein_coding
ENST00000467786.1 FGD3 nonsense_mediated_decay
ENST00000467790.4 IFI44 retained_intron
ENST00000467799.5 BLNK nonsense_mediated_decay
ENST00000467815.4 SULF2 protein_coding
ENST00000467833.1 KLF7 processed_transcript
ENST00000467838.2 SEC16A processed_transcript
ENST00000467853.4 PSMD6 retained_intron
ENST00000467867.1 PGAM1 processed_transcript
ENST00000467883.3 RN7SL128P misc_RNA
ENST00000467894.4 PHC2 nonsense_mediated_decay
ENST00000467900.1 OXSR1 retained_intron
ENST00000467901.1 SLMAP retained_intron
ENST00000467903.4 FCGR2C processed_transcript
ENST00000467908.1 PDXK protein_coding
ENST00000467911.1 NCK1 protein_coding
ENST00000467917.4 SCAI nonsense_mediated_decay
ENST00000467930.1 processed_pseudogene
ENST00000467937.1 CABIN1 retained_intron
ENST00000467944.4 OR2A1-AS1 lncRNA
ENST00000467956.1 CLASP2 retained_intron
ENST00000467957.1 YBX1 processed_transcript
ENST00000467966.1 TBC1D14 retained_intron
ENST00000467975.2 ZNF25 processed_transcript
ENST00000467978.1 HEATR5B retained_intron
ENST00000467979.1 STIMATE nonsense_mediated_decay
ENST00000468001.1 KLHL24 protein_coding

ENST00000468010.1 ARL8B nonsense_mediated_decay
ENST00000468027.4 PTMA retained_intron
ENST00000468036.1 PIK3CA protein_coding
ENST00000468038.1 FGR processed_transcript
ENST00000468040.1 SNIP1 nonsense_mediated_decay
ENST00000468059.1 BACH1 protein_coding
ENST00000468069.1 RPS6KC1 processed_transcript
ENST00000468075.1 MAP4 protein_coding
ENST00000468085.4 DUSP10 nonsense_mediated_decay
ENST00000468095.2 NHSL1 retained_intron
ENST00000468107.4 LYST processed_transcript
ENST00000468108.1 CCDC66 retained_intron
ENST00000468111.1 KAT2B retained_intron
ENST00000468115.1 INTS1 retained_intron
ENST00000468116.1 HBP1 processed_transcript
ENST00000468119.3 DIAPH1 protein_coding
ENST00000468147.4 EIF4E3 processed_transcript
ENST00000468164.1 AOPEP processed_transcript
ENST00000468166.4 SND1 processed_transcript
ENST00000468173.1 DYSF processed_transcript
ENST00000468200.2 C12orf40 nonsense_mediated_decay
ENST00000468206.4 FGD3 protein_coding
ENST00000468208.1 SETD5 protein_coding
ENST00000468234.1 SERINC3 processed_transcript
ENST00000468235.1 ZGPAT processed_transcript
ENST00000468243.4 PIM1 processed_transcript
ENST00000468247.1 RERE protein_coding
ENST00000468249.1 ODF3B retained_intron
ENST00000468259.1 XPO1 processed_transcript
ENST00000468286.4 SGIP1 retained_intron
ENST00000468289.1 RNF13 protein_coding
ENST00000468296.4 ZNF717 protein_coding
ENST00000468305.1 MED12L processed_transcript
ENST00000468311.1 PFDN2 processed_transcript
ENST00000468312.1 STAT5B retained_intron
ENST00000468316.1 PRKRIP1 retained_intron
ENST00000468344.4 RSRC1 processed_transcript
ENST00000468345.1 ADD3 processed_transcript
ENST00000468355.4 SLC23A2 processed_transcript
ENST00000468371.4 SHQ1 processed_transcript
ENST00000468376.2 ZMYND8 processed_transcript
ENST00000468379.1 NIBAN2 processed_transcript
ENST00000468384.1 HMGB1 processed_transcript
ENST00000468388.4 HMGN2 processed_transcript
ENST00000468391.4 CREB5 retained_intron
ENST00000468399.2 DISC1 retained_intron
ENST00000468401.1 HBP1 protein_coding
ENST00000468409.1 MFSD1 retained_intron
ENST00000468417.1 CBWD2 retained_intron
ENST00000468422.1 GNAI2 retained_intron
ENST00000468425.2 ATP5IF1 protein_coding
ENST00000468442.1 LRP5L processed_transcript
ENST00000468485.4 IRF4 processed_transcript
ENST00000468511.4 SERPINB1 processed_transcript

ENST00000468513.4 MLLT3 processed_transcript
ENST00000468520.1 BCKDHB processed_transcript
ENST00000468523.2 ELK4 protein_coding
ENST00000468527.1 KRT8P12 processed_transcript
ENST00000468560.1 ARMC8 protein_coding
ENST00000468564.5 PARVG retained_intron
ENST00000468577.4 FOXP1 protein_coding
ENST00000468598.4 SFPQ processed_transcript
ENST00000468601.1 NAGK retained_intron
ENST00000468618.5 GPR89B protein_coding
ENST00000468626.2 LYST processed_transcript
ENST00000468628.2 SNRK processed_transcript
ENST00000468639.4 ANXA1 processed_transcript
ENST00000468642.4 SLC35A5 protein_coding
ENST00000468644.4 RHBDD2 processed_transcript
ENST00000468646.5 LINC00877 lncRNA
ENST00000468658.1 CACNA2D3 nonsense_mediated_decay
ENST00000468665.1 EYA3 processed_transcript
ENST00000468677.4 ZMYM2 processed_transcript
ENST00000468700.1 NR1D2 retained_intron
ENST00000468704.1 TMEM50A processed_transcript
ENST00000468715.1 RPL7P9 processed_pseudogene
ENST00000468722.1 NRDC processed_transcript
ENST00000468734.1 KLHL6 nonsense_mediated_decay
ENST00000468744.4 SH2D2A processed_transcript
ENST00000468749.1 DUSP5 processed_transcript
ENST00000468757.1 TNIK protein_coding
ENST00000468761.1 STX12 processed_transcript
ENST00000468762.2 XACT lncRNA
ENST00000468769.1 ZBTB20 processed_transcript
ENST00000468783.1 PIK3AP1 processed_transcript
ENST00000468804.4 CUL2 protein_coding
ENST00000468819.1 NA NA
ENST00000468830.1 MKLN1 retained_intron
ENST00000468831.4 TANK retained_intron
ENST00000468844.1 RPL39 processed_transcript
ENST00000468852.2 MKNK1 retained_intron
ENST00000468860.1 ABCD3 processed_transcript
ENST00000468864.1 TRAPPC10 retained_intron
ENST00000468869.2 PELI1 processed_transcript
ENST00000468888.5 CLASP2 protein_coding
ENST00000468894.1 XRN1 processed_transcript
ENST00000468896.1 MAPKAP1 protein_coding
ENST00000468921.4 RNF185 retained_intron
ENST00000468928.4 DNAJB6 retained_intron
ENST00000468950.4 LRRFIP1 processed_transcript
ENST00000468958.1 ABCF1 protein_coding
ENST00000468986.5 CASK retained_intron
ENST00000468996.2 TGFBR3 processed_transcript
ENST00000469000.4 WDR82 protein_coding
ENST00000469013.1 SLC15A2 protein_coding
ENST00000469043.1 SCFD1 protein_coding
ENST00000469056.1 PARL retained_intron
ENST00000469059.1 EPC1 protein_coding

ENST00000469063.1 ADA2 processed_transcript
ENST00000469067.4 DAPK1 retained_intron
ENST00000469079.4 EVA1C retained_intron
ENST00000469085.1 KAT2B processed_transcript
ENST00000469086.4 SUN2 retained_intron
ENST00000469087.1 RUNX1 retained_intron
ENST00000469089.4 NAP1L4 processed_transcript
ENST00000469109.1 LUC7L retained_intron
ENST00000469113.4 CCNL2 processed_transcript
ENST00000469117.4 ARHGAP15 processed_transcript
ENST00000469120.1 HLA-DPB1 retained_intron
ENST00000469122.4 RAB5A retained_intron
ENST00000469129.1 SMC1A retained_intron
ENST00000469131.4 ARHGAP26 protein_coding
ENST00000469135.1 STX6 processed_transcript
ENST00000469141.5 THRAP3 protein_coding
ENST00000469149.1 TSPAN14 processed_transcript
ENST00000469152.4 JMJD1C processed_transcript
ENST00000469157.4 FAM217A processed_transcript
ENST00000469163.1 NUP50 retained_intron
ENST00000469170.4 SRSF11 processed_transcript
ENST00000469176.1 NDUFS6 protein_coding
ENST00000469188.1 MAP3K1 retained_intron
ENST00000469197.4 DOCK8 nonsense_mediated_decay
ENST00000469225.1 PARK7 protein_coding
ENST00000469227.4 LCP1 processed_transcript
ENST00000469251.1 RERE processed_transcript
ENST00000469257.1 EIF1 protein_coding
ENST00000469265.1 NA NA
ENST00000469266.1 PLSCR1 retained_intron
ENST00000469272.1 GIMAP5 retained_intron
ENST00000469280.1 NCF2 processed_transcript
ENST00000469295.1 SLC2A3 retained_intron
ENST00000469308.1 EIF1 processed_transcript
ENST00000469315.1 BCAR3 processed_transcript
ENST00000469317.1 HACD2 protein_coding
ENST00000469323.4 THADA processed_transcript
ENST00000469328.4 IMPDH1 nonsense_mediated_decay
ENST00000469336.1 UBE3C retained_intron
ENST00000469338.1 STXBP3 retained_intron
ENST00000469366.1 MACF1 processed_transcript
ENST00000469375.1 RNF216 processed_transcript
ENST00000469380.1 CSF3R retained_intron
ENST00000469381.1 GSE1 retained_intron
ENST00000469387.1 CDC14A retained_intron
ENST00000469388.1 CFP protein_coding
ENST00000469400.1 TRAPPC12 processed_transcript
ENST00000469412.4 TIAM1 retained_intron
ENST00000469428.1 RPL27AP5 processed_pseudogene
ENST00000469438.1 MBD5 processed_transcript
ENST00000469449.1 SLC11A1 retained_intron
ENST00000469470.1 TNS3 processed_transcript
ENST00000469479.1 VPS8 retained_intron
ENST00000469481.1 STAG2 processed_transcript

ENST00000469482.1 SETD4 processed_transcript
ENST00000469490.1 MACF1 processed_transcript
ENST00000469491.4 FNDC3B retained_intron
ENST00000469506.3 TRAK1 retained_intron
ENST00000469507.1 CYRIA retained_intron
ENST00000469522.1 SNU13 protein_coding
ENST00000469524.1 EIF4E3 protein_coding
ENST00000469525.1 SLC44A5 processed_transcript
ENST00000469528.4 GAPVD1 processed_transcript
ENST00000469532.1 RAC2 retained_intron
ENST00000469537.1 SLC25A44 processed_transcript
ENST00000469543.4 VIM nonsense_mediated_decay
ENST00000469573.1 GRB14 retained_intron
ENST00000469575.1 RGS10 processed_transcript
ENST00000469577.4 PRRC2A processed_transcript
ENST00000469605.1 RPL24 protein_coding
ENST00000469611.1 MYO5A retained_intron
ENST00000469617.3 RN7SL795P misc_RNA
ENST00000469630.1 TBXAS1 retained_intron
ENST00000469637.1 CASP9 protein_coding
ENST00000469653.4 SEMA4D processed_transcript
ENST00000469671.1 CDYL retained_intron
ENST00000469673.1 DRG1 retained_intron
ENST00000469702.4 SLC35F5 retained_intron
ENST00000469707.4 MBNL2 nonsense_mediated_decay
ENST00000469712.1 PIBF1 processed_transcript
ENST00000469714.1 REPS2 processed_transcript
ENST00000469735.1 SYTL3 processed_transcript
ENST00000469737.1 YAE1 processed_transcript
ENST00000469774.1 GLS retained_intron
ENST00000469778.1 SPTLC1 processed_transcript
ENST00000469790.1 KPNA6 processed_transcript
ENST00000469796.5 ACSL4 miRNA
ENST00000469811.1 RPL22P2 miRNA
ENST00000469838.4 RBM5 miRNA
ENST00000469850.4 NCOA1 miRNA
ENST00000469873.1 GYG1 miRNA
ENST00000469930.1 BRAF miRNA
ENST00000469931.2 miRNA
ENST00000469944.1 ZZZ3 miRNA
ENST00000469947.4 SH3BP1 miRNA
ENST00000469967.1 FAM162A miRNA
ENST00000469976.2 LDHA miRNA
ENST00000469979.5 PHF20L1 miRNA
ENST00000469989.1 STAB1 miRNA
ENST00000470017.1 PRDX6 miRNA
ENST00000470025.1 CCNY miRNA
ENST00000470032.1 ARHGAP26 miRNA
ENST00000470035.1 MACO1 miRNA
ENST00000470036.4 NIPSNAP2 miRNA
ENST00000470050.1 HK1 miRNA
ENST00000470052.4 WDR11 miRNA
ENST00000470063.4 MBD5 miRNA
ENST00000470067.4 CUL4A miRNA

ENST00000470087.1 DIS3L2 miRNA
ENST00000470092.1 HCK miRNA
ENST00000470093.4 FBXW4 miRNA
ENST00000470099.1 LDAH miRNA
ENST00000470112.1 FOXP1 miRNA
ENST00000470116.2 PIGL miRNA
ENST00000470139.1 SZT2 miRNA
ENST00000470143.1 NOP56 miRNA
ENST00000470145.2 ASXL1 miRNA
ENST00000470186.1 ZBTB20 miRNA
ENST00000470212.1 TUT4 miRNA
ENST00000470215.1 PHTF2 miRNA
ENST00000470216.1 CEBPZOS miRNA
ENST00000470229.4 NUB1 miRNA
ENST00000470238.1 SCYL3 miRNA
ENST00000470250.1 BIRC6 miRNA
ENST00000470268.1 SF3B1 miRNA
ENST00000470273.4 FGD2 miRNA
ENST00000470278.4 CDC42SE1 miRNA
ENST00000470280.1 LDHB miRNA
ENST00000470281.1 AAK1 miRNA
ENST00000470286.1 DYNC1I2 miRNA
ENST00000470287.1 FUBP1 miRNA
ENST00000470299.1 NT5C2 miRNA
ENST00000470300.4 AHCTF1 miRNA
ENST00000470305.1 SECISBP2 miRNA
ENST00000470313.4 miRNA
ENST00000470317.2 VSIR miRNA
ENST00000470340.1 TLK1 miRNA
ENST00000470352.1 RPN2 miRNA
ENST00000470354.1 GNB2 miRNA
ENST00000470362.1 NA miRNA
ENST00000470363.4 HLA-C miRNA
ENST00000470369.1 C11orf21 miRNA
ENST00000470370.1 RHEB miRNA
ENST00000470394.1 ECE1 miRNA
ENST00000470408.4 UBE3C miRNA
ENST00000470430.1 HSPA14 miRNA
ENST00000470441.1 IFRD1 miRNA
ENST00000470455.4 ATP6V1A miRNA
ENST00000470458.1 RFTN1 miRNA
ENST00000470463.1 SND1 miRNA
ENST00000470466.4 TAB2 miRNA
ENST00000470470.1 CHD6 miRNA
ENST00000470474.1 ATG7 miRNA
ENST00000470475.1 RPS8 miRNA
ENST00000470481.2 ANAPC16 miRNA
ENST00000470490.1 PCNP miRNA
ENST00000470496.1 PLSCR1 miRNA
ENST00000470515.1 PARP12 miRNA
ENST00000470521.1 MIA3 miRNA
ENST00000470531.4 CHD3 miRNA
ENST00000470537.1 XRN1 miRNA
ENST00000470541.5 ARHGEF2 miRNA

ENST00000470551.1 DNAJC5 miRNA
ENST00000470554.4 GSTO1 miRNA
ENST00000470556.1 ZBTB20 miRNA
ENST00000470557.2 PTRH2 miRNA
ENST00000470567.4 MVB12B miRNA
ENST00000470571.1 SKIL miRNA
ENST00000470574.1 KAT6A miRNA
ENST00000470579.4 ARID5A miRNA
ENST00000470600.1 TGFBR3 miRNA
ENST00000470622.1 IPCEF1 miRNA
ENST00000470625.1 EIPR1 miRNA
ENST00000470626.1 TUT4 miRNA
ENST00000470633.1 ST6GAL1 miRNA
ENST00000470635.1 TIAL1 miRNA
ENST00000470638.3 PRIM2 miRNA
ENST00000470642.1 SUN2 miRNA
ENST00000470649.1 TTC19 miRNA
ENST00000470660.1 LRP10 miRNA
ENST00000470673.4 PRKACB miRNA
ENST00000470687.1 UBXN4 miRNA
ENST00000470696.1 ICA1 miRNA
ENST00000470698.1 ANKS1A miRNA
ENST00000470704.1 ALG13 miRNA
ENST00000470707.1 ASB3 miRNA
ENST00000470710.1 HDLBP miRNA
ENST00000470718.4 PSME1 miRNA
ENST00000470721.4 DCAF6 miRNA
ENST00000470723.4 SND1 miRNA
ENST00000470726.5 DLEU1 miRNA
ENST00000470729.4 ANKRD30BL miRNA
ENST00000470741.1 CYB5R3 miRNA
ENST00000470742.4 ITSN1 miRNA
ENST00000470743.4 SDHC miRNA
ENST00000470745.4 VDAC2 miRNA
ENST00000470750.1 SASH1 miRNA
ENST00000470751.4 SUMF1 miRNA
ENST00000470752.4 WIPF1 miRNA
ENST00000470756.4 ALCAM miRNA
ENST00000470797.4 MDM4 miRNA
ENST00000470800.1 MAP3K7CL miRNA
ENST00000470801.1 PPP4R3B miRNA
ENST00000470809.1 MAST2 miRNA
ENST00000470815.1 HERC3 miRNA
ENST00000470836.1 GTPBP1 miRNA
ENST00000470844.4 CC2D1B miRNA
ENST00000470863.1 SMYD3 processed_transcript
ENST00000470874.4 ARHGEF2 nonsense_mediated_decay
ENST00000470876.1 JADE2 trna
ENST00000470877.1 MINDY1 trna
ENST00000470886.1 RRP1B trna
ENST00000470899.1 FBXO11 trna
ENST00000470904.4 KPNA1 trna
ENST00000470940.4 SP100 trna
ENST00000470951.4 SRGAP3 trna

ENST00000470960.1 RIC8B trna
ENST00000470961.1 RPL24 trna
ENST00000470967.1 DNAJC8 trna
ENST00000470972.4 SLC44A1 trna
ENST00000470975.2 ARHGEF2 trna
ENST00000470978.1 PDSS1 trna
ENST00000470990.4 MAGI1 trna
ENST00000470998.1 HDAC8 trna
ENST00000471007.1 CBFA2T2 trna
ENST00000471010.1 NPL trna
ENST00000471014.1 CTNNB1 trna
ENST00000471036.1 IBTK trna
ENST00000471060.1 RNF144A trna
ENST00000471074.1 FBXL13 trna
ENST00000471086.3 RN7SL396P trna
ENST00000471094.1 ACO2 trna
ENST00000471095.4 TFDP2 trna
ENST00000471098.1 TBC1D23 trna
ENST00000471099.1 AGO3 trna
ENST00000471101.4 DNAJB14 trna
ENST00000471102.1 WASHC2C trna
ENST00000471107.1 STAG2 trna
ENST00000471110.4 ST7-OT4 trna
ENST00000471115.4 C1orf52 trna
ENST00000471135.2 RAI1 trna
ENST00000471152.1 RPL23AP42 trna
ENST00000471166.1 TNPO3 trna
ENST00000471184.4 HLA-DPB1 trna
ENST00000471196.1 ZNF148 trna
ENST00000471197.1 ZFAND5 trna
ENST00000471198.1 WDR49 trna
ENST00000471206.1 TMEM268 trna
ENST00000471216.1 LILRB2 trna
ENST00000471217.4 BTAF1 trna
ENST00000471218.4 PTPRE trna
ENST00000471220.4 CD53 trna
ENST00000471232.4 BIRC6 trna
ENST00000471236.1 HACD2 trna
ENST00000471246.1 UBXN4 trna
ENST00000471255.4 TMEM164 trna
ENST00000471257.4 ARID4B trna
ENST00000471273.1 TBC1D23 trna
ENST00000471281.4 GNA12 trna
ENST00000471289.2 CMPK1 trna
ENST00000471290.1 RPL3 trna
ENST00000471296.4 GTF2B trna
ENST00000471301.3 BNC2 trna
ENST00000471335.4 EXOC6B trna
ENST00000471341.4 GON4L trna
ENST00000471350.1 COMMD3 trna
ENST00000471354.1 GNB1 trna
ENST00000471355.4 ARPC2 trna
ENST00000471364.1 RUBCN trna
ENST00000471374.1 YWHAH trna

ENST00000471378.1 NDUFA10 trna
ENST00000471419.5 NONO trna
ENST00000471471.1 GTF2B trna
ENST00000471473.1 JAK1 trna
ENST00000471475.1 DDX50 trna
ENST00000471492.2 ARHGAP30 trna
ENST00000471498.4 EYA3 trna
ENST00000471538.1 SPEN trna
ENST00000471552.4 ACTR2 trna
ENST00000471555.4 ATP11A trna
ENST00000471572.5 NF1 trna
ENST00000471593.1 ACAP2 trna
ENST00000471599.4 KPNA6 trna
ENST00000471632.1 TMEM87B trna
ENST00000471635.4 RBM39 trna
ENST00000471649.1 XRCC5 trna
ENST00000471652.1 ZC3HAV1 trna
ENST00000471675.1 B4GALT4 trna
ENST00000471679.1 TBC1D5 trna
ENST00000471694.1 CD47 trna
ENST00000471698.4 NFKB2 trna
ENST00000471706.1 POLR3GL trna
ENST00000471710.2 TDRD3 trna
ENST00000471722.1 trna
ENST00000471727.2 APLF trna
ENST00000471737.1 MTCH1 trna
ENST00000471739.1 FYCO1 trna
ENST00000471744.1 SNX13 trna
ENST00000471746.1 NA trna
ENST00000471758.1 APEX2 trna
ENST00000471760.4 UMAD1 trna
ENST00000471787.1 BARD1 trna
ENST00000471793.1 IKZF1 trna
ENST00000471810.4 NA trna
ENST00000471815.4 FAM107B trna
ENST00000471821.1 MPP1 trna
ENST00000471826.1 P4HA2 trna
ENST00000471830.1 PANK2 trna
ENST00000471834.1 SYNE1 trna
ENST00000471837.1 PTPRZ1 trna
ENST00000471849.4 LUZP1 trna
ENST00000471856.1 PDIA3P1 trna
ENST00000471865.1 CACNA2D3 trna
ENST00000471866.4 ATXN2 trna
ENST00000471875.4 SLC11A1 trna
ENST00000471887.1 HDAC9 trna
ENST00000471894.1 PUM1 trna
ENST00000471897.1 NCOA6 trna
ENST00000471937.1 PACC1 trna
ENST00000471938.1 GBP4 trna
ENST00000471939.4 SH2D3C trna
ENST00000471946.1 MDM2 trna
ENST00000471947.1 CCDC88A trna
ENST00000471959.2 FYN trna

ENST00000471981.1 SFT2D2 trna
ENST00000471996.1 SORT1 trna
ENST00000472020.1 EXOC4 trna
ENST00000472021.1 PRKCE trna
ENST00000472022.1 KLF12 trna
ENST00000472024.2 CHMP2B trna
ENST00000472025.1 CTSZ trna
ENST00000472030.1 RNF103 trna
ENST00000472032.1 BBX trna
ENST00000472035.4 ZMIZ1 trna
ENST00000472038.1 RCSD1 trna
ENST00000472045.1 IRF1 trna
ENST00000472046.1 PSMD6-AS2 trna
ENST00000472066.1 PRKCB trna
ENST00000472067.1 CASP2 trna
ENST00000472077.1 PPM1G trna
ENST00000472080.1 DNHD1 trna
ENST00000472083.1 CUL4A trna
ENST00000472084.2 MTSS1 trna
ENST00000472099.1 STXBP3 trna
ENST00000472129.3 CLTC trna
ENST00000472142.1 ID2 trna
ENST00000472143.2 RBM26 trna
ENST00000472145.4 PLGRKT trna
ENST00000472150.1 ARHGAP21 trna
ENST00000472162.2 SLC25A13 trna
ENST00000472166.1 NEPRO trna
ENST00000472169.1 CIR1 trna
ENST00000472173.4 LONRF3 trna
ENST00000472179.2 POLR1D trna
ENST00000472180.1 TP53BP2 trna
ENST00000472195.1 ATXN7L1 trna
ENST00000472202.1 ATP5F1C trna
ENST00000472207.4 TMEM245 trna
ENST00000472210.4 MYH9 trna
ENST00000472226.1 ARGLU1 trna
ENST00000472246.4 NUDCD3 trna
ENST00000472270.1 OGT trna
ENST00000472283.1 CAMTA1 trna
ENST00000472287.1 trna
ENST00000472298.1 N4BP2L1 trna
ENST00000472302.1 HK2 trna
ENST00000472304.1 TLR4 trna
ENST00000472305.1 SEC16A trna
ENST00000472311.5 COX7A2 trna
ENST00000472321.1 LGALS1 trna
ENST00000472333.1 MAPK14 trna
ENST00000472344.1 DAPK1 trna
ENST00000472359.1 ELMO1 trna
ENST00000472373.1 U2SURP trna
ENST00000472374.5 CENPM trna
ENST00000472378.1 TSPO trna
ENST00000472379.1 DENND10 trna
ENST00000472382.4 FOXP1 trna

ENST00000472467.1 SSR2 trna
ENST00000472475.1 GOLGB1 trna
ENST00000472477.1 TSC22D1 trna
ENST00000472479.1 UBE2F trna
ENST00000472498.2 RPL37P2 trna
ENST00000472517.1 INPP5D trna
ENST00000472545.5 DLG2 trna
ENST00000472564.1 LAMP1 trna
ENST00000472569.1 ATXN7 trna
ENST00000472614.2 GNB1 trna
ENST00000472616.1 KDM7A trna
ENST00000472625.1 XRN1 trna
ENST00000472627.1 SMURF1 trna
ENST00000472650.1 PNKD trna
ENST00000472666.1 PRKY trna
ENST00000472677.1 TRABD trna
ENST00000472680.1 RNF103 trna
ENST00000472697.4 XRN1 trna
ENST00000472700.5 KCNAB2 trna
ENST00000472701.4 GRM8 trna
ENST00000472704.1 CASK trna
ENST00000472706.4 USP34 trna
ENST00000472709.5 EIF2D trna
ENST00000472713.1 CEP85L trna
ENST00000472720.4 THSD7B trna
ENST00000472725.4 SF1 trna
ENST00000472730.1 RPL10AP6 trna
ENST00000472732.4 DGKD trna
ENST00000472754.1 LATS2 trna
ENST00000472776.1 CLASP1 trna
ENST00000472778.4 HNRNPK trna
ENST00000472783.4 FGGY trna
ENST00000472786.1 TBC1D2B trna
ENST00000472791.4 TBC1D22A trna
ENST00000472800.1 AUP1 trna
ENST00000472804.1 TNFSF10 trna
ENST00000472808.1 FAF1 trna
ENST00000472832.2 PTEN trna
ENST00000472835.1 FTO trna
ENST00000472836.1 NA trna
ENST00000472842.1 ARNTL trna
ENST00000472851.1 PEX14 trna
ENST00000472859.2 SGK1 trna
ENST00000472907.5 PIP5K1B trna
ENST00000472910.4 ATXN7L1 trna
ENST00000472949.1 FBXL3 trna
ENST00000472951.1 STN1 trna
ENST00000472989.4 TM2D1 trna
ENST00000473002.1 ITGA4 trna
ENST00000473004.4 THADA trna
ENST00000473022.1 MYH9 trna
ENST00000473025.1 N4BP2L2 trna
ENST00000473036.2 TPM3 trna
ENST00000473037.4 LYST trna

ENST00000473039.4 DENND1A trna
ENST00000473040.5 ABHD18 trna
ENST00000473050.1 PHF14 trna
ENST00000473062.1 CLCC1 protein_coding
ENST00000473070.1 RIPOR2 processed_transcript
ENST00000473073.1 PCCB retained_intron
ENST00000473075.2 DLEU1 lncRNA
ENST00000473076.1 DHX9 processed_transcript
ENST00000473078.1 CDK6 retained_intron
ENST00000473081.1 ANKRD44 processed_transcript
ENST00000473088.1 IQSEC1 processed_transcript
ENST00000473091.4 UBAC2 processed_transcript
ENST00000473093.1 MSL2 protein_coding
ENST00000473109.2 processed_pseudogene
ENST00000473136.3 ZNF654 retained_intron
ENST00000473155.1 DDIT4 retained_intron
ENST00000473184.1 GATAD2A processed_transcript
ENST00000473186.4 KMT2C retained_intron
ENST00000473194.4 UBAC2 processed_transcript
ENST00000473200.1 XXYLT1 processed_transcript
ENST00000473201.1 SEPTIN7 retained_intron
ENST00000473205.1 ANP32B processed_transcript
ENST00000473208.4 RYK retained_intron
ENST00000473231.4 FCRL3 processed_transcript
ENST00000473252.4 C9orf85 nonsense_mediated_decay
ENST00000473272.1 PLB1 retained_intron
ENST00000473299.1 lncRNA
ENST00000473306.1 ASB1 processed_transcript
ENST00000473321.1 NDUFS2 processed_transcript
ENST00000473338.1 UXS1 retained_intron
ENST00000473349.1 BRD3 processed_transcript
ENST00000473352.1 LINC00886 lncRNA
ENST00000473361.4 SPOCD1 retained_intron
ENST00000473371.1 NHLRC3 retained_intron
ENST00000473378.1 IL2RG protein_coding
ENST00000473387.1 PRCC processed_transcript
ENST00000473388.5 ATXN1 processed_transcript
ENST00000473413.1 ATP6V1G1 nonsense_mediated_decay
ENST00000473417.4 SNX4 processed_transcript
ENST00000473426.1 lncRNA
ENST00000473428.1 RHOQ nonsense_mediated_decay
ENST00000473435.1 PIK3CB nonsense_mediated_decay
ENST00000473444.1 MKRN1 protein_coding
ENST00000473453.2 TXNDC5 protein_coding
ENST00000473462.4 PCID2 processed_transcript
ENST00000473470.1 CNOT4 processed_transcript
ENST00000473474.4 CEPT1 processed_transcript
ENST00000473478.4 MBD5 processed_transcript
ENST00000473481.1 ABI1 processed_transcript
ENST00000473505.4 ECE1 protein_coding
ENST00000473519.4 STIM2 protein_coding
ENST00000473525.1 NONO retained_intron
ENST00000473541.4 PIK3CG protein_coding
ENST00000473546.1 ARF1 processed_transcript

ENST00000473558.4 RPL21 retained_intron
ENST00000473592.1 ZNF37BP processed_transcript
ENST00000473601.1 ZNF680 processed_transcript
ENST00000473606.1 AP1B1 retained_intron
ENST00000473608.1 RAB5A retained_intron
ENST00000473612.1 TBC1D5 processed_transcript
ENST00000473613.4 TAB1 retained_intron
ENST00000473631.1 UST processed_transcript
ENST00000473662.1 AMMECR1 processed_transcript
ENST00000473669.4 ADD3 processed_transcript
ENST00000473691.1 BRWD3 processed_transcript
ENST00000473702.4 TIPARP protein_coding
ENST00000473710.4 ACOT9 protein_coding
ENST00000473719.4 ADGRL2 processed_transcript
ENST00000473733.4 STK40 processed_transcript
ENST00000473748.1 RPS28P7 processed_pseudogene
ENST00000473752.4 DIP2A processed_transcript
ENST00000473758.4 ACIN1 nonsense_mediated_decay
ENST00000473767.4 ROBO2 nonsense_mediated_decay
ENST00000473776.1 WEE2-AS1 retained_intron
ENST00000473791.1 CDK5RAP1 retained_intron
ENST00000473796.1 PDK4 retained_intron
ENST00000473797.1 GPCPD1 retained_intron
ENST00000473813.1 BRWD1 retained_intron
ENST00000473815.1 LRRFIP1 processed_transcript
ENST00000473819.1 REV1 retained_intron
ENST00000473832.1 DGCR2 processed_transcript
ENST00000473837.4 MAPKAP1 retained_intron
ENST00000473842.1 CWF19L1 protein_coding
ENST00000473860.1 UBE2V1 processed_transcript
ENST00000473877.1 PLGRKT processed_transcript
ENST00000473883.4 HCLS1 processed_transcript
ENST00000473886.1 GSK3B processed_transcript
ENST00000473891.1 MAN1C1 processed_transcript
ENST00000473894.1 RAB4A processed_transcript
ENST00000473914.1 LNPEP retained_intron
ENST00000473922.1 SIRT1 processed_transcript
ENST00000473954.1 PPP1R10 processed_transcript
ENST00000473967.1 BRI3 processed_transcript
ENST00000473986.1 ARHGAP25 nonsense_mediated_decay
ENST00000473989.6 UBN2 protein_coding
ENST00000474002.1 IFI44L retained_intron
ENST00000474007.4 ABHD16A retained_intron
ENST00000474011.4 BAGE2 processed_transcript
ENST00000474017.4 PISD retained_intron
ENST00000474041.4 FCMR retained_intron
ENST00000474046.1 PBX1 processed_transcript
ENST00000474050.1 GLIPR2 processed_transcript
ENST00000474063.4 DIRC3 lncRNA
ENST00000474071.4 EXOG retained_intron
ENST00000474072.1 FLNA retained_intron
ENST00000474077.1 DYNC1LI1 retained_intron
ENST00000474086.4 RBM34 nonsense_mediated_decay
ENST00000474102.1 DOCK10 processed_transcript

ENST00000474113.1 SH3BGRL processed_transcript
ENST00000474116.4 CUL4A processed_transcript
ENST00000474117.1 NA NA
ENST00000474131.4 CAMK2G processed_transcript
ENST00000474143.4 FAM107B retained_intron
ENST00000474153.1 UBE3C retained_intron
ENST00000474167.1 RPS3AP20 processed_pseudogene
ENST00000474176.2 TPD52L2 retained_intron
ENST00000474194.1 COP1 nonsense_mediated_decay
ENST00000474202.1 LRRK2 retained_intron
ENST00000474203.4 KMT2E protein_coding
ENST00000474211.1 STK17A retained_intron
ENST00000474214.2 RUBCN protein_coding
ENST00000474223.1 SAT1 retained_intron
ENST00000474240.1 ECHDC1 retained_intron
ENST00000474241.1 DPYD retained_intron
ENST00000474258.1 SEMA4D retained_intron
ENST00000474260.1 SLK processed_transcript
ENST00000474266.1 RPS6KA3 retained_intron
ENST00000474281.1 PGK1 processed_transcript
ENST00000474288.1 HUWE1 retained_intron
ENST00000474301.1 TNFSF8 processed_transcript
ENST00000474312.4 GIGYF2 retained_intron
ENST00000474314.5 MTF2 processed_transcript
ENST00000474320.1 DNAJC15 processed_transcript
ENST00000474321.5 TTC7A processed_transcript
ENST00000474332.4 WIPF1 retained_intron
ENST00000474358.4 FLNA retained_intron
ENST00000474363.1 TBL1XR1 retained_intron
ENST00000474368.1 MX2 retained_intron
ENST00000474374.4 ELP4 nonsense_mediated_decay
ENST00000474375.4 RABGAP1L protein_coding
ENST00000474381.1 HLA-B retained_intron
ENST00000474383.1 PRKAG2 processed_transcript
ENST00000474384.2 SNRPB nonsense_mediated_decay
ENST00000474386.1 DENND4B processed_transcript
ENST00000474405.1 ZNF644 processed_transcript
ENST00000474426.1 UBR3 retained_intron
ENST00000474429.4 MAPK14 nonsense_mediated_decay
ENST00000474441.1 CGGBP1 processed_transcript
ENST00000474446.4 DHX9 processed_transcript
ENST00000474450.4 SULF2 processed_transcript
ENST00000474453.5 TUT4 protein_coding
ENST00000474465.4 GIGYF2 processed_transcript
ENST00000474466.4 HM13 processed_transcript
ENST00000474467.1 IQSEC1 processed_transcript
ENST00000474472.4 LPP processed_transcript
ENST00000474473.1 IFI16 protein_coding
ENST00000474474.4 ARHGAP15 processed_transcript
ENST00000474488.1 DGKD retained_intron
ENST00000474495.1 CEMIP2 retained_intron
ENST00000474503.1 GTPBP10 processed_transcript
ENST00000474510.2 UBAC2 protein_coding
ENST00000474513.5 ATXN7 protein_coding

ENST00000474521.1 FGFR3 retained_intron
ENST00000474522.4 ZFAND3 protein_coding
ENST00000474532.1 HIVEP2 processed_transcript
ENST00000474535.2 ATL2 retained_intron
ENST00000474542.2 REEP5 processed_transcript
ENST00000474545.1 ANXA11 processed_transcript
ENST00000474574.1 GLUD1 retained_intron
ENST00000474582.1 RPS8 processed_transcript
ENST00000474586.4 TRA2A processed_transcript
ENST00000474624.4 CA5B retained_intron
ENST00000474632.4 FUBP1 processed_transcript
ENST00000474637.4 GAPVD1 retained_intron
ENST00000474640.1 RPL35A retained_intron
ENST00000474653.4 MORF4L2 processed_transcript
ENST00000474657.4 FKBP1A processed_transcript
ENST00000474662.1 PIGA retained_intron
ENST00000474665.1 LIMD1 retained_intron
ENST00000474669.1 PARP14 retained_intron
ENST00000474676.1 DENND1A processed_transcript
ENST00000474682.1 MPP7 processed_transcript
ENST00000474694.1 DDX18 processed_transcript
ENST00000474699.1 TIA1 retained_intron
ENST00000474710.4 ZBTB20 protein_coding
ENST00000474715.1 YWHAQ processed_transcript
ENST00000474717.2 STK4 protein_coding
ENST00000474725.4 EDEM3 retained_intron
ENST00000474727.2 SLC9A9 nonsense_mediated_decay
ENST00000474731.1 MPP7 processed_transcript
ENST00000474732.1 EPHB1 protein_coding
ENST00000474737.4 PTTG1IP retained_intron
ENST00000474742.1 HDAC9 processed_transcript
ENST00000474756.1 ICMT nonsense_mediated_decay
ENST00000474770.1 SRPK2 protein_coding
ENST00000474772.1 DOCK8 retained_intron
ENST00000474788.1 PLEK retained_intron
ENST00000474789.1 ECHDC2 retained_intron
ENST00000474792.1 NLRP3 protein_coding
ENST00000474806.1 EVI5 processed_transcript
ENST00000474808.1 DPYSL2 processed_transcript
ENST00000474815.2 COMMD7 protein_coding
ENST00000474828.1 SCAF11 retained_intron
ENST00000474830.1 GSK3B retained_intron
ENST00000474835.4 USP16 retained_intron
ENST00000474837.4 SFMBT1 processed_transcript
ENST00000474842.1 CFLAR retained_intron
ENST00000474850.2 AKAP7 protein_coding
ENST00000474851.1 LSAMP protein_coding
ENST00000474854.1 JARID2 processed_transcript
ENST00000474865.4 ASMTL processed_transcript
ENST00000474868.1 MKNK1 protein_coding
ENST00000474882.2 MYCBP2 processed_transcript
ENST00000474903.1 MFN1 protein_coding
ENST00000474906.2 SENP6 retained_intron
ENST00000474925.2 PTAR1 retained_intron

ENST00000474948.4 TTLL3 nonsense_mediated_decay
ENST00000474953.4 ARID4B nonsense_mediated_decay
ENST00000474988.4 KCTD20 processed_transcript
ENST00000474990.4 FUS processed_transcript
ENST00000475004.4 DDX17 nonsense_mediated_decay
ENST00000475005.1 ERMP1 retained_intron
ENST00000475010.4 MKRN1 retained_intron
ENST00000475023.1 AUH processed_transcript
ENST00000475028.2 EXOC2 processed_transcript
ENST00000475034.1 ROBO2 processed_transcript
ENST00000475038.2 FMR1 nonsense_mediated_decay
ENST00000475045.5 RUNX1 protein_coding
ENST00000475046.4 IVNS1ABP retained_intron
ENST00000475055.1 SLMAP protein_coding
ENST00000475059.4 PIGC processed_transcript
ENST00000475072.1 SON retained_intron
ENST00000475073.4 SRBD1 processed_transcript
ENST00000475081.1 AHCYL1 processed_transcript
ENST00000475083.4 SATB1 protein_coding
ENST00000475094.1 CCNT2 retained_intron
ENST00000475102.1 lncRNA
ENST00000475123.1 XPNPEP1 processed_transcript
ENST00000475134.1 TBC1D23 protein_coding
ENST00000475146.1 CPNE1 processed_transcript
ENST00000475148.1 ANKHD1 retained_intron
ENST00000475162.2 SLC2A1 protein_coding
ENST00000475164.2 LUZP1 protein_coding
ENST00000475168.1 TSNAX retained_intron
ENST00000475170.4 ITGB2 retained_intron
ENST00000475178.1 NR6A1 protein_coding
ENST00000475200.4 BTRC processed_transcript
ENST00000475213.1 SSR1 processed_transcript
ENST00000475233.1 KDM6A retained_intron
ENST00000475243.4 VAPB protein_coding
ENST00000475275.4 ESRRG protein_coding
ENST00000475277.1 LYST processed_transcript
ENST00000475283.2 SUSD1 nonsense_mediated_decay
ENST00000475287.1 ARHGAP26 protein_coding
ENST00000475288.4 PCGF3 retained_intron
ENST00000475289.1 S1PR1 protein_coding
ENST00000475302.1 ITGA6 retained_intron
ENST00000475319.1 ECHDC1 retained_intron
ENST00000475321.4 RBBP4 protein_coding
ENST00000475338.2 ACBD6 processed_transcript
ENST00000475360.5 SLC25A12 nonsense_mediated_decay
ENST00000475365.1 EEF1A1P10 processed_pseudogene
ENST00000475366.4 LRIG1 processed_transcript
ENST00000475375.1 VPS36 retained_intron
ENST00000475394.4 DLG1 retained_intron
ENST00000475401.1 TM9SF3 processed_transcript
ENST00000475405.2 TET3 retained_intron
ENST00000475407.4 GOLGA1 nonsense_mediated_decay
ENST00000475440.1 AHR processed_transcript
ENST00000475446.4 TANGO2 processed_transcript

ENST00000475455.1 INHCAP transcribed_unprocessed_pseudogene
ENST00000475463.5 PCNX2 nonsense_mediated_decay
ENST00000475464.1 PTP4A2 processed_transcript
ENST00000475465.1 COX7B protein_coding
ENST00000475468.4 RPS24 processed_transcript
ENST00000475472.4 FGR processed_transcript
ENST00000475483.4 ATP1B3 protein_coding
ENST00000475490.1 PLEKHG1 nonsense_mediated_decay
ENST00000475506.1 SDHB retained_intron
ENST00000475527.1 PTPRG processed_transcript
ENST00000475539.4 ZNF385B processed_transcript
ENST00000475540.1 SMC5 retained_intron
ENST00000475556.1 RBM41 processed_transcript
ENST00000475564.4 EHMT1 retained_intron
ENST00000475584.1 IGF2R processed_transcript
ENST00000475607.1 RABGAP1 processed_transcript
ENST00000475608.1 RPL23AP65 processed_pseudogene
ENST00000475610.1 GNAS retained_intron
ENST00000475613.5 DLG5 retained_intron
ENST00000475615.1 DENND1A processed_transcript
ENST00000475627.1 HLA-DMA retained_intron
ENST00000475636.1 ATP11B processed_transcript
ENST00000475638.5 COG5 processed_transcript
ENST00000475640.1 PARP14 processed_transcript
ENST00000475645.1 ATG5 processed_transcript
ENST00000475651.4 RBM39 retained_intron
ENST00000475660.1 AUTS2 retained_intron
ENST00000475683.1 TUBA4A retained_intron
ENST00000475685.1 GAB3 processed_transcript
ENST00000475708.1 MPC1 processed_transcript
ENST00000475715.1 NRDC processed_transcript
ENST00000475724.1 LYPLAL1 processed_transcript
ENST00000475729.4 PHC3 protein_coding
ENST00000475743.1 ZNF638 protein_coding
ENST00000475769.1 RAB3GAP2 processed_transcript
ENST00000475770.4 KRIT1 retained_intron
ENST00000475777.2 ARPC3 retained_intron
ENST00000475795.4 NA NA
ENST00000475808.4 GLUL processed_transcript
ENST00000475809.1 ANKRD10 processed_transcript
ENST00000475820.1 KANSL3 processed_transcript
ENST00000475821.1 S1PR1 protein_coding
ENST00000475834.1 IGF2R protein_coding
ENST00000475846.5 AHI1 nonsense_mediated_decay
ENST00000475848.1 CACNB4 retained_intron
ENST00000475851.1 CEP68 processed_transcript
ENST00000475855.1 AADACL2-AS1 lncRNA
ENST00000475873.2 USP4 processed_transcript
ENST00000475877.4 BRD7 processed_transcript
ENST00000475879.1 STX7 retained_intron
ENST00000475881.4 PFKFB3 nonsense_mediated_decay
ENST00000475895.4 MRPS5 retained_intron
ENST00000475897.1 ATXN7 retained_intron
ENST00000475905.4 ACAP2 retained_intron

ENST00000475922.1 LPIN1 retained_intron
ENST00000475927.4 SERF2 retained_intron
ENST00000475949.4 FGGY processed_transcript
ENST00000475952.1 NUDCD3 retained_intron
ENST00000475957.1 MLLT3 retained_intron
ENST00000475962.4 CBX7 processed_transcript
ENST00000475971.4 SUN1 retained_intron
ENST00000475983.1 PLD3 retained_intron
ENST00000475993.1 ABCF1 nonsense_mediated_decay
ENST00000475996.1 HLA-F protein_coding
ENST00000476009.4 PHF14 processed_transcript
ENST00000476032.1 ZC4H2 processed_transcript
ENST00000476046.1 NA NA
ENST00000476047.1 CAP1P1 processed_pseudogene
ENST00000476068.1 C1GALT1 retained_intron
ENST00000476084.1 PDXK processed_transcript
ENST00000476089.1 ITGA4 retained_intron
ENST00000476106.4 SOD1 processed_transcript
ENST00000476110.1 RBX1 retained_intron
ENST00000476111.4 USP22 protein_coding
ENST00000476117.1 SRPK2 retained_intron
ENST00000476126.1 SP140 retained_intron
ENST00000476128.1 POGZ retained_intron
ENST00000476134.1 MAPK8 protein_coding
ENST00000476135.4 HDAC9 processed_transcript
ENST00000476149.1 DDX18 retained_intron
ENST00000476151.4 nonsense_mediated_decay
ENST00000476168.1 MAGT1 retained_intron
ENST00000476170.2 PHYKPL protein_coding
ENST00000476176.4 MCCC1 protein_coding
ENST00000476189.1 EZR processed_transcript
ENST00000476196.4 GNAS retained_intron
ENST00000476199.1 PPP1R21 processed_transcript
ENST00000476203.2 MIGA1 retained_intron
ENST00000476233.1 HNRNPA2B1 retained_intron
ENST00000476241.1 HNRNPU retained_intron
ENST00000476251.1 CLASP2 nonsense_mediated_decay
ENST00000476275.4 ZZZ3 processed_transcript
ENST00000476286.1 NCK1 protein_coding
ENST00000476300.1 RPL13A retained_intron
ENST00000476312.1 ZNF124 processed_transcript
ENST00000476317.2 SIPA1L3 processed_transcript
ENST00000476329.1 KIAA0319L processed_transcript
ENST00000476339.1 DIAPH1 retained_intron
ENST00000476343.1 ARL8B retained_intron
ENST00000476345.1 RNF216 processed_transcript
ENST00000476350.1 MACF1 processed_transcript
ENST00000476352.4 NIN protein_coding
ENST00000476360.1 XRCC5 retained_intron
ENST00000476364.4 PPP1R12B nonsense_mediated_decay
ENST00000476370.1 CBLB retained_intron
ENST00000476375.4 AGAP3 processed_transcript
ENST00000476376.4 WDR49 processed_transcript
ENST00000476377.1 SPART processed_transcript

ENST00000476408.1 CD53 processed_transcript
ENST00000476419.4 PTBP2 nonsense_mediated_decay
ENST00000476423.4 SZRD1 nonsense_mediated_decay
ENST00000476433.4 CLASP2 processed_transcript
ENST00000476437.1 ATF6 processed_transcript
ENST00000476451.1 HNRNPR protein_coding
ENST00000476463.1 SEM1 processed_transcript
ENST00000476481.1 NA NA
ENST00000476492.1 LAPTM5 processed_transcript
ENST00000476504.4 ZNF33A processed_transcript
ENST00000476505.2 PPP4R2 protein_coding
ENST00000476517.1 CDKAL1 processed_transcript
ENST00000476518.1 ATG5 processed_transcript
ENST00000476519.1 SYNE1 processed_transcript
ENST00000476531.1 PGK1 processed_transcript
ENST00000476532.1 SASH3 processed_transcript
ENST00000476562.4 MIDEAS retained_intron
ENST00000476571.1 NKAIN2 processed_transcript
ENST00000476574.4 JAK2 protein_coding
ENST00000476581.5 XPC nonsense_mediated_decay
ENST00000476590.1 CD55 retained_intron
ENST00000476592.4 PNPLA8 processed_transcript
ENST00000476594.1 CCM2 processed_transcript
ENST00000476598.1 APOO retained_intron
ENST00000476599.2 INIP processed_transcript
ENST00000476609.1 MAP4K4 retained_intron
ENST00000476613.1 IRF1 protein_coding
ENST00000476618.4 MAP3K20 retained_intron
ENST00000476620.1 EPDR1 protein_coding
ENST00000476637.4 TBXAS1 retained_intron
ENST00000476645.1 IP6K2 processed_transcript
ENST00000476646.4 NFIA protein_coding
ENST00000476650.2 NA NA
ENST00000476660.1 HNRNPR processed_transcript
ENST00000476664.1 LRCH3 retained_intron
ENST00000476666.4 INTS6 processed_transcript
ENST00000476675.4 PRKCE processed_transcript
ENST00000476695.1 AUTS2 processed_transcript
ENST00000476708.1 SYK processed_transcript
ENST00000476717.1 EGLN1 processed_transcript
ENST00000476729.4 FLOT1 retained_intron
ENST00000476733.4 CD247 processed_transcript
ENST00000476740.1 SETD5 nonsense_mediated_decay
ENST00000476756.1 INSIG1 protein_coding
ENST00000476762.4 KRBOX4 protein_coding
ENST00000476765.1 CHST15 processed_transcript
ENST00000476769.5 FYN processed_transcript
ENST00000476784.4 TTC3 retained_intron
ENST00000476787.1 SLC66A3 processed_transcript
ENST00000476794.1 FNDC3B processed_transcript
ENST00000476798.1 SCML4 retained_intron
ENST00000476805.2 SERTAD2 processed_transcript
ENST00000476808.1 KLHL24 protein_coding
ENST00000476824.1 ITGA4 retained_intron

ENST00000476842.1 SMIM4 protein_coding
ENST00000476846.4 DOCK4 processed_transcript
ENST00000476869.1 RAP2A nonsense_mediated_decay
ENST00000476895.4 MICU2 processed_transcript
ENST00000476897.4 XXYLT1 processed_transcript
ENST00000476904.4 ANGEL2 processed_transcript
ENST00000476933.1 OMA1 processed_transcript
ENST00000476936.4 CAPZA1 processed_transcript
ENST00000476953.5 PHF11 processed_transcript
ENST00000476958.4 GTDC1 processed_transcript
ENST00000476980.4 NAA16 processed_transcript
ENST00000477030.2 EPRS1 nonsense_mediated_decay
ENST00000477034.2 MASTL protein_coding
ENST00000477043.4 TASOR2 processed_transcript
ENST00000477049.1 SETX processed_transcript
ENST00000477051.1 PHF20L1 retained_intron
ENST00000477063.4 SEC22A processed_transcript
ENST00000477066.4 SRC processed_transcript
ENST00000477068.1 SP110 retained_intron
ENST00000477091.4 MRPS6 processed_transcript
ENST00000477096.1 ZRANB2 processed_transcript
ENST00000477102.1 SH3KBP1 processed_transcript
ENST00000477104.1 GSN protein_coding
ENST00000477112.1 DDX17 retained_intron
ENST00000477129.1 KBTBD2 retained_intron
ENST00000477132.1 PRKD3 processed_transcript
ENST00000477147.4 MACROD2 processed_transcript
ENST00000477171.1 MBNL1 processed_transcript
ENST00000477179.4 RPS6KB1 processed_transcript
ENST00000477183.1 UHRF2 processed_transcript
ENST00000477186.4 SCAI nonsense_mediated_decay
ENST00000477189.1 MYH9 retained_intron
ENST00000477192.4 NUTM2B-AS1 lncRNA
ENST00000477223.1 ORC5 retained_intron
ENST00000477232.4 PSMD14 retained_intron
ENST00000477237.1 XRN1 protein_coding
ENST00000477255.1 LILRA1 retained_intron
ENST00000477258.1 FILIP1L protein_coding
ENST00000477264.1 CLSTN1 processed_transcript
ENST00000477265.4 IL15 protein_coding
ENST00000477309.1 PLEKHO1 miRNA
ENST00000477322.1 RPAP2 miRNA
ENST00000477344.1 DENND6A miRNA
ENST00000477369.4 AFF4 miRNA
ENST00000477373.1 ZYX miRNA
ENST00000477398.1 IL1B miRNA
ENST00000477410.1 PREPL miRNA
ENST00000477413.1 CNIH4 miRNA
ENST00000477425.1 WDR26 miRNA
ENST00000477428.4 NRF1 miRNA
ENST00000477432.1 YME1L1 miRNA
ENST00000477460.5 SGK1 miRNA
ENST00000477468.1 MRTFA miRNA
ENST00000477474.2 STIM2 miRNA

ENST00000477486.1 RBMS1 miRNA
ENST00000477513.1 C21orf62-AS1 miRNA
ENST00000477514.1 LINC02478 miRNA
ENST00000477516.1 ATXN7 miRNA
ENST00000477534.1 NFE2L2 miRNA
ENST00000477538.1 VHL miRNA
ENST00000477543.1 BNIP2 miRNA
ENST00000477544.4 SARS1 miRNA
ENST00000477559.2 GPR137B miRNA
ENST00000477573.1 RTF2 miRNA
ENST00000477575.1 MMS19 miRNA
ENST00000477577.1 ITPR1 miRNA
ENST00000477583.1 CACUL1 miRNA
ENST00000477605.1 CCM2 miRNA
ENST00000477624.1 TMA7 miRNA
ENST00000477627.2 NA miRNA
ENST00000477647.1 SLC49A4 miRNA
ENST00000477665.1 PLCXD2 miRNA
ENST00000477670.1 RPS4XP6 miRNA
ENST00000477680.4 AP5Z1 miRNA
ENST00000477723.1 WDR12 miRNA
ENST00000477763.1 SMNDC1 miRNA
ENST00000477788.4 RSRC1 miRNA
ENST00000477790.1 ZNF37A miRNA
ENST00000477794.2 PRKCD miRNA
ENST00000477801.1 S100A8 miRNA
ENST00000477812.1 ACTB miRNA
ENST00000477816.1 SAMD9L miRNA
ENST00000477823.1 CASK miRNA
ENST00000477830.1 CAMKMT miRNA
ENST00000477852.1 ANKRD44 miRNA
ENST00000477860.1 UTP6 miRNA
ENST00000477861.1 PITPNB miRNA
ENST00000477867.4 ARHGAP26 miRNA
ENST00000477868.1 CDC73 miRNA
ENST00000477874.1 IL17RA miRNA
ENST00000477876.1 NUP58 miRNA
ENST00000477886.4 PRKRIP1 miRNA
ENST00000477888.1 SPTLC1 miRNA
ENST00000477889.4 SELENOT miRNA
ENST00000477890.5 CPSF7 miRNA
ENST00000477891.1 MINDY3 miRNA
ENST00000477893.1 ERCC8 miRNA
ENST00000477896.1 YWHAB miRNA
ENST00000477925.4 SRPK2 miRNA
ENST00000477930.2 NFKBIE miRNA
ENST00000477939.1 TRA2B miRNA
ENST00000477943.1 RPL7AP6 miRNA
ENST00000477958.4 PGD miRNA
ENST00000477964.1 miRNA
ENST00000477965.1 RBMS1 miRNA
ENST00000477973.2 RYBP miRNA
ENST00000477974.1 PLSCR1 miRNA
ENST00000477982.2 FBXL3 miRNA

ENST00000477983.1 LMBR1 miRNA
ENST00000477992.1 ARPC2 miRNA
ENST00000478004.1 PSMF1 miRNA
ENST00000478015.4 LCLAT1 miRNA
ENST00000478016.1 FNDC3B miRNA
ENST00000478026.1 RBM6 miRNA
ENST00000478039.1 ZNF701 miRNA
ENST00000478044.1 CUL2 miRNA
ENST00000478049.1 STIM2 miRNA
ENST00000478052.1 TASOR miRNA
ENST00000478059.1 ADAM17 miRNA
ENST00000478068.1 TEX261 miRNA
ENST00000478089.1 FBXO42 miRNA
ENST00000478129.4 FNBP1 miRNA
ENST00000478138.1 ITGB3BP miRNA
ENST00000478152.1 CAB39 miRNA
ENST00000478157.1 IFT57 miRNA
ENST00000478168.1 NSFL1C miRNA
ENST00000478169.4 CCM2 miRNA
ENST00000478188.4 POLR2B miRNA
ENST00000478189.1 HLA-DPB1 miRNA
ENST00000478190.2 MBD5 miRNA
ENST00000478193.4 ANAPC16 miRNA
ENST00000478197.1 C2orf88 miRNA
ENST00000478207.1 GCC2 miRNA
ENST00000478216.1 RC3H2 miRNA
ENST00000478222.1 CFP miRNA
ENST00000478223.2 SKI miRNA
ENST00000478263.4 FRMD4B miRNA
ENST00000478265.1 EXOC4 miRNA
ENST00000478274.5 SCP2 miRNA
ENST00000478285.1 TDP2 miRNA
ENST00000478290.2 TLE4 miRNA
ENST00000478318.5 STEAP2-AS1 miRNA
ENST00000478329.1 USP37 miRNA
ENST00000478333.1 IFNGR1 miRNA
ENST00000478348.1 S100A10 miRNA
ENST00000478353.1 CRBN miRNA
ENST00000478357.1 ZMIZ1 miRNA
ENST00000478359.4 TRAF3IP3 miRNA
ENST00000478361.5 IDE miRNA
ENST00000478380.4 DOCK8 miRNA
ENST00000478390.1 CD86 miRNA
ENST00000478391.1 PIGK miRNA
ENST00000478396.1 MARCHF7 miRNA
ENST00000478400.2 ARL13B miRNA
ENST00000478401.1 TRIM14 miRNA
ENST00000478404.1 RNF149 miRNA
ENST00000478412.1 LRCH1 miRNA
ENST00000478415.1 BRWD3 miRNA
ENST00000478419.4 NBPF15 miRNA
ENST00000478422.1 TRMT2B miRNA
ENST00000478440.1 ITGA4 miRNA
ENST00000478465.4 AUH processed_transcript

ENST00000478500.3 PIP5K1B nonsense_mediated_decay
ENST00000478504.1 COA1 trna
ENST00000478507.1 TDP2 trna
ENST00000478515.1 ITPR1 trna
ENST00000478519.4 HLA-G trna
ENST00000478530.1 TRIM14 trna
ENST00000478535.1 MBNL1 trna
ENST00000478537.1 TUT1 trna
ENST00000478541.1 CYTH3 trna
ENST00000478545.1 HIVEP1 trna
ENST00000478553.1 RASSF2 trna
ENST00000478556.1 ZNF33A trna
ENST00000478568.1 NA trna
ENST00000478572.1 NR2C2 trna
ENST00000478576.4 GFM1 trna
ENST00000478578.1 RPL22L1 trna
ENST00000478582.4 CCM2 trna
ENST00000478596.4 USP11 trna
ENST00000478601.1 HSPBAP1 trna
ENST00000478611.2 ADK trna
ENST00000478643.1 MTHFD1L trna
ENST00000478657.1 EPS15 trna
ENST00000478659.1 NAGK trna
ENST00000478660.5 RUNX2 trna
ENST00000478662.1 SLCO2A1 trna
ENST00000478669.1 ZDHHC3 trna
ENST00000478679.4 ARHGEF7 trna
ENST00000478698.1 HNRNPH3 trna
ENST00000478705.4 SFT2D1 trna
ENST00000478732.1 TMEM14B trna
ENST00000478741.1 CD86 trna
ENST00000478746.1 VIM trna
ENST00000478753.2 SEPHS2 trna
ENST00000478761.2 ARID1B trna
ENST00000478766.1 SULF2 trna
ENST00000478800.1 CDKN1A trna
ENST00000478825.4 DHRSX trna
ENST00000478830.1 TRAT1 trna
ENST00000478831.4 MED15 trna
ENST00000478845.2 THBS1-IT1 trna
ENST00000478849.1 OPN3 trna
ENST00000478853.1 NA trna
ENST00000478864.1 NOTCH2 trna
ENST00000478878.1 EIF4E2 trna
ENST00000478886.1 CMTM6 trna
ENST00000478887.1 SFI1 trna
ENST00000478896.2 VCL trna
ENST00000478900.4 UTY trna
ENST00000478905.1 ATF2 trna
ENST00000478915.1 ATXN7L1 trna
ENST00000478916.4 SYNE1 trna
ENST00000478928.1 ACTR3 trna
ENST00000478930.4 HBP1 trna
ENST00000478935.1 ARL6IP5 trna

ENST00000478937.3 DOT1L trna
ENST00000478938.1 SDF4 trna
ENST00000478941.1 NOP58 trna
ENST00000478943.4 TOM1L2 trna
ENST00000478958.4 LRRFIP1 trna
ENST00000478976.1 RAB3GAP2 trna
ENST00000478981.1 BNIP2 trna
ENST00000478989.4 PRKAG2 trna
ENST00000479023.4 FGD4 trna
ENST00000479025.1 GNAS trna
ENST00000479037.4 TRIP12 trna
ENST00000479049.5 DYSF trna
ENST00000479067.1 MIER1 trna
ENST00000479101.1 ERI3 trna
ENST00000479103.1 IKZF5 trna
ENST00000479107.1 HLA-DPA1 trna
ENST00000479114.2 STRBP trna
ENST00000479121.4 PDCD10 trna
ENST00000479158.1 CNKSR2 trna
ENST00000479164.1 CYB5R4 trna
ENST00000479165.1 PHIP trna
ENST00000479169.4 HDLBP trna
ENST00000479173.1 OSBPL10 trna
ENST00000479184.1 AP1S2 trna
ENST00000479187.4 LRRK2 trna
ENST00000479188.4 BCR trna
ENST00000479201.1 MEPCE trna
ENST00000479202.4 ITGB2 trna
ENST00000479204.1 CSTA trna
ENST00000479213.1 MED23 trna
ENST00000479220.4 DLGAP4 trna
ENST00000479223.1 PIK3CD trna
ENST00000479232.1 GIMAP4 trna
ENST00000479235.4 GPBP1L1 trna
ENST00000479252.1 LRRC8C trna
ENST00000479257.4 CALU trna
ENST00000479267.2 trna
ENST00000479282.4 ZBTB17 trna
ENST00000479300.1 VCP trna
ENST00000479310.4 WDR27 trna
ENST00000479320.4 HLA-A trna
ENST00000479325.1 RUNX1 trna
ENST00000479338.4 EIF4H trna
ENST00000479341.1 RUNX3 trna
ENST00000479345.2 DHX57 trna
ENST00000479347.1 HADHB trna
ENST00000479350.1 HIVEP3 trna
ENST00000479366.1 RPL10 trna
ENST00000479380.1 EPC1 trna
ENST00000479389.1 RIPK1 trna
ENST00000479402.1 ACTR3B trna
ENST00000479412.2 NOTCH2 trna
ENST00000479421.4 YWHAB trna
ENST00000479423.1 KDM6A trna

ENST00000479428.3 RN7SL546P trna
ENST00000479435.1 TNFSF13B trna
ENST00000479443.1 CBLL1 trna
ENST00000479446.4 ABCA2 trna
ENST00000479447.1 ELMO1 trna
ENST00000479449.1 QRICH1 trna
ENST00000479456.1 STARD7 trna
ENST00000479464.1 IP6K1 trna
ENST00000479485.4 SSR1 trna
ENST00000479505.4 KLHL20 trna
ENST00000479515.1 PHTF2 trna
ENST00000479534.4 PADI2 trna
ENST00000479537.4 BRAF trna
ENST00000479541.1 RNF216 trna
ENST00000479547.2 DOCK5 trna
ENST00000479556.1 GIMAP5 trna
ENST00000479584.1 CDK5RAP2 trna
ENST00000479614.1 NF1 trna
ENST00000479629.4 ZCCHC17 trna
ENST00000479630.1 MAP3K7 trna
ENST00000479654.1 DIP2A trna
ENST00000479657.4 SELL trna
ENST00000479672.4 TFG trna
ENST00000479680.1 ATXN1 trna
ENST00000479722.1 SLC25A28 trna
ENST00000479727.1 WDR26 trna
ENST00000479729.1 TIAL1 trna
ENST00000479734.1 DDX17 trna
ENST00000479735.1 ZEB2 trna
ENST00000479769.1 DOCK9 trna
ENST00000479774.4 UXS1 trna
ENST00000479778.1 WDR26 trna
ENST00000479796.4 TRAF3IP3 trna
ENST00000479798.1 ZNF644 trna
ENST00000479802.1 RPS18 trna
ENST00000479803.1 SCML4 trna
ENST00000479806.1 DYNC1I2 trna
ENST00000479809.1 RPS6KA3 trna
ENST00000479811.4 OGA trna
ENST00000479818.4 PLXNB2 trna
ENST00000479828.1 FAM102A trna
ENST00000479832.1 SETD2 trna
ENST00000479844.1 ARHGAP25 trna
ENST00000479853.1 SDHAF3 trna
ENST00000479896.1 PTPRE trna
ENST00000479900.4 GUSBP2 trna
ENST00000479907.1 ZNF282 trna
ENST00000479928.1 GDI2 trna
ENST00000479933.2 TNFRSF8 trna
ENST00000479936.1 NA trna
ENST00000479944.4 TRABD2A trna
ENST00000479955.4 TRAPPC12 trna
ENST00000479956.1 ARPC4 trna
ENST00000479970.1 SULF2 trna

ENST00000479971.1 TBC1D15 trna
ENST00000479979.1 CD247 trna
ENST00000479994.1 HCLS1 trna
ENST00000479995.2 NOTCH2NLA trna
ENST00000480033.2 AHDC1 trna
ENST00000480038.1 FBXO11 trna
ENST00000480055.4 SMURF1 trna
ENST00000480057.1 VTI1A trna
ENST00000480083.4 GPBP1L1 trna
ENST00000480109.2 PDE4B trna
ENST00000480117.1 PPM1L trna
ENST00000480125.1 POLA1 trna
ENST00000480128.1 ARID2 trna
ENST00000480155.1 SHOC2 trna
ENST00000480158.1 HERC4 trna
ENST00000480195.1 ZFX trna
ENST00000480198.1 TUBGCP2 trna
ENST00000480201.1 AOAH trna
ENST00000480212.1 CD302 trna
ENST00000480218.1 BTN2A1 trna
ENST00000480222.1 SIK3 trna
ENST00000480226.4 ITGB1 trna
ENST00000480234.1 PTTG1IP trna
ENST00000480242.1 SNRNP200 trna
ENST00000480245.4 UBE2H trna
ENST00000480249.4 CRBN trna
ENST00000480266.4 ENG trna
ENST00000480276.1 ADA2 trna
ENST00000480277.2 NAA15 trna
ENST00000480281.4 TPST1 trna
ENST00000480283.4 ATRX trna
ENST00000480288.1 VGLL4 trna
ENST00000480301.1 ACTB trna
ENST00000480302.4 IQCG trna
ENST00000480307.1 SUN2 trna
ENST00000480310.1 FCRL1 trna
ENST00000480311.4 GCNT1 trna
ENST00000480317.1 CFP trna
ENST00000480320.1 EML4 trna
ENST00000480324.4 CEPT1 trna
ENST00000480327.1 VCP trna
ENST00000480332.1 CARD11 trna
ENST00000480342.4 RERE trna
ENST00000480368.4 KMT2E trna
ENST00000480381.1 RYK trna
ENST00000480385.1 CLASP2 trna
ENST00000480388.1 PALM2AKAP2 trna
ENST00000480400.1 WIPF1 trna
ENST00000480436.4 TASP1 trna
ENST00000480454.1 GMEB1 trna
ENST00000480464.2 TARDBP trna
ENST00000480468.1 SIK3 trna
ENST00000480494.1 SPAG16 trna
ENST00000480503.4 MYO1G trna

ENST00000480510.4 CYTH4 trna
ENST00000480522.4 ASB3 trna
ENST00000480528.1 ATP5F1C trna
ENST00000480540.1 trna
ENST00000480553.1 DIP2A trna
ENST00000480581.1 CAPN2 trna
ENST00000480592.4 DDX3X trna
ENST00000480602.1 PUM1 trna
ENST00000480603.1 PPIA trna
ENST00000480606.1 MAP3K20 trna
ENST00000480615.1 SNRNP200 trna
ENST00000480633.1 PRKCE processed_transcript
ENST00000480662.1 RPS24 retained_intron
ENST00000480676.2 WDR26 protein_coding
ENST00000480707.1 MALRD1 processed_transcript
ENST00000480733.1 STAG1 protein_coding
ENST00000480738.1 UBAC2 processed_transcript
ENST00000480746.4 SIDT1 retained_intron
ENST00000480748.2 CRYL1 protein_coding
ENST00000480755.1 RPS4XP22 processed_pseudogene
ENST00000480760.1 CTSS retained_intron
ENST00000480769.4 IVNS1ABP retained_intron
ENST00000480793.1 R3HDM1 processed_transcript
ENST00000480798.1 CST7 protein_coding
ENST00000480809.4 DGKG retained_intron
ENST00000480824.5 C6orf89 protein_coding
ENST00000480825.5 CSF3R retained_intron
ENST00000480863.1 GCC2 retained_intron
ENST00000480881.1 HSDL2 processed_transcript
ENST00000480887.4 RSF1 protein_coding
ENST00000480896.4 PCNT processed_transcript
ENST00000480911.4 CACNA1C nonsense_mediated_decay
ENST00000480927.1 NCKAP5L processed_transcript
ENST00000480936.1 SLBP protein_coding
ENST00000480940.1 PHF20 processed_transcript
ENST00000480942.1 RPS10 retained_intron
ENST00000480945.4 DDI2 protein_coding
ENST00000480948.4 DUSP11 processed_transcript
ENST00000480961.1 CTSA retained_intron
ENST00000480984.1 SLC20A1 processed_transcript
ENST00000480991.1 SLC35E2B retained_intron
ENST00000480996.1 KDM4C processed_transcript
ENST00000480999.1 TRIM26 retained_intron
ENST00000481014.5 TRIM66 retained_intron
ENST00000481037.4 RBM39 retained_intron
ENST00000481038.4 GPCPD1 retained_intron
ENST00000481048.4 LRCH3 retained_intron
ENST00000481049.4 UHRF2 processed_transcript
ENST00000481051.1 DOCK9 processed_transcript
ENST00000481061.2 COX7A2 retained_intron
ENST00000481073.4 XPO1 retained_intron
ENST00000481100.4 DOCK5 protein_coding
ENST00000481115.2 PKP4 nonsense_mediated_decay
ENST00000481118.1 LARP4B processed_transcript

ENST00000481126.1 KLHL24 retained_intron
ENST00000481135.1 CUL3 retained_intron
ENST00000481136.1 PDCD10 retained_intron
ENST00000481145.1 GBP5 retained_intron
ENST00000481152.1 CHCHD3 protein_coding
ENST00000481154.1 PTPN12 retained_intron
ENST00000481160.1 FBXL17 retained_intron
ENST00000481161.4 GCA retained_intron
ENST00000481164.1 ADAM10 retained_intron
ENST00000481168.1 RPN1 retained_intron
ENST00000481195.4 PPP2CA protein_coding
ENST00000481197.4 VOPP1 retained_intron
ENST00000481203.1 TFG retained_intron
ENST00000481209.4 FAM107B nonsense_mediated_decay
ENST00000481210.4 SEC61A1 protein_coding
ENST00000481215.1 RAC2 retained_intron
ENST00000481218.1 OXA1L retained_intron
ENST00000481229.1 PNISR protein_coding
ENST00000481244.4 MPP7 processed_transcript
ENST00000481245.4 CSF2RA processed_transcript
ENST00000481256.1 LRRK2 processed_transcript
ENST00000481260.1 RAPGEF1 processed_transcript
ENST00000481263.1 GRAP2 retained_intron
ENST00000481266.1 CDK5RAP2 nonsense_mediated_decay
ENST00000481276.1 SLC35E2B retained_intron
ENST00000481288.1 STK24 processed_transcript
ENST00000481319.1 AGPAT3 processed_transcript
ENST00000481337.4 ALCAM processed_transcript
ENST00000481360.1 CACUL1 retained_intron
ENST00000481367.4 IAH1 protein_coding
ENST00000481374.1 ACADM processed_transcript
ENST00000481402.4 AKAP4 processed_transcript
ENST00000481412.4 MAT2A retained_intron
ENST00000481418.4 PHF14 processed_transcript
ENST00000481423.1 AHCYL1 processed_transcript
ENST00000481425.4 TMEM243 processed_transcript
ENST00000481434.4 PRKAG2 retained_intron
ENST00000481437.4 PPIA retained_intron
ENST00000481451.1 PLEKHA1 retained_intron
ENST00000481452.1 LHPP processed_transcript
ENST00000481463.4 ACAP2 processed_transcript
ENST00000481470.4 FLNB retained_intron
ENST00000481474.4 TRIM27 retained_intron
ENST00000481515.1 SIMC1 retained_intron
ENST00000481517.1 STAT5B retained_intron
ENST00000481518.1 PSME4 processed_transcript
ENST00000481546.1 UBE2G2 retained_intron
ENST00000481549.1 NT5C2 processed_transcript
ENST00000481550.5 ZNF169 protein_coding
ENST00000481558.1 ZNF618 processed_transcript
ENST00000481566.1 ASB1 processed_transcript
ENST00000481568.2 VSIR retained_intron
ENST00000481569.1 RAB2A retained_intron
ENST00000481579.4 USP33 protein_coding

ENST00000481585.1 ANKUB1 processed_transcript
ENST00000481607.1 STAB1 retained_intron
ENST00000481610.1 ZNF337 processed_transcript
ENST00000481628.1 CLIP4 retained_intron
ENST00000481632.4 ZBTB20 protein_coding
ENST00000481633.1 MAD1L1 processed_transcript
ENST00000481638.1 EVA1C processed_transcript
ENST00000481641.1 CLK1 retained_intron
ENST00000481648.4 SRSF11 nonsense_mediated_decay
ENST00000481650.1 TIA1 retained_intron
ENST00000481658.1 FAM117B retained_intron
ENST00000481661.1 GPR135 nonsense_mediated_decay
ENST00000481674.1 CTNNBL1 processed_transcript
ENST00000481677.1 CD244 processed_transcript
ENST00000481682.1 LRRFIP2 processed_transcript
ENST00000481687.1 CD81 protein_coding
ENST00000481690.1 GPCPD1 nonsense_mediated_decay
ENST00000481700.1 KIAA1217 processed_transcript
ENST00000481707.4 RGS18 processed_transcript
ENST00000481727.1 ONECUT2 processed_transcript
ENST00000481728.2 DOCK5 retained_intron
ENST00000481749.4 PIK3CB retained_intron
ENST00000481762.4 ZNF767P processed_transcript
ENST00000481769.1 AXIN1 processed_transcript
ENST00000481773.1 lncRNA
ENST00000481784.1 NA NA
ENST00000481789.1 KCNAB2 retained_intron
ENST00000481792.1 REPS2 processed_transcript
ENST00000481793.1 RNLS processed_transcript
ENST00000481801.4 MGAT5 processed_transcript
ENST00000481819.4 HNRNPH3 processed_transcript
ENST00000481827.4 GLIS3 protein_coding
ENST00000481847.4 WFDC3 processed_transcript
ENST00000481849.4 HLA-B retained_intron
ENST00000481861.1 PRMT2 retained_intron
ENST00000481868.1 IL13RA1 processed_transcript
ENST00000481880.4 ATXN7L1 retained_intron
ENST00000481892.1 SNRK retained_intron
ENST00000481893.1 NA NA
ENST00000481937.4 OSBPL9 protein_coding
ENST00000481941.4 DHX36 protein_coding
ENST00000481958.1 APOBEC3G retained_intron
ENST00000481980.1 NUP58 nonsense_mediated_decay
ENST00000481996.1 SLC17A5 processed_transcript
ENST00000481997.4 ARPC1B retained_intron
ENST00000482000.1 PRKCB processed_transcript
ENST00000482010.5 PPP2R2D nonsense_mediated_decay
ENST00000482028.4 PABPC4 retained_intron
ENST00000482034.4 MED14 processed_transcript
ENST00000482035.2 NA NA
ENST00000482041.4 RINT1 nonsense_mediated_decay
ENST00000482059.5 CD36 protein_coding
ENST00000482063.1 LRRC8C nonsense_mediated_decay
ENST00000482067.2 UMAD1 protein_coding

ENST00000482070.1 ATP11B protein_coding
ENST00000482081.1 RPL12P8 processed_pseudogene
ENST00000482083.6 RNF13 protein_coding
ENST00000482087.1 NAA15 processed_transcript
ENST00000482103.1 EEF1B2 retained_intron
ENST00000482107.1 FNBP1 processed_transcript
ENST00000482110.4 PBX1 protein_coding
ENST00000482111.1 EIF1 retained_intron
ENST00000482114.1 KIAA1109 retained_intron
ENST00000482125.1 FXR1 protein_coding
ENST00000482128.1 SEMA4D processed_transcript
ENST00000482131.1 ZMYM4 processed_transcript
ENST00000482134.4 ASB3 processed_transcript
ENST00000482142.4 lncRNA
ENST00000482150.2 KLF3 retained_intron
ENST00000482155.1 STIMATE protein_coding
ENST00000482158.1 SF3B1 retained_intron
ENST00000482190.1 RBBP4 protein_coding
ENST00000482218.2 CLCN5 protein_coding
ENST00000482224.4 ABHD16A nonsense_mediated_decay
ENST00000482225.1 PLEKHB2 retained_intron
ENST00000482226.2 FCGR2C processed_transcript
ENST00000482230.4 TBCE processed_transcript
ENST00000482236.4 SORT1 protein_coding
ENST00000482258.1 PAPSS2 processed_transcript
ENST00000482265.1 DHRS3 retained_intron
ENST00000482274.2 OMA1 protein_coding
ENST00000482320.4 TNPO3 protein_coding
ENST00000482340.4 EHMT1 protein_coding
ENST00000482345.1 MTMR6 processed_transcript
ENST00000482386.4 GON4L processed_transcript
ENST00000482393.1 TPP2 processed_transcript
ENST00000482419.1 TASOR2 protein_coding
ENST00000482426.1 ROR1 processed_transcript
ENST00000482429.1 LTB retained_intron
ENST00000482449.2 RHOQ protein_coding
ENST00000482459.1 IFITM10 processed_transcript
ENST00000482460.1 CYTH3 processed_transcript
ENST00000482464.4 EPB41 processed_transcript
ENST00000482466.4 LRRFIP2 processed_transcript
ENST00000482484.1 CDC73 processed_transcript
ENST00000482493.1 DENND11 protein_coding
ENST00000482496.4 PDZD8 processed_transcript
ENST00000482499.1 CMC1 processed_transcript
ENST00000482501.4 BAZ2B processed_transcript
ENST00000482503.4 BAZ2B processed_transcript
ENST00000482504.1 SH3GLB1 protein_coding
ENST00000482512.4 GOLGB1 nonsense_mediated_decay
ENST00000482517.4 NA NA
ENST00000482518.5 PID1 processed_transcript
ENST00000482519.1 VAMP4 processed_transcript
ENST00000482525.4 RAB7A protein_coding
ENST00000482532.4 CALM2 retained_intron
ENST00000482540.1 GLCCI1 retained_intron

ENST00000482542.4 SFXN5 processed_transcript
ENST00000482544.4 TAF1 processed_transcript
ENST00000482547.1 MGMT retained_intron
ENST00000482548.1 STPG4 processed_transcript
ENST00000482556.4 PREX1 nonsense_mediated_decay
ENST00000482558.4 SNX13 retained_intron
ENST00000482561.1 PDCD6IP processed_transcript
ENST00000482570.4 EIPR1 retained_intron
ENST00000482590.1 CLK1 retained_intron
ENST00000482600.4 EIF3L retained_intron
ENST00000482601.1 GTDC1 processed_transcript
ENST00000482632.4 SPTLC1 retained_intron
ENST00000482635.1 ATP1B3 retained_intron
ENST00000482646.4 CREM processed_transcript
ENST00000482651.4 HDGF processed_transcript
ENST00000482653.1 MGMT processed_transcript
ENST00000482660.1 EML4 processed_transcript
ENST00000482677.4 SUCLG2-AS1 lncRNA
ENST00000482679.1 MRPS6 processed_transcript
ENST00000482689.4 ZBTB20 protein_coding
ENST00000482693.1 SF1 retained_intron
ENST00000482706.1 SIAH2 protein_coding
ENST00000482713.1 BOD1L1 nonsense_mediated_decay
ENST00000482720.1 XBP1 retained_intron
ENST00000482743.1 ARHGAP31 protein_coding
ENST00000482746.1 SLC35F6 processed_transcript
ENST00000482750.4 IL2RG protein_coding
ENST00000482752.1 MAP4 retained_intron
ENST00000482786.4 PIGF processed_transcript
ENST00000482794.1 ATP11B processed_transcript
ENST00000482808.1 CAPZB retained_intron
ENST00000482811.1 MAN1A2 processed_transcript
ENST00000482818.1 AP1B1 retained_intron
ENST00000482820.5 CHN2 processed_transcript
ENST00000482822.2 RSRC1 protein_coding
ENST00000482828.1 THEMIS2 processed_transcript
ENST00000482829.4 ASB3 processed_transcript
ENST00000482835.1 MFSD1 protein_coding
ENST00000482838.1 BRD2 retained_intron
ENST00000482851.1 SUSD1 processed_transcript
ENST00000482853.4 OARD1 nonsense_mediated_decay
ENST00000482860.1 LUC7L2 processed_transcript
ENST00000482865.1 OPA1 retained_intron
ENST00000482881.1 MAST2 retained_intron
ENST00000482915.1 C21orf91 retained_intron
ENST00000482934.1 CAMTA1 nonsense_mediated_decay
ENST00000482945.4 ADAM10 retained_intron
ENST00000482952.4 GIGYF2 retained_intron
ENST00000482979.4 ZKSCAN1 retained_intron
ENST00000482985.4 lncRNA
ENST00000483012.1 EZH2 nonsense_mediated_decay
ENST00000483019.1 CDYL processed_transcript
ENST00000483021.4 R3HDM1 processed_transcript
ENST00000483033.1 TMED5 processed_transcript

ENST00000483061.1 WDFY1 retained_intron
ENST00000483072.4 SMYD3 processed_transcript
ENST00000483081.1 CHN2 processed_transcript
ENST00000483083.2 SRCAP protein_coding
ENST00000483084.1 SP3 processed_transcript
ENST00000483085.1 DNAJC1 processed_transcript
ENST00000483088.1 N4BP2L2 retained_intron
ENST00000483118.4 ZNF341 nonsense_mediated_decay
ENST00000483124.1 SLC9A9 processed_transcript
ENST00000483135.1 HNRNPK retained_intron
ENST00000483142.4 HDAC9 processed_transcript
ENST00000483150.1 RREB1 protein_coding
ENST00000483168.4 ITGB5 protein_coding
ENST00000483176.2 CLTC nonsense_mediated_decay
ENST00000483189.1 ARHGEF7 retained_intron
ENST00000483194.1 BIRC6 retained_intron
ENST00000483213.4 ENTPD1 nonsense_mediated_decay
ENST00000483215.1 NAA16 processed_transcript
ENST00000483221.1 ANP32A processed_transcript
ENST00000483226.1 ZBTB7B processed_transcript
ENST00000483264.1 CCAR1 processed_transcript
ENST00000483268.1 RHBDD1 processed_transcript
ENST00000483270.1 DNAJC16 processed_transcript
ENST00000483279.1 LZTFL1 processed_transcript
ENST00000483281.4 RXRB nonsense_mediated_decay
ENST00000483293.1 PMS1 processed_transcript
ENST00000483295.1 RGS2 processed_transcript
ENST00000483296.4 TIMM23B processed_transcript
ENST00000483300.4 PLSCR1 protein_coding
ENST00000483326.1 LTN1 nonsense_mediated_decay
ENST00000483350.1 RBM6 retained_intron
ENST00000483371.1 FAM102B retained_intron
ENST00000483378.1 NSUN3 nonsense_mediated_decay
ENST00000483387.1 GNAS retained_intron
ENST00000483398.4 TP53BP2 nonsense_mediated_decay
ENST00000483407.1 CSDE1 processed_transcript
ENST00000483409.1 SSR1 processed_transcript
ENST00000483412.4 CDK5RAP2 retained_intron
ENST00000483416.1 DHX9 processed_transcript
ENST00000483421.6 ZNF638 retained_intron
ENST00000483426.4 UXS1 protein_coding
ENST00000483437.1 KPNA4 protein_coding
ENST00000483441.4 INTS6 retained_intron
ENST00000483442.4 LRCH3 retained_intron
ENST00000483443.1 LRRFIP1 retained_intron
ENST00000483457.1 EEFSEC protein_coding
ENST00000483487.2 SLC11A1 retained_intron
ENST00000483493.5 CAAP1 nonsense_mediated_decay
ENST00000483497.5 NUP214 protein_coding
ENST00000483500.1 CXCL8 retained_intron
ENST00000483532.1 TUBGCP3 processed_transcript
ENST00000483539.2 OBSCN protein_coding
ENST00000483549.4 ATXN10 retained_intron
ENST00000483551.5 ANP32A processed_transcript

ENST00000483573.1 IL2RB retained_intron
ENST00000483581.1 TOMM7 retained_intron
ENST00000483583.1 NA NA
ENST00000483591.5 ATXN1 processed_transcript
ENST00000483597.1 TLE4 processed_transcript
ENST00000483600.1 AFF3 processed_transcript
ENST00000483611.1 PARP14 processed_transcript
ENST00000483617.4 PTPN18 retained_intron
ENST00000483623.2 ALOX5 processed_transcript
ENST00000483630.4 NBPF9 protein_coding
ENST00000483668.4 FRMD4B retained_intron
ENST00000483670.4 PDCD4 processed_transcript
ENST00000483671.1 RFTN1 processed_transcript
ENST00000483695.1 OXSR1 retained_intron
ENST00000483706.1 TKT retained_intron
ENST00000483727.4 ITCH processed_transcript
ENST00000483728.4 SP140L retained_intron
ENST00000483733.1 RABL3 protein_coding
ENST00000483743.4 COBLL1 protein_coding
ENST00000483754.1 nonsense_mediated_decay
ENST00000483757.4 DOCK8 nonsense_mediated_decay
ENST00000483758.1 SVIL processed_transcript
ENST00000483763.4 CDC42SE1 retained_intron
ENST00000483775.1 PRKAG2 retained_intron
ENST00000483779.1 TMEM63A retained_intron
ENST00000483781.4 ZAP70 retained_intron
ENST00000483789.4 CCNL1 retained_intron
ENST00000483809.1 HBP1 retained_intron
ENST00000483826.1 ZNF44 retained_intron
ENST00000483842.1 PAN3 processed_transcript
ENST00000483847.1 SMARCC1 retained_intron
ENST00000483848.4 ARF4 processed_transcript
ENST00000483853.1 FUS retained_intron
ENST00000483859.5 SENP6 protein_coding
ENST00000483871.5 SRSF6 nonsense_mediated_decay
ENST00000483900.1 AHCTF1 processed_transcript
ENST00000483904.4 NCOA1 retained_intron
ENST00000483923.4 PYROXD2 processed_transcript
ENST00000483925.1 PTCD3 retained_intron
ENST00000483929.1 CYTIP retained_intron
ENST00000483930.1 CTSS nonsense_mediated_decay
ENST00000483935.1 GPR107 processed_transcript
ENST00000483937.4 MAPKAP1 processed_transcript
ENST00000483938.1 LPP processed_transcript
ENST00000483946.1 SIDT1 retained_intron
ENST00000483949.1 CD86 processed_transcript
ENST00000483954.1 ATXN1 processed_transcript
ENST00000483956.1 SEC61A1 processed_transcript
ENST00000483958.4 ESYT2 processed_transcript
ENST00000483966.2 HNRNPU protein_coding
ENST00000483968.4 PIK3CB protein_coding
ENST00000483990.1 TYW3 protein_coding
ENST00000484003.1 PSMB8 retained_intron
ENST00000484024.4 RGPD6 nonsense_mediated_decay

ENST00000484025.4 CCR3 retained_intron
ENST00000484026.4 SLC25A6 processed_transcript
ENST00000484035.4 CDK14 processed_transcript
ENST00000484041.4 PCNX2 retained_intron
ENST00000484042.1 FXR1 protein_coding
ENST00000484047.4 TTC3 processed_transcript
ENST00000484058.1 SLC25A43 processed_transcript
ENST00000484061.4 TTC7A retained_intron
ENST00000484063.5 PRDM2 protein_coding
ENST00000484067.5 FYN protein_coding
ENST00000484068.4 PHC3 protein_coding
ENST00000484070.1 WAPL retained_intron
ENST00000484073.1 WDPCP processed_transcript
ENST00000484078.4 SUPT20H retained_intron
ENST00000484081.1 CUL3 retained_intron
ENST00000484088.4 ITGB1 processed_transcript
ENST00000484106.2 RYK nonsense_mediated_decay
ENST00000484123.1 SFXN5 processed_transcript
ENST00000484130.1 RPL13AP25 processed_pseudogene
ENST00000484135.1 PTPRC retained_intron
ENST00000484147.4 PTPN22 retained_intron
ENST00000484150.1 LBH processed_transcript
ENST00000484153.1 C1orf167 processed_transcript
ENST00000484158.1 RPAP2 processed_transcript
ENST00000484165.1 ACTR3 retained_intron
ENST00000484179.4 USP34 retained_intron
ENST00000484188.1 SLC35C2 processed_transcript
ENST00000484194.1 HLA-E retained_intron
ENST00000484210.1 HIVEP1 protein_coding
ENST00000484213.1 ABCD3 processed_transcript
ENST00000484219.1 ANXA4 processed_transcript
ENST00000484220.1 PI4KA retained_intron
ENST00000484221.1 SLAMF7 retained_intron
ENST00000484235.1 SMDT1 retained_intron
ENST00000484237.4 COG5 processed_transcript
ENST00000484256.1 XBP1 retained_intron
ENST00000484259.1 FXN protein_coding
ENST00000484265.1 PSIP1 processed_transcript
ENST00000484270.2 KHDRBS1 processed_transcript
ENST00000484274.1 MAP4K3 protein_coding
ENST00000484275.1 SCAF11 processed_transcript
ENST00000484282.1 DOP1A processed_transcript
ENST00000484296.1 ACAP2 retained_intron
ENST00000484303.4 GTDC1 retained_intron
ENST00000484305.1 HDAC1 retained_intron
ENST00000484313.2 ZEB2 nonsense_mediated_decay
ENST00000484322.4 lncRNA
ENST00000484337.4 TTC7A processed_transcript
ENST00000484373.4 TANGO2 retained_intron
ENST00000484378.1 HLA-C retained_intron
ENST00000484383.1 CREB5 processed_transcript
ENST00000484389.5 TDRD3 nonsense_mediated_decay
ENST00000484398.1 CAB39 processed_transcript
ENST00000484404.1 ITGA4 retained_intron

ENST00000484430.1 RPS3AP6 processed_pseudogene
ENST00000484432.1 ZC3HC1 nonsense_mediated_decay
ENST00000484438.1 EEFSEC processed_transcript
ENST00000484449.1 PPP1R10 processed_transcript
ENST00000484452.1 THAP5 retained_intron
ENST00000484458.2 RNF216 processed_transcript
ENST00000484461.1 EFCAB14 retained_intron
ENST00000484465.1 MX1 retained_intron
ENST00000484468.1 LPP processed_transcript
ENST00000484474.1 ERBB4 processed_transcript
ENST00000484475.4 ATXN7L1 protein_coding
ENST00000484483.1 PTP4A2 retained_intron
ENST00000484498.1 SLTM retained_intron
ENST00000484499.4 OTUD5 processed_transcript
ENST00000484501.1 IL20RB processed_transcript
ENST00000484504.4 GNAS protein_coding
ENST00000484505.1 TAB2 processed_transcript
ENST00000484513.1 MYD88 retained_intron
ENST00000484523.1 TSPAN32 processed_transcript
ENST00000484527.4 NAMPT processed_transcript
ENST00000484529.4 DLEU1 lncRNA
ENST00000484547.5 RABGEF1 nonsense_mediated_decay
ENST00000484559.1 LY75 retained_intron
ENST00000484560.1 PLSCR1 processed_transcript
ENST00000484563.1 PSMA5 processed_transcript
ENST00000484566.4 DDX39B nonsense_mediated_decay
ENST00000484568.1 RPL14P1 processed_pseudogene
ENST00000484589.1 CDK13 protein_coding
ENST00000484599.1 RPS8 processed_transcript
ENST00000484616.2 RPS12 retained_intron
ENST00000484621.1 RNF38 processed_transcript
ENST00000484638.1 RIN2 processed_transcript
ENST00000484646.1 ATRAID retained_intron
ENST00000484647.1 TIMM17A processed_transcript
ENST00000484658.1 RAB3GAP2 processed_transcript
ENST00000484668.1 ATXN7 retained_intron
ENST00000484674.4 FRMD5 protein_coding
ENST00000484687.1 GBE1 retained_intron
ENST00000484689.1 SETD2 retained_intron
ENST00000484698.4 LINC00882 lncRNA
ENST00000484728.4 ST3GAL5 retained_intron
ENST00000484729.2 HLA-DQB1 nonsense_mediated_decay
ENST00000484734.1 THBS1 retained_intron
ENST00000484746.1 ATP2B4 nonsense_mediated_decay
ENST00000484747.4 ZNF467 protein_coding
ENST00000484749.4 IBA57 processed_transcript
ENST00000484751.1 TMEM248 retained_intron
ENST00000484768.1 SLC25A26 processed_transcript
ENST00000484774.1 NAB1 miRNA
ENST00000484786.4 TRAK1 miRNA
ENST00000484791.1 SNRK miRNA
ENST00000484812.1 FBXO9 miRNA
ENST00000484815.1 AKAP9 miRNA
ENST00000484822.1 RXRA miRNA

ENST00000484825.1 RBM8A miRNA
ENST00000484827.1 IPCEF1 miRNA
ENST00000484840.4 GTF2I miRNA
ENST00000484842.1 OPHN1 miRNA
ENST00000484846.1 EHD1 miRNA
ENST00000484847.4 IKZF1 miRNA
ENST00000484851.1 PIK3CD miRNA
ENST00000484855.2 CTSA miRNA
ENST00000484865.1 AGPAT3 miRNA
ENST00000484868.1 ST3GAL3 miRNA
ENST00000484869.5 DLEU1 miRNA
ENST00000484880.1 SERBP1 miRNA
ENST00000484890.4 OSBPL9 miRNA
ENST00000484895.4 LSP1 miRNA
ENST00000484907.1 CDC16 miRNA
ENST00000484925.1 PPM1G miRNA
ENST00000484932.4 ZNF76 miRNA
ENST00000484934.1 PILRA miRNA
ENST00000484936.4 MGAT4A miRNA
ENST00000484937.4 PATJ miRNA
ENST00000484961.1 ARPC2 miRNA
ENST00000484976.5 ECD miRNA
ENST00000484989.1 PARP4 miRNA
ENST00000485005.1 NIN miRNA
ENST00000485009.1 TES miRNA
ENST00000485016.2 ZNF292 miRNA
ENST00000485021.1 BCAS2 miRNA
ENST00000485037.4 EWSR1 miRNA
ENST00000485041.4 TUBA4B miRNA
ENST00000485049.1 BCAS4 miRNA
ENST00000485055.4 miRNA
ENST00000485061.2 MYCBP2 miRNA
ENST00000485089.4 CD247 miRNA
ENST00000485093.1 TM9SF3 miRNA
ENST00000485105.1 ST6GAL1 miRNA
ENST00000485112.1 CLASP1 miRNA
ENST00000485114.1 RABGAP1L miRNA
ENST00000485140.1 VPS50 miRNA
ENST00000485145.1 ST3GAL6 miRNA
ENST00000485148.1 TRIM62 miRNA
ENST00000485167.1 STXBP3 miRNA
ENST00000485176.1 PRKCE miRNA
ENST00000485178.4 INTS6 miRNA
ENST00000485186.1 RCOR3 miRNA
ENST00000485192.1 TNFAIP3 miRNA
ENST00000485194.1 ATP13A3 miRNA
ENST00000485219.1 C3orf38 miRNA
ENST00000485249.1 KDM4A miRNA
ENST00000485254.2 DNM3 miRNA
ENST00000485273.1 ARPC4 miRNA
ENST00000485274.1 GPATCH2 miRNA
ENST00000485282.4 miRNA
ENST00000485299.1 MORC3 miRNA
ENST00000485310.1 CRTAP miRNA

ENST00000485325.1 ANAPC1 miRNA
ENST00000485326.5 FOXP1 miRNA
ENST00000485329.1 GNA12 miRNA
ENST00000485337.1 GOLGA1 miRNA
ENST00000485341.4 LSP1 miRNA
ENST00000485353.4 THADA miRNA
ENST00000485362.1 MICOS10 miRNA
ENST00000485363.1 SIK3 miRNA
ENST00000485364.1 NOL4L miRNA
ENST00000485382.3 miRNA
ENST00000485383.1 HNRNPKP4 miRNA
ENST00000485386.1 ST20 miRNA
ENST00000485408.1 CASP10 miRNA
ENST00000485409.4 DLG1 miRNA
ENST00000485414.1 SELENOK miRNA
ENST00000485421.1 TSC22D2 miRNA
ENST00000485431.1 PIM2 miRNA
ENST00000485435.5 SURF4 miRNA
ENST00000485438.1 GALNT2 miRNA
ENST00000485444.1 ARL6IP5 miRNA
ENST00000485446.1 BMT2 miRNA
ENST00000485455.1 SRPK2 miRNA
ENST00000485465.1 CHMP3 miRNA
ENST00000485491.5 ADAM8 miRNA
ENST00000485497.2 SENP6 miRNA
ENST00000485513.1 HLA-F miRNA
ENST00000485525.4 PSD4 miRNA
ENST00000485542.4 CAPZA1 miRNA
ENST00000485549.1 LRRC29 miRNA
ENST00000485551.4 KIAA0319L miRNA
ENST00000485560.4 ZNF248 miRNA
ENST00000485568.4 KAT6A miRNA
ENST00000485576.1 ZNF394 miRNA
ENST00000485577.1 ZNF800 miRNA
ENST00000485583.1 LRCH4 miRNA
ENST00000485595.4 PHF20L1 miRNA
ENST00000485617.5 UHRF2 miRNA
ENST00000485619.1 KMT2E miRNA
ENST00000485621.1 TRAPPC10 miRNA
ENST00000485623.4 ATG16L1 miRNA
ENST00000485626.1 ATP11C miRNA
ENST00000485631.1 BCL2L13 miRNA
ENST00000485636.1 FAM126B miRNA
ENST00000485649.3 SLC19A1 miRNA
ENST00000485652.1 ATXN7L1 miRNA
ENST00000485653.1 HNMT miRNA
ENST00000485662.1 IFI44 miRNA
ENST00000485687.4 TBC1D23 miRNA
ENST00000485692.1 GTF3C5 miRNA
ENST00000485709.1 C1D miRNA
ENST00000485720.1 FGGY miRNA
ENST00000485722.1 EIF4G3 miRNA
ENST00000485723.1 HIP1 miRNA
ENST00000485737.1 SMARCC1 miRNA

ENST00000485760.4 DAB1 miRNA
ENST00000485765.4 NISCH miRNA
ENST00000485775.4 STRIP1 miRNA
ENST00000485778.1 FCGR2B miRNA
ENST00000485790.1 SCAF4 miRNA
ENST00000485793.4 NSUN3 miRNA
ENST00000485800.5 SEC31B miRNA
ENST00000485842.4 HPS4 retained_intron
ENST00000485845.4 DTNB retained_intron
ENST00000485851.1 TBC1D8 trna
ENST00000485853.4 NAALADL2 trna
ENST00000485854.4 WIPI2 trna
ENST00000485881.1 HSPA8P9 trna
ENST00000485886.1 MVB12B trna
ENST00000485917.1 BAZ2B trna
ENST00000485919.4 PHF11 trna
ENST00000485947.1 VIM trna
ENST00000485963.4 PI4KA trna
ENST00000485967.1 SCAP trna
ENST00000485978.1 SLC2A6 trna
ENST00000485985.1 LMBR1 trna
ENST00000486002.1 ATP5PF trna
ENST00000486006.1 ANKRD44 trna
ENST00000486011.1 TSPAN32 trna
ENST00000486014.1 ADD3 trna
ENST00000486021.4 CPNE1 trna
ENST00000486037.1 SND1 trna
ENST00000486042.1 PHC3 trna
ENST00000486044.1 PDE6D trna
ENST00000486058.4 RNPEPL1 trna
ENST00000486076.4 GABPB2 trna
ENST00000486085.4 RTN4 trna
ENST00000486100.1 PRPF40A trna
ENST00000486113.1 VAV2 trna
ENST00000486115.1 CYREN trna
ENST00000486123.1 MFSD6 trna
ENST00000486146.2 SP110 trna
ENST00000486152.5 ZBTB20 trna
ENST00000486166.1 RBBP7 trna
ENST00000486181.4 HSPD1 trna
ENST00000486184.1 NUP50 trna
ENST00000486193.1 PSMA7 trna
ENST00000486194.1 HLA-F trna
ENST00000486218.1 MYH9 trna
ENST00000486220.4 RABGAP1L trna
ENST00000486224.4 TBC1D5 trna
ENST00000486229.4 NBPF3 trna
ENST00000486256.4 PMS2CL trna
ENST00000486275.1 MX1 trna
ENST00000486280.1 BET1L trna
ENST00000486283.1 UBE2H trna
ENST00000486313.1 AKAP9 trna
ENST00000486331.1 ZC3H7B trna
ENST00000486332.1 TAP1 trna

ENST00000486365.4 DIRC3 trna
ENST00000486371.1 ZNF212 trna
ENST00000486374.4 ARHGAP21 trna
ENST00000486409.5 EXOC4 trna
ENST00000486437.1 STX5 trna
ENST00000486444.1 NFKBIZ trna
ENST00000486446.2 PUDP trna
ENST00000486453.1 MR1 trna
ENST00000486456.1 MAGI3 trna
ENST00000486458.1 STAMBP trna
ENST00000486468.1 SESTD1 trna
ENST00000486483.4 TIPARP trna
ENST00000486492.4 ZNF767P trna
ENST00000486498.1 GORASP2 trna
ENST00000486500.1 CALM3 trna
ENST00000486502.1 TNFSF13B trna
ENST00000486506.4 MECP2 trna
ENST00000486520.1 PRMT2 trna
ENST00000486530.1 HPS3 trna
ENST00000486554.1 TSC22D3 trna
ENST00000486555.4 DTNB trna
ENST00000486593.4 LAMP2 trna
ENST00000486597.1 TXNIP trna
ENST00000486601.4 STXBP3 trna
ENST00000486608.1 FCGR2A trna
ENST00000486622.1 RNF144B trna
ENST00000486633.1 IMMT trna
ENST00000486640.4 KIAA0930 trna
ENST00000486650.1 ARHGAP26 trna
ENST00000486663.4 UBN2 trna
ENST00000486708.1 SFI1 trna
ENST00000486733.1 RPS4X trna
ENST00000486735.4 THADA trna
ENST00000486739.1 MIDEAS trna
ENST00000486747.1 RBM33 trna
ENST00000486749.4 SLC2A3 trna
ENST00000486750.1 SP140 trna
ENST00000486752.4 DNAH1 trna
ENST00000486764.1 ABHD5 trna
ENST00000486769.1 ANP32B trna
ENST00000486774.1 LGALS9 trna
ENST00000486776.4 OVOL2 trna
ENST00000486780.3 RN7SL97P trna
ENST00000486788.2 TLN1 trna
ENST00000486796.1 CLASP2 trna
ENST00000486798.4 DNAJC13 trna
ENST00000486825.5 TRIT1 trna
ENST00000486832.1 ARMC8 trna
ENST00000486837.1 LMBR1 trna
ENST00000486848.1 CRCP trna
ENST00000486857.4 TLK1 trna
ENST00000486868.1 PRKCB trna
ENST00000486875.1 CDS2 trna
ENST00000486883.4 ITCH trna

ENST00000486895.4 DLEU1 trna
ENST00000486897.4 PHC2 trna
ENST00000486930.4 RPL13A trna
ENST00000486932.1 AGFG1 trna
ENST00000486935.2 SEMA4D trna
ENST00000486940.2 COG3 trna
ENST00000486950.4 NIN trna
ENST00000486960.4 SF1 trna
ENST00000486967.4 FAM228B trna
ENST00000486973.1 ELOVL5 trna
ENST00000487007.1 CMTM7 trna
ENST00000487016.1 ITPR1 trna
ENST00000487028.1 TRMT1L trna
ENST00000487031.4 NA trna
ENST00000487034.4 RPL13 trna
ENST00000487035.1 ARHGAP19 trna
ENST00000487045.5 FUS trna
ENST00000487065.4 STAG1 trna
ENST00000487074.4 NIBAN1 trna
ENST00000487084.1 CDHR3 trna
ENST00000487086.1 NSFL1C trna
ENST00000487088.4 ERMP1 trna
ENST00000487093.4 RAD54L2 trna
ENST00000487116.4 RNPEP trna
ENST00000487117.1 HP1BP3 trna
ENST00000487143.4 STK39 trna
ENST00000487145.1 CDK14 trna
ENST00000487159.4 TRAK1 trna
ENST00000487160.1 PRPF38A trna
ENST00000487162.1 TYMP trna
ENST00000487178.1 TRIP12 trna
ENST00000487188.1 VPS13D trna
ENST00000487189.4 CLEC16A trna
ENST00000487215.1 SMPDL3A trna
ENST00000487223.4 CAPN2 trna
ENST00000487230.4 DOCK8 trna
ENST00000487231.4 PIGS trna
ENST00000487234.1 TMEM50A trna
ENST00000487242.1 TOX4 trna
ENST00000487252.1 TRIM56 trna
ENST00000487257.1 PDE12 trna
ENST00000487266.1 GABPA trna
ENST00000487271.4 TRAF3IP3 trna
ENST00000487273.5 ADAM9 trna
ENST00000487282.4 FEZ2 trna
ENST00000487283.4 ZAP70 trna
ENST00000487286.1 PPM1B trna
ENST00000487296.1 TAP1 trna
ENST00000487304.5 RPLP1 trna
ENST00000487321.1 ARPC2 trna
ENST00000487330.1 ZFAND5 trna
ENST00000487336.1 ELMO1 trna
ENST00000487347.1 LPP trna
ENST00000487375.1 PRKAG2 trna

ENST00000487392.1 TBC1D8 trna
ENST00000487397.1 TTC14 trna
ENST00000487399.4 GRHPR trna
ENST00000487402.1 WDR82 trna
ENST00000487413.1 CACNA2D2 trna
ENST00000487422.1 TMLHE trna
ENST00000487428.1 PTPRE trna
ENST00000487433.5 PHF11 trna
ENST00000487451.1 TFE3 trna
ENST00000487453.1 PRDM2 trna
ENST00000487468.4 TTLL11 trna
ENST00000487470.1 CHRM3 trna
ENST00000487476.4 NF1 trna
ENST00000487480.1 DNAJB6 trna
ENST00000487502.1 NAPB trna
ENST00000487530.2 LYST trna
ENST00000487533.1 EIF1P7 trna
ENST00000487603.4 WSB1 trna
ENST00000487604.1 RBM39 trna
ENST00000487614.1 INPP5A trna
ENST00000487617.4 HDAC4 trna
ENST00000487621.4 SEMA3C trna
ENST00000487635.1 NCOA7 trna
ENST00000487639.1 IL1B trna
ENST00000487643.1 KLHL6 trna
ENST00000487651.4 SLC12A9 trna
ENST00000487660.1 TKT trna
ENST00000487661.1 HEG1 trna
ENST00000487662.1 DENND6A trna
ENST00000487674.1 PRKCB trna
ENST00000487679.1 BCR trna
ENST00000487681.1 CRYBG1 trna
ENST00000487682.4 ARHGEF11 trna
ENST00000487684.1 FUBP1 trna
ENST00000487695.5 PLCL1 trna
ENST00000487696.1 CAMK1D trna
ENST00000487707.1 ZNF638 trna
ENST00000487713.1 SAT1 trna
ENST00000487717.4 ATXN7 trna
ENST00000487720.1 SKAP2 trna
ENST00000487733.4 IMMP2L trna
ENST00000487736.4 SEC62 trna
ENST00000487752.1 CLEC2D trna
ENST00000487761.4 GPSM3 trna
ENST00000487769.1 AFTPH trna
ENST00000487778.4 MBP trna
ENST00000487810.4 NT5C2 trna
ENST00000487824.2 FYN trna
ENST00000487829.1 TRIM26 trna
ENST00000487876.1 RPSA trna
ENST00000487880.2 USP48 trna
ENST00000487883.1 IL2RG trna
ENST00000487895.1 RPL32P18 trna
ENST00000487898.1 FAF1 trna

ENST00000487903.4 ATP11A trna
ENST00000487906.4 PIGK trna
ENST00000487907.1 MTMR3 trna
ENST00000487909.4 MORC3 trna
ENST00000487920.1 FGD2 trna
ENST00000487927.4 TRIM8 trna
ENST00000487959.4 ACVR2A trna
ENST00000487973.1 SPAG6 trna
ENST00000487977.1 MIR29B2CHG trna
ENST00000487986.1 NA trna
ENST00000487990.4 RCAN1 trna
ENST00000487996.4 HLA-DMB trna
ENST00000487999.1 CEP162 trna
ENST00000488015.4 SRI trna
ENST00000488024.1 RASSF1 trna
ENST00000488032.1 PCGF3 trna
ENST00000488048.1 CD36 trna
ENST00000488054.1 CD96 trna
ENST00000488055.1 ZNF512 trna
ENST00000488058.1 RNF38 processed_transcript
ENST00000488060.1 SPATA13 protein_coding
ENST00000488066.4 FCAR nonsense_mediated_decay
ENST00000488071.1 RASGRP2 retained_intron
ENST00000488073.1 C7orf50 processed_transcript
ENST00000488077.1 RIN2 processed_transcript
ENST00000488084.1 STARD7 retained_intron
ENST00000488086.1 MTHFD2 retained_intron
ENST00000488087.1 ECHDC1 processed_transcript
ENST00000488101.4 HSDL2 processed_transcript
ENST00000488107.5 TFDP2 protein_coding
ENST00000488117.4 C9orf72 processed_transcript
ENST00000488122.2 RPLP1 retained_intron
ENST00000488123.2 NA NA
ENST00000488127.1 LDLRAP1 processed_transcript
ENST00000488147.1 WASH7P unprocessed_pseudogene
ENST00000488150.1 ELMOD3 retained_intron
ENST00000488151.1 STT3B retained_intron
ENST00000488153.4 SMYD3 processed_transcript
ENST00000488164.4 ZFAND5 processed_transcript
ENST00000488171.4 CD55 retained_intron
ENST00000488174.4 OGT retained_intron
ENST00000488180.1 SP100 retained_intron
ENST00000488228.1 UBE4B nonsense_mediated_decay
ENST00000488234.1 SUCLG1 processed_transcript
ENST00000488242.2 EHMT1 processed_transcript
ENST00000488251.1 GON4L processed_transcript
ENST00000488263.4 CRBN retained_intron
ENST00000488266.4 LAT2 nonsense_mediated_decay
ENST00000488269.4 ARHGAP4 protein_coding
ENST00000488277.1 EPB41 processed_transcript
ENST00000488281.1 RPL13AP2 processed_pseudogene
ENST00000488293.3 MECP2 processed_transcript
ENST00000488331.1 MORF4L2 processed_transcript
ENST00000488337.4 SIK3 processed_transcript

ENST00000488347.4 ZMAT1 processed_transcript
ENST00000488354.1 PSMD1 processed_transcript
ENST00000488357.1 MTMR1 nonsense_mediated_decay
ENST00000488373.4 RAC1 processed_transcript
ENST00000488380.4 NISCH protein_coding
ENST00000488386.4 CDHR3 nonsense_mediated_decay
ENST00000488390.1 SIDT1 retained_intron
ENST00000488395.1 CTSZ retained_intron
ENST00000488398.3 RN7SL521P misc_RNA
ENST00000488400.1 SNRPG retained_intron
ENST00000488407.1 FGD3 processed_transcript
ENST00000488410.4 TM2D1 processed_transcript
ENST00000488418.2 UBE2J2 protein_coding
ENST00000488427.1 ITGB1 protein_coding
ENST00000488434.1 G6PD retained_intron
ENST00000488436.1 HACD4 processed_transcript
ENST00000488439.1 UGGT1 retained_intron
ENST00000488441.2 RPS11P5 processed_pseudogene
ENST00000488449.1 TMCO4 processed_transcript
ENST00000488467.4 FHIT protein_coding
ENST00000488470.4 TNIK protein_coding
ENST00000488500.1 EEF1A1 retained_intron
ENST00000488520.4 USP4 processed_transcript
ENST00000488527.4 ADAP1 processed_transcript
ENST00000488535.2 ABCA2 nonsense_mediated_decay
ENST00000488537.1 TXNIP retained_intron
ENST00000488545.1 LINC00877 lncRNA
ENST00000488554.4 MYO1G retained_intron
ENST00000488564.2 TAX1BP1 protein_coding
ENST00000488587.1 U2SURP retained_intron
ENST00000488592.1 SCMH1 retained_intron
ENST00000488596.4 CD46 processed_transcript
ENST00000488607.4 ZCCHC7 processed_transcript
ENST00000488618.1 STK4 processed_transcript
ENST00000488621.1 ZNF736 retained_intron
ENST00000488632.1 LBR processed_transcript
ENST00000488645.1 MYOF processed_transcript
ENST00000488655.4 LRMDA processed_transcript
ENST00000488663.1 ZBTB20 processed_transcript
ENST00000488683.1 CLIC4 nonsense_mediated_decay
ENST00000488685.1 SH2D3C processed_transcript
ENST00000488687.4 PSME4 retained_intron
ENST00000488702.1 TRAF3IP3 processed_transcript
ENST00000488722.4 HP1BP3 processed_transcript
ENST00000488723.1 CDK5RAP1 processed_transcript
ENST00000488733.1 ATP1A1 retained_intron
ENST00000488742.4 GCNT2 processed_transcript
ENST00000488751.1 ZNF655 retained_intron
ENST00000488758.1 APOBEC3A retained_intron
ENST00000488759.4 LRMDA processed_transcript
ENST00000488778.1 AGO4 processed_transcript
ENST00000488797.1 PSIP1 processed_transcript
ENST00000488799.4 ADD3 processed_transcript
ENST00000488805.4 NUTM2B-AS1 lncRNA

ENST00000488807.2 RBM6 processed_transcript
ENST00000488816.1 SLX4IP nonsense_mediated_decay
ENST00000488831.4 ZC4H2 processed_transcript
ENST00000488837.1 ADD3 processed_transcript
ENST00000488849.1 ZNF92 retained_intron
ENST00000488864.4 CHD1L nonsense_mediated_decay
ENST00000488865.2 RNF220 nonsense_mediated_decay
ENST00000488868.1 DNAJC8 processed_transcript
ENST00000488903.1 GALNT2 processed_transcript
ENST00000488910.1 ATG3 retained_intron
ENST00000488914.1 CTNNB1 processed_transcript
ENST00000488924.4 ATG7 processed_transcript
ENST00000488946.1 RPL13A retained_intron
ENST00000488949.1 RAB22A processed_transcript
ENST00000488971.1 KDM3A retained_intron
ENST00000488978.1 AZI2 retained_intron
ENST00000488998.1 CCDC146 processed_transcript
ENST00000489025.1 GBA2 processed_transcript
ENST00000489044.1 COQ8A processed_transcript
ENST00000489049.4 PDCD4 processed_transcript
ENST00000489057.1 MEF2D protein_coding
ENST00000489076.1 UBAP2L processed_transcript
ENST00000489077.1 ARHGEF35-AS1 lncRNA
ENST00000489085.1 GK5 retained_intron
ENST00000489092.4 AP4B1 processed_transcript
ENST00000489100.4 FAM107B protein_coding
ENST00000489103.4 HIVEP3 processed_transcript
ENST00000489108.1 USP48 processed_transcript
ENST00000489109.1 ANXA1 processed_transcript
ENST00000489142.4 VTI1A processed_transcript
ENST00000489143.5 C1orf21 processed_transcript
ENST00000489169.1 CACUL1 retained_intron
ENST00000489196.1 CASD1 retained_intron
ENST00000489203.2 NSFL1C nonsense_mediated_decay
ENST00000489241.1 XRN1 nonsense_mediated_decay
ENST00000489243.4 KDM4C processed_transcript
ENST00000489245.4 KLHL6 retained_intron
ENST00000489255.2 TLN1 processed_transcript
ENST00000489265.1 AGTPBP1 processed_transcript
ENST00000489271.4 NGLY1 retained_intron
ENST00000489274.1 NINJ1 processed_transcript
ENST00000489282.1 MYSM1 processed_transcript
ENST00000489287.1 YWHAE retained_intron
ENST00000489291.4 DAPK1 nonsense_mediated_decay
ENST00000489294.1 UHMK1 protein_coding
ENST00000489313.1 PTP4A2 retained_intron
ENST00000489320.1 ZNF655 retained_intron
ENST00000489327.1 AAK1 retained_intron
ENST00000489345.4 CRLS1 processed_transcript
ENST00000489355.1 UGCG processed_transcript
ENST00000489356.1 FGD2 retained_intron
ENST00000489358.4 NAMPT protein_coding
ENST00000489365.1 PRDM1 retained_intron
ENST00000489372.3 JMJD1C processed_transcript

ENST00000489384.1 RALY processed_transcript
ENST00000489385.4 GABBR1 nonsense_mediated_decay
ENST00000489393.1 TANK processed_transcript
ENST00000489399.1 MAL processed_transcript
ENST00000489400.1 GOLGB1 protein_coding
ENST00000489405.4 GLCCI1 retained_intron
ENST00000489412.1 EPM2A processed_transcript
ENST00000489415.1 MADD processed_transcript
ENST00000489417.4 SND1 retained_intron
ENST00000489451.1 ACTN4 retained_intron
ENST00000489480.1 MTF2 processed_transcript
ENST00000489491.1 PDZD8 processed_transcript
ENST00000489500.1 TNRC6B retained_intron
ENST00000489507.1 TMEM131 retained_intron
ENST00000489515.1 MFNG retained_intron
ENST00000489542.5 DLEU1 lncRNA
ENST00000489552.1 DENND2A protein_coding
ENST00000489570.1 MVB12B processed_transcript
ENST00000489577.1 ATP11A processed_transcript
ENST00000489585.4 LYST processed_transcript
ENST00000489603.4 LRRFIP1 processed_transcript
ENST00000489607.4 CAPZB processed_transcript
ENST00000489615.4 RABGAP1L protein_coding
ENST00000489634.2 KAT14 protein_coding
ENST00000489639.1 MFSD1 retained_intron
ENST00000489645.1 FKBP15 processed_transcript
ENST00000489651.4 MED15 retained_intron
ENST00000489652.1 PARP9 retained_intron
ENST00000489662.1 PDIA6 processed_transcript
ENST00000489665.1 AMZ1 processed_transcript
ENST00000489666.2 MICU1 protein_coding
ENST00000489687.1 CNOT9 processed_transcript
ENST00000489705.2 HNRNPU nonsense_mediated_decay
ENST00000489715.1 GBE1 protein_coding
ENST00000489731.1 NOTCH2 processed_transcript
ENST00000489732.1 NAMPT retained_intron
ENST00000489741.1 PRPF40A retained_intron
ENST00000489742.1 CALM2 protein_coding
ENST00000489758.1 CLIC4 processed_transcript
ENST00000489769.1 SERPINA1 nonsense_mediated_decay
ENST00000489770.1 SETD3 retained_intron
ENST00000489773.4 IPO13 processed_transcript
ENST00000489779.1 ACTR3 retained_intron
ENST00000489783.1 SHOC2 processed_transcript
ENST00000489787.1 SLC9A8 processed_transcript
ENST00000489793.1 MFSD6 processed_transcript
ENST00000489798.4 TPK1 nonsense_mediated_decay
ENST00000489814.4 TMCO4 processed_transcript
ENST00000489818.4 GLUL processed_transcript
ENST00000489820.4 ZNF862 retained_intron
ENST00000489824.1 RPS6KB1 nonsense_mediated_decay
ENST00000489828.4 SRPK2 protein_coding
ENST00000489867.1 ENO1 protein_coding
ENST00000489869.1 PDCD6IP retained_intron

ENST00000489886.1 SLC15A2 processed_transcript
ENST00000489895.4 NISCH retained_intron
ENST00000489901.1 SESTD1 retained_intron
ENST00000489909.1 MCCC1 processed_transcript
ENST00000489920.1 GSAP retained_intron
ENST00000489921.1 TNFRSF1B processed_transcript
ENST00000489924.1 ARHGAP26 retained_intron
ENST00000489949.4 DTNB retained_intron
ENST00000489961.1 VPS13D processed_transcript
ENST00000489977.4 SKAP2 processed_transcript
ENST00000489982.1 PIK3AP1 processed_transcript
ENST00000489988.4 DENND10 processed_transcript
ENST00000489990.4 OSBPL9 nonsense_mediated_decay
ENST00000490010.1 TPP2 processed_transcript
ENST00000490012.5 CCNY protein_coding
ENST00000490018.1 AKT3 processed_transcript
ENST00000490031.1 SVIL processed_transcript
ENST00000490044.4 NONO processed_transcript
ENST00000490056.4 SFXN5 processed_transcript
ENST00000490065.4 SDCCAG8 processed_transcript
ENST00000490072.4 SLC25A13 processed_transcript
ENST00000490093.4 RAB7A nonsense_mediated_decay
ENST00000490104.4 TSN nonsense_mediated_decay
ENST00000490106.1 ALKBH5 retained_intron
ENST00000490110.4 UGCG processed_transcript
ENST00000490121.1 TANGO2 processed_transcript
ENST00000490122.4 GNAI2 retained_intron
ENST00000490130.1 KMT2C retained_intron
ENST00000490133.4 SRBD1 processed_transcript
ENST00000490159.1 TPST1 processed_transcript
ENST00000490163.4 UBXN4 processed_transcript
ENST00000490179.2 TSC1 protein_coding
ENST00000490182.1 SP3 processed_transcript
ENST00000490183.4 QTRT2 retained_intron
ENST00000490185.1 ANKHD1 retained_intron
ENST00000490191.4 RPS18 retained_intron
ENST00000490192.1 TM9SF3 processed_transcript
ENST00000490210.5 ATP9B retained_intron
ENST00000490211.5 YPEL5 processed_transcript
ENST00000490232.3 RN7SL2 misc_RNA
ENST00000490238.4 IMMT processed_transcript
ENST00000490247.1 THBS1 retained_intron
ENST00000490253.1 PLCG1 retained_intron
ENST00000490262.1 SREBF2 retained_intron
ENST00000490278.1 CD46 retained_intron
ENST00000490279.4 ARHGAP30 nonsense_mediated_decay
ENST00000490291.1 RETSAT retained_intron
ENST00000490294.4 CBWD6 processed_transcript
ENST00000490296.1 PCGF6 processed_transcript
ENST00000490299.4 RPS6KC1 processed_transcript
ENST00000490303.4 ATP11B retained_intron
ENST00000490313.1 PRIM2 processed_transcript
ENST00000490320.2 C3orf80 nonsense_mediated_decay
ENST00000490331.2 SMIM19 protein_coding

ENST00000490335.4 RPL10A processed_transcript
ENST00000490365.1 KAT6B retained_intron
ENST00000490368.4 DCAF8 nonsense_mediated_decay
ENST00000490390.4 TRMT61B retained_intron
ENST00000490412.4 CASP8 processed_transcript
ENST00000490420.1 TPP2 retained_intron
ENST00000490434.4 EAF2 nonsense_mediated_decay
ENST00000490435.4 ITGA4 retained_intron
ENST00000490444.2 PUM3 nonsense_mediated_decay
ENST00000490449.1 TBC1D10A processed_transcript
ENST00000490452.4 DGKG retained_intron
ENST00000490455.1 TAX1BP1 retained_intron
ENST00000490458.1 SNAP29 retained_intron
ENST00000490477.1 EAF2 nonsense_mediated_decay
ENST00000490481.1 CFAP44 nonsense_mediated_decay
ENST00000490484.5 RBM39 nonsense_mediated_decay
ENST00000490530.1 MTM1 processed_transcript
ENST00000490536.1 SLC11A1 retained_intron
ENST00000490547.1 PATJ protein_coding
ENST00000490550.1 KIFAP3 processed_transcript
ENST00000490553.1 RNF149 retained_intron
ENST00000490564.1 NINJ1 processed_transcript
ENST00000490568.1 GBP5 processed_transcript
ENST00000490586.1 LAT2 retained_intron
ENST00000490613.1 PSMB8 retained_intron
ENST00000490627.1 WAS processed_transcript
ENST00000490631.4 RNF13 protein_coding
ENST00000490646.1 DIS3 nonsense_mediated_decay
ENST00000490659.4 ENTPD1 retained_intron
ENST00000490668.4 SFPQ processed_transcript
ENST00000490674.1 SLC20A1 retained_intron
ENST00000490682.4 CASP8 processed_transcript
ENST00000490684.1 ST3GAL6 retained_intron
ENST00000490697.1 HDLBP processed_transcript
ENST00000490705.1 ARF1 processed_transcript
ENST00000490713.4 DNAH1 nonsense_mediated_decay
ENST00000490725.4 FAM126B retained_intron
ENST00000490736.2 UEVLD processed_transcript
ENST00000490740.5 XPNPEP1 processed_transcript
ENST00000490745.4 PLSCR1 retained_intron
ENST00000490751.1 NA NA
ENST00000490759.1 RPL31P49 processed_pseudogene
ENST00000490763.1 SLC2A3 retained_intron
ENST00000490767.1 AKNA processed_transcript
ENST00000490775.4 IDS processed_transcript
ENST00000490777.3 PPP2R2D retained_intron
ENST00000490791.4 SETD5 nonsense_mediated_decay
ENST00000490806.4 KDM4C processed_transcript
ENST00000490809.1 SLC31A2 processed_transcript
ENST00000490837.1 ILKAP processed_transcript
ENST00000490851.4 HDAC9 retained_intron
ENST00000490856.4 lncRNA
ENST00000490869.1 UBA1 processed_transcript
ENST00000490872.1 SLC11A1 retained_intron

ENST00000490882.4 FLNB protein_coding
ENST00000490886.4 EXD3 processed_transcript
ENST00000490887.1 WIPF1 processed_transcript
ENST00000490891.1 IARS2 processed_transcript
ENST00000490936.4 FLNA retained_intron
ENST00000490939.1 CMAHP retained_intron
ENST00000490952.1 FHIT retained_intron
ENST00000490953.4 SBDS retained_intron
ENST00000490955.1 GPR89B retained_intron
ENST00000490956.1 ZNF813 protein_coding
ENST00000490959.2 ZNF362 processed_transcript
ENST00000490969.4 SNAPC3 nonsense_mediated_decay
ENST00000490974.1 UBE2H protein_coding
ENST00000490982.1 TRPM2 processed_transcript
ENST00000490997.4 lncRNA
ENST00000491011.1 DHX36 retained_intron
ENST00000491017.1 TAFA1 processed_transcript
ENST00000491021.1 POLDIP3 processed_transcript
ENST00000491027.4 NAMPT protein_coding
ENST00000491047.4 processed_transcript
ENST00000491053.4 USP20 processed_transcript
ENST00000491056.4 IL1B processed_transcript
ENST00000491065.1 IDH3B retained_intron
ENST00000491067.1 DCAF6 processed_transcript
ENST00000491099.1 EPC2 retained_intron
ENST00000491102.2 SLC25A16 nonsense_mediated_decay
ENST00000491114.1 nonsense_mediated_decay
ENST00000491133.1 AKNA processed_transcript
ENST00000491137.4 MLLT3 protein_coding
ENST00000491160.1 PBX3 processed_transcript
ENST00000491164.1 PXK protein_coding
ENST00000491165.4 USF3 protein_coding
ENST00000491179.1 FAAH2 processed_transcript
ENST00000491192.1 ANXA1 processed_transcript
ENST00000491207.4 DLGAP4 processed_transcript
ENST00000491210.1 MNDA processed_transcript
ENST00000491217.1 AHSA2P processed_transcript
ENST00000491236.1 RBM3 processed_transcript
ENST00000491239.2 WDTC1 retained_intron
ENST00000491257.1 SLC22A4 processed_transcript
ENST00000491258.1 PHC3 retained_intron
ENST00000491281.1 NFKBIZ protein_coding
ENST00000491290.1 RAF1 retained_intron
ENST00000491294.1 ARPC1B retained_intron
ENST00000491298.1 PFKL processed_transcript
ENST00000491302.1 PTPRC retained_intron
ENST00000491322.1 GLUL processed_transcript
ENST00000491331.4 NABP1 retained_intron
ENST00000491345.1 ANTXR2 processed_transcript
ENST00000491349.4 RNF38 processed_transcript
ENST00000491366.4 SEC22A protein_coding
ENST00000491378.1 RSRP1 retained_intron
ENST00000491380.4 TTC14 protein_coding
ENST00000491388.5 ALCAM retained_intron

ENST00000491395.4 APP processed_transcript
ENST00000491404.1 EEF1A1 retained_intron
ENST00000491410.1 NRDC processed_transcript
ENST00000491415.5 UTP25 protein_coding
ENST00000491416.4 FAM204A processed_transcript
ENST00000491418.4 SPATA21 protein_coding
ENST00000491430.1 IRF2BP2 processed_transcript
ENST00000491431.1 ZNF786 protein_coding
ENST00000491442.4 HIVEP3 processed_transcript
ENST00000491443.2 AGO3 retained_intron
ENST00000491444.4 AOAH processed_transcript
ENST00000491451.3 RN7SL242P misc_RNA
ENST00000491453.1 WDFY1 retained_intron
ENST00000491456.1 CYP1B1 processed_transcript
ENST00000491458.1 FAM107B processed_transcript
ENST00000491462.2 SLC46A2 nonsense_mediated_decay
ENST00000491463.4 BRI3 nonsense_mediated_decay
ENST00000491473.4 EIF4A2 retained_intron
ENST00000491481.1 DARS1 retained_intron
ENST00000491489.5 GOSR1 retained_intron
ENST00000491491.1 MYCBP2 processed_transcript
ENST00000491495.2 CEP350 protein_coding
ENST00000491519.4 SATB1 protein_coding
ENST00000491541.1 SREBF2 processed_transcript
ENST00000491555.4 IPO5 retained_intron
ENST00000491562.4 PI4KAP2 retained_intron
ENST00000491574.1 SMARCA2 processed_transcript
ENST00000491582.1 DNAJB9 retained_intron
ENST00000491588.1 MAPK1 processed_transcript
ENST00000491592.1 RTN4 retained_intron
ENST00000491600.4 COP1 nonsense_mediated_decay
ENST00000491616.1 RPS6KC1 processed_transcript
ENST00000491618.1 CBFA2T2 processed_transcript
ENST00000491623.2 TBL1XR1 retained_intron
ENST00000491626.4 GDAP2 processed_transcript
ENST00000491635.1 GTPBP4 processed_transcript
ENST00000491650.4 DENND1A processed_transcript
ENST00000491651.1 SMARCD3 protein_coding
ENST00000491660.1 SERP1 protein_coding
ENST00000491661.2 PEX14 protein_coding
ENST00000491667.1 TFDP2 retained_intron
ENST00000491674.1 FXR1 protein_coding
ENST00000491681.1 RAB7A retained_intron
ENST00000491701.1 CLIP4 retained_intron
ENST00000491710.4 HIVEP1 protein_coding
ENST00000491718.4 TNRC6A nonsense_mediated_decay
ENST00000491720.4 ITPRID2 retained_intron
ENST00000491730.4 SIDT1 retained_intron
ENST00000491737.4 THOC2 protein_coding
ENST00000491775.4 ARHGEF7 protein_coding
ENST00000491786.4 TTC7A retained_intron
ENST00000491787.6 PBX3 protein_coding
ENST00000491788.1 SMYD4 protein_coding
ENST00000491789.1 VPS45 nonsense_mediated_decay

ENST00000491798.1 DIPK2A processed_transcript
ENST00000491815.1 PRDM2 retained_intron
ENST00000491825.1 CDC42SE1 processed_transcript
ENST00000491841.1 FCGR2A processed_transcript
ENST00000491843.2 ZNF638 retained_intron
ENST00000491847.1 CISH retained_intron
ENST00000491848.1 ZNF124 processed_transcript
ENST00000491854.1 TMEM245 nonsense_mediated_decay
ENST00000491858.4 MAD1L1 processed_transcript
ENST00000491868.1 ITPR1 processed_transcript
ENST00000491877.1 CD99L2 processed_transcript
ENST00000491881.1 VTA1 processed_transcript
ENST00000491885.5 FYN processed_transcript
ENST00000491894.3 SLC9A7 processed_transcript
ENST00000491905.1 FNBP1 processed_transcript
ENST00000491922.1 CCDC6 retained_intron
ENST00000491937.4 RPS7 retained_intron
ENST00000491940.4 EVI5 protein_coding
ENST00000491987.1 TOM1 retained_intron
ENST00000492002.1 EGFL7 processed_transcript
ENST00000492021.5 PDPK1 nonsense_mediated_decay
ENST00000492028.4 ARHGAP12 processed_transcript
ENST00000492063.4 CD244 nonsense_mediated_decay
ENST00000492066.4 STX5 nonsense_mediated_decay
ENST00000492073.1 CRTC2 processed_transcript
ENST00000492081.1 ACOT9 protein_coding
ENST00000492092.1 SELENOO processed_transcript
ENST00000492115.2 ZNF169 retained_intron
ENST00000492119.1 IKZF1 retained_intron
ENST00000492120.1 AHR processed_transcript
ENST00000492125.1 KLF6 processed_transcript
ENST00000492145.1 CEP170 protein_coding
ENST00000492163.4 MGAT4A processed_transcript
ENST00000492178.1 CRBN retained_intron
ENST00000492190.4 LMNB1 protein_coding
ENST00000492193.1 ZRANB3 retained_intron
ENST00000492202.1 MALRD1 processed_transcript
ENST00000492204.1 OGA processed_transcript
ENST00000492222.1 RPS27AP16 transcribed_processed_pseudogene
ENST00000492232.4 EHMT1 protein_coding
ENST00000492236.4 RAD51B processed_transcript
ENST00000492251.1 DOCK10 retained_intron
ENST00000492257.1 EXOC6B processed_transcript
ENST00000492263.1 ENOX2 processed_transcript
ENST00000492272.4 BCL11A processed_transcript
ENST00000492274.1 ST7L processed_transcript
ENST00000492283.1 RBMS1 processed_transcript
ENST00000492288.1 CYBB processed_transcript
ENST00000492304.1 PLEKHO1 processed_transcript
ENST00000492318.1 STAG1 protein_coding
ENST00000492336.4 MORC3 retained_intron
ENST00000492343.1 ENO1 retained_intron
ENST00000492351.5 PPP3CA nonsense_mediated_decay
ENST00000492354.1 SZRD1 protein_coding

ENST00000492361.1 TNFRSF1B processed_transcript
ENST00000492363.4 CASP10 processed_transcript
ENST00000492369.4 DOCK10 miRNA
ENST00000492378.1 GOT2 miRNA
ENST00000492380.1 ANXA7 miRNA
ENST00000492383.1 GNAI2 miRNA
ENST00000492386.4 SEMA4D miRNA
ENST00000492397.1 SETD2 miRNA
ENST00000492404.4 TAF1 miRNA
ENST00000492409.2 KLHL24 miRNA
ENST00000492449.4 VPS8 miRNA
ENST00000492452.2 DIPK2A miRNA
ENST00000492456.1 ZMYM4 miRNA
ENST00000492460.4 CACNA2D3 miRNA
ENST00000492466.2 SS18L1 miRNA
ENST00000492478.4 CCNY miRNA
ENST00000492479.1 PTPRE miRNA
ENST00000492514.1 VPS26C miRNA
ENST00000492546.1 SP100 miRNA
ENST00000492552.4 NR1D2 miRNA
ENST00000492560.5 TBXAS1 miRNA
ENST00000492563.1 DCUN1D1 miRNA
ENST00000492565.1 CAPN2 miRNA
ENST00000492566.1 CBWD2 miRNA
ENST00000492572.4 RPL10 miRNA
ENST00000492578.1 PLXNB2 miRNA
ENST00000492585.1 DALRD3 miRNA
ENST00000492601.2 SGMS1 miRNA
ENST00000492613.1 EVI5 miRNA
ENST00000492630.1 ADAR miRNA
ENST00000492639.4 RWDD3 miRNA
ENST00000492641.1 RPL37P23 miRNA
ENST00000492648.2 TAF1B miRNA
ENST00000492664.4 CAPN2 miRNA
ENST00000492693.4 AGK miRNA
ENST00000492702.1 BABAM2 miRNA
ENST00000492714.1 OXSR1 miRNA
ENST00000492727.1 FTH1P23 miRNA
ENST00000492728.1 CHD1L miRNA
ENST00000492736.1 YWHAZ miRNA
ENST00000492740.4 NOP58 miRNA
ENST00000492741.4 PSPC1 miRNA
ENST00000492751.1 EDEM1 miRNA
ENST00000492760.1 DOP1B miRNA
ENST00000492782.4 IKZF1 miRNA
ENST00000492795.1 SUCLG2 miRNA
ENST00000492796.4 CDC42SE1 miRNA
ENST00000492797.4 GLCCI1 miRNA
ENST00000492803.1 PIBF1 miRNA
ENST00000492808.4 CD52 miRNA
ENST00000492811.1 PRRC2C miRNA
ENST00000492832.2 GUSBP2 miRNA
ENST00000492840.1 SND1 miRNA
ENST00000492843.4 PRKAG2 miRNA

ENST00000492853.1 UHRF2 miRNA
ENST00000492857.1 ATXN1 miRNA
ENST00000492869.1 SLC25A13 miRNA
ENST00000492875.1 AKNA miRNA
ENST00000492877.1 THEMIS2 miRNA
ENST00000492886.4 ATG3 miRNA
ENST00000492896.1 SMARCC1 miRNA
ENST00000492914.1 WASHC2A miRNA
ENST00000492915.1 NLRP12 miRNA
ENST00000492919.1 TFDP2 miRNA
ENST00000492932.4 PDCD4 miRNA
ENST00000492967.4 RGS18 miRNA
ENST00000492974.2 miRNA
ENST00000492980.1 CCDC88B miRNA
ENST00000492985.1 DNAJC27 miRNA
ENST00000492989.1 KHDRBS1 miRNA
ENST00000492996.5 HERC4 miRNA
ENST00000493010.2 FOXP1 miRNA
ENST00000493013.3 RN7SL767P miRNA
ENST00000493034.1 PIK3IP1 miRNA
ENST00000493055.1 DGUOK miRNA
ENST00000493072.1 RPS4XP14 miRNA
ENST00000493078.1 INPP5D miRNA
ENST00000493110.1 DNAJB14 miRNA
ENST00000493113.1 ALDH1A1 miRNA
ENST00000493114.1 IRF4 miRNA
ENST00000493116.4 SOX2-OT miRNA
ENST00000493127.1 FRMD4B miRNA
ENST00000493133.1 PBXIP1 miRNA
ENST00000493143.1 PSAP miRNA
ENST00000493163.1 TLE4 miRNA
ENST00000493173.1 BTN2A1 miRNA
ENST00000493175.1 FAM102A miRNA
ENST00000493181.1 TYW5 miRNA
ENST00000493186.4 RAB7A miRNA
ENST00000493201.4 BACH2 miRNA
ENST00000493207.1 RER1 miRNA
ENST00000493208.1 IRF1 miRNA
ENST00000493214.2 LINC02006 miRNA
ENST00000493216.1 MED15 miRNA
ENST00000493217.1 TSC22D4 miRNA
ENST00000493220.4 NF1 miRNA
ENST00000493224.4 RPS27 miRNA
ENST00000493227.1 MIOS miRNA
ENST00000493238.1 RNF13 miRNA
ENST00000493239.1 miRNA
ENST00000493240.1 LHPP miRNA
ENST00000493246.4 UMAD1 miRNA
ENST00000493247.1 MBP miRNA
ENST00000493261.4 ING5 miRNA
ENST00000493262.1 PARP12 miRNA
ENST00000493278.1 CALU miRNA
ENST00000493315.1 WDPCP miRNA
ENST00000493334.1 SDCCAG8 miRNA

ENST00000493362.1 HNRNPK miRNA
ENST00000493364.1 HM13 miRNA
ENST00000493372.1 LMTK2 miRNA
ENST00000493378.1 ARF4 miRNA
ENST00000493385.4 CUTC miRNA
ENST00000493396.1 DIPK2A miRNA
ENST00000493403.1 ARPC1B miRNA
ENST00000493417.4 GPR107 miRNA
ENST00000493438.4 RALGDS miRNA
ENST00000493442.1 FAM199X miRNA
ENST00000493448.1 WDR44 miRNA
ENST00000493452.4 FLNB miRNA
ENST00000493454.4 NAA50 miRNA
ENST00000493470.2 ATRX miRNA
ENST00000493486.1 LCOR miRNA
ENST00000493494.1 TRIM22 retained_intron
ENST00000493499.1 STAG3L5P processed_transcript
ENST00000493503.1 NA trna
ENST00000493510.1 KPNA1 trna
ENST00000493514.1 C1orf52 trna
ENST00000493552.1 CTBP2 trna
ENST00000493559.1 FBXW2 trna
ENST00000493566.1 UBXN7 trna
ENST00000493568.4 PIK3CB trna
ENST00000493583.4 TFE3 trna
ENST00000493589.2 TBC1D2 trna
ENST00000493595.1 TRIM24 trna
ENST00000493609.1 SMG7 trna
ENST00000493613.4 NUDCD3 trna
ENST00000493626.4 SEPTIN7 trna
ENST00000493632.4 INPP5D trna
ENST00000493637.5 INTS6L trna
ENST00000493638.1 SRPK2 trna
ENST00000493640.1 SAMSN1 trna
ENST00000493645.1 PARP15 trna
ENST00000493658.1 ARID1B trna
ENST00000493661.2 SERPINB8 trna
ENST00000493666.2 DOCK8 trna
ENST00000493676.1 HENMT1 trna
ENST00000493685.1 NA trna
ENST00000493699.1 HLA-E trna
ENST00000493703.1 NOTCH2 trna
ENST00000493707.1 UBE2E1 trna
ENST00000493737.4 RNFT1 trna
ENST00000493759.2 NA trna
ENST00000493767.1 DYSF trna
ENST00000493768.4 NRBP1 trna
ENST00000493789.5 DPYSL2 trna
ENST00000493790.1 NEK7 trna
ENST00000493793.1 EPS15 trna
ENST00000493796.4 CD46 trna
ENST00000493802.4 GBP2 trna
ENST00000493807.1 KCNAB2 trna
ENST00000493808.1 NA trna

ENST00000493826.1 ATP11B trna
ENST00000493835.4 ZEB1 trna
ENST00000493850.1 LFNG trna
ENST00000493857.1 APBB1IP trna
ENST00000493880.4 FRMD4B trna
ENST00000493886.4 VCP trna
ENST00000493889.1 KIF5B trna
ENST00000493898.1 PLAGL1 trna
ENST00000493899.2 trna
ENST00000493903.1 GTF3A trna
ENST00000493906.4 CHN2 trna
ENST00000493934.1 SLF1 trna
ENST00000493941.1 LINGO2 trna
ENST00000493945.4 ACTB trna
ENST00000493946.1 GSTO1 trna
ENST00000493947.4 ZNF655 trna
ENST00000493952.2 SATB1 trna
ENST00000493953.1 ST3GAL6 trna
ENST00000493958.4 GNAS trna
ENST00000493959.5 SENP6 trna
ENST00000493960.5 TASOR trna
ENST00000493962.5 FBXO11 trna
ENST00000493969.2 GIMAP6 trna
ENST00000493974.1 TESK2 trna
ENST00000494002.4 RSRC1 trna
ENST00000494011.1 TBC1D8 trna
ENST00000494060.1 DEPDC5 trna
ENST00000494061.5 ZMYM2 trna
ENST00000494066.1 NMRK1 trna
ENST00000494069.1 LRCH3 trna
ENST00000494073.4 SH2D1A trna
ENST00000494089.1 HS6ST1 trna
ENST00000494097.4 BID trna
ENST00000494098.4 SMOX trna
ENST00000494106.1 GALNT2 trna
ENST00000494113.1 PI4KA trna
ENST00000494114.1 CAP1 trna
ENST00000494124.2 DENND4C trna
ENST00000494142.1 MBOAT7 trna
ENST00000494144.4 CCSER2 trna
ENST00000494145.1 RASSF1 trna
ENST00000494180.1 CALHM2 trna
ENST00000494185.1 LMNB1 trna
ENST00000494186.1 ZNF789 trna
ENST00000494188.1 RAB1A trna
ENST00000494201.1 GFPT1 trna
ENST00000494203.1 PELI1 trna
ENST00000494211.4 NHEJ1 trna
ENST00000494224.1 GDAP2 trna
ENST00000494233.4 LPP trna
ENST00000494241.4 ZNF638 trna
ENST00000494252.1 MX2 trna
ENST00000494253.1 HK1 trna
ENST00000494258.4 CFLAR trna

ENST00000494260.1 ARID1B trna
ENST00000494282.5 AIG1 trna
ENST00000494284.5 PHF3 trna
ENST00000494286.4 MKLN1 trna
ENST00000494296.1 CCT8 trna
ENST00000494322.4 SLC11A1 trna
ENST00000494355.1 TRAF5 trna
ENST00000494366.4 LUC7L trna
ENST00000494367.1 HLA-J trna
ENST00000494371.4 COG2 trna
ENST00000494380.4 ZNF138 trna
ENST00000494395.1 ITGB1 trna
ENST00000494400.4 FAF1 trna
ENST00000494401.4 SNX5 trna
ENST00000494402.4 SNX13 trna
ENST00000494411.4 PCMT1 trna
ENST00000494433.1 PRDX3 trna
ENST00000494437.1 AGTRAP trna
ENST00000494454.4 TXNRD2 trna
ENST00000494461.1 ZER1 trna
ENST00000494482.1 PARD3B trna
ENST00000494506.1 RAB5IF trna
ENST00000494513.1 WTAP trna
ENST00000494516.5 GATAD2A trna
ENST00000494528.2 SGK3 trna
ENST00000494531.1 LCP1 trna
ENST00000494532.1 UBE3C trna
ENST00000494536.4 UGP2 trna
ENST00000494571.1 ATG3 trna
ENST00000494580.1 CTNNA3 trna
ENST00000494586.4 CCSER2 trna
ENST00000494587.1 CYBRD1 trna
ENST00000494589.1 STAM2 trna
ENST00000494598.4 PLCH1 trna
ENST00000494604.1 MCUB trna
ENST00000494606.1 FAM102A trna
ENST00000494607.4 SLC66A2 trna
ENST00000494618.1 NCL trna
ENST00000494681.4 ITPR1 trna
ENST00000494688.1 AGK trna
ENST00000494707.4 RPGR trna
ENST00000494731.4 ZDHHC20 trna
ENST00000494735.1 GSTK1 trna
ENST00000494747.2 BACH2 trna
ENST00000494750.1 GIMAP4 trna
ENST00000494772.4 SPATA13 trna
ENST00000494785.4 MKLN1 trna
ENST00000494798.1 CD96 trna
ENST00000494800.1 HDAC4 trna
ENST00000494811.1 PARP14 trna
ENST00000494851.4 TNFSF10 trna
ENST00000494853.1 CLEC16A trna
ENST00000494859.4 ANKRD10 trna
ENST00000494866.1 ADAR trna

ENST00000494873.1 RNF11 trna
ENST00000494876.1 TBXAS1 trna
ENST00000494880.4 IVNS1ABP trna
ENST00000494901.4 SP100 trna
ENST00000494903.2 CCAR1 trna
ENST00000494904.1 JAK1 trna
ENST00000494910.1 IFI44L trna
ENST00000494915.1 LINC01322 trna
ENST00000494921.1 DTD1 trna
ENST00000494924.2 PLCB1 trna
ENST00000494947.4 RNF216P1 trna
ENST00000494948.1 KIAA0319L trna
ENST00000494958.1 EHBP1 trna
ENST00000494967.4 DIAPH1 trna
ENST00000494973.1 APTX trna
ENST00000494977.1 FGD4 trna
ENST00000494982.1 RAP1A trna
ENST00000494983.1 CREB1 trna
ENST00000494985.4 CUL4A trna
ENST00000495001.1 ARMH3 trna
ENST00000495003.1 XPO1 trna
ENST00000495013.4 MED4 trna
ENST00000495015.4 DNAJA1 trna
ENST00000495019.4 ZNF148 trna
ENST00000495025.2 PBLD trna
ENST00000495034.4 RALGPS2 trna
ENST00000495037.1 LRIG1 trna
ENST00000495044.4 CCDC30 trna
ENST00000495089.1 NFKBIZ trna
ENST00000495097.1 PTK2B trna
ENST00000495098.4 MTMR3 trna
ENST00000495110.1 MOSPD2 trna
ENST00000495130.4 MLLT10 trna
ENST00000495132.4 EXOC6 trna
ENST00000495146.4 RAB10 trna
ENST00000495162.4 MBP trna
ENST00000495174.4 SH3BP1 trna
ENST00000495177.1 CMTM6 trna
ENST00000495178.1 ATXN1 trna
ENST00000495182.4 DAPK1 trna
ENST00000495183.4 HLA-A trna
ENST00000495187.1 EIF4H trna
ENST00000495188.2 RNF146 trna
ENST00000495196.1 PSAP trna
ENST00000495216.1 ATP1B3 trna
ENST00000495244.5 RNASEH2B trna
ENST00000495245.1 STXBP3 trna
ENST00000495250.2 PRR5-ARHGAP8 trna
ENST00000495260.4 KIAA1109 trna
ENST00000495274.1 PFKL trna
ENST00000495286.4 MOB1A trna
ENST00000495288.4 PSMB4 trna
ENST00000495292.1 FAM107B trna
ENST00000495306.1 SH2D2A trna

ENST00000495321.1 SMG7 trna
ENST00000495326.1 STAT4 trna
ENST00000495333.4 EEF1A1 trna
ENST00000495338.4 PHF20 trna
ENST00000495354.1 ARF4 trna
ENST00000495355.1 YIPF4 trna
ENST00000495373.4 ARHGEF3 trna
ENST00000495390.1 DNAJB11 trna
ENST00000495394.1 RPSA trna
ENST00000495403.1 CRYBG3 trna
ENST00000495407.1 MCMBP trna
ENST00000495420.4 ANP32A trna
ENST00000495422.1 TAF12 trna
ENST00000495446.1 UPP1 trna
ENST00000495451.1 H6PD trna
ENST00000495457.1 RPRD1B trna
ENST00000495476.1 OPA1 trna
ENST00000495497.1 HDAC4 trna
ENST00000495501.2 RRBP1 trna
ENST00000495516.1 FOXP2 trna
ENST00000495528.1 VIM trna
ENST00000495530.4 PTPRE trna
ENST00000495539.4 ACSL5 trna
ENST00000495550.4 DENND1B trna
ENST00000495553.1 ACTN4 trna
ENST00000495554.1 CHTOP trna
ENST00000495557.4 PXK trna
ENST00000495562.4 ADIPOR1 retained_intron
ENST00000495566.1 PPP4R2 protein_coding
ENST00000495585.1 PRRC2C protein_coding
ENST00000495595.1 STRN retained_intron
ENST00000495596.4 ATP5MC2 retained_intron
ENST00000495601.1 ORC4 retained_intron
ENST00000495607.4 DENND6B retained_intron
ENST00000495623.1 processed_pseudogene
ENST00000495657.4 EHMT1 processed_transcript
ENST00000495666.4 RFTN1 processed_transcript
ENST00000495673.1 YPEL5 processed_transcript
ENST00000495686.1 ADAP1 retained_intron
ENST00000495692.4 PANK2 protein_coding
ENST00000495705.4 LMO4 processed_transcript
ENST00000495713.4 ANXA1 processed_transcript
ENST00000495716.1 PPP2R5CP processed_pseudogene
ENST00000495717.4 FMR1 protein_coding
ENST00000495719.1 NFKBIZ retained_intron
ENST00000495733.4 BRD2 nonsense_mediated_decay
ENST00000495743.1 SYAP1 processed_transcript
ENST00000495746.4 WARS2 processed_transcript
ENST00000495749.1 TM9SF4 retained_intron
ENST00000495752.1 UQCC1 processed_transcript
ENST00000495754.1 ZAP70 retained_intron
ENST00000495764.1 ANP32A processed_transcript
ENST00000495786.1 LILRA2 processed_transcript
ENST00000495790.1 EPC1 retained_intron

ENST00000495792.1 HIBCH processed_transcript
ENST00000495799.4 PPP1R3F protein_coding
ENST00000495806.1 TFB1M processed_transcript
ENST00000495809.1 ADAP1 retained_intron
ENST00000495810.1 HNRNPA2B1 retained_intron
ENST00000495827.2 HACD4 protein_coding
ENST00000495828.1 ERI3 processed_transcript
ENST00000495836.1 AAK1 retained_intron
ENST00000495849.4 STAT4 retained_intron
ENST00000495851.4 GPD2 processed_transcript
ENST00000495876.4 PPP2R1A processed_transcript
ENST00000495880.1 DUSP7 protein_coding
ENST00000495887.1 DCAF8 protein_coding
ENST00000495890.1 KDM4C processed_transcript
ENST00000495891.1 TIPARP protein_coding
ENST00000495892.1 MX2 retained_intron
ENST00000495903.1 ATAD1 protein_coding
ENST00000495910.5 NF1 nonsense_mediated_decay
ENST00000495923.4 EYA3 processed_transcript
ENST00000495927.4 FYN retained_intron
ENST00000495928.1 MYH9 retained_intron
ENST00000495929.3 ZNF48 processed_transcript
ENST00000495935.1 FYN retained_intron
ENST00000495958.4 CAAP1 processed_transcript
ENST00000495971.1 TYW1 retained_intron
ENST00000495980.1 PALM2AKAP2 nonsense_mediated_decay
ENST00000495985.4 ERCC8 processed_transcript
ENST00000495990.4 TRABD2A retained_intron
ENST00000496035.1 SLC25A28 processed_transcript
ENST00000496057.1 LRRN2 processed_transcript
ENST00000496063.1 MAPKAP1 processed_transcript
ENST00000496099.1 ZCCHC7 processed_transcript
ENST00000496104.1 XPA processed_transcript
ENST00000496110.1 MTARC1 processed_transcript
ENST00000496116.1 CMSS1 processed_transcript
ENST00000496144.4 VPS13B nonsense_mediated_decay
ENST00000496167.1 TBC1D23 processed_transcript
ENST00000496170.1 GLS retained_intron
ENST00000496189.1 SF3A1 retained_intron
ENST00000496202.4 PRPF3 processed_transcript
ENST00000496250.4 MAPK14 retained_intron
ENST00000496257.1 AGO4 processed_transcript
ENST00000496266.1 ARHGAP25 processed_transcript
ENST00000496268.1 RSRC1 processed_transcript
ENST00000496278.1 GPBP1L1 processed_transcript
ENST00000496282.4 TP53BP2 processed_transcript
ENST00000496294.1 processed_pseudogene
ENST00000496310.1 UBP1 processed_transcript
ENST00000496318.4 HLA-DQA1 protein_coding
ENST00000496335.1 PDAP1 processed_transcript
ENST00000496347.1 HDAC4 retained_intron
ENST00000496355.1 TATDN2 processed_transcript
ENST00000496360.4 MACF1 processed_transcript
ENST00000496361.1 SDCCAG8 processed_transcript

ENST00000496382.1 EIF4A2 retained_intron
ENST00000496383.4 KIDINS220 protein_coding
ENST00000496384.5 BRAF protein_coding
ENST00000496393.2 NVL processed_transcript
ENST00000496395.2 UBE2G2 processed_transcript
ENST00000496402.2 INPP5D retained_intron
ENST00000496427.4 CHCHD3 processed_transcript
ENST00000496440.1 CEP350 processed_transcript
ENST00000496459.2 RBMX processed_transcript
ENST00000496462.1 U2AF1 retained_intron
ENST00000496464.1 KDM4C processed_transcript
ENST00000496466.1 UBXN11 retained_intron
ENST00000496492.4 TNIK retained_intron
ENST00000496522.4 DAPK1 processed_transcript
ENST00000496532.4 MAN1C1 processed_transcript
ENST00000496537.1 RGPD8 protein_coding
ENST00000496543.1 ORMDL1 processed_transcript
ENST00000496555.1 BIRC6 retained_intron
ENST00000496569.1 HDLBP processed_transcript
ENST00000496571.4 CRY2 retained_intron
ENST00000496582.4 RPL35A retained_intron
ENST00000496617.2 GLCCI1 processed_transcript
ENST00000496637.5 MPP7 nonsense_mediated_decay
ENST00000496670.5 ORC4 processed_transcript
ENST00000496672.1 DNTTIP2 processed_transcript
ENST00000496687.1 TAFA1 protein_coding
ENST00000496689.1 ALG5 processed_transcript
ENST00000496694.1 RBM14 processed_transcript
ENST00000496706.1 YWHAE protein_coding
ENST00000496707.4 KLHL21 protein_coding
ENST00000496712.1 NFIA protein_coding
ENST00000496714.1 FBXL17 protein_coding
ENST00000496718.1 PGD retained_intron
ENST00000496722.1 UBE2L3 retained_intron
ENST00000496723.1 CD46 retained_intron
ENST00000496727.1 FLII retained_intron
ENST00000496729.2 TXNRD2 retained_intron
ENST00000496747.4 ATP6V1A protein_coding
ENST00000496772.1 SLC33A1 protein_coding
ENST00000496773.1 BAGE2 transcribed_unprocessed_pseudogene
ENST00000496774.4 MX2 retained_intron
ENST00000496776.4 LYPLAL1 processed_transcript
ENST00000496785.4 CTDSP1 processed_transcript
ENST00000496791.1 GPX1 protein_coding
ENST00000496792.3 NRXN1 protein_coding
ENST00000496804.4 MACF1 nonsense_mediated_decay
ENST00000496805.4 PEF1 processed_transcript
ENST00000496806.2 ZNF292 protein_coding
ENST00000496808.1 NLK nonsense_mediated_decay
ENST00000496817.4 S100A6 protein_coding
ENST00000496823.1 BCL6 processed_transcript
ENST00000496826.1 USF3 retained_intron
ENST00000496827.1 SEC24C processed_transcript
ENST00000496830.1 THOC2 retained_intron

ENST00000496835.5 PIN4 protein_coding
ENST00000496850.1 STK26 protein_coding
ENST00000496855.1 SNX13 processed_transcript
ENST00000496857.4 EHBP1 processed_transcript
ENST00000496864.5 FYN retained_intron
ENST00000496870.1 SP140L retained_intron
ENST00000496875.1 EIF3D retained_intron
ENST00000496878.4 MTERF4 retained_intron
ENST00000496887.5 KCNQ1 protein_coding
ENST00000496897.4 USP13 protein_coding
ENST00000496903.4 IDE protein_coding
ENST00000496906.1 RGP1 retained_intron
ENST00000496912.1 TES retained_intron
ENST00000496920.1 PTK2B processed_transcript
ENST00000496935.1 DENND1B retained_intron
ENST00000496951.1 BABAM2 retained_intron
ENST00000496967.1 RUNX3 processed_transcript
ENST00000496969.1 SF1 processed_transcript
ENST00000496989.4 NA NA
ENST00000496993.4 ACBD6 processed_transcript
ENST00000497024.1 ELMO1 retained_intron
ENST00000497029.4 STXBP5L protein_coding
ENST00000497044.4 SHTN1 processed_transcript
ENST00000497048.4 KLF4 retained_intron
ENST00000497049.4 SIK3 processed_transcript
ENST00000497091.1 PBX3 processed_transcript
ENST00000497092.1 ASXL2 protein_coding
ENST00000497100.4 KDM5C retained_intron
ENST00000497103.1 ARHGAP12 processed_transcript
ENST00000497111.1 ESYT2 processed_transcript
ENST00000497112.1 AIDA processed_transcript
ENST00000497135.4 DENND1A processed_transcript
ENST00000497151.1 DHRS12 processed_transcript
ENST00000497165.4 ARF1 processed_transcript
ENST00000497171.1 PHC3 retained_intron
ENST00000497184.4 DBNL processed_transcript
ENST00000497187.4 WLS processed_transcript
ENST00000497216.2 SPTAN1 processed_transcript
ENST00000497222.1 NA NA
ENST00000497244.1 CASS4 retained_intron
ENST00000497247.1 TMEM131L retained_intron
ENST00000497259.4 ARHGAP25 protein_coding
ENST00000497267.4 ARHGEF3 protein_coding
ENST00000497268.1 ZEB2 retained_intron
ENST00000497277.1 SEC62 retained_intron
ENST00000497295.1 SELL protein_coding
ENST00000497305.1 SHOC2 processed_transcript
ENST00000497322.1 RPL12 processed_transcript
ENST00000497326.1 OGDH retained_intron
ENST00000497329.5 NA NA
ENST00000497343.4 PPM1L protein_coding
ENST00000497351.4 PHLPP1 processed_transcript
ENST00000497355.4 FOXP1 protein_coding
ENST00000497358.1 NRDC processed_transcript

ENST00000497362.4 AIF1 retained_intron
ENST00000497391.4 HCLS1 nonsense_mediated_decay
ENST00000497402.1 WARS2 processed_transcript
ENST00000497404.1 CD53 processed_transcript
ENST00000497412.4 ELMO2 processed_transcript
ENST00000497423.1 LCLAT1 protein_coding
ENST00000497429.4 ARNTL processed_transcript
ENST00000497443.4 MOB4 processed_transcript
ENST00000497451.1 AGBL4 processed_transcript
ENST00000497452.4 IL12A-AS1 lncRNA
ENST00000497460.4 RAD51B processed_transcript
ENST00000497491.1 BANP processed_transcript
ENST00000497493.1 VPS26C retained_intron
ENST00000497511.4 SKAP2 processed_transcript
ENST00000497513.1 GNB4 protein_coding
ENST00000497516.4 TRIM24 processed_transcript
ENST00000497519.1 RPL5 retained_intron
ENST00000497544.1 CTSD retained_intron
ENST00000497553.2 FOXP1 protein_coding
ENST00000497559.1 TNRC6B processed_transcript
ENST00000497564.4 VPS26A processed_transcript
ENST00000497602.1 PRKCE processed_transcript
ENST00000497606.4 DCUN1D1 protein_coding
ENST00000497629.1 BBX retained_intron
ENST00000497639.4 SIRT1 processed_transcript
ENST00000497648.1 RABGGTB processed_transcript
ENST00000497674.4 GORASP2 retained_intron
ENST00000497690.1 NAGK retained_intron
ENST00000497692.1 CCNY processed_transcript
ENST00000497715.1 CUL3 retained_intron
ENST00000497717.4 UQCC1 retained_intron
ENST00000497727.4 NAA16 processed_transcript
ENST00000497734.4 SRC processed_transcript
ENST00000497741.4 RAC1 processed_transcript
ENST00000497755.1 CLIC4 processed_transcript
ENST00000497757.1 FRMD4B processed_transcript
ENST00000497759.1 MEPCE retained_intron
ENST00000497770.1 THADA processed_transcript
ENST00000497784.1 BRAF nonsense_mediated_decay
ENST00000497829.1 TFEC processed_transcript
ENST00000497838.4 EIF4E3 protein_coding
ENST00000497842.5 TTC19 processed_transcript
ENST00000497845.1 POLR2B retained_intron
ENST00000497849.1 VIM protein_coding
ENST00000497861.4 SELENOF protein_coding
ENST00000497873.1 PAXBP1 retained_intron
ENST00000497875.1 DNAH1 retained_intron
ENST00000497880.4 FRMD4B protein_coding
ENST00000497885.1 lncRNA
ENST00000497920.1 ARID5A retained_intron
ENST00000497929.1 ZNF148 processed_transcript
ENST00000497936.4 CEP162 processed_transcript
ENST00000497943.1 SUN1 retained_intron
ENST00000497961.1 lncRNA

ENST00000497964.1 MACF1 retained_intron
ENST00000497969.5 SUZ12P1 processed_transcript
ENST00000497971.1 MBNL1 retained_intron
ENST00000497976.4 MTF2 processed_transcript
ENST00000497987.1 NUB1 retained_intron
ENST00000497989.4 INTS6 protein_coding
ENST00000498004.4 CA5B protein_coding
ENST00000498020.1 HLA-DMB retained_intron
ENST00000498026.1 INPP4A processed_transcript
ENST00000498034.4 LMBR1 processed_transcript
ENST00000498035.4 HM13 protein_coding
ENST00000498038.1 HLA-DPB1 processed_transcript
ENST00000498039.4 RNF6 processed_transcript
ENST00000498049.1 C2orf76 processed_transcript
ENST00000498058.1 PRKCB processed_transcript
ENST00000498070.4 PPP1R12B processed_transcript
ENST00000498073.2 NA NA
ENST00000498077.5 XRN1 protein_coding
ENST00000498086.4 ATP11B protein_coding
ENST00000498103.1 processed_pseudogene
ENST00000498104.1 IQCB1 protein_coding
ENST00000498123.4 PPP2R5A processed_transcript
ENST00000498126.4 CNIH3 processed_transcript
ENST00000498127.4 TAMM41 retained_intron
ENST00000498128.1 GPBP1L1 processed_transcript
ENST00000498129.5 PPP2R5A processed_transcript
ENST00000498147.4 PDCD6IP processed_transcript
ENST00000498149.1 IP6K1 processed_transcript
ENST00000498153.4 CSF2RA processed_transcript
ENST00000498154.4 NA NA
ENST00000498161.1 FTH1P8 processed_pseudogene
ENST00000498176.1 HNRNPF processed_transcript
ENST00000498177.1 XPR1 processed_transcript
ENST00000498189.1 BBS9 retained_intron
ENST00000498196.1 MDFIC protein_coding
ENST00000498201.1 RNF2 retained_intron
ENST00000498236.1 CDC42 nonsense_mediated_decay
ENST00000498255.5 DGKH processed_transcript
ENST00000498271.1 C4A protein_coding
ENST00000498280.1 RBM39 processed_transcript
ENST00000498289.4 C1orf112 processed_transcript
ENST00000498294.4 TGDS processed_transcript
ENST00000498303.4 ZNF644 processed_transcript
ENST00000498313.4 TSPAN32 retained_intron
ENST00000498316.3 CREB5 protein_coding
ENST00000498318.1 CHIC1 nonsense_mediated_decay
ENST00000498319.2 DIS3L2 processed_transcript
ENST00000498337.1 INPP5A processed_transcript
ENST00000498351.1 NA NA
ENST00000498367.1 PDCD4 processed_transcript
ENST00000498371.1 IL10RB retained_intron
ENST00000498396.2 SLFN11 retained_intron
ENST00000498402.2 LRRC41 nonsense_mediated_decay
ENST00000498411.1 FLNA processed_transcript

ENST00000498430.4 TRPM2 processed_transcript
ENST00000498440.4 QRICH1 retained_intron
ENST00000498444.4 NA NA
ENST00000498452.1 HBEGF retained_intron
ENST00000498453.1 processed_pseudogene
ENST00000498454.1 TTYH3 retained_intron
ENST00000498458.2 NA NA
ENST00000498474.2 PUDP processed_transcript
ENST00000498482.4 TBC1D15 nonsense_mediated_decay
ENST00000498485.4 PSMB7 processed_transcript
ENST00000498516.1 HNRNPLL processed_transcript
ENST00000498517.4 ARHGEF3 processed_transcript
ENST00000498524.4 ANKRD28 retained_intron
ENST00000498530.4 PMPCB retained_intron
ENST00000498550.4 PDS5B retained_intron
ENST00000498557.4 DLEU1 lncRNA
ENST00000498562.2 CUL4A processed_transcript
ENST00000498566.2 OGT processed_transcript
ENST00000498596.4 PRRC2C protein_coding
ENST00000498625.1 PLSCR4 protein_coding
ENST00000498626.1 CAPZA1 processed_transcript
ENST00000498633.1 BTBD9 protein_coding
ENST00000498650.1 ANGEL2 processed_transcript
ENST00000498660.1 TUBA4A processed_transcript
ENST00000498690.1 LPGAT1 retained_intron
ENST00000498703.1 PTEN retained_intron
ENST00000498706.4 AFTPH retained_intron
ENST00000498711.1 CLTC retained_intron
ENST00000498730.4 NKTR retained_intron
ENST00000498735.1 ANKRD13C processed_transcript
ENST00000498739.1 PRKCB protein_coding
ENST00000498768.1 SLC35F5 retained_intron
ENST00000498774.1 LRRFIP1P1 processed_pseudogene
ENST00000498778.4 MKLN1 processed_transcript
ENST00000498815.1 RPS6 processed_transcript
ENST00000498819.1 SECISBP2 processed_transcript
ENST00000498824.3 ELF1 nonsense_mediated_decay
ENST00000498828.1 RNF7 retained_intron
ENST00000498833.1 FAM126A processed_transcript
ENST00000498907.2 CEBPA protein_coding
ENST00000498971.5 CD14 protein_coding
ENST00000498999.2 MTND4P12 processed_pseudogene
ENST00000499045.2 CHFR processed_transcript
ENST00000499096.2 lncRNA
ENST00000499098.1 CYP2U1-AS1 lncRNA
ENST00000499247.2 TOB1 protein_coding
ENST00000499481.2 lncRNA
ENST00000499587.2 lncRNA
ENST00000499676.4 TFDP2 protein_coding
ENST00000499732.2 NEAT1 lncRNA
ENST00000499762.2 A2M-AS1 lncRNA
ENST00000499847.5 PPA2 retained_intron
ENST00000499869.5 WDR1 protein_coding
ENST00000499871.2 lncRNA

ENST00000499879.5 STK4 protein_coding
ENST00000499986.7 lncRNA
ENST00000500036.2 PRKCH-AS1 lncRNA
ENST00000500323.2 DOK3 retained_intron
ENST00000500365.2 PCED1B-AS1 retained_intron
ENST00000500450.5 CNBP protein_coding
ENST00000500538.5 UBA6-DT lncRNA
ENST00000500559.5 SEPTIN7P14 processed_transcript
ENST00000500563.2 MRFAP1L1 retained_intron
ENST00000500576.4 LYRM4 protein_coding
ENST00000500610.1 TAFA2 retained_intron
ENST00000500626.2 unprocessed_pseudogene
ENST00000500636.2 lncRNA
ENST00000500682.1 KLRK1-AS1 lncRNA
ENST00000500728.2 NA NA
ENST00000500765.1 FAM13A-AS1 lncRNA
ENST00000500779.2 STARD4-AS1 lncRNA
ENST00000500893.3 ZCCHC3 protein_coding
ENST00000500949.5 OIP5-AS1 lncRNA
ENST00000500989.2 LINC00861 lncRNA
ENST00000501008.2 BTG1-DT lncRNA
ENST00000501056.2 MTCYBP18 processed_pseudogene
ENST00000501122.2 NEAT1 lncRNA
ENST00000501271.2 CLIP1 retained_intron
ENST00000501338.4 lncRNA
ENST00000501579.5 RORA-AS1 lncRNA
ENST00000501695.3 FBXO38-DT lncRNA
ENST00000501740.5 ACSM3 processed_transcript
ENST00000501748.3 ZNF623 protein_coding
ENST00000501825.2 FRG1-DT lncRNA
ENST00000501897.1 lncRNA
ENST00000501917.5 HSPA9 processed_transcript
ENST00000502213.5 TLR1 protein_coding
ENST00000502247.1 SLC38A9 protein_coding
ENST00000502249.5 CEP170P1 transcribed_unprocessed_pseudogene
ENST00000502261.4 MAN2A1 processed_transcript
ENST00000502269.1 TENT2 processed_transcript
ENST00000502273.5 processed_transcript
ENST00000502289.1 MARCHF3 processed_transcript
ENST00000502297.4 KIF13A protein_coding
ENST00000502307.1 AREG protein_coding
ENST00000502344.4 C1QTNF7-AS1 lncRNA
ENST00000502367.1 NFKB1 retained_intron
ENST00000502371.2 APC nonsense_mediated_decay
ENST00000502372.1 CNBP retained_intron
ENST00000502375.1 SMG7 protein_coding
ENST00000502386.1 ADH5 retained_intron
ENST00000502399.6 MARK2 protein_coding
ENST00000502400.4 lncRNA
ENST00000502422.4 MATR3 retained_intron
ENST00000502423.4 NDUFS4 nonsense_mediated_decay
ENST00000502431.4 PAPSS1 processed_transcript
ENST00000502453.1 SUB1 retained_intron
ENST00000502462.5 SLC25A46 retained_intron

ENST00000502483.4 SPON2 protein_coding
ENST00000502485.1 RAPGEF2 processed_transcript
ENST00000502489.4 PDZD2 retained_intron
ENST00000502495.1 SCLT1 processed_transcript
ENST00000502503.1 FAM172A nonsense_mediated_decay
ENST00000502521.1 NUDT16-DT lncRNA
ENST00000502524.4 PARP8 protein_coding
ENST00000502537.1 ARHGAP24 processed_transcript
ENST00000502562.4 ANAPC10 processed_transcript
ENST00000502574.1 retained_intron
ENST00000502582.1 CDC42SE2 protein_coding
ENST00000502596.4 PPA2 protein_coding
ENST00000502610.1 PPA2 retained_intron
ENST00000502613.1 BMP2K protein_coding
ENST00000502635.1 DCP2 nonsense_mediated_decay
ENST00000502645.2 lncRNA
ENST00000502658.1 NDUFAF2 protein_coding
ENST00000502660.4 CSF1R retained_intron
ENST00000502677.1 RASGRF2 retained_intron
ENST00000502679.1 IL6R retained_intron
ENST00000502693.1 STX18-AS1 lncRNA
ENST00000502696.1 MAML3 protein_coding
ENST00000502699.4 GRID2 nonsense_mediated_decay
ENST00000502704.2 KIF13A protein_coding
ENST00000502713.1 WDFY3 protein_coding
ENST00000502717.4 HMGXB3 protein_coding
ENST00000502747.1 SERINC5 retained_intron
ENST00000502749.1 ZNF451 nonsense_mediated_decay
ENST00000502756.1 SLC25A36 retained_intron
ENST00000502758.1 UBA6-DT lncRNA
ENST00000502761.1 CALCOCO2 protein_coding
ENST00000502767.1 PLEKHO1 processed_transcript
ENST00000502768.1 TTC37 retained_intron
ENST00000502790.1 ANKRD17-DT lncRNA
ENST00000502797.1 TMEM165 retained_intron
ENST00000502798.5 SV2C protein_coding
ENST00000502801.1 CCDC149 protein_coding
ENST00000502810.4 PKD2L2 protein_coding
ENST00000502813.1 lncRNA
ENST00000502847.1 ANAPC10 protein_coding
ENST00000502851.1 MTND5P12 processed_pseudogene
ENST00000502869.4 MOB1B protein_coding
ENST00000502873.4 TBC1D19 retained_intron
ENST00000502908.1 SCARB2 retained_intron
ENST00000502913.1 HERC5 retained_intron
ENST00000502914.1 FAM13A retained_intron
ENST00000502916.4 CAMK4 processed_transcript
ENST00000502925.1 FRYL retained_intron
ENST00000502949.4 SEL1L3 protein_coding
ENST00000502953.1 MTREX retained_intron
ENST00000502962.4 WDR1 processed_transcript
ENST00000502972.1 OCIAD1 retained_intron
ENST00000502984.4 RUFY1 protein_coding
ENST00000502988.4 ZFR retained_intron

ENST00000503000.1 ZFYVE28 protein_coding
ENST00000503002.4 RPS3A protein_coding
ENST00000503010.1 TMED7 processed_transcript
ENST00000503050.4 NA NA
ENST00000503059.1 NNT nonsense_mediated_decay
ENST00000503073.4 MAD2L1-DT lncRNA
ENST00000503080.1 UBE2B retained_intron
ENST00000503084.1 TNPO1 retained_intron
ENST00000503118.2 LRRC59 protein_coding
ENST00000503152.1 lncRNA
ENST00000503170.1 RACK1 miRNA
ENST00000503171.4 PPA2 miRNA
ENST00000503183.1 ETF1 miRNA
ENST00000503192.4 ARRDC3 miRNA
ENST00000503194.1 TMEM161B miRNA
ENST00000503225.4 ARAP2 miRNA
ENST00000503238.4 FRYL miRNA
ENST00000503252.1 LPCAT1 miRNA
ENST00000503256.4 TMCC1 miRNA
ENST00000503273.1 UIMC1 miRNA
ENST00000503279.1 TTC37 miRNA
ENST00000503280.4 SLC2A9 miRNA
ENST00000503282.1 UBE2D3 miRNA
ENST00000503288.5 ATP10D miRNA
ENST00000503291.4 CDC42SE2 miRNA
ENST00000503299.1 miRNA
ENST00000503303.1 CANX miRNA
ENST00000503322.1 LY6G6F-LY6G6D miRNA
ENST00000503336.1 SLC26A2 miRNA
ENST00000503339.4 FRYL miRNA
ENST00000503350.5 CSNK1A1 miRNA
ENST00000503362.1 SPCS3 miRNA
ENST00000503369.1 AFF1 miRNA
ENST00000503388.1 NDFIP1 miRNA
ENST00000503396.4 PDS5A miRNA
ENST00000503398.2 RAPGEF6 miRNA
ENST00000503401.1 SCLT1 miRNA
ENST00000503407.4 ACSL1 miRNA
ENST00000503424.1 COMMD10 miRNA
ENST00000503425.4 ZNF346 miRNA
ENST00000503441.1 miRNA
ENST00000503449.1 miRNA
ENST00000503501.1 SENP6 miRNA
ENST00000503503.4 APBB2 miRNA
ENST00000503515.1 TSC22D3 miRNA
ENST00000503537.1 EEF1A1P13 miRNA
ENST00000503554.2 MEF2C miRNA
ENST00000503562.1 DHX15 miRNA
ENST00000503565.4 SCLT1 miRNA
ENST00000503573.4 FAM117A miRNA
ENST00000503574.1 RPL34 miRNA
ENST00000503599.4 ZNF131 miRNA
ENST00000503608.4 SNX14 miRNA
ENST00000503636.1 NAAA miRNA

ENST00000503638.1 PGGT1B miRNA
ENST00000503649.4 ZNF330 miRNA
ENST00000503676.4 SPOP miRNA
ENST00000503687.1 miRNA
ENST00000503699.1 ZNF141 miRNA
ENST00000503716.4 LRBA miRNA
ENST00000503743.4 KAZN miRNA
ENST00000503745.1 RAP1GDS1 miRNA
ENST00000503750.3 NA miRNA
ENST00000503784.1 RFC1 miRNA
ENST00000503788.4 MARCHF6 miRNA
ENST00000503791.4 PAN3 miRNA
ENST00000503795.1 RASGRF2 miRNA
ENST00000503798.1 LUC7L3 miRNA
ENST00000503805.4 ANAPC4 miRNA
ENST00000503814.1 N4BP2L2 miRNA
ENST00000503816.1 MGST2 miRNA
ENST00000503828.4 CAST miRNA
ENST00000503833.5 CTSZ miRNA
ENST00000503857.4 YTHDC2 miRNA
ENST00000503876.4 RUFY3 miRNA
ENST00000503883.1 CFAP299 miRNA
ENST00000503889.2 EXOC3 miRNA
ENST00000503906.1 miRNA
ENST00000503916.1 SPOCK1 miRNA
ENST00000503923.1 VCAN miRNA
ENST00000503925.1 ANK3 miRNA
ENST00000503927.4 INPP4B miRNA
ENST00000503943.4 TMEM161B miRNA
ENST00000503945.2 MED28 miRNA
ENST00000503946.1 DDX46 miRNA
ENST00000503968.1 HIPK1 miRNA
ENST00000503970.2 MAN2A1 miRNA
ENST00000503978.1 RHOH miRNA
ENST00000503988.1 DCTD miRNA
ENST00000503991.1 SCARNA22 miRNA
ENST00000504005.4 WWC2 miRNA
ENST00000504012.1 LINC01340 miRNA
ENST00000504013.4 CEP63 miRNA
ENST00000504023.1 MATR3 miRNA
ENST00000504024.1 ATP8A1 miRNA
ENST00000504028.4 PPA2 miRNA
ENST00000504029.1 KLHL8 miRNA
ENST00000504032.1 UGDH-AS1 miRNA
ENST00000504033.4 ZNF331 miRNA
ENST00000504039.1 RAPGEF6 miRNA
ENST00000504042.4 TET2 miRNA
ENST00000504054.1 SPEF2 miRNA
ENST00000504064.1 TMEM154 miRNA
ENST00000504090.1 CAMK4 miRNA
ENST00000504094.1 FIP1L1 miRNA
ENST00000504101.1 POLR2J3 miRNA
ENST00000504106.4 MAD2L1-DT miRNA
ENST00000504110.1 C4orf3 miRNA

ENST00000504123.4 CNOT6L miRNA
ENST00000504131.5 NA miRNA
ENST00000504143.5 FER miRNA
ENST00000504145.4 RIPK2-DT miRNA
ENST00000504185.4 TGFBI miRNA
ENST00000504186.4 ANKRD13D miRNA
ENST00000504196.1 PLK2 miRNA
ENST00000504197.4 MACROH2A1 miRNA
ENST00000504220.5 PCDH11X miRNA
ENST00000504221.4 CAST miRNA
ENST00000504227.4 CYSTM1 miRNA
ENST00000504233.4 TENT2 miRNA
ENST00000504248.4 MAN2B2 miRNA
ENST00000504254.4 IQGAP2 miRNA
ENST00000504293.4 RPS23 miRNA
ENST00000504295.1 COPB2 miRNA
ENST00000504298.1 BACH1-IT1 miRNA
ENST00000504314.1 ELF2 miRNA
ENST00000504324.1 ABL2 miRNA
ENST00000504336.1 NR3C1 miRNA
ENST00000504340.1 IRF2 miRNA
ENST00000504342.4 ACSL1 miRNA
ENST00000504343.1 CNOT6 miRNA
ENST00000504358.1 ARNT nonsense_mediated_decay
ENST00000504383.1 ACSL4 retained_intron
ENST00000504388.4 MTREX trna
ENST00000504403.1 BANK1 trna
ENST00000504411.1 TGFBI trna
ENST00000504430.4 NIPBL trna
ENST00000504431.1 UBE2B trna
ENST00000504435.1 GAK trna
ENST00000504445.1 ATP10D trna
ENST00000504455.4 ELOVL7 trna
ENST00000504469.1 trna
ENST00000504472.1 EIF4E trna
ENST00000504475.5 ANXA2 trna
ENST00000504477.4 IQGAP2 trna
ENST00000504500.4 PRLR trna
ENST00000504510.4 ATP8A1 trna
ENST00000504526.1 TRAPPC11 trna
ENST00000504542.1 FYB1 trna
ENST00000504546.4 NAIPP1 trna
ENST00000504564.1 WDR70 trna
ENST00000504571.4 ATP2C1 trna
ENST00000504578.2 LIX1-AS1 trna
ENST00000504592.4 BANK1 trna
ENST00000504604.4 RAPGEF2 trna
ENST00000504610.2 LINC02762 trna
ENST00000504612.4 ATP2C1 trna
ENST00000504614.4 LAP3 trna
ENST00000504634.4 NEDD9 trna
ENST00000504637.4 SEPTIN11 trna
ENST00000504642.1 TNFAIP8 trna
ENST00000504644.1 RAB28 trna

ENST00000504661.1 DCUN1D4 trna
ENST00000504666.4 SIL1 trna
ENST00000504671.1 MGAT1 trna
ENST00000504677.4 POLR2J3 trna
ENST00000504701.4 CDC42SE2 trna
ENST00000504704.5 FNIP2 trna
ENST00000504715.1 FNIP2 trna
ENST00000504743.1 ZFYVE28 trna
ENST00000504745.1 ALPK1 trna
ENST00000504753.1 NR3C2 trna
ENST00000504757.2 SRP72 trna
ENST00000504768.2 FAM172A trna
ENST00000504769.4 TMEM161B-DT trna
ENST00000504771.2 TNFAIP8 trna
ENST00000504782.4 RNF4 trna
ENST00000504788.4 LMNB1 trna
ENST00000504804.4 CNOT6L trna
ENST00000504805.5 RUFY3 trna
ENST00000504815.1 IQGAP2 trna
ENST00000504827.1 trna
ENST00000504837.1 FBXL5 trna
ENST00000504861.2 FLJ46284 trna
ENST00000504863.1 RASGEF1B trna
ENST00000504867.4 CDV3 trna
ENST00000504875.4 CSF1R trna
ENST00000504881.1 SRFBP1 trna
ENST00000504900.4 ACSL1 trna
ENST00000504901.2 MRPL1 trna
ENST00000504910.1 HK3 trna
ENST00000504915.2 APC trna
ENST00000504919.1 RAPGEF6 trna
ENST00000504938.1 RBPJ trna
ENST00000504945.1 ACSF2 trna
ENST00000504952.1 DCK trna
ENST00000504959.4 SNX25 trna
ENST00000504961.1 DCP2 trna
ENST00000504971.1 FBXO38 trna
ENST00000504982.1 TENT2 trna
ENST00000505009.4 UBE2D3 trna
ENST00000505010.4 TPM3 trna
ENST00000505064.1 CCND3 trna
ENST00000505067.4 IRF2 trna
ENST00000505073.4 CCSER1 trna
ENST00000505075.1 ACSL4 trna
ENST00000505078.4 MCTP1 trna
ENST00000505082.1 TNPO1 trna
ENST00000505091.1 trna
ENST00000505093.1 IL7R trna
ENST00000505095.1 TMEM192 trna
ENST00000505104.4 SLC2A9 trna
ENST00000505123.4 PRR16 trna
ENST00000505131.1 SLAIN2 trna
ENST00000505133.4 LINC02615 trna
ENST00000505134.4 SEC24D trna

ENST00000505138.1 PHF3 trna
ENST00000505176.4 ALPK1 trna
ENST00000505181.4 NA trna
ENST00000505204.1 ZFR trna
ENST00000505219.1 SNORA58 trna
ENST00000505225.1 RERE trna
ENST00000505230.1 CCNH trna
ENST00000505236.1 RELB trna
ENST00000505239.1 MANBA trna
ENST00000505252.1 ATG12 trna
ENST00000505253.1 MARCHF6 trna
ENST00000505279.4 SPAG9 trna
ENST00000505284.4 MFSD8 trna
ENST00000505285.4 TCERG1 trna
ENST00000505295.1 CKMT2-AS1 trna
ENST00000505303.4 WDR36 trna
ENST00000505314.1 SRP72 trna
ENST00000505323.1 FER trna
ENST00000505328.1 LEF1 trna
ENST00000505350.1 APC trna
ENST00000505366.1 ZFR trna
ENST00000505372.1 PAM trna
ENST00000505379.4 LEF1 trna
ENST00000505383.1 ARL15 trna
ENST00000505399.2 FBXO38 trna
ENST00000505412.1 ZCCHC4 trna
ENST00000505423.1 AP3S1 trna
ENST00000505426.1 ZNF331 trna
ENST00000505434.4 SEC31A trna
ENST00000505445.1 FNIP2 trna
ENST00000505452.4 TEC trna
ENST00000505453.1 PDE4D trna
ENST00000505464.1 ICE1 trna
ENST00000505478.4 RAPGEF2 trna
ENST00000505492.1 ACSL1 trna
ENST00000505514.4 RCHY1 trna
ENST00000505516.2 GUSBP16 trna
ENST00000505517.1 MARCHF1 trna
ENST00000505526.1 PDCD6 trna
ENST00000505569.4 FAM193B trna
ENST00000505588.1 KIF13A trna
ENST00000505600.1 F2R trna
ENST00000505602.1 ACAD9 trna
ENST00000505614.1 NDFIP1 trna
ENST00000505615.1 VCAN trna
ENST00000505616.4 TMCC1 trna
ENST00000505630.4 ARL15 trna
ENST00000505635.1 trna
ENST00000505645.1 trna
ENST00000505655.2 NA trna
ENST00000505658.4 LUC7L3 trna
ENST00000505673.2 UBA6 trna
ENST00000505678.5 NNT trna
ENST00000505682.1 MGAT1 trna

ENST00000505687.4 GRID2 trna
ENST00000505690.1 SPOCK1 trna
ENST00000505691.2 FAM110A trna
ENST00000505720.1 GRAMD2B trna
ENST00000505725.1 BMP2K trna
ENST00000505729.1 SMIM14 trna
ENST00000505744.4 TLR1 trna
ENST00000505755.4 ARNT trna
ENST00000505759.1 FRYL trna
ENST00000505761.2 CD6 trna
ENST00000505763.1 CAMK4 trna
ENST00000505766.4 NA trna
ENST00000505774.4 POLK trna
ENST00000505785.4 BST1 trna
ENST00000505801.1 TET2 trna
ENST00000505823.4 PRDM2 trna
ENST00000505827.1 MACROH2A1 trna
ENST00000505854.4 SNX2 trna
ENST00000505855.1 ACSL4 trna
ENST00000505856.1 ARHGAP24 trna
ENST00000505863.1 DDX60L trna
ENST00000505868.1 FAM135A trna
ENST00000505875.1 IL7R trna
ENST00000505883.1 MBD4 trna
ENST00000505890.4 DDX60L trna
ENST00000505892.4 NSUN2 trna
ENST00000505896.4 EMB trna
ENST00000505900.1 ZNF721 trna
ENST00000505907.1 MAN2B2 trna
ENST00000505923.4 WDFY3 trna
ENST00000505941.4 SH3BP2 trna
ENST00000505945.1 SIL1 trna
ENST00000505946.2 GUSBP17 trna
ENST00000505958.4 RBPJ trna
ENST00000505970.2 ANKRD55 trna
ENST00000505971.4 TRIO trna
ENST00000505983.1 CNOT6L trna
ENST00000505987.1 HSP90AB3P trna
ENST00000505997.1 LINC01950 trna
ENST00000505998.4 NIPBL trna
ENST00000506002.1 MTND6P4 trna
ENST00000506020.4 ARHGAP10 trna
ENST00000506024.4 PDE4D trna
ENST00000506045.1 PHYKPL trna
ENST00000506061.1 FAF2 trna
ENST00000506067.1 MFSD14C trna
ENST00000506098.4 NUP54 trna
ENST00000506103.2 TMEM165 trna
ENST00000506127.1 PAM trna
ENST00000506146.4 TLR1 trna
ENST00000506156.1 FAM117A trna
ENST00000506157.1 ADD1 trna
ENST00000506166.1 CNOT6L trna
ENST00000506175.1 YTHDC1 trna

ENST00000506182.1 SNX14 trna
ENST00000506189.1 ARAP2 trna
ENST00000506190.1 EMB trna
ENST00000506191.1 CD38 trna
ENST00000506198.4 TMEM165 trna
ENST00000506202.1 CEP135 trna
ENST00000506217.4 INPP4B trna
ENST00000506233.4 SCLT1 trna
ENST00000506241.1 IL6ST trna
ENST00000506246.4 WDR1 trna
ENST00000506252.1 SREK1IP1 trna
ENST00000506258.2 OTULINL trna
ENST00000506260.1 PAM trna
ENST00000506271.1 UBE2Z trna
ENST00000506280.1 TBCK trna
ENST00000506297.4 INPP4B trna
ENST00000506298.1 NA trna
ENST00000506303.1 RO60 trna
ENST00000506310.1 LMAN2 trna
ENST00000506327.5 DACH2 trna
ENST00000506333.1 YTHDC2 trna
ENST00000506347.2 PABPC1P1 trna
ENST00000506348.1 CDHR2 trna
ENST00000506351.5 TNPO1 trna
ENST00000506365.1 RASA2 trna
ENST00000506398.5 FAM172A trna
ENST00000506432.1 FAM193B trna
ENST00000506435.1 CCNO-DT trna
ENST00000506441.1 RETREG1 trna
ENST00000506453.1 GRSF1 trna
ENST00000506461.1 ELOVL6 trna
ENST00000506480.4 trna
ENST00000506490.2 GUSBP13 trna
ENST00000506515.1 HERC4 trna
ENST00000506523.5 RBM4 trna
ENST00000506533.4 KAT7 protein_coding
ENST00000506548.1 METAP1 processed_transcript
ENST00000506555.4 CCND3 processed_transcript
ENST00000506560.4 LIN54 protein_coding
ENST00000506565.1 RPS6KA2 protein_coding
ENST00000506568.7 MCTP1 protein_coding
ENST00000506571.1 UBA6 retained_intron
ENST00000506578.4 LEMD2 protein_coding
ENST00000506589.1 COMMD10 protein_coding
ENST00000506602.4 ATP8A1 nonsense_mediated_decay
ENST00000506614.1 CCNI retained_intron
ENST00000506632.2 PDLIM5 nonsense_mediated_decay
ENST00000506643.4 CC2D2A protein_coding
ENST00000506649.4 CD164 processed_transcript
ENST00000506674.1 MRPL1 processed_transcript
ENST00000506685.1 FRYL retained_intron
ENST00000506727.1 PKD2 retained_intron
ENST00000506733.4 ACSL1 nonsense_mediated_decay
ENST00000506742.2 RACK1P1 processed_pseudogene

ENST00000506747.4 CLOCK retained_intron
ENST00000506750.4 MTREX nonsense_mediated_decay
ENST00000506755.1 ANKHD1 retained_intron
ENST00000506780.2 METTL14 protein_coding
ENST00000506787.4 UBE2B protein_coding
ENST00000506793.4 GUF1 processed_transcript
ENST00000506816.1 SGTB protein_coding
ENST00000506850.4 IL7R protein_coding
ENST00000506857.4 KIF2A protein_coding
ENST00000506860.1 TMEM267 protein_coding
ENST00000506880.4 PDGFC nonsense_mediated_decay
ENST00000506891.1 TTC39B protein_coding
ENST00000506896.1 SNX25 processed_transcript
ENST00000506904.1 FAM193A retained_intron
ENST00000506929.1 CDC42SE2 processed_transcript
ENST00000506960.4 SSBP2 retained_intron
ENST00000506982.1 lncRNA
ENST00000507001.1 SPCS3 processed_transcript
ENST00000507012.1 RERE processed_transcript
ENST00000507016.1 CNOT6 processed_transcript
ENST00000507020.1 PDS5A nonsense_mediated_decay
ENST00000507023.1 AGA-DT lncRNA
ENST00000507030.4 SLC10A7 protein_coding
ENST00000507038.1 lncRNA
ENST00000507053.1 DDX46 nonsense_mediated_decay
ENST00000507063.1 NEDD4 nonsense_mediated_decay
ENST00000507064.2 CEP192 nonsense_mediated_decay
ENST00000507071.4 CPEB2 protein_coding
ENST00000507073.1 POLK processed_transcript
ENST00000507075.1 ARRDC3 retained_intron
ENST00000507078.1 ZNF721 processed_transcript
ENST00000507090.2 HSP90AB2P transcribed_processed_pseudogene
ENST00000507114.1 ANKRD40 retained_intron
ENST00000507123.1 SH3TC1 retained_intron
ENST00000507126.2 SRP72 retained_intron
ENST00000507131.4 NFXL1 retained_intron
ENST00000507148.1 ATR nonsense_mediated_decay
ENST00000507154.1 ERAP1 protein_coding
ENST00000507162.1 VCAN processed_transcript
ENST00000507166.4 protein_coding
ENST00000507167.1 TSPAN5 retained_intron
ENST00000507175.4 TCERG1 retained_intron
ENST00000507176.4 PPP3CA protein_coding
ENST00000507180.4 RBM47 protein_coding
ENST00000507185.1 PFDN1 retained_intron
ENST00000507224.4 LRBA protein_coding
ENST00000507254.5 CEP192 processed_transcript
ENST00000507259.4 LARP1B nonsense_mediated_decay
ENST00000507273.4 RACK1 retained_intron
ENST00000507274.1 transcribed_unprocessed_pseudogene
ENST00000507282.1 LPCAT1 retained_intron
ENST00000507285.4 TTC39B protein_coding
ENST00000507287.4 RPL32P3 processed_transcript
ENST00000507307.4 NA NA

ENST00000507310.1 PPIP5K2 protein_coding
ENST00000507345.5 FCHO2 protein_coding
ENST00000507352.1 FAM13A nonsense_mediated_decay
ENST00000507358.1 MANBA retained_intron
ENST00000507364.4 SNX24 processed_transcript
ENST00000507366.1 processed_pseudogene
ENST00000507371.4 RPS6KA2 protein_coding
ENST00000507374.4 FCGR2C processed_transcript
ENST00000507377.1 LARP1B protein_coding
ENST00000507384.1 MGAT1 protein_coding
ENST00000507406.1 SMG7 processed_transcript
ENST00000507412.1 GLRX retained_intron
ENST00000507415.1 PAIP2 retained_intron
ENST00000507434.1 TRIM52-AS1 lncRNA
ENST00000507446.1 lncRNA
ENST00000507462.4 INPP4B nonsense_mediated_decay
ENST00000507465.1 ZFR nonsense_mediated_decay
ENST00000507470.4 LEF1 retained_intron
ENST00000507481.2 DELE1 protein_coding
ENST00000507490.1 ERBIN processed_transcript
ENST00000507503.1 LUC7L3 retained_intron
ENST00000507514.1 lncRNA
ENST00000507518.4 HSD17B11 processed_transcript
ENST00000507531.4 UVSSA protein_coding
ENST00000507538.1 RASGEF1B retained_intron
ENST00000507545.1 CHSY3 processed_transcript
ENST00000507548.1 ZFYVE16 nonsense_mediated_decay
ENST00000507560.4 SLC10A7 processed_transcript
ENST00000507573.1 CNBP retained_intron
ENST00000507576.1 KIF13A processed_transcript
ENST00000507586.1 PACSIN2 nonsense_mediated_decay
ENST00000507598.4 DTHD1 protein_coding
ENST00000507600.1 LINC02228 lncRNA
ENST00000507603.1 EREG retained_intron
ENST00000507605.1 GLRX processed_transcript
ENST00000507606.2 STMP1 protein_coding
ENST00000507608.1 PPWD1 retained_intron
ENST00000507632.2 CDH18 processed_transcript
ENST00000507634.4 PACRGL protein_coding
ENST00000507640.1 IPO11 retained_intron
ENST00000507641.4 PITX1-AS1 lncRNA
ENST00000507646.2 ARL15 protein_coding
ENST00000507660.4 THAP9-AS1 lncRNA
ENST00000507661.1 ARHGAP10 protein_coding
ENST00000507668.5 SERINC5 protein_coding
ENST00000507670.1 BMP2K retained_intron
ENST00000507695.1 HSD17B4 retained_intron
ENST00000507696.1 TBCK protein_coding
ENST00000507701.1 G3BP2 retained_intron
ENST00000507704.4 AFDN protein_coding
ENST00000507747.1 protein_coding
ENST00000507766.1 PDS5A retained_intron
ENST00000507768.1 DCAF16 processed_transcript
ENST00000507788.1 CCNI protein_coding

ENST00000507794.2 SEPSECS-AS1 lncRNA
ENST00000507801.1 SH3TC1 processed_transcript
ENST00000507826.1 USP38-DT lncRNA
ENST00000507863.1 MARCHF6 processed_transcript
ENST00000507870.1 LINC01098 lncRNA
ENST00000507874.4 CLDND1 protein_coding
ENST00000507875.4 CLCN3 protein_coding
ENST00000507886.1 NA NA
ENST00000507909.1 SRPK1 protein_coding
ENST00000507921.4 PPIP5K2 protein_coding
ENST00000507922.4 FIP1L1 protein_coding
ENST00000507937.1 RMND5B retained_intron
ENST00000507939.4 ETF1 protein_coding
ENST00000507984.4 MEF2C protein_coding
ENST00000507991.1 GAK retained_intron
ENST00000507994.1 DAPP1 retained_intron
ENST00000507996.4 KDM3B nonsense_mediated_decay
ENST00000507997.1 MIR583HG lncRNA
ENST00000508020.2 lncRNA
ENST00000508032.4 DHX15 processed_transcript
ENST00000508043.1 MAN2A1 processed_transcript
ENST00000508065.6 GTF2H2B transcribed_unprocessed_pseudogene
ENST00000508066.1 ARAP2 retained_intron
ENST00000508077.1 ERAP2 protein_coding
ENST00000508096.1 lncRNA
ENST00000508115.1 NFXL1 retained_intron
ENST00000508117.5 CAST nonsense_mediated_decay
ENST00000508119.4 HNRNPD processed_transcript
ENST00000508137.5 KLHL5 protein_coding
ENST00000508141.1 MANBA retained_intron
ENST00000508157.3 BDP1 retained_intron
ENST00000508163.1 lncRNA
ENST00000508184.4 ZFYVE28 protein_coding
ENST00000508235.1 RNF4 nonsense_mediated_decay
ENST00000508237.4 ZNF652 nonsense_mediated_decay
ENST00000508250.4 COMMD10 processed_transcript
ENST00000508253.4 DAP retained_intron
ENST00000508256.4 PPM1K protein_coding
ENST00000508260.4 GUSBP1 processed_transcript
ENST00000508294.1 RASGEF1B processed_transcript
ENST00000508302.1 ACOX3 processed_transcript
ENST00000508309.1 LINC00847 lncRNA
ENST00000508359.1 DCP2 retained_intron
ENST00000508360.4 FAM13A retained_intron
ENST00000508371.1 CNOT6L retained_intron
ENST00000508405.1 CPLANE1 processed_transcript
ENST00000508413.1 HSD17B11 processed_transcript
ENST00000508416.1 SKI processed_transcript
ENST00000508423.1 KIAA0232 protein_coding
ENST00000508424.4 SEMA6A-AS1 lncRNA
ENST00000508425.4 CLTB protein_coding
ENST00000508426.5 NAIP protein_coding
ENST00000508431.1 FCHO2 retained_intron
ENST00000508439.2 GUSBP15 unprocessed_pseudogene

ENST00000508441.1 MFSD8 retained_intron
ENST00000508442.5 CEP120 protein_coding
ENST00000508474.4 UBE2D3 retained_intron
ENST00000508493.1 RPL37 protein_coding
ENST00000508497.4 LAP3 retained_intron
ENST00000508515.1 ERBIN protein_coding
ENST00000508519.4 SEPTIN7P14 processed_transcript
ENST00000508521.1 MEF2C-AS1 lncRNA
ENST00000508531.1 PDLIM5 processed_transcript
ENST00000508539.2 CEP192 processed_transcript
ENST00000508546.4 NEDD9 protein_coding
ENST00000508572.1 lncRNA
ENST00000508575.1 MROH2B retained_intron
ENST00000508584.1 NFKB1 protein_coding
ENST00000508585.4 SLC2A9 processed_transcript
ENST00000508606.1 LRBA protein_coding
ENST00000508614.4 WWC2 nonsense_mediated_decay
ENST00000508620.4 TENT2 processed_transcript
ENST00000508628.5 RNF213 protein_coding
ENST00000508634.1 MAEA retained_intron
ENST00000508645.4 SLC27A6 protein_coding
ENST00000508650.2 unprocessed_pseudogene
ENST00000508651.1 FCGR2C processed_transcript
ENST00000508658.4 SNX14 protein_coding
ENST00000508680.4 NECAP2 processed_transcript
ENST00000508685.1 SH3RF1 processed_transcript
ENST00000508691.1 KLHL2P1 unprocessed_pseudogene
ENST00000508692.1 CERT1 protein_coding
ENST00000508694.1 ELL2 retained_intron
ENST00000508705.1 GRK6 nonsense_mediated_decay
ENST00000508708.1 CSNK1G3 processed_transcript
ENST00000508730.1 WDR70 retained_intron
ENST00000508738.4 CAMK2D protein_coding
ENST00000508745.1 lncRNA
ENST00000508752.1 GASK1B-AS1 lncRNA
ENST00000508762.4 TNPO1 processed_transcript
ENST00000508772.4 THAP9-AS1 lncRNA
ENST00000508778.1 CEP63 protein_coding
ENST00000508787.1 NA NA
ENST00000508794.5 NAIP retained_intron
ENST00000508795.1 ZNF131 processed_transcript
ENST00000508800.1 NEDD9 processed_transcript
ENST00000508810.1 TXNDC15 protein_coding
ENST00000508814.4 ATG10 processed_transcript
ENST00000508832.2 MALAT1 lncRNA
ENST00000508867.4 POLK retained_intron
ENST00000508878.4 LINC01184 lncRNA
ENST00000508903.5 UNKL protein_coding
ENST00000508908.1 USP45 protein_coding
ENST00000508912.1 SSBP2 retained_intron
ENST00000508962.1 MACROH2A1 processed_transcript
ENST00000508963.1 RACK1 retained_intron
ENST00000508982.1 PPWD1 retained_intron
ENST00000508989.4 FBN2 protein_coding

ENST00000509024.2 SRP19 retained_intron
ENST00000509028.1 KLHL2 processed_transcript
ENST00000509032.4 RASA2 processed_transcript
ENST00000509073.1 RNASET2 processed_transcript
ENST00000509097.1 GFM2 protein_coding
ENST00000509102.4 UBE3D nonsense_mediated_decay
ENST00000509107.1 HNRNPD protein_coding
ENST00000509108.1 CBR4 processed_transcript
ENST00000509109.4 CCSER1 nonsense_mediated_decay
ENST00000509116.1 processed_pseudogene
ENST00000509124.4 KAT7 processed_transcript
ENST00000509127.2 ENC1 protein_coding
ENST00000509168.4 TSPAN5 processed_transcript
ENST00000509176.4 CCSER1 protein_coding
ENST00000509198.1 LRPAP1 retained_intron
ENST00000509207.1 KCNIP4 protein_coding
ENST00000509227.1 TENT2 retained_intron
ENST00000509238.1 CXXC5 protein_coding
ENST00000509247.1 RAI14 processed_transcript
ENST00000509249.1 IL15 retained_intron
ENST00000509251.1 ZNF451 processed_transcript
ENST00000509268.1 XRCC4 processed_transcript
ENST00000509274.1 IRF2 nonsense_mediated_decay
ENST00000509293.1 SPOCK1 processed_transcript
ENST00000509300.4 ARHGAP24 protein_coding
ENST00000509330.4 PHF3 protein_coding
ENST00000509341.4 ZNF131 protein_coding
ENST00000509348.2 GUSBP3 transcribed_unprocessed_pseudogene
ENST00000509349.1 MEF2C processed_transcript
ENST00000509354.1 TRIO retained_intron
ENST00000509368.5 PDE4D nonsense_mediated_decay
ENST00000509373.1 MEF2C protein_coding
ENST00000509384.4 LYSMD3 protein_coding
ENST00000509385.1 TOB1 processed_transcript
ENST00000509386.2 AMN1 processed_transcript
ENST00000509413.1 ARFIP1 processed_transcript
ENST00000509418.1 SMARCAD1 protein_coding
ENST00000509434.4 NAF1 protein_coding
ENST00000509436.1 NDFIP1 processed_transcript
ENST00000509443.1 NDUFS4 retained_intron
ENST00000509448.4 STMP1 nonsense_mediated_decay
ENST00000509475.4 APBB2 retained_intron
ENST00000509479.5 MAML3 protein_coding
ENST00000509480.4 RELB nonsense_mediated_decay
ENST00000509485.4 TGFBI nonsense_mediated_decay
ENST00000509497.1 lncRNA
ENST00000509501.4 RAP1GDS1 protein_coding
ENST00000509523.1 PAM protein_coding
ENST00000509529.1 CAST retained_intron
ENST00000509535.4 RACK1 protein_coding
ENST00000509539.2 MCCC2 nonsense_mediated_decay
ENST00000509543.1 PTGER4 processed_transcript
ENST00000509549.4 C22orf39 nonsense_mediated_decay
ENST00000509562.1 ZFYVE16 protein_coding

ENST00000509565.1 TMEM131L retained_intron
ENST00000509567.4 RICTOR processed_transcript
ENST00000509575.1 TMEM165 retained_intron
ENST00000509594.1 CAMK2D protein_coding
ENST00000509620.5 OTUD4 protein_coding
ENST00000509637.5 RAB40C nonsense_mediated_decay
ENST00000509648.4 ANXA5 retained_intron
ENST00000509662.4 ATP2C1 protein_coding
ENST00000509663.2 nonsense_mediated_decay
ENST00000509667.4 PLPP1 nonsense_mediated_decay
ENST00000509668.1 IL7R retained_intron
ENST00000509674.1 DTNBP1 protein_coding
ENST00000509678.1 CHIC2 retained_intron
ENST00000509681.1 TXK nonsense_mediated_decay
ENST00000509683.4 SLC30A9 retained_intron
ENST00000509688.4 ALPK1 retained_intron
ENST00000509695.1 WDR1 processed_transcript
ENST00000509704.4 KLHL2 protein_coding
ENST00000509708.2 BTF3 protein_coding
ENST00000509709.1 ARHGAP24 retained_intron
ENST00000509717.4 HK3 nonsense_mediated_decay
ENST00000509739.4 FAM172A protein_coding
ENST00000509741.1 IQGAP2 retained_intron
ENST00000509743.5 SSBP2 processed_transcript
ENST00000509748.1 LIN54 processed_transcript
ENST00000509749.1 TGFBI retained_intron
ENST00000509761.1 TBC1D1 processed_transcript
ENST00000509765.1 SPOP retained_intron
ENST00000509769.4 USP53 nonsense_mediated_decay
ENST00000509770.1 TMEM167A processed_transcript
ENST00000509797.1 RUFY1 retained_intron
ENST00000509818.4 SEC24D nonsense_mediated_decay
ENST00000509825.1 WDFY3 retained_intron
ENST00000509835.4 LRBA protein_coding
ENST00000509839.4 PRLR protein_coding
ENST00000509867.5 ANKRD17 protein_coding
ENST00000509868.1 CTNND2 processed_transcript
ENST00000509869.4 SPOP nonsense_mediated_decay
ENST00000509912.4 NEK1 retained_intron
ENST00000509913.1 C5orf15 processed_transcript
ENST00000509932.1 LINC02232 lncRNA
ENST00000509953.1 RASA1 retained_intron
ENST00000509983.1 processed_transcript
ENST00000509992.1 GAB1 protein_coding
ENST00000509994.1 SCARB2 nonsense_mediated_decay
ENST00000510013.1 PCBD2 processed_transcript
ENST00000510030.2 SCAMP1 protein_coding
ENST00000510040.1 PARP8 retained_intron
ENST00000510071.4 RAPGEF6 protein_coding
ENST00000510075.1 MARCHF1 processed_transcript
ENST00000510080.1 PAIP2 protein_coding
ENST00000510083.1 RNASET2 retained_intron
ENST00000510084.2 PGM2 nonsense_mediated_decay
ENST00000510085.1 ATG10 protein_coding

ENST00000510087.4 TMEM161B-DT lncRNA
ENST00000510092.4 ANAPC4 protein_coding
ENST00000510095.4 lncRNA
ENST00000510098.1 CAST nonsense_mediated_decay
ENST00000510099.4 PDLIM5 protein_coding
ENST00000510105.4 SMARCAD1 nonsense_mediated_decay
ENST00000510108.1 NEK1 nonsense_mediated_decay
ENST00000510121.4 LCORL processed_transcript
ENST00000510124.4 CDHR2 nonsense_mediated_decay
ENST00000510138.2 GUSBP9 unprocessed_pseudogene
ENST00000510173.5 RBM4 protein_coding
ENST00000510189.4 CENPC retained_intron
ENST00000510207.4 AIMP1 protein_coding
ENST00000510231.1 processed_pseudogene
ENST00000510245.1 CAST retained_intron
ENST00000510252.1 TMEM154 retained_intron
ENST00000510259.4 PRELID2 processed_transcript
ENST00000510264.5 NAIPP3 processed_transcript
ENST00000510284.1 MIR3945HG lncRNA
ENST00000510289.1 ATP8A1 protein_coding
ENST00000510292.1 PPP3CA processed_transcript
ENST00000510304.2 processed_transcript
ENST00000510309.1 ERAP2 protein_coding
ENST00000510327.1 lncRNA
ENST00000510355.1 POLR2B processed_transcript
ENST00000510365.4 ACOX3 retained_intron
ENST00000510369.4 ZFR processed_transcript
ENST00000510372.4 SNX2 processed_transcript
ENST00000510377.4 USP38 protein_coding
ENST00000510379.1 ARHGAP10 retained_intron
ENST00000510382.1 ANK3 processed_transcript
ENST00000510383.1 TMEM33 retained_intron
ENST00000510391.1 lncRNA
ENST00000510405.4 SPOCK1 processed_transcript
ENST00000510407.1 INSL6 processed_transcript
ENST00000510433.1 lncRNA
ENST00000510445.1 FAM172A retained_intron
ENST00000510446.1 FAF2 processed_transcript
ENST00000510447.1 COX7C processed_transcript
ENST00000510448.4 SEL1L3 protein_coding
ENST00000510457.1 TXK processed_transcript
ENST00000510460.1 SLC30A9 retained_intron
ENST00000510461.4 FNIP1 protein_coding
ENST00000510470.1 UBE2D2 retained_intron
ENST00000510476.4 SPOP protein_coding
ENST00000510490.1 UGDH protein_coding
ENST00000510492.1 PRR7 protein_coding
ENST00000510510.4 RAPGEF2 protein_coding
ENST00000510521.1 TMCC1 processed_transcript
ENST00000510528.1 RAB28 protein_coding
ENST00000510531.4 SRD5A1 nonsense_mediated_decay
ENST00000510534.1 DKK2 retained_intron
ENST00000510546.1 DAP processed_transcript
ENST00000510552.1 TLR1 processed_transcript

ENST00000510573.5 TBC1D1 protein_coding
ENST00000510582.3 NA NA
ENST00000510590.1 DDX60L retained_intron
ENST00000510592.1 lncRNA
ENST00000510595.1 SLAIN2 protein_coding
ENST00000510599.4 UBE2D3 retained_intron
ENST00000510626.4 HTT retained_intron
ENST00000510638.1 NFKB1 processed_transcript
ENST00000510645.4 DHX15 processed_transcript
ENST00000510657.1 EMCN processed_transcript
ENST00000510661.1 NHSL2 protein_coding
ENST00000510662.1 FBXL7 protein_coding
ENST00000510674.1 CD38 protein_coding
ENST00000510685.1 FSTL4 protein_coding
ENST00000510692.4 processed_transcript
ENST00000510699.1 WDR70 processed_transcript
ENST00000510705.3 lncRNA
ENST00000510711.4 RICTOR retained_intron
ENST00000510721.1 TENT2 retained_intron
ENST00000510737.1 USP53 processed_transcript
ENST00000510739.1 CTBP1 nonsense_mediated_decay
ENST00000510746.1 YTHDC1 protein_coding
ENST00000510774.1 ATP2C1 retained_intron
ENST00000510784.5 RIPOR2 protein_coding
ENST00000510786.1 MARCHF1 nonsense_mediated_decay
ENST00000510792.1 MARCHF6 protein_coding
ENST00000510799.1 GAK protein_coding
ENST00000510821.1 SULT1B1 protein_coding
ENST00000510822.1 MXD4 nonsense_mediated_decay
ENST00000510842.5 CSNK1G3 protein_coding
ENST00000510844.4 SEPTIN7P14 processed_transcript
ENST00000510852.1 USP53 retained_intron
ENST00000510855.1 SPAG9 protein_coding
ENST00000510894.1 CHIC2 protein_coding
ENST00000510895.5 MCCC2 retained_intron
ENST00000510896.1 HNRNPKP1 processed_pseudogene
ENST00000510909.1 GABRB1 nonsense_mediated_decay
ENST00000510924.1 DMXL1 processed_transcript
ENST00000510927.4 TBCK retained_intron
ENST00000510933.1 ZNF346 retained_intron
ENST00000510934.4 UBE2K protein_coding
ENST00000510950.1 BANK1 processed_transcript
ENST00000510954.4 NSD1 processed_transcript
ENST00000510985.1 HELQ protein_coding
ENST00000510989.1 ZNF451 processed_transcript
ENST00000511006.1 ELF2 retained_intron
ENST00000511011.4 TBCK processed_transcript
ENST00000511024.4 FAM53C retained_intron
ENST00000511027.1 MAML1 retained_intron
ENST00000511034.1 RPS6KA2 protein_coding
ENST00000511037.1 MTND5P11 processed_pseudogene
ENST00000511038.4 TMEM144 protein_coding
ENST00000511067.2 CHD1 protein_coding
ENST00000511088.1 NADK2 protein_coding

ENST00000511114.1 RBM14-RBM4 processed_transcript
ENST00000511118.1 TMEM167A retained_intron
ENST00000511124.1 CLOCK retained_intron
ENST00000511139.1 FAM172A retained_intron
ENST00000511151.4 ANKHD1 protein_coding
ENST00000511155.1 XPC protein_coding
ENST00000511156.1 TAPT1 processed_transcript
ENST00000511164.1 RAC1P2 processed_pseudogene
ENST00000511184.4 ELF2 nonsense_mediated_decay
ENST00000511199.1 SORCS2 processed_transcript
ENST00000511209.4 GPBP1 protein_coding
ENST00000511213.1 LINC02432 lncRNA
ENST00000511218.4 TMEM161B protein_coding
ENST00000511229.4 GAK protein_coding
ENST00000511238.4 TBC1D1 retained_intron
ENST00000511305.4 KLHL2 protein_coding
ENST00000511331.4 CTC-338M12.4 lncRNA
ENST00000511333.4 MATR3 retained_intron
ENST00000511338.1 SEC31A miRNA
ENST00000511365.1 SNX2 miRNA
ENST00000511373.1 UBE2Z miRNA
ENST00000511374.4 SLC10A7 miRNA
ENST00000511391.1 ARRDC3 miRNA
ENST00000511421.4 SH3RF1 miRNA
ENST00000511429.1 PAM miRNA
ENST00000511431.1 miRNA
ENST00000511440.1 ZCCHC10 miRNA
ENST00000511442.1 AFF1 miRNA
ENST00000511450.4 TMEM167A miRNA
ENST00000511458.1 ZBTB49 miRNA
ENST00000511467.1 PTPN13 miRNA
ENST00000511480.4 N4BP2 miRNA
ENST00000511484.1 miRNA
ENST00000511494.4 MACROH2A1 miRNA
ENST00000511501.4 FHIP1A miRNA
ENST00000511516.4 RICTOR miRNA
ENST00000511524.1 CLSTN2 miRNA
ENST00000511527.4 POLK miRNA
ENST00000511528.1 NR3C2 miRNA
ENST00000511543.1 FAM13A-AS1 miRNA
ENST00000511584.2 NDUFA13 miRNA
ENST00000511586.4 PDLIM5 miRNA
ENST00000511600.4 RNF4 miRNA
ENST00000511606.1 NAAA miRNA
ENST00000511621.1 HEXB miRNA
ENST00000511622.4 DMXL1 miRNA
ENST00000511630.5 WDR41 miRNA
ENST00000511640.1 MSNP1 miRNA
ENST00000511643.4 RPL9 miRNA
ENST00000511649.4 RAB28 miRNA
ENST00000511650.1 miRNA
ENST00000511661.2 LINC01948 miRNA
ENST00000511665.1 HMCES miRNA
ENST00000511671.4 ERBIN miRNA

ENST00000511691.1 UBE2D2 miRNA
ENST00000511710.1 TMEM165 miRNA
ENST00000511713.4 IPO11 miRNA
ENST00000511722.4 AFF1 miRNA
ENST00000511724.1 PPIP5K2 miRNA
ENST00000511730.2 TTC33 miRNA
ENST00000511735.1 miRNA
ENST00000511762.2 DTNBP1 miRNA
ENST00000511767.3 PDLIM5 miRNA
ENST00000511775.1 miRNA
ENST00000511805.4 RGS12 miRNA
ENST00000511813.1 MANBA miRNA
ENST00000511830.2 NAIPP2 miRNA
ENST00000511833.2 ZNF721 miRNA
ENST00000511836.1 GAB1 miRNA
ENST00000511844.1 RPS23 miRNA
ENST00000511848.1 FNIP1 miRNA
ENST00000511893.1 miRNA
ENST00000511906.4 WDR70 miRNA
ENST00000511926.4 NFKB1 miRNA
ENST00000511951.1 miRNA
ENST00000511982.1 IL7R miRNA
ENST00000511985.1 PDGFC miRNA
ENST00000511987.4 SPAG9 miRNA
ENST00000511988.4 SUB1 miRNA
ENST00000511996.4 AFF1 miRNA
ENST00000512006.2 KIF2A miRNA
ENST00000512040.1 MATR3 miRNA
ENST00000512042.1 SPOCK3 miRNA
ENST00000512046.1 MAPK10 miRNA
ENST00000512052.4 RAPGEF6 miRNA
ENST00000512055.4 CPNE4 miRNA
ENST00000512066.4 FBXL5 miRNA
ENST00000512069.5 PDE4D miRNA
ENST00000512076.1 DROSHA miRNA
ENST00000512088.1 NDUFA2 miRNA
ENST00000512090.1 VCAN-AS1 miRNA
ENST00000512093.4 SLAIN2 miRNA
ENST00000512114.1 RELL1 miRNA
ENST00000512121.1 PAQR8 miRNA
ENST00000512129.1 LEF1-AS1 miRNA
ENST00000512138.1 FYB1 miRNA
ENST00000512145.1 DEK miRNA
ENST00000512172.1 LEF1 miRNA
ENST00000512191.1 CAST miRNA
ENST00000512193.4 NEK1 miRNA
ENST00000512194.1 miRNA
ENST00000512195.4 STX18 miRNA
ENST00000512201.4 ARHGAP24 miRNA
ENST00000512211.5 APC miRNA
ENST00000512212.1 CD164 miRNA
ENST00000512214.1 MARCHF1 miRNA
ENST00000512223.5 AEBP2 miRNA
ENST00000512256.1 IQGAP2 miRNA

ENST00000512258.1 MSH3 miRNA
ENST00000512281.1 DMXL1 miRNA
ENST00000512283.1 RBM14 miRNA
ENST00000512285.4 GALNT7 miRNA
ENST00000512315.4 ZNF346 miRNA
ENST00000512342.4 SLC2A9 miRNA
ENST00000512348.4 FCHO2 miRNA
ENST00000512354.1 ERBIN miRNA
ENST00000512371.1 DDX60L miRNA
ENST00000512376.2 LCORL miRNA
ENST00000512381.1 CCND3 miRNA
ENST00000512395.4 EPB41L4A miRNA
ENST00000512432.1 CCDC149 miRNA
ENST00000512438.4 STX18-AS1 miRNA
ENST00000512441.4 SMIM14 miRNA
ENST00000512457.4 PXYLP1 miRNA
ENST00000512463.1 ACP3 miRNA
ENST00000512466.1 PDCD6 miRNA
ENST00000512469.2 GLRX miRNA
ENST00000512471.1 IL6R miRNA
ENST00000512476.1 TRAPPC11 miRNA
ENST00000512480.4 TENM3 miRNA
ENST00000512506.4 SLC25A36 miRNA
ENST00000512507.4 MACROH2A1 miRNA
ENST00000512512.3 IGFBP7 miRNA
ENST00000512541.4 KIF2A miRNA
ENST00000512545.1 CD14 miRNA
ENST00000512559.4 LINC02899 miRNA
ENST00000512567.4 RCHY1 miRNA
ENST00000512568.4 MCTP1 miRNA
ENST00000512608.4 ICE1 miRNA
ENST00000512611.4 RAPGEF6 retained_intron
ENST00000512617.5 PVT1 lncRNA
ENST00000512623.1 NDUFAF2 trna
ENST00000512639.4 PPP2R2B trna
ENST00000512657.4 SLC39A8 trna
ENST00000512665.4 NEDD9 trna
ENST00000512668.1 GOLPH3 trna
ENST00000512684.1 GRK6 trna
ENST00000512686.4 SLC4A4 trna
ENST00000512693.1 trna
ENST00000512701.5 TTC39B trna
ENST00000512707.1 PFDN1 trna
ENST00000512716.1 RASGEF1B trna
ENST00000512721.2 NA trna
ENST00000512755.1 N4BP2L2 trna
ENST00000512760.4 PAQR3 trna
ENST00000512763.4 RASA1 trna
ENST00000512780.2 STX18 trna
ENST00000512782.1 trna
ENST00000512783.4 PCBD2 trna
ENST00000512804.1 ARAP2 trna
ENST00000512810.1 FRYL trna
ENST00000512813.4 CLCN3 trna

ENST00000512822.1 PJA2 trna
ENST00000512838.1 LINC02196 trna
ENST00000512852.1 ERAP1 trna
ENST00000512870.1 SULT1B1 trna
ENST00000512885.1 DROSHA trna
ENST00000512890.1 CAMK4 trna
ENST00000512907.4 ZFYVE16 trna
ENST00000512908.2 NAIPP4 trna
ENST00000512914.1 MRFAP1 trna
ENST00000512921.4 PI4K2B trna
ENST00000512923.4 SSBP2 trna
ENST00000512928.1 KDM3B trna
ENST00000512960.4 JADE1 trna
ENST00000512972.5 SERINC5 trna
ENST00000512978.1 LINC02100 trna
ENST00000512990.4 RGS12 trna
ENST00000513000.4 INPP4B trna
ENST00000513001.4 ACSL1 trna
ENST00000513010.4 S100Z trna
ENST00000513013.4 SUB1 trna
ENST00000513016.4 VCAN trna
ENST00000513020.4 SH3BP2 trna
ENST00000513021.1 LRBA trna
ENST00000513033.1 CLOCK trna
ENST00000513044.1 RBM47 trna
ENST00000513045.4 NAAA trna
ENST00000513054.4 ANAPC10 trna
ENST00000513069.1 SH3BP2 trna
ENST00000513078.1 SPEF2 trna
ENST00000513081.4 OXCT1 trna
ENST00000513084.4 ERAP2 trna
ENST00000513091.1 RHOBTB3 trna
ENST00000513098.4 UBE2D3 trna
ENST00000513115.1 ANKH trna
ENST00000513139.1 SPCS3 trna
ENST00000513148.1 HMGCL trna
ENST00000513149.1 MGAT1 trna
ENST00000513162.4 RMND5B trna
ENST00000513163.4 FBXL5 trna
ENST00000513175.1 CHD1-DT trna
ENST00000513200.6 KIAA0825 trna
ENST00000513210.4 MACROH2A1 trna
ENST00000513211.1 LINC02506 trna
ENST00000513216.4 CENPC trna
ENST00000513223.1 SLC12A7 trna
ENST00000513230.1 HNRNPH1 trna
ENST00000513241.2 ANKRD55 trna
ENST00000513250.1 CLPTM1L trna
ENST00000513253.1 CHCHD6 trna
ENST00000513268.1 MACROH2A1 trna
ENST00000513269.1 N4BP2 trna
ENST00000513314.1 DHFR trna
ENST00000513317.4 ACSL1 trna
ENST00000513341.4 PDLIM5 trna

ENST00000513342.4 UBE2Z trna
ENST00000513350.2 FAM193A trna
ENST00000513355.2 KIAA2026 trna
ENST00000513361.1 ARFIP1 trna
ENST00000513364.1 SEL1L3 trna
ENST00000513371.1 ABHD18 trna
ENST00000513372.4 MXD4 trna
ENST00000513374.1 TNFAIP8 trna
ENST00000513384.1 C1QTNF7-AS1 trna
ENST00000513401.4 FRYL trna
ENST00000513405.1 LINC02714 trna
ENST00000513411.4 TMCC1 trna
ENST00000513418.1 SMAD5 trna
ENST00000513420.1 CTBP1 trna
ENST00000513443.4 ATG10 trna
ENST00000513450.1 RNF4 trna
ENST00000513453.4 SIL1 trna
ENST00000513463.1 HPSE trna
ENST00000513473.4 RBM47 trna
ENST00000513499.4 CCNH trna
ENST00000513511.4 ABCC3 trna
ENST00000513524.4 GPBP1 trna
ENST00000513534.1 IQGAP2 trna
ENST00000513558.1 TMEM33 trna
ENST00000513570.1 ZBTB38 trna
ENST00000513579.1 PPP1R9B trna
ENST00000513583.1 SLC10A7 trna
ENST00000513585.4 DCP2 trna
ENST00000513597.4 RUFY3 trna
ENST00000513605.4 PPA2 trna
ENST00000513613.4 SNX24 trna
ENST00000513615.4 QDPR trna
ENST00000513622.1 MTMR12 trna
ENST00000513635.1 PTGER4 trna
ENST00000513636.1 ATP2C1 trna
ENST00000513637.1 EEF1A1P19 trna
ENST00000513639.4 HTT trna
ENST00000513651.4 ADTRP trna
ENST00000513696.1 FBXO8 trna
ENST00000513699.4 SLC30A9 trna
ENST00000513705.4 STK32B trna
ENST00000513710.3 WDR36 trna
ENST00000513728.1 ANXA5 trna
ENST00000513729.1 TICAM2 trna
ENST00000513738.4 PARP8 trna
ENST00000513739.4 AGTRAP trna
ENST00000513750.4 ANKRD13D trna
ENST00000513757.4 LPCAT1 trna
ENST00000513769.4 TPM3 trna
ENST00000513775.1 GUF1 trna
ENST00000513785.4 SSBP2 trna
ENST00000513789.1 ZFYVE16 trna
ENST00000513798.1 PDS5A trna
ENST00000513854.1 HSD17B11 trna

ENST00000513857.1 MCTP1 trna
ENST00000513869.1 SNX14 trna
ENST00000513899.1 VCAN-AS1 trna
ENST00000513907.1 SERINC5 trna
ENST00000513931.2 PITX1-AS1 trna
ENST00000513939.4 GCLC trna
ENST00000513944.4 TNPO1 trna
ENST00000513965.4 COPG1 trna
ENST00000513984.4 VCAN trna
ENST00000513996.4 HERC4 trna
ENST00000514001.4 IQGAP2 trna
ENST00000514005.1 ETF1 trna
ENST00000514030.1 SNX2 trna
ENST00000514033.1 KLF3 trna
ENST00000514040.1 MCTP1 trna
ENST00000514048.4 trna
ENST00000514051.1 METAP1 trna
ENST00000514057.1 MTATP6P1 trna
ENST00000514062.2 IGFBP7 trna
ENST00000514111.1 EMB trna
ENST00000514123.4 CYB5B trna
ENST00000514128.1 SIMC1 trna
ENST00000514139.2 RAP1GDS1 trna
ENST00000514143.1 RHOH trna
ENST00000514151.1 DMXL1 trna
ENST00000514178.4 PGGT1B trna
ENST00000514179.4 RAPGEF6 trna
ENST00000514200.4 NA trna
ENST00000514213.5 USO1 trna
ENST00000514238.2 RUFY1 trna
ENST00000514250.1 THOC3 trna
ENST00000514252.1 ANKRD17 trna
ENST00000514253.2 ATG10 trna
ENST00000514271.2 CLIP1 trna
ENST00000514277.5 YJEFN3 trna
ENST00000514301.1 DAPP1 trna
ENST00000514310.4 FAM13B trna
ENST00000514318.1 RACK1 trna
ENST00000514319.4 WDR1 trna
ENST00000514322.1 RWDD4 trna
ENST00000514325.1 HNRNPD trna
ENST00000514335.1 NIPBL trna
ENST00000514342.5 PARP8 trna
ENST00000514343.1 PTGER4 trna
ENST00000514350.4 IQGAP2 trna
ENST00000514356.4 ZFR trna
ENST00000514372.4 ATP8A1 trna
ENST00000514399.1 KLHL5 trna
ENST00000514402.1 MATR3 trna
ENST00000514424.4 PHYKPL trna
ENST00000514428.1 RERE trna
ENST00000514429.4 CPLANE1 trna
ENST00000514440.1 THAP9 trna
ENST00000514451.4 WDR41 trna

ENST00000514463.1 ZXDC trna
ENST00000514484.5 LPCAT1 trna
ENST00000514498.1 HADHAP1 trna
ENST00000514499.1 ARFIP1 trna
ENST00000514500.1 ACSL4 trna
ENST00000514511.1 HNRNPDL trna
ENST00000514522.2 GUSBP14 trna
ENST00000514525.1 INPP4B trna
ENST00000514541.1 FBXL5 trna
ENST00000514552.4 PDE4D trna
ENST00000514558.4 TMEM144 trna
ENST00000514565.1 RAPGEF2 trna
ENST00000514579.1 IQGAP2 trna
ENST00000514580.4 DDX60L trna
ENST00000514587.1 LHFPL2 trna
ENST00000514588.1 CCND3 trna
ENST00000514595.1 DMXL1 trna
ENST00000514604.4 ERAP1 trna
ENST00000514606.1 ELF2 trna
ENST00000514612.4 ANAPC13 trna
ENST00000514613.4 SPAG9 trna
ENST00000514616.4 PKD2L2-DT trna
ENST00000514617.4 FRYL trna
ENST00000514633.4 HNRNPAB trna
ENST00000514635.1 trna
ENST00000514639.4 GAB1 trna
ENST00000514642.1 CCDC30 trna
ENST00000514655.1 TLR6 trna
ENST00000514660.1 NEDD9 trna
ENST00000514678.1 CEP63 trna
ENST00000514710.1 SDAD1 trna
ENST00000514711.1 WDFY3 trna
ENST00000514712.4 CEP44 trna
ENST00000514720.1 YTHDC2 trna
ENST00000514723.1 OXCT1 trna
ENST00000514729.1 COPB2-DT trna
ENST00000514735.1 RICTOR trna
ENST00000514740.1 JADE1 trna
ENST00000514742.1 FBN2 trna
ENST00000514756.1 CCNG2 trna
ENST00000514780.4 MCTP1 trna
ENST00000514782.4 RBM47 trna
ENST00000514805.1 TRIM52 trna
ENST00000514815.1 PAPSS1 processed_transcript
ENST00000514826.4 ZNF330 retained_intron
ENST00000514839.1 RHOH processed_transcript
ENST00000514852.1 EEF1A1P20 processed_pseudogene
ENST00000514857.2 NAIP protein_coding
ENST00000514863.1 lncRNA
ENST00000514870.1 TET2 protein_coding
ENST00000514876.4 LINC02241 lncRNA
ENST00000514886.1 PRMT9 nonsense_mediated_decay
ENST00000514889.1 RASGEF1B processed_transcript
ENST00000514928.1 MRPL27 retained_intron

ENST00000514946.1 RASGRF2 processed_transcript
ENST00000514959.1 ANTXR2 processed_transcript
ENST00000514961.1 MARCHF6 retained_intron
ENST00000514962.4 CALCOCO2 retained_intron
ENST00000514970.2 AFF1 protein_coding
ENST00000514975.1 EEF1A1P9 processed_pseudogene
ENST00000514989.1 BST1 protein_coding
ENST00000514992.2 SMURF2P1 transcribed_unprocessed_pseudogene
ENST00000515007.5 LHFPL2 protein_coding
ENST00000515010.4 FYB1 protein_coding
ENST00000515011.4 PDE4D retained_intron
ENST00000515016.1 THOC3 protein_coding
ENST00000515018.1 WDR1 processed_transcript
ENST00000515020.1 ELL2 retained_intron
ENST00000515031.4 LINC00504 lncRNA
ENST00000515045.1 processed_transcript
ENST00000515055.1 lncRNA
ENST00000515063.1 CAST retained_intron
ENST00000515067.1 MGST2 processed_transcript
ENST00000515068.1 CSF1R nonsense_mediated_decay
ENST00000515100.1 LAMTOR3 processed_transcript
ENST00000515101.1 SETD7 retained_intron
ENST00000515114.2 MCUB processed_transcript
ENST00000515127.1 lncRNA
ENST00000515140.1 CENPC nonsense_mediated_decay
ENST00000515144.4 TRIO retained_intron
ENST00000515151.1 PPWD1 retained_intron
ENST00000515155.1 FAM13A retained_intron
ENST00000515156.2 unprocessed_pseudogene
ENST00000515160.4 CAST retained_intron
ENST00000515169.4 ZFYVE28 protein_coding
ENST00000515175.4 PARP8 protein_coding
ENST00000515187.4 RAP1GDS1 processed_transcript
ENST00000515190.1 IL6R protein_coding
ENST00000515195.1 UNKL processed_transcript
ENST00000515203.4 TCERG1 processed_transcript
ENST00000515239.4 CSF1R processed_transcript
ENST00000515240.4 FAM117A processed_transcript
ENST00000515247.4 COPB2-DT lncRNA
ENST00000515280.4 FAM135A protein_coding
ENST00000515295.4 POLK protein_coding
ENST00000515312.4 ZFYVE28 protein_coding
ENST00000515322.2 CSNK1G3 protein_coding
ENST00000515332.4 SGMS2 processed_transcript
ENST00000515338.1 PAIP1 protein_coding
ENST00000515365.1 HERC6 retained_intron
ENST00000515387.1 ERAP2 retained_intron
ENST00000515388.1 GAB1 processed_transcript
ENST00000515397.1 VCAN retained_intron
ENST00000515408.4 DCP2 protein_coding
ENST00000515420.1 CLCN3 protein_coding
ENST00000515421.1 CDV3 protein_coding
ENST00000515426.1 TCF20 protein_coding
ENST00000515433.1 TGFBI retained_intron

ENST00000515439.6 DTWD2 protein_coding
ENST00000515441.1 NA NA
ENST00000515457.4 G3BP2 nonsense_mediated_decay
ENST00000515467.2 SDHAP3 transcribed_unprocessed_pseudogene
ENST00000515468.1 CCNI protein_coding
ENST00000515483.4 TNPO1 processed_transcript
ENST00000515484.2 GPBP1 retained_intron
ENST00000515489.1 ELF2 processed_transcript
ENST00000515495.1 lncRNA
ENST00000515499.4 TRIM41 protein_coding
ENST00000515506.4 CNOT6L protein_coding
ENST00000515528.1 HEXB retained_intron
ENST00000515545.4 ZXDC nonsense_mediated_decay
ENST00000515549.1 RASA2 nonsense_mediated_decay
ENST00000515550.1 N4BP2 protein_coding
ENST00000515554.1 JADE2 processed_transcript
ENST00000515565.1 HNRNPH1P3 processed_pseudogene
ENST00000515573.4 RBPJ protein_coding
ENST00000515576.1 NAA15 protein_coding
ENST00000515612.1 KLHL5 protein_coding
ENST00000515641.1 ANKRD50 protein_coding
ENST00000515663.4 CAST protein_coding
ENST00000515665.1 IL7R protein_coding
ENST00000515666.4 GRK6 retained_intron
ENST00000515670.1 lncRNA
ENST00000515684.1 FRYL processed_transcript
ENST00000515690.1 CTBP1 processed_transcript
ENST00000515693.1 CCSER1 processed_transcript
ENST00000515705.4 TBCK processed_transcript
ENST00000515710.1 TRIO retained_intron
ENST00000515712.1 ACTBP2 processed_pseudogene
ENST00000515715.2 MEF2C retained_intron
ENST00000515717.1 ANXA5 retained_intron
ENST00000515721.1 TENT4A protein_coding
ENST00000515731.1 SDHC processed_transcript
ENST00000515742.1 DEK protein_coding
ENST00000515748.2 CSNK1A1 protein_coding
ENST00000515749.1 SEC31A retained_intron
ENST00000515766.1 MAEA retained_intron
ENST00000515768.4 CSNK1A1 protein_coding
ENST00000515790.1 CCNI retained_intron
ENST00000515809.1 RBM47 processed_transcript
ENST00000515835.2 PDE4D protein_coding
ENST00000515838.2 RBM4 processed_transcript
ENST00000515845.4 PPIP5K2 protein_coding
ENST00000515846.1 RICTOR retained_intron
ENST00000515854.4 ATP2C1 protein_coding
ENST00000515867.1 CHCHD6 processed_transcript
ENST00000515883.4 YTHDC2 protein_coding
ENST00000515896.1 RNA5-8SP6 rRNA_pseudogene
ENST00000515924.1 SCARNA8 scaRNA
ENST00000515932.1 RNU4ATAC13P snRNA
ENST00000515939.1 RNU4ATAC11P snRNA
ENST00000515945.1 RNU6-999P snRNA

ENST00000515961.1 RNA5SP486 rRNA_pseudogene
ENST00000515981.1 SCARNA16 scaRNA
ENST00000515982.1 SCARNA6 scaRNA
ENST00000515983.1 Y_RNA misc_RNA
ENST00000515994.1 Y_RNA misc_RNA
ENST00000516002.1 Y_RNA misc_RNA
ENST00000516006.1 SNORD116-26 snoRNA
ENST00000516014.2 RNA5SP162 rRNA_pseudogene
ENST00000516043.1 Y_RNA misc_RNA
ENST00000516075.1 RNA5SP288 rRNA_pseudogene
ENST00000516087.1 SNORD116-27 snoRNA
ENST00000516089.1 SCARNA11 scaRNA
ENST00000516111.1 RNA5SP216 rRNA_pseudogene
ENST00000516146.1 RNU6-27P snRNA
ENST00000516177.1 Y_RNA misc_RNA
ENST00000516192.1 RNA5SP303 rRNA_pseudogene
ENST00000516201.1 SCARNA5 scaRNA
ENST00000516209.1 RNU2-30P snRNA
ENST00000516225.1 RNY4P37 misc_RNA
ENST00000516238.1 Y_RNA misc_RNA
ENST00000516327.1 snoRNA
ENST00000516330.1 scaRNA
ENST00000516336.1 RNU6-1225P snRNA
ENST00000516362.1 Y_RNA misc_RNA
ENST00000516370.1 RNA5SP255 rRNA_pseudogene
ENST00000516404.1 SNORA74D snoRNA
ENST00000516408.1 Y_RNA misc_RNA
ENST00000516441.1 Y_RNA misc_RNA
ENST00000516449.1 SNORA70 snoRNA
ENST00000516461.1 NA NA
ENST00000516468.1 SNORD116-30 snoRNA
ENST00000516480.1 RNA5SP519 rRNA_pseudogene
ENST00000516507.1 RNY4 misc_RNA
ENST00000516508.1 Y_RNA misc_RNA
ENST00000516517.1 SNORD116-25 snoRNA
ENST00000516540.1 snoRNA
ENST00000516564.1 RNU6-358P snRNA
ENST00000516659.1 RNU2-57P snRNA
ENST00000516672.1 SCARNA13 scaRNA
ENST00000516678.1 RNY4P20 misc_RNA
ENST00000516733.1 SNORD36C snoRNA
ENST00000516747.1 RNU6-256P snRNA
ENST00000516752.1 RNU1-125P snRNA
ENST00000516775.1 NA NA
ENST00000516814.1 RNA5SP481 rRNA_pseudogene
ENST00000516816.1 RNU1-40P snRNA
ENST00000516826.1 RNU2-56P snRNA
ENST00000516843.1 Y_RNA misc_RNA
ENST00000516851.1 Y_RNA misc_RNA
ENST00000516862.1 Y_RNA misc_RNA
ENST00000516869.1 RPPH1 ribozyme
ENST00000516872.1 RNU1-97P snRNA
ENST00000516881.1 SCARNA15 scaRNA
ENST00000516903.1 SCARNA14 scaRNA

ENST00000516905.1 RNA5SP392 rRNA_pseudogene
ENST00000516933.1 Y_RNA misc_RNA
ENST00000516935.1 Y_RNA misc_RNA
ENST00000516950.1 Y_RNA misc_RNA
ENST00000516967.1 RNU4ATAC10P snRNA
ENST00000516989.1 scaRNA
ENST00000517013.1 Y_RNA misc_RNA
ENST00000517026.1 SCARNA21 scaRNA
ENST00000517038.1 RNU2-46P snRNA
ENST00000517041.1 RNU1-117P snRNA
ENST00000517055.1 RNA5SP278 rRNA_pseudogene
ENST00000517057.1 RNA5SP243 rRNA_pseudogene
ENST00000517083.1 RNU6-548P snRNA
ENST00000517097.1 SCARNA3 scaRNA
ENST00000517110.1 Y_RNA misc_RNA
ENST00000517138.1 SCARNA1 scaRNA
ENST00000517146.1 RNU4ATAC9P snRNA
ENST00000517226.1 RNU4ATAC5P snRNA
ENST00000517238.1 snoRNA
ENST00000517242.1 SNORA31B snoRNA
ENST00000517285.1 SNORD36 snoRNA
ENST00000517293.4 AGO2 processed_transcript
ENST00000517306.4 DGLUCY nonsense_mediated_decay
ENST00000517307.4 ZFAT processed_transcript
ENST00000517310.4 EFCAB13 protein_coding
ENST00000517315.1 NSMCE2 protein_coding
ENST00000517319.1 HGSNAT nonsense_mediated_decay
ENST00000517323.2 TRPS1 protein_coding
ENST00000517339.4 PTK2B protein_coding
ENST00000517360.1 STK10 retained_intron
ENST00000517366.1 SLC20A2 protein_coding
ENST00000517371.4 YTHDF3 protein_coding
ENST00000517374.4 ADAM19 protein_coding
ENST00000517378.1 CDH12 retained_intron
ENST00000517381.1 STK10 processed_transcript
ENST00000517386.1 HMBOX1 processed_transcript
ENST00000517391.4 CPNE3 protein_coding
ENST00000517403.4 PABPC1 protein_coding
ENST00000517409.1 ASAH1 retained_intron
ENST00000517412.1 PIK3R1 retained_intron
ENST00000517419.4 FYN protein_coding
ENST00000517421.1 DCTN4 nonsense_mediated_decay
ENST00000517437.1 CFAP418-AS1 lncRNA
ENST00000517455.1 OXR1 processed_transcript
ENST00000517479.1 CDK16 processed_transcript
ENST00000517516.1 ZHX1 processed_transcript
ENST00000517517.1 RRM2B retained_intron
ENST00000517523.1 UQCRB protein_coding
ENST00000517524.4 STK10 retained_intron
ENST00000517534.4 CTNNA1 retained_intron
ENST00000517545.4 PCM1 retained_intron
ENST00000517551.2 XPO7 protein_coding
ENST00000517557.4 PTDSS1 retained_intron
ENST00000517561.1 AP3B1 processed_transcript

ENST00000517577.2 FTH1P11 processed_pseudogene
ENST00000517584.4 RNF19A protein_coding
ENST00000517594.1 MSRA processed_transcript
ENST00000517615.1 SLA processed_transcript
ENST00000517639.4 NCALD retained_intron
ENST00000517643.1 PIK3R1 processed_transcript
ENST00000517651.4 CSGALNACT1 processed_transcript
ENST00000517655.1 CFAP418-AS1 lncRNA
ENST00000517662.1 PCBP2P2 processed_pseudogene
ENST00000517667.4 TRAPPC9 processed_transcript
ENST00000517669.1 ATP6V0E1 protein_coding
ENST00000517672.4 CYRIB protein_coding
ENST00000517691.1 SKP1 retained_intron
ENST00000517696.1 RIPK2 nonsense_mediated_decay
ENST00000517707.4 ANXA6 retained_intron
ENST00000517742.1 CPQ protein_coding
ENST00000517770.1 TNKS protein_coding
ENST00000517775.1 STK10 processed_transcript
ENST00000517799.4 TCF7 protein_coding
ENST00000517801.4 CYRIB retained_intron
ENST00000517812.4 IKBKB nonsense_mediated_decay
ENST00000517832.1 STK3 processed_transcript
ENST00000517838.4 PVT1 lncRNA
ENST00000517845.4 WASHC5 protein_coding
ENST00000517855.4 TCF7 protein_coding
ENST00000517869.1 LINC00861 lncRNA
ENST00000517870.1 SHISAL2A protein_coding
ENST00000517877.1 DGLUCY protein_coding
ENST00000517878.4 SLC45A4 protein_coding
ENST00000517904.1 CTNNA1 retained_intron
ENST00000517905.1 ADAM19 protein_coding
ENST00000517913.4 SGCD protein_coding
ENST00000517915.1 PCAT1 lncRNA
ENST00000517928.1 ASPH processed_transcript
ENST00000517946.1 CAAP1 processed_transcript
ENST00000517955.1 ARFGEF1 retained_intron
ENST00000517958.1 GALNT10 retained_intron
ENST00000517975.1 ELP3 protein_coding
ENST00000517983.1 MAILR lncRNA
ENST00000517985.4 DENND3 protein_coding
ENST00000517990.4 PABPC1 protein_coding
ENST00000517992.1 SNTB1 protein_coding
ENST00000518013.4 NSMCE2 protein_coding
ENST00000518018.1 TRPS1 protein_coding
ENST00000518019.1 AGO2 processed_transcript
ENST00000518023.4 DCSTAMP processed_transcript
ENST00000518027.4 TNKS processed_transcript
ENST00000518033.1 ITGB2 retained_intron
ENST00000518047.4 DIAPH1 protein_coding
ENST00000518057.1 DEPTOR processed_transcript
ENST00000518071.4 ZNF706 nonsense_mediated_decay
ENST00000518076.1 FUT10 processed_transcript
ENST00000518079.1 MYBL1 processed_transcript
ENST00000518080.2 HMBOX1 processed_transcript

ENST00000518084.4 NRG1 protein_coding
ENST00000518105.1 lncRNA
ENST00000518139.4 RPS14 nonsense_mediated_decay
ENST00000518143.1 ZFHX4-AS1 lncRNA
ENST00000518162.1 IPCEF1 retained_intron
ENST00000518180.1 ZFPM2 processed_transcript
ENST00000518190.3 MIR2052HG lncRNA
ENST00000518191.1 ZFAT protein_coding
ENST00000518192.4 LEPROTL1 protein_coding
ENST00000518196.4 PABPC1 protein_coding
ENST00000518205.4 UBR5 protein_coding
ENST00000518206.4 NRG1 protein_coding
ENST00000518211.1 RB1CC1 nonsense_mediated_decay
ENST00000518229.4 NSMAF nonsense_mediated_decay
ENST00000518230.4 ARFGEF1 protein_coding
ENST00000518240.4 FBXO25 protein_coding
ENST00000518277.1 ERICH1 processed_transcript
ENST00000518281.4 TNKS protein_coding
ENST00000518285.1 CYRIB processed_transcript
ENST00000518287.5 NCOA2 nonsense_mediated_decay
ENST00000518293.4 PABPC1 protein_coding
ENST00000518296.4 SARAF protein_coding
ENST00000518313.1 ERICH1 processed_transcript
ENST00000518344.1 CYRIB retained_intron
ENST00000518346.1 NDST1 processed_transcript
ENST00000518347.4 DENND3 protein_coding
ENST00000518352.1 MTFR1 retained_intron
ENST00000518360.4 UBE2V2 nonsense_mediated_decay
ENST00000518363.2 NCOA2 protein_coding
ENST00000518364.1 MRPL22 processed_transcript
ENST00000518371.2 MRPS28 protein_coding
ENST00000518388.4 SGK3 protein_coding
ENST00000518400.4 TRIQK processed_transcript
ENST00000518402.4 HACE1 protein_coding
ENST00000518416.1 lncRNA
ENST00000518419.4 ZFAND1 protein_coding
ENST00000518437.1 THUMPD3-AS1 lncRNA
ENST00000518445.1 GTF2E2 protein_coding
ENST00000518450.4 CARD8 nonsense_mediated_decay
ENST00000518484.1 DIAPH1 processed_transcript
ENST00000518490.1 MYL12AP1 processed_pseudogene
ENST00000518531.4 TNFRSF10B retained_intron
ENST00000518537.4 PCM1 protein_coding
ENST00000518542.1 CSGALNACT1 processed_transcript
ENST00000518549.1 CPA6 retained_intron
ENST00000518552.2 lncRNA
ENST00000518560.1 TTC1 protein_coding
ENST00000518569.1 VPS13B processed_transcript
ENST00000518570.1 lncRNA
ENST00000518582.4 STK3 processed_transcript
ENST00000518585.1 CTNNA1 processed_transcript
ENST00000518594.1 SLA retained_intron
ENST00000518607.1 EFCAB2 retained_intron
ENST00000518611.4 BNIP3L protein_coding

ENST00000518630.4 FYN protein_coding
ENST00000518634.1 EIF3E processed_transcript
ENST00000518667.1 PDE7A processed_transcript
ENST00000518678.1 TRAM1 protein_coding
ENST00000518683.4 WWP1 retained_intron
ENST00000518692.1 SPIDR processed_transcript
ENST00000518707.1 DMGDH processed_transcript
ENST00000518708.1 ZNF674 protein_coding
ENST00000518716.1 PABPC1 retained_intron
ENST00000518717.1 SLC20A2 protein_coding
ENST00000518718.1 ENTPD4 protein_coding
ENST00000518726.4 G3BP1 retained_intron
ENST00000518727.4 NCALD protein_coding
ENST00000518730.4 HCK protein_coding
ENST00000518746.1 ASAH1 retained_intron
ENST00000518752.1 FBXW11 protein_coding
ENST00000518776.1 PTDSS1 processed_transcript
ENST00000518782.4 CNOT8 processed_transcript
ENST00000518789.4 ARFGEF1 protein_coding
ENST00000518797.4 CD74 nonsense_mediated_decay
ENST00000518839.1 TRAPPC9 processed_transcript
ENST00000518845.1 ZNF292 protein_coding
ENST00000518869.1 PIP4P2 nonsense_mediated_decay
ENST00000518877.1 PCM1 retained_intron
ENST00000518890.4 PPP2R2A retained_intron
ENST00000518892.1 LARP1 protein_coding
ENST00000518893.1 LY96 protein_coding
ENST00000518909.1 DCTN4 retained_intron
ENST00000518927.1 lncRNA
ENST00000518928.1 TSNARE1 processed_transcript
ENST00000518930.1 PCM1 processed_transcript
ENST00000518936.4 PCM1 processed_transcript
ENST00000518939.1 PLEKHA2 processed_transcript
ENST00000518940.4 NA NA
ENST00000518957.1 ASAP1 processed_transcript
ENST00000518959.1 ATP6V1C1 retained_intron
ENST00000518991.4 POMK protein_coding
ENST00000519001.1 VIRMA processed_transcript
ENST00000519014.1 NAIP nonsense_mediated_decay
ENST00000519018.4 DERL1 protein_coding
ENST00000519028.1 TNFRSF10B retained_intron
ENST00000519037.4 TCF7 protein_coding
ENST00000519046.4 EIF3H nonsense_mediated_decay
ENST00000519070.4 CYRIB protein_coding
ENST00000519076.4 TRPS1 protein_coding
ENST00000519077.3 TUG1 processed_transcript
ENST00000519091.4 IPCEF1 processed_transcript
ENST00000519093.4 TACC1 retained_intron
ENST00000519103.1 ZNF706 protein_coding
ENST00000519124.4 ATAD2 nonsense_mediated_decay
ENST00000519132.4 CLK4 retained_intron
ENST00000519136.4 NDUFAF6 protein_coding
ENST00000519149.1 LCP2 retained_intron
ENST00000519152.4 CPEB4 protein_coding

ENST00000519163.4 CSPP1 nonsense_mediated_decay
ENST00000519165.1 TCF7 retained_intron
ENST00000519169.4 ASAP1 retained_intron
ENST00000519177.4 SNTB1 processed_transcript
ENST00000519187.4 TMEM71 protein_coding
ENST00000519190.1 IPCEF1 protein_coding
ENST00000519223.1 DOCK2 retained_intron
ENST00000519231.4 PDE7A processed_transcript
ENST00000519247.4 RMDN1 protein_coding
ENST00000519255.2 UBE2W nonsense_mediated_decay
ENST00000519259.4 SREK1 retained_intron
ENST00000519269.1 STK10 retained_intron
ENST00000519295.4 AP3B1 protein_coding
ENST00000519298.1 SNTB1 processed_transcript
ENST00000519307.1 ZC2HC1A protein_coding
ENST00000519319.1 PCAT1 lncRNA
ENST00000519326.1 PAG1 processed_transcript
ENST00000519339.4 TNIP1 nonsense_mediated_decay
ENST00000519342.1 RNF19A processed_transcript
ENST00000519355.4 TAF2 processed_transcript
ENST00000519362.4 SPIDR retained_intron
ENST00000519363.1 PABPC1 protein_coding
ENST00000519369.1 RPS20 retained_intron
ENST00000519375.1 lncRNA
ENST00000519381.1 VPS37A nonsense_mediated_decay
ENST00000519392.4 TNKS processed_transcript
ENST00000519394.4 CNOT8 protein_coding
ENST00000519396.4 SGK3 protein_coding
ENST00000519401.4 SPIDR protein_coding
ENST00000519402.4 ITK retained_intron
ENST00000519413.1 EIF3E processed_transcript
ENST00000519415.5 OXR1 protein_coding
ENST00000519428.4 YTHDF3 retained_intron
ENST00000519435.4 ST3GAL1 processed_transcript
ENST00000519436.1 ARFGEF1 protein_coding
ENST00000519441.4 STK10 retained_intron
ENST00000519448.1 CCDC69 retained_intron
ENST00000519449.4 RNF19A protein_coding
ENST00000519450.2 EMC2 retained_intron
ENST00000519467.1 CPEB4 protein_coding
ENST00000519482.1 TRAPPC9 processed_transcript
ENST00000519483.4 ASAP1 protein_coding
ENST00000519484.1 CPQ protein_coding
ENST00000519487.4 ELOC protein_coding
ENST00000519499.1 protein_coding
ENST00000519504.4 SLA retained_intron
ENST00000519512.4 PTK2B protein_coding
ENST00000519516.1 PLEKHF2 protein_coding
ENST00000519534.1 CHTF8 nonsense_mediated_decay
ENST00000519541.1 nonsense_mediated_decay
ENST00000519543.4 TG protein_coding
ENST00000519544.4 GALNT10 processed_transcript
ENST00000519545.4 ASAH1 retained_intron
ENST00000519554.4 PCMTD1 protein_coding

ENST00000519571.1 GALNT10 processed_transcript
ENST00000519576.1 TRIB1 protein_coding
ENST00000519594.4 LCP2 retained_intron
ENST00000519622.4 PABPC1 nonsense_mediated_decay
ENST00000519628.1 DOCK2 protein_coding
ENST00000519640.4 PLEKHA2 protein_coding
ENST00000519651.4 TSNARE1 protein_coding
ENST00000519656.1 EFR3A protein_coding
ENST00000519666.1 SLC2A3P1 processed_pseudogene
ENST00000519671.1 MTSS1 processed_transcript
ENST00000519674.1 TRPS1 protein_coding
ENST00000519675.1 LRP12 retained_intron
ENST00000519689.1 lncRNA
ENST00000519704.1 TCEA1 nonsense_mediated_decay
ENST00000519708.1 RNF130 retained_intron
ENST00000519710.1 STK10 processed_transcript
ENST00000519712.1 NSMCE2 nonsense_mediated_decay
ENST00000519719.1 MAT2B retained_intron
ENST00000519724.1 NCOA2 protein_coding
ENST00000519726.2 FAM85B lncRNA
ENST00000519728.4 LYN protein_coding
ENST00000519740.1 CCDC69 nonsense_mediated_decay
ENST00000519749.1 ITK processed_transcript
ENST00000519759.1 ITK retained_intron
ENST00000519827.1 ZFAT nonsense_mediated_decay
ENST00000519829.2 SLC36A1 protein_coding
ENST00000519839.1 ENTPD4 miRNA
ENST00000519848.1 PABPC1 miRNA
ENST00000519855.1 RPS14 miRNA
ENST00000519858.1 NSMAF miRNA
ENST00000519860.2 ERGIC1 miRNA
ENST00000519868.1 DOCK2 miRNA
ENST00000519871.1 NSMAF miRNA
ENST00000519888.4 AP3B1 miRNA
ENST00000519890.4 EBF1 miRNA
ENST00000519900.1 CPQ miRNA
ENST00000519909.1 ERICH1 miRNA
ENST00000519910.1 TNFRSF10B miRNA
ENST00000519912.1 RB1CC1 miRNA
ENST00000519920.1 EFR3A miRNA
ENST00000519924.4 ST3GAL1 miRNA
ENST00000519934.4 MTDH miRNA
ENST00000519938.4 IKBKB miRNA
ENST00000519943.1 HNRNPH1 miRNA
ENST00000519948.3 ATXN7L3B miRNA
ENST00000519975.1 PCMTD1 miRNA
ENST00000519984.1 CHMP7 miRNA
ENST00000519985.1 RNF145 miRNA
ENST00000520003.4 CSGALNACT1 miRNA
ENST00000520011.1 ELP3 miRNA
ENST00000520013.4 RARS1 miRNA
ENST00000520020.4 ST3GAL1 miRNA
ENST00000520021.4 GLI4 miRNA
ENST00000520033.1 ANGPT1 miRNA

ENST00000520039.1 TBCA miRNA
ENST00000520048.1 CCDC26 miRNA
ENST00000520050.1 LYN miRNA
ENST00000520051.1 ASAH1 miRNA
ENST00000520054.1 ANXA6 miRNA
ENST00000520060.1 LINC02055 miRNA
ENST00000520064.2 LARP4 miRNA
ENST00000520071.1 RNF19A miRNA
ENST00000520083.1 PAX5 miRNA
ENST00000520110.4 ELP3 miRNA
ENST00000520122.1 AP3B1 miRNA
ENST00000520132.1 RBM22 miRNA
ENST00000520142.1 PABPC1 miRNA
ENST00000520146.1 miRNA
ENST00000520148.1 DECR1 miRNA
ENST00000520173.1 ITK miRNA
ENST00000520177.4 G3BP1 miRNA
ENST00000520181.4 DOCK2 miRNA
ENST00000520189.1 ASAP1 miRNA
ENST00000520203.1 PEX2 miRNA
ENST00000520220.5 LYN miRNA
ENST00000520228.1 SDCBP miRNA
ENST00000520231.1 PPP2R2B miRNA
ENST00000520244.4 HSD17B4 miRNA
ENST00000520261.1 IPCEF1 miRNA
ENST00000520269.4 IL7 miRNA
ENST00000520289.1 EIF3H miRNA
ENST00000520322.1 LCP2 miRNA
ENST00000520334.4 PPP2CB miRNA
ENST00000520339.4 CTNNA1 miRNA
ENST00000520342.4 ASAP1 miRNA
ENST00000520344.1 LCP2 miRNA
ENST00000520374.4 WWP1 miRNA
ENST00000520381.4 ARFGEF1 miRNA
ENST00000520392.1 BPNT2 miRNA
ENST00000520399.1 ERGIC1 miRNA
ENST00000520401.1 miRNA
ENST00000520407.4 NRG1 miRNA
ENST00000520408.4 TNKS miRNA
ENST00000520409.4 BNIP3L miRNA
ENST00000520415.1 HNRNPH1 miRNA
ENST00000520416.1 NCOA2 miRNA
ENST00000520417.1 SKP1 miRNA
ENST00000520420.4 CREBRF miRNA
ENST00000520424.1 CYFIP2 miRNA
ENST00000520437.1 INTS9 miRNA
ENST00000520438.1 NA miRNA
ENST00000520450.4 DOCK2 miRNA
ENST00000520462.4 TSNARE1 miRNA
ENST00000520463.1 PCNX2 miRNA
ENST00000520464.1 CREBRF miRNA
ENST00000520482.1 DENND3 miRNA
ENST00000520497.1 VAPB miRNA
ENST00000520500.1 PPP2CB miRNA

ENST00000520511.1 FBXO32 miRNA
ENST00000520512.1 PCAT1 miRNA
ENST00000520531.4 LY6E miRNA
ENST00000520548.1 RNF145 miRNA
ENST00000520555.4 ITK miRNA
ENST00000520611.1 TACC1 miRNA
ENST00000520638.1 RNF145 miRNA
ENST00000520659.1 OSGIN2 miRNA
ENST00000520662.1 PWWP2A miRNA
ENST00000520664.1 SLU7 miRNA
ENST00000520668.1 CDH12 miRNA
ENST00000520675.1 PIK3R1 miRNA
ENST00000520677.1 MRPL13 miRNA
ENST00000520682.4 LEPROTL1 miRNA
ENST00000520687.1 SPARC miRNA
ENST00000520695.4 TNIP1 miRNA
ENST00000520699.1 TCF7 miRNA
ENST00000520715.4 MFHAS1 miRNA
ENST00000520751.1 MYC miRNA
ENST00000520754.1 XPO7 miRNA
ENST00000520771.1 MTSS1 miRNA
ENST00000520781.4 ASAH1 miRNA
ENST00000520798.4 WWP1 miRNA
ENST00000520813.1 EIF3H miRNA
ENST00000520819.4 miRNA
ENST00000520823.5 SMG1P3 miRNA
ENST00000520830.1 ATP6V1B2 miRNA
ENST00000520831.1 INTS9 miRNA
ENST00000520836.4 DOCK2 miRNA
ENST00000520837.4 TSPAN18 miRNA
ENST00000520857.4 TRAPPC9 miRNA
ENST00000520859.4 DECR1 miRNA
ENST00000520868.4 PABPC1 miRNA
ENST00000520886.5 ANKRD6 miRNA
ENST00000520887.1 CYRIB miRNA
ENST00000520896.4 LYPLA1 miRNA
ENST00000520898.4 UBR5 miRNA
ENST00000520899.1 MFAP3 miRNA
ENST00000520911.4 RNF130 miRNA
ENST00000520913.1 PVT1 lncRNA
ENST00000520946.1 MRPS28 protein_coding
ENST00000520947.1 PTPN12 trna
ENST00000520949.1 SLC25A37 trna
ENST00000521000.1 PTK2B trna
ENST00000521001.1 TNIP1 trna
ENST00000521007.1 TAF2 trna
ENST00000521030.1 SIRLNT trna
ENST00000521037.1 VPS13B trna
ENST00000521038.4 PHF20L1 trna
ENST00000521039.4 TNKS trna
ENST00000521046.1 PCMTD1 trna
ENST00000521047.1 CLINT1 trna
ENST00000521049.4 TRAM1 trna
ENST00000521057.1 ASAP1 trna

ENST00000521064.4 DGLUCY trna
ENST00000521079.1 WWP1 trna
ENST00000521081.4 DGLUCY trna
ENST00000521083.1 SARAF trna
ENST00000521109.1 WASHC5 trna
ENST00000521112.1 RPL30 trna
ENST00000521120.1 LARP4 trna
ENST00000521122.1 PVT1 trna
ENST00000521123.1 PLEKHA2 trna
ENST00000521127.1 SNHG6 trna
ENST00000521129.1 MCPH1 trna
ENST00000521134.4 KCNQ3 trna
ENST00000521140.1 BIN3 trna
ENST00000521142.1 CPQ trna
ENST00000521154.1 TACC1 trna
ENST00000521166.4 FAM91A1 trna
ENST00000521167.1 TRAPPC9 trna
ENST00000521174.4 CNOT8 trna
ENST00000521175.1 MCPH1 trna
ENST00000521180.4 ST3GAL1 trna
ENST00000521209.5 MSRA trna
ENST00000521254.1 BNIP3L trna
ENST00000521265.4 SARAF trna
ENST00000521266.1 RNF145 trna
ENST00000521282.1 BAG4 trna
ENST00000521288.1 NUDT16 trna
ENST00000521297.1 EIF3E trna
ENST00000521308.4 CCDC69 trna
ENST00000521324.1 CSPP1 trna
ENST00000521328.4 YWHAZ trna
ENST00000521361.4 FYN trna
ENST00000521368.4 CTNNA1 trna
ENST00000521377.4 ATP6V1H trna
ENST00000521381.4 PIK3R1 trna
ENST00000521382.1 PLEKHA2 trna
ENST00000521392.4 ERGIC1 trna
ENST00000521416.4 LCP2 trna
ENST00000521419.4 STAU2 trna
ENST00000521420.4 CYFIP2 trna
ENST00000521425.4 TRAM1 trna
ENST00000521426.4 ASAP1 trna
ENST00000521432.2 trna
ENST00000521438.1 NDRG1 trna
ENST00000521439.1 NUDCD1 trna
ENST00000521442.1 ATP6V1B2 trna
ENST00000521447.4 STAU2 trna
ENST00000521467.4 MSC-AS1 trna
ENST00000521477.4 DENND3 trna
ENST00000521479.1 GSR trna
ENST00000521483.1 LINC00968 trna
ENST00000521496.4 ATAD2 trna
ENST00000521514.4 ATP6V1C1 trna
ENST00000521527.4 MFAP3 trna
ENST00000521534.1 RPL30 trna

ENST00000521559.1 VPS13B trna
ENST00000521561.1 TPD52 trna
ENST00000521568.1 TACC1 trna
ENST00000521592.4 OXR1 trna
ENST00000521611.1 RB1CC1 trna
ENST00000521615.1 CLINT1 trna
ENST00000521620.4 WRN trna
ENST00000521651.4 PPP3CC trna
ENST00000521657.4 PIK3R1 trna
ENST00000521664.1 NDRG1 trna
ENST00000521667.4 TRAPPC9 trna
ENST00000521668.1 DECR1 trna
ENST00000521673.4 ZFAT trna
ENST00000521686.1 MSRA trna
ENST00000521696.1 VPS13B-DT trna
ENST00000521700.4 TRAPPC9 trna
ENST00000521731.1 trna
ENST00000521737.4 CEP57L1 trna
ENST00000521749.4 ANXA6 trna
ENST00000521764.2 NACA2 trna
ENST00000521776.2 UTP14C trna
ENST00000521781.4 GALNT10 trna
ENST00000521786.4 GALNT10 trna
ENST00000521790.4 HNRNPH1 trna
ENST00000521796.1 UBXN2B trna
ENST00000521804.1 SLC45A4 trna
ENST00000521806.4 GPAT4 trna
ENST00000521835.1 DENND3 trna
ENST00000521838.2 MAT2B trna
ENST00000521881.4 MFHAS1 trna
ENST00000521889.4 C8orf44 trna
ENST00000521900.4 ATP6V1H trna
ENST00000521901.4 RNF130 trna
ENST00000521918.5 SPIDR trna
ENST00000521922.4 UBR5 trna
ENST00000521932.1 VPS13B trna
ENST00000521933.1 MTDH trna
ENST00000521934.1 SARAF trna
ENST00000521940.1 EFR3A trna
ENST00000521941.1 CTNNA1 trna
ENST00000521944.1 TRAPPC9 trna
ENST00000522000.1 PPP3CC trna
ENST00000522010.1 VDAC3 trna
ENST00000522020.1 MCPH1 trna
ENST00000522034.4 PPP3CC trna
ENST00000522042.1 MRPS27 trna
ENST00000522061.4 STAU2 trna
ENST00000522068.4 CARD8 trna
ENST00000522072.1 PTDSS1 trna
ENST00000522082.4 HGSNAT trna
ENST00000522084.4 PIK3R1 trna
ENST00000522104.1 MTDH trna
ENST00000522118.1 MTSS1 trna
ENST00000522138.1 DOCK2 trna

ENST00000522144.1 LRRC69 trna
ENST00000522153.1 CD74 trna
ENST00000522159.4 ATP6V1H trna
ENST00000522162.1 MTSS1 trna
ENST00000522173.1 LINC02267 trna
ENST00000522176.1 PANK3 trna
ENST00000522179.1 CCDC69 trna
ENST00000522184.1 ZDHHC2 trna
ENST00000522189.1 trna
ENST00000522204.1 ST3GAL1 trna
ENST00000522208.5 RNF130 trna
ENST00000522221.1 THUMPD3-AS1 trna
ENST00000522222.4 SPIDR trna
ENST00000522243.4 SDCBP trna
ENST00000522246.4 CD74 trna
ENST00000522253.1 PHF20L1 trna
ENST00000522257.4 ZFAT trna
ENST00000522268.1 BIN3 trna
ENST00000522275.4 PCM1 trna
ENST00000522280.1 TATDN1 trna
ENST00000522282.1 YTHDF3 trna
ENST00000522285.1 ST3GAL1 trna
ENST00000522286.4 RGPD8 trna
ENST00000522311.1 NA trna
ENST00000522313.1 MTDH trna
ENST00000522338.4 PTK2B trna
ENST00000522344.1 CPEB4 trna
ENST00000522345.1 ATXN7 trna
ENST00000522352.4 EIF3E trna
ENST00000522370.1 TBCA trna
ENST00000522396.4 ADGB trna
ENST00000522397.4 TCEA1 trna
ENST00000522431.4 CARD8 trna
ENST00000522468.1 HMBOX1 trna
ENST00000522469.4 RBM22 trna
ENST00000522477.1 C8orf76 trna
ENST00000522480.4 SMG1P3 trna
ENST00000522501.1 CDKL3 trna
ENST00000522504.4 TRAPPC9 trna
ENST00000522507.1 FBXW11 trna
ENST00000522532.4 NDUFB9 trna
ENST00000522537.4 MOK trna
ENST00000522538.4 DST trna
ENST00000522540.4 DCTN6 trna
ENST00000522551.1 RPL10P9 trna
ENST00000522573.4 CSGALNACT1 trna
ENST00000522580.4 PHF20L1 trna
ENST00000522586.4 NCK2 trna
ENST00000522590.1 IPCEF1 trna
ENST00000522593.4 HAVCR2 trna
ENST00000522594.1 TMEM71 trna
ENST00000522616.1 ITK trna
ENST00000522617.3 CPQ trna
ENST00000522621.1 CLVS1 trna

ENST00000522650.4 ASCC3 trna
ENST00000522652.4 ST3GAL1 trna
ENST00000522664.4 ANXA6 trna
ENST00000522672.4 MSR1 trna
ENST00000522695.4 STAU2 trna
ENST00000522698.1 EXTL3 trna
ENST00000522709.4 EFR3A trna
ENST00000522710.1 ATOX1 trna
ENST00000522712.5 C11orf49 trna
ENST00000522722.1 MTSS1 trna
ENST00000522727.4 RAB11FIP1 trna
ENST00000522732.1 CSGALNACT1 trna
ENST00000522761.4 G3BP1 trna
ENST00000522780.4 TMEM71 trna
ENST00000522792.1 CTNNA1 trna
ENST00000522793.4 TTC1 trna
ENST00000522795.1 ZNF395 trna
ENST00000522802.4 VPS13B trna
ENST00000522811.1 PAG1 trna
ENST00000522816.1 DGLUCY trna
ENST00000522819.4 YWHAZ trna
ENST00000522823.4 LCP2 trna
ENST00000522833.4 GTF2E2 trna
ENST00000522845.2 TPT1-AS1 trna
ENST00000522846.1 KIF13B trna
ENST00000522849.1 ATP6V1H trna
ENST00000522878.4 ARFGEF1 trna
ENST00000522879.1 STK10 trna
ENST00000522884.4 CYFIP2 trna
ENST00000522891.1 FBXW11 trna
ENST00000522892.1 CYFIP2 trna
ENST00000522905.1 MCPH1 trna
ENST00000522907.4 MSRA trna
ENST00000522955.4 TACC1 trna
ENST00000522957.1 RB1CC1 trna
ENST00000522964.1 CCDC69 trna
ENST00000522965.1 RIPK2 trna
ENST00000522978.1 UBXN2B trna
ENST00000522987.1 MRPS28 trna
ENST00000522994.1 DOCK2 trna
ENST00000522998.1 PCM1 trna
ENST00000523005.1 trna
ENST00000523015.1 DENND3 trna
ENST00000523016.1 COX6C trna
ENST00000523020.1 DPY19L4 trna
ENST00000523027.1 DPYSL2 trna
ENST00000523028.4 SMG1P4 trna
ENST00000523039.4 IL6ST trna
ENST00000523041.1 NRG1 trna
ENST00000523053.1 ERICH1 trna
ENST00000523063.4 trna
ENST00000523082.1 PPP2CA protein_coding
ENST00000523086.4 COPS5 processed_transcript
ENST00000523091.4 CHMP7 retained_intron

ENST00000523093.4 DPYSL2 retained_intron
ENST00000523100.4 DIAPH1 nonsense_mediated_decay
ENST00000523126.1 ITGB2 processed_transcript
ENST00000523127.4 SARAF protein_coding
ENST00000523130.1 KIF13B protein_coding
ENST00000523132.1 ZDHHC2 protein_coding
ENST00000523136.1 HNRNPH1 protein_coding
ENST00000523147.4 PTP4A3 protein_coding
ENST00000523160.1 STAU2 processed_transcript
ENST00000523161.1 CREBRF protein_coding
ENST00000523182.4 RAB11FIP1 processed_transcript
ENST00000523190.4 PVT1 lncRNA
ENST00000523203.4 CSNK1A1 nonsense_mediated_decay
ENST00000523204.1 AP3B1 processed_transcript
ENST00000523207.1 KCNB2 protein_coding
ENST00000523208.4 CD74 nonsense_mediated_decay
ENST00000523212.1 PLAA protein_coding
ENST00000523215.1 ERGIC1 retained_intron
ENST00000523219.1 SLU7 retained_intron
ENST00000523221.1 INPP4A protein_coding
ENST00000523227.4 CSGALNACT1 processed_transcript
ENST00000523253.1 PDE7A protein_coding
ENST00000523271.1 EXTL3 retained_intron
ENST00000523278.4 UBE2W protein_coding
ENST00000523295.4 GSR nonsense_mediated_decay
ENST00000523321.4 INTS8 retained_intron
ENST00000523339.1 NPM1 retained_intron
ENST00000523351.4 DOCK2 processed_transcript
ENST00000523369.1 LCP2 retained_intron
ENST00000523376.4 CALCB protein_coding
ENST00000523381.1 ST8SIA4 nonsense_mediated_decay
ENST00000523389.4 LARP4 protein_coding
ENST00000523401.4 SGK3 processed_transcript
ENST00000523415.4 ERICH1 nonsense_mediated_decay
ENST00000523420.4 GOLGA7 protein_coding
ENST00000523441.1 SDCBP retained_intron
ENST00000523463.1 PAG1 processed_transcript
ENST00000523464.1 EBF1 processed_transcript
ENST00000523466.4 GM2A protein_coding
ENST00000523468.4 ZDHHC14 retained_intron
ENST00000523473.4 PPP2R2A processed_transcript
ENST00000523482.4 ATP6V1B2 retained_intron
ENST00000523513.1 DNM3 protein_coding
ENST00000523533.4 STAU2 protein_coding
ENST00000523547.1 RAD21 processed_transcript
ENST00000523549.1 NSMCE2 retained_intron
ENST00000523555.4 PABPC1 protein_coding
ENST00000523574.4 FYN protein_coding
ENST00000523576.1 DGLUCY processed_transcript
ENST00000523581.1 CEP170 retained_intron
ENST00000523583.1 MAPK9 protein_coding
ENST00000523586.1 AGPAT5 nonsense_mediated_decay
ENST00000523609.4 AGO2 nonsense_mediated_decay
ENST00000523620.1 PPP3CC protein_coding

ENST00000523627.1 lncRNA
ENST00000523648.4 lncRNA
ENST00000523654.4 MRPS27 processed_transcript
ENST00000523655.1 SREK1 nonsense_mediated_decay
ENST00000523663.4 ITGB2 protein_coding
ENST00000523668.1 CARD8 retained_intron
ENST00000523674.1 EIF3E retained_intron
ENST00000523700.4 lncRNA
ENST00000523714.4 ANXA6 protein_coding
ENST00000523750.1 CARD8 processed_transcript
ENST00000523752.4 TNFRSF10B retained_intron
ENST00000523761.1 SARAF protein_coding
ENST00000523797.1 PTK2 processed_transcript
ENST00000523813.1 CD74 retained_intron
ENST00000523825.1 CASC8 lncRNA
ENST00000523829.4 TMEM71 protein_coding
ENST00000523836.4 CD74 retained_intron
ENST00000523848.4 YWHAZ nonsense_mediated_decay
ENST00000523851.1 SREK1 processed_transcript
ENST00000523863.4 WWP1 processed_transcript
ENST00000523866.1 SLC25A32 nonsense_mediated_decay
ENST00000523867.4 HDAC9 retained_intron
ENST00000523892.4 NDRG1 protein_coding
ENST00000523908.4 CLINT1 protein_coding
ENST00000523921.1 HNRNPH1 protein_coding
ENST00000523922.1 ZNF706 protein_coding
ENST00000523929.1 NA NA
ENST00000523931.1 NDRG1 retained_intron
ENST00000523932.1 DOK2 protein_coding
ENST00000523938.1 YWHAZ protein_coding
ENST00000523948.4 TGS1 nonsense_mediated_decay
ENST00000523949.4 BNIP3L protein_coding
ENST00000523952.4 PTPN12 nonsense_mediated_decay
ENST00000523959.4 IL7 processed_transcript
ENST00000523968.1 KIF13B nonsense_mediated_decay
ENST00000523970.1 INSYN2B processed_transcript
ENST00000524003.1 OTUD6B-AS1 lncRNA
ENST00000524013.1 MYC protein_coding
ENST00000524015.1 FBXO25 retained_intron
ENST00000524018.1 ASAP1 retained_intron
ENST00000524050.4 PWWP2A nonsense_mediated_decay
ENST00000524061.1 PINX1 nonsense_mediated_decay
ENST00000524075.1 LOXL2 retained_intron
ENST00000524079.4 TMEM71 processed_transcript
ENST00000524081.4 INTS9 protein_coding
ENST00000524087.4 NPIPB11 protein_coding
ENST00000524097.1 TAGLN2P1 processed_pseudogene
ENST00000524106.1 THAP8 processed_transcript
ENST00000524112.1 CSGALNACT1 processed_transcript
ENST00000524118.1 RAB11FIP1 protein_coding
ENST00000524119.1 DERL1 retained_intron
ENST00000524120.4 MOK processed_transcript
ENST00000524124.4 ASAP1 protein_coding
ENST00000524137.4 NCALD protein_coding

ENST00000524138.4 ERICH1 processed_transcript
ENST00000524143.4 EMC2 protein_coding
ENST00000524158.4 TNFRSF10A protein_coding
ENST00000524161.1 NDST1 processed_transcript
ENST00000524162.4 TRAPPC9 processed_transcript
ENST00000524165.4 PVT1 lncRNA
ENST00000524170.4 MAPK9 processed_transcript
ENST00000524176.2 MYBL1 protein_coding
ENST00000524179.1 HNRNPH1 retained_intron
ENST00000524185.4 DOCK2 nonsense_mediated_decay
ENST00000524189.4 KIF13B protein_coding
ENST00000524193.1 TACC1 protein_coding
ENST00000524195.1 GDAP1 protein_coding
ENST00000524201.1 NKAIN3 protein_coding
ENST00000524202.4 PTK2 nonsense_mediated_decay
ENST00000524203.1 PCM1 processed_transcript
ENST00000524204.1 NPM1 retained_intron
ENST00000524205.3 CDRT4 protein_coding
ENST00000524210.1 THUMPD3-AS1 lncRNA
ENST00000524219.1 HAVCR2 protein_coding
ENST00000524235.4 ZFPM2 processed_transcript
ENST00000524238.3 HMBOX1 protein_coding
ENST00000524264.4 SAP30L-AS1 lncRNA
ENST00000524288.1 SKP1 retained_intron
ENST00000524301.1 DIAPH1 processed_transcript
ENST00000524306.1 CLINT1 retained_intron
ENST00000524310.4 FYN protein_coding
ENST00000524315.4 CD74 retained_intron
ENST00000524321.1 ATP5MF retained_intron
ENST00000524326.1 DECR1 processed_transcript
ENST00000524328.1 AGO2 protein_coding
ENST00000524330.1 VPS13B processed_transcript
ENST00000524336.4 lncRNA
ENST00000524340.4 PAX5 protein_coding
ENST00000524342.4 TCF7 retained_intron
ENST00000524344.4 CCDC69 retained_intron
ENST00000524347.2 SGCD nonsense_mediated_decay
ENST00000524348.1 lncRNA
ENST00000524404.4 ARHGAP27 nonsense_mediated_decay
ENST00000524416.1 FHIP1B protein_coding
ENST00000524422.1 ATP5MG protein_coding
ENST00000524448.1 DGKZ protein_coding
ENST00000524451.1 EMSY protein_coding
ENST00000524463.4 CTSC protein_coding
ENST00000524480.4 DERA protein_coding
ENST00000524516.1 PUM1 retained_intron
ENST00000524523.1 CTR9 protein_coding
ENST00000524546.4 FNTA processed_transcript
ENST00000524548.4 PPFIBP2 protein_coding
ENST00000524556.1 CSTF3 nonsense_mediated_decay
ENST00000524563.1 NUP98 nonsense_mediated_decay
ENST00000524572.1 ARID1A protein_coding
ENST00000524574.4 VAV3 processed_transcript
ENST00000524579.1 NA NA

ENST00000524589.4 SERGEF processed_transcript
ENST00000524596.1 LIN7C protein_coding
ENST00000524602.4 CHD7 protein_coding
ENST00000524606.1 PSMA1 processed_transcript
ENST00000524607.4 CACNA1E protein_coding
ENST00000524620.1 OSBPL9 processed_transcript
ENST00000524633.1 SORL1 retained_intron
ENST00000524648.1 CELF1 processed_transcript
ENST00000524652.1 CSDE1 retained_intron
ENST00000524655.4 ARHGAP32 protein_coding
ENST00000524657.1 KDM2A processed_transcript
ENST00000524670.4 CENATAC retained_intron
ENST00000524678.1 QSER1 protein_coding
ENST00000524686.1 MADD processed_transcript
ENST00000524691.1 CCDC190 processed_transcript
ENST00000524696.1 FNBP4 processed_transcript
ENST00000524717.4 MAML2 protein_coding
ENST00000524726.1 TBCEL protein_coding
ENST00000524752.1 POLD3 protein_coding
ENST00000524764.1 SGK1 retained_intron
ENST00000524788.1 TPP1 retained_intron
ENST00000524792.4 ATM retained_intron
ENST00000524793.1 NAP1L1P1 processed_pseudogene
ENST00000524801.5 POU2F2 protein_coding
ENST00000524822.4 STIM1 protein_coding
ENST00000524827.4 CSTF3 protein_coding
ENST00000524830.1 SYTL2 processed_transcript
ENST00000524864.1 RPS25 retained_intron
ENST00000524873.1 SORL1 retained_intron
ENST00000524875.1 SLC36A4 nonsense_mediated_decay
ENST00000524899.1 GRK2 processed_transcript
ENST00000524902.4 MTA2 protein_coding
ENST00000524915.4 FPGT processed_transcript
ENST00000524922.1 CD44 protein_coding
ENST00000524933.1 FAR1 retained_intron
ENST00000525003.1 IL32 retained_intron
ENST00000525013.1 CAPN1 retained_intron
ENST00000525015.1 CYP2R1 nonsense_mediated_decay
ENST00000525018.4 TMEM135 protein_coding
ENST00000525029.1 TTC17 non_stop_decay
ENST00000525036.1 RB1 retained_intron
ENST00000525041.4 KDM2A processed_transcript
ENST00000525048.4 NPEPPS nonsense_mediated_decay
ENST00000525052.1 RBL1 protein_coding
ENST00000525054.1 EIF3M retained_intron
ENST00000525056.1 ATM retained_intron
ENST00000525067.1 HBS1L protein_coding
ENST00000525070.1 SIGIRR retained_intron
ENST00000525081.1 NSD3 processed_transcript
ENST00000525082.1 DDX6 retained_intron
ENST00000525091.4 EDRF1 retained_intron
ENST00000525099.1 FNTA retained_intron
ENST00000525106.1 C11orf58 retained_intron
ENST00000525134.5 DIS3L protein_coding

ENST00000525141.1 SMCO4 protein_coding
ENST00000525161.4 RBM25 protein_coding
ENST00000525168.1 SERGEF processed_transcript
ENST00000525178.4 ATM processed_transcript
ENST00000525183.1 UVRAG nonsense_mediated_decay
ENST00000525184.1 APOL3 processed_transcript
ENST00000525205.4 SLC43A3 nonsense_mediated_decay
ENST00000525217.1 lncRNA
ENST00000525223.1 EEF1D protein_coding
ENST00000525234.1 ARHGAP32 protein_coding
ENST00000525244.4 EED retained_intron
ENST00000525256.1 C11orf58 retained_intron
ENST00000525257.1 USP47 processed_transcript
ENST00000525259.1 SOX6 processed_transcript
ENST00000525265.1 TXNRD1 retained_intron
ENST00000525287.4 MROH1 retained_intron
ENST00000525309.1 lncRNA
ENST00000525310.1 CPQ processed_transcript
ENST00000525315.4 CTSB retained_intron
ENST00000525325.1 ACER3 retained_intron
ENST00000525340.4 EEF1G retained_intron
ENST00000525345.4 NARS2 nonsense_mediated_decay
ENST00000525348.1 CD44 retained_intron
ENST00000525349.1 NFYC retained_intron
ENST00000525357.1 OSBP nonsense_mediated_decay
ENST00000525377.5 IL32 protein_coding
ENST00000525389.1 BLK processed_transcript
ENST00000525399.2 PPM1A protein_coding
ENST00000525403.4 STIM1 protein_coding
ENST00000525421.4 PPP6R3 nonsense_mediated_decay
ENST00000525434.4 DGKZ processed_transcript
ENST00000525439.1 PDE3B processed_transcript
ENST00000525444.1 MICAL2 retained_intron
ENST00000525460.4 VAV3 processed_transcript
ENST00000525467.1 IL10RA retained_intron
ENST00000525469.1 CD44 protein_coding
ENST00000525490.4 ZDHHC13 retained_intron
ENST00000525508.1 CHD7 protein_coding
ENST00000525529.4 RAB2A retained_intron
ENST00000525532.4 SORL1 protein_coding
ENST00000525559.1 EXT2 retained_intron
ENST00000525562.4 NPIPB14P processed_transcript
ENST00000525565.1 JAML protein_coding
ENST00000525570.1 RSRC2 retained_intron
ENST00000525594.1 lncRNA
ENST00000525604.1 APLP2 retained_intron
ENST00000525605.1 CLEC7A protein_coding
ENST00000525635.1 ARFGAP2 retained_intron
ENST00000525637.1 NUCB2 processed_transcript
ENST00000525645.1 PTS retained_intron
ENST00000525661.4 BTBD10 retained_intron
ENST00000525679.1 PHF21A processed_transcript
ENST00000525681.4 EIF4G2 protein_coding
ENST00000525711.1 UBASH3B processed_transcript

ENST00000525729.4 C11orf65 protein_coding
ENST00000525774.4 LRRC8D protein_coding
ENST00000525793.4 FCMR protein_coding
ENST00000525799.1 PTPN22 protein_coding
ENST00000525811.4 GMDS-DT lncRNA
ENST00000525819.1 PPP3CA protein_coding
ENST00000525844.1 BDP1 nonsense_mediated_decay
ENST00000525847.1 STX17 processed_transcript
ENST00000525875.1 AHNAK processed_transcript
ENST00000525904.4 DEAF1 processed_transcript
ENST00000525946.4 STT3A retained_intron
ENST00000525962.4 TGFBR3 protein_coding
ENST00000525964.5 NCAPD3 nonsense_mediated_decay
ENST00000525973.4 NCAM1 processed_transcript
ENST00000525975.4 HIPK3 protein_coding
ENST00000525980.4 NUP214 retained_intron
ENST00000525990.1 SSBP3 protein_coding
ENST00000525997.1 PUM1 protein_coding
ENST00000526004.1 GZMB protein_coding
ENST00000526006.1 TXNRD1 processed_transcript
ENST00000526018.1 GNLY protein_coding
ENST00000526019.4 L3MBTL3 protein_coding
ENST00000526025.1 CD44 protein_coding
ENST00000526070.2 DDX6 protein_coding
ENST00000526076.5 MAP2K3 protein_coding
ENST00000526087.4 L3MBTL3 protein_coding
ENST00000526095.1 KCNQ1 protein_coding
ENST00000526104.4 RIC8A protein_coding
ENST00000526117.4 LMX1B protein_coding
ENST00000526128.1 PUM1 processed_transcript
ENST00000526134.1 UBQLN1 protein_coding
ENST00000526137.1 SIPA1 protein_coding
ENST00000526145.5 ETS1 protein_coding
ENST00000526149.1 CKLF retained_intron
ENST00000526153.4 MS4A4E nonsense_mediated_decay
ENST00000526156.1 STIM1 retained_intron
ENST00000526163.4 NXF1 processed_transcript
ENST00000526192.1 VNN2 processed_transcript
ENST00000526197.4 PDE4B protein_coding
ENST00000526228.1 BCLAF1 retained_intron
ENST00000526245.1 NA NA
ENST00000526253.1 RABGAP1L processed_transcript
ENST00000526257.1 RELA protein_coding
ENST00000526267.1 EIF3M protein_coding
ENST00000526274.1 lncRNA
ENST00000526277.1 CELF1 protein_coding
ENST00000526285.1 GRK2 protein_coding
ENST00000526292.1 MTMR9 protein_coding
ENST00000526295.1 RNH1 processed_transcript
ENST00000526303.1 CUL5 retained_intron
ENST00000526307.4 PPP6R3 retained_intron
ENST00000526309.1 PDHX protein_coding
ENST00000526324.4 RSF1 protein_coding
ENST00000526328.4 SAMD12 processed_transcript

ENST00000526346.4 NUP214 nonsense_mediated_decay
ENST00000526363.1 LRRIQ1 protein_coding
ENST00000526372.1 RAB38 protein_coding
ENST00000526386.4 UBASH3B processed_transcript
ENST00000526394.1 API5 protein_coding
ENST00000526404.1 ABCB7 protein_coding
ENST00000526419.1 CELF1 protein_coding
ENST00000526423.1 ARHGAP1 retained_intron
ENST00000526442.4 NA NA
ENST00000526460.1 BANP processed_transcript
ENST00000526475.1 MICAL2 processed_transcript
ENST00000526478.4 PDXDC2P-NPIPB14P retained_intron
ENST00000526481.5 CTSB nonsense_mediated_decay
ENST00000526482.1 SLC24A4 retained_intron
ENST00000526484.4 ARHGAP27 retained_intron
ENST00000526532.4 TESPA1 protein_coding
ENST00000526533.1 lncRNA
ENST00000526535.1 POU2AF1 processed_transcript
ENST00000526536.1 GRIK4 processed_transcript
ENST00000526539.2 SLC38A6 nonsense_mediated_decay
ENST00000526544.4 IL10RA nonsense_mediated_decay
ENST00000526545.4 AMBRA1 protein_coding
ENST00000526547.1 PPT1 nonsense_mediated_decay
ENST00000526548.2 PICALM retained_intron
ENST00000526550.1 PTPRJ processed_transcript
ENST00000526553.4 CD44 protein_coding
ENST00000526567.4 ATM protein_coding
ENST00000526574.1 PPP6R3 retained_intron
ENST00000526578.4 VPS51 protein_coding
ENST00000526579.4 SNX19 processed_transcript
ENST00000526586.5 RPS2 protein_coding
ENST00000526593.1 PPP6R3 nonsense_mediated_decay
ENST00000526595.4 JAML nonsense_mediated_decay
ENST00000526604.1 MICAL2 processed_transcript
ENST00000526606.1 AMBRA1 protein_coding
ENST00000526627.1 SMIM3 protein_coding
ENST00000526635.1 RAD51-AS1 lncRNA
ENST00000526672.1 MICAL2 processed_transcript
ENST00000526676.1 TMEM123 protein_coding
ENST00000526686.1 HSPA8 protein_coding
ENST00000526696.4 SSRP1 protein_coding
ENST00000526697.1 MS4A6A retained_intron
ENST00000526698.1 MOB2 retained_intron
ENST00000526700.4 COG4 retained_intron
ENST00000526704.5 LINC01001 lncRNA
ENST00000526707.4 DENND5A nonsense_mediated_decay
ENST00000526713.1 TRPC6 processed_transcript
ENST00000526717.1 RRAS2 nonsense_mediated_decay
ENST00000526725.1 C11orf65 processed_transcript
ENST00000526735.1 SLC36A4 retained_intron
ENST00000526761.4 CLNS1A nonsense_mediated_decay
ENST00000526769.3 GVINP1 processed_transcript
ENST00000526771.4 STIM1 processed_transcript
ENST00000526774.4 TTC17 processed_transcript

ENST00000526794.1 DDX10 protein_coding
ENST00000526831.1 POU2F2 protein_coding
ENST00000526841.1 BTBD10 protein_coding
ENST00000526846.1 CHD7 protein_coding
ENST00000526862.1 HSPA8 processed_transcript
ENST00000526872.2 ATXN3 protein_coding
ENST00000526882.4 HOOK3 nonsense_mediated_decay
ENST00000526886.4 SAMD3 retained_intron
ENST00000526890.4 CARS1 retained_intron
ENST00000526903.1 RNF121 retained_intron
ENST00000526905.1 ADAR processed_transcript
ENST00000526907.1 PICALM retained_intron
ENST00000526910.1 PAK1 retained_intron
ENST00000526950.1 TXNRD1 protein_coding
ENST00000526952.1 FBXO3 processed_transcript
ENST00000526968.1 PAK1 protein_coding
ENST00000526975.1 CFL1 protein_coding
ENST00000526980.4 CSF3R processed_transcript
ENST00000526981.1 CD58 protein_coding
ENST00000526988.1 BEST1 protein_coding
ENST00000526990.1 EHBP1L1 retained_intron
ENST00000526991.2 CHMP1B protein_coding
ENST00000527005.4 HBS1L retained_intron
ENST00000527018.4 CTSC protein_coding
ENST00000527019.4 SBF2 processed_transcript
ENST00000527064.1 NXF1 retained_intron
ENST00000527065.1 CARD16 retained_intron
ENST00000527069.4 PPP6R3 retained_intron
ENST00000527071.1 MROH1 processed_transcript
ENST00000527088.1 DLG2 protein_coding
ENST00000527095.4 IQCJ-SCHIP1 protein_coding
ENST00000527116.4 SNX19 retained_intron
ENST00000527128.4 SUGT1P4-STRA6LP retained_intron
ENST00000527133.2 CLNS1A nonsense_mediated_decay
ENST00000527140.1 MSANTD2 nonsense_mediated_decay
ENST00000527146.1 IFITM2 nonsense_mediated_decay
ENST00000527149.4 SMCO4 protein_coding
ENST00000527155.4 MTFR1 protein_coding
ENST00000527156.1 LRRC8D protein_coding
ENST00000527157.1 KDM2A processed_transcript
ENST00000527181.1 ATM retained_intron
ENST00000527202.1 FAR1 processed_transcript
ENST00000527213.4 HSD17B12 processed_transcript
ENST00000527224.1 PACS1 retained_intron
ENST00000527225.1 SPCS2 processed_transcript
ENST00000527226.2 TPT1 protein_coding
ENST00000527243.4 CTSB protein_coding
ENST00000527250.4 QSER1 protein_coding
ENST00000527253.2 PRPF40B processed_transcript
ENST00000527263.1 AK5 nonsense_mediated_decay
ENST00000527266.1 BCL9L processed_transcript
ENST00000527268.4 C11orf49 processed_transcript
ENST00000527290.1 SPCS2 retained_intron
ENST00000527297.1 lncRNA

ENST00000527301.1 RNF169 protein_coding
ENST00000527303.1 PLEC protein_coding
ENST00000527304.1 MAP3K11 retained_intron
ENST00000527306.1 HOOK3 retained_intron
ENST00000527326.1 CD44 retained_intron
ENST00000527366.1 H2AZ1 retained_intron
ENST00000527380.1 PACS1 protein_coding
ENST00000527385.1 ARRB1 nonsense_mediated_decay
ENST00000527397.1 RAB1B protein_coding
ENST00000527401.4 PHF21A retained_intron
ENST00000527403.5 PPP6R3 protein_coding
ENST00000527426.1 lncRNA
ENST00000527433.4 HSD17B12 nonsense_mediated_decay
ENST00000527449.1 RBM25 retained_intron
ENST00000527484.4 STIM1 processed_transcript
ENST00000527487.1 PRR5L protein_coding
ENST00000527490.1 MCPH1-AS1 lncRNA
ENST00000527498.1 PUM1 nonsense_mediated_decay
ENST00000527502.4 NSD3 protein_coding
ENST00000527507.1 HBS1L protein_coding
ENST00000527508.1 ACER3 retained_intron
ENST00000527517.1 RPLP2 retained_intron
ENST00000527529.5 CREBZF nonsense_mediated_decay
ENST00000527570.1 INKA2 processed_transcript
ENST00000527625.4 CASP1 retained_intron
ENST00000527672.1 lncRNA
ENST00000527674.4 DGKZ retained_intron
ENST00000527683.1 LINC01001 lncRNA
ENST00000527690.1 TAF1D protein_coding
ENST00000527698.1 LRRK1 retained_intron
ENST00000527700.4 DENND5A retained_intron
ENST00000527706.4 DSCAML1 protein_coding
ENST00000527715.4 CTSH retained_intron
ENST00000527733.4 USP47 protein_coding
ENST00000527735.1 NUCB2 miRNA
ENST00000527743.1 SLC36A4 miRNA
ENST00000527750.1 NUP160 miRNA
ENST00000527752.1 CFL1 miRNA
ENST00000527753.4 PHF21A miRNA
ENST00000527767.6 FLI1 miRNA
ENST00000527788.4 QSER1 miRNA
ENST00000527805.4 ATM miRNA
ENST00000527808.1 BIRC2 miRNA
ENST00000527816.4 NA miRNA
ENST00000527825.1 CHD7 miRNA
ENST00000527830.1 APIP miRNA
ENST00000527839.1 KMT2A miRNA
ENST00000527849.4 FMO5 miRNA
ENST00000527858.1 BTBD10 miRNA
ENST00000527867.4 TOR1AIP1 miRNA
ENST00000527869.5 KMT2A miRNA
ENST00000527886.4 TOLLIP miRNA
ENST00000527891.4 NA miRNA
ENST00000527896.1 DENND5A miRNA

ENST00000527934.1 SORL1 miRNA
ENST00000527941.4 TUT4 miRNA
ENST00000527958.4 PAFAH1B2 miRNA
ENST00000527991.2 ECE1 miRNA
ENST00000528012.4 ZNF517 miRNA
ENST00000528018.1 PSMA1 miRNA
ENST00000528028.1 ZNF250 miRNA
ENST00000528042.1 TMX2 miRNA
ENST00000528056.4 PRKY miRNA
ENST00000528066.1 RCOR3 miRNA
ENST00000528071.4 NUP160 miRNA
ENST00000528074.1 RPS13 miRNA
ENST00000528079.5 TXNRD1 miRNA
ENST00000528081.4 RBM25 miRNA
ENST00000528088.1 CENATAC miRNA
ENST00000528124.1 OSBPL5 miRNA
ENST00000528128.2 TTYH2 miRNA
ENST00000528131.1 NA miRNA
ENST00000528159.1 EXT2 miRNA
ENST00000528191.4 CTSH miRNA
ENST00000528206.1 ASRGL1 miRNA
ENST00000528215.1 MS4A7 miRNA
ENST00000528229.1 BCLAF1 miRNA
ENST00000528240.1 TESPA1 miRNA
ENST00000528261.1 NAP1L4 miRNA
ENST00000528264.1 UVRAG miRNA
ENST00000528268.4 ANKRD36C miRNA
ENST00000528275.1 UBE2L6 miRNA
ENST00000528278.1 KMT2A miRNA
ENST00000528293.1 JAK3 miRNA
ENST00000528294.1 ECE1 miRNA
ENST00000528322.5 MYO18A miRNA
ENST00000528329.1 GAB2 miRNA
ENST00000528337.1 WAC-AS1 miRNA
ENST00000528339.4 SORL1 miRNA
ENST00000528344.1 BIRC2 miRNA
ENST00000528379.1 RAB30 miRNA
ENST00000528380.1 KDM2A miRNA
ENST00000528384.4 ARHGAP27 miRNA
ENST00000528401.1 ZC3H3 miRNA
ENST00000528408.4 RCOR3 miRNA
ENST00000528411.1 PICALM miRNA
ENST00000528420.4 UVRAG miRNA
ENST00000528424.1 CASP1 miRNA
ENST00000528433.2 RPL38 miRNA
ENST00000528436.1 CTSH miRNA
ENST00000528466.1 miRNA
ENST00000528489.1 LPXN miRNA
ENST00000528500.1 MS4A7 miRNA
ENST00000528501.4 NUP160 miRNA
ENST00000528503.1 GANAB miRNA
ENST00000528508.4 AHNAK miRNA
ENST00000528511.5 miRNA
ENST00000528512.4 NA miRNA

ENST00000528517.4 NME7 miRNA
ENST00000528540.4 CD3G miRNA
ENST00000528547.1 RPS25 miRNA
ENST00000528561.4 CREBZF miRNA
ENST00000528607.1 miRNA
ENST00000528619.4 TPT1 miRNA
ENST00000528627.1 NSD3 miRNA
ENST00000528633.1 PAK1 miRNA
ENST00000528644.4 NUCB2 miRNA
ENST00000528653.1 EIF3F miRNA
ENST00000528672.1 CD44 miRNA
ENST00000528673.4 ZC3H12C miRNA
ENST00000528688.4 TENM4 miRNA
ENST00000528697.4 LRRC4C miRNA
ENST00000528727.4 CNTN5 miRNA
ENST00000528729.1 FDFT1 miRNA
ENST00000528773.1 SUSD1 miRNA
ENST00000528787.1 SSBP3 miRNA
ENST00000528790.1 FLI1 miRNA
ENST00000528799.1 CLEC7A miRNA
ENST00000528811.1 CARD18 miRNA
ENST00000528819.1 XRRA1 miRNA
ENST00000528847.1 RPS3 miRNA
ENST00000528869.1 CD44-AS1 miRNA
ENST00000528889.1 CREBZF miRNA
ENST00000528893.1 PHF21A miRNA
ENST00000528922.1 CD44 miRNA
ENST00000528940.1 BIRC3 miRNA
ENST00000528947.4 PPFIBP2 miRNA
ENST00000528948.1 CAPRIN1 miRNA
ENST00000528962.1 TCP11L1 miRNA
ENST00000528969.4 TMEM123 miRNA
ENST00000528973.1 PCSK7 miRNA
ENST00000528988.1 HSF1 miRNA
ENST00000529028.1 MICAL2 miRNA
ENST00000529033.4 VAV3 miRNA
ENST00000529038.4 OPCML miRNA
ENST00000529045.1 PTPN22 miRNA
ENST00000529046.4 CSDE1 miRNA
ENST00000529050.4 ARNTL miRNA
ENST00000529051.4 CCDC15 miRNA
ENST00000529087.1 PGGHG miRNA
ENST00000529099.4 EHBP1L1 miRNA
ENST00000529119.1 SAMD3 miRNA
ENST00000529145.4 RABGAP1L miRNA
ENST00000529162.1 DDX6 miRNA
ENST00000529168.1 ADAR miRNA
ENST00000529172.1 PPP6R3 retained_intron
ENST00000529194.4 APOL2 protein_coding
ENST00000529213.1 PLEKHA7 trna
ENST00000529223.1 NSD3 trna
ENST00000529227.4 RPL27A trna
ENST00000529268.1 PDGFD trna
ENST00000529307.1 CAPRIN1 trna

ENST00000529336.1 XKR6 trna
ENST00000529348.1 ZBTB44 trna
ENST00000529364.1 TCIRG1 trna
ENST00000529379.1 NUP98 trna
ENST00000529380.1 MOB2 trna
ENST00000529390.1 ARNTL trna
ENST00000529428.4 SMG1P5 trna
ENST00000529445.1 SORL1 trna
ENST00000529447.1 ZDHHC5 trna
ENST00000529463.4 MTSS1 trna
ENST00000529483.1 APLP2 trna
ENST00000529500.1 PSMC3 trna
ENST00000529507.4 AMPD3 trna
ENST00000529508.1 TAF1D trna
ENST00000529543.4 TBRG1 trna
ENST00000529548.1 FTH1 trna
ENST00000529553.1 AMBRA1 trna
ENST00000529558.1 AMBRA1 trna
ENST00000529569.1 MTCO1P15 trna
ENST00000529588.4 ATM trna
ENST00000529592.1 PRMT3 trna
ENST00000529613.4 IL15 trna
ENST00000529624.4 HERC2P9 trna
ENST00000529660.4 DGKZ trna
ENST00000529666.1 AAMDC trna
ENST00000529669.1 POGZ trna
ENST00000529679.1 PSMD13 trna
ENST00000529698.1 DGKZ trna
ENST00000529701.1 APLP2 trna
ENST00000529714.4 SMCO4 trna
ENST00000529716.4 NPIPB8 trna
ENST00000529723.1 SAMD3 trna
ENST00000529724.1 PPP1CA trna
ENST00000529728.4 SERGEF trna
ENST00000529738.1 GRK2 trna
ENST00000529742.1 MUS81 trna
ENST00000529759.1 LDLRAD3 trna
ENST00000529807.4 INTS4 trna
ENST00000529809.4 VAV3 trna
ENST00000529814.1 CR1 trna
ENST00000529828.4 PTPRC trna
ENST00000529835.5 AMPD3 trna
ENST00000529851.4 APLF trna
ENST00000529863.1 NUP160 trna
ENST00000529880.1 NARS2 trna
ENST00000529894.1 BLK trna
ENST00000529900.1 TAF1D trna
ENST00000529902.2 LINC01588 trna
ENST00000529905.4 PPT1 trna
ENST00000529906.4 MS4A6A trna
ENST00000529915.1 LPXN trna
ENST00000529923.1 UBQLN1 trna
ENST00000529924.4 IL10RA trna
ENST00000529933.4 SUSD1 trna

ENST00000529998.4 UBASH3B trna
ENST00000530019.4 FTH1 trna
ENST00000530045.4 CCDC90B-AS1 trna
ENST00000530068.4 CTNND1 trna
ENST00000530086.1 ARRB1 trna
ENST00000530115.1 HSP90AA2P trna
ENST00000530116.1 DDX10 trna
ENST00000530170.1 RPS3 trna
ENST00000530178.1 IL10RA trna
ENST00000530182.4 ACER3 trna
ENST00000530201.1 trna
ENST00000530217.2 NPIPA7 trna
ENST00000530221.1 STX3 trna
ENST00000530245.4 TPT1 trna
ENST00000530254.5 MYO18A trna
ENST00000530255.1 REPS1 trna
ENST00000530260.4 DLG2 trna
ENST00000530271.5 WNK1 trna
ENST00000530275.2 MACF1 trna
ENST00000530285.4 AHNAK trna
ENST00000530290.4 CTSB trna
ENST00000530291.4 GRK2 trna
ENST00000530293.1 BCL9L trna
ENST00000530307.1 SLCO5A1 trna
ENST00000530321.1 EDA trna
ENST00000530326.4 NUP160 trna
ENST00000530330.1 SNX19 trna
ENST00000530332.1 COMMD5 trna
ENST00000530336.2 PYGL trna
ENST00000530342.2 KDM2A trna
ENST00000530358.1 PAK1 trna
ENST00000530365.1 SORL1 trna
ENST00000530391.1 HSPA8 trna
ENST00000530413.1 CFL1 trna
ENST00000530420.4 HERC2P9 trna
ENST00000530422.1 SCARNA9 trna
ENST00000530425.1 CEP295 trna
ENST00000530427.4 PPP6R3 trna
ENST00000530459.1 GMDS trna
ENST00000530463.4 ZNF143 trna
ENST00000530469.1 TTC17 trna
ENST00000530498.1 STX3 trna
ENST00000530501.1 UVRAG trna
ENST00000530505.1 FCMR trna
ENST00000530526.1 FEZ1 trna
ENST00000530534.1 NADSYN1 trna
ENST00000530542.1 PICALM trna
ENST00000530554.4 STIM1 trna
ENST00000530561.4 LPXN trna
ENST00000530562.4 XRRA1 trna
ENST00000530567.1 CAPN1 trna
ENST00000530570.1 ZBED5 trna
ENST00000530575.1 REPS1 trna
ENST00000530589.1 DLG2 trna

ENST00000530600.1 trna
ENST00000530604.1 RSF1 trna
ENST00000530611.1 trna
ENST00000530622.5 N4BP2L1 trna
ENST00000530624.4 NA trna
ENST00000530627.1 PRR5L trna
ENST00000530669.1 PUM1 trna
ENST00000530671.1 VAV3 trna
ENST00000530691.4 MICAL2 trna
ENST00000530693.1 LONRF1 trna
ENST00000530709.4 USP33 trna
ENST00000530715.4 POLG trna
ENST00000530727.4 PTPRC trna
ENST00000530729.1 MAP7D1 trna
ENST00000530739.4 NME7 trna
ENST00000530741.1 SBF2 trna
ENST00000530758.1 BRK1 trna
ENST00000530780.1 DENND5A trna
ENST00000530784.1 CSDE1 trna
ENST00000530801.4 AP2A2 trna
ENST00000530819.1 MED17 trna
ENST00000530832.4 IDNK trna
ENST00000530833.4 GMDS-DT trna
ENST00000530835.1 TUBAP2 trna
ENST00000530849.1 RNF214 trna
ENST00000530853.4 NA trna
ENST00000530858.4 GRM5P1 trna
ENST00000530863.1 NUP214 trna
ENST00000530865.4 WAC trna
ENST00000530881.4 ZNF720 trna
ENST00000530884.1 CTBP2 trna
ENST00000530896.4 MIR4300HG trna
ENST00000530900.1 BSCL2 trna
ENST00000530906.1 PCF11 trna
ENST00000530907.4 BTBD10 trna
ENST00000530909.1 ARL14EP trna
ENST00000530913.1 RPL27A trna
ENST00000530924.2 ETS1 trna
ENST00000530927.4 GMDS trna
ENST00000530930.1 CTBP2 trna
ENST00000530931.1 CD82 trna
ENST00000530935.1 TMX4 trna
ENST00000530945.1 CFL1 trna
ENST00000530958.4 ATM trna
ENST00000530965.4 NFYC trna
ENST00000530967.5 TBC1D10C trna
ENST00000530997.5 NIN trna
ENST00000531008.4 RPS13 trna
ENST00000531012.1 ARRB1 trna
ENST00000531026.4 RSF1 trna
ENST00000531030.4 FOXK2 trna
ENST00000531050.4 PTPRK trna
ENST00000531060.1 DENND2B trna
ENST00000531061.1 OSBPL9 trna

ENST00000531063.1 HSPA8 trna
ENST00000531095.1 APOL3 trna
ENST00000531098.1 RAB2A trna
ENST00000531103.4 TMEM123 trna
ENST00000531118.4 CD44 trna
ENST00000531131.1 NXF1 trna
ENST00000531134.4 FGD4 trna
ENST00000531141.1 CD44 trna
ENST00000531143.5 PPM1A trna
ENST00000531147.1 FAM111A trna
ENST00000531153.4 ANKRD36C trna
ENST00000531162.4 DICER1 trna
ENST00000531180.1 EIF4G2 trna
ENST00000531220.1 OAF trna
ENST00000531224.4 BCLAF1 trna
ENST00000531270.4 LRRK1 trna
ENST00000531298.4 PACS1 trna
ENST00000531305.1 ALG9-IT1 trna
ENST00000531324.1 AHNAK trna
ENST00000531325.4 MCOLN2 trna
ENST00000531331.1 IMMP1L trna
ENST00000531341.1 CHKA trna
ENST00000531358.1 PDE4B trna
ENST00000531365.1 IL10RA trna
ENST00000531384.1 NPAT trna
ENST00000531421.4 RRAS2 trna
ENST00000531427.4 CUL5 trna
ENST00000531431.1 PRDM10 trna
ENST00000531432.1 PPP6R3 trna
ENST00000531433.4 SLC24A4 trna
ENST00000531440.4 RNF170 trna
ENST00000531498.4 TRIM66 trna
ENST00000531500.4 RBM25 trna
ENST00000531504.4 HIPK3 trna
ENST00000531525.2 ATM trna
ENST00000531530.4 JAML trna
ENST00000531536.1 JAML trna
ENST00000531608.1 SNX19 trna
ENST00000531611.4 ETS1 trna
ENST00000531647.4 EIF4G2 trna
ENST00000531648.4 C11orf49 trna
ENST00000531658.1 C11orf58 trna
ENST00000531665.4 ARNTL trna
ENST00000531668.1 CAPRIN1 trna
ENST00000531673.4 PTS trna
ENST00000531675.1 REPS1 trna
ENST00000531685.1 GNLY trna
ENST00000531687.1 THUMPD2 trna
ENST00000531688.1 IFITM3 trna
ENST00000531690.4 GMDS trna
ENST00000531696.4 KDM2A trna
ENST00000531706.1 LYVE1 trna
ENST00000531744.4 trna
ENST00000531767.1 RPL8 trna

ENST00000531770.4 NA trna
ENST00000531771.4 PICALM trna
ENST00000531779.1 LAMTOR5 trna
ENST00000531800.1 TMEM135 trna
ENST00000531814.4 SWAP70 trna
ENST00000531822.1 AKIRIN1 protein_coding
ENST00000531871.3 GVINP1 transcribed_unprocessed_pseudogene
ENST00000531874.1 MCOLN2 processed_transcript
ENST00000531883.4 RNPC3 protein_coding
ENST00000531901.4 EYA4 protein_coding
ENST00000531913.1 CD3E retained_intron
ENST00000531914.4 MS4A6A retained_intron
ENST00000531937.1 PPM1A protein_coding
ENST00000531949.1 MS4A14 nonsense_mediated_decay
ENST00000531957.1 NA NA
ENST00000531959.4 PHF21A protein_coding
ENST00000531989.1 DPF2 protein_coding
ENST00000532000.4 SLC37A2 protein_coding
ENST00000532009.1 IL10RA retained_intron
ENST00000532020.2 APBB1 protein_coding
ENST00000532024.1 PPFIA1 protein_coding
ENST00000532026.4 ZDHHC13 processed_transcript
ENST00000532027.4 DNHD1 nonsense_mediated_decay
ENST00000532028.1 PHF21A protein_coding
ENST00000532029.5 LRRK1 protein_coding
ENST00000532053.4 VNN2 nonsense_mediated_decay
ENST00000532054.1 EIF3M processed_transcript
ENST00000532066.1 TRIM44 processed_transcript
ENST00000532083.1 NLRP3 retained_intron
ENST00000532090.2 AP5B1 protein_coding
ENST00000532118.4 RDX protein_coding
ENST00000532130.1 UVRAG protein_coding
ENST00000532154.4 PPP2R2B nonsense_mediated_decay
ENST00000532159.1 PDHX retained_intron
ENST00000532160.1 VPS26B retained_intron
ENST00000532176.2 POU2F2 protein_coding
ENST00000532178.1 HSD17B12 processed_transcript
ENST00000532180.1 TMEM126A protein_coding
ENST00000532192.1 RBM25 protein_coding
ENST00000532201.4 LRRC8D protein_coding
ENST00000532204.4 KMT2A protein_coding
ENST00000532206.1 NOD2 processed_transcript
ENST00000532226.1 TRAF3IP2-AS1 lncRNA
ENST00000532251.1 CWF19L2 nonsense_mediated_decay
ENST00000532252.4 JAM3 nonsense_mediated_decay
ENST00000532254.1 NPIPB6 protein_coding
ENST00000532256.1 PSMA1 protein_coding
ENST00000532259.1 SRPRA protein_coding
ENST00000532261.1 BTBD10 processed_transcript
ENST00000532270.4 HIKESHI processed_transcript
ENST00000532279.1 PPP1CA retained_intron
ENST00000532298.4 NPIPB14P transcribed_unprocessed_pseudogene
ENST00000532317.4 PICALM protein_coding
ENST00000532337.1 SMG1P6 unprocessed_pseudogene

ENST00000532339.1 CD44 retained_intron
ENST00000532346.1 PLEC retained_intron
ENST00000532348.1 SUSD1 retained_intron
ENST00000532365.1 CTR9 nonsense_mediated_decay
ENST00000532392.1 CTSB protein_coding
ENST00000532405.4 SLC24A4 protein_coding
ENST00000532420.1 MICAL2 protein_coding
ENST00000532424.4 NA NA
ENST00000532436.4 KDM2A processed_transcript
ENST00000532442.4 MS4A4E nonsense_mediated_decay
ENST00000532447.4 ARRB1 protein_coding
ENST00000532451.1 SORL1 processed_transcript
ENST00000532458.1 NA NA
ENST00000532462.4 PYGL protein_coding
ENST00000532475.1 NUP98 protein_coding
ENST00000532483.4 RBM25 processed_transcript
ENST00000532490.1 LDLRAD3 processed_transcript
ENST00000532502.1 FAR1 protein_coding
ENST00000532520.1 CASP1 retained_intron
ENST00000532528.4 CPQ processed_transcript
ENST00000532556.1 RSF1 protein_coding
ENST00000532572.1 PRSS23 retained_intron
ENST00000532573.1 DERA processed_transcript
ENST00000532578.1 PRR5L processed_transcript
ENST00000532601.1 FTH1 protein_coding
ENST00000532611.1 GRK2 retained_intron
ENST00000532678.1 PUM1 protein_coding
ENST00000532685.1 TALDO1 retained_intron
ENST00000532693.4 SLC35A3 nonsense_mediated_decay
ENST00000532696.1 DENND5A retained_intron
ENST00000532701.1 FAR1 protein_coding
ENST00000532711.1 PAK1 retained_intron
ENST00000532739.1 PKD1P2 unprocessed_pseudogene
ENST00000532755.4 CAPRIN1 processed_transcript
ENST00000532763.4 SAMD3 protein_coding
ENST00000532765.1 ATM processed_transcript
ENST00000532769.1 FAR1 retained_intron
ENST00000532781.1 ARID1A nonsense_mediated_decay
ENST00000532782.1 CUL5 protein_coding
ENST00000532809.1 PRCP protein_coding
ENST00000532826.4 LGALS8 retained_intron
ENST00000532843.1 PRR5L processed_transcript
ENST00000532852.4 protein_coding
ENST00000532857.1 ZNF516 protein_coding
ENST00000532899.2 NA NA
ENST00000532939.2 DICER1 protein_coding
ENST00000532947.1 METTL15 nonsense_mediated_decay
ENST00000532959.4 TMEM135 protein_coding
ENST00000532965.4 BDNF-AS lncRNA
ENST00000532968.1 RBM4 protein_coding
ENST00000532980.3 processed_pseudogene
ENST00000532983.1 SMG1P5 processed_transcript
ENST00000532986.1 EEF1G processed_transcript
ENST00000532990.1 STIM1 protein_coding

ENST00000533018.1 PCF11 protein_coding
ENST00000533022.4 CLEC7A protein_coding
ENST00000533030.1 SPI1 protein_coding
ENST00000533072.1 TTC17 retained_intron
ENST00000533077.4 GRK2 retained_intron
ENST00000533079.4 CAPN1 retained_intron
ENST00000533096.4 COPB1 processed_transcript
ENST00000533157.4 FRG1 nonsense_mediated_decay
ENST00000533169.1 TBCEL retained_intron
ENST00000533180.1 INTS4 retained_intron
ENST00000533190.1 IRF7 nonsense_mediated_decay
ENST00000533206.1 PAFAH1B2 retained_intron
ENST00000533209.4 SSBP3 retained_intron
ENST00000533217.1 RHOG protein_coding
ENST00000533233.1 IPO7 nonsense_mediated_decay
ENST00000533237.4 EHBP1L1 protein_coding
ENST00000533239.1 DDX6 retained_intron
ENST00000533255.1 ARRB1 retained_intron
ENST00000533262.1 PDHX protein_coding
ENST00000533276.5 RAB30 protein_coding
ENST00000533279.1 GMDS processed_transcript
ENST00000533305.4 LMO7 processed_transcript
ENST00000533338.1 HOOK3 protein_coding
ENST00000533350.1 PICALM retained_intron
ENST00000533365.4 AHNAK protein_coding
ENST00000533370.1 TGFBR3 processed_transcript
ENST00000533371.4 TPP1 protein_coding
ENST00000533389.1 MICAL2 protein_coding
ENST00000533398.4 VAV3 processed_transcript
ENST00000533402.4 CSRP1 retained_intron
ENST00000533422.4 BCLAF1 nonsense_mediated_decay
ENST00000533423.4 AASDHPPT protein_coding
ENST00000533431.1 NA NA
ENST00000533445.1 STIM1 processed_transcript
ENST00000533453.4 DCBLD1 retained_intron
ENST00000533459.1 lncRNA
ENST00000533461.4 POGZ protein_coding
ENST00000533464.4 PHRF1 protein_coding
ENST00000533468.4 PAK1 retained_intron
ENST00000533509.1 ARHGAP32 retained_intron
ENST00000533528.1 RAB30-DT lncRNA
ENST00000533532.4 APLP2 retained_intron
ENST00000533551.4 PRKY transcribed_unitary_pseudogene
ENST00000533554.1 TTC17 processed_transcript
ENST00000533562.1 CAPRIN1 processed_transcript
ENST00000533571.1 MTCH2 processed_transcript
ENST00000533573.4 NPEPPS processed_transcript
ENST00000533584.1 SBF2 retained_intron
ENST00000533591.1 LINC02718 lncRNA
ENST00000533607.1 TESPA1 protein_coding
ENST00000533609.4 ARRB1 nonsense_mediated_decay
ENST00000533616.4 APLP2 nonsense_mediated_decay
ENST00000533621.1 BCLAF1 nonsense_mediated_decay
ENST00000533640.4 NPIPB6 protein_coding

ENST00000533645.1 PIK3C2A nonsense_mediated_decay
ENST00000533657.4 CAPRIN1 processed_transcript
ENST00000533659.1 lncRNA
ENST00000533661.1 SBF2 processed_transcript
ENST00000533690.4 ATM retained_intron
ENST00000533705.4 UBQLN1 retained_intron
ENST00000533723.1 TMEM41B protein_coding
ENST00000533737.4 DENND5A protein_coding
ENST00000533756.4 PACS1 protein_coding
ENST00000533770.4 SBF2 protein_coding
ENST00000533788.1 NPEPL1 nonsense_mediated_decay
ENST00000533796.1 TALDO1 retained_intron
ENST00000533825.1 OSBPL9 processed_transcript
ENST00000533835.4 VNN2 nonsense_mediated_decay
ENST00000533836.1 BTF3L4 processed_transcript
ENST00000533849.1 MS4A7 retained_intron
ENST00000533860.4 APLP2 retained_intron
ENST00000533865.4 CTSC retained_intron
ENST00000533866.1 SIGLEC14 retained_intron
ENST00000533875.1 processed_transcript
ENST00000533890.1 L3MBTL3 retained_intron
ENST00000533897.1 CTSC retained_intron
ENST00000533921.1 IMMP1L processed_transcript
ENST00000533928.1 USP33 retained_intron
ENST00000533968.1 SPI1 protein_coding
ENST00000533972.4 EMSY protein_coding
ENST00000533978.1 lncRNA
ENST00000533988.4 EMSY protein_coding
ENST00000534003.4 FNBP4 processed_transcript
ENST00000534010.1 SDHD nonsense_mediated_decay
ENST00000534048.1 EXT2 retained_intron
ENST00000534067.4 NOD2 nonsense_mediated_decay
ENST00000534080.4 SERHL2 retained_intron
ENST00000534082.1 CD44 retained_intron
ENST00000534091.1 LDLRAD3 processed_transcript
ENST00000534113.5 SECISBP2 protein_coding
ENST00000534130.1 BIRC2 retained_intron
ENST00000534143.4 CMTM1 nonsense_mediated_decay
ENST00000534157.1 OSBPL5 protein_coding
ENST00000534160.1 GMDS-DT lncRNA
ENST00000534164.4 PKD1P3 transcribed_unprocessed_pseudogene
ENST00000534168.1 lncRNA
ENST00000534197.4 GNPTG retained_intron
ENST00000534202.4 CR1 nonsense_mediated_decay
ENST00000534210.5 DNHD1 retained_intron
ENST00000534213.4 GTF2H1 processed_transcript
ENST00000534219.4 PTPRJ protein_coding
ENST00000534234.4 COPB1 protein_coding
ENST00000534246.1 EIF4G2 retained_intron
ENST00000534247.1 ZHX2 protein_coding
ENST00000534259.1 ASCC1 retained_intron
ENST00000534261.3 SIGLEC5 protein_coding
ENST00000534262.1 HIPK3 processed_transcript
ENST00000534279.1 ADAR retained_intron

ENST00000534281.1 RNF141 retained_intron
ENST00000534286.4 SORL1 protein_coding
ENST00000534291.1 lncRNA
ENST00000534310.4 MS4A7 retained_intron
ENST00000534317.1 PDE3B processed_transcript
ENST00000534319.4 HSPA8 protein_coding
ENST00000534335.1 IL10RA processed_transcript
ENST00000534336.1 MALAT1 lncRNA
ENST00000534347.4 TTC17 nonsense_mediated_decay
ENST00000534351.4 GNLY processed_transcript
ENST00000534358.4 KMT2A protein_coding
ENST00000534372.1 NAP1L4 protein_coding
ENST00000534375.1 PICALM retained_intron
ENST00000534389.2 CSDE1 protein_coding
ENST00000534399.4 NFYC protein_coding
ENST00000534412.4 PICALM nonsense_mediated_decay
ENST00000534417.1 TTC17 retained_intron
ENST00000534419.1 GANAB processed_transcript
ENST00000534439.4 DDX10 processed_transcript
ENST00000534444.1 JAK3 protein_coding
ENST00000534454.4 OSBPL5 protein_coding
ENST00000534463.4 PDE4B retained_intron
ENST00000534467.1 NA NA
ENST00000534468.1 GMDS-DT lncRNA
ENST00000534478.4 RCOR3 protein_coding
ENST00000534529.4 PCSK7 retained_intron
ENST00000534539.1 NSD3 protein_coding
ENST00000534555.4 RPS3 processed_transcript
ENST00000534577.1 LRRC4C processed_transcript
ENST00000534579.4 CTNND1 protein_coding
ENST00000534580.4 PDXDC2P processed_transcript
ENST00000534590.4 PSMD13 retained_intron
ENST00000534625.1 NA NA
ENST00000534643.4 ZDHHC18 protein_coding
ENST00000534647.1 retained_intron
ENST00000534662.1 SWAP70 protein_coding
ENST00000534700.4 PDXDC2P transcribed_unprocessed_pseudogene
ENST00000534706.1 ILK processed_transcript
ENST00000534727.1 NPEPPS retained_intron
ENST00000534733.5 ST3GAL4 nonsense_mediated_decay
ENST00000534743.1 MAP2K3 processed_transcript
ENST00000534748.5 IL32 retained_intron
ENST00000534754.4 SORL1 retained_intron
ENST00000534761.4 APLP2 processed_transcript
ENST00000534764.1 GRAMD1B protein_coding
ENST00000534766.1 PHF21A retained_intron
ENST00000534779.4 GANAB protein_coding
ENST00000534781.1 RPL8 retained_intron
ENST00000534794.1 RSF1 processed_transcript
ENST00000534823.1 GAB2 retained_intron
ENST00000534834.1 ZNF26 retained_intron
ENST00000534864.4 lncRNA
ENST00000534870.4 ADD1 retained_intron
ENST00000534891.4 lncRNA

ENST00000534903.1 NPIPB3 protein_coding
ENST00000534905.4 ATG16L2 protein_coding
ENST00000534923.1 PRH1 processed_transcript
ENST00000534927.4 NLRC5 retained_intron
ENST00000534930.1 TMBIM4 nonsense_mediated_decay
ENST00000534931.1 NLRC5 retained_intron
ENST00000534948.2 ZNF518A retained_intron
ENST00000534952.1 PID1 nonsense_mediated_decay
ENST00000534966.1 SPPL3 retained_intron
ENST00000534978.1 EEPD1 protein_coding
ENST00000535009.4 TPCN2 retained_intron
ENST00000535021.4 LPCAT3 processed_transcript
ENST00000535064.1 TIAM2 processed_transcript
ENST00000535132.1 ATF7IP protein_coding
ENST00000535146.1 CAND1 retained_intron
ENST00000535164.4 VPS35L retained_intron
ENST00000535179.1 ATF7IP retained_intron
ENST00000535191.4 NEK4 protein_coding
ENST00000535193.2 NA NA
ENST00000535202.1 SFSWAP retained_intron
ENST00000535206.4 B4GALT1 protein_coding
ENST00000535212.1 CCDC91 protein_coding
ENST00000535230.4 PZP nonsense_mediated_decay
ENST00000535272.1 SLC15A4 processed_transcript
ENST00000535290.4 CLIP1 protein_coding
ENST00000535292.1 CRACR2A protein_coding
ENST00000535324.1 lncRNA
ENST00000535335.1 KLF11 protein_coding
ENST00000535388.2 ANGEL2 protein_coding
ENST00000535391.4 EIF3J protein_coding
ENST00000535399.1 SFSWAP retained_intron
ENST00000535413.1 MLEC processed_transcript
ENST00000535432.2 E2F3 protein_coding
ENST00000535459.4 SOD2 retained_intron
ENST00000535489.4 ZNF426 protein_coding
ENST00000535493.4 HDAC4 retained_intron
ENST00000535507.1 CRACR2A processed_transcript
ENST00000535549.4 ETS1 protein_coding
ENST00000535555.1 C2CD5 protein_coding
ENST00000535559.4 ZNF423 protein_coding
ENST00000535560.1 RHOF protein_coding
ENST00000535561.4 SOD2 protein_coding
ENST00000535570.4 TAOK3 protein_coding
ENST00000535572.4 WNK1 protein_coding
ENST00000535589.3 ACTR3 protein_coding
ENST00000535598.1 IPO8 protein_coding
ENST00000535606.1 RESF1 processed_transcript
ENST00000535610.4 CMAS nonsense_mediated_decay
ENST00000535614.4 LINC01089 lncRNA
ENST00000535621.5 PDXDC1 protein_coding
ENST00000535638.4 WBP11 nonsense_mediated_decay
ENST00000535649.1 NOL8 retained_intron
ENST00000535663.1 DOCK10 protein_coding
ENST00000535677.4 EPSTI1 processed_transcript

ENST00000535698.1 WNK1 protein_coding
ENST00000535721.1 GRIP1 processed_transcript
ENST00000535738.1 ATF7IP protein_coding
ENST00000535752.4 EPS8 protein_coding
ENST00000535765.4 VPS37B protein_coding
ENST00000535784.5 PTER protein_coding
ENST00000535786.4 NA NA
ENST00000535810.1 NA NA
ENST00000535812.1 TMBIM4 protein_coding
ENST00000535814.4 TEX10 protein_coding
ENST00000535818.1 VPS37C retained_intron
ENST00000535836.1 C10orf53 protein_coding
ENST00000535838.4 NUMA1 protein_coding
ENST00000535928.4 P2RX7 nonsense_mediated_decay
ENST00000535943.1 INTS4 protein_coding
ENST00000535947.4 NUMA1 protein_coding
ENST00000535982.1 CELF1 protein_coding
ENST00000536014.4 KDM5A protein_coding
ENST00000536028.2 ZKSCAN8 nonsense_mediated_decay
ENST00000536042.1 EPSTI1 processed_transcript
ENST00000536078.1 ADD1 retained_intron
ENST00000536133.2 STEEP1 protein_coding
ENST00000536163.4 DENND11 protein_coding
ENST00000536168.2 NA NA
ENST00000536189.5 TIMP2 protein_coding
ENST00000536196.1 CHFR processed_transcript
ENST00000536197.4 WASHC3 retained_intron
ENST00000536202.1 TAOK1 protein_coding
ENST00000536224.2 GCH1 protein_coding
ENST00000536226.1 CRNKL1 protein_coding
ENST00000536231.1 NLRC5 retained_intron
ENST00000536236.1 DUSP16 processed_transcript
ENST00000536271.4 NXPE1 retained_intron
ENST00000536272.4 KIAA1958 protein_coding
ENST00000536295.1 RTN3P1 processed_pseudogene
ENST00000536355.4 CLEC2D nonsense_mediated_decay
ENST00000536368.1 RPL12 protein_coding
ENST00000536413.2 MAP2K4 retained_intron
ENST00000536420.4 ACVR1B protein_coding
ENST00000536441.4 SESN3 protein_coding
ENST00000536444.4 ATF7IP protein_coding
ENST00000536495.1 lncRNA
ENST00000536521.4 PTPN6 retained_intron
ENST00000536537.1 CCND2 processed_transcript
ENST00000536539.1 LINC00937 lncRNA
ENST00000536549.4 ASB8 protein_coding
ENST00000536569.1 MRPS35 nonsense_mediated_decay
ENST00000536573.5 ERC1 processed_transcript
ENST00000536584.1 TAOK3 retained_intron
ENST00000536590.1 CD4 processed_transcript
ENST00000536592.4 ARHGDIB protein_coding
ENST00000536657.1 WASF2 protein_coding
ENST00000536661.1 UBC processed_transcript
ENST00000536662.4 LINC01089 lncRNA

ENST00000536670.4 retained_intron
ENST00000536684.2 MTRNR2L8 protein_coding
ENST00000536702.1 CUL3 processed_transcript
ENST00000536703.4 GLIPR1 nonsense_mediated_decay
ENST00000536709.1 CD69 protein_coding
ENST00000536711.4 MED21 nonsense_mediated_decay
ENST00000536717.4 TNFRSF1A retained_intron
ENST00000536782.1 TNFRSF1B protein_coding
ENST00000536795.1 CCND2 retained_intron
ENST00000536821.4 TMED3 protein_coding
ENST00000536836.1 SINHCAF processed_transcript
ENST00000536914.1 IFNG-AS1 lncRNA
ENST00000536957.4 PXN protein_coding
ENST00000536985.4 PFKFB3 protein_coding
ENST00000537019.4 lncRNA
ENST00000537030.3 PPP2R5A protein_coding
ENST00000537056.4 NLRC5 nonsense_mediated_decay
ENST00000537066.1 GLTP nonsense_mediated_decay
ENST00000537093.4 CEP57 protein_coding
ENST00000537098.6 CMIP protein_coding
ENST00000537111.5 LTA4H retained_intron
ENST00000537114.2 ZADH2 protein_coding
ENST00000537124.4 QKI protein_coding
ENST00000537143.4 ATG16L2 retained_intron
ENST00000537157.4 LINC01089 lncRNA
ENST00000537164.1 SFSWAP protein_coding
ENST00000537169.1 ARMCX3 protein_coding
ENST00000537175.1 SUPT16HP1 processed_pseudogene
ENST00000537184.5 ITFG1 processed_transcript
ENST00000537196.1 ELP1 protein_coding
ENST00000537226.2 ZNF891 protein_coding
ENST00000537265.4 RHOF protein_coding
ENST00000537285.1 C2CD3 processed_transcript
ENST00000537288.1 A2ML1-AS1 lncRNA
ENST00000537317.1 GOLGA3 protein_coding
ENST00000537329.1 CEMIP2 retained_intron
ENST00000537336.1 INTS13 protein_coding
ENST00000537350.4 RCSD1 protein_coding
ENST00000537386.1 CARS2 retained_intron
ENST00000537387.2 TLE3 nonsense_mediated_decay
ENST00000537403.4 SEC23A protein_coding
ENST00000537413.4 SIPA1L1 protein_coding
ENST00000537416.1 NINJ2 processed_transcript
ENST00000537446.4 RAB6A nonsense_mediated_decay
ENST00000537452.2 NA NA
ENST00000537468.1 GLT1D1 protein_coding
ENST00000537473.2 PTGES3 processed_transcript
ENST00000537480.1 SESN3 processed_transcript
ENST00000537495.2 protein_coding
ENST00000537502.4 ACSS1 protein_coding
ENST00000537505.4 RABEP1 protein_coding
ENST00000537533.1 PTPN6 processed_transcript
ENST00000537545.1 ADIPOR2 processed_transcript
ENST00000537556.1 SRGAP1 retained_intron

ENST00000537570.1 processed_pseudogene
ENST00000537616.1 lncRNA
ENST00000537626.4 CD163 protein_coding
ENST00000537634.1 CHD4 retained_intron
ENST00000537645.4 ZNF460 protein_coding
ENST00000537657.4 SOD2 protein_coding
ENST00000537659.4 LINC00937 lncRNA
ENST00000537662.1 NUP107 retained_intron
ENST00000537668.1 transcribed_unprocessed_pseudogene
ENST00000537698.1 CLEC4E protein_coding
ENST00000537710.4 ITFG2 processed_transcript
ENST00000537743.1 CARS2 protein_coding
ENST00000537756.2 CPT1A processed_transcript
ENST00000537764.1 processed_transcript
ENST00000537784.4 CACNA2D4 nonsense_mediated_decay
ENST00000537796.1 NECAP1 retained_intron
ENST00000537821.2 lncRNA
ENST00000537822.1 TAOK3 protein_coding
ENST00000537823.1 GNS retained_intron
ENST00000537824.2 NA NA
ENST00000537828.4 EPSTI1 protein_coding
ENST00000537874.1 PIWIL4-AS1 lncRNA
ENST00000537942.4 NA NA
ENST00000537952.1 TAOK3 protein_coding
ENST00000537997.1 RNF10 protein_coding
ENST00000538004.4 NEK7 protein_coding
ENST00000538021.4 CLPB nonsense_mediated_decay
ENST00000538028.1 AAGAB processed_transcript
ENST00000538030.2 MAK nonsense_mediated_decay
ENST00000538059.4 NLRC5 protein_coding
ENST00000538065.4 NA NA
ENST00000538095.1 CEP57 nonsense_mediated_decay
ENST00000538098.2 WDR74 protein_coding
ENST00000538104.4 IST1 protein_coding
ENST00000538134.1 RBM7 retained_intron
ENST00000538144.4 PXN processed_transcript
ENST00000538167.2 EFHC1 protein_coding
ENST00000538173.1 EIF2S3B protein_coding
ENST00000538183.5 SOD2 protein_coding
ENST00000538186.4 MED21 processed_transcript
ENST00000538204.4 MBNL3 protein_coding
ENST00000538209.1 ZW10 nonsense_mediated_decay
ENST00000538217.1 TMBIM4 miRNA
ENST00000538218.1 ETNK1 miRNA
ENST00000538219.1 LINC00987 miRNA
ENST00000538223.1 ANAPC5 miRNA
ENST00000538243.1 KDM2B miRNA
ENST00000538273.4 NLRC5 miRNA
ENST00000538274.4 KIAA0513 miRNA
ENST00000538290.2 SLAMF1 miRNA
ENST00000538304.1 LINC00937 miRNA
ENST00000538307.1 SH2B3 miRNA
ENST00000538316.1 FAM76B miRNA
ENST00000538332.2 PRH1 miRNA

ENST00000538340.4 ACSF3 miRNA
ENST00000538351.5 ZNF438 miRNA
ENST00000538363.1 TNFRSF1A miRNA
ENST00000538372.5 NDUFA12 miRNA
ENST00000538422.2 LRCH2 miRNA
ENST00000538423.4 BCKDHA miRNA
ENST00000538439.1 NPIPB3 miRNA
ENST00000538451.1 LDHA miRNA
ENST00000538456.3 SMARCA4 miRNA
ENST00000538502.4 SORT1 miRNA
ENST00000538571.5 KIAA0586 miRNA
ENST00000538590.1 NPIPB5 miRNA
ENST00000538591.4 ANKLE2 miRNA
ENST00000538596.5 CPNE8 miRNA
ENST00000538601.1 TAOK3 miRNA
ENST00000538610.4 PSMA5 miRNA
ENST00000538611.1 CD6 miRNA
ENST00000538635.4 VWF miRNA
ENST00000538669.1 CEMIP2 miRNA
ENST00000538698.4 MLXIP miRNA
ENST00000538739.1 SLC15A3 miRNA
ENST00000538765.2 ZNF263 miRNA
ENST00000538778.4 NLRC5 miRNA
ENST00000538784.2 NOC4L miRNA
ENST00000538786.1 ATL3 miRNA
ENST00000538787.1 NA miRNA
ENST00000538822.1 ITFG2 miRNA
ENST00000538842.1 ATG16L2 miRNA
ENST00000538857.1 MGST1 miRNA
ENST00000538860.1 ADD1 miRNA
ENST00000538864.5 HEATR5A miRNA
ENST00000538899.2 RNGTT miRNA
ENST00000538910.1 LPCAT3 miRNA
ENST00000538941.5 SCAPER miRNA
ENST00000538946.4 NA miRNA
ENST00000538984.1 ITPR2 miRNA
ENST00000538994.1 CPT1A miRNA
ENST00000538997.1 KLRD1 miRNA
ENST00000538998.1 PBX3 miRNA
ENST00000539007.4 ERC1 miRNA
ENST00000539028.1 CLEC2B miRNA
ENST00000539044.4 GLT1D1 miRNA
ENST00000539047.1 VAMP1 miRNA
ENST00000539054.2 NA miRNA
ENST00000539065.4 MARCHF7 miRNA
ENST00000539066.4 POLR3B miRNA
ENST00000539068.4 FKBP5 miRNA
ENST00000539080.1 CLIP1 miRNA
ENST00000539108.4 ADD1 miRNA
ENST00000539131.1 ARHGDIB miRNA
ENST00000539134.1 RELT miRNA
ENST00000539149.1 ADD1 miRNA
ENST00000539158.1 MED23 miRNA
ENST00000539167.2 MYRIP miRNA

ENST00000539191.1 NPIPB4 miRNA
ENST00000539197.4 CCDC7 miRNA
ENST00000539225.1 TJP2 miRNA
ENST00000539240.4 KLRG1 miRNA
ENST00000539254.1 CELF1 miRNA
ENST00000539269.4 CARS2 miRNA
ENST00000539284.2 VAV1 miRNA
ENST00000539289.4 GABARAPL1 miRNA
ENST00000539317.2 SMAP2 miRNA
ENST00000539330.1 UCP2 miRNA
ENST00000539332.1 DDB1 miRNA
ENST00000539345.5 TBCD miRNA
ENST00000539368.2 RNF115 miRNA
ENST00000539370.4 KLRC4-KLRK1 miRNA
ENST00000539374.1 KLRD1 miRNA
ENST00000539380.1 CAMKK2 miRNA
ENST00000539381.4 SLC8A2 miRNA
ENST00000539394.4 KDM2B miRNA
ENST00000539399.1 TM7SF3 miRNA
ENST00000539404.1 LINC02384 miRNA
ENST00000539463.1 DENR miRNA
ENST00000539479.4 MDM2 miRNA
ENST00000539492.1 CD4 miRNA
ENST00000539493.2 RAB3GAP1 miRNA
ENST00000539506.4 SFSWAP miRNA
ENST00000539550.4 NA miRNA
ENST00000539594.4 ESRRA miRNA
ENST00000539605.4 ANKLE2 miRNA
ENST00000539613.4 RHBDD1 miRNA
ENST00000539615.1 C2CD5 miRNA
ENST00000539666.4 ZNF518A miRNA
ENST00000539672.1 RECQL miRNA
ENST00000539677.1 CPPED1 miRNA
ENST00000539683.2 KAT8 miRNA
ENST00000539687.4 SPARC miRNA
ENST00000539695.4 P2RX7 miRNA
ENST00000539697.4 BROX miRNA
ENST00000539703.1 SLC15A4 miRNA
ENST00000539722.4 PRKCQ miRNA
ENST00000539743.4 CPT1A miRNA
ENST00000539745.1 H4-16 miRNA
ENST00000539764.1 UCP2 miRNA
ENST00000539778.5 CMIP miRNA
ENST00000539802.2 ERC1 miRNA
ENST00000539810.1 TBK1 miRNA
ENST00000539815.4 NA miRNA
ENST00000539869.2 PDE10A miRNA
ENST00000539872.4 TAOK3 miRNA
ENST00000539881.4 NLRC5 miRNA
ENST00000539904.1 CCDC91 miRNA
ENST00000539974.1 ETNK1 miRNA
ENST00000539977.1 PABPC1P4 miRNA
ENST00000540033.2 EVI5 miRNA
ENST00000540040.2 MTRNR2L1 miRNA

ENST00000540091.1 SPPL3 miRNA
ENST00000540097.1 ERP27 protein_coding
ENST00000540103.1 NPIPB4 processed_transcript
ENST00000540168.1 KDM5A trna
ENST00000540196.4 GNS trna
ENST00000540224.1 CREBL2 trna
ENST00000540264.2 NFE2 trna
ENST00000540267.4 KLRK1 trna
ENST00000540360.4 WNK1 trna
ENST00000540367.4 CPT1A trna
ENST00000540429.1 ITPR2 trna
ENST00000540469.4 SFSWAP trna
ENST00000540475.4 SEMA4D trna
ENST00000540494.4 PDIA6 trna
ENST00000540499.2 IDH2 trna
ENST00000540522.4 FAM157B trna
ENST00000540525.1 CAND1 trna
ENST00000540534.4 STMN3 trna
ENST00000540541.1 ADD1 trna
ENST00000540557.1 trna
ENST00000540572.1 PLBD1 trna
ENST00000540586.1 ZCCHC8 trna
ENST00000540589.2 OAS1 trna
ENST00000540608.4 NPC1 trna
ENST00000540641.1 trna
ENST00000540663.4 SYNE1 trna
ENST00000540703.1 C2CD5 trna
ENST00000540714.1 KIAA2026 trna
ENST00000540717.1 DAGLA trna
ENST00000540719.1 QKI trna
ENST00000540750.4 NA trna
ENST00000540791.1 ITPR2 trna
ENST00000540793.4 ATF7IP trna
ENST00000540798.4 RTN3 trna
ENST00000540801.4 SSH2 trna
ENST00000540803.1 RAB35 trna
ENST00000540815.2 LGR5 trna
ENST00000540847.5 CEP192 trna
ENST00000540874.4 NA trna
ENST00000540885.1 WNK1 trna
ENST00000540898.1 FOXO3 trna
ENST00000540918.2 TTC39C trna
ENST00000540921.4 ZMAT1 trna
ENST00000540930.4 P2RX4 trna
ENST00000540957.1 FERMT3 trna
ENST00000540960.1 CHD4 trna
ENST00000540963.1 CHFR trna
ENST00000540964.4 TLR5 trna
ENST00000540972.4 PLEKHA5 trna
ENST00000540974.1 ADIPOR2 trna
ENST00000540998.4 CDC42SE1 trna
ENST00000541036.4 TIAM1 trna
ENST00000541051.4 ADD1 trna
ENST00000541082.2 FAM110A trna

ENST00000541108.1 CLIP1 trna
ENST00000541118.1 GNA13 trna
ENST00000541160.4 ZNF826P trna
ENST00000541171.1 LEMD3 trna
ENST00000541175.1 PRH1 trna
ENST00000541182.1 ARF1 trna
ENST00000541190.4 ELP2 trna
ENST00000541196.2 HCP5 trna
ENST00000541233.1 CD27 trna
ENST00000541244.1 PITPNM2 trna
ENST00000541252.1 FERMT3 trna
ENST00000541272.1 UBC trna
ENST00000541292.4 PEX7 trna
ENST00000541294.1 ELP2 trna
ENST00000541310.4 C2CD5 trna
ENST00000541352.4 DICER1 trna
ENST00000541366.1 COPA trna
ENST00000541372.1 SLC3A2 trna
ENST00000541386.4 RAP1B trna
ENST00000541410.1 CLIP1 trna
ENST00000541428.3 ASPH trna
ENST00000541456.1 PRH1 trna
ENST00000541458.4 ZNF101 trna
ENST00000541463.5 KIF21A trna
ENST00000541492.1 SPECC1L trna
ENST00000541503.1 ERC1 trna
ENST00000541517.4 TPCN1 trna
ENST00000541548.4 CUL3 trna
ENST00000541549.2 EIF3A trna
ENST00000541573.4 SOD2 trna
ENST00000541602.4 ZBTB16 trna
ENST00000541618.1 PLBD1 trna
ENST00000541623.4 ARAP1 trna
ENST00000541671.1 SLC2A3 trna
ENST00000541674.4 NPIPB4 trna
ENST00000541696.1 QKI trna
ENST00000541713.4 PTPN1 trna
ENST00000541715.4 IFNG-AS1 trna
ENST00000541777.5 ZNF83 trna
ENST00000541790.2 H2BC8 trna
ENST00000541795.1 RAB6A trna
ENST00000541800.5 PLBD1 trna
ENST00000541810.4 NPIPA8 trna
ENST00000541868.1 ST8SIA1 trna
ENST00000541972.4 CD163 trna
ENST00000541973.4 RAB6A trna
ENST00000541981.4 RESF1 trna
ENST00000542072.1 TMBIM4 trna
ENST00000542075.4 SLC38A10 trna
ENST00000542104.4 RASSF3 trna
ENST00000542112.4 SNHG1 trna
ENST00000542128.4 NA trna
ENST00000542187.1 PPFIBP1 trna
ENST00000542194.1 GOLT1B trna

ENST00000542215.4 DDX3X trna
ENST00000542219.1 LINC02421 trna
ENST00000542254.4 CD6 trna
ENST00000542276.1 ARHGDIB trna
ENST00000542424.1 WNK1 trna
ENST00000542440.4 SETD1B trna
ENST00000542451.1 SLFN5 trna
ENST00000542452.1 C2CD3 trna
ENST00000542481.1 ATG16L2 trna
ENST00000542488.4 TSG101 trna
ENST00000542546.4 SLC2A14 trna
ENST00000542565.1 NCOR2 trna
ENST00000542575.5 SLC1A5 trna
ENST00000542579.4 CELF2 trna
ENST00000542596.4 ARAP1 trna
ENST00000542598.4 trna
ENST00000542612.1 ARAP1 trna
ENST00000542651.2 RASA3 trna
ENST00000542652.5 EPB41L3 trna
ENST00000542666.4 CDK17 trna
ENST00000542675.1 PDE3A trna
ENST00000542683.1 C2CD5 trna
ENST00000542685.4 XRCC4 trna
ENST00000542691.4 ITFG1 trna
ENST00000542692.1 TAOK3 trna
ENST00000542701.1 RNF10 trna
ENST00000542709.4 CARS2 trna
ENST00000542713.1 FKBP5 trna
ENST00000542720.1 LATS1 trna
ENST00000542747.4 LATS1 trna
ENST00000542749.4 NA trna
ENST00000542761.4 PTPN6 trna
ENST00000542801.4 CCDC91 trna
ENST00000542807.1 RAP1GAP2 trna
ENST00000542814.4 CCDC91 trna
ENST00000542818.1 ZNF516 trna
ENST00000542845.2 LIMS1 trna
ENST00000542850.2 NA trna
ENST00000542866.2 KDM3B trna
ENST00000542876.1 PPP1CA trna
ENST00000542921.4 JMJD1C trna
ENST00000542935.4 CEMIP2 trna
ENST00000542952.4 MAP3K4 trna
ENST00000542953.1 NPIPB3 trna
ENST00000542973.4 KDM2B trna
ENST00000542975.1 FNBP4 trna
ENST00000542977.4 NUMA1 trna
ENST00000542991.1 ATF7IP trna
ENST00000543030.4 NLRC5 trna
ENST00000543038.1 ADA2 trna
ENST00000543049.1 NLRC5 trna
ENST00000543087.4 TSG101 trna
ENST00000543093.1 CSF1R trna
ENST00000543123.1 RTN3 trna

ENST00000543147.1 CD69 trna
ENST00000543151.1 ERC1 trna
ENST00000543152.4 VPS35L trna
ENST00000543165.1 CEMIP2 trna
ENST00000543181.4 SPPL3 trna
ENST00000543185.4 HDAC4 trna
ENST00000543220.4 MARK2 trna
ENST00000543225.4 CCAR1 trna
ENST00000543233.2 CDH4 trna
ENST00000543263.2 ERC1 trna
ENST00000543271.4 ZNF10 trna
ENST00000543278.1 FBXL14 trna
ENST00000543283.2 CCSER2 trna
ENST00000543316.1 WBP11 trna
ENST00000543360.1 FBRSL1 trna
ENST00000543363.4 EPS8 trna
ENST00000543368.1 NPIPB3 trna
ENST00000543374.1 MRPL48 trna
ENST00000543390.2 NA trna
ENST00000543392.4 NOMO2 trna
ENST00000543402.1 NLRC5 trna
ENST00000543406.1 SLC15A3 trna
ENST00000543407.4 NPIPB5 trna
ENST00000543422.4 MACROD1 trna
ENST00000543436.2 A2M trna
ENST00000543449.1 NPIPB4 trna
ENST00000543464.5 CPD trna
ENST00000543473.1 SUDS3 trna
ENST00000543476.4 PYROXD1 trna
ENST00000543482.4 MCTP2 trna
ENST00000543500.2 MTRNR2L3 trna
ENST00000543507.1 KDM5A trna
ENST00000543534.4 CCDC91 trna
ENST00000543571.4 LATS1 trna
ENST00000543578.1 RNF10 trna
ENST00000543628.4 NA trna
ENST00000543632.4 MYO3A trna
ENST00000543644.1 FCHSD2 trna
ENST00000543646.4 GNS trna
ENST00000543674.1 MARK2 trna
ENST00000543677.1 OASL trna
ENST00000543680.4 SOS2 trna
ENST00000543709.4 TAOK3 trna
ENST00000543710.4 VPS13D trna
ENST00000543715.1 DOCK10 trna
ENST00000543742.1 AOAH trna
ENST00000543744.1 PTPN6 trna
ENST00000543763.1 RESF1 trna
ENST00000543766.2 VPS13D trna
ENST00000543776.1 SPTY2D1 trna
ENST00000543780.4 NA trna
ENST00000543801.4 NPIPA5 trna
ENST00000543805.4 DHRS4L2 trna
ENST00000543815.4 AKAP7 trna

ENST00000543839.1 CLEC12A trna
ENST00000543854.4 SPPL3 trna
ENST00000543855.4 C2CD5 trna
ENST00000543879.5 AQR trna
ENST00000543895.1 KLRG1 trna
ENST00000543926.5 ZNF236 trna
ENST00000543969.1 trna
ENST00000543976.2 NA trna
ENST00000543990.1 BORCS5 trna
ENST00000544014.1 CD5 trna
ENST00000544039.4 ABCC9 trna
ENST00000544041.4 MAP3K4 trna
ENST00000544079.2 CCT4 trna
ENST00000544085.4 AFF1 protein_coding
ENST00000544111.4 FGFR1OP2 protein_coding
ENST00000544151.1 LDHB retained_intron
ENST00000544180.5 PYGL protein_coding
ENST00000544189.1 NLRC5 retained_intron
ENST00000544191.1 ETNK1 retained_intron
ENST00000544193.1 M6PR protein_coding
ENST00000544199.4 NA NA
ENST00000544277.1 ERC1 processed_transcript
ENST00000544278.2 SNRNP70 retained_intron
ENST00000544291.1 SLC2A3 protein_coding
ENST00000544292.4 PARD3 protein_coding
ENST00000544305.4 EPHB2 protein_coding
ENST00000544322.1 CLEC2D protein_coding
ENST00000544382.4 CLPB processed_transcript
ENST00000544392.4 CACUL1 processed_transcript
ENST00000544405.5 RBL2 protein_coding
ENST00000544436.4 QKI protein_coding
ENST00000544440.5 MYCBP2 protein_coding
ENST00000544461.1 LINC00937 lncRNA
ENST00000544464.4 NA NA
ENST00000544481.1 UBC processed_transcript
ENST00000544484.4 CHD4 protein_coding
ENST00000544485.1 CAMKK2 protein_coding
ENST00000544491.2 BPTF protein_coding
ENST00000544500.4 METTL3 retained_intron
ENST00000544513.1 SEMA4D retained_intron
ENST00000544545.2 NA NA
ENST00000544576.1 MROH1 processed_transcript
ENST00000544594.4 LAMTOR1 protein_coding
ENST00000544615.4 UCP2 retained_intron
ENST00000544619.1 CAND1 protein_coding
ENST00000544622.4 YBX3 protein_coding
ENST00000544642.2 NA NA
ENST00000544681.1 C12orf57 protein_coding
ENST00000544693.4 NPIPB4 protein_coding
ENST00000544698.1 DENND5B nonsense_mediated_decay
ENST00000544715.1 ETV6 processed_transcript
ENST00000544760.1 KDM5A protein_coding
ENST00000544778.5 BPTF protein_coding
ENST00000544780.4 CCDC91 processed_transcript

ENST00000544786.1 MAPK1 protein_coding
ENST00000544802.4 GTF2IP4 processed_transcript
ENST00000544824.2 MTRNR2L7 protein_coding
ENST00000544829.4 IPO8 protein_coding
ENST00000544868.2 MALAT1 lncRNA
ENST00000544897.4 PPP1R37 processed_transcript
ENST00000544920.2 RPL41P2 processed_pseudogene
ENST00000544932.1 NA NA
ENST00000544936.1 SLC2A3 nonsense_mediated_decay
ENST00000544939.1 lncRNA
ENST00000544944.5 CLASRP protein_coding
ENST00000544989.1 NA NA
ENST00000544991.6 NUMB protein_coding
ENST00000545000.1 CWC27 processed_transcript
ENST00000545006.1 ZNF506 protein_coding
ENST00000545027.1 ETV6 protein_coding
ENST00000545031.4 SLC15A4 retained_intron
ENST00000545037.1 CCDC92 protein_coding
ENST00000545046.4 CHFR retained_intron
ENST00000545055.1 WNK1 retained_intron
ENST00000545068.4 FOXJ3 protein_coding
ENST00000545075.2 MTRNR2L10 protein_coding
ENST00000545077.1 IPO8 protein_coding
ENST00000545101.1 ZNF26 processed_transcript
ENST00000545132.4 ZYG11B protein_coding
ENST00000545134.1 HELB nonsense_mediated_decay
ENST00000545156.4 WIZ protein_coding
ENST00000545196.1 KLRF1 nonsense_mediated_decay
ENST00000545232.5 UHRF1BP1L protein_coding
ENST00000545237.1 PIAS1 protein_coding
ENST00000545243.1 RIC1 nonsense_mediated_decay
ENST00000545249.4 LAMTOR1 protein_coding
ENST00000545250.4 TSC1 protein_coding
ENST00000545273.1 GNS protein_coding
ENST00000545284.1 COPA retained_intron
ENST00000545312.1 PLXNC1 protein_coding
ENST00000545320.1 CD6 processed_transcript
ENST00000545335.4 PIP4K2A protein_coding
ENST00000545344.4 TM7SF3 protein_coding
ENST00000545346.1 QKI nonsense_mediated_decay
ENST00000545349.1 NLRC5 nonsense_mediated_decay
ENST00000545354.1 UBE4A protein_coding
ENST00000545369.2 IFNGR2 nonsense_mediated_decay
ENST00000545424.4 PLBD1-AS1 lncRNA
ENST00000545433.2 NKAIN2 protein_coding
ENST00000545518.1 IST1 retained_intron
ENST00000545538.4 CAMKK2 protein_coding
ENST00000545606.4 CAND1 protein_coding
ENST00000545609.2 PLEKHA8P1 processed_transcript
ENST00000545614.1 nonsense_mediated_decay
ENST00000545616.2 RNF24 protein_coding
ENST00000545638.2 NCKAP1L protein_coding
ENST00000545641.4 RIC1 protein_coding
ENST00000545650.4 NA NA

ENST00000545673.4 PIAS2 protein_coding
ENST00000545687.4 RELT protein_coding
ENST00000545694.1 SYNE1 retained_intron
ENST00000545698.1 TDG retained_intron
ENST00000545718.2 NA NA
ENST00000545719.1 CEMIP2 protein_coding
ENST00000545723.1 ATF7IP protein_coding
ENST00000545781.2 HHAT protein_coding
ENST00000545822.2 HADHB protein_coding
ENST00000545829.1 PRB2 processed_transcript
ENST00000545837.1 IRAK3 protein_coding
ENST00000545851.4 ZBTB16 processed_transcript
ENST00000545864.1 DUSP16 processed_transcript
ENST00000545885.4 LINC01089 lncRNA
ENST00000545889.4 CLIP1 protein_coding
ENST00000545895.4 ARHGDIB protein_coding
ENST00000545919.1 PHACTR2 retained_intron
ENST00000545920.1 SNHG1 lncRNA
ENST00000545942.4 CHD4 protein_coding
ENST00000545951.4 FAM193A protein_coding
ENST00000545981.4 CENPJ nonsense_mediated_decay
ENST00000546010.5 STAT5A protein_coding
ENST00000546038.1 PDE2A nonsense_mediated_decay
ENST00000546084.4 TRAF1 protein_coding
ENST00000546087.4 SOD2 protein_coding
ENST00000546097.4 STXBP5 protein_coding
ENST00000546102.1 CUL3 processed_transcript
ENST00000546120.2 NA NA
ENST00000546160.4 NA NA
ENST00000546168.4 NPIPB5 processed_transcript
ENST00000546181.1 NECAP1 retained_intron
ENST00000546190.4 ADGRB3 protein_coding
ENST00000546206.5 ARL6IP1 protein_coding
ENST00000546241.1 C3AR1 protein_coding
ENST00000546265.1 PRH1 processed_transcript
ENST00000546283.4 NDUFS7 protein_coding
ENST00000546305.5 FAM214A protein_coding
ENST00000546309.5 KDM1B protein_coding
ENST00000546311.4 EPS8 protein_coding
ENST00000546317.1 PRH1 processed_transcript
ENST00000546339.1 lncRNA
ENST00000546352.1 SLC38A10 processed_transcript
ENST00000546394.1 MAPKAPK5 processed_transcript
ENST00000546410.1 NACA retained_intron
ENST00000546415.1 NELL2 retained_intron
ENST00000546420.4 BICDL1 nonsense_mediated_decay
ENST00000546421.1 TMPO-AS1 lncRNA
ENST00000546433.4 SSH1 nonsense_mediated_decay
ENST00000546450.1 TBC1D15 retained_intron
ENST00000546455.4 PCED1B protein_coding
ENST00000546476.1 ANKRD13A retained_intron
ENST00000546477.1 ERP29 protein_coding
ENST00000546483.1 ATXN2 retained_intron
ENST00000546490.1 CHPT1 retained_intron

ENST00000546491.1 APAF1 processed_transcript
ENST00000546519.1 SLC38A1 processed_transcript
ENST00000546525.4 WDR27 processed_transcript
ENST00000546556.1 CPM nonsense_mediated_decay
ENST00000546566.4 lncRNA
ENST00000546584.1 TGFBR1 protein_coding
ENST00000546630.1 MYL6 retained_intron
ENST00000546678.1 lncRNA
ENST00000546679.1 GPR132 processed_transcript
ENST00000546685.4 POU6F1 processed_transcript
ENST00000546689.1 CHST11 protein_coding
ENST00000546694.1 USP15 protein_coding
ENST00000546695.4 BAZ2A protein_coding
ENST00000546705.4 CORO1C protein_coding
ENST00000546718.4 USP15 retained_intron
ENST00000546719.1 DIP2B retained_intron
ENST00000546732.1 DIP2B nonsense_mediated_decay
ENST00000546733.1 PLXNC1 retained_intron
ENST00000546740.4 POC1B processed_transcript
ENST00000546780.4 IRAK4 retained_intron
ENST00000546788.1 NDUFA12 nonsense_mediated_decay
ENST00000546806.1 TPK1 processed_transcript
ENST00000546822.1 TFCP2 retained_intron
ENST00000546830.1 POC1B nonsense_mediated_decay
ENST00000546840.2 protein_coding
ENST00000546873.1 CHPT1 nonsense_mediated_decay
ENST00000546885.4 TMBIM6 retained_intron
ENST00000546893.4 SLC38A1 protein_coding
ENST00000546904.1 PPP1CC retained_intron
ENST00000546915.1 PACS2 processed_transcript
ENST00000546916.1 OS9 retained_intron
ENST00000546925.1 NUDT4 protein_coding
ENST00000546942.1 SRSF9 retained_intron
ENST00000546946.4 OSBPL8 protein_coding
ENST00000546972.4 YAF2 retained_intron
ENST00000547018.4 SCAF11 non_stop_decay
ENST00000547019.1 R3HDM2 retained_intron
ENST00000547034.4 TMTC3 nonsense_mediated_decay
ENST00000547043.1 BRAP retained_intron
ENST00000547048.4 PCBP2 retained_intron
ENST00000547116.4 FAR2 protein_coding
ENST00000547149.4 CNOT2 processed_transcript
ENST00000547169.4 CTDSP2 retained_intron
ENST00000547170.1 CORO1C retained_intron
ENST00000547172.1 NELL2 processed_transcript
ENST00000547235.4 NPIPB12 nonsense_mediated_decay
ENST00000547249.1 ELK3 protein_coding
ENST00000547252.4 SLC38A2 processed_transcript
ENST00000547260.4 ADCY6 retained_intron
ENST00000547267.2 GIT2 retained_intron
ENST00000547291.1 DUSP6 protein_coding
ENST00000547317.4 USP15 processed_transcript
ENST00000547361.1 CORO1C retained_intron
ENST00000547404.1 WASHC4 nonsense_mediated_decay

ENST00000547411.1 FAR2 retained_intron
ENST00000547417.1 CPNE8 processed_transcript
ENST00000547427.4 RBFOX1 processed_transcript
ENST00000547428.1 UHRF1BP1L nonsense_mediated_decay
ENST00000547484.4 VEZT nonsense_mediated_decay
ENST00000547486.1 CPSF6 retained_intron
ENST00000547500.1 NCKAP1L retained_intron
ENST00000547505.2 EEF1A1P17 processed_pseudogene
ENST00000547519.1 HECTD4 retained_intron
ENST00000547526.1 DDIT3 protein_coding
ENST00000547562.4 BRF1 retained_intron
ENST00000547625.1 CHURC1 protein_coding
ENST00000547626.4 PCED1B-AS1 lncRNA
ENST00000547639.4 ANKRD13A protein_coding
ENST00000547654.4 SCAF11 processed_transcript
ENST00000547666.1 APAF1 processed_transcript
ENST00000547667.4 ESYT1 retained_intron
ENST00000547691.5 CEP290 protein_coding
ENST00000547694.2 GIT2 retained_intron
ENST00000547697.5 SLC4A8 retained_intron
ENST00000547701.4 CTDSP2 protein_coding
ENST00000547717.1 lncRNA
ENST00000547765.4 TUBA1B nonsense_mediated_decay
ENST00000547772.1 PXN processed_transcript
ENST00000547798.1 TMBIM6 protein_coding
ENST00000547833.4 EEA1 processed_transcript
ENST00000547848.4 POTEG protein_coding
ENST00000547860.1 ELK3 protein_coding
ENST00000547867.4 CNOT2 protein_coding
ENST00000547869.1 SLC25A3 retained_intron
ENST00000547870.1 HNRNPA1 retained_intron
ENST00000547879.4 YLPM1 protein_coding
ENST00000547887.1 HVCN1 nonsense_mediated_decay
ENST00000547893.4 CASP8AP2 retained_intron
ENST00000547919.1 TAFA2 processed_transcript
ENST00000547928.1 IRAK4 retained_intron
ENST00000547950.1 SCAF11 retained_intron
ENST00000547966.4 TPK1 processed_transcript
ENST00000547982.1 LTA4H retained_intron
ENST00000547985.1 METTL25 processed_transcript
ENST00000547993.4 NAP1L1 retained_intron
ENST00000548084.1 NACA nonsense_mediated_decay
ENST00000548092.4 TRAFD1 protein_coding
ENST00000548108.1 TFCP2 protein_coding
ENST00000548118.5 ATF7 protein_coding
ENST00000548143.1 DRG1 nonsense_mediated_decay
ENST00000548144.1 lncRNA
ENST00000548150.1 SLC11A2 protein_coding
ENST00000548169.2 ATP2A2 protein_coding
ENST00000548181.1 NAA25 retained_intron
ENST00000548190.1 PCBP2 retained_intron
ENST00000548195.4 WASHC4 protein_coding
ENST00000548216.2 MARF1 retained_intron
ENST00000548241.1 ANKRD52 processed_transcript

ENST00000548242.4 ARHGAP15 processed_transcript
ENST00000548265.1 PACS2 retained_intron
ENST00000548304.1 KANSL2 protein_coding
ENST00000548305.4 TMTC2 protein_coding
ENST00000548318.1 PPP1R12A protein_coding
ENST00000548336.1 C12orf75 processed_transcript
ENST00000548341.4 OSBPL8 protein_coding
ENST00000548344.1 lncRNA
ENST00000548346.1 NPIPB12 retained_intron
ENST00000548375.4 LTA4H processed_transcript
ENST00000548388.5 SPATS2 retained_intron
ENST00000548414.4 PPM1H processed_transcript
ENST00000548421.2 BRF1 protein_coding
ENST00000548425.1 APPL2 retained_intron
ENST00000548432.4 METTL25 retained_intron
ENST00000548440.1 BAZ2B retained_intron
ENST00000548441.1 ERGIC2 protein_coding
ENST00000548467.1 BACH1 protein_coding
ENST00000548468.2 lncRNA
ENST00000548470.1 TUBA1C retained_intron
ENST00000548483.4 CRADD protein_coding
ENST00000548492.1 ATXN2 processed_transcript
ENST00000548522.4 SSH1 nonsense_mediated_decay
ENST00000548531.1 NELL2 protein_coding
ENST00000548552.2 LINC01619 lncRNA
ENST00000548559.1 AK2 processed_transcript
ENST00000548560.1 SP1 protein_coding
ENST00000548583.1 NOPCHAP1 protein_coding
ENST00000548590.1 RPS26 retained_intron
ENST00000548610.4 SLC16A7 protein_coding
ENST00000548620.1 USP15 nonsense_mediated_decay
ENST00000548643.4 GIT2 retained_intron
ENST00000548647.1 ATP5F1B processed_transcript
ENST00000548664.1 VDR protein_coding
ENST00000548712.4 UHRF1BP1L protein_coding
ENST00000548713.4 TMBIM6 protein_coding
ENST00000548721.1 PPTC7 retained_intron
ENST00000548734.1 CDK17 protein_coding
ENST00000548743.1 MED13L protein_coding
ENST00000548746.4 CS protein_coding
ENST00000548749.4 RBFOX1 processed_transcript
ENST00000548755.1 DUSP6 protein_coding
ENST00000548792.1 SRSF9 retained_intron
ENST00000548800.1 ARHGAP15 retained_intron
ENST00000548830.1 ASH1L nonsense_mediated_decay
ENST00000548831.1 TPK1 processed_transcript
ENST00000548836.1 USP15 protein_coding
ENST00000548839.1 LYZ protein_coding
ENST00000548852.4 LTA4H nonsense_mediated_decay
ENST00000548863.1 CNOT2 retained_intron
ENST00000548870.4 SLC38A2 processed_transcript
ENST00000548900.1 lncRNA
ENST00000548911.1 TMPO processed_transcript
ENST00000548917.1 YAF2 protein_coding

ENST00000548929.1 ARHGAP15 retained_intron
ENST00000548937.4 NUBPL processed_transcript
ENST00000548941.1 NA NA
ENST00000548983.1 LMBR1L processed_transcript
ENST00000548985.1 COX14 protein_coding
ENST00000549007.1 APAF1 protein_coding
ENST00000549016.1 CHST11 protein_coding
ENST00000549021.4 TGFBR1 protein_coding
ENST00000549023.2 lncRNA
ENST00000549032.4 lncRNA
ENST00000549041.1 DAZAP2 retained_intron
ENST00000549049.4 SLC38A1 protein_coding
ENST00000549060.1 ARHGAP15 processed_transcript
ENST00000549063.1 P2RX5 retained_intron
ENST00000549080.1 FAR2 processed_transcript
ENST00000549101.1 USP15 processed_transcript
ENST00000549106.1 ALDH2 nonsense_mediated_decay
ENST00000549133.1 MARS1 processed_transcript
ENST00000549137.1 FMNL3 retained_intron
ENST00000549165.1 GNPTAB protein_coding
ENST00000549183.1 TUBA1C protein_coding
ENST00000549187.1 PLXNC1 retained_intron
ENST00000549203.2 lncRNA
ENST00000549220.4 EFCAB2 processed_transcript
ENST00000549268.1 USP15 retained_intron
ENST00000549272.1 PCBP2 retained_intron
ENST00000549278.1 LINC02458 lncRNA
ENST00000549279.1 TPCN1 protein_coding
ENST00000549286.1 MON2 nonsense_mediated_decay
ENST00000549293.4 YLPM1 nonsense_mediated_decay
ENST00000549305.1 SLC16A7 nonsense_mediated_decay
ENST00000549362.1 USP25 processed_transcript
ENST00000549365.1 DRAM1 nonsense_mediated_decay
ENST00000549373.1 lncRNA
ENST00000549378.4 MON2 processed_transcript
ENST00000549407.1 ZFC3H1 processed_transcript
ENST00000549411.1 OVCH1-AS1 lncRNA
ENST00000549415.1 USP15 protein_coding
ENST00000549417.4 LIN7A protein_coding
ENST00000549419.4 PRANCR lncRNA
ENST00000549436.4 ARHGAP15 retained_intron
ENST00000549451.1 NCKAP1L retained_intron
ENST00000549456.4 TAFA2 processed_transcript
ENST00000549465.4 SLC16A7 protein_coding
ENST00000549466.1 CMKLR1 protein_coding
ENST00000549477.1 lncRNA
ENST00000549479.1 NAP1L1 retained_intron
ENST00000549499.1 FGD6 protein_coding
ENST00000549500.1 PCED1B protein_coding
ENST00000549524.1 TCHP retained_intron
ENST00000549529.1 ELK3 processed_transcript
ENST00000549533.1 RPL18 retained_intron
ENST00000549585.1 ATP2B1 retained_intron
ENST00000549595.4 SENP1 protein_coding

ENST00000549612.4 ATF1 nonsense_mediated_decay
ENST00000549615.4 CRADD processed_transcript
ENST00000549620.4 DIP2B retained_intron
ENST00000549641.1 FICD protein_coding
ENST00000549645.4 EIF4B processed_transcript
ENST00000549668.1 NELL2 retained_intron
ENST00000549682.4 ZDHHC17 protein_coding
ENST00000549689.4 FBXO21 processed_transcript
ENST00000549690.1 LYZ protein_coding
ENST00000549705.1 CNOT2 processed_transcript
ENST00000549727.1 ATP2B1 retained_intron
ENST00000549755.1 MED13L retained_intron
ENST00000549757.4 SMARCC2 retained_intron
ENST00000549770.1 NPAS3 retained_intron
ENST00000549774.4 PPHLN1 retained_intron
ENST00000549780.4 TGIF1 protein_coding
ENST00000549781.1 CPM protein_coding
ENST00000549787.4 BAZ2A protein_coding
ENST00000549818.4 TUBA1C nonsense_mediated_decay
ENST00000549833.1 UBE2N protein_coding
ENST00000549842.1 CPNE8 processed_transcript
ENST00000549866.4 ANKS1B protein_coding
ENST00000549870.4 TUBA1B protein_coding
ENST00000549897.1 OS9 retained_intron
ENST00000549915.4 RAB5B protein_coding
ENST00000549919.1 TMTC2 protein_coding
ENST00000549926.4 DNM1L processed_transcript
ENST00000549939.1 PYM1 protein_coding
ENST00000549940.4 GNPTAB protein_coding
ENST00000549944.1 ZDHHC17 nonsense_mediated_decay
ENST00000549947.1 CNOT2 processed_transcript
ENST00000549958.4 TAFA2 protein_coding
ENST00000549982.4 MRPL42 protein_coding
ENST00000549992.4 NUDT4 protein_coding
ENST00000549999.1 GIT2 retained_intron
ENST00000550007.1 PPP1R12A retained_intron
ENST00000550011.4 DNM1L retained_intron
ENST00000550013.1 RAB5C retained_intron
ENST00000550021.1 YLPM1 retained_intron
ENST00000550040.1 TMBIM6 retained_intron
ENST00000550067.1 SCYL2 processed_transcript
ENST00000550069.1 PFDN5 retained_intron
ENST00000550075.4 CPSF6 nonsense_mediated_decay
ENST00000550078.1 ZBED6 protein_coding
ENST00000550080.4 PLXNC1 protein_coding
ENST00000550091.4 FGD4 processed_transcript
ENST00000550097.1 METTL7A nonsense_mediated_decay
ENST00000550103.2 EFCAB11 processed_transcript
ENST00000550114.4 EGLN3 processed_transcript
ENST00000550124.4 nonsense_mediated_decay
ENST00000550162.1 ATP5F1B processed_transcript
ENST00000550169.4 FRS2 protein_coding
ENST00000550173.1 SLC38A1 protein_coding
ENST00000550178.1 DYNLL1 protein_coding

ENST00000550180.4 FBXO21 protein_coding
ENST00000550192.4 PCBP2 protein_coding
ENST00000550205.1 PXN processed_transcript
ENST00000550215.4 RIC8B nonsense_mediated_decay
ENST00000550248.2 ATP2A2 processed_transcript
ENST00000550253.1 TGFBR1 protein_coding
ENST00000550254.1 TUBA1A retained_intron
ENST00000550267.1 VPS29 retained_intron
ENST00000550290.2 MIRLET7IHG lncRNA
ENST00000550298.1 METTL25 protein_coding
ENST00000550306.1 lncRNA
ENST00000550313.4 NELL2 protein_coding
ENST00000550314.4 VDR protein_coding
ENST00000550324.4 lncRNA
ENST00000550351.4 PPP1R12A retained_intron
ENST00000550355.1 NUBPL processed_transcript
ENST00000550366.4 HEATR5A protein_coding
ENST00000550367.1 TUBA1B protein_coding
ENST00000550372.4 OS9 protein_coding
ENST00000550375.1 BRF1 protein_coding
ENST00000550376.4 PRDM4 nonsense_mediated_decay
ENST00000550388.1 AKT3 processed_transcript
ENST00000550396.1 TAFA2 processed_transcript
ENST00000550404.1 ANKRD13A processed_transcript
ENST00000550424.1 FMNL3 protein_coding
ENST00000550461.1 CSRNP2 processed_transcript
ENST00000550462.4 NELL2 protein_coding
ENST00000550475.2 PRIM2 protein_coding
ENST00000550491.1 GLIPR1 protein_coding
ENST00000550495.1 TMEM117 protein_coding
ENST00000550497.4 C12orf42 processed_transcript
ENST00000550504.4 RAB5C protein_coding
ENST00000550510.4 PPP1R12A protein_coding
ENST00000550516.1 lncRNA
ENST00000550544.4 UHRF1BP1L protein_coding
ENST00000550578.1 EIF4A1P4 processed_pseudogene
ENST00000550582.2 NR4A1 protein_coding
ENST00000550589.1 CCDC59 retained_intron
ENST00000550592.1 LIMA1 protein_coding
ENST00000550613.1 WASHC4 retained_intron
ENST00000550623.1 TMEM117 processed_transcript
ENST00000550630.4 ANO6 nonsense_mediated_decay
ENST00000550632.4 USP15 processed_transcript
ENST00000550633.1 CRY1 processed_transcript
ENST00000550639.1 MYL6 processed_transcript
ENST00000550671.1 RPL18 retained_intron
ENST00000550699.4 OS9 retained_intron
ENST00000550712.1 ZFC3H1 processed_transcript
ENST00000550716.1 ATP2B1 protein_coding
ENST00000550722.4 HECTD4 protein_coding
ENST00000550724.1 HECTD4 miRNA
ENST00000550733.1 PCBP2 miRNA
ENST00000550767.4 TUBA1A miRNA
ENST00000550780.1 TCHP miRNA

ENST00000550826.1 ZNF844 miRNA
ENST00000550847.1 RAB3IP miRNA
ENST00000550863.1 CPNE8 miRNA
ENST00000550883.1 OAS1 miRNA
ENST00000550889.4 ATXN2 miRNA
ENST00000550896.1 UNC13A miRNA
ENST00000550899.1 CERS5 miRNA
ENST00000550905.4 LINC01234 miRNA
ENST00000550906.1 OVCH1-AS1 miRNA
ENST00000550920.4 NACA miRNA
ENST00000550925.2 SH2B3 miRNA
ENST00000550948.1 SELPLG miRNA
ENST00000550968.4 HECTD4 miRNA
ENST00000550983.1 NPIPB9 miRNA
ENST00000550986.1 ESYT1 miRNA
ENST00000551007.1 OAS3 miRNA
ENST00000551009.4 ATP2B1 miRNA
ENST00000551015.1 NUBPL miRNA
ENST00000551025.3 CASP8AP2 miRNA
ENST00000551059.1 CORO1C miRNA
ENST00000551062.4 TPK1 miRNA
ENST00000551076.4 DNM1L miRNA
ENST00000551087.1 ATF7 miRNA
ENST00000551093.4 CHURC1 miRNA
ENST00000551179.1 CNOT2 miRNA
ENST00000551193.1 FAR2 miRNA
ENST00000551197.1 MED13L miRNA
ENST00000551214.4 PPM1H miRNA
ENST00000551216.2 SCN8A miRNA
ENST00000551228.1 TCP11L2 miRNA
ENST00000551229.2 miRNA
ENST00000551236.4 MTA1 miRNA
ENST00000551265.4 SLC25A3 miRNA
ENST00000551307.4 MON2 miRNA
ENST00000551310.1 ATP2B1 miRNA
ENST00000551313.1 DAZAP2 miRNA
ENST00000551336.1 RPLP0 miRNA
ENST00000551338.1 RAB5C miRNA
ENST00000551361.1 SNHG14 miRNA
ENST00000551374.4 SLC38A2 miRNA
ENST00000551402.1 TGIF1 miRNA
ENST00000551405.1 SLC38A2 miRNA
ENST00000551457.1 TMCC3 miRNA
ENST00000551458.1 FBXO21 miRNA
ENST00000551466.1 LRP10 miRNA
ENST00000551468.1 DDX23 miRNA
ENST00000551484.1 CDK17 miRNA
ENST00000551486.1 LIMA1 miRNA
ENST00000551487.1 ZFC3H1 miRNA
ENST00000551495.1 PLXNC1 miRNA
ENST00000551500.1 NAP1L1 miRNA
ENST00000551503.4 miRNA
ENST00000551504.4 BAZ2B miRNA
ENST00000551516.1 CPSF6 miRNA

ENST00000551519.1 PPM1H miRNA
ENST00000551524.4 NAP1L1 miRNA
ENST00000551528.4 YAF2 miRNA
ENST00000551585.1 DGKA miRNA
ENST00000551597.5 PSMA3-AS1 miRNA
ENST00000551667.1 ANO6 miRNA
ENST00000551673.4 YARS2 miRNA
ENST00000551681.1 RRN3P1 miRNA
ENST00000551690.1 PPP1CC miRNA
ENST00000551707.4 DGKA miRNA
ENST00000551721.1 GIT2 miRNA
ENST00000551722.1 METTL25 miRNA
ENST00000551766.4 RRN3P3 miRNA
ENST00000551777.1 PCED1B miRNA
ENST00000551792.4 VEZT miRNA
ENST00000551812.4 BAZ2A miRNA
ENST00000551840.4 METAP2 miRNA
ENST00000551850.1 PLXNC1 miRNA
ENST00000551855.1 CPNE8 miRNA
ENST00000551858.1 NAA25 miRNA
ENST00000551864.1 ZNF385A miRNA
ENST00000551869.1 GPR132 miRNA
ENST00000551877.4 SLC16A7 miRNA
ENST00000551914.1 RPLP0 miRNA
ENST00000551915.4 TMTC2 miRNA
ENST00000551983.1 HSP90B1 miRNA
ENST00000551996.1 BAZ2A miRNA
ENST00000552002.5 CHURC1 miRNA
ENST00000552013.1 NAP1L1 miRNA
ENST00000552023.1 MED13L miRNA
ENST00000552026.1 HLX-AS1 miRNA
ENST00000552033.4 CRADD miRNA
ENST00000552051.1 HSP90B1 miRNA
ENST00000552080.1 SARNP miRNA
ENST00000552091.1 LTA4H miRNA
ENST00000552093.1 LIN7A miRNA
ENST00000552111.4 MAPKAPK5 miRNA
ENST00000552115.4 MON2 miRNA
ENST00000552137.1 FAR2 miRNA
ENST00000552140.4 NPIPA3 miRNA
ENST00000552151.2 CNOT2 miRNA
ENST00000552173.4 DAZAP2 miRNA
ENST00000552178.1 OSBPL8 miRNA
ENST00000552200.1 PRICKLE1 miRNA
ENST00000552203.1 WASHC4 miRNA
ENST00000552211.4 NCKAP1L miRNA
ENST00000552220.1 miRNA
ENST00000552262.1 CDK17 miRNA
ENST00000552277.3 LINC01619 miRNA
ENST00000552289.1 ARHGAP15 miRNA
ENST00000552301.1 PRPF40B miRNA
ENST00000552314.1 RPL41 miRNA
ENST00000552315.1 BTG1 miRNA
ENST00000552327.1 BAZ2B miRNA

ENST00000552329.1 CHPT1 miRNA
ENST00000552346.1 USP15 miRNA
ENST00000552372.1 C12orf42 miRNA
ENST00000552382.4 ZNF385A miRNA
ENST00000552384.1 RBM19 miRNA
ENST00000552421.4 YLPM1 miRNA
ENST00000552428.4 R3HDM2 miRNA
ENST00000552447.1 NA miRNA
ENST00000552459.2 DAZAP2 miRNA
ENST00000552488.1 G2E3 processed_transcript
ENST00000552516.4 TGFBR1 protein_coding
ENST00000552542.4 TPCN1 trna
ENST00000552553.4 MARF1 trna
ENST00000552564.4 ITGA5 trna
ENST00000552573.5 TGFBR1 trna
ENST00000552581.4 MORC3 trna
ENST00000552586.1 OSBPL8 trna
ENST00000552613.1 SLC8B1 trna
ENST00000552631.1 AKT3 trna
ENST00000552632.4 CEP83 trna
ENST00000552636.1 ATP2A2 trna
ENST00000552657.1 PRKAG1 trna
ENST00000552674.4 SMARCC2 trna
ENST00000552738.4 MON2 trna
ENST00000552761.4 PPHLN1 trna
ENST00000552771.4 MARF1 trna
ENST00000552778.2 trna
ENST00000552781.2 SNHG14 trna
ENST00000552784.1 trna
ENST00000552785.1 trna
ENST00000552790.4 CRY1 trna
ENST00000552794.1 PPHLN1 trna
ENST00000552831.1 TMPO trna
ENST00000552846.1 PRR13 trna
ENST00000552856.1 trna
ENST00000552875.2 PRKCH trna
ENST00000552881.1 TPK1 trna
ENST00000552924.1 TUBA1A trna
ENST00000552929.1 APAF1 trna
ENST00000552977.4 C12orf42 trna
ENST00000552980.5 PCBP2 trna
ENST00000552984.1 TUBA1B trna
ENST00000552994.4 ZFC3H1 trna
ENST00000552997.1 USP15 trna
ENST00000553002.1 LRP10 trna
ENST00000553009.4 COPZ1 trna
ENST00000553019.1 EEA1 trna
ENST00000553022.1 TMBIM6 trna
ENST00000553039.1 CKAP4 trna
ENST00000553042.1 CDK17 trna
ENST00000553053.4 MAPKAPK5 trna
ENST00000553071.1 ITGA5 trna
ENST00000553076.1 trna
ENST00000553078.4 CNOT2 trna

ENST00000553081.4 PPP1R12A trna
ENST00000553105.1 trna
ENST00000553108.1 SNHG14 trna
ENST00000553134.4 SNHG14 trna
ENST00000553143.4 TCP11L2 trna
ENST00000553144.1 ATP2A2 trna
ENST00000553145.1 HDAC4 trna
ENST00000553152.1 OAS1 trna
ENST00000553189.4 TMTC1 trna
ENST00000553201.1 NPIPA2 trna
ENST00000553205.1 RPL6 trna
ENST00000553207.4 LINC01619 trna
ENST00000553209.1 EIF4B trna
ENST00000553251.4 ANKRD13A trna
ENST00000553252.1 SLC38A2 trna
ENST00000553265.4 PRKCH trna
ENST00000553274.1 KLHDC1 trna
ENST00000553289.4 SYNE2 trna
ENST00000553293.4 DCAF5 trna
ENST00000553335.4 TMEM260 trna
ENST00000553340.1 SYNE3 trna
ENST00000553341.4 FLVCR2 trna
ENST00000553342.1 NFKBIA trna
ENST00000553346.1 LINC02316 trna
ENST00000553352.1 MIA2 trna
ENST00000553353.4 FOXN3 trna
ENST00000553354.4 MNAT1 trna
ENST00000553365.4 TEP1 trna
ENST00000553371.1 LGMN trna
ENST00000553372.1 PTGDR trna
ENST00000553375.1 ZFP36L1 trna
ENST00000553381.1 YLPM1 trna
ENST00000553385.1 BAZ1A trna
ENST00000553422.1 CALM1 trna
ENST00000553427.4 CPSF2 trna
ENST00000553428.2 PCNX1 trna
ENST00000553437.1 CCDC88C trna
ENST00000553438.1 IFT43 trna
ENST00000553446.1 LRP1 trna
ENST00000553452.4 ITPK1 trna
ENST00000553453.4 SIPA1L1 trna
ENST00000553480.1 DOCK11P1 trna
ENST00000553497.1 SUSD6 trna
ENST00000553508.4 PCNX1 trna
ENST00000553510.1 trna
ENST00000553547.4 AKAP6 trna
ENST00000553571.4 NSFL1C trna
ENST00000553573.1 BAZ1A trna
ENST00000553577.1 ATP10A trna
ENST00000553588.1 GPATCH2L trna
ENST00000553612.4 GTF2A1 trna
ENST00000553616.1 HECTD1 trna
ENST00000553624.4 KTN1 trna
ENST00000553625.4 YY1 trna

ENST00000553630.1 CALM1 trna
ENST00000553636.1 CCNK trna
ENST00000553653.4 SLCO3A1 trna
ENST00000553655.1 ITPK1 trna
ENST00000553657.4 PSMA3-AS1 trna
ENST00000553659.1 ACTN1 trna
ENST00000553668.2 LINC02328 trna
ENST00000553676.1 CATSPERB trna
ENST00000553695.4 ITPK1 trna
ENST00000553733.1 CLMN trna
ENST00000553739.2 PPP1R13B trna
ENST00000553743.4 CHURC1-FNTB trna
ENST00000553754.1 trna
ENST00000553755.4 LGALS3 trna
ENST00000553779.4 ACTN1 trna
ENST00000553788.4 ETFRF1 trna
ENST00000553817.1 KLHL28 trna
ENST00000553827.1 LINC02307 trna
ENST00000553829.1 CHASERR trna
ENST00000553842.1 PPP2R5C trna
ENST00000553843.4 RHOXF1-AS1 trna
ENST00000553846.1 PRKCH trna
ENST00000553853.1 BAZ1A trna
ENST00000553854.1 EEF1A1P2 trna
ENST00000553865.1 CCNK trna
ENST00000553891.4 ZFYVE1 trna
ENST00000553892.2 RALGAPA1 trna
ENST00000553898.1 PCNX4 trna
ENST00000553910.1 EVL trna
ENST00000553914.5 LINC01588 trna
ENST00000553916.4 MYO5A trna
ENST00000553917.4 RALGAPA1 trna
ENST00000553924.4 FUT8 trna
ENST00000553943.1 DCAF5 trna
ENST00000553945.1 NEK9 trna
ENST00000553946.1 trna
ENST00000553951.1 MAX trna
ENST00000553952.1 GMFB trna
ENST00000553957.4 HECTD1 trna
ENST00000553959.1 RIN3 trna
ENST00000553974.1 ATP6V1D trna
ENST00000553984.1 TEP1 trna
ENST00000553986.1 DHRS7 trna
ENST00000554005.1 FOXN3 trna
ENST00000554011.4 EXOC5 trna
ENST00000554015.4 ZBTB1 trna
ENST00000554018.1 TRAPPC6B trna
ENST00000554031.4 EVL trna
ENST00000554059.1 SLC39A9 trna
ENST00000554061.4 SLC7A7 trna
ENST00000554070.1 AREL1 trna
ENST00000554076.4 LIN52 trna
ENST00000554084.4 WARS1 trna
ENST00000554087.4 ATP6V1D trna

ENST00000554090.1 ARHGAP5 trna
ENST00000554093.1 MIS18BP1 trna
ENST00000554107.2 YLPM1 trna
ENST00000554109.1 NKAPP1 trna
ENST00000554118.1 LRP1 trna
ENST00000554124.2 STRN3 trna
ENST00000554137.4 PPP2R5C trna
ENST00000554147.1 PPP2R5C trna
ENST00000554158.1 ACTN1 trna
ENST00000554163.4 GMFB trna
ENST00000554165.1 CCDC88C trna
ENST00000554169.1 SNHG10 trna
ENST00000554174.1 LRP1 trna
ENST00000554175.4 WDR25 trna
ENST00000554183.1 RAD51B trna
ENST00000554190.1 FBXO33 trna
ENST00000554203.1 C14orf119 trna
ENST00000554210.4 FNTB trna
ENST00000554215.4 DCAF5 trna
ENST00000554216.1 SYNJ2BP trna
ENST00000554218.1 ARMH4 trna
ENST00000554237.1 VASH1 trna
ENST00000554243.1 MTCO1P2 trna
ENST00000554245.1 trna
ENST00000554246.1 H2AZ2P1 trna
ENST00000554251.4 ERO1A trna
ENST00000554253.1 trna
ENST00000554254.1 HIF1A-AS3 trna
ENST00000554259.4 RALGAPA1 trna
ENST00000554273.1 NA trna
ENST00000554292.1 PCNX1 trna
ENST00000554293.1 SEL1L trna
ENST00000554308.4 PPP4R3A trna
ENST00000554309.1 PSMA3-AS1 trna
ENST00000554357.4 TRIP11 trna
ENST00000554360.4 PSMA3-AS1 trna
ENST00000554364.1 MAPK1IP1L trna
ENST00000554375.4 GPATCH2L trna
ENST00000554378.4 PSMA3-AS1 trna
ENST00000554399.1 TXNDC16 trna
ENST00000554403.1 ZFHX2-AS1 trna
ENST00000554417.1 HNRNPC trna
ENST00000554437.4 SCFD1 trna
ENST00000554461.4 SLC24A4 trna
ENST00000554464.1 NRDE2 trna
ENST00000554480.4 TMEM229B trna
ENST00000554486.1 SCFD1 trna
ENST00000554487.1 TTLL5 trna
ENST00000554496.1 FLVCR2 trna
ENST00000554502.4 CEP128 trna
ENST00000554510.4 TTLL5 trna
ENST00000554511.1 PPP4R3A trna
ENST00000554512.1 KLHDC1 trna
ENST00000554514.1 ARHGEF40 trna

ENST00000554518.1 EVL trna
ENST00000554525.1 AREL1 trna
ENST00000554537.1 trna
ENST00000554567.1 KTN1 trna
ENST00000554573.4 RALGAPA1 trna
ENST00000554574.1 PPP4R3A trna
ENST00000554575.2 RAD51B trna
ENST00000554584.4 SYNE2 trna
ENST00000554585.4 AKT1 trna
ENST00000554601.1 WARS1 trna
ENST00000554617.1 FOS trna
ENST00000554626.4 NEMF trna
ENST00000554641.1 MNAT1 trna
ENST00000554644.1 BTBD7 trna
ENST00000554659.4 VTI1B trna
ENST00000554667.1 FUT8 trna
ENST00000554669.4 CHASERR trna
ENST00000554675.1 TMEM229B trna
ENST00000554681.1 DCAF5 trna
ENST00000554691.4 PCNX1 trna
ENST00000554700.1 GOLGA5 trna
ENST00000554703.1 NAA30 protein_coding
ENST00000554709.1 MAX retained_intron
ENST00000554717.1 WDR89 protein_coding
ENST00000554719.4 NRXN3 protein_coding
ENST00000554726.1 SNHG14 lncRNA
ENST00000554744.1 SEL1L retained_intron
ENST00000554765.1 FUT8 processed_transcript
ENST00000554792.1 EAPP protein_coding
ENST00000554814.1 lncRNA
ENST00000554819.4 SCFD1 processed_transcript
ENST00000554822.4 LINC01550 lncRNA
ENST00000554859.1 lncRNA
ENST00000554862.1 LINC02325 lncRNA
ENST00000554873.4 SYNE3 protein_coding
ENST00000554874.4 SIPA1L1 processed_transcript
ENST00000554875.1 GNG2 protein_coding
ENST00000554879.4 PCNX1 retained_intron
ENST00000554888.1 RIN3 processed_transcript
ENST00000554894.4 CHASERR lncRNA
ENST00000554901.1 SPTLC2 protein_coding
ENST00000554902.4 PNN retained_intron
ENST00000554911.1 NA NA
ENST00000554916.4 GALC processed_transcript
ENST00000554918.1 HSPA2-AS1 lncRNA
ENST00000554926.1 lncRNA
ENST00000554929.1 SRSF5 retained_intron
ENST00000554938.1 LIN52 protein_coding
ENST00000554942.4 IRAG2 protein_coding
ENST00000554945.1 SRP54-AS1 processed_transcript
ENST00000554950.1 WARS1 retained_intron
ENST00000554960.1 SIPA1L1 retained_intron
ENST00000554968.1 BTBD7 protein_coding
ENST00000554979.1 ACIN1 retained_intron

ENST00000554987.1 HNRNPUP1 processed_pseudogene
ENST00000554990.4 MAP4K5 retained_intron
ENST00000555008.4 TEP1 nonsense_mediated_decay
ENST00000555010.1 FOXN3 processed_transcript
ENST00000555014.1 HIF1A processed_transcript
ENST00000555015.4 SRP54-AS1 processed_transcript
ENST00000555034.4 FOXN3 protein_coding
ENST00000555036.1 TMED10 retained_intron
ENST00000555046.1 TMEM260 protein_coding
ENST00000555053.4 ACIN1 protein_coding
ENST00000555054.1 DDX24 protein_coding
ENST00000555058.4 FLVCR2 protein_coding
ENST00000555066.1 SIPA1L1 protein_coding
ENST00000555070.1 lncRNA
ENST00000555085.1 TMED10 retained_intron
ENST00000555087.1 FBXO34 processed_transcript
ENST00000555100.1 ALKBH1 nonsense_mediated_decay
ENST00000555105.1 TRIP11 processed_transcript
ENST00000555110.1 PRKCH processed_transcript
ENST00000555111.1 SLCO3A1 processed_transcript
ENST00000555112.1 SAMD4A processed_transcript
ENST00000555127.1 HNRNPC retained_intron
ENST00000555132.4 CALM1 processed_transcript
ENST00000555141.4 IRAG2 processed_transcript
ENST00000555152.1 STRN3 protein_coding
ENST00000555162.1 PSMA3-AS1 lncRNA
ENST00000555168.1 NKAPP1 processed_transcript
ENST00000555171.1 DHRS7 retained_intron
ENST00000555179.1 GALC nonsense_mediated_decay
ENST00000555204.1 NA NA
ENST00000555210.1 SLCO3A1 nonsense_mediated_decay
ENST00000555227.1 CHASERR retained_intron
ENST00000555232.4 ARHGEF40 retained_intron
ENST00000555233.4 PRKCH processed_transcript
ENST00000555235.1 MARK3 retained_intron
ENST00000555246.4 LINC00871 lncRNA
ENST00000555280.1 FBXO34 nonsense_mediated_decay
ENST00000555296.1 MED6 retained_intron
ENST00000555302.1 TC2N processed_transcript
ENST00000555321.1 ZBTB1 protein_coding
ENST00000555324.1 DDX24 retained_intron
ENST00000555336.5 CLMN protein_coding
ENST00000555343.2 ASXL1 nonsense_mediated_decay
ENST00000555347.1 FOS protein_coding
ENST00000555349.4 SRSF5 protein_coding
ENST00000555351.1 VRK1 retained_intron
ENST00000555352.2 ACIN1 nonsense_mediated_decay
ENST00000555358.4 STRN3 nonsense_mediated_decay
ENST00000555371.1 NFKBIA retained_intron
ENST00000555372.4 FNTB processed_transcript
ENST00000555373.1 ERH protein_coding
ENST00000555375.4 STAT6 retained_intron
ENST00000555379.1 LINC00641 retained_intron
ENST00000555397.1 KIAA0586 protein_coding

ENST00000555405.4 NEK9 retained_intron
ENST00000555411.1 NEMF processed_transcript
ENST00000555412.1 SRSF5 retained_intron
ENST00000555417.4 AP4S1 protein_coding
ENST00000555424.1 ZBTB25 protein_coding
ENST00000555429.1 G2E3 protein_coding
ENST00000555443.4 KLHDC2 nonsense_mediated_decay
ENST00000555452.1 ZFYVE26 protein_coding
ENST00000555456.1 GPHN protein_coding
ENST00000555464.1 WARS1 processed_transcript
ENST00000555467.1 KLC1 retained_intron
ENST00000555495.4 ITPK1 protein_coding
ENST00000555498.4 KTN1 protein_coding
ENST00000555513.1 SLCO3A1 processed_transcript
ENST00000555520.1 CHASERR lncRNA
ENST00000555525.1 BTBD7 protein_coding
ENST00000555537.1 NEK9 retained_intron
ENST00000555543.1 VTI1B nonsense_mediated_decay
ENST00000555549.4 SLCO3A1 processed_transcript
ENST00000555557.4 SRP54 protein_coding
ENST00000555574.1 TMX1 retained_intron
ENST00000555589.4 RIN3 nonsense_mediated_decay
ENST00000555592.1 NPC2 protein_coding
ENST00000555601.1 COX16 processed_transcript
ENST00000555604.1 PRKCH processed_transcript
ENST00000555615.1 CLMN protein_coding
ENST00000555616.4 ACTN1 protein_coding
ENST00000555628.4 PRKCH processed_transcript
ENST00000555629.1 NFKBIA retained_intron
ENST00000555646.1 STAT2 nonsense_mediated_decay
ENST00000555648.1 SNX6 protein_coding
ENST00000555658.4 FOXN3 processed_transcript
ENST00000555672.1 FOS protein_coding
ENST00000555678.1 SLC7A7 retained_intron
ENST00000555683.1 EAPP retained_intron
ENST00000555686.1 FOS protein_coding
ENST00000555688.1 LINC00641 lncRNA
ENST00000555691.1 RBM23 protein_coding
ENST00000555718.4 PPP4R3A processed_transcript
ENST00000555727.4 TEP1 nonsense_mediated_decay
ENST00000555759.1 SYNE3 retained_intron
ENST00000555761.4 SNW1 protein_coding
ENST00000555771.2 SIPA1L1-AS1 lncRNA
ENST00000555776.1 lncRNA
ENST00000555794.2 SOS2 protein_coding
ENST00000555795.1 BATF processed_transcript
ENST00000555843.4 HECTD1 retained_intron
ENST00000555846.1 SOCS4 protein_coding
ENST00000555853.1 lncRNA
ENST00000555855.4 FOXN3 protein_coding
ENST00000555856.1 KLC1 protein_coding
ENST00000555867.1 PSEN1 retained_intron
ENST00000555891.1 RIN3 retained_intron
ENST00000555892.4 SLCO3A1 nonsense_mediated_decay

ENST00000555905.4 TMEM260 nonsense_mediated_decay
ENST00000555909.4 lncRNA
ENST00000555912.1 PAPOLA retained_intron
ENST00000555925.4 PALS1 protein_coding
ENST00000555935.1 CHD8 protein_coding
ENST00000555937.1 lncRNA
ENST00000555941.1 LRP1 retained_intron
ENST00000555945.1 TOGARAM1 retained_intron
ENST00000555947.4 CRAT37 lncRNA
ENST00000555962.4 CHD8 nonsense_mediated_decay
ENST00000555968.1 AP1G2-AS1 lncRNA
ENST00000555973.1 WDR20 processed_transcript
ENST00000555997.1 ZFP36L1 processed_transcript
ENST00000555999.1 EVL processed_transcript
ENST00000556010.2 NA NA
ENST00000556011.4 PSEN1 protein_coding
ENST00000556018.4 TC2N protein_coding
ENST00000556019.2 LINC01588 lncRNA
ENST00000556020.1 GPHN processed_transcript
ENST00000556027.4 DDHD1 retained_intron
ENST00000556045.4 DICER1 nonsense_mediated_decay
ENST00000556048.4 ADCK1 nonsense_mediated_decay
ENST00000556050.1 SPAG5-AS1 lncRNA
ENST00000556061.4 CEP128 protein_coding
ENST00000556063.1 TDP1 protein_coding
ENST00000556066.1 PSEN1 protein_coding
ENST00000556068.4 PPP2R5C processed_transcript
ENST00000556089.1 CHURC1 retained_intron
ENST00000556125.4 SLC39A9 processed_transcript
ENST00000556131.1 KRAS protein_coding
ENST00000556150.1 PPP2R5E processed_transcript
ENST00000556162.4 SNX6 nonsense_mediated_decay
ENST00000556164.4 PRKCH protein_coding
ENST00000556167.1 PSMA6 retained_intron
ENST00000556178.4 RPS6KA5 nonsense_mediated_decay
ENST00000556185.4 ITPK1 processed_transcript
ENST00000556190.4 DLST retained_intron
ENST00000556196.4 MYO5A nonsense_mediated_decay
ENST00000556223.1 ERO1A nonsense_mediated_decay
ENST00000556224.4 HECTD1 protein_coding
ENST00000556225.1 PSMA3-AS1 lncRNA
ENST00000556245.1 PRKCH processed_transcript
ENST00000556248.1 PAPOLA processed_transcript
ENST00000556251.1 RAD51B processed_transcript
ENST00000556254.1 PPP2R3C retained_intron
ENST00000556255.1 KHNYN retained_intron
ENST00000556260.5 PPP2R5C protein_coding
ENST00000556261.4 GALC processed_transcript
ENST00000556268.1 GTF2A1 retained_intron
ENST00000556272.1 PCNX1 processed_transcript
ENST00000556275.4 ESR2 protein_coding
ENST00000556278.1 protein_coding
ENST00000556292.1 FUT8 processed_transcript
ENST00000556307.4 PPP2R5C nonsense_mediated_decay

ENST00000556321.1 PSMA3 retained_intron
ENST00000556324.2 FOS processed_transcript
ENST00000556330.4 SRSF5 retained_intron
ENST00000556347.1 protein_coding
ENST00000556356.1 LRP1 retained_intron
ENST00000556360.1 PCNX4 nonsense_mediated_decay
ENST00000556363.4 MCTP2 retained_intron
ENST00000556373.1 PPP2R5C processed_transcript
ENST00000556375.4 SLIRP protein_coding
ENST00000556377.1 AP5M1 nonsense_mediated_decay
ENST00000556385.4 RIN3 retained_intron
ENST00000556418.1 RIN3 protein_coding
ENST00000556452.1 SOS2 nonsense_mediated_decay
ENST00000556454.1 CLMN retained_intron
ENST00000556462.1 LINC01550 lncRNA
ENST00000556463.4 MARK3 retained_intron
ENST00000556469.4 SOS2 processed_transcript
ENST00000556471.1 GNG2 processed_transcript
ENST00000556474.1 HECTD1 processed_transcript
ENST00000556484.4 PPP2R5E processed_transcript
ENST00000556496.2 NRXN3 retained_intron
ENST00000556501.1 GPHN retained_intron
ENST00000556515.1 MAPK1IP1L processed_transcript
ENST00000556516.1 PCNX1 processed_transcript
ENST00000556530.1 PNN protein_coding
ENST00000556534.1 SCFD1 retained_intron
ENST00000556539.4 STAT2 processed_transcript
ENST00000556554.1 HSP90AA1 retained_intron
ENST00000556562.1 RNF34 retained_intron
ENST00000556569.3 lncRNA
ENST00000556570.4 PPP2R5C processed_transcript
ENST00000556580.4 NGDN retained_intron
ENST00000556590.1 TC2N protein_coding
ENST00000556594.1 RPS6KA5 nonsense_mediated_decay
ENST00000556596.1 DAAM1 retained_intron
ENST00000556600.1 NUMB processed_transcript
ENST00000556607.1 SPTLC2 nonsense_mediated_decay
ENST00000556646.1 lncRNA
ENST00000556649.1 SLCO3A1 processed_transcript
ENST00000556663.4 GPATCH2L protein_coding
ENST00000556664.1 NFKBIA retained_intron
ENST00000556675.2 GPATCH2L processed_transcript
ENST00000556678.1 TMPO protein_coding
ENST00000556683.1 TMX1 nonsense_mediated_decay
ENST00000556691.4 NEMF processed_transcript
ENST00000556694.4 ACTR10 retained_intron
ENST00000556695.4 WARS1 protein_coding
ENST00000556700.4 lncRNA
ENST00000556712.4 SNX6 nonsense_mediated_decay
ENST00000556724.1 EIF2S1 retained_intron
ENST00000556725.1 SYNE2 processed_transcript
ENST00000556726.4 CCDC88C protein_coding
ENST00000556734.1 lncRNA
ENST00000556739.4 SLC24A4 processed_transcript

ENST00000556752.2 GNG2 protein_coding
ENST00000556754.1 PNP retained_intron
ENST00000556767.1 CCDC88C retained_intron
ENST00000556769.1 ERO1A retained_intron
ENST00000556772.4 NUMB protein_coding
ENST00000556787.4 PAPOLA retained_intron
ENST00000556791.1 NA NA
ENST00000556805.1 PPP2R5C retained_intron
ENST00000556810.4 TMEM260 protein_coding
ENST00000556818.1 ZBTB1 processed_transcript
ENST00000556827.1 HIF1A retained_intron
ENST00000556833.1 CHD8 processed_transcript
ENST00000556842.4 KHNYN protein_coding
ENST00000556846.1 PCNX1 retained_intron
ENST00000556847.4 DCAF5 protein_coding
ENST00000556849.2 PCNX1 protein_coding
ENST00000556861.1 STYX processed_transcript
ENST00000556874.1 lncRNA
ENST00000556878.1 PPP2R5E retained_intron
ENST00000556883.1 MOAP1 protein_coding
ENST00000556886.1 LINC00871 lncRNA
ENST00000556910.1 DDHD1 protein_coding
ENST00000556916.4 FOXN3 protein_coding
ENST00000556918.4 NRG3 protein_coding
ENST00000556921.1 EVL processed_transcript
ENST00000556975.4 TMEM260 processed_transcript
ENST00000556989.1 NUMB retained_intron
ENST00000556990.1 MIA2 processed_transcript
ENST00000556993.1 SUSD6 retained_intron
ENST00000557005.1 SYNE2 retained_intron
ENST00000557017.1 TRIP11 nonsense_mediated_decay
ENST00000557023.1 EVL retained_intron
ENST00000557026.4 NEK9 retained_intron
ENST00000557033.1 HNRNPC retained_intron
ENST00000557045.4 RAD51B processed_transcript
ENST00000557060.1 SYNE2 retained_intron
ENST00000557070.1 LINC02296 lncRNA
ENST00000557089.1 HSP90AA1 retained_intron
ENST00000557108.4 SNHG14 retained_intron
ENST00000557111.4 RPS29 protein_coding
ENST00000557123.4 CALM1 retained_intron
ENST00000557135.4 WARS1 protein_coding
ENST00000557139.1 FOS protein_coding
ENST00000557143.1 KLC1 retained_intron
ENST00000557195.4 LINC02328 lncRNA
ENST00000557199.1 STAT2 retained_intron
ENST00000557206.1 HIF1A processed_transcript
ENST00000557208.1 GNG2 retained_intron
ENST00000557222.4 VRK1 protein_coding
ENST00000557238.4 MDGA2 nonsense_mediated_decay
ENST00000557275.4 SYNE3 protein_coding
ENST00000557294.4 PRKCH protein_coding
ENST00000557303.1 DGLUCY processed_transcript
ENST00000557314.4 TEP1 nonsense_mediated_decay

ENST00000557316.4 GALC nonsense_mediated_decay
ENST00000557362.1 NOP9 protein_coding
ENST00000557367.2 RPS29 retained_intron
ENST00000557369.1 NA NA
ENST00000557372.1 SEL1L protein_coding
ENST00000557374.1 TXNDC16 protein_coding
ENST00000557384.4 EVL protein_coding
ENST00000557385.1 ZBTB33 protein_coding
ENST00000557389.1 NFKBIA protein_coding
ENST00000557394.4 SUPT16H retained_intron
ENST00000557404.3 PRORP protein_coding
ENST00000557407.1 ZFYVE26 retained_intron
ENST00000557422.1 RTN1 processed_transcript
ENST00000557428.1 PCNX1 processed_transcript
ENST00000557430.1 SLC35F4 processed_transcript
ENST00000557435.1 SRSF5 retained_intron
ENST00000557436.1 PTGER2 protein_coding
ENST00000557445.1 DDHD1 retained_intron
ENST00000557446.4 HIF1A processed_transcript
ENST00000557468.1 KLHL28 protein_coding
ENST00000557469.1 SIPA1L1 processed_transcript
ENST00000557472.1 IRAG2 processed_transcript
ENST00000557473.1 PRKCH protein_coding
ENST00000557486.1 NUMB retained_intron
ENST00000557534.1 PPP2R5C nonsense_mediated_decay
ENST00000557538.4 HIF1A processed_transcript
ENST00000557553.4 RTRAF nonsense_mediated_decay
ENST00000557561.2 YBX2P2 transcribed_processed_pseudogene
ENST00000557563.4 STAT6 retained_intron
ENST00000557566.1 SPTLC2 retained_intron
ENST00000557572.1 FOXN3 protein_coding
ENST00000557578.1 MAP4K5 processed_transcript
ENST00000557585.4 PRKCH protein_coding
ENST00000557614.1 WARS1 retained_intron
ENST00000557625.1 FRMD6-AS2 lncRNA
ENST00000557643.1 ARHGAP5 nonsense_mediated_decay
ENST00000557663.1 SNW1 retained_intron
ENST00000557680.1 PNN processed_transcript
ENST00000557682.5 CHASERR lncRNA
ENST00000557686.1 KLC1 nonsense_mediated_decay
ENST00000557712.1 SIPA1L1 processed_transcript
ENST00000557713.4 SCFD1 nonsense_mediated_decay
ENST00000557714.1 PPP2R5C protein_coding
ENST00000557742.1 MCTP2 protein_coding
ENST00000557768.1 HNRNPC protein_coding
ENST00000557775.1 NA NA
ENST00000557778.1 LINC02320 lncRNA
ENST00000557781.4 STAT6 retained_intron
ENST00000557815.1 TLE3 retained_intron
ENST00000557819.5 PDE8A processed_transcript
ENST00000557849.1 INO80 nonsense_mediated_decay
ENST00000557866.1 SPG11 non_stop_decay
ENST00000557869.2 MAP2K5 protein_coding
ENST00000557873.4 IGF1R processed_transcript

ENST00000557875.1 RASGRP1 nonsense_mediated_decay
ENST00000557885.1 TGIF2 protein_coding
ENST00000557927.1 AEN retained_intron
ENST00000557938.4 IGF1R processed_transcript
ENST00000557939.1 EFL1 protein_coding
ENST00000557954.1 PDE8A retained_intron
ENST00000557961.4 MORF4L1 retained_intron
ENST00000557967.4 ALDH1A2 protein_coding
ENST00000557972.1 LACTB protein_coding
ENST00000557983.1 ZFAND6 retained_intron
ENST00000557984.4 TLE3 nonsense_mediated_decay
ENST00000557987.4 BNIP2 retained_intron
ENST00000558009.1 AKAP13 retained_intron
ENST00000558021.1 MAP2K5 retained_intron
ENST00000558024.1 ATP8B4 protein_coding
ENST00000558031.4 CRNDE lncRNA
ENST00000558039.1 lncRNA
ENST00000558051.2 SEMA4B processed_transcript
ENST00000558056.1 TNFAIP2 protein_coding
ENST00000558067.4 ZNF280D nonsense_mediated_decay
ENST00000558068.1 JDP2 processed_transcript
ENST00000558088.1 VPS13C processed_transcript
ENST00000558093.1 SPG11 retained_intron
ENST00000558113.4 CCDC32 protein_coding
ENST00000558116.4 RNF213 retained_intron
ENST00000558120.4 LINC02284 lncRNA
ENST00000558131.1 RPL28 protein_coding
ENST00000558145.4 GALK2 protein_coding
ENST00000558162.1 TRIP4 protein_coding
ENST00000558166.2 AKAP13 protein_coding
ENST00000558170.5 ZEB2 protein_coding
ENST00000558188.1 lncRNA
ENST00000558189.1 ATG14 retained_intron
ENST00000558194.4 OAZ2 retained_intron
ENST00000558196.1 SEC11A protein_coding
ENST00000558202.1 AQR retained_intron
ENST00000558206.1 APBA2 protein_coding
ENST00000558209.1 lncRNA
ENST00000558210.4 TCF12 processed_transcript
ENST00000558218.4 USP3 processed_transcript
ENST00000558222.1 PBX4 nonsense_mediated_decay
ENST00000558234.1 RORA retained_intron
ENST00000558235.4 RORA-AS1 lncRNA
ENST00000558236.1 ISG20 processed_transcript
ENST00000558240.1 CDK12 processed_transcript
ENST00000558253.4 SPG11 processed_transcript
ENST00000558259.4 APBA2 protein_coding
ENST00000558266.4 DPH6 protein_coding
ENST00000558274.1 MAP2K5 processed_transcript
ENST00000558280.1 NRL protein_coding
ENST00000558334.1 ZNF710-AS1 lncRNA
ENST00000558338.1 VPS13C retained_intron
ENST00000558353.1 EIF3J nonsense_mediated_decay
ENST00000558355.1 IGF1R protein_coding

ENST00000558375.1 lncRNA
ENST00000558414.1 SPPL2A protein_coding
ENST00000558418.4 RASGRP1 protein_coding
ENST00000558428.4 SMAD3 protein_coding
ENST00000558432.4 RASGRP1 protein_coding
ENST00000558444.4 TRPM7 processed_transcript
ENST00000558448.4 WDR20 processed_transcript
ENST00000558455.1 TMOD3 protein_coding
ENST00000558469.4 CUX1 processed_transcript
ENST00000558491.1 IQGAP1 retained_intron
ENST00000558500.4 LRRC28 processed_transcript
ENST00000558504.1 ALDH1A2 processed_transcript
ENST00000558506.1 EIF5 protein_coding
ENST00000558507.1 DMXL2 retained_intron
ENST00000558510.1 TINF2 processed_transcript
ENST00000558529.1 DPP8 protein_coding
ENST00000558533.4 TARS3 nonsense_mediated_decay
ENST00000558539.4 MORF4L1 processed_transcript
ENST00000558545.1 COPS2 retained_intron
ENST00000558551.1 EIF5 retained_intron
ENST00000558559.1 DPP8 protein_coding
ENST00000558569.1 TSHZ3 protein_coding
ENST00000558570.4 IREB2 nonsense_mediated_decay
ENST00000558573.1 PATL2 processed_transcript
ENST00000558580.1 SORD processed_transcript
ENST00000558593.1 GABPB1-AS1 lncRNA
ENST00000558608.1 WDR73 retained_intron
ENST00000558644.2 AKAP13 retained_intron
ENST00000558646.1 MYO1E retained_intron
ENST00000558665.1 KMT2C protein_coding
ENST00000558673.1 KLF13 processed_transcript
ENST00000558678.1 TECPR2 protein_coding
ENST00000558680.4 ZNF609 processed_transcript
ENST00000558700.4 TRAF3 nonsense_mediated_decay
ENST00000558724.1 ZFAND6 protein_coding
ENST00000558739.1 SMAD3 protein_coding
ENST00000558747.4 ASB7 protein_coding
ENST00000558752.1 RPL28 protein_coding
ENST00000558762.4 NA NA
ENST00000558763.1 SMAD3 retained_intron
ENST00000558771.1 LRRC28 processed_transcript
ENST00000558772.4 AQP9 protein_coding
ENST00000558783.1 nonsense_mediated_decay
ENST00000558787.1 MARK3 retained_intron
ENST00000558790.4 SPG11 processed_transcript
ENST00000558804.4 APBA2 protein_coding
ENST00000558811.2 AKAP13 protein_coding
ENST00000558812.4 MEF2A protein_coding
ENST00000558814.1 MYO1E processed_transcript
ENST00000558832.4 ZEB1 processed_transcript
ENST00000558836.4 CUX1 processed_transcript
ENST00000558844.1 KLF13 protein_coding
ENST00000558847.1 GOLM2 protein_coding
ENST00000558856.4 MEF2A processed_transcript

ENST00000558870.1 PSTPIP1 protein_coding
ENST00000558873.1 PARP16 processed_transcript
ENST00000558877.1 CERS4 protein_coding
ENST00000558882.1 FMN1 processed_transcript
ENST00000558894.4 SMAD3 miRNA
ENST00000558908.4 TCF12 miRNA
ENST00000558919.1 VPS13C miRNA
ENST00000558921.1 KLF13 miRNA
ENST00000558925.1 USP3 miRNA
ENST00000558934.1 SPPL2A miRNA
ENST00000558943.4 SPG21 miRNA
ENST00000558959.4 ATP8B4 miRNA
ENST00000558968.1 CTDSPL2 miRNA
ENST00000558970.2 GABPB1 miRNA
ENST00000558973.1 DPH6 miRNA
ENST00000558980.1 miRNA
ENST00000558983.4 MEF2A miRNA
ENST00000558990.4 RAB8B miRNA
ENST00000559000.4 miRNA
ENST00000559031.1 IQGAP1 miRNA
ENST00000559037.1 GOLM2 miRNA
ENST00000559038.4 POU2F1 miRNA
ENST00000559047.4 NA miRNA
ENST00000559059.1 DMXL2 miRNA
ENST00000559070.4 RNF213 miRNA
ENST00000559086.4 PDE8A miRNA
ENST00000559089.4 UBAP1L miRNA
ENST00000559090.4 AQR miRNA
ENST00000559091.1 IREB2 miRNA
ENST00000559092.1 SMAD3 miRNA
ENST00000559111.1 HSP90B2P miRNA
ENST00000559119.1 VPS13C miRNA
ENST00000559160.1 RNF111 miRNA
ENST00000559162.4 AP3S2 miRNA
ENST00000559176.4 ANXA2 miRNA
ENST00000559193.4 SPG11 miRNA
ENST00000559199.4 SPG21 miRNA
ENST00000559209.4 RNF111 miRNA
ENST00000559210.1 DPH6-DT miRNA
ENST00000559216.4 TCF12 miRNA
ENST00000559218.1 CLPX miRNA
ENST00000559220.1 B2M miRNA
ENST00000559244.4 MORF4L1 miRNA
ENST00000559255.1 TNFAIP2 miRNA
ENST00000559262.4 MAP2K5 miRNA
ENST00000559273.1 RPS17 miRNA
ENST00000559274.1 MARK3 miRNA
ENST00000559276.4 USP3 miRNA
ENST00000559278.1 AKAP13 miRNA
ENST00000559285.1 IQCH-AS1 miRNA
ENST00000559303.2 miRNA
ENST00000559314.4 PPP1R3E miRNA
ENST00000559318.1 miRNA
ENST00000559342.1 IL16 miRNA

ENST00000559343.1 RORA miRNA
ENST00000559352.1 ZNF280D miRNA
ENST00000559362.4 AKAP13 miRNA
ENST00000559364.1 ZNF609 miRNA
ENST00000559367.1 ANKRD17 miRNA
ENST00000559385.1 EEF1A1P22 miRNA
ENST00000559388.4 NA miRNA
ENST00000559391.1 AKAP13 miRNA
ENST00000559419.1 ZNF710 miRNA
ENST00000559424.1 SECISBP2L miRNA
ENST00000559434.1 MYO5C miRNA
ENST00000559443.1 AQP9 miRNA
ENST00000559447.5 RFX7 miRNA
ENST00000559458.2 miRNA
ENST00000559460.4 SMAD3 miRNA
ENST00000559464.1 KATNBL1 miRNA
ENST00000559468.1 SYNM-AS1 miRNA
ENST00000559478.1 WDR20 miRNA
ENST00000559484.1 SLC12A6 miRNA
ENST00000559485.1 NA miRNA
ENST00000559486.2 AKAP13 miRNA
ENST00000559488.4 ITGB3 miRNA
ENST00000559498.1 DMXL2 miRNA
ENST00000559527.1 SPPL2A miRNA
ENST00000559531.1 CRTC3-AS1 miRNA
ENST00000559571.1 NIPA2 miRNA
ENST00000559580.4 GALK2 miRNA
ENST00000559587.1 RORA miRNA
ENST00000559592.1 RNF111 miRNA
ENST00000559610.1 FMN1 miRNA
ENST00000559624.4 EIF2AK4 miRNA
ENST00000559627.1 PTGS2 miRNA
ENST00000559634.1 PLCB2 miRNA
ENST00000559652.4 BTBD1 miRNA
ENST00000559656.1 DET1 miRNA
ENST00000559663.1 CDK12 miRNA
ENST00000559681.1 CNOT6LP1 miRNA
ENST00000559686.2 SNRPA1 miRNA
ENST00000559706.1 GTF2A2 miRNA
ENST00000559709.4 APBA2 miRNA
ENST00000559711.4 USP3 miRNA
ENST00000559718.1 USP3 miRNA
ENST00000559726.4 ATP8B4 miRNA
ENST00000559745.2 MINDY2 miRNA
ENST00000559746.1 THBS1 miRNA
ENST00000559757.1 RNF111 miRNA
ENST00000559763.1 RPS27L miRNA
ENST00000559769.1 DMXL2 miRNA
ENST00000559771.4 USP3 miRNA
ENST00000559783.2 miRNA
ENST00000559795.4 PSTPIP1 miRNA
ENST00000559800.1 NA miRNA
ENST00000559809.2 IQGAP1 miRNA
ENST00000559822.1 NA miRNA

ENST00000559845.4 LIPC miRNA
ENST00000559847.5 RFX7 miRNA
ENST00000559859.4 PSTPIP1 miRNA
ENST00000559861.1 USP3-AS1 miRNA
ENST00000559864.1 RNF213 miRNA
ENST00000559884.4 WASH3P miRNA
ENST00000559886.1 HERC1 miRNA
ENST00000559891.1 TM2D3 miRNA
ENST00000559895.4 LHX6 miRNA
ENST00000559903.1 MEF2A miRNA
ENST00000559916.1 B2M miRNA
ENST00000559925.4 IGF1R miRNA
ENST00000559937.1 SMAD3 miRNA
ENST00000559950.1 CIAO2A miRNA
ENST00000559961.1 SNX1 miRNA
ENST00000559976.1 ADAMTS17 miRNA
ENST00000559995.1 INO80 miRNA
ENST00000560004.4 RORA processed_transcript
ENST00000560018.1 FURIN retained_intron
ENST00000560023.1 SLC12A6 trna
ENST00000560034.1 trna
ENST00000560054.4 trna
ENST00000560059.4 trna
ENST00000560064.1 PSTPIP1 trna
ENST00000560067.1 USP3-AS1 trna
ENST00000560076.2 DCP1A trna
ENST00000560079.5 ZNF592 trna
ENST00000560083.1 RNF213 trna
ENST00000560091.4 CFAP161 trna
ENST00000560097.1 trna
ENST00000560098.4 CRTC3 trna
ENST00000560105.1 NIPA1 trna
ENST00000560116.1 GNB5 trna
ENST00000560130.1 HSP90AA1 trna
ENST00000560137.1 ANPEP trna
ENST00000560161.1 ATP8B4 trna
ENST00000560164.4 SLC12A6 trna
ENST00000560179.1 ETFA trna
ENST00000560185.1 AKAP13 trna
ENST00000560188.1 SPESP1 trna
ENST00000560196.4 ZEB1 trna
ENST00000560197.4 trna
ENST00000560202.1 USP3 trna
ENST00000560218.1 IQGAP1 trna
ENST00000560228.4 ZFAND6 trna
ENST00000560230.1 IL16 trna
ENST00000560236.1 LRRC28 trna
ENST00000560240.4 COPS2 trna
ENST00000560251.1 AP3S2 trna
ENST00000560255.2 ST20-AS1 trna
ENST00000560256.1 AKAP13 trna
ENST00000560269.1 HMBOX1 trna
ENST00000560274.1 RPLP1 trna
ENST00000560277.4 IGF1R trna

ENST00000560283.4 APBA2 trna
ENST00000560289.4 MINDY2 trna
ENST00000560302.4 AKAP13 trna
ENST00000560307.4 SNRPA1 trna
ENST00000560316.1 HERC1 trna
ENST00000560324.1 trna
ENST00000560328.1 NEO1 trna
ENST00000560332.1 NA trna
ENST00000560335.4 NUMB trna
ENST00000560340.4 AKAP13 trna
ENST00000560354.1 ATP8B4 trna
ENST00000560373.1 IQGAP1 trna
ENST00000560386.4 DPH6 trna
ENST00000560387.1 trna
ENST00000560418.1 IQGAP1 trna
ENST00000560420.1 PSME1 trna
ENST00000560421.4 DMXL2 trna
ENST00000560425.1 RASGRP1 trna
ENST00000560428.1 TNFAIP2 trna
ENST00000560436.1 DPP8 trna
ENST00000560437.1 ATP8B4 trna
ENST00000560440.4 IREB2 trna
ENST00000560454.1 IREB2 trna
ENST00000560463.4 TRAF3 trna
ENST00000560470.4 ZFAND6 trna
ENST00000560472.1 RCOR1 trna
ENST00000560473.1 KLF13 trna
ENST00000560482.4 AKAP13 trna
ENST00000560491.2 LYSMD2 trna
ENST00000560502.4 LDLR trna
ENST00000560514.1 TSPAN3 trna
ENST00000560519.1 HERC1 trna
ENST00000560520.1 ICE2 trna
ENST00000560527.1 USP8 trna
ENST00000560532.1 SLTM trna
ENST00000560546.4 ANXA2 trna
ENST00000560556.1 B2M trna
ENST00000560561.1 ADNP2 trna
ENST00000560579.4 AKAP13 trna
ENST00000560598.1 OTUD7A trna
ENST00000560603.4 MARK3 trna
ENST00000560620.1 CTDSPL2 trna
ENST00000560624.4 DCP1A trna
ENST00000560629.1 trna
ENST00000560637.4 VPS13C trna
ENST00000560638.1 TRPM7 trna
ENST00000560661.1 TSHZ1 trna
ENST00000560665.1 DPP8 trna
ENST00000560676.4 AKAP13 trna
ENST00000560681.1 B2M trna
ENST00000560694.1 RNF213 trna
ENST00000560701.1 PLCB2 trna
ENST00000560707.1 TSHZ3 trna
ENST00000560716.1 APH1B trna

ENST00000560725.1 TMOD3 trna
ENST00000560731.4 MARK3 trna
ENST00000560738.1 IQGAP1 trna
ENST00000560763.4 EIF5 trna
ENST00000560774.1 MAPK6 trna
ENST00000560776.1 BNIP2 trna
ENST00000560783.1 LINS1 trna
ENST00000560792.3 RFX7 trna
ENST00000560799.4 INO80 trna
ENST00000560802.1 MAPK6 trna
ENST00000560816.4 ETFA trna
ENST00000560861.1 SNX1 trna
ENST00000560873.1 trna
ENST00000560887.1 TCF12 trna
ENST00000560891.4 DMXL2 trna
ENST00000560894.1 THBS1 trna
ENST00000560900.1 trna
ENST00000560909.2 ZEB1 trna
ENST00000560918.2 TSHZ1 trna
ENST00000560919.4 MTHFS trna
ENST00000560927.1 CRTC3 trna
ENST00000560929.1 RASGRP1 trna
ENST00000560955.4 TRPM7 trna
ENST00000560956.1 WASH3P trna
ENST00000560964.1 WHAMM trna
ENST00000560982.4 USP8 trna
ENST00000560986.1 HMG20A trna
ENST00000561000.1 LINC01220 trna
ENST00000561006.4 PPP2R5C trna
ENST00000561007.1 trna
ENST00000561014.1 GALK2 trna
ENST00000561019.1 PELI2 trna
ENST00000561024.1 PDE8A trna
ENST00000561029.1 ANKRD17 trna
ENST00000561048.1 PPIB trna
ENST00000561054.1 trna
ENST00000561074.4 GALK2 trna
ENST00000561079.1 DMXL2 trna
ENST00000561086.1 IQGAP1 trna
ENST00000561092.1 ETFA trna
ENST00000561093.1 RORA trna
ENST00000561106.1 SEMA6D trna
ENST00000561119.4 CRTC3 trna
ENST00000561125.2 MEF2A trna
ENST00000561139.1 B2M trna
ENST00000561149.1 ADAM10 trna
ENST00000561152.1 TCF12 trna
ENST00000561154.1 WDR20 trna
ENST00000561156.1 TNFAIP2 trna
ENST00000561164.1 MARK3 trna
ENST00000561186.4 RNF111 trna
ENST00000561205.1 SPRED1 trna
ENST00000561217.1 TNFAIP2 trna
ENST00000561228.1 TECPR2 trna

ENST00000561235.4 TCF12 trna
ENST00000561237.1 TRIM69 trna
ENST00000561245.1 CEP152 trna
ENST00000561258.1 VPS13C trna
ENST00000561267.4 TRPM7 trna
ENST00000561280.4 CHP1 trna
ENST00000561315.4 PSTPIP1 trna
ENST00000561328.1 ICE2 trna
ENST00000561346.4 TCF12 trna
ENST00000561356.1 TM2D3 trna
ENST00000561377.1 HMBOX1 trna
ENST00000561393.4 AP4E1 trna
ENST00000561397.1 AP4E1 trna
ENST00000561400.1 HERC1 trna
ENST00000561406.1 EIF5 trna
ENST00000561407.1 TMOD2 trna
ENST00000561412.1 IRF9 trna
ENST00000561428.1 SECISBP2L trna
ENST00000561430.4 ANP32A trna
ENST00000561438.4 TMOD3 trna
ENST00000561440.1 trna
ENST00000561441.4 AP4E1 trna
ENST00000561442.4 USP3 trna
ENST00000561488.1 XPO6 trna
ENST00000561494.4 POLR3E trna
ENST00000561545.1 RAB27A trna
ENST00000561551.1 TM6SF1 trna
ENST00000561596.4 DISC1FP1 trna
ENST00000561608.4 MCTP2 trna
ENST00000561618.4 MYO9A trna
ENST00000561630.1 CDR2 trna
ENST00000561650.4 ARPP19 trna
ENST00000561664.1 FBXO31 trna
ENST00000561674.1 trna
ENST00000561678.4 TENT4B trna
ENST00000561707.1 COTL1 trna
ENST00000561708.1 ST3GAL2 trna
ENST00000561714.4 NFATC3 trna
ENST00000561721.5 SEC14L1 trna
ENST00000561728.1 trna
ENST00000561733.4 RBMX trna
ENST00000561742.4 IL4R trna
ENST00000561765.1 MAPK8IP3 trna
ENST00000561770.1 ARIH1 trna
ENST00000561798.1 UQCRC2 trna
ENST00000561810.1 MYO5A trna
ENST00000561849.1 CORO1A trna
ENST00000561867.1 RSRP1 trna
ENST00000561869.1 CHD9 trna
ENST00000561885.1 FBXO22 trna
ENST00000561903.5 EME2 trna
ENST00000561920.4 PDPR trna
ENST00000561921.1 trna
ENST00000561931.1 TANGO6 trna

ENST00000561932.4 ATF7IP2 trna
ENST00000561933.1 SLC7A6OS trna
ENST00000561942.1 GLG1 trna
ENST00000561945.1 SPG7 trna
ENST00000561962.1 PDPK1 trna
ENST00000561972.1 CYBA trna
ENST00000561996.1 CPT1A trna
ENST00000561999.1 LINC02256 trna
ENST00000562015.4 CNN2 trna
ENST00000562046.1 CNOT1 trna
ENST00000562066.4 CSK trna
ENST00000562073.1 ABHD2 trna
ENST00000562129.1 CORO1A trna
ENST00000562153.4 TXNL4B trna
ENST00000562169.1 GSPT1 trna
ENST00000562171.1 NFATC3 trna
ENST00000562174.4 EDC3 trna
ENST00000562190.1 PIAS1 trna
ENST00000562193.1 TCF25 trna
ENST00000562194.1 ANKRD11 trna
ENST00000562211.1 ANKRD11 trna
ENST00000562217.1 trna
ENST00000562221.1 trna
ENST00000562225.1 IFI16 trna
ENST00000562254.1 ABHD2 trna
ENST00000562286.1 ADPGK trna
ENST00000562304.1 trna
ENST00000562351.2 FAM214A trna
ENST00000562367.1 CSNK2A2 trna
ENST00000562370.4 CAPN15 protein_coding
ENST00000562373.1 NR4A1 protein_coding
ENST00000562380.4 LMF1 protein_coding
ENST00000562388.1 KIAA0513 retained_intron
ENST00000562408.1 XPO6 retained_intron
ENST00000562415.2 PDPK2P transcribed_unprocessed_pseudogene
ENST00000562417.1 ZFPM1 retained_intron
ENST00000562429.1 NFAT5 retained_intron
ENST00000562430.4 RBBP6 retained_intron
ENST00000562484.2 TK2 protein_coding
ENST00000562510.1 SNX29 protein_coding
ENST00000562522.2 ITGAX protein_coding
ENST00000562564.1 KIAA0513 protein_coding
ENST00000562593.4 GALNS retained_intron
ENST00000562621.1 ADPGK retained_intron
ENST00000562639.4 WWOX processed_transcript
ENST00000562646.4 RBMX protein_coding
ENST00000562695.1 MOSMO protein_coding
ENST00000562753.1 CDYL2 processed_transcript
ENST00000562784.1 PKM retained_intron
ENST00000562816.4 ANKRD11 processed_transcript
ENST00000562829.1 TNRC6A processed_transcript
ENST00000562837.1 RBL2 retained_intron
ENST00000562840.1 C16orf95 processed_transcript
ENST00000562850.1 RBL2 retained_intron

ENST00000562866.1 VPS9D1-AS1 lncRNA
ENST00000562880.4 TTBK2 protein_coding
ENST00000562890.1 SCAPER processed_transcript
ENST00000562896.1 PARN protein_coding
ENST00000562902.1 processed_transcript
ENST00000562913.4 KLHDC4 retained_intron
ENST00000562914.1 HERPUD1 retained_intron
ENST00000562929.1 COX4I1 processed_transcript
ENST00000562934.1 AP1G1 processed_transcript
ENST00000562937.4 NPIPB13 nonsense_mediated_decay
ENST00000562955.1 SZT2 protein_coding
ENST00000562961.1 METTL9 protein_coding
ENST00000562970.1 lncRNA
ENST00000562971.1 ZNF267 protein_coding
ENST00000563010.1 CSK retained_intron
ENST00000563036.2 lncRNA
ENST00000563092.1 CKLF processed_transcript
ENST00000563111.2 WDR59 protein_coding
ENST00000563128.4 lncRNA
ENST00000563158.1 DNAJA2 nonsense_mediated_decay
ENST00000563180.1 IRF8 protein_coding
ENST00000563190.1 lncRNA
ENST00000563192.1 SNHG19 lncRNA
ENST00000563194.1 RPS2 protein_coding
ENST00000563269.1 PLCG2 retained_intron
ENST00000563279.4 MON1B protein_coding
ENST00000563281.1 RBM15B protein_coding
ENST00000563291.1 ANKRD11 protein_coding
ENST00000563296.1 SQOR protein_coding
ENST00000563298.1 PPCDC processed_transcript
ENST00000563305.1 ATP6V0D1 retained_intron
ENST00000563307.1 CSNK2A2 retained_intron
ENST00000563308.1 SNX29 protein_coding
ENST00000563310.1 ARIH1 retained_intron
ENST00000563328.4 lncRNA
ENST00000563333.1 SNAP23 retained_intron
ENST00000563343.4 HERPUD1 protein_coding
ENST00000563350.1 ITFG1 processed_transcript
ENST00000563357.1 FAM157C lncRNA
ENST00000563358.4 WWOX processed_transcript
ENST00000563376.4 PHKB protein_coding
ENST00000563403.1 XYLT1 processed_transcript
ENST00000563406.1 TCF25 processed_transcript
ENST00000563448.1 SMG1 retained_intron
ENST00000563474.2 NFAT5 nonsense_mediated_decay
ENST00000563477.1 lncRNA
ENST00000563523.4 PECAM1 protein_coding
ENST00000563542.4 MYO9A processed_transcript
ENST00000563556.1 AMDHD2 protein_coding
ENST00000563579.1 RPS15A protein_coding
ENST00000563580.4 RAB11A processed_transcript
ENST00000563592.4 ADPGK-AS1 lncRNA
ENST00000563614.1 DCTN5 retained_intron
ENST00000563618.4 WWP2 processed_transcript

ENST00000563625.1 ZFHX3 retained_intron
ENST00000563629.1 CYLD retained_intron
ENST00000563640.1 CBFA2T3 protein_coding
ENST00000563648.1 MYO9A retained_intron
ENST00000563662.2 VAC14 non_stop_decay
ENST00000563681.1 DISC1FP1 lncRNA
ENST00000563688.4 SCAPER retained_intron
ENST00000563783.4 SPG7 processed_transcript
ENST00000563798.1 VPS9D1 nonsense_mediated_decay
ENST00000563836.1 SMG1 protein_coding
ENST00000563840.1 SIRPB1 protein_coding
ENST00000563872.4 STARD9 retained_intron
ENST00000563886.1 IL4R retained_intron
ENST00000563892.4 USP10 protein_coding
ENST00000563894.1 CSK retained_intron
ENST00000563908.1 HEXA retained_intron
ENST00000563919.1 SCAPER protein_coding
ENST00000563920.1 CBFA2T3 retained_intron
ENST00000563924.4 PECAM1 protein_coding
ENST00000563939.2 CBFB nonsense_mediated_decay
ENST00000563952.1 CMTM4 protein_coding
ENST00000563966.1 UBE2Q2 processed_transcript
ENST00000563986.1 PKM retained_intron
ENST00000563992.1 ABAT retained_intron
ENST00000563996.1 PIAS1 nonsense_mediated_decay
ENST00000564009.1 PIAS1 retained_intron
ENST00000564056.1 IRF8 retained_intron
ENST00000564067.4 TRAF7 nonsense_mediated_decay
ENST00000564084.1 LPCAT2 protein_coding
ENST00000564089.4 IL21R protein_coding
ENST00000564118.1 NA NA
ENST00000564131.1 RRN3 protein_coding
ENST00000564143.1 UBE2I retained_intron
ENST00000564155.4 AP1G1 protein_coding
ENST00000564161.1 ZC3H18 retained_intron
ENST00000564172.1 GDE1 nonsense_mediated_decay
ENST00000564177.1 SCAPER protein_coding
ENST00000564178.4 PKM protein_coding
ENST00000564189.1 ZNF646 protein_coding
ENST00000564190.1 MGA protein_coding
ENST00000564247.1 CMTM3 retained_intron
ENST00000564250.1 C16orf87 processed_transcript
ENST00000564255.4 CHD9 retained_intron
ENST00000564259.1 LONP2 processed_transcript
ENST00000564308.1 ITGAX retained_intron
ENST00000564352.1 lncRNA
ENST00000564416.1 CBFA2T3 protein_coding
ENST00000564419.1 COG8 processed_transcript
ENST00000564444.1 MBTPS1 processed_transcript
ENST00000564446.1 CORO1A processed_transcript
ENST00000564466.4 ZDHHC7 protein_coding
ENST00000564489.1 lncRNA
ENST00000564502.1 NPIPB13 nonsense_mediated_decay
ENST00000564526.1 ZDHHC7 retained_intron

ENST00000564553.4 ANKRD11 retained_intron
ENST00000564559.5 DYNC1LI2 nonsense_mediated_decay
ENST00000564566.1 USP10 processed_transcript
ENST00000564572.1 CNN2 retained_intron
ENST00000564582.2 CHD9 processed_transcript
ENST00000564586.4 CDIN1 protein_coding
ENST00000564615.4 ATP6V0D1 protein_coding
ENST00000564632.1 ITGAL retained_intron
ENST00000564634.4 CYLD protein_coding
ENST00000564643.1 MBTPS1 retained_intron
ENST00000564647.4 RPL4 retained_intron
ENST00000564662.1 COTL1 retained_intron
ENST00000564663.1 CCPG1 protein_coding
ENST00000564666.1 CMIP processed_transcript
ENST00000564670.1 LINC02256 lncRNA
ENST00000564671.2 TERF2IP protein_coding
ENST00000564674.4 DENND4A protein_coding
ENST00000564682.4 SGF29 retained_intron
ENST00000564685.4 VAC14 retained_intron
ENST00000564702.1 retained_intron
ENST00000564710.1 N4BP1 protein_coding
ENST00000564725.1 PML retained_intron
ENST00000564733.1 METTL9 retained_intron
ENST00000564734.4 HERC2 nonsense_mediated_decay
ENST00000564744.1 RPL4 retained_intron
ENST00000564757.4 SCAPER nonsense_mediated_decay
ENST00000564766.1 FUS retained_intron
ENST00000564768.1 CORO1A retained_intron
ENST00000564787.1 MTMR10 nonsense_mediated_decay
ENST00000564791.4 SNX29 protein_coding
ENST00000564793.1 CFDP1 processed_transcript
ENST00000564831.4 APOBR protein_coding
ENST00000564848.1 C15orf39 protein_coding
ENST00000564859.1 WDR59 nonsense_mediated_decay
ENST00000564866.4 PECAM1 protein_coding
ENST00000564873.1 PHKB protein_coding
ENST00000564882.4 PARN processed_transcript
ENST00000564904.4 PARN processed_transcript
ENST00000564905.1 XPO6 nonsense_mediated_decay
ENST00000564935.1 ITGAL retained_intron
ENST00000564970.1 PTPN9 processed_transcript
ENST00000564973.1 ATP6V0C protein_coding
ENST00000564983.4 ADGRG5 retained_intron
ENST00000564988.4 TM6SF1 processed_transcript
ENST00000565029.4 CMIP retained_intron
ENST00000565056.1 LPCAT2 processed_transcript
ENST00000565058.2 lncRNA
ENST00000565066.4 ABHD2 protein_coding
ENST00000565071.1 CTU2 retained_intron
ENST00000565074.1 C15orf39 protein_coding
ENST00000565079.4 MEAK7 protein_coding
ENST00000565099.1 EIF3C processed_transcript
ENST00000565117.1 POLR3E retained_intron
ENST00000565138.4 lncRNA

ENST00000565143.1 PKM retained_intron
ENST00000565164.1 MVP protein_coding
ENST00000565165.1 ARSB protein_coding
ENST00000565181.1 TMEM202-AS1 lncRNA
ENST00000565223.1 ATP6V0C protein_coding
ENST00000565242.1 BANP retained_intron
ENST00000565246.4 SMG1P7 transcribed_unprocessed_pseudogene
ENST00000565260.1 GLG1 protein_coding
ENST00000565262.1 ITFG1 protein_coding
ENST00000565264.1 SIN3A protein_coding
ENST00000565267.4 GSPT1 protein_coding
ENST00000565276.4 LMF1 nonsense_mediated_decay
ENST00000565280.4 FAN1 nonsense_mediated_decay
ENST00000565282.4 NPTN protein_coding
ENST00000565301.1 NFAT5 protein_coding
ENST00000565324.4 SMG1 protein_coding
ENST00000565352.1 IL4R protein_coding
ENST00000565353.1 PSME3IP1 processed_transcript
ENST00000565355.1 ALDOA protein_coding
ENST00000565364.1 GALNS processed_transcript
ENST00000565391.1 ADGRG1 protein_coding
ENST00000565400.4 PLCG2 processed_transcript
ENST00000565412.4 AP1G1 protein_coding
ENST00000565417.4 BOLA2-SMG1P6 nonsense_mediated_decay
ENST00000565455.4 USP7 nonsense_mediated_decay
ENST00000565474.1 GLG1 protein_coding
ENST00000565493.1 NORAD lncRNA
ENST00000565499.1 IGSF6 retained_intron
ENST00000565500.4 ZNF106 protein_coding
ENST00000565514.1 CMC2 processed_transcript
ENST00000565552.1 IRF8 protein_coding
ENST00000565588.4 CYBA protein_coding
ENST00000565638.1 N4BP1 processed_transcript
ENST00000565644.4 SLC7A5 protein_coding
ENST00000565653.1 ADAMTSL3 processed_transcript
ENST00000565680.1 CMIP retained_intron
ENST00000565690.4 FBXL19 protein_coding
ENST00000565697.1 CNOT1 protein_coding
ENST00000565723.1 RPL4 processed_transcript
ENST00000565750.2 NFATC3 protein_coding
ENST00000565776.1 RAB27A retained_intron
ENST00000565791.1 WWOX processed_transcript
ENST00000565803.1 CHD9 protein_coding
ENST00000565820.4 PEAK1 protein_coding
ENST00000565825.4 ABHD2 processed_transcript
ENST00000565857.1 BMERB1 protein_coding
ENST00000565867.2 LONP2 protein_coding
ENST00000565907.1 RBMX retained_intron
ENST00000565915.4 IL4R processed_transcript
ENST00000565932.4 EIF3C retained_intron
ENST00000565934.1 PMM2 retained_intron
ENST00000565948.1 ZNF106 protein_coding
ENST00000565950.1 ARIH1 retained_intron
ENST00000565956.4 PSME3IP1 protein_coding

ENST00000565958.1 CCNDBP1 retained_intron
ENST00000565965.1 lncRNA
ENST00000565970.4 SCAPER protein_coding
ENST00000566004.4 USP7 protein_coding
ENST00000566021.1 PARN processed_transcript
ENST00000566024.1 CYLD protein_coding
ENST00000566037.5 PHKB protein_coding
ENST00000566038.1 transcribed_unprocessed_pseudogene
ENST00000566044.4 PHKB protein_coding
ENST00000566073.1 XPO6 protein_coding
ENST00000566078.1 CTCF retained_intron
ENST00000566093.1 CNEP1R1 protein_coding
ENST00000566095.5 SF3B3 protein_coding
ENST00000566104.1 TRADD processed_transcript
ENST00000566127.1 SMG1P2 transcribed_unprocessed_pseudogene
ENST00000566146.1 ALDOA retained_intron
ENST00000566147.1 ARID3B protein_coding
ENST00000566149.1 ITGAL retained_intron
ENST00000566151.1 CRISPLD2 protein_coding
ENST00000566165.1 CRISPLD2 nonsense_mediated_decay
ENST00000566173.3 CDYL2 protein_coding
ENST00000566175.1 XPO6 nonsense_mediated_decay
ENST00000566179.1 AP1G1 nonsense_mediated_decay
ENST00000566224.1 USP7 processed_transcript
ENST00000566228.4 SNX29 protein_coding
ENST00000566240.1 CNOT1 nonsense_mediated_decay
ENST00000566264.1 TMEM204 protein_coding
ENST00000566267.1 ITGAL processed_transcript
ENST00000566273.4 USP7 protein_coding
ENST00000566275.2 PHKB protein_coding
ENST00000566277.2 processed_pseudogene
ENST00000566278.5 A2MP1 transcribed_unprocessed_pseudogene
ENST00000566315.4 NUP93 protein_coding
ENST00000566326.1 MAP2K1 protein_coding
ENST00000566334.1 AMFR retained_intron
ENST00000566338.4 MTMR10 retained_intron
ENST00000566343.4 ERVK13-1 lncRNA
ENST00000566352.1 RSPRY1 retained_intron
ENST00000566391.1 ARL6IP1 retained_intron
ENST00000566394.1 lncRNA
ENST00000566426.1 PDXDC1 protein_coding
ENST00000566428.4 KIAA0513 protein_coding
ENST00000566454.4 SLC7A6 protein_coding
ENST00000566455.1 MON1B retained_intron
ENST00000566462.2 CMIP protein_coding
ENST00000566463.4 WWP2 retained_intron
ENST00000566480.4 SCAMP2 protein_coding
ENST00000566488.1 ATMIN protein_coding
ENST00000566491.1 RPL4 retained_intron
ENST00000566509.4 ADPGK retained_intron
ENST00000566512.1 USP10 retained_intron
ENST00000566513.4 CMIP protein_coding
ENST00000566547.1 GGA2 retained_intron
ENST00000566560.4 KARS1 retained_intron

ENST00000566579.4 SLC7A6 nonsense_mediated_decay
ENST00000566601.1 GLG1 retained_intron
ENST00000566609.1 LMF1 processed_transcript
ENST00000566633.1 PDXDC1 retained_intron
ENST00000566670.2 GRIN2A processed_transcript
ENST00000566677.1 CDIN1 retained_intron
ENST00000566678.1 NUP93 protein_coding
ENST00000566679.5 CYLD protein_coding
ENST00000566681.1 PSME3IP1 protein_coding
ENST00000566695.4 CNN2 protein_coding
ENST00000566718.4 MGA protein_coding
ENST00000566744.4 MYO9A processed_transcript
ENST00000566748.1 MPHOSPH6 retained_intron
ENST00000566751.4 TCF25 processed_transcript
ENST00000566753.4 BLOC1S6 retained_intron
ENST00000566756.4 CMTM3 nonsense_mediated_decay
ENST00000566761.2 ADCY7 protein_coding
ENST00000566772.1 KARS1 protein_coding
ENST00000566775.1 UBE2I nonsense_mediated_decay
ENST00000566833.1 CCNDBP1 retained_intron
ENST00000566858.4 ANKRD11 protein_coding
ENST00000566877.4 RAB27A protein_coding
ENST00000566911.1 LPCAT2 retained_intron
ENST00000566934.1 SQOR protein_coding
ENST00000566942.1 lncRNA
ENST00000566973.1 ANKRD11 processed_transcript
ENST00000567028.4 protein_coding
ENST00000567031.1 ITGAM protein_coding
ENST00000567041.1 ZNF106 protein_coding
ENST00000567124.4 KIAA0040 processed_transcript
ENST00000567133.1 CNOT1 protein_coding
ENST00000567165.4 LYRM1 nonsense_mediated_decay
ENST00000567188.4 CNOT1 nonsense_mediated_decay
ENST00000567198.1 NPTN protein_coding
ENST00000567213.2 HEXA protein_coding
ENST00000567216.4 CAPN15 processed_transcript
ENST00000567232.1 TNRC6A retained_intron
ENST00000567274.4 TTBK2 protein_coding
ENST00000567277.5 ADCY7 nonsense_mediated_decay
ENST00000567278.1 COTL1 processed_transcript
ENST00000567285.1 CNOT1 protein_coding
ENST00000567287.2 UTP4 processed_transcript
ENST00000567323.2 DENND4A processed_transcript
ENST00000567328.4 KIAA0513 protein_coding
ENST00000567356.1 PLCG2 retained_intron
ENST00000567417.1 PIAS1 processed_transcript
ENST00000567428.2 lncRNA
ENST00000567468.4 GGA2 protein_coding
ENST00000567485.1 TTBK2 processed_transcript
ENST00000567513.1 KLHDC4 processed_transcript
ENST00000567526.1 USP10 protein_coding
ENST00000567555.1 ALDOA retained_intron
ENST00000567571.4 CSK protein_coding
ENST00000567573.4 CDIN1 processed_transcript

ENST00000567579.4 WWP2 processed_transcript
ENST00000567586.1 ST3GAL2 processed_transcript
ENST00000567597.1 UQCRC2 retained_intron
ENST00000567617.1 C15orf39 protein_coding
ENST00000567658.1 EEF1A1P38 processed_pseudogene
ENST00000567661.4 IFI16 protein_coding
ENST00000567671.1 RAB11A protein_coding
ENST00000567688.1 transcribed_unprocessed_pseudogene
ENST00000567697.1 PMM2 retained_intron
ENST00000567730.5 CSNK2A2 protein_coding
ENST00000567732.1 NA NA
ENST00000567733.1 ADPGK processed_transcript
ENST00000567736.4 ANKRD11 protein_coding
ENST00000567741.1 RSRP1 processed_transcript
ENST00000567762.1 ARIH1 processed_transcript
ENST00000567772.2 ZNF106 protein_coding
ENST00000567810.1 UQCRC2 retained_intron
ENST00000567826.1 BRD7 retained_intron
ENST00000567830.1 ARPP19 protein_coding
ENST00000567842.4 XPO6 retained_intron
ENST00000567844.1 RNF166 protein_coding
ENST00000567849.1 MAPK8IP3 nonsense_mediated_decay
ENST00000567869.1 HERC2 retained_intron
ENST00000567913.2 processed_transcript
ENST00000567947.1 CBFB retained_intron
ENST00000567957.1 ITFG1 retained_intron
ENST00000567964.5 RBL2 protein_coding
ENST00000567980.4 PLCG2 retained_intron
ENST00000567986.4 WWP2 retained_intron
ENST00000567990.4 NFAT5 nonsense_mediated_decay
ENST00000568020.4 ZCCHC14 nonsense_mediated_decay
ENST00000568024.1 SLC7A6 protein_coding
ENST00000568033.1 GOLGA8M lncRNA
ENST00000568038.1 SMG1 nonsense_mediated_decay
ENST00000568053.4 NPIPB13 protein_coding
ENST00000568070.1 FAM157C lncRNA
ENST00000568072.1 ZC3H18 retained_intron
ENST00000568107.2 NA NA
ENST00000568171.1 PHKB retained_intron
ENST00000568212.1 RSRP1 retained_intron
ENST00000568226.4 XYLT1 processed_transcript
ENST00000568258.4 KATNIP protein_coding
ENST00000568269.4 EEF2K nonsense_mediated_decay
ENST00000568294.4 HDGFL3 protein_coding
ENST00000568298.1 ATP6V0D1 retained_intron
ENST00000568312.1 KIF22 nonsense_mediated_decay
ENST00000568314.1 lncRNA
ENST00000568315.1 SLC7A6OS nonsense_mediated_decay
ENST00000568357.1 VPS39 processed_transcript
ENST00000568359.1 SNX29 processed_transcript
ENST00000568361.4 TANGO6 retained_intron
ENST00000568372.4 CCPG1 retained_intron
ENST00000568439.1 PHKB protein_coding
ENST00000568440.1 LAT retained_intron

ENST00000568455.1 GABARAPL2 protein_coding
ENST00000568457.1 lncRNA
ENST00000568466.5 NFATC3 nonsense_mediated_decay
ENST00000568469.4 TMC7 retained_intron
ENST00000568479.1 PDK3 protein_coding
ENST00000568487.1 ADGRG1 processed_transcript
ENST00000568511.1 ZNRF1 protein_coding
ENST00000568512.4 ANKRD11 processed_transcript
ENST00000568514.4 SNAP23 retained_intron
ENST00000568515.1 DENND4A retained_intron
ENST00000568530.4 PDPR protein_coding
ENST00000568543.1 CCPG1 protein_coding
ENST00000568559.1 TMEM170A nonsense_mediated_decay
ENST00000568562.1 ATP6V0C protein_coding
ENST00000568592.1 CCPG1 protein_coding
ENST00000568611.4 MTMR10 retained_intron
ENST00000568637.4 FAM214A protein_coding
ENST00000568641.1 protein_coding
ENST00000568642.4 VPS35 processed_transcript
ENST00000568657.1 AMFR processed_transcript
ENST00000568684.1 WWP2 protein_coding
ENST00000568695.1 DDX59-AS1 lncRNA
ENST00000568702.4 PECAM1 processed_transcript
ENST00000568704.2 CYLD protein_coding
ENST00000568743.1 PKM retained_intron
ENST00000568762.1 AMFR retained_intron
ENST00000568777.4 HEXA retained_intron
ENST00000568784.5 VPS35 nonsense_mediated_decay
ENST00000568803.1 RAB27A protein_coding
ENST00000568816.4 BLOC1S6 protein_coding
ENST00000568826.1 METTL9 protein_coding
ENST00000568841.4 SNAP23 protein_coding
ENST00000568845.4 WWP2 processed_transcript
ENST00000568847.4 ABAT protein_coding
ENST00000568855.1 SMG1P7 processed_transcript
ENST00000568863.1 FAM214A protein_coding
ENST00000568870.1 TCF25 retained_intron
ENST00000568886.4 VAC14 nonsense_mediated_decay
ENST00000568914.1 NA NA
ENST00000568917.1 CNOT1 protein_coding
ENST00000568924.1 ANKRD11 nonsense_mediated_decay
ENST00000568930.1 ADCY7 retained_intron
ENST00000568949.1 SNX29 retained_intron
ENST00000568956.1 ZNF865 protein_coding
ENST00000568980.1 HNRNPLP2 unprocessed_pseudogene
ENST00000568987.1 ITGAL retained_intron
ENST00000569017.1 ZDHHC7 protein_coding
ENST00000569045.1 CDR2 processed_transcript
ENST00000569050.1 PKM protein_coding
ENST00000569086.4 ZFPM1 protein_coding
ENST00000569122.1 SMG1 protein_coding
ENST00000569124.1 SCAMP2 retained_intron
ENST00000569145.4 IRF8 protein_coding
ENST00000569159.1 PEAK1 processed_transcript

ENST00000569179.1 RIPOR1 protein_coding
ENST00000569216.2 XPO6 processed_transcript
ENST00000569223.1 EHD4 retained_intron
ENST00000569225.4 CHD9 retained_intron
ENST00000569234.1 TERF2IP nonsense_mediated_decay
ENST00000569251.4 SCAMP2 retained_intron
ENST00000569257.4 CPEB1 protein_coding
ENST00000569263.1 CNOT1 processed_transcript
ENST00000569265.1 ADCY7 protein_coding
ENST00000569276.1 TMEM170A protein_coding
ENST00000569280.1 TERF2 nonsense_mediated_decay
ENST00000569289.4 DUS2 protein_coding
ENST00000569290.1 METTL9 miRNA
ENST00000569304.1 RAB11A miRNA
ENST00000569314.1 MYO9A miRNA
ENST00000569332.4 WWOX miRNA
ENST00000569341.1 CFDP1 miRNA
ENST00000569395.4 SCAPER miRNA
ENST00000569435.4 ZC3H18 miRNA
ENST00000569478.1 RNF166 miRNA
ENST00000569488.1 ZDHHC7 miRNA
ENST00000569511.4 USP10 miRNA
ENST00000569515.1 MCMBP miRNA
ENST00000569523.1 PLCG2 miRNA
ENST00000569530.1 PECAM1 miRNA
ENST00000569551.5 ITFG1 miRNA
ENST00000569569.4 HERPUD1 miRNA
ENST00000569591.2 MRC1 miRNA
ENST00000569607.1 IRF8 miRNA
ENST00000569648.4 ZNF106 miRNA
ENST00000569653.1 GTF3C1 miRNA
ENST00000569680.1 ARID3B miRNA
ENST00000569684.1 PHKG2 miRNA
ENST00000569689.1 KCTD5 miRNA
ENST00000569693.4 ADPGK miRNA
ENST00000569710.1 CDK13-DT miRNA
ENST00000569725.1 ITGAL miRNA
ENST00000569730.1 miRNA
ENST00000569764.1 SMG1 miRNA
ENST00000569774.5 BRD7 miRNA
ENST00000569784.1 SCAPER miRNA
ENST00000569801.4 SNX29 miRNA
ENST00000569819.4 PEAK1 miRNA
ENST00000569839.1 ADGRG5 miRNA
ENST00000569858.1 miRNA
ENST00000569880.1 TCF12 miRNA
ENST00000569882.1 CNOT1 miRNA
ENST00000569895.3 RBFOX1 miRNA
ENST00000569896.1 RAB11A miRNA
ENST00000569912.1 TNRC6B-DT miRNA
ENST00000569929.4 PLCG2 miRNA
ENST00000569955.2 miRNA
ENST00000569967.1 PECAM1 miRNA
ENST00000569970.1 CORO1A miRNA

ENST00000569971.4 UBR1 miRNA
ENST00000569973.1 XPO6 miRNA
ENST00000570005.4 KCTD5 miRNA
ENST00000570012.1 MBTPS1 miRNA
ENST00000570033.1 XPO6 miRNA
ENST00000570034.1 NSFP1 miRNA
ENST00000570046.1 CBFA2T3 miRNA
ENST00000570047.2 PHKB miRNA
ENST00000570054.3 miRNA
ENST00000570061.1 MVP miRNA
ENST00000570064.4 MBTPS1 miRNA
ENST00000570074.1 METTL9 miRNA
ENST00000570085.4 ARIH1 miRNA
ENST00000570090.1 FUS miRNA
ENST00000570104.1 WWP2 miRNA
ENST00000570106.5 NA miRNA
ENST00000570108.4 ZFYVE19 miRNA
ENST00000570134.4 PMM2 miRNA
ENST00000570140.1 miRNA
ENST00000570149.1 RPL13 miRNA
ENST00000570161.4 MGA miRNA
ENST00000570174.1 LONP2 miRNA
ENST00000570187.1 ADCY7 miRNA
ENST00000570198.1 PLCG2 miRNA
ENST00000570199.1 miRNA
ENST00000570202.1 miRNA
ENST00000570215.1 KARS1 miRNA
ENST00000570219.1 BFAR miRNA
ENST00000570230.1 FAM157C miRNA
ENST00000570231.1 THUMPD1 miRNA
ENST00000570259.1 HSBP1 miRNA
ENST00000570272.4 CCPG1 miRNA
ENST00000570294.4 XPO6 miRNA
ENST00000570297.4 AP1G1 miRNA
ENST00000570311.1 LRRC37A17P miRNA
ENST00000570382.1 ACTG1 miRNA
ENST00000570395.1 FTO miRNA
ENST00000570403.1 TRAP1 miRNA
ENST00000570439.1 ACAP1 miRNA
ENST00000570478.4 LRRC37A17P miRNA
ENST00000570479.1 SCPEP1 miRNA
ENST00000570525.4 ABR miRNA
ENST00000570539.1 SRRM2 miRNA
ENST00000570555.1 NAA38 miRNA
ENST00000570564.4 URI1 miRNA
ENST00000570583.4 STX8 miRNA
ENST00000570585.1 FOXK2 miRNA
ENST00000570618.4 ARL17B miRNA
ENST00000570632.1 KDM6B miRNA
ENST00000570650.1 VPS53 miRNA
ENST00000570668.1 SMG6 miRNA
ENST00000570704.4 URI1 miRNA
ENST00000570727.4 NDE1 miRNA
ENST00000570749.4 TRIM25 miRNA

ENST00000570771.1 VPS53 miRNA
ENST00000570776.1 RNF213 miRNA
ENST00000570778.4 METRNL miRNA
ENST00000570818.4 CDC27 miRNA
ENST00000570841.4 ARHGAP17 miRNA
ENST00000570874.1 SMG6 miRNA
ENST00000570891.4 RPTOR miRNA
ENST00000570913.4 BAIAP2 miRNA
ENST00000570928.4 CORO7 miRNA
ENST00000570934.4 ANKFY1 miRNA
ENST00000570939.1 CREBBP miRNA
ENST00000570981.1 LCMT1 miRNA
ENST00000570987.1 STX8 miRNA
ENST00000571022.4 ABR miRNA
ENST00000571047.4 KDM6B miRNA
ENST00000571058.4 RPA1 miRNA
ENST00000571081.5 DUS2 miRNA
ENST00000571177.4 ULK2 miRNA
ENST00000571206.1 ARRB2 miRNA
ENST00000571207.4 MINK1 miRNA
ENST00000571218.1 B3GNTL1 miRNA
ENST00000571246.4 ARL17B miRNA
ENST00000571277.1 LITAF miRNA
ENST00000571289.1 PAFAH1B1 miRNA
ENST00000571298.4 METTL16 miRNA
ENST00000571303.1 MLKL nonsense_mediated_decay
ENST00000571307.1 NLRP1 processed_transcript
ENST00000571346.1 PRPF8 trna
ENST00000571376.1 SLC43A2 trna
ENST00000571378.4 SRRM2 trna
ENST00000571383.4 ABR trna
ENST00000571406.1 ARHGAP17 trna
ENST00000571457.1 CHMP6 trna
ENST00000571495.1 PAFAH1B1 trna
ENST00000571507.4 P4HB trna
ENST00000571545.1 CIITA trna
ENST00000571555.1 RAP1GAP2 trna
ENST00000571575.4 ARHGAP17 trna
ENST00000571578.1 MMD trna
ENST00000571618.4 TBCD trna
ENST00000571637.1 P2RX1 trna
ENST00000571644.1 ITGAX trna
ENST00000571680.1 ITGB3 trna
ENST00000571691.4 ACTG1 trna
ENST00000571698.1 KANSL1 trna
ENST00000571702.1 RPS7P1 trna
ENST00000571720.2 ZFP90 trna
ENST00000571722.3 SNORD3B-2 trna
ENST00000571763.4 CREBBP trna
ENST00000571814.1 METRNL trna
ENST00000571835.4 WDR45B trna
ENST00000571858.1 AKAP10 trna
ENST00000571882.1 TXNDC11 trna
ENST00000571889.1 ADCY9 trna

ENST00000571908.1 RNF213 trna
ENST00000571945.4 ABR trna
ENST00000571953.4 UBE2G1 trna
ENST00000571975.1 trna
ENST00000571980.1 UBE2G1 trna
ENST00000572004.1 PITPNA trna
ENST00000572123.4 LINC00921 trna
ENST00000572134.1 CREBBP trna
ENST00000572143.1 NLRP1 trna
ENST00000572151.1 RNF213-AS1 trna
ENST00000572218.4 KANSL1 trna
ENST00000572288.1 ADCY9 trna
ENST00000572334.5 VPS53 trna
ENST00000572383.1 PFN1 trna
ENST00000572412.4 ANKFY1 trna
ENST00000572418.1 P2RX1 trna
ENST00000572426.4 ZZEF1 trna
ENST00000572518.1 CORO7 trna
ENST00000572547.1 trna
ENST00000572554.1 RNF167 trna
ENST00000572569.1 CREBBP trna
ENST00000572585.4 ABR trna
ENST00000572621.4 PRPF8 trna
ENST00000572622.1 RNF213 trna
ENST00000572624.4 DPEP2 trna
ENST00000572628.4 BCL7C trna
ENST00000572638.1 LRRC37A2 trna
ENST00000572681.5 CIC trna
ENST00000572694.1 ATP2A3 trna
ENST00000572721.1 SRRM2 trna
ENST00000572733.1 RPTOR trna
ENST00000572782.1 ARRB2 trna
ENST00000572798.1 AATK trna
ENST00000572844.1 POLR2A trna
ENST00000572870.4 ZC3H7A trna
ENST00000572875.1 SGSM2 trna
ENST00000572882.2 ABCC1 trna
ENST00000572888.4 DPEP2 trna
ENST00000572902.4 SIRT7 trna
ENST00000572915.5 PAFAH1B1 trna
ENST00000572952.1 SRRM2 trna
ENST00000572967.1 NDE1 trna
ENST00000572977.4 B3GNTL1 trna
ENST00000572984.4 TBCD trna
ENST00000572991.4 ARL17B trna
ENST00000573016.2 STX8 trna
ENST00000573051.1 MRTFB trna
ENST00000573056.1 PITPNA trna
ENST00000573108.1 TRIM25 trna
ENST00000573113.3 ZFP90 trna
ENST00000573161.1 ZFP90 trna
ENST00000573165.1 CORO7 trna
ENST00000573169.1 NUP88 trna
ENST00000573174.1 TXNDC11 trna

ENST00000573177.1 DLGAP1-AS2 trna
ENST00000573185.4 ARL17A trna
ENST00000573224.1 GOSR2 trna
ENST00000573245.1 CORO7 trna
ENST00000573250.2 ANKFY1 trna
ENST00000573308.4 TAPT1-AS1 trna
ENST00000573325.1 ABR trna
ENST00000573366.4 SPAG7 trna
ENST00000573394.1 RNF213-AS1 trna
ENST00000573434.1 MRTFB trna
ENST00000573517.4 CREBBP trna
ENST00000573518.1 XAF1 trna
ENST00000573548.1 RNF213 trna
ENST00000573554.1 RPS15A trna
ENST00000573645.1 XPO6 trna
ENST00000573681.1 PRPF8 trna
ENST00000573703.1 ARHGAP17 trna
ENST00000573746.1 RPTOR trna
ENST00000573772.1 ZNF594-DT trna
ENST00000573773.4 CORO7 trna
ENST00000573850.1 KATNIP trna
ENST00000573866.2 trna
ENST00000573928.1 GABARAP trna
ENST00000573930.1 SAT2 trna
ENST00000573939.1 ALOX12-AS1 trna
ENST00000573950.4 TAPT1-AS1 trna
ENST00000573984.1 CYB5D2 trna
ENST00000574016.4 MIR22HG trna
ENST00000574035.4 trna
ENST00000574049.1 RPA1 trna
ENST00000574059.1 UBE2G1 trna
ENST00000574093.1 TMEM105 trna
ENST00000574139.4 ABR trna
ENST00000574163.1 NDE1 trna
ENST00000574202.1 ATP2A3 trna
ENST00000574224.1 ABCC1 trna
ENST00000574234.1 TRIM25 trna
ENST00000574245.1 trna
ENST00000574257.4 ABR trna
ENST00000574266.1 ABR trna
ENST00000574285.2 C16orf72 trna
ENST00000574287.1 RSL1D1 trna
ENST00000574295.1 CRK trna
ENST00000574297.1 SCIMP trna
ENST00000574334.4 NUBP1 trna
ENST00000574340.1 SRRM2 trna
ENST00000574364.1 RSL1D1-DT trna
ENST00000574371.1 MTCO1P40 trna
ENST00000574401.4 PLSCR3 trna
ENST00000574406.1 NLRP1 trna
ENST00000574446.1 CISD2 trna
ENST00000574474.1 ZZEF1 trna
ENST00000574512.1 NLRP1 trna
ENST00000574515.1 RAP1GAP2 trna

ENST00000574544.1 ABR trna
ENST00000574568.1 RABEP1 trna
ENST00000574594.4 HMOX2 trna
ENST00000574616.2 trna
ENST00000574632.4 ABR trna
ENST00000574671.4 ACTG1 trna
ENST00000574679.1 WNK1 trna
ENST00000574694.1 FOXK2 trna
ENST00000574740.1 CREBBP trna
ENST00000574741.1 trna
ENST00000574752.4 METTL16 trna
ENST00000574775.1 STX8 trna
ENST00000574799.4 CCDC40 trna
ENST00000574828.1 WDR45B trna
ENST00000574866.1 SRRM2 trna
ENST00000574867.4 NUP88 trna
ENST00000574872.1 PFN1 trna
ENST00000574888.4 ARRB2 trna
ENST00000574897.4 NPLOC4 trna
ENST00000574997.1 TRIM25 trna
ENST00000575001.1 EIF5A trna
ENST00000575068.4 NSF trna
ENST00000575129.4 HMOX2 trna
ENST00000575173.1 trna
ENST00000575176.4 SMG6 trna
ENST00000575207.4 VPS53 trna
ENST00000575270.4 NFATC3 trna
ENST00000575288.4 PITPNA trna
ENST00000575318.4 KANSL1 trna
ENST00000575337.2 PRPSAP2 trna
ENST00000575338.1 SMG6 trna
ENST00000575354.5 CIC trna
ENST00000575422.4 ABCC1 trna
ENST00000575426.1 LITAF trna
ENST00000575447.1 ARHGAP17 trna
ENST00000575471.2 ZNF267 trna
ENST00000575475.2 RABEP1 trna
ENST00000575477.4 PAFAH1B1 trna
ENST00000575521.1 KDM6B trna
ENST00000575542.4 RPTOR trna
ENST00000575550.4 ADCY9 trna
ENST00000575556.1 PSMB10 trna
ENST00000575656.4 ARHGAP17 trna
ENST00000575674.1 XYLT1 trna
ENST00000575695.4 MLKL trna
ENST00000575709.4 USP6 trna
ENST00000575714.4 CORO7 trna
ENST00000575822.4 RNASEK trna
ENST00000575839.2 CIC trna
ENST00000575889.4 ALOX12-AS1 trna
ENST00000575917.4 trna
ENST00000575938.1 CEP20 trna
ENST00000575944.1 SLC43A2 trna
ENST00000575953.1 trna

ENST00000575955.1 ANKFY1 trna
ENST00000575957.1 EIF4A3 trna
ENST00000575960.4 ARL17B trna
ENST00000575979.1 RAP1GAP2 trna
ENST00000575991.1 RABEP1 trna
ENST00000576018.4 ZC3H7A trna
ENST00000576037.1 MINK1 trna
ENST00000576040.4 NSF trna
ENST00000576076.1 SRRM2 trna
ENST00000576149.4 VPS53 trna
ENST00000576153.4 CXCL16 trna
ENST00000576248.1 KANSL1 trna
ENST00000576302.1 trna
ENST00000576346.4 NSF trna
ENST00000576382.1 LCMT1 trna
ENST00000576387.1 ARHGAP17 trna
ENST00000576407.1 PRPF8 trna
ENST00000576442.1 URI1 trna
ENST00000576462.1 SLC12A4 trna
ENST00000576479.3 SPON1 trna
ENST00000576502.4 NDE1 trna
ENST00000576557.1 ABCC1 trna
ENST00000576590.1 ZNF213-AS1 trna
ENST00000576601.1 CIITA trna
ENST00000576628.1 ACAP1 trna
ENST00000576668.1 ABR trna
ENST00000576674.1 SRRM2 trna
ENST00000576721.4 SLC43A2 trna
ENST00000576739.1 KANSL1 trna
ENST00000576789.1 NA trna
ENST00000576870.4 KANSL1 trna
ENST00000576936.4 ADCY9 trna
ENST00000576938.1 trna
ENST00000576958.1 PRPF8 trna
ENST00000576976.2 METTL16 trna
ENST00000577001.1 PRPF8 trna
ENST00000577021.1 MINK1 trna
ENST00000577035.4 GABARAP protein_coding
ENST00000577054.1 ARRB2 retained_intron
ENST00000577075.5 CYB5D2 protein_coding
ENST00000577114.1 KANSL1 processed_transcript
ENST00000577119.4 NLRP1 protein_coding
ENST00000577148.1 METTL16 retained_intron
ENST00000577169.1 NCBP3 processed_transcript
ENST00000577221.1 RALBP1 protein_coding
ENST00000577241.1 MSI2 retained_intron
ENST00000577300.1 SMCHD1 retained_intron
ENST00000577352.4 MYO1D retained_intron
ENST00000577363.1 IL6ST processed_transcript
ENST00000577371.1 RAB31 processed_transcript
ENST00000577468.2 PTPRM protein_coding
ENST00000577474.2 TRAPPC8 retained_intron
ENST00000577487.1 LRRC37A3 processed_transcript
ENST00000577489.1 TEX2 retained_intron

ENST00000577510.4 MYL12A retained_intron
ENST00000577511.1 CD300A protein_coding
ENST00000577514.4 MPRIP protein_coding
ENST00000577538.4 ZNF407 protein_coding
ENST00000577539.1 VAPA retained_intron
ENST00000577552.4 THOC1 retained_intron
ENST00000577563.1 MTAP protein_coding
ENST00000577564.1 ABHD3 retained_intron
ENST00000577567.4 ERN1 processed_transcript
ENST00000577583.1 TAOK1 retained_intron
ENST00000577597.4 MAP3K3 nonsense_mediated_decay
ENST00000577598.4 BRIP1 protein_coding
ENST00000577606.1 OGFOD3 processed_transcript
ENST00000577610.4 USP22 processed_transcript
ENST00000577616.1 TLK2 processed_transcript
ENST00000577698.1 lncRNA
ENST00000577708.1 miRNA
ENST00000577711.1 GRB2 retained_intron
ENST00000577725.1 CRLF3 processed_transcript
ENST00000577734.1 DYM protein_coding
ENST00000577741.1 RPL19 retained_intron
ENST00000577759.1 NA NA
ENST00000577798.5 COX10-AS1 lncRNA
ENST00000577806.1 ZNF407-AS1 lncRNA
ENST00000577827.4 PTPRM retained_intron
ENST00000577831.4 MED1 nonsense_mediated_decay
ENST00000577836.1 DYM processed_transcript
ENST00000577840.4 CUEDC1 protein_coding
ENST00000577846.1 lncRNA
ENST00000577887.1 SNORD53B snoRNA
ENST00000577889.1 GARS1-DT retained_intron
ENST00000577894.1 EVI2B protein_coding
ENST00000577908.4 ZNF207 protein_coding
ENST00000577914.1 CLASP1-AS1 lncRNA
ENST00000577918.4 KPNB1 processed_transcript
ENST00000577929.1 EIF4A1 protein_coding
ENST00000577949.4 SNURF protein_coding
ENST00000577958.1 UBB protein_coding
ENST00000577961.4 YES1 protein_coding
ENST00000577981.1 PIK3R5 processed_transcript
ENST00000577988.2 SNORD3B-1 snoRNA
ENST00000577991.1 SSH2 protein_coding
ENST00000577992.1 ANKRD12 protein_coding
ENST00000578015.1 PSMD12 retained_intron
ENST00000578038.1 MYL12A processed_transcript
ENST00000578049.3 SEC22B protein_coding
ENST00000578063.4 PRKCA nonsense_mediated_decay
ENST00000578067.4 LIMD2 retained_intron
ENST00000578085.1 CCR7 protein_coding
ENST00000578115.1 RPL26 retained_intron
ENST00000578121.4 TAF4B protein_coding
ENST00000578150.1 TTC39C processed_transcript
ENST00000578179.1 SNORA77B snoRNA
ENST00000578181.1 RPL23A protein_coding

ENST00000578183.1 SNORA59B snoRNA
ENST00000578186.2 H4C14 protein_coding
ENST00000578189.1 NOTCH2NLC protein_coding
ENST00000578200.1 SMURF2 processed_transcript
ENST00000578207.4 NA NA
ENST00000578209.4 MPRIP protein_coding
ENST00000578238.2 SUMO2 protein_coding
ENST00000578250.5 DLGAP1 processed_transcript
ENST00000578260.1 MIB1 retained_intron
ENST00000578269.4 ACADVL retained_intron
ENST00000578272.1 MBD2 nonsense_mediated_decay
ENST00000578314.1 VAPA processed_transcript
ENST00000578396.1 DYM protein_coding
ENST00000578411.1 SSH2 retained_intron
ENST00000578430.1 SRSF1 retained_intron
ENST00000578443.1 lncRNA
ENST00000578446.1 USP22 retained_intron
ENST00000578457.4 PIK3R5 processed_transcript
ENST00000578527.1 PITPNC1 retained_intron
ENST00000578539.1 lncRNA
ENST00000578596.4 DYM processed_transcript
ENST00000578599.4 GAS7 protein_coding
ENST00000578618.4 EPB41L3 retained_intron
ENST00000578653.1 TAOK1 retained_intron
ENST00000578692.1 CRLF3 nonsense_mediated_decay
ENST00000578697.1 NA NA
ENST00000578699.4 NA NA
ENST00000578706.4 UBB protein_coding
ENST00000578737.4 RAB31 processed_transcript
ENST00000578748.5 PIPOX processed_transcript
ENST00000578764.4 TMEM104 protein_coding
ENST00000578765.1 TMX3 retained_intron
ENST00000578783.1 HELZ protein_coding
ENST00000578793.2 RN7SL665P misc_RNA
ENST00000578794.1 nonsense_mediated_decay
ENST00000578804.4 DDX5 protein_coding
ENST00000578824.4 ACADVL retained_intron
ENST00000578825.4 TTYH2 retained_intron
ENST00000578847.4 SLC16A5 protein_coding
ENST00000578904.1 CSNK1D processed_transcript
ENST00000578918.1 ZNF207 protein_coding
ENST00000578921.4 RAB31 protein_coding
ENST00000578928.1 CD226 nonsense_mediated_decay
ENST00000578938.1 HELZ processed_transcript
ENST00000578944.1 LGALS9 protein_coding
ENST00000578948.2 RN7SL444P misc_RNA
ENST00000579003.1 lncRNA
ENST00000579017.1 NA NA
ENST00000579024.4 GARS1-DT lncRNA
ENST00000579039.2 NCOA4 protein_coding
ENST00000579089.4 MAP2K4 processed_transcript
ENST00000579099.1 DHRS7B retained_intron
ENST00000579125.1 ARHGAP27P1-BPTFP1-KPNA2P3 lncRNA
ENST00000579150.1 NDEL1 processed_transcript

ENST00000579205.4 MSI2 processed_transcript
ENST00000579214.1 TTC39C protein_coding
ENST00000579225.2 NA NA
ENST00000579280.1 ATG13 processed_transcript
ENST00000579289.1 SUPT4H1 nonsense_mediated_decay
ENST00000579290.1 nonsense_mediated_decay
ENST00000579308.1 CSNK1D retained_intron
ENST00000579309.1 KSR1 processed_transcript
ENST00000579319.1 SLC39A11 nonsense_mediated_decay
ENST00000579344.1 CCR7 protein_coding
ENST00000579365.4 PSMD12 nonsense_mediated_decay
ENST00000579369.1 NA NA
ENST00000579381.1 FAM222B nonsense_mediated_decay
ENST00000579392.4 LLGL2 protein_coding
ENST00000579427.1 NA NA
ENST00000579475.6 NA NA
ENST00000579476.4 SKAP1 retained_intron
ENST00000579483.1 MSI2 retained_intron
ENST00000579505.4 MSI2 processed_transcript
ENST00000579512.2 H4C15 protein_coding
ENST00000579526.4 SUZ12P1 processed_transcript
ENST00000579573.2 NCOR1 protein_coding
ENST00000579586.1 TOM1L2 protein_coding
ENST00000579589.1 RAB11FIP4 nonsense_mediated_decay
ENST00000579590.4 MSI2 protein_coding
ENST00000579606.1 NCOR1 protein_coding
ENST00000579618.1 SNRPD1 protein_coding
ENST00000579645.4 USP22 nonsense_mediated_decay
ENST00000579665.1 NUFIP2 protein_coding
ENST00000579688.1 SPECC1 processed_transcript
ENST00000579690.4 YTHDC1 protein_coding
ENST00000579692.1 processed_transcript
ENST00000579700.1 DTX2P1-UPK3BP1-PMS2P11 lncRNA
ENST00000579701.1 RDM1P1 transcribed_unprocessed_pseudogene
ENST00000579727.4 RARA processed_transcript
ENST00000579793.5 NOTCH2NLA protein_coding
ENST00000579810.2 ZNF207 retained_intron
ENST00000579814.1 LIMD2 retained_intron
ENST00000579815.1 CLTC processed_transcript
ENST00000579833.1 lncRNA
ENST00000579861.1 HELZ protein_coding
ENST00000579872.1 FAM104A protein_coding
ENST00000579875.4 ABHD3 processed_transcript
ENST00000579879.1 SNORD95 snoRNA
ENST00000579913.4 LRRC37A4P processed_transcript
ENST00000579925.4 MTMR4 protein_coding
ENST00000579942.2 RASSF5 retained_intron
ENST00000579953.4 HELZ nonsense_mediated_decay
ENST00000579955.1 ZSWIM7 nonsense_mediated_decay
ENST00000579961.4 KSR1 processed_transcript
ENST00000579969.1 SNORD53 snoRNA
ENST00000579974.1 NCOR1 retained_intron
ENST00000579988.1 SLC39A11 processed_transcript
ENST00000579991.2 DYNLL2 protein_coding

ENST00000580003.1 SS18 retained_intron
ENST00000580012.1 NDEL1 protein_coding
ENST00000580018.3 TIMM23 protein_coding
ENST00000580043.1 GAS7 protein_coding
ENST00000580045.1 KPNB1-DT lncRNA
ENST00000580061.4 CSNK1D retained_intron
ENST00000580081.1 CLTC protein_coding
ENST00000580091.1 DCAF7 nonsense_mediated_decay
ENST00000580094.4 BMS1P1 processed_transcript
ENST00000580098.4 SLC16A3 protein_coding
ENST00000580101.1 ESCO1 processed_transcript
ENST00000580108.1 DDX42 retained_intron
ENST00000580112.1 LGALS9DP unprocessed_pseudogene
ENST00000580158.1 KPNB1 retained_intron
ENST00000580162.1 L3MBTL4 processed_transcript
ENST00000580170.4 PTPRM protein_coding
ENST00000580179.2 EPB41L3 protein_coding
ENST00000580243.1 TSHZ1 protein_coding
ENST00000580283.1 PPP4R1 retained_intron
ENST00000580398.1 SUZ12 protein_coding
ENST00000580413.1 NA NA
ENST00000580444.2 RNF135 protein_coding
ENST00000580449.4 RASSF5 protein_coding
ENST00000580464.1 LRRC37A3 protein_coding
ENST00000580465.4 BPTF protein_coding
ENST00000580466.1 TANC2 protein_coding
ENST00000580499.1 RNF138 protein_coding
ENST00000580519.1 RNF138 processed_transcript
ENST00000580524.1 lncRNA
ENST00000580526.1 ADAP2 nonsense_mediated_decay
ENST00000580533.1 SNORD3C snoRNA
ENST00000580573.1 KPNB1 retained_intron
ENST00000580615.1 DYM processed_transcript
ENST00000580617.4 NCOR1 retained_intron
ENST00000580623.1 lncRNA
ENST00000580633.4 TSPOAP1-AS1 lncRNA
ENST00000580662.4 HELZ processed_transcript
ENST00000580675.1 MTAP retained_intron
ENST00000580710.4 DMTF1 processed_transcript
ENST00000580755.1 RPL23A retained_intron
ENST00000580782.1 lncRNA
ENST00000580828.1 SNHG25 lncRNA
ENST00000580835.2 RN7SL230P misc_RNA
ENST00000580863.1 RNF125 retained_intron
ENST00000580865.4 GAS7 protein_coding
ENST00000580886.4 EIF4A1 retained_intron
ENST00000580904.1 PSMD11 protein_coding
ENST00000580919.4 PLEKHM1P1 processed_transcript
ENST00000580972.1 RNU4ATAC snRNA
ENST00000580976.1 RHOT1 processed_transcript
ENST00000580989.4 EPB41L3 protein_coding
ENST00000581002.1 BRD2 processed_transcript
ENST00000581039.1 RFFL protein_coding
ENST00000581041.4 TLK2 protein_coding

ENST00000581056.4 CEP95 protein_coding
ENST00000581066.2 MRPS21 protein_coding
ENST00000581082.4 PER1 protein_coding
ENST00000581108.4 CSNK1D retained_intron
ENST00000581109.1 RAB31 nonsense_mediated_decay
ENST00000581112.4 GAS7 processed_transcript
ENST00000581113.5 NF1 protein_coding
ENST00000581135.4 DDX42 protein_coding
ENST00000581147.1 TEX14 nonsense_mediated_decay
ENST00000581159.4 HELZ protein_coding
ENST00000581220.1 RIOK3 retained_intron
ENST00000581226.1 SMCHD1 retained_intron
ENST00000581232.1 lncRNA
ENST00000581237.2 DDX5 processed_transcript
ENST00000581273.4 TVP23C nonsense_mediated_decay
ENST00000581296.1 LRRC37A4P transcribed_unprocessed_pseudogene
ENST00000581305.1 RPL17 retained_intron
ENST00000581328.1 TBX21 retained_intron
ENST00000581332.1 ENOSF1 processed_transcript
ENST00000581356.1 SLC9A3R1 nonsense_mediated_decay
ENST00000581368.4 LRRC37A3 protein_coding
ENST00000581370.1 LRRC37B processed_transcript
ENST00000581383.1 SMCHD1 retained_intron
ENST00000581392.2 RN7SL49P misc_RNA
ENST00000581399.4 SPECC1 protein_coding
ENST00000581400.2 SKAP1 nonsense_mediated_decay
ENST00000581401.1 FLII nonsense_mediated_decay
ENST00000581419.1 SKAP1 nonsense_mediated_decay
ENST00000581421.1 lncRNA
ENST00000581438.4 ATG13 protein_coding
ENST00000581454.4 EPB41L3 processed_transcript
ENST00000581457.1 CXADRP3 processed_transcript
ENST00000581458.2 RN7SL5P misc_RNA
ENST00000581467.1 TIMM21 retained_intron
ENST00000581474.1 ABHD15-AS1 lncRNA
ENST00000581485.1 LASP1 retained_intron
ENST00000581487.1 CMTM4 retained_intron
ENST00000581497.1 SRSF1 processed_transcript
ENST00000581500.1 CD300LF protein_coding
ENST00000581505.4 STRADA nonsense_mediated_decay
ENST00000581525.1 SNORD55 snoRNA
ENST00000581527.4 DLGAP1 protein_coding
ENST00000581531.4 ZNF207 nonsense_mediated_decay
ENST00000581551.1 DDX5 retained_intron
ENST00000581552.4 PIK3R5 protein_coding
ENST00000581568.1 LPIN2 processed_transcript
ENST00000581580.4 DCC protein_coding
ENST00000581583.1 RTTN retained_intron
ENST00000581593.1 CDK12 protein_coding
ENST00000581631.1 SMCHD1 retained_intron
ENST00000581639.1 NA NA
ENST00000581641.1 TWSG1 protein_coding
ENST00000581655.1 CSNK1D processed_transcript
ENST00000581661.1 NA NA

ENST00000581671.1 CTC1 processed_transcript
ENST00000581679.1 NDEL1 protein_coding
ENST00000581703.1 PER1 protein_coding
ENST00000581711.1 SMCHD1 retained_intron
ENST00000581758.1 ANKRD12 processed_transcript
ENST00000581775.1 NA NA
ENST00000581776.4 MSI2 nonsense_mediated_decay
ENST00000581792.1 MIR3648-2 miRNA
ENST00000581805.4 lncRNA
ENST00000581816.1 MIR17HG lncRNA
ENST00000581871.4 GAS7 processed_transcript
ENST00000581897.5 NBPF15 protein_coding
ENST00000581912.1 CNDP2 protein_coding
ENST00000581923.4 PITPNC1 retained_intron
ENST00000581931.4 COX10 nonsense_mediated_decay
ENST00000581952.2 NA NA
ENST00000581959.1 GRB2 protein_coding
ENST00000581960.1 YES1 processed_transcript
ENST00000581973.5 SPECC1 protein_coding
ENST00000581989.1 MPRIP processed_transcript
ENST00000582037.2 NLK protein_coding
ENST00000582055.1 LIMD2 protein_coding
ENST00000582059.4 FAM222B protein_coding
ENST00000582078.2 processed_pseudogene
ENST00000582092.1 SS18 processed_transcript
ENST00000582097.4 KPNB1 protein_coding
ENST00000582151.1 MPDU1 protein_coding
ENST00000582154.4 ARSG processed_transcript
ENST00000582174.4 FLOT2 retained_intron
ENST00000582179.4 SLC39A11 retained_intron
ENST00000582192.1 YPEL2 retained_intron
ENST00000582201.4 PLEKHM1P1 transcribed_unprocessed_pseudogene
ENST00000582277.4 NDEL1 protein_coding
ENST00000582308.1 RFFL protein_coding
ENST00000582311.1 KSR1 processed_transcript
ENST00000582322.1 SOCS6 protein_coding
ENST00000582329.1 SUZ12P1 processed_transcript
ENST00000582330.1 TMEM104 protein_coding
ENST00000582337.4 ZNF407 protein_coding
ENST00000582348.1 TSPOAP1-AS1 lncRNA
ENST00000582376.1 GGA3 nonsense_mediated_decay
ENST00000582377.1 MAP2K4 retained_intron
ENST00000582394.1 transcribed_unprocessed_pseudogene
ENST00000582399.1 NA NA
ENST00000582401.4 TXNIP protein_coding
ENST00000582438.1 CYBC1 protein_coding
ENST00000582463.4 RPL36 protein_coding
ENST00000582467.1 BPTF protein_coding
ENST00000582513.4 TRAPPC8 protein_coding
ENST00000582520.1 ARHGDIA processed_transcript
ENST00000582522.2 RN7SL300P misc_RNA
ENST00000582539.4 TRAPPC8 protein_coding
ENST00000582549.1 GARS1-DT lncRNA
ENST00000582582.1 GRB2 protein_coding

ENST00000582660.1 TLK2 retained_intron
ENST00000582693.4 RNF115 protein_coding
ENST00000582724.1 CEP95 protein_coding
ENST00000582736.1 RPL23A retained_intron
ENST00000582772.1 MSI2 processed_transcript
ENST00000582813.1 YPEL2 retained_intron
ENST00000582835.1 processed_transcript
ENST00000582867.1 SLC16A6 protein_coding
ENST00000582884.4 MSL1 nonsense_mediated_decay
ENST00000582897.4 MAP2K4 processed_transcript
ENST00000582913.4 PIEZO2 nonsense_mediated_decay
ENST00000582933.4 PPP4R1 processed_transcript
ENST00000582935.1 RPL17 retained_intron
ENST00000582986.4 PLEKHM1P1 processed_transcript
ENST00000582994.1 TTC39B processed_transcript
ENST00000583025.1 SLC16A3 protein_coding
ENST00000583028.1 NA NA
ENST00000583043.4 RTTN nonsense_mediated_decay
ENST00000583046.1 MBD2 protein_coding
ENST00000583054.4 L3MBTL4 protein_coding
ENST00000583062.1 lncRNA
ENST00000583065.1 RPL6P27 processed_transcript
ENST00000583081.1 NDUFV2-AS1 lncRNA
ENST00000583086.1 lncRNA
ENST00000583093.4 SECTM1 protein_coding
ENST00000583095.1 ZNF236 processed_transcript
ENST00000583096.1 WSB1 retained_intron
ENST00000583119.4 USP14 nonsense_mediated_decay
ENST00000583121.4 TAOK1 protein_coding
ENST00000583137.1 RAB31 nonsense_mediated_decay
ENST00000583170.4 SAP30BP nonsense_mediated_decay
ENST00000583184.1 lncRNA
ENST00000583186.1 ICAM2 retained_intron
ENST00000583212.1 DDX5 protein_coding
ENST00000583217.1 EIF4A1 retained_intron
ENST00000583222.1 PIK3R5 processed_transcript
ENST00000583226.1 NCOR1 processed_transcript
ENST00000583266.1 MBP processed_transcript
ENST00000583268.1 WIPF2 protein_coding
ENST00000583270.1 DYM retained_intron
ENST00000583271.5 NBPF8 transcribed_unprocessed_pseudogene
ENST00000583275.1 CPD retained_intron
ENST00000583301.4 NSRP1 processed_transcript
ENST00000583313.5 RBM8A protein_coding
ENST00000583344.1 SMCHD1 retained_intron
ENST00000583356.4 TANC2 protein_coding
ENST00000583361.1 PRKCA processed_transcript
ENST00000583367.1 NAPG retained_intron
ENST00000583368.1 IKZF3 protein_coding
ENST00000583369.4 SLC9A3R1 protein_coding
ENST00000583375.4 NDUFV2 processed_transcript
ENST00000583389.4 EIF4A1 protein_coding
ENST00000583444.1 SLC16A3 retained_intron
ENST00000583475.1 VAPA retained_intron

ENST00000583527.1 CRLF3 protein_coding
ENST00000583540.1 GRAPL nonsense_mediated_decay
ENST00000583544.1 CD300LF retained_intron
ENST00000583546.1 lncRNA
ENST00000583556.1 ROCK1 retained_intron
ENST00000583562.1 NA NA
ENST00000583565.4 NCOA4 protein_coding
ENST00000583593.2 CCDC57 nonsense_mediated_decay
ENST00000583595.1 SS18 processed_transcript
ENST00000583621.1 MYO1D protein_coding
ENST00000583648.4 KPNB1 protein_coding
ENST00000583687.1 DSEL-AS1 lncRNA
ENST00000583696.1 GAREM1 protein_coding
ENST00000583715.4 SLC39A11 protein_coding
ENST00000583718.4 STARD3 protein_coding
ENST00000583738.1 TEX2 protein_coding
ENST00000583741.1 SRSF1 nonsense_mediated_decay
ENST00000583747.1 PHF12 protein_coding
ENST00000583776.1 EMILIN2 retained_intron
ENST00000583786.4 WSB1 processed_transcript
ENST00000583791.1 ARHGDIA retained_intron
ENST00000583798.1 MBP protein_coding
ENST00000583800.1 SMCHD1 processed_transcript
ENST00000583805.1 CRLF3 processed_transcript
ENST00000583812.1 NA NA
ENST00000583826.4 TSPOAP1-AS1 lncRNA
ENST00000583841.1 TSPOAP1-AS1 lncRNA
ENST00000583843.4 TLK2 protein_coding
ENST00000583844.4 SKAP1 nonsense_mediated_decay
ENST00000583861.1 SNORD43 snoRNA
ENST00000583896.1 ERN1 retained_intron
ENST00000583912.1 GRB2 processed_transcript
ENST00000583922.1 TEX2 protein_coding
ENST00000583945.4 TRIM37 protein_coding
ENST00000583951.1 AKAP10 protein_coding
ENST00000583955.4 CD226 protein_coding
ENST00000583958.1 MED13 protein_coding
ENST00000584041.1 ERN1 retained_intron
ENST00000584052.4 RIOK3 retained_intron
ENST00000584056.1 RPL23 retained_intron
ENST00000584058.2 RN7SL4P misc_RNA
ENST00000584106.1 LASP1 retained_intron
ENST00000584112.1 CCDC47 retained_intron
ENST00000584129.1 SLC39A11 nonsense_mediated_decay
ENST00000584130.1 RIOK3 nonsense_mediated_decay
ENST00000584180.1 CD68 protein_coding
ENST00000584188.1 NLK processed_transcript
ENST00000584202.1 PER1 protein_coding
ENST00000584204.1 lncRNA
ENST00000584205.4 ATPAF2 nonsense_mediated_decay
ENST00000584217.1 TSHZ1 retained_intron
ENST00000584246.1 TMEM104 retained_intron
ENST00000584254.1 NA NA
ENST00000584275.1 SNORD84 snoRNA

ENST00000584289.4 PSMD12 retained_intron
ENST00000584294.1 NA NA
ENST00000584296.1 WIPF2 protein_coding
ENST00000584302.1 SNORA4 snoRNA
ENST00000584356.1 FAM182B lncRNA
ENST00000584416.1 ZNF207 processed_transcript
ENST00000584423.4 NSRP1 protein_coding
ENST00000584424.1 TTC39C retained_intron
ENST00000584441.4 RPL26 protein_coding
ENST00000584464.1 ABHD3 protein_coding
ENST00000584470.4 THOC1 retained_intron
ENST00000584471.4 PITPNC1 protein_coding
ENST00000584502.1 CD68 protein_coding
ENST00000584516.4 MYO15B nonsense_mediated_decay
ENST00000584527.1 SPECC1 protein_coding
ENST00000584539.1 MYL12B protein_coding
ENST00000584554.1 PITPNC1 protein_coding
ENST00000584569.1 FLOT2 processed_transcript
ENST00000584583.1 RPL23 retained_intron
ENST00000584585.1 PLEKHM1P1 processed_transcript
ENST00000584632.4 CDK12 miRNA
ENST00000584659.4 NA miRNA
ENST00000584672.1 CSNK1D miRNA
ENST00000584692.1 RHOT1 miRNA
ENST00000584732.1 TMEM11 miRNA
ENST00000584798.1 EIF4A1 miRNA
ENST00000584808.1 BRD2 miRNA
ENST00000584822.1 PHF12 miRNA
ENST00000584858.2 C8orf34-AS1 miRNA
ENST00000584861.4 SAP30BP miRNA
ENST00000584897.4 SMCHD1 miRNA
ENST00000584915.1 LPIN2 miRNA
ENST00000584923.1 SNORD3A miRNA
ENST00000584924.4 SKAP1 miRNA
ENST00000584958.2 miRNA
ENST00000584960.1 RIOK3 miRNA
ENST00000584968.4 SNRPN miRNA
ENST00000584992.1 RIOK3 miRNA
ENST00000584998.4 IKBKE miRNA
ENST00000585042.4 VAPA miRNA
ENST00000585078.1 SNORD92 miRNA
ENST00000585081.1 miRNA
ENST00000585132.4 NCOA4 miRNA
ENST00000585166.1 YPEL2 miRNA
ENST00000585198.1 CLTC miRNA
ENST00000585199.1 RPL19 miRNA
ENST00000585223.1 NA miRNA
ENST00000585237.2 RN7SL296P miRNA
ENST00000585247.4 OSBPL1A miRNA
ENST00000585258.1 ZNF236-DT miRNA
ENST00000585302.1 MAP3K3 miRNA
ENST00000585317.1 DDX5 miRNA
ENST00000585348.1 GRN miRNA
ENST00000585350.1 ZNF562 miRNA

ENST00000585368.1 APPBP2 miRNA
ENST00000585377.4 ZNF559 miRNA
ENST00000585379.4 FBXL12 miRNA
ENST00000585388.1 LSM12 miRNA
ENST00000585429.1 JMJD6 miRNA
ENST00000585444.1 SUGP1 miRNA
ENST00000585448.1 SMAD4 miRNA
ENST00000585482.4 SARM1 miRNA
ENST00000585485.2 DAZAP1 miRNA
ENST00000585505.1 NBR1 miRNA
ENST00000585509.4 CYTH1 miRNA
ENST00000585512.4 GRN miRNA
ENST00000585562.4 miRNA
ENST00000585592.1 MAPRE2 miRNA
ENST00000585626.1 TYROBP miRNA
ENST00000585665.2 RPS15 miRNA
ENST00000585669.1 WDR7 miRNA
ENST00000585680.1 ME2 miRNA
ENST00000585745.4 LINC01409 miRNA
ENST00000585799.4 SMARCA4 miRNA
ENST00000585801.1 miRNA
ENST00000585833.1 GARRE1 miRNA
ENST00000585847.4 VMP1 miRNA
ENST00000585856.2 PTBP1 miRNA
ENST00000585867.2 RBFOX1 miRNA
ENST00000585886.4 TNPO2 miRNA
ENST00000585918.4 TMEM150B miRNA
ENST00000585933.2 CEBPG miRNA
ENST00000585949.1 ZCCHC2 miRNA
ENST00000585951.1 CTDP1 miRNA
ENST00000585958.4 MFSD11 miRNA
ENST00000585978.1 SMAD2 miRNA
ENST00000585983.4 ZNF529 miRNA
ENST00000586014.4 VASP miRNA
ENST00000586033.1 ARHGAP45 miRNA
ENST00000586037.4 GPATCH8 miRNA
ENST00000586040.4 SMAD2 miRNA
ENST00000586043.4 CYTH1 miRNA
ENST00000586057.4 TIMP2 miRNA
ENST00000586066.3 USP36 miRNA
ENST00000586084.1 PLEKHM1 miRNA
ENST00000586086.1 DNMT1 miRNA
ENST00000586094.4 ZNF146 miRNA
ENST00000586106.1 miRNA
ENST00000586113.1 FOSB miRNA
ENST00000586124.2 WDR7 miRNA
ENST00000586128.4 SEPTIN9 miRNA
ENST00000586135.1 KIAA1328 miRNA
ENST00000586157.2 LSM14A miRNA
ENST00000586171.1 NACC1 miRNA
ENST00000586175.4 CYTH1 miRNA
ENST00000586189.6 MAU2 miRNA
ENST00000586207.4 LDLRAD4 miRNA
ENST00000586222.1 LDLRAD4 miRNA

ENST00000586231.1 LINC00910 miRNA
ENST00000586241.1 BCAS3 miRNA
ENST00000586242.1 GRN miRNA
ENST00000586262.4 TXNL1 miRNA
ENST00000586265.4 GPATCH8 miRNA
ENST00000586287.1 TREM1 miRNA
ENST00000586299.1 CYTH1 miRNA
ENST00000586318.1 TCF3 miRNA
ENST00000586325.4 PGS1 miRNA
ENST00000586335.1 DNAJC7 miRNA
ENST00000586355.1 PGS1 miRNA
ENST00000586376.4 FMNL1-DT miRNA
ENST00000586394.1 miRNA
ENST00000586409.4 UBE2O miRNA
ENST00000586425.1 GPI miRNA
ENST00000586430.1 CYTH1 miRNA
ENST00000586434.1 NFATC1 miRNA
ENST00000586456.1 SEPTIN9 miRNA
ENST00000586461.1 AP1M1 miRNA
ENST00000586466.4 ZNF451-AS1 miRNA
ENST00000586474.1 CHMP1B-AS1 miRNA
ENST00000586487.2 SMAD2 miRNA
ENST00000586515.4 miRNA
ENST00000586521.4 SEPTIN9 miRNA
ENST00000586538.1 NA miRNA
ENST00000586543.1 AP1M1 miRNA
ENST00000586544.1 ILF3 miRNA
ENST00000586560.1 ATXN7L3-AS1 miRNA
ENST00000586594.1 RAB27B miRNA
ENST00000586602.4 ZNF121 miRNA
ENST00000586619.1 VASP miRNA
ENST00000586641.4 MAP2K6 miRNA
ENST00000586643.4 FMNL1 miRNA
ENST00000586646.1 ZNF345 protein_coding
ENST00000586657.1 lncRNA
ENST00000586697.4 TMC6 trna
ENST00000586699.1 EIF1 trna
ENST00000586713.4 CEP295NL trna
ENST00000586722.4 ATP9B trna
ENST00000586725.1 GALNT1 trna
ENST00000586757.4 NA trna
ENST00000586759.1 GADD45B trna
ENST00000586768.1 CDIP1 trna
ENST00000586787.1 ZNF787 trna
ENST00000586796.1 RETREG3 trna
ENST00000586803.1 CALR trna
ENST00000586812.1 SEPTIN9 trna
ENST00000586815.1 WIPI1 trna
ENST00000586825.1 TXNL4A trna
ENST00000586829.1 SLC39A6 trna
ENST00000586843.2 NA trna
ENST00000586845.1 GP6-AS1 trna
ENST00000586849.4 ADGRE5 trna
ENST00000586872.4 HSH2D trna

ENST00000586880.1 PGS1 trna
ENST00000586896.2 USP36 trna
ENST00000586907.4 NA trna
ENST00000586925.1 FXYD5 trna
ENST00000586937.2 ARHGAP45 trna
ENST00000586939.4 DNM2 trna
ENST00000586961.1 GP6-AS1 trna
ENST00000586963.1 CAPNS1 trna
ENST00000586967.1 CALR trna
ENST00000587002.1 BCAS3 trna
ENST00000587024.4 SBNO2 trna
ENST00000587042.1 PSTPIP2 trna
ENST00000587044.1 DHX8 trna
ENST00000587052.1 CEP295NL trna
ENST00000587082.4 ZNF420 trna
ENST00000587083.1 TLE5 trna
ENST00000587084.4 ZNF507 trna
ENST00000587135.1 HDAC5 trna
ENST00000587181.1 RFX2 trna
ENST00000587186.4 ARHGAP45 trna
ENST00000587205.1 GMIP trna
ENST00000587223.1 NPC1 trna
ENST00000587238.4 GMIP trna
ENST00000587251.4 LGALS3BP trna
ENST00000587259.4 VMP1 trna
ENST00000587261.4 PIK3C3 trna
ENST00000587263.4 RANBP3 trna
ENST00000587275.1 U2AF2 trna
ENST00000587277.1 TAOK1 trna
ENST00000587279.1 ZNF787 trna
ENST00000587294.4 BCAS3 trna
ENST00000587329.1 DNM2 trna
ENST00000587344.1 LDLRAD4 trna
ENST00000587353.4 SMAD2 trna
ENST00000587358.1 FOSB trna
ENST00000587393.4 TLE5 trna
ENST00000587402.2 PIK3C3 trna
ENST00000587403.4 WDR7 trna
ENST00000587410.1 ATP9B trna
ENST00000587424.2 SRSF8 trna
ENST00000587444.1 VASP trna
ENST00000587457.1 PPP6R1 trna
ENST00000587482.1 PIP5K1C trna
ENST00000587485.1 DNM2 trna
ENST00000587486.5 CALR trna
ENST00000587489.4 FMNL1 trna
ENST00000587510.4 ATP6V0A1 trna
ENST00000587524.1 REXO1 trna
ENST00000587560.4 H3-3B trna
ENST00000587578.1 SOCS3 trna
ENST00000587591.4 C18orf25 trna
ENST00000587600.1 LDLRAD4 trna
ENST00000587606.4 ADGRE5 trna
ENST00000587614.1 ZSCAN5A trna

ENST00000587616.1 trna
ENST00000587637.2 SF3A2 trna
ENST00000587640.1 RHBDF2 trna
ENST00000587655.2 SBNO2 trna
ENST00000587663.1 ACTBP9 trna
ENST00000587703.4 PTPN2 trna
ENST00000587725.4 RELCH trna
ENST00000587740.4 NCLN trna
ENST00000587755.1 ZNF532 trna
ENST00000587764.4 RELCH trna
ENST00000587769.1 CTIF trna
ENST00000587776.1 HDAC5 trna
ENST00000587783.4 USP36 trna
ENST00000587802.4 SAFB2 trna
ENST00000587805.1 AP2B1 trna
ENST00000587820.1 SEC14L1 trna
ENST00000587822.1 ZNF506 trna
ENST00000587830.2 DNM2 trna
ENST00000587833.4 DAZAP1 trna
ENST00000587856.1 FMNL1 trna
ENST00000587860.1 CTIF trna
ENST00000587867.1 GNG7 trna
ENST00000587878.1 ATP9B trna
ENST00000587885.1 IER2 trna
ENST00000587902.1 ATP5F1A trna
ENST00000587919.1 ATP9B trna
ENST00000587927.4 ACOX1 trna
ENST00000587935.1 PTRH2 trna
ENST00000587936.4 GLYR1 trna
ENST00000587939.4 ZNF627 trna
ENST00000587942.1 PIGN trna
ENST00000587966.2 MIER2 trna
ENST00000587974.1 EPG5 trna
ENST00000588015.4 MGRN1 trna
ENST00000588048.2 MOB3A trna
ENST00000588065.1 NA trna
ENST00000588085.1 MAPRE2 trna
ENST00000588116.1 AP2B1 trna
ENST00000588131.1 VMP1 trna
ENST00000588150.4 LINC00662 trna
ENST00000588173.1 IER2 trna
ENST00000588178.4 PRKAR1A trna
ENST00000588188.5 PRKAR1A trna
ENST00000588250.1 ZNF383 trna
ENST00000588251.1 SEH1L trna
ENST00000588295.1 DCAKD trna
ENST00000588337.4 KDM4B trna
ENST00000588338.5 GARRE1 trna
ENST00000588344.2 CIRBP trna
ENST00000588345.1 CTIF trna
ENST00000588361.1 KDM4B trna
ENST00000588393.4 SLC44A2 trna
ENST00000588410.1 TPM4 trna
ENST00000588412.4 ERC1 trna

ENST00000588422.2 EIF3K trna
ENST00000588428.4 NCLN trna
ENST00000588439.1 TYROBP trna
ENST00000588446.1 RELCH trna
ENST00000588452.4 PHLPP1 trna
ENST00000588454.4 CALR trna
ENST00000588459.1 RPRD1A trna
ENST00000588470.4 GARRE1 trna
ENST00000588482.4 VASP trna
ENST00000588483.1 TPM4 trna
ENST00000588554.1 GPATCH8 trna
ENST00000588569.1 BCAS3 trna
ENST00000588582.3 LSM14A trna
ENST00000588595.4 UBL5 trna
ENST00000588598.4 ABHD17A trna
ENST00000588600.1 ATP9B trna
ENST00000588617.1 VMP1 trna
ENST00000588618.4 ACTN4 trna
ENST00000588645.1 ICAM1 trna
ENST00000588708.4 RASGRP4 trna
ENST00000588725.1 DHX40P1 trna
ENST00000588737.4 RPRD1A trna
ENST00000588772.1 ATP9B trna
ENST00000588786.2 NPIPB1P trna
ENST00000588801.4 PSTPIP2 trna
ENST00000588835.1 trna
ENST00000588847.4 TNRC6C trna
ENST00000588850.4 U2AF2 trna
ENST00000588852.1 SAFB trna
ENST00000588861.1 HDHD2 trna
ENST00000588868.4 STAT5A trna
ENST00000588885.1 RFX1 trna
ENST00000588898.1 USP32 trna
ENST00000588910.4 MAPRE2 trna
ENST00000588925.4 trna
ENST00000588936.4 CLASRP trna
ENST00000588939.1 MPPE1 trna
ENST00000588947.4 CARM1 trna
ENST00000588954.4 ZNF440 trna
ENST00000588961.1 KDM4B trna
ENST00000588975.1 NA trna
ENST00000589002.4 MAP4K1 trna
ENST00000589020.1 SSC5D trna
ENST00000589076.4 SMAD4 trna
ENST00000589086.1 PTPN2 trna
ENST00000589100.2 RASGRP4 trna
ENST00000589104.4 KDM4B trna
ENST00000589118.4 SF3A2 trna
ENST00000589123.4 NFIC trna
ENST00000589140.1 SEPTIN9 trna
ENST00000589162.1 CAPNS1 trna
ENST00000589163.4 PLIN3 trna
ENST00000589166.1 SLC39A3 trna
ENST00000589175.1 IGFLR1 trna

ENST00000589200.2 BTBD2 trna
ENST00000589216.1 PTPN2 trna
ENST00000589239.4 RFX1 trna
ENST00000589240.1 UBALD2 trna
ENST00000589246.1 SEPTIN9 trna
ENST00000589250.4 SEPTIN9 trna
ENST00000589284.1 PRKACA trna
ENST00000589301.1 ACOX1 trna
ENST00000589309.4 PRKAR1A trna
ENST00000589314.1 NARS1 trna
ENST00000589341.4 APPBP2 trna
ENST00000589361.2 OAZ1 trna
ENST00000589414.4 PIGN trna
ENST00000589417.1 H3-3B trna
ENST00000589441.4 MKNK2 trna
ENST00000589452.1 SLC66A2 trna
ENST00000589484.4 DAZAP1 trna
ENST00000589496.2 trna
ENST00000589499.1 SNHG22 trna
ENST00000589517.1 TYROBP trna
ENST00000589534.2 MKNK2 trna
ENST00000589537.1 NFIC trna
ENST00000589551.1 ZNF433-AS1 trna
ENST00000589583.4 GARRE1 trna
ENST00000589585.1 CTIF trna
ENST00000589596.4 CEP192 trna
ENST00000589604.4 VPS4B trna
ENST00000589609.1 ABCA5 trna
ENST00000589611.1 ATP5F1A trna
ENST00000589614.4 TREM1 trna
ENST00000589627.1 VASP trna
ENST00000589657.1 ZNF56P trna
ENST00000589668.1 ZNF253 trna
ENST00000589691.1 TMC8 trna
ENST00000589695.1 TREM1 trna
ENST00000589699.1 MAPRE2 trna
ENST00000589704.1 EPN1 trna
ENST00000589706.1 SMAD4 trna
ENST00000589726.1 ATF7 trna
ENST00000589732.1 ATP9B trna
ENST00000589739.3 OAZ1 trna
ENST00000589761.1 USP32 trna
ENST00000589765.1 HOOK2 trna
ENST00000589768.4 CYTH1 trna
ENST00000589782.2 AP1M1 trna
ENST00000589790.1 UNK retained_intron
ENST00000589799.4 ZNF235 protein_coding
ENST00000589859.1 MPPE1 protein_coding
ENST00000589866.4 RNMT protein_coding
ENST00000589872.1 NBR1 protein_coding
ENST00000589873.4 MALT1 retained_intron
ENST00000589874.2 DAZAP1 retained_intron
ENST00000589877.4 SMAD2 protein_coding
ENST00000589878.4 LSM14A nonsense_mediated_decay

ENST00000589881.4 ZNF24 protein_coding
ENST00000589882.1 TREM1 retained_intron
ENST00000589916.4 BCAS3 retained_intron
ENST00000589920.4 SEPTIN9 retained_intron
ENST00000589923.1 GRN processed_transcript
ENST00000589935.1 WDR7 protein_coding
ENST00000589950.1 lncRNA
ENST00000589978.1 UBE2S protein_coding
ENST00000590013.2 PIK3C3 processed_transcript
ENST00000590048.5 UBA2 protein_coding
ENST00000590060.4 POLR2E nonsense_mediated_decay
ENST00000590067.4 TLE5 retained_intron
ENST00000590082.1 EZH1 processed_transcript
ENST00000590115.1 LDLRAD4 retained_intron
ENST00000590123.1 NARS1 nonsense_mediated_decay
ENST00000590128.4 BCAS3 protein_coding
ENST00000590168.4 RHBDF2 retained_intron
ENST00000590180.1 TPM4 processed_transcript
ENST00000590211.4 CAPNS1 protein_coding
ENST00000590217.4 PSMG2 protein_coding
ENST00000590219.4 lncRNA
ENST00000590220.1 PIK3C3 processed_transcript
ENST00000590282.4 NFIC protein_coding
ENST00000590300.1 CYTH1 protein_coding
ENST00000590306.4 ZNF266 protein_coding
ENST00000590322.1 RHBDF2 protein_coding
ENST00000590335.1 FOSB protein_coding
ENST00000590371.4 LDLRAD4 processed_transcript
ENST00000590382.4 SLC44A2 protein_coding
ENST00000590386.4 ZNF875 processed_transcript
ENST00000590413.4 LAMTOR5-AS1 lncRNA
ENST00000590416.1 LSM14A retained_intron
ENST00000590418.1 PPM1D processed_transcript
ENST00000590419.4 DAZAP1 processed_transcript
ENST00000590456.4 KIAA1328 processed_transcript
ENST00000590478.1 lncRNA
ENST00000590485.1 SAFB retained_intron
ENST00000590505.1 ERVK3-1 retained_intron
ENST00000590512.1 ARHGAP45 processed_transcript
ENST00000590515.1 BCL2 protein_coding
ENST00000590519.2 ANKRD27 protein_coding
ENST00000590541.1 RETREG3 nonsense_mediated_decay
ENST00000590551.1 U2AF2 protein_coding
ENST00000590574.4 SMARCA4 protein_coding
ENST00000590577.2 ARHGAP45 protein_coding
ENST00000590596.1 PMAIP1 retained_intron
ENST00000590608.1 ATP9B retained_intron
ENST00000590617.4 KIAA1328 nonsense_mediated_decay
ENST00000590648.4 GASK1B protein_coding
ENST00000590652.4 TPGS2 nonsense_mediated_decay
ENST00000590654.1 GALNT1 nonsense_mediated_decay
ENST00000590683.2 AP3D1 nonsense_mediated_decay
ENST00000590726.5 STAT5A protein_coding
ENST00000590731.4 PEPD processed_transcript

ENST00000590735.1 ACLY processed_transcript
ENST00000590755.5 PEPD nonsense_mediated_decay
ENST00000590776.1 STAT3 processed_transcript
ENST00000590787.1 DNM2 retained_intron
ENST00000590802.1 SLFN12L retained_intron
ENST00000590810.4 TCF4 protein_coding
ENST00000590811.1 AFG3L2 protein_coding
ENST00000590822.1 RFX2 processed_transcript
ENST00000590828.4 NFKBID nonsense_mediated_decay
ENST00000590854.4 EPG5 protein_coding
ENST00000590856.1 UNC13D retained_intron
ENST00000590857.4 SLC44A2 protein_coding
ENST00000590861.1 NFATC1 processed_transcript
ENST00000590864.1 RPL27 nonsense_mediated_decay
ENST00000590870.4 RMC1 nonsense_mediated_decay
ENST00000590886.4 DNAJC7 protein_coding
ENST00000590895.1 SLC66A2 processed_transcript
ENST00000590917.4 SEPTIN9 protein_coding
ENST00000590935.1 DUSP3 protein_coding
ENST00000590998.4 SBNO2 protein_coding
ENST00000591020.4 SEPTIN9 protein_coding
ENST00000591035.1 protein_coding
ENST00000591048.2 UNC45B protein_coding
ENST00000591051.1 NPC1 protein_coding
ENST00000591095.1 CYTH1 retained_intron
ENST00000591107.5 NPC1 protein_coding
ENST00000591120.1 SAFB2 nonsense_mediated_decay
ENST00000591144.1 TMC8 processed_transcript
ENST00000591145.1 ZCCHC2 nonsense_mediated_decay
ENST00000591154.1 HSH2D nonsense_mediated_decay
ENST00000591192.1 RHBDF2 protein_coding
ENST00000591226.1 TPM4 retained_intron
ENST00000591227.1 RELCH processed_transcript
ENST00000591254.1 TRIR nonsense_mediated_decay
ENST00000591258.1 MTCO2P2 processed_pseudogene
ENST00000591269.1 FAM210A protein_coding
ENST00000591284.2 AP3D1 nonsense_mediated_decay
ENST00000591293.2 ARHGAP45 retained_intron
ENST00000591295.4 ATXN7L3 protein_coding
ENST00000591306.4 RNF152 protein_coding
ENST00000591333.4 RANBP3 protein_coding
ENST00000591340.4 ZNF529 protein_coding
ENST00000591344.1 ZNF850 protein_coding
ENST00000591348.4 CROCCP3 processed_transcript
ENST00000591383.1 VPS4B processed_transcript
ENST00000591413.1 SEC14L1 retained_intron
ENST00000591434.1 FMNL1 retained_intron
ENST00000591436.4 TMC6 protein_coding
ENST00000591446.5 MIDN protein_coding
ENST00000591449.1 WDR7 nonsense_mediated_decay
ENST00000591456.1 HS2ST1 protein_coding
ENST00000591467.1 RAD23A retained_intron
ENST00000591472.5 SEPTIN9 protein_coding
ENST00000591482.1 lncRNA

ENST00000591487.1 LINC01478 lncRNA
ENST00000591494.4 WIPI1 processed_transcript
ENST00000591501.1 ILF3-DT lncRNA
ENST00000591508.1 ZNF317 protein_coding
ENST00000591519.1 VPS4B protein_coding
ENST00000591524.1 WDR7 retained_intron
ENST00000591545.4 SMARCA4 protein_coding
ENST00000591549.1 lncRNA
ENST00000591561.4 AP2B1 processed_transcript
ENST00000591567.1 lncRNA
ENST00000591629.1 MIR924HG lncRNA
ENST00000591638.1 MOB3A protein_coding
ENST00000591659.4 CIRBP protein_coding
ENST00000591666.4 SAFB retained_intron
ENST00000591671.1 HDHD2 processed_transcript
ENST00000591701.4 DNM2 retained_intron
ENST00000591714.4 HDAC5 protein_coding
ENST00000591729.1 PSTPIP2 retained_intron
ENST00000591732.1 CANT1 processed_transcript
ENST00000591752.4 CSNK1G2 protein_coding
ENST00000591776.4 EIF1 protein_coding
ENST00000591792.1 MALT1 protein_coding
ENST00000591818.1 DNM2 protein_coding
ENST00000591829.4 R3HDM4 processed_transcript
ENST00000591851.4 TNRC6C retained_intron
ENST00000591877.1 VMP1 protein_coding
ENST00000591884.1 ZNF56P processed_transcript
ENST00000591895.4 MGRN1 protein_coding
ENST00000591901.4 PTPN2 protein_coding
ENST00000591911.1 KIAA1328 protein_coding
ENST00000592003.1 KLF2 protein_coding
ENST00000592006.4 FMNL1 processed_transcript
ENST00000592030.1 lncRNA
ENST00000592038.4 ZNF451-AS1 lncRNA
ENST00000592048.4 ERC1 protein_coding
ENST00000592069.1 MAU2 processed_transcript
ENST00000592083.4 ERCC1 protein_coding
ENST00000592086.1 ADGRL1-AS1 lncRNA
ENST00000592098.1 SEPTIN9 processed_transcript
ENST00000592099.4 PTPRS protein_coding
ENST00000592113.4 PTBP1 retained_intron
ENST00000592119.2 RMC1 processed_transcript
ENST00000592120.4 UBN1 protein_coding
ENST00000592165.1 GPATCH1 nonsense_mediated_decay
ENST00000592175.4 KDM4B processed_transcript
ENST00000592212.4 PIAS2 protein_coding
ENST00000592222.4 SBNO2 retained_intron
ENST00000592223.4 NFATC1 protein_coding
ENST00000592242.1 PPP6R1 protein_coding
ENST00000592261.5 ADGRE5 protein_coding
ENST00000592266.1 RANBP3 retained_intron
ENST00000592267.1 MAP3K14 retained_intron
ENST00000592271.1 RNF157 protein_coding
ENST00000592272.4 EPG5 nonsense_mediated_decay

ENST00000592280.1 MOB3A protein_coding
ENST00000592304.1 NBR1 retained_intron
ENST00000592325.1 SLFN5 protein_coding
ENST00000592334.1 RAB27B protein_coding
ENST00000592335.2 ARHGAP45 processed_transcript
ENST00000592339.4 USP32 protein_coding
ENST00000592351.1 GEMIN7 processed_transcript
ENST00000592396.1 SAFB retained_intron
ENST00000592397.1 SSH2 protein_coding
ENST00000592414.1 TLE5 retained_intron
ENST00000592420.1 SEPTIN9 retained_intron
ENST00000592440.1 lncRNA
ENST00000592441.1 lncRNA
ENST00000592456.1 SYNGR2 processed_transcript
ENST00000592483.4 CAPNS1 protein_coding
ENST00000592497.1 CYTH1 retained_intron
ENST00000592521.4 KIAA1328 protein_coding
ENST00000592580.4 ERMARD retained_intron
ENST00000592626.2 RNF126 retained_intron
ENST00000592629.1 UNK nonsense_mediated_decay
ENST00000592674.1 RPRD1A nonsense_mediated_decay
ENST00000592725.1 ZNF253 protein_coding
ENST00000592761.2 TIMP2 nonsense_mediated_decay
ENST00000592787.2 OAZ1 retained_intron
ENST00000592790.1 VMP1 retained_intron
ENST00000592791.1 UBA2 protein_coding
ENST00000592802.1 WBP2 retained_intron
ENST00000592811.4 FOSB protein_coding
ENST00000592827.1 BCAS3 processed_transcript
ENST00000592841.4 UBA2 retained_intron
ENST00000592860.2 CFD protein_coding
ENST00000592874.1 U2AF2 nonsense_mediated_decay
ENST00000592881.1 ZNF580 protein_coding
ENST00000592904.4 ZNF266 protein_coding
ENST00000592908.1 lncRNA
ENST00000592945.1 ICAM3 protein_coding
ENST00000592965.2 MBD3 protein_coding
ENST00000593007.1 RNMT protein_coding
ENST00000593103.1 RHBDF2 retained_intron
ENST00000593143.4 HOOK2 retained_intron
ENST00000593168.4 VMP1 nonsense_mediated_decay
ENST00000593189.5 SEPTIN9 protein_coding
ENST00000593199.4 ILF3 protein_coding
ENST00000593220.1 DNM2 retained_intron
ENST00000593223.1 SMAD4 protein_coding
ENST00000593224.1 MGRN1 protein_coding
ENST00000593230.4 RNF165 protein_coding
ENST00000593236.1 LDLRAD4 protein_coding
ENST00000593259.1 MBTD1 processed_transcript
ENST00000593293.1 HNRNPM retained_intron
ENST00000593345.1 NOSIP retained_intron
ENST00000593381.4 FCGRT protein_coding
ENST00000593396.1 PLAUR retained_intron
ENST00000593427.1 lncRNA

ENST00000593460.1 ARRDC2 retained_intron
ENST00000593495.2 NOTCH2NLB protein_coding
ENST00000593531.4 NA NA
ENST00000593533.1 MYO9B retained_intron
ENST00000593551.1 ZNF43 protein_coding
ENST00000593612.1 ZNF766 protein_coding
ENST00000593655.4 lncRNA
ENST00000593682.1 nonsense_mediated_decay
ENST00000593708.1 USF2 retained_intron
ENST00000593748.1 processed_pseudogene
ENST00000593795.4 SUGP2 nonsense_mediated_decay
ENST00000593817.1 LRMDA processed_transcript
ENST00000593832.1 COLGALT1 retained_intron
ENST00000593837.1 lncRNA
ENST00000593848.4 SLC8A1-AS1 lncRNA
ENST00000593939.4 PLAUR protein_coding
ENST00000593959.1 SYMPK retained_intron
ENST00000594044.1 ARHGEF1 retained_intron
ENST00000594045.1 ZFP36 protein_coding
ENST00000594085.4 PLD3 processed_transcript
ENST00000594192.2 KCNN1 processed_transcript
ENST00000594199.2 SLC25A53 protein_coding
ENST00000594328.1 ZNF587B protein_coding
ENST00000594396.1 ZNF417 protein_coding
ENST00000594401.1 ZNF100 protein_coding
ENST00000594442.1 ZFP36 protein_coding
ENST00000594479.6 NA NA
ENST00000594527.1 UBA52 protein_coding
ENST00000594665.1 ARHGEF18 protein_coding
ENST00000594708.1 SMC5-DT lncRNA
ENST00000594729.4 SUPT5H protein_coding
ENST00000594743.1 CD37 protein_coding
ENST00000594824.4 MYO9B protein_coding
ENST00000594866.1 BICRA protein_coding
ENST00000594885.1 EEF2 retained_intron
ENST00000594900.1 FPR1 protein_coding
ENST00000594901.1 ZNF587B protein_coding
ENST00000594975.4 EPS15L1 protein_coding
ENST00000594996.4 TBC1D17 nonsense_mediated_decay
ENST00000595038.4 PLAUR protein_coding
ENST00000595042.4 FPR1 protein_coding
ENST00000595132.4 PRKD2 protein_coding
ENST00000595157.1 NKG7 protein_coding
ENST00000595191.1 MYO1F retained_intron
ENST00000595201.1 lncRNA
ENST00000595288.4 XAB2 retained_intron
ENST00000595310.1 lncRNA
ENST00000595464.2 C5AR2 protein_coding
ENST00000595491.1 processed_pseudogene
ENST00000595510.1 FLT3LG protein_coding
ENST00000595533.1 ZNF91 protein_coding
ENST00000595538.4 nonsense_mediated_decay
ENST00000595552.2 PGPEP1 protein_coding
ENST00000595600.1 ARHGEF18 retained_intron

ENST00000595641.4 MYO9B protein_coding
ENST00000595677.4 FCGRT processed_transcript
ENST00000595804.4 HNRNPL retained_intron
ENST00000595806.1 HNRNPUL1 retained_intron
ENST00000595822.1 ARHGAP35 retained_intron
ENST00000595833.1 ZNF430 retained_intron
ENST00000595839.4 ADGRE2 protein_coding
ENST00000595840.1 LRRC25 protein_coding
ENST00000595845.1 EPS15L1 retained_intron
ENST00000595926.1 BRD4 retained_intron
ENST00000595936.1 LRMDA processed_transcript
ENST00000596032.1 ITGB1P1 processed_pseudogene
ENST00000596085.1 ZNF551 protein_coding
ENST00000596116.1 LIPE-AS1 lncRNA
ENST00000596124.3 ELL protein_coding
ENST00000596154.4 ELAVL1 protein_coding
ENST00000596295.1 HNRNPM protein_coding
ENST00000596315.4 EMP3 protein_coding
ENST00000596388.1 ZNF160 processed_transcript
ENST00000596417.1 EEF2 retained_intron
ENST00000596431.4 RYR1 processed_transcript
ENST00000596459.4 ELAVL1 protein_coding
ENST00000596573.4 PCBP1-AS1 lncRNA
ENST00000596583.4 GMFG nonsense_mediated_decay
ENST00000596591.1 MAN2B1 nonsense_mediated_decay
ENST00000596638.1 SIN3B protein_coding
ENST00000596645.1 MYO1F retained_intron
ENST00000596802.4 SIN3B protein_coding
ENST00000596811.1 EEF1A1P7 processed_pseudogene
ENST00000596815.1 ZNF493 nonsense_mediated_decay
ENST00000596915.1 ELL retained_intron
ENST00000596952.4 ZRANB2-AS2 lncRNA
ENST00000596957.1 ARHGEF1 processed_transcript
ENST00000597001.1 LIPE protein_coding
ENST00000597024.1 DPP9 processed_transcript
ENST00000597026.4 COPE retained_intron
ENST00000597057.1 EMP3 retained_intron
ENST00000597069.1 ZC3H4 processed_transcript
ENST00000597073.4 MYO9B protein_coding
ENST00000597160.1 NAPA processed_transcript
ENST00000597203.1 LIPE-AS1 lncRNA
ENST00000597222.1 MYO1F retained_intron
ENST00000597230.2 ZNF8-DT lncRNA
ENST00000597270.1 HNRNPM protein_coding
ENST00000597346.1 KCNQ1OT1 lncRNA
ENST00000597366.4 lncRNA
ENST00000597372.1 ADGRE4P transcribed_unprocessed_pseudogene
ENST00000597430.2 CD70 protein_coding
ENST00000597447.4 ZNF264 protein_coding
ENST00000597453.1 TGFB1 retained_intron
ENST00000597467.1 STXBP2 retained_intron
ENST00000597529.1 EMP3 retained_intron
ENST00000597598.1 SMG9 nonsense_mediated_decay
ENST00000597602.1 CD37 protein_coding

ENST00000597618.4 TRIM28 retained_intron
ENST00000597638.1 lncRNA
ENST00000597661.4 SLC25A42 retained_intron
ENST00000597683.1 lncRNA
ENST00000597695.1 NA NA
ENST00000597748.4 ZNF415 protein_coding
ENST00000597766.1 PAK4 processed_transcript
ENST00000597785.1 RAB11B-AS1 lncRNA
ENST00000597802.2 IFI30 protein_coding
ENST00000597813.4 HNRNPM retained_intron
ENST00000597870.1 BLVRB nonsense_mediated_decay
ENST00000597936.4 SNRNP70 retained_intron
ENST00000597967.4 VAV1 retained_intron
ENST00000598005.1 MYO1F processed_transcript
ENST00000598015.1 ALDH16A1 processed_transcript
ENST00000598076.1 FCGRT protein_coding
ENST00000598101.1 MYO9B retained_intron
ENST00000598118.1 GRAMD1A retained_intron
ENST00000598149.4 SATB1-AS1 lncRNA
ENST00000598183.1 ZNF586 protein_coding
ENST00000598190.1 ZNF83 retained_intron
ENST00000598200.1 DEDD2 protein_coding
ENST00000598216.1 INSR retained_intron
ENST00000598218.1 GMFG retained_intron
ENST00000598221.1 SLC6A16 retained_intron
ENST00000598240.1 SUGP2 nonsense_mediated_decay
ENST00000598261.1 RPS19 protein_coding
ENST00000598278.4 SMIM7 nonsense_mediated_decay
ENST00000598301.1 NUP62 processed_transcript
ENST00000598367.4 HNRNPM protein_coding
ENST00000598436.1 EEF2 retained_intron
ENST00000598466.4 RPS19 retained_intron
ENST00000598500.1 ADGRE2 retained_intron
ENST00000598529.4 MYO1F retained_intron
ENST00000598585.1 EMC10 protein_coding
ENST00000598597.5 AKAP8 nonsense_mediated_decay
ENST00000598607.1 GTF2F1 protein_coding
ENST00000598614.1 SIGLEC12 protein_coding
ENST00000598706.1 RAB11B retained_intron
ENST00000598758.4 TGFB1 processed_transcript
ENST00000598776.1 FPR2 protein_coding
ENST00000598797.1 MYO1F retained_intron
ENST00000598885.4 ZNF586 protein_coding
ENST00000598997.1 SYT3 protein_coding
ENST00000599123.1 MYO1F retained_intron
ENST00000599168.1 ZNF675 protein_coding
ENST00000599232.1 RPS5 retained_intron
ENST00000599256.4 UBA52 protein_coding
ENST00000599281.1 ZNF91 processed_transcript
ENST00000599296.4 ZNF431 protein_coding
ENST00000599335.4 SUPT5H retained_intron
ENST00000599345.1 MAP2K2 processed_transcript
ENST00000599528.1 IQCN protein_coding
ENST00000599546.1 PLAUR protein_coding

ENST00000599589.4 ARHGEF1 protein_coding
ENST00000599614.4 HNRNPUL1 protein_coding
ENST00000599625.1 USF2 protein_coding
ENST00000599648.1 STXBP2 retained_intron
ENST00000599704.4 EMP3 protein_coding
ENST00000599712.1 SAMD4B protein_coding
ENST00000599729.1 ZNF160 processed_transcript
ENST00000599752.4 NA NA
ENST00000599766.4 FCHO1 retained_intron
ENST00000599790.1 EPS15L1 protein_coding
ENST00000599798.1 ZNF611 processed_transcript
ENST00000599806.4 VAV1 protein_coding
ENST00000599814.4 SYMPK protein_coding
ENST00000599870.1 RPL18A protein_coding
ENST00000599883.1 AKAP8 nonsense_mediated_decay
ENST00000599962.1 H2BS1 protein_coding
ENST00000600033.1 ZFP36 processed_transcript
ENST00000600059.4 LMTK3 protein_coding
ENST00000600065.4 AKAP8L processed_transcript
ENST00000600076.4 TECR retained_intron
ENST00000600078.4 RAB4B retained_intron
ENST00000600196.1 TGFB1 protein_coding
ENST00000600199.1 AP2A1 processed_transcript
ENST00000600213.3 MTRNR2L12 protein_coding
ENST00000600233.4 HNRNPL protein_coding
ENST00000600238.1 RPL18A retained_intron
ENST00000600273.4 FCGRT protein_coding
ENST00000600281.1 MAN2B1 retained_intron
ENST00000600297.1 SLC27A1 nonsense_mediated_decay
ENST00000600343.4 NFKB1 protein_coding
ENST00000600376.4 NUTM2B-AS1 lncRNA
ENST00000600387.4 ARHGEF1 retained_intron
ENST00000600406.1 PPP1R15A protein_coding
ENST00000600408.1 KCNN4 nonsense_mediated_decay
ENST00000600463.1 IFI30 retained_intron
ENST00000600517.1 ARHGEF1 nonsense_mediated_decay
ENST00000600596.1 HNRNPUL1 nonsense_mediated_decay
ENST00000600608.4 MAP1S nonsense_mediated_decay
ENST00000600689.1 UPF1 retained_intron
ENST00000600710.1 STRN4 processed_transcript
ENST00000600714.4 ZNF83 protein_coding
ENST00000600741.1 HNRNPL retained_intron
ENST00000600764.1 CCDC200 processed_transcript
ENST00000600794.1 EEF2 protein_coding
ENST00000600810.1 nonsense_mediated_decay
ENST00000600815.1 FPR1 protein_coding
ENST00000600972.1 JUND protein_coding
ENST00000600973.4 MED29 nonsense_mediated_decay
ENST00000601071.1 BRD4 processed_transcript
ENST00000601079.4 MIR663AHG lncRNA
ENST00000601116.4 lncRNA
ENST00000601216.1 RPS11 retained_intron
ENST00000601237.4 ZNF83 retained_intron
ENST00000601241.4 SCGB2B2 protein_coding

ENST00000601249.4 ZNF611 nonsense_mediated_decay
ENST00000601306.1 RPS11 nonsense_mediated_decay
ENST00000601309.4 HNRNPUL1 protein_coding
ENST00000601336.4 HNRNPUL1 protein_coding
ENST00000601346.4 BLVRB retained_intron
ENST00000601421.4 ZNF160 protein_coding
ENST00000601453.2 ATM protein_coding
ENST00000601478.1 MED26 protein_coding
ENST00000601502.1 MYO1F retained_intron
ENST00000601564.4 HAUS8 nonsense_mediated_decay
ENST00000601581.3 ZNF638 processed_transcript
ENST00000601588.1 ZBTB7A protein_coding
ENST00000601627.1 nonsense_mediated_decay
ENST00000601664.1 HNRNPL retained_intron
ENST00000601682.1 SIGLEC7 protein_coding
ENST00000601711.4 ZNF766 protein_coding
ENST00000601715.4 ZNF780A non_stop_decay
ENST00000601801.2 NEAT1 lncRNA
ENST00000601813.1 HNRNPL protein_coding
ENST00000601857.4 NA NA
ENST00000601860.4 lncRNA
ENST00000601876.1 PLAUR retained_intron
ENST00000601916.1 CRTC1 protein_coding
ENST00000601941.1 BRD4 protein_coding
ENST00000601973.1 ZC3H4 protein_coding
ENST00000601981.4 UPF1 processed_transcript
ENST00000601995.1 ZNF274 processed_transcript
ENST00000602009.4 EPS15L1 protein_coding
ENST00000602023.1 ZNF708 miRNA
ENST00000602029.1 VRK3 miRNA
ENST00000602101.5 RASAL3 miRNA
ENST00000602136.1 MYO1F miRNA
ENST00000602142.4 VAV1 miRNA
ENST00000602153.4 RPS16 miRNA
ENST00000602158.1 MYO9B miRNA
ENST00000602198.4 TEX101 miRNA
ENST00000602201.1 DEDD2 miRNA
ENST00000602219.1 HNRNPM miRNA
ENST00000602230.1 BRD4 miRNA
ENST00000602243.4 NA miRNA
ENST00000602301.1 VTRNA2-1 miRNA
ENST00000602312.2 SDR42E2 miRNA
ENST00000602355.1 STXBP2 miRNA
ENST00000602366.4 ARMCX5-GPRASP2 miRNA
ENST00000602398.2 SBNO1 miRNA
ENST00000602402.4 DNAJC3 miRNA
ENST00000602404.4 NA miRNA
ENST00000602420.4 FTX miRNA
ENST00000602447.4 ECPAS miRNA
ENST00000602477.1 H6PD miRNA
ENST00000602478.1 miRNA
ENST00000602490.1 ARPC5 miRNA
ENST00000602498.1 HCG18 miRNA
ENST00000602499.4 UBE2E3 miRNA

ENST00000602500.1 miRNA
ENST00000602539.1 COX10-AS1 miRNA
ENST00000602579.1 LINC02893 miRNA
ENST00000602619.1 SGMS1 miRNA
ENST00000602624.5 DDOST miRNA
ENST00000602629.1 MYO3B miRNA
ENST00000602632.1 UBE2E3 miRNA
ENST00000602637.1 H2AC6 miRNA
ENST00000602645.1 NA miRNA
ENST00000602663.1 miRNA
ENST00000602666.1 miRNA
ENST00000602675.4 C16orf74 miRNA
ENST00000602691.1 FARS2 miRNA
ENST00000602703.1 MIRLET7A1HG miRNA
ENST00000602708.1 DENND4C miRNA
ENST00000602723.4 CSMD1 miRNA
ENST00000602725.4 PTP4A2 miRNA
ENST00000602743.1 COX10-AS1 miRNA
ENST00000602751.4 MYNN miRNA
ENST00000602756.1 miRNA
ENST00000602772.4 JPX miRNA
ENST00000602776.4 FTX miRNA
ENST00000602779.1 miRNA
ENST00000602782.4 NAAA miRNA
ENST00000602788.4 NPEPPS miRNA
ENST00000602791.1 miRNA
ENST00000602819.4 SNHG8 miRNA
ENST00000602849.1 RBM15 miRNA
ENST00000602863.1 XIST miRNA
ENST00000602873.4 DISC1 miRNA
ENST00000602875.4 SRA1 miRNA
ENST00000602887.1 RANBP10 miRNA
ENST00000602932.1 NA miRNA
ENST00000602938.4 JPX miRNA
ENST00000602941.5 MGAT4C miRNA
ENST00000602962.4 TSNAX-DISC1 miRNA
ENST00000602978.1 ECPAS miRNA
ENST00000602997.4 AKR1C3 miRNA
ENST00000603005.4 DUSP22 miRNA
ENST00000603037.1 FTX miRNA
ENST00000603055.1 NLRC3 miRNA
ENST00000603061.1 MOB3B miRNA
ENST00000603067.4 TAF15 miRNA
ENST00000603118.4 MCU miRNA
ENST00000603173.4 ALOX15P1 miRNA
ENST00000603197.4 CCL5 miRNA
ENST00000603199.1 USP34-DT miRNA
ENST00000603274.1 miRNA
ENST00000603290.1 DUSP22 miRNA
ENST00000603310.4 MIR4435-2HG miRNA
ENST00000603319.1 MTATP6P2 miRNA
ENST00000603346.1 TAF15 miRNA
ENST00000603492.1 FOCAD miRNA
ENST00000603504.1 7SK miRNA

ENST00000603649.4 MCU miRNA
ENST00000603669.4 GRAMD1A miRNA
ENST00000603672.4 FTX miRNA
ENST00000603678.1 miRNA
ENST00000603713.4 BNC2 miRNA
ENST00000603719.1 MTND5P10 miRNA
ENST00000603731.1 HERPUD2 miRNA
ENST00000603767.1 miRNA
ENST00000603841.1 FBXW7 miRNA
ENST00000603844.1 FOCAD miRNA
ENST00000603993.1 ANKRD10 miRNA
ENST00000604000.3 LIX1L miRNA
ENST00000604011.4 RNF103-CHMP3 miRNA
ENST00000604025.1 MICU1 miRNA
ENST00000604036.1 NA miRNA
ENST00000604046.1 GOLGA8S miRNA
ENST00000604052.1 miRNA
ENST00000604069.1 FBXW7 miRNA
ENST00000604070.1 miRNA
ENST00000604095.1 FBXW7 miRNA
ENST00000604103.1 FOCAD miRNA
ENST00000604123.4 TMEM91 miRNA
ENST00000604133.1 SRGAP2 miRNA
ENST00000604152.1 MCU miRNA
ENST00000604164.4 CCDC12 miRNA
ENST00000604220.4 WDR11 miRNA
ENST00000604238.1 MICU1 miRNA
ENST00000604257.1 MTND4P35 miRNA
ENST00000604301.1 miRNA
ENST00000604348.4 TP53 miRNA
ENST00000604369.4 NA miRNA
ENST00000604411.1 TSIX miRNA
ENST00000604419.1 SRGAP2 miRNA
ENST00000604430.1 miRNA
ENST00000604529.1 MICU1 miRNA
ENST00000604560.4 BIN2 miRNA
ENST00000604619.1 MTCO1P22 miRNA
ENST00000604646.1 MTRNR2L11 miRNA
ENST00000604669.4 miRNA
ENST00000604694.1 TAF15 miRNA
ENST00000604702.1 BIN2 miRNA
ENST00000604751.1 SNX30 processed_transcript
ENST00000604759.1 YTHDF2P1 processed_pseudogene
ENST00000604872.4 FBXW7 trna
ENST00000604879.4 TAF15 trna
ENST00000604882.2 MTRNR2L2 trna
ENST00000604912.1 PIP4K2A trna
ENST00000604914.4 DUSP22 trna
ENST00000604922.1 SH3D19 trna
ENST00000604926.1 CERT1 trna
ENST00000604929.1 TUBB4B trna
ENST00000604932.1 BABAM2 trna
ENST00000604933.2 ANTXRLP1 trna
ENST00000604952.1 MTRNR2L6 trna

ENST00000604988.1 DUSP22 trna
ENST00000605011.1 PIP4K2A trna
ENST00000605086.4 FOCAD trna
ENST00000605120.1 RASSF5 trna
ENST00000605204.1 SRSF4 trna
ENST00000605210.1 TNPO1 trna
ENST00000605233.2 POC1B-AS1 trna
ENST00000605242.4 SRGAP2 trna
ENST00000605265.1 BBIP1 trna
ENST00000605292.4 trna
ENST00000605342.1 trna
ENST00000605386.1 ATP2B1-AS1 trna
ENST00000605423.1 BIN2 trna
ENST00000605476.4 SRGAP2 trna
ENST00000605499.1 CCNY-AS1 trna
ENST00000605509.1 CCL5 trna
ENST00000605551.2 SRGAP2D trna
ENST00000605653.1 RASSF5 trna
ENST00000605656.2 TSHZ2 trna
ENST00000605806.1 RNVU1-31 trna
ENST00000605819.1 BIN2 trna
ENST00000605833.1 VIM-AS1 trna
ENST00000605860.4 UPP2 trna
ENST00000605862.4 trna
ENST00000605888.1 COG5 trna
ENST00000605920.1 trna
ENST00000605930.2 ITPR3 trna
ENST00000605990.4 SLC4A10 trna
ENST00000606059.1 BRD2 trna
ENST00000606098.1 OXNAD1 trna
ENST00000606161.1 ZEB1 trna
ENST00000606173.1 ZNF731P trna
ENST00000606190.1 RNU2-4P trna
ENST00000606202.1 TAB2 trna
ENST00000606212.1 ZNF731P trna
ENST00000606225.1 CSNK1G1 trna
ENST00000606251.1 LINC01184 trna
ENST00000606277.1 NFYC-AS1 trna
ENST00000606296.1 SATB1 trna
ENST00000606336.4 CASC15 trna
ENST00000606347.1 UCHL3 trna
ENST00000606367.1 trna
ENST00000606385.1 RIPOR2 trna
ENST00000606389.4 AAK1 trna
ENST00000606420.1 SNORA51 trna
ENST00000606425.2 NSUN6 trna
ENST00000606497.1 MARCHF6 trna
ENST00000606526.1 SNORD14A trna
ENST00000606574.1 RNU1-29P trna
ENST00000606577.1 SNORD96A trna
ENST00000606599.1 ZNF318 trna
ENST00000606623.1 RNU6-6P trna
ENST00000606712.1 DSE trna
ENST00000606749.1 CUX1 trna

ENST00000606752.1 USP6NL trna
ENST00000606757.1 trna
ENST00000606769.1 SNORA28 trna
ENST00000606775.2 NBPF25P trna
ENST00000606789.1 STK17B trna
ENST00000606797.4 TAB2 trna
ENST00000606828.2 MED16 trna
ENST00000606855.1 LINC01184 trna
ENST00000606865.1 ACYP2 trna
ENST00000606868.4 IRAK1BP1 trna
ENST00000606877.2 NBPF14 trna
ENST00000606893.4 CDC40 trna
ENST00000606895.1 ERN1 trna
ENST00000606932.1 trna
ENST00000606950.1 NA trna
ENST00000606986.1 trna
ENST00000606998.1 WAKMAR2 trna
ENST00000607003.4 SAP18 trna
ENST00000607016.1 NUDT3 trna
ENST00000607047.1 trna
ENST00000607092.1 CUX1 trna
ENST00000607197.4 HEBP2 trna
ENST00000607203.1 trna
ENST00000607242.1 PTMAP5 trna
ENST00000607313.1 SNORD58B trna
ENST00000607315.1 DCTN6-DT trna
ENST00000607318.1 RNA5SP357 trna
ENST00000607320.4 RFTN1 trna
ENST00000607353.1 trna
ENST00000607355.2 H2AC19 trna
ENST00000607367.4 POM121C trna
ENST00000607427.1 CTSS trna
ENST00000607453.1 trna
ENST00000607545.4 GUSBP1 trna
ENST00000607639.1 RNA5SP386 trna
ENST00000607661.1 trna
ENST00000607671.1 WAKMAR2 trna
ENST00000607681.1 HBP1 trna
ENST00000607692.1 SLC44A1 trna
ENST00000607707.1 SNORD11B trna
ENST00000607746.1 LINC01215 trna
ENST00000607778.1 GTF2H5 trna
ENST00000607796.4 TAGAP-AS1 trna
ENST00000607833.4 BRD2 trna
ENST00000607841.4 CTSA trna
ENST00000607866.1 ITM2B trna
ENST00000607874.1 BCORL1 trna
ENST00000607882.4 RABGEF1 trna
ENST00000607997.1 CDC37L1-DT trna
ENST00000608023.4 MIR29B2CHG trna
ENST00000608055.1 FLI1 trna
ENST00000608072.1 BACH1-IT2 trna
ENST00000608113.1 PPP4R3B-DT trna
ENST00000608176.4 trna

ENST00000608183.1 LINC01344 trna
ENST00000608184.1 STX18-AS1 trna
ENST00000608210.4 STAG3L3 trna
ENST00000608254.1 NA trna
ENST00000608287.1 SGMS1 trna
ENST00000608367.1 trna
ENST00000608382.4 ZBTB25 trna
ENST00000608389.1 trna
ENST00000608420.1 trna
ENST00000608453.1 PRKCQ-AS1 trna
ENST00000608459.2 SNORD91B trna
ENST00000608487.4 MIR663AHG trna
ENST00000608521.4 MIR663AHG trna
ENST00000608548.1 DGCR2 trna
ENST00000608566.1 SUCO trna
ENST00000608617.1 trna
ENST00000608648.1 MAST3 trna
ENST00000608663.4 RAB33B-AS1 trna
ENST00000608703.1 HSPA1A trna
ENST00000608747.1 SIRPB2 trna
ENST00000608804.1 SUCO trna
ENST00000608830.4 CELF2 trna
ENST00000608887.1 TAF4 trna
ENST00000608928.1 NA trna
ENST00000608933.4 PPM1K trna
ENST00000608978.1 ETS1 trna
ENST00000609078.1 PAFAH1B1 trna
ENST00000609132.1 LINC00649 trna
ENST00000609154.1 CRLS1 trna
ENST00000609199.1 ELOA trna
ENST00000609220.1 MIR4435-2HG trna
ENST00000609274.4 RASAL3 trna
ENST00000609284.1 NUP50-DT trna
ENST00000609285.4 SDCBP2-AS1 trna
ENST00000609297.1 PCMTD2 trna
ENST00000609309.2 PILRB trna
ENST00000609318.4 LARP4B trna
ENST00000609375.1 MKKS trna
ENST00000609401.4 LINC01278 trna
ENST00000609438.1 GPRIN3 trna
ENST00000609445.4 SGMS1 trna
ENST00000609492.1 RIT1 trna
ENST00000609518.4 ZNF595 trna
ENST00000609525.1 CSNK2A1 trna
ENST00000609620.2 SNORD91A trna
ENST00000609683.4 ZNF438 trna
ENST00000609687.4 MIR663AHG trna
ENST00000609712.1 GDI2 trna
ENST00000609714.1 ZNF718 trna
ENST00000609741.2 NBPF26 trna
ENST00000609745.1 SDCBP2-AS1 trna
ENST00000609762.1 UBN2 trna
ENST00000609796.1 SIRPB2 trna
ENST00000609799.4 LARGE1 trna

ENST00000609803.2 trna
ENST00000609928.4 TOR1AIP2 trna
ENST00000609944.4 RNF146 trna
ENST00000609981.4 PLCH2 trna
ENST00000610020.1 RPAP2 trna
ENST00000610032.1 trna
ENST00000610033.4 NFATC2 trna
ENST00000610051.4 SUCO trna
ENST00000610127.1 TXNDC12 trna
ENST00000610128.2 LINC02035 trna
ENST00000610213.4 DCP1A trna
ENST00000610229.1 trna
ENST00000610253.4 NPAT trna
ENST00000610256.1 ECD trna
ENST00000610281.1 DNAJB14 trna
ENST00000610315.1 NA trna
ENST00000610323.2 TLR6 trna
ENST00000610324.1 LINC01145 trna
ENST00000610347.1 LENG8 trna
ENST00000610363.3 NA trna
ENST00000610365.3 CYFIP1 trna
ENST00000610416.1 NA trna
ENST00000610434.3 PSMB3 trna
ENST00000610442.1 trna
ENST00000610457.1 PRKACB trna
ENST00000610460.1 5_8S_rRNA trna
ENST00000610470.3 PRKN trna
ENST00000610481.1 MALAT1 trna
ENST00000610485.1 NA trna
ENST00000610489.1 SYNE1 trna
ENST00000610537.3 NA trna
ENST00000610547.1 NA trna
ENST00000610553.1 EWSR1 trna
ENST00000610559.3 DDX3X trna
ENST00000610563.1 NA trna
ENST00000610589.1 NA trna
ENST00000610600.1 TYW1B trna
ENST00000610605.3 NA trna
ENST00000610634.1 NA trna
ENST00000610637.1 NA trna
ENST00000610639.1 NA trna
ENST00000610642.3 SLC6A6 trna
ENST00000610674.1 RN7SL3 trna
ENST00000610681.1 NUTM2B-AS1 trna
ENST00000610682.1 PHF2 trna
ENST00000610686.1 NA trna
ENST00000610690.1 PARGP1 trna
ENST00000610705.1 trna
ENST00000610727.1 NA trna
ENST00000610729.1 ZNF831 trna
ENST00000610732.3 NA trna
ENST00000610760.1 SAMD9L trna
ENST00000610798.3 AATF trna
ENST00000610803.3 FRG1HP processed_transcript

ENST00000610810.3 NA NA
ENST00000610838.2 NA NA
ENST00000610884.3 SNAPC3 protein_coding
ENST00000610889.1 NA NA
ENST00000610895.2 ZBTB10 protein_coding
ENST00000610898.1 NA NA
ENST00000610916.3 NA NA
ENST00000610925.3 NA NA
ENST00000610929.2 NA NA
ENST00000610976.1 RNVU1-28 snRNA
ENST00000610978.1 NA NA
ENST00000611049.3 GRID2 protein_coding
ENST00000611060.3 NA NA
ENST00000611066.1 miRNA
ENST00000611068.1 NA NA
ENST00000611075.3 CYFIP2 protein_coding
ENST00000611107.1 SNCA protein_coding
ENST00000611109.1 NA NA
ENST00000611116.1 TRAC TR_C_gene
ENST00000611118.1 EP400 retained_intron
ENST00000611124.1 NA NA
ENST00000611132.3 VPS36 protein_coding
ENST00000611152.1 TUBGCP3 protein_coding
ENST00000611189.1 SPG7 processed_transcript
ENST00000611197.1 C5orf67 lncRNA
ENST00000611204.3 NA NA
ENST00000611212.1 NPEPPSP1 unprocessed_pseudogene
ENST00000611219.1 GGNBP2 protein_coding
ENST00000611222.1 NA NA
ENST00000611243.1 lncRNA
ENST00000611255.3 LRMDA protein_coding
ENST00000611270.1 PIAS1 processed_transcript
ENST00000611281.3 NA NA
ENST00000611300.1 sRNA
ENST00000611351.1 NA NA
ENST00000611390.1 NA NA
ENST00000611392.3 protein_coding
ENST00000611393.1 NA NA
ENST00000611468.1 MECP2 processed_transcript
ENST00000611479.1 NA NA
ENST00000611481.1 LINC00540 lncRNA
ENST00000611494.3 NA NA
ENST00000611510.3 RNASEH2B protein_coding
ENST00000611544.1 U2 snRNA
ENST00000611551.1 NATD1 protein_coding
ENST00000611563.1 NA NA
ENST00000611587.3 NA NA
ENST00000611593.1 NBPF9 retained_intron
ENST00000611606.3 FRG1HP processed_transcript
ENST00000611683.1 ZRANB2 protein_coding
ENST00000611718.1 ORAI1 processed_transcript
ENST00000611722.1 IFIT2 protein_coding
ENST00000611764.1 POLA1 retained_intron
ENST00000611769.3 lncRNA

ENST00000611785.1 lncRNA
ENST00000611795.1 NA NA
ENST00000611815.1 SETDB2 protein_coding
ENST00000611827.1 RCBTB2 processed_transcript
ENST00000611835.3 GTF2IRD2B retained_intron
ENST00000611841.1 EP400 retained_intron
ENST00000611848.1 SMAD4 nonsense_mediated_decay
ENST00000611877.1 lncRNA
ENST00000611882.1 ABHD18 protein_coding
ENST00000611899.3 PDE1B nonsense_mediated_decay
ENST00000611910.3 SYNRG processed_transcript
ENST00000611927.1 H4C12 protein_coding
ENST00000611959.1 RNASET2 nonsense_mediated_decay
ENST00000611968.1 DDX3X protein_coding
ENST00000611974.3 PARG processed_transcript
ENST00000612013.1 lncRNA
ENST00000612036.3 NA NA
ENST00000612046.3 NA NA
ENST00000612090.1 NA NA
ENST00000612128.1 PHF10 protein_coding
ENST00000612135.1 lncRNA
ENST00000612145.1 NDOR1 retained_intron
ENST00000612161.3 SLC38A1 protein_coding
ENST00000612187.3 NA NA
ENST00000612199.3 SRGAP2B protein_coding
ENST00000612268.1 NA NA
ENST00000612303.1 NEAT1 lncRNA
ENST00000612313.1 PDE4DIPP4 processed_transcript
ENST00000612320.1 unprocessed_pseudogene
ENST00000612338.3 GFOD1 protein_coding
ENST00000612340.1 INTS7 protein_coding
ENST00000612404.3 SREK1 protein_coding
ENST00000612418.1 AKAP13 protein_coding
ENST00000612421.2 SERPINB6 protein_coding
ENST00000612429.3 NA NA
ENST00000612463.1 RNA5-8SN2 rRNA
ENST00000612477.1 WDFY2 protein_coding
ENST00000612480.1 PDE4DIPP2 transcribed_unprocessed_pseudogene
ENST00000612484.3 HUWE1 protein_coding
ENST00000612500.3 PI4KAP1 processed_transcript
ENST00000612501.1 PPP1R9B protein_coding
ENST00000612520.1 NBPF10 protein_coding
ENST00000612558.3 NA NA
ENST00000612592.1 lncRNA
ENST00000612635.3 NA NA
ENST00000612661.1 MARCKS protein_coding
ENST00000612665.1 RNA5SP440 rRNA_pseudogene
ENST00000612688.1 PPP4R3B processed_transcript
ENST00000612692.3 DDHD1 protein_coding
ENST00000612732.1 5_8S_rRNA rRNA
ENST00000612782.1 NA NA
ENST00000612797.3 NA NA
ENST00000612800.1 CIB1 protein_coding
ENST00000612822.1 NA NA

ENST00000612873.1 NA NA
ENST00000612898.1 H2BC15 protein_coding
ENST00000612932.3 SOCS7 protein_coding
ENST00000612936.1 NA NA
ENST00000612946.3 TTC28 protein_coding
ENST00000612956.3 NA NA
ENST00000612957.3 TPTE protein_coding
ENST00000613014.1 NA NA
ENST00000613023.1 RNVU1-19 snRNA
ENST00000613039.1 S100Z protein_coding
ENST00000613047.3 DIPK1A protein_coding
ENST00000613065.3 ZNF254 protein_coding
ENST00000613067.1 lncRNA
ENST00000613093.1 lncRNA
ENST00000613119.1 U2 snRNA
ENST00000613139.1 NA NA
ENST00000613141.1 PGM5P2 processed_transcript
ENST00000613146.3 ACACA retained_intron
ENST00000613151.1 TPR protein_coding
ENST00000613171.3 NA NA
ENST00000613196.3 NA NA
ENST00000613206.1 IQSEC1 protein_coding
ENST00000613214.3 TNXB protein_coding
ENST00000613246.3 NA NA
ENST00000613284.1 FAM156A nonsense_mediated_decay
ENST00000613290.3 processed_transcript
ENST00000613338.3 MAP2K3 protein_coding
ENST00000613347.1 misc_RNA
ENST00000613359.1 RNA5-8SN3 rRNA
ENST00000613376.1 MALAT1 lncRNA
ENST00000613390.1 TULP4 nonsense_mediated_decay
ENST00000613419.3 ZNF33B protein_coding
ENST00000613449.3 RNASEH2B retained_intron
ENST00000613452.1 LINC01138 lncRNA
ENST00000613473.1 NA NA
ENST00000613477.1 NA NA
ENST00000613486.1 LINC00623 lncRNA
ENST00000613488.2 SIK1B protein_coding
ENST00000613501.1 COL26A1 protein_coding
ENST00000613507.3 RIPOR2 protein_coding
ENST00000613513.1 GTF2I processed_transcript
ENST00000613525.3 MYO1F protein_coding
ENST00000613531.1 NBPF11 protein_coding
ENST00000613538.1 LEPROT protein_coding
ENST00000613545.1 UBE2E2 protein_coding
ENST00000613575.3 CDKAL1 protein_coding
ENST00000613578.3 TMEM154 protein_coding
ENST00000613624.1 NA NA
ENST00000613626.3 CDK13 protein_coding
ENST00000613640.1 NA NA
ENST00000613664.1 KYNU protein_coding
ENST00000613733.3 lncRNA
ENST00000613761.3 NA NA
ENST00000613778.1 U2 snRNA

ENST00000613788.1 CD48 protein_coding
ENST00000613823.1 UGP2 protein_coding
ENST00000613834.1 NA NA
ENST00000613840.1 AATF nonsense_mediated_decay
ENST00000613854.1 H3C1 protein_coding
ENST00000613863.3 NA NA
ENST00000613865.3 RPS24 protein_coding
ENST00000613873.3 MAP2K6 protein_coding
ENST00000613925.3 NA NA
ENST00000613928.1 CCL3 retained_intron
ENST00000613930.3 SLC6A6 retained_intron
ENST00000613956.1 U2 snRNA
ENST00000613960.1 NA NA
ENST00000613993.1 ATG5 protein_coding
ENST00000614035.3 NA NA
ENST00000614046.1 processed_transcript
ENST00000614048.1 GTF2I retained_intron
ENST00000614079.1 ARHGAP35 protein_coding
ENST00000614136.1 SRSF3 retained_intron
ENST00000614170.1 ATP11A retained_intron
ENST00000614176.3 FOXP1 protein_coding
ENST00000614183.1 FOXP1 processed_transcript
ENST00000614189.3 PCGF5 protein_coding
ENST00000614196.1 SYNRG protein_coding
ENST00000614245.1 BICRA protein_coding
ENST00000614254.1 AOAH retained_intron
ENST00000614292.1 LINC01138 lncRNA
ENST00000614365.1 5_8S_rRNA rRNA
ENST00000614377.1 CBWD3 retained_intron
ENST00000614381.3 NA NA
ENST00000614382.1 NA NA
ENST00000614386.1 GTF2IRD2 protein_coding
ENST00000614404.1 UBB protein_coding
ENST00000614407.1 NA NA
ENST00000614410.3 ALMS1 protein_coding
ENST00000614467.3 BICRAL protein_coding
ENST00000614492.1 miRNA
ENST00000614506.3 NBPF11 nonsense_mediated_decay
ENST00000614531.1 TASOR protein_coding
ENST00000614549.3 SMAP2 protein_coding
ENST00000614583.1 POM121C retained_intron
ENST00000614585.3 CPEB3 protein_coding
ENST00000614616.3 CHD1 protein_coding
ENST00000614654.1 ZSWIM9 protein_coding
ENST00000614659.1 SNPH protein_coding
ENST00000614678.1 CPNE3 retained_intron
ENST00000614681.1 NA NA
ENST00000614707.1 CSNK1G2 processed_transcript
ENST00000614730.1 NA NA
ENST00000614735.3 lncRNA
ENST00000614785.3 NBPF15 protein_coding
ENST00000614789.3 ACACA protein_coding
ENST00000614800.3 SNX9 protein_coding
ENST00000614805.1 NA NA

ENST00000614856.1 FKBP1A protein_coding
ENST00000614870.3 FCGR3B protein_coding
ENST00000614899.3 lncRNA
ENST00000614908.3 LARP4B protein_coding
ENST00000614925.1 NA NA
ENST00000614927.1 NPIPB11 protein_coding
ENST00000614931.1 PCDH9 nonsense_mediated_decay
ENST00000614987.3 RPS6KA5 protein_coding
ENST00000615008.3 TMEM230 protein_coding
ENST00000615041.1 NA NA
ENST00000615137.1 LINC00598 lncRNA
ENST00000615164.1 H4C5 protein_coding
ENST00000615188.1 SLC6A6 processed_transcript
ENST00000615191.3 NA NA
ENST00000615259.3 PDE7B protein_coding
ENST00000615277.3 PLCL2 protein_coding
ENST00000615292.1 NA NA
ENST00000615294.1 NA NA
ENST00000615321.1 RSBN1 protein_coding
ENST00000615339.1 BCOR protein_coding
ENST00000615353.1 H4C9 protein_coding
ENST00000615374.3 ST20-MTHFS protein_coding
ENST00000615377.3 NA NA
ENST00000615392.3 FBXO8 protein_coding
ENST00000615422.1 NHS retained_intron
ENST00000615424.3 LINC00623 lncRNA
ENST00000615427.1 U2 snRNA
ENST00000615441.1 MLLT6 processed_transcript
ENST00000615443.1 CHM protein_coding
ENST00000615445.3 NA NA
ENST00000615452.1 NA NA
ENST00000615466.1 ZNF189 protein_coding
ENST00000615491.3 IKZF1 protein_coding
ENST00000615497.3 NA NA
ENST00000615512.1 DCAF7 protein_coding
ENST00000615519.3 DIPK1A protein_coding
ENST00000615525.1 NCAM1 processed_transcript
ENST00000615550.3 PRIM2 protein_coding
ENST00000615553.1 C18orf25 protein_coding
ENST00000615612.1 GNAZ protein_coding
ENST00000615635.1 lncRNA
ENST00000615647.1 NA NA
ENST00000615671.1 GABPB1-IT1 lncRNA
ENST00000615673.3 NA NA
ENST00000615689.1 NA NA
ENST00000615695.1 DDB2 retained_intron
ENST00000615716.1 LINC02391 lncRNA
ENST00000615722.1 lncRNA
ENST00000615727.1 NA NA
ENST00000615747.1 NA NA
ENST00000615792.1 NA NA
ENST00000615793.1 UBE2D1 protein_coding
ENST00000615825.1 MME protein_coding
ENST00000615842.1 RNVU1-29 snRNA

ENST00000615871.3 AUTS2 protein_coding
ENST00000615892.3 PPP1R18 protein_coding
ENST00000615899.1 NA NA
ENST00000615927.1 NA NA
ENST00000615932.1 NA NA
ENST00000615959.1 MIR3648-1 miRNA
ENST00000615993.1 TFG protein_coding
ENST00000616019.1 GGNBP2 protein_coding
ENST00000616023.1 SEC22B3P unprocessed_pseudogene
ENST00000616050.1 DDX3X protein_coding
ENST00000616065.3 PLEKHO2 protein_coding
ENST00000616084.1 PCIF1 processed_transcript
ENST00000616146.3 GMFB protein_coding
ENST00000616153.1 MIR6718 miRNA
ENST00000616167.1 NCOA6 protein_coding
ENST00000616170.3 SH3GLB1 protein_coding
ENST00000616200.1 NEXMIF protein_coding
ENST00000616235.1 FAM156A processed_transcript
ENST00000616236.1 RFC3 protein_coding
ENST00000616246.3 NA NA
ENST00000616286.1 NA NA
ENST00000616305.1 CASTOR2 protein_coding
ENST00000616315.1 NEAT1 lncRNA
ENST00000616345.1 U2 snRNA
ENST00000616356.3 NA NA
ENST00000616365.1 H3C11 protein_coding
ENST00000616381.3 NA NA
ENST00000616392.1 AATF retained_intron
ENST00000616406.1 TSPAN14 protein_coding
ENST00000616407.1 PPP4R3B protein_coding
ENST00000616441.2 PTCRA protein_coding
ENST00000616448.1 PARG protein_coding
ENST00000616528.1 NA NA
ENST00000616535.1 U2 snRNA
ENST00000616568.3 PHF19 protein_coding
ENST00000616577.3 TPT1 protein_coding
ENST00000616588.3 lncRNA
ENST00000616646.3 DDX52 nonsense_mediated_decay
ENST00000616721.3 FCGBP protein_coding
ENST00000616733.1 NA NA
ENST00000616760.1 CLIC1 protein_coding
ENST00000616768.3 MLH1 protein_coding
ENST00000616778.3 NA NA
ENST00000616814.3 NA NA
ENST00000616829.3 NA NA
ENST00000616833.3 CLN5 protein_coding
ENST00000616844.1 NA NA
ENST00000616856.1 TULP4 retained_intron
ENST00000616860.1 NA NA
ENST00000616875.1 NA NA
ENST00000616902.3 NA NA
ENST00000616907.1 RNASEH2B protein_coding
ENST00000616921.1 NA NA
ENST00000616927.3 PLEKHA2 protein_coding

ENST00000616974.1 LINC00540 lncRNA
ENST00000616976.1 NA NA
ENST00000617005.3 PLCB1 protein_coding
ENST00000617010.2 NBPF10 protein_coding
ENST00000617017.1 NA NA
ENST00000617047.1 RBM15 protein_coding
ENST00000617077.1 lncRNA
ENST00000617118.1 NA NA
ENST00000617141.1 AATF retained_intron
ENST00000617166.1 NCAM1 processed_transcript
ENST00000617179.3 NA NA
ENST00000617236.1 NA NA
ENST00000617238.1 NA NA
ENST00000617246.3 LPP protein_coding
ENST00000617257.1 ESF1 protein_coding
ENST00000617267.3 AOAH protein_coding
ENST00000617290.1 NA NA
ENST00000617311.1 NPIPB12 protein_coding
ENST00000617318.3 processed_transcript
ENST00000617331.1 MAP3K14 protein_coding
ENST00000617343.3 NA NA
ENST00000617352.1 MCL1 processed_transcript
ENST00000617377.1 ZC3H12B retained_intron
ENST00000617381.3 NA NA
ENST00000617432.3 NA NA
ENST00000617444.3 SLC35E2B protein_coding
ENST00000617474.1 PTPRT nonsense_mediated_decay
ENST00000617489.1 MALAT1 lncRNA
ENST00000617493.1 COG3 protein_coding
ENST00000617499.1 PIP4K2B protein_coding
ENST00000617523.1 COPG2 retained_intron
ENST00000617560.1 PHF20 processed_transcript
ENST00000617585.1 ARHGEF1 retained_intron
ENST00000617586.1 NA NA
ENST00000617611.1 NA NA
ENST00000617626.1 RNVU1-2A snRNA
ENST00000617629.3 CYFIP2 protein_coding
ENST00000617677.1 EIF5B protein_coding
ENST00000617689.3 PIBF1 protein_coding
ENST00000617694.3 RPS15 protein_coding
ENST00000617702.1 LINC00623 lncRNA
ENST00000617712.1 lncRNA
ENST00000617721.1 NA NA
ENST00000617773.1 NA NA
ENST00000617779.1 BNC2 protein_coding
ENST00000617785.1 U2 snRNA
ENST00000617791.1 MALAT1 lncRNA
ENST00000617797.1 NA NA
ENST00000617804.1 nonsense_mediated_decay
ENST00000617821.3 NA NA
ENST00000617840.1 ZNF516 protein_coding
ENST00000617844.3 NBPF12 protein_coding
ENST00000617859.3 IKZF5 protein_coding
ENST00000617860.3 GGNBP2 retained_intron

ENST00000617864.2 NA NA
ENST00000617878.1 PDE4DIPP7 processed_transcript
ENST00000617914.1 lncRNA
ENST00000617931.3 NBPF12 protein_coding
ENST00000617933.1 CBWD6 protein_coding
ENST00000617940.1 FRG1HP transcribed_unprocessed_pseudogene
ENST00000617952.3 YTHDF3 protein_coding
ENST00000618008.3 ZNF714 protein_coding
ENST00000618025.3 AGAP6 processed_transcript
ENST00000618050.3 NA NA
ENST00000618076.3 ADORA2A protein_coding
ENST00000618078.1 CBX5 processed_transcript
ENST00000618099.3 FURIN protein_coding
ENST00000618106.3 lncRNA
ENST00000618112.1 BSG nonsense_mediated_decay
ENST00000618123.3 CNOT6 protein_coding
ENST00000618132.1 MALAT1 lncRNA
ENST00000618137.1 NLRC3 protein_coding
ENST00000618148.1 VPS45 retained_intron
ENST00000618163.1 NA NA
ENST00000618168.1 lncRNA
ENST00000618169.3 NA NA
ENST00000618203.1 NA NA
ENST00000618227.1 MALAT1 lncRNA
ENST00000618249.1 misc_RNA
ENST00000618253.1 EMBP1 processed_transcript
ENST00000618282.3 MAGT1 protein_coding
ENST00000618312.3 MTHFD1L protein_coding
ENST00000618353.1 FER protein_coding
ENST00000618400.3 NA NA
ENST00000618406.1 SEC22B4P transcribed_unprocessed_pseudogene
ENST00000618439.1 NA NA
ENST00000618471.3 NA NA
ENST00000618509.3 OSBPL2 protein_coding
ENST00000618527.3 NA NA
ENST00000618553.1 APOBEC3A protein_coding
ENST00000618573.3 NA NA
ENST00000618577.3 CEACAM21 processed_transcript
ENST00000618589.1 lncRNA
ENST00000618590.1 NUDT2 protein_coding
ENST00000618602.1 U2 snRNA
ENST00000618646.3 NA NA
ENST00000618652.1 MLLT6 retained_intron
ENST00000618664.1 RNU2-1 snRNA
ENST00000618680.3 NA NA
ENST00000618702.1 SEC22B2P unprocessed_pseudogene
ENST00000618714.3 NA NA
ENST00000618718.1 CITED2 protein_coding
ENST00000618741.1 LYPLA1 protein_coding
ENST00000618756.3 NA NA
ENST00000618765.1 RRP12 protein_coding
ENST00000618775.3 UBE2Q2P2 processed_transcript
ENST00000618778.3 GTDC1 protein_coding
ENST00000618786.1 RN7SL1 misc_RNA

ENST00000618808.3 NA NA
ENST00000618828.1 COL5A2 protein_coding
ENST00000618829.3 SYNRG retained_intron
ENST00000618836.3 UBE2D3 protein_coding
ENST00000618844.1 lncRNA
ENST00000618849.1 MAML2 protein_coding
ENST00000618852.3 WASF2 protein_coding
ENST00000618887.2 POLDIP2 protein_coding
ENST00000618923.1 ADAM17 nonsense_mediated_decay
ENST00000618925.1 MALAT1 lncRNA
ENST00000618940.3 AP2B1 protein_coding
ENST00000618947.1 TPT1-AS1 lncRNA
ENST00000618951.1 NA NA
ENST00000618959.1 GALNT17 protein_coding
ENST00000618962.3 GTF2IRD2P1 retained_intron
ENST00000618978.1 U2 snRNA
ENST00000619021.3 HERC2P2 processed_transcript
ENST00000619023.3 NA NA
ENST00000619029.1 NA NA
ENST00000619035.3 NA NA
ENST00000619037.1 CYFIP1 nonsense_mediated_decay
ENST00000619039.3 PIP4K2B protein_coding
ENST00000619046.3 ATP6AP1 protein_coding
ENST00000619061.1 NA NA
ENST00000619101.3 REST protein_coding
ENST00000619168.3 CAMK1D protein_coding
ENST00000619178.1 SNORD3D snoRNA
ENST00000619199.3 NA NA
ENST00000619225.1 U2 snRNA
ENST00000619230.1 C18orf25 retained_intron
ENST00000619232.1 DACH1 protein_coding
ENST00000619235.1 MIR7849 miRNA
ENST00000619300.3 PSPC1 protein_coding
ENST00000619301.3 C18orf25 protein_coding
ENST00000619321.1 SLC16A3 protein_coding
ENST00000619348.3 CYFIP1 retained_intron
ENST00000619355.1 HERC2P7 miRNA
ENST00000619366.1 NA miRNA
ENST00000619404.1 NA miRNA
ENST00000619412.3 NA miRNA
ENST00000619420.3 SNX10 miRNA
ENST00000619433.1 PANK2 miRNA
ENST00000619449.1 MALAT1 miRNA
ENST00000619456.3 AGAP1 miRNA
ENST00000619464.1 miRNA
ENST00000619465.1 U2 miRNA
ENST00000619471.1 RNA5-8SN1 miRNA
ENST00000619499.3 NA miRNA
ENST00000619513.1 KIAA0040 miRNA
ENST00000619548.1 MRPL45 miRNA
ENST00000619573.1 GGNBP2 miRNA
ENST00000619590.1 AMD1 miRNA
ENST00000619601.1 GAPDH miRNA
ENST00000619610.2 U2AF1L5 miRNA

ENST00000619650.3 ZRANB3 miRNA
ENST00000619653.1 miRNA
ENST00000619666.1 miRNA
ENST00000619678.1 SRGAP2B miRNA
ENST00000619682.1 MX1 miRNA
ENST00000619706.3 MCM9 miRNA
ENST00000619713.1 miRNA
ENST00000619732.3 NA miRNA
ENST00000619760.1 DNAAF9 miRNA
ENST00000619790.3 SRP9 miRNA
ENST00000619791.1 SETD1B miRNA
ENST00000619801.3 CSNK2A1 miRNA
ENST00000619859.1 HM13 miRNA
ENST00000619862.1 ERVK13-1 miRNA
ENST00000619867.3 LINC00624 miRNA
ENST00000619899.1 miRNA
ENST00000619906.3 NA miRNA
ENST00000619914.1 SYNRG miRNA
ENST00000619932.3 NBPF25P miRNA
ENST00000620015.3 GATD3B miRNA
ENST00000620026.1 TULP4 miRNA
ENST00000620041.3 FTH1 miRNA
ENST00000620068.1 MIR7854 miRNA
ENST00000620081.1 NA miRNA
ENST00000620097.1 OAS2 miRNA
ENST00000620121.3 ASXL1 miRNA
ENST00000620123.3 NA miRNA
ENST00000620125.1 NA miRNA
ENST00000620145.3 MIAT miRNA
ENST00000620148.1 NPEPPSP1 miRNA
ENST00000620157.3 DDX6 miRNA
ENST00000620184.3 NA miRNA
ENST00000620186.3 LINC01297 miRNA
ENST00000620194.1 NA miRNA
ENST00000620195.3 ZNF778 miRNA
ENST00000620242.1 SRSF3 miRNA
ENST00000620248.1 BCL10 miRNA
ENST00000620266.1 miRNA
ENST00000620268.1 U2 miRNA
ENST00000620272.3 miRNA
ENST00000620285.1 NPIPB2 miRNA
ENST00000620300.3 miRNA
ENST00000620308.1 LUZP2 miRNA
ENST00000620315.1 DDX21 miRNA
ENST00000620348.1 miRNA
ENST00000620353.1 ANXA8L1 miRNA
ENST00000620355.1 NA miRNA
ENST00000620364.3 COL19A1 miRNA
ENST00000620383.1 NA miRNA
ENST00000620399.1 miRNA
ENST00000620406.1 NA miRNA
ENST00000620424.1 SYNRG miRNA
ENST00000620426.3 NA miRNA
ENST00000620430.3 WASHC4 miRNA

ENST00000620436.3 NA miRNA
ENST00000620438.1 H2BU1 miRNA
ENST00000620447.1 ZMYM2 miRNA
ENST00000620465.3 MALAT1 miRNA
ENST00000620494.1 LINC02340 miRNA
ENST00000620510.1 ZKSCAN1 miRNA
ENST00000620511.1 NA miRNA
ENST00000620525.1 miRNA
ENST00000620529.1 PCGF3 miRNA
ENST00000620572.1 FOXP1 miRNA
ENST00000620612.3 NBPF26 miRNA
ENST00000620633.3 LPAR6 miRNA
ENST00000620651.3 MRTFA miRNA
ENST00000620670.1 RNPEP miRNA
ENST00000620676.3 VPS13D miRNA
ENST00000620715.3 NA miRNA
ENST00000620717.3 NA miRNA
ENST00000620725.1 NA miRNA
ENST00000620747.3 NA miRNA
ENST00000620771.3 HPCAL1 miRNA
ENST00000620806.3 NA miRNA
ENST00000620828.3 FMR1 miRNA
ENST00000620831.3 NA miRNA
ENST00000620838.3 TADA2A miRNA
ENST00000620854.3 NEDD9 miRNA
ENST00000620902.1 MALAT1 miRNA
ENST00000620915.3 CANT1 miRNA
ENST00000620954.1 WDR89 miRNA
ENST00000620955.1 NA miRNA
ENST00000620969.3 NA miRNA
ENST00000620995.3 TYW1B miRNA
ENST00000620996.3 VTA1 miRNA
ENST00000620998.3 NA miRNA
ENST00000621010.1 PPEF2 miRNA
ENST00000621032.3 NA miRNA
ENST00000621048.3 ZCCHC2 miRNA
ENST00000621066.3 NBPF14 miRNA
ENST00000621101.2 NA miRNA
ENST00000621106.3 NA miRNA
ENST00000621147.3 KDM6A miRNA
ENST00000621152.3 SMC6 miRNA
ENST00000621161.3 RAB11FIP4 miRNA
ENST00000621208.3 FRMD3 miRNA
ENST00000621220.3 MLLT10 miRNA
ENST00000621235.1 miRNA
ENST00000621237.1 IRF1-AS1 miRNA
ENST00000621244.1 NA miRNA
ENST00000621272.1 GARS1-DT miRNA
ENST00000621282.3 DLEU2 miRNA
ENST00000621309.3 F11R protein_coding
ENST00000621332.3 MLLT6 protein_coding
ENST00000621359.2 NA trna
ENST00000621366.1 KIZ trna
ENST00000621375.3 UGGT2 trna

ENST00000621386.1 NA trna
ENST00000621442.3 NA trna
ENST00000621473.3 NA trna
ENST00000621507.1 PANK2 trna
ENST00000621510.1 AOAH trna
ENST00000621516.1 CYFIP2 trna
ENST00000621526.3 CPNE3 trna
ENST00000621530.1 ST6GALNAC3 trna
ENST00000621533.3 WTAP trna
ENST00000621537.3 NA trna
ENST00000621582.1 SRGAP2B trna
ENST00000621605.1 SYNRG trna
ENST00000621615.1 trna
ENST00000621645.3 NBPF9 trna
ENST00000621650.1 ZNF8 trna
ENST00000621655.1 NA trna
ENST00000621667.1 MIR941-5 trna
ENST00000621744.3 trna
ENST00000621766.3 NA trna
ENST00000621800.3 NA trna
ENST00000621814.3 NA trna
ENST00000621820.3 YTHDF3 trna
ENST00000621840.3 TDRD3 trna
ENST00000621856.1 SPAST trna
ENST00000621876.1 NA trna
ENST00000621933.1 MIR223HG trna
ENST00000621948.3 GTF2IP1 trna
ENST00000621956.3 NA trna
ENST00000621973.1 DDX1 trna
ENST00000622018.3 HPCAL1 trna
ENST00000622020.3 NPHP4 trna
ENST00000622037.1 RYR3 trna
ENST00000622089.3 NA trna
ENST00000622090.3 DDB2 trna
ENST00000622132.3 ABCB1 trna
ENST00000622151.3 FOXP1 trna
ENST00000622186.3 SLC6A6 trna
ENST00000622198.3 DDX3X trna
ENST00000622241.1 PRAG1 trna
ENST00000622254.1 MAPK1IP1L trna
ENST00000622285.1 RNVU1-27 trna
ENST00000622328.1 LINC01138 trna
ENST00000622332.3 OSBPL2 trna
ENST00000622333.1 ESCO1 trna
ENST00000622341.1 NA trna
ENST00000622371.1 TMCO3 trna
ENST00000622373.1 DDX3X trna
ENST00000622375.3 NA trna
ENST00000622383.1 NA trna
ENST00000622386.1 KMT2E trna
ENST00000622426.1 NA trna
ENST00000622427.3 ANK3 trna
ENST00000622512.1 ZBTB18 trna
ENST00000622514.3 DAPK1 trna

ENST00000622552.3 NA trna
ENST00000622559.3 CYSLTR2 trna
ENST00000622577.2 NA trna
ENST00000622583.3 RAD9A trna
ENST00000622663.1 NA trna
ENST00000622671.1 trna
ENST00000622695.1 LINC00869 trna
ENST00000622699.2 PARD3B trna
ENST00000622704.1 NA trna
ENST00000622724.3 KIF1B trna
ENST00000622744.3 NA trna
ENST00000622766.1 MYSM1 trna
ENST00000622775.3 NA trna
ENST00000622786.3 NA trna
ENST00000622787.3 EMBP1 trna
ENST00000622810.3 SLC6A6 trna
ENST00000622811.1 XKR4 trna
ENST00000622827.3 NA trna
ENST00000622863.3 REST trna
ENST00000622865.1 GNAT2 trna
ENST00000622870.3 NA trna
ENST00000622875.3 EBF1 trna
ENST00000622893.1 NA trna
ENST00000622931.1 trna
ENST00000622961.2 trna
ENST00000622968.1 trna
ENST00000622986.2 ALG13 trna
ENST00000623033.1 NA trna
ENST00000623083.2 WASH9P trna
ENST00000623087.1 NA trna
ENST00000623091.1 HEIH trna
ENST00000623127.1 NA trna
ENST00000623170.1 RANBP6 trna
ENST00000623177.1 trna
ENST00000623180.1 LINC02887 trna
ENST00000623210.1 trna
ENST00000623213.1 DOCK1 trna
ENST00000623316.1 ATRX trna
ENST00000623317.2 AAK1 trna
ENST00000623318.1 ERCC6 trna
ENST00000623354.1 NHSL2 trna
ENST00000623375.2 U2AF1L5 trna
ENST00000623400.2 WASHC2C trna
ENST00000623464.1 PRCP trna
ENST00000623535.1 CDKL5 trna
ENST00000623550.1 B2M trna
ENST00000623596.1 ZNF654 trna
ENST00000623629.1 NA trna
ENST00000623664.1 trna
ENST00000623674.1 trna
ENST00000623678.1 trna
ENST00000623706.2 ATRX trna
ENST00000623724.2 IKZF3 trna
ENST00000623733.1 NA trna

ENST00000623785.1 trna
ENST00000623788.1 ERCC6 trna
ENST00000623860.1 trna
ENST00000623924.1 EPM2AIP1 trna
ENST00000623932.1 NA trna
ENST00000623935.1 SOX9-AS1 trna
ENST00000623936.1 trna
ENST00000623998.1 trna
ENST00000624028.1 PPHLN1 trna
ENST00000624029.1 trna
ENST00000624076.1 trna
ENST00000624098.2 UTY trna
ENST00000624128.1 SNHG5 trna
ENST00000624166.2 ATRX trna
ENST00000624193.1 ATRX trna
ENST00000624349.1 trna
ENST00000624357.1 NA trna
ENST00000624419.1 NOTCH2NLR trna
ENST00000624491.1 trna
ENST00000624538.1 ARHGEF9 trna
ENST00000624575.1 trna
ENST00000624586.1 EPM2AIP1 trna
ENST00000624620.1 RNF114 trna
ENST00000624624.1 trna
ENST00000624666.1 RNF114 trna
ENST00000624699.1 TAF7 trna
ENST00000624705.1 trna
ENST00000624735.1 NA trna
ENST00000624829.2 LINC01572 trna
ENST00000624900.1 AAK1 trna
ENST00000624929.1 trna
ENST00000624931.1 PCF11 trna
ENST00000624936.1 trna
ENST00000624945.1 trna
ENST00000624953.1 CDKL5 trna
ENST00000624956.1 BCLAF1P2 trna
ENST00000625012.1 trna
ENST00000625036.2 GATD3B trna
ENST00000625054.1 trna
ENST00000625063.2 ATRX trna
ENST00000625084.1 PLCL1 trna
ENST00000625138.1 OSBPL9 trna
ENST00000625147.2 PRKCQ-AS1 trna
ENST00000625153.2 trna
ENST00000625158.1 NA trna
ENST00000625161.1 ZEB2 trna
ENST00000625223.1 NA trna
ENST00000625244.1 SNX1 trna
ENST00000625245.1 SMN1 trna
ENST00000625260.1 PFKFB3 trna
ENST00000625269.1 snoZ196 trna
ENST00000625282.1 SPTAN1 trna
ENST00000625312.1 PGM2 trna
ENST00000625349.1 LUC7L3 trna

ENST00000625359.1 ELF1 trna
ENST00000625364.1 PPA1 trna
ENST00000625365.1 CNTNAP2 trna
ENST00000625402.1 SCARNA4 trna
ENST00000625444.1 PSMD5 trna
ENST00000625445.1 EBLN3P trna
ENST00000625449.1 trna
ENST00000625458.1 MTRES1 trna
ENST00000625463.1 CHD2 trna
ENST00000625585.1 MEF2C trna
ENST00000625598.1 trna
ENST00000625626.1 FAM120B trna
ENST00000625662.1 CHD2 trna
ENST00000625670.1 POU2F2 trna
ENST00000625713.1 ARRDC3-AS1 trna
ENST00000625716.1 TCF4 trna
ENST00000625812.1 trna
ENST00000625924.1 RUNX2 trna
ENST00000625926.1 SSBP4 trna
ENST00000625943.1 SNORD38B trna
ENST00000625990.1 CHD2 trna
ENST00000626021.1 PTP4A1 trna
ENST00000626040.1 THEMIS trna
ENST00000626066.1 EHMT1 trna
ENST00000626114.1 PLCB1 trna
ENST00000626176.1 UBE3A trna
ENST00000626216.1 EHMT1 trna
ENST00000626236.1 SGMS1 trna
ENST00000626245.1 GARS1-DT trna
ENST00000626247.1 DGKH trna
ENST00000626262.1 UBAP1 trna
ENST00000626355.1 NA trna
ENST00000626464.1 ZDHHC20 trna
ENST00000626466.1 TCF4 trna
ENST00000626479.1 SLFNL1-AS1 trna
ENST00000626518.1 LINC01163 trna
ENST00000626530.1 NA trna
ENST00000626547.1 RPS9 trna
ENST00000626572.1 LAMTOR5-AS1 trna
ENST00000626589.1 UBE3A trna
ENST00000626620.1 KCNMA1 trna
ENST00000626660.1 SQSTM1 trna
ENST00000626677.1 MUC20-OT1 trna
ENST00000626716.1 R3HDM4 trna
ENST00000626725.1 NSL1 trna
ENST00000626826.1 HELLPAR trna
ENST00000626874.1 CHD2 trna
ENST00000626886.1 SNORA17B trna
ENST00000626902.1 GARS1-DT trna
ENST00000626963.1 NA trna
ENST00000626966.1 PLCB1 trna
ENST00000627002.1 PTP4A1 trna
ENST00000627018.1 UBE3A trna
ENST00000627019.1 PCBP1-AS1 trna

ENST00000627024.1 DOCK9 trna
ENST00000627032.1 GLG1 trna
ENST00000627092.1 NA trna
ENST00000627200.1 CHD2 trna
ENST00000627215.1 LINC01127 trna
ENST00000627241.1 NA trna
ENST00000627264.1 BBS9 trna
ENST00000627286.1 ZMYND11 protein_coding
ENST00000627296.1 RNGTT retained_intron
ENST00000627368.1 DCTN4 protein_coding
ENST00000627393.1 PCNP protein_coding
ENST00000627409.1 MYCBP2-AS1 lncRNA
ENST00000627435.1 NA NA
ENST00000627440.1 SNAP23 protein_coding
ENST00000627441.1 SPTAN1 protein_coding
ENST00000627460.1 CHD2 nonsense_mediated_decay
ENST00000627498.1 LINC01347 processed_transcript
ENST00000627526.1 NA NA
ENST00000627527.1 TTN-AS1 lncRNA
ENST00000627532.1 ZEB2 protein_coding
ENST00000627546.1 SATB1-AS1 lncRNA
ENST00000627564.1 TTN-AS1 lncRNA
ENST00000627568.1 TCF4 processed_transcript
ENST00000627604.1 POLR1D protein_coding
ENST00000627622.1 CHD2 protein_coding
ENST00000627631.1 MAPKBP1 protein_coding
ENST00000627640.1 NA NA
ENST00000627651.1 MBD5 protein_coding
ENST00000627700.1 lncRNA
ENST00000627802.1 DNAJC17 protein_coding
ENST00000627856.1 NA NA
ENST00000627864.1 MECP2 processed_transcript
ENST00000627916.1 PPIP5K2 protein_coding
ENST00000627960.1 UBR1 protein_coding
ENST00000627981.1 lncRNA
ENST00000628013.1 LINC02614 lncRNA
ENST00000628092.1 NA NA
ENST00000628102.1 PCBP1-AS1 lncRNA
ENST00000628121.1 LINC01176 lncRNA
ENST00000628146.1 UTRN protein_coding
ENST00000628181.1 CHD2 processed_transcript
ENST00000628298.1 EIF1B-AS1 lncRNA
ENST00000628311.1 UBE2E2 protein_coding
ENST00000628324.1 LINC01170 lncRNA
ENST00000628388.1 Y_RNA misc_RNA
ENST00000628403.1 SLC12A2 protein_coding
ENST00000628426.1 CHST15 protein_coding
ENST00000628453.1 SLFN12L protein_coding
ENST00000628473.1 ZEB2 retained_intron
ENST00000628477.1 DDX46 protein_coding
ENST00000628514.1 SUGCT protein_coding
ENST00000628531.1 Y_RNA misc_RNA
ENST00000628590.1 snoRNA
ENST00000628603.1 NA NA

ENST00000628689.1 TCF4 nonsense_mediated_decay
ENST00000628694.1 NA NA
ENST00000628696.1 SMN2 protein_coding
ENST00000628752.1 NCOA6 protein_coding
ENST00000628758.1 NA NA
ENST00000628857.1 NA NA
ENST00000628861.1 NA NA
ENST00000628900.1 PLCB1 processed_transcript
ENST00000628924.1 EBLN3P lncRNA
ENST00000628959.1 PPP2R1A protein_coding
ENST00000628962.1 VPS8 protein_coding
ENST00000628997.1 MAGI2 retained_intron
ENST00000629069.1 NCBP1 protein_coding
ENST00000629083.1 Metazoa_SRP misc_RNA
ENST00000629086.1 NA NA
ENST00000629104.1 CHD2 nonsense_mediated_decay
ENST00000629117.1 TTN-AS1 lncRNA
ENST00000629124.1 STAG1 protein_coding
ENST00000629136.1 CHD2 processed_transcript
ENST00000629145.1 SCHLAP1 lncRNA
ENST00000629203.1 SH2D3C protein_coding
ENST00000629257.1 CYTOR lncRNA
ENST00000629272.1 ATL2 protein_coding
ENST00000629277.1 MECP2 retained_intron
ENST00000629304.1 PCBP1-AS1 lncRNA
ENST00000629357.1 TRG-AS1 lncRNA
ENST00000629385.1 ABCC4 protein_coding
ENST00000629393.1 N4BP2L2-IT2 lncRNA
ENST00000629399.1 MDN1 protein_coding
ENST00000629417.1 EHMT1 protein_coding
ENST00000629504.1 GABARAPL1 protein_coding
ENST00000629531.1 NA NA
ENST00000629536.1 SNORA50A snoRNA
ENST00000629629.1 snoRNA
ENST00000629650.1 PPM1D protein_coding
ENST00000629688.1 DRG1 protein_coding
ENST00000629705.1 NA NA
ENST00000629728.1 RANBP2 protein_coding
ENST00000629751.1 HSPH1 retained_intron
ENST00000629789.1 NA NA
ENST00000629819.1 lncRNA
ENST00000629853.1 lncRNA
ENST00000629893.1 NASP protein_coding
ENST00000629955.1 ZEB2 processed_transcript
ENST00000629969.1 lncRNA
ENST00000630016.1 CHD2 nonsense_mediated_decay
ENST00000630027.1 PSME2 protein_coding
ENST00000630164.1 PIP4P2 protein_coding
ENST00000630242.1 FAM30A lncRNA
ENST00000630369.1 THEMIS protein_coding
ENST00000630477.1 PSMB2 processed_transcript
ENST00000630479.1 NOCT nonsense_mediated_decay
ENST00000630499.1 SRRM2 protein_coding
ENST00000630585.1 UBA3 protein_coding

ENST00000630598.1 EBLN3P lncRNA
ENST00000630607.1 UBE3A retained_intron
ENST00000630615.1 snoRNA
ENST00000630639.1 HNRNPH1 protein_coding
ENST00000630717.1 lncRNA
ENST00000630741.1 NA NA
ENST00000630757.1 PLCB1 retained_intron
ENST00000630798.1 DPYD-AS1 lncRNA
ENST00000630813.1 CHD2 retained_intron
ENST00000630839.1 UQCRC2 protein_coding
ENST00000630856.1 MIR325HG lncRNA
ENST00000630869.1 SFT2D2 protein_coding
ENST00000630925.1 SDCBP protein_coding
ENST00000631044.1 POLG nonsense_mediated_decay
ENST00000631047.1 WDR75 protein_coding
ENST00000631057.1 GPC3 protein_coding
ENST00000631118.1 GLUD1P2 processed_transcript
ENST00000631148.1 PWWP2B protein_coding
ENST00000631162.1 NA NA
ENST00000631184.1 PLAGL1 processed_transcript
ENST00000631198.1 lncRNA
ENST00000631211.1 lncRNA
ENST00000631253.1 TBL1XR1 nonsense_mediated_decay
ENST00000631292.1 NA NA
ENST00000631308.1 NA NA
ENST00000631312.1 RALB protein_coding
ENST00000631321.1 LINC00540 lncRNA
ENST00000631335.1 SCRN1 protein_coding
ENSTR0000381177.4 NA NA
ENSTR0000381297.7 NA NA
ENSTR0000381317.6 NA NA
ENSTR0000381401.8 NA NA
ENSTR0000381625.7 NA NA
ENSTR0000414513.5 NA NA
ENSTR0000430536.5 NA NA
ENSTR0000432757.4 NA NA
ENSTR0000435581.5 NA NA
ENSTR0000449611.4 NA NA
ENSTR0000460672.4 NA NA
ENSTR0000461691.4 NA NA
ENSTR0000463317.4 NA NA
ENSTR0000474865.4 NA NA
ENSTR0000478825.4 NA NA
ENSTR0000481245.4 NA NA
ENSTR0000484026.4 NA NA
ENSTR0000498153.4 NA NA
ENSTR0000578699.4 NA NA
MIMAT0000062_2 NA NA
MIMAT0000063 NA NA
MIMAT0000064 NA NA
MIMAT0000065 NA NA
MIMAT0000066 NA NA
MIMAT0000067_1 NA NA
MIMAT0000068 NA NA

MIMAT0000069_1 NA NA
MIMAT0000070 NA NA
MIMAT0000071 NA NA
MIMAT0000072 NA NA
MIMAT0000073 NA NA
MIMAT0000074_1 NA NA
MIMAT0000075 NA NA
MIMAT0000076 NA NA
MIMAT0000077 NA NA
MIMAT0000078 NA NA
MIMAT0000079 NA NA
MIMAT0000080_1 NA NA
MIMAT0000081 NA NA
MIMAT0000082_1 NA NA
MIMAT0000083 NA NA
MIMAT0000084 NA NA
MIMAT0000085 NA NA
MIMAT0000086 NA NA
MIMAT0000087 NA NA
MIMAT0000088 NA NA
MIMAT0000089 NA NA
MIMAT0000090 NA NA
MIMAT0000091 NA NA
MIMAT0000092_1 NA NA
MIMAT0000093 NA NA
MIMAT0000094 NA NA
MIMAT0000095 NA NA
MIMAT0000096 NA NA
MIMAT0000097 NA NA
MIMAT0000098 NA NA
MIMAT0000099_1 NA NA
MIMAT0000100_1 NA NA
MIMAT0000101_1 NA NA
MIMAT0000103 NA NA
MIMAT0000104 NA NA
MIMAT0000222 NA NA
MIMAT0000226_1 NA NA
MIMAT0000227 NA NA
MIMAT0000231_1 NA NA
MIMAT0000232_1 NA NA
MIMAT0000243 NA NA
MIMAT0000244_1 NA NA
MIMAT0000245 NA NA
MIMAT0000250 NA NA
MIMAT0000252_2 NA NA
MIMAT0000253 NA NA
MIMAT0000254 NA NA
MIMAT0000255 NA NA
MIMAT0000256_1 NA NA
MIMAT0000257_1 NA NA
MIMAT0000258 NA NA
MIMAT0000259 NA NA
MIMAT0000261 NA NA
MIMAT0000262 NA NA

MIMAT0000263 NA NA
MIMAT0000265 NA NA
MIMAT0000267 NA NA
MIMAT0000269 NA NA
MIMAT0000270 NA NA
MIMAT0000272 NA NA
MIMAT0000276_1 NA NA
MIMAT0000278 NA NA
MIMAT0000279 NA NA
MIMAT0000280 NA NA
MIMAT0000281 NA NA
MIMAT0000318 NA NA
MIMAT0000414 NA NA
MIMAT0000415 NA NA
MIMAT0000416_1 NA NA
MIMAT0000417 NA NA
MIMAT0000418 NA NA
MIMAT0000419 NA NA
MIMAT0000420 NA NA
MIMAT0000422_2 NA NA
MIMAT0000423_1 NA NA
MIMAT0000424_1 NA NA
MIMAT0000425 NA NA
MIMAT0000426 NA NA
MIMAT0000427_1 NA NA
MIMAT0000428_1 NA NA
MIMAT0000430_1 NA NA
MIMAT0000431 NA NA
MIMAT0000432 NA NA
MIMAT0000433 NA NA
MIMAT0000434 NA NA
MIMAT0000435 NA NA
MIMAT0000436 NA NA
MIMAT0000437 NA NA
MIMAT0000438 NA NA
MIMAT0000439_1 NA NA
MIMAT0000440 NA NA
MIMAT0000441_2 NA NA
MIMAT0000442_2 NA NA
MIMAT0000443 NA NA
MIMAT0000444 NA NA
MIMAT0000445 NA NA
MIMAT0000446 NA NA
MIMAT0000447 NA NA
MIMAT0000448 NA NA
MIMAT0000449 NA NA
MIMAT0000450 NA NA
MIMAT0000451 NA NA
MIMAT0000452 NA NA
MIMAT0000453 NA NA
MIMAT0000455 NA NA
MIMAT0000456 NA NA
MIMAT0000457 NA NA
MIMAT0000458 NA NA

MIMAT0000460_1 NA NA
MIMAT0000461 NA NA
MIMAT0000510 NA NA
MIMAT0000617 NA NA
MIMAT0000646 NA NA
MIMAT0000680 NA NA
MIMAT0000681 NA NA
MIMAT0000682 NA NA
MIMAT0000686 NA NA
MIMAT0000687 NA NA
MIMAT0000688 NA NA
MIMAT0000689 NA NA
MIMAT0000690 NA NA
MIMAT0000691 NA NA
MIMAT0000692 NA NA
MIMAT0000693 NA NA
MIMAT0000703 NA NA
MIMAT0000705 NA NA
MIMAT0000707 NA NA
MIMAT0000710 NA NA
MIMAT0000720 NA NA
MIMAT0000721 NA NA
MIMAT0000722 NA NA
MIMAT0000723 NA NA
MIMAT0000727 NA NA
MIMAT0000729_1 NA NA
MIMAT0000730 NA NA
MIMAT0000731 NA NA
MIMAT0000732 NA NA
MIMAT0000733 NA NA
MIMAT0000735 NA NA
MIMAT0000736 NA NA
MIMAT0000737 NA NA
MIMAT0000750 NA NA
MIMAT0000751 NA NA
MIMAT0000752 NA NA
MIMAT0000753 NA NA
MIMAT0000754 NA NA
MIMAT0000755 NA NA
MIMAT0000756 NA NA
MIMAT0000757 NA NA
MIMAT0000759 NA NA
MIMAT0000760 NA NA
MIMAT0000761 NA NA
MIMAT0000762 NA NA
MIMAT0000763 NA NA
MIMAT0000764 NA NA
MIMAT0000765 NA NA
MIMAT0000772 NA NA
MIMAT0001080 NA NA
MIMAT0001340 NA NA
MIMAT0001341 NA NA
MIMAT0001343 NA NA
MIMAT0001412 NA NA

MIMAT0001413 NA NA
MIMAT0001541 NA NA
MIMAT0001545_1 NA NA
MIMAT0001618 NA NA
MIMAT0001621 NA NA
MIMAT0001625 NA NA
MIMAT0001627 NA NA
MIMAT0001629_1 NA NA
MIMAT0001631 NA NA
MIMAT0001635 NA NA
MIMAT0001638 NA NA
MIMAT0001639 NA NA
MIMAT0002170 NA NA
MIMAT0002171 NA NA
MIMAT0002172 NA NA
MIMAT0002174 NA NA
MIMAT0002175 NA NA
MIMAT0002176 NA NA
MIMAT0002177_1 NA NA
MIMAT0002178 NA NA
MIMAT0002806 NA NA
MIMAT0002807 NA NA
MIMAT0002809 NA NA
MIMAT0002813 NA NA
MIMAT0002814 NA NA
MIMAT0002815 NA NA
MIMAT0002816 NA NA
MIMAT0002817 NA NA
MIMAT0002818 NA NA
MIMAT0002819 NA NA
MIMAT0002820 NA NA
MIMAT0002821 NA NA
MIMAT0002870 NA NA
MIMAT0002871 NA NA
MIMAT0002872 NA NA
MIMAT0002873 NA NA
MIMAT0002874 NA NA
MIMAT0002875 NA NA
MIMAT0002876 NA NA
MIMAT0002880 NA NA
MIMAT0002881_2 NA NA
MIMAT0002883_2 NA NA
MIMAT0002888 NA NA
MIMAT0002890 NA NA
MIMAT0002891 NA NA
MIMAT0003161 NA NA
MIMAT0003163 NA NA
MIMAT0003180 NA NA
MIMAT0003218 NA NA
MIMAT0003220 NA NA
MIMAT0003239 NA NA
MIMAT0003241 NA NA
MIMAT0003242 NA NA
MIMAT0003244 NA NA

MIMAT0003247 NA NA
MIMAT0003249 NA NA
MIMAT0003257_2 NA NA
MIMAT0003266 NA NA
MIMAT0003273 NA NA
MIMAT0003283 NA NA
MIMAT0003284 NA NA
MIMAT0003287 NA NA
MIMAT0003293 NA NA
MIMAT0003294 NA NA
MIMAT0003296 NA NA
MIMAT0003297 NA NA
MIMAT0003298 NA NA
MIMAT0003301 NA NA
MIMAT0003311 NA NA
MIMAT0003312 NA NA
MIMAT0003313 NA NA
MIMAT0003321 NA NA
MIMAT0003322 NA NA
MIMAT0003329 NA NA
MIMAT0003330 NA NA
MIMAT0003331 NA NA
MIMAT0003332 NA NA
MIMAT0003338 NA NA
MIMAT0003339 NA NA
MIMAT0003340 NA NA
MIMAT0003385 NA NA
MIMAT0003389 NA NA
MIMAT0003393 NA NA
MIMAT0003879 NA NA
MIMAT0003880 NA NA
MIMAT0003881 NA NA
MIMAT0003884 NA NA
MIMAT0003885 NA NA
MIMAT0003886 NA NA
MIMAT0003887 NA NA
MIMAT0003888 NA NA
MIMAT0003948 NA NA
MIMAT0004481_1 NA NA
MIMAT0004482 NA NA
MIMAT0004484 NA NA
MIMAT0004485 NA NA
MIMAT0004486 NA NA
MIMAT0004493 NA NA
MIMAT0004494 NA NA
MIMAT0004495 NA NA
MIMAT0004496 NA NA
MIMAT0004497 NA NA
MIMAT0004498 NA NA
MIMAT0004499 NA NA
MIMAT0004500 NA NA
MIMAT0004501 NA NA
MIMAT0004502 NA NA
MIMAT0004503 NA NA

MIMAT0004505 NA NA
MIMAT0004506 NA NA
MIMAT0004507 NA NA
MIMAT0004509 NA NA
MIMAT0004513 NA NA
MIMAT0004514 NA NA
MIMAT0004515 NA NA
MIMAT0004517 NA NA
MIMAT0004518 NA NA
MIMAT0004549 NA NA
MIMAT0004551 NA NA
MIMAT0004552 NA NA
MIMAT0004553 NA NA
MIMAT0004555 NA NA
MIMAT0004558 NA NA
MIMAT0004559 NA NA
MIMAT0004563 NA NA
MIMAT0004568 NA NA
MIMAT0004569 NA NA
MIMAT0004570 NA NA
MIMAT0004571 NA NA
MIMAT0004585 NA NA
MIMAT0004586 NA NA
MIMAT0004587 NA NA
MIMAT0004588 NA NA
MIMAT0004589 NA NA
MIMAT0004593 NA NA
MIMAT0004594 NA NA
MIMAT0004597 NA NA
MIMAT0004599 NA NA
MIMAT0004600 NA NA
MIMAT0004601 NA NA
MIMAT0004602 NA NA
MIMAT0004603 NA NA
MIMAT0004604 NA NA
MIMAT0004606 NA NA
MIMAT0004610 NA NA
MIMAT0004611 NA NA
MIMAT0004612 NA NA
MIMAT0004613 NA NA
MIMAT0004614 NA NA
MIMAT0004672 NA NA
MIMAT0004673 NA NA
MIMAT0004674 NA NA
MIMAT0004678 NA miRNA
MIMAT0004680 NA miRNA
MIMAT0004681 NA miRNA
MIMAT0004682 NA miRNA
MIMAT0004688 NA miRNA
MIMAT0004689 NA miRNA
MIMAT0004690 NA miRNA
MIMAT0004692 NA miRNA
MIMAT0004693 NA miRNA
MIMAT0004694 NA miRNA

MIMAT0004695 NA miRNA
MIMAT0004696 NA miRNA
MIMAT0004697 NA miRNA
MIMAT0004699 NA miRNA
MIMAT0004700 NA miRNA
MIMAT0004701 NA miRNA
MIMAT0004702 NA miRNA
MIMAT0004703 NA miRNA
MIMAT0004748 NA miRNA
MIMAT0004749 NA miRNA
MIMAT0004752 NA miRNA
MIMAT0004757 NA miRNA
MIMAT0004762_1 NA miRNA
MIMAT0004764 NA miRNA
MIMAT0004766 NA miRNA
MIMAT0004767 NA miRNA
MIMAT0004773 NA miRNA
MIMAT0004774 NA miRNA
MIMAT0004775 NA miRNA
MIMAT0004776 NA miRNA
MIMAT0004780 NA miRNA
MIMAT0004784 NA miRNA
MIMAT0004792 NA miRNA
MIMAT0004793 NA miRNA
MIMAT0004795 NA miRNA
MIMAT0004796 NA miRNA
MIMAT0004797 NA miRNA
MIMAT0004799 NA miRNA
MIMAT0004800_1 NA miRNA
MIMAT0004801 NA miRNA
MIMAT0004807 NA miRNA
MIMAT0004808 NA miRNA
MIMAT0004809 NA miRNA
MIMAT0004810 NA miRNA
MIMAT0004811 NA miRNA
MIMAT0004813 NA miRNA
MIMAT0004814 NA miRNA
MIMAT0004819 NA miRNA
MIMAT0004909 NA miRNA
MIMAT0004911 NA miRNA
MIMAT0004921 NA miRNA
MIMAT0004925 NA miRNA
MIMAT0004926 NA miRNA
MIMAT0004929 NA miRNA
MIMAT0004945 NA miRNA
MIMAT0004946 NA miRNA
MIMAT0004949 NA miRNA
MIMAT0004950 NA miRNA
MIMAT0004952 NA miRNA
MIMAT0004953 NA miRNA
MIMAT0004954 NA miRNA
MIMAT0004955 NA miRNA
MIMAT0004956 NA miRNA
MIMAT0004957 NA miRNA

MIMAT0004958 NA miRNA
MIMAT0004983 NA miRNA
MIMAT0004984_4 NA miRNA
MIMAT0004985 NA miRNA
MIMAT0005577 NA miRNA
MIMAT0005583 NA miRNA
MIMAT0005584 NA miRNA
MIMAT0005592 NA miRNA
MIMAT0005792_1 NA miRNA
MIMAT0005793_1 NA miRNA
MIMAT0005794 NA miRNA
MIMAT0005796 NA miRNA
MIMAT0005797 NA miRNA
MIMAT0005798_1 NA miRNA
MIMAT0005824 NA miRNA
MIMAT0005825 NA miRNA
MIMAT0005867 NA miRNA
MIMAT0005874 NA miRNA
MIMAT0005875 NA miRNA
MIMAT0005876_1 NA miRNA
MIMAT0005878 NA miRNA
MIMAT0005881 NA miRNA
MIMAT0005882 NA miRNA
MIMAT0005884 NA miRNA
MIMAT0005889 NA miRNA
MIMAT0005892 NA miRNA
MIMAT0005900 NA miRNA
MIMAT0005901 NA miRNA
MIMAT0005902 NA miRNA
MIMAT0005905_1 NA miRNA
MIMAT0005906 NA miRNA
MIMAT0005911 NA miRNA
MIMAT0005919_1 NA miRNA
MIMAT0005924 NA miRNA
MIMAT0005929 NA miRNA
MIMAT0005933 NA miRNA
MIMAT0005941 NA miRNA
MIMAT0005943 NA miRNA
MIMAT0005948 NA miRNA
MIMAT0005949 NA miRNA
MIMAT0005950 NA miRNA
MIMAT0005951 NA miRNA
MIMAT0005955 NA miRNA
MIMAT0006764_1 NA miRNA
MIMAT0006789 NA miRNA
MIMAT0007400 NA miRNA
MIMAT0007881 NA miRNA
MIMAT0007885 NA miRNA
MIMAT0007887 NA miRNA
MIMAT0007889 NA miRNA
MIMAT0009196 NA miRNA
MIMAT0009198 NA miRNA
MIMAT0009451 NA miRNA
MIMAT0010133 NA miRNA

MIMAT0010214 NA miRNA
MIMAT0011161 NA miRNA
MIMAT0011163 NA miRNA
MIMAT0011775 NA NA
MIMAT0011778 NA NA
MIMAT0014977 NA trna
MIMAT0014982 NA trna
MIMAT0014987 NA trna
MIMAT0014994_1 NA trna
MIMAT0015003 NA trna
MIMAT0015004 NA trna
MIMAT0015006 NA trna
MIMAT0015008 NA trna
MIMAT0015017 NA trna
MIMAT0015027 NA trna
MIMAT0015031 NA trna
MIMAT0015032_1 NA trna
MIMAT0015035 NA trna
MIMAT0015041 NA trna
MIMAT0015050 NA trna
MIMAT0015051 NA trna
MIMAT0015053 NA trna
MIMAT0015054 NA trna
MIMAT0015066 NA trna
MIMAT0015069 NA trna
MIMAT0015070 NA trna
MIMAT0015084_1 NA trna
MIMAT0015089_1 NA trna
MIMAT0015378 NA trna
MIMAT0016847 NA trna
MIMAT0016888 NA trna
MIMAT0016895 NA trna
MIMAT0016916 NA trna
MIMAT0016925 NA trna
MIMAT0017352 NA trna
MIMAT0017950 NA trna
MIMAT0017981 NA trna
MIMAT0017982 NA trna
MIMAT0017984 NA trna
MIMAT0017985 NA trna
MIMAT0017986 NA trna
MIMAT0017990 NA trna
MIMAT0017991 NA trna
MIMAT0017992 NA trna
MIMAT0017994 NA trna
MIMAT0017997 NA trna
MIMAT0018001 NA trna
MIMAT0018071 NA trna
MIMAT0018073 NA trna
MIMAT0018089 NA trna
MIMAT0018104 NA trna
MIMAT0018115_1 NA trna
MIMAT0018116_1 NA trna
MIMAT0018119_1 NA trna

MIMAT0018183 NA trna
MIMAT0018186 NA trna
MIMAT0018187_1 NA trna
MIMAT0018194 NA trna
MIMAT0018197 NA trna
MIMAT0018205 NA trna
MIMAT0018349 NA trna
MIMAT0018356 NA trna
MIMAT0018443 NA trna
MIMAT0018926_1 NA trna
MIMAT0018935 NA trna
MIMAT0018951_1 NA trna
MIMAT0018965 NA trna
MIMAT0018968 NA trna
MIMAT0018976 NA trna
MIMAT0019000 NA trna
MIMAT0019019 NA trna
MIMAT0019055 NA trna
MIMAT0019058 NA trna
MIMAT0019061 NA trna
MIMAT0019198 NA trna
MIMAT0019206 NA trna
MIMAT0019208 NA trna
MIMAT0019210 NA trna
MIMAT0019211_1 NA trna
MIMAT0019214 NA trna
MIMAT0019706 NA trna
MIMAT0019727 NA trna
MIMAT0019729 NA trna
MIMAT0019731 NA trna
MIMAT0019732 NA trna
MIMAT0019733 NA trna
MIMAT0019734 NA trna
MIMAT0019739 NA trna
MIMAT0019758 NA trna
MIMAT0019759 NA trna
MIMAT0019761 NA trna
MIMAT0019774 NA trna
MIMAT0019776 NA trna
MIMAT0019777 NA trna
MIMAT0019830 NA trna
MIMAT0019864 NA trna
MIMAT0019872 NA trna
MIMAT0019880 NA trna
MIMAT0019892 NA trna
MIMAT0019895 NA trna
MIMAT0019926 NA trna
MIMAT0019927 NA trna
MIMAT0019942 NA trna
MIMAT0019943 NA trna
MIMAT0019976 NA trna
MIMAT0020924 NA trna
MIMAT0020925 NA trna
MIMAT0021017 NA trna

MIMAT0021043 NA trna
MIMAT0021044 NA trna
MIMAT0021082 NA trna
MIMAT0021083 NA trna
MIMAT0021086 NA trna
MIMAT0021117 NA trna
MIMAT0021120 NA trna
MIMAT0021124 NA trna
MIMAT0021129 NA trna
MIMAT0022271 NA trna
MIMAT0022272 NA trna
MIMAT0022277 NA trna
MIMAT0022290 NA trna
MIMAT0022472 NA trna
MIMAT0022474 NA trna
MIMAT0022691 NA trna
MIMAT0022695 NA trna
MIMAT0022696 NA trna
MIMAT0022697 NA trna
MIMAT0022705 NA trna
MIMAT0022709 NA trna
MIMAT0022710 NA trna
MIMAT0022711 NA trna
MIMAT0022713 NA trna
MIMAT0022714 NA trna
MIMAT0022726 NA trna
MIMAT0022727 NA trna
MIMAT0022834 NA trna
MIMAT0022838 NA trna
MIMAT0022929 NA trna
MIMAT0023712 NA trna
MIMAT0025453 NA trna
MIMAT0025458 NA trna
MIMAT0025459 NA trna
MIMAT0025460 NA trna
MIMAT0025463 NA trna
MIMAT0025479_3 NA trna
MIMAT0025482 NA trna
MIMAT0025483 NA trna
MIMAT0025486 NA trna
MIMAT0025848_1 NA trna
MIMAT0025849 NA trna
MIMAT0025852 NA trna
MIMAT0025856_3 NA trna
MIMAT0026477 NA trna
MIMAT0026480 NA trna
MIMAT0026481 NA trna
MIMAT0026483 NA trna
MIMAT0026557 NA trna
MIMAT0026559 NA trna
MIMAT0026614 NA trna
MIMAT0026619 NA trna
MIMAT0026620 NA trna
MIMAT0026621 NA trna

MIMAT0026623 NA trna
MIMAT0026718 NA trna
MIMAT0026734 NA trna
MIMAT0026736 NA trna
MIMAT0027032 NA trna
MIMAT0027088 NA trna
MIMAT0027369 NA trna
MIMAT0027371 NA trna
MIMAT0027384 NA trna
MIMAT0027440_2 NA trna
MIMAT0027441_2 NA trna
MIMAT0027449 NA trna
MIMAT0027475 NA trna
MIMAT0027487 NA trna
MIMAT0027497 NA trna
MIMAT0027507 NA trna
MIMAT0027520 NA trna
MIMAT0027521 NA trna
MIMAT0027536 NA trna
MIMAT0027576 NA trna
MIMAT0027577 NA trna
MIMAT0027586 NA trna
MIMAT0027587 NA trna
MIMAT0027594 NA trna
MIMAT0027604 NA trna
MIMAT0027621 NA trna
MIMAT0027654 NA trna
MIMAT0027664 NA trna
MIMAT0027682 NA trna
MIMAT0028118 NA trna
MIMAT0028123 NA trna
MIMAT0029782_1 NA trna
MIMAT0030021 NA trna
MIMAT0030413 NA trna
MIMAT0030414 NA trna
MIMAT0030415 NA trna
MIMAT0030416 NA trna
MIMAT0030417 NA trna
MIMAT0030418 NA trna
MIMAT0030423 NA trna
MIMAT0030424 NA trna
MIMAT0030429 NA trna
MIMAT0031179 NA trna
MIMAT0032110 NA trna
MIMAT0032116 NA trna
RNA5−8S5_ShortStack NA trna
rRNA45S NA trna
trna1−ArgCCG_1 NA trna
trna1−AsnGTT_1 NA trna
trna1−GlnCTG_1 NA trna
trna1−GlyCCC_1 NA trna
trna1−HisGTG_1 NA trna
trna1−LeuAAG_1 NA trna
trna1−LeuCAA_1 NA trna

trna1-PheGAA_1 NA trna
trna1-SeC(e)TCA_1 NA trna
trna1-TrpCCA_1 NA trna
trna10-AlaCGC_1 NA trna
trna10-AspGTC_1 NA trna
trna10-GluCTC_1 NA trna
trna10-GlyTCC_1 NA trna
trna10-IleAAT_1 NA trna
trna10-IleTAT_1 NA trna
trna10-LysCTT_1 NA trna
trna10-MetCAT_1 NA trna
trna10-SerGCT_1 NA trna
trna10-ValCAC_1 NA trna
trna100-LeuCAA_1 NA trna
trna100-PheGAA_1 NA trna
trna101-AlaAGC_1 NA NA
trna102-AlaAGC_1 NA NA
trna102-GluTTC_1 NA NA
trna103-AsnGTT_1 NA NA
trna103-PheGAA_1 NA NA
trna104-AlaTGC_1 NA NA
trna106-HisGTG_1 NA NA
trna106-PheGAA_1 NA NA
trna107-AlaTGC_1 NA NA
trna108-AlaAGC_1 NA NA
trna109-PheGAA_1 NA NA
trna11-ArgACG_1 NA NA
trna11-GlnTTG_1 NA NA
trna11-GluTTC_1 NA NA
trna11-IleAAT_1 NA NA
trna11-LysCTT_1 NA NA
trna11-LysTTT_1 NA NA
trna11-PheGAA_1 NA NA
trna11-ProAGG_1 NA NA
trna11-SerAGA_1 NA NA
trna110-AlaTGC_1 NA NA
trna111-HisGTG_1 NA NA
trna112-GlnCTG_1 NA NA
trna113-AlaTGC_1 NA NA
trna114-ArgCCG_1 NA NA
trna119-AlaCGC_1 NA NA
trna12-ArgCCT_1 NA NA
trna12-AspGTC_1 NA NA
trna12-CysGCA_1 NA NA
trna12-LysTTT_1 NA NA
trna12-ProAGG_1 NA NA
trna12-ProTGG_1 NA NA
trna12-TrpCCA_1 NA NA
trna12-ValAAC_1 NA NA
trna120-AlaAGC_1 NA NA
trna121-ThrCGT_1 NA NA
trna123-SerGCT_1 NA NA
trna124-ArgTCG_1 NA NA
trna125-ThrCGT_1 NA NA

trna126-LeuAAG_1 NA NA
trna127-CysGCA_1 NA NA
trna127-ThrTGT_1 NA NA
trna128-GlyGCC_1 NA NA
trna128-LysCTT_1 NA NA
trna129-MetCAT_1 NA NA
trna13-AlaCGC_1 NA NA
trna13-AlaTGC_1 NA NA
trna13-GlyCCC_1 NA NA
trna13-LysCTT_2 NA NA
trna13-LysCTT_3 NA NA
trna13-PheGAA_1 NA NA
trna13-ThrTGT_1 NA NA
trna13-ValCAC_1 NA NA
trna130-GlnTTG_1 NA NA
trna131-GlnCTG_1 NA NA
trna131-GlyCCC_1 NA NA
trna132-ValAAC_1 NA NA
trna133-GlyCCC_1 NA NA
trna133-ValCAC_1 NA NA
trna134-GluTTC_1 NA NA
trna135-ThrAGT_1 NA NA
trna136-ValAAC_1 NA NA
trna137-SerCGA_1 NA NA
trna137-Undet???_1 NA NA
trna138-ArgACG_1 NA NA
trna139-ValAAC_1 NA NA
trna14-CysGCA_1 NA NA
trna14-GluTTC_1 NA NA
trna14-LysTTT_1 NA NA
trna14-PheGAA_1 NA NA
trna14-ProTGG_1 NA NA
trna14-ThrCGT_1 NA NA
trna14-TyrGTA_1 NA NA
trna140-LeuCAA_1 NA NA
trna141-LeuCAA_1 NA NA
trna142-MetCAT_1 NA NA
trna143-LysTTT_1 NA NA
trna144-AspGTC_1 NA NA
trna145-SerAGA_1 NA NA
trna146-GlnCTG_1 NA NA
trna147-SerAGA_1 NA NA
trna148-SerTGA_1 NA NA
trna149-LysTTT_1 NA NA
trna15-CysGCA_1 NA NA
trna15-CysGCA_2 NA NA
trna15-GlnCTG_2 NA NA
trna15-PheGAA_1 NA NA
trna15-ThrCGT_1 NA NA
trna15-TyrGTA_1 NA NA
trna15-ValAAC_1 NA NA
trna150-MetCAT_1 NA NA
trna151-ThrCGT_1 NA NA
trna152-ValCAC_1 NA NA

trna153-IleAAT_1 NA NA
trna154-IleAAT_1 NA NA
trna155-LeuTAA_1 NA NA
trna156-ArgACG_1 NA NA
trna157-ValCAC_1 NA NA
trna158-IleAAT_1 NA NA
trna16-CysGCA_1 NA NA
trna16-GlnTTG_2 NA NA
trna16-HisGTG_1 NA NA
trna16-LeuAAG_1 NA NA
trna16-LeuAAG_2 NA NA
trna16-TyrGTA_1 NA NA
trna16-ValTAC_1 NA NA
trna161-AlaAGC_1 NA NA
trna164-MetCAT_1 NA NA
trna165-IleAAT_1 NA NA
trna166-AlaAGC_1 NA NA
trna167-ThrAGT_1 NA NA
trna168-TrpCCA_1 NA NA
trna169-MetCAT_1 NA NA
trna17-GluTTC_1 NA NA
trna17-LeuCAG_1 NA NA
trna17-SupTTA_1 NA NA
trna17-ValTAC_1 NA NA
trna170-TrpCCA_1 NA NA
trna171-MetCAT_1 NA NA
trna172-SerTGA_1 NA NA
trna173-GlnTTG_1 NA NA
trna174-GlnTTG_1 NA NA
trna175-SerGCT_1 NA NA
trna18-ArgCCT_1 NA NA
trna18-GluCTC_1 NA NA
trna18-GlyGCC_1 NA NA
trna18-ValCAC_1 NA NA
trna19-ArgTCG_1 NA NA
trna19-CysGCA_1 NA NA
trna19-GlnCTG_1 NA NA
trna19-GlyGCC_1 NA NA
trna19-GlyGCC_2 NA NA
trna19-LeuAAG_1 NA NA
trna2-ArgCCT_1 NA NA
trna2-GlyCCC_2 NA NA
trna2-GlyGCC_1 NA NA
trna2-GlyTCC_1 NA NA
trna2-GlyTCC_2 NA NA
trna2-LeuTAA_2 NA NA
trna2-LeuTAG_1 NA NA
trna2-LysCTT_1 NA NA
trna2-LysTTT_2 NA NA
trna2-MetCAT_1 NA NA
trna2-ProAGG_1 NA NA
trna2-SerCGA_1 NA NA
trna2-SerTGA_1 NA NA
trna2-TyrGTA_1 NA NA

trna2-ValAAC_1 NA NA
trna2-ValCAC_1 NA NA
trna20-GluTTC_1 NA NA
trna20-MetCAT_1 NA NA
trna20-MetCAT_2 NA NA
trna20-ThrTGT_1 NA NA
trna21-ArgCCT_1 NA NA
trna21-CysGCA_1 NA NA
trna21-HisGTG_1 NA NA
trna21-MetCAT_1 NA NA
trna21-ThrTGT_1 NA NA
trna22-AspGTC_1 NA NA
trna22-GlnCTG_1 NA NA
trna22-MetCAT_1 NA NA
trna22-ProAGG_1 NA NA
trna23-ArgCCG_1 NA NA
trna23-LysTTT_1 NA NA
trna23-ProAGG_1 NA NA
trna24-GlyGCC_1 NA NA
trna25-GluCTC_1 NA NA
trna25-GlyGCC_1 NA NA
trna25-SeCTCA_1 NA NA
trna26-AsnGTT_1 NA NA
trna26-CysGCA_1 NA NA
trna26-LeuCAG_1 NA NA
trna27-CysGCA_1 NA NA
trna27-GlyCCC_1 NA NA
trna27-LeuTAG_1 NA NA
trna27-MetCAT_1 NA NA
trna28-CysGCA_1 NA NA
trna28-GlnCTG_1 NA NA
trna28-IleAAT_1 NA NA
trna28-ProTGG_1 NA NA
trna29-IleTAT_1 NA NA
trna29-ProAGG_1 NA NA
trna3-AlaAGC_1 NA NA
trna3-ArgCCT_1 NA NA
trna3-ArgTCG_1 NA NA
trna3-ArgTCT_1 NA NA
trna3-CysGCA_1 NA NA
trna3-CysGCA_2 NA NA
trna3-GlnCTG_1 NA NA
trna3-GluTTC_1 NA NA
trna3-GlyTCC_1 NA NA
trna3-LeuAAG_1 NA NA
trna3-LysTTT_1 NA NA
trna3-ProTGG_1 NA NA
trna3-ProTGG_2 NA NA
trna3-ValCAC_2 NA NA
trna30-CysGCA_1 NA NA
trna30-LysCTT_1 NA NA
trna30-ProCGG_1 NA NA
trna31-AsnGTT_1 NA NA
trna31-ProTGG_1 NA NA

trna31-SerGCT_1 NA NA
trna32-LysCTT_1 NA NA
trna32-MetCAT_1 NA NA
trna33-HisGTG_1 NA NA
trna33-ProAGG_1 NA NA
trna33-Undet???_1 NA NA
trna34-GlyCCC_1 NA NA
trna34-IleAAT_1 NA NA
trna34-LeuCAG_1 NA NA
trna34-ThrAGT_1 NA NA
trna35-GlyGCC_1 NA NA
trna35-SerAGA_1 NA NA
trna35-SerCGA_1 NA NA
trna36-ArgACG_1 NA NA
trna36-LeuCAG_1 NA NA
trna36-ThrAGT_1 NA NA
trna37-GlyGCC_1 NA NA
trna37-ProCGG_1 NA NA
trna37-ValAAC_1 NA NA
trna38-AspGTC_1 NA NA
trna38-LeuCAG_1 NA NA
trna39-GlyGCC_1 NA NA
trna39-TrpCCA_1 NA NA
trna4-ArgTCG_1 NA NA
trna4-ArgTCG_2 NA NA
trna4-ArgTCT_1 NA NA
trna4-ArgTCT_2 NA NA
trna4-AsnGTT_1 NA NA
trna4-AspGTC_1 NA NA
trna4-GluCTC_1 NA NA
trna4-GlyCCC_1 NA NA
trna4-LeuTAA_1 NA NA
trna4-LeuTAA_2 NA NA
trna4-LysCTT_1 NA NA
trna4-ThrAGT_1 NA NA
trna4-ThrTGT_1 NA NA
trna4-TyrGTA_1 NA NA
trna4-ValAAC_1 NA NA
trna4-ValTAC_1 NA NA
trna40-LeuCAG_1 NA NA
trna40-ThrAGT_1 NA NA
trna40-ValTAC_1 NA NA
trna41-GlyGCC_1 NA NA
trna41-SerCGA_1 NA NA
trna42-GlnCTG_1 NA NA
trna42-LeuCAG_1 NA NA
trna42-LeuTAG_1 NA NA
trna43-GlyGCC_1 NA NA
trna43-SerGCT_1 NA NA
trna44-AspGTC_1 NA NA
trna44-SerAGA_1 NA NA
trna45-AspGTC_1 NA NA
trna45-GlyTCC_1 NA NA
trna46-SerAGA_1 NA NA

trna47-AsnGTT_1 NA NA
trna47-SerAGA_1 NA NA
trna48-AspGTC_1 NA NA
trna49-GlnCTG_1 NA NA
trna49-GluTTC_1 NA NA
trna5-AspGTC_1 NA NA
trna5-CysGCA_1 NA NA
trna5-GluTTC_1 NA NA
trna5-GluTTC_2 NA NA
trna5-GlyGCC_1 NA NA
trna5-IleTAT_1 NA NA
trna5-LysTTT_1 NA NA
trna5-SerAGA_1 NA NA
trna5-TyrGTA_1 NA NA
trna5-TyrGTA_2 NA NA
trna5-ValAAC_1 NA NA
trna50-SerAGA_1 NA NA
trna51-SerTGA_1 NA NA
trna52-ArgTCT_1 NA NA
trna52-ProCGG_1 NA NA
trna54-LysTTT_1 NA NA
trna55-IleTAT_1 NA NA
trna55-LysTTT_1 NA NA
trna56-ThrTGT_1 NA NA
trna57-IleAAT_1 NA NA
trna58-LeuCAA_1 NA NA
trna59-GluCTC_1 NA NA
trna59-IleAAT_1 NA NA
trna6-AlaAGC_1 NA NA
trna6-ArgACG_1 NA NA
trna6-AspGTC_1 NA NA
trna6-CysGCA_2 NA NA
trna6-LysCTT_1 NA NA
trna6-LysCTT_2 NA NA
trna6-ProCGG_1 NA NA
trna6-ProTGG_1 NA NA
trna6-TrpCCA_1 NA NA
trna6-TrpCCA_2 NA NA
trna6-ValCAC_1 NA NA
trna6-ValTAC_1 NA NA
trna60-LeuTAA_1 NA NA
trna60-ThrAGT_1 NA NA
trna61-MetCAT_1 NA NA
trna62-LysTTT_1 NA NA
trna62-SerGCT_1 NA NA
trna63-IleTAT_1 NA NA
trna64-GlnTTG_1 NA NA
trna64-GluTTC_1 NA NA
trna65-AlaAGC_1 NA NA
trna65-ProAGG_1 NA NA
trna66-AlaTGC_1 NA NA
trna67-AlaAGC_1 NA NA
trna67-LeuCAG_1 NA NA
trna68-AlaAGC_1 NA NA

trna68−GlyGCC_1 NA NA
trna69−AspGTC_1 NA NA
trna69−ThrAGT_1 NA NA
trna7−ArgACG_1 NA NA
trna7−AsnGTT_1 NA NA
trna7−AsnGTT_2 NA NA
trna7−CysGCA_1 NA NA
trna7−GlnCTG_1 NA NA
trna7−HisGTG_1 NA NA
trna7−LeuAAG_1 NA NA
trna7−LeuCAG_1 NA NA
trna7−LysCTT_1 NA NA
trna7−SerGCT_1 NA NA
trna70−AlaCGC_1 NA NA
trna70−GlyTCC_1 NA NA
trna71−GluCTC_1 NA NA
trna72−AspGTC_1 NA NA
trna72−PheGAA_1 NA NA
trna73−ArgCCG_1 NA NA
trna73−GlyTCC_1 NA NA
trna74−GluCTC_1 NA NA
trna74−LeuCAA_1 NA NA
trna75−AspGTC_1 NA NA
trna75−MetCAT_1 NA NA
trna76−GlyTCC_1 NA NA
trna76−LysTTT_1 NA NA
trna77−GluCTC_1 NA NA
trna77−GluCTC_2 NA NA
trna78−AspGTC_1 NA NA
trna78−LeuAAG_1 NA NA
trna79−GlyTCC_1 NA NA
trna8−AlaTGC_1 NA NA
trna8−AlaTGC_2 NA NA
trna8−ArgACG_1 NA NA
trna8−CysGCA_1 NA NA
trna8−HisGTG_1 NA NA
trna8−ProTGG_1 NA NA
trna8−SeC(e)TCA_1 NA NA
trna8−SerGCT_1 NA NA
trna8−ThrAGT_1 NA NA
trna80−GluCTC_1 NA NA
trna80−IleAAT_1 NA NA
trna81−AspGTC_1 NA NA
trna82−GlyTCC_1 NA NA
trna83−AsnGTT_1 NA NA
trna83−LeuTAA_1 NA NA
trna84−GlnTTG_1 NA NA
trna84−GluTTC_1 NA NA
trna85−GluTTC_1 NA NA
trna85−ValCAC_1 NA NA
trna87−GluCTC_1 NA NA
trna9−ArgTCT_1 NA NA
trna9−HisGTG_1 NA NA
trna9−IleAAT_2 NA NA

trna9-LeuTAA_1 NA NA
trna9-LysCTT_1 NA NA
trna9-ProAGG_1 NA NA
trna9-ProAGG_2 NA NA
trna9-ValCAC_1 NA NA
trna90-ValCAC_1 NA NA
trna91-GlyCCC_1 NA NA
trna92-PheGAA_1 NA NA
trna94-GluTTC_1 NA NA
trna96-PheGAA_1 NA NA
trna97-MetCAT_1 NA NA
trna98-LeuAAG_1 NA NA
trna98-ValCAC_1 NA NA
trna99-GlnCTG_1 NA NA
trna99-ValCAC_1 NA NA
