# Supplementary figures and images for: Integration of small RNAs from plasma and cerebrospinal fluid for classification of multiple sclerosis
Source: Front Genet. 2022 Nov 17;13:1042483. doi: 10.3389/fgene.2022.1042483 (PMC9713411; doi:10.3389/fgene.2022.1042483)

# Supplementary figure 2

A)

Full model

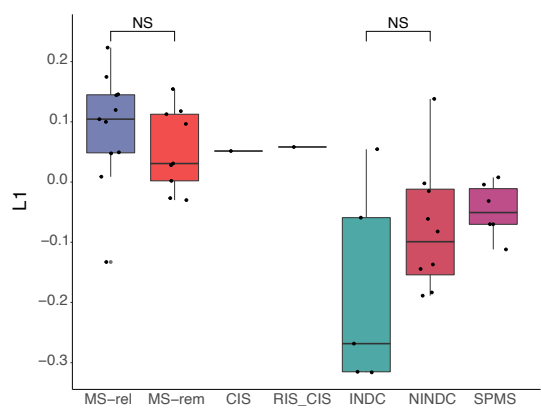

B)

Reduced model

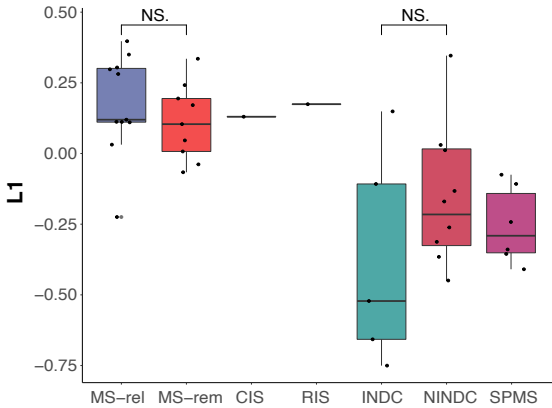

Supplement: Supplementary file 7 [file Image2.pdf]

Supplementary figure 3

Small-seq pipeline

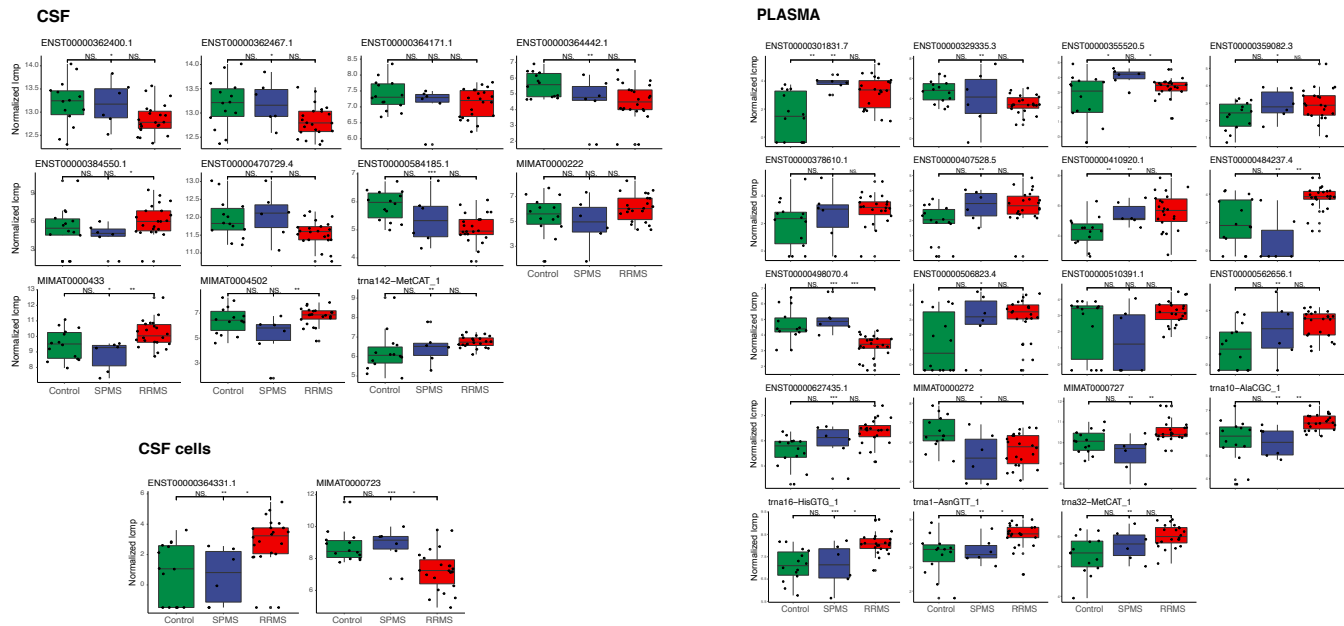

MINTmap

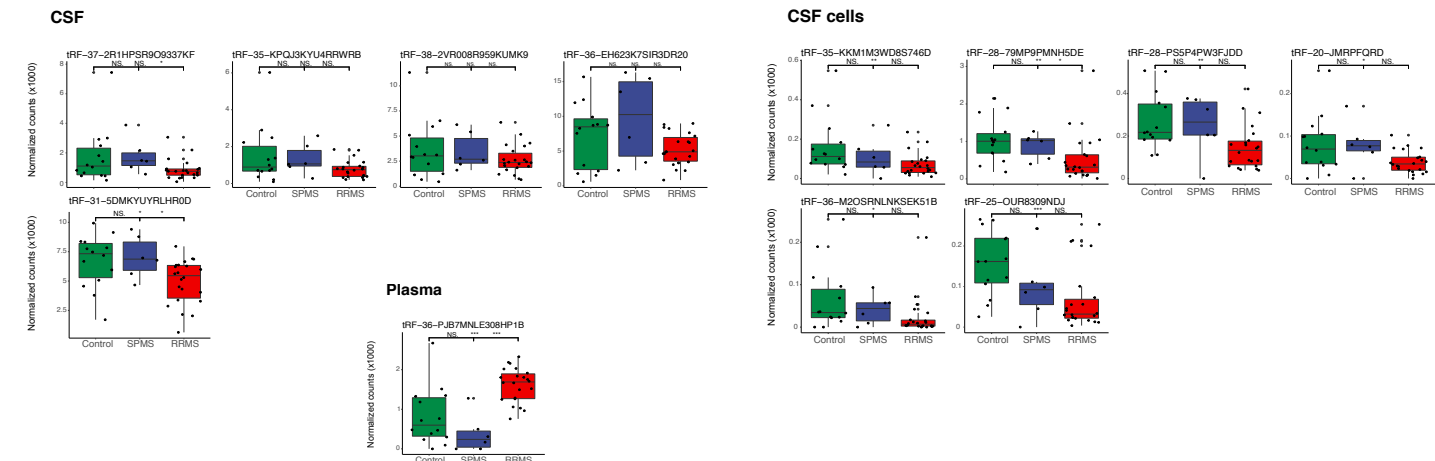

Supplement: Supplementary file 10 [file Image3.pdf]

Supplementary figure 1

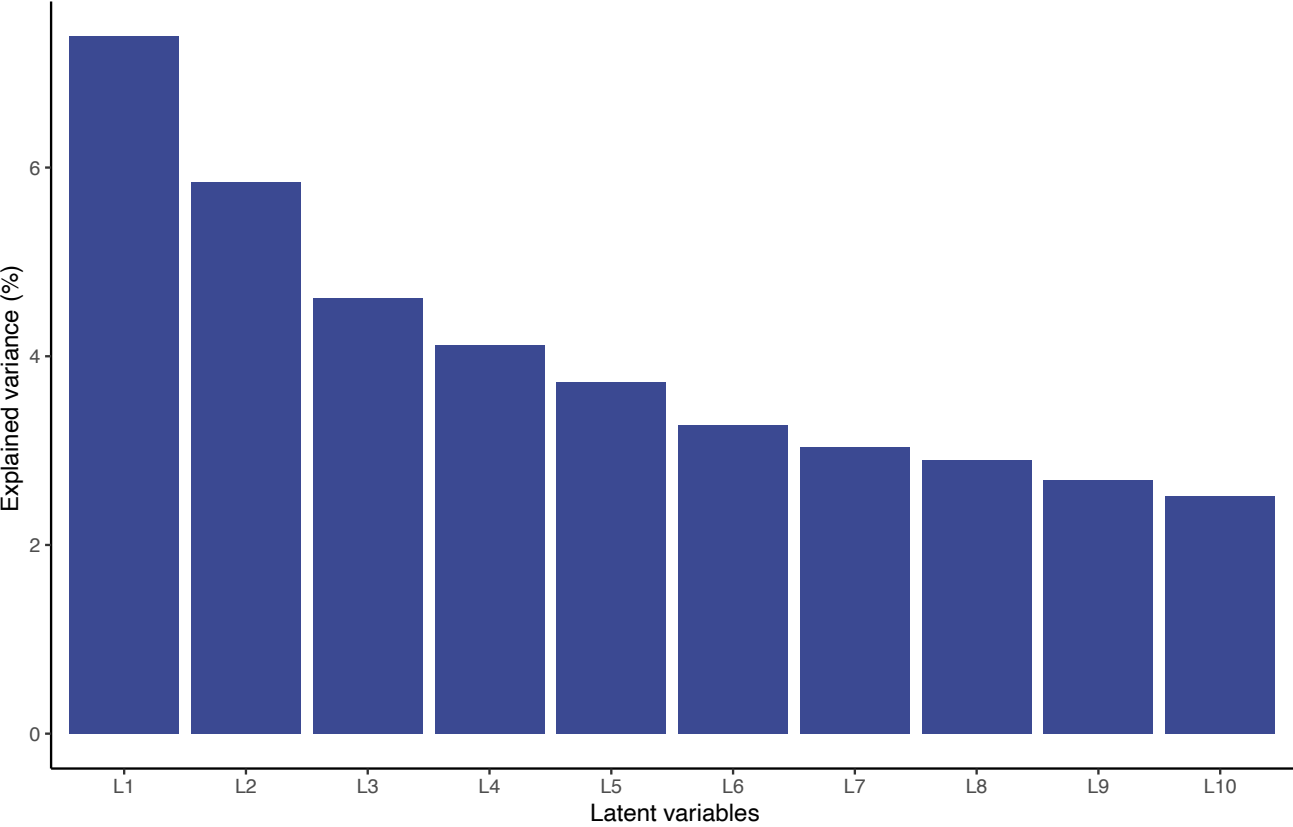

Supplement: Supplementary file 14 [file Image1.pdf]
