# Supplementary material for: Integration of small RNAs from plasma and cerebrospinal fluid for classification of multiple sclerosis
Source: Front Genet. 2022 Nov 17;13:1042483. doi: 10.3389/fgene.2022.1042483 (PMC9713411; doi:10.3389/fgene.2022.1042483)
Supplement: Supplementary file 11 [file DataSheet12.PDF]

3trna26-CysGCA_1 NA trna
3trna27-LeuTAG_1 NA trna
3trna4-ThrAGT_1 NA trna
ENST00000085079.10 EPN1 protein_coding
ENST00000217133.1 TUBB1 protein_coding
ENST00000230449.7 EXOC2 protein_coding
ENST00000242351.8 ZC3HAV1 protein_coding
ENST00000251691.4 ARFGEF3 protein_coding
ENST00000257336.4 BIVM protein_coding
ENST00000260810.8 TOPBP1 protein_coding
ENST00000262426.5 FOXF1 protein_coding
ENST00000262719.8 PHLPP1 protein_coding
ENST00000263321.5 TYR protein_coding
ENST00000263563.6 PALD1 protein_coding
ENST00000264033.4 CBL protein_coding
ENST00000264790.5 MMRN1 protein_coding
ENST00000264870.6 F13A1 protein_coding
ENST00000265361.6 SEMA3C protein_coding
ENST00000266037.9 DOCK3 protein_coding
ENST00000268489.8 ZFHX3 protein_coding
ENST00000275034.4 PHIP protein_coding
ENST00000277942.6 NPFFR1 protein_coding
ENST00000281828.6 FARSB protein_coding
ENST00000284384.6 PRKCA nonsense_mediated_decay
ENST00000295268.3 STPG2 protein_coding
ENST00000295874.13 PTPRG protein_coding
ENST00000301831.7 ULK4 protein_coding
ENST00000302277.6 ZNF804A protein_coding
ENST00000303230.4 HCN1 protein_coding
ENST00000303635.10 CAMTA1 protein_coding
ENST00000305447.4 GRM5 protein_coding
ENST00000306010.7 MGMT protein_coding
ENST00000306675.3 ADRA1B protein_coding
ENST00000309415.7 SH3RF3 protein_coding
ENST00000310085.5 CCDC96 protein_coding
ENST00000310924.5 TBC1D16 protein_coding
ENST00000315765.9 HEPHL1 protein_coding
ENST00000318407.4 BOK protein_coding
ENST00000321949.11 CRTC1 protein_coding
ENST00000321990.4 ATAD5 protein_coding
ENST00000324093.7 PLXND1 protein_coding
ENST00000326005.9 OAZ2 protein_coding
ENST00000328252.3 PAPPA protein_coding
ENST00000328299.3 ST6GALNAC3 protein_coding
ENST00000329335.3 RP1L1 processed_transcript
ENST00000329378.7 KIAA0825 protein_coding
ENST00000331351.5 HS3ST4 protein_coding
ENST00000335327.5 WASF3 protein_coding
ENST00000343508.6 CSMD3 protein_coding
ENST00000348120.5 RGS7 protein_coding
ENST00000354069.6 PID1 protein_coding
ENST00000355327.6 THSD4 protein_coding
ENST00000355520.5 OPHN1 protein_coding
ENST00000356956.4 IGF2R protein_coding

ENST00000357277.6 REPS2 protein_coding
ENST00000358991.4 NCKAP5 protein_coding
ENST00000359082.3 SHANK1 protein_coding
ENST00000359872.6 ASIC2 protein_coding
ENST00000360064.7 NA NA
ENST00000360256.7 F8 protein_coding
ENST00000360270.6 MSN protein_coding
ENST00000360286.2 SUDS3 processed_transcript
ENST00000360655.7 NAV2 protein_coding
ENST00000361421.1 TOX protein_coding
ENST00000361441.4 APOBEC3C protein_coding
ENST00000361567.2 MT-ND5 protein_coding
ENST00000361727.6 CNTNAP2 protein_coding
ENST00000362330.2 Y_RNA misc_RNA
ENST00000362352.1 Y_RNA misc_RNA
ENST00000362375.1 Y_RNA misc_RNA
ENST00000362400.1 RNA5S12 rRNA
ENST00000362415.1 Y_RNA misc_RNA
ENST00000362421.1 Y_RNA misc_RNA
ENST00000362452.1 RNA5SP183 rRNA_pseudogene
ENST00000362464.1 RNA5S11 rRNA
ENST00000362467.1 RNA5S3 rRNA
ENST00000362482.1 RNA5S1 rRNA
ENST00000362484.1 RNA5SP273 rRNA_pseudogene
ENST00000362487.1 Y_RNA misc_RNA
ENST00000362512.1 RNU12 snRNA
ENST00000362526.1 RNA5S5 rRNA
ENST00000362530.1 RNY4P19 misc_RNA
ENST00000362540.1 Y_RNA misc_RNA
ENST00000362554.1 Y_RNA misc_RNA
ENST00000362591.1 Y_RNA misc_RNA
ENST00000362645.1 Y_RNA misc_RNA
ENST00000362686.1 RNA5SP361 rRNA_pseudogene
ENST00000362697.1 Y_RNA misc_RNA
ENST00000362698.1 RNU5A-1 snRNA
ENST00000362710.1 Y_RNA misc_RNA
ENST00000362735.1 RNY4P24 misc_RNA
ENST00000362739.1 RNA5SP124 rRNA_pseudogene
ENST00000362862.1 Y_RNA misc_RNA
ENST00000362883.1 SNORD104 snoRNA
ENST00000362901.1 RNA5SP426 rRNA_pseudogene
ENST00000362911.1 Y_RNA misc_RNA
ENST00000362918.1 RNY3P2 misc_RNA
ENST00000362969.1 RNA5SP150 rRNA
ENST00000362973.1 RNA5SP358 rRNA_pseudogene
ENST00000363004.1 RNA5SP452 rRNA_pseudogene
ENST00000363022.2 RNU1-17P snRNA
ENST00000363030.1 NA NA
ENST00000363040.1 RNA5S10 rRNA
ENST00000363046.1 RMRP ribozyme
ENST00000363062.1 RNU1-18P snRNA
ENST00000363079.2 Y_RNA misc_RNA
ENST00000363120.1 VTRNA1-1 misc_RNA
ENST00000363171.1 Y_RNA misc_RNA

ENST00000363194.1 RNY4P9 misc_RNA
ENST00000363220.1 Y_RNA misc_RNA
ENST00000363248.1 Y_RNA misc_RNA
ENST00000363251.2 Y_RNA misc_RNA
ENST00000363286.1 RNU5B-1 snRNA
ENST00000363294.1 Y_RNA misc_RNA
ENST00000363301.1 Y_RNA misc_RNA
ENST00000363331.1 Y_RNA misc_RNA
ENST00000363341.1 RNY4P27 misc_RNA
ENST00000363376.1 RNA5SP263 rRNA_pseudogene
ENST00000363421.1 Y_RNA misc_RNA
ENST00000363439.1 Y_RNA misc_RNA
ENST00000363444.1 Y_RNA misc_RNA
ENST00000363473.1 RNA5S8 rRNA
ENST00000363500.1 RNA5S17 rRNA
ENST00000363511.1 RNA5S4 rRNA
ENST00000363538.1 RNA5SP74 rRNA_pseudogene
ENST00000363564.1 RNA5-8SP2 rRNA_pseudogene
ENST00000363624.1 Y_RNA misc_RNA
ENST00000363636.1 Y_RNA misc_RNA
ENST00000363651.1 Y_RNA misc_RNA
ENST00000363656.1 RNY4P14 misc_RNA
ENST00000363667.1 RNY4P6 misc_RNA
ENST00000363688.1 RNA5SP429 rRNA_pseudogene
ENST00000363745.1 Y_RNA misc_RNA
ENST00000363754.1 RNA5S6 rRNA
ENST00000363781.1 Y_RNA misc_RNA
ENST00000363872.1 Y_RNA misc_RNA
ENST00000363894.1 Y_RNA misc_RNA
ENST00000363964.1 Y_RNA misc_RNA
ENST00000363981.1 SNORD27 snoRNA
ENST00000363985.1 Y_RNA misc_RNA
ENST00000364003.1 RNA5SP399 rRNA_pseudogene
ENST00000364018.1 Y_RNA misc_RNA
ENST00000364161.1 Y_RNA misc_RNA
ENST00000364165.1 RNA5SP469 rRNA_pseudogene
ENST00000364171.1 RNA5SP242 rRNA_pseudogene
ENST00000364189.1 RNA5SP336 rRNA_pseudogene
ENST00000364201.1 Y_RNA misc_RNA
ENST00000364228.1 RNY1 misc_RNA
ENST00000364275.1 RNA5SP175 rRNA_pseudogene
ENST00000364315.1 RNA5SP202 rRNA_pseudogene
ENST00000364326.1 Y_RNA misc_RNA
ENST00000364338.1 Y_RNA misc_RNA
ENST00000364369.1 Y_RNA misc_RNA
ENST00000364407.1 Y_RNA misc_RNA
ENST00000364409.1 Y_RNA misc_RNA
ENST00000364442.1 RNA5SP72 rRNA_pseudogene
ENST00000364451.1 RNA5S9 rRNA
ENST00000364469.1 Y_RNA misc_RNA
ENST00000364473.1 Y_RNA misc_RNA
ENST00000364481.1 Y_RNA misc_RNA
ENST00000364485.1 RNA5S14 rRNA
ENST00000364486.1 RNA5SP500 rRNA_pseudogene

ENST00000364507.1 RNY4P23 misc_RNA
ENST00000364535.1 Y_RNA misc_RNA
ENST00000364581.1 Y_RNA misc_RNA
ENST00000364596.1 Y_RNA misc_RNA
ENST00000364600.1 RNY4P7 misc_RNA
ENST00000364613.1 Y_RNA misc_RNA
ENST00000364648.1 RNA5SP513 rRNA_pseudogene
ENST00000364659.1 Y_RNA misc_RNA
ENST00000364662.1 RNA5SP194 rRNA
ENST00000364678.1 Y_RNA misc_RNA
ENST00000364685.1 Y_RNA misc_RNA
ENST00000364718.1 RNA5S2 rRNA
ENST00000364725.1 RNA5SP132 rRNA_pseudogene
ENST00000364768.2 RNY4P16 misc_RNA
ENST00000364774.2 Y_RNA misc_RNA
ENST00000364853.2 Y_RNA misc_RNA
ENST00000364908.1 Y_RNA misc_RNA
ENST00000364916.1 Y_RNA misc_RNA
ENST00000364930.1 Y_RNA misc_RNA
ENST00000364948.1 RNY4P17 misc_RNA
ENST00000364950.1 Y_RNA misc_RNA
ENST00000364995.1 SNORD50B snoRNA
ENST00000365055.1 RNA5S15 rRNA
ENST00000365068.1 Y_RNA misc_RNA
ENST00000365138.1 Y_RNA misc_RNA
ENST00000365174.1 RNA5SP223 rRNA_pseudogene
ENST00000365184.1 RNA5SP152 rRNA_pseudogene
ENST00000365186.1 RNA5SP19 rRNA_pseudogene
ENST00000365241.1 VTRNA1-2 misc_RNA
ENST00000365274.1 Y_RNA misc_RNA
ENST00000365277.1 RNA5SP329 rRNA_pseudogene
ENST00000365281.1 RNY4P28 misc_RNA
ENST00000365329.1 RNU1-115P snRNA
ENST00000365387.1 RNA5S16 rRNA
ENST00000365393.1 RNA5SP22 rRNA_pseudogene
ENST00000365403.1 Y_RNA misc_RNA
ENST00000365436.1 Y_RNA misc_RNA
ENST00000365462.1 Y_RNA misc_RNA
ENST00000365484.1 RNY3 misc_RNA
ENST00000365498.1 Y_RNA misc_RNA
ENST00000365571.2 RNY4P10 misc_RNA
ENST00000365602.1 RNA5SP248 rRNA_pseudogene
ENST00000365651.1 RNA5S7 rRNA
ENST00000365656.1 RNA5S13 rRNA
ENST00000365668.1 RNU4-2 snRNA
ENST00000366595.6 NID1 protein_coding
ENST00000366789.5 STUM protein_coding
ENST00000366892.4 PRKN protein_coding
ENST00000367940.2 OLFML2B protein_coding
ENST00000368553.4 NUP210L protein_coding
ENST00000369448.3 TENT5C protein_coding
ENST00000369699.7 NA NA
ENST00000369780.7 NEURL1 protein_coding
ENST00000370192.6 DPYD protein_coding

ENST00000370618.6 NA NA
ENST00000371230.1 DAB1 protein_coding
ENST00000371332.7 HELLS protein_coding
ENST00000371836.1 AGBL4 protein_coding
ENST00000371951.4 HTR2C protein_coding
ENST00000372582.4 IL1RAPL2 protein_coding
ENST00000372734.3 ADK protein_coding
ENST00000372801.4 STK4 protein_coding
ENST00000373054.5 NA NA
ENST00000373381.7 CSMD2 protein_coding
ENST00000373955.4 PCDH15 protein_coding
ENST00000373976.6 PRKG1 protein_coding
ENST00000373986.6 ASTN2 protein_coding
ENST00000374466.3 CSGALNACT2 protein_coding
ENST00000374773.4 PARD3 protein_coding
ENST00000374778.4 OPCML protein_coding
ENST00000374998.3 MGAT5B processed_transcript
ENST00000375167.1 H2AX nonsense_mediated_decay
ENST00000375915.3 NALF1 protein_coding
ENST00000376705.3 HS6ST3 protein_coding
ENST00000377047.7 GPC6 protein_coding
ENST00000377067.6 GPC5 protein_coding
ENST00000377259.4 TJP2 retained_intron
ENST00000377284.4 PIP5K1B protein_coding
ENST00000377315.4 CACNB2 protein_coding
ENST00000377474.3 KCTD12 protein_coding
ENST00000377560.8 HECTD4 protein_coding
ENST00000377977.3 lncRNA
ENST00000378539.5 PLXNA4 protein_coding
ENST00000378610.1 CDKAL1 protein_coding
ENST00000378616.3 XK protein_coding
ENST00000378993.4 IL1RAPL1 protein_coding
ENST00000379162.7 PDK3 protein_coding
ENST00000380599.7 ZBTB4 protein_coding
ENST00000380701.6 CCDC171 protein_coding
ENST00000380874.3 NA NA
ENST00000381196.7 PTPRD protein_coding
ENST00000381312.4 ADARB2 protein_coding
ENST00000382080.4 SGCZ protein_coding
ENST00000383858.1 RNVU1-7 snRNA
ENST00000383861.1 RNU1-28P snRNA
ENST00000383869.1 RNU1-27P snRNA
ENST00000383884.1 SNORD24 snoRNA
ENST00000383897.1 RNU1-39P snRNA
ENST00000383925.1 RNU1-1 snRNA
ENST00000383975.1 RNU1-11P snRNA
ENST00000383978.1 Y_RNA misc_RNA
ENST00000383990.1 Y_RNA misc_RNA
ENST00000384010.1 RNVU1-18 snRNA
ENST00000384048.1 SNORD37 snoRNA
ENST00000384095.1 Y_RNA misc_RNA
ENST00000384097.1 Y_RNA misc_RNA
ENST00000384113.1 Y_RNA misc_RNA
ENST00000384119.1 Y_RNA misc_RNA

ENST00000384123.1 Y_RNA misc_RNA
ENST00000384138.1 Y_RNA misc_RNA
ENST00000384187.1 Y_RNA misc_RNA
ENST00000384198.1 Y_RNA misc_RNA
ENST00000384251.1 Y_RNA misc_RNA
ENST00000384252.1 SNORD61 snoRNA
ENST00000384268.1 Y_RNA misc_RNA
ENST00000384278.1 RNU1-2 snRNA
ENST00000384290.1 Y_RNA misc_RNA
ENST00000384297.1 Y_RNA misc_RNA
ENST00000384341.1 Y_RNA misc_RNA
ENST00000384413.1 Y_RNA misc_RNA
ENST00000384432.1 Y_RNA misc_RNA
ENST00000384476.1 RNVU1-15 snRNA
ENST00000384550.1 SNORD20 snoRNA
ENST00000384587.1 Y_RNA misc_RNA
ENST00000384619.1 RNVU1-17 snRNA
ENST00000384653.1 Y_RNA misc_RNA
ENST00000384656.1 Y_RNA misc_RNA
ENST00000384659.1 RNU1-4 snRNA
ENST00000384665.1 Y_RNA misc_RNA
ENST00000384753.1 Y_RNA misc_RNA
ENST00000384756.1 SNORD22 snoRNA
ENST00000384763.1 Y_RNA misc_RNA
ENST00000384766.1 Y_RNA misc_RNA
ENST00000384770.1 RNVU1-14 snRNA
ENST00000384782.1 RNU1-3 snRNA
ENST00000385059.1 MIR451A miRNA
ENST00000386347.1 MT-TL1 Mt_tRNA
ENST00000387314.1 MT-TF Mt_tRNA
ENST00000387342.1 MT-TV Mt_tRNA
ENST00000387347.2 MT-RNR2 Mt_rRNA
ENST00000387365.1 MT-TI Mt_tRNA
ENST00000387372.1 MT-TQ Mt_tRNA
ENST00000387377.1 MT-TM Mt_tRNA
ENST00000387382.1 MT-TW Mt_tRNA
ENST00000387392.1 MT-TA Mt_tRNA
ENST00000387400.1 MT-TN Mt_tRNA
ENST00000387405.1 MT-TC Mt_tRNA
ENST00000387409.1 MT-TY Mt_tRNA
ENST00000387416.2 MT-TS1 Mt_tRNA
ENST00000387419.1 MT-TD Mt_tRNA
ENST00000387421.1 MT-TK Mt_tRNA
ENST00000387429.1 MT-TG Mt_tRNA
ENST00000387439.1 MT-TR Mt_tRNA
ENST00000387441.1 MT-TH Mt_tRNA
ENST00000387449.1 MT-TS2 Mt_tRNA
ENST00000387456.1 MT-TL2 Mt_tRNA
ENST00000387459.1 MT-TE Mt_tRNA
ENST00000387460.2 MT-TT Mt_tRNA
ENST00000387461.2 MT-TP Mt_tRNA
ENST00000388940.7 SCFD2 protein_coding
ENST00000389418.7 PTPRN2 protein_coding
ENST00000389531.6 SDK1 protein_coding

ENST00000389680.2 MT-RNR1 Mt_rRNA
ENST00000390136.2 NA NA
ENST00000390382.3 TRBV7-5 TR_V_pseudogene
ENST00000390851.1 RNU1-82P snRNA
ENST00000390880.2 U3 snoRNA
ENST00000390930.1 SNORD17 snoRNA
ENST00000391004.1 Y_RNA misc_RNA
ENST00000391017.1 Y_RNA misc_RNA
ENST00000391023.1 Y_RNA misc_RNA
ENST00000391079.1 SNORD65 snoRNA
ENST00000391107.1 RNY4P18 misc_RNA
ENST00000391131.1 RNA5SP252 rRNA_pseudogene
ENST00000391146.1 Y_RNA misc_RNA
ENST00000391152.1 RNA5SP286 rRNA_pseudogene
ENST00000391269.1 RNA5SP482 rRNA_pseudogene
ENST00000392043.3 ABL2 protein_coding
ENST00000392687.4 CERS6 protein_coding
ENST00000392771.1 PLA2R1 protein_coding
ENST00000395720.2 ANKDD1A protein_coding
ENST00000396193.4 PDE1C protein_coding
ENST00000397077.4 ATP2B2 protein_coding
ENST00000398319.5 CABIN1 protein_coding
ENST00000398580.3 EYS protein_coding
ENST00000399391.5 OTOG protein_coding
ENST00000399469.2 HIVEP1 nonsense_mediated_decay
ENST00000399816.3 UBE2QL1 protein_coding
ENST00000400135.4 CHODL protein_coding
ENST00000400454.4 DSCAM protein_coding
ENST00000400457.3 PCDH11Y protein_coding
ENST00000401095.6 TTC34 protein_coding
ENST00000401376.3 MIR941-4 miRNA
ENST00000401970.2 LHFPL3 protein_coding
ENST00000402357.4 TAFA5 protein_coding
ENST00000402739.7 CTNNA2 protein_coding
ENST00000402914.4 MACROD2 protein_coding
ENST00000404019.2 DSCAM protein_coding
ENST00000406453.3 SLC29A4 protein_coding
ENST00000406533.6 KCNMA1 protein_coding
ENST00000406696.4 HS6ST2 protein_coding
ENST00000406733.1 TBC1D22A protein_coding
ENST00000407528.5 CADM2 protein_coding
ENST00000408534.2 U3 snoRNA
ENST00000408876.1 SNORD23 snoRNA
ENST00000408984.6 WWOX protein_coding
ENST00000409283.5 ACVR1 protein_coding
ENST00000409758.1 lncRNA
ENST00000409884.4 LRRTM4 protein_coding
ENST00000410216.1 RNY4P30 misc_RNA
ENST00000410344.1 RNU2-33P snRNA
ENST00000410361.1 RNU2-36P snRNA
ENST00000410396.1 RNU2-2P snRNA
ENST00000410423.1 RNU2-29P snRNA
ENST00000410508.1 RNU2-26P snRNA
ENST00000410535.1 Y_RNA misc_RNA

ENST00000410577.1 Y_RNA misc_RNA
ENST00000410597.1 Y_RNA misc_RNA
ENST00000410647.1 Y_RNA misc_RNA
ENST00000410669.1 Y_RNA misc_RNA
ENST00000410694.1 RNU2-48P snRNA
ENST00000410695.1 RNU2-37P snRNA
ENST00000410717.1 Y_RNA misc_RNA
ENST00000410766.1 Y_RNA misc_RNA
ENST00000410794.1 RNU2-7P snRNA
ENST00000410801.1 RNY4P29 misc_RNA
ENST00000410809.1 RNA5SP229 rRNA_pseudogene
ENST00000410878.1 RNU2-68P snRNA
ENST00000410920.1 Y_RNA misc_RNA
ENST00000411069.1 RNU2-61P snRNA
ENST00000411222.1 Y_RNA misc_RNA
ENST00000411288.1 Y_RNA misc_RNA
ENST00000411317.1 NA NA
ENST00000411366.1 RNY3P8 misc_RNA
ENST00000411404.1 RNU2-6P snRNA
ENST00000411542.1 lncRNA
ENST00000414318.2 TBC1D5 processed_transcript
ENST00000415045.1 NLGN1 protein_coding
ENST00000415555.4 DDR2 protein_coding
ENST00000415947.1 PARD3B nonsense_mediated_decay
ENST00000417403.4 ABCA13 nonsense_mediated_decay
ENST00000417751.4 LINC00276 lncRNA
ENST00000418236.4 ARHGAP26 protein_coding
ENST00000418253.2 NA NA
ENST00000419035.1 EPCAM-DT lncRNA
ENST00000419706.5 BTBD9 protein_coding
ENST00000419883.1 LINC01392 lncRNA
ENST00000419940.4 TGFA protein_coding
ENST00000420084.1 unprocessed_pseudogene
ENST00000420877.1 LINC01426 lncRNA
ENST00000420921.5 RNF150 protein_coding
ENST00000420922.5 FTCDNL1 protein_coding
ENST00000421109.5 NR2F2 protein_coding
ENST00000421505.1 RASAL2-AS1 lncRNA
ENST00000421686.5 lncRNA
ENST00000421768.1 LARGE1 nonsense_mediated_decay
ENST00000421865.2 LAMA2 protein_coding
ENST00000422452.2 TENM1 protein_coding
ENST00000422558.1 LINC01320 lncRNA
ENST00000423686.1 MRPS31P5 transcribed_unprocessed_pseudogene
ENST00000425666.1 LINC01508 lncRNA
ENST00000426886.1 SMIM12 nonsense_mediated_decay
ENST00000427391.1 FIRRE lncRNA
ENST00000427444.1 HFM1 protein_coding
ENST00000427802.2 FSTL5 protein_coding
ENST00000428068.4 ADGRA2 protein_coding
ENST00000428546.1 KCNMA1 processed_transcript
ENST00000428762.4 RELN protein_coding
ENST00000429636.1 lncRNA
ENST00000429656.1 unprocessed_pseudogene

ENST00000430736.4 RGPD5 protein_coding
ENST00000431078.1 CNTNAP5 protein_coding
ENST00000431824.2 IGF2BP1 protein_coding
ENST00000433211.5 CTNNA3 protein_coding
ENST00000434794.1 LRP1B protein_coding
ENST00000435159.2 TMEM132C protein_coding
ENST00000435576.2 TRABD2B processed_transcript
ENST00000435946.1 lncRNA
ENST00000436732.4 DPP10 protein_coding
ENST00000436949.4 SEMA3A protein_coding
ENST00000437857.2 CCDC181 retained_intron
ENST00000440200.4 processed_transcript
ENST00000440698.1 lncRNA
ENST00000441693.2 CCDC127 protein_coding
ENST00000441760.2 lncRNA
ENST00000442430.1 PIM2 protein_coding
ENST00000442578.4 LINC00174 lncRNA
ENST00000445072.1 LINC01035 lncRNA
ENST00000445220.4 SHANK3 protein_coding
ENST00000445741.4 SAMD12 nonsense_mediated_decay
ENST00000446781.3 KIF5C-AS1 lncRNA
ENST00000447585.1 OFD1P5Y unprocessed_pseudogene
ENST00000447745.4 CTDSPL protein_coding
ENST00000447845.1 NA NA
ENST00000448413.4 SUMF1 nonsense_mediated_decay
ENST00000448863.1 ACOXL protein_coding
ENST00000449068.1 SLC1A2 protein_coding
ENST00000449684.5 LIMS1 processed_transcript
ENST00000449842.1 LINC01344 lncRNA
ENST00000450123.5 BCL6 protein_coding
ENST00000450287.2 TMEM72-AS1 lncRNA
ENST00000450446.5 FAM168A protein_coding
ENST00000451061.4 OFD1P6Y transcribed_unprocessed_pseudogene
ENST00000451391.4 IDH1 protein_coding
ENST00000451853.1 VDAC1P12 processed_pseudogene
ENST00000453395.4 PSLNR lncRNA
ENST00000455399.1 NA NA
ENST00000456197.1 TRRAP protein_coding
ENST00000457478.1 LINC01250 lncRNA
ENST00000457843.1 NALCN-AS1 lncRNA
ENST00000458207.4 PPIEL processed_transcript
ENST00000458220.1 GAS5 retained_intron
ENST00000458902.1 Y_RNA misc_RNA
ENST00000459006.1 Y_RNA misc_RNA
ENST00000459091.1 Y_RNA misc_RNA
ENST00000459174.1 SNORD4A snoRNA
ENST00000459254.1 RNY4P25 misc_RNA
ENST00000459548.1 MIR1976 miRNA
ENST00000459864.1 PLD5 protein_coding
ENST00000460085.1 ATP6V1E1 processed_transcript
ENST00000460467.1 PLCL2 processed_transcript
ENST00000460805.4 FOXP1 retained_intron
ENST00000461520.4 USP54 processed_transcript
ENST00000463477.4 PTPRD protein_coding

ENST00000463779.3 RN7SL752P misc_RNA
ENST00000464170.1 CATSPERE processed_transcript
ENST00000464233.4 ROBO1 protein_coding
ENST00000464741.2 GRID1 nonsense_mediated_decay
ENST00000464976.1 RAPGEF4 processed_transcript
ENST00000465084.1 PAX8 protein_coding
ENST00000465127.1 protein_coding
ENST00000465249.1 ZBTB20-AS5 lncRNA
ENST00000465614.1 LMO2 retained_intron
ENST00000466278.1 MRTFA processed_transcript
ENST00000468425.2 ATP5IF1 protein_coding
ENST00000469129.1 SMC1A retained_intron
ENST00000469481.1 STAG2 processed_transcript
ENST00000469524.1 EIF4E3 protein_coding
ENST00000469774.1 GLS retained_intron
ENST00000470495.1 HOXB3 protein_coding
ENST00000470721.4 DCAF6 nonsense_mediated_decay
ENST00000470729.4 ANKRD30BL processed_transcript
ENST00000471074.1 FBXL13 processed_transcript
ENST00000471086.3 RN7SL396P misc_RNA
ENST00000471727.2 APLF processed_transcript
ENST00000471894.1 PUM1 retained_intron
ENST00000472020.1 EXOC4 processed_transcript
ENST00000474344.1 VANGL1 processed_transcript
ENST00000474384.2 SNRPB nonsense_mediated_decay
ENST00000474495.1 CEMIP2 retained_intron
ENST00000474521.1 FGFR3 retained_intron
ENST00000474522.4 ZFAND3 protein_coding
ENST00000474732.1 EPHB1 protein_coding
ENST00000474851.1 LSAMP protein_coding
ENST00000474953.4 ARID4B nonsense_mediated_decay
ENST00000475034.1 ROBO2 processed_transcript
ENST00000475045.5 RUNX1 protein_coding
ENST00000475638.5 COG5 processed_transcript
ENST00000475839.1 FEZF2 protein_coding
ENST00000475855.1 AADACL2-AS1 lncRNA
ENST00000475962.4 CBX7 processed_transcript
ENST00000476423.4 SZRD1 nonsense_mediated_decay
ENST00000476571.1 NKAIN2 processed_transcript
ENST00000477373.1 ZYX processed_transcript
ENST00000478541.1 CYTH3 retained_intron
ENST00000479173.1 OSBPL10 processed_transcript
ENST00000480494.1 SPAG16 processed_transcript
ENST00000480693.1 PNCK processed_transcript
ENST00000480707.1 MALRD1 processed_transcript
ENST00000481402.4 AKAP4 processed_transcript
ENST00000481762.4 ZNF767P processed_transcript
ENST00000481793.1 RNLS processed_transcript
ENST00000481847.4 WFDC3 processed_transcript
ENST00000483779.1 TMEM63A retained_intron
ENST00000484216.1 HBA2 protein_coding
ENST00000484237.4 COG5 processed_transcript
ENST00000484842.1 OPHN1 processed_transcript
ENST00000485431.1 PIM2 processed_transcript

ENST00000485542.4 CAPZA1 processed_transcript
ENST00000485743.1 HBB protein_coding
ENST00000485760.4 DAB1 processed_transcript
ENST00000486764.1 ABHD5 retained_intron
ENST00000487696.1 CAMK1D retained_intron
ENST00000487791.1 HBA1 retained_intron
ENST00000488123.2 NA NA
ENST00000488467.4 FHIT protein_coding
ENST00000488663.1 ZBTB20 processed_transcript
ENST00000488831.4 ZC4H2 processed_transcript
ENST00000489243.4 KDM4C processed_transcript
ENST00000490232.3 RN7SL2 misc_RNA
ENST00000490952.1 FHIT retained_intron
ENST00000491228.1 LRP2 retained_intron
ENST00000491418.4 SPATA21 protein_coding
ENST00000492002.1 EGFL7 processed_transcript
ENST00000492310.4 HECW1 processed_transcript
ENST00000493214.2 LINC02006 lncRNA
ENST00000494482.1 PARD3B processed_transcript
ENST00000494580.1 CTNNA3 protein_coding
ENST00000495034.4 RALGPS2 retained_intron
ENST00000496393.2 NVL processed_transcript
ENST00000497407.2 SIRPG processed_transcript
ENST00000497566.4 MAP4K3 retained_intron
ENST00000498070.4 PPP1R12B processed_transcript
ENST00000498088.1 IL17D protein_coding
ENST00000498517.4 ARHGEF3 processed_transcript
ENST00000499481.2 lncRNA
ENST00000503396.4 PDS5A protein_coding
ENST00000503743.4 KAZN protein_coding
ENST00000504220.5 PCDH11X protein_coding
ENST00000504592.4 BANK1 protein_coding
ENST00000505383.1 ARL15 retained_intron
ENST00000505687.4 GRID2 processed_transcript
ENST00000506174.4 ATP13A2 protein_coding
ENST00000506823.4 GALNTL6 protein_coding
ENST00000507023.1 AGA-DT lncRNA
ENST00000507166.4 protein_coding
ENST00000507651.1 processed_pseudogene
ENST00000507863.1 MARCHF6 processed_transcript
ENST00000509667.4 PLPP1 nonsense_mediated_decay
ENST00000510013.1 PCBD2 processed_transcript
ENST00000510391.1 lncRNA
ENST00000510463.1 DKK2 protein_coding
ENST00000510504.1 TENM3 protein_coding
ENST00000510534.1 DKK2 retained_intron
ENST00000510626.4 HTT retained_intron
ENST00000511199.1 SORCS2 processed_transcript
ENST00000511496.4 LIMCH1 protein_coding
ENST00000511524.1 CLSTN2 retained_intron
ENST00000511919.4 LINC01091 lncRNA
ENST00000512069.5 PDE4D processed_transcript
ENST00000512223.5 AEBP2 protein_coding
ENST00000512870.1 SULT1B1 protein_coding

ENST00000513241.2 ANKRD55 protein_coding
ENST00000513314.1 DHFR retained_intron
ENST00000515031.4 LINC00504 lncRNA
ENST00000515364.1 lncRNA
ENST00000515847.1 LIMCH1 processed_transcript
ENST00000515896.1 RNA5-8SP6 rRNA_pseudogene
ENST00000515924.1 SCARNA8 scaRNA
ENST00000515932.1 RNU4ATAC13P snRNA
ENST00000515939.1 RNU4ATAC11P snRNA
ENST00000515961.1 RNA5SP486 rRNA_pseudogene
ENST00000515983.1 Y_RNA misc_RNA
ENST00000516014.2 RNA5SP162 rRNA_pseudogene
ENST00000516043.1 Y_RNA misc_RNA
ENST00000516177.1 Y_RNA misc_RNA
ENST00000516225.1 RNY4P37 misc_RNA
ENST00000516362.1 Y_RNA misc_RNA
ENST00000516441.1 Y_RNA misc_RNA
ENST00000516507.1 RNY4 misc_RNA
ENST00000516508.1 Y_RNA misc_RNA
ENST00000516659.1 RNU2-57P snRNA
ENST00000516678.1 RNY4P20 misc_RNA
ENST00000516733.1 SNORD36C snoRNA
ENST00000516843.1 Y_RNA misc_RNA
ENST00000516869.1 RPPH1 ribozyme
ENST00000516881.1 SCARNA15 scaRNA
ENST00000516950.1 Y_RNA misc_RNA
ENST00000516967.1 RNU4ATAC10P snRNA
ENST00000517097.1 SCARNA3 scaRNA
ENST00000517110.1 Y_RNA misc_RNA
ENST00000517146.1 RNU4ATAC9P snRNA
ENST00000517226.1 RNU4ATAC5P snRNA
ENST00000517758.1 lncRNA
ENST00000517869.1 LINC00861 lncRNA
ENST00000517937.1 DST retained_intron
ENST00000518180.1 ZFPM2 processed_transcript
ENST00000518549.1 CPA6 retained_intron
ENST00000518634.1 EIF3E processed_transcript
ENST00000518764.1 CCNJL processed_transcript
ENST00000518928.1 TSNARE1 processed_transcript
ENST00000520426.1 lncRNA
ENST00000522153.1 CD74 retained_intron
ENST00000522165.4 COL13A1 protein_coding
ENST00000522537.4 MOK protein_coding
ENST00000523207.1 KCNB2 protein_coding
ENST00000524120.4 MOK processed_transcript
ENST00000524176.2 MYBL1 protein_coding
ENST00000524201.1 NKAIN3 protein_coding
ENST00000524202.4 PTK2 nonsense_mediated_decay
ENST00000524235.4 ZFPM2 processed_transcript
ENST00000524422.1 ATP5MG protein_coding
ENST00000524607.4 CACNA1E protein_coding
ENST00000527088.1 DLG2 protein_coding
ENST00000528315.1 CRACR2B protein_coding
ENST00000529038.4 OPCML processed_transcript

ENST00000529666.1 AAMDC nonsense_mediated_decay
ENST00000529979.4 IRAG1-AS1 lncRNA
ENST00000529993.4 LRP5 nonsense_mediated_decay
ENST00000530307.1 SLCO5A1 protein_coding
ENST00000530321.1 EDA processed_transcript
ENST00000530858.4 GRM5P1 transcribed_unprocessed_pseudogene
ENST00000531305.1 ALG9-IT1 lncRNA
ENST00000531901.4 EYA4 protein_coding
ENST00000534291.1 lncRNA
ENST00000534577.1 LRRC4C processed_transcript
ENST00000535229.1 PDE10A processed_transcript
ENST00000535391.4 EIF3J protein_coding
ENST00000536331.4 NA NA
ENST00000536684.2 MTRNR2L8 protein_coding
ENST00000536795.1 CCND2 retained_intron
ENST00000537116.4 ACSF3 processed_transcript
ENST00000537824.2 NA NA
ENST00000538899.2 RNGTT protein_coding
ENST00000539240.4 KLRG1 protein_coding
ENST00000539977.1 PABPC1P4 processed_pseudogene
ENST00000540040.2 MTRNR2L1 protein_coding
ENST00000540663.4 SYNE1 protein_coding
ENST00000540815.2 LGR5 protein_coding
ENST00000542675.1 PDE3A retained_intron
ENST00000543233.2 CDH4 protein_coding
ENST00000543854.4 SPPL3 protein_coding
ENST00000545433.2 NKAIN2 protein_coding
ENST00000545609.2 PLEKHA8P1 processed_transcript
ENST00000545650.4 NA NA
ENST00000545829.1 PRB2 processed_transcript
ENST00000545920.1 SNHG1 lncRNA
ENST00000546190.4 ADGRB3 protein_coding
ENST00000546426.4 RPH3A protein_coding
ENST00000547427.4 RBFOX1 processed_transcript
ENST00000548458.5 C12orf75 processed_transcript
ENST00000548749.4 RBFOX1 processed_transcript
ENST00000549419.4 PRANCR lncRNA
ENST00000549866.4 ANKS1B protein_coding
ENST00000550103.2 EFCAB11 processed_transcript
ENST00000550114.4 EGLN3 processed_transcript
ENST00000550905.4 LINC01234 lncRNA
ENST00000552200.1 PRICKLE1 processed_transcript
ENST00000552630.1 PAN2 retained_intron
ENST00000553371.1 LGMN protein_coding
ENST00000553547.4 AKAP6 protein_coding
ENST00000553653.4 SLCO3A1 retained_intron
ENST00000553827.1 LINC02307 lncRNA
ENST00000554318.2 lncRNA
ENST00000554814.1 lncRNA
ENST00000555015.4 SRP54-AS1 processed_transcript
ENST00000556810.4 TMEM260 protein_coding
ENST00000556918.4 NRG3 protein_coding
ENST00000557430.1 SLC35F4 processed_transcript
ENST00000558031.4 CRNDE lncRNA

ENST00000558187.4 EFL1P1 processed_transcript
ENST00000558469.4 CUX1 processed_transcript
ENST00000558607.1 lncRNA
ENST00000558616.1 lncRNA
ENST00000560036.1 WDR72 protein_coding
ENST00000560161.1 ATP8B4 processed_transcript
ENST00000561106.1 SEMA6D retained_intron
ENST00000562611.1 GSG1L nonsense_mediated_decay
ENST00000562656.1 lncRNA
ENST00000563716.4 DUX4 processed_transcript
ENST00000564033.1 SLC9A3R2 retained_intron
ENST00000564731.4 MYLK3 retained_intron
ENST00000565066.4 ABHD2 protein_coding
ENST00000566670.2 GRIN2A processed_transcript
ENST00000568642.4 VPS35 processed_transcript
ENST00000568845.4 WWP2 processed_transcript
ENST00000569050.1 PKM protein_coding
ENST00000569314.1 MYO9A protein_coding
ENST00000569332.4 WWOX nonsense_mediated_decay
ENST00000569895.3 RBFOX1 processed_transcript
ENST00000571722.3 SNORD3B-2 snoRNA
ENST00000574245.1 lncRNA
ENST00000577597.4 MAP3K3 nonsense_mediated_decay
ENST00000578189.1 NOTCH2NLC protein_coding
ENST00000578207.4 NA NA
ENST00000579879.1 SNORD95 snoRNA
ENST00000580524.1 lncRNA
ENST00000580533.1 SNORD3C snoRNA
ENST00000580972.1 RNU4ATAC snRNA
ENST00000581135.4 DDX42 protein_coding
ENST00000581147.1 TEX14 nonsense_mediated_decay
ENST00000581457.1 CXADRP3 processed_transcript
ENST00000581458.2 RN7SL5P misc_RNA
ENST00000581912.1 CNDP2 protein_coding
ENST00000581940.1 lncRNA
ENST00000582320.2 lncRNA
ENST00000582913.4 PIEZO2 nonsense_mediated_decay
ENST00000583289.4 PTPRM protein_coding
ENST00000583687.1 DSEL-AS1 lncRNA
ENST00000584058.2 RN7SL4P misc_RNA
ENST00000584072.1 NA NA
ENST00000584296.1 WIPF2 protein_coding
ENST00000584858.2 C8orf34-AS1 lncRNA
ENST00000584923.1 SNORD3A snoRNA
ENST00000585867.2 RBFOX1 protein_coding
ENST00000586448.4 SLC14A2 protein_coding
ENST00000586594.1 RAB27B processed_transcript
ENST00000587591.4 C18orf25 protein_coding
ENST00000588655.1 lncRNA
ENST00000590399.1 lncRNA
ENST00000591219.4 EPS8L1 retained_intron
ENST00000591683.1 HEATR6 nonsense_mediated_decay
ENST00000592483.4 CAPNS1 protein_coding
ENST00000593495.2 NOTCH2NLB protein_coding

ENST00000593817.1 LRMDA processed_transcript
ENST00000596431.4 RYR1 processed_transcript
ENST00000598623.1 lncRNA
ENST00000600213.3 MTRNR2L12 protein_coding
ENST00000601860.4 lncRNA
ENST00000602198.4 TEX101 protein_coding
ENST00000602723.4 CSMD1 protein_coding
ENST00000603274.1 unprocessed_pseudogene
ENST00000605806.1 RNVU1-31 snRNA
ENST00000606950.1 NA NA
ENST00000607318.1 RNA5SP357 rRNA_pseudogene
ENST00000607639.1 RNA5SP386 rRNA_pseudogene
ENST00000608721.1 MAP3K4-AS1 lncRNA
ENST00000610460.1 5_8S_rRNA rRNA
ENST00000610674.1 RN7SL3 misc_RNA
ENST00000610976.1 RNVU1-28 snRNA
ENST00000610978.1 NA NA
ENST00000611049.3 GRID2 protein_coding
ENST00000611393.1 NA NA
ENST00000611544.1 U2 snRNA
ENST00000611785.1 lncRNA
ENST00000612313.1 PDE4DIPP4 processed_transcript
ENST00000612463.1 RNA5-8SN2 rRNA
ENST00000612665.1 RNA5SP440 rRNA_pseudogene
ENST00000612732.1 5_8S_rRNA rRNA
ENST00000612822.1 NA NA
ENST00000613119.1 U2 snRNA
ENST00000613214.3 TNXB protein_coding
ENST00000613359.1 RNA5-8SN3 rRNA
ENST00000613778.1 U2 snRNA
ENST00000613834.1 NA NA
ENST00000613956.1 U2 snRNA
ENST00000614931.1 PCDH9 nonsense_mediated_decay
ENST00000615294.1 NA NA
ENST00000615427.1 U2 snRNA
ENST00000615825.1 MME protein_coding
ENST00000615833.1 LINC01378 lncRNA
ENST00000615842.1 RNVU1-29 snRNA
ENST00000616345.1 U2 snRNA
ENST00000616528.1 NA NA
ENST00000616535.1 U2 snRNA
ENST00000616588.3 lncRNA
ENST00000616860.1 NA NA
ENST00000617226.3 NA NA
ENST00000617236.1 NA NA
ENST00000617238.1 NA NA
ENST00000617474.1 PTPRT nonsense_mediated_decay
ENST00000617489.1 MALAT1 lncRNA
ENST00000617785.1 U2 snRNA
ENST00000617878.1 PDE4DIPP7 processed_transcript
ENST00000617940.1 FRG1HP transcribed_unprocessed_pseudogene
ENST00000618119.3 ALK protein_coding
ENST00000618602.1 U2 snRNA
ENST00000618664.1 RNU2-1 snRNA

ENST00000618786.1 RN7SL1 misc_RNA
ENST00000618808.3 NA NA
ENST00000618828.1 COL5A2 protein_coding
ENST00000618938.2 NA NA
ENST00000618951.1 NA NA
ENST00000618959.1 GALNT17 protein_coding
ENST00000619178.1 SNORD3D snoRNA
ENST00000619225.1 U2 snRNA
ENST00000619232.1 DACH1 protein_coding
ENST00000619366.1 NA NA
ENST00000619465.1 U2 snRNA
ENST00000619471.1 RNA5-8SN1 rRNA
ENST00000620186.3 LINC01297 lncRNA
ENST00000620268.1 U2 snRNA
ENST00000620651.3 MRTFA protein_coding
ENST00000620683.3 NA NA
ENST00000620725.1 NA NA
ENST00000621235.1 processed_transcript
ENST00000621667.1 MIR941-5 miRNA
ENST00000622018.3 HPCAL1 protein_coding
ENST00000623177.1 TEC
ENST00000623213.1 DOCK1 protein_coding
ENST00000623664.1 lncRNA
ENST00000623678.1 lncRNA
ENST00000623860.1 lncRNA
ENST00000624098.2 UTY protein_coding
ENST00000624419.1 NOTCH2NLR protein_coding
ENST00000625012.1 processed_transcript
ENST00000625365.1 CNTNAP2 protein_coding
ENST00000626530.1 NA NA
ENST00000626963.1 NA NA
ENST00000627092.1 NA NA
ENST00000627435.1 NA NA
ENST00000627526.1 NA NA
ENST00000627640.1 NA NA
ENST00000628388.1 Y_RNA misc_RNA
ENST00000628531.1 Y_RNA misc_RNA
ENST00000628603.1 NA NA
ENST00000628689.1 TCF4 nonsense_mediated_decay
ENST00000628694.1 NA NA
ENST00000629083.1 Metazoa_SRP misc_RNA
ENST00000629705.1 NA NA
ENST00000629789.1 NA NA
ENST00000631047.1 WDR75 protein_coding
ENST00000631057.1 GPC3 protein_coding
ENST00000631162.1 NA NA
ENST00000631292.1 NA NA
MIMAT0000062_2 NA miRNA
MIMAT0000063 NA miRNA
MIMAT0000064 NA miRNA
MIMAT0000065 NA miRNA
MIMAT0000066 NA miRNA
MIMAT0000067_1 NA miRNA
MIMAT0000068 NA miRNA

MIMAT0000069_1 NA miRNA
MIMAT0000070 NA miRNA
MIMAT0000071 NA miRNA
MIMAT0000072 NA miRNA
MIMAT0000073 NA miRNA
MIMAT0000074_1 NA miRNA
MIMAT0000075 NA miRNA
MIMAT0000076 NA miRNA
MIMAT0000077 NA miRNA
MIMAT0000078 NA miRNA
MIMAT0000080_1 NA miRNA
MIMAT0000081 NA miRNA
MIMAT0000082_1 NA miRNA
MIMAT0000083 NA miRNA
MIMAT0000084 NA miRNA
MIMAT0000085 NA miRNA
MIMAT0000086 NA miRNA
MIMAT0000087 NA miRNA
MIMAT0000088 NA miRNA
MIMAT0000090 NA miRNA
MIMAT0000092_1 NA miRNA
MIMAT0000093 NA miRNA
MIMAT0000094 NA miRNA
MIMAT0000095 NA miRNA
MIMAT0000096 NA miRNA
MIMAT0000097 NA miRNA
MIMAT0000098 NA miRNA
MIMAT0000099_1 NA miRNA
MIMAT0000100_1 NA miRNA
MIMAT0000101_1 NA miRNA
MIMAT0000103 NA miRNA
MIMAT0000104 NA miRNA
MIMAT0000222 NA miRNA
MIMAT0000227 NA miRNA
MIMAT0000231_1 NA miRNA
MIMAT0000232_1 NA miRNA
MIMAT0000243 NA miRNA
MIMAT0000244_1 NA miRNA
MIMAT0000245 NA miRNA
MIMAT0000250 NA miRNA
MIMAT0000252_2 NA miRNA
MIMAT0000253 NA miRNA
MIMAT0000254 NA miRNA
MIMAT0000256_1 NA miRNA
MIMAT0000257_1 NA miRNA
MIMAT0000258 NA miRNA
MIMAT0000259 NA miRNA
MIMAT0000261 NA miRNA
MIMAT0000263 NA miRNA
MIMAT0000265 NA miRNA
MIMAT0000266 NA miRNA
MIMAT0000267 NA miRNA
MIMAT0000270 NA miRNA
MIMAT0000272 NA miRNA

MIMAT0000278 NA miRNA
MIMAT0000279 NA miRNA
MIMAT0000280 NA miRNA
MIMAT0000281 NA miRNA
MIMAT0000318 NA miRNA
MIMAT0000414 NA miRNA
MIMAT0000415 NA miRNA
MIMAT0000416_1 NA miRNA
MIMAT0000417 NA miRNA
MIMAT0000418 NA miRNA
MIMAT0000419 NA miRNA
MIMAT0000420 NA miRNA
MIMAT0000421 NA miRNA
MIMAT0000423_1 NA miRNA
MIMAT0000424_1 NA miRNA
MIMAT0000425 NA miRNA
MIMAT0000426 NA miRNA
MIMAT0000427_1 NA miRNA
MIMAT0000430_1 NA miRNA
MIMAT0000431 NA miRNA
MIMAT0000432 NA miRNA
MIMAT0000433 NA miRNA
MIMAT0000434 NA miRNA
MIMAT0000435 NA miRNA
MIMAT0000436 NA miRNA
MIMAT0000437 NA miRNA
MIMAT0000438 NA miRNA
MIMAT0000439_1 NA miRNA
MIMAT0000440 NA miRNA
MIMAT0000442_2 NA miRNA
MIMAT0000443 NA miRNA
MIMAT0000444 NA miRNA
MIMAT0000445 NA miRNA
MIMAT0000446 NA miRNA
MIMAT0000447 NA miRNA
MIMAT0000448 NA miRNA
MIMAT0000449 NA miRNA
MIMAT0000451 NA miRNA
MIMAT0000455 NA miRNA
MIMAT0000456 NA miRNA
MIMAT0000457 NA miRNA
MIMAT0000458 NA miRNA
MIMAT0000460_1 NA miRNA
MIMAT0000461 NA miRNA
MIMAT0000462 NA miRNA
MIMAT0000510 NA miRNA
MIMAT0000617 NA miRNA
MIMAT0000646 NA miRNA
MIMAT0000680 NA miRNA
MIMAT0000681 NA miRNA
MIMAT0000682 NA miRNA
MIMAT0000688 NA miRNA
MIMAT0000689 NA miRNA
MIMAT0000691 NA miRNA

MIMAT0000692 NA miRNA
MIMAT0000693 NA miRNA
MIMAT0000703 NA miRNA
MIMAT0000705 NA miRNA
MIMAT0000707 NA miRNA
MIMAT0000720 NA miRNA
MIMAT0000721 NA miRNA
MIMAT0000722 NA miRNA
MIMAT0000723 NA miRNA
MIMAT0000727 NA miRNA
MIMAT0000728 NA miRNA
MIMAT0000729_1 NA miRNA
MIMAT0000730 NA miRNA
MIMAT0000732 NA miRNA
MIMAT0000733 NA miRNA
MIMAT0000735 NA miRNA
MIMAT0000736 NA miRNA
MIMAT0000737 NA miRNA
MIMAT0000750 NA miRNA
MIMAT0000751 NA miRNA
MIMAT0000752 NA miRNA
MIMAT0000753 NA miRNA
MIMAT0000754 NA miRNA
MIMAT0000755 NA miRNA
MIMAT0000757 NA miRNA
MIMAT0000759 NA miRNA
MIMAT0000760 NA miRNA
MIMAT0000761 NA miRNA
MIMAT0000763 NA miRNA
MIMAT0000764 NA miRNA
MIMAT0000765 NA miRNA
MIMAT0000772 NA miRNA
MIMAT0001080 NA miRNA
MIMAT0001340 NA miRNA
MIMAT0001341 NA miRNA
MIMAT0001343 NA miRNA
MIMAT0001412 NA miRNA
MIMAT0001413 NA miRNA
MIMAT0001545_1 NA miRNA
MIMAT0001618 NA miRNA
MIMAT0001625 NA miRNA
MIMAT0001627 NA miRNA
MIMAT0001629_1 NA miRNA
MIMAT0001631 NA miRNA
MIMAT0001635 NA miRNA
MIMAT0001638 NA miRNA
MIMAT0001639 NA miRNA
MIMAT0002171 NA miRNA
MIMAT0002172 NA miRNA
MIMAT0002173 NA miRNA
MIMAT0002174 NA miRNA
MIMAT0002176 NA miRNA
MIMAT0002177_1 NA miRNA
MIMAT0002178 NA miRNA

MIMAT0002807 NA miRNA
MIMAT0002809 NA miRNA
MIMAT0002813 NA miRNA
MIMAT0002814 NA miRNA
MIMAT0002816 NA miRNA
MIMAT0002817 NA miRNA
MIMAT0002820 NA miRNA
MIMAT0002821 NA miRNA
MIMAT0002870 NA miRNA
MIMAT0002871 NA miRNA
MIMAT0002872 NA miRNA
MIMAT0002876 NA miRNA
MIMAT0002888 NA miRNA
MIMAT0002891 NA miRNA
MIMAT0003161 NA miRNA
MIMAT0003163 NA miRNA
MIMAT0003180 NA miRNA
MIMAT0003218 NA miRNA
MIMAT0003220 NA miRNA
MIMAT0003239 NA miRNA
MIMAT0003241 NA miRNA
MIMAT0003244 NA miRNA
MIMAT0003249 NA miRNA
MIMAT0003266 NA miRNA
MIMAT0003283 NA miRNA
MIMAT0003293 NA miRNA
MIMAT0003294 NA miRNA
MIMAT0003297 NA miRNA
MIMAT0003321 NA miRNA
MIMAT0003322 NA miRNA
MIMAT0003329 NA miRNA
MIMAT0003330 NA miRNA
MIMAT0003332 NA miRNA
MIMAT0003338 NA miRNA
MIMAT0003339 NA miRNA
MIMAT0003340 NA miRNA
MIMAT0003393 NA miRNA
MIMAT0003879 NA miRNA
MIMAT0003880 NA miRNA
MIMAT0003884 NA miRNA
MIMAT0003885 NA miRNA
MIMAT0003886 NA miRNA
MIMAT0003887 NA miRNA
MIMAT0003888 NA miRNA
MIMAT0004481_1 NA miRNA
MIMAT0004482 NA miRNA
MIMAT0004484 NA miRNA
MIMAT0004486 NA miRNA
MIMAT0004494 NA miRNA
MIMAT0004495 NA miRNA
MIMAT0004496 NA miRNA
MIMAT0004497 NA miRNA
MIMAT0004498 NA miRNA
MIMAT0004499 NA miRNA

MIMAT0004500 NA miRNA
MIMAT0004502 NA miRNA
MIMAT0004505 NA miRNA
MIMAT0004509 NA miRNA
MIMAT0004515 NA miRNA
MIMAT0004517 NA miRNA
MIMAT0004518 NA miRNA
MIMAT0004549 NA miRNA
MIMAT0004551 NA miRNA
MIMAT0004552 NA miRNA
MIMAT0004555 NA miRNA
MIMAT0004556 NA miRNA
MIMAT0004558 NA miRNA
MIMAT0004559 NA miRNA
MIMAT0004563 NA miRNA
MIMAT0004568 NA miRNA
MIMAT0004570 NA miRNA
MIMAT0004585 NA miRNA
MIMAT0004586 NA miRNA
MIMAT0004587 NA miRNA
MIMAT0004588 NA miRNA
MIMAT0004594 NA miRNA
MIMAT0004597 NA miRNA
MIMAT0004600 NA miRNA
MIMAT0004601 NA miRNA
MIMAT0004602 NA miRNA
MIMAT0004603 NA miRNA
MIMAT0004606 NA miRNA
MIMAT0004610 NA miRNA
MIMAT0004611 NA miRNA
MIMAT0004614 NA miRNA
MIMAT0004672 NA miRNA
MIMAT0004673 NA miRNA
MIMAT0004674 NA miRNA
MIMAT0004678 NA miRNA
MIMAT0004680 NA miRNA
MIMAT0004681 NA miRNA
MIMAT0004682 NA miRNA
MIMAT0004688 NA miRNA
MIMAT0004692 NA miRNA
MIMAT0004694 NA miRNA
MIMAT0004697 NA miRNA
MIMAT0004700 NA miRNA
MIMAT0004701 NA miRNA
MIMAT0004702 NA miRNA
MIMAT0004703 NA miRNA
MIMAT0004748 NA miRNA
MIMAT0004749 NA miRNA
MIMAT0004757 NA miRNA
MIMAT0004761 NA miRNA
MIMAT0004762_1 NA miRNA
MIMAT0004766 NA miRNA
MIMAT0004767 NA miRNA
MIMAT0004773 NA miRNA

MIMAT0004774 NA miRNA
MIMAT0004775 NA miRNA
MIMAT0004776 NA miRNA
MIMAT0004780 NA miRNA
MIMAT0004795 NA miRNA
MIMAT0004796 NA miRNA
MIMAT0004797 NA miRNA
MIMAT0004799 NA miRNA
MIMAT0004800_1 NA miRNA
MIMAT0004801 NA miRNA
MIMAT0004807 NA miRNA
MIMAT0004810 NA miRNA
MIMAT0004813 NA miRNA
MIMAT0004814 NA miRNA
MIMAT0004819 NA miRNA
MIMAT0004909 NA miRNA
MIMAT0004911 NA miRNA
MIMAT0004921 NA miRNA
MIMAT0004945 NA miRNA
MIMAT0004947 NA miRNA
MIMAT0004949 NA miRNA
MIMAT0004952 NA miRNA
MIMAT0004953 NA miRNA
MIMAT0004954 NA miRNA
MIMAT0004955 NA miRNA
MIMAT0004956 NA miRNA
MIMAT0004957 NA miRNA
MIMAT0004984_4 NA miRNA
MIMAT0004985 NA miRNA
MIMAT0005577 NA miRNA
MIMAT0005582 NA miRNA
MIMAT0005792_1 NA miRNA
MIMAT0005793_1 NA miRNA
MIMAT0005794 NA miRNA
MIMAT0005796 NA miRNA
MIMAT0005797 NA miRNA
MIMAT0005825 NA miRNA
MIMAT0005874 NA miRNA
MIMAT0005878 NA miRNA
MIMAT0005882 NA miRNA
MIMAT0005892 NA miRNA
MIMAT0005919_1 NA miRNA
MIMAT0005933 NA miRNA
MIMAT0005943 NA miRNA
MIMAT0005948 NA miRNA
MIMAT0005951 NA miRNA
MIMAT0006764_1 NA miRNA
MIMAT0006789 NA miRNA
MIMAT0007881 NA miRNA
MIMAT0009198 NA miRNA
MIMAT0010133 NA miRNA
MIMAT0010214 NA miRNA
MIMAT0014982 NA miRNA
MIMAT0014994_1 NA miRNA

MIMAT0015032_1 NA miRNA
MIMAT0015050 NA miRNA
MIMAT0015069 NA miRNA
MIMAT0016895 NA miRNA
MIMAT0016925 NA miRNA
MIMAT0017392 NA miRNA
MIMAT0017950 NA miRNA
MIMAT0017991 NA miRNA
MIMAT0017994 NA miRNA
MIMAT0018104 NA miRNA
MIMAT0018116_1 NA miRNA
MIMAT0018356 NA miRNA
MIMAT0018443 NA miRNA
MIMAT0018965 NA miRNA
MIMAT0019211_1 NA miRNA
MIMAT0019727 NA miRNA
MIMAT0019731 NA miRNA
MIMAT0019734 NA miRNA
MIMAT0019776 NA miRNA
MIMAT0019855 NA miRNA
MIMAT0019856 NA miRNA
MIMAT0020924 NA miRNA
MIMAT0020925 NA miRNA
MIMAT0021043 NA miRNA
MIMAT0022472 NA miRNA
MIMAT0022474 NA miRNA
MIMAT0022697 NA miRNA
MIMAT0022705 NA miRNA
MIMAT0022709 NA miRNA
MIMAT0022711 NA miRNA
MIMAT0022714 NA miRNA
MIMAT0022724 NA miRNA
MIMAT0022726 NA miRNA
MIMAT0022727 NA miRNA
MIMAT0022838 NA miRNA
MIMAT0023712 NA miRNA
MIMAT0025479_3 NA miRNA
MIMAT0027587 NA miRNA
MIMAT0027604 NA miRNA
MIMAT0027682 NA miRNA
MIMAT0030021 NA miRNA
MIMAT0030413 NA miRNA
MIMAT0030414 NA miRNA
MIMAT0031179 NA miRNA
rRNA45S NA NA
trna1−ArgCCG_1 NA trna
trna1−AsnGTT_1 NA trna
trna1−GlnCTG_1 NA trna
trna1−GlyCCC_1 NA trna
trna1−HisGTG_1 NA trna
trna1−PheGAA_1 NA trna
trna1−SeC(e)TCA_1 NA trna
trna1−TrpCCA_1 NA trna
trna10−AlaCGC_1 NA trna

trna10-AspGTC_1 NA trna
trna10-GluCTC_1 NA trna
trna10-GlyTCC_1 NA trna
trna10-IleAAT_1 NA trna
trna10-LysCTT_1 NA trna
trna10-MetCAT_1 NA trna
trna10-SerGCT_1 NA trna
trna10-ValCAC_1 NA trna
trna100-LeuCAA_1 NA trna
trna101-AlaAGC_1 NA trna
trna102-AlaAGC_1 NA trna
trna103-PheGAA_1 NA trna
trna106-PheGAA_1 NA trna
trna109-PheGAA_1 NA trna
trna11-ArgACG_1 NA trna
trna11-GluTTC_1 NA trna
trna11-IleAAT_1 NA trna
trna11-LysCTT_1 NA trna
trna11-LysTTT_1 NA trna
trna11-PheGAA_1 NA trna
trna11-ProAGG_1 NA trna
trna110-AlaTGC_1 NA trna
trna111-HisGTG_1 NA trna
trna113-AlaTGC_1 NA trna
trna118-LysTTT_1 NA trna
trna119-AlaCGC_1 NA trna
trna12-AspGTC_1 NA trna
trna12-ProAGG_1 NA trna
trna12-ProTGG_1 NA trna
trna12-TrpCCA_1 NA trna
trna12-ValAAC_1 NA trna
trna120-AlaAGC_1 NA trna
trna121-ThrCGT_1 NA trna
trna123-SerGCT_1 NA trna
trna125-ThrCGT_1 NA trna
trna127-ThrTGT_1 NA trna
trna128-GlyGCC_1 NA trna
trna128-LysCTT_1 NA trna
trna129-MetCAT_1 NA trna
trna13-AlaCGC_1 NA trna
trna13-AlaTGC_1 NA trna
trna13-LysCTT_2 NA trna
trna13-LysCTT_3 NA trna
trna13-PheGAA_1 NA trna
trna13-ValCAC_1 NA trna
trna130-GlnTTG_1 NA trna
trna132-ValAAC_1 NA trna
trna133-GlyCCC_1 NA trna
trna134-GluTTC_1 NA trna
trna135-ThrAGT_1 NA trna
trna136-ValAAC_1 NA trna
trna138-ArgACG_1 NA trna
trna139-ValAAC_1 NA trna
trna14-LysTTT_1 NA trna

trna14-PheGAA_1 NA trna
trna14-ProTGG_1 NA trna
trna14-ThrCGT_1 NA trna
trna14-TyrGTA_1 NA trna
trna140-LeuCAA_1 NA trna
trna141-LeuCAA_1 NA trna
trna142-MetCAT_1 NA trna
trna143-LysTTT_1 NA trna
trna144-AspGTC_1 NA trna
trna146-GlnCTG_1 NA trna
trna149-LysTTT_1 NA trna
trna15-CysGCA_1 NA trna
trna15-PheGAA_1 NA trna
trna15-ThrCGT_1 NA trna
trna15-TyrGTA_1 NA trna
trna15-ValAAC_1 NA trna
trna150-MetCAT_1 NA trna
trna152-ValCAC_1 NA trna
trna156-ArgACG_1 NA trna
trna16-GlnTTG_2 NA trna
trna16-HisGTG_1 NA trna
trna16-ValTAC_1 NA trna
trna167-ThrAGT_1 NA trna
trna169-MetCAT_1 NA trna
trna17-GluTTC_1 NA trna
trna17-LeuCAG_1 NA trna
trna17-ValTAC_1 NA trna
trna171-MetCAT_1 NA trna
trna173-GlnTTG_1 NA trna
trna174-GlnTTG_1 NA trna
trna18-ArgCCT_1 NA trna
trna18-GluCTC_1 NA trna
trna18-GlyGCC_1 NA trna
trna18-ValCAC_1 NA trna
trna19-ArgTCG_1 NA trna
trna19-GlyGCC_1 NA trna
trna19-GlyGCC_2 NA trna
trna2-ArgCCT_1 NA trna
trna2-GlyGCC_1 NA trna
trna2-GlyTCC_2 NA trna
trna2-LysCTT_1 NA trna
trna2-LysTTT_2 NA trna
trna2-MetCAT_1 NA trna
trna2-ProAGG_1 NA trna
trna2-SerCGA_1 NA trna
trna2-SerTGA_1 NA trna
trna2-TyrGTA_1 NA trna
trna2-ValAAC_1 NA trna
trna2-ValCAC_1 NA trna
trna20-GluTTC_1 NA trna
trna20-MetCAT_2 NA trna
trna21-ArgCCT_1 NA trna
trna21-HisGTG_1 NA trna
trna22-AspGTC_1 NA trna

trna22-ProAGG_1 NA trna
trna23-ArgCCG_1 NA trna
trna23-LysTTT_1 NA trna
trna23-ProAGG_1 NA trna
trna24-GlyGCC_1 NA trna
trna25-GluCTC_1 NA trna
trna25-GlyGCC_1 NA trna
trna26-AsnGTT_1 NA trna
trna26-LeuCAG_1 NA trna
trna27-GlyCCC_1 NA trna
trna28-IleAAT_1 NA trna
trna28-ProTGG_1 NA trna
trna29-ProAGG_1 NA trna
trna3-AlaAGC_1 NA trna
trna3-ArgCCT_1 NA trna
trna3-ArgTCT_1 NA trna
trna3-CysGCA_1 NA trna
trna3-CysGCA_2 NA trna
trna3-GlnCTG_1 NA trna
trna3-GluTTC_1 NA trna
trna3-ProTGG_1 NA trna
trna3-ProTGG_2 NA trna
trna30-ProCGG_1 NA trna
trna31-ProTGG_1 NA trna
trna31-SerGCT_1 NA trna
trna32-LysCTT_1 NA trna
trna32-MetCAT_1 NA trna
trna33-HisGTG_1 NA trna
trna34-GlyCCC_1 NA trna
trna34-LeuCAG_1 NA trna
trna34-ThrAGT_1 NA trna
trna35-GlyGCC_1 NA trna
trna35-SerAGA_1 NA trna
trna36-ArgACG_1 NA trna
trna36-LeuCAG_1 NA trna
trna36-ThrAGT_1 NA trna
trna37-GlyGCC_1 NA trna
trna37-ProCGG_1 NA trna
trna37-ValAAC_1 NA trna
trna38-AspGTC_1 NA trna
trna38-LeuCAG_1 NA trna
trna39-GlyGCC_1 NA trna
trna4-ArgTCG_1 NA trna
trna4-ArgTCT_1 NA trna
trna4-ArgTCT_2 NA trna
trna4-AspGTC_1 NA trna
trna4-GlyCCC_1 NA trna
trna4-LysCTT_1 NA trna
trna4-ProAGG_1 NA trna
trna4-ThrAGT_1 NA trna
trna4-ThrTGT_1 NA trna
trna4-TyrGTA_1 NA trna
trna4-ValAAC_1 NA trna
trna4-ValTAC_1 NA trna

trna40-LeuCAG_1 NA trna
trna40-ThrAGT_1 NA trna
trna41-GlyGCC_1 NA trna
trna41-SerCGA_1 NA trna
trna42-GlnCTG_1 NA trna
trna42-LeuCAG_1 NA trna
trna43-SerGCT_1 NA trna
trna44-SerAGA_1 NA trna
trna45-AspGTC_1 NA trna
trna45-GlyTCC_1 NA trna
trna46-SerAGA_1 NA trna
trna47-SerAGA_1 NA trna
trna48-AspGTC_1 NA trna
trna49-GlnCTG_1 NA trna
trna5-AspGTC_1 NA trna
trna5-GluTTC_1 NA trna
trna5-GluTTC_2 NA trna
trna5-GlyGCC_1 NA trna
trna5-IleTAT_1 NA trna
trna5-LysTTT_1 NA trna
trna5-SerAGA_1 NA trna
trna5-TyrGTA_1 NA trna
trna5-TyrGTA_2 NA trna
trna5-ValAAC_1 NA trna
trna51-SerTGA_1 NA trna
trna52-ArgTCT_1 NA trna
trna52-ProCGG_1 NA trna
trna54-LysTTT_1 NA trna
trna55-IleTAT_1 NA trna
trna57-IleAAT_1 NA trna
trna58-LeuCAA_1 NA trna
trna59-GluCTC_1 NA trna
trna59-IleAAT_1 NA trna
trna6-AlaAGC_1 NA trna
trna6-ArgACG_1 NA trna
trna6-AspGTC_1 NA trna
trna6-ProCGG_1 NA trna
trna6-ProTGG_1 NA trna
trna6-TrpCCA_1 NA trna
trna6-TrpCCA_2 NA trna
trna6-ValCAC_1 NA trna
trna6-ValTAC_1 NA trna
trna61-MetCAT_1 NA trna
trna62-LysTTT_1 NA trna
trna62-SerGCT_1 NA trna
trna64-GlnTTG_1 NA trna
trna64-GluTTC_1 NA trna
trna65-AlaAGC_1 NA trna
trna65-ProAGG_1 NA trna
trna66-AlaTGC_1 NA trna
trna67-LeuCAG_1 NA trna
trna68-AlaAGC_1 NA trna
trna68-GlyGCC_1 NA trna
trna69-AspGTC_1 NA trna

trna7-ArgACG_1 NA trna
trna7-CysGCA_1 NA trna
trna7-GlnCTG_1 NA trna
trna7-HisGTG_1 NA trna
trna7-LeuCAG_1 NA trna
trna7-LysCTT_1 NA trna
trna7-SerGCT_1 NA trna
trna70-GlyTCC_1 NA trna
trna71-GluCTC_1 NA trna
trna72-AspGTC_1 NA trna
trna73-ArgCCG_1 NA trna
trna73-GlyTCC_1 NA trna
trna74-GluCTC_1 NA trna
trna74-LeuCAA_1 NA trna
trna75-AspGTC_1 NA trna
trna75-MetCAT_1 NA trna
trna76-GlyTCC_1 NA trna
trna76-LysTTT_1 NA trna
trna77-GluCTC_1 NA trna
trna77-GluCTC_2 NA trna
trna78-AspGTC_1 NA trna
trna79-GlyTCC_1 NA trna
trna8-AlaTGC_1 NA trna
trna8-AlaTGC_2 NA trna
trna8-ArgACG_1 NA trna
trna8-CysGCA_1 NA trna
trna8-HisGTG_1 NA trna
trna8-ProTGG_1 NA trna
trna8-SeC(e)TCA_1 NA trna
trna8-SerGCT_1 NA trna
trna8-ThrAGT_1 NA trna
trna80-GluCTC_1 NA trna
trna80-IleAAT_1 NA trna
trna81-AspGTC_1 NA trna
trna83-LeuTAA_1 NA trna
trna84-GluTTC_1 NA trna
trna85-ValCAC_1 NA trna
trna87-GluCTC_1 NA trna
trna88-PheGAA_1 NA trna
trna9-ArgTCT_1 NA trna
trna9-HisGTG_1 NA trna
trna9-IleAAT_2 NA trna
trna9-LysCTT_1 NA trna
trna9-ProAGG_1 NA trna
trna9-ProAGG_2 NA trna
trna9-ValCAC_1 NA trna
trna90-ValCAC_1 NA trna
trna91-GlyCCC_1 NA trna
trna94-GluTTC_1 NA trna
trna96-PheGAA_1 NA trna
trna98-ValCAC_1 NA trna
trna99-GlnCTG_1 NA trna
UniSP100 NA NA
UniSP103 NA NA

UniSP104 NA NA
UniSP105 NA NA
UniSP107 NA NA
UniSP109 NA NA
UniSP111 NA NA
UniSP116 NA NA
UniSP118 NA NA
UniSP119 NA NA
UniSP121 NA NA
UniSP123 NA NA
UniSP127 NA NA
UniSP132 NA NA
UniSP133 NA NA
UniSP136 NA NA
UniSP137 NA NA
UniSP138 NA NA
UniSP139 NA NA
UniSP140 NA NA
UniSP142 NA NA
UniSP143 NA NA
UniSP144 NA NA
UniSP145 NA NA
UniSP151 NA NA
