# Supplementary material for: Integration of small RNAs from plasma and cerebrospinal fluid for classification of multiple sclerosis
Source: Front Genet. 2022 Nov 17;13:1042483. doi: 10.3389/fgene.2022.1042483 (PMC9713411; doi:10.3389/fgene.2022.1042483)
Supplement: Supplementary file 1 [file DataSheet13.PDF]

chr5 42138
chr5 101446
chr5 217684
chr5 217725
chr5 217766
chr5 217803
chr5 217922
chr5 217931
chr5 217936
chr5 220052
chr5 220193
chr5 220266
chr5 271611
chr5 271642
chr5 271651
chr5 271657
chr5 271663
chr5 271680
chr5 271683
chr5 271692
chr5 271706
chr5 271708
chr5 271726
chr5 271739
chr5 271744
chr5 271756
chr5 271777
chr5 271779
chr5 271782
chr5 271788
chr5 271810
chr5 271825
chr5 271836
chr5 271840
chr5 271851
chr5 271857
chr5 271860
chr5 271862
chr5 271865
chr5 271871
chr5 272104
chr5 272107
chr5 272132
chr5 272146
chr5 272152
chr5 272154
chr5 272161
chr5 272187
chr5 272301
chr5 272309
chr5 272330
chr5 272333
chr5 272350
chr5 272361

chr5 272369
chr5 272446
chr5 474185
chr5 474212
chr5 474235
chr5 474237
chr5 474239
chr5 474241
chr5 474246
chr5 474270
chr5 474285
chr5 474337
chr5 474339
chr5 474341
chr5 474343
chr5 474348
chr5 474373
chr5 474388
chr5 474438
chr5 474440
chr5 474442
chr5 474444
chr5 474449
chr5 474473
chr5 474488
chr5 474540
chr5 474542
chr5 474544
chr5 474546
chr5 474551
chr5 474576
chr5 474591
chr5 474637
chr5 474639
chr5 474641
chr5 474643
chr5 474648
chr5 474673
chr5 474688
chr5 474703
chr5 474738
chr5 474740
chr5 474742
chr5 474744
chr5 474749
chr5 474774
chr5 474789
chr5 474808
chr5 474810
chr5 474812
chr5 474814
chr5 474819
chr5 474844
chr5 553754

chr5 690177
chr5 690195
chr5 690996
chr5 691002
chr5 691004
chr5 691046
chr5 691057
chr5 691060
chr5 691081
chr5 691114
chr5 691122
chr5 691143
chr5 691156
chr5 691161
chr5 691222
chr5 691226
chr5 691237
chr5 691239
chr5 691257
chr5 691260
chr5 691309
chr5 691421
chr5 691435
chr5 691444
chr5 691465
chr5 691521
chr5 691540
chr5 691930
chr5 692530
chr5 692540
chr5 692567
chr5 692573
chr5 692591
chr5 692913
chr5 693021
chr5 693023
chr5 693027
chr5 693030
chr5 694096
chr5 694146
chr5 694157
chr5 694177
chr5 694193
chr5 694197
chr5 694207
chr5 694215
chr5 694242
chr5 694248
chr5 694261
chr5 694323
chr5 694328
chr5 694420
chr5 694462
chr5 783055

chr5 783061
chr5 783063
chr5 783105
chr5 783116
chr5 783119
chr5 783173
chr5 783181
chr5 783202
chr5 783215
chr5 783220
chr5 783228
chr5 783250
chr5 783259
chr5 783272
chr5 783281
chr5 783285
chr5 783296
chr5 783298
chr5 783316
chr5 783319
chr5 783368
chr5 783480
chr5 783494
chr5 783503
chr5 783524
chr5 783545
chr5 783580
chr5 783599
chr5 783890
chr5 783911
chr5 783921
chr5 783948
chr5 783954
chr5 783960
chr5 783966
chr5 783972
chr5 783999
chr5 784003
chr5 784020
chr5 784033
chr5 784039
chr5 784050
chr5 784061
chr5 784063
chr5 784081
chr5 785585
chr5 785593
chr5 785655
chr5 785660
chr5 785702
chr5 785717
chr5 785752
chr5 785794
chr5 789202

chr5 789264
chr5 984246
chr5 984390
chr5 1191077
chr5 1191081
chr5 1191162
chr5 1191189
chr5 1191197
chr5 1191491
chr5 1191512
chr5 1191528
chr5 1191565
chr5 1191600
chr5 1191636
chr5 1191657
chr5 1191673
chr5 1191694
chr5 1191710
chr5 1191731
chr5 1191768
chr5 1191879
chr5 1191895
chr5 1191916
chr5 1191953
chr5 1191969
chr5 1192007
chr5 1192041
chr5 1192606
chr5 1192705
chr5 1192761
chr5 1192812
chr5 1192834
chr5 1192867
chr5 1192901
chr5 1192928
chr5 1193013
chr5 1193050
chr5 1193088
chr5 1536180
chr5 1536201
chr5 1536349
chr5 1536376
chr5 1536384
chr5 1536434
chr5 1536458
chr5 1536479
chr5 1536485
chr5 1536647
chr5 1537036
chr5 1711909
chr5 1711939
chr5 1712786
chr5 1712918
chr5 1712964

chr5 1932088
chr5 1932157
chr5 1932283
chr5 1968664
chr5 1968706
chr5 2312291
chr5 2312315
chr5 2312329
chr5 2741938
chr5 2741946
chr5 2742052
chr5 2742106
chr5 2742264
chr5 2742458
chr5 2742509
chr5 2742540
chr5 2742547
chr5 2742874
chr5 2742948
chr5 2742954
chr5 2742956
chr5 2742960
chr5 2742967
chr5 2742998
chr5 2743010
chr5 2744073
chr5 2744137
chr5 2744179
chr5 2744214
chr5 2744308
chr5 2744467
chr5 2744495
chr5 2746854
chr5 2746919
chr5 2746961
chr5 2747036
chr5 2747041
chr5 3117352
chr5 3117495
chr5 3117498
chr5 3117630
chr5 3119015
chr5 3119050
chr5 3119172
chr5 3119308
chr5 3119488
chr5 3174670
chr5 3427410
chr5 3475386
chr5 3475522
chr5 3492964
chr5 3493190
chr5 3589579
chr5 3589588

chr5 3701842
chr5 3701899
chr5 3701938
chr5 3702036
chr5 3702316
chr5 3828042
chr5 3828438
chr5 3886617
chr5 3886646
chr5 3886773
chr5 3887178
chr5 3887191
chr5 3887199
chr5 3887262
chr5 3979830
chr5 4249681
chr5 4840119
chr5 5035836
chr5 5055874
chr5 5056252
chr5 5141086
chr5 5141115
chr5 5141133
chr5 5141147
chr5 5141200
chr5 5141234
chr5 5141278
chr5 5141339
chr5 5141493
chr5 5141504
chr5 5141593
chr5 5172105
chr5 5172203
chr5 5421568
chr5 5421697
chr5 5421754
chr5 5421766
chr5 5421792
chr5 5421795
chr5 5421820
chr5 5421841
chr5 5421866
chr5 5421875
chr5 5421901
chr5 5421907
chr5 5421932
chr5 5421948
chr5 5422013
chr5 5422017
chr5 5422042
chr5 5422053
chr5 5422059
chr5 5422083
chr5 5422086

chr5 5422090
chr5 5422107
chr5 5422109
chr5 5422112
chr5 5422116
chr5 5422119
chr5 5422133
chr5 5422140
chr5 5422184
chr5 5422203
chr5 5422214
chr5 5422217
chr5 5422222
chr5 5422226
chr5 5422233
chr5 5422237
chr5 5422242
chr5 5422247
chr5 5422249
chr5 5422252
chr5 5423476
chr5 5423505
chr5 5423613
chr5 5423616
chr5 5481017
chr5 5481387
chr5 5481393
chr5 5481410
chr5 5898609
chr5 6715391
chr5 6715406
chr5 6715425
chr5 6715474
chr5 6715507
chr5 6715763
chr5 6715844
chr5 6729524
chr5 6729532
chr5 6729549
chr5 6729551
chr5 6729560
chr5 6729582
chr5 6729636
chr5 7172213
chr5 7172348
chr5 7172612
chr5 7172641
chr5 7172674
chr5 7172958
chr5 7363894
chr5 7809995
chr5 7869913
chr5 7869938
chr5 7869947

chr5 7869959
chr5 7870065
chr5 7870093
chr5 8019305
chr5 8019377
chr5 8019956
chr5 8020047
chr5 8020163
chr5 8020186
chr5 8020189
chr5 8020365
chr5 8020464
chr5 8333968
chr5 8334041
chr5 8376679
chr5 8376700
chr5 8377555
chr5 8378015
chr5 9019239
chr5 9459618
chr5 10055577
chr5 10055688
chr5 10249463
chr5 10249486
chr5 10249536
chr5 10249547
chr5 10249594
chr5 10353352
chr5 10353362
chr5 10353364
chr5 10353367
chr5 10353381
chr5 10353385
chr5 10353388
chr5 10353397
chr5 10353428
chr5 10353435
chr5 10353459
chr5 10354833
chr5 10354842
chr5 10354852
chr5 10354870
chr5 10354873
chr5 10354934
chr5 10355009
chr5 10355011
chr5 10355022
chr5 10355081
chr5 10355100
chr5 10355214
chr5 10355226
chr5 10355249
chr5 10355267
chr5 10355298

chr5 10355393
chr5 10355397
chr5 10355489
chr5 10355493
chr5 10355500
chr5 10913176
chr5 10913661
chr5 10940240
chr5 11234899
chr5 11234955
chr5 11253673
chr5 11254043
chr5 11385779
chr5 11385781
chr5 11385816
chr5 11385847
chr5 11385852
chr5 11386027
chr5 11386142
chr5 11386207
chr5 11386344
chr5 11386413
chr5 11587295
chr5 11587784
chr5 11588306
chr5 11588311
chr5 11588705
chr5 11588707
chr5 11642849
chr5 11642874
chr5 11642965
chr5 11642980
chr5 11643146
chr5 11643188
chr5 11643341
chr5 11933324
chr5 12002288
chr5 12002314
chr5 12044865
chr5 12044950
chr5 12045372
chr5 12552058
chr5 12576588
chr5 12634210
chr5 12644637
chr5 12646185
chr5 12646188
chr5 12763519
chr5 12763645
chr5 12763843
chr5 12791819
chr5 12791877
chr5 12792042
chr5 12792841

chr5 12793455
chr5 13766402
chr5 13766512
chr5 13766612
chr5 13766702
chr5 13988187
chr5 14264428
chr5 14264687
chr5 14264721
chr5 14264731
chr5 14264735
chr5 14264739
chr5 14264767
chr5 14264774
chr5 14264788
chr5 14264793
chr5 14264805
chr5 14264820
chr5 14264822
chr5 14264826
chr5 14264832
chr5 14264837
chr5 14264843
chr5 14264894
chr5 14264898
chr5 14264900
chr5 14264902
chr5 14264904
chr5 14264939
chr5 14265113
chr5 14265147
chr5 14265235
chr5 14265252
chr5 14356516
chr5 14447905
chr5 14559948
chr5 14560072
chr5 14560183
chr5 14560225
chr5 14560250
chr5 14560275
chr5 14595090
chr5 14595381
chr5 14648718
chr5 14665483
chr5 14665547
chr5 14665550
chr5 14665593
chr5 14665595
chr5 14665616
chr5 14665952
chr5 14666070
chr5 14666075
chr5 14666091

chr5 14666132
chr5 14666156
chr5 14666172
chr5 14675921
chr5 14675973
chr5 14676048
chr5 14676109
chr5 14676134
chr5 14676298
chr5 14676351
chr5 14706525
chr5 14727539
chr5 14727548
chr5 14808598
chr5 14809077
chr5 14809503
chr5 14809509
chr5 14809527
chr5 14809846
chr5 14809851
chr5 14809867
chr5 14809959
chr5 14809985
chr5 14810050
chr5 14870101
chr5 14870138
chr5 14870140
chr5 14870304
chr5 14870311
chr5 14870332
chr5 14870415
chr5 14870474
chr5 14870478
chr5 14870557
chr5 14870568
chr5 14870581
chr5 14870601
chr5 14870640
chr5 14870657
chr5 14870674
chr5 14870690
chr5 14870708
chr5 14870716
chr5 14870850
chr5 14870869
chr5 14870898
chr5 14870944
chr5 14870970
chr5 14871061
chr5 14871081
chr5 14871233
chr5 14871263
chr5 14871283
chr5 14871289

chr5 14871295
chr5 14871299
chr5 14925815
chr5 14925855
chr5 14925861
chr5 14925945
chr5 14992891
chr5 14992969
chr5 14992985
chr5 14993029
chr5 14993031
chr5 14993041
chr5 14993066
chr5 14993069
chr5 14993108
chr5 14993134
chr5 14993142
chr5 14993150
chr5 14993164
chr5 14993172
chr5 14993174
chr5 14993198
chr5 14993201
chr5 14993203
chr5 15275488
chr5 15275814
chr5 15402902
chr5 15403291
chr5 15748401
chr5 15750073
chr5 15750727
chr5 15750736
chr5 15824778
chr5 15906605
chr5 15906965
chr5 15906978
chr5 15907735
chr5 16464904
chr5 16464969
chr5 16465021
chr5 16465041
chr5 16465071
chr5 16465105
chr5 16465130
chr5 16465147
chr5 16465154
chr5 16465158
chr5 16465169
chr5 16465185
chr5 16934172
chr5 16934192
chr5 16934200
chr5 16934202
chr5 16934263

chr5 16934307
chr5 16934411
chr5 16934442
chr5 16934459
chr5 16934483
chr5 16934516
chr5 16934555
chr5 16934559
chr5 16934822
chr5 16934876
chr5 16934912
chr5 16935043
chr5 16935099
chr5 16935118
chr5 16935155
chr5 16935172
chr5 16965455
chr5 16965489
chr5 17028954
chr5 17029094
chr5 17215977
chr5 17216027
chr5 17607067
chr5 17780905
chr5 18032656
chr5 18111981
chr5 18112050
chr5 18112214
chr5 18112281
chr5 18112366
chr5 18112579
chr5 18394673
chr5 18394940
chr5 18698517
chr5 18698523
chr5 18698562
chr5 18698575
chr5 18698666
chr5 18698677
chr5 18698699
chr5 18698705
chr5 18699006
chr5 18699018
chr5 18699037
chr5 18699338
chr5 18745175
chr5 18745187
chr5 18745403
chr5 18745519
chr5 18745697
chr5 18745713
chr5 18745736
chr5 18745752
chr5 18745872

chr5 18745949
chr5 18746014
chr5 18746091
chr5 18746120
chr5 18746142
chr5 18746169
chr5 18746179
chr5 18746183
chr5 18746219
chr5 18746236
chr5 18803692
chr5 19066047
chr5 19066249
chr5 19066301
chr5 19143773
chr5 19143941
chr5 19144072
chr5 19144258
chr5 19363025
chr5 19363058
chr5 19406725
chr5 19407026
chr5 19430131
chr5 19772079
chr5 19772101
chr5 19773206
chr5 20121882
chr5 20121959
chr5 20122188
chr5 20243575
chr5 20244185
chr5 20244396
chr5 20244405
chr5 20245269
chr5 20245445
chr5 20245723
chr5 20248435
chr5 20248749
chr5 20248753
chr5 20335087
chr5 21085589
chr5 21336253
chr5 21336370
chr5 21459489
chr5 21459491
chr5 21459544
chr5 21459547
chr5 21459551
chr5 21459566
chr5 21459580
chr5 21459600
chr5 21459610
chr5 21459620
chr5 21459625

chr5 21459639
chr5 21459642
chr5 21459656
chr5 21459691
chr5 21459696
chr5 21459702
chr5 21459708
chr5 21459717
chr5 21459735
chr5 21459742
chr5 21459798
chr5 21459818
chr5 21460163
chr5 21460239
chr5 21803379
chr5 21803381
chr5 21990289
chr5 22200398
chr5 22211339
chr5 22211540
chr5 22212100
chr5 22212350
chr5 22212353
chr5 22212479
chr5 22212522
chr5 22212536
chr5 22212548
chr5 22212568
chr5 22212676
chr5 22285283
chr5 22285331
chr5 22285617
chr5 22327042
chr5 22327099
chr5 22327130
chr5 22327199
chr5 22467557
chr5 22667890
chr5 22852280
chr5 22852618
chr5 22852646
chr5 22852829
chr5 22852841
chr5 22852875
chr5 22852898
chr5 22852940
chr5 22852957
chr5 22853011
chr5 22853023
chr5 22853025
chr5 22853131
chr5 22853133
chr5 22853206
chr5 22853235

chr5 22853350
chr5 22853355
chr5 22853375
chr5 22853378
chr5 22853382
chr5 22853385
chr5 22853425
chr5 22853446
chr5 22853531
chr5 22853560
chr5 22853594
chr5 22853606
chr5 22853621
chr5 22853654
chr5 22853755
chr5 22853792
chr5 22853831
chr5 22897430
chr5 22897545
chr5 23208130
chr5 23208248
chr5 23208744
chr5 23208893
chr5 23209000
chr5 23305994
chr5 23764443
chr5 23765320
chr5 23765442
chr5 23766105
chr5 23766309
chr5 23866089
chr5 24360605
chr5 24489818
chr5 24489988
chr5 24490103
chr5 24582741
chr5 24644400
chr5 24644683
chr5 24644769
chr5 24644778
chr5 24644784
chr5 24644879
chr5 24644915
chr5 24645001
chr5 25132902
chr5 25132958
chr5 25133747
chr5 25134495
chr5 25201692
chr5 25202480
chr5 25615960
chr5 25616685
chr5 25763703
chr5 25847019

chr5 25847074
chr5 25847321
chr5 25847867
chr5 25848013
chr5 25854037
chr5 25854243
chr5 25856098
chr5 26168801
chr5 26168867
chr5 26329642
chr5 26329671
chr5 26329732
chr5 26329985
chr5 26330559
chr5 26590634
chr5 26591068
chr5 26784137
chr5 26784514
chr5 26810511
chr5 26810532
chr5 27062300
chr5 27097424
chr5 27097917
chr5 27097981
chr5 27098014
chr5 27123788
chr5 27213541
chr5 27213603
chr5 27214630
chr5 27214897
chr5 27215098
chr5 27215110
chr5 27219347
chr5 27219464
chr5 27219806
chr5 27219815
chr5 27219833
chr5 27266525
chr5 27283221
chr5 27283537
chr5 27284602
chr5 27290624
chr5 27579052
chr5 27813781
chr5 27813979
chr5 27814680
chr5 27814690
chr5 27814891
chr5 27814935
chr5 27815090
chr5 28406723
chr5 28634923
chr5 28635803
chr5 28635923

chr5 28851766
chr5 29124722
chr5 29124763
chr5 29170267
chr5 29170399
chr5 29170912
chr5 29171242
chr5 29292689
chr5 29292899
chr5 29293348
chr5 29293360
chr5 29465907
chr5 29468797
chr5 29931537
chr5 30045042
chr5 30045323
chr5 30045391
chr5 30045794
chr5 30083850
chr5 30107530
chr5 30107711
chr5 30692905
chr5 30692977
chr5 30693455
chr5 31018019
chr5 31851326
chr5 32138961
chr5 32139006
chr5 32139012
chr5 32139020
chr5 32139029
chr5 32139148
chr5 32159855
chr5 32172462
chr5 32172471
chr5 32172492
chr5 32172523
chr5 32172560
chr5 32172563
chr5 32172567
chr5 32172579
chr5 32172587
chr5 32172609
chr5 32172619
chr5 32172626
chr5 32172649
chr5 32172651
chr5 32172673
chr5 32172689
chr5 32172728
chr5 32172740
chr5 32172775
chr5 32173174
chr5 32211402

chr5 32211485
chr5 32211534
chr5 32280684
chr5 32280780
chr5 32280844
chr5 32280965
chr5 32442900
chr5 32442946
chr5 32442971
chr5 32442996
chr5 32443126
chr5 32443203
chr5 32443212
chr5 32443218
chr5 32443359
chr5 32443420
chr5 32443437
chr5 32542859
chr5 32586024
chr5 32586044
chr5 32586100
chr5 32586105
chr5 32586121
chr5 32586152
chr5 32586208
chr5 32586212
chr5 32586216
chr5 32586792
chr5 32586801
chr5 32587076
chr5 32587123
chr5 32613177
chr5 32613203
chr5 32613217
chr5 32613236
chr5 32613265
chr5 32613284
chr5 32613299
chr5 32613385
chr5 32613460
chr5 32693994
chr5 32707114
chr5 32707660
chr5 32707930
chr5 32708758
chr5 32708931
chr5 32708936
chr5 32708965
chr5 32709011
chr5 32709034
chr5 32709045
chr5 32709067
chr5 32709094
chr5 32709125

chr5 32709134
chr5 32709160
chr5 32709231
chr5 32709315
chr5 32709348
chr5 32709443
chr5 32709601
chr5 32709639
chr5 32710042
chr5 32710102
chr5 32710107
chr5 32710110
chr5 32710112
chr5 32710114
chr5 32710117
chr5 32710125
chr5 32710127
chr5 32710144
chr5 32710160
chr5 32710170
chr5 32710180
chr5 32713434
chr5 32713459
chr5 32713472
chr5 32713480
chr5 32713483
chr5 32713521
chr5 32713535
chr5 32713639
chr5 32713654
chr5 32713657
chr5 32727026
chr5 33160669
chr5 33161134
chr5 33161149
chr5 33161633
chr5 33161679
chr5 33299765
chr5 33299984
chr5 33300141
chr5 33300149
chr5 33300195
chr5 33300408
chr5 33300466
chr5 33300673
chr5 33300782
chr5 33441478
chr5 33441498
chr5 33441582
chr5 33441590
chr5 33441642
chr5 33441657
chr5 33441693
chr5 33441846

chr5 33441858
chr5 33441890
chr5 33441908
chr5 33763155
chr5 33850040
chr5 33850052
chr5 33850081
chr5 33850123
chr5 33850131
chr5 33850172
chr5 33850176
chr5 33850188
chr5 33850202
chr5 33934905
chr5 33997913
chr5 34008358
chr5 34008383
chr5 34008423
chr5 34008431
chr5 34008444
chr5 34008450
chr5 34008486
chr5 34008531
chr5 34008538
chr5 34008558
chr5 34008577
chr5 34008579
chr5 34008681
chr5 34008707
chr5 34008781
chr5 34008973
chr5 34244202
chr5 34244325
chr5 34244407
chr5 34244432
chr5 34244731
chr5 34244735
chr5 34244737
chr5 34244741
chr5 34244751
chr5 34244764
chr5 34244766
chr5 34244772
chr5 34244785
chr5 34244792
chr5 34244799
chr5 34244805
chr5 34244812
chr5 34244814
chr5 34244829
chr5 34244833
chr5 34244841
chr5 34244849
chr5 34244851

chr5 34244853
chr5 34244861
chr5 34244863
chr5 34244874
chr5 34244888
chr5 34244894
chr5 34244937
chr5 34244957
chr5 34244959
chr5 34244981
chr5 34244989
chr5 34244993
chr5 34244995
chr5 34244998
chr5 34245001
chr5 34245004
chr5 34245039
chr5 34245053
chr5 34245082
chr5 34245085
chr5 34245088
chr5 34245099
chr5 34245110
chr5 34245115
chr5 34245117
chr5 34245120
chr5 34245126
chr5 34245128
chr5 34245136
chr5 34245143
chr5 34245145
chr5 34245210
chr5 34245227
chr5 34245263
chr5 34245274
chr5 34245295
chr5 34245297
chr5 34245317
chr5 34245325
chr5 34245328
chr5 34245338
chr5 34245396
chr5 34245735
chr5 34246152
chr5 34246352
chr5 34291839
chr5 34291868
chr5 34291940
chr5 34486063
chr5 34486285
chr5 34486295
chr5 34655178
chr5 34655232
chr5 34655277

chr5 34655337
chr5 34655388
chr5 34655531
chr5 34655545
chr5 34655581
chr5 34655610
chr5 34655638
chr5 34655648
chr5 34655656
chr5 34655716
chr5 34655784
chr5 34655791
chr5 34916126
chr5 34916134
chr5 34916159
chr5 34916237
chr5 34916313
chr5 34924106
chr5 34924188
chr5 34928938
chr5 34929061
chr5 34929126
chr5 34929143
chr5 34929248
chr5 34929278
chr5 34929297
chr5 34929299
chr5 34929301
chr5 34929307
chr5 34929314
chr5 34929319
chr5 34929322
chr5 34929330
chr5 34929350
chr5 34929370
chr5 34929394
chr5 34929396
chr5 34929401
chr5 34929437
chr5 34929439
chr5 34929467
chr5 34930316
chr5 34930331
chr5 34930335
chr5 35575926
chr5 35617414
chr5 35617420
chr5 35617437
chr5 35617460
chr5 35617491
chr5 35617495
chr5 35617503
chr5 35617507
chr5 35617533

chr5 35617705
chr5 35617754
chr5 35618046
chr5 35618051
chr5 35618067
chr5 35618077
chr5 35618081
chr5 35618096
chr5 35618101
chr5 35618115
chr5 35618120
chr5 35618125
chr5 35618143
chr5 35618152
chr5 35618154
chr5 35618178
chr5 35618184
chr5 35618209
chr5 35618214
chr5 35618217
chr5 35618231
chr5 35618236
chr5 35618281
chr5 35618295
chr5 35618301
chr5 35618317
chr5 35618331
chr5 35618441
chr5 35618491
chr5 35672481
chr5 35672853
chr5 35777231
chr5 35785382
chr5 35796980
chr5 35833015
chr5 35833129
chr5 35833191
chr5 35833386
chr5 35833413
chr5 35834091
chr5 36150942
chr5 36151034
chr5 36606000
chr5 36606112
chr5 36606171
chr5 36606186
chr5 36606191
chr5 36606208
chr5 36606245
chr5 36606318
chr5 36606344
chr5 36606354
chr5 36606382
chr5 36606418

chr5 36606499
chr5 36606523
chr5 36606551
chr5 36606645
chr5 36606647
chr5 36606649
chr5 36606654
chr5 36606667
chr5 36606693
chr5 36606792
chr5 36606876
chr5 36606879
chr5 36606905
chr5 36606911
chr5 36606922
chr5 36606937
chr5 36606963
chr5 36606965
chr5 36606993
chr5 36607074
chr5 36607217
chr5 36607231
chr5 36607269
chr5 36607275
chr5 36607288
chr5 36607297
chr5 36607315
chr5 36607330
chr5 36607452
chr5 36607548
chr5 36607562
chr5 36607574
chr5 36607637
chr5 36625798
chr5 36690319
chr5 36690355
chr5 36690360
chr5 36690367
chr5 36690370
chr5 36690393
chr5 36690547
chr5 36690553
chr5 36690555
chr5 36690613
chr5 36690634
chr5 36690890
chr5 36690915
chr5 36874807
chr5 36874854
chr5 36874892
chr5 36875013
chr5 36875099
chr5 36875125
chr5 36875150

chr5 36875157
chr5 36875312
chr5 36875314
chr5 36875323
chr5 36875332
chr5 36875371
chr5 36875374
chr5 36875395
chr5 36875417
chr5 36875432
chr5 36878110
chr5 36878383
chr5 36878401
chr5 36878490
chr5 36878514
chr5 36878561
chr5 36878671
chr5 36878710
chr5 36878714
chr5 36878754
chr5 36878785
chr5 36878802
chr5 36878808
chr5 36878877
chr5 36878950
chr5 36878969
chr5 36878985
chr5 36879018
chr5 37111852
chr5 37111908
chr5 37112045
chr5 37112130
chr5 37112587
chr5 37197897
chr5 37198187
chr5 37198295
chr5 37198309
chr5 37369742
chr5 37369819
chr5 37369889
chr5 37370286
chr5 37370303
chr5 37370320
chr5 37370326
chr5 37370328
chr5 37370362
chr5 37370367
chr5 37370929
chr5 37370945
chr5 37371342
chr5 37371388
chr5 37371407
chr5 37371439
chr5 37371451

chr5 37371453
chr5 37371481
chr5 37371483
chr5 37371486
chr5 37371493
chr5 37371516
chr5 37371518
chr5 37379294
chr5 37379359
chr5 37379374
chr5 37379388
chr5 37379408
chr5 37379414
chr5 37379418
chr5 37379428
chr5 37379433
chr5 37379447
chr5 37379450
chr5 37379464
chr5 37379502
chr5 37379504
chr5 37379507
chr5 37379513
chr5 37379519
chr5 37379528
chr5 37379546
chr5 37379553
chr5 37379609
chr5 37379629
chr5 37379642
chr5 37379658
chr5 37672181
chr5 37672192
chr5 37672268
chr5 37672309
chr5 37672316
chr5 37672382
chr5 37672405
chr5 37672426
chr5 37672430
chr5 37672438
chr5 37672481
chr5 37672494
chr5 37722390
chr5 38170979
chr5 38171106
chr5 38172108
chr5 38172243
chr5 38172320
chr5 38172369
chr5 38634859
chr5 38743867
chr5 39072736
chr5 39072970

chr5 39073023
chr5 39073076
chr5 39073085
chr5 39073193
chr5 39073217
chr5 39073234
chr5 39073237
chr5 39075276
chr5 39075408
chr5 39075722
chr5 39177279
chr5 39177337
chr5 39177564
chr5 39177738
chr5 39177760
chr5 39177896
chr5 39178356
chr5 39178461
chr5 39178473
chr5 39178603
chr5 39178846
chr5 39178891
chr5 39178959
chr5 39178961
chr5 39178987
chr5 39179008
chr5 39179158
chr5 39187333
chr5 39187419
chr5 39187709
chr5 39188008
chr5 39188039
chr5 39188061
chr5 39188129
chr5 39189218
chr5 39189338
chr5 39191358
chr5 39191374
chr5 39191464
chr5 39191481
chr5 39191513
chr5 39191778
chr5 39192037
chr5 39192203
chr5 39195146
chr5 39195241
chr5 39195246
chr5 39197727
chr5 39197795
chr5 39197844
chr5 39198137
chr5 39198168
chr5 39198189
chr5 39198217

chr5 39198232
chr5 39198763
chr5 39198817
chr5 39198914
chr5 39199023
chr5 39199067
chr5 39199095
chr5 39199424
chr5 39199458
chr5 39199646
chr5 39200187
chr5 39200308
chr5 39200316
chr5 39200363
chr5 39200480
chr5 39200699
chr5 39200737
chr5 39200763
chr5 39200810
chr5 39200827
chr5 39201012
chr5 39201564
chr5 39201810
chr5 39201854
chr5 39201935
chr5 39202454
chr5 39202502
chr5 39202646
chr5 39202651
chr5 39202684
chr5 39202718
chr5 39202905
chr5 39202931
chr5 39202943
chr5 39202955
chr5 39203088
chr5 39203149
chr5 39203209
chr5 39203653
chr5 39204284
chr5 39207446
chr5 39207604
chr5 39207786
chr5 39207837
chr5 39207900
chr5 39208142
chr5 39208413
chr5 39208450
chr5 39208489
chr5 39208515
chr5 39208630
chr5 39208651
chr5 39208672
chr5 39208689

chr5 39208707
chr5 39208717
chr5 39211995
chr5 39212232
chr5 39212993
chr5 39213012
chr5 39217574
chr5 39217656
chr5 39217712
chr5 39217740
chr5 39217746
chr5 39217753
chr5 39217868
chr5 39217937
chr5 39217994
chr5 39218400
chr5 39218423
chr5 39270613
chr5 39271891
chr5 39283562
chr5 39455943
chr5 39456025
chr5 40067983
chr5 40223713
chr5 40223858
chr5 40224140
chr5 40333124
chr5 40333420
chr5 40384084
chr5 40384157
chr5 40384525
chr5 40385157
chr5 40385369
chr5 40385387
chr5 40409584
chr5 40409796
chr5 40409812
chr5 40409852
chr5 40409933
chr5 40410273
chr5 40410282
chr5 40410349
chr5 40410416
chr5 40410462
chr5 40410523
chr5 40437044
chr5 40437155
chr5 40437199
chr5 40437565
chr5 40437667
chr5 40437811
chr5 40437832
chr5 40441705
chr5 40441778

chr5 40441959
chr5 40442035
chr5 40442087
chr5 40442418
chr5 40442458
chr5 40579652
chr5 40579981
chr5 40580236
chr5 40675821
chr5 40675848
chr5 40675987
chr5 40678222
chr5 40678308
chr5 40678526
chr5 40678601
chr5 40678607
chr5 40678914
chr5 40678916
chr5 40684372
chr5 40684910
chr5 40685086
chr5 40685798
chr5 40685887
chr5 40686259
chr5 40686496
chr5 40686536
chr5 40687928
chr5 40688222
chr5 40688256
chr5 40688263
chr5 40688357
chr5 40688367
chr5 40688371
chr5 40688422
chr5 40688449
chr5 40688813
chr5 40688840
chr5 40688988
chr5 40689427
chr5 40754676
chr5 40754866
chr5 40754870
chr5 40755418
chr5 40755436
chr5 40755440
chr5 40755449
chr5 40755451
chr5 40755457
chr5 40755462
chr5 40755467
chr5 40755472
chr5 40755489
chr5 40755496
chr5 40755500

chr5 40755505
chr5 40755510
chr5 40755514
chr5 40755522
chr5 40755538
chr5 40755542
chr5 40755544
chr5 40797017
chr5 40797182
chr5 40797189
chr5 40797236
chr5 40798075
chr5 40798084
chr5 40798094
chr5 40798117
chr5 40798128
chr5 40798132
chr5 40798135
chr5 40798150
chr5 40798157
chr5 40798710
chr5 40798728
chr5 40798747
chr5 40798754
chr5 40798762
chr5 40798772
chr5 40798785
chr5 40798796
chr5 40798818
chr5 40798833
chr5 40798839
chr5 40798895
chr5 40798952
chr5 40799039
chr5 40799052
chr5 40799437
chr5 40833915
chr5 40833931
chr5 40833939
chr5 40833943
chr5 40833993
chr5 40833999
chr5 40834126
chr5 40834163
chr5 40834190
chr5 40834217
chr5 40834227
chr5 40834238
chr5 40834335
chr5 40834355
chr5 40834370
chr5 40834482
chr5 40834514
chr5 40834548

chr5 40834551
chr5 40834559
chr5 40834565
chr5 40834575
chr5 40834578
chr5 40834589
chr5 40834595
chr5 40834604
chr5 40834660
chr5 40834669
chr5 40834704
chr5 40834713
chr5 40834721
chr5 40834740
chr5 40834766
chr5 40834775
chr5 40834792
chr5 40834838
chr5 40834908
chr5 40834931
chr5 40834956
chr5 40834975
chr5 40834995
chr5 40834998
chr5 40835055
chr5 40835091
chr5 40835140
chr5 40835151
chr5 40835153
chr5 40835188
chr5 40835196
chr5 40835200
chr5 40835215
chr5 40835233
chr5 40835243
chr5 40835257
chr5 40835278
chr5 40835283
chr5 40835285
chr5 40835293
chr5 40835299
chr5 40835303
chr5 40835307
chr5 40835310
chr5 40835313
chr5 41412156
chr5 41490677
chr5 41490770
chr5 41491520
chr5 41491523
chr5 41511048
chr5 41511395
chr5 41511985
chr5 41869855

chr5 41869861
chr5 41869873
chr5 41869908
chr5 41869922
chr5 41869953
chr5 41869972
chr5 41869975
chr5 41904592
chr5 41904602
chr5 41904620
chr5 41904626
chr5 41904661
chr5 41904670
chr5 41904677
chr5 41904714
chr5 41904885
chr5 41904897
chr5 41904904
chr5 41925815
chr5 41925821
chr5 41925872
chr5 41925887
chr5 41925899
chr5 41925996
chr5 41972285
chr5 42139133
chr5 42139325
chr5 42589897
chr5 42591089
chr5 42591096
chr5 42720417
chr5 42720419
chr5 42720438
chr5 42720477
chr5 42720567
chr5 42757314
chr5 42757325
chr5 42757329
chr5 42757332
chr5 42757365
chr5 42757372
chr5 42757411
chr5 42757422
chr5 42757498
chr5 42757538
chr5 42757584
chr5 42908236
chr5 42908581
chr5 42908629
chr5 42908643
chr5 42908699
chr5 42908741
chr5 42908744
chr5 42908765

chr5 42908772
chr5 42908781
chr5 42908784
chr5 42908799
chr5 42908814
chr5 42908853
chr5 42908858
chr5 42908903
chr5 42908931
chr5 42908966
chr5 42909011
chr5 42909017
chr5 42909036
chr5 42909071
chr5 42909078
chr5 42909099
chr5 42909132
chr5 42909144
chr5 42909149
chr5 42949395
chr5 42950008
chr5 42950042
chr5 42950191
chr5 42950200
chr5 42950303
chr5 42950322
chr5 42950443
chr5 42950449
chr5 42950452
chr5 42950498
chr5 42950556
chr5 42950563
chr5 42950620
chr5 42950646
chr5 42950652
chr5 42993250
chr5 42993323
chr5 42993326
chr5 42993338
chr5 42993348
chr5 42993356
chr5 42993403
chr5 42993432
chr5 42993517
chr5 42993539
chr5 42993547
chr5 42993550
chr5 42993556
chr5 42993580
chr5 43007732
chr5 43007743
chr5 43007745
chr5 43007751
chr5 43007779

chr5 43007788
chr5 43007850
chr5 43007864
chr5 43007906
chr5 43007912
chr5 43007942
chr5 43007949
chr5 43007951
chr5 43007971
chr5 43007986
chr5 43008034
chr5 43008045
chr5 43008052
chr5 43008064
chr5 43008112
chr5 43008115
chr5 43008131
chr5 43008575
chr5 43009389
chr5 43009393
chr5 43009435
chr5 43009441
chr5 43009449
chr5 43009481
chr5 43009509
chr5 43009552
chr5 43009571
chr5 43009583
chr5 43009591
chr5 43009618
chr5 43009659
chr5 43009688
chr5 43009694
chr5 43017637
chr5 43017666
chr5 43017685
chr5 43017697
chr5 43017706
chr5 43017867
chr5 43017872
chr5 43017877
chr5 43017880
chr5 43017883
chr5 43017900
chr5 43018565
chr5 43018573
chr5 43018577
chr5 43018581
chr5 43018583
chr5 43018593
chr5 43018621
chr5 43018626
chr5 43018630
chr5 43018651

chr5 43018671
chr5 43018682
chr5 43018712
chr5 43018716
chr5 43018724
chr5 43018748
chr5 43018772
chr5 43018780
chr5 43018787
chr5 43018804
chr5 43018810
chr5 43018812
chr5 43018834
chr5 43018842
chr5 43018904
chr5 43018906
chr5 43018912
chr5 43018931
chr5 43018980
chr5 43019049
chr5 43019055
chr5 43019057
chr5 43019078
chr5 43019101
chr5 43019105
chr5 43019107
chr5 43019152
chr5 43019164
chr5 43019184
chr5 43019231
chr5 43019264
chr5 43019691
chr5 43019700
chr5 43019712
chr5 43019820
chr5 43019831
chr5 43019837
chr5 43019844
chr5 43019866
chr5 43019876
chr5 43019903
chr5 43019922
chr5 43019994
chr5 43020013
chr5 43020030
chr5 43020041
chr5 43020059
chr5 43020126
chr5 43020136
chr5 43020139
chr5 43020156
chr5 43020159
chr5 43020174
chr5 43020178

chr5 43020201
chr5 43020250
chr5 43020264
chr5 43020269
chr5 43020349
chr5 43020395
chr5 43020525
chr5 43020541
chr5 43020568
chr5 43020579
chr5 43020595
chr5 43020614
chr5 43020625
chr5 43020672
chr5 43039638
chr5 43039707
chr5 43039737
chr5 43039765
chr5 43064699
chr5 43064730
chr5 43064759
chr5 43064775
chr5 43064806
chr5 43064814
chr5 43064832
chr5 43064850
chr5 43064885
chr5 43064913
chr5 43064926
chr5 43067285
chr5 43067313
chr5 43067340
chr5 43067350
chr5 43067355
chr5 43067384
chr5 43067433
chr5 43067440
chr5 43067482
chr5 43067522
chr5 43067528
chr5 43067574
chr5 43067606
chr5 43067625
chr5 43105979
chr5 43106106
chr5 43122453
chr5 43122689
chr5 43122711
chr5 43304687
chr5 43484529
chr5 43484574
chr5 43484623
chr5 43484798
chr5 43514594

chr5 43514639
chr5 43514645
chr5 43514668
chr5 43514813
chr5 43514819
chr5 43514829
chr5 43514848
chr5 43514886
chr5 43514891
chr5 43514894
chr5 43514897
chr5 43555938
chr5 43555992
chr5 43556018
chr5 43556023
chr5 43556085
chr5 43556134
chr5 43556137
chr5 43556356
chr5 43556358
chr5 43556385
chr5 43556441
chr5 43556447
chr5 43556449
chr5 43556458
chr5 43556471
chr5 43556479
chr5 43556492
chr5 43562008
chr5 43602014
chr5 43602037
chr5 43602174
chr5 43602278
chr5 43602403
chr5 43602417
chr5 43602442
chr5 43602493
chr5 43602503
chr5 43602564
chr5 43602597
chr5 43602646
chr5 43602659
chr5 43602680
chr5 43603541
chr5 43603547
chr5 43603585
chr5 43603606
chr5 43603649
chr5 43603656
chr5 43603660
chr5 43603671
chr5 43603684
chr5 43603719
chr5 43603726

chr5 43603788
chr5 43603801
chr5 43603823
chr5 43603852
chr5 43603860
chr5 43603912
chr5 43603921
chr5 44389330
chr5 44389333
chr5 44389384
chr5 44389746
chr5 44389752
chr5 44389763
chr5 44389770
chr5 44389837
chr5 44389988
chr5 44390182
chr5 44390207
chr5 44390210
chr5 44390238
chr5 44390278
chr5 44390296
chr5 44390308
chr5 44390312
chr5 44808148
chr5 44808278
chr5 44808485
chr5 44808690
chr5 44808736
chr5 44808777
chr5 44808789
chr5 44809514
chr5 44809562
chr5 44809579
chr5 44809589
chr5 44809727
chr5 44809761
chr5 44973852
chr5 44974399
chr5 45171349
chr5 45266007
chr5 45661115
chr5 45661161
chr5 45661292
chr5 45661367
chr5 45661724
chr5 46791167
chr5 46791215
chr5 46791727
chr5 46791801
chr5 46791861
chr5 46791897
chr5 46791990
chr5 46792345

chr5 46792409
chr5 46792748
chr5 46792847
chr5 46794797
chr5 46794802
chr5 46795151
chr5 46795160
chr5 46795599
chr5 46795658
chr5 46796283
chr5 46796428
chr5 46797014
chr5 49685975
chr5 49685983
chr5 49686070
chr5 49686204
chr5 49686329
chr5 49686866
chr5 49686902
chr5 49687414
chr5 49687428
chr5 49687438
chr5 49687663
chr5 50415889
chr5 50416015
chr5 50440145
chr5 50440169
chr5 50440251
chr5 50440271
chr5 50440299
chr5 50440304
chr5 50440308
chr5 50440331
chr5 50440390
chr5 50440393
chr5 50440401
chr5 50440447
chr5 50440457
chr5 50440473
chr5 50440481
chr5 50440489
chr5 50440528
chr5 50440609
chr5 50440622
chr5 50441378
chr5 50441388
chr5 50441394
chr5 50441402
chr5 50441408
chr5 50441421
chr5 50441436
chr5 50441443
chr5 50441451
chr5 50441465

chr5 50441502
chr5 50441515
chr5 50441610
chr5 50441617
chr5 50441639
chr5 50441658
chr5 50441773
chr5 50441807
chr5 50441939
chr5 50442031
chr5 50445382
chr5 50445585
chr5 50445600
chr5 50445711
chr5 50669082
chr5 50669332
chr5 50669365
chr5 50711079
chr5 50711149
chr5 50744953
chr5 50745112
chr5 50745180
chr5 50745186
chr5 50745224
chr5 50962901
chr5 50962961
chr5 50962983
chr5 50962985
chr5 50962996
chr5 50963038
chr5 50963059
chr5 50963078
chr5 50963081
chr5 50963088
chr5 50963090
chr5 50963111
chr5 50963119
chr5 50963130
chr5 50963147
chr5 50963179
chr5 50963194
chr5 50963205
chr5 50963245
chr5 50963252
chr5 50963255
chr5 50963264
chr5 50963299
chr5 50963363
chr5 50967547
chr5 50967568
chr5 50967848
chr5 51242627
chr5 51376374
chr5 51376447

chr5 51379435
chr5 51379516
chr5 51379723
chr5 51379733
chr5 51379761
chr5 51379765
chr5 51379767
chr5 51379789
chr5 51379805
chr5 51379904
chr5 51379922
chr5 51380715
chr5 51380762
chr5 51380796
chr5 51380815
chr5 51380836
chr5 51381069
chr5 51381101
chr5 51381233
chr5 51381576
chr5 51381731
chr5 51381743
chr5 51381745
chr5 51381872
chr5 51381892
chr5 51381912
chr5 51381931
chr5 51381935
chr5 51384270
chr5 51384282
chr5 51384291
chr5 51384455
chr5 51384470
chr5 51384649
chr5 51384671
chr5 51385318
chr5 51385432
chr5 51385505
chr5 51385613
chr5 51385788
chr5 51386005
chr5 51386036
chr5 51386080
chr5 51386132
chr5 51386182
chr5 51386214
chr5 51386535
chr5 51386663
chr5 51386758
chr5 51386944
chr5 51396447
chr5 51396567
chr5 51396624
chr5 51396668

chr5 51396807
chr5 51396965
chr5 51396981
chr5 51397160
chr5 51397341
chr5 51397359
chr5 51397494
chr5 51397595
chr5 51397692
chr5 51397848
chr5 51397851
chr5 51397951
chr5 51398033
chr5 51398063
chr5 51398142
chr5 51398182
chr5 51398253
chr5 51398270
chr5 51398376
chr5 51398396
chr5 51400328
chr5 51400372
chr5 51400388
chr5 51400614
chr5 51400912
chr5 51495766
chr5 51495918
chr5 51881889
chr5 51949972
chr5 51950268
chr5 51950302
chr5 52085597
chr5 52085773
chr5 52665902
chr5 52665911
chr5 52666026
chr5 52786731
chr5 52786739
chr5 52786874
chr5 52786982
chr5 52787012
chr5 52787024
chr5 52787182
chr5 52787330
chr5 52787369
chr5 52787397
chr5 52787484
chr5 52787510
chr5 52787584
chr5 52787598
chr5 52787626
chr5 52787654
chr5 52787668
chr5 52787678

chr5 52787687
chr5 52787695
chr5 52787713
chr5 52787745
chr5 52787759
chr5 52787775
chr5 52787786
chr5 52787802
chr5 52787811
chr5 52787830
chr5 52787843
chr5 52787848
chr5 52787872
chr5 52787900
chr5 52787919
chr5 52787949
chr5 52787956
chr5 52788012
chr5 52788032
chr5 52788038
chr5 52788040
chr5 52788067
chr5 52799725
chr5 52799741
chr5 52915345
chr5 52915464
chr5 52915520
chr5 52915544
chr5 52915947
chr5 52915988
chr5 52915990
chr5 52988931
chr5 52988938
chr5 52988951
chr5 52988955
chr5 52988970
chr5 52988981
chr5 52988989
chr5 52989010
chr5 52989018
chr5 52989028
chr5 52989034
chr5 52989050
chr5 53109039
chr5 53109057
chr5 53109081
chr5 53109096
chr5 53109106
chr5 53109173
chr5 53109231
chr5 53109234
chr5 53109260
chr5 53109278
chr5 53109401

chr5 53109440
chr5 53109451
chr5 53109457
chr5 53109469
chr5 53109822
chr5 53110015
chr5 53110026
chr5 53110034
chr5 53110056
chr5 53110062
chr5 53110147
chr5 53110161
chr5 53477852
chr5 53477930
chr5 53478212
chr5 53478460
chr5 53478502
chr5 53478606
chr5 53478982
chr5 53479023
chr5 53479034
chr5 53479173
chr5 53479269
chr5 53481096
chr5 53481222
chr5 53481352
chr5 53481460
chr5 53481712
chr5 53481761
chr5 53481772
chr5 53481848
chr5 53481910
chr5 53481959
chr5 53481962
chr5 53481978
chr5 53482379
chr5 53482424
chr5 53482455
chr5 53482458
chr5 53482522
chr5 53482565
chr5 53511896
chr5 53512181
chr5 53560143
chr5 53560158
chr5 53560171
chr5 53560344
chr5 53560387
chr5 53560480
chr5 53560513
chr5 53560572
chr5 53560582
chr5 53560602
chr5 53560622

chr5 53560630
chr5 54310466
chr5 54310481
chr5 54310498
chr5 54310503
chr5 54310509
chr5 54310515
chr5 54310531
chr5 54310543
chr5 54310547
chr5 54310576
chr5 54310586
chr5 54310596
chr5 54311781
chr5 54311894
chr5 54311899
chr5 54311942
chr5 54311981
chr5 54312074
chr5 54610513
chr5 54610672
chr5 54610832
chr5 54646012
chr5 54646094
chr5 54646371
chr5 54646402
chr5 54646408
chr5 54646460
chr5 54646471
chr5 54646478
chr5 54646485
chr5 54646868
chr5 54646943
chr5 54646948
chr5 54646953
chr5 54647014
chr5 54647020
chr5 54647057
chr5 55028145
chr5 55028251
chr5 55037064
chr5 55037253
chr5 55037327
chr5 55037579
chr5 55037594
chr5 55037626
chr5 55037634
chr5 55062580
chr5 55146590
chr5 55146600
chr5 55146611
chr5 55146653
chr5 55146673
chr5 55146776

chr5 55146793
chr5 55146806
chr5 55146833
chr5 55146850
chr5 55147517
chr5 55172534
chr5 55172587
chr5 55172593
chr5 55172637
chr5 55172825
chr5 55172844
chr5 55172879
chr5 55172888
chr5 55173896
chr5 55173936
chr5 55173985
chr5 55174051
chr5 55175009
chr5 55175412
chr5 55175580
chr5 55175800
chr5 55175938
chr5 55175947
chr5 55176016
chr5 55176019
chr5 55176087
chr5 55176171
chr5 55176179
chr5 55176227
chr5 55176251
chr5 55176258
chr5 55308596
chr5 55308654
chr5 55308708
chr5 55308953
chr5 55309010
chr5 55440389
chr5 55538709
chr5 55540707
chr5 55540807
chr5 55822066
chr5 55822102
chr5 55822104
chr5 55822107
chr5 55822124
chr5 55822126
chr5 55822137
chr5 55903458
chr5 55903461
chr5 55903466
chr5 55903510
chr5 55903568
chr5 55903571
chr5 55903576

chr5 55903591
chr5 55903614
chr5 55903625
chr5 55903822
chr5 55923365
chr5 55923396
chr5 55923399
chr5 55923483
chr5 55923497
chr5 55993492
chr5 55993678
chr5 55993847
chr5 56012712
chr5 56051229
chr5 56058786
chr5 56058953
chr5 56058959
chr5 56059045
chr5 56059051
chr5 56059057
chr5 56080886
chr5 56152586
chr5 56152700
chr5 56327979
chr5 56327999
chr5 56328004
chr5 56328023
chr5 56328119
chr5 56328146
chr5 56328751
chr5 56791468
chr5 56814400
chr5 56814431
chr5 56814436
chr5 56817014
chr5 56817025
chr5 56817027
chr5 56817032
chr5 56817036
chr5 56817053
chr5 56817060
chr5 56817070
chr5 56817081
chr5 56817131
chr5 56817145
chr5 56817243
chr5 56817309
chr5 56817352
chr5 56817369
chr5 56817457
chr5 56817515
chr5 56817785
chr5 56817950
chr5 56817961

chr5 56818061
chr5 56818200
chr5 56818527
chr5 56818606
chr5 56818790
chr5 56819043
chr5 56910667
chr5 56910674
chr5 56910702
chr5 56910811
chr5 56910829
chr5 56950828
chr5 56950840
chr5 56950869
chr5 56950874
chr5 56950881
chr5 56950906
chr5 56950959
chr5 57030287
chr5 57030321
chr5 57030330
chr5 57030370
chr5 57030379
chr5 57030573
chr5 57172409
chr5 57172492
chr5 57172515
chr5 57172524
chr5 57172597
chr5 57172602
chr5 57172645
chr5 57172983
chr5 57172994
chr5 57173031
chr5 57173056
chr5 57176202
chr5 57176276
chr5 57176303
chr5 57176479
chr5 57176585
chr5 57176640
chr5 57176665
chr5 57176673
chr5 57176752
chr5 57176784
chr5 57176956
chr5 57177240
chr5 57177270
chr5 57187958
chr5 57298257
chr5 57298410
chr5 57298429
chr5 57298444
chr5 57298546

chr5 57298645
chr5 57298713
chr5 57299083
chr5 57299112
chr5 57299120
chr5 57299144
chr5 57299166
chr5 58294505
chr5 58295154
chr5 58380083
chr5 58554435
chr5 58554708
chr5 58561823
chr5 58561858
chr5 58562224
chr5 58584008
chr5 58584031
chr5 58584204
chr5 58584214
chr5 58584807
chr5 58751683
chr5 58752447
chr5 58752509
chr5 58891602
chr5 58891655
chr5 58891686
chr5 58891697
chr5 58891772
chr5 58891774
chr5 58891830
chr5 58891871
chr5 58891897
chr5 58891912
chr5 58891948
chr5 58934538
chr5 58934567
chr5 59038361
chr5 59038404
chr5 59038450
chr5 59038532
chr5 59038649
chr5 59038681
chr5 59038779
chr5 59042242
chr5 59042330
chr5 59042333
chr5 59127491
chr5 59127495
chr5 59127512
chr5 59127542
chr5 59127575
chr5 59127644
chr5 59127730
chr5 59127759

chr5 59127828
chr5 59127840
chr5 59127854
chr5 59127867
chr5 59127898
chr5 59127931
chr5 59127986
chr5 59127995
chr5 59128015
chr5 59128225
chr5 59656685
chr5 59657034
chr5 59657180
chr5 59768627
chr5 59768675
chr5 59768679
chr5 59768689
chr5 59768691
chr5 59768697
chr5 59768706
chr5 59768753
chr5 59768784
chr5 59768786
chr5 59768790
chr5 59768794
chr5 59768804
chr5 59768843
chr5 59768846
chr5 59768854
chr5 59768856
chr5 59769006
chr5 59769035
chr5 59769263
chr5 59810119
chr5 59810134
chr5 59810148
chr5 59810173
chr5 59810178
chr5 59810190
chr5 59810196
chr5 59810207
chr5 59810212
chr5 59810235
chr5 59810248
chr5 59810298
chr5 59810371
chr5 59810451
chr5 60294395
chr5 60677955
chr5 60699477
chr5 60699566
chr5 60945157
chr5 60945171
chr5 60945176

chr5 60945189
chr5 60945192
chr5 60945215
chr5 60945222
chr5 60945234
chr5 60945252
chr5 60945280
chr5 60945282
chr5 60945296
chr5 60945321
chr5 60945329
chr5 60945339
chr5 60945362
chr5 60945392
chr5 60945395
chr5 60945404
chr5 60945410
chr5 60945425
chr5 60945470
chr5 60945479
chr5 60945497
chr5 60945511
chr5 60945517
chr5 60945530
chr5 60945550
chr5 60945562
chr5 60945567
chr5 60945577
chr5 60945587
chr5 60945593
chr5 60945597
chr5 60945601
chr5 60945603
chr5 60945620
chr5 60945630
chr5 60945633
chr5 60945640
chr5 60945651
chr5 60945662
chr5 60945687
chr5 60945724
chr5 60945795
chr5 60945799
chr5 60945809
chr5 60945859
chr5 60945983
chr5 60945998
chr5 60946021
chr5 60946042
chr5 60946076
chr5 60946093
chr5 60946117
chr5 60946149
chr5 60946152

chr5 60946155
chr5 60946180
chr5 60946183
chr5 60946210
chr5 60946218
chr5 60946254
chr5 60946263
chr5 60960796
chr5 61161535
chr5 61161541
chr5 61161783
chr5 61161819
chr5 61161849
chr5 61161889
chr5 61162468
chr5 61162475
chr5 61162477
chr5 61162486
chr5 61162491
chr5 61162501
chr5 61162548
chr5 61162624
chr5 61407741
chr5 61408298
chr5 61408424
chr5 61408614
chr5 61408621
chr5 61408782
chr5 61408829
chr5 61408831
chr5 61408835
chr5 61408845
chr5 61408894
chr5 61408901
chr5 61408924
chr5 61408933
chr5 61408947
chr5 61408949
chr5 61408955
chr5 61408960
chr5 61408963
chr5 61408980
chr5 61408984
chr5 61408986
chr5 61408994
chr5 61409013
chr5 61409097
chr5 61534570
chr5 61534698
chr5 61534751
chr5 61534881
chr5 61658129
chr5 62223236
chr5 62223261

chr5 62223333
chr5 62223342
chr5 62223464
chr5 62223492
chr5 62223576
chr5 62223585
chr5 62223614
chr5 62223630
chr5 62223699
chr5 62223714
chr5 62223729
chr5 62223816
chr5 62223850
chr5 62224098
chr5 62224100
chr5 62224102
chr5 62224104
chr5 62224106
chr5 62224108
chr5 62224114
chr5 62224116
chr5 62224120
chr5 62224122
chr5 62224129
chr5 62224134
chr5 62224136
chr5 62224593
chr5 62277495
chr5 62402772
chr5 62403187
chr5 62403205
chr5 62403278
chr5 62403330
chr5 62403360
chr5 62403444
chr5 62403459
chr5 62403464
chr5 62403469
chr5 62403573
chr5 62404041
chr5 62404122
chr5 62404130
chr5 62404337
chr5 62413416
chr5 62413451
chr5 62413470
chr5 62778745
chr5 62799862
chr5 62800288
chr5 62800402
chr5 62801140
chr5 62801283
chr5 62805828
chr5 62805865

chr5 62806514
chr5 62806587
chr5 62806631
chr5 62878292
chr5 62878489
chr5 62878647
chr5 63005770
chr5 63006064
chr5 63081068
chr5 63081950
chr5 63082331
chr5 63082443
chr5 63574079
chr5 63574395
chr5 63692955
chr5 63693314
chr5 63693365
chr5 63958511
chr5 63963534
chr5 63963680
chr5 63985902
chr5 63985941
chr5 63986010
chr5 63986187
chr5 64637153
chr5 64637240
chr5 64637269
chr5 64637271
chr5 64637334
chr5 64637343
chr5 64637361
chr5 64637372
chr5 64637443
chr5 64768139
chr5 64768202
chr5 64768206
chr5 64768399
chr5 64768417
chr5 64768425
chr5 64768431
chr5 64768434
chr5 64768458
chr5 64768467
chr5 64768474
chr5 64768478
chr5 64768520
chr5 64768540
chr5 64768555
chr5 64768563
chr5 64768574
chr5 64768605
chr5 64768608
chr5 64768625
chr5 64768639

chr5 64768644
chr5 64768648
chr5 64768663
chr5 64768880
chr5 64768891
chr5 64769077
chr5 64769102
chr5 64769115
chr5 64769178
chr5 64769244
chr5 64769277
chr5 64769295
chr5 64769338
chr5 64769413
chr5 64769449
chr5 64769555
chr5 64769561
chr5 64769585
chr5 64769588
chr5 64769691
chr5 64784575
chr5 64784596
chr5 65035612
chr5 65035620
chr5 65035622
chr5 65035633
chr5 65035638
chr5 65035643
chr5 65035646
chr5 65035651
chr5 65035659
chr5 65035664
chr5 65035668
chr5 65035681
chr5 65035685
chr5 65035687
chr5 65035690
chr5 65035708
chr5 65035723
chr5 65035725
chr5 65035730
chr5 65035740
chr5 65035807
chr5 65035852
chr5 65035869
chr5 65035875
chr5 65035888
chr5 65035897
chr5 65035902
chr5 65035912
chr5 65035924
chr5 65035926
chr5 65035932
chr5 65035940

chr5 65035944
chr5 65035965
chr5 65035967
chr5 65035987
chr5 65036031
chr5 65036077
chr5 65036131
chr5 65036200
chr5 65036352
chr5 65079660
chr5 65079968
chr5 65080549
chr5 65167997
chr5 65465489
chr5 65479967
chr5 65480093
chr5 65480202
chr5 65480319
chr5 65480423
chr5 65480445
chr5 65480447
chr5 65480480
chr5 65480482
chr5 65480490
chr5 65480546
chr5 65480562
chr5 65480722
chr5 65480737
chr5 65480758
chr5 65480792
chr5 65480844
chr5 65480917
chr5 65481006
chr5 65481096
chr5 65481149
chr5 65481262
chr5 65481269
chr5 65481343
chr5 65481402
chr5 65481409
chr5 65483403
chr5 65483597
chr5 65484047
chr5 65548616
chr5 65562238
chr5 65562442
chr5 65562482
chr5 65562485
chr5 65562541
chr5 65562725
chr5 65562877
chr5 65562879
chr5 65562949
chr5 65562955

chr5 65562957
chr5 65562959
chr5 65562967
chr5 65562976
chr5 65562990
chr5 65563018
chr5 65563021
chr5 65563066
chr5 65563083
chr5 65563106
chr5 65563123
chr5 65563131
chr5 65563136
chr5 65563145
chr5 65563158
chr5 65563168
chr5 65563170
chr5 65563172
chr5 65563303
chr5 65563992
chr5 65564010
chr5 65596523
chr5 65623731
chr5 65623818
chr5 65623842
chr5 65721050
chr5 65722833
chr5 65722848
chr5 65722883
chr5 65722950
chr5 65722988
chr5 65722998
chr5 65723106
chr5 65723112
chr5 65723122
chr5 65723134
chr5 65723146
chr5 65928224
chr5 65928362
chr5 65928513
chr5 65928647
chr5 65928661
chr5 65928729
chr5 65928762
chr5 66002485
chr5 66002598
chr5 66145252
chr5 66145254
chr5 66145299
chr5 66145303
chr5 66145322
chr5 66145407
chr5 66145693
chr5 66145865

chr5 66145890
chr5 66263276
chr5 66828868
chr5 66828973
chr5 66829009
chr5 66829016
chr5 66829192
chr5 66829215
chr5 66829247
chr5 66829267
chr5 66829280
chr5 66829284
chr5 66829287
chr5 66829302
chr5 66829339
chr5 66829867
chr5 66830086
chr5 66830523
chr5 67003941
chr5 67003958
chr5 67003962
chr5 67003964
chr5 67003986
chr5 67003989
chr5 67003997
chr5 67004047
chr5 67004049
chr5 67004064
chr5 67004076
chr5 67004078
chr5 67004091
chr5 67004100
chr5 67004102
chr5 67004122
chr5 67004127
chr5 67004138
chr5 67004145
chr5 67004158
chr5 67004161
chr5 67004173
chr5 67004184
chr5 67004190
chr5 67004200
chr5 67004202
chr5 67004205
chr5 67004207
chr5 67004211
chr5 67004227
chr5 67004237
chr5 67004248
chr5 67004250
chr5 67004254
chr5 67004294
chr5 67004296

chr5 67208962
chr5 67209055
chr5 67209181
chr5 67209253
chr5 67209326
chr5 67209420
chr5 67215914
chr5 67215941
chr5 67215972
chr5 67216009
chr5 67216094
chr5 67216200
chr5 67216211
chr5 67216262
chr5 67216320
chr5 67221915
chr5 67221962
chr5 67221981
chr5 67222036
chr5 67701755
chr5 68070789
chr5 68071041
chr5 68071348
chr5 68071396
chr5 68071473
chr5 68214905
chr5 68214913
chr5 68214922
chr5 68214926
chr5 68214928
chr5 68214944
chr5 68214953
chr5 68214959
chr5 68214965
chr5 68214967
chr5 68214971
chr5 68214974
chr5 68217028
chr5 68217041
chr5 68217128
chr5 68217154
chr5 68217291
chr5 68217523
chr5 68217655
chr5 68217657
chr5 68217659
chr5 68217661
chr5 68217669
chr5 68239922
chr5 68240154
chr5 68255680
chr5 68255695
chr5 68255764
chr5 68255866

chr5 68280180
chr5 68280363
chr5 68280487
chr5 68280541
chr5 68280579
chr5 68280609
chr5 68280991
chr5 68281189
chr5 68287770
chr5 68287781
chr5 68303344
chr5 68434244
chr5 68434248
chr5 68434274
chr5 68434278
chr5 68434282
chr5 68434301
chr5 68434307
chr5 68434313
chr5 68434327
chr5 68434364
chr5 68434386
chr5 68434404
chr5 68434432
chr5 68884322
chr5 68884334
chr5 68884474
chr5 68884484
chr5 69042691
chr5 69042979
chr5 69042997
chr5 69043011
chr5 69043073
chr5 69043121
chr5 69043173
chr5 69043220
chr5 69043262
chr5 69043268
chr5 69043289
chr5 69043291
chr5 69043315
chr5 69043335
chr5 69043470
chr5 69043482
chr5 69043492
chr5 69106293
chr5 69167346
chr5 69167352
chr5 69167363
chr5 69167367
chr5 69167378
chr5 69167386
chr5 69167426
chr5 69167429

chr5 69167466
chr5 69167504
chr5 69167540
chr5 69167587
chr5 69167646
chr5 69167751
chr5 69167755
chr5 69189927
chr5 69190011
chr5 69190049
chr5 69190139
chr5 69190186
chr5 69190190
chr5 69190214
chr5 69190232
chr5 69190249
chr5 69218349
chr5 69218351
chr5 69218358
chr5 69218364
chr5 69218367
chr5 69218372
chr5 69218396
chr5 69218505
chr5 69218754
chr5 69332306
chr5 69332331
chr5 69332413
chr5 69332528
chr5 69332596
chr5 69332604
chr5 69332611
chr5 69332639
chr5 69332665
chr5 69332684
chr5 69332689
chr5 69332696
chr5 69332703
chr5 69332718
chr5 69332736
chr5 69332741
chr5 69332744
chr5 69332758
chr5 69332785
chr5 69332789
chr5 69332791
chr5 69332795
chr5 69332798
chr5 69332804
chr5 69332807
chr5 69332809
chr5 69332811
chr5 69332818
chr5 69332828

chr5 69368609
chr5 69368719
chr5 69368758
chr5 69368775
chr5 69370327
chr5 69370385
chr5 69370402
chr5 69370425
chr5 69370484
chr5 69370496
chr5 69370500
chr5 69370528
chr5 69370584
chr5 69370588
chr5 69370593
chr5 69370631
chr5 69370633
chr5 69370637
chr5 69370663
chr5 69370895
chr5 69558122
chr5 69560171
chr5 69560176
chr5 69560187
chr5 69560190
chr5 69560203
chr5 69560216
chr5 69560235
chr5 69560238
chr5 69560241
chr5 69560250
chr5 69560252
chr5 69560271
chr5 69560289
chr5 69560305
chr5 69560315
chr5 69560321
chr5 69560326
chr5 69560333
chr5 70050405
chr5 70050424
chr5 70050438
chr5 70050440
chr5 70050575
chr5 70050579
chr5 70050583
chr5 70050585
chr5 70050623
chr5 70050628
chr5 70050632
chr5 70050653
chr5 70050673
chr5 70050684
chr5 70050713

chr5 70050717
chr5 70050725
chr5 70050729
chr5 70050749
chr5 70050781
chr5 70050788
chr5 70050805
chr5 70050811
chr5 70050813
chr5 70399820
chr5 70401056
chr5 70401063
chr5 70401066
chr5 70401074
chr5 70401091
chr5 70401590
chr5 70401615
chr5 70401678
chr5 70403836
chr5 70404368
chr5 70404486
chr5 70409825
chr5 70410143
chr5 70410175
chr5 70410207
chr5 70410332
chr5 70410360
chr5 70410471
chr5 70410473
chr5 70410616
chr5 70410634
chr5 70410666
chr5 70414547
chr5 70414569
chr5 70414589
chr5 70414601
chr5 70414605
chr5 71287430
chr5 71288062
chr5 71290075
chr5 71290078
chr5 71290081
chr5 71290088
chr5 71290092
chr5 71290104
chr5 71290109
chr5 71290111
chr5 71290114
chr5 71290120
chr5 71290122
chr5 71290130
chr5 71290137
chr5 71290139
chr5 71290148

chr5 71290150
chr5 71290158
chr5 71290165
chr5 71290167
chr5 71290178
chr5 71290185
chr5 71444686
chr5 71445246
chr5 71445497
chr5 71445500
chr5 71445626
chr5 71445669
chr5 71445683
chr5 71445695
chr5 71445714
chr5 71445822
chr5 71445853
chr5 71445866
chr5 71445878
chr5 71445884
chr5 71445891
chr5 71445936
chr5 71445964
chr5 71445969
chr5 71445975
chr5 71445988
chr5 71445991
chr5 71446010
chr5 71455809
chr5 71455817
chr5 71455820
chr5 71455827
chr5 71455839
chr5 71455843
chr5 71455859
chr5 71455874
chr5 71455884
chr5 71455893
chr5 71455901
chr5 71455909
chr5 71455931
chr5 71455962
chr5 71455968
chr5 71456126
chr5 71456134
chr5 71456182
chr5 71456188
chr5 71456286
chr5 71456302
chr5 71456341
chr5 71456508
chr5 71456574
chr5 71456606
chr5 71587379

chr5 71587393
chr5 71587400
chr5 71587408
chr5 71601843
chr5 71601976
chr5 71717324
chr5 71758914
chr5 71759244
chr5 71759303
chr5 71782890
chr5 72137777
chr5 72137939
chr5 72138270
chr5 72138487
chr5 72138583
chr5 72138707
chr5 72139015
chr5 72139058
chr5 72319325
chr5 72319413
chr5 72319466
chr5 72319574
chr5 72319604
chr5 72319606
chr5 72319629
chr5 72319636
chr5 72319646
chr5 72815697
chr5 72815747
chr5 72815892
chr5 72815922
chr5 72816035
chr5 72816083
chr5 72816099
chr5 72816124
chr5 72816139
chr5 72816142
chr5 72816153
chr5 72816178
chr5 72816181
chr5 72816236
chr5 72847423
chr5 72847617
chr5 72847758
chr5 72847772
chr5 72847904
chr5 72847963
chr5 72849127
chr5 72849207
chr5 72849238
chr5 72849282
chr5 72849308
chr5 72849385
chr5 72849501

chr5 72849555
chr5 72849564
chr5 72849583
chr5 72849756
chr5 72849983
chr5 73037233
chr5 73160716
chr5 73312660
chr5 73312696
chr5 73312730
chr5 73312830
chr5 73313115
chr5 73433612
chr5 73433686
chr5 73433818
chr5 73434063
chr5 73434832
chr5 73435284
chr5 73435423
chr5 73435755
chr5 73435761
chr5 73435816
chr5 73438167
chr5 73438169
chr5 73438410
chr5 73438513
chr5 73438555
chr5 73438758
chr5 73438978
chr5 73439011
chr5 73439086
chr5 73439180
chr5 73442465
chr5 73442509
chr5 73442530
chr5 73442625
chr5 73442680
chr5 73442699
chr5 73443057
chr5 73443162
chr5 73443484
chr5 73443592
chr5 73445267
chr5 73445388
chr5 73445664
chr5 73445753
chr5 73445885
chr5 73445994
chr5 73446377
chr5 73446402
chr5 73446538
chr5 73446599
chr5 73450008
chr5 73450029

chr5 73450103
chr5 73498282
chr5 73498297
chr5 73498304
chr5 73498334
chr5 73498351
chr5 73498357
chr5 73498359
chr5 73498361
chr5 73498365
chr5 73498378
chr5 73498384
chr5 73498386
chr5 73498389
chr5 73498436
chr5 73498477
chr5 73498500
chr5 73498531
chr5 73498533
chr5 73498535
chr5 73498547
chr5 73498552
chr5 73499276
chr5 73499291
chr5 73499311
chr5 73499324
chr5 73499340
chr5 73499347
chr5 73499356
chr5 73499382
chr5 73499396
chr5 73499503
chr5 73499655
chr5 73500047
chr5 73519448
chr5 73564880
chr5 73564948
chr5 73564966
chr5 73565006
chr5 73565063
chr5 73565135
chr5 73566283
chr5 73566532
chr5 73566636
chr5 73687568
chr5 73751646
chr5 73751895
chr5 73751921
chr5 73881625
chr5 73881668
chr5 73881698
chr5 73901814
chr5 73902396
chr5 73902713

chr5 73902769
chr5 73902771
chr5 73902821
chr5 73902823
chr5 74201838
chr5 74202315
chr5 74221249
chr5 74223684
chr5 74223899
chr5 74327123
chr5 74327154
chr5 74327383
chr5 74579267
chr5 74579270
chr5 74579277
chr5 74579354
chr5 74639300
chr5 74639381
chr5 74639500
chr5 74639505
chr5 74639589
chr5 74639666
chr5 74639706
chr5 74639779
chr5 74639840
chr5 74639843
chr5 74685576
chr5 74685580
chr5 74767432
chr5 74767451
chr5 74767461
chr5 74767622
chr5 74935662
chr5 74935686
chr5 74935706
chr5 74935727
chr5 74935736
chr5 75236937
chr5 75236940
chr5 75236950
chr5 75236955
chr5 75236959
chr5 75236965
chr5 75236978
chr5 75236984
chr5 75236998
chr5 75237000
chr5 75237002
chr5 75237004
chr5 75237006
chr5 75237008
chr5 75237010
chr5 75237027
chr5 75237037

chr5 75237042
chr5 75237055
chr5 75237069
chr5 75237073
chr5 75237125
chr5 75237130
chr5 75237148
chr5 75237171
chr5 75237217
chr5 75237241
chr5 75237276
chr5 75237383
chr5 75237454
chr5 75336652
chr5 75510265
chr5 75716637
chr5 75716641
chr5 75716821
chr5 75716855
chr5 75716899
chr5 75716901
chr5 75716991
chr5 75717062
chr5 75717075
chr5 75717097
chr5 75717138
chr5 75717143
chr5 75717224
chr5 75717258
chr5 75717275
chr5 75717288
chr5 75717293
chr5 75717306
chr5 75717309
chr5 75717312
chr5 75717316
chr5 75717318
chr5 75717329
chr5 75717337
chr5 75717344
chr5 75717355
chr5 75717357
chr5 75717360
chr5 75717402
chr5 75717413
chr5 75854716
chr5 75855953
chr5 75856036
chr5 76038131
chr5 76038166
chr5 76256381
chr5 76338062
chr5 76390798
chr5 76391148

chr5 76391216
chr5 76391218
chr5 76391220
chr5 76391230
chr5 76391258
chr5 76391284
chr5 76391302
chr5 76404334
chr5 76404367
chr5 76404485
chr5 76404608
chr5 76404677
chr5 76404683
chr5 76404850
chr5 76451214
chr5 76473926
chr5 76542879
chr5 76543060
chr5 76543117
chr5 76543156
chr5 76543172
chr5 76543243
chr5 76547638
chr5 76547690
chr5 76547995
chr5 76738875
chr5 76739062
chr5 76739118
chr5 76739270
chr5 76739281
chr5 76739309
chr5 76739314
chr5 76739333
chr5 76739491
chr5 76739524
chr5 76774631
chr5 76774705
chr5 77086314
chr5 77086498
chr5 77086556
chr5 77086569
chr5 77086598
chr5 77086605
chr5 77086614
chr5 77086691
chr5 77086704
chr5 77086714
chr5 77086758
chr5 77086830
chr5 77086845
chr5 77086858
chr5 77379679
chr5 77379703
chr5 77491980

chr5 77491996
chr5 77492003
chr5 77492005
chr5 77492012
chr5 77492021
chr5 77492024
chr5 77492202
chr5 77492213
chr5 77492222
chr5 77492229
chr5 77492232
chr5 77492235
chr5 77492244
chr5 77492271
chr5 77492282
chr5 77492301
chr5 77492310
chr5 77626784
chr5 77626832
chr5 77627224
chr5 77627315
chr5 77627433
chr5 77633549
chr5 77633710
chr5 77633898
chr5 77633910
chr5 77633924
chr5 77634057
chr5 77634538
chr5 77635048
chr5 77635413
chr5 77635569
chr5 77635604
chr5 77635627
chr5 77635638
chr5 77635642
chr5 77635760
chr5 77635777
chr5 77718508
chr5 77719015
chr5 77719148
chr5 77719282
chr5 77775441
chr5 77775467
chr5 77775515
chr5 77775607
chr5 77775614
chr5 77852191
chr5 77852262
chr5 77852279
chr5 77852281
chr5 77852294
chr5 77852353
chr5 77852357

chr5 77852394
chr5 77852578
chr5 77852585
chr5 77852600
chr5 77852602
chr5 77852611
chr5 77852690
chr5 77852697
chr5 77852703
chr5 77852714
chr5 77852723
chr5 77852725
chr5 77852728
chr5 77852733
chr5 77852738
chr5 77852740
chr5 77852744
chr5 77852754
chr5 77852795
chr5 77852826
chr5 77852830
chr5 77852841
chr5 77852862
chr5 77852864
chr5 77852866
chr5 77852868
chr5 77852870
chr5 77852874
chr5 77853051
chr5 77853676
chr5 78293754
chr5 78293797
chr5 78294002
chr5 78294036
chr5 78294100
chr5 78294103
chr5 78294107
chr5 78294615
chr5 78294652
chr5 78294670
chr5 78294674
chr5 78294682
chr5 78294684
chr5 78294686
chr5 78294692
chr5 78294698
chr5 78295438
chr5 78295460
chr5 78295498
chr5 78295506
chr5 78295565
chr5 78295585
chr5 78295596
chr5 78295628

chr5 78295630
chr5 78295632
chr5 78295639
chr5 78295674
chr5 78295708
chr5 78295845
chr5 78295887
chr5 78295889
chr5 78295902
chr5 78295906
chr5 78295920
chr5 78295928
chr5 78295991
chr5 78295999
chr5 78296026
chr5 78296043
chr5 78296094
chr5 78361296
chr5 78361391
chr5 78361397
chr5 78361435
chr5 78361585
chr5 78361601
chr5 78361607
chr5 78361685
chr5 78361733
chr5 78985795
chr5 78985840
chr5 78985861
chr5 78985996
chr5 78986004
chr5 79024860
chr5 79024910
chr5 79024927
chr5 79025011
chr5 79025027
chr5 79025034
chr5 79109659
chr5 79109678
chr5 79109691
chr5 79109695
chr5 79109758
chr5 79110001
chr5 79110004
chr5 79110042
chr5 79110065
chr5 79133398
chr5 79133740
chr5 79133768
chr5 79133791
chr5 79133824
chr5 79133951
chr5 79134042
chr5 79229716

chr5 79229726
chr5 79235435
chr5 79514753
chr5 79514764
chr5 79514776
chr5 79514779
chr5 79514831
chr5 79515105
chr5 79515185
chr5 79517729
chr5 79612296
chr5 79612328
chr5 79612338
chr5 79612397
chr5 79612404
chr5 79612420
chr5 79612427
chr5 79612444
chr5 79612452
chr5 79612455
chr5 79612463
chr5 79612488
chr5 79612491
chr5 79612499
chr5 79612502
chr5 79612518
chr5 79612531
chr5 79612595
chr5 79612618
chr5 79612624
chr5 79612647
chr5 79612672
chr5 79612685
chr5 79612695
chr5 79612704
chr5 79612724
chr5 79612733
chr5 79612787
chr5 79612797
chr5 79612818
chr5 79612821
chr5 79612824
chr5 79612831
chr5 79612844
chr5 79612849
chr5 79612855
chr5 79612867
chr5 79612913
chr5 79612953
chr5 79612970
chr5 79612984
chr5 79612986
chr5 79612996
chr5 79613079

chr5 79613199
chr5 79613237
chr5 79613257
chr5 79613539
chr5 79613599
chr5 79613677
chr5 79613838
chr5 79685979
chr5 79686036
chr5 79686510
chr5 79736571
chr5 79736675
chr5 79736692
chr5 79736810
chr5 79736817
chr5 79736887
chr5 79990207
chr5 79990234
chr5 79990382
chr5 79990401
chr5 79990426
chr5 79990488
chr5 79990703
chr5 79990762
chr5 79990778
chr5 79990790
chr5 79990808
chr5 79990810
chr5 79990814
chr5 79990825
chr5 79990894
chr5 79990942
chr5 79990969
chr5 79990972
chr5 79990975
chr5 79990982
chr5 79990985
chr5 79991265
chr5 79991302
chr5 79991304
chr5 79991310
chr5 79991365
chr5 79991587
chr5 80136175
chr5 80136233
chr5 80136271
chr5 80136361
chr5 80136394
chr5 80136498
chr5 80136572
chr5 80234596
chr5 80234658
chr5 80234709
chr5 80254925

chr5 80254961
chr5 80254990
chr5 80254994
chr5 80255023
chr5 80255028
chr5 80255088
chr5 80255140
chr5 80255212
chr5 80255232
chr5 80517026
chr5 80517033
chr5 80517079
chr5 80517124
chr5 80655137
chr5 80655157
chr5 80655266
chr5 80655306
chr5 80655422
chr5 80655572
chr5 80655583
chr5 80655717
chr5 80961789
chr5 80961791
chr5 80961876
chr5 80961983
chr5 80961995
chr5 80962039
chr5 80962083
chr5 80962154
chr5 80962158
chr5 81163969
chr5 81183284
chr5 81287474
chr5 81287540
chr5 81287546
chr5 81287823
chr5 81288096
chr5 81288249
chr5 81301464
chr5 81301469
chr5 81301472
chr5 81779238
chr5 81779241
chr5 81779297
chr5 81779568
chr5 81779579
chr5 81851388
chr5 81851397
chr5 81851619
chr5 81851666
chr5 81851759
chr5 81851763
chr5 81852321
chr5 81971128

chr5 81971189
chr5 81971286
chr5 81971395
chr5 81971489
chr5 81972029
chr5 81972033
chr5 81972038
chr5 81972043
chr5 81972047
chr5 81972052
chr5 81972072
chr5 81972074
chr5 81972116
chr5 81972143
chr5 81972149
chr5 81972179
chr5 81972190
chr5 81972193
chr5 81972195
chr5 81972207
chr5 81972214
chr5 81972252
chr5 81972255
chr5 81972260
chr5 81972266
chr5 81972269
chr5 81972281
chr5 81972294
chr5 81972298
chr5 81972529
chr5 81972536
chr5 81972539
chr5 81972541
chr5 81972547
chr5 81972588
chr5 81972803
chr5 81972813
chr5 81972862
chr5 82277372
chr5 82277394
chr5 82277487
chr5 82277554
chr5 82277632
chr5 82277647
chr5 82277675
chr5 82277706
chr5 82277804
chr5 82277807
chr5 82277816
chr5 82277834
chr5 82277843
chr5 82312392
chr5 82356730
chr5 82356965

chr5 82357038
chr5 82357143
chr5 82357162
chr5 82357191
chr5 82357211
chr5 82357260
chr5 82357273
chr5 82357303
chr5 82357344
chr5 82357349
chr5 82357411
chr5 83076422
chr5 83076523
chr5 83077457
chr5 83077489
chr5 83077498
chr5 83077512
chr5 83077517
chr5 83077524
chr5 83077527
chr5 83077576
chr5 83077580
chr5 83077595
chr5 83077609
chr5 83077624
chr5 83077736
chr5 83077764
chr5 83077766
chr5 83077805
chr5 83077824
chr5 83077853
chr5 83077882
chr5 83077928
chr5 83078037
chr5 83078048
chr5 83078109
chr5 83078115
chr5 83078125
chr5 83305876
chr5 83306288
chr5 83306743
chr5 83306781
chr5 83307025
chr5 83471132
chr5 83471142
chr5 83591395
chr5 83662660
chr5 83662893
chr5 83662896
chr5 84253510
chr5 84253700
chr5 84254232
chr5 84380820
chr5 84381438

chr5 84381593
chr5 84381734
chr5 84381787
chr5 84381856
chr5 84381925
chr5 84381974
chr5 84385238
chr5 84385362
chr5 84385383
chr5 84584769
chr5 84603980
chr5 84604144
chr5 84653259
chr5 84653276
chr5 84653383
chr5 84653414
chr5 84653437
chr5 84653446
chr5 84653486
chr5 84659498
chr5 84659507
chr5 84659966
chr5 84936349
chr5 84936573
chr5 84936749
chr5 85364029
chr5 85364288
chr5 85365327
chr5 85365332
chr5 86149057
chr5 86149220
chr5 86195645
chr5 86195735
chr5 86195814
chr5 86195845
chr5 86227638
chr5 86467997
chr5 86618028
chr5 86618035
chr5 86618052
chr5 86618457
chr5 86618463
chr5 86690698
chr5 86690772
chr5 86690780
chr5 86690791
chr5 86691463
chr5 87019948
chr5 87019967
chr5 87020182
chr5 87020343
chr5 87020721
chr5 87020872
chr5 87075459

chr5 87081197
chr5 87081245
chr5 87081689
chr5 87081697
chr5 87082353
chr5 87120596
chr5 87120736
chr5 87121482
chr5 87267198
chr5 87267284
chr5 87267354
chr5 87267383
chr5 87268589
chr5 87268673
chr5 87268733
chr5 87268816
chr5 87268835
chr5 87268844
chr5 87268847
chr5 87268894
chr5 87268901
chr5 87268913
chr5 87268943
chr5 87268966
chr5 87268973
chr5 87269174
chr5 87269258
chr5 87269315
chr5 87269409
chr5 87282270
chr5 87282603
chr5 87282674
chr5 87282689
chr5 87282946
chr5 87282956
chr5 87282995
chr5 87411704
chr5 87411711
chr5 87411724
chr5 87411894
chr5 87411956
chr5 87411986
chr5 87412621
chr5 87412645
chr5 87412661
chr5 87412683
chr5 87412692
chr5 87412712
chr5 87412719
chr5 87412727
chr5 87412749
chr5 87412769
chr5 87412803
chr5 87413309

chr5 87413411
chr5 87413452
chr5 87570503
chr5 87571109
chr5 87780174
chr5 87780204
chr5 87780379
chr5 87781491
chr5 87781522
chr5 88050733
chr5 88050859
chr5 88140733
chr5 88140768
chr5 88140774
chr5 88140784
chr5 88140793
chr5 88140810
chr5 88140853
chr5 88140858
chr5 88140865
chr5 88140882
chr5 88140891
chr5 88140911
chr5 88140938
chr5 88141097
chr5 88141112
chr5 88141126
chr5 88141136
chr5 88141331
chr5 88141369
chr5 88141475
chr5 88141493
chr5 88141505
chr5 88141507
chr5 88141520
chr5 88141541
chr5 88141563
chr5 88141583
chr5 88141735
chr5 88141741
chr5 88141747
chr5 88141753
chr5 88141759
chr5 88141788
chr5 88146427
chr5 88146614
chr5 88147188
chr5 88204557
chr5 88204702
chr5 88204720
chr5 88204796
chr5 88205059
chr5 88268364
chr5 88268366

chr5 88268382
chr5 88268402
chr5 88268430
chr5 88268500
chr5 88268512
chr5 88268517
chr5 88268540
chr5 88268612
chr5 88269171
chr5 88269182
chr5 88269213
chr5 88269215
chr5 88269225
chr5 88269272
chr5 88269297
chr5 88269329
chr5 88269369
chr5 88269404
chr5 88269423
chr5 88661442
chr5 88661462
chr5 88661520
chr5 88661532
chr5 88661584
chr5 88661685
chr5 88661784
chr5 88661888
chr5 88661911
chr5 88662841
chr5 88662859
chr5 88662878
chr5 88663097
chr5 88663111
chr5 88663204
chr5 88663214
chr5 88663483
chr5 88663596
chr5 88664471
chr5 88664864
chr5 88664892
chr5 88665072
chr5 88665193
chr5 88665214
chr5 88665450
chr5 88665636
chr5 88665784
chr5 88665866
chr5 88665872
chr5 88665890
chr5 88665894
chr5 88665903
chr5 88665908
chr5 88665916
chr5 88665936

chr5 88665943
chr5 88666011
chr5 88666090
chr5 88666096
chr5 88666108
chr5 88666139
chr5 88666187
chr5 88666193
chr5 88666400
chr5 88666813
chr5 88666837
chr5 88666844
chr5 88666847
chr5 88666871
chr5 88666933
chr5 88670385
chr5 88670507
chr5 88670969
chr5 88671250
chr5 88671525
chr5 88671600
chr5 88671727
chr5 88671883
chr5 88671898
chr5 88676304
chr5 88676306
chr5 88676309
chr5 88681744
chr5 88681760
chr5 88681893
chr5 88681964
chr5 88682202
chr5 88682813
chr5 88682974
chr5 88683096
chr5 88683156
chr5 88687728
chr5 88687780
chr5 88687803
chr5 88687819
chr5 88687865
chr5 88688226
chr5 88688257
chr5 88688487
chr5 88688731
chr5 88688740
chr5 88688766
chr5 88688776
chr5 88688806
chr5 88688817
chr5 88688826
chr5 88688843
chr5 88688856
chr5 88695539

chr5 88695554
chr5 88695562
chr5 88695571
chr5 88695586
chr5 88695610
chr5 88695616
chr5 88695631
chr5 88695732
chr5 88695775
chr5 88695862
chr5 88696161
chr5 88696192
chr5 88696312
chr5 88697492
chr5 88697514
chr5 88844922
chr5 88845092
chr5 88880617
chr5 88880971
chr5 88881043
chr5 88881139
chr5 88881584
chr5 88881594
chr5 88881724
chr5 88881859
chr5 88881894
chr5 88881975
chr5 88882203
chr5 88882322
chr5 88882326
chr5 88882406
chr5 88882583
chr5 88882641
chr5 88882669
chr5 88882715
chr5 88882749
chr5 88882755
chr5 88882808
chr5 88882825
chr5 88882878
chr5 88882968
chr5 88883404
chr5 88883418
chr5 88883443
chr5 88883471
chr5 88883483
chr5 88883485
chr5 88883512
chr5 88883544
chr5 88883562
chr5 88883569
chr5 88883594
chr5 88883598
chr5 88883660

chr5 88883662
chr5 88883679
chr5 88883707
chr5 88883722
chr5 88883724
chr5 88883766
chr5 88883779
chr5 88883996
chr5 88884021
chr5 88884050
chr5 88884066
chr5 88884069
chr5 88884076
chr5 88884093
chr5 88884095
chr5 88884098
chr5 88884106
chr5 88884113
chr5 88884117
chr5 88884136
chr5 88884141
chr5 88884144
chr5 88884146
chr5 88884150
chr5 88884161
chr5 88884167
chr5 88884172
chr5 88884204
chr5 88884231
chr5 88884233
chr5 88884276
chr5 88884278
chr5 88884301
chr5 88884305
chr5 88884310
chr5 88884318
chr5 88884327
chr5 88884329
chr5 88884340
chr5 88884363
chr5 88884372
chr5 88884387
chr5 88884398
chr5 88884400
chr5 88884425
chr5 88884435
chr5 88884440
chr5 88884469
chr5 88884504
chr5 88884513
chr5 88884515
chr5 88884554
chr5 88884571
chr5 88884682

chr5 88884854
chr5 88885123
chr5 88888740
chr5 88888870
chr5 88889052
chr5 88890010
chr5 88890013
chr5 88890037
chr5 88890074
chr5 88890168
chr5 88890170
chr5 88890176
chr5 88890307
chr5 88890401
chr5 88890415
chr5 88890447
chr5 88890581
chr5 88890593
chr5 89330003
chr5 89330135
chr5 90265438
chr5 90265447
chr5 90265557
chr5 90409020
chr5 90409050
chr5 90409058
chr5 90409188
chr5 90409266
chr5 90409275
chr5 90409300
chr5 90409320
chr5 90409322
chr5 90409354
chr5 90409356
chr5 90409375
chr5 90409379
chr5 90409417
chr5 90409419
chr5 90409460
chr5 90409500
chr5 90409527
chr5 90409529
chr5 90409553
chr5 90409558
chr5 90409568
chr5 90409602
chr5 90409607
chr5 90409627
chr5 90409736
chr5 90409749
chr5 90410093
chr5 90410121
chr5 90410124
chr5 90410161

chr5 90410198
chr5 90410280
chr5 90410316
chr5 90410467
chr5 90410581
chr5 90410614
chr5 90410633
chr5 90410666
chr5 90410676
chr5 90410741
chr5 90410776
chr5 90410873
chr5 90528976
chr5 90529036
chr5 90529061
chr5 90529067
chr5 90529076
chr5 90529219
chr5 90529246
chr5 90530274
chr5 90530473
chr5 90559069
chr5 90559071
chr5 90559078
chr5 90559100
chr5 90559102
chr5 90559105
chr5 90559123
chr5 90559138
chr5 90559162
chr5 90559255
chr5 90559311
chr5 90559316
chr5 90559393
chr5 90559396
chr5 90559561
chr5 90597214
chr5 90597697
chr5 90604572
chr5 90604627
chr5 90604676
chr5 90956648
chr5 90956758
chr5 90963089
chr5 90963562
chr5 91266603
chr5 91311969
chr5 91378958
chr5 91379089
chr5 91379133
chr5 91379287
chr5 91379423
chr5 91379468
chr5 91379484

chr5 91379643
chr5 91379688
chr5 91379744
chr5 91379821
chr5 91379844
chr5 91379873
chr5 91379876
chr5 91379906
chr5 91379932
chr5 91379957
chr5 91380006
chr5 91380015
chr5 91380021
chr5 91380025
chr5 91380032
chr5 91380059
chr5 91383519
chr5 91383571
chr5 91383581
chr5 91383584
chr5 91383600
chr5 91383606
chr5 91383610
chr5 91383807
chr5 91383819
chr5 91383823
chr5 91383844
chr5 91383861
chr5 91383872
chr5 91383968
chr5 91384000
chr5 91384082
chr5 91384114
chr5 91384148
chr5 91384183
chr5 91384247
chr5 91384321
chr5 91384350
chr5 91384422
chr5 91384426
chr5 91384447
chr5 91384530
chr5 91384698
chr5 91384708
chr5 91384725
chr5 91384930
chr5 91385162
chr5 91385283
chr5 91419354
chr5 91920620
chr5 91920641
chr5 91920730
chr5 91921299
chr5 92196077

chr5 92196373
chr5 92196384
chr5 92196775
chr5 92304375
chr5 92304961
chr5 92305173
chr5 92305221
chr5 92518425
chr5 92579024
chr5 92579090
chr5 92579212
chr5 92627907
chr5 92628333
chr5 92864136
chr5 93042903
chr5 93043538
chr5 93043632
chr5 93215599
chr5 93216386
chr5 93217003
chr5 93217093
chr5 93263163
chr5 93264196
chr5 93264229
chr5 93264987
chr5 93265264
chr5 93265458
chr5 93265745
chr5 93266142
chr5 93574789
chr5 93574828
chr5 93575063
chr5 93575448
chr5 93575836
chr5 93575883
chr5 93575987
chr5 93576055
chr5 93576256
chr5 93576263
chr5 93581500
chr5 93581520
chr5 93581531
chr5 93581535
chr5 93581541
chr5 93581548
chr5 93581552
chr5 93581566
chr5 93581575
chr5 93581591
chr5 93581594
chr5 93581596
chr5 93581603
chr5 93581612
chr5 93581628

chr5 93581630
chr5 93581658
chr5 93581666
chr5 93581815
chr5 93581886
chr5 93582116
chr5 93582362
chr5 93582366
chr5 93582644
chr5 93582719
chr5 93582726
chr5 93582738
chr5 93582767
chr5 93582811
chr5 93582847
chr5 93582854
chr5 93582872
chr5 93582948
chr5 93583004
chr5 93583050
chr5 93583075
chr5 93583123
chr5 93583142
chr5 93583157
chr5 93583204
chr5 93583206
chr5 93583221
chr5 93583237
chr5 93583247
chr5 93583255
chr5 93583566
chr5 93583580
chr5 93583629
chr5 93583677
chr5 93583770
chr5 93586245
chr5 93586309
chr5 93586319
chr5 93586337
chr5 93586502
chr5 93586561
chr5 93599420
chr5 93599486
chr5 93599490
chr5 93599529
chr5 93599535
chr5 93599562
chr5 93599577
chr5 93599591
chr5 93599603
chr5 93599668
chr5 93599692
chr5 93599751
chr5 93599795

chr5 93600038
chr5 93600091
chr5 93600364
chr5 93600584
chr5 93600640
chr5 93600681
chr5 93600728
chr5 93600752
chr5 93600801
chr5 93600822
chr5 93601035
chr5 93601228
chr5 93601456
chr5 93601659
chr5 93601905
chr5 93605043
chr5 93605063
chr5 93605770
chr5 93605947
chr5 93606031
chr5 93606073
chr5 93606114
chr5 93606119
chr5 93606207
chr5 93606311
chr5 93607103
chr5 93607473
chr5 93607489
chr5 93607503
chr5 93607543
chr5 93607561
chr5 93607566
chr5 93607578
chr5 93607715
chr5 93607750
chr5 93607787
chr5 93607828
chr5 93607836
chr5 93608002
chr5 93608006
chr5 93608040
chr5 93608054
chr5 93608155
chr5 93608352
chr5 93608355
chr5 93608411
chr5 93608691
chr5 93608770
chr5 93608856
chr5 93608998
chr5 93609049
chr5 93609089
chr5 93609380
chr5 93609516

chr5 93609757
chr5 93609853
chr5 93609875
chr5 93609881
chr5 93609883
chr5 93609921
chr5 93609947
chr5 93610016
chr5 93610235
chr5 93610294
chr5 93610311
chr5 93610473
chr5 93610621
chr5 93610678
chr5 93610720
chr5 93612707
chr5 93612718
chr5 93612762
chr5 93612777
chr5 93612781
chr5 93612806
chr5 93613493
chr5 93613570
chr5 93613731
chr5 93614619
chr5 93614670
chr5 93614675
chr5 93620312
chr5 93620364
chr5 93620445
chr5 93620575
chr5 93620615
chr5 93620621
chr5 93620626
chr5 93620629
chr5 93620633
chr5 93620642
chr5 93620644
chr5 93620647
chr5 93620650
chr5 93620653
chr5 93620656
chr5 93620662
chr5 93620671
chr5 93620673
chr5 93620675
chr5 93620709
chr5 93621089
chr5 93623046
chr5 93623223
chr5 93623290
chr5 93874181
chr5 94111340
chr5 94111369

chr5 94111383
chr5 94617979
chr5 94618004
chr5 94618111
chr5 94618127
chr5 94618161
chr5 94618186
chr5 94618209
chr5 94746189
chr5 94746242
chr5 94746355
chr5 94746394
chr5 95080460
chr5 95080482
chr5 95080514
chr5 95080546
chr5 95081328
chr5 95081562
chr5 95081608
chr5 95081635
chr5 95082037
chr5 95082147
chr5 95082188
chr5 95240874
chr5 95283316
chr5 95283357
chr5 95283425
chr5 95283441
chr5 95425340
chr5 95555658
chr5 95555663
chr5 95555681
chr5 95555709
chr5 95555758
chr5 95555893
chr5 95555919
chr5 95556029
chr5 95556031
chr5 95556117
chr5 95622139
chr5 95622200
chr5 95622311
chr5 95622370
chr5 95622577
chr5 95622914
chr5 95646973
chr5 95646981
chr5 95646989
chr5 95647011
chr5 95647069
chr5 95647071
chr5 95647076
chr5 95647146
chr5 95647336

chr5 95647514
chr5 95821372
chr5 95821767
chr5 95821959
chr5 95822083
chr5 95822186
chr5 95822775
chr5 95822798
chr5 95822819
chr5 95823286
chr5 95823318
chr5 95823423
chr5 95823446
chr5 95823661
chr5 95823686
chr5 95823719
chr5 95823754
chr5 95823816
chr5 95823874
chr5 95823901
chr5 95823910
chr5 95823935
chr5 95823994
chr5 95824042
chr5 95824108
chr5 95824122
chr5 95824315
chr5 95824355
chr5 95834529
chr5 95835410
chr5 95835584
chr5 95835690
chr5 95835784
chr5 95835889
chr5 95835920
chr5 95835970
chr5 95835995
chr5 95835999
chr5 95836151
chr5 95836209
chr5 95836251
chr5 95843151
chr5 95843185
chr5 95843250
chr5 95843288
chr5 95843337
chr5 95843378
chr5 95869624
chr5 95870513
chr5 96431213
chr5 96431298
chr5 96431340
chr5 96431385
chr5 96434475

chr5 96434505
chr5 96434534
chr5 96434562
chr5 96434565
chr5 96435158
chr5 96435246
chr5 96661232
chr5 96661245
chr5 96661295
chr5 96661297
chr5 96661339
chr5 96661356
chr5 96661368
chr5 96661379
chr5 96661381
chr5 96661403
chr5 96661482
chr5 96661494
chr5 96661608
chr5 96661651
chr5 96661704
chr5 96661752
chr5 96662675
chr5 96662683
chr5 96662687
chr5 96662698
chr5 96662705
chr5 96662710
chr5 96662717
chr5 96662738
chr5 96662740
chr5 96662765
chr5 96662791
chr5 96662818
chr5 96662847
chr5 96662862
chr5 96662864
chr5 96662873
chr5 96662878
chr5 96662885
chr5 96662901
chr5 96662927
chr5 96662941
chr5 96662945
chr5 96662955
chr5 96662964
chr5 96662967
chr5 96663003
chr5 96663017
chr5 96663035
chr5 96663041
chr5 96663049
chr5 96663062
chr5 96663065

chr5 96663074
chr5 96663084
chr5 96663086
chr5 96663092
chr5 96663095
chr5 96663104
chr5 96663107
chr5 96663111
chr5 96663116
chr5 96663126
chr5 96806652
chr5 96806660
chr5 96806692
chr5 96806888
chr5 96807009
chr5 96807025
chr5 96807098
chr5 96807194
chr5 96807241
chr5 96807314
chr5 96807319
chr5 96807321
chr5 96816233
chr5 96816256
chr5 96816430
chr5 96816501
chr5 96816590
chr5 96816673
chr5 96816811
chr5 96816904
chr5 96816967
chr5 96817144
chr5 96877115
chr5 96877135
chr5 96877194
chr5 96877270
chr5 96877423
chr5 96877650
chr5 96877674
chr5 96877791
chr5 96896433
chr5 96896452
chr5 96896461
chr5 96896648
chr5 96933554
chr5 96933577
chr5 96933780
chr5 96933833
chr5 96933924
chr5 96934156
chr5 96934346
chr5 96934410
chr5 96934417
chr5 96934457

chr5 96934464
chr5 96937028
chr5 96937035
chr5 96937097
chr5 96937482
chr5 96937561
chr5 96957459
chr5 96957685
chr5 96957807
chr5 96958224
chr5 96958229
chr5 96958424
chr5 97182113
chr5 97182286
chr5 97183697
chr5 97183716
chr5 97183725
chr5 97183734
chr5 97183741
chr5 97183761
chr5 97183801
chr5 97183808
chr5 97183814
chr5 97183844
chr5 97183853
chr5 97183855
chr5 97183858
chr5 97183862
chr5 97183900
chr5 97183912
chr5 97183914
chr5 97183926
chr5 97183940
chr5 97246418
chr5 97300257
chr5 97300328
chr5 97318849
chr5 97319211
chr5 97319239
chr5 97319463
chr5 97319480
chr5 97409744
chr5 97409779
chr5 97410054
chr5 97410355
chr5 97491136
chr5 97491888
chr5 97581876
chr5 97581963
chr5 98123619
chr5 98337586
chr5 98337718
chr5 98337809
chr5 98549448

chr5 98549487
chr5 98549783
chr5 98549862
chr5 98549955
chr5 98550304
chr5 98659329
chr5 98659628
chr5 98767642
chr5 98767665
chr5 98768091
chr5 98768104
chr5 98768192
chr5 98768261
chr5 98768294
chr5 98768301
chr5 98768359
chr5 98768442
chr5 98768455
chr5 98768462
chr5 98768464
chr5 98768478
chr5 98768572
chr5 98768581
chr5 98768588
chr5 98768593
chr5 98768597
chr5 98768607
chr5 98768616
chr5 98771037
chr5 98771120
chr5 98771129
chr5 98771153
chr5 98771312
chr5 98771344
chr5 98771370
chr5 98771390
chr5 98771409
chr5 98771418
chr5 98926845
chr5 98926881
chr5 98926905
chr5 98927009
chr5 98927101
chr5 98927212
chr5 98927309
chr5 98927354
chr5 98927367
chr5 98927396
chr5 98927428
chr5 98927456
chr5 98933656
chr5 98933690
chr5 99033303
chr5 99033331

chr5 99033389
chr5 99033464
chr5 99033711
chr5 99413018
chr5 99413340
chr5 99413521
chr5 99413850
chr5 99413907
chr5 99414018
chr5 99414127
chr5 99516837
chr5 99517013
chr5 99645007
chr5 99680699
chr5 99680903
chr5 99856987
chr5 99857113
chr5 99857149
chr5 99857154
chr5 99857813
chr5 100073054
chr5 100245762
chr5 100245773
chr5 100270073
chr5 100270245
chr5 100270309
chr5 100270333
chr5 100270483
chr5 100270579
chr5 100270607
chr5 100466129
chr5 100534762
chr5 100534794
chr5 100535171
chr5 100535183
chr5 100535222
chr5 100535228
chr5 100535234
chr5 100535251
chr5 100535253
chr5 100535257
chr5 100535266
chr5 100535280
chr5 100535296
chr5 100535316
chr5 100535322
chr5 100535681
chr5 100535727
chr5 100535732
chr5 100535751
chr5 100535757
chr5 100535759
chr5 100535767
chr5 100535797

chr5 100535808
chr5 100535958
chr5 100535979
chr5 100535993
chr5 100536008
chr5 100536015
chr5 100536211
chr5 100536835
chr5 100731466
chr5 100731598
chr5 100731841
chr5 100731886
chr5 100731980
chr5 100732080
chr5 100732095
chr5 100732124
chr5 100853196
chr5 100853257
chr5 100853321
chr5 100889354
chr5 100889551
chr5 100889956
chr5 100890073
chr5 100890249
chr5 100890515
chr5 100890593
chr5 100898735
chr5 100898876
chr5 100898889
chr5 100899071
chr5 100899121
chr5 100899398
chr5 100899990
chr5 100900067
chr5 100900131
chr5 100900145
chr5 100900277
chr5 100900635
chr5 100900647
chr5 100900657
chr5 100900659
chr5 100900662
chr5 100900666
chr5 100900668
chr5 100900701
chr5 100900733
chr5 100900864
chr5 100900867
chr5 100900870
chr5 100900873
chr5 100900897
chr5 100900902
chr5 100900904
chr5 100900933

chr5 100900945
chr5 100904627
chr5 100904667
chr5 100904716
chr5 100904747
chr5 100904751
chr5 100904757
chr5 100904791
chr5 100904795
chr5 100904833
chr5 100904966
chr5 100904985
chr5 100905116
chr5 100905171
chr5 100905366
chr5 100905381
chr5 100905479
chr5 100905585
chr5 100905787
chr5 100905792
chr5 101627142
chr5 101627930
chr5 101627994
chr5 102015602
chr5 102153661
chr5 102153811
chr5 102295964
chr5 102295998
chr5 102296019
chr5 102296026
chr5 102296036
chr5 102296049
chr5 102296172
chr5 102296185
chr5 102296188
chr5 102296198
chr5 102296201
chr5 102296253
chr5 102296280
chr5 102296286
chr5 102296346
chr5 102499960
chr5 102500354
chr5 102500391
chr5 102755848
chr5 102755892
chr5 102756339
chr5 102756344
chr5 102866560
chr5 102866596
chr5 103119237
chr5 103120979
chr5 103121000
chr5 103121015

chr5 103121063
chr5 103121093
chr5 103121174
chr5 103121185
chr5 103121275
chr5 103121308
chr5 103121451
chr5 103121526
chr5 103139188
chr5 103139463
chr5 103139576
chr5 103143471
chr5 103258257
chr5 103258267
chr5 103258288
chr5 103258296
chr5 103258422
chr5 103260050
chr5 103260164
chr5 103260237
chr5 103260999
chr5 103562555
chr5 103562578
chr5 103615439
chr5 103615572
chr5 103710167
chr5 103947079
chr5 103947706
chr5 103948331
chr5 103948457
chr5 103948661
chr5 103948759
chr5 103948768
chr5 104082102
chr5 104287221
chr5 104287262
chr5 104287305
chr5 104287364
chr5 104287818
chr5 104287941
chr5 104288070
chr5 104402744
chr5 104615011
chr5 104615392
chr5 104930818
chr5 105386844
chr5 105386893
chr5 105387208
chr5 105387221
chr5 105387267
chr5 105387427
chr5 105388288
chr5 105388717
chr5 105388812

chr5 105395246
chr5 105633152
chr5 105633165
chr5 105633441
chr5 105633718
chr5 105634680
chr5 105656625
chr5 105656672
chr5 105856357
chr5 106152996
chr5 106153249
chr5 106156316
chr5 106156423
chr5 106157960
chr5 106158192
chr5 106158360
chr5 106158755
chr5 106158785
chr5 106158876
chr5 106159185
chr5 106207392
chr5 106408172
chr5 106510229
chr5 106717384
chr5 106717769
chr5 106718006
chr5 106870261
chr5 106870373
chr5 107388837
chr5 107542063
chr5 107571672
chr5 107571710
chr5 107571750
chr5 107571775
chr5 107571801
chr5 107571822
chr5 107571885
chr5 107571964
chr5 107572023
chr5 107572052
chr5 107572124
chr5 107572134
chr5 107572138
chr5 107572161
chr5 107572189
chr5 107572203
chr5 107572205
chr5 107572218
chr5 107572233
chr5 107572247
chr5 107572267
chr5 107673521
chr5 107673528
chr5 107673545

chr5 107673548
chr5 107673559
chr5 107673575
chr5 107673582
chr5 107673597
chr5 107673599
chr5 107673605
chr5 107673614
chr5 107673756
chr5 107673770
chr5 107673788
chr5 107673801
chr5 107673822
chr5 107673830
chr5 107673856
chr5 107673881
chr5 107673890
chr5 107673936
chr5 107673966
chr5 107674009
chr5 107674056
chr5 107674173
chr5 108379616
chr5 108380129
chr5 108380143
chr5 108380196
chr5 108380201
chr5 108380242
chr5 108380280
chr5 108380378
chr5 108380412
chr5 108380577
chr5 108380609
chr5 108380611
chr5 108380638
chr5 108380644
chr5 108382816
chr5 108382832
chr5 108382857
chr5 108382883
chr5 108382950
chr5 108382981
chr5 108382993
chr5 108383015
chr5 108594553
chr5 108728396
chr5 108728442
chr5 108728453
chr5 108728472
chr5 108728484
chr5 108728486
chr5 108728525
chr5 108728577
chr5 108728615

chr5 108728637
chr5 108728677
chr5 108728723
chr5 108728737
chr5 108728882
chr5 108728944
chr5 108728952
chr5 108728963
chr5 108728980
chr5 108729007
chr5 108747746
chr5 108747754
chr5 108747788
chr5 108747832
chr5 108747842
chr5 108747930
chr5 108747941
chr5 108747964
chr5 108747979
chr5 108747994
chr5 108748959
chr5 108748975
chr5 108749001
chr5 108749006
chr5 108749008
chr5 108749011
chr5 108749014
chr5 108749017
chr5 108749024
chr5 108749034
chr5 108749042
chr5 108749044
chr5 108749083
chr5 108749103
chr5 108749113
chr5 108749117
chr5 108749124
chr5 108749133
chr5 108749148
chr5 108749162
chr5 108749175
chr5 108749191
chr5 108749213
chr5 108749225
chr5 108749229
chr5 108749261
chr5 108749282
chr5 108749289
chr5 108749306
chr5 108749313
chr5 108749328
chr5 108749335
chr5 108749338
chr5 108749340

chr5 108749354
chr5 108749358
chr5 108749369
chr5 108749374
chr5 108749377
chr5 108749379
chr5 108749400
chr5 108749407
chr5 108749467
chr5 108749470
chr5 108749485
chr5 108749488
chr5 108749491
chr5 108749496
chr5 108749501
chr5 108749556
chr5 108749573
chr5 108749577
chr5 108749616
chr5 108994073
chr5 108994402
chr5 109403182
chr5 109407752
chr5 109407795
chr5 109408208
chr5 109409580
chr5 109409587
chr5 109409590
chr5 109409594
chr5 109409596
chr5 109409600
chr5 109409605
chr5 109411012
chr5 109411017
chr5 109411020
chr5 109411045
chr5 109411059
chr5 109411061
chr5 109411104
chr5 109411253
chr5 109411303
chr5 109691334
chr5 109691336
chr5 109691338
chr5 109691347
chr5 109691381
chr5 109691399
chr5 109691441
chr5 109691501
chr5 109691520
chr5 109691725
chr5 110867881
chr5 110867912
chr5 110874218

chr5 110874292
chr5 110895852
chr5 110895999
chr5 110955324
chr5 111223733
chr5 111224889
chr5 111224925
chr5 111224965
chr5 111225020
chr5 111225080
chr5 111225106
chr5 111225417
chr5 111225571
chr5 111225969
chr5 111226015
chr5 111226205
chr5 111226230
chr5 111226376
chr5 111226411
chr5 111511662
chr5 111512943
chr5 111512951
chr5 111512990
chr5 111513008
chr5 111513075
chr5 111513117
chr5 111513153
chr5 111513271
chr5 111529720
chr5 111529787
chr5 111530027
chr5 111530272
chr5 111530313
chr5 111530366
chr5 111530368
chr5 111530385
chr5 111530464
chr5 111530468
chr5 111530472
chr5 111550771
chr5 111550793
chr5 111550830
chr5 111551013
chr5 111757689
chr5 111757702
chr5 111757708
chr5 111757718
chr5 111757723
chr5 111757729
chr5 111757734
chr5 111757742
chr5 111757745
chr5 111757747
chr5 111757749

chr5 111757759
chr5 111757791
chr5 111757796
chr5 111757799
chr5 111757803
chr5 111969192
chr5 111969496
chr5 112160853
chr5 112160882
chr5 112160922
chr5 112160936
chr5 112160938
chr5 112160950
chr5 112160958
chr5 112160961
chr5 112160965
chr5 112160973
chr5 112160975
chr5 112160998
chr5 112161004
chr5 112161007
chr5 112161018
chr5 112161027
chr5 112161033
chr5 112161048
chr5 112161067
chr5 112161075
chr5 112161085
chr5 112161095
chr5 112161098
chr5 112161117
chr5 112161160
chr5 112161180
chr5 112161211
chr5 112161234
chr5 112161307
chr5 112161311
chr5 112161325
chr5 112161337
chr5 112161342
chr5 112161367
chr5 112161379
chr5 112161540
chr5 112161551
chr5 112161628
chr5 112161636
chr5 112161638
chr5 112161874
chr5 112205444
chr5 112418147
chr5 112418152
chr5 112418205
chr5 112418211
chr5 112418213

chr5 112418216
chr5 112737519
chr5 112737651
chr5 112737653
chr5 112737667
chr5 112737676
chr5 112737701
chr5 112737709
chr5 112737729
chr5 112737736
chr5 112737741
chr5 112737748
chr5 112737773
chr5 112737782
chr5 112737792
chr5 112737805
chr5 112737808
chr5 112737816
chr5 112737819
chr5 112737837
chr5 112737841
chr5 112737847
chr5 112737873
chr5 112861622
chr5 112861652
chr5 112861654
chr5 112861677
chr5 112861741
chr5 112923507
chr5 112923680
chr5 112923684
chr5 112923782
chr5 112924153
chr5 112924173
chr5 112929574
chr5 112929812
chr5 112976328
chr5 112976359
chr5 112976361
chr5 112976382
chr5 112976396
chr5 112976425
chr5 112976427
chr5 112977642
chr5 112977644
chr5 112977731
chr5 112977761
chr5 112977791
chr5 112977800
chr5 112979022
chr5 112979058
chr5 112979488
chr5 112979928
chr5 112979935

chr5 112979947
chr5 112996220
chr5 112996239
chr5 112996248
chr5 112996310
chr5 113188355
chr5 113293807
chr5 113293809
chr5 113293853
chr5 113293863
chr5 113293868
chr5 113293872
chr5 113489435
chr5 113489525
chr5 113489569
chr5 113490497
chr5 113513787
chr5 113513803
chr5 113616125
chr5 113616224
chr5 113616263
chr5 113626435
chr5 113626578
chr5 113626629
chr5 113692333
chr5 113692442
chr5 113692477
chr5 113692495
chr5 113692596
chr5 113692763
chr5 113692831
chr5 113692980
chr5 113693017
chr5 114359905
chr5 114359968
chr5 114359986
chr5 114360128
chr5 114360161
chr5 114360295
chr5 114360303
chr5 114360352
chr5 114360421
chr5 114360450
chr5 114360516
chr5 114694268
chr5 115262141
chr5 115262155
chr5 115262178
chr5 115262202
chr5 115262221
chr5 115262279
chr5 115262372
chr5 115262375
chr5 115262405

chr5 115262440
chr5 115262491
chr5 115262516
chr5 115262544
chr5 115262548
chr5 115262551
chr5 115262605
chr5 115262609
chr5 115262611
chr5 115262624
chr5 115262627
chr5 115262638
chr5 115262643
chr5 115262649
chr5 115262652
chr5 115262654
chr5 115262662
chr5 115262672
chr5 115262704
chr5 115262722
chr5 115262736
chr5 115262741
chr5 115262760
chr5 115296201
chr5 115296224
chr5 115296230
chr5 115296246
chr5 115296268
chr5 115296290
chr5 115296297
chr5 115296299
chr5 115296311
chr5 115296387
chr5 115296394
chr5 115296469
chr5 115296471
chr5 115296475
chr5 115296492
chr5 115296499
chr5 115296501
chr5 115296508
chr5 115296514
chr5 115296526
chr5 115296529
chr5 115296537
chr5 115296551
chr5 115296554
chr5 115296563
chr5 115296568
chr5 115296570
chr5 115296573
chr5 115296580
chr5 115296595
chr5 115296604

chr5 115296634
chr5 115296639
chr5 115296649
chr5 115296679
chr5 115296685
chr5 115296697
chr5 115296710
chr5 115296726
chr5 115296734
chr5 115296743
chr5 115296761
chr5 115296767
chr5 115296774
chr5 115296779
chr5 115296786
chr5 115296797
chr5 115296807
chr5 115296812
chr5 115296831
chr5 115296835
chr5 115296841
chr5 115296859
chr5 115296878
chr5 115544009
chr5 115544027
chr5 115544079
chr5 115544102
chr5 115544109
chr5 115544128
chr5 115544144
chr5 115544167
chr5 115544198
chr5 115544214
chr5 115544238
chr5 115544242
chr5 115544252
chr5 115602217
chr5 115602219
chr5 115602222
chr5 115602224
chr5 115602227
chr5 115626602
chr5 115626606
chr5 115626763
chr5 115626838
chr5 115634413
chr5 115634516
chr5 115634518
chr5 115634655
chr5 115634800
chr5 115634809
chr5 115634830
chr5 115634884
chr5 115673654

chr5 115839054
chr5 115839071
chr5 115840959
chr5 115841785
chr5 115841797
chr5 115841801
chr5 115841807
chr5 115841825
chr5 115841833
chr5 115841851
chr5 115841872
chr5 115841883
chr5 115841897
chr5 115841901
chr5 115841904
chr5 115841909
chr5 115841915
chr5 115841917
chr5 115841919
chr5 115841921
chr5 115841925
chr5 115841927
chr5 115841931
chr5 115841941
chr5 115841956
chr5 115841962
chr5 115841970
chr5 115841975
chr5 115841982
chr5 115841984
chr5 115841986
chr5 115841990
chr5 115841996
chr5 115842019
chr5 115842023
chr5 115842028
chr5 115842048
chr5 115842070
chr5 115842078
chr5 115842080
chr5 115842106
chr5 115842120
chr5 115842123
chr5 115842131
chr5 115842135
chr5 115842146
chr5 115842152
chr5 115842155
chr5 115842162
chr5 115842169
chr5 115842171
chr5 115842185
chr5 115842195
chr5 115842198

chr5 115842200
chr5 115842205
chr5 115842217
chr5 115842222
chr5 115842226
chr5 115842233
chr5 115842237
chr5 115842288
chr5 115842290
chr5 115842292
chr5 115842303
chr5 115842306
chr5 115842318
chr5 115842322
chr5 115842335
chr5 115842917
chr5 115843067
chr5 115843113
chr5 116135631
chr5 116136015
chr5 116904167
chr5 116904216
chr5 116904278
chr5 117068329
chr5 117068369
chr5 117068611
chr5 117068713
chr5 117563400
chr5 117563587
chr5 117564185
chr5 118036147
chr5 118036671
chr5 118036968
chr5 118211017
chr5 118280507
chr5 119031304
chr5 119031390
chr5 119031448
chr5 119031497
chr5 119063741
chr5 119063845
chr5 119063861
chr5 119063949
chr5 119071731
chr5 119071734
chr5 119071744
chr5 119071746
chr5 119071830
chr5 119071935
chr5 119071963
chr5 119072181
chr5 119270090
chr5 119270214
chr5 119270220

chr5 119270351
chr5 119270373
chr5 119270386
chr5 119270934
chr5 119271059
chr5 119271072
chr5 119274128
chr5 119274188
chr5 119274270
chr5 119274543
chr5 119274568
chr5 119287695
chr5 119287720
chr5 119287966
chr5 119288390
chr5 119299403
chr5 119299428
chr5 119299435
chr5 119299437
chr5 119299455
chr5 119299489
chr5 119299493
chr5 119299589
chr5 119299768
chr5 119299814
chr5 119299818
chr5 119299835
chr5 119299890
chr5 119308199
chr5 119308253
chr5 119308914
chr5 119309482
chr5 119315662
chr5 119315768
chr5 119318402
chr5 119318418
chr5 119318484
chr5 119318540
chr5 119318671
chr5 119318759
chr5 119324266
chr5 119328957
chr5 119329050
chr5 119329083
chr5 119329090
chr5 119329129
chr5 119329166
chr5 119329196
chr5 119333000
chr5 119333015
chr5 119333152
chr5 119340174
chr5 119340193
chr5 119340246

chr5 119340591
chr5 119340619
chr5 119340707
chr5 119340800
chr5 119340872
chr5 119340885
chr5 119340897
chr5 119340951
chr5 119340967
chr5 119341020
chr5 119341032
chr5 119341146
chr5 119341220
chr5 119341346
chr5 119341455
chr5 119341477
chr5 119341544
chr5 119341696
chr5 119342105
chr5 119342290
chr5 119355999
chr5 119356002
chr5 119356008
chr5 119356015
chr5 119356025
chr5 119356033
chr5 119356038
chr5 119356042
chr5 119356056
chr5 119356063
chr5 119356099
chr5 119356148
chr5 119356161
chr5 119356215
chr5 119356231
chr5 119356242
chr5 119356248
chr5 119356259
chr5 119356273
chr5 119356280
chr5 119356310
chr5 119356316
chr5 119356342
chr5 119356394
chr5 119356474
chr5 119356536
chr5 119356549
chr5 119356580
chr5 119356648
chr5 119356731
chr5 119356812
chr5 119357178
chr5 119357243
chr5 119357328

chr5 119357495
chr5 119357619
chr5 119357746
chr5 119357764
chr5 119452830
chr5 119452848
chr5 120143896
chr5 120150488
chr5 120184321
chr5 120184819
chr5 120458388
chr5 120458435
chr5 120458441
chr5 120463195
chr5 120463198
chr5 120463361
chr5 120463512
chr5 120463525
chr5 120463689
chr5 120463836
chr5 120464685
chr5 120464694
chr5 120464708
chr5 120464959
chr5 120464972
chr5 120464983
chr5 120464992
chr5 120514890
chr5 120833511
chr5 120834008
chr5 120834213
chr5 121109593
chr5 121109978
chr5 121615304
chr5 121887530
chr5 121919265
chr5 121919552
chr5 121919738
chr5 121951856
chr5 121951859
chr5 121951884
chr5 121961467
chr5 121961506
chr5 121961586
chr5 121961624
chr5 121961627
chr5 121961634
chr5 121961668
chr5 121962464
chr5 121962543
chr5 121962581
chr5 121962683
chr5 121962701
chr5 121962707

chr5 121962809
chr5 121962855
chr5 121963024
chr5 121963263
chr5 121963375
chr5 122076397
chr5 122076441
chr5 122076489
chr5 122076506
chr5 122264489
chr5 122690425
chr5 122723540
chr5 122723689
chr5 122723750
chr5 122724118
chr5 122775384
chr5 122775412
chr5 122775417
chr5 122775419
chr5 122775710
chr5 122775890
chr5 122775945
chr5 122775956
chr5 122776060
chr5 123037189
chr5 123037198
chr5 123037217
chr5 123037247
chr5 123037296
chr5 123091652
chr5 123091698
chr5 123091769
chr5 123091854
chr5 123091875
chr5 123091928
chr5 123091967
chr5 123092047
chr5 123092468
chr5 123092496
chr5 123092732
chr5 123092785
chr5 123092805
chr5 123092895
chr5 123092958
chr5 123093032
chr5 123093201
chr5 123093250
chr5 123093264
chr5 123093271
chr5 123093442
chr5 123107921
chr5 123285475
chr5 123285490
chr5 123285492

chr5 123285549
chr5 123285551
chr5 123423608
chr5 123423624
chr5 123423635
chr5 123423719
chr5 123423799
chr5 123423807
chr5 123423809
chr5 123423825
chr5 123423853
chr5 123512652
chr5 123512670
chr5 123512674
chr5 123512690
chr5 123512693
chr5 123512699
chr5 123512702
chr5 123512705
chr5 123512711
chr5 123512713
chr5 123512720
chr5 123512735
chr5 123512747
chr5 123513215
chr5 123513264
chr5 123513331
chr5 124163159
chr5 124163291
chr5 124163321
chr5 124163347
chr5 124163555
chr5 124164415
chr5 124165086
chr5 124519704
chr5 124519792
chr5 124519816
chr5 124519973
chr5 124560397
chr5 124560696
chr5 124649899
chr5 124649903
chr5 124649905
chr5 124649909
chr5 124650051
chr5 124650079
chr5 124650122
chr5 124650255
chr5 124650316
chr5 124650436
chr5 124650484
chr5 124650981
chr5 124651222
chr5 124746497

chr5 124746561
chr5 124746589
chr5 124746626
chr5 124746630
chr5 124746639
chr5 124746660
chr5 124746724
chr5 124746735
chr5 124746753
chr5 124746757
chr5 124746933
chr5 124746937
chr5 124746942
chr5 124746949
chr5 124746963
chr5 124746983
chr5 124746986
chr5 124747008
chr5 124747084
chr5 124747106
chr5 124747135
chr5 124747188
chr5 124747198
chr5 124747221
chr5 124747230
chr5 124747272
chr5 124747306
chr5 124747338
chr5 124747391
chr5 124747408
chr5 124747431
chr5 124747463
chr5 124747495
chr5 124747523
chr5 124747547
chr5 124747639
chr5 124747647
chr5 124747656
chr5 124747689
chr5 124747706
chr5 124747713
chr5 124747722
chr5 124747804
chr5 124747827
chr5 124747833
chr5 124747860
chr5 124748046
chr5 124748109
chr5 124748140
chr5 124748175
chr5 124748326
chr5 124748466
chr5 124748491
chr5 124748518

chr5 124748524
chr5 124748532
chr5 124748547
chr5 124748595
chr5 124748669
chr5 124748871
chr5 124748876
chr5 124748968
chr5 124748990
chr5 124749029
chr5 124749068
chr5 124749094
chr5 124749162
chr5 124749176
chr5 124749227
chr5 124749231
chr5 124749252
chr5 124749257
chr5 124749263
chr5 124749265
chr5 124749270
chr5 124749308
chr5 124749320
chr5 124749348
chr5 125613363
chr5 125613392
chr5 125613506
chr5 125613606
chr5 125614492
chr5 125617169
chr5 125617347
chr5 126051476
chr5 126051713
chr5 126052035
chr5 126153104
chr5 126423099
chr5 126423117
chr5 126423130
chr5 126423142
chr5 126423155
chr5 126423187
chr5 126423647
chr5 126423657
chr5 126423733
chr5 126423755
chr5 126423760
chr5 126423777
chr5 126424005
chr5 126424075
chr5 126424539
chr5 126449757
chr5 126449774
chr5 126449809
chr5 126449957

chr5 126449987
chr5 126450141
chr5 126450211
chr5 126450219
chr5 126450273
chr5 126450490
chr5 126624534
chr5 126624732
chr5 126747949
chr5 126748067
chr5 126748126
chr5 126748184
chr5 126748322
chr5 126748385
chr5 126748426
chr5 126758084
chr5 126758482
chr5 126758535
chr5 126758603
chr5 126758642
chr5 126758775
chr5 126778848
chr5 126778875
chr5 126778881
chr5 126779034
chr5 126779124
chr5 126779140
chr5 126845879
chr5 126845889
chr5 126845898
chr5 126845901
chr5 126845906
chr5 126845911
chr5 126845916
chr5 126845919
chr5 126846282
chr5 126855736
chr5 126972403
chr5 126972545
chr5 126972699
chr5 126972924
chr5 126973005
chr5 127072961
chr5 127073064
chr5 127073114
chr5 127073119
chr5 127073127
chr5 127073130
chr5 127073174
chr5 127073222
chr5 127073226
chr5 127073249
chr5 127073271
chr5 127073273

chr5 127073315
chr5 127073331
chr5 127073338
chr5 127073359
chr5 127073361
chr5 127073369
chr5 127073377
chr5 127073387
chr5 127073389
chr5 127073403
chr5 127073411
chr5 127073413
chr5 127073417
chr5 127073427
chr5 127073430
chr5 127073432
chr5 127073461
chr5 127073464
chr5 127073470
chr5 127073482
chr5 127073489
chr5 127073492
chr5 127073502
chr5 127073506
chr5 127073519
chr5 127073522
chr5 127073535
chr5 127073543
chr5 127073550
chr5 127073553
chr5 127289899
chr5 127290050
chr5 127290096
chr5 127290148
chr5 127290194
chr5 127290250
chr5 127290297
chr5 127290333
chr5 127290362
chr5 127290365
chr5 127290486
chr5 127290510
chr5 127290523
chr5 127290548
chr5 127290550
chr5 127290565
chr5 127290582
chr5 127394069
chr5 127394463
chr5 127423252
chr5 127423346
chr5 127423509
chr5 127423748
chr5 127518040

chr5 127518137
chr5 127518143
chr5 127518146
chr5 127518149
chr5 127518161
chr5 127518182
chr5 127518205
chr5 127518228
chr5 127518232
chr5 127518262
chr5 127567076
chr5 127567337
chr5 127567545
chr5 127567589
chr5 127567595
chr5 127567684
chr5 127629762
chr5 128502210
chr5 128535610
chr5 128740542
chr5 128964645
chr5 128964652
chr5 128964700
chr5 128964721
chr5 128967444
chr5 129044134
chr5 129308848
chr5 129910271
chr5 130731248
chr5 131069384
chr5 131110347
chr5 131110371
chr5 131163436
chr5 131163462
chr5 131163540
chr5 131163795
chr5 131163920
chr5 131163938
chr5 131163987
chr5 131164097
chr5 131164112
chr5 131170235
chr5 131170369
chr5 131170382
chr5 131170418
chr5 131170428
chr5 131170474
chr5 131171302
chr5 131171336
chr5 131171386
chr5 131171479
chr5 131171580
chr5 131171628
chr5 131171645

chr5 131171655
chr5 131171658
chr5 131171689
chr5 131209632
chr5 131209715
chr5 131209726
chr5 131209990
chr5 131210037
chr5 131210081
chr5 131252825
chr5 131252857
chr5 131252967
chr5 131252981
chr5 131253003
chr5 131253124
chr5 131253213
chr5 131253243
chr5 131253371
chr5 131253530
chr5 131253535
chr5 131253585
chr5 131253679
chr5 131253696
chr5 131254884
chr5 131263133
chr5 131263266
chr5 131263311
chr5 131263335
chr5 131263354
chr5 131265547
chr5 131265602
chr5 131266194
chr5 131266439
chr5 131266477
chr5 131266482
chr5 131266490
chr5 131266517
chr5 131266528
chr5 131266545
chr5 131266558
chr5 131266608
chr5 131266646
chr5 131266677
chr5 131266704
chr5 131266709
chr5 131270510
chr5 131270661
chr5 131270845
chr5 131271021
chr5 131272300
chr5 131272764
chr5 131272766
chr5 131274559
chr5 131274626

chr5 131274684
chr5 131274692
chr5 131281058
chr5 131281148
chr5 131281186
chr5 131281780
chr5 131281814
chr5 131281830
chr5 131281970
chr5 131289307
chr5 131289394
chr5 131289410
chr5 131289438
chr5 131289443
chr5 131289454
chr5 131289468
chr5 131289482
chr5 131289533
chr5 131289543
chr5 131289545
chr5 131289627
chr5 131289659
chr5 131378526
chr5 131378572
chr5 131379557
chr5 131379759
chr5 131379804
chr5 131379819
chr5 131379834
chr5 131382683
chr5 131382832
chr5 131383249
chr5 131389747
chr5 131389810
chr5 131416826
chr5 131416832
chr5 131416990
chr5 131417390
chr5 131417449
chr5 131417454
chr5 131417482
chr5 131514432
chr5 131514532
chr5 131514772
chr5 131514803
chr5 131514809
chr5 131515047
chr5 131595299
chr5 131634037
chr5 131634058
chr5 131634271
chr5 131634331
chr5 131634372
chr5 131634628

chr5 131634650
chr5 131634668
chr5 131636045
chr5 131636084
chr5 131636138
chr5 131636303
chr5 131636357
chr5 131636369
chr5 131636482
chr5 131636546
chr5 131795932
chr5 131795976
chr5 131810749
chr5 132179523
chr5 132179675
chr5 132179685
chr5 132180169
chr5 132189911
chr5 132190273
chr5 132190419
chr5 132190579
chr5 132410266
chr5 132410318
chr5 132410370
chr5 132411535
chr5 132411567
chr5 132411660
chr5 132411701
chr5 132411765
chr5 132411788
chr5 132411870
chr5 132412379
chr5 132412474
chr5 132412481
chr5 132412504
chr5 132412506
chr5 132412525
chr5 132412545
chr5 132412725
chr5 132412762
chr5 132412811
chr5 132420983
chr5 132421057
chr5 132421064
chr5 132421084
chr5 132421118
chr5 132421229
chr5 132455962
chr5 132456111
chr5 132456302
chr5 132456341
chr5 132456366
chr5 132456507
chr5 132456531

chr5 132456586
chr5 132466462
chr5 132466469
chr5 132466490
chr5 132466495
chr5 132466531
chr5 132466558
chr5 132466576
chr5 132466620
chr5 132466671
chr5 132466705
chr5 132466831
chr5 132466862
chr5 132466902
chr5 132466928
chr5 132467037
chr5 132467049
chr5 132467129
chr5 132467165
chr5 132473098
chr5 132473106
chr5 132473118
chr5 132473123
chr5 132473200
chr5 132473278
chr5 132473304
chr5 132473320
chr5 132487759
chr5 132487866
chr5 132487946
chr5 132488548
chr5 132488689
chr5 132488810
chr5 132488993
chr5 132489018
chr5 132489061
chr5 132489104
chr5 132489242
chr5 132489293
chr5 132489413
chr5 132489459
chr5 132489465
chr5 132489554
chr5 132495670
chr5 132495789
chr5 132495805
chr5 132495810
chr5 132495831
chr5 132495841
chr5 132495846
chr5 132495870
chr5 132495873
chr5 132495920
chr5 132495926

chr5 132515895
chr5 132515924
chr5 132516300
chr5 132517077
chr5 132517598
chr5 132518917
chr5 132519839
chr5 132519889
chr5 132519906
chr5 132520731
chr5 132520834
chr5 132557331
chr5 132557336
chr5 132557609
chr5 132684725
chr5 132684785
chr5 132684809
chr5 132684964
chr5 132685171
chr5 132746731
chr5 132746850
chr5 132746941
chr5 132853901
chr5 132962216
chr5 132962226
chr5 132962245
chr5 132962387
chr5 132962419
chr5 132962433
chr5 132962442
chr5 132962475
chr5 132962512
chr5 132962608
chr5 132962617
chr5 132962672
chr5 132962705
chr5 132962733
chr5 132962750
chr5 132962776
chr5 132962783
chr5 132962789
chr5 132962822
chr5 132962847
chr5 132962855
chr5 132962857
chr5 132962869
chr5 132962970
chr5 132962978
chr5 132963015
chr5 133062552
chr5 133515703
chr5 133515876
chr5 133516033
chr5 133516058

chr5 133516174
chr5 133853449
chr5 133853694
chr5 133967912
chr5 133967917
chr5 133968031
chr5 133968053
chr5 133968086
chr5 133968116
chr5 133968128
chr5 133968146
chr5 133968157
chr5 133968180
chr5 133968237
chr5 133968296
chr5 133968308
chr5 133968310
chr5 133968335
chr5 133968338
chr5 133968361
chr5 134175598
chr5 134175602
chr5 134175713
chr5 134175729
chr5 134175912
chr5 134175949
chr5 134223800
chr5 134223907
chr5 134224039
chr5 134224060
chr5 134224083
chr5 134224161
chr5 134224266
chr5 134224301
chr5 134224458
chr5 134224534
chr5 134224582
chr5 134224858
chr5 134302616
chr5 134366383
chr5 134366478
chr5 134366583
chr5 134366780
chr5 134367021
chr5 134367024
chr5 134367050
chr5 134367066
chr5 134367086
chr5 134370582
chr5 134370779
chr5 134370783
chr5 134370862
chr5 134370966
chr5 134372354

chr5 134372445
chr5 134372594
chr5 134372670
chr5 134372700
chr5 134372734
chr5 134372934
chr5 134372990
chr5 134373092
chr5 134524368
chr5 134524372
chr5 134648018
chr5 134649457
chr5 134649489
chr5 134649556
chr5 134649582
chr5 134649883
chr5 134738822
chr5 134738921
chr5 134738929
chr5 134738976
chr5 134738994
chr5 134739002
chr5 134739005
chr5 134739092
chr5 134739108
chr5 134739112
chr5 134739280
chr5 134739295
chr5 134739344
chr5 134739362
chr5 134739367
chr5 134739423
chr5 134739505
chr5 134739564
chr5 134739587
chr5 134739589
chr5 134739649
chr5 134759169
chr5 134759182
chr5 134759201
chr5 134759225
chr5 134759294
chr5 134759296
chr5 134759313
chr5 134759347
chr5 134759349
chr5 134759382
chr5 134759403
chr5 134759408
chr5 134759423
chr5 134759461
chr5 134759476
chr5 134759489
chr5 134759761

chr5 134759802
chr5 134759864
chr5 134759880
chr5 135373158
chr5 135373197
chr5 135373207
chr5 135373391
chr5 135373449
chr5 135397829
chr5 135397868
chr5 135397925
chr5 135398215
chr5 135398223
chr5 135398296
chr5 135398326
chr5 135398346
chr5 135398407
chr5 135398416
chr5 135837443
chr5 135837512
chr5 135838348
chr5 135887176
chr5 135887203
chr5 135887215
chr5 135887222
chr5 135887230
chr5 135887233
chr5 135887332
chr5 135977114
chr5 135977344
chr5 135977446
chr5 135977495
chr5 136036950
chr5 136037010
chr5 136037159
chr5 136037244
chr5 136037286
chr5 136037396
chr5 136037454
chr5 136037565
chr5 136037597
chr5 136133472
chr5 136133503
chr5 136133629
chr5 136133656
chr5 136133663
chr5 136133755
chr5 136133795
chr5 136133797
chr5 136133801
chr5 136133855
chr5 136133859
chr5 136133882
chr5 136133889

chr5 136133910
chr5 136134059
chr5 136134107
chr5 136134238
chr5 136134284
chr5 136134298
chr5 136292912
chr5 136292989
chr5 136293395
chr5 136364177
chr5 136364466
chr5 136364582
chr5 136365018
chr5 136365032
chr5 136919304
chr5 137516996
chr5 137517026
chr5 137555558
chr5 137555740
chr5 137555790
chr5 137555849
chr5 137556142
chr5 137751220
chr5 137751459
chr5 137751609
chr5 137751787
chr5 137995497
chr5 138011808
chr5 138069207
chr5 138178035
chr5 138178039
chr5 138331862
chr5 138331866
chr5 138331879
chr5 138337648
chr5 138337654
chr5 138337692
chr5 138337697
chr5 138337727
chr5 138337743
chr5 138337745
chr5 138337775
chr5 138337778
chr5 138337780
chr5 138337784
chr5 138337816
chr5 138337828
chr5 138337837
chr5 138337876
chr5 138337881
chr5 138337883
chr5 138337888
chr5 138337893
chr5 138337916

chr5 138337940
chr5 138337963
chr5 138338010
chr5 138353847
chr5 138353858
chr5 138408562
chr5 138541870
chr5 138541877
chr5 138574405
chr5 138574511
chr5 138574576
chr5 138574756
chr5 138574802
chr5 138574817
chr5 138574854
chr5 138611111
chr5 138611199
chr5 138611278
chr5 138611343
chr5 138611423
chr5 138611427
chr5 138611469
chr5 138611480
chr5 138633368
chr5 138633542
chr5 138653887
chr5 138654234
chr5 138654347
chr5 138654354
chr5 138654415
chr5 138654473
chr5 138654502
chr5 138654554
chr5 138792658
chr5 139246439
chr5 139246454
chr5 139273772
chr5 139273776
chr5 139273782
chr5 139273791
chr5 139273796
chr5 139273868
chr5 139273877
chr5 139274076
chr5 139274088
chr5 139274095
chr5 139274113
chr5 139274123
chr5 139274134
chr5 139274141
chr5 139274149
chr5 139274155
chr5 139274239
chr5 139274246

chr5 139274251
chr5 139274315
chr5 139274321
chr5 139274386
chr5 139274395
chr5 139274450
chr5 139274478
chr5 139274526
chr5 139294993
chr5 139295070
chr5 139295117
chr5 139295204
chr5 139295214
chr5 139295217
chr5 139295264
chr5 139295275
chr5 139295323
chr5 139295490
chr5 139295519
chr5 139295642
chr5 139295687
chr5 139295696
chr5 139295714
chr5 139295766
chr5 139295846
chr5 139305590
chr5 139305657
chr5 139342720
chr5 139342862
chr5 139342947
chr5 139342959
chr5 139343003
chr5 139343076
chr5 139343097
chr5 139403946
chr5 139403988
chr5 139403993
chr5 139404009
chr5 139454239
chr5 139454259
chr5 139454307
chr5 139470890
chr5 139470956
chr5 139470972
chr5 139470982
chr5 139471021
chr5 139471027
chr5 139471038
chr5 139481929
chr5 139482020
chr5 139482064
chr5 139482151
chr5 139482182
chr5 139482221

chr5 139482247
chr5 139482262
chr5 139482265
chr5 139482270
chr5 139482324
chr5 139482356
chr5 139482369
chr5 139482550
chr5 139482571
chr5 139482592
chr5 139482664
chr5 139482675
chr5 139482768
chr5 139482856
chr5 139562607
chr5 139562623
chr5 139562645
chr5 139562918
chr5 139562966
chr5 139562977
chr5 139563078
chr5 139563226
chr5 139563439
chr5 139563654
chr5 139563658
chr5 139563660
chr5 139563670
chr5 139563714
chr5 139563728
chr5 139563748
chr5 139563759
chr5 139563791
chr5 139563803
chr5 139563851
chr5 139563858
chr5 139563866
chr5 139563883
chr5 139563889
chr5 139563891
chr5 139565234
chr5 139565692
chr5 139636480
chr5 139636682
chr5 139636964
chr5 139637124
chr5 139637274
chr5 139637285
chr5 139652143
chr5 139652146
chr5 139652149
chr5 139652151
chr5 139652153
chr5 139652155
chr5 139652157

chr5 139652159
chr5 139652161
chr5 139652163
chr5 139652165
chr5 139652167
chr5 139652169
chr5 139652171
chr5 139652293
chr5 139652302
chr5 139652304
chr5 139652378
chr5 139652402
chr5 139746962
chr5 139747091
chr5 140038760
chr5 140038811
chr5 140038968
chr5 140043728
chr5 140043768
chr5 140043882
chr5 140043888
chr5 140044255
chr5 140113372
chr5 140113375
chr5 140113380
chr5 140113424
chr5 140113454
chr5 140113468
chr5 140113487
chr5 140113494
chr5 140113537
chr5 140113555
chr5 140113562
chr5 140113593
chr5 140113596
chr5 140115046
chr5 140115099
chr5 140115102
chr5 140115352
chr5 140115517
chr5 140144289
chr5 140159921
chr5 140251604
chr5 140251677
chr5 140251699
chr5 140251775
chr5 140251923
chr5 140302279
chr5 140302403
chr5 140302409
chr5 140302494
chr5 140302566
chr5 140302602
chr5 140534158

chr5 140534163
chr5 140534188
chr5 140534219
chr5 140534346
chr5 140534386
chr5 140556291
chr5 140556324
chr5 140556561
chr5 140556585
chr5 140556647
chr5 140556703
chr5 140556782
chr5 140556991
chr5 140557054
chr5 140557115
chr5 140569408
chr5 140648214
chr5 140648216
chr5 140648231
chr5 140710635
chr5 140710641
chr5 140710698
chr5 140710700
chr5 140710715
chr5 140710750
chr5 140710819
chr5 140710850
chr5 140710933
chr5 140710944
chr5 140710974
chr5 140710989
chr5 140711004
chr5 140711009
chr5 140711053
chr5 140711470
chr5 140711517
chr5 140711655
chr5 140711764
chr5 140711811
chr5 140755153
chr5 140755280
chr5 140755946
chr5 140756181
chr5 140756493
chr5 140785353
chr5 140785369
chr5 140785476
chr5 140785711
chr5 140785854
chr5 140789735
chr5 140789747
chr5 140789770
chr5 140789802
chr5 140789849

chr5 140790209
chr5 140790236
chr5 140798609
chr5 140798768
chr5 140798854
chr5 140799905
chr5 140799908
chr5 140800112
chr5 140800290
chr5 140803738
chr5 140803913
chr5 140804211
chr5 140804249
chr5 140804856
chr5 140818805
chr5 140819080
chr5 140820200
chr5 140820605
chr5 140820889
chr5 140820903
chr5 140821010
chr5 140821030
chr5 140821150
chr5 140827256
chr5 140827662
chr5 140827773
chr5 140827788
chr5 140827803
chr5 140827875
chr5 140831239
chr5 140831444
chr5 140831498
chr5 140839763
chr5 140840023
chr5 140840028
chr5 140840106
chr5 140840145
chr5 140844172
chr5 140844241
chr5 140844451
chr5 140844720
chr5 140844828
chr5 140844883
chr5 140844933
chr5 140845157
chr5 140845890
chr5 140847774
chr5 140851722
chr5 140854895
chr5 140854993
chr5 140855277
chr5 140855334
chr5 140855442
chr5 140855628

chr5 140855699
chr5 140855737
chr5 140855776
chr5 140855829
chr5 140855850
chr5 140858899
chr5 140859043
chr5 140859652
chr5 140860101
chr5 140866026
chr5 140866542
chr5 140866632
chr5 140867182
chr5 140868730
chr5 140868805
chr5 140868839
chr5 140868889
chr5 140868899
chr5 140872189
chr5 140877946
chr5 140878252
chr5 140878501
chr5 140879468
chr5 140879634
chr5 140879659
chr5 140885011
chr5 140965569
chr5 140965748
chr5 140965876
chr5 140965928
chr5 140984173
chr5 140984243
chr5 141001811
chr5 141008346
chr5 141008837
chr5 141009034
chr5 141041232
chr5 141041245
chr5 141041254
chr5 141041260
chr5 141041267
chr5 141041292
chr5 141041327
chr5 141041335
chr5 141041341
chr5 141041347
chr5 141041349
chr5 141041354
chr5 141041471
chr5 141041551
chr5 141041573
chr5 141041759
chr5 141042537
chr5 141042728

chr5 141043385
chr5 141043645
chr5 141057637
chr5 141058069
chr5 141058438
chr5 141058590
chr5 141058615
chr5 141080002
chr5 141099441
chr5 141099679
chr5 141103245
chr5 141103259
chr5 141103342
chr5 141103356
chr5 141109026
chr5 141109321
chr5 141119357
chr5 141119496
chr5 141119785
chr5 141119844
chr5 141119915
chr5 141124332
chr5 141124410
chr5 141124414
chr5 141125355
chr5 141138899
chr5 141149204
chr5 141149260
chr5 141149458
chr5 141149620
chr5 141171337
chr5 141171646
chr5 141172322
chr5 141172387
chr5 141172413
chr5 141172486
chr5 141172524
chr5 141181450
chr5 141181486
chr5 141181664
chr5 141181670
chr5 141181731
chr5 141185512
chr5 141185588
chr5 141185739
chr5 141186099
chr5 141191574
chr5 141191597
chr5 141191944
chr5 141192210
chr5 141192309
chr5 141192315
chr5 141192333
chr5 141192539

chr5 141195351
chr5 141196148
chr5 141196295
chr5 141196439
chr5 141196465
chr5 141196631
chr5 141197283
chr5 141208273
chr5 141208508
chr5 141208567
chr5 141212475
chr5 141212932
chr5 141213295
chr5 141216429
chr5 141216615
chr5 141216956
chr5 141217051
chr5 141232584
chr5 141232657
chr5 141233811
chr5 141233907
chr5 141234056
chr5 141234168
chr5 141234248
chr5 141237678
chr5 141237691
chr5 141237725
chr5 141237758
chr5 141237763
chr5 141237930
chr5 141238592
chr5 141238689
chr5 141238767
chr5 141239060
chr5 141321052
chr5 141321071
chr5 141321081
chr5 141321095
chr5 141321109
chr5 141321132
chr5 141330416
chr5 141330419
chr5 141330503
chr5 141330527
chr5 141335305
chr5 141335526
chr5 141335647
chr5 141336918
chr5 141337480
chr5 141337910
chr5 141338210
chr5 141338365
chr5 141338394
chr5 141338403

chr5 141342132
chr5 141342527
chr5 141342765
chr5 141343020
chr5 141343244
chr5 141354696
chr5 141354773
chr5 141359491
chr5 141359668
chr5 141359693
chr5 141359934
chr5 141359937
chr5 141359955
chr5 141363452
chr5 141363598
chr5 141363768
chr5 141363891
chr5 141363897
chr5 141367850
chr5 141367962
chr5 141368452
chr5 141368503
chr5 141368992
chr5 141369294
chr5 141369429
chr5 141369758
chr5 141369791
chr5 141369924
chr5 141369929
chr5 141370097
chr5 141387211
chr5 141387344
chr5 141392059
chr5 141396922
chr5 141397238
chr5 141397699
chr5 141397777
chr5 141397841
chr5 141397876
chr5 141397879
chr5 141397906
chr5 141402157
chr5 141402220
chr5 141402710
chr5 141402727
chr5 141402730
chr5 141407199
chr5 141407406
chr5 141407446
chr5 141407628
chr5 141412285
chr5 141412724
chr5 141412944
chr5 141412947

chr5 141412973
chr5 141412991
chr5 141413029
chr5 141413117
chr5 141413133
chr5 141415577
chr5 141415677
chr5 141417325
chr5 141417525
chr5 141417596
chr5 141417599
chr5 141417602
chr5 141417605
chr5 141417623
chr5 141417629
chr5 141417632
chr5 141417667
chr5 141429712
chr5 141429726
chr5 141430100
chr5 141430420
chr5 141430426
chr5 141430484
chr5 141430539
chr5 141430542
chr5 141430556
chr5 141430570
chr5 141604490
chr5 141604493
chr5 141604620
chr5 141604762
chr5 141604816
chr5 141604843
chr5 141605391
chr5 141605654
chr5 141605738
chr5 141605850
chr5 141605873
chr5 141605901
chr5 141605914
chr5 141616803
chr5 141616987
chr5 141617197
chr5 141617512
chr5 141617719
chr5 141617758
chr5 141617776
chr5 141618458
chr5 141618464
chr5 141618511
chr5 141618524
chr5 141618528
chr5 141618538
chr5 141618542

chr5 141618571
chr5 141618583
chr5 141618592
chr5 141618604
chr5 141618616
chr5 141618626
chr5 141618634
chr5 141618665
chr5 141618670
chr5 141618674
chr5 141618679
chr5 141619359
chr5 141619361
chr5 141619506
chr5 141619539
chr5 141619564
chr5 141878931
chr5 141878935
chr5 141878941
chr5 141878948
chr5 141885085
chr5 141885376
chr5 141885807
chr5 141924105
chr5 141924335
chr5 141924394
chr5 141924407
chr5 141968678
chr5 141968687
chr5 141968691
chr5 141968714
chr5 141968723
chr5 141968753
chr5 141968777
chr5 141969528
chr5 141969538
chr5 141969563
chr5 141970066
chr5 141970141
chr5 142015328
chr5 142015882
chr5 142015909
chr5 142016013
chr5 142016082
chr5 142016356
chr5 142016484
chr5 142016686
chr5 142024702
chr5 142024767
chr5 142024788
chr5 142024843
chr5 142024894
chr5 142024916
chr5 142024977

chr5 142024999
chr5 142025061
chr5 142025075
chr5 142025081
chr5 142025088
chr5 142025094
chr5 142025097
chr5 142025100
chr5 142025108
chr5 142025134
chr5 142025141
chr5 142025151
chr5 142025159
chr5 142025170
chr5 142025180
chr5 142025196
chr5 142025226
chr5 142025235
chr5 142025238
chr5 142025258
chr5 142025290
chr5 142025345
chr5 142025402
chr5 142025451
chr5 142025506
chr5 142110448
chr5 142223857
chr5 142223866
chr5 142713485
chr5 142713519
chr5 142713571
chr5 142977477
chr5 143008976
chr5 143009090
chr5 143009099
chr5 143009248
chr5 143009294
chr5 143009309
chr5 143009312
chr5 143009327
chr5 143009391
chr5 143009515
chr5 143009637
chr5 143009712
chr5 143054607
chr5 143054618
chr5 143054660
chr5 143054686
chr5 143054813
chr5 143055390
chr5 143055655
chr5 143055876
chr5 143055928
chr5 143056182

chr5 143182966
chr5 143183004
chr5 143183358
chr5 143183385
chr5 143183394
chr5 143222302
chr5 143222333
chr5 143222401
chr5 143222431
chr5 143222448
chr5 143222511
chr5 143222545
chr5 143222705
chr5 143222752
chr5 143222761
chr5 143222853
chr5 143223029
chr5 143223073
chr5 143223092
chr5 143223145
chr5 143223158
chr5 143402225
chr5 143402252
chr5 143402278
chr5 143402284
chr5 143402296
chr5 143402417
chr5 143402441
chr5 143402459
chr5 143404739
chr5 143404758
chr5 143404777
chr5 143404804
chr5 143404815
chr5 143404817
chr5 143404829
chr5 143404847
chr5 143404870
chr5 143404880
chr5 143404897
chr5 143404957
chr5 143405020
chr5 143405027
chr5 143405048
chr5 143405055
chr5 143405066
chr5 143405070
chr5 143405081
chr5 143405104
chr5 143405107
chr5 143405109
chr5 143405112
chr5 143405126
chr5 143405132

chr5 143405138
chr5 143405594
chr5 143405600
chr5 143405607
chr5 143405614
chr5 143405636
chr5 143405663
chr5 143405671
chr5 143405693
chr5 143405703
chr5 143405739
chr5 143405793
chr5 143435369
chr5 143435405
chr5 143435445
chr5 143435898
chr5 143435904
chr5 143436242
chr5 143605833
chr5 144169901
chr5 144169929
chr5 144170020
chr5 144170077
chr5 144170094
chr5 144170103
chr5 144170116
chr5 144170119
chr5 144170125
chr5 144170163
chr5 144170216
chr5 144170222
chr5 144170759
chr5 144170768
chr5 144170791
chr5 144170823
chr5 144170829
chr5 144170849
chr5 144170903
chr5 144170920
chr5 144170965
chr5 144171178
chr5 144187937
chr5 144187952
chr5 144189632
chr5 144189656
chr5 144189974
chr5 144190004
chr5 144190026
chr5 144190091
chr5 144190134
chr5 144205181
chr5 144205191
chr5 144205197
chr5 144205209

chr5 144205211
chr5 144205213
chr5 144205224
chr5 144205278
chr5 144279546
chr5 144282586
chr5 144472279
chr5 144472407
chr5 144472668
chr5 145423523
chr5 145423690
chr5 145423711
chr5 145423736
chr5 145423767
chr5 145423994
chr5 145424047
chr5 145424110
chr5 145830869
chr5 145831028
chr5 145946665
chr5 146173782
chr5 146173806
chr5 146182249
chr5 146182297
chr5 146182320
chr5 146182358
chr5 146182367
chr5 146182376
chr5 146182853
chr5 146182915
chr5 146182935
chr5 146182950
chr5 146182967
chr5 146202863
chr5 146204273
chr5 146204333
chr5 146205748
chr5 146205758
chr5 146205795
chr5 146238799
chr5 146336624
chr5 146336911
chr5 146336924
chr5 146447244
chr5 146447268
chr5 146447277
chr5 146447290
chr5 146447313
chr5 146447316
chr5 146447321
chr5 146447324
chr5 146447336
chr5 146447338
chr5 146447354

chr5 146447359
chr5 146447364
chr5 146447370
chr5 146447376
chr5 146447386
chr5 146447396
chr5 146447417
chr5 146447444
chr5 146447464
chr5 146447488
chr5 146447493
chr5 146447497
chr5 146447507
chr5 146447513
chr5 146447517
chr5 146447522
chr5 146447527
chr5 146447533
chr5 146447581
chr5 146448288
chr5 146448534
chr5 146448675
chr5 146542790
chr5 146542840
chr5 146610430
chr5 146610437
chr5 146610997
chr5 146611015
chr5 146611078
chr5 146611104
chr5 146611371
chr5 146611381
chr5 146611414
chr5 146612671
chr5 146612675
chr5 146612726
chr5 146613028
chr5 146613117
chr5 147189424
chr5 147189511
chr5 147224186
chr5 147369775
chr5 147781906
chr5 147782361
chr5 147782468
chr5 147782524
chr5 147782529
chr5 147782542
chr5 147782567
chr5 147782598
chr5 147782606
chr5 147782654
chr5 147782657
chr5 147782660

chr5 147782663
chr5 147782710
chr5 147782728
chr5 147782812
chr5 147805285
chr5 147805351
chr5 147805371
chr5 147805392
chr5 147997694
chr5 147997721
chr5 147999144
chr5 147999288
chr5 147999332
chr5 147999364
chr5 148270340
chr5 148383640
chr5 148384091
chr5 148384098
chr5 148384102
chr5 148384142
chr5 148384146
chr5 148384156
chr5 148384232
chr5 148384240
chr5 148384368
chr5 148384372
chr5 148384377
chr5 148384403
chr5 148384411
chr5 148384443
chr5 148384497
chr5 148384550
chr5 148384624
chr5 148384741
chr5 148655304
chr5 148826215
chr5 148826222
chr5 148826235
chr5 148826245
chr5 148826262
chr5 148826295
chr5 148826297
chr5 148826303
chr5 148826323
chr5 148826348
chr5 148826408
chr5 148826426
chr5 148826451
chr5 148826455
chr5 148826480
chr5 148826513
chr5 148826521
chr5 148826523
chr5 148826530

chr5 148826533
chr5 148826545
chr5 148826555
chr5 148826559
chr5 148826564
chr5 148826567
chr5 148826601
chr5 148826610
chr5 148826617
chr5 148826622
chr5 149133062
chr5 149240349
chr5 149240500
chr5 149240528
chr5 149240533
chr5 149240546
chr5 149240554
chr5 149240612
chr5 149240614
chr5 149240653
chr5 149240666
chr5 149240680
chr5 149240690
chr5 149344934
chr5 149344947
chr5 149344953
chr5 149344994
chr5 149345153
chr5 149345155
chr5 149345182
chr5 149345206
chr5 149345223
chr5 149345964
chr5 149345991
chr5 149345998
chr5 149346111
chr5 149346117
chr5 149346233
chr5 149346254
chr5 149346263
chr5 149346266
chr5 149346283
chr5 149346361
chr5 149549684
chr5 149549698
chr5 149549703
chr5 149549708
chr5 149549759
chr5 149549762
chr5 149724613
chr5 149724922
chr5 149828908
chr5 149828921
chr5 150001910

chr5 150001939
chr5 150001950
chr5 150410037
chr5 150410059
chr5 150410062
chr5 150410069
chr5 150410076
chr5 150410145
chr5 150410249
chr5 150410327
chr5 150410367
chr5 150410418
chr5 150410548
chr5 150410596
chr5 150410733
chr5 150411178
chr5 150411309
chr5 150411311
chr5 150411524
chr5 150411708
chr5 150411850
chr5 150412184
chr5 150412211
chr5 150412244
chr5 150412385
chr5 150412455
chr5 150412549
chr5 150412571
chr5 150412575
chr5 150412594
chr5 150412767
chr5 150412772
chr5 150412785
chr5 150412808
chr5 150412827
chr5 150412900
chr5 150412917
chr5 150412952
chr5 150436138
chr5 150436224
chr5 150436226
chr5 150436291
chr5 150436297
chr5 150436329
chr5 150436369
chr5 150436386
chr5 150436446
chr5 150436458
chr5 150436479
chr5 150448119
chr5 150448474
chr5 150448521
chr5 150448598
chr5 150448682

chr5 150448856
chr5 150700246
chr5 150700277
chr5 150700283
chr5 150700317
chr5 150700329
chr5 150700331
chr5 150700403
chr5 150700422
chr5 150700893
chr5 150700916
chr5 150700932
chr5 150700980
chr5 150700994
chr5 150700998
chr5 150701017
chr5 150701035
chr5 150701046
chr5 150701056
chr5 150701065
chr5 150701068
chr5 150701070
chr5 150701073
chr5 150701077
chr5 150701080
chr5 150701091
chr5 150701093
chr5 150701837
chr5 150701937
chr5 150758326
chr5 150758344
chr5 150758347
chr5 150758363
chr5 150758475
chr5 150758481
chr5 150758497
chr5 150758525
chr5 150758568
chr5 150758596
chr5 150758608
chr5 150758641
chr5 150758661
chr5 150758675
chr5 150758722
chr5 150758734
chr5 150758741
chr5 150758788
chr5 150783868
chr5 150783879
chr5 150846745
chr5 150846761
chr5 150846783
chr5 150846792
chr5 150846841

chr5 150846847
chr5 150846875
chr5 150846877
chr5 150846885
chr5 150846913
chr5 150846931
chr5 150847016
chr5 150847137
chr5 150847166
chr5 150847198
chr5 150847210
chr5 150847290
chr5 150905555
chr5 150905615
chr5 150919286
chr5 150963494
chr5 150963605
chr5 151080105
chr5 151080129
chr5 151087106
chr5 151087127
chr5 151087150
chr5 151087218
chr5 151087232
chr5 151087248
chr5 151087278
chr5 151087342
chr5 151094141
chr5 151094151
chr5 151094168
chr5 151094247
chr5 151094261
chr5 151150591
chr5 151150758
chr5 151150783
chr5 151151191
chr5 151151386
chr5 151151464
chr5 151155310
chr5 151155547
chr5 151155601
chr5 151155628
chr5 151155647
chr5 151155834
chr5 151155870
chr5 151155917
chr5 151157027
chr5 151157081
chr5 151157089
chr5 151202669
chr5 151202745
chr5 151211815
chr5 151211823
chr5 151211984

chr5 151212002
chr5 151212099
chr5 151212141
chr5 151212179
chr5 151212630
chr5 151212644
chr5 151213939
chr5 151214123
chr5 151214238
chr5 151214300
chr5 151214323
chr5 151214361
chr5 151214374
chr5 151214421
chr5 151214678
chr5 151214686
chr5 151214694
chr5 151214745
chr5 151224288
chr5 151224311
chr5 151252567
chr5 151252575
chr5 151252622
chr5 151252654
chr5 151252685
chr5 151252687
chr5 151252716
chr5 151252722
chr5 151252727
chr5 151252748
chr5 151252764
chr5 151252770
chr5 151252793
chr5 151252798
chr5 151252804
chr5 151252810
chr5 151252814
chr5 151252825
chr5 151252827
chr5 151252832
chr5 151252838
chr5 151252847
chr5 151252849
chr5 151252851
chr5 151252853
chr5 151252861
chr5 151252866
chr5 151252872
chr5 151252880
chr5 151252891
chr5 151252908
chr5 151252917
chr5 151253031
chr5 151253039

chr5 151253045
chr5 151258976
chr5 151259314
chr5 151613018
chr5 151613031
chr5 151613112
chr5 151613124
chr5 151613145
chr5 151613177
chr5 151613206
chr5 151613241
chr5 151687003
chr5 151773118
chr5 151773124
chr5 151773139
chr5 151773149
chr5 151773251
chr5 151773254
chr5 151773292
chr5 151773315
chr5 151773352
chr5 151773413
chr5 151773445
chr5 151773641
chr5 151773733
chr5 151773739
chr5 151773939
chr5 151774024
chr5 151774149
chr5 151774174
chr5 151774361
chr5 151774389
chr5 151774437
chr5 151781685
chr5 152080080
chr5 152080122
chr5 152403819
chr5 152481480
chr5 152731575
chr5 152731839
chr5 152732237
chr5 152732544
chr5 152732718
chr5 153352592
chr5 153352613
chr5 153352634
chr5 153352756
chr5 153352774
chr5 153352790
chr5 153352798
chr5 153352802
chr5 153352813
chr5 153352836
chr5 153352938

chr5 153352964
chr5 153353107
chr5 153353181
chr5 153353236
chr5 153353289
chr5 153353344
chr5 153353681
chr5 153379971
chr5 153380393
chr5 153492339
chr5 153492387
chr5 153493111
chr5 153601573
chr5 154071973
chr5 154072414
chr5 154072492
chr5 154072503
chr5 154072522
chr5 154075380
chr5 154221953
chr5 154273783
chr5 154444650
chr5 154444653
chr5 154444667
chr5 154444794
chr5 154444876
chr5 154444901
chr5 154444921
chr5 154444928
chr5 154444952
chr5 154445001
chr5 154445298
chr5 154445311
chr5 154445343
chr5 154445345
chr5 154445348
chr5 154445387
chr5 154445405
chr5 154445439
chr5 154445453
chr5 154445486
chr5 154445503
chr5 154447255
chr5 154447275
chr5 154447289
chr5 154447372
chr5 154486920
chr5 154757965
chr5 154758072
chr5 154758154
chr5 154758223
chr5 154758242
chr5 154859798
chr5 154859808

chr5 154859814
chr5 154859954
chr5 154860014
chr5 154860053
chr5 154860178
chr5 154860496
chr5 154860692
chr5 154860818
chr5 154860902
chr5 154860950
chr5 154861074
chr5 154861165
chr5 154937601
chr5 154937662
chr5 154937673
chr5 154937675
chr5 154937777
chr5 154937781
chr5 154937889
chr5 154937900
chr5 154937908
chr5 154937911
chr5 154937924
chr5 154937945
chr5 154937970
chr5 154937975
chr5 154937995
chr5 154938001
chr5 154938005
chr5 154941220
chr5 154941246
chr5 154941276
chr5 154941281
chr5 154941289
chr5 154941336
chr5 154941342
chr5 154941380
chr5 154941394
chr5 154941477
chr5 154941489
chr5 154941496
chr5 154941500
chr5 154941554
chr5 154941606
chr5 154941630
chr5 154941633
chr5 154941654
chr5 154941691
chr5 154941711
chr5 154941744
chr5 154941855
chr5 154941883
chr5 154942066
chr5 155012274

chr5 155012564
chr5 155012568
chr5 155012714
chr5 155587740
chr5 155587881
chr5 156942061
chr5 156942137
chr5 156942325
chr5 157109368
chr5 157109385
chr5 157109944
chr5 157143179
chr5 157143410
chr5 157143479
chr5 157143490
chr5 157143497
chr5 157143512
chr5 157143579
chr5 157143587
chr5 157143599
chr5 157172153
chr5 157172366
chr5 157178370
chr5 157178512
chr5 157178577
chr5 157179083
chr5 157179111
chr5 157179160
chr5 157179561
chr5 157179849
chr5 157180752
chr5 157180782
chr5 157180825
chr5 157180842
chr5 157181048
chr5 157181062
chr5 157181107
chr5 157181145
chr5 157181423
chr5 157181597
chr5 157181631
chr5 157181634
chr5 157181777
chr5 157182455
chr5 157182557
chr5 157182634
chr5 157182757
chr5 157183446
chr5 157183755
chr5 157183990
chr5 157184044
chr5 157184240
chr5 157184384
chr5 157184399

chr5 157184413
chr5 157184416
chr5 157184494
chr5 157184508
chr5 157184607
chr5 157220017
chr5 157247916
chr5 157248395
chr5 157266681
chr5 157266694
chr5 157266710
chr5 157266717
chr5 157266722
chr5 157266725
chr5 157266738
chr5 157266773
chr5 157266804
chr5 157266822
chr5 157266828
chr5 157266933
chr5 157267075
chr5 157267282
chr5 157267325
chr5 157271780
chr5 157271798
chr5 157271805
chr5 157271844
chr5 157271915
chr5 157271917
chr5 157272018
chr5 157272111
chr5 157272179
chr5 157272212
chr5 157272231
chr5 157272291
chr5 157272352
chr5 157272430
chr5 157273629
chr5 157607282
chr5 157607561
chr5 157607564
chr5 157607784
chr5 157670566
chr5 157670589
chr5 157670663
chr5 157731166
chr5 157761880
chr5 157857642
chr5 157858128
chr5 157858137
chr5 157858231
chr5 157858293
chr5 157858490
chr5 157858576

chr5 157858600
chr5 157858620
chr5 157858632
chr5 157858650
chr5 157858653
chr5 157858684
chr5 157858738
chr5 157858762
chr5 157858768
chr5 157858781
chr5 157858787
chr5 157858797
chr5 157858802
chr5 157858805
chr5 157858835
chr5 157858840
chr5 157858894
chr5 157858903
chr5 157858920
chr5 157858929
chr5 157858946
chr5 157858948
chr5 157858971
chr5 157858978
chr5 157858980
chr5 157858982
chr5 157858987
chr5 157858992
chr5 157858995
chr5 157859017
chr5 157859024
chr5 157859035
chr5 157859040
chr5 157859056
chr5 157859065
chr5 157859068
chr5 157859072
chr5 157859075
chr5 157859080
chr5 157859118
chr5 157859121
chr5 157859124
chr5 157859127
chr5 157859135
chr5 157859138
chr5 157859143
chr5 157859145
chr5 157871212
chr5 157871402
chr5 157871508
chr5 157871643
chr5 157871964
chr5 158354926
chr5 158435478

chr5 158435606
chr5 159094110
chr5 159094426
chr5 159094539
chr5 159094877
chr5 159094898
chr5 159094957
chr5 159094959
chr5 159094983
chr5 159099606
chr5 159099615
chr5 159099634
chr5 159099685
chr5 159099693
chr5 159099721
chr5 159099853
chr5 159099991
chr5 159099993
chr5 159099999
chr5 159100106
chr5 159100123
chr5 159100133
chr5 159203038
chr5 159206132
chr5 159206139
chr5 159206195
chr5 159206240
chr5 159206252
chr5 159206281
chr5 159206336
chr5 159206569
chr5 159206766
chr5 159207077
chr5 159209099
chr5 159209118
chr5 159209145
chr5 159209148
chr5 159209150
chr5 159209172
chr5 159209181
chr5 159209185
chr5 159209192
chr5 159209194
chr5 159263174
chr5 159263193
chr5 159263221
chr5 159263238
chr5 159263258
chr5 159263270
chr5 159263276
chr5 159367724
chr5 159412379
chr5 159412498
chr5 159412560

chr5 159412627
chr5 159469376
chr5 159515828
chr5 159515897
chr5 159646672
chr5 159879335
chr5 159970397
chr5 160009176
chr5 160009205
chr5 160009217
chr5 160009263
chr5 160009265
chr5 160009328
chr5 160009357
chr5 160009366
chr5 160009432
chr5 160009446
chr5 160009452
chr5 160009465
chr5 160009481
chr5 160009498
chr5 160009559
chr5 160009637
chr5 160009639
chr5 160009733
chr5 160009762
chr5 160009787
chr5 160009832
chr5 160009865
chr5 160118239
chr5 160118256
chr5 160118364
chr5 160118377
chr5 160118403
chr5 160118419
chr5 160118427
chr5 160118435
chr5 160118447
chr5 160118489
chr5 160119419
chr5 160119428
chr5 160119431
chr5 160119434
chr5 160119437
chr5 160119443
chr5 160165749
chr5 160165768
chr5 160165830
chr5 160369937
chr5 160370046
chr5 160370157
chr5 160370228
chr5 160370250
chr5 160370268

chr5 160399425
chr5 160399446
chr5 160419536
chr5 160419546
chr5 160419644
chr5 160419741
chr5 160419816
chr5 160419902
chr5 160419996
chr5 160421292
chr5 160421357
chr5 160421366
chr5 160421401
chr5 160421640
chr5 160421662
chr5 160421689
chr5 160421929
chr5 160421935
chr5 160421938
chr5 160421950
chr5 160421957
chr5 160421963
chr5 160422003
chr5 160422020
chr5 160422026
chr5 160422034
chr5 160422042
chr5 160422062
chr5 160422094
chr5 160422096
chr5 160422098
chr5 160422105
chr5 160422171
chr5 160422175
chr5 160422186
chr5 160422750
chr5 160422763
chr5 160422767
chr5 160438141
chr5 160438369
chr5 160438401
chr5 160438576
chr5 160438799
chr5 160438857
chr5 160438862
chr5 160438885
chr5 160438905
chr5 160438996
chr5 160439001
chr5 160439050
chr5 160439053
chr5 160439126
chr5 160467904
chr5 160467920

chr5 160467930
chr5 160467932
chr5 160468106
chr5 160468114
chr5 160468153
chr5 160468260
chr5 160468268
chr5 160468301
chr5 160468325
chr5 160468375
chr5 160468393
chr5 160468437
chr5 160469119
chr5 160469206
chr5 160469214
chr5 160469218
chr5 160470567
chr5 160470788
chr5 160471018
chr5 160471049
chr5 160471069
chr5 160471188
chr5 160508760
chr5 160508782
chr5 160508821
chr5 160595718
chr5 160596386
chr5 160596414
chr5 160711549
chr5 160712356
chr5 160712449
chr5 160712788
chr5 160712824
chr5 160713710
chr5 160713974
chr5 160713983
chr5 160714020
chr5 160874173
chr5 160874193
chr5 160874394
chr5 160874467
chr5 160874515
chr5 160874546
chr5 160874682
chr5 160874727
chr5 160876031
chr5 160877152
chr5 161072343
chr5 161072374
chr5 161072543
chr5 161072552
chr5 161072639
chr5 161072833
chr5 161073233

chr5 161543588
chr5 161543879
chr5 161544027
chr5 161544256
chr5 161544400
chr5 161544409
chr5 161544419
chr5 161544467
chr5 161544490
chr5 161544546
chr5 161544626
chr5 161544788
chr5 161545186
chr5 161545286
chr5 161545308
chr5 161545318
chr5 161547080
chr5 161547088
chr5 161547102
chr5 161608846
chr5 161608891
chr5 161608924
chr5 161609166
chr5 161609305
chr5 161689666
chr5 161689862
chr5 161846007
chr5 161846338
chr5 161846481
chr5 161846833
chr5 161846847
chr5 161846971
chr5 161847017
chr5 161847075
chr5 161848291
chr5 161848297
chr5 161848313
chr5 161848315
chr5 161848319
chr5 161848325
chr5 161848331
chr5 161848339
chr5 161848341
chr5 161848518
chr5 161848528
chr5 161848562
chr5 161848566
chr5 161848570
chr5 161848582
chr5 161848623
chr5 161848650
chr5 161848686
chr5 161848739
chr5 161848823

chr5 161848826
chr5 161848851
chr5 161848859
chr5 161848900
chr5 161848948
chr5 161848995
chr5 161849040
chr5 161849049
chr5 161849335
chr5 161849481
chr5 161849513
chr5 161849585
chr5 161849812
chr5 162066846
chr5 162067009
chr5 162363407
chr5 162365478
chr5 162533597
chr5 162534378
chr5 162683479
chr5 162683654
chr5 162683747
chr5 163332105
chr5 163332169
chr5 163332621
chr5 163436841
chr5 163437074
chr5 163437262
chr5 163437265
chr5 163437274
chr5 163437285
chr5 163437870
chr5 163437888
chr5 163437890
chr5 163437915
chr5 163437945
chr5 163437959
chr5 163437965
chr5 163437971
chr5 163437991
chr5 163438000
chr5 163438150
chr5 163438230
chr5 163438240
chr5 163438250
chr5 163458749
chr5 163458791
chr5 163458821
chr5 163458908
chr5 163459022
chr5 163459121
chr5 163459260
chr5 163459557
chr5 163459612

chr5 163459656
chr5 163459706
chr5 163461980
chr5 163461995
chr5 163462018
chr5 163462123
chr5 163462349
chr5 163462440
chr5 163503534
chr5 163503566
chr5 163503604
chr5 163507013
chr5 163507016
chr5 163507159
chr5 163507350
chr5 163507360
chr5 163507452
chr5 163507486
chr5 163509372
chr5 163509408
chr5 163509433
chr5 163509447
chr5 163509558
chr5 163509602
chr5 163509682
chr5 164795048
chr5 164970178
chr5 165220890
chr5 165221080
chr5 165228493
chr5 165228820
chr5 165228896
chr5 165321485
chr5 165325383
chr5 165617256
chr5 165865962
chr5 165866030
chr5 165866449
chr5 165866458
chr5 166041575
chr5 166142933
chr5 166142989
chr5 166143201
chr5 166143255
chr5 166143520
chr5 166143780
chr5 166143813
chr5 166143846
chr5 166144044
chr5 166144053
chr5 166266351
chr5 166431012
chr5 166431070
chr5 166431231

chr5 166431554
chr5 166431574
chr5 166431579
chr5 166431705
chr5 166840952
chr5 166841109
chr5 166841138
chr5 166841390
chr5 166876214
chr5 166876627
chr5 166877255
chr5 166877259
chr5 166968831
chr5 166969526
chr5 166969568
chr5 166979420
chr5 166979446
chr5 166979457
chr5 166979501
chr5 166979514
chr5 166979619
chr5 166979656
chr5 166979745
chr5 166979753
chr5 166979840
chr5 166979861
chr5 166979898
chr5 166979922
chr5 166980074
chr5 166980292
chr5 166980461
chr5 167366468
chr5 167366580
chr5 167366919
chr5 167700371
chr5 168074729
chr5 168074743
chr5 168074841
chr5 168329152
chr5 168329349
chr5 168329398
chr5 168409829
chr5 168409858
chr5 168409876
chr5 168409924
chr5 168409967
chr5 168409973
chr5 168410018
chr5 168410087
chr5 168486454
chr5 168486460
chr5 168486466
chr5 168486477
chr5 168486486

chr5 168486504
chr5 168486525
chr5 168486527
chr5 168486529
chr5 168486551
chr5 168486561
chr5 168486564
chr5 168486610
chr5 168486616
chr5 168486622
chr5 168486635
chr5 168486666
chr5 168486671
chr5 168486727
chr5 168486747
chr5 168486792
chr5 168486798
chr5 168486868
chr5 168486870
chr5 168487042
chr5 168487045
chr5 168487080
chr5 168487084
chr5 168487088
chr5 168487090
chr5 168487128
chr5 168487137
chr5 168487144
chr5 168487158
chr5 168578307
chr5 168578314
chr5 168578333
chr5 168578413
chr5 168578440
chr5 168578569
chr5 168578623
chr5 168852161
chr5 168852185
chr5 168852194
chr5 168852213
chr5 168852248
chr5 168852405
chr5 168852528
chr5 168852541
chr5 168852602
chr5 169583137
chr5 169583263
chr5 169583285
chr5 169583314
chr5 169583371
chr5 169583404
chr5 169583408
chr5 169583434
chr5 169583446

chr5 169583473
chr5 169583498
chr5 169583521
chr5 169583596
chr5 169583602
chr5 169583608
chr5 169583619
chr5 169583622
chr5 169583624
chr5 169583712
chr5 169583716
chr5 169583721
chr5 169583728
chr5 169583740
chr5 169583742
chr5 169583768
chr5 169583776
chr5 169583791
chr5 169583929
chr5 169583945
chr5 169583973
chr5 169583981
chr5 169583985
chr5 169584015
chr5 169584045
chr5 169584050
chr5 169584063
chr5 169584076
chr5 169584079
chr5 169584109
chr5 169584117
chr5 169584124
chr5 169584144
chr5 169584158
chr5 169584165
chr5 169584170
chr5 169584185
chr5 169623142
chr5 169637943
chr5 169637979
chr5 169638031
chr5 169638087
chr5 169638169
chr5 169638203
chr5 169638325
chr5 169638507
chr5 170254205
chr5 170254279
chr5 170254286
chr5 170254329
chr5 170254345
chr5 170254358
chr5 170262237
chr5 170262340

chr5 170267594
chr5 170267614
chr5 170267621
chr5 170267669
chr5 170267743
chr5 170267952
chr5 170267965
chr5 170267980
chr5 170268005
chr5 170268041
chr5 170268086
chr5 170275823
chr5 170277953
chr5 170278043
chr5 170278093
chr5 170278115
chr5 170278386
chr5 170278541
chr5 170278602
chr5 170278628
chr5 170278710
chr5 170279106
chr5 170279234
chr5 170279411
chr5 170279587
chr5 170279628
chr5 170279637
chr5 170293210
chr5 170293267
chr5 170293317
chr5 170293327
chr5 170293405
chr5 170293464
chr5 170293537
chr5 170293596
chr5 170293702
chr5 170293715
chr5 170293721
chr5 170293968
chr5 170293971
chr5 170293982
chr5 170294282
chr5 170294335
chr5 170294359
chr5 170294970
chr5 170295156
chr5 170295257
chr5 170295285
chr5 170295363
chr5 170295553
chr5 170295570
chr5 170295684
chr5 170295693
chr5 170295946

chr5 170296063
chr5 170296225
chr5 170298096
chr5 170298780
chr5 170313382
chr5 170313415
chr5 170331015
chr5 170331051
chr5 170331312
chr5 170331336
chr5 170331387
chr5 170331479
chr5 170331495
chr5 170331535
chr5 170331557
chr5 170331669
chr5 170331690
chr5 170331846
chr5 170333006
chr5 170333010
chr5 170333029
chr5 170333265
chr5 170333300
chr5 170333338
chr5 170333377
chr5 170333398
chr5 170333433
chr5 170333587
chr5 170333693
chr5 170333918
chr5 170334216
chr5 170334247
chr5 170334275
chr5 170334277
chr5 170393624
chr5 170393685
chr5 170393719
chr5 170633105
chr5 170633114
chr5 170633133
chr5 170633159
chr5 170633175
chr5 170633185
chr5 170633195
chr5 170633217
chr5 170633222
chr5 170633232
chr5 170633248
chr5 170633271
chr5 170633273
chr5 170633279
chr5 170633285
chr5 170633529
chr5 170633668

chr5 170633675
chr5 170633688
chr5 170633693
chr5 170633705
chr5 170633713
chr5 171388980
chr5 171388991
chr5 171389029
chr5 171389040
chr5 171389072
chr5 171389100
chr5 171389107
chr5 171389221
chr5 171389246
chr5 171389269
chr5 171389306
chr5 171389488
chr5 171389538
chr5 171389594
chr5 171389652
chr5 171389663
chr5 171389818
chr5 171390067
chr5 171390129
chr5 171390399
chr5 171601104
chr5 171601244
chr5 171601324
chr5 171601423
chr5 171721954
chr5 171722250
chr5 171722512
chr5 171728859
chr5 171770206
chr5 171941843
chr5 171989564
chr5 171989738
chr5 171989925
chr5 172004585
chr5 172004661
chr5 172004732
chr5 172004864
chr5 172004892
chr5 172005127
chr5 172005289
chr5 172005416
chr5 172005549
chr5 172005572
chr5 172005577
chr5 172005622
chr5 172005631
chr5 172111389
chr5 172111404
chr5 172111432

chr5 172111481
chr5 172111553
chr5 172111692
chr5 172111695
chr5 172111722
chr5 172111783
chr5 172111818
chr5 172113249
chr5 172113261
chr5 172113352
chr5 172113394
chr5 172114568
chr5 172114575
chr5 172114583
chr5 172114607
chr5 172114627
chr5 172114631
chr5 172114684
chr5 172114718
chr5 172114725
chr5 172114729
chr5 172114734
chr5 172114762
chr5 172114772
chr5 172114778
chr5 172114782
chr5 172114802
chr5 172114823
chr5 172114825
chr5 172114847
chr5 172114854
chr5 172114873
chr5 172114896
chr5 172114931
chr5 172114940
chr5 172115017
chr5 172115049
chr5 172115073
chr5 172115128
chr5 172115134
chr5 172115147
chr5 172115157
chr5 172115172
chr5 172115195
chr5 172115264
chr5 172115323
chr5 172115349
chr5 172115407
chr5 172115444
chr5 172115456
chr5 172115474
chr5 172115486
chr5 172174678
chr5 172174685

chr5 172174725
chr5 172174784
chr5 172174790
chr5 172174806
chr5 172174825
chr5 172174939
chr5 172175171
chr5 172175234
chr5 172175252
chr5 172175293
chr5 172175785
chr5 172175928
chr5 172178802
chr5 172178870
chr5 172178965
chr5 172178988
chr5 172179027
chr5 172179056
chr5 172179145
chr5 172179495
chr5 172179514
chr5 172179527
chr5 172179540
chr5 172179567
chr5 172185156
chr5 172185203
chr5 172185240
chr5 172185300
chr5 172185308
chr5 172185329
chr5 172185371
chr5 172185398
chr5 172185409
chr5 172185429
chr5 172185546
chr5 172185558
chr5 172185562
chr5 172185591
chr5 172185758
chr5 172185813
chr5 172185885
chr5 172186649
chr5 172186724
chr5 172186746
chr5 172186806
chr5 172186826
chr5 172186837
chr5 172186905
chr5 172238487
chr5 172765128
chr5 172765159
chr5 172765179
chr5 172765244
chr5 172765260

chr5 172765318
chr5 172765338
chr5 172765341
chr5 172765345
chr5 172765347
chr5 172765358
chr5 172765363
chr5 172765401
chr5 172765429
chr5 172765515
chr5 172765517
chr5 172772300
chr5 172959114
chr5 172959256
chr5 172959296
chr5 172959304
chr5 172959310
chr5 172959345
chr5 172959354
chr5 172959361
chr5 172959367
chr5 172959375
chr5 172959402
chr5 172959408
chr5 172959410
chr5 172959415
chr5 172959429
chr5 172959437
chr5 172959466
chr5 172959492
chr5 172959509
chr5 172959514
chr5 172959536
chr5 172959538
chr5 172959580
chr5 172959652
chr5 172959665
chr5 172959673
chr5 172959686
chr5 172959716
chr5 172959802
chr5 172959830
chr5 172959843
chr5 172959853
chr5 172959891
chr5 172959899
chr5 172959904
chr5 172984338
chr5 172984403
chr5 172984405
chr5 172984411
chr5 172984430
chr5 172984465
chr5 172984475

chr5 172984496
chr5 172984542
chr5 172984561
chr5 172984566
chr5 173057804
chr5 173402584
chr5 173403624
chr5 173404269
chr5 173404274
chr5 173404279
chr5 173404818
chr5 173404848
chr5 173405543
chr5 173572979
chr5 173572981
chr5 173572986
chr5 173573087
chr5 173573100
chr5 173573113
chr5 173888260
chr5 173888305
chr5 173888310
chr5 173888352
chr5 173888357
chr5 173888381
chr5 173888383
chr5 173888389
chr5 173888396
chr5 173888403
chr5 173888410
chr5 173888416
chr5 173888419
chr5 173888422
chr5 173888425
chr5 173888428
chr5 173888437
chr5 173888440
chr5 173888506
chr5 173888512
chr5 173888521
chr5 173888537
chr5 173888540
chr5 173888543
chr5 173888546
chr5 173888549
chr5 173888552
chr5 173888564
chr5 173888634
chr5 173888668
chr5 173888711
chr5 173888719
chr5 173888742
chr5 173888773
chr5 173888779

chr5 173888804
chr5 173888821
chr5 173888829
chr5 173888836
chr5 173888849
chr5 173888853
chr5 173917758
chr5 173917779
chr5 173917843
chr5 173917890
chr5 173917894
chr5 173917907
chr5 173917953
chr5 173917985
chr5 173918063
chr5 173918134
chr5 173918173
chr5 173918283
chr5 173918374
chr5 173918441
chr5 173918500
chr5 173918556
chr5 173918562
chr5 173918573
chr5 173918667
chr5 173918697
chr5 173918736
chr5 173918836
chr5 174009654
chr5 174045734
chr5 174045929
chr5 174046116
chr5 174063774
chr5 174318297
chr5 174318506
chr5 174419121
chr5 174419145
chr5 174419152
chr5 174419167
chr5 174419169
chr5 174419177
chr5 174419209
chr5 174419225
chr5 174419248
chr5 174419305
chr5 174419319
chr5 174419326
chr5 174419395
chr5 174419410
chr5 174419503
chr5 174419533
chr5 174419554
chr5 174419577
chr5 174419673

chr5 174693208
chr5 174722612
chr5 174722669
chr5 174722813
chr5 174733938
chr5 174899843
chr5 174951449
chr5 175059033
chr5 175059660
chr5 175059785
chr5 175059811
chr5 175059872
chr5 175059900
chr5 175059925
chr5 175060005
chr5 175060010
chr5 175060014
chr5 175060021
chr5 175060036
chr5 175060045
chr5 175060052
chr5 175060054
chr5 175060061
chr5 175060079
chr5 175060088
chr5 175060129
chr5 175060146
chr5 175060150
chr5 175060154
chr5 175060167
chr5 175060190
chr5 175060362
chr5 175065459
chr5 175065756
chr5 175168512
chr5 175254490
chr5 175325640
chr5 175440772
chr5 175464966
chr5 175464986
chr5 175465023
chr5 175479048
chr5 175479053
chr5 175479055
chr5 175479061
chr5 175479087
chr5 175479122
chr5 175548246
chr5 175548308
chr5 175653058
chr5 175653114
chr5 175653153
chr5 175653169
chr5 175653394

chr5 175653444
chr5 175653502
chr5 175822075
chr5 175822141
chr5 175822222
chr5 175967531
chr5 175967542
chr5 175967556
chr5 175967620
chr5 175967646
chr5 175967684
chr5 175967690
chr5 175967848
chr5 175967850
chr5 175967856
chr5 175967860
chr5 175967870
chr5 175967884
chr5 175967901
chr5 175967924
chr5 175967931
chr5 175967968
chr5 175967975
chr5 175967983
chr5 175967993
chr5 175968003
chr5 175968006
chr5 175968010
chr5 175968013
chr5 175968029
chr5 175968043
chr5 175968050
chr5 175968081
chr5 175968285
chr5 175968291
chr5 175968295
chr5 175968303
chr5 175968306
chr5 175968317
chr5 175968323
chr5 175968325
chr5 175968335
chr5 175968340
chr5 175968359
chr5 175968369
chr5 175968371
chr5 175968377
chr5 175968379
chr5 175968512
chr5 175968577
chr5 176060995
chr5 176061001
chr5 176061004
chr5 176061020

chr5 176061033
chr5 176061040
chr5 176061091
chr5 176061100
chr5 176061108
chr5 176061126
chr5 176061136
chr5 176061138
chr5 176061140
chr5 176061146
chr5 176061154
chr5 176061158
chr5 176061176
chr5 176061195
chr5 176061197
chr5 176061206
chr5 176061209
chr5 176061221
chr5 176061228
chr5 176061235
chr5 176061239
chr5 176061247
chr5 176061271
chr5 176061334
chr5 176061348
chr5 176061350
chr5 176061367
chr5 176061388
chr5 176061405
chr5 176061407
chr5 176061424
chr5 176061426
chr5 176061443
chr5 176061445
chr5 176066942
chr5 176067053
chr5 176067276
chr5 176079656
chr5 176234950
chr5 176235152
chr5 176235291
chr5 176238199
chr5 176238203
chr5 176238223
chr5 176238229
chr5 176238235
chr5 176238272
chr5 176238281
chr5 176238283
chr5 176238286
chr5 176238291
chr5 176238301
chr5 176238307
chr5 176238323

chr5 176238327
chr5 176238330
chr5 176238333
chr5 176238340
chr5 176238350
chr5 176238355
chr5 176238358
chr5 176238363
chr5 176238370
chr5 176238375
chr5 176238381
chr5 176238384
chr5 176238389
chr5 176238397
chr5 176238408
chr5 176238411
chr5 176238414
chr5 176238420
chr5 176238423
chr5 176238428
chr5 176238431
chr5 176238434
chr5 176238455
chr5 176238467
chr5 176238470
chr5 176238473
chr5 176238491
chr5 176238495
chr5 176238502
chr5 176238523
chr5 176238531
chr5 176238535
chr5 176238541
chr5 176238579
chr5 176238581
chr5 176238588
chr5 176238590
chr5 176238593
chr5 176238603
chr5 176238605
chr5 176238613
chr5 176238616
chr5 176238618
chr5 176238626
chr5 176238629
chr5 176238634
chr5 176238647
chr5 176238650
chr5 176238655
chr5 176238665
chr5 176238667
chr5 176238669
chr5 176238675
chr5 176238677

chr5 176279409
chr5 176279723
chr5 176279925
chr5 176361562
chr5 176361617
chr5 176361619
chr5 176361631
chr5 176361636
chr5 176361643
chr5 176361660
chr5 176387758
chr5 176387774
chr5 176387825
chr5 176388469
chr5 176388478
chr5 176388484
chr5 176388514
chr5 176388540
chr5 176388542
chr5 176388552
chr5 176389149
chr5 176389197
chr5 176389202
chr5 176389213
chr5 176389216
chr5 176389227
chr5 176535386
chr5 176535478
chr5 176535521
chr5 176535567
chr5 176541423
chr5 176541578
chr5 176541647
chr5 176541677
chr5 176541731
chr5 176542108
chr5 176542166
chr5 176647272
chr5 176647279
chr5 176647286
chr5 176647304
chr5 176647315
chr5 176647339
chr5 176647357
chr5 176647389
chr5 176647396
chr5 176647407
chr5 176647424
chr5 176647451
chr5 176647465
chr5 176647469
chr5 176647476
chr5 176870881
chr5 176870915

chr5 176870953
chr5 176870983
chr5 176870987
chr5 176871021
chr5 176871123
chr5 176871153
chr5 176871326
chr5 176871360
chr5 176871394
chr5 176871428
chr5 176871458
chr5 176871767
chr5 176871834
chr5 176871869
chr5 176871903
chr5 176871938
chr5 176871973
chr5 176872142
chr5 176872176
chr5 176872278
chr5 176872341
chr5 176872411
chr5 176872446
chr5 176872480
chr5 176872514
chr5 176872549
chr5 176872583
chr5 176872614
chr5 176872820
chr5 177022830
chr5 177022834
chr5 177022841
chr5 177022850
chr5 177022852
chr5 177022860
chr5 177022862
chr5 177022865
chr5 177022879
chr5 177022885
chr5 177022947
chr5 177022951
chr5 177022956
chr5 177022958
chr5 177022963
chr5 177022965
chr5 177022975
chr5 177022981
chr5 177022984
chr5 177022987
chr5 177023005
chr5 177023027
chr5 177023059
chr5 177023061
chr5 177023077

chr5 177023088
chr5 177023090
chr5 177023107
chr5 177023113
chr5 177351471
chr5 177351481
chr5 177351604
chr5 177351607
chr5 177351622
chr5 177351639
chr5 177351642
chr5 177351685
chr5 177351692
chr5 177351715
chr5 177351719
chr5 177351742
chr5 177351769
chr5 177351778
chr5 177351783
chr5 177351799
chr5 177351858
chr5 177351861
chr5 177357718
chr5 177357746
chr5 177357756
chr5 177357762
chr5 177357776
chr5 177357778
chr5 177357798
chr5 177357802
chr5 177357881
chr5 177357904
chr5 177357913
chr5 177357940
chr5 177357946
chr5 177357961
chr5 177357964
chr5 177357996
chr5 177357998
chr5 177358001
chr5 177358015
chr5 177358021
chr5 177358051
chr5 177358060
chr5 177358064
chr5 177358084
chr5 177358097
chr5 177358143
chr5 177358166
chr5 177358181
chr5 177358259
chr5 177358295
chr5 177358376
chr5 177358430

chr5 177358507
chr5 177358521
chr5 177358571
chr5 177426429
chr5 177426445
chr5 177426505
chr5 177426511
chr5 177426525
chr5 177426545
chr5 177427655
chr5 177427956
chr5 177427978
chr5 177428036
chr5 177428057
chr5 177429046
chr5 177429204
chr5 177429419
chr5 177429549
chr5 177429616
chr5 177517495
chr5 177517506
chr5 177517551
chr5 177517592
chr5 177517606
chr5 177517622
chr5 177517636
chr5 177517651
chr5 177517673
chr5 177517712
chr5 177517762
chr5 177671341
chr5 177671355
chr5 177671385
chr5 177671394
chr5 177671567
chr5 177671569
chr5 177671634
chr5 177671644
chr5 177671646
chr5 177671652
chr5 177671665
chr5 177671670
chr5 177671683
chr5 177671693
chr5 177671698
chr5 177671701
chr5 177671709
chr5 177671715
chr5 177671721
chr5 177672202
chr5 177672235
chr5 177672238
chr5 177672244
chr5 177672247

chr5 177672250
chr5 177672261
chr5 177672269
chr5 177672274
chr5 177672277
chr5 177672283
chr5 177672288
chr5 177672303
chr5 177672308
chr5 177672318
chr5 177672325
chr5 177672328
chr5 177672331
chr5 177672335
chr5 177672357
chr5 177672367
chr5 177672372
chr5 177672375
chr5 177672377
chr5 177672386
chr5 177672422
chr5 177672428
chr5 177672466
chr5 177672472
chr5 177672474
chr5 177773271
chr5 177773296
chr5 177773317
chr5 177773866
chr5 177773912
chr5 177774701
chr5 177777132
chr5 177778027
chr5 177778117
chr5 177778184
chr5 177778887
chr5 177780832
chr5 177780840
chr5 177782659
chr5 177782661
chr5 177782678
chr5 177782697
chr5 177782718
chr5 177782735
chr5 177782737
chr5 177782751
chr5 177782838
chr5 177782846
chr5 177782850
chr5 177782857
chr5 177782864
chr5 177782876
chr5 177782879
chr5 177782888

chr5 177782890
chr5 177782928
chr5 177782932
chr5 177782940
chr5 177782946
chr5 177782948
chr5 177782950
chr5 177782960
chr5 177782978
chr5 177782986
chr5 177782995
chr5 177783044
chr5 177783046
chr5 177783053
chr5 177783066
chr5 177783082
chr5 177783085
chr5 177783091
chr5 177783130
chr5 177783137
chr5 177783153
chr5 177783169
chr5 177783178
chr5 177783185
chr5 177783195
chr5 177783197
chr5 177783227
chr5 177783229
chr5 177783237
chr5 177783256
chr5 177783319
chr5 177783336
chr5 177783505
chr5 177783513
chr5 177783515
chr5 177783521
chr5 177810184
chr5 177810273
chr5 177810347
chr5 177810631
chr5 177810695
chr5 177871215
chr5 177875131
chr5 177875290
chr5 177875662
chr5 177875681
chr5 177875686
chr5 177875689
chr5 177875691
chr5 177875696
chr5 177875721
chr5 177875737
chr5 177875739
chr5 177875741

chr5 177875751
chr5 177875758
chr5 177876117
chr5 177876155
chr5 177876171
chr5 177876181
chr5 177876245
chr5 177876256
chr5 177876267
chr5 177876753
chr5 177939636
chr5 177939651
chr5 177939655
chr5 177939664
chr5 177939667
chr5 177939674
chr5 177939677
chr5 177939690
chr5 177939702
chr5 177939704
chr5 177939713
chr5 177939735
chr5 177939742
chr5 177939747
chr5 177939750
chr5 177939788
chr5 177939792
chr5 177939797
chr5 177939829
chr5 177939832
chr5 177939838
chr5 177939866
chr5 177939876
chr5 177939883
chr5 177939905
chr5 177939915
chr5 177939926
chr5 177939930
chr5 177939933
chr5 177939958
chr5 177939962
chr5 177939964
chr5 177939971
chr5 177939994
chr5 177940012
chr5 177940036
chr5 177943962
chr5 177943972
chr5 177943976
chr5 177944010
chr5 177944021
chr5 177944027
chr5 177944047
chr5 177944051

chr5 177944067
chr5 177944069
chr5 177944074
chr5 177944085
chr5 177944090
chr5 177944111
chr5 177944118
chr5 177944153
chr5 177944158
chr5 177944179
chr5 177944181
chr5 177944199
chr5 177944363
chr5 177944367
chr5 177944376
chr5 177944379
chr5 177944386
chr5 177944391
chr5 177944399
chr5 177944402
chr5 177944415
chr5 177944426
chr5 177944428
chr5 177944434
chr5 177944439
chr5 177944447
chr5 177944460
chr5 178006649
chr5 178006665
chr5 178006672
chr5 178006711
chr5 178006717
chr5 178006736
chr5 178006749
chr5 178006756
chr5 178006758
chr5 178006790
chr5 178006802
chr5 178006807
chr5 178006816
chr5 178006824
chr5 178006842
chr5 178006852
chr5 178006854
chr5 178006856
chr5 178006862
chr5 178006870
chr5 178006874
chr5 178006892
chr5 178006912
chr5 178006914
chr5 178006923
chr5 178006926
chr5 178006938

chr5 178006945
chr5 178006952
chr5 178006956
chr5 178006964
chr5 178006988
chr5 178006995
chr5 178007004
chr5 178007017
chr5 178007026
chr5 178007032
chr5 178007034
chr5 178007065
chr5 178007067
chr5 178008134
chr5 178008178
chr5 178008189
chr5 178008208
chr5 178008334
chr5 178008424
chr5 178008426
chr5 178008436
chr5 178008449
chr5 178008464
chr5 178008473
chr5 178008480
chr5 178008520
chr5 178008570
chr5 178008574
chr5 178008586
chr5 178008607
chr5 178009177
chr5 178009212
chr5 178009330
chr5 178009414
chr5 178625577
chr5 178625581
chr5 178625747
chr5 178625754
chr5 178625886
chr5 178625893
chr5 178625900
chr5 178625916
chr5 178626038
chr5 178683910
chr5 178683971
chr5 178684014
chr5 178684028
chr5 178833432
chr5 178833484
chr5 178833534
chr5 178833563
chr5 179059900
chr5 179059927
chr5 179060002

chr5 179060076
chr5 179530734
chr5 179530742
chr5 179530758
chr5 179530767
chr5 179530777
chr5 179530785
chr5 179530787
chr5 179530794
chr5 179530804
chr5 179530810
chr5 179530819
chr5 179530828
chr5 179530841
chr5 179530850
chr5 179530869
chr5 179530879
chr5 179530882
chr5 179530893
chr5 179530898
chr5 179530924
chr5 179530929
chr5 179530931
chr5 179531006
chr5 179531050
chr5 179531132
chr5 179559466
chr5 179559480
chr5 179619336
chr5 179619349
chr5 179619373
chr5 179619396
chr5 179619973
chr5 179619993
chr5 179620069
chr5 179620147
chr5 179620208
chr5 179620243
chr5 179620439
chr5 179620498
chr5 179620550
chr5 179621329
chr5 179621364
chr5 179621414
chr5 179621526
chr5 179621728
chr5 179621742
chr5 179621747
chr5 179621775
chr5 179621802
chr5 179621924
chr5 179621934
chr5 179621959
chr5 179621966

chr5 179621975
chr5 179622008
chr5 179634156
chr5 179634166
chr5 179634317
chr5 179634352
chr5 179634357
chr5 179634359
chr5 179634381
chr5 179634386
chr5 179634393
chr5 179634481
chr5 179634483
chr5 179634492
chr5 179634501
chr5 179634503
chr5 179634508
chr5 179634510
chr5 179634518
chr5 179634525
chr5 179634527
chr5 179634529
chr5 179634539
chr5 179634543
chr5 179634547
chr5 179634552
chr5 179634554
chr5 179634559
chr5 179634566
chr5 179634699
chr5 179634759
chr5 179634770
chr5 179634778
chr5 179634787
chr5 179634794
chr5 179634814
chr5 179634826
chr5 179634841
chr5 179635128
chr5 179657704
chr5 179657719
chr5 179657731
chr5 179657751
chr5 179657758
chr5 179657767
chr5 179657775
chr5 179657786
chr5 179657846
chr5 179658095
chr5 179658145
chr5 179658152
chr5 179658164
chr5 179658186
chr5 179658272

chr5 179658275
chr5 179658307
chr5 179658320
chr5 179658323
chr5 179658325
chr5 179658336
chr5 179658426
chr5 179658428
chr5 179658453
chr5 179658466
chr5 179658519
chr5 179700165
chr5 179700182
chr5 180006651
chr5 180292917
chr5 180292934
chr5 180292951
chr5 180292959
chr5 180292963
chr5 180293213
chr5 180293254
chr5 180293266
chr5 180293285
chr5 180293365
chr5 180293375
chr5 180411510
chr5 180411850
chr5 180688465
chr5 180688501
chr5 180688599
chr5 180688716
chr5 180689041
chr5 180689095
chr5 180689156
chr5 180809780
chr5 180809862
chr5 180809867
chr5 180809921
chr5 180809929
chr5 180809936
chr5 180832043
chr5 180832076
chr5 180832212
chr5 180832285
chr5 180832444
chr5 180861955
chr5 180861961
chr5 180861995
chr5 180862021
chr5 180868671
chr5 180868699
chr5 180906528
chr5 180906589
chr5 180906618

chr5 180906661
chr5 180907017
chr5 180907035
chr5 180907061
chr5 180907168
chr5 180907284
chr5 180907310
chr5 180907477
chr5 180907523
chr5 180907554
chr5 180907573
chr5 180907595
chr5 180907777
chr5 181097334
chr5 181097352
chr5 181097390
chr5 181097396
chr5 181097404
chr5 181097418
chr5 181097881
chr5 181097886
chr5 181097890
chr5 181097896
chr5 181097912
chr5 181098064
chr5 181098067
chr5 181098076
chr5 181098078
chr5 181098089
chr5 181098099
chr5 181098102
chr5 181098108
chr5 181098114
chr5 181098117
chr5 181098135
chr5 181098146
chr5 181098181
chr5 181098183
chr5 181098189
chr5 181098203
chr5 181098211
chr5 181098237
chr5 181098242
chr5 181098257
chr5 181098264
chr5 181098269
chr5 181098273
chr5 181098275
chr5 181098316
chr5 181098335
chr5 181098728
chr5 181098748
chr5 181098763
chr5 181098828

chr5 181098890
chr5 181101183
chr5 181101205
chr5 181101211
chr5 181101213
chr5 181101242
chr5 181101248
chr5 181101257
chr5 181101259
chr5 181101277
chr5 181101280
chr5 181101286
chr5 181101292
chr5 181101295
chr5 181101305
chr5 181101316
chr5 181101318
chr5 181101327
chr5 181101330
chr5 181101343
chr5 181101348
chr5 181101353
chr5 181101359
chr5 181101368
chr5 181101372
chr5 181101389
chr5 181101402
chr5 181101409
chr5 181101413
chr5 181101420
chr5 181101422
chr5 181101427
chr5 181101429
chr5 181101438
chr5 181101444
chr5 181101446
chr5 181102121
chr5 181102124
chr5 181102135
chr5 181160836
chr5 181160843
chr5 181160866
chr5 181160873
chr5 181160876
chr5 181160896
chr5 181160903
chr5 181160906
chr5 181161106
chr5 181161113
chr5 181161116
chr5 181161136
chr5 181161143
chr5 181161146
chr5 181161166

chr5 181161173
chr5 181161196
chr5 181161203
chr5 181183326
chr5 181183335
chr5 181183351
chr5 181183356
chr5 181183363
chr5 181183368
chr5 181183393
chr5 181183406
chr5 181183410
chr5 181183496
chr5 181183498
chr5 181183508
chr5 181183536
chr5 181183541
chr5 181183545
chr5 181183552
chr5 181183566
chr5 181183586
chr5 181183597
chr5 181183629
chr5 181183645
chr5 181183664
chr5 181183666
chr5 181183672
chr5 181183676
chr5 181183696
chr5 181183718
chr5 181183735
chr5 181183751
chr5 181183760
chr5 181183781
chr5 181183789
chr5 181184034
chr5 181184054
chr5 181184082
chr5 181184085
chr5 181184122
chr5 181184129
chr5 181184134
chr5 181184141
chr5 181184155
chr5 181188976
chr5 181188984
chr5 181188992
chr5 181189000
chr5 181189059
chr5 181189072
chr5 181189074
chr5 181189095
chr5 181189120
chr5 181189158

chr5 181189166
chr5 181207043
chr5 181207147
chr5 181207175
chr5 181207180
chr5 181207191
chr5 181216555
chr5 181216598
chr5 181216612
chr5 181216623
chr5 181216626
chr5 181216713
chr5 181216727
chr5 181216741
chr5 181216772
chr5 181216814
chr5 181216825
chr5 181216850
chr5 181216869
chr5 181216978
chr5 181216980
chr5 181217765
chr5 181217772
chr5 181217779
chr5 181217901
chr5 181217913
chr5 181217930
chr5 181217933
chr5 181217949
chr5 181217952
chr5 181217980
chr5 181218003
chr5 181218019
chr5 181218031
chr5 181218034
chr5 181218053
chr5 181218056
chr5 181218124
chr5 181218139
chr5 181218176
chr5 181218180
chr5 181218209
chr5 181224296
chr5 181242669
chr5 181242723
chr5 181242751
chr5 181242805
chr5 181242838
chr5 181242848
chr5 181242850
chr5 181242919
chr5 181243016
chr5 181243058
chr5 181243110

chr5 181243169
chr5 181243184
chr5 181243192
chr5 181243218
chr5 181243248
chr5 181243262
chr5 181246543
chr5 181246554
chr5 181246565
chr5 181246568
chr5 181246575
chr5 181246580
chr5 181246604
chr5 181246616
chr5 181260254
chr5 181260301
chr3 10720
chr3 10726
chr3 313196
chr3 313477
chr3 352993
chr3 353094
chr3 480507
chr3 582762
chr3 582804
chr3 582931
chr3 583171
chr3 896799
chr3 896810
chr3 897001
chr3 1103438
chr3 1103553
chr3 1103631
chr3 1103638
chr3 1103907
chr3 1103919
chr3 1103957
chr3 1104129
chr3 1104197
chr3 1390657
chr3 1390825
chr3 1597907
chr3 1598268
chr3 1623055
chr3 1623339
chr3 1625803
chr3 1626509
chr3 1695353
chr3 1988221
chr3 1988227
chr3 1988304
chr3 2082294
chr3 3141609
chr3 3173088

chr3 3173093
chr3 3173304
chr3 3173309
chr3 3179182
chr3 3179198
chr3 3179209
chr3 3179214
chr3 3179491
chr3 3180044
chr3 3547693
chr3 3547956
chr3 3691348
chr3 3691429
chr3 3800127
chr3 3800169
chr3 3800220
chr3 3800226
chr3 3800249
chr3 3800270
chr3 3800276
chr3 3800296
chr3 3800308
chr3 3800337
chr3 3800339
chr3 3800395
chr3 3800399
chr3 3800402
chr3 3800432
chr3 3800518
chr3 3800526
chr3 3800529
chr3 3800536
chr3 3800538
chr3 3800556
chr3 3800574
chr3 3800576
chr3 3800594
chr3 3800612
chr3 3800614
chr3 3800670
chr3 3800688
chr3 3800690
chr3 3800708
chr3 3800726
chr3 3800728
chr3 3800746
chr3 4025760
chr3 4025941
chr3 4027348
chr3 4112995
chr3 4113203
chr3 4351034
chr3 4351054
chr3 4351314

chr3 4351379
chr3 4351442
chr3 4351461
chr3 4351504
chr3 4351547
chr3 4351624
chr3 4351627
chr3 4494250
chr3 4494269
chr3 4494277
chr3 4494310
chr3 4494390
chr3 4494392
chr3 4494465
chr3 4494581
chr3 4494619
chr3 4494691
chr3 4494776
chr3 4494779
chr3 4494790
chr3 4494915
chr3 4504458
chr3 4504552
chr3 4504608
chr3 4504684
chr3 4504793
chr3 4504801
chr3 4504820
chr3 4504865
chr3 4505007
chr3 4584616
chr3 4584625
chr3 4584645
chr3 4584682
chr3 4584780
chr3 4584917
chr3 4585057
chr3 4585076
chr3 4585091
chr3 4643400
chr3 4646044
chr3 4711670
chr3 4711736
chr3 4822056
chr3 4822073
chr3 4822076
chr3 4822097
chr3 4822133
chr3 4822135
chr3 4822145
chr3 4822158
chr3 4822227
chr3 4822275
chr3 4822352

chr3 4830983
chr3 4831058
chr3 4831091
chr3 4831253
chr3 4831324
chr3 4831354
chr3 4831378
chr3 4831410
chr3 4831435
chr3 4850520
chr3 4850523
chr3 4850563
chr3 4850970
chr3 4851116
chr3 4851131
chr3 4889381
chr3 4987028
chr3 4987038
chr3 4987045
chr3 4987062
chr3 4987107
chr3 4987124
chr3 4987128
chr3 4987154
chr3 5022379
chr3 5122891
chr3 5122990
chr3 5122997
chr3 5123001
chr3 5123009
chr3 5123069
chr3 5123086
chr3 5123108
chr3 5123144
chr3 5123155
chr3 5123157
chr3 5123236
chr3 5136864
chr3 5181800
chr3 5188710
chr3 5188718
chr3 5188744
chr3 5188777
chr3 5188820
chr3 5188843
chr3 5188981
chr3 5188987
chr3 5188996
chr3 5189016
chr3 5189028
chr3 5189038
chr3 5189263
chr3 5189348
chr3 5189425

chr3 5189623
chr3 5189631
chr3 5189661
chr3 5189663
chr3 5189670
chr3 5189686
chr3 5189725
chr3 5189747
chr3 5189757
chr3 5189790
chr3 5189878
chr3 5189888
chr3 5189894
chr3 5194565
chr3 5194645
chr3 5194828
chr3 5195507
chr3 5195603
chr3 6070051
chr3 6487304
chr3 6672231
chr3 6948572
chr3 7659065
chr3 7659436
chr3 8501516
chr3 8613622
chr3 8614274
chr3 8614313
chr3 8716791
chr3 8716844
chr3 8718431
chr3 8718745
chr3 8719226
chr3 8719310
chr3 8719487
chr3 8719664
chr3 8719701
chr3 8962743
chr3 8962759
chr3 8962860
chr3 8962869
chr3 8962931
chr3 8962980
chr3 8963035
chr3 8963089
chr3 8963181
chr3 8963183
chr3 8963187
chr3 8963208
chr3 8963230
chr3 8963234
chr3 8963236
chr3 8963259
chr3 8963294

chr3 8963304
chr3 9170089
chr3 9170136
chr3 9170178
chr3 9170190
chr3 9170261
chr3 9170276
chr3 9170360
chr3 9249429
chr3 9249436
chr3 9249438
chr3 9249441
chr3 9249453
chr3 9249496
chr3 9362914
chr3 9362964
chr3 9363000
chr3 9363041
chr3 9363077
chr3 9363116
chr3 9363122
chr3 9363126
chr3 9363136
chr3 9363139
chr3 9363149
chr3 9363153
chr3 9363219
chr3 9363222
chr3 9363225
chr3 9363230
chr3 9363241
chr3 9363253
chr3 9363353
chr3 9363461
chr3 9363464
chr3 9363492
chr3 9363499
chr3 9395717
chr3 9399392
chr3 9399424
chr3 9399957
chr3 9400012
chr3 9400052
chr3 9642959
chr3 9649724
chr3 9649730
chr3 9649733
chr3 9727373
chr3 9727385
chr3 9727642
chr3 9727649
chr3 9792022
chr3 9793355
chr3 9793394

chr3 9793429
chr3 9793437
chr3 9793443
chr3 9793503
chr3 9793510
chr3 9793560
chr3 9793626
chr3 9793778
chr3 9793827
chr3 9793850
chr3 9793869
chr3 9843829
chr3 9843833
chr3 9843858
chr3 9843863
chr3 9890666
chr3 9890672
chr3 9890677
chr3 9890689
chr3 9890691
chr3 9890702
chr3 9890727
chr3 9890732
chr3 9890742
chr3 9890744
chr3 9890748
chr3 9890752
chr3 9890758
chr3 9890761
chr3 9890767
chr3 9890780
chr3 9890786
chr3 9890788
chr3 9890791
chr3 9890825
chr3 9890834
chr3 9890850
chr3 9890862
chr3 9890876
chr3 9890879
chr3 9890882
chr3 9890887
chr3 9890919
chr3 9890945
chr3 9890950
chr3 9890956
chr3 9890984
chr3 9890993
chr3 9890995
chr3 9891014
chr3 9891018
chr3 9891042
chr3 9891044
chr3 9891054

chr3 9891061
chr3 9891071
chr3 9891078
chr3 9891081
chr3 9891093
chr3 9891107
chr3 9891113
chr3 9952418
chr3 9952421
chr3 9952427
chr3 9952431
chr3 9952433
chr3 9952439
chr3 9952451
chr3 9987130
chr3 9987162
chr3 9987192
chr3 9987208
chr3 9987249
chr3 9987255
chr3 9987263
chr3 9987286
chr3 9987296
chr3 9987304
chr3 9987742
chr3 9987791
chr3 9987921
chr3 10115663
chr3 10115668
chr3 10115674
chr3 10115680
chr3 10115694
chr3 10115697
chr3 10115706
chr3 10115721
chr3 10115729
chr3 10115756
chr3 10115797
chr3 10115814
chr3 10115818
chr3 10115825
chr3 10115859
chr3 10115909
chr3 10115918
chr3 10115988
chr3 10116021
chr3 10116081
chr3 10116083
chr3 10116102
chr3 10116107
chr3 10116161
chr3 10116192
chr3 10142105
chr3 10223895

chr3 10223947
chr3 10249482
chr3 10249488
chr3 10249510
chr3 10249519
chr3 10249552
chr3 10249563
chr3 10249629
chr3 10249640
chr3 10249763
chr3 10249786
chr3 10249788
chr3 10249826
chr3 10249959
chr3 10249969
chr3 10250099
chr3 10250290
chr3 10319909
chr3 10320460
chr3 10320468
chr3 10320485
chr3 10320492
chr3 10320524
chr3 10320565
chr3 10320599
chr3 10320639
chr3 10320645
chr3 10320715
chr3 10320724
chr3 10320742
chr3 10320771
chr3 10320793
chr3 10320823
chr3 10320831
chr3 10320834
chr3 10320836
chr3 10320841
chr3 10320862
chr3 10320885
chr3 10320895
chr3 10320903
chr3 10320931
chr3 10320949
chr3 10320979
chr3 10320983
chr3 10320992
chr3 10552126
chr3 10552148
chr3 10552152
chr3 10552155
chr3 10552160
chr3 10552166
chr3 10552189
chr3 10552196

chr3 10552202
chr3 10552338
chr3 10552356
chr3 10552390
chr3 10552402
chr3 10552493
chr3 10552679
chr3 10603847
chr3 10787643
chr3 10787945
chr3 10788228
chr3 10788689
chr3 10789019
chr3 10789044
chr3 11074208
chr3 11074247
chr3 11272600
chr3 11272607
chr3 11272614
chr3 11272624
chr3 11272626
chr3 11272649
chr3 11272672
chr3 11272744
chr3 11272803
chr3 11272856
chr3 11272913
chr3 11272962
chr3 11273070
chr3 11273084
chr3 11273088
chr3 11273117
chr3 11273157
chr3 11291427
chr3 11291457
chr3 11641471
chr3 11641755
chr3 11641817
chr3 11641828
chr3 11641835
chr3 11641856
chr3 11642119
chr3 11642190
chr3 11642251
chr3 11643776
chr3 11643833
chr3 11643835
chr3 11643842
chr3 11643859
chr3 11643862
chr3 12092140
chr3 12092241
chr3 12092256
chr3 12092371

chr3 12187486
chr3 12187497
chr3 12187501
chr3 12187505
chr3 12187520
chr3 12187906
chr3 12289284
chr3 12289335
chr3 12289526
chr3 12289604
chr3 12289655
chr3 12336347
chr3 12553747
chr3 12553767
chr3 12553881
chr3 12553914
chr3 12553921
chr3 12553938
chr3 12553954
chr3 12553974
chr3 12553977
chr3 12553981
chr3 12553987
chr3 12554010
chr3 12554034
chr3 12554080
chr3 12554107
chr3 12554232
chr3 12554234
chr3 12556346
chr3 12556396
chr3 12556419
chr3 12556443
chr3 12557705
chr3 12557708
chr3 12557713
chr3 12607289
chr3 12662499
chr3 12662707
chr3 12662740
chr3 12662757
chr3 12662869
chr3 12841322
chr3 12841340
chr3 12841343
chr3 12841349
chr3 12841366
chr3 12841407
chr3 12841409
chr3 12841427
chr3 12841439
chr3 12841442
chr3 12841459
chr3 12841469

chr3 12841479
chr3 12841494
chr3 12841518
chr3 12841527
chr3 12841530
chr3 12841548
chr3 12841554
chr3 12841556
chr3 12841573
chr3 13015355
chr3 13015424
chr3 13015490
chr3 13015510
chr3 13015555
chr3 13015597
chr3 13015637
chr3 13015643
chr3 13015684
chr3 13015708
chr3 13015762
chr3 13015902
chr3 13015952
chr3 13015996
chr3 13016126
chr3 13016186
chr3 13016218
chr3 13016253
chr3 13016294
chr3 13016358
chr3 13016362
chr3 13016376
chr3 13016395
chr3 13016440
chr3 13016447
chr3 13016484
chr3 13016495
chr3 13182368
chr3 13413988
chr3 13414023
chr3 13414202
chr3 13414220
chr3 13414222
chr3 13414239
chr3 13414266
chr3 13414278
chr3 13420965
chr3 13451479
chr3 13451486
chr3 13451535
chr3 13451563
chr3 13451584
chr3 13451590
chr3 13451690
chr3 13451702

chr3 13451757
chr3 13451780
chr3 13451790
chr3 13451807
chr3 13451833
chr3 13451865
chr3 13451877
chr3 13451897
chr3 13451899
chr3 13451908
chr3 13451918
chr3 13451948
chr3 13451988
chr3 13451994
chr3 13452008
chr3 13544461
chr3 13544552
chr3 13544596
chr3 13544620
chr3 14025749
chr3 14026015
chr3 14039307
chr3 14175970
chr3 14178827
chr3 14178829
chr3 14178836
chr3 14178846
chr3 14178865
chr3 14178869
chr3 14178872
chr3 14178903
chr3 14178907
chr3 14178962
chr3 14179011
chr3 14179025
chr3 14179035
chr3 14179094
chr3 14179204
chr3 14179292
chr3 14179335
chr3 14378012
chr3 14403210
chr3 14403224
chr3 14403264
chr3 14403344
chr3 14403380
chr3 14403406
chr3 14403410
chr3 14403458
chr3 14403468
chr3 14403535
chr3 14403538
chr3 14403566
chr3 14403614

chr3 14403620
chr3 14403683
chr3 14431638
chr3 14431690
chr3 14431697
chr3 14431769
chr3 14431788
chr3 14431802
chr3 14431834
chr3 14432005
chr3 14432038
chr3 14432072
chr3 14432083
chr3 14432409
chr3 14432602
chr3 14432683
chr3 14432685
chr3 14432710
chr3 14651863
chr3 14651868
chr3 14651877
chr3 14651905
chr3 14651912
chr3 14652109
chr3 14652115
chr3 14652135
chr3 14652152
chr3 14946726
chr3 14946757
chr3 14946901
chr3 14948396
chr3 14948402
chr3 14948407
chr3 14948409
chr3 14948415
chr3 14948421
chr3 14948424
chr3 14948444
chr3 14948461
chr3 14948476
chr3 15055130
chr3 15055168
chr3 15055197
chr3 15055205
chr3 15055219
chr3 15098171
chr3 15098183
chr3 15098246
chr3 15098249
chr3 15098319
chr3 15180077
chr3 15180117
chr3 15205737
chr3 15291992

chr3 15292006
chr3 15292040
chr3 15292050
chr3 15332157
chr3 15332167
chr3 15332214
chr3 15332221
chr3 15332233
chr3 15332235
chr3 15332242
chr3 15332248
chr3 15332253
chr3 15332259
chr3 15332261
chr3 15332263
chr3 15332278
chr3 15332342
chr3 15332347
chr3 15332355
chr3 15332366
chr3 15332379
chr3 15332383
chr3 15332389
chr3 15332400
chr3 15332402
chr3 15332418
chr3 15332423
chr3 15332425
chr3 15332427
chr3 15332430
chr3 15332432
chr3 15332444
chr3 15332449
chr3 15332454
chr3 15332461
chr3 15332463
chr3 15332488
chr3 15332493
chr3 15332502
chr3 15332508
chr3 15332512
chr3 15332520
chr3 15332523
chr3 15332529
chr3 15332536
chr3 15332543
chr3 15332550
chr3 15332553
chr3 15332558
chr3 15332568
chr3 15332572
chr3 15332574
chr3 15332577
chr3 15332595

chr3 15332597
chr3 15332601
chr3 15332615
chr3 15332617
chr3 15332627
chr3 15332632
chr3 15332636
chr3 15332641
chr3 15332647
chr3 15332652
chr3 15332657
chr3 15332663
chr3 15332665
chr3 15332670
chr3 15332673
chr3 15361399
chr3 15361586
chr3 15361659
chr3 15461091
chr3 15461197
chr3 15461302
chr3 15557912
chr3 15630109
chr3 15630277
chr3 15630296
chr3 15630332
chr3 15630371
chr3 15630531
chr3 15630822
chr3 15630838
chr3 15630875
chr3 15630877
chr3 15630911
chr3 15630917
chr3 15647609
chr3 15647675
chr3 15647787
chr3 15647837
chr3 16247420
chr3 16247442
chr3 16247455
chr3 16247458
chr3 16247481
chr3 16247559
chr3 16247591
chr3 16264040
chr3 16264132
chr3 16264197
chr3 16264205
chr3 16264236
chr3 16264273
chr3 16264369
chr3 16264379
chr3 16264401

chr3 16264434
chr3 16264441
chr3 16264445
chr3 16264473
chr3 16264486
chr3 16264814
chr3 16264820
chr3 16264828
chr3 16265634
chr3 16265637
chr3 16286076
chr3 16301487
chr3 16301690
chr3 16301709
chr3 16301730
chr3 16301758
chr3 16301763
chr3 16311993
chr3 16312026
chr3 16312122
chr3 16338448
chr3 16338456
chr3 16338903
chr3 16339058
chr3 16673337
chr3 16674177
chr3 16824346
chr3 16841487
chr3 16841523
chr3 16841673
chr3 16841728
chr3 16841820
chr3 16842033
chr3 16842038
chr3 16842266
chr3 16842354
chr3 16842392
chr3 16842438
chr3 16842450
chr3 16842481
chr3 16842577
chr3 16842611
chr3 16842655
chr3 16842701
chr3 16842832
chr3 16843015
chr3 16843069
chr3 16843075
chr3 16843084
chr3 16848967
chr3 16848972
chr3 16849348
chr3 16849382
chr3 16849386

chr3 16849414
chr3 16849453
chr3 16849486
chr3 16849505
chr3 16849543
chr3 16885504
chr3 16885647
chr3 16885773
chr3 16885776
chr3 16886404
chr3 16886592
chr3 16886666
chr3 16886729
chr3 16888491
chr3 16888495
chr3 16888547
chr3 17741041
chr3 17741352
chr3 17741408
chr3 17741510
chr3 17741543
chr3 17741625
chr3 17741683
chr3 17741697
chr3 17741725
chr3 17743191
chr3 17743379
chr3 17743423
chr3 17743477
chr3 17749812
chr3 17819008
chr3 17819839
chr3 17820221
chr3 17842147
chr3 18423199
chr3 18423364
chr3 18423387
chr3 18423402
chr3 18423434
chr3 18423443
chr3 18423503
chr3 18423508
chr3 18423612
chr3 18423685
chr3 18423779
chr3 18423821
chr3 18423833
chr3 18423854
chr3 18423904
chr3 18423959
chr3 18424027
chr3 18424041
chr3 18424061
chr3 18424097

chr3 18424100
chr3 18425958
chr3 18425961
chr3 18425975
chr3 18425996
chr3 18426001
chr3 18426007
chr3 18426020
chr3 18426022
chr3 18426028
chr3 18426041
chr3 18426092
chr3 18426109
chr3 18426117
chr3 18426119
chr3 18426134
chr3 18426244
chr3 18426262
chr3 18426275
chr3 18426318
chr3 18426324
chr3 18442521
chr3 18443147
chr3 18443180
chr3 18443216
chr3 18443220
chr3 18443250
chr3 18443453
chr3 18740997
chr3 18741208
chr3 18741234
chr3 18741255
chr3 18741257
chr3 18741266
chr3 18741299
chr3 18741355
chr3 18741398
chr3 18741492
chr3 18741610
chr3 18741671
chr3 18988679
chr3 18988684
chr3 18988839
chr3 18988869
chr3 18989396
chr3 18989431
chr3 18989556
chr3 19146508
chr3 19146531
chr3 19146629
chr3 19146669
chr3 19146674
chr3 19146769
chr3 19146785

chr3 19146795
chr3 19146802
chr3 19146809
chr3 19147211
chr3 19147239
chr3 19147244
chr3 19147248
chr3 19147274
chr3 19147277
chr3 19147279
chr3 19147318
chr3 19147342
chr3 19147417
chr3 19147458
chr3 19147701
chr3 19147765
chr3 19147791
chr3 19149547
chr3 19149552
chr3 19149587
chr3 19149721
chr3 19183678
chr3 19183715
chr3 19183788
chr3 19183815
chr3 19183936
chr3 19184516
chr3 19184561
chr3 19184579
chr3 19184592
chr3 19185535
chr3 19427132
chr3 19700705
chr3 19748086
chr3 19947726
chr3 19947871
chr3 19947905
chr3 19947924
chr3 19948006
chr3 19948039
chr3 19948055
chr3 19948111
chr3 19948138
chr3 19948182
chr3 19948240
chr3 19948289
chr3 19948602
chr3 20039020
chr3 20039041
chr3 20039181
chr3 20039188
chr3 20039206
chr3 20039323
chr3 20039371

chr3 20039375
chr3 20039377
chr3 20039400
chr3 20039405
chr3 20039429
chr3 20039570
chr3 20039669
chr3 20039716
chr3 20039756
chr3 20039852
chr3 20039856
chr3 20039863
chr3 20039875
chr3 20039877
chr3 20054854
chr3 20054878
chr3 20055127
chr3 20055231
chr3 20055273
chr3 20055424
chr3 20055512
chr3 20055517
chr3 20055553
chr3 20055586
chr3 20104281
chr3 20104368
chr3 20104382
chr3 20104447
chr3 20184826
chr3 20184894
chr3 20185002
chr3 20185197
chr3 20185201
chr3 20185270
chr3 20185324
chr3 20185402
chr3 20185462
chr3 20185464
chr3 20185472
chr3 20185494
chr3 20185502
chr3 20185538
chr3 20185567
chr3 20185599
chr3 20186216
chr3 20186221
chr3 20186463
chr3 20186580
chr3 20186600
chr3 20186712
chr3 20186740
chr3 20186771
chr3 20186791
chr3 20186829

chr3 20186837
chr3 20186845
chr3 20186954
chr3 20339845
chr3 20340177
chr3 20342745
chr3 20342879
chr3 20660805
chr3 20777066
chr3 20955345
chr3 21933705
chr3 21934094
chr3 22006559
chr3 22010697
chr3 22564347
chr3 22564418
chr3 22565056
chr3 22565060
chr3 22565083
chr3 22565100
chr3 23203909
chr3 23203916
chr3 23442464
chr3 23442656
chr3 23442788
chr3 23805638
chr3 23805654
chr3 23805669
chr3 23805718
chr3 23805729
chr3 23805733
chr3 23805737
chr3 23805746
chr3 23805804
chr3 23805806
chr3 23805840
chr3 23805842
chr3 23805856
chr3 23805861
chr3 23805882
chr3 23805905
chr3 23805908
chr3 23805911
chr3 23805930
chr3 23805934
chr3 23806034
chr3 23806040
chr3 23806052
chr3 23806055
chr3 23806057
chr3 23806060
chr3 23806065
chr3 23806092
chr3 23806107

chr3 23806112
chr3 23806115
chr3 23806122
chr3 23806126
chr3 23806128
chr3 23806131
chr3 23806134
chr3 23806141
chr3 23806154
chr3 23806157
chr3 23806166
chr3 23806171
chr3 23806173
chr3 23806176
chr3 23806179
chr3 23806185
chr3 23806188
chr3 23806193
chr3 23806202
chr3 23806208
chr3 23806211
chr3 23806213
chr3 23806224
chr3 23806253
chr3 23806260
chr3 23806264
chr3 23806266
chr3 23806286
chr3 23806291
chr3 23806293
chr3 23806302
chr3 23806309
chr3 23806313
chr3 23806324
chr3 23806341
chr3 23806352
chr3 23806381
chr3 23806390
chr3 23806396
chr3 23806424
chr3 23810873
chr3 23810882
chr3 23810887
chr3 23810903
chr3 23810909
chr3 23810919
chr3 23810928
chr3 23810943
chr3 23810958
chr3 23810962
chr3 23810967
chr3 23810978
chr3 23810986
chr3 23810994

chr3 23811002
chr3 23811008
chr3 23811016
chr3 23944917
chr3 23944920
chr3 23944931
chr3 23944968
chr3 23944983
chr3 23944993
chr3 23945006
chr3 23999125
chr3 23999128
chr3 23999221
chr3 23999254
chr3 23999459
chr3 23999654
chr3 23999660
chr3 24000002
chr3 24004503
chr3 24522594
chr3 24678472
chr3 24678521
chr3 24678551
chr3 24678618
chr3 24678809
chr3 24678818
chr3 24678823
chr3 24679037
chr3 24881317
chr3 25428070
chr3 25428073
chr3 25428082
chr3 25428086
chr3 25428203
chr3 25428217
chr3 25428229
chr3 25428345
chr3 25428369
chr3 25428375
chr3 25428377
chr3 25428381
chr3 25428386
chr3 25428408
chr3 25428411
chr3 25428423
chr3 25428428
chr3 25428434
chr3 25428495
chr3 25428500
chr3 25428513
chr3 25663053
chr3 25663126
chr3 25663249
chr3 25663307

chr3 25663320
chr3 25663361
chr3 25663410
chr3 25663429
chr3 25663432
chr3 25663440
chr3 25663448
chr3 25663477
chr3 25663513
chr3 25663516
chr3 25663584
chr3 25663595
chr3 25663604
chr3 25663648
chr3 25663688
chr3 25663702
chr3 25665413
chr3 25665420
chr3 25665445
chr3 25665454
chr3 25665493
chr3 25782044
chr3 25782246
chr3 25782354
chr3 25782598
chr3 25782610
chr3 25783557
chr3 25783566
chr3 25783570
chr3 25783766
chr3 25783788
chr3 25783800
chr3 25783814
chr3 25783851
chr3 25784270
chr3 25789517
chr3 25789559
chr3 25789604
chr3 25789700
chr3 25789705
chr3 25789774
chr3 25789791
chr3 26398588
chr3 26449886
chr3 26449903
chr3 26450019
chr3 26461048
chr3 26461096
chr3 26461110
chr3 26461738
chr3 26771361
chr3 27369214
chr3 27369225
chr3 27369227

chr3 27369229
chr3 27369238
chr3 27369572
chr3 27369574
chr3 27467453
chr3 27467581
chr3 27467675
chr3 27467699
chr3 27479874
chr3 27596526
chr3 27597121
chr3 27632578
chr3 27632661
chr3 27632699
chr3 27632719
chr3 27632725
chr3 27632734
chr3 27632737
chr3 27632740
chr3 27632745
chr3 27632749
chr3 27632753
chr3 27632762
chr3 27632767
chr3 27632770
chr3 27632805
chr3 27632872
chr3 27632882
chr3 27632886
chr3 27632890
chr3 27632893
chr3 27632902
chr3 27632904
chr3 27632907
chr3 27632910
chr3 27632919
chr3 27632921
chr3 27632924
chr3 27632927
chr3 27632936
chr3 27632938
chr3 27632941
chr3 27632944
chr3 27632953
chr3 27632955
chr3 27632958
chr3 27632961
chr3 27632970
chr3 27632972
chr3 27632981
chr3 27633009
chr3 27633037
chr3 27633041
chr3 27633046

chr3 27633054
chr3 27633056
chr3 27633070
chr3 27633081
chr3 27633094
chr3 27633097
chr3 27633099
chr3 27633123
chr3 27633133
chr3 27633136
chr3 27633147
chr3 27633188
chr3 27633194
chr3 27633262
chr3 27633329
chr3 27633343
chr3 27633364
chr3 27633409
chr3 27633494
chr3 27633577
chr3 27712847
chr3 27712860
chr3 27712864
chr3 27712879
chr3 27712886
chr3 27712893
chr3 27712896
chr3 27712898
chr3 27712900
chr3 27712911
chr3 27712928
chr3 27712936
chr3 27712949
chr3 27712971
chr3 27712990
chr3 27712992
chr3 27712995
chr3 27713003
chr3 27725515
chr3 27725946
chr3 27728054
chr3 27728057
chr3 27728281
chr3 27728314
chr3 27728373
chr3 27728388
chr3 27881484
chr3 27881654
chr3 27881812
chr3 27881997
chr3 27882180
chr3 27882226
chr3 27882301
chr3 27882645

chr3 27883001
chr3 27883013
chr3 27883587
chr3 27883798
chr3 27903977
chr3 28013930
chr3 28050095
chr3 28050198
chr3 28050327
chr3 28050388
chr3 28050817
chr3 28916605
chr3 28916762
chr3 28916823
chr3 29110174
chr3 29110203
chr3 29110236
chr3 29280563
chr3 29280576
chr3 29280601
chr3 29280683
chr3 29280808
chr3 29280812
chr3 29280842
chr3 29280851
chr3 29280866
chr3 29280877
chr3 29280899
chr3 29280979
chr3 29280983
chr3 29281007
chr3 29281020
chr3 29281063
chr3 29281287
chr3 29281328
chr3 29281332
chr3 29281399
chr3 29281471
chr3 29281551
chr3 29281585
chr3 29281620
chr3 29281660
chr3 29281691
chr3 29281772
chr3 29281780
chr3 29281787
chr3 29281795
chr3 29281826
chr3 29482217
chr3 30012726
chr3 30013092
chr3 30013133
chr3 30289495
chr3 30289899

chr3 30608179
chr3 30608216
chr3 30608300
chr3 30608410
chr3 30608415
chr3 30608514
chr3 30608582
chr3 30608589
chr3 30608768
chr3 30608777
chr3 30609027
chr3 30609080
chr3 30609256
chr3 30609322
chr3 30609418
chr3 30609543
chr3 30609812
chr3 30609825
chr3 30635384
chr3 30635919
chr3 30643789
chr3 30644554
chr3 30644754
chr3 30644778
chr3 30645057
chr3 30658403
chr3 30658564
chr3 30658575
chr3 30658792
chr3 30658794
chr3 30658874
chr3 30658916
chr3 30658944
chr3 30658954
chr3 30659145
chr3 30768215
chr3 30768585
chr3 30768602
chr3 30985138
chr3 30985188
chr3 30985265
chr3 31449070
chr3 31449539
chr3 31531090
chr3 31531104
chr3 31531203
chr3 31531477
chr3 31531483
chr3 31531486
chr3 31531495
chr3 31531528
chr3 31531600
chr3 31534599
chr3 31534839

chr3 31535129
chr3 31535445
chr3 31535449
chr3 31535451
chr3 31535461
chr3 31535481
chr3 31535489
chr3 31535521
chr3 31535580
chr3 31535592
chr3 31535596
chr3 31535641
chr3 31535647
chr3 31535654
chr3 31536480
chr3 31536579
chr3 31550739
chr3 31979125
chr3 31979185
chr3 31979215
chr3 31979424
chr3 31979663
chr3 32091271
chr3 32091338
chr3 32091378
chr3 32091698
chr3 32114695
chr3 32114697
chr3 32114707
chr3 32114774
chr3 32114799
chr3 32114807
chr3 32114868
chr3 32114870
chr3 32114910
chr3 32428849
chr3 32428859
chr3 32428957
chr3 32443638
chr3 32443880
chr3 32444099
chr3 32460470
chr3 32460532
chr3 32460550
chr3 32460562
chr3 32460585
chr3 32460593
chr3 32460596
chr3 32460643
chr3 32460684
chr3 32460718
chr3 32501551
chr3 32501567
chr3 32501575

chr3 32501633
chr3 32501753
chr3 32501861
chr3 32501910
chr3 32501992
chr3 32502103
chr3 32502134
chr3 32502136
chr3 32502144
chr3 32502185
chr3 32502681
chr3 32502694
chr3 32502701
chr3 32503050
chr3 32503058
chr3 32503075
chr3 32510309
chr3 32510453
chr3 32569893
chr3 32569966
chr3 32569978
chr3 32570050
chr3 32570069
chr3 32570073
chr3 32570121
chr3 32577742
chr3 32577918
chr3 32684557
chr3 32684678
chr3 32684711
chr3 32684830
chr3 32684844
chr3 32684860
chr3 33055253
chr3 33055332
chr3 33055345
chr3 33063737
chr3 33063832
chr3 33217568
chr3 33217591
chr3 33217641
chr3 33217650
chr3 33217732
chr3 33217769
chr3 33217784
chr3 33217815
chr3 33217898
chr3 33438343
chr3 33438372
chr3 33438419
chr3 33438617
chr3 33438949
chr3 33439031
chr3 33441034

chr3 33441141
chr3 33441157
chr3 33441174
chr3 33441182
chr3 33441190
chr3 33441192
chr3 33441200
chr3 33718383
chr3 33718399
chr3 33718404
chr3 33718418
chr3 33718440
chr3 33718459
chr3 33718522
chr3 33718534
chr3 33718657
chr3 33718672
chr3 33718690
chr3 33718707
chr3 33797847
chr3 33797897
chr3 33797992
chr3 33798071
chr3 33798115
chr3 33798170
chr3 33798211
chr3 34786369
chr3 34786933
chr3 35058347
chr3 35058925
chr3 35101754
chr3 35101777
chr3 35102375
chr3 35103098
chr3 35214456
chr3 35214499
chr3 35224441
chr3 35224633
chr3 35627171
chr3 35627245
chr3 35628540
chr3 35628657
chr3 35640859
chr3 35641597
chr3 35825977
chr3 36451870
chr3 36876577
chr3 36876610
chr3 36876715
chr3 36876790
chr3 36876907
chr3 36907978
chr3 36907989
chr3 36908034

chr3 36908048
chr3 36908095
chr3 36908363
chr3 36908414
chr3 36908435
chr3 36908466
chr3 36908475
chr3 36908491
chr3 36908513
chr3 36908546
chr3 36908963
chr3 36912461
chr3 36958593
chr3 36958609
chr3 36958615
chr3 36958744
chr3 36958891
chr3 36958897
chr3 36958901
chr3 36958988
chr3 36990220
chr3 36990715
chr3 36991139
chr3 36991298
chr3 36991324
chr3 36991381
chr3 36991681
chr3 37174726
chr3 37174816
chr3 37174886
chr3 37175663
chr3 37176637
chr3 37176659
chr3 37177062
chr3 37177181
chr3 37177263
chr3 37177280
chr3 37177468
chr3 37177586
chr3 37746393
chr3 37907896
chr3 37907912
chr3 37907952
chr3 37908139
chr3 37908179
chr3 37908223
chr3 37993972
chr3 37993979
chr3 37994016
chr3 37994063
chr3 37994078
chr3 37994103
chr3 37994110
chr3 37994151

chr3 37994153
chr3 37994170
chr3 37994211
chr3 37994219
chr3 37994228
chr3 37994251
chr3 37994274
chr3 37994283
chr3 37994292
chr3 37994299
chr3 37994328
chr3 37994334
chr3 37994340
chr3 37994347
chr3 37994352
chr3 37994369
chr3 37994398
chr3 37994408
chr3 37994410
chr3 37994415
chr3 37994425
chr3 37994431
chr3 37994569
chr3 37994818
chr3 37994865
chr3 38024075
chr3 38024078
chr3 38024253
chr3 38024255
chr3 38024273
chr3 38024276
chr3 38024306
chr3 38024315
chr3 38024327
chr3 38024345
chr3 38024356
chr3 38024359
chr3 38024375
chr3 38024402
chr3 38024408
chr3 38024414
chr3 38024458
chr3 38024460
chr3 38024466
chr3 38024472
chr3 38024483
chr3 38024525
chr3 38024532
chr3 38024541
chr3 38024563
chr3 38024597
chr3 38024609
chr3 38024616
chr3 38024624

chr3 38024629
chr3 38024663
chr3 38024673
chr3 38024686
chr3 38024690
chr3 38024693
chr3 38038506
chr3 38038559
chr3 38038712
chr3 38038832
chr3 38038859
chr3 38039138
chr3 38039167
chr3 38039263
chr3 38039268
chr3 38039276
chr3 38039299
chr3 38496738
chr3 38496748
chr3 38496771
chr3 38496774
chr3 38496778
chr3 38496804
chr3 38496806
chr3 39052869
chr3 39052961
chr3 39107898
chr3 39107908
chr3 39107926
chr3 39107956
chr3 39107978
chr3 39108017
chr3 39108026
chr3 39108038
chr3 39108066
chr3 39108153
chr3 39108159
chr3 39108414
chr3 39108485
chr3 39108753
chr3 39178067
chr3 39178071
chr3 39178075
chr3 39178089
chr3 39178096
chr3 39178103
chr3 39226186
chr3 39226229
chr3 39226560
chr3 39267864
chr3 39267899
chr3 39267923
chr3 39267936
chr3 39267944

chr3 39268037
chr3 39268046
chr3 39268060
chr3 39268147
chr3 39347023
chr3 39406751
chr3 39406754
chr3 39406773
chr3 39406780
chr3 39406789
chr3 39407130
chr3 39407143
chr3 39407145
chr3 39407148
chr3 39407152
chr3 39407173
chr3 39407207
chr3 39407227
chr3 39407333
chr3 39407386
chr3 39407395
chr3 39407412
chr3 39407424
chr3 39407453
chr3 39407461
chr3 39407470
chr3 39407626
chr3 39407629
chr3 39407659
chr3 39448221
chr3 39448284
chr3 39656609
chr3 39707819
chr3 39708091
chr3 39708256
chr3 39709256
chr3 40133270
chr3 40191906
chr3 40310100
chr3 40310114
chr3 40310117
chr3 40310129
chr3 40310141
chr3 40310637
chr3 40310759
chr3 40452506
chr3 40452701
chr3 40452710
chr3 40452806
chr3 40452814
chr3 40452838
chr3 40452856
chr3 40452874
chr3 40452876

chr3 40452924
chr3 40452927
chr3 40452931
chr3 40452949
chr3 40452979
chr3 40452981
chr3 40452994
chr3 40452998
chr3 40453001
chr3 40453018
chr3 40453085
chr3 40453091
chr3 40453109
chr3 40453113
chr3 40453117
chr3 40453147
chr3 40453153
chr3 40453162
chr3 40453180
chr3 40453242
chr3 40453247
chr3 40453268
chr3 40453293
chr3 40453324
chr3 40453339
chr3 40453349
chr3 40453358
chr3 40453367
chr3 40453582
chr3 40453642
chr3 40453662
chr3 40453673
chr3 40477251
chr3 40477254
chr3 40506213
chr3 40506252
chr3 40506259
chr3 40506279
chr3 40506315
chr3 40506345
chr3 40506364
chr3 40506371
chr3 40506660
chr3 40757726
chr3 40758316
chr3 41200723
chr3 41200733
chr3 41200814
chr3 41200838
chr3 41200891
chr3 41200943
chr3 41200957
chr3 41201034
chr3 41201079

chr3 41201139
chr3 41201166
chr3 41201286
chr3 41201305
chr3 41201461
chr3 41961318
chr3 41961337
chr3 41961352
chr3 41961355
chr3 41961385
chr3 41961406
chr3 41961418
chr3 41961441
chr3 41961452
chr3 41961458
chr3 41961460
chr3 41961462
chr3 41961495
chr3 41961498
chr3 41961534
chr3 41961539
chr3 41961570
chr3 41961610
chr3 41961618
chr3 41961623
chr3 41961627
chr3 42012703
chr3 42013034
chr3 42013088
chr3 42013114
chr3 42013189
chr3 42013195
chr3 42013228
chr3 42013249
chr3 42013267
chr3 42013290
chr3 42013300
chr3 42013305
chr3 42013310
chr3 42013312
chr3 42013354
chr3 42013378
chr3 42013382
chr3 42247204
chr3 42289240
chr3 42404011
chr3 42529876
chr3 42581113
chr3 42581290
chr3 42581458
chr3 42581505
chr3 42581514
chr3 42601392
chr3 42601397

chr3 42601404
chr3 42601467
chr3 42601530
chr3 42601722
chr3 42601764
chr3 42601878
chr3 42601932
chr3 42804237
chr3 42804244
chr3 42804258
chr3 42804268
chr3 42863973
chr3 43023962
chr3 43024010
chr3 43024019
chr3 43024030
chr3 43024178
chr3 43024319
chr3 43024363
chr3 43024410
chr3 43106133
chr3 43106135
chr3 43106160
chr3 43106436
chr3 43106499
chr3 43106501
chr3 43106510
chr3 43287811
chr3 43287855
chr3 43287911
chr3 43288302
chr3 43288503
chr3 43288763
chr3 43389523
chr3 43389821
chr3 43893038
chr3 43893245
chr3 44022547
chr3 44022597
chr3 44022684
chr3 44183533
chr3 44184357
chr3 44184391
chr3 44184401
chr3 44319027
chr3 44319070
chr3 44319102
chr3 44319142
chr3 44319187
chr3 44319545
chr3 44338976
chr3 44338981
chr3 44339044
chr3 44339059

chr3 44339075
chr3 44339088
chr3 44339107
chr3 44339239
chr3 44339281
chr3 44476265
chr3 44476354
chr3 44476480
chr3 44476609
chr3 44476655
chr3 44476712
chr3 44476887
chr3 44476905
chr3 44477072
chr3 44477166
chr3 44477177
chr3 44477264
chr3 44477293
chr3 44477321
chr3 44477347
chr3 44477357
chr3 44477367
chr3 44477388
chr3 44477392
chr3 44477420
chr3 44477429
chr3 44477434
chr3 44477440
chr3 44477450
chr3 44477461
chr3 44477466
chr3 44477470
chr3 44477484
chr3 44477491
chr3 44477495
chr3 44477498
chr3 44477502
chr3 44477512
chr3 44477521
chr3 44477523
chr3 44477530
chr3 44477540
chr3 44477542
chr3 44477546
chr3 44477554
chr3 44477569
chr3 44477571
chr3 44510824
chr3 44510840
chr3 44510842
chr3 44510858
chr3 44510867
chr3 44510891
chr3 44510901

chr3 44510954
chr3 44510972
chr3 44510980
chr3 44510982
chr3 44510992
chr3 44511020
chr3 44511029
chr3 44511036
chr3 44511050
chr3 44555421
chr3 44555425
chr3 44555432
chr3 44555438
chr3 44555448
chr3 44555457
chr3 44555473
chr3 44555487
chr3 44555490
chr3 44555610
chr3 44555852
chr3 44555933
chr3 44555999
chr3 44556014
chr3 44556194
chr3 44585939
chr3 44625292
chr3 44625295
chr3 44625298
chr3 44625306
chr3 44625322
chr3 44625327
chr3 44625354
chr3 44625518
chr3 44648073
chr3 44648229
chr3 44648396
chr3 44649136
chr3 44649171
chr3 44649181
chr3 44649189
chr3 44649288
chr3 44762184
chr3 44762205
chr3 44762244
chr3 44762269
chr3 44762424
chr3 44762428
chr3 44762448
chr3 44762500
chr3 44762525
chr3 44762691
chr3 44976634
chr3 44976637
chr3 44976641

chr3 44976643
chr3 44976658
chr3 44976677
chr3 44976727
chr3 44976741
chr3 44976747
chr3 44976756
chr3 44976779
chr3 44976812
chr3 44976821
chr3 44976849
chr3 45037869
chr3 45037900
chr3 45038010
chr3 45038260
chr3 45118241
chr3 45167829
chr3 45167906
chr3 45567890
chr3 45567900
chr3 45567908
chr3 45568061
chr3 45568078
chr3 45568394
chr3 45568451
chr3 45568467
chr3 45568489
chr3 45568491
chr3 45568498
chr3 45568514
chr3 45568521
chr3 45568529
chr3 45568552
chr3 45568573
chr3 45568585
chr3 45568589
chr3 45568618
chr3 45568629
chr3 45568649
chr3 45568663
chr3 45568717
chr3 45568719
chr3 45568723
chr3 45568735
chr3 45665220
chr3 45688209
chr3 45688219
chr3 45688234
chr3 45688253
chr3 45688264
chr3 45688284
chr3 45688289
chr3 45688304
chr3 45688310

chr3 45688315
chr3 45688317
chr3 45690966
chr3 45691075
chr3 45841503
chr3 45841527
chr3 45841529
chr3 45841531
chr3 45841533
chr3 45841535
chr3 45841537
chr3 45841652
chr3 45841666
chr3 45864418
chr3 45865006
chr3 45865024
chr3 45865061
chr3 45865080
chr3 45870480
chr3 45870577
chr3 45870758
chr3 45870775
chr3 45870780
chr3 45994683
chr3 45994740
chr3 45994786
chr3 45994814
chr3 45994981
chr3 45994983
chr3 45994990
chr3 45995031
chr3 45995050
chr3 45995061
chr3 45995079
chr3 45995102
chr3 45995128
chr3 45995154
chr3 45995164
chr3 45995264
chr3 45995274
chr3 45995307
chr3 45995346
chr3 45995361
chr3 45995380
chr3 45995408
chr3 45995411
chr3 45995435
chr3 45995444
chr3 45995451
chr3 45995491
chr3 46068762
chr3 46068781
chr3 46069902
chr3 46078878

chr3 46078882
chr3 46078907
chr3 46078922
chr3 46078947
chr3 46078957
chr3 46079013
chr3 46079063
chr3 46079130
chr3 46079156
chr3 46079246
chr3 46097770
chr3 46097810
chr3 46097813
chr3 46097834
chr3 46097868
chr3 46097928
chr3 46098354
chr3 46098757
chr3 46098935
chr3 46150803
chr3 46186862
chr3 46186999
chr3 46299139
chr3 46299488
chr3 46299492
chr3 46299536
chr3 46299936
chr3 46299951
chr3 46299981
chr3 46369708
chr3 46369777
chr3 46369956
chr3 46369983
chr3 46370050
chr3 46370312
chr3 46370354
chr3 46370421
chr3 46370757
chr3 46370842
chr3 46370963
chr3 46370987
chr3 46371157
chr3 46378388
chr3 46378403
chr3 46488698
chr3 46538732
chr3 46538853
chr3 46538858
chr3 46538861
chr3 46538916
chr3 46538959
chr3 46539019
chr3 46539045
chr3 46539100

chr3 46539242
chr3 46539256
chr3 46539305
chr3 46558565
chr3 46558571
chr3 46558604
chr3 46558661
chr3 46558678
chr3 46558723
chr3 46882655
chr3 46882683
chr3 46882695
chr3 46882700
chr3 46882713
chr3 46882737
chr3 46882752
chr3 46882773
chr3 46882781
chr3 46882784
chr3 46882790
chr3 46882795
chr3 46882797
chr3 46882801
chr3 46882806
chr3 46882814
chr3 46882820
chr3 46882851
chr3 46882858
chr3 46882865
chr3 46882889
chr3 46882893
chr3 46882897
chr3 46882907
chr3 46882911
chr3 46882951
chr3 46882968
chr3 46882973
chr3 46882975
chr3 46882977
chr3 46882987
chr3 46882989
chr3 46883000
chr3 46883016
chr3 46883019
chr3 46883026
chr3 46883028
chr3 46953982
chr3 46954069
chr3 47057389
chr3 47057419
chr3 47057422
chr3 47057446
chr3 47057458
chr3 47162162

chr3 47162273
chr3 47162557
chr3 47164720
chr3 47164725
chr3 47164737
chr3 47164744
chr3 47164752
chr3 47164765
chr3 47164777
chr3 47164788
chr3 47164808
chr3 47164811
chr3 47164813
chr3 47164816
chr3 47164831
chr3 47164845
chr3 47164969
chr3 47165001
chr3 47165003
chr3 47475212
chr3 47475237
chr3 47475243
chr3 47475258
chr3 47475528
chr3 47475563
chr3 47475565
chr3 47475580
chr3 47475584
chr3 47475597
chr3 47475601
chr3 47475618
chr3 47475620
chr3 47475622
chr3 47475639
chr3 47475646
chr3 47475659
chr3 47475664
chr3 47780240
chr3 47780296
chr3 47780332
chr3 47780359
chr3 47780390
chr3 47780417
chr3 47780421
chr3 47780434
chr3 47780436
chr3 47780440
chr3 47780471
chr3 47780648
chr3 47780654
chr3 47780765
chr3 47780892
chr3 47780895
chr3 47780904

chr3 47788902
chr3 47788908
chr3 47788910
chr3 47788932
chr3 47788940
chr3 47788948
chr3 47788984
chr3 47789045
chr3 47789049
chr3 47789113
chr3 47789120
chr3 47789132
chr3 47789145
chr3 47789276
chr3 47789317
chr3 47789321
chr3 47789327
chr3 47789365
chr3 47789370
chr3 47789374
chr3 47789381
chr3 47789395
chr3 47789415
chr3 47789426
chr3 47789457
chr3 47789469
chr3 47789473
chr3 47789517
chr3 47789525
chr3 47789532
chr3 47789549
chr3 47789578
chr3 47789586
chr3 47884189
chr3 47884779
chr3 47974758
chr3 47974983
chr3 48087526
chr3 48087540
chr3 48087687
chr3 48087694
chr3 48087696
chr3 48087698
chr3 48087700
chr3 48087702
chr3 48087704
chr3 48087722
chr3 48087726
chr3 48087728
chr3 48087738
chr3 48087742
chr3 48087744
chr3 48087821
chr3 48087913

chr3 48087915
chr3 48087924
chr3 48087935
chr3 48088016
chr3 48088088
chr3 48088091
chr3 48088200
chr3 48088222
chr3 48089437
chr3 48112720
chr3 48112776
chr3 48170347
chr3 48321128
chr3 48383451
chr3 48383486
chr3 48466581
chr3 48472697
chr3 48472816
chr3 48472865
chr3 48472894
chr3 48472910
chr3 48472926
chr3 48472930
chr3 48472938
chr3 48472987
chr3 48472997
chr3 48473074
chr3 48473204
chr3 48479404
chr3 48557405
chr3 48557416
chr3 48557419
chr3 48557443
chr3 48557535
chr3 48557558
chr3 48557566
chr3 48557597
chr3 48557622
chr3 48557629
chr3 48557653
chr3 48557681
chr3 48557693
chr3 48557725
chr3 48557738
chr3 48557745
chr3 48557756
chr3 48557779
chr3 48557816
chr3 48557826
chr3 48557828
chr3 48557832
chr3 48557836
chr3 48557840
chr3 48557898

chr3 48614236
chr3 48614241
chr3 48685313
chr3 48685329
chr3 48685343
chr3 48685349
chr3 48715852
chr3 48717278
chr3 48717292
chr3 48717296
chr3 48717298
chr3 48717319
chr3 48717343
chr3 48717367
chr3 48812189
chr3 48812395
chr3 48812504
chr3 48812521
chr3 48887466
chr3 48919871
chr3 48919895
chr3 48919906
chr3 48920001
chr3 48920039
chr3 48920042
chr3 48920064
chr3 48920126
chr3 48920169
chr3 48920227
chr3 48920260
chr3 48952650
chr3 49165853
chr3 49165863
chr3 49165883
chr3 49165890
chr3 49165905
chr3 49165913
chr3 49165935
chr3 49165947
chr3 49165958
chr3 49165974
chr3 49165991
chr3 49166015
chr3 49166040
chr3 49166091
chr3 49166097
chr3 49166118
chr3 49166153
chr3 49166162
chr3 49166165
chr3 49166172
chr3 49166174
chr3 49166180
chr3 49166194

chr3 49166202
chr3 49166206
chr3 49166214
chr3 49166219
chr3 49166221
chr3 49166226
chr3 49166234
chr3 49166236
chr3 49276695
chr3 49276702
chr3 49276714
chr3 49276717
chr3 49276719
chr3 49276722
chr3 49276784
chr3 49276849
chr3 49276937
chr3 49276983
chr3 49277005
chr3 49277012
chr3 49277019
chr3 49277021
chr3 49277031
chr3 49410000
chr3 49410079
chr3 49410632
chr3 49410666
chr3 49415784
chr3 49416041
chr3 49416210
chr3 49444054
chr3 49444242
chr3 49586583
chr3 49689512
chr3 49689514
chr3 49689520
chr3 49689524
chr3 49689526
chr3 49690118
chr3 49723932
chr3 49723945
chr3 49723956
chr3 49724666
chr3 49724688
chr3 49724690
chr3 49724699
chr3 49724705
chr3 49785790
chr3 49785799
chr3 49785809
chr3 49785818
chr3 49785828
chr3 49785832
chr3 49785888

chr3 49785896
chr3 49785910
chr3 49785912
chr3 49785931
chr3 49785951
chr3 49785967
chr3 49785975
chr3 49786262
chr3 49786272
chr3 49786274
chr3 49813909
chr3 49813920
chr3 49814006
chr3 49906136
chr3 49940135
chr3 49940137
chr3 49940139
chr3 49940151
chr3 49940158
chr3 49940160
chr3 49940163
chr3 49940171
chr3 49940216
chr3 49940219
chr3 49940235
chr3 50090012
chr3 50090027
chr3 50090127
chr3 50090183
chr3 50090215
chr3 50090224
chr3 50091674
chr3 50226391
chr3 50226405
chr3 50226416
chr3 50226423
chr3 50226442
chr3 50226462
chr3 50226472
chr3 50226475
chr3 50226513
chr3 50226539
chr3 50226557
chr3 50226571
chr3 50226587
chr3 50226652
chr3 50226661
chr3 50226674
chr3 50226676
chr3 50226682
chr3 50226699
chr3 50226706
chr3 50226714
chr3 50226738

chr3 50226758
chr3 50226762
chr3 50226768
chr3 50226770
chr3 50226774
chr3 50226971
chr3 50227608
chr3 50237799
chr3 50237807
chr3 50237846
chr3 50237896
chr3 50237913
chr3 50237962
chr3 50237968
chr3 50237975
chr3 50238044
chr3 50238054
chr3 50238057
chr3 50238082
chr3 50238089
chr3 50238120
chr3 50246210
chr3 50246242
chr3 50246272
chr3 50246306
chr3 50246351
chr3 50246358
chr3 50246362
chr3 50246378
chr3 50246408
chr3 50246420
chr3 50260458
chr3 50260461
chr3 50260474
chr3 50260481
chr3 50260494
chr3 50299639
chr3 50299661
chr3 50299670
chr3 50299697
chr3 50299704
chr3 50299707
chr3 50299736
chr3 50299741
chr3 50590470
chr3 50590480
chr3 50590487
chr3 50590585
chr3 50590633
chr3 50590650
chr3 50590695
chr3 50591010
chr3 50601409
chr3 50601751

chr3 50601759
chr3 50601765
chr3 50601891
chr3 50601900
chr3 50610749
chr3 50611060
chr3 50615966
chr3 50615976
chr3 50616007
chr3 50616027
chr3 50616055
chr3 50616071
chr3 50616223
chr3 50616346
chr3 50616373
chr3 50950851
chr3 50950950
chr3 50971525
chr3 50971539
chr3 51231772
chr3 51383899
chr3 51384188
chr3 51384252
chr3 51384255
chr3 51384314
chr3 51384321
chr3 51384374
chr3 51384413
chr3 51384437
chr3 51384443
chr3 51384453
chr3 51384465
chr3 51384502
chr3 51384529
chr3 51384556
chr3 51384570
chr3 51384574
chr3 51384587
chr3 51384591
chr3 51539317
chr3 51539329
chr3 51539337
chr3 51539379
chr3 51539497
chr3 51539595
chr3 51539609
chr3 51672163
chr3 51686680
chr3 51686796
chr3 51899189
chr3 51968014
chr3 51968037
chr3 51968044
chr3 51982812

chr3 51982822
chr3 51982831
chr3 51982838
chr3 51995343
chr3 51995505
chr3 51995579
chr3 51995586
chr3 51995600
chr3 51995608
chr3 51995666
chr3 52054881
chr3 52054906
chr3 52055051
chr3 52055070
chr3 52055107
chr3 52055123
chr3 52055127
chr3 52055136
chr3 52055157
chr3 52055161
chr3 52055166
chr3 52055209
chr3 52055223
chr3 52055230
chr3 52055258
chr3 52055288
chr3 52055297
chr3 52055337
chr3 52055350
chr3 52055361
chr3 52055380
chr3 52197908
chr3 52197927
chr3 52197942
chr3 52197946
chr3 52197953
chr3 52197960
chr3 52239654
chr3 52239657
chr3 52253122
chr3 52253150
chr3 52275599
chr3 52275607
chr3 52275656
chr3 52275667
chr3 52275840
chr3 52275862
chr3 52275870
chr3 52276091
chr3 52276204
chr3 52276303
chr3 52276345
chr3 52276355
chr3 52276385

chr3 52276387
chr3 52276409
chr3 52276412
chr3 52276425
chr3 52276436
chr3 52276505
chr3 52276708
chr3 52276867
chr3 52277149
chr3 52277203
chr3 52277254
chr3 52277258
chr3 52277319
chr3 52277456
chr3 52277476
chr3 52277505
chr3 52277515
chr3 52282137
chr3 52282218
chr3 52282280
chr3 52537412
chr3 52670631
chr3 52670860
chr3 52670944
chr3 52671307
chr3 52671333
chr3 52671394
chr3 52671426
chr3 52671520
chr3 52685084
chr3 52685125
chr3 52685252
chr3 52685327
chr3 52685338
chr3 52685366
chr3 52685371
chr3 52685410
chr3 52685432
chr3 52686627
chr3 52686649
chr3 52686663
chr3 52686713
chr3 52686789
chr3 52686807
chr3 52686894
chr3 52933912
chr3 52934515
chr3 52934713
chr3 52934746
chr3 52934812
chr3 52934825
chr3 52983198
chr3 52983360
chr3 52983464

chr3 52983661
chr3 52983665
chr3 52983728
chr3 53073014
chr3 53073066
chr3 53073112
chr3 53073114
chr3 53073128
chr3 53073140
chr3 53346647
chr3 53346776
chr3 53346846
chr3 53346856
chr3 53346886
chr3 53346929
chr3 53346948
chr3 53347015
chr3 53887228
chr3 53891010
chr3 53891027
chr3 53891041
chr3 53891101
chr3 53891186
chr3 53891191
chr3 53891204
chr3 53891352
chr3 53891374
chr3 53891412
chr3 53891417
chr3 53891426
chr3 53891429
chr3 53891462
chr3 53891469
chr3 53891490
chr3 53891508
chr3 53891560
chr3 53891563
chr3 53891571
chr3 53891665
chr3 53891674
chr3 53891679
chr3 53891695
chr3 53891699
chr3 53891702
chr3 53891723
chr3 53891739
chr3 53891743
chr3 53891756
chr3 53891770
chr3 53891774
chr3 53891804
chr3 53891809
chr3 53925999
chr3 53926536

chr3 53926541
chr3 53926749
chr3 53927007
chr3 53927123
chr3 54124156
chr3 54262727
chr3 54262794
chr3 54262801
chr3 54262831
chr3 54342926
chr3 54342936
chr3 54342949
chr3 54342964
chr3 54639729
chr3 54639734
chr3 54639758
chr3 54639760
chr3 54639764
chr3 54639802
chr3 55830104
chr3 55830369
chr3 55830447
chr3 55846020
chr3 55846037
chr3 56172840
chr3 56467785
chr3 56467789
chr3 56467841
chr3 56467847
chr3 56467849
chr3 56467865
chr3 56467880
chr3 56467916
chr3 56467938
chr3 56467951
chr3 56467963
chr3 56467971
chr3 56467980
chr3 56467984
chr3 56467987
chr3 56467991
chr3 56467993
chr3 56467995
chr3 56468008
chr3 56556510
chr3 56556543
chr3 56556559
chr3 56556584
chr3 56556586
chr3 56556654
chr3 56556706
chr3 56556912
chr3 56556914
chr3 56556935

chr3 56556942
chr3 56556952
chr3 56556958
chr3 56556979
chr3 56556996
chr3 56557003
chr3 56557017
chr3 56557026
chr3 56557137
chr3 56557140
chr3 56557167
chr3 56557178
chr3 56557181
chr3 56557184
chr3 56557615
chr3 56557624
chr3 56557714
chr3 56557746
chr3 56557802
chr3 56557860
chr3 56557947
chr3 56557994
chr3 56558013
chr3 56558045
chr3 56558123
chr3 56558132
chr3 56558269
chr3 56682043
chr3 56682077
chr3 56682183
chr3 56682234
chr3 56682304
chr3 56682320
chr3 56682355
chr3 56682390
chr3 56682393
chr3 56692931
chr3 56692960
chr3 56692970
chr3 56692972
chr3 56693016
chr3 56693355
chr3 56693474
chr3 56693949
chr3 56783726
chr3 56783776
chr3 56799182
chr3 56799335
chr3 56799357
chr3 56799398
chr3 56799589
chr3 56799620
chr3 56799698
chr3 56799793

chr3 56799967
chr3 56800288
chr3 56800438
chr3 56800496
chr3 56800591
chr3 56800647
chr3 56802266
chr3 56802272
chr3 56802279
chr3 56915978
chr3 56916045
chr3 56916063
chr3 56916085
chr3 56916125
chr3 56916136
chr3 56916252
chr3 56916257
chr3 56916275
chr3 56916312
chr3 56916316
chr3 56916346
chr3 56916365
chr3 56916374
chr3 56916455
chr3 56916521
chr3 56916690
chr3 56916699
chr3 56916854
chr3 56916878
chr3 56916880
chr3 56916907
chr3 56942819
chr3 57100115
chr3 57100218
chr3 57100239
chr3 57100268
chr3 57159112
chr3 57159342
chr3 57159354
chr3 57159447
chr3 57159475
chr3 57159494
chr3 57159516
chr3 57197274
chr3 57197276
chr3 57224852
chr3 57224874
chr3 57225000
chr3 57225005
chr3 57225101
chr3 57228441
chr3 57228448
chr3 57228461
chr3 57228470

chr3 57228473
chr3 57228483
chr3 57228488
chr3 57228490
chr3 57228494
chr3 57228506
chr3 57228516
chr3 57228527
chr3 57228530
chr3 57228541
chr3 57228550
chr3 57228553
chr3 57228558
chr3 57228560
chr3 57228569
chr3 57228630
chr3 57228633
chr3 57228786
chr3 57228795
chr3 57228807
chr3 57228817
chr3 57228895
chr3 57229163
chr3 57229359
chr3 57339728
chr3 57544481
chr3 57596110
chr3 57596164
chr3 57596260
chr3 57596310
chr3 57596344
chr3 57596438
chr3 57596461
chr3 57596475
chr3 57596485
chr3 57596511
chr3 57596533
chr3 57596560
chr3 57596575
chr3 57598503
chr3 57598547
chr3 57598552
chr3 57598563
chr3 57598577
chr3 57598637
chr3 57598641
chr3 57598649
chr3 57598674
chr3 57598697
chr3 57598705
chr3 57598735
chr3 57598737
chr3 57598759
chr3 57692086

chr3 57692096
chr3 57692120
chr3 57692152
chr3 57692168
chr3 57692182
chr3 57692184
chr3 57692307
chr3 57692326
chr3 57692389
chr3 57692458
chr3 57692468
chr3 57692511
chr3 57692593
chr3 57692613
chr3 57692623
chr3 57692631
chr3 57692638
chr3 57692646
chr3 57692648
chr3 57692667
chr3 57692671
chr3 57692677
chr3 57692680
chr3 57692682
chr3 57692698
chr3 57692707
chr3 57692709
chr3 57692714
chr3 57692732
chr3 57692737
chr3 57692740
chr3 57692753
chr3 57692756
chr3 57692766
chr3 57692772
chr3 57692787
chr3 57692808
chr3 57692826
chr3 57692841
chr3 57755736
chr3 57755748
chr3 57755771
chr3 57755787
chr3 57755877
chr3 57755965
chr3 58035272
chr3 58187713
chr3 58187810
chr3 58187888
chr3 58187901
chr3 58237981
chr3 58238033
chr3 58238036
chr3 58238060

chr3 58238067
chr3 58238069
chr3 58238071
chr3 58238075
chr3 58238091
chr3 58238117
chr3 58238124
chr3 58238127
chr3 58238163
chr3 58238181
chr3 58238186
chr3 58238211
chr3 58238228
chr3 58238232
chr3 58333186
chr3 58333193
chr3 58333209
chr3 58333219
chr3 58333308
chr3 58333317
chr3 58333320
chr3 58333323
chr3 58333325
chr3 58333431
chr3 58333464
chr3 58333488
chr3 58333542
chr3 58333616
chr3 58333631
chr3 58333647
chr3 58333663
chr3 58333670
chr3 58333675
chr3 58333760
chr3 58333827
chr3 58333851
chr3 58433784
chr3 58433801
chr3 58433804
chr3 58433816
chr3 58433829
chr3 58433839
chr3 58433854
chr3 58433861
chr3 58433874
chr3 58568765
chr3 58568779
chr3 58568784
chr3 58568790
chr3 58813913
chr3 58814061
chr3 58814350
chr3 59386325
chr3 59386355

chr3 59386459
chr3 59386519
chr3 59386581
chr3 59451497
chr3 59451528
chr3 59451575
chr3 59451601
chr3 59451821
chr3 59451841
chr3 59772844
chr3 59772851
chr3 59772943
chr3 59772964
chr3 60048763
chr3 60048783
chr3 60048795
chr3 60925627
chr3 61249862
chr3 61249880
chr3 61250053
chr3 61250066
chr3 61250093
chr3 61250102
chr3 61250118
chr3 61250128
chr3 61250136
chr3 61250155
chr3 61250158
chr3 61250172
chr3 61250177
chr3 61250195
chr3 61250198
chr3 61250212
chr3 61251431
chr3 61251445
chr3 61251473
chr3 61251482
chr3 61251498
chr3 61251518
chr3 61251530
chr3 61251532
chr3 61251543
chr3 61251549
chr3 61251552
chr3 61251596
chr3 61251665
chr3 61251716
chr3 61251723
chr3 61251736
chr3 61251767
chr3 61251769
chr3 61251787
chr3 61251814
chr3 61251933

chr3 61251939
chr3 61251981
chr3 62366834
chr3 62366958
chr3 62367134
chr3 62367178
chr3 62367261
chr3 62367347
chr3 62367402
chr3 62375491
chr3 62375516
chr3 62375774
chr3 62375787
chr3 62375805
chr3 62375863
chr3 62375873
chr3 62375954
chr3 62375983
chr3 62376143
chr3 62439222
chr3 62673692
chr3 63094682
chr3 63215013
chr3 63217083
chr3 63217243
chr3 63217289
chr3 63217293
chr3 63217357
chr3 63217365
chr3 63217371
chr3 63217383
chr3 63217407
chr3 63217417
chr3 63217429
chr3 63344025
chr3 63344046
chr3 63736031
chr3 63736041
chr3 63736087
chr3 63736500
chr3 63736943
chr3 63737024
chr3 63737289
chr3 63864963
chr3 63864979
chr3 63878443
chr3 63878642
chr3 63911145
chr3 63911320
chr3 63911365
chr3 63911400
chr3 63911465
chr3 63911598
chr3 63911615

chr3 63911643
chr3 63967495
chr3 63967701
chr3 63967928
chr3 63967952
chr3 63968307
chr3 63968314
chr3 63968420
chr3 63968430
chr3 63968572
chr3 63968797
chr3 63968875
chr3 63969031
chr3 63969387
chr3 63969595
chr3 63969861
chr3 63970001
chr3 63970051
chr3 63979225
chr3 63979270
chr3 63979280
chr3 64030727
chr3 64030730
chr3 64030736
chr3 64030752
chr3 64030770
chr3 64030777
chr3 64030807
chr3 64030862
chr3 64030869
chr3 64030878
chr3 64030881
chr3 64030885
chr3 64030903
chr3 64030913
chr3 64030947
chr3 64030950
chr3 64030963
chr3 64030980
chr3 64031082
chr3 64031164
chr3 64031169
chr3 64505458
chr3 64505471
chr3 64505486
chr3 64505512
chr3 64505565
chr3 64505678
chr3 64505715
chr3 64790224
chr3 64790314
chr3 64790639
chr3 64790677
chr3 64902991

chr3 64903325
chr3 65512533
chr3 66221238
chr3 66472765
chr3 66472880
chr3 66816421
chr3 66971724
chr3 66971787
chr3 66971880
chr3 66971959
chr3 66972001
chr3 66972022
chr3 66972030
chr3 66972061
chr3 66972067
chr3 66972123
chr3 66972128
chr3 66972131
chr3 66972176
chr3 66972314
chr3 66972378
chr3 66972393
chr3 66972395
chr3 66972399
chr3 66972428
chr3 66972430
chr3 66972441
chr3 66972450
chr3 66972458
chr3 66972466
chr3 66972482
chr3 66998378
chr3 66998394
chr3 66998397
chr3 66998426
chr3 66998432
chr3 66998456
chr3 66998461
chr3 66998466
chr3 66998489
chr3 66998582
chr3 66998587
chr3 66998740
chr3 66998746
chr3 66998749
chr3 66998756
chr3 66998787
chr3 66998790
chr3 66998794
chr3 66998796
chr3 66998801
chr3 66998828
chr3 66998830
chr3 66998838

chr3 66998844
chr3 66998848
chr3 66998924
chr3 66998926
chr3 66998932
chr3 66998943
chr3 66998963
chr3 66999005
chr3 66999036
chr3 66999135
chr3 66999162
chr3 67000275
chr3 67653683
chr3 67653771
chr3 67653820
chr3 67653901
chr3 67653947
chr3 67653973
chr3 67654067
chr3 67654090
chr3 67654101
chr3 67654205
chr3 67654212
chr3 67654224
chr3 67654230
chr3 67655821
chr3 67655836
chr3 67655877
chr3 67655888
chr3 67655896
chr3 67655924
chr3 67655928
chr3 67655969
chr3 67655990
chr3 67655998
chr3 67656007
chr3 67656062
chr3 67656074
chr3 67656076
chr3 67656107
chr3 67656112
chr3 67656126
chr3 67656143
chr3 68007253
chr3 68007300
chr3 68007366
chr3 68007370
chr3 68086937
chr3 69014083
chr3 69014094
chr3 69014145
chr3 69014177
chr3 69014184
chr3 69014257

chr3 69051389
chr3 69051406
chr3 69051453
chr3 69051456
chr3 69051469
chr3 69051498
chr3 69051500
chr3 69051510
chr3 69051512
chr3 69051534
chr3 69051563
chr3 69051594
chr3 69051599
chr3 69051601
chr3 69051611
chr3 69051784
chr3 69051814
chr3 69051819
chr3 69051911
chr3 69052113
chr3 69052251
chr3 69052255
chr3 69052261
chr3 69052274
chr3 69052312
chr3 69052333
chr3 69052346
chr3 69052350
chr3 69052357
chr3 69052373
chr3 69052379
chr3 69052472
chr3 69052493
chr3 69052503
chr3 69052512
chr3 69052533
chr3 69079333
chr3 69079400
chr3 69079405
chr3 69079484
chr3 69079508
chr3 69079738
chr3 69079855
chr3 69079867
chr3 69079908
chr3 69079911
chr3 69079915
chr3 69079920
chr3 69079931
chr3 69079937
chr3 69079942
chr3 69079953
chr3 69079961
chr3 69079966

chr3 69080395
chr3 69080405
chr3 69080419
chr3 69080429
chr3 69080438
chr3 69080443
chr3 69080453
chr3 69080471
chr3 69080484
chr3 69080496
chr3 69080554
chr3 69080578
chr3 69080600
chr3 69080626
chr3 69080651
chr3 69083412
chr3 69083509
chr3 69083558
chr3 69083617
chr3 69084112
chr3 69084158
chr3 69084476
chr3 69084608
chr3 69086250
chr3 69086424
chr3 69086492
chr3 69086588
chr3 69086753
chr3 69086761
chr3 69086783
chr3 69086791
chr3 69086815
chr3 69086832
chr3 69086869
chr3 69086944
chr3 69086977
chr3 69092026
chr3 69092089
chr3 69092214
chr3 69092395
chr3 69092510
chr3 69199515
chr3 69199739
chr3 69199752
chr3 69206465
chr3 69206473
chr3 69206520
chr3 69206570
chr3 69206576
chr3 69306508
chr3 69306541
chr3 69478776
chr3 69479302
chr3 69738744

chr3 69738747
chr3 69738782
chr3 69738895
chr3 69738920
chr3 69738936
chr3 69738954
chr3 69738967
chr3 69738986
chr3 69739008
chr3 69739045
chr3 69739108
chr3 69739116
chr3 69797701
chr3 70230139
chr3 70292787
chr3 70293483
chr3 70336400
chr3 70336454
chr3 70336548
chr3 70336934
chr3 70608934
chr3 70608999
chr3 70703179
chr3 70703269
chr3 70703299
chr3 70703355
chr3 70703367
chr3 70703493
chr3 70703519
chr3 70703527
chr3 70703566
chr3 70703575
chr3 71062772
chr3 71062810
chr3 71062856
chr3 71063098
chr3 71063104
chr3 71063286
chr3 71065659
chr3 71065669
chr3 71065689
chr3 71065886
chr3 71065895
chr3 71065918
chr3 71065921
chr3 71066306
chr3 71066308
chr3 71066351
chr3 71066358
chr3 71066516
chr3 71066563
chr3 71066588
chr3 71066662
chr3 71087360

chr3 71087374
chr3 71087416
chr3 71087422
chr3 71087427
chr3 71087475
chr3 71129085
chr3 71129089
chr3 71129166
chr3 71129269
chr3 71129334
chr3 71129492
chr3 71129504
chr3 71129770
chr3 71129895
chr3 71130311
chr3 71130408
chr3 71130436
chr3 71130518
chr3 71130529
chr3 71130552
chr3 71130588
chr3 71130593
chr3 71130603
chr3 71130645
chr3 71130660
chr3 71130881
chr3 71130901
chr3 71130913
chr3 71130920
chr3 71130962
chr3 71226727
chr3 71226765
chr3 71226792
chr3 71226796
chr3 71226810
chr3 71226842
chr3 71226852
chr3 71226912
chr3 71226926
chr3 71226962
chr3 71226971
chr3 71227014
chr3 71227025
chr3 71227031
chr3 71227063
chr3 71227072
chr3 71227183
chr3 71227185
chr3 71227200
chr3 71227230
chr3 71227260
chr3 71227274
chr3 71227280
chr3 71227291

chr3 71227315
chr3 71227402
chr3 71227480
chr3 71360556
chr3 71360588
chr3 71360724
chr3 71360734
chr3 71360842
chr3 71360875
chr3 71422259
chr3 71435314
chr3 71435334
chr3 71435354
chr3 71435362
chr3 71435407
chr3 71435427
chr3 71435469
chr3 71435489
chr3 71435530
chr3 71435550
chr3 71435571
chr3 71435591
chr3 71435611
chr3 71435631
chr3 71435651
chr3 71435671
chr3 71444148
chr3 71444169
chr3 71444177
chr3 71444218
chr3 71444262
chr3 71444404
chr3 71444436
chr3 71444459
chr3 71444499
chr3 71444527
chr3 71444622
chr3 71444708
chr3 71444750
chr3 71444825
chr3 71456385
chr3 71456416
chr3 71456485
chr3 71456672
chr3 71456675
chr3 71456750
chr3 71493152
chr3 71493269
chr3 71493455
chr3 71493488
chr3 71493506
chr3 71493520
chr3 71493666
chr3 71493719

chr3 71493763
chr3 71584404
chr3 71584413
chr3 71584439
chr3 71584453
chr3 71584577
chr3 71584621
chr3 71584631
chr3 71584675
chr3 71584681
chr3 71584738
chr3 71584748
chr3 71584772
chr3 71584799
chr3 71584843
chr3 71584893
chr3 71584911
chr3 71723443
chr3 71723756
chr3 71723788
chr3 71723854
chr3 71724455
chr3 71724483
chr3 71724594
chr3 71724685
chr3 71724689
chr3 71724695
chr3 71724706
chr3 71724710
chr3 71724713
chr3 71724735
chr3 71777616
chr3 71784010
chr3 71784258
chr3 71784377
chr3 71784379
chr3 71784392
chr3 71784431
chr3 71784454
chr3 71784473
chr3 71784480
chr3 71784485
chr3 71784538
chr3 71784569
chr3 71784586
chr3 71784661
chr3 71784674
chr3 72445998
chr3 72446110
chr3 72446180
chr3 72446256
chr3 72446308
chr3 72446370
chr3 72446388

chr3 72446521
chr3 72446569
chr3 72446590
chr3 72446596
chr3 72446606
chr3 72446626
chr3 72446678
chr3 72446703
chr3 72446705
chr3 72446714
chr3 72446717
chr3 72446900
chr3 72446902
chr3 72446905
chr3 72446909
chr3 72446918
chr3 72446922
chr3 72446940
chr3 72483040
chr3 72483056
chr3 72483074
chr3 72483121
chr3 72483135
chr3 72483146
chr3 72483156
chr3 72483162
chr3 72483167
chr3 72483211
chr3 72483334
chr3 72483386
chr3 72483476
chr3 72756863
chr3 72756972
chr3 72757154
chr3 72757297
chr3 72849036
chr3 72849069
chr3 72849073
chr3 72965544
chr3 72977016
chr3 72977226
chr3 72977335
chr3 72996814
chr3 72996839
chr3 72996856
chr3 72996864
chr3 72996878
chr3 72996882
chr3 72996891
chr3 72996901
chr3 72996914
chr3 72996918
chr3 72996924
chr3 72996929

chr3 72996940
chr3 72996956
chr3 72996959
chr3 72996977
chr3 72996982
chr3 72996998
chr3 72997000
chr3 72997010
chr3 72997029
chr3 72997043
chr3 72997046
chr3 72997063
chr3 72997113
chr3 72997118
chr3 72997139
chr3 72997149
chr3 72997156
chr3 72997162
chr3 72997166
chr3 72997168
chr3 72997171
chr3 72997189
chr3 72997221
chr3 72997248
chr3 72997253
chr3 72997269
chr3 72997273
chr3 72997276
chr3 72997279
chr3 72997302
chr3 72997316
chr3 72997341
chr3 72997365
chr3 72997412
chr3 72997416
chr3 72997423
chr3 72997460
chr3 72998182
chr3 73029936
chr3 73086065
chr3 73086130
chr3 73086159
chr3 73086174
chr3 73086203
chr3 73086305
chr3 73086395
chr3 73086414
chr3 73086444
chr3 74161059
chr3 74613601
chr3 74613618
chr3 75290915
chr3 75291148
chr3 75435770

chr3 75435776
chr3 75435802
chr3 75435806
chr3 75435847
chr3 75435910
chr3 75640708
chr3 75651550
chr3 75657283
chr3 75672782
chr3 75673188
chr3 75673211
chr3 75673222
chr3 75673237
chr3 75673313
chr3 75689026
chr3 75689083
chr3 75689487
chr3 75710522
chr3 75710577
chr3 75711296
chr3 75711380
chr3 76007971
chr3 76008153
chr3 76008189
chr3 76008271
chr3 76330716
chr3 76379693
chr3 76379846
chr3 76380438
chr3 76380539
chr3 76380634
chr3 76380716
chr3 76466863
chr3 76655533
chr3 76656041
chr3 76656343
chr3 76656573
chr3 76656658
chr3 77094202
chr3 77094242
chr3 77100111
chr3 77100391
chr3 77110754
chr3 77319632
chr3 77319662
chr3 77319932
chr3 77320008
chr3 77320427
chr3 77320506
chr3 78343349
chr3 78613545
chr3 78613555
chr3 79017421
chr3 79017442

chr3 79017510
chr3 79017516
chr3 79017564
chr3 79017663
chr3 79017745
chr3 79017778
chr3 79017791
chr3 79017815
chr3 79175581
chr3 79175805
chr3 79374156
chr3 79497538
chr3 79497582
chr3 79497655
chr3 79497798
chr3 79520686
chr3 79521447
chr3 79521451
chr3 79521479
chr3 79521485
chr3 79521526
chr3 79521576
chr3 79768327
chr3 79768819
chr3 79768883
chr3 79769050
chr3 79769284
chr3 79769355
chr3 79769505
chr3 80060327
chr3 80060586
chr3 80060807
chr3 80061329
chr3 80207847
chr3 80208442
chr3 80288705
chr3 80288768
chr3 80289009
chr3 80289229
chr3 80305141
chr3 80305175
chr3 80306085
chr3 80306094
chr3 80306261
chr3 80403222
chr3 80403455
chr3 80403829
chr3 80404110
chr3 80404157
chr3 80404675
chr3 80404820
chr3 80629234
chr3 80629386
chr3 80629801

chr3 80680222
chr3 80680263
chr3 80824351
chr3 80824360
chr3 80824751
chr3 80863723
chr3 80863986
chr3 82097104
chr3 82312512
chr3 82581847
chr3 82792412
chr3 82792730
chr3 82863443
chr3 82935877
chr3 82936970
chr3 82937076
chr3 82937462
chr3 82937679
chr3 82938099
chr3 82938161
chr3 82949023
chr3 82949150
chr3 82949459
chr3 82949853
chr3 82950148
chr3 83174666
chr3 83174721
chr3 83215902
chr3 83216213
chr3 83216360
chr3 83216378
chr3 83221946
chr3 83242345
chr3 83242524
chr3 83309371
chr3 83309443
chr3 83430819
chr3 83430924
chr3 83431340
chr3 83653100
chr3 83653371
chr3 83729912
chr3 83730074
chr3 83730268
chr3 83730888
chr3 83731500
chr3 83837207
chr3 83920687
chr3 83921616
chr3 83922684
chr3 83922712
chr3 83922729
chr3 84244841
chr3 84245596

chr3 84245664
chr3 84245956
chr3 84246490
chr3 84282569
chr3 84283401
chr3 84284042
chr3 84284288
chr3 84284504
chr3 84302359
chr3 84302395
chr3 84302587
chr3 84302706
chr3 84547259
chr3 84547281
chr3 84547440
chr3 84588976
chr3 84836805
chr3 84836839
chr3 84837085
chr3 84837102
chr3 84837596
chr3 84837656
chr3 84838006
chr3 84838293
chr3 84838345
chr3 84838587
chr3 85028660
chr3 85147936
chr3 85148124
chr3 85148201
chr3 85148238
chr3 85342544
chr3 85342849
chr3 85342859
chr3 85343094
chr3 85346147
chr3 85502128
chr3 85502375
chr3 85502699
chr3 85541901
chr3 85542222
chr3 86124896
chr3 87024489
chr3 87024492
chr3 87024612
chr3 87024812
chr3 87024822
chr3 87024974
chr3 87226369
chr3 87226373
chr3 87226479
chr3 87226507
chr3 87226685
chr3 87226691

chr3 87226723
chr3 87226733
chr3 87226798
chr3 87226800
chr3 87226842
chr3 87227776
chr3 87227791
chr3 87227817
chr3 87227822
chr3 87227896
chr3 87227918
chr3 87227961
chr3 87297954
chr3 87297965
chr3 87298099
chr3 87298186
chr3 87298229
chr3 87298359
chr3 87675783
chr3 87676162
chr3 87792228
chr3 87792263
chr3 87792337
chr3 87792387
chr3 87792402
chr3 87792412
chr3 87792435
chr3 87792479
chr3 87792491
chr3 87792518
chr3 87792628
chr3 87793553
chr3 87793561
chr3 87793564
chr3 87793642
chr3 87793749
chr3 87793831
chr3 87793835
chr3 87794022
chr3 87794029
chr3 87794163
chr3 87794186
chr3 87794200
chr3 87794218
chr3 87794303
chr3 87794326
chr3 87796475
chr3 87796949
chr3 87829396
chr3 87947617
chr3 87997097
chr3 87997328
chr3 87997448
chr3 87997513

chr3 87997524
chr3 88000183
chr3 88057645
chr3 88057652
chr3 88057792
chr3 88057855
chr3 88057869
chr3 88057965
chr3 88058019
chr3 88058041
chr3 88058075
chr3 88058087
chr3 88059821
chr3 88059830
chr3 88059908
chr3 88059911
chr3 88059922
chr3 88059942
chr3 88060025
chr3 88060033
chr3 88060048
chr3 88060054
chr3 88060064
chr3 88060085
chr3 88060098
chr3 88060137
chr3 88060148
chr3 88060206
chr3 88060282
chr3 88060308
chr3 88060312
chr3 88060434
chr3 88060526
chr3 88060691
chr3 88151166
chr3 88151369
chr3 88151460
chr3 88180522
chr3 88301867
chr3 89107935
chr3 89108440
chr3 89108793
chr3 89109016
chr3 89129453
chr3 89129832
chr3 89538781
chr3 89569809
chr3 89570093
chr3 90182665
chr3 90182724
chr3 90182916
chr3 90183347
chr3 90183416
chr3 90183669

chr3 90233111
chr3 90233312
chr3 90564381
chr3 90564437
chr3 90564507
chr3 90564536
chr3 90564720
chr3 91499263
chr3 91499317
chr3 93459404
chr3 93766333
chr3 93840459
chr3 93841412
chr3 93841437
chr3 93841443
chr3 93841696
chr3 93841702
chr3 93841709
chr3 93841713
chr3 93841718
chr3 93841756
chr3 93841762
chr3 93841772
chr3 93841793
chr3 93841802
chr3 93841861
chr3 93842171
chr3 93842335
chr3 93842368
chr3 93842482
chr3 93842997
chr3 93973265
chr3 93973286
chr3 93973471
chr3 93973525
chr3 93973539
chr3 93973553
chr3 93973619
chr3 93973630
chr3 93980532
chr3 93980543
chr3 93980548
chr3 93980551
chr3 93980562
chr3 93980564
chr3 93980572
chr3 93980588
chr3 93980619
chr3 93980659
chr3 93980672
chr3 93980860
chr3 94004410
chr3 94004439
chr3 94004514

chr3 94004586
chr3 94062140
chr3 94062247
chr3 94062262
chr3 94062264
chr3 94062348
chr3 94709205
chr3 95187214
chr3 95187450
chr3 95188079
chr3 95188102
chr3 95571109
chr3 95571938
chr3 95952049
chr3 95982056
chr3 95982942
chr3 95983207
chr3 96021416
chr3 96021553
chr3 96022411
chr3 96022433
chr3 96022848
chr3 96022905
chr3 96207452
chr3 96210988
chr3 96211289
chr3 96211309
chr3 96334692
chr3 96334716
chr3 96334837
chr3 96475043
chr3 96659920
chr3 96662697
chr3 96663352
chr3 96663871
chr3 96812685
chr3 96812711
chr3 97247944
chr3 97248394
chr3 97248451
chr3 97248468
chr3 97249533
chr3 97249622
chr3 97477541
chr3 97477652
chr3 97478131
chr3 97478705
chr3 97478748
chr3 97479084
chr3 97479162
chr3 97765157
chr3 97765200
chr3 97765289
chr3 97765372

chr3 97821715
chr3 97821725
chr3 97910006
chr3 97910102
chr3 97910282
chr3 98039591
chr3 98039616
chr3 98039627
chr3 98039685
chr3 98039762
chr3 98039772
chr3 98039833
chr3 98039992
chr3 98040022
chr3 98040129
chr3 98040401
chr3 98522197
chr3 98522201
chr3 98522468
chr3 98522566
chr3 98522569
chr3 98522574
chr3 98522583
chr3 98522587
chr3 98522616
chr3 98522619
chr3 98522625
chr3 98522646
chr3 98522655
chr3 98522661
chr3 98522667
chr3 98522682
chr3 98522689
chr3 98522700
chr3 98522714
chr3 98522731
chr3 98522734
chr3 98522738
chr3 98522745
chr3 98522754
chr3 98522760
chr3 98522764
chr3 98522788
chr3 98522791
chr3 98522805
chr3 98522807
chr3 98522810
chr3 98522812
chr3 98522823
chr3 98522826
chr3 98522829
chr3 98523239
chr3 98557248
chr3 98557416

chr3 98733423
chr3 98733457
chr3 98733459
chr3 98733499
chr3 98733513
chr3 98733565
chr3 98733590
chr3 98733664
chr3 98733667
chr3 98733673
chr3 98733682
chr3 98733745
chr3 98733748
chr3 98733802
chr3 98733812
chr3 98733904
chr3 98733936
chr3 98733948
chr3 98733968
chr3 98734035
chr3 98734050
chr3 98734058
chr3 98734075
chr3 98734085
chr3 98734096
chr3 98734119
chr3 98734135
chr3 98734242
chr3 98734257
chr3 98734311
chr3 98734332
chr3 98734339
chr3 98902340
chr3 98930159
chr3 99139111
chr3 99178029
chr3 99179202
chr3 99179285
chr3 99179954
chr3 99180314
chr3 99180445
chr3 99180448
chr3 99180874
chr3 99505634
chr3 99638041
chr3 99638072
chr3 99638076
chr3 99818431
chr3 99818505
chr3 99875514
chr3 99875525
chr3 99875533
chr3 99875577
chr3 99875619

chr3 99875672
chr3 99875696
chr3 99875708
chr3 99875811
chr3 99875919
chr3 99875947
chr3 99875954
chr3 99876049
chr3 100261310
chr3 100261324
chr3 100261379
chr3 100261470
chr3 100261489
chr3 100261496
chr3 100261520
chr3 100261576
chr3 100261601
chr3 100316997
chr3 100317014
chr3 100396582
chr3 100400229
chr3 100400267
chr3 100400274
chr3 100400293
chr3 100400454
chr3 100400520
chr3 100400534
chr3 100400567
chr3 100400585
chr3 100400605
chr3 100400643
chr3 100400753
chr3 100400756
chr3 100400759
chr3 100402064
chr3 100402117
chr3 100402180
chr3 100402422
chr3 100402534
chr3 100492682
chr3 100492696
chr3 100492714
chr3 100492730
chr3 100492773
chr3 100492778
chr3 100492802
chr3 100492865
chr3 100492943
chr3 100492969
chr3 100493046
chr3 100493063
chr3 100493078
chr3 100656407
chr3 100664835

chr3 100665101
chr3 100665221
chr3 100665280
chr3 100665289
chr3 100665358
chr3 100665413
chr3 100665485
chr3 100665488
chr3 100665592
chr3 100893290
chr3 100893292
chr3 100893324
chr3 101512610
chr3 101512614
chr3 101512683
chr3 101512691
chr3 101512726
chr3 101512737
chr3 101512767
chr3 101512776
chr3 101512800
chr3 101512817
chr3 101512825
chr3 101512840
chr3 101512842
chr3 101518595
chr3 101562107
chr3 101573629
chr3 101573705
chr3 101573708
chr3 101575177
chr3 101575181
chr3 101575252
chr3 101575281
chr3 101575419
chr3 101575652
chr3 101644198
chr3 101644201
chr3 101685586
chr3 101685601
chr3 101685631
chr3 101685693
chr3 101685717
chr3 101685757
chr3 101685804
chr3 101685818
chr3 101685870
chr3 101685896
chr3 101685901
chr3 101685908
chr3 101686115
chr3 101686130
chr3 101686173
chr3 101686203

chr3 101686229
chr3 101686254
chr3 101686288
chr3 101686345
chr3 101686361
chr3 101686367
chr3 101720896
chr3 101720934
chr3 101720962
chr3 101721095
chr3 101722589
chr3 101722764
chr3 101722799
chr3 101722802
chr3 101722805
chr3 101722814
chr3 101722837
chr3 101725729
chr3 101725743
chr3 101725759
chr3 101725851
chr3 101726030
chr3 101726065
chr3 101726077
chr3 101827461
chr3 101827481
chr3 101827523
chr3 101827540
chr3 101827564
chr3 101827642
chr3 101827694
chr3 101827718
chr3 101827725
chr3 101827738
chr3 101827745
chr3 101827750
chr3 101827811
chr3 101827853
chr3 101827860
chr3 101827865
chr3 101828079
chr3 101828098
chr3 101828115
chr3 101828151
chr3 101828452
chr3 101828528
chr3 101828589
chr3 101828845
chr3 101828889
chr3 101829048
chr3 101829139
chr3 101829167
chr3 101829222
chr3 101829234

chr3 101829293
chr3 101829336
chr3 101851503
chr3 101851524
chr3 101851573
chr3 101851921
chr3 101852029
chr3 101852138
chr3 101852177
chr3 101936137
chr3 101936319
chr3 101936372
chr3 101936382
chr3 101936410
chr3 101936415
chr3 101936434
chr3 101936462
chr3 101936504
chr3 101936584
chr3 101936598
chr3 101936888
chr3 101936916
chr3 102226493
chr3 102466106
chr3 103028007
chr3 103028795
chr3 103028818
chr3 103028908
chr3 103028958
chr3 103029324
chr3 103029743
chr3 103107189
chr3 103107223
chr3 103107253
chr3 103411869
chr3 103530371
chr3 103530379
chr3 103530387
chr3 103530389
chr3 103530397
chr3 103530405
chr3 103727170
chr3 103727289
chr3 103727327
chr3 103925132
chr3 103968629
chr3 103968998
chr3 103969150
chr3 103969838
chr3 103970566
chr3 104188802
chr3 104189011
chr3 104189061
chr3 104189543

chr3 104335133
chr3 104407332
chr3 104407374
chr3 104407459
chr3 104507362
chr3 104507369
chr3 104507457
chr3 104507895
chr3 105298729
chr3 105298817
chr3 105299332
chr3 105299378
chr3 105299411
chr3 105299639
chr3 105299764
chr3 105299853
chr3 105299934
chr3 105367994
chr3 105368005
chr3 105368028
chr3 105368039
chr3 105368051
chr3 105368058
chr3 105368073
chr3 105368096
chr3 105369229
chr3 105369235
chr3 105369244
chr3 105369251
chr3 105369254
chr3 105369303
chr3 105369322
chr3 105369337
chr3 105518928
chr3 105519002
chr3 105519005
chr3 105519092
chr3 105686814
chr3 105764916
chr3 105764940
chr3 105866829
chr3 105867382
chr3 105867393
chr3 105867442
chr3 105867450
chr3 105867457
chr3 105867516
chr3 105867525
chr3 105867540
chr3 105867693
chr3 105867717
chr3 105867840
chr3 105868020
chr3 105869482

chr3 105869491
chr3 105869500
chr3 105869571
chr3 105869692
chr3 105869770
chr3 105869913
chr3 106068776
chr3 106206084
chr3 106206287
chr3 106206304
chr3 106206315
chr3 106206356
chr3 106206448
chr3 106206510
chr3 106206546
chr3 106206554
chr3 106206628
chr3 106206671
chr3 106206709
chr3 106206893
chr3 106206948
chr3 106207130
chr3 106338541
chr3 106338568
chr3 106338571
chr3 106338588
chr3 106338629
chr3 106338769
chr3 106711589
chr3 106712047
chr3 107064629
chr3 107064656
chr3 107064669
chr3 107064677
chr3 107064683
chr3 107064848
chr3 107065010
chr3 107065095
chr3 107065123
chr3 107065183
chr3 107065365
chr3 107108146
chr3 107240190
chr3 107240219
chr3 107240328
chr3 107240343
chr3 107240387
chr3 107240389
chr3 107421310
chr3 107421329
chr3 107421467
chr3 107421843
chr3 107422413
chr3 107522417

chr3 107522552
chr3 107522570
chr3 107522613
chr3 107523725
chr3 107523740
chr3 107523756
chr3 107523761
chr3 107523767
chr3 107523777
chr3 107599636
chr3 107599667
chr3 107927912
chr3 107927915
chr3 107927918
chr3 107927930
chr3 108088237
chr3 108088245
chr3 108088454
chr3 108088468
chr3 108088583
chr3 108088944
chr3 108088958
chr3 108089267
chr3 108089284
chr3 108089309
chr3 108089399
chr3 108089515
chr3 108089533
chr3 108089606
chr3 108124589
chr3 108124726
chr3 108124781
chr3 108124809
chr3 108124922
chr3 108124940
chr3 108125268
chr3 108125272
chr3 108125461
chr3 108125518
chr3 108125567
chr3 108125635
chr3 108125746
chr3 108125803
chr3 108125902
chr3 108125914
chr3 108126235
chr3 108221625
chr3 108221720
chr3 108221731
chr3 108221840
chr3 108221875
chr3 108221888
chr3 108221937
chr3 108221948

chr3 108221957
chr3 108221997
chr3 108222019
chr3 108222027
chr3 108223172
chr3 108223463
chr3 108223606
chr3 108223902
chr3 108224188
chr3 108224213
chr3 108224245
chr3 108224275
chr3 108293900
chr3 108294005
chr3 108294067
chr3 108590149
chr3 108590179
chr3 108806322
chr3 108806348
chr3 108806437
chr3 108806663
chr3 108806757
chr3 108806838
chr3 108823431
chr3 108823614
chr3 108823932
chr3 108823974
chr3 108824016
chr3 108824018
chr3 108824028
chr3 108824091
chr3 108824112
chr3 108824116
chr3 108824149
chr3 108824161
chr3 108862909
chr3 108862938
chr3 108862977
chr3 108862994
chr3 108862999
chr3 108863603
chr3 109058777
chr3 109059145
chr3 109257494
chr3 109257505
chr3 109284512
chr3 109337195
chr3 109806333
chr3 109806404
chr3 109806488
chr3 109806887
chr3 109806976
chr3 109807018
chr3 109807084

chr3 109828866
chr3 109829119
chr3 110743428
chr3 111541614
chr3 111541909
chr3 111541936
chr3 111542046
chr3 111542050
chr3 111542089
chr3 111542133
chr3 111542149
chr3 111542163
chr3 111542337
chr3 111542346
chr3 111542357
chr3 111542456
chr3 111542650
chr3 111542680
chr3 111542787
chr3 111542858
chr3 111543419
chr3 111543852
chr3 111543915
chr3 111546697
chr3 111547083
chr3 111547465
chr3 111547697
chr3 111547714
chr3 111547729
chr3 111547909
chr3 111609744
chr3 111609783
chr3 111610306
chr3 111610386
chr3 111610419
chr3 111616125
chr3 111616505
chr3 111668134
chr3 111668140
chr3 111674452
chr3 111674527
chr3 111674601
chr3 111674606
chr3 111674608
chr3 111674613
chr3 111674619
chr3 111674629
chr3 111674634
chr3 111674643
chr3 111674656
chr3 111674664
chr3 111674693
chr3 111674696
chr3 111674933

chr3 111675011
chr3 111675045
chr3 111675057
chr3 111675110
chr3 111675146
chr3 111675155
chr3 111675158
chr3 111675168
chr3 111675311
chr3 111675328
chr3 111675344
chr3 111675358
chr3 111675414
chr3 111690608
chr3 111755281
chr3 111859746
chr3 111859748
chr3 111859762
chr3 111859764
chr3 111859779
chr3 111859782
chr3 111859809
chr3 111859826
chr3 111859834
chr3 111859837
chr3 111859840
chr3 111859846
chr3 111859853
chr3 111859856
chr3 111859877
chr3 111859891
chr3 111859941
chr3 111859952
chr3 111859969
chr3 111859992
chr3 111860015
chr3 111967681
chr3 111967702
chr3 111967723
chr3 111967726
chr3 111967729
chr3 111967747
chr3 111967831
chr3 111968069
chr3 111978929
chr3 111978947
chr3 111978969
chr3 111978986
chr3 111978992
chr3 111979000
chr3 111979006
chr3 111979011
chr3 111979016
chr3 111979045

chr3 111979057
chr3 111979066
chr3 111979072
chr3 111979074
chr3 111979090
chr3 111979107
chr3 111979127
chr3 111979136
chr3 111979146
chr3 111979157
chr3 111979170
chr3 111979182
chr3 111979186
chr3 111979191
chr3 111979213
chr3 111979220
chr3 111979233
chr3 111979241
chr3 111979245
chr3 111979250
chr3 111979257
chr3 111979267
chr3 111979282
chr3 111979291
chr3 111979295
chr3 111979341
chr3 111979383
chr3 111979385
chr3 111979393
chr3 111979410
chr3 111979418
chr3 111979424
chr3 111979611
chr3 111979639
chr3 111979828
chr3 111979875
chr3 111979954
chr3 112043766
chr3 112043822
chr3 112132782
chr3 112133149
chr3 112133233
chr3 112133309
chr3 112133380
chr3 112133395
chr3 112133465
chr3 112133478
chr3 112185747
chr3 112185768
chr3 112185772
chr3 112185776
chr3 112185781
chr3 112185787
chr3 112185802

chr3 112185811
chr3 112185833
chr3 112185846
chr3 112185854
chr3 112185856
chr3 112185872
chr3 112185879
chr3 112185905
chr3 112185910
chr3 112185915
chr3 112185918
chr3 112185933
chr3 112185959
chr3 112185977
chr3 112185995
chr3 112185997
chr3 112186045
chr3 112332961
chr3 112333094
chr3 112333108
chr3 112333117
chr3 112333135
chr3 112333144
chr3 112333494
chr3 112333502
chr3 112333517
chr3 112333519
chr3 112333525
chr3 112333534
chr3 112333543
chr3 112333553
chr3 112333558
chr3 112333667
chr3 112333670
chr3 112333692
chr3 112333701
chr3 112333715
chr3 112333853
chr3 112333886
chr3 112333911
chr3 112334092
chr3 112334160
chr3 112334179
chr3 112334400
chr3 112334426
chr3 112358666
chr3 112358954
chr3 112395775
chr3 112396236
chr3 112459969
chr3 112460364
chr3 112491102
chr3 112491339
chr3 112495846

chr3 112498331
chr3 112498542
chr3 112498712
chr3 112498752
chr3 112498848
chr3 112498916
chr3 112499063
chr3 112499117
chr3 112499126
chr3 112499159
chr3 112499172
chr3 112499180
chr3 112499188
chr3 112499410
chr3 112499561
chr3 112499663
chr3 112499673
chr3 112499914
chr3 112560378
chr3 112560437
chr3 112560840
chr3 112560869
chr3 112601721
chr3 112602156
chr3 112604726
chr3 112604772
chr3 112604887
chr3 112643093
chr3 112643133
chr3 112643136
chr3 112643139
chr3 112643255
chr3 112643303
chr3 112643346
chr3 112643397
chr3 112643828
chr3 112643842
chr3 112643873
chr3 112677050
chr3 112734561
chr3 112734683
chr3 112734801
chr3 112734812
chr3 112773796
chr3 112773820
chr3 112773859
chr3 112773867
chr3 112773942
chr3 112773958
chr3 112773964
chr3 112773986
chr3 112773998
chr3 112972824
chr3 112973503

chr3 112973944
chr3 112974001
chr3 112974050
chr3 112974118
chr3 112974305
chr3 112974386
chr3 112990749
chr3 112990778
chr3 112990789
chr3 112990820
chr3 112990830
chr3 112990851
chr3 112990853
chr3 112990878
chr3 112990883
chr3 112990887
chr3 112990919
chr3 112990922
chr3 112990924
chr3 112990930
chr3 112990940
chr3 112990944
chr3 112990946
chr3 112990981
chr3 112991783
chr3 112991831
chr3 112991852
chr3 112991907
chr3 113018921
chr3 113018988
chr3 113018991
chr3 113019000
chr3 113019038
chr3 113019095
chr3 113019132
chr3 113019139
chr3 113019146
chr3 113019180
chr3 113019200
chr3 113019208
chr3 113019243
chr3 113019269
chr3 113019280
chr3 113019284
chr3 113019354
chr3 113019398
chr3 113019402
chr3 113019411
chr3 113019418
chr3 113019420
chr3 113019433
chr3 113019440
chr3 113019445
chr3 113019455

chr3 113019467
chr3 113019477
chr3 113019480
chr3 113019493
chr3 113019503
chr3 113019509
chr3 113019549
chr3 113019551
chr3 113019554
chr3 113019575
chr3 113019580
chr3 113019588
chr3 113051076
chr3 113051124
chr3 113051164
chr3 113051238
chr3 113051268
chr3 113051306
chr3 113051473
chr3 113514424
chr3 113514493
chr3 113514530
chr3 113514553
chr3 113531945
chr3 113532074
chr3 113532214
chr3 113532231
chr3 113532334
chr3 113532342
chr3 113533119
chr3 113533141
chr3 113533144
chr3 113533156
chr3 113533159
chr3 113533165
chr3 113533180
chr3 113533201
chr3 113533237
chr3 113533254
chr3 113533263
chr3 113697470
chr3 113697526
chr3 113697554
chr3 113697605
chr3 113697667
chr3 113697748
chr3 113697781
chr3 113697825
chr3 113697907
chr3 113744834
chr3 113744884
chr3 113744942
chr3 113744963
chr3 113744975

chr3 113745001
chr3 113745018
chr3 113745063
chr3 113745081
chr3 113745094
chr3 113747794
chr3 113747803
chr3 113747819
chr3 113747901
chr3 113747977
chr3 113748008
chr3 113748080
chr3 114057087
chr3 114057133
chr3 114057158
chr3 114057250
chr3 114057413
chr3 114057540
chr3 114057553
chr3 114057593
chr3 114057633
chr3 114057689
chr3 114057872
chr3 114101608
chr3 114152554
chr3 114152568
chr3 114152574
chr3 114152591
chr3 114213424
chr3 114213500
chr3 114213530
chr3 114213652
chr3 114213708
chr3 114213747
chr3 114213760
chr3 114213884
chr3 114214008
chr3 114214176
chr3 114214196
chr3 114214376
chr3 114214406
chr3 114214436
chr3 114214457
chr3 114214467
chr3 114214807
chr3 114214828
chr3 114215218
chr3 114215225
chr3 114215232
chr3 114215303
chr3 114221797
chr3 114221853
chr3 114221967
chr3 114223196

chr3 114223349
chr3 114223403
chr3 114234981
chr3 114235009
chr3 114235024
chr3 114291316
chr3 114291333
chr3 114291354
chr3 114291387
chr3 114291396
chr3 114291412
chr3 114291419
chr3 114291493
chr3 114291501
chr3 114291547
chr3 114500216
chr3 114500351
chr3 114500430
chr3 114721512
chr3 114721798
chr3 114721978
chr3 114722049
chr3 114809342
chr3 114809925
chr3 114809966
chr3 114810019
chr3 114810065
chr3 114810084
chr3 115145900
chr3 115145961
chr3 115145966
chr3 115146105
chr3 115148442
chr3 115148505
chr3 115148545
chr3 115148720
chr3 115512519
chr3 115512522
chr3 115512524
chr3 115512543
chr3 115512591
chr3 115512607
chr3 115512654
chr3 115512659
chr3 115512661
chr3 115512702
chr3 115512708
chr3 115512713
chr3 115512729
chr3 115512809
chr3 115512813
chr3 115512850
chr3 115512872
chr3 115512874

chr3 115512898
chr3 115512923
chr3 115512936
chr3 115512954
chr3 115513021
chr3 115513109
chr3 115513128
chr3 115513173
chr3 115513179
chr3 115513291
chr3 115513306
chr3 115513377
chr3 115513466
chr3 115513530
chr3 115513537
chr3 115623191
chr3 115623260
chr3 115623326
chr3 115623337
chr3 115623419
chr3 115623460
chr3 115623476
chr3 115623486
chr3 115623571
chr3 115623573
chr3 115623579
chr3 115623581
chr3 115623589
chr3 115623601
chr3 115623680
chr3 115623763
chr3 115623780
chr3 115623819
chr3 115623828
chr3 115623830
chr3 115624025
chr3 115658808
chr3 115658834
chr3 115658836
chr3 115658857
chr3 115658862
chr3 115658865
chr3 115658879
chr3 115658978
chr3 115659031
chr3 115659056
chr3 115784726
chr3 115784849
chr3 115784867
chr3 115784873
chr3 115784875
chr3 115785009
chr3 115785117
chr3 115785188

chr3 115785226
chr3 115792730
chr3 115813099
chr3 115813223
chr3 115814522
chr3 116044917
chr3 116045011
chr3 116045164
chr3 116443375
chr3 116443489
chr3 116443509
chr3 116443601
chr3 116444416
chr3 116444620
chr3 116444835
chr3 116444843
chr3 116444845
chr3 116444847
chr3 116444865
chr3 116444905
chr3 116444918
chr3 116444925
chr3 116444938
chr3 116444943
chr3 116445564
chr3 116445639
chr3 116445651
chr3 116445748
chr3 116445794
chr3 116446070
chr3 116446096
chr3 116769881
chr3 116770999
chr3 116771028
chr3 116771038
chr3 116771071
chr3 116771141
chr3 116771153
chr3 116864706
chr3 116864729
chr3 117055596
chr3 117055678
chr3 117055748
chr3 117056370
chr3 117101360
chr3 117411596
chr3 117412043
chr3 117412221
chr3 117553786
chr3 117554853
chr3 117604581
chr3 117626838
chr3 117627007
chr3 117885012

chr3 117885174
chr3 117885263
chr3 118202304
chr3 118203023
chr3 118203081
chr3 118203363
chr3 118244349
chr3 118267889
chr3 118267916
chr3 118267968
chr3 118268839
chr3 118319011
chr3 118319324
chr3 118319465
chr3 118319608
chr3 118396250
chr3 118396270
chr3 118661044
chr3 118661128
chr3 118661320
chr3 118674440
chr3 118674597
chr3 118836266
chr3 118836376
chr3 118863175
chr3 118998501
chr3 118999154
chr3 118999320
chr3 119240513
chr3 119240519
chr3 119240525
chr3 119293837
chr3 119293841
chr3 119293871
chr3 119293874
chr3 119295406
chr3 119295439
chr3 119295483
chr3 119295598
chr3 119323351
chr3 119323364
chr3 119323366
chr3 119323640
chr3 119462644
chr3 119462747
chr3 119462807
chr3 119462926
chr3 119463012
chr3 119463047
chr3 119463087
chr3 119463906
chr3 119463953
chr3 119463993
chr3 119464011

chr3 119464056
chr3 119464094
chr3 119464176
chr3 119464332
chr3 119469314
chr3 119469350
chr3 119469358
chr3 119469367
chr3 119469374
chr3 119469415
chr3 119469434
chr3 119469438
chr3 119469440
chr3 119469442
chr3 119469446
chr3 119469460
chr3 119469550
chr3 119469552
chr3 119469626
chr3 119469647
chr3 119469653
chr3 119469687
chr3 119469692
chr3 119582070
chr3 119677460
chr3 119677462
chr3 119677466
chr3 119677494
chr3 119677514
chr3 119677520
chr3 119677532
chr3 119677539
chr3 119677618
chr3 119677653
chr3 119677662
chr3 119677682
chr3 119677687
chr3 119677793
chr3 119702622
chr3 119702660
chr3 119702669
chr3 119702706
chr3 119702757
chr3 119702767
chr3 119702820
chr3 120091700
chr3 120091750
chr3 120091797
chr3 120091996
chr3 120092410
chr3 120092428
chr3 120092446
chr3 120348052
chr3 120348134

chr3 120348177
chr3 120348248
chr3 120348360
chr3 120348365
chr3 120348377
chr3 120348407
chr3 120348480
chr3 120348517
chr3 120348564
chr3 120413333
chr3 120596771
chr3 120596785
chr3 120596807
chr3 120596830
chr3 120596909
chr3 120596913
chr3 120596920
chr3 120597302
chr3 120597355
chr3 120597481
chr3 120678903
chr3 120742701
chr3 120742713
chr3 120742715
chr3 120742724
chr3 120742740
chr3 120742752
chr3 120742788
chr3 120742821
chr3 120742835
chr3 120743338
chr3 120743386
chr3 120743462
chr3 120743607
chr3 120869510
chr3 120909244
chr3 120909288
chr3 120909419
chr3 120909444
chr3 120909448
chr3 120909523
chr3 120909597
chr3 120909686
chr3 120909694
chr3 120909697
chr3 120909745
chr3 120909934
chr3 120910073
chr3 120980856
chr3 120980918
chr3 121218921
chr3 121219307
chr3 121219448
chr3 121432115

chr3 121432339
chr3 121545431
chr3 121545436
chr3 121545623
chr3 121545635
chr3 121545655
chr3 121545667
chr3 121545670
chr3 121545682
chr3 121545691
chr3 121545699
chr3 121545748
chr3 121545752
chr3 121545755
chr3 121545766
chr3 121545772
chr3 121545805
chr3 121545808
chr3 121545815
chr3 121545821
chr3 121545843
chr3 121545846
chr3 121545849
chr3 121545861
chr3 121545864
chr3 121545891
chr3 121545895
chr3 121546511
chr3 121546514
chr3 121546516
chr3 121660489
chr3 121660848
chr3 121660852
chr3 121660860
chr3 121660862
chr3 121660871
chr3 121660898
chr3 121748965
chr3 121749029
chr3 121749037
chr3 121749043
chr3 121749115
chr3 121749151
chr3 121749180
chr3 121749182
chr3 121749193
chr3 121749246
chr3 121749249
chr3 121749255
chr3 121750303
chr3 121750321
chr3 121750333
chr3 121834558
chr3 121835612

chr3 121835678
chr3 121835747
chr3 121835754
chr3 121835949
chr3 121836163
chr3 121836199
chr3 121836315
chr3 121836339
chr3 122041756
chr3 122258830
chr3 122258874
chr3 122258938
chr3 122258967
chr3 122258980
chr3 122259023
chr3 122259183
chr3 122285431
chr3 122294805
chr3 122295522
chr3 122295544
chr3 122295559
chr3 122298453
chr3 122298468
chr3 122298948
chr3 122384496
chr3 122384548
chr3 122384550
chr3 122384572
chr3 122384868
chr3 122415475
chr3 122415625
chr3 122415692
chr3 122415706
chr3 122415773
chr3 122415801
chr3 122415809
chr3 122415839
chr3 122415842
chr3 122415844
chr3 122415878
chr3 122415898
chr3 122415908
chr3 122415961
chr3 122415964
chr3 122415973
chr3 122415997
chr3 122416006
chr3 122416065
chr3 122416067
chr3 122416086
chr3 122416102
chr3 122416135
chr3 122416140
chr3 122416159

chr3 122416169
chr3 122416175
chr3 122416186
chr3 122416202
chr3 122416212
chr3 122416217
chr3 122416230
chr3 122416236
chr3 122416281
chr3 122416315
chr3 122416371
chr3 122416411
chr3 122416428
chr3 122416438
chr3 122416454
chr3 122416457
chr3 122416463
chr3 122416492
chr3 122416538
chr3 122416549
chr3 122416554
chr3 122416616
chr3 122416632
chr3 122416658
chr3 122416678
chr3 122416686
chr3 122416704
chr3 122416728
chr3 122416786
chr3 122416891
chr3 122564493
chr3 122564533
chr3 122564537
chr3 122564540
chr3 122564551
chr3 122564570
chr3 122564578
chr3 122564589
chr3 122577562
chr3 122577570
chr3 122577588
chr3 122577594
chr3 122577616
chr3 122577641
chr3 122577658
chr3 122577675
chr3 122577694
chr3 122577717
chr3 122577739
chr3 122577766
chr3 122577770
chr3 122577776
chr3 122577783
chr3 122577790

chr3 122577798
chr3 122577802
chr3 122577809
chr3 122577816
chr3 122577818
chr3 122577830
chr3 122577833
chr3 122577845
chr3 122577862
chr3 122577864
chr3 122577871
chr3 122577877
chr3 122577910
chr3 122577927
chr3 122577931
chr3 122577935
chr3 122577948
chr3 122577953
chr3 122577977
chr3 122577980
chr3 122578003
chr3 122578018
chr3 122578120
chr3 122578258
chr3 122578490
chr3 122578552
chr3 122578589
chr3 122609982
chr3 122610195
chr3 122610256
chr3 122793716
chr3 122793721
chr3 122794218
chr3 122794227
chr3 122975545
chr3 122975591
chr3 122975704
chr3 122975727
chr3 123201549
chr3 123201660
chr3 123201663
chr3 123201667
chr3 123201698
chr3 123201735
chr3 123201743
chr3 123201771
chr3 123201793
chr3 123201831
chr3 123201857
chr3 123201863
chr3 123201876
chr3 123201878
chr3 123201882
chr3 123201884

chr3 123201891
chr3 123201898
chr3 123201900
chr3 123201903
chr3 123201908
chr3 123201911
chr3 123201914
chr3 123201952
chr3 123201954
chr3 123201961
chr3 123552245
chr3 123552401
chr3 123585578
chr3 123585584
chr3 123585604
chr3 123585661
chr3 123585679
chr3 123585683
chr3 123585728
chr3 123585730
chr3 123585738
chr3 123585745
chr3 123585753
chr3 123585781
chr3 123585798
chr3 123585968
chr3 123586073
chr3 123586124
chr3 123586154
chr3 123586183
chr3 123586189
chr3 123586287
chr3 123586308
chr3 123586333
chr3 123884752
chr3 123884771
chr3 123884777
chr3 123960485
chr3 123960537
chr3 123960652
chr3 123960704
chr3 123960716
chr3 123960869
chr3 123960872
chr3 123960921
chr3 123960939
chr3 123960942
chr3 124269819
chr3 124550291
chr3 124550357
chr3 124761564
chr3 124761566
chr3 124761576
chr3 124834813

chr3 124835081
chr3 124835119
chr3 124835152
chr3 124835166
chr3 124835179
chr3 124835211
chr3 124835235
chr3 124835246
chr3 124835293
chr3 125051874
chr3 125052003
chr3 125052113
chr3 125052135
chr3 125052220
chr3 125052238
chr3 125052289
chr3 125052332
chr3 125052365
chr3 125054929
chr3 125054940
chr3 125054952
chr3 125055053
chr3 125055055
chr3 125055074
chr3 125055087
chr3 125055104
chr3 125055132
chr3 125055175
chr3 125374454
chr3 125374486
chr3 125375472
chr3 125375478
chr3 125375500
chr3 125511940
chr3 125512283
chr3 125585779
chr3 125594263
chr3 125594294
chr3 125594489
chr3 125594507
chr3 125594565
chr3 125594607
chr3 125594665
chr3 125594667
chr3 125594717
chr3 125594726
chr3 125594755
chr3 125594762
chr3 125594806
chr3 125594833
chr3 125594901
chr3 125594917
chr3 125594920
chr3 125594929

chr3 125595904
chr3 125595906
chr3 125595992
chr3 125596000
chr3 125822302
chr3 125822330
chr3 125822383
chr3 125822425
chr3 125822509
chr3 125915559
chr3 125915570
chr3 125915580
chr3 125915603
chr3 125915609
chr3 125915619
chr3 125915628
chr3 125915641
chr3 125915660
chr3 125915666
chr3 125915670
chr3 125915678
chr3 125915692
chr3 125915695
chr3 125915704
chr3 125915706
chr3 125915709
chr3 125915717
chr3 125915725
chr3 125915751
chr3 125915760
chr3 125915767
chr3 125917047
chr3 125917067
chr3 125917079
chr3 125917081
chr3 125917091
chr3 125917103
chr3 125917105
chr3 125917132
chr3 125917176
chr3 125917237
chr3 125917322
chr3 125972283
chr3 125972349
chr3 125972377
chr3 125972399
chr3 125972415
chr3 125972447
chr3 125972449
chr3 125972460
chr3 125972487
chr3 125972503
chr3 125972529
chr3 125972589

chr3 125972602
chr3 125972873
chr3 125990388
chr3 125990395
chr3 125990432
chr3 125990437
chr3 125990445
chr3 125990453
chr3 125990473
chr3 125990482
chr3 125990518
chr3 125990521
chr3 125990537
chr3 125990544
chr3 125990547
chr3 125990559
chr3 125990565
chr3 125990573
chr3 125990580
chr3 125990595
chr3 125990605
chr3 125990608
chr3 125990640
chr3 125990663
chr3 125990680
chr3 125990696
chr3 125990757
chr3 125990784
chr3 125990797
chr3 125990801
chr3 125990804
chr3 125990854
chr3 125990865
chr3 125990897
chr3 125990900
chr3 125990915
chr3 125990918
chr3 125990925
chr3 125990935
chr3 125990956
chr3 125990959
chr3 125991032
chr3 126086165
chr3 126086190
chr3 126260780
chr3 126323574
chr3 126323599
chr3 126323781
chr3 126324634
chr3 126324802
chr3 126335191
chr3 126335979
chr3 126337126
chr3 126337263

chr3 126337611
chr3 126384037
chr3 126911575
chr3 126911635
chr3 126911673
chr3 127189320
chr3 127190388
chr3 127626305
chr3 127626526
chr3 127626554
chr3 127626564
chr3 128051781
chr3 128122803
chr3 128122827
chr3 128122838
chr3 128123040
chr3 128123096
chr3 128123144
chr3 128123150
chr3 128123215
chr3 128123222
chr3 128123264
chr3 128123280
chr3 128123282
chr3 128123298
chr3 128123311
chr3 128123358
chr3 128123379
chr3 128123399
chr3 128123422
chr3 128123425
chr3 128123435
chr3 128649553
chr3 128649761
chr3 128649844
chr3 128649895
chr3 128649962
chr3 128650016
chr3 128715309
chr3 128715326
chr3 128726889
chr3 128726898
chr3 128726903
chr3 128726924
chr3 128726933
chr3 128726971
chr3 128727004
chr3 128727046
chr3 128727050
chr3 128727143
chr3 128727164
chr3 128788514
chr3 128788645
chr3 128788682

chr3 128788804
chr3 128788937
chr3 128789008
chr3 128789040
chr3 128789059
chr3 128789278
chr3 128789366
chr3 128789388
chr3 128789398
chr3 128789404
chr3 128789460
chr3 128803853
chr3 128804062
chr3 128804123
chr3 128804232
chr3 128804490
chr3 128923168
chr3 128923193
chr3 128923248
chr3 128923276
chr3 128923294
chr3 128923306
chr3 128923377
chr3 128923498
chr3 128923619
chr3 128923752
chr3 129016155
chr3 129016181
chr3 129088156
chr3 129088692
chr3 129160255
chr3 129160496
chr3 129160545
chr3 129160579
chr3 129160840
chr3 129160876
chr3 129160891
chr3 129161997
chr3 129162014
chr3 129162059
chr3 129162077
chr3 129162129
chr3 129162140
chr3 129162150
chr3 129162201
chr3 129162425
chr3 129181598
chr3 129181604
chr3 129181606
chr3 129181628
chr3 129181644
chr3 129181831
chr3 129182178
chr3 129224917

chr3 129224945
chr3 129276636
chr3 129282195
chr3 129282469
chr3 129282903
chr3 129282955
chr3 129399215
chr3 129399227
chr3 129399230
chr3 129399269
chr3 129399280
chr3 129399286
chr3 129399309
chr3 129399381
chr3 129399427
chr3 129399430
chr3 129399484
chr3 129399490
chr3 129399539
chr3 129399671
chr3 129399695
chr3 129399710
chr3 129399730
chr3 129399737
chr3 129399747
chr3 129399767
chr3 129399818
chr3 129399824
chr3 129399830
chr3 129399834
chr3 129439440
chr3 129440735
chr3 129440853
chr3 129440856
chr3 129440958
chr3 129650265
chr3 129888132
chr3 129888269
chr3 129888370
chr3 129892174
chr3 130002416
chr3 130002430
chr3 130002487
chr3 130002496
chr3 130002526
chr3 130002528
chr3 130002556
chr3 130002568
chr3 130002577
chr3 130002587
chr3 130002590
chr3 130002609
chr3 130002623
chr3 130002640

chr3 130002645
chr3 130002652
chr3 130002657
chr3 130002670
chr3 130002672
chr3 130002676
chr3 130002679
chr3 130002688
chr3 130002710
chr3 130002716
chr3 130002722
chr3 130002763
chr3 130002771
chr3 130178919
chr3 130179020
chr3 130179199
chr3 130179267
chr3 130179442
chr3 130179500
chr3 130179515
chr3 130179520
chr3 130179649
chr3 130179700
chr3 130179736
chr3 130179757
chr3 130179767
chr3 130179803
chr3 130346217
chr3 130346329
chr3 130747008
chr3 130747012
chr3 130747051
chr3 130747217
chr3 130747311
chr3 130747319
chr3 130747343
chr3 130747349
chr3 130747373
chr3 130747383
chr3 130747405
chr3 130747425
chr3 130747429
chr3 130747437
chr3 130747441
chr3 130747471
chr3 130747482
chr3 130747502
chr3 130747516
chr3 130747523
chr3 130747560
chr3 130747567
chr3 130747570
chr3 130747572
chr3 130747576

chr3 130747646
chr3 130747670
chr3 130747680
chr3 130747724
chr3 130747734
chr3 130747770
chr3 130747778
chr3 130747787
chr3 130747809
chr3 130747811
chr3 130747817
chr3 130747834
chr3 130747841
chr3 130747849
chr3 130747873
chr3 130747893
chr3 130747897
chr3 130747903
chr3 130747905
chr3 130747908
chr3 130747943
chr3 130850190
chr3 130850208
chr3 130850316
chr3 130850343
chr3 130850443
chr3 130850528
chr3 130850609
chr3 130850716
chr3 130850897
chr3 130850900
chr3 130850919
chr3 130850962
chr3 130850988
chr3 130850991
chr3 130851136
chr3 130892887
chr3 130892891
chr3 130892910
chr3 130892932
chr3 130893013
chr3 130893132
chr3 130893150
chr3 130893191
chr3 130893210
chr3 130893263
chr3 130893276
chr3 130893287
chr3 130893340
chr3 130894867
chr3 130895062
chr3 130895102
chr3 130895157
chr3 130895169

chr3 130895357
chr3 130895398
chr3 131381263
chr3 131381286
chr3 131381313
chr3 131381316
chr3 131381322
chr3 131381328
chr3 131381355
chr3 131381375
chr3 131381380
chr3 131381383
chr3 131381437
chr3 131381498
chr3 131381506
chr3 131381520
chr3 131381522
chr3 131381540
chr3 131381548
chr3 131382130
chr3 131382132
chr3 131382220
chr3 131382222
chr3 131382282
chr3 131382285
chr3 131382292
chr3 131382294
chr3 131382306
chr3 131502216
chr3 131502421
chr3 131502459
chr3 131502477
chr3 131502513
chr3 131502517
chr3 131502541
chr3 131502577
chr3 131502595
chr3 131502619
chr3 131503023
chr3 131503031
chr3 131503036
chr3 131503043
chr3 131503067
chr3 131503122
chr3 131503197
chr3 131503232
chr3 131503262
chr3 131503297
chr3 132029679
chr3 132029823
chr3 132030121
chr3 132030384
chr3 132031413
chr3 132031600

chr3 132034774
chr3 132034783
chr3 132034804
chr3 132034852
chr3 132138063
chr3 132138072
chr3 132138353
chr3 132138357
chr3 132138397
chr3 132138523
chr3 132138532
chr3 132294804
chr3 132375881
chr3 132417490
chr3 132417496
chr3 132417506
chr3 132417508
chr3 132417512
chr3 132417527
chr3 132417536
chr3 132417604
chr3 132417618
chr3 132417620
chr3 132417626
chr3 132418471
chr3 132418478
chr3 132418487
chr3 132418515
chr3 132418561
chr3 132418614
chr3 132418663
chr3 132418951
chr3 132419014
chr3 132419049
chr3 132419118
chr3 132419536
chr3 132659213
chr3 132659220
chr3 132659232
chr3 132659389
chr3 132659397
chr3 132659409
chr3 132659437
chr3 132659443
chr3 132659451
chr3 132659480
chr3 132659575
chr3 132660736
chr3 132660749
chr3 132660768
chr3 132660777
chr3 132660782
chr3 132660794
chr3 132660798

chr3 132660819
chr3 132660863
chr3 132660939
chr3 132660980
chr3 132660985
chr3 132661098
chr3 132661103
chr3 132661113
chr3 132661139
chr3 132661167
chr3 132661309
chr3 132661346
chr3 133442224
chr3 133506132
chr3 133506142
chr3 133572720
chr3 133572825
chr3 133572926
chr3 133573081
chr3 133575041
chr3 133575393
chr3 133575419
chr3 133575933
chr3 133576010
chr3 133576237
chr3 133595210
chr3 133595464
chr3 133660858
chr3 133661017
chr3 133661046
chr3 133661104
chr3 133661256
chr3 133661264
chr3 133661270
chr3 133661359
chr3 133661468
chr3 133661478
chr3 133661485
chr3 133662261
chr3 133662289
chr3 133662331
chr3 133662364
chr3 133662411
chr3 133662454
chr3 133662573
chr3 133662576
chr3 133662698
chr3 133662711
chr3 134249799
chr3 134249851
chr3 134249875
chr3 134249916
chr3 134249964
chr3 134250022

chr3 134250031
chr3 134250046
chr3 134364297
chr3 134364316
chr3 134364319
chr3 134364327
chr3 134364334
chr3 134364341
chr3 134364347
chr3 134364354
chr3 134364358
chr3 134364399
chr3 134364444
chr3 134364473
chr3 134364578
chr3 134365059
chr3 134485713
chr3 134485726
chr3 134485757
chr3 134485765
chr3 134485771
chr3 134485792
chr3 134485810
chr3 134486762
chr3 134486875
chr3 134486950
chr3 134486959
chr3 134487002
chr3 134487023
chr3 134487027
chr3 134487173
chr3 134487191
chr3 134487461
chr3 134802958
chr3 135440826
chr3 135583376
chr3 135583833
chr3 136192810
chr3 136192941
chr3 136193034
chr3 136193236
chr3 136596299
chr3 136596377
chr3 136596406
chr3 136750163
chr3 136750381
chr3 136750396
chr3 136750567
chr3 136750611
chr3 136750661
chr3 136750668
chr3 136750693
chr3 136750874
chr3 136750948

chr3 136750973
chr3 136861622
chr3 136861649
chr3 136861675
chr3 136861798
chr3 136861805
chr3 136861821
chr3 136861924
chr3 136861927
chr3 136861966
chr3 136861997
chr3 136862005
chr3 136862034
chr3 136862037
chr3 136862039
chr3 136919965
chr3 136920711
chr3 136921055
chr3 137032440
chr3 137121849
chr3 137121900
chr3 137196783
chr3 137351508
chr3 137505281
chr3 137506409
chr3 137506492
chr3 137567620
chr3 137612460
chr3 137612481
chr3 137612548
chr3 137766155
chr3 137766235
chr3 137766271
chr3 137766300
chr3 137766394
chr3 137766400
chr3 137766424
chr3 137766458
chr3 137766481
chr3 137766510
chr3 137772184
chr3 137772322
chr3 137772390
chr3 137772407
chr3 137772414
chr3 137772439
chr3 137772620
chr3 137772644
chr3 137772982
chr3 137773831
chr3 137773938
chr3 137774087
chr3 137840960
chr3 137840965

chr3 137874777
chr3 137971602
chr3 138174569
chr3 138174578
chr3 138174584
chr3 138174588
chr3 138174590
chr3 138174620
chr3 138174631
chr3 138174636
chr3 138174647
chr3 138174655
chr3 138174660
chr3 138174665
chr3 138174670
chr3 138174681
chr3 138174684
chr3 138174702
chr3 138174712
chr3 138174717
chr3 138174719
chr3 138174722
chr3 138174733
chr3 138174739
chr3 138174762
chr3 138174765
chr3 138174791
chr3 138174801
chr3 138174818
chr3 138174830
chr3 138174857
chr3 138174865
chr3 138174876
chr3 138186788
chr3 138188207
chr3 138188223
chr3 138188225
chr3 138188270
chr3 138188350
chr3 138188419
chr3 138188487
chr3 138188508
chr3 138188517
chr3 138188655
chr3 138188683
chr3 138234738
chr3 138235066
chr3 138329611
chr3 138329617
chr3 138611297
chr3 138611467
chr3 138833729
chr3 138833964
chr3 138834085

chr3 138915679
chr3 138915683
chr3 138915688
chr3 138915716
chr3 138915721
chr3 138915728
chr3 138915753
chr3 138915765
chr3 138915768
chr3 138915860
chr3 138915873
chr3 138915875
chr3 138915892
chr3 138915910
chr3 138915917
chr3 138916116
chr3 138916132
chr3 138916142
chr3 138916146
chr3 138960619
chr3 138960787
chr3 138960813
chr3 138960827
chr3 139111973
chr3 139111975
chr3 139112006
chr3 139112036
chr3 139112047
chr3 139112060
chr3 139112080
chr3 139112082
chr3 139112108
chr3 139112123
chr3 139112343
chr3 139112702
chr3 139149101
chr3 139192227
chr3 139344102
chr3 139344108
chr3 139344125
chr3 139344142
chr3 139344158
chr3 139344286
chr3 139344288
chr3 139344293
chr3 139344303
chr3 139344373
chr3 139344405
chr3 139344410
chr3 139344518
chr3 139344535
chr3 140495931
chr3 141011586
chr3 141011629

chr3 141011650
chr3 141011661
chr3 141011689
chr3 141011697
chr3 141011701
chr3 141011714
chr3 141011724
chr3 141011865
chr3 141011997
chr3 141012016
chr3 141012171
chr3 141266854
chr3 141266970
chr3 141267038
chr3 141267092
chr3 141267101
chr3 141267152
chr3 141267181
chr3 141267206
chr3 141267253
chr3 141267322
chr3 141267329
chr3 141267355
chr3 141267453
chr3 141294400
chr3 141294551
chr3 141361526
chr3 141370262
chr3 141370355
chr3 141411616
chr3 141411657
chr3 141411766
chr3 141411972
chr3 141412226
chr3 141412262
chr3 141412290
chr3 141412347
chr3 141412363
chr3 141412406
chr3 141441379
chr3 141486449
chr3 141486548
chr3 141486698
chr3 141486700
chr3 141486704
chr3 141486776
chr3 141487991
chr3 141488015
chr3 141488038
chr3 141488061
chr3 141488083
chr3 141488143
chr3 141488255
chr3 141488342

chr3 141488485
chr3 141488879
chr3 141489030
chr3 141489491
chr3 141498666
chr3 141498972
chr3 141499009
chr3 141499022
chr3 141499078
chr3 141499204
chr3 141519191
chr3 141519603
chr3 141552414
chr3 141552555
chr3 141552741
chr3 141553326
chr3 141738940
chr3 141739012
chr3 141739031
chr3 141739038
chr3 141739069
chr3 141739082
chr3 141739085
chr3 141739092
chr3 141739124
chr3 141739142
chr3 141739223
chr3 141739548
chr3 141739561
chr3 141739670
chr3 141796936
chr3 141796956
chr3 141796968
chr3 141797020
chr3 141797034
chr3 141797079
chr3 141797214
chr3 141877575
chr3 141877674
chr3 141877727
chr3 141877764
chr3 141877948
chr3 141878065
chr3 141878238
chr3 142053891
chr3 142096023
chr3 142096079
chr3 142225015
chr3 142225049
chr3 142225053
chr3 142225070
chr3 142446190
chr3 142446219
chr3 142446594

chr3 142446605
chr3 142446732
chr3 142446894
chr3 142446903
chr3 142447071
chr3 142447115
chr3 142447121
chr3 142447138
chr3 142447197
chr3 142447249
chr3 142447361
chr3 142447364
chr3 142447380
chr3 142447387
chr3 142447396
chr3 142447398
chr3 142448364
chr3 142448440
chr3 142448456
chr3 142448466
chr3 142448506
chr3 142448513
chr3 142448541
chr3 142498618
chr3 142498807
chr3 142578001
chr3 142578055
chr3 142578060
chr3 142578071
chr3 142578080
chr3 142578176
chr3 142578238
chr3 142578495
chr3 142579202
chr3 142579211
chr3 142579313
chr3 142723692
chr3 142723769
chr3 142723785
chr3 142843735
chr3 142994170
chr3 142994178
chr3 142994399
chr3 143002920
chr3 143002940
chr3 143002985
chr3 143003014
chr3 143003028
chr3 143122877
chr3 143123045
chr3 143123442
chr3 143123459
chr3 143123474
chr3 143674308

chr3 143674528
chr3 143674533
chr3 143674549
chr3 143674560
chr3 143847423
chr3 143847593
chr3 143847636
chr3 143848183
chr3 143848189
chr3 143848260
chr3 143848538
chr3 143848650
chr3 143848737
chr3 143970742
chr3 143973582
chr3 143973628
chr3 143973667
chr3 143973744
chr3 143973774
chr3 143973783
chr3 143973796
chr3 143973899
chr3 143973943
chr3 143974011
chr3 143974236
chr3 144025974
chr3 144026045
chr3 144026914
chr3 144345323
chr3 144345341
chr3 144501388
chr3 144501436
chr3 144501733
chr3 144570412
chr3 144755836
chr3 144756226
chr3 144901181
chr3 144902053
chr3 144902093
chr3 144989422
chr3 144989563
chr3 144989853
chr3 145330646
chr3 145332039
chr3 145332044
chr3 145332112
chr3 145332280
chr3 145426655
chr3 145682376
chr3 145682544
chr3 145708451
chr3 145709010
chr3 145858245
chr3 145858581

chr3 145858981
chr3 145902409
chr3 145902776
chr3 145916628
chr3 145916759
chr3 145917175
chr3 145920738
chr3 145920875
chr3 146159844
chr3 146159881
chr3 146160180
chr3 146160217
chr3 146160244
chr3 146160257
chr3 146160337
chr3 146250092
chr3 146250421
chr3 146250455
chr3 146250507
chr3 146250517
chr3 146250548
chr3 146250560
chr3 146250580
chr3 146469423
chr3 146469431
chr3 146469471
chr3 146469484
chr3 146469496
chr3 146469514
chr3 146469528
chr3 146469533
chr3 146469544
chr3 146469557
chr3 146469566
chr3 146469601
chr3 146469608
chr3 146469611
chr3 146469716
chr3 146469718
chr3 146469733
chr3 146469750
chr3 146469752
chr3 146469756
chr3 146469767
chr3 146469769
chr3 146498361
chr3 146498386
chr3 146498412
chr3 146498429
chr3 146498441
chr3 146498456
chr3 146498640
chr3 146543560
chr3 146543724

chr3 146543847
chr3 146543864
chr3 146543876
chr3 146543911
chr3 146543923
chr3 146543978
chr3 146543981
chr3 146544056
chr3 146544102
chr3 146544147
chr3 146544154
chr3 146544183
chr3 146544204
chr3 146544211
chr3 146544219
chr3 146544236
chr3 146544340
chr3 146544348
chr3 146544350
chr3 146544353
chr3 146544387
chr3 146544402
chr3 146544411
chr3 146544413
chr3 146760757
chr3 146761066
chr3 146761180
chr3 146770644
chr3 146770802
chr3 146770884
chr3 146930199
chr3 146972950
chr3 147302678
chr3 147302731
chr3 147322886
chr3 147322912
chr3 147323850
chr3 147353777
chr3 147354354
chr3 147354470
chr3 147354547
chr3 147354556
chr3 147354615
chr3 147354654
chr3 147357517
chr3 147357534
chr3 147357546
chr3 147357571
chr3 147357824
chr3 147357899
chr3 147358523
chr3 147358672
chr3 147358737
chr3 147358780

chr3 147358823
chr3 147358828
chr3 147358842
chr3 147358848
chr3 147358930
chr3 147359013
chr3 147359039
chr3 147361188
chr3 147361197
chr3 147361248
chr3 147361263
chr3 147361266
chr3 147361282
chr3 147361315
chr3 147361318
chr3 147361324
chr3 147361327
chr3 147361331
chr3 147361338
chr3 147361357
chr3 147361377
chr3 147361387
chr3 147361445
chr3 147361485
chr3 147361506
chr3 147361615
chr3 147361705
chr3 147361952
chr3 147361978
chr3 147362014
chr3 147362024
chr3 147362026
chr3 147362251
chr3 147366261
chr3 147366923
chr3 147367363
chr3 147367463
chr3 147367640
chr3 147367964
chr3 147368221
chr3 147368378
chr3 147368663
chr3 147368681
chr3 147368775
chr3 147368834
chr3 147368879
chr3 147368904
chr3 147368929
chr3 147368989
chr3 147369024
chr3 147369030
chr3 147369051
chr3 147369073
chr3 147369106

chr3 147371780
chr3 147371792
chr3 147371842
chr3 147373174
chr3 147373359
chr3 147373811
chr3 147375893
chr3 147375950
chr3 147375987
chr3 147376080
chr3 147376164
chr3 147376244
chr3 147376311
chr3 147376434
chr3 147376747
chr3 147377089
chr3 147377254
chr3 147377434
chr3 147377523
chr3 147377591
chr3 147377616
chr3 147377631
chr3 147377648
chr3 147378924
chr3 147378936
chr3 147378946
chr3 147379068
chr3 147379098
chr3 147379191
chr3 147379227
chr3 147379230
chr3 147379253
chr3 147379267
chr3 147379535
chr3 147379655
chr3 147379688
chr3 147379758
chr3 147379776
chr3 147379812
chr3 147379830
chr3 147379854
chr3 147379964
chr3 147379997
chr3 147380020
chr3 147381192
chr3 147381689
chr3 147381760
chr3 147381826
chr3 147381865
chr3 147381903
chr3 147381923
chr3 147382077
chr3 147382474
chr3 147385957

chr3 147386003
chr3 147386010
chr3 147386105
chr3 147386701
chr3 147386739
chr3 147386849
chr3 147387158
chr3 147387322
chr3 147387336
chr3 147387372
chr3 147387392
chr3 147387403
chr3 147387414
chr3 147387477
chr3 147400619
chr3 147401174
chr3 147401664
chr3 147413741
chr3 147413789
chr3 147413800
chr3 147413830
chr3 147413839
chr3 147413857
chr3 147413867
chr3 147413884
chr3 147413969
chr3 147413978
chr3 147413997
chr3 147414033
chr3 147414041
chr3 147414069
chr3 147414073
chr3 147414146
chr3 147414161
chr3 147414164
chr3 147414223
chr3 147414283
chr3 147414292
chr3 147414306
chr3 147414370
chr3 147414432
chr3 147414446
chr3 147414493
chr3 147414534
chr3 147414562
chr3 147414684
chr3 147414692
chr3 147414724
chr3 147414772
chr3 147414776
chr3 147414849
chr3 147414870
chr3 147414894
chr3 147414925

chr3 147414934
chr3 147414958
chr3 147415051
chr3 147415106
chr3 147415293
chr3 147415468
chr3 147415480
chr3 147415517
chr3 147415536
chr3 147415542
chr3 147416020
chr3 147416097
chr3 147416394
chr3 147416693
chr3 147416799
chr3 147417290
chr3 147417634
chr3 147417865
chr3 147417949
chr3 147418057
chr3 147418065
chr3 147424901
chr3 147424950
chr3 147425086
chr3 147425118
chr3 147425252
chr3 147425263
chr3 147425308
chr3 147425399
chr3 147992864
chr3 147992874
chr3 147992881
chr3 147993357
chr3 148344404
chr3 148344719
chr3 148476307
chr3 148476619
chr3 148527338
chr3 148527664
chr3 148527751
chr3 148568321
chr3 148568546
chr3 148618254
chr3 148618270
chr3 148618303
chr3 148618309
chr3 148618337
chr3 148618339
chr3 148618349
chr3 148618409
chr3 148618801
chr3 148619013
chr3 148620833
chr3 148672580

chr3 148673012
chr3 148990930
chr3 148990966
chr3 148991034
chr3 148991112
chr3 148991131
chr3 148991142
chr3 148991168
chr3 148991250
chr3 148991290
chr3 149085856
chr3 149085866
chr3 149085889
chr3 149085905
chr3 149085983
chr3 149085994
chr3 149086010
chr3 149086056
chr3 149086088
chr3 149086118
chr3 149086125
chr3 149086144
chr3 149086150
chr3 149086173
chr3 149086176
chr3 149086188
chr3 149086200
chr3 149086219
chr3 149086230
chr3 149086240
chr3 149086242
chr3 149086268
chr3 149086279
chr3 149086287
chr3 149086291
chr3 149086305
chr3 149086339
chr3 149086363
chr3 149086368
chr3 149086382
chr3 149086388
chr3 149086393
chr3 149086398
chr3 149086403
chr3 149086420
chr3 149086434
chr3 149086447
chr3 149086451
chr3 149086454
chr3 149086465
chr3 149086469
chr3 149086485
chr3 149086488
chr3 149086490

chr3 149086504
chr3 149086507
chr3 149086510
chr3 149086513
chr3 149086544
chr3 149086547
chr3 149086580
chr3 149120476
chr3 149120496
chr3 149120598
chr3 149120647
chr3 149120902
chr3 149121227
chr3 149128948
chr3 149128952
chr3 149129140
chr3 149129220
chr3 149204000
chr3 149204064
chr3 149225063
chr3 149225141
chr3 149225240
chr3 149225315
chr3 149225329
chr3 149225390
chr3 149225416
chr3 149752048
chr3 149812838
chr3 149812842
chr3 149813245
chr3 149813259
chr3 149813264
chr3 149813302
chr3 149813307
chr3 149813310
chr3 149813312
chr3 149813537
chr3 149813545
chr3 149813854
chr3 149813950
chr3 149813971
chr3 149813997
chr3 149814086
chr3 149814109
chr3 149814123
chr3 149814153
chr3 149814167
chr3 149814398
chr3 149886529
chr3 149886779
chr3 149967194
chr3 150052096
chr3 150052481
chr3 150184019

chr3 150184044
chr3 150184055
chr3 150184088
chr3 150184106
chr3 150184665
chr3 150385383
chr3 150385438
chr3 150407445
chr3 150407450
chr3 150407484
chr3 150407573
chr3 150407578
chr3 150407713
chr3 150407771
chr3 150407874
chr3 150411203
chr3 150411395
chr3 150411484
chr3 150411501
chr3 150411655
chr3 150411826
chr3 150411881
chr3 150411999
chr3 150412344
chr3 150412650
chr3 150412666
chr3 150412988
chr3 150413019
chr3 150413103
chr3 150413743
chr3 150543719
chr3 150543763
chr3 150544707
chr3 150544979
chr3 150547153
chr3 150547226
chr3 150547562
chr3 150547579
chr3 150547692
chr3 150547694
chr3 150602919
chr3 150602978
chr3 150603136
chr3 150603193
chr3 150603216
chr3 150603218
chr3 150603222
chr3 150603225
chr3 150603230
chr3 150603232
chr3 150603238
chr3 150610209
chr3 150611004
chr3 150760488

chr3 150760501
chr3 150760705
chr3 150760710
chr3 150760712
chr3 150760995
chr3 150763622
chr3 150763637
chr3 150764070
chr3 150764082
chr3 150764090
chr3 150764100
chr3 150764167
chr3 150764173
chr3 150764207
chr3 150764366
chr3 150787943
chr3 150787966
chr3 150787969
chr3 150787986
chr3 150787994
chr3 150788002
chr3 150788009
chr3 150788012
chr3 150865379
chr3 150865418
chr3 150865529
chr3 150865531
chr3 150865533
chr3 150865535
chr3 150865537
chr3 150865541
chr3 150865543
chr3 150865545
chr3 150865549
chr3 151062903
chr3 151146254
chr3 151146311
chr3 151146367
chr3 151146770
chr3 151171199
chr3 151171363
chr3 151183654
chr3 151199943
chr3 151200101
chr3 151200240
chr3 151200719
chr3 151200757
chr3 151202624
chr3 151202864
chr3 151203062
chr3 151203177
chr3 151203264
chr3 151204407
chr3 151204845

chr3 151205197
chr3 151230922
chr3 151230993
chr3 151231026
chr3 151267905
chr3 151267944
chr3 151268023
chr3 151268078
chr3 151268876
chr3 151268937
chr3 151268948
chr3 151680791
chr3 151788238
chr3 151788269
chr3 151895707
chr3 151976568
chr3 152193269
chr3 152193365
chr3 152193619
chr3 152193714
chr3 152193791
chr3 152193813
chr3 152193861
chr3 152193921
chr3 152193929
chr3 152193936
chr3 152194169
chr3 152194173
chr3 152194175
chr3 152194185
chr3 152194218
chr3 152194222
chr3 152194381
chr3 152194389
chr3 152194396
chr3 152196670
chr3 152196685
chr3 152197000
chr3 152197108
chr3 152197517
chr3 152197527
chr3 152197555
chr3 152197925
chr3 152198197
chr3 152198259
chr3 152198267
chr3 152198775
chr3 152199263
chr3 152199570
chr3 152199585
chr3 152199856
chr3 152200075
chr3 152200229
chr3 152200715

chr3 152200882
chr3 152201090
chr3 152203090
chr3 152203560
chr3 152203617
chr3 152203750
chr3 152204124
chr3 152204361
chr3 152204564
chr3 152205051
chr3 152205412
chr3 152205417
chr3 152242525
chr3 152242550
chr3 152242689
chr3 152242708
chr3 152242735
chr3 152242889
chr3 152242921
chr3 152242949
chr3 152243866
chr3 152243868
chr3 152243891
chr3 152243908
chr3 152243949
chr3 152243993
chr3 152244051
chr3 152244084
chr3 152244094
chr3 152244567
chr3 152244690
chr3 152244897
chr3 152245491
chr3 152245712
chr3 152245844
chr3 152245885
chr3 152257865
chr3 152257949
chr3 152258743
chr3 152258923
chr3 152258978
chr3 152259024
chr3 152259190
chr3 152259330
chr3 152272475
chr3 152272713
chr3 152272948
chr3 152272990
chr3 152273103
chr3 152273294
chr3 152273303
chr3 152273333
chr3 152273452
chr3 152273652

chr3 152273902
chr3 152274145
chr3 152274255
chr3 152274374
chr3 152274420
chr3 152274624
chr3 152274687
chr3 152283322
chr3 152298805
chr3 152298840
chr3 152298848
chr3 152298856
chr3 152299017
chr3 152299083
chr3 152299094
chr3 152299096
chr3 152453217
chr3 152472138
chr3 152472174
chr3 152472192
chr3 152472201
chr3 152472203
chr3 152472266
chr3 152472393
chr3 152472419
chr3 152498845
chr3 152499032
chr3 152499780
chr3 152499893
chr3 152499911
chr3 152557651
chr3 152557740
chr3 152594420
chr3 152594433
chr3 152594614
chr3 152594798
chr3 152596117
chr3 152596339
chr3 152796363
chr3 153043014
chr3 153043035
chr3 153043061
chr3 153043227
chr3 153160638
chr3 153160642
chr3 153160683
chr3 153160689
chr3 153160848
chr3 153160856
chr3 153160861
chr3 153160867
chr3 153160873
chr3 153160913
chr3 153160922

chr3 153163737
chr3 153163817
chr3 153163864
chr3 153164114
chr3 153164203
chr3 153164315
chr3 153164664
chr3 153164917
chr3 153221060
chr3 153221611
chr3 153221657
chr3 153221772
chr3 153221796
chr3 153221830
chr3 153221886
chr3 153221970
chr3 153222017
chr3 153222033
chr3 153222062
chr3 153222098
chr3 153319924
chr3 153320320
chr3 153463024
chr3 154120831
chr3 154120881
chr3 154120949
chr3 154120999
chr3 154121012
chr3 154121024
chr3 154121045
chr3 154121048
chr3 154121050
chr3 154121052
chr3 154121058
chr3 154121067
chr3 154253244
chr3 154253247
chr3 154253258
chr3 154253394
chr3 154253706
chr3 154323753
chr3 154323755
chr3 154323783
chr3 154323888
chr3 154323897
chr3 154324115
chr3 154324186
chr3 154324216
chr3 154324586
chr3 154335670
chr3 154336087
chr3 154336287
chr3 154429532
chr3 154429547

chr3 154429679
chr3 154429837
chr3 154430160
chr3 154430164
chr3 154430166
chr3 154430328
chr3 154453670
chr3 154652755
chr3 154758369
chr3 154758435
chr3 154758440
chr3 154891196
chr3 154903081
chr3 154903737
chr3 154903890
chr3 154904088
chr3 155312950
chr3 155313062
chr3 155524315
chr3 155554321
chr3 155554341
chr3 155554731
chr3 155554735
chr3 155752581
chr3 155752908
chr3 155862658
chr3 155871477
chr3 155871487
chr3 155871966
chr3 155871974
chr3 155871991
chr3 155872024
chr3 155872071
chr3 155872093
chr3 155872114
chr3 155872220
chr3 155872245
chr3 155872397
chr3 155872408
chr3 155873187
chr3 155873190
chr3 155887790
chr3 155888264
chr3 156121565
chr3 156524723
chr3 156524852
chr3 156524855
chr3 156524944
chr3 156554054
chr3 156554103
chr3 156554130
chr3 156554162
chr3 156554230
chr3 156554241

chr3 156554346
chr3 156554510
chr3 156554598
chr3 156554603
chr3 156554672
chr3 156554678
chr3 156554722
chr3 156554728
chr3 156554732
chr3 156554756
chr3 156554782
chr3 156554811
chr3 156554821
chr3 156554848
chr3 156554855
chr3 156554859
chr3 156554868
chr3 156554881
chr3 156554884
chr3 156554896
chr3 156554902
chr3 156554911
chr3 156554919
chr3 156554922
chr3 156554936
chr3 156554941
chr3 156647954
chr3 156648239
chr3 156674540
chr3 156674546
chr3 156674548
chr3 156674551
chr3 156674556
chr3 156674559
chr3 156674569
chr3 156674573
chr3 156674608
chr3 156674623
chr3 156674628
chr3 156674630
chr3 156674634
chr3 156674646
chr3 156674650
chr3 156674652
chr3 156674655
chr3 156674658
chr3 156674668
chr3 156674672
chr3 156674674
chr3 156674679
chr3 156674682
chr3 156674684
chr3 156674687
chr3 156674689

chr3 156826738
chr3 156826766
chr3 156826797
chr3 157008593
chr3 157008688
chr3 157008977
chr3 157009156
chr3 157137626
chr3 157137662
chr3 157137698
chr3 157137702
chr3 157158678
chr3 157158756
chr3 157158772
chr3 157158818
chr3 157158884
chr3 157158936
chr3 157159091
chr3 157159210
chr3 157159231
chr3 157159237
chr3 157174623
chr3 157174640
chr3 157175745
chr3 157175843
chr3 157175846
chr3 157175848
chr3 157175853
chr3 157175867
chr3 157175870
chr3 157175880
chr3 157175883
chr3 157175893
chr3 157176299
chr3 157176380
chr3 157436672
chr3 157436684
chr3 157436717
chr3 157436763
chr3 157436769
chr3 157436865
chr3 157436898
chr3 157436921
chr3 157436947
chr3 157436984
chr3 157436992
chr3 157437029
chr3 157437063
chr3 157437235
chr3 157437348
chr3 157437377
chr3 157437413
chr3 157437434
chr3 157437479

chr3 157437490
chr3 157437503
chr3 157437516
chr3 157437519
chr3 157437523
chr3 157437529
chr3 157499286
chr3 157499338
chr3 157499345
chr3 157499376
chr3 157499378
chr3 157499386
chr3 157499398
chr3 157499416
chr3 157499486
chr3 157499497
chr3 157499530
chr3 157499536
chr3 157499542
chr3 157500053
chr3 157500069
chr3 157500103
chr3 157500190
chr3 157727808
chr3 157727938
chr3 157727997
chr3 157728329
chr3 157729040
chr3 157729116
chr3 157729403
chr3 157730262
chr3 157730455
chr3 157730919
chr3 157730976
chr3 157731378
chr3 157882255
chr3 157882294
chr3 157882761
chr3 157882894
chr3 157883106
chr3 157883187
chr3 157883201
chr3 157883296
chr3 157970991
chr3 157971011
chr3 157971189
chr3 157971296
chr3 157995401
chr3 158096714
chr3 158096735
chr3 158096740
chr3 158097089
chr3 158097192
chr3 158097261

chr3 158097275
chr3 158097296
chr3 158097304
chr3 158097313
chr3 158097356
chr3 158097419
chr3 158097467
chr3 158097474
chr3 158097611
chr3 158097622
chr3 158097686
chr3 158097699
chr3 158097714
chr3 158097716
chr3 158099191
chr3 158099400
chr3 158099694
chr3 158099889
chr3 158099904
chr3 158099931
chr3 158099986
chr3 158100029
chr3 158100295
chr3 158100399
chr3 158100738
chr3 158101018
chr3 158101493
chr3 158101577
chr3 158107541
chr3 158107584
chr3 158107640
chr3 158107659
chr3 158107663
chr3 158107705
chr3 158107711
chr3 158107744
chr3 158107749
chr3 158107759
chr3 158107805
chr3 158108050
chr3 158108101
chr3 158108279
chr3 158108348
chr3 158108439
chr3 158108599
chr3 158108676
chr3 158109213
chr3 158109242
chr3 158109280
chr3 158109326
chr3 158109380
chr3 158109574
chr3 158109581
chr3 158109586

chr3 158109694
chr3 158109743
chr3 158571377
chr3 158571379
chr3 158571381
chr3 158571396
chr3 158571424
chr3 158571438
chr3 158571547
chr3 158571557
chr3 158571586
chr3 158571588
chr3 158571599
chr3 158571617
chr3 158571672
chr3 158571727
chr3 158571782
chr3 158571834
chr3 158571860
chr3 158571886
chr3 158571912
chr3 158637873
chr3 158638491
chr3 158638516
chr3 158639220
chr3 158639223
chr3 158645012
chr3 158672285
chr3 158672287
chr3 158672304
chr3 158672395
chr3 158672440
chr3 158672464
chr3 158672467
chr3 158672481
chr3 158672523
chr3 158672540
chr3 158672543
chr3 158672547
chr3 158672568
chr3 158672575
chr3 158672580
chr3 158672589
chr3 158672628
chr3 158672634
chr3 158672669
chr3 158672701
chr3 158672721
chr3 158672736
chr3 158791448
chr3 158791457
chr3 158791475
chr3 158791501
chr3 158791579

chr3 158791585
chr3 158802404
chr3 158802465
chr3 159186068
chr3 159186557
chr3 159841249
chr3 159841375
chr3 159841402
chr3 159973260
chr3 159973468
chr3 159979450
chr3 160006584
chr3 160006630
chr3 160006703
chr3 160027627
chr3 160027859
chr3 160028055
chr3 160028065
chr3 160028135
chr3 160028196
chr3 160028239
chr3 160038953
chr3 160038958
chr3 160070061
chr3 160070183
chr3 160222519
chr3 160222796
chr3 160227005
chr3 160227088
chr3 160227172
chr3 160227201
chr3 160227213
chr3 160227328
chr3 160227610
chr3 160227622
chr3 160227719
chr3 160227864
chr3 160227912
chr3 160398597
chr3 160398677
chr3 160398691
chr3 160398771
chr3 160398806
chr3 160398811
chr3 160398888
chr3 160398903
chr3 160398917
chr3 160398931
chr3 160398988
chr3 160399008
chr3 160399016
chr3 160399022
chr3 160401374
chr3 160401390

chr3 160401404
chr3 160401470
chr3 160401481
chr3 160401526
chr3 160401531
chr3 160401601
chr3 160401674
chr3 160401736
chr3 160401843
chr3 160401875
chr3 160401878
chr3 160401888
chr3 160401981
chr3 160402016
chr3 160402151
chr3 160402254
chr3 160402344
chr3 160402452
chr3 160402458
chr3 160402529
chr3 160402647
chr3 160402676
chr3 160402693
chr3 160402754
chr3 160402971
chr3 160403000
chr3 160403232
chr3 160403487
chr3 160403555
chr3 160403869
chr3 160447586
chr3 160447686
chr3 160447743
chr3 160447943
chr3 160448183
chr3 160448269
chr3 160448577
chr3 160448585
chr3 160448612
chr3 160448737
chr3 160449602
chr3 160449605
chr3 160449607
chr3 160449623
chr3 160449690
chr3 160449696
chr3 160449717
chr3 160449719
chr3 160449752
chr3 160449763
chr3 160449768
chr3 160563893
chr3 160563922
chr3 160564041

chr3 160564046
chr3 160564097
chr3 160564203
chr3 160564251
chr3 160564308
chr3 160564521
chr3 160564567
chr3 160564607
chr3 160564610
chr3 160564628
chr3 160666036
chr3 160666354
chr3 160847461
chr3 160932783
chr3 160932839
chr3 161035856
chr3 161035944
chr3 161371140
chr3 161371155
chr3 161371459
chr3 161371472
chr3 161371475
chr3 161371503
chr3 161371517
chr3 161371520
chr3 161371524
chr3 161371539
chr3 161371553
chr3 161371559
chr3 161371605
chr3 161371641
chr3 161371659
chr3 161371673
chr3 161371747
chr3 161371968
chr3 161371973
chr3 162125820
chr3 162704263
chr3 162845997
chr3 162846029
chr3 162846066
chr3 162846112
chr3 162846145
chr3 162846225
chr3 162846785
chr3 162846869
chr3 162847348
chr3 163022625
chr3 163210672
chr3 163210819
chr3 163211268
chr3 163211389
chr3 163211727
chr3 163211896

chr3 163481144
chr3 163481818
chr3 163497186
chr3 163497458
chr3 163893523
chr3 163893862
chr3 163951054
chr3 163951126
chr3 163951222
chr3 163951444
chr3 163951571
chr3 164286855
chr3 164286883
chr3 164698108
chr3 164698177
chr3 164698936
chr3 164699628
chr3 164700320
chr3 164700691
chr3 164813175
chr3 164813652
chr3 164954382
chr3 164954690
chr3 164955861
chr3 165191960
chr3 165192226
chr3 165192263
chr3 165192523
chr3 165197852
chr3 165206887
chr3 165206900
chr3 165206912
chr3 165206916
chr3 165206918
chr3 165206928
chr3 165206932
chr3 165206971
chr3 165206986
chr3 165206988
chr3 165206990
chr3 165207015
chr3 165207017
chr3 165207090
chr3 165207094
chr3 165207153
chr3 165207163
chr3 165700863
chr3 165701145
chr3 165702162
chr3 165702332
chr3 165703109
chr3 165703139
chr3 165800342
chr3 165801103

chr3 165801269
chr3 165801290
chr3 166313849
chr3 166321852
chr3 166322134
chr3 166385159
chr3 166385257
chr3 166385434
chr3 166385457
chr3 166422146
chr3 166422149
chr3 166422535
chr3 166432858
chr3 166433142
chr3 166433342
chr3 166433493
chr3 166433979
chr3 166434097
chr3 166729814
chr3 166729876
chr3 166729886
chr3 166729956
chr3 166730713
chr3 166730850
chr3 166830876
chr3 166831101
chr3 166831487
chr3 166982962
chr3 166983017
chr3 166983576
chr3 167019831
chr3 167118064
chr3 167192282
chr3 167192561
chr3 167193023
chr3 167380448
chr3 167380479
chr3 167380808
chr3 167380821
chr3 167691109
chr3 167691536
chr3 167691558
chr3 167691679
chr3 167733589
chr3 167733655
chr3 167733823
chr3 167733957
chr3 167734739
chr3 167734762
chr3 167735798
chr3 167735829
chr3 167735843
chr3 167735848
chr3 167735871

chr3 167735914
chr3 167735916
chr3 167735932
chr3 167735947
chr3 167735969
chr3 167736020
chr3 167736105
chr3 167736139
chr3 167736242
chr3 167736269
chr3 167736530
chr3 168094838
chr3 168094849
chr3 168094856
chr3 168094861
chr3 168094877
chr3 168094895
chr3 168094911
chr3 168094951
chr3 168096407
chr3 168096442
chr3 168096468
chr3 168096526
chr3 168096536
chr3 168096552
chr3 168250688
chr3 168250693
chr3 168250720
chr3 168250761
chr3 168250776
chr3 168250787
chr3 168250865
chr3 168250877
chr3 168250917
chr3 168250934
chr3 168545080
chr3 168774004
chr3 168890635
chr3 168890781
chr3 168891058
chr3 168893041
chr3 168893334
chr3 169143748
chr3 169143823
chr3 169144150
chr3 169144198
chr3 169144395
chr3 169144449
chr3 169144804
chr3 169145092
chr3 169145118
chr3 169145240
chr3 169145658
chr3 169145788

chr3 169145882
chr3 169145968
chr3 169146041
chr3 169146046
chr3 169146055
chr3 169146072
chr3 169146154
chr3 169146243
chr3 169146258
chr3 169146260
chr3 169146272
chr3 169146313
chr3 169146317
chr3 169146323
chr3 169146335
chr3 169146337
chr3 169146371
chr3 169146382
chr3 169146388
chr3 169146394
chr3 169146397
chr3 169146411
chr3 169146437
chr3 169146453
chr3 169146461
chr3 169146477
chr3 169146484
chr3 169147038
chr3 169147082
chr3 169147486
chr3 169147531
chr3 169147552
chr3 169147624
chr3 169149599
chr3 169149602
chr3 169149607
chr3 169149609
chr3 169149617
chr3 169149624
chr3 169149686
chr3 169149725
chr3 169149736
chr3 169149814
chr3 169218413
chr3 169228179
chr3 169239979
chr3 169241482
chr3 169241810
chr3 169242040
chr3 169242128
chr3 169242176
chr3 169242292
chr3 169242454
chr3 169242463

chr3 169242491
chr3 169242511
chr3 169242540
chr3 169242544
chr3 169242560
chr3 169242563
chr3 169242587
chr3 169657989
chr3 169658123
chr3 169663393
chr3 169663453
chr3 169663712
chr3 169663715
chr3 169663734
chr3 169663775
chr3 169663778
chr3 169663786
chr3 169663800
chr3 169663802
chr3 169663811
chr3 169663814
chr3 169663821
chr3 169663828
chr3 169663832
chr3 169663861
chr3 169663926
chr3 169664002
chr3 169664093
chr3 169664151
chr3 169664184
chr3 169664227
chr3 169669383
chr3 169669440
chr3 169669464
chr3 169762925
chr3 169763047
chr3 169764564
chr3 169764570
chr3 169764574
chr3 169764578
chr3 169764581
chr3 169765332
chr3 169765334
chr3 169765344
chr3 169770065
chr3 169770137
chr3 169863552
chr3 169863561
chr3 169863563
chr3 169863641
chr3 169863648
chr3 169863750
chr3 169954199
chr3 169954362

chr3 169967198
chr3 169967204
chr3 169967217
chr3 169967379
chr3 169967420
chr3 169967475
chr3 169967486
chr3 169967490
chr3 169967493
chr3 169967516
chr3 169967530
chr3 169967577
chr3 169967585
chr3 169967616
chr3 169967642
chr3 169967718
chr3 170007208
chr3 170007243
chr3 170007359
chr3 170033378
chr3 170033509
chr3 170033551
chr3 170033676
chr3 170033718
chr3 170132659
chr3 170154658
chr3 170178786
chr3 170178828
chr3 170178896
chr3 170179170
chr3 170179184
chr3 170179214
chr3 170179510
chr3 170179847
chr3 170179865
chr3 170179918
chr3 170180053
chr3 170180093
chr3 170180144
chr3 170180187
chr3 170180239
chr3 170180363
chr3 170180403
chr3 170180443
chr3 170180494
chr3 170180669
chr3 170180695
chr3 170180700
chr3 170180843
chr3 170180861
chr3 170180869
chr3 170180887
chr3 170180899
chr3 170180937

chr3 170181022
chr3 170181038
chr3 170181046
chr3 170181066
chr3 170181068
chr3 170181083
chr3 170181100
chr3 170181159
chr3 170181162
chr3 170181176
chr3 170181179
chr3 170181190
chr3 170181195
chr3 170181204
chr3 170181214
chr3 170181216
chr3 170181221
chr3 170181231
chr3 170181252
chr3 170181255
chr3 170181260
chr3 170181277
chr3 170181298
chr3 170181319
chr3 170181321
chr3 170181331
chr3 170181341
chr3 170181356
chr3 170181358
chr3 170181383
chr3 170181400
chr3 170181406
chr3 170181413
chr3 170181430
chr3 170222609
chr3 170222612
chr3 170222616
chr3 170222621
chr3 170222629
chr3 170222637
chr3 170222961
chr3 170222976
chr3 170222981
chr3 170222989
chr3 170223009
chr3 170223051
chr3 170223060
chr3 170223119
chr3 170223177
chr3 170223200
chr3 170223224
chr3 170223257
chr3 170353258
chr3 170506467

chr3 170868607
chr3 170868654
chr3 170868772
chr3 170868865
chr3 170868902
chr3 170869026
chr3 170869045
chr3 170869052
chr3 170869144
chr3 170869387
chr3 170869524
chr3 170869616
chr3 170869671
chr3 170869717
chr3 170869724
chr3 170869726
chr3 170869737
chr3 170870010
chr3 170870012
chr3 170870072
chr3 170870082
chr3 170870098
chr3 170870106
chr3 170870148
chr3 170870159
chr3 170870162
chr3 170870172
chr3 170870177
chr3 170870181
chr3 170870183
chr3 170870203
chr3 170870207
chr3 170870209
chr3 170870211
chr3 170870232
chr3 170870239
chr3 170870244
chr3 170870246
chr3 170870255
chr3 170870356
chr3 170870358
chr3 170870366
chr3 170870368
chr3 170870394
chr3 170879006
chr3 170879019
chr3 170879626
chr3 170879698
chr3 170880034
chr3 170908043
chr3 170908055
chr3 170908074
chr3 170908083
chr3 170908091

chr3 170908112
chr3 170908122
chr3 170908130
chr3 170908139
chr3 170908142
chr3 170908153
chr3 170908156
chr3 170908167
chr3 170908171
chr3 170908174
chr3 170908179
chr3 170908191
chr3 170908210
chr3 170908220
chr3 170908222
chr3 170908231
chr3 170908260
chr3 170908278
chr3 170908280
chr3 170908287
chr3 170908304
chr3 170908307
chr3 170908310
chr3 170908313
chr3 170908315
chr3 170908319
chr3 170908327
chr3 170908342
chr3 170908361
chr3 170908366
chr3 170908371
chr3 170908394
chr3 170908397
chr3 170908404
chr3 170908406
chr3 170908410
chr3 170908446
chr3 170908453
chr3 170908467
chr3 170908476
chr3 170908561
chr3 170908568
chr3 170908574
chr3 170908580
chr3 170908582
chr3 170945416
chr3 170945796
chr3 170946684
chr3 171254782
chr3 171254787
chr3 171254901
chr3 171457918
chr3 171457924
chr3 171457928

chr3 171457946
chr3 171457971
chr3 171457998
chr3 171458027
chr3 171458077
chr3 171458085
chr3 171458102
chr3 171461332
chr3 171461346
chr3 171461348
chr3 171461361
chr3 171461369
chr3 171461458
chr3 171461474
chr3 171461476
chr3 171461493
chr3 171461876
chr3 171461897
chr3 171461946
chr3 171461961
chr3 171461978
chr3 171461981
chr3 171462058
chr3 171462205
chr3 171562100
chr3 171562397
chr3 171562803
chr3 171709605
chr3 171709671
chr3 171771835
chr3 171771975
chr3 171772596
chr3 171772663
chr3 171772746
chr3 171772775
chr3 172040172
chr3 172040510
chr3 172040517
chr3 172040520
chr3 172040524
chr3 172040526
chr3 172040530
chr3 172040539
chr3 172107184
chr3 172107221
chr3 172107337
chr3 172107350
chr3 172107462
chr3 172107649
chr3 172107736
chr3 172130116
chr3 172130146
chr3 172132074
chr3 172132169

chr3 172416157
chr3 172521075
chr3 172522041
chr3 172522173
chr3 172522184
chr3 172522346
chr3 172522534
chr3 172522688
chr3 172523218
chr3 172523223
chr3 172523264
chr3 172523279
chr3 172523327
chr3 172523336
chr3 172523426
chr3 172523481
chr3 172523594
chr3 172524001
chr3 172562935
chr3 172563023
chr3 172563047
chr3 172563065
chr3 172563136
chr3 172563423
chr3 172563450
chr3 172563736
chr3 172592304
chr3 172592330
chr3 172592380
chr3 172592433
chr3 172592525
chr3 172592564
chr3 172592574
chr3 172592603
chr3 172592636
chr3 172592685
chr3 172592702
chr3 172596106
chr3 172596158
chr3 172596252
chr3 172596273
chr3 172596291
chr3 172596301
chr3 172596331
chr3 172596457
chr3 172622816
chr3 172622965
chr3 172622988
chr3 172623378
chr3 172623395
chr3 172623399
chr3 172623402
chr3 172623417
chr3 172709621

chr3 172710064
chr3 172710119
chr3 172710208
chr3 172710278
chr3 172710287
chr3 172710343
chr3 172710349
chr3 172710354
chr3 172710377
chr3 172710445
chr3 172710475
chr3 172710492
chr3 172710544
chr3 172710582
chr3 172710627
chr3 172710636
chr3 172710642
chr3 172710663
chr3 172710829
chr3 172710863
chr3 172710873
chr3 172750042
chr3 172750367
chr3 172750399
chr3 172751542
chr3 172751551
chr3 172751917
chr3 173283876
chr3 173284103
chr3 173284326
chr3 173394818
chr3 173395003
chr3 173395017
chr3 173395053
chr3 173395100
chr3 173395158
chr3 173397211
chr3 173397218
chr3 173453680
chr3 173584305
chr3 173584352
chr3 173584395
chr3 173584434
chr3 173584513
chr3 173584552
chr3 173584583
chr3 173584625
chr3 173584637
chr3 173584672
chr3 173584715
chr3 173584748
chr3 173584753
chr3 173584768
chr3 173584812

chr3 173584845
chr3 173584872
chr3 173584880
chr3 173584896
chr3 173584942
chr3 173584945
chr3 173584962
chr3 173584989
chr3 173585088
chr3 173585090
chr3 173585102
chr3 173585106
chr3 173585108
chr3 173585153
chr3 173585155
chr3 173585163
chr3 173585172
chr3 173585192
chr3 173585218
chr3 173585221
chr3 173585286
chr3 173585367
chr3 173585388
chr3 173585399
chr3 173585403
chr3 173585406
chr3 173585414
chr3 173585421
chr3 174148584
chr3 174243338
chr3 174243419
chr3 174243649
chr3 174243775
chr3 174440025
chr3 174440292
chr3 174440384
chr3 174440504
chr3 174440583
chr3 174440592
chr3 174554063
chr3 174554410
chr3 174626200
chr3 174626530
chr3 174834430
chr3 174835121
chr3 175889288
chr3 176013542
chr3 176013795
chr3 176014868
chr3 176014993
chr3 176421482
chr3 176421557
chr3 176421561
chr3 176422268

chr3 176422281
chr3 176824843
chr3 176824931
chr3 176825150
chr3 177145097
chr3 177145161
chr3 177145225
chr3 177145482
chr3 177194586
chr3 177194753
chr3 177194800
chr3 177194835
chr3 177194949
chr3 177194976
chr3 177195059
chr3 177195068
chr3 177195104
chr3 177195123
chr3 177195163
chr3 177195264
chr3 177198117
chr3 177198137
chr3 177198142
chr3 177198174
chr3 177198200
chr3 177198220
chr3 177198346
chr3 177198477
chr3 177198684
chr3 177198708
chr3 177198864
chr3 177198912
chr3 177199204
chr3 177199263
chr3 177199407
chr3 177199453
chr3 177201271
chr3 177201335
chr3 177201359
chr3 177201389
chr3 177201473
chr3 177201522
chr3 177201759
chr3 177201768
chr3 177201782
chr3 177201809
chr3 177201852
chr3 177201925
chr3 177202110
chr3 177202336
chr3 177345490
chr3 177345496
chr3 177345527
chr3 177345614

chr3 177345644
chr3 177345739
chr3 177345829
chr3 177359851
chr3 177359883
chr3 177359896
chr3 177359902
chr3 177359908
chr3 177359911
chr3 177359914
chr3 177359916
chr3 177359919
chr3 177360717
chr3 177360759
chr3 177427151
chr3 177427234
chr3 177427262
chr3 177427339
chr3 177427419
chr3 177427427
chr3 177427436
chr3 177449107
chr3 177449183
chr3 177449185
chr3 177449405
chr3 177449412
chr3 177449531
chr3 177551523
chr3 177551530
chr3 177598039
chr3 177598094
chr3 177598123
chr3 177982077
chr3 178759572
chr3 178907552
chr3 179071179
chr3 179071263
chr3 179071266
chr3 179071993
chr3 179148225
chr3 179149531
chr3 179149627
chr3 179149658
chr3 179149663
chr3 179149691
chr3 179149758
chr3 179149844
chr3 179149873
chr3 179149911
chr3 179261554
chr3 179261558
chr3 179261570
chr3 179261575
chr3 179261679

chr3 179262019
chr3 179316149
chr3 179324385
chr3 179324399
chr3 179324624
chr3 179324642
chr3 179324646
chr3 179324679
chr3 179324729
chr3 179324746
chr3 179324834
chr3 179348219
chr3 179348269
chr3 179348363
chr3 179348517
chr3 179348588
chr3 179348678
chr3 179464211
chr3 179464323
chr3 179464326
chr3 179464396
chr3 179464421
chr3 179464437
chr3 179464505
chr3 179464537
chr3 179464544
chr3 179464573
chr3 179558737
chr3 179563354
chr3 179563991
chr3 179603727
chr3 179603818
chr3 179604004
chr3 179604207
chr3 179604220
chr3 179604891
chr3 179604896
chr3 179604918
chr3 179604928
chr3 179604962
chr3 179604966
chr3 179604973
chr3 179604979
chr3 179604984
chr3 179604999
chr3 179605026
chr3 179605036
chr3 179609350
chr3 179609507
chr3 179619768
chr3 179652234
chr3 179652334
chr3 179652397
chr3 179652400

chr3 179652489
chr3 179652524
chr3 179652539
chr3 179652576
chr3 179652609
chr3 179652632
chr3 179653980
chr3 179653984
chr3 179653989
chr3 179654128
chr3 179654137
chr3 179956011
chr3 179956050
chr3 179956368
chr3 179956376
chr3 179956665
chr3 179956753
chr3 179956758
chr3 179956761
chr3 179956772
chr3 179956797
chr3 179956806
chr3 179956859
chr3 179957144
chr3 179957150
chr3 180035775
chr3 180035867
chr3 180035933
chr3 180035946
chr3 180035977
chr3 180036113
chr3 180036146
chr3 180036270
chr3 180036278
chr3 180036305
chr3 180036369
chr3 180602371
chr3 180602375
chr3 180602378
chr3 180602388
chr3 180602395
chr3 180602398
chr3 180602410
chr3 180602432
chr3 180602446
chr3 180602451
chr3 180602463
chr3 180602484
chr3 180602492
chr3 180602500
chr3 180602519
chr3 180602521
chr3 180602525
chr3 180602538

chr3 180602542
chr3 180602544
chr3 180602546
chr3 180602570
chr3 180602577
chr3 180602597
chr3 180602604
chr3 180602641
chr3 180602692
chr3 180602712
chr3 180602799
chr3 180602808
chr3 180679153
chr3 180679161
chr3 180679239
chr3 180679269
chr3 180679317
chr3 180679321
chr3 180679327
chr3 180679335
chr3 180679904
chr3 180679962
chr3 180679965
chr3 180679978
chr3 180680155
chr3 180680163
chr3 180680229
chr3 180680240
chr3 180680392
chr3 180680405
chr3 180680462
chr3 180680499
chr3 180865909
chr3 180866258
chr3 180866487
chr3 180866930
chr3 180913589
chr3 180913602
chr3 180913643
chr3 180913695
chr3 180913717
chr3 180913724
chr3 180913861
chr3 180913898
chr3 180990654
chr3 181448130
chr3 181699697
chr3 181699710
chr3 181699729
chr3 181699767
chr3 181699817
chr3 181699825
chr3 181700064
chr3 181700143

chr3 181700391
chr3 181700394
chr3 181700420
chr3 181700461
chr3 181700469
chr3 181700565
chr3 181700592
chr3 181700600
chr3 181700604
chr3 181700631
chr3 181700668
chr3 181700728
chr3 181700912
chr3 181700929
chr3 181700933
chr3 181700954
chr3 181700995
chr3 181701104
chr3 181701135
chr3 181701274
chr3 181701276
chr3 181701434
chr3 181701924
chr3 181701940
chr3 181701991
chr3 181702026
chr3 181702028
chr3 181702030
chr3 181702032
chr3 181702034
chr3 181702156
chr3 181702177
chr3 181702183
chr3 181702185
chr3 181702189
chr3 181702196
chr3 181702203
chr3 181702216
chr3 181702232
chr3 181702243
chr3 181702248
chr3 181702256
chr3 181702265
chr3 181702293
chr3 181702298
chr3 181702314
chr3 181702372
chr3 181702379
chr3 181702443
chr3 181702510
chr3 181702544
chr3 181702572
chr3 181702598
chr3 181702628

chr3 181702656
chr3 181702684
chr3 181702696
chr3 181702729
chr3 181702743
chr3 181702751
chr3 181702931
chr3 181702941
chr3 181702992
chr3 181703340
chr3 181703342
chr3 181703365
chr3 181703372
chr3 181707349
chr3 181707356
chr3 181707923
chr3 181708060
chr3 181708138
chr3 181708210
chr3 181708557
chr3 181708606
chr3 181708765
chr3 181708932
chr3 181708958
chr3 181709027
chr3 181709203
chr3 181709231
chr3 181709357
chr3 181709404
chr3 181709488
chr3 181709548
chr3 181709692
chr3 181709711
chr3 181709738
chr3 181709740
chr3 181709751
chr3 181709758
chr3 181709812
chr3 181709871
chr3 181709874
chr3 181709915
chr3 181709968
chr3 181710116
chr3 181710165
chr3 181710190
chr3 181713614
chr3 181713656
chr3 181713685
chr3 181713750
chr3 181713753
chr3 181713801
chr3 181713922
chr3 181714006
chr3 181714108

chr3 181714135
chr3 181714265
chr3 181714287
chr3 181714315
chr3 181714491
chr3 181714592
chr3 181714674
chr3 181715053
chr3 181715092
chr3 181715185
chr3 181715187
chr3 181715212
chr3 181715312
chr3 181715546
chr3 181715566
chr3 181717722
chr3 181717886
chr3 181717942
chr3 181718012
chr3 181718058
chr3 181718081
chr3 181718228
chr3 181718242
chr3 181718423
chr3 181718535
chr3 181721400
chr3 181721921
chr3 181721975
chr3 181722027
chr3 181722260
chr3 181722390
chr3 181722495
chr3 181722521
chr3 181722556
chr3 181729849
chr3 181729879
chr3 181729904
chr3 181730504
chr3 181730549
chr3 181730616
chr3 181730675
chr3 181730858
chr3 181782956
chr3 182242417
chr3 182243086
chr3 182258629
chr3 182258806
chr3 182384367
chr3 182384395
chr3 182384417
chr3 182384458
chr3 182682155
chr3 182682186
chr3 182682226

chr3 182682291
chr3 182682303
chr3 182682310
chr3 182682313
chr3 182682326
chr3 182682329
chr3 182682334
chr3 182682346
chr3 182682348
chr3 182682355
chr3 182682358
chr3 182682367
chr3 182682389
chr3 182682391
chr3 182682401
chr3 182682406
chr3 182682409
chr3 182682426
chr3 182683399
chr3 182683622
chr3 182683706
chr3 182683731
chr3 182683810
chr3 182683857
chr3 182795546
chr3 182795567
chr3 182795876
chr3 182795983
chr3 182795997
chr3 182914061
chr3 182968071
chr3 182968105
chr3 182968132
chr3 182968136
chr3 183099033
chr3 183099042
chr3 183099085
chr3 183099135
chr3 183099145
chr3 183099151
chr3 183099190
chr3 183099193
chr3 183099196
chr3 183099205
chr3 183099219
chr3 183099243
chr3 183099257
chr3 183099270
chr3 183099273
chr3 183099354
chr3 183099357
chr3 183099372
chr3 183099626
chr3 183099638

chr3 183099641
chr3 183099660
chr3 183099666
chr3 183099691
chr3 183099777
chr3 183099790
chr3 183099796
chr3 183099838
chr3 183235340
chr3 183285993
chr3 183285998
chr3 183286010
chr3 183286027
chr3 183286071
chr3 183404341
chr3 183447636
chr3 183447642
chr3 183447648
chr3 183447662
chr3 183447666
chr3 183447671
chr3 183447684
chr3 183447699
chr3 183447714
chr3 183447734
chr3 183447750
chr3 183447770
chr3 183447783
chr3 183447785
chr3 183447793
chr3 183447798
chr3 183447806
chr3 183447831
chr3 183447838
chr3 183447842
chr3 183447856
chr3 183447858
chr3 183447862
chr3 183447999
chr3 183554219
chr3 183554244
chr3 183554371
chr3 183554539
chr3 183554886
chr3 183554890
chr3 183554895
chr3 183554929
chr3 183554935
chr3 183554954
chr3 183555020
chr3 183555057
chr3 183555079
chr3 183555105
chr3 183555113

chr3 183555121
chr3 183555160
chr3 183555380
chr3 183555392
chr3 183555394
chr3 183555446
chr3 183555457
chr3 183555498
chr3 183555500
chr3 183555503
chr3 183698733
chr3 183698753
chr3 183698772
chr3 183698882
chr3 184017661
chr3 184017664
chr3 184017690
chr3 184017693
chr3 184017718
chr3 184134087
chr3 184134092
chr3 184134102
chr3 184134271
chr3 184134291
chr3 184134304
chr3 184134343
chr3 184134361
chr3 184134369
chr3 184134379
chr3 184134385
chr3 184134456
chr3 184134481
chr3 184134485
chr3 184134541
chr3 184135900
chr3 184135906
chr3 184135928
chr3 184135950
chr3 184135972
chr3 184136027
chr3 184136083
chr3 184136122
chr3 184136188
chr3 184136225
chr3 184156469
chr3 184156506
chr3 184156801
chr3 184156816
chr3 184174948
chr3 184174966
chr3 184174985
chr3 184174997
chr3 184175002
chr3 184175007

chr3 184314430
chr3 184314442
chr3 184314448
chr3 184314464
chr3 184314469
chr3 184314474
chr3 184314495
chr3 184314498
chr3 184314501
chr3 184314508
chr3 184314512
chr3 184314515
chr3 184314517
chr3 184314523
chr3 184314532
chr3 184314535
chr3 184314541
chr3 184314549
chr3 184314552
chr3 184314558
chr3 184314564
chr3 184314572
chr3 184314579
chr3 184314582
chr3 184314586
chr3 184525145
chr3 184525158
chr3 184525174
chr3 184525248
chr3 184525298
chr3 184525302
chr3 184525327
chr3 184525344
chr3 184525346
chr3 184525356
chr3 184525359
chr3 184525376
chr3 184525399
chr3 184525412
chr3 184525423
chr3 184525430
chr3 184544088
chr3 184544116
chr3 184544120
chr3 184544122
chr3 184544197
chr3 184544219
chr3 184544237
chr3 184544251
chr3 184544269
chr3 184544359
chr3 184544463
chr3 184711409
chr3 184711445

chr3 184711458
chr3 184711467
chr3 184711477
chr3 184711506
chr3 184711522
chr3 184711526
chr3 184711579
chr3 184711585
chr3 184711589
chr3 184711598
chr3 184711601
chr3 184711613
chr3 184711633
chr3 184711645
chr3 184711647
chr3 184712498
chr3 184712665
chr3 184712689
chr3 184712845
chr3 185216090
chr3 185253973
chr3 185253979
chr3 185253987
chr3 185253995
chr3 185254006
chr3 185254008
chr3 185254016
chr3 185254037
chr3 185254056
chr3 185254059
chr3 185254079
chr3 185254083
chr3 185254085
chr3 185254089
chr3 185254110
chr3 185254127
chr3 185254132
chr3 185254137
chr3 185254140
chr3 185254147
chr3 185254150
chr3 185254152
chr3 185254180
chr3 185254192
chr3 185254195
chr3 185254206
chr3 185254213
chr3 185254292
chr3 185254300
chr3 185254357
chr3 185254365
chr3 185254383
chr3 185254385
chr3 185354424

chr3 185354438
chr3 185363028
chr3 185363117
chr3 185363152
chr3 185363163
chr3 185363209
chr3 185363235
chr3 185363362
chr3 185363473
chr3 185363479
chr3 185363504
chr3 185363516
chr3 185363524
chr3 185363586
chr3 185363605
chr3 185363615
chr3 185363623
chr3 185363728
chr3 185363735
chr3 185363781
chr3 185363785
chr3 185363797
chr3 185363853
chr3 185363911
chr3 185364004
chr3 185537485
chr3 185537703
chr3 185575666
chr3 185575706
chr3 185575729
chr3 185575750
chr3 185575841
chr3 185575845
chr3 185586348
chr3 185586351
chr3 185586354
chr3 185586372
chr3 185586377
chr3 185586387
chr3 185586391
chr3 185586394
chr3 185773834
chr3 185822900
chr3 185822922
chr3 185822930
chr3 185823073
chr3 185823094
chr3 185823109
chr3 185823121
chr3 185823261
chr3 185823263
chr3 185823282
chr3 185823286
chr3 185823296

chr3 185823300
chr3 185823307
chr3 185823313
chr3 185823317
chr3 185823339
chr3 185823344
chr3 185823353
chr3 185823584
chr3 185823591
chr3 185823603
chr3 185823630
chr3 185823639
chr3 185823647
chr3 185823669
chr3 185823672
chr3 185823677
chr3 185823696
chr3 185823720
chr3 185823723
chr3 185823726
chr3 185823731
chr3 185823746
chr3 185823749
chr3 185823767
chr3 185823770
chr3 185823775
chr3 185823782
chr3 185823789
chr3 185823792
chr3 185823796
chr3 185823803
chr3 185823811
chr3 185823813
chr3 185823815
chr3 185823825
chr3 185824214
chr3 185824217
chr3 185824242
chr3 185824249
chr3 185824364
chr3 185824437
chr3 185824455
chr3 185928531
chr3 185929158
chr3 185929163
chr3 185929991
chr3 185930019
chr3 185930102
chr3 185931821
chr3 185931828
chr3 185932095
chr3 185932250
chr3 185932277
chr3 185932333

chr3 185932342
chr3 185932386
chr3 185932919
chr3 185977361
chr3 185977578
chr3 185977608
chr3 185977622
chr3 185977894
chr3 185977939
chr3 185977972
chr3 186068315
chr3 186068520
chr3 186068616
chr3 186361286
chr3 186361298
chr3 186361319
chr3 186361325
chr3 186519183
chr3 186540522
chr3 186540659
chr3 186540664
chr3 186540686
chr3 186540721
chr3 186540725
chr3 186570806
chr3 186570948
chr3 186570953
chr3 186570963
chr3 186570982
chr3 186570991
chr3 186571058
chr3 186571076
chr3 186571089
chr3 186571097
chr3 186571144
chr3 186571273
chr3 186571291
chr3 186571371
chr3 186571447
chr3 186784934
chr3 186785134
chr3 186785312
chr3 186785317
chr3 186785415
chr3 186785422
chr3 186785598
chr3 186785635
chr3 186785662
chr3 186785738
chr3 186785854
chr3 186785958
chr3 186786014
chr3 186786574
chr3 186787159

chr3 186832651
chr3 186832758
chr3 186871528
chr3 186882349
chr3 186882507
chr3 186882649
chr3 187028019
chr3 187101928
chr3 187101992
chr3 187102010
chr3 187130414
chr3 187130447
chr3 187130450
chr3 187130544
chr3 187130884
chr3 187368399
chr3 187368505
chr3 187368518
chr3 187368742
chr3 187368755
chr3 187368816
chr3 187368886
chr3 187369003
chr3 187369298
chr3 187534535
chr3 187562512
chr3 187563460
chr3 187679559
chr3 187679576
chr3 187679620
chr3 187733037
chr3 187737678
chr3 187737683
chr3 187737728
chr3 187737783
chr3 187737786
chr3 187737833
chr3 187737838
chr3 187737850
chr3 187737858
chr3 187737889
chr3 187737924
chr3 187737930
chr3 187737937
chr3 187737946
chr3 187737955
chr3 187737960
chr3 187737967
chr3 187737970
chr3 187737990
chr3 187737996
chr3 187738010
chr3 187738012
chr3 187738045

chr3 187738049
chr3 187738051
chr3 187738054
chr3 187738176
chr3 187738287
chr3 187738343
chr3 187738405
chr3 187738430
chr3 187738463
chr3 187738475
chr3 187738481
chr3 187738490
chr3 187738496
chr3 187738517
chr3 187738527
chr3 187738535
chr3 187739026
chr3 187740245
chr3 187740253
chr3 187740295
chr3 187740307
chr3 187740314
chr3 187740358
chr3 187740368
chr3 187740375
chr3 187740410
chr3 187740421
chr3 187740476
chr3 187740539
chr3 187740581
chr3 187740670
chr3 187740673
chr3 187740708
chr3 187742889
chr3 187742935
chr3 187743014
chr3 187743103
chr3 187743112
chr3 187743254
chr3 187743320
chr3 187743335
chr3 187743342
chr3 187743345
chr3 187743356
chr3 187743358
chr3 187743494
chr3 187743544
chr3 187743615
chr3 187743634
chr3 187743651
chr3 187743681
chr3 187743694
chr3 187746056
chr3 187746083

chr3 187746246
chr3 187746261
chr3 187746267
chr3 187746285
chr3 187746318
chr3 187746593
chr3 187918852
chr3 187918906
chr3 187919082
chr3 187919216
chr3 187919230
chr3 187919253
chr3 187919261
chr3 187919308
chr3 187919504
chr3 187919678
chr3 187919879
chr3 187919899
chr3 187920020
chr3 187935864
chr3 187935882
chr3 187935916
chr3 187935939
chr3 187936076
chr3 187936079
chr3 187936085
chr3 187959080
chr3 188050357
chr3 188148469
chr3 188309777
chr3 188309844
chr3 188788633
chr3 188788764
chr3 188788786
chr3 188788794
chr3 189021360
chr3 189021370
chr3 189021378
chr3 189021393
chr3 189021444
chr3 189021591
chr3 189021726
chr3 189021744
chr3 189021770
chr3 189021877
chr3 189021926
chr3 189022019
chr3 189022186
chr3 189022207
chr3 189022232
chr3 189022263
chr3 189022282
chr3 189132348
chr3 189132361

chr3 189132371
chr3 189132373
chr3 189132403
chr3 189132420
chr3 189132557
chr3 189132561
chr3 189132573
chr3 189132687
chr3 189132716
chr3 189331295
chr3 189630278
chr3 189630484
chr3 189630553
chr3 189630750
chr3 189961379
chr3 189961467
chr3 189961534
chr3 189961570
chr3 189961630
chr3 189961650
chr3 189961734
chr3 189961739
chr3 189961790
chr3 189961796
chr3 190001828
chr3 190001990
chr3 190035617
chr3 190035819
chr3 190036482
chr3 190036505
chr3 190036787
chr3 190036930
chr3 190115269
chr3 190175829
chr3 190201753
chr3 190201760
chr3 190201777
chr3 190201783
chr3 190201785
chr3 190201807
chr3 190201815
chr3 190201823
chr3 190201928
chr3 190202057
chr3 190202068
chr3 190202182
chr3 190423618
chr3 190424255
chr3 190424262
chr3 190446945
chr3 190446962
chr3 190474764
chr3 190514749
chr3 190514769

chr3 190514771
chr3 190514785
chr3 190514792
chr3 190514796
chr3 190514834
chr3 190514843
chr3 190514992
chr3 190515066
chr3 190515071
chr3 190515073
chr3 190515201
chr3 190863443
chr3 190884442
chr3 190884478
chr3 190884602
chr3 190884846
chr3 190964295
chr3 190968056
chr3 191054350
chr3 191054561
chr3 191123710
chr3 191124151
chr3 191124225
chr3 191142470
chr3 191143404
chr3 191143740
chr3 191170057
chr3 191170232
chr3 191263854
chr3 191410902
chr3 191411646
chr3 191411782
chr3 191411962
chr3 191617395
chr3 191631420
chr3 191657555
chr3 192382913
chr3 192382965
chr3 192383840
chr3 192513651
chr3 192513717
chr3 192513927
chr3 192628089
chr3 192709155
chr3 193447044
chr3 193461284
chr3 193461544
chr3 193461755
chr3 193461791
chr3 193461799
chr3 193461817
chr3 193461827
chr3 193518671
chr3 193593062

chr3 193593070
chr3 193593079
chr3 193593097
chr3 193593103
chr3 193593106
chr3 193593115
chr3 193593121
chr3 193593132
chr3 193593134
chr3 193593139
chr3 193593143
chr3 193593152
chr3 193593180
chr3 193593185
chr3 193593196
chr3 193593217
chr3 193593229
chr3 193593246
chr3 193593250
chr3 193593252
chr3 193593302
chr3 193593309
chr3 193593325
chr3 193593335
chr3 193593337
chr3 193593343
chr3 193593348
chr3 193593351
chr3 193593357
chr3 193593368
chr3 193593371
chr3 193593374
chr3 193593384
chr3 193593390
chr3 193593393
chr3 193593398
chr3 193593435
chr3 193593514
chr3 193593517
chr3 193593582
chr3 193593586
chr3 193593592
chr3 193593663
chr3 193594043
chr3 193594049
chr3 193594138
chr3 193735831
chr3 193735842
chr3 194058528
chr3 194058535
chr3 194058540
chr3 194058547
chr3 194058578
chr3 194058597

chr3 194058619
chr3 194058643
chr3 194058658
chr3 194071104
chr3 194071115
chr3 194071123
chr3 194071125
chr3 194071128
chr3 194071132
chr3 194071134
chr3 194071140
chr3 194071144
chr3 194071152
chr3 194071155
chr3 194071157
chr3 194071159
chr3 194071163
chr3 194071175
chr3 194071179
chr3 194071186
chr3 194135326
chr3 194135328
chr3 194135350
chr3 194135386
chr3 194135437
chr3 194135462
chr3 194135480
chr3 194135515
chr3 194135636
chr3 194135654
chr3 194135676
chr3 194135692
chr3 194135858
chr3 194135931
chr3 194135943
chr3 194135961
chr3 194135963
chr3 194135965
chr3 194135975
chr3 194135994
chr3 194136009
chr3 194136011
chr3 194136018
chr3 194136022
chr3 194136028
chr3 194136030
chr3 194136092
chr3 194136097
chr3 194136101
chr3 194136103
chr3 194136130
chr3 194136158
chr3 194136179
chr3 194136189

chr3 194136219
chr3 194136325
chr3 194136412
chr3 194136418
chr3 194136448
chr3 194136454
chr3 194136496
chr3 194136568
chr3 194136644
chr3 194136733
chr3 194255246
chr3 194255397
chr3 194255428
chr3 194255433
chr3 194255525
chr3 194255536
chr3 194255553
chr3 194255575
chr3 194255594
chr3 194255607
chr3 194255641
chr3 194255665
chr3 194255725
chr3 194255732
chr3 194255738
chr3 194255743
chr3 194306356
chr3 194306420
chr3 194306443
chr3 194306564
chr3 194306612
chr3 194306639
chr3 194306656
chr3 194306661
chr3 194306703
chr3 194306830
chr3 194306901
chr3 194306905
chr3 194306936
chr3 194307028
chr3 194307096
chr3 194307254
chr3 194307418
chr3 194307441
chr3 194307451
chr3 194485663
chr3 194485697
chr3 194485768
chr3 194485885
chr3 194485922
chr3 194486032
chr3 194486044
chr3 194486103
chr3 194487041

chr3 194487043
chr3 194487045
chr3 194487052
chr3 194487060
chr3 194487062
chr3 194487066
chr3 194487069
chr3 194487184
chr3 194487190
chr3 194487200
chr3 194487215
chr3 194487234
chr3 194583925
chr3 194583927
chr3 194583936
chr3 194583943
chr3 194583945
chr3 194583952
chr3 194583961
chr3 194671395
chr3 194671438
chr3 194671472
chr3 194671496
chr3 194671634
chr3 194769319
chr3 194769380
chr3 194769431
chr3 194769433
chr3 194769437
chr3 194769442
chr3 194769453
chr3 194769455
chr3 194769490
chr3 194769585
chr3 194769594
chr3 194769656
chr3 194769680
chr3 194769757
chr3 194810803
chr3 194893362
chr3 194893370
chr3 194893832
chr3 195088386
chr3 195088416
chr3 195088434
chr3 195088638
chr3 195194149
chr3 195194216
chr3 195194657
chr3 195194679
chr3 195440796
chr3 195441323
chr3 195441398
chr3 195441489

chr3 195441498
chr3 195441554
chr3 195441659
chr3 195441671
chr3 195441713
chr3 195441779
chr3 195441861
chr3 195441872
chr3 195441876
chr3 195441913
chr3 195458581
chr3 195458616
chr3 195459072
chr3 195540708
chr3 195540777
chr3 195540828
chr3 195541064
chr3 195541232
chr3 195541420
chr3 195541576
chr3 195541588
chr3 195541708
chr3 195541774
chr3 195541825
chr3 195542074
chr3 195542086
chr3 195542132
chr3 195542142
chr3 195542191
chr3 195542196
chr3 195550269
chr3 195550293
chr3 195550375
chr3 195550461
chr3 195550474
chr3 195550827
chr3 195624344
chr3 195624352
chr3 195624640
chr3 195624684
chr3 195624686
chr3 195624701
chr3 195624897
chr3 195624912
chr3 195624916
chr3 195624935
chr3 195624946
chr3 195624961
chr3 195624989
chr3 195625008
chr3 195625018
chr3 195625020
chr3 195625027
chr3 195625030

chr3 195625048
chr3 195625080
chr3 195625085
chr3 195625089
chr3 195625111
chr3 195625138
chr3 195625151
chr3 195625172
chr3 195625187
chr3 195625191
chr3 195625195
chr3 195625210
chr3 195625220
chr3 195625224
chr3 195625240
chr3 195625261
chr3 195625279
chr3 195625297
chr3 195625362
chr3 195625395
chr3 195625419
chr3 195625442
chr3 195625502
chr3 195625509
chr3 195634025
chr3 195634044
chr3 195634073
chr3 195634085
chr3 195634096
chr3 195634118
chr3 195634122
chr3 195634129
chr3 195634131
chr3 195634144
chr3 195634146
chr3 195634152
chr3 195634188
chr3 195634200
chr3 195634207
chr3 195634228
chr3 195634230
chr3 195634274
chr3 195634322
chr3 195634339
chr3 195634346
chr3 195634373
chr3 195634378
chr3 195634398
chr3 195634402
chr3 195634410
chr3 195634414
chr3 195634472
chr3 195634498
chr3 195634504

chr3 195634530
chr3 195634532
chr3 195634542
chr3 195634548
chr3 195634588
chr3 195634598
chr3 195634620
chr3 195634630
chr3 195634636
chr3 195634644
chr3 195634790
chr3 195634794
chr3 195634800
chr3 195634812
chr3 195634837
chr3 195634839
chr3 195634844
chr3 195634857
chr3 195634867
chr3 195634882
chr3 195634920
chr3 195634949
chr3 195634954
chr3 195634956
chr3 195634959
chr3 195634961
chr3 195635481
chr3 195635485
chr3 195635503
chr3 195635510
chr3 195635512
chr3 195635516
chr3 195635532
chr3 195635541
chr3 195635543
chr3 195635553
chr3 195635557
chr3 195635564
chr3 195635578
chr3 195635595
chr3 195635611
chr3 195635614
chr3 195635618
chr3 195635639
chr3 195635653
chr3 195635659
chr3 195635661
chr3 195635668
chr3 195635687
chr3 195635694
chr3 195635699
chr3 195635704
chr3 195635707
chr3 195635835

chr3 195635838
chr3 195635855
chr3 195635878
chr3 195653549
chr3 195653695
chr3 195653773
chr3 195653791
chr3 195653796
chr3 195653824
chr3 195653836
chr3 195653862
chr3 195653893
chr3 195653908
chr3 195653918
chr3 195653946
chr3 195654010
chr3 195654024
chr3 195654036
chr3 195654068
chr3 195658455
chr3 195658458
chr3 195658464
chr3 195658466
chr3 195658484
chr3 195658497
chr3 195658507
chr3 195658518
chr3 195658537
chr3 195658559
chr3 195658570
chr3 195658593
chr3 195658599
chr3 195658603
chr3 195658610
chr3 195658622
chr3 195658625
chr3 195658630
chr3 195658640
chr3 195658654
chr3 195658665
chr3 195658671
chr3 195658675
chr3 195658687
chr3 195658697
chr3 195658699
chr3 195658702
chr3 195658704
chr3 195658708
chr3 195658929
chr3 195658941
chr3 195658948
chr3 195658954
chr3 195659030
chr3 195659048

chr3 195659076
chr3 195776440
chr3 195776455
chr3 195776485
chr3 195776530
chr3 195776560
chr3 195776817
chr3 195786908
chr3 195786911
chr3 195787119
chr3 195787199
chr3 195787292
chr3 195787295
chr3 195787316
chr3 195787343
chr3 195787364
chr3 195787407
chr3 195787532
chr3 195787535
chr3 195787700
chr3 195787743
chr3 195787775
chr3 195895087
chr3 195895109
chr3 195895156
chr3 195895159
chr3 195895180
chr3 195895194
chr3 195895274
chr3 195895317
chr3 195895323
chr3 195895342
chr3 195895346
chr3 195895355
chr3 195895360
chr3 195895365
chr3 195895371
chr3 195895377
chr3 195895390
chr3 195895403
chr3 195895410
chr3 195895435
chr3 195895443
chr3 195895467
chr3 195895473
chr3 195907162
chr3 195907214
chr3 195907224
chr3 195907247
chr3 195907272
chr3 195907274
chr3 195907301
chr3 195907324
chr3 195907333

chr3 195907403
chr3 195907444
chr3 195907557
chr3 195907593
chr3 195907613
chr3 195907618
chr3 195907647
chr3 195907759
chr3 195907838
chr3 195907849
chr3 195953934
chr3 195953937
chr3 195953942
chr3 195953947
chr3 195953954
chr3 195953973
chr3 195953980
chr3 195953982
chr3 195953988
chr3 195954002
chr3 195954023
chr3 195954027
chr3 195954030
chr3 195954046
chr3 195954063
chr3 195954077
chr3 195954084
chr3 195954129
chr3 195954131
chr3 195954138
chr3 195954156
chr3 195954160
chr3 195954162
chr3 195954165
chr3 195954169
chr3 195954181
chr3 195954187
chr3 195954191
chr3 195954193
chr3 195954201
chr3 195954597
chr3 195955004
chr3 195955010
chr3 195955020
chr3 195989480
chr3 195989520
chr3 195989552
chr3 196081004
chr3 196081045
chr3 196081052
chr3 196081225
chr3 196081902
chr3 196081912
chr3 196081964

chr3 196099856
chr3 196099886
chr3 196099916
chr3 196099946
chr3 196099976
chr3 196179565
chr3 196179614
chr3 196179665
chr3 196179755
chr3 196179912
chr3 196179922
chr3 196179976
chr3 196180096
chr3 196431440
chr3 196431455
chr3 196431466
chr3 196431505
chr3 196431514
chr3 196431587
chr3 196431593
chr3 196431599
chr3 196431633
chr3 196469145
chr3 196469278
chr3 196501221
chr3 196501284
chr3 196501289
chr3 196501306
chr3 196501366
chr3 196501390
chr3 196501441
chr3 196501550
chr3 196501591
chr3 196501738
chr3 196501805
chr3 196501843
chr3 196501855
chr3 196501880
chr3 196501886
chr3 196501894
chr3 196503629
chr3 196503637
chr3 196503646
chr3 196503648
chr3 196503650
chr3 196503657
chr3 196503670
chr3 196503674
chr3 196503681
chr3 196503699
chr3 196503701
chr3 196503707
chr3 196503718
chr3 196609080

chr3 196609304
chr3 196609433
chr3 196609470
chr3 196609598
chr3 196609649
chr3 196609740
chr3 196632134
chr3 196632250
chr3 196632289
chr3 196632331
chr3 196632336
chr3 196632360
chr3 196632371
chr3 196632390
chr3 196632408
chr3 196632415
chr3 196632427
chr3 196632433
chr3 196632437
chr3 196632444
chr3 196632641
chr3 196632644
chr3 196632646
chr3 196640344
chr3 196640416
chr3 196640422
chr3 196640491
chr3 196640584
chr3 196640587
chr3 196640595
chr3 196640599
chr3 196640607
chr3 196640616
chr3 196640636
chr3 196640655
chr3 196640667
chr3 196640684
chr3 196640743
chr3 196640761
chr3 196640775
chr3 196640780
chr3 196640806
chr3 196640820
chr3 196640888
chr3 196640919
chr3 196640961
chr3 196641024
chr3 196641070
chr3 196641276
chr3 196685009
chr3 196685099
chr3 196685141
chr3 196685168
chr3 196685170

chr3 196685174
chr3 196685179
chr3 196711858
chr3 196711879
chr3 196711897
chr3 196711954
chr3 196711968
chr3 196727415
chr3 196727457
chr3 197297018
chr3 197297078
chr3 197297105
chr3 197297116
chr3 197297138
chr3 197297156
chr3 197297170
chr3 197297194
chr3 197297199
chr3 197297251
chr3 197297396
chr3 197297452
chr3 197463616
chr3 197517613
chr3 197517666
chr3 197517770
chr3 197518148
chr3 197518152
chr3 197518311
chr3 197518365
chr3 197518419
chr3 197518428
chr3 197518482
chr3 197518527
chr3 197518536
chr3 197518634
chr3 197518740
chr3 197518795
chr3 197518849
chr3 197626569
chr3 197626572
chr3 197627044
chr3 197627048
chr3 197627069
chr3 197627109
chr3 197627179
chr3 197627202
chr3 197627300
chr3 197627304
chr3 197627314
chr3 197627319
chr3 197627334
chr3 197627341
chr3 197627345
chr3 197627351

chr3 197632027
chr3 197632052
chr3 197632092
chr3 197632097
chr3 197632123
chr3 197632135
chr3 197632163
chr3 197632168
chr3 197632186
chr3 197632275
chr3 197632410
chr3 197632431
chr3 197654762
chr3 197654767
chr3 197654780
chr3 197654796
chr3 197654798
chr3 197654800
chr3 197654820
chr3 197654826
chr3 197654830
chr3 197654841
chr3 197655060
chr3 197655075
chr3 197655085
chr3 197655098
chr3 197655103
chr3 197655105
chr3 197655130
chr3 197655142
chr3 197655148
chr3 197655152
chr3 197655175
chr3 197655192
chr3 197655233
chr3 197655237
chr3 197655267
chr3 197655279
chr3 197655288
chr3 197655298
chr3 197655306
chr3 197655312
chr3 197655322
chr3 197655344
chr3 197655354
chr3 197655391
chr3 197655397
chr3 197655407
chr3 197655409
chr3 197655435
chr3 197655441
chr3 197655525
chr3 197655529
chr3 197655537

chr3 197655541
chr3 197655561
chr3 197655566
chr3 197655593
chr3 197655600
chr3 197655617
chr3 197655665
chr3 197655714
chr3 197664670
chr3 197664693
chr3 197664717
chr3 197664811
chr3 197664829
chr3 197664847
chr3 197664868
chr3 197664884
chr3 197664888
chr3 197664898
chr3 197664913
chr3 197664917
chr3 197664921
chr3 197664936
chr3 197664957
chr3 197664970
chr3 197664997
chr3 197665019
chr3 197665023
chr3 197665028
chr3 197665060
chr3 197665078
chr3 197665081
chr3 197665088
chr3 197665090
chr3 197665100
chr3 197665119
chr3 197665147
chr3 197665162
chr3 197665173
chr3 197665192
chr3 197665196
chr3 197665520
chr3 197665549
chr3 197665555
chr3 197665559
chr3 197665574
chr3 197665597
chr3 197665601
chr3 197711795
chr3 197711935
chr3 197712303
chr3 197712371
chr3 197712438
chr3 197712463
chr3 197712472

chr3 197712527
chr3 197712545
chr3 197712598
chr3 197712603
chr3 197712889
chr3 197751050
chr3 197751083
chr3 197751088
chr3 197751105
chr3 197751143
chr3 197751239
chr3 197751366
chr3 197751368
chr3 197751378
chr3 197791649
chr3 197791655
chr3 197791661
chr3 197791668
chr3 197791679
chr3 197791682
chr3 197791685
chr3 197791696
chr3 197791704
chr3 197791730
chr3 197791733
chr3 197791737
chr3 197791740
chr3 197791763
chr3 197791818
chr3 197791971
chr3 197791994
chr3 197792013
chr3 197792032
chr3 197793394
chr3 197793587
chr3 197793772
chr3 197793892
chr3 197793971
chr3 197848214
chr3 197848333
chr3 197848376
chr3 197848477
chr3 197848496
chr3 197848575
chr3 197878561
chr3 197878608
chr3 197878715
chr3 197878725
chr3 197906223
chr3 197906243
chr3 197906247
chr3 197906265
chr3 197906279
chr3 197906281

chr3 197906308
chr3 197906463
chr3 197906563
chr3 197906582
chr3 197906840
chr3 197921734
chr3 197921739
chr3 197921790
chr3 197921795
chr3 197921851
chr3 197921907
chr3 197921989
chr3 197922014
chr3 197922019
chr3 197950656
chr3 198090649
chr3 198099139
chr3 198099189
chr3 198099191
chr3 198099239
chr3 198099241
chr3 198099263
chr3 198099289
chr3 198099313
chr3 198099315
chr3 198099339
chr3 198099341
chr3 198099363
chr3 198099365
chr3 198099413
chr3 198099415
chr3 198099439
chr3 198099539
chr3 198099541
chr3 198099589
chr3 198099591
chr3 198099615
chr3 198099737
chr3 198099756
chr3 198099787
chr3 198099789
chr3 198099837
chr3 198099839
chr3 198099856
chr3 198099887
chr3 198099937
chr3 198099939
chr3 198099956
chr3 198099987
chr3 198099989
chr3 198101206
chr3 198101237
chr3 198101239
chr3 198101287

chr3 198101289
chr3 198101337
chr3 198101339
chr3 198101387
chr3 198101406
chr3 198101437
chr3 198101439
chr3 198101487
chr3 198101489
chr3 198101537
chr3 198101539
chr3 198101561
chr3 198101563
chr3 198101587
chr3 198101589
chr3 198101637
chr3 198101687
chr3 198101689
chr3 198101861
chr3 198101887
chr3 198101911
chr3 198101913
chr3 198101937
chr3 198101956
chr3 198101987
chr3 198101989
chr3 198102037
chr3 198102056
chr3 198102087
chr3 198102089
chr3 198102106
chr3 198102137
chr3 198102139
chr3 198102187
chr3 198102206
chr3 198102237
chr3 198102239
chr3 198102256
chr3 198102287
chr3 198102306
chr3 198102337
chr3 198102339
chr3 198102387
chr3 198102389
chr3 198102437
chr3 198102439
chr3 198102461
chr3 198102463
chr3 198102487
chr3 198102489
chr3 198102537
chr3 198102539
chr3 198102587
chr3 198102589

chr3 198102637
chr3 198102687
chr3 198102689
chr3 198102737
chr3 198102887
chr3 198102937
chr3 198102939
chr3 198102963
chr3 198102987
chr3 198102989
chr3 198103037
chr3 198103039
chr3 198103087
chr3 198103089
chr3 198103137
chr3 198103237
chr3 198103337
chr3 198103339
chr3 198103387
chr3 198103437
chr3 198103439
chr3 198103463
chr3 198103537
chr3 198103539
chr3 198103561
chr3 198108074
chr3 198108150
chr3 198108178
chr3 198142321
chr3 198157306
chr3 198157319
chr3 198157323
chr3 198157325
chr3 198167099
chr3 198167235
chr3 198167393
chr3 198167709
chr3 198167712
chr3 198167732
chr3 198167751
chr3 198167764
chr3 198167766
chr3 198167768
chr3 198167774
chr3 198167791
chr3 198167811
chr3 198167894
chr14 16000423
chr14 16001155
chr14 16003495
chr14 16004543
chr14 16004557
chr14 16005394
chr14 16006384

chr14 16006480
chr14 16007397
chr14 16007439
chr14 16008372
chr14 16009944
chr14 16010521
chr14 16010558
chr14 16010827
chr14 16010851
chr14 16011544
chr14 16013036
chr14 16013078
chr14 16020085
chr14 16020509
chr14 16020969
chr14 16040582
chr14 16040586
chr14 16040598
chr14 16041131
chr14 16041605
chr14 16041628
chr14 16041641
chr14 16041658
chr14 16041763
chr14 16041778
chr14 16041785
chr14 16041798
chr14 16041820
chr14 16041845
chr14 16041849
chr14 16041851
chr14 16041861
chr14 16041889
chr14 16041894
chr14 16042195
chr14 16042239
chr14 16042249
chr14 16042265
chr14 16042274
chr14 16042420
chr14 16042474
chr14 16042479
chr14 16042498
chr14 16042559
chr14 16042589
chr14 16042611
chr14 16042641
chr14 16042644
chr14 16042676
chr14 16042730
chr14 16042760
chr14 16042780
chr14 16042792
chr14 16042796

chr14 16042910
chr14 16042916
chr14 16043777
chr14 16043848
chr14 16043905
chr14 16044038
chr14 16063185
chr14 16063327
chr14 16063933
chr14 16064274
chr14 16064869
chr14 16064887
chr14 16065473
chr14 16066675
chr14 16067189
chr14 16067316
chr14 16069043
chr14 16069099
chr14 16069174
chr14 16069347
chr14 16069807
chr14 16070723
chr14 16073052
chr14 16073658
chr14 16074172
chr14 16074329
chr14 16074781
chr14 16074829
chr14 16075221
chr14 16075784
chr14 16075891
chr14 16076081
chr14 16076093
chr14 16076312
chr14 16076779
chr14 16076801
chr14 16078797
chr14 16078841
chr14 16079044
chr14 16079136
chr14 16082261
chr14 16083288
chr14 16083523
chr14 16085322
chr14 16085341
chr14 16113592
chr14 16113986
chr14 16113989
chr14 16114456
chr14 16114532
chr14 16115902
chr14 16116076
chr14 16118858
chr14 16119164

chr14 16119426
chr14 16119507
chr14 16120307
chr14 16120796
chr14 16121061
chr14 16121144
chr14 16122046
chr14 16122265
chr14 16125774
chr14 16129279
chr14 16129426
chr14 16130135
chr14 16130182
chr14 16130338
chr14 16130347
chr14 16130387
chr14 16133417
chr14 16133739
chr14 16133786
chr14 16134182
chr14 16135046
chr14 16135594
chr14 16135644
chr14 16136072
chr14 16136570
chr14 16136572
chr14 16137928
chr14 16138711
chr14 16139021
chr14 16139118
chr14 16139186
chr14 16139310
chr14 16139381
chr14 16142489
chr14 16142783
chr14 16142827
chr14 16143296
chr14 16144328
chr14 16144335
chr14 16144747
chr14 16145512
chr14 16150624
chr14 16150672
chr14 16151099
chr14 16151136
chr14 16151913
chr14 16152241
chr14 16153014
chr14 16154112
chr14 16155752
chr14 16156086
chr14 16156930
chr14 16157004
chr14 16157103

chr14 16158031
chr14 16158504
chr14 16160364
chr14 16160419
chr14 16161563
chr14 16161623
chr14 16161660
chr14 16161950
chr14 16165626
chr14 16165636
chr14 16165864
chr14 16166336
chr14 16166683
chr14 16166894
chr14 16166913
chr14 16166934
chr14 16167828
chr14 16169090
chr14 16169320
chr14 16172159
chr14 16174119
chr14 16174163
chr14 16176323
chr14 16176837
chr14 16179070
chr14 16179124
chr14 16180891
chr14 16181178
chr14 16181231
chr14 16182251
chr14 16182288
chr14 16182801
chr14 16183905
chr14 16184166
chr14 16185665
chr14 16185691
chr14 16185739
chr14 16185812
chr14 16186080
chr14 16186082
chr14 16186166
chr14 16186203
chr14 16186971
chr14 16186980
chr14 16188034
chr14 16188081
chr14 16191227
chr14 16193081
chr14 16193787
chr14 16193827
chr14 16193864
chr14 16194561
chr14 16195740
chr14 16196152

chr14 16196655
chr14 16196666
chr14 16198119
chr14 16198630
chr14 16198970
chr14 16199049
chr14 16200742
chr14 16202624
chr14 16202890
chr14 16203217
chr14 16203734
chr14 16203913
chr14 16207556
chr14 16208231
chr14 16209294
chr14 16209528
chr14 16210898
chr14 16211537
chr14 16211583
chr14 16211593
chr14 16211753
chr14 16214374
chr14 16215219
chr14 16215408
chr14 16217877
chr14 16217951
chr14 16218807
chr14 16223338
chr14 16223825
chr14 16223969
chr14 16223995
chr14 16224431
chr14 16225021
chr14 16228786
chr14 16229299
chr14 16229416
chr14 16229464
chr14 16229475
chr14 16229736
chr14 16230341
chr14 16230387
chr14 16231172
chr14 16231316
chr14 16231326
chr14 16231530
chr14 16231581
chr14 16231677
chr14 16231683
chr14 16231688
chr14 16231698
chr14 16231835
chr14 16231852
chr14 16231873
chr14 16231977

chr14 16231990
chr14 16232029
chr14 16232050
chr14 16232476
chr14 16232708
chr14 16232892
chr14 16232903
chr14 16233425
chr14 16233768
chr14 16233814
chr14 18157102
chr14 18157117
chr14 18157161
chr14 18157163
chr14 18157686
chr14 18157851
chr14 18157857
chr14 18157866
chr14 18157967
chr14 18158346
chr14 18158350
chr14 18158358
chr14 18158364
chr14 18158971
chr14 18158986
chr14 18160177
chr14 18160208
chr14 18160212
chr14 18160220
chr14 18160226
chr14 18160662
chr14 18160677
chr14 18161911
chr14 18161917
chr14 18162540
chr14 18162567
chr14 18162586
chr14 18163221
chr14 18163267
chr14 18165340
chr14 18165380
chr14 18165472
chr14 18165476
chr14 18165484
chr14 18165490
chr14 18166678
chr14 18167174
chr14 18167178
chr14 18167186
chr14 18167503
chr14 18167518
chr14 18167532
chr14 18167563
chr14 18167799

chr14 18168164
chr14 18168383
chr14 18168392
chr14 18168548
chr14 18168563
chr14 18169045
chr14 18169049
chr14 18169057
chr14 18169063
chr14 18169245
chr14 18169389
chr14 18169403
chr14 18169434
chr14 18170654
chr14 18170822
chr14 18170932
chr14 18171258
chr14 18171272
chr14 18171312
chr14 18171539
chr14 18171554
chr14 18172694
chr14 18172786
chr14 18172790
chr14 18172975
chr14 18172984
chr14 18173128
chr14 18173142
chr14 18173173
chr14 18340413
chr14 18340784
chr14 18575204
chr14 18576700
chr14 18585812
chr14 18586014
chr14 18640129
chr14 18864218
chr14 18864262
chr14 18865231
chr14 18907868
chr14 18907987
chr14 18908434
chr14 18908840
chr14 18974155
chr14 18974824
chr14 18974832
chr14 18975667
chr14 18976653
chr14 19018735
chr14 19018796
chr14 19018860
chr14 19019296
chr14 19019423
chr14 19019429

chr14 19019461
chr14 19022442
chr14 19023221
chr14 19023406
chr14 19023501
chr14 19023598
chr14 19044793
chr14 19044925
chr14 19045334
chr14 19046573
chr14 19046755
chr14 19048599
chr14 19048730
chr14 19093394
chr14 19093416
chr14 19094070
chr14 19094915
chr14 19095592
chr14 19095701
chr14 19095772
chr14 19095837
chr14 19096353
chr14 19096378
chr14 19096417
chr14 19096419
chr14 19134838
chr14 19134879
chr14 19137019
chr14 19152134
chr14 19156355
chr14 19159600
chr14 19160076
chr14 19243113
chr14 19243209
chr14 19271682
chr14 19305787
chr14 19305802
chr14 19305954
chr14 19306210
chr14 19306610
chr14 19306632
chr14 19306694
chr14 19307593
chr14 19347312
chr14 19347406
chr14 19348213
chr14 19348772
chr14 19349340
chr14 19351688
chr14 19352828
chr14 19353264
chr14 19353445
chr14 19353945
chr14 19354427

chr14 19355092
chr14 19355288
chr14 19355331
chr14 19355405
chr14 19355449
chr14 19356178
chr14 19356348
chr14 19356478
chr14 19356937
chr14 19356966
chr14 19357095
chr14 19357264
chr14 19357267
chr14 19357402
chr14 19357419
chr14 19357421
chr14 19357536
chr14 19357673
chr14 19357949
chr14 19358260
chr14 19358328
chr14 19358348
chr14 19358427
chr14 19365261
chr14 19365930
chr14 19367736
chr14 19368084
chr14 19368710
chr14 19368741
chr14 19368850
chr14 19368918
chr14 19381209
chr14 19381270
chr14 19381797
chr14 19381936
chr14 19382359
chr14 19382388
chr14 19382405
chr14 19382508
chr14 19382522
chr14 19382762
chr14 19382804
chr14 19383390
chr14 19383413
chr14 19431207
chr14 19431271
chr14 19431646
chr14 19431661
chr14 19431667
chr14 19432130
chr14 19432209
chr14 19432221
chr14 19432296
chr14 19432302

chr14 19494084
chr14 19611838
chr14 19612379
chr14 19613067
chr14 19613209
chr14 19613220
chr14 19613649
chr14 19613676
chr14 19613690
chr14 19613698
chr14 19613703
chr14 19613926
chr14 19670494
chr14 19670604
chr14 19671297
chr14 19671319
chr14 19675038
chr14 19675730
chr14 19676101
chr14 19676136
chr14 19676196
chr14 19676547
chr14 19676623
chr14 19677071
chr14 19677092
chr14 19677117
chr14 19677127
chr14 19677670
chr14 19677759
chr14 19686144
chr14 19687151
chr14 19687279
chr14 19692297
chr14 19692727
chr14 19692990
chr14 19693038
chr14 19694076
chr14 19694326
chr14 19694403
chr14 19694986
chr14 19695514
chr14 19699195
chr14 19699832
chr14 19699855
chr14 19699889
chr14 19700745
chr14 19701543
chr14 19701572
chr14 19826846
chr14 20025543
chr14 20025670
chr14 20026032
chr14 20100828
chr14 20101022

chr14 20124755
chr14 20234180
chr14 20234408
chr14 20305250
chr14 20305322
chr14 20305481
chr14 20305592
chr14 20306007
chr14 20306017
chr14 20306060
chr14 20306265
chr14 20327348
chr14 20327373
chr14 20327513
chr14 20327552
chr14 20332593
chr14 20332754
chr14 20332768
chr14 20332793
chr14 20332844
chr14 20332871
chr14 20332881
chr14 20332944
chr14 20332962
chr14 20332965
chr14 20332982
chr14 20333031
chr14 20333037
chr14 20333068
chr14 20333123
chr14 20333140
chr14 20333162
chr14 20333243
chr14 20333267
chr14 20333386
chr14 20333458
chr14 20333519
chr14 20333531
chr14 20333653
chr14 20342015
chr14 20342211
chr14 20342249
chr14 20342257
chr14 20342274
chr14 20342281
chr14 20342291
chr14 20342298
chr14 20342333
chr14 20342337
chr14 20342339
chr14 20342345
chr14 20342393
chr14 20342401
chr14 20343836

chr14 20343882
chr14 20412212
chr14 20412422
chr14 20412453
chr14 20412707
chr14 20412751
chr14 20412761
chr14 20412783
chr14 20412803
chr14 20455938
chr14 20455942
chr14 20456072
chr14 20456174
chr14 20456210
chr14 20469679
chr14 20469690
chr14 20469703
chr14 20469713
chr14 20469715
chr14 20469725
chr14 20469729
chr14 20469731
chr14 20469738
chr14 20469751
chr14 20469757
chr14 20469778
chr14 20469783
chr14 20469798
chr14 20469810
chr14 20469823
chr14 20469909
chr14 20469950
chr14 20469974
chr14 20470061
chr14 20470088
chr14 20470102
chr14 20470142
chr14 20470163
chr14 20531607
chr14 20532159
chr14 20612569
chr14 20612622
chr14 20612719
chr14 20612780
chr14 20613155
chr14 20613327
chr14 20613354
chr14 20613398
chr14 20613406
chr14 20613410
chr14 20613423
chr14 20613433
chr14 20613444
chr14 20613467

chr14 20613645
chr14 20613655
chr14 20613673
chr14 20613681
chr14 20613684
chr14 20613707
chr14 20613925
chr14 20614157
chr14 20614180
chr14 20614192
chr14 20614205
chr14 20614234
chr14 20614241
chr14 20614269
chr14 20614292
chr14 20630721
chr14 20630776
chr14 20630788
chr14 20630799
chr14 20630840
chr14 20630850
chr14 20630939
chr14 20630962
chr14 20630974
chr14 20631000
chr14 20631014
chr14 20631017
chr14 20631030
chr14 20631102
chr14 20631158
chr14 20631169
chr14 20631182
chr14 20631184
chr14 20631232
chr14 20631358
chr14 20631391
chr14 20631571
chr14 20631589
chr14 20631635
chr14 20631685
chr14 20631696
chr14 20631739
chr14 20631762
chr14 20631814
chr14 20631874
chr14 20631895
chr14 20631904
chr14 20631929
chr14 20631940
chr14 20631974
chr14 20631981
chr14 20632080
chr14 20632086
chr14 20632129

chr14 20632162
chr14 20632165
chr14 20632168
chr14 20632186
chr14 20632268
chr14 20632283
chr14 20632320
chr14 20632347
chr14 20652979
chr14 20653059
chr14 20653072
chr14 20653076
chr14 20653092
chr14 20653104
chr14 20653109
chr14 20653115
chr14 20653123
chr14 20653167
chr14 20653187
chr14 20653194
chr14 20653198
chr14 20653257
chr14 20653287
chr14 20653308
chr14 20653311
chr14 20653759
chr14 20653771
chr14 20653776
chr14 20653812
chr14 20653822
chr14 20653921
chr14 20654361
chr14 20654375
chr14 20654413
chr14 20654474
chr14 20654503
chr14 20654571
chr14 20654582
chr14 20663144
chr14 20663184
chr14 20663197
chr14 20663202
chr14 20663208
chr14 20663216
chr14 20663231
chr14 20663260
chr14 20681737
chr14 20681739
chr14 20681752
chr14 20681765
chr14 20681898
chr14 20681945
chr14 20681948
chr14 20681966

chr14 20681979
chr14 20681995
chr14 20682007
chr14 20682015
chr14 20682096
chr14 20682143
chr14 20682163
chr14 20682168
chr14 20682176
chr14 20682195
chr14 20682247
chr14 20682259
chr14 20682494
chr14 20782240
chr14 20782273
chr14 20782350
chr14 20782487
chr14 20782508
chr14 20881772
chr14 20881867
chr14 20881949
chr14 20882952
chr14 20882974
chr14 20883043
chr14 20945876
chr14 20961155
chr14 20961294
chr14 20961324
chr14 20961326
chr14 20961332
chr14 20961349
chr14 20961356
chr14 20961362
chr14 20961364
chr14 20961388
chr14 20961408
chr14 20961418
chr14 20961420
chr14 20961424
chr14 20961456
chr14 20961465
chr14 20961499
chr14 20961506
chr14 20961510
chr14 20961515
chr14 20961734
chr14 20962115
chr14 21068092
chr14 21068100
chr14 21068124
chr14 21068144
chr14 21068148
chr14 21068234
chr14 21068241

chr14 21068245
chr14 21068250
chr14 21068278
chr14 21068288
chr14 21068290
chr14 21068294
chr14 21068298
chr14 21070505
chr14 21070674
chr14 21070681
chr14 21070741
chr14 21070754
chr14 21070756
chr14 21070769
chr14 21070785
chr14 21070802
chr14 21070810
chr14 21070866
chr14 21070888
chr14 21070923
chr14 21070930
chr14 21070957
chr14 21071024
chr14 21071041
chr14 21071054
chr14 21071139
chr14 21071144
chr14 21071192
chr14 21071199
chr14 21071211
chr14 21071285
chr14 21071290
chr14 21071315
chr14 21071333
chr14 21071353
chr14 21071357
chr14 21071404
chr14 21071442
chr14 21071452
chr14 21087597
chr14 21087745
chr14 21087768
chr14 21087845
chr14 21097893
chr14 21097925
chr14 21097927
chr14 21097936
chr14 21097958
chr14 21097960
chr14 21097971
chr14 21098102
chr14 21098108
chr14 21098122
chr14 21098143

chr14 21098160
chr14 21098165
chr14 21098168
chr14 21098179
chr14 21192035
chr14 21192068
chr14 21192078
chr14 21192081
chr14 21192086
chr14 21192098
chr14 21192112
chr14 21192146
chr14 21192158
chr14 21229889
chr14 21229891
chr14 21264411
chr14 21264528
chr14 21264806
chr14 21264848
chr14 21266045
chr14 21266584
chr14 21266806
chr14 21266812
chr14 21266818
chr14 21266828
chr14 21266888
chr14 21266908
chr14 21266919
chr14 21266948
chr14 21266953
chr14 21266963
chr14 21266983
chr14 21266991
chr14 21267005
chr14 21267015
chr14 21267022
chr14 21267039
chr14 21267052
chr14 21267074
chr14 21267082
chr14 21267090
chr14 21267407
chr14 21267435
chr14 21267474
chr14 21267525
chr14 21267629
chr14 21267673
chr14 21267688
chr14 21300857
chr14 21300887
chr14 21308658
chr14 21308697
chr14 21308920
chr14 21308939

chr14 21308980
chr14 21309005
chr14 21309012
chr14 21309074
chr14 21309112
chr14 21382593
chr14 21382655
chr14 21382883
chr14 21384815
chr14 21436724
chr14 21436747
chr14 21436825
chr14 21436861
chr14 21436863
chr14 21436889
chr14 21436914
chr14 21436949
chr14 21437018
chr14 21437051
chr14 21437061
chr14 21437085
chr14 21437107
chr14 21437121
chr14 21437130
chr14 21437152
chr14 21439684
chr14 21439824
chr14 21449581
chr14 21449850
chr14 21449930
chr14 21450235
chr14 21487021
chr14 21487197
chr14 21510100
chr14 21510138
chr14 21510303
chr14 21510518
chr14 21510525
chr14 21526305
chr14 21533716
chr14 21748261
chr14 21791452
chr14 21791465
chr14 21847836
chr14 21847917
chr14 21847948
chr14 22069999
chr14 22070303
chr14 22070327
chr14 22070338
chr14 22070423
chr14 22070471
chr14 22070493
chr14 22070496

chr14 22070668
chr14 22448332
chr14 22448427
chr14 22448437
chr14 22448460
chr14 22448472
chr14 22448518
chr14 22448580
chr14 22448805
chr14 22448817
chr14 22449077
chr14 22449124
chr14 22490693
chr14 22490766
chr14 22490937
chr14 22490942
chr14 22491008
chr14 22491035
chr14 22491305
chr14 22491412
chr14 22491658
chr14 22491847
chr14 22491868
chr14 22492198
chr14 22492219
chr14 22492241
chr14 22492253
chr14 22492269
chr14 22492278
chr14 22492413
chr14 22492425
chr14 22492573
chr14 22496697
chr14 22497359
chr14 22497362
chr14 22497648
chr14 22497669
chr14 22497687
chr14 22497701
chr14 22497718
chr14 22499543
chr14 22499569
chr14 22499915
chr14 22499955
chr14 22500112
chr14 22500712
chr14 22503583
chr14 22503632
chr14 22503857
chr14 22504380
chr14 22504405
chr14 22504411
chr14 22504421
chr14 22504449

chr14 22504454
chr14 22504465
chr14 22504479
chr14 22504510
chr14 22504547
chr14 22504552
chr14 22504556
chr14 22504639
chr14 22504641
chr14 22505082
chr14 22505148
chr14 22505158
chr14 22505310
chr14 22506671
chr14 22506677
chr14 22506702
chr14 22506742
chr14 22506933
chr14 22507293
chr14 22507677
chr14 22509973
chr14 22509996
chr14 22510179
chr14 22510265
chr14 22510635
chr14 22510677
chr14 22511070
chr14 22511508
chr14 22512253
chr14 22517902
chr14 22520447
chr14 22520458
chr14 22520602
chr14 22520605
chr14 22520979
chr14 22520986
chr14 22520996
chr14 22521176
chr14 22522425
chr14 22522759
chr14 22523092
chr14 22523099
chr14 22524605
chr14 22530590
chr14 22531191
chr14 22531544
chr14 22531589
chr14 22531730
chr14 22531840
chr14 22531967
chr14 22536447
chr14 22536488
chr14 22536508
chr14 22544680

chr14 22544689
chr14 22544882
chr14 22550038
chr14 22550223
chr14 22550252
chr14 22550332
chr14 22550482
chr14 22550533
chr14 22550560
chr14 22550598
chr14 22550621
chr14 22550641
chr14 22550646
chr14 22550701
chr14 22550703
chr14 22555562
chr14 22555709
chr14 22555982
chr14 22556058
chr14 22556103
chr14 22556124
chr14 22556192
chr14 22556214
chr14 22556221
chr14 22556247
chr14 22556249
chr14 22556252
chr14 22557349
chr14 22557362
chr14 22557369
chr14 22557373
chr14 22557379
chr14 22557388
chr14 22557397
chr14 22557436
chr14 22557454
chr14 22558381
chr14 22558411
chr14 22558416
chr14 22558459
chr14 22558504
chr14 22558527
chr14 22558544
chr14 22558546
chr14 22560974
chr14 22560985
chr14 22561095
chr14 22561112
chr14 22561142
chr14 22561146
chr14 22561245
chr14 22561317
chr14 22561553
chr14 22561595

chr14 22561646
chr14 22561701
chr14 22561788
chr14 22561947
chr14 22562110
chr14 22562177
chr14 22566395
chr14 22566403
chr14 22566411
chr14 22566420
chr14 22566427
chr14 22566437
chr14 22566478
chr14 22566553
chr14 22566580
chr14 22567101
chr14 22567209
chr14 22567718
chr14 22567882
chr14 22568315
chr14 22568455
chr14 22570092
chr14 22570189
chr14 22570218
chr14 22570277
chr14 22570288
chr14 22570373
chr14 22570537
chr14 22570609
chr14 22570647
chr14 22583174
chr14 22583300
chr14 22583423
chr14 22583611
chr14 22583691
chr14 22583716
chr14 22588545
chr14 22588553
chr14 22588676
chr14 22588716
chr14 22588721
chr14 22588759
chr14 22588765
chr14 22598208
chr14 22598210
chr14 22598216
chr14 22598231
chr14 22598233
chr14 22598241
chr14 22598259
chr14 22598266
chr14 22598273
chr14 22598279
chr14 22598312

chr14 22598426
chr14 22598445
chr14 22598452
chr14 22598461
chr14 22598474
chr14 22598508
chr14 22598535
chr14 22598537
chr14 22598541
chr14 22598545
chr14 22598552
chr14 22598581
chr14 22598583
chr14 22598643
chr14 22598662
chr14 22598664
chr14 22598670
chr14 22598687
chr14 22598693
chr14 22598698
chr14 22598715
chr14 22598722
chr14 22598750
chr14 22598754
chr14 22598794
chr14 22598796
chr14 22598818
chr14 22598843
chr14 22598850
chr14 22598857
chr14 22598863
chr14 22598898
chr14 22704110
chr14 22704160
chr14 22704170
chr14 22704492
chr14 22713206
chr14 22713214
chr14 22713241
chr14 22713244
chr14 22713246
chr14 22713252
chr14 22713269
chr14 22713276
chr14 22713284
chr14 22713295
chr14 22713308
chr14 22713328
chr14 22713338
chr14 22713340
chr14 22713344
chr14 22766646
chr14 22766668
chr14 22766674

chr14 22766694
chr14 22766706
chr14 22767516
chr14 22830252
chr14 22830260
chr14 22830309
chr14 22830319
chr14 22830347
chr14 22830380
chr14 22830428
chr14 22830650
chr14 22853404
chr14 22853448
chr14 22919130
chr14 22919140
chr14 22919152
chr14 22919178
chr14 22919184
chr14 22919197
chr14 22919224
chr14 22919226
chr14 22919233
chr14 22919294
chr14 22919309
chr14 22919356
chr14 22919370
chr14 22919403
chr14 22919410
chr14 22919419
chr14 22919431
chr14 22919468
chr14 22919470
chr14 22919487
chr14 22919494
chr14 22919503
chr14 22919530
chr14 22919540
chr14 22919560
chr14 22919563
chr14 22919567
chr14 22919595
chr14 22919598
chr14 22919609
chr14 22919613
chr14 22919628
chr14 22919637
chr14 22919647
chr14 22919668
chr14 22928437
chr14 22928523
chr14 22928536
chr14 22928541
chr14 22928580
chr14 22928582

chr14 22928623
chr14 22928625
chr14 22928938
chr14 22929001
chr14 22929003
chr14 22929092
chr14 22929104
chr14 22929155
chr14 22929178
chr14 22929202
chr14 22929857
chr14 22929881
chr14 22929918
chr14 22929959
chr14 22930125
chr14 22930338
chr14 22930364
chr14 22930381
chr14 22930387
chr14 22930390
chr14 22930410
chr14 22930486
chr14 22930538
chr14 22930549
chr14 22930578
chr14 22930605
chr14 22930625
chr14 22956487
chr14 22956516
chr14 22956688
chr14 22956700
chr14 22956752
chr14 22956774
chr14 22956783
chr14 22956786
chr14 22956809
chr14 22956829
chr14 22956832
chr14 22956835
chr14 22956842
chr14 22956853
chr14 22956891
chr14 22956895
chr14 22956949
chr14 22956955
chr14 22956964
chr14 22956973
chr14 22956988
chr14 22957006
chr14 22957010
chr14 22957012
chr14 22957015
chr14 22957023
chr14 22957033

chr14 22957037
chr14 22957042
chr14 22957046
chr14 22957064
chr14 22957071
chr14 22957094
chr14 22957111
chr14 22957129
chr14 22957131
chr14 22957134
chr14 22957149
chr14 22957161
chr14 22957166
chr14 22957172
chr14 22957182
chr14 22957189
chr14 22957192
chr14 22957198
chr14 22957200
chr14 22957214
chr14 22957225
chr14 22957233
chr14 22957247
chr14 23006347
chr14 23006419
chr14 23006425
chr14 23006444
chr14 23006462
chr14 23006559
chr14 23006633
chr14 23006641
chr14 23006714
chr14 23006720
chr14 23006744
chr14 23006753
chr14 23006784
chr14 23009066
chr14 23009327
chr14 23009676
chr14 23009682
chr14 23009720
chr14 23009761
chr14 23009788
chr14 23009878
chr14 23009884
chr14 23009894
chr14 23010102
chr14 23010109
chr14 23010127
chr14 23010151
chr14 23010153
chr14 23010160
chr14 23010165
chr14 23010175

chr14 23010185
chr14 23010189
chr14 23010197
chr14 23010206
chr14 23010210
chr14 23010212
chr14 23010216
chr14 23010218
chr14 23010223
chr14 23010228
chr14 23010232
chr14 23010242
chr14 23010244
chr14 23010247
chr14 23010249
chr14 23010253
chr14 23010258
chr14 23010267
chr14 23010279
chr14 23010298
chr14 23010301
chr14 23010309
chr14 23010322
chr14 23010375
chr14 23010448
chr14 23010455
chr14 23010527
chr14 23010541
chr14 23010570
chr14 23010592
chr14 23010610
chr14 23028695
chr14 23028936
chr14 23028949
chr14 23034719
chr14 23034753
chr14 23034755
chr14 23034811
chr14 23034824
chr14 23034833
chr14 23034835
chr14 23034846
chr14 23034852
chr14 23034866
chr14 23034876
chr14 23034916
chr14 23034934
chr14 23034941
chr14 23034946
chr14 23034949
chr14 23034981
chr14 23035013
chr14 23035038
chr14 23035186

chr14 23035197
chr14 23035214
chr14 23035233
chr14 23035267
chr14 23070723
chr14 23070848
chr14 23070888
chr14 23071031
chr14 23071038
chr14 23071070
chr14 23071085
chr14 23071088
chr14 23071297
chr14 23071330
chr14 23071375
chr14 23071413
chr14 23071426
chr14 23071436
chr14 23071442
chr14 23071515
chr14 23071520
chr14 23071526
chr14 23071534
chr14 23071683
chr14 23094341
chr14 23094375
chr14 23096149
chr14 23096202
chr14 23096238
chr14 23096242
chr14 23096244
chr14 23096254
chr14 23096282
chr14 23096287
chr14 23096291
chr14 23182692
chr14 23182742
chr14 23182744
chr14 23182767
chr14 23182794
chr14 23182818
chr14 23182827
chr14 23182839
chr14 23182848
chr14 23182881
chr14 23182892
chr14 23182963
chr14 23182969
chr14 23183005
chr14 23183015
chr14 23183069
chr14 23183087
chr14 23183110
chr14 23183112

chr14 23183205
chr14 23183211
chr14 23183401
chr14 23183467
chr14 23183520
chr14 23183533
chr14 23183561
chr14 23285197
chr14 23285344
chr14 23285414
chr14 23285913
chr14 23285925
chr14 23285929
chr14 23285932
chr14 23285945
chr14 23285957
chr14 23286018
chr14 23286022
chr14 23286027
chr14 23286237
chr14 23286246
chr14 23286269
chr14 23286301
chr14 23286360
chr14 23286363
chr14 23286371
chr14 23469847
chr14 23469865
chr14 23469875
chr14 23469902
chr14 23469916
chr14 23469931
chr14 23469948
chr14 23469976
chr14 23470021
chr14 23470056
chr14 23470071
chr14 23470113
chr14 23551237
chr14 23551249
chr14 23551254
chr14 23551391
chr14 23551393
chr14 23551398
chr14 23551414
chr14 23551436
chr14 23551640
chr14 23551644
chr14 23551707
chr14 23551721
chr14 23551748
chr14 23551780
chr14 23551784
chr14 23551811

chr14 23551816
chr14 23551820
chr14 23551823
chr14 23551825
chr14 23551828
chr14 23551835
chr14 23567581
chr14 23568114
chr14 23568455
chr14 23577812
chr14 23577848
chr14 23577878
chr14 23577886
chr14 23577934
chr14 23577945
chr14 23577979
chr14 23610209
chr14 23610240
chr14 23610859
chr14 23610871
chr14 23630947
chr14 23631777
chr14 23735212
chr14 23735374
chr14 23735828
chr14 23969492
chr14 23969531
chr14 23969568
chr14 23969580
chr14 23969585
chr14 23969597
chr14 23969632
chr14 23969775
chr14 23969789
chr14 23969792
chr14 23969795
chr14 23969806
chr14 23969809
chr14 23969815
chr14 24006621
chr14 24006660
chr14 24006697
chr14 24006709
chr14 24006714
chr14 24006726
chr14 24006761
chr14 24006829
chr14 24006835
chr14 24006863
chr14 24007042
chr14 24007044
chr14 24007056
chr14 24007071
chr14 24007077

chr14 24007083
chr14 24007098
chr14 24007104
chr14 24007112
chr14 24007115
chr14 24007125
chr14 24007129
chr14 24007144
chr14 24007160
chr14 24007174
chr14 24007186
chr14 24007189
chr14 24007209
chr14 24007224
chr14 24007240
chr14 24007269
chr14 24007280
chr14 24007292
chr14 24007297
chr14 24007323
chr14 24007332
chr14 24007345
chr14 24007350
chr14 24007365
chr14 24007419
chr14 24007440
chr14 24007478
chr14 24007491
chr14 24013781
chr14 24014017
chr14 24014028
chr14 24014154
chr14 24036519
chr14 24036534
chr14 24036547
chr14 24036552
chr14 24036566
chr14 24036576
chr14 24036579
chr14 24036586
chr14 24036613
chr14 24036623
chr14 24036643
chr14 24036689
chr14 24036731
chr14 24095056
chr14 24095081
chr14 24095158
chr14 24095178
chr14 24095187
chr14 24095229
chr14 24095279
chr14 24095344
chr14 24095438

chr14 24095480
chr14 24108807
chr14 24108870
chr14 24115352
chr14 24115602
chr14 24115604
chr14 24115645
chr14 24115658
chr14 24115676
chr14 24115679
chr14 24115745
chr14 24115846
chr14 24145938
chr14 24146002
chr14 24146021
chr14 24146069
chr14 24146187
chr14 24146260
chr14 24161196
chr14 24161264
chr14 24161725
chr14 24161777
chr14 24161808
chr14 24215540
chr14 24215563
chr14 24215568
chr14 24215591
chr14 24215624
chr14 24215628
chr14 24215639
chr14 24215648
chr14 24215681
chr14 24215707
chr14 24215751
chr14 24215762
chr14 24215766
chr14 24215771
chr14 24215801
chr14 24215834
chr14 24215851
chr14 24215892
chr14 24215909
chr14 24215941
chr14 24215948
chr14 24215950
chr14 24215960
chr14 24215965
chr14 24215974
chr14 24215976
chr14 24215999
chr14 24216016
chr14 24216025
chr14 24216066
chr14 24216075

chr14 24216078
chr14 24216085
chr14 24216090
chr14 24231727
chr14 24231746
chr14 24231810
chr14 24231828
chr14 24231834
chr14 24231841
chr14 24231892
chr14 24231916
chr14 24231930
chr14 24231955
chr14 24231963
chr14 24231979
chr14 24231988
chr14 24232053
chr14 24232074
chr14 24232594
chr14 24232600
chr14 24232604
chr14 24232637
chr14 24232660
chr14 24232686
chr14 24232694
chr14 24232697
chr14 24232700
chr14 24232713
chr14 24232716
chr14 24232730
chr14 24232733
chr14 24232756
chr14 24232775
chr14 24232785
chr14 24232864
chr14 24232888
chr14 24232895
chr14 24232898
chr14 24232912
chr14 24232974
chr14 24233066
chr14 24233074
chr14 24233084
chr14 24233113
chr14 24233124
chr14 24233143
chr14 24233314
chr14 24233332
chr14 24233378
chr14 24233384
chr14 24233432
chr14 24271192
chr14 24271200
chr14 24271215

chr14 24271231
chr14 24271244
chr14 24271298
chr14 24271320
chr14 24271338
chr14 24271359
chr14 24271364
chr14 24271368
chr14 24271378
chr14 24271380
chr14 24271385
chr14 24271398
chr14 24271430
chr14 24271437
chr14 24271441
chr14 24271448
chr14 24271455
chr14 24271465
chr14 24271472
chr14 24271474
chr14 24271478
chr14 24271490
chr14 24271503
chr14 24271545
chr14 24271557
chr14 24271560
chr14 24271565
chr14 24271570
chr14 24272118
chr14 24310834
chr14 24310840
chr14 24310858
chr14 24310865
chr14 24310868
chr14 24310871
chr14 24310873
chr14 24310888
chr14 24310891
chr14 24310914
chr14 24310927
chr14 24310939
chr14 24310951
chr14 24310961
chr14 24310967
chr14 24310969
chr14 24310982
chr14 24310988
chr14 24311000
chr14 24311019
chr14 24311027
chr14 24311037
chr14 24311050
chr14 24311055
chr14 24311061

chr14 24311068
chr14 24311079
chr14 24311082
chr14 24311095
chr14 24311108
chr14 24311120
chr14 24311123
chr14 24311128
chr14 24311132
chr14 24311135
chr14 24311142
chr14 24311160
chr14 24311162
chr14 24311182
chr14 24311185
chr14 24311188
chr14 24311195
chr14 24311198
chr14 24311201
chr14 24311228
chr14 24311234
chr14 24311246
chr14 24311258
chr14 24311270
chr14 24311275
chr14 24311283
chr14 24311289
chr14 24311294
chr14 24311298
chr14 24311309
chr14 24311313
chr14 24311318
chr14 24311323
chr14 24311325
chr14 24311334
chr14 24311345
chr14 24311351
chr14 24311363
chr14 24311387
chr14 24311443
chr14 24311453
chr14 24311459
chr14 24311467
chr14 24311470
chr14 24311472
chr14 24311476
chr14 24311485
chr14 24311510
chr14 24311518
chr14 24368348
chr14 24368358
chr14 24368391
chr14 24368414
chr14 24368421

chr14 24368423
chr14 24368488
chr14 24368520
chr14 24368554
chr14 24368564
chr14 24368574
chr14 24368584
chr14 24368604
chr14 24368630
chr14 24368667
chr14 24368670
chr14 24368677
chr14 24368686
chr14 24368708
chr14 24368712
chr14 24368721
chr14 24368740
chr14 24368743
chr14 24368766
chr14 24368785
chr14 24368790
chr14 24368802
chr14 24368806
chr14 24368814
chr14 24368818
chr14 24430533
chr14 24430545
chr14 24430627
chr14 24430749
chr14 24430754
chr14 24430756
chr14 24430767
chr14 24430774
chr14 24430783
chr14 24430803
chr14 24430827
chr14 24430838
chr14 24430850
chr14 24430858
chr14 24430870
chr14 24430916
chr14 24430937
chr14 24430972
chr14 24430987
chr14 24431003
chr14 24441757
chr14 24441764
chr14 24441982
chr14 24442983
chr14 24442992
chr14 24443066
chr14 24443110
chr14 24443140
chr14 24443164

chr14 24443191
chr14 24481067
chr14 24673634
chr14 24673661
chr14 24673746
chr14 24673788
chr14 24673810
chr14 24673831
chr14 24673845
chr14 24674027
chr14 24674134
chr14 24674218
chr14 24674305
chr14 24674369
chr14 24674523
chr14 24674736
chr14 25132421
chr14 25132450
chr14 25132785
chr14 25132795
chr14 25132824
chr14 25132830
chr14 25132835
chr14 25132866
chr14 25132897
chr14 25132917
chr14 25132941
chr14 25132962
chr14 25132968
chr14 25233920
chr14 25301190
chr14 25301846
chr14 25583953
chr14 25786078
chr14 25786375
chr14 25786413
chr14 25787528
chr14 26199327
chr14 26199372
chr14 26199600
chr14 26199616
chr14 26199905
chr14 26205567
chr14 26205715
chr14 26205997
chr14 26206008
chr14 26206110
chr14 26424721
chr14 26424899
chr14 26433251
chr14 26433316
chr14 26433366
chr14 26433438
chr14 26433505

chr14 26434314
chr14 26434587
chr14 26434971
chr14 26435162
chr14 26435282
chr14 27309161
chr14 27349855
chr14 27349913
chr14 27349952
chr14 27350345
chr14 27353580
chr14 27353696
chr14 27353709
chr14 27481133
chr14 27481382
chr14 27671979
chr14 27672219
chr14 27672231
chr14 27672237
chr14 27672465
chr14 27672480
chr14 27676631
chr14 27676695
chr14 27676921
chr14 27824716
chr14 27824901
chr14 27825048
chr14 27825879
chr14 27826206
chr14 27826229
chr14 27826299
chr14 27826364
chr14 28159378
chr14 28159500
chr14 28185543
chr14 28187070
chr14 28191171
chr14 28191217
chr14 28191256
chr14 28191631
chr14 28191724
chr14 28275080
chr14 28275239
chr14 28275530
chr14 28275738
chr14 28316761
chr14 28425080
chr14 28609403
chr14 28609414
chr14 28609422
chr14 28609738
chr14 28610150
chr14 28710280
chr14 28754236

chr14 28754310
chr14 28754422
chr14 28754914
chr14 28754941
chr14 28755178
chr14 28755269
chr14 28755322
chr14 28755329
chr14 28755441
chr14 28755521
chr14 28755532
chr14 28755552
chr14 28755571
chr14 28755574
chr14 28755587
chr14 28755626
chr14 28755706
chr14 28755734
chr14 28755843
chr14 28755871
chr14 28755966
chr14 28756016
chr14 28756261
chr14 28756341
chr14 28756368
chr14 28756375
chr14 28757576
chr14 28757686
chr14 28757829
chr14 28757838
chr14 28757943
chr14 28757962
chr14 28757992
chr14 28758173
chr14 28758176
chr14 28758256
chr14 28758308
chr14 28758461
chr14 28758564
chr14 28758569
chr14 28758587
chr14 28762446
chr14 28762506
chr14 28762616
chr14 28762669
chr14 28762705
chr14 28762809
chr14 28763052
chr14 28763104
chr14 28763276
chr14 28763385
chr14 28763742
chr14 28763842
chr14 28763883

chr14 28763988
chr14 28764488
chr14 28764552
chr14 28764626
chr14 28764652
chr14 28764660
chr14 28765015
chr14 28765076
chr14 28768863
chr14 28768917
chr14 28769045
chr14 28769099
chr14 28769175
chr14 28769387
chr14 28769525
chr14 28769534
chr14 28769602
chr14 28770150
chr14 28770525
chr14 28770581
chr14 28771485
chr14 28771698
chr14 28771786
chr14 28771937
chr14 28771941
chr14 28771982
chr14 28772471
chr14 28772738
chr14 28772834
chr14 28772967
chr14 28773060
chr14 28773214
chr14 28773316
chr14 28775545
chr14 28775617
chr14 28775629
chr14 28775635
chr14 28775647
chr14 28775660
chr14 28775819
chr14 28776138
chr14 28776813
chr14 28776926
chr14 28776963
chr14 28777547
chr14 28777588
chr14 28777595
chr14 28777802
chr14 28777824
chr14 28778499
chr14 28778520
chr14 28778522
chr14 28778566
chr14 28778570

chr14 28778640
chr14 28778908
chr14 28778960
chr14 28779959
chr14 28780462
chr14 28781203
chr14 28781210
chr14 28781216
chr14 28781230
chr14 28781706
chr14 28781805
chr14 28782337
chr14 28782503
chr14 28782847
chr14 28783255
chr14 28783401
chr14 28783484
chr14 28783698
chr14 28783916
chr14 28783926
chr14 28783928
chr14 28784169
chr14 28784193
chr14 28784207
chr14 28784218
chr14 28784424
chr14 28784435
chr14 28784441
chr14 28784519
chr14 28784639
chr14 28784666
chr14 28784721
chr14 28784740
chr14 28828493
chr14 28870372
chr14 28870452
chr14 28974318
chr14 29341589
chr14 29341696
chr14 29522894
chr14 29523297
chr14 29527117
chr14 29527912
chr14 29528179
chr14 29528623
chr14 29528742
chr14 29528746
chr14 30069913
chr14 30070185
chr14 30559874
chr14 30559895
chr14 30559905
chr14 30559923
chr14 30559938

chr14 30559996
chr14 30560009
chr14 30560274
chr14 30560297
chr14 30560499
chr14 30621749
chr14 30621760
chr14 30621768
chr14 30621806
chr14 30621900
chr14 30621910
chr14 30622108
chr14 30622133
chr14 30622145
chr14 30622168
chr14 30622186
chr14 30622220
chr14 30622828
chr14 30622844
chr14 30623087
chr14 31025174
chr14 31025235
chr14 31025321
chr14 31025388
chr14 31025408
chr14 31025410
chr14 31026849
chr14 31026979
chr14 31027000
chr14 31123984
chr14 31124251
chr14 31124390
chr14 31124398
chr14 31205999
chr14 31206098
chr14 31206106
chr14 31206145
chr14 31206157
chr14 31206540
chr14 31206682
chr14 31206688
chr14 31206692
chr14 31206770
chr14 31356785
chr14 31419569
chr14 31419751
chr14 31419794
chr14 31419797
chr14 31419799
chr14 31419806
chr14 31421056
chr14 31421265
chr14 31421268
chr14 31457055

chr14 31457067
chr14 31457083
chr14 31457093
chr14 31457107
chr14 31457119
chr14 31457137
chr14 31457139
chr14 31457159
chr14 31457168
chr14 31457170
chr14 31457174
chr14 31457179
chr14 31457182
chr14 31457205
chr14 31457209
chr14 31457217
chr14 31457225
chr14 31457227
chr14 31457237
chr14 31457242
chr14 31457246
chr14 31457249
chr14 31457259
chr14 31457269
chr14 31457277
chr14 31457298
chr14 31457300
chr14 31457303
chr14 31457312
chr14 31457320
chr14 31457332
chr14 31457336
chr14 31457358
chr14 31457362
chr14 31457377
chr14 31457406
chr14 31457409
chr14 31457411
chr14 31457415
chr14 31457438
chr14 31457453
chr14 31457456
chr14 31457458
chr14 31457464
chr14 31457473
chr14 31457478
chr14 31457485
chr14 31457488
chr14 31457499
chr14 31457502
chr14 31457519
chr14 31457533
chr14 31457541
chr14 31457553

chr14 31561434
chr14 31561436
chr14 32075793
chr14 32075802
chr14 32075861
chr14 32075886
chr14 32076007
chr14 32076037
chr14 32076071
chr14 32076078
chr14 32076088
chr14 32078292
chr14 32078320
chr14 32078335
chr14 32078355
chr14 32078385
chr14 32078398
chr14 32078467
chr14 32078489
chr14 32078570
chr14 32078617
chr14 32078624
chr14 32078689
chr14 32078703
chr14 32078778
chr14 32078815
chr14 32078864
chr14 32078872
chr14 32078912
chr14 32200421
chr14 32200486
chr14 32200660
chr14 32200784
chr14 32200849
chr14 32200854
chr14 32200862
chr14 32200962
chr14 32200988
chr14 32201109
chr14 32202331
chr14 32202336
chr14 32202346
chr14 32202375
chr14 32202383
chr14 32202475
chr14 32202489
chr14 32202536
chr14 32202676
chr14 32202740
chr14 32202753
chr14 32202905
chr14 32203008
chr14 32203023
chr14 32927530

chr14 32940743
chr14 33792381
chr14 33792387
chr14 33792648
chr14 33792678
chr14 33792811
chr14 33792838
chr14 33792841
chr14 33792880
chr14 33792891
chr14 33792893
chr14 33792898
chr14 33792901
chr14 33792911
chr14 33792916
chr14 33792952
chr14 33947243
chr14 33947247
chr14 34461495
chr14 34461499
chr14 34461779
chr14 34461783
chr14 34461794
chr14 34461820
chr14 34461845
chr14 34461865
chr14 34461882
chr14 34461899
chr14 34461925
chr14 34461927
chr14 34538838
chr14 34538896
chr14 34539036
chr14 34539238
chr14 34539804
chr14 34539815
chr14 34539825
chr14 34539831
chr14 34539836
chr14 34539842
chr14 34539887
chr14 34539895
chr14 34540764
chr14 34540836
chr14 34542349
chr14 34542361
chr14 34542394
chr14 34542398
chr14 34542405
chr14 34542425
chr14 34542439
chr14 34542450
chr14 34542483
chr14 34542491

chr14 34542515
chr14 34542539
chr14 34542547
chr14 34542648
chr14 34542656
chr14 34542662
chr14 34542680
chr14 34542742
chr14 34542744
chr14 34542775
chr14 34543046
chr14 34543077
chr14 34543081
chr14 34545441
chr14 34545463
chr14 34545473
chr14 34545480
chr14 34545489
chr14 34545879
chr14 34546326
chr14 34628971
chr14 34629047
chr14 34629080
chr14 34629083
chr14 34629212
chr14 34629414
chr14 34629489
chr14 34629501
chr14 34629515
chr14 34629535
chr14 34629548
chr14 34629578
chr14 34629594
chr14 34629606
chr14 34629623
chr14 34629637
chr14 34629642
chr14 34629644
chr14 34629672
chr14 34629679
chr14 34629682
chr14 34629697
chr14 34666235
chr14 34666329
chr14 34700263
chr14 34700482
chr14 34742598
chr14 34875420
chr14 34875461
chr14 34875507
chr14 34875567
chr14 34875636
chr14 34875660
chr14 34875926

chr14 34891444
chr14 34930644
chr14 34930869
chr14 34931035
chr14 34931100
chr14 34931181
chr14 34931328
chr14 34981377
chr14 34981405
chr14 34981408
chr14 34981430
chr14 34981486
chr14 34981510
chr14 34981515
chr14 34981566
chr14 34981586
chr14 34981633
chr14 34981735
chr14 34983534
chr14 34983584
chr14 34983595
chr14 34983606
chr14 35045693
chr14 35045705
chr14 35045712
chr14 35045728
chr14 35045744
chr14 35045750
chr14 35121619
chr14 35121631
chr14 35122835
chr14 35122872
chr14 35122910
chr14 35122926
chr14 35122931
chr14 35123009
chr14 35123016
chr14 35123019
chr14 35123032
chr14 35123148
chr14 35123159
chr14 35123162
chr14 35123270
chr14 35123280
chr14 35123334
chr14 35123351
chr14 35123421
chr14 35133872
chr14 35156264
chr14 35204176
chr14 35204690
chr14 35286381
chr14 35286410
chr14 35286499

chr14 35286548
chr14 35287010
chr14 35287247
chr14 35287454
chr14 35287628
chr14 35287659
chr14 35291991
chr14 35292090
chr14 35292121
chr14 35292150
chr14 35292195
chr14 35292200
chr14 35292214
chr14 35292242
chr14 35292248
chr14 35292269
chr14 35292298
chr14 35292530
chr14 35292569
chr14 35292604
chr14 35292615
chr14 35292623
chr14 35292625
chr14 35292630
chr14 35292987
chr14 35293087
chr14 35293098
chr14 35293353
chr14 35293361
chr14 35293454
chr14 35293490
chr14 35293793
chr14 35293817
chr14 35293826
chr14 35293919
chr14 35294105
chr14 35294107
chr14 35294113
chr14 35294121
chr14 35294130
chr14 35294137
chr14 35294189
chr14 35294197
chr14 35294228
chr14 35294250
chr14 35294282
chr14 35294310
chr14 35294319
chr14 35294638
chr14 35314499
chr14 35315059
chr14 35356899
chr14 35402751
chr14 35402812

chr14 35402852
chr14 35403101
chr14 35403115
chr14 35403187
chr14 35403219
chr14 35403269
chr14 35403278
chr14 35403364
chr14 35403414
chr14 35403430
chr14 35406780
chr14 35416262
chr14 35416311
chr14 35416440
chr14 35416540
chr14 35416786
chr14 35416856
chr14 35416890
chr14 35417048
chr14 35417093
chr14 35417098
chr14 35422491
chr14 35422543
chr14 35422567
chr14 35422593
chr14 35422616
chr14 35532912
chr14 35533206
chr14 35533232
chr14 35533235
chr14 35533273
chr14 35808238
chr14 35808268
chr14 35808438
chr14 35808915
chr14 35808960
chr14 35809026
chr14 35809028
chr14 35809045
chr14 35809065
chr14 35809078
chr14 35809088
chr14 35809093
chr14 35809104
chr14 35809352
chr14 35809378
chr14 35809401
chr14 35809413
chr14 35809478
chr14 35809608
chr14 35825732
chr14 35825868
chr14 36221740
chr14 36271158

chr14 36271339
chr14 36271967
chr14 36272036
chr14 36272097
chr14 36272178
chr14 36272257
chr14 36272282
chr14 36272284
chr14 36272292
chr14 36272345
chr14 36272352
chr14 36272354
chr14 36272363
chr14 36272371
chr14 36272739
chr14 36319622
chr14 36319652
chr14 36319763
chr14 36319780
chr14 36319834
chr14 36319873
chr14 36319885
chr14 36319901
chr14 36319963
chr14 36319975
chr14 36320055
chr14 36320057
chr14 36320059
chr14 36320135
chr14 36320201
chr14 36320207
chr14 36320264
chr14 36320276
chr14 36410853
chr14 36411142
chr14 36411188
chr14 36441106
chr14 36441256
chr14 36441775
chr14 36441837
chr14 36446096
chr14 36446497
chr14 36446688
chr14 36494699
chr14 36494743
chr14 36502334
chr14 36502465
chr14 36502496
chr14 36502617
chr14 36503190
chr14 36503517
chr14 36503570
chr14 36503614
chr14 36503662

chr14 36503684
chr14 36503693
chr14 36509390
chr14 36509411
chr14 36509428
chr14 36509469
chr14 36510975
chr14 36511117
chr14 36511132
chr14 36511158
chr14 36511180
chr14 36511226
chr14 36512118
chr14 36512134
chr14 36512151
chr14 36512161
chr14 36512184
chr14 36512303
chr14 36512496
chr14 36515500
chr14 36515579
chr14 36515743
chr14 36515784
chr14 36515858
chr14 36515875
chr14 36516149
chr14 36516271
chr14 36516347
chr14 36516503
chr14 36516506
chr14 36585508
chr14 36585510
chr14 36585544
chr14 36585749
chr14 36586052
chr14 36586142
chr14 36586531
chr14 36587209
chr14 36587253
chr14 36587946
chr14 36588258
chr14 36588420
chr14 36588433
chr14 36588454
chr14 36588474
chr14 36588736
chr14 36588942
chr14 36588994
chr14 36589031
chr14 36589051
chr14 36589092
chr14 36589129
chr14 36589152
chr14 36589373

chr14 36589395
chr14 36589404
chr14 36589458
chr14 36589729
chr14 36649000
chr14 36649071
chr14 36649142
chr14 36649194
chr14 36649663
chr14 36649839
chr14 36650007
chr14 36650040
chr14 36650183
chr14 36650282
chr14 36650664
chr14 36651501
chr14 36651677
chr14 36652055
chr14 36652470
chr14 36652481
chr14 36653396
chr14 36653441
chr14 36664363
chr14 36664371
chr14 36664379
chr14 36664479
chr14 36664509
chr14 36664528
chr14 36664535
chr14 36664590
chr14 36665049
chr14 36665896
chr14 36667853
chr14 36667869
chr14 36667915
chr14 36667930
chr14 36668996
chr14 36669173
chr14 36669386
chr14 36669969
chr14 36670840
chr14 36671077
chr14 36671240
chr14 36671354
chr14 36671500
chr14 36677115
chr14 36677353
chr14 36942038
chr14 37173128
chr14 37173142
chr14 37195314
chr14 37195438
chr14 37195764
chr14 37549373

chr14 37549419
chr14 37586074
chr14 37586080
chr14 37586177
chr14 37586215
chr14 37586632
chr14 37586675
chr14 37586789
chr14 37587051
chr14 37587223
chr14 37587339
chr14 37587587
chr14 37587606
chr14 37587815
chr14 37587821
chr14 37589362
chr14 37589412
chr14 37589434
chr14 37589442
chr14 37589453
chr14 37589513
chr14 37589550
chr14 37589925
chr14 37589946
chr14 37590044
chr14 37590232
chr14 37590297
chr14 37590351
chr14 37590603
chr14 37590674
chr14 37590935
chr14 37591122
chr14 37593662
chr14 37594031
chr14 37594055
chr14 37594131
chr14 37594133
chr14 37594171
chr14 37731170
chr14 37731425
chr14 37731466
chr14 37731664
chr14 37733899
chr14 37770866
chr14 37770899
chr14 37770932
chr14 37773906
chr14 38173722
chr14 38256814
chr14 38256984
chr14 38257002
chr14 38284845
chr14 38284887
chr14 38414207

chr14 38414351
chr14 38415433
chr14 38549764
chr14 38726708
chr14 38839927
chr14 38840228
chr14 38840324
chr14 38840436
chr14 38919991
chr14 38920207
chr14 38920393
chr14 38920406
chr14 39015367
chr14 39097232
chr14 39097243
chr14 39102634
chr14 39102640
chr14 39102717
chr14 39102772
chr14 39102781
chr14 39102789
chr14 39102834
chr14 39102842
chr14 39113831
chr14 39113894
chr14 39114072
chr14 39114086
chr14 39114123
chr14 39114125
chr14 39114129
chr14 39114486
chr14 39114496
chr14 39114537
chr14 39114551
chr14 39114566
chr14 39114605
chr14 39114611
chr14 39114626
chr14 39114636
chr14 39114759
chr14 39114807
chr14 39114809
chr14 39114832
chr14 39115005
chr14 39115023
chr14 39169527
chr14 39169716
chr14 39169761
chr14 39169774
chr14 39169778
chr14 39169815
chr14 39169827
chr14 39169840
chr14 39170423

chr14 39170430
chr14 39176026
chr14 39176130
chr14 39176253
chr14 39266445
chr14 39266477
chr14 39266533
chr14 39266547
chr14 39266560
chr14 39266599
chr14 39266604
chr14 39266630
chr14 39268268
chr14 39268346
chr14 39268587
chr14 39268645
chr14 39269053
chr14 39269108
chr14 39269150
chr14 39430763
chr14 39430812
chr14 39430897
chr14 39430904
chr14 39430908
chr14 39430917
chr14 39430921
chr14 39431044
chr14 39431063
chr14 39431114
chr14 39431180
chr14 39431244
chr14 39431274
chr14 39431294
chr14 39587664
chr14 39587843
chr14 39628652
chr14 39628772
chr14 39641204
chr14 40215772
chr14 40453163
chr14 40453406
chr14 40453687
chr14 40453696
chr14 40454197
chr14 40810974
chr14 40811272
chr14 40811370
chr14 41151728
chr14 41173445
chr14 41173662
chr14 41214030
chr14 41214068
chr14 41214164
chr14 41309158

chr14 41309247
chr14 41309344
chr14 41348229
chr14 41348276
chr14 41602565
chr14 41603286
chr14 41603655
chr14 41603969
chr14 41604265
chr14 41604276
chr14 41604380
chr14 41604471
chr14 41604491
chr14 41604588
chr14 41604799
chr14 41604813
chr14 41604856
chr14 41604870
chr14 41604954
chr14 41604978
chr14 41605018
chr14 41605042
chr14 41605095
chr14 41610211
chr14 41610220
chr14 41610270
chr14 41610272
chr14 41610430
chr14 41610454
chr14 41610524
chr14 41610619
chr14 41610859
chr14 41677281
chr14 41826604
chr14 41827519
chr14 41827675
chr14 41828158
chr14 41828281
chr14 41828529
chr14 41829470
chr14 42046643
chr14 42047131
chr14 42047152
chr14 42047271
chr14 42047622
chr14 42442612
chr14 42442703
chr14 42442722
chr14 42442743
chr14 42443129
chr14 42745499
chr14 42745628
chr14 42748769
chr14 42959949

chr14 42960022
chr14 43058829
chr14 43059004
chr14 43059435
chr14 43104408
chr14 43104552
chr14 43104929
chr14 43368611
chr14 43525823
chr14 43596637
chr14 43813777
chr14 43813890
chr14 43813977
chr14 43926753
chr14 44386818
chr14 44388140
chr14 44598329
chr14 44598529
chr14 44598589
chr14 44598596
chr14 44598768
chr14 44599069
chr14 44600221
chr14 44630971
chr14 44631056
chr14 44726386
chr14 44960226
chr14 44960246
chr14 44960611
chr14 44960757
chr14 45084981
chr14 45085076
chr14 45085110
chr14 45085181
chr14 45136529
chr14 45136628
chr14 45136748
chr14 45136939
chr14 45251775
chr14 45251894
chr14 45251938
chr14 45252811
chr14 45252996
chr14 45252998
chr14 45253012
chr14 45253019
chr14 45253021
chr14 45253075
chr14 45253080
chr14 45712539
chr14 45713161
chr14 45769350
chr14 45810289
chr14 45888018

chr14 45888023
chr14 45888059
chr14 45888185
chr14 45888281
chr14 46386393
chr14 46386894
chr14 46387150
chr14 46541024
chr14 46541345
chr14 46552610
chr14 46552815
chr14 46821322
chr14 46842510
chr14 46842609
chr14 46847631
chr14 46847739
chr14 46847791
chr14 46847858
chr14 47015359
chr14 47016566
chr14 47016828
chr14 47016861
chr14 47016927
chr14 47017497
chr14 47017572
chr14 47121463
chr14 47156944
chr14 47157123
chr14 47157197
chr14 47935594
chr14 47935597
chr14 47936308
chr14 48289195
chr14 48297197
chr14 48298102
chr14 48298502
chr14 48299329
chr14 48299973
chr14 48806677
chr14 48806701
chr14 48806855
chr14 48806965
chr14 48807611
chr14 49038390
chr14 49038862
chr14 49585240
chr14 49585324
chr14 49585330
chr14 49585352
chr14 49585358
chr14 49585394
chr14 49585436
chr14 49585438
chr14 49585440

chr14 49585472
chr14 49585477
chr14 49585494
chr14 49585515
chr14 49585531
chr14 49585539
chr14 49587068
chr14 49587076
chr14 49587111
chr14 49587341
chr14 49600259
chr14 49600307
chr14 49600323
chr14 49600369
chr14 49622631
chr14 49622998
chr14 49631577
chr14 49631601
chr14 49631604
chr14 49632049
chr14 49632052
chr14 49632087
chr14 49687739
chr14 49687919
chr14 49687933
chr14 49687940
chr14 49693402
chr14 49693405
chr14 49693450
chr14 49693452
chr14 49693465
chr14 49693468
chr14 49693473
chr14 49693476
chr14 49693505
chr14 49693511
chr14 49693530
chr14 49693560
chr14 49693589
chr14 49693591
chr14 49693618
chr14 49693636
chr14 49693642
chr14 49693645
chr14 49853763
chr14 49853769
chr14 49854090
chr14 49854360
chr14 49862429
chr14 49863232
chr14 49863259
chr14 49863354
chr14 49863379
chr14 49863383

chr14 49863423
chr14 49863436
chr14 49863463
chr14 49863514
chr14 49863566
chr14 49863583
chr14 49863672
chr14 49863681
chr14 49863699
chr14 49863770
chr14 49864107
chr14 49864213
chr14 49868033
chr14 49868038
chr14 49868040
chr14 49868050
chr14 49868057
chr14 49868090
chr14 49868139
chr14 49868142
chr14 49868145
chr14 49868151
chr14 49868154
chr14 49868164
chr14 49868173
chr14 49868176
chr14 49868192
chr14 49868197
chr14 49868208
chr14 49868213
chr14 49868224
chr14 49868244
chr14 49868248
chr14 49868255
chr14 49868261
chr14 49868315
chr14 49868321
chr14 49868324
chr14 49868327
chr14 49868332
chr14 49868334
chr14 49868366
chr14 49868381
chr14 49868389
chr14 49868406
chr14 49868424
chr14 49868434
chr14 49868437
chr14 49868441
chr14 49868474
chr14 49868481
chr14 49868489
chr14 49868525
chr14 49868528

chr14 49868559
chr14 49868594
chr14 49868598
chr14 49868610
chr14 49868635
chr14 49868646
chr14 49868659
chr14 49868703
chr14 49868817
chr14 49868872
chr14 49868918
chr14 49868931
chr14 49868948
chr14 49897063
chr14 49897075
chr14 49897097
chr14 49897109
chr14 49897228
chr14 49897479
chr14 49898231
chr14 49899252
chr14 49899256
chr14 49899288
chr14 49899341
chr14 49899347
chr14 49899380
chr14 49899388
chr14 49899420
chr14 49899522
chr14 49899661
chr14 49899715
chr14 49899811
chr14 49971401
chr14 49971406
chr14 49971433
chr14 49971637
chr14 49971651
chr14 49971750
chr14 49972058
chr14 49972372
chr14 49972416
chr14 49972441
chr14 49972475
chr14 49972623
chr14 49972688
chr14 49972876
chr14 49972991
chr14 49973025
chr14 50000632
chr14 50000637
chr14 50000649
chr14 50000718
chr14 50000752
chr14 50000808

chr14 50000878
chr14 50000911
chr14 50000934
chr14 50001223
chr14 50001300
chr14 50001328
chr14 50001333
chr14 50001338
chr14 50001344
chr14 50001350
chr14 50001356
chr14 50001378
chr14 50001387
chr14 50001391
chr14 50001462
chr14 50001523
chr14 50001542
chr14 50001808
chr14 50002174
chr14 50044130
chr14 50044139
chr14 50044388
chr14 50044559
chr14 50044868
chr14 50044893
chr14 50061227
chr14 50061263
chr14 50061294
chr14 50061348
chr14 50061407
chr14 50061518
chr14 50061692
chr14 50061751
chr14 50061881
chr14 50061923
chr14 50061948
chr14 50062052
chr14 50062235
chr14 50062303
chr14 50062314
chr14 50062371
chr14 50062496
chr14 50062638
chr14 50062645
chr14 50064970
chr14 50065302
chr14 50096134
chr14 50096438
chr14 50096484
chr14 50186849
chr14 50230791
chr14 50230837
chr14 50230882
chr14 50230899

chr14 50321091
chr14 50321102
chr14 50321184
chr14 50321211
chr14 50321253
chr14 50321262
chr14 50321443
chr14 50321495
chr14 50321526
chr14 50321805
chr14 50363164
chr14 50363187
chr14 50363193
chr14 50363196
chr14 50363221
chr14 50363223
chr14 50363283
chr14 50363497
chr14 50363720
chr14 50363762
chr14 50363770
chr14 50364031
chr14 50406174
chr14 50521666
chr14 50521857
chr14 50560736
chr14 50560743
chr14 50560751
chr14 50560767
chr14 50560769
chr14 50560777
chr14 50560780
chr14 50560783
chr14 50560787
chr14 50560790
chr14 50560794
chr14 50560797
chr14 50560799
chr14 50560815
chr14 50560820
chr14 50560822
chr14 50560825
chr14 50560832
chr14 50560836
chr14 50560840
chr14 50560851
chr14 50600015
chr14 50667624
chr14 50739832
chr14 50740006
chr14 50741661
chr14 50741679
chr14 50741707
chr14 50786426

chr14 50813423
chr14 50816430
chr14 50821369
chr14 50860081
chr14 50860089
chr14 50860115
chr14 50860173
chr14 50860199
chr14 50860239
chr14 50860315
chr14 50860326
chr14 50860357
chr14 50860370
chr14 50860774
chr14 50871833
chr14 50875295
chr14 50875319
chr14 50967936
chr14 50984574
chr14 51056002
chr14 51056116
chr14 51057005
chr14 51092218
chr14 51242624
chr14 51242640
chr14 51242701
chr14 51242733
chr14 51242763
chr14 51242807
chr14 51242931
chr14 51243562
chr14 51243678
chr14 51367846
chr14 51458205
chr14 51758062
chr14 51758079
chr14 51758108
chr14 51758192
chr14 51847462
chr14 51847497
chr14 51847660
chr14 51847681
chr14 51847767
chr14 51847824
chr14 51860522
chr14 51860560
chr14 51860581
chr14 51861540
chr14 51861584
chr14 51861586
chr14 51861808
chr14 51862073
chr14 51862127
chr14 51862344

chr14 51862439
chr14 51862674
chr14 51862677
chr14 51862767
chr14 51863227
chr14 51905720
chr14 51905759
chr14 51905906
chr14 51905938
chr14 51906034
chr14 51979257
chr14 51979276
chr14 51979304
chr14 51979315
chr14 51979317
chr14 51979324
chr14 51979354
chr14 51989435
chr14 51989473
chr14 51989476
chr14 51989483
chr14 51989496
chr14 51989503
chr14 51989510
chr14 51989513
chr14 51989521
chr14 51989526
chr14 51989534
chr14 51989542
chr14 51989550
chr14 51989553
chr14 51989555
chr14 51989558
chr14 51989561
chr14 51989570
chr14 51989577
chr14 51989587
chr14 51989601
chr14 51989605
chr14 51989612
chr14 51989625
chr14 51989632
chr14 51990351
chr14 51990392
chr14 51990563
chr14 51990727
chr14 52055219
chr14 52131495
chr14 52131749
chr14 52131851
chr14 52131854
chr14 52328715
chr14 52329045
chr14 52329529

chr14 52352349
chr14 52352424
chr14 52352629
chr14 52352656
chr14 52352668
chr14 52401018
chr14 52413921
chr14 52413929
chr14 52414132
chr14 52414236
chr14 52414258
chr14 52553468
chr14 52553487
chr14 52553491
chr14 52553495
chr14 52553560
chr14 52553587
chr14 52553598
chr14 52553607
chr14 52553614
chr14 52553621
chr14 52553625
chr14 52553631
chr14 52553633
chr14 52553639
chr14 52553642
chr14 52553655
chr14 52553668
chr14 52553674
chr14 52553688
chr14 52553695
chr14 52553753
chr14 52554029
chr14 52554067
chr14 52554120
chr14 52554141
chr14 52554145
chr14 52554239
chr14 52554249
chr14 52694550
chr14 52694703
chr14 52706779
chr14 52707052
chr14 52707084
chr14 52707094
chr14 52707115
chr14 52707141
chr14 52707148
chr14 52707153
chr14 52707165
chr14 52707169
chr14 52707173
chr14 52707189
chr14 52707208

chr14 52707224
chr14 52707242
chr14 52707256
chr14 52707285
chr14 52707288
chr14 52707292
chr14 52707341
chr14 52707346
chr14 52707377
chr14 52707380
chr14 52707386
chr14 52707393
chr14 52707433
chr14 52707445
chr14 52707475
chr14 52707502
chr14 52707517
chr14 52707520
chr14 52707572
chr14 52707623
chr14 52707644
chr14 52707689
chr14 52707706
chr14 52707713
chr14 52707717
chr14 52707719
chr14 52707764
chr14 52707776
chr14 52707958
chr14 52708003
chr14 52708015
chr14 52708087
chr14 52708226
chr14 52708245
chr14 52708250
chr14 52730954
chr14 52730960
chr14 52741582
chr14 52741678
chr14 52741728
chr14 52741861
chr14 52742331
chr14 52742374
chr14 52742395
chr14 52895117
chr14 52895201
chr14 52949365
chr14 52949404
chr14 52949684
chr14 53109065
chr14 53109078
chr14 53109117
chr14 53109539
chr14 53109645

chr14 53126942
chr14 53126962
chr14 53127070
chr14 53127274
chr14 53151693
chr14 53151834
chr14 53217579
chr14 53217583
chr14 53217666
chr14 53217695
chr14 53217697
chr14 53217726
chr14 53217729
chr14 53217738
chr14 53217752
chr14 53217775
chr14 53217780
chr14 53217783
chr14 53217789
chr14 53217792
chr14 53217808
chr14 53217816
chr14 53217825
chr14 53217833
chr14 53217843
chr14 53217845
chr14 53217862
chr14 53217868
chr14 53217877
chr14 53217946
chr14 53217954
chr14 53217990
chr14 53218007
chr14 53218010
chr14 53924604
chr14 54175449
chr14 54175460
chr14 54220821
chr14 54220912
chr14 54220965
chr14 54298564
chr14 54298594
chr14 54329049
chr14 54441789
chr14 54441917
chr14 54441956
chr14 54442078
chr14 54489041
chr14 54489043
chr14 54489052
chr14 54489057
chr14 54489062
chr14 54509244
chr14 54509331

chr14 54509416
chr14 54509493
chr14 54509500
chr14 54509594
chr14 54509597
chr14 54509625
chr14 54509634
chr14 54509638
chr14 54509967
chr14 54509982
chr14 54509997
chr14 54510000
chr14 54510013
chr14 54510015
chr14 54510037
chr14 54510046
chr14 54510087
chr14 54510095
chr14 54510102
chr14 54510116
chr14 54510118
chr14 54510121
chr14 54510138
chr14 54510232
chr14 54510243
chr14 54510262
chr14 54510271
chr14 54510276
chr14 54510300
chr14 54510335
chr14 54510410
chr14 54510416
chr14 54510481
chr14 54510562
chr14 54564366
chr14 54564406
chr14 54564601
chr14 54564660
chr14 54564666
chr14 54564711
chr14 54564722
chr14 54564731
chr14 54564793
chr14 54564895
chr14 54564897
chr14 54627407
chr14 54627602
chr14 54627654
chr14 54627728
chr14 54627819
chr14 54628033
chr14 54628067
chr14 54628192
chr14 54730407

chr14 54730449
chr14 54730498
chr14 54730535
chr14 54731075
chr14 54731122
chr14 54881299
chr14 54881465
chr14 54881516
chr14 54881611
chr14 54881623
chr14 54881671
chr14 54881963
chr14 54881991
chr14 54882056
chr14 54882147
chr14 54882151
chr14 54901045
chr14 54901082
chr14 54901178
chr14 54901230
chr14 54901260
chr14 54901307
chr14 55026484
chr14 55026511
chr14 55026526
chr14 55026548
chr14 55026553
chr14 55026617
chr14 55026688
chr14 55027641
chr14 55027676
chr14 55027692
chr14 55027734
chr14 55027797
chr14 55027811
chr14 55028076
chr14 55028127
chr14 55028207
chr14 55028210
chr14 55028221
chr14 55028242
chr14 55028282
chr14 55028371
chr14 55051832
chr14 55051845
chr14 55051861
chr14 55051868
chr14 55051870
chr14 55051874
chr14 55051876
chr14 55051884
chr14 55102271
chr14 55102316
chr14 55102350

chr14 55102384
chr14 55102484
chr14 55102507
chr14 55102519
chr14 55191868
chr14 55213276
chr14 55270878
chr14 55270892
chr14 55270911
chr14 55271094
chr14 55271136
chr14 55271223
chr14 55272345
chr14 55272350
chr14 55272452
chr14 55297929
chr14 55333053
chr14 55333911
chr14 55333927
chr14 55374121
chr14 55374133
chr14 55374356
chr14 55374758
chr14 55374784
chr14 55374833
chr14 55386358
chr14 55411023
chr14 55411080
chr14 55411230
chr14 55411304
chr14 55411752
chr14 55411764
chr14 55411771
chr14 55412081
chr14 55412144
chr14 55412155
chr14 55581377
chr14 55581390
chr14 55581422
chr14 55581507
chr14 55864580
chr14 56184826
chr14 56184981
chr14 56185300
chr14 56580443
chr14 56670947
chr14 56671161
chr14 56671175
chr14 56672480
chr14 56672732
chr14 56673470
chr14 56673588
chr14 56673747
chr14 56796297

chr14 56805833
chr14 56805876
chr14 56806140
chr14 56806159
chr14 56806208
chr14 56806245
chr14 56806289
chr14 56806349
chr14 56806490
chr14 56806558
chr14 56806589
chr14 56806800
chr14 56806949
chr14 56806988
chr14 56807004
chr14 56807254
chr14 56807302
chr14 56807376
chr14 56807419
chr14 56813369
chr14 56813462
chr14 56813692
chr14 56813806
chr14 56813853
chr14 56813967
chr14 56814065
chr14 56814112
chr14 56814436
chr14 56814803
chr14 56815116
chr14 56815146
chr14 56815225
chr14 56815247
chr14 56815708
chr14 56815952
chr14 56815968
chr14 56815997
chr14 56816039
chr14 56816066
chr14 56816169
chr14 56816207
chr14 56816278
chr14 56816298
chr14 56816339
chr14 56816430
chr14 56837039
chr14 56837134
chr14 56855386
chr14 56855772
chr14 56855935
chr14 56855999
chr14 56856037
chr14 56856155
chr14 56856241

chr14 56856271
chr14 56856302
chr14 56856343
chr14 56993909
chr14 57067031
chr14 57067041
chr14 57067044
chr14 57067051
chr14 57067056
chr14 57067073
chr14 57067089
chr14 57067095
chr14 57067114
chr14 57067247
chr14 57067382
chr14 57067524
chr14 57067777
chr14 57068419
chr14 57267663
chr14 57267737
chr14 57267746
chr14 57267779
chr14 57267783
chr14 57267903
chr14 57267912
chr14 57267949
chr14 57267960
chr14 57268037
chr14 57268165
chr14 57268185
chr14 57269319
chr14 57269324
chr14 57269347
chr14 57269352
chr14 57269374
chr14 57269432
chr14 57269461
chr14 57269479
chr14 57269489
chr14 57269526
chr14 57269605
chr14 57269647
chr14 57269684
chr14 57269744
chr14 57269831
chr14 57269839
chr14 57269871
chr14 57269878
chr14 57269894
chr14 57269901
chr14 58109417
chr14 58171536
chr14 58171676
chr14 58171992

chr14 58172206
chr14 58200528
chr14 58200531
chr14 58200534
chr14 58200904
chr14 58239509
chr14 58239544
chr14 58239710
chr14 58239719
chr14 58239735
chr14 58239760
chr14 58239863
chr14 58239893
chr14 58239895
chr14 58239902
chr14 58239911
chr14 58239919
chr14 58239950
chr14 58239963
chr14 58239967
chr14 58239976
chr14 58240033
chr14 58240035
chr14 58240064
chr14 58240077
chr14 58240103
chr14 58240176
chr14 58240301
chr14 58240324
chr14 58240375
chr14 58240377
chr14 58240443
chr14 58240471
chr14 58240499
chr14 58240613
chr14 58240714
chr14 58240718
chr14 58240720
chr14 58245445
chr14 58245509
chr14 58245537
chr14 58245556
chr14 58245562
chr14 58245665
chr14 58245839
chr14 58246178
chr14 58275376
chr14 58296387
chr14 58296470
chr14 58296478
chr14 58296497
chr14 58296611
chr14 58296640
chr14 58296697

chr14 58296881
chr14 58297008
chr14 58299744
chr14 58299751
chr14 58299804
chr14 58299810
chr14 58299821
chr14 58299883
chr14 58299890
chr14 58299892
chr14 58299925
chr14 58299982
chr14 58300034
chr14 58300102
chr14 58300210
chr14 58300222
chr14 58300244
chr14 58330937
chr14 58331082
chr14 58331112
chr14 58331177
chr14 58331218
chr14 58331246
chr14 58331392
chr14 58331564
chr14 58428044
chr14 58428152
chr14 58428228
chr14 58428328
chr14 58428398
chr14 58428418
chr14 58428434
chr14 58428460
chr14 58428540
chr14 58428592
chr14 58428652
chr14 58428763
chr14 58718295
chr14 58718748
chr14 58718953
chr14 58719012
chr14 58795194
chr14 58795261
chr14 59029215
chr14 59029787
chr14 59030140
chr14 59030238
chr14 59172122
chr14 59172295
chr14 59187420
chr14 59187490
chr14 59187562
chr14 59187679
chr14 59187684

chr14 59187810
chr14 59187841
chr14 59187843
chr14 59187883
chr14 59408090
chr14 59408795
chr14 59444353
chr14 59485205
chr14 59576402
chr14 59576419
chr14 59576454
chr14 59576485
chr14 59576525
chr14 59576540
chr14 59576553
chr14 59576571
chr14 59576588
chr14 59576600
chr14 59576604
chr14 59576611
chr14 59576641
chr14 59576646
chr14 59576650
chr14 59576652
chr14 59576656
chr14 59576712
chr14 59576719
chr14 59576736
chr14 59576742
chr14 59868177
chr14 59868204
chr14 59918462
chr14 59918688
chr14 59918782
chr14 59918991
chr14 59919073
chr14 59919174
chr14 59919218
chr14 59919262
chr14 59919277
chr14 59919292
chr14 59919312
chr14 59919327
chr14 59919332
chr14 59919341
chr14 59919499
chr14 59919505
chr14 59919520
chr14 59919554
chr14 59919558
chr14 59919566
chr14 59919568
chr14 59919571
chr14 60093444

chr14 60093474
chr14 60164606
chr14 60164676
chr14 60164748
chr14 60164790
chr14 60164800
chr14 60164822
chr14 60164859
chr14 60165680
chr14 60165713
chr14 60165814
chr14 60166153
chr14 60247985
chr14 60248013
chr14 60248035
chr14 60248051
chr14 60248092
chr14 60248121
chr14 60248340
chr14 60248367
chr14 60331165
chr14 60331201
chr14 60331261
chr14 60331280
chr14 60331284
chr14 60331286
chr14 60331288
chr14 60331294
chr14 60331369
chr14 60331474
chr14 60331500
chr14 60331544
chr14 60331557
chr14 60331696
chr14 60331708
chr14 60331713
chr14 60331730
chr14 60331739
chr14 60331755
chr14 60331844
chr14 60331915
chr14 60331939
chr14 60331947
chr14 60332126
chr14 60486180
chr14 60486190
chr14 60486201
chr14 60486203
chr14 60486213
chr14 60486215
chr14 60486227
chr14 60486760
chr14 60487016
chr14 60487033

chr14 60487062
chr14 60487246
chr14 60487492
chr14 60487740
chr14 60487826
chr14 60487856
chr14 60487871
chr14 60487900
chr14 60505437
chr14 60505529
chr14 60505675
chr14 60505905
chr14 60505960
chr14 60506008
chr14 60506111
chr14 60511988
chr14 60512000
chr14 60512074
chr14 60512112
chr14 60512134
chr14 60512177
chr14 60512595
chr14 60512648
chr14 60512688
chr14 60512739
chr14 60513032
chr14 60513091
chr14 60513750
chr14 60514402
chr14 60514455
chr14 60514500
chr14 60514503
chr14 60514524
chr14 60514536
chr14 60514570
chr14 60514591
chr14 60514633
chr14 60514715
chr14 60638393
chr14 60638450
chr14 60638478
chr14 60638528
chr14 60639666
chr14 60639980
chr14 60640554
chr14 60640626
chr14 60641010
chr14 60641051
chr14 60644309
chr14 60644421
chr14 60644840
chr14 60645370
chr14 60645599
chr14 60645963

chr14 60650547
chr14 60650553
chr14 60650559
chr14 60650884
chr14 60651648
chr14 60651653
chr14 60658476
chr14 60658539
chr14 60658630
chr14 60682050
chr14 60682203
chr14 60723811
chr14 60723832
chr14 60723835
chr14 60723838
chr14 60723843
chr14 60723847
chr14 60723850
chr14 60723853
chr14 60723856
chr14 60723876
chr14 60723888
chr14 60723894
chr14 60723898
chr14 60723900
chr14 60723903
chr14 60723910
chr14 60723937
chr14 60723955
chr14 60723958
chr14 60723960
chr14 60723967
chr14 60723977
chr14 60723982
chr14 60723997
chr14 60724002
chr14 60734376
chr14 60734471
chr14 60734572
chr14 60734679
chr14 60734695
chr14 60734700
chr14 60734714
chr14 60734719
chr14 60734740
chr14 60734742
chr14 60735046
chr14 60735089
chr14 60735115
chr14 60735124
chr14 60735128
chr14 60735151
chr14 60735214
chr14 60735259

chr14 60735272
chr14 60735324
chr14 60753523
chr14 60753587
chr14 60753620
chr14 60753874
chr14 60753995
chr14 60754017
chr14 60754049
chr14 60971390
chr14 60981643
chr14 60981661
chr14 60981729
chr14 60981767
chr14 60981823
chr14 60981861
chr14 60981896
chr14 60981908
chr14 60981942
chr14 60981992
chr14 61189240
chr14 61189313
chr14 61189391
chr14 61189416
chr14 61189589
chr14 61269396
chr14 61269425
chr14 61269546
chr14 61269563
chr14 61269611
chr14 61269701
chr14 61269755
chr14 61269783
chr14 61269807
chr14 61269831
chr14 61269836
chr14 61269841
chr14 61269846
chr14 61269862
chr14 61269875
chr14 61269887
chr14 61269895
chr14 61269898
chr14 61323216
chr14 61323228
chr14 61323310
chr14 61323319
chr14 61323378
chr14 61323444
chr14 61323616
chr14 61323703
chr14 61323900
chr14 61323995
chr14 61324103

chr14 61324141
chr14 61324217
chr14 61326936
chr14 61327039
chr14 61327058
chr14 61327636
chr14 61327972
chr14 61327976
chr14 61327978
chr14 61327988
chr14 61328016
chr14 61328021
chr14 61328046
chr14 61328166
chr14 61328174
chr14 61328181
chr14 61328198
chr14 61328206
chr14 61328214
chr14 61328219
chr14 61328226
chr14 61328243
chr14 61328431
chr14 61328524
chr14 61328644
chr14 61328709
chr14 61328711
chr14 61328725
chr14 61331001
chr14 61332691
chr14 61332749
chr14 61332769
chr14 61332820
chr14 61332825
chr14 61332878
chr14 61332925
chr14 61332959
chr14 61333053
chr14 61333070
chr14 61333223
chr14 61343549
chr14 61343883
chr14 61343978
chr14 61343990
chr14 61355708
chr14 61355773
chr14 61385322
chr14 61385334
chr14 61385422
chr14 61385523
chr14 61385537
chr14 61385576
chr14 61452496
chr14 61452530

chr14 61452560
chr14 61452891
chr14 61452896
chr14 61453117
chr14 61471812
chr14 61471909
chr14 61471974
chr14 61472084
chr14 61472096
chr14 61472102
chr14 61472109
chr14 61472174
chr14 61472485
chr14 61472614
chr14 61472636
chr14 61473013
chr14 61473227
chr14 61473230
chr14 61525128
chr14 61578949
chr14 61578964
chr14 61662187
chr14 61662206
chr14 61662272
chr14 61662340
chr14 61662490
chr14 61671169
chr14 61671188
chr14 61671304
chr14 61695224
chr14 61695227
chr14 61695240
chr14 61695246
chr14 61695252
chr14 61695255
chr14 61695262
chr14 61695286
chr14 61695307
chr14 61695310
chr14 61695333
chr14 61695339
chr14 61695342
chr14 61695346
chr14 61695359
chr14 61695368
chr14 61695388
chr14 61695396
chr14 61695402
chr14 61695404
chr14 61695406
chr14 61695409
chr14 61695422
chr14 61695426
chr14 61695430

chr14 61695432
chr14 61695438
chr14 61695456
chr14 61695458
chr14 61695460
chr14 61695464
chr14 61695468
chr14 61695470
chr14 61695476
chr14 61697530
chr14 61697813
chr14 61697866
chr14 61697981
chr14 61698035
chr14 61698686
chr14 61698965
chr14 61699054
chr14 61699194
chr14 61699215
chr14 61750598
chr14 61750689
chr14 61750716
chr14 61750760
chr14 61750822
chr14 61750909
chr14 61750924
chr14 61750931
chr14 61750948
chr14 61750981
chr14 61751008
chr14 61751014
chr14 61751088
chr14 61751404
chr14 61751450
chr14 61751456
chr14 61751477
chr14 61751493
chr14 61867018
chr14 61867313
chr14 61867399
chr14 61867419
chr14 61867472
chr14 61867526
chr14 62472010
chr14 63044234
chr14 63044374
chr14 63044455
chr14 63084260
chr14 63084495
chr14 63383499
chr14 63383832
chr14 63383944
chr14 63477915
chr14 63478185

chr14 63478238
chr14 63484577
chr14 63510817
chr14 63542128
chr14 63542172
chr14 63542188
chr14 63542198
chr14 63542226
chr14 63542239
chr14 63542245
chr14 63542271
chr14 63542290
chr14 63633582
chr14 63642826
chr14 63650438
chr14 63650676
chr14 63650702
chr14 63759535
chr14 63759541
chr14 63759676
chr14 63759790
chr14 63864241
chr14 63864512
chr14 63864891
chr14 63865094
chr14 63959024
chr14 64294648
chr14 64337509
chr14 64337562
chr14 64337932
chr14 64337934
chr14 64337964
chr14 64338011
chr14 64338014
chr14 64338039
chr14 64338068
chr14 64338074
chr14 64338085
chr14 64338095
chr14 64338127
chr14 64338129
chr14 64338279
chr14 64339104
chr14 64388941
chr14 64388983
chr14 64389036
chr14 64389103
chr14 64389134
chr14 64389313
chr14 64389414
chr14 64501672
chr14 64501902
chr14 64501923
chr14 64501943

chr14 64502002
chr14 64502025
chr14 64502129
chr14 64502179
chr14 64505725
chr14 64505757
chr14 64505791
chr14 64505802
chr14 64505824
chr14 64505859
chr14 64505900
chr14 64505918
chr14 64505936
chr14 64506018
chr14 64506254
chr14 64506258
chr14 64506270
chr14 64506298
chr14 64506303
chr14 64506316
chr14 64506330
chr14 64506353
chr14 64506393
chr14 64506401
chr14 64506405
chr14 64506447
chr14 64506464
chr14 64506481
chr14 64506489
chr14 64506520
chr14 64506619
chr14 64506655
chr14 64506765
chr14 64506984
chr14 64507004
chr14 64507349
chr14 64507511
chr14 64510553
chr14 64511163
chr14 64511214
chr14 64511283
chr14 64549991
chr14 64550004
chr14 64550012
chr14 64671661
chr14 64914155
chr14 64983850
chr14 64983883
chr14 64983896
chr14 64983902
chr14 64983970
chr14 64984003
chr14 65098696
chr14 65098780

chr14 65101026
chr14 65101086
chr14 65101120
chr14 65101127
chr14 65170880
chr14 65170887
chr14 65170896
chr14 65170928
chr14 65170957
chr14 65170996
chr14 65171041
chr14 65171087
chr14 65171089
chr14 65171092
chr14 65171100
chr14 65171105
chr14 65171120
chr14 65171131
chr14 65171192
chr14 65171347
chr14 65171442
chr14 65180071
chr14 65180109
chr14 65180244
chr14 65206076
chr14 65206134
chr14 65206227
chr14 65206255
chr14 65206284
chr14 65206317
chr14 65206345
chr14 65206532
chr14 65206928
chr14 65206943
chr14 65321446
chr14 65321531
chr14 65321560
chr14 65411319
chr14 65411378
chr14 65411610
chr14 65411613
chr14 65411640
chr14 65411701
chr14 65411712
chr14 65411719
chr14 65411769
chr14 65411785
chr14 65411801
chr14 65411806
chr14 65411819
chr14 65411821
chr14 65413882
chr14 65413933
chr14 65413978

chr14 65414042
chr14 65414166
chr14 65414209
chr14 65414295
chr14 65414298
chr14 65414688
chr14 65414896
chr14 65847193
chr14 65847216
chr14 65847283
chr14 65847375
chr14 65847593
chr14 65847650
chr14 65927901
chr14 65927985
chr14 65928060
chr14 66359057
chr14 66359450
chr14 66359499
chr14 67242004
chr14 67242016
chr14 67242098
chr14 67242134
chr14 67242157
chr14 67242233
chr14 67242247
chr14 67242346
chr14 67242417
chr14 67242549
chr14 67242625
chr14 67361054
chr14 67361064
chr14 67361152
chr14 67361240
chr14 67361413
chr14 67488394
chr14 67488471
chr14 67488508
chr14 67488526
chr14 67488533
chr14 67488554
chr14 67488585
chr14 67488596
chr14 67488705
chr14 67488756
chr14 67488768
chr14 67593658
chr14 67599605
chr14 67599652
chr14 67599702
chr14 67599704
chr14 67599746
chr14 67599749
chr14 67599751

chr14 67599783
chr14 67599801
chr14 67599843
chr14 67599868
chr14 67599876
chr14 67599889
chr14 67599899
chr14 67599930
chr14 67599942
chr14 67599948
chr14 67599979
chr14 67599993
chr14 67599997
chr14 67599999
chr14 67600010
chr14 67600026
chr14 67600044
chr14 67600051
chr14 67600063
chr14 67600077
chr14 67600084
chr14 67600088
chr14 67600093
chr14 67600100
chr14 67600124
chr14 67600132
chr14 67600145
chr14 67600148
chr14 67600156
chr14 67600163
chr14 67600167
chr14 67619478
chr14 67669292
chr14 67674778
chr14 67674780
chr14 67674787
chr14 67674797
chr14 67674803
chr14 67674819
chr14 67674824
chr14 67694834
chr14 67694879
chr14 67695514
chr14 67695641
chr14 67695683
chr14 67695710
chr14 67695719
chr14 67695730
chr14 67695743
chr14 67695750
chr14 67695753
chr14 67695778
chr14 67695795
chr14 67695803

chr14 67695860
chr14 67695868
chr14 67695874
chr14 67695880
chr14 67739953
chr14 67816709
chr14 67816714
chr14 67816725
chr14 67816750
chr14 67816778
chr14 67816807
chr14 67816810
chr14 67816835
chr14 67816839
chr14 67816930
chr14 67816958
chr14 67816960
chr14 67816971
chr14 67820157
chr14 67820162
chr14 67820222
chr14 67820238
chr14 67820251
chr14 67820257
chr14 68182576
chr14 68182712
chr14 68182745
chr14 68182789
chr14 68182799
chr14 68210792
chr14 68210821
chr14 68210830
chr14 68210956
chr14 68283510
chr14 68283959
chr14 68283974
chr14 68283997
chr14 68628957
chr14 68628962
chr14 68628970
chr14 68628982
chr14 68628986
chr14 68663798
chr14 68664025
chr14 68685673
chr14 68685707
chr14 68685754
chr14 68685832
chr14 68711968
chr14 68712151
chr14 68734749
chr14 68734773
chr14 68779792
chr14 68779889

chr14 68780042
chr14 68780058
chr14 68780143
chr14 68780171
chr14 68780221
chr14 68780498
chr14 68780650
chr14 68780709
chr14 68781018
chr14 68781039
chr14 68781230
chr14 68787566
chr14 68787675
chr14 68787707
chr14 68787767
chr14 68787770
chr14 68787909
chr14 68788182
chr14 68788229
chr14 68788303
chr14 68788354
chr14 68788468
chr14 68788493
chr14 68788538
chr14 68788778
chr14 68788782
chr14 68788851
chr14 68789001
chr14 68789035
chr14 68789060
chr14 68789073
chr14 68789091
chr14 68789102
chr14 68789116
chr14 68789227
chr14 68789573
chr14 68789593
chr14 68789650
chr14 68789700
chr14 68789711
chr14 68789728
chr14 68789742
chr14 68789907
chr14 68789910
chr14 68789919
chr14 68789960
chr14 68789966
chr14 68789973
chr14 68789984
chr14 68789991
chr14 68790002
chr14 68790005
chr14 68790015
chr14 68790035

chr14 68790042
chr14 68790057
chr14 68790074
chr14 68790085
chr14 68790104
chr14 68790116
chr14 68790138
chr14 68790156
chr14 68790209
chr14 68790228
chr14 68790234
chr14 68790260
chr14 68790264
chr14 68790274
chr14 68790284
chr14 68790290
chr14 68790296
chr14 68790305
chr14 68790307
chr14 68790318
chr14 68790398
chr14 68790577
chr14 68790581
chr14 68790611
chr14 68790681
chr14 68790859
chr14 68790866
chr14 68790872
chr14 68790878
chr14 68790882
chr14 68790884
chr14 68790928
chr14 68791151
chr14 68791211
chr14 68814985
chr14 68815120
chr14 68844416
chr14 68844468
chr14 68844479
chr14 68862942
chr14 68863423
chr14 68863427
chr14 68978042
chr14 69191411
chr14 69191423
chr14 69191430
chr14 69191440
chr14 69191455
chr14 69191471
chr14 69191479
chr14 69191491
chr14 69191506
chr14 69191516
chr14 69191518

chr14 69191524
chr14 69191528
chr14 69191550
chr14 69191558
chr14 69191571
chr14 69191581
chr14 69191611
chr14 69191614
chr14 69191637
chr14 69191639
chr14 69191677
chr14 69191684
chr14 69191698
chr14 69191704
chr14 69397104
chr14 69397288
chr14 69397312
chr14 69398709
chr14 69398730
chr14 69398733
chr14 69398739
chr14 69398751
chr14 69398755
chr14 69398779
chr14 69398837
chr14 69398876
chr14 69535495
chr14 69535498
chr14 69584853
chr14 69584863
chr14 69585052
chr14 69585177
chr14 69612523
chr14 69612565
chr14 69612626
chr14 69612665
chr14 69612723
chr14 69612747
chr14 69612893
chr14 69612927
chr14 69613286
chr14 69613480
chr14 69613547
chr14 69615712
chr14 69615846
chr14 69615935
chr14 69615995
chr14 69616121
chr14 69616539
chr14 69625215
chr14 69625442
chr14 69625451
chr14 69625512
chr14 69625523

chr14 69625557
chr14 69625584
chr14 69883418
chr14 70254585
chr14 70254596
chr14 70254605
chr14 70254620
chr14 70254683
chr14 70254689
chr14 70254694
chr14 70254705
chr14 70289178
chr14 70289270
chr14 70289278
chr14 70289290
chr14 70289300
chr14 70359246
chr14 70359248
chr14 70359252
chr14 70359255
chr14 70359267
chr14 70359272
chr14 70359313
chr14 70359329
chr14 70359332
chr14 70359390
chr14 70359393
chr14 70359412
chr14 70359493
chr14 70359539
chr14 70359556
chr14 70359565
chr14 70359573
chr14 70359575
chr14 70359604
chr14 70359677
chr14 70359717
chr14 70416358
chr14 70416462
chr14 70416490
chr14 70416514
chr14 70416538
chr14 70416572
chr14 70416721
chr14 70416767
chr14 70416772
chr14 70416779
chr14 70416804
chr14 70416809
chr14 70416825
chr14 70416891
chr14 70599831
chr14 70599866
chr14 70600101

chr14 70600128
chr14 70600132
chr14 70600148
chr14 70600193
chr14 70600200
chr14 70600202
chr14 70600618
chr14 70600643
chr14 70600649
chr14 70600671
chr14 70600682
chr14 70600695
chr14 70600698
chr14 70600707
chr14 70600723
chr14 70600741
chr14 70600748
chr14 70600760
chr14 70600765
chr14 70600795
chr14 70600814
chr14 70600816
chr14 70600824
chr14 70600831
chr14 70600840
chr14 70600842
chr14 70600849
chr14 70600856
chr14 70600901
chr14 70600906
chr14 70600970
chr14 70600978
chr14 70601035
chr14 70601042
chr14 70601075
chr14 70601085
chr14 70601153
chr14 70601186
chr14 70640322
chr14 70640596
chr14 70640724
chr14 70640809
chr14 70640827
chr14 70640976
chr14 70641017
chr14 70641026
chr14 70641044
chr14 70641143
chr14 70641214
chr14 70641218
chr14 70641222
chr14 70641230
chr14 70655702
chr14 70655920

chr14 70655947
chr14 70818170
chr14 70818188
chr14 70818233
chr14 70822184
chr14 70822199
chr14 70822235
chr14 70822237
chr14 70822239
chr14 70822262
chr14 70822266
chr14 70822268
chr14 70822272
chr14 70822282
chr14 70822366
chr14 70822370
chr14 70822377
chr14 70822395
chr14 70822411
chr14 70822421
chr14 70822431
chr14 70822441
chr14 70822467
chr14 70822498
chr14 70822509
chr14 70822589
chr14 70822593
chr14 70822612
chr14 70887292
chr14 70908665
chr14 70908671
chr14 70908677
chr14 70908684
chr14 70908718
chr14 70908729
chr14 70908827
chr14 70908832
chr14 70908887
chr14 70908918
chr14 70908931
chr14 70909096
chr14 70909231
chr14 70909358
chr14 70909447
chr14 70981507
chr14 70981530
chr14 70981784
chr14 70981799
chr14 70981882
chr14 70982419
chr14 71319859
chr14 71319869
chr14 71319941
chr14 71320076

chr14 71320094
chr14 71322038
chr14 71322090
chr14 71322121
chr14 71322166
chr14 71322408
chr14 71322427
chr14 71322533
chr14 71322883
chr14 71478672
chr14 71554520
chr14 71554928
chr14 71555045
chr14 71555330
chr14 71555731
chr14 71566160
chr14 71566383
chr14 71566443
chr14 72128569
chr14 72128708
chr14 72183308
chr14 72476651
chr14 72476744
chr14 72476753
chr14 72476782
chr14 72476832
chr14 72476877
chr14 72477006
chr14 72477064
chr14 72518688
chr14 72518709
chr14 72518954
chr14 72519014
chr14 72519063
chr14 72594249
chr14 72594253
chr14 72594318
chr14 72594340
chr14 72594358
chr14 72594607
chr14 72594625
chr14 72594648
chr14 72594668
chr14 72594769
chr14 72594800
chr14 72594838
chr14 72706932
chr14 72894672
chr14 72894688
chr14 72894703
chr14 73026869
chr14 73026871
chr14 73026879
chr14 73026890

chr14 73026904
chr14 73026907
chr14 73026909
chr14 73026914
chr14 73027398
chr14 73136520
chr14 73136547
chr14 73136552
chr14 73136554
chr14 73136557
chr14 73136563
chr14 73136568
chr14 73136571
chr14 73136579
chr14 73136587
chr14 73136601
chr14 73136621
chr14 73136623
chr14 73136633
chr14 73136637
chr14 73136639
chr14 73136650
chr14 73136664
chr14 73136668
chr14 73136673
chr14 73136675
chr14 73318154
chr14 73455430
chr14 73457556
chr14 73457566
chr14 73457951
chr14 73644923
chr14 73644937
chr14 73644943
chr14 73644958
chr14 73644961
chr14 73644967
chr14 73644996
chr14 73644998
chr14 73645033
chr14 73645036
chr14 73645055
chr14 73645060
chr14 73645062
chr14 73645140
chr14 73645151
chr14 73645161
chr14 73645186
chr14 73645191
chr14 73645209
chr14 73645231
chr14 73645246
chr14 73645294
chr14 73645322

chr14 73645404
chr14 73645407
chr14 73645451
chr14 73645468
chr14 73645508
chr14 73696441
chr14 73696703
chr14 73712150
chr14 73712152
chr14 73712210
chr14 73712271
chr14 73712273
chr14 73712312
chr14 73712323
chr14 73712370
chr14 73712385
chr14 73712391
chr14 73712398
chr14 73712654
chr14 73712706
chr14 73712726
chr14 73712736
chr14 73712751
chr14 73712771
chr14 73712840
chr14 73712869
chr14 73712922
chr14 73712944
chr14 73712958
chr14 73712971
chr14 73712973
chr14 73712996
chr14 73713009
chr14 73713012
chr14 73713038
chr14 73713041
chr14 73713057
chr14 73713244
chr14 73713250
chr14 73713278
chr14 73713294
chr14 73713394
chr14 73713412
chr14 73713414
chr14 73713417
chr14 73713429
chr14 73713457
chr14 73713494
chr14 73713514
chr14 73713534
chr14 73713608
chr14 73741713
chr14 73741835
chr14 73743659

chr14 73758527
chr14 73758556
chr14 73758558
chr14 73758566
chr14 73758575
chr14 73758604
chr14 73758611
chr14 73758613
chr14 73758615
chr14 73758646
chr14 73758676
chr14 73771823
chr14 73771983
chr14 73772002
chr14 73772030
chr14 73772045
chr14 73772088
chr14 73772099
chr14 73772163
chr14 73787926
chr14 73788044
chr14 73788079
chr14 73788135
chr14 73788177
chr14 73788232
chr14 73825570
chr14 73825637
chr14 73825688
chr14 73825737
chr14 73825801
chr14 73887111
chr14 73887113
chr14 73887127
chr14 73887202
chr14 73887210
chr14 73887215
chr14 73887236
chr14 73887242
chr14 73887255
chr14 73887400
chr14 73887414
chr14 73887418
chr14 73887441
chr14 73887469
chr14 73887485
chr14 73887490
chr14 73887636
chr14 73887646
chr14 73950273
chr14 73950277
chr14 73950284
chr14 73950298
chr14 73950313
chr14 73950316

chr14 73950319
chr14 73950337
chr14 73950342
chr14 73950354
chr14 73950359
chr14 73950369
chr14 73950386
chr14 74083853
chr14 74083860
chr14 74083862
chr14 74083893
chr14 74083938
chr14 74083944
chr14 74084022
chr14 74084029
chr14 74084068
chr14 74085659
chr14 74085826
chr14 74167161
chr14 74302370
chr14 74302624
chr14 74302644
chr14 74302664
chr14 74302674
chr14 74302684
chr14 74302686
chr14 74302690
chr14 74302695
chr14 74302700
chr14 74302710
chr14 74302713
chr14 74302721
chr14 74302725
chr14 74302733
chr14 74401586
chr14 74401660
chr14 74401678
chr14 74401688
chr14 74401740
chr14 74492420
chr14 74492427
chr14 74492524
chr14 74492537
chr14 74492554
chr14 74492621
chr14 74492624
chr14 74492785
chr14 74492825
chr14 74492910
chr14 74712617
chr14 74712660
chr14 74712673
chr14 74712684
chr14 74763827

chr14 74763883
chr14 74763888
chr14 74763947
chr14 74763998
chr14 74764019
chr14 74764055
chr14 74764064
chr14 74764094
chr14 74764130
chr14 74764189
chr14 74764238
chr14 74764242
chr14 74764326
chr14 74764349
chr14 74764489
chr14 74764555
chr14 74764712
chr14 74764752
chr14 74764943
chr14 74764961
chr14 74765045
chr14 75063666
chr14 75063677
chr14 75063703
chr14 75063745
chr14 75063748
chr14 75063763
chr14 75063768
chr14 75063778
chr14 75063813
chr14 75063837
chr14 75063843
chr14 75247483
chr14 75247608
chr14 75247665
chr14 75247706
chr14 75247709
chr14 75249252
chr14 75249261
chr14 75249583
chr14 75249604
chr14 75249655
chr14 75249666
chr14 75249674
chr14 75249707
chr14 75249822
chr14 75249874
chr14 75294725
chr14 75294728
chr14 75294753
chr14 75294806
chr14 75294811
chr14 75295063
chr14 75295090

chr14 75295094
chr14 75295462
chr14 75295492
chr14 75295528
chr14 75295603
chr14 75295706
chr14 75295717
chr14 75295731
chr14 75295853
chr14 75488885
chr14 75488892
chr14 75488910
chr14 75488938
chr14 75521908
chr14 75522013
chr14 75522034
chr14 75522090
chr14 75522150
chr14 75522211
chr14 75523121
chr14 75542777
chr14 75542808
chr14 75542865
chr14 75542914
chr14 75542927
chr14 75542950
chr14 75542985
chr14 75543059
chr14 75543094
chr14 75543108
chr14 75543135
chr14 75543157
chr14 75543224
chr14 75543264
chr14 75543290
chr14 75543505
chr14 75543547
chr14 75543885
chr14 75578071
chr14 75578075
chr14 75578089
chr14 75578098
chr14 75578131
chr14 75578142
chr14 75660443
chr14 75660483
chr14 75660853
chr14 75661898
chr14 75661950
chr14 75981811
chr14 75981816
chr14 75981825
chr14 75981848
chr14 75981852

chr14 75981879
chr14 75981946
chr14 75982040
chr14 75985647
chr14 75985690
chr14 75985719
chr14 75985737
chr14 75985739
chr14 75985752
chr14 75985769
chr14 75985776
chr14 75985780
chr14 75985782
chr14 75985801
chr14 75985810
chr14 75985820
chr14 75985840
chr14 76152104
chr14 76152108
chr14 76152711
chr14 76152735
chr14 76682978
chr14 76768282
chr14 76768373
chr14 76768379
chr14 76768405
chr14 76768442
chr14 76768445
chr14 76768471
chr14 76768474
chr14 76768509
chr14 76768550
chr14 76956172
chr14 76956184
chr14 76956231
chr14 76956249
chr14 76956256
chr14 76956330
chr14 76956347
chr14 76956368
chr14 76956375
chr14 76956377
chr14 76956395
chr14 76956418
chr14 76956500
chr14 76956507
chr14 76956514
chr14 76956551
chr14 76957091
chr14 76957544
chr14 76961839
chr14 76961858
chr14 76961867
chr14 76961872

chr14 76961883
chr14 76961885
chr14 76961931
chr14 76962123
chr14 76977959
chr14 76977973
chr14 76978029
chr14 76978041
chr14 76978080
chr14 76978088
chr14 76978091
chr14 76978099
chr14 76978124
chr14 77032998
chr14 77033001
chr14 77033046
chr14 77033048
chr14 77033055
chr14 77033077
chr14 77033084
chr14 77033108
chr14 77033114
chr14 77033137
chr14 77033151
chr14 77033154
chr14 77033156
chr14 77033158
chr14 77033176
chr14 77033204
chr14 77033278
chr14 77033283
chr14 77033317
chr14 77033362
chr14 77033372
chr14 77033385
chr14 77033408
chr14 77033415
chr14 77033428
chr14 77033448
chr14 77033476
chr14 77033478
chr14 77033480
chr14 77033488
chr14 77033497
chr14 77033507
chr14 77033905
chr14 77033934
chr14 77033938
chr14 77033955
chr14 77033965
chr14 77033979
chr14 77033982
chr14 77033986
chr14 77033988

chr14 77033990
chr14 77034044
chr14 77034062
chr14 77034066
chr14 77034072
chr14 77034080
chr14 77034086
chr14 77034089
chr14 77034092
chr14 77034119
chr14 77034132
chr14 77034155
chr14 77034165
chr14 77039901
chr14 77039955
chr14 77040064
chr14 77040102
chr14 77040104
chr14 77040112
chr14 77040162
chr14 77040187
chr14 77040197
chr14 77040200
chr14 77040267
chr14 77040290
chr14 77040324
chr14 77040343
chr14 77040357
chr14 77040362
chr14 77040371
chr14 77040388
chr14 77040460
chr14 77040530
chr14 77040552
chr14 77040601
chr14 77040614
chr14 77040616
chr14 77040627
chr14 77040641
chr14 77040706
chr14 77040717
chr14 77040771
chr14 77040788
chr14 77040791
chr14 77321501
chr14 77321504
chr14 77321526
chr14 77321600
chr14 77321605
chr14 77321609
chr14 77321611
chr14 77321621
chr14 77321649
chr14 77321654

chr14 77321658
chr14 77321699
chr14 77321743
chr14 77321751
chr14 77321755
chr14 77321775
chr14 77321799
chr14 77321814
chr14 77321831
chr14 77321837
chr14 77321839
chr14 77321861
chr14 77321869
chr14 77321877
chr14 77321904
chr14 77321908
chr14 77321947
chr14 77321967
chr14 77322038
chr14 77322057
chr14 77339032
chr14 77372529
chr14 77372670
chr14 77372699
chr14 77456810
chr14 77456888
chr14 77456956
chr14 77459117
chr14 77459173
chr14 77459195
chr14 77581295
chr14 77593659
chr14 77615341
chr14 77615449
chr14 77615478
chr14 77615507
chr14 77615533
chr14 77615557
chr14 77615736
chr14 77617181
chr14 77708532
chr14 77708575
chr14 77708595
chr14 77708651
chr14 77708656
chr14 77708683
chr14 77708816
chr14 77708868
chr14 77708879
chr14 77708913
chr14 77708983
chr14 77709105
chr14 77709127
chr14 77709254

chr14 77709389
chr14 77760365
chr14 77760424
chr14 77760434
chr14 77760471
chr14 77760489
chr14 77760505
chr14 77760537
chr14 77800197
chr14 77800200
chr14 77800209
chr14 77800214
chr14 77800232
chr14 77800237
chr14 77800239
chr14 77800365
chr14 77800372
chr14 77800580
chr14 77800604
chr14 78158418
chr14 78171197
chr14 78202682
chr14 78202790
chr14 78713714
chr14 78809876
chr14 78810216
chr14 78810230
chr14 79280844
chr14 79281052
chr14 79281263
chr14 79281327
chr14 79281413
chr14 79281431
chr14 79281715
chr14 79281780
chr14 79282220
chr14 79282298
chr14 80193174
chr14 80193260
chr14 80193500
chr14 80193718
chr14 80194219
chr14 80194869
chr14 80212270
chr14 80212330
chr14 80212587
chr14 80289595
chr14 80289647
chr14 80289694
chr14 80289956
chr14 80289971
chr14 80313275
chr14 80313350
chr14 80931132

chr14 80931139
chr14 80931247
chr14 80931269
chr14 80931385
chr14 80931700
chr14 80931708
chr14 80941452
chr14 80941470
chr14 80941472
chr14 80941475
chr14 80941500
chr14 80941521
chr14 80941535
chr14 80941547
chr14 80941587
chr14 80941614
chr14 80941622
chr14 80941637
chr14 80941644
chr14 80941646
chr14 80941682
chr14 80941687
chr14 80941696
chr14 80941717
chr14 80941742
chr14 80941771
chr14 80941777
chr14 80941780
chr14 80941811
chr14 80941849
chr14 80941892
chr14 80941905
chr14 80941929
chr14 80941931
chr14 80942082
chr14 80942117
chr14 80942220
chr14 80942224
chr14 80942306
chr14 80942430
chr14 80942597
chr14 80988452
chr14 80988783
chr14 81170168
chr14 81170182
chr14 81170239
chr14 81170245
chr14 81170271
chr14 81170277
chr14 81170312
chr14 81170343
chr14 81170351
chr14 81170706
chr14 81170731

chr14 81170749
chr14 81170766
chr14 81170784
chr14 81170787
chr14 81170974
chr14 81218753
chr14 81218841
chr14 81218909
chr14 81220757
chr14 81220823
chr14 81220830
chr14 81220839
chr14 81220842
chr14 81220845
chr14 81220851
chr14 81474320
chr14 81474382
chr14 81532768
chr14 81532786
chr14 81532880
chr14 81533030
chr14 81533119
chr14 81533159
chr14 81533178
chr14 81533902
chr14 81533906
chr14 81533909
chr14 81533912
chr14 81533914
chr14 81533945
chr14 81533963
chr14 81533968
chr14 81534122
chr14 81534144
chr14 81534162
chr14 81534182
chr14 81534192
chr14 81534214
chr14 81550185
chr14 81550573
chr14 81680164
chr14 81680345
chr14 81680675
chr14 81680897
chr14 82084417
chr14 82084824
chr14 82086220
chr14 82086231
chr14 82086355
chr14 83158366
chr14 83158398
chr14 83231534
chr14 83424677
chr14 83424814

chr14 84128671
chr14 84128769
chr14 84128850
chr14 84129044
chr14 84129057
chr14 84129207
chr14 84129306
chr14 84129328
chr14 84129510
chr14 85308311
chr14 85308716
chr14 85308840
chr14 85309028
chr14 85309053
chr14 85320472
chr14 85320490
chr14 85416987
chr14 85417021
chr14 85417273
chr14 85620099
chr14 85620205
chr14 85620382
chr14 85699341
chr14 85934637
chr14 85934654
chr14 85934698
chr14 85934715
chr14 85934719
chr14 85934745
chr14 85934780
chr14 85934789
chr14 85934793
chr14 85934834
chr14 85934970
chr14 85935095
chr14 85935102
chr14 85935427
chr14 85935671
chr14 86072333
chr14 86072380
chr14 86072383
chr14 86072427
chr14 86072654
chr14 86073415
chr14 86073447
chr14 86393777
chr14 86764575
chr14 86867923
chr14 86867981
chr14 87022736
chr14 87088605
chr14 87088607
chr14 87156665
chr14 87418175

chr14 87418201
chr14 87418250
chr14 87418288
chr14 87418307
chr14 87421352
chr14 87421486
chr14 87426053
chr14 87426905
chr14 87427081
chr14 87427790
chr14 87427803
chr14 87427808
chr14 87427852
chr14 87609489
chr14 87662914
chr14 87663867
chr14 87914999
chr14 88004527
chr14 88004810
chr14 88004824
chr14 88005060
chr14 88005209
chr14 88005297
chr14 88005803
chr14 88006093
chr14 88006251
chr14 88006297
chr14 88006415
chr14 88006435
chr14 88006466
chr14 88006657
chr14 88006694
chr14 88006703
chr14 88006777
chr14 88006805
chr14 88006807
chr14 88006833
chr14 88007052
chr14 88007074
chr14 88007351
chr14 88384791
chr14 88384903
chr14 88385040
chr14 88385167
chr14 88385193
chr14 88385206
chr14 88385307
chr14 88385388
chr14 88385402
chr14 88385443
chr14 88553639
chr14 88553793
chr14 88553934
chr14 88553951

chr14 88553983
chr14 88553988
chr14 88554016
chr14 88555298
chr14 88557508
chr14 88557539
chr14 88557571
chr14 88823868
chr14 88823968
chr14 88823986
chr14 88824145
chr14 88824160
chr14 88824170
chr14 88824254
chr14 88824266
chr14 88824281
chr14 88824285
chr14 88824575
chr14 88824577
chr14 88824582
chr14 88824593
chr14 88824609
chr14 88824612
chr14 88824614
chr14 88824622
chr14 88824632
chr14 88824636
chr14 88824639
chr14 88824657
chr14 88824677
chr14 88824688
chr14 88824695
chr14 88824700
chr14 88824715
chr14 88824727
chr14 88824756
chr14 88824776
chr14 88824779
chr14 88824790
chr14 88824893
chr14 88977096
chr14 88977637
chr14 88977778
chr14 89351448
chr14 89351512
chr14 89351561
chr14 89351618
chr14 89351635
chr14 89351638
chr14 89351646
chr14 89351658
chr14 89351676
chr14 89351757
chr14 89365071

chr14 89365089
chr14 89365102
chr14 89365110
chr14 89365132
chr14 89365185
chr14 89365248
chr14 89365354
chr14 89365363
chr14 89416039
chr14 89416044
chr14 89416078
chr14 89429647
chr14 89429686
chr14 89429723
chr14 89429725
chr14 89429742
chr14 89429879
chr14 89429968
chr14 89430470
chr14 89430638
chr14 89430640
chr14 89430708
chr14 89430805
chr14 89430907
chr14 89430939
chr14 89431065
chr14 89510525
chr14 89510572
chr14 89510670
chr14 89510867
chr14 89510874
chr14 89616549
chr14 89616644
chr14 89616929
chr14 89616931
chr14 89617102
chr14 89617133
chr14 89617236
chr14 89619827
chr14 89619854
chr14 89619896
chr14 89641608
chr14 89641654
chr14 89641703
chr14 89641728
chr14 89641783
chr14 89641938
chr14 89681107
chr14 89681139
chr14 89681159
chr14 89681193
chr14 89681227
chr14 89681254
chr14 89681775

chr14 89681833
chr14 89720821
chr14 89720873
chr14 89720916
chr14 89724865
chr14 89725050
chr14 89725066
chr14 89725108
chr14 89725172
chr14 89751553
chr14 89751718
chr14 89954082
chr14 89954105
chr14 89954216
chr14 89954225
chr14 89954235
chr14 89954329
chr14 89954402
chr14 89954414
chr14 89954458
chr14 89954498
chr14 89954519
chr14 89954531
chr14 89955174
chr14 89955270
chr14 89955275
chr14 89955280
chr14 89955371
chr14 89955415
chr14 89955422
chr14 89955448
chr14 89955454
chr14 89960058
chr14 90384110
chr14 90384118
chr14 90384149
chr14 90384167
chr14 90384199
chr14 90384226
chr14 90384235
chr14 90384242
chr14 90384245
chr14 90384338
chr14 90384363
chr14 90384528
chr14 90384555
chr14 90384702
chr14 90384827
chr14 90384927
chr14 90398693
chr14 90398735
chr14 90398748
chr14 90398782
chr14 90398787

chr14 90398797
chr14 90398805
chr14 90398855
chr14 90399017
chr14 90399038
chr14 90399048
chr14 90399100
chr14 90399121
chr14 90399442
chr14 90399537
chr14 90399554
chr14 90399572
chr14 90399581
chr14 90399673
chr14 90399697
chr14 90399720
chr14 90399799
chr14 90399930
chr14 90400028
chr14 90483776
chr14 90483827
chr14 90483861
chr14 90483951
chr14 90484015
chr14 90484165
chr14 90677907
chr14 90970059
chr14 90970286
chr14 91078488
chr14 91078527
chr14 91078584
chr14 91078630
chr14 91114686
chr14 91114720
chr14 91115446
chr14 91115462
chr14 91241897
chr14 91241919
chr14 91241962
chr14 91242002
chr14 91242048
chr14 91242081
chr14 91242133
chr14 91242479
chr14 91242483
chr14 91242758
chr14 91242935
chr14 91242960
chr14 91242993
chr14 91243607
chr14 91243659
chr14 91243671
chr14 91243685
chr14 91243690

chr14 91243728
chr14 91243915
chr14 91243934
chr14 91244002
chr14 91244130
chr14 91244202
chr14 91244204
chr14 91244311
chr14 91244403
chr14 91250219
chr14 91250247
chr14 91250321
chr14 91250491
chr14 91250530
chr14 91350842
chr14 91350882
chr14 91350888
chr14 91350963
chr14 91351033
chr14 91351045
chr14 91351056
chr14 91351074
chr14 91351127
chr14 91351160
chr14 91351165
chr14 91351283
chr14 91351325
chr14 91367548
chr14 91367569
chr14 91367584
chr14 91372828
chr14 91372855
chr14 91372893
chr14 91372928
chr14 91372966
chr14 91372971
chr14 91373089
chr14 91373133
chr14 91373245
chr14 91373293
chr14 91373345
chr14 91373497
chr14 91373676
chr14 91373695
chr14 91373744
chr14 91373841
chr14 91373857
chr14 91373920
chr14 91373923
chr14 91373970
chr14 91374123
chr14 91374175
chr14 91374215
chr14 91415580

chr14 91415588
chr14 91415612
chr14 91415634
chr14 91415644
chr14 91415651
chr14 91415845
chr14 91415903
chr14 91415919
chr14 91415925
chr14 91415988
chr14 91416058
chr14 91416083
chr14 91416126
chr14 91416346
chr14 91416365
chr14 91418921
chr14 91418930
chr14 91418962
chr14 91418973
chr14 91418989
chr14 91419009
chr14 91419015
chr14 91419022
chr14 91419071
chr14 91419101
chr14 91419131
chr14 91419148
chr14 91419210
chr14 91419214
chr14 91419217
chr14 91419219
chr14 91419229
chr14 91419235
chr14 91419237
chr14 91419345
chr14 91419464
chr14 91419469
chr14 91431690
chr14 91432004
chr14 91432082
chr14 91432100
chr14 91432118
chr14 91432182
chr14 91432345
chr14 91432363
chr14 91433474
chr14 91434934
chr14 91434963
chr14 91434996
chr14 91435822
chr14 91435843
chr14 91435868
chr14 91435899
chr14 91507702

chr14 91507879
chr14 91507887
chr14 91507892
chr14 91507903
chr14 91507992
chr14 91508436
chr14 91508461
chr14 91573963
chr14 91573967
chr14 91574037
chr14 91574068
chr14 91574073
chr14 91574077
chr14 91574081
chr14 91574094
chr14 91574132
chr14 91574139
chr14 91574155
chr14 91835578
chr14 91835736
chr14 91835782
chr14 91835804
chr14 91865987
chr14 91866029
chr14 91866165
chr14 91866224
chr14 91866512
chr14 91866553
chr14 91866569
chr14 91866589
chr14 91867043
chr14 91867159
chr14 91867295
chr14 91867301
chr14 91867305
chr14 91867319
chr14 91867421
chr14 91867427
chr14 91867685
chr14 91872335
chr14 91872337
chr14 91872539
chr14 91872619
chr14 91872649
chr14 92039013
chr14 92039073
chr14 92039249
chr14 92039264
chr14 92039276
chr14 92039284
chr14 92105837
chr14 92105846
chr14 92105888
chr14 92105908

chr14 92105929
chr14 92105932
chr14 92105976
chr14 92105996
chr14 92106005
chr14 92106007
chr14 92106031
chr14 92106053
chr14 92106089
chr14 92106211
chr14 92106218
chr14 92108665
chr14 92108681
chr14 92108709
chr14 92108727
chr14 92108738
chr14 92108776
chr14 92681357
chr14 92681380
chr14 92681431
chr14 92681447
chr14 92719289
chr14 92719292
chr14 92719295
chr14 92719298
chr14 92719304
chr14 92719307
chr14 92719313
chr14 92719316
chr14 92719319
chr14 92719322
chr14 92719328
chr14 92719334
chr14 92719343
chr14 92748049
chr14 92748052
chr14 92748071
chr14 92748123
chr14 92748170
chr14 92748188
chr14 92748198
chr14 92748200
chr14 92748247
chr14 92748249
chr14 92748258
chr14 92748314
chr14 92748344
chr14 92748348
chr14 92748377
chr14 92748407
chr14 93066131
chr14 93206074
chr14 93206108
chr14 93206130

chr14 93206167
chr14 93206230
chr14 93206244
chr14 93206260
chr14 93206288
chr14 93206303
chr14 93206439
chr14 93206722
chr14 93206756
chr14 93206784
chr14 93206788
chr14 93206793
chr14 93206800
chr14 93206815
chr14 93206830
chr14 93206842
chr14 93206852
chr14 93206855
chr14 93206862
chr14 93207571
chr14 93207677
chr14 93207682
chr14 93207715
chr14 93207755
chr14 93207766
chr14 93207768
chr14 93207844
chr14 93207892
chr14 93208034
chr14 93333440
chr14 93333455
chr14 93333457
chr14 93333488
chr14 93333523
chr14 93333669
chr14 93333674
chr14 93333690
chr14 93333737
chr14 93333776
chr14 93333809
chr14 93333947
chr14 93416922
chr14 93549189
chr14 93549386
chr14 93549490
chr14 93731103
chr14 93959989
chr14 93960005
chr14 93960018
chr14 93960033
chr14 93963103
chr14 93970920
chr14 93970942
chr14 93970971

chr14 93970982
chr14 93971048
chr14 93971061
chr14 93971165
chr14 93971167
chr14 93971191
chr14 93971211
chr14 93971220
chr14 93971232
chr14 93971289
chr14 93971299
chr14 93976267
chr14 93976280
chr14 93976304
chr14 93976344
chr14 93976367
chr14 93978378
chr14 93978448
chr14 94080135
chr14 94129529
chr14 94129954
chr14 94130000
chr14 94130326
chr14 94130350
chr14 94130419
chr14 94130687
chr14 94136450
chr14 94423328
chr14 94423398
chr14 94423421
chr14 94423501
chr14 94423535
chr14 94423561
chr14 94423574
chr14 94423579
chr14 94774555
chr14 94774623
chr14 94774633
chr14 94774702
chr14 94774924
chr14 94775125
chr14 94775204
chr14 94775271
chr14 94775343
chr14 94775429
chr14 94775443
chr14 94775461
chr14 95516162
chr14 95516164
chr14 95516168
chr14 95516170
chr14 95516174
chr14 95516183
chr14 95516196

chr14 95516199
chr14 95516201
chr14 95516203
chr14 95516228
chr14 95516256
chr14 95516268
chr14 95516273
chr14 95516281
chr14 95516283
chr14 95516290
chr14 95516318
chr14 95516322
chr14 95516332
chr14 95516352
chr14 95516361
chr14 95516377
chr14 95516402
chr14 95516406
chr14 95516410
chr14 95516421
chr14 95516424
chr14 95516433
chr14 95516439
chr14 95516441
chr14 95516444
chr14 95516448
chr14 95516450
chr14 95516475
chr14 95516478
chr14 95516484
chr14 95516491
chr14 95520217
chr14 95533172
chr14 95533184
chr14 95533224
chr14 95533277
chr14 95533411
chr14 95533438
chr14 95533472
chr14 95533530
chr14 95533588
chr14 95533610
chr14 95533659
chr14 95533674
chr14 95533695
chr14 95700443
chr14 95700636
chr14 95917627
chr14 95918267
chr14 95918519
chr14 95918584
chr14 96126293
chr14 96126296
chr14 96126373

chr14 96126414
chr14 96126441
chr14 96126501
chr14 96362147
chr14 96362170
chr14 96362180
chr14 96362224
chr14 96362291
chr14 96362333
chr14 96362340
chr14 96362366
chr14 96362484
chr14 96362499
chr14 96362528
chr14 96362558
chr14 96362616
chr14 96362623
chr14 96362688
chr14 96362699
chr14 96362763
chr14 96362780
chr14 96362787
chr14 96362791
chr14 96362796
chr14 96362799
chr14 96362802
chr14 96362838
chr14 96362847
chr14 96392012
chr14 96392017
chr14 96392027
chr14 96392031
chr14 96424265
chr14 96424268
chr14 96424271
chr14 96424277
chr14 96424280
chr14 96424285
chr14 96424295
chr14 96424374
chr14 96424553
chr14 96424564
chr14 96424600
chr14 96424624
chr14 96424740
chr14 96424775
chr14 96424844
chr14 96425010
chr14 96503229
chr14 96503268
chr14 96503413
chr14 96503512
chr14 96503596
chr14 96503625

chr14 96503655
chr14 96503724
chr14 96503790
chr14 96503879
chr14 96627600
chr14 96627607
chr14 96627639
chr14 96627664
chr14 96662082
chr14 96798306
chr14 96798339
chr14 96974254
chr14 97412957
chr14 97458964
chr14 97458967
chr14 97458997
chr14 97459015
chr14 97459036
chr14 97459069
chr14 97459072
chr14 97459081
chr14 97459083
chr14 97459089
chr14 97459092
chr14 97459098
chr14 97459101
chr14 97459172
chr14 97459300
chr14 97459310
chr14 97602678
chr14 97602700
chr14 97602753
chr14 97602821
chr14 97602871
chr14 97602882
chr14 97602960
chr14 97603015
chr14 97603044
chr14 97603047
chr14 97603073
chr14 97634389
chr14 97634688
chr14 97634754
chr14 97634883
chr14 97634910
chr14 97634957
chr14 97634963
chr14 97634993
chr14 98173229
chr14 98198415
chr14 98198426
chr14 98198451
chr14 98210450
chr14 98210512

chr14 98210527
chr14 98210536
chr14 98210558
chr14 98210567
chr14 98210587
chr14 98210590
chr14 98210619
chr14 98210678
chr14 98210726
chr14 98210770
chr14 98211101
chr14 98211542
chr14 98227421
chr14 98227462
chr14 98227638
chr14 99191630
chr14 99191637
chr14 99191707
chr14 99191739
chr14 99191834
chr14 99191947
chr14 99191967
chr14 99191997
chr14 99192035
chr14 99192047
chr14 99192062
chr14 99192073
chr14 99235050
chr14 99235063
chr14 99235069
chr14 99235076
chr14 99235079
chr14 99235086
chr14 99235102
chr14 99235121
chr14 99235139
chr14 99235170
chr14 99235250
chr14 99235367
chr14 99235369
chr14 99235410
chr14 99235412
chr14 99235452
chr14 99235566
chr14 99235712
chr14 99235729
chr14 99235739
chr14 99235747
chr14 99236238
chr14 99236240
chr14 99237136
chr14 99239821
chr14 99239874
chr14 99239883

chr14 99239939
chr14 99239979
chr14 99240415
chr14 99240591
chr14 99240691
chr14 99240700
chr14 99240724
chr14 99240730
chr14 99240739
chr14 99240772
chr14 99240830
chr14 99240845
chr14 99240847
chr14 99240849
chr14 99240854
chr14 99240857
chr14 99240996
chr14 99241379
chr14 99241427
chr14 99242938
chr14 99242961
chr14 99242982
chr14 99243413
chr14 99243605
chr14 99243615
chr14 99243644
chr14 99243656
chr14 99243661
chr14 99244384
chr14 99244410
chr14 99244439
chr14 99244464
chr14 99244480
chr14 99244520
chr14 99244542
chr14 99244579
chr14 99244594
chr14 99244681
chr14 99244754
chr14 99244799
chr14 99244807
chr14 99244813
chr14 99244828
chr14 99244886
chr14 99245028
chr14 99245038
chr14 99259441
chr14 99259444
chr14 99259454
chr14 99259461
chr14 99259463
chr14 99259467
chr14 99259493
chr14 99259496

chr14 99259505
chr14 99259569
chr14 99259615
chr14 99259620
chr14 99259626
chr14 99259704
chr14 99259779
chr14 99259852
chr14 99259889
chr14 99262366
chr14 99262479
chr14 99262531
chr14 99262544
chr14 99262553
chr14 99262570
chr14 99262602
chr14 99262606
chr14 99262619
chr14 99262647
chr14 99262668
chr14 99262693
chr14 99262718
chr14 99262751
chr14 99262756
chr14 99262762
chr14 99262772
chr14 99262780
chr14 99262783
chr14 99262849
chr14 99262932
chr14 99263681
chr14 99263944
chr14 99264052
chr14 99264063
chr14 99264141
chr14 99264248
chr14 99264287
chr14 99264387
chr14 99264697
chr14 99264798
chr14 99264825
chr14 99264827
chr14 99264848
chr14 99264864
chr14 99264906
chr14 99264909
chr14 99264943
chr14 99264984
chr14 99265112
chr14 99265135
chr14 99265223
chr14 99265247
chr14 99265343
chr14 99265443

chr14 99265588
chr14 99265613
chr14 99265617
chr14 99265630
chr14 99265639
chr14 99265673
chr14 99265701
chr14 99265788
chr14 99265794
chr14 99265858
chr14 99266136
chr14 99266159
chr14 99266274
chr14 99266297
chr14 99266343
chr14 99266367
chr14 99266377
chr14 99266388
chr14 99266407
chr14 99266418
chr14 99266422
chr14 99266542
chr14 99266593
chr14 99266650
chr14 99266660
chr14 99266663
chr14 99266680
chr14 99266699
chr14 99266705
chr14 99266720
chr14 99266727
chr14 99266752
chr14 99266759
chr14 99266761
chr14 99266766
chr14 99266769
chr14 99266863
chr14 99266907
chr14 99266954
chr14 99266977
chr14 99266996
chr14 99267128
chr14 99267143
chr14 99267232
chr14 99267382
chr14 99267451
chr14 99267604
chr14 99267681
chr14 99267801
chr14 99267813
chr14 99267882
chr14 99267892
chr14 99267988
chr14 99268010

chr14 99268020
chr14 99268041
chr14 99268046
chr14 99268261
chr14 99268289
chr14 99268313
chr14 99268498
chr14 99268508
chr14 99268546
chr14 99268664
chr14 99268758
chr14 99268780
chr14 99268894
chr14 99268916
chr14 99269009
chr14 99269055
chr14 99269105
chr14 99269108
chr14 99269154
chr14 99269209
chr14 99269257
chr14 99269303
chr14 99269328
chr14 99269401
chr14 99269407
chr14 99269410
chr14 99269432
chr14 99269435
chr14 99269493
chr14 99269606
chr14 99272110
chr14 99272119
chr14 99272145
chr14 99272156
chr14 99272163
chr14 99272801
chr14 99272803
chr14 99272807
chr14 99272810
chr14 99272938
chr14 99273087
chr14 99415823
chr14 99482180
chr14 99482276
chr14 99482286
chr14 99482332
chr14 99482371
chr14 99482385
chr14 99482404
chr14 99482572
chr14 99482615
chr14 99683317
chr14 99683326
chr14 99683341

chr14 99683365
chr14 99684215
chr14 99684281
chr14 99684285
chr14 99821988
chr14 99822239
chr14 99896450
chr14 99896479
chr14 99995870
chr14 99995939
chr14 100064994
chr14 100065254
chr14 100065260
chr14 100065463
chr14 100065572
chr14 100065620
chr14 100066717
chr14 100066792
chr14 100067012
chr14 100067057
chr14 100067064
chr14 100067108
chr14 100067119
chr14 100067190
chr14 100067194
chr14 100067237
chr14 100067272
chr14 100067277
chr14 100067439
chr14 100067576
chr14 100067999
chr14 100068071
chr14 100068110
chr14 100068152
chr14 100068243
chr14 100068365
chr14 100068377
chr14 100068396
chr14 100068398
chr14 100068557
chr14 100068618
chr14 100068648
chr14 100068693
chr14 100068748
chr14 100068864
chr14 100068866
chr14 100068884
chr14 100068937
chr14 100068987
chr14 100068994
chr14 100069009
chr14 100069088
chr14 100072859
chr14 100072862

chr14 100072892
chr14 100072895
chr14 100072900
chr14 100072930
chr14 100072932
chr14 100072947
chr14 100072975
chr14 100073021
chr14 100073037
chr14 100073050
chr14 100073075
chr14 100081283
chr14 100081300
chr14 100166988
chr14 100166996
chr14 100167000
chr14 100167035
chr14 100167043
chr14 100167067
chr14 100167073
chr14 100167075
chr14 100167083
chr14 100167105
chr14 100167113
chr14 100167208
chr14 100167226
chr14 100167244
chr14 100167437
chr14 100221766
chr14 100221825
chr14 100221886
chr14 100221891
chr14 100221893
chr14 100221916
chr14 100222066
chr14 100222077
chr14 100222110
chr14 100222126
chr14 100222240
chr14 100237328
chr14 100237380
chr14 100237481
chr14 100237575
chr14 100237583
chr14 100237591
chr14 100240762
chr14 100240783
chr14 100240882
chr14 100240953
chr14 100240966
chr14 100241011
chr14 100241191
chr14 100241220
chr14 100241240

chr14 100241336
chr14 100241346
chr14 100900031
chr14 100900061
chr14 100900070
chr14 100900181
chr14 101706087
chr14 101706094
chr14 101706097
chr14 101706099
chr14 101706108
chr14 101706128
chr14 101706131
chr14 101706144
chr14 101706162
chr14 101706164
chr14 101706167
chr14 101722009
chr14 101722028
chr14 101722063
chr14 101722080
chr14 101722106
chr14 101722133
chr14 101722210
chr14 101722310
chr14 101722375
chr14 101722382
chr14 101722393
chr14 101722480
chr14 101731308
chr14 101731344
chr14 101731366
chr14 101731439
chr14 101731657
chr14 101781743
chr14 101781746
chr14 101781789
chr14 101781793
chr14 101781795
chr14 101781800
chr14 101781802
chr14 101781810
chr14 101781821
chr14 101781826
chr14 101781831
chr14 101781851
chr14 101781857
chr14 101781863
chr14 101781878
chr14 101781883
chr14 101781898
chr14 101781910
chr14 101781916
chr14 101781927

chr14 101781933
chr14 101781941
chr14 101781973
chr14 101792148
chr14 101792440
chr14 101809205
chr14 101809218
chr14 101809234
chr14 101809471
chr14 101811063
chr14 101811433
chr14 101811487
chr14 101811580
chr14 101811595
chr14 101811660
chr14 101819779
chr14 101819903
chr14 101819913
chr14 101819939
chr14 101819944
chr14 101819968
chr14 101819995
chr14 101820000
chr14 101820010
chr14 101820209
chr14 101820368
chr14 101820416
chr14 101820420
chr14 101820436
chr14 101820473
chr14 101820584
chr14 101820629
chr14 101820713
chr14 101820722
chr14 101820782
chr14 101822997
chr14 101823097
chr14 101823642
chr14 101823717
chr14 101823745
chr14 101823823
chr14 101823937
chr14 101824098
chr14 101824105
chr14 101824114
chr14 101824166
chr14 101824175
chr14 101824253
chr14 101824279
chr14 101824342
chr14 101824432
chr14 101824572
chr14 101824752
chr14 101824774

chr14 101824782
chr14 101825032
chr14 101825058
chr14 101825076
chr14 101825085
chr14 101825211
chr14 101843356
chr14 101843678
chr14 101876214
chr14 101876414
chr14 101876484
chr14 101876566
chr14 101876714
chr14 102087797
chr14 102087838
chr14 102087861
chr14 102087869
chr14 102087873
chr14 102087880
chr14 102087977
chr14 102087985
chr14 102088002
chr14 102088004
chr14 102088007
chr14 102088015
chr14 102088031
chr14 102088040
chr14 102088059
chr14 102088085
chr14 102088089
chr14 102088109
chr14 102088111
chr14 102088153
chr14 102088208
chr14 102088262
chr14 102088311
chr14 102088329
chr14 102088360
chr14 102138853
chr14 102138861
chr14 102138997
chr14 102139044
chr14 102139056
chr14 102139064
chr14 102139069
chr14 102139102
chr14 102139112
chr14 102139119
chr14 102139565
chr14 102139568
chr14 102139570
chr14 102139584
chr14 102139592
chr14 102139604

chr14 102139610
chr14 102139615
chr14 102316778
chr14 102316827
chr14 102316877
chr14 102316883
chr14 102316896
chr14 102316940
chr14 102317028
chr14 102317065
chr14 102317071
chr14 102362187
chr14 102362265
chr14 102362325
chr14 102362439
chr14 102362505
chr14 102362585
chr14 102362607
chr14 102362614
chr14 102362619
chr14 102362649
chr14 102362657
chr14 102362673
chr14 102362682
chr14 102362693
chr14 102362704
chr14 102362754
chr14 102363124
chr14 102363127
chr14 102363130
chr14 102524750
chr14 102524759
chr14 102524770
chr14 102524859
chr14 102524886
chr14 102524920
chr14 102524940
chr14 102524961
chr14 102524975
chr14 102524978
chr14 102524987
chr14 102524989
chr14 102524994
chr14 102525045
chr14 102525057
chr14 102525070
chr14 102525086
chr14 102525100
chr14 102525122
chr14 102525134
chr14 102525341
chr14 102525364
chr14 102525427
chr14 102525552

chr14 102594690
chr14 102594753
chr14 102795237
chr14 103386495
chr14 103386515
chr14 103386532
chr14 103386538
chr14 103386604
chr14 103386633
chr14 103386635
chr14 103386761
chr14 103387110
chr14 103391231
chr14 103391256
chr14 103391302
chr14 103630398
chr14 103630547
chr14 103806543
chr14 104665685
chr14 104665688
chr14 104665758
chr14 104665763
chr14 104827470
chr14 104827491
chr14 105065119
chr14 105065168
chr14 105065211
chr14 105065295
chr14 105065359
chr14 105065371
chr14 105065388
chr14 105065394
chr14 105065406
chr14 105186168
chr14 105186192
chr14 105481732
chr14 105481737
chr14 105481744
chr14 105481809
chr14 105481853
chr14 105481856
chr14 105481893
chr14 105481899
chr14 105481948
chr14 105481969
chr14 105482005
chr14 105482011
chr14 105482025
chr14 105482036
chr14 105578571
chr14 105579013
chr14 105579046
chr14 105579058
chr14 105680127

chr14 105680134
chr14 105680140
chr14 105680148
chr14 105680210
chr14 105680235
chr14 105680241
chr14 105680248
chr14 105680251
chr14 105680253
chr14 105680259
chr14 105680262
chr14 105680265
chr14 105680269
chr14 105680281
chr14 105680289
chr14 105680547
chr14 105680551
chr14 105680578
chr14 105680600
chr14 105680610
chr14 105680628
chr14 105680791
chr14 105680796
chr14 105680802
chr14 105862061
chr14 105862071
chr14 105862087
chr14 105862216
chr14 105862218
chr14 105862252
chr14 105862305
chr14 105862350
chr14 105862358
chr14 105862363
chr14 105862369
chr14 105998977
chr14 105999242
chr14 106004368
chr14 106004523
chr14 106004566
chr14 106005100
chr14 106005118
chr14 106005163
chr14 106005165
chr14 106005234
chr14 106005267
chr14 106005278
chr14 106005448
chr14 106005540
chr14 106005642
chr14 106013793
chr14 106013828
chr14 106013904
chr14 106013965

chr14 106014022
chr14 106025057
chr14 106025150
chr14 106025164
chr14 106085613
chr14 106085680
chr14 106085702
chr14 106085719
chr14 106085736
chr14 106086053
chr14 106086180
chr14 106086530
chr14 106086555
chr14 106086590
chr14 106086643
chr14 106086693
chr14 106086735
chr14 106086769
chr14 106094570
chr14 106094733
chr14 106094773
chr14 106094813
chr14 106094911
chr14 106094941
chr14 106107148
chr14 106107228
chr14 106139349
chr14 106140544
chr14 106153457
chr14 106153504
chr14 106153529
chr14 106173710
chr14 106173739
chr14 106173755
chr14 106173774
chr14 106224133
chr14 106224176
chr14 106224462
chr14 106349099
chr14 106349134
chr14 106349160
chr14 106349182
chr14 106349184
chr14 106349186
chr14 106349215
chr14 106349219
chr14 106349228
chr14 106373841
chr14 106437169
chr14 106510636
chr14 106510643
chr14 106510647
chr14 106510962
chr14 106511003

chr14 106511120
chr14 106511134
chr14 106511138
chr14 106511183
chr14 106511189
chr14 106511230
chr14 106511298
chr14 106621943
chr14 106627856
chr14 106639373
chr14 106639397
chr14 106694375
chr14 106694515
chr14 106694681
chr14 106694700
chr14 106694708
chr14 106698553
chr14 106698681
chr14 106698686
chr14 106698783
chr14 106698830
chr14 106698909
chr14 106698980
chr14 106699037
chr14 106699085
chr14 106699087
chr14 106699093
chr14 106699123
chr14 106699129
chr14 106699150
chr14 106699163
chr14 106699169
chr14 106699208
chr14 106714443
chr14 106714490
chr14 106714619
chr14 106714701
chr14 106714705
chr14 106714750
chr14 106714762
chr14 106714764
chr14 106714771
chr14 106714797
chr14 106715595
chr14 106715739
chr14 106720098
chr14 106722127
chr14 106722236
chr14 106722260
chr14 106722658
chr14 106741185
chr14 106741204
chr14 106741212
chr14 106741236

chr14 106741238
chr14 106741248
chr14 106741267
chr14 106741288
chr14 106741384
chr14 106741391
chr14 106741425
chr14 106744406
chr14 106744481
chr14 106744518
chr14 106744744
chr14 106745024
chr14 106745152
chr14 106745157
chr14 106745380
chr14 106745508
chr14 106745556
chr14 106745558
chr14 106745564
chr14 106745593
chr14 106745600
chr14 106745612
chr14 106745621
chr14 106745634
chr14 106745640
chr14 106757297
chr14 106757319
chr14 106757523
chr14 106761897
chr14 106762029
chr14 106762097
chr14 106762111
chr14 106762115
chr14 106762160
chr14 106762168
chr14 106762172
chr14 106762174
chr14 106762181
chr14 106762207
chr14 106788739
chr14 106788744
chr14 106788760
chr14 106788871
chr14 106788961
chr14 106789179
chr14 106789508
chr14 106789526
chr14 106789572
chr14 106789978
chr14 106790110
chr14 106790544
chr14 106790568
chr14 106790599
chr14 106844086

chr14 106844150
chr14 106844168
chr14 106844238
chr14 106844246
chr14 106844257
chr14 106844290
chr14 106844325
chr14 106844370
chr14 106844390
chr14 106844423
chr14 106844427
chr14 106844434
chr14 106844506
chr14 106844563
chr14 106844590
chr14 106844593
chr14 106844622
chr14 106844631
chr14 106844653
chr14 106844659
chr2 265310
chr2 265349
chr2 265465
chr2 265471
chr2 420376
chr2 420430
chr2 420439
chr2 420457
chr2 420465
chr2 420467
chr2 420469
chr2 420484
chr2 420493
chr2 529256
chr2 529502
chr2 529516
chr2 530060
chr2 530146
chr2 572121
chr2 664529
chr2 677898
chr2 677908
chr2 677918
chr2 677955
chr2 705707
chr2 705710
chr2 946279
chr2 994913
chr2 995125
chr2 1163154
chr2 1163183
chr2 1343743
chr2 1343832
chr2 1343856

chr2 1344172
chr2 1344196
chr2 1344309
chr2 1344398
chr2 1344511
chr2 1344851
chr2 1468779
chr2 1725665
chr2 1725667
chr2 1725972
chr2 1726128
chr2 1726141
chr2 1726321
chr2 1726344
chr2 1726434
chr2 1726485
chr2 2060247
chr2 2060645
chr2 2164094
chr2 2164195
chr2 2164489
chr2 2164498
chr2 2329036
chr2 2329048
chr2 2329380
chr2 2686208
chr2 2686227
chr2 2686286
chr2 2686293
chr2 3091559
chr2 3091761
chr2 3091957
chr2 3092204
chr2 3092919
chr2 3222860
chr2 3222916
chr2 3222920
chr2 3222938
chr2 3223134
chr2 3223153
chr2 3223210
chr2 3374251
chr2 3377447
chr2 3377615
chr2 3377629
chr2 3518596
chr2 3518609
chr2 3518626
chr2 3518630
chr2 3590605
chr2 3590609
chr2 3590619
chr2 3590633
chr2 3590637

chr2 3590642
chr2 3590654
chr2 3590683
chr2 3590687
chr2 3590692
chr2 3590829
chr2 3590838
chr2 3614411
chr2 3614426
chr2 3614470
chr2 3614497
chr2 3614501
chr2 3651488
chr2 3651508
chr2 3651536
chr2 3651705
chr2 3651716
chr2 3651720
chr2 3651728
chr2 3651741
chr2 3651744
chr2 3651763
chr2 3651782
chr2 3651784
chr2 3651797
chr2 3651807
chr2 3651809
chr2 3651825
chr2 3651858
chr2 3651860
chr2 3651871
chr2 3651874
chr2 3738238
chr2 3740008
chr2 3740706
chr2 3740995
chr2 3741144
chr2 3741267
chr2 3741500
chr2 3741548
chr2 4317572
chr2 4317929
chr2 4317951
chr2 4644166
chr2 4644202
chr2 4644207
chr2 4644233
chr2 5141935
chr2 5142103
chr2 5694332
chr2 5694425
chr2 5694451
chr2 5694456
chr2 5694477

chr2 5694482
chr2 5694489
chr2 5694506
chr2 5694527
chr2 5694730
chr2 5694780
chr2 5695014
chr2 5695136
chr2 5695366
chr2 5695415
chr2 5695551
chr2 5726032
chr2 5726065
chr2 5726153
chr2 5726188
chr2 5726216
chr2 5726255
chr2 5726338
chr2 5726374
chr2 5726444
chr2 5726484
chr2 5726518
chr2 5726667
chr2 5726703
chr2 5726715
chr2 6169163
chr2 6261829
chr2 6270071
chr2 6270427
chr2 6270725
chr2 6909998
chr2 6910322
chr2 7097400
chr2 7097414
chr2 7097419
chr2 7097424
chr2 7097438
chr2 7097440
chr2 7097442
chr2 7097449
chr2 7097451
chr2 7097472
chr2 7097474
chr2 7097540
chr2 7437573
chr2 7567658
chr2 7567769
chr2 7567895
chr2 7567939
chr2 8303465
chr2 8303491
chr2 8303551
chr2 8303563
chr2 8303644

chr2 8303655
chr2 8303672
chr2 8303729
chr2 8303734
chr2 8303789
chr2 8303874
chr2 8304029
chr2 8304031
chr2 8337670
chr2 8337712
chr2 8337962
chr2 8442747
chr2 8442862
chr2 8477355
chr2 8477422
chr2 8477622
chr2 8477685
chr2 8477694
chr2 8477748
chr2 8477767
chr2 8477826
chr2 8486227
chr2 8486258
chr2 8486263
chr2 8486290
chr2 8515194
chr2 8515200
chr2 8515292
chr2 8515334
chr2 8515544
chr2 8515567
chr2 8515633
chr2 8515644
chr2 8515653
chr2 8515658
chr2 8515734
chr2 8683883
chr2 8683963
chr2 8683969
chr2 8684029
chr2 8684465
chr2 8684525
chr2 8684703
chr2 8684804
chr2 8684812
chr2 8684908
chr2 8836989
chr2 8837030
chr2 8837061
chr2 8837066
chr2 8837102
chr2 9538319
chr2 9538449
chr2 9552691

chr2 9554650
chr2 9554698
chr2 9554821
chr2 9554883
chr2 9554904
chr2 9554960
chr2 9554975
chr2 9555013
chr2 9555023
chr2 9555074
chr2 9555219
chr2 9555239
chr2 9555273
chr2 9555295
chr2 9555356
chr2 9555394
chr2 9555396
chr2 9556767
chr2 9629432
chr2 9629497
chr2 9629619
chr2 9629777
chr2 9629809
chr2 9629846
chr2 9629857
chr2 9629876
chr2 9629889
chr2 9629923
chr2 9629950
chr2 9629979
chr2 9630001
chr2 9712400
chr2 9712416
chr2 9712424
chr2 9712428
chr2 9712435
chr2 9712441
chr2 9712495
chr2 9712556
chr2 9712618
chr2 9712651
chr2 9782502
chr2 9782598
chr2 9782640
chr2 9782660
chr2 9799667
chr2 9799719
chr2 9799831
chr2 9799833
chr2 9799865
chr2 9799876
chr2 9799889
chr2 9799891
chr2 9799906

chr2 9799935
chr2 9799958
chr2 9979961
chr2 9980043
chr2 9980219
chr2 10080342
chr2 10080349
chr2 10080353
chr2 10080429
chr2 10080433
chr2 10080465
chr2 10080472
chr2 10080488
chr2 10080494
chr2 10080502
chr2 10080505
chr2 10080515
chr2 10080521
chr2 10080525
chr2 10080527
chr2 10080540
chr2 10080569
chr2 10080575
chr2 10080585
chr2 10080592
chr2 10080595
chr2 10080598
chr2 10080614
chr2 10080640
chr2 10080649
chr2 10080675
chr2 10080686
chr2 10080689
chr2 10080693
chr2 10080696
chr2 10080702
chr2 10080712
chr2 10080714
chr2 10080754
chr2 10080759
chr2 10080761
chr2 10080770
chr2 10080793
chr2 10080795
chr2 10080797
chr2 10080802
chr2 10080806
chr2 10080808
chr2 10080815
chr2 10080819
chr2 10080827
chr2 10080832
chr2 10080847
chr2 10080861

chr2 10080866
chr2 10080880
chr2 10080888
chr2 10080908
chr2 10080937
chr2 10120680
chr2 10120685
chr2 10120699
chr2 10120722
chr2 10120914
chr2 10120928
chr2 10120933
chr2 10120946
chr2 10121100
chr2 10121110
chr2 10121196
chr2 10121220
chr2 10124466
chr2 10298293
chr2 10298360
chr2 10298495
chr2 10298736
chr2 10408753
chr2 10408904
chr2 10408957
chr2 10409006
chr2 10515837
chr2 10668657
chr2 10668688
chr2 10689103
chr2 10689349
chr2 10689451
chr2 10689462
chr2 10689474
chr2 10689481
chr2 10689521
chr2 10689599
chr2 10689601
chr2 10689607
chr2 10689632
chr2 10689644
chr2 10689658
chr2 10689674
chr2 10689679
chr2 10811313
chr2 10811489
chr2 10811780
chr2 10811783
chr2 10811785
chr2 10811795
chr2 10976657
chr2 10976685
chr2 10977146
chr2 10977155

chr2 10977158
chr2 10977174
chr2 10977273
chr2 10977293
chr2 10977303
chr2 10977331
chr2 10977353
chr2 10977465
chr2 11343046
chr2 11343108
chr2 11343195
chr2 11343246
chr2 11343309
chr2 11343337
chr2 11343353
chr2 11343412
chr2 11343482
chr2 11343550
chr2 11343581
chr2 11343615
chr2 11343619
chr2 11343634
chr2 11343715
chr2 11343766
chr2 11344782
chr2 11344796
chr2 11344807
chr2 11344811
chr2 11344817
chr2 11344833
chr2 11693087
chr2 11753692
chr2 11753695
chr2 11753740
chr2 11753915
chr2 11753918
chr2 11753934
chr2 11753965
chr2 11754025
chr2 11754057
chr2 11754084
chr2 11754146
chr2 11754157
chr2 11754316
chr2 11754338
chr2 11754403
chr2 11754462
chr2 11754558
chr2 11754696
chr2 11757323
chr2 11945594
chr2 11945635
chr2 11945668
chr2 11945728

chr2 11945791
chr2 12005956
chr2 12006053
chr2 12036583
chr2 12044913
chr2 12044960
chr2 12044966
chr2 12045039
chr2 12430890
chr2 12430934
chr2 12430963
chr2 12455840
chr2 12455864
chr2 12456381
chr2 12456389
chr2 12456441
chr2 12456685
chr2 12456717
chr2 12501657
chr2 12501667
chr2 12501674
chr2 12502018
chr2 12719269
chr2 12728260
chr2 12857440
chr2 13170862
chr2 13344472
chr2 13344828
chr2 13344886
chr2 13345429
chr2 13531322
chr2 13531403
chr2 13531553
chr2 13762991
chr2 13764436
chr2 13764495
chr2 13779907
chr2 13780209
chr2 13780576
chr2 13780859
chr2 13781108
chr2 13781236
chr2 13955801
chr2 13970138
chr2 13970206
chr2 13970857
chr2 14114261
chr2 14114298
chr2 14508225
chr2 14508328
chr2 14635578
chr2 14635589
chr2 14635612
chr2 14635618

chr2 14635632
chr2 14635637
chr2 14635643
chr2 14635656
chr2 14635662
chr2 14635668
chr2 14635680
chr2 14635684
chr2 14635722
chr2 14635757
chr2 14635972
chr2 14636306
chr2 14636354
chr2 14820260
chr2 15142093
chr2 15142119
chr2 15142204
chr2 15142221
chr2 15142276
chr2 15142294
chr2 15142365
chr2 15142437
chr2 15142498
chr2 15142504
chr2 15176878
chr2 15177250
chr2 15177269
chr2 15560787
chr2 15560789
chr2 15560831
chr2 15560840
chr2 15560870
chr2 15561512
chr2 15561618
chr2 15561716
chr2 15561750
chr2 15561767
chr2 15561819
chr2 15561878
chr2 15561883
chr2 15561896
chr2 15561978
chr2 15561994
chr2 15561999
chr2 15940224
chr2 16305293
chr2 16609111
chr2 16609113
chr2 16609152
chr2 16609198
chr2 16609206
chr2 16609244
chr2 16609258
chr2 16609480

chr2 17123000
chr2 17123008
chr2 17123019
chr2 17123061
chr2 17123299
chr2 17123453
chr2 17123488
chr2 17123519
chr2 17239729
chr2 17430281
chr2 17430431
chr2 17518554
chr2 17518560
chr2 17518588
chr2 17518593
chr2 17518632
chr2 17518637
chr2 17518659
chr2 17518674
chr2 17518695
chr2 17518713
chr2 17518754
chr2 17518852
chr2 17518866
chr2 17518871
chr2 17518892
chr2 17518957
chr2 17519071
chr2 17755157
chr2 17755221
chr2 17755263
chr2 18016536
chr2 18016643
chr2 18016785
chr2 18016790
chr2 18016826
chr2 18017077
chr2 18020223
chr2 18020647
chr2 18215030
chr2 18272937
chr2 18272948
chr2 18272957
chr2 18273823
chr2 18559657
chr2 18559690
chr2 18559725
chr2 18559870
chr2 18559875
chr2 18560969
chr2 18561124
chr2 19362232
chr2 19362295
chr2 19362345

chr2 19362370
chr2 19362401
chr2 19362445
chr2 19362453
chr2 19362475
chr2 19362537
chr2 19362555
chr2 19362695
chr2 19362702
chr2 19474132
chr2 19474190
chr2 19474246
chr2 19474309
chr2 19474344
chr2 19474448
chr2 19560713
chr2 19561594
chr2 19561923
chr2 19562060
chr2 19562176
chr2 19562687
chr2 19900068
chr2 19900265
chr2 19900417
chr2 19900603
chr2 19900652
chr2 19900724
chr2 19900864
chr2 19900898
chr2 19900922
chr2 19902344
chr2 19902354
chr2 19902378
chr2 19902423
chr2 19902443
chr2 19902521
chr2 19902530
chr2 19902573
chr2 19902604
chr2 19902607
chr2 19902614
chr2 19902652
chr2 19902703
chr2 20045557
chr2 20050032
chr2 20050078
chr2 20050087
chr2 20050145
chr2 20050179
chr2 20050230
chr2 20050283
chr2 20050420
chr2 20050441
chr2 20050443

chr2 20050466
chr2 20050488
chr2 20050494
chr2 20052142
chr2 20052153
chr2 20052212
chr2 20052232
chr2 20052431
chr2 20052436
chr2 20052464
chr2 20052493
chr2 20052506
chr2 20052549
chr2 20052642
chr2 20052671
chr2 20052710
chr2 20349196
chr2 20349312
chr2 20349389
chr2 20349416
chr2 20349600
chr2 20349636
chr2 20349706
chr2 20349754
chr2 20507434
chr2 20821986
chr2 20822163
chr2 20822185
chr2 20822193
chr2 20822217
chr2 20822225
chr2 20822249
chr2 20822269
chr2 20822273
chr2 20822281
chr2 20822285
chr2 20822344
chr2 20822364
chr2 20822633
chr2 20822636
chr2 20822649
chr2 20822652
chr2 20823399
chr2 20823627
chr2 20823667
chr2 20878155
chr2 20878221
chr2 20878663
chr2 21090844
chr2 21090955
chr2 21101114
chr2 21165273
chr2 21165312
chr2 21165677

chr2 21165937
chr2 21454977
chr2 21455065
chr2 21455560
chr2 21910223
chr2 22158565
chr2 23319737
chr2 23319766
chr2 23319780
chr2 23319902
chr2 23892017
chr2 23925850
chr2 23925873
chr2 23925876
chr2 23925985
chr2 23925991
chr2 23927721
chr2 23927752
chr2 23927761
chr2 23927764
chr2 23927777
chr2 23927782
chr2 24046855
chr2 24046996
chr2 24047003
chr2 24047019
chr2 24047024
chr2 24047098
chr2 24047105
chr2 24047140
chr2 24047282
chr2 24047291
chr2 24047357
chr2 24047369
chr2 24047372
chr2 24047375
chr2 24047402
chr2 24047411
chr2 24047437
chr2 24047446
chr2 24047487
chr2 24047492
chr2 24047503
chr2 24047542
chr2 24359315
chr2 24359429
chr2 24359520
chr2 24359528
chr2 24359549
chr2 24512088
chr2 24520945
chr2 24521034
chr2 24578054
chr2 24578330

chr2 24792809
chr2 24792815
chr2 24793952
chr2 24794129
chr2 24794148
chr2 24794176
chr2 24794223
chr2 24794256
chr2 24919329
chr2 24919336
chr2 24919360
chr2 24975526
chr2 25210343
chr2 25277256
chr2 25340944
chr2 25340949
chr2 25340951
chr2 25340971
chr2 25340977
chr2 25340991
chr2 25340996
chr2 25340999
chr2 25341007
chr2 25341030
chr2 25341034
chr2 25341043
chr2 25341049
chr2 25341052
chr2 25341061
chr2 25341067
chr2 25341070
chr2 25341073
chr2 25341078
chr2 25341100
chr2 25341111
chr2 25341113
chr2 25341116
chr2 25341118
chr2 25341121
chr2 25341128
chr2 25341133
chr2 25341139
chr2 25341152
chr2 25341157
chr2 25341163
chr2 25341174
chr2 25341183
chr2 25341188
chr2 25341190
chr2 25341196
chr2 25341206
chr2 25341210
chr2 25341213
chr2 25341225

chr2 25341233
chr2 25341238
chr2 25341261
chr2 25341264
chr2 25341267
chr2 25341269
chr2 25341278
chr2 25341280
chr2 25341287
chr2 25341294
chr2 25341306
chr2 25352824
chr2 25352853
chr2 25352908
chr2 25352918
chr2 25362196
chr2 25362215
chr2 25362243
chr2 25362257
chr2 25362269
chr2 25362297
chr2 25362520
chr2 25362537
chr2 25362613
chr2 25522212
chr2 25522372
chr2 25712224
chr2 25788279
chr2 25788577
chr2 25788654
chr2 25788691
chr2 25788884
chr2 25789576
chr2 25789660
chr2 25794436
chr2 25794816
chr2 25822232
chr2 25822265
chr2 25822269
chr2 25822278
chr2 25822289
chr2 25822291
chr2 25822308
chr2 25822320
chr2 25822323
chr2 25822329
chr2 25822335
chr2 25822348
chr2 25822359
chr2 25822361
chr2 25822365
chr2 25822375
chr2 25822383
chr2 25822389

chr2 25822400
chr2 25822418
chr2 25822421
chr2 25822432
chr2 25822444
chr2 25822448
chr2 25822499
chr2 25822831
chr2 25822920
chr2 25822942
chr2 25824339
chr2 25824476
chr2 25824600
chr2 25824659
chr2 25824792
chr2 25832253
chr2 25832311
chr2 25852343
chr2 25877152
chr2 25877158
chr2 25877169
chr2 25877228
chr2 25877265
chr2 25877399
chr2 25877480
chr2 25877489
chr2 25877502
chr2 25877738
chr2 25877755
chr2 25877769
chr2 25877831
chr2 25877888
chr2 25877900
chr2 25877905
chr2 25916619
chr2 25916629
chr2 25916637
chr2 25916651
chr2 25916653
chr2 25916655
chr2 25916712
chr2 25916778
chr2 25916784
chr2 25916787
chr2 25916812
chr2 25916814
chr2 25916824
chr2 25916868
chr2 25916876
chr2 26035625
chr2 26035659
chr2 26035664
chr2 26035707
chr2 26035766

chr2 26035773
chr2 26035829
chr2 26086473
chr2 26086497
chr2 26086769
chr2 26086802
chr2 26086829
chr2 26086833
chr2 26087006
chr2 26145539
chr2 26145583
chr2 26178827
chr2 26178829
chr2 26243198
chr2 26243218
chr2 26243235
chr2 26243287
chr2 26243402
chr2 26243611
chr2 26243617
chr2 26243710
chr2 26243758
chr2 26329531
chr2 26330122
chr2 26330167
chr2 26330210
chr2 26330215
chr2 26330224
chr2 26330237
chr2 26330288
chr2 26346850
chr2 26346852
chr2 26346860
chr2 26346904
chr2 26346915
chr2 26346929
chr2 26347006
chr2 26347042
chr2 26347078
chr2 26347117
chr2 26347165
chr2 26347219
chr2 26347244
chr2 26347253
chr2 26347276
chr2 26347283
chr2 26347430
chr2 26347496
chr2 26347521
chr2 26347583
chr2 26347861
chr2 26433312
chr2 26786130
chr2 26832915

chr2 27081554
chr2 27098612
chr2 27217526
chr2 27217575
chr2 27217580
chr2 27217588
chr2 27218232
chr2 27264808
chr2 27264826
chr2 27264891
chr2 27264894
chr2 27264900
chr2 27264903
chr2 27264906
chr2 27264909
chr2 27264921
chr2 27264924
chr2 27264975
chr2 27264980
chr2 27264987
chr2 27264989
chr2 27264996
chr2 27264998
chr2 27265003
chr2 27265009
chr2 27265022
chr2 27265036
chr2 27265046
chr2 27265065
chr2 27265077
chr2 27265098
chr2 27356929
chr2 27356931
chr2 27356944
chr2 27356953
chr2 27356963
chr2 27356975
chr2 27356984
chr2 27356987
chr2 27356996
chr2 27357040
chr2 27357043
chr2 27357049
chr2 27357051
chr2 27357058
chr2 27357068
chr2 27357070
chr2 27357076
chr2 27357079
chr2 27357086
chr2 27357096
chr2 27357109
chr2 27357115
chr2 27357117

chr2 27357136
chr2 27357145
chr2 27357149
chr2 27357157
chr2 27357159
chr2 27357169
chr2 27357213
chr2 27357216
chr2 27357220
chr2 27357231
chr2 27357257
chr2 27357260
chr2 27357263
chr2 27357267
chr2 27408368
chr2 27408377
chr2 27408646
chr2 27408696
chr2 27408751
chr2 27408822
chr2 27583187
chr2 27583193
chr2 27583200
chr2 27583202
chr2 27583210
chr2 27583250
chr2 27583301
chr2 27583342
chr2 27583396
chr2 27583459
chr2 27583481
chr2 27629141
chr2 27629148
chr2 27629174
chr2 27629202
chr2 27629218
chr2 27629225
chr2 27629269
chr2 27629272
chr2 27629283
chr2 27629315
chr2 27629329
chr2 27629364
chr2 27629383
chr2 27629398
chr2 27629431
chr2 27629438
chr2 27629522
chr2 27629554
chr2 27629580
chr2 27663888
chr2 27663895
chr2 27663906
chr2 27663949

chr2 27663952
chr2 27663991
chr2 27664003
chr2 27664006
chr2 27664008
chr2 27664021
chr2 27664072
chr2 27664100
chr2 27664120
chr2 27664127
chr2 27664159
chr2 27664161
chr2 27664168
chr2 27664174
chr2 27664190
chr2 27664200
chr2 27664274
chr2 27664293
chr2 27664309
chr2 27664325
chr2 27703796
chr2 27771850
chr2 27771867
chr2 27771877
chr2 27771891
chr2 27771900
chr2 27771961
chr2 27771964
chr2 27771966
chr2 27771976
chr2 27771978
chr2 27772008
chr2 27772036
chr2 27772041
chr2 27772061
chr2 27772076
chr2 27772097
chr2 27772130
chr2 27772152
chr2 27772158
chr2 27772188
chr2 27772212
chr2 27772220
chr2 27772345
chr2 27772347
chr2 27772720
chr2 27890630
chr2 27890632
chr2 27890638
chr2 27890646
chr2 27890650
chr2 27890654
chr2 27890659
chr2 27890662

chr2 27890664
chr2 27890667
chr2 27890669
chr2 27890675
chr2 27890677
chr2 27890681
chr2 27890684
chr2 27890694
chr2 27890700
chr2 27890711
chr2 27890716
chr2 27890721
chr2 27890725
chr2 27890734
chr2 27890738
chr2 27890749
chr2 27890751
chr2 27890754
chr2 27890756
chr2 27890758
chr2 27890760
chr2 28134969
chr2 28252475
chr2 28391375
chr2 28391385
chr2 28391462
chr2 28391531
chr2 28458425
chr2 28681326
chr2 28681346
chr2 28681374
chr2 28681416
chr2 28681589
chr2 28753148
chr2 28753226
chr2 28753229
chr2 28753236
chr2 28753373
chr2 28753756
chr2 28753772
chr2 28753796
chr2 28753819
chr2 28821260
chr2 28821285
chr2 28821292
chr2 28821356
chr2 28821360
chr2 28821447
chr2 28821452
chr2 28821492
chr2 28821496
chr2 28821597
chr2 28821689
chr2 28869481

chr2 28869571
chr2 28869662
chr2 28895820
chr2 28895928
chr2 28896130
chr2 28896220
chr2 28896242
chr2 28896506
chr2 28896595
chr2 28896853
chr2 28896900
chr2 29014683
chr2 29014723
chr2 29014775
chr2 29014848
chr2 29014870
chr2 29014879
chr2 29014883
chr2 29014917
chr2 29014922
chr2 29014985
chr2 29015007
chr2 29015063
chr2 29092579
chr2 29093034
chr2 29093364
chr2 29093491
chr2 29116342
chr2 29116405
chr2 29116428
chr2 29116437
chr2 29116441
chr2 29116483
chr2 29116519
chr2 29116560
chr2 29116581
chr2 29116588
chr2 29116651
chr2 29116697
chr2 29116736
chr2 29119320
chr2 29119330
chr2 29119457
chr2 29314888
chr2 29314910
chr2 29314912
chr2 29314927
chr2 29314932
chr2 29314938
chr2 29314993
chr2 29315102
chr2 29315140
chr2 29315164
chr2 29315387

chr2 29315403
chr2 29315462
chr2 29359959
chr2 29360483
chr2 29360494
chr2 29360570
chr2 29496048
chr2 29496052
chr2 30209001
chr2 30232997
chr2 30233043
chr2 30233193
chr2 30233510
chr2 30233581
chr2 30233628
chr2 30233720
chr2 30265749
chr2 30265903
chr2 30266303
chr2 30266309
chr2 30266350
chr2 30266472
chr2 30266493
chr2 30266545
chr2 30337337
chr2 30337355
chr2 30337368
chr2 30337370
chr2 30337437
chr2 30337541
chr2 30337739
chr2 30337753
chr2 30337755
chr2 30337762
chr2 30337769
chr2 30337800
chr2 30446731
chr2 30446891
chr2 30446893
chr2 30446982
chr2 30446997
chr2 30447013
chr2 30447040
chr2 30447045
chr2 30447074
chr2 30447086
chr2 30447766
chr2 30447813
chr2 30447831
chr2 30447835
chr2 30447843
chr2 30447846
chr2 30447853
chr2 30447886

chr2 30447899
chr2 30447937
chr2 30447939
chr2 30447967
chr2 30447997
chr2 30448283
chr2 30448306
chr2 30448397
chr2 30775373
chr2 30775626
chr2 30775660
chr2 30775681
chr2 31214078
chr2 31214244
chr2 31225968
chr2 31226157
chr2 31226178
chr2 31226256
chr2 31351468
chr2 31903901
chr2 32008971
chr2 32008981
chr2 32009202
chr2 32009224
chr2 32009258
chr2 32009274
chr2 32009282
chr2 32009319
chr2 32040258
chr2 32118387
chr2 32118493
chr2 32118502
chr2 32165713
chr2 32165722
chr2 32166210
chr2 32166290
chr2 32166296
chr2 32166302
chr2 32166345
chr2 32166393
chr2 32356475
chr2 32356483
chr2 32356526
chr2 32356540
chr2 32356591
chr2 32356620
chr2 32356624
chr2 32356643
chr2 32358112
chr2 32358115
chr2 32358131
chr2 32358135
chr2 32358138
chr2 32358144

chr2 32358167
chr2 32358628
chr2 32358634
chr2 32358705
chr2 32592072
chr2 32592108
chr2 32628443
chr2 32715536
chr2 32715632
chr2 32715662
chr2 32715822
chr2 32756227
chr2 32756241
chr2 32756250
chr2 32756279
chr2 32756312
chr2 32887257
chr2 32887314
chr2 32916183
chr2 32916186
chr2 32916222
chr2 32916252
chr2 32916581
chr2 32916587
chr2 32946263
chr2 32946267
chr2 32946274
chr2 32946280
chr2 32946292
chr2 33271385
chr2 33271453
chr2 33272179
chr2 33321344
chr2 33321362
chr2 33321368
chr2 33321396
chr2 33321505
chr2 33321547
chr2 33321571
chr2 33321753
chr2 33322292
chr2 33322327
chr2 33322361
chr2 33322738
chr2 33322787
chr2 33436505
chr2 33475610
chr2 33476053
chr2 33476074
chr2 33476162
chr2 33476924
chr2 33476930
chr2 33598114
chr2 33598306

chr2 33598374
chr2 33598509
chr2 33598520
chr2 33598532
chr2 33598556
chr2 33598606
chr2 33598631
chr2 33598643
chr2 33598657
chr2 33598677
chr2 33598855
chr2 33598869
chr2 33598885
chr2 33598911
chr2 33598926
chr2 33598931
chr2 33598940
chr2 33599032
chr2 33599048
chr2 33774240
chr2 33774349
chr2 33774587
chr2 33900642
chr2 33900914
chr2 33901051
chr2 34143431
chr2 34354269
chr2 34354479
chr2 34387981
chr2 34391640
chr2 34707772
chr2 34733619
chr2 34733638
chr2 34734419
chr2 34734620
chr2 34735231
chr2 34735403
chr2 35214738
chr2 35214775
chr2 35214939
chr2 35214956
chr2 35215096
chr2 35215420
chr2 36240507
chr2 36240556
chr2 36240573
chr2 36240614
chr2 36240630
chr2 36240632
chr2 36240648
chr2 36240656
chr2 36240688
chr2 36240811
chr2 36240964

chr2 36241322
chr2 36597576
chr2 36597603
chr2 36597623
chr2 36597650
chr2 36664366
chr2 36664464
chr2 36859501
chr2 36863753
chr2 36965646
chr2 36965728
chr2 36965739
chr2 36965744
chr2 37005654
chr2 37008181
chr2 37080868
chr2 37080883
chr2 37081030
chr2 37085031
chr2 37085060
chr2 37085088
chr2 37085263
chr2 37085461
chr2 37085498
chr2 37085555
chr2 37085573
chr2 37085969
chr2 37156320
chr2 37156330
chr2 37156425
chr2 37156445
chr2 37156480
chr2 37156644
chr2 37156674
chr2 37156681
chr2 37156717
chr2 37156815
chr2 37156822
chr2 37156827
chr2 37230181
chr2 37230281
chr2 37230453
chr2 37230672
chr2 37390458
chr2 37390462
chr2 37395760
chr2 37395857
chr2 37395870
chr2 37395988
chr2 37396011
chr2 37396067
chr2 37396069
chr2 37396091
chr2 37582675

chr2 37582819
chr2 37582867
chr2 37582879
chr2 37582935
chr2 37583055
chr2 37600271
chr2 37600280
chr2 37600328
chr2 37600997
chr2 37601227
chr2 37601349
chr2 37601568
chr2 37601734
chr2 37602048
chr2 37602052
chr2 37635618
chr2 37670573
chr2 37670579
chr2 37670600
chr2 37670759
chr2 37670790
chr2 37672914
chr2 37672960
chr2 37673062
chr2 37673069
chr2 37673110
chr2 37673158
chr2 37852129
chr2 37852243
chr2 37877513
chr2 37877533
chr2 37877566
chr2 37877581
chr2 37877673
chr2 37877699
chr2 37877715
chr2 37877717
chr2 37892750
chr2 37925060
chr2 37934717
chr2 37934850
chr2 37935145
chr2 37935166
chr2 37935233
chr2 37935397
chr2 37935553
chr2 38098854
chr2 38098918
chr2 38146633
chr2 38146635
chr2 38146638
chr2 38146640
chr2 38527844
chr2 38528065

chr2 38528129
chr2 38528132
chr2 38528138
chr2 38528141
chr2 38528156
chr2 38528158
chr2 38542878
chr2 38542906
chr2 38543007
chr2 38543078
chr2 38543228
chr2 38598760
chr2 38598873
chr2 38599207
chr2 38599979
chr2 38600008
chr2 38600038
chr2 38600045
chr2 38600289
chr2 38600292
chr2 38600322
chr2 38600345
chr2 38600454
chr2 38600464
chr2 38601014
chr2 38601028
chr2 38601037
chr2 38601167
chr2 38601198
chr2 38601280
chr2 38601327
chr2 38601351
chr2 38601418
chr2 38601495
chr2 38626372
chr2 38666456
chr2 38666471
chr2 38666486
chr2 38666505
chr2 38666572
chr2 38666595
chr2 38666630
chr2 38666641
chr2 38667014
chr2 38667056
chr2 38667250
chr2 38667323
chr2 38667328
chr2 38667440
chr2 38667501
chr2 38667526
chr2 38667542
chr2 38667556
chr2 38700614

chr2 38701680
chr2 38701697
chr2 38701796
chr2 38709511
chr2 38709600
chr2 38709837
chr2 38709892
chr2 38709933
chr2 38710020
chr2 38749622
chr2 38749625
chr2 38749829
chr2 38749858
chr2 38749877
chr2 38750029
chr2 38750060
chr2 38750094
chr2 38750159
chr2 38750206
chr2 38750224
chr2 38750229
chr2 38754681
chr2 38754748
chr2 38754854
chr2 38778309
chr2 38778348
chr2 38778396
chr2 38778407
chr2 38778454
chr2 38778467
chr2 38778473
chr2 38778483
chr2 38778504
chr2 38778506
chr2 38872910
chr2 39119263
chr2 39119341
chr2 39119604
chr2 39119650
chr2 39119742
chr2 39119746
chr2 39119808
chr2 39119816
chr2 39119851
chr2 39128294
chr2 39216661
chr2 39216701
chr2 39216797
chr2 39216961
chr2 39216988
chr2 39217040
chr2 39217078
chr2 39217081
chr2 39217083

chr2 39217086
chr2 39217108
chr2 39217236
chr2 39217255
chr2 39217289
chr2 39217469
chr2 39228162
chr2 39228224
chr2 39228271
chr2 39228274
chr2 39228276
chr2 39228498
chr2 39228501
chr2 39228585
chr2 39228596
chr2 39228618
chr2 39228669
chr2 39228718
chr2 39228729
chr2 39228764
chr2 39228768
chr2 39416824
chr2 39416993
chr2 39439917
chr2 39440045
chr2 39492679
chr2 39778343
chr2 39778555
chr2 39778648
chr2 39778672
chr2 39778785
chr2 39778951
chr2 39778955
chr2 39778960
chr2 39778966
chr2 39778983
chr2 39778990
chr2 39778993
chr2 39778997
chr2 39779000
chr2 39779002
chr2 39779019
chr2 39779025
chr2 39779028
chr2 39779036
chr2 39779038
chr2 39779044
chr2 40013591
chr2 40013641
chr2 40013663
chr2 40449977
chr2 40450022
chr2 40450245
chr2 40450310

chr2 40450373
chr2 40450375
chr2 40450377
chr2 40450379
chr2 40450381
chr2 40450582
chr2 40985557
chr2 41314495
chr2 41314916
chr2 41314997
chr2 41315043
chr2 41315120
chr2 41376466
chr2 41376740
chr2 41576597
chr2 41576834
chr2 41576857
chr2 41576874
chr2 41756159
chr2 42101797
chr2 42101809
chr2 42101834
chr2 42101851
chr2 42101853
chr2 42101877
chr2 42101894
chr2 42101925
chr2 42101948
chr2 42101965
chr2 42101974
chr2 42102014
chr2 42102667
chr2 42102683
chr2 42102748
chr2 42102776
chr2 42102786
chr2 42102805
chr2 42102817
chr2 42105723
chr2 42105739
chr2 42105783
chr2 42105856
chr2 42105908
chr2 42105949
chr2 42106138
chr2 42106155
chr2 42132863
chr2 42132903
chr2 42132958
chr2 42133024
chr2 42133030
chr2 42133088
chr2 42133120
chr2 42133132

chr2 42133167
chr2 42133190
chr2 42133194
chr2 42169491
chr2 42169498
chr2 42169503
chr2 42169507
chr2 42169519
chr2 42169523
chr2 42169537
chr2 42169540
chr2 42169552
chr2 42169557
chr2 42169573
chr2 42169587
chr2 42169594
chr2 42169597
chr2 42169606
chr2 42169617
chr2 42169623
chr2 42169626
chr2 42169635
chr2 42169720
chr2 42169731
chr2 42169742
chr2 42169775
chr2 42169783
chr2 42169785
chr2 42169792
chr2 42169855
chr2 42170174
chr2 42170269
chr2 42170580
chr2 42170617
chr2 42170696
chr2 42170766
chr2 42170845
chr2 42170866
chr2 42170993
chr2 42171358
chr2 42171533
chr2 42171582
chr2 42229069
chr2 42232182
chr2 42256515
chr2 42256839
chr2 42257005
chr2 42267697
chr2 42359573
chr2 42359610
chr2 42359816
chr2 42359825
chr2 42359846
chr2 42359963

chr2 42359970
chr2 42359976
chr2 42360005
chr2 42360014
chr2 42360152
chr2 42360162
chr2 42360304
chr2 42360307
chr2 42360370
chr2 42360377
chr2 42360397
chr2 42360472
chr2 42360507
chr2 42360533
chr2 42360549
chr2 42360575
chr2 42360649
chr2 42360678
chr2 42360782
chr2 42361704
chr2 42361719
chr2 42361734
chr2 42361756
chr2 42361827
chr2 42474929
chr2 42510743
chr2 42568918
chr2 42568958
chr2 42568960
chr2 42568966
chr2 42568968
chr2 42568976
chr2 42568994
chr2 42569001
chr2 42569038
chr2 42569062
chr2 42569074
chr2 42569086
chr2 42569089
chr2 42775948
chr2 42776056
chr2 42859086
chr2 42859283
chr2 43059402
chr2 43059405
chr2 43059532
chr2 43059575
chr2 43059616
chr2 43059657
chr2 43131151
chr2 43131193
chr2 43131200
chr2 43131204
chr2 43131289

chr2 43131345
chr2 43131382
chr2 43131445
chr2 43131474
chr2 43131537
chr2 43131556
chr2 43131564
chr2 43131569
chr2 43131600
chr2 43131756
chr2 43131922
chr2 43134033
chr2 43174357
chr2 43174381
chr2 43174416
chr2 43174419
chr2 43174494
chr2 43174532
chr2 43174651
chr2 43174666
chr2 43174719
chr2 43174816
chr2 43175158
chr2 43175499
chr2 43218773
chr2 43218795
chr2 43218810
chr2 43218820
chr2 43218881
chr2 43218927
chr2 43219217
chr2 43219225
chr2 43220072
chr2 43220110
chr2 43220124
chr2 43220135
chr2 43220567
chr2 43220572
chr2 43220627
chr2 43220683
chr2 43220773
chr2 43220805
chr2 43220874
chr2 43220886
chr2 43220902
chr2 43221165
chr2 43221272
chr2 43221299
chr2 43221305
chr2 43221320
chr2 43221330
chr2 43221344
chr2 43221387
chr2 43221403

chr2 43221413
chr2 43221421
chr2 43221524
chr2 43221527
chr2 43221564
chr2 43221637
chr2 43221643
chr2 43228303
chr2 43228634
chr2 43228723
chr2 43229024
chr2 43229243
chr2 43229341
chr2 43229348
chr2 43229418
chr2 43229567
chr2 43229688
chr2 43229700
chr2 43293927
chr2 43294027
chr2 43294177
chr2 43426025
chr2 43426646
chr2 43426668
chr2 43559752
chr2 43559860
chr2 43559897
chr2 43560293
chr2 43560337
chr2 43566257
chr2 43566286
chr2 43566759
chr2 43566859
chr2 43577477
chr2 43577580
chr2 43595586
chr2 43595595
chr2 43612677
chr2 43676668
chr2 43676703
chr2 43676761
chr2 43774214
chr2 43774272
chr2 43774311
chr2 43774428
chr2 43774442
chr2 43774445
chr2 43774497
chr2 43774505
chr2 43774517
chr2 43774574
chr2 43905373
chr2 43905399
chr2 43995656

chr2 43995663
chr2 43995670
chr2 43995691
chr2 43995706
chr2 43995723
chr2 43995733
chr2 43995742
chr2 43995748
chr2 43995761
chr2 43995764
chr2 43995800
chr2 43995839
chr2 43995855
chr2 43995860
chr2 43995874
chr2 43995885
chr2 43996009
chr2 43996024
chr2 43996026
chr2 43996043
chr2 43996047
chr2 43996100
chr2 43996110
chr2 43996145
chr2 43996166
chr2 43996182
chr2 43996189
chr2 43996195
chr2 43996207
chr2 43996217
chr2 43996250
chr2 43996278
chr2 43996314
chr2 43996324
chr2 43996334
chr2 44120551
chr2 44167928
chr2 44168123
chr2 44170448
chr2 44170460
chr2 44170611
chr2 44170849
chr2 44236879
chr2 44237169
chr2 44237284
chr2 44237363
chr2 44243698
chr2 44244041
chr2 44244244
chr2 44261308
chr2 44261313
chr2 44261357
chr2 44261471
chr2 44360902

chr2 44360927
chr2 44360932
chr2 44360935
chr2 44360941
chr2 44360966
chr2 44360983
chr2 44361019
chr2 44361050
chr2 44361086
chr2 44361191
chr2 44361200
chr2 44361228
chr2 44361264
chr2 44361268
chr2 44361276
chr2 44361295
chr2 44361329
chr2 44361379
chr2 44361400
chr2 44479446
chr2 44479669
chr2 44479699
chr2 44479862
chr2 44927173
chr2 44927214
chr2 44927250
chr2 44927589
chr2 45168623
chr2 45220900
chr2 45220921
chr2 45220951
chr2 45610550
chr2 45611471
chr2 45860695
chr2 45860856
chr2 46098833
chr2 46246934
chr2 46248049
chr2 46499506
chr2 46499515
chr2 46499544
chr2 46499636
chr2 46499698
chr2 46499752
chr2 46499764
chr2 46499785
chr2 46499790
chr2 46499816
chr2 46499820
chr2 46499822
chr2 46499838
chr2 46499853
chr2 46499864
chr2 46499872

chr2 46499882
chr2 46499884
chr2 46499886
chr2 46499889
chr2 46500177
chr2 46500217
chr2 46500233
chr2 46500236
chr2 46500239
chr2 46500247
chr2 46500258
chr2 46500262
chr2 46500269
chr2 46500289
chr2 46500296
chr2 46500302
chr2 46500308
chr2 46500345
chr2 46617778
chr2 46617871
chr2 46776265
chr2 46776581
chr2 46776695
chr2 46942115
chr2 46942124
chr2 46942240
chr2 46944087
chr2 47173698
chr2 47174039
chr2 47174151
chr2 47174219
chr2 47174344
chr2 47174395
chr2 47174413
chr2 47174551
chr2 47174661
chr2 47174695
chr2 47174744
chr2 47174890
chr2 47174900
chr2 47174924
chr2 47345211
chr2 47345219
chr2 47345278
chr2 47345350
chr2 47345369
chr2 47345399
chr2 47345420
chr2 47345478
chr2 47345593
chr2 47345695
chr2 47569145
chr2 47783937
chr2 47783945

chr2 47783949
chr2 47783960
chr2 47783975
chr2 47783990
chr2 47783994
chr2 47783997
chr2 47783999
chr2 47784028
chr2 47784032
chr2 47784062
chr2 47784064
chr2 47784068
chr2 47784071
chr2 47784076
chr2 47784087
chr2 47784098
chr2 47784115
chr2 47784118
chr2 47784133
chr2 47784140
chr2 47784144
chr2 47784146
chr2 47784171
chr2 47784173
chr2 47784188
chr2 47784193
chr2 47784199
chr2 47784209
chr2 47784224
chr2 47784237
chr2 47784253
chr2 47784260
chr2 47867464
chr2 47867559
chr2 47888930
chr2 47889209
chr2 47898088
chr2 47898162
chr2 47898178
chr2 47898447
chr2 47903839
chr2 47904077
chr2 47904135
chr2 47904213
chr2 47904236
chr2 47904250
chr2 47904295
chr2 47904309
chr2 47904333
chr2 47904468
chr2 47907406
chr2 47907424
chr2 47907442
chr2 47907532

chr2 47907596
chr2 47907648
chr2 47907658
chr2 47907732
chr2 48111626
chr2 48111641
chr2 48111646
chr2 48111666
chr2 48111689
chr2 48111694
chr2 48111699
chr2 48111701
chr2 48111716
chr2 48111724
chr2 48313532
chr2 48313539
chr2 48313651
chr2 48313696
chr2 48313754
chr2 48313760
chr2 48313811
chr2 48313843
chr2 48316177
chr2 48316228
chr2 48316231
chr2 48316241
chr2 48316493
chr2 48316604
chr2 48316751
chr2 48317184
chr2 48356928
chr2 48409288
chr2 48409304
chr2 48440122
chr2 48441192
chr2 48441196
chr2 48441205
chr2 48441209
chr2 48441454
chr2 48441464
chr2 48441486
chr2 48441610
chr2 48448874
chr2 48448914
chr2 48448998
chr2 49480664
chr2 49812647
chr2 49975173
chr2 49975200
chr2 50018570
chr2 50021162
chr2 50021296
chr2 50278725
chr2 50278797

chr2 50348096
chr2 50348186
chr2 50348188
chr2 50529966
chr2 50590395
chr2 50590728
chr2 51025163
chr2 51025168
chr2 51025933
chr2 51032865
chr2 51032993
chr2 51032996
chr2 51236629
chr2 51346998
chr2 51448109
chr2 51448508
chr2 51448849
chr2 51449388
chr2 51594033
chr2 51777943
chr2 51777966
chr2 51778009
chr2 51778451
chr2 51818862
chr2 51818867
chr2 51819002
chr2 51819510
chr2 51820567
chr2 51820802
chr2 51969570
chr2 51970078
chr2 52042024
chr2 52042627
chr2 52042666
chr2 52108276
chr2 52108826
chr2 52240944
chr2 52240959
chr2 52240968
chr2 52241430
chr2 52241478
chr2 52241571
chr2 52241832
chr2 52261299
chr2 52404662
chr2 52404760
chr2 52805643
chr2 52807146
chr2 52966709
chr2 53331660
chr2 53331729
chr2 53331889
chr2 53359527
chr2 53359814

chr2 53360074
chr2 53360311
chr2 53767132
chr2 53767283
chr2 53768573
chr2 53788038
chr2 53788040
chr2 53788073
chr2 53788095
chr2 53788181
chr2 53964136
chr2 53964710
chr2 53964871
chr2 53969525
chr2 53969541
chr2 53969620
chr2 53969686
chr2 53969822
chr2 53969940
chr2 53969972
chr2 53969982
chr2 53969992
chr2 53970072
chr2 53970078
chr2 53970086
chr2 53970094
chr2 53970101
chr2 53970119
chr2 53970176
chr2 53970189
chr2 53970201
chr2 53994411
chr2 53994588
chr2 53994599
chr2 53994925
chr2 53994931
chr2 53994995
chr2 53995025
chr2 53995098
chr2 54103901
chr2 54115137
chr2 54115217
chr2 54115361
chr2 54115447
chr2 54115480
chr2 54116102
chr2 54522779
chr2 54522831
chr2 54558204
chr2 54558208
chr2 54558210
chr2 54558215
chr2 54558219
chr2 54558225

chr2 54558236
chr2 54558238
chr2 54558243
chr2 54558257
chr2 54558259
chr2 54558269
chr2 54558325
chr2 54558336
chr2 54560880
chr2 54561155
chr2 54561177
chr2 54561213
chr2 54561380
chr2 54561662
chr2 54568925
chr2 54569006
chr2 54569145
chr2 54569178
chr2 54569230
chr2 54569405
chr2 54569441
chr2 54569515
chr2 54569542
chr2 54571210
chr2 54571258
chr2 54571403
chr2 54571412
chr2 54571431
chr2 54571458
chr2 54571707
chr2 54572148
chr2 54572403
chr2 54572422
chr2 54572424
chr2 54572537
chr2 54572760
chr2 54574292
chr2 54574298
chr2 54574324
chr2 54574361
chr2 54574447
chr2 54574491
chr2 54574699
chr2 54575048
chr2 54575076
chr2 54575085
chr2 54575105
chr2 54575184
chr2 54580141
chr2 54580149
chr2 54580279
chr2 54942372
chr2 55047927
chr2 55047978

chr2 55048189
chr2 55048244
chr2 55048314
chr2 55048423
chr2 55048538
chr2 55048575
chr2 55048578
chr2 55048585
chr2 55048627
chr2 55048638
chr2 55048658
chr2 55048661
chr2 55048673
chr2 55048679
chr2 55051291
chr2 55051346
chr2 55051386
chr2 55051688
chr2 55051738
chr2 55051766
chr2 55052271
chr2 55052492
chr2 55052519
chr2 55052607
chr2 55133708
chr2 55133761
chr2 55133863
chr2 55134092
chr2 55134123
chr2 55134174
chr2 55134212
chr2 55134247
chr2 55227778
chr2 55228212
chr2 55231558
chr2 55231605
chr2 55231713
chr2 55231823
chr2 55231827
chr2 55231878
chr2 55231937
chr2 55231940
chr2 55231958
chr2 55231986
chr2 55231992
chr2 55232033
chr2 55269223
chr2 55269235
chr2 55269248
chr2 55269254
chr2 55269259
chr2 55269266
chr2 55269269
chr2 55269277

chr2 55269283
chr2 55269302
chr2 55269323
chr2 55269326
chr2 55269347
chr2 55269354
chr2 55269356
chr2 55269359
chr2 55269363
chr2 55269379
chr2 55269414
chr2 55269416
chr2 55269418
chr2 55269428
chr2 55269563
chr2 55269770
chr2 55269795
chr2 55269841
chr2 55269876
chr2 55269886
chr2 55404400
chr2 55420762
chr2 55420847
chr2 55420870
chr2 55519295
chr2 55519326
chr2 55519349
chr2 55519358
chr2 55519386
chr2 55519396
chr2 55519398
chr2 55519402
chr2 55519411
chr2 55519413
chr2 55519436
chr2 55519457
chr2 55519509
chr2 55519540
chr2 55519551
chr2 55519564
chr2 55519573
chr2 55520450
chr2 55520497
chr2 55615898
chr2 55615905
chr2 55615911
chr2 55615935
chr2 55615963
chr2 55616153
chr2 55616447
chr2 55693110
chr2 55693196
chr2 55693208
chr2 55693219

chr2 55693237
chr2 55693268
chr2 55693300
chr2 55693313
chr2 55693323
chr2 55693334
chr2 55693342
chr2 55693388
chr2 55693404
chr2 55693419
chr2 55693429
chr2 55693448
chr2 55693470
chr2 55693478
chr2 55693480
chr2 55693926
chr2 55693929
chr2 55693937
chr2 55693959
chr2 55694072
chr2 55718272
chr2 55718374
chr2 55718412
chr2 55751321
chr2 55751465
chr2 55752181
chr2 55752619
chr2 55796737
chr2 56183431
chr2 56185495
chr2 56185693
chr2 56185733
chr2 56185736
chr2 56185744
chr2 56185782
chr2 56185789
chr2 56185800
chr2 56185833
chr2 56185899
chr2 56185934
chr2 56186002
chr2 56186111
chr2 56186118
chr2 56186124
chr2 56192079
chr2 56772676
chr2 56773184
chr2 56886661
chr2 56887020
chr2 57303020
chr2 57303206
chr2 57303483
chr2 57376944
chr2 57377046

chr2 57377435
chr2 57497665
chr2 57497899
chr2 57498009
chr2 57720714
chr2 57720799
chr2 57760030
chr2 57760033
chr2 57760040
chr2 57760045
chr2 57760074
chr2 57760080
chr2 57760097
chr2 57760114
chr2 57760128
chr2 57760136
chr2 57760145
chr2 57760190
chr2 57760214
chr2 57760290
chr2 57760294
chr2 57760444
chr2 57760654
chr2 57760668
chr2 57760764
chr2 58047479
chr2 58047496
chr2 58047514
chr2 58240600
chr2 58240748
chr2 58240807
chr2 58240830
chr2 58240850
chr2 58240874
chr2 58240922
chr2 58240934
chr2 58240953
chr2 58240979
chr2 58427711
chr2 58427721
chr2 58427723
chr2 58427729
chr2 58427731
chr2 58427735
chr2 58427737
chr2 58427739
chr2 58427741
chr2 58427752
chr2 58559961
chr2 59068180
chr2 59068438
chr2 59068591
chr2 59594124
chr2 60105188

chr2 60548320
chr2 60548504
chr2 60552892
chr2 60552924
chr2 60552941
chr2 60552944
chr2 60553003
chr2 60553027
chr2 60553081
chr2 60553088
chr2 60553101
chr2 60553145
chr2 60553165
chr2 60553167
chr2 60553173
chr2 60553215
chr2 60553217
chr2 60553227
chr2 60553260
chr2 60555875
chr2 60556041
chr2 60556051
chr2 60556054
chr2 60556063
chr2 60556214
chr2 60556255
chr2 60556321
chr2 60556426
chr2 60556434
chr2 60556677
chr2 60556680
chr2 60556688
chr2 60556746
chr2 60556752
chr2 60557549
chr2 60557566
chr2 60557568
chr2 60557581
chr2 60557657
chr2 60557681
chr2 60557713
chr2 60557718
chr2 60557725
chr2 60557732
chr2 60557734
chr2 60557743
chr2 60565645
chr2 60565673
chr2 60565822
chr2 60569077
chr2 60569081
chr2 60569207
chr2 60569218
chr2 60569227

chr2 60569230
chr2 60569263
chr2 60569271
chr2 60569410
chr2 60569412
chr2 60569468
chr2 60569481
chr2 60569483
chr2 60569485
chr2 60569497
chr2 60569536
chr2 60569540
chr2 60569589
chr2 60569596
chr2 60569599
chr2 60569636
chr2 60569669
chr2 60569690
chr2 60569803
chr2 60569871
chr2 60569919
chr2 60569959
chr2 60569992
chr2 60570018
chr2 60570024
chr2 60570064
chr2 60570078
chr2 60570083
chr2 60570085
chr2 60570107
chr2 60570130
chr2 60570132
chr2 60570178
chr2 60570193
chr2 60570434
chr2 60570440
chr2 60570510
chr2 60570516
chr2 60570523
chr2 60735864
chr2 60735882
chr2 60735886
chr2 60735900
chr2 60735916
chr2 60735921
chr2 60736068
chr2 60736075
chr2 60755880
chr2 60755946
chr2 60755952
chr2 60756019
chr2 60756059
chr2 60756067
chr2 60756082

chr2 60756084
chr2 60756117
chr2 60756125
chr2 60757028
chr2 60757074
chr2 60757201
chr2 60757221
chr2 60757235
chr2 60757245
chr2 60757258
chr2 60807084
chr2 60807352
chr2 60807398
chr2 60841312
chr2 60884862
chr2 60885053
chr2 60885416
chr2 60885764
chr2 60886035
chr2 60886171
chr2 60890948
chr2 60971680
chr2 60993055
chr2 61016381
chr2 61016386
chr2 61016409
chr2 61016452
chr2 61016555
chr2 61016607
chr2 61018737
chr2 61018799
chr2 61018862
chr2 61018936
chr2 61019035
chr2 61019056
chr2 61080925
chr2 61081242
chr2 61177521
chr2 61177532
chr2 61177535
chr2 61177574
chr2 61177584
chr2 61177592
chr2 61177601
chr2 61177756
chr2 61177763
chr2 61177770
chr2 61177775
chr2 61177793
chr2 61177800
chr2 61177861
chr2 61177883
chr2 61177916
chr2 61177937

chr2 61369093
chr2 61369193
chr2 61469795
chr2 61469857
chr2 61469890
chr2 61469968
chr2 61470020
chr2 61470103
chr2 61493674
chr2 61535433
chr2 61535497
chr2 61535604
chr2 61535738
chr2 61535743
chr2 61535763
chr2 61535979
chr2 61671311
chr2 61671403
chr2 61691060
chr2 61694668
chr2 61694677
chr2 61694700
chr2 61694725
chr2 61694733
chr2 61694739
chr2 61694745
chr2 61694752
chr2 61694758
chr2 61694772
chr2 61694784
chr2 61694801
chr2 61694809
chr2 61694825
chr2 61694835
chr2 61694841
chr2 61694843
chr2 61694863
chr2 61694903
chr2 61694945
chr2 61694957
chr2 61695003
chr2 61695009
chr2 61695031
chr2 61695049
chr2 61695057
chr2 61695077
chr2 61695079
chr2 61695085
chr2 61695102
chr2 61695138
chr2 61763367
chr2 61763429
chr2 61763547
chr2 61763549

chr2 61763555
chr2 61763588
chr2 61853483
chr2 61853556
chr2 61853675
chr2 61853697
chr2 61853703
chr2 61905295
chr2 61905401
chr2 61905479
chr2 61905489
chr2 61906211
chr2 61906302
chr2 61958210
chr2 61958240
chr2 62198100
chr2 62198163
chr2 62198455
chr2 62198834
chr2 62309512
chr2 62309761
chr2 62309768
chr2 62309794
chr2 62309799
chr2 62309823
chr2 62309852
chr2 62309868
chr2 62309879
chr2 62309889
chr2 62309903
chr2 62309912
chr2 62309946
chr2 62309956
chr2 62309998
chr2 62310017
chr2 62310056
chr2 62310154
chr2 62314177
chr2 62314337
chr2 62314393
chr2 62506482
chr2 62506513
chr2 62506798
chr2 62506854
chr2 62704961
chr2 62704967
chr2 62705006
chr2 62705092
chr2 62705099
chr2 62705103
chr2 62705111
chr2 62705188
chr2 62705212
chr2 62705224

chr2 62705226
chr2 62705240
chr2 62705367
chr2 63060219
chr2 63060287
chr2 63060405
chr2 63060419
chr2 63060551
chr2 63060599
chr2 63060648
chr2 63060772
chr2 63060956
chr2 63061051
chr2 63061650
chr2 63061909
chr2 63587543
chr2 63587594
chr2 63587618
chr2 63587769
chr2 63587830
chr2 63589529
chr2 63589542
chr2 63589550
chr2 63589817
chr2 63589832
chr2 63589875
chr2 63842089
chr2 63842130
chr2 63842161
chr2 63842190
chr2 63842230
chr2 63842318
chr2 63842330
chr2 63842371
chr2 63842380
chr2 63842382
chr2 63842449
chr2 63842452
chr2 63856359
chr2 63856465
chr2 63977690
chr2 63977995
chr2 63978041
chr2 63978355
chr2 64018774
chr2 64018801
chr2 64018815
chr2 64018822
chr2 64018827
chr2 64019582
chr2 64019604
chr2 64019618
chr2 64019691
chr2 64019700

chr2 64019711
chr2 64019713
chr2 64019725
chr2 64019734
chr2 64019753
chr2 64019758
chr2 64019778
chr2 64019805
chr2 64019832
chr2 64019896
chr2 64021706
chr2 64021807
chr2 64035768
chr2 64035863
chr2 64036069
chr2 64036076
chr2 64036217
chr2 64036604
chr2 64141303
chr2 64141564
chr2 64142312
chr2 64214028
chr2 64214080
chr2 64214086
chr2 64214175
chr2 64214286
chr2 64214699
chr2 64214848
chr2 64228198
chr2 64228252
chr2 64228263
chr2 64228305
chr2 64228347
chr2 64228375
chr2 64228381
chr2 64228389
chr2 64228395
chr2 64228397
chr2 64228417
chr2 64228750
chr2 64325864
chr2 64326059
chr2 64326067
chr2 64326077
chr2 64326247
chr2 64454295
chr2 64454301
chr2 64522287
chr2 64522363
chr2 64522611
chr2 64522745
chr2 64522747
chr2 64522883
chr2 64522935

chr2 64522955
chr2 64525472
chr2 64525500
chr2 64525590
chr2 64525747
chr2 64525810
chr2 64525965
chr2 64526129
chr2 64526178
chr2 64526191
chr2 64532984
chr2 64533331
chr2 64533656
chr2 64645268
chr2 64645272
chr2 64645274
chr2 64645276
chr2 64645284
chr2 64645294
chr2 64645336
chr2 64645361
chr2 64645408
chr2 64645423
chr2 64646236
chr2 64646311
chr2 64646458
chr2 64646516
chr2 64646524
chr2 64646530
chr2 64646544
chr2 64646694
chr2 64646854
chr2 64646934
chr2 64646964
chr2 64647130
chr2 64652346
chr2 64652363
chr2 64652377
chr2 64652391
chr2 64652396
chr2 64652554
chr2 64652621
chr2 64711506
chr2 64711693
chr2 64727388
chr2 64727446
chr2 64730578
chr2 64730669
chr2 64730753
chr2 64730778
chr2 64730807
chr2 64730881
chr2 64730947
chr2 64730965

chr2 64749732
chr2 64749828
chr2 64749839
chr2 64749844
chr2 64749916
chr2 64749925
chr2 64750040
chr2 64750053
chr2 64750116
chr2 64750125
chr2 64750131
chr2 64750156
chr2 64752000
chr2 64752071
chr2 64752074
chr2 64752082
chr2 64767146
chr2 64768654
chr2 64768668
chr2 64946207
chr2 64946329
chr2 64946347
chr2 64946389
chr2 64946406
chr2 64946449
chr2 64946549
chr2 64946556
chr2 64946559
chr2 64946600
chr2 64946677
chr2 64946684
chr2 64947254
chr2 65057128
chr2 65057204
chr2 65057254
chr2 65057267
chr2 65128793
chr2 65128992
chr2 65129070
chr2 65129074
chr2 65129114
chr2 65129259
chr2 65129262
chr2 65129579
chr2 65129602
chr2 65187702
chr2 65187804
chr2 65226819
chr2 65226919
chr2 65227073
chr2 65227085
chr2 65227090
chr2 65227192
chr2 65227202

chr2 65227230
chr2 65227296
chr2 65228535
chr2 65228541
chr2 65228546
chr2 65228595
chr2 65228642
chr2 65228727
chr2 65228849
chr2 65228876
chr2 65228894
chr2 65228907
chr2 65228936
chr2 65228940
chr2 65229011
chr2 65229028
chr2 65229036
chr2 65229058
chr2 65229075
chr2 65382303
chr2 65382546
chr2 65382798
chr2 65382874
chr2 65382882
chr2 65382938
chr2 65382978
chr2 65970487
chr2 66429369
chr2 66429555
chr2 66429578
chr2 66434818
chr2 66434821
chr2 66435174
chr2 66435178
chr2 66435191
chr2 66435201
chr2 66435205
chr2 66435366
chr2 66435370
chr2 66435432
chr2 66435453
chr2 66435465
chr2 66435615
chr2 66435632
chr2 66435637
chr2 66435657
chr2 66435661
chr2 66435678
chr2 66435685
chr2 66435709
chr2 66435721
chr2 66435855
chr2 66435861
chr2 66435872

chr2 66435919
chr2 66435925
chr2 66435991
chr2 66436002
chr2 66436681
chr2 66436768
chr2 66437213
chr2 66437253
chr2 66437262
chr2 66437277
chr2 66437334
chr2 66437358
chr2 66437403
chr2 66437421
chr2 66437535
chr2 66437542
chr2 66574267
chr2 66574291
chr2 66574356
chr2 66574618
chr2 66574652
chr2 66574654
chr2 66574677
chr2 66574747
chr2 66574761
chr2 66574774
chr2 66574792
chr2 66578607
chr2 66578634
chr2 66578651
chr2 66578705
chr2 66578724
chr2 66578802
chr2 66578807
chr2 66578922
chr2 66578946
chr2 66578948
chr2 66579004
chr2 66579072
chr2 66579076
chr2 66579223
chr2 66579368
chr2 66579514
chr2 66580016
chr2 66580150
chr2 66580333
chr2 66580366
chr2 66580404
chr2 66580631
chr2 66580657
chr2 66580737
chr2 66580786
chr2 66580878
chr2 66580923

chr2 66581029
chr2 66581057
chr2 66581062
chr2 66581097
chr2 66581126
chr2 66581129
chr2 66581134
chr2 66581152
chr2 66581189
chr2 66581212
chr2 66581217
chr2 66583183
chr2 66583209
chr2 66583254
chr2 66583270
chr2 66583339
chr2 66583346
chr2 66583355
chr2 66583369
chr2 66583395
chr2 66583419
chr2 66583438
chr2 66583474
chr2 66583492
chr2 66583507
chr2 66583530
chr2 66583641
chr2 66583671
chr2 66583711
chr2 66583713
chr2 66583720
chr2 66583737
chr2 66583798
chr2 66583819
chr2 66583823
chr2 66583904
chr2 66584025
chr2 66584112
chr2 66584182
chr2 66584192
chr2 66584491
chr2 66585681
chr2 66660243
chr2 66823617
chr2 66823706
chr2 66823794
chr2 67397705
chr2 67397708
chr2 67397711
chr2 67397830
chr2 67397892
chr2 67397927
chr2 67397935
chr2 67398003

chr2 67398023
chr2 67398145
chr2 67825184
chr2 67825195
chr2 67846825
chr2 67846832
chr2 67846953
chr2 67847003
chr2 68025595
chr2 68061916
chr2 68062155
chr2 68156567
chr2 68156804
chr2 68156810
chr2 68156839
chr2 68156857
chr2 68156970
chr2 68157020
chr2 68157053
chr2 68157061
chr2 68157063
chr2 68157077
chr2 68157079
chr2 68157123
chr2 68157215
chr2 68157221
chr2 68157230
chr2 68157238
chr2 68157296
chr2 68157311
chr2 68157314
chr2 68157322
chr2 68157326
chr2 68157353
chr2 68157384
chr2 68157413
chr2 68157431
chr2 68157438
chr2 68158494
chr2 68189216
chr2 68189247
chr2 68204265
chr2 68206535
chr2 68206698
chr2 68235907
chr2 68249748
chr2 68250003
chr2 68250245
chr2 68250257
chr2 68250364
chr2 68250374
chr2 68250409
chr2 68253827
chr2 68253834

chr2 68253909
chr2 68254132
chr2 68254179
chr2 68362321
chr2 68362372
chr2 68362377
chr2 68362397
chr2 68385709
chr2 68385838
chr2 68385949
chr2 68465488
chr2 68465505
chr2 68465512
chr2 68465573
chr2 68465687
chr2 68466283
chr2 68466362
chr2 68466383
chr2 68466400
chr2 68466589
chr2 68466620
chr2 68466638
chr2 68653678
chr2 68653683
chr2 68653698
chr2 68653723
chr2 68653733
chr2 68653759
chr2 68653886
chr2 68653910
chr2 68654264
chr2 68721595
chr2 68721691
chr2 68721798
chr2 68721858
chr2 68734363
chr2 68734465
chr2 68734555
chr2 68734606
chr2 68734823
chr2 68734855
chr2 68734880
chr2 68735165
chr2 68735252
chr2 68735279
chr2 68735299
chr2 68735307
chr2 68735363
chr2 68735505
chr2 68735752
chr2 68735957
chr2 68736026
chr2 68736219
chr2 68736310

chr2 68737380
chr2 68737900
chr2 68751810
chr2 68751821
chr2 68751890
chr2 68751905
chr2 68751928
chr2 68751960
chr2 68751966
chr2 68752025
chr2 68752027
chr2 68752033
chr2 68752061
chr2 68752169
chr2 68766163
chr2 68766268
chr2 68766412
chr2 68766433
chr2 68766460
chr2 68766544
chr2 68766615
chr2 68767024
chr2 68767077
chr2 68767121
chr2 68767228
chr2 68767271
chr2 68767306
chr2 68767373
chr2 68767435
chr2 68767440
chr2 68767456
chr2 68767473
chr2 68767542
chr2 68767578
chr2 68767585
chr2 68767621
chr2 68767627
chr2 68774284
chr2 68774292
chr2 68774377
chr2 68774642
chr2 68774667
chr2 68774683
chr2 68837613
chr2 68837730
chr2 68838183
chr2 68908523
chr2 68908620
chr2 68943451
chr2 68943513
chr2 68943615
chr2 68943647
chr2 68943681
chr2 68943683

chr2 69013507
chr2 69014320
chr2 69014393
chr2 69014555
chr2 69014622
chr2 69014648
chr2 69014691
chr2 69014830
chr2 69015009
chr2 69015026
chr2 69385928
chr2 69385959
chr2 69386070
chr2 69386168
chr2 69386211
chr2 69386417
chr2 69386445
chr2 69386556
chr2 69386572
chr2 69386675
chr2 69386679
chr2 69386832
chr2 69386846
chr2 69386872
chr2 69386876
chr2 69386891
chr2 69386895
chr2 69386904
chr2 69386911
chr2 69436796
chr2 69436947
chr2 69436992
chr2 69437035
chr2 69437048
chr2 69437068
chr2 69437102
chr2 69437107
chr2 69437160
chr2 69437164
chr2 69437182
chr2 69437185
chr2 69437193
chr2 69437226
chr2 69437229
chr2 69437237
chr2 69437243
chr2 69437246
chr2 69437272
chr2 69437277
chr2 69437296
chr2 69437299
chr2 69437303
chr2 69437308
chr2 69437351

chr2 69642090
chr2 69642163
chr2 69642236
chr2 69642396
chr2 69642402
chr2 69642409
chr2 69642594
chr2 69642662
chr2 69642674
chr2 69644839
chr2 69644856
chr2 69644925
chr2 69645055
chr2 69645100
chr2 69645197
chr2 69645279
chr2 69645310
chr2 69645370
chr2 69645694
chr2 69646056
chr2 69646076
chr2 69648338
chr2 69648348
chr2 69648497
chr2 69648565
chr2 69648589
chr2 69648652
chr2 69648680
chr2 69650705
chr2 69650840
chr2 69725071
chr2 69725091
chr2 69893804
chr2 69902331
chr2 69902417
chr2 69902462
chr2 69902818
chr2 69902827
chr2 69902833
chr2 69902870
chr2 69902877
chr2 69903067
chr2 69914680
chr2 69914708
chr2 69914725
chr2 69914806
chr2 69915664
chr2 69915676
chr2 69915702
chr2 69915713
chr2 69915717
chr2 69915734
chr2 69915737
chr2 69915765

chr2 69915772
chr2 69915779
chr2 69915786
chr2 69915798
chr2 69915806
chr2 69915830
chr2 69915872
chr2 69915938
chr2 69915946
chr2 69916183
chr2 70069244
chr2 70069257
chr2 70085360
chr2 70085483
chr2 70085574
chr2 70085703
chr2 70085721
chr2 70085754
chr2 70085816
chr2 70085860
chr2 70085869
chr2 70086031
chr2 70086069
chr2 70086071
chr2 70086077
chr2 70086101
chr2 70086103
chr2 70086120
chr2 70086163
chr2 70086165
chr2 70086181
chr2 70086753
chr2 70086758
chr2 70086784
chr2 70089149
chr2 70089279
chr2 70089315
chr2 70089454
chr2 70089501
chr2 70089637
chr2 70089675
chr2 70089737
chr2 70089746
chr2 70089751
chr2 70089755
chr2 70095447
chr2 70104304
chr2 70104337
chr2 70104364
chr2 70104385
chr2 70104390
chr2 70104694
chr2 70104745
chr2 70104768

chr2 70104776
chr2 70104837
chr2 70104870
chr2 70124184
chr2 70124186
chr2 70124191
chr2 70124203
chr2 70124210
chr2 70124212
chr2 70124216
chr2 70124245
chr2 70124293
chr2 70124299
chr2 70124311
chr2 70124315
chr2 70124331
chr2 70124335
chr2 70124380
chr2 70124386
chr2 70124392
chr2 70124415
chr2 70124417
chr2 70124486
chr2 70124543
chr2 70124574
chr2 70124610
chr2 70124667
chr2 70124693
chr2 70124697
chr2 70124699
chr2 70131902
chr2 70131937
chr2 70131957
chr2 70131966
chr2 70132049
chr2 70132407
chr2 70132459
chr2 70132479
chr2 70132767
chr2 70132889
chr2 70132935
chr2 70133018
chr2 70133078
chr2 70133246
chr2 70133485
chr2 70133627
chr2 70133650
chr2 70133665
chr2 70133698
chr2 70133725
chr2 70135560
chr2 70135584
chr2 70135596
chr2 70135639

chr2 70135671
chr2 70135687
chr2 70135715
chr2 70135725
chr2 70135830
chr2 70135892
chr2 70135987
chr2 70136084
chr2 70136300
chr2 70136417
chr2 70143387
chr2 70143389
chr2 70190513
chr2 70190519
chr2 70190546
chr2 70190560
chr2 70190615
chr2 70190621
chr2 70190629
chr2 70190640
chr2 70190643
chr2 70190660
chr2 70190665
chr2 70190669
chr2 70190673
chr2 70190679
chr2 70190684
chr2 70190695
chr2 70190699
chr2 70190706
chr2 70190722
chr2 70190754
chr2 70190766
chr2 70190768
chr2 70190770
chr2 70190774
chr2 70190784
chr2 70190818
chr2 70190821
chr2 70190828
chr2 70190848
chr2 70190859
chr2 70190882
chr2 70190885
chr2 70190890
chr2 70246834
chr2 70246840
chr2 70247019
chr2 70247093
chr2 70247173
chr2 70247347
chr2 70247514
chr2 70257583
chr2 70257628

chr2 70257666
chr2 70257674
chr2 70257691
chr2 70257698
chr2 70257706
chr2 70257730
chr2 70257750
chr2 70257754
chr2 70257765
chr2 70257795
chr2 70257806
chr2 70257826
chr2 70257856
chr2 70257894
chr2 70257896
chr2 70257900
chr2 70257917
chr2 70257944
chr2 70257949
chr2 70258013
chr2 70258016
chr2 70258021
chr2 70258027
chr2 70258032
chr2 70258039
chr2 70258106
chr2 70258108
chr2 70293096
chr2 70293183
chr2 70293200
chr2 70293270
chr2 70293295
chr2 70766496
chr2 70766575
chr2 70766746
chr2 70766755
chr2 70766796
chr2 70766830
chr2 70785230
chr2 70785445
chr2 70785464
chr2 70785471
chr2 70789323
chr2 70789429
chr2 70789511
chr2 70789522
chr2 70884277
chr2 70884304
chr2 70884358
chr2 70884489
chr2 70884947
chr2 70884949
chr2 70884970
chr2 70885174

chr2 70885667
chr2 70907722
chr2 70907728
chr2 70907744
chr2 70907792
chr2 70907806
chr2 70907817
chr2 70907829
chr2 70908053
chr2 70908123
chr2 70908322
chr2 70908781
chr2 70908942
chr2 70995518
chr2 70995525
chr2 70995529
chr2 70995536
chr2 70995709
chr2 70995733
chr2 71071822
chr2 71072082
chr2 71072224
chr2 71082612
chr2 71083036
chr2 71083097
chr2 71083256
chr2 71094614
chr2 71094767
chr2 71129392
chr2 71129401
chr2 71129539
chr2 71130163
chr2 71130170
chr2 71130254
chr2 71130268
chr2 71130270
chr2 71130272
chr2 71130282
chr2 71130287
chr2 71130303
chr2 71130454
chr2 71226459
chr2 71226461
chr2 71226489
chr2 71226492
chr2 71226494
chr2 71226516
chr2 71226528
chr2 71226538
chr2 71226540
chr2 71226542
chr2 71226601
chr2 71226606
chr2 71226621

chr2 71226653
chr2 71226676
chr2 71226691
chr2 71226711
chr2 71226734
chr2 71226738
chr2 71226748
chr2 71226751
chr2 71226758
chr2 71226760
chr2 71226763
chr2 71226851
chr2 71226858
chr2 71226870
chr2 71226878
chr2 71226881
chr2 71226890
chr2 71226911
chr2 71332642
chr2 71332696
chr2 71332831
chr2 71332878
chr2 71332906
chr2 71332933
chr2 71333097
chr2 71333269
chr2 71353031
chr2 71353051
chr2 71353077
chr2 71353193
chr2 71353343
chr2 71467163
chr2 71467173
chr2 71467217
chr2 71467401
chr2 71467591
chr2 71467640
chr2 71467802
chr2 71467827
chr2 71871694
chr2 71871721
chr2 71871725
chr2 71871727
chr2 71871756
chr2 71871761
chr2 71871773
chr2 71871777
chr2 71871779
chr2 72068384
chr2 72068511
chr2 72068516
chr2 72068671
chr2 72068674
chr2 72554278

chr2 72813842
chr2 72861643
chr2 72892892
chr2 72892903
chr2 72892905
chr2 72926376
chr2 72926423
chr2 72926498
chr2 72926575
chr2 72979990
chr2 73214867
chr2 73214878
chr2 73214986
chr2 73294022
chr2 73294070
chr2 73385174
chr2 73385266
chr2 73385283
chr2 73385339
chr2 73385385
chr2 73385400
chr2 73385414
chr2 73386902
chr2 73736451
chr2 73736519
chr2 73736556
chr2 73736596
chr2 73736638
chr2 73736657
chr2 73736670
chr2 73736734
chr2 73736823
chr2 73736840
chr2 73736872
chr2 73736875
chr2 73736917
chr2 73736931
chr2 73736963
chr2 73736970
chr2 73737017
chr2 73737029
chr2 73737037
chr2 73737100
chr2 73737108
chr2 73737306
chr2 73737318
chr2 73737323
chr2 73737335
chr2 73737340
chr2 73737390
chr2 73737504
chr2 73779496
chr2 73779705
chr2 73779723

chr2 73779730
chr2 73779757
chr2 73779760
chr2 73779777
chr2 73779786
chr2 73779789
chr2 73779809
chr2 73779834
chr2 73779850
chr2 73780293
chr2 73780298
chr2 73780305
chr2 73780310
chr2 73780344
chr2 73780556
chr2 73784692
chr2 73785090
chr2 73785172
chr2 73829063
chr2 73829067
chr2 73829070
chr2 73829101
chr2 73829114
chr2 73829124
chr2 73829128
chr2 73829137
chr2 73829140
chr2 73829153
chr2 73829155
chr2 73829163
chr2 73829184
chr2 73829192
chr2 73829215
chr2 73829253
chr2 73829293
chr2 73829317
chr2 73829326
chr2 73829357
chr2 73829370
chr2 73829380
chr2 73829383
chr2 73829447
chr2 73829450
chr2 73829453
chr2 73829559
chr2 73829606
chr2 73829633
chr2 73927335
chr2 73927346
chr2 73927353
chr2 74032062
chr2 74032230
chr2 74032361
chr2 74032397

chr2 74032417
chr2 74032429
chr2 74032554
chr2 74032556
chr2 74119940
chr2 74120032
chr2 74120063
chr2 74120080
chr2 74120088
chr2 74120147
chr2 74120155
chr2 74120163
chr2 74120174
chr2 74120194
chr2 74120198
chr2 74120205
chr2 74120214
chr2 74120218
chr2 74120228
chr2 74120233
chr2 74120264
chr2 74120270
chr2 74120274
chr2 74120283
chr2 74120286
chr2 74120295
chr2 74120298
chr2 74120308
chr2 74120316
chr2 74120329
chr2 74120332
chr2 74120345
chr2 74120358
chr2 74120369
chr2 74120373
chr2 74120376
chr2 74120422
chr2 74120431
chr2 74120489
chr2 74120501
chr2 74120540
chr2 74120542
chr2 74120552
chr2 74147349
chr2 74147351
chr2 74147383
chr2 74147427
chr2 74177514
chr2 74177538
chr2 74177568
chr2 74199640
chr2 74199656
chr2 74199686
chr2 74199694

chr2 74199852
chr2 74199864
chr2 74199932
chr2 74199967
chr2 74200030
chr2 74200032
chr2 74200052
chr2 74200081
chr2 74200117
chr2 74200174
chr2 74205911
chr2 74205917
chr2 74206058
chr2 74206315
chr2 74206371
chr2 74206447
chr2 74305433
chr2 74391029
chr2 74391048
chr2 74391164
chr2 74391171
chr2 74391242
chr2 74391329
chr2 74391366
chr2 74391430
chr2 74391443
chr2 74391452
chr2 74391508
chr2 74391517
chr2 74391521
chr2 74391529
chr2 74455106
chr2 74455132
chr2 74458553
chr2 74458609
chr2 74458620
chr2 74458626
chr2 74458632
chr2 74458654
chr2 74458658
chr2 74458661
chr2 74458699
chr2 74458763
chr2 74458827
chr2 74458892
chr2 74459001
chr2 74459007
chr2 74459054
chr2 74459056
chr2 74459181
chr2 74459200
chr2 74459202
chr2 74459242
chr2 74459249

chr2 74482726
chr2 74482730
chr2 74482734
chr2 74482768
chr2 74482775
chr2 74482784
chr2 74482792
chr2 74482798
chr2 74482821
chr2 74482826
chr2 74482842
chr2 74530868
chr2 74530924
chr2 74530938
chr2 74530953
chr2 74531005
chr2 74531015
chr2 74531037
chr2 74531109
chr2 74554627
chr2 74554629
chr2 74554641
chr2 74554648
chr2 74554651
chr2 74554658
chr2 74554668
chr2 74554689
chr2 74554695
chr2 74554702
chr2 74554707
chr2 74554714
chr2 74554717
chr2 74554732
chr2 74554734
chr2 74554744
chr2 74554747
chr2 74554760
chr2 74554780
chr2 74554800
chr2 74554824
chr2 74588107
chr2 74588150
chr2 74606463
chr2 74653735
chr2 74653814
chr2 74653841
chr2 74653869
chr2 74653881
chr2 74653883
chr2 74653885
chr2 74653887
chr2 74653889
chr2 74788649
chr2 74788835

chr2 74789552
chr2 74789560
chr2 74840833
chr2 74840857
chr2 74840955
chr2 74849406
chr2 74849412
chr2 74849443
chr2 74849456
chr2 74849490
chr2 74849828
chr2 74849840
chr2 74849844
chr2 74896684
chr2 74896910
chr2 75709530
chr2 75709634
chr2 75709898
chr2 75710006
chr2 75710340
chr2 75710362
chr2 75710409
chr2 75710421
chr2 75710434
chr2 75710489
chr2 75710532
chr2 75710534
chr2 75710545
chr2 75710555
chr2 75710566
chr2 75710574
chr2 75710576
chr2 75710600
chr2 75710604
chr2 75710606
chr2 75710608
chr2 75710616
chr2 75722172
chr2 75722295
chr2 75879910
chr2 75880045
chr2 75880149
chr2 75880333
chr2 75880476
chr2 75880595
chr2 76433801
chr2 76434395
chr2 76649314
chr2 76649472
chr2 76649477
chr2 76649932
chr2 76696616
chr2 76696798
chr2 76699097

chr2 76699471
chr2 76749847
chr2 76750356
chr2 77087869
chr2 77088662
chr2 77088666
chr2 77088668
chr2 77088685
chr2 77088727
chr2 77088766
chr2 77088821
chr2 77088828
chr2 77116242
chr2 77116596
chr2 77132037
chr2 77361428
chr2 77435569
chr2 77435715
chr2 77435977
chr2 77436124
chr2 77436216
chr2 77436258
chr2 77441786
chr2 77442066
chr2 77485257
chr2 77516292
chr2 77516508
chr2 77582869
chr2 77583338
chr2 77588858
chr2 77589155
chr2 77596907
chr2 77816835
chr2 78093259
chr2 78093370
chr2 78093861
chr2 78384474
chr2 78384533
chr2 78385032
chr2 78385494
chr2 78549062
chr2 78549249
chr2 78549251
chr2 78553116
chr2 78553882
chr2 78554298
chr2 78554985
chr2 78670240
chr2 78670272
chr2 78670408
chr2 78670424
chr2 78670684
chr2 78670753
chr2 78670941

chr2 78838481
chr2 78838683
chr2 78839223
chr2 78839578
chr2 78839602
chr2 78839615
chr2 78858260
chr2 78858269
chr2 78858295
chr2 79291941
chr2 79292169
chr2 79514369
chr2 79738668
chr2 79738811
chr2 79739734
chr2 79873410
chr2 79875827
chr2 79875940
chr2 79876669
chr2 79876714
chr2 79876719
chr2 79935069
chr2 79935151
chr2 79935328
chr2 80244982
chr2 80244986
chr2 80245049
chr2 80245079
chr2 80245132
chr2 80245259
chr2 80245466
chr2 80300673
chr2 80300793
chr2 80301286
chr2 80301486
chr2 80301755
chr2 80302070
chr2 80323637
chr2 80546727
chr2 80721628
chr2 80721722
chr2 80721896
chr2 80873131
chr2 80873161
chr2 81181496
chr2 81181538
chr2 81183785
chr2 81483080
chr2 81775691
chr2 82230381
chr2 82230447
chr2 82232021
chr2 82232033
chr2 82232183

chr2 82471261
chr2 82471734
chr2 82471827
chr2 82580111
chr2 82580404
chr2 82580483
chr2 82580965
chr2 82812156
chr2 82812646
chr2 83088002
chr2 83125473
chr2 83125785
chr2 83669665
chr2 83669961
chr2 83799473
chr2 83953946
chr2 83954230
chr2 83955005
chr2 83955663
chr2 83959237
chr2 83959276
chr2 83959483
chr2 83959941
chr2 84206281
chr2 84206631
chr2 84447362
chr2 84448151
chr2 84458501
chr2 84458539
chr2 84458545
chr2 84458562
chr2 84458631
chr2 84458640
chr2 84458921
chr2 84458927
chr2 84458966
chr2 84458985
chr2 84458992
chr2 84459094
chr2 84459096
chr2 84459120
chr2 84459123
chr2 84459137
chr2 84459139
chr2 84459143
chr2 84459172
chr2 84460112
chr2 84460121
chr2 84460128
chr2 84513284
chr2 84581400
chr2 84581650
chr2 84694256
chr2 84694265

chr2 84694326
chr2 84694348
chr2 84694379
chr2 84694535
chr2 84694700
chr2 84695015
chr2 84846285
chr2 84881395
chr2 84881633
chr2 84881650
chr2 84881722
chr2 84881732
chr2 84881816
chr2 84881823
chr2 84881846
chr2 84881877
chr2 84881883
chr2 84881895
chr2 84881907
chr2 84881922
chr2 84881948
chr2 84881979
chr2 84882028
chr2 84882384
chr2 84882528
chr2 84882548
chr2 84882642
chr2 84888641
chr2 84888906
chr2 84888989
chr2 84889110
chr2 84889161
chr2 84889274
chr2 84889464
chr2 84889509
chr2 84897963
chr2 84898204
chr2 84898525
chr2 84926249
chr2 84938209
chr2 84938309
chr2 84938450
chr2 84938667
chr2 84939727
chr2 84939799
chr2 84939836
chr2 84939977
chr2 84939981
chr2 84940025
chr2 84940033
chr2 84946237
chr2 84946242
chr2 84946297
chr2 84946487

chr2 84969346
chr2 84969416
chr2 84969438
chr2 84969491
chr2 84969736
chr2 84969831
chr2 84970074
chr2 84970112
chr2 84970117
chr2 84970133
chr2 84970135
chr2 84970156
chr2 84970165
chr2 84970170
chr2 84970263
chr2 84972236
chr2 84972259
chr2 84972266
chr2 84972298
chr2 84972374
chr2 84972511
chr2 84972575
chr2 84972677
chr2 84972747
chr2 84972761
chr2 84972764
chr2 84972795
chr2 85326141
chr2 85326172
chr2 85326338
chr2 85326418
chr2 85326432
chr2 85326486
chr2 85326494
chr2 85326500
chr2 85326956
chr2 85327012
chr2 85327018
chr2 85327032
chr2 85327059
chr2 85327074
chr2 85327096
chr2 85327102
chr2 85327116
chr2 85327138
chr2 85328367
chr2 85328384
chr2 85328394
chr2 85328436
chr2 85328451
chr2 85328460
chr2 85328483
chr2 85328491
chr2 85328497

chr2 85328513
chr2 85328589
chr2 85328687
chr2 85328742
chr2 85418571
chr2 85418593
chr2 85418708
chr2 85418713
chr2 85418720
chr2 85418740
chr2 85418744
chr2 85537890
chr2 85540641
chr2 85540712
chr2 85541335
chr2 85577353
chr2 85577480
chr2 85577518
chr2 85577523
chr2 85577530
chr2 85577539
chr2 85577601
chr2 85577609
chr2 85577626
chr2 85577727
chr2 85577891
chr2 85577899
chr2 85577902
chr2 85577914
chr2 85577960
chr2 85577965
chr2 85578027
chr2 85578046
chr2 85578227
chr2 85578286
chr2 85578341
chr2 85578374
chr2 85578393
chr2 85578398
chr2 85584697
chr2 85584711
chr2 85584713
chr2 85584720
chr2 85584724
chr2 85584731
chr2 85584758
chr2 85584771
chr2 85584783
chr2 85584790
chr2 85584816
chr2 85584853
chr2 85584855
chr2 85637487
chr2 85707441

chr2 85707451
chr2 85707480
chr2 85707492
chr2 85707793
chr2 85853362
chr2 85853372
chr2 85853507
chr2 85853527
chr2 85853572
chr2 85853629
chr2 85853640
chr2 85853698
chr2 85853867
chr2 85853898
chr2 85854041
chr2 85854138
chr2 85854199
chr2 85993959
chr2 85993961
chr2 85993981
chr2 86036094
chr2 86036101
chr2 86036136
chr2 86036147
chr2 86036149
chr2 86036245
chr2 86036379
chr2 86036414
chr2 86036615
chr2 86036675
chr2 86106672
chr2 86106705
chr2 86106714
chr2 86106751
chr2 86106757
chr2 86106792
chr2 86107050
chr2 86107192
chr2 86107209
chr2 86107266
chr2 86107381
chr2 86107522
chr2 86185216
chr2 86185837
chr2 86186379
chr2 86199324
chr2 86199334
chr2 86199337
chr2 86199344
chr2 86199602
chr2 86199658
chr2 86199677
chr2 86199686
chr2 86199694

chr2 86199708
chr2 86199713
chr2 86199721
chr2 86199723
chr2 86199967
chr2 86199974
chr2 86200038
chr2 86200092
chr2 86200109
chr2 86200114
chr2 86440643
chr2 86442751
chr2 86442802
chr2 86442902
chr2 86442936
chr2 86552931
chr2 86553007
chr2 86553020
chr2 86553120
chr2 86553122
chr2 86553162
chr2 86553219
chr2 86622145
chr2 86622263
chr2 86622438
chr2 86622506
chr2 86622533
chr2 86622556
chr2 86718844
chr2 86718864
chr2 86718872
chr2 86718907
chr2 86718955
chr2 86719055
chr2 86719242
chr2 86719279
chr2 86719291
chr2 86719309
chr2 86719323
chr2 86719328
chr2 86719333
chr2 86719374
chr2 86719627
chr2 86721794
chr2 86837356
chr2 86837361
chr2 86837387
chr2 86837390
chr2 86837471
chr2 86837486
chr2 86837491
chr2 86837512
chr2 86837674
chr2 86837679

chr2 86837749
chr2 86837944
chr2 87074271
chr2 87074280
chr2 87074318
chr2 87074464
chr2 87074587
chr2 87074592
chr2 87074635
chr2 87074932
chr2 87077526
chr2 87077671
chr2 87077771
chr2 87077854
chr2 87077862
chr2 87128789
chr2 87128801
chr2 87128901
chr2 87255247
chr2 87255432
chr2 87255553
chr2 87255642
chr2 87255651
chr2 87255661
chr2 87255681
chr2 87255685
chr2 87255712
chr2 87255719
chr2 87255733
chr2 87255799
chr2 87255825
chr2 87256358
chr2 87290919
chr2 87290945
chr2 87324001
chr2 87324167
chr2 87324249
chr2 87338742
chr2 87339142
chr2 87339609
chr2 87339640
chr2 87339789
chr2 87364082
chr2 87364113
chr2 87454763
chr2 87455024
chr2 87455458
chr2 87455485
chr2 87455531
chr2 87455569
chr2 87455582
chr2 87455721
chr2 87455740
chr2 87456628

chr2 87456639
chr2 87456647
chr2 87456671
chr2 87456684
chr2 87456821
chr2 87457287
chr2 87457335
chr2 87457344
chr2 87457380
chr2 87459371
chr2 87459412
chr2 87459446
chr2 87459630
chr2 87459659
chr2 87459702
chr2 87459728
chr2 87459898
chr2 87460182
chr2 87460596
chr2 87470916
chr2 87470940
chr2 87470968
chr2 87471085
chr2 87471123
chr2 87471151
chr2 87471163
chr2 87471173
chr2 87471203
chr2 87497372
chr2 87497449
chr2 87497555
chr2 87497705
chr2 87497902
chr2 87497921
chr2 87497960
chr2 87498355
chr2 87498361
chr2 87510812
chr2 87511544
chr2 87511573
chr2 87511576
chr2 87512627
chr2 87512719
chr2 87512808
chr2 87512857
chr2 87513412
chr2 87513686
chr2 87514173
chr2 87514223
chr2 87514641
chr2 87544688
chr2 87544746
chr2 87544805
chr2 87545103

chr2 87545203
chr2 88016783
chr2 88016790
chr2 88016807
chr2 88016821
chr2 88053714
chr2 88200390
chr2 88200414
chr2 88200427
chr2 88200433
chr2 88200453
chr2 88200468
chr2 88200473
chr2 88200475
chr2 88200481
chr2 88200495
chr2 88200501
chr2 88200545
chr2 88200919
chr2 88201071
chr2 88201080
chr2 88201141
chr2 88201278
chr2 88201328
chr2 88628037
chr2 88628096
chr2 88628101
chr2 88628116
chr2 88628159
chr2 88628164
chr2 88628171
chr2 88628185
chr2 88628198
chr2 88628214
chr2 88628233
chr2 88628293
chr2 88638242
chr2 88638798
chr2 88639003
chr2 88639122
chr2 88639359
chr2 88692177
chr2 88692187
chr2 88692345
chr2 88692375
chr2 88692382
chr2 88692385
chr2 88692421
chr2 88692444
chr2 88692446
chr2 88692449
chr2 88692486
chr2 88692560
chr2 88858895

chr2 88859008
chr2 88880668
chr2 88880670
chr2 88880678
chr2 88880702
chr2 88880720
chr2 88880752
chr2 88880828
chr2 88880881
chr2 88880889
chr2 89149325
chr2 89149393
chr2 89149451
chr2 89149481
chr2 89327271
chr2 89327287
chr2 89328214
chr2 89329823
chr2 89330240
chr2 89330251
chr2 89585865
chr2 89585879
chr2 89585900
chr2 89585915
chr2 89585921
chr2 89585926
chr2 89585929
chr2 89586007
chr2 89586041
chr2 89586067
chr2 89586071
chr2 89586078
chr2 89586090
chr2 89586098
chr2 89586295
chr2 89586303
chr2 89587216
chr2 89587230
chr2 89587480
chr2 89587947
chr2 89588028
chr2 89589187
chr2 89589225
chr2 89591229
chr2 89609015
chr2 89609287
chr2 89651043
chr2 90166600
chr2 90166659
chr2 90166950
chr2 90166960
chr2 90167347
chr2 90167642
chr2 90235480

chr2 90247111
chr2 90254953
chr2 90255437
chr2 90255485
chr2 90321954
chr2 90322305
chr2 90322423
chr2 90322688
chr2 90323602
chr2 90323611
chr2 90323619
chr2 90323621
chr2 90323624
chr2 90323638
chr2 90323641
chr2 90323644
chr2 90323662
chr2 90323664
chr2 90323667
chr2 90323680
chr2 90323683
chr2 90323686
chr2 90323700
chr2 90323702
chr2 90323705
chr2 90323718
chr2 90323721
chr2 90323724
chr2 90323727
chr2 90323743
chr2 90323745
chr2 90323759
chr2 90323761
chr2 90323764
chr2 90323783
chr2 90323785
chr2 90323788
chr2 90323807
chr2 90323809
chr2 90323812
chr2 90323815
chr2 90323831
chr2 90323833
chr2 90323836
chr2 90323848
chr2 90323850
chr2 90323856
chr2 90323872
chr2 90323874
chr2 90323877
chr2 90323880
chr2 90323896
chr2 90323898
chr2 90323901

chr2 90323904
chr2 90323919
chr2 90323924
chr2 90323936
chr2 90323938
chr2 90323952
chr2 90323954
chr2 90323957
chr2 90323975
chr2 90323977
chr2 90323980
chr2 90323992
chr2 90324000
chr2 90324015
chr2 90324020
chr2 90324032
chr2 90324037
chr2 90324049
chr2 90324051
chr2 90324054
chr2 90324072
chr2 90324077
chr2 90324079
chr2 90324089
chr2 90324091
chr2 90324094
chr2 90324097
chr2 90324100
chr2 90324103
chr2 90324106
chr2 90324119
chr2 90324124
chr2 90324136
chr2 90324138
chr2 90324141
chr2 90324186
chr2 90324189
chr2 90324201
chr2 90324203
chr2 90324209
chr2 90324214
chr2 90324230
chr2 90324233
chr2 90324248
chr2 90324250
chr2 90324253
chr2 90324256
chr2 90324272
chr2 90324277
chr2 90324289
chr2 90324291
chr2 90324294
chr2 90324297
chr2 90324314

chr2 90324329
chr2 90324331
chr2 90324334
chr2 90324351
chr2 90324353
chr2 90324356
chr2 90324370
chr2 90324373
chr2 90324376
chr2 90324394
chr2 90324397
chr2 90324416
chr2 90324421
chr2 90324433
chr2 90324435
chr2 90324449
chr2 90324451
chr2 90324465
chr2 90324467
chr2 90324481
chr2 90324483
chr2 90324497
chr2 90324499
chr2 90324536
chr2 90324538
chr2 90324541
chr2 90324553
chr2 90324561
chr2 90324581
chr2 90324593
chr2 90324598
chr2 90324610
chr2 90324615
chr2 90324618
chr2 90324633
chr2 90324635
chr2 90324638
chr2 90324641
chr2 90324644
chr2 90324647
chr2 90324650
chr2 90324653
chr2 90324656
chr2 90324668
chr2 90324670
chr2 90324673
chr2 90324676
chr2 90324679
chr2 90324691
chr2 90324694
chr2 90324696
chr2 90324699
chr2 90324702
chr2 90324977

chr2 90324979
chr2 90325047
chr2 90325058
chr2 90325060
chr2 90325063
chr2 90325074
chr2 90325076
chr2 90325079
chr2 90325082
chr2 90325097
chr2 90325099
chr2 90325102
chr2 90325114
chr2 90325116
chr2 90325119
chr2 90325135
chr2 90325155
chr2 90325160
chr2 90325174
chr2 90325187
chr2 90325189
chr2 90325192
chr2 90325210
chr2 90325212
chr2 90325215
chr2 90325227
chr2 90325235
chr2 90325255
chr2 90325267
chr2 90325272
chr2 90325275
chr2 90325290
chr2 90325295
chr2 90325297
chr2 90325304
chr2 90325307
chr2 90325309
chr2 90325312
chr2 90325315
chr2 90325318
chr2 90325321
chr2 90325324
chr2 90325327
chr2 90325330
chr2 90325342
chr2 90325345
chr2 90325347
chr2 90325350
chr2 90325353
chr2 90325356
chr2 90325359
chr2 90325362
chr2 90325365
chr2 90325368

chr2 90325380
chr2 90325383
chr2 90325385
chr2 90325388
chr2 90325391
chr2 90325394
chr2 90325397
chr2 90325400
chr2 90325403
chr2 90325406
chr2 90325409
chr2 90325418
chr2 90325421
chr2 90325423
chr2 90325426
chr2 90325429
chr2 90325432
chr2 90325444
chr2 90325447
chr2 90325449
chr2 90325452
chr2 90325455
chr2 90325458
chr2 90325461
chr2 90325464
chr2 90325467
chr2 90325470
chr2 90325473
chr2 90325476
chr2 90325479
chr2 90325482
chr2 90325485
chr2 90325497
chr2 90325500
chr2 90325502
chr2 90325505
chr2 90325508
chr2 90325521
chr2 90325524
chr2 90325526
chr2 90325529
chr2 90325532
chr2 90325535
chr2 90325548
chr2 90325553
chr2 90325565
chr2 90325567
chr2 90325570
chr2 90325573
chr2 90325589
chr2 90325591
chr2 90325594
chr2 90325606
chr2 90325614

chr2 90325619
chr2 90325638
chr2 90325640
chr2 90325643
chr2 90325680
chr2 90325682
chr2 90325685
chr2 90325703
chr2 90325705
chr2 90325708
chr2 90325720
chr2 90325722
chr2 90325728
chr2 90325746
chr2 90325748
chr2 90325754
chr2 90325773
chr2 90325775
chr2 90325778
chr2 90325781
chr2 90325797
chr2 90325799
chr2 90325821
chr2 90325826
chr2 90325838
chr2 90325840
chr2 90325843
chr2 90325859
chr2 90325862
chr2 90325878
chr2 90325880
chr2 90325883
chr2 90325894
chr2 90325896
chr2 90325899
chr2 90325902
chr2 90325917
chr2 90325922
chr2 90325936
chr2 90325952
chr2 90325954
chr2 90325957
chr2 90325971
chr2 90325992
chr2 90325998
chr2 90326010
chr2 90326012
chr2 90326015
chr2 90326032
chr2 90326034
chr2 90326037
chr2 90326041
chr2 90326047
chr2 90326065

chr2 90326070
chr2 90326082
chr2 90326084
chr2 90326087
chr2 90326105
chr2 90326107
chr2 90326113
chr2 90326132
chr2 90326135
chr2 90326146
chr2 90326148
chr2 90326151
chr2 90326154
chr2 90326169
chr2 90326171
chr2 90326174
chr2 90326186
chr2 90326188
chr2 90326191
chr2 90326194
chr2 90326210
chr2 90326212
chr2 90326218
chr2 90326239
chr2 90326241
chr2 90326244
chr2 90326256
chr2 90326261
chr2 90326281
chr2 90326283
chr2 90326286
chr2 90326307
chr2 90326310
chr2 90326313
chr2 90326328
chr2 90326330
chr2 90326333
chr2 90326345
chr2 90326347
chr2 90326350
chr2 90326353
chr2 90326368
chr2 90326373
chr2 90326385
chr2 90326390
chr2 90326410
chr2 90326412
chr2 90326415
chr2 90326418
chr2 90326425
chr2 90326435
chr2 90326438
chr2 90326475
chr2 90326720

chr2 90327731
chr2 90327985
chr2 90327990
chr2 90328197
chr2 90330003
chr2 90330568
chr2 90330627
chr2 90331024
chr2 90331117
chr2 90331371
chr2 90331487
chr2 90331580
chr2 90331881
chr2 90332324
chr2 90332346
chr2 90360024
chr2 90360027
chr2 90360036
chr2 90360057
chr2 90360503
chr2 90360505
chr2 90360508
chr2 90360519
chr2 90360529
chr2 90360534
chr2 90360581
chr2 90360589
chr2 90360631
chr2 90360633
chr2 90360636
chr2 90360639
chr2 90360653
chr2 90360658
chr2 90360668
chr2 90360670
chr2 90360676
chr2 90360695
chr2 90360698
chr2 90360712
chr2 90360714
chr2 90360717
chr2 90360720
chr2 90360768
chr2 90360771
chr2 90360782
chr2 90360784
chr2 90360790
chr2 90360814
chr2 90360816
chr2 90360836
chr2 90360838
chr2 90360844
chr2 90360858
chr2 90360860

chr2 90360866
chr2 90360882
chr2 90360888
chr2 90360902
chr2 90360907
chr2 90360910
chr2 90360924
chr2 90360934
chr2 90360942
chr2 90360961
chr2 90360972
chr2 90360974
chr2 90360977
chr2 90360980
chr2 90361219
chr2 90362423
chr2 90362578
chr2 90362600
chr2 90362602
chr2 90364088
chr2 90364375
chr2 90364427
chr2 90364999
chr2 90365426
chr2 90366523
chr2 90366574
chr2 90366783
chr2 90375506
chr2 90375634
chr2 90376139
chr2 90376171
chr2 90376234
chr2 90376490
chr2 90376755
chr2 90376763
chr2 90377222
chr2 90377602
chr2 90378016
chr2 90378036
chr2 90378255
chr2 90378336
chr2 90378888
chr2 90379122
chr2 90379198
chr2 90379256
chr2 91404406
chr2 91404439
chr2 91404478
chr2 91404580
chr2 91404601
chr2 91404650
chr2 91404736
chr2 91404748
chr2 91404758

chr2 91404810
chr2 91404823
chr2 91404849
chr2 91424713
chr2 91441756
chr2 91475634
chr2 91476092
chr2 91476392
chr2 91476784
chr2 91477025
chr2 91477062
chr2 91477065
chr2 91477067
chr2 91477087
chr2 91477104
chr2 91477109
chr2 91477124
chr2 91477127
chr2 91477130
chr2 91477132
chr2 91477144
chr2 91477147
chr2 91477149
chr2 91477164
chr2 91477167
chr2 91477170
chr2 91659693
chr2 91659696
chr2 91660098
chr2 91660102
chr2 91660162
chr2 91660178
chr2 91660192
chr2 91660213
chr2 91660228
chr2 91660234
chr2 91660239
chr2 91660242
chr2 91660354
chr2 91660380
chr2 91660391
chr2 91660403
chr2 91660411
chr2 91660608
chr2 91660616
chr2 91663527
chr2 91664701
chr2 91665530
chr2 91665950
chr2 91666249
chr2 91755295
chr2 91759495
chr2 91808733
chr2 91808785

chr2 91809015
chr2 91809017
chr2 91809020
chr2 91809023
chr2 91809025
chr2 91809047
chr2 91809050
chr2 91809055
chr2 91809070
chr2 91809073
chr2 91809075
chr2 91809078
chr2 91809096
chr2 91809099
chr2 91809101
chr2 91809116
chr2 91809119
chr2 91809122
chr2 91809124
chr2 91809142
chr2 91809145
chr2 91809162
chr2 91809165
chr2 91809168
chr2 91809170
chr2 91809194
chr2 91809196
chr2 91809206
chr2 91809208
chr2 91809229
chr2 91809232
chr2 91809235
chr2 91809237
chr2 91809252
chr2 91809254
chr2 91809271
chr2 91809274
chr2 91809279
chr2 91809314
chr2 91809317
chr2 91809319
chr2 91809331
chr2 91809336
chr2 91809352
chr2 91809358
chr2 91809372
chr2 91809375
chr2 91809377
chr2 91809399
chr2 91809442
chr2 91809445
chr2 91809447
chr2 91809462
chr2 91809465

chr2 91809468
chr2 91809470
chr2 91809485
chr2 91809487
chr2 91809501
chr2 91809503
chr2 91809518
chr2 91809521
chr2 91809524
chr2 91809526
chr2 91809542
chr2 91809548
chr2 91809550
chr2 91809565
chr2 91809568
chr2 91809571
chr2 91809573
chr2 91809585
chr2 91809590
chr2 91809606
chr2 91809609
chr2 91809612
chr2 91809614
chr2 91809625
chr2 91809631
chr2 91809637
chr2 91809639
chr2 91809651
chr2 91809654
chr2 91809656
chr2 91809672
chr2 91809675
chr2 91809678
chr2 91809680
chr2 91809695
chr2 91809698
chr2 91809701
chr2 91809703
chr2 91809714
chr2 91809718
chr2 91809720
chr2 91809737
chr2 91809752
chr2 91809754
chr2 91809769
chr2 91809772
chr2 91809775
chr2 91809788
chr2 91809791
chr2 91809793
chr2 91809810
chr2 91809829
chr2 91809832
chr2 91809834

chr2 91809849
chr2 91809852
chr2 91809855
chr2 91809857
chr2 91809873
chr2 91809876
chr2 91809879
chr2 91809881
chr2 91809893
chr2 91809896
chr2 91809898
chr2 91809910
chr2 91809913
chr2 91809916
chr2 91809919
chr2 91809922
chr2 91809925
chr2 91809928
chr2 91809931
chr2 91809934
chr2 91809937
chr2 91809940
chr2 91809942
chr2 91809959
chr2 91809978
chr2 91809981
chr2 91809998
chr2 91810001
chr2 91810004
chr2 91810006
chr2 91810017
chr2 91810232
chr2 91810235
chr2 91810240
chr2 91810259
chr2 91810262
chr2 91810264
chr2 91810275
chr2 91810281
chr2 91810297
chr2 91810299
chr2 91810313
chr2 91810315
chr2 91810321
chr2 91810323
chr2 91810335
chr2 91810397
chr2 91810400
chr2 91810403
chr2 91810419
chr2 91810422
chr2 91810425
chr2 91810427
chr2 91810442

chr2 91810445
chr2 91810448
chr2 91810481
chr2 91814411
chr2 91814517
chr2 91814567
chr2 91815224
chr2 91815345
chr2 91815511
chr2 91815516
chr2 91815520
chr2 91816218
chr2 91817638
chr2 91817739
chr2 91822568
chr2 91824241
chr2 91824559
chr2 91824623
chr2 91826438
chr2 91826799
chr2 91826907
chr2 91826921
chr2 91826965
chr2 91827024
chr2 91827593
chr2 91827926
chr2 91828113
chr2 91828709
chr2 91828745
chr2 91828762
chr2 91829413
chr2 91829800
chr2 91829867
chr2 91829889
chr2 91830132
chr2 91836068
chr2 91836349
chr2 91836355
chr2 91836379
chr2 91836385
chr2 91836410
chr2 91836419
chr2 91836463
chr2 91836467
chr2 91836470
chr2 91836479
chr2 91836539
chr2 91836553
chr2 91836560
chr2 91836569
chr2 91836597
chr2 91836607
chr2 91837182
chr2 91837609

chr2 91838962
chr2 91839093
chr2 91839099
chr2 91839109
chr2 91839137
chr2 91839153
chr2 91839185
chr2 91839575
chr2 91839694
chr2 91839722
chr2 91839806
chr2 91839814
chr2 91839828
chr2 91839831
chr2 91839834
chr2 91839848
chr2 91839853
chr2 91839882
chr2 91839917
chr2 91839920
chr2 91839922
chr2 91839924
chr2 91839935
chr2 91853092
chr2 91853192
chr2 91978582
chr2 91978634
chr2 91978921
chr2 91979010
chr2 91979289
chr2 92025170
chr2 92025798
chr2 92025842
chr2 92029586
chr2 92029633
chr2 92029640
chr2 92030273
chr2 92031192
chr2 92031885
chr2 92031887
chr2 92032073
chr2 92034992
chr2 92034996
chr2 92037258
chr2 92037424
chr2 92038143
chr2 92038202
chr2 92038252
chr2 92042323
chr2 92042326
chr2 92042329
chr2 92042344
chr2 92042346
chr2 92042349

chr2 92042352
chr2 92042368
chr2 92042371
chr2 92042374
chr2 92042385
chr2 92042387
chr2 92042448
chr2 92042450
chr2 92042456
chr2 92042472
chr2 92042474
chr2 92042490
chr2 92042496
chr2 92042507
chr2 92042509
chr2 92042512
chr2 92042531
chr2 92042536
chr2 92042539
chr2 92042554
chr2 92042561
chr2 92042571
chr2 92042573
chr2 92042585
chr2 92042601
chr2 92042603
chr2 92042606
chr2 92042609
chr2 92042624
chr2 92042626
chr2 92042629
chr2 92042641
chr2 92042643
chr2 92042649
chr2 92042653
chr2 92042655
chr2 92042660
chr2 92042672
chr2 92042790
chr2 92042793
chr2 92042812
chr2 92042829
chr2 92042831
chr2 92042834
chr2 92042837
chr2 92042840
chr2 92042843
chr2 92042846
chr2 92042849
chr2 92042852
chr2 92042855
chr2 92042858
chr2 92042861
chr2 92042873

chr2 92042875
chr2 92042878
chr2 92042890
chr2 92042892
chr2 92042895
chr2 92042898
chr2 92042914
chr2 92042916
chr2 92042919
chr2 92042922
chr2 92042937
chr2 92042939
chr2 92042942
chr2 92042961
chr2 92042978
chr2 92042980
chr2 92042983
chr2 92042996
chr2 92042999
chr2 92043002
chr2 92043017
chr2 92043019
chr2 92043034
chr2 92043051
chr2 92043053
chr2 92043057
chr2 92043068
chr2 92043070
chr2 92043073
chr2 92043076
chr2 92043091
chr2 92043093
chr2 92043096
chr2 92043099
chr2 92043115
chr2 92043117
chr2 92043120
chr2 92043132
chr2 92043134
chr2 92043140
chr2 92043146
chr2 92043157
chr2 92043159
chr2 92043162
chr2 92043165
chr2 92043181
chr2 92043183
chr2 92043186
chr2 92043198
chr2 92043200
chr2 92043203
chr2 92043206
chr2 92043210
chr2 92043221

chr2 92043223
chr2 92043229
chr2 92043245
chr2 92043247
chr2 92043250
chr2 92043253
chr2 92043268
chr2 92043270
chr2 92043284
chr2 92043286
chr2 92043301
chr2 92043303
chr2 92043306
chr2 92043309
chr2 92043324
chr2 92043326
chr2 92043329
chr2 92043347
chr2 92043349
chr2 92043352
chr2 92043355
chr2 92043359
chr2 92043372
chr2 92043394
chr2 92043396
chr2 92043399
chr2 92043413
chr2 92043419
chr2 92043435
chr2 92043440
chr2 92043452
chr2 92043454
chr2 92043457
chr2 92043492
chr2 92043497
chr2 92043500
chr2 92043517
chr2 92043519
chr2 92043536
chr2 92043601
chr2 92043603
chr2 92043606
chr2 92043609
chr2 92043626
chr2 92043629
chr2 92043647
chr2 92043649
chr2 92043652
chr2 92043655
chr2 92043670
chr2 92043672
chr2 92043675
chr2 92043693
chr2 92043696

chr2 92043698
chr2 92043701
chr2 92043716
chr2 92043721
chr2 92056930
chr2 92057007
chr2 92057025
chr2 92057044
chr2 92076697
chr2 92076944
chr2 92077063
chr2 92077107
chr2 92122622
chr2 92122705
chr2 92122807
chr2 92122816
chr2 92122964
chr2 92123996
chr2 92124005
chr2 94217517
chr2 94218243
chr2 94219195
chr2 94220404
chr2 94220424
chr2 94272157
chr2 94272363
chr2 94272682
chr2 94272766
chr2 94272834
chr2 94272836
chr2 94272839
chr2 94272858
chr2 94272863
chr2 94272875
chr2 94272877
chr2 94272880
chr2 94272883
chr2 94272886
chr2 94272901
chr2 94272903
chr2 94272918
chr2 94272920
chr2 94272926
chr2 94272943
chr2 94273711
chr2 94273909
chr2 94274000
chr2 94274790
chr2 94274867
chr2 94670011
chr2 94674079
chr2 94721305
chr2 94721404
chr2 94722100

chr2 94722165
chr2 94723413
chr2 94732568
chr2 94732721
chr2 94733345
chr2 94733750
chr2 94896476
chr2 94896509
chr2 95164860
chr2 95165040
chr2 95165167
chr2 95165198
chr2 95193304
chr2 95193308
chr2 95206925
chr2 95207022
chr2 95207028
chr2 95207051
chr2 95207080
chr2 95207144
chr2 95207152
chr2 95402328
chr2 95402356
chr2 95402405
chr2 95402450
chr2 95402470
chr2 95402476
chr2 95402489
chr2 95402494
chr2 95402512
chr2 95402519
chr2 95402529
chr2 95427605
chr2 95427633
chr2 95427647
chr2 95427684
chr2 95427687
chr2 95427806
chr2 95428965
chr2 95559093
chr2 95559247
chr2 95614846
chr2 95616270
chr2 95616352
chr2 95616844
chr2 95616875
chr2 95617689
chr2 95617745
chr2 95617778
chr2 95617782
chr2 95617845
chr2 95617853
chr2 95617865
chr2 95617870

chr2 95617876
chr2 95620786
chr2 95620927
chr2 95620931
chr2 95620983
chr2 95621012
chr2 95621048
chr2 95621096
chr2 95621104
chr2 95682714
chr2 95682716
chr2 95682762
chr2 95682773
chr2 95682786
chr2 95778848
chr2 95778850
chr2 95779070
chr2 95779088
chr2 95779118
chr2 95779122
chr2 95779168
chr2 95779545
chr2 95779564
chr2 95779583
chr2 95779806
chr2 95779828
chr2 95779833
chr2 95780249
chr2 95780908
chr2 95781683
chr2 95782122
chr2 95782140
chr2 95782184
chr2 95782217
chr2 95782256
chr2 95782316
chr2 95942488
chr2 95990059
chr2 95990337
chr2 95990355
chr2 95990365
chr2 95990553
chr2 95992604
chr2 95992631
chr2 95992633
chr2 95992675
chr2 95992690
chr2 95992693
chr2 95992700
chr2 95992714
chr2 95992933
chr2 95992953
chr2 95992957
chr2 95992983

chr2 95993008
chr2 96042897
chr2 96043867
chr2 96043871
chr2 96044331
chr2 96044368
chr2 96044747
chr2 96044752
chr2 96044997
chr2 96045035
chr2 96045412
chr2 96045458
chr2 96045462
chr2 96045492
chr2 96045510
chr2 96045716
chr2 96045730
chr2 96045732
chr2 96045782
chr2 96045943
chr2 96045953
chr2 96046166
chr2 96046405
chr2 96046452
chr2 96138508
chr2 96138586
chr2 96138613
chr2 96138640
chr2 96138694
chr2 96138712
chr2 96138718
chr2 96145794
chr2 96145829
chr2 96145914
chr2 96146102
chr2 96146128
chr2 96146281
chr2 96146298
chr2 96146304
chr2 96146310
chr2 96146328
chr2 96146413
chr2 96146416
chr2 96146448
chr2 96146482
chr2 96146504
chr2 96146528
chr2 96146607
chr2 96146615
chr2 96146630
chr2 96161961
chr2 96161965
chr2 96162182
chr2 96162184

chr2 96162284
chr2 96174667
chr2 96174802
chr2 96207286
chr2 96207301
chr2 96207328
chr2 96207336
chr2 96207483
chr2 96207521
chr2 96207574
chr2 96207577
chr2 96207647
chr2 96207672
chr2 96207679
chr2 96207854
chr2 96207900
chr2 96242189
chr2 96242434
chr2 96242586
chr2 96321067
chr2 96321081
chr2 96324926
chr2 96324976
chr2 96325589
chr2 96325601
chr2 96325629
chr2 96325637
chr2 96325661
chr2 96325697
chr2 96325704
chr2 96325762
chr2 96325770
chr2 96486384
chr2 96486395
chr2 96528026
chr2 96528080
chr2 96528109
chr2 96528173
chr2 96538210
chr2 96538238
chr2 96538265
chr2 96538320
chr2 96538380
chr2 96538800
chr2 96538815
chr2 96594544
chr2 96594570
chr2 96594664
chr2 96594839
chr2 96963063
chr2 96963070
chr2 96963115
chr2 96963182
chr2 97048907

chr2 97049174
chr2 97049345
chr2 97112198
chr2 97112227
chr2 97112425
chr2 97112437
chr2 97112462
chr2 97112488
chr2 97112492
chr2 97112512
chr2 97112545
chr2 97112608
chr2 97112626
chr2 97112979
chr2 97112989
chr2 97112991
chr2 97112993
chr2 97113000
chr2 97113015
chr2 97113017
chr2 97113062
chr2 97113065
chr2 97114121
chr2 97285958
chr2 97286515
chr2 97543097
chr2 97543639
chr2 97544468
chr2 97544479
chr2 97544698
chr2 97589234
chr2 97589280
chr2 97589339
chr2 97589413
chr2 97589424
chr2 97589431
chr2 97589440
chr2 97589445
chr2 97589497
chr2 97589505
chr2 97589512
chr2 97591014
chr2 97591026
chr2 97591223
chr2 97625149
chr2 97625155
chr2 97663455
chr2 97663478
chr2 97663485
chr2 97663487
chr2 97663522
chr2 97663545
chr2 97663550
chr2 97663552

chr2 97663558
chr2 97663562
chr2 97663566
chr2 97663570
chr2 97663621
chr2 97663637
chr2 97663640
chr2 97663642
chr2 97663646
chr2 97697119
chr2 97697294
chr2 97697337
chr2 97697349
chr2 97713834
chr2 97713886
chr2 97718636
chr2 97718728
chr2 97718734
chr2 97718764
chr2 97719254
chr2 97719264
chr2 97719268
chr2 97719343
chr2 97719459
chr2 97994527
chr2 97994591
chr2 97994751
chr2 97994846
chr2 97994886
chr2 97994893
chr2 98081891
chr2 98081903
chr2 98081907
chr2 98081914
chr2 98081941
chr2 98081973
chr2 98081975
chr2 98081977
chr2 98081998
chr2 98082026
chr2 98082114
chr2 98082123
chr2 98082144
chr2 98082169
chr2 98082315
chr2 98086560
chr2 98086566
chr2 98086587
chr2 98086611
chr2 98138017
chr2 98412982
chr2 98412990
chr2 98413063
chr2 98413066

chr2 98413403
chr2 98445995
chr2 98448304
chr2 98448417
chr2 98448442
chr2 98448537
chr2 98448557
chr2 98448655
chr2 98448749
chr2 98448938
chr2 98448951
chr2 98448958
chr2 98448982
chr2 98448998
chr2 98464218
chr2 98464253
chr2 98464278
chr2 98464327
chr2 98464333
chr2 98464336
chr2 98464370
chr2 98464384
chr2 98464404
chr2 98464607
chr2 98464833
chr2 98464887
chr2 98464896
chr2 98464928
chr2 98465029
chr2 98465042
chr2 98465054
chr2 98465126
chr2 98465139
chr2 98465233
chr2 98465264
chr2 98465271
chr2 98482977
chr2 98638067
chr2 98650263
chr2 98729728
chr2 98729746
chr2 98729750
chr2 98729853
chr2 98729917
chr2 98729947
chr2 98729958
chr2 98729967
chr2 98729973
chr2 98729983
chr2 98730070
chr2 98730100
chr2 98730108
chr2 98730112
chr2 98730172

chr2 98730221
chr2 98730241
chr2 98730264
chr2 98730277
chr2 98730294
chr2 98730316
chr2 98730322
chr2 98730355
chr2 98761513
chr2 98761522
chr2 98771965
chr2 98772002
chr2 98772020
chr2 98772022
chr2 98772031
chr2 98772172
chr2 98772174
chr2 98772198
chr2 98772212
chr2 98772249
chr2 98837683
chr2 99066068
chr2 99141902
chr2 99141921
chr2 99141926
chr2 99141936
chr2 99141943
chr2 99142140
chr2 99155498
chr2 99155521
chr2 99155559
chr2 99155670
chr2 99155694
chr2 99155709
chr2 99155721
chr2 99155776
chr2 99155804
chr2 99181738
chr2 99181838
chr2 99335906
chr2 99338246
chr2 99338252
chr2 99338305
chr2 99338352
chr2 99338422
chr2 99338426
chr2 99338457
chr2 99338570
chr2 99338585
chr2 99488952
chr2 99488962
chr2 100104241
chr2 100104263
chr2 100104272

chr2 100142524
chr2 100142531
chr2 100142552
chr2 100142568
chr2 100142574
chr2 100142672
chr2 100142697
chr2 100427942
chr2 100427967
chr2 100428208
chr2 100428311
chr2 100428332
chr2 100428343
chr2 100554915
chr2 100554931
chr2 100555026
chr2 100555034
chr2 100555075
chr2 100555088
chr2 100562318
chr2 100562373
chr2 100562381
chr2 100580894
chr2 100580908
chr2 100580921
chr2 100580928
chr2 100581044
chr2 100581053
chr2 100581103
chr2 100581151
chr2 100581239
chr2 100588283
chr2 100588397
chr2 100644189
chr2 100644208
chr2 101002094
chr2 101002120
chr2 101002131
chr2 101002133
chr2 101002139
chr2 101002144
chr2 101002147
chr2 101002169
chr2 101002172
chr2 101002174
chr2 101002176
chr2 101002179
chr2 101002195
chr2 101002232
chr2 101002237
chr2 101002258
chr2 101002283
chr2 101002285
chr2 101002307

chr2 101002330
chr2 101002389
chr2 101002403
chr2 101002413
chr2 101002421
chr2 101002502
chr2 101002507
chr2 101002520
chr2 101002525
chr2 101002542
chr2 101002550
chr2 101002552
chr2 101002558
chr2 101002576
chr2 101002611
chr2 101002616
chr2 101002628
chr2 101003005
chr2 101003082
chr2 101003107
chr2 101003167
chr2 101003180
chr2 101134035
chr2 101272282
chr2 101272522
chr2 101306414
chr2 101306428
chr2 101306460
chr2 101306469
chr2 101306535
chr2 101306558
chr2 101306617
chr2 101307051
chr2 101307063
chr2 101307221
chr2 101307267
chr2 101307322
chr2 101307326
chr2 101307330
chr2 101307338
chr2 101307344
chr2 101307346
chr2 101307416
chr2 101307430
chr2 101307458
chr2 101307474
chr2 101307524
chr2 101328673
chr2 101328697
chr2 101329134
chr2 101329321
chr2 101329328
chr2 101517391
chr2 101517431

chr2 101517493
chr2 101517510
chr2 101623268
chr2 101623328
chr2 101623379
chr2 101623382
chr2 101623410
chr2 101623586
chr2 101652168
chr2 101699006
chr2 101699060
chr2 101703779
chr2 101703784
chr2 101703867
chr2 101703871
chr2 101703879
chr2 101703881
chr2 101703963
chr2 101704112
chr2 102010937
chr2 102010954
chr2 102010969
chr2 102010978
chr2 102011042
chr2 102011102
chr2 102025187
chr2 102082579
chr2 102082647
chr2 102082717
chr2 102082786
chr2 102082815
chr2 102082826
chr2 102082978
chr2 102083072
chr2 102083096
chr2 102083308
chr2 102083395
chr2 102094548
chr2 102094571
chr2 102095062
chr2 102095123
chr2 102104687
chr2 102104741
chr2 102104865
chr2 102104871
chr2 102355218
chr2 102355230
chr2 102355380
chr2 102355559
chr2 102355578
chr2 102355580
chr2 102355598
chr2 102355608
chr2 102356277

chr2 102356291
chr2 102356298
chr2 102356332
chr2 102356338
chr2 102356340
chr2 102356348
chr2 102356365
chr2 102356371
chr2 102356382
chr2 102356388
chr2 102356415
chr2 102356425
chr2 102356468
chr2 102356531
chr2 102356626
chr2 102566307
chr2 102567280
chr2 102686960
chr2 102687033
chr2 102736680
chr2 102736683
chr2 102736690
chr2 102736693
chr2 102736699
chr2 102736705
chr2 102736715
chr2 102736720
chr2 102736746
chr2 102736753
chr2 102736760
chr2 102736772
chr2 102736778
chr2 102736814
chr2 102736816
chr2 102736819
chr2 102736823
chr2 102736830
chr2 102736834
chr2 102736846
chr2 102736848
chr2 102736859
chr2 102736873
chr2 102736878
chr2 102736882
chr2 102736895
chr2 102736897
chr2 102736899
chr2 102736905
chr2 102736920
chr2 102736940
chr2 102736942
chr2 102736948
chr2 102736954
chr2 102737012

chr2 102737089
chr2 103134690
chr2 104300438
chr2 104434018
chr2 104434036
chr2 104434227
chr2 104434265
chr2 104434471
chr2 104434476
chr2 104434500
chr2 104434637
chr2 104434670
chr2 104434681
chr2 104434686
chr2 104434718
chr2 104434720
chr2 104661166
chr2 104661173
chr2 104661326
chr2 104661397
chr2 104661659
chr2 104661770
chr2 104846219
chr2 104846243
chr2 104846359
chr2 104847495
chr2 104847615
chr2 104847646
chr2 104849232
chr2 104849541
chr2 104849610
chr2 104849636
chr2 104849707
chr2 104849715
chr2 104849725
chr2 104849786
chr2 104850119
chr2 104850131
chr2 104850155
chr2 104850196
chr2 104850206
chr2 104850329
chr2 104850378
chr2 104850439
chr2 104850569
chr2 104850663
chr2 104850686
chr2 104850737
chr2 104850760
chr2 104850814
chr2 104851010
chr2 104851312
chr2 104851425
chr2 104851430

chr2 104851456
chr2 104851461
chr2 104851595
chr2 104851610
chr2 104851627
chr2 104851647
chr2 104851654
chr2 104851659
chr2 104851672
chr2 104851675
chr2 104858887
chr2 104858899
chr2 104859048
chr2 104859148
chr2 104859414
chr2 104859425
chr2 104859482
chr2 104859595
chr2 104859715
chr2 104860005
chr2 104860498
chr2 104860660
chr2 104860663
chr2 104860698
chr2 104860812
chr2 104860826
chr2 104860841
chr2 104860979
chr2 104861088
chr2 104861177
chr2 104861444
chr2 104861520
chr2 104861718
chr2 104861758
chr2 104861771
chr2 104861809
chr2 104861892
chr2 104861904
chr2 104864927
chr2 104864931
chr2 104864947
chr2 104864949
chr2 104864957
chr2 104865074
chr2 104865138
chr2 104865598
chr2 104866286
chr2 104866299
chr2 104866303
chr2 104866339
chr2 104871580
chr2 104871911
chr2 104871938
chr2 104871951

chr2 105037755
chr2 105037760
chr2 105037853
chr2 105037864
chr2 105037886
chr2 105037902
chr2 105037908
chr2 105037913
chr2 105037915
chr2 105038251
chr2 105038265
chr2 105038267
chr2 105038269
chr2 105336996
chr2 105337081
chr2 105337137
chr2 105337174
chr2 105416354
chr2 105416419
chr2 105738530
chr2 105738599
chr2 105738723
chr2 105738843
chr2 105738884
chr2 105738934
chr2 105738953
chr2 105745850
chr2 105745862
chr2 105746106
chr2 105746275
chr2 105746282
chr2 105746318
chr2 105746366
chr2 105746375
chr2 105746382
chr2 105746492
chr2 105746510
chr2 105746520
chr2 105746596
chr2 105746605
chr2 105746626
chr2 105746632
chr2 105746688
chr2 105746747
chr2 105776113
chr2 105776358
chr2 105790138
chr2 105790146
chr2 105790261
chr2 105790285
chr2 105795453
chr2 105795579
chr2 105795645
chr2 105795702

chr2 105795719
chr2 105795733
chr2 105795738
chr2 105795840
chr2 105795843
chr2 105795862
chr2 105795953
chr2 105796183
chr2 105796783
chr2 105856508
chr2 105856618
chr2 105856704
chr2 105856733
chr2 105857006
chr2 105857116
chr2 105857229
chr2 105857264
chr2 105857278
chr2 105857310
chr2 105857314
chr2 105857325
chr2 105857337
chr2 105857339
chr2 105857343
chr2 105857349
chr2 105857378
chr2 105857383
chr2 105857386
chr2 105857391
chr2 105857415
chr2 106094551
chr2 106094592
chr2 106094607
chr2 106094635
chr2 106094695
chr2 106131741
chr2 106131839
chr2 106131846
chr2 106131868
chr2 106131887
chr2 106144729
chr2 106144739
chr2 106144754
chr2 106144802
chr2 106160098
chr2 106160164
chr2 106160198
chr2 106160241
chr2 106160272
chr2 106160349
chr2 106160366
chr2 106160377
chr2 106160389
chr2 106160397

chr2 106160406
chr2 106160457
chr2 106160483
chr2 106160487
chr2 106160519
chr2 106160521
chr2 106160553
chr2 106160559
chr2 106160582
chr2 106160684
chr2 106160693
chr2 106160752
chr2 106160759
chr2 106160767
chr2 106160804
chr2 106160830
chr2 106462706
chr2 106462714
chr2 106511570
chr2 106511575
chr2 106511632
chr2 106511638
chr2 106511640
chr2 106511737
chr2 106511758
chr2 106511763
chr2 106511778
chr2 106511786
chr2 106582198
chr2 106582211
chr2 106582229
chr2 106582238
chr2 106582240
chr2 106582258
chr2 106582265
chr2 106582267
chr2 106582271
chr2 106582284
chr2 106582316
chr2 106582331
chr2 106582342
chr2 106582362
chr2 106582470
chr2 106582550
chr2 106582588
chr2 106582640
chr2 106582646
chr2 106582680
chr2 106582684
chr2 106582732
chr2 106582767
chr2 106582788
chr2 106582805
chr2 106884904

chr2 106885191
chr2 106885194
chr2 106885228
chr2 106885277
chr2 106885289
chr2 106885567
chr2 106885580
chr2 106890203
chr2 106890246
chr2 106890377
chr2 106890393
chr2 106890609
chr2 106890769
chr2 106890771
chr2 106890789
chr2 106890819
chr2 107472497
chr2 107802989
chr2 107833760
chr2 107833767
chr2 107833776
chr2 107833826
chr2 107833834
chr2 107833904
chr2 107833908
chr2 107833931
chr2 107833936
chr2 107833961
chr2 107833969
chr2 107834048
chr2 107834171
chr2 107834532
chr2 107835140
chr2 107835164
chr2 107835174
chr2 107835215
chr2 107836219
chr2 107836234
chr2 107836258
chr2 107836297
chr2 107836318
chr2 107836364
chr2 107836399
chr2 107836910
chr2 107838812
chr2 107937236
chr2 107937242
chr2 107987273
chr2 108449677
chr2 108449711
chr2 108449773
chr2 108449828
chr2 108449834
chr2 108449856

chr2 108449865
chr2 108449933
chr2 108612316
chr2 108612322
chr2 108612362
chr2 108612400
chr2 108612433
chr2 108612694
chr2 108612720
chr2 108612739
chr2 108612825
chr2 108612834
chr2 108612839
chr2 108612848
chr2 108612881
chr2 108620975
chr2 108621116
chr2 108621138
chr2 108621269
chr2 108621279
chr2 108621409
chr2 108621415
chr2 108621420
chr2 108621545
chr2 108622284
chr2 108622551
chr2 108622804
chr2 108622916
chr2 108637244
chr2 108652307
chr2 108652353
chr2 108720207
chr2 108720210
chr2 108720213
chr2 108767803
chr2 108785723
chr2 109031962
chr2 109031966
chr2 109031992
chr2 109031994
chr2 109032040
chr2 109032079
chr2 109032128
chr2 109032145
chr2 109032269
chr2 109032289
chr2 109032404
chr2 109032471
chr2 109032475
chr2 109032496
chr2 109032568
chr2 109076057
chr2 109143920
chr2 109143927

chr2 109143985
chr2 109144036
chr2 109144041
chr2 109144044
chr2 109144153
chr2 109213898
chr2 109213993
chr2 109213998
chr2 109214078
chr2 109214081
chr2 109238549
chr2 109336189
chr2 109546517
chr2 109547009
chr2 109616521
chr2 109616534
chr2 109754923
chr2 109756461
chr2 109756668
chr2 109756783
chr2 109756831
chr2 109757496
chr2 109758225
chr2 109758268
chr2 109758356
chr2 109758655
chr2 109758753
chr2 109758801
chr2 109759405
chr2 109760332
chr2 109760346
chr2 109760349
chr2 109760352
chr2 109760355
chr2 109760358
chr2 109760361
chr2 109760369
chr2 109760375
chr2 109760381
chr2 109760392
chr2 109760397
chr2 109760403
chr2 109760406
chr2 109760409
chr2 109760412
chr2 109760415
chr2 109760418
chr2 109760421
chr2 109760424
chr2 109760427
chr2 109760430
chr2 109760438
chr2 109760444
chr2 109760447

chr2 109760450
chr2 109760453
chr2 109760456
chr2 109760459
chr2 109760461
chr2 109760464
chr2 109760470
chr2 109760495
chr2 109760497
chr2 109760504
chr2 109760511
chr2 109760521
chr2 109760524
chr2 109760528
chr2 109760597
chr2 109760600
chr2 109760604
chr2 109760696
chr2 109760699
chr2 109760702
chr2 109760730
chr2 109760781
chr2 109760786
chr2 109760790
chr2 109760795
chr2 109760799
chr2 109760803
chr2 109760809
chr2 109760812
chr2 109760819
chr2 109760823
chr2 109760825
chr2 109760828
chr2 109760833
chr2 109760835
chr2 109760837
chr2 109760852
chr2 109760861
chr2 109760865
chr2 109760869
chr2 109760873
chr2 109760876
chr2 109760894
chr2 109760900
chr2 109760909
chr2 109760931
chr2 109760942
chr2 109760948
chr2 109760979
chr2 109760988
chr2 109760990
chr2 109760993
chr2 109760998
chr2 109761105

chr2 109761108
chr2 109761140
chr2 109761146
chr2 109761238
chr2 109761242
chr2 109761248
chr2 109761285
chr2 109761311
chr2 109761318
chr2 109761330
chr2 109761335
chr2 109761375
chr2 109761446
chr2 109761450
chr2 109761474
chr2 109761485
chr2 109761492
chr2 109761511
chr2 109761518
chr2 109761657
chr2 109761754
chr2 109761814
chr2 109793739
chr2 109794498
chr2 109794501
chr2 109794506
chr2 109794524
chr2 109794527
chr2 109794532
chr2 109794550
chr2 109794559
chr2 109794562
chr2 109794565
chr2 109794571
chr2 109794576
chr2 109794580
chr2 109794587
chr2 109794590
chr2 109794593
chr2 109794596
chr2 109794599
chr2 109794602
chr2 109794605
chr2 109794608
chr2 109794617
chr2 109794620
chr2 109794623
chr2 109794626
chr2 109794630
chr2 109794635
chr2 109794647
chr2 109794659
chr2 109794666
chr2 109794674

chr2 109794679
chr2 109794681
chr2 109794694
chr2 109794697
chr2 109794854
chr2 109794864
chr2 109794877
chr2 109794881
chr2 109794883
chr2 109794890
chr2 109794899
chr2 109794901
chr2 109794903
chr2 109794915
chr2 109794931
chr2 109795945
chr2 109796027
chr2 109796088
chr2 109796116
chr2 109796120
chr2 109796131
chr2 109796151
chr2 109796215
chr2 109796668
chr2 109796676
chr2 109796684
chr2 109796706
chr2 109796708
chr2 109796714
chr2 109796737
chr2 109796746
chr2 109796789
chr2 109796801
chr2 109796831
chr2 109796842
chr2 109796862
chr2 109796883
chr2 109796887
chr2 109796920
chr2 109796930
chr2 109797696
chr2 109797705
chr2 109797744
chr2 109797751
chr2 109797767
chr2 109797779
chr2 109800367
chr2 109800397
chr2 109800407
chr2 109800435
chr2 109800448
chr2 109800523
chr2 109800550
chr2 109800562

chr2 109800593
chr2 109802472
chr2 109802489
chr2 109802624
chr2 109805540
chr2 109805548
chr2 109805565
chr2 109805582
chr2 109805596
chr2 109805604
chr2 109805641
chr2 110204639
chr2 110204651
chr2 110204668
chr2 110204685
chr2 110204729
chr2 110204765
chr2 110205110
chr2 110205124
chr2 110205145
chr2 110205164
chr2 110205188
chr2 110205195
chr2 110205198
chr2 110205460
chr2 110212135
chr2 110212165
chr2 110212169
chr2 110212233
chr2 110212246
chr2 110212276
chr2 110212279
chr2 110212282
chr2 110212287
chr2 110212295
chr2 110212298
chr2 110212300
chr2 110212339
chr2 110212346
chr2 110212379
chr2 110212382
chr2 110212385
chr2 110212428
chr2 110212434
chr2 110212440
chr2 110212445
chr2 110212474
chr2 110212489
chr2 110212520
chr2 110212532
chr2 110212535
chr2 110212538
chr2 110212545
chr2 110212547

chr2 110212554
chr2 110212557
chr2 110212559
chr2 110212562
chr2 110212565
chr2 110212578
chr2 110212581
chr2 110212583
chr2 110212585
chr2 110212600
chr2 110212618
chr2 110212624
chr2 110212627
chr2 110212632
chr2 110212644
chr2 110212672
chr2 110212778
chr2 110212793
chr2 110212811
chr2 110212975
chr2 110293536
chr2 110294333
chr2 110294616
chr2 110294633
chr2 110294808
chr2 110294854
chr2 110294867
chr2 110294870
chr2 110295025
chr2 110295212
chr2 110295331
chr2 110295889
chr2 110297810
chr2 110297866
chr2 110298327
chr2 110298369
chr2 110298504
chr2 110298629
chr2 110298925
chr2 110299189
chr2 110299193
chr2 110299195
chr2 110299200
chr2 110299202
chr2 110299204
chr2 110299211
chr2 110299227
chr2 110299251
chr2 110299259
chr2 110299264
chr2 110299269
chr2 110299292
chr2 110299313
chr2 110299326

chr2 110299925
chr2 110299934
chr2 110300009
chr2 110300068
chr2 110300137
chr2 110300355
chr2 110339311
chr2 110340327
chr2 110340436
chr2 110340904
chr2 110572291
chr2 110572306
chr2 110572314
chr2 110572339
chr2 110572344
chr2 110572367
chr2 110572510
chr2 110572516
chr2 110573728
chr2 110573740
chr2 110573756
chr2 110573763
chr2 110573802
chr2 110573811
chr2 110574665
chr2 110574676
chr2 110574706
chr2 110574718
chr2 110574799
chr2 110574801
chr2 110574823
chr2 110574831
chr2 110574839
chr2 110575262
chr2 110575292
chr2 110575356
chr2 110575376
chr2 110575387
chr2 110575391
chr2 110575419
chr2 110575469
chr2 110575480
chr2 110575831
chr2 110576799
chr2 110576806
chr2 110576810
chr2 110576813
chr2 110576826
chr2 110576828
chr2 110576833
chr2 110576841
chr2 110576848
chr2 110576860
chr2 110576872

chr2 110576877
chr2 110576881
chr2 110576884
chr2 110576887
chr2 110576890
chr2 110576909
chr2 110576913
chr2 110576918
chr2 110576924
chr2 110576927
chr2 110576930
chr2 110576939
chr2 110576957
chr2 110576962
chr2 110576965
chr2 110576983
chr2 110576988
chr2 110576991
chr2 110577152
chr2 110577156
chr2 110577160
chr2 110577163
chr2 110577166
chr2 110577172
chr2 110577188
chr2 110577196
chr2 110577222
chr2 110577225
chr2 110577228
chr2 110577231
chr2 110577234
chr2 110577249
chr2 110577253
chr2 110577259
chr2 110577264
chr2 110577267
chr2 110578066
chr2 110578342
chr2 110578977
chr2 110579738
chr2 110579746
chr2 110580179
chr2 110580253
chr2 110580549
chr2 110580616
chr2 110580760
chr2 110580825
chr2 110580897
chr2 110581239
chr2 110581256
chr2 110581599
chr2 110581637
chr2 110581825
chr2 110581894

chr2 110582347
chr2 110582352
chr2 110609967
chr2 110609974
chr2 110609993
chr2 110610000
chr2 110610011
chr2 110610030
chr2 110610035
chr2 110610039
chr2 110610072
chr2 110610110
chr2 110610155
chr2 110610167
chr2 110610174
chr2 110610200
chr2 110610237
chr2 110610243
chr2 110610247
chr2 110610487
chr2 110610492
chr2 110610495
chr2 110610497
chr2 110610506
chr2 110610529
chr2 110610537
chr2 110610543
chr2 110610554
chr2 110610725
chr2 110610755
chr2 110611024
chr2 110611030
chr2 110611033
chr2 110611035
chr2 110611038
chr2 110611041
chr2 110611044
chr2 110611050
chr2 110611056
chr2 110611064
chr2 110611067
chr2 110611076
chr2 110611079
chr2 110611082
chr2 110611085
chr2 110611088
chr2 110611091
chr2 110611097
chr2 110611102
chr2 110611113
chr2 110611119
chr2 110611125
chr2 110611133
chr2 110611136

chr2 110611139
chr2 110611142
chr2 110611145
chr2 110611148
chr2 110611162
chr2 110611343
chr2 110611674
chr2 110611784
chr2 110612692
chr2 110612740
chr2 110612838
chr2 110613139
chr2 110613227
chr2 110613270
chr2 110613707
chr2 110613999
chr2 110677187
chr2 110677287
chr2 110677299
chr2 110677317
chr2 110677410
chr2 110677924
chr2 110678159
chr2 110678211
chr2 110678220
chr2 110678229
chr2 110678240
chr2 110678251
chr2 110678255
chr2 110678257
chr2 110678273
chr2 110678278
chr2 110678281
chr2 110678302
chr2 110678567
chr2 110678572
chr2 110678579
chr2 110678588
chr2 110678625
chr2 110678638
chr2 110678663
chr2 110678669
chr2 110678851
chr2 110678966
chr2 110678982
chr2 110695890
chr2 110695963
chr2 110695986
chr2 110696008
chr2 110858569
chr2 110858686
chr2 110858715
chr2 110858727
chr2 110858775

chr2 110858949
chr2 110858953
chr2 110858958
chr2 110858988
chr2 110859005
chr2 111115646
chr2 111115652
chr2 111115955
chr2 111116031
chr2 111116137
chr2 111116172
chr2 111116273
chr2 111493088
chr2 111493091
chr2 111493135
chr2 111493171
chr2 111493180
chr2 111493228
chr2 111493534
chr2 111493543
chr2 111493555
chr2 111493558
chr2 111493567
chr2 111493575
chr2 111493694
chr2 111493844
chr2 111493855
chr2 111493881
chr2 111493889
chr2 111493900
chr2 111494102
chr2 111494182
chr2 111495044
chr2 111495070
chr2 111495100
chr2 111495504
chr2 111529947
chr2 111529951
chr2 111530069
chr2 111545939
chr2 111545947
chr2 111545976
chr2 111546017
chr2 111546226
chr2 111546251
chr2 111546491
chr2 111698743
chr2 111698785
chr2 111698900
chr2 111698916
chr2 111698921
chr2 111698955
chr2 111699013
chr2 111699132

chr2 111699152
chr2 111719925
chr2 111720077
chr2 111727950
chr2 111747525
chr2 111747546
chr2 111747552
chr2 111747598
chr2 111747614
chr2 111747766
chr2 111747785
chr2 111747791
chr2 111747827
chr2 111747863
chr2 111747865
chr2 111747883
chr2 111747888
chr2 111747892
chr2 111747895
chr2 111747933
chr2 111748022
chr2 111748079
chr2 111748087
chr2 111748119
chr2 111748153
chr2 112048895
chr2 112049160
chr2 112049173
chr2 112049318
chr2 112049407
chr2 112054094
chr2 112054118
chr2 112054142
chr2 112054192
chr2 112054212
chr2 112054233
chr2 112054235
chr2 112054239
chr2 112054245
chr2 112054248
chr2 112054255
chr2 112054268
chr2 112054291
chr2 112054310
chr2 112054328
chr2 112054347
chr2 112054355
chr2 112054365
chr2 112054371
chr2 112054403
chr2 112054426
chr2 112054448
chr2 112054458
chr2 112054489

chr2 112054502
chr2 112054526
chr2 112054534
chr2 112054545
chr2 112054553
chr2 112054556
chr2 112054576
chr2 112054582
chr2 112054590
chr2 112054592
chr2 112054598
chr2 112054606
chr2 112054608
chr2 112054614
chr2 112054622
chr2 112054626
chr2 112054708
chr2 112054714
chr2 112054724
chr2 112054730
chr2 112054738
chr2 112054740
chr2 112054746
chr2 112054754
chr2 112054758
chr2 112054800
chr2 112054807
chr2 112054818
chr2 112054825
chr2 112054830
chr2 112054840
chr2 112054846
chr2 112054872
chr2 112056401
chr2 112056464
chr2 112056564
chr2 112056745
chr2 112187996
chr2 112188081
chr2 112188219
chr2 112188405
chr2 112188483
chr2 112254360
chr2 112254623
chr2 112254630
chr2 112254641
chr2 112254652
chr2 112254660
chr2 112254672
chr2 112254681
chr2 112254690
chr2 112254708
chr2 112254710
chr2 112254731

chr2 112254738
chr2 112254742
chr2 112254760
chr2 112254766
chr2 112254770
chr2 112254774
chr2 112254781
chr2 112254792
chr2 112254800
chr2 112254802
chr2 112254805
chr2 112254807
chr2 112254812
chr2 112254816
chr2 112254824
chr2 112254857
chr2 112254865
chr2 112254912
chr2 112254922
chr2 112259528
chr2 112259547
chr2 112259566
chr2 112259574
chr2 112259612
chr2 112259687
chr2 112259700
chr2 112276628
chr2 112276651
chr2 112276753
chr2 112346469
chr2 112432442
chr2 112432478
chr2 112432495
chr2 112432515
chr2 112432539
chr2 112432542
chr2 112432548
chr2 112432562
chr2 112432570
chr2 112432608
chr2 112432615
chr2 112432618
chr2 112432620
chr2 112432659
chr2 112432662
chr2 112432674
chr2 112432690
chr2 112432702
chr2 112432704
chr2 112432706
chr2 112432715
chr2 112432730
chr2 112432740
chr2 112432748

chr2 112432752
chr2 112432759
chr2 112432762
chr2 112432773
chr2 112432792
chr2 112432795
chr2 112432809
chr2 112432813
chr2 112432816
chr2 112432818
chr2 112432841
chr2 112432851
chr2 112432856
chr2 112432859
chr2 112432873
chr2 112432880
chr2 112432887
chr2 112432900
chr2 112432902
chr2 112432915
chr2 112432921
chr2 112432945
chr2 112432951
chr2 112432954
chr2 112432957
chr2 112432962
chr2 112432967
chr2 112432973
chr2 112432976
chr2 112432989
chr2 112432991
chr2 112433004
chr2 112433010
chr2 112433034
chr2 112433040
chr2 112433046
chr2 112433051
chr2 112433056
chr2 112433062
chr2 112433065
chr2 112433078
chr2 112433080
chr2 112433093
chr2 112433099
chr2 112433123
chr2 112433129
chr2 112433132
chr2 112433135
chr2 112433140
chr2 112433145
chr2 112433151
chr2 112433154
chr2 112433167
chr2 112433169

chr2 112433182
chr2 112433186
chr2 112433189
chr2 112433213
chr2 112433219
chr2 112433222
chr2 112433225
chr2 112433230
chr2 112433241
chr2 112433244
chr2 112433257
chr2 112433259
chr2 112433272
chr2 112433276
chr2 112433279
chr2 112433303
chr2 112433309
chr2 112433312
chr2 112433315
chr2 112433320
chr2 112433325
chr2 112433331
chr2 112433334
chr2 112433337
chr2 112433341
chr2 112433345
chr2 112433350
chr2 112433356
chr2 112433359
chr2 112433386
chr2 112433391
chr2 112433394
chr2 112433412
chr2 112433417
chr2 112433420
chr2 112433425
chr2 112433435
chr2 112433446
chr2 112433454
chr2 112433456
chr2 112481008
chr2 112481015
chr2 112481028
chr2 112481036
chr2 112481045
chr2 112481049
chr2 112481056
chr2 112481076
chr2 112481093
chr2 112481165
chr2 112481183
chr2 112481203
chr2 112481208
chr2 112481219

chr2 112481227
chr2 112481234
chr2 112481498
chr2 112481524
chr2 112481527
chr2 112481544
chr2 112481551
chr2 112481580
chr2 112481601
chr2 112481624
chr2 112506990
chr2 112507717
chr2 112508790
chr2 112508815
chr2 112508865
chr2 112509126
chr2 112509169
chr2 112509189
chr2 112541652
chr2 112541654
chr2 112541695
chr2 112541793
chr2 112541827
chr2 112541832
chr2 112541853
chr2 112541859
chr2 112541863
chr2 112541911
chr2 112541920
chr2 112541923
chr2 112541932
chr2 112541939
chr2 112541973
chr2 112542002
chr2 112542009
chr2 112542016
chr2 112542024
chr2 112542035
chr2 112542045
chr2 112542063
chr2 112542072
chr2 112542121
chr2 112542131
chr2 112542851
chr2 112542856
chr2 112542874
chr2 112542905
chr2 112542922
chr2 112542979
chr2 112543015
chr2 112543022
chr2 112543032
chr2 112543036
chr2 112543254

chr2 112599945
chr2 112599999
chr2 112600118
chr2 112600149
chr2 112600196
chr2 112600199
chr2 112600213
chr2 112600248
chr2 112600259
chr2 112600272
chr2 112624567
chr2 112624598
chr2 112624686
chr2 112631147
chr2 112631162
chr2 112631170
chr2 112631187
chr2 112646788
chr2 112646836
chr2 112646864
chr2 112646867
chr2 112646942
chr2 112706377
chr2 112706471
chr2 112706651
chr2 112708769
chr2 112708828
chr2 112708885
chr2 112708946
chr2 112749096
chr2 112749854
chr2 112749925
chr2 112765144
chr2 112997395
chr2 112997910
chr2 113138700
chr2 113138704
chr2 113138734
chr2 113138752
chr2 113173645
chr2 113173687
chr2 113173821
chr2 113173900
chr2 113173903
chr2 113174496
chr2 113174647
chr2 113174929
chr2 113175011
chr2 113175041
chr2 113175238
chr2 113175277
chr2 113175378
chr2 113175431
chr2 113175447

chr2 113175546
chr2 113175557
chr2 113175602
chr2 113175665
chr2 113175719
chr2 113175853
chr2 113176149
chr2 113176158
chr2 113176230
chr2 113176234
chr2 113176299
chr2 113251495
chr2 113251503
chr2 113251540
chr2 113251579
chr2 113274871
chr2 113274949
chr2 113275528
chr2 113275572
chr2 113390058
chr2 113390225
chr2 113390432
chr2 113390444
chr2 113390486
chr2 113390495
chr2 113390499
chr2 113390552
chr2 113391588
chr2 113436964
chr2 113437068
chr2 113437083
chr2 113437205
chr2 113437428
chr2 113438078
chr2 113438086
chr2 113438205
chr2 113438217
chr2 113438275
chr2 113438285
chr2 113438337
chr2 113438392
chr2 113438427
chr2 113438442
chr2 113438447
chr2 113438458
chr2 113438481
chr2 113438514
chr2 113438563
chr2 113499869
chr2 113499928
chr2 113499931
chr2 113499947
chr2 113499981
chr2 113500012

chr2 113500018
chr2 113500025
chr2 113500325
chr2 113500455
chr2 113500465
chr2 113500492
chr2 113500511
chr2 113500543
chr2 113500549
chr2 113500571
chr2 113500607
chr2 113500612
chr2 113500634
chr2 113500636
chr2 113500650
chr2 113500683
chr2 113500730
chr2 113500753
chr2 113500762
chr2 113500769
chr2 113500793
chr2 113500816
chr2 113500847
chr2 113500912
chr2 113501174
chr2 113501445
chr2 113501628
chr2 113501631
chr2 113501641
chr2 113501667
chr2 113501719
chr2 113501734
chr2 113501747
chr2 113501753
chr2 113501758
chr2 113501899
chr2 113501910
chr2 113501929
chr2 113501972
chr2 113501996
chr2 113502008
chr2 113503867
chr2 113503873
chr2 113503886
chr2 113503901
chr2 113503904
chr2 113503914
chr2 113503929
chr2 113503931
chr2 113503938
chr2 113503943
chr2 113503945
chr2 113503959
chr2 113504145

chr2 113504154
chr2 113504166
chr2 113504175
chr2 113504177
chr2 113504184
chr2 113504207
chr2 113504216
chr2 113504271
chr2 113504277
chr2 113504302
chr2 113504306
chr2 113504338
chr2 113504360
chr2 113504363
chr2 113504385
chr2 113504402
chr2 113504405
chr2 113504436
chr2 113504487
chr2 113504499
chr2 113504558
chr2 113504575
chr2 113504878
chr2 113540953
chr2 113541049
chr2 113541073
chr2 113541150
chr2 113541197
chr2 113541253
chr2 113541303
chr2 113541308
chr2 113541312
chr2 113541319
chr2 113541328
chr2 113541356
chr2 113541390
chr2 113541418
chr2 113551135
chr2 113551504
chr2 113551515
chr2 113551700
chr2 113551706
chr2 113551746
chr2 113551836
chr2 113551868
chr2 113551960
chr2 113552176
chr2 113552242
chr2 113552258
chr2 113552288
chr2 113552306
chr2 113552432
chr2 113552788
chr2 113584435

chr2 113584445
chr2 113584454
chr2 113584462
chr2 113584468
chr2 113584548
chr2 113584565
chr2 113584591
chr2 113584593
chr2 113584604
chr2 113584616
chr2 113584631
chr2 113584704
chr2 113584706
chr2 113584720
chr2 113584751
chr2 113584779
chr2 113584796
chr2 113603440
chr2 113603483
chr2 113603509
chr2 113603547
chr2 113603555
chr2 113603558
chr2 113603561
chr2 113603577
chr2 113603581
chr2 113603585
chr2 113603613
chr2 113603619
chr2 113603630
chr2 113603651
chr2 113603659
chr2 113603665
chr2 113603667
chr2 113603677
chr2 113603697
chr2 113603705
chr2 113603711
chr2 113603719
chr2 113603722
chr2 113603724
chr2 113603727
chr2 113603730
chr2 113603736
chr2 113603738
chr2 113603746
chr2 113603752
chr2 113603754
chr2 113603757
chr2 113603762
chr2 113603768
chr2 113603770
chr2 113603778
chr2 113603784

chr2 113603786
chr2 113603789
chr2 113603794
chr2 113603800
chr2 113603802
chr2 113603810
chr2 113603812
chr2 113603815
chr2 113603829
chr2 113603831
chr2 113603834
chr2 113603847
chr2 113603851
chr2 113603854
chr2 113603857
chr2 113603865
chr2 113603879
chr2 113603886
chr2 113603890
chr2 113603901
chr2 113603903
chr2 113603906
chr2 113603909
chr2 113603912
chr2 113603920
chr2 113603924
chr2 113603927
chr2 113603944
chr2 113603960
chr2 113603968
chr2 113603970
chr2 113603972
chr2 113603975
chr2 113603978
chr2 113603982
chr2 113603989
chr2 113603991
chr2 113604011
chr2 113604029
chr2 113604032
chr2 113604035
chr2 113604043
chr2 113604072
chr2 113604077
chr2 113604081
chr2 113604085
chr2 113604098
chr2 113604124
chr2 113604140
chr2 113604153
chr2 113604198
chr2 113604211
chr2 113604214
chr2 113604221

chr2 113604239
chr2 113604322
chr2 113604348
chr2 113604362
chr2 113604370
chr2 113604380
chr2 113626516
chr2 113626525
chr2 113626537
chr2 113626600
chr2 113626643
chr2 113626647
chr2 113626651
chr2 113626695
chr2 113626708
chr2 113626735
chr2 113626750
chr2 113626774
chr2 113627029
chr2 113627038
chr2 113627056
chr2 113627060
chr2 113627067
chr2 113627111
chr2 113627117
chr2 113627121
chr2 113627697
chr2 113627731
chr2 113627737
chr2 113627739
chr2 113627761
chr2 113627799
chr2 113627941
chr2 113627997
chr2 113628335
chr2 113693056
chr2 113693307
chr2 113886974
chr2 113889131
chr2 113889168
chr2 113889208
chr2 113889213
chr2 113889219
chr2 113889402
chr2 113889458
chr2 113889483
chr2 113889505
chr2 113889526
chr2 113889533
chr2 113889556
chr2 113892050
chr2 113892160
chr2 113892264
chr2 113892312

chr2 113892660
chr2 114028520
chr2 114222682
chr2 114222838
chr2 114222900
chr2 114441770
chr2 114543692
chr2 114660186
chr2 114660630
chr2 115103700
chr2 115694818
chr2 115818693
chr2 115818698
chr2 115818808
chr2 115819015
chr2 115819026
chr2 115819090
chr2 115952836
chr2 116024560
chr2 116550429
chr2 117040128
chr2 117040579
chr2 117041488
chr2 117041515
chr2 117041542
chr2 117150552
chr2 117156742
chr2 117621760
chr2 117621881
chr2 117621891
chr2 117625138
chr2 117625148
chr2 117625376
chr2 117650380
chr2 117650389
chr2 117650416
chr2 117650441
chr2 117650470
chr2 117650557
chr2 117650664
chr2 117650668
chr2 117650690
chr2 117650708
chr2 117650834
chr2 117650853
chr2 117651041
chr2 117651064
chr2 117651074
chr2 117651136
chr2 117651552
chr2 117651903
chr2 117652034
chr2 117652219
chr2 117652425

chr2 117652602
chr2 117652814
chr2 117652831
chr2 117652895
chr2 117804228
chr2 117804232
chr2 117804269
chr2 117804758
chr2 117804833
chr2 117804854
chr2 117815191
chr2 117815216
chr2 117815234
chr2 117815273
chr2 118011034
chr2 118011319
chr2 118011350
chr2 118014522
chr2 118014533
chr2 118014578
chr2 118014596
chr2 118014598
chr2 118014614
chr2 118014649
chr2 118014691
chr2 118014719
chr2 118014722
chr2 118014724
chr2 118059565
chr2 118088169
chr2 118088173
chr2 118088175
chr2 118088182
chr2 118088193
chr2 118088221
chr2 118088242
chr2 118088254
chr2 118088285
chr2 118088292
chr2 118088304
chr2 118088307
chr2 118088314
chr2 118088321
chr2 118088339
chr2 118088346
chr2 118088363
chr2 118088368
chr2 118088378
chr2 118088399
chr2 118088416
chr2 118088420
chr2 118088426
chr2 118088428
chr2 118088430

chr2 118088436
chr2 118088443
chr2 118088451
chr2 118088463
chr2 118088467
chr2 118088537
chr2 118088559
chr2 118088562
chr2 118088567
chr2 118088569
chr2 118088576
chr2 118088593
chr2 118088670
chr2 118088713
chr2 118088732
chr2 118088754
chr2 118088760
chr2 118088783
chr2 118088793
chr2 118088816
chr2 118088835
chr2 118088838
chr2 118088847
chr2 118088878
chr2 118088947
chr2 118088977
chr2 118089077
chr2 118089202
chr2 118089204
chr2 118089214
chr2 118089252
chr2 118089409
chr2 118089437
chr2 118089445
chr2 118089593
chr2 118089673
chr2 118089729
chr2 118089785
chr2 118089858
chr2 118139388
chr2 118139417
chr2 118141058
chr2 118141120
chr2 118141724
chr2 118141753
chr2 118141779
chr2 118141811
chr2 118141820
chr2 118141946
chr2 118141967
chr2 118141992
chr2 118142023
chr2 118142251
chr2 118142304

chr2 118185558
chr2 118185561
chr2 118185583
chr2 118185608
chr2 118185623
chr2 118185713
chr2 118185753
chr2 118185807
chr2 118185902
chr2 118185929
chr2 118185946
chr2 118185949
chr2 118185952
chr2 118185980
chr2 118185982
chr2 118186001
chr2 118186009
chr2 118186015
chr2 118186021
chr2 118186025
chr2 118186027
chr2 118186064
chr2 118186100
chr2 118186117
chr2 118186150
chr2 118186160
chr2 118186248
chr2 118186254
chr2 118186274
chr2 118222223
chr2 118222664
chr2 118222890
chr2 118223166
chr2 118223189
chr2 118223240
chr2 118223246
chr2 118223265
chr2 118223343
chr2 118223372
chr2 118223487
chr2 118223494
chr2 118223532
chr2 118223541
chr2 118223548
chr2 118223574
chr2 118223591
chr2 118223614
chr2 118223635
chr2 118223639
chr2 118223701
chr2 118223799
chr2 118223803
chr2 118223815
chr2 118223830

chr2 118223844
chr2 118223877
chr2 118223889
chr2 118223899
chr2 118223923
chr2 118310422
chr2 118470576
chr2 118470702
chr2 118832268
chr2 118832279
chr2 118832304
chr2 118832516
chr2 118832547
chr2 118832595
chr2 118832726
chr2 118832739
chr2 118832747
chr2 118832887
chr2 118898679
chr2 118900720
chr2 118900741
chr2 118900766
chr2 119543989
chr2 119544022
chr2 119678799
chr2 119678842
chr2 119678846
chr2 119678854
chr2 119678884
chr2 119678894
chr2 119678914
chr2 119678955
chr2 119678958
chr2 119678962
chr2 119678974
chr2 119679018
chr2 119679030
chr2 119679033
chr2 119679037
chr2 119679060
chr2 119679065
chr2 119679074
chr2 119679078
chr2 119679081
chr2 119679088
chr2 119679116
chr2 119679122
chr2 119679125
chr2 119679132
chr2 119702804
chr2 119702967
chr2 119702979
chr2 119702996
chr2 119703036

chr2 119703048
chr2 119760489
chr2 119760497
chr2 119760558
chr2 119760595
chr2 119760630
chr2 119760656
chr2 119760768
chr2 119760779
chr2 119760923
chr2 119761051
chr2 119761073
chr2 119761124
chr2 119821778
chr2 119821941
chr2 119821965
chr2 119929627
chr2 119929819
chr2 119929941
chr2 119930121
chr2 119930215
chr2 120177073
chr2 120177118
chr2 120177199
chr2 120177260
chr2 120177354
chr2 120177377
chr2 120177383
chr2 120177388
chr2 120177392
chr2 120177399
chr2 120177410
chr2 120177524
chr2 120231853
chr2 120232042
chr2 120232084
chr2 120232095
chr2 120232148
chr2 120232166
chr2 120232210
chr2 120232311
chr2 120232317
chr2 120232323
chr2 120232384
chr2 120232397
chr2 120232447
chr2 120232467
chr2 120232481
chr2 120232559
chr2 120232729
chr2 120232746
chr2 120232936
chr2 120232967
chr2 120233120

chr2 120233124
chr2 120233129
chr2 121080304
chr2 121176632
chr2 121176827
chr2 121176855
chr2 121176887
chr2 121277120
chr2 121529836
chr2 121529932
chr2 121529947
chr2 121530944
chr2 121530961
chr2 121531119
chr2 121531145
chr2 121531156
chr2 121605091
chr2 121648483
chr2 121648595
chr2 121648602
chr2 121648616
chr2 121648647
chr2 121648671
chr2 121648679
chr2 121648750
chr2 121648779
chr2 121648788
chr2 121649004
chr2 121650206
chr2 121650222
chr2 121650230
chr2 121650238
chr2 121650241
chr2 121650243
chr2 121650247
chr2 121650309
chr2 121650312
chr2 121650315
chr2 121650324
chr2 121650341
chr2 121650343
chr2 121650349
chr2 121650365
chr2 121650405
chr2 121699623
chr2 121699664
chr2 121699743
chr2 121735638
chr2 121735684
chr2 121735707
chr2 121735749
chr2 121735758
chr2 121735823
chr2 121735994

chr2 121736059
chr2 121974652
chr2 121974836
chr2 121975254
chr2 122347287
chr2 122347450
chr2 122347577
chr2 122347654
chr2 122571217
chr2 122571467
chr2 123185968
chr2 123701408
chr2 123702443
chr2 123760497
chr2 123760620
chr2 123760697
chr2 123760907
chr2 123909531
chr2 123909628
chr2 123909722
chr2 123909991
chr2 123910260
chr2 123910394
chr2 123910427
chr2 123910926
chr2 123912697
chr2 123912715
chr2 123912730
chr2 123912778
chr2 123913116
chr2 123913144
chr2 123913205
chr2 123913347
chr2 123913369
chr2 123913378
chr2 124024150
chr2 124024451
chr2 124027282
chr2 124027347
chr2 124027472
chr2 124027638
chr2 124027786
chr2 124028139
chr2 124028359
chr2 124409823
chr2 124409992
chr2 124410320
chr2 124410374
chr2 124410642
chr2 124410681
chr2 124410717
chr2 124410977
chr2 124411056
chr2 124411577

chr2 124412359
chr2 124412411
chr2 125151454
chr2 125384800
chr2 125421315
chr2 125421716
chr2 125425194
chr2 125441765
chr2 125690182
chr2 125690224
chr2 125690519
chr2 125691039
chr2 125691846
chr2 125691912
chr2 125692474
chr2 125692651
chr2 126656594
chr2 126657048
chr2 126657050
chr2 126657206
chr2 126657226
chr2 126657244
chr2 126657251
chr2 126657261
chr2 126657291
chr2 126657307
chr2 126657332
chr2 126657716
chr2 126657722
chr2 126657806
chr2 126657846
chr2 126657877
chr2 126657902
chr2 126657989
chr2 126658006
chr2 126658014
chr2 126658066
chr2 126658068
chr2 126658135
chr2 126658168
chr2 126658307
chr2 126659170
chr2 126659252
chr2 126659263
chr2 126659304
chr2 126659314
chr2 126659353
chr2 127081497
chr2 127081685
chr2 127081695
chr2 127081816
chr2 127081836
chr2 127081873
chr2 127081897

chr2 127081922
chr2 127081926
chr2 127081935
chr2 127081956
chr2 127081963
chr2 127081966
chr2 127081977
chr2 127082020
chr2 127082050
chr2 127082077
chr2 127082098
chr2 127082133
chr2 127386711
chr2 127386722
chr2 127386807
chr2 127386827
chr2 127386864
chr2 127387011
chr2 127387015
chr2 127387053
chr2 127410685
chr2 127410919
chr2 127410980
chr2 127411222
chr2 127411411
chr2 127411445
chr2 127411487
chr2 127416243
chr2 127416267
chr2 127416314
chr2 127416324
chr2 127416329
chr2 127416335
chr2 127416376
chr2 127416440
chr2 127416475
chr2 127416499
chr2 127416502
chr2 127416572
chr2 127416578
chr2 127416581
chr2 127494766
chr2 127495642
chr2 127495667
chr2 127495783
chr2 127495826
chr2 127525827
chr2 127525835
chr2 127525959
chr2 127525962
chr2 127526003
chr2 127526014
chr2 127526017
chr2 127527061

chr2 127527113
chr2 127675100
chr2 127675104
chr2 127675108
chr2 127675116
chr2 127675121
chr2 127675123
chr2 127675136
chr2 127675157
chr2 127675162
chr2 127675166
chr2 127675168
chr2 127675316
chr2 127675319
chr2 127810134
chr2 127810309
chr2 127810322
chr2 127810330
chr2 127810333
chr2 127810336
chr2 127810352
chr2 127875139
chr2 127875189
chr2 127875191
chr2 127885320
chr2 127885325
chr2 127885344
chr2 127885352
chr2 127885355
chr2 127885374
chr2 127885379
chr2 127885381
chr2 127885398
chr2 127885401
chr2 127885405
chr2 127885417
chr2 127885420
chr2 127885425
chr2 127885428
chr2 127885433
chr2 127885454
chr2 127885457
chr2 127885460
chr2 127885482
chr2 127885486
chr2 127885527
chr2 127885534
chr2 127885541
chr2 127885543
chr2 127885548
chr2 127885555
chr2 127885558
chr2 127885561
chr2 127885565

chr2 127885575
chr2 127885588
chr2 127885603
chr2 127886732
chr2 127886736
chr2 127886746
chr2 127886767
chr2 127886773
chr2 127886777
chr2 127886779
chr2 127886787
chr2 127886795
chr2 127886802
chr2 127886826
chr2 127886832
chr2 127886859
chr2 127886893
chr2 128026500
chr2 128026509
chr2 128026718
chr2 128026750
chr2 128026770
chr2 128026799
chr2 128026818
chr2 128026897
chr2 128026926
chr2 128026934
chr2 128026939
chr2 128026943
chr2 128026959
chr2 128026961
chr2 128090729
chr2 128090736
chr2 128090754
chr2 128318296
chr2 128318351
chr2 128318371
chr2 128861156
chr2 128861731
chr2 128862227
chr2 128862294
chr2 128863056
chr2 129145818
chr2 129146337
chr2 129146555
chr2 129146979
chr2 129146999
chr2 129147263
chr2 129147281
chr2 129147866
chr2 129203153
chr2 129203521
chr2 129203579
chr2 129203633

chr2 129849659
chr2 129849702
chr2 129849713
chr2 129849915
chr2 129849941
chr2 129850128
chr2 129850406
chr2 129850447
chr2 129979458
chr2 129979472
chr2 129979579
chr2 129979633
chr2 129979643
chr2 129979650
chr2 129979661
chr2 129979663
chr2 129980043
chr2 129980050
chr2 129980065
chr2 129980070
chr2 129980091
chr2 129980094
chr2 129980121
chr2 129980123
chr2 129980127
chr2 129980136
chr2 129980144
chr2 129980394
chr2 129980480
chr2 129980567
chr2 129980617
chr2 129980661
chr2 129980721
chr2 129980840
chr2 129980870
chr2 129980913
chr2 129980963
chr2 129981078
chr2 130005854
chr2 130005911
chr2 130132929
chr2 130144162
chr2 130144170
chr2 130144181
chr2 130144208
chr2 130144225
chr2 130144232
chr2 130144240
chr2 130144264
chr2 130144284
chr2 130144329
chr2 130144340
chr2 130144360
chr2 130144374

chr2 130144376
chr2 130144381
chr2 130144385
chr2 130144390
chr2 130144418
chr2 130144425
chr2 130144428
chr2 130144430
chr2 130144434
chr2 130144498
chr2 130144548
chr2 130144558
chr2 130144567
chr2 130144569
chr2 130144602
chr2 130144612
chr2 130144621
chr2 130144647
chr2 130144682
chr2 130144688
chr2 130144721
chr2 130144736
chr2 130144739
chr2 130144758
chr2 130144771
chr2 130144802
chr2 130144851
chr2 130144859
chr2 130144864
chr2 130144880
chr2 130144889
chr2 130144906
chr2 130210211
chr2 130341579
chr2 130341798
chr2 130341800
chr2 130341804
chr2 130341808
chr2 130426248
chr2 130426259
chr2 130426365
chr2 130427233
chr2 130427264
chr2 130427295
chr2 130427301
chr2 130427311
chr2 130427333
chr2 130427661
chr2 130427669
chr2 130427671
chr2 130427676
chr2 130427689
chr2 130427704
chr2 130427711

chr2 130427727
chr2 130427735
chr2 130427756
chr2 130427760
chr2 130427953
chr2 130427964
chr2 130427967
chr2 130427980
chr2 130427997
chr2 130428012
chr2 130428014
chr2 130428103
chr2 130428125
chr2 130428127
chr2 130428133
chr2 130428165
chr2 130428188
chr2 130428209
chr2 130428213
chr2 130428219
chr2 130428225
chr2 130428257
chr2 130428611
chr2 130428613
chr2 130428615
chr2 130428633
chr2 130428638
chr2 130428646
chr2 130428660
chr2 130428668
chr2 130428684
chr2 130428697
chr2 130428713
chr2 130428718
chr2 130428722
chr2 130428733
chr2 130428744
chr2 130428836
chr2 130428843
chr2 130428849
chr2 130564131
chr2 130564135
chr2 130564167
chr2 130564375
chr2 130564412
chr2 130564432
chr2 130565194
chr2 130565257
chr2 130565292
chr2 130565304
chr2 130597798
chr2 130597810
chr2 130597825
chr2 130597828

chr2 130597838
chr2 130597849
chr2 130597851
chr2 130597871
chr2 130597887
chr2 130597895
chr2 130597899
chr2 130597920
chr2 130597938
chr2 130597947
chr2 130597950
chr2 130598250
chr2 130598262
chr2 130598274
chr2 130598286
chr2 130598289
chr2 130598307
chr2 130598309
chr2 130598432
chr2 130598593
chr2 130598608
chr2 130598613
chr2 130598629
chr2 130598648
chr2 130598656
chr2 130598670
chr2 130598679
chr2 130598693
chr2 130598696
chr2 130598714
chr2 130598739
chr2 130598749
chr2 130599201
chr2 130599391
chr2 130599408
chr2 130599457
chr2 130599849
chr2 130599867
chr2 130600535
chr2 130600855
chr2 130600944
chr2 130600990
chr2 130601007
chr2 130601014
chr2 130601046
chr2 130601066
chr2 130601112
chr2 130601123
chr2 130601157
chr2 130601168
chr2 130601173
chr2 130601213
chr2 130601217
chr2 130601221

chr2 130601737
chr2 130601781
chr2 130601784
chr2 130692933
chr2 130692942
chr2 130692977
chr2 130692988
chr2 130693001
chr2 130693014
chr2 130693018
chr2 130693030
chr2 130693063
chr2 130693068
chr2 130693074
chr2 130693088
chr2 130693108
chr2 130693153
chr2 130693157
chr2 130693165
chr2 130693169
chr2 130693202
chr2 130693206
chr2 130693213
chr2 130693257
chr2 130693289
chr2 130693295
chr2 130693301
chr2 130693326
chr2 130693349
chr2 130693381
chr2 130693387
chr2 130693389
chr2 130693411
chr2 130693485
chr2 130693487
chr2 130693502
chr2 130693519
chr2 130693532
chr2 130693535
chr2 130693640
chr2 130693673
chr2 130693679
chr2 130693682
chr2 130693684
chr2 130693693
chr2 130693708
chr2 130693714
chr2 130693719
chr2 130693739
chr2 130693743
chr2 130693764
chr2 130693772
chr2 130693788
chr2 130693795

chr2 130693810
chr2 130693823
chr2 130693828
chr2 130693830
chr2 130693838
chr2 130694166
chr2 130694266
chr2 130694293
chr2 130777217
chr2 130777247
chr2 131105829
chr2 131105841
chr2 131105846
chr2 131105849
chr2 131105863
chr2 131105866
chr2 131105870
chr2 131105978
chr2 131105980
chr2 131105985
chr2 131105990
chr2 131105996
chr2 131106113
chr2 131106142
chr2 131106147
chr2 131106155
chr2 131106177
chr2 131106318
chr2 131106412
chr2 131106437
chr2 131106530
chr2 131216290
chr2 131216998
chr2 131273542
chr2 131273973
chr2 131331103
chr2 131331112
chr2 131331115
chr2 131331119
chr2 131331121
chr2 131331127
chr2 131331136
chr2 131331139
chr2 131331158
chr2 131331161
chr2 131331164
chr2 131331167
chr2 131331170
chr2 131331174
chr2 131331183
chr2 131331185
chr2 131331187
chr2 131331193
chr2 131331202

chr2 131331213
chr2 131331228
chr2 131331241
chr2 131331247
chr2 131331249
chr2 131331251
chr2 131331269
chr2 131331289
chr2 131331294
chr2 131331302
chr2 131331318
chr2 131331331
chr2 131331333
chr2 131331339
chr2 131331357
chr2 131331367
chr2 131331370
chr2 131331374
chr2 131331379
chr2 131331390
chr2 131331567
chr2 131353385
chr2 131353393
chr2 131353402
chr2 131353404
chr2 131362683
chr2 131362696
chr2 131362879
chr2 131362922
chr2 131362965
chr2 131362995
chr2 131363218
chr2 131363268
chr2 131363355
chr2 131363441
chr2 131363651
chr2 131363691
chr2 131363699
chr2 131363708
chr2 131363712
chr2 131363714
chr2 131363741
chr2 131363744
chr2 131363765
chr2 131363770
chr2 131363785
chr2 131363792
chr2 131363801
chr2 131363805
chr2 131363813
chr2 131363817
chr2 131363820
chr2 131363831
chr2 131363846

chr2 131363859
chr2 131363897
chr2 131363900
chr2 131363903
chr2 131363910
chr2 131363922
chr2 131363929
chr2 131363933
chr2 131363940
chr2 131363942
chr2 131363963
chr2 131363969
chr2 131364012
chr2 131364031
chr2 131364046
chr2 131364057
chr2 131364067
chr2 131364073
chr2 131364079
chr2 131364085
chr2 131364103
chr2 131364105
chr2 131364107
chr2 131364110
chr2 131364115
chr2 131364128
chr2 131364131
chr2 131364134
chr2 131364137
chr2 131364171
chr2 131364173
chr2 131364178
chr2 131364188
chr2 131364242
chr2 131364349
chr2 131413376
chr2 131413396
chr2 131413470
chr2 131413475
chr2 131491626
chr2 131491663
chr2 131491672
chr2 131491689
chr2 131491710
chr2 131491732
chr2 131491763
chr2 131491767
chr2 131491796
chr2 131491799
chr2 131491802
chr2 131491810
chr2 131491812
chr2 131491827
chr2 131491831

chr2 131491840
chr2 131527836
chr2 131527846
chr2 131527852
chr2 131527859
chr2 131527864
chr2 131527866
chr2 131527873
chr2 131527876
chr2 131527880
chr2 131527895
chr2 131527900
chr2 131527913
chr2 131527930
chr2 131527946
chr2 131528060
chr2 131528077
chr2 131528086
chr2 131528102
chr2 131528107
chr2 131528115
chr2 131528164
chr2 131528195
chr2 131528208
chr2 131528227
chr2 131528230
chr2 131528232
chr2 131528345
chr2 131528354
chr2 131528364
chr2 131528397
chr2 131528399
chr2 131528408
chr2 131528418
chr2 131528437
chr2 131528468
chr2 131528532
chr2 131528536
chr2 131528592
chr2 131682779
chr2 131682784
chr2 131682786
chr2 131682803
chr2 131682822
chr2 131682830
chr2 131682832
chr2 131682848
chr2 131682920
chr2 131683053
chr2 131683109
chr2 131683147
chr2 131683155
chr2 131683159
chr2 131683169

chr2 131683228
chr2 131683231
chr2 131683281
chr2 131728205
chr2 131728248
chr2 131802251
chr2 131802312
chr2 131802335
chr2 131802356
chr2 131802361
chr2 131802367
chr2 131802380
chr2 131802412
chr2 131802416
chr2 131802441
chr2 131802477
chr2 131802489
chr2 131802536
chr2 131802548
chr2 131802599
chr2 131802602
chr2 131802618
chr2 131802624
chr2 131802630
chr2 131802638
chr2 131802640
chr2 131802646
chr2 131802649
chr2 131802652
chr2 131802668
chr2 131802674
chr2 131802682
chr2 131811026
chr2 131814030
chr2 131814177
chr2 131852369
chr2 131853112
chr2 131853201
chr2 131853487
chr2 131893261
chr2 131964168
chr2 132009692
chr2 132009772
chr2 132009791
chr2 132009808
chr2 132009810
chr2 132009813
chr2 132009816
chr2 132009832
chr2 132009857
chr2 132009859
chr2 132009862
chr2 132009865
chr2 132009879

chr2 132009881
chr2 132009884
chr2 132010303
chr2 132010317
chr2 132010319
chr2 132010322
chr2 132010341
chr2 132010344
chr2 132010355
chr2 132010360
chr2 132010363
chr2 132010444
chr2 132038548
chr2 132038550
chr2 132038553
chr2 132038564
chr2 132038587
chr2 132038593
chr2 132038646
chr2 132038648
chr2 132038651
chr2 132038654
chr2 132038670
chr2 132038673
chr2 132038686
chr2 132038689
chr2 132038692
chr2 132038695
chr2 132038699
chr2 132038701
chr2 132038709
chr2 132038719
chr2 132038721
chr2 132038724
chr2 132038727
chr2 132038733
chr2 132038741
chr2 132038753
chr2 132038756
chr2 132038759
chr2 132038773
chr2 132038775
chr2 132038778
chr2 132038782
chr2 132038787
chr2 132038795
chr2 132038805
chr2 132038807
chr2 132038810
chr2 132039189
chr2 132039469
chr2 132039485
chr2 132039771
chr2 132122270

chr2 132267251
chr2 132267511
chr2 132267544
chr2 132267556
chr2 132267562
chr2 132267569
chr2 132267621
chr2 132267627
chr2 132300795
chr2 132300799
chr2 132336195
chr2 132336229
chr2 132346928
chr2 132346960
chr2 132353881
chr2 132353959
chr2 132384285
chr2 132384320
chr2 132384561
chr2 133267143
chr2 133267186
chr2 133267268
chr2 133267290
chr2 133267327
chr2 133267511
chr2 133267515
chr2 133267608
chr2 133267648
chr2 133544288
chr2 133544554
chr2 133544564
chr2 133779935
chr2 134104955
chr2 134127811
chr2 134127899
chr2 134245557
chr2 134677040
chr2 134677125
chr2 134677128
chr2 134677143
chr2 134677153
chr2 134677162
chr2 134677173
chr2 134677214
chr2 134677222
chr2 134677256
chr2 134677279
chr2 134918163
chr2 134920234
chr2 134920385
chr2 134920400
chr2 134920441
chr2 134920467
chr2 134920593

chr2 134920618
chr2 134989228
chr2 135004277
chr2 135004292
chr2 135004418
chr2 135052123
chr2 135052148
chr2 135052174
chr2 135052746
chr2 135052785
chr2 135052800
chr2 135052815
chr2 135052820
chr2 135052833
chr2 135052848
chr2 135052876
chr2 135052888
chr2 135052894
chr2 135053030
chr2 135053039
chr2 135053052
chr2 135053070
chr2 135053076
chr2 135053103
chr2 135053114
chr2 135053138
chr2 135053140
chr2 135053148
chr2 135053173
chr2 135053221
chr2 135053245
chr2 135053255
chr2 135053459
chr2 135053524
chr2 135053578
chr2 135530530
chr2 135530536
chr2 135530539
chr2 135530557
chr2 135530573
chr2 135530683
chr2 135531433
chr2 135531446
chr2 135531579
chr2 135531582
chr2 135531585
chr2 135531590
chr2 135531593
chr2 135531595
chr2 135531598
chr2 135531600
chr2 135531609
chr2 135531629
chr2 135531631

chr2 135531657
chr2 135531669
chr2 135531678
chr2 135531680
chr2 135531698
chr2 135531702
chr2 135531709
chr2 135531715
chr2 135531721
chr2 135531775
chr2 135531778
chr2 135531919
chr2 135531929
chr2 135531956
chr2 135531966
chr2 135532081
chr2 135532097
chr2 135532108
chr2 135532111
chr2 135532119
chr2 135532129
chr2 135532133
chr2 135532140
chr2 135532144
chr2 135532460
chr2 135590004
chr2 135590893
chr2 135591196
chr2 135742897
chr2 135742909
chr2 135743014
chr2 135743106
chr2 135743126
chr2 135743169
chr2 135743230
chr2 135743339
chr2 135983882
chr2 135984474
chr2 135984716
chr2 135984792
chr2 135984823
chr2 136118941
chr2 136118977
chr2 136118994
chr2 136119001
chr2 136119008
chr2 136119022
chr2 136131741
chr2 136131839
chr2 136131918
chr2 136131991
chr2 136132029
chr2 136132065
chr2 136132138

chr2 136132154
chr2 136132173
chr2 136132242
chr2 136132275
chr2 136132284
chr2 136132323
chr2 136132412
chr2 136132474
chr2 136132489
chr2 136132508
chr2 136136109
chr2 136136259
chr2 136136294
chr2 136136312
chr2 136136350
chr2 136136760
chr2 136136838
chr2 136238571
chr2 136238785
chr2 136238789
chr2 136238810
chr2 136238825
chr2 136239061
chr2 136281504
chr2 136327229
chr2 136327286
chr2 136327326
chr2 136327332
chr2 136327385
chr2 136327395
chr2 136327408
chr2 136327496
chr2 136327512
chr2 136327514
chr2 136327574
chr2 136423681
chr2 136423685
chr2 136423691
chr2 136423696
chr2 136423704
chr2 136423710
chr2 136423728
chr2 136423730
chr2 136423782
chr2 136423839
chr2 136423848
chr2 136423852
chr2 136423885
chr2 136423899
chr2 136423907
chr2 136424071
chr2 136424198
chr2 136424215
chr2 136424320

chr2 136424344
chr2 136636692
chr2 136637472
chr2 136637620
chr2 136637887
chr2 136764002
chr2 136764294
chr2 136764373
chr2 136764488
chr2 136764516
chr2 136764526
chr2 136764535
chr2 137056056
chr2 137056147
chr2 137218534
chr2 137218861
chr2 137447625
chr2 137969834
chr2 138074001
chr2 138502526
chr2 138502528
chr2 138502533
chr2 138502581
chr2 138502619
chr2 138502626
chr2 138502644
chr2 138502725
chr2 138502727
chr2 138502729
chr2 138502734
chr2 138502918
chr2 138503123
chr2 138503296
chr2 138503860
chr2 138503903
chr2 138504003
chr2 138515755
chr2 138516207
chr2 138516252
chr2 138516292
chr2 138778942
chr2 138778960
chr2 138898610
chr2 138899594
chr2 138899615
chr2 138903333
chr2 138903373
chr2 138903567
chr2 138903846
chr2 138904080
chr2 139409227
chr2 140637927
chr2 140638058
chr2 140685613

chr2 140685949
chr2 140685984
chr2 140961374
chr2 141143585
chr2 141207107
chr2 141207251
chr2 141287677
chr2 141287809
chr2 141332297
chr2 141332689
chr2 141419689
chr2 142126152
chr2 142126203
chr2 142126377
chr2 142126451
chr2 142126505
chr2 142131082
chr2 142131103
chr2 142131106
chr2 142131174
chr2 142131181
chr2 142439977
chr2 142440467
chr2 142511229
chr2 142511246
chr2 142511343
chr2 142511373
chr2 142809844
chr2 142810170
chr2 143128715
chr2 143128757
chr2 143128760
chr2 143128803
chr2 143128806
chr2 143129313
chr2 143129371
chr2 143129394
chr2 143129458
chr2 143129515
chr2 143129570
chr2 143130864
chr2 143131375
chr2 143802417
chr2 143802435
chr2 143802466
chr2 143802520
chr2 143802779
chr2 143929636
chr2 144183664
chr2 144183759
chr2 144183900
chr2 144183905
chr2 144183975
chr2 144183985

chr2 144183995
chr2 144184003
chr2 144184007
chr2 144184012
chr2 144184430
chr2 144233506
chr2 144233629
chr2 144233783
chr2 144234204
chr2 144234237
chr2 144331087
chr2 144331188
chr2 144331220
chr2 144331304
chr2 144358510
chr2 144358571
chr2 144490417
chr2 144515594
chr2 144515679
chr2 144515684
chr2 144518262
chr2 144518279
chr2 144518301
chr2 144518310
chr2 144518359
chr2 144518396
chr2 144518424
chr2 144518505
chr2 144518524
chr2 144518833
chr2 144519041
chr2 144519083
chr2 144519264
chr2 144519370
chr2 144519419
chr2 144519465
chr2 144519559
chr2 144519749
chr2 144519814
chr2 144519885
chr2 144519942
chr2 144519999
chr2 144520001
chr2 144520029
chr2 144520044
chr2 144520067
chr2 144520079
chr2 144520092
chr2 144520129
chr2 144520159
chr2 144520167
chr2 144520174
chr2 144520223
chr2 144520240

chr2 144520242
chr2 144520276
chr2 144520285
chr2 144520304
chr2 144520310
chr2 144574706
chr2 145187249
chr2 145187426
chr2 146222086
chr2 146222131
chr2 146388955
chr2 146389020
chr2 146389307
chr2 146389496
chr2 146409373
chr2 147500095
chr2 147500226
chr2 147500272
chr2 147500298
chr2 147500523
chr2 147845501
chr2 147845524
chr2 147845618
chr2 147845637
chr2 147845649
chr2 147845734
chr2 147845752
chr2 148020098
chr2 148020480
chr2 148020565
chr2 148020578
chr2 148509361
chr2 148509374
chr2 148509444
chr2 148509507
chr2 148509521
chr2 148509547
chr2 148509554
chr2 148509559
chr2 148509599
chr2 148509657
chr2 148509718
chr2 148509744
chr2 148509764
chr2 148509836
chr2 148509941
chr2 148509943
chr2 148509965
chr2 148510165
chr2 148510368
chr2 148510372
chr2 148603521
chr2 148603546
chr2 148603578

chr2 148603610
chr2 148603630
chr2 148604088
chr2 148643428
chr2 148643472
chr2 148643668
chr2 148643748
chr2 148643793
chr2 148643879
chr2 148643923
chr2 148644027
chr2 148644103
chr2 148722145
chr2 148729340
chr2 148874720
chr2 148874998
chr2 148875024
chr2 149586592
chr2 149586824
chr2 149586843
chr2 149587022
chr2 149587049
chr2 149587786
chr2 149587790
chr2 149587792
chr2 149587795
chr2 149587800
chr2 150487557
chr2 150487559
chr2 150487562
chr2 150487568
chr2 150634885
chr2 151238944
chr2 151238957
chr2 151239156
chr2 151239227
chr2 151260767
chr2 151260821
chr2 151260849
chr2 151260857
chr2 151261031
chr2 151261034
chr2 151261043
chr2 151261063
chr2 151261083
chr2 151261136
chr2 151261258
chr2 151289659
chr2 151374107
chr2 151374296
chr2 151374590
chr2 151374629
chr2 151379234
chr2 151379356

chr2 151379692
chr2 151379738
chr2 151410836
chr2 151410978
chr2 151411048
chr2 151411261
chr2 151411318
chr2 151701259
chr2 151701528
chr2 151701608
chr2 151701650
chr2 151702171
chr2 151705689
chr2 151827485
chr2 151827608
chr2 151827679
chr2 152025339
chr2 152025353
chr2 152025380
chr2 152025445
chr2 152025731
chr2 152025762
chr2 152025798
chr2 152025909
chr2 152025939
chr2 152025963
chr2 152175156
chr2 152176347
chr2 152176358
chr2 152176370
chr2 152176380
chr2 152263679
chr2 152469020
chr2 152573369
chr2 152573432
chr2 152573455
chr2 152573534
chr2 152573620
chr2 152694393
chr2 152694542
chr2 152694605
chr2 152694773
chr2 152716067
chr2 152716212
chr2 152716278
chr2 152716300
chr2 152716315
chr2 152716352
chr2 152716583
chr2 152716722
chr2 152716760
chr2 152716791
chr2 152716878
chr2 152716881

chr2 152716908
chr2 152716961
chr2 152716980
chr2 152716989
chr2 152717014
chr2 152998300
chr2 153286958
chr2 153287072
chr2 153287163
chr2 153801878
chr2 153802061
chr2 153802403
chr2 153805052
chr2 153811533
chr2 154238804
chr2 154239222
chr2 154239274
chr2 154301469
chr2 154301718
chr2 154301874
chr2 154302029
chr2 154375741
chr2 154375771
chr2 154375789
chr2 154375906
chr2 154375977
chr2 154375995
chr2 154376353
chr2 154376767
chr2 154376871
chr2 154575024
chr2 154696917
chr2 154714334
chr2 154714419
chr2 154847879
chr2 155579081
chr2 155965185
chr2 156008179
chr2 156008579
chr2 156008810
chr2 156331807
chr2 156331903
chr2 156331917
chr2 156331978
chr2 156332010
chr2 156332020
chr2 156332022
chr2 156332024
chr2 156332035
chr2 156332037
chr2 156334818
chr2 156334841
chr2 156334862
chr2 156334864

chr2 156334880
chr2 156335220
chr2 156335226
chr2 156335281
chr2 156335471
chr2 156335495
chr2 156335549
chr2 156335616
chr2 156335661
chr2 156335678
chr2 156335732
chr2 156336250
chr2 156336385
chr2 156336391
chr2 156336924
chr2 156340612
chr2 156340802
chr2 156340918
chr2 156341094
chr2 156341121
chr2 156343088
chr2 156343124
chr2 156343148
chr2 156343212
chr2 156401287
chr2 156436373
chr2 156436378
chr2 156436391
chr2 156436393
chr2 156436395
chr2 156436400
chr2 156436413
chr2 156436418
chr2 156436443
chr2 156436481
chr2 156436492
chr2 156436494
chr2 156436496
chr2 156436901
chr2 156436909
chr2 156437356
chr2 156437364
chr2 156437403
chr2 156489350
chr2 156489750
chr2 156489764
chr2 156489787
chr2 156892568
chr2 156892629
chr2 156892799
chr2 156892841
chr2 156892977
chr2 156892983
chr2 157207082

chr2 157207147
chr2 157327680
chr2 157327801
chr2 157404282
chr2 157404474
chr2 157404516
chr2 157405320
chr2 157413519
chr2 157413535
chr2 157413559
chr2 157413667
chr2 157414253
chr2 157417032
chr2 157417138
chr2 157417875
chr2 157417947
chr2 157418032
chr2 157418183
chr2 157418280
chr2 157418312
chr2 157418322
chr2 157436126
chr2 157436268
chr2 157436277
chr2 157437395
chr2 157437529
chr2 157437614
chr2 157437624
chr2 157437628
chr2 157437631
chr2 157437677
chr2 157438006
chr2 157438055
chr2 157438617
chr2 157438746
chr2 157438896
chr2 157438940
chr2 157439206
chr2 157439276
chr2 157439293
chr2 157439300
chr2 157439332
chr2 157439350
chr2 157439378
chr2 157439485
chr2 157439499
chr2 157439590
chr2 157440091
chr2 157440255
chr2 157440313
chr2 157440324
chr2 157440333
chr2 157440346
chr2 157440544

chr2 157440566
chr2 157440575
chr2 157440823
chr2 157440918
chr2 157444299
chr2 157444448
chr2 157444493
chr2 157444512
chr2 157444730
chr2 157444740
chr2 157444986
chr2 157450986
chr2 157451042
chr2 157451142
chr2 157465004
chr2 157627647
chr2 157627791
chr2 157627876
chr2 157627948
chr2 157627990
chr2 157627997
chr2 157628010
chr2 157628044
chr2 157628066
chr2 157628085
chr2 157877110
chr2 158455878
chr2 158456087
chr2 158456183
chr2 158456207
chr2 158456215
chr2 158456292
chr2 158456371
chr2 158456387
chr2 158456479
chr2 158456495
chr2 158456513
chr2 158456516
chr2 158456558
chr2 158681698
chr2 158901248
chr2 158970255
chr2 159226346
chr2 159226449
chr2 159227616
chr2 159340189
chr2 159340804
chr2 159409344
chr2 159409513
chr2 159409953
chr2 159410061
chr2 159460442
chr2 159461132
chr2 159494589

chr2 159515708
chr2 159516320
chr2 159516357
chr2 159516604
chr2 159516699
chr2 159516711
chr2 159516753
chr2 159615081
chr2 159615240
chr2 159615267
chr2 159615269
chr2 159615279
chr2 159615301
chr2 159616065
chr2 159616078
chr2 159616081
chr2 159616088
chr2 159616096
chr2 159616099
chr2 159616104
chr2 159616108
chr2 159616116
chr2 159616127
chr2 159616137
chr2 159616145
chr2 159616149
chr2 159616160
chr2 159616175
chr2 159616209
chr2 159616220
chr2 159616224
chr2 159616251
chr2 159616265
chr2 159616401
chr2 159616413
chr2 159616429
chr2 159616436
chr2 159616528
chr2 159711847
chr2 159711856
chr2 159711877
chr2 159712068
chr2 159712167
chr2 159712175
chr2 159712178
chr2 159712182
chr2 159713478
chr2 159713591
chr2 159713820
chr2 159714038
chr2 159714080
chr2 159714132
chr2 159714183
chr2 159903563

chr2 159903570
chr2 159903685
chr2 159903701
chr2 159903771
chr2 159903802
chr2 159903885
chr2 159903916
chr2 159903931
chr2 159903956
chr2 159904009
chr2 159904054
chr2 159904094
chr2 159904099
chr2 159904105
chr2 159904117
chr2 159904124
chr2 159904140
chr2 160220539
chr2 160394268
chr2 160411393
chr2 160411956
chr2 160412326
chr2 160428061
chr2 160428254
chr2 160428298
chr2 160428308
chr2 160428344
chr2 160428457
chr2 160428606
chr2 160428631
chr2 160428743
chr2 160428845
chr2 160428877
chr2 160429475
chr2 160482824
chr2 160482898
chr2 160485372
chr2 160485470
chr2 160485513
chr2 160485589
chr2 160493068
chr2 160493070
chr2 160493085
chr2 160493092
chr2 160493099
chr2 160493114
chr2 160493117
chr2 160493124
chr2 160493126
chr2 160493129
chr2 160493133
chr2 160757996
chr2 160758059
chr2 160759664

chr2 160759790
chr2 160769074
chr2 160769391
chr2 160769541
chr2 160970203
chr2 160970311
chr2 160970754
chr2 160971046
chr2 160994648
chr2 160994684
chr2 160994765
chr2 160994818
chr2 160994830
chr2 160994833
chr2 160994989
chr2 161063818
chr2 161063906
chr2 161064060
chr2 161064261
chr2 161064370
chr2 161077658
chr2 161077725
chr2 161077828
chr2 161078201
chr2 161078241
chr2 161078248
chr2 161090605
chr2 161090665
chr2 161090672
chr2 161090674
chr2 161090682
chr2 161139652
chr2 161139824
chr2 161139874
chr2 161159730
chr2 161159948
chr2 161159968
chr2 161160037
chr2 161160132
chr2 161160143
chr2 161160208
chr2 161160269
chr2 161160276
chr2 161160281
chr2 161160291
chr2 161160653
chr2 161160665
chr2 161160667
chr2 161160680
chr2 161160685
chr2 161160708
chr2 161160714
chr2 161160716
chr2 161160733

chr2 161160736
chr2 161160751
chr2 161160770
chr2 161160775
chr2 161160788
chr2 161160813
chr2 161160819
chr2 161160852
chr2 161160863
chr2 161160898
chr2 161160912
chr2 161161407
chr2 161161627
chr2 161161705
chr2 161161767
chr2 161163000
chr2 161163173
chr2 161167211
chr2 161200258
chr2 161238095
chr2 161238107
chr2 161238120
chr2 161238185
chr2 161238433
chr2 161238435
chr2 161238448
chr2 161238451
chr2 161238460
chr2 161238475
chr2 161238530
chr2 161238551
chr2 161238584
chr2 161238642
chr2 161238649
chr2 161238653
chr2 161238670
chr2 161238706
chr2 161238710
chr2 161238721
chr2 161238735
chr2 161238737
chr2 161238770
chr2 161238774
chr2 161238776
chr2 161238778
chr2 161238782
chr2 161238811
chr2 161238822
chr2 161238824
chr2 161244531
chr2 161244535
chr2 161244552
chr2 161244563
chr2 161244572

chr2 161244587
chr2 161244612
chr2 161244626
chr2 161244682
chr2 161244690
chr2 161244692
chr2 161244696
chr2 161244748
chr2 161244751
chr2 161244766
chr2 161244776
chr2 161244827
chr2 161244834
chr2 161244868
chr2 161244929
chr2 161244934
chr2 161244945
chr2 161244949
chr2 161244995
chr2 161244998
chr2 161245001
chr2 161245014
chr2 161245021
chr2 161245026
chr2 161245035
chr2 161245037
chr2 161245039
chr2 161245064
chr2 161245067
chr2 161245075
chr2 161245084
chr2 161245097
chr2 161245101
chr2 161245107
chr2 161245117
chr2 161245127
chr2 161245134
chr2 161245138
chr2 161245168
chr2 161245172
chr2 161245174
chr2 161245178
chr2 161245180
chr2 161245183
chr2 161245190
chr2 161245199
chr2 161245203
chr2 161245322
chr2 161245326
chr2 161245348
chr2 161245386
chr2 161245432
chr2 161308686
chr2 161308736

chr2 161308755
chr2 161308797
chr2 161308828
chr2 161308918
chr2 161308948
chr2 161308994
chr2 161419235
chr2 161419263
chr2 161419266
chr2 161419277
chr2 161419414
chr2 161425087
chr2 161425164
chr2 161425695
chr2 161425751
chr2 161425803
chr2 161425819
chr2 161425828
chr2 161425865
chr2 161858162
chr2 161979443
chr2 161979505
chr2 161992627
chr2 161992780
chr2 161992805
chr2 162123763
chr2 162123903
chr2 162123949
chr2 162301278
chr2 162316877
chr2 162317229
chr2 162317353
chr2 162317628
chr2 162317790
chr2 162317856
chr2 162317908
chr2 162318381
chr2 162318386
chr2 162318393
chr2 162318422
chr2 162318442
chr2 162318468
chr2 162318471
chr2 162318473
chr2 162318475
chr2 162318487
chr2 162318509
chr2 162318524
chr2 162318529
chr2 162318532
chr2 162318534
chr2 162318536
chr2 162318542
chr2 162318544

chr2 162318557
chr2 162318568
chr2 162318570
chr2 162318587
chr2 162318594
chr2 162318606
chr2 162318611
chr2 162318890
chr2 162318893
chr2 162319022
chr2 162319026
chr2 162319046
chr2 162319090
chr2 162319233
chr2 162344072
chr2 162727484
chr2 162727513
chr2 162742246
chr2 162837864
chr2 162840534
chr2 162840541
chr2 162840649
chr2 162840684
chr2 162915053
chr2 162915373
chr2 163065346
chr2 163065415
chr2 163098565
chr2 163154053
chr2 163345430
chr2 163345507
chr2 163345861
chr2 163346021
chr2 163346057
chr2 163346078
chr2 163346104
chr2 163346209
chr2 163794273
chr2 163794424
chr2 163852716
chr2 163852790
chr2 163852911
chr2 163853192
chr2 163853469
chr2 163906242
chr2 163906377
chr2 163906593
chr2 164045361
chr2 164532181
chr2 164532679
chr2 164761594
chr2 164761685
chr2 164761716
chr2 164762043

chr2 164834998
chr2 164840317
chr2 164840586
chr2 164840641
chr2 164840643
chr2 164840650
chr2 164840654
chr2 164840660
chr2 164840673
chr2 164840701
chr2 164840712
chr2 164890541
chr2 165015572
chr2 165015948
chr2 165016396
chr2 165326885
chr2 165472258
chr2 165610461
chr2 165610642
chr2 165610727
chr2 165610770
chr2 165610840
chr2 165610903
chr2 165611020
chr2 165611357
chr2 165611834
chr2 165611894
chr2 165611967
chr2 165612150
chr2 165612558
chr2 165767015
chr2 165767388
chr2 165767483
chr2 165795061
chr2 165795308
chr2 165953071
chr2 165953112
chr2 165953179
chr2 165953268
chr2 165953299
chr2 165953358
chr2 165953364
chr2 165953370
chr2 165984812
chr2 166181882
chr2 166182079
chr2 166182093
chr2 166182173
chr2 166374436
chr2 166374908
chr2 166375024
chr2 166375986
chr2 166375994
chr2 166376006

chr2 166376009
chr2 166376035
chr2 166376037
chr2 166376041
chr2 166376089
chr2 166376093
chr2 166376096
chr2 166376103
chr2 166376121
chr2 166376123
chr2 166376132
chr2 166376135
chr2 166376156
chr2 166376169
chr2 166376186
chr2 166376200
chr2 166376220
chr2 166449578
chr2 166551576
chr2 166552169
chr2 166552271
chr2 167212239
chr2 167212276
chr2 167212303
chr2 167291909
chr2 167292182
chr2 167292338
chr2 167292362
chr2 167292381
chr2 167292409
chr2 167292420
chr2 167292450
chr2 167292477
chr2 167292524
chr2 167292530
chr2 167292548
chr2 167292550
chr2 167292566
chr2 167292569
chr2 167292587
chr2 167292598
chr2 167292600
chr2 167292603
chr2 167292607
chr2 167292613
chr2 167292620
chr2 167292622
chr2 167292644
chr2 167294897
chr2 167294962
chr2 167295021
chr2 167295101
chr2 167295630
chr2 167315702

chr2 167316225
chr2 167541498
chr2 167596220
chr2 168136587
chr2 168194805
chr2 168195142
chr2 168195165
chr2 168195225
chr2 168195314
chr2 168195350
chr2 168210542
chr2 168210622
chr2 168210680
chr2 168210747
chr2 168210799
chr2 168210811
chr2 168210835
chr2 168346729
chr2 168346853
chr2 168346876
chr2 168346878
chr2 168346881
chr2 168346891
chr2 168457355
chr2 168457404
chr2 168457463
chr2 168457469
chr2 168457472
chr2 168457474
chr2 168490586
chr2 168490720
chr2 168490807
chr2 168490869
chr2 168490922
chr2 168491024
chr2 168491115
chr2 168890094
chr2 168890097
chr2 168890150
chr2 168890161
chr2 168890210
chr2 168890223
chr2 168890226
chr2 168890229
chr2 168890246
chr2 168890273
chr2 168890299
chr2 168890302
chr2 168890321
chr2 168890340
chr2 168890343
chr2 168890363
chr2 168890365
chr2 168890717

chr2 169107735
chr2 169107798
chr2 169108331
chr2 169363579
chr2 169363610
chr2 169363619
chr2 169363623
chr2 169363638
chr2 169363696
chr2 169363732
chr2 169363779
chr2 169363781
chr2 169363841
chr2 169363850
chr2 169363855
chr2 169363878
chr2 169363882
chr2 169363888
chr2 169364776
chr2 169364805
chr2 169364862
chr2 169364892
chr2 169364939
chr2 169364958
chr2 169365030
chr2 169365035
chr2 169365061
chr2 169442791
chr2 169480029
chr2 169572956
chr2 169573138
chr2 169573176
chr2 169573210
chr2 169573213
chr2 169573215
chr2 169573388
chr2 169573476
chr2 169573479
chr2 169573544
chr2 169573568
chr2 169573581
chr2 169573612
chr2 169573618
chr2 169573645
chr2 169573661
chr2 169584818
chr2 169584824
chr2 169584830
chr2 169584850
chr2 169584973
chr2 169585026
chr2 169585057
chr2 169585128
chr2 169585130

chr2 169585141
chr2 169585148
chr2 169585166
chr2 169585218
chr2 169585284
chr2 169693420
chr2 169693524
chr2 169693602
chr2 169693711
chr2 169693913
chr2 169693917
chr2 169693958
chr2 169694059
chr2 169694095
chr2 169694142
chr2 169798444
chr2 169800028
chr2 169802439
chr2 170409239
chr2 170409267
chr2 170409299
chr2 170409329
chr2 170409332
chr2 170409337
chr2 170409449
chr2 170718718
chr2 170718930
chr2 170719075
chr2 170770648
chr2 170770690
chr2 170770716
chr2 170770719
chr2 170770735
chr2 170770740
chr2 170770742
chr2 170770746
chr2 170770750
chr2 170770758
chr2 170770770
chr2 170770781
chr2 170770784
chr2 170770787
chr2 170770789
chr2 170770824
chr2 170770828
chr2 170770890
chr2 170770894
chr2 170770899
chr2 170770904
chr2 170770906
chr2 170770916
chr2 170770923
chr2 170770934
chr2 170770953

chr2 170770963
chr2 170770980
chr2 170770986
chr2 170770992
chr2 170771004
chr2 170771007
chr2 170771010
chr2 170930184
chr2 170930211
chr2 170930236
chr2 170930285
chr2 170930289
chr2 170930366
chr2 170930371
chr2 170930378
chr2 170930434
chr2 170930577
chr2 170930585
chr2 170930665
chr2 170930694
chr2 171069756
chr2 171069791
chr2 171070025
chr2 171158863
chr2 171158889
chr2 171158906
chr2 171158941
chr2 171159101
chr2 171159128
chr2 171159133
chr2 171159149
chr2 171159183
chr2 171159223
chr2 171159229
chr2 171159251
chr2 171159264
chr2 171159275
chr2 171159301
chr2 171161519
chr2 171161679
chr2 171161736
chr2 171161746
chr2 171161954
chr2 171163469
chr2 171163554
chr2 171163886
chr2 171164017
chr2 171248870
chr2 171248902
chr2 171248910
chr2 171260363
chr2 171260442
chr2 171260528
chr2 171260538

chr2 171260717
chr2 171260909
chr2 171319268
chr2 171319337
chr2 171319542
chr2 171319564
chr2 171319581
chr2 171319597
chr2 171433443
chr2 171433457
chr2 171433488
chr2 171433593
chr2 171433596
chr2 171433613
chr2 171573715
chr2 171574033
chr2 171574162
chr2 171574201
chr2 171574213
chr2 171574216
chr2 171574233
chr2 171574291
chr2 171574302
chr2 171574306
chr2 171574318
chr2 171687392
chr2 171687395
chr2 171687402
chr2 171687420
chr2 171687429
chr2 171687432
chr2 171687436
chr2 171687455
chr2 171687474
chr2 171687478
chr2 171687495
chr2 171687501
chr2 171687534
chr2 171687559
chr2 171687608
chr2 171687622
chr2 171687636
chr2 171687655
chr2 171687687
chr2 171687693
chr2 171687725
chr2 171687728
chr2 171687737
chr2 171687740
chr2 171687771
chr2 171687773
chr2 171687813
chr2 171687820
chr2 171687826

chr2 171687840
chr2 171687919
chr2 171687922
chr2 171687929
chr2 171687936
chr2 171687963
chr2 171687967
chr2 171687991
chr2 171688007
chr2 171688020
chr2 171688025
chr2 171688028
chr2 171688055
chr2 171688069
chr2 171688095
chr2 171688098
chr2 171688115
chr2 171688119
chr2 171688263
chr2 171688269
chr2 171688321
chr2 171688332
chr2 171688427
chr2 171688540
chr2 171706069
chr2 171893688
chr2 171893690
chr2 171893766
chr2 171893788
chr2 171893836
chr2 171894067
chr2 171894077
chr2 171894151
chr2 171894223
chr2 171894230
chr2 171894236
chr2 171894240
chr2 171894248
chr2 171894258
chr2 171894271
chr2 171894273
chr2 171894275
chr2 171894277
chr2 171894279
chr2 171894282
chr2 171894290
chr2 171894294
chr2 171894296
chr2 171894300
chr2 171894312
chr2 171894315
chr2 171894318
chr2 171894321
chr2 171894323

chr2 171894325
chr2 171894327
chr2 171894347
chr2 171894352
chr2 171894354
chr2 171894357
chr2 171894359
chr2 171894363
chr2 171894375
chr2 172011863
chr2 172099304
chr2 172099311
chr2 172099313
chr2 172099319
chr2 172099327
chr2 172099341
chr2 172099350
chr2 172099354
chr2 172099366
chr2 172099378
chr2 172099396
chr2 172099406
chr2 172099430
chr2 172099564
chr2 172099861
chr2 172103412
chr2 172103423
chr2 172103439
chr2 172103478
chr2 172103571
chr2 172103576
chr2 172103578
chr2 172103600
chr2 172103635
chr2 172103651
chr2 172103686
chr2 172103697
chr2 172104053
chr2 172104060
chr2 172104080
chr2 172104088
chr2 172235329
chr2 172235335
chr2 172235592
chr2 172235607
chr2 172235639
chr2 172235645
chr2 172462198
chr2 172462202
chr2 173149723
chr2 173149732
chr2 173149734
chr2 173149755
chr2 173210718

chr2 173210748
chr2 173210755
chr2 173210772
chr2 173210779
chr2 173210829
chr2 173288773
chr2 173288779
chr2 173353916
chr2 173353935
chr2 173354022
chr2 173354038
chr2 173354056
chr2 173354101
chr2 173354105
chr2 173354198
chr2 173354242
chr2 173358947
chr2 173359185
chr2 173359231
chr2 173359332
chr2 173359410
chr2 173359419
chr2 173622818
chr2 173962048
chr2 173962450
chr2 173962477
chr2 173966238
chr2 173966249
chr2 173966315
chr2 173966328
chr2 173966335
chr2 174248121
chr2 174248128
chr2 174248156
chr2 174248199
chr2 174248212
chr2 174248216
chr2 174248224
chr2 174248227
chr2 174248334
chr2 174248366
chr2 174248373
chr2 174248375
chr2 174248402
chr2 174248405
chr2 174248416
chr2 174248423
chr2 174248425
chr2 174248431
chr2 174248452
chr2 174248458
chr2 174248467
chr2 174248475
chr2 174248486

chr2 174248492
chr2 174248509
chr2 174248522
chr2 174248543
chr2 174248551
chr2 174248560
chr2 174248563
chr2 174248567
chr2 174248572
chr2 174248576
chr2 174248579
chr2 174248582
chr2 174248585
chr2 174248591
chr2 174248594
chr2 174248599
chr2 174248603
chr2 174248608
chr2 174248612
chr2 174248615
chr2 174248619
chr2 174248628
chr2 174248630
chr2 174248646
chr2 174248656
chr2 174248665
chr2 174248667
chr2 174248669
chr2 174248674
chr2 174248690
chr2 174248694
chr2 174248702
chr2 174248713
chr2 174248739
chr2 174248741
chr2 174248743
chr2 174248746
chr2 174329462
chr2 174330120
chr2 174330175
chr2 174330395
chr2 174330497
chr2 174337893
chr2 174337906
chr2 174337910
chr2 174338037
chr2 174338043
chr2 174338183
chr2 174338268
chr2 174338398
chr2 174338430
chr2 174338545
chr2 174338552
chr2 174338564

chr2 174338643
chr2 174338709
chr2 174338769
chr2 174338901
chr2 174338971
chr2 174338988
chr2 174339163
chr2 174339165
chr2 174339255
chr2 174339286
chr2 174339376
chr2 174339419
chr2 174339421
chr2 174339424
chr2 174339438
chr2 174339462
chr2 174341912
chr2 174341916
chr2 174394731
chr2 174394841
chr2 174394981
chr2 174395113
chr2 174395179
chr2 174395219
chr2 174395607
chr2 174395668
chr2 174395684
chr2 174395692
chr2 174395700
chr2 174395726
chr2 174395748
chr2 174395783
chr2 174395799
chr2 174485903
chr2 174485935
chr2 174485974
chr2 174486016
chr2 174486027
chr2 174486101
chr2 174486158
chr2 174486192
chr2 174486228
chr2 174486251
chr2 174486290
chr2 174486307
chr2 174486320
chr2 174486921
chr2 174486950
chr2 174486960
chr2 174486976
chr2 174486979
chr2 174486983
chr2 174486989
chr2 174487016

chr2 174487021
chr2 174487044
chr2 174487049
chr2 174487071
chr2 174487074
chr2 174487078
chr2 174487080
chr2 174487088
chr2 174487094
chr2 174487099
chr2 174489781
chr2 174489958
chr2 174490202
chr2 174490256
chr2 174490329
chr2 174490481
chr2 174490486
chr2 174490679
chr2 174490696
chr2 174493055
chr2 174493057
chr2 174493116
chr2 174493159
chr2 174493253
chr2 174493263
chr2 174493315
chr2 174493672
chr2 174493998
chr2 174494017
chr2 174494041
chr2 174494066
chr2 174494103
chr2 174592668
chr2 174592730
chr2 174592750
chr2 174592841
chr2 174592852
chr2 174593453
chr2 174593479
chr2 174593501
chr2 174593672
chr2 174593750
chr2 174593752
chr2 174593839
chr2 174594054
chr2 174594140
chr2 174594245
chr2 174594292
chr2 174594440
chr2 174594486
chr2 174594628
chr2 174594941
chr2 174594978
chr2 174595459

chr2 174595570
chr2 174595625
chr2 174596103
chr2 174596205
chr2 174596653
chr2 174596658
chr2 174596823
chr2 174597022
chr2 174597080
chr2 174597184
chr2 174597301
chr2 174597601
chr2 174597657
chr2 174597710
chr2 174597724
chr2 174597794
chr2 174597812
chr2 174606203
chr2 174606226
chr2 174606512
chr2 174632657
chr2 174632662
chr2 174632687
chr2 174632893
chr2 174632989
chr2 174633100
chr2 174634555
chr2 174634637
chr2 174634649
chr2 174634911
chr2 174635002
chr2 174635191
chr2 174901802
chr2 174901902
chr2 174902032
chr2 175004356
chr2 175004418
chr2 175004518
chr2 175004555
chr2 175004558
chr2 175004627
chr2 175054478
chr2 175166713
chr2 175166735
chr2 175166837
chr2 175167202
chr2 175167210
chr2 175167214
chr2 175167229
chr2 175167247
chr2 175168746
chr2 175168759
chr2 175168769
chr2 175168794

chr2 175169173
chr2 175169276
chr2 175180770
chr2 175180882
chr2 175180903
chr2 175180913
chr2 175180944
chr2 175180975
chr2 175181001
chr2 175181333
chr2 175181372
chr2 175181381
chr2 175181387
chr2 175181405
chr2 175181413
chr2 175181415
chr2 175181439
chr2 175181442
chr2 175181457
chr2 175181470
chr2 175227491
chr2 175366839
chr2 175366878
chr2 175366880
chr2 175366896
chr2 175366907
chr2 175485180
chr2 175485219
chr2 175486066
chr2 175760620
chr2 175760645
chr2 175760801
chr2 175760833
chr2 175760859
chr2 175761215
chr2 175761252
chr2 175761459
chr2 175761482
chr2 175761492
chr2 175761554
chr2 175761659
chr2 175762050
chr2 175762104
chr2 175762374
chr2 175762755
chr2 175762881
chr2 175763456
chr2 175763688
chr2 175763715
chr2 176001130
chr2 176001262
chr2 176001333
chr2 176001347
chr2 176001374

chr2 176001393
chr2 176001481
chr2 176073455
chr2 176073457
chr2 176073495
chr2 176073507
chr2 176073589
chr2 176073597
chr2 176073633
chr2 176073805
chr2 176073917
chr2 176074052
chr2 176074192
chr2 176074227
chr2 176074255
chr2 176074628
chr2 176074957
chr2 176077181
chr2 176077186
chr2 176077205
chr2 176077256
chr2 176077422
chr2 176077518
chr2 176077600
chr2 176077690
chr2 176078035
chr2 176078274
chr2 176078359
chr2 176078380
chr2 176088702
chr2 176088784
chr2 176088879
chr2 176088985
chr2 176089067
chr2 176089092
chr2 176089110
chr2 176089805
chr2 176089836
chr2 176090677
chr2 176090859
chr2 176090864
chr2 176091040
chr2 176091048
chr2 176091122
chr2 176091232
chr2 176094518
chr2 176094577
chr2 176094677
chr2 176094769
chr2 176095041
chr2 176095163
chr2 176095253
chr2 176095460
chr2 176095527

chr2 176095529
chr2 176095629
chr2 176095691
chr2 176095994
chr2 176096412
chr2 176096444
chr2 176096453
chr2 176096509
chr2 176096569
chr2 176096733
chr2 176096762
chr2 176096804
chr2 176096808
chr2 176096824
chr2 176096873
chr2 176096875
chr2 176096903
chr2 176096941
chr2 176096951
chr2 176096957
chr2 176096999
chr2 176097025
chr2 176097063
chr2 176097096
chr2 176097108
chr2 176110386
chr2 176110832
chr2 176110937
chr2 176111173
chr2 176111183
chr2 176111218
chr2 176111250
chr2 176111317
chr2 176111353
chr2 176111359
chr2 176111374
chr2 176111400
chr2 176111406
chr2 176111408
chr2 176111414
chr2 176111426
chr2 176111428
chr2 176111444
chr2 176111446
chr2 176111482
chr2 176111506
chr2 176126019
chr2 176126193
chr2 176126310
chr2 176126312
chr2 176126614
chr2 176126674
chr2 176126690
chr2 176126943

chr2 176127142
chr2 176127216
chr2 176127240
chr2 176127256
chr2 176127259
chr2 176127305
chr2 176127384
chr2 176127505
chr2 176127507
chr2 176127533
chr2 176127588
chr2 176127655
chr2 176127799
chr2 176127921
chr2 176127925
chr2 176127931
chr2 176131904
chr2 176132113
chr2 176132119
chr2 176132149
chr2 176132172
chr2 176132194
chr2 176132353
chr2 176132367
chr2 176146378
chr2 176146416
chr2 176146551
chr2 176146606
chr2 176154227
chr2 176154292
chr2 176155943
chr2 176165807
chr2 176165847
chr2 176165898
chr2 176166316
chr2 176166438
chr2 176173322
chr2 176173355
chr2 176173397
chr2 176173561
chr2 176173563
chr2 176173810
chr2 176173840
chr2 176173889
chr2 176173936
chr2 176173944
chr2 176174006
chr2 176174210
chr2 176174421
chr2 176174468
chr2 176174490
chr2 176174513
chr2 176174536
chr2 176174567

chr2 176179079
chr2 176179279
chr2 176192757
chr2 176192889
chr2 176193224
chr2 176193587
chr2 176193725
chr2 176200681
chr2 176200783
chr2 176200893
chr2 176269866
chr2 176491548
chr2 176491648
chr2 176491880
chr2 176491910
chr2 176553766
chr2 176553784
chr2 176553792
chr2 176553821
chr2 176553828
chr2 176553833
chr2 176553864
chr2 176553871
chr2 176553882
chr2 176553891
chr2 176553897
chr2 176553925
chr2 176553936
chr2 176553957
chr2 176554026
chr2 176554039
chr2 176554132
chr2 176554173
chr2 176554177
chr2 176554212
chr2 176554284
chr2 176554296
chr2 176554307
chr2 176554321
chr2 176554337
chr2 176554339
chr2 176554401
chr2 176554445
chr2 176554503
chr2 176554623
chr2 176554629
chr2 176554884
chr2 176555046
chr2 176555151
chr2 177009040
chr2 177009102
chr2 177143328
chr2 177143455
chr2 177148190

chr2 177148197
chr2 177148232
chr2 177148250
chr2 177148312
chr2 177148458
chr2 177148487
chr2 177148552
chr2 177148584
chr2 177148633
chr2 177148695
chr2 177148713
chr2 177148783
chr2 177148801
chr2 177148810
chr2 177148851
chr2 177148866
chr2 177149131
chr2 177149137
chr2 177149139
chr2 177149205
chr2 177149633
chr2 177163192
chr2 177163319
chr2 177163858
chr2 177164273
chr2 177164394
chr2 177164441
chr2 177164577
chr2 177164605
chr2 177164690
chr2 177164692
chr2 177164701
chr2 177164704
chr2 177164724
chr2 177164732
chr2 177164759
chr2 177164791
chr2 177166305
chr2 177166366
chr2 177193481
chr2 177193506
chr2 177193594
chr2 177211784
chr2 177211885
chr2 177211914
chr2 177211928
chr2 177211951
chr2 177212000
chr2 177212127
chr2 177212156
chr2 177212158
chr2 177213914
chr2 177213977
chr2 177214015

chr2 177214090
chr2 177214222
chr2 177214359
chr2 177216278
chr2 177262095
chr2 177262265
chr2 177262600
chr2 177262644
chr2 177262670
chr2 177262686
chr2 177262765
chr2 177262862
chr2 177263391
chr2 177266068
chr2 177266406
chr2 177392240
chr2 177392333
chr2 177392968
chr2 177392970
chr2 177392978
chr2 177392980
chr2 177392983
chr2 177392986
chr2 177392992
chr2 177392998
chr2 177393004
chr2 177393014
chr2 177393017
chr2 177393019
chr2 177393028
chr2 177393048
chr2 177393058
chr2 177393078
chr2 177393081
chr2 177393094
chr2 177393121
chr2 177393143
chr2 177393151
chr2 177393163
chr2 177393195
chr2 177393236
chr2 177393293
chr2 177393419
chr2 177393471
chr2 177393473
chr2 177553078
chr2 177553083
chr2 177553132
chr2 177553156
chr2 177553168
chr2 177553170
chr2 177553173
chr2 177553187
chr2 177553204

chr2 177553223
chr2 177553232
chr2 177553274
chr2 177553289
chr2 177553325
chr2 177553345
chr2 177553366
chr2 177553376
chr2 177553379
chr2 177553462
chr2 177553485
chr2 177553498
chr2 177553530
chr2 177553555
chr2 177553589
chr2 177618934
chr2 177618940
chr2 177618950
chr2 177618961
chr2 177618964
chr2 177618969
chr2 177618975
chr2 177618981
chr2 177619000
chr2 177619017
chr2 177619022
chr2 177619027
chr2 177619029
chr2 177619034
chr2 177619042
chr2 177619046
chr2 177619054
chr2 177619109
chr2 177619119
chr2 177619124
chr2 177619138
chr2 177619154
chr2 177619198
chr2 177619228
chr2 177628076
chr2 177628143
chr2 177628150
chr2 177632208
chr2 177946979
chr2 177946983
chr2 177946991
chr2 177946996
chr2 177947000
chr2 177947029
chr2 177947033
chr2 177947041
chr2 177947046
chr2 177947050
chr2 177947064

chr2 178112441
chr2 178112447
chr2 178112455
chr2 178112460
chr2 178112488
chr2 178112501
chr2 178112534
chr2 178112552
chr2 178112569
chr2 178112573
chr2 178112576
chr2 178112586
chr2 178112590
chr2 178112594
chr2 178112609
chr2 178112614
chr2 178112625
chr2 178112662
chr2 178112676
chr2 178112683
chr2 178112693
chr2 178112718
chr2 178112756
chr2 178112765
chr2 178112769
chr2 178112819
chr2 178112828
chr2 178112850
chr2 178112858
chr2 178112863
chr2 178112874
chr2 178112922
chr2 178112947
chr2 178112953
chr2 178113019
chr2 178113027
chr2 178113030
chr2 178113049
chr2 178113056
chr2 178113072
chr2 178113078
chr2 178113084
chr2 178113091
chr2 178113093
chr2 178113140
chr2 178113182
chr2 178113252
chr2 178113288
chr2 178113342
chr2 178113360
chr2 178113404
chr2 178195509
chr2 178195600
chr2 178195667

chr2 178404167
chr2 178404483
chr2 178404505
chr2 178404559
chr2 178404596
chr2 178413273
chr2 178413280
chr2 178413290
chr2 178413348
chr2 178413414
chr2 178413422
chr2 178413441
chr2 178413448
chr2 178413475
chr2 178413927
chr2 178413933
chr2 178413936
chr2 178413939
chr2 178413941
chr2 178413999
chr2 178414006
chr2 178414020
chr2 178414128
chr2 178414272
chr2 178414404
chr2 178414592
chr2 178414601
chr2 178414643
chr2 178414661
chr2 178414713
chr2 178414716
chr2 178414728
chr2 178414800
chr2 178414809
chr2 178414840
chr2 178414866
chr2 178414932
chr2 178414942
chr2 178415054
chr2 178415077
chr2 178415084
chr2 178415107
chr2 178450593
chr2 178450605
chr2 178450614
chr2 178450642
chr2 178450682
chr2 178450685
chr2 178450709
chr2 178450720
chr2 178450724
chr2 178450726
chr2 178450729
chr2 178450733

chr2 178450737
chr2 178450746
chr2 178451193
chr2 178451207
chr2 178451215
chr2 178451221
chr2 178451229
chr2 178477932
chr2 178478050
chr2 178478172
chr2 178478214
chr2 178478251
chr2 178478255
chr2 178478264
chr2 178478293
chr2 178478298
chr2 178478316
chr2 178480921
chr2 178701043
chr2 178701065
chr2 179030891
chr2 179031232
chr2 179031360
chr2 179031867
chr2 179050120
chr2 179050137
chr2 179050141
chr2 179050157
chr2 179050306
chr2 179096289
chr2 179677302
chr2 179677335
chr2 179677486
chr2 179677795
chr2 179828681
chr2 179860008
chr2 179860028
chr2 179860043
chr2 179860208
chr2 179860249
chr2 179860269
chr2 180006552
chr2 180006594
chr2 180006679
chr2 180006740
chr2 180006901
chr2 180007098
chr2 180007476
chr2 180055541
chr2 180552192
chr2 180552941
chr2 180689195
chr2 180689362
chr2 180690077

chr2 180690289
chr2 180690365
chr2 180981477
chr2 180981523
chr2 180981541
chr2 180981565
chr2 180981590
chr2 180981641
chr2 180981786
chr2 180981822
chr2 180981837
chr2 180981843
chr2 180981871
chr2 180981886
chr2 181123623
chr2 181123821
chr2 181124062
chr2 181124584
chr2 181124681
chr2 181124746
chr2 181142514
chr2 181142711
chr2 181142771
chr2 181142822
chr2 181143282
chr2 181143329
chr2 181144021
chr2 181144191
chr2 181144365
chr2 181163437
chr2 181164252
chr2 181164631
chr2 181165384
chr2 181165444
chr2 181165501
chr2 181165505
chr2 181165609
chr2 181275526
chr2 181275740
chr2 181281160
chr2 181281324
chr2 181281830
chr2 181305399
chr2 181305455
chr2 181305461
chr2 181305670
chr2 181305764
chr2 181305770
chr2 181456542
chr2 181456555
chr2 181456627
chr2 181458289
chr2 181458297
chr2 181458301

chr2 181458354
chr2 181458402
chr2 181460911
chr2 181461124
chr2 181461225
chr2 181461861
chr2 181461996
chr2 181462147
chr2 181674249
chr2 181674321
chr2 181675295
chr2 181675298
chr2 181675332
chr2 181675509
chr2 181676234
chr2 181676565
chr2 181677071
chr2 181677487
chr2 181677504
chr2 181685514
chr2 181685731
chr2 181891109
chr2 181891312
chr2 181891405
chr2 181891450
chr2 181891501
chr2 181893029
chr2 181893061
chr2 181954119
chr2 181954233
chr2 181954235
chr2 181954247
chr2 181954254
chr2 181954261
chr2 181954299
chr2 181954301
chr2 181954303
chr2 181954305
chr2 181954307
chr2 181954337
chr2 181954358
chr2 181954390
chr2 181954392
chr2 181954410
chr2 181954425
chr2 181954435
chr2 181954475
chr2 182097956
chr2 182098071
chr2 182098583
chr2 182213118
chr2 182213171
chr2 182213379
chr2 182213398

chr2 182213454
chr2 182213475
chr2 182213669
chr2 182213698
chr2 182213736
chr2 182213892
chr2 182213918
chr2 182213936
chr2 182214025
chr2 182214062
chr2 182214071
chr2 182214176
chr2 182214331
chr2 182379142
chr2 182379491
chr2 182531195
chr2 182566653
chr2 182567155
chr2 182567240
chr2 182567272
chr2 182715467
chr2 182715535
chr2 182715656
chr2 182715736
chr2 182715796
chr2 182715822
chr2 182715847
chr2 182715857
chr2 182715864
chr2 182716707
chr2 182716754
chr2 182716807
chr2 182716817
chr2 182716835
chr2 182716849
chr2 182716857
chr2 182716864
chr2 182716873
chr2 182716885
chr2 182716905
chr2 182717132
chr2 182717139
chr2 182717161
chr2 182717611
chr2 182717674
chr2 183035876
chr2 183036014
chr2 183036466
chr2 183036797
chr2 183036886
chr2 183036898
chr2 183036960
chr2 183063018
chr2 183063251

chr2 183063338
chr2 183078007
chr2 183078073
chr2 183078254
chr2 183078278
chr2 183078329
chr2 183078346
chr2 183078430
chr2 183078433
chr2 183078447
chr2 183078453
chr2 183078470
chr2 183078475
chr2 183078491
chr2 183078566
chr2 183078591
chr2 183078651
chr2 183078660
chr2 183078709
chr2 183078741
chr2 183078756
chr2 183078768
chr2 183078778
chr2 183078807
chr2 183078823
chr2 183078879
chr2 183078970
chr2 183079007
chr2 183079076
chr2 183079118
chr2 183079134
chr2 183079170
chr2 183079210
chr2 183079233
chr2 183079263
chr2 183079320
chr2 183094273
chr2 183094384
chr2 183123869
chr2 183123873
chr2 183123912
chr2 183123915
chr2 183123926
chr2 183123978
chr2 183124010
chr2 183125235
chr2 183125299
chr2 183125313
chr2 183125316
chr2 183125330
chr2 183125342
chr2 183125596
chr2 183125857
chr2 183302495

chr2 183303103
chr2 183884301
chr2 183884563
chr2 183884607
chr2 184326744
chr2 184332101
chr2 184333362
chr2 184521519
chr2 184556848
chr2 184557762
chr2 184557778
chr2 184596655
chr2 184596709
chr2 184596855
chr2 184596865
chr2 184597130
chr2 184597223
chr2 184597410
chr2 184597426
chr2 184597438
chr2 184597542
chr2 184597611
chr2 184597626
chr2 184597645
chr2 184597670
chr2 184597681
chr2 184597685
chr2 184597695
chr2 184597713
chr2 184597757
chr2 184597766
chr2 184888972
chr2 184889328
chr2 184889619
chr2 184889754
chr2 184889884
chr2 184890272
chr2 184896413
chr2 184896793
chr2 184896857
chr2 184897850
chr2 184897879
chr2 185124779
chr2 185125249
chr2 185168139
chr2 185168510
chr2 185243060
chr2 185259982
chr2 185260291
chr2 185260442
chr2 185260619
chr2 185261115
chr2 185487135
chr2 185487163

chr2 186119837
chr2 186485462
chr2 186485492
chr2 186485592
chr2 186487205
chr2 186487247
chr2 186487256
chr2 186487332
chr2 186487357
chr2 186487374
chr2 186487458
chr2 186487671
chr2 186487705
chr2 186487751
chr2 186487760
chr2 186487778
chr2 186487993
chr2 186589572
chr2 186589594
chr2 186589598
chr2 186589631
chr2 186590728
chr2 186590761
chr2 186590796
chr2 186590832
chr2 186590841
chr2 186590859
chr2 186590883
chr2 186590948
chr2 186590952
chr2 186590995
chr2 186591222
chr2 186591283
chr2 186591288
chr2 186591290
chr2 186591302
chr2 186591351
chr2 186591401
chr2 186591497
chr2 186694149
chr2 186694152
chr2 186694154
chr2 186694178
chr2 186694189
chr2 186694192
chr2 186694215
chr2 186694224
chr2 186694241
chr2 186694243
chr2 186694254
chr2 186694256
chr2 186694260
chr2 186694268
chr2 186694279

chr2 186694284
chr2 186694403
chr2 186694433
chr2 186694447
chr2 186694451
chr2 186694454
chr2 186694470
chr2 186694473
chr2 186694482
chr2 186694509
chr2 186694513
chr2 186694534
chr2 186694586
chr2 186694601
chr2 186694636
chr2 186694655
chr2 186694795
chr2 186848612
chr2 186848685
chr2 186848804
chr2 186848827
chr2 186848837
chr2 186848843
chr2 186848865
chr2 186848888
chr2 186848924
chr2 186849697
chr2 186849706
chr2 186849709
chr2 186849932
chr2 187249227
chr2 187249676
chr2 187249984
chr2 187462896
chr2 187463303
chr2 187553610
chr2 187553622
chr2 187553724
chr2 187554079
chr2 187554140
chr2 187554244
chr2 187554336
chr2 187554360
chr2 187554390
chr2 187554425
chr2 187554463
chr2 187554623
chr2 187554841
chr2 187554989
chr2 187555103
chr2 187555206
chr2 187950911
chr2 188063621
chr2 188113960

chr2 188114107
chr2 188125394
chr2 188125404
chr2 188181425
chr2 188181523
chr2 188181638
chr2 188181677
chr2 188181683
chr2 188604752
chr2 188607595
chr2 188607879
chr2 188733674
chr2 188893881
chr2 188894005
chr2 188894222
chr2 188894497
chr2 188894879
chr2 188950750
chr2 188951212
chr2 188951422
chr2 188951657
chr2 188978852
chr2 188979060
chr2 188979073
chr2 189178183
chr2 189178265
chr2 189178811
chr2 189178822
chr2 189178921
chr2 189186484
chr2 189186509
chr2 189186518
chr2 189186572
chr2 189440383
chr2 189440521
chr2 189440622
chr2 189440669
chr2 189658038
chr2 189658058
chr2 189658201
chr2 189658220
chr2 189660617
chr2 189660966
chr2 189661025
chr2 189661040
chr2 189661087
chr2 189661794
chr2 189661902
chr2 189661963
chr2 189662043
chr2 189662075
chr2 189662088
chr2 189662103
chr2 189662121

chr2 189662158
chr2 189662186
chr2 189662290
chr2 189674868
chr2 189674883
chr2 189674885
chr2 189674900
chr2 189674909
chr2 189674927
chr2 189674949
chr2 189674987
chr2 189675052
chr2 189762144
chr2 189762305
chr2 189762337
chr2 189782537
chr2 189782552
chr2 189782589
chr2 189782688
chr2 189783014
chr2 189818754
chr2 189818794
chr2 190344296
chr2 190344331
chr2 190344389
chr2 190344654
chr2 190344662
chr2 190344683
chr2 190344726
chr2 190360043
chr2 190360118
chr2 190360160
chr2 190360164
chr2 190360201
chr2 190360254
chr2 190409008
chr2 190409010
chr2 190409045
chr2 190409071
chr2 190409128
chr2 190409270
chr2 190409285
chr2 190409339
chr2 190409347
chr2 190409351
chr2 190409805
chr2 190534016
chr2 190534022
chr2 190534030
chr2 190534043
chr2 190534080
chr2 190534085
chr2 190534167
chr2 190534192

chr2 190535269
chr2 190535302
chr2 190535308
chr2 190535333
chr2 190535884
chr2 190627873
chr2 190627889
chr2 190627913
chr2 190627928
chr2 190627930
chr2 190627940
chr2 190627942
chr2 190627948
chr2 190627954
chr2 190627961
chr2 190627976
chr2 190627978
chr2 190627985
chr2 190627987
chr2 190650332
chr2 190650452
chr2 190650474
chr2 190650508
chr2 190650814
chr2 190722565
chr2 190722913
chr2 190722921
chr2 190876014
chr2 190876088
chr2 190876541
chr2 190879955
chr2 190879965
chr2 190880038
chr2 190880044
chr2 190880080
chr2 190880123
chr2 190880137
chr2 190880153
chr2 190880158
chr2 190880165
chr2 190880168
chr2 190883537
chr2 190883829
chr2 190883890
chr2 190883970
chr2 190883995
chr2 190883999
chr2 191012799
chr2 191012832
chr2 191012929
chr2 191012963
chr2 191013009
chr2 191013012
chr2 191013108

chr2 191013147
chr2 191013352
chr2 191013480
chr2 191013578
chr2 191013587
chr2 191013606
chr2 191013627
chr2 191013631
chr2 191013650
chr2 191014616
chr2 191014638
chr2 191014657
chr2 191014784
chr2 191014815
chr2 191014885
chr2 191020218
chr2 191020220
chr2 191020231
chr2 191020671
chr2 191020677
chr2 191023424
chr2 191052383
chr2 191052388
chr2 191056273
chr2 191056486
chr2 191063262
chr2 191064551
chr2 191064562
chr2 191064602
chr2 191064731
chr2 191065183
chr2 191065221
chr2 191065784
chr2 191148579
chr2 191148784
chr2 191148999
chr2 191149078
chr2 191149361
chr2 191149599
chr2 191149607
chr2 191149683
chr2 191149703
chr2 191149855
chr2 191150170
chr2 191150202
chr2 191150284
chr2 191150359
chr2 191150788
chr2 191150796
chr2 191150800
chr2 191571214
chr2 191571255
chr2 191617749
chr2 191617778

chr2 191617864
chr2 191675938
chr2 191676225
chr2 191676456
chr2 191677001
chr2 191677012
chr2 191677222
chr2 191677224
chr2 191677415
chr2 191677451
chr2 191680084
chr2 191680588
chr2 191680597
chr2 191680737
chr2 192196893
chr2 192196913
chr2 192197013
chr2 192317207
chr2 192417208
chr2 192417278
chr2 192417291
chr2 192417412
chr2 192417477
chr2 192418515
chr2 192625173
chr2 192826701
chr2 192896187
chr2 193060195
chr2 193275007
chr2 193279527
chr2 193279629
chr2 193279981
chr2 193427897
chr2 193500748
chr2 193500893
chr2 193712015
chr2 193827459
chr2 193827659
chr2 193827675
chr2 193827720
chr2 193827784
chr2 193827806
chr2 193828823
chr2 193829019
chr2 193829953
chr2 193830203
chr2 193830453
chr2 193830774
chr2 193830951
chr2 193831138
chr2 193831258
chr2 193831331
chr2 193831696
chr2 193831897

chr2 193832352
chr2 193833060
chr2 193833168
chr2 193833297
chr2 193833477
chr2 193833537
chr2 193833567
chr2 193833612
chr2 193833635
chr2 193833783
chr2 193869923
chr2 193879853
chr2 193906921
chr2 193907354
chr2 193907728
chr2 193956391
chr2 193956661
chr2 193957119
chr2 193960427
chr2 194091649
chr2 194091720
chr2 194091764
chr2 194178329
chr2 194323477
chr2 194323955
chr2 194905784
chr2 194906157
chr2 195532883
chr2 195532968
chr2 195533026
chr2 195533074
chr2 195533331
chr2 195533723
chr2 195534032
chr2 195534087
chr2 195534102
chr2 195534226
chr2 195534243
chr2 195534251
chr2 195559463
chr2 195559516
chr2 195559546
chr2 195559657
chr2 195559870
chr2 195560235
chr2 195560336
chr2 195560341
chr2 195560505
chr2 195560548
chr2 195560595
chr2 195560649
chr2 195560659
chr2 195560709
chr2 195560714

chr2 195562430
chr2 195562490
chr2 195562609
chr2 195562617
chr2 195562667
chr2 195562960
chr2 195562998
chr2 195563004
chr2 195563012
chr2 195563062
chr2 195563079
chr2 195576292
chr2 195601286
chr2 195659411
chr2 195659725
chr2 195659843
chr2 195660047
chr2 195660371
chr2 195682296
chr2 196067273
chr2 196067298
chr2 196068056
chr2 196068367
chr2 196068380
chr2 196068456
chr2 196068458
chr2 196068461
chr2 196068468
chr2 196068587
chr2 196068589
chr2 196158782
chr2 196158838
chr2 196158867
chr2 196159088
chr2 196159340
chr2 196159387
chr2 196159457
chr2 196159549
chr2 196159701
chr2 196159937
chr2 196160180
chr2 196160254
chr2 196160282
chr2 196161119
chr2 196161158
chr2 196161183
chr2 196161215
chr2 196163019
chr2 196163243
chr2 196163358
chr2 196163378
chr2 196164109
chr2 196164200
chr2 196164233

chr2 196165286
chr2 196165324
chr2 196165351
chr2 196165362
chr2 196165654
chr2 196165684
chr2 196165811
chr2 196165999
chr2 196166319
chr2 196166415
chr2 196166539
chr2 196167024
chr2 196167341
chr2 196168352
chr2 196168373
chr2 196168414
chr2 196168460
chr2 196169099
chr2 196169137
chr2 196169145
chr2 196169200
chr2 196169220
chr2 196169222
chr2 196169224
chr2 196169236
chr2 196169264
chr2 196169286
chr2 196169290
chr2 196169307
chr2 196169311
chr2 196169316
chr2 196169356
chr2 196169360
chr2 196169448
chr2 196169631
chr2 196169689
chr2 196172237
chr2 196176478
chr2 196177018
chr2 196177134
chr2 196177592
chr2 196177900
chr2 196210738
chr2 196211117
chr2 196211155
chr2 196211177
chr2 196211282
chr2 196211405
chr2 196211409
chr2 196211411
chr2 196249446
chr2 196263406
chr2 196263456
chr2 196263513

chr2 196263591
chr2 196263656
chr2 196263701
chr2 196268145
chr2 196268639
chr2 196268661
chr2 196268938
chr2 196269011
chr2 196291997
chr2 196403713
chr2 196459772
chr2 196459831
chr2 196459861
chr2 196459917
chr2 196459971
chr2 196528920
chr2 196529442
chr2 196640094
chr2 196640097
chr2 196751064
chr2 196926149
chr2 196926520
chr2 196926526
chr2 196926581
chr2 196926622
chr2 196926631
chr2 196926634
chr2 196926637
chr2 196926640
chr2 196926643
chr2 196926646
chr2 196926667
chr2 196926670
chr2 196926672
chr2 196927123
chr2 196927134
chr2 196927140
chr2 196927145
chr2 196927195
chr2 196927201
chr2 196927213
chr2 196927227
chr2 196927234
chr2 196927262
chr2 196927270
chr2 196927282
chr2 196927361
chr2 196927388
chr2 196927401
chr2 196927443
chr2 196927450
chr2 196927453
chr2 196927502
chr2 196927546

chr2 196927565
chr2 196927579
chr2 196927587
chr2 196927631
chr2 196927653
chr2 196927674
chr2 196927690
chr2 196927705
chr2 196927746
chr2 196927829
chr2 196927842
chr2 196988041
chr2 196988089
chr2 196988108
chr2 196988162
chr2 196988180
chr2 196988267
chr2 196988391
chr2 197130359
chr2 197130397
chr2 197130438
chr2 197130456
chr2 197130599
chr2 197130693
chr2 197146898
chr2 197147072
chr2 197147092
chr2 197147119
chr2 197198661
chr2 197198685
chr2 197198707
chr2 197198717
chr2 197198747
chr2 197198771
chr2 197198790
chr2 197198893
chr2 197198929
chr2 197198938
chr2 197198946
chr2 197198952
chr2 197198955
chr2 197198966
chr2 197199035
chr2 197199057
chr2 197199136
chr2 197199233
chr2 197199238
chr2 197199347
chr2 197199524
chr2 197199567
chr2 197199677
chr2 197199803
chr2 197199897
chr2 197205989

chr2 197206092
chr2 197206112
chr2 197239525
chr2 197239662
chr2 197239917
chr2 197240112
chr2 197240154
chr2 197240192
chr2 197240220
chr2 197240235
chr2 197240282
chr2 197240329
chr2 197240801
chr2 197255731
chr2 197255748
chr2 197255801
chr2 197255804
chr2 197255849
chr2 197255863
chr2 197255892
chr2 197255929
chr2 197256422
chr2 197256777
chr2 197256793
chr2 197257448
chr2 197257866
chr2 197293698
chr2 197293739
chr2 197305047
chr2 197305095
chr2 197305353
chr2 197305443
chr2 197306909
chr2 197307021
chr2 197307046
chr2 197307349
chr2 197307990
chr2 197308042
chr2 197308044
chr2 197308133
chr2 197308278
chr2 197308282
chr2 197309142
chr2 197309385
chr2 197309476
chr2 197309526
chr2 197309704
chr2 197328032
chr2 197452844
chr2 197452850
chr2 197452888
chr2 197452915
chr2 197452932
chr2 197452999

chr2 197454287
chr2 197454294
chr2 197454311
chr2 197454398
chr2 197454461
chr2 197454643
chr2 197497765
chr2 197497800
chr2 197497818
chr2 197498075
chr2 197498610
chr2 197498644
chr2 197498692
chr2 197498704
chr2 197498739
chr2 197501084
chr2 197501113
chr2 197501211
chr2 197501222
chr2 197501317
chr2 197501340
chr2 197501486
chr2 197501492
chr2 197515157
chr2 197515255
chr2 197515391
chr2 197515413
chr2 197516693
chr2 197516776
chr2 197516958
chr2 197516993
chr2 197517055
chr2 197517126
chr2 197704358
chr2 197704362
chr2 197782469
chr2 197782538
chr2 197782700
chr2 197782736
chr2 197803963
chr2 197804181
chr2 197804196
chr2 197804208
chr2 197804328
chr2 197804356
chr2 197804361
chr2 197804367
chr2 197804379
chr2 197804381
chr2 197804383
chr2 197804385
chr2 197804387
chr2 198296827
chr2 198296855

chr2 198296888
chr2 198296891
chr2 198296925
chr2 198296963
chr2 198296981
chr2 198297048
chr2 198297484
chr2 198297578
chr2 198372847
chr2 198373094
chr2 198373574
chr2 198373728
chr2 198374047
chr2 198374150
chr2 198374167
chr2 198866610
chr2 198880875
chr2 199461077
chr2 199461152
chr2 199461193
chr2 199461214
chr2 199461259
chr2 199461305
chr2 199461330
chr2 199461364
chr2 199461437
chr2 199461488
chr2 199461511
chr2 199461639
chr2 199461648
chr2 199461722
chr2 199465219
chr2 199465365
chr2 199465456
chr2 199465490
chr2 199465573
chr2 199465640
chr2 199465728
chr2 199465832
chr2 199465938
chr2 199485842
chr2 199485965
chr2 199486038
chr2 199598387
chr2 199598749
chr2 199598793
chr2 199598821
chr2 199605353
chr2 199605507
chr2 199659279
chr2 199659281
chr2 199659284
chr2 199659289
chr2 199659293

chr2 199659296
chr2 199659304
chr2 199659309
chr2 199659318
chr2 199662016
chr2 199850516
chr2 199850531
chr2 199850562
chr2 199851550
chr2 199851570
chr2 199872301
chr2 199912368
chr2 199912406
chr2 199955114
chr2 200373375
chr2 200373401
chr2 200373516
chr2 200510364
chr2 200510375
chr2 200510626
chr2 200510708
chr2 200525571
chr2 200525582
chr2 200525646
chr2 200525709
chr2 200525721
chr2 200525765
chr2 200525801
chr2 200712742
chr2 200712811
chr2 200713013
chr2 200713044
chr2 200713155
chr2 200713159
chr2 200713161
chr2 200713244
chr2 200713292
chr2 200713298
chr2 200713335
chr2 200713341
chr2 200713350
chr2 200713352
chr2 200713374
chr2 200713378
chr2 200713401
chr2 200713506
chr2 200713595
chr2 200812855
chr2 200812862
chr2 200812876
chr2 200812938
chr2 200812965
chr2 200813029
chr2 200813071

chr2 200813246
chr2 200813260
chr2 200813293
chr2 200860576
chr2 200860608
chr2 200860723
chr2 200861077
chr2 200861229
chr2 200861248
chr2 200861251
chr2 200861341
chr2 200861362
chr2 200861416
chr2 200861419
chr2 200888160
chr2 200888278
chr2 200888425
chr2 200888577
chr2 200888588
chr2 200888605
chr2 200888611
chr2 200900584
chr2 200901427
chr2 200901512
chr2 200901611
chr2 200901632
chr2 200901670
chr2 200962525
chr2 200962585
chr2 200962740
chr2 200962747
chr2 200962767
chr2 200962778
chr2 200962811
chr2 200962862
chr2 200962948
chr2 200963027
chr2 200963029
chr2 200963072
chr2 200963115
chr2 201069239
chr2 201069366
chr2 201069374
chr2 201069442
chr2 201069589
chr2 201069616
chr2 201072453
chr2 201072679
chr2 201117547
chr2 201117587
chr2 201117607
chr2 201117647
chr2 201117659
chr2 201117678

chr2 201117716
chr2 201117832
chr2 201117841
chr2 201117848
chr2 201117858
chr2 201117865
chr2 201117916
chr2 201117943
chr2 201117974
chr2 201117997
chr2 201118001
chr2 201118006
chr2 201118034
chr2 201118050
chr2 201118181
chr2 201118202
chr2 201118217
chr2 201118256
chr2 201118279
chr2 201118309
chr2 201118330
chr2 201118341
chr2 201118343
chr2 201118348
chr2 201118358
chr2 201118412
chr2 201120381
chr2 201120440
chr2 201121069
chr2 201121235
chr2 201121350
chr2 201121435
chr2 201125014
chr2 201125163
chr2 201125461
chr2 201125753
chr2 201125832
chr2 201125896
chr2 201125943
chr2 201126027
chr2 201126051
chr2 201126065
chr2 201126093
chr2 201126105
chr2 201126108
chr2 201126126
chr2 201126147
chr2 201126319
chr2 201126321
chr2 201126333
chr2 201126347
chr2 201126364
chr2 201126447
chr2 201126567

chr2 201126821
chr2 201126899
chr2 201127283
chr2 201127412
chr2 201127588
chr2 201127679
chr2 201127718
chr2 201127807
chr2 201127854
chr2 201128276
chr2 201182946
chr2 201182952
chr2 201183031
chr2 201183271
chr2 201183377
chr2 201183470
chr2 201183496
chr2 201183594
chr2 201194211
chr2 201194221
chr2 201194270
chr2 201212228
chr2 201212237
chr2 201212240
chr2 201212275
chr2 201212306
chr2 201212385
chr2 201212564
chr2 201232248
chr2 201232271
chr2 201232339
chr2 201232370
chr2 201232406
chr2 201232417
chr2 201232450
chr2 201232761
chr2 201232778
chr2 201232802
chr2 201232884
chr2 201233174
chr2 201233293
chr2 201234162
chr2 201234322
chr2 201234353
chr2 201234378
chr2 201234399
chr2 201234488
chr2 201234510
chr2 201234512
chr2 201234535
chr2 201234542
chr2 201234559
chr2 201234754
chr2 201234941

chr2 201235123
chr2 201235277
chr2 201235306
chr2 201235462
chr2 201235507
chr2 201235778
chr2 201235817
chr2 201236029
chr2 201236041
chr2 201236079
chr2 201236156
chr2 201236250
chr2 201253015
chr2 201253020
chr2 201257436
chr2 201257463
chr2 201257468
chr2 201257476
chr2 201257586
chr2 201257649
chr2 201257748
chr2 201257751
chr2 201257864
chr2 201257926
chr2 201257930
chr2 201257946
chr2 201258030
chr2 201258109
chr2 201258740
chr2 201258743
chr2 201260268
chr2 201260365
chr2 201260369
chr2 201260452
chr2 201260489
chr2 201260525
chr2 201260539
chr2 201260587
chr2 201260602
chr2 201260704
chr2 201260735
chr2 201260804
chr2 201260811
chr2 201261034
chr2 201261118
chr2 201261130
chr2 201261158
chr2 201261163
chr2 201261181
chr2 201261220
chr2 201261247
chr2 201261259
chr2 201261283
chr2 201261305

chr2 201261339
chr2 201261345
chr2 201261378
chr2 201261386
chr2 201261595
chr2 201261606
chr2 201261629
chr2 201261656
chr2 201261666
chr2 201261717
chr2 201261741
chr2 201261769
chr2 201261794
chr2 201262138
chr2 201262229
chr2 201262787
chr2 201262819
chr2 201263098
chr2 201263287
chr2 201263978
chr2 201264102
chr2 201604116
chr2 201604218
chr2 201604228
chr2 201781108
chr2 201781127
chr2 201781163
chr2 201781185
chr2 201781199
chr2 201781212
chr2 201781230
chr2 201781234
chr2 201781252
chr2 201858471
chr2 201858573
chr2 201858726
chr2 201858902
chr2 202173068
chr2 202238193
chr2 202238203
chr2 202238216
chr2 202238222
chr2 202238232
chr2 202238236
chr2 202238242
chr2 202238251
chr2 202238263
chr2 202238272
chr2 202238276
chr2 202238282
chr2 202238296
chr2 202238314
chr2 202238318
chr2 202238335

chr2 202238337
chr2 202238350
chr2 202238352
chr2 202238364
chr2 202238378
chr2 202238412
chr2 202238415
chr2 202238434
chr2 202238457
chr2 202238470
chr2 202238480
chr2 202238490
chr2 202238496
chr2 202238512
chr2 202238517
chr2 202238558
chr2 202238560
chr2 202238576
chr2 202238581
chr2 202238586
chr2 202238588
chr2 202238597
chr2 202238602
chr2 202238604
chr2 202238644
chr2 202238700
chr2 202238707
chr2 202238709
chr2 202238711
chr2 202238719
chr2 202238756
chr2 202267762
chr2 202267796
chr2 202267803
chr2 202267867
chr2 202267965
chr2 202268014
chr2 202268041
chr2 202268186
chr2 202268300
chr2 202268306
chr2 202268309
chr2 202278166
chr2 202278565
chr2 202278582
chr2 202375153
chr2 202375155
chr2 202375169
chr2 202375196
chr2 202375205
chr2 202375210
chr2 202375214
chr2 202375230
chr2 202375233

chr2 202375253
chr2 202375256
chr2 202375266
chr2 202375271
chr2 202375284
chr2 202375296
chr2 202375312
chr2 202375338
chr2 202375364
chr2 202375373
chr2 202375505
chr2 202375517
chr2 202375519
chr2 202375526
chr2 202375534
chr2 202375536
chr2 202375538
chr2 202375542
chr2 202375547
chr2 202375552
chr2 202377721
chr2 202377943
chr2 202529789
chr2 202582718
chr2 202588757
chr2 202588769
chr2 202588803
chr2 202588824
chr2 202588830
chr2 202642782
chr2 202642940
chr2 202642990
chr2 202669737
chr2 202832014
chr2 202832048
chr2 202832361
chr2 202832371
chr2 202832378
chr2 202832414
chr2 202832430
chr2 202910850
chr2 202911034
chr2 202911141
chr2 202912583
chr2 202912585
chr2 202912606
chr2 202912623
chr2 202912630
chr2 202912637
chr2 202912642
chr2 202912647
chr2 202912654
chr2 202912826
chr2 202912852

chr2 202912891
chr2 202913039
chr2 202913101
chr2 202913140
chr2 202913172
chr2 202965866
chr2 202965881
chr2 202965888
chr2 202966035
chr2 203251967
chr2 203328918
chr2 203706280
chr2 203706296
chr2 203706423
chr2 203706492
chr2 203706573
chr2 203706586
chr2 203706595
chr2 203706621
chr2 203706645
chr2 203706654
chr2 203706684
chr2 203707139
chr2 203707165
chr2 203707402
chr2 203707550
chr2 203707598
chr2 203707648
chr2 203707791
chr2 203707879
chr2 203707953
chr2 203707981
chr2 203708205
chr2 203708224
chr2 203708260
chr2 203708310
chr2 203708806
chr2 203708865
chr2 203708875
chr2 203708882
chr2 203708890
chr2 203708924
chr2 203708927
chr2 203708994
chr2 203709046
chr2 203709051
chr2 203709053
chr2 203709297
chr2 203709595
chr2 203710347
chr2 203710547
chr2 203718530
chr2 203718716
chr2 203718739

chr2 203719963
chr2 203719982
chr2 203720073
chr2 203720363
chr2 203720572
chr2 203720679
chr2 203729002
chr2 203729679
chr2 203750678
chr2 203750793
chr2 203750850
chr2 203750856
chr2 203750860
chr2 203871387
chr2 203871435
chr2 203871626
chr2 203871637
chr2 203871940
chr2 203871998
chr2 203872216
chr2 203936748
chr2 203936785
chr2 203936787
chr2 203936854
chr2 203936950
chr2 203937552
chr2 203937939
chr2 203938275
chr2 203938314
chr2 203938335
chr2 203939599
chr2 203939739
chr2 203939795
chr2 203940040
chr2 203940057
chr2 203940261
chr2 203940374
chr2 203940460
chr2 203940806
chr2 203940822
chr2 203940844
chr2 203940852
chr2 203940876
chr2 203940886
chr2 203940932
chr2 203940940
chr2 203940966
chr2 203940974
chr2 203940985
chr2 203941019
chr2 203941026
chr2 203941036
chr2 203941042
chr2 203941048

chr2 203941075
chr2 203941085
chr2 203941087
chr2 203941092
chr2 203942007
chr2 203942057
chr2 203951439
chr2 203951559
chr2 203951596
chr2 203951636
chr2 203951654
chr2 203951687
chr2 203951779
chr2 203951808
chr2 203951821
chr2 203951890
chr2 203951982
chr2 203952689
chr2 204108020
chr2 204108393
chr2 204108420
chr2 204108637
chr2 204108967
chr2 204108987
chr2 204109018
chr2 204109030
chr2 204110185
chr2 204110313
chr2 204110336
chr2 204110371
chr2 204110427
chr2 204110432
chr2 204110453
chr2 204110458
chr2 204110581
chr2 204110613
chr2 204110702
chr2 204145586
chr2 204145668
chr2 204145690
chr2 204145692
chr2 204145697
chr2 204145840
chr2 204145890
chr2 204342109
chr2 204342176
chr2 204342679
chr2 204680313
chr2 204680322
chr2 205803713
chr2 206083422
chr2 206083579
chr2 206083624
chr2 206083674

chr2 206083684
chr2 206083704
chr2 206083826
chr2 206083898
chr2 206083923
chr2 206084015
chr2 206084048
chr2 206084090
chr2 206084151
chr2 206084223
chr2 206084304
chr2 206084388
chr2 206086922
chr2 206087047
chr2 206087347
chr2 206087361
chr2 206087435
chr2 206160312
chr2 206160517
chr2 206160535
chr2 206160541
chr2 206160627
chr2 206160641
chr2 206160648
chr2 206160667
chr2 206160693
chr2 206160753
chr2 206275426
chr2 206275430
chr2 206275451
chr2 206275485
chr2 206275488
chr2 206275513
chr2 206275520
chr2 206275526
chr2 206313666
chr2 206442417
chr2 206442426
chr2 206442557
chr2 206442566
chr2 206442597
chr2 206442683
chr2 206442699
chr2 206442755
chr2 206442766
chr2 206442778
chr2 206442820
chr2 206442824
chr2 206442859
chr2 206442878
chr2 206442888
chr2 206442896
chr2 206442898
chr2 206442917

chr2 206442919
chr2 206442929
chr2 206442932
chr2 206442938
chr2 206442951
chr2 206442963
chr2 206442989
chr2 206442991
chr2 206443008
chr2 206530805
chr2 206530921
chr2 206530952
chr2 206530959
chr2 206530962
chr2 206530965
chr2 206531019
chr2 206531030
chr2 206531122
chr2 206700161
chr2 206700191
chr2 206700204
chr2 206700265
chr2 206700271
chr2 206700302
chr2 206700336
chr2 206700374
chr2 206765573
chr2 206765583
chr2 206765592
chr2 206765602
chr2 206765638
chr2 206765667
chr2 206765790
chr2 206765884
chr2 206765890
chr2 206765914
chr2 206766088
chr2 206766116
chr2 206766120
chr2 206766132
chr2 206766214
chr2 206766216
chr2 206766337
chr2 207046841
chr2 207046918
chr2 207046997
chr2 207047091
chr2 207047108
chr2 207074049
chr2 207074134
chr2 207074318
chr2 207074326
chr2 207074335
chr2 207074348

chr2 207074408
chr2 207164339
chr2 207164354
chr2 207164390
chr2 207164443
chr2 207164453
chr2 207164504
chr2 207164529
chr2 207164628
chr2 207164647
chr2 207164731
chr2 207164788
chr2 207164867
chr2 207164958
chr2 207164980
chr2 207165005
chr2 207165016
chr2 207165087
chr2 207165093
chr2 207165112
chr2 207165143
chr2 207165152
chr2 207165167
chr2 207165197
chr2 207165203
chr2 207165209
chr2 207165227
chr2 207165236
chr2 207165247
chr2 207165252
chr2 207165265
chr2 207165275
chr2 207165303
chr2 207165407
chr2 207165418
chr2 207165424
chr2 207165517
chr2 207165562
chr2 207165580
chr2 207165589
chr2 207165596
chr2 207165609
chr2 207165764
chr2 207165798
chr2 207165813
chr2 207165841
chr2 207165942
chr2 207336907
chr2 207531436
chr2 207531507
chr2 207531511
chr2 207531570
chr2 207531647
chr2 207531668

chr2 207531697
chr2 207533447
chr2 207533513
chr2 207711297
chr2 207737312
chr2 207737731
chr2 208024654
chr2 208024993
chr2 208025003
chr2 208025019
chr2 208025088
chr2 208025112
chr2 208025632
chr2 208025641
chr2 208253957
chr2 208254053
chr2 208254081
chr2 208254108
chr2 208254155
chr2 208254161
chr2 208254810
chr2 208254844
chr2 208254877
chr2 208254910
chr2 208254959
chr2 208255026
chr2 208255071
chr2 208255090
chr2 208255107
chr2 208255130
chr2 208255158
chr2 208255164
chr2 208255169
chr2 208255179
chr2 208255186
chr2 208255196
chr2 208255207
chr2 208255216
chr2 208255225
chr2 208255268
chr2 208255277
chr2 208255296
chr2 208255300
chr2 208255317
chr2 208265576
chr2 208265718
chr2 208265779
chr2 208265883
chr2 208265892
chr2 208265907
chr2 208265959
chr2 208265969
chr2 208265976
chr2 208266892

chr2 208373798
chr2 208373900
chr2 208799572
chr2 208799735
chr2 209146423
chr2 209146490
chr2 209146604
chr2 209146888
chr2 209208238
chr2 209208246
chr2 209208312
chr2 209208321
chr2 209208323
chr2 209208366
chr2 209333037
chr2 209333099
chr2 209333104
chr2 209333440
chr2 209333598
chr2 209333613
chr2 209333883
chr2 209334006
chr2 209334407
chr2 209334566
chr2 209334574
chr2 209334637
chr2 209334670
chr2 209334713
chr2 209334726
chr2 209424570
chr2 209424585
chr2 209424592
chr2 209424611
chr2 209424664
chr2 209424851
chr2 209424858
chr2 209424892
chr2 209424908
chr2 209425027
chr2 209425182
chr2 209425234
chr2 209425264
chr2 209425299
chr2 209425316
chr2 209425652
chr2 209473052
chr2 209473220
chr2 209773222
chr2 209773610
chr2 209773616
chr2 209773695
chr2 209774203
chr2 209870119
chr2 209870321

chr2 210002335
chr2 210002383
chr2 210002397
chr2 210002403
chr2 210002449
chr2 210002456
chr2 210002465
chr2 210002470
chr2 210002479
chr2 210002483
chr2 210003488
chr2 210169569
chr2 210169670
chr2 210169720
chr2 210169992
chr2 210171718
chr2 210171754
chr2 210171770
chr2 210171774
chr2 210171776
chr2 210171779
chr2 210171787
chr2 210171800
chr2 210171804
chr2 210171806
chr2 210171813
chr2 210171817
chr2 210171820
chr2 210171831
chr2 210171865
chr2 210171875
chr2 210171879
chr2 210171898
chr2 210171902
chr2 210171912
chr2 210316111
chr2 210422091
chr2 210422095
chr2 210422258
chr2 210422322
chr2 210422603
chr2 210422609
chr2 210475985
chr2 210476191
chr2 210476200
chr2 210476203
chr2 210476265
chr2 210476272
chr2 210476321
chr2 210476342
chr2 210476376
chr2 210476399
chr2 210476402
chr2 210476458

chr2 210476462
chr2 210476469
chr2 210535359
chr2 210655766
chr2 210927011
chr2 210927208
chr2 210927249
chr2 210927691
chr2 210991959
chr2 210992279
chr2 210994771
chr2 210994938
chr2 210995280
chr2 210995386
chr2 210995875
chr2 210997153
chr2 211158039
chr2 211158049
chr2 211158101
chr2 211158142
chr2 211158373
chr2 211211298
chr2 211218529
chr2 211218780
chr2 211219022
chr2 211683172
chr2 211683236
chr2 211799412
chr2 211799685
chr2 211799917
chr2 212209490
chr2 212535286
chr2 212535450
chr2 212535531
chr2 212535631
chr2 212535867
chr2 212535973
chr2 212535987
chr2 212536009
chr2 212536057
chr2 212536085
chr2 212536145
chr2 212536147
chr2 212674469
chr2 212686310
chr2 212686380
chr2 212686464
chr2 212686490
chr2 212686496
chr2 212686562
chr2 213148597
chr2 213149147
chr2 213149392
chr2 213149479

chr2 213149647
chr2 213149933
chr2 213149962
chr2 213150035
chr2 213150048
chr2 213150130
chr2 213150196
chr2 213150244
chr2 213150259
chr2 213150413
chr2 213150562
chr2 213150624
chr2 213150653
chr2 213150668
chr2 213150714
chr2 213150835
chr2 213151066
chr2 213151102
chr2 213151136
chr2 213151148
chr2 213151275
chr2 213151389
chr2 213151402
chr2 213151506
chr2 213151596
chr2 213151614
chr2 213151642
chr2 213151647
chr2 213151657
chr2 213153192
chr2 213153264
chr2 213153401
chr2 213153502
chr2 213153644
chr2 213233642
chr2 213234290
chr2 213234770
chr2 213235468
chr2 213246766
chr2 213246861
chr2 213246876
chr2 213246921
chr2 213283940
chr2 213284234
chr2 213284248
chr2 213284252
chr2 213284260
chr2 213284262
chr2 213284279
chr2 213284287
chr2 213284301
chr2 213284303
chr2 213284311
chr2 213284330

chr2 213579679
chr2 213657383
chr2 213658260
chr2 213803006
chr2 213803552
chr2 213868548
chr2 214006823
chr2 214006900
chr2 214007331
chr2 214104049
chr2 214409173
chr2 214409835
chr2 214683822
chr2 214683828
chr2 214683831
chr2 214683840
chr2 214683906
chr2 214683929
chr2 214683933
chr2 214683935
chr2 214683940
chr2 214683950
chr2 214683967
chr2 214683974
chr2 214684024
chr2 214684039
chr2 214684054
chr2 214684063
chr2 214684071
chr2 214684075
chr2 214684087
chr2 214684090
chr2 214684110
chr2 214684133
chr2 214684136
chr2 214684191
chr2 214684214
chr2 214684218
chr2 214684540
chr2 214684544
chr2 214684727
chr2 214684749
chr2 214684768
chr2 214684799
chr2 214684824
chr2 214684838
chr2 214684845
chr2 214807730
chr2 214807821
chr2 214807864
chr2 214808062
chr2 214834339
chr2 214834416
chr2 214834441

chr2 214834449
chr2 214834504
chr2 215210408
chr2 215210457
chr2 215210687
chr2 215211292
chr2 215211563
chr2 215211704
chr2 215212229
chr2 215222232
chr2 215269605
chr2 215269650
chr2 215269805
chr2 215436575
chr2 215436618
chr2 215436641
chr2 215436674
chr2 215436710
chr2 215436730
chr2 215436763
chr2 215436767
chr2 215436771
chr2 215437143
chr2 215437451
chr2 215437544
chr2 215571906
chr2 215619606
chr2 215650054
chr2 215650364
chr2 215681125
chr2 215681385
chr2 215681503
chr2 215681520
chr2 215920298
chr2 215920312
chr2 216006181
chr2 216006235
chr2 216006322
chr2 216006324
chr2 216012789
chr2 216012795
chr2 216012861
chr2 216012865
chr2 216012883
chr2 216015892
chr2 216015949
chr2 216016055
chr2 216016209
chr2 216016707
chr2 216056993
chr2 216057738
chr2 216057974
chr2 216058015
chr2 216058093

chr2 216058136
chr2 216058139
chr2 216058145
chr2 216081423
chr2 216081478
chr2 216081486
chr2 216081501
chr2 216081536
chr2 216081539
chr2 216081556
chr2 216109538
chr2 216109540
chr2 216109665
chr2 216110550
chr2 216110581
chr2 216182736
chr2 216236110
chr2 216250853
chr2 216371038
chr2 216371119
chr2 216371138
chr2 216371188
chr2 216371509
chr2 216371512
chr2 216371516
chr2 216371526
chr2 216371531
chr2 216373064
chr2 216373214
chr2 216373265
chr2 216373517
chr2 216412529
chr2 216412541
chr2 216412552
chr2 216412560
chr2 216412568
chr2 216412574
chr2 216412578
chr2 216412594
chr2 216412596
chr2 216412612
chr2 216412618
chr2 216412629
chr2 216412631
chr2 216412677
chr2 216412680
chr2 216412686
chr2 216412690
chr2 216412753
chr2 216412772
chr2 216412777
chr2 216412781
chr2 216412807
chr2 216412812

chr2 216412819
chr2 216412824
chr2 216412833
chr2 216412870
chr2 216412881
chr2 216412925
chr2 216499368
chr2 216499485
chr2 216499518
chr2 216499574
chr2 216499595
chr2 216499603
chr2 216499752
chr2 216499782
chr2 216499964
chr2 216500014
chr2 216500025
chr2 216500170
chr2 216500337
chr2 216693022
chr2 216693039
chr2 216693052
chr2 216693116
chr2 216693459
chr2 216693474
chr2 216693479
chr2 216693519
chr2 216693635
chr2 216693692
chr2 216693700
chr2 216693844
chr2 216693911
chr2 216693923
chr2 216693995
chr2 216694057
chr2 216694062
chr2 216694064
chr2 216694150
chr2 216694175
chr2 216694197
chr2 216694204
chr2 216694231
chr2 216694249
chr2 216694266
chr2 216694297
chr2 217090020
chr2 217090063
chr2 217090096
chr2 217090180
chr2 217090202
chr2 217090268
chr2 217222686
chr2 217222709
chr2 217222718

chr2 217222738
chr2 217222809
chr2 217222951
chr2 217223174
chr2 217223321
chr2 217223356
chr2 217223399
chr2 217510029
chr2 217757193
chr2 218137474
chr2 218137479
chr2 218137484
chr2 218218844
chr2 218219112
chr2 218219241
chr2 218219304
chr2 218219375
chr2 218219410
chr2 218219412
chr2 218219434
chr2 218219466
chr2 218219592
chr2 218219596
chr2 218219601
chr2 218219722
chr2 218219919
chr2 218220267
chr2 218220287
chr2 218220402
chr2 218221058
chr2 218221080
chr2 218286680
chr2 218286721
chr2 218286845
chr2 218286908
chr2 218286932
chr2 218287020
chr2 218291980
chr2 218291995
chr2 218291997
chr2 218292019
chr2 218292108
chr2 218292134
chr2 218292150
chr2 218292156
chr2 218292170
chr2 218292195
chr2 218292216
chr2 218292255
chr2 218292259
chr2 218292267
chr2 218292269
chr2 218292271
chr2 218292284

chr2 218292289
chr2 218323522
chr2 218323527
chr2 218323563
chr2 218323571
chr2 218396097
chr2 218396103
chr2 218396106
chr2 218396110
chr2 218396118
chr2 218396123
chr2 218396139
chr2 218396146
chr2 218396151
chr2 218396154
chr2 218396156
chr2 218396164
chr2 218396168
chr2 218396172
chr2 218396179
chr2 218396181
chr2 218396203
chr2 218396235
chr2 218396261
chr2 218396277
chr2 218396306
chr2 218396353
chr2 218396356
chr2 218396386
chr2 218396394
chr2 218396399
chr2 218396442
chr2 218396467
chr2 218396495
chr2 218396506
chr2 218396508
chr2 218396518
chr2 218396522
chr2 218396564
chr2 218396569
chr2 218396603
chr2 218396613
chr2 218396626
chr2 218396655
chr2 218396659
chr2 218396727
chr2 218396734
chr2 218396780
chr2 218396857
chr2 218396868
chr2 218396908
chr2 218396911
chr2 218396950
chr2 218396960

chr2 218396967
chr2 218396972
chr2 218397014
chr2 218397017
chr2 218397080
chr2 218397120
chr2 218397144
chr2 218397151
chr2 218397185
chr2 218397193
chr2 218397240
chr2 218397242
chr2 218397273
chr2 218397338
chr2 218397405
chr2 218397411
chr2 218397449
chr2 218397461
chr2 218397543
chr2 218397586
chr2 218397683
chr2 218400136
chr2 218400143
chr2 218400148
chr2 218400162
chr2 218400170
chr2 218400182
chr2 218400188
chr2 218400193
chr2 218400196
chr2 218406475
chr2 218406485
chr2 218406495
chr2 218406612
chr2 218406615
chr2 218407092
chr2 218407111
chr2 218569712
chr2 218569714
chr2 218569890
chr2 218569935
chr2 218569980
chr2 218569997
chr2 218570056
chr2 218570067
chr2 218570293
chr2 218570328
chr2 218570559
chr2 218570678
chr2 218570691
chr2 218570836
chr2 218659215
chr2 218659227
chr2 218659270

chr2 218659291
chr2 218659298
chr2 218659319
chr2 218659328
chr2 218711398
chr2 218711413
chr2 218896375
chr2 218896386
chr2 218896454
chr2 218896458
chr2 218896485
chr2 218896550
chr2 218896620
chr2 218896633
chr2 218896637
chr2 218896715
chr2 218896741
chr2 218896745
chr2 218896760
chr2 218896768
chr2 218896780
chr2 218896797
chr2 218896806
chr2 218896813
chr2 218896837
chr2 218896903
chr2 218896905
chr2 218896970
chr2 219155126
chr2 219155187
chr2 219155271
chr2 219160656
chr2 219176446
chr2 219176463
chr2 219176465
chr2 219176477
chr2 219176529
chr2 219228944
chr2 219249207
chr2 219249296
chr2 219249317
chr2 219249348
chr2 219249567
chr2 219249820
chr2 219249854
chr2 219250186
chr2 219294541
chr2 219294549
chr2 219294562
chr2 219294582
chr2 219294605
chr2 219294717
chr2 219294723
chr2 219294750

chr2 219294754
chr2 219294757
chr2 219294759
chr2 219294772
chr2 219294774
chr2 219330522
chr2 219387864
chr2 219387866
chr2 219388110
chr2 219388117
chr2 219388130
chr2 219388134
chr2 219388146
chr2 219388159
chr2 219388163
chr2 219388168
chr2 219388175
chr2 219388188
chr2 219388192
chr2 219388197
chr2 219388204
chr2 219388217
chr2 219388226
chr2 219388236
chr2 219388261
chr2 219388301
chr2 219388312
chr2 219388317
chr2 219388324
chr2 219388339
chr2 219388346
chr2 219388361
chr2 219388366
chr2 219388375
chr2 219388380
chr2 219388387
chr2 219441838
chr2 219441849
chr2 219441859
chr2 219441862
chr2 219441865
chr2 219441868
chr2 219441871
chr2 219441880
chr2 219441883
chr2 219441886
chr2 219441889
chr2 219441892
chr2 219476493
chr2 219509567
chr2 219509806
chr2 219509815
chr2 219509822
chr2 219509827

chr2 219509937
chr2 219509941
chr2 219509947
chr2 219509954
chr2 219509962
chr2 219510002
chr2 219510084
chr2 219510087
chr2 219510122
chr2 219510331
chr2 219513423
chr2 219513504
chr2 219513516
chr2 219513545
chr2 219513610
chr2 219513663
chr2 219513937
chr2 219752871
chr2 219752874
chr2 220686911
chr2 220687284
chr2 220687371
chr2 220687374
chr2 221037678
chr2 221299175
chr2 221299415
chr2 221299479
chr2 221299638
chr2 221299737
chr2 221299760
chr2 221517465
chr2 221517565
chr2 221517570
chr2 221517577
chr2 221517787
chr2 221517912
chr2 221517932
chr2 221517944
chr2 221518044
chr2 221518340
chr2 221518351
chr2 221518457
chr2 221518511
chr2 221518548
chr2 221518646
chr2 221518800
chr2 221574381
chr2 221574406
chr2 221574504
chr2 221574558
chr2 221574648
chr2 221574849
chr2 221672658
chr2 222152816

chr2 222152855
chr2 222154339
chr2 222154353
chr2 222154394
chr2 222154399
chr2 222154426
chr2 222154435
chr2 222154607
chr2 222154638
chr2 222155141
chr2 222241159
chr2 222241209
chr2 222241298
chr2 222241300
chr2 222241439
chr2 222241448
chr2 222241481
chr2 222288067
chr2 222288146
chr2 222288468
chr2 222288724
chr2 222288882
chr2 222288938
chr2 222288940
chr2 222288946
chr2 222289199
chr2 222309467
chr2 222310036
chr2 222310147
chr2 222310178
chr2 222310258
chr2 222310280
chr2 222310314
chr2 222310413
chr2 222310421
chr2 222310615
chr2 222310665
chr2 222310704
chr2 222310713
chr2 222313625
chr2 222313651
chr2 222314239
chr2 222314521
chr2 222317094
chr2 222317381
chr2 222317443
chr2 222317753
chr2 222317917
chr2 222317934
chr2 222318056
chr2 222655150
chr2 222655152
chr2 222655185
chr2 222655444

chr2 222655461
chr2 222655496
chr2 222655507
chr2 222655512
chr2 222655566
chr2 222655588
chr2 222655606
chr2 222655618
chr2 222656443
chr2 222656476
chr2 222656527
chr2 222656530
chr2 222656590
chr2 222656627
chr2 222701630
chr2 222701633
chr2 222701732
chr2 222701764
chr2 222701865
chr2 222701893
chr2 222701928
chr2 223601921
chr2 223602108
chr2 223602182
chr2 223602331
chr2 223602338
chr2 223602342
chr2 223602351
chr2 223602356
chr2 223602368
chr2 223602395
chr2 223602400
chr2 223602410
chr2 223602426
chr2 223602432
chr2 223602460
chr2 223602558
chr2 223602622
chr2 223602641
chr2 223602762
chr2 223602765
chr2 223603177
chr2 223603200
chr2 223929575
chr2 223929696
chr2 223929730
chr2 223929779
chr2 223944714
chr2 223944727
chr2 223944729
chr2 223944832
chr2 223944859
chr2 223944862
chr2 223944864

chr2 223944872
chr2 223944881
chr2 223944894
chr2 223944896
chr2 223944913
chr2 223944926
chr2 223944928
chr2 223944931
chr2 223944937
chr2 223956867
chr2 223956895
chr2 223957003
chr2 223957027
chr2 223957122
chr2 223957152
chr2 223957956
chr2 223957978
chr2 223957985
chr2 223958009
chr2 223958332
chr2 223958334
chr2 223958345
chr2 223958437
chr2 224025955
chr2 224025997
chr2 224357696
chr2 224579151
chr2 224584107
chr2 224584117
chr2 224584125
chr2 224584162
chr2 224584181
chr2 224584200
chr2 224584207
chr2 224584261
chr2 224584291
chr2 224584302
chr2 224584309
chr2 224584366
chr2 224584403
chr2 224584413
chr2 224586142
chr2 224586304
chr2 224586362
chr2 224586421
chr2 224586429
chr2 224586462
chr2 224586557
chr2 224894225
chr2 224922655
chr2 224922983
chr2 224925532
chr2 224925578
chr2 224926438

chr2 224926518
chr2 224926631
chr2 224943488
chr2 224944833
chr2 224944856
chr2 224944859
chr2 224944936
chr2 224944938
chr2 224944951
chr2 224945077
chr2 224945091
chr2 224945805
chr2 224945811
chr2 224945912
chr2 224945966
chr2 224945990
chr2 224946065
chr2 224946652
chr2 224946666
chr2 224946893
chr2 224946923
chr2 224946941
chr2 224946952
chr2 224946998
chr2 224947021
chr2 224947221
chr2 224947272
chr2 224947324
chr2 224947460
chr2 224953799
chr2 224954495
chr2 225052522
chr2 225266461
chr2 225266484
chr2 225266623
chr2 225266768
chr2 225266785
chr2 225267150
chr2 225464772
chr2 225581116
chr2 225585445
chr2 225585472
chr2 225585656
chr2 225585747
chr2 225586152
chr2 225983189
chr2 225983576
chr2 225983984
chr2 225984324
chr2 226030438
chr2 226030445
chr2 226030483
chr2 226030635
chr2 226030637

chr2 226030639
chr2 226030753
chr2 226030755
chr2 226030786
chr2 226030821
chr2 226030827
chr2 226030861
chr2 226030878
chr2 226030884
chr2 226031140
chr2 226031158
chr2 226031180
chr2 226031200
chr2 226031217
chr2 226031220
chr2 226031231
chr2 226031235
chr2 226031239
chr2 226031247
chr2 226031254
chr2 226031282
chr2 226031292
chr2 226031300
chr2 226031380
chr2 226031420
chr2 226031459
chr2 226031465
chr2 226031476
chr2 226031487
chr2 226031489
chr2 226031491
chr2 226031503
chr2 226031509
chr2 226031545
chr2 226031551
chr2 226031603
chr2 226031746
chr2 226031789
chr2 226031818
chr2 226031887
chr2 226032020
chr2 226032079
chr2 226423001
chr2 226801510
chr2 226801536
chr2 226801540
chr2 226802011
chr2 226802121
chr2 226802257
chr2 226802519
chr2 226802521
chr2 226802652
chr2 226802720
chr2 226802796

chr2 226802853
chr2 226803087
chr2 227025646
chr2 227165151
chr2 227165167
chr2 227165175
chr2 227165208
chr2 227165250
chr2 227165269
chr2 227165396
chr2 227165537
chr2 227325326
chr2 227325332
chr2 227325360
chr2 227325368
chr2 227325372
chr2 227325377
chr2 227325391
chr2 227325401
chr2 227325404
chr2 227325439
chr2 227325447
chr2 227325452
chr2 227325454
chr2 227325462
chr2 227325466
chr2 227325487
chr2 227325490
chr2 227325493
chr2 227325496
chr2 227325506
chr2 227325521
chr2 227325523
chr2 227325532
chr2 227325541
chr2 227325560
chr2 227325567
chr2 227325573
chr2 227325611
chr2 227325627
chr2 227325636
chr2 227325649
chr2 227325672
chr2 227325696
chr2 227325699
chr2 227325721
chr2 227325731
chr2 227325760
chr2 227325769
chr2 227325775
chr2 227325781
chr2 227325784
chr2 227325954
chr2 227325968

chr2 227325988
chr2 227326016
chr2 227326043
chr2 227326056
chr2 227326068
chr2 227326071
chr2 227326097
chr2 227326116
chr2 227326170
chr2 227326298
chr2 227460254
chr2 227460267
chr2 227460302
chr2 227460356
chr2 227472649
chr2 227472655
chr2 227472666
chr2 227472683
chr2 227472690
chr2 227472699
chr2 227472703
chr2 227472709
chr2 227472713
chr2 227472720
chr2 227472723
chr2 227472730
chr2 227472734
chr2 227472736
chr2 227472739
chr2 227472751
chr2 227472762
chr2 227472771
chr2 227472779
chr2 227472821
chr2 227472837
chr2 227472848
chr2 227472854
chr2 227473128
chr2 227473158
chr2 227473242
chr2 227473257
chr2 227473313
chr2 227473423
chr2 227473436
chr2 227473504
chr2 227473582
chr2 227473596
chr2 227473671
chr2 227474093
chr2 227608719
chr2 227608780
chr2 227608810
chr2 227608817
chr2 227766372

chr2 227766387
chr2 227871994
chr2 227872000
chr2 228599686
chr2 228599768
chr2 229368189
chr2 229716083
chr2 229716111
chr2 229716145
chr2 229716650
chr2 229716682
chr2 229822664
chr2 229850079
chr2 229852184
chr2 229852733
chr2 229852768
chr2 229852771
chr2 229920070
chr2 229920083
chr2 229920195
chr2 229920289
chr2 229920334
chr2 229920346
chr2 229920353
chr2 229920380
chr2 229920387
chr2 229920389
chr2 229920424
chr2 229920437
chr2 229920448
chr2 229920458
chr2 229920464
chr2 229920474
chr2 229920479
chr2 229920558
chr2 229920646
chr2 229920662
chr2 229920720
chr2 229920747
chr2 229920757
chr2 229920800
chr2 229920803
chr2 229920925
chr2 229920972
chr2 229920981
chr2 229920998
chr2 229921115
chr2 229921147
chr2 229921179
chr2 229921197
chr2 229921199
chr2 230067172
chr2 230067180
chr2 230067204

chr2 230067206
chr2 230067223
chr2 230067270
chr2 230067284
chr2 230067315
chr2 230067375
chr2 230067461
chr2 230067590
chr2 230067596
chr2 230067600
chr2 230067688
chr2 230067753
chr2 230067770
chr2 230067780
chr2 230067789
chr2 230067795
chr2 230125163
chr2 230125198
chr2 230125238
chr2 230125279
chr2 230125347
chr2 230125720
chr2 230125734
chr2 230225614
chr2 230225622
chr2 230225661
chr2 230225708
chr2 230225758
chr2 230225801
chr2 230225816
chr2 230225821
chr2 230226030
chr2 230226088
chr2 230226195
chr2 230226297
chr2 230226340
chr2 230318605
chr2 230318654
chr2 230319199
chr2 230319237
chr2 230319240
chr2 230319322
chr2 230319408
chr2 230319552
chr2 230327560
chr2 230327579
chr2 230411407
chr2 230411444
chr2 230411462
chr2 230411490
chr2 230411499
chr2 230411540
chr2 230411560
chr2 230411599

chr2 230411626
chr2 230411648
chr2 230411661
chr2 230411665
chr2 230411707
chr2 230411724
chr2 230411733
chr2 230411753
chr2 230411756
chr2 230411761
chr2 230411771
chr2 230411823
chr2 230411845
chr2 230411866
chr2 230411909
chr2 230415966
chr2 230415983
chr2 230416890
chr2 230417219
chr2 230417654
chr2 230417712
chr2 230417803
chr2 230417827
chr2 230417989
chr2 230418060
chr2 230418069
chr2 230418180
chr2 230418347
chr2 230441251
chr2 230441664
chr2 230441681
chr2 230441752
chr2 230602730
chr2 230602939
chr2 230602979
chr2 230603030
chr2 230603078
chr2 230603292
chr2 230636774
chr2 230636783
chr2 230637016
chr2 230637168
chr2 230637194
chr2 230643756
chr2 230643849
chr2 230643949
chr2 230643955
chr2 230644171
chr2 230644221
chr2 230644271
chr2 230658894
chr2 230658971
chr2 230658976
chr2 230659095

chr2 230659118
chr2 230660713
chr2 230660771
chr2 230660789
chr2 230660810
chr2 230660854
chr2 230661012
chr2 230661146
chr2 230661522
chr2 230661599
chr2 230661683
chr2 230661866
chr2 230714140
chr2 230714257
chr2 230714286
chr2 230714521
chr2 230714525
chr2 230714564
chr2 230714684
chr2 230714826
chr2 230714842
chr2 230714894
chr2 230715060
chr2 230723180
chr2 230723287
chr2 230723509
chr2 230793941
chr2 230794039
chr2 230794185
chr2 230794193
chr2 230794258
chr2 230794264
chr2 230956993
chr2 230957032
chr2 230957050
chr2 230957072
chr2 230957231
chr2 230957271
chr2 230957290
chr2 230957340
chr2 230957348
chr2 230957363
chr2 230957373
chr2 230957377
chr2 230957400
chr2 230957622
chr2 230957624
chr2 230957683
chr2 230974065
chr2 230974079
chr2 230974145
chr2 230974194
chr2 230978887
chr2 230979414

chr2 230979436
chr2 231104436
chr2 231462441
chr2 231462588
chr2 231462641
chr2 231463006
chr2 231463395
chr2 231463407
chr2 231545294
chr2 231545307
chr2 231545477
chr2 231545592
chr2 231545648
chr2 231545741
chr2 231545815
chr2 231711282
chr2 231711398
chr2 231711410
chr2 231711457
chr2 231711546
chr2 231711566
chr2 231711571
chr2 231711573
chr2 231711608
chr2 231711615
chr2 231711647
chr2 231711796
chr2 231711817
chr2 231711833
chr2 231711883
chr2 231711931
chr2 231712809
chr2 231712815
chr2 231712891
chr2 231712894
chr2 231712919
chr2 231712935
chr2 231712947
chr2 231712961
chr2 231712965
chr2 231712972
chr2 231712988
chr2 231712999
chr2 231713001
chr2 231713181
chr2 231713201
chr2 231713206
chr2 231769698
chr2 231769891
chr2 231770083
chr2 231770118
chr2 231782126
chr2 231782157
chr2 231782185

chr2 231782271
chr2 231782334
chr2 232154253
chr2 232219667
chr2 232363922
chr2 232363931
chr2 232363953
chr2 232363971
chr2 232364151
chr2 232364179
chr2 232412832
chr2 232412956
chr2 232413044
chr2 232413101
chr2 232413119
chr2 232413130
chr2 232413192
chr2 232554430
chr2 232697383
chr2 232697388
chr2 232697402
chr2 232697407
chr2 232697412
chr2 232697424
chr2 232697543
chr2 232697548
chr2 232697552
chr2 232697562
chr2 232697572
chr2 232697594
chr2 232697615
chr2 232697622
chr2 232697637
chr2 232697639
chr2 232697644
chr2 232697648
chr2 232697650
chr2 232697658
chr2 232697922
chr2 232697938
chr2 232697943
chr2 232697974
chr2 232697994
chr2 232698008
chr2 232698124
chr2 232698144
chr2 232698172
chr2 232698177
chr2 232698185
chr2 232698255
chr2 232698287
chr2 233060320
chr2 233060636
chr2 233060733

chr2 233061043
chr2 233061071
chr2 233061400
chr2 233061453
chr2 233061471
chr2 233061546
chr2 233061640
chr2 233061770
chr2 233061842
chr2 233061938
chr2 233061948
chr2 233062031
chr2 233062078
chr2 233062216
chr2 233076060
chr2 233076082
chr2 233076394
chr2 233076706
chr2 233076901
chr2 233077040
chr2 233077048
chr2 233233972
chr2 233234031
chr2 233268343
chr2 233268373
chr2 233268390
chr2 233268407
chr2 233399360
chr2 233418363
chr2 233418474
chr2 233418802
chr2 233418831
chr2 233418853
chr2 233418875
chr2 233418886
chr2 233565854
chr2 233565920
chr2 233565983
chr2 233566122
chr2 233566136
chr2 233566175
chr2 233566182
chr2 233566247
chr2 233566324
chr2 233566331
chr2 233566935
chr2 233566947
chr2 233566949
chr2 233566959
chr2 233566966
chr2 233566979
chr2 233578662
chr2 233579313
chr2 233579318

chr2 233855000
chr2 233855035
chr2 233855066
chr2 233865379
chr2 233865403
chr2 233865502
chr2 233865599
chr2 233865780
chr2 233868347
chr2 233868350
chr2 233868353
chr2 233868360
chr2 233868364
chr2 233868366
chr2 233868371
chr2 233868375
chr2 233868381
chr2 233868386
chr2 233868389
chr2 233868395
chr2 233868397
chr2 233868405
chr2 233868407
chr2 233868409
chr2 233868411
chr2 233868414
chr2 233868420
chr2 233868423
chr2 233868426
chr2 233868432
chr2 233868434
chr2 233868439
chr2 233868447
chr2 233868451
chr2 233868561
chr2 233868581
chr2 233868659
chr2 233868753
chr2 233990539
chr2 233990628
chr2 233990704
chr2 233990717
chr2 233990842
chr2 234025600
chr2 234025656
chr2 234025665
chr2 234025689
chr2 234068727
chr2 234292328
chr2 234292418
chr2 234292538
chr2 234292543
chr2 234292572
chr2 234292616

chr2 234292629
chr2 234292657
chr2 234292663
chr2 234292710
chr2 234425921
chr2 234426106
chr2 234426120
chr2 234426147
chr2 234426200
chr2 234426285
chr2 234426614
chr2 234426636
chr2 234446504
chr2 234446513
chr2 234459852
chr2 234459862
chr2 234460330
chr2 234460547
chr2 234483605
chr2 234483750
chr2 234483753
chr2 234483827
chr2 234483832
chr2 234484001
chr2 234484107
chr2 234488891
chr2 234489018
chr2 234490652
chr2 234490903
chr2 234490952
chr2 234490967
chr2 234490993
chr2 234491025
chr2 234491039
chr2 234491056
chr2 234491064
chr2 234491099
chr2 234491107
chr2 234491150
chr2 234491176
chr2 234491408
chr2 234491435
chr2 234491507
chr2 234491571
chr2 234493573
chr2 234493737
chr2 234493841
chr2 234493945
chr2 234494062
chr2 234494125
chr2 234494431
chr2 234494497
chr2 234494525
chr2 234494574

chr2 234494638
chr2 234494835
chr2 234494837
chr2 234494986
chr2 234495136
chr2 234495309
chr2 234495311
chr2 234495338
chr2 234495343
chr2 234495349
chr2 234495357
chr2 234495449
chr2 234495529
chr2 234495535
chr2 234495574
chr2 234495591
chr2 234495634
chr2 234495648
chr2 234495682
chr2 234495695
chr2 234495714
chr2 234495716
chr2 234495724
chr2 234545009
chr2 234545041
chr2 234646400
chr2 234647304
chr2 234648035
chr2 234648436
chr2 234648487
chr2 234648519
chr2 234648535
chr2 234649321
chr2 234892934
chr2 234892939
chr2 234893491
chr2 234893589
chr2 234893846
chr2 235136539
chr2 235211240
chr2 235217646
chr2 235218231
chr2 235218233
chr2 235218235
chr2 235218237
chr2 235218241
chr2 235218251
chr2 235218255
chr2 235218257
chr2 235218259
chr2 235218263
chr2 235218265
chr2 235218267
chr2 235218273

chr2 235218277
chr2 235666128
chr2 235666130
chr2 235666152
chr2 235666165
chr2 235666404
chr2 235666428
chr2 235666657
chr2 235666664
chr2 235921395
chr2 235921547
chr2 235921600
chr2 236050065
chr2 236050199
chr2 236050211
chr2 236174974
chr2 236175150
chr2 236175290
chr2 236175596
chr2 236175681
chr2 236175708
chr2 236338926
chr2 236338962
chr2 236338974
chr2 236339155
chr2 236339173
chr2 236366475
chr2 236366549
chr2 236366720
chr2 236366852
chr2 236366872
chr2 236366894
chr2 236366902
chr2 236366970
chr2 236381321
chr2 236381361
chr2 236381429
chr2 236381470
chr2 236381477
chr2 236381538
chr2 236383036
chr2 236383121
chr2 236512963
chr2 236513048
chr2 236513089
chr2 236513141
chr2 236513241
chr2 236513272
chr2 236513382
chr2 236537153
chr2 236537186
chr2 236546096
chr2 236546101
chr2 236546213

chr2 236546294
chr2 236546563
chr2 236546574
chr2 236546591
chr2 236546665
chr2 236546732
chr2 236546748
chr2 236546797
chr2 236547005
chr2 236569728
chr2 236676407
chr2 237086208
chr2 237086214
chr2 237086223
chr2 237086229
chr2 237086232
chr2 237086269
chr2 237086282
chr2 237086303
chr2 237086344
chr2 237086416
chr2 237086427
chr2 237086484
chr2 237086699
chr2 237086734
chr2 237291445
chr2 237291447
chr2 237291463
chr2 237291508
chr2 237291535
chr2 237291558
chr2 237291561
chr2 237291563
chr2 237291576
chr2 237291580
chr2 237291594
chr2 237291658
chr2 237291695
chr2 237291995
chr2 237543996
chr2 237544082
chr2 237544116
chr2 237673537
chr2 237673548
chr2 237673581
chr2 237673591
chr2 237673595
chr2 237673599
chr2 237673617
chr2 237673631
chr2 237673634
chr2 237673649
chr2 237673709
chr2 237673721

chr2 237673795
chr2 237673824
chr2 237673830
chr2 237673834
chr2 237673846
chr2 237673864
chr2 237694487
chr2 237694497
chr2 237694591
chr2 237694616
chr2 237694637
chr2 237694658
chr2 237694783
chr2 237694788
chr2 237694842
chr2 237694860
chr2 237694941
chr2 237695049
chr2 237695054
chr2 237695093
chr2 237695134
chr2 237695274
chr2 237695366
chr2 237695377
chr2 237695506
chr2 237695587
chr2 237695739
chr2 237696799
chr2 237696821
chr2 237697013
chr2 237752976
chr2 237752979
chr2 237753063
chr2 237998606
chr2 237998639
chr2 237998888
chr2 237998904
chr2 238377208
chr2 238776686
chr2 238776722
chr2 238776766
chr2 238776806
chr2 238776842
chr2 238776846
chr2 238776886
chr2 238776926
chr2 238776966
chr2 238777006
chr2 238777046
chr2 238777086
chr2 238777126
chr2 238798930
chr2 239218312
chr2 239218882

chr2 239289242
chr2 239289336
chr2 239289383
chr2 239289464
chr2 239289665
chr2 239289711
chr2 239289741
chr2 239289768
chr2 239289818
chr2 239381437
chr2 239623258
chr2 239623270
chr2 239623291
chr2 239623299
chr2 239623303
chr2 239623440
chr2 239623894
chr2 239659373
chr2 239659376
chr2 239659382
chr2 239659404
chr2 239659551
chr2 239707869
chr2 239708010
chr2 239708025
chr2 240024310
chr2 240024356
chr2 240024520
chr2 240024554
chr2 240024622
chr2 240024649
chr2 240024725
chr2 240121356
chr2 240121389
chr2 240233156
chr2 240233160
chr2 240233170
chr2 240233173
chr2 240233184
chr2 240233192
chr2 240233209
chr2 240233246
chr2 240233255
chr2 240233286
chr2 240233303
chr2 240233338
chr2 240233344
chr2 240233372
chr2 240233399
chr2 240233433
chr2 240233436
chr2 240233449
chr2 240233455
chr2 240561609

chr2 240565660
chr2 240565698
chr2 240565743
chr2 240565759
chr2 240565771
chr2 240565784
chr2 240565856
chr2 240565909
chr2 240565923
chr2 240565926
chr2 240565930
chr2 240565984
chr2 240565994
chr2 240565996
chr2 240566033
chr2 240566116
chr2 240566215
chr2 240566217
chr2 240566278
chr2 240566330
chr2 240566364
chr2 240566366
chr2 240566382
chr2 240566443
chr2 240566445
chr2 240566448
chr2 240566506
chr2 240732381
chr2 240732411
chr2 240732453
chr2 240732673
chr2 240732686
chr2 240732815
chr2 240732820
chr2 240979929
chr2 240981300
chr2 240984454
chr2 241036339
chr2 241102839
chr2 241102886
chr2 241218793
chr2 241218822
chr2 241218831
chr2 241218860
chr2 241218877
chr2 241218895
chr2 241218959
chr2 241218978
chr2 241316712
chr2 241316756
chr2 241316766
chr2 241316893
chr2 241317016
chr2 241317073

chr2 241317104
chr2 241336529
chr2 241355897
chr2 241443934
chr2 241444079
chr2 241444245
chr2 241687620
chr2 241687630
chr2 241687652
chr2 241687672
chr2 241687676
chr2 241687681
chr2 241687684
chr2 241687688
chr2 241687717
chr2 241687728
chr2 241687759
chr2 241687770
chr2 241687775
chr2 241687803
chr2 241687813
chr2 241687815
chr2 241687823
chr2 241687832
chr2 241687865
chr2 241687876
chr2 241741372
chr2 241741431
chr2 241804912
chr2 241804998
chr2 241805025
chr2 241805044
chr2 241805047
chr2 241805057
chr2 241805080
chr2 241805145
chr2 241805149
chr2 241805194
chr2 241805236
chr2 241805275
chr2 241806107
chr2 241806146
chr2 241806181
chr2 241806369
chr2 241806446
chr2 241806598
chr2 241806637
chr2 241806941
chr2 241807128
chr2 241881296
chr2 241881300
chr2 241881312
chr2 241881323
chr2 241881334

chr2 241881341
chr2 241881349
chr2 241881358
chr2 241881392
chr2 241881394
chr2 241881411
chr2 241881461
chr2 241881471
chr2 241881486
chr2 241881496
chr2 241881498
chr2 241881513
chr2 241881528
chr2 241881538
chr2 241881551
chr2 241881561
chr2 241881584
chr2 241881594
chr2 241888738
chr2 241965617
chr2 241965952
chr2 241965980
chr2 241966017
chr2 241966067
chr2 241966183
chr2 241966210
chr2 241966235
chr2 241966243
chr2 241966266
chr2 242000475
chr2 242000535
chr2 242085416
chr2 242085419
chr2 242085613
chr2 242085644
chr2 242085650
chr2 242085705
chr2 242085906
chr2 242085987
chr2 242086047
chr2 242086057
chr2 242086090
chr2 242086533
chr2 242086576
chr2 242089166
chr2 242089170
chr2 242089173
chr2 242089257
chr2 242089279
chr2 242089290
chr2 242089309
chr2 242089322
chr2 242089334
chr2 242089339

chr2 242089356
chr2 242089362
chr2 242089372
chr2 242089375
chr2 242089386
chr2 242089392
chr2 242089407
chr2 242089410
chr2 242089461
chr2 242154774
chr2 242154781
chr2 242155609
chr2 242183233
chr2 242183318
chr2 242183322
chr2 242183328
chr2 242183334
chr2 242183340
chr2 242183346
chr2 242183383
chr2 242183389
chr2 242183395
chr18 18000
chr18 19189
chr18 20064
chr18 20096
chr18 20686
chr18 20873
chr18 39522
chr18 39802
chr18 40681
chr18 537561
chr18 537584
chr18 537822
chr18 650116
chr18 650438
chr18 650461
chr18 650509
chr18 650547
chr18 650558
chr18 711283
chr18 711403
chr18 711415
chr18 711466
chr18 711531
chr18 711533
chr18 711556
chr18 711632
chr18 711641
chr18 712032
chr18 712036
chr18 712061
chr18 712132
chr18 715830

chr18 716220
chr18 810225
chr18 810399
chr18 810515
chr18 810691
chr18 810855
chr18 810935
chr18 811003
chr18 811035
chr18 811156
chr18 811176
chr18 811183
chr18 811278
chr18 811321
chr18 811501
chr18 811505
chr18 811510
chr18 811517
chr18 813306
chr18 813493
chr18 813496
chr18 813533
chr18 813603
chr18 813614
chr18 813620
chr18 813645
chr18 820828
chr18 820870
chr18 820920
chr18 821029
chr18 830369
chr18 830391
chr18 830593
chr18 830600
chr18 830765
chr18 831146
chr18 864910
chr18 864948
chr18 864964
chr18 864977
chr18 864988
chr18 865004
chr18 865008
chr18 865011
chr18 865027
chr18 865034
chr18 865040
chr18 865053
chr18 865064
chr18 865070
chr18 865122
chr18 903004
chr18 903422
chr18 903528

chr18 903557
chr18 903630
chr18 903659
chr18 903677
chr18 909933
chr18 909954
chr18 911317
chr18 911548
chr18 911601
chr18 960547
chr18 961063
chr18 961404
chr18 961733
chr18 961746
chr18 961766
chr18 961778
chr18 961789
chr18 961849
chr18 961871
chr18 961916
chr18 961920
chr18 961922
chr18 961969
chr18 1408247
chr18 1408385
chr18 1585755
chr18 1585757
chr18 1585968
chr18 1585986
chr18 1586011
chr18 1586025
chr18 1586125
chr18 1586127
chr18 1680615
chr18 1680910
chr18 1681062
chr18 1681441
chr18 1681540
chr18 1681932
chr18 1774469
chr18 1775335
chr18 2338591
chr18 2338664
chr18 2338755
chr18 2570852
chr18 2570951
chr18 2570983
chr18 2572411
chr18 2572662
chr18 2572687
chr18 2572698
chr18 2606214
chr18 2636321
chr18 2636365

chr18 2636426
chr18 2636606
chr18 2636633
chr18 2636637
chr18 2636743
chr18 2636751
chr18 2637138
chr18 2637230
chr18 2637466
chr18 2637575
chr18 2641048
chr18 2641285
chr18 2641291
chr18 2641315
chr18 2641423
chr18 2641455
chr18 2641515
chr18 2641809
chr18 2641872
chr18 2644611
chr18 2657391
chr18 2657404
chr18 2657490
chr18 2657572
chr18 2657724
chr18 2657781
chr18 2657875
chr18 2657931
chr18 2657963
chr18 2657972
chr18 2657980
chr18 2658012
chr18 2658039
chr18 2658066
chr18 2658111
chr18 2658195
chr18 2658267
chr18 2658343
chr18 2658560
chr18 2869369
chr18 2956349
chr18 2956421
chr18 2956427
chr18 2956450
chr18 2956482
chr18 2956996
chr18 2957167
chr18 2958718
chr18 2958742
chr18 2959424
chr18 2959544
chr18 2960431
chr18 2960486
chr18 2962135

chr18 2962174
chr18 2962176
chr18 2962223
chr18 2962317
chr18 2962970
chr18 2971365
chr18 2971681
chr18 2971775
chr18 2971785
chr18 2971947
chr18 2971954
chr18 2971961
chr18 2972001
chr18 2972003
chr18 2972025
chr18 2972226
chr18 2972258
chr18 2972308
chr18 2972321
chr18 2972323
chr18 2972368
chr18 2972388
chr18 2972466
chr18 2972626
chr18 2972671
chr18 2972719
chr18 2973485
chr18 2980428
chr18 2980509
chr18 2980517
chr18 2980724
chr18 2980730
chr18 2980758
chr18 2981331
chr18 2981400
chr18 2981419
chr18 2981558
chr18 2981680
chr18 2981718
chr18 2982225
chr18 2994793
chr18 2994796
chr18 2994799
chr18 2994809
chr18 2994925
chr18 2998649
chr18 2998789
chr18 2998813
chr18 3058910
chr18 3060634
chr18 3060668
chr18 3060727
chr18 3060730
chr18 3060813

chr18 3061069
chr18 3061091
chr18 3061203
chr18 3061298
chr18 3061317
chr18 3246241
chr18 3246260
chr18 3246266
chr18 3246268
chr18 3246274
chr18 3246290
chr18 3246293
chr18 3246308
chr18 3246319
chr18 3246325
chr18 3246328
chr18 3246393
chr18 3246415
chr18 3246536
chr18 3246547
chr18 3246566
chr18 3246583
chr18 3246588
chr18 3246594
chr18 3246618
chr18 3246641
chr18 3246643
chr18 3246646
chr18 3246665
chr18 3246687
chr18 3246703
chr18 3246746
chr18 3246786
chr18 3246791
chr18 3246869
chr18 3246880
chr18 3248799
chr18 3248816
chr18 3248856
chr18 3248958
chr18 3249014
chr18 3249090
chr18 3249958
chr18 3249989
chr18 3250129
chr18 3250184
chr18 3250276
chr18 3250543
chr18 3250693
chr18 3250742
chr18 3250862
chr18 3251036
chr18 3260946
chr18 3260986

chr18 3262888
chr18 3262947
chr18 3262996
chr18 3263002
chr18 3263027
chr18 3263073
chr18 3263084
chr18 3263087
chr18 3263120
chr18 3263233
chr18 3263265
chr18 3263273
chr18 3263374
chr18 3263422
chr18 3263515
chr18 3263522
chr18 3263631
chr18 3264604
chr18 3297054
chr18 3297072
chr18 3297145
chr18 3297164
chr18 3297178
chr18 3297188
chr18 3297200
chr18 3297221
chr18 3297240
chr18 3297320
chr18 3297367
chr18 3297386
chr18 3297407
chr18 3297471
chr18 3297555
chr18 3297579
chr18 3297697
chr18 3298104
chr18 3447405
chr18 3447456
chr18 3447465
chr18 3447538
chr18 3447543
chr18 3447585
chr18 3451075
chr18 3451081
chr18 3451144
chr18 3451198
chr18 3451216
chr18 3453872
chr18 3453893
chr18 3453911
chr18 3454019
chr18 3454050
chr18 3454177
chr18 3454179

chr18 3454432
chr18 3454510
chr18 3454955
chr18 3454981
chr18 3458702
chr18 3458714
chr18 3458749
chr18 3458780
chr18 3458807
chr18 3495810
chr18 3495849
chr18 3495942
chr18 3495967
chr18 3512190
chr18 3512299
chr18 3593655
chr18 3593694
chr18 3593717
chr18 3593757
chr18 3593800
chr18 3593814
chr18 3594000
chr18 3594062
chr18 3594100
chr18 3594652
chr18 3594710
chr18 3594724
chr18 3595031
chr18 3595044
chr18 3595056
chr18 3595059
chr18 3595115
chr18 3595154
chr18 3595291
chr18 3595734
chr18 3595778
chr18 3596455
chr18 3596815
chr18 3596865
chr18 3596887
chr18 3602771
chr18 3602813
chr18 3602826
chr18 3602860
chr18 3602876
chr18 3602891
chr18 3602928
chr18 3602982
chr18 3603036
chr18 3603068
chr18 3603123
chr18 3603143
chr18 3603228
chr18 3603241

chr18 3604245
chr18 3604449
chr18 3604455
chr18 3604488
chr18 3604557
chr18 3604575
chr18 3604850
chr18 3604909
chr18 3604917
chr18 3604983
chr18 3605213
chr18 3605221
chr18 3605260
chr18 3605268
chr18 3605272
chr18 3605290
chr18 3605473
chr18 3605816
chr18 3605862
chr18 3605887
chr18 3606005
chr18 3606031
chr18 3606228
chr18 3606238
chr18 3606261
chr18 3606282
chr18 3606302
chr18 3606338
chr18 3606357
chr18 3715603
chr18 3715657
chr18 3730593
chr18 3730622
chr18 3771541
chr18 3771545
chr18 3771549
chr18 3771554
chr18 3771559
chr18 3771570
chr18 3771625
chr18 3771657
chr18 3771736
chr18 3771799
chr18 3771899
chr18 3771925
chr18 3771941
chr18 3771982
chr18 3771994
chr18 3989959
chr18 4306156
chr18 4306360
chr18 4306671
chr18 4306788
chr18 4329605

chr18 4329644
chr18 4329893
chr18 4453265
chr18 4453496
chr18 4453514
chr18 5004788
chr18 5004808
chr18 5004897
chr18 5057918
chr18 5057923
chr18 5057961
chr18 5058069
chr18 5058097
chr18 5058165
chr18 5058224
chr18 5058389
chr18 5058464
chr18 5058878
chr18 5058895
chr18 5058921
chr18 5058949
chr18 5059085
chr18 5059101
chr18 5400537
chr18 5400659
chr18 5400989
chr18 6004154
chr18 6730026
chr18 6730030
chr18 6730034
chr18 6730037
chr18 6730122
chr18 6730126
chr18 6730154
chr18 6730200
chr18 6730246
chr18 6730270
chr18 6730697
chr18 6730804
chr18 6730903
chr18 6937973
chr18 6937983
chr18 6938082
chr18 6938091
chr18 6938108
chr18 6938155
chr18 6938194
chr18 6938250
chr18 6938386
chr18 6938480
chr18 6938730
chr18 7000768
chr18 7000791
chr18 7000888

chr18 7000981
chr18 7572013
chr18 7572154
chr18 7572811
chr18 7572925
chr18 7572927
chr18 8574692
chr18 8610534
chr18 8610544
chr18 8610571
chr18 8610580
chr18 8610613
chr18 8610640
chr18 8610785
chr18 8610814
chr18 8610845
chr18 8610956
chr18 8696630
chr18 8696863
chr18 8696950
chr18 8696958
chr18 8696960
chr18 8696969
chr18 8697234
chr18 9073844
chr18 9083067
chr18 9083095
chr18 9083157
chr18 9083376
chr18 9083396
chr18 9084010
chr18 9084036
chr18 9084047
chr18 9102064
chr18 9102083
chr18 9102113
chr18 9102141
chr18 9102200
chr18 9102755
chr18 9102757
chr18 9102760
chr18 9102765
chr18 9102771
chr18 9102775
chr18 9102788
chr18 9102820
chr18 9102823
chr18 9102829
chr18 9102832
chr18 9102850
chr18 9102871
chr18 9102895
chr18 9104695
chr18 9104815

chr18 9104822
chr18 9105237
chr18 9105294
chr18 9117006
chr18 9117059
chr18 9117091
chr18 9117097
chr18 9117099
chr18 9117105
chr18 9117139
chr18 9117161
chr18 9117181
chr18 9117185
chr18 9117193
chr18 9117256
chr18 9117270
chr18 9117330
chr18 9117350
chr18 9117564
chr18 9117622
chr18 9118016
chr18 9118316
chr18 9118600
chr18 9118663
chr18 9118795
chr18 9120063
chr18 9120172
chr18 9139637
chr18 9139652
chr18 9139912
chr18 9139966
chr18 9140065
chr18 9140238
chr18 9140310
chr18 9140612
chr18 9140660
chr18 9140801
chr18 9140929
chr18 9141050
chr18 9141065
chr18 9185362
chr18 9334114
chr18 9334116
chr18 9334377
chr18 9337930
chr18 9476320
chr18 9476363
chr18 9476473
chr18 9476487
chr18 9476510
chr18 9476518
chr18 9476559
chr18 9476644
chr18 9673543

chr18 9673647
chr18 9673665
chr18 9898760
chr18 9898819
chr18 9898829
chr18 9915240
chr18 9915246
chr18 9915253
chr18 9915381
chr18 9915537
chr18 9915774
chr18 9915890
chr18 9916001
chr18 9916370
chr18 9916614
chr18 10213698
chr18 10213774
chr18 10213851
chr18 10401648
chr18 10577973
chr18 10578009
chr18 10578029
chr18 10822638
chr18 10822668
chr18 10822683
chr18 10917031
chr18 10917104
chr18 10917135
chr18 10917158
chr18 10917548
chr18 10917648
chr18 10918523
chr18 10918583
chr18 10918646
chr18 10996193
chr18 10996467
chr18 10997119
chr18 10997330
chr18 11069659
chr18 11240791
chr18 11383436
chr18 11690245
chr18 11690268
chr18 11690276
chr18 11690313
chr18 11690380
chr18 11719083
chr18 11756560
chr18 11757125
chr18 11850118
chr18 11850122
chr18 11850140
chr18 11850147
chr18 11850154

chr18 11850181
chr18 11850198
chr18 11850212
chr18 11850214
chr18 11850221
chr18 11850260
chr18 11850355
chr18 11850399
chr18 11850470
chr18 11850542
chr18 11850574
chr18 11850737
chr18 11850742
chr18 11850895
chr18 11850897
chr18 11850908
chr18 11852036
chr18 11852044
chr18 11852048
chr18 11852056
chr18 11852059
chr18 11852091
chr18 11852097
chr18 11852112
chr18 11852122
chr18 11852206
chr18 11852317
chr18 11852366
chr18 11852385
chr18 11852568
chr18 11852665
chr18 11937948
chr18 12049569
chr18 12147169
chr18 12147199
chr18 12147226
chr18 12147932
chr18 12148578
chr18 12159665
chr18 12180841
chr18 12203926
chr18 12204760
chr18 12204841
chr18 12391971
chr18 12607223
chr18 12607272
chr18 12837649
chr18 12838281
chr18 12838301
chr18 12838486
chr18 12838624
chr18 12838671
chr18 12839042
chr18 12839236

chr18 12839242
chr18 12840153
chr18 12840212
chr18 12840292
chr18 12840327
chr18 12857690
chr18 12857704
chr18 12857729
chr18 12896449
chr18 12896513
chr18 12896530
chr18 12896576
chr18 12896864
chr18 12896874
chr18 12896882
chr18 12896886
chr18 12896893
chr18 12896896
chr18 12896907
chr18 12896926
chr18 12896929
chr18 12896943
chr18 12896950
chr18 12896952
chr18 12896975
chr18 12896985
chr18 12896990
chr18 12897007
chr18 12897014
chr18 12897018
chr18 12897021
chr18 12897034
chr18 12897036
chr18 12897049
chr18 12897053
chr18 12897056
chr18 12897068
chr18 12897080
chr18 12897102
chr18 12897108
chr18 12897111
chr18 12897114
chr18 12897117
chr18 12897120
chr18 12897123
chr18 12897126
chr18 12897129
chr18 12897132
chr18 12897135
chr18 12897137
chr18 12897153
chr18 12946781
chr18 12946954
chr18 12946965

chr18 12947004
chr18 12947043
chr18 12947048
chr18 12947067
chr18 12947140
chr18 12947157
chr18 12947181
chr18 12947185
chr18 12947518
chr18 12947521
chr18 12947541
chr18 12947548
chr18 12947654
chr18 12947657
chr18 12947660
chr18 12947664
chr18 12947739
chr18 12947750
chr18 12947926
chr18 12947931
chr18 12947943
chr18 12947958
chr18 12947973
chr18 12947993
chr18 12947998
chr18 12948004
chr18 12948020
chr18 12948024
chr18 12948030
chr18 12948037
chr18 12948055
chr18 12948062
chr18 12948064
chr18 12948067
chr18 12948070
chr18 12948073
chr18 12948079
chr18 12948082
chr18 12948097
chr18 12948103
chr18 12948105
chr18 12948111
chr18 12948114
chr18 12948134
chr18 12948142
chr18 12948144
chr18 12948147
chr18 12948169
chr18 12948181
chr18 12948190
chr18 12948194
chr18 12948197
chr18 12948217
chr18 12948226

chr18 12948236
chr18 12948238
chr18 12948240
chr18 12948243
chr18 12948249
chr18 12948253
chr18 12948259
chr18 12948265
chr18 12991505
chr18 12991510
chr18 12991517
chr18 12992191
chr18 12992198
chr18 12992353
chr18 12992368
chr18 12992371
chr18 12992411
chr18 12992720
chr18 13067270
chr18 13067282
chr18 13067296
chr18 13067368
chr18 13279341
chr18 13279357
chr18 13279374
chr18 13279377
chr18 13279383
chr18 13279409
chr18 13279512
chr18 13464015
chr18 13464271
chr18 13464438
chr18 13464622
chr18 13464741
chr18 13464869
chr18 13465130
chr18 13465213
chr18 13465269
chr18 13465293
chr18 13465495
chr18 13465561
chr18 13465681
chr18 13562289
chr18 13562372
chr18 13577584
chr18 13577617
chr18 13612307
chr18 13612354
chr18 13612363
chr18 13612380
chr18 13612388
chr18 13612392
chr18 13612403
chr18 13612457

chr18 13612461
chr18 13612527
chr18 13612561
chr18 13612631
chr18 13612655
chr18 13612668
chr18 13612816
chr18 13612933
chr18 13725713
chr18 13725732
chr18 13727543
chr18 13824330
chr18 13824336
chr18 13824355
chr18 13824360
chr18 13824377
chr18 13824397
chr18 13824430
chr18 14131861
chr18 14131917
chr18 14131933
chr18 14131953
chr18 14131965
chr18 14131984
chr18 14132002
chr18 14132007
chr18 14132010
chr18 14132112
chr18 14132115
chr18 14132135
chr18 14132139
chr18 14132153
chr18 14132183
chr18 14132188
chr18 14132202
chr18 14132208
chr18 14132282
chr18 14132343
chr18 14132346
chr18 14132367
chr18 14132377
chr18 14132413
chr18 14132415
chr18 14132441
chr18 14132443
chr18 14132453
chr18 14132460
chr18 14132480
chr18 14132482
chr18 14132537
chr18 14132539
chr18 14132554
chr18 14132583
chr18 14132593

chr18 14132651
chr18 14132698
chr18 14132716
chr18 14132737
chr18 14132805
chr18 14132808
chr18 14376125
chr18 14376500
chr18 14376644
chr18 14376738
chr18 14376938
chr18 14376968
chr18 14376999
chr18 14394879
chr18 14395005
chr18 14395343
chr18 14395737
chr18 14395860
chr18 14395897
chr18 14395986
chr18 14431352
chr18 14431367
chr18 14431373
chr18 14431376
chr18 14431379
chr18 14431384
chr18 14431526
chr18 14431536
chr18 14431552
chr18 14431554
chr18 14431567
chr18 14431601
chr18 14431612
chr18 14450343
chr18 14450356
chr18 14450362
chr18 14450364
chr18 14450368
chr18 14450370
chr18 14450374
chr18 14450376
chr18 14450380
chr18 14450382
chr18 14450386
chr18 14450392
chr18 14450394
chr18 14450396
chr18 14450411
chr18 14450416
chr18 14450427
chr18 14450435
chr18 14450451
chr18 14450469
chr18 14450486

chr18 14450488
chr18 14450493
chr18 14450495
chr18 14450498
chr18 14450502
chr18 14450511
chr18 14450533
chr18 14450536
chr18 14450540
chr18 14450549
chr18 14450562
chr18 14450566
chr18 14450568
chr18 14450575
chr18 14450590
chr18 14450594
chr18 14450615
chr18 14450619
chr18 14450624
chr18 14450638
chr18 14450646
chr18 14450651
chr18 14450653
chr18 14450761
chr18 14450800
chr18 14450814
chr18 14450821
chr18 14450833
chr18 14450846
chr18 14450857
chr18 14450860
chr18 14450863
chr18 14450869
chr18 14450887
chr18 14450889
chr18 14450901
chr18 14450905
chr18 14450916
chr18 14450922
chr18 14450924
chr18 14450935
chr18 14450960
chr18 14450963
chr18 14450969
chr18 14450971
chr18 14450984
chr18 14451004
chr18 14451036
chr18 14458568
chr18 14458611
chr18 14458636
chr18 14458649
chr18 14458674
chr18 14458682

chr18 14458693
chr18 14458700
chr18 14458704
chr18 14458707
chr18 14540781
chr18 14540976
chr18 14541080
chr18 14541092
chr18 14541124
chr18 14541367
chr18 14579153
chr18 14829921
chr18 14851756
chr18 14851768
chr18 14852103
chr18 14852241
chr18 14852325
chr18 14852336
chr18 14852382
chr18 14852591
chr18 15029951
chr18 15110528
chr18 15110586
chr18 15111026
chr18 15112055
chr18 15198707
chr18 15198730
chr18 15198961
chr18 15199599
chr18 15199670
chr18 15199725
chr18 15199737
chr18 15335007
chr18 15337332
chr18 15337476
chr18 15337914
chr18 15337924
chr18 15338048
chr18 15338139
chr18 15625656
chr18 15626031
chr18 15626067
chr18 15626082
chr18 15626107
chr18 15626800
chr18 15626833
chr18 15627054
chr18 15627159
chr18 15627163
chr18 15627213
chr18 15627231
chr18 15627357
chr18 15627469
chr18 15627477

chr18 15627528
chr18 15628309
chr18 15628363
chr18 15628369
chr18 15628378
chr18 17030093
chr18 17030102
chr18 17031268
chr18 17031282
chr18 17032627
chr18 17032641
chr18 17035692
chr18 17037720
chr18 17041797
chr18 17791294
chr18 17791354
chr18 17791405
chr18 17791414
chr18 20560086
chr18 20560201
chr18 20560312
chr18 20560387
chr18 20560396
chr18 20571509
chr18 20571760
chr18 20571823
chr18 20572591
chr18 20572621
chr18 20572998
chr18 20573079
chr18 20573298
chr18 20573307
chr18 20609036
chr18 20609251
chr18 20609330
chr18 20609339
chr18 20609543
chr18 20609619
chr18 20610190
chr18 20610338
chr18 20610346
chr18 20610523
chr18 20610532
chr18 20695719
chr18 20695730
chr18 20695738
chr18 20695759
chr18 20695783
chr18 20695834
chr18 20695859
chr18 20700751
chr18 20700755
chr18 21109698
chr18 21109744

chr18 21109790
chr18 21109881
chr18 21110200
chr18 21112411
chr18 21112470
chr18 21112497
chr18 21112501
chr18 21112544
chr18 21112557
chr18 21112649
chr18 21112657
chr18 21112703
chr18 21112869
chr18 21112884
chr18 21112915
chr18 21163790
chr18 21163815
chr18 21164005
chr18 21164161
chr18 21164410
chr18 21164473
chr18 21164579
chr18 21604960
chr18 21605015
chr18 21605094
chr18 21605116
chr18 21629100
chr18 21629106
chr18 21629133
chr18 21629285
chr18 21682744
chr18 21682858
chr18 21682892
chr18 21682904
chr18 21682965
chr18 21682971
chr18 21683249
chr18 21703100
chr18 21703180
chr18 21703307
chr18 21703368
chr18 21703377
chr18 22190892
chr18 22190920
chr18 22190933
chr18 22190941
chr18 22190963
chr18 22191032
chr18 22323983
chr18 22324102
chr18 22324192
chr18 22324251
chr18 22324295
chr18 22324297

chr18 22324306
chr18 22324309
chr18 22534249
chr18 22534350
chr18 22534528
chr18 22534744
chr18 22534972
chr18 22799322
chr18 22799325
chr18 22799509
chr18 22799522
chr18 22847808
chr18 22848311
chr18 22932722
chr18 22932738
chr18 22932826
chr18 22932851
chr18 22933117
chr18 22933136
chr18 22933157
chr18 22980385
chr18 23071240
chr18 23071246
chr18 23071331
chr18 23071337
chr18 23071515
chr18 23071532
chr18 23071673
chr18 23231417
chr18 23231444
chr18 23231499
chr18 23231627
chr18 23231651
chr18 23231659
chr18 23231756
chr18 23437386
chr18 23437406
chr18 23437458
chr18 23437525
chr18 23437569
chr18 23437590
chr18 23437641
chr18 23437652
chr18 23437668
chr18 23437708
chr18 23437717
chr18 23437730
chr18 23437764
chr18 23437766
chr18 23437770
chr18 23437787
chr18 23452613
chr18 23452778
chr18 23452801

chr18 23452810
chr18 23452815
chr18 23453859
chr18 23453979
chr18 23453994
chr18 23453997
chr18 23454013
chr18 23454020
chr18 23454066
chr18 23487909
chr18 23574129
chr18 23574232
chr18 23574296
chr18 23574361
chr18 23574373
chr18 23585691
chr18 23585725
chr18 23585834
chr18 23712559
chr18 23712575
chr18 23712634
chr18 23918183
chr18 23918204
chr18 23918615
chr18 23918621
chr18 23994248
chr18 23994542
chr18 24013891
chr18 24014015
chr18 24014030
chr18 24014050
chr18 24014057
chr18 24014080
chr18 24014085
chr18 24014087
chr18 24014095
chr18 24015809
chr18 24015889
chr18 24015918
chr18 24015932
chr18 24015945
chr18 24015970
chr18 24015992
chr18 24016048
chr18 24016142
chr18 24016198
chr18 24016241
chr18 24016407
chr18 24016444
chr18 24394801
chr18 24394879
chr18 24660213
chr18 24660251
chr18 24660285

chr18 24660288
chr18 24660290
chr18 24660297
chr18 24660352
chr18 24660372
chr18 24660383
chr18 24660453
chr18 24660496
chr18 24660511
chr18 24660524
chr18 24660533
chr18 24660571
chr18 24660703
chr18 25177508
chr18 25177702
chr18 25228229
chr18 25228370
chr18 25228791
chr18 25228940
chr18 25229158
chr18 25352675
chr18 25352709
chr18 25352715
chr18 25352738
chr18 25352811
chr18 25352837
chr18 25353030
chr18 25353053
chr18 25353089
chr18 25353189
chr18 25353275
chr18 25353346
chr18 25353732
chr18 25504245
chr18 25504325
chr18 25504543
chr18 25504738
chr18 25770211
chr18 25770456
chr18 26088974
chr18 26089164
chr18 26089294
chr18 26089302
chr18 26089384
chr18 26089436
chr18 26089462
chr18 26089478
chr18 26089675
chr18 26089696
chr18 26089750
chr18 26089770
chr18 26091095
chr18 26091115
chr18 26091120

chr18 26091130
chr18 26091138
chr18 26190979
chr18 26215650
chr18 26487730
chr18 26487763
chr18 26551493
chr18 26551507
chr18 26551533
chr18 26551567
chr18 26551640
chr18 26551839
chr18 26551898
chr18 26551922
chr18 26551931
chr18 26552174
chr18 26552204
chr18 26574940
chr18 26574980
chr18 26575213
chr18 26575243
chr18 26575271
chr18 26575285
chr18 26575288
chr18 26575293
chr18 26575303
chr18 26575309
chr18 26575311
chr18 26575334
chr18 26575337
chr18 26575339
chr18 26575351
chr18 26575374
chr18 26575387
chr18 26575396
chr18 26575400
chr18 26575411
chr18 26575431
chr18 26575443
chr18 26575455
chr18 26575460
chr18 26656306
chr18 26656314
chr18 26656317
chr18 26656334
chr18 26656358
chr18 26656383
chr18 26656436
chr18 27142853
chr18 27142964
chr18 27168898
chr18 27169442
chr18 27282002
chr18 27282056

chr18 27426207
chr18 27426597
chr18 28189904
chr18 28190554
chr18 28190706
chr18 28190766
chr18 28420059
chr18 28420443
chr18 28782198
chr18 28782211
chr18 28905026
chr18 29244184
chr18 29244597
chr18 29244843
chr18 29359934
chr18 29360340
chr18 29360451
chr18 29401586
chr18 29401592
chr18 29929515
chr18 29972184
chr18 29972452
chr18 29972612
chr18 30052828
chr18 30052846
chr18 30351832
chr18 30351956
chr18 30354767
chr18 30596335
chr18 30596407
chr18 30840540
chr18 30840876
chr18 30840900
chr18 31040238
chr18 31040515
chr18 31040565
chr18 31040782
chr18 31040816
chr18 31040881
chr18 31040886
chr18 31040949
chr18 31040991
chr18 31041022
chr18 31041056
chr18 31041068
chr18 31266222
chr18 31357682
chr18 31498652
chr18 31498736
chr18 31498739
chr18 31634546
chr18 31634551
chr18 31634572
chr18 31687740

chr18 31944157
chr18 31944177
chr18 31944354
chr18 31944366
chr18 31944397
chr18 31944438
chr18 31944458
chr18 31944476
chr18 31944642
chr18 31944735
chr18 31944804
chr18 31944819
chr18 32018604
chr18 32018609
chr18 32018616
chr18 32018622
chr18 32018628
chr18 32018630
chr18 32018644
chr18 32018652
chr18 32018667
chr18 32018669
chr18 32018673
chr18 32018675
chr18 32018677
chr18 32018681
chr18 32018706
chr18 32018711
chr18 32018714
chr18 32018734
chr18 32018736
chr18 32018757
chr18 32018760
chr18 32018764
chr18 32018775
chr18 32018794
chr18 32018798
chr18 32018817
chr18 32018836
chr18 32018850
chr18 32019713
chr18 32019720
chr18 32019796
chr18 32019810
chr18 32019813
chr18 32019847
chr18 32019886
chr18 32019892
chr18 32020054
chr18 32026698
chr18 32026931
chr18 32040549
chr18 32040561
chr18 32040747

chr18 32040872
chr18 32041024
chr18 32041054
chr18 32041085
chr18 32085164
chr18 32085344
chr18 32085557
chr18 32085566
chr18 32085569
chr18 32085615
chr18 32085692
chr18 32085748
chr18 32085784
chr18 32093283
chr18 32093302
chr18 32093315
chr18 32093339
chr18 32093343
chr18 32093348
chr18 32093350
chr18 32093393
chr18 32093401
chr18 32093855
chr18 32158060
chr18 32158070
chr18 32158111
chr18 32158130
chr18 32158160
chr18 32158192
chr18 32158224
chr18 32158234
chr18 32158239
chr18 32158254
chr18 32158261
chr18 32158292
chr18 32158316
chr18 32158335
chr18 32158356
chr18 32192171
chr18 32552705
chr18 32577185
chr18 32772317
chr18 32772320
chr18 32772334
chr18 32772338
chr18 32772400
chr18 32772426
chr18 32772431
chr18 32772444
chr18 32772542
chr18 32772565
chr18 32772593
chr18 32772819
chr18 32772947

chr18 32773012
chr18 32773017
chr18 32773037
chr18 32773262
chr18 32773320
chr18 32773326
chr18 32863771
chr18 32864330
chr18 32864552
chr18 32945743
chr18 32945790
chr18 32948238
chr18 32948251
chr18 33201523
chr18 33201581
chr18 33201645
chr18 33201657
chr18 33201711
chr18 33203590
chr18 33203925
chr18 33204201
chr18 33204395
chr18 33204532
chr18 33476922
chr18 33576659
chr18 33576765
chr18 33577245
chr18 33577397
chr18 33577511
chr18 34062162
chr18 34062165
chr18 34062460
chr18 34062888
chr18 34062978
chr18 34063556
chr18 34063567
chr18 34064147
chr18 34220865
chr18 34220976
chr18 34221146
chr18 34221862
chr18 34221897
chr18 34221957
chr18 34222009
chr18 34222020
chr18 34222104
chr18 34222107
chr18 34222115
chr18 34222144
chr18 34222184
chr18 34222186
chr18 34222189
chr18 34222260
chr18 34222266

chr18 34222306
chr18 34222351
chr18 34225389
chr18 34225454
chr18 34225545
chr18 34231498
chr18 34231667
chr18 34232251
chr18 34491992
chr18 34492332
chr18 34492524
chr18 34493052
chr18 34493148
chr18 34493152
chr18 34493182
chr18 34493248
chr18 34493252
chr18 34493254
chr18 34493258
chr18 34493260
chr18 34493272
chr18 34493281
chr18 34493382
chr18 34493388
chr18 34559306
chr18 34559395
chr18 34559522
chr18 34559533
chr18 34604872
chr18 34644138
chr18 34644291
chr18 34860701
chr18 34860731
chr18 34860813
chr18 34860848
chr18 34866579
chr18 34893900
chr18 35040565
chr18 35043366
chr18 35043380
chr18 35043436
chr18 35043475
chr18 35043548
chr18 35231112
chr18 35231130
chr18 35231183
chr18 35231207
chr18 35231218
chr18 35231221
chr18 35231587
chr18 35231833
chr18 35241709
chr18 35241742
chr18 35241804

chr18 35241820
chr18 35241883
chr18 35269460
chr18 35289616
chr18 35289630
chr18 35289690
chr18 35289733
chr18 35289844
chr18 35289876
chr18 35289931
chr18 35289933
chr18 35290000
chr18 35290013
chr18 35290043
chr18 35290104
chr18 35290121
chr18 35290140
chr18 35290167
chr18 35290212
chr18 35290214
chr18 35290217
chr18 35290236
chr18 35290247
chr18 35290254
chr18 35290268
chr18 35290275
chr18 35290281
chr18 35290283
chr18 35290291
chr18 35290328
chr18 35290753
chr18 35343952
chr18 35343955
chr18 35343963
chr18 35343972
chr18 35343978
chr18 35343982
chr18 35344009
chr18 35344011
chr18 35344020
chr18 35344046
chr18 35344063
chr18 35344065
chr18 35344070
chr18 35344081
chr18 35344097
chr18 35344101
chr18 35344129
chr18 35344202
chr18 35344220
chr18 35344222
chr18 35344229
chr18 35344231
chr18 35344252

chr18 35376919
chr18 35376969
chr18 35376971
chr18 35376974
chr18 35376976
chr18 35376979
chr18 35376981
chr18 35377002
chr18 35377005
chr18 35377010
chr18 35377015
chr18 35377025
chr18 35377027
chr18 35581524
chr18 35581528
chr18 35581534
chr18 35581538
chr18 35581545
chr18 35581548
chr18 35756702
chr18 35757109
chr18 35758803
chr18 35758917
chr18 35762038
chr18 35762093
chr18 35762269
chr18 35762273
chr18 35762306
chr18 35762831
chr18 35763494
chr18 35970200
chr18 35970440
chr18 35970474
chr18 35970530
chr18 35972115
chr18 36017803
chr18 36017939
chr18 36018023
chr18 36066709
chr18 36066862
chr18 36067022
chr18 36067701
chr18 36067866
chr18 36084010
chr18 36240089
chr18 36240110
chr18 37385751
chr18 38377867
chr18 38378011
chr18 38378058
chr18 38940147
chr18 38940263
chr18 38940967
chr18 38941033

chr18 38941264
chr18 38941355
chr18 38941367
chr18 38946252
chr18 38946551
chr18 38946590
chr18 38946716
chr18 38946905
chr18 38947055
chr18 39360764
chr18 39360854
chr18 39361186
chr18 39361474
chr18 39472413
chr18 39472531
chr18 39693144
chr18 39800069
chr18 39800091
chr18 39800093
chr18 39800358
chr18 39800367
chr18 39925412
chr18 40060995
chr18 40061275
chr18 40279685
chr18 40279753
chr18 40279817
chr18 40279915
chr18 40280130
chr18 40280619
chr18 40280909
chr18 40280963
chr18 41285669
chr18 41286241
chr18 41286710
chr18 41287013
chr18 41287979
chr18 41287987
chr18 41287989
chr18 41288043
chr18 41288050
chr18 41288104
chr18 41288184
chr18 41288449
chr18 41288711
chr18 41288945
chr18 41390312
chr18 41390318
chr18 41393082
chr18 41906624
chr18 41906650
chr18 41906705
chr18 41906830
chr18 41955345

chr18 41955373
chr18 41955399
chr18 41955407
chr18 41955448
chr18 41955455
chr18 41955478
chr18 41955505
chr18 41955507
chr18 41955520
chr18 41955555
chr18 41955564
chr18 41955601
chr18 41955688
chr18 41955691
chr18 41955705
chr18 41955722
chr18 41955822
chr18 42028585
chr18 42028650
chr18 42595959
chr18 42794207
chr18 42794726
chr18 42855200
chr18 43117224
chr18 43138688
chr18 43138786
chr18 43223555
chr18 43223612
chr18 43277166
chr18 43277219
chr18 43277259
chr18 43277273
chr18 43277291
chr18 43277302
chr18 43277355
chr18 43277358
chr18 43277365
chr18 43277370
chr18 43277385
chr18 43277443
chr18 43277449
chr18 43277469
chr18 43277499
chr18 43277505
chr18 43277517
chr18 43277530
chr18 43277538
chr18 43279160
chr18 43279204
chr18 43279440
chr18 43279523
chr18 43279535
chr18 43279655
chr18 43279849

chr18 43280567
chr18 43280610
chr18 43281047
chr18 43288176
chr18 43424888
chr18 43425274
chr18 43997954
chr18 43998237
chr18 44322259
chr18 44322292
chr18 44502114
chr18 44502284
chr18 44680829
chr18 44680971
chr18 44680976
chr18 44681014
chr18 44681017
chr18 44725183
chr18 44759241
chr18 44759330
chr18 44759336
chr18 44759349
chr18 44759360
chr18 44759424
chr18 44759514
chr18 44759881
chr18 44839211
chr18 44839325
chr18 44839333
chr18 44839433
chr18 44839450
chr18 44839473
chr18 44839517
chr18 44909278
chr18 44909379
chr18 45436083
chr18 45436095
chr18 45620688
chr18 45621014
chr18 45621172
chr18 45687000
chr18 45687069
chr18 45687105
chr18 45687116
chr18 45687122
chr18 45687326
chr18 45687449
chr18 45755881
chr18 45755934
chr18 45755956
chr18 45755968
chr18 45756177
chr18 45775270
chr18 45775287

chr18 45775294
chr18 45775326
chr18 45775354
chr18 45775357
chr18 45775388
chr18 45775396
chr18 45775401
chr18 45775407
chr18 45775411
chr18 45775449
chr18 45775475
chr18 45775492
chr18 45775497
chr18 45775502
chr18 45775513
chr18 45775516
chr18 45775521
chr18 45775523
chr18 45775526
chr18 45775531
chr18 45874585
chr18 45874657
chr18 45874897
chr18 45875073
chr18 45875114
chr18 45967190
chr18 45967211
chr18 45967214
chr18 45967228
chr18 45967233
chr18 45967257
chr18 45967263
chr18 45967304
chr18 45967317
chr18 45967333
chr18 45967341
chr18 45967350
chr18 45967361
chr18 45967369
chr18 45967372
chr18 45967380
chr18 45967385
chr18 45967390
chr18 45967412
chr18 45967439
chr18 46073887
chr18 46073889
chr18 46097145
chr18 46097338
chr18 46097380
chr18 46097496
chr18 46097526
chr18 46097853
chr18 46097864

chr18 46097906
chr18 46097916
chr18 46097955
chr18 46097991
chr18 46098038
chr18 46098049
chr18 46098063
chr18 46098070
chr18 46098083
chr18 46098100
chr18 46098104
chr18 46098109
chr18 46098112
chr18 46098137
chr18 46098141
chr18 46098155
chr18 46098162
chr18 46098177
chr18 46098182
chr18 46098185
chr18 46098195
chr18 46098197
chr18 46098204
chr18 46098208
chr18 46098216
chr18 46098218
chr18 46098222
chr18 46098246
chr18 46098252
chr18 46098267
chr18 46098276
chr18 46104562
chr18 46104575
chr18 46104578
chr18 46104599
chr18 46104637
chr18 46104664
chr18 46104673
chr18 46104685
chr18 46104726
chr18 46104733
chr18 46104738
chr18 46104750
chr18 46104753
chr18 46175238
chr18 46249978
chr18 46249990
chr18 46249999
chr18 46250163
chr18 46250195
chr18 46250489
chr18 46250683
chr18 46251010
chr18 46251090

chr18 46308959
chr18 46440724
chr18 46598876
chr18 46599048
chr18 46916725
chr18 46916738
chr18 46968012
chr18 46968029
chr18 47023772
chr18 47023793
chr18 47023807
chr18 47023822
chr18 47023856
chr18 47024510
chr18 47024525
chr18 47024538
chr18 47030425
chr18 47030440
chr18 47030453
chr18 47150713
chr18 47150751
chr18 47150755
chr18 47150768
chr18 47150814
chr18 47150865
chr18 47150932
chr18 47150939
chr18 47150942
chr18 47244123
chr18 47244265
chr18 47244284
chr18 47244410
chr18 47245300
chr18 47245598
chr18 47245689
chr18 47245736
chr18 47252961
chr18 47253039
chr18 47253044
chr18 47253064
chr18 47253166
chr18 47253244
chr18 47253246
chr18 47253282
chr18 47253379
chr18 47253420
chr18 47253428
chr18 47253455
chr18 47253465
chr18 47253527
chr18 47253543
chr18 47254125
chr18 47254264
chr18 47254267

chr18 47254273
chr18 47254288
chr18 47254294
chr18 47254318
chr18 47254339
chr18 47254358
chr18 47256540
chr18 47256752
chr18 47256806
chr18 47256824
chr18 47256903
chr18 47257420
chr18 47912324
chr18 47912366
chr18 47928682
chr18 47928739
chr18 47928813
chr18 47928926
chr18 47928955
chr18 47928970
chr18 47928976
chr18 47929026
chr18 47929084
chr18 47929331
chr18 47929344
chr18 47929483
chr18 47929573
chr18 47953617
chr18 47953646
chr18 48021412
chr18 48021434
chr18 48834242
chr18 48834255
chr18 48834261
chr18 48834296
chr18 48834448
chr18 48834463
chr18 48834479
chr18 48834504
chr18 48834597
chr18 48834687
chr18 48834764
chr18 48834773
chr18 48834812
chr18 48834841
chr18 48834848
chr18 48834880
chr18 48928971
chr18 48929065
chr18 48929137
chr18 48929149
chr18 48929381
chr18 48929598
chr18 48940737

chr18 48940745
chr18 48940758
chr18 49459927
chr18 49460046
chr18 49487001
chr18 49491088
chr18 49491135
chr18 49491238
chr18 49491308
chr18 49491536
chr18 49491548
chr18 49491564
chr18 49491617
chr18 49491658
chr18 49491725
chr18 49491895
chr18 49491995
chr18 49492013
chr18 49492046
chr18 49492065
chr18 49492072
chr18 49492082
chr18 49492112
chr18 49492117
chr18 49492141
chr18 49492149
chr18 49492156
chr18 49492160
chr18 49492171
chr18 49492176
chr18 49492185
chr18 49492188
chr18 49492193
chr18 49492196
chr18 49820427
chr18 49820457
chr18 49820533
chr18 50280873
chr18 50280905
chr18 50280955
chr18 50281092
chr18 50281108
chr18 50281133
chr18 50308457
chr18 50375028
chr18 50375033
chr18 50375049
chr18 50375055
chr18 50375060
chr18 50375068
chr18 50375070
chr18 50375073
chr18 50375077
chr18 50375103

chr18 50375160
chr18 50375163
chr18 50375181
chr18 50375185
chr18 50375270
chr18 50375272
chr18 50375275
chr18 50375291
chr18 50375341
chr18 50375344
chr18 50375379
chr18 50375391
chr18 50375499
chr18 50375544
chr18 50375612
chr18 50375643
chr18 50375649
chr18 50375756
chr18 50375793
chr18 50375838
chr18 50561992
chr18 50562257
chr18 50777776
chr18 50777895
chr18 50778396
chr18 50819856
chr18 50819910
chr18 50880174
chr18 50880188
chr18 51031092
chr18 51031139
chr18 51031277
chr18 51031440
chr18 51031508
chr18 51031566
chr18 51031682
chr18 51083191
chr18 51193542
chr18 51193753
chr18 51193793
chr18 51194102
chr18 51194151
chr18 51194162
chr18 51194466
chr18 51194661
chr18 51194709
chr18 51194778
chr18 51195119
chr18 51195231
chr18 51195279
chr18 51197739
chr18 51197785
chr18 51197792
chr18 51197846

chr18 51741282
chr18 51741479
chr18 51741606
chr18 51741625
chr18 51741656
chr18 51741681
chr18 51741702
chr18 51790252
chr18 53310069
chr18 53310181
chr18 53373505
chr18 53374565
chr18 53374699
chr18 53386014
chr18 53386089
chr18 53386137
chr18 53386347
chr18 53514158
chr18 54129594
chr18 54129682
chr18 54129688
chr18 54129930
chr18 54223051
chr18 54223141
chr18 54223215
chr18 54223340
chr18 54223411
chr18 54223707
chr18 54223880
chr18 54263528
chr18 54263550
chr18 54263573
chr18 54263596
chr18 54269938
chr18 54269958
chr18 54269961
chr18 54270016
chr18 54270041
chr18 54270083
chr18 54270095
chr18 54270106
chr18 54270112
chr18 54270149
chr18 54270180
chr18 54270245
chr18 54270271
chr18 54270285
chr18 54270290
chr18 54270312
chr18 54270399
chr18 54270419
chr18 54270451
chr18 54270591
chr18 54270625

chr18 54270711
chr18 54357067
chr18 54357070
chr18 54357301
chr18 54357305
chr18 54357378
chr18 54357419
chr18 54357491
chr18 54357508
chr18 54357526
chr18 54357578
chr18 54357582
chr18 54358565
chr18 54358569
chr18 54358582
chr18 54358587
chr18 54358601
chr18 54358605
chr18 54358612
chr18 54358619
chr18 54358623
chr18 54358648
chr18 54358660
chr18 54358723
chr18 54358817
chr18 54358819
chr18 54398052
chr18 54647398
chr18 54647428
chr18 54717788
chr18 54717803
chr18 54717814
chr18 54717827
chr18 54717856
chr18 54717899
chr18 54717926
chr18 54717978
chr18 54718072
chr18 54718131
chr18 54718149
chr18 54718187
chr18 54718212
chr18 54718288
chr18 54718420
chr18 54719524
chr18 54828678
chr18 54828692
chr18 54828715
chr18 54828734
chr18 54828736
chr18 54828750
chr18 54828762
chr18 54828765
chr18 54828772

chr18 54828775
chr18 54828821
chr18 54828833
chr18 54828837
chr18 54828839
chr18 54828890
chr18 54828909
chr18 54829097
chr18 54829215
chr18 54830014
chr18 54870104
chr18 54873229
chr18 54873370
chr18 54946233
chr18 54946241
chr18 54946283
chr18 54946295
chr18 54946333
chr18 54946411
chr18 54946560
chr18 55320969
chr18 55321057
chr18 55321412
chr18 55321489
chr18 55321565
chr18 55321845
chr18 55321892
chr18 55321906
chr18 55321908
chr18 55321911
chr18 55321924
chr18 55321959
chr18 55321975
chr18 55321992
chr18 55321994
chr18 55322000
chr18 55322014
chr18 55322036
chr18 55322046
chr18 55322290
chr18 55322309
chr18 55322373
chr18 55322391
chr18 55322396
chr18 55489455
chr18 55489532
chr18 55489746
chr18 55489819
chr18 55489877
chr18 55489882
chr18 55489899
chr18 55489982
chr18 55489998
chr18 55490037

chr18 55587032
chr18 55587045
chr18 55587081
chr18 55587100
chr18 55587156
chr18 55587199
chr18 55587234
chr18 55587242
chr18 55587376
chr18 55587398
chr18 55587455
chr18 55587465
chr18 55587494
chr18 55587537
chr18 55587542
chr18 55587581
chr18 55587595
chr18 55587601
chr18 55587613
chr18 55587632
chr18 55587669
chr18 55587688
chr18 55588333
chr18 55588336
chr18 55588344
chr18 55588346
chr18 55588356
chr18 55588361
chr18 55588386
chr18 55588397
chr18 55588431
chr18 55588436
chr18 55588452
chr18 55588463
chr18 55588487
chr18 55588528
chr18 55588540
chr18 55588562
chr18 55588601
chr18 55588709
chr18 55588747
chr18 55588752
chr18 55588763
chr18 55588772
chr18 55588839
chr18 55588906
chr18 55588921
chr18 55589034
chr18 55589039
chr18 55589051
chr18 55589062
chr18 55589089
chr18 55589144
chr18 55589228

chr18 55589234
chr18 55589278
chr18 55589291
chr18 55589727
chr18 55589788
chr18 55589797
chr18 55589821
chr18 55589825
chr18 55589827
chr18 55589860
chr18 55589865
chr18 55589875
chr18 55589898
chr18 55589910
chr18 55589922
chr18 55589932
chr18 55589938
chr18 55589947
chr18 55589953
chr18 55589959
chr18 55778713
chr18 55778727
chr18 55778803
chr18 55778837
chr18 55779137
chr18 55779297
chr18 55779321
chr18 55779334
chr18 55779371
chr18 55779399
chr18 55779420
chr18 55779454
chr18 55779488
chr18 55779553
chr18 55779561
chr18 55779612
chr18 55779697
chr18 55890280
chr18 55890617
chr18 55890848
chr18 55891506
chr18 55891817
chr18 55891950
chr18 56637190
chr18 56637373
chr18 56637398
chr18 56637431
chr18 56637469
chr18 56637490
chr18 56637493
chr18 56637563
chr18 56637644
chr18 56637663
chr18 56637703

chr18 56637723
chr18 56637760
chr18 56637764
chr18 56637810
chr18 56637823
chr18 56637859
chr18 56637875
chr18 56637937
chr18 56638024
chr18 56638056
chr18 56651671
chr18 56651680
chr18 56651686
chr18 56651704
chr18 56651709
chr18 56651728
chr18 56651744
chr18 56651747
chr18 56651759
chr18 56651772
chr18 56651875
chr18 56651899
chr18 56651928
chr18 56652078
chr18 56652119
chr18 56652258
chr18 56652309
chr18 56652518
chr18 56652645
chr18 56652830
chr18 56652957
chr18 56653010
chr18 56653050
chr18 56716918
chr18 56717010
chr18 57017897
chr18 57017916
chr18 57431946
chr18 57431995
chr18 57432078
chr18 57432175
chr18 57432185
chr18 57432275
chr18 57432666
chr18 57432861
chr18 57432917
chr18 57432940
chr18 57432953
chr18 57432963
chr18 57433011
chr18 57433019
chr18 57433057
chr18 57433333
chr18 57545083

chr18 57545362
chr18 57545368
chr18 57630416
chr18 57630418
chr18 57630427
chr18 57630517
chr18 57630544
chr18 57630548
chr18 57630561
chr18 57630580
chr18 57720179
chr18 57720186
chr18 57720208
chr18 57720258
chr18 57720304
chr18 57833596
chr18 57833608
chr18 57833629
chr18 57833641
chr18 57833966
chr18 58043287
chr18 58043347
chr18 58529604
chr18 58542024
chr18 58542129
chr18 58542155
chr18 58542196
chr18 58615569
chr18 58615803
chr18 58615996
chr18 58616003
chr18 58654117
chr18 58672278
chr18 58672284
chr18 58672354
chr18 58672357
chr18 58672394
chr18 58672429
chr18 58672495
chr18 58672786
chr18 58672814
chr18 58687720
chr18 58687723
chr18 58687852
chr18 58687879
chr18 58687895
chr18 58688005
chr18 58688037
chr18 58688048
chr18 58688146
chr18 58688327
chr18 58697245
chr18 58715779
chr18 58716199

chr18 58716218
chr18 58716325
chr18 58818780
chr18 58818984
chr18 58887514
chr18 58887531
chr18 58887694
chr18 58887710
chr18 58887796
chr18 58887801
chr18 58887806
chr18 58887813
chr18 58887851
chr18 58887880
chr18 58887887
chr18 58887931
chr18 58887982
chr18 59070653
chr18 59070723
chr18 59070751
chr18 59070807
chr18 59070961
chr18 59140884
chr18 59140940
chr18 59141136
chr18 59141233
chr18 59141555
chr18 59142074
chr18 59142147
chr18 59270411
chr18 59270432
chr18 59270435
chr18 59270540
chr18 59270674
chr18 59270714
chr18 59271555
chr18 59357224
chr18 59357285
chr18 59357611
chr18 59357709
chr18 59357933
chr18 59358062
chr18 59358112
chr18 59358175
chr18 59395770
chr18 59395897
chr18 59834319
chr18 59901284
chr18 59901365
chr18 59901369
chr18 59901634
chr18 59901649
chr18 59901720
chr18 59901783

chr18 59902084
chr18 59902405
chr18 59902449
chr18 59902459
chr18 60012493
chr18 60031992
chr18 60032073
chr18 60032515
chr18 60032548
chr18 60033251
chr18 60372760
chr18 60372788
chr18 60372837
chr18 60372968
chr18 60372980
chr18 60549542
chr18 60549587
chr18 60979506
chr18 61361009
chr18 61361196
chr18 61361201
chr18 61555373
chr18 61555393
chr18 61555853
chr18 61833053
chr18 61833241
chr18 61833500
chr18 61833513
chr18 61833612
chr18 61833619
chr18 61833632
chr18 62188143
chr18 62188163
chr18 62188173
chr18 62188313
chr18 62188481
chr18 62188520
chr18 62188532
chr18 62420727
chr18 62420906
chr18 62420932
chr18 62420951
chr18 62421024
chr18 62421131
chr18 62506384
chr18 62506432
chr18 62521832
chr18 62522025
chr18 62522061
chr18 62522174
chr18 62524998
chr18 62525010
chr18 62525035
chr18 62525046

chr18 62525068
chr18 62525070
chr18 62525093
chr18 62525100
chr18 62525102
chr18 62525112
chr18 62525144
chr18 62525160
chr18 62525166
chr18 62525173
chr18 62525220
chr18 62525244
chr18 62526117
chr18 62526187
chr18 62526194
chr18 62526264
chr18 62526290
chr18 62526326
chr18 62526328
chr18 62526362
chr18 62526404
chr18 62526438
chr18 62526444
chr18 62526458
chr18 62526472
chr18 62599967
chr18 62717445
chr18 62717539
chr18 62717623
chr18 62717636
chr18 62717703
chr18 62717724
chr18 62784664
chr18 62784672
chr18 62784824
chr18 62784833
chr18 62784978
chr18 62869825
chr18 62910573
chr18 63074675
chr18 63074826
chr18 63074849
chr18 63075325
chr18 63075331
chr18 63075365
chr18 63075403
chr18 63107247
chr18 63107344
chr18 63107375
chr18 63107450
chr18 63155104
chr18 63155139
chr18 63155144
chr18 63155162

chr18 63155176
chr18 63155186
chr18 63155265
chr18 63155374
chr18 63155385
chr18 63155395
chr18 63155407
chr18 63155415
chr18 63155436
chr18 63155486
chr18 63155541
chr18 63155543
chr18 63155558
chr18 63155922
chr18 63161175
chr18 63161195
chr18 63161415
chr18 63161446
chr18 63161460
chr18 63161509
chr18 63161750
chr18 63161760
chr18 63161775
chr18 63161789
chr18 63161937
chr18 63162024
chr18 63162061
chr18 63162092
chr18 63162097
chr18 63200152
chr18 63200192
chr18 63216733
chr18 63216817
chr18 63216849
chr18 63216975
chr18 63216987
chr18 63217038
chr18 63217048
chr18 63217217
chr18 63217272
chr18 63253879
chr18 63253982
chr18 63303238
chr18 63317111
chr18 63317252
chr18 63317356
chr18 63317423
chr18 63317559
chr18 63317604
chr18 63317632
chr18 63363938
chr18 63364109
chr18 63364145
chr18 63366073

chr18 63366088
chr18 63366107
chr18 63366117
chr18 63366137
chr18 63366152
chr18 63366419
chr18 63366427
chr18 63366482
chr18 63366492
chr18 63366499
chr18 63366521
chr18 63366531
chr18 63366534
chr18 63366541
chr18 63368195
chr18 63368205
chr18 63368221
chr18 63368230
chr18 63368232
chr18 63379240
chr18 63400070
chr18 63400083
chr18 63400087
chr18 63400663
chr18 63421985
chr18 63422030
chr18 63422046
chr18 63422057
chr18 63422066
chr18 63422076
chr18 63422106
chr18 63422112
chr18 63422122
chr18 63422127
chr18 63422137
chr18 63422139
chr18 63422143
chr18 63422161
chr18 63422172
chr18 63422179
chr18 63422181
chr18 63422245
chr18 63422510
chr18 63422516
chr18 63422527
chr18 63422529
chr18 63422583
chr18 63422608
chr18 63422618
chr18 63423104
chr18 63423110
chr18 63423114
chr18 63423228
chr18 63423409

chr18 63578769
chr18 63578873
chr18 63579396
chr18 63579417
chr18 63579574
chr18 63618013
chr18 63830299
chr18 63844394
chr18 63844416
chr18 63844569
chr18 63845131
chr18 63845272
chr18 63935987
chr18 63936167
chr18 63936170
chr18 63936174
chr18 63936184
chr18 63936186
chr18 63936188
chr18 63936204
chr18 63936214
chr18 63936234
chr18 63936241
chr18 63936260
chr18 63936266
chr18 63936283
chr18 63936285
chr18 63936288
chr18 63936310
chr18 63936318
chr18 63936320
chr18 63936330
chr18 63936364
chr18 63936373
chr18 63950650
chr18 63950665
chr18 63951262
chr18 63951402
chr18 63951510
chr18 63951637
chr18 63951684
chr18 63951737
chr18 63970424
chr18 63970431
chr18 63970438
chr18 63970450
chr18 64157261
chr18 64157269
chr18 64276295
chr18 64276840
chr18 64276924
chr18 64276997
chr18 64277023
chr18 64277029

chr18 64779466
chr18 64779549
chr18 65016176
chr18 65019314
chr18 65234589
chr18 65234787
chr18 65235156
chr18 65248302
chr18 65365151
chr18 65712107
chr18 65712251
chr18 65712567
chr18 65720933
chr18 65721417
chr18 65721465
chr18 65721739
chr18 65721948
chr18 65725676
chr18 65725872
chr18 65726793
chr18 65727000
chr18 65753696
chr18 65753738
chr18 65753978
chr18 65881796
chr18 65882042
chr18 66257012
chr18 66257615
chr18 66311354
chr18 66311832
chr18 66313167
chr18 66393743
chr18 66394014
chr18 66394512
chr18 66394951
chr18 66395201
chr18 66525858
chr18 66525869
chr18 66540716
chr18 66540823
chr18 66541138
chr18 66541169
chr18 66541730
chr18 66683795
chr18 66684344
chr18 66864361
chr18 66867896
chr18 66867938
chr18 66868962
chr18 66869189
chr18 66869360
chr18 67217581
chr18 67217619
chr18 67217755

chr18 67217781
chr18 67217804
chr18 67217915
chr18 67217938
chr18 67360476
chr18 67360639
chr18 67361160
chr18 67361727
chr18 67361833
chr18 67361908
chr18 67361917
chr18 67362050
chr18 67404615
chr18 67404723
chr18 67404898
chr18 67495079
chr18 67517050
chr18 67517057
chr18 67517059
chr18 67517061
chr18 67517067
chr18 67517076
chr18 67517106
chr18 67517120
chr18 67517134
chr18 67517161
chr18 67517250
chr18 67517363
chr18 67517547
chr18 67517588
chr18 67517597
chr18 67517621
chr18 67634742
chr18 67634774
chr18 67634839
chr18 67635142
chr18 67668517
chr18 67668576
chr18 67784111
chr18 67853286
chr18 67862722
chr18 68102669
chr18 68103414
chr18 68103736
chr18 68226874
chr18 68226897
chr18 68227769
chr18 68227947
chr18 68227950
chr18 68228105
chr18 68228237
chr18 68228431
chr18 68228668
chr18 68228805

chr18 68392860
chr18 68392983
chr18 68396625
chr18 68396661
chr18 68623036
chr18 68713845
chr18 68713880
chr18 68713954
chr18 68714100
chr18 68714106
chr18 68714181
chr18 68714269
chr18 68714316
chr18 68714330
chr18 68714357
chr18 68714403
chr18 68714438
chr18 68714486
chr18 68714496
chr18 68714499
chr18 68714521
chr18 68721868
chr18 68722022
chr18 68722149
chr18 68864090
chr18 68881821
chr18 68882155
chr18 68990982
chr18 69024106
chr18 69141984
chr18 69142068
chr18 69897322
chr18 69935050
chr18 69935053
chr18 69935454
chr18 69947202
chr18 69947519
chr18 69947621
chr18 69947633
chr18 69947644
chr18 69947907
chr18 69947920
chr18 69947927
chr18 69948467
chr18 69948517
chr18 69949021
chr18 69949031
chr18 69949082
chr18 69949144
chr18 69949160
chr18 69949162
chr18 69949165
chr18 69957204
chr18 69957237

chr18 70194533
chr18 70194564
chr18 70464872
chr18 70464886
chr18 70621090
chr18 70621134
chr18 70621663
chr18 70621874
chr18 70621975
chr18 70781942
chr18 70781954
chr18 70782045
chr18 70782058
chr18 70782078
chr18 70782244
chr18 70782256
chr18 71067494
chr18 71067603
chr18 71187429
chr18 71280008
chr18 71280077
chr18 71280099
chr18 71280155
chr18 71280537
chr18 71280557
chr18 71280938
chr18 71281165
chr18 71281402
chr18 71281424
chr18 71281487
chr18 71290027
chr18 71290863
chr18 71290985
chr18 71638047
chr18 71638298
chr18 71638329
chr18 72339118
chr18 72757640
chr18 72969740
chr18 72969835
chr18 72970101
chr18 72970626
chr18 72970659
chr18 72970669
chr18 72970692
chr18 72970899
chr18 72970936
chr18 72971025
chr18 72971069
chr18 73009989
chr18 73010189
chr18 73031322
chr18 73031992
chr18 73032018

chr18 73767031
chr18 73767061
chr18 73767111
chr18 73767136
chr18 73767185
chr18 73767345
chr18 73767678
chr18 73767803
chr18 73842415
chr18 73842918
chr18 73842939
chr18 73842949
chr18 73842961
chr18 73842990
chr18 73842997
chr18 73843014
chr18 73975711
chr18 73976001
chr18 74149025
chr18 74149087
chr18 74149090
chr18 74149229
chr18 74196237
chr18 74196256
chr18 74196290
chr18 74196409
chr18 74196440
chr18 74196509
chr18 74196545
chr18 74196578
chr18 74196743
chr18 74342746
chr18 74373566
chr18 74373693
chr18 74373723
chr18 74373832
chr18 74485147
chr18 74485249
chr18 74485257
chr18 74485265
chr18 74485281
chr18 74485294
chr18 74485302
chr18 74485319
chr18 74485327
chr18 74485335
chr18 74485359
chr18 74485379
chr18 74485383
chr18 74485389
chr18 74485391
chr18 74485395
chr18 74485424
chr18 74485455

chr18 74485480
chr18 74485485
chr18 74485513
chr18 74485523
chr18 74485525
chr18 74485529
chr18 74496109
chr18 74496112
chr18 74496117
chr18 74496138
chr18 74496151
chr18 74496156
chr18 74496180
chr18 74496190
chr18 74496264
chr18 74496269
chr18 74496275
chr18 74496280
chr18 74496294
chr18 74496303
chr18 74496321
chr18 74496325
chr18 74496327
chr18 74496332
chr18 74496340
chr18 74496343
chr18 74496350
chr18 74496356
chr18 74496363
chr18 74496370
chr18 74496376
chr18 74496379
chr18 74496382
chr18 74496384
chr18 74496460
chr18 74496468
chr18 74496484
chr18 74496493
chr18 74496504
chr18 74496512
chr18 74496515
chr18 74499068
chr18 74499089
chr18 74499148
chr18 74499167
chr18 74499191
chr18 74499301
chr18 74499345
chr18 74499347
chr18 74499469
chr18 74499696
chr18 74499784
chr18 74499802
chr18 74499884

chr18 74599014
chr18 74917246
chr18 74917361
chr18 74917467
chr18 74994932
chr18 74994948
chr18 74994983
chr18 74995189
chr18 75210014
chr18 75210025
chr18 75210049
chr18 75210079
chr18 75210102
chr18 75210110
chr18 75210116
chr18 75210133
chr18 75210281
chr18 75210357
chr18 75210419
chr18 75210427
chr18 75210545
chr18 75210655
chr18 75210733
chr18 75851741
chr18 76237857
chr18 76374812
chr18 76374832
chr18 76374847
chr18 76374897
chr18 76375099
chr18 76375112
chr18 76493342
chr18 76493349
chr18 76493352
chr18 76493379
chr18 76493409
chr18 76493422
chr18 76493455
chr18 76493474
chr18 76493477
chr18 76493503
chr18 76493513
chr18 76493559
chr18 76493619
chr18 76493664
chr18 76493826
chr18 76493927
chr18 76493949
chr18 76493957
chr18 76493974
chr18 76494012
chr18 76494022
chr18 76494037
chr18 76494078

chr18 76494113
chr18 76494172
chr18 76495775
chr18 76495790
chr18 76495805
chr18 76495808
chr18 76495813
chr18 76495819
chr18 76495909
chr18 76495930
chr18 76495943
chr18 76495973
chr18 76495982
chr18 76589016
chr18 76589046
chr18 76589066
chr18 76589096
chr18 76589155
chr18 76589160
chr18 76589197
chr18 76658103
chr18 76658145
chr18 76658686
chr18 76658721
chr18 76727825
chr18 76761829
chr18 76803802
chr18 76803811
chr18 76803818
chr18 76803850
chr18 76803857
chr18 76803863
chr18 76803914
chr18 76803940
chr18 76803958
chr18 76803978
chr18 76804027
chr18 76804093
chr18 76804098
chr18 77054482
chr18 77054735
chr18 77054738
chr18 77054784
chr18 77054787
chr18 77054794
chr18 77054833
chr18 77054972
chr18 77055065
chr18 77055092
chr18 77055099
chr18 77055119
chr18 77055125
chr18 77055128
chr18 77055150

chr18 77055189
chr18 77055476
chr18 77065279
chr18 77065294
chr18 77065428
chr18 77065472
chr18 77067179
chr18 77067192
chr18 77067214
chr18 77067401
chr18 77101728
chr18 77101739
chr18 77101826
chr18 77101858
chr18 77101938
chr18 77101987
chr18 77102040
chr18 77102084
chr18 77102188
chr18 77102223
chr18 77102369
chr18 77102387
chr18 77102695
chr18 77106444
chr18 77106459
chr18 77106468
chr18 77124406
chr18 77124432
chr18 77124451
chr18 77124492
chr18 77124525
chr18 77124542
chr18 77124544
chr18 77124559
chr18 77124592
chr18 77124629
chr18 77124702
chr18 77124742
chr18 77125011
chr18 77125022
chr18 77125032
chr18 77125100
chr18 77125148
chr18 77125479
chr18 77125528
chr18 77125699
chr18 77208673
chr18 77208751
chr18 77208819
chr18 77208855
chr18 77208868
chr18 77209176
chr18 77209196
chr18 77209216

chr18 77419229
chr18 77419238
chr18 77419497
chr18 77650324
chr18 77650721
chr18 77650741
chr18 77650844
chr18 77650847
chr18 77650883
chr18 77650892
chr18 77650910
chr18 77650912
chr18 77650917
chr18 77691712
chr18 77691826
chr18 77691989
chr18 77692392
chr18 77765792
chr18 77765820
chr18 77766041
chr18 77766096
chr18 77766130
chr18 77900372
chr18 77900385
chr18 77900387
chr18 77900391
chr18 77900399
chr18 77900412
chr18 77900414
chr18 77900461
chr18 77900470
chr18 77900555
chr18 77900591
chr18 77900601
chr18 77900603
chr18 77900628
chr18 77900641
chr18 77900658
chr18 77900661
chr18 77900675
chr18 77900683
chr18 78004380
chr18 78004498
chr18 78004657
chr18 78004696
chr18 78370240
chr18 78370790
chr18 78371474
chr18 78371664
chr18 78407260
chr18 78407263
chr18 78407326
chr18 78407338
chr18 78407571

chr18 78407623
chr18 78407662
chr18 78407667
chr18 78407854
chr18 78408052
chr18 78408126
chr18 78633160
chr18 78633207
chr18 78633254
chr18 78633301
chr18 78633348
chr18 78643184
chr18 78643209
chr18 78744223
chr18 78744469
chr18 78842736
chr18 78843128
chr18 78843249
chr18 78843763
chr18 78975738
chr18 78975783
chr18 78975891
chr18 78982269
chr18 78982373
chr18 78982430
chr18 78982441
chr18 78982516
chr18 78982556
chr18 78982593
chr18 78982597
chr18 78982609
chr18 78982733
chr18 78982738
chr18 78982780
chr18 78982807
chr18 78983068
chr18 78983226
chr18 78983256
chr18 78983997
chr18 78986317
chr18 78986337
chr18 78986339
chr18 78986374
chr18 78986381
chr18 78986400
chr18 78986429
chr18 78986664
chr18 78986687
chr18 78986943
chr18 79393447
chr18 79393476
chr18 79393502
chr18 79520568
chr18 79520586

chr18 79520648
chr18 79520976
chr18 79521086
chr18 79521119
chr18 79521272
chr18 79521306
chr18 79521597
chr18 79521637
chr18 79521673
chr18 79521852
chr18 79522182
chr18 79522389
chr18 79522395
chr18 79522418
chr18 79522460
chr18 79522519
chr18 79522585
chr18 79522592
chr18 79523531
chr18 79523700
chr18 79523736
chr18 79523745
chr18 79523870
chr18 79523883
chr18 79523925
chr18 79537183
chr18 79906260
chr18 79964762
chr18 79964765
chr18 79964769
chr18 79964771
chr18 79964773
chr18 79964778
chr18 79964783
chr18 79964785
chr18 79964800
chr18 79964827
chr18 79964835
chr18 79964874
chr18 79964893
chr18 79964900
chr18 79964929
chr18 79965020
chr18 79965076
chr18 79965083
chr18 79965136
chr18 79965173
chr18 79965187
chr18 79965194
chr18 79965199
chr18 79965211
chr18 79965221
chr18 79965234
chr16 10035

chr16 23021
chr16 23025
chr16 23027
chr16 23044
chr16 23058
chr16 23076
chr16 138293
chr16 138313
chr16 138319
chr16 138325
chr16 138345
chr16 138351
chr16 138357
chr16 154051
chr16 154060
chr16 154073
chr16 154075
chr16 154083
chr16 154100
chr16 154102
chr16 154107
chr16 154109
chr16 154114
chr16 154118
chr16 154123
chr16 154128
chr16 154133
chr16 154138
chr16 154140
chr16 154145
chr16 154149
chr16 154154
chr16 154159
chr16 154164
chr16 154169
chr16 154179
chr16 163863
chr16 163892
chr16 164641
chr16 164650
chr16 164663
chr16 164665
chr16 164673
chr16 164690
chr16 164692
chr16 164697
chr16 164699
chr16 164704
chr16 164708
chr16 164713
chr16 164718
chr16 164724
chr16 164726
chr16 164731

chr16 164735
chr16 164741
chr16 164743
chr16 164748
chr16 164754
chr16 164756
chr16 164761
chr16 164766
chr16 164777
chr16 164779
chr16 164784
chr16 164789
chr16 164795
chr16 164797
chr16 164802
chr16 164807
chr16 164812
chr16 164817
chr16 164822
chr16 164826
chr16 164831
chr16 164835
chr16 164850
chr16 164855
chr16 164860
chr16 164875
chr16 164880
chr16 164885
chr16 164887
chr16 164892
chr16 172075
chr16 172173
chr16 172199
chr16 172216
chr16 172227
chr16 172249
chr16 172282
chr16 172310
chr16 172859
chr16 172861
chr16 172865
chr16 172867
chr16 172872
chr16 172930
chr16 172942
chr16 173293
chr16 173295
chr16 173306
chr16 173975
chr16 174020
chr16 174040
chr16 174180
chr16 174243
chr16 174435

chr16 174447
chr16 175562
chr16 175667
chr16 175779
chr16 175879
chr16 175961
chr16 175977
chr16 176003
chr16 176020
chr16 176031
chr16 176053
chr16 176086
chr16 176114
chr16 176175
chr16 176179
chr16 176185
chr16 176187
chr16 176199
chr16 176214
chr16 176224
chr16 176229
chr16 176235
chr16 176239
chr16 176243
chr16 176254
chr16 176260
chr16 176269
chr16 176274
chr16 176276
chr16 176283
chr16 176285
chr16 176295
chr16 176300
chr16 176308
chr16 228642
chr16 228789
chr16 228809
chr16 228840
chr16 228853
chr16 228864
chr16 228873
chr16 228900
chr16 228928
chr16 228942
chr16 229082
chr16 234696
chr16 234699
chr16 234902
chr16 234905
chr16 234912
chr16 234974
chr16 234977
chr16 235069
chr16 235091

chr16 235108
chr16 235159
chr16 235174
chr16 235189
chr16 235192
chr16 235197
chr16 235202
chr16 235213
chr16 235217
chr16 235223
chr16 235229
chr16 235241
chr16 235299
chr16 235306
chr16 235314
chr16 235326
chr16 235331
chr16 235350
chr16 235354
chr16 235362
chr16 235375
chr16 235379
chr16 235385
chr16 235395
chr16 528806
chr16 528839
chr16 528848
chr16 528881
chr16 528887
chr16 528917
chr16 528929
chr16 528931
chr16 807304
chr16 807342
chr16 807344
chr16 807356
chr16 807367
chr16 807395
chr16 807409
chr16 807422
chr16 807425
chr16 807439
chr16 807454
chr16 807466
chr16 807479
chr16 807482
chr16 807485
chr16 807495
chr16 807502
chr16 807519
chr16 807523
chr16 807525
chr16 807532
chr16 807534

chr16 807542
chr16 807545
chr16 807565
chr16 807570
chr16 807576
chr16 807580
chr16 807625
chr16 807631
chr16 807667
chr16 807688
chr16 807693
chr16 807701
chr16 807710
chr16 807723
chr16 807727
chr16 807733
chr16 807737
chr16 807746
chr16 807750
chr16 807755
chr16 807771
chr16 807775
chr16 807778
chr16 807780
chr16 807782
chr16 807785
chr16 807795
chr16 807814
chr16 807821
chr16 807839
chr16 807841
chr16 807845
chr16 807852
chr16 807856
chr16 807858
chr16 807863
chr16 807869
chr16 807873
chr16 807876
chr16 1308031
chr16 1308324
chr16 1308344
chr16 1308562
chr16 1308600
chr16 1308667
chr16 2266767
chr16 2266798
chr16 2267001
chr16 2267042
chr16 2267115
chr16 2267119
chr16 2267133
chr16 2267137
chr16 2267189

chr16 2267202
chr16 2267222
chr16 2267232
chr16 2267234
chr16 2267276
chr16 2267287
chr16 2267292
chr16 2267351
chr16 2267378
chr16 2267407
chr16 2267413
chr16 2532665
chr16 2532745
chr16 2532785
chr16 2532812
chr16 2532838
chr16 2532997
chr16 2533015
chr16 2533037
chr16 2533060
chr16 2533067
chr16 2533077
chr16 2533085
chr16 2533118
chr16 2533122
chr16 2533218
chr16 2533222
chr16 2533255
chr16 2533299
chr16 2560738
chr16 2560850
chr16 2560917
chr16 2560944
chr16 2561146
chr16 2561176
chr16 2561185
chr16 2561219
chr16 2561308
chr16 2580658
chr16 2580675
chr16 2580703
chr16 2581184
chr16 2581241
chr16 2604159
chr16 2604171
chr16 2604177
chr16 2604195
chr16 2604236
chr16 2604261
chr16 2604293
chr16 2604319
chr16 2604427
chr16 2606964
chr16 2607000

chr16 2619705
chr16 2639247
chr16 2682601
chr16 2682614
chr16 2682624
chr16 2682627
chr16 2682635
chr16 2682644
chr16 2682647
chr16 2682660
chr16 2682671
chr16 2682687
chr16 2682695
chr16 2682698
chr16 2682701
chr16 2682723
chr16 2682738
chr16 2682745
chr16 2682762
chr16 2682776
chr16 2682782
chr16 2682813
chr16 2682823
chr16 2682842
chr16 2682849
chr16 2683216
chr16 2683235
chr16 2683247
chr16 2683253
chr16 2683271
chr16 2683312
chr16 2683337
chr16 2683369
chr16 2683373
chr16 2683395
chr16 2683503
chr16 2686836
chr16 2686848
chr16 2686873
chr16 2686963
chr16 2686966
chr16 3004209
chr16 3004213
chr16 3004219
chr16 3004227
chr16 3004231
chr16 3004245
chr16 3004248
chr16 3004250
chr16 3004259
chr16 3004269
chr16 3004275
chr16 3004277
chr16 3004282

chr16 3004286
chr16 3004288
chr16 3004292
chr16 3004294
chr16 3004302
chr16 3004304
chr16 3004313
chr16 3004319
chr16 3004323
chr16 3004326
chr16 3004332
chr16 3004341
chr16 3004350
chr16 3004353
chr16 3004362
chr16 3004368
chr16 3004371
chr16 3004378
chr16 3004386
chr16 3004390
chr16 3004407
chr16 3004410
chr16 3004413
chr16 3020304
chr16 3020309
chr16 3020316
chr16 3020367
chr16 3020376
chr16 3020379
chr16 3020383
chr16 3020390
chr16 3020404
chr16 3020411
chr16 3020416
chr16 3020419
chr16 3020428
chr16 3020436
chr16 3020456
chr16 3020464
chr16 3020469
chr16 3020475
chr16 3021086
chr16 3021105
chr16 3021107
chr16 3021173
chr16 3021180
chr16 3021198
chr16 3058753
chr16 3058783
chr16 3058786
chr16 3058791
chr16 3058802
chr16 3058849
chr16 3058862

chr16 3072649
chr16 3072936
chr16 3074738
chr16 3074822
chr16 3074860
chr16 3074907
chr16 3075042
chr16 3075127
chr16 3075191
chr16 3075276
chr16 3075318
chr16 3075432
chr16 3077167
chr16 3077177
chr16 3077185
chr16 3077221
chr16 3077243
chr16 3077249
chr16 3077254
chr16 3077267
chr16 3101820
chr16 3101840
chr16 3101856
chr16 3101912
chr16 3101914
chr16 3101937
chr16 3101941
chr16 3101946
chr16 3101959
chr16 3101962
chr16 3101984
chr16 3101986
chr16 3101990
chr16 3101997
chr16 3102015
chr16 3102029
chr16 3102135
chr16 3102143
chr16 3102154
chr16 3102159
chr16 3102201
chr16 3102235
chr16 3102265
chr16 3102272
chr16 3134136
chr16 3235216
chr16 3235230
chr16 3235252
chr16 3235369
chr16 3235400
chr16 3235403
chr16 3235425
chr16 3235464
chr16 3235485

chr16 3235489
chr16 3235496
chr16 3235498
chr16 3235501
chr16 3235510
chr16 3235528
chr16 3235540
chr16 3503697
chr16 3503861
chr16 3504194
chr16 3504284
chr16 3576298
chr16 3576352
chr16 3576362
chr16 3576365
chr16 3576395
chr16 3576499
chr16 3576512
chr16 3576539
chr16 3576693
chr16 3576725
chr16 3576731
chr16 3576748
chr16 3576755
chr16 3576765
chr16 3576787
chr16 3576811
chr16 3576912
chr16 3576949
chr16 3576959
chr16 3577030
chr16 3577153
chr16 3577160
chr16 3577201
chr16 3577242
chr16 3577314
chr16 3577329
chr16 3577340
chr16 3577357
chr16 3577376
chr16 3577385
chr16 3577403
chr16 3578551
chr16 3578917
chr16 4488077
chr16 4488100
chr16 4488108
chr16 4488761
chr16 4514889
chr16 4515406
chr16 4515671
chr16 4516058
chr16 4516083
chr16 4516088

chr16 4566713
chr16 5098511
chr16 5098516
chr16 5098530
chr16 5098539
chr16 5098563
chr16 5098707
chr16 5098714
chr16 5098720
chr16 5348645
chr16 5616498
chr16 5616506
chr16 5616523
chr16 5870752
chr16 6558679
chr16 6558738
chr16 6558822
chr16 6642933
chr16 6642978
chr16 6643019
chr16 6643052
chr16 6750080
chr16 6750506
chr16 6750560
chr16 6750706
chr16 7091075
chr16 7091123
chr16 7091334
chr16 7091467
chr16 7091488
chr16 7091547
chr16 7188556
chr16 7304987
chr16 7304992
chr16 7305002
chr16 7305132
chr16 7420135
chr16 7420180
chr16 7420216
chr16 7567056
chr16 8621866
chr16 8621889
chr16 8621904
chr16 8621926
chr16 8621949
chr16 8621993
chr16 8622024
chr16 8622073
chr16 8622084
chr16 8622101
chr16 8622113
chr16 8622120
chr16 8622126
chr16 8622149

chr16 8622154
chr16 8622167
chr16 8622228
chr16 8622239
chr16 8622241
chr16 8622313
chr16 8622380
chr16 8622422
chr16 8798222
chr16 8798232
chr16 8798289
chr16 8798320
chr16 8798359
chr16 8798409
chr16 8880443
chr16 8880455
chr16 9093216
chr16 9093304
chr16 9093309
chr16 9093323
chr16 9093370
chr16 9093385
chr16 9093456
chr16 9093467
chr16 9093508
chr16 9093552
chr16 9093564
chr16 9093566
chr16 9093576
chr16 9093756
chr16 9093786
chr16 9094025
chr16 9094089
chr16 9094194
chr16 9094353
chr16 9094363
chr16 9094659
chr16 9094665
chr16 9348299
chr16 9586542
chr16 9587102
chr16 9587114
chr16 9588026
chr16 9588066
chr16 9588396
chr16 9588426
chr16 10039021
chr16 10039087
chr16 10039102
chr16 10039106
chr16 10039260
chr16 10039288
chr16 10039299
chr16 10039305

chr16 10039317
chr16 10039322
chr16 10039412
chr16 10256882
chr16 10256902
chr16 10256991
chr16 10266931
chr16 10266984
chr16 10267031
chr16 10267076
chr16 10267079
chr16 10386346
chr16 10386367
chr16 10386380
chr16 10386392
chr16 10386396
chr16 10386417
chr16 10386432
chr16 10386465
chr16 10386475
chr16 10386496
chr16 10386599
chr16 10386606
chr16 10386683
chr16 10386700
chr16 10386730
chr16 10386752
chr16 10386953
chr16 10386969
chr16 10387099
chr16 10552896
chr16 10552979
chr16 10553049
chr16 10743484
chr16 10743776
chr16 10743778
chr16 10871487
chr16 10871553
chr16 10871597
chr16 10877103
chr16 10877178
chr16 10877248
chr16 10877414
chr16 10878591
chr16 10878613
chr16 10878730
chr16 10878749
chr16 10878777
chr16 10954122
chr16 10954128
chr16 10954214
chr16 10954226
chr16 10954229
chr16 10954320

chr16	10954336
chr16	10954372
chr16	10987841
chr16	10988027
chr16	10988074
chr16	10988112
chr16	11105164
chr16	11105189
chr16	11105210
chr16	11105286
chr16	11105501
chr16	11224723
chr16	11224748
chr16	11232728
chr16	11232960
chr16	11233039
chr16	11233062
chr16	11233152
chr16	11233159
chr16	11233165
chr16	11233169
chr16	11233196
chr16	11233221
chr16	11233255
chr16	11233261
chr16	11233281
chr16	11233284
chr16	11233293
chr16	11233300
chr16	11233313
chr16	11233317
chr16	11233324
chr16	11233331
chr16	11584723
chr16	11584726
chr16	11584755
chr16	11584800
chr16	11584812
chr16	11584847
chr16	11585005
chr16	11585093
chr16	11585181
chr16	11585187
chr16	11585211
chr16	11585266
chr16	11585519
chr16	11586516
chr16	11586518
chr16	11586544
chr16	11586553
chr16	11586565
chr16	11586567
chr16	11586570
chr16	11586575

chr16 11586577
chr16 11586584
chr16 11586588
chr16 11586592
chr16 11586603
chr16 11586607
chr16 11586822
chr16 11586828
chr16 11586836
chr16 11586846
chr16 11783052
chr16 11783056
chr16 11783133
chr16 11783143
chr16 11783356
chr16 11783363
chr16 11783582
chr16 11783671
chr16 11783870
chr16 11850389
chr16 11850400
chr16 11850572
chr16 11850704
chr16 11850751
chr16 11850814
chr16 11850834
chr16 11850838
chr16 11850846
chr16 11850878
chr16 11850909
chr16 11850936
chr16 11850941
chr16 11913972
chr16 11913984
chr16 11914108
chr16 11914136
chr16 11914160
chr16 11914344
chr16 11914359
chr16 11914386
chr16 11914408
chr16 12646541
chr16 12646707
chr16 12646776
chr16 12646813
chr16 12646878
chr16 12646894
chr16 12646964
chr16 12646972
chr16 13425808
chr16 13548289
chr16 13918825
chr16 13918942
chr16 13918959

chr16 13919194
chr16 13919199
chr16 13919213
chr16 13919271
chr16 13919435
chr16 13919590
chr16 13919631
chr16 13919685
chr16 13919722
chr16 13919728
chr16 13919821
chr16 13919853
chr16 13919863
chr16 13919895
chr16 13919905
chr16 13919916
chr16 13919926
chr16 13919933
chr16 13919951
chr16 13919961
chr16 13919975
chr16 13919977
chr16 14007521
chr16 14007588
chr16 14071590
chr16 14071592
chr16 14071596
chr16 14071600
chr16 14071602
chr16 14071608
chr16 14071614
chr16 14071773
chr16 14071787
chr16 14071853
chr16 14071883
chr16 14354379
chr16 14354428
chr16 14354607
chr16 14354612
chr16 14354742
chr16 14354815
chr16 14354821
chr16 14354892
chr16 14354961
chr16 14355053
chr16 14355167
chr16 14355239
chr16 14355441
chr16 14487689
chr16 14487742
chr16 14487871
chr16 14692266
chr16 14692608
chr16 14692636

chr16 14692641
chr16 14692706
chr16 14692741
chr16 14692783
chr16 14692793
chr16 14692817
chr16 14692823
chr16 14693284
chr16 14693354
chr16 14693385
chr16 14693578
chr16 14824733
chr16 14824737
chr16 14824739
chr16 14824748
chr16 14824759
chr16 14824774
chr16 14824789
chr16 14824800
chr16 14824813
chr16 14824823
chr16 14824840
chr16 14824842
chr16 14824845
chr16 14824859
chr16 14824876
chr16 14833509
chr16 14833585
chr16 14833590
chr16 14833604
chr16 14833606
chr16 14833608
chr16 14833645
chr16 14833694
chr16 14833702
chr16 14833707
chr16 14833709
chr16 14833711
chr16 14833715
chr16 14833717
chr16 14833720
chr16 14833729
chr16 14833732
chr16 14833735
chr16 14833743
chr16 14833762
chr16 14833787
chr16 14834491
chr16 14834493
chr16 14834569
chr16 14834592
chr16 14834744
chr16 14834772
chr16 14911944

chr16 14911949
chr16 14911953
chr16 14911957
chr16 14911963
chr16 14911965
chr16 14911977
chr16 14911982
chr16 14911994
chr16 14911996
chr16 14912006
chr16 14912017
chr16 14912025
chr16 14912028
chr16 14912038
chr16 14912058
chr16 14912103
chr16 14912119
chr16 14912128
chr16 14912143
chr16 14912149
chr16 14912155
chr16 14912160
chr16 14912162
chr16 14912169
chr16 14912171
chr16 14912195
chr16 14912220
chr16 14912233
chr16 14912242
chr16 15014444
chr16 15014463
chr16 15153839
chr16 15153876
chr16 15153908
chr16 15154014
chr16 15154023
chr16 15229908
chr16 15229943
chr16 15230217
chr16 15249855
chr16 15249875
chr16 15249977
chr16 15259455
chr16 15259976
chr16 15261358
chr16 15261407
chr16 15261441
chr16 15261452
chr16 15261466
chr16 15261471
chr16 15328581
chr16 15328649
chr16 15372643
chr16 15372898

chr16 15390092
chr16 15390159
chr16 15390178
chr16 15590550
chr16 15590558
chr16 15590606
chr16 15590654
chr16 15590703
chr16 15590805
chr16 15591067
chr16 15591090
chr16 15591182
chr16 15591320
chr16 15591330
chr16 15591358
chr16 15591363
chr16 15591374
chr16 15591465
chr16 15591507
chr16 15591541
chr16 15591549
chr16 15636873
chr16 15643601
chr16 15643998
chr16 15644014
chr16 15650920
chr16 15650931
chr16 15650940
chr16 15650947
chr16 15897982
chr16 15897993
chr16 15897999
chr16 15961275
chr16 15961290
chr16 15961528
chr16 15961572
chr16 15961612
chr16 15961618
chr16 15961802
chr16 16006161
chr16 16006656
chr16 16223498
chr16 16223502
chr16 16223504
chr16 16223513
chr16 16223524
chr16 16223539
chr16 16223554
chr16 16223565
chr16 16223572
chr16 16223578
chr16 16223588
chr16 16223605
chr16 16223607

chr16 16223610
chr16 16223624
chr16 16223641
chr16 16223845
chr16 16223871
chr16 16223879
chr16 16223887
chr16 16223889
chr16 16223899
chr16 16223904
chr16 16223910
chr16 16223925
chr16 16223927
chr16 16223941
chr16 16224160
chr16 16224193
chr16 16232098
chr16 16232124
chr16 16232147
chr16 16232165
chr16 16232207
chr16 16232222
chr16 16232261
chr16 16232313
chr16 16232323
chr16 16232399
chr16 16232418
chr16 16232420
chr16 16232422
chr16 16232459
chr16 16232470
chr16 16232478
chr16 16232508
chr16 16232516
chr16 16232521
chr16 16232523
chr16 16232525
chr16 16232529
chr16 16232531
chr16 16232534
chr16 16232537
chr16 16232544
chr16 16232547
chr16 16232550
chr16 16232577
chr16 16232602
chr16 16232605
chr16 16232621
chr16 16233275
chr16 16233306
chr16 16233308
chr16 16233384
chr16 16233407
chr16 16233560

chr16 16360462
chr16 16360504
chr16 16360546
chr16 16369713
chr16 16369719
chr16 16369727
chr16 16369752
chr16 16414728
chr16 16415457
chr16 16416243
chr16 16416364
chr16 16417598
chr16 16417657
chr16 16494546
chr16 16494566
chr16 16494617
chr16 16494642
chr16 16495180
chr16 16495230
chr16 16495250
chr16 16495255
chr16 16495269
chr16 16495280
chr16 16495314
chr16 16495363
chr16 16495437
chr16 16590046
chr16 16590062
chr16 16590304
chr16 16590424
chr16 16593363
chr16 16593462
chr16 16593483
chr16 16593558
chr16 16593571
chr16 16840232
chr16 16841678
chr16 16842476
chr16 16985044
chr16 16985092
chr16 16993163
chr16 16993179
chr16 16993523
chr16 16993562
chr16 17013775
chr16 17013796
chr16 17013799
chr16 17013848
chr16 17013903
chr16 17013911
chr16 17013914
chr16 17013929
chr16 17013955
chr16 17013968

chr16 17014015
chr16 17014025
chr16 17014069
chr16 17369170
chr16 17369190
chr16 17369198
chr16 17369222
chr16 17369232
chr16 17369380
chr16 17369595
chr16 17369618
chr16 17369626
chr16 17369741
chr16 17369763
chr16 17369824
chr16 17369876
chr16 17369901
chr16 17369918
chr16 17369932
chr16 17369969
chr16 17449053
chr16 17449112
chr16 17449277
chr16 17477018
chr16 17607490
chr16 17732033
chr16 17909038
chr16 18560939
chr16 18560960
chr16 18560969
chr16 18561010
chr16 18561057
chr16 18561107
chr16 18561135
chr16 18561299
chr16 18561322
chr16 18561398
chr16 18561400
chr16 18561679
chr16 18561738
chr16 18561753
chr16 18561785
chr16 18561878
chr16 18561885
chr16 18561902
chr16 18561906
chr16 18561945
chr16 18561950
chr16 18561954
chr16 18561957
chr16 18561965
chr16 18561968
chr16 18562283
chr16 18562444

chr16 18562483
chr16 18562540
chr16 18562552
chr16 18562558
chr16 18571003
chr16 18571018
chr16 18571025
chr16 18571038
chr16 18571042
chr16 18571068
chr16 18571085
chr16 18571099
chr16 18571102
chr16 18571104
chr16 18571121
chr16 18571131
chr16 18571137
chr16 18571144
chr16 18571155
chr16 18571170
chr16 18571185
chr16 18571196
chr16 18571205
chr16 18571207
chr16 18571211
chr16 18571235
chr16 18571246
chr16 18571258
chr16 18571261
chr16 18571271
chr16 18571274
chr16 18571281
chr16 18571283
chr16 18571299
chr16 18571301
chr16 18571327
chr16 18571329
chr16 18571373
chr16 18571380
chr16 18571419
chr16 18571426
chr16 18760130
chr16 18760143
chr16 18763045
chr16 18763297
chr16 18763623
chr16 18767536
chr16 18767538
chr16 18788009
chr16 18788871
chr16 18788966
chr16 18789007
chr16 18789011
chr16 18789027

chr16 18789046
chr16 18789062
chr16 18789106
chr16 18789190
chr16 18789224
chr16 18789293
chr16 18789314
chr16 18789319
chr16 18789368
chr16 18789373
chr16 18789388
chr16 18789437
chr16 18789466
chr16 18789480
chr16 18789490
chr16 18789524
chr16 18789738
chr16 18789834
chr16 18789877
chr16 18789882
chr16 18789930
chr16 18789932
chr16 18789936
chr16 18789956
chr16 18789970
chr16 18789980
chr16 18789983
chr16 18789987
chr16 18789998
chr16 18790028
chr16 18790033
chr16 18790038
chr16 18790043
chr16 18790052
chr16 18790068
chr16 18790077
chr16 18790084
chr16 18790088
chr16 18790090
chr16 18790095
chr16 18790109
chr16 18790114
chr16 18790142
chr16 18790180
chr16 18790198
chr16 18790223
chr16 18790233
chr16 18790239
chr16 18800328
chr16 18800338
chr16 18800472
chr16 18800500
chr16 18804206
chr16 18804660

chr16 18924077
chr16 18924089
chr16 18924173
chr16 18924315
chr16 18924323
chr16 18924643
chr16 18924653
chr16 18924674
chr16 18924831
chr16 18924934
chr16 18924936
chr16 18926650
chr16 18926680
chr16 18926690
chr16 18926698
chr16 18926720
chr16 18926738
chr16 18926764
chr16 18926769
chr16 18926771
chr16 18926773
chr16 18926783
chr16 18969125
chr16 18969157
chr16 18969204
chr16 18969358
chr16 18969360
chr16 18969395
chr16 18969407
chr16 18969427
chr16 18969442
chr16 18969457
chr16 18969460
chr16 18969478
chr16 19521244
chr16 19521270
chr16 19521276
chr16 19521311
chr16 19521326
chr16 19521336
chr16 19523965
chr16 19523985
chr16 19523988
chr16 19523991
chr16 19523996
chr16 19524003
chr16 19524030
chr16 19524047
chr16 19524052
chr16 19524076
chr16 19524121
chr16 19524127
chr16 19524147
chr16 19524152

chr16 19524161
chr16 19524180
chr16 19524200
chr16 19524207
chr16 19524224
chr16 19524227
chr16 19524241
chr16 19524249
chr16 19524265
chr16 19524269
chr16 19524285
chr16 19524297
chr16 19524312
chr16 19524330
chr16 19524359
chr16 19524381
chr16 19524401
chr16 19524412
chr16 19524425
chr16 19524448
chr16 19524468
chr16 19524492
chr16 19524548
chr16 19717873
chr16 19717875
chr16 19717877
chr16 19717898
chr16 19717931
chr16 19717956
chr16 19717972
chr16 19718016
chr16 19718021
chr16 19718025
chr16 19718032
chr16 19718045
chr16 19718068
chr16 20400083
chr16 20400124
chr16 20400140
chr16 20530744
chr16 20530765
chr16 20531078
chr16 20531162
chr16 20531176
chr16 20531178
chr16 20531186
chr16 20531198
chr16 20532958
chr16 20602727
chr16 20763689
chr16 20763797
chr16 20763844
chr16 20763894
chr16 20763990

chr16 20764030
chr16 20874485
chr16 20899838
chr16 20899893
chr16 20899982
chr16 20900085
chr16 20900087
chr16 20911249
chr16 20911339
chr16 20911498
chr16 21158805
chr16 21158808
chr16 21158812
chr16 21158829
chr16 21158831
chr16 21158837
chr16 21158840
chr16 21158843
chr16 21158849
chr16 21158851
chr16 21158857
chr16 21158867
chr16 21158871
chr16 21158886
chr16 21158904
chr16 21158922
chr16 21158925
chr16 21158930
chr16 21158942
chr16 21158946
chr16 21158956
chr16 21158990
chr16 21159071
chr16 21159107
chr16 21159110
chr16 21159140
chr16 21159144
chr16 21159177
chr16 21159195
chr16 21159260
chr16 21159307
chr16 21159309
chr16 21159374
chr16 21159391
chr16 21159405
chr16 21159420
chr16 21159425
chr16 21159493
chr16 21300647
chr16 21300893
chr16 21300915
chr16 21300927
chr16 21300930
chr16 21300942

chr16 21300955
chr16 21300959
chr16 21300970
chr16 21300979
chr16 21300986
chr16 21300988
chr16 21300996
chr16 21301001
chr16 21301012
chr16 21301022
chr16 21301041
chr16 21301043
chr16 21301052
chr16 21301054
chr16 21301056
chr16 21301061
chr16 21301063
chr16 21301080
chr16 21301099
chr16 21301122
chr16 21301382
chr16 21301395
chr16 21301404
chr16 21301417
chr16 21301426
chr16 21301462
chr16 21301478
chr16 21301488
chr16 21301496
chr16 21301518
chr16 21301546
chr16 21301559
chr16 21301594
chr16 21499890
chr16 21499902
chr16 21500458
chr16 21500468
chr16 21501051
chr16 21501362
chr16 21501367
chr16 21501387
chr16 21501441
chr16 21518663
chr16 21518703
chr16 21518713
chr16 21518791
chr16 21518797
chr16 21518800
chr16 21518807
chr16 21518835
chr16 21518840
chr16 21518844
chr16 21518865
chr16 21518885

chr16 21518896
chr16 21518927
chr16 21518931
chr16 21518939
chr16 21518943
chr16 21518963
chr16 21518987
chr16 21518995
chr16 21519002
chr16 21519019
chr16 21519025
chr16 21519027
chr16 21519049
chr16 21519317
chr16 21600662
chr16 21600695
chr16 21600706
chr16 21600743
chr16 21626827
chr16 21626905
chr16 21627123
chr16 21627125
chr16 21627130
chr16 21638575
chr16 21638682
chr16 21638845
chr16 21638907
chr16 21640810
chr16 21640885
chr16 21641042
chr16 21641131
chr16 21657003
chr16 21657332
chr16 21657413
chr16 21657477
chr16 21835864
chr16 21835879
chr16 21835886
chr16 21835892
chr16 21835899
chr16 21835905
chr16 21835909
chr16 21835974
chr16 21835978
chr16 21836003
chr16 21836018
chr16 21836025
chr16 21836031
chr16 21836035
chr16 21836478
chr16 21836482
chr16 21836507
chr16 21836585
chr16 21836589

chr16 21836614
chr16 21837281
chr16 21837301
chr16 21837357
chr16 21837382
chr16 21837439
chr16 21837471
chr16 21837496
chr16 21837802
chr16 21839637
chr16 21839676
chr16 21839701
chr16 21839731
chr16 21935196
chr16 21935199
chr16 21935222
chr16 21935227
chr16 21935265
chr16 21953847
chr16 21953852
chr16 21953867
chr16 21958536
chr16 21958568
chr16 22191500
chr16 22191510
chr16 22191578
chr16 22191596
chr16 22191602
chr16 22191718
chr16 22191847
chr16 22191886
chr16 22191976
chr16 22192026
chr16 22192057
chr16 22192291
chr16 22192375
chr16 22192384
chr16 22192406
chr16 22192451
chr16 22192468
chr16 22192482
chr16 22192491
chr16 22192509
chr16 22192533
chr16 22195766
chr16 22195780
chr16 22195786
chr16 22195796
chr16 22195800
chr16 22195817
chr16 22206692
chr16 22206694
chr16 22206698
chr16 22206725

chr16 22206727
chr16 22206742
chr16 22206746
chr16 22206759
chr16 22206768
chr16 22206827
chr16 22241183
chr16 22241367
chr16 22241382
chr16 22296624
chr16 22296627
chr16 22296644
chr16 22296705
chr16 22296707
chr16 22296782
chr16 22296829
chr16 22296844
chr16 22296849
chr16 22358094
chr16 22373252
chr16 22373275
chr16 22373304
chr16 22373338
chr16 22373353
chr16 22436132
chr16 22436195
chr16 22437603
chr16 22437608
chr16 22437636
chr16 22437726
chr16 22437750
chr16 22437764
chr16 22437783
chr16 22437799
chr16 22437853
chr16 22437873
chr16 22437878
chr16 22438753
chr16 22438772
chr16 22438782
chr16 22811018
chr16 22811874
chr16 22898899
chr16 23036253
chr16 23036496
chr16 23036743
chr16 23452433
chr16 23452435
chr16 23452443
chr16 23452445
chr16 23452460
chr16 23452465
chr16 23452472
chr16 23452481

chr16 23452499
chr16 23452521
chr16 23803620
chr16 23803648
chr16 23803652
chr16 23803660
chr16 23803665
chr16 23803815
chr16 23803829
chr16 23803896
chr16 23803925
chr16 23826541
chr16 23826598
chr16 23826701
chr16 23826717
chr16 23826860
chr16 23826953
chr16 23827096
chr16 23827166
chr16 23827278
chr16 23827331
chr16 23863285
chr16 23863307
chr16 23897208
chr16 23897244
chr16 23897316
chr16 23950051
chr16 23950054
chr16 23950061
chr16 23950175
chr16 23950464
chr16 23950510
chr16 23950631
chr16 24014345
chr16 24014404
chr16 24014458
chr16 24042102
chr16 24042168
chr16 24042204
chr16 24042206
chr16 24042253
chr16 24042338
chr16 24541769
chr16 24542358
chr16 24542402
chr16 24542420
chr16 24542435
chr16 24662924
chr16 24663143
chr16 24729256
chr16 25031041
chr16 25031051
chr16 25031130
chr16 25031191

chr16 25031221
chr16 25031259
chr16 25031283
chr16 25031313
chr16 25031341
chr16 25031422
chr16 25031437
chr16 25031486
chr16 25031491
chr16 25031552
chr16 25031554
chr16 25031562
chr16 25048615
chr16 25048762
chr16 25048894
chr16 25048914
chr16 25048925
chr16 25048953
chr16 25110587
chr16 25110658
chr16 25110749
chr16 25110828
chr16 25110887
chr16 25110993
chr16 25111001
chr16 25111009
chr16 25111018
chr16 25111031
chr16 25111056
chr16 25111063
chr16 25111073
chr16 25111114
chr16 25111118
chr16 25111126
chr16 25111130
chr16 25111158
chr16 25111189
chr16 25111211
chr16 25111216
chr16 25111221
chr16 25111261
chr16 25111265
chr16 25111284
chr16 25111320
chr16 25111374
chr16 25111432
chr16 25111436
chr16 25111460
chr16 25111490
chr16 25111492
chr16 25111510
chr16 25111558
chr16 25111593
chr16 25111604

chr16 25111617
chr16 25111649
chr16 25111660
chr16 25111684
chr16 25111689
chr16 25111708
chr16 25111729
chr16 25111732
chr16 25111738
chr16 25111741
chr16 25111752
chr16 25111754
chr16 25320220
chr16 26705326
chr16 26834740
chr16 27199608
chr16 27199674
chr16 27199928
chr16 27235167
chr16 27235181
chr16 27235208
chr16 27235288
chr16 27235389
chr16 27235442
chr16 27235453
chr16 27235508
chr16 27237215
chr16 27237243
chr16 27237246
chr16 27237310
chr16 27237348
chr16 27237400
chr16 27237447
chr16 27237456
chr16 27237466
chr16 27237526
chr16 27237570
chr16 27237579
chr16 27237668
chr16 27314766
chr16 27314925
chr16 27367609
chr16 27367615
chr16 27367624
chr16 27367640
chr16 27367651
chr16 27367661
chr16 27367695
chr16 27367731
chr16 27367772
chr16 27367786
chr16 27367802
chr16 27367809
chr16 27367835

chr16 27367849
chr16 27367890
chr16 27367967
chr16 27368093
chr16 27368123
chr16 27402121
chr16 27402129
chr16 27402145
chr16 27402186
chr16 27402204
chr16 27402226
chr16 27402245
chr16 27402253
chr16 27402256
chr16 27402951
chr16 27402960
chr16 27402970
chr16 27403075
chr16 27403097
chr16 27403120
chr16 27403137
chr16 27403147
chr16 27403180
chr16 27403203
chr16 27403210
chr16 27403215
chr16 27426622
chr16 28141216
chr16 28141379
chr16 28141452
chr16 28141540
chr16 28141593
chr16 28141726
chr16 28141731
chr16 28141756
chr16 28141776
chr16 28141787
chr16 28141816
chr16 28141820
chr16 28141828
chr16 28141852
chr16 28141891
chr16 28141903
chr16 28141914
chr16 28141916
chr16 28187626
chr16 28187896
chr16 28188478
chr16 28188509
chr16 28188538
chr16 28188557
chr16 28192987
chr16 28193060
chr16 28193261

chr16 28193303
chr16 28193320
chr16 28193344
chr16 28210809
chr16 28361645
chr16 28361699
chr16 28363609
chr16 28363866
chr16 28363901
chr16 28363921
chr16 28363925
chr16 28363933
chr16 28363966
chr16 28364005
chr16 28364066
chr16 28364069
chr16 28364071
chr16 28364075
chr16 28364358
chr16 28364374
chr16 28364647
chr16 28364661
chr16 28364671
chr16 28364679
chr16 28364683
chr16 28364690
chr16 28364692
chr16 28364704
chr16 28364711
chr16 28364714
chr16 28364718
chr16 28364729
chr16 28364732
chr16 28364835
chr16 28364854
chr16 28364858
chr16 28364860
chr16 28364863
chr16 28364870
chr16 28364876
chr16 28364885
chr16 28364899
chr16 28364902
chr16 28364947
chr16 28364954
chr16 28364968
chr16 28364970
chr16 28364974
chr16 28364979
chr16 28364992
chr16 28365007
chr16 28365009
chr16 28365017
chr16 28365032

chr16 28365041
chr16 28365063
chr16 28365071
chr16 28365085
chr16 28365118
chr16 28365126
chr16 28374392
chr16 28374482
chr16 28374503
chr16 28374512
chr16 28374523
chr16 28374547
chr16 28374554
chr16 28374594
chr16 28374597
chr16 28374624
chr16 28374635
chr16 28374640
chr16 28374726
chr16 28374822
chr16 28374860
chr16 28374916
chr16 28374918
chr16 28374994
chr16 28375184
chr16 28375200
chr16 28375289
chr16 28375327
chr16 28375358
chr16 28375475
chr16 28375489
chr16 28375516
chr16 28375765
chr16 28419922
chr16 28419954
chr16 28419978
chr16 28420038
chr16 28420049
chr16 28420123
chr16 28420183
chr16 28420213
chr16 28494166
chr16 28494176
chr16 28494200
chr16 28494203
chr16 28494338
chr16 28494344
chr16 28494505
chr16 28494541
chr16 28494565
chr16 28494638
chr16 28494694
chr16 28494796
chr16 28739921

chr16 28739924
chr16 28739952
chr16 28739990
chr16 28740361
chr16 28740363
chr16 28740384
chr16 28740395
chr16 28740419
chr16 28740457
chr16 28740553
chr16 28740639
chr16 28740644
chr16 28740655
chr16 28740682
chr16 28740685
chr16 28740731
chr16 28740738
chr16 28740762
chr16 28740773
chr16 28740803
chr16 28740865
chr16 28740870
chr16 28740936
chr16 28740980
chr16 28740988
chr16 28740990
chr16 28740995
chr16 28741010
chr16 28746639
chr16 28746648
chr16 28746650
chr16 28746661
chr16 28746775
chr16 28746786
chr16 28746791
chr16 28746798
chr16 28746801
chr16 28746844
chr16 28746846
chr16 28746851
chr16 28746853
chr16 28746855
chr16 28746861
chr16 28746997
chr16 28747008
chr16 28747019
chr16 28749444
chr16 28750168
chr16 28750209
chr16 28750223
chr16 28750231
chr16 28750253
chr16 28750262
chr16 28750277

chr16 28750285
chr16 28750287
chr16 28750302
chr16 28750315
chr16 28750320
chr16 28750324
chr16 28750326
chr16 28750340
chr16 28750347
chr16 28750481
chr16 28750490
chr16 28750504
chr16 28750507
chr16 28750515
chr16 28750517
chr16 28750522
chr16 28750528
chr16 28750544
chr16 28750547
chr16 28750734
chr16 28750737
chr16 28750755
chr16 28750765
chr16 28751155
chr16 28751223
chr16 28751225
chr16 28751228
chr16 28751289
chr16 28802682
chr16 28802685
chr16 28802741
chr16 28802761
chr16 28863150
chr16 28863182
chr16 28863190
chr16 28863222
chr16 28863226
chr16 28985720
chr16 28985748
chr16 28985757
chr16 28985882
chr16 28985888
chr16 29074671
chr16 29074795
chr16 29074844
chr16 29074900
chr16 29074906
chr16 29074916
chr16 29074942
chr16 29074950
chr16 29074952
chr16 29074961
chr16 29074997
chr16 29075007

chr16 29075012
chr16 29075016
chr16 29075018
chr16 29075026
chr16 29075037
chr16 29075043
chr16 29075055
chr16 29075057
chr16 29075064
chr16 29075073
chr16 29075077
chr16 29075082
chr16 29075172
chr16 29082086
chr16 29082092
chr16 29082120
chr16 29082126
chr16 29082150
chr16 29082159
chr16 29082161
chr16 29082169
chr16 29082179
chr16 29082181
chr16 29082184
chr16 29082192
chr16 29082197
chr16 29082199
chr16 29082203
chr16 29082207
chr16 29082245
chr16 29082254
chr16 29082257
chr16 29386072
chr16 29386150
chr16 29386182
chr16 29386221
chr16 29386246
chr16 29386276
chr16 29386620
chr16 29386660
chr16 29402504
chr16 29402627
chr16 29402634
chr16 29402681
chr16 29593102
chr16 29593244
chr16 29593252
chr16 29593568
chr16 29593578
chr16 29593598
chr16 29594834
chr16 29594838
chr16 29600371
chr16 29600382

chr16 29600408
chr16 29600437
chr16 29600444
chr16 29600453
chr16 29600464
chr16 29600553
chr16 29600575
chr16 29600721
chr16 29600747
chr16 29600761
chr16 29600773
chr16 29600799
chr16 29611695
chr16 29611819
chr16 29611933
chr16 29611973
chr16 29611983
chr16 29612061
chr16 29612067
chr16 29612070
chr16 29612077
chr16 29612105
chr16 29612110
chr16 29612114
chr16 29612135
chr16 29612155
chr16 29612197
chr16 29612201
chr16 29612209
chr16 29612213
chr16 29612233
chr16 29612257
chr16 29612265
chr16 29612272
chr16 29612289
chr16 29612295
chr16 29612297
chr16 29612319
chr16 29612474
chr16 29612478
chr16 29612520
chr16 29612592
chr16 29662752
chr16 29662792
chr16 29662809
chr16 29662863
chr16 29663061
chr16 29663106
chr16 29663143
chr16 29663166
chr16 29663263
chr16 29663297
chr16 29663310
chr16 29663315

chr16 29663582
chr16 29663590
chr16 29663607
chr16 29663638
chr16 29663651
chr16 29663661
chr16 29663663
chr16 29663678
chr16 29663736
chr16 29663776
chr16 29663815
chr16 29663839
chr16 29663893
chr16 29664115
chr16 29664138
chr16 29664186
chr16 29664376
chr16 29664403
chr16 29664432
chr16 29664445
chr16 29664464
chr16 29664485
chr16 29664525
chr16 29664538
chr16 29664560
chr16 29664563
chr16 29664566
chr16 29664575
chr16 29664578
chr16 29664883
chr16 29664904
chr16 29664916
chr16 29664936
chr16 29664959
chr16 29665012
chr16 29665030
chr16 29665049
chr16 29665086
chr16 29665094
chr16 29665117
chr16 29665157
chr16 29665179
chr16 29666286
chr16 29666304
chr16 29666413
chr16 29666501
chr16 29666557
chr16 29666559
chr16 29666561
chr16 29666563
chr16 29666565
chr16 29666567
chr16 29666614
chr16 29701917

chr16 29701977
chr16 29701986
chr16 29701993
chr16 29702010
chr16 29702036
chr16 29702049
chr16 29702101
chr16 29702109
chr16 29702130
chr16 29702188
chr16 29702218
chr16 29702275
chr16 29702278
chr16 29702319
chr16 29702322
chr16 29702341
chr16 29702359
chr16 29702429
chr16 29738635
chr16 29738713
chr16 29745391
chr16 29745429
chr16 29745548
chr16 29745630
chr16 29745646
chr16 29745653
chr16 29745665
chr16 29745687
chr16 29745706
chr16 29745735
chr16 29745748
chr16 29745753
chr16 29745997
chr16 29746002
chr16 29746054
chr16 29746084
chr16 29746140
chr16 29746371
chr16 29746420
chr16 29746448
chr16 29746467
chr16 29746477
chr16 29746484
chr16 29746507
chr16 29811567
chr16 29811573
chr16 29811591
chr16 29811843
chr16 29811845
chr16 29811876
chr16 29811884
chr16 29811894
chr16 29811918
chr16 29811926

chr16 29811940
chr16 29811975
chr16 29811982
chr16 29811988
chr16 29811998
chr16 29812017
chr16 29812032
chr16 29812034
chr16 29812036
chr16 29820355
chr16 29820384
chr16 29820437
chr16 29820481
chr16 29820747
chr16 29820859
chr16 29821020
chr16 29898603
chr16 29898660
chr16 29898666
chr16 29898782
chr16 29899083
chr16 29899089
chr16 29899136
chr16 29899205
chr16 29899251
chr16 29899253
chr16 29899273
chr16 29899275
chr16 29899281
chr16 29899300
chr16 29899309
chr16 29899318
chr16 29899348
chr16 29899431
chr16 29899438
chr16 29899463
chr16 29899473
chr16 29899511
chr16 29899518
chr16 29899533
chr16 29899559
chr16 29899567
chr16 29899583
chr16 29899587
chr16 29899615
chr16 29899662
chr16 29899673
chr16 29899688
chr16 29899690
chr16 29899746
chr16 29973761
chr16 29973774
chr16 29973776
chr16 29973795

chr16 29973812
chr16 29973818
chr16 29973832
chr16 29973835
chr16 29973844
chr16 29973846
chr16 29973852
chr16 29973854
chr16 29973860
chr16 29973864
chr16 29973906
chr16 29973911
chr16 29973917
chr16 29973930
chr16 29973946
chr16 29973971
chr16 29973973
chr16 29973980
chr16 29974004
chr16 29974018
chr16 29974046
chr16 29974051
chr16 29974073
chr16 29974080
chr16 29974083
chr16 29974095
chr16 29974099
chr16 29974103
chr16 29974107
chr16 29974113
chr16 29974130
chr16 29974137
chr16 29974207
chr16 29974236
chr16 29974243
chr16 29974311
chr16 29974325
chr16 29974339
chr16 29974343
chr16 30021464
chr16 30021473
chr16 30021476
chr16 30021494
chr16 30021509
chr16 30021518
chr16 30021525
chr16 30021527
chr16 30064190
chr16 30064196
chr16 30064220
chr16 30064226
chr16 30064235
chr16 30064325
chr16 30064387

chr16 30064394
chr16 30064467
chr16 30064504
chr16 30184675
chr16 30184702
chr16 30184739
chr16 30184775
chr16 30184810
chr16 30184835
chr16 30184856
chr16 30184883
chr16 30184938
chr16 30185216
chr16 30185228
chr16 30185243
chr16 30185248
chr16 30185262
chr16 30185272
chr16 30185294
chr16 30185296
chr16 30185371
chr16 30185446
chr16 30185482
chr16 30185591
chr16 30185746
chr16 30186023
chr16 30186041
chr16 30186043
chr16 30186091
chr16 30186201
chr16 30186287
chr16 30186345
chr16 30186348
chr16 30186372
chr16 30186393
chr16 30186456
chr16 30186462
chr16 30186604
chr16 30186620
chr16 30186626
chr16 30186660
chr16 30186671
chr16 30186863
chr16 30186866
chr16 30192162
chr16 30192167
chr16 30192184
chr16 30194637
chr16 30194647
chr16 30194662
chr16 30194668
chr16 30194689
chr16 30194691
chr16 30194700

chr16 30194710
chr16 30194725
chr16 30194752
chr16 30194755
chr16 30194761
chr16 30194769
chr16 30194776
chr16 30194782
chr16 30194789
chr16 30194806
chr16 30194813
chr16 30195526
chr16 30195548
chr16 30195550
chr16 30195573
chr16 30195588
chr16 30195612
chr16 30195632
chr16 30195636
chr16 30195644
chr16 30195677
chr16 30195688
chr16 30195708
chr16 30195722
chr16 30195778
chr16 30195782
chr16 30204380
chr16 30205882
chr16 30205891
chr16 30205937
chr16 30205966
chr16 30206022
chr16 30206029
chr16 30206049
chr16 30206051
chr16 30206064
chr16 30206070
chr16 30206078
chr16 30206080
chr16 30206083
chr16 30206131
chr16 30206134
chr16 30206140
chr16 30206152
chr16 30206155
chr16 30206157
chr16 30206161
chr16 30206168
chr16 30206185
chr16 30206189
chr16 30206192
chr16 30207358
chr16 30207361
chr16 30207713

chr16 30207720
chr16 30207896
chr16 30207913
chr16 30208692
chr16 30208701
chr16 30208755
chr16 30208783
chr16 30209085
chr16 30209110
chr16 30209134
chr16 30209140
chr16 30209608
chr16 30209620
chr16 30209812
chr16 30209827
chr16 30209855
chr16 30210361
chr16 30210382
chr16 30210606
chr16 30210740
chr16 30210858
chr16 30210870
chr16 30211176
chr16 30211183
chr16 30211190
chr16 30212206
chr16 30212277
chr16 30212292
chr16 30333657
chr16 30333667
chr16 30333686
chr16 30333951
chr16 30333976
chr16 30334250
chr16 30355128
chr16 30355151
chr16 30355177
chr16 30355185
chr16 30355189
chr16 30355201
chr16 30355203
chr16 30355221
chr16 30355223
chr16 30355230
chr16 30355257
chr16 30355272
chr16 30355295
chr16 30355308
chr16 30355394
chr16 30355400
chr16 30355418
chr16 30368215
chr16 30368264
chr16 30368339

chr16 30368504
chr16 30368559
chr16 30368577
chr16 30368603
chr16 30368685
chr16 30368837
chr16 30368862
chr16 30368953
chr16 30369086
chr16 30369094
chr16 30369096
chr16 30369108
chr16 30369157
chr16 30369163
chr16 30369168
chr16 30369193
chr16 30369198
chr16 30369204
chr16 30369236
chr16 30371034
chr16 30371056
chr16 30371061
chr16 30371075
chr16 30378370
chr16 30378372
chr16 30378391
chr16 30378401
chr16 30378407
chr16 30378418
chr16 30378422
chr16 30378427
chr16 30378442
chr16 30378454
chr16 30378513
chr16 30378516
chr16 30378531
chr16 30378543
chr16 30378554
chr16 30378580
chr16 30378584
chr16 30378594
chr16 30378601
chr16 30378615
chr16 30378624
chr16 30378626
chr16 30378639
chr16 30378646
chr16 30378667
chr16 30378681
chr16 30378689
chr16 30378704
chr16 30382288
chr16 30382319
chr16 30382353

chr16 30382491
chr16 30382533
chr16 30382555
chr16 30382596
chr16 30382611
chr16 30382730
chr16 30382792
chr16 30382895
chr16 30382911
chr16 30382997
chr16 30472160
chr16 30472392
chr16 30472640
chr16 30472675
chr16 30472786
chr16 30472798
chr16 30472824
chr16 30472872
chr16 30472897
chr16 30473157
chr16 30473159
chr16 30473169
chr16 30473259
chr16 30473293
chr16 30473325
chr16 30473387
chr16 30473516
chr16 30473518
chr16 30473623
chr16 30473641
chr16 30473668
chr16 30473719
chr16 30473732
chr16 30473797
chr16 30473814
chr16 30473838
chr16 30473844
chr16 30535309
chr16 30535417
chr16 30535446
chr16 30535493
chr16 30535522
chr16 30535638
chr16 30535844
chr16 30535851
chr16 30535855
chr16 30535899
chr16 30535912
chr16 30535914
chr16 30571590
chr16 30571597
chr16 30571600
chr16 30571609
chr16 30571621

chr16 30571624
chr16 30571637
chr16 30571645
chr16 30571649
chr16 30571652
chr16 30571656
chr16 30571673
chr16 30571676
chr16 30571684
chr16 30571690
chr16 30571703
chr16 30571721
chr16 30571727
chr16 30571735
chr16 30571740
chr16 30658498
chr16 30660161
chr16 30660186
chr16 30660290
chr16 30660302
chr16 30660323
chr16 30660352
chr16 30660377
chr16 30660389
chr16 30660404
chr16 30660410
chr16 30876392
chr16 30893265
chr16 30893282
chr16 30893468
chr16 30893515
chr16 30893541
chr16 30893560
chr16 30893588
chr16 30893623
chr16 30893644
chr16 30893659
chr16 30893672
chr16 30893701
chr16 30893705
chr16 30893756
chr16 30893764
chr16 30893780
chr16 30893789
chr16 30893795
chr16 30893816
chr16 30893826
chr16 30893839
chr16 30893846
chr16 30893859
chr16 30893869
chr16 30893876
chr16 30921827
chr16 30921850

chr16 30921875
chr16 30921886
chr16 30921915
chr16 30921954
chr16 30921956
chr16 30921963
chr16 30922027
chr16 30922096
chr16 30922101
chr16 30922106
chr16 30922114
chr16 30922139
chr16 30922365
chr16 30922369
chr16 30922372
chr16 30923957
chr16 30924041
chr16 30924070
chr16 30942110
chr16 30942129
chr16 30942141
chr16 30942156
chr16 30942159
chr16 31033514
chr16 31033516
chr16 31033526
chr16 31033535
chr16 31033546
chr16 31033554
chr16 31033568
chr16 31033580
chr16 31033626
chr16 31033802
chr16 31033809
chr16 31033823
chr16 31033842
chr16 31033853
chr16 31033865
chr16 31033869
chr16 31033873
chr16 31033883
chr16 31033901
chr16 31033942
chr16 31033961
chr16 31033973
chr16 31033978
chr16 31033983
chr16 31034001
chr16 31034026
chr16 31034030
chr16 31034046
chr16 31034051
chr16 31034053
chr16 31034068

chr16 31034074
chr16 31034079
chr16 31034092
chr16 31034096
chr16 31034133
chr16 31105501
chr16 31118194
chr16 31118223
chr16 31118257
chr16 31118444
chr16 31142311
chr16 31142317
chr16 31142333
chr16 31142355
chr16 31142361
chr16 31142368
chr16 31142377
chr16 31179236
chr16 31179257
chr16 31179316
chr16 31179333
chr16 31179390
chr16 31179441
chr16 31181242
chr16 31181252
chr16 31181256
chr16 31181260
chr16 31181670
chr16 31181703
chr16 31181706
chr16 31301932
chr16 31354589
chr16 31354797
chr16 31354821
chr16 31402897
chr16 31476552
chr16 31476576
chr16 31476597
chr16 31476607
chr16 31476610
chr16 31476615
chr16 31476629
chr16 31476636
chr16 31476702
chr16 31476708
chr16 31476719
chr16 31476729
chr16 31476740
chr16 31476766
chr16 31476782
chr16 31476813
chr16 31505813
chr16 31508170
chr16 31508184

chr16 31508227
chr16 31665900
chr16 31695623
chr16 31695936
chr16 31701282
chr16 31701384
chr16 31701634
chr16 31713516
chr16 31713538
chr16 31713540
chr16 31713544
chr16 31713551
chr16 31713568
chr16 31713571
chr16 31713581
chr16 31713585
chr16 31713590
chr16 31713597
chr16 31713605
chr16 31713607
chr16 31713609
chr16 31713637
chr16 31713653
chr16 31713659
chr16 31868960
chr16 31868976
chr16 31869107
chr16 31869114
chr16 31869228
chr16 31869544
chr16 31874688
chr16 31874696
chr16 31874749
chr16 31874760
chr16 31874769
chr16 31874848
chr16 31875006
chr16 31875098
chr16 31875104
chr16 31941152
chr16 31941306
chr16 31989326
chr16 31989331
chr16 31989341
chr16 31989394
chr16 31989399
chr16 31989403
chr16 31989539
chr16 32031484
chr16 32086206
chr16 32086224
chr16 32086247
chr16 32087009
chr16 32088273

chr16 32111261
chr16 32111457
chr16 32112024
chr16 32112662
chr16 32114884
chr16 32114918
chr16 32115559
chr16 32115594
chr16 32115597
chr16 32115600
chr16 32115618
chr16 32115621
chr16 32115624
chr16 32115626
chr16 32115646
chr16 32115649
chr16 32115651
chr16 32115662
chr16 32115667
chr16 32115670
chr16 32115673
chr16 32115675
chr16 32115692
chr16 32115694
chr16 32115700
chr16 32115711
chr16 32115714
chr16 32115716
chr16 32115735
chr16 32115755
chr16 32115757
chr16 32115772
chr16 32115780
chr16 32115790
chr16 32115793
chr16 32115807
chr16 32115810
chr16 32115812
chr16 32115837
chr16 32115855
chr16 32115869
chr16 32115872
chr16 32115888
chr16 32115891
chr16 32115894
chr16 32115896
chr16 32115905
chr16 32115934
chr16 32115936
chr16 32115948
chr16 32115951
chr16 32115953
chr16 32115961
chr16 32115969

chr16 32116009
chr16 32116012
chr16 32116015
chr16 32116017
chr16 32116029
chr16 32116034
chr16 32116123
chr16 32116125
chr16 32116137
chr16 32116140
chr16 32116142
chr16 32116161
chr16 32116166
chr16 32116181
chr16 32116184
chr16 32116189
chr16 32116213
chr16 32116245
chr16 32116248
chr16 32116251
chr16 32116253
chr16 32116261
chr16 32116268
chr16 32116271
chr16 32116274
chr16 32116276
chr16 32184723
chr16 32184728
chr16 32184753
chr16 32184784
chr16 32185406
chr16 32185410
chr16 32185912
chr16 32185920
chr16 32185952
chr16 32186015
chr16 32186022
chr16 32186304
chr16 32186387
chr16 32186945
chr16 32203212
chr16 32203232
chr16 32203234
chr16 32203277
chr16 32203288
chr16 32203329
chr16 32208269
chr16 32219509
chr16 32220076
chr16 32220085
chr16 32220098
chr16 32220100
chr16 32220106
chr16 32220182

chr16 32220194
chr16 32220227
chr16 32220238
chr16 32220258
chr16 32220279
chr16 32251700
chr16 32252081
chr16 32273913
chr16 32274478
chr16 32277478
chr16 32284565
chr16 32284643
chr16 32284997
chr16 32290913
chr16 32291037
chr16 32291318
chr16 32292079
chr16 32293793
chr16 32293822
chr16 32294205
chr16 32294229
chr16 32294235
chr16 32294311
chr16 32294327
chr16 32294388
chr16 32294402
chr16 32294418
chr16 32295378
chr16 32295464
chr16 32307559
chr16 32307569
chr16 32308044
chr16 32308118
chr16 32308214
chr16 32308323
chr16 32308369
chr16 32308378
chr16 32323845
chr16 32332041
chr16 32370869
chr16 32639099
chr16 32639419
chr16 32645103
chr16 32645430
chr16 32645446
chr16 32645532
chr16 32645835
chr16 32645874
chr16 32646280
chr16 32646381
chr16 32652173
chr16 32652248
chr16 32652264
chr16 32652272

chr16 32653626
chr16 32655839
chr16 32657841
chr16 32662968
chr16 32667282
chr16 32792487
chr16 32812821
chr16 32813365
chr16 33055323
chr16 33057782
chr16 33058859
chr16 33058894
chr16 33058897
chr16 33058900
chr16 33058902
chr16 33058925
chr16 33058927
chr16 33058950
chr16 33058952
chr16 33058975
chr16 33058977
chr16 33059001
chr16 33059003
chr16 33064298
chr16 33064345
chr16 33064392
chr16 33064724
chr16 33065251
chr16 33067794
chr16 33067809
chr16 33067856
chr16 33067892
chr16 33067894
chr16 33067928
chr16 33067958
chr16 33068707
chr16 33068765
chr16 33120039
chr16 33123167
chr16 33123177
chr16 33123222
chr16 33123287
chr16 33123292
chr16 33123319
chr16 33123326
chr16 33123335
chr16 33123343
chr16 33267090
chr16 33267094
chr16 33267126
chr16 33267165
chr16 33268461
chr16 33271170
chr16 33271222

chr16 33271234
chr16 33271267
chr16 33271278
chr16 33271298
chr16 33271319
chr16 33271356
chr16 33271366
chr16 33271368
chr16 33446936
chr16 33447571
chr16 33449810
chr16 33449829
chr16 33450854
chr16 33454490
chr16 33454527
chr16 33464750
chr16 33475650
chr16 33475661
chr16 33475675
chr16 33475695
chr16 33475739
chr16 33475747
chr16 33475751
chr16 33475771
chr16 33477934
chr16 33479938
chr16 33479971
chr16 33483602
chr16 33484509
chr16 33484512
chr16 33484525
chr16 33484528
chr16 33484544
chr16 33484550
chr16 33484552
chr16 33484566
chr16 33484569
chr16 33484572
chr16 33484574
chr16 33484584
chr16 33484600
chr16 33484614
chr16 33484617
chr16 33484620
chr16 33484623
chr16 33484786
chr16 33484792
chr16 33484799
chr16 33484819
chr16 33484824
chr16 33484843
chr16 33484849
chr16 33484854
chr16 33484863

chr16 33484872
chr16 33497572
chr16 33498333
chr16 33500085
chr16 33500574
chr16 33500590
chr16 33500651
chr16 33500665
chr16 33500681
chr16 33513833
chr16 33514324
chr16 33514398
chr16 33514494
chr16 33518637
chr16 33806886
chr16 33806895
chr16 33825764
chr16 33826448
chr16 33877918
chr16 33877948
chr16 33878636
chr16 33878771
chr16 33878906
chr16 33879512
chr16 33879605
chr16 33879609
chr16 33940646
chr16 33940877
chr16 33940979
chr16 33941049
chr16 33973931
chr16 33973961
chr16 33973972
chr16 33973974
chr16 33973985
chr16 33974397
chr16 33974427
chr16 34007266
chr16 34007329
chr16 34007397
chr16 34009025
chr16 34009488
chr16 34009953
chr16 34010445
chr16 34035494
chr16 34035521
chr16 34035714
chr16 34036516
chr16 34048460
chr16 34048563
chr16 34054179
chr16 34054223
chr16 34054914
chr16 34055086

chr16 34055190
chr16 34055249
chr16 34055342
chr16 34056026
chr16 34056039
chr16 34056070
chr16 34131831
chr16 34132060
chr16 34132109
chr16 34138524
chr16 34138597
chr16 34155525
chr16 34155649
chr16 34508456
chr16 34508526
chr16 34509049
chr16 34509221
chr16 34509275
chr16 34509364
chr16 34509489
chr16 34509623
chr16 34509665
chr16 34511408
chr16 34511559
chr16 34511775
chr16 34511789
chr16 34512189
chr16 34512950
chr16 34514888
chr16 34514927
chr16 34515330
chr16 34515587
chr16 34515637
chr16 34515663
chr16 34518096
chr16 34518185
chr16 34518190
chr16 34518206
chr16 34518220
chr16 34518306
chr16 34518492
chr16 34518616
chr16 34519025
chr16 34519387
chr16 34520121
chr16 34520258
chr16 34520789
chr16 34520939
chr16 34521338
chr16 34521385
chr16 34633318
chr16 34633344
chr16 34656468
chr16 34793061

chr16 34793303
chr16 34793348
chr16 34793512
chr16 34793682
chr16 34800743
chr16 34808800
chr16 34808849
chr16 34808996
chr16 34809309
chr16 34809425
chr16 34809438
chr16 34809717
chr16 34809844
chr16 34810145
chr16 34810769
chr16 34810779
chr16 34823567
chr16 34823867
chr16 34824357
chr16 34824696
chr16 34824813
chr16 34827206
chr16 34827652
chr16 34827716
chr16 34827802
chr16 34828197
chr16 34828333
chr16 34828470
chr16 34828493
chr16 34828504
chr16 34828635
chr16 34828655
chr16 34829062
chr16 34829204
chr16 34829646
chr16 34829655
chr16 34839765
chr16 34840056
chr16 34840223
chr16 34840289
chr16 34840625
chr16 34841304
chr16 34841474
chr16 34842548
chr16 34842975
chr16 34843177
chr16 34843664
chr16 34845173
chr16 34852643
chr16 34852914
chr16 34869387
chr16 34869665
chr16 34869764
chr16 34874470

chr16 34875085
chr16 34875267
chr16 34876099
chr16 34876145
chr16 34876251
chr16 34877830
chr16 34877964
chr16 34879365
chr16 34879635
chr16 34880330
chr16 34881906
chr16 34882992
chr16 34883049
chr16 34883544
chr16 34884103
chr16 34884124
chr16 34884161
chr16 34886649
chr16 34886663
chr16 34886879
chr16 34886918
chr16 34887959
chr16 34892168
chr16 34892181
chr16 34892187
chr16 34892191
chr16 34892217
chr16 34892243
chr16 34892353
chr16 34892379
chr16 34892431
chr16 34892441
chr16 34893418
chr16 34893444
chr16 34893454
chr16 34893594
chr16 34893604
chr16 34893615
chr16 34893630
chr16 34893646
chr16 34893656
chr16 34893672
chr16 34893682
chr16 34893708
chr16 34893724
chr16 34893734
chr16 34893747
chr16 34893773
chr16 34893818
chr16 34893939
chr16 34893965
chr16 34893975
chr16 34893988
chr16 34894112

chr16 34894122
chr16 34894144
chr16 34894209
chr16 34895200
chr16 34895804
chr16 34895820
chr16 34895826
chr16 34895843
chr16 34895853
chr16 34895892
chr16 34912098
chr16 34912124
chr16 34912137
chr16 34912170
chr16 34912699
chr16 34912712
chr16 34912912
chr16 34912928
chr16 34912954
chr16 34912964
chr16 34923282
chr16 34923370
chr16 34923531
chr16 34923577
chr16 34923851
chr16 34923856
chr16 34923950
chr16 34923959
chr16 34924021
chr16 34925058
chr16 34925162
chr16 34925790
chr16 34925796
chr16 34925982
chr16 34926085
chr16 34926208
chr16 34926574
chr16 34960090
chr16 34960106
chr16 34960116
chr16 34961152
chr16 34961518
chr16 34961528
chr16 34961580
chr16 34961590
chr16 34961606
chr16 34963229
chr16 34963265
chr16 34965958
chr16 34966002
chr16 34966528
chr16 34966568
chr16 34967332
chr16 35006717

chr16 35007036
chr16 35007047
chr16 35007122
chr16 35007126
chr16 35007134
chr16 35007159
chr16 35007182
chr16 35007190
chr16 35007197
chr16 35007244
chr16 35007289
chr16 35007306
chr16 35007865
chr16 35008243
chr16 35008774
chr16 35008790
chr16 35008874
chr16 35012418
chr16 35012514
chr16 35012572
chr16 35012617
chr16 35014478
chr16 35014677
chr16 35015553
chr16 35015591
chr16 35015647
chr16 35015938
chr16 35015968
chr16 35016020
chr16 35016024
chr16 35016064
chr16 35016095
chr16 35017193
chr16 35017493
chr16 35017646
chr16 35017662
chr16 35023785
chr16 35023793
chr16 35052099
chr16 35052704
chr16 35054239
chr16 35056479
chr16 35056518
chr16 35056574
chr16 35056606
chr16 35056985
chr16 35056996
chr16 35057797
chr16 35057801
chr16 35057807
chr16 35057845
chr16 35057861
chr16 35057875
chr16 35057947

chr16 35057951
chr16 35058153
chr16 35058872
chr16 35058907
chr16 35200303
chr16 35307189
chr16 35348674
chr16 35348995
chr16 35349084
chr16 35349158
chr16 35349254
chr16 35353537
chr16 35354080
chr16 35360190
chr16 35360332
chr16 35483846
chr16 35483958
chr16 35487642
chr16 35488025
chr16 35490799
chr16 35490810
chr16 35491330
chr16 35551709
chr16 35551729
chr16 35552058
chr16 35562159
chr16 35562218
chr16 35681421
chr16 35681429
chr16 35807271
chr16 35807523
chr16 35825623
chr16 36075233
chr16 36109202
chr16 36109634
chr16 36109870
chr16 36110103
chr16 36110172
chr16 36110190
chr16 36110281
chr16 36110676
chr16 36110686
chr16 36110697
chr16 36145410
chr16 36145498
chr16 36145982
chr16 36165819
chr16 36166060
chr16 36201575
chr16 36201604
chr16 36201676
chr16 36201775
chr16 36201789
chr16 36201828

chr16 36202265
chr16 36202382
chr16 36202831
chr16 36202910
chr16 36203075
chr16 36203466
chr16 36203591
chr16 36203679
chr16 36203886
chr16 36260711
chr16 36260968
chr16 46422877
chr16 46422968
chr16 46423092
chr16 46423154
chr16 46423537
chr16 46456683
chr16 46457026
chr16 46457235
chr16 46457352
chr16 46457383
chr16 46457691
chr16 46557864
chr16 46558031
chr16 46620451
chr16 46620656
chr16 46620668
chr16 46620760
chr16 46620779
chr16 46620810
chr16 46659432
chr16 46789920
chr16 46789934
chr16 46789952
chr16 46789970
chr16 46789988
chr16 46790006
chr16 46790021
chr16 46790023
chr16 46790040
chr16 46790049
chr16 46790059
chr16 46790065
chr16 46790067
chr16 46790132
chr16 46790141
chr16 46790143
chr16 46790145
chr16 46790149
chr16 46790169
chr16 46790185
chr16 46790201
chr16 46790222
chr16 46790231

chr16 46829535
chr16 46829613
chr16 46972333
chr16 47038116
chr16 47038132
chr16 47038134
chr16 47038141
chr16 47038151
chr16 47038154
chr16 47038179
chr16 47038185
chr16 47038191
chr16 47038193
chr16 47038197
chr16 47038207
chr16 47038219
chr16 47038226
chr16 47038236
chr16 47038243
chr16 47038258
chr16 47392349
chr16 47392368
chr16 47392689
chr16 47392698
chr16 48365964
chr16 48365969
chr16 48365988
chr16 48365990
chr16 48365992
chr16 48365995
chr16 48366004
chr16 48366011
chr16 48366020
chr16 48366022
chr16 48366036
chr16 48366042
chr16 48366051
chr16 48366069
chr16 48366075
chr16 48366089
chr16 48366097
chr16 48366100
chr16 48366122
chr16 48366128
chr16 48366174
chr16 48366183
chr16 48366200
chr16 48366207
chr16 48366226
chr16 48366243
chr16 48366258
chr16 48366272
chr16 48366277
chr16 48366291

chr16 48366295
chr16 48366342
chr16 48366353
chr16 48366564
chr16 48366622
chr16 48366652
chr16 48500312
chr16 48611895
chr16 48613029
chr16 48613144
chr16 48613391
chr16 48613490
chr16 48687581
chr16 49856070
chr16 49856073
chr16 49856075
chr16 49856084
chr16 49856095
chr16 49856099
chr16 49856104
chr16 49856146
chr16 49856190
chr16 49856219
chr16 49856256
chr16 49856266
chr16 49856291
chr16 49856295
chr16 49856301
chr16 50152182
chr16 50152313
chr16 50152444
chr16 50152455
chr16 50152458
chr16 50152460
chr16 50155601
chr16 50155718
chr16 50155767
chr16 50155769
chr16 50155865
chr16 50266411
chr16 50266454
chr16 50266493
chr16 50266496
chr16 50266499
chr16 50266502
chr16 50266511
chr16 50266517
chr16 50266566
chr16 50266581
chr16 50266599
chr16 50266601
chr16 50266619
chr16 50266626
chr16 50266652

chr16 50266665
chr16 50266740
chr16 50266773
chr16 50266831
chr16 50266863
chr16 50266888
chr16 50266927
chr16 50266958
chr16 50267012
chr16 50267234
chr16 50267267
chr16 50267430
chr16 50267470
chr16 50267481
chr16 50267510
chr16 50267555
chr16 50267626
chr16 50267638
chr16 50267779
chr16 50368160
chr16 50368188
chr16 50584315
chr16 50584320
chr16 50584339
chr16 50680976
chr16 50681224
chr16 50681249
chr16 50681270
chr16 50681273
chr16 50681281
chr16 50681308
chr16 50681349
chr16 50681427
chr16 50681434
chr16 50681451
chr16 50681500
chr16 50693757
chr16 50693766
chr16 50693791
chr16 50693835
chr16 50693851
chr16 50694089
chr16 50743790
chr16 50743808
chr16 50744053
chr16 50744278
chr16 50744302
chr16 50744835
chr16 50744965
chr16 50745032
chr16 50746317
chr16 50746337
chr16 50746386
chr16 50746391

chr16 50747099
chr16 50747225
chr16 50747290
chr16 50747295
chr16 50747319
chr16 50747410
chr16 50747412
chr16 50806408
chr16 50806437
chr16 50806442
chr16 51218936
chr16 51219094
chr16 51219844
chr16 51220229
chr16 51220307
chr16 51441035
chr16 51441042
chr16 51441059
chr16 51441248
chr16 51441454
chr16 51441752
chr16 51441766
chr16 51635275
chr16 51635335
chr16 51635364
chr16 51635429
chr16 52103339
chr16 52103669
chr16 52502975
chr16 52549150
chr16 52549172
chr16 52549213
chr16 53055439
chr16 53055445
chr16 53055473
chr16 53055493
chr16 53055624
chr16 53055690
chr16 53098871
chr16 53098885
chr16 53098948
chr16 53099016
chr16 53099086
chr16 53099219
chr16 53099244
chr16 53099254
chr16 53099384
chr16 53099406
chr16 53099410
chr16 53099453
chr16 53435605
chr16 53435613
chr16 53435681
chr16 53435897

chr16 53435912
chr16 53518653
chr16 53518655
chr16 53518787
chr16 53518809
chr16 53518904
chr16 53518919
chr16 53518937
chr16 53519019
chr16 53519160
chr16 53519174
chr16 53519267
chr16 53544990
chr16 53545507
chr16 53545522
chr16 53545590
chr16 53545600
chr16 53545616
chr16 53545623
chr16 53545649
chr16 53704148
chr16 53704157
chr16 53704163
chr16 53704169
chr16 53704191
chr16 53704198
chr16 53704244
chr16 54592626
chr16 54655728
chr16 54918091
chr16 55421101
chr16 55421225
chr16 55421284
chr16 55421307
chr16 55421313
chr16 55421318
chr16 55479801
chr16 55479875
chr16 55479946
chr16 55479978
chr16 55479991
chr16 55480049
chr16 55480085
chr16 55480113
chr16 55480147
chr16 55480149
chr16 55480180
chr16 55480208
chr16 55480219
chr16 55480246
chr16 55480293
chr16 55480344
chr16 55480357
chr16 55480375

chr16 55480401
chr16 55480429
chr16 55509446
chr16 55509450
chr16 55509459
chr16 55509485
chr16 55509490
chr16 55509494
chr16 55509553
chr16 55509575
chr16 55509603
chr16 55509613
chr16 55509617
chr16 55509624
chr16 55509626
chr16 55509641
chr16 55509717
chr16 55509734
chr16 55509781
chr16 55509787
chr16 55509815
chr16 55509865
chr16 55509968
chr16 55510224
chr16 55510434
chr16 55510445
chr16 55510706
chr16 55944019
chr16 55990838
chr16 55991262
chr16 55999057
chr16 56000064
chr16 56191907
chr16 56191973
chr16 56191988
chr16 56191998
chr16 56192003
chr16 56192123
chr16 56262247
chr16 56262249
chr16 56262264
chr16 56262487
chr16 56262525
chr16 56262594
chr16 56262602
chr16 56356688
chr16 56356875
chr16 56356899
chr16 56356912
chr16 56356943
chr16 56356970
chr16 56356984
chr16 56357110
chr16 56357113

chr16 56357177
chr16 56424428
chr16 56451855
chr16 56451861
chr16 56452351
chr16 56452359
chr16 56452361
chr16 56452397
chr16 56452399
chr16 56452418
chr16 56452424
chr16 56520359
chr16 56576895
chr16 56576901
chr16 56577035
chr16 56608639
chr16 56608652
chr16 56608676
chr16 56608679
chr16 56608682
chr16 56608718
chr16 56608832
chr16 56608849
chr16 56608894
chr16 56608914
chr16 56608920
chr16 56608933
chr16 56608936
chr16 56608961
chr16 56609008
chr16 56609011
chr16 56609160
chr16 56609162
chr16 56609218
chr16 56609224
chr16 56609243
chr16 56609726
chr16 56609869
chr16 56610147
chr16 56610198
chr16 56610241
chr16 56610336
chr16 56610352
chr16 56610436
chr16 56610455
chr16 56741605
chr16 56861082
chr16 56917123
chr16 56917190
chr16 56917211
chr16 56933088
chr16 56933106
chr16 56933191
chr16 56933226

chr16 56933249
chr16 56933271
chr16 56933539
chr16 56933576
chr16 56933845
chr16 56933921
chr16 56933956
chr16 56933967
chr16 56934131
chr16 56934167
chr16 56934239
chr16 56989076
chr16 56989097
chr16 56989110
chr16 56989114
chr16 56989159
chr16 56990127
chr16 56990160
chr16 56990179
chr16 56990201
chr16 56990236
chr16 56990437
chr16 56990484
chr16 56990539
chr16 56990548
chr16 56990573
chr16 56990579
chr16 56990686
chr16 56990714
chr16 56990758
chr16 56992308
chr16 56992545
chr16 57041425
chr16 57041436
chr16 57041557
chr16 57041572
chr16 57041761
chr16 57041765
chr16 57041797
chr16 57041837
chr16 57042019
chr16 57042076
chr16 57185789
chr16 57185792
chr16 57185806
chr16 57185812
chr16 57185825
chr16 57185828
chr16 57187146
chr16 57187173
chr16 57187209
chr16 57187222
chr16 57187262
chr16 57187309

chr16 57187328
chr16 57187338
chr16 57187343
chr16 57187347
chr16 57187359
chr16 57187449
chr16 57187465
chr16 57187552
chr16 57187583
chr16 57187589
chr16 57187617
chr16 57349311
chr16 57349337
chr16 57349404
chr16 57349427
chr16 57349612
chr16 57349648
chr16 57349680
chr16 57349705
chr16 57349714
chr16 57349745
chr16 57349814
chr16 57462412
chr16 57462527
chr16 57462565
chr16 57462574
chr16 57462578
chr16 57462594
chr16 57475950
chr16 57475966
chr16 57476183
chr16 57476232
chr16 57476257
chr16 57476543
chr16 57486310
chr16 57486313
chr16 57486320
chr16 57486331
chr16 57486342
chr16 57486345
chr16 57486348
chr16 57486350
chr16 57486367
chr16 57486369
chr16 57486374
chr16 57486377
chr16 57486379
chr16 57486386
chr16 57486392
chr16 57486403
chr16 57486406
chr16 57486409
chr16 57601607
chr16 57601721

chr16 57601837
chr16 57601954
chr16 57601972
chr16 57602046
chr16 57602078
chr16 57602232
chr16 57602251
chr16 57602283
chr16 57602291
chr16 57602440
chr16 57609663
chr16 57609707
chr16 57609733
chr16 57609757
chr16 57609790
chr16 57609799
chr16 57609998
chr16 57610020
chr16 57610061
chr16 57736166
chr16 57736168
chr16 57736192
chr16 57736203
chr16 57736217
chr16 57736229
chr16 57736289
chr16 57736311
chr16 57736333
chr16 57736343
chr16 57736435
chr16 57736478
chr16 57736493
chr16 57736534
chr16 57736540
chr16 57736571
chr16 57808119
chr16 57808161
chr16 57808287
chr16 57808303
chr16 57808319
chr16 57808325
chr16 57808330
chr16 57808342
chr16 57808371
chr16 57808374
chr16 57808401
chr16 57808435
chr16 57808456
chr16 57808472
chr16 57999723
chr16 57999745
chr16 57999806
chr16 57999840
chr16 57999852

chr16 57999994
chr16 58090240
chr16 58090262
chr16 58090356
chr16 58177516
chr16 58197725
chr16 58197730
chr16 58197739
chr16 58197743
chr16 58197749
chr16 58197756
chr16 58197759
chr16 58197761
chr16 58197766
chr16 58515075
chr16 58515091
chr16 58515124
chr16 58515138
chr16 58628393
chr16 58628549
chr16 58628553
chr16 58628635
chr16 58628651
chr16 58628789
chr16 58628939
chr16 58863931
chr16 58951210
chr16 59259269
chr16 59259278
chr16 59259281
chr16 59259362
chr16 59259539
chr16 59259564
chr16 59259613
chr16 59259690
chr16 59259867
chr16 59259921
chr16 59259942
chr16 59260404
chr16 59314340
chr16 59314474
chr16 59314477
chr16 59562464
chr16 59562861
chr16 59563191
chr16 59838104
chr16 59838249
chr16 60018744
chr16 60019002
chr16 60019251
chr16 60087285
chr16 60129360
chr16 60129762
chr16 61084158

chr16 61084214
chr16 61084743
chr16 61084994
chr16 61130330
chr16 61130464
chr16 61133335
chr16 61133525
chr16 61133591
chr16 61222652
chr16 61222753
chr16 61222775
chr16 61223012
chr16 61554323
chr16 61554580
chr16 61554757
chr16 61658443
chr16 61785444
chr16 61785492
chr16 61941947
chr16 61952728
chr16 61952781
chr16 62122808
chr16 62282663
chr16 62282694
chr16 62282992
chr16 62283123
chr16 62450496
chr16 62450615
chr16 62597263
chr16 62655992
chr16 62723165
chr16 62957064
chr16 63289398
chr16 63289888
chr16 63293129
chr16 63293133
chr16 63293250
chr16 63293698
chr16 63405383
chr16 63973890
chr16 63974155
chr16 64371230
chr16 64371496
chr16 64371713
chr16 65118192
chr16 65118194
chr16 65118276
chr16 65118317
chr16 65119229
chr16 65119249
chr16 65119366
chr16 65119480
chr16 65119601
chr16 65119941

chr16 65120031
chr16 65236134
chr16 65236856
chr16 65237157
chr16 65237179
chr16 65373737
chr16 65373779
chr16 66406600
chr16 66406604
chr16 66406606
chr16 66406645
chr16 66406647
chr16 66406676
chr16 66406685
chr16 66406692
chr16 66406694
chr16 66406698
chr16 66406722
chr16 66406912
chr16 66474894
chr16 66552842
chr16 66552874
chr16 66552895
chr16 66552954
chr16 66553015
chr16 66553020
chr16 66553023
chr16 66553028
chr16 66553060
chr16 66553065
chr16 66553074
chr16 66553092
chr16 66553107
chr16 66553114
chr16 66553138
chr16 66553241
chr16 66553261
chr16 66554189
chr16 66751425
chr16 66751428
chr16 66751433
chr16 66751445
chr16 66751466
chr16 66751470
chr16 66751474
chr16 66751476
chr16 66751522
chr16 66751525
chr16 66751528
chr16 66751532
chr16 66751538
chr16 66751540
chr16 66751546
chr16 66751583

chr16 66751586
chr16 66751626
chr16 66751632
chr16 66751635
chr16 66751643
chr16 66751645
chr16 66751653
chr16 66751658
chr16 66751663
chr16 66751670
chr16 66831209
chr16 66873487
chr16 66873557
chr16 66873589
chr16 66874504
chr16 66881363
chr16 67063000
chr16 67063143
chr16 67063247
chr16 67158769
chr16 67158796
chr16 67158930
chr16 67158969
chr16 67159067
chr16 67159109
chr16 67159112
chr16 67164020
chr16 67164029
chr16 67164033
chr16 67164077
chr16 67164092
chr16 67164094
chr16 67164100
chr16 67164103
chr16 67164122
chr16 67164126
chr16 67164128
chr16 67164133
chr16 67164181
chr16 67164195
chr16 67164201
chr16 67164227
chr16 67164229
chr16 67227244
chr16 67227251
chr16 67248080
chr16 67248144
chr16 67248164
chr16 67248174
chr16 67248212
chr16 67248422
chr16 67248440
chr16 67248449
chr16 67248451

chr16 67248455
chr16 67248468
chr16 67248479
chr16 67248504
chr16 67248510
chr16 67248517
chr16 67290074
chr16 67290079
chr16 67363676
chr16 67365260
chr16 67365262
chr16 67365290
chr16 67365572
chr16 67521086
chr16 67521127
chr16 67521181
chr16 67521206
chr16 67521223
chr16 67521251
chr16 67521257
chr16 67521292
chr16 67521299
chr16 67521324
chr16 67521331
chr16 67521339
chr16 67521366
chr16 67555890
chr16 67556076
chr16 67564000
chr16 67564043
chr16 67564052
chr16 67564067
chr16 67564073
chr16 67564096
chr16 67564174
chr16 67564211
chr16 67564221
chr16 67719300
chr16 67719302
chr16 67719305
chr16 67719347
chr16 67719352
chr16 67719366
chr16 67719380
chr16 67719394
chr16 67719407
chr16 67719413
chr16 67719422
chr16 67719430
chr16 67719443
chr16 67719487
chr16 67719490
chr16 67719493
chr16 67719499

chr16 67719527
chr16 67719532
chr16 67719539
chr16 67719542
chr16 67719599
chr16 67719605
chr16 67719645
chr16 67719651
chr16 67719654
chr16 67719837
chr16 67719854
chr16 67719924
chr16 67720046
chr16 67720104
chr16 67806143
chr16 67833524
chr16 67833530
chr16 67833600
chr16 68006005
chr16 68006084
chr16 68006187
chr16 68006214
chr16 68006247
chr16 68063292
chr16 68079755
chr16 68079762
chr16 68079785
chr16 68079841
chr16 68079848
chr16 68079869
chr16 68079885
chr16 68079920
chr16 68080164
chr16 68080188
chr16 68081395
chr16 68084703
chr16 68084711
chr16 68084851
chr16 68084856
chr16 68084916
chr16 68084919
chr16 68084979
chr16 68084984
chr16 68084998
chr16 68085034
chr16 68085046
chr16 68085079
chr16 68085113
chr16 68085124
chr16 68087264
chr16 68087396
chr16 68087819
chr16 68087860
chr16 68089980

chr16 68090291
chr16 68090340
chr16 68090352
chr16 68090839
chr16 68090880
chr16 68090889
chr16 68091042
chr16 68261665
chr16 68261706
chr16 68261741
chr16 68276043
chr16 68276099
chr16 68284318
chr16 68284349
chr16 68284359
chr16 68284856
chr16 68284956
chr16 68284970
chr16 68289750
chr16 68418092
chr16 68418124
chr16 68418217
chr16 68418225
chr16 68418240
chr16 68418255
chr16 68418422
chr16 68445115
chr16 68470140
chr16 68470192
chr16 68470205
chr16 68521196
chr16 68539694
chr16 68539699
chr16 68539704
chr16 68539715
chr16 68539726
chr16 68539767
chr16 68539773
chr16 68539783
chr16 68539800
chr16 68539834
chr16 68539836
chr16 68540038
chr16 68540068
chr16 68540105
chr16 68668598
chr16 68844205
chr16 68844225
chr16 69131412
chr16 69131451
chr16 69131454
chr16 69385000
chr16 69385220
chr16 69385240

chr16 69385261
chr16 69385880
chr16 69385884
chr16 69385889
chr16 69385897
chr16 69385906
chr16 69385909
chr16 69385919
chr16 69385923
chr16 69385926
chr16 69385930
chr16 69385935
chr16 69385941
chr16 69385948
chr16 69385951
chr16 69385956
chr16 69385963
chr16 69385965
chr16 69385983
chr16 69385988
chr16 69385993
chr16 69385997
chr16 69385999
chr16 69396222
chr16 69396242
chr16 69424362
chr16 69424375
chr16 69424390
chr16 69424392
chr16 69424406
chr16 69424426
chr16 69425212
chr16 69425277
chr16 69425290
chr16 69425326
chr16 69444917
chr16 69445263
chr16 69521231
chr16 69530618
chr16 69530645
chr16 69530661
chr16 69530722
chr16 69530760
chr16 69530829
chr16 69530832
chr16 69530846
chr16 69530888
chr16 69530962
chr16 69530997
chr16 69531170
chr16 69531232
chr16 69564022
chr16 69564115
chr16 69564160

chr16 69564213
chr16 69564225
chr16 69564267
chr16 69564424
chr16 69564514
chr16 69564625
chr16 69564676
chr16 69564740
chr16 69565153
chr16 69565230
chr16 69565321
chr16 69565330
chr16 69565357
chr16 69567395
chr16 69567404
chr16 69567419
chr16 69567466
chr16 69726391
chr16 69726398
chr16 69726402
chr16 69726414
chr16 69726433
chr16 69726455
chr16 69726460
chr16 69726477
chr16 69726486
chr16 69726491
chr16 69726501
chr16 69726515
chr16 69726527
chr16 69726533
chr16 69726554
chr16 69726556
chr16 69726569
chr16 69726597
chr16 69726602
chr16 69726628
chr16 69726638
chr16 69726661
chr16 69726736
chr16 69726739
chr16 69726772
chr16 69726855
chr16 69726896
chr16 69762655
chr16 69762707
chr16 69762725
chr16 69762743
chr16 70026310
chr16 70026330
chr16 70026335
chr16 70026369
chr16 70064932
chr16 70064986

chr16 70064996
chr16 70065010
chr16 70065016
chr16 70065051
chr16 70065058
chr16 70065070
chr16 70065079
chr16 70065115
chr16 70065126
chr16 70065152
chr16 70065159
chr16 70065175
chr16 70065191
chr16 70065200
chr16 70065206
chr16 70065227
chr16 70113911
chr16 70113966
chr16 70113973
chr16 70188104
chr16 70188106
chr16 70188146
chr16 70188148
chr16 70188282
chr16 70188284
chr16 70188303
chr16 70188310
chr16 70188321
chr16 70188327
chr16 70188329
chr16 70188333
chr16 70188338
chr16 70188342
chr16 70188351
chr16 70188358
chr16 70188366
chr16 70188368
chr16 70188384
chr16 70188390
chr16 70188392
chr16 70188399
chr16 70188405
chr16 70188412
chr16 70188430
chr16 70188435
chr16 70188439
chr16 70188546
chr16 70188551
chr16 70188562
chr16 70188572
chr16 70188599
chr16 70188612
chr16 70188615
chr16 70188617

chr16 70188622
chr16 70188627
chr16 70188630
chr16 70188655
chr16 70188658
chr16 70188671
chr16 70188673
chr16 70188708
chr16 70188725
chr16 70188729
chr16 70188759
chr16 70188766
chr16 70188769
chr16 70298975
chr16 70299074
chr16 70299219
chr16 70299221
chr16 70299229
chr16 70299238
chr16 70299460
chr16 70299506
chr16 70299512
chr16 70299518
chr16 70299520
chr16 70299525
chr16 70299531
chr16 70299550
chr16 70299558
chr16 70299719
chr16 70299766
chr16 70299771
chr16 70454105
chr16 70454142
chr16 70520580
chr16 70520586
chr16 70520624
chr16 70520826
chr16 71288678
chr16 71288771
chr16 71288822
chr16 71288916
chr16 71288950
chr16 71288982
chr16 71289042
chr16 71289087
chr16 71289098
chr16 71289110
chr16 71289116
chr16 71461546
chr16 71461588
chr16 71461596
chr16 71461636
chr16 71461704
chr16 71461721

chr16 71461743
chr16 71461753
chr16 71461795
chr16 71461806
chr16 71461883
chr16 71461959
chr16 71461976
chr16 71461980
chr16 71461999
chr16 71462035
chr16 71462042
chr16 71462044
chr16 71462062
chr16 71462065
chr16 71462405
chr16 71462446
chr16 71484626
chr16 71484659
chr16 71484661
chr16 71484678
chr16 71484683
chr16 71484715
chr16 71484732
chr16 71484781
chr16 71484795
chr16 71484846
chr16 71484864
chr16 71484931
chr16 71484963
chr16 71484999
chr16 71565186
chr16 71565216
chr16 71565224
chr16 71565247
chr16 71565258
chr16 71565300
chr16 71565302
chr16 71565304
chr16 71565312
chr16 71565344
chr16 71565389
chr16 71565396
chr16 71565400
chr16 71565405
chr16 71565421
chr16 71565433
chr16 71565443
chr16 71565445
chr16 71565449
chr16 71565453
chr16 71565459
chr16 71611146
chr16 71611164
chr16 71611166

chr16 71611243
chr16 71611249
chr16 71611355
chr16 71611357
chr16 71611606
chr16 71611639
chr16 71776296
chr16 71795131
chr16 71795626
chr16 71846827
chr16 71846861
chr16 71846895
chr16 71882538
chr16 71882559
chr16 71882678
chr16 71882683
chr16 71882766
chr16 71882989
chr16 71883069
chr16 71883082
chr16 71883131
chr16 71883169
chr16 71883202
chr16 71883234
chr16 71883250
chr16 71883313
chr16 71883353
chr16 71883367
chr16 71885877
chr16 71886240
chr16 71896479
chr16 72008513
chr16 72008523
chr16 72008526
chr16 72008534
chr16 72008583
chr16 72008587
chr16 72008663
chr16 72008666
chr16 72008681
chr16 72008692
chr16 72008708
chr16 72008743
chr16 72008755
chr16 72008769
chr16 72008794
chr16 72008796
chr16 72008828
chr16 72008837
chr16 72008843
chr16 72008856
chr16 72008860
chr16 72008872
chr16 72008878

chr16 72008884
chr16 72008907
chr16 72094211
chr16 72094220
chr16 72094266
chr16 72094272
chr16 72094326
chr16 72094352
chr16 72094358
chr16 72094460
chr16 72585622
chr16 72664159
chr16 72787493
chr16 72787505
chr16 73059098
chr16 73059122
chr16 73059125
chr16 73059352
chr16 73059363
chr16 73059367
chr16 73059369
chr16 73059371
chr16 73059397
chr16 73059410
chr16 73062419
chr16 73062751
chr16 73062779
chr16 73062787
chr16 73062839
chr16 73062851
chr16 73062887
chr16 73070827
chr16 73070893
chr16 73070910
chr16 73070917
chr16 73070919
chr16 73070921
chr16 73070923
chr16 73070925
chr16 73070927
chr16 73070929
chr16 73070931
chr16 73070933
chr16 73070935
chr16 73070937
chr16 73071007
chr16 73071027
chr16 73071034
chr16 73071204
chr16 73071242
chr16 73071250
chr16 73071326
chr16 73071408
chr16 73071431

chr16 73122748
chr16 73122783
chr16 73122791
chr16 73122843
chr16 73122872
chr16 73122876
chr16 73122988
chr16 73123179
chr16 73123255
chr16 73123301
chr16 73349586
chr16 73549098
chr16 73549156
chr16 73810565
chr16 73890958
chr16 73890982
chr16 73891015
chr16 73891018
chr16 73891042
chr16 73891260
chr16 73891309
chr16 73891349
chr16 73891360
chr16 73891362
chr16 73891400
chr16 73891461
chr16 73891590
chr16 74368624
chr16 74368626
chr16 74368659
chr16 74407141
chr16 74407148
chr16 74407150
chr16 74601252
chr16 74601289
chr16 74601318
chr16 74601337
chr16 74601359
chr16 74601397
chr16 74601665
chr16 74601698
chr16 74601711
chr16 74606062
chr16 74606200
chr16 74606249
chr16 74606298
chr16 74606330
chr16 74606333
chr16 74606375
chr16 74606385
chr16 74606397
chr16 74606400
chr16 74606407
chr16 74606427

chr16 74606439
chr16 74606463
chr16 74667083
chr16 74667087
chr16 74667188
chr16 74667211
chr16 74667270
chr16 74667315
chr16 74667350
chr16 74667367
chr16 74667417
chr16 74667457
chr16 74699989
chr16 74700156
chr16 74700218
chr16 74700235
chr16 74700280
chr16 74700287
chr16 74700391
chr16 74700696
chr16 74700770
chr16 74700814
chr16 74700829
chr16 74700832
chr16 74709500
chr16 74709561
chr16 74709563
chr16 74709619
chr16 74709657
chr16 75018334
chr16 75074983
chr16 75075265
chr16 75075315
chr16 75075346
chr16 75075365
chr16 75075373
chr16 75075398
chr16 75075415
chr16 75075434
chr16 75075458
chr16 75075791
chr16 75075829
chr16 75148137
chr16 75148153
chr16 75148293
chr16 75148346
chr16 75148359
chr16 75148371
chr16 75148399
chr16 75148401
chr16 75148407
chr16 75152163
chr16 75333892
chr16 75333935

chr16 75380855
chr16 75380933
chr16 75385210
chr16 75385383
chr16 75385608
chr16 75402211
chr16 75402762
chr16 75403092
chr16 75472092
chr16 75505548
chr16 75505948
chr16 75517143
chr16 75517162
chr16 75517170
chr16 75517176
chr16 75517179
chr16 75517183
chr16 75517189
chr16 75517194
chr16 75517205
chr16 75517214
chr16 75616563
chr16 75616749
chr16 75616795
chr16 75622385
chr16 75622461
chr16 75622567
chr16 75622591
chr16 75622658
chr16 75622791
chr16 75622815
chr16 75649688
chr16 75649715
chr16 75649749
chr16 75649847
chr16 75649873
chr16 75649898
chr16 75649945
chr16 75650296
chr16 75650370
chr16 75763777
chr16 75763832
chr16 75764226
chr16 75764368
chr16 75764481
chr16 75764494
chr16 75764583
chr16 75764692
chr16 75764729
chr16 75764788
chr16 75853717
chr16 75853728
chr16 75853913
chr16 76502864

chr16 76502968
chr16 76503023
chr16 76504386
chr16 76504659
chr16 76504755
chr16 76504891
chr16 76505242
chr16 76505318
chr16 76505704
chr16 76505832
chr16 76611186
chr16 76611418
chr16 76611676
chr16 76612244
chr16 76875527
chr16 76875717
chr16 77191598
chr16 77191601
chr16 77191608
chr16 77191614
chr16 77212616
chr16 77212622
chr16 77212628
chr16 77212634
chr16 77212637
chr16 77212657
chr16 77212670
chr16 77212675
chr16 77212686
chr16 77212701
chr16 77212705
chr16 77212720
chr16 77212727
chr16 77212754
chr16 77212760
chr16 77212776
chr16 77212778
chr16 77432668
chr16 77571547
chr16 77585757
chr16 77585770
chr16 77585777
chr16 77722161
chr16 77722167
chr16 77722283
chr16 77722316
chr16 77722329
chr16 77722334
chr16 77722336
chr16 77722358
chr16 77722377
chr16 77722382
chr16 77722387
chr16 77722393

chr16 77722400
chr16 77722413
chr16 77722418
chr16 77722423
chr16 77722436
chr16 77722453
chr16 77722458
chr16 77722472
chr16 77722495
chr16 77722520
chr16 77722526
chr16 77722528
chr16 77722532
chr16 77722536
chr16 77722546
chr16 77722555
chr16 77722559
chr16 77722589
chr16 77722603
chr16 77722630
chr16 77722645
chr16 77722661
chr16 77722671
chr16 77722676
chr16 77722686
chr16 77722700
chr16 78235079
chr16 78235091
chr16 78742648
chr16 78742672
chr16 78742683
chr16 78742695
chr16 78742720
chr16 78742750
chr16 78742805
chr16 78742843
chr16 78742862
chr16 78742967
chr16 78743030
chr16 78743049
chr16 78743074
chr16 78743085
chr16 78743098
chr16 78750011
chr16 78896203
chr16 78896364
chr16 79093605
chr16 79093644
chr16 79093659
chr16 79093673
chr16 79093807
chr16 79093851
chr16 79093880
chr16 79093902

chr16 79278665
chr16 79278710
chr16 79278722
chr16 79278740
chr16 79278761
chr16 79278848
chr16 79416187
chr16 79416324
chr16 79416413
chr16 79416442
chr16 79416592
chr16 79416638
chr16 79596867
chr16 79597014
chr16 79597191
chr16 79597438
chr16 79597472
chr16 79597648
chr16 79597727
chr16 79597731
chr16 79598062
chr16 79598072
chr16 79598077
chr16 79598185
chr16 79598386
chr16 79598434
chr16 79598445
chr16 79598567
chr16 79598618
chr16 79598622
chr16 79598661
chr16 79598690
chr16 79602134
chr16 79602198
chr16 79602230
chr16 79602285
chr16 79602310
chr16 79602333
chr16 79602343
chr16 79602368
chr16 79602383
chr16 79602466
chr16 79602486
chr16 79602576
chr16 79602666
chr16 79602828
chr16 80027041
chr16 80027166
chr16 80027224
chr16 80027228
chr16 80027239
chr16 80027302
chr16 80027305
chr16 80027367

chr16 80027393
chr16 80027402
chr16 80540483
chr16 80540487
chr16 80540525
chr16 80540575
chr16 80540656
chr16 80540727
chr16 80540752
chr16 80540789
chr16 80540820
chr16 80540845
chr16 80540860
chr16 80541096
chr16 80541102
chr16 80541107
chr16 80541110
chr16 80541121
chr16 80541124
chr16 80541128
chr16 80541141
chr16 80541296
chr16 80541298
chr16 80541384
chr16 80541415
chr16 80541423
chr16 80564567
chr16 80570251
chr16 80570329
chr16 80570367
chr16 80570400
chr16 80570502
chr16 80580305
chr16 80580322
chr16 80586184
chr16 80591166
chr16 80591242
chr16 80605248
chr16 80656739
chr16 80656971
chr16 80657010
chr16 80657044
chr16 80657051
chr16 80657123
chr16 80700670
chr16 80759413
chr16 80759423
chr16 80759474
chr16 80759577
chr16 80759741
chr16 80862274
chr16 80862566
chr16 80862623
chr16 80964217

chr16 80964374
chr16 80964395
chr16 80996023
chr16 80996164
chr16 80996283
chr16 80996438
chr16 80996709
chr16 80996911
chr16 80996996
chr16 80997090
chr16 80997167
chr16 80997222
chr16 80997238
chr16 80997295
chr16 80997349
chr16 80997475
chr16 80997686
chr16 81007935
chr16 81007963
chr16 81007967
chr16 81007975
chr16 81008085
chr16 81008093
chr16 81008107
chr16 81076911
chr16 81076918
chr16 81076951
chr16 81076969
chr16 81076986
chr16 81076990
chr16 81077006
chr16 81077029
chr16 81077039
chr16 81077049
chr16 81077053
chr16 81077068
chr16 81077076
chr16 81077093
chr16 81077123
chr16 81096113
chr16 81096119
chr16 81096122
chr16 81096124
chr16 81096132
chr16 81096159
chr16 81096163
chr16 81096167
chr16 81096170
chr16 81096273
chr16 81096360
chr16 81096370
chr16 81096398
chr16 81096400
chr16 81096406

chr16 81314186
chr16 81314192
chr16 81314253
chr16 81314279
chr16 81314293
chr16 81314334
chr16 81314417
chr16 81314458
chr16 81314479
chr16 81314493
chr16 81366581
chr16 81445711
chr16 81445740
chr16 81445744
chr16 81445779
chr16 81445787
chr16 81446051
chr16 81446121
chr16 81446186
chr16 81446193
chr16 81446232
chr16 81446259
chr16 81470195
chr16 81798875
chr16 81798889
chr16 81799008
chr16 81799044
chr16 81799049
chr16 81799060
chr16 81799065
chr16 81839084
chr16 81839198
chr16 82011133
chr16 82011157
chr16 83277380
chr16 83642641
chr16 83642652
chr16 83643362
chr16 83643396
chr16 83643437
chr16 83688723
chr16 83689027
chr16 83689063
chr16 84277184
chr16 84277237
chr16 84380336
chr16 84457679
chr16 84599735
chr16 84599812
chr16 84599830
chr16 84616427
chr16 84616434
chr16 84616546
chr16 84616564

chr16 84616567
chr16 84616576
chr16 84616589
chr16 84616596
chr16 84616638
chr16 84616698
chr16 84616712
chr16 84616724
chr16 84616935
chr16 84616966
chr16 84617029
chr16 84617137
chr16 84617237
chr16 84700724
chr16 84700746
chr16 84700776
chr16 84700827
chr16 84700863
chr16 84700924
chr16 84700952
chr16 84701091
chr16 84701105
chr16 84701199
chr16 84701219
chr16 84701240
chr16 84701268
chr16 84701331
chr16 84701379
chr16 84751477
chr16 84751493
chr16 84751537
chr16 84751771
chr16 84751777
chr16 85028882
chr16 85028904
chr16 85028909
chr16 85028923
chr16 85028990
chr16 85028995
chr16 85029034
chr16 85029071
chr16 85029152
chr16 85029170
chr16 85029247
chr16 85029269
chr16 85029275
chr16 85029314
chr16 85235785
chr16 85553916
chr16 85555141
chr16 85555155
chr16 85555302
chr16 85555314
chr16 85555316

chr16 85555368
chr16 85555371
chr16 85555417
chr16 85555435
chr16 85555461
chr16 85555479
chr16 85555482
chr16 85555497
chr16 85555516
chr16 85555564
chr16 85819209
chr16 85819266
chr16 85819293
chr16 85819320
chr16 85882416
chr16 85882541
chr16 85882599
chr16 85888820
chr16 85888851
chr16 86007592
chr16 86007761
chr16 86161529
chr16 86161698
chr16 86239539
chr16 86239543
chr16 86239558
chr16 86239579
chr16 86239597
chr16 86239672
chr16 86263655
chr16 86263721
chr16 86263787
chr16 86514548
chr16 86564045
chr16 86564052
chr16 86564060
chr16 86564074
chr16 86564080
chr16 86564085
chr16 86564094
chr16 86564099
chr16 86564101
chr16 86564110
chr16 86564117
chr16 86564142
chr16 86564145
chr16 86564165
chr16 86564167
chr16 86564184
chr16 86564190
chr16 86564192
chr16 86564196
chr16 86564328
chr16 86564361

chr16 86564442
chr16 86604715
chr16 86882738
chr16 86882768
chr16 86882812
chr16 86882837
chr16 86882855
chr16 86883074
chr16 87060768
chr16 87060825
chr16 87388157
chr16 87388159
chr16 87388186
chr16 87388188
chr16 87388425
chr16 87388436
chr16 87388471
chr16 87388480
chr16 87388526
chr16 87388557
chr16 87388559
chr16 87388569
chr16 87388600
chr16 87388743
chr16 87388926
chr16 87868767
chr16 87896175
chr16 88239470
chr16 88239511
chr16 88239519
chr16 88239548
chr16 88239550
chr16 88239556
chr16 88239573
chr16 88239588
chr16 88239612
chr16 88239644
chr16 88239648
chr16 88239795
chr16 88634048
chr16 88634057
chr16 88634072
chr16 88634099
chr16 88634103
chr16 88634105
chr16 88634108
chr16 88634140
chr16 88634150
chr16 88634164
chr16 88634179
chr16 88634195
chr16 88634208
chr16 88634214
chr16 88634216

chr16 88634236
chr16 88634269
chr16 88634279
chr16 88634317
chr16 88634325
chr16 88634334
chr16 88634352
chr16 88634360
chr16 88634367
chr16 88634372
chr16 88634383
chr16 88634390
chr16 88634392
chr16 88634406
chr16 88634410
chr16 88634418
chr16 88634436
chr16 88634441
chr16 88634449
chr16 88634461
chr16 88634467
chr16 88634483
chr16 88634489
chr16 88634508
chr16 88634512
chr16 88634529
chr16 88634535
chr16 88634540
chr16 88686186
chr16 88686192
chr16 88686197
chr16 88686205
chr16 88686252
chr16 88686279
chr16 88686292
chr16 88686299
chr16 88686310
chr16 88686317
chr16 88686338
chr16 88686348
chr16 88686351
chr16 88686380
chr16 88686399
chr16 88686401
chr16 88703652
chr16 88703708
chr16 88703723
chr16 88703726
chr16 88703749
chr16 88703753
chr16 88703755
chr16 88703780
chr16 88703810
chr16 88703835

chr16 88703840
chr16 88703905
chr16 88703943
chr16 88703951
chr16 88703953
chr16 88790889
chr16 88803332
chr16 88803334
chr16 88803373
chr16 88803377
chr16 88803382
chr16 88803384
chr16 88803393
chr16 88803407
chr16 88803428
chr16 89026823
chr16 89027069
chr16 89171013
chr16 89171015
chr16 89171025
chr16 89171034
chr16 89171043
chr16 89192330
chr16 89192379
chr16 89192404
chr16 89192416
chr16 89192504
chr16 89192524
chr16 89192527
chr16 89192544
chr16 89192561
chr16 89192581
chr16 89192584
chr16 89192601
chr16 89192618
chr16 89192623
chr16 89192638
chr16 89192641
chr16 89192658
chr16 89192675
chr16 89192695
chr16 89192698
chr16 89192715
chr16 89192732
chr16 89192755
chr16 89192772
chr16 89192789
chr16 89192809
chr16 89192812
chr16 89192846
chr16 89192865
chr16 89192868
chr16 89192894
chr16 89192902

chr16 89192922
chr16 89192951
chr16 89192979
chr16 89192982
chr16 89192997
chr16 89193008
chr16 89193017
chr16 89193037
chr16 89193057
chr16 89193074
chr16 89193094
chr16 89193111
chr16 89216523
chr16 89216525
chr16 89216532
chr16 89216538
chr16 89216578
chr16 89216604
chr16 89216643
chr16 89216649
chr16 89216687
chr16 89216695
chr16 89241054
chr16 89241065
chr16 89241084
chr16 89241095
chr16 89241114
chr16 89241215
chr16 89241234
chr16 89241245
chr16 89241264
chr16 89241275
chr16 89241290
chr16 89241302
chr16 89241310
chr16 89241322
chr16 89241333
chr16 89241356
chr16 89241359
chr16 89241366
chr16 89241368
chr16 89241372
chr16 89241378
chr16 89241393
chr16 89241407
chr16 89241424
chr16 89361197
chr16 89361242
chr16 89361272
chr16 89361322
chr16 89361325
chr16 89361331
chr16 89484326
chr16 89484493

chr16 89488521
chr16 89488600
chr16 89488610
chr16 89488658
chr16 89488916
chr16 89489098
chr16 89489120
chr16 89489226
chr16 89489228
chr16 89489230
chr16 89489232
chr16 89489234
chr16 89489236
chr16 89489238
chr16 89918211
chr16 89918216
chr16 89918229
chr16 89918267
chr16 89918283
chr16 89918307
chr16 89918377
chr16 89918385
chr16 89918399
chr16 89918416
chr16 89918418
chr16 89918420
chr16 89918505
chr16 89918511
chr16 89918538
chr16 89918588
chr16 90082154
chr16 90082157
chr16 90082162
chr16 90082202
chr16 90082204
chr16 90082220
chr16 90082226
chr16 90082251
chr16 90082253
chr16 90082275
chr16 90082289
chr16 90082304
chr16 90082312
chr16 90082340
chr16 90082352
chr16 90082355
chr16 90082361
chr16 90082363
chr16 90082367
chr16 90082381
chr16 90082384
chr16 90082420
chr16 90082456
chr16 90082514

chr16 90082526
chr16 90082528
chr16 90082546
chr16 90082554
chr16 90082570
chr16 90082582
chr16 90082588
chr16 90082600
chr16 90107974
chr16 90108004
chr16 90108007
chr16 90108066
chr16 90108093
chr16 90109402
chr16 90109440
chr16 90109448
chr16 90109456
chr16 90109478
chr16 90109480
chr16 90109503
chr16 90109510
chr21 5065705
chr21 5065710
chr21 5065719
chr21 5065740
chr21 5065747
chr21 5065792
chr21 5065811
chr21 5065821
chr21 5089570
chr21 5101024
chr21 5101334
chr21 5101387
chr21 5101405
chr21 5101450
chr21 5101455
chr21 5101462
chr21 5101476
chr21 5101525
chr21 5101544
chr21 5101581
chr21 5101592
chr21 5101622
chr21 5101814
chr21 5101885
chr21 5101888
chr21 5101900
chr21 5101932
chr21 5101937
chr21 5101973
chr21 5101996
chr21 5102032
chr21 5102044
chr21 5102338

chr21 5102348
chr21 5102353
chr21 5102552
chr21 5102859
chr21 5102886
chr21 5103831
chr21 5103859
chr21 5103872
chr21 5103956
chr21 5103983
chr21 5103994
chr21 5104000
chr21 5104024
chr21 5104047
chr21 5104058
chr21 5104061
chr21 5104073
chr21 5104078
chr21 5104128
chr21 5104362
chr21 5104430
chr21 5104471
chr21 5104636
chr21 5104639
chr21 5104722
chr21 5104729
chr21 5104734
chr21 5104778
chr21 5104784
chr21 5104792
chr21 5105563
chr21 5105592
chr21 5105621
chr21 5106028
chr21 5106038
chr21 5106043
chr21 5106051
chr21 5106060
chr21 5106068
chr21 5106072
chr21 5106078
chr21 5106080
chr21 5106124
chr21 5106133
chr21 5106151
chr21 5106165
chr21 5106177
chr21 5106193
chr21 5106247
chr21 5106254
chr21 5106268
chr21 5106709
chr21 5106719
chr21 5106726

chr21 5115759
chr21 5115768
chr21 5115772
chr21 5115778
chr21 5115782
chr21 5115802
chr21 5115844
chr21 5115877
chr21 5115892
chr21 5115925
chr21 5115938
chr21 5116019
chr21 5116054
chr21 5116096
chr21 5116098
chr21 5116109
chr21 5116128
chr21 5116140
chr21 5116178
chr21 5116406
chr21 5116420
chr21 5117295
chr21 5117316
chr21 5117318
chr21 5117361
chr21 5117382
chr21 5118354
chr21 5118363
chr21 5118427
chr21 5118442
chr21 5118455
chr21 5118464
chr21 5121408
chr21 5121572
chr21 5121628
chr21 5121655
chr21 5121657
chr21 5121665
chr21 5121687
chr21 5123674
chr21 5123678
chr21 5123684
chr21 5123688
chr21 5123733
chr21 5123738
chr21 5123748
chr21 5123832
chr21 5123912
chr21 5123918
chr21 5123929
chr21 5123950
chr21 5123973
chr21 5123981
chr21 5126792

chr21 5126840
chr21 5127298
chr21 5127396
chr21 5127423
chr21 5127445
chr21 5127501
chr21 5127550
chr21 5127608
chr21 5127672
chr21 5127694
chr21 5127711
chr21 5127718
chr21 5127734
chr21 5127736
chr21 5127758
chr21 5127954
chr21 5127963
chr21 5127975
chr21 5127989
chr21 5127998
chr21 5128001
chr21 5128027
chr21 5128217
chr21 5128219
chr21 5128229
chr21 5128231
chr21 5128236
chr21 5128238
chr21 5128243
chr21 5128261
chr21 5128263
chr21 5128274
chr21 5128278
chr21 5128282
chr21 5128285
chr21 5128298
chr21 5128313
chr21 5128318
chr21 5128325
chr21 5128349
chr21 5128422
chr21 5128424
chr21 5128481
chr21 5128505
chr21 5128508
chr21 5128515
chr21 5128522
chr21 5128524
chr21 5128530
chr21 5128541
chr21 5128553
chr21 5128573
chr21 5128591
chr21 5128983

chr21 5128986
chr21 5129023
chr21 5129044
chr21 5129087
chr21 5129104
chr21 5129150
chr21 5153891
chr21 5153913
chr21 5153932
chr21 5153953
chr21 5153958
chr21 5153986
chr21 5153998
chr21 5154015
chr21 5154035
chr21 5154041
chr21 5154043
chr21 5154070
chr21 5154125
chr21 5154129
chr21 5154386
chr21 5154408
chr21 5154416
chr21 5154419
chr21 5154422
chr21 5154428
chr21 5154462
chr21 5154464
chr21 5154470
chr21 5154482
chr21 5154484
chr21 5154494
chr21 5154497
chr21 5154506
chr21 5154508
chr21 5154516
chr21 5154519
chr21 5154534
chr21 5154536
chr21 5154547
chr21 5154551
chr21 5154576
chr21 5154585
chr21 5154587
chr21 5154590
chr21 5154603
chr21 5154607
chr21 5154610
chr21 5154614
chr21 5154616
chr21 5154624
chr21 5154631
chr21 5154643
chr21 5154650

chr21 5154654
chr21 5154661
chr21 5154673
chr21 5154676
chr21 5154680
chr21 5154699
chr21 5154707
chr21 5154709
chr21 5154711
chr21 5154717
chr21 5154736
chr21 5392238
chr21 5392387
chr21 5392717
chr21 5393358
chr21 5393396
chr21 5393441
chr21 5966613
chr21 5966629
chr21 5966655
chr21 5968301
chr21 5968741
chr21 5968773
chr21 5968890
chr21 5969625
chr21 5969658
chr21 5970122
chr21 5970128
chr21 5970281
chr21 5970310
chr21 5971329
chr21 5971920
chr21 5971967
chr21 5974015
chr21 5974043
chr21 5974185
chr21 5974751
chr21 5974883
chr21 5975771
chr21 5975840
chr21 5976001
chr21 5976626
chr21 5977512
chr21 5978979
chr21 5979040
chr21 5979578
chr21 5980178
chr21 5983458
chr21 5983940
chr21 5983952
chr21 5984015
chr21 6011144
chr21 6011168
chr21 6011178

chr21 6011201
chr21 6011264
chr21 6011363
chr21 6011368
chr21 6011412
chr21 6011501
chr21 6011518
chr21 6011560
chr21 6012069
chr21 6012179
chr21 6013844
chr21 6013893
chr21 6015037
chr21 6016665
chr21 6016688
chr21 6016934
chr21 6016983
chr21 6017127
chr21 6017207
chr21 6029596
chr21 6029618
chr21 6029635
chr21 6030344
chr21 6030357
chr21 6030380
chr21 6036065
chr21 6036089
chr21 6036093
chr21 6037095
chr21 6037101
chr21 6037140
chr21 6046369
chr21 6046430
chr21 6046459
chr21 6046463
chr21 6046471
chr21 6046495
chr21 6046519
chr21 6046527
chr21 6046551
chr21 6046581
chr21 6046597
chr21 6110880
chr21 6110882
chr21 6110886
chr21 6110897
chr21 6110908
chr21 6110910
chr21 6110912
chr21 6110914
chr21 6110923
chr21 6110941
chr21 6110953
chr21 6110964

chr21 6110971
chr21 6111453
chr21 6111470
chr21 6111472
chr21 6111494
chr21 6111512
chr21 6111516
chr21 6111518
chr21 6111522
chr21 6111531
chr21 6111544
chr21 6111550
chr21 6111552
chr21 6111559
chr21 6111576
chr21 6111589
chr21 6111593
chr21 6111598
chr21 6111600
chr21 6111604
chr21 6111607
chr21 6111854
chr21 6111878
chr21 6111887
chr21 6111891
chr21 6111894
chr21 6111900
chr21 6112073
chr21 6112075
chr21 6112077
chr21 6112097
chr21 6112107
chr21 6112109
chr21 6112116
chr21 6114301
chr21 6114314
chr21 6114339
chr21 6114341
chr21 6114347
chr21 6122883
chr21 6122919
chr21 6127428
chr21 6127454
chr21 6127460
chr21 6127469
chr21 6127503
chr21 6127656
chr21 6127662
chr21 6127666
chr21 6127750
chr21 6128147
chr21 6128550
chr21 6128561
chr21 6128581

chr21 6128588
chr21 6128616
chr21 6128627
chr21 6152935
chr21 6153260
chr21 6153381
chr21 6153544
chr21 6153598
chr21 6153605
chr21 6153955
chr21 6153994
chr21 6154023
chr21 6299243
chr21 6299812
chr21 6299888
chr21 6299917
chr21 6299934
chr21 6300263
chr21 6300269
chr21 6437005
chr21 6437028
chr21 6437072
chr21 6437099
chr21 6437138
chr21 6437148
chr21 6465571
chr21 6465610
chr21 6465616
chr21 6465640
chr21 6465732
chr21 6465763
chr21 6465786
chr21 6465790
chr21 6467562
chr21 6467572
chr21 6467580
chr21 6467586
chr21 6467593
chr21 6467596
chr21 6467599
chr21 6467611
chr21 6467625
chr21 6467629
chr21 6467659
chr21 6468465
chr21 6468472
chr21 6468478
chr21 6468483
chr21 6468489
chr21 6468504
chr21 6468512
chr21 6468524
chr21 6468529
chr21 6468531

chr21 6468539
chr21 6468542
chr21 6468544
chr21 6468556
chr21 6468587
chr21 6468739
chr21 6468747
chr21 6468813
chr21 6468827
chr21 6468829
chr21 6469085
chr21 6469095
chr21 6469117
chr21 6469139
chr21 6469181
chr21 6469212
chr21 6495627
chr21 6496446
chr21 6496471
chr21 6498732
chr21 6498748
chr21 6498756
chr21 6498769
chr21 6498792
chr21 6498794
chr21 6498799
chr21 6498804
chr21 6498811
chr21 6498818
chr21 6498821
chr21 6498826
chr21 6498834
chr21 6498838
chr21 6498841
chr21 6498845
chr21 6498858
chr21 6498871
chr21 6498882
chr21 6498886
chr21 6498888
chr21 6498891
chr21 6498896
chr21 6498922
chr21 6498935
chr21 6498938
chr21 6498940
chr21 6498942
chr21 6498949
chr21 6498954
chr21 6498957
chr21 6498963
chr21 6498970
chr21 6498972
chr21 6498982

chr21 6498994
chr21 6499145
chr21 6499167
chr21 6499199
chr21 6499211
chr21 6499214
chr21 6499217
chr21 6499220
chr21 6499229
chr21 6499232
chr21 6499236
chr21 6499238
chr21 6499245
chr21 6499290
chr21 6499319
chr21 6499321
chr21 6499418
chr21 6499422
chr21 6499437
chr21 6499445
chr21 6499449
chr21 6499453
chr21 6499456
chr21 6548775
chr21 6548834
chr21 6548846
chr21 6548902
chr21 6549024
chr21 6549047
chr21 6549077
chr21 6549092
chr21 6549153
chr21 6549157
chr21 6549165
chr21 6549539
chr21 6552082
chr21 6552123
chr21 6564641
chr21 6564713
chr21 6564719
chr21 6564732
chr21 6564845
chr21 6564854
chr21 6564869
chr21 6564882
chr21 6564900
chr21 6564923
chr21 6564946
chr21 6564979
chr21 6564990
chr21 6564998
chr21 6565013
chr21 6565216
chr21 6565257

chr21 6565269
chr21 6565282
chr21 6565308
chr21 6565340
chr21 6656930
chr21 6657040
chr21 6657167
chr21 6658119
chr21 6660404
chr21 6660410
chr21 6660444
chr21 6662578
chr21 6664024
chr21 6664420
chr21 6667030
chr21 6667045
chr21 6667069
chr21 6667093
chr21 6667105
chr21 6667145
chr21 6667179
chr21 6670638
chr21 6670679
chr21 6671136
chr21 6671177
chr21 6672602
chr21 6673706
chr21 6673729
chr21 6675037
chr21 6677576
chr21 6683748
chr21 6685971
chr21 6689115
chr21 6689191
chr21 6689208
chr21 6689956
chr21 6692046
chr21 6693206
chr21 6695759
chr21 6698768
chr21 6698802
chr21 6703343
chr21 6703397
chr21 6703401
chr21 6704515
chr21 6704531
chr21 6705094
chr21 6705122
chr21 6705238
chr21 6708696
chr21 6710465
chr21 6710467
chr21 6821127
chr21 6821267

chr21 6823023
chr21 6838295
chr21 6838576
chr21 6839824
chr21 6839826
chr21 6839889
chr21 6839893
chr21 6839914
chr21 6839917
chr21 6839927
chr21 6839935
chr21 6839953
chr21 6839963
chr21 6839970
chr21 6839978
chr21 6839985
chr21 6839992
chr21 6839996
chr21 6839998
chr21 6840842
chr21 6840878
chr21 6842406
chr21 6842417
chr21 6844999
chr21 6845036
chr21 6845231
chr21 6861254
chr21 6862006
chr21 6862167
chr21 6862172
chr21 6862581
chr21 6875377
chr21 6875409
chr21 6875485
chr21 6875499
chr21 6878092
chr21 6878258
chr21 6911529
chr21 6913115
chr21 6915516
chr21 6915867
chr21 6915872
chr21 6916162
chr21 6916310
chr21 6916663
chr21 6918092
chr21 7095492
chr21 7095509
chr21 7096683
chr21 7096701
chr21 7096721
chr21 7096789
chr21 7105373
chr21 7105389

chr21 7105412
chr21 7105435
chr21 7106094
chr21 7142603
chr21 7142630
chr21 7143297
chr21 7146605
chr21 7149383
chr21 7149427
chr21 7149439
chr21 7149443
chr21 7149503
chr21 7149523
chr21 7675998
chr21 7676012
chr21 7676020
chr21 7676083
chr21 7676141
chr21 7678600
chr21 7678659
chr21 7679413
chr21 7752154
chr21 7752483
chr21 7752492
chr21 7752499
chr21 7752505
chr21 7752523
chr21 7752584
chr21 7753437
chr21 7753527
chr21 7753536
chr21 7753547
chr21 7768171
chr21 7768235
chr21 7768250
chr21 7768737
chr21 7768763
chr21 7769463
chr21 7771196
chr21 7771202
chr21 7771219
chr21 7771226
chr21 7771294
chr21 7771334
chr21 7771354
chr21 7771375
chr21 7771379
chr21 7772779
chr21 7772793
chr21 7772839
chr21 7772905
chr21 7773404
chr21 7773448
chr21 7773498

chr21 7773521
chr21 7774289
chr21 7774305
chr21 7775531
chr21 7775677
chr21 7775737
chr21 7776067
chr21 7776823
chr21 7776865
chr21 7777075
chr21 7777165
chr21 7777275
chr21 7777495
chr21 7777527
chr21 7777542
chr21 7777558
chr21 7777573
chr21 7777586
chr21 7780646
chr21 7780654
chr21 7781131
chr21 7781142
chr21 7782666
chr21 7782799
chr21 8204885
chr21 8204908
chr21 8204910
chr21 8204913
chr21 8206099
chr21 8206106
chr21 8206113
chr21 8206115
chr21 8206132
chr21 8206134
chr21 8206136
chr21 8206140
chr21 8206144
chr21 8206149
chr21 8206156
chr21 8206162
chr21 8206167
chr21 8206173
chr21 8206190
chr21 8206196
chr21 8206199
chr21 8206208
chr21 8206211
chr21 8206221
chr21 8206229
chr21 8206233
chr21 8206240
chr21 8206245
chr21 8206249
chr21 8206253

chr21 8206258
chr21 8206262
chr21 8206264
chr21 8206278
chr21 8206466
chr21 8206470
chr21 8206477
chr21 8206481
chr21 8206485
chr21 8206490
chr21 8206494
chr21 8206500
chr21 8206509
chr21 8206514
chr21 8206519
chr21 8206521
chr21 8206533
chr21 8206548
chr21 8206552
chr21 8206555
chr21 8206558
chr21 8206563
chr21 8206804
chr21 8206818
chr21 8206823
chr21 8206843
chr21 8206849
chr21 8206853
chr21 8206864
chr21 8206870
chr21 8206876
chr21 8206879
chr21 8206891
chr21 8206895
chr21 8206898
chr21 8206901
chr21 8206903
chr21 8206910
chr21 8206912
chr21 8206914
chr21 8206933
chr21 8206936
chr21 8206948
chr21 8207224
chr21 8207226
chr21 8207251
chr21 8207260
chr21 8207262
chr21 8207266
chr21 8207270
chr21 8207279
chr21 8207281
chr21 8207283
chr21 8207297

chr21 8207302
chr21 8207327
chr21 8207334
chr21 8207337
chr21 8207345
chr21 8207351
chr21 8207361
chr21 8207368
chr21 8207394
chr21 8207416
chr21 8207428
chr21 8207453
chr21 8207458
chr21 8207461
chr21 8207479
chr21 8207490
chr21 8207496
chr21 8207501
chr21 8207506
chr21 8207510
chr21 8207527
chr21 8207538
chr21 8207541
chr21 8207560
chr21 8207569
chr21 8207572
chr21 8207579
chr21 8207581
chr21 8207591
chr21 8207597
chr21 8207602
chr21 8207605
chr21 8207619
chr21 8207621
chr21 8207634
chr21 8208130
chr21 8208136
chr21 8208139
chr21 8208147
chr21 8208155
chr21 8208171
chr21 8208173
chr21 8208180
chr21 8208187
chr21 8208191
chr21 8208209
chr21 8208213
chr21 8208216
chr21 8208220
chr21 8208223
chr21 8208241
chr21 8208245
chr21 8208253
chr21 8208261

chr21 8208265
chr21 8208277
chr21 8208287
chr21 8208293
chr21 8208299
chr21 8208304
chr21 8208311
chr21 8208314
chr21 8208317
chr21 8208389
chr21 8208398
chr21 8208410
chr21 8208420
chr21 8208427
chr21 8208434
chr21 8208437
chr21 8208439
chr21 8208443
chr21 8208455
chr21 8208461
chr21 8208469
chr21 8208473
chr21 8208475
chr21 8208481
chr21 8208484
chr21 8208487
chr21 8208503
chr21 8208505
chr21 8208512
chr21 8208515
chr21 8208522
chr21 8208526
chr21 8208542
chr21 8208544
chr21 8208546
chr21 8208552
chr21 8208555
chr21 8208558
chr21 8208561
chr21 8208572
chr21 8208574
chr21 8208576
chr21 8208585
chr21 8208591
chr21 8208595
chr21 8208599
chr21 8208601
chr21 8208605
chr21 8208616
chr21 8209343
chr21 8209345
chr21 8209349
chr21 8209354
chr21 8209357

chr21 8209365
chr21 8209368
chr21 8209370
chr21 8209381
chr21 8209383
chr21 8209391
chr21 8209398
chr21 8209403
chr21 8209405
chr21 8209414
chr21 8209427
chr21 8209431
chr21 8209438
chr21 8209440
chr21 8209445
chr21 8209450
chr21 8209459
chr21 8209465
chr21 8209469
chr21 8209472
chr21 8209478
chr21 8209480
chr21 8209484
chr21 8209488
chr21 8209498
chr21 8209502
chr21 8209506
chr21 8209510
chr21 8209512
chr21 8209517
chr21 8209521
chr21 8209525
chr21 8209529
chr21 8209533
chr21 8209536
chr21 8209545
chr21 8209551
chr21 8209556
chr21 8209559
chr21 8209565
chr21 8209571
chr21 8209573
chr21 8209583
chr21 8209587
chr21 8209591
chr21 8209595
chr21 8209599
chr21 8209601
chr21 8209604
chr21 8209612
chr21 8209615
chr21 8209617
chr21 8209755
chr21 8209759

chr21 8209809
chr21 8209812
chr21 8209816
chr21 8209829
chr21 8209831
chr21 8209869
chr21 8209884
chr21 8209890
chr21 8209894
chr21 8209901
chr21 8209905
chr21 8209908
chr21 8209911
chr21 8209936
chr21 8209941
chr21 8209945
chr21 8209949
chr21 8209956
chr21 8209961
chr21 8209964
chr21 8209967
chr21 8209976
chr21 8209980
chr21 8209999
chr21 8210009
chr21 8210012
chr21 8210032
chr21 8210039
chr21 8210054
chr21 8210060
chr21 8210080
chr21 8210109
chr21 8210111
chr21 8210147
chr21 8210173
chr21 8210215
chr21 8210306
chr21 8210325
chr21 8210329
chr21 8210333
chr21 8210338
chr21 8210341
chr21 8210343
chr21 8210348
chr21 8210356
chr21 8210360
chr21 8210366
chr21 8210380
chr21 8210383
chr21 8210391
chr21 8210413
chr21 8210415
chr21 8210793
chr21 8210864

chr21 8211458
chr21 8211472
chr21 8211501
chr21 8211508
chr21 8211515
chr21 8211522
chr21 8211524
chr21 8211527
chr21 8211530
chr21 8211533
chr21 8211536
chr21 8211539
chr21 8211541
chr21 8211543
chr21 8211553
chr21 8211572
chr21 8211574
chr21 8211577
chr21 8211579
chr21 8211582
chr21 8211584
chr21 8211588
chr21 8211590
chr21 8211594
chr21 8211601
chr21 8211603
chr21 8211613
chr21 8211617
chr21 8211621
chr21 8211625
chr21 8211629
chr21 8211633
chr21 8211636
chr21 8211640
chr21 8211646
chr21 8211649
chr21 8211653
chr21 8211655
chr21 8211662
chr21 8211671
chr21 8211682
chr21 8211721
chr21 8211824
chr21 8211827
chr21 8211830
chr21 8211833
chr21 8211835
chr21 8211838
chr21 8211853
chr21 8211868
chr21 8211871
chr21 8211878
chr21 8211881
chr21 8211885

chr21 8211891
chr21 8211894
chr21 8211896
chr21 8211899
chr21 8211907
chr21 8211913
chr21 8211958
chr21 8211961
chr21 8211967
chr21 8211973
chr21 8211977
chr21 8211986
chr21 8211995
chr21 8212003
chr21 8212008
chr21 8212015
chr21 8212022
chr21 8212026
chr21 8212029
chr21 8212042
chr21 8212050
chr21 8212054
chr21 8212062
chr21 8212065
chr21 8212080
chr21 8212082
chr21 8212087
chr21 8212090
chr21 8212092
chr21 8212225
chr21 8212231
chr21 8212233
chr21 8212236
chr21 8212239
chr21 8212242
chr21 8212245
chr21 8212248
chr21 8212250
chr21 8212253
chr21 8212257
chr21 8212260
chr21 8212277
chr21 8212280
chr21 8212284
chr21 8212383
chr21 8212386
chr21 8212388
chr21 8212390
chr21 8212398
chr21 8212415
chr21 8212426
chr21 8212433
chr21 8212439
chr21 8212451

chr21 8212459
chr21 8212495
chr21 8212503
chr21 8212508
chr21 8212510
chr21 8212530
chr21 8212535
chr21 8212538
chr21 8212541
chr21 8212547
chr21 8212553
chr21 8212561
chr21 8212567
chr21 8212571
chr21 8212663
chr21 8212671
chr21 8212683
chr21 8212689
chr21 8212700
chr21 8212708
chr21 8212720
chr21 8212723
chr21 8212738
chr21 8212744
chr21 8212813
chr21 8212825
chr21 8212833
chr21 8212836
chr21 8212839
chr21 8212843
chr21 8212857
chr21 8212860
chr21 8212862
chr21 8212866
chr21 8212885
chr21 8212887
chr21 8212896
chr21 8212904
chr21 8212906
chr21 8212909
chr21 8212916
chr21 8212935
chr21 8212939
chr21 8212952
chr21 8212961
chr21 8212964
chr21 8212967
chr21 8212970
chr21 8212973
chr21 8212977
chr21 8212979
chr21 8212985
chr21 8213005
chr21 8213010

chr21 8213015
chr21 8213023
chr21 8213029
chr21 8213032
chr21 8213038
chr21 8213043
chr21 8213056
chr21 8213126
chr21 8213132
chr21 8213135
chr21 8213138
chr21 8213141
chr21 8213144
chr21 8213147
chr21 8213149
chr21 8213151
chr21 8213154
chr21 8213157
chr21 8213160
chr21 8213169
chr21 8213176
chr21 8213180
chr21 8213207
chr21 8213213
chr21 8213230
chr21 8213233
chr21 8213247
chr21 8213255
chr21 8213257
chr21 8213262
chr21 8213266
chr21 8213269
chr21 8213271
chr21 8213276
chr21 8213279
chr21 8213283
chr21 8213289
chr21 8213905
chr21 8213910
chr21 8213915
chr21 8213940
chr21 8213976
chr21 8213979
chr21 8214002
chr21 8214005
chr21 8214013
chr21 8214018
chr21 8214020
chr21 8214023
chr21 8214028
chr21 8214042
chr21 8214046
chr21 8214144
chr21 8214147

chr21 8214153
chr21 8214163
chr21 8214169
chr21 8214196
chr21 8214227
chr21 8214232
chr21 8214238
chr21 8214255
chr21 8214301
chr21 8214309
chr21 8214323
chr21 8214334
chr21 8214336
chr21 8214343
chr21 8214347
chr21 8214385
chr21 8214390
chr21 8214404
chr21 8214411
chr21 8214420
chr21 8214433
chr21 8214448
chr21 8214451
chr21 8214470
chr21 8214566
chr21 8214570
chr21 8214581
chr21 8214583
chr21 8214591
chr21 8214594
chr21 8214596
chr21 8214600
chr21 8214606
chr21 8214611
chr21 8214625
chr21 8214628
chr21 8214642
chr21 8214652
chr21 8214656
chr21 8214660
chr21 8214664
chr21 8214668
chr21 8214684
chr21 8214690
chr21 8214697
chr21 8214703
chr21 8214705
chr21 8214715
chr21 8214723
chr21 8214725
chr21 8214727
chr21 8214732
chr21 8214737
chr21 8214740

```
chr21 8214743
chr21 8214746
chr21 8214752
chr21 8214755
chr21 8214758
chr21 8214761
chr21 8214764
chr21 8214767
chr21 8214770
chr21 8214776
chr21 8214788
chr21 8214806
chr21 8214819
chr21 8214822
chr21 8214829
chr21 8214835
chr21 8214843
chr21 8214850
chr21 8214853
chr21 8214856
chr21 8214858
chr21 8214873
chr21 8214876
chr21 8214886
chr21 8214888
chr21 8214900
chr21 8214902
chr21 8214918
chr21 8214920
chr21 8215091
chr21 8215097
chr21 8215113
chr21 8215121
chr21 8215123
chr21 8215125
chr21 8215127
chr21 8215134
chr21 8215146
chr21 8215149
chr21 8215154
chr21 8215158
chr21 8215162
chr21 8215168
chr21 8215171
chr21 8215174
chr21 8215284
chr21 8215287
chr21 8215289
chr21 8215293
chr21 8215296
chr21 8215300
chr21 8215313
chr21 8215329
chr21 8215332
```

chr21 8215338
chr21 8215346
chr21 8215351
chr21 8215356
chr21 8215586
chr21 8215589
chr21 8215597
chr21 8215602
chr21 8215614
chr21 8215622
chr21 8215645
chr21 8215650
chr21 8215691
chr21 8215696
chr21 8215702
chr21 8215710
chr21 8215714
chr21 8215723
chr21 8215793
chr21 8215797
chr21 8215803
chr21 8215806
chr21 8215853
chr21 8215869
chr21 8215876
chr21 8215905
chr21 8215933
chr21 8215941
chr21 8215944
chr21 8215956
chr21 8215960
chr21 8215963
chr21 8215966
chr21 8215973
chr21 8215983
chr21 8215989
chr21 8215992
chr21 8215998
chr21 8216001
chr21 8216003
chr21 8216005
chr21 8216007
chr21 8216009
chr21 8216011
chr21 8216013
chr21 8216015
chr21 8216071
chr21 8216084
chr21 8216102
chr21 8216108
chr21 8216113
chr21 8216120
chr21 8216126
chr21 8216134

chr21 8216136
chr21 8216140
chr21 8216145
chr21 8216148
chr21 8216150
chr21 8216201
chr21 8216211
chr21 8216214
chr21 8216253
chr21 8216269
chr21 8216317
chr21 8216354
chr21 8216358
chr21 8216368
chr21 8216377
chr21 8216381
chr21 8216385
chr21 8216396
chr21 8216410
chr21 8216416
chr21 8216424
chr21 8216434
chr21 8216437
chr21 8216439
chr21 8216444
chr21 8216453
chr21 8216459
chr21 8216461
chr21 8216465
chr21 8216471
chr21 8216480
chr21 8216483
chr21 8216492
chr21 8216527
chr21 8216543
chr21 8216548
chr21 8216561
chr21 8216575
chr21 8216578
chr21 8216580
chr21 8216586
chr21 8216589
chr21 8216592
chr21 8216596
chr21 8216607
chr21 8216625
chr21 8216646
chr21 8216648
chr21 8216651
chr21 8216656
chr21 8216668
chr21 8216812
chr21 8216814
chr21 8216816

chr21 8216819
chr21 8216821
chr21 8216829
chr21 8216834
chr21 8216837
chr21 8216840
chr21 8216851
chr21 8216855
chr21 8216869
chr21 8216871
chr21 8216882
chr21 8216892
chr21 8216894
chr21 8216896
chr21 8216898
chr21 8216901
chr21 8217171
chr21 8217175
chr21 8217181
chr21 8217190
chr21 8217193
chr21 8217197
chr21 8217200
chr21 8217203
chr21 8217205
chr21 8217224
chr21 8217229
chr21 8217232
chr21 8217234
chr21 8217236
chr21 8217238
chr21 8217240
chr21 8217244
chr21 8217253
chr21 8217260
chr21 8217264
chr21 8217268
chr21 8217271
chr21 8217274
chr21 8217278
chr21 8217281
chr21 8217285
chr21 8217288
chr21 8217293
chr21 8217298
chr21 8217303
chr21 8217311
chr21 8217324
chr21 8217330
chr21 8217334
chr21 8217341
chr21 8217358
chr21 8217361
chr21 8217363

chr21 8217365
chr21 8217368
chr21 8217371
chr21 8217621
chr21 8217624
chr21 8217663
chr21 8217680
chr21 8217682
chr21 8217698
chr21 8217730
chr21 8217750
chr21 8217759
chr21 8217768
chr21 8217809
chr21 8217851
chr21 8217854
chr21 8217862
chr21 8217871
chr21 8217873
chr21 8217936
chr21 8217952
chr21 8217968
chr21 8217975
chr21 8218475
chr21 8218485
chr21 8218597
chr21 8218602
chr21 8218608
chr21 8218611
chr21 8218613
chr21 8218621
chr21 8218631
chr21 8218639
chr21 8218647
chr21 8218657
chr21 8218660
chr21 8218663
chr21 8218668
chr21 8218682
chr21 8218684
chr21 8218689
chr21 8218691
chr21 8218693
chr21 8218695
chr21 8218697
chr21 8218705
chr21 8218707
chr21 8218714
chr21 8218717
chr21 8218719
chr21 8218721
chr21 8218724
chr21 8218730
chr21 8218737

chr21 8218742
chr21 8218754
chr21 8218766
chr21 8218771
chr21 8218773
chr21 8218785
chr21 8218789
chr21 8218797
chr21 8218801
chr21 8218806
chr21 8218811
chr21 8218815
chr21 8218819
chr21 8218925
chr21 8218942
chr21 8218944
chr21 8218946
chr21 8218948
chr21 8218950
chr21 8218952
chr21 8218954
chr21 8218956
chr21 8218958
chr21 8218962
chr21 8218966
chr21 8218975
chr21 8218978
chr21 8218983
chr21 8218987
chr21 8218989
chr21 8218993
chr21 8218996
chr21 8218999
chr21 8219003
chr21 8219011
chr21 8219015
chr21 8219030
chr21 8219040
chr21 8219043
chr21 8219047
chr21 8219049
chr21 8219053
chr21 8219079
chr21 8219081
chr21 8219083
chr21 8219089
chr21 8219093
chr21 8219096
chr21 8219098
chr21 8219103
chr21 8219111
chr21 8219118
chr21 8219129
chr21 8219136

chr21 8219139
chr21 8219144
chr21 8219149
chr21 8219153
chr21 8219158
chr21 8219163
chr21 8219168
chr21 8219175
chr21 8219182
chr21 8219241
chr21 8219246
chr21 8219257
chr21 8219259
chr21 8219261
chr21 8219268
chr21 8219276
chr21 8219284
chr21 8219292
chr21 8219299
chr21 8219301
chr21 8219303
chr21 8219316
chr21 8219324
chr21 8219328
chr21 8219333
chr21 8219345
chr21 8219347
chr21 8219358
chr21 8219361
chr21 8219365
chr21 8219368
chr21 8219378
chr21 8219383
chr21 8219387
chr21 8219389
chr21 8219395
chr21 8219399
chr21 8219406
chr21 8219410
chr21 8219423
chr21 8219427
chr21 8219435
chr21 8219715
chr21 8219719
chr21 8219721
chr21 8219731
chr21 8219737
chr21 8219742
chr21 8219744
chr21 8219761
chr21 8219763
chr21 8219798
chr21 8219800
chr21 8219843

chr21 8220509
chr21 8220511
chr21 8220515
chr21 8220517
chr21 8220527
chr21 8220533
chr21 8220538
chr21 8220541
chr21 8220557
chr21 8220559
chr21 8220594
chr21 8220596
chr21 8220608
chr21 8220610
chr21 8220639
chr21 8220761
chr21 8220767
chr21 8220775
chr21 8220787
chr21 8220791
chr21 8220845
chr21 8220851
chr21 8220985
chr21 8221003
chr21 8221011
chr21 8221014
chr21 8249658
chr21 8249671
chr21 8249680
chr21 8249683
chr21 8249687
chr21 8249699
chr21 8249707
chr21 8249717
chr21 8249724
chr21 8249729
chr21 8249735
chr21 8249742
chr21 8249749
chr21 8249756
chr21 8249763
chr21 8249768
chr21 8249773
chr21 8249775
chr21 8249787
chr21 8249789
chr21 8249802
chr21 8249810
chr21 8249814
chr21 8249820
chr21 8249827
chr21 8250293
chr21 8250308
chr21 8250315

chr21 8250322
chr21 8250324
chr21 8250341
chr21 8250343
chr21 8250345
chr21 8250349
chr21 8250353
chr21 8250358
chr21 8250365
chr21 8250371
chr21 8250376
chr21 8250382
chr21 8250399
chr21 8250405
chr21 8250408
chr21 8250417
chr21 8250420
chr21 8250430
chr21 8250438
chr21 8250442
chr21 8250449
chr21 8250454
chr21 8250458
chr21 8250462
chr21 8250467
chr21 8250471
chr21 8250473
chr21 8250487
chr21 8250496
chr21 8250501
chr21 8250505
chr21 8250514
chr21 8250520
chr21 8250526
chr21 8250528
chr21 8250675
chr21 8250679
chr21 8250686
chr21 8250690
chr21 8250694
chr21 8250699
chr21 8250703
chr21 8250709
chr21 8250718
chr21 8250723
chr21 8250728
chr21 8250730
chr21 8250742
chr21 8250757
chr21 8250761
chr21 8250764
chr21 8250767
chr21 8250772
chr21 8251013

chr21 8251027
chr21 8251032
chr21 8251052
chr21 8251058
chr21 8251062
chr21 8251073
chr21 8251079
chr21 8251085
chr21 8251088
chr21 8251100
chr21 8251104
chr21 8251107
chr21 8251110
chr21 8251112
chr21 8251119
chr21 8251121
chr21 8251123
chr21 8251142
chr21 8251145
chr21 8251157
chr21 8251162
chr21 8251176
chr21 8251181
chr21 8251186
chr21 8251188
chr21 8251199
chr21 8251320
chr21 8251324
chr21 8251327
chr21 8251338
chr21 8251349
chr21 8251353
chr21 8251357
chr21 8251374
chr21 8251380
chr21 8251390
chr21 8251452
chr21 8251460
chr21 8251469
chr21 8251471
chr21 8251475
chr21 8251479
chr21 8251488
chr21 8251490
chr21 8251492
chr21 8251506
chr21 8251511
chr21 8251536
chr21 8251543
chr21 8251546
chr21 8251554
chr21 8251560
chr21 8251570
chr21 8251577

chr21 8251603
chr21 8251625
chr21 8251637
chr21 8251662
chr21 8251667
chr21 8251670
chr21 8251688
chr21 8251699
chr21 8251705
chr21 8251710
chr21 8251715
chr21 8251719
chr21 8251736
chr21 8251747
chr21 8251750
chr21 8251769
chr21 8251778
chr21 8251781
chr21 8251788
chr21 8251790
chr21 8251800
chr21 8251806
chr21 8251811
chr21 8251814
chr21 8251828
chr21 8251830
chr21 8251843
chr21 8252081
chr21 8252089
chr21 8252094
chr21 8252100
chr21 8252105
chr21 8252116
chr21 8252125
chr21 8252129
chr21 8252132
chr21 8252144
chr21 8252149
chr21 8252155
chr21 8252163
chr21 8252167
chr21 8252172
chr21 8252181
chr21 8252187
chr21 8252195
chr21 8252202
chr21 8252216
chr21 8252237
chr21 8252240
chr21 8252249
chr21 8252254
chr21 8252257
chr21 8252259
chr21 8252265

chr21 8252271
chr21 8252282
chr21 8252293
chr21 8252295
chr21 8252299
chr21 8252317
chr21 8252321
chr21 8252340
chr21 8252345
chr21 8252348
chr21 8252356
chr21 8252364
chr21 8252380
chr21 8252382
chr21 8252389
chr21 8252396
chr21 8252400
chr21 8252418
chr21 8252422
chr21 8252425
chr21 8252429
chr21 8252432
chr21 8252450
chr21 8252454
chr21 8252462
chr21 8252470
chr21 8252474
chr21 8252486
chr21 8252496
chr21 8252502
chr21 8252508
chr21 8252512
chr21 8252518
chr21 8252521
chr21 8252523
chr21 8252528
chr21 8252537
chr21 8252542
chr21 8252545
chr21 8252550
chr21 8252552
chr21 8252559
chr21 8252562
chr21 8252564
chr21 8252566
chr21 8252578
chr21 8252583
chr21 8252589
chr21 8252594
chr21 8252596
chr21 8252605
chr21 8252646
chr21 8252650
chr21 8252667

chr21 8252673
chr21 8253965
chr21 8253969
chr21 8254019
chr21 8254022
chr21 8254026
chr21 8254039
chr21 8254041
chr21 8254052
chr21 8254079
chr21 8254094
chr21 8254100
chr21 8254104
chr21 8254111
chr21 8254115
chr21 8254118
chr21 8254121
chr21 8254146
chr21 8254151
chr21 8254155
chr21 8254159
chr21 8254166
chr21 8254171
chr21 8254174
chr21 8254177
chr21 8254186
chr21 8254190
chr21 8254209
chr21 8254219
chr21 8254222
chr21 8254242
chr21 8254249
chr21 8254264
chr21 8254270
chr21 8254290
chr21 8254319
chr21 8254338
chr21 8254343
chr21 8254357
chr21 8254423
chr21 8254460
chr21 8254462
chr21 8254483
chr21 8254514
chr21 8254533
chr21 8254537
chr21 8254541
chr21 8254546
chr21 8254549
chr21 8254551
chr21 8254564
chr21 8254568
chr21 8254574
chr21 8254588

chr21 8254591
chr21 8254715
chr21 8254717
chr21 8254738
chr21 8254765
chr21 8254769
chr21 8254779
chr21 8254786
chr21 8254789
chr21 8255001
chr21 8255043
chr21 8255072
chr21 8255193
chr21 8255212
chr21 8255218
chr21 8255222
chr21 8255248
chr21 8255250
chr21 8255257
chr21 8255261
chr21 8255265
chr21 8255268
chr21 8255299
chr21 8255311
chr21 8255352
chr21 8255362
chr21 8255364
chr21 8255366
chr21 8255386
chr21 8255401
chr21 8255404
chr21 8255407
chr21 8255421
chr21 8255435
chr21 8255447
chr21 8255473
chr21 8255493
chr21 8255508
chr21 8255539
chr21 8255543
chr21 8255546
chr21 8255555
chr21 8255580
chr21 8255585
chr21 8255591
chr21 8255594
chr21 8255599
chr21 8255606
chr21 8255614
chr21 8255618
chr21 8255629
chr21 8255633
chr21 8255663
chr21 8255677

chr21 8255691
chr21 8255706
chr21 8255713
chr21 8255725
chr21 8255727
chr21 8255730
chr21 8255733
chr21 8255736
chr21 8255739
chr21 8255742
chr21 8255745
chr21 8255747
chr21 8255749
chr21 8255759
chr21 8255778
chr21 8255780
chr21 8255783
chr21 8255785
chr21 8255788
chr21 8255790
chr21 8255794
chr21 8255796
chr21 8255800
chr21 8255807
chr21 8255809
chr21 8255815
chr21 8255819
chr21 8255823
chr21 8255827
chr21 8255831
chr21 8255835
chr21 8255839
chr21 8255842
chr21 8255846
chr21 8255852
chr21 8255855
chr21 8255859
chr21 8255861
chr21 8255868
chr21 8255877
chr21 8255888
chr21 8255927
chr21 8256030
chr21 8256033
chr21 8256036
chr21 8256039
chr21 8256041
chr21 8256044
chr21 8256061
chr21 8256076
chr21 8256079
chr21 8256086
chr21 8256089
chr21 8256093

chr21 8256099
chr21 8256102
chr21 8256104
chr21 8256107
chr21 8256115
chr21 8256121
chr21 8256127
chr21 8256131
chr21 8256139
chr21 8256142
chr21 8256148
chr21 8256151
chr21 8256153
chr21 8256156
chr21 8256161
chr21 8256163
chr21 8256166
chr21 8256169
chr21 8256175
chr21 8256181
chr21 8256185
chr21 8256194
chr21 8256203
chr21 8256211
chr21 8256216
chr21 8256223
chr21 8256230
chr21 8256234
chr21 8256237
chr21 8256250
chr21 8256258
chr21 8256262
chr21 8256270
chr21 8256273
chr21 8256288
chr21 8256290
chr21 8256295
chr21 8256298
chr21 8256300
chr21 8256347
chr21 8256353
chr21 8256365
chr21 8256367
chr21 8256378
chr21 8256381
chr21 8256392
chr21 8256402
chr21 8256413
chr21 8256415
chr21 8256422
chr21 8256429
chr21 8256583
chr21 8256585
chr21 8256591

chr21 8256594
chr21 8256596
chr21 8256598
chr21 8256606
chr21 8256623
chr21 8256634
chr21 8256641
chr21 8256647
chr21 8257022
chr21 8257034
chr21 8257042
chr21 8257045
chr21 8257048
chr21 8257052
chr21 8257066
chr21 8257069
chr21 8257071
chr21 8257075
chr21 8257094
chr21 8257096
chr21 8257105
chr21 8257113
chr21 8257115
chr21 8257118
chr21 8257125
chr21 8257142
chr21 8257144
chr21 8257148
chr21 8257161
chr21 8257170
chr21 8257173
chr21 8257176
chr21 8257179
chr21 8257182
chr21 8257186
chr21 8257188
chr21 8257194
chr21 8257214
chr21 8257219
chr21 8257224
chr21 8257232
chr21 8257238
chr21 8257241
chr21 8257247
chr21 8257252
chr21 8257265
chr21 8257279
chr21 8257281
chr21 8257284
chr21 8257286
chr21 8257288
chr21 8257290
chr21 8257295
chr21 8257302

chr21 8257309
chr21 8257312
chr21 8257318
chr21 8257322
chr21 8257336
chr21 8257342
chr21 8257345
chr21 8257348
chr21 8257351
chr21 8257354
chr21 8257357
chr21 8257360
chr21 8257362
chr21 8257364
chr21 8257367
chr21 8257370
chr21 8257373
chr21 8257382
chr21 8257389
chr21 8257393
chr21 8257420
chr21 8257426
chr21 8257443
chr21 8257446
chr21 8257460
chr21 8257468
chr21 8257470
chr21 8257475
chr21 8257479
chr21 8257482
chr21 8257484
chr21 8257489
chr21 8257492
chr21 8257496
chr21 8257502
chr21 8257508
chr21 8257513
chr21 8257516
chr21 8257520
chr21 8257524
chr21 8257528
chr21 8257531
chr21 8257537
chr21 8257539
chr21 8257555
chr21 8257558
chr21 8257569
chr21 8257577
chr21 8257580
chr21 8257590
chr21 8257599
chr21 8257601
chr21 8257603
chr21 8257607

chr21 8257609
chr21 8257611
chr21 8257613
chr21 8257615
chr21 8257619
chr21 8257623
chr21 8257627
chr21 8257632
chr21 8257637
chr21 8257639
chr21 8257642
chr21 8257644
chr21 8257647
chr21 8257651
chr21 8257657
chr21 8257665
chr21 8257670
chr21 8257672
chr21 8257677
chr21 8257681
chr21 8257685
chr21 8257687
chr21 8257691
chr21 8257697
chr21 8257699
chr21 8257702
chr21 8257705
chr21 8257712
chr21 8257714
chr21 8257722
chr21 8257725
chr21 8257728
chr21 8257733
chr21 8257735
chr21 8257740
chr21 8257743
chr21 8257745
chr21 8257751
chr21 8257755
chr21 8257758
chr21 8257761
chr21 8257763
chr21 8257765
chr21 8257769
chr21 8257775
chr21 8257780
chr21 8257782
chr21 8257786
chr21 8257788
chr21 8257791
chr21 8257793
chr21 8257796
chr21 8257798
chr21 8257801

chr21 8257809
chr21 8257814
chr21 8257822
chr21 8257824
chr21 8257827
chr21 8257832
chr21 8257843
chr21 8257846
chr21 8257849
chr21 8257851
chr21 8257857
chr21 8257863
chr21 8257871
chr21 8257878
chr21 8257880
chr21 8257889
chr21 8257894
chr21 8257899
chr21 8257926
chr21 8257940
chr21 8257944
chr21 8257949
chr21 8257954
chr21 8257957
chr21 8257962
chr21 8257965
chr21 8257967
chr21 8257976
chr21 8257979
chr21 8257983
chr21 8257985
chr21 8257987
chr21 8257999
chr21 8258002
chr21 8258015
chr21 8258018
chr21 8258024
chr21 8258031
chr21 8258034
chr21 8258039
chr21 8258042
chr21 8258046
chr21 8258050
chr21 8258054
chr21 8258059
chr21 8258062
chr21 8258064
chr21 8258082
chr21 8258085
chr21 8258089
chr21 8258094
chr21 8258104
chr21 8258109
chr21 8258111

chr21 8258126
chr21 8258131
chr21 8258136
chr21 8258161
chr21 8258197
chr21 8258200
chr21 8258223
chr21 8258226
chr21 8258234
chr21 8258239
chr21 8258241
chr21 8258244
chr21 8258249
chr21 8258251
chr21 8258448
chr21 8258453
chr21 8258459
chr21 8258476
chr21 8258522
chr21 8258530
chr21 8258544
chr21 8258555
chr21 8258557
chr21 8258570
chr21 8258584
chr21 8258587
chr21 8258590
chr21 8258596
chr21 8258600
chr21 8258605
chr21 8258608
chr21 8258613
chr21 8258616
chr21 8258627
chr21 8258634
chr21 8258643
chr21 8258656
chr21 8258671
chr21 8258674
chr21 8258693
chr21 8258702
chr21 8258709
chr21 8258712
chr21 8258718
chr21 8258728
chr21 8258732
chr21 8258738
chr21 8258750
chr21 8258753
chr21 8258761
chr21 8258806
chr21 8258809
chr21 8258814
chr21 8258817

chr21 8258819
chr21 8258823
chr21 8258829
chr21 8258834
chr21 8258848
chr21 8258851
chr21 8258865
chr21 8258875
chr21 8258879
chr21 8258883
chr21 8258887
chr21 8258891
chr21 8258907
chr21 8258913
chr21 8258920
chr21 8258926
chr21 8258928
chr21 8258938
chr21 8258946
chr21 8258948
chr21 8258950
chr21 8258955
chr21 8258960
chr21 8258963
chr21 8258966
chr21 8258969
chr21 8258972
chr21 8258975
chr21 8258981
chr21 8258984
chr21 8258987
chr21 8258990
chr21 8258993
chr21 8258996
chr21 8258999
chr21 8259005
chr21 8259017
chr21 8259035
chr21 8259048
chr21 8259051
chr21 8259058
chr21 8259064
chr21 8259072
chr21 8259079
chr21 8259082
chr21 8259085
chr21 8259087
chr21 8259102
chr21 8259105
chr21 8259115
chr21 8259277
chr21 8259282
chr21 8259298
chr21 8259303

chr21 8259307
chr21 8259312
chr21 8259314
chr21 8259317
chr21 8259375
chr21 8259378
chr21 8259383
chr21 8259387
chr21 8259391
chr21 8259397
chr21 8259400
chr21 8259403
chr21 8259406
chr21 8259409
chr21 8259421
chr21 8259426
chr21 8259438
chr21 8259457
chr21 8259597
chr21 8259612
chr21 8259616
chr21 8259620
chr21 8259662
chr21 8259815
chr21 8259818
chr21 8259826
chr21 8259831
chr21 8259843
chr21 8259851
chr21 8259874
chr21 8259879
chr21 8259920
chr21 8259925
chr21 8259931
chr21 8259939
chr21 8259943
chr21 8259952
chr21 8260162
chr21 8260170
chr21 8260173
chr21 8260185
chr21 8260189
chr21 8260192
chr21 8260195
chr21 8260202
chr21 8260212
chr21 8260218
chr21 8260221
chr21 8260227
chr21 8260230
chr21 8260232
chr21 8260234
chr21 8260236
chr21 8260238

chr21 8260240
chr21 8260242
chr21 8260244
chr21 8260255
chr21 8260258
chr21 8260265
chr21 8260268
chr21 8260271
chr21 8260274
chr21 8260277
chr21 8260300
chr21 8260313
chr21 8260331
chr21 8260337
chr21 8260342
chr21 8260349
chr21 8260355
chr21 8260430
chr21 8260440
chr21 8260443
chr21 8260482
chr21 8260498
chr21 8260546
chr21 8260563
chr21 8260567
chr21 8260570
chr21 8260575
chr21 8260583
chr21 8260587
chr21 8260653
chr21 8260663
chr21 8260666
chr21 8260668
chr21 8260673
chr21 8260682
chr21 8260688
chr21 8260690
chr21 8260694
chr21 8260700
chr21 8260709
chr21 8260712
chr21 8260721
chr21 8260756
chr21 8260772
chr21 8260777
chr21 8260790
chr21 8260804
chr21 8260807
chr21 8260809
chr21 8260815
chr21 8260818
chr21 8260821
chr21 8260825
chr21 8260836

chr21 8260854
chr21 8260877
chr21 8260880
chr21 8260885
chr21 8321991
chr21 8322099
chr21 8337160
chr21 8337164
chr21 8347000
chr21 8347063
chr21 8347077
chr21 8354667
chr21 8354713
chr21 8354731
chr21 8356876
chr21 8375522
chr21 8375660
chr21 8375743
chr21 8387960
chr21 8388000
chr21 8388002
chr21 8388014
chr21 8388044
chr21 8388050
chr21 8388056
chr21 8388070
chr21 8388077
chr21 8388083
chr21 8388089
chr21 8388091
chr21 8388098
chr21 8388362
chr21 8388367
chr21 8388381
chr21 8388384
chr21 8388388
chr21 8388400
chr21 8388405
chr21 8388409
chr21 8388416
chr21 8388418
chr21 8388421
chr21 8388449
chr21 8388451
chr21 8388459
chr21 8388462
chr21 8388464
chr21 8388466
chr21 8388471
chr21 8388473
chr21 8388487
chr21 8388496
chr21 8388501
chr21 8388505

chr21 8389146
chr21 8389153
chr21 8389160
chr21 8389162
chr21 8389179
chr21 8389181
chr21 8389183
chr21 8389187
chr21 8389191
chr21 8389196
chr21 8389203
chr21 8389209
chr21 8389214
chr21 8389220
chr21 8389237
chr21 8389243
chr21 8389246
chr21 8389255
chr21 8389258
chr21 8389268
chr21 8389276
chr21 8389280
chr21 8389287
chr21 8389292
chr21 8389296
chr21 8389300
chr21 8389305
chr21 8389309
chr21 8389311
chr21 8389325
chr21 8389334
chr21 8389339
chr21 8389343
chr21 8389352
chr21 8389358
chr21 8389364
chr21 8389366
chr21 8389376
chr21 8389379
chr21 8389390
chr21 8389407
chr21 8389413
chr21 8389417
chr21 8389422
chr21 8389429
chr21 8389433
chr21 8389444
chr21 8389462
chr21 8389468
chr21 8389479
chr21 8389513
chr21 8389517
chr21 8389524
chr21 8389528

chr21 8389532
chr21 8389537
chr21 8389541
chr21 8389547
chr21 8389556
chr21 8389561
chr21 8389566
chr21 8389568
chr21 8389580
chr21 8389595
chr21 8389599
chr21 8389602
chr21 8389605
chr21 8389610
chr21 8389851
chr21 8389865
chr21 8389870
chr21 8389890
chr21 8389896
chr21 8389900
chr21 8389911
chr21 8389917
chr21 8389923
chr21 8389926
chr21 8389938
chr21 8389942
chr21 8389945
chr21 8389948
chr21 8389950
chr21 8389957
chr21 8389959
chr21 8389961
chr21 8389980
chr21 8389983
chr21 8390063
chr21 8390070
chr21 8390074
chr21 8390079
chr21 8390094
chr21 8390100
chr21 8390102
chr21 8390104
chr21 8390109
chr21 8390117
chr21 8390121
chr21 8390123
chr21 8390195
chr21 8390212
chr21 8390218
chr21 8390228
chr21 8390250
chr21 8390271
chr21 8390273
chr21 8390277

chr21 8390279
chr21 8390281
chr21 8390286
chr21 8390290
chr21 8390298
chr21 8390307
chr21 8390309
chr21 8390313
chr21 8390317
chr21 8390326
chr21 8390328
chr21 8390330
chr21 8390344
chr21 8390349
chr21 8390374
chr21 8390381
chr21 8390384
chr21 8390392
chr21 8390398
chr21 8390408
chr21 8390415
chr21 8390441
chr21 8390463
chr21 8390475
chr21 8390491
chr21 8390500
chr21 8390505
chr21 8390508
chr21 8390526
chr21 8390537
chr21 8390543
chr21 8390548
chr21 8390553
chr21 8390557
chr21 8390574
chr21 8390585
chr21 8390588
chr21 8390607
chr21 8390616
chr21 8390619
chr21 8390626
chr21 8390628
chr21 8390638
chr21 8390644
chr21 8390649
chr21 8390652
chr21 8390666
chr21 8390668
chr21 8390681
chr21 8390736
chr21 8390739
chr21 8390743
chr21 8390746
chr21 8390750

chr21 8390761
chr21 8390763
chr21 8390767
chr21 8390770
chr21 8390779
chr21 8390782
chr21 8390784
chr21 8390796
chr21 8390799
chr21 8390801
chr21 8390806
chr21 8390814
chr21 8390818
chr21 8390824
chr21 8390829
chr21 8390834
chr21 8390840
chr21 8390846
chr21 8390849
chr21 8390853
chr21 8390855
chr21 8390861
chr21 8391177
chr21 8391183
chr21 8391186
chr21 8391194
chr21 8391202
chr21 8391218
chr21 8391220
chr21 8391227
chr21 8391234
chr21 8391238
chr21 8391256
chr21 8391260
chr21 8391263
chr21 8391267
chr21 8391270
chr21 8391288
chr21 8391292
chr21 8391300
chr21 8391308
chr21 8391312
chr21 8391324
chr21 8391334
chr21 8391340
chr21 8391346
chr21 8391351
chr21 8391358
chr21 8391361
chr21 8391364
chr21 8391436
chr21 8391445
chr21 8391457
chr21 8391467

chr21 8391474
chr21 8391481
chr21 8391484
chr21 8391486
chr21 8391490
chr21 8391502
chr21 8391508
chr21 8391516
chr21 8391520
chr21 8391522
chr21 8391528
chr21 8391531
chr21 8391534
chr21 8391550
chr21 8391552
chr21 8391559
chr21 8391562
chr21 8391569
chr21 8391573
chr21 8391577
chr21 8391582
chr21 8391589
chr21 8391591
chr21 8391593
chr21 8391599
chr21 8391602
chr21 8391605
chr21 8391608
chr21 8391619
chr21 8391621
chr21 8391623
chr21 8391632
chr21 8391638
chr21 8391642
chr21 8391646
chr21 8391648
chr21 8391652
chr21 8391661
chr21 8391665
chr21 8391678
chr21 8391681
chr21 8391685
chr21 8391694
chr21 8391696
chr21 8391705
chr21 8391708
chr21 8391713
chr21 8391718
chr21 8391721
chr21 8391731
chr21 8391776
chr21 8391890
chr21 8391896
chr21 8391901

chr21 8391906
chr21 8391919
chr21 8391927
chr21 8391931
chr21 8391935
chr21 8391938
chr21 8391945
chr21 8391952
chr21 8391959
chr21 8391967
chr21 8391971
chr21 8391975
chr21 8392060
chr21 8392070
chr21 8392074
chr21 8392098
chr21 8392157
chr21 8392190
chr21 8392212
chr21 8392217
chr21 8392224
chr21 8392226
chr21 8392231
chr21 8392238
chr21 8392242
chr21 8392244
chr21 8392248
chr21 8392252
chr21 8392255
chr21 8392307
chr21 8392314
chr21 8392316
chr21 8392318
chr21 8392320
chr21 8392322
chr21 8392328
chr21 8392331
chr21 8392341
chr21 8392345
chr21 8392348
chr21 8392352
chr21 8392354
chr21 8392359
chr21 8392364
chr21 8392368
chr21 8392376
chr21 8392378
chr21 8392382
chr21 8392386
chr21 8392388
chr21 8392391
chr21 8392399
chr21 8392402
chr21 8392404

chr21 8392415
chr21 8392417
chr21 8392425
chr21 8392432
chr21 8392437
chr21 8392439
chr21 8392448
chr21 8392461
chr21 8392465
chr21 8392470
chr21 8392473
chr21 8392475
chr21 8392480
chr21 8392485
chr21 8392494
chr21 8392500
chr21 8392504
chr21 8392507
chr21 8392513
chr21 8392515
chr21 8392519
chr21 8392523
chr21 8392533
chr21 8392537
chr21 8392541
chr21 8392545
chr21 8392547
chr21 8392552
chr21 8392556
chr21 8392560
chr21 8392564
chr21 8392568
chr21 8392571
chr21 8392580
chr21 8392586
chr21 8392591
chr21 8392594
chr21 8392600
chr21 8392606
chr21 8392608
chr21 8392618
chr21 8392622
chr21 8392626
chr21 8392630
chr21 8392634
chr21 8392636
chr21 8392639
chr21 8392647
chr21 8392650
chr21 8392652
chr21 8392790
chr21 8392794
chr21 8392844
chr21 8392847

chr21 8392851
chr21 8392864
chr21 8392866
chr21 8392877
chr21 8392904
chr21 8392919
chr21 8392925
chr21 8392929
chr21 8392936
chr21 8392940
chr21 8392943
chr21 8392946
chr21 8392971
chr21 8392976
chr21 8392980
chr21 8392984
chr21 8392991
chr21 8393115
chr21 8393144
chr21 8393146
chr21 8393163
chr21 8393168
chr21 8393182
chr21 8393360
chr21 8393364
chr21 8393373
chr21 8393376
chr21 8393378
chr21 8393383
chr21 8393391
chr21 8393395
chr21 8393401
chr21 8393415
chr21 8393418
chr21 8393426
chr21 8393542
chr21 8393544
chr21 8393565
chr21 8393592
chr21 8393596
chr21 8393606
chr21 8393613
chr21 8393616
chr21 8393639
chr21 8393642
chr21 8393649
chr21 8393659
chr21 8393790
chr21 8393793
chr21 8393828
chr21 8393870
chr21 8393899
chr21 8394521
chr21 8394536

chr21 8394543
chr21 8394550
chr21 8394557
chr21 8394559
chr21 8394562
chr21 8394565
chr21 8394568
chr21 8394571
chr21 8394574
chr21 8394576
chr21 8394578
chr21 8394588
chr21 8394607
chr21 8394609
chr21 8394612
chr21 8394614
chr21 8394617
chr21 8394619
chr21 8394623
chr21 8394625
chr21 8394629
chr21 8394636
chr21 8394638
chr21 8394644
chr21 8394648
chr21 8394652
chr21 8394656
chr21 8394660
chr21 8394664
chr21 8394668
chr21 8394671
chr21 8394675
chr21 8394681
chr21 8394684
chr21 8394688
chr21 8394690
chr21 8394697
chr21 8394706
chr21 8394717
chr21 8394852
chr21 8394859
chr21 8394862
chr21 8394865
chr21 8394868
chr21 8394870
chr21 8394873
chr21 8394888
chr21 8394903
chr21 8394906
chr21 8394913
chr21 8394916
chr21 8394920
chr21 8394926
chr21 8394929

chr21 8394931
chr21 8394934
chr21 8394942
chr21 8394948
chr21 8394969
chr21 8394975
chr21 8394978
chr21 8394980
chr21 8394983
chr21 8394988
chr21 8394990
chr21 8394993
chr21 8394996
chr21 8395002
chr21 8395008
chr21 8395012
chr21 8395021
chr21 8395030
chr21 8395038
chr21 8395043
chr21 8395050
chr21 8395057
chr21 8395061
chr21 8395064
chr21 8395077
chr21 8395085
chr21 8395089
chr21 8395097
chr21 8395100
chr21 8395115
chr21 8395117
chr21 8395122
chr21 8395125
chr21 8395127
chr21 8395229
chr21 8395231
chr21 8395240
chr21 8395242
chr21 8395249
chr21 8395256
chr21 8395260
chr21 8395266
chr21 8395268
chr21 8395271
chr21 8395274
chr21 8395277
chr21 8395280
chr21 8395283
chr21 8395285
chr21 8395288
chr21 8395292
chr21 8395366
chr21 8395376
chr21 8395380

chr21 8395398
chr21 8395400
chr21 8395404
chr21 8395410
chr21 8395412
chr21 8395418
chr21 8395421
chr21 8395423
chr21 8395425
chr21 8395433
chr21 8395461
chr21 8395468
chr21 8395474
chr21 8395486
chr21 8395530
chr21 8395538
chr21 8395543
chr21 8395545
chr21 8395573
chr21 8395576
chr21 8395582
chr21 8395588
chr21 8395848
chr21 8395860
chr21 8395868
chr21 8395871
chr21 8395874
chr21 8395878
chr21 8395892
chr21 8395895
chr21 8395897
chr21 8395901
chr21 8395920
chr21 8395922
chr21 8395931
chr21 8395939
chr21 8395944
chr21 8395951
chr21 8395968
chr21 8395974
chr21 8395987
chr21 8395996
chr21 8395999
chr21 8396002
chr21 8396005
chr21 8396008
chr21 8396012
chr21 8396014
chr21 8396020
chr21 8396045
chr21 8396050
chr21 8396058
chr21 8396091
chr21 8396104

chr21 8396106
chr21 8396109
chr21 8396111
chr21 8396113
chr21 8396115
chr21 8396120
chr21 8396127
chr21 8396134
chr21 8396137
chr21 8396143
chr21 8396147
chr21 8396161
chr21 8396167
chr21 8396170
chr21 8396173
chr21 8396176
chr21 8396179
chr21 8396182
chr21 8396184
chr21 8396186
chr21 8396189
chr21 8396192
chr21 8396195
chr21 8396204
chr21 8396211
chr21 8396215
chr21 8396242
chr21 8396248
chr21 8396265
chr21 8396268
chr21 8396282
chr21 8396290
chr21 8396292
chr21 8396297
chr21 8396301
chr21 8396304
chr21 8396306
chr21 8396311
chr21 8396314
chr21 8396318
chr21 8396324
chr21 8396330
chr21 8396334
chr21 8396337
chr21 8396341
chr21 8396344
chr21 8396348
chr21 8396351
chr21 8396357
chr21 8396359
chr21 8396376
chr21 8396379
chr21 8396390
chr21 8396398

chr21 8396412
chr21 8396421
chr21 8396423
chr21 8396425
chr21 8396429
chr21 8396431
chr21 8396433
chr21 8396437
chr21 8396441
chr21 8396445
chr21 8396450
chr21 8396455
chr21 8396457
chr21 8396460
chr21 8396462
chr21 8396465
chr21 8396469
chr21 8396475
chr21 8396483
chr21 8396488
chr21 8396490
chr21 8396675
chr21 8396681
chr21 8396689
chr21 8396696
chr21 8396698
chr21 8396707
chr21 8396712
chr21 8396717
chr21 8396721
chr21 8396724
chr21 8396728
chr21 8396733
chr21 8396739
chr21 8396744
chr21 8396758
chr21 8396762
chr21 8396767
chr21 8396772
chr21 8396775
chr21 8396780
chr21 8396783
chr21 8396785
chr21 8396797
chr21 8396801
chr21 8396803
chr21 8396816
chr21 8396819
chr21 8396832
chr21 8396835
chr21 8396841
chr21 8396848
chr21 8396851
chr21 8396856

chr21 8396859
chr21 8396902
chr21 8396906
chr21 8396911
chr21 8396921
chr21 8396926
chr21 8396928
chr21 8396943
chr21 8396948
chr21 8396953
chr21 8396978
chr21 8397014
chr21 8397017
chr21 8397040
chr21 8397043
chr21 8397051
chr21 8397056
chr21 8397058
chr21 8397061
chr21 8397066
chr21 8397068
chr21 8397080
chr21 8397084
chr21 8397093
chr21 8397100
chr21 8397103
chr21 8397106
chr21 8397110
chr21 8397135
chr21 8397147
chr21 8397265
chr21 8397270
chr21 8397276
chr21 8397293
chr21 8397339
chr21 8397347
chr21 8397361
chr21 8397372
chr21 8397374
chr21 8397381
chr21 8397442
chr21 8397449
chr21 8397458
chr21 8397471
chr21 8397486
chr21 8397489
chr21 8397508
chr21 8397576
chr21 8397583
chr21 8397587
chr21 8397590
chr21 8397594
chr21 8397619
chr21 8397621

chr21 8397624
chr21 8397632
chr21 8397634
chr21 8397638
chr21 8397693
chr21 8397697
chr21 8397701
chr21 8397705
chr21 8397720
chr21 8397726
chr21 8397731
chr21 8397737
chr21 8397739
chr21 8397749
chr21 8397757
chr21 8397759
chr21 8397761
chr21 8397766
chr21 8397771
chr21 8397774
chr21 8397777
chr21 8397780
chr21 8397786
chr21 8397789
chr21 8397792
chr21 8397795
chr21 8397798
chr21 8397801
chr21 8397804
chr21 8397810
chr21 8397840
chr21 8397853
chr21 8397856
chr21 8397863
chr21 8397869
chr21 8397877
chr21 8397884
chr21 8397887
chr21 8397890
chr21 8397892
chr21 8397907
chr21 8397910
chr21 8397920
chr21 8398402
chr21 8398417
chr21 8398421
chr21 8398425
chr21 8398437
chr21 8398467
chr21 8398544
chr21 8398556
chr21 8398582
chr21 8398606
chr21 8398612

chr21 8398620
chr21 8398623
chr21 8398631
chr21 8398636
chr21 8398648
chr21 8398656
chr21 8398679
chr21 8398684
chr21 8398725
chr21 8398730
chr21 8398736
chr21 8398744
chr21 8398748
chr21 8398887
chr21 8398903
chr21 8398910
chr21 8398939
chr21 8398967
chr21 8398975
chr21 8398978
chr21 8398990
chr21 8398994
chr21 8398997
chr21 8399000
chr21 8399007
chr21 8399017
chr21 8399023
chr21 8399026
chr21 8399032
chr21 8399035
chr21 8399037
chr21 8399039
chr21 8399041
chr21 8399043
chr21 8399045
chr21 8399047
chr21 8399049
chr21 8399060
chr21 8399063
chr21 8399070
chr21 8399073
chr21 8399076
chr21 8399079
chr21 8399082
chr21 8399105
chr21 8399118
chr21 8399136
chr21 8399142
chr21 8399147
chr21 8399154
chr21 8399160
chr21 8399168
chr21 8399170
chr21 8399174

chr21 8399179
chr21 8399182
chr21 8399235
chr21 8399245
chr21 8399248
chr21 8399287
chr21 8399303
chr21 8399351
chr21 8399368
chr21 8399372
chr21 8399458
chr21 8399468
chr21 8399471
chr21 8399473
chr21 8399478
chr21 8399487
chr21 8399493
chr21 8399495
chr21 8399499
chr21 8399505
chr21 8399514
chr21 8399517
chr21 8399526
chr21 8399561
chr21 8399577
chr21 8399582
chr21 8399595
chr21 8399609
chr21 8399612
chr21 8399614
chr21 8399620
chr21 8399623
chr21 8399626
chr21 8399630
chr21 8399641
chr21 8399659
chr21 8399680
chr21 8399682
chr21 8399685
chr21 8399690
chr21 8399702
chr21 8399763
chr21 8399771
chr21 8399785
chr21 8399815
chr21 8399819
chr21 8399950
chr21 8399955
chr21 8399957
chr21 8399981
chr21 8399988
chr21 8400004
chr21 8400012
chr21 8400015

chr21 8400017
chr21 8400025
chr21 8400028
chr21 8400031
chr21 8400046
chr21 8400051
chr21 8400061
chr21 8400064
chr21 8400067
chr21 8400072
chr21 8400075
chr21 8400077
chr21 8403042
chr21 8403058
chr21 8403112
chr21 8403118
chr21 8403252
chr21 8403270
chr21 8403278
chr21 8403281
chr21 8403288
chr21 8403501
chr21 8403508
chr21 8403510
chr21 8403513
chr21 8403516
chr21 8403526
chr21 8403532
chr21 8403537
chr21 8403539
chr21 8403556
chr21 8403558
chr21 8403760
chr21 8403766
chr21 8403774
chr21 8403786
chr21 8403790
chr21 8403844
chr21 8403850
chr21 8403984
chr21 8404002
chr21 8404010
chr21 8404013
chr21 8404218
chr21 8404224
chr21 8404227
chr21 8404233
chr21 8404240
chr21 8404242
chr21 8404245
chr21 8404248
chr21 8404258
chr21 8404264
chr21 8404269

chr21 8404271
chr21 8404288
chr21 8404290
chr21 8409744
chr21 8409754
chr21 8409760
chr21 8409762
chr21 8432100
chr21 8432123
chr21 8432125
chr21 8432128
chr21 8432151
chr21 8432153
chr21 8432381
chr21 8432394
chr21 8432400
chr21 8432422
chr21 8432433
chr21 8432530
chr21 8432535
chr21 8432547
chr21 8432549
chr21 8432552
chr21 8432556
chr21 8432573
chr21 8432577
chr21 8432584
chr21 8432586
chr21 8432589
chr21 8432742
chr21 8432749
chr21 8432754
chr21 8432760
chr21 8432767
chr21 8432774
chr21 8432781
chr21 8432788
chr21 8432793
chr21 8432798
chr21 8432800
chr21 8432812
chr21 8432814
chr21 8432827
chr21 8433366
chr21 8433368
chr21 8433370
chr21 8433374
chr21 8433378
chr21 8433383
chr21 8433390
chr21 8433396
chr21 8433401
chr21 8433407
chr21 8433424

chr21 8433430
chr21 8433433
chr21 8433442
chr21 8433445
chr21 8433455
chr21 8433463
chr21 8433467
chr21 8433474
chr21 8433479
chr21 8433483
chr21 8433487
chr21 8433492
chr21 8433496
chr21 8433498
chr21 8433512
chr21 8433521
chr21 8433526
chr21 8433530
chr21 8433539
chr21 8433545
chr21 8433551
chr21 8433563
chr21 8433566
chr21 8433594
chr21 8436737
chr21 8436747
chr21 8436751
chr21 8436755
chr21 8436759
chr21 8436761
chr21 8436766
chr21 8436770
chr21 8436774
chr21 8436778
chr21 8436781
chr21 8436790
chr21 8436796
chr21 8436801
chr21 8436804
chr21 8436810
chr21 8436816
chr21 8436818
chr21 8436828
chr21 8436832
chr21 8436836
chr21 8436840
chr21 8436844
chr21 8436846
chr21 8436849
chr21 8436857
chr21 8436860
chr21 8436862
chr21 8436940
chr21 8436944

chr21 8436948
chr21 8436964
chr21 8437000
chr21 8437004
chr21 8437054
chr21 8437057
chr21 8437061
chr21 8437074
chr21 8437076
chr21 8437087
chr21 8437244
chr21 8437254
chr21 8437257
chr21 8437277
chr21 8437284
chr21 8437299
chr21 8437305
chr21 8437325
chr21 8437354
chr21 8437356
chr21 8437373
chr21 8437378
chr21 8437392
chr21 8437418
chr21 8437551
chr21 8437570
chr21 8437574
chr21 8437578
chr21 8437583
chr21 8437586
chr21 8437588
chr21 8437593
chr21 8437601
chr21 8437605
chr21 8437611
chr21 8437625
chr21 8437628
chr21 8437636
chr21 8437658
chr21 8437660
chr21 8437752
chr21 8437754
chr21 8437775
chr21 8437802
chr21 8437806
chr21 8437816
chr21 8437823
chr21 8437869
chr21 8437907
chr21 8437914
chr21 8437922
chr21 8437928
chr21 8437939
chr21 8437942

chr21 8437950
chr21 8438038
chr21 8438080
chr21 8438658
chr21 8438669
chr21 8438673
chr21 8438969
chr21 8439050
chr21 8439053
chr21 8439057
chr21 8439059
chr21 8439065
chr21 8439072
chr21 8439075
chr21 8439078
chr21 8439081
chr21 8439083
chr21 8439086
chr21 8439103
chr21 8439118
chr21 8439121
chr21 8439128
chr21 8439131
chr21 8439135
chr21 8439141
chr21 8439144
chr21 8439146
chr21 8439149
chr21 8439157
chr21 8439163
chr21 8439169
chr21 8439173
chr21 8439181
chr21 8439184
chr21 8439190
chr21 8439193
chr21 8439195
chr21 8439198
chr21 8439203
chr21 8439205
chr21 8439208
chr21 8439211
chr21 8439217
chr21 8439223
chr21 8439227
chr21 8439236
chr21 8439245
chr21 8439253
chr21 8439258
chr21 8439265
chr21 8439272
chr21 8439276
chr21 8439279
chr21 8439292

chr21 8439300
chr21 8439304
chr21 8439312
chr21 8439315
chr21 8439330
chr21 8439332
chr21 8439337
chr21 8439340
chr21 8439342
chr21 8439423
chr21 8439434
chr21 8439444
chr21 8439446
chr21 8439455
chr21 8439457
chr21 8439464
chr21 8439471
chr21 8439475
chr21 8439481
chr21 8439483
chr21 8439648
chr21 8439676
chr21 8439683
chr21 8439689
chr21 8439701
chr21 8439709
chr21 8439866
chr21 8439878
chr21 8439914
chr21 8439922
chr21 8439926
chr21 8439934
chr21 8439940
chr21 8439951
chr21 8440160
chr21 8440167
chr21 8440184
chr21 8440190
chr21 8440203
chr21 8443522
chr21 8443538
chr21 8443586
chr21 8443693
chr21 8443703
chr21 8443706
chr21 8443708
chr21 8443713
chr21 8443722
chr21 8443728
chr21 8443730
chr21 8443734
chr21 8443740
chr21 8443749
chr21 8443752

chr21 8443761
chr21 8443796
chr21 8443812
chr21 8443817
chr21 8443830
chr21 8443844
chr21 8443847
chr21 8443849
chr21 8443855
chr21 8443858
chr21 8443861
chr21 8443865
chr21 8443876
chr21 8443894
chr21 8443915
chr21 8443917
chr21 8443920
chr21 8444083
chr21 8444085
chr21 8444088
chr21 8444090
chr21 8444098
chr21 8444103
chr21 8444120
chr21 8444124
chr21 8444138
chr21 8444140
chr21 8444151
chr21 8444161
chr21 8444163
chr21 8444165
chr21 8444167
chr21 8444170
chr21 8444174
chr21 8444185
chr21 8444190
chr21 8444192
chr21 8444216
chr21 8444223
chr21 8444239
chr21 8444247
chr21 8444250
chr21 8444252
chr21 8444260
chr21 8444263
chr21 8444266
chr21 8444281
chr21 8444286
chr21 8444296
chr21 8444299
chr21 8444302
chr21 8444307
chr21 8444310
chr21 8444312

chr21 8444320
chr21 8444323
chr21 8444326
chr21 8444334
chr21 8444508
chr21 8444510
chr21 8444514
chr21 8444523
chr21 8444530
chr21 8444534
chr21 8444538
chr21 8444541
chr21 8444544
chr21 8444548
chr21 8444551
chr21 8444555
chr21 8444558
chr21 8444563
chr21 8444568
chr21 8444573
chr21 8444581
chr21 8444594
chr21 8444600
chr21 8444604
chr21 8444611
chr21 8444628
chr21 8444631
chr21 8444633
chr21 8444635
chr21 8444638
chr21 8444641
chr21 8445020
chr21 8445029
chr21 8445038
chr21 8445079
chr21 8445121
chr21 8445124
chr21 8445206
chr21 8445222
chr21 8445230
chr21 8445238
chr21 8445245
chr21 8445256
chr21 8445265
chr21 8445272
chr21 8445862
chr21 8445867
chr21 8445872
chr21 8445878
chr21 8445881
chr21 8445883
chr21 8445891
chr21 8445901
chr21 8445909

chr21 8445917
chr21 8445927
chr21 8445930
chr21 8445933
chr21 8445938
chr21 8445952
chr21 8445954
chr21 8445959
chr21 8445961
chr21 8445963
chr21 8445965
chr21 8446195
chr21 8446212
chr21 8446214
chr21 8446216
chr21 8446218
chr21 8446220
chr21 8446222
chr21 8446224
chr21 8446226
chr21 8446228
chr21 8446232
chr21 8446236
chr21 8446245
chr21 8446248
chr21 8446253
chr21 8446257
chr21 8446259
chr21 8446263
chr21 8446266
chr21 8446269
chr21 8446273
chr21 8446281
chr21 8446285
chr21 8446300
chr21 8446310
chr21 8446313
chr21 8446317
chr21 8446319
chr21 8446323
chr21 8446349
chr21 8446351
chr21 8446353
chr21 8446359
chr21 8446363
chr21 8446366
chr21 8446368
chr21 8446373
chr21 8446381
chr21 8446399
chr21 8446406
chr21 8446504
chr21 8446511
chr21 8446516

chr21 8446527
chr21 8446529
chr21 8446531
chr21 8446538
chr21 8446546
chr21 8446554
chr21 8446562
chr21 8446569
chr21 8446571
chr21 8446573
chr21 8446586
chr21 8446594
chr21 8446598
chr21 8446603
chr21 8446615
chr21 8446617
chr21 8446628
chr21 8446631
chr21 8446635
chr21 8446638
chr21 8446648
chr21 8446653
chr21 8446659
chr21 8446665
chr21 8446669
chr21 8446676
chr21 8447235
chr21 8447241
chr21 8447249
chr21 8447265
chr21 8447319
chr21 8447325
chr21 8447459
chr21 8447477
chr21 8447485
chr21 8447488
chr21 8447495
chr21 8447708
chr21 8447715
chr21 8447717
chr21 8447720
chr21 8447723
chr21 8447733
chr21 8447739
chr21 8447744
chr21 8447746
chr21 8447763
chr21 8447765
chr21 8447967
chr21 8447973
chr21 8447981
chr21 8447993
chr21 8447997
chr21 8448051

chr21 8448057
chr21 8448191
chr21 8448209
chr21 8448217
chr21 8448220
chr21 8448227
chr21 8448423
chr21 8448425
chr21 8448431
chr21 8448434
chr21 8448440
chr21 8448447
chr21 8448449
chr21 8448452
chr21 8448455
chr21 8448465
chr21 8448471
chr21 8448476
chr21 8448478
chr21 8448497
chr21 8448625
chr21 8522719
chr21 8522951
chr21 8523266
chr21 8534389
chr21 8534506
chr21 8542553
chr21 8542569
chr21 8542704
chr21 8542721
chr21 8542763
chr21 8543356
chr21 8543595
chr21 8544888
chr21 8545136
chr21 8545344
chr21 8545433
chr21 8545453
chr21 8545543
chr21 8545601
chr21 8545641
chr21 8545917
chr21 8545930
chr21 8546167
chr21 8546854
chr21 8553342
chr21 8553876
chr21 8554348
chr21 8554587
chr21 8555800
chr21 8583950
chr21 8583964
chr21 8583971
chr21 8584006

chr21 8584016
chr21 8584018
chr21 8584215
chr21 8584327
chr21 8591198
chr21 8599681
chr21 8599966
chr21 8599985
chr21 8600340
chr21 8600764
chr21 8602836
chr21 8603811
chr21 8604288
chr21 8605658
chr21 8605705
chr21 8605879
chr21 8606929
chr21 8606984
chr21 8608421
chr21 8608460
chr21 8608670
chr21 8609858
chr21 8610488
chr21 8610605
chr21 8611011
chr21 8611026
chr21 8611194
chr21 8611232
chr21 8648302
chr21 8648438
chr21 8648535
chr21 8648660
chr21 8649703
chr21 8986610
chr21 8986612
chr21 8986616
chr21 8986634
chr21 8986638
chr21 8986657
chr21 8986662
chr21 8986665
chr21 8986673
chr21 8986681
chr21 8986699
chr21 8986706
chr21 8986713
chr21 8986717
chr21 8986735
chr21 8986739
chr21 8986742
chr21 8986746
chr21 8986749
chr21 8986767
chr21 8986771

chr21 8986779
chr21 8986787
chr21 8986791
chr21 8986803
chr21 8986813
chr21 8986819
chr21 8986825
chr21 8986830
chr21 8986837
chr21 8986840
chr21 8986843
chr21 8986846
chr21 8986857
chr21 8986862
chr21 8986865
chr21 8986869
chr21 8986871
chr21 8986878
chr21 8986881
chr21 8986883
chr21 8986885
chr21 8986897
chr21 8986908
chr21 8986913
chr21 8986915
chr21 8986924
chr21 8986936
chr21 8986946
chr21 8986953
chr21 8986960
chr21 8986963
chr21 8986965
chr21 8986969
chr21 8986981
chr21 8986987
chr21 8986995
chr21 8986999
chr21 8987001
chr21 8987007
chr21 8987010
chr21 8987013
chr21 8987029
chr21 8987031
chr21 8987038
chr21 8987041
chr21 8987048
chr21 8987052
chr21 8987056
chr21 8987081
chr21 8987084
chr21 8987087
chr21 8987098
chr21 8987100
chr21 8987102

chr21 8987111
chr21 8987117
chr21 8987121
chr21 8987125
chr21 8987127
chr21 8987131
chr21 8987142
chr21 8987147
chr21 8987159
chr21 8987162
chr21 8987166
chr21 8987175
chr21 8987177
chr21 8987186
chr21 8987189
chr21 8987194
chr21 8987200
chr21 8987204
chr21 8987214
chr21 8987224
chr21 8987229
chr21 8987237
chr21 8987242
chr21 8987247
chr21 8987250
chr21 8987252
chr21 8987259
chr21 8987265
chr21 8987270
chr21 8987277
chr21 8987286
chr21 8987288
chr21 8987299
chr21 8987304
chr21 8987308
chr21 8987313
chr21 8987317
chr21 8987346
chr21 8987348
chr21 8987368
chr21 8987370
chr21 8987372
chr21 8987374
chr21 8987378
chr21 8987530
chr21 8987540
chr21 9830065
chr21 9830067
chr21 9830091
chr21 9830099
chr21 9830103
chr21 9830105
chr21 9830237
chr21 9830240

chr21 9840565
chr21 9840662
chr21 9841321
chr21 9841423
chr21 9841959
chr21 9842169
chr21 9918855
chr21 9919120
chr21 9919549
chr21 9945529
chr21 9946346
chr21 9946824
chr21 10036928
chr21 10037181
chr21 10037318
chr21 10037587
chr21 10037747
chr21 10038024
chr21 10038730
chr21 10042601
chr21 10043351
chr21 10043951
chr21 10114722
chr21 10116452
chr21 10116543
chr21 10116962
chr21 10492120
chr21 10492870
chr21 10492933
chr21 10493048
chr21 10494344
chr21 10494745
chr21 10495062
chr21 10495067
chr21 10495152
chr21 10495227
chr21 10495677
chr21 10507199
chr21 10507208
chr21 10507708
chr21 10508326
chr21 10508379
chr21 10508434
chr21 10508437
chr21 10508512
chr21 10508552
chr21 10508651
chr21 10618886
chr21 10618983
chr21 10619049
chr21 10619056
chr21 10619390
chr21 10619811
chr21 10625342

chr21 10625716
chr21 10625759
chr21 10625819
chr21 10626000
chr21 10628216
chr21 10628336
chr21 10628908
chr21 10870944
chr21 10870972
chr21 10871040
chr21 10871957
chr21 10874504
chr21 10874592
chr21 10875301
chr21 10875387
chr21 10887907
chr21 10889353
chr21 10889397
chr21 10889475
chr21 10889525
chr21 10889829
chr21 10890905
chr21 10893789
chr21 10898483
chr21 10899149
chr21 10899348
chr21 10899400
chr21 10899996
chr21 10900184
chr21 10901588
chr21 10903068
chr21 10903500
chr21 10903673
chr21 10903915
chr21 10905543
chr21 10905652
chr21 10905673
chr21 10906989
chr21 10907160
chr21 10907238
chr21 10908230
chr21 10908874
chr21 10910663
chr21 10911978
chr21 10912434
chr21 10915890
chr21 10916021
chr21 10916230
chr21 10918562
chr21 10919749
chr21 10920733
chr21 10921165
chr21 10921202
chr21 10921382

chr21 10922644
chr21 10923076
chr21 10923407
chr21 10923887
chr21 10924159
chr21 10924943
chr21 10929371
chr21 10930303
chr21 10931604
chr21 10932309
chr21 10932382
chr21 10932736
chr21 10932773
chr21 10934049
chr21 10937545
chr21 10938350
chr21 10939377
chr21 10940947
chr21 10941131
chr21 10941798
chr21 10942126
chr21 10942263
chr21 10942310
chr21 10943664
chr21 10943708
chr21 10943713
chr21 10944689
chr21 10945619
chr21 10948096
chr21 10948793
chr21 10950483
chr21 12896680
chr21 12897063
chr21 12899106
chr21 12899120
chr21 12899151
chr21 12899387
chr21 12899402
chr21 12899582
chr21 12899633
chr21 12899642
chr21 12900136
chr21 12900142
chr21 12900151
chr21 12900252
chr21 12900326
chr21 12900975
chr21 12900989
chr21 12901020
chr21 12901256
chr21 12901271
chr21 12901502
chr21 12902505
chr21 12902511

chr21 12902947
chr21 12902962
chr21 12904196
chr21 12904202
chr21 12906877
chr21 12906910
chr21 12906924
chr21 12906955
chr21 12907094
chr21 12907625
chr21 12907665
chr21 13001339
chr21 13001618
chr21 13002227
chr21 13033006
chr21 13033188
chr21 13046286
chr21 13047564
chr21 13047622
chr21 13047646
chr21 13048693
chr21 13096142
chr21 13096462
chr21 13096839
chr21 13097127
chr21 13097273
chr21 13120087
chr21 13137814
chr21 13194391
chr21 13199228
chr21 13199370
chr21 13199636
chr21 13200203
chr21 13200391
chr21 13200428
chr21 13200662
chr21 13201200
chr21 13201316
chr21 13348399
chr21 13348448
chr21 13358730
chr21 13358739
chr21 13358814
chr21 13382081
chr21 13382514
chr21 13382701
chr21 13383988
chr21 13384343
chr21 13384354
chr21 13384427
chr21 13384565
chr21 13384585
chr21 13384900
chr21 13385060

chr21 13399970
chr21 13400250
chr21 13400262
chr21 13401033
chr21 13401288
chr21 13401760
chr21 13401858
chr21 13402261
chr21 13406704
chr21 13407034
chr21 13568569
chr21 13568867
chr21 13569416
chr21 13569787
chr21 13570484
chr21 13570686
chr21 13574738
chr21 13575028
chr21 13575360
chr21 13611907
chr21 13612012
chr21 13612253
chr21 13612297
chr21 13612401
chr21 13612594
chr21 13613321
chr21 13613514
chr21 13613964
chr21 13615187
chr21 13615262
chr21 13615447
chr21 13615454
chr21 13615483
chr21 13655350
chr21 13655455
chr21 13655513
chr21 13656908
chr21 13656985
chr21 13657116
chr21 13667642
chr21 13668964
chr21 13669505
chr21 13669698
chr21 13669754
chr21 13669862
chr21 13670498
chr21 13672193
chr21 13685213
chr21 13686160
chr21 13686300
chr21 13686350
chr21 13699185
chr21 13699275
chr21 13703321

chr21 13703598
chr21 13704846
chr21 13704848
chr21 13704866
chr21 13705864
chr21 13705900
chr21 13705931
chr21 13828910
chr21 13829496
chr21 13939273
chr21 14027362
chr21 14027389
chr21 14027391
chr21 14027411
chr21 14027418
chr21 14027428
chr21 14027430
chr21 14027458
chr21 14027494
chr21 14027504
chr21 14027525
chr21 14027528
chr21 14027589
chr21 14027617
chr21 14027622
chr21 14027650
chr21 14027663
chr21 14027669
chr21 14027683
chr21 14027688
chr21 14027721
chr21 14027735
chr21 14027739
chr21 14027759
chr21 14027762
chr21 14027856
chr21 14027864
chr21 14027867
chr21 14027872
chr21 14027890
chr21 14027909
chr21 14027921
chr21 14028013
chr21 14127059
chr21 14127829
chr21 14164516
chr21 14216404
chr21 14216417
chr21 14216437
chr21 14216459
chr21 14216461
chr21 14216478
chr21 14216480
chr21 14216656

chr21 14280687
chr21 14382661
chr21 14382702
chr21 14382711
chr21 14382759
chr21 14382765
chr21 14382831
chr21 14382950
chr21 14382957
chr21 14382988
chr21 14383051
chr21 14383059
chr21 14383063
chr21 14383084
chr21 14383102
chr21 14383128
chr21 14383131
chr21 14383235
chr21 14383247
chr21 14383382
chr21 14383385
chr21 14383390
chr21 14383401
chr21 14383425
chr21 14383427
chr21 14383466
chr21 14383471
chr21 14383473
chr21 14383492
chr21 14383515
chr21 14383517
chr21 14383546
chr21 14383569
chr21 14383594
chr21 14383610
chr21 14383630
chr21 14383632
chr21 14383636
chr21 14383665
chr21 14383681
chr21 14383700
chr21 14383717
chr21 14383851
chr21 14482243
chr21 14482263
chr21 14482274
chr21 14482430
chr21 14526763
chr21 14526800
chr21 14527146
chr21 14527264
chr21 14527290
chr21 14527367
chr21 14527412

chr21 14527443
chr21 14527449
chr21 14527458
chr21 14527523
chr21 14527633
chr21 14542082
chr21 14542135
chr21 14542154
chr21 14543434
chr21 14602314
chr21 14762565
chr21 14762872
chr21 14763121
chr21 14763142
chr21 15034440
chr21 15034758
chr21 15066257
chr21 15066322
chr21 15066335
chr21 15066344
chr21 15066379
chr21 15066458
chr21 15731312
chr21 15731343
chr21 15731367
chr21 15731593
chr21 15731644
chr21 15731864
chr21 15732115
chr21 15732134
chr21 15732341
chr21 15732353
chr21 15732422
chr21 15732445
chr21 15732467
chr21 15732802
chr21 16064110
chr21 16307865
chr21 16307910
chr21 16308168
chr21 16308180
chr21 16308498
chr21 17089251
chr21 17089525
chr21 17294485
chr21 17514062
chr21 17514076
chr21 17514123
chr21 17514157
chr21 17514178
chr21 17514359
chr21 17586143
chr21 17611485
chr21 17611626

chr21 17611643
chr21 17780923
chr21 17784785
chr21 17784825
chr21 17784878
chr21 17817791
chr21 17817827
chr21 17817924
chr21 17818015
chr21 17818312
chr21 17818460
chr21 17819618
chr21 17819628
chr21 17819634
chr21 17819674
chr21 17820194
chr21 17820388
chr21 17820396
chr21 17979711
chr21 17979923
chr21 18520399
chr21 18859196
chr21 18859527
chr21 18859739
chr21 18859785
chr21 19018597
chr21 19018860
chr21 19018912
chr21 19019289
chr21 19019410
chr21 19019886
chr21 19020177
chr21 19129773
chr21 19164986
chr21 19284302
chr21 19353237
chr21 19353426
chr21 19353474
chr21 19353905
chr21 19353909
chr21 19353949
chr21 19898524
chr21 19898807
chr21 20001000
chr21 20001135
chr21 20001163
chr21 20047189
chr21 20047298
chr21 20059034
chr21 20145107
chr21 20145338
chr21 20145766
chr21 20256044
chr21 20256047

chr21 20256413
chr21 20256662
chr21 20256685
chr21 20256889
chr21 20541774
chr21 20541795
chr21 20542319
chr21 20542370
chr21 20542512
chr21 20917074
chr21 21176006
chr21 21177190
chr21 21177502
chr21 22421422
chr21 22421638
chr21 22421689
chr21 22421740
chr21 22575008
chr21 22575130
chr21 22575202
chr21 22575206
chr21 22575275
chr21 22575351
chr21 22575478
chr21 22595578
chr21 23066439
chr21 23066447
chr21 23067280
chr21 23067940
chr21 23068022
chr21 23068188
chr21 23105901
chr21 23388066
chr21 23388579
chr21 23453261
chr21 23453321
chr21 23453660
chr21 23454049
chr21 23454242
chr21 23551472
chr21 23551759
chr21 23552002
chr21 23552301
chr21 23572503
chr21 23619017
chr21 23619050
chr21 23657626
chr21 23657690
chr21 23862286
chr21 23862330
chr21 23862488
chr21 23921980
chr21 23922012
chr21 23977501

chr21 23978090
chr21 23978136
chr21 24128024
chr21 24129032
chr21 24129089
chr21 24207120
chr21 24250800
chr21 24250847
chr21 24275869
chr21 24276928
chr21 24429197
chr21 24429459
chr21 24648078
chr21 24648434
chr21 25054716
chr21 25054864
chr21 25088843
chr21 25089233
chr21 25361249
chr21 25363244
chr21 25363306
chr21 25363315
chr21 25363416
chr21 25363465
chr21 25363521
chr21 25363539
chr21 25366017
chr21 25385444
chr21 25385489
chr21 25385644
chr21 25385940
chr21 25385961
chr21 25396444
chr21 25396527
chr21 25396561
chr21 25396576
chr21 25396802
chr21 25397203
chr21 25563577
chr21 25563597
chr21 25563997
chr21 25564480
chr21 25564529
chr21 25564797
chr21 25572888
chr21 25572939
chr21 25573300
chr21 25573322
chr21 25573597
chr21 25573604
chr21 25573648
chr21 25573855
chr21 25573994
chr21 25574089

chr21 25607903
chr21 25607937
chr21 25607939
chr21 25607962
chr21 25607979
chr21 25608001
chr21 25608034
chr21 25608041
chr21 25608064
chr21 25608141
chr21 25608183
chr21 25608202
chr21 25608211
chr21 25608302
chr21 25608395
chr21 25608443
chr21 25608446
chr21 25608465
chr21 25608490
chr21 25608495
chr21 25608503
chr21 25608507
chr21 25608544
chr21 25638827
chr21 25638916
chr21 25639150
chr21 25639156
chr21 25717536
chr21 25736220
chr21 25736225
chr21 25736324
chr21 25736361
chr21 25736373
chr21 25736378
chr21 25736394
chr21 25736425
chr21 25736519
chr21 25736644
chr21 25801713
chr21 25801715
chr21 25801745
chr21 25801804
chr21 25801831
chr21 25922943
chr21 25923000
chr21 25923315
chr21 25923332
chr21 25924124
chr21 26168960
chr21 26169021
chr21 26169068
chr21 26169075
chr21 26169167
chr21 26169182

chr21 26320320
chr21 26320406
chr21 26571776
chr21 26572053
chr21 26572080
chr21 26572097
chr21 26572179
chr21 26572267
chr21 26572292
chr21 26572353
chr21 26572460
chr21 26572610
chr21 26572692
chr21 26572694
chr21 26572698
chr21 26572710
chr21 26572712
chr21 26572723
chr21 26572726
chr21 26572731
chr21 26572733
chr21 26769721
chr21 26769773
chr21 26847115
chr21 26847119
chr21 26847182
chr21 26847202
chr21 26847205
chr21 26847240
chr21 26847305
chr21 26847311
chr21 26847318
chr21 26847354
chr21 27498739
chr21 27498782
chr21 27499733
chr21 27499859
chr21 27500235
chr21 27580600
chr21 27580681
chr21 27580815
chr21 27722800
chr21 28056868
chr21 28056965
chr21 28057022
chr21 28057058
chr21 28739274
chr21 28745038
chr21 28745148
chr21 28745243
chr21 28745294
chr21 28745300
chr21 28745372
chr21 28745440

chr21 28745444
chr21 28745467
chr21 28745533
chr21 28745547
chr21 28831878
chr21 28884547
chr21 28884677
chr21 28991968
chr21 28991999
chr21 28992032
chr21 28992152
chr21 28992186
chr21 28992196
chr21 28992381
chr21 28992437
chr21 28992440
chr21 28992459
chr21 28992574
chr21 28992870
chr21 28992880
chr21 28992959
chr21 28992965
chr21 28993144
chr21 28993264
chr21 28993286
chr21 29019062
chr21 29019065
chr21 29019072
chr21 29019077
chr21 29019081
chr21 29019088
chr21 29019107
chr21 29019113
chr21 29019121
chr21 29019127
chr21 29019150
chr21 29019158
chr21 29019171
chr21 29019176
chr21 29019189
chr21 29019201
chr21 29019218
chr21 29019242
chr21 29025336
chr21 29025377
chr21 29025398
chr21 29025412
chr21 29025538
chr21 29025588
chr21 29072314
chr21 29072435
chr21 29072582
chr21 29072647
chr21 29072654

chr21 29072796
chr21 29072804
chr21 29072920
chr21 29072982
chr21 29073002
chr21 29073032
chr21 29074243
chr21 29074284
chr21 29074438
chr21 29196129
chr21 29196185
chr21 29196304
chr21 29196363
chr21 29196369
chr21 29196443
chr21 29196460
chr21 29196592
chr21 29196676
chr21 29196813
chr21 29299793
chr21 29299808
chr21 29300025
chr21 29300061
chr21 29300174
chr21 29348244
chr21 29348528
chr21 29476564
chr21 29476572
chr21 29476638
chr21 29476644
chr21 29476657
chr21 29476662
chr21 29476811
chr21 29476813
chr21 29656824
chr21 29656838
chr21 29656941
chr21 29657065
chr21 30213392
chr21 30214088
chr21 30463922
chr21 30464058
chr21 30464112
chr21 30465311
chr21 30465342
chr21 30465382
chr21 30504662
chr21 30504754
chr21 30504860
chr21 30527908
chr21 30528519
chr21 30819053
chr21 30819161
chr21 30819174

chr21 30819455
chr21 30819463
chr21 30819808
chr21 30839964
chr21 30840020
chr21 30840844
chr21 30840854
chr21 30840877
chr21 31195405
chr21 31195438
chr21 31195484
chr21 31195766
chr21 31195807
chr21 31196112
chr21 31196173
chr21 31198409
chr21 31198610
chr21 31268724
chr21 31268736
chr21 31326629
chr21 31326644
chr21 31326675
chr21 31326757
chr21 31326886
chr21 31326921
chr21 31326959
chr21 31327008
chr21 31327012
chr21 31327014
chr21 31327026
chr21 31354622
chr21 31354645
chr21 31354716
chr21 31354845
chr21 31354891
chr21 31355195
chr21 31426689
chr21 31660327
chr21 31660558
chr21 31660675
chr21 31660762
chr21 31660776
chr21 31660801
chr21 31660840
chr21 31660865
chr21 31660890
chr21 31730560
chr21 31730569
chr21 31730587
chr21 31730729
chr21 31730815
chr21 31730866
chr21 31730879
chr21 31730895

chr21 31730907
chr21 31730931
chr21 31730945
chr21 31730955
chr21 31730960
chr21 31768920
chr21 31815711
chr21 31816622
chr21 31889090
chr21 31889123
chr21 31889141
chr21 31889210
chr21 31889254
chr21 31889519
chr21 31974928
chr21 32241257
chr21 32538802
chr21 32569214
chr21 32569223
chr21 32569266
chr21 32569280
chr21 32569372
chr21 32569427
chr21 32569455
chr21 32569457
chr21 32569473
chr21 32569513
chr21 32569516
chr21 32569527
chr21 32569530
chr21 32569536
chr21 32569539
chr21 32569553
chr21 32624756
chr21 32726684
chr21 32726781
chr21 32770017
chr21 32770199
chr21 32770276
chr21 32770282
chr21 32770536
chr21 32770577
chr21 32770723
chr21 32770728
chr21 32770753
chr21 32770811
chr21 32770901
chr21 32770914
chr21 32772154
chr21 32772160
chr21 32772168
chr21 32772170
chr21 32772175
chr21 32772196

chr21 32772245
chr21 32772252
chr21 32772514
chr21 32772556
chr21 32772690
chr21 32772704
chr21 32772732
chr21 32772751
chr21 32772772
chr21 32772792
chr21 32772812
chr21 32772831
chr21 32772849
chr21 32824404
chr21 32929768
chr21 32929795
chr21 33205358
chr21 33205450
chr21 33231095
chr21 33231275
chr21 33231328
chr21 33231575
chr21 33231705
chr21 33231772
chr21 33231780
chr21 33231782
chr21 33231844
chr21 33231886
chr21 33231898
chr21 33232239
chr21 33232456
chr21 33232493
chr21 33232853
chr21 33244110
chr21 33324125
chr21 33324171
chr21 33362096
chr21 33374758
chr21 33374965
chr21 33375015
chr21 33383656
chr21 33383710
chr21 33383785
chr21 33384846
chr21 33384869
chr21 33384901
chr21 33384903
chr21 33384906
chr21 33385009
chr21 33385052
chr21 33492045
chr21 33544908
chr21 33544959
chr21 33545081

chr21 33545510
chr21 33589433
chr21 33589455
chr21 33820987
chr21 33821090
chr21 33924176
chr21 33924181
chr21 33931129
chr21 33931166
chr21 33931187
chr21 33931234
chr21 33931242
chr21 33931248
chr21 33931260
chr21 33931284
chr21 33931291
chr21 33931294
chr21 33931307
chr21 33931310
chr21 33931318
chr21 33931327
chr21 33931342
chr21 33931356
chr21 33931360
chr21 33931380
chr21 33931904
chr21 33931908
chr21 33931931
chr21 33932086
chr21 33932096
chr21 33932103
chr21 33932110
chr21 33932338
chr21 33932356
chr21 33932469
chr21 33932548
chr21 33947986
chr21 33948037
chr21 33948043
chr21 33948122
chr21 33948239
chr21 33948258
chr21 33948292
chr21 33948295
chr21 33948311
chr21 33948363
chr21 33948373
chr21 33948457
chr21 33948502
chr21 33948514
chr21 33948553
chr21 33948585
chr21 33948694
chr21 33948802

chr21 33948851
chr21 33976324
chr21 33976334
chr21 33976424
chr21 33976463
chr21 33976581
chr21 33976590
chr21 33976621
chr21 33976657
chr21 33976741
chr21 33976811
chr21 33976827
chr21 34032224
chr21 34032289
chr21 34362194
chr21 34362212
chr21 34362239
chr21 34379914
chr21 34381813
chr21 34381821
chr21 34381886
chr21 34381942
chr21 34381972
chr21 34382682
chr21 34383011
chr21 34383020
chr21 34383027
chr21 34383033
chr21 34383051
chr21 34383112
chr21 34418095
chr21 34418331
chr21 34418449
chr21 34420920
chr21 34424593
chr21 34425495
chr21 34425570
chr21 34427258
chr21 34427271
chr21 34431955
chr21 34431975
chr21 34433086
chr21 34433131
chr21 34433157
chr21 34433169
chr21 34433173
chr21 34433197
chr21 34433202
chr21 34433227
chr21 34433247
chr21 34433258
chr21 34433326
chr21 34433350
chr21 34433358

chr21 34433382
chr21 34433390
chr21 34434996
chr21 34435053
chr21 34435338
chr21 34435574
chr21 34436016
chr21 34436037
chr21 34436255
chr21 34437623
chr21 34437683
chr21 34437714
chr21 34437752
chr21 34437813
chr21 34438445
chr21 34438476
chr21 34438518
chr21 34438548
chr21 34438626
chr21 34440803
chr21 34442940
chr21 34526643
chr21 34526677
chr21 34526771
chr21 34526784
chr21 34526999
chr21 34527150
chr21 34527157
chr21 34527161
chr21 34527170
chr21 34527287
chr21 34527586
chr21 34974202
chr21 34974239
chr21 34974347
chr21 34974353
chr21 34974389
chr21 34974395
chr21 34983266
chr21 34984487
chr21 34984500
chr21 35027655
chr21 35027806
chr21 35045072
chr21 35045117
chr21 35045140
chr21 35045465
chr21 35045620
chr21 35045761
chr21 35045769
chr21 35045858
chr21 35045881
chr21 35045887
chr21 35045917

chr21 35046007
chr21 35046012
chr21 35046052
chr21 35046091
chr21 35046105
chr21 35046115
chr21 35046144
chr21 35046173
chr21 35046209
chr21 35046261
chr21 35046437
chr21 35046451
chr21 35046469
chr21 35046499
chr21 35046513
chr21 35046616
chr21 35046650
chr21 35046878
chr21 35046906
chr21 35046919
chr21 35046940
chr21 35046962
chr21 35046987
chr21 35047035
chr21 35047056
chr21 35047181
chr21 35047299
chr21 35047323
chr21 35047348
chr21 35047351
chr21 35049644
chr21 35049658
chr21 35049666
chr21 35049711
chr21 35049734
chr21 35049815
chr21 35050889
chr21 35050910
chr21 35051071
chr21 35051105
chr21 35051245
chr21 35051359
chr21 35051377
chr21 35051389
chr21 35051440
chr21 35051453
chr21 35051466
chr21 35051481
chr21 35051488
chr21 35051501
chr21 35139266
chr21 35222728
chr21 35222814
chr21 35222877

chr21 35234763
chr21 35234804
chr21 35234844
chr21 35234849
chr21 35235021
chr21 35497858
chr21 35497860
chr21 35497893
chr21 35497903
chr21 35498170
chr21 35498219
chr21 35498259
chr21 35498277
chr21 35498303
chr21 35511495
chr21 35528630
chr21 35528640
chr21 35528646
chr21 35528674
chr21 35528686
chr21 35528691
chr21 35528735
chr21 35528839
chr21 35528841
chr21 35528859
chr21 35528876
chr21 35528920
chr21 35528948
chr21 35528959
chr21 35528966
chr21 35529025
chr21 35529034
chr21 35529062
chr21 35529078
chr21 35529094
chr21 35529140
chr21 35529158
chr21 35529163
chr21 35529325
chr21 35529460
chr21 35529496
chr21 35631353
chr21 35631446
chr21 36069806
chr21 36113698
chr21 36113714
chr21 36113745
chr21 36113751
chr21 36113760
chr21 36113798
chr21 36113806
chr21 36113846
chr21 36113852
chr21 36113898

chr21 36113901
chr21 36113943
chr21 36114041
chr21 36114058
chr21 36130293
chr21 36130318
chr21 36130321
chr21 36130351
chr21 36130368
chr21 36130374
chr21 36130460
chr21 36130473
chr21 36130498
chr21 36141752
chr21 36298251
chr21 36298273
chr21 36298326
chr21 36298459
chr21 36298470
chr21 36321619
chr21 36321748
chr21 36321792
chr21 36321842
chr21 36321935
chr21 36430255
chr21 36430267
chr21 36430297
chr21 36430334
chr21 36430340
chr21 36430417
chr21 36710882
chr21 36711033
chr21 36711049
chr21 36711053
chr21 36711074
chr21 36711102
chr21 36711286
chr21 36711431
chr21 36712047
chr21 36712065
chr21 37009503
chr21 37009679
chr21 37009687
chr21 37009741
chr21 37037703
chr21 37257709
chr21 37257713
chr21 37257752
chr21 37257767
chr21 37257777
chr21 37257802
chr21 37257805
chr21 37257818
chr21 37257821

chr21 37257853
chr21 37257872
chr21 37257888
chr21 37257895
chr21 37257903
chr21 37257933
chr21 37257944
chr21 37257950
chr21 37257964
chr21 37257969
chr21 37257985
chr21 37257987
chr21 37258011
chr21 37258026
chr21 37258041
chr21 37258093
chr21 37258106
chr21 37258136
chr21 37258149
chr21 37258183
chr21 37258187
chr21 37258193
chr21 37258198
chr21 37258201
chr21 37258205
chr21 37258217
chr21 37258223
chr21 37258258
chr21 37258260
chr21 37258277
chr21 37258287
chr21 37258303
chr21 37258314
chr21 37258320
chr21 37258341
chr21 37258350
chr21 37258381
chr21 37258397
chr21 37258405
chr21 37258418
chr21 37258424
chr21 37258427
chr21 37258440
chr21 37258445
chr21 37273616
chr21 37368402
chr21 37368484
chr21 37368832
chr21 37368873
chr21 37368887
chr21 37368911
chr21 37369428
chr21 37369616
chr21 37369671

chr21 37369991
chr21 37370047
chr21 37370246
chr21 37370344
chr21 37370417
chr21 37370512
chr21 37580803
chr21 38372585
chr21 38384898
chr21 38384903
chr21 38384908
chr21 38384939
chr21 38494900
chr21 38494947
chr21 38495003
chr21 38495037
chr21 38497360
chr21 38498473
chr21 38506271
chr21 38506315
chr21 38506429
chr21 38506454
chr21 38506704
chr21 38506776
chr21 38506783
chr21 38506787
chr21 38506815
chr21 38506842
chr21 38506865
chr21 38506942
chr21 38734228
chr21 38734241
chr21 38734364
chr21 38734378
chr21 38734395
chr21 38734430
chr21 38734434
chr21 38734437
chr21 38734440
chr21 38734453
chr21 38734487
chr21 38734489
chr21 38734498
chr21 38734544
chr21 38734634
chr21 38755842
chr21 38755847
chr21 38755907
chr21 38767098
chr21 38767120
chr21 38767122
chr21 38767200
chr21 38767219
chr21 38767226

chr21 38767262
chr21 38767363
chr21 38767402
chr21 38767541
chr21 38767617
chr21 38767790
chr21 38767852
chr21 38767888
chr21 38767910
chr21 38768029
chr21 38768105
chr21 38768365
chr21 38777579
chr21 38777686
chr21 38777717
chr21 38777756
chr21 38777817
chr21 38985402
chr21 39184169
chr21 39184174
chr21 39184190
chr21 39184247
chr21 39184401
chr21 39184483
chr21 39184551
chr21 39312037
chr21 39312042
chr21 39312085
chr21 39312182
chr21 39312265
chr21 39312343
chr21 39312449
chr21 39312974
chr21 39312978
chr21 39312981
chr21 39312985
chr21 39312988
chr21 39312992
chr21 39347162
chr21 39347251
chr21 39347578
chr21 39347580
chr21 39347686
chr21 39347726
chr21 39347741
chr21 39347769
chr21 39347810
chr21 39347858
chr21 39347879
chr21 39347917
chr21 39347983
chr21 39348015
chr21 39348020
chr21 39348275

chr21 39348277
chr21 39616649
chr21 40026943
chr21 40027015
chr21 40027852
chr21 40027908
chr21 40027949
chr21 40028236
chr21 40033653
chr21 40033871
chr21 40033879
chr21 40034104
chr21 40034145
chr21 40034201
chr21 40035081
chr21 40035110
chr21 40035735
chr21 40386454
chr21 40386457
chr21 40386463
chr21 40386466
chr21 40386471
chr21 40386476
chr21 40386488
chr21 40386510
chr21 40386518
chr21 40386523
chr21 40386542
chr21 40386547
chr21 40386549
chr21 40386577
chr21 40386605
chr21 40386635
chr21 40386714
chr21 40386783
chr21 40386801
chr21 40387734
chr21 40440770
chr21 40476896
chr21 40477398
chr21 40477414
chr21 40477475
chr21 40477497
chr21 40478062
chr21 40877202
chr21 41112887
chr21 41112903
chr21 41112910
chr21 41113060
chr21 41113158
chr21 41113244
chr21 41113286
chr21 41362331
chr21 41362339

chr21 41362374
chr21 41362415
chr21 41362423
chr21 41362428
chr21 41362476
chr21 41362490
chr21 41362523
chr21 41362601
chr21 41420433
chr21 41420460
chr21 41420463
chr21 41420471
chr21 41420498
chr21 41420558
chr21 41420574
chr21 41420589
chr21 41420598
chr21 41420600
chr21 41420609
chr21 41420616
chr21 41420654
chr21 41420674
chr21 41420682
chr21 41555454
chr21 41767450
chr21 41767469
chr21 41767478
chr21 41767532
chr21 41767563
chr21 41767575
chr21 41767582
chr21 41767668
chr21 41767700
chr21 41767726
chr21 41767743
chr21 41767949
chr21 41768011
chr21 41768016
chr21 41768071
chr21 41768097
chr21 41768128
chr21 41768156
chr21 41768168
chr21 41768203
chr21 41768207
chr21 41768209
chr21 41768233
chr21 41768241
chr21 41768243
chr21 41768272
chr21 41768276
chr21 41768311
chr21 41768322
chr21 41768372

chr21 41768489
chr21 41768574
chr21 41768618
chr21 41768631
chr21 41768662
chr21 41768676
chr21 42062547
chr21 42062612
chr21 42062615
chr21 42062682
chr21 42062693
chr21 42062726
chr21 42062737
chr21 42062744
chr21 42062752
chr21 42062768
chr21 42062816
chr21 42062846
chr21 42062849
chr21 42062917
chr21 42062924
chr21 42062945
chr21 42062959
chr21 42062981
chr21 42062993
chr21 42063040
chr21 42063293
chr21 42063372
chr21 42063397
chr21 42063558
chr21 42063601
chr21 42063682
chr21 42063695
chr21 42063718
chr21 42063733
chr21 42063755
chr21 42063777
chr21 42063827
chr21 42063837
chr21 42063911
chr21 42063921
chr21 42063975
chr21 42064000
chr21 42064038
chr21 42188139
chr21 42188160
chr21 42188162
chr21 42188300
chr21 42188303
chr21 42188423
chr21 42188504
chr21 42188508
chr21 42219997
chr21 42220278

chr21 42220306
chr21 42220311
chr21 42220323
chr21 42227936
chr21 42227962
chr21 42227983
chr21 42228252
chr21 42228255
chr21 42228412
chr21 42228627
chr21 42234424
chr21 42234490
chr21 42238254
chr21 42238466
chr21 42238468
chr21 42238503
chr21 42238563
chr21 42258586
chr21 42258698
chr21 42406328
chr21 42406340
chr21 42406343
chr21 42406393
chr21 42406456
chr21 42406460
chr21 42406486
chr21 42424924
chr21 42425003
chr21 42425039
chr21 42425048
chr21 42425075
chr21 42425099
chr21 42425257
chr21 42425321
chr21 42425384
chr21 42425389
chr21 42425412
chr21 42425518
chr21 42425693
chr21 42425740
chr21 42425747
chr21 42496343
chr21 42496384
chr21 42496398
chr21 42614837
chr21 42614880
chr21 42614909
chr21 42614924
chr21 42653426
chr21 42653441
chr21 42653445
chr21 42653522
chr21 42653557
chr21 42653561

chr21 42653565
chr21 42653569
chr21 42653581
chr21 42653583
chr21 42653585
chr21 42653590
chr21 42653596
chr21 42653603
chr21 42653609
chr21 42653613
chr21 42653615
chr21 42653620
chr21 42653624
chr21 42653638
chr21 42653641
chr21 42653643
chr21 42653645
chr21 42653648
chr21 42653651
chr21 42653658
chr21 42653662
chr21 42653667
chr21 42653673
chr21 42653679
chr21 42653792
chr21 42653802
chr21 42738188
chr21 42738195
chr21 42738211
chr21 42738295
chr21 42738310
chr21 42738312
chr21 42975941
chr21 42976094
chr21 42976110
chr21 42976130
chr21 42976164
chr21 43045421
chr21 43045460
chr21 43075744
chr21 43075751
chr21 43075760
chr21 43075775
chr21 43075796
chr21 43075800
chr21 43075809
chr21 43075864
chr21 43075867
chr21 43075880
chr21 43075884
chr21 43075894
chr21 43075902
chr21 43075908
chr21 43075915

chr21 43075918
chr21 43075921
chr21 43075933
chr21 43076746
chr21 43076755
chr21 43076759
chr21 43076787
chr21 43076794
chr21 43076800
chr21 43076805
chr21 43076811
chr21 43076826
chr21 43076834
chr21 43076846
chr21 43076851
chr21 43076853
chr21 43076861
chr21 43076864
chr21 43076866
chr21 43076878
chr21 43076909
chr21 43077031
chr21 43077037
chr21 43077039
chr21 43077061
chr21 43077069
chr21 43104768
chr21 43104793
chr21 43105108
chr21 43105133
chr21 43107054
chr21 43107070
chr21 43107078
chr21 43107091
chr21 43107114
chr21 43107116
chr21 43107121
chr21 43107126
chr21 43107133
chr21 43107140
chr21 43107143
chr21 43107148
chr21 43107218
chr21 43107257
chr21 43107260
chr21 43107262
chr21 43107264
chr21 43107271
chr21 43107276
chr21 43107279
chr21 43107285
chr21 43107292
chr21 43107294
chr21 43107304

chr21 43107316
chr21 43107580
chr21 43107583
chr21 43107586
chr21 43107596
chr21 43107601
chr21 43107604
chr21 43107612
chr21 43107627
chr21 43107641
chr21 43107643
chr21 43107649
chr21 43173170
chr21 43173185
chr21 43173198
chr21 43173216
chr21 43173239
chr21 43173262
chr21 43173295
chr21 43173306
chr21 43173314
chr21 43173329
chr21 43173573
chr21 43173585
chr21 43173598
chr21 43173624
chr21 43173656
chr21 43173662
chr21 43173674
chr21 43383513
chr21 43383529
chr21 43383535
chr21 43383541
chr21 43383571
chr21 43384112
chr21 43384120
chr21 43384128
chr21 43384150
chr21 43384175
chr21 43384182
chr21 43384214
chr21 43384246
chr21 43384275
chr21 43384314
chr21 43509528
chr21 43509531
chr21 43509558
chr21 43511635
chr21 43511672
chr21 43511919
chr21 43513937
chr21 43515110
chr21 43535535
chr21 43535589

chr21 43535611
chr21 43535617
chr21 43536573
chr21 43536604
chr21 43536627
chr21 43537006
chr21 43538522
chr21 43538554
chr21 43538563
chr21 43538602
chr21 43541648
chr21 43541703
chr21 43542485
chr21 43542524
chr21 43543667
chr21 43547132
chr21 43548242
chr21 43548264
chr21 43569682
chr21 43569724
chr21 43569728
chr21 43570271
chr21 43646023
chr21 43646038
chr21 43646043
chr21 43646115
chr21 43646526
chr21 43646621
chr21 43646637
chr21 43646730
chr21 43646785
chr21 43646832
chr21 43646870
chr21 43660353
chr21 43660361
chr21 43660371
chr21 43660419
chr21 43660431
chr21 43660454
chr21 43660476
chr21 43660493
chr21 43660502
chr21 43660510
chr21 43660516
chr21 43660531
chr21 43660549
chr21 43660557
chr21 43660698
chr21 43660705
chr21 43660734
chr21 43660782
chr21 43842958
chr21 43894304
chr21 44013482

chr21 44013548
chr21 44013582
chr21 44013753
chr21 44013979
chr21 44014044
chr21 44025682
chr21 44083835
chr21 44083931
chr21 44084044
chr21 44084052
chr21 44084054
chr21 44084060
chr21 44084217
chr21 44084220
chr21 44084247
chr21 44084255
chr21 44106684
chr21 44106760
chr21 44106762
chr21 44106785
chr21 44106824
chr21 44107190
chr21 44107192
chr21 44107203
chr21 44107208
chr21 44107213
chr21 44107218
chr21 44107224
chr21 44107227
chr21 44107251
chr21 44107256
chr21 44107260
chr21 44107268
chr21 44107270
chr21 44107276
chr21 44107281
chr21 44107285
chr21 44107287
chr21 44107289
chr21 44107291
chr21 44107300
chr21 44107320
chr21 44107328
chr21 44107520
chr21 44107522
chr21 44107537
chr21 44107540
chr21 44107548
chr21 44107550
chr21 44107559
chr21 44107562
chr21 44107572
chr21 44107574
chr21 44107578

chr21 44107586
chr21 44107592
chr21 44107594
chr21 44107628
chr21 44107634
chr21 44107637
chr21 44107640
chr21 44107648
chr21 44107986
chr21 44108013
chr21 44108021
chr21 44108041
chr21 44108058
chr21 44108231
chr21 44109037
chr21 44109061
chr21 44109212
chr21 44109300
chr21 44133478
chr21 44133498
chr21 44133510
chr21 44133521
chr21 44133527
chr21 44133529
chr21 44133536
chr21 44133543
chr21 44133546
chr21 44133570
chr21 44133576
chr21 44133589
chr21 44133603
chr21 44133609
chr21 44133617
chr21 44133620
chr21 44133627
chr21 44133629
chr21 44133649
chr21 44133653
chr21 44133666
chr21 44133676
chr21 44133682
chr21 44133694
chr21 44133702
chr21 44133726
chr21 44133733
chr21 44133738
chr21 44133753
chr21 44133766
chr21 44133769
chr21 44133773
chr21 44133777
chr21 44133788
chr21 44133790
chr21 44133870

chr21 44133890
chr21 44133910
chr21 44133929
chr21 44133935
chr21 44133938
chr21 44133945
chr21 44133948
chr21 44134012
chr21 44134014
chr21 44134024
chr21 44134050
chr21 44134053
chr21 44134062
chr21 44134076
chr21 44134088
chr21 44134097
chr21 44134293
chr21 44134315
chr21 44134317
chr21 44134333
chr21 44134340
chr21 44134357
chr21 44134379
chr21 44134443
chr21 44134501
chr21 44134550
chr21 44134628
chr21 44134655
chr21 44135211
chr21 44135259
chr21 44136293
chr21 44136369
chr21 44136373
chr21 44136378
chr21 44136513
chr21 44138220
chr21 44138314
chr21 44138319
chr21 44138364
chr21 44138368
chr21 44138374
chr21 44138378
chr21 44138556
chr21 44138578
chr21 44143949
chr21 44144167
chr21 44144191
chr21 44144213
chr21 44144266
chr21 44144341
chr21 44144397
chr21 44144669
chr21 44144690
chr21 44144733

chr21 44144735
chr21 44144756
chr21 44145873
chr21 44145911
chr21 44145923
chr21 44145942
chr21 44145953
chr21 44146099
chr21 44146113
chr21 44146126
chr21 44146159
chr21 44146174
chr21 44146185
chr21 44146207
chr21 44146242
chr21 44152640
chr21 44153142
chr21 44153317
chr21 44153321
chr21 44153384
chr21 44153431
chr21 44153463
chr21 44153477
chr21 44153490
chr21 44153527
chr21 44153911
chr21 44153917
chr21 44153950
chr21 44153957
chr21 44153961
chr21 44154001
chr21 44154013
chr21 44154041
chr21 44154058
chr21 44154191
chr21 44154194
chr21 44154263
chr21 44154652
chr21 44154701
chr21 44154751
chr21 44154768
chr21 44154786
chr21 44154908
chr21 44154917
chr21 44154939
chr21 44154948
chr21 44154959
chr21 44154969
chr21 44154980
chr21 44154992
chr21 44155280
chr21 44155296
chr21 44155307
chr21 44155339

chr21 44155575
chr21 44155588
chr21 44155598
chr21 44155609
chr21 44155631
chr21 44155641
chr21 44155676
chr21 44155690
chr21 44155697
chr21 44155744
chr21 44155751
chr21 44155767
chr21 44155779
chr21 44155793
chr21 44155811
chr21 44155820
chr21 44155833
chr21 44155864
chr21 44155866
chr21 44155872
chr21 44155876
chr21 44155884
chr21 44155893
chr21 44155901
chr21 44155906
chr21 44155916
chr21 44156410
chr21 44156439
chr21 44156468
chr21 44157152
chr21 44157160
chr21 44157166
chr21 44157210
chr21 44157215
chr21 44157222
chr21 44157305
chr21 44157308
chr21 44157473
chr21 44157514
chr21 44157581
chr21 44157815
chr21 44157865
chr21 44157870
chr21 44157882
chr21 44157885
chr21 44157896
chr21 44157919
chr21 44157943
chr21 44157949
chr21 44157960
chr21 44158071
chr21 44158084
chr21 44158112
chr21 44158214

chr21 44158217
chr21 44158278
chr21 44158292
chr21 44158478
chr21 44158489
chr21 44158501
chr21 44158577
chr21 44158698
chr21 44159035
chr21 44159062
chr21 44159070
chr21 44159239
chr21 44159266
chr21 44159274
chr21 44159278
chr21 44159284
chr21 44159369
chr21 44159467
chr21 44159568
chr21 44159573
chr21 44159877
chr21 44159889
chr21 44159926
chr21 44159949
chr21 44159985
chr21 44159990
chr21 44159998
chr21 44160020
chr21 44160032
chr21 44160035
chr21 44160132
chr21 44160199
chr21 44160228
chr21 44160298
chr21 44160328
chr21 44160339
chr21 44160376
chr21 44160395
chr21 44160444
chr21 44160458
chr21 44160465
chr21 44160470
chr21 44171118
chr21 44196143
chr21 44196165
chr21 44196172
chr21 44196326
chr21 44196336
chr21 44196355
chr21 44196400
chr21 44196407
chr21 44196428
chr21 44196437
chr21 44196442

chr21 44196568
chr21 44196576
chr21 44196581
chr21 44196691
chr21 44196743
chr21 44196757
chr21 44243713
chr21 44243726
chr21 44243731
chr21 44243737
chr21 44243779
chr21 44244255
chr21 44244306
chr21 44244327
chr21 44244812
chr21 44244885
chr21 44246127
chr21 44246171
chr21 44246173
chr21 44246183
chr21 44246205
chr21 44247651
chr21 44247674
chr21 44247687
chr21 44248969
chr21 44249002
chr21 44249525
chr21 44249599
chr21 44249935
chr21 44250064
chr21 44250092
chr21 44250219
chr21 44250247
chr21 44250398
chr21 44250820
chr21 44250827
chr21 44250830
chr21 44250866
chr21 44250907
chr21 44338733
chr21 44338760
chr21 44338766
chr21 44338771
chr21 44338776
chr21 44338781
chr21 44338794
chr21 44338800
chr21 44338806
chr21 44338811
chr21 44338816
chr21 44338829
chr21 44338835
chr21 44338841
chr21 44338846

chr21 44338851
chr21 44338864
chr21 44338870
chr21 44338876
chr21 44338881
chr21 44338886
chr21 44338899
chr21 44455537
chr21 44455539
chr21 44455543
chr21 44455569
chr21 44455572
chr21 44455574
chr21 44455581
chr21 44455598
chr21 44455605
chr21 44588535
chr21 44588547
chr21 44588576
chr21 44588599
chr21 44588644
chr21 44618981
chr21 44619367
chr21 44822989
chr21 44823129
chr21 44875198
chr21 44912063
chr21 44912132
chr21 44912153
chr21 44912177
chr21 44914499
chr21 44914536
chr21 44914542
chr21 44914545
chr21 44914570
chr21 44914603
chr21 44914660
chr21 44914851
chr21 44914853
chr21 44914919
chr21 44915017
chr21 44915049
chr21 44915057
chr21 44915066
chr21 44915084
chr21 44915086
chr21 44915109
chr21 44919280
chr21 44919344
chr21 44919604
chr21 44919610
chr21 44919615
chr21 44919621
chr21 44919626

chr21 44919635
chr21 44919645
chr21 44919671
chr21 44919691
chr21 44919771
chr21 44919841
chr21 44919946
chr21 44920216
chr21 44920224
chr21 44920259
chr21 44920273
chr21 44920311
chr21 44920326
chr21 44920362
chr21 44920367
chr21 44920384
chr21 44920397
chr21 44920436
chr21 44920461
chr21 44920485
chr21 44920507
chr21 44920551
chr21 44920580
chr21 44920596
chr21 44920602
chr21 44920655
chr21 44920683
chr21 44920720
chr21 44920790
chr21 44920801
chr21 44920824
chr21 44920841
chr21 44920844
chr21 44920861
chr21 44920895
chr21 44920918
chr21 44920920
chr21 44920925
chr21 44920990
chr21 44921016
chr21 44921045
chr21 44921091
chr21 44921094
chr21 44921139
chr21 44921152
chr21 44921252
chr21 44921299
chr21 44921355
chr21 44921361
chr21 44921389
chr21 44927451
chr21 44927470
chr21 44927477
chr21 44927516

chr21 44927586
chr21 44927665
chr21 44927698
chr21 44927704
chr21 44927766
chr21 44927781
chr21 44927825
chr21 44927932
chr21 44928321
chr21 44928341
chr21 44930633
chr21 44930655
chr21 44930693
chr21 44930764
chr21 44930793
chr21 44930802
chr21 44930897
chr21 44930957
chr21 44931113
chr21 44931133
chr21 44931153
chr21 44931167
chr21 44931184
chr21 44931203
chr21 44931209
chr21 44931224
chr21 44931272
chr21 44931274
chr21 44931283
chr21 44931326
chr21 44931357
chr21 45109858
chr21 45109866
chr21 45109899
chr21 45109920
chr21 45109952
chr21 45109973
chr21 45109990
chr21 45196598
chr21 45196633
chr21 45550923
chr21 45550976
chr21 45551065
chr21 45551178
chr21 45551202
chr21 45551226
chr21 45551235
chr21 45554013
chr21 45554015
chr21 45554034
chr21 45554036
chr21 45554054
chr21 45554056
chr21 45554075

chr21 45554077
chr21 45554200
chr21 45554218
chr21 45554220
chr21 45554240
chr21 45554276
chr21 45554278
chr21 45554297
chr21 45554299
chr21 45554319
chr21 45554339
chr21 45554357
chr21 45554359
chr21 45554378
chr21 45554380
chr21 45554401
chr21 45554421
chr21 45554439
chr21 45554441
chr21 45554478
chr21 45554480
chr21 45554501
chr21 45554523
chr21 45554536
chr21 45554538
chr21 45555738
chr21 45555742
chr21 45555755
chr21 45555762
chr21 45555772
chr21 45555774
chr21 45555781
chr21 45555790
chr21 45555797
chr21 45555805
chr21 45555807
chr21 45555822
chr21 45555833
chr21 45555835
chr21 45555856
chr21 45555867
chr21 45555889
chr21 45555891
chr21 45555894
chr21 45555899
chr21 45555921
chr21 45555934
chr21 45555939
chr21 45555961
chr21 45555963
chr21 45555974
chr21 45555983
chr21 45556012
chr21 45556032

chr21 45818927
chr21 45885849
chr21 45886060
chr21 45886089
chr21 45886119
chr21 45886165
chr21 45886171
chr21 45886173
chr21 45886193
chr21 45886222
chr21 45886272
chr21 45886274
chr21 45886324
chr21 45886346
chr21 45886373
chr21 45886375
chr21 45886424
chr21 45886474
chr21 45886476
chr21 45980577
chr21 45981165
chr21 45981195
chr21 45981201
chr21 45981226
chr21 45981239
chr21 45981241
chr21 45981272
chr21 45981275
chr21 45981294
chr21 45981311
chr21 45981325
chr21 45981329
chr21 45981334
chr21 45981344
chr21 45981748
chr21 45981782
chr21 45981793
chr21 45981796
chr21 45981799
chr21 45981802
chr21 46143698
chr21 46143710
chr21 46143722
chr21 46143726
chr21 46143736
chr21 46143779
chr21 46143794
chr21 46143870
chr21 46143881
chr21 46143892
chr21 46143967
chr21 46144102
chr21 46144120
chr21 46144196

chr21 46144222
chr21 46144229
chr21 46144253
chr21 46144320
chr21 46184138
chr21 46184142
chr21 46184146
chr21 46184193
chr21 46184205
chr21 46184224
chr21 46184232
chr21 46184237
chr21 46184252
chr21 46184279
chr21 46184298
chr21 46184302
chr21 46184377
chr21 46184401
chr21 46184443
chr21 46184447
chr21 46184472
chr21 46184495
chr21 46184525
chr21 46184548
chr21 46184631
chr21 46184654
chr21 46184684
chr21 46184740
chr21 46184763
chr21 46184832
chr21 46184836
chr21 46184919
chr21 46184984
chr21 46228915
chr21 46228930
chr21 46228933
chr21 46228935
chr21 46228939
chr21 46228947
chr21 46228961
chr21 46228978
chr21 46228994
chr21 46228997
chr21 46229004
chr21 46229019
chr21 46229038
chr21 46229041
chr21 46229046
chr21 46229055
chr21 46229073
chr21 46229087
chr21 46229114
chr21 46229141
chr21 46229193

chr21 46229199
chr21 46229209
chr21 46229211
chr21 46229213
chr21 46229230
chr21 46229235
chr21 46229243
chr21 46229249
chr21 46229253
chr21 46229256
chr21 46229258
chr21 46459862
chr21 46459923
chr21 46500459
chr21 46500658
chr21 46500722
chr21 46501212
chr21 46501464
chr21 46559433
chr21 46559445
chr21 46559514
chr21 46559529
chr21 46559533
chr21 46559552
chr21 46559578
chr21 46559587
chr21 46559602
chr21 46559610
chr21 46559640
chr21 46559643
chr21 46559698
chr21 46559933
chr21 46636042
chr21 46636049
chr21 46636051
chr21 46636057
chr21 46636072
chr21 46636084
chr21 46636091
chr21 46636093
chr21 46636096
chr21 46636118
chr21 46636128
chr21 46636134
chr21 46636136
chr21 46636147
chr21 46636216
chr21 46636245
chr21 46636247
chr21 46636254
chr21 46636266
chr21 46636272
chr21 46636340
chr21 46636474

chr21 46636622
chr21 46692706
chr21 46694443
chr21 46694585
chr21 46694602
chr21 46695009
chr21 46695174
chr21 46696106
chr21 46699198
chr21 46699201
chr21 46699204
chr21 46699206
chr21 46699209
chr21 46699211
chr21 46699221
chr21 46699227
chr21 46699230
chr21 46699233
chr21 46699235
chr21 46699238
chr21 46699240
chr21 46699250
chr21 46699256
chr21 46699259
chr21 46699262
chr21 46699264
chr21 46699267
chr21 46699269
chr21 46699279
chr21 46699285
chr21 46699288
chr21 46699291
chr21 46699293
chr21 46699296
chr21 46699298
chr21 46699308
chr21 46699317
chr21 46699320
chr21 46699322
chr21 46699325
chr21 46699333
chr21 46699339
chr21 46699347
chr11 181405
chr11 181768
chr11 182762
chr11 182892
chr11 183093
chr11 183334
chr11 188098
chr11 188193
chr11 188744
chr11 188837
chr11 237677

chr11 237694
chr11 237697
chr11 267438
chr11 267454
chr11 267473
chr11 267570
chr11 267581
chr11 267603
chr11 267613
chr11 267619
chr11 267685
chr11 267710
chr11 269001
chr11 269010
chr11 269024
chr11 269041
chr11 269152
chr11 269168
chr11 269712
chr11 308442
chr11 308455
chr11 308473
chr11 308554
chr11 308575
chr11 308688
chr11 310077
chr11 310132
chr11 310223
chr11 310251
chr11 310263
chr11 310367
chr11 310391
chr11 310395
chr11 310397
chr11 310405
chr11 310710
chr11 310729
chr11 310736
chr11 310757
chr11 311220
chr11 311235
chr11 311247
chr11 311251
chr11 311266
chr11 311268
chr11 311277
chr11 311282
chr11 311284
chr11 311298
chr11 311303
chr11 311338
chr11 311344
chr11 311350
chr11 311354

chr11 311364
chr11 311398
chr11 311400
chr11 311409
chr11 311416
chr11 311418
chr11 311454
chr11 311512
chr11 311542
chr11 311552
chr11 311565
chr11 311570
chr11 311584
chr11 311607
chr11 311615
chr11 311621
chr11 314341
chr11 314350
chr11 314360
chr11 314373
chr11 314391
chr11 314481
chr11 314493
chr11 314783
chr11 314807
chr11 318015
chr11 318165
chr11 318173
chr11 318196
chr11 318210
chr11 318215
chr11 318228
chr11 318238
chr11 318268
chr11 318326
chr11 318362
chr11 318364
chr11 318371
chr11 318380
chr11 318382
chr11 318416
chr11 318426
chr11 318430
chr11 318899
chr11 318906
chr11 318911
chr11 318921
chr11 318935
chr11 318940
chr11 318954
chr11 318968
chr11 318984
chr11 318994
chr11 319009

chr11 319012
chr11 319023
chr11 319044
chr11 319051
chr11 319070
chr11 413452
chr11 413465
chr11 413506
chr11 413512
chr11 413594
chr11 413721
chr11 413967
chr11 414483
chr11 415088
chr11 415111
chr11 415120
chr11 415147
chr11 1336179
chr11 1336191
chr11 1336201
chr11 1336219
chr11 1336248
chr11 1336256
chr11 1729056
chr11 1729072
chr11 1729076
chr11 1729097
chr11 1729115
chr11 1729123
chr11 1772569
chr11 1772604
chr11 1772647
chr11 1772675
chr11 1844562
chr11 1844698
chr11 1844738
chr11 1844746
chr11 1844770
chr11 1844782
chr11 1844801
chr11 1844827
chr11 1844829
chr11 1845419
chr11 1845475
chr11 1845490
chr11 1852986
chr11 1853016
chr11 1853036
chr11 1853061
chr11 1853090
chr11 1853149
chr11 1853158
chr11 1853167
chr11 1853174

chr11 1853181
chr11 1853209
chr11 1853212
chr11 1853215
chr11 1853219
chr11 1853222
chr11 1853232
chr11 1853330
chr11 1853414
chr11 1853449
chr11 1853451
chr11 1853485
chr11 1853501
chr11 2077630
chr11 2077662
chr11 2077675
chr11 2142537
chr11 2142578
chr11 2142633
chr11 2143166
chr11 2143259
chr11 2143301
chr11 2143337
chr11 2143347
chr11 2143349
chr11 2143368
chr11 2143370
chr11 2143380
chr11 2143384
chr11 2489273
chr11 2786291
chr11 2786492
chr11 2786773
chr11 2786942
chr11 2892409
chr11 2892437
chr11 2892454
chr11 2892464
chr11 2892486
chr11 2892495
chr11 2892499
chr11 2892535
chr11 2892542
chr11 2892558
chr11 2892566
chr11 2892675
chr11 2892683
chr11 2892687
chr11 2892695
chr11 2892698
chr11 2892711
chr11 2892717
chr11 2892726
chr11 2892736

chr11 2892762
chr11 2892782
chr11 2892801
chr11 2892803
chr11 2892822
chr11 2892875
chr11 2988115
chr11 2988196
chr11 2988263
chr11 2988422
chr11 2988472
chr11 2988561
chr11 2988704
chr11 2988747
chr11 2988841
chr11 2989107
chr11 2989214
chr11 2989226
chr11 2989390
chr11 2989410
chr11 2989422
chr11 2989687
chr11 2989715
chr11 2989820
chr11 3216666
chr11 3216689
chr11 3216806
chr11 3217160
chr11 3217171
chr11 3217180
chr11 3217196
chr11 3217208
chr11 3217245
chr11 3217269
chr11 3217272
chr11 3217279
chr11 3378996
chr11 3379421
chr11 3454947
chr11 3455211
chr11 3455265
chr11 3455267
chr11 3781588
chr11 3781618
chr11 3781922
chr11 3785275
chr11 3785486
chr11 3785587
chr11 3785621
chr11 3785651
chr11 3785694
chr11 3785713
chr11 3786068
chr11 3786145

chr11 3786157
chr11 3796094
chr11 3796106
chr11 3796120
chr11 3796184
chr11 3796260
chr11 3796280
chr11 3796361
chr11 3796432
chr11 3796443
chr11 3796456
chr11 3796462
chr11 3796519
chr11 3796537
chr11 3796563
chr11 3796570
chr11 3796590
chr11 3796614
chr11 3796619
chr11 3796736
chr11 3796743
chr11 3839001
chr11 3839062
chr11 3839065
chr11 3839078
chr11 3839226
chr11 3839260
chr11 3839286
chr11 3839339
chr11 3841128
chr11 3841287
chr11 3841336
chr11 3854260
chr11 3854277
chr11 3854309
chr11 3854355
chr11 3854393
chr11 3854485
chr11 3854544
chr11 3854593
chr11 3854613
chr11 3854630
chr11 3854702
chr11 3854800
chr11 3854806
chr11 3856282
chr11 3856286
chr11 3856362
chr11 3856377
chr11 3856424
chr11 3856468
chr11 3856621
chr11 3856763
chr11 3856884

chr11 3856937
chr11 3856956
chr11 3857290
chr11 3857376
chr11 3857569
chr11 3990397
chr11 4035309
chr11 4035315
chr11 4035491
chr11 4058218
chr11 4058326
chr11 4234887
chr11 4235089
chr11 4235271
chr11 4336613
chr11 4336683
chr11 4336870
chr11 4336914
chr11 4337444
chr11 4337570
chr11 4337647
chr11 4393038
chr11 4393227
chr11 4393650
chr11 4393686
chr11 4393732
chr11 4762795
chr11 4763203
chr11 4763218
chr11 4959731
chr11 4960035
chr11 4960258
chr11 4960519
chr11 5033493
chr11 5033802
chr11 5034325
chr11 5410781
chr11 5412003
chr11 5475094
chr11 5475255
chr11 5475378
chr11 5476087
chr11 5596582
chr11 5596601
chr11 5596621
chr11 5596626
chr11 5596662
chr11 5596685
chr11 5596689
chr11 5683986
chr11 5683988
chr11 5684018
chr11 5684026
chr11 5684061

chr11 5684084
chr11 5684093
chr11 5684124
chr11 5684128
chr11 5684130
chr11 5684174
chr11 5684259
chr11 5684711
chr11 5684765
chr11 5684772
chr11 5684838
chr11 5684996
chr11 5685023
chr11 5685061
chr11 5685132
chr11 5685151
chr11 5685171
chr11 5685176
chr11 5685205
chr11 5689424
chr11 5689507
chr11 5689759
chr11 5689838
chr11 5690208
chr11 5690282
chr11 5690332
chr11 5690373
chr11 5690439
chr11 5690539
chr11 5690721
chr11 5690776
chr11 5690879
chr11 5691009
chr11 5691029
chr11 5691173
chr11 5691199
chr11 5691236
chr11 5691245
chr11 5691295
chr11 5691297
chr11 5691308
chr11 5691314
chr11 5691335
chr11 5691357
chr11 5691369
chr11 5691488
chr11 5691757
chr11 5691766
chr11 5691874
chr11 5692048
chr11 5692115
chr11 5695025
chr11 5808955
chr11 5808957

chr11 5808971
chr11 5973456
chr11 5973655
chr11 6296482
chr11 6296542
chr11 6315632
chr11 6316085
chr11 6316091
chr11 6316229
chr11 6322359
chr11 6390164
chr11 6390166
chr11 6390195
chr11 6390256
chr11 6402429
chr11 6402828
chr11 6604412
chr11 6604691
chr11 6604910
chr11 6604988
chr11 6607087
chr11 6607121
chr11 6607143
chr11 6607148
chr11 6607160
chr11 6607181
chr11 6618224
chr11 6618425
chr11 6618435
chr11 6618447
chr11 6618553
chr11 6618609
chr11 6618702
chr11 6618740
chr11 6618776
chr11 6618798
chr11 6618819
chr11 6618882
chr11 6618886
chr11 6619095
chr11 6619112
chr11 6619195
chr11 6619202
chr11 6619209
chr11 6619216
chr11 6619303
chr11 6619347
chr11 6619363
chr11 6619411
chr11 6619413
chr11 6619432
chr11 6619489
chr11 6619544
chr11 6619551

chr11 6619606
chr11 6619765
chr11 6656117
chr11 6682188
chr11 6682236
chr11 6682276
chr11 6682296
chr11 6682308
chr11 6682359
chr11 6682520
chr11 6682556
chr11 6682591
chr11 6744546
chr11 6744859
chr11 6744870
chr11 6745027
chr11 6745078
chr11 6745101
chr11 6745113
chr11 6745707
chr11 6745743
chr11 6746393
chr11 6746434
chr11 6746437
chr11 6746535
chr11 6746595
chr11 7024305
chr11 7032306
chr11 7032936
chr11 7033125
chr11 7087582
chr11 7087676
chr11 7087833
chr11 7087882
chr11 7154995
chr11 7155126
chr11 7225304
chr11 7225352
chr11 7225527
chr11 7225748
chr11 7257169
chr11 7743834
chr11 7744032
chr11 7744168
chr11 7744271
chr11 7753760
chr11 7753804
chr11 7834211
chr11 7834358
chr11 7959043
chr11 7986807
chr11 7986886
chr11 7986901
chr11 7986919

chr11 7986928
chr11 7986933
chr11 7986941
chr11 7986961
chr11 7986971
chr11 7986973
chr11 7987052
chr11 7987054
chr11 7987126
chr11 7987162
chr11 7987271
chr11 7987325
chr11 7987331
chr11 7987346
chr11 7987363
chr11 7987366
chr11 7987387
chr11 7987393
chr11 7987408
chr11 7987411
chr11 7987414
chr11 7987435
chr11 7987441
chr11 7987450
chr11 7987456
chr11 7987462
chr11 7987469
chr11 7987474
chr11 7987501
chr11 7987510
chr11 7987522
chr11 7987540
chr11 7987549
chr11 7987561
chr11 7987573
chr11 7987577
chr11 7987670
chr11 7987677
chr11 7987682
chr11 7987695
chr11 7987703
chr11 7987814
chr11 7987918
chr11 7987938
chr11 7988083
chr11 7988086
chr11 8911723
chr11 8911741
chr11 8911746
chr11 8911777
chr11 8911825
chr11 8914123
chr11 8914272
chr11 8914280

chr11 8914325
chr11 8914327
chr11 8914425
chr11 8914463
chr11 8962645
chr11 8962663
chr11 8963656
chr11 8963805
chr11 8963823
chr11 8963830
chr11 8963875
chr11 9264039
chr11 9264169
chr11 9264254
chr11 9264312
chr11 9264415
chr11 9264453
chr11 9264455
chr11 9264505
chr11 9280907
chr11 9281037
chr11 9281136
chr11 9281364
chr11 9364577
chr11 9364579
chr11 9364589
chr11 9364591
chr11 9364593
chr11 9364612
chr11 9364614
chr11 9364616
chr11 9364619
chr11 9364624
chr11 9385052
chr11 9385078
chr11 9385487
chr11 9385581
chr11 9385624
chr11 9385756
chr11 9385842
chr11 9565930
chr11 9566035
chr11 9566043
chr11 9566196
chr11 9575149
chr11 9575159
chr11 9575177
chr11 9575184
chr11 9575206
chr11 9575272
chr11 9575505
chr11 9575789
chr11 9601140
chr11 9601204

chr11 9601375
chr11 9601440
chr11 9601540
chr11 9601631
chr11 9601776
chr11 9601898
chr11 9613624
chr11 9613639
chr11 9613649
chr11 9613655
chr11 9613672
chr11 9613674
chr11 9613679
chr11 9613681
chr11 9613697
chr11 9613699
chr11 9613715
chr11 9613744
chr11 9613764
chr11 9613784
chr11 9613816
chr11 9613825
chr11 9613875
chr11 9613892
chr11 9613903
chr11 9613939
chr11 9613961
chr11 9613981
chr11 9663657
chr11 9663672
chr11 9663685
chr11 9663718
chr11 9663752
chr11 9663789
chr11 9663794
chr11 9663807
chr11 9663832
chr11 9663849
chr11 9663851
chr11 9663877
chr11 9663895
chr11 9663932
chr11 9663974
chr11 9663986
chr11 9664005
chr11 9664015
chr11 9664017
chr11 9664028
chr11 9664031
chr11 9664034
chr11 9664071
chr11 9664086
chr11 9664088
chr11 9664091

chr11 9664104
chr11 9664118
chr11 9664122
chr11 9664127
chr11 9664129
chr11 9664159
chr11 9664173
chr11 9664175
chr11 9664296
chr11 9664308
chr11 9664359
chr11 10302807
chr11 10302817
chr11 10302820
chr11 10302822
chr11 10302928
chr11 10302934
chr11 10302936
chr11 10302938
chr11 10302940
chr11 10303315
chr11 10303352
chr11 10303394
chr11 10303396
chr11 10303502
chr11 10303504
chr11 10303506
chr11 10303522
chr11 10303589
chr11 10303608
chr11 10303627
chr11 10303644
chr11 10303652
chr11 10303691
chr11 10303708
chr11 10303711
chr11 10303716
chr11 10303735
chr11 10303778
chr11 10303793
chr11 10303873
chr11 10303884
chr11 10303893
chr11 10303989
chr11 10303992
chr11 10303994
chr11 10304003
chr11 10304007
chr11 10304020
chr11 10304025
chr11 10304224
chr11 10304231
chr11 10304479
chr11 10304502

chr11 10304507
chr11 10304510
chr11 10304623
chr11 10304634
chr11 10304636
chr11 10306012
chr11 10306018
chr11 10306024
chr11 10306033
chr11 10442067
chr11 10442119
chr11 10455938
chr11 10455942
chr11 10455974
chr11 10456017
chr11 10456047
chr11 10456050
chr11 10456191
chr11 10456274
chr11 10456300
chr11 10456304
chr11 10456350
chr11 10456411
chr11 10456459
chr11 10456466
chr11 10456596
chr11 10456710
chr11 10507468
chr11 10604742
chr11 10604762
chr11 10743045
chr11 10743371
chr11 10743523
chr11 10743632
chr11 10743646
chr11 10743729
chr11 10743862
chr11 10743871
chr11 10743891
chr11 10743901
chr11 10743904
chr11 10743912
chr11 10743929
chr11 10743950
chr11 10751417
chr11 10751419
chr11 10807063
chr11 10807178
chr11 10807261
chr11 10807284
chr11 10807301
chr11 10807312
chr11 10807388
chr11 10807453

chr11 10807561
chr11 10807587
chr11 10809666
chr11 10809671
chr11 10809706
chr11 10809713
chr11 10809720
chr11 10809749
chr11 10809751
chr11 10809769
chr11 10809817
chr11 10809893
chr11 10809951
chr11 10809957
chr11 10856825
chr11 10856951
chr11 10856976
chr11 10856981
chr11 10859088
chr11 10859099
chr11 10859110
chr11 10859249
chr11 10931763
chr11 10931781
chr11 10931810
chr11 10933826
chr11 10933868
chr11 10933956
chr11 10933961
chr11 10933972
chr11 10933983
chr11 10933988
chr11 10933992
chr11 10934006
chr11 10934057
chr11 10934079
chr11 10934121
chr11 10934129
chr11 10934213
chr11 10934267
chr11 10934279
chr11 10934289
chr11 10934306
chr11 10934421
chr11 10934437
chr11 10934454
chr11 10934466
chr11 10979082
chr11 10979174
chr11 10979585
chr11 10979701
chr11 10979857
chr11 11244293
chr11 11244323

chr11 11244625
chr11 11416384
chr11 11416396
chr11 11843771
chr11 11843816
chr11 11844196
chr11 11844373
chr11 11856941
chr11 12157719
chr11 12225032
chr11 12225083
chr11 12236280
chr11 12236377
chr11 12236478
chr11 12827844
chr11 12828390
chr11 12828464
chr11 12828469
chr11 12828723
chr11 12828757
chr11 12828859
chr11 12828995
chr11 12829053
chr11 12829069
chr11 12829139
chr11 12829170
chr11 12829251
chr11 12829420
chr11 12842563
chr11 12842665
chr11 12842690
chr11 13276888
chr11 13276914
chr11 13276920
chr11 13276922
chr11 13276988
chr11 13276993
chr11 13276995
chr11 13276999
chr11 13277020
chr11 13277063
chr11 13277072
chr11 13277093
chr11 13277128
chr11 13277136
chr11 13463186
chr11 13463216
chr11 13463222
chr11 13463224
chr11 13463229
chr11 13463235
chr11 13463240
chr11 13463255
chr11 13463274

chr11 13463276
chr11 13463291
chr11 13463299
chr11 13463311
chr11 13463317
chr11 13463319
chr11 13463325
chr11 13463335
chr11 13463349
chr11 13463357
chr11 13466138
chr11 13466151
chr11 13466226
chr11 13466255
chr11 13466279
chr11 13669275
chr11 13669277
chr11 13669283
chr11 13669359
chr11 13669404
chr11 13669437
chr11 13669474
chr11 13669537
chr11 13669539
chr11 13669615
chr11 13669621
chr11 14320111
chr11 14320198
chr11 14320418
chr11 14342939
chr11 14414617
chr11 14414712
chr11 14499094
chr11 14499174
chr11 14499181
chr11 14499210
chr11 14500673
chr11 14500692
chr11 14501071
chr11 14501134
chr11 14518884
chr11 14518921
chr11 14519037
chr11 14519221
chr11 14519421
chr11 14519492
chr11 14537235
chr11 14544768
chr11 14544932
chr11 14633137
chr11 14633187
chr11 14633209
chr11 14633492
chr11 14633512

chr11 14633672
chr11 14636283
chr11 14645525
chr11 14645538
chr11 14645563
chr11 14645947
chr11 14891718
chr11 14891738
chr11 14891743
chr11 14891751
chr11 14891756
chr11 14891769
chr11 14891933
chr11 14905540
chr11 14905575
chr11 14905585
chr11 14905601
chr11 14905622
chr11 16608368
chr11 16608375
chr11 16608384
chr11 16608467
chr11 16608580
chr11 16608595
chr11 16739122
chr11 16739185
chr11 16739222
chr11 16739233
chr11 16739242
chr11 16739248
chr11 16739331
chr11 16739348
chr11 16739362
chr11 16739403
chr11 16739432
chr11 16739447
chr11 16739461
chr11 16739479
chr11 16739522
chr11 16739544
chr11 16739566
chr11 16739581
chr11 16820433
chr11 16820451
chr11 17078247
chr11 17078283
chr11 17078298
chr11 17207337
chr11 17207474
chr11 17208095
chr11 17230398
chr11 17230409
chr11 17230425
chr11 17230573

chr11 17230686
chr11 17230709
chr11 17230754
chr11 17230759
chr11 17230942
chr11 17230956
chr11 17277040
chr11 17277051
chr11 17277057
chr11 17277080
chr11 17277109
chr11 17277120
chr11 17277131
chr11 17277146
chr11 17277198
chr11 17277214
chr11 17277233
chr11 17277252
chr11 17277304
chr11 17277340
chr11 17277448
chr11 17277544
chr11 17285668
chr11 17285675
chr11 17285700
chr11 17285738
chr11 17285920
chr11 17285922
chr11 17285926
chr11 17285932
chr11 17351999
chr11 17352001
chr11 17352004
chr11 17352025
chr11 17352031
chr11 17352035
chr11 17352048
chr11 17352067
chr11 17352080
chr11 17352094
chr11 17352096
chr11 17352101
chr11 17352117
chr11 17352203
chr11 17352211
chr11 17352241
chr11 17352243
chr11 17352249
chr11 17352266
chr11 17352281
chr11 17352305
chr11 17352325
chr11 17352329
chr11 17352341

chr11 17352370
chr11 17352421
chr11 17352426
chr11 17352441
chr11 17352454
chr11 17352464
chr11 17352466
chr11 17352470
chr11 17352494
chr11 17352514
chr11 17352523
chr11 17353006
chr11 17353012
chr11 17353014
chr11 17353023
chr11 17353028
chr11 17353053
chr11 17353055
chr11 17353069
chr11 17353083
chr11 17353099
chr11 17353115
chr11 17353122
chr11 17353128
chr11 17353141
chr11 17353874
chr11 17353892
chr11 17353952
chr11 17354010
chr11 17354040
chr11 17354096
chr11 17354299
chr11 17354371
chr11 17354379
chr11 17354394
chr11 17354526
chr11 17354587
chr11 18187071
chr11 18211165
chr11 18211605
chr11 18321453
chr11 18321550
chr11 18321552
chr11 18321586
chr11 18321656
chr11 18321803
chr11 18322810
chr11 18322817
chr11 18322820
chr11 18322844
chr11 18322847
chr11 18322853
chr11 18322856
chr11 18322867

chr11 18322905
chr11 18322916
chr11 18323042
chr11 18332894
chr11 18332945
chr11 18333120
chr11 18383944
chr11 18383962
chr11 18383979
chr11 18384206
chr11 18384239
chr11 18384515
chr11 18396585
chr11 18396588
chr11 18396604
chr11 18525751
chr11 18525838
chr11 18525910
chr11 18525919
chr11 18525967
chr11 18526067
chr11 18526162
chr11 18526199
chr11 18526201
chr11 18526300
chr11 18527342
chr11 18527431
chr11 18527506
chr11 18527556
chr11 18579371
chr11 18579492
chr11 18579505
chr11 18579528
chr11 18587959
chr11 18588151
chr11 18588189
chr11 18588326
chr11 18588336
chr11 18588388
chr11 18588422
chr11 18588464
chr11 18588466
chr11 18588477
chr11 18588492
chr11 18588497
chr11 18588520
chr11 18588527
chr11 18588534
chr11 18588568
chr11 18588599
chr11 18588608
chr11 18588626
chr11 18588639
chr11 18588645

chr11 18588663
chr11 18588669
chr11 18588696
chr11 18588708
chr11 18588724
chr11 18588731
chr11 18588750
chr11 18588753
chr11 18588766
chr11 18588794
chr11 18616173
chr11 18616464
chr11 18616673
chr11 18616689
chr11 18618489
chr11 18618536
chr11 18633309
chr11 18633339
chr11 18633363
chr11 18633448
chr11 18633482
chr11 18633490
chr11 18633500
chr11 18633620
chr11 18633691
chr11 18633712
chr11 18633738
chr11 18633745
chr11 18633761
chr11 18633881
chr11 18633896
chr11 18633927
chr11 18633953
chr11 18633984
chr11 18633987
chr11 18634198
chr11 18634309
chr11 18634319
chr11 18634324
chr11 18634352
chr11 18634372
chr11 18634380
chr11 18634392
chr11 18634395
chr11 18634453
chr11 18634461
chr11 18634465
chr11 18634467
chr11 18634477
chr11 18634486
chr11 18634497
chr11 18634504
chr11 18634512
chr11 18634519

chr11 18634531
chr11 18634534
chr11 18634539
chr11 18706380
chr11 18706402
chr11 18706418
chr11 18706435
chr11 18706458
chr11 18706477
chr11 18706488
chr11 18706528
chr11 18706537
chr11 18706540
chr11 18706542
chr11 18706590
chr11 18706623
chr11 18706649
chr11 18706651
chr11 18706657
chr11 18706672
chr11 18706686
chr11 18706747
chr11 18706774
chr11 18706779
chr11 18706795
chr11 18722033
chr11 18722092
chr11 18722094
chr11 18722124
chr11 18722200
chr11 18722244
chr11 18722529
chr11 18933370
chr11 18957401
chr11 19117230
chr11 19117260
chr11 19117272
chr11 19117284
chr11 19117288
chr11 19117292
chr11 19117311
chr11 19117319
chr11 19240112
chr11 19240185
chr11 19240302
chr11 19240316
chr11 19240332
chr11 19240366
chr11 19240377
chr11 19240389
chr11 19240396
chr11 19240429
chr11 19240434
chr11 19240437

chr11 19240462
chr11 19240503
chr11 19240515
chr11 19240525
chr11 19240553
chr11 19240568
chr11 19240577
chr11 19240581
chr11 19240602
chr11 19240606
chr11 19240608
chr11 19240670
chr11 19240694
chr11 19240704
chr11 20157927
chr11 20157982
chr11 20158261
chr11 20158326
chr11 20214486
chr11 20241376
chr11 20242065
chr11 20354114
chr11 20362831
chr11 20363891
chr11 20363897
chr11 20363923
chr11 20363925
chr11 20363935
chr11 20363958
chr11 20363963
chr11 20363972
chr11 20603383
chr11 20603484
chr11 20603563
chr11 20603614
chr11 20603747
chr11 20603992
chr11 20668126
chr11 20668221
chr11 20668250
chr11 20668273
chr11 20668280
chr11 20668358
chr11 20668470
chr11 20668561
chr11 20668595
chr11 20668677
chr11 20754780
chr11 20913023
chr11 20913042
chr11 20913071
chr11 20957489
chr11 20957967
chr11 21612353

chr11 21612432
chr11 21701330
chr11 21701412
chr11 21701421
chr11 21809165
chr11 21933574
chr11 22337504
chr11 22337518
chr11 22337642
chr11 22337659
chr11 22337679
chr11 22337684
chr11 22337759
chr11 22337780
chr11 22337787
chr11 22337795
chr11 22337799
chr11 22337809
chr11 22337831
chr11 22338752
chr11 22338949
chr11 22338976
chr11 22339599
chr11 22339743
chr11 22339760
chr11 22339776
chr11 22339789
chr11 22339855
chr11 22339873
chr11 22339889
chr11 22339921
chr11 22339983
chr11 22340047
chr11 22340059
chr11 22340080
chr11 22340161
chr11 22340227
chr11 22340261
chr11 22340285
chr11 22340298
chr11 22340310
chr11 22345130
chr11 22368504
chr11 22368748
chr11 22369046
chr11 22369390
chr11 22369628
chr11 22369873
chr11 22431919
chr11 22432133
chr11 22432212
chr11 22668644
chr11 22828617
chr11 22828695

chr11 22829048
chr11 22829060
chr11 22829084
chr11 22829120
chr11 22829170
chr11 22829220
chr11 22829234
chr11 22829242
chr11 22829250
chr11 22829290
chr11 22829305
chr11 22829320
chr11 22829340
chr11 22829342
chr11 22829345
chr11 22829368
chr11 22829380
chr11 22829382
chr11 22829387
chr11 22829396
chr11 22829400
chr11 22829403
chr11 22829406
chr11 22829421
chr11 22829424
chr11 22830347
chr11 22830359
chr11 22830425
chr11 23034322
chr11 23034415
chr11 23035044
chr11 23263877
chr11 23398723
chr11 23399058
chr11 23569212
chr11 23569702
chr11 23569769
chr11 23569814
chr11 23570188
chr11 23933307
chr11 23933352
chr11 23933656
chr11 23933851
chr11 24721588
chr11 24721818
chr11 24722052
chr11 24722386
chr11 24784032
chr11 24784061
chr11 24834299
chr11 24834847
chr11 24895478
chr11 25051722
chr11 25052251

chr11 25052334
chr11 25514805
chr11 26111270
chr11 26111370
chr11 26123467
chr11 26285034
chr11 26520002
chr11 26520487
chr11 26588959
chr11 26588981
chr11 26968306
chr11 26968357
chr11 26968399
chr11 26968522
chr11 26974507
chr11 26993814
chr11 26995762
chr11 27145520
chr11 27145936
chr11 27146177
chr11 27251835
chr11 27252072
chr11 27301439
chr11 27301520
chr11 27301707
chr11 27499304
chr11 27505929
chr11 27505956
chr11 27505974
chr11 27507061
chr11 27507156
chr11 27507169
chr11 27507217
chr11 27507293
chr11 27507507
chr11 27507509
chr11 27697908
chr11 27698094
chr11 27698398
chr11 27698471
chr11 27698665
chr11 27698693
chr11 27698793
chr11 27698802
chr11 27698894
chr11 27698901
chr11 27698925
chr11 27698927
chr11 27698959
chr11 27698977
chr11 27698980
chr11 27698983
chr11 27698988
chr11 27698992

chr11 27699000
chr11 27699059
chr11 27699099
chr11 27699130
chr11 27699162
chr11 27699188
chr11 27699204
chr11 27699218
chr11 27701780
chr11 27701838
chr11 27701862
chr11 27701926
chr11 27701964
chr11 27701987
chr11 27702012
chr11 27702111
chr11 27702212
chr11 27702242
chr11 27702362
chr11 27702485
chr11 27702525
chr11 27702769
chr11 27702789
chr11 27702818
chr11 27717902
chr11 27717988
chr11 27718006
chr11 27718061
chr11 27718104
chr11 27718112
chr11 27718128
chr11 27718130
chr11 27796153
chr11 27796171
chr11 27796295
chr11 27796471
chr11 27796620
chr11 28108726
chr11 28108907
chr11 28108915
chr11 28343440
chr11 28343490
chr11 28343539
chr11 28565917
chr11 28566533
chr11 28566560
chr11 28890273
chr11 29002384
chr11 29002908
chr11 29081521
chr11 29082373
chr11 29082582
chr11 29082712
chr11 29118616

chr11 29118786
chr11 29119247
chr11 29119619
chr11 29715396
chr11 29715957
chr11 29932054
chr11 30016062
chr11 30016083
chr11 30016087
chr11 30322754
chr11 30322795
chr11 30322826
chr11 30322852
chr11 30323052
chr11 30323430
chr11 30323452
chr11 30323455
chr11 30323489
chr11 30323498
chr11 30323533
chr11 30323601
chr11 30323638
chr11 30323670
chr11 30323703
chr11 30400056
chr11 30588755
chr11 30589007
chr11 30951489
chr11 30952186
chr11 30953226
chr11 31317705
chr11 31317825
chr11 31318830
chr11 31318832
chr11 31318868
chr11 31318907
chr11 31370506
chr11 31508719
chr11 31508892
chr11 31509004
chr11 31509056
chr11 31509118
chr11 31509231
chr11 31509246
chr11 31509318
chr11 31509329
chr11 31510256
chr11 31510266
chr11 31510378
chr11 31510468
chr11 31510524
chr11 31510579
chr11 31510602
chr11 31510680

chr11 31808304
chr11 31808415
chr11 31821372
chr11 31821376
chr11 31821427
chr11 31821566
chr11 31828141
chr11 31828161
chr11 31828341
chr11 31828355
chr11 31828385
chr11 31828445
chr11 31828487
chr11 31828543
chr11 31828557
chr11 31828835
chr11 32323859
chr11 32323867
chr11 32324057
chr11 32420778
chr11 32443947
chr11 32444364
chr11 32584046
chr11 32584048
chr11 32584063
chr11 32584075
chr11 32584077
chr11 32584079
chr11 32584091
chr11 32584096
chr11 32584106
chr11 32584109
chr11 32584113
chr11 32584116
chr11 32584125
chr11 32584127
chr11 32584224
chr11 32584228
chr11 32584247
chr11 32584324
chr11 32584329
chr11 32584333
chr11 32584335
chr11 32584345
chr11 32584370
chr11 32584373
chr11 32584378
chr11 32584389
chr11 32584403
chr11 32584423
chr11 32584464
chr11 32584468
chr11 32584470
chr11 32584478

chr11 32584480
chr11 32584502
chr11 32584522
chr11 32584532
chr11 32584534
chr11 32584539
chr11 32584562
chr11 32584564
chr11 32584586
chr11 32584594
chr11 32584637
chr11 32584641
chr11 32584663
chr11 32584674
chr11 32584733
chr11 32596169
chr11 32642403
chr11 32642978
chr11 32643213
chr11 32643495
chr11 32643708
chr11 32792188
chr11 32792399
chr11 32792598
chr11 32792796
chr11 32792882
chr11 32792941
chr11 32792996
chr11 32793015
chr11 32793024
chr11 32793033
chr11 32793060
chr11 32793075
chr11 32793097
chr11 32828733
chr11 32829250
chr11 32829288
chr11 32829441
chr11 32829503
chr11 32829541
chr11 32829547
chr11 32892142
chr11 32892207
chr11 32892226
chr11 32892315
chr11 32893864
chr11 32893885
chr11 32893893
chr11 32893895
chr11 32893899
chr11 32893901
chr11 32893913
chr11 32893930
chr11 32893951

chr11 32893965
chr11 32893967
chr11 32894045
chr11 32894076
chr11 32894104
chr11 32894141
chr11 32894155
chr11 32894171
chr11 32894243
chr11 32894245
chr11 32894277
chr11 32894305
chr11 32894332
chr11 33011363
chr11 33011407
chr11 33011486
chr11 33040381
chr11 33040476
chr11 33040514
chr11 33040619
chr11 33040677
chr11 33158066
chr11 33160198
chr11 33160226
chr11 33160258
chr11 33160300
chr11 33160347
chr11 33160373
chr11 33160392
chr11 33160491
chr11 33160753
chr11 33160779
chr11 33160835
chr11 33160923
chr11 33259008
chr11 33259036
chr11 33259048
chr11 33259254
chr11 33259259
chr11 33433349
chr11 33433472
chr11 33434028
chr11 33773690
chr11 33773937
chr11 33773958
chr11 33773961
chr11 33773971
chr11 33773980
chr11 33803171
chr11 33858072
chr11 33868240
chr11 33868269
chr11 33893768
chr11 33893812

chr11 33893826
chr11 33893982
chr11 33894042
chr11 33894092
chr11 33894111
chr11 33894199
chr11 33894203
chr11 33894205
chr11 33894214
chr11 33894273
chr11 33894288
chr11 33894343
chr11 33894502
chr11 33894548
chr11 33894627
chr11 33894657
chr11 33894875
chr11 34018508
chr11 34018657
chr11 34053836
chr11 34053848
chr11 34053859
chr11 34054175
chr11 34056211
chr11 34056439
chr11 34056842
chr11 34057018
chr11 34248668
chr11 34248852
chr11 34248856
chr11 34248858
chr11 34248868
chr11 34360175
chr11 34360688
chr11 34360776
chr11 34467380
chr11 34467556
chr11 34467571
chr11 34557948
chr11 34585729
chr11 34586077
chr11 34586100
chr11 34586104
chr11 34650558
chr11 34650613
chr11 34650647
chr11 34688108
chr11 34688286
chr11 34689454
chr11 34690220
chr11 34690483
chr11 34691097
chr11 34691211
chr11 34691310

chr11 34691315
chr11 34691328
chr11 34691354
chr11 34691461
chr11 34691548
chr11 34691577
chr11 34691603
chr11 34691816
chr11 34691847
chr11 35007010
chr11 35007038
chr11 35007353
chr11 35030089
chr11 35030110
chr11 35030212
chr11 35030217
chr11 35030360
chr11 35030727
chr11 35040217
chr11 35040264
chr11 35040272
chr11 35040280
chr11 35040541
chr11 35040547
chr11 35040578
chr11 35040597
chr11 35040602
chr11 35040699
chr11 35044847
chr11 35045098
chr11 35045126
chr11 35045168
chr11 35045238
chr11 35045257
chr11 35045384
chr11 35045400
chr11 35045412
chr11 35140262
chr11 35140283
chr11 35140341
chr11 35140353
chr11 35140462
chr11 35140918
chr11 35141007
chr11 35141191
chr11 35141302
chr11 35141593
chr11 35141663
chr11 35141682
chr11 35141692
chr11 35141703
chr11 35141755
chr11 35141762
chr11 35144350

chr11 35144418
chr11 35144467
chr11 35144589
chr11 35144618
chr11 35144683
chr11 35166278
chr11 35166284
chr11 35173727
chr11 35173966
chr11 35174521
chr11 35174736
chr11 35174800
chr11 35217230
chr11 35217308
chr11 35217409
chr11 35272325
chr11 35272672
chr11 35272674
chr11 35272747
chr11 35433637
chr11 35485443
chr11 35485552
chr11 35485758
chr11 35485842
chr11 35485856
chr11 35485872
chr11 35485880
chr11 35662757
chr11 35662762
chr11 35662765
chr11 35662767
chr11 35662774
chr11 35662777
chr11 35662784
chr11 35662786
chr11 35662824
chr11 35662827
chr11 35662829
chr11 35662836
chr11 35662839
chr11 35662845
chr11 35662848
chr11 35662856
chr11 35662858
chr11 35662861
chr11 35662874
chr11 35662876
chr11 35662888
chr11 35662901
chr11 35662911
chr11 35662958
chr11 35662960
chr11 35662973
chr11 35662978

chr11 35662983
chr11 35662985
chr11 35662988
chr11 35662990
chr11 35662999
chr11 35663006
chr11 35821946
chr11 35821950
chr11 35821958
chr11 35822083
chr11 35822211
chr11 35822257
chr11 35822274
chr11 35822346
chr11 35822466
chr11 36091206
chr11 36091287
chr11 36091343
chr11 36091393
chr11 36091406
chr11 36091464
chr11 36347078
chr11 36347244
chr11 36376377
chr11 36377918
chr11 36377968
chr11 36378027
chr11 36378071
chr11 36378162
chr11 36378179
chr11 36378191
chr11 36378277
chr11 36378367
chr11 36378984
chr11 36380942
chr11 36380951
chr11 36409266
chr11 36409298
chr11 36409326
chr11 36409343
chr11 36409539
chr11 36483066
chr11 36487869
chr11 36487912
chr11 36487960
chr11 36509355
chr11 36509358
chr11 36509371
chr11 36509375
chr11 36509383
chr11 36509395
chr11 36509411
chr11 36509444
chr11 36509511

chr11 36509517
chr11 36509526
chr11 36509572
chr11 36509618
chr11 36509633
chr11 36509648
chr11 36509651
chr11 36509653
chr11 36509723
chr11 36509732
chr11 36509746
chr11 36509763
chr11 36509768
chr11 36509782
chr11 36509796
chr11 36509805
chr11 36509812
chr11 36509819
chr11 36509833
chr11 36509836
chr11 36509842
chr11 36509847
chr11 36509860
chr11 36509870
chr11 36509873
chr11 36509893
chr11 36509910
chr11 36509916
chr11 36509930
chr11 36509947
chr11 36509953
chr11 36509961
chr11 36509967
chr11 36509975
chr11 36509982
chr11 36509995
chr11 36510005
chr11 36510010
chr11 36558841
chr11 36558911
chr11 36564105
chr11 36564553
chr11 36564601
chr11 36564641
chr11 36564644
chr11 36564750
chr11 36564760
chr11 36564771
chr11 36564774
chr11 36564790
chr11 36564816
chr11 36564824
chr11 36564827
chr11 36564872

chr11 36594588
chr11 36594596
chr11 36594659
chr11 36594667
chr11 36594671
chr11 36594679
chr11 36594692
chr11 36594696
chr11 36594701
chr11 36594707
chr11 36594716
chr11 36594768
chr11 36594800
chr11 36594815
chr11 36594834
chr11 36594846
chr11 36594851
chr11 36594881
chr11 36594883
chr11 36594905
chr11 36594926
chr11 36594946
chr11 36594961
chr11 36594980
chr11 36595085
chr11 36595422
chr11 36684835
chr11 36684866
chr11 36685053
chr11 36685087
chr11 36685132
chr11 36693249
chr11 36693877
chr11 36693947
chr11 36941594
chr11 36941603
chr11 36942326
chr11 36949192
chr11 37809614
chr11 37809623
chr11 38337865
chr11 38479990
chr11 38480063
chr11 38969433
chr11 38969493
chr11 38969516
chr11 39074370
chr11 39074509
chr11 39074713
chr11 39076476
chr11 39076652
chr11 39076688
chr11 39076748
chr11 39077307

chr11 39077403
chr11 39078061
chr11 39164540
chr11 39189630
chr11 39189791
chr11 39190194
chr11 39190325
chr11 39190641
chr11 39195472
chr11 39269188
chr11 39352238
chr11 39371310
chr11 39371348
chr11 39371377
chr11 39371400
chr11 39371417
chr11 39372054
chr11 39386714
chr11 39526302
chr11 39526375
chr11 39526461
chr11 39650891
chr11 39651184
chr11 39662964
chr11 39753415
chr11 39753566
chr11 39875057
chr11 39875219
chr11 39878262
chr11 39878691
chr11 40019455
chr11 40019871
chr11 40292623
chr11 40292716
chr11 40292718
chr11 40292850
chr11 40292854
chr11 40292873
chr11 40314993
chr11 40315259
chr11 40511661
chr11 40593485
chr11 40593751
chr11 40594111
chr11 40722567
chr11 40909876
chr11 40912547
chr11 40912642
chr11 40913499
chr11 40913653
chr11 41460792
chr11 41460962
chr11 41460998
chr11 41498834

chr11 41499296
chr11 41624533
chr11 42311672
chr11 42311693
chr11 42952131
chr11 42952917
chr11 43215355
chr11 43215545
chr11 43215670
chr11 43215688
chr11 43311582
chr11 43311595
chr11 43311649
chr11 43311674
chr11 43311751
chr11 43311777
chr11 43311860
chr11 43312705
chr11 43312739
chr11 43312743
chr11 43312768
chr11 43312770
chr11 43312831
chr11 43312846
chr11 43359764
chr11 43359818
chr11 43359833
chr11 43359876
chr11 43359913
chr11 43359940
chr11 43359946
chr11 43359959
chr11 43359990
chr11 43360078
chr11 43360154
chr11 43360185
chr11 43360239
chr11 43360284
chr11 43360302
chr11 43360486
chr11 43556790
chr11 43556800
chr11 43556811
chr11 43556918
chr11 43556955
chr11 43557205
chr11 43557208
chr11 43557356
chr11 43557429
chr11 43557452
chr11 43557557
chr11 43557590
chr11 43557684
chr11 43557763

chr11 43557769
chr11 43557787
chr11 43557866
chr11 43557927
chr11 43557941
chr11 43557961
chr11 43557985
chr11 43558013
chr11 43574349
chr11 43574803
chr11 43574811
chr11 43574835
chr11 43574955
chr11 43574979
chr11 43574992
chr11 43575010
chr11 43575020
chr11 43575032
chr11 43575038
chr11 43575044
chr11 43579754
chr11 43579756
chr11 43579783
chr11 43579797
chr11 43579826
chr11 43579829
chr11 43579836
chr11 43579845
chr11 43579849
chr11 43579858
chr11 43579878
chr11 43579880
chr11 43579882
chr11 43579884
chr11 43579886
chr11 43579888
chr11 43579892
chr11 43579896
chr11 43579900
chr11 43579903
chr11 43580033
chr11 43580106
chr11 43580116
chr11 43580167
chr11 43580171
chr11 43580276
chr11 43580295
chr11 43580298
chr11 43580323
chr11 43580329
chr11 43580381
chr11 43580419
chr11 43580450
chr11 43580456

chr11 43580467
chr11 43580604
chr11 43580859
chr11 43580870
chr11 43643677
chr11 43643828
chr11 43643878
chr11 43644025
chr11 43644036
chr11 43644064
chr11 43644112
chr11 43644139
chr11 43644147
chr11 43644150
chr11 43644773
chr11 43644785
chr11 43881324
chr11 44093963
chr11 44094367
chr11 44094379
chr11 44094458
chr11 44094530
chr11 44094570
chr11 44096318
chr11 44096351
chr11 44096366
chr11 44096372
chr11 44212846
chr11 44213396
chr11 44213703
chr11 44213833
chr11 44322111
chr11 44322414
chr11 44322436
chr11 44322490
chr11 44322500
chr11 44322528
chr11 44322541
chr11 44322656
chr11 44322662
chr11 44322703
chr11 44565409
chr11 44565418
chr11 44565437
chr11 44565442
chr11 44565447
chr11 44565471
chr11 44565477
chr11 44565494
chr11 44565507
chr11 44565518
chr11 44565521
chr11 44565527
chr11 44565532

chr11 44565549
chr11 44565555
chr11 44565561
chr11 44565564
chr11 44565573
chr11 44565588
chr11 44565598
chr11 44565604
chr11 44565606
chr11 44565609
chr11 44565616
chr11 44565626
chr11 44565637
chr11 44565655
chr11 44565669
chr11 44565672
chr11 44565675
chr11 44565686
chr11 44565690
chr11 44565697
chr11 44565711
chr11 44565713
chr11 44565732
chr11 44566081
chr11 44566099
chr11 44566101
chr11 44566148
chr11 44566178
chr11 44566189
chr11 44566242
chr11 44568995
chr11 44569023
chr11 44569107
chr11 44569140
chr11 44569159
chr11 44569233
chr11 44569276
chr11 44569310
chr11 44569339
chr11 44611945
chr11 44612028
chr11 44612065
chr11 44612104
chr11 44612196
chr11 44612282
chr11 45103468
chr11 45123776
chr11 45123787
chr11 45123820
chr11 45125645
chr11 45125857
chr11 45355487
chr11 45355528
chr11 45355530

chr11 45355547
chr11 45355571
chr11 45355575
chr11 45355591
chr11 45355593
chr11 45355601
chr11 45355626
chr11 45355631
chr11 45355633
chr11 45355653
chr11 45355660
chr11 45355673
chr11 45355678
chr11 45355691
chr11 45355737
chr11 45355773
chr11 45355779
chr11 45355866
chr11 45843200
chr11 45917846
chr11 45917850
chr11 45917860
chr11 45917863
chr11 45918035
chr11 45918068
chr11 45918075
chr11 45918086
chr11 45918093
chr11 45918096
chr11 45918115
chr11 45918122
chr11 45918139
chr11 45918145
chr11 45918159
chr11 45918168
chr11 45918172
chr11 45918215
chr11 45918233
chr11 45918236
chr11 45918245
chr11 45918249
chr11 45918258
chr11 45918283
chr11 45918306
chr11 45918326
chr11 45918331
chr11 46100120
chr11 46118942
chr11 46119016
chr11 46119045
chr11 46119212
chr11 46119234
chr11 46119348
chr11 46119424

chr11 46119482
chr11 46119526
chr11 46119540
chr11 46122230
chr11 46122245
chr11 46122282
chr11 46122436
chr11 46122442
chr11 46122451
chr11 46122458
chr11 46122468
chr11 46122594
chr11 46122600
chr11 46122609
chr11 46122612
chr11 46122735
chr11 46122740
chr11 46126970
chr11 46127244
chr11 46127367
chr11 46127431
chr11 46127472
chr11 46127489
chr11 46347757
chr11 46347766
chr11 46348006
chr11 46348013
chr11 46348017
chr11 46348023
chr11 46348027
chr11 46348044
chr11 46348068
chr11 46348081
chr11 46348098
chr11 46348109
chr11 46348153
chr11 46348191
chr11 46348259
chr11 46348274
chr11 46348302
chr11 46348318
chr11 46348326
chr11 46348335
chr11 46348649
chr11 46348657
chr11 46348718
chr11 46348725
chr11 46348744
chr11 46348763
chr11 46348798
chr11 46348812
chr11 46348969
chr11 46349028
chr11 46349042

chr11 46349060
chr11 46349086
chr11 46349095
chr11 46349179
chr11 46349199
chr11 46560010
chr11 46560275
chr11 46560470
chr11 46560508
chr11 46560594
chr11 46560660
chr11 46593194
chr11 46593259
chr11 46593317
chr11 46593358
chr11 46593374
chr11 46593481
chr11 46593490
chr11 46593531
chr11 46593544
chr11 46593565
chr11 46616947
chr11 46700776
chr11 46700781
chr11 46700784
chr11 46700817
chr11 46700833
chr11 46700852
chr11 46700859
chr11 46700866
chr11 46700877
chr11 46700978
chr11 46701021
chr11 46701024
chr11 46701044
chr11 46701058
chr11 46701088
chr11 46701098
chr11 46701112
chr11 46701119
chr11 46701121
chr11 46701152
chr11 46701190
chr11 46701222
chr11 46701297
chr11 46701304
chr11 46701324
chr11 46756417
chr11 46846266
chr11 46846452
chr11 46846458
chr11 46846473
chr11 46846491
chr11 46846511

chr11 46846525
chr11 46846578
chr11 46846659
chr11 46846673
chr11 46846778
chr11 46846855
chr11 46846891
chr11 46916991
chr11 46916998
chr11 46917018
chr11 46917020
chr11 46917032
chr11 46917034
chr11 47214449
chr11 47214462
chr11 47214468
chr11 47214484
chr11 47214487
chr11 47214489
chr11 47214504
chr11 47214531
chr11 47214555
chr11 47214560
chr11 47214572
chr11 47214580
chr11 47214627
chr11 47214631
chr11 47214639
chr11 47214643
chr11 47214685
chr11 47214725
chr11 47214749
chr11 47214798
chr11 47214834
chr11 47214854
chr11 47214863
chr11 47214872
chr11 47214878
chr11 47214887
chr11 47214911
chr11 47214913
chr11 47214919
chr11 47214936
chr11 47214945
chr11 47214950
chr11 47214960
chr11 47215002
chr11 47215591
chr11 47215606
chr11 47215612
chr11 47215707
chr11 47215722
chr11 47215784
chr11 47215787

chr11 47215928
chr11 47216009
chr11 47216095
chr11 47216187
chr11 47216215
chr11 47216243
chr11 47216248
chr11 47216270
chr11 47216295
chr11 47714980
chr11 47715016
chr11 47715018
chr11 47715021
chr11 47715027
chr11 47715032
chr11 47715042
chr11 47715151
chr11 47715188
chr11 47715197
chr11 47715201
chr11 47715207
chr11 47715209
chr11 47715214
chr11 47715219
chr11 47715233
chr11 47715243
chr11 47715260
chr11 47715269
chr11 47715276
chr11 47715306
chr11 47715324
chr11 47715333
chr11 47715337
chr11 47715343
chr11 47715360
chr11 47715376
chr11 47715389
chr11 47715400
chr11 47715405
chr11 47715409
chr11 47715413
chr11 47715419
chr11 47715434
chr11 47715451
chr11 47766084
chr11 47766097
chr11 47766115
chr11 47766126
chr11 47766142
chr11 47766170
chr11 47766219
chr11 47766228
chr11 47766238
chr11 47766269

chr11 47766271
chr11 47766293
chr11 47766314
chr11 47766438
chr11 47766456
chr11 47766464
chr11 47766486
chr11 47766490
chr11 47766492
chr11 47766501
chr11 47766504
chr11 47768146
chr11 47768194
chr11 47768217
chr11 47768299
chr11 47768346
chr11 47768366
chr11 47768461
chr11 47768472
chr11 47768483
chr11 47768535
chr11 47934502
chr11 47934595
chr11 47934629
chr11 47934642
chr11 47934677
chr11 48109831
chr11 48109874
chr11 48109912
chr11 48110237
chr11 48110275
chr11 48110292
chr11 48110299
chr11 48307962
chr11 48308000
chr11 48308005
chr11 48308029
chr11 48308194
chr11 48606092
chr11 48606828
chr11 48882589
chr11 48889893
chr11 48890421
chr11 48890566
chr11 48890586
chr11 48895560
chr11 48895724
chr11 48895758
chr11 48895773
chr11 48895775
chr11 48898822
chr11 48898845
chr11 48904479
chr11 48905289

chr11 48905547
chr11 48953159
chr11 48953758
chr11 48954042
chr11 48954075
chr11 48958655
chr11 48958892
chr11 49037447
chr11 49037631
chr11 49037783
chr11 49037840
chr11 49050351
chr11 49050796
chr11 49264133
chr11 49264153
chr11 49264255
chr11 49264316
chr11 49454413
chr11 49555704
chr11 49555949
chr11 49556864
chr11 49557812
chr11 49660331
chr11 49664022
chr11 49664193
chr11 49664389
chr11 49664727
chr11 49722013
chr11 49722162
chr11 49722297
chr11 49723212
chr11 49723541
chr11 49796730
chr11 49797255
chr11 49797288
chr11 49799726
chr11 49799851
chr11 49799871
chr11 49802531
chr11 49802727
chr11 49943945
chr11 49945565
chr11 50120429
chr11 50121800
chr11 50121823
chr11 50121964
chr11 50121981
chr11 50122753
chr11 50122776
chr11 50122975
chr11 50128659
chr11 50128698
chr11 50128700
chr11 50128706

chr11 50128708
chr11 50131644
chr11 50131808
chr11 50131882
chr11 50131932
chr11 50133937
chr11 50165304
chr11 50165698
chr11 50165739
chr11 50171390
chr11 50171668
chr11 50249718
chr11 50295312
chr11 50295332
chr11 50295411
chr11 50295800
chr11 50821118
chr11 50821323
chr11 52578824
chr11 52582083
chr11 54847891
chr11 54847942
chr11 54848021
chr11 54848079
chr11 54848317
chr11 54848356
chr11 54848678
chr11 55458004
chr11 55682134
chr11 55682810
chr11 55683038
chr11 56330741
chr11 56333761
chr11 57123546
chr11 57123587
chr11 57123684
chr11 57231360
chr11 57231469
chr11 57231472
chr11 57231562
chr11 57231642
chr11 57232685
chr11 57232814
chr11 57232817
chr11 57289937
chr11 57290018
chr11 57457717
chr11 57457822
chr11 57457835
chr11 57457927
chr11 57457952
chr11 57458125
chr11 57458140
chr11 57458216

chr11 57458236
chr11 57458257
chr11 57458281
chr11 57458285
chr11 57458302
chr11 57458309
chr11 57458340
chr11 57458348
chr11 57458365
chr11 57458388
chr11 57458395
chr11 57458397
chr11 57458404
chr11 57458411
chr11 57458467
chr11 57458484
chr11 57458491
chr11 57458509
chr11 57458514
chr11 57458525
chr11 57458541
chr11 57458554
chr11 57458556
chr11 57458566
chr11 57458576
chr11 57458614
chr11 57458617
chr11 57458620
chr11 57458643
chr11 57458656
chr11 57458676
chr11 57458730
chr11 57458770
chr11 57458776
chr11 57514505
chr11 57514507
chr11 57514519
chr11 57514527
chr11 57514529
chr11 57514586
chr11 57514636
chr11 57514640
chr11 57514663
chr11 57514679
chr11 57514695
chr11 57514717
chr11 57514736
chr11 57514747
chr11 57514763
chr11 57514765
chr11 57514767
chr11 57514783
chr11 57514792
chr11 57514814

chr11 57514821
chr11 57514841
chr11 57514984
chr11 57514987
chr11 57530062
chr11 57530132
chr11 57530157
chr11 57530203
chr11 57530320
chr11 57530333
chr11 57530372
chr11 57530403
chr11 57530412
chr11 57530421
chr11 57530434
chr11 57530441
chr11 57530489
chr11 57530496
chr11 57531394
chr11 57531409
chr11 57531475
chr11 57531676
chr11 57566139
chr11 57566466
chr11 57566501
chr11 57566528
chr11 57638692
chr11 57638702
chr11 57638708
chr11 57638715
chr11 57638798
chr11 57638854
chr11 57638898
chr11 57638924
chr11 57638933
chr11 57638937
chr11 57638972
chr11 57638978
chr11 57638990
chr11 57639000
chr11 57639007
chr11 57639068
chr11 57639094
chr11 57639125
chr11 57639148
chr11 57639152
chr11 57639258
chr11 57639262
chr11 57639281
chr11 57639296
chr11 57639328
chr11 57639412
chr11 57639415
chr11 57639426

chr11 57639555
chr11 57639601
chr11 57649401
chr11 57649485
chr11 57649498
chr11 57649505
chr11 57649534
chr11 57649547
chr11 57649556
chr11 57649621
chr11 57649709
chr11 57649774
chr11 57649883
chr11 57649895
chr11 57649983
chr11 57650013
chr11 57650018
chr11 57668951
chr11 57668958
chr11 57712180
chr11 57712189
chr11 57712194
chr11 57712197
chr11 57712206
chr11 57712215
chr11 57712259
chr11 57742071
chr11 57742080
chr11 57742096
chr11 57792428
chr11 57792436
chr11 57792438
chr11 57792440
chr11 57792446
chr11 57792448
chr11 57792493
chr11 57792612
chr11 57792616
chr11 57800833
chr11 57855870
chr11 57905760
chr11 57905797
chr11 57996772
chr11 57996949
chr11 57997079
chr11 58025950
chr11 58291283
chr11 58388317
chr11 58525429
chr11 58525439
chr11 58525502
chr11 58525542
chr11 58525792
chr11 58525876

chr11 58572966
chr11 58573011
chr11 58573074
chr11 58573155
chr11 58573160
chr11 58573186
chr11 58573194
chr11 58573200
chr11 58573241
chr11 58573729
chr11 58573871
chr11 58573923
chr11 58574027
chr11 58574144
chr11 58574363
chr11 58574552
chr11 58574653
chr11 58574838
chr11 58575335
chr11 58575413
chr11 58575459
chr11 58575524
chr11 58575526
chr11 58575807
chr11 58575845
chr11 58577276
chr11 58577540
chr11 58580282
chr11 58580353
chr11 58580404
chr11 58580836
chr11 58580994
chr11 58865169
chr11 58865547
chr11 58865564
chr11 58879350
chr11 58962028
chr11 58962115
chr11 59058268
chr11 59058270
chr11 59058358
chr11 59058362
chr11 59058397
chr11 59058422
chr11 59058441
chr11 59058558
chr11 59102285
chr11 59107554
chr11 59107560
chr11 59107564
chr11 59107566
chr11 59107589
chr11 59107872
chr11 59107884

chr11 59108043
chr11 59108054
chr11 59108105
chr11 59108179
chr11 59142311
chr11 59142915
chr11 59142930
chr11 59142955
chr11 59142958
chr11 59142966
chr11 59142992
chr11 59142994
chr11 59142997
chr11 59143010
chr11 59143032
chr11 59143034
chr11 59143042
chr11 59143074
chr11 59143093
chr11 59143103
chr11 59143110
chr11 59143177
chr11 59143179
chr11 59143259
chr11 59143269
chr11 59143289
chr11 59143360
chr11 59143408
chr11 59144372
chr11 59144495
chr11 59144528
chr11 59145097
chr11 59145102
chr11 59145105
chr11 59145139
chr11 59145187
chr11 59145535
chr11 59172698
chr11 59172700
chr11 59172703
chr11 59172754
chr11 59172756
chr11 59172829
chr11 59172834
chr11 59172902
chr11 59172947
chr11 59172957
chr11 59172968
chr11 59173048
chr11 59173083
chr11 59173135
chr11 59173165
chr11 59173189
chr11 59173194

chr11 59173198
chr11 59207907
chr11 59208003
chr11 59208100
chr11 59208113
chr11 59511322
chr11 59537346
chr11 59537386
chr11 59537425
chr11 59537440
chr11 59537485
chr11 59537521
chr11 59537543
chr11 59537554
chr11 59537561
chr11 59537578
chr11 59537586
chr11 59537608
chr11 59550044
chr11 59550073
chr11 59550081
chr11 59551378
chr11 59551444
chr11 59551471
chr11 59551529
chr11 59551541
chr11 59551552
chr11 59551597
chr11 59561003
chr11 59561011
chr11 59561021
chr11 59561028
chr11 59561033
chr11 59561059
chr11 59561113
chr11 59561117
chr11 59566427
chr11 59566449
chr11 59566456
chr11 59566465
chr11 59566511
chr11 59566740
chr11 59566744
chr11 59614823
chr11 59614841
chr11 59616526
chr11 59616533
chr11 59616554
chr11 59616597
chr11 59616611
chr11 59668396
chr11 59668461
chr11 59755615
chr11 59755635

chr11 59810817
chr11 59810820
chr11 59810826
chr11 59810837
chr11 59810841
chr11 59810844
chr11 59810858
chr11 59810878
chr11 59810888
chr11 59810891
chr11 59810895
chr11 59810905
chr11 59810974
chr11 59810977
chr11 59810982
chr11 59811017
chr11 59811041
chr11 59811067
chr11 59811187
chr11 59811202
chr11 59823218
chr11 59823473
chr11 59858164
chr11 59858215
chr11 59858277
chr11 59858335
chr11 60367146
chr11 60367344
chr11 60447593
chr11 60477151
chr11 60477159
chr11 60477275
chr11 60477316
chr11 60477352
chr11 60514185
chr11 60514297
chr11 60514670
chr11 60533105
chr11 60533112
chr11 60533153
chr11 60533271
chr11 60533365
chr11 60533392
chr11 60533539
chr11 60533825
chr11 60619283
chr11 60646317
chr11 60842388
chr11 60842393
chr11 60842404
chr11 60842429
chr11 60842467
chr11 60842477
chr11 60842531

chr11 60842564
chr11 60842588
chr11 60842591
chr11 60842619
chr11 60842634
chr11 60842639
chr11 60842692
chr11 60842708
chr11 60842735
chr11 60914939
chr11 60915007
chr11 60915011
chr11 60915020
chr11 60915084
chr11 60915114
chr11 60915121
chr11 60915125
chr11 60915170
chr11 60915232
chr11 60915295
chr11 60915335
chr11 60915357
chr11 60915379
chr11 60915459
chr11 60915542
chr11 60915574
chr11 60915598
chr11 60915601
chr11 60915627
chr11 60915630
chr11 60915645
chr11 60915665
chr11 60915758
chr11 60915760
chr11 60915813
chr11 60978976
chr11 61061079
chr11 61061105
chr11 61061147
chr11 61061188
chr11 61061203
chr11 61061208
chr11 61061231
chr11 61061264
chr11 61061339
chr11 61061344
chr11 61061449
chr11 61061508
chr11 61061605
chr11 61061629
chr11 61206334
chr11 61207327
chr11 61207374
chr11 61207402

chr11 61429067
chr11 61429075
chr11 61429077
chr11 61429196
chr11 61947814
chr11 61948116
chr11 61948131
chr11 61966525
chr11 61966599
chr11 61967326
chr11 61967335
chr11 61967358
chr11 61967368
chr11 61967397
chr11 61967405
chr11 61967414
chr11 61967416
chr11 61967420
chr11 61967428
chr11 61967431
chr11 61967445
chr11 61967448
chr11 61967451
chr11 61967454
chr11 61967457
chr11 61967466
chr11 61967468
chr11 61967471
chr11 61967479
chr11 61967482
chr11 61967485
chr11 61967504
chr11 61967512
chr11 61967526
chr11 61967544
chr11 61967548
chr11 61967552
chr11 61967564
chr11 61967568
chr11 61967571
chr11 61967578
chr11 61967607
chr11 61967610
chr11 61967617
chr11 61967620
chr11 61967627
chr11 61967639
chr11 61967644
chr11 61967664
chr11 61967666
chr11 61967669
chr11 61967672
chr11 61967681
chr11 61967686

chr11 61967697
chr11 61967700
chr11 61967704
chr11 61967709
chr11 61967715
chr11 61967724
chr11 61967731
chr11 61967733
chr11 61967741
chr11 61967763
chr11 61967767
chr11 61967780
chr11 61967782
chr11 61967784
chr11 62025524
chr11 62025803
chr11 62025807
chr11 62025813
chr11 62025841
chr11 62025847
chr11 62025852
chr11 62337912
chr11 62337914
chr11 62337916
chr11 62337989
chr11 62338001
chr11 62338004
chr11 62338007
chr11 62338013
chr11 62338032
chr11 62338041
chr11 62380811
chr11 62380844
chr11 62380862
chr11 62380961
chr11 62380976
chr11 62541320
chr11 62544939
chr11 62544970
chr11 62544992
chr11 62544998
chr11 62545018
chr11 62545025
chr11 62555590
chr11 62555744
chr11 62555792
chr11 62556016
chr11 62556222
chr11 62556227
chr11 62556310
chr11 62556382
chr11 62556440
chr11 62557086
chr11 62557559

chr11 62557651
chr11 62574170
chr11 62574182
chr11 62574184
chr11 62574198
chr11 62574203
chr11 62574205
chr11 62574327
chr11 62574343
chr11 62574345
chr11 62574413
chr11 62574429
chr11 62574440
chr11 62574501
chr11 62574513
chr11 62574521
chr11 62590774
chr11 62591033
chr11 62591075
chr11 62599334
chr11 62599347
chr11 62599679
chr11 62599683
chr11 62599695
chr11 62599715
chr11 62599797
chr11 62599830
chr11 62599910
chr11 62599969
chr11 62600213
chr11 62600216
chr11 62600292
chr11 62600516
chr11 62600588
chr11 62600641
chr11 62600684
chr11 62600718
chr11 62600741
chr11 62652267
chr11 62652275
chr11 62652305
chr11 62652374
chr11 62652379
chr11 62652686
chr11 62652795
chr11 62652914
chr11 62652968
chr11 62652995
chr11 62664722
chr11 62664782
chr11 62709054
chr11 62709070
chr11 62709072
chr11 62709081

chr11 62709090
chr11 62709299
chr11 62709309
chr11 62709325
chr11 62709335
chr11 62709339
chr11 62709341
chr11 62709349
chr11 62709380
chr11 62709394
chr11 62709419
chr11 62709426
chr11 62709453
chr11 62709480
chr11 62709492
chr11 62709525
chr11 62709545
chr11 62709547
chr11 62709557
chr11 62709580
chr11 62709610
chr11 62709632
chr11 62709637
chr11 62709647
chr11 62709671
chr11 62709686
chr11 62709689
chr11 62709710
chr11 62709755
chr11 62709762
chr11 62709764
chr11 62709777
chr11 62709791
chr11 62709802
chr11 62709811
chr11 62709821
chr11 62709825
chr11 62709834
chr11 62709872
chr11 62709878
chr11 62709881
chr11 62709888
chr11 62709890
chr11 62709901
chr11 62725042
chr11 62725228
chr11 62725236
chr11 62725258
chr11 62725260
chr11 62725274
chr11 62725283
chr11 62725290
chr11 62725721
chr11 62725800

chr11 62725837
chr11 62725875
chr11 62725889
chr11 62729094
chr11 62729163
chr11 62729328
chr11 62729341
chr11 62753899
chr11 62753903
chr11 62753946
chr11 62753965
chr11 62753971
chr11 62753978
chr11 62753986
chr11 62754024
chr11 62754035
chr11 62754045
chr11 62754071
chr11 62754110
chr11 62754138
chr11 62754153
chr11 62754160
chr11 62754214
chr11 62754224
chr11 62761681
chr11 62761718
chr11 62761721
chr11 62761724
chr11 62761726
chr11 62761741
chr11 62761759
chr11 62761772
chr11 62761790
chr11 62761809
chr11 62761826
chr11 62761828
chr11 62761836
chr11 62761856
chr11 62761878
chr11 62761913
chr11 62761922
chr11 62761927
chr11 62761935
chr11 62761939
chr11 62761944
chr11 62761950
chr11 62761955
chr11 62761988
chr11 62762062
chr11 62762165
chr11 62804811
chr11 62804828
chr11 62804846
chr11 62804923

chr11 62831577
chr11 62831598
chr11 62831604
chr11 62831606
chr11 62831640
chr11 62831665
chr11 62831672
chr11 62842228
chr11 62842230
chr11 62842233
chr11 62842235
chr11 62853632
chr11 62853702
chr11 62853706
chr11 62853786
chr11 62853895
chr11 62853934
chr11 62854125
chr11 62854319
chr11 62854504
chr11 62854701
chr11 62854707
chr11 62854755
chr11 62905645
chr11 63024096
chr11 63024124
chr11 63024156
chr11 63024210
chr11 63024214
chr11 63024228
chr11 63024232
chr11 63024269
chr11 63024308
chr11 63024338
chr11 63024423
chr11 63024617
chr11 63024669
chr11 63241399
chr11 63242240
chr11 63242356
chr11 63242376
chr11 63242484
chr11 63352884
chr11 63353615
chr11 63353876
chr11 63436063
chr11 63436290
chr11 63436321
chr11 63436346
chr11 63436367
chr11 63436493
chr11 63436502
chr11 63436511
chr11 63439386

chr11 63439716
chr11 63439881
chr11 63440003
chr11 63440862
chr11 63440875
chr11 63441192
chr11 63441235
chr11 63441922
chr11 63479129
chr11 63479158
chr11 63479261
chr11 63518708
chr11 63536865
chr11 63536876
chr11 63536985
chr11 63537083
chr11 63537099
chr11 63537108
chr11 63537142
chr11 63537232
chr11 63537269
chr11 63537279
chr11 63537297
chr11 63567068
chr11 63567136
chr11 63567211
chr11 63567391
chr11 63567789
chr11 63664053
chr11 63682086
chr11 63682142
chr11 63682358
chr11 63682378
chr11 63682396
chr11 63682411
chr11 63682471
chr11 63682506
chr11 63682511
chr11 63682527
chr11 63682537
chr11 63682616
chr11 63738826
chr11 63738841
chr11 63738973
chr11 63738976
chr11 63813625
chr11 63813631
chr11 63813633
chr11 63813658
chr11 63813671
chr11 63813677
chr11 63813681
chr11 63813701
chr11 63813707

chr11 63813710
chr11 63813716
chr11 63813725
chr11 63813733
chr11 63813743
chr11 63839909
chr11 63839916
chr11 63839931
chr11 63839933
chr11 63839967
chr11 63839981
chr11 63839992
chr11 63839996
chr11 63840001
chr11 63840052
chr11 63840058
chr11 63840107
chr11 63840179
chr11 63840237
chr11 63840250
chr11 63840256
chr11 63840279
chr11 63840301
chr11 63840363
chr11 63840466
chr11 63840506
chr11 63840537
chr11 63840541
chr11 63840583
chr11 63840675
chr11 63840691
chr11 63840735
chr11 63840835
chr11 63840845
chr11 63840930
chr11 63840971
chr11 63856061
chr11 63856096
chr11 63866767
chr11 63887925
chr11 63888189
chr11 63888193
chr11 63888238
chr11 63888276
chr11 63888402
chr11 63888423
chr11 63888431
chr11 63888483
chr11 63888528
chr11 63888616
chr11 63888618
chr11 63888657
chr11 63888716
chr11 63888722

chr11 63888728
chr11 63888751
chr11 63888755
chr11 63888803
chr11 63888839
chr11 63888849
chr11 63917481
chr11 63917490
chr11 63917572
chr11 63917584
chr11 63917716
chr11 63917891
chr11 63917901
chr11 63917925
chr11 63917934
chr11 64206681
chr11 64206690
chr11 64206757
chr11 64206777
chr11 64206890
chr11 64206897
chr11 64206922
chr11 64206975
chr11 64207129
chr11 64207160
chr11 64207162
chr11 64207171
chr11 64207177
chr11 64207232
chr11 64207451
chr11 64207456
chr11 64207467
chr11 64207483
chr11 64207490
chr11 64207493
chr11 64207534
chr11 64207541
chr11 64207554
chr11 64207566
chr11 64207570
chr11 64207575
chr11 64226620
chr11 64226643
chr11 64226691
chr11 64226721
chr11 64226733
chr11 64226743
chr11 64226746
chr11 64226959
chr11 64226976
chr11 64226990
chr11 64227005
chr11 64227024
chr11 64227042

chr11 64227064
chr11 64227067
chr11 64227073
chr11 64227105
chr11 64245874
chr11 64245904
chr11 64245919
chr11 64245983
chr11 64246088
chr11 64270836
chr11 64270932
chr11 64271248
chr11 64271335
chr11 64284686
chr11 64284693
chr11 64284715
chr11 64284728
chr11 64340262
chr11 64340346
chr11 64340391
chr11 64340398
chr11 64340424
chr11 64359444
chr11 64359468
chr11 64359476
chr11 64359485
chr11 64359487
chr11 64359560
chr11 64359584
chr11 64359594
chr11 64359653
chr11 64359663
chr11 64359710
chr11 64359790
chr11 64359800
chr11 64359878
chr11 64359897
chr11 64359910
chr11 64359987
chr11 64722913
chr11 64722917
chr11 64722924
chr11 64722931
chr11 64722935
chr11 64722941
chr11 64722944
chr11 64722949
chr11 64722979
chr11 64722984
chr11 64723022
chr11 64723027
chr11 64723033
chr11 64723036
chr11 64777068

chr11 64777076
chr11 64777275
chr11 64777370
chr11 64777382
chr11 64777505
chr11 64777554
chr11 64777591
chr11 64777598
chr11 64777619
chr11 64777648
chr11 64777655
chr11 64777666
chr11 64777681
chr11 64779039
chr11 64810397
chr11 64810402
chr11 64810415
chr11 64810427
chr11 64810453
chr11 64810497
chr11 64810507
chr11 64810517
chr11 64810519
chr11 64810542
chr11 64810544
chr11 64810547
chr11 64810551
chr11 64810554
chr11 64810558
chr11 64810583
chr11 64810595
chr11 64810604
chr11 64810607
chr11 64810638
chr11 64810659
chr11 64810664
chr11 64810669
chr11 64810674
chr11 64810701
chr11 64810703
chr11 64810709
chr11 64810724
chr11 64810758
chr11 64810773
chr11 64810779
chr11 64810805
chr11 64810826
chr11 64810839
chr11 64810842
chr11 64810853
chr11 64810867
chr11 64810872
chr11 64810876
chr11 64851057

chr11 64858932
chr11 64858963
chr11 64858993
chr11 64859074
chr11 64859084
chr11 64859100
chr11 64859105
chr11 64859110
chr11 64859143
chr11 64859200
chr11 64859210
chr11 64859390
chr11 64859412
chr11 64859468
chr11 64859506
chr11 64859514
chr11 64916541
chr11 64916552
chr11 64916582
chr11 64916626
chr11 64916653
chr11 64916687
chr11 64916690
chr11 64916752
chr11 64916774
chr11 64916815
chr11 64916829
chr11 64916864
chr11 64916868
chr11 64916888
chr11 64916897
chr11 64916914
chr11 64916919
chr11 64916932
chr11 64916948
chr11 64916987
chr11 64916997
chr11 64917015
chr11 64917083
chr11 64917092
chr11 64917128
chr11 64917138
chr11 65027707
chr11 65027734
chr11 65027774
chr11 65027808
chr11 65027824
chr11 65027844
chr11 65027850
chr11 65027854
chr11 65027860
chr11 65027873
chr11 65027885
chr11 65027889

chr11 65027977
chr11 65028031
chr11 65111907
chr11 65112037
chr11 65112044
chr11 65112054
chr11 65112108
chr11 65112124
chr11 65112139
chr11 65112145
chr11 65112177
chr11 65112193
chr11 65112206
chr11 65112242
chr11 65112262
chr11 65112278
chr11 65112303
chr11 65112318
chr11 65112335
chr11 65112361
chr11 65112377
chr11 65112390
chr11 65112397
chr11 65112403
chr11 65112419
chr11 65112430
chr11 65112438
chr11 65112446
chr11 65112450
chr11 65112454
chr11 65112459
chr11 65112462
chr11 65141222
chr11 65141268
chr11 65181532
chr11 65181537
chr11 65181546
chr11 65181554
chr11 65181556
chr11 65181558
chr11 65181567
chr11 65181571
chr11 65181575
chr11 65181588
chr11 65181623
chr11 65181637
chr11 65181644
chr11 65181646
chr11 65181657
chr11 65181660
chr11 65181662
chr11 65181686
chr11 65181688
chr11 65181702

chr11 65262417
chr11 65262421
chr11 65262430
chr11 65262433
chr11 65262443
chr11 65262454
chr11 65262490
chr11 65262560
chr11 65262567
chr11 65262977
chr11 65279962
chr11 65334359
chr11 65334371
chr11 65334402
chr11 65334469
chr11 65334476
chr11 65334520
chr11 65334556
chr11 65334562
chr11 65334617
chr11 65334680
chr11 65355023
chr11 65355037
chr11 65355048
chr11 65355055
chr11 65355062
chr11 65355077
chr11 65355081
chr11 65355085
chr11 65355126
chr11 65355128
chr11 65382028
chr11 65382061
chr11 65382096
chr11 65382116
chr11 65382127
chr11 65382153
chr11 65382172
chr11 65382181
chr11 65382186
chr11 65382210
chr11 65382216
chr11 65382230
chr11 65382267
chr11 65382365
chr11 65382389
chr11 65382395
chr11 65382404
chr11 65382431
chr11 65382440
chr11 65382445
chr11 65382517
chr11 65382526
chr11 65382535

chr11 65382562
chr11 65382612
chr11 65382752
chr11 65417173
chr11 65417241
chr11 65417268
chr11 65417288
chr11 65417310
chr11 65417438
chr11 65417511
chr11 65417518
chr11 65417603
chr11 65417658
chr11 65417688
chr11 65417737
chr11 65417758
chr11 65417787
chr11 65417811
chr11 65417835
chr11 65417842
chr11 65417869
chr11 65418012
chr11 65418066
chr11 65418093
chr11 65418113
chr11 65418142
chr11 65418213
chr11 65418228
chr11 65418235
chr11 65418243
chr11 65418294
chr11 65418309
chr11 65418371
chr11 65418455
chr11 65418478
chr11 65418598
chr11 65418666
chr11 65418875
chr11 65418889
chr11 65418908
chr11 65418911
chr11 65419668
chr11 65419670
chr11 65419689
chr11 65419846
chr11 65420086
chr11 65420113
chr11 65420377
chr11 65420386
chr11 65421179
chr11 65421267
chr11 65421275
chr11 65421313
chr11 65421391

chr11 65421453
chr11 65421459
chr11 65421484
chr11 65421604
chr11 65421712
chr11 65421723
chr11 65426585
chr11 65428524
chr11 65428563
chr11 65428581
chr11 65428751
chr11 65428756
chr11 65428759
chr11 65429151
chr11 65429225
chr11 65429497
chr11 65429679
chr11 65429795
chr11 65429822
chr11 65430062
chr11 65430142
chr11 65430226
chr11 65430245
chr11 65430305
chr11 65430312
chr11 65430331
chr11 65430405
chr11 65430552
chr11 65430666
chr11 65430730
chr11 65430965
chr11 65431182
chr11 65431189
chr11 65431449
chr11 65431514
chr11 65431542
chr11 65431770
chr11 65432000
chr11 65477525
chr11 65477527
chr11 65477539
chr11 65477562
chr11 65477565
chr11 65477570
chr11 65477580
chr11 65477584
chr11 65477595
chr11 65477614
chr11 65477618
chr11 65477650
chr11 65477710
chr11 65477783
chr11 65477801
chr11 65477853

chr11 65477876
chr11 65477882
chr11 65477927
chr11 65477933
chr11 65477970
chr11 65497918
chr11 65497923
chr11 65497926
chr11 65498056
chr11 65498197
chr11 65498213
chr11 65498249
chr11 65498261
chr11 65498307
chr11 65498344
chr11 65498359
chr11 65504404
chr11 65505134
chr11 65505159
chr11 65505207
chr11 65505264
chr11 65546470
chr11 65546472
chr11 65546495
chr11 65546506
chr11 65546517
chr11 65546530
chr11 65546547
chr11 65546550
chr11 65546556
chr11 65546562
chr11 65546568
chr11 65546571
chr11 65546587
chr11 65569891
chr11 65569901
chr11 65569922
chr11 65575226
chr11 65575238
chr11 65575275
chr11 65575328
chr11 65575349
chr11 65575442
chr11 65575555
chr11 65575613
chr11 65575625
chr11 65575647
chr11 65614866
chr11 65615000
chr11 65638455
chr11 65638496
chr11 65638536
chr11 65638582
chr11 65638584

chr11 65638598
chr11 65639742
chr11 65639747
chr11 65639753
chr11 65639845
chr11 65639928
chr11 65640263
chr11 65640409
chr11 65640464
chr11 65640491
chr11 65640557
chr11 65640729
chr11 65640739
chr11 65640751
chr11 65711879
chr11 65711881
chr11 65711976
chr11 65711981
chr11 65711989
chr11 65761522
chr11 65872859
chr11 65872862
chr11 65872904
chr11 65872912
chr11 65872918
chr11 65872940
chr11 65872948
chr11 65872950
chr11 65872953
chr11 65872956
chr11 65872966
chr11 65872973
chr11 65872976
chr11 65872979
chr11 65873001
chr11 65873015
chr11 65873045
chr11 65873059
chr11 65873106
chr11 65873138
chr11 65873170
chr11 65873177
chr11 65873183
chr11 65873267
chr11 65873314
chr11 65873324
chr11 65873356
chr11 65873385
chr11 65873395
chr11 65873417
chr11 65873462
chr11 65873468
chr11 65873530
chr11 65873534

chr11 65873538
chr11 65873552
chr11 65873564
chr11 65873572
chr11 65873581
chr11 65873589
chr11 65873596
chr11 65873600
chr11 65873607
chr11 65873613
chr11 65873617
chr11 65873628
chr11 65873641
chr11 65873652
chr11 65873674
chr11 65873693
chr11 65873703
chr11 65873713
chr11 65873726
chr11 65888719
chr11 65888734
chr11 65888987
chr11 65888998
chr11 65889018
chr11 65889043
chr11 65889052
chr11 65889059
chr11 65889084
chr11 65889152
chr11 65889196
chr11 65889209
chr11 65889244
chr11 65889260
chr11 65889314
chr11 65889355
chr11 65889387
chr11 65889396
chr11 65919746
chr11 65919749
chr11 65919775
chr11 65919781
chr11 65919783
chr11 65919805
chr11 65919809
chr11 65919829
chr11 65919832
chr11 65919835
chr11 65919862
chr11 65919868
chr11 65919873
chr11 65919878
chr11 65919885
chr11 65919903
chr11 65919908

chr11 66001450
chr11 66001466
chr11 66001536
chr11 66001592
chr11 66001608
chr11 66001675
chr11 66001697
chr11 66001755
chr11 66001779
chr11 66001824
chr11 66001839
chr11 66001880
chr11 66001927
chr11 66001946
chr11 66001957
chr11 66001960
chr11 66001973
chr11 66071316
chr11 66071351
chr11 66071365
chr11 66071663
chr11 66071795
chr11 66071840
chr11 66071931
chr11 66071958
chr11 66081785
chr11 66081810
chr11 66100689
chr11 66100736
chr11 66100768
chr11 66100852
chr11 66100873
chr11 66100878
chr11 66100919
chr11 66100927
chr11 66100956
chr11 66113327
chr11 66113523
chr11 66113609
chr11 66126253
chr11 66126333
chr11 66126889
chr11 66126892
chr11 66126914
chr11 66126992
chr11 66268911
chr11 66268916
chr11 66268943
chr11 66268951
chr11 66344914
chr11 66344924
chr11 66344932
chr11 66386434
chr11 66386444

chr11 66386448
chr11 66386552
chr11 66386614
chr11 66386642
chr11 66466826
chr11 66466840
chr11 66466849
chr11 66466860
chr11 66466910
chr11 66466915
chr11 66466923
chr11 66466925
chr11 66466931
chr11 66466934
chr11 66466936
chr11 66466941
chr11 66618510
chr11 66618518
chr11 66618524
chr11 66618857
chr11 66618862
chr11 66618927
chr11 66618990
chr11 66619051
chr11 66619121
chr11 66619159
chr11 66619178
chr11 66638856
chr11 66638869
chr11 66638875
chr11 66638882
chr11 66638890
chr11 66638892
chr11 66638900
chr11 66638921
chr11 66639210
chr11 66639215
chr11 66639217
chr11 66639219
chr11 66639273
chr11 66639300
chr11 66639449
chr11 66639523
chr11 66639592
chr11 66639634
chr11 66639650
chr11 66639729
chr11 66639742
chr11 66639766
chr11 66639777
chr11 66678309
chr11 66678362
chr11 66678440
chr11 66678463

chr11 66678473
chr11 66678533
chr11 66678564
chr11 67024684
chr11 67024826
chr11 67120827
chr11 67121023
chr11 67121028
chr11 67121040
chr11 67121171
chr11 67121325
chr11 67121341
chr11 67121459
chr11 67239771
chr11 67239804
chr11 67239824
chr11 67241257
chr11 67241272
chr11 67241288
chr11 67241345
chr11 67241518
chr11 67241605
chr11 67241776
chr11 67241993
chr11 67242000
chr11 67242005
chr11 67242022
chr11 67242030
chr11 67242166
chr11 67268076
chr11 67268125
chr11 67268256
chr11 67268304
chr11 67268348
chr11 67268444
chr11 67268482
chr11 67268494
chr11 67272165
chr11 67272191
chr11 67272300
chr11 67272336
chr11 67277149
chr11 67277228
chr11 67277276
chr11 67277306
chr11 67277311
chr11 67277378
chr11 67353309
chr11 67353339
chr11 67353364
chr11 67353386
chr11 67353392
chr11 67353402
chr11 67353436

chr11 67353453
chr11 67353464
chr11 67353466
chr11 67353495
chr11 67353509
chr11 67353512
chr11 67353539
chr11 67353628
chr11 67353636
chr11 67353641
chr11 67364445
chr11 67364465
chr11 67364490
chr11 67403720
chr11 67403729
chr11 67403895
chr11 67403952
chr11 67404005
chr11 67404114
chr11 67404136
chr11 67404186
chr11 67404195
chr11 67404241
chr11 67404274
chr11 67404289
chr11 67404305
chr11 67404313
chr11 67404317
chr11 67404379
chr11 67404432
chr11 67404523
chr11 67404539
chr11 67404559
chr11 67404896
chr11 67404899
chr11 67404923
chr11 67405055
chr11 67405087
chr11 67405113
chr11 67405119
chr11 67405147
chr11 67405170
chr11 67405173
chr11 67405284
chr11 67405438
chr11 67405440
chr11 67409214
chr11 67436624
chr11 67436659
chr11 67436680
chr11 67436802
chr11 67436873
chr11 67437143
chr11 67437155

chr11 67437186
chr11 67437196
chr11 67437222
chr11 67437226
chr11 67437248
chr11 67437252
chr11 67437276
chr11 67437328
chr11 67437355
chr11 67437411
chr11 67437567
chr11 67437571
chr11 67437597
chr11 67437607
chr11 67437625
chr11 67437637
chr11 67437642
chr11 67437671
chr11 67437675
chr11 67437695
chr11 67437724
chr11 67437820
chr11 67438100
chr11 67438139
chr11 67468756
chr11 67468794
chr11 67468800
chr11 67468818
chr11 67468833
chr11 67468845
chr11 67468881
chr11 67468885
chr11 67468887
chr11 67468892
chr11 67468900
chr11 67468906
chr11 67468908
chr11 67482406
chr11 67482412
chr11 67482428
chr11 67482446
chr11 67482455
chr11 67482460
chr11 67482493
chr11 67482495
chr11 67482499
chr11 67482502
chr11 67482531
chr11 67482575
chr11 67482581
chr11 67484411
chr11 67484419
chr11 67484879
chr11 67484925

chr11 67484937
chr11 67484970
chr11 67485160
chr11 67485182
chr11 67485189
chr11 67485193
chr11 67485209
chr11 67485225
chr11 67505784
chr11 67505824
chr11 67505838
chr11 67505908
chr11 67505922
chr11 67505938
chr11 67505960
chr11 67506025
chr11 67506036
chr11 67579770
chr11 67579787
chr11 67579826
chr11 67579910
chr11 67579996
chr11 67714078
chr11 67714128
chr11 67805454
chr11 67805465
chr11 67805480
chr11 67805490
chr11 67805517
chr11 67805528
chr11 67805555
chr11 67805558
chr11 67805592
chr11 67805604
chr11 67806017
chr11 68039143
chr11 68039184
chr11 68039201
chr11 68039220
chr11 68039238
chr11 68039246
chr11 68039265
chr11 68039275
chr11 68039330
chr11 68039336
chr11 68039349
chr11 68039351
chr11 68039363
chr11 68039497
chr11 68039630
chr11 68039643
chr11 68039689
chr11 68039747
chr11 68039760

chr11 68039780
chr11 68039830
chr11 68039837
chr11 68039845
chr11 68040065
chr11 68040112
chr11 68040190
chr11 68040297
chr11 68040329
chr11 68040384
chr11 68040426
chr11 68040487
chr11 68040525
chr11 68040609
chr11 68040648
chr11 68040666
chr11 68040678
chr11 68040726
chr11 68040736
chr11 68040740
chr11 68040745
chr11 68219853
chr11 68219911
chr11 68219957
chr11 68234508
chr11 68468620
chr11 68468642
chr11 68468887
chr11 68468965
chr11 68495506
chr11 68495587
chr11 68550587
chr11 68843401
chr11 68843408
chr11 68843411
chr11 68843477
chr11 68843481
chr11 68843515
chr11 68843544
chr11 68843546
chr11 68843548
chr11 68843556
chr11 68843570
chr11 68843696
chr11 68843748
chr11 68843783
chr11 68843792
chr11 68843794
chr11 68843796
chr11 68843815
chr11 68843819
chr11 69297408
chr11 69337496
chr11 69337619

chr11 69337663
chr11 69639928
chr11 69639959
chr11 69639970
chr11 69640000
chr11 69640134
chr11 69640260
chr11 69640272
chr11 69640277
chr11 69640308
chr11 69640317
chr11 69640553
chr11 69640563
chr11 69640629
chr11 69640646
chr11 69640654
chr11 69640658
chr11 69640670
chr11 69640675
chr11 69640689
chr11 69640701
chr11 69642721
chr11 69642734
chr11 69642747
chr11 69642754
chr11 69642762
chr11 69642764
chr11 69642767
chr11 69642770
chr11 69642772
chr11 69642776
chr11 69642779
chr11 69642793
chr11 69642799
chr11 69642808
chr11 69642819
chr11 69642821
chr11 69642843
chr11 69642865
chr11 69642867
chr11 69642869
chr11 69642881
chr11 69642888
chr11 69642902
chr11 69642914
chr11 69642922
chr11 69642925
chr11 69642941
chr11 69642954
chr11 69642956
chr11 69642968
chr11 69642974
chr11 69642978
chr11 69642986

chr11 69642989
chr11 69642991
chr11 69642999
chr11 69643002
chr11 69643005
chr11 69643011
chr11 69643018
chr11 69643026
chr11 69643036
chr11 69643051
chr11 69643068
chr11 69643091
chr11 69643101
chr11 69643111
chr11 69643124
chr11 69643156
chr11 69643191
chr11 69643195
chr11 69643216
chr11 69643229
chr11 69643234
chr11 69643262
chr11 69643265
chr11 69643272
chr11 69643279
chr11 69643281
chr11 69643286
chr11 69643290
chr11 69643300
chr11 69643306
chr11 69643321
chr11 69643327
chr11 69643331
chr11 69917619
chr11 69917640
chr11 69925435
chr11 69925545
chr11 69925549
chr11 69925561
chr11 69925598
chr11 69925670
chr11 69925702
chr11 69925763
chr11 69925796
chr11 69925852
chr11 69926063
chr11 69926075
chr11 69926081
chr11 69926101
chr11 69926294
chr11 70122032
chr11 70122090
chr11 70122306
chr11 70122364

chr11 70122503
chr11 70122711
chr11 70195820
chr11 70271530
chr11 70271544
chr11 70365229
chr11 70365258
chr11 70365273
chr11 70365302
chr11 70365399
chr11 70365413
chr11 70365417
chr11 70365425
chr11 70365428
chr11 70365434
chr11 70365444
chr11 70365470
chr11 70365472
chr11 70365489
chr11 70365542
chr11 70398183
chr11 70398187
chr11 70398193
chr11 70398203
chr11 70398205
chr11 70398207
chr11 70398209
chr11 70398241
chr11 70398247
chr11 70398260
chr11 70457731
chr11 70469588
chr11 70469594
chr11 70469863
chr11 70470091
chr11 70470187
chr11 70470404
chr11 70470412
chr11 70470419
chr11 70470430
chr11 70470440
chr11 70513535
chr11 70825001
chr11 70825048
chr11 70825054
chr11 70825070
chr11 70825081
chr11 70825094
chr11 70825099
chr11 71449414
chr11 71449439
chr11 71449537
chr11 71449539
chr11 71580306

chr11 71580318
chr11 71580323
chr11 71580341
chr11 71580795
chr11 71604817
chr11 71604974
chr11 71604989
chr11 71605009
chr11 71629800
chr11 71630249
chr11 71787332
chr11 71787370
chr11 71787396
chr11 71787415
chr11 71787434
chr11 71787450
chr11 71813431
chr11 71813454
chr11 71813465
chr11 71813507
chr11 71813514
chr11 71813530
chr11 71813536
chr11 71813605
chr11 71813608
chr11 71813619
chr11 71813700
chr11 71813707
chr11 71813716
chr11 71813719
chr11 71813724
chr11 71813742
chr11 71813754
chr11 71813763
chr11 71813774
chr11 71813854
chr11 71813859
chr11 71813862
chr11 71813884
chr11 71813897
chr11 71813949
chr11 71813962
chr11 71814046
chr11 71814115
chr11 71814144
chr11 71814175
chr11 71870306
chr11 71870363
chr11 71928024
chr11 71928033
chr11 71928064
chr11 71928184
chr11 71928186
chr11 71928323

chr11 71928355
chr11 71928366
chr11 71928392
chr11 71928400
chr11 71928407
chr11 71929110
chr11 71929130
chr11 71929143
chr11 71929189
chr11 71929252
chr11 71929258
chr11 71929267
chr11 71929368
chr11 71929426
chr11 71929459
chr11 71929543
chr11 71929612
chr11 71929665
chr11 71929704
chr11 71929751
chr11 71929791
chr11 71929911
chr11 71929931
chr11 72040340
chr11 72040435
chr11 72040437
chr11 72041184
chr11 72041195
chr11 72041294
chr11 72041312
chr11 72041322
chr11 72041325
chr11 72080128
chr11 72080156
chr11 72080164
chr11 72080179
chr11 72080200
chr11 72111817
chr11 72111950
chr11 72111963
chr11 72112005
chr11 72112020
chr11 72112057
chr11 72112075
chr11 72112104
chr11 72112141
chr11 72112156
chr11 72112637
chr11 72112675
chr11 72144552
chr11 72144708
chr11 72225330
chr11 72225424
chr11 72225433

chr11 72225444
chr11 72519464
chr11 72519636
chr11 72519672
chr11 72519723
chr11 72519867
chr11 72721808
chr11 72721832
chr11 72721835
chr11 72721843
chr11 72721857
chr11 72721895
chr11 72721903
chr11 72721910
chr11 72721927
chr11 72721939
chr11 72721956
chr11 72752150
chr11 72752171
chr11 72752185
chr11 72815410
chr11 72815470
chr11 72815495
chr11 72815517
chr11 72815522
chr11 72815616
chr11 73034276
chr11 73034401
chr11 73034490
chr11 73034664
chr11 73034805
chr11 73140780
chr11 73184528
chr11 73184970
chr11 73184974
chr11 73184996
chr11 73185008
chr11 73185050
chr11 73185183
chr11 73185198
chr11 73185216
chr11 73185254
chr11 73307785
chr11 73307787
chr11 73520932
chr11 73521169
chr11 73521702
chr11 73759117
chr11 73759303
chr11 73759358
chr11 73759575
chr11 73759711
chr11 73759867
chr11 73759915

chr11 73759918
chr11 73759956
chr11 73760054
chr11 73760068
chr11 73760096
chr11 73760137
chr11 73760143
chr11 73779246
chr11 73779294
chr11 73779305
chr11 73779331
chr11 73779731
chr11 73779743
chr11 73779880
chr11 73779883
chr11 73779885
chr11 73779907
chr11 73979728
chr11 73980006
chr11 73980224
chr11 73980453
chr11 73980509
chr11 73980580
chr11 73980724
chr11 73981110
chr11 73981128
chr11 73981495
chr11 73981534
chr11 73981567
chr11 73981604
chr11 73981777
chr11 73981940
chr11 73981959
chr11 73981982
chr11 74170215
chr11 74170297
chr11 74170344
chr11 74171857
chr11 74296132
chr11 74296136
chr11 74296599
chr11 74388544
chr11 74388590
chr11 74388844
chr11 74389158
chr11 74398557
chr11 74398563
chr11 74398565
chr11 74398580
chr11 74398603
chr11 74398607
chr11 74398629
chr11 74398671
chr11 74398732

chr11 74398734
chr11 74398736
chr11 74398738
chr11 74398766
chr11 74398768
chr11 74398865
chr11 74398873
chr11 74398926
chr11 74398947
chr11 74398972
chr11 74399035
chr11 74493998
chr11 74494038
chr11 74494084
chr11 74494116
chr11 74536353
chr11 74593606
chr11 74739666
chr11 74739934
chr11 74739936
chr11 74739978
chr11 74739993
chr11 74749572
chr11 74749581
chr11 74749589
chr11 74749612
chr11 74749620
chr11 74749808
chr11 74749861
chr11 74749971
chr11 74783408
chr11 74949967
chr11 74950022
chr11 74950028
chr11 74950180
chr11 74950190
chr11 74989104
chr11 74989107
chr11 74989122
chr11 74989131
chr11 74989140
chr11 74989160
chr11 74989186
chr11 74989230
chr11 74989276
chr11 74989327
chr11 74989342
chr11 74989424
chr11 74989498
chr11 74989523
chr11 75103713
chr11 75103902
chr11 75103985
chr11 75214565

chr11 75214584
chr11 75214586
chr11 75214595
chr11 75214683
chr11 75214730
chr11 75214821
chr11 75275426
chr11 75275678
chr11 75275682
chr11 75275686
chr11 75275789
chr11 75275853
chr11 75275864
chr11 75326676
chr11 75383001
chr11 75383023
chr11 75383027
chr11 75383053
chr11 75399879
chr11 75399907
chr11 75400008
chr11 75400021
chr11 75400128
chr11 75400141
chr11 75400169
chr11 75400201
chr11 75400236
chr11 75400394
chr11 75400411
chr11 75400462
chr11 75400639
chr11 75400656
chr11 75400699
chr11 75400742
chr11 75400781
chr11 75582923
chr11 75582950
chr11 75582978
chr11 75583023
chr11 75583036
chr11 75583045
chr11 75583055
chr11 75583087
chr11 75583095
chr11 75583127
chr11 75583136
chr11 75583139
chr11 75583151
chr11 75583182
chr11 75583184
chr11 75583207
chr11 75749096
chr11 75749105
chr11 75749560

chr11 75749567
chr11 75814551
chr11 75814577
chr11 75814589
chr11 75814592
chr11 75814618
chr11 75814667
chr11 75814673
chr11 75814676
chr11 75814716
chr11 75814720
chr11 75814738
chr11 75814748
chr11 75815901
chr11 75815950
chr11 75815956
chr11 75815979
chr11 75816050
chr11 75816103
chr11 75816117
chr11 75816250
chr11 75816348
chr11 75816492
chr11 75816676
chr11 75816863
chr11 75816949
chr11 75816971
chr11 75886162
chr11 75886224
chr11 75886392
chr11 75886842
chr11 76440291
chr11 76443922
chr11 76443950
chr11 76443956
chr11 76444058
chr11 76444062
chr11 76444071
chr11 76444077
chr11 76444171
chr11 76444219
chr11 76444280
chr11 76444283
chr11 76446019
chr11 76446051
chr11 76495470
chr11 76495854
chr11 76587820
chr11 76588002
chr11 76588305
chr11 76588386
chr11 76588417
chr11 76588459
chr11 76588548

chr11 76588554
chr11 76673048
chr11 76673077
chr11 76673096
chr11 76857857
chr11 77047265
chr11 77047345
chr11 77047391
chr11 77047413
chr11 77047423
chr11 77047431
chr11 77047440
chr11 77047453
chr11 77047706
chr11 77047727
chr11 77047771
chr11 77047775
chr11 77047805
chr11 77243275
chr11 77243357
chr11 77243380
chr11 77411432
chr11 77411461
chr11 77411483
chr11 77411516
chr11 77411530
chr11 77411692
chr11 77411707
chr11 77411714
chr11 77411717
chr11 77411735
chr11 77411765
chr11 77411794
chr11 77411806
chr11 77411816
chr11 77411819
chr11 77411827
chr11 77411952
chr11 77412027
chr11 77412038
chr11 77412040
chr11 77412042
chr11 77412075
chr11 77412286
chr11 77412336
chr11 77474036
chr11 77474042
chr11 77474052
chr11 77474054
chr11 77474072
chr11 77474082
chr11 77474084
chr11 77474094
chr11 77474097

chr11 77474101
chr11 77474114
chr11 77474150
chr11 77474212
chr11 77474215
chr11 77474217
chr11 77474220
chr11 77474223
chr11 77474226
chr11 77474232
chr11 77474238
chr11 77474247
chr11 77474253
chr11 77474258
chr11 77474265
chr11 77474281
chr11 77474296
chr11 77474301
chr11 77474309
chr11 77474327
chr11 77474337
chr11 77474341
chr11 77474353
chr11 77474359
chr11 77474362
chr11 77474364
chr11 77474369
chr11 77474391
chr11 77474400
chr11 77474405
chr11 77474447
chr11 77474458
chr11 77474489
chr11 77474532
chr11 77474543
chr11 77474546
chr11 77474581
chr11 77630578
chr11 77631233
chr11 77637102
chr11 77637116
chr11 77637156
chr11 77637194
chr11 77637198
chr11 77637210
chr11 77637214
chr11 77637216
chr11 77637238
chr11 77637379
chr11 77637383
chr11 77637409
chr11 77637429
chr11 77637442
chr11 77637462

chr11 77637465
chr11 77637471
chr11 77637479
chr11 77637501
chr11 77637512
chr11 77637521
chr11 77819545
chr11 77819622
chr11 77819668
chr11 77819687
chr11 77819696
chr11 77819706
chr11 77819754
chr11 77819839
chr11 77819845
chr11 77819856
chr11 77819880
chr11 77819941
chr11 77820009
chr11 77820019
chr11 78039839
chr11 78188144
chr11 78188261
chr11 78188304
chr11 78188318
chr11 78188328
chr11 78188342
chr11 78188365
chr11 78188391
chr11 78188401
chr11 78188406
chr11 78202806
chr11 78203021
chr11 78203186
chr11 78203200
chr11 78203202
chr11 78203264
chr11 78203295
chr11 78292286
chr11 78453511
chr11 78453614
chr11 78573784
chr11 78573792
chr11 78573865
chr11 78573917
chr11 78573993
chr11 78575145
chr11 78575162
chr11 78575182
chr11 78575212
chr11 78806874
chr11 79442275
chr11 80856544
chr11 80856707

chr11 81041362
chr11 81041608
chr11 81207026
chr11 81746056
chr11 81746208
chr11 81746465
chr11 82180182
chr11 82289057
chr11 82289164
chr11 82309393
chr11 82309484
chr11 82309492
chr11 82310571
chr11 82310676
chr11 82590716
chr11 82750982
chr11 82751014
chr11 82751032
chr11 82751124
chr11 82751260
chr11 82751285
chr11 82920290
chr11 82971637
chr11 82971726
chr11 82971835
chr11 82971841
chr11 82971844
chr11 82971869
chr11 82971879
chr11 82972044
chr11 82979358
chr11 83070440
chr11 83070720
chr11 83070803
chr11 83070857
chr11 83070871
chr11 83071209
chr11 83071239
chr11 83071247
chr11 83071273
chr11 83072481
chr11 83072488
chr11 83072498
chr11 83117383
chr11 83117400
chr11 83117408
chr11 83117436
chr11 83117518
chr11 83137145
chr11 83137233
chr11 83156009
chr11 83156016
chr11 83156020
chr11 83156025

chr11 83156053
chr11 83156063
chr11 83156065
chr11 83156074
chr11 83156115
chr11 83156136
chr11 83156155
chr11 83156165
chr11 83156197
chr11 83156254
chr11 83156256
chr11 83156272
chr11 83156333
chr11 83156484
chr11 83156513
chr11 83156516
chr11 83156548
chr11 83156556
chr11 83159612
chr11 83159697
chr11 83159719
chr11 83159795
chr11 83159853
chr11 83160023
chr11 83160292
chr11 83160616
chr11 83160619
chr11 83160701
chr11 83160759
chr11 83194656
chr11 83194667
chr11 83194676
chr11 83194762
chr11 83194786
chr11 83231719
chr11 83285091
chr11 83285110
chr11 83285122
chr11 83285175
chr11 83285201
chr11 83285249
chr11 83285252
chr11 83285430
chr11 83285473
chr11 83285557
chr11 83285570
chr11 83285609
chr11 83285733
chr11 83285736
chr11 83285739
chr11 83285753
chr11 83285761
chr11 83285772
chr11 83285790

chr11 83285794
chr11 83285798
chr11 83285806
chr11 83285808
chr11 83285816
chr11 83285823
chr11 83285857
chr11 83285860
chr11 83285878
chr11 83285886
chr11 83285889
chr11 83285900
chr11 83285902
chr11 83285920
chr11 83285950
chr11 83285962
chr11 83674065
chr11 83724726
chr11 83724962
chr11 83724987
chr11 83933222
chr11 83933448
chr11 83934444
chr11 84305432
chr11 84306134
chr11 84329153
chr11 84329362
chr11 84329854
chr11 84437309
chr11 84686715
chr11 84686799
chr11 84687291
chr11 84720546
chr11 84720549
chr11 84720773
chr11 84720807
chr11 84720875
chr11 84720927
chr11 84720934
chr11 84720977
chr11 84721010
chr11 84721024
chr11 84721039
chr11 84721047
chr11 84721049
chr11 84721060
chr11 84721062
chr11 84721068
chr11 84721076
chr11 84721098
chr11 84721111
chr11 84721128
chr11 84721134
chr11 84721142

chr11 84721157
chr11 84721161
chr11 84721166
chr11 84721168
chr11 84721213
chr11 84721269
chr11 84721282
chr11 84721303
chr11 84721332
chr11 84721732
chr11 85131897
chr11 85132129
chr11 85627208
chr11 85627335
chr11 85627429
chr11 85627477
chr11 85627499
chr11 85627502
chr11 85627523
chr11 85627563
chr11 85627566
chr11 85627604
chr11 85627609
chr11 85627611
chr11 85627727
chr11 85627751
chr11 85627793
chr11 85627795
chr11 85627851
chr11 85627888
chr11 85627898
chr11 85627927
chr11 85627941
chr11 85627968
chr11 85627975
chr11 85627996
chr11 85627998
chr11 85628023
chr11 85629001
chr11 85629029
chr11 85629044
chr11 85629093
chr11 85629156
chr11 85629199
chr11 85629219
chr11 85629239
chr11 85629331
chr11 85629386
chr11 85647462
chr11 85647675
chr11 85648341
chr11 85648457
chr11 85648481
chr11 85648484

chr11 85648516
chr11 85662185
chr11 85662429
chr11 85662491
chr11 85662630
chr11 85662738
chr11 85662915
chr11 85663128
chr11 85663157
chr11 85663237
chr11 85663287
chr11 85665426
chr11 85665510
chr11 85823638
chr11 85823791
chr11 85823857
chr11 85824073
chr11 85824282
chr11 85948192
chr11 85948306
chr11 85948337
chr11 85948397
chr11 86042040
chr11 86046313
chr11 86047386
chr11 86047418
chr11 86047653
chr11 86047811
chr11 86047817
chr11 86062882
chr11 86063092
chr11 86063250
chr11 86063486
chr11 86063637
chr11 86064008
chr11 86064105
chr11 86065677
chr11 86065684
chr11 86065881
chr11 86065924
chr11 86066152
chr11 86066358
chr11 86066411
chr11 86066451
chr11 86069336
chr11 86069373
chr11 86069378
chr11 86069383
chr11 86069397
chr11 86069403
chr11 86069414
chr11 86069425
chr11 86069430
chr11 86138905

chr11 86139138
chr11 86139157
chr11 86139188
chr11 86139244
chr11 86176305
chr11 86176642
chr11 86176657
chr11 86176715
chr11 86193042
chr11 86193602
chr11 86193608
chr11 86193615
chr11 86193648
chr11 86193752
chr11 86202360
chr11 86202401
chr11 86202412
chr11 86202427
chr11 86202603
chr11 86202637
chr11 86202653
chr11 86202675
chr11 86202677
chr11 86202684
chr11 86202691
chr11 86202700
chr11 86202707
chr11 86202738
chr11 86202744
chr11 86202748
chr11 86202756
chr11 86202792
chr11 86202803
chr11 86202837
chr11 86202844
chr11 86202848
chr11 86202857
chr11 86202885
chr11 86202897
chr11 86202901
chr11 86218992
chr11 86243847
chr11 86243899
chr11 86243906
chr11 86243920
chr11 86244123
chr11 86244177
chr11 86244344
chr11 86244357
chr11 86244362
chr11 86244378
chr11 86244384
chr11 86246084
chr11 86246093

chr11 86246095
chr11 86246139
chr11 86246149
chr11 86246187
chr11 86246200
chr11 86246259
chr11 86246377
chr11 86246437
chr11 86246504
chr11 86246634
chr11 86246642
chr11 86246708
chr11 86246725
chr11 86246821
chr11 86286966
chr11 86301834
chr11 86302442
chr11 86302445
chr11 86302468
chr11 86302492
chr11 86302521
chr11 86302527
chr11 86302537
chr11 86302551
chr11 86302557
chr11 86302568
chr11 86302615
chr11 86302663
chr11 86302739
chr11 86302768
chr11 86302804
chr11 86302856
chr11 86302990
chr11 86303036
chr11 86303045
chr11 86303072
chr11 86329179
chr11 86329213
chr11 86329517
chr11 86374324
chr11 86427274
chr11 86428235
chr11 86916251
chr11 87037716
chr11 87037728
chr11 87037747
chr11 87037759
chr11 87037761
chr11 87037764
chr11 87037767
chr11 87037769
chr11 87037771
chr11 87037777
chr11 87037779

chr11 87037787
chr11 87037807
chr11 87037809
chr11 87037813
chr11 87037815
chr11 87037818
chr11 87037828
chr11 87037836
chr11 87037841
chr11 87037843
chr11 87037848
chr11 87037863
chr11 87037868
chr11 87037871
chr11 87037875
chr11 87037886
chr11 87037911
chr11 87037917
chr11 87037938
chr11 87038483
chr11 87038551
chr11 87038946
chr11 87049189
chr11 87049232
chr11 87081517
chr11 87081598
chr11 87081627
chr11 87081841
chr11 87082257
chr11 87082272
chr11 87082324
chr11 87352038
chr11 87352195
chr11 87352303
chr11 87352387
chr11 87352416
chr11 87352640
chr11 87352742
chr11 87352811
chr11 87352833
chr11 87352862
chr11 87352871
chr11 87352874
chr11 87352882
chr11 87352939
chr11 87352948
chr11 87352952
chr11 87352959
chr11 87739455
chr11 87739473
chr11 88174761
chr11 88174797
chr11 88174802
chr11 88174812

chr11 88174820
chr11 88174824
chr11 88174839
chr11 88174858
chr11 88174860
chr11 88174871
chr11 88175186
chr11 88175206
chr11 88175255
chr11 88175258
chr11 88175269
chr11 88175277
chr11 88175297
chr11 88175302
chr11 88175327
chr11 88175336
chr11 88175373
chr11 88336453
chr11 88336463
chr11 88336470
chr11 88336487
chr11 88336495
chr11 88336994
chr11 88337018
chr11 88337033
chr11 88337095
chr11 88337167
chr11 88337178
chr11 88337195
chr11 88338525
chr11 88338592
chr11 88338614
chr11 88338681
chr11 88338694
chr11 88338803
chr11 88359724
chr11 88414405
chr11 88414409
chr11 88414648
chr11 88414677
chr11 88414706
chr11 88415266
chr11 88415279
chr11 88415327
chr11 88415416
chr11 88421772
chr11 88422003
chr11 88568922
chr11 88953870
chr11 88953988
chr11 88954482
chr11 88954868
chr11 88955052
chr11 88955060

chr11 89063764
chr11 89063917
chr11 89064267
chr11 89066337
chr11 89066372
chr11 89066433
chr11 89066449
chr11 89066466
chr11 89066606
chr11 89069162
chr11 89069279
chr11 89069566
chr11 89293845
chr11 89293956
chr11 89296864
chr11 89490637
chr11 89490813
chr11 89490932
chr11 89786922
chr11 89786938
chr11 89786951
chr11 89786956
chr11 89786965
chr11 89786987
chr11 89786999
chr11 89787012
chr11 89787022
chr11 89787045
chr11 89787049
chr11 89787073
chr11 89787082
chr11 89787088
chr11 89787147
chr11 89787186
chr11 89787195
chr11 89787197
chr11 89787203
chr11 89787206
chr11 89787210
chr11 89787213
chr11 89787217
chr11 89787232
chr11 89787307
chr11 89787316
chr11 89787331
chr11 89863851
chr11 89863877
chr11 89863881
chr11 89863894
chr11 89863900
chr11 89863913
chr11 89863916
chr11 89863921
chr11 89864044

chr11 89864052
chr11 89864054
chr11 89864070
chr11 89864087
chr11 89864094
chr11 89864099
chr11 89864103
chr11 89864112
chr11 89864134
chr11 89864136
chr11 89864139
chr11 89864157
chr11 89864175
chr11 89864182
chr11 89864195
chr11 89864219
chr11 89865535
chr11 89865541
chr11 89865554
chr11 89865556
chr11 89865561
chr11 89865571
chr11 90053709
chr11 90053713
chr11 90053738
chr11 90053744
chr11 90053750
chr11 90053758
chr11 90053760
chr11 90053766
chr11 90053770
chr11 90053787
chr11 90053838
chr11 90053849
chr11 90053852
chr11 90053927
chr11 90134808
chr11 90134865
chr11 90134871
chr11 90134878
chr11 90134936
chr11 90135124
chr11 90135171
chr11 90135197
chr11 90135229
chr11 90135316
chr11 90137541
chr11 90137862
chr11 90223240
chr11 90223255
chr11 90223277
chr11 90223349
chr11 90223354
chr11 90223371

chr11 90223507
chr11 90223536
chr11 90223540
chr11 90223550
chr11 90223565
chr11 90223609
chr11 90223638
chr11 90223753
chr11 90223865
chr11 90918994
chr11 90919279
chr11 90919461
chr11 90919566
chr11 90919807
chr11 90974750
chr11 90975039
chr11 91267183
chr11 91267651
chr11 91267868
chr11 92024445
chr11 92024681
chr11 92024913
chr11 92025090
chr11 92025458
chr11 92065023
chr11 92766642
chr11 93197045
chr11 93197229
chr11 93197237
chr11 93197331
chr11 93198308
chr11 93198417
chr11 93198424
chr11 93198554
chr11 93198579
chr11 93198592
chr11 93536063
chr11 93661744
chr11 93661764
chr11 93661804
chr11 93661809
chr11 93661827
chr11 93661854
chr11 93661878
chr11 93661888
chr11 93661892
chr11 93661900
chr11 93661905
chr11 93661907
chr11 93661921
chr11 93661928
chr11 93661941
chr11 93661943
chr11 93661947

chr11 93661955
chr11 93661972
chr11 93661983
chr11 93661996
chr11 93662008
chr11 93662010
chr11 93662024
chr11 93662029
chr11 93662031
chr11 93662033
chr11 93662044
chr11 93662059
chr11 93662061
chr11 93662070
chr11 93662075
chr11 93662086
chr11 93662104
chr11 93662106
chr11 93662154
chr11 93662172
chr11 93662293
chr11 93662309
chr11 93662367
chr11 93662505
chr11 93737685
chr11 93738115
chr11 93738138
chr11 93738251
chr11 93738387
chr11 93738407
chr11 93738419
chr11 93738723
chr11 93738923
chr11 93738967
chr11 93738972
chr11 93739027
chr11 93739030
chr11 93739240
chr11 93739403
chr11 93739432
chr11 93739547
chr11 93739571
chr11 93739642
chr11 93739682
chr11 93741656
chr11 93741671
chr11 93741686
chr11 93741694
chr11 93741716
chr11 93741722
chr11 93741754
chr11 93741775
chr11 93741779
chr11 93741827

chr11 93741832
chr11 93741838
chr11 93741847
chr11 93741849
chr11 93741864
chr11 93741888
chr11 93741899
chr11 93741908
chr11 93742060
chr11 93742094
chr11 93785218
chr11 93785248
chr11 93785257
chr11 93801499
chr11 93801509
chr11 93801766
chr11 94128042
chr11 94128117
chr11 94240008
chr11 94284119
chr11 94492953
chr11 94492975
chr11 94492993
chr11 94493148
chr11 94493204
chr11 94493259
chr11 94493314
chr11 94493551
chr11 94493562
chr11 94493571
chr11 94493663
chr11 94493669
chr11 94493802
chr11 94493810
chr11 94493872
chr11 94493894
chr11 94493911
chr11 94493915
chr11 94494386
chr11 94494417
chr11 94494510
chr11 94494549
chr11 94494665
chr11 94494675
chr11 94544317
chr11 94544322
chr11 94544326
chr11 94544354
chr11 94544356
chr11 94544376
chr11 94544392
chr11 94544400
chr11 94544403
chr11 94544407

chr11 94544413
chr11 94544416
chr11 94544426
chr11 94544446
chr11 94544452
chr11 94544462
chr11 94544468
chr11 94544472
chr11 94544474
chr11 94544479
chr11 94544483
chr11 94544492
chr11 94544503
chr11 94544505
chr11 94972784
chr11 94972836
chr11 94973000
chr11 94973029
chr11 94973058
chr11 95066659
chr11 95066856
chr11 95066891
chr11 95068151
chr11 95068883
chr11 95068904
chr11 95069042
chr11 95069590
chr11 95069710
chr11 95069767
chr11 95069835
chr11 95069848
chr11 95069850
chr11 95069864
chr11 95069898
chr11 95069907
chr11 95069913
chr11 95153295
chr11 95153298
chr11 95153317
chr11 95153437
chr11 95153454
chr11 95153544
chr11 95153595
chr11 95153842
chr11 95228829
chr11 95229498
chr11 95230068
chr11 95230091
chr11 95231799
chr11 95232414
chr11 95232446
chr11 95232494
chr11 95232504
chr11 95232543

chr11 95232570
chr11 95232598
chr11 95232627
chr11 95232664
chr11 95232760
chr11 95232823
chr11 95698892
chr11 95699143
chr11 95699166
chr11 95700180
chr11 95700340
chr11 95700477
chr11 95700547
chr11 95700675
chr11 95700737
chr11 95700756
chr11 95700795
chr11 95720450
chr11 95720545
chr11 95744560
chr11 95788556
chr11 95788684
chr11 95788771
chr11 95788859
chr11 95923357
chr11 95923551
chr11 95924421
chr11 95924512
chr11 95924552
chr11 95924579
chr11 96011638
chr11 96012023
chr11 96012119
chr11 96012220
chr11 96012243
chr11 96012341
chr11 96012375
chr11 96012397
chr11 96012476
chr11 96012611
chr11 96014640
chr11 96127491
chr11 96127713
chr11 96156903
chr11 96157148
chr11 96157418
chr11 96157455
chr11 96188677
chr11 96189132
chr11 96189313
chr11 96189473
chr11 96207651
chr11 96207785
chr11 96207854

chr11 96208375
chr11 96215683
chr11 96215745
chr11 96243211
chr11 96243265
chr11 96243278
chr11 96243282
chr11 96243321
chr11 96243330
chr11 96243343
chr11 96243367
chr11 96243415
chr11 96243437
chr11 96243464
chr11 96243476
chr11 96243478
chr11 96243480
chr11 96243497
chr11 96243499
chr11 96243501
chr11 96243513
chr11 96243515
chr11 96254167
chr11 96254218
chr11 96254293
chr11 96254297
chr11 96254314
chr11 96254360
chr11 96254372
chr11 96254546
chr11 96254711
chr11 96256730
chr11 96256811
chr11 96256986
chr11 96257600
chr11 96257804
chr11 96266905
chr11 96266927
chr11 96267359
chr11 96267554
chr11 96267600
chr11 96339014
chr11 96339060
chr11 96339287
chr11 96341521
chr11 96341554
chr11 96341575
chr11 96341607
chr11 96341693
chr11 96341706
chr11 96341717
chr11 96341726
chr11 96341753
chr11 96341763

chr11 96341768
chr11 96341774
chr11 96341780
chr11 96341787
chr11 96341806
chr11 96341836
chr11 96341865
chr11 96341875
chr11 96341881
chr11 96341901
chr11 96342041
chr11 96342246
chr11 96343105
chr11 96343124
chr11 96343126
chr11 96343130
chr11 96343138
chr11 96343141
chr11 96343166
chr11 96343250
chr11 96343261
chr11 96343271
chr11 96343275
chr11 96343307
chr11 96343309
chr11 96343321
chr11 96343334
chr11 96343348
chr11 96343393
chr11 96343431
chr11 96343439
chr11 96343509
chr11 96343547
chr11 96343552
chr11 96343614
chr11 96343630
chr11 96388868
chr11 96389017
chr11 96389207
chr11 96390646
chr11 96390662
chr11 96390668
chr11 96390721
chr11 96778242
chr11 97746617
chr11 97746789
chr11 97746841
chr11 97747370
chr11 98043377
chr11 98043461
chr11 98043928
chr11 98289984
chr11 98290082
chr11 98297596

chr11 98586891
chr11 98587587
chr11 98589065
chr11 98873171
chr11 98873538
chr11 98873775
chr11 98874068
chr11 98874160
chr11 98956135
chr11 98956615
chr11 99149163
chr11 99149540
chr11 99185275
chr11 99185396
chr11 99185826
chr11 99423334
chr11 99423404
chr11 99465058
chr11 99465272
chr11 99479701
chr11 99749881
chr11 99750060
chr11 100450821
chr11 100451112
chr11 100452884
chr11 100452909
chr11 100452930
chr11 100453097
chr11 100453123
chr11 100453147
chr11 100687753
chr11 100687824
chr11 100687847
chr11 100687863
chr11 100687880
chr11 100687882
chr11 100687889
chr11 100687895
chr11 100687916
chr11 100688076
chr11 100688091
chr11 100790917
chr11 100791651
chr11 100863363
chr11 100876842
chr11 101126118
chr11 101126155
chr11 101126321
chr11 101126426
chr11 101126552
chr11 101126804
chr11 101126824
chr11 102008321
chr11 102008729

chr11 102087174
chr11 102087261
chr11 102087628
chr11 102087670
chr11 102112769
chr11 102112831
chr11 102113038
chr11 102162033
chr11 102268817
chr11 102268846
chr11 102268865
chr11 102268882
chr11 102268885
chr11 102268889
chr11 102268905
chr11 102268912
chr11 102268920
chr11 102268972
chr11 102269012
chr11 102269050
chr11 102269100
chr11 102269151
chr11 102269242
chr11 102269316
chr11 102269557
chr11 102279779
chr11 102279810
chr11 102288212
chr11 102288220
chr11 102288229
chr11 102293958
chr11 102294012
chr11 102294094
chr11 102294143
chr11 102294246
chr11 102306854
chr11 102306949
chr11 102306978
chr11 102307031
chr11 102307212
chr11 102316673
chr11 102317024
chr11 102317093
chr11 102317109
chr11 102317167
chr11 102317210
chr11 102317981
chr11 102318069
chr11 102318092
chr11 102318205
chr11 102318315
chr11 102318340
chr11 102318456
chr11 102318467

chr11 102318572
chr11 102318892
chr11 102319112
chr11 102319136
chr11 102319146
chr11 102319204
chr11 102319236
chr11 102319247
chr11 102319281
chr11 102319292
chr11 102319341
chr11 102319345
chr11 102322447
chr11 102322478
chr11 102322737
chr11 102322842
chr11 102345854
chr11 102346088
chr11 102346169
chr11 102346178
chr11 102346189
chr11 102346205
chr11 102346209
chr11 102346241
chr11 102346277
chr11 102346420
chr11 102346595
chr11 102348373
chr11 102348386
chr11 102374345
chr11 102374426
chr11 102427064
chr11 102451534
chr11 102453246
chr11 102453377
chr11 102453431
chr11 102560555
chr11 102560607
chr11 102560680
chr11 102560859
chr11 102560932
chr11 102560940
chr11 102561361
chr11 102920226
chr11 102920426
chr11 102920465
chr11 102941194
chr11 102941197
chr11 102941208
chr11 102941260
chr11 102941273
chr11 102941303
chr11 102941363
chr11 103035967

chr11 103036027
chr11 103036188
chr11 103091977
chr11 103091991
chr11 103091999
chr11 103092004
chr11 103092007
chr11 103092009
chr11 103092019
chr11 103092023
chr11 103092026
chr11 103092030
chr11 103092046
chr11 103092049
chr11 103092063
chr11 103092082
chr11 103092215
chr11 103092217
chr11 103092223
chr11 103092229
chr11 103092247
chr11 103092285
chr11 103092300
chr11 103092303
chr11 103092309
chr11 103092317
chr11 103092319
chr11 103109045
chr11 103332525
chr11 103537818
chr11 104039182
chr11 104039368
chr11 104040256
chr11 104040418
chr11 104041486
chr11 104163471
chr11 104163474
chr11 104163490
chr11 104163499
chr11 104163629
chr11 104163809
chr11 104163822
chr11 104163827
chr11 104163846
chr11 104163853
chr11 104163872
chr11 104210135
chr11 104210286
chr11 104560387
chr11 104653525
chr11 104653537
chr11 104653593
chr11 104653618
chr11 104653842

chr11 104653879
chr11 104653886
chr11 104967639
chr11 104967689
chr11 104967691
chr11 104967744
chr11 104967823
chr11 104967978
chr11 104968047
chr11 104968112
chr11 104968153
chr11 104968216
chr11 104968347
chr11 104968426
chr11 104968554
chr11 104968616
chr11 104968622
chr11 105044714
chr11 105045032
chr11 105045291
chr11 105045348
chr11 105045368
chr11 105608792
chr11 105608977
chr11 105609116
chr11 105609230
chr11 105609240
chr11 105609440
chr11 105609489
chr11 105609565
chr11 105609620
chr11 105609648
chr11 105962592
chr11 105962665
chr11 105962725
chr11 106021803
chr11 106021829
chr11 106021831
chr11 106021842
chr11 106021878
chr11 106021888
chr11 106021936
chr11 106021972
chr11 106021999
chr11 106022007
chr11 106022014
chr11 106022016
chr11 106022018
chr11 106022032
chr11 106022036
chr11 106022046
chr11 106022051
chr11 106022054
chr11 106022074

chr11 106022084
chr11 106022089
chr11 106022099
chr11 106022111
chr11 106022128
chr11 106022147
chr11 106022153
chr11 106022156
chr11 106022168
chr11 106022188
chr11 106022198
chr11 106022204
chr11 106022207
chr11 106022220
chr11 106022224
chr11 106022266
chr11 106022283
chr11 106022294
chr11 106022321
chr11 106022555
chr11 106022583
chr11 106022600
chr11 106076507
chr11 106076530
chr11 106076555
chr11 106076566
chr11 106076574
chr11 106076700
chr11 106076741
chr11 106076857
chr11 106076905
chr11 106076913
chr11 106076949
chr11 106076961
chr11 106076971
chr11 106076985
chr11 106077009
chr11 106077016
chr11 106078259
chr11 106078322
chr11 106078372
chr11 106078379
chr11 106078453
chr11 106078552
chr11 106111543
chr11 106111573
chr11 106111992
chr11 106156379
chr11 106156409
chr11 106156430
chr11 106156810
chr11 106178127
chr11 106178615
chr11 106178794

chr11 106179327
chr11 106315400
chr11 106315460
chr11 106315955
chr11 106431998
chr11 106745995
chr11 107457169
chr11 107457171
chr11 107457323
chr11 107457374
chr11 107457956
chr11 107458027
chr11 107458050
chr11 107458103
chr11 107458172
chr11 107458188
chr11 107479444
chr11 107479490
chr11 107479694
chr11 107565156
chr11 107565177
chr11 107565253
chr11 107565267
chr11 107565277
chr11 107565298
chr11 107565311
chr11 107565327
chr11 107565373
chr11 107565379
chr11 107565383
chr11 107565432
chr11 107565466
chr11 107565518
chr11 107565532
chr11 107565555
chr11 107565570
chr11 107565578
chr11 107565604
chr11 107565609
chr11 107565619
chr11 107565634
chr11 107565681
chr11 107565686
chr11 107565698
chr11 107565714
chr11 107565716
chr11 107565757
chr11 107565759
chr11 107565767
chr11 107565774
chr11 107565792
chr11 107565806
chr11 107565823
chr11 107566003

chr11 107589688
chr11 107589755
chr11 107589795
chr11 107590033
chr11 107590068
chr11 107590070
chr11 107590073
chr11 107590105
chr11 107590718
chr11 107590913
chr11 107590938
chr11 107840263
chr11 107840464
chr11 107840506
chr11 107840526
chr11 107840647
chr11 107840734
chr11 107840841
chr11 107840900
chr11 107840927
chr11 107840933
chr11 107840936
chr11 107841016
chr11 107841110
chr11 107841120
chr11 107841176
chr11 107859414
chr11 107859442
chr11 107859446
chr11 107859454
chr11 107859478
chr11 107859500
chr11 107859517
chr11 107859520
chr11 107859537
chr11 107859539
chr11 107859574
chr11 107859577
chr11 107859617
chr11 107859633
chr11 107859681
chr11 107859780
chr11 107965265
chr11 108009738
chr11 108009788
chr11 108009817
chr11 108009830
chr11 108009838
chr11 108009889
chr11 108010003
chr11 108010012
chr11 108010022
chr11 108010202
chr11 108010258

chr11 108010589
chr11 108010726
chr11 108011090
chr11 108011113
chr11 108136481
chr11 108221061
chr11 108221204
chr11 108223595
chr11 108223603
chr11 108223659
chr11 108223665
chr11 108223816
chr11 108223868
chr11 108223909
chr11 108224012
chr11 108224038
chr11 108224052
chr11 108224341
chr11 108224403
chr11 108224419
chr11 108224447
chr11 108224568
chr11 108225084
chr11 108225357
chr11 108225504
chr11 108225512
chr11 108225534
chr11 108225559
chr11 108225662
chr11 108225672
chr11 108225697
chr11 108225711
chr11 108225718
chr11 108225727
chr11 108225767
chr11 108225777
chr11 108225907
chr11 108376665
chr11 108376727
chr11 108376806
chr11 108376899
chr11 108376916
chr11 108377159
chr11 108377329
chr11 108467475
chr11 108467486
chr11 108467490
chr11 108467493
chr11 108467511
chr11 108467514
chr11 108467520
chr11 108467529
chr11 108467541
chr11 108467551

chr11 108467573
chr11 108467587
chr11 108467602
chr11 108467604
chr11 108467637
chr11 108467670
chr11 108467676
chr11 108467734
chr11 108593799
chr11 108593812
chr11 108593836
chr11 108593848
chr11 108593853
chr11 108593858
chr11 108593865
chr11 108593877
chr11 108593886
chr11 108593899
chr11 108593974
chr11 109085223
chr11 109180241
chr11 109180283
chr11 109181859
chr11 109181868
chr11 109181905
chr11 109196241
chr11 109196253
chr11 109196517
chr11 109196607
chr11 109425226
chr11 109425234
chr11 109425348
chr11 109425598
chr11 110173983
chr11 110174275
chr11 110174278
chr11 110174281
chr11 110209269
chr11 110209277
chr11 110209280
chr11 110209293
chr11 110209296
chr11 110209303
chr11 110209315
chr11 110209327
chr11 110209339
chr11 110209345
chr11 110209353
chr11 110209394
chr11 110209400
chr11 110209417
chr11 110209421
chr11 110209458
chr11 110209467

chr11 110209950
chr11 110209969
chr11 110210000
chr11 110210025
chr11 110295252
chr11 110295376
chr11 110295453
chr11 110295468
chr11 110346037
chr11 110346049
chr11 110430935
chr11 110431045
chr11 110431071
chr11 111222977
chr11 111223000
chr11 111223029
chr11 111223070
chr11 111223079
chr11 111223105
chr11 111223132
chr11 111223134
chr11 111223161
chr11 111223163
chr11 111223171
chr11 111223173
chr11 111223278
chr11 111223373
chr11 111223531
chr11 111376691
chr11 111378895
chr11 111378911
chr11 111378934
chr11 111379001
chr11 111379025
chr11 111379054
chr11 111379107
chr11 111379117
chr11 111379127
chr11 111379161
chr11 111379214
chr11 111603185
chr11 111603193
chr11 111603196
chr11 111603391
chr11 111604373
chr11 111604553
chr11 111765073
chr11 111765094
chr11 111765320
chr11 111765380
chr11 111765425
chr11 111765559
chr11 111765572
chr11 111765575

chr11 111765610
chr11 111841526
chr11 111841590
chr11 111841615
chr11 111841623
chr11 111841645
chr11 111841700
chr11 111870974
chr11 111871000
chr11 111871040
chr11 111878873
chr11 111878890
chr11 111926978
chr11 111926995
chr11 111926997
chr11 111927007
chr11 111927044
chr11 111927065
chr11 111927079
chr11 111936574
chr11 111936582
chr11 111936764
chr11 111936824
chr11 111936884
chr11 111936897
chr11 111936907
chr11 111936932
chr11 111936934
chr11 111936936
chr11 111936938
chr11 111936940
chr11 111936950
chr11 111936960
chr11 111936967
chr11 111936972
chr11 111936986
chr11 111939237
chr11 111939247
chr11 111939281
chr11 112024261
chr11 112024312
chr11 112024406
chr11 112024446
chr11 112024510
chr11 112024555
chr11 112024579
chr11 112024617
chr11 112024731
chr11 112024761
chr11 112024826
chr11 112024853
chr11 112024855
chr11 112024860
chr11 112024862

chr11 112024882
chr11 112024888
chr11 112024908
chr11 112024911
chr11 112024920
chr11 112024929
chr11 112024931
chr11 112024935
chr11 112025052
chr11 112025059
chr11 112025081
chr11 112025085
chr11 112025096
chr11 112025098
chr11 112025106
chr11 112025129
chr11 112025133
chr11 112025158
chr11 112025171
chr11 112025212
chr11 112025236
chr11 112025240
chr11 112025270
chr11 112025282
chr11 112025288
chr11 112025293
chr11 112025306
chr11 112025310
chr11 112025315
chr11 112025317
chr11 112025337
chr11 112025354
chr11 112025368
chr11 112025374
chr11 112025378
chr11 112025386
chr11 112025976
chr11 112073629
chr11 112073696
chr11 112073724
chr11 112073766
chr11 112073882
chr11 112073898
chr11 112073948
chr11 112074010
chr11 112074253
chr11 112074269
chr11 112074276
chr11 112074335
chr11 112074340
chr11 112074342
chr11 112074350
chr11 112074353
chr11 112074357

chr11 112074395
chr11 112074414
chr11 112074423
chr11 112074429
chr11 112074448
chr11 112074502
chr11 112074538
chr11 112074545
chr11 112074560
chr11 112074595
chr11 112074602
chr11 112074615
chr11 112074621
chr11 112074641
chr11 112074650
chr11 112074657
chr11 112074662
chr11 112074674
chr11 112074685
chr11 112074705
chr11 112074724
chr11 112074726
chr11 112074777
chr11 112074785
chr11 112074794
chr11 112074846
chr11 112075097
chr11 112075123
chr11 112075220
chr11 112086683
chr11 112086694
chr11 112086702
chr11 112086708
chr11 112086718
chr11 112086728
chr11 112086739
chr11 112086749
chr11 112086764
chr11 112086770
chr11 112086772
chr11 112086775
chr11 112086786
chr11 112086793
chr11 112086800
chr11 112086836
chr11 112086839
chr11 112086842
chr11 112086858
chr11 112086872
chr11 112086904
chr11 112086912
chr11 112086934
chr11 112086940
chr11 112086956

chr11 112086991
chr11 112087037
chr11 112087070
chr11 112087118
chr11 112087148
chr11 112174918
chr11 112175218
chr11 112175242
chr11 112175259
chr11 112175316
chr11 112175365
chr11 112175413
chr11 112175437
chr11 112175459
chr11 112280220
chr11 112280250
chr11 112280385
chr11 112280398
chr11 112280400
chr11 112280424
chr11 112280470
chr11 112280485
chr11 112280498
chr11 112280524
chr11 112280532
chr11 112280580
chr11 112280582
chr11 112280594
chr11 112280598
chr11 112280626
chr11 112280664
chr11 112280703
chr11 112280708
chr11 112280792
chr11 112290568
chr11 113289780
chr11 113304773
chr11 113305325
chr11 113305346
chr11 113305777
chr11 113305835
chr11 113305893
chr11 113773298
chr11 113773330
chr11 113774106
chr11 113774134
chr11 113774196
chr11 113774626
chr11 113774760
chr11 113774858
chr11 114160082
chr11 114160086
chr11 114315975
chr11 114399627

chr11 114399668
chr11 114399721
chr11 114399761
chr11 114399841
chr11 114399882
chr11 114399957
chr11 114399973
chr11 114400034
chr11 114401234
chr11 114401566
chr11 114401598
chr11 114401759
chr11 114401880
chr11 114439991
chr11 114440000
chr11 114440062
chr11 114440070
chr11 114440149
chr11 114440216
chr11 114440237
chr11 114762627
chr11 114762638
chr11 114762919
chr11 114762955
chr11 114763942
chr11 114763984
chr11 115194885
chr11 115194899
chr11 115194936
chr11 115194940
chr11 115256952
chr11 115502317
chr11 115502391
chr11 115502401
chr11 115502460
chr11 115707546
chr11 115707674
chr11 115707677
chr11 116158853
chr11 116194253
chr11 116348085
chr11 116348179
chr11 116348329
chr11 116772210
chr11 116772373
chr11 116773348
chr11 116773422
chr11 116773434
chr11 116773439
chr11 116773475
chr11 116773482
chr11 116773578
chr11 116773887
chr11 116773923

chr11 116773991
chr11 116774076
chr11 116774220
chr11 116925149
chr11 116925151
chr11 116925205
chr11 116925248
chr11 116925355
chr11 116925811
chr11 116929553
chr11 116929901
chr11 116929946
chr11 116930583
chr11 116930599
chr11 116986764
chr11 116987078
chr11 117020336
chr11 117022669
chr11 117022708
chr11 117022755
chr11 117096632
chr11 117096687
chr11 117096916
chr11 117097060
chr11 117097117
chr11 117097215
chr11 117097245
chr11 117097262
chr11 117097341
chr11 117097536
chr11 117097546
chr11 117097558
chr11 117097590
chr11 117097649
chr11 117097666
chr11 117143818
chr11 117143900
chr11 117143984
chr11 117144000
chr11 117144008
chr11 117144013
chr11 117144033
chr11 117144041
chr11 117178645
chr11 117178684
chr11 117178690
chr11 117178694
chr11 117178718
chr11 117178725
chr11 117178728
chr11 117178749
chr11 117178753
chr11 117178756
chr11 117178761

chr11 117178764
chr11 117178766
chr11 117178770
chr11 117178774
chr11 117178789
chr11 117178817
chr11 117230835
chr11 117230838
chr11 117230844
chr11 117230849
chr11 117230899
chr11 117230917
chr11 117231213
chr11 117231332
chr11 117231383
chr11 117231489
chr11 117232926
chr11 117232956
chr11 117232964
chr11 117280189
chr11 117280273
chr11 117327440
chr11 117327450
chr11 117327553
chr11 117327581
chr11 117327591
chr11 117327593
chr11 117876046
chr11 117876050
chr11 117876072
chr11 117876143
chr11 117876175
chr11 117876181
chr11 117876185
chr11 117876197
chr11 117950430
chr11 117950481
chr11 117950494
chr11 117951002
chr11 117951189
chr11 117951196
chr11 117986731
chr11 117986757
chr11 117986778
chr11 117986813
chr11 117986915
chr11 117986979
chr11 117987104
chr11 117987158
chr11 117987200
chr11 117987257
chr11 117987480
chr11 117987492
chr11 117987550

chr11 117987723
chr11 118002921
chr11 118003086
chr11 118003466
chr11 118008016
chr11 118008026
chr11 118008030
chr11 118008116
chr11 118008149
chr11 118008537
chr11 118053576
chr11 118053624
chr11 118053879
chr11 118053979
chr11 118198847
chr11 118223969
chr11 118224115
chr11 118224374
chr11 118224649
chr11 118224690
chr11 118224757
chr11 118224829
chr11 118225024
chr11 118225060
chr11 118241397
chr11 118241406
chr11 118241440
chr11 118241481
chr11 118241594
chr11 118241617
chr11 118241888
chr11 118251242
chr11 118251694
chr11 118251712
chr11 118252384
chr11 118252403
chr11 118252408
chr11 118252485
chr11 118252528
chr11 118252611
chr11 118252619
chr11 118252624
chr11 118304457
chr11 118304669
chr11 118304691
chr11 118305175
chr11 118305327
chr11 118305366
chr11 118305422
chr11 118305427
chr11 118305607
chr11 118305779
chr11 118305847
chr11 118305899

chr11 118305918
chr11 118305955
chr11 118306042
chr11 118306055
chr11 118306105
chr11 118306233
chr11 118316822
chr11 118316857
chr11 118316868
chr11 118316876
chr11 118316886
chr11 118317032
chr11 118317055
chr11 118317128
chr11 118317276
chr11 118317342
chr11 118339871
chr11 118340370
chr11 118340377
chr11 118340393
chr11 118340446
chr11 118340461
chr11 118340508
chr11 118341599
chr11 118341610
chr11 118341612
chr11 118341717
chr11 118341807
chr11 118341988
chr11 118344047
chr11 118344095
chr11 118344130
chr11 118344212
chr11 118344397
chr11 118344406
chr11 118344733
chr11 118344830
chr11 118353314
chr11 118353407
chr11 118353467
chr11 118359296
chr11 118359333
chr11 118359338
chr11 118359380
chr11 118359417
chr11 118359450
chr11 118359707
chr11 118359718
chr11 118359830
chr11 118359929
chr11 118359934
chr11 118359975
chr11 118359982
chr11 118359986

chr11 118360007
chr11 118360009
chr11 118360089
chr11 118360096
chr11 118360121
chr11 118400699
chr11 118400780
chr11 118400847
chr11 118400849
chr11 118400859
chr11 118400882
chr11 118400909
chr11 118400940
chr11 118400972
chr11 118401026
chr11 118401036
chr11 118401060
chr11 118401066
chr11 118401090
chr11 118401098
chr11 118401281
chr11 118401283
chr11 118427978
chr11 118434304
chr11 118434332
chr11 118434402
chr11 118434428
chr11 118434659
chr11 118434708
chr11 118434710
chr11 118434721
chr11 118439569
chr11 118440125
chr11 118488670
chr11 118488674
chr11 118488710
chr11 118488713
chr11 118488730
chr11 118488995
chr11 118489018
chr11 118565711
chr11 118571815
chr11 118571817
chr11 118571827
chr11 118571858
chr11 118571862
chr11 118571900
chr11 118571916
chr11 118571944
chr11 118571948
chr11 118571958
chr11 118571960
chr11 118571983
chr11 118572004

chr11 118572038
chr11 118572046
chr11 118572068
chr11 118572122
chr11 118572184
chr11 118572209
chr11 118572226
chr11 118572251
chr11 118572276
chr11 118572302
chr11 118572308
chr11 118572313
chr11 118572347
chr11 118573302
chr11 118573352
chr11 118573378
chr11 118573478
chr11 118573639
chr11 118573913
chr11 118785562
chr11 118785703
chr11 118785728
chr11 118786243
chr11 118786418
chr11 118786448
chr11 118786512
chr11 118789320
chr11 118789367
chr11 118789382
chr11 118789415
chr11 118789641
chr11 118789661
chr11 118789669
chr11 118789735
chr11 118789739
chr11 118789960
chr11 118789987
chr11 118790014
chr11 118792581
chr11 118792828
chr11 118792859
chr11 118792937
chr11 118792939
chr11 118792981
chr11 118793095
chr11 118793097
chr11 118793147
chr11 118793164
chr11 118793220
chr11 118870502
chr11 118870523
chr11 118870552
chr11 118870599
chr11 118870678

chr11 118870691
chr11 118870754
chr11 118892403
chr11 118913624
chr11 118913627
chr11 118913681
chr11 118913694
chr11 118913878
chr11 118913977
chr11 118914003
chr11 118914006
chr11 118914265
chr11 118914354
chr11 118914361
chr11 118914379
chr11 118914383
chr11 118914387
chr11 118914505
chr11 118914561
chr11 118914573
chr11 118914640
chr11 118924053
chr11 118924266
chr11 118924305
chr11 118924401
chr11 118924576
chr11 118924620
chr11 118924665
chr11 118924765
chr11 118924884
chr11 118924942
chr11 118924965
chr11 118924980
chr11 118925024
chr11 118926161
chr11 118926163
chr11 118926252
chr11 118926254
chr11 118926256
chr11 118926258
chr11 118926297
chr11 118926564
chr11 118926568
chr11 118926622
chr11 118926653
chr11 118926655
chr11 118926695
chr11 118926753
chr11 118926783
chr11 118926796
chr11 118926811
chr11 118926871
chr11 118926881
chr11 118926883

chr11 118926887
chr11 118926897
chr11 118928055
chr11 118928091
chr11 118928112
chr11 118929827
chr11 118929881
chr11 118929884
chr11 118929954
chr11 118929961
chr11 118929993
chr11 118930022
chr11 118930055
chr11 118930065
chr11 118930130
chr11 118930143
chr11 118930165
chr11 118930228
chr11 118930244
chr11 118930284
chr11 118930295
chr11 118930320
chr11 118930331
chr11 118930335
chr11 118930344
chr11 118930460
chr11 118930474
chr11 118930490
chr11 118930492
chr11 119017523
chr11 119017607
chr11 119017618
chr11 119067323
chr11 119067338
chr11 119067372
chr11 119067420
chr11 119067483
chr11 119067496
chr11 119067500
chr11 119067503
chr11 119067549
chr11 119067556
chr11 119067558
chr11 119067598
chr11 119067620
chr11 119067638
chr11 119067647
chr11 119067673
chr11 119067675
chr11 119067677
chr11 119067683
chr11 119067701
chr11 119067706
chr11 119067717

chr11 119067738
chr11 119067740
chr11 119067748
chr11 119067758
chr11 119067764
chr11 119067771
chr11 119067904
chr11 119067910
chr11 119106846
chr11 119106941
chr11 119106948
chr11 119120929
chr11 119207022
chr11 119207166
chr11 119207228
chr11 119207231
chr11 119207409
chr11 119315498
chr11 119315550
chr11 119315595
chr11 119315667
chr11 119315730
chr11 119315744
chr11 119315753
chr11 119315792
chr11 119315802
chr11 119315804
chr11 119315893
chr11 119315908
chr11 119315942
chr11 119315992
chr11 119321262
chr11 119321264
chr11 119321402
chr11 119321426
chr11 119321433
chr11 119321445
chr11 119321463
chr11 119321471
chr11 119321544
chr11 119440851
chr11 120108326
chr11 120108331
chr11 120108352
chr11 120108390
chr11 120108419
chr11 120108521
chr11 120334918
chr11 120335099
chr11 120335114
chr11 120335192
chr11 120335222
chr11 120335523
chr11 120335531

chr11 120335556
chr11 120335616
chr11 120335628
chr11 120335717
chr11 120335752
chr11 120335762
chr11 120335768
chr11 120335780
chr11 120335791
chr11 120335797
chr11 120344489
chr11 120432170
chr11 120432213
chr11 120432597
chr11 120986139
chr11 120986147
chr11 120986149
chr11 121235768
chr11 121291877
chr11 121292047
chr11 121293177
chr11 121293181
chr11 121293183
chr11 121293196
chr11 121293198
chr11 121293204
chr11 121293232
chr11 121293236
chr11 121293242
chr11 121293323
chr11 121293344
chr11 121293401
chr11 121293441
chr11 121293444
chr11 121293648
chr11 121450805
chr11 121464460
chr11 121464470
chr11 121464607
chr11 121464622
chr11 121464668
chr11 121464680
chr11 121464685
chr11 121467922
chr11 121467996
chr11 121467999
chr11 121655900
chr11 121655905
chr11 121655915
chr11 121655950
chr11 121929839
chr11 121929842
chr11 121930189
chr11 121930285

chr11 121930313
chr11 121930320
chr11 121930329
chr11 121930345
chr11 121930347
chr11 122101800
chr11 122101843
chr11 122101896
chr11 122440664
chr11 122440667
chr11 122440676
chr11 122440688
chr11 122440697
chr11 122440854
chr11 122440901
chr11 122440918
chr11 122440950
chr11 122440977
chr11 122440980
chr11 122440993
chr11 122441036
chr11 122441041
chr11 122441087
chr11 122441109
chr11 122441240
chr11 122441242
chr11 122441248
chr11 122441257
chr11 122441259
chr11 122441280
chr11 122441319
chr11 122441477
chr11 122441487
chr11 122441496
chr11 122441504
chr11 122441506
chr11 122441517
chr11 122441530
chr11 122441541
chr11 122441545
chr11 122441568
chr11 122580632
chr11 122580699
chr11 122580702
chr11 122580718
chr11 122580746
chr11 122580770
chr11 122580879
chr11 122580990
chr11 122581000
chr11 122581038
chr11 122581094
chr11 122581105
chr11 122581161

chr11 122581228
chr11 122581230
chr11 122746097
chr11 122746103
chr11 122808565
chr11 122808753
chr11 122843953
chr11 122843996
chr11 122844041
chr11 122844116
chr11 122844126
chr11 122856672
chr11 122856678
chr11 122856732
chr11 122856759
chr11 122856869
chr11 122856929
chr11 122857039
chr11 122883319
chr11 122883323
chr11 122883343
chr11 122883365
chr11 122883381
chr11 122883435
chr11 122975729
chr11 122975832
chr11 122984754
chr11 122984766
chr11 122984769
chr11 122984808
chr11 122984810
chr11 122984812
chr11 122984845
chr11 122984850
chr11 122984862
chr11 122984946
chr11 122984959
chr11 122984962
chr11 122984984
chr11 122985014
chr11 122985016
chr11 122985096
chr11 122985142
chr11 122985145
chr11 123058814
chr11 123058818
chr11 123058910
chr11 123058965
chr11 123059135
chr11 123059281
chr11 123059336
chr11 123059393
chr11 123059420
chr11 123059568

chr11 123059628
chr11 123059661
chr11 123059691
chr11 123059697
chr11 123059719
chr11 123059740
chr11 123059778
chr11 123059787
chr11 123059802
chr11 123059808
chr11 123059853
chr11 123059886
chr11 123060010
chr11 123060033
chr11 123060060
chr11 123060089
chr11 123060130
chr11 123060216
chr11 123060470
chr11 123060513
chr11 123060532
chr11 123060542
chr11 123063406
chr11 123063472
chr11 123063474
chr11 123063549
chr11 123063589
chr11 123063668
chr11 123063689
chr11 123063827
chr11 123064054
chr11 123064173
chr11 123064274
chr11 123064323
chr11 123069157
chr11 123069169
chr11 123069189
chr11 123069213
chr11 123069221
chr11 123069228
chr11 123069275
chr11 123069290
chr11 123069461
chr11 123069749
chr11 123069776
chr11 123070058
chr11 123070178
chr11 123070211
chr11 123070644
chr11 123301422
chr11 123301726
chr11 123301734
chr11 123301747
chr11 123301752

chr11 123301762
chr11 123301768
chr11 123301782
chr11 123301790
chr11 123301811
chr11 123301813
chr11 123301817
chr11 123301826
chr11 123301830
chr11 123301837
chr11 123301841
chr11 123301844
chr11 123301853
chr11 123301864
chr11 123301882
chr11 123301885
chr11 123301907
chr11 123301910
chr11 123301913
chr11 123301928
chr11 123301938
chr11 123301951
chr11 123301954
chr11 123301967
chr11 123301978
chr11 123301981
chr11 123357931
chr11 123357944
chr11 123407283
chr11 123407294
chr11 123407341
chr11 123407363
chr11 123407380
chr11 123407638
chr11 123454376
chr11 123454387
chr11 123454445
chr11 123454467
chr11 123454480
chr11 124040401
chr11 124040474
chr11 124040775
chr11 124040848
chr11 124041051
chr11 124041388
chr11 124068706
chr11 124068747
chr11 124069021
chr11 124069100
chr11 124069246
chr11 124069353
chr11 124069493
chr11 124069509
chr11 124069517

chr11 124069558
chr11 124069576
chr11 124069590
chr11 124069629
chr11 124069643
chr11 124069660
chr11 124069664
chr11 124071038
chr11 124071071
chr11 124071199
chr11 124071482
chr11 124071501
chr11 124071526
chr11 124071530
chr11 124076315
chr11 124076318
chr11 124076351
chr11 124076363
chr11 124076373
chr11 124076379
chr11 124076406
chr11 124076441
chr11 124076450
chr11 124076456
chr11 124076466
chr11 124076471
chr11 124076483
chr11 124076503
chr11 124076508
chr11 124076514
chr11 124076523
chr11 124076532
chr11 124076538
chr11 124076547
chr11 124076562
chr11 124076565
chr11 124076664
chr11 124076979
chr11 124115280
chr11 124115291
chr11 124115319
chr11 124115327
chr11 124115350
chr11 124115373
chr11 124115387
chr11 124115404
chr11 124115414
chr11 124115444
chr11 124115459
chr11 124115464
chr11 124115500
chr11 124115603
chr11 124151654
chr11 124169697

chr11 124402329
chr11 124402354
chr11 124402524
chr11 124402546
chr11 124402564
chr11 124402622
chr11 124481533
chr11 124481562
chr11 124481567
chr11 124481815
chr11 124481821
chr11 124481823
chr11 124481850
chr11 124481866
chr11 124622515
chr11 124622647
chr11 124622769
chr11 124622778
chr11 124622782
chr11 124622815
chr11 124622832
chr11 124622838
chr11 124622843
chr11 124622852
chr11 124622880
chr11 124622896
chr11 124622899
chr11 124622907
chr11 124622911
chr11 124622917
chr11 124622935
chr11 124622951
chr11 124622956
chr11 124622980
chr11 124622986
chr11 124622994
chr11 124623012
chr11 124623018
chr11 124623022
chr11 124623029
chr11 124623041
chr11 124623047
chr11 124623053
chr11 124623067
chr11 124623072
chr11 124623098
chr11 124623104
chr11 124623112
chr11 124623115
chr11 124623117
chr11 124623124
chr11 124623160
chr11 124623186
chr11 124623201

chr11 124623207
chr11 124623214
chr11 124623226
chr11 124623248
chr11 124623256
chr11 124623346
chr11 124623352
chr11 124623390
chr11 124623480
chr11 124623511
chr11 124623550
chr11 124623670
chr11 124644504
chr11 124644520
chr11 124644523
chr11 124644577
chr11 124740061
chr11 124740075
chr11 124740152
chr11 124740174
chr11 124740197
chr11 124740234
chr11 124740239
chr11 124740262
chr11 124740266
chr11 124740270
chr11 124740305
chr11 124740307
chr11 124740313
chr11 124740321
chr11 124740329
chr11 124740359
chr11 124740386
chr11 124740488
chr11 124800902
chr11 124836894
chr11 124836905
chr11 124836925
chr11 124836939
chr11 124836950
chr11 124836955
chr11 124836983
chr11 124836993
chr11 124836995
chr11 124837261
chr11 124837275
chr11 124837305
chr11 124837311
chr11 124840556
chr11 124841064
chr11 124841297
chr11 124841467
chr11 124841563
chr11 124841691

chr11 124841952
chr11 124842197
chr11 125110933
chr11 125111006
chr11 125111029
chr11 125111089
chr11 125111108
chr11 125111121
chr11 125111130
chr11 125111218
chr11 125111246
chr11 125111302
chr11 125111403
chr11 125111424
chr11 125111440
chr11 125111447
chr11 125111454
chr11 125111457
chr11 125111461
chr11 125111467
chr11 125111491
chr11 125111516
chr11 125111522
chr11 125111527
chr11 125111596
chr11 125111599
chr11 125111601
chr11 125111613
chr11 125111635
chr11 125111684
chr11 125111698
chr11 125111724
chr11 125111731
chr11 125111733
chr11 125111740
chr11 125111746
chr11 125111749
chr11 125111753
chr11 125111755
chr11 125111768
chr11 125111778
chr11 125111783
chr11 125111790
chr11 125111798
chr11 125111820
chr11 125111831
chr11 125111845
chr11 125111850
chr11 125111864
chr11 125111872
chr11 125514414
chr11 125514671
chr11 125514712
chr11 125514851

chr11 125514863
chr11 125514891
chr11 125570183
chr11 125570206
chr11 125591856
chr11 125591858
chr11 125591895
chr11 125591899
chr11 125592256
chr11 125592283
chr11 125593211
chr11 125593213
chr11 125625687
chr11 125625691
chr11 125625698
chr11 125625706
chr11 125625718
chr11 125626597
chr11 125626661
chr11 125626683
chr11 125626750
chr11 125626759
chr11 125874836
chr11 125874937
chr11 125887446
chr11 125887452
chr11 125903862
chr11 126107938
chr11 126211170
chr11 126211172
chr11 126211175
chr11 126211181
chr11 126211221
chr11 126211226
chr11 126211229
chr11 126211275
chr11 126211286
chr11 126211311
chr11 126211337
chr11 126211341
chr11 126211358
chr11 126211392
chr11 126211411
chr11 126211450
chr11 126211475
chr11 126211487
chr11 126211498
chr11 126211522
chr11 126211533
chr11 126211560
chr11 126211578
chr11 126211590
chr11 126211598
chr11 126211653

chr11 126211658
chr11 126211671
chr11 126211683
chr11 126211697
chr11 126212085
chr11 126212098
chr11 126212103
chr11 126212105
chr11 126212108
chr11 126212124
chr11 126212132
chr11 126212186
chr11 126212188
chr11 126212220
chr11 126480763
chr11 126480780
chr11 126480798
chr11 126480802
chr11 126480820
chr11 126480854
chr11 126480873
chr11 126480875
chr11 126480892
chr11 126480903
chr11 126480915
chr11 126481004
chr11 126481025
chr11 126481045
chr11 126481227
chr11 127294888
chr11 127295747
chr11 127479163
chr11 127479703
chr11 127649263
chr11 127649277
chr11 127649562
chr11 127776802
chr11 127776852
chr11 127776942
chr11 127777523
chr11 127778018
chr11 127779440
chr11 127779482
chr11 127779528
chr11 128031218
chr11 128031254
chr11 128031339
chr11 128031461
chr11 128031521
chr11 128039388
chr11 128039578
chr11 128039639
chr11 128039662
chr11 128194683

chr11 128195139
chr11 128195264
chr11 128195280
chr11 128195287
chr11 128238937
chr11 128239362
chr11 128289198
chr11 128289207
chr11 128289236
chr11 128289361
chr11 128289386
chr11 128289515
chr11 128290133
chr11 128290197
chr11 128290252
chr11 128290379
chr11 128290388
chr11 128290407
chr11 128290415
chr11 128290445
chr11 128290488
chr11 128290523
chr11 128290532
chr11 128290539
chr11 128290581
chr11 128290674
chr11 128290709
chr11 128290742
chr11 128305092
chr11 128305117
chr11 128305347
chr11 128305368
chr11 128319138
chr11 128319255
chr11 128367726
chr11 128367739
chr11 128367761
chr11 128367775
chr11 128367810
chr11 128367816
chr11 128367830
chr11 128367862
chr11 128367874
chr11 128367888
chr11 128471627
chr11 128471994
chr11 128472044
chr11 128472100
chr11 128472129
chr11 128472156
chr11 128472218
chr11 128472439
chr11 128472456
chr11 128476761

chr11 128476806
chr11 128476845
chr11 128476906
chr11 128476919
chr11 128481842
chr11 128481857
chr11 128481954
chr11 128482278
chr11 128482394
chr11 128482404
chr11 128482619
chr11 128482643
chr11 128482679
chr11 128482740
chr11 128482804
chr11 128482807
chr11 128482871
chr11 128482902
chr11 128482956
chr11 128482971
chr11 128483026
chr11 128498677
chr11 128498711
chr11 128499013
chr11 128499132
chr11 128500314
chr11 128500436
chr11 128500521
chr11 128500561
chr11 128507218
chr11 128507223
chr11 128507259
chr11 128507288
chr11 128507319
chr11 128507354
chr11 128507357
chr11 128507439
chr11 128507507
chr11 128507525
chr11 128507651
chr11 128507704
chr11 128507776
chr11 128508055
chr11 128508059
chr11 128508070
chr11 128508110
chr11 128508192
chr11 128508209
chr11 128514348
chr11 128514465
chr11 128514896
chr11 128515154
chr11 128515217
chr11 128515273

chr11 128515275
chr11 128515277
chr11 128515279
chr11 128515501
chr11 128515573
chr11 128515599
chr11 128515713
chr11 128515956
chr11 128515985
chr11 128516009
chr11 128516141
chr11 128516232
chr11 128516348
chr11 128516459
chr11 128516495
chr11 128517258
chr11 128517522
chr11 128517784
chr11 128517798
chr11 128517825
chr11 128518218
chr11 128519201
chr11 128519296
chr11 128519496
chr11 128519556
chr11 128519617
chr11 128519724
chr11 128519841
chr11 128520019
chr11 128520104
chr11 128520116
chr11 128520205
chr11 128520353
chr11 128520358
chr11 128520539
chr11 128520623
chr11 128520658
chr11 128520732
chr11 128520816
chr11 128521257
chr11 128523168
chr11 128523172
chr11 128523310
chr11 128523651
chr11 128624452
chr11 128624499
chr11 128624501
chr11 128624515
chr11 128624522
chr11 128624562
chr11 128624571
chr11 128624598
chr11 128624716
chr11 128624902

chr11 128624920
chr11 128630464
chr11 128630535
chr11 128630552
chr11 128630569
chr11 128630618
chr11 128630624
chr11 128630669
chr11 128696019
chr11 128696114
chr11 128696365
chr11 128713692
chr11 128713705
chr11 128713765
chr11 128713786
chr11 128734846
chr11 128734971
chr11 128734987
chr11 128735030
chr11 128735043
chr11 128735047
chr11 128736070
chr11 128736178
chr11 128736215
chr11 128757365
chr11 128757385
chr11 128757522
chr11 128757722
chr11 128830647
chr11 128830680
chr11 128830734
chr11 128830945
chr11 128831100
chr11 128831174
chr11 128831285
chr11 128831329
chr11 128831347
chr11 128831373
chr11 129331868
chr11 129331917
chr11 129331964
chr11 129331969
chr11 129331981
chr11 129332012
chr11 129332016
chr11 129332052
chr11 129332069
chr11 129332111
chr11 129364723
chr11 129364768
chr11 129364881
chr11 129364965
chr11 129365091
chr11 129365535

chr11 129365941
chr11 129386409
chr11 129750945
chr11 129750947
chr11 129750949
chr11 129750953
chr11 129751122
chr11 129751190
chr11 129751201
chr11 129751225
chr11 129751282
chr11 129751286
chr11 129751301
chr11 129751349
chr11 129828589
chr11 129828699
chr11 129828729
chr11 129828788
chr11 129828961
chr11 129923832
chr11 129923945
chr11 129923966
chr11 129923972
chr11 130069213
chr11 130069232
chr11 130069295
chr11 130313221
chr11 130313405
chr11 130313569
chr11 130313584
chr11 130313594
chr11 130315213
chr11 130315258
chr11 130431350
chr11 130431359
chr11 130431424
chr11 130431428
chr11 130431606
chr11 130431634
chr11 130431694
chr11 130431727
chr11 130434687
chr11 130434693
chr11 130434697
chr11 130434701
chr11 130434707
chr11 130434715
chr11 130434719
chr11 130434731
chr11 130434762
chr11 130434844
chr11 130434886
chr11 130434893
chr11 130434897

chr11 130435022
chr11 130435046
chr11 131245898
chr11 131246064
chr11 131246347
chr11 131246445
chr11 131246542
chr11 131246627
chr11 131836124
chr11 131836308
chr11 131986461
chr11 132126512
chr11 132693210
chr11 132719389
chr11 132719414
chr11 132719447
chr11 132719477
chr11 132719499
chr11 132806016
chr11 133141209
chr11 133141228
chr11 133141236
chr11 134068691
chr11 134068781
chr11 134068862
chr11 134068893
chr11 134068906
chr11 134068915
chr11 134068956
chr11 134068961
chr11 134068971
chr11 134068974
chr11 134068978
chr11 134068995
chr11 134069000
chr11 134223195
chr11 134223515
chr11 134223647
chr11 134223671
chr11 134223686
chr11 134223703
chr11 134223714
chr11 134223721
chr11 134223743
chr11 134223746
chr11 134223753
chr11 134223757
chr11 134223768
chr11 134252789
chr11 134253533
chr11 134253541
chr11 134253545
chr11 134253550
chr11 134253557

chr11 134253563
chr11 134253583
chr11 134253873
chr11 134253891
chr11 134253895
chr11 134254011
chr11 134254015
chr11 134254040
chr11 134254044
chr11 134254056
chr11 134254070
chr11 134254075
chr11 134254087
chr11 134254091
chr11 134254101
chr11 134270442
chr11 134275191
chr11 134275263
chr11 134275421
chr11 134275444
chr11 134275476
chr11 134275596
chr11 134275627
chr11 134275647
chr11 134275666
chr11 134275678
chr11 134275687
chr11 134275702
chr11 134275728
chr11 134275742
chr11 134275748
chr11 134675817
chr11 134868937
chr11 134868953
chr11 134869102
chr12 24087
chr12 24557
chr12 24631
chr12 67732
chr12 67755
chr12 67964
chr12 68379
chr12 252436
chr12 387528
chr12 387619
chr12 387732
chr12 387804
chr12 387827
chr12 387832
chr12 387863
chr12 387964
chr12 387981
chr12 388302
chr12 388322

chr12 388418
chr12 388437
chr12 388478
chr12 389238
chr12 389251
chr12 401428
chr12 401431
chr12 401436
chr12 401438
chr12 401484
chr12 401486
chr12 401490
chr12 401499
chr12 401501
chr12 401511
chr12 401522
chr12 401555
chr12 401584
chr12 401586
chr12 401594
chr12 401606
chr12 401616
chr12 401619
chr12 401621
chr12 401624
chr12 401656
chr12 401668
chr12 401684
chr12 401767
chr12 401778
chr12 401790
chr12 401811
chr12 401829
chr12 401881
chr12 401904
chr12 401908
chr12 401938
chr12 401945
chr12 401949
chr12 401962
chr12 401967
chr12 401985
chr12 402043
chr12 402078
chr12 402196
chr12 402255
chr12 402257
chr12 402277
chr12 402281
chr12 402298
chr12 455110
chr12 576237
chr12 576248
chr12 576259

chr12 576262
chr12 576265
chr12 576268
chr12 576285
chr12 576295
chr12 576298
chr12 576300
chr12 576303
chr12 576321
chr12 576325
chr12 576327
chr12 576329
chr12 576339
chr12 576361
chr12 630405
chr12 630443
chr12 630467
chr12 630477
chr12 630499
chr12 630522
chr12 630531
chr12 630566
chr12 630596
chr12 630623
chr12 630628
chr12 630656
chr12 630666
chr12 630673
chr12 630676
chr12 630766
chr12 630770
chr12 630779
chr12 630784
chr12 630787
chr12 630798
chr12 630814
chr12 630820
chr12 630843
chr12 630851
chr12 630868
chr12 630870
chr12 630905
chr12 630921
chr12 630934
chr12 631038
chr12 631089
chr12 631094
chr12 631101
chr12 631172
chr12 674167
chr12 674317
chr12 674591
chr12 674689
chr12 674708

chr12 674894
chr12 674915
chr12 674917
chr12 674952
chr12 685438
chr12 685554
chr12 685573
chr12 728431
chr12 728471
chr12 728475
chr12 728844
chr12 728849
chr12 743597
chr12 743714
chr12 743795
chr12 750854
chr12 750919
chr12 755125
chr12 755409
chr12 755642
chr12 755649
chr12 755704
chr12 755714
chr12 755747
chr12 755758
chr12 755772
chr12 755803
chr12 755831
chr12 755843
chr12 755845
chr12 755847
chr12 755867
chr12 755922
chr12 755929
chr12 755937
chr12 755947
chr12 755960
chr12 755968
chr12 756014
chr12 756074
chr12 756166
chr12 756365
chr12 756407
chr12 756475
chr12 756796
chr12 949608
chr12 949615
chr12 949617
chr12 949637
chr12 949680
chr12 949682
chr12 949705
chr12 949707
chr12 949711

chr12 949730
chr12 949738
chr12 949745
chr12 949748
chr12 949787
chr12 949799
chr12 949815
chr12 949829
chr12 949836
chr12 949854
chr12 949856
chr12 949871
chr12 949881
chr12 949885
chr12 949893
chr12 949903
chr12 949905
chr12 949926
chr12 949939
chr12 949944
chr12 949946
chr12 949950
chr12 949953
chr12 949983
chr12 949989
chr12 1118027
chr12 1118046
chr12 1118119
chr12 1118198
chr12 1122959
chr12 1122983
chr12 1278550
chr12 1278651
chr12 1278881
chr12 1533451
chr12 1533479
chr12 1533488
chr12 1533497
chr12 1533532
chr12 1533620
chr12 1533668
chr12 1533674
chr12 1533713
chr12 1533782
chr12 1533814
chr12 1533848
chr12 1533854
chr12 1533874
chr12 1533896
chr12 1583542
chr12 1591255
chr12 1591329
chr12 1591435
chr12 1591575

chr12 1592063
chr12 1592168
chr12 1601508
chr12 1601633
chr12 1601639
chr12 1601656
chr12 1601662
chr12 1601683
chr12 1601692
chr12 1605366
chr12 1605382
chr12 1661906
chr12 1661909
chr12 1661916
chr12 1691875
chr12 2003310
chr12 2003489
chr12 2003665
chr12 2003682
chr12 2004027
chr12 2004043
chr12 2004052
chr12 2004100
chr12 2004122
chr12 2004144
chr12 2004178
chr12 2004184
chr12 2004201
chr12 2004225
chr12 2004232
chr12 2004264
chr12 2004267
chr12 2004269
chr12 2004273
chr12 2004277
chr12 2004287
chr12 2004295
chr12 2004302
chr12 2004332
chr12 2004338
chr12 2004343
chr12 2004359
chr12 2004364
chr12 2004375
chr12 2004389
chr12 2004391
chr12 2004410
chr12 2004417
chr12 2004422
chr12 2004457
chr12 2004487
chr12 2004494
chr12 2004498
chr12 2004508

chr12 2004514
chr12 2063985
chr12 2064170
chr12 2064185
chr12 2064217
chr12 2064234
chr12 2135357
chr12 2135661
chr12 2135679
chr12 2135681
chr12 2135841
chr12 2210059
chr12 2358181
chr12 2358188
chr12 2358548
chr12 2691951
chr12 2813479
chr12 2813531
chr12 2813583
chr12 3129213
chr12 3395080
chr12 3395098
chr12 3395126
chr12 3572241
chr12 3572384
chr12 3572554
chr12 3752796
chr12 3752802
chr12 3838757
chr12 3838764
chr12 3838772
chr12 3838775
chr12 3838785
chr12 3838798
chr12 3838806
chr12 3838808
chr12 3838830
chr12 3838837
chr12 3838840
chr12 3838849
chr12 3838851
chr12 3838853
chr12 3838860
chr12 3838869
chr12 3873810
chr12 3873954
chr12 4094730
chr12 4142729
chr12 4142731
chr12 4142781
chr12 4164868
chr12 4164904
chr12 4268227
chr12 4272704

chr12 4272712
chr12 4272747
chr12 4272775
chr12 4272786
chr12 4320660
chr12 4320669
chr12 4320700
chr12 4506494
chr12 4506546
chr12 4506632
chr12 4506889
chr12 4507045
chr12 4507059
chr12 4507230
chr12 4507253
chr12 4507279
chr12 4539168
chr12 4539171
chr12 4539174
chr12 4539322
chr12 4563548
chr12 4604384
chr12 4604397
chr12 4604412
chr12 4604673
chr12 4604685
chr12 4604695
chr12 4604747
chr12 4604818
chr12 4604824
chr12 4604858
chr12 4604886
chr12 4604909
chr12 4604920
chr12 4605090
chr12 4605092
chr12 4605103
chr12 4605116
chr12 4605123
chr12 4605144
chr12 4605260
chr12 4605270
chr12 4605353
chr12 4605391
chr12 4648339
chr12 4648484
chr12 4648549
chr12 4648551
chr12 4648562
chr12 4648593
chr12 4648599
chr12 4648602
chr12 4648661
chr12 4648708

chr12 4648710
chr12 4648720
chr12 4648736
chr12 4648818
chr12 4649408
chr12 4649422
chr12 4649431
chr12 4649441
chr12 4649505
chr12 4649546
chr12 4649703
chr12 4649711
chr12 4649728
chr12 4649738
chr12 4649942
chr12 4807395
chr12 4807526
chr12 4807538
chr12 4807747
chr12 4807896
chr12 4807983
chr12 4809287
chr12 4809299
chr12 4809338
chr12 4809346
chr12 4809351
chr12 4809362
chr12 4809365
chr12 4809368
chr12 4809374
chr12 4809387
chr12 4809405
chr12 4809407
chr12 4809411
chr12 4809415
chr12 4809418
chr12 4809429
chr12 4809440
chr12 4809449
chr12 4809452
chr12 4809454
chr12 4809475
chr12 4809484
chr12 4809488
chr12 4809494
chr12 4809496
chr12 4809506
chr12 4809518
chr12 4809520
chr12 4809527
chr12 4809534
chr12 4809541
chr12 4809548
chr12 4809564

chr12 4809569
chr12 4809579
chr12 4809588
chr12 4809593
chr12 4809602
chr12 4809609
chr12 4809617
chr12 4809622
chr12 4809634
chr12 4809661
chr12 4809672
chr12 4809682
chr12 4809686
chr12 4809764
chr12 4809770
chr12 4809796
chr12 4809800
chr12 4809802
chr12 4809809
chr12 4809816
chr12 4809835
chr12 4829438
chr12 4829783
chr12 4829841
chr12 5119130
chr12 5119157
chr12 5288511
chr12 5402152
chr12 5402170
chr12 5989077
chr12 6167811
chr12 6167891
chr12 6167902
chr12 6285112
chr12 6285123
chr12 6285141
chr12 6285169
chr12 6285218
chr12 6285252
chr12 6285262
chr12 6285499
chr12 6285528
chr12 6285717
chr12 6335805
chr12 6335813
chr12 6335839
chr12 6335858
chr12 6335901
chr12 6335913
chr12 6335952
chr12 6335967
chr12 6336073
chr12 6336162
chr12 6336264

chr12 6336308
chr12 6336338
chr12 6336590
chr12 6336621
chr12 6336638
chr12 6336651
chr12 6336712
chr12 6336731
chr12 6336783
chr12 6336864
chr12 6337037
chr12 6337062
chr12 6337223
chr12 6337368
chr12 6337424
chr12 6337427
chr12 6337436
chr12 6337455
chr12 6337545
chr12 6337593
chr12 6337610
chr12 6337631
chr12 6340017
chr12 6340235
chr12 6340342
chr12 6340413
chr12 6340469
chr12 6340502
chr12 6340562
chr12 6340585
chr12 6340596
chr12 6340626
chr12 6340745
chr12 6340761
chr12 6340824
chr12 6340834
chr12 6340843
chr12 6340900
chr12 6340984
chr12 6341069
chr12 6341124
chr12 6341279
chr12 6341331
chr12 6341341
chr12 6341373
chr12 6341394
chr12 6341407
chr12 6342095
chr12 6433117
chr12 6433146
chr12 6433151
chr12 6451363
chr12 6451370
chr12 6451404

chr12 6451442
chr12 6451445
chr12 6451523
chr12 6451541
chr12 6451556
chr12 6451609
chr12 6451615
chr12 6451628
chr12 6451662
chr12 6452421
chr12 6452453
chr12 6452653
chr12 6452673
chr12 6452742
chr12 6452919
chr12 6461180
chr12 6461404
chr12 6461529
chr12 6461598
chr12 6461653
chr12 6461703
chr12 6461782
chr12 6461808
chr12 6461836
chr12 6462002
chr12 6462066
chr12 6462120
chr12 6462127
chr12 6462129
chr12 6462157
chr12 6462181
chr12 6469739
chr12 6469751
chr12 6469774
chr12 6469879
chr12 6469904
chr12 6469927
chr12 6493115
chr12 6493195
chr12 6493201
chr12 6493214
chr12 6493226
chr12 6494609
chr12 6494714
chr12 6494873
chr12 6495024
chr12 6567973
chr12 6567981
chr12 6567985
chr12 6567992
chr12 6607515
chr12 6607582
chr12 6607591
chr12 6607596

chr12 6607600
chr12 6607602
chr12 6607604
chr12 6607606
chr12 6607608
chr12 6607623
chr12 6607654
chr12 6607931
chr12 6608066
chr12 6608074
chr12 6608096
chr12 6608290
chr12 6608401
chr12 6608493
chr12 6608543
chr12 6662894
chr12 6662908
chr12 6662923
chr12 6662953
chr12 6662975
chr12 6663020
chr12 6663093
chr12 6663104
chr12 6663126
chr12 6663131
chr12 6663136
chr12 6663151
chr12 6663153
chr12 6663163
chr12 6663176
chr12 6688359
chr12 6688398
chr12 6688440
chr12 6688456
chr12 6688497
chr12 6688680
chr12 6688689
chr12 6688704
chr12 6688743
chr12 6688787
chr12 6689954
chr12 6689970
chr12 6723492
chr12 6723571
chr12 6723690
chr12 6723718
chr12 6723777
chr12 6723803
chr12 6723826
chr12 6724141
chr12 6724157
chr12 6724162
chr12 6724183
chr12 6724186

chr12 6724219
chr12 6724240
chr12 6724248
chr12 6724276
chr12 6724288
chr12 6724290
chr12 6724294
chr12 6724321
chr12 6724324
chr12 6724343
chr12 6724394
chr12 6724447
chr12 6724489
chr12 6724511
chr12 6724519
chr12 6724567
chr12 6724578
chr12 6724664
chr12 6724718
chr12 6724733
chr12 6724779
chr12 6752929
chr12 6752946
chr12 6752951
chr12 6752960
chr12 6752969
chr12 6752973
chr12 6752979
chr12 6752986
chr12 6752991
chr12 6753008
chr12 6753010
chr12 6753015
chr12 6753020
chr12 6753034
chr12 6753049
chr12 6753059
chr12 6753062
chr12 6753087
chr12 6753091
chr12 6753100
chr12 6753107
chr12 6753114
chr12 6753121
chr12 6753131
chr12 6753135
chr12 6753144
chr12 6753149
chr12 6753158
chr12 6753166
chr12 6753172
chr12 6753177
chr12 6754074
chr12 6821447

chr12 6821458
chr12 6821503
chr12 6821526
chr12 6821541
chr12 6821563
chr12 6821574
chr12 6821597
chr12 6821604
chr12 6821614
chr12 6821667
chr12 6821673
chr12 6821711
chr12 6821720
chr12 6827775
chr12 6827856
chr12 6828003
chr12 6828015
chr12 6828018
chr12 6828048
chr12 6828109
chr12 6828159
chr12 6828269
chr12 6852990
chr12 6853019
chr12 6853070
chr12 6853135
chr12 6853185
chr12 6853452
chr12 6853454
chr12 6853467
chr12 6872219
chr12 6872247
chr12 6872258
chr12 6872264
chr12 6872289
chr12 6872299
chr12 6872325
chr12 6872327
chr12 6872512
chr12 6872517
chr12 6872946
chr12 6873013
chr12 6873033
chr12 6873041
chr12 6873081
chr12 6873087
chr12 6873101
chr12 6873137
chr12 6873156
chr12 6873169
chr12 6873171
chr12 6873177
chr12 6904448
chr12 6904520

chr12 6904627
chr12 6904635
chr12 6927467
chr12 6927471
chr12 6937405
chr12 6937423
chr12 6937433
chr12 6937438
chr12 6937456
chr12 6937478
chr12 6951833
chr12 6951921
chr12 6951953
chr12 6951966
chr12 6952008
chr12 6952060
chr12 6952116
chr12 6952118
chr12 6952121
chr12 6952147
chr12 6952160
chr12 6952188
chr12 6952196
chr12 6952220
chr12 6952264
chr12 6952267
chr12 6952388
chr12 6952412
chr12 6952422
chr12 6952491
chr12 6952537
chr12 6952655
chr12 6952660
chr12 6952699
chr12 6952726
chr12 6952760
chr12 6952768
chr12 6952813
chr12 6952900
chr12 6952938
chr12 6952946
chr12 6952982
chr12 6952996
chr12 6953019
chr12 6953035
chr12 6953076
chr12 6953138
chr12 6953144
chr12 6953188
chr12 6953239
chr12 6953271
chr12 6953297
chr12 6953356
chr12 6953477

chr12 6953482
chr12 6953505
chr12 6953531
chr12 6953620
chr12 6953634
chr12 6953655
chr12 6953666
chr12 6970960
chr12 6970965
chr12 6971008
chr12 6971014
chr12 6971037
chr12 6971044
chr12 6971126
chr12 6971142
chr12 6971366
chr12 6971430
chr12 6971434
chr12 6971486
chr12 6971495
chr12 6971518
chr12 6971522
chr12 6971527
chr12 6971555
chr12 7108427
chr12 7108435
chr12 7108631
chr12 7129708
chr12 7129812
chr12 7129816
chr12 7129822
chr12 7129831
chr12 7129835
chr12 7129837
chr12 7129848
chr12 7129851
chr12 7129881
chr12 7129887
chr12 7129894
chr12 7129899
chr12 7129915
chr12 7129990
chr12 7129997
chr12 7130050
chr12 7130058
chr12 7130060
chr12 7130079
chr12 7130092
chr12 7130098
chr12 7130142
chr12 7130167
chr12 7130196
chr12 7130206
chr12 7130258

chr12 7130261
chr12 7130288
chr12 7130332
chr12 7130335
chr12 7130348
chr12 7130355
chr12 7130367
chr12 7130383
chr12 7131128
chr12 7131177
chr12 7131184
chr12 7131197
chr12 7131276
chr12 7131294
chr12 7131303
chr12 7131311
chr12 7131324
chr12 7131340
chr12 7131367
chr12 7131412
chr12 7131453
chr12 7131464
chr12 7131566
chr12 7131594
chr12 7131603
chr12 7131685
chr12 7131706
chr12 7131744
chr12 7131774
chr12 7188906
chr12 7188984
chr12 7189022
chr12 7189033
chr12 7189048
chr12 7189070
chr12 7189131
chr12 7189137
chr12 7189165
chr12 7189173
chr12 7189193
chr12 7189224
chr12 7189353
chr12 7189375
chr12 7189393
chr12 7195859
chr12 7346197
chr12 7351280
chr12 7351456
chr12 7371486
chr12 7411727
chr12 7588973
chr12 7588999
chr12 7589018
chr12 7589329

chr12 7589334
chr12 7589383
chr12 7847821
chr12 7847840
chr12 7933353
chr12 7933475
chr12 7933482
chr12 7933487
chr12 7933511
chr12 7933860
chr12 7935219
chr12 7935225
chr12 7935308
chr12 7935314
chr12 7935366
chr12 7935376
chr12 7935383
chr12 7935402
chr12 7935406
chr12 7935527
chr12 7935580
chr12 7935725
chr12 7935750
chr12 7935807
chr12 7935869
chr12 7935891
chr12 7936035
chr12 7936145
chr12 7936194
chr12 7936222
chr12 7936246
chr12 7936307
chr12 7936335
chr12 7936341
chr12 7936358
chr12 7936550
chr12 7936611
chr12 7938300
chr12 7938337
chr12 7959690
chr12 7959731
chr12 7959804
chr12 7959916
chr12 7960176
chr12 7960205
chr12 7960399
chr12 7960403
chr12 7960407
chr12 7960409
chr12 7960419
chr12 7960452
chr12 7960456
chr12 7960477
chr12 7960508

chr12 7960538
chr12 7960550
chr12 7960574
chr12 7960606
chr12 7960613
chr12 7960630
chr12 7960638
chr12 7960660
chr12 7960668
chr12 7960708
chr12 7960814
chr12 7960870
chr12 7960880
chr12 7960903
chr12 7960919
chr12 7960946
chr12 7971031
chr12 7971215
chr12 7971224
chr12 7971302
chr12 7971369
chr12 7971444
chr12 7971467
chr12 7971502
chr12 7971513
chr12 7971531
chr12 7971547
chr12 7971582
chr12 7972002
chr12 7972029
chr12 7972471
chr12 7972519
chr12 7972559
chr12 8026696
chr12 8026698
chr12 8026702
chr12 8026852
chr12 8026856
chr12 8026861
chr12 8026874
chr12 8026887
chr12 8027106
chr12 8027127
chr12 8027212
chr12 8027261
chr12 8027274
chr12 8027294
chr12 8027387
chr12 8027447
chr12 8027451
chr12 8027472
chr12 8027492
chr12 8027503
chr12 8029704

chr12 8029713
chr12 8037452
chr12 8037621
chr12 8081136
chr12 8081237
chr12 8081311
chr12 8081418
chr12 8081525
chr12 8087586
chr12 8087666
chr12 8087675
chr12 8088146
chr12 8088176
chr12 8088212
chr12 8088734
chr12 8088745
chr12 8088834
chr12 8108358
chr12 8135927
chr12 8179915
chr12 8179975
chr12 8242810
chr12 8242812
chr12 8242819
chr12 8242821
chr12 8242831
chr12 8242835
chr12 8242840
chr12 8242843
chr12 8242866
chr12 8242875
chr12 8242877
chr12 8242880
chr12 8242890
chr12 8242893
chr12 8242897
chr12 8242918
chr12 8242924
chr12 8242926
chr12 8242935
chr12 8242939
chr12 8242947
chr12 8242953
chr12 8242959
chr12 8242964
chr12 8242970
chr12 8242989
chr12 8243010
chr12 8243016
chr12 8243026
chr12 8243028
chr12 8243045
chr12 8243074
chr12 8243085

chr12 8243123
chr12 8243343
chr12 8319948
chr12 8320010
chr12 8320253
chr12 8320311
chr12 8320326
chr12 8320460
chr12 8320482
chr12 8320511
chr12 8320528
chr12 8320547
chr12 8320578
chr12 8320679
chr12 8320682
chr12 8390852
chr12 8390857
chr12 8390872
chr12 8390938
chr12 8390955
chr12 8390967
chr12 8406033
chr12 8406507
chr12 8406854
chr12 8406884
chr12 8406898
chr12 8406909
chr12 8406918
chr12 8406981
chr12 8406986
chr12 8406993
chr12 8406998
chr12 8407025
chr12 8407032
chr12 8407034
chr12 8407047
chr12 8407059
chr12 8407067
chr12 8407092
chr12 8407099
chr12 8407102
chr12 8407105
chr12 8407110
chr12 8407125
chr12 8407159
chr12 8407183
chr12 8407186
chr12 8407210
chr12 8407214
chr12 8407276
chr12 8407699
chr12 8407770
chr12 8407789
chr12 8407917

chr12 8407965
chr12 8408033
chr12 8408121
chr12 8408263
chr12 8408265
chr12 8408277
chr12 8408332
chr12 8408460
chr12 8408465
chr12 8408499
chr12 8408557
chr12 8408613
chr12 8408624
chr12 8408758
chr12 8408763
chr12 8408914
chr12 8409150
chr12 8409200
chr12 8409261
chr12 8409270
chr12 8409338
chr12 8409341
chr12 8409537
chr12 8409643
chr12 8409799
chr12 8409806
chr12 8409818
chr12 8409840
chr12 8409914
chr12 8409932
chr12 8410014
chr12 8410287
chr12 8410341
chr12 8410397
chr12 8410451
chr12 8410514
chr12 8410710
chr12 8411053
chr12 8411070
chr12 8411078
chr12 8411100
chr12 8411112
chr12 8411134
chr12 8411158
chr12 8411166
chr12 8411559
chr12 8411576
chr12 8411586
chr12 8411603
chr12 8411613
chr12 8411639
chr12 8412353
chr12 8412357
chr12 8412363

chr12 8412369
chr12 8412397
chr12 8412444
chr12 8412461
chr12 8412492
chr12 8412524
chr12 8412572
chr12 8412662
chr12 8413009
chr12 8413016
chr12 8413026
chr12 8413072
chr12 8413084
chr12 8413105
chr12 8413113
chr12 8413137
chr12 8413144
chr12 8413176
chr12 8413218
chr12 8413446
chr12 8413505
chr12 8413605
chr12 8413638
chr12 8413644
chr12 8413648
chr12 8413663
chr12 8413748
chr12 8413804
chr12 8413828
chr12 8413834
chr12 8413842
chr12 8413913
chr12 8414051
chr12 8414066
chr12 8414202
chr12 8414205
chr12 8414489
chr12 8414503
chr12 8414535
chr12 8414656
chr12 8415021
chr12 8415053
chr12 8425172
chr12 8425179
chr12 8425203
chr12 8425230
chr12 8425267
chr12 8425307
chr12 8425343
chr12 8425370
chr12 8425393
chr12 8425467
chr12 8425493
chr12 8425496

chr12 8425555
chr12 8425705
chr12 8425921
chr12 8426233
chr12 8426292
chr12 8426296
chr12 8426321
chr12 8426342
chr12 8426372
chr12 8426714
chr12 8426774
chr12 8426802
chr12 8426854
chr12 8426890
chr12 8426907
chr12 8427045
chr12 8427072
chr12 8427088
chr12 8427312
chr12 8427387
chr12 8427486
chr12 8427499
chr12 8427551
chr12 8427673
chr12 8427677
chr12 8427681
chr12 8427684
chr12 8427791
chr12 8427808
chr12 8427823
chr12 8427874
chr12 8427952
chr12 8427975
chr12 8428191
chr12 8428210
chr12 8428377
chr12 8428395
chr12 8428464
chr12 8428527
chr12 8428530
chr12 8428534
chr12 8428563
chr12 8428611
chr12 8428867
chr12 8428902
chr12 8428934
chr12 8429312
chr12 8429316
chr12 8429318
chr12 8429328
chr12 8429331
chr12 8429356
chr12 8429365
chr12 8430317

chr12 8430321
chr12 8430329
chr12 8430394
chr12 8430415
chr12 8430519
chr12 8430603
chr12 8430652
chr12 8430686
chr12 8430800
chr12 8433241
chr12 8433258
chr12 8433288
chr12 8433300
chr12 8433322
chr12 8433342
chr12 8433346
chr12 8433386
chr12 8433417
chr12 8433440
chr12 8433479
chr12 8433533
chr12 8433563
chr12 8433619
chr12 8433665
chr12 8433712
chr12 8433719
chr12 8433729
chr12 8518397
chr12 8518459
chr12 8518707
chr12 8518798
chr12 8609204
chr12 8644380
chr12 8644467
chr12 8644573
chr12 8644640
chr12 8645146
chr12 8645273
chr12 8648753
chr12 8648794
chr12 8648850
chr12 8648854
chr12 8648930
chr12 8687561
chr12 8698784
chr12 8698792
chr12 8698806
chr12 8699070
chr12 8699077
chr12 8914759
chr12 8914772
chr12 8914788
chr12 8914794
chr12 8914821

chr12 8914833
chr12 8914837
chr12 8914841
chr12 8914848
chr12 8914881
chr12 8914883
chr12 8914918
chr12 8914959
chr12 8914962
chr12 8914974
chr12 8914982
chr12 8914996
chr12 8915023
chr12 8915041
chr12 8915112
chr12 8915143
chr12 8915150
chr12 8915237
chr12 8915240
chr12 8915321
chr12 8915335
chr12 8915364
chr12 8948284
chr12 8948457
chr12 8948462
chr12 8948664
chr12 8948833
chr12 8949585
chr12 8949613
chr12 8949625
chr12 8949630
chr12 8951733
chr12 8951934
chr12 8954113
chr12 8954273
chr12 8954311
chr12 8954319
chr12 9239541
chr12 9239659
chr12 9239664
chr12 9239684
chr12 9239703
chr12 9239864
chr12 9239866
chr12 9239877
chr12 9239893
chr12 9239900
chr12 9239904
chr12 9239918
chr12 9239930
chr12 9239946
chr12 9239971
chr12 9239974
chr12 9240011

chr12 9240025
chr12 9240028
chr12 9240049
chr12 9240055
chr12 9240081
chr12 9240089
chr12 9240181
chr12 9240194
chr12 9240223
chr12 9240235
chr12 9240251
chr12 9240289
chr12 9240301
chr12 9240316
chr12 9240322
chr12 9240341
chr12 9240344
chr12 9240350
chr12 9240352
chr12 9240376
chr12 9240396
chr12 9240408
chr12 9240415
chr12 9240417
chr12 9240420
chr12 9240423
chr12 9284228
chr12 9284245
chr12 9284260
chr12 9284278
chr12 9284283
chr12 9284290
chr12 9284295
chr12 9284312
chr12 9284350
chr12 9284413
chr12 9284428
chr12 9284466
chr12 9284546
chr12 9284615
chr12 9284655
chr12 9284662
chr12 9284789
chr12 9284973
chr12 9285007
chr12 9285110
chr12 9329616
chr12 9444067
chr12 9447003
chr12 9447049
chr12 9447088
chr12 9447222
chr12 9447262
chr12 9447331

chr12 9447411
chr12 9447449
chr12 9447464
chr12 9447527
chr12 9447565
chr12 9447582
chr12 9447587
chr12 9447594
chr12 9447617
chr12 9447632
chr12 9447649
chr12 9447764
chr12 9447820
chr12 9447822
chr12 9447847
chr12 9447852
chr12 9447854
chr12 9447857
chr12 9447865
chr12 9447893
chr12 9447913
chr12 9447926
chr12 9647628
chr12 9651050
chr12 9651588
chr12 9651600
chr12 9651686
chr12 9651948
chr12 9651998
chr12 9652083
chr12 9652363
chr12 9652391
chr12 9667502
chr12 9667625
chr12 9668243
chr12 9668880
chr12 9668908
chr12 9668926
chr12 9669157
chr12 9669254
chr12 9669390
chr12 9669457
chr12 9669617
chr12 9669633
chr12 9669642
chr12 9669691
chr12 9670543
chr12 9670614
chr12 9671010
chr12 9671062
chr12 9671134
chr12 9671284
chr12 9671286
chr12 9671292

chr12 9671324
chr12 9671365
chr12 9671369
chr12 9671377
chr12 9671381
chr12 9671410
chr12 9671421
chr12 9671441
chr12 9671462
chr12 9671466
chr12 9671471
chr12 9671499
chr12 9671509
chr12 9671511
chr12 9671515
chr12 9671519
chr12 9671641
chr12 9671775
chr12 9672227
chr12 9672520
chr12 9672714
chr12 9672889
chr12 9673025
chr12 9673032
chr12 9673705
chr12 9673907
chr12 9674104
chr12 9674328
chr12 9674490
chr12 9674604
chr12 9674925
chr12 9675048
chr12 9675216
chr12 9732654
chr12 9732662
chr12 9732698
chr12 9733228
chr12 9733277
chr12 9733402
chr12 9733485
chr12 9733760
chr12 9737790
chr12 9748736
chr12 9748738
chr12 9750767
chr12 9750795
chr12 9750797
chr12 9750799
chr12 9750817
chr12 9750821
chr12 9750833
chr12 9750858
chr12 9750909
chr12 9750957

chr12 9751148
chr12 9751157
chr12 9751160
chr12 9751417
chr12 9751444
chr12 9751478
chr12 9751584
chr12 9757567
chr12 9757859
chr12 9757877
chr12 9757893
chr12 9758012
chr12 9758124
chr12 9758211
chr12 9758267
chr12 9758579
chr12 9758619
chr12 9758628
chr12 9758669
chr12 9758693
chr12 9758720
chr12 9759180
chr12 9759361
chr12 9759520
chr12 9759534
chr12 9759548
chr12 9759650
chr12 9759677
chr12 9759751
chr12 9760104
chr12 9760490
chr12 9760773
chr12 9760799
chr12 9760949
chr12 9760966
chr12 9761274
chr12 9761281
chr12 9761434
chr12 9761461
chr12 9761490
chr12 9764757
chr12 9764778
chr12 9764870
chr12 9765006
chr12 9765023
chr12 9765221
chr12 9765254
chr12 9765523
chr12 9765653
chr12 9865350
chr12 9865676
chr12 9865900
chr12 9866241
chr12 9866845

chr12 9866915
chr12 9867002
chr12 9867035
chr12 9867107
chr12 9867421
chr12 9867631
chr12 9867646
chr12 9867682
chr12 9867726
chr12 9868044
chr12 9868055
chr12 9868321
chr12 9868362
chr12 9868373
chr12 9868428
chr12 9868437
chr12 9868463
chr12 9868513
chr12 9868617
chr12 9868729
chr12 9868779
chr12 9868799
chr12 9869133
chr12 9869173
chr12 9869205
chr12 9869223
chr12 9869456
chr12 9870077
chr12 9870083
chr12 9870089
chr12 9951213
chr12 10113000
chr12 10113108
chr12 10113854
chr12 10143370
chr12 10143770
chr12 10182832
chr12 10182913
chr12 10183196
chr12 10212771
chr12 10212786
chr12 10212790
chr12 10212810
chr12 10212817
chr12 10212828
chr12 10212833
chr12 10212840
chr12 10212842
chr12 10212850
chr12 10212860
chr12 10212863
chr12 10212865
chr12 10212899
chr12 10212907

chr12 10212910
chr12 10212956
chr12 10212965
chr12 10213260
chr12 10213262
chr12 10213268
chr12 10213274
chr12 10213708
chr12 10213901
chr12 10213903
chr12 10213922
chr12 10213933
chr12 10213945
chr12 10364631
chr12 10364651
chr12 10364701
chr12 10364727
chr12 10364869
chr12 10364893
chr12 10364920
chr12 10364977
chr12 10365011
chr12 10365020
chr12 10365023
chr12 10365051
chr12 10365090
chr12 10365314
chr12 10365335
chr12 10365361
chr12 10409426
chr12 10410023
chr12 10410102
chr12 10410404
chr12 10410806
chr12 10411040
chr12 10411052
chr12 10411348
chr12 10420448
chr12 10420745
chr12 10612880
chr12 10612901
chr12 10612975
chr12 10612997
chr12 10613038
chr12 10613129
chr12 10613176
chr12 10613179
chr12 10613883
chr12 10613886
chr12 10613954
chr12 10613967
chr12 10701258
chr12 10701540
chr12 10701613

chr12 10715886
chr12 10721784
chr12 10721809
chr12 10721827
chr12 10721831
chr12 10721917
chr12 10721926
chr12 10722023
chr12 10722059
chr12 10722076
chr12 10722104
chr12 10722108
chr12 10722121
chr12 10722135
chr12 10722174
chr12 10722200
chr12 10722224
chr12 10722230
chr12 10722275
chr12 10722303
chr12 10722360
chr12 10722373
chr12 10722435
chr12 10722446
chr12 10722451
chr12 10722521
chr12 10722539
chr12 10722541
chr12 10722547
chr12 10722559
chr12 10722598
chr12 10722621
chr12 10722625
chr12 10722634
chr12 10722641
chr12 10722646
chr12 10722648
chr12 10722662
chr12 10722665
chr12 10722677
chr12 10723927
chr12 10724065
chr12 10749897
chr12 10749949
chr12 10749952
chr12 10750101
chr12 10750122
chr12 10750139
chr12 10750148
chr12 10750150
chr12 10750155
chr12 10750266
chr12 10750279
chr12 10750317

chr12 10750330
chr12 10750348
chr12 10750380
chr12 10750397
chr12 10750412
chr12 10750451
chr12 10750489
chr12 10750839
chr12 11172050
chr12 11172062
chr12 11172132
chr12 11172179
chr12 11172199
chr12 11172208
chr12 11172263
chr12 11172368
chr12 11172400
chr12 11172522
chr12 11488497
chr12 11488610
chr12 11489498
chr12 11489883
chr12 11545824
chr12 11545834
chr12 11546122
chr12 11546263
chr12 11546336
chr12 11554017
chr12 11565943
chr12 11565967
chr12 11651291
chr12 11651362
chr12 11651379
chr12 11651588
chr12 11651649
chr12 11651690
chr12 11651716
chr12 11651792
chr12 11652842
chr12 11652846
chr12 12010256
chr12 12010302
chr12 12010314
chr12 12010423
chr12 12010429
chr12 12010439
chr12 12010487
chr12 12010495
chr12 12010507
chr12 12010531
chr12 12010590
chr12 12010629
chr12 12010640
chr12 12010696

chr12 12010711
chr12 12010753
chr12 12010789
chr12 12033134
chr12 12033153
chr12 12033171
chr12 12033183
chr12 12033203
chr12 12033217
chr12 12033223
chr12 12033234
chr12 12033251
chr12 12033263
chr12 12033321
chr12 12033351
chr12 12033428
chr12 12033457
chr12 12033508
chr12 12033519
chr12 12033622
chr12 12033626
chr12 12033639
chr12 12033849
chr12 12172300
chr12 12172772
chr12 12249471
chr12 12350437
chr12 12350446
chr12 12350455
chr12 12350457
chr12 12350462
chr12 12350474
chr12 12350495
chr12 12350521
chr12 12350523
chr12 12350528
chr12 12350530
chr12 12350610
chr12 12350819
chr12 12357625
chr12 12357668
chr12 12357688
chr12 12357711
chr12 12357717
chr12 12357748
chr12 12357782
chr12 12357888
chr12 12358003
chr12 12358093
chr12 12358209
chr12 12358243
chr12 12358255
chr12 12358289
chr12 12358292

chr12	12384857
chr12	12431237
chr12	12442102
chr12	12442127
chr12	12456758
chr12	12456765
chr12	12475224
chr12	12475429
chr12	12475464
chr12	12475467
chr12	12475474
chr12	12475556
chr12	12560839
chr12	12560971
chr12	12561095
chr12	12561185
chr12	12561278
chr12	12561289
chr12	12561546
chr12	12607080
chr12	12607136
chr12	12607191
chr12	12611818
chr12	12611820
chr12	12611828
chr12	12611835
chr12	12611849
chr12	12611858
chr12	12611864
chr12	12611875
chr12	12611881
chr12	12611884
chr12	12611897
chr12	12611941
chr12	12611944
chr12	12611947
chr12	12611950
chr12	12611960
chr12	12611970
chr12	12611983
chr12	12612010
chr12	12612016
chr12	12612038
chr12	12612050
chr12	12612090
chr12	12612095
chr12	12612102
chr12	12612112
chr12	12612121
chr12	12612134
chr12	12612157
chr12	12612169
chr12	12612171
chr12	12612207

chr12 12612209
chr12 12612212
chr12 12612215
chr12 12612226
chr12 12612242
chr12 12612244
chr12 12612256
chr12 12612464
chr12 12612542
chr12 12655730
chr12 12655774
chr12 12667199
chr12 12696454
chr12 12696465
chr12 12696624
chr12 12696638
chr12 12696654
chr12 12697207
chr12 12697365
chr12 12714149
chr12 12714273
chr12 12714300
chr12 12714328
chr12 12714338
chr12 12714375
chr12 12714394
chr12 12714397
chr12 12714403
chr12 12714528
chr12 12714582
chr12 12714642
chr12 12714703
chr12 12714735
chr12 12714749
chr12 12714790
chr12 12714792
chr12 12714795
chr12 12714798
chr12 12714813
chr12 12714819
chr12 12714847
chr12 12714891
chr12 12714962
chr12 12714982
chr12 12715036
chr12 12720094
chr12 12720107
chr12 12720189
chr12 12720239
chr12 12720543
chr12 12720649
chr12 12720798
chr12 12720985
chr12 12721016

chr12 12721123
chr12 12721222
chr12 12721354
chr12 12721361
chr12 12721754
chr12 12721842
chr12 12721881
chr12 12722511
chr12 12726377
chr12 12726405
chr12 12726539
chr12 12726569
chr12 12726628
chr12 12726632
chr12 12726830
chr12 12726930
chr12 12727010
chr12 12727178
chr12 12727230
chr12 12737021
chr12 12737028
chr12 12737138
chr12 12788917
chr12 12788942
chr12 12788993
chr12 12813414
chr12 12813469
chr12 12813530
chr12 12813602
chr12 12813606
chr12 12813668
chr12 12813687
chr12 12813877
chr12 12813893
chr12 12909520
chr12 12909574
chr12 12910013
chr12 13165691
chr12 13179195
chr12 13179369
chr12 13179639
chr12 13179699
chr12 13179722
chr12 13179752
chr12 13196635
chr12 13196704
chr12 13196764
chr12 13196790
chr12 13255559
chr12 13255605
chr12 13255700
chr12 13255728
chr12 13255782
chr12 13255865

chr12 13255868
chr12 13255896
chr12 13255910
chr12 13392001
chr12 13392681
chr12 13392745
chr12 13393081
chr12 13393086
chr12 13393655
chr12 13394001
chr12 13394350
chr12 13394392
chr12 13395292
chr12 13434826
chr12 13739768
chr12 13739798
chr12 13739862
chr12 13739878
chr12 13739882
chr12 13739951
chr12 14023581
chr12 14065749
chr12 14256242
chr12 14256284
chr12 14256305
chr12 14256471
chr12 14256513
chr12 14256586
chr12 14256631
chr12 14256652
chr12 14367644
chr12 14367754
chr12 14384951
chr12 14385066
chr12 14385810
chr12 14386251
chr12 14386331
chr12 14386437
chr12 14386625
chr12 14386736
chr12 14386742
chr12 14386858
chr12 14387377
chr12 14387445
chr12 14390915
chr12 14394295
chr12 14394505
chr12 14394589
chr12 14394989
chr12 14395141
chr12 14395375
chr12 14395616
chr12 14399980
chr12 14400138

chr12 14400173
chr12 14403549
chr12 14403998
chr12 14404559
chr12 14716226
chr12 14771563
chr12 14771613
chr12 14771667
chr12 14771701
chr12 14771708
chr12 14771734
chr12 14771996
chr12 14772019
chr12 14772368
chr12 14775212
chr12 14775243
chr12 14775248
chr12 14775263
chr12 14775297
chr12 14775347
chr12 14775350
chr12 14775355
chr12 14775366
chr12 14775384
chr12 14775417
chr12 14775444
chr12 14775558
chr12 14775590
chr12 14775625
chr12 14775635
chr12 14775731
chr12 14802798
chr12 14804117
chr12 14804262
chr12 14881083
chr12 14881096
chr12 14884538
chr12 14884821
chr12 14948761
chr12 14948839
chr12 14948873
chr12 14949148
chr12 14951271
chr12 14951276
chr12 14951520
chr12 14951982
chr12 14952540
chr12 14952602
chr12 14952720
chr12 14952754
chr12 14952761
chr12 14952769
chr12 14952842
chr12 14952990

chr12 14953121
chr12 14956348
chr12 14956726
chr12 14956849
chr12 14957236
chr12 14957303
chr12 14957639
chr12 14957730
chr12 14957819
chr12 14958043
chr12 14958163
chr12 14958209
chr12 14958231
chr12 14958277
chr12 14958316
chr12 14958418
chr12 14958540
chr12 14958563
chr12 14958730
chr12 14958785
chr12 14958877
chr12 14958940
chr12 14959055
chr12 14959073
chr12 14959079
chr12 14959097
chr12 14959156
chr12 14959193
chr12 14959247
chr12 14959278
chr12 14959288
chr12 14959290
chr12 14959310
chr12 14959323
chr12 14959334
chr12 14959367
chr12 14959566
chr12 14959641
chr12 14962213
chr12 14962385
chr12 14962455
chr12 14962567
chr12 14962664
chr12 14962689
chr12 15096402
chr12 15096637
chr12 15227347
chr12 15227607
chr12 15227838
chr12 15269253
chr12 15269767
chr12 15269783
chr12 15269795
chr12 15323151

chr12 15323167
chr12 15323177
chr12 15323278
chr12 15323464
chr12 15323606
chr12 15323609
chr12 15323693
chr12 15323715
chr12 15342094
chr12 15342115
chr12 15404598
chr12 15510964
chr12 15701226
chr12 15788323
chr12 15788338
chr12 15881686
chr12 15881722
chr12 15881784
chr12 15881831
chr12 15881855
chr12 15881861
chr12 15881863
chr12 15881870
chr12 15881879
chr12 15881885
chr12 15881893
chr12 15881899
chr12 15881962
chr12 15881998
chr12 15882011
chr12 15882015
chr12 15882028
chr12 15882045
chr12 15882051
chr12 15882070
chr12 15882096
chr12 15882114
chr12 15882156
chr12 15882160
chr12 15882162
chr12 15882175
chr12 15882185
chr12 15882195
chr12 15882215
chr12 15882227
chr12 15882240
chr12 15882243
chr12 15882253
chr12 15882267
chr12 15882282
chr12 15882288
chr12 15910837
chr12 15910944
chr12 16511368

chr12 16606637
chr12 16606657
chr12 16606667
chr12 16606711
chr12 16606753
chr12 16606768
chr12 16606797
chr12 16606848
chr12 16606859
chr12 16607064
chr12 16607106
chr12 16607114
chr12 16607122
chr12 16608996
chr12 16611375
chr12 16611472
chr12 16611620
chr12 16611756
chr12 16612086
chr12 16709645
chr12 16709667
chr12 16710323
chr12 16710346
chr12 16710700
chr12 16710704
chr12 16710913
chr12 16711217
chr12 16711494
chr12 16711518
chr12 16711702
chr12 16711751
chr12 16895203
chr12 17511974
chr12 17629256
chr12 17630049
chr12 17748833
chr12 17771885
chr12 17858653
chr12 17858825
chr12 17858960
chr12 17859085
chr12 17859174
chr12 17860094
chr12 17980597
chr12 17980681
chr12 17980774
chr12 17981106
chr12 17981231
chr12 18074349
chr12 18074971
chr12 18075410
chr12 18075453
chr12 18075547
chr12 18249551

chr12 18249889
chr12 18250132
chr12 18250310
chr12 18250314
chr12 18250343
chr12 18250462
chr12 18250510
chr12 18250530
chr12 18250889
chr12 18251134
chr12 18286092
chr12 18286165
chr12 18322820
chr12 19125000
chr12 19125335
chr12 19196748
chr12 19439498
chr12 19439508
chr12 19439510
chr12 19439516
chr12 19439518
chr12 19439520
chr12 19439530
chr12 19439532
chr12 19439534
chr12 19439541
chr12 19439546
chr12 19439548
chr12 19439562
chr12 19439569
chr12 19439571
chr12 19439594
chr12 19439597
chr12 19439600
chr12 19439603
chr12 19439615
chr12 19439630
chr12 19439645
chr12 19439658
chr12 19439661
chr12 19439693
chr12 19439696
chr12 19439705
chr12 19439708
chr12 19439717
chr12 19439726
chr12 19439745
chr12 19439765
chr12 19439773
chr12 19439779
chr12 19439782
chr12 19439785
chr12 19439787
chr12 19439793

chr12 19439807
chr12 19439845
chr12 19439852
chr12 19439857
chr12 19439863
chr12 19439866
chr12 19439879
chr12 19439882
chr12 19439888
chr12 19439897
chr12 19439900
chr12 19439906
chr12 19439909
chr12 19439912
chr12 19439927
chr12 19439930
chr12 19439941
chr12 19439947
chr12 19439953
chr12 19439960
chr12 19439966
chr12 19439978
chr12 19439987
chr12 19439993
chr12 19440005
chr12 19440008
chr12 19440947
chr12 19441008
chr12 19441064
chr12 19441084
chr12 19441113
chr12 19441122
chr12 19441199
chr12 19441265
chr12 19441373
chr12 19441415
chr12 19441502
chr12 19441570
chr12 19679957
chr12 19679999
chr12 19736592
chr12 19736611
chr12 19901286
chr12 19901670
chr12 19901733
chr12 20257980
chr12 20258116
chr12 20368116
chr12 20368133
chr12 20368141
chr12 20368372
chr12 20368503
chr12 20368529
chr12 20404218

chr12 20404782
chr12 20480862
chr12 20480934
chr12 20667270
chr12 20670611
chr12 20670686
chr12 20670992
chr12 20671025
chr12 20671041
chr12 20671076
chr12 20671372
chr12 20805322
chr12 20805463
chr12 20805880
chr12 20806156
chr12 20858018
chr12 20858493
chr12 20968010
chr12 20969887
chr12 20970040
chr12 20970072
chr12 20970121
chr12 20970796
chr12 20971233
chr12 20971441
chr12 21019638
chr12 21020130
chr12 21020132
chr12 21020190
chr12 21142177
chr12 21215927
chr12 21216386
chr12 21393222
chr12 21437371
chr12 21437741
chr12 21437743
chr12 21437753
chr12 21437756
chr12 21437769
chr12 21437778
chr12 21437781
chr12 21437784
chr12 21437790
chr12 21437792
chr12 21437796
chr12 21437807
chr12 21437821
chr12 21437832
chr12 21437838
chr12 21437850
chr12 21437909
chr12 21437927
chr12 21437937
chr12 21438008

chr12 21438038
chr12 21438061
chr12 21438163
chr12 21438203
chr12 21438243
chr12 21501110
chr12 21501114
chr12 21501122
chr12 21501312
chr12 21501324
chr12 21501338
chr12 21502226
chr12 21502228
chr12 21502258
chr12 21502295
chr12 21502304
chr12 21502306
chr12 21502317
chr12 21502323
chr12 21502331
chr12 21502345
chr12 21502387
chr12 21502436
chr12 21502462
chr12 21502584
chr12 21502633
chr12 21502694
chr12 21528158
chr12 21528201
chr12 21528203
chr12 21528239
chr12 21528341
chr12 21528361
chr12 21528396
chr12 21615040
chr12 21656028
chr12 21656138
chr12 21656319
chr12 21656451
chr12 21656509
chr12 21656513
chr12 21656588
chr12 21656645
chr12 21656792
chr12 21656799
chr12 21656873
chr12 21656920
chr12 21657787
chr12 21657791
chr12 21657812
chr12 21657816
chr12 21657825
chr12 21657828
chr12 21657831

chr12 21657857
chr12 21657874
chr12 21657911
chr12 21657918
chr12 21657934
chr12 21657939
chr12 21658026
chr12 21658049
chr12 21658065
chr12 21658100
chr12 21658119
chr12 21679077
chr12 21680648
chr12 21774171
chr12 21774185
chr12 21774256
chr12 21775037
chr12 21775039
chr12 21775050
chr12 21775056
chr12 21775124
chr12 21775245
chr12 21775422
chr12 22046645
chr12 22046818
chr12 22046847
chr12 22046860
chr12 22046873
chr12 22046898
chr12 22046946
chr12 22046992
chr12 22047032
chr12 22163977
chr12 22164075
chr12 22164098
chr12 22164336
chr12 22288123
chr12 22288263
chr12 22288295
chr12 22288395
chr12 22288415
chr12 22332988
chr12 22333006
chr12 22333095
chr12 22333141
chr12 22333513
chr12 22333520
chr12 22333547
chr12 22333550
chr12 22374725
chr12 22374796
chr12 22396412
chr12 22421963
chr12 22422503

chr12 22423133
chr12 22424230
chr12 22424232
chr12 22424270
chr12 22424325
chr12 22425039
chr12 22443258
chr12 22443312
chr12 22443334
chr12 22543461
chr12 22543508
chr12 22543702
chr12 22543742
chr12 22543751
chr12 22543753
chr12 22543759
chr12 22543821
chr12 22543847
chr12 22543864
chr12 22543866
chr12 22543881
chr12 22543892
chr12 22544066
chr12 22544092
chr12 22544113
chr12 22544117
chr12 22544123
chr12 22544156
chr12 22544162
chr12 22627169
chr12 22627173
chr12 22627297
chr12 22627360
chr12 22627698
chr12 22627728
chr12 22628055
chr12 22738156
chr12 22738424
chr12 22738683
chr12 23924993
chr12 23925396
chr12 23948969
chr12 23949008
chr12 23949041
chr12 23949098
chr12 23949128
chr12 23949213
chr12 23949287
chr12 23949343
chr12 23949371
chr12 23949402
chr12 23949472
chr12 23949647
chr12 23949873

chr12 23949896
chr12 23949898
chr12 23949908
chr12 23949913
chr12 23950010
chr12 23950063
chr12 23950088
chr12 23950135
chr12 23950241
chr12 23950251
chr12 23950261
chr12 24109373
chr12 24109599
chr12 24109903
chr12 24109947
chr12 24561507
chr12 24561557
chr12 24561573
chr12 24561575
chr12 24561594
chr12 24561666
chr12 24561718
chr12 24561722
chr12 24561763
chr12 24561776
chr12 24561785
chr12 24561827
chr12 24561894
chr12 24561896
chr12 24561925
chr12 24561938
chr12 24561956
chr12 24561990
chr12 24562023
chr12 24563384
chr12 24563417
chr12 24563435
chr12 24601134
chr12 24740849
chr12 24741013
chr12 24839028
chr12 24839289
chr12 24839322
chr12 24839328
chr12 24839336
chr12 24839343
chr12 24839349
chr12 24839381
chr12 24839512
chr12 24839721
chr12 24839735
chr12 24839749
chr12 24839776
chr12 24839782

chr12 24839818
chr12 24947710
chr12 24947712
chr12 24947912
chr12 24947927
chr12 24947973
chr12 24948048
chr12 24948079
chr12 24948193
chr12 24948206
chr12 24948253
chr12 24948372
chr12 24948674
chr12 24948686
chr12 24948702
chr12 24948712
chr12 24948719
chr12 24948735
chr12 24948755
chr12 24948762
chr12 24948771
chr12 24948795
chr12 24948801
chr12 24948826
chr12 24948868
chr12 24948871
chr12 24948876
chr12 24948883
chr12 24948904
chr12 24948938
chr12 24948941
chr12 24948947
chr12 24948958
chr12 24948967
chr12 24948988
chr12 24949001
chr12 24949010
chr12 24949018
chr12 24949029
chr12 24949031
chr12 24949037
chr12 24949065
chr12 24949070
chr12 24949073
chr12 24949076
chr12 24949084
chr12 24949089
chr12 25051372
chr12 25051398
chr12 25051434
chr12 25051603
chr12 25051689
chr12 25051715
chr12 25051731

chr12 25051738
chr12 25052041
chr12 25052195
chr12 25052326
chr12 25052912
chr12 25053005
chr12 25053364
chr12 25053953
chr12 25053997
chr12 25054512
chr12 25054562
chr12 25054621
chr12 25054646
chr12 25155044
chr12 25155489
chr12 25186650
chr12 25187153
chr12 25187170
chr12 25187182
chr12 25194651
chr12 25194729
chr12 25194761
chr12 25194818
chr12 25194832
chr12 25194856
chr12 25194891
chr12 25194932
chr12 25195448
chr12 25195458
chr12 25195464
chr12 25195507
chr12 25195526
chr12 25195542
chr12 25195550
chr12 25195572
chr12 25195605
chr12 25195668
chr12 25195708
chr12 25195712
chr12 25195714
chr12 25195732
chr12 25195794
chr12 25195824
chr12 25195892
chr12 25195895
chr12 25195908
chr12 25195951
chr12 25249980
chr12 25250090
chr12 25250168
chr12 25250204
chr12 25250222
chr12 25250233
chr12 25250237

chr12 25250258
chr12 25320239
chr12 25320366
chr12 25320483
chr12 25320635
chr12 25320683
chr12 25364936
chr12 25364947
chr12 25365073
chr12 25365138
chr12 25365538
chr12 25365593
chr12 25365719
chr12 25366044
chr12 25377638
chr12 25377669
chr12 25387181
chr12 25387495
chr12 25387500
chr12 25387522
chr12 25387580
chr12 25387617
chr12 25387666
chr12 25387668
chr12 25387677
chr12 25387846
chr12 25387852
chr12 25387919
chr12 25388124
chr12 25388220
chr12 25388366
chr12 25388421
chr12 25388430
chr12 25490076
chr12 25490208
chr12 25490552
chr12 25496422
chr12 25496436
chr12 25496531
chr12 25516186
chr12 25516251
chr12 25516319
chr12 25516893
chr12 25517270
chr12 25517915
chr12 25554048
chr12 25728463
chr12 25729462
chr12 25729592
chr12 25729636
chr12 25860094
chr12 25860125
chr12 25860173
chr12 25860186

chr12 25860206
chr12 25860243
chr12 25860390
chr12 25887246
chr12 25960085
chr12 25960327
chr12 25960355
chr12 25960403
chr12 25960417
chr12 25960473
chr12 25960986
chr12 25986151
chr12 26125648
chr12 26125656
chr12 26125659
chr12 26125684
chr12 26125693
chr12 26341020
chr12 26341058
chr12 26729188
chr12 26831951
chr12 26885071
chr12 26885083
chr12 26885102
chr12 26885116
chr12 26885132
chr12 26885169
chr12 26885252
chr12 26885278
chr12 26885313
chr12 26885486
chr12 26936719
chr12 26940441
chr12 26940485
chr12 26940840
chr12 26941066
chr12 26941148
chr12 26941214
chr12 26941412
chr12 26972035
chr12 26972162
chr12 27013700
chr12 27013763
chr12 27013863
chr12 27013912
chr12 27013931
chr12 27013936
chr12 27013958
chr12 27013968
chr12 27015887
chr12 27015994
chr12 27022265
chr12 27022323
chr12 27022347

chr12 27022410
chr12 27022424
chr12 27022429
chr12 27022451
chr12 27022456
chr12 27022470
chr12 27022480
chr12 27022491
chr12 27022514
chr12 27022516
chr12 27022535
chr12 27022537
chr12 27022557
chr12 27022570
chr12 27022584
chr12 27022589
chr12 27022596
chr12 27022609
chr12 27022620
chr12 27022637
chr12 27244351
chr12 27244355
chr12 27244367
chr12 27244373
chr12 27244376
chr12 27244378
chr12 27244395
chr12 27244446
chr12 27244603
chr12 27244610
chr12 27244619
chr12 27244624
chr12 27244632
chr12 27244662
chr12 27244664
chr12 27244675
chr12 27244688
chr12 27244694
chr12 27244699
chr12 27244712
chr12 27244735
chr12 27244774
chr12 27244792
chr12 27244854
chr12 27244900
chr12 27244955
chr12 27244961
chr12 27244965
chr12 27245004
chr12 27245019
chr12 27245130
chr12 27245313
chr12 27460118
chr12 27460272

chr12 27460324
chr12 27460412
chr12 27460561
chr12 27460592
chr12 27460644
chr12 27460677
chr12 27460721
chr12 27460734
chr12 27460921
chr12 27462197
chr12 27462205
chr12 27462208
chr12 27523467
chr12 27524755
chr12 27703932
chr12 27711452
chr12 27711486
chr12 27711513
chr12 27711541
chr12 27711547
chr12 27711564
chr12 27711583
chr12 27821415
chr12 27821417
chr12 27821430
chr12 27821464
chr12 27821478
chr12 27821965
chr12 27822007
chr12 27822048
chr12 27822102
chr12 27824488
chr12 29103557
chr12 29103645
chr12 29103703
chr12 29103711
chr12 29148546
chr12 29379939
chr12 29380027
chr12 29380101
chr12 29380417
chr12 29380448
chr12 29390656
chr12 29390684
chr12 29390730
chr12 29391251
chr12 29412880
chr12 29413279
chr12 29438438
chr12 29629963
chr12 29630389
chr12 29782155
chr12 29782188
chr12 29782293

chr12 29782321
chr12 29782441
chr12 30384885
chr12 30695119
chr12 30695129
chr12 30695224
chr12 30695226
chr12 30695290
chr12 30695426
chr12 30695433
chr12 30695443
chr12 30695450
chr12 30695464
chr12 30695479
chr12 30695486
chr12 30695489
chr12 30695504
chr12 30695512
chr12 30695521
chr12 30695592
chr12 30695600
chr12 30754216
chr12 30754218
chr12 30754257
chr12 30754292
chr12 30754303
chr12 30754326
chr12 30754334
chr12 30754340
chr12 30754347
chr12 30754362
chr12 30822212
chr12 30822564
chr12 30822573
chr12 30822845
chr12 30822867
chr12 30822871
chr12 30822879
chr12 30822881
chr12 30822884
chr12 31074607
chr12 31074622
chr12 31074660
chr12 31074701
chr12 31074740
chr12 31074809
chr12 31074849
chr12 31075022
chr12 31075068
chr12 31075079
chr12 31075303
chr12 31117500
chr12 31117511
chr12 31117515

chr12 31117522
chr12 31117558
chr12 31117569
chr12 31117580
chr12 31117602
chr12 31117624
chr12 31117631
chr12 31117681
chr12 31117703
chr12 31117705
chr12 31117710
chr12 31173596
chr12 31173640
chr12 31174236
chr12 31174282
chr12 31174509
chr12 31316938
chr12 31317072
chr12 31317159
chr12 31317386
chr12 31317429
chr12 31317774
chr12 31317843
chr12 31317988
chr12 31318000
chr12 31318002
chr12 31318152
chr12 31318184
chr12 31318186
chr12 31322843
chr12 31575049
chr12 31575398
chr12 31658886
chr12 31658953
chr12 31658983
chr12 31658998
chr12 31659082
chr12 31659112
chr12 31659125
chr12 31659127
chr12 31659165
chr12 31659183
chr12 31659189
chr12 31659191
chr12 31659199
chr12 31659202
chr12 31659206
chr12 31659211
chr12 31659221
chr12 31659226
chr12 31659228
chr12 31659231
chr12 31659244
chr12 31659263

chr12 31659271
chr12 31659290
chr12 31659300
chr12 31659308
chr12 31659319
chr12 31659339
chr12 31659345
chr12 31659381
chr12 31659402
chr12 31659413
chr12 31659432
chr12 31659435
chr12 31659438
chr12 31659446
chr12 31659456
chr12 31659547
chr12 31659558
chr12 31659571
chr12 31659574
chr12 31728734
chr12 31728741
chr12 31728743
chr12 31728748
chr12 31728770
chr12 31728812
chr12 31728864
chr12 31728867
chr12 31728916
chr12 31728942
chr12 31728952
chr12 31728992
chr12 31728995
chr12 31729001
chr12 31729009
chr12 31729017
chr12 31763082
chr12 31763106
chr12 31763138
chr12 31763168
chr12 31763208
chr12 31790770
chr12 31832936
chr12 31833037
chr12 31833129
chr12 31833140
chr12 31833152
chr12 31833316
chr12 31859966
chr12 31875241
chr12 31875305
chr12 31896585
chr12 31896588
chr12 31896633
chr12 31896658

chr12 31896756
chr12 31897020
chr12 31928639
chr12 31928863
chr12 31928892
chr12 31960555
chr12 31960569
chr12 31960813
chr12 31960841
chr12 31960870
chr12 31960897
chr12 31960915
chr12 31960955
chr12 31960987
chr12 31961223
chr12 31961253
chr12 32060671
chr12 32060684
chr12 32060709
chr12 32060725
chr12 32060843
chr12 32060937
chr12 32060971
chr12 32060976
chr12 32061352
chr12 32061360
chr12 32061365
chr12 32107779
chr12 32107826
chr12 32107871
chr12 32107898
chr12 32107907
chr12 32108147
chr12 32108152
chr12 32134643
chr12 32134645
chr12 32422919
chr12 32422997
chr12 32589306
chr12 32589470
chr12 32756209
chr12 32756227
chr12 32756334
chr12 32756354
chr12 32756373
chr12 32756389
chr12 32893208
chr12 32893423
chr12 32893468
chr12 32896492
chr12 32896495
chr12 32896509
chr12 32896518
chr12 32896526

chr12 32896535
chr12 32896539
chr12 32896568
chr12 33036433
chr12 33225891
chr12 33227095
chr12 33451495
chr12 33451621
chr12 33616830
chr12 33846207
chr12 33850360
chr12 33850763
chr12 33850906
chr12 33851240
chr12 33851315
chr12 34021802
chr12 34021942
chr12 34021973
chr12 34021982
chr12 34022041
chr12 34022106
chr12 34023533
chr12 34167716
chr12 34167725
chr12 34428701
chr12 34429441
chr12 34429886
chr12 36908585
chr12 36908620
chr12 36910624
chr12 37424430
chr12 37425432
chr12 37425510
chr12 37426226
chr12 37426902
chr12 37510968
chr12 37511331
chr12 37512351
chr12 37512420
chr12 37536749
chr12 37536832
chr12 37537685
chr12 37538608
chr12 37538722
chr12 37538899
chr12 37539044
chr12 37539157
chr12 38101341
chr12 38101501
chr12 38316030
chr12 38316253
chr12 38316284
chr12 38316293
chr12 38316352

chr12 38316417
chr12 38316490
chr12 38316500
chr12 38316503
chr12 38317115
chr12 38317151
chr12 38327527
chr12 38327614
chr12 38327736
chr12 38328355
chr12 38328422
chr12 38328452
chr12 38336272
chr12 38336356
chr12 38337048
chr12 38440013
chr12 38440381
chr12 38798572
chr12 38906031
chr12 38906042
chr12 38906091
chr12 38906106
chr12 38906144
chr12 38906150
chr12 38906172
chr12 38906192
chr12 38906234
chr12 38906255
chr12 38906271
chr12 38906291
chr12 38906306
chr12 38906332
chr12 38906340
chr12 38906416
chr12 38906434
chr12 38906437
chr12 38906475
chr12 38906544
chr12 38906699
chr12 38906704
chr12 38906854
chr12 38906858
chr12 38906866
chr12 38906923
chr12 38907024
chr12 38907090
chr12 38907152
chr12 38907389
chr12 38951453
chr12 38990311
chr12 38991221
chr12 38991346
chr12 39199253
chr12 39199494

chr12 39199538
chr12 39199544
chr12 39199615
chr12 39200004
chr12 39441953
chr12 39442064
chr12 39442075
chr12 39442083
chr12 39442099
chr12 39442163
chr12 39442168
chr12 39442410
chr12 39442418
chr12 39442420
chr12 39442422
chr12 39442425
chr12 39442434
chr12 39442438
chr12 39442441
chr12 39442444
chr12 39442483
chr12 39617098
chr12 39618147
chr12 39618386
chr12 39618592
chr12 39618725
chr12 39618738
chr12 39618983
chr12 39619069
chr12 39619083
chr12 39619138
chr12 39619194
chr12 39619222
chr12 39619341
chr12 39619385
chr12 39619408
chr12 39619468
chr12 39619488
chr12 39619529
chr12 39619565
chr12 39619584
chr12 39619634
chr12 39619651
chr12 39619710
chr12 39619727
chr12 39619794
chr12 39619806
chr12 39620490
chr12 39620550
chr12 39620589
chr12 39620726
chr12 39621162
chr12 39621185
chr12 39621213

chr12 39621229
chr12 39621237
chr12 39621357
chr12 39621368
chr12 39621380
chr12 39621464
chr12 39621488
chr12 39622526
chr12 39629135
chr12 39629278
chr12 39701465
chr12 39701796
chr12 40201810
chr12 40201918
chr12 40354539
chr12 40691925
chr12 40691996
chr12 40692058
chr12 40692082
chr12 40692099
chr12 40692115
chr12 41061492
chr12 41061541
chr12 41186356
chr12 41190382
chr12 41190433
chr12 41190615
chr12 41242705
chr12 41242779
chr12 41575246
chr12 41575562
chr12 41575755
chr12 41930754
chr12 41930851
chr12 41930973
chr12 41931041
chr12 41931080
chr12 41931216
chr12 41931234
chr12 41931306
chr12 41932695
chr12 41932698
chr12 41932703
chr12 41932709
chr12 41932715
chr12 41932723
chr12 41932726
chr12 41932728
chr12 41932809
chr12 41932821
chr12 41932829
chr12 41932861
chr12 41932863
chr12 41932868

chr12 41932878
chr12 41932905
chr12 41932922
chr12 41932928
chr12 41932930
chr12 41932960
chr12 41933039
chr12 41933072
chr12 42024399
chr12 42024411
chr12 42024422
chr12 42024433
chr12 42024456
chr12 42024531
chr12 42024539
chr12 42024741
chr12 42098262
chr12 42098272
chr12 42098321
chr12 42098333
chr12 42098400
chr12 42237202
chr12 42237327
chr12 42237332
chr12 42237342
chr12 42237395
chr12 42237423
chr12 42237433
chr12 42237444
chr12 42326534
chr12 42326637
chr12 42326750
chr12 42326810
chr12 42326885
chr12 42326948
chr12 42326966
chr12 42326988
chr12 42327020
chr12 42327026
chr12 42327037
chr12 42327114
chr12 42327184
chr12 42327208
chr12 42343793
chr12 42343820
chr12 42343862
chr12 42484341
chr12 42484364
chr12 42484374
chr12 42484376
chr12 42484378
chr12 42484385
chr12 42484390
chr12 42484537

chr12 42484559
chr12 42484613
chr12 42484615
chr12 43468842
chr12 43469031
chr12 43469243
chr12 43469296
chr12 43692176
chr12 43758314
chr12 43758370
chr12 43758392
chr12 43758407
chr12 43758413
chr12 43758650
chr12 43758704
chr12 43758706
chr12 43758717
chr12 43758723
chr12 43758732
chr12 43758762
chr12 43758769
chr12 43758777
chr12 43758802
chr12 43758811
chr12 43758829
chr12 43758835
chr12 43758843
chr12 43758845
chr12 43758852
chr12 43758859
chr12 43758884
chr12 43758912
chr12 43758925
chr12 43758936
chr12 43758946
chr12 43758954
chr12 43758956
chr12 43758959
chr12 43758971
chr12 43758987
chr12 43758990
chr12 43759004
chr12 43759006
chr12 43759010
chr12 43759030
chr12 43759043
chr12 43759051
chr12 43759054
chr12 43759058
chr12 43759065
chr12 43759078
chr12 43759107
chr12 43759113
chr12 43759118

chr12 43759137
chr12 43759226
chr12 43759229
chr12 43805173
chr12 43805184
chr12 43805284
chr12 43805295
chr12 43805342
chr12 43805346
chr12 43805364
chr12 43805385
chr12 43805427
chr12 43805465
chr12 43805476
chr12 43805478
chr12 43805487
chr12 43805594
chr12 43805660
chr12 43805670
chr12 43806594
chr12 43806601
chr12 43806715
chr12 43806721
chr12 43806801
chr12 43806888
chr12 43806940
chr12 43807293
chr12 43836503
chr12 43836528
chr12 43836531
chr12 43836565
chr12 43836630
chr12 43836692
chr12 43836806
chr12 43836829
chr12 43909763
chr12 43909864
chr12 43910570
chr12 43910597
chr12 43912240
chr12 43912330
chr12 43912403
chr12 43912443
chr12 43912460
chr12 43912462
chr12 43957902
chr12 44285624
chr12 44286392
chr12 44286647
chr12 44488085
chr12 44490601
chr12 44490812
chr12 44828201
chr12 44828434

chr12 44829299
chr12 44874174
chr12 44874830
chr12 44874910
chr12 44874915
chr12 44875535
chr12 44875550
chr12 44875605
chr12 44875622
chr12 44875629
chr12 44875634
chr12 44875636
chr12 44875683
chr12 44876924
chr12 44877284
chr12 44931194
chr12 45130993
chr12 45200474
chr12 45200590
chr12 45200786
chr12 45200797
chr12 45200821
chr12 45215315
chr12 45215444
chr12 45215452
chr12 45215463
chr12 45215482
chr12 45215484
chr12 45215486
chr12 45215490
chr12 45215502
chr12 45215517
chr12 45215519
chr12 45215534
chr12 45215560
chr12 45215594
chr12 45215598
chr12 45215603
chr12 45215653
chr12 45215662
chr12 45215768
chr12 45215774
chr12 45217102
chr12 45217106
chr12 45217233
chr12 45217301
chr12 45217306
chr12 45217449
chr12 45217459
chr12 45518479
chr12 45572420
chr12 45572733
chr12 45573092
chr12 45669824

chr12 45669965
chr12 45725134
chr12 45725290
chr12 45725298
chr12 45725305
chr12 45725373
chr12 45725627
chr12 45725630
chr12 45725637
chr12 45731282
chr12 45731313
chr12 45731508
chr12 45731593
chr12 45732003
chr12 45732070
chr12 45732327
chr12 45732360
chr12 45732419
chr12 45732572
chr12 45732618
chr12 45878754
chr12 45878772
chr12 45878870
chr12 45984502
chr12 45984618
chr12 45984687
chr12 45988928
chr12 45989167
chr12 45989247
chr12 45989288
chr12 45989306
chr12 45989468
chr12 45989510
chr12 45992100
chr12 45992103
chr12 45992199
chr12 45992232
chr12 45992241
chr12 45992350
chr12 45992865
chr12 46209968
chr12 46210097
chr12 46210101
chr12 46210108
chr12 46214834
chr12 46214944
chr12 46215010
chr12 46215048
chr12 46215212
chr12 46215280
chr12 46215306
chr12 46215597
chr12 46216029
chr12 46216348

chr12 46216351
chr12 46217293
chr12 46217423
chr12 46217440
chr12 46217747
chr12 46217806
chr12 46217859
chr12 46218139
chr12 46218243
chr12 46218275
chr12 46218390
chr12 46218415
chr12 46218498
chr12 46264843
chr12 46265844
chr12 46266020
chr12 46266094
chr12 46266160
chr12 46266225
chr12 46266472
chr12 46266523
chr12 46266555
chr12 46266564
chr12 46269851
chr12 46269859
chr12 46269863
chr12 46269900
chr12 46269911
chr12 46269918
chr12 46269926
chr12 46269973
chr12 46270051
chr12 46270070
chr12 46270096
chr12 46270098
chr12 46270117
chr12 46270119
chr12 46270218
chr12 46270313
chr12 46270336
chr12 46370809
chr12 46371043
chr12 46371064
chr12 46371146
chr12 46371221
chr12 46371255
chr12 46373133
chr12 46373157
chr12 46373339
chr12 46373346
chr12 46373349
chr12 46373364
chr12 46373382
chr12 46373403

chr12 46373465
chr12 46373468
chr12 46373480
chr12 46373506
chr12 46373514
chr12 46373537
chr12 46373540
chr12 46373545
chr12 46373573
chr12 46373584
chr12 46373593
chr12 46373664
chr12 46373686
chr12 46373846
chr12 46373882
chr12 46373900
chr12 46373903
chr12 46373907
chr12 46384334
chr12 46547103
chr12 46547197
chr12 46547594
chr12 47078949
chr12 47078957
chr12 47078960
chr12 47078992
chr12 47078997
chr12 47079009
chr12 47079027
chr12 47079042
chr12 47079341
chr12 47081108
chr12 47081132
chr12 47081153
chr12 47172331
chr12 47172417
chr12 47207530
chr12 47207631
chr12 47207983
chr12 47213545
chr12 47214145
chr12 47214535
chr12 47214599
chr12 47214858
chr12 47214919
chr12 47214947
chr12 47214955
chr12 47214972
chr12 47214989
chr12 47215027
chr12 47215179
chr12 47215316
chr12 47215339
chr12 47215356

chr12 47215378
chr12 47215410
chr12 47215442
chr12 47215473
chr12 47215500
chr12 47215505
chr12 47215545
chr12 47215552
chr12 47215574
chr12 47215730
chr12 47215765
chr12 47215825
chr12 47215852
chr12 47215910
chr12 47215946
chr12 47215967
chr12 47216001
chr12 47216048
chr12 47216082
chr12 47216223
chr12 47216951
chr12 47217130
chr12 47217144
chr12 47217355
chr12 47276861
chr12 47277171
chr12 47307728
chr12 47356029
chr12 47356327
chr12 47360853
chr12 47360869
chr12 47360929
chr12 47361316
chr12 47361449
chr12 47361457
chr12 47652334
chr12 47652373
chr12 47706503
chr12 47706515
chr12 47706632
chr12 47706664
chr12 47706680
chr12 47706702
chr12 47706810
chr12 47706868
chr12 47707022
chr12 47741542
chr12 47741575
chr12 47741621
chr12 47741649
chr12 47741716
chr12 47741766
chr12 47741774
chr12 47742142

chr12 47742304
chr12 47742318
chr12 47808317
chr12 47808391
chr12 47808510
chr12 47808535
chr12 47808546
chr12 47808631
chr12 47808641
chr12 47813533
chr12 47813632
chr12 47817296
chr12 47817317
chr12 47817468
chr12 47817488
chr12 47817556
chr12 47817656
chr12 47817722
chr12 47819588
chr12 47819594
chr12 47819598
chr12 47819607
chr12 47819613
chr12 47904592
chr12 47904603
chr12 47904638
chr12 47904643
chr12 47904699
chr12 47904717
chr12 47904720
chr12 47904839
chr12 47904863
chr12 47904869
chr12 47904873
chr12 47904884
chr12 47904896
chr12 47904901
chr12 47904907
chr12 47904986
chr12 47904988
chr12 47904994
chr12 47963719
chr12 47963723
chr12 47963760
chr12 47964068
chr12 47964078
chr12 47964106
chr12 47964125
chr12 47964152
chr12 47964162
chr12 47964178
chr12 47964184
chr12 47964232
chr12 47964254

chr12 47964264
chr12 47964282
chr12 47990596
chr12 48106621
chr12 48106738
chr12 48119332
chr12 48119381
chr12 48119384
chr12 48119471
chr12 48119481
chr12 48119524
chr12 48119543
chr12 48119567
chr12 48119601
chr12 48119654
chr12 48119658
chr12 48197687
chr12 48197751
chr12 48197824
chr12 48197880
chr12 48197890
chr12 48197912
chr12 48197920
chr12 48197934
chr12 48197950
chr12 48197956
chr12 48198528
chr12 48198540
chr12 48262076
chr12 48262168
chr12 48330767
chr12 48334618
chr12 48334626
chr12 48334835
chr12 48334838
chr12 48334847
chr12 48335028
chr12 48335051
chr12 48350674
chr12 48350676
chr12 48350679
chr12 48350723
chr12 48350728
chr12 48350740
chr12 48350756
chr12 48350768
chr12 48350773
chr12 48350789
chr12 48350803
chr12 48350805
chr12 48350810
chr12 48350824
chr12 48350828
chr12 48350831

chr12 48350848
chr12 48431664
chr12 48503152
chr12 48504144
chr12 48504233
chr12 48682604
chr12 48682608
chr12 48682610
chr12 48682620
chr12 48682672
chr12 48682680
chr12 48682708
chr12 48682711
chr12 48682738
chr12 48682742
chr12 48682754
chr12 48682774
chr12 48682813
chr12 48682822
chr12 48682830
chr12 48682836
chr12 48682838
chr12 48682869
chr12 48682877
chr12 48766397
chr12 48789609
chr12 48789623
chr12 48789631
chr12 48789639
chr12 48789641
chr12 48789685
chr12 48789698
chr12 48818190
chr12 48818245
chr12 48818330
chr12 48818334
chr12 48818339
chr12 48818341
chr12 48818361
chr12 48853300
chr12 48853364
chr12 48853420
chr12 48853428
chr12 48853432
chr12 48865704
chr12 48865710
chr12 48865719
chr12 48865737
chr12 48865754
chr12 48865780
chr12 48865782
chr12 48865796
chr12 48865816
chr12 48865825

chr12 48865835
chr12 48865882
chr12 48865891
chr12 48865931
chr12 48865943
chr12 48866041
chr12 48917820
chr12 48918030
chr12 48918102
chr12 48918383
chr12 48925021
chr12 48925026
chr12 48925046
chr12 48925064
chr12 48925081
chr12 48925087
chr12 48925198
chr12 48925215
chr12 48925228
chr12 48999244
chr12 48999263
chr12 48999305
chr12 48999325
chr12 48999357
chr12 48999360
chr12 48999375
chr12 48999377
chr12 48999383
chr12 48999407
chr12 48999415
chr12 48999441
chr12 48999443
chr12 48999445
chr12 48999457
chr12 48999459
chr12 48999461
chr12 48999471
chr12 48999473
chr12 48999496
chr12 48999500
chr12 48999514
chr12 48999524
chr12 48999565
chr12 48999569
chr12 48999577
chr12 48999914
chr12 49018223
chr12 49018255
chr12 49058269
chr12 49058278
chr12 49058302
chr12 49058807
chr12 49058851
chr12 49058870

chr12 49058945
chr12 49059019
chr12 49059158
chr12 49059306
chr12 49059322
chr12 49059384
chr12 49059493
chr12 49059539
chr12 49059595
chr12 49059599
chr12 49059625
chr12 49059644
chr12 49059663
chr12 49059668
chr12 49059675
chr12 49110027
chr12 49110078
chr12 49110081
chr12 49110099
chr12 49110151
chr12 49110175
chr12 49110192
chr12 49110213
chr12 49110215
chr12 49110253
chr12 49110268
chr12 49110274
chr12 49110277
chr12 49110291
chr12 49110325
chr12 49110332
chr12 49110340
chr12 49110352
chr12 49110356
chr12 49110366
chr12 49110369
chr12 49110373
chr12 49110376
chr12 49110398
chr12 49110422
chr12 49131974
chr12 49132003
chr12 49132257
chr12 49132343
chr12 49132348
chr12 49188120
chr12 49188134
chr12 49189260
chr12 49201038
chr12 49201255
chr12 49201288
chr12 49201344
chr12 49234672
chr12 49234675

chr12 49234679
chr12 49234681
chr12 49234687
chr12 49234692
chr12 49234710
chr12 49234728
chr12 49234735
chr12 49234737
chr12 49234839
chr12 49234878
chr12 49234880
chr12 49234886
chr12 49234903
chr12 49234920
chr12 49234942
chr12 49234966
chr12 49234974
chr12 49235006
chr12 49235037
chr12 49235051
chr12 49235058
chr12 49235062
chr12 49235067
chr12 49235095
chr12 49235101
chr12 49235107
chr12 49235111
chr12 49235146
chr12 49235193
chr12 49235215
chr12 49323176
chr12 49323183
chr12 49323185
chr12 49323194
chr12 49323210
chr12 49323212
chr12 49323220
chr12 49323222
chr12 49323228
chr12 49323230
chr12 49323234
chr12 49347275
chr12 49347299
chr12 49347301
chr12 49421982
chr12 49514292
chr12 49514565
chr12 49514575
chr12 49567540
chr12 49567547
chr12 49567561
chr12 49567592
chr12 49567602
chr12 49567611

chr12 49567647
chr12 49567698
chr12 49621856
chr12 49621905
chr12 49621928
chr12 49621979
chr12 49622070
chr12 49622126
chr12 49622137
chr12 49622146
chr12 49622148
chr12 49622158
chr12 49622174
chr12 49622186
chr12 49622189
chr12 49622197
chr12 49622211
chr12 49622215
chr12 49622240
chr12 49622242
chr12 49622245
chr12 49622248
chr12 49622254
chr12 49622259
chr12 49622261
chr12 49622263
chr12 49623331
chr12 49623338
chr12 49623345
chr12 49623352
chr12 49623449
chr12 49623452
chr12 49623455
chr12 49623473
chr12 49623479
chr12 49623489
chr12 49623497
chr12 49623500
chr12 49623506
chr12 49623514
chr12 49623520
chr12 49623538
chr12 49623551
chr12 49699292
chr12 49741461
chr12 49741485
chr12 49741497
chr12 49741509
chr12 49741516
chr12 49741520
chr12 49741523
chr12 49741535
chr12 49741541
chr12 49741551

chr12 49741567
chr12 49741579
chr12 49741600
chr12 49741615
chr12 49741635
chr12 49741708
chr12 49741732
chr12 49741744
chr12 49741757
chr12 49741799
chr12 49741801
chr12 49741805
chr12 49741821
chr12 49742448
chr12 49742459
chr12 49742708
chr12 49742740
chr12 49742856
chr12 49750175
chr12 49750286
chr12 49750303
chr12 49750613
chr12 49828271
chr12 49828279
chr12 49828285
chr12 49828288
chr12 49828322
chr12 49828355
chr12 49828361
chr12 49828376
chr12 49828378
chr12 49828389
chr12 49828413
chr12 49828419
chr12 49828449
chr12 49828459
chr12 49828477
chr12 49828506
chr12 49828521
chr12 49828523
chr12 49828557
chr12 49828559
chr12 49828574
chr12 49828578
chr12 49828581
chr12 49828583
chr12 49828585
chr12 49828588
chr12 49828594
chr12 49828647
chr12 49828652
chr12 49828672
chr12 50025153
chr12 50075195

chr12 50075222
chr12 50075319
chr12 50075423
chr12 50088696
chr12 50088758
chr12 50112626
chr12 50112638
chr12 50112647
chr12 50112672
chr12 50112750
chr12 50166547
chr12 50220819
chr12 50220919
chr12 50220930
chr12 50220951
chr12 50221228
chr12 50221240
chr12 50221383
chr12 50221436
chr12 50221468
chr12 50221628
chr12 50221764
chr12 50221931
chr12 50222007
chr12 50222072
chr12 50222099
chr12 50222209
chr12 50222229
chr12 50222239
chr12 50222317
chr12 50222379
chr12 50222479
chr12 50222651
chr12 50222779
chr12 50400888
chr12 50400900
chr12 50400917
chr12 50400948
chr12 50400950
chr12 50400975
chr12 50400978
chr12 50400981
chr12 50400987
chr12 50401388
chr12 50401390
chr12 50401395
chr12 50401402
chr12 50401406
chr12 50401408
chr12 50401414
chr12 50401416
chr12 50401438
chr12 50401457
chr12 50401478

chr12 50401540
chr12 50401583
chr12 50401598
chr12 50401638
chr12 50401677
chr12 50401703
chr12 50401754
chr12 50401764
chr12 50401818
chr12 50401879
chr12 50401923
chr12 50401990
chr12 50402015
chr12 50402078
chr12 50402103
chr12 50402133
chr12 50402156
chr12 50402192
chr12 50402229
chr12 50402232
chr12 50402323
chr12 50442561
chr12 50476361
chr12 50476409
chr12 50476690
chr12 50505496
chr12 50505517
chr12 50505530
chr12 50505538
chr12 50505552
chr12 50505585
chr12 50505628
chr12 50505642
chr12 50505695
chr12 50505729
chr12 50505731
chr12 50505765
chr12 50505827
chr12 50505889
chr12 50505894
chr12 50505903
chr12 50505954
chr12 50506017
chr12 50506112
chr12 50644714
chr12 50765273
chr12 50765325
chr12 50765362
chr12 50765389
chr12 50765441
chr12 50825883
chr12 51024867
chr12 51024885
chr12 51025005

chr12 51025077
chr12 51025097
chr12 51025177
chr12 51025180
chr12 51025381
chr12 51025445
chr12 51025461
chr12 51025610
chr12 51025640
chr12 51048361
chr12 51048384
chr12 51048388
chr12 51048403
chr12 51048432
chr12 51048450
chr12 51048477
chr12 51048494
chr12 51048512
chr12 51048575
chr12 51048689
chr12 51048691
chr12 51172452
chr12 51172469
chr12 51172471
chr12 51172500
chr12 51172535
chr12 51172563
chr12 51172596
chr12 51172612
chr12 51172663
chr12 51172719
chr12 51172744
chr12 51172786
chr12 51172801
chr12 51172804
chr12 51172812
chr12 51172835
chr12 51172873
chr12 51172898
chr12 51172904
chr12 51172916
chr12 51172926
chr12 51172948
chr12 51172973
chr12 51172981
chr12 51172989
chr12 51173008
chr12 51173045
chr12 51173060
chr12 51173076
chr12 51173080
chr12 51173083
chr12 51173091
chr12 51173107

chr12 51173120
chr12 51173128
chr12 51173131
chr12 51173140
chr12 51173145
chr12 51173151
chr12 51173155
chr12 51218075
chr12 51218087
chr12 51218091
chr12 51218093
chr12 51218100
chr12 51218103
chr12 51218109
chr12 51218112
chr12 51218125
chr12 51218148
chr12 51218161
chr12 51218200
chr12 51218204
chr12 51218214
chr12 51218228
chr12 51238591
chr12 51246490
chr12 51246502
chr12 51246517
chr12 51246521
chr12 51246539
chr12 51246590
chr12 51318943
chr12 51319267
chr12 51319436
chr12 51322658
chr12 51322672
chr12 51322702
chr12 51322776
chr12 51322791
chr12 51322922
chr12 51323068
chr12 51323121
chr12 51323159
chr12 51323193
chr12 51323446
chr12 51323489
chr12 51323588
chr12 51323596
chr12 51323754
chr12 51323811
chr12 51323818
chr12 51323825
chr12 51323868
chr12 51323874
chr12 51323890
chr12 51323904

chr12 51323921
chr12 51323943
chr12 51323956
chr12 51323960
chr12 51323966
chr12 51323972
chr12 51323978
chr12 51324004
chr12 51324011
chr12 51324063
chr12 51324072
chr12 51324076
chr12 51324078
chr12 51324081
chr12 51324123
chr12 51324126
chr12 51324137
chr12 51324139
chr12 51324163
chr12 51369102
chr12 51369117
chr12 51369136
chr12 51369213
chr12 51565939
chr12 51566205
chr12 51566535
chr12 51567589
chr12 51567610
chr12 51567635
chr12 51567685
chr12 51567707
chr12 51567714
chr12 51821015
chr12 51821018
chr12 51821021
chr12 51821025
chr12 51821037
chr12 51821039
chr12 51821042
chr12 51821044
chr12 51821050
chr12 51821058
chr12 51821063
chr12 51821073
chr12 51821078
chr12 51821086
chr12 51821097
chr12 51821106
chr12 51821108
chr12 51821113
chr12 51821157
chr12 51821165
chr12 51821169
chr12 51821205

chr12 51821208
chr12 51821217
chr12 51821219
chr12 51821229
chr12 51821238
chr12 51821250
chr12 51821258
chr12 51821277
chr12 51821306
chr12 51821325
chr12 51821328
chr12 51821333
chr12 51821336
chr12 51821347
chr12 51821432
chr12 51821451
chr12 51821454
chr12 51821463
chr12 51821465
chr12 51821475
chr12 51821485
chr12 51821492
chr12 51821500
chr12 51821507
chr12 51821510
chr12 51821517
chr12 51821519
chr12 51821522
chr12 51821537
chr12 51821546
chr12 51821558
chr12 51821564
chr12 51821568
chr12 51821570
chr12 51821578
chr12 51821584
chr12 51821591
chr12 51821600
chr12 51821603
chr12 51821606
chr12 51821614
chr12 51821622
chr12 51821630
chr12 51821636
chr12 51821643
chr12 51821646
chr12 51821648
chr12 51821652
chr12 51821669
chr12 51821676
chr12 51821682
chr12 51821685
chr12 51821687
chr12 51821697

chr12 51847422
chr12 51847425
chr12 51847435
chr12 51847439
chr12 51951130
chr12 51951185
chr12 51951187
chr12 51951192
chr12 51951194
chr12 51951215
chr12 51951217
chr12 51971151
chr12 51971164
chr12 51971285
chr12 51971438
chr12 51971459
chr12 51971470
chr12 51971510
chr12 51971615
chr12 52008028
chr12 52008038
chr12 52008043
chr12 52023773
chr12 52023778
chr12 52372399
chr12 52610323
chr12 52741299
chr12 52741396
chr12 52741458
chr12 52874016
chr12 52874036
chr12 52874048
chr12 52874076
chr12 52874091
chr12 52874107
chr12 52874113
chr12 52874117
chr12 52879766
chr12 52879797
chr12 52879838
chr12 52879867
chr12 52879891
chr12 52879968
chr12 52880059
chr12 52880073
chr12 52880154
chr12 52880159
chr12 52880173
chr12 52884597
chr12 52884613
chr12 52884655
chr12 53007217
chr12 53007249
chr12 53007255

chr12 53007271
chr12 53007337
chr12 53007363
chr12 53007369
chr12 53007372
chr12 53007726
chr12 53053998
chr12 53054042
chr12 53054056
chr12 53054059
chr12 53054090
chr12 53054105
chr12 53079108
chr12 53079124
chr12 53079134
chr12 53079164
chr12 53079201
chr12 53079211
chr12 53079232
chr12 53079245
chr12 53079252
chr12 53079264
chr12 53079274
chr12 53079283
chr12 53079291
chr12 53079297
chr12 53079304
chr12 53079311
chr12 53079317
chr12 53079320
chr12 53079322
chr12 53079352
chr12 53079398
chr12 53200749
chr12 53200797
chr12 53200860
chr12 53200877
chr12 53201072
chr12 53201085
chr12 53201237
chr12 53205862
chr12 53205874
chr12 53205890
chr12 53205921
chr12 53205989
chr12 53206022
chr12 53206029
chr12 53209061
chr12 53209134
chr12 53209255
chr12 53209287
chr12 53209338
chr12 53209380
chr12 53219478

chr12 53219484
chr12 53219788
chr12 53219799
chr12 53219813
chr12 53219887
chr12 53219933
chr12 53219940
chr12 53219962
chr12 53219969
chr12 53219992
chr12 53219995
chr12 53220007
chr12 53220010
chr12 53220021
chr12 53220031
chr12 53220037
chr12 53220043
chr12 53220047
chr12 53220052
chr12 53220060
chr12 53220063
chr12 53220065
chr12 53220071
chr12 53220080
chr12 53220094
chr12 53220101
chr12 53220103
chr12 53220108
chr12 53232323
chr12 53232356
chr12 53232372
chr12 53232388
chr12 53232393
chr12 53232398
chr12 53232403
chr12 53232439
chr12 53232462
chr12 53232468
chr12 53232499
chr12 53232590
chr12 53232606
chr12 53321163
chr12 53321217
chr12 53321239
chr12 53321294
chr12 53321300
chr12 53321331
chr12 53321350
chr12 53321357
chr12 53321364
chr12 53321385
chr12 53321396
chr12 53321428
chr12 53321433

chr12 53321471
chr12 53321476
chr12 53321483
chr12 53321493
chr12 53321508
chr12 53321513
chr12 53321523
chr12 53321531
chr12 53321538
chr12 53321545
chr12 53321556
chr12 53321562
chr12 53321566
chr12 53321573
chr12 53324611
chr12 53324632
chr12 53324643
chr12 53324670
chr12 53324730
chr12 53324734
chr12 53441312
chr12 53441318
chr12 53441368
chr12 53441478
chr12 53441512
chr12 53441524
chr12 53441531
chr12 53441541
chr12 53451693
chr12 53453730
chr12 53454093
chr12 53454132
chr12 53454214
chr12 53498738
chr12 53498855
chr12 53499173
chr12 53499200
chr12 53499216
chr12 53499218
chr12 53499229
chr12 53499261
chr12 53499264
chr12 53499312
chr12 53499323
chr12 53499811
chr12 53499825
chr12 53499887
chr12 53499911
chr12 53499944
chr12 53499972
chr12 53500040
chr12 53500120
chr12 53500291
chr12 53500293

chr12 53500331
chr12 53500427
chr12 53500470
chr12 53500497
chr12 53500549
chr12 53500581
chr12 53500634
chr12 53500667
chr12 53500680
chr12 53500690
chr12 53500693
chr12 53500721
chr12 53500738
chr12 53500741
chr12 53500753
chr12 53618127
chr12 53618171
chr12 53618298
chr12 53626207
chr12 53626241
chr12 53626279
chr12 53626292
chr12 53626698
chr12 53626720
chr12 53695795
chr12 53695847
chr12 53695864
chr12 53695876
chr12 53695916
chr12 53695987
chr12 53696003
chr12 53696129
chr12 53696135
chr12 53696146
chr12 53701275
chr12 53701852
chr12 53726860
chr12 53726875
chr12 53726888
chr12 53726954
chr12 53726961
chr12 53727049
chr12 53727052
chr12 53727072
chr12 53727092
chr12 53727105
chr12 53727120
chr12 53727167
chr12 53727189
chr12 53727192
chr12 53727200
chr12 53727252
chr12 53727272
chr12 53727293

chr12 53727311
chr12 53727325
chr12 53727377
chr12 53727412
chr12 53929716
chr12 53929813
chr12 53929916
chr12 53930229
chr12 53930344
chr12 53931328
chr12 53931421
chr12 53940742
chr12 53940810
chr12 53940828
chr12 53940840
chr12 53940921
chr12 53941760
chr12 53941823
chr12 53941930
chr12 53942070
chr12 53942141
chr12 53942554
chr12 53942636
chr12 53943331
chr12 53943407
chr12 53943587
chr12 53958777
chr12 53958864
chr12 53958879
chr12 53959390
chr12 53959433
chr12 53959470
chr12 53959550
chr12 53962306
chr12 53964519
chr12 53964629
chr12 53964846
chr12 53964858
chr12 53965045
chr12 53965574
chr12 53977259
chr12 53977462
chr12 53977469
chr12 53977474
chr12 53977542
chr12 53977556
chr12 53977917
chr12 53978116
chr12 53978193
chr12 53978205
chr12 53978222
chr12 53978230
chr12 53978253
chr12 53978276

chr12 53978285
chr12 53978318
chr12 53978485
chr12 53988004
chr12 53988014
chr12 53988374
chr12 53989036
chr12 53989111
chr12 53989126
chr12 53989132
chr12 53989218
chr12 53989256
chr12 53989298
chr12 53989300
chr12 53989306
chr12 53989309
chr12 53989372
chr12 53989399
chr12 53989408
chr12 53989414
chr12 53989450
chr12 53989452
chr12 53989473
chr12 53989489
chr12 53989552
chr12 53989605
chr12 53989612
chr12 53989650
chr12 53989731
chr12 53989774
chr12 53989784
chr12 53989836
chr12 53989855
chr12 53989908
chr12 53989919
chr12 53998345
chr12 53998413
chr12 53998433
chr12 53998448
chr12 53998492
chr12 53998498
chr12 54011595
chr12 54011615
chr12 54011710
chr12 54011719
chr12 54011786
chr12 54016848
chr12 54016873
chr12 54016878
chr12 54016882
chr12 54016887
chr12 54016943
chr12 54017035
chr12 54017111

chr12 54017182
chr12 54017326
chr12 54017332
chr12 54017352
chr12 54017409
chr12 54017413
chr12 54017456
chr12 54017461
chr12 54017484
chr12 54017518
chr12 54017530
chr12 54017532
chr12 54017560
chr12 54017574
chr12 54017593
chr12 54017611
chr12 54017689
chr12 54017692
chr12 54017702
chr12 54027249
chr12 54027327
chr12 54027339
chr12 54027551
chr12 54027592
chr12 54027748
chr12 54028343
chr12 54028376
chr12 54028433
chr12 54081418
chr12 54081440
chr12 54081450
chr12 54081476
chr12 54081484
chr12 54081486
chr12 54081625
chr12 54081663
chr12 54081681
chr12 54081711
chr12 54081729
chr12 54081743
chr12 54081751
chr12 54081768
chr12 54081774
chr12 54081785
chr12 54081788
chr12 54081791
chr12 54081801
chr12 54081805
chr12 54081823
chr12 54081847
chr12 54081852
chr12 54081857
chr12 54081863
chr12 54081871

chr12 54081882
chr12 54081889
chr12 54081920
chr12 54081939
chr12 54081973
chr12 54081977
chr12 54081995
chr12 54081999
chr12 54082007
chr12 54082026
chr12 54082031
chr12 54082121
chr12 54082248
chr12 54082365
chr12 54082384
chr12 54082456
chr12 54082499
chr12 54082508
chr12 54082536
chr12 54082547
chr12 54082551
chr12 54082555
chr12 54082558
chr12 54082563
chr12 54082568
chr12 54082574
chr12 54082581
chr12 54082587
chr12 54082593
chr12 54082599
chr12 54082605
chr12 54082611
chr12 54082630
chr12 54082636
chr12 54082648
chr12 54082654
chr12 54082669
chr12 54082690
chr12 54082693
chr12 54082791
chr12 54082900
chr12 54083035
chr12 54083135
chr12 54083146
chr12 54083202
chr12 54090770
chr12 54090813
chr12 54115785
chr12 54115809
chr12 54115812
chr12 54115889
chr12 54191667
chr12 54191704
chr12 54191734

chr12 54191744
chr12 54191792
chr12 54192047
chr12 54216419
chr12 54216432
chr12 54216597
chr12 54216635
chr12 54216644
chr12 54216651
chr12 54216654
chr12 54216791
chr12 54278483
chr12 54278504
chr12 54278713
chr12 54278901
chr12 54282228
chr12 54282272
chr12 54282284
chr12 54282575
chr12 54282605
chr12 54282619
chr12 54282637
chr12 54282641
chr12 54282662
chr12 54282732
chr12 54282817
chr12 54283930
chr12 54283958
chr12 54284063
chr12 54300109
chr12 54300342
chr12 54300531
chr12 54300698
chr12 54300750
chr12 54300767
chr12 54300844
chr12 54301066
chr12 54301094
chr12 54301118
chr12 54301141
chr12 54301165
chr12 54301232
chr12 54301425
chr12 54301663
chr12 54302426
chr12 54304157
chr12 54304162
chr12 54304174
chr12 54358364
chr12 54358387
chr12 54358469
chr12 54358548
chr12 54358616
chr12 54358662

chr12 54358669
chr12 54358688
chr12 54358739
chr12 54358864
chr12 54358876
chr12 54358918
chr12 54358944
chr12 54358952
chr12 54358994
chr12 54359030
chr12 54417329
chr12 54418266
chr12 54418301
chr12 54418307
chr12 54418320
chr12 54418330
chr12 54418351
chr12 54418392
chr12 54418395
chr12 54418405
chr12 54418417
chr12 54418470
chr12 54418505
chr12 54418516
chr12 54418527
chr12 54418535
chr12 54418538
chr12 54418554
chr12 54418561
chr12 54418593
chr12 54418602
chr12 54418613
chr12 54418623
chr12 54418680
chr12 54418682
chr12 54418702
chr12 54418732
chr12 54418748
chr12 54418777
chr12 54418797
chr12 54418802
chr12 54418822
chr12 54418824
chr12 54418919
chr12 54418921
chr12 54418938
chr12 54418958
chr12 54418982
chr12 54418992
chr12 54418996
chr12 54419018
chr12 54419031
chr12 54419040
chr12 54419061

chr12 54419063
chr12 54419081
chr12 54419094
chr12 54419097
chr12 54419118
chr12 54419127
chr12 54419134
chr12 54419137
chr12 54419140
chr12 54419152
chr12 54419162
chr12 54419165
chr12 54419184
chr12 54419188
chr12 54419201
chr12 54419205
chr12 54419229
chr12 54419234
chr12 54419240
chr12 54419262
chr12 54419274
chr12 54419276
chr12 54419299
chr12 54419447
chr12 54497707
chr12 54497850
chr12 54497852
chr12 54497871
chr12 54497956
chr12 54498117
chr12 54498619
chr12 54498746
chr12 54498763
chr12 54498846
chr12 54498948
chr12 54603581
chr12 54603604
chr12 54603693
chr12 54617120
chr12 54617139
chr12 54617224
chr12 54935121
chr12 54935134
chr12 54935415
chr12 54935441
chr12 54935569
chr12 54935791
chr12 54936045
chr12 54936139
chr12 54936529
chr12 54983113
chr12 54983160
chr12 54983196
chr12 54983253

chr12 54983292
chr12 54983976
chr12 54984020
chr12 54985316
chr12 54985342
chr12 54985444
chr12 54985477
chr12 54985508
chr12 55187823
chr12 55187915
chr12 55188299
chr12 55188660
chr12 55188804
chr12 55188988
chr12 55189139
chr12 55391536
chr12 55477962
chr12 55478254
chr12 55482454
chr12 55482896
chr12 55482977
chr12 55483489
chr12 55591816
chr12 55620096
chr12 55646068
chr12 55646075
chr12 55646205
chr12 55646217
chr12 55646226
chr12 55646238
chr12 55646241
chr12 55646262
chr12 55646267
chr12 55646273
chr12 55646308
chr12 55646312
chr12 55646322
chr12 55646330
chr12 55646333
chr12 55681305
chr12 55681377
chr12 55681392
chr12 55681403
chr12 55681537
chr12 55681583
chr12 55681671
chr12 55681681
chr12 55681687
chr12 55708105
chr12 55708143
chr12 55708158
chr12 55708161
chr12 55708173
chr12 55708250

chr12 55708308
chr12 55708328
chr12 55708397
chr12 55708427
chr12 55708438
chr12 55708468
chr12 55708520
chr12 55708529
chr12 55708630
chr12 55708760
chr12 55708965
chr12 55709023
chr12 55709245
chr12 55729729
chr12 55729733
chr12 55729735
chr12 55729738
chr12 55729745
chr12 55729806
chr12 55729818
chr12 55729838
chr12 55729874
chr12 55729914
chr12 55729990
chr12 55730003
chr12 55730007
chr12 55730032
chr12 55730145
chr12 55730177
chr12 55730186
chr12 55730188
chr12 55730209
chr12 55730256
chr12 55738299
chr12 55738402
chr12 55738425
chr12 55738468
chr12 55738510
chr12 55738593
chr12 55738652
chr12 55738665
chr12 55738821
chr12 55738867
chr12 55738898
chr12 55738924
chr12 55742575
chr12 55742599
chr12 55742778
chr12 55816615
chr12 55816901
chr12 55829582
chr12 55829593
chr12 55829600
chr12 55830344

chr12 55830354
chr12 55830360
chr12 55830368
chr12 55830388
chr12 55830393
chr12 55830396
chr12 55830415
chr12 55830434
chr12 55830444
chr12 55830477
chr12 55830484
chr12 55830513
chr12 55830519
chr12 55830527
chr12 55830594
chr12 55830627
chr12 55830649
chr12 55830764
chr12 55926338
chr12 55926409
chr12 55926481
chr12 55926494
chr12 55926595
chr12 55926623
chr12 55926650
chr12 55926668
chr12 55926956
chr12 55929175
chr12 55929236
chr12 55929295
chr12 55929336
chr12 55929426
chr12 55929547
chr12 55929555
chr12 55929599
chr12 55929643
chr12 55929702
chr12 55929715
chr12 55929855
chr12 55929943
chr12 55930032
chr12 55930400
chr12 55930435
chr12 55930469
chr12 55930489
chr12 55930491
chr12 55930567
chr12 55931053
chr12 55931131
chr12 55931231
chr12 55931365
chr12 55931487
chr12 55931528
chr12 55931533

chr12 55931586
chr12 55931675
chr12 55931697
chr12 55931713
chr12 55931718
chr12 55931722
chr12 55932657
chr12 55932677
chr12 55932708
chr12 55932851
chr12 55932935
chr12 55933001
chr12 55933033
chr12 55933253
chr12 55933324
chr12 55933343
chr12 55967143
chr12 55967191
chr12 55967195
chr12 55967203
chr12 55967214
chr12 55967354
chr12 55967359
chr12 55967375
chr12 55967387
chr12 55967454
chr12 55967457
chr12 55967464
chr12 55972929
chr12 55973125
chr12 55973214
chr12 55973232
chr12 55973248
chr12 55973272
chr12 55973275
chr12 55973443
chr12 55973478
chr12 55973512
chr12 55973541
chr12 55973545
chr12 55973547
chr12 55973607
chr12 55973620
chr12 55973670
chr12 55973678
chr12 55973708
chr12 56042068
chr12 56042087
chr12 56042094
chr12 56042096
chr12 56042137
chr12 56042150
chr12 56042155
chr12 56042183

chr12 56042202
chr12 56042246
chr12 56042262
chr12 56042291
chr12 56042294
chr12 56042303
chr12 56042320
chr12 56042324
chr12 56042327
chr12 56042345
chr12 56042350
chr12 56042361
chr12 56042389
chr12 56042392
chr12 56042420
chr12 56042449
chr12 56042464
chr12 56042466
chr12 56042472
chr12 56042485
chr12 56042503
chr12 56042508
chr12 56069739
chr12 56069795
chr12 56103737
chr12 56105603
chr12 56116070
chr12 56116079
chr12 56116095
chr12 56116268
chr12 56116270
chr12 56116330
chr12 56116343
chr12 56116361
chr12 56116401
chr12 56116417
chr12 56116453
chr12 56116468
chr12 56116476
chr12 56116515
chr12 56116530
chr12 56116547
chr12 56116569
chr12 56116577
chr12 56116586
chr12 56116593
chr12 56116596
chr12 56116612
chr12 56116618
chr12 56116626
chr12 56116637
chr12 56116646
chr12 56116681
chr12 56116692

chr12 56116719
chr12 56116784
chr12 56117313
chr12 56117360
chr12 56117369
chr12 56117410
chr12 56117489
chr12 56117527
chr12 56117543
chr12 56118051
chr12 56126380
chr12 56127600
chr12 56127635
chr12 56127810
chr12 56127890
chr12 56127908
chr12 56128010
chr12 56151965
chr12 56151986
chr12 56151998
chr12 56152010
chr12 56152026
chr12 56159346
chr12 56159487
chr12 56159616
chr12 56159690
chr12 56159693
chr12 56188394
chr12 56188407
chr12 56188521
chr12 56188650
chr12 56188707
chr12 56188720
chr12 56188730
chr12 56188754
chr12 56188803
chr12 56188806
chr12 56189130
chr12 56189144
chr12 56189172
chr12 56220780
chr12 56220846
chr12 56220850
chr12 56220995
chr12 56221066
chr12 56221099
chr12 56221238
chr12 56221295
chr12 56221326
chr12 56221358
chr12 56221363
chr12 56221369
chr12 56221387
chr12 56221409

chr12 56221438
chr12 56221465
chr12 56221491
chr12 56221509
chr12 56221520
chr12 56221567
chr12 56221595
chr12 56221603
chr12 56221679
chr12 56221740
chr12 56221742
chr12 56221748
chr12 56333997
chr12 56334000
chr12 56334010
chr12 56334017
chr12 56334022
chr12 56334046
chr12 56334077
chr12 56334081
chr12 56334112
chr12 56334115
chr12 56334131
chr12 56334138
chr12 56334142
chr12 56334144
chr12 56334147
chr12 56334162
chr12 56334167
chr12 56334170
chr12 56334182
chr12 56334192
chr12 56334195
chr12 56334211
chr12 56334583
chr12 56334595
chr12 56334651
chr12 56358691
chr12 56359349
chr12 56449063
chr12 56449065
chr12 56449105
chr12 56449107
chr12 56449111
chr12 56449164
chr12 56449166
chr12 56449170
chr12 56449172
chr12 56449183
chr12 56449190
chr12 56449208
chr12 56449215
chr12 56449232
chr12 56449239

chr12 56449259
chr12 56449271
chr12 56449277
chr12 56449284
chr12 56449292
chr12 56449311
chr12 56449348
chr12 56449359
chr12 56449361
chr12 56449367
chr12 56449399
chr12 56449406
chr12 56449412
chr12 56449448
chr12 56449456
chr12 56449460
chr12 56488009
chr12 56488013
chr12 56488019
chr12 56488036
chr12 56488041
chr12 56488060
chr12 56488064
chr12 56488081
chr12 56488114
chr12 56488134
chr12 56488148
chr12 56488152
chr12 56488184
chr12 56488189
chr12 56488193
chr12 56488205
chr12 56488226
chr12 56488242
chr12 56488257
chr12 56496891
chr12 56496897
chr12 56496903
chr12 56496915
chr12 56496919
chr12 56496934
chr12 56496944
chr12 56496973
chr12 56496988
chr12 56496995
chr12 56497090
chr12 56497099
chr12 56497112
chr12 56497142
chr12 56569966
chr12 56570107
chr12 56570244
chr12 56570264
chr12 56581980

chr12 56628692
chr12 56628801
chr12 56628872
chr12 56628879
chr12 56628936
chr12 56628984
chr12 56632019
chr12 56632425
chr12 56632455
chr12 56632459
chr12 56632466
chr12 56632478
chr12 56632526
chr12 56632535
chr12 56635261
chr12 56635290
chr12 56635292
chr12 56635316
chr12 56635333
chr12 56635401
chr12 56635419
chr12 56635421
chr12 56635426
chr12 56635451
chr12 56635482
chr12 56635513
chr12 56635552
chr12 56635635
chr12 56635829
chr12 56635922
chr12 56635927
chr12 56635930
chr12 56636329
chr12 56636405
chr12 56636438
chr12 56636518
chr12 56636572
chr12 56636579
chr12 56636589
chr12 56636659
chr12 56636666
chr12 56636875
chr12 56636897
chr12 56636949
chr12 56636951
chr12 56637015
chr12 56637047
chr12 56637225
chr12 56644973
chr12 56645016
chr12 56645069
chr12 56645286
chr12 56645720
chr12 56645746

chr12 56645761
chr12 56683815
chr12 56683819
chr12 56683827
chr12 56683851
chr12 56683875
chr12 56683891
chr12 56683908
chr12 56683914
chr12 56683916
chr12 56683938
chr12 56683944
chr12 56683952
chr12 56684012
chr12 56684093
chr12 56684121
chr12 56684182
chr12 56684227
chr12 56684465
chr12 56684514
chr12 56684562
chr12 56684674
chr12 56684678
chr12 56684682
chr12 56684706
chr12 56684738
chr12 56684795
chr12 56684865
chr12 56684871
chr12 56685013
chr12 56685036
chr12 56685046
chr12 56685278
chr12 56686201
chr12 56686203
chr12 56686596
chr12 56686604
chr12 56686619
chr12 56686623
chr12 56686628
chr12 56686666
chr12 56686668
chr12 56686705
chr12 56686716
chr12 56686783
chr12 56686796
chr12 56687059
chr12 56687061
chr12 56689091
chr12 56689126
chr12 56689156
chr12 56689185
chr12 56723273
chr12 56723366

chr12 56723412
chr12 56723727
chr12 56723838
chr12 56723847
chr12 56751841
chr12 56751942
chr12 56751956
chr12 56751959
chr12 56752165
chr12 56877555
chr12 56880189
chr12 56985546
chr12 56999092
chr12 56999095
chr12 56999139
chr12 57063736
chr12 57063870
chr12 57064037
chr12 57064058
chr12 57064170
chr12 57078740
chr12 57078772
chr12 57079227
chr12 57079240
chr12 57086307
chr12 57086352
chr12 57086587
chr12 57086643
chr12 57086684
chr12 57086702
chr12 57086717
chr12 57086724
chr12 57086730
chr12 57086746
chr12 57086858
chr12 57086886
chr12 57086929
chr12 57086945
chr12 57086954
chr12 57087346
chr12 57087394
chr12 57087404
chr12 57087413
chr12 57087425
chr12 57087462
chr12 57089665
chr12 57089681
chr12 57089696
chr12 57089717
chr12 57089725
chr12 57089730
chr12 57089732
chr12 57089734
chr12 57089742

chr12 57089770
chr12 57089789
chr12 57089813
chr12 57089848
chr12 57089897
chr12 57089922
chr12 57112038
chr12 57112099
chr12 57112178
chr12 57112370
chr12 57112387
chr12 57112417
chr12 57123735
chr12 57124502
chr12 57124537
chr12 57124560
chr12 57127669
chr12 57127700
chr12 57127742
chr12 57127744
chr12 57127758
chr12 57127767
chr12 57127771
chr12 57127796
chr12 57127805
chr12 57127807
chr12 57127811
chr12 57127822
chr12 57127825
chr12 57127835
chr12 57127854
chr12 57127860
chr12 57127866
chr12 57127877
chr12 57127885
chr12 57127887
chr12 57128140
chr12 57128152
chr12 57240110
chr12 57243318
chr12 57243322
chr12 57243338
chr12 57243372
chr12 57243381
chr12 57243386
chr12 57243417
chr12 57280295
chr12 57280400
chr12 57280473
chr12 57280575
chr12 57459312
chr12 57459319
chr12 57459327
chr12 57459345

chr12 57459350
chr12 57459358
chr12 57459406
chr12 57459438
chr12 57459516
chr12 57459529
chr12 57459533
chr12 57459613
chr12 57459637
chr12 57459643
chr12 57459692
chr12 57459713
chr12 57460180
chr12 57460189
chr12 57460220
chr12 57460294
chr12 57460437
chr12 57460453
chr12 57460490
chr12 57460534
chr12 57460567
chr12 57460569
chr12 57460642
chr12 57460879
chr12 57461368
chr12 57462325
chr12 57462330
chr12 57462340
chr12 57462342
chr12 57462349
chr12 57462356
chr12 57462399
chr12 57476119
chr12 57476124
chr12 57476126
chr12 57476136
chr12 57476145
chr12 57476150
chr12 57476158
chr12 57476195
chr12 57476227
chr12 57476281
chr12 57476290
chr12 57476296
chr12 57476310
chr12 57476317
chr12 57476327
chr12 57476331
chr12 57476344
chr12 57476351
chr12 57476379
chr12 57476385
chr12 57476388
chr12 57476398

chr12 57476421
chr12 57476439
chr12 57476458
chr12 57476486
chr12 57476510
chr12 57476599
chr12 57476637
chr12 57476689
chr12 57476707
chr12 57476879
chr12 57476892
chr12 57476902
chr12 57476934
chr12 57476941
chr12 57476998
chr12 57477002
chr12 57478072
chr12 57478226
chr12 57478298
chr12 57478340
chr12 57478362
chr12 57478424
chr12 57479921
chr12 57479949
chr12 57480097
chr12 57480203
chr12 57480211
chr12 57480242
chr12 57480250
chr12 57480267
chr12 57480274
chr12 57480306
chr12 57487998
chr12 57488013
chr12 57488016
chr12 57488023
chr12 57488025
chr12 57488032
chr12 57488047
chr12 57488055
chr12 57488061
chr12 57488073
chr12 57488084
chr12 57488087
chr12 57488102
chr12 57488114
chr12 57488119
chr12 57488131
chr12 57488141
chr12 57488144
chr12 57488147
chr12 57488157
chr12 57488194
chr12 57488205

chr12 57488223
chr12 57488238
chr12 57488244
chr12 57488251
chr12 57488293
chr12 57488309
chr12 57488342
chr12 57488429
chr12 57488437
chr12 57488444
chr12 57488595
chr12 57488629
chr12 57488743
chr12 57519728
chr12 57519754
chr12 57519849
chr12 57519882
chr12 57519927
chr12 57520025
chr12 57520034
chr12 57520039
chr12 57520042
chr12 57520048
chr12 57520057
chr12 57520079
chr12 57520569
chr12 57520603
chr12 57520606
chr12 57520618
chr12 57520623
chr12 57520630
chr12 57520636
chr12 57520652
chr12 57520654
chr12 57520660
chr12 57520666
chr12 57520669
chr12 57520679
chr12 57520690
chr12 57520693
chr12 57520701
chr12 57520707
chr12 57520726
chr12 57520736
chr12 57520758
chr12 57520760
chr12 57520762
chr12 57520764
chr12 57520766
chr12 57520781
chr12 57547271
chr12 57547286
chr12 57547318
chr12 57547321

chr12 57547367
chr12 57547374
chr12 57547383
chr12 57547526
chr12 57547606
chr12 57547608
chr12 57694087
chr12 57694245
chr12 57741285
chr12 57741405
chr12 57741452
chr12 57741914
chr12 57741954
chr12 57741960
chr12 57741989
chr12 57742019
chr12 57742050
chr12 57742180
chr12 57742190
chr12 57742198
chr12 57752741
chr12 57754105
chr12 57754533
chr12 57782392
chr12 57782447
chr12 57782509
chr12 57782565
chr12 57782623
chr12 57782637
chr12 57782660
chr12 57782709
chr12 57782714
chr12 57782736
chr12 57782739
chr12 57782741
chr12 57782744
chr12 57782768
chr12 57782772
chr12 57782825
chr12 57782837
chr12 57782839
chr12 57782841
chr12 57782846
chr12 57782857
chr12 57782882
chr12 57898713
chr12 57899027
chr12 57899096
chr12 57899317
chr12 57899334
chr12 57899355
chr12 57899378
chr12 57899460
chr12 57899464

chr12 57899644
chr12 57899650
chr12 57935794
chr12 57935811
chr12 57935871
chr12 57936153
chr12 57936173
chr12 57936177
chr12 57936185
chr12 57936196
chr12 57936202
chr12 57936210
chr12 57936220
chr12 57936248
chr12 57936286
chr12 57936374
chr12 57936393
chr12 57936411
chr12 57936630
chr12 57940709
chr12 57940907
chr12 57940961
chr12 57941050
chr12 57941125
chr12 57941136
chr12 57941164
chr12 57941193
chr12 57941205
chr12 57941398
chr12 57941719
chr12 57941723
chr12 57941726
chr12 57941729
chr12 57941737
chr12 57941739
chr12 57941767
chr12 57941788
chr12 57941792
chr12 57941877
chr12 58291303
chr12 58291673
chr12 58292246
chr12 58292327
chr12 58292620
chr12 58292623
chr12 59027595
chr12 59027700
chr12 59027730
chr12 59028159
chr12 59028290
chr12 59186078
chr12 59230844
chr12 59231120
chr12 59231652

chr12 59595397
chr12 59595559
chr12 59595693
chr12 59596993
chr12 59597002
chr12 59597013
chr12 59597025
chr12 59597031
chr12 59597037
chr12 59597041
chr12 59597061
chr12 59597073
chr12 59597075
chr12 59597078
chr12 59597111
chr12 59597113
chr12 59597144
chr12 59597148
chr12 59597168
chr12 59597270
chr12 59597289
chr12 59597341
chr12 59597377
chr12 59597396
chr12 59597775
chr12 59597923
chr12 59993249
chr12 60656667
chr12 60656678
chr12 60657012
chr12 60657322
chr12 60657505
chr12 60657539
chr12 60657982
chr12 60658667
chr12 60866906
chr12 60867066
chr12 60867441
chr12 60867618
chr12 60868288
chr12 61191389
chr12 61191666
chr12 61192468
chr12 61192987
chr12 61856762
chr12 61857669
chr12 61869093
chr12 61869303
chr12 61869411
chr12 61869506
chr12 61869747
chr12 61870175
chr12 61870183
chr12 61870214

chr12 62064556
chr12 62065243
chr12 62065302
chr12 62065410
chr12 62066126
chr12 62066376
chr12 62192644
chr12 62192711
chr12 62192715
chr12 62192718
chr12 62192726
chr12 62192771
chr12 62192787
chr12 62192806
chr12 62192874
chr12 62192884
chr12 62192993
chr12 62258881
chr12 62259004
chr12 62259119
chr12 62259159
chr12 62259180
chr12 62259246
chr12 62261329
chr12 62261662
chr12 62261977
chr12 62262004
chr12 62434762
chr12 62435572
chr12 62437924
chr12 62438205
chr12 62466179
chr12 62466192
chr12 62466194
chr12 62466312
chr12 62466319
chr12 62466460
chr12 62466470
chr12 62466482
chr12 62466545
chr12 62466571
chr12 62466604
chr12 62466609
chr12 62466619
chr12 62466650
chr12 62466722
chr12 62466727
chr12 62466730
chr12 62466733
chr12 62466741
chr12 62466744
chr12 62466754
chr12 62466756
chr12 62466759

chr12 62466765
chr12 62466774
chr12 62466781
chr12 62466789
chr12 62466796
chr12 62467180
chr12 62467213
chr12 62467228
chr12 62467233
chr12 62467264
chr12 62467271
chr12 62467273
chr12 62467334
chr12 62467442
chr12 62467660
chr12 62467665
chr12 62467703
chr12 62467784
chr12 62467901
chr12 62467952
chr12 62604264
chr12 62604309
chr12 62604311
chr12 62604354
chr12 62604720
chr12 62604793
chr12 62604848
chr12 62605035
chr12 62605054
chr12 62605136
chr12 62605195
chr12 62641261
chr12 62641291
chr12 62813233
chr12 62813236
chr12 62813310
chr12 62813325
chr12 62813355
chr12 62813358
chr12 62813371
chr12 62813399
chr12 62813413
chr12 62813419
chr12 62813421
chr12 62813428
chr12 62813443
chr12 62813445
chr12 62817903
chr12 62818079
chr12 62818096
chr12 62818152
chr12 62818171
chr12 63667429
chr12 63667508

chr12 63667846
chr12 63667935
chr12 63668014
chr12 63668021
chr12 63668057
chr12 63668063
chr12 63668086
chr12 63668089
chr12 63780401
chr12 63780404
chr12 63780426
chr12 63780454
chr12 63780457
chr12 63780475
chr12 63780587
chr12 63780621
chr12 63781124
chr12 64221560
chr12 64221586
chr12 64221670
chr12 64221736
chr12 64221758
chr12 64221798
chr12 64221815
chr12 64221822
chr12 64221829
chr12 64221862
chr12 64221886
chr12 64288825
chr12 64289287
chr12 64289744
chr12 64290006
chr12 64291720
chr12 64294939
chr12 64385183
chr12 64385246
chr12 64385593
chr12 64404624
chr12 64404634
chr12 64404636
chr12 64404638
chr12 64404644
chr12 64404654
chr12 64404693
chr12 64404696
chr12 64453021
chr12 64453030
chr12 64453054
chr12 64453132
chr12 64453377
chr12 64453393
chr12 64453408
chr12 64453492
chr12 64455943

chr12 64456088
chr12 64495156
chr12 64583991
chr12 64584057
chr12 64584112
chr12 64609473
chr12 64609647
chr12 64612124
chr12 64612233
chr12 64625778
chr12 64625796
chr12 64668958
chr12 64669098
chr12 64669125
chr12 64669358
chr12 64669404
chr12 64669556
chr12 64669624
chr12 64669670
chr12 64669694
chr12 64669712
chr12 64669728
chr12 64669751
chr12 64669775
chr12 64670110
chr12 64670131
chr12 64697857
chr12 64697889
chr12 64698113
chr12 64698133
chr12 64698146
chr12 64824059
chr12 64824239
chr12 64824241
chr12 65123581
chr12 65123586
chr12 65123617
chr12 65123647
chr12 65123746
chr12 65156449
chr12 65156791
chr12 65168884
chr12 65168961
chr12 65168996
chr12 65169042
chr12 65171909
chr12 65276328
chr12 65276454
chr12 65279413
chr12 65279416
chr12 65279420
chr12 65279431
chr12 65279442
chr12 65279463

chr12 65279469
chr12 65279494
chr12 65279506
chr12 65279592
chr12 65279656
chr12 65492755
chr12 65492790
chr12 65493457
chr12 65494010
chr12 65557941
chr12 65728276
chr12 65728338
chr12 65728573
chr12 65728655
chr12 65728717
chr12 65728743
chr12 65728776
chr12 65728843
chr12 65728868
chr12 65728879
chr12 65729009
chr12 65729024
chr12 65729036
chr12 65729041
chr12 65729054
chr12 65729060
chr12 65811758
chr12 65811998
chr12 65822535
chr12 65822581
chr12 65822740
chr12 65822797
chr12 65822827
chr12 65822943
chr12 65822981
chr12 65823027
chr12 65823086
chr12 65823088
chr12 65823100
chr12 65823106
chr12 65823108
chr12 65823128
chr12 65823130
chr12 65823484
chr12 65823567
chr12 65823575
chr12 65823601
chr12 65823652
chr12 65823686
chr12 65823706
chr12 65824392
chr12 65824398
chr12 65824400
chr12 65824406

chr12 65824498
chr12 65824505
chr12 65824507
chr12 65824509
chr12 65824540
chr12 65826475
chr12 65826495
chr12 65826498
chr12 65826533
chr12 65826630
chr12 65826655
chr12 65826668
chr12 65826681
chr12 65826744
chr12 65826887
chr12 65826902
chr12 65826954
chr12 65826974
chr12 65826991
chr12 65827030
chr12 65827097
chr12 65827208
chr12 65827594
chr12 65827639
chr12 65827887
chr12 66072324
chr12 66072495
chr12 66131245
chr12 66131304
chr12 66131412
chr12 66131440
chr12 66131456
chr12 66131547
chr12 66169206
chr12 66169259
chr12 66169357
chr12 66169374
chr12 66169403
chr12 66169972
chr12 66176875
chr12 66235716
chr12 66235789
chr12 66235803
chr12 66235942
chr12 66287738
chr12 66288270
chr12 66288287
chr12 66288465
chr12 66288488
chr12 66301644
chr12 66301853
chr12 66302063
chr12 66302110
chr12 66302139

chr12 66302756
chr12 66302777
chr12 66302787
chr12 66302789
chr12 66302801
chr12 66302809
chr12 66302839
chr12 66302879
chr12 66303037
chr12 66303073
chr12 66303077
chr12 66303131
chr12 66303172
chr12 66303218
chr12 66304495
chr12 67270708
chr12 67270713
chr12 67270735
chr12 67270798
chr12 67270959
chr12 67270982
chr12 67271078
chr12 67271145
chr12 67271275
chr12 67271288
chr12 67271345
chr12 67382645
chr12 67382690
chr12 67382712
chr12 67630929
chr12 67631000
chr12 67650933
chr12 67651055
chr12 67651477
chr12 67651530
chr12 67651607
chr12 67651622
chr12 67651675
chr12 67652055
chr12 68032947
chr12 68040671
chr12 68040710
chr12 68159448
chr12 68159573
chr12 68159616
chr12 68159622
chr12 68159797
chr12 68163525
chr12 68175911
chr12 68176072
chr12 68190241
chr12 68223257
chr12 68223259
chr12 68223267

chr12 68223279
chr12 68223302
chr12 68223360
chr12 68223363
chr12 68223473
chr12 68223492
chr12 68364811
chr12 68364895
chr12 68364997
chr12 68365011
chr12 68365037
chr12 68365048
chr12 68365057
chr12 68365060
chr12 68365063
chr12 68365077
chr12 68365110
chr12 68365120
chr12 68365150
chr12 68365164
chr12 68365186
chr12 68365199
chr12 68403605
chr12 68451208
chr12 68451242
chr12 68451256
chr12 68539240
chr12 68539264
chr12 68539287
chr12 68539330
chr12 68539478
chr12 68612432
chr12 68612549
chr12 68612580
chr12 68612627
chr12 68612796
chr12 68612864
chr12 68612898
chr12 68612912
chr12 68613080
chr12 68613092
chr12 68613120
chr12 68613125
chr12 68613134
chr12 68613157
chr12 68613188
chr12 68613216
chr12 68613230
chr12 68613232
chr12 68613267
chr12 68614189
chr12 68614252
chr12 68614317
chr12 68614508

chr12 68615014
chr12 68641199
chr12 68641219
chr12 68641355
chr12 68641363
chr12 68641483
chr12 68641630
chr12 68641760
chr12 68673894
chr12 68673988
chr12 68674032
chr12 68674079
chr12 68675299
chr12 68675348
chr12 68675653
chr12 68675662
chr12 68686634
chr12 68687508
chr12 68687547
chr12 68687607
chr12 68687699
chr12 68687749
chr12 68687875
chr12 68745838
chr12 68745870
chr12 68807420
chr12 68830382
chr12 68830404
chr12 68932339
chr12 68932347
chr12 68932545
chr12 68932551
chr12 68958140
chr12 69155204
chr12 69238844
chr12 69238955
chr12 69239010
chr12 69239040
chr12 69240731
chr12 69240760
chr12 69240763
chr12 69240779
chr12 69240781
chr12 69240824
chr12 69240840
chr12 69240864
chr12 69241030
chr12 69284089
chr12 69284215
chr12 69284220
chr12 69284223
chr12 69284226
chr12 69284232
chr12 69284254

chr12 69284258
chr12 69284264
chr12 69284290
chr12 69284316
chr12 69284322
chr12 69284327
chr12 69284339
chr12 69284354
chr12 69284365
chr12 69284367
chr12 69284391
chr12 69330719
chr12 69330871
chr12 69330936
chr12 69331043
chr12 69331099
chr12 69331102
chr12 69331114
chr12 69331140
chr12 69331181
chr12 69331189
chr12 69331218
chr12 69331226
chr12 69331233
chr12 69331289
chr12 69331337
chr12 69331421
chr12 69331438
chr12 69357594
chr12 69357600
chr12 69357719
chr12 69357736
chr12 69357755
chr12 69357794
chr12 69358002
chr12 69359502
chr12 69360160
chr12 69360178
chr12 69360186
chr12 69360189
chr12 69360212
chr12 69360214
chr12 69360236
chr12 69360276
chr12 69360280
chr12 69360297
chr12 69360301
chr12 69360307
chr12 69497707
chr12 69585632
chr12 69585640
chr12 69585651
chr12 69585660
chr12 69585943

chr12 69739954
chr12 69740000
chr12 69740010
chr12 69740233
chr12 69856840
chr12 69856935
chr12 69857225
chr12 69900811
chr12 69900841
chr12 69900873
chr12 69900941
chr12 69901146
chr12 69941179
chr12 70012452
chr12 70241970
chr12 70242014
chr12 70242426
chr12 70242485
chr12 70244410
chr12 70244529
chr12 70244602
chr12 70244636
chr12 70244711
chr12 70244765
chr12 70577703
chr12 70785031
chr12 70785049
chr12 70785750
chr12 70785782
chr12 70786127
chr12 70786281
chr12 71408551
chr12 71468801
chr12 71573295
chr12 71662147
chr12 71662262
chr12 71662277
chr12 71662301
chr12 71662525
chr12 71662600
chr12 71665068
chr12 71665273
chr12 71665279
chr12 71665348
chr12 71665401
chr12 71665513
chr12 71665797
chr12 71754959
chr12 71754987
chr12 71755020
chr12 71755023
chr12 71755029
chr12 71755034
chr12 71755049

chr12 71755053
chr12 71755067
chr12 71755073
chr12 71755075
chr12 71755082
chr12 71755090
chr12 71755093
chr12 71755095
chr12 71755102
chr12 71755108
chr12 71755112
chr12 71755116
chr12 71755123
chr12 71755126
chr12 71755137
chr12 71755141
chr12 71755144
chr12 71755147
chr12 71755150
chr12 71755153
chr12 71755156
chr12 71755161
chr12 71755164
chr12 71755167
chr12 71755170
chr12 71755175
chr12 71755185
chr12 71755216
chr12 71755227
chr12 71755230
chr12 71840068
chr12 71840364
chr12 71840474
chr12 71840492
chr12 71840502
chr12 71840520
chr12 71840524
chr12 71840533
chr12 71840536
chr12 71840551
chr12 71840553
chr12 71840558
chr12 72187435
chr12 72187477
chr12 72755157
chr12 72755401
chr12 73155995
chr12 73156671
chr12 73157950
chr12 73238643
chr12 73238828
chr12 73239038
chr12 73239105
chr12 73239117

chr12 73290786
chr12 73291314
chr12 73291957
chr12 73336252
chr12 73336338
chr12 73336960
chr12 73337381
chr12 73667812
chr12 73668331
chr12 73700561
chr12 73700988
chr12 73701635
chr12 74019226
chr12 74019365
chr12 74019763
chr12 74020783
chr12 74021727
chr12 74022002
chr12 74022005
chr12 74022010
chr12 74022037
chr12 74022043
chr12 74022053
chr12 74022075
chr12 74022230
chr12 74169948
chr12 74169990
chr12 74170183
chr12 74173483
chr12 74173636
chr12 74290849
chr12 74291143
chr12 74291220
chr12 74295702
chr12 74404832
chr12 74490565
chr12 74490616
chr12 74490826
chr12 74491261
chr12 74491436
chr12 74492077
chr12 74492661
chr12 74510654
chr12 74510742
chr12 74537310
chr12 74537361
chr12 74537439
chr12 74537482
chr12 74538163
chr12 74538318
chr12 74538323
chr12 74538330
chr12 74538346
chr12 74538383

chr12 74538393
chr12 74538465
chr12 74538636
chr12 74538680
chr12 74863305
chr12 74872280
chr12 74872284
chr12 74872313
chr12 74876493
chr12 74876530
chr12 75209805
chr12 75209892
chr12 75209968
chr12 75210077
chr12 75329894
chr12 75329987
chr12 75329989
chr12 75330005
chr12 75330050
chr12 75330052
chr12 75330067
chr12 75330077
chr12 75330080
chr12 75330085
chr12 75330120
chr12 75330129
chr12 75330132
chr12 75330150
chr12 75330157
chr12 75330167
chr12 75330173
chr12 75330195
chr12 75330210
chr12 75330228
chr12 75330237
chr12 75330242
chr12 75330300
chr12 75330303
chr12 75330321
chr12 75330327
chr12 75330329
chr12 75330332
chr12 75330345
chr12 75330348
chr12 75330356
chr12 75330361
chr12 75330375
chr12 75330377
chr12 75333732
chr12 75333756
chr12 75333927
chr12 75334133
chr12 75334146
chr12 75334304

chr12 75334313
chr12 75334324
chr12 75334346
chr12 75334369
chr12 75334430
chr12 75334432
chr12 75334501
chr12 75334572
chr12 75334595
chr12 75334597
chr12 75334606
chr12 75334616
chr12 75334640
chr12 75334647
chr12 75334653
chr12 75334664
chr12 75334678
chr12 75334689
chr12 75334691
chr12 75334709
chr12 75334854
chr12 75334861
chr12 75334881
chr12 75334883
chr12 75334887
chr12 75334972
chr12 75335115
chr12 75390761
chr12 75390791
chr12 75390837
chr12 75391020
chr12 75391027
chr12 75391045
chr12 75391052
chr12 75391075
chr12 75391082
chr12 75391084
chr12 75391088
chr12 75391104
chr12 75391108
chr12 75391125
chr12 75391137
chr12 75391141
chr12 75391182
chr12 75391195
chr12 75391239
chr12 75391257
chr12 75391266
chr12 75391281
chr12 75391287
chr12 75391309
chr12 75391314
chr12 75391317
chr12 75391327

chr12 75391342
chr12 75391358
chr12 75391365
chr12 75391373
chr12 75391388
chr12 75391399
chr12 75391414
chr12 75391450
chr12 75391452
chr12 75391460
chr12 75391489
chr12 75391515
chr12 75391609
chr12 75391641
chr12 75391755
chr12 75391833
chr12 75480360
chr12 75480380
chr12 75480481
chr12 75480522
chr12 75480634
chr12 75480719
chr12 75480776
chr12 75480834
chr12 75480837
chr12 75480884
chr12 75480964
chr12 75480991
chr12 75480995
chr12 75481013
chr12 75481080
chr12 75481132
chr12 75481577
chr12 75481666
chr12 75510532
chr12 75510632
chr12 75510672
chr12 75510731
chr12 75510778
chr12 75510935
chr12 75510955
chr12 75511007
chr12 75511090
chr12 75511183
chr12 75511204
chr12 75511224
chr12 75511227
chr12 75511268
chr12 75511275
chr12 75511286
chr12 75511344
chr12 75511352
chr12 75511384
chr12 75511440

chr12 75511447
chr12 75511463
chr12 75511473
chr12 75511493
chr12 75511523
chr12 75511529
chr12 75511542
chr12 75511561
chr12 75511575
chr12 75511583
chr12 75511592
chr12 75511612
chr12 75511621
chr12 75511649
chr12 75511658
chr12 75562295
chr12 75562580
chr12 75959131
chr12 75959580
chr12 75959820
chr12 76060185
chr12 76060256
chr12 76082362
chr12 76082750
chr12 76082874
chr12 76083042
chr12 76083053
chr12 76083075
chr12 76083266
chr12 76083372
chr12 76083389
chr12 76085665
chr12 76085674
chr12 76085735
chr12 76085748
chr12 76085771
chr12 76086072
chr12 76158611
chr12 76158625
chr12 76158687
chr12 76158759
chr12 76158767
chr12 76158790
chr12 76158907
chr12 76158956
chr12 76159005
chr12 76347511
chr12 76347558
chr12 76347580
chr12 76347611
chr12 76347953
chr12 76348037
chr12 76348048
chr12 76348058

chr12 76348079
chr12 76558364
chr12 76558396
chr12 76558497
chr12 76558600
chr12 76558657
chr12 76558667
chr12 76558738
chr12 76865106
chr12 76865164
chr12 76877958
chr12 76877995
chr12 76878085
chr12 76878125
chr12 76878128
chr12 76878142
chr12 76878148
chr12 76878167
chr12 76878211
chr12 76878223
chr12 76901641
chr12 76901714
chr12 76902191
chr12 76902379
chr12 76902416
chr12 77008738
chr12 77011042
chr12 77011098
chr12 77011126
chr12 77011139
chr12 77011151
chr12 77011197
chr12 77011212
chr12 77064804
chr12 77064806
chr12 77064923
chr12 77064985
chr12 77065006
chr12 77065027
chr12 77065042
chr12 77065046
chr12 77065067
chr12 77065074
chr12 77176961
chr12 77656603
chr12 77656774
chr12 77656815
chr12 77999426
chr12 77999506
chr12 77999784
chr12 77999951
chr12 77999960
chr12 78701744
chr12 78862744

chr12 78862841
chr12 78863118
chr12 78863134
chr12 78863458
chr12 78863495
chr12 78863533
chr12 78863557
chr12 78863563
chr12 78863577
chr12 78863579
chr12 78863581
chr12 78863598
chr12 78863615
chr12 78863623
chr12 78865580
chr12 78865607
chr12 78866129
chr12 79688323
chr12 79688515
chr12 79688571
chr12 79688611
chr12 79688625
chr12 79688988
chr12 79688993
chr12 79689076
chr12 79689084
chr12 79848517
chr12 79848601
chr12 79848632
chr12 79848636
chr12 79927645
chr12 79928027
chr12 79928810
chr12 79933563
chr12 79933604
chr12 79933646
chr12 79933681
chr12 80250631
chr12 80305663
chr12 80306041
chr12 80306141
chr12 80401943
chr12 80402805
chr12 80403070
chr12 80403653
chr12 80474491
chr12 80474890
chr12 80475232
chr12 80475313
chr12 80475429
chr12 80476748
chr12 80713703
chr12 80713712
chr12 80713796

chr12 80904380
chr12 80936134
chr12 81623230
chr12 81623584
chr12 81623691
chr12 81634270
chr12 81757574
chr12 81757831
chr12 81758026
chr12 81758089
chr12 81758283
chr12 81758327
chr12 81758338
chr12 81758362
chr12 81886505
chr12 82357694
chr12 82357740
chr12 82357829
chr12 82357846
chr12 82357871
chr12 82357924
chr12 82358010
chr12 82358022
chr12 82358047
chr12 82358069
chr12 82358087
chr12 82358874
chr12 82358886
chr12 82358920
chr12 82358925
chr12 82358928
chr12 82358934
chr12 82358939
chr12 82358944
chr12 82358955
chr12 82358966
chr12 82359108
chr12 82359331
chr12 82359341
chr12 82359389
chr12 82359460
chr12 82359629
chr12 82685711
chr12 82685772
chr12 82685815
chr12 82685820
chr12 82685874
chr12 82685892
chr12 82685894
chr12 82685902
chr12 82685936
chr12 82685946
chr12 82688192
chr12 82845882

chr12 83224401
chr12 83224410
chr12 83224779
chr12 83651041
chr12 84002598
chr12 84168851
chr12 84169231
chr12 84169593
chr12 84301524
chr12 84301566
chr12 84318732
chr12 84318799
chr12 84319016
chr12 84396668
chr12 84397232
chr12 84552400
chr12 84552672
chr12 84553003
chr12 84553129
chr12 84566276
chr12 84566316
chr12 84566383
chr12 84664264
chr12 84792340
chr12 84792533
chr12 84792579
chr12 84792631
chr12 84945579
chr12 84945707
chr12 84946077
chr12 85035474
chr12 85036215
chr12 85036247
chr12 85036251
chr12 85036267
chr12 85036307
chr12 85036345
chr12 85036357
chr12 85036367
chr12 85036396
chr12 85036414
chr12 85036437
chr12 85036472
chr12 85036477
chr12 85036521
chr12 85036523
chr12 85036543
chr12 85036551
chr12 85036558
chr12 85036585
chr12 85036636
chr12 85036654
chr12 85036675
chr12 85037614

chr12 85037745
chr12 85038472
chr12 85039751
chr12 85039907
chr12 85039984
chr12 85040241
chr12 85272683
chr12 85272758
chr12 85272845
chr12 85272858
chr12 85272973
chr12 85273100
chr12 85273104
chr12 85273154
chr12 85273200
chr12 85273210
chr12 85273282
chr12 85274334
chr12 85274360
chr12 85274398
chr12 85274417
chr12 85274432
chr12 85274548
chr12 85274552
chr12 85274634
chr12 85274681
chr12 85274787
chr12 85274793
chr12 85274830
chr12 85275531
chr12 85275655
chr12 85275807
chr12 85275993
chr12 85276205
chr12 85276420
chr12 85276487
chr12 85276628
chr12 85276961
chr12 85277075
chr12 85281092
chr12 85281108
chr12 85281110
chr12 85281151
chr12 85281166
chr12 85281194
chr12 85281365
chr12 85281367
chr12 85281381
chr12 85281446
chr12 85281468
chr12 85281626
chr12 85281700
chr12 85281727
chr12 85281731

chr12 85281778
chr12 85281828
chr12 85281922
chr12 85281953
chr12 85282038
chr12 85282158
chr12 85282211
chr12 85282226
chr12 85282339
chr12 85282405
chr12 85282459
chr12 85764062
chr12 85819828
chr12 85964626
chr12 85964702
chr12 86260236
chr12 86260344
chr12 86277006
chr12 86277054
chr12 86277121
chr12 86300352
chr12 86300869
chr12 86301018
chr12 86301133
chr12 86348623
chr12 86348695
chr12 86348765
chr12 86515076
chr12 86519050
chr12 86519094
chr12 86519522
chr12 86598749
chr12 86726566
chr12 86726704
chr12 86726815
chr12 86726971
chr12 86761641
chr12 87130047
chr12 87131233
chr12 87136559
chr12 87344582
chr12 88141508
chr12 88141531
chr12 88141549
chr12 88141626
chr12 88141628
chr12 88141630
chr12 88141652
chr12 88141654
chr12 88141672
chr12 88141720
chr12 88141809
chr12 88141832
chr12 88141858

chr12 88142899
chr12 88142910
chr12 88142996
chr12 88143025
chr12 88143049
chr12 88143175
chr12 88143223
chr12 88716355
chr12 88847641
chr12 88847704
chr12 88847984
chr12 88848040
chr12 88848477
chr12 88848503
chr12 88848521
chr12 88848558
chr12 88848610
chr12 88848877
chr12 88890287
chr12 89017394
chr12 89017405
chr12 89017456
chr12 89017480
chr12 89017538
chr12 89017670
chr12 89017960
chr12 89019255
chr12 89019369
chr12 89019454
chr12 89019477
chr12 89019529
chr12 89019621
chr12 89019656
chr12 89019671
chr12 89019674
chr12 89019683
chr12 89019715
chr12 89019771
chr12 89019794
chr12 89019822
chr12 89019875
chr12 89019889
chr12 89235755
chr12 89235812
chr12 89235967
chr12 89346383
chr12 89352835
chr12 89352864
chr12 89352866
chr12 89352869
chr12 89352915
chr12 89352955
chr12 89352994
chr12 89353059

chr12 89353102
chr12 89353135
chr12 89353183
chr12 89353305
chr12 89353310
chr12 89353313
chr12 89353316
chr12 89353340
chr12 89353364
chr12 89353381
chr12 89353396
chr12 89353398
chr12 89353428
chr12 89353438
chr12 89353447
chr12 89398371
chr12 89398457
chr12 89398585
chr12 89427457
chr12 89427744
chr12 89427764
chr12 89427814
chr12 89468317
chr12 89468773
chr12 89469134
chr12 89469144
chr12 89469208
chr12 89469404
chr12 89526326
chr12 89526341
chr12 89526486
chr12 89526547
chr12 89526578
chr12 89526675
chr12 89526873
chr12 89618369
chr12 89618710
chr12 89663337
chr12 89663686
chr12 89663897
chr12 89710287
chr12 89710400
chr12 89710412
chr12 89710420
chr12 89710437
chr12 89710547
chr12 89710587
chr12 89710589
chr12 89710624
chr12 89710627
chr12 89710793
chr12 89863829
chr12 89863872
chr12 89880779

chr12 90412556
chr12 90853424
chr12 90853681
chr12 91280370
chr12 91280378
chr12 91280439
chr12 91382216
chr12 91382229
chr12 91382233
chr12 91382293
chr12 91383167
chr12 91577776
chr12 91577827
chr12 91578026
chr12 91578079
chr12 91778695
chr12 91778773
chr12 91778954
chr12 91779008
chr12 91779551
chr12 91978382
chr12 92034303
chr12 92034778
chr12 92034990
chr12 92034998
chr12 92035031
chr12 92035058
chr12 92035080
chr12 92035091
chr12 92035150
chr12 92035171
chr12 92094794
chr12 92131423
chr12 92131522
chr12 92131794
chr12 92132412
chr12 92133033
chr12 92133468
chr12 92134113
chr12 92134320
chr12 92134563
chr12 92134735
chr12 92134989
chr12 92135137
chr12 92135147
chr12 92135194
chr12 92135300
chr12 92135310
chr12 92135322
chr12 92137405
chr12 92137777
chr12 92137998
chr12 92138397
chr12 92138586

chr12 92139032
chr12 92139584
chr12 92139960
chr12 92140034
chr12 92140198
chr12 92140323
chr12 92140691
chr12 92141031
chr12 92141115
chr12 92141123
chr12 92141347
chr12 92141651
chr12 92147016
chr12 92147158
chr12 92147282
chr12 92147312
chr12 92147492
chr12 92147599
chr12 92147678
chr12 92185557
chr12 92208740
chr12 92403946
chr12 92403992
chr12 92404172
chr12 92404211
chr12 92462145
chr12 92462327
chr12 92462353
chr12 92462838
chr12 92593829
chr12 92593834
chr12 92593848
chr12 92593898
chr12 92593991
chr12 92594084
chr12 92594237
chr12 92594367
chr12 92652495
chr12 92920934
chr12 92921075
chr12 92921097
chr12 92921672
chr12 92921782
chr12 92921816
chr12 92921845
chr12 92928367
chr12 92929797
chr12 92929815
chr12 92929825
chr12 93167959
chr12 93168071
chr12 93168143
chr12 93168154
chr12 93168182

chr12 93168294
chr12 93168334
chr12 93168356
chr12 93377850
chr12 93377856
chr12 93377864
chr12 93377866
chr12 93377868
chr12 93377877
chr12 93377882
chr12 93377917
chr12 93377920
chr12 93377922
chr12 93377924
chr12 93377928
chr12 93377931
chr12 93377933
chr12 93377955
chr12 93377963
chr12 93377975
chr12 93377977
chr12 93377982
chr12 93377984
chr12 93377986
chr12 93377996
chr12 93378010
chr12 93378097
chr12 93379067
chr12 93379183
chr12 93379198
chr12 93379264
chr12 93379414
chr12 93379537
chr12 93379548
chr12 93379646
chr12 93379668
chr12 93379686
chr12 93379814
chr12 93379962
chr12 93380444
chr12 93436815
chr12 93436900
chr12 93440088
chr12 93440178
chr12 93440290
chr12 93440297
chr12 93440370
chr12 93440461
chr12 93440563
chr12 93440637
chr12 93440692
chr12 93440737
chr12 93440786
chr12 93440801

chr12 93440836
chr12 93440906
chr12 93440935
chr12 93467582
chr12 93467594
chr12 93467622
chr12 93467642
chr12 93467649
chr12 93467655
chr12 93467663
chr12 93467708
chr12 93467721
chr12 93467723
chr12 93467735
chr12 93467754
chr12 93467758
chr12 93467762
chr12 93467807
chr12 93467892
chr12 93468214
chr12 93468321
chr12 93530609
chr12 93591256
chr12 93591290
chr12 93591304
chr12 93591335
chr12 93591351
chr12 93591401
chr12 93591412
chr12 93591420
chr12 93591424
chr12 93591452
chr12 93591475
chr12 93591504
chr12 93591531
chr12 93591537
chr12 93591735
chr12 93591753
chr12 93591761
chr12 93591862
chr12 93591887
chr12 93592087
chr12 93677550
chr12 93677555
chr12 93677568
chr12 93677576
chr12 93677581
chr12 93677602
chr12 93677611
chr12 93677619
chr12 93677635
chr12 93742215
chr12 93742429
chr12 93742439

chr12 93742442
chr12 93742454
chr12 93742461
chr12 93742477
chr12 93742479
chr12 93742565
chr12 93742570
chr12 93742654
chr12 93742677
chr12 93742704
chr12 93742725
chr12 93742733
chr12 93742746
chr12 93742760
chr12 93742773
chr12 93742794
chr12 93742804
chr12 93742806
chr12 93742826
chr12 93767469
chr12 93768096
chr12 93768133
chr12 93784886
chr12 93784981
chr12 93785333
chr12 93785341
chr12 93785432
chr12 93785440
chr12 93881397
chr12 94101718
chr12 94101749
chr12 94101815
chr12 94101866
chr12 94101900
chr12 94101943
chr12 94101947
chr12 94101965
chr12 94102002
chr12 94102004
chr12 94102016
chr12 94102021
chr12 94102058
chr12 94102065
chr12 94102072
chr12 94102078
chr12 94102096
chr12 94102114
chr12 94102116
chr12 94102123
chr12 94102137
chr12 94102152
chr12 94102159
chr12 94102169
chr12 94102195

chr12 94206925
chr12 94206942
chr12 94207683
chr12 94207706
chr12 94207911
chr12 94207966
chr12 94208024
chr12 94208039
chr12 94208077
chr12 94208106
chr12 94208134
chr12 94208186
chr12 94208296
chr12 94208331
chr12 94208515
chr12 94208519
chr12 94208530
chr12 94208540
chr12 94262695
chr12 94262994
chr12 94263011
chr12 94263059
chr12 94263089
chr12 94263093
chr12 94263098
chr12 94263109
chr12 94263325
chr12 94459024
chr12 94459245
chr12 94459308
chr12 94459334
chr12 94640463
chr12 94640644
chr12 94640679
chr12 94640689
chr12 94640739
chr12 94640764
chr12 94640783
chr12 94640874
chr12 94674254
chr12 95002809
chr12 95002880
chr12 95003130
chr12 95003344
chr12 95003380
chr12 95003411
chr12 95003422
chr12 95003475
chr12 95003516
chr12 95003549
chr12 95003588
chr12 95103241
chr12 95216761
chr12 95216811

chr12 95216838
chr12 95216913
chr12 95216922
chr12 95216942
chr12 95216946
chr12 95216960
chr12 95218951
chr12 95219029
chr12 95219054
chr12 95219095
chr12 95219138
chr12 95762634
chr12 95762678
chr12 95859539
chr12 95859541
chr12 95859597
chr12 95859620
chr12 95859652
chr12 95859682
chr12 95859686
chr12 95859739
chr12 95859741
chr12 95859765
chr12 95859777
chr12 95859845
chr12 95942754
chr12 95942767
chr12 95942787
chr12 95942789
chr12 95942986
chr12 96035632
chr12 96035634
chr12 96035641
chr12 96035644
chr12 96035671
chr12 96035675
chr12 96035715
chr12 96035717
chr12 96035719
chr12 96035726
chr12 96035730
chr12 96035748
chr12 96035780
chr12 96035834
chr12 96047604
chr12 96195922
chr12 96195966
chr12 96196005
chr12 96196147
chr12 96196154
chr12 96196198
chr12 96210139
chr12 96210235
chr12 96210254

chr12 96210397
chr12 96210454
chr12 96210459
chr12 96210545
chr12 96210552
chr12 96210554
chr12 96210556
chr12 96210560
chr12 96210564
chr12 96210601
chr12 96215020
chr12 96215130
chr12 96215281
chr12 96215333
chr12 96215354
chr12 96215357
chr12 96215361
chr12 96215375
chr12 96215419
chr12 96215463
chr12 96294884
chr12 96295061
chr12 96295100
chr12 96315963
chr12 96315979
chr12 96315996
chr12 96316002
chr12 96316005
chr12 96316014
chr12 96316045
chr12 96316080
chr12 96316098
chr12 96316103
chr12 96316114
chr12 96316124
chr12 96316148
chr12 96316174
chr12 96316194
chr12 96377756
chr12 96377762
chr12 96377786
chr12 96377794
chr12 96377894
chr12 96397951
chr12 96397979
chr12 96398136
chr12 96398369
chr12 96401446
chr12 96401531
chr12 96401583
chr12 96401700
chr12 96401704
chr12 96401708
chr12 96401752

chr12 96401778
chr12 96401780
chr12 96401834
chr12 96401848
chr12 96434275
chr12 96434334
chr12 96434421
chr12 96434606
chr12 96434719
chr12 96434920
chr12 96441339
chr12 96445769
chr12 96445948
chr12 96445983
chr12 96446181
chr12 96446361
chr12 96485324
chr12 96485603
chr12 96485658
chr12 96489279
chr12 96489295
chr12 96489307
chr12 96489379
chr12 96489385
chr12 96516716
chr12 96516764
chr12 96516801
chr12 96516889
chr12 96516917
chr12 96906557
chr12 96906632
chr12 96906639
chr12 96906651
chr12 96906691
chr12 96906723
chr12 96906725
chr12 96906744
chr12 96906804
chr12 96906861
chr12 96906908
chr12 96907025
chr12 96907033
chr12 96907036
chr12 96907044
chr12 96907056
chr12 96907061
chr12 96907066
chr12 96907075
chr12 96987066
chr12 97462421
chr12 97462461
chr12 97462733
chr12 97462814
chr12 97462854

chr12 97462949
chr12 97462984
chr12 97463079
chr12 97463111
chr12 97463144
chr12 97463430
chr12 97463451
chr12 97463454
chr12 97463471
chr12 97463526
chr12 97463590
chr12 97463733
chr12 97463999
chr12 97464026
chr12 97464030
chr12 97464179
chr12 97464342
chr12 97464369
chr12 97464419
chr12 97464665
chr12 97464734
chr12 97464813
chr12 97464842
chr12 97465661
chr12 97465668
chr12 97465717
chr12 97465766
chr12 97466117
chr12 97466169
chr12 97514228
chr12 97686365
chr12 97686635
chr12 97722845
chr12 97723201
chr12 97723280
chr12 97723292
chr12 98399865
chr12 98457291
chr12 98457307
chr12 98503018
chr12 98503067
chr12 98503073
chr12 98503094
chr12 98503172
chr12 98503217
chr12 98503239
chr12 98503241
chr12 98503253
chr12 98503298
chr12 98503300
chr12 98503358
chr12 98503881
chr12 98503887
chr12 98503889

chr12 98503948
chr12 98503953
chr12 98503964
chr12 98503974
chr12 98504358
chr12 98504372
chr12 98504388
chr12 98504482
chr12 98514370
chr12 98514392
chr12 98514425
chr12 98514702
chr12 98514710
chr12 98514822
chr12 98514837
chr12 98514857
chr12 98514883
chr12 98514927
chr12 98515030
chr12 98517495
chr12 98517558
chr12 98517627
chr12 98517658
chr12 98517669
chr12 98517753
chr12 98517804
chr12 98517810
chr12 98517818
chr12 98517991
chr12 98518107
chr12 98518132
chr12 98518140
chr12 98518260
chr12 98518314
chr12 98518322
chr12 98518339
chr12 98518392
chr12 98518433
chr12 98518445
chr12 98518454
chr12 98566695
chr12 98566724
chr12 98566770
chr12 98595224
chr12 98595749
chr12 98595751
chr12 98595840
chr12 98643738
chr12 98643782
chr12 98645999
chr12 98646009
chr12 98646013
chr12 98646030
chr12 98646117

chr12 98646252
chr12 98646304
chr12 98646339
chr12 98646417
chr12 98646421
chr12 98646535
chr12 98646686
chr12 98646815
chr12 98743515
chr12 98743572
chr12 98743664
chr12 98743958
chr12 99180656
chr12 99181160
chr12 99620635
chr12 99766864
chr12 99772383
chr12 99772531
chr12 99772584
chr12 99772763
chr12 99772782
chr12 99772824
chr12 99772910
chr12 99773018
chr12 99773080
chr12 99773460
chr12 99774129
chr12 99775826
chr12 99776098
chr12 100141637
chr12 100141675
chr12 100141711
chr12 100141767
chr12 100141828
chr12 100141830
chr12 100142751
chr12 100142755
chr12 100142774
chr12 100142790
chr12 100142793
chr12 100142796
chr12 100142804
chr12 100142814
chr12 100142823
chr12 100142828
chr12 100142832
chr12 100142835
chr12 100142837
chr12 100142841
chr12 100142874
chr12 100142884
chr12 100142890
chr12 100142895
chr12 100142905

chr12 100142911
chr12 100142916
chr12 100142921
chr12 100142926
chr12 100142935
chr12 100142938
chr12 100142948
chr12 100142959
chr12 100142992
chr12 100142998
chr12 100143018
chr12 100143037
chr12 100143039
chr12 100163725
chr12 100163893
chr12 100163936
chr12 100163938
chr12 100163957
chr12 100163960
chr12 100163967
chr12 100163972
chr12 100163975
chr12 100163986
chr12 100163998
chr12 100164052
chr12 100164059
chr12 100164067
chr12 100164073
chr12 100199794
chr12 100199801
chr12 100199807
chr12 100199822
chr12 100199824
chr12 100199831
chr12 100199842
chr12 100199849
chr12 100199851
chr12 100199857
chr12 100199859
chr12 100199886
chr12 100199888
chr12 100199902
chr12 100199905
chr12 100199910
chr12 100199925
chr12 100199953
chr12 100199989
chr12 100200005
chr12 100200009
chr12 100200081
chr12 100200089
chr12 100266615
chr12 100267990
chr12 100268000

chr12 100268021
chr12 100268070
chr12 100268115
chr12 100268145
chr12 100268194
chr12 100268275
chr12 100433279
chr12 100574407
chr12 100574409
chr12 100574419
chr12 100574449
chr12 100576950
chr12 100576973
chr12 100718107
chr12 100718242
chr12 100718244
chr12 100718275
chr12 100718403
chr12 100718499
chr12 101143877
chr12 101143921
chr12 101273136
chr12 101279479
chr12 101279487
chr12 101279489
chr12 101279527
chr12 101279532
chr12 101279536
chr12 101280640
chr12 101280649
chr12 101280654
chr12 101280661
chr12 101280696
chr12 101280790
chr12 101408536
chr12 101408600
chr12 101408614
chr12 101408634
chr12 101408678
chr12 101408686
chr12 101408690
chr12 101408766
chr12 101408772
chr12 101408796
chr12 101408804
chr12 101408812
chr12 101414187
chr12 101414190
chr12 101414257
chr12 101414638
chr12 101414681
chr12 101415480
chr12 101456077
chr12 101456198

chr12 101634935
chr12 101634966
chr12 101635195
chr12 101635329
chr12 101829901
chr12 101831532
chr12 101917875
chr12 101917882
chr12 101917918
chr12 101917943
chr12 101917963
chr12 101918062
chr12 102061387
chr12 102061426
chr12 102061510
chr12 102061564
chr12 102061643
chr12 102061649
chr12 102061652
chr12 102061659
chr12 102061662
chr12 102061665
chr12 102061680
chr12 102061692
chr12 102061713
chr12 102917716
chr12 102917722
chr12 102917757
chr12 102917797
chr12 102917818
chr12 102917983
chr12 102948186
chr12 102948471
chr12 102948481
chr12 102948519
chr12 102948895
chr12 102948911
chr12 102959748
chr12 102959786
chr12 102959801
chr12 102959899
chr12 102960021
chr12 102960479
chr12 102961134
chr12 102961159
chr12 102961178
chr12 102962348
chr12 102962625
chr12 102962680
chr12 102962699
chr12 102962703
chr12 102962709
chr12 102962712
chr12 102962722

chr12 102962731
chr12 102962758
chr12 102962760
chr12 102962900
chr12 102963010
chr12 102963028
chr12 102963276
chr12 103043365
chr12 103043504
chr12 103043687
chr12 103101590
chr12 103101824
chr12 103435320
chr12 103499361
chr12 103505497
chr12 103548818
chr12 103548917
chr12 103548962
chr12 103928829
chr12 103930858
chr12 103930877
chr12 103930881
chr12 103930894
chr12 103930901
chr12 103930910
chr12 103930928
chr12 103930950
chr12 103930958
chr12 103930966
chr12 103930991
chr12 103930997
chr12 103931015
chr12 103931032
chr12 103931059
chr12 103931203
chr12 103931210
chr12 103931212
chr12 103931219
chr12 103931275
chr12 103931309
chr12 103931505
chr12 103931524
chr12 103931537
chr12 103931602
chr12 103931680
chr12 103931747
chr12 103931909
chr12 103932078
chr12 103956483
chr12 103956507
chr12 103956622
chr12 103956637
chr12 103957612
chr12 103957683

chr12 103957760
chr12 103957766
chr12 103957776
chr12 103957813
chr12 103957817
chr12 103965249
chr12 103965335
chr12 103965365
chr12 103965467
chr12 103965470
chr12 103965541
chr12 103965546
chr12 103965553
chr12 103965585
chr12 103965629
chr12 103965635
chr12 103965637
chr12 103965651
chr12 103965676
chr12 103965679
chr12 103965687
chr12 103965700
chr12 103965724
chr12 103965736
chr12 103965739
chr12 103965750
chr12 103965756
chr12 103965771
chr12 103965794
chr12 103965799
chr12 103965808
chr12 103965810
chr12 103965825
chr12 103965833
chr12 103965859
chr12 103965868
chr12 103965926
chr12 103965954
chr12 103965968
chr12 103965970
chr12 103965985
chr12 103965993
chr12 103966001
chr12 103966014
chr12 103966018
chr12 103966026
chr12 103966032
chr12 103966045
chr12 103966052
chr12 103966054
chr12 103966068
chr12 103966077
chr12 103966080
chr12 103966093

chr12 103966122
chr12 103966124
chr12 103966126
chr12 103966128
chr12 103966130
chr12 103966132
chr12 103966136
chr12 103966156
chr12 103966160
chr12 103966174
chr12 103966180
chr12 103966182
chr12 103966184
chr12 103966188
chr12 103966204
chr12 103966225
chr12 103966228
chr12 103966244
chr12 103966295
chr12 103966297
chr12 103966392
chr12 103966410
chr12 103966435
chr12 103966486
chr12 103966534
chr12 103966574
chr12 103966624
chr12 103966638
chr12 103966714
chr12 103966934
chr12 104064086
chr12 104064088
chr12 104064101
chr12 104064105
chr12 104064115
chr12 104064119
chr12 104064128
chr12 104064135
chr12 104064149
chr12 104064161
chr12 104064194
chr12 104064228
chr12 104138281
chr12 104138284
chr12 104138286
chr12 104138300
chr12 104138302
chr12 104138305
chr12 104138309
chr12 104138311
chr12 104138313
chr12 104138317
chr12 104138321
chr12 104138324

chr12 104138326
chr12 104138332
chr12 104138337
chr12 104138342
chr12 104138347
chr12 104138353
chr12 104138359
chr12 104138364
chr12 104138370
chr12 104138381
chr12 104215775
chr12 104215811
chr12 104215814
chr12 104215831
chr12 104215834
chr12 104215838
chr12 104215846
chr12 104215858
chr12 104215871
chr12 104215874
chr12 104215881
chr12 104215884
chr12 104215897
chr12 104215901
chr12 104287657
chr12 104287690
chr12 104287693
chr12 104287696
chr12 104287699
chr12 104287749
chr12 104287751
chr12 104287754
chr12 104287783
chr12 104288013
chr12 104288029
chr12 104288053
chr12 104288076
chr12 104288093
chr12 104288145
chr12 104288147
chr12 104288151
chr12 104288235
chr12 104288240
chr12 104288278
chr12 104288392
chr12 104288462
chr12 104288483
chr12 104288571
chr12 104288608
chr12 104288618
chr12 104288631
chr12 104288644
chr12 104456008
chr12 104456362

chr12 104456379
chr12 104456388
chr12 104456395
chr12 104456441
chr12 104456476
chr12 104456482
chr12 104456496
chr12 104456499
chr12 104456543
chr12 104456548
chr12 104456551
chr12 104459040
chr12 104459066
chr12 104459070
chr12 104459141
chr12 104459168
chr12 104459217
chr12 104459294
chr12 104459301
chr12 104459322
chr12 104459353
chr12 104459380
chr12 104459419
chr12 104459428
chr12 104459452
chr12 104459467
chr12 104459496
chr12 104459750
chr12 104459982
chr12 104478375
chr12 104478414
chr12 104478512
chr12 104478523
chr12 104478602
chr12 104478654
chr12 104494602
chr12 104494618
chr12 104494636
chr12 104494642
chr12 104494665
chr12 104494697
chr12 104494715
chr12 104494866
chr12 104494931
chr12 104495008
chr12 104495300
chr12 104570016
chr12 104570104
chr12 104682779
chr12 104682878
chr12 104682884
chr12 104682893
chr12 104682988
chr12 104683103

chr12 104683208
chr12 104695730
chr12 104695754
chr12 104695809
chr12 104695827
chr12 104695832
chr12 104695840
chr12 104695898
chr12 104695908
chr12 104720812
chr12 104720819
chr12 104720830
chr12 104720863
chr12 104720874
chr12 104720877
chr12 104740748
chr12 104740810
chr12 104740829
chr12 104740878
chr12 104740902
chr12 104740945
chr12 104741226
chr12 104957292
chr12 104957563
chr12 104957587
chr12 104958362
chr12 104958368
chr12 104958370
chr12 104958373
chr12 104958376
chr12 104958379
chr12 104958389
chr12 104958409
chr12 104958411
chr12 104958416
chr12 104958419
chr12 104958424
chr12 104958429
chr12 104958438
chr12 104958443
chr12 104958447
chr12 104958454
chr12 104986731
chr12 104986734
chr12 104986776
chr12 104986822
chr12 104986848
chr12 105031016
chr12 105036941
chr12 105037153
chr12 105053913
chr12 105054142
chr12 105057031
chr12 105057041

chr12 105057048
chr12 105057272
chr12 105107343
chr12 105107349
chr12 105107382
chr12 105107393
chr12 105107402
chr12 105107510
chr12 105107524
chr12 105107556
chr12 105108348
chr12 105108350
chr12 105108397
chr12 105108409
chr12 105108488
chr12 105108545
chr12 105110163
chr12 105110406
chr12 105110421
chr12 105235515
chr12 105306850
chr12 105306955
chr12 105307375
chr12 105307830
chr12 105444730
chr12 105445006
chr12 105445118
chr12 105445280
chr12 105445548
chr12 105457761
chr12 105476923
chr12 105476932
chr12 105777447
chr12 105777996
chr12 105778025
chr12 106358508
chr12 106358535
chr12 106358554
chr12 106594091
chr12 106594308
chr12 106594311
chr12 106594315
chr12 106594343
chr12 106760485
chr12 106760628
chr12 106774490
chr12 106774494
chr12 106774517
chr12 106774521
chr12 106774528
chr12 106774531
chr12 106774546
chr12 106774552
chr12 106774555

chr12 106774557
chr12 106774576
chr12 106774582
chr12 106774585
chr12 106774590
chr12 106774595
chr12 106774599
chr12 106774609
chr12 106774625
chr12 106774666
chr12 106774670
chr12 106774676
chr12 106774678
chr12 106774683
chr12 106774700
chr12 106774709
chr12 106774711
chr12 106774718
chr12 106774722
chr12 106774724
chr12 106774733
chr12 106774741
chr12 106774758
chr12 106774760
chr12 106774772
chr12 106774776
chr12 106774781
chr12 106774784
chr12 106774799
chr12 106774803
chr12 106774823
chr12 106774847
chr12 106774851
chr12 106774855
chr12 106774861
chr12 106774869
chr12 106774874
chr12 106774881
chr12 106774894
chr12 106774920
chr12 106774928
chr12 106774943
chr12 106774949
chr12 106774955
chr12 106774968
chr12 106903874
chr12 106903989
chr12 106957388
chr12 106957403
chr12 106957505
chr12 106957529
chr12 106957772
chr12 106986721
chr12 106986761

chr12 106986781
chr12 106986791
chr12 106986808
chr12 106986820
chr12 106986842
chr12 106986860
chr12 106986877
chr12 106986884
chr12 106986889
chr12 106987377
chr12 106987397
chr12 106987436
chr12 107287917
chr12 107288015
chr12 107288098
chr12 107288123
chr12 107320659
chr12 107320672
chr12 107320732
chr12 107320828
chr12 107320860
chr12 107320896
chr12 107320899
chr12 107320921
chr12 107320973
chr12 107321002
chr12 107321007
chr12 107321017
chr12 107321025
chr12 107321027
chr12 107321033
chr12 107321043
chr12 107321049
chr12 107321056
chr12 107321105
chr12 107321126
chr12 107321150
chr12 107321153
chr12 107321171
chr12 107321173
chr12 107321526
chr12 107321553
chr12 107321598
chr12 107321643
chr12 107321704
chr12 107322236
chr12 107368713
chr12 107368715
chr12 107368842
chr12 107369137
chr12 107369319
chr12 107369428
chr12 107369442
chr12 107369492

chr12 107369618
chr12 107369636
chr12 107859450
chr12 107859671
chr12 107885023
chr12 107885241
chr12 107885248
chr12 107885261
chr12 107885265
chr12 107885296
chr12 107903888
chr12 107903892
chr12 107903895
chr12 107903898
chr12 107903901
chr12 107903909
chr12 107903930
chr12 107903933
chr12 107903939
chr12 107903955
chr12 107903957
chr12 107903965
chr12 107904196
chr12 107904277
chr12 107904325
chr12 107904333
chr12 107904341
chr12 108047104
chr12 108047114
chr12 108047126
chr12 108047197
chr12 108047423
chr12 108047835
chr12 108209137
chr12 108209270
chr12 108210829
chr12 108560278
chr12 108561710
chr12 108561759
chr12 108561872
chr12 108561990
chr12 108562001
chr12 108562015
chr12 108563598
chr12 108563665
chr12 108563808
chr12 108568819
chr12 108568856
chr12 108631930
chr12 108631954
chr12 108631990
chr12 108632070
chr12 108632137
chr12 108632207

chr12 108632213
chr12 108632265
chr12 108632378
chr12 108632557
chr12 108632583
chr12 108632593
chr12 108632704
chr12 108632767
chr12 108632973
chr12 108633079
chr12 108633310
chr12 108633363
chr12 108633366
chr12 108633373
chr12 108633593
chr12 108633646
chr12 108633656
chr12 108633743
chr12 108633773
chr12 108633825
chr12 108633855
chr12 108633864
chr12 108633870
chr12 108633907
chr12 108634094
chr12 108634156
chr12 108634215
chr12 108634240
chr12 108634326
chr12 108634567
chr12 108635318
chr12 108635323
chr12 108635482
chr12 108635526
chr12 108635611
chr12 108635618
chr12 108635626
chr12 108635697
chr12 108635705
chr12 108635752
chr12 108635759
chr12 108635830
chr12 108635985
chr12 108636113
chr12 108636210
chr12 108689969
chr12 108730666
chr12 108730683
chr12 108730813
chr12 108730841
chr12 108730922
chr12 108730941
chr12 108730955
chr12 108730967

chr12 108731000
chr12 108731009
chr12 108731014
chr12 108731018
chr12 108731094
chr12 108731104
chr12 108731109
chr12 108731111
chr12 108731117
chr12 108731123
chr12 108731133
chr12 108731138
chr12 108803359
chr12 108803543
chr12 108838773
chr12 108857190
chr12 108857197
chr12 108951054
chr12 108951116
chr12 108951248
chr12 108951277
chr12 108951283
chr12 108951308
chr12 109053215
chr12 109053223
chr12 109053230
chr12 109053232
chr12 109053246
chr12 109053267
chr12 109053289
chr12 109053412
chr12 109053431
chr12 109053565
chr12 109053625
chr12 109053655
chr12 109053726
chr12 109053747
chr12 109053785
chr12 109053889
chr12 109098685
chr12 109098711
chr12 109098729
chr12 109098833
chr12 109098838
chr12 109098840
chr12 109098897
chr12 109098951
chr12 109099405
chr12 109099459
chr12 109099546
chr12 109099548
chr12 109154767
chr12 109154774
chr12 109154779

chr12 109154781
chr12 109154805
chr12 109154812
chr12 109154817
chr12 109154827
chr12 109155069
chr12 109155102
chr12 109155220
chr12 109155230
chr12 109155239
chr12 109155253
chr12 109155315
chr12 109155318
chr12 109155367
chr12 109155599
chr12 109155642
chr12 109357960
chr12 109358139
chr12 109358165
chr12 109358342
chr12 109358384
chr12 109358537
chr12 109358543
chr12 109358609
chr12 109358682
chr12 109358719
chr12 109358731
chr12 109888489
chr12 109888568
chr12 109888582
chr12 109888617
chr12 109888647
chr12 109994197
chr12 109994392
chr12 109994398
chr12 109994959
chr12 110100407
chr12 110199867
chr12 110200194
chr12 110282584
chr12 110282610
chr12 110283128
chr12 110283249
chr12 110283267
chr12 110283566
chr12 110283697
chr12 110283717
chr12 110283781
chr12 110283872
chr12 110289607
chr12 110289751
chr12 110317159
chr12 110403932
chr12 110577653

chr12 110577941
chr12 110578018
chr12 110578297
chr12 110578358
chr12 110578365
chr12 110578482
chr12 110578487
chr12 110578676
chr12 110581876
chr12 110581894
chr12 110581898
chr12 110581929
chr12 110581968
chr12 110582033
chr12 110582125
chr12 110689423
chr12 110689448
chr12 110689470
chr12 110689473
chr12 110689482
chr12 110689487
chr12 110689498
chr12 110689511
chr12 110689535
chr12 110689541
chr12 110740307
chr12 110740313
chr12 110740351
chr12 110740358
chr12 110740557
chr12 110741025
chr12 110741249
chr12 110776612
chr12 110776710
chr12 110888038
chr12 110888050
chr12 110888052
chr12 110888060
chr12 110888074
chr12 110888083
chr12 110888095
chr12 110888134
chr12 110888146
chr12 110888177
chr12 110888179
chr12 110888198
chr12 110888208
chr12 110888231
chr12 110888267
chr12 110888285
chr12 110888445
chr12 111057154
chr12 111057356
chr12 111430709

chr12 111451396
chr12 111451698
chr12 111451719
chr12 111451882
chr12 111451952
chr12 111451963
chr12 111452125
chr12 111505870
chr12 111529480
chr12 111529609
chr12 111529647
chr12 111529654
chr12 111530065
chr12 111530111
chr12 111568724
chr12 111597383
chr12 111597386
chr12 111597394
chr12 111597397
chr12 111597405
chr12 111597408
chr12 111597447
chr12 111597481
chr12 111597502
chr12 111597511
chr12 111597526
chr12 111597553
chr12 111597593
chr12 111597602
chr12 111834459
chr12 111834474
chr12 111834484
chr12 111834491
chr12 111834511
chr12 111834545
chr12 111834549
chr12 111834665
chr12 111993727
chr12 111994113
chr12 111994329
chr12 111994350
chr12 111994360
chr12 111994389
chr12 111994397
chr12 111994443
chr12 111994449
chr12 111994470
chr12 111994518
chr12 111994607
chr12 111994797
chr12 111994858
chr12 111994874
chr12 111994907
chr12 111995024

chr12 111995028
chr12 112001566
chr12 112002341
chr12 112002380
chr12 112002427
chr12 112002493
chr12 112002519
chr12 112002540
chr12 112002543
chr12 112075945
chr12 112096755
chr12 112107605
chr12 112107639
chr12 112107646
chr12 112107657
chr12 112107717
chr12 112107842
chr12 112107936
chr12 112108059
chr12 112108081
chr12 112108111
chr12 112108120
chr12 112109071
chr12 112109101
chr12 112109138
chr12 112109159
chr12 112109193
chr12 112109230
chr12 112109239
chr12 112109243
chr12 112109282
chr12 112109287
chr12 112109291
chr12 112109312
chr12 112109332
chr12 112109373
chr12 112109378
chr12 112109386
chr12 112109390
chr12 112109434
chr12 112109449
chr12 112109466
chr12 112109472
chr12 112109474
chr12 112130553
chr12 112130751
chr12 112131028
chr12 112183235
chr12 112183256
chr12 112303249
chr12 112387359
chr12 112387412
chr12 112387521
chr12 112387593

chr12 112387633
chr12 112387637
chr12 112387639
chr12 112387649
chr12 112387677
chr12 112387682
chr12 112387693
chr12 112387727
chr12 112387738
chr12 112408771
chr12 112408925
chr12 112408937
chr12 112408968
chr12 112409054
chr12 112409094
chr12 112409108
chr12 112409118
chr12 112409152
chr12 112409168
chr12 112409175
chr12 112409179
chr12 112409185
chr12 112409195
chr12 112409197
chr12 112409218
chr12 112409229
chr12 112409237
chr12 112409287
chr12 112409289
chr12 112409292
chr12 112409300
chr12 112409329
chr12 112409337
chr12 112409348
chr12 112409372
chr12 112409380
chr12 112409395
chr12 112409427
chr12 112409453
chr12 112409489
chr12 112409505
chr12 112409526
chr12 112409533
chr12 112409575
chr12 112409577
chr12 112409617
chr12 112409624
chr12 112409650
chr12 112409667
chr12 112419822
chr12 112419842
chr12 112420021
chr12 112573648
chr12 112573681

chr12 112573925
chr12 112574332
chr12 112574574
chr12 112574589
chr12 112575054
chr12 112575087
chr12 112575148
chr12 112575166
chr12 112575229
chr12 112575231
chr12 112625744
chr12 112625899
chr12 112625902
chr12 112626080
chr12 112626651
chr12 112798323
chr12 112798446
chr12 112798526
chr12 112798531
chr12 112798568
chr12 112798575
chr12 112798625
chr12 112798667
chr12 112902508
chr12 112902526
chr12 112902536
chr12 112978590
chr12 112978637
chr12 112979763
chr12 112979824
chr12 112979843
chr12 112979999
chr12 112980649
chr12 112980804
chr12 112980875
chr12 113086525
chr12 113086572
chr12 113086845
chr12 113086877
chr12 113086885
chr12 113086990
chr12 113087001
chr12 113087006
chr12 113087018
chr12 113087047
chr12 113087077
chr12 113087089
chr12 113089426
chr12 113089513
chr12 113089522
chr12 113089531
chr12 113089535
chr12 113089569
chr12 113089581

chr12 113089604
chr12 113089617
chr12 113089628
chr12 113089655
chr12 113089695
chr12 113208706
chr12 113208732
chr12 113208815
chr12 113208818
chr12 113221131
chr12 113334506
chr12 113334559
chr12 113334564
chr12 113334655
chr12 113334658
chr12 113334660
chr12 113404129
chr12 113404273
chr12 113474063
chr12 113474126
chr12 113474181
chr12 113474242
chr12 113474269
chr12 113474297
chr12 113474331
chr12 113474366
chr12 113474511
chr12 113474548
chr12 113474592
chr12 113477301
chr12 113477336
chr12 113477507
chr12 113477520
chr12 113477556
chr12 113477606
chr12 113480751
chr12 113480758
chr12 113480797
chr12 113481099
chr12 113610141
chr12 113610196
chr12 113610258
chr12 113610345
chr12 113610384
chr12 113610386
chr12 113610418
chr12 113610433
chr12 113610466
chr12 113610527
chr12 113610536
chr12 113610550
chr12 113610613
chr12 113610619
chr12 113610648

chr12 113610667
chr12 113610735
chr12 113610770
chr12 113610841
chr12 113610845
chr12 113966762
chr12 113966795
chr12 113966799
chr12 113966852
chr12 113966891
chr12 114012974
chr12 114397374
chr12 114397378
chr12 114397393
chr12 114397415
chr12 114397463
chr12 114397478
chr12 114397510
chr12 114398064
chr12 114398149
chr12 114398180
chr12 114398209
chr12 114398241
chr12 114398376
chr12 114401852
chr12 114401863
chr12 114402018
chr12 114402226
chr12 114402247
chr12 114402462
chr12 114406681
chr12 114406686
chr12 114406721
chr12 114411369
chr12 114411389
chr12 114411642
chr12 114411831
chr12 114411968
chr12 114412061
chr12 114412190
chr12 114412200
chr12 114412232
chr12 114412249
chr12 114412275
chr12 114412285
chr12 114412299
chr12 114412320
chr12 114412333
chr12 114412370
chr12 114412495
chr12 114412499
chr12 114412526
chr12 114412533
chr12 114412929

chr12 114412934
chr12 114412945
chr12 114412964
chr12 114413005
chr12 114413023
chr12 114413039
chr12 114413047
chr12 114413481
chr12 114413535
chr12 114413547
chr12 114413567
chr12 114413615
chr12 114413663
chr12 114413666
chr12 114413690
chr12 114413715
chr12 114413720
chr12 114413841
chr12 114437807
chr12 114437832
chr12 114438394
chr12 114438467
chr12 114438487
chr12 114438499
chr12 114438504
chr12 114438554
chr12 114438669
chr12 114438711
chr12 114438864
chr12 114439065
chr12 114439092
chr12 114439128
chr12 114441981
chr12 114442037
chr12 114442042
chr12 114442063
chr12 114442087
chr12 114442183
chr12 114442369
chr12 114442855
chr12 114443065
chr12 114443488
chr12 114443615
chr12 114443648
chr12 114443735
chr12 114443753
chr12 114443758
chr12 114443845
chr12 114443854
chr12 114443861
chr12 114443863
chr12 114449214
chr12 114449290
chr12 114449338

chr12 114449414
chr12 114449446
chr12 114449494
chr12 114449529
chr12 114449625
chr12 114449643
chr12 114449852
chr12 114450038
chr12 114450042
chr12 114450225
chr12 114450233
chr12 114450347
chr12 114450475
chr12 114450521
chr12 114450962
chr12 114451032
chr12 114451043
chr12 114501418
chr12 114501471
chr12 114501855
chr12 114664023
chr12 114664118
chr12 114664260
chr12 114664332
chr12 114670149
chr12 114670447
chr12 114670477
chr12 114670539
chr12 114671016
chr12 114671175
chr12 114671232
chr12 114671349
chr12 114671418
chr12 114688068
chr12 114688075
chr12 114688094
chr12 114688147
chr12 114688220
chr12 114688256
chr12 114688316
chr12 114688368
chr12 114688409
chr12 114688876
chr12 114689103
chr12 114689470
chr12 114689548
chr12 114689649
chr12 114689667
chr12 114698441
chr12 114698505
chr12 114701189
chr12 114701344
chr12 114701722
chr12 114702095

chr12 114702191
chr12 114702232
chr12 114702239
chr12 114702259
chr12 114702471
chr12 114702570
chr12 114702683
chr12 114702750
chr12 114702763
chr12 114702782
chr12 114702801
chr12 114702826
chr12 114702844
chr12 114702855
chr12 114702895
chr12 114703086
chr12 114703237
chr12 114703252
chr12 114703367
chr12 114703369
chr12 114703435
chr12 114703689
chr12 114703746
chr12 114734002
chr12 114734033
chr12 114734051
chr12 114813731
chr12 114813748
chr12 114813809
chr12 114813820
chr12 115631963
chr12 115632855
chr12 115964863
chr12 115964883
chr12 116060815
chr12 116060831
chr12 116061211
chr12 116087453
chr12 116087600
chr12 116087628
chr12 116236471
chr12 116276081
chr12 116276123
chr12 116276139
chr12 116276142
chr12 116276162
chr12 116276204
chr12 116276215
chr12 116276250
chr12 116276252
chr12 116276261
chr12 116276277
chr12 116276291
chr12 116276353

chr12 116276359
chr12 116276434
chr12 116276437
chr12 116276442
chr12 116276458
chr12 116277412
chr12 116277421
chr12 116277427
chr12 116277430
chr12 116277452
chr12 116277463
chr12 116277470
chr12 116277473
chr12 116277476
chr12 116277478
chr12 116277480
chr12 116277490
chr12 116277497
chr12 116277503
chr12 116277506
chr12 116277509
chr12 116277539
chr12 116277541
chr12 116277547
chr12 116277549
chr12 116277563
chr12 116277567
chr12 116277576
chr12 116277580
chr12 116277586
chr12 116277593
chr12 116277597
chr12 116277600
chr12 116277652
chr12 116277669
chr12 116277675
chr12 116277696
chr12 116277700
chr12 116277703
chr12 116277707
chr12 116277709
chr12 116277714
chr12 116277719
chr12 116277782
chr12 116277795
chr12 116277803
chr12 116277806
chr12 116277809
chr12 116277812
chr12 116277815
chr12 116277820
chr12 116316795
chr12 116359131
chr12 116359158

chr12 116359208
chr12 116359216
chr12 116359237
chr12 116359395
chr12 116359563
chr12 116533251
chr12 116533282
chr12 116533289
chr12 116533311
chr12 116533329
chr12 116533334
chr12 116533343
chr12 116533346
chr12 116533355
chr12 116533360
chr12 116533382
chr12 116533388
chr12 116533397
chr12 116533406
chr12 116533435
chr12 116533457
chr12 116533486
chr12 116533492
chr12 116533505
chr12 116533553
chr12 116533635
chr12 116533647
chr12 116533695
chr12 116533757
chr12 116533770
chr12 116533841
chr12 116533846
chr12 116533855
chr12 116534012
chr12 116534057
chr12 116534099
chr12 116534114
chr12 116534128
chr12 116534130
chr12 116534167
chr12 116534171
chr12 116534182
chr12 116534207
chr12 116534258
chr12 116559264
chr12 116559267
chr12 116559274
chr12 116559290
chr12 116559295
chr12 116559315
chr12 116559339
chr12 116559380
chr12 116559389
chr12 116559402

chr12 116559431
chr12 116559503
chr12 116559631
chr12 116559634
chr12 116559663
chr12 116559730
chr12 116594460
chr12 116594515
chr12 116594580
chr12 116737348
chr12 116737427
chr12 116737454
chr12 116737708
chr12 116737779
chr12 116737819
chr12 116737823
chr12 116737831
chr12 116737843
chr12 116737860
chr12 116737869
chr12 116737872
chr12 116737886
chr12 116737903
chr12 116737906
chr12 116737908
chr12 116737912
chr12 116737919
chr12 116737921
chr12 116737924
chr12 116737928
chr12 116737933
chr12 116737935
chr12 116737940
chr12 116737942
chr12 116737951
chr12 116737958
chr12 116737962
chr12 116737976
chr12 116737978
chr12 116737982
chr12 116738007
chr12 116738010
chr12 116738016
chr12 116738019
chr12 116738023
chr12 116738041
chr12 116738054
chr12 116738064
chr12 116738072
chr12 116738079
chr12 116738090
chr12 116738095
chr12 116738100
chr12 116881620

chr12 116881627
chr12 116881629
chr12 116881632
chr12 116881634
chr12 116881639
chr12 116881642
chr12 116881644
chr12 116881657
chr12 116881662
chr12 116881687
chr12 116881698
chr12 116881700
chr12 116881706
chr12 116881708
chr12 116881713
chr12 116881715
chr12 116910641
chr12 116910655
chr12 116910761
chr12 116922173
chr12 116922365
chr12 116924688
chr12 116925051
chr12 117063442
chr12 117134001
chr12 117134081
chr12 117134115
chr12 117134119
chr12 117134142
chr12 117134188
chr12 117134204
chr12 117134243
chr12 117134259
chr12 117134334
chr12 117134366
chr12 117314264
chr12 117314280
chr12 117968086
chr12 117968108
chr12 117968183
chr12 117968228
chr12 117968235
chr12 117968256
chr12 117968471
chr12 117968676
chr12 117968694
chr12 118061994
chr12 118061999
chr12 118062028
chr12 118062045
chr12 118062057
chr12 118062068
chr12 118062073
chr12 118062102

chr12 118062105
chr12 118062114
chr12 118062124
chr12 118062158
chr12 118062180
chr12 118062193
chr12 118062277
chr12 118062361
chr12 118062365
chr12 118062400
chr12 118062402
chr12 118062438
chr12 118062459
chr12 118062469
chr12 118062499
chr12 118062520
chr12 118062525
chr12 118062561
chr12 118062579
chr12 118062595
chr12 118062621
chr12 118182382
chr12 118182388
chr12 118182411
chr12 118183624
chr12 118274363
chr12 118307180
chr12 118307212
chr12 118307333
chr12 118307548
chr12 118307699
chr12 118307730
chr12 118307782
chr12 118307880
chr12 118357320
chr12 118357411
chr12 118357496
chr12 118357623
chr12 118357673
chr12 118357844
chr12 118357918
chr12 118358248
chr12 118358610
chr12 118358936
chr12 118358984
chr12 118359008
chr12 118359115
chr12 118359191
chr12 118359222
chr12 118359344
chr12 118359660
chr12 118359720
chr12 118371902
chr12 118371920

chr12 118371941
chr12 118371949
chr12 118371981
chr12 118372005
chr12 118372105
chr12 118372160
chr12 118372176
chr12 118372198
chr12 118979902
chr12 118980180
chr12 118980189
chr12 118980199
chr12 118980255
chr12 118980305
chr12 119008681
chr12 119008690
chr12 119061825
chr12 119595963
chr12 119596057
chr12 119596064
chr12 119596067
chr12 119596073
chr12 119596083
chr12 119596129
chr12 119666611
chr12 119667119
chr12 119667466
chr12 119667527
chr12 119667557
chr12 119667567
chr12 119901758
chr12 119902138
chr12 119902204
chr12 119902215
chr12 119980000
chr12 119980200
chr12 119980276
chr12 120007335
chr12 120007423
chr12 120007462
chr12 120007734
chr12 120015552
chr12 120015642
chr12 120086849
chr12 120086883
chr12 120086891
chr12 120086893
chr12 120086912
chr12 120086937
chr12 120086971
chr12 120086974
chr12 120086979
chr12 120086982
chr12 120086984

chr12	120087013
chr12	120087016
chr12	120087022
chr12	120087049
chr12	120087058
chr12	120087061
chr12	120087071
chr12	120087078
chr12	120087081
chr12	120087106
chr12	120194098
chr12	120194338
chr12	120194346
chr12	120194359
chr12	120201373
chr12	120201379
chr12	120201387
chr12	120201407
chr12	120201433
chr12	120201503
chr12	120201511
chr12	120201519
chr12	120201533
chr12	120201541
chr12	120201549
chr12	120201566
chr12	120201573
chr12	120201581
chr12	120201604
chr12	120201625
chr12	120201629
chr12	120201635
chr12	120201641
chr12	120201674
chr12	120201705
chr12	120201719
chr12	120201730
chr12	120201735
chr12	120201763
chr12	120201770
chr12	120201773
chr12	120201775
chr12	120201779
chr12	120201798
chr12	120201816
chr12	120201856
chr12	120201858
chr12	120252319
chr12	120252329
chr12	120252411
chr12	120291309
chr12	120291333
chr12	120291339
chr12	120291341

chr12 120291369
chr12 120291378
chr12 120293484
chr12 120293568
chr12 120325586
chr12 120325643
chr12 120325659
chr12 120325662
chr12 120325877
chr12 120325913
chr12 120325947
chr12 120325955
chr12 120439861
chr12 120439875
chr12 120439890
chr12 120439911
chr12 120439962
chr12 120468964
chr12 120538441
chr12 120538451
chr12 120581116
chr12 120581118
chr12 120581124
chr12 120581939
chr12 120821810
chr12 120902893
chr12 120902973
chr12 120903016
chr12 120903023
chr12 120903033
chr12 120903112
chr12 120903179
chr12 120903263
chr12 120903278
chr12 120903301
chr12 120903333
chr12 120903355
chr12 120903358
chr12 120903362
chr12 120903432
chr12 120903471
chr12 120903500
chr12 120903511
chr12 120903523
chr12 120904153
chr12 120904156
chr12 120904159
chr12 120904162
chr12 120904164
chr12 120904187
chr12 120904191
chr12 120904195
chr12 120904203
chr12 120904209

chr12 120904211
chr12 120939381
chr12 120939729
chr12 120939770
chr12 120939803
chr12 121015896
chr12 121015934
chr12 121016018
chr12 121016027
chr12 121016052
chr12 121016060
chr12 121016140
chr12 121016221
chr12 121016234
chr12 121016293
chr12 121016330
chr12 121016351
chr12 121016355
chr12 121016359
chr12 121016379
chr12 121016388
chr12 121016551
chr12 121016553
chr12 121016590
chr12 121016648
chr12 121038717
chr12 121038746
chr12 121038793
chr12 121038814
chr12 121038854
chr12 121038859
chr12 121038866
chr12 121038892
chr12 121038917
chr12 121038980
chr12 121038989
chr12 121060226
chr12 121060517
chr12 121060540
chr12 121074338
chr12 121074344
chr12 121074518
chr12 121095834
chr12 121096057
chr12 121096089
chr12 121096100
chr12 121096109
chr12 121096115
chr12 121096119
chr12 121096140
chr12 121096187
chr12 121096210
chr12 121096225
chr12 121096252

chr12 121129224
chr12 121129267
chr12 121129296
chr12 121129515
chr12 121129566
chr12 121129641
chr12 121129650
chr12 121334617
chr12 121399578
chr12 121399580
chr12 121399588
chr12 121399601
chr12 121399611
chr12 121400996
chr12 121401057
chr12 121401120
chr12 121401136
chr12 121401210
chr12 121401221
chr12 121530729
chr12 121579571
chr12 121579576
chr12 121579588
chr12 121579593
chr12 121579607
chr12 121579638
chr12 121579640
chr12 121579642
chr12 121579644
chr12 121579683
chr12 121579709
chr12 121579716
chr12 121579720
chr12 121579743
chr12 121579747
chr12 121579754
chr12 121579760
chr12 121579777
chr12 121579787
chr12 121579932
chr12 121589997
chr12 121590002
chr12 121590088
chr12 121590092
chr12 121590100
chr12 121590102
chr12 121590126
chr12 121590146
chr12 121590156
chr12 121590163
chr12 121590180
chr12 121590186
chr12 121590188
chr12 121590218

chr12 121590362
chr12 121590369
chr12 121590482
chr12 121590530
chr12 121590590
chr12 121590599
chr12 121590608
chr12 121590689
chr12 121590805
chr12 121590934
chr12 121591002
chr12 121591033
chr12 121773034
chr12 121773280
chr12 121789355
chr12 121789360
chr12 121789373
chr12 121789391
chr12 121789522
chr12 121789545
chr12 121789548
chr12 121789630
chr12 121789731
chr12 121789847
chr12 121789854
chr12 121789928
chr12 121789957
chr12 121789966
chr12 121789991
chr12 121791946
chr12 121791953
chr12 121791958
chr12 121792012
chr12 121792074
chr12 121792093
chr12 121792132
chr12 121799741
chr12 121799747
chr12 121801573
chr12 121801582
chr12 121801614
chr12 121801629
chr12 121801729
chr12 121805632
chr12 121805645
chr12 121805652
chr12 121805658
chr12 121805662
chr12 121805664
chr12 121805740
chr12 121805748
chr12 121805755
chr12 121805759
chr12 121805764

chr12 121805798
chr12 121805822
chr12 121805837
chr12 121805849
chr12 121805857
chr12 121805866
chr12 121805904
chr12 121806019
chr12 121806024
chr12 121806041
chr12 122099979
chr12 122245228
chr12 122501176
chr12 122501356
chr12 122501369
chr12 122501393
chr12 122501417
chr12 122524111
chr12 122524170
chr12 122525330
chr12 122525332
chr12 122525475
chr12 122525556
chr12 122525612
chr12 122525675
chr12 122525713
chr12 122525739
chr12 122525770
chr12 122525795
chr12 122525844
chr12 122525852
chr12 122525882
chr12 122525890
chr12 122525907
chr12 122525914
chr12 122525922
chr12 122525966
chr12 122525970
chr12 122525978
chr12 122525982
chr12 122526023
chr12 122526032
chr12 122526043
chr12 122526057
chr12 122526064
chr12 122526068
chr12 122526073
chr12 122526101
chr12 122526111
chr12 122526113
chr12 122526117
chr12 122526124
chr12 122526175
chr12 122526182

chr12 122526205
chr12 122526215
chr12 122526222
chr12 122526224
chr12 122526300
chr12 122526652
chr12 122526675
chr12 122528023
chr12 122528032
chr12 122528170
chr12 122528174
chr12 122646967
chr12 122647048
chr12 122726176
chr12 122726255
chr12 122753148
chr12 122753152
chr12 122774740
chr12 122774746
chr12 122774760
chr12 122774783
chr12 122774811
chr12 122774815
chr12 122774817
chr12 122774827
chr12 122774842
chr12 122774854
chr12 122774860
chr12 122774864
chr12 122774871
chr12 122774885
chr12 122774916
chr12 122774950
chr12 122774954
chr12 122774959
chr12 122774962
chr12 122774966
chr12 122774986
chr12 122775010
chr12 122775018
chr12 122775042
chr12 122775048
chr12 122775050
chr12 122826514
chr12 122826695
chr12 122835014
chr12 122836872
chr12 122974778
chr12 122974806
chr12 122974835
chr12 122974846
chr12 122974854
chr12 122974879
chr12 122974893

chr12 122974900
chr12 122974908
chr12 122974911
chr12 122974923
chr12 122974926
chr12 122975407
chr12 122975612
chr12 122975626
chr12 122975647
chr12 122975677
chr12 123223278
chr12 123223280
chr12 123229165
chr12 123229209
chr12 123229231
chr12 123232244
chr12 123232250
chr12 123232451
chr12 123232503
chr12 123232538
chr12 123232543
chr12 123232551
chr12 123353429
chr12 123353459
chr12 123383949
chr12 123383952
chr12 123383962
chr12 123383970
chr12 123384226
chr12 123384232
chr12 123384237
chr12 123384253
chr12 123384456
chr12 123384472
chr12 123384513
chr12 123448342
chr12 123448348
chr12 123448350
chr12 123448372
chr12 123448380
chr12 123448434
chr12 123448442
chr12 123448516
chr12 123448550
chr12 123448552
chr12 123448563
chr12 123448634
chr12 123448672
chr12 123458188
chr12 123458228
chr12 123458242
chr12 123458251
chr12 123458254
chr12 123458276

chr12 123458285
chr12 123458294
chr12 123458296
chr12 123458309
chr12 123458336
chr12 123458351
chr12 123458356
chr12 123458369
chr12 123458392
chr12 123458406
chr12 123458429
chr12 123458437
chr12 123458443
chr12 123458453
chr12 123458516
chr12 123458528
chr12 123458556
chr12 123458634
chr12 123458638
chr12 123458658
chr12 123458664
chr12 123458681
chr12 123458698
chr12 123458720
chr12 123458740
chr12 123458744
chr12 123458752
chr12 123458784
chr12 123585361
chr12 123671261
chr12 123937905
chr12 123937994
chr12 123938008
chr12 123938031
chr12 123938309
chr12 123938316
chr12 123938339
chr12 123972113
chr12 123972119
chr12 123972151
chr12 123972153
chr12 123972229
chr12 123972231
chr12 123972237
chr12 123972241
chr12 123972243
chr12 123972250
chr12 123972253
chr12 123972277
chr12 123972283
chr12 123972286
chr12 123972293
chr12 123972304
chr12 123972308

chr12 123972338
chr12 124466040
chr12 124466061
chr12 124466160
chr12 124466174
chr12 124466181
chr12 124466184
chr12 124466207
chr12 124466215
chr12 124466220
chr12 124466227
chr12 124466230
chr12 124466238
chr12 124466253
chr12 124466257
chr12 124466305
chr12 124466326
chr12 124466330
chr12 124466356
chr12 124466358
chr12 124466379
chr12 124466386
chr12 124466390
chr12 124466392
chr12 124466444
chr12 124466450
chr12 124466519
chr12 124566634
chr12 124588804
chr12 124588841
chr12 124588908
chr12 124589396
chr12 124774382
chr12 124774399
chr12 124774402
chr12 124774413
chr12 124774535
chr12 124774550
chr12 124774647
chr12 124774697
chr12 124774729
chr12 124774763
chr12 124774797
chr12 124774823
chr12 124774827
chr12 124774855
chr12 124916464
chr12 124916608
chr12 124916832
chr12 124917130
chr12 124917189
chr12 124917224
chr12 124917248
chr12 124917267

chr12 124917695
chr12 124917806
chr12 124917839
chr12 124917849
chr12 124917927
chr12 124917930
chr12 124918064
chr12 124921240
chr12 124921248
chr12 124921279
chr12 124921330
chr12 124921420
chr12 124921433
chr12 124921463
chr12 124921718
chr12 124921728
chr12 124921764
chr12 124921771
chr12 124921777
chr12 124921802
chr12 124921881
chr12 124921898
chr12 124921938
chr12 124927551
chr12 124927555
chr12 124927577
chr12 124927652
chr12 124927686
chr12 124927725
chr12 124927727
chr12 124927738
chr12 124927912
chr12 124927919
chr12 124927924
chr12 124927930
chr12 124927953
chr12 124927961
chr12 124927966
chr12 124927983
chr12 124928012
chr12 124938289
chr12 124938334
chr12 124938651
chr12 124938681
chr12 124938721
chr12 124938730
chr12 124938805
chr12 124938880
chr12 124938999
chr12 124939019
chr12 124939128
chr12 124939169
chr12 124939184
chr12 124939186

chr12 124939190
chr12 124988609
chr12 125984011
chr12 125984353
chr12 125984356
chr12 125984567
chr12 126421100
chr12 126421525
chr12 126421583
chr12 126451000
chr12 126451331
chr12 126566229
chr12 126566274
chr12 126566405
chr12 126884152
chr12 126884756
chr12 126957442
chr12 126957763
chr12 127098660
chr12 127098672
chr12 127098734
chr12 127098965
chr12 127099011
chr12 127099144
chr12 127099338
chr12 127099393
chr12 127099398
chr12 127099425
chr12 127099434
chr12 127099560
chr12 127099581
chr12 127099606
chr12 127099637
chr12 127099656
chr12 127165944
chr12 127165949
chr12 127165968
chr12 127165972
chr12 127165980
chr12 127165996
chr12 127166002
chr12 127166014
chr12 127166028
chr12 127166032
chr12 127166036
chr12 127166041
chr12 127166046
chr12 127166050
chr12 127166058
chr12 127166062
chr12 127166070
chr12 127166074
chr12 127166076
chr12 127166081

chr12 127166105
chr12 127166110
chr12 127166126
chr12 127166128
chr12 127166133
chr12 127166138
chr12 127166143
chr12 127166146
chr12 127166149
chr12 127166156
chr12 127166164
chr12 127166173
chr12 127166183
chr12 127166193
chr12 127166198
chr12 127166202
chr12 127166212
chr12 127166222
chr12 127166228
chr12 127166232
chr12 127166238
chr12 127166242
chr12 127559475
chr12 127900157
chr12 127900163
chr12 127900191
chr12 127900200
chr12 127900222
chr12 128137333
chr12 128137432
chr12 128137452
chr12 128269215
chr12 128269234
chr12 128269240
chr12 128269250
chr12 128269289
chr12 128269528
chr12 128269533
chr12 128269557
chr12 128269636
chr12 128353321
chr12 128841007
chr12 128841063
chr12 128841086
chr12 128841232
chr12 128936912
chr12 128937271
chr12 129205729
chr12 129320558
chr12 129320887
chr12 129320934
chr12 129771779
chr12 129771980
chr12 129772188

chr12 129772361
chr12 129772495
chr12 129790736
chr12 129803130
chr12 129803452
chr12 129803491
chr12 129803787
chr12 129803917
chr12 129804308
chr12 129804471
chr12 129805096
chr12 129805117
chr12 129805223
chr12 129805383
chr12 129805386
chr12 129923498
chr12 129923581
chr12 129923611
chr12 130165146
chr12 130165175
chr12 130165182
chr12 130165225
chr12 130165292
chr12 130165470
chr12 130415628
chr12 130625521
chr12 130838841
chr12 130838845
chr12 130838868
chr12 130838877
chr12 130838904
chr12 130838930
chr12 130838939
chr12 130838949
chr12 130838952
chr12 130838962
chr12 130838979
chr12 130838987
chr12 130838996
chr12 130839001
chr12 130839009
chr12 130839013
chr12 130839024
chr12 130839027
chr12 130839032
chr12 130839036
chr12 130839047
chr12 130839054
chr12 130839056
chr12 130839063
chr12 130839070
chr12 130839073
chr12 130839089
chr12 130839095

chr12 130839103
chr12 130839106
chr12 130839110
chr12 130839117
chr12 130839119
chr12 130839124
chr12 130839127
chr12 130839142
chr12 130839153
chr12 130839157
chr12 130839164
chr12 130839173
chr12 130839180
chr12 130839192
chr12 130839194
chr12 130839200
chr12 130839205
chr12 130839208
chr12 130839230
chr12 130839233
chr12 130839241
chr12 130839248
chr12 130839250
chr12 130839255
chr12 130839257
chr12 130839259
chr12 130839266
chr12 130873082
chr12 130873097
chr12 130873152
chr12 130873257
chr12 130873689
chr12 130873695
chr12 130919346
chr12 131171195
chr12 131171267
chr12 131171280
chr12 131171357
chr12 131298470
chr12 131298511
chr12 131298517
chr12 131298549
chr12 131298552
chr12 131298573
chr12 131298585
chr12 131298602
chr12 131299129
chr12 131299131
chr12 131299139
chr12 131299141
chr12 131299155
chr12 131299158
chr12 131299185
chr12 131299203

chr12 131299211
chr12 131299232
chr12 131299236
chr12 131299247
chr12 131299249
chr12 131299254
chr12 131299284
chr12 131299295
chr12 131299342
chr12 131299391
chr12 131299395
chr12 131299402
chr12 131299408
chr12 131299415
chr12 131299422
chr12 131299424
chr12 131299430
chr12 131299432
chr12 131299455
chr12 131299457
chr12 131299468
chr12 131475300
chr12 131475329
chr12 131475464
chr12 131604154
chr12 131604245
chr12 131604262
chr12 131604295
chr12 131604317
chr12 131604348
chr12 131604371
chr12 131604374
chr12 131604382
chr12 131604648
chr12 131604651
chr12 131604659
chr12 131626850
chr12 131626880
chr12 131626884
chr12 131626914
chr12 131626918
chr12 131626952
chr12 131626986
chr12 131627084
chr12 131627088
chr12 131627152
chr12 131627156
chr12 131627186
chr12 131627190
chr12 131627220
chr12 131627224
chr12 131627288
chr12 131627292
chr12 131627322

chr12 131627326
chr12 131627394
chr12 131627424
chr12 131627458
chr12 131627462
chr12 131627492
chr12 131627496
chr12 131627526
chr12 131627530
chr12 131627564
chr12 131627598
chr12 131627632
chr12 131627666
chr12 131627700
chr12 131627734
chr12 131627768
chr12 131627802
chr12 131627899
chr12 131627975
chr12 131661345
chr12 131661356
chr12 131661358
chr12 131661381
chr12 131661383
chr12 131661389
chr12 131661391
chr12 131661398
chr12 131661405
chr12 131661411
chr12 131661418
chr12 131661422
chr12 131661471
chr12 131661493
chr12 131661501
chr12 131661503
chr12 131661513
chr12 131661518
chr12 131661529
chr12 131661559
chr12 131661564
chr12 131661566
chr12 131661577
chr12 131661581
chr12 131661602
chr12 131661610
chr12 131661628
chr12 131661655
chr12 131661658
chr12 131661672
chr12 131661674
chr12 131661682
chr12 131661684
chr12 131661686
chr12 131661693

chr12 131661712
chr12 131661726
chr12 131661964
chr12 131661973
chr12 131661983
chr12 131661996
chr12 131662010
chr12 131662012
chr12 131662018
chr12 131662021
chr12 131662045
chr12 132222970
chr12 132223029
chr12 132223109
chr12 132376924
chr12 132376979
chr12 132377186
chr12 132377268
chr12 132597522
chr12 132597919
chr12 132597924
chr12 132597945
chr12 132597947
chr12 132597952
chr12 132597963
chr12 132597965
chr12 132597967
chr12 132597980
chr12 132597982
chr12 132597984
chr12 132598000
chr12 132598004
chr12 132598017
chr12 132598019
chr12 132598021
chr12 132598037
chr12 132598039
chr12 132598041
chr12 132598046
chr12 132598057
chr12 132598059
chr12 132598061
chr12 132598077
chr12 132598079
chr12 132598081
chr12 132598097
chr12 132598101
chr12 132598114
chr12 132598116
chr12 132598118
chr12 132598134
chr12 132598138
chr12 132598172
chr12 132598188

chr12 132598190
chr12 132598208
chr12 132598212
chr12 132598225
chr12 132598229
chr12 132598242
chr12 132598244
chr12 132598246
chr12 132598262
chr12 132598264
chr12 132598266
chr12 132598282
chr12 132598284
chr12 132598286
chr12 132598302
chr12 132598304
chr12 132598306
chr12 132598319
chr12 132598321
chr12 132598323
chr12 132598339
chr12 132598343
chr12 132598356
chr12 132598358
chr12 132598360
chr12 132598373
chr12 132598375
chr12 132598377
chr12 132598393
chr12 132598395
chr12 132598397
chr12 132598413
chr12 132598415
chr12 132598417
chr12 132598433
chr12 132598435
chr12 132598437
chr12 132598450
chr12 132598452
chr12 132598454
chr12 132598459
chr12 132598470
chr12 132598472
chr12 132598474
chr12 132598488
chr12 132598490
chr12 132598492
chr12 132598508
chr12 132598510
chr12 132598512
chr12 132598526
chr12 132598528
chr12 132598530
chr12 132598544

chr12 132598546
chr12 132598548
chr12 132598564
chr12 132598566
chr12 132598568
chr12 132598581
chr12 132598585
chr12 132598598
chr12 132598602
chr12 132598615
chr12 132598617
chr12 132598619
chr12 132598632
chr12 132598636
chr12 132598649
chr12 132598651
chr12 132598653
chr12 132598669
chr12 132598671
chr12 132598673
chr12 132598764
chr12 132598816
chr12 132598824
chr12 132688239
chr12 132688241
chr12 132688256
chr12 132688265
chr12 132688273
chr12 132688278
chr12 132688280
chr12 132688304
chr12 132688312
chr12 132688319
chr12 132688343
chr12 132887844
chr12 132887876
chr12 132887910
chr12 133068892
chr12 133181851
chr12 133212197
chr12 133261767
chr12 133262536
chr12 133262782
chr12 133263391
chr12 133263558
chr12 133263635
chr12 133263837
chr8 110305
chr8 110408
chr8 111152
chr8 111860
chr8 112054
chr8 112066
chr8 112078

chr8 113585
chr8 113760
chr8 113876
chr8 114038
chr8 114841
chr8 114883
chr8 114921
chr8 115869
chr8 116247
chr8 116359
chr8 116374
chr8 116825
chr8 117810
chr8 117831
chr8 118460
chr8 118462
chr8 123357
chr8 123391
chr8 124617
chr8 125284
chr8 125295
chr8 125343
chr8 166709
chr8 170404
chr8 171856
chr8 171861
chr8 231624
chr8 231662
chr8 231733
chr8 232156
chr8 232164
chr8 232172
chr8 232176
chr8 232193
chr8 232196
chr8 233097
chr8 233101
chr8 233163
chr8 233272
chr8 266104
chr8 317448
chr8 317628
chr8 317636
chr8 407484
chr8 407491
chr8 407506
chr8 407508
chr8 407510
chr8 407520
chr8 407542
chr8 407553
chr8 407572
chr8 407585
chr8 407597

chr8 407602
chr8 407619
chr8 407625
chr8 407635
chr8 407638
chr8 407649
chr8 407655
chr8 407670
chr8 407673
chr8 407724
chr8 580556
chr8 580598
chr8 664048
chr8 664054
chr8 664098
chr8 664126
chr8 664479
chr8 664753
chr8 730420
chr8 730871
chr8 730877
chr8 730916
chr8 764169
chr8 764174
chr8 764222
chr8 764344
chr8 764383
chr8 764416
chr8 764440
chr8 821873
chr8 821882
chr8 821894
chr8 953741
chr8 953754
chr8 979650
chr8 987646
chr8 991573
chr8 991604
chr8 991627
chr8 991658
chr8 991681
chr8 991735
chr8 991766
chr8 991789
chr8 991843
chr8 991897
chr8 991951
chr8 992167
chr8 992221
chr8 992437
chr8 992468
chr8 992491
chr8 992545
chr8 992653

chr8 992684
chr8 992707
chr8 992761
chr8 992869
chr8 992923
chr8 992977
chr8 993031
chr8 993085
chr8 993139
chr8 993170
chr8 995210
chr8 995479
chr8 995843
chr8 1106430
chr8 1106492
chr8 1405586
chr8 1405597
chr8 1405621
chr8 1405634
chr8 1405643
chr8 1405700
chr8 1405713
chr8 1405937
chr8 1405943
chr8 1572438
chr8 1572459
chr8 1607778
chr8 1745850
chr8 1745853
chr8 1745880
chr8 1745978
chr8 1788298
chr8 1788301
chr8 1788367
chr8 1822825
chr8 1822946
chr8 1822991
chr8 1823070
chr8 1823101
chr8 1892195
chr8 1892224
chr8 1892339
chr8 1892429
chr8 1892530
chr8 1895512
chr8 2044718
chr8 2044751
chr8 2044782
chr8 2044793
chr8 2044829
chr8 2127376
chr8 2127386
chr8 2127410
chr8 2127540

chr8 2127581
chr8 2127584
chr8 2127589
chr8 2127591
chr8 2127597
chr8 2127603
chr8 2127621
chr8 2127624
chr8 2127629
chr8 2127631
chr8 2127637
chr8 2127643
chr8 2127669
chr8 2127671
chr8 2127677
chr8 2127683
chr8 2127701
chr8 2127704
chr8 2127709
chr8 2127711
chr8 2127717
chr8 2127723
chr8 2127741
chr8 2127744
chr8 2127749
chr8 2127751
chr8 2127757
chr8 2127763
chr8 2127781
chr8 2127784
chr8 2127789
chr8 2127797
chr8 2127803
chr8 2127817
chr8 2127832
chr8 2127853
chr8 2127985
chr8 2128000
chr8 2131225
chr8 2145383
chr8 2145546
chr8 2145621
chr8 2145628
chr8 2238463
chr8 2238474
chr8 2238490
chr8 2238501
chr8 2238508
chr8 2238565
chr8 2238585
chr8 2368889
chr8 2369544
chr8 2481024
chr8 2481044

chr8 2481101
chr8 2481108
chr8 2481110
chr8 2481119
chr8 2481135
chr8 2481146
chr8 2481165
chr8 2481172
chr8 2481174
chr8 2481180
chr8 2481183
chr8 2481225
chr8 2481250
chr8 2481287
chr8 2481291
chr8 2481357
chr8 2481383
chr8 2481386
chr8 2481424
chr8 2481460
chr8 2502098
chr8 2502146
chr8 2502320
chr8 2623493
chr8 2623504
chr8 2623552
chr8 2623613
chr8 2623641
chr8 2623656
chr8 2733559
chr8 2733690
chr8 2733693
chr8 2733706
chr8 2733717
chr8 2733734
chr8 2814948
chr8 2815060
chr8 2815299
chr8 3285140
chr8 3285152
chr8 3285182
chr8 3285204
chr8 3420717
chr8 3420793
chr8 3420842
chr8 3421074
chr8 3572415
chr8 3572465
chr8 3572691
chr8 3572713
chr8 4065520
chr8 4194570
chr8 4194945
chr8 4262244

chr8 4262302
chr8 4742803
chr8 4742897
chr8 4881562
chr8 5186415
chr8 5186757
chr8 5193523
chr8 5193938
chr8 5208633
chr8 5208895
chr8 5963864
chr8 6291356
chr8 6291492
chr8 6291515
chr8 6291610
chr8 6291661
chr8 6291720
chr8 6291795
chr8 6291839
chr8 6291891
chr8 6291909
chr8 6291915
chr8 6291926
chr8 6405598
chr8 6405605
chr8 6405729
chr8 6405731
chr8 6405747
chr8 6405761
chr8 6405763
chr8 6405911
chr8 6405913
chr8 6405965
chr8 6406017
chr8 6406033
chr8 6406062
chr8 6406071
chr8 6406073
chr8 6406102
chr8 6406119
chr8 6406135
chr8 6406141
chr8 6406149
chr8 6406157
chr8 6406171
chr8 6406176
chr8 6406721
chr8 6406736
chr8 6689730
chr8 6690227
chr8 6690255
chr8 6690336
chr8 6690573
chr8 6690682

chr8 6690786
chr8 6709313
chr8 6709352
chr8 6709377
chr8 6709491
chr8 6709697
chr8 6709802
chr8 6709945
chr8 6729463
chr8 6729470
chr8 6729583
chr8 7157621
chr8 7157721
chr8 7158006
chr8 7158137
chr8 7158157
chr8 7221355
chr8 7221372
chr8 7221412
chr8 7221421
chr8 7221451
chr8 7221453
chr8 7221481
chr8 7221512
chr8 7221515
chr8 7221548
chr8 7221555
chr8 7221570
chr8 7221577
chr8 7221582
chr8 7221584
chr8 7221595
chr8 7221597
chr8 7221604
chr8 7221635
chr8 7221641
chr8 7221647
chr8 7221657
chr8 7221660
chr8 7221669
chr8 7221672
chr8 7221678
chr8 7221724
chr8 7221742
chr8 7221762
chr8 7221764
chr8 7221767
chr8 7221769
chr8 7221778
chr8 7221784
chr8 7221793
chr8 7221802
chr8 7221825
chr8 7221842

chr8 7221850
chr8 7221853
chr8 7221873
chr8 7221888
chr8 7221907
chr8 7221916
chr8 7221936
chr8 7221973
chr8 7221976
chr8 7221978
chr8 7221982
chr8 7221990
chr8 7221993
chr8 7222004
chr8 7222012
chr8 7222014
chr8 7222020
chr8 7222029
chr8 7222031
chr8 7222033
chr8 7222036
chr8 7222038
chr8 7222057
chr8 7222060
chr8 7222062
chr8 7222072
chr8 7222074
chr8 7222116
chr8 7222118
chr8 7222126
chr8 7222131
chr8 7222134
chr8 7222148
chr8 7222179
chr8 7222239
chr8 7222255
chr8 7222294
chr8 7222299
chr8 7222655
chr8 7222686
chr8 7222772
chr8 7225331
chr8 7225415
chr8 7226359
chr8 7226437
chr8 7226459
chr8 7226929
chr8 7226943
chr8 7227757
chr8 7227763
chr8 7227769
chr8 7227779
chr8 7227782
chr8 7227790

chr8 7227793
chr8 7227795
chr8 7227801
chr8 7227810
chr8 7227812
chr8 7227827
chr8 7227835
chr8 7227837
chr8 7227841
chr8 7227843
chr8 7227861
chr8 7227864
chr8 7227882
chr8 7227892
chr8 7227898
chr8 7227904
chr8 7227907
chr8 7227981
chr8 7227990
chr8 7228016
chr8 7228031
chr8 7228050
chr8 7228080
chr8 7228117
chr8 7228120
chr8 7228122
chr8 7228126
chr8 7228134
chr8 7228137
chr8 7228148
chr8 7228156
chr8 7228158
chr8 7228164
chr8 7228173
chr8 7228175
chr8 7228180
chr8 7228182
chr8 7228195
chr8 7228201
chr8 7228206
chr8 7228216
chr8 7228218
chr8 7228222
chr8 7228240
chr8 7228254
chr8 7228260
chr8 7228262
chr8 7228270
chr8 7228275
chr8 7228278
chr8 7228292
chr8 7228323
chr8 7228349
chr8 7228378

chr8 7228383
chr8 7303705
chr8 7303806
chr8 7303915
chr8 7355008
chr8 7355011
chr8 7355018
chr8 7355020
chr8 7355026
chr8 7355032
chr8 7355038
chr8 7355040
chr8 7355057
chr8 7355076
chr8 7355079
chr8 7355090
chr8 7355099
chr8 7355109
chr8 7355128
chr8 7355138
chr8 7355148
chr8 7355159
chr8 7355161
chr8 7355171
chr8 7355178
chr8 7355187
chr8 7355190
chr8 7355195
chr8 7355212
chr8 7355233
chr8 7355244
chr8 7355270
chr8 7355276
chr8 7355324
chr8 7355329
chr8 7355332
chr8 7355334
chr8 7355346
chr8 7355348
chr8 7355354
chr8 7355367
chr8 7355395
chr8 7355419
chr8 7355432
chr8 7355585
chr8 7355614
chr8 7355645
chr8 7397917
chr8 7397938
chr8 7397965
chr8 7397967
chr8 7397984
chr8 7397989
chr8 7398013

chr8 7398102
chr8 7398147
chr8 7398200
chr8 7398287
chr8 7398293
chr8 7398521
chr8 7502854
chr8 7503740
chr8 7562894
chr8 7563396
chr8 7563402
chr8 7563675
chr8 7563691
chr8 7611381
chr8 7611387
chr8 7611397
chr8 7611408
chr8 7611411
chr8 7611419
chr8 7611428
chr8 7611463
chr8 7611465
chr8 7611495
chr8 7611514
chr8 7611552
chr8 7611561
chr8 7611600
chr8 7611609
chr8 7611618
chr8 7611624
chr8 7611635
chr8 7611638
chr8 7611660
chr8 7611678
chr8 7611681
chr8 7611701
chr8 7611705
chr8 7611707
chr8 7612053
chr8 7612059
chr8 7612089
chr8 7612117
chr8 7612120
chr8 7612173
chr8 7612207
chr8 7612244
chr8 7612260
chr8 7612433
chr8 7612530
chr8 7612599
chr8 7612613
chr8 7612831
chr8 7613587
chr8 7613600

chr8 7667338
chr8 7667419
chr8 7667451
chr8 7667745
chr8 7669812
chr8 7669830
chr8 7669840
chr8 7669886
chr8 7670278
chr8 7670295
chr8 7670431
chr8 7670465
chr8 7670649
chr8 7671115
chr8 7671491
chr8 7671508
chr8 7671523
chr8 7673391
chr8 7673443
chr8 7673523
chr8 7674257
chr8 7675204
chr8 7675227
chr8 7675247
chr8 7675252
chr8 7676439
chr8 7677531
chr8 7677550
chr8 7678880
chr8 7678896
chr8 7678898
chr8 7678933
chr8 7678940
chr8 7678967
chr8 7679020
chr8 7679023
chr8 7679650
chr8 7679668
chr8 7679682
chr8 7679701
chr8 7679706
chr8 7679764
chr8 7679842
chr8 7679867
chr8 7679870
chr8 7679878
chr8 7679913
chr8 7679951
chr8 7680017
chr8 7680047
chr8 7680049
chr8 7680077
chr8 7680105
chr8 7680108

chr8 7680111
chr8 7680144
chr8 7680151
chr8 7680161
chr8 7680166
chr8 7680173
chr8 7680178
chr8 7680180
chr8 7680191
chr8 7680193
chr8 7680200
chr8 7680209
chr8 7680231
chr8 7680237
chr8 7680243
chr8 7680253
chr8 7680256
chr8 7680265
chr8 7680268
chr8 7680274
chr8 7680283
chr8 7680338
chr8 7680358
chr8 7680360
chr8 7680363
chr8 7680365
chr8 7680374
chr8 7680380
chr8 7680389
chr8 7680398
chr8 7680437
chr8 7680446
chr8 7680449
chr8 7680469
chr8 7680484
chr8 7680503
chr8 7680512
chr8 7680532
chr8 7680569
chr8 7680574
chr8 7680578
chr8 7680586
chr8 7680600
chr8 7680608
chr8 7680610
chr8 7680616
chr8 7680625
chr8 7680627
chr8 7680629
chr8 7680632
chr8 7680634
chr8 7680744
chr8 7680775
chr8 7680801

chr8 7680830
chr8 7680835
chr8 7680851
chr8 7680890
chr8 7680910
chr8 7680976
chr8 7680993
chr8 7681787
chr8 7681792
chr8 7681842
chr8 7682563
chr8 7685524
chr8 7685538
chr8 7686352
chr8 7686358
chr8 7686364
chr8 7686374
chr8 7686377
chr8 7686385
chr8 7686388
chr8 7686390
chr8 7686396
chr8 7686405
chr8 7686407
chr8 7686411
chr8 7686413
chr8 7686431
chr8 7686434
chr8 7686452
chr8 7686462
chr8 7686468
chr8 7686474
chr8 7686477
chr8 7686551
chr8 7686560
chr8 7686586
chr8 7686601
chr8 7686620
chr8 7686650
chr8 7686687
chr8 7686690
chr8 7686692
chr8 7686696
chr8 7686718
chr8 7686728
chr8 7686734
chr8 7686743
chr8 7686745
chr8 7686747
chr8 7686750
chr8 7686752
chr8 7686765
chr8 7686771
chr8 7686776

chr8 7686786
chr8 7686788
chr8 7686792
chr8 7686824
chr8 7686830
chr8 7686832
chr8 7686840
chr8 7686845
chr8 7686848
chr8 7686862
chr8 7686893
chr8 7686919
chr8 7686948
chr8 7689995
chr8 7690012
chr8 7690691
chr8 7691043
chr8 7691073
chr8 7909273
chr8 7909296
chr8 7909470
chr8 7910360
chr8 7910751
chr8 7911640
chr8 7912207
chr8 7912213
chr8 7912230
chr8 7912237
chr8 7912245
chr8 7912289
chr8 7912293
chr8 7912305
chr8 7912346
chr8 7912380
chr8 7912387
chr8 7912643
chr8 7912716
chr8 7913307
chr8 7913394
chr8 7913492
chr8 7913581
chr8 7913605
chr8 7913610
chr8 7913627
chr8 7913629
chr8 7913656
chr8 7952379
chr8 7952559
chr8 7955172
chr8 7955177
chr8 7955180
chr8 7955189
chr8 7955196
chr8 7955206

chr8 7955208
chr8 7955219
chr8 7955249
chr8 7955258
chr8 7955268
chr8 7955277
chr8 7955289
chr8 7955292
chr8 7955311
chr8 7955328
chr8 7955330
chr8 7955336
chr8 7955342
chr8 7955348
chr8 7955350
chr8 7955357
chr8 7955360
chr8 7955366
chr8 7955382
chr8 7955389
chr8 7955397
chr8 7955402
chr8 7955415
chr8 8003489
chr8 8004370
chr8 8005097
chr8 8005143
chr8 8021185
chr8 8021187
chr8 8021213
chr8 8021243
chr8 8021262
chr8 8021288
chr8 8021293
chr8 8021327
chr8 8021338
chr8 8058503
chr8 8058616
chr8 8058702
chr8 8059074
chr8 8059089
chr8 8059094
chr8 8059133
chr8 8059154
chr8 8059183
chr8 8059452
chr8 8059482
chr8 8059501
chr8 8059536
chr8 8059545
chr8 8059584
chr8 8059593
chr8 8059602
chr8 8059608

chr8 8059619
chr8 8059622
chr8 8059628
chr8 8059644
chr8 8059662
chr8 8059665
chr8 8059685
chr8 8059689
chr8 8059691
chr8 8059699
chr8 8059708
chr8 8059833
chr8 8059859
chr8 8063833
chr8 8064339
chr8 8064758
chr8 8065275
chr8 8065325
chr8 8065475
chr8 8065490
chr8 8065492
chr8 8065640
chr8 8065655
chr8 8065675
chr8 8065678
chr8 8065687
chr8 8065703
chr8 8065726
chr8 8065735
chr8 8065744
chr8 8065750
chr8 8065759
chr8 8065761
chr8 8065764
chr8 8065766
chr8 8065786
chr8 8065804
chr8 8065825
chr8 8065827
chr8 8065831
chr8 8065834
chr8 8065841
chr8 8065850
chr8 8065856
chr8 8065859
chr8 8065868
chr8 8065871
chr8 8065887
chr8 8065893
chr8 8065915
chr8 8065924
chr8 8065931
chr8 8065933
chr8 8065944

chr8 8065946
chr8 8065951
chr8 8065958
chr8 8065963
chr8 8066035
chr8 8066047
chr8 8066075
chr8 8066077
chr8 8066107
chr8 8066116
chr8 8066156
chr8 8066173
chr8 8066210
chr8 8069361
chr8 8124889
chr8 8124893
chr8 8124994
chr8 8125745
chr8 8126153
chr8 8126160
chr8 8142695
chr8 8142761
chr8 8142841
chr8 8142866
chr8 8142870
chr8 8143020
chr8 8143117
chr8 8143410
chr8 8143416
chr8 8143517
chr8 8143544
chr8 8143606
chr8 8143761
chr8 8144061
chr8 8198869
chr8 8198892
chr8 8198894
chr8 8198984
chr8 8199005
chr8 8199012
chr8 8199026
chr8 8199029
chr8 8199059
chr8 8199064
chr8 8199101
chr8 8199104
chr8 8199110
chr8 8199122
chr8 8199209
chr8 8199224
chr8 8199230
chr8 8199234
chr8 8199287
chr8 8199700

chr8 8227495
chr8 8227497
chr8 8227503
chr8 8227537
chr8 8227548
chr8 8227560
chr8 8227583
chr8 8227599
chr8 8227614
chr8 8227621
chr8 8227634
chr8 8227640
chr8 8227646
chr8 8227650
chr8 8227658
chr8 8227672
chr8 8229059
chr8 8229061
chr8 8229083
chr8 8229085
chr8 8229138
chr8 8229164
chr8 8229178
chr8 8229302
chr8 8272955
chr8 8273078
chr8 8273125
chr8 8273152
chr8 8273159
chr8 8273308
chr8 8273355
chr8 8333762
chr8 8333785
chr8 8333934
chr8 8334054
chr8 8334165
chr8 8334218
chr8 8334418
chr8 8345861
chr8 8346129
chr8 8346336
chr8 8346382
chr8 8346647
chr8 8346692
chr8 8346698
chr8 8346791
chr8 8346843
chr8 8346865
chr8 8347173
chr8 8347204
chr8 8347286
chr8 8548305
chr8 8548393
chr8 8701340

chr8 8701667
chr8 8701763
chr8 8701783
chr8 8783416
chr8 8783468
chr8 8797466
chr8 8797513
chr8 9030249
chr8 9082110
chr8 9082329
chr8 9151780
chr8 9151783
chr8 9151798
chr8 9151805
chr8 9151809
chr8 9151837
chr8 9151842
chr8 9151953
chr8 9151963
chr8 9151987
chr8 9151989
chr8 9152306
chr8 9153134
chr8 9164323
chr8 9556786
chr8 9556951
chr8 9557134
chr8 9557264
chr8 9557317
chr8 9577154
chr8 9577165
chr8 9577361
chr8 9592395
chr8 9592487
chr8 9592606
chr8 9592651
chr8 9592696
chr8 9723420
chr8 10055424
chr8 10055426
chr8 10055438
chr8 10055449
chr8 10055471
chr8 10055496
chr8 10055528
chr8 10055547
chr8 10055610
chr8 10055787
chr8 10055852
chr8 10055875
chr8 10332630
chr8 10332855
chr8 10332966
chr8 10333185

chr8 10333284
chr8 10333453
chr8 10333467
chr8 10333492
chr8 10510604
chr8 10510606
chr8 10510617
chr8 10805610
chr8 10805624
chr8 10805650
chr8 10805664
chr8 10805704
chr8 10805730
chr8 10805744
chr8 10805850
chr8 10805864
chr8 10805944
chr8 10805984
chr8 10806024
chr8 10937220
chr8 10937229
chr8 10937355
chr8 10937376
chr8 10937432
chr8 10937473
chr8 10937480
chr8 10937935
chr8 10937944
chr8 10937981
chr8 10937985
chr8 11015309
chr8 11015352
chr8 11123849
chr8 11123936
chr8 11123949
chr8 11123981
chr8 11124076
chr8 11124347
chr8 11124361
chr8 11124421
chr8 11254930
chr8 11255020
chr8 11259336
chr8 11284016
chr8 11284021
chr8 11284023
chr8 11284026
chr8 11284032
chr8 11284083
chr8 11284100
chr8 11284171
chr8 11400845
chr8 11401101
chr8 11408949

chr8 11409329
chr8 11418378
chr8 11418437
chr8 11418440
chr8 11456626
chr8 11456739
chr8 11456756
chr8 11456783
chr8 11456821
chr8 11456909
chr8 11456943
chr8 11456966
chr8 11457081
chr8 11457104
chr8 11457127
chr8 11457174
chr8 11457217
chr8 11524782
chr8 11524832
chr8 11524850
chr8 11524857
chr8 11524876
chr8 11524954
chr8 11525147
chr8 11525192
chr8 11677038
chr8 11677101
chr8 11682188
chr8 11682224
chr8 11682230
chr8 11682252
chr8 11682399
chr8 11682427
chr8 11682652
chr8 11682768
chr8 11682780
chr8 11682805
chr8 11682810
chr8 11684007
chr8 11684080
chr8 11684171
chr8 11684532
chr8 11684637
chr8 11684690
chr8 11684932
chr8 11685080
chr8 11685176
chr8 11685225
chr8 11685273
chr8 11685349
chr8 11685390
chr8 11685484
chr8 11685502
chr8 11685508

chr8 11685774
chr8 11685959
chr8 11685983
chr8 11686043
chr8 11686276
chr8 11688586
chr8 11689064
chr8 11689080
chr8 11689119
chr8 11695808
chr8 11695812
chr8 11696004
chr8 11696025
chr8 11696240
chr8 11696411
chr8 11696536
chr8 11696697
chr8 11696753
chr8 11699013
chr8 11699173
chr8 11705620
chr8 11705665
chr8 11706498
chr8 11706597
chr8 11706675
chr8 11706961
chr8 11707117
chr8 11707169
chr8 11707210
chr8 11707218
chr8 11707281
chr8 11722958
chr8 11803619
chr8 11803623
chr8 11803625
chr8 11803736
chr8 11803761
chr8 11803866
chr8 11803939
chr8 11804051
chr8 11804170
chr8 11804188
chr8 11944234
chr8 11944327
chr8 12047574
chr8 12107340
chr8 12107346
chr8 12107521
chr8 12107581
chr8 12107597
chr8 12107632
chr8 12107719
chr8 12109463
chr8 12109492

chr8 12113146
chr8 12113171
chr8 12115477
chr8 12115489
chr8 12115520
chr8 12115549
chr8 12115701
chr8 12115714
chr8 12115766
chr8 12115779
chr8 12115785
chr8 12115787
chr8 12115799
chr8 12115801
chr8 12115804
chr8 12115809
chr8 12115889
chr8 12115900
chr8 12115909
chr8 12115921
chr8 12115939
chr8 12115944
chr8 12115947
chr8 12115956
chr8 12115963
chr8 12115973
chr8 12115975
chr8 12115986
chr8 12116016
chr8 12116035
chr8 12116044
chr8 12116056
chr8 12116059
chr8 12116078
chr8 12116095
chr8 12116097
chr8 12116103
chr8 12116115
chr8 12116117
chr8 12116124
chr8 12116127
chr8 12116133
chr8 12116149
chr8 12116156
chr8 12116164
chr8 12116169
chr8 12116213
chr8 12116236
chr8 12183108
chr8 12183130
chr8 12183160
chr8 12183212
chr8 12193509
chr8 12193518

chr8 12193535
chr8 12193562
chr8 12193588
chr8 12193590
chr8 12193612
chr8 12193614
chr8 12193626
chr8 12193646
chr8 12193675
chr8 12193696
chr8 12193708
chr8 12193715
chr8 12193726
chr8 12193731
chr8 12193733
chr8 12193735
chr8 12193751
chr8 12193760
chr8 12193773
chr8 12193776
chr8 12193784
chr8 12193786
chr8 12193802
chr8 12193806
chr8 12195182
chr8 12195189
chr8 12195200
chr8 12195260
chr8 12195282
chr8 12195314
chr8 12195363
chr8 12195415
chr8 12195421
chr8 12195433
chr8 12195456
chr8 12195690
chr8 12195736
chr8 12220687
chr8 12221766
chr8 12221905
chr8 12223045
chr8 12223052
chr8 12223086
chr8 12223192
chr8 12223927
chr8 12223931
chr8 12223937
chr8 12223952
chr8 12224039
chr8 12224051
chr8 12224057
chr8 12224060
chr8 12224100
chr8 12224105

chr8 12224135
chr8 12224138
chr8 12224152
chr8 12358098
chr8 12359408
chr8 12359532
chr8 12359563
chr8 12362181
chr8 12362184
chr8 12362193
chr8 12362200
chr8 12362210
chr8 12362212
chr8 12362223
chr8 12362253
chr8 12362262
chr8 12362272
chr8 12362281
chr8 12362293
chr8 12362296
chr8 12362315
chr8 12362332
chr8 12362334
chr8 12362340
chr8 12362346
chr8 12362352
chr8 12362354
chr8 12362361
chr8 12362364
chr8 12362370
chr8 12362386
chr8 12362393
chr8 12362401
chr8 12362406
chr8 12362473
chr8 12435782
chr8 12435791
chr8 12435808
chr8 12435835
chr8 12435863
chr8 12435885
chr8 12435887
chr8 12435899
chr8 12435919
chr8 12435948
chr8 12435969
chr8 12435981
chr8 12435988
chr8 12436000
chr8 12436004
chr8 12436006
chr8 12436008
chr8 12436024
chr8 12436033

chr8 12436046
chr8 12436049
chr8 12436057
chr8 12436059
chr8 12436075
chr8 12436079
chr8 12436338
chr8 12436984
chr8 12437160
chr8 12437167
chr8 12437176
chr8 12437202
chr8 12437218
chr8 12437221
chr8 12437223
chr8 12437235
chr8 12437249
chr8 12437257
chr8 12437261
chr8 12437267
chr8 12437286
chr8 12437293
chr8 12437308
chr8 12437324
chr8 12437327
chr8 12437347
chr8 12437359
chr8 12437370
chr8 12437455
chr8 12437462
chr8 12437473
chr8 12437533
chr8 12437555
chr8 12437587
chr8 12437636
chr8 12437706
chr8 12437729
chr8 12437787
chr8 12437819
chr8 12437963
chr8 12459676
chr8 12459686
chr8 12460012
chr8 12460033
chr8 12461775
chr8 12461806
chr8 12461990
chr8 12461995
chr8 12462005
chr8 12462011
chr8 12462028
chr8 12464354
chr8 12464377
chr8 12464427

chr8 12465316
chr8 12465519
chr8 12465540
chr8 12465566
chr8 12465573
chr8 12465607
chr8 12465702
chr8 12465725
chr8 12466437
chr8 12466441
chr8 12466462
chr8 12466549
chr8 12466561
chr8 12466567
chr8 12466570
chr8 12466610
chr8 12466615
chr8 12466645
chr8 12467204
chr8 12467382
chr8 12467388
chr8 12467399
chr8 12467402
chr8 12467421
chr8 12467430
chr8 12467458
chr8 12467531
chr8 12468057
chr8 12468091
chr8 12468453
chr8 12522247
chr8 12522253
chr8 12522477
chr8 12522546
chr8 12522643
chr8 12523154
chr8 12523258
chr8 12523288
chr8 12523297
chr8 12600500
chr8 12600518
chr8 12600532
chr8 12600551
chr8 12600556
chr8 12600692
chr8 12600697
chr8 12600717
chr8 12600720
chr8 12600728
chr8 12600763
chr8 12600857
chr8 12600866
chr8 12600896
chr8 12600898

chr8 12600926
chr8 12600938
chr8 12601027
chr8 12601029
chr8 12601040
chr8 12601042
chr8 12601049
chr8 12601058
chr8 12601080
chr8 12601086
chr8 12601102
chr8 12601105
chr8 12601114
chr8 12601117
chr8 12601123
chr8 12601132
chr8 12601139
chr8 12601142
chr8 12601146
chr8 12601148
chr8 12601169
chr8 12601187
chr8 12601207
chr8 12601209
chr8 12601212
chr8 12601214
chr8 12601223
chr8 12601229
chr8 12601238
chr8 12601247
chr8 12601270
chr8 12601286
chr8 12601295
chr8 12601298
chr8 12601318
chr8 12601333
chr8 12601418
chr8 12601421
chr8 12601423
chr8 12601427
chr8 12601435
chr8 12601438
chr8 12601449
chr8 12601457
chr8 12601459
chr8 12601465
chr8 12601474
chr8 12601478
chr8 12601481
chr8 12601498
chr8 12601502
chr8 12601505
chr8 12601507
chr8 12601517

chr8 12601519
chr8 12601524
chr8 12601537
chr8 12601539
chr8 12601553
chr8 12601557
chr8 12601559
chr8 12601561
chr8 12601566
chr8 12601568
chr8 12606450
chr8 12607105
chr8 12607131
chr8 12607145
chr8 12607195
chr8 12607201
chr8 12607207
chr8 12607217
chr8 12607220
chr8 12607228
chr8 12607233
chr8 12607239
chr8 12607248
chr8 12607256
chr8 12607265
chr8 12607273
chr8 12607275
chr8 12607279
chr8 12607299
chr8 12607302
chr8 12607320
chr8 12607336
chr8 12607342
chr8 12607345
chr8 12607356
chr8 12607362
chr8 12607371
chr8 12607380
chr8 12608331
chr8 12608351
chr8 12608408
chr8 12608770
chr8 12608775
chr8 12608947
chr8 12726683
chr8 12726796
chr8 13042079
chr8 13042395
chr8 13042412
chr8 13042448
chr8 13042483
chr8 13042533
chr8 13042542
chr8 13168832

chr8 13168868
chr8 13168916
chr8 13383112
chr8 13383193
chr8 13383273
chr8 13565147
chr8 13662639
chr8 13887953
chr8 13888050
chr8 13888207
chr8 13888283
chr8 13938123
chr8 13938883
chr8 13938902
chr8 13939675
chr8 13939861
chr8 14124242
chr8 14233079
chr8 14326414
chr8 14326513
chr8 14326667
chr8 14327455
chr8 14477236
chr8 14477657
chr8 14478224
chr8 14478294
chr8 14478402
chr8 14478561
chr8 14478581
chr8 14565510
chr8 14565625
chr8 15187027
chr8 15187049
chr8 15187300
chr8 15187467
chr8 15187764
chr8 15187969
chr8 15188066
chr8 15234197
chr8 15234238
chr8 15238085
chr8 15342251
chr8 15342471
chr8 15525169
chr8 15615785
chr8 16101453
chr8 16101532
chr8 16101538
chr8 16252413
chr8 16457762
chr8 16458342
chr8 16458488
chr8 16458531
chr8 16458535

chr8 16458537
chr8 16458625
chr8 16546142
chr8 16546229
chr8 16546573
chr8 16547081
chr8 16548032
chr8 16548472
chr8 16548734
chr8 16604025
chr8 17016336
chr8 17016592
chr8 17016699
chr8 17016870
chr8 17022835
chr8 17027308
chr8 17027316
chr8 17155268
chr8 17155313
chr8 17155536
chr8 17155590
chr8 17155640
chr8 17155657
chr8 17155699
chr8 17155712
chr8 17155724
chr8 17155765
chr8 17246093
chr8 17246202
chr8 17246246
chr8 17246269
chr8 17246277
chr8 17246283
chr8 17247931
chr8 17247978
chr8 17429096
chr8 17429259
chr8 17429301
chr8 17429425
chr8 17531219
chr8 17531289
chr8 17531361
chr8 17690357
chr8 17708131
chr8 17708342
chr8 17791763
chr8 17791961
chr8 17799798
chr8 17800075
chr8 17800254
chr8 17800281
chr8 17800317
chr8 17800322
chr8 17800433

chr8 17800519
chr8 17800541
chr8 17800748
chr8 17924467
chr8 17926201
chr8 17926390
chr8 17926410
chr8 17926634
chr8 17926644
chr8 17926759
chr8 17928041
chr8 18196907
chr8 18196969
chr8 18196991
chr8 18196997
chr8 18197037
chr8 18197040
chr8 18197073
chr8 18197234
chr8 18197245
chr8 18197842
chr8 18209907
chr8 18209925
chr8 18210051
chr8 18210165
chr8 18747972
chr8 18747982
chr8 18769401
chr8 18769516
chr8 18957486
chr8 18957654
chr8 18957729
chr8 18957916
chr8 18957957
chr8 18958201
chr8 19013209
chr8 19013236
chr8 19013251
chr8 19209440
chr8 19209540
chr8 19209568
chr8 19209673
chr8 19209708
chr8 19267655
chr8 19267658
chr8 19268240
chr8 19696602
chr8 19696633
chr8 19696777
chr8 19696803
chr8 19818454
chr8 20436998
chr8 20437029
chr8 20437101

chr8 20437123
chr8 20437135
chr8 20493552
chr8 20493606
chr8 20516111
chr8 20516117
chr8 20830287
chr8 20830358
chr8 20830612
chr8 20831075
chr8 21103364
chr8 21103431
chr8 21484131
chr8 21484170
chr8 21484194
chr8 21484215
chr8 21484258
chr8 21484461
chr8 21484489
chr8 21672306
chr8 21672308
chr8 21672467
chr8 21672490
chr8 21672556
chr8 21828170
chr8 21858071
chr8 21858348
chr8 21858359
chr8 21858482
chr8 21858515
chr8 21911747
chr8 21911767
chr8 21911945
chr8 21911968
chr8 21912001
chr8 21912006
chr8 21912023
chr8 21912100
chr8 21913548
chr8 21913592
chr8 21913615
chr8 21913673
chr8 21913741
chr8 21913747
chr8 21913767
chr8 21913935
chr8 21914029
chr8 21914133
chr8 21914157
chr8 21949012
chr8 21949021
chr8 21949275
chr8 21949291
chr8 21959869

chr8 21959878
chr8 21959888
chr8 21959903
chr8 21959949
chr8 21960103
chr8 21960284
chr8 21960294
chr8 21960357
chr8 21960636
chr8 21960695
chr8 21960783
chr8 21960804
chr8 22442832
chr8 22442837
chr8 22442867
chr8 22442888
chr8 22442987
chr8 22444170
chr8 22444234
chr8 22540630
chr8 22540647
chr8 22540663
chr8 22540665
chr8 22540677
chr8 22540689
chr8 22540701
chr8 22540718
chr8 22540765
chr8 22540775
chr8 22540816
chr8 22540900
chr8 22541203
chr8 22541374
chr8 22541423
chr8 22579829
chr8 22579861
chr8 22579872
chr8 22579946
chr8 22579971
chr8 22579981
chr8 22580004
chr8 22580038
chr8 22580113
chr8 22580187
chr8 22580220
chr8 22580357
chr8 22580429
chr8 22598970
chr8 22598979
chr8 22598984
chr8 22598994
chr8 22599050
chr8 22599205
chr8 22599261

chr8 22599276
chr8 22639883
chr8 22639891
chr8 22695192
chr8 22695200
chr8 22695206
chr8 22695208
chr8 22695213
chr8 22695235
chr8 22695242
chr8 22695248
chr8 22705103
chr8 22705162
chr8 22705173
chr8 22705196
chr8 22705198
chr8 22705439
chr8 22705457
chr8 22980885
chr8 23018513
chr8 23018541
chr8 23018558
chr8 23018586
chr8 23018636
chr8 23018644
chr8 23018665
chr8 23018761
chr8 23018920
chr8 23018999
chr8 23019012
chr8 23019065
chr8 23019120
chr8 23019183
chr8 23019276
chr8 23019291
chr8 23019309
chr8 23069171
chr8 23069177
chr8 23069179
chr8 23103494
chr8 23103610
chr8 23103660
chr8 23103677
chr8 23103722
chr8 23103754
chr8 23103782
chr8 23163185
chr8 23163192
chr8 23163227
chr8 23163247
chr8 23163251
chr8 23163259
chr8 23163263
chr8 23163291

chr8 23163345
chr8 23163349
chr8 23163354
chr8 23163365
chr8 23163420
chr8 23163424
chr8 23163428
chr8 23218309
chr8 23218450
chr8 23218476
chr8 23218554
chr8 23218666
chr8 23218757
chr8 23218921
chr8 23219074
chr8 23219326
chr8 23219371
chr8 23219487
chr8 23219537
chr8 23219560
chr8 23224048
chr8 23224052
chr8 23224054
chr8 23224064
chr8 23224092
chr8 23224097
chr8 23224101
chr8 23224127
chr8 23224156
chr8 23224160
chr8 23224192
chr8 23224214
chr8 23224231
chr8 23224248
chr8 23224254
chr8 23224256
chr8 23224278
chr8 23224294
chr8 23224342
chr8 23230402
chr8 23230514
chr8 23230723
chr8 23230749
chr8 23230756
chr8 23230764
chr8 23230920
chr8 23231144
chr8 23242233
chr8 23243026
chr8 23243130
chr8 23243350
chr8 23243403
chr8 23245875
chr8 23246263

chr8 23287919
chr8 23287947
chr8 23287966
chr8 23287988
chr8 23288016
chr8 23288026
chr8 23288034
chr8 23288051
chr8 23288053
chr8 23288055
chr8 23288061
chr8 23288066
chr8 23288123
chr8 23288126
chr8 23288132
chr8 23288138
chr8 23288141
chr8 23288143
chr8 23288146
chr8 23288154
chr8 23288156
chr8 23288186
chr8 23313328
chr8 23313978
chr8 23314235
chr8 23314343
chr8 23314356
chr8 23314368
chr8 23314603
chr8 23314650
chr8 23314699
chr8 23314716
chr8 23456812
chr8 23711270
chr8 23711766
chr8 23711966
chr8 23712107
chr8 23712393
chr8 23712395
chr8 23712412
chr8 23712624
chr8 23712931
chr8 23712975
chr8 23745229
chr8 23747306
chr8 23747368
chr8 23747729
chr8 23747922
chr8 23747979
chr8 23747989
chr8 23853501
chr8 23853562
chr8 23853720
chr8 23853757

chr8 23853795
chr8 23853808
chr8 23853889
chr8 23854076
chr8 23854774
chr8 23854851
chr8 23855037
chr8 23855249
chr8 23855503
chr8 23855522
chr8 24229303
chr8 24229403
chr8 24229484
chr8 24229527
chr8 24229535
chr8 24229541
chr8 24229606
chr8 24229626
chr8 24229830
chr8 24299599
chr8 24299620
chr8 24299884
chr8 24452221
chr8 24452493
chr8 24452551
chr8 24452746
chr8 24915691
chr8 24915718
chr8 24915723
chr8 24915733
chr8 24915745
chr8 24915749
chr8 24915755
chr8 24915767
chr8 24915786
chr8 24916119
chr8 24954520
chr8 24955118
chr8 24955183
chr8 24955192
chr8 24955205
chr8 24955249
chr8 24955254
chr8 24955257
chr8 24955270
chr8 24955296
chr8 24955300
chr8 24955308
chr8 24958015
chr8 24958570
chr8 24958746
chr8 24958774
chr8 24958812
chr8 24958865

chr8 24958867
chr8 24959156
chr8 24977018
chr8 24977346
chr8 24977842
chr8 25220084
chr8 25220165
chr8 25220170
chr8 25220273
chr8 25220343
chr8 25345162
chr8 25345283
chr8 25345306
chr8 25345394
chr8 25345518
chr8 25347181
chr8 25759509
chr8 26019377
chr8 26038606
chr8 26038680
chr8 26038894
chr8 26039123
chr8 26039146
chr8 26039152
chr8 26039160
chr8 26052227
chr8 26052234
chr8 26052246
chr8 26052263
chr8 26052405
chr8 26053083
chr8 26053201
chr8 26053223
chr8 26053284
chr8 26053363
chr8 26053382
chr8 26053388
chr8 26053391
chr8 26053403
chr8 26088602
chr8 26088624
chr8 26088634
chr8 26088685
chr8 26088706
chr8 26088823
chr8 26088851
chr8 26088856
chr8 26088858
chr8 26088860
chr8 26088877
chr8 26088887
chr8 26088933
chr8 26089061
chr8 26089153

chr8 26089178
chr8 26291474
chr8 26291477
chr8 26291483
chr8 26291497
chr8 26291516
chr8 26291519
chr8 26291539
chr8 26291552
chr8 26291558
chr8 26291561
chr8 26291564
chr8 26291567
chr8 26291570
chr8 26291576
chr8 26291582
chr8 26291585
chr8 26291588
chr8 26291591
chr8 26291597
chr8 26291610
chr8 26291614
chr8 26291648
chr8 26291661
chr8 26291683
chr8 26291690
chr8 26291703
chr8 26291716
chr8 26291719
chr8 26291726
chr8 26291775
chr8 26291784
chr8 26291814
chr8 26291846
chr8 26291864
chr8 26291897
chr8 26291901
chr8 26291905
chr8 26291907
chr8 26291912
chr8 26291914
chr8 26291929
chr8 26291936
chr8 26291976
chr8 26291989
chr8 26292006
chr8 26293004
chr8 26293115
chr8 26293254
chr8 26293268
chr8 26327588
chr8 26327623
chr8 26327626
chr8 26327940

chr8 26327950
chr8 26374309
chr8 26374357
chr8 26449536
chr8 26449568
chr8 26449604
chr8 26449670
chr8 26449675
chr8 26449689
chr8 26449693
chr8 26449729
chr8 26449740
chr8 26449769
chr8 26449783
chr8 26449787
chr8 26449789
chr8 26449801
chr8 26449840
chr8 26449842
chr8 26576300
chr8 26576310
chr8 26576495
chr8 26576508
chr8 26576538
chr8 26576553
chr8 26576635
chr8 26579441
chr8 26579456
chr8 26579485
chr8 26579538
chr8 26579578
chr8 26579590
chr8 26579612
chr8 26579614
chr8 26863164
chr8 26863384
chr8 26863789
chr8 26863871
chr8 26864007
chr8 26864035
chr8 26864126
chr8 26867227
chr8 26867265
chr8 26867271
chr8 26867300
chr8 26867319
chr8 26867331
chr8 26867349
chr8 26867399
chr8 26867441
chr8 26867443
chr8 26867445
chr8 26867447
chr8 26867449

chr8 26867451
chr8 26867453
chr8 26867455
chr8 26867516
chr8 26868048
chr8 27008272
chr8 27008603
chr8 27206191
chr8 27274256
chr8 27274303
chr8 27274354
chr8 27274367
chr8 27274436
chr8 27274495
chr8 27274497
chr8 27274513
chr8 27274522
chr8 27274542
chr8 27274569
chr8 27274738
chr8 27274765
chr8 27274789
chr8 27274850
chr8 27274854
chr8 27274860
chr8 27274888
chr8 27274960
chr8 27274971
chr8 27274973
chr8 27274998
chr8 27275026
chr8 27275053
chr8 27312194
chr8 27315508
chr8 27315511
chr8 27315568
chr8 27325599
chr8 27325603
chr8 27325624
chr8 27325629
chr8 27325641
chr8 27325648
chr8 27325659
chr8 27325669
chr8 27325671
chr8 27325694
chr8 27325696
chr8 27325711
chr8 27325716
chr8 27325720
chr8 27325742
chr8 27325768
chr8 27325799
chr8 27325807

chr8 27325825
chr8 27325831
chr8 27325852
chr8 27325859
chr8 27325870
chr8 27325872
chr8 27325982
chr8 27326035
chr8 27326219
chr8 27326653
chr8 27326857
chr8 27326928
chr8 27327027
chr8 27327151
chr8 27327155
chr8 27327202
chr8 27327212
chr8 27362236
chr8 27362295
chr8 27366691
chr8 27378957
chr8 27378963
chr8 27378989
chr8 27378994
chr8 27379011
chr8 27379066
chr8 27379111
chr8 27380513
chr8 27380561
chr8 27380592
chr8 27380696
chr8 27380715
chr8 27380722
chr8 27380805
chr8 27380859
chr8 27380940
chr8 27597275
chr8 27597311
chr8 27597338
chr8 27597378
chr8 27597385
chr8 27597424
chr8 27597427
chr8 27614123
chr8 27614130
chr8 27614156
chr8 27614258
chr8 27614266
chr8 27614294
chr8 27614334
chr8 27614339
chr8 27614341
chr8 27614376
chr8 27614438

chr8 27614450
chr8 27614453
chr8 27614459
chr8 27614462
chr8 27727601
chr8 27774200
chr8 27774216
chr8 27775115
chr8 27775150
chr8 27775160
chr8 27775203
chr8 27775238
chr8 27775375
chr8 27864270
chr8 27864287
chr8 28078057
chr8 28078133
chr8 28078380
chr8 28078541
chr8 28078687
chr8 28078708
chr8 28093227
chr8 28093237
chr8 28093271
chr8 28093280
chr8 28093284
chr8 28093308
chr8 28093319
chr8 28093321
chr8 28093329
chr8 28093345
chr8 28093350
chr8 28093384
chr8 28093394
chr8 28093455
chr8 28093507
chr8 28093518
chr8 28093521
chr8 28093529
chr8 28093567
chr8 28093579
chr8 28093735
chr8 28093885
chr8 28094031
chr8 28341256
chr8 28341426
chr8 28348432
chr8 28363472
chr8 28363555
chr8 28363667
chr8 28363784
chr8 28363788
chr8 28363879
chr8 28363885

chr8 28490220
chr8 28490226
chr8 28490260
chr8 28490375
chr8 28490377
chr8 28490411
chr8 28490435
chr8 28490445
chr8 28490489
chr8 28490491
chr8 28490494
chr8 28490513
chr8 28490535
chr8 28490564
chr8 28490571
chr8 28490598
chr8 28490606
chr8 28490680
chr8 28594402
chr8 28594432
chr8 28594482
chr8 28657728
chr8 28657910
chr8 28658005
chr8 28658063
chr8 28658111
chr8 28658124
chr8 28890945
chr8 28890952
chr8 28890983
chr8 28891097
chr8 28891125
chr8 28891127
chr8 28891135
chr8 28891139
chr8 28891162
chr8 28891166
chr8 29085591
chr8 29229315
chr8 29229447
chr8 29229656
chr8 29250117
chr8 29250181
chr8 29250207
chr8 29250233
chr8 29263073
chr8 29263075
chr8 29263082
chr8 29263085
chr8 29263093
chr8 29263104
chr8 29263107
chr8 29263118
chr8 29263124

chr8 29263130
chr8 29263143
chr8 29263154
chr8 29263160
chr8 29263163
chr8 29263165
chr8 29263172
chr8 29263176
chr8 29263183
chr8 29263193
chr8 29263216
chr8 29263219
chr8 29263225
chr8 29263231
chr8 29263236
chr8 29263242
chr8 29263248
chr8 29263258
chr8 29263262
chr8 29263264
chr8 29263269
chr8 29263298
chr8 29289777
chr8 29289794
chr8 29314546
chr8 29314636
chr8 29314674
chr8 29314694
chr8 29314713
chr8 29314818
chr8 29314843
chr8 29314868
chr8 29314879
chr8 29314884
chr8 29314947
chr8 29314951
chr8 29314993
chr8 29314995
chr8 29315030
chr8 29315090
chr8 29315092
chr8 29315119
chr8 29315129
chr8 29315163
chr8 29315172
chr8 29315174
chr8 29315318
chr8 29320571
chr8 29320610
chr8 29320622
chr8 29320657
chr8 29320740
chr8 29320940
chr8 29321171

chr8 29321206
chr8 29321214
chr8 29490537
chr8 29490583
chr8 29490812
chr8 29490822
chr8 29490887
chr8 29499780
chr8 29499809
chr8 29500127
chr8 29500137
chr8 29500215
chr8 29500285
chr8 29500293
chr8 29500301
chr8 29529648
chr8 29529700
chr8 29529705
chr8 29529719
chr8 29529721
chr8 29529730
chr8 29529831
chr8 29529833
chr8 29529836
chr8 29529860
chr8 29529868
chr8 29529878
chr8 29530066
chr8 29530135
chr8 29656160
chr8 29656181
chr8 29656377
chr8 29665322
chr8 29665408
chr8 29665431
chr8 29665501
chr8 29681088
chr8 30080493
chr8 30080624
chr8 30080784
chr8 30080861
chr8 30080867
chr8 30080877
chr8 30080910
chr8 30080914
chr8 30080995
chr8 30081031
chr8 30081053
chr8 30081079
chr8 30081093
chr8 30081095
chr8 30081117
chr8 30081262
chr8 30081374

chr8 30081482
chr8 30081554
chr8 30081639
chr8 30081705
chr8 30081771
chr8 30081826
chr8 30081839
chr8 30081949
chr8 30081979
chr8 30082008
chr8 30082010
chr8 30082012
chr8 30082031
chr8 30091218
chr8 30093070
chr8 30093169
chr8 30093175
chr8 30093253
chr8 30093268
chr8 30093454
chr8 30093472
chr8 30093598
chr8 30096479
chr8 30096674
chr8 30096714
chr8 30097322
chr8 30155507
chr8 30155520
chr8 30155549
chr8 30155554
chr8 30155564
chr8 30155585
chr8 30155605
chr8 30155693
chr8 30155751
chr8 30155754
chr8 30155787
chr8 30155793
chr8 30155795
chr8 30155822
chr8 30155824
chr8 30155869
chr8 30155892
chr8 30155899
chr8 30155918
chr8 30155942
chr8 30155951
chr8 30155970
chr8 30155980
chr8 30157005
chr8 30157101
chr8 30363989
chr8 30385888
chr8 30385893

chr8 30385901
chr8 30386017
chr8 30386048
chr8 30386130
chr8 30386145
chr8 30386199
chr8 30386211
chr8 30386371
chr8 30386414
chr8 30386481
chr8 30386587
chr8 30386752
chr8 30387007
chr8 30600886
chr8 30666655
chr8 30722523
chr8 30723047
chr8 30723113
chr8 30727664
chr8 30727668
chr8 30727671
chr8 30727680
chr8 30727686
chr8 31277930
chr8 31288068
chr8 32027517
chr8 32027896
chr8 32028194
chr8 32028216
chr8 32221237
chr8 32221328
chr8 32221346
chr8 32221351
chr8 32221454
chr8 32221752
chr8 32221758
chr8 32534599
chr8 32534766
chr8 32545744
chr8 32545825
chr8 32546055
chr8 32546173
chr8 32546574
chr8 32554181
chr8 32554336
chr8 32554372
chr8 32646591
chr8 32646595
chr8 32646710
chr8 32646881
chr8 32646931
chr8 32646985
chr8 32647035
chr8 32647057

chr8 32647073
chr8 32647100
chr8 33117622
chr8 33233966
chr8 33234038
chr8 33472071
chr8 33472096
chr8 33472135
chr8 33472309
chr8 33472451
chr8 33472502
chr8 33472509
chr8 33472543
chr8 33472546
chr8 33472580
chr8 33472647
chr8 33472649
chr8 33472654
chr8 33472683
chr8 33472687
chr8 33472689
chr8 33472707
chr8 33472711
chr8 33472720
chr8 33472728
chr8 33472731
chr8 33472739
chr8 33472741
chr8 33472752
chr8 33472771
chr8 33472776
chr8 33472783
chr8 33472790
chr8 33472836
chr8 33472885
chr8 33472896
chr8 33472909
chr8 33472945
chr8 33472966
chr8 33472982
chr8 33472990
chr8 33473048
chr8 33473051
chr8 33473055
chr8 33473066
chr8 33473068
chr8 33473070
chr8 33473082
chr8 33473087
chr8 33473099
chr8 33473135
chr8 33473138
chr8 33473148
chr8 33473156

chr8 33473166
chr8 33473175
chr8 33473179
chr8 33473181
chr8 33473183
chr8 33473187
chr8 33513409
chr8 33513842
chr8 33513868
chr8 33513888
chr8 33513890
chr8 33513892
chr8 33513900
chr8 33513941
chr8 33513955
chr8 33513962
chr8 33513966
chr8 33513971
chr8 33513999
chr8 33514009
chr8 33514011
chr8 33514070
chr8 33514128
chr8 33514186
chr8 33514228
chr8 33554193
chr8 33554235
chr8 33554561
chr8 33554716
chr8 33554817
chr8 33554875
chr8 33554893
chr8 33599262
chr8 33599288
chr8 33599319
chr8 33599336
chr8 33625272
chr8 33625451
chr8 33968250
chr8 33968761
chr8 34016941
chr8 34017145
chr8 34017989
chr8 34018256
chr8 34213749
chr8 34213943
chr8 34214354
chr8 34216676
chr8 34216684
chr8 34216722
chr8 34216944
chr8 34217291
chr8 34234664
chr8 34235013

chr8 34235150
chr8 34235205
chr8 34532785
chr8 34532869
chr8 35093373
chr8 35093469
chr8 35174266
chr8 35174999
chr8 35175091
chr8 35175578
chr8 35175611
chr8 35175621
chr8 35175644
chr8 35202099
chr8 35216893
chr8 35255122
chr8 35255179
chr8 35255326
chr8 35415543
chr8 35529524
chr8 35529529
chr8 35657675
chr8 35657993
chr8 36123207
chr8 36142257
chr8 36142300
chr8 36142425
chr8 36502993
chr8 36503088
chr8 37088453
chr8 37088706
chr8 37229119
chr8 37574410
chr8 37574413
chr8 37574509
chr8 37574516
chr8 37574592
chr8 37705061
chr8 37705066
chr8 37737078
chr8 37737084
chr8 37737092
chr8 37737096
chr8 37737144
chr8 37737164
chr8 37737166
chr8 37737182
chr8 37737184
chr8 37737210
chr8 37737360
chr8 37737369
chr8 37737393
chr8 37737647
chr8 37737666

chr8 37737676
chr8 37737698
chr8 37737736
chr8 37737749
chr8 37737760
chr8 37738053
chr8 37738182
chr8 37779811
chr8 37779814
chr8 37881010
chr8 37881043
chr8 37881083
chr8 37881131
chr8 37881166
chr8 37881171
chr8 37881497
chr8 37881661
chr8 37881685
chr8 37881759
chr8 38177497
chr8 38177506
chr8 38177532
chr8 38177538
chr8 38177554
chr8 38177573
chr8 38177822
chr8 38177879
chr8 38177902
chr8 38177949
chr8 38184581
chr8 38184585
chr8 38184590
chr8 38184616
chr8 38184637
chr8 38269797
chr8 38359563
chr8 38359632
chr8 38359725
chr8 38359943
chr8 38360020
chr8 38360140
chr8 38362429
chr8 38362603
chr8 38362760
chr8 38362812
chr8 38362961
chr8 38363016
chr8 38380026
chr8 38381469
chr8 38381486
chr8 38381641
chr8 38384668
chr8 38384794
chr8 38384877

chr8 38385094
chr8 38405348
chr8 38405387
chr8 38405473
chr8 38467248
chr8 38527783
chr8 38527907
chr8 38543812
chr8 38543815
chr8 38543842
chr8 38768205
chr8 38768300
chr8 38768448
chr8 38768461
chr8 38768488
chr8 38768495
chr8 38768524
chr8 38768587
chr8 38768887
chr8 38769004
chr8 38769011
chr8 38770037
chr8 38770078
chr8 38770096
chr8 38770107
chr8 38770113
chr8 38770150
chr8 38770179
chr8 38770181
chr8 38770253
chr8 38770262
chr8 38770286
chr8 38770352
chr8 38770361
chr8 38770371
chr8 38770376
chr8 38770379
chr8 38770388
chr8 38770413
chr8 38770416
chr8 38770418
chr8 38770431
chr8 38770439
chr8 38770471
chr8 38770548
chr8 38770557
chr8 38770564
chr8 38770567
chr8 38770569
chr8 38770587
chr8 38900292
chr8 38900341
chr8 38900353
chr8 38900360

chr8 38900381
chr8 38900384
chr8 38900403
chr8 38900411
chr8 38900418
chr8 38900420
chr8 38900449
chr8 38900458
chr8 38900534
chr8 38900544
chr8 38900558
chr8 38900564
chr8 38900573
chr8 38900593
chr8 38900615
chr8 38900632
chr8 38900656
chr8 38900662
chr8 38900678
chr8 38900755
chr8 38900761
chr8 38900843
chr8 38900865
chr8 38900872
chr8 38900893
chr8 38900940
chr8 38900942
chr8 38900952
chr8 38902109
chr8 38902175
chr8 38902246
chr8 38902273
chr8 38902287
chr8 38902296
chr8 38902301
chr8 38902305
chr8 38902329
chr8 38966596
chr8 38966605
chr8 38966611
chr8 38966642
chr8 38966697
chr8 38966734
chr8 38966830
chr8 38966907
chr8 38966911
chr8 38966939
chr8 38996052
chr8 38996088
chr8 38996093
chr8 38996182
chr8 38996190
chr8 38996195
chr8 38996233

chr8 39340626
chr8 39430823
chr8 39471530
chr8 39471641
chr8 39471695
chr8 39471794
chr8 39471863
chr8 39853020
chr8 39853936
chr8 39929412
chr8 39929439
chr8 39939830
chr8 39939836
chr8 39939978
chr8 39940054
chr8 39940080
chr8 40346954
chr8 40346959
chr8 41264061
chr8 41264112
chr8 41264116
chr8 41264369
chr8 41361554
chr8 41361597
chr8 41361607
chr8 41361686
chr8 41361689
chr8 41361896
chr8 41490584
chr8 41490589
chr8 41490615
chr8 41490630
chr8 41490635
chr8 41490640
chr8 41490654
chr8 41490656
chr8 41490672
chr8 41490686
chr8 41490688
chr8 41490713
chr8 41490741
chr8 41490744
chr8 41490747
chr8 41490757
chr8 41490768
chr8 41490781
chr8 41490792
chr8 41490805
chr8 41490807
chr8 41490822
chr8 41490828
chr8 41490833
chr8 41490835
chr8 41529376

chr8 41529394
chr8 41529402
chr8 41529405
chr8 41529476
chr8 41529489
chr8 41529514
chr8 41578848
chr8 41578919
chr8 41578929
chr8 41792787
chr8 41792815
chr8 41956541
chr8 41957055
chr8 41957095
chr8 41957110
chr8 41957118
chr8 41957174
chr8 41957178
chr8 41957225
chr8 41957243
chr8 41957250
chr8 41957264
chr8 41957280
chr8 41957336
chr8 41957492
chr8 41957498
chr8 41963888
chr8 42050054
chr8 42050115
chr8 42050135
chr8 42050139
chr8 42050229
chr8 42050496
chr8 42140963
chr8 42140995
chr8 42140998
chr8 42141043
chr8 42141075
chr8 42141124
chr8 42141271
chr8 42141323
chr8 42141405
chr8 42141629
chr8 42141656
chr8 42141713
chr8 42141770
chr8 42141802
chr8 42141905
chr8 42141921
chr8 42141942
chr8 42154902
chr8 42154940
chr8 42154964
chr8 42154995

chr8 42155171
chr8 42155193
chr8 42155624
chr8 42155659
chr8 42155748
chr8 42155909
chr8 42156017
chr8 42156039
chr8 42177465
chr8 42177836
chr8 42177839
chr8 42177855
chr8 42177945
chr8 42177949
chr8 42337567
chr8 42337661
chr8 42337691
chr8 42337724
chr8 42337744
chr8 42337907
chr8 42338356
chr8 42338362
chr8 42338370
chr8 42338379
chr8 42338401
chr8 42338409
chr8 42338423
chr8 42338428
chr8 42338433
chr8 42338442
chr8 42338444
chr8 42338449
chr8 42338461
chr8 42338464
chr8 42338468
chr8 42338473
chr8 42338483
chr8 42338501
chr8 42338504
chr8 42338508
chr8 42338510
chr8 42338513
chr8 42338536
chr8 42338610
chr8 42338621
chr8 42338634
chr8 42338641
chr8 42338644
chr8 42338660
chr8 42338672
chr8 42338694
chr8 42338699
chr8 42338706
chr8 42338762

chr8 42355852
chr8 42355954
chr8 42392756
chr8 42392977
chr8 42393055
chr8 42537799
chr8 42537864
chr8 42537900
chr8 42537934
chr8 42537939
chr8 42538053
chr8 42538170
chr8 42538723
chr8 42767864
chr8 42767927
chr8 42768543
chr8 42768575
chr8 42768587
chr8 42768628
chr8 42768643
chr8 42768749
chr8 42790414
chr8 42794208
chr8 42842121
chr8 42842224
chr8 42842350
chr8 42842398
chr8 42842469
chr8 42842479
chr8 42891719
chr8 42893508
chr8 42893558
chr8 43055743
chr8 43055765
chr8 43055834
chr8 43239477
chr8 43239927
chr8 43239970
chr8 43240130
chr8 43240260
chr8 43278306
chr8 43278442
chr8 43278780
chr8 43283004
chr8 43283129
chr8 43293279
chr8 43315280
chr8 43315307
chr8 43689475
chr8 43689760
chr8 43690035
chr8 44366719
chr8 44727783
chr8 44727796

chr8 45180876
chr8 45180912
chr8 45180991
chr8 45181000
chr8 45643074
chr8 45644430
chr8 45644953
chr8 45644962
chr8 45645176
chr8 45645300
chr8 45875587
chr8 45875625
chr8 45875685
chr8 45955308
chr8 47245363
chr8 47245864
chr8 47261628
chr8 47261647
chr8 47261766
chr8 47514086
chr8 47514192
chr8 47514456
chr8 47515000
chr8 47573924
chr8 47600855
chr8 47600893
chr8 47600903
chr8 47680504
chr8 47680572
chr8 47687122
chr8 47687322
chr8 47687351
chr8 47687390
chr8 47945082
chr8 47945302
chr8 47959400
chr8 47959406
chr8 47959416
chr8 47959444
chr8 47959449
chr8 47959460
chr8 47959474
chr8 47959535
chr8 47959539
chr8 47959547
chr8 47959549
chr8 47959551
chr8 47959571
chr8 47959603
chr8 47959610
chr8 48008672
chr8 48008674
chr8 48259328
chr8 48259423

chr8 48273896
chr8 48273942
chr8 48274085
chr8 48514515
chr8 48514521
chr8 48514531
chr8 48514534
chr8 48514551
chr8 48514556
chr8 48514562
chr8 48514575
chr8 48514579
chr8 48514585
chr8 48514596
chr8 48514604
chr8 48514606
chr8 48514608
chr8 48514626
chr8 48514634
chr8 48514648
chr8 48514653
chr8 48514665
chr8 48514677
chr8 48514684
chr8 48514715
chr8 48514723
chr8 48514726
chr8 48514740
chr8 48514744
chr8 48514759
chr8 48514776
chr8 48514784
chr8 48514793
chr8 48514801
chr8 48514807
chr8 48514823
chr8 48514836
chr8 48514838
chr8 48514855
chr8 48514878
chr8 48514918
chr8 48515013
chr8 48672397
chr8 48922142
chr8 48922223
chr8 48979099
chr8 48979130
chr8 48979139
chr8 48979171
chr8 48979379
chr8 48979504
chr8 48979570
chr8 48995795
chr8 48995902

chr8 48996635
chr8 48996761
chr8 49023695
chr8 49023844
chr8 49024110
chr8 49226915
chr8 49227208
chr8 49227327
chr8 49227367
chr8 49227478
chr8 49307099
chr8 49307122
chr8 49307200
chr8 49307261
chr8 49307296
chr8 49307303
chr8 49307366
chr8 49307374
chr8 49307402
chr8 49307408
chr8 49307411
chr8 49307438
chr8 49307448
chr8 49307469
chr8 49307502
chr8 49307508
chr8 49307571
chr8 49307579
chr8 49307625
chr8 49307636
chr8 49742216
chr8 49882611
chr8 49909089
chr8 49909247
chr8 49909344
chr8 49909375
chr8 49909429
chr8 49981970
chr8 49982568
chr8 49983113
chr8 49983265
chr8 50399719
chr8 50621330
chr8 50621556
chr8 50622237
chr8 50720134
chr8 50720700
chr8 50720844
chr8 50786778
chr8 51182895
chr8 51273190
chr8 51273238
chr8 51307427
chr8 51308279

chr8 51308413
chr8 51308512
chr8 51308535
chr8 51309342
chr8 51309614
chr8 51309626
chr8 51316346
chr8 51897738
chr8 51897779
chr8 51897880
chr8 51898026
chr8 51898028
chr8 51898151
chr8 51898201
chr8 51899625
chr8 51899641
chr8 51899661
chr8 51899665
chr8 51899669
chr8 51899672
chr8 51899678
chr8 51899686
chr8 51899688
chr8 51899701
chr8 51899706
chr8 51899712
chr8 51899730
chr8 51899734
chr8 51899751
chr8 51899832
chr8 51899844
chr8 51899859
chr8 51899887
chr8 51899896
chr8 51899950
chr8 51899990
chr8 51899994
chr8 51900014
chr8 51900036
chr8 51900046
chr8 51900053
chr8 51900089
chr8 51900098
chr8 51900150
chr8 51900155
chr8 51962418
chr8 51963226
chr8 51963532
chr8 52008629
chr8 52008632
chr8 52008665
chr8 52008675
chr8 52008679
chr8 52008683

chr8 52009080
chr8 52111901
chr8 52158748
chr8 52158785
chr8 52169008
chr8 52169011
chr8 52169055
chr8 52169118
chr8 52169231
chr8 52169412
chr8 52177075
chr8 52177118
chr8 52177200
chr8 52177262
chr8 52177326
chr8 52407774
chr8 52407793
chr8 52407848
chr8 52407854
chr8 52408020
chr8 52408073
chr8 52466630
chr8 52466655
chr8 52466824
chr8 52467192
chr8 52467273
chr8 52467278
chr8 52467313
chr8 52467357
chr8 52467361
chr8 52467395
chr8 52467425
chr8 52467562
chr8 52467639
chr8 52467661
chr8 52467663
chr8 52467693
chr8 52494544
chr8 52496197
chr8 52496232
chr8 52496250
chr8 52496275
chr8 52496283
chr8 52496290
chr8 52496322
chr8 52496333
chr8 52496341
chr8 52496356
chr8 52496528
chr8 52496725
chr8 52496742
chr8 52712982
chr8 52713198
chr8 52713206

chr8 52713304
chr8 52713315
chr8 52713352
chr8 52737597
chr8 52737622
chr8 52938675
chr8 52938689
chr8 52938719
chr8 52938726
chr8 53165570
chr8 53618775
chr8 53619182
chr8 53662402
chr8 53662437
chr8 53662480
chr8 53662512
chr8 53662881
chr8 53767945
chr8 53768288
chr8 53875524
chr8 53875663
chr8 53876301
chr8 53876317
chr8 53876324
chr8 54101026
chr8 54101031
chr8 54101040
chr8 54101065
chr8 54101068
chr8 54101083
chr8 54135146
chr8 54135156
chr8 54135163
chr8 54135171
chr8 54135173
chr8 54135175
chr8 54135177
chr8 54135186
chr8 54135188
chr8 54135197
chr8 54135205
chr8 54135240
chr8 54135254
chr8 54136070
chr8 54169387
chr8 54369930
chr8 54455326
chr8 54455330
chr8 54455334
chr8 54455336
chr8 54455346
chr8 54455349
chr8 54554206
chr8 54554321

chr8 54554342
chr8 54554409
chr8 54554414
chr8 54554425
chr8 54554449
chr8 54554468
chr8 54554475
chr8 54554478
chr8 54554529
chr8 54554599
chr8 54554615
chr8 54554621
chr8 54554635
chr8 54622621
chr8 54622704
chr8 54933808
chr8 54933838
chr8 55521108
chr8 55521127
chr8 55521133
chr8 55521153
chr8 55521156
chr8 55521158
chr8 55521201
chr8 55521238
chr8 55521260
chr8 55521263
chr8 55521322
chr8 55521353
chr8 55521439
chr8 55521707
chr8 55771509
chr8 55771565
chr8 55771567
chr8 55771571
chr8 55771573
chr8 55771937
chr8 55772047
chr8 55772104
chr8 55772150
chr8 55773875
chr8 55773884
chr8 55773949
chr8 55774090
chr8 55774274
chr8 55774320
chr8 55774360
chr8 55774486
chr8 55774514
chr8 55774560
chr8 55843823
chr8 55843902
chr8 55843950
chr8 55860761

chr8 55860795
chr8 56073471
chr8 56073555
chr8 56073568
chr8 56073581
chr8 56073619
chr8 56073654
chr8 56073682
chr8 56073708
chr8 56073745
chr8 56073800
chr8 56073815
chr8 56096006
chr8 56096041
chr8 56096227
chr8 56112876
chr8 56112947
chr8 56154140
chr8 56209557
chr8 56209849
chr8 56209852
chr8 56209871
chr8 56209952
chr8 56210065
chr8 56210088
chr8 56210098
chr8 56210112
chr8 56210141
chr8 56210214
chr8 56210236
chr8 56210256
chr8 56210416
chr8 56210847
chr8 56212770
chr8 56270246
chr8 56270549
chr8 56270809
chr8 56376540
chr8 56376657
chr8 56416710
chr8 56416889
chr8 56417348
chr8 56417675
chr8 56479539
chr8 56480146
chr8 56480726
chr8 56992625
chr8 56992700
chr8 56992707
chr8 56992709
chr8 56992713
chr8 56992721
chr8 56992738
chr8 56992766

chr8 56992819
chr8 56992908
chr8 58145306
chr8 58145471
chr8 58145474
chr8 58145483
chr8 58145503
chr8 58145565
chr8 58247122
chr8 58321913
chr8 58322156
chr8 58322176
chr8 58322206
chr8 58323298
chr8 58411315
chr8 58545733
chr8 58545843
chr8 58554380
chr8 58554392
chr8 58554650
chr8 58554692
chr8 58554775
chr8 58657965
chr8 58658008
chr8 58658037
chr8 58658078
chr8 58658377
chr8 58658391
chr8 58658426
chr8 58658496
chr8 58658555
chr8 58658593
chr8 58658698
chr8 58658749
chr8 58658878
chr8 58658884
chr8 58658889
chr8 58658891
chr8 58658897
chr8 58808008
chr8 58808021
chr8 58808048
chr8 58808062
chr8 58808080
chr8 58808100
chr8 58808107
chr8 58808111
chr8 58808134
chr8 58808150
chr8 58808177
chr8 58808181
chr8 58973275
chr8 59000778
chr8 59001114

chr8 59001340
chr8 59106605
chr8 59106675
chr8 59106705
chr8 59106747
chr8 59106752
chr8 59106765
chr8 59106812
chr8 59107061
chr8 59119176
chr8 59119196
chr8 59119198
chr8 59119201
chr8 59119203
chr8 59119209
chr8 59119212
chr8 59119215
chr8 59119218
chr8 59119222
chr8 59119224
chr8 59119239
chr8 59119242
chr8 59119255
chr8 59119258
chr8 59119261
chr8 59119264
chr8 59119270
chr8 59119273
chr8 59119276
chr8 59119279
chr8 59119297
chr8 59119313
chr8 59119317
chr8 59119331
chr8 59119338
chr8 59119340
chr8 59119347
chr8 59119354
chr8 59119361
chr8 59119363
chr8 59119367
chr8 59119445
chr8 59119472
chr8 59119494
chr8 59119505
chr8 59119509
chr8 59119514
chr8 59119519
chr8 59119523
chr8 59119535
chr8 59119537
chr8 59119541
chr8 59119545
chr8 59119547

chr8 59119553
chr8 59142455
chr8 59142457
chr8 59142670
chr8 59170593
chr8 59170710
chr8 59171442
chr8 59171469
chr8 59171487
chr8 59171493
chr8 59171526
chr8 59171573
chr8 59171908
chr8 59188076
chr8 59188091
chr8 59188111
chr8 59188132
chr8 59188135
chr8 59188160
chr8 59274474
chr8 59274551
chr8 59282773
chr8 59282779
chr8 59282796
chr8 59282811
chr8 59282814
chr8 59282833
chr8 59548995
chr8 59549090
chr8 59600172
chr8 59763235
chr8 59763594
chr8 59763973
chr8 59764369
chr8 59764450
chr8 59764636
chr8 60154215
chr8 60238552
chr8 60238581
chr8 60238645
chr8 60280303
chr8 60280507
chr8 60280528
chr8 60280535
chr8 60281635
chr8 60281656
chr8 60281677
chr8 60516449
chr8 60516616
chr8 60516629
chr8 60516633
chr8 60516636
chr8 60653155
chr8 60653172

chr8 60653174
chr8 60653200
chr8 60653207
chr8 60653213
chr8 60653222
chr8 60653234
chr8 60653238
chr8 60653250
chr8 60653255
chr8 60653264
chr8 60653268
chr8 60653270
chr8 60653284
chr8 60653293
chr8 60653360
chr8 60653388
chr8 60653390
chr8 60653461
chr8 60653491
chr8 60653502
chr8 60653654
chr8 60654258
chr8 60654339
chr8 60660435
chr8 60677935
chr8 60677940
chr8 60793206
chr8 60793235
chr8 60793292
chr8 60793312
chr8 60816460
chr8 60903578
chr8 60903685
chr8 60903707
chr8 60903995
chr8 60904008
chr8 60904042
chr8 60904279
chr8 60904339
chr8 60904377
chr8 60904414
chr8 60904471
chr8 60909476
chr8 60909483
chr8 60909498
chr8 60909548
chr8 60911642
chr8 60911749
chr8 60911796
chr8 60911810
chr8 60911911
chr8 60911943
chr8 60912180
chr8 60912243

chr8 60912608
chr8 60912649
chr8 60927500
chr8 60929388
chr8 60929393
chr8 60929417
chr8 60929538
chr8 61004630
chr8 61004651
chr8 61004671
chr8 61004688
chr8 61004690
chr8 61076320
chr8 61076417
chr8 61096029
chr8 61096112
chr8 61096174
chr8 61096219
chr8 61096223
chr8 61096234
chr8 61096300
chr8 61096308
chr8 61710651
chr8 61711074
chr8 61711295
chr8 61713563
chr8 61713606
chr8 61713610
chr8 61713634
chr8 61713671
chr8 61713675
chr8 61713717
chr8 61713811
chr8 61713814
chr8 61713850
chr8 61713913
chr8 61713924
chr8 61713960
chr8 61714042
chr8 61714056
chr8 61749130
chr8 61810053
chr8 61810141
chr8 62250341
chr8 62250892
chr8 62396342
chr8 62396349
chr8 62727300
chr8 62949966
chr8 62950247
chr8 63111368
chr8 63111376
chr8 63111737
chr8 63167474

chr8 63167782
chr8 63200136
chr8 63200165
chr8 63200373
chr8 63200378
chr8 63226270
chr8 63226685
chr8 63226703
chr8 63391408
chr8 63491697
chr8 64082358
chr8 64100780
chr8 64101211
chr8 64101403
chr8 64101485
chr8 64144709
chr8 64145228
chr8 64367660
chr8 64367957
chr8 64374667
chr8 64374680
chr8 64374724
chr8 64374728
chr8 64374764
chr8 64374826
chr8 64374907
chr8 64375106
chr8 64375425
chr8 64375728
chr8 64375915
chr8 64375934
chr8 64376508
chr8 64376551
chr8 64376582
chr8 64376592
chr8 64376621
chr8 64376642
chr8 64376666
chr8 64376670
chr8 64376682
chr8 64376718
chr8 64475550
chr8 64476667
chr8 64575162
chr8 64575214
chr8 64575244
chr8 64575261
chr8 64575285
chr8 64575287
chr8 64575327
chr8 64575365
chr8 64582090
chr8 64582122
chr8 64582226

chr8 64582228
chr8 64582519
chr8 64582605
chr8 64583097
chr8 64583376
chr8 64583502
chr8 64584566
chr8 64584605
chr8 64584639
chr8 64584688
chr8 64584724
chr8 64584770
chr8 64584783
chr8 64623875
chr8 64695695
chr8 64695727
chr8 64955025
chr8 65017590
chr8 65017599
chr8 65192362
chr8 65192807
chr8 65562219
chr8 65562276
chr8 65633546
chr8 65633567
chr8 65633581
chr8 65633663
chr8 65633678
chr8 65633831
chr8 65837875
chr8 65837880
chr8 65837964
chr8 65838361
chr8 65838610
chr8 65838884
chr8 65838895
chr8 65838921
chr8 65838947
chr8 65839308
chr8 65842714
chr8 65842733
chr8 65842871
chr8 65842879
chr8 65842885
chr8 65842968
chr8 65931578
chr8 65931799
chr8 65932343
chr8 65932402
chr8 65945454
chr8 65945462
chr8 65945505
chr8 65945926
chr8 65951788

chr8 65951911
chr8 65951956
chr8 65952008
chr8 66114329
chr8 66114351
chr8 66114399
chr8 66176216
chr8 66176476
chr8 66176606
chr8 66176660
chr8 66176671
chr8 66176682
chr8 66176707
chr8 66176809
chr8 66176878
chr8 66176883
chr8 66176946
chr8 66176972
chr8 66177015
chr8 66177020
chr8 66177034
chr8 66177051
chr8 66177054
chr8 66430203
chr8 66430581
chr8 66430627
chr8 66430635
chr8 66431901
chr8 66431955
chr8 66432066
chr8 66432263
chr8 66432272
chr8 66432290
chr8 66432314
chr8 66432318
chr8 66432321
chr8 66432336
chr8 66432353
chr8 66533456
chr8 66533591
chr8 66533617
chr8 66533728
chr8 66533815
chr8 66535481
chr8 66535492
chr8 66535635
chr8 66535685
chr8 66536001
chr8 66536043
chr8 66609337
chr8 66609365
chr8 66609409
chr8 66609881
chr8 66610138

chr8 66610156
chr8 66610236
chr8 66610320
chr8 66610454
chr8 66610508
chr8 66611950
chr8 66612339
chr8 66612373
chr8 66612386
chr8 66612388
chr8 66612411
chr8 66612429
chr8 66612433
chr8 66612456
chr8 66612502
chr8 66613721
chr8 66613726
chr8 66613729
chr8 66614052
chr8 66614087
chr8 66614089
chr8 66614117
chr8 66614197
chr8 66614212
chr8 66614248
chr8 66663954
chr8 66666834
chr8 66666944
chr8 66666947
chr8 66666972
chr8 66666979
chr8 66666984
chr8 66666986
chr8 66666995
chr8 66667033
chr8 66667038
chr8 66667059
chr8 66667101
chr8 66667107
chr8 66667113
chr8 66667116
chr8 66667149
chr8 66667163
chr8 66667241
chr8 66667258
chr8 66667264
chr8 66667273
chr8 66667361
chr8 66667373
chr8 66667381
chr8 66667896
chr8 66674713
chr8 66674727
chr8 66674818

chr8 66674981
chr8 66675023
chr8 66688748
chr8 66870552
chr8 66923116
chr8 66923139
chr8 66923183
chr8 66923213
chr8 66923385
chr8 66923618
chr8 66923624
chr8 66923634
chr8 66923662
chr8 66923667
chr8 66923780
chr8 66923802
chr8 66923842
chr8 66923844
chr8 66923866
chr8 66924060
chr8 66924233
chr8 66924264
chr8 66924307
chr8 66924348
chr8 66924352
chr8 66924354
chr8 66924364
chr8 66924397
chr8 66924408
chr8 66924463
chr8 66924491
chr8 66924495
chr8 66924507
chr8 66924527
chr8 66924559
chr8 66924589
chr8 66924591
chr8 66924613
chr8 66924697
chr8 66924754
chr8 66924797
chr8 66924832
chr8 66924834
chr8 66924841
chr8 66924878
chr8 66925194
chr8 66925203
chr8 66925246
chr8 66925248
chr8 66925254
chr8 66925278
chr8 66925296
chr8 66925302
chr8 66925319

chr8 66925328
chr8 66925332
chr8 66925337
chr8 66925342
chr8 66925346
chr8 66925364
chr8 66925366
chr8 66925384
chr8 66925394
chr8 66925399
chr8 67028405
chr8 67062331
chr8 67062336
chr8 67062354
chr8 67062396
chr8 67062404
chr8 67062411
chr8 67062555
chr8 67062597
chr8 67062627
chr8 67062639
chr8 67062645
chr8 67062687
chr8 67062692
chr8 67062728
chr8 67062752
chr8 67062860
chr8 67342522
chr8 67342551
chr8 67342612
chr8 67342618
chr8 67342634
chr8 67342714
chr8 67342721
chr8 67342747
chr8 67342918
chr8 67342920
chr8 67342936
chr8 67342938
chr8 67342945
chr8 67344539
chr8 67344674
chr8 67344705
chr8 67344787
chr8 67353621
chr8 67353634
chr8 67602736
chr8 67602741
chr8 67602745
chr8 67602839
chr8 67603000
chr8 67643124
chr8 67643275
chr8 67952952

chr8 67952999
chr8 67953011
chr8 67953037
chr8 67953095
chr8 67953142
chr8 67953179
chr8 67953186
chr8 67953191
chr8 67953265
chr8 67953273
chr8 67953302
chr8 67953338
chr8 67953358
chr8 67953721
chr8 68321936
chr8 69127490
chr8 69127621
chr8 69128183
chr8 69252500
chr8 69420576
chr8 69466119
chr8 69466121
chr8 69466145
chr8 69466239
chr8 69466684
chr8 69467183
chr8 69467185
chr8 69467207
chr8 69467393
chr8 69467523
chr8 69643440
chr8 69643925
chr8 69764572
chr8 69858856
chr8 70136695
chr8 70136928
chr8 70152559
chr8 70152595
chr8 70152735
chr8 70152771
chr8 70213920
chr8 70214046
chr8 70214052
chr8 70214138
chr8 70214302
chr8 70401577
chr8 70401678
chr8 70401759
chr8 70401775
chr8 70401814
chr8 70401818
chr8 70668564
chr8 70668582
chr8 70825822

chr8 71361104
chr8 71361262
chr8 71361429
chr8 71361566
chr8 71361649
chr8 71361689
chr8 71361821
chr8 71361940
chr8 71361968
chr8 71361979
chr8 71361998
chr8 71362021
chr8 71362028
chr8 71362052
chr8 71362105
chr8 71362146
chr8 71362192
chr8 71362311
chr8 71362313
chr8 71362320
chr8 71362363
chr8 71362378
chr8 71362380
chr8 71548739
chr8 71552660
chr8 71552895
chr8 71553812
chr8 71553818
chr8 71553942
chr8 71553947
chr8 71553957
chr8 71553996
chr8 71554007
chr8 71554012
chr8 71554049
chr8 71554066
chr8 71554086
chr8 71554088
chr8 71554129
chr8 71554301
chr8 71554398
chr8 71554401
chr8 71554420
chr8 71554443
chr8 71554461
chr8 71554529
chr8 71554531
chr8 71554672
chr8 71554678
chr8 71554714
chr8 71554765
chr8 71554845
chr8 71554869
chr8 71555055

chr8 71555294
chr8 71555300
chr8 71555518
chr8 71555545
chr8 71555595
chr8 71555622
chr8 71555733
chr8 71559177
chr8 71559206
chr8 71559314
chr8 71559829
chr8 71560126
chr8 71560459
chr8 71560808
chr8 71561101
chr8 71561110
chr8 71561243
chr8 71561248
chr8 71561365
chr8 71561425
chr8 71561786
chr8 71561807
chr8 71804747
chr8 71804785
chr8 71804788
chr8 71804790
chr8 71804819
chr8 71804828
chr8 71804849
chr8 71842563
chr8 71842584
chr8 71842602
chr8 71842619
chr8 71842628
chr8 71842657
chr8 71842666
chr8 71842693
chr8 71842718
chr8 71842727
chr8 71842735
chr8 71842765
chr8 71842785
chr8 71842799
chr8 71842815
chr8 71842817
chr8 71842927
chr8 71842943
chr8 71842949
chr8 71842970
chr8 71843013
chr8 71843049
chr8 72462441
chr8 72878810
chr8 72878866

chr8 72879967
chr8 72880294
chr8 72880562
chr8 72880612
chr8 72880634
chr8 72880641
chr8 72880873
chr8 72880920
chr8 73008949
chr8 73008988
chr8 73008991
chr8 73009003
chr8 73009048
chr8 73009075
chr8 73009077
chr8 73009090
chr8 73009096
chr8 73009102
chr8 73009111
chr8 73009340
chr8 73009413
chr8 73009513
chr8 73009575
chr8 73293105
chr8 73293254
chr8 73293289
chr8 73293311
chr8 73293320
chr8 73293335
chr8 73293347
chr8 73293358
chr8 73293361
chr8 73293384
chr8 73293392
chr8 73346760
chr8 73346912
chr8 73346971
chr8 73347024
chr8 73347042
chr8 73347093
chr8 73653648
chr8 73747853
chr8 73747890
chr8 73747899
chr8 73747958
chr8 73813815
chr8 73813838
chr8 73813934
chr8 73877793
chr8 73877878
chr8 73877919
chr8 73879215
chr8 73879299
chr8 73879339

chr8 73879384
chr8 73879408
chr8 73879428
chr8 73895134
chr8 73895376
chr8 73895489
chr8 73895546
chr8 73895839
chr8 73895906
chr8 73896011
chr8 73896032
chr8 73896049
chr8 73896066
chr8 73896088
chr8 73896112
chr8 73896115
chr8 73896120
chr8 73896124
chr8 73896154
chr8 73896212
chr8 73896217
chr8 73896245
chr8 73896255
chr8 73972043
chr8 73972051
chr8 73972057
chr8 73991160
chr8 73991526
chr8 73991566
chr8 73991614
chr8 73991621
chr8 73991646
chr8 74349628
chr8 74349798
chr8 74350033
chr8 74350037
chr8 74350129
chr8 74350234
chr8 74350287
chr8 74350296
chr8 74350298
chr8 74350304
chr8 74350316
chr8 74350319
chr8 74350322
chr8 74350340
chr8 74350345
chr8 74350347
chr8 74350349
chr8 74350360
chr8 74350381
chr8 74350394
chr8 74350424
chr8 74554016

chr8 74596397
chr8 74596623
chr8 74596837
chr8 74597065
chr8 74965424
chr8 74965591
chr8 74984814
chr8 74984816
chr8 74984820
chr8 74984872
chr8 74984876
chr8 74984880
chr8 74984944
chr8 74984952
chr8 74985040
chr8 74985042
chr8 74985062
chr8 74985072
chr8 74985075
chr8 74985088
chr8 74985141
chr8 74985143
chr8 74985145
chr8 74985216
chr8 74985236
chr8 74985356
chr8 74985558
chr8 75073207
chr8 75236516
chr8 75236640
chr8 75236724
chr8 75236743
chr8 75402795
chr8 75402865
chr8 75402969
chr8 75403102
chr8 75403195
chr8 75403329
chr8 75405338
chr8 75405418
chr8 75405425
chr8 75405447
chr8 75405506
chr8 75405556
chr8 75405633
chr8 75405636
chr8 75405839
chr8 75405857
chr8 75405928
chr8 75405984
chr8 75484062
chr8 75484131
chr8 75484281
chr8 75484947

chr8 75531935
chr8 75532292
chr8 75532424
chr8 75533370
chr8 75534213
chr8 75709128
chr8 75713118
chr8 75713517
chr8 75714036
chr8 75714060
chr8 75714121
chr8 75728143
chr8 75728149
chr8 75852080
chr8 75852472
chr8 76311515
chr8 76311727
chr8 76311953
chr8 76312639
chr8 76334620
chr8 76373912
chr8 76374276
chr8 76374667
chr8 76598806
chr8 76599028
chr8 76599202
chr8 76599423
chr8 76599490
chr8 76599657
chr8 76599818
chr8 76673211
chr8 76673213
chr8 76673226
chr8 76673240
chr8 76673359
chr8 76673384
chr8 76673391
chr8 76673406
chr8 76673426
chr8 76673449
chr8 76673456
chr8 76673483
chr8 76673493
chr8 76673498
chr8 76673507
chr8 76673513
chr8 76673554
chr8 76673591
chr8 76673692
chr8 76673881
chr8 76673883
chr8 76674249
chr8 76674257
chr8 76674368

chr8 76674411
chr8 76674442
chr8 76674479
chr8 76674619
chr8 76674622
chr8 76674634
chr8 76674671
chr8 76674698
chr8 76674767
chr8 76674878
chr8 76674911
chr8 76675008
chr8 76675020
chr8 76675024
chr8 76675041
chr8 76675093
chr8 76675168
chr8 76675340
chr8 76675369
chr8 76675432
chr8 76675973
chr8 76676000
chr8 76676822
chr8 76676885
chr8 76677219
chr8 76677335
chr8 76677421
chr8 76677442
chr8 76677471
chr8 76677477
chr8 76677479
chr8 76677626
chr8 76677645
chr8 76677670
chr8 76677673
chr8 76677785
chr8 76677805
chr8 76677879
chr8 76677884
chr8 76677933
chr8 76677938
chr8 76677946
chr8 76677949
chr8 76677952
chr8 76677958
chr8 76677960
chr8 76678317
chr8 76678358
chr8 76678405
chr8 76678455
chr8 76678470
chr8 76678548
chr8 76678609
chr8 76678642

chr8 76678702
chr8 76678784
chr8 76678897
chr8 76678907
chr8 76683507
chr8 76683758
chr8 76683834
chr8 76683841
chr8 76683852
chr8 76683887
chr8 76683896
chr8 76683898
chr8 76683909
chr8 76683916
chr8 76683920
chr8 76683953
chr8 76683970
chr8 76684147
chr8 76685937
chr8 76999443
chr8 76999464
chr8 77049633
chr8 77050042
chr8 77050142
chr8 77050489
chr8 77622289
chr8 78083024
chr8 78347677
chr8 78347817
chr8 78348133
chr8 78348450
chr8 78516249
chr8 78516255
chr8 78516261
chr8 78516270
chr8 78516461
chr8 78516479
chr8 78516621
chr8 78666308
chr8 78666318
chr8 78666329
chr8 78666334
chr8 78666346
chr8 78666362
chr8 78666371
chr8 78666392
chr8 78666396
chr8 78666423
chr8 78666431
chr8 78694369
chr8 78719432
chr8 78762707
chr8 78804983
chr8 78805470

chr8 78840712
chr8 78840838
chr8 78840857
chr8 78858298
chr8 78858552
chr8 78860047
chr8 78934824
chr8 78957219
chr8 78984651
chr8 78985824
chr8 78986314
chr8 79052057
chr8 79052151
chr8 79165922
chr8 79166054
chr8 79539183
chr8 79765109
chr8 79765199
chr8 79765206
chr8 79765248
chr8 79765298
chr8 79765317
chr8 79783758
chr8 79783760
chr8 79783785
chr8 79783805
chr8 79783807
chr8 79783814
chr8 79783816
chr8 79783819
chr8 79783824
chr8 79783842
chr8 79783847
chr8 79783867
chr8 79783876
chr8 79783878
chr8 79783933
chr8 79783947
chr8 79783979
chr8 79783981
chr8 79783995
chr8 79784014
chr8 79805647
chr8 79828448
chr8 79828481
chr8 79828590
chr8 79828619
chr8 79828631
chr8 79828644
chr8 79828811
chr8 79828815
chr8 80016756
chr8 80017188
chr8 80017330

chr8 80085191
chr8 80085260
chr8 80085283
chr8 80085289
chr8 80085308
chr8 80230837
chr8 80346992
chr8 80347023
chr8 80347184
chr8 80347397
chr8 80347552
chr8 80347607
chr8 80347621
chr8 80347883
chr8 80348097
chr8 80377618
chr8 80377772
chr8 80378090
chr8 80484988
chr8 80485032
chr8 80485058
chr8 80485238
chr8 80485313
chr8 80485360
chr8 80485366
chr8 80485375
chr8 80485379
chr8 80485399
chr8 80487697
chr8 80487699
chr8 80487708
chr8 80487743
chr8 80487752
chr8 80487767
chr8 80487776
chr8 80487863
chr8 80487903
chr8 80487921
chr8 80487934
chr8 80488017
chr8 80488022
chr8 80488113
chr8 80488168
chr8 80488184
chr8 80488195
chr8 80488257
chr8 80488265
chr8 80488399
chr8 80534232
chr8 80534673
chr8 80578313
chr8 80578315
chr8 80578322
chr8 80578331

chr8 80578338
chr8 80578340
chr8 80578374
chr8 80578392
chr8 80578400
chr8 80578424
chr8 80578428
chr8 80578434
chr8 80578436
chr8 80578448
chr8 80578458
chr8 80578466
chr8 80578471
chr8 80578473
chr8 80578514
chr8 80578517
chr8 81106700
chr8 81106751
chr8 81106824
chr8 81106858
chr8 81106890
chr8 81108948
chr8 81108975
chr8 81109015
chr8 81109370
chr8 81109520
chr8 81109985
chr8 81110339
chr8 81110466
chr8 81110656
chr8 81111163
chr8 81111170
chr8 81111172
chr8 81111203
chr8 81111218
chr8 81111332
chr8 81111341
chr8 81131331
chr8 81131522
chr8 81132355
chr8 81132359
chr8 81132745
chr8 81132755
chr8 81132761
chr8 81138854
chr8 81138905
chr8 81281589
chr8 81281646
chr8 81281651
chr8 81329426
chr8 81329698
chr8 81329922
chr8 81329951
chr8 81427864

chr8 81427920
chr8 81427973
chr8 81428159
chr8 81428181
chr8 81428200
chr8 81428231
chr8 81428256
chr8 81428863
chr8 81492647
chr8 81492665
chr8 81492718
chr8 81640523
chr8 81640746
chr8 81641281
chr8 81641300
chr8 81641493
chr8 81685749
chr8 81685833
chr8 81685877
chr8 81780343
chr8 81780499
chr8 81780604
chr8 81780610
chr8 81780660
chr8 81780664
chr8 81780680
chr8 81780697
chr8 81780705
chr8 81780712
chr8 81780732
chr8 81780745
chr8 81780783
chr8 81780817
chr8 81842317
chr8 81842326
chr8 81842461
chr8 81842539
chr8 81842543
chr8 81842555
chr8 81842561
chr8 81842581
chr8 81842601
chr8 81842615
chr8 81842624
chr8 81842628
chr8 81842635
chr8 81842643
chr8 81842657
chr8 81842665
chr8 81842676
chr8 81842707
chr8 82188764
chr8 82188829
chr8 82188839

chr8 82188957
chr8 82189036
chr8 82189686
chr8 82189888
chr8 82189934
chr8 82190030
chr8 82190192
chr8 82190537
chr8 82190574
chr8 82190604
chr8 82190656
chr8 82191117
chr8 82191195
chr8 82191234
chr8 82191361
chr8 82191371
chr8 82191373
chr8 82191403
chr8 82191422
chr8 82191430
chr8 82191436
chr8 82191469
chr8 82191500
chr8 82191526
chr8 82256988
chr8 82257432
chr8 82257486
chr8 82257843
chr8 82257882
chr8 82878048
chr8 82878176
chr8 82878273
chr8 83137466
chr8 83138369
chr8 83138864
chr8 83139574
chr8 83139584
chr8 83139586
chr8 83139653
chr8 83139743
chr8 83139747
chr8 83139757
chr8 83139804
chr8 83139845
chr8 83253496
chr8 83288527
chr8 83288562
chr8 83288576
chr8 83288673
chr8 83288869
chr8 83289174
chr8 83289433
chr8 83289499
chr8 83305016

chr8 83305187
chr8 83374134
chr8 83400087
chr8 83400098
chr8 83400116
chr8 83400805
chr8 83446204
chr8 83446212
chr8 83449863
chr8 83450561
chr8 83450686
chr8 83450775
chr8 84003503
chr8 84003833
chr8 84003979
chr8 84004072
chr8 84004107
chr8 84022070
chr8 84181036
chr8 84181082
chr8 84181488
chr8 84181546
chr8 84181597
chr8 84181784
chr8 84181889
chr8 84181929
chr8 84181959
chr8 84182019
chr8 84182117
chr8 84182170
chr8 84182198
chr8 84465281
chr8 84465459
chr8 84466311
chr8 84466751
chr8 84587253
chr8 84776549
chr8 84776975
chr8 84777025
chr8 84976691
chr8 85106510
chr8 85106666
chr8 85106701
chr8 85106728
chr8 85107031
chr8 85107035
chr8 85107071
chr8 85107076
chr8 85107093
chr8 85107115
chr8 85107132
chr8 85107138
chr8 85107146
chr8 85107158

chr8 85107165
chr8 85107184
chr8 85107189
chr8 85107194
chr8 85107202
chr8 85107207
chr8 85107215
chr8 85107217
chr8 85107223
chr8 85107245
chr8 85107254
chr8 85107264
chr8 85107276
chr8 85107283
chr8 85107286
chr8 85107303
chr8 85107306
chr8 85107309
chr8 85107312
chr8 85107321
chr8 85107330
chr8 85107343
chr8 85107349
chr8 85221059
chr8 85221062
chr8 85221192
chr8 85221226
chr8 85245700
chr8 85245706
chr8 85245717
chr8 85245720
chr8 85245723
chr8 85245728
chr8 85245730
chr8 85245736
chr8 85245756
chr8 85245768
chr8 85245785
chr8 85245820
chr8 85245833
chr8 85245864
chr8 85245871
chr8 85245902
chr8 85245909
chr8 85245978
chr8 85246052
chr8 85246076
chr8 85246128
chr8 85246246
chr8 85462908
chr8 85462945
chr8 85463060
chr8 85463167
chr8 85463181

chr8 85514315
chr8 85728289
chr8 85728298
chr8 85731374
chr8 85731383
chr8 85743042
chr8 85743219
chr8 85743281
chr8 85743519
chr8 85743556
chr8 85743815
chr8 85743842
chr8 85747244
chr8 85747314
chr8 85749060
chr8 85749078
chr8 85749136
chr8 85750449
chr8 85750458
chr8 85753534
chr8 85753543
chr8 85756853
chr8 86344068
chr8 86344338
chr8 86344356
chr8 86344409
chr8 86344500
chr8 86344698
chr8 86344713
chr8 86507684
chr8 86507951
chr8 86507994
chr8 86508010
chr8 86508026
chr8 86508229
chr8 86508242
chr8 86508265
chr8 86508267
chr8 86508345
chr8 86508349
chr8 86508369
chr8 86508382
chr8 86508406
chr8 86508408
chr8 86508414
chr8 86508440
chr8 86508468
chr8 86508483
chr8 86508494
chr8 86508500
chr8 86508502
chr8 86508508
chr8 86508527
chr8 86508529

chr8 86508537
chr8 86508546
chr8 86508556
chr8 86508569
chr8 86508577
chr8 86508580
chr8 86508595
chr8 86508604
chr8 86508615
chr8 86508634
chr8 86508665
chr8 86508671
chr8 86508675
chr8 86508701
chr8 86508711
chr8 86508713
chr8 86508717
chr8 86508721
chr8 86508744
chr8 86523344
chr8 86523434
chr8 86601700
chr8 86758726
chr8 86758853
chr8 87146425
chr8 87147975
chr8 87329126
chr8 87566970
chr8 87567222
chr8 87845845
chr8 87852482
chr8 87969987
chr8 88324807
chr8 88325618
chr8 88326087
chr8 88326168
chr8 88326209
chr8 88326323
chr8 88326375
chr8 88326385
chr8 88326438
chr8 88326455
chr8 88326736
chr8 88326782
chr8 88326829
chr8 88326851
chr8 88326853
chr8 88326861
chr8 88326876
chr8 88326885
chr8 88326903
chr8 88326909
chr8 88328441
chr8 88328460

chr8 88328471
chr8 88328473
chr8 88328487
chr8 88328521
chr8 88328614
chr8 88328636
chr8 88328654
chr8 88328661
chr8 88328665
chr8 88328680
chr8 88328715
chr8 88328731
chr8 88328735
chr8 88328739
chr8 88328741
chr8 88328794
chr8 88328874
chr8 88328953
chr8 88328970
chr8 88329012
chr8 88329476
chr8 88329710
chr8 88388555
chr8 88434911
chr8 88683952
chr8 88684037
chr8 88689420
chr8 88690127
chr8 88690242
chr8 88690254
chr8 88690259
chr8 88882989
chr8 88883039
chr8 88883379
chr8 89469877
chr8 89469987
chr8 89689172
chr8 89903020
chr8 89903032
chr8 89903048
chr8 89903058
chr8 89903060
chr8 89903062
chr8 89903069
chr8 89903077
chr8 89903083
chr8 89903092
chr8 89903102
chr8 89903108
chr8 89903111
chr8 89903159
chr8 89903305
chr8 89903336
chr8 89982765

chr8 89982773
chr8 89982820
chr8 89982829
chr8 89982912
chr8 89983380
chr8 89983407
chr8 89983426
chr8 90000477
chr8 90000779
chr8 90002373
chr8 90005638
chr8 90005814
chr8 90005900
chr8 90005936
chr8 90006052
chr8 90080949
chr8 90080977
chr8 90081219
chr8 90081624
chr8 90081871
chr8 90081901
chr8 90081914
chr8 90081934
chr8 90081941
chr8 90083037
chr8 90083227
chr8 90345588
chr8 90345760
chr8 90644036
chr8 90644240
chr8 90644516
chr8 90644524
chr8 90644701
chr8 90895930
chr8 90984402
chr8 90984453
chr8 90984494
chr8 90984627
chr8 90984693
chr8 90984736
chr8 90984754
chr8 90984773
chr8 90984834
chr8 90984849
chr8 90984865
chr8 90984868
chr8 90984907
chr8 90984910
chr8 90984916
chr8 90984977
chr8 91039743
chr8 91039799
chr8 91039803
chr8 91039819

chr8 91040243
chr8 91041007
chr8 91041037
chr8 91041090
chr8 91041092
chr8 91068739
chr8 91068826
chr8 91069276
chr8 91069295
chr8 91069302
chr8 91069381
chr8 91070732
chr8 91070756
chr8 91278018
chr8 91278222
chr8 91278299
chr8 91530705
chr8 91551249
chr8 91551338
chr8 91551592
chr8 91551693
chr8 91551772
chr8 91601966
chr8 91602079
chr8 92094588
chr8 92094607
chr8 92094706
chr8 92106454
chr8 92106486
chr8 92178555
chr8 92367496
chr8 92367708
chr8 92373101
chr8 92808141
chr8 92808165
chr8 92808638
chr8 92808984
chr8 92809207
chr8 92810434
chr8 92811262
chr8 92964832
chr8 92965124
chr8 92965318
chr8 92965320
chr8 92965376
chr8 92965398
chr8 92965460
chr8 92965532
chr8 92965566
chr8 92965570
chr8 92965572
chr8 92965580
chr8 93589475
chr8 93589489

chr8 93739881
chr8 93739886
chr8 93740046
chr8 93740055
chr8 93740097
chr8 93740149
chr8 93740244
chr8 93740284
chr8 93740295
chr8 93740304
chr8 93754575
chr8 93754620
chr8 93754646
chr8 93755008
chr8 93803289
chr8 93803339
chr8 93803606
chr8 93803652
chr8 93803660
chr8 93915956
chr8 93991283
chr8 93991320
chr8 93991480
chr8 93991536
chr8 93991553
chr8 93991561
chr8 93991563
chr8 93991566
chr8 93991582
chr8 93991585
chr8 93991600
chr8 93991613
chr8 93991617
chr8 93991626
chr8 93991638
chr8 93991644
chr8 93991663
chr8 93991674
chr8 93991687
chr8 94171121
chr8 94171177
chr8 94171290
chr8 94171708
chr8 94171768
chr8 94172021
chr8 94272512
chr8 94272521
chr8 94272544
chr8 94272687
chr8 94272723
chr8 94272779
chr8 94420139
chr8 94420216
chr8 94420235

chr8 94474472
chr8 94474482
chr8 94474526
chr8 94552566
chr8 94552590
chr8 94552692
chr8 94552801
chr8 94552814
chr8 94552851
chr8 94552883
chr8 94552893
chr8 94552939
chr8 94552978
chr8 94552985
chr8 94552994
chr8 94553003
chr8 94553021
chr8 94553067
chr8 94553953
chr8 94554042
chr8 94554057
chr8 94554113
chr8 94554138
chr8 94554152
chr8 94554219
chr8 94554486
chr8 94554495
chr8 94554536
chr8 94554544
chr8 94554651
chr8 94554771
chr8 94623701
chr8 94623741
chr8 94623776
chr8 94624187
chr8 94640507
chr8 94640514
chr8 94640520
chr8 94640523
chr8 94640695
chr8 94640701
chr8 94640731
chr8 94640741
chr8 94640763
chr8 94640783
chr8 94640810
chr8 94640840
chr8 94670421
chr8 94670721
chr8 94670844
chr8 94823818
chr8 94823854
chr8 94823859
chr8 94823881

chr8 94823898
chr8 94823965
chr8 94823970
chr8 94824222
chr8 94947268
chr8 94947303
chr8 94947381
chr8 94947386
chr8 94947452
chr8 94960326
chr8 94960362
chr8 94960416
chr8 94960448
chr8 94960600
chr8 94960792
chr8 94960840
chr8 94960864
chr8 94960907
chr8 94961098
chr8 94961129
chr8 94961181
chr8 95100586
chr8 95100613
chr8 95100667
chr8 95134739
chr8 95134782
chr8 95134805
chr8 95134807
chr8 95134832
chr8 95134846
chr8 95134991
chr8 95134993
chr8 95627802
chr8 95628103
chr8 95628266
chr8 95628745
chr8 95628978
chr8 95631827
chr8 95692186
chr8 95692456
chr8 95692945
chr8 96125933
chr8 96132885
chr8 96132948
chr8 96132992
chr8 96133142
chr8 96133234
chr8 96133240
chr8 96133245
chr8 96133250
chr8 96133252
chr8 96133257
chr8 96133271
chr8 96133304

chr8 96133312
chr8 96133365
chr8 96133436
chr8 96149094
chr8 96149355
chr8 96155701
chr8 96155744
chr8 96155787
chr8 96155826
chr8 96155837
chr8 96155851
chr8 96155887
chr8 96155910
chr8 96155950
chr8 96155952
chr8 96155986
chr8 96155992
chr8 96156035
chr8 96156134
chr8 96156265
chr8 96234493
chr8 96262769
chr8 96262793
chr8 96262861
chr8 96262897
chr8 96262964
chr8 96262976
chr8 96262978
chr8 96263012
chr8 96263159
chr8 96263219
chr8 96263285
chr8 96263483
chr8 96284112
chr8 96284120
chr8 96284141
chr8 96854391
chr8 96854399
chr8 96854403
chr8 96854447
chr8 96854455
chr8 96854462
chr8 96854479
chr8 96854487
chr8 96854509
chr8 97640126
chr8 97640219
chr8 97643576
chr8 97643579
chr8 97645135
chr8 97645147
chr8 97645176
chr8 97645182
chr8 97645239

chr8 97645284
chr8 97645288
chr8 97645344
chr8 97645398
chr8 97645400
chr8 97645476
chr8 97645481
chr8 97645503
chr8 97645516
chr8 97645554
chr8 97645563
chr8 97645567
chr8 97645590
chr8 97645646
chr8 97645882
chr8 97645927
chr8 97645987
chr8 97646256
chr8 97646611
chr8 97646618
chr8 97646660
chr8 97646663
chr8 97646730
chr8 97646767
chr8 97649371
chr8 97649487
chr8 97736692
chr8 98044501
chr8 98044697
chr8 98044770
chr8 98044786
chr8 98044794
chr8 98044821
chr8 98044827
chr8 98046065
chr8 98046086
chr8 98046181
chr8 98046184
chr8 98046190
chr8 98046196
chr8 98046208
chr8 98046233
chr8 98046235
chr8 98117612
chr8 98117615
chr8 98117691
chr8 98117697
chr8 98117711
chr8 98117723
chr8 98117772
chr8 98117943
chr8 98117948
chr8 98117964
chr8 98118156

chr8 98293127
chr8 98293256
chr8 98293283
chr8 98293347
chr8 98293399
chr8 98293433
chr8 98293485
chr8 98293489
chr8 98293527
chr8 98293540
chr8 98293542
chr8 98293676
chr8 98293689
chr8 98293693
chr8 98364944
chr8 98364953
chr8 98365003
chr8 98365024
chr8 98365056
chr8 98365108
chr8 98365143
chr8 98365255
chr8 98365275
chr8 98612655
chr8 98903833
chr8 98938109
chr8 98938211
chr8 98938309
chr8 98945438
chr8 98945467
chr8 98945492
chr8 98945512
chr8 98945519
chr8 98945524
chr8 98945532
chr8 98945595
chr8 98945641
chr8 98945684
chr8 98945706
chr8 98945712
chr8 98945871
chr8 98945979
chr8 98946237
chr8 98946242
chr8 98946270
chr8 98946326
chr8 98946603
chr8 98946691
chr8 98946760
chr8 98946777
chr8 98950959
chr8 98951031
chr8 98951088
chr8 98951281

chr8 98951292
chr8 98951304
chr8 98951322
chr8 98951344
chr8 98951376
chr8 98951429
chr8 98951441
chr8 98951620
chr8 98951629
chr8 98951645
chr8 98951660
chr8 98951685
chr8 98951687
chr8 98974697
chr8 98974714
chr8 98974736
chr8 98974741
chr8 98974754
chr8 98974772
chr8 98974932
chr8 98975000
chr8 99013603
chr8 99013623
chr8 99013688
chr8 99013701
chr8 99013705
chr8 99013725
chr8 99013728
chr8 99013738
chr8 99013764
chr8 99013770
chr8 99224721
chr8 99224825
chr8 99498903
chr8 99499193
chr8 99499419
chr8 100143960
chr8 100144062
chr8 100144064
chr8 100144068
chr8 100144078
chr8 100145986
chr8 100146036
chr8 100146054
chr8 100146220
chr8 100150008
chr8 100150015
chr8 100150030
chr8 100150082
chr8 100150115
chr8 100150124
chr8 100150265
chr8 100150286
chr8 100150301

chr8 100150305
chr8 100150312
chr8 100150331
chr8 100150346
chr8 100151289
chr8 100151373
chr8 100151552
chr8 100151576
chr8 100151617
chr8 100158299
chr8 100158301
chr8 100158308
chr8 100158314
chr8 100158321
chr8 100158326
chr8 100158328
chr8 100158337
chr8 100158349
chr8 100158358
chr8 100158361
chr8 100158363
chr8 100158693
chr8 100212627
chr8 100212687
chr8 100212741
chr8 100218885
chr8 100258457
chr8 100258517
chr8 100258633
chr8 100258773
chr8 100258815
chr8 100258964
chr8 100259006
chr8 100259131
chr8 100259145
chr8 100259366
chr8 100259375
chr8 100306763
chr8 100306905
chr8 100307214
chr8 100307248
chr8 100307322
chr8 100307440
chr8 100307542
chr8 100307559
chr8 100307756
chr8 100307878
chr8 100385933
chr8 100386039
chr8 100386166
chr8 100386239
chr8 100386303
chr8 100386498
chr8 100386510

chr8 100386529
chr8 100386551
chr8 100386580
chr8 100386586
chr8 100386645
chr8 100443524
chr8 100443602
chr8 100444014
chr8 100494999
chr8 100495060
chr8 100502315
chr8 100502362
chr8 100559858
chr8 100559867
chr8 100559875
chr8 100559899
chr8 100559978
chr8 100559989
chr8 100564342
chr8 100564405
chr8 100564417
chr8 100718991
chr8 100719028
chr8 100719190
chr8 100719205
chr8 100719286
chr8 100719663
chr8 100719686
chr8 100719728
chr8 100827325
chr8 100827339
chr8 100908302
chr8 100908350
chr8 100908432
chr8 100908444
chr8 100908448
chr8 100909001
chr8 100909110
chr8 100939008
chr8 100944510
chr8 100944649
chr8 100944926
chr8 100948117
chr8 100948162
chr8 100948423
chr8 100948626
chr8 100948651
chr8 100948683
chr8 100948706
chr8 100948726
chr8 100948837
chr8 100948850
chr8 100949368
chr8 100949614

chr8 100949701
chr8 100949815
chr8 100949840
chr8 100949890
chr8 100950024
chr8 100950095
chr8 100953577
chr8 100953646
chr8 100953675
chr8 100953723
chr8 100953740
chr8 101137242
chr8 101137267
chr8 101137350
chr8 101137369
chr8 101137374
chr8 101137418
chr8 101203138
chr8 101203149
chr8 101203239
chr8 101203676
chr8 101203693
chr8 101203712
chr8 101203868
chr8 101203877
chr8 101203936
chr8 101203948
chr8 101204057
chr8 101204212
chr8 101204342
chr8 101301735
chr8 101301904
chr8 101301967
chr8 101327002
chr8 101327020
chr8 101327118
chr8 101327145
chr8 101327165
chr8 101327185
chr8 101327220
chr8 101327290
chr8 101327303
chr8 101327306
chr8 101334731
chr8 101501316
chr8 101501327
chr8 101501366
chr8 101501586
chr8 101501620
chr8 101679037
chr8 101679056
chr8 101679087
chr8 101679112
chr8 101679133

chr8 102122980
chr8 102122986
chr8 102123269
chr8 102123282
chr8 102123306
chr8 102123315
chr8 102123340
chr8 102123345
chr8 102123351
chr8 102123358
chr8 102123367
chr8 102123492
chr8 102123518
chr8 102123573
chr8 102237758
chr8 102237917
chr8 102238178
chr8 102238258
chr8 102238282
chr8 102287341
chr8 102287371
chr8 102287466
chr8 102287474
chr8 102287640
chr8 102287904
chr8 102530386
chr8 102535840
chr8 102535907
chr8 102535917
chr8 102535941
chr8 102535948
chr8 102536094
chr8 102583673
chr8 102653306
chr8 102653444
chr8 102653510
chr8 102653559
chr8 102653611
chr8 102653632
chr8 102653654
chr8 102657545
chr8 102657573
chr8 102657597
chr8 102662541
chr8 102810971
chr8 102810973
chr8 102810977
chr8 102810999
chr8 102811001
chr8 102811006
chr8 102811012
chr8 102811016
chr8 102811165
chr8 102811237

chr8 102890087
chr8 102890158
chr8 102890195
chr8 103079360
chr8 103298323
chr8 103298378
chr8 103298438
chr8 103373357
chr8 103373416
chr8 103373428
chr8 103373661
chr8 103373667
chr8 103373669
chr8 103373684
chr8 103413861
chr8 103413943
chr8 103414266
chr8 103414364
chr8 103414376
chr8 103415900
chr8 103416043
chr8 103416108
chr8 103416220
chr8 103416235
chr8 103416448
chr8 103461614
chr8 103461653
chr8 103461856
chr8 103461907
chr8 103498526
chr8 103498603
chr8 103498643
chr8 103498645
chr8 103498647
chr8 103498670
chr8 103498678
chr8 103498692
chr8 103501823
chr8 103501837
chr8 103501883
chr8 103501890
chr8 103501965
chr8 103502136
chr8 103502152
chr8 103904985
chr8 103905516
chr8 103905760
chr8 104542301
chr8 104634314
chr8 104634592
chr8 104635009
chr8 105320132
chr8 105439967
chr8 105860116

chr8 106270588
chr8 106270603
chr8 106270626
chr8 106270628
chr8 106270630
chr8 106270633
chr8 106270646
chr8 106270650
chr8 106270653
chr8 106270666
chr8 106270670
chr8 106270690
chr8 106270692
chr8 106270700
chr8 106270714
chr8 106270736
chr8 106270739
chr8 106270760
chr8 106270780
chr8 106270798
chr8 106270814
chr8 106270841
chr8 106270843
chr8 106270852
chr8 106270862
chr8 106270875
chr8 106270878
chr8 106270900
chr8 106270908
chr8 106270918
chr8 106270988
chr8 106271000
chr8 106278671
chr8 106330172
chr8 106657154
chr8 106657176
chr8 106657240
chr8 106657288
chr8 106657303
chr8 106657306
chr8 106657445
chr8 106657966
chr8 106658144
chr8 106745499
chr8 106745656
chr8 106745711
chr8 106745851
chr8 106745958
chr8 107334562
chr8 107334590
chr8 107334593
chr8 107334623
chr8 107439537
chr8 107439920

chr8 107439982
chr8 107440595
chr8 107493840
chr8 107494574
chr8 107494839
chr8 107495264
chr8 107495437
chr8 107495547
chr8 107495570
chr8 107495623
chr8 107495681
chr8 107496127
chr8 107496271
chr8 107496395
chr8 107496471
chr8 107496543
chr8 107496598
chr8 107496604
chr8 107496619
chr8 107496850
chr8 107496876
chr8 107497049
chr8 107497126
chr8 107497230
chr8 107497331
chr8 107497339
chr8 107497363
chr8 107497395
chr8 107497408
chr8 107497455
chr8 107497488
chr8 107497491
chr8 107497528
chr8 107497581
chr8 107497590
chr8 107497684
chr8 107497775
chr8 107497837
chr8 107497868
chr8 107498055
chr8 107498058
chr8 107498064
chr8 107498086
chr8 107498096
chr8 107498115
chr8 107498266
chr8 107754140
chr8 107759385
chr8 107759611
chr8 107759693
chr8 107761486
chr8 108071052
chr8 108074034
chr8 108247173

chr8 108247386
chr8 108247506
chr8 108247516
chr8 108247585
chr8 108247603
chr8 108247618
chr8 108247746
chr8 108319541
chr8 108319673
chr8 108320125
chr8 108320134
chr8 108442678
chr8 108442767
chr8 108442830
chr8 108442904
chr8 108442933
chr8 108442966
chr8 108442968
chr8 108443022
chr8 108443025
chr8 108443029
chr8 108443080
chr8 108443127
chr8 108443135
chr8 108443150
chr8 108444019
chr8 108444024
chr8 108444033
chr8 108444149
chr8 108444204
chr8 109072677
chr8 109332882
chr8 109333032
chr8 109333067
chr8 109333087
chr8 109333262
chr8 109333271
chr8 109334331
chr8 109334362
chr8 109539349
chr8 109688418
chr8 109752892
chr8 109976019
chr8 109976027
chr8 110205133
chr8 110381469
chr8 110381550
chr8 110382003
chr8 110503194
chr8 110503817
chr8 110594348
chr8 110594350
chr8 110594543
chr8 110594608

chr8 110599169
chr8 110599468
chr8 110796569
chr8 111018381
chr8 111018476
chr8 111018791
chr8 111019090
chr8 111019228
chr8 111174524
chr8 111312554
chr8 111469570
chr8 111469593
chr8 111577751
chr8 111577896
chr8 111578622
chr8 111578743
chr8 111647249
chr8 111648284
chr8 111648293
chr8 111759749
chr8 111760032
chr8 111760350
chr8 111760426
chr8 111760710
chr8 112118425
chr8 112118456
chr8 112118560
chr8 112118805
chr8 112130638
chr8 112131558
chr8 112132060
chr8 112497355
chr8 112497883
chr8 112497926
chr8 112498153
chr8 112512570
chr8 112512778
chr8 112512900
chr8 112513699
chr8 112591226
chr8 112592616
chr8 112592805
chr8 112593045
chr8 112593153
chr8 112594769
chr8 112597716
chr8 112598354
chr8 112754239
chr8 112854529
chr8 112854742
chr8 112956657
chr8 113045059
chr8 113374367
chr8 113378515

chr8 113378648
chr8 113402537
chr8 113402860
chr8 113404943
chr8 113405610
chr8 113405946
chr8 113406141
chr8 113406522
chr8 113428074
chr8 113428275
chr8 113435114
chr8 113435117
chr8 113435298
chr8 113435303
chr8 113435452
chr8 113435502
chr8 113435504
chr8 113435509
chr8 113435550
chr8 113435576
chr8 113435578
chr8 113435607
chr8 113435609
chr8 113435617
chr8 113435625
chr8 113435627
chr8 113435642
chr8 113435670
chr8 113435682
chr8 113435690
chr8 113435793
chr8 113435957
chr8 113435988
chr8 113436065
chr8 113436240
chr8 113436243
chr8 113436287
chr8 113436430
chr8 113436436
chr8 113436483
chr8 113436492
chr8 113436540
chr8 113436583
chr8 113436650
chr8 113436923
chr8 113436974
chr8 113436997
chr8 113437189
chr8 113437246
chr8 113437258
chr8 113437292
chr8 113437321
chr8 113437326
chr8 113437337

chr8 113437347
chr8 113437356
chr8 113437359
chr8 113437489
chr8 113437537
chr8 113437686
chr8 113437750
chr8 113437787
chr8 113437836
chr8 113437872
chr8 113437886
chr8 113437892
chr8 113437897
chr8 113525761
chr8 113787299
chr8 113787437
chr8 113787915
chr8 113788214
chr8 113788364
chr8 113788434
chr8 113788466
chr8 113808911
chr8 113876924
chr8 113877499
chr8 113877549
chr8 113880339
chr8 113886060
chr8 113886366
chr8 113886402
chr8 113886484
chr8 114004189
chr8 114004384
chr8 114004417
chr8 114004441
chr8 114004450
chr8 114004694
chr8 114004934
chr8 114005021
chr8 114005243
chr8 114005264
chr8 114005289
chr8 114005320
chr8 114005368
chr8 114007055
chr8 114009845
chr8 114201042
chr8 114201300
chr8 114201496
chr8 114208228
chr8 114208278
chr8 114208319
chr8 114211379
chr8 114211438
chr8 114384663

chr8 114384770
chr8 114391532
chr8 114503940
chr8 114630258
chr8 114630676
chr8 114631375
chr8 114631392
chr8 114631734
chr8 114631747
chr8 114631855
chr8 115184879
chr8 115185445
chr8 115224264
chr8 115451735
chr8 115451739
chr8 115451829
chr8 115588613
chr8 115588915
chr8 115589021
chr8 115589249
chr8 115647672
chr8 115666434
chr8 115666461
chr8 115666505
chr8 115666561
chr8 115666579
chr8 115667472
chr8 115667480
chr8 115667496
chr8 115667517
chr8 115667536
chr8 115667538
chr8 115667540
chr8 115667550
chr8 115667559
chr8 115667564
chr8 115667597
chr8 115667603
chr8 115667607
chr8 115667634
chr8 115667650
chr8 115667654
chr8 115667673
chr8 115667683
chr8 115667701
chr8 115667708
chr8 115667822
chr8 115667830
chr8 115667847
chr8 115667856
chr8 115667862
chr8 115667900
chr8 115667905
chr8 115667914

chr8 115667939
chr8 115667942
chr8 115667945
chr8 115667948
chr8 115667951
chr8 115667959
chr8 115667984
chr8 115667989
chr8 115667993
chr8 115668030
chr8 115668048
chr8 115668065
chr8 115668116
chr8 115668119
chr8 115668167
chr8 115668265
chr8 115669517
chr8 115669523
chr8 115669535
chr8 115669593
chr8 115669602
chr8 115669656
chr8 115669821
chr8 116447892
chr8 116448052
chr8 116754976
chr8 116754999
chr8 116755188
chr8 116755203
chr8 116755206
chr8 116755255
chr8 116755343
chr8 116755352
chr8 116755396
chr8 116756167
chr8 116756205
chr8 116756226
chr8 116756256
chr8 116766994
chr8 116766999
chr8 116767010
chr8 116767040
chr8 116767046
chr8 116767111
chr8 116767171
chr8 116767197
chr8 116767215
chr8 116767376
chr8 116767523
chr8 116767663
chr8 116872753
chr8 116872778
chr8 116872902
chr8 116872946

chr8 116873050
chr8 116873199
chr8 116873353
chr8 116873397
chr8 116875122
chr8 116875273
chr8 116875391
chr8 117044607
chr8 117044666
chr8 117193380
chr8 117193436
chr8 117193479
chr8 117194270
chr8 117194365
chr8 117194833
chr8 117194934
chr8 117229009
chr8 117229294
chr8 117304233
chr8 117304236
chr8 117304264
chr8 117304272
chr8 117304274
chr8 117304322
chr8 117304515
chr8 117305169
chr8 117305376
chr8 117316183
chr8 117316244
chr8 117316449
chr8 117317174
chr8 117520320
chr8 117520329
chr8 117520349
chr8 117520381
chr8 117520386
chr8 117520412
chr8 117520486
chr8 117520515
chr8 117520536
chr8 117520574
chr8 117520591
chr8 117520617
chr8 117520621
chr8 117520631
chr8 117520664
chr8 117521063
chr8 117521067
chr8 117521069
chr8 117521071
chr8 117521123
chr8 117521132
chr8 117521144
chr8 117521213

chr8 117521284
chr8 117521337
chr8 118089670
chr8 118089801
chr8 118089837
chr8 118089839
chr8 118099444
chr8 118099561
chr8 118164797
chr8 118419288
chr8 118419554
chr8 118919581
chr8 118951364
chr8 119638726
chr8 119638737
chr8 119638754
chr8 119638763
chr8 119638783
chr8 119638810
chr8 119638829
chr8 119638950
chr8 119638996
chr8 119639077
chr8 119639158
chr8 119639239
chr8 119639301
chr8 119639344
chr8 119639412
chr8 119639512
chr8 119639543
chr8 119639578
chr8 119639600
chr8 119639604
chr8 119639609
chr8 119639621
chr8 119648555
chr8 119648587
chr8 119648606
chr8 119874412
chr8 119874472
chr8 119874575
chr8 119874595
chr8 119874605
chr8 120008605
chr8 120008693
chr8 120008698
chr8 120008861
chr8 120008912
chr8 120008986
chr8 120008996
chr8 120124214
chr8 120242598
chr8 120242616
chr8 120242676

chr8 120242850
chr8 120242915
chr8 120242953
chr8 120242998
chr8 120243060
chr8 120243148
chr8 120243158
chr8 120243209
chr8 120243211
chr8 120243217
chr8 120444294
chr8 120444322
chr8 120444549
chr8 120444568
chr8 120444573
chr8 120444654
chr8 120444706
chr8 120444807
chr8 120731433
chr8 120731531
chr8 120731759
chr8 120731769
chr8 120731897
chr8 120732171
chr8 120732180
chr8 120732240
chr8 120732570
chr8 120808705
chr8 120808801
chr8 120808812
chr8 120808830
chr8 120809034
chr8 120809209
chr8 120809230
chr8 120809599
chr8 120809606
chr8 120809654
chr8 120810949
chr8 120811122
chr8 120868506
chr8 120868512
chr8 120868578
chr8 120868658
chr8 120868743
chr8 120868746
chr8 120868776
chr8 120868785
chr8 120868792
chr8 120868797
chr8 120868834
chr8 120868842
chr8 120868908
chr8 120868950
chr8 120868972

chr8 120868974
chr8 120869115
chr8 120869339
chr8 120869708
chr8 121158718
chr8 121158935
chr8 121419970
chr8 121420136
chr8 121420520
chr8 121420788
chr8 121420818
chr8 121420827
chr8 121420833
chr8 121626875
chr8 121641084
chr8 121641202
chr8 121641224
chr8 121641349
chr8 121641379
chr8 121641399
chr8 121641402
chr8 121641473
chr8 121641476
chr8 121641551
chr8 121641553
chr8 121641557
chr8 121641561
chr8 121641565
chr8 121641569
chr8 121641576
chr8 121641637
chr8 121641678
chr8 121641739
chr8 121641742
chr8 121641758
chr8 121641783
chr8 121641785
chr8 121641805
chr8 121641822
chr8 121641872
chr8 121641908
chr8 121641936
chr8 121641961
chr8 121641968
chr8 121641989
chr8 121641991
chr8 121642023
chr8 121642049
chr8 121642088
chr8 121642153
chr8 121642172
chr8 121642174
chr8 121642285
chr8 121642323

chr8 121642326
chr8 121642359
chr8 121642390
chr8 121642518
chr8 121642615
chr8 121822783
chr8 121822790
chr8 121822807
chr8 121822849
chr8 121946690
chr8 121946738
chr8 122780131
chr8 122780137
chr8 122780164
chr8 122780180
chr8 122780188
chr8 122780203
chr8 122780212
chr8 122780232
chr8 122780234
chr8 122780244
chr8 122780255
chr8 122780259
chr8 122780269
chr8 122780297
chr8 122780307
chr8 122780310
chr8 122780312
chr8 122780315
chr8 122780317
chr8 122780336
chr8 122780340
chr8 122780349
chr8 122780386
chr8 122780391
chr8 122780395
chr8 122780505
chr8 122780507
chr8 122780510
chr8 122780524
chr8 122780536
chr8 122780554
chr8 122780595
chr8 122780597
chr8 122780600
chr8 122780604
chr8 122780783
chr8 122780790
chr8 122780796
chr8 122783402
chr8 122919093
chr8 122919163
chr8 122919302
chr8 122919350

chr8 122919409
chr8 122919415
chr8 122919442
chr8 123017668
chr8 123017731
chr8 123041413
chr8 123041430
chr8 123041479
chr8 123041544
chr8 123041597
chr8 123041636
chr8 123041646
chr8 123096252
chr8 123096261
chr8 123096303
chr8 123156668
chr8 123156683
chr8 123156745
chr8 123156765
chr8 123156800
chr8 123156811
chr8 123156814
chr8 123156824
chr8 123156860
chr8 123156872
chr8 123156876
chr8 123156878
chr8 123156882
chr8 123156887
chr8 123156890
chr8 123156910
chr8 123156938
chr8 123156940
chr8 123156960
chr8 123156962
chr8 123156965
chr8 123157000
chr8 123157011
chr8 123157019
chr8 123157043
chr8 123157063
chr8 123157068
chr8 123157075
chr8 123157090
chr8 123157195
chr8 123157198
chr8 123157208
chr8 123157235
chr8 123157246
chr8 123157254
chr8 123160790
chr8 123160804
chr8 123160809
chr8 123160871

chr8 123160887
chr8 123160891
chr8 123160905
chr8 123160908
chr8 123160919
chr8 123160935
chr8 123160944
chr8 123160952
chr8 123160963
chr8 123160976
chr8 123160991
chr8 123160996
chr8 123161012
chr8 123161022
chr8 123161028
chr8 123161048
chr8 123161050
chr8 123161071
chr8 123161091
chr8 123161110
chr8 123161118
chr8 123161129
chr8 123161145
chr8 123161151
chr8 123161154
chr8 123161164
chr8 123161178
chr8 123161198
chr8 123161247
chr8 123161269
chr8 123161276
chr8 123161281
chr8 123161298
chr8 123161300
chr8 123240619
chr8 123240730
chr8 123240843
chr8 123240940
chr8 123240960
chr8 123240973
chr8 123240988
chr8 123241015
chr8 123241024
chr8 123241026
chr8 123241036
chr8 123241048
chr8 123386642
chr8 123386651
chr8 123386855
chr8 123387402
chr8 123395051
chr8 123395238
chr8 123395248
chr8 123395369

chr8 123445280
chr8 123743581
chr8 123743727
chr8 123766153
chr8 123766266
chr8 123766292
chr8 124022172
chr8 124022249
chr8 124022520
chr8 124197865
chr8 124197875
chr8 124197940
chr8 124197953
chr8 124197970
chr8 124198218
chr8 124198226
chr8 124262155
chr8 124262172
chr8 124262242
chr8 124262280
chr8 124262872
chr8 124299943
chr8 124370116
chr8 124370534
chr8 124370544
chr8 124370548
chr8 124370696
chr8 124370790
chr8 124370973
chr8 124371098
chr8 124371291
chr8 124372790
chr8 124372795
chr8 124372818
chr8 124372826
chr8 124372828
chr8 124372838
chr8 124372840
chr8 124372848
chr8 124372873
chr8 124372889
chr8 124372905
chr8 124372919
chr8 124372935
chr8 124372939
chr8 124425796
chr8 124425874
chr8 124425940
chr8 124450230
chr8 124450286
chr8 124450316
chr8 124450359
chr8 124450390
chr8 124450467

chr8 124450486
chr8 124450564
chr8 124450737
chr8 124450741
chr8 124450760
chr8 124450766
chr8 124450783
chr8 124450803
chr8 124450816
chr8 124450825
chr8 124450831
chr8 124450840
chr8 124450871
chr8 124450896
chr8 124450918
chr8 124450978
chr8 124451069
chr8 124451085
chr8 124451094
chr8 124451100
chr8 124451106
chr8 124451121
chr8 124451156
chr8 124451196
chr8 124476654
chr8 124538396
chr8 124538420
chr8 124538437
chr8 124538451
chr8 124564371
chr8 124564373
chr8 124564430
chr8 124564445
chr8 124564504
chr8 124564518
chr8 124564547
chr8 124564627
chr8 124564722
chr8 124564779
chr8 124564824
chr8 124564849
chr8 124565045
chr8 124565164
chr8 124637731
chr8 124637734
chr8 124638017
chr8 124660225
chr8 124660264
chr8 124727705
chr8 124727714
chr8 124727722
chr8 124727727
chr8 124727747
chr8 124727751

chr8 124727761
chr8 124727771
chr8 124727796
chr8 124727804
chr8 124727809
chr8 124727820
chr8 124727825
chr8 124727827
chr8 124727830
chr8 124727833
chr8 124727844
chr8 124727846
chr8 124727849
chr8 124727853
chr8 124727856
chr8 124727859
chr8 124727865
chr8 124727873
chr8 124727876
chr8 124727892
chr8 124727961
chr8 124727967
chr8 124727975
chr8 124727987
chr8 124727989
chr8 124727995
chr8 124728005
chr8 124728007
chr8 124728009
chr8 124728015
chr8 124728022
chr8 124757249
chr8 124757266
chr8 124757297
chr8 124757598
chr8 124757713
chr8 124790587
chr8 125090775
chr8 125090842
chr8 125090984
chr8 125090995
chr8 125091025
chr8 125091083
chr8 125091101
chr8 125091194
chr8 125091196
chr8 125091199
chr8 125091257
chr8 125091323
chr8 125091326
chr8 125091349
chr8 125091372
chr8 125091389
chr8 125091404

chr8 125091439
chr8 125091445
chr8 125091467
chr8 125273201
chr8 125429665
chr8 125429675
chr8 125429682
chr8 125429685
chr8 125429688
chr8 125429694
chr8 125429704
chr8 125429745
chr8 125429778
chr8 125429807
chr8 125429812
chr8 125429880
chr8 125429888
chr8 125429897
chr8 125429909
chr8 125429919
chr8 125429924
chr8 125429939
chr8 125429951
chr8 125429960
chr8 125434681
chr8 125434711
chr8 125608032
chr8 125608066
chr8 125608128
chr8 125608150
chr8 125608290
chr8 125608294
chr8 125608327
chr8 125648312
chr8 125648356
chr8 125648380
chr8 125648390
chr8 125648590
chr8 125648631
chr8 125950078
chr8 125950512
chr8 125971623
chr8 125972093
chr8 125972140
chr8 125972169
chr8 125972186
chr8 125972330
chr8 126344087
chr8 126344430
chr8 126780973
chr8 126781388
chr8 127088109
chr8 127088427
chr8 127307416

chr8 127734005
chr8 127734020
chr8 127734072
chr8 127734078
chr8 127734185
chr8 127734210
chr8 127734225
chr8 127734227
chr8 127734233
chr8 127734508
chr8 127734535
chr8 127739055
chr8 127739096
chr8 127739317
chr8 127739342
chr8 127739422
chr8 127794677
chr8 127794692
chr8 127794716
chr8 127794758
chr8 127794774
chr8 127794799
chr8 127794812
chr8 127794816
chr8 127794833
chr8 127794858
chr8 127794895
chr8 127794905
chr8 127794938
chr8 127794946
chr8 127795071
chr8 127795128
chr8 127795158
chr8 127795188
chr8 127795218
chr8 127795314
chr8 127795387
chr8 127795506
chr8 127795595
chr8 127887268
chr8 127975479
chr8 127975522
chr8 127975544
chr8 127975634
chr8 127975637
chr8 127975781
chr8 127975801
chr8 127976615
chr8 127976749
chr8 127976992
chr8 127977039
chr8 127981784
chr8 127981892
chr8 127992923

chr8 127992931
chr8 127993006
chr8 127993016
chr8 127993263
chr8 127993280
chr8 127993292
chr8 127993321
chr8 127993337
chr8 127993353
chr8 127993420
chr8 127993504
chr8 128128455
chr8 128128464
chr8 128128473
chr8 128222099
chr8 128222274
chr8 128240828
chr8 128540483
chr8 128540558
chr8 128540581
chr8 128540668
chr8 128540690
chr8 128540930
chr8 128541062
chr8 128541451
chr8 128653429
chr8 128653493
chr8 128682057
chr8 128682323
chr8 128682491
chr8 128682556
chr8 128682585
chr8 128859148
chr8 129938943
chr8 129938945
chr8 129938998
chr8 129939023
chr8 129940496
chr8 130008768
chr8 130008860
chr8 130008863
chr8 130068445
chr8 130068507
chr8 130129410
chr8 130129619
chr8 130129757
chr8 130129784
chr8 130130002
chr8 130130050
chr8 130310063
chr8 130443997
chr8 130443999
chr8 130444020
chr8 130444028

chr8 130444038
chr8 130444084
chr8 130444257
chr8 130444303
chr8 130444413
chr8 130444610
chr8 130444667
chr8 130444758
chr8 130444812
chr8 130444847
chr8 130444863
chr8 130444968
chr8 130445022
chr8 130445092
chr8 130445170
chr8 130445214
chr8 130445241
chr8 130519172
chr8 130519376
chr8 130912310
chr8 130912429
chr8 131223895
chr8 131341134
chr8 132251400
chr8 132251480
chr8 132758266
chr8 132758480
chr8 132758491
chr8 132758618
chr8 132758798
chr8 132758815
chr8 132758872
chr8 132758891
chr8 132759297
chr8 132759661
chr8 132760018
chr8 132760257
chr8 132760291
chr8 132760361
chr8 132760411
chr8 132760496
chr8 132760539
chr8 132760643
chr8 132773281
chr8 132775747
chr8 132775759
chr8 132775766
chr8 132775769
chr8 132775786
chr8 132775789
chr8 132775953
chr8 132775984
chr8 132776035
chr8 132776101

chr8 132776201
chr8 132901700
chr8 132901731
chr8 132901818
chr8 132901866
chr8 133057546
chr8 133057586
chr8 133057618
chr8 133057922
chr8 133058027
chr8 133058057
chr8 133058149
chr8 133058301
chr8 133058489
chr8 133058531
chr8 133058547
chr8 133058591
chr8 133058672
chr8 133058713
chr8 133058720
chr8 133058735
chr8 133059196
chr8 133059671
chr8 133059904
chr8 133059907
chr8 133059925
chr8 133059990
chr8 133060020
chr8 133060033
chr8 133060104
chr8 133060124
chr8 133060131
chr8 133060182
chr8 133060220
chr8 133060234
chr8 133060281
chr8 133060318
chr8 133060366
chr8 133060461
chr8 133060931
chr8 133060993
chr8 133061034
chr8 133061050
chr8 133061074
chr8 133061080
chr8 133061103
chr8 133061113
chr8 133063960
chr8 133067198
chr8 133067214
chr8 133067229
chr8 133067273
chr8 133067346
chr8 133067482

chr8 133067591
chr8 133067700
chr8 133067730
chr8 133067735
chr8 133072187
chr8 133072300
chr8 133072350
chr8 133072385
chr8 133072796
chr8 133072803
chr8 133072845
chr8 133073121
chr8 133073168
chr8 133073201
chr8 133073385
chr8 133073455
chr8 133073465
chr8 133073479
chr8 133073486
chr8 133073510
chr8 133073619
chr8 133073759
chr8 133073769
chr8 133073827
chr8 133073966
chr8 133075299
chr8 133075336
chr8 133075373
chr8 133075446
chr8 133075569
chr8 133075577
chr8 133075599
chr8 133075619
chr8 133075629
chr8 133075824
chr8 133075922
chr8 133076013
chr8 133076274
chr8 133076278
chr8 133076455
chr8 133171115
chr8 133217420
chr8 133217493
chr8 133217546
chr8 133217640
chr8 133217642
chr8 133217710
chr8 133217858
chr8 133217914
chr8 133217979
chr8 133218032
chr8 133282663
chr8 133283096
chr8 133283100

chr8 133283200
chr8 133444933
chr8 133444960
chr8 133498646
chr8 133498657
chr8 133498702
chr8 133498746
chr8 133498749
chr8 133498810
chr8 133498864
chr8 133498882
chr8 133498893
chr8 133498904
chr8 133498910
chr8 133499035
chr8 133499112
chr8 133499135
chr8 133499212
chr8 133499237
chr8 133499394
chr8 133499440
chr8 133499527
chr8 133499730
chr8 133499751
chr8 133499758
chr8 133499765
chr8 133499774
chr8 133499777
chr8 133499866
chr8 133499876
chr8 133499985
chr8 133500001
chr8 133526698
chr8 133526738
chr8 133526833
chr8 133526924
chr8 133526953
chr8 133564690
chr8 134324917
chr8 134325205
chr8 134333061
chr8 134333148
chr8 134333173
chr8 134333186
chr8 134333222
chr8 134333358
chr8 134691110
chr8 134691345
chr8 134691378
chr8 134691386
chr8 134691390
chr8 134691398
chr8 134831825
chr8 134831965

chr8 134831988
chr8 134831994
chr8 134832001
chr8 134832005
chr8 134832014
chr8 134832017
chr8 134832022
chr8 134832025
chr8 134832027
chr8 134832042
chr8 134832051
chr8 134832056
chr8 134832058
chr8 134832063
chr8 134832069
chr8 134832074
chr8 134832076
chr8 134832085
chr8 134832089
chr8 134832101
chr8 134832103
chr8 134832109
chr8 134832111
chr8 134832117
chr8 134832132
chr8 134832134
chr8 134832136
chr8 134832143
chr8 134832157
chr8 134832159
chr8 134832162
chr8 134832176
chr8 134832179
chr8 134832182
chr8 134832186
chr8 134832206
chr8 134832209
chr8 134832211
chr8 134832215
chr8 134832223
chr8 134832225
chr8 134832227
chr8 134832230
chr8 134832233
chr8 134832239
chr8 135060791
chr8 136194982
chr8 136194987
chr8 136195014
chr8 136195267
chr8 136529156
chr8 137116934
chr8 137314156
chr8 137314410

chr8 137314871
chr8 137582822
chr8 137582908
chr8 137745674
chr8 137745825
chr8 137745917
chr8 138590151
chr8 138665732
chr8 138847069
chr8 138847313
chr8 139232939
chr8 139292542
chr8 140393907
chr8 140393910
chr8 140393936
chr8 140393959
chr8 140393963
chr8 140457978
chr8 140457991
chr8 140457997
chr8 140458056
chr8 140458173
chr8 140458192
chr8 140458238
chr8 140458241
chr8 140458271
chr8 140458274
chr8 140458294
chr8 140458296
chr8 140458343
chr8 140458345
chr8 140458368
chr8 140458389
chr8 140458399
chr8 140458410
chr8 140458414
chr8 140458425
chr8 140458439
chr8 140458444
chr8 140458446
chr8 140458448
chr8 140458450
chr8 140458461
chr8 140458473
chr8 140458482
chr8 140458491
chr8 140458500
chr8 140465461
chr8 140465547
chr8 140465564
chr8 140467191
chr8 140467273
chr8 140467307
chr8 140467323

chr8 140467335
chr8 140591181
chr8 140591183
chr8 140591201
chr8 140591297
chr8 140591388
chr8 140591393
chr8 140591402
chr8 140591418
chr8 140591526
chr8 140591552
chr8 140591593
chr8 140591615
chr8 140591726
chr8 140596829
chr8 140596837
chr8 140596843
chr8 140596859
chr8 140596862
chr8 140596868
chr8 140790810
chr8 140790944
chr8 140790983
chr8 140791208
chr8 140791224
chr8 140791279
chr8 140830952
chr8 140831126
chr8 140831189
chr8 140831287
chr8 140831319
chr8 140831332
chr8 140831458
chr8 140831479
chr8 140831610
chr8 141129321
chr8 141129345
chr8 141129348
chr8 141129354
chr8 141129356
chr8 141129379
chr8 141129410
chr8 141129454
chr8 141129496
chr8 141129515
chr8 141129555
chr8 141129603
chr8 141129619
chr8 141129648
chr8 141129651
chr8 141129665
chr8 141129679
chr8 141129740
chr8 141129839

chr8 141129860
chr8 141129880
chr8 141129922
chr8 141129951
chr8 141129993
chr8 141203452
chr8 141206250
chr8 141206264
chr8 141206333
chr8 141206348
chr8 141206362
chr8 141206398
chr8 141206415
chr8 141206431
chr8 141206453
chr8 141206485
chr8 141206500
chr8 141245454
chr8 142714901
chr8 142715279
chr8 142731876
chr8 142731955
chr8 142731958
chr8 142732105
chr8 142777347
chr8 142777352
chr8 142777361
chr8 142777370
chr8 142777375
chr8 142777381
chr8 142777405
chr8 142777407
chr8 142777423
chr8 142777434
chr8 142777449
chr8 142777452
chr8 142777457
chr8 142777466
chr8 142777475
chr8 142777480
chr8 142777486
chr8 142777501
chr8 142777504
chr8 142777509
chr8 142777518
chr8 142777553
chr8 142777556
chr8 142777561
chr8 142777570
chr8 142777579
chr8 142777584
chr8 142844411
chr8 142844514
chr8 142844523

chr8 142844707
chr8 143026379
chr8 143026397
chr8 143026418
chr8 143026439
chr8 143026482
chr8 143026685
chr8 143026791
chr8 143026801
chr8 143026862
chr8 143026912
chr8 143026927
chr8 143166731
chr8 143166812
chr8 143166833
chr8 143166867
chr8 143166934
chr8 143166981
chr8 143167002
chr8 143167036
chr8 143167083
chr8 143167104
chr8 143167138
chr8 143167171
chr8 143167204
chr8 143167239
chr8 143167307
chr8 143167341
chr8 143167409
chr8 143167422
chr8 143167477
chr8 143168021
chr8 143168068
chr8 143168123
chr8 143168157
chr8 143168259
chr8 143168293
chr8 143168327
chr8 143168340
chr8 143168374
chr8 143168395
chr8 143168430
chr8 143168464
chr8 143168498
chr8 143191175
chr8 143191179
chr8 143191214
chr8 143191261
chr8 143191346
chr8 143191468
chr8 143191520
chr8 143191525
chr8 143191537
chr8 143246202

chr8 143246210
chr8 143246223
chr8 143246229
chr8 143246232
chr8 143246234
chr8 143246240
chr8 143246254
chr8 143246257
chr8 143246263
chr8 143246277
chr8 143246280
chr8 143246297
chr8 143246304
chr8 143246330
chr8 143246332
chr8 143246336
chr8 143246338
chr8 143246347
chr8 143246374
chr8 143246376
chr8 143246378
chr8 143246381
chr8 143246387
chr8 143246403
chr8 143246412
chr8 143246423
chr8 143246428
chr8 143246434
chr8 143246458
chr8 143246463
chr8 143246469
chr8 143246478
chr8 143246494
chr8 143246499
chr8 143246504
chr8 143246520
chr8 143246532
chr8 143246545
chr8 143246580
chr8 143246586
chr8 143246593
chr8 143246599
chr8 143246614
chr8 143246632
chr8 143246637
chr8 143246651
chr8 143246657
chr8 143246664
chr8 143246668
chr8 143246686
chr8 143246698
chr8 143246706
chr8 143246719
chr8 143553817

chr8 143553821
chr8 143568982
chr8 143568992
chr8 143569002
chr8 143569011
chr8 143569017
chr8 143569042
chr8 143569059
chr8 143569173
chr8 143569177
chr8 143569191
chr8 143569220
chr8 143569225
chr8 143577818
chr8 143577827
chr8 143577897
chr8 143577906
chr8 143577924
chr8 143577941
chr8 143577951
chr8 143577962
chr8 143577974
chr8 143577976
chr8 143577987
chr8 143577991
chr8 143578012
chr8 143578033
chr8 143578056
chr8 143578060
chr8 143578071
chr8 143578074
chr8 143578076
chr8 143578078
chr8 143578082
chr8 143578093
chr8 143578096
chr8 143578101
chr8 143578107
chr8 143578114
chr8 143578116
chr8 143578125
chr8 143578135
chr8 143578137
chr8 143578144
chr8 143578147
chr8 143578156
chr8 143578162
chr8 143578165
chr8 143578168
chr8 143578172
chr8 143578179
chr8 143578182
chr8 143578195
chr8 143578201

chr8 143578204
chr8 143578207
chr8 143578212
chr8 143578214
chr8 143578218
chr8 143578223
chr8 143578225
chr8 143578227
chr8 143578241
chr8 143578274
chr8 143578278
chr8 143578280
chr8 143578283
chr8 143578286
chr8 143578288
chr8 143578290
chr8 143578292
chr8 143578297
chr8 143578310
chr8 143578313
chr8 143578327
chr8 143578330
chr8 143578334
chr8 143578341
chr8 143578346
chr8 143578351
chr8 143578357
chr8 143578362
chr8 143578364
chr8 143578369
chr8 143578379
chr8 143578400
chr8 143578405
chr8 143578415
chr8 143578420
chr8 143578427
chr8 143578437
chr8 143578456
chr8 143578459
chr8 143578461
chr8 143578464
chr8 143578489
chr8 143856347
chr8 143856423
chr8 143856437
chr8 143856565
chr8 143943256
chr8 143978122
chr8 143978133
chr8 143978136
chr8 143978250
chr8 143978275
chr8 143978304
chr8 143978316

chr8 143978320
chr8 143978343
chr8 144103165
chr8 144103182
chr8 144103255
chr8 144103263
chr8 144103273
chr8 144103277
chr8 144103321
chr8 144103338
chr8 144103400
chr8 144103404
chr8 144103420
chr8 144103424
chr8 144413354
chr8 144413393
chr8 144413397
chr8 144413407
chr8 144413415
chr8 144413417
chr8 144413427
chr8 144413434
chr8 144413491
chr8 144413497
chr8 144878655
chr8 144878836
chr8 144879017
chr8 144900377
chr8 144900416
chr8 144900420
chr8 144900437
chr8 144900498
chr8 144900673
chr8 144900704
chr8 144900720
chr8 144900736
chr8 144900747
chr8 144900753
chr8 144900761
chr8 144900763
chr8 144900794
chr8 144900800
chr8 144900805
chr8 144900808
chr8 144900811
chr8 144950300
chr8 144950306
chr8 144950327
chr8 144950334
chr8 144950394
chr8 144950450
chr8 144950453
chr8 144950458
chr8 144950462

chr8 144950468
chr8 144950481
chr8 144950488
chr8 144950494
chr8 144950511
chr8 144950524
chr8 144950532
chr8 144950534
chr8 144950537
chr8 144950548
chr8 144950559
chr8 144950573
chr8 144950612
chr8 144950623
chr8 144950628
chr8 144950637
chr8 144950639
chr8 144950655
chr8 144950657
chr8 144950659
chr8 144950665
chr8 144950671
chr8 144950675
chr8 144950689
chr8 145078546
chr1 10609
chr1 10617
chr1 10620
chr1 10631
chr1 10633
chr1 10636
chr1 10638
chr1 10641
chr1 10644
chr1 10650
chr1 10660
chr1 10662
chr1 10665
chr1 10667
chr1 10670
chr1 10673
chr1 10679
chr1 10708
chr1 10718
chr1 10720
chr1 10723
chr1 10725
chr1 10728
chr1 10731
chr1 10737
chr1 10747
chr1 10749
chr1 10752
chr1 10754

chr1 10757
chr1 10760
chr1 10766
chr1 10776
chr1 10778
chr1 10781
chr1 10783
chr1 10786
chr1 10789
chr1 10795
chr1 10811
chr1 10813
chr1 10816
chr1 10827
chr1 10832
chr1 10838
chr1 10848
chr1 10850
chr1 10853
chr1 10855
chr1 10858
chr1 10861
chr1 10867
chr1 10885
chr1 10887
chr1 11842
chr1 11868
chr1 11879
chr1 26862
chr1 26870
chr1 26932
chr1 26957
chr1 26964
chr1 27026
chr1 27028
chr1 28512
chr1 28660
chr1 28925
chr1 28934
chr1 28936
chr1 28938
chr1 28955
chr1 28964
chr1 28972
chr1 28988
chr1 28994
chr1 29002
chr1 29011
chr1 29021
chr1 29215
chr1 29221
chr1 29230
chr1 29234
chr1 29253

chr1 29267
chr1 29272
chr1 29285
chr1 29298
chr1 29306
chr1 29310
chr1 29324
chr1 29329
chr1 29337
chr1 29347
chr1 29350
chr1 29353
chr1 29359
chr1 29367
chr1 29373
chr1 29389
chr1 29392
chr1 29428
chr1 29431
chr1 29435
chr1 29447
chr1 30922
chr1 30984
chr1 31028
chr1 31623
chr1 31650
chr1 31696
chr1 31792
chr1 34014
chr1 35428
chr1 36375
chr1 39676
chr1 39703
chr1 39734
chr1 39778
chr1 147241
chr1 147313
chr1 147337
chr1 147425
chr1 158040
chr1 191943
chr1 191948
chr1 192989
chr1 193019
chr1 193093
chr1 193364
chr1 193389
chr1 193423
chr1 193933
chr1 194025
chr1 194032
chr1 194073
chr1 194108
chr1 257683

chr1 257715
chr1 257741
chr1 257871
chr1 586000
chr1 586154
chr1 586191
chr1 586197
chr1 586205
chr1 586222
chr1 586280
chr1 586566
chr1 605428
chr1 605437
chr1 605445
chr1 605447
chr1 605457
chr1 605459
chr1 605462
chr1 605471
chr1 605475
chr1 605487
chr1 605489
chr1 605501
chr1 605506
chr1 605509
chr1 605517
chr1 605531
chr1 605540
chr1 605581
chr1 605599
chr1 605611
chr1 623359
chr1 623671
chr1 624100
chr1 779203
chr1 816392
chr1 817074
chr1 826624
chr1 826972
chr1 827925
chr1 827935
chr1 827938
chr1 827943
chr1 827945
chr1 827947
chr1 827951
chr1 827958
chr1 827960
chr1 827964
chr1 827966
chr1 827969
chr1 827980
chr1 827992
chr1 844615

chr1 996103
chr1 996159
chr1 996186
chr1 996213
chr1 996277
chr1 996341
chr1 996343
chr1 996407
chr1 1040218
chr1 1040231
chr1 1040237
chr1 1040287
chr1 1040298
chr1 1040303
chr1 1040316
chr1 1040329
chr1 1140474
chr1 1140479
chr1 1157688
chr1 1157690
chr1 1157832
chr1 1157849
chr1 1157861
chr1 1157868
chr1 1350011
chr1 1350014
chr1 1350040
chr1 1350051
chr1 1350054
chr1 1350200
chr1 1350211
chr1 1350214
chr1 1350240
chr1 1350251
chr1 1350254
chr1 1350291
chr1 1350320
chr1 1350331
chr1 1350334
chr1 1350360
chr1 1350371
chr1 1350374
chr1 1350401
chr1 1350411
chr1 1350414
chr1 1350931
chr1 1350934
chr1 1350960
chr1 1350971
chr1 1350974
chr1 1351001
chr1 1351011
chr1 1351014
chr1 1351041

chr1 1351051
chr1 1351054
chr1 1351120
chr1 1351131
chr1 1351134
chr1 1351160
chr1 1351171
chr1 1351174
chr1 1351200
chr1 1351211
chr1 1351214
chr1 1351240
chr1 1427167
chr1 1427182
chr1 1427195
chr1 1427222
chr1 1427240
chr1 1427256
chr1 1427274
chr1 1427278
chr1 1427288
chr1 1629643
chr1 1629645
chr1 1629647
chr1 1629682
chr1 1629698
chr1 1629720
chr1 1629731
chr1 1629754
chr1 1629756
chr1 1629764
chr1 1629793
chr1 1629871
chr1 1632346
chr1 1632349
chr1 1632355
chr1 1632365
chr1 1632370
chr1 1632380
chr1 1632383
chr1 1632402
chr1 1632421
chr1 1632441
chr1 1632450
chr1 1632457
chr1 1632463
chr1 1632472
chr1 1632489
chr1 1632497
chr1 1632502
chr1 1632508
chr1 1632520
chr1 1658356
chr1 1658373

chr1 1658405
chr1 1658430
chr1 1658455
chr1 1658466
chr1 1658470
chr1 1658472
chr1 1658488
chr1 1658803
chr1 1658807
chr1 1658813
chr1 1658819
chr1 1658825
chr1 1658830
chr1 1658835
chr1 1658846
chr1 1658860
chr1 1658865
chr1 1658877
chr1 1658879
chr1 1658904
chr1 1658919
chr1 1658922
chr1 1691377
chr1 1691388
chr1 1691594
chr1 1691674
chr1 1691722
chr1 1692315
chr1 1692321
chr1 1692587
chr1 1692608
chr1 1692614
chr1 1692699
chr1 1692702
chr1 1692725
chr1 1692772
chr1 1692781
chr1 1692785
chr1 1692790
chr1 1692859
chr1 1693163
chr1 1693178
chr1 1693180
chr1 1693188
chr1 1693197
chr1 1693201
chr1 1693205
chr1 1693211
chr1 1693215
chr1 1693231
chr1 1693244
chr1 1693250
chr1 1693270
chr1 1693284

chr1 1713396
chr1 1713469
chr1 1723557
chr1 1723561
chr1 1723700
chr1 1723717
chr1 1723749
chr1 1723774
chr1 1723799
chr1 1723810
chr1 1723814
chr1 1723816
chr1 1723832
chr1 1724512
chr1 1724514
chr1 1724519
chr1 1724524
chr1 1724527
chr1 1724536
chr1 1724539
chr1 1724542
chr1 1724561
chr1 1724567
chr1 1724569
chr1 1724574
chr1 1724576
chr1 1724582
chr1 1724589
chr1 1724600
chr1 1724611
chr1 1724617
chr1 1724624
chr1 1724626
chr1 1744652
chr1 1744663
chr1 1744834
chr1 1744869
chr1 1744917
chr1 1744949
chr1 1745590
chr1 1745596
chr1 1745611
chr1 1745615
chr1 1745619
chr1 1745639
chr1 1745659
chr1 1745666
chr1 1745816
chr1 1745818
chr1 1745830
chr1 1745834
chr1 1745836
chr1 1745842
chr1 1745844

chr1 1746468
chr1 1746477
chr1 1746481
chr1 1746497
chr1 1746510
chr1 1746516
chr1 1746536
chr1 1746550
chr1 1747060
chr1 1747070
chr1 1747073
chr1 1747101
chr1 1747105
chr1 1747113
chr1 1747117
chr1 1747138
chr1 1747169
chr1 1747176
chr1 1747193
chr1 1747201
chr1 1747222
chr1 1747238
chr1 1747292
chr1 1747449
chr1 1777574
chr1 1777580
chr1 1777670
chr1 1777682
chr1 1777711
chr1 1777714
chr1 1777732
chr1 1777764
chr1 1777919
chr1 2121969
chr1 2121987
chr1 2121990
chr1 2122005
chr1 2122021
chr1 2122054
chr1 2122071
chr1 2122087
chr1 2122122
chr1 2122138
chr1 2122153
chr1 2122168
chr1 2122213
chr1 2122218
chr1 2122231
chr1 2122236
chr1 2122266
chr1 2122284
chr1 2122302
chr1 2122463
chr1 2122468

chr1 2122498
chr1 2122516
chr1 2122568
chr1 2122598
chr1 2122603
chr1 2122618
chr1 2123358
chr1 2123388
chr1 2123393
chr1 2123408
chr1 2123439
chr1 2123444
chr1 2204979
chr1 2204984
chr1 2204998
chr1 2205010
chr1 2205021
chr1 2205026
chr1 2205050
chr1 2205055
chr1 2205068
chr1 2205079
chr1 2205091
chr1 2205096
chr1 2205110
chr1 2205121
chr1 2205128
chr1 2205147
chr1 2205158
chr1 2205171
chr1 2205176
chr1 2205188
chr1 2255756
chr1 2255858
chr1 2256018
chr1 2300624
chr1 2300636
chr1 2300658
chr1 2300668
chr1 2300693
chr1 2300705
chr1 2300712
chr1 2300727
chr1 2300742
chr1 2300756
chr1 2300764
chr1 2300790
chr1 2300854
chr1 2300872
chr1 2300883
chr1 2300891
chr1 2300899
chr1 2300908
chr1 2300919

chr1 2300924
chr1 2416187
chr1 2416218
chr1 2416228
chr1 2548160
chr1 2548164
chr1 2548206
chr1 2548369
chr1 2548498
chr1 2656986
chr1 2656994
chr1 2657156
chr1 2661473
chr1 2661507
chr1 2661509
chr1 2661729
chr1 2661771
chr1 2662084
chr1 2662095
chr1 2662164
chr1 2662430
chr1 2662446
chr1 2662561
chr1 2662582
chr1 2662606
chr1 2662636
chr1 2662646
chr1 2662658
chr1 2662686
chr1 2662833
chr1 2662999
chr1 2663047
chr1 2663055
chr1 2663208
chr1 2663240
chr1 2663431
chr1 2663449
chr1 2663537
chr1 2663550
chr1 2663559
chr1 2663569
chr1 2663590
chr1 2663627
chr1 2663636
chr1 2663681
chr1 2663732
chr1 2663770
chr1 2663781
chr1 2663864
chr1 2664018
chr1 2664086
chr1 2664267
chr1 2664283
chr1 2664307

chr1 2664343
chr1 2664439
chr1 2664488
chr1 2664598
chr1 2664600
chr1 2664607
chr1 2667252
chr1 2667262
chr1 2667274
chr1 2667302
chr1 2667358
chr1 2667374
chr1 2667539
chr1 2667615
chr1 2667663
chr1 2667671
chr1 2667769
chr1 2667824
chr1 2667856
chr1 2668185
chr1 2668206
chr1 2668243
chr1 2668252
chr1 2668297
chr1 2668348
chr1 2668386
chr1 2668397
chr1 2668480
chr1 2668634
chr1 2668702
chr1 2668723
chr1 2668747
chr1 2668777
chr1 2668787
chr1 2668799
chr1 2668803
chr1 2668827
chr1 2668883
chr1 2668899
chr1 2668923
chr1 2668959
chr1 2669014
chr1 2669055
chr1 2669104
chr1 2669216
chr1 2669223
chr1 2669268
chr1 2669316
chr1 2669348
chr1 2669369
chr1 2669399
chr1 2669442
chr1 2669558
chr1 2669590

chr1 2669598
chr1 2669677
chr1 2669694
chr1 2669710
chr1 2669726
chr1 2669745
chr1 2669964
chr1 2670364
chr1 2670392
chr1 2670448
chr1 2670464
chr1 2670539
chr1 2677948
chr1 2677980
chr1 2677988
chr1 2678067
chr1 2678084
chr1 2678100
chr1 2678116
chr1 2678267
chr1 2678300
chr1 2678354
chr1 2678475
chr1 2678589
chr1 2678782
chr1 2678838
chr1 2678854
chr1 2678878
chr1 2678929
chr1 2679095
chr1 2679129
chr1 2679266
chr1 2679270
chr1 2679304
chr1 2679315
chr1 2679325
chr1 2679344
chr1 2679351
chr1 2679393
chr1 2679465
chr1 2679956
chr1 2680052
chr1 2680068
chr1 2680183
chr1 2680204
chr1 2680228
chr1 2680364
chr1 2680476
chr1 2680508
chr1 2680529
chr1 2680559
chr1 2680602
chr1 2681964
chr1 2682163

chr1 2682204
chr1 2682283
chr1 2688862
chr1 2688866
chr1 2688874
chr1 2688988
chr1 2689021
chr1 2689093
chr1 2689115
chr1 2689174
chr1 2689263
chr1 2689298
chr1 2689311
chr1 2689391
chr1 2689435
chr1 2689470
chr1 2689487
chr1 2689503
chr1 2689623
chr1 2689639
chr1 2689663
chr1 2689671
chr1 2689705
chr1 2689890
chr1 2689933
chr1 2689945
chr1 2690028
chr1 2690059
chr1 2690066
chr1 2690077
chr1 2690160
chr1 2693073
chr1 2693081
chr1 2694284
chr1 2694357
chr1 2694364
chr1 2700744
chr1 2700987
chr1 2701027
chr1 2701051
chr1 2701091
chr1 2701103
chr1 2701107
chr1 2701122
chr1 2701168
chr1 2701171
chr1 2701203
chr1 2701211
chr1 2701331
chr1 2701342
chr1 2701371
chr1 2701401
chr1 2701411
chr1 2701452

chr1 2701900
chr1 2701989
chr1 2702227
chr1 2702233
chr1 2702297
chr1 2702337
chr1 2702535
chr1 2702553
chr1 2702645
chr1 2702681
chr1 2702725
chr1 2702761
chr1 2746318
chr1 2746334
chr1 2746355
chr1 2746358
chr1 2746366
chr1 2746400
chr1 2746503
chr1 2746531
chr1 2746559
chr1 2746642
chr1 2746646
chr1 2746654
chr1 2746768
chr1 2746801
chr1 2746841
chr1 2746873
chr1 2746895
chr1 2746954
chr1 2747005
chr1 2747078
chr1 2747091
chr1 2762042
chr1 2762054
chr1 2762058
chr1 2762279
chr1 2762282
chr1 2762314
chr1 2762322
chr1 2762442
chr1 2762453
chr1 2762482
chr1 2762512
chr1 2762522
chr1 2762563
chr1 2763011
chr1 2763338
chr1 2763344
chr1 2763408
chr1 2763448
chr1 2763646
chr1 2763664
chr1 2764573

chr1 2764757
chr1 2765132
chr1 2765160
chr1 2765191
chr1 2765551
chr1 2765651
chr1 2765683
chr1 2765723
chr1 2765747
chr1 2765787
chr1 2765799
chr1 2765803
chr1 2765864
chr1 2765867
chr1 2765899
chr1 2765907
chr1 2766027
chr1 2766038
chr1 2766067
chr1 2766097
chr1 2766107
chr1 2766148
chr1 2766923
chr1 2766929
chr1 2766993
chr1 2767033
chr1 2769743
chr1 2769773
chr1 2769783
chr1 2769824
chr1 2770086
chr1 2770272
chr1 2770361
chr1 2770600
chr1 2770606
chr1 2770670
chr1 2770710
chr1 2770908
chr1 2770926
chr1 2771018
chr1 2771054
chr1 2771301
chr1 2997017
chr1 2998053
chr1 3899717
chr1 3899738
chr1 3899768
chr1 3899793
chr1 3899799
chr1 3899806
chr1 3899823
chr1 3899825
chr1 3899854
chr1 3899883

chr1 3900758
chr1 3900768
chr1 3900800
chr1 3900809
chr1 3900832
chr1 3900850
chr1 3900854
chr1 3900887
chr1 3900897
chr1 3900915
chr1 3900919
chr1 3900924
chr1 3900928
chr1 3900978
chr1 3901000
chr1 3901007
chr1 3901024
chr1 3901031
chr1 3901044
chr1 3901046
chr1 3901067
chr1 3901074
chr1 3901080
chr1 3901095
chr1 3901097
chr1 3901104
chr1 3901121
chr1 3901123
chr1 3901131
chr1 3901151
chr1 3901155
chr1 3901181
chr1 3901186
chr1 3901190
chr1 3901213
chr1 3901216
chr1 3901240
chr1 3901243
chr1 3901246
chr1 3901258
chr1 3901265
chr1 3901272
chr1 3901277
chr1 3901286
chr1 4007356
chr1 4007376
chr1 4007420
chr1 4007428
chr1 4007432
chr1 4007452
chr1 4007476
chr1 4007491
chr1 4007508
chr1 4007514

chr1 4538679
chr1 4538985
chr1 4539062
chr1 4539183
chr1 4539522
chr1 4556610
chr1 4556770
chr1 5290372
chr1 6026360
chr1 6026388
chr1 6026404
chr1 6026423
chr1 6026427
chr1 6026433
chr1 6026461
chr1 6026470
chr1 6026498
chr1 6026540
chr1 6026584
chr1 6026592
chr1 6026648
chr1 6026655
chr1 6026723
chr1 6026727
chr1 6026780
chr1 6026783
chr1 6026924
chr1 6026926
chr1 6026954
chr1 6027122
chr1 6027277
chr1 6027295
chr1 6027311
chr1 6027332
chr1 6027392
chr1 6027411
chr1 6027416
chr1 6027421
chr1 6027465
chr1 6027475
chr1 6027481
chr1 6027622
chr1 6027652
chr1 6027712
chr1 6027740
chr1 6027744
chr1 6027766
chr1 6027817
chr1 6027820
chr1 6027837
chr1 6027948
chr1 6027993
chr1 6028099
chr1 6028149

chr1 6028271
chr1 6108872
chr1 6198750
chr1 6485967
chr1 6486023
chr1 6486030
chr1 6486081
chr1 6486089
chr1 6486093
chr1 6486154
chr1 6486159
chr1 6486271
chr1 6486402
chr1 6486436
chr1 6486457
chr1 6486467
chr1 6486481
chr1 6486501
chr1 6486514
chr1 6486586
chr1 6962485
chr1 6962505
chr1 6962528
chr1 6962556
chr1 6962604
chr1 6962613
chr1 6962618
chr1 6962681
chr1 6962752
chr1 6962765
chr1 6962799
chr1 6962914
chr1 6962953
chr1 6962977
chr1 6963015
chr1 6963091
chr1 6963199
chr1 6963204
chr1 6963242
chr1 6963287
chr1 6963299
chr1 6963322
chr1 6963353
chr1 6963355
chr1 6963358
chr1 6963388
chr1 6963423
chr1 6963430
chr1 6963435
chr1 6963440
chr1 6963447
chr1 6963461
chr1 6963470
chr1 6963565

chr1 6963571
chr1 6963595
chr1 7549708
chr1 7549816
chr1 7549866
chr1 7602185
chr1 7602263
chr1 7602398
chr1 7602524
chr1 7602570
chr1 7931164
chr1 7931220
chr1 7931255
chr1 7931303
chr1 7931319
chr1 7931337
chr1 7931375
chr1 7940211
chr1 7940254
chr1 7940279
chr1 7940803
chr1 7940844
chr1 7940874
chr1 7949089
chr1 7949225
chr1 7949263
chr1 7949293
chr1 7949392
chr1 7949418
chr1 7949439
chr1 7949446
chr1 7949467
chr1 7949568
chr1 7949604
chr1 7949613
chr1 7961258
chr1 7961267
chr1 7961271
chr1 7961277
chr1 7961289
chr1 7961304
chr1 7961340
chr1 7961345
chr1 7961353
chr1 7961355
chr1 7962625
chr1 8395331
chr1 8395335
chr1 8395347
chr1 8395367
chr1 8395399
chr1 8395431
chr1 8395727
chr1 8395740

chr1 8422777
chr1 8423301
chr1 8423330
chr1 8423338
chr1 8423348
chr1 8423362
chr1 8423366
chr1 8423384
chr1 8423415
chr1 8423423
chr1 8423426
chr1 8423433
chr1 8423436
chr1 8423463
chr1 8423480
chr1 8472911
chr1 8510391
chr1 8510478
chr1 8510587
chr1 8510619
chr1 8525944
chr1 8525951
chr1 8604226
chr1 8604252
chr1 8604400
chr1 8604500
chr1 8651366
chr1 8651388
chr1 8701003
chr1 8701187
chr1 8701267
chr1 8701323
chr1 8701399
chr1 8701443
chr1 8701502
chr1 8701573
chr1 8701599
chr1 8701678
chr1 8701684
chr1 8701691
chr1 8701705
chr1 8703847
chr1 8703868
chr1 8705188
chr1 8816246
chr1 8816258
chr1 8816280
chr1 8816554
chr1 8816570
chr1 8816796
chr1 8816954
chr1 8816962
chr1 8816975
chr1 8817004

chr1 8876602
chr1 8876619
chr1 8876623
chr1 8876625
chr1 8876635
chr1 8876663
chr1 8876679
chr1 8876722
chr1 8876746
chr1 8876752
chr1 8876754
chr1 8876776
chr1 8876790
chr1 8876817
chr1 8876866
chr1 8876874
chr1 8876883
chr1 8876905
chr1 8876950
chr1 8876970
chr1 8876982
chr1 8877012
chr1 8877018
chr1 8877020
chr1 8877028
chr1 8877052
chr1 8877058
chr1 8877075
chr1 8877102
chr1 8877118
chr1 8877173
chr1 8877187
chr1 8877203
chr1 8877231
chr1 8877241
chr1 8877243
chr1 8877251
chr1 8877275
chr1 8877288
chr1 8877314
chr1 8877345
chr1 8877362
chr1 8877373
chr1 8877383
chr1 8877385
chr1 8877389
chr1 8877429
chr1 8877442
chr1 8877469
chr1 9413544
chr1 9413654
chr1 9413675
chr1 9413692
chr1 9413731

chr1 9413736
chr1 9413799
chr1 9628899
chr1 9628917
chr1 9628953
chr1 9628960
chr1 9628990
chr1 9629019
chr1 9629045
chr1 9629109
chr1 9629120
chr1 9629151
chr1 9629157
chr1 9629259
chr1 9629306
chr1 9629318
chr1 9629369
chr1 9629411
chr1 9629466
chr1 9629481
chr1 9629540
chr1 9629550
chr1 9629559
chr1 9629607
chr1 9629708
chr1 9629728
chr1 9652764
chr1 9652832
chr1 9652841
chr1 9652847
chr1 9652849
chr1 9653278
chr1 9653305
chr1 9653381
chr1 9653457
chr1 9653460
chr1 9653466
chr1 9653468
chr1 9653489
chr1 9653832
chr1 9653933
chr1 9653976
chr1 9654035
chr1 9654247
chr1 9654275
chr1 9654277
chr1 9654825
chr1 9654834
chr1 9655186
chr1 9655278
chr1 9800400
chr1 9800457
chr1 9800485
chr1 9800501

chr1 9917951
chr1 9918600
chr1 9926651
chr1 10033669
chr1 10033685
chr1 10033737
chr1 10033743
chr1 10033766
chr1 10033803
chr1 10033828
chr1 10033838
chr1 10033913
chr1 10033984
chr1 10033997
chr1 10170423
chr1 10170509
chr1 10475622
chr1 10475631
chr1 10475696
chr1 10475702
chr1 10475716
chr1 10475720
chr1 10495144
chr1 10495200
chr1 10510583
chr1 10510663
chr1 10510742
chr1 10510790
chr1 10510841
chr1 10510864
chr1 10511023
chr1 10511042
chr1 10511058
chr1 10511068
chr1 10511418
chr1 10511451
chr1 10511668
chr1 10511767
chr1 10792562
chr1 10792584
chr1 10792678
chr1 10792688
chr1 10792795
chr1 10793311
chr1 10793351
chr1 10793362
chr1 10793382
chr1 10793402
chr1 10793632
chr1 10793838
chr1 10793840
chr1 10793861
chr1 11262424
chr1 11262445

chr1 11262447
chr1 11262449
chr1 11262455
chr1 11262474
chr1 11262477
chr1 11262486
chr1 11262492
chr1 11262495
chr1 11262517
chr1 11262521
chr1 11262530
chr1 11262586
chr1 11262655
chr1 11262671
chr1 11262735
chr1 11407242
chr1 11407425
chr1 11407497
chr1 11407532
chr1 11407560
chr1 11736297
chr1 11736434
chr1 11736448
chr1 11736463
chr1 11736477
chr1 11736487
chr1 11736506
chr1 11736513
chr1 11736516
chr1 11736542
chr1 11736575
chr1 11736577
chr1 11736594
chr1 11736608
chr1 11736610
chr1 11736612
chr1 11736638
chr1 11857181
chr1 11857194
chr1 11857204
chr1 11857223
chr1 11857438
chr1 11857493
chr1 11857587
chr1 11857662
chr1 11857742
chr1 11857775
chr1 11907914
chr1 11907955
chr1 11908015
chr1 11908021
chr1 11908081
chr1 11908102
chr1 11908116

chr1 11908138
chr1 11908141
chr1 11908174
chr1 11908187
chr1 11908207
chr1 11908219
chr1 11908255
chr1 12063048
chr1 12063081
chr1 12063182
chr1 12063189
chr1 12063202
chr1 12063205
chr1 12065467
chr1 12065938
chr1 12065968
chr1 12066006
chr1 12066048
chr1 12066134
chr1 12066141
chr1 12150687
chr1 12150738
chr1 12150747
chr1 12150787
chr1 12150920
chr1 12161997
chr1 12162033
chr1 12162047
chr1 12167818
chr1 12167841
chr1 12167863
chr1 12167870
chr1 12167920
chr1 12167940
chr1 12167944
chr1 12168153
chr1 12168175
chr1 12168188
chr1 12168208
chr1 12168256
chr1 12230281
chr1 12230295
chr1 12230310
chr1 12230323
chr1 12230369
chr1 12230371
chr1 12230392
chr1 12230419
chr1 12230422
chr1 12230426
chr1 12230442
chr1 12230450
chr1 12230460
chr1 12230472

chr1 12230491
chr1 12230517
chr1 12230519
chr1 12230527
chr1 12230564
chr1 12230580
chr1 12230583
chr1 12230641
chr1 12230655
chr1 12230657
chr1 12230709
chr1 12230794
chr1 12230831
chr1 12230834
chr1 12230837
chr1 12296868
chr1 12345458
chr1 12345471
chr1 12345508
chr1 12345542
chr1 12434389
chr1 12434421
chr1 12434569
chr1 12449628
chr1 12449651
chr1 12472904
chr1 12472935
chr1 12473253
chr1 12618694
chr1 12618736
chr1 12618755
chr1 12618794
chr1 12618812
chr1 12618874
chr1 12618988
chr1 12620302
chr1 12620354
chr1 12620369
chr1 12620449
chr1 12620510
chr1 12900827
chr1 12901046
chr1 12901188
chr1 12901228
chr1 12901359
chr1 12901505
chr1 12901956
chr1 12901977
chr1 12902128
chr1 13117734
chr1 13117889
chr1 13730477
chr1 13730865
chr1 13730896

chr1 13731019
chr1 13731026
chr1 13731047
chr1 13731060
chr1 13731070
chr1 13731111
chr1 13731137
chr1 14599818
chr1 14599833
chr1 14599877
chr1 14599920
chr1 14599937
chr1 14600025
chr1 14600441
chr1 14600456
chr1 14600489
chr1 14600549
chr1 14600637
chr1 14778332
chr1 14778467
chr1 14778472
chr1 15411124
chr1 15411149
chr1 15411166
chr1 15411196
chr1 15411359
chr1 15411444
chr1 15411614
chr1 15524014
chr1 15524021
chr1 15524031
chr1 15524035
chr1 15524042
chr1 15524044
chr1 15524058
chr1 15524064
chr1 15524080
chr1 15524104
chr1 15524106
chr1 15524119
chr1 15525684
chr1 15526394
chr1 15526460
chr1 15527695
chr1 15527711
chr1 15579396
chr1 15579482
chr1 15579592
chr1 15579633
chr1 15585033
chr1 15585037
chr1 15585040
chr1 15585045
chr1 15585055

chr1 15585057
chr1 15618716
chr1 15618728
chr1 15618881
chr1 15618924
chr1 15833480
chr1 15833485
chr1 15833536
chr1 15833582
chr1 15833592
chr1 15833594
chr1 15833653
chr1 15833677
chr1 15833748
chr1 15833865
chr1 15833993
chr1 15834039
chr1 15834103
chr1 15834189
chr1 15834197
chr1 15849678
chr1 15849719
chr1 15849738
chr1 15849763
chr1 15849770
chr1 15849888
chr1 15849897
chr1 15849910
chr1 15849926
chr1 15849930
chr1 15849955
chr1 15850057
chr1 15850091
chr1 15850099
chr1 15850133
chr1 15850169
chr1 15850200
chr1 15850209
chr1 15850226
chr1 15850259
chr1 15850282
chr1 15850778
chr1 15850969
chr1 15919436
chr1 15919562
chr1 15919621
chr1 15965825
chr1 15965912
chr1 15966064
chr1 15966147
chr1 15966180
chr1 15966251
chr1 15966258
chr1 15966276

chr1 15966329
chr1 15966432
chr1 15966443
chr1 15966448
chr1 16073711
chr1 16073717
chr1 16073726
chr1 16073742
chr1 16073747
chr1 16073749
chr1 16073751
chr1 16073774
chr1 16187887
chr1 16187891
chr1 16187901
chr1 16187925
chr1 16187971
chr1 16187982
chr1 16351184
chr1 16351491
chr1 16351722
chr1 16351794
chr1 16351855
chr1 16351989
chr1 16352001
chr1 16352135
chr1 16352137
chr1 16352146
chr1 16352155
chr1 16367455
chr1 16367458
chr1 16367480
chr1 16367482
chr1 16367514
chr1 16367524
chr1 16367534
chr1 16367536
chr1 16367538
chr1 16367540
chr1 16367547
chr1 16367552
chr1 16367561
chr1 16367568
chr1 16367591
chr1 16367593
chr1 16367608
chr1 16367630
chr1 16367647
chr1 16367680
chr1 16367685
chr1 16368530
chr1 16420795
chr1 16420830
chr1 16420841

chr1 16420940
chr1 16440958
chr1 16440999
chr1 16441025
chr1 16441029
chr1 16441039
chr1 16441042
chr1 16441046
chr1 16441058
chr1 16441061
chr1 16441070
chr1 16441079
chr1 16441088
chr1 16441101
chr1 16441112
chr1 16441131
chr1 16441152
chr1 16441166
chr1 16441173
chr1 16441194
chr1 16441208
chr1 16441230
chr1 16441240
chr1 16441266
chr1 16441279
chr1 16441285
chr1 16441303
chr1 16441307
chr1 16591588
chr1 16591948
chr1 16591958
chr1 16591977
chr1 16591991
chr1 16592027
chr1 16669804
chr1 16670466
chr1 16670468
chr1 16671517
chr1 16671749
chr1 16671843
chr1 16672021
chr1 16678339
chr1 16678410
chr1 16678426
chr1 16678490
chr1 16678519
chr1 16683665
chr1 16684065
chr1 16684282
chr1 16685018
chr1 16685057
chr1 16685070
chr1 16685272
chr1 16744101

chr1 16744109
chr1 16744204
chr1 16744527
chr1 16744557
chr1 16744581
chr1 16744677
chr1 16895495
chr1 16895512
chr1 16895563
chr1 16895626
chr1 16895651
chr1 16895711
chr1 16895744
chr1 16895753
chr1 16895813
chr1 16895838
chr1 16895850
chr1 16895852
chr1 16895857
chr1 16895900
chr1 16895908
chr1 16895910
chr1 16895923
chr1 16896071
chr1 16896095
chr1 16896126
chr1 16896130
chr1 16896132
chr1 16896174
chr1 16896220
chr1 16896254
chr1 16896273
chr1 16896281
chr1 16896298
chr1 16896309
chr1 16896314
chr1 16896339
chr1 16896348
chr1 16896397
chr1 16896493
chr1 16903903
chr1 16903943
chr1 16904004
chr1 16904042
chr1 16904050
chr1 16904058
chr1 16904081
chr1 16904104
chr1 16904111
chr1 16904119
chr1 16904144
chr1 16904164
chr1 16904168
chr1 16904176

chr1 16904213
chr1 16904224
chr1 16904232
chr1 16904246
chr1 16904252
chr1 16904257
chr1 16904290
chr1 16904300
chr1 16904302
chr1 16904306
chr1 16904384
chr1 16904510
chr1 16904652
chr1 16904669
chr1 16904674
chr1 16905131
chr1 16905144
chr1 16905148
chr1 16905164
chr1 16905179
chr1 16905184
chr1 16905298
chr1 16960597
chr1 16960614
chr1 16960629
chr1 17011139
chr1 17011200
chr1 17011240
chr1 17011278
chr1 17011290
chr1 17011335
chr1 17011347
chr1 17011368
chr1 17011375
chr1 17011387
chr1 17011399
chr1 17011415
chr1 17011421
chr1 17011425
chr1 17053195
chr1 17053219
chr1 17053243
chr1 17053287
chr1 17053398
chr1 17053408
chr1 17201743
chr1 17201812
chr1 17201831
chr1 17201979
chr1 17439382
chr1 17439393
chr1 17439401
chr1 17915855
chr1 17915879

chr1 17915890
chr1 17915908
chr1 18101003
chr1 18101163
chr1 18135839
chr1 18384726
chr1 18384903
chr1 18385221
chr1 18477649
chr1 18635222
chr1 18635259
chr1 18635635
chr1 18639118
chr1 18639122
chr1 18639807
chr1 18641929
chr1 18641934
chr1 18641943
chr1 18641970
chr1 18641972
chr1 18642331
chr1 18642579
chr1 18642619
chr1 18642690
chr1 18642712
chr1 18642722
chr1 18642759
chr1 18642772
chr1 18642787
chr1 18642809
chr1 18642844
chr1 18642870
chr1 18642892
chr1 18645592
chr1 18645602
chr1 18645652
chr1 18645668
chr1 18645858
chr1 18645865
chr1 18645926
chr1 18645936
chr1 18645943
chr1 18645945
chr1 18645972
chr1 18926600
chr1 18926630
chr1 18926639
chr1 18926775
chr1 18926799
chr1 18926878
chr1 18926887
chr1 18926941
chr1 18926993
chr1 18927027

chr1 18927106
chr1 18927194
chr1 18927200
chr1 18927208
chr1 18927268
chr1 18927276
chr1 18927280
chr1 18927284
chr1 18927289
chr1 18927298
chr1 18927378
chr1 18927432
chr1 18927435
chr1 18927475
chr1 18927537
chr1 18927585
chr1 19124100
chr1 19124306
chr1 19124313
chr1 19208822
chr1 19208836
chr1 19208900
chr1 19208978
chr1 19209103
chr1 19209173
chr1 19209377
chr1 19251112
chr1 19463201
chr1 19463203
chr1 19463295
chr1 19463339
chr1 19463346
chr1 19463392
chr1 19477027
chr1 19477108
chr1 19477438
chr1 19477545
chr1 19477573
chr1 19477600
chr1 19477603
chr1 19477671
chr1 19477680
chr1 19477822
chr1 19477926
chr1 19477990
chr1 19478075
chr1 19484889
chr1 19484916
chr1 19484935
chr1 19484991
chr1 19485021
chr1 19485024
chr1 19485039
chr1 19485047

chr1 19485106
chr1 19485130
chr1 19485135
chr1 19858837
chr1 20185695
chr1 20185773
chr1 20185802
chr1 20185817
chr1 20185868
chr1 20185874
chr1 20185902
chr1 20185904
chr1 20185915
chr1 20185946
chr1 20185956
chr1 20185970
chr1 20185977
chr1 20185993
chr1 20186004
chr1 20186014
chr1 20186024
chr1 20186034
chr1 20186040
chr1 20186046
chr1 20186049
chr1 20186057
chr1 20186074
chr1 20186077
chr1 20186084
chr1 20186142
chr1 20186147
chr1 20186161
chr1 20186283
chr1 20186292
chr1 20186303
chr1 20186330
chr1 20186383
chr1 20186399
chr1 20186416
chr1 20186448
chr1 20186451
chr1 20186493
chr1 20186507
chr1 20186562
chr1 20186573
chr1 20186583
chr1 20186635
chr1 20186688
chr1 20186909
chr1 20186920
chr1 20588917
chr1 20634096
chr1 20634168
chr1 20634184

chr1 20634226
chr1 20634270
chr1 20634318
chr1 20634374
chr1 20634377
chr1 20634414
chr1 20634432
chr1 20634435
chr1 20660754
chr1 20660838
chr1 20660845
chr1 20660864
chr1 20660867
chr1 20660869
chr1 21022396
chr1 21022413
chr1 21022478
chr1 21022731
chr1 21290350
chr1 21290352
chr1 21290438
chr1 21290445
chr1 21290453
chr1 21290477
chr1 21292978
chr1 21292991
chr1 21293047
chr1 21293078
chr1 21293089
chr1 21293204
chr1 21293222
chr1 21293420
chr1 21293484
chr1 21293505
chr1 21293540
chr1 21293578
chr1 21293639
chr1 21293672
chr1 21293699
chr1 21293713
chr1 21293778
chr1 21293848
chr1 21293939
chr1 21293966
chr1 21294059
chr1 21294095
chr1 21294113
chr1 21294120
chr1 21294122
chr1 21294125
chr1 21294149
chr1 21294319
chr1 21294329
chr1 21294398

chr1 21294502
chr1 21294515
chr1 21294623
chr1 21294638
chr1 21294653
chr1 21294706
chr1 21294733
chr1 21294751
chr1 21294886
chr1 21294902
chr1 21295282
chr1 21389563
chr1 21389617
chr1 21389625
chr1 21389650
chr1 21389846
chr1 21389853
chr1 21389897
chr1 21440522
chr1 21440529
chr1 21440535
chr1 21440553
chr1 21440559
chr1 21440693
chr1 21440705
chr1 21440718
chr1 21440724
chr1 21440728
chr1 21440730
chr1 21440768
chr1 21440785
chr1 21737455
chr1 22019260
chr1 22019403
chr1 22024645
chr1 22024649
chr1 22024667
chr1 22024675
chr1 22024692
chr1 22024699
chr1 22024731
chr1 22024751
chr1 22024763
chr1 22024767
chr1 22024826
chr1 22024832
chr1 22024853
chr1 22024886
chr1 22024896
chr1 22024898
chr1 22024902
chr1 22024932
chr1 22024940
chr1 22025007

chr1 22025031
chr1 22025055
chr1 22025067
chr1 22025110
chr1 22025112
chr1 22025117
chr1 22025649
chr1 22025684
chr1 22025690
chr1 22025698
chr1 22025700
chr1 22025728
chr1 22025778
chr1 22025787
chr1 22025828
chr1 22025855
chr1 22025861
chr1 22025874
chr1 22025880
chr1 22025882
chr1 22025903
chr1 22025929
chr1 22025944
chr1 22025956
chr1 22025994
chr1 22026001
chr1 22026058
chr1 22026101
chr1 22026127
chr1 22026198
chr1 22056705
chr1 22075148
chr1 22075203
chr1 22089262
chr1 22089523
chr1 22428336
chr1 22452521
chr1 22452590
chr1 22452596
chr1 22452765
chr1 22452767
chr1 22452775
chr1 22547044
chr1 22547158
chr1 22547488
chr1 22547518
chr1 22547528
chr1 22547574
chr1 22547617
chr1 22547649
chr1 23119060
chr1 23119072
chr1 23119232
chr1 23148191

chr1 23148196
chr1 23148378
chr1 23148432
chr1 23167966
chr1 23167972
chr1 23167976
chr1 23167979
chr1 23167982
chr1 23168046
chr1 23168055
chr1 23168067
chr1 23168075
chr1 23168086
chr1 23168100
chr1 23168104
chr1 23168109
chr1 23168111
chr1 23168116
chr1 23168119
chr1 23168121
chr1 23168125
chr1 23168134
chr1 23168137
chr1 23168141
chr1 23168143
chr1 23168156
chr1 23168160
chr1 23168165
chr1 23168167
chr1 23168172
chr1 23168174
chr1 23168179
chr1 23168181
chr1 23168185
chr1 23168197
chr1 23168201
chr1 23168204
chr1 23168209
chr1 23168212
chr1 23168214
chr1 23168225
chr1 23168254
chr1 23168286
chr1 23168293
chr1 23168296
chr1 23168303
chr1 23168305
chr1 23168319
chr1 23168323
chr1 23168328
chr1 23168331
chr1 23168338
chr1 23168347
chr1 23168352

chr1 23168354
chr1 23168385
chr1 23168395
chr1 23168398
chr1 23168406
chr1 23168421
chr1 23168423
chr1 23184336
chr1 23184393
chr1 23184636
chr1 23184647
chr1 23184699
chr1 23221253
chr1 23340982
chr1 23341247
chr1 23341370
chr1 23341486
chr1 23341563
chr1 23341614
chr1 23341659
chr1 23341817
chr1 23342198
chr1 23342209
chr1 23404117
chr1 23404125
chr1 23404151
chr1 23452164
chr1 23484459
chr1 23484461
chr1 23484543
chr1 23484599
chr1 23484602
chr1 23484646
chr1 23484651
chr1 23484668
chr1 23484693
chr1 23484723
chr1 23484741
chr1 23484746
chr1 23484751
chr1 23484756
chr1 23484777
chr1 23484781
chr1 23484814
chr1 23484838
chr1 23484841
chr1 23484846
chr1 23484861
chr1 23528340
chr1 23528355
chr1 23528395
chr1 23528435
chr1 23528448
chr1 23528472

chr1 23528627
chr1 23528657
chr1 23528832
chr1 23558717
chr1 23558952
chr1 23558956
chr1 23558983
chr1 23559002
chr1 23559013
chr1 23559048
chr1 23559050
chr1 23559059
chr1 23559068
chr1 23559077
chr1 23559111
chr1 23559117
chr1 23559171
chr1 23559192
chr1 23744710
chr1 23744758
chr1 23744763
chr1 23744809
chr1 23744829
chr1 23744933
chr1 23745025
chr1 23745067
chr1 23745082
chr1 23745178
chr1 23745303
chr1 23772545
chr1 23779202
chr1 23779481
chr1 23791481
chr1 23791607
chr1 23791736
chr1 23825652
chr1 23825657
chr1 23825684
chr1 23825707
chr1 23825716
chr1 23825771
chr1 23825775
chr1 23825787
chr1 23825839
chr1 23825846
chr1 23825857
chr1 23825861
chr1 23825868
chr1 23825892
chr1 23896576
chr1 23896754
chr1 23896762
chr1 23961284
chr1 23978247

chr1 23978489
chr1 23978556
chr1 23978737
chr1 23978740
chr1 23978788
chr1 23978800
chr1 23978803
chr1 24085513
chr1 24319643
chr1 24319652
chr1 24319658
chr1 24319701
chr1 24319715
chr1 24319801
chr1 24391335
chr1 24391388
chr1 24391409
chr1 24391434
chr1 24391436
chr1 24391496
chr1 24391507
chr1 24391532
chr1 24391552
chr1 24391817
chr1 24391823
chr1 24391860
chr1 24391871
chr1 24391940
chr1 24392023
chr1 24392028
chr1 24392030
chr1 24392048
chr1 24392093
chr1 24392179
chr1 24415834
chr1 24415843
chr1 24415852
chr1 24415984
chr1 24416028
chr1 24416043
chr1 24416076
chr1 24416091
chr1 24416182
chr1 24416187
chr1 24416191
chr1 24416196
chr1 24502402
chr1 24502405
chr1 24502433
chr1 24502438
chr1 24502441
chr1 24502448
chr1 24502460
chr1 24502471

chr1 24502474
chr1 24502476
chr1 24502478
chr1 24502480
chr1 24502485
chr1 24502517
chr1 24502521
chr1 24506821
chr1 24506830
chr1 24506934
chr1 24535408
chr1 24535425
chr1 24535429
chr1 24536392
chr1 24536566
chr1 24536630
chr1 24536676
chr1 24536701
chr1 24536720
chr1 24536756
chr1 24536980
chr1 24540029
chr1 24540062
chr1 24540080
chr1 24540091
chr1 24545435
chr1 24545501
chr1 24545503
chr1 24635380
chr1 24635413
chr1 24635736
chr1 24635824
chr1 24635907
chr1 24636507
chr1 24644368
chr1 24644402
chr1 24644418
chr1 24644525
chr1 24644572
chr1 24644581
chr1 24644684
chr1 24644689
chr1 24770405
chr1 24801362
chr1 24848121
chr1 24848161
chr1 24848186
chr1 24848196
chr1 24848202
chr1 24848241
chr1 24848243
chr1 24848248
chr1 24848254
chr1 24848260

chr1 24848285
chr1 24848289
chr1 24848293
chr1 24848300
chr1 24848303
chr1 24848306
chr1 24848311
chr1 24848316
chr1 24848325
chr1 24848327
chr1 24848336
chr1 24848339
chr1 24848357
chr1 24848395
chr1 24848409
chr1 24848428
chr1 24848430
chr1 24848441
chr1 24848445
chr1 24848454
chr1 24848457
chr1 24848497
chr1 24848513
chr1 24848518
chr1 24848522
chr1 24848524
chr1 24848541
chr1 24848544
chr1 24848553
chr1 24848555
chr1 24848563
chr1 24848572
chr1 24848577
chr1 24848587
chr1 24848591
chr1 24848594
chr1 24848605
chr1 24848615
chr1 24925239
chr1 24925245
chr1 24925271
chr1 24925324
chr1 24925365
chr1 24925449
chr1 24925612
chr1 24925653
chr1 24925672
chr1 24925698
chr1 24928255
chr1 24928367
chr1 24928390
chr1 25001856
chr1 25023071
chr1 25023103

chr1 25023190
chr1 25099809
chr1 25099936
chr1 25101686
chr1 25101705
chr1 25232446
chr1 25232462
chr1 25232538
chr1 25232543
chr1 25232547
chr1 25232568
chr1 25232577
chr1 25232588
chr1 25232629
chr1 25232641
chr1 25232647
chr1 25232649
chr1 25232659
chr1 25232674
chr1 25232686
chr1 25232718
chr1 25232722
chr1 25233073
chr1 25233083
chr1 25233119
chr1 25233125
chr1 25233168
chr1 25233202
chr1 25233220
chr1 25233252
chr1 25239840
chr1 25239847
chr1 25239851
chr1 25239866
chr1 25239888
chr1 25240124
chr1 25240128
chr1 25240137
chr1 25240146
chr1 25240150
chr1 25240155
chr1 25240158
chr1 25240178
chr1 25265438
chr1 25265592
chr1 25267621
chr1 25267632
chr1 25267660
chr1 25267662
chr1 25267669
chr1 25267674
chr1 25267705
chr1 25267707
chr1 25267711

chr1 25267734
chr1 25267751
chr1 25267757
chr1 25267767
chr1 25267771
chr1 25267786
chr1 25267812
chr1 25267829
chr1 25267901
chr1 25267909
chr1 25267915
chr1 25267917
chr1 25267932
chr1 25267944
chr1 25267950
chr1 25267953
chr1 25267958
chr1 25267965
chr1 25267970
chr1 25267976
chr1 25267982
chr1 25267984
chr1 25267993
chr1 25267995
chr1 25268016
chr1 25268032
chr1 25268064
chr1 25336623
chr1 25336655
chr1 25431609
chr1 25431621
chr1 25431623
chr1 25431671
chr1 25431814
chr1 25431885
chr1 25431933
chr1 25431980
chr1 25432036
chr1 25432047
chr1 25467527
chr1 25608859
chr1 25608911
chr1 25608918
chr1 25642616
chr1 25642788
chr1 25642811
chr1 25642817
chr1 25642839
chr1 25643020
chr1 25643039
chr1 25676093
chr1 25676126
chr1 25676163
chr1 25676293

chr1 25676337
chr1 25676350
chr1 25676369
chr1 25676386
chr1 25676485
chr1 25676610
chr1 25905049
chr1 25905093
chr1 25905165
chr1 25905182
chr1 25997252
chr1 25997278
chr1 25997476
chr1 25997561
chr1 25997576
chr1 25997591
chr1 25997593
chr1 25997598
chr1 25997629
chr1 25997697
chr1 25997737
chr1 25997778
chr1 25997797
chr1 25997830
chr1 25997841
chr1 25997846
chr1 25997850
chr1 25997869
chr1 25997898
chr1 25997907
chr1 25997941
chr1 25997944
chr1 25997957
chr1 25998059
chr1 25998068
chr1 25998071
chr1 25998084
chr1 25998097
chr1 25998111
chr1 25998114
chr1 25998139
chr1 25998156
chr1 25998183
chr1 25998267
chr1 25998271
chr1 25998277
chr1 25998332
chr1 25998335
chr1 25998337
chr1 26126641
chr1 26145075
chr1 26145084
chr1 26145189
chr1 26145207

chr1 26145212
chr1 26276160
chr1 26276239
chr1 26288788
chr1 26288853
chr1 26289028
chr1 26306602
chr1 26306609
chr1 26306614
chr1 26306619
chr1 26306622
chr1 26306638
chr1 26306640
chr1 26306652
chr1 26306663
chr1 26306715
chr1 26306731
chr1 26306741
chr1 26306764
chr1 26306774
chr1 26306776
chr1 26306780
chr1 26306797
chr1 26306819
chr1 26306824
chr1 26306847
chr1 26306865
chr1 26307023
chr1 26307043
chr1 26307137
chr1 26307141
chr1 26307149
chr1 26307274
chr1 26307385
chr1 26307417
chr1 26307436
chr1 26307474
chr1 26317743
chr1 26317778
chr1 26317908
chr1 26318219
chr1 26318229
chr1 26318271
chr1 26318329
chr1 26318331
chr1 26318602
chr1 26318670
chr1 26336409
chr1 26336444
chr1 26336453
chr1 26336455
chr1 26336459
chr1 26336461
chr1 26336467

chr1 26336473
chr1 26336491
chr1 26336500
chr1 26336522
chr1 26377822
chr1 26377858
chr1 26378145
chr1 26378286
chr1 26378483
chr1 26378713
chr1 26409573
chr1 26409652
chr1 26409663
chr1 26409800
chr1 26412614
chr1 26412635
chr1 26412795
chr1 26412841
chr1 26473388
chr1 26473524
chr1 26473584
chr1 26473595
chr1 26473659
chr1 26473688
chr1 26473717
chr1 26473719
chr1 26473756
chr1 26474258
chr1 26474262
chr1 26474276
chr1 26545673
chr1 26545727
chr1 26545804
chr1 26545823
chr1 26545834
chr1 26545846
chr1 26545855
chr1 26545883
chr1 26545898
chr1 26545920
chr1 26545925
chr1 26545931
chr1 26545970
chr1 26546027
chr1 26546034
chr1 26546047
chr1 26546408
chr1 26647675
chr1 26647697
chr1 26647717
chr1 26647969
chr1 26677598
chr1 26692238
chr1 26692240

chr1 26692263
chr1 26692284
chr1 26692301
chr1 26692317
chr1 26692328
chr1 26692356
chr1 26692375
chr1 26692406
chr1 26692438
chr1 26694861
chr1 26695038
chr1 26698894
chr1 26743943
chr1 26744063
chr1 26744154
chr1 26744184
chr1 26744308
chr1 26744382
chr1 26744497
chr1 26744528
chr1 26744595
chr1 26744787
chr1 26786728
chr1 26786763
chr1 26786968
chr1 26787017
chr1 26787069
chr1 26787082
chr1 26787149
chr1 26787209
chr1 26787262
chr1 26789648
chr1 26789741
chr1 26789774
chr1 26833456
chr1 26833485
chr1 26833536
chr1 26833549
chr1 26833627
chr1 26833635
chr1 26833700
chr1 26833719
chr1 26833729
chr1 26833734
chr1 26899797
chr1 26899811
chr1 26899958
chr1 26900582
chr1 26985661
chr1 27112254
chr1 27112415
chr1 27112457
chr1 27235331
chr1 27235334

chr1 27235360
chr1 27235706
chr1 27235769
chr1 27322235
chr1 27322238
chr1 27322241
chr1 27322250
chr1 27322262
chr1 27322274
chr1 27322277
chr1 27322280
chr1 27322283
chr1 27322287
chr1 27322292
chr1 27322317
chr1 27322320
chr1 27322323
chr1 27322326
chr1 27322342
chr1 27322348
chr1 27322362
chr1 27322373
chr1 27322382
chr1 27322391
chr1 27322404
chr1 27322411
chr1 27322421
chr1 27322453
chr1 27322465
chr1 27322474
chr1 27322476
chr1 27322494
chr1 27341567
chr1 27341607
chr1 27341621
chr1 27341626
chr1 27341834
chr1 27341851
chr1 27341878
chr1 27341930
chr1 27341936
chr1 27341942
chr1 27341950
chr1 27341954
chr1 27341971
chr1 27341992
chr1 27342065
chr1 27342430
chr1 27342495
chr1 27342499
chr1 27342507
chr1 27342638
chr1 27342641
chr1 27342754

chr1 27342759
chr1 27342780
chr1 27342818
chr1 27395385
chr1 27395418
chr1 27395465
chr1 27395690
chr1 27395714
chr1 27395774
chr1 27488141
chr1 27488214
chr1 27488291
chr1 27488478
chr1 27488615
chr1 27488862
chr1 27489080
chr1 27489186
chr1 27489415
chr1 27489426
chr1 27489451
chr1 27489631
chr1 27489634
chr1 27490109
chr1 27490181
chr1 27490218
chr1 27490226
chr1 27490253
chr1 27490270
chr1 27490279
chr1 27490287
chr1 27490312
chr1 27490316
chr1 27490323
chr1 27490329
chr1 27490341
chr1 27490369
chr1 27606347
chr1 27632844
chr1 27662885
chr1 27662918
chr1 27663002
chr1 27663020
chr1 27663055
chr1 27663213
chr1 27663230
chr1 27663279
chr1 27663353
chr1 27672155
chr1 27672184
chr1 27672192
chr1 27672200
chr1 27672243
chr1 27672271
chr1 27726610

chr1 27726660
chr1 27726676
chr1 27726731
chr1 27726767
chr1 27772377
chr1 27772397
chr1 27772421
chr1 27772445
chr1 27772470
chr1 27772502
chr1 27772513
chr1 27772534
chr1 27772597
chr1 27772605
chr1 27772643
chr1 27772682
chr1 27772821
chr1 27772828
chr1 27772839
chr1 27772855
chr1 27772942
chr1 27772975
chr1 27773028
chr1 27773039
chr1 27830369
chr1 27830436
chr1 27830451
chr1 27830455
chr1 27830467
chr1 27869588
chr1 27869606
chr1 27869634
chr1 27869642
chr1 27979981
chr1 28056314
chr1 28056376
chr1 28056428
chr1 28056513
chr1 28057217
chr1 28088103
chr1 28088137
chr1 28088142
chr1 28088157
chr1 28091051
chr1 28091239
chr1 28091250
chr1 28091499
chr1 28091522
chr1 28091529
chr1 28115268
chr1 28115391
chr1 28115400
chr1 28115433
chr1 28199371

chr1 28199447
chr1 28199466
chr1 28199485
chr1 28199498
chr1 28199753
chr1 28199853
chr1 28200085
chr1 28200275
chr1 28245656
chr1 28245660
chr1 28245687
chr1 28245741
chr1 28245788
chr1 28245969
chr1 28246210
chr1 28246406
chr1 28246521
chr1 28246529
chr1 28246541
chr1 28248556
chr1 28248593
chr1 28248659
chr1 28248678
chr1 28248729
chr1 28248758
chr1 28248797
chr1 28248805
chr1 28248968
chr1 28249093
chr1 28249350
chr1 28256356
chr1 28329147
chr1 28329155
chr1 28329207
chr1 28329221
chr1 28329308
chr1 28329316
chr1 28329347
chr1 28329349
chr1 28329355
chr1 28329363
chr1 28329371
chr1 28329378
chr1 28329388
chr1 28329430
chr1 28329434
chr1 28329475
chr1 28329506
chr1 28329530
chr1 28329533
chr1 28329539
chr1 28369761
chr1 28369779
chr1 28369809

chr1 28369812
chr1 28369815
chr1 28369836
chr1 28369859
chr1 28369861
chr1 28369878
chr1 28369896
chr1 28417464
chr1 28417525
chr1 28507382
chr1 28507399
chr1 28507417
chr1 28507468
chr1 28507490
chr1 28507498
chr1 28507533
chr1 28507633
chr1 28507663
chr1 28507686
chr1 28507755
chr1 28507763
chr1 28507847
chr1 28507885
chr1 28507989
chr1 28508055
chr1 28508073
chr1 28508307
chr1 28508528
chr1 28508563
chr1 28519045
chr1 28552891
chr1 28552898
chr1 28552900
chr1 28552905
chr1 28552916
chr1 28552941
chr1 28552960
chr1 28552965
chr1 28552970
chr1 28552975
chr1 28552979
chr1 28552989
chr1 28552998
chr1 28553001
chr1 28553007
chr1 28553021
chr1 28553028
chr1 28553043
chr1 28553047
chr1 28553049
chr1 28553051
chr1 28553059
chr1 28553066
chr1 28553070

chr1 28553080
chr1 28553084
chr1 28553099
chr1 28553104
chr1 28553106
chr1 28553115
chr1 28553134
chr1 28553137
chr1 28553153
chr1 28553157
chr1 28553183
chr1 28553189
chr1 28553191
chr1 28580302
chr1 28580428
chr1 28580593
chr1 28580603
chr1 28580803
chr1 28580843
chr1 28582236
chr1 28582243
chr1 28582258
chr1 28582279
chr1 28582286
chr1 28582334
chr1 28582336
chr1 28582393
chr1 28582440
chr1 28582446
chr1 28582493
chr1 28582551
chr1 28582659
chr1 28582683
chr1 28582745
chr1 28582791
chr1 28582797
chr1 28582807
chr1 28582840
chr1 28582851
chr1 28582865
chr1 28582885
chr1 28582888
chr1 28582895
chr1 28582932
chr1 28642276
chr1 28642444
chr1 28642459
chr1 28642466
chr1 28643475
chr1 28643576
chr1 28643593
chr1 28643603
chr1 28643608
chr1 28643747

chr1 28643923
chr1 28644037
chr1 28648102
chr1 28648715
chr1 28648724
chr1 28648727
chr1 28648755
chr1 28648765
chr1 28648767
chr1 28648859
chr1 28648933
chr1 28648941
chr1 28649008
chr1 28669617
chr1 28669619
chr1 28669653
chr1 28669664
chr1 28669732
chr1 28669736
chr1 28669809
chr1 28669885
chr1 28785475
chr1 28785516
chr1 28785527
chr1 28785556
chr1 28785560
chr1 28785563
chr1 28785572
chr1 28785592
chr1 28785616
chr1 28785624
chr1 28785631
chr1 28785648
chr1 28785656
chr1 28785686
chr1 28785694
chr1 28915552
chr1 28915596
chr1 28927003
chr1 28927251
chr1 28927294
chr1 28927413
chr1 28927476
chr1 28928148
chr1 28928433
chr1 28928595
chr1 28962824
chr1 29017259
chr1 29179763
chr1 29179987
chr1 29180146
chr1 29180297
chr1 29180305
chr1 29180335

chr1 29180405
chr1 29180422
chr1 29180479
chr1 29180502
chr1 29180572
chr1 29180730
chr1 29180807
chr1 29180882
chr1 30743873
chr1 30743890
chr1 30744148
chr1 30744327
chr1 30744359
chr1 30744393
chr1 30744407
chr1 30753918
chr1 30754100
chr1 30754259
chr1 30754284
chr1 30754286
chr1 30756148
chr1 30756275
chr1 30756377
chr1 30756401
chr1 30756408
chr1 30756604
chr1 30756628
chr1 30756691
chr1 30756722
chr1 30757344
chr1 30757380
chr1 30757392
chr1 30757401
chr1 30757456
chr1 30757648
chr1 30757688
chr1 30757693
chr1 30759435
chr1 31063038
chr1 31063050
chr1 31063927
chr1 31063980
chr1 31155148
chr1 31155160
chr1 31155215
chr1 31155254
chr1 31155261
chr1 31155268
chr1 31155277
chr1 31155281
chr1 31155289
chr1 31155292
chr1 31155300
chr1 31155326

chr1 31155352
chr1 31155367
chr1 31155386
chr1 31155406
chr1 31155412
chr1 31155415
chr1 31155426
chr1 31155428
chr1 31155448
chr1 31155450
chr1 31155459
chr1 31155469
chr1 31155479
chr1 31155488
chr1 31155491
chr1 31155494
chr1 31155502
chr1 31155513
chr1 31155515
chr1 31181886
chr1 31181889
chr1 31181901
chr1 31181912
chr1 31181927
chr1 31181930
chr1 31181945
chr1 31181956
chr1 31181959
chr1 31181973
chr1 31181976
chr1 31181979
chr1 31181985
chr1 31182049
chr1 31182081
chr1 31182095
chr1 31182111
chr1 31182119
chr1 31182159
chr1 31182162
chr1 31182168
chr1 31295879
chr1 31295918
chr1 31295928
chr1 31296012
chr1 31296030
chr1 31296075
chr1 31296119
chr1 31296944
chr1 31297009
chr1 31297011
chr1 31297028
chr1 31297031
chr1 31297043
chr1 31297125

chr1 31297130
chr1 31297630
chr1 31297652
chr1 31297757
chr1 31394614
chr1 31394643
chr1 31394647
chr1 31394661
chr1 31394705
chr1 31394707
chr1 31394716
chr1 31394734
chr1 31394743
chr1 31394752
chr1 31394761
chr1 31394774
chr1 31394791
chr1 31394808
chr1 31763342
chr1 31763353
chr1 31763355
chr1 31763406
chr1 31763441
chr1 31763453
chr1 31763458
chr1 31827017
chr1 31827045
chr1 31827293
chr1 31827325
chr1 31827360
chr1 31827365
chr1 31827455
chr1 31873206
chr1 31873233
chr1 31889411
chr1 31889483
chr1 31889492
chr1 31925163
chr1 31925286
chr1 31926271
chr1 31926375
chr1 31927237
chr1 31927245
chr1 31927268
chr1 31927423
chr1 31927445
chr1 31927454
chr1 31927458
chr1 31927476
chr1 31928421
chr1 31928425
chr1 31928427
chr1 31928437
chr1 31928474

chr1 31928568
chr1 31928628
chr1 31928651
chr1 31928932
chr1 31929032
chr1 31929042
chr1 31929261
chr1 31929264
chr1 31930020
chr1 31930055
chr1 31930060
chr1 31930064
chr1 31930076
chr1 31931530
chr1 31931594
chr1 31932024
chr1 31932535
chr1 31932656
chr1 31932963
chr1 31933070
chr1 31933219
chr1 31933246
chr1 31934479
chr1 31934834
chr1 31934911
chr1 31935063
chr1 31935202
chr1 31935235
chr1 31935285
chr1 31936344
chr1 31936363
chr1 31936388
chr1 31936473
chr1 31936480
chr1 31936902
chr1 31936908
chr1 31940237
chr1 31940260
chr1 31943113
chr1 31943178
chr1 31943495
chr1 31944333
chr1 31944347
chr1 31944369
chr1 31944436
chr1 31944471
chr1 31944500
chr1 31944505
chr1 31944519
chr1 31944549
chr1 31944588
chr1 31944595
chr1 31944600
chr1 31944610

chr1 31944619
chr1 31985350
chr1 31985363
chr1 32015082
chr1 32015095
chr1 32015097
chr1 32015111
chr1 32015121
chr1 32015254
chr1 32015291
chr1 32015324
chr1 32015836
chr1 32020812
chr1 32072281
chr1 32072341
chr1 32072352
chr1 32072374
chr1 32072433
chr1 32072451
chr1 32072461
chr1 32107942
chr1 32200927
chr1 32200944
chr1 32200969
chr1 32200978
chr1 32249848
chr1 32249882
chr1 32249884
chr1 32249924
chr1 32250144
chr1 32250215
chr1 32250249
chr1 32250377
chr1 32250450
chr1 32250543
chr1 32250611
chr1 32250777
chr1 32250856
chr1 32250906
chr1 32250956
chr1 32252276
chr1 32253799
chr1 32253910
chr1 32254465
chr1 32255451
chr1 32255464
chr1 32255518
chr1 32255540
chr1 32255589
chr1 32255601
chr1 32273892
chr1 32274062
chr1 32274108
chr1 32274151

chr1 32394469
chr1 32394484
chr1 32394490
chr1 32394495
chr1 32394499
chr1 32394600
chr1 32394613
chr1 32394636
chr1 32394689
chr1 32394695
chr1 32394698
chr1 32394704
chr1 32394720
chr1 32500207
chr1 32500221
chr1 32520703
chr1 32520725
chr1 32520727
chr1 32622994
chr1 32623343
chr1 32623602
chr1 32623902
chr1 32623944
chr1 32623950
chr1 32623981
chr1 32624028
chr1 32647833
chr1 32647873
chr1 32647912
chr1 32647934
chr1 32647971
chr1 32801333
chr1 32801365
chr1 32801391
chr1 32801411
chr1 32801454
chr1 32801629
chr1 32816329
chr1 32816465
chr1 32816476
chr1 32816479
chr1 32981105
chr1 32981305
chr1 32981310
chr1 32981418
chr1 32981488
chr1 32981544
chr1 32981697
chr1 32981716
chr1 33036423
chr1 33036529
chr1 33036559
chr1 33036582
chr1 33036601

chr1 33036609
chr1 33036616
chr1 33036623
chr1 33036639
chr1 33036644
chr1 33036649
chr1 33036676
chr1 33036680
chr1 33036708
chr1 33036711
chr1 33036729
chr1 33036750
chr1 33036756
chr1 33036774
chr1 33036779
chr1 33036798
chr1 33036808
chr1 33036816
chr1 33036832
chr1 33036835
chr1 33036859
chr1 33036863
chr1 33099417
chr1 33099497
chr1 33099516
chr1 33099539
chr1 33126970
chr1 33127269
chr1 33127291
chr1 33127299
chr1 33127305
chr1 33127319
chr1 33127325
chr1 33127343
chr1 33127350
chr1 33127352
chr1 33127358
chr1 33127360
chr1 33127370
chr1 33127387
chr1 33127400
chr1 33127437
chr1 33127445
chr1 33127481
chr1 33127489
chr1 33127492
chr1 33127498
chr1 33127509
chr1 33127526
chr1 33127537
chr1 33127540
chr1 33127549
chr1 33127553
chr1 33127596

chr1 33127614
chr1 33127629
chr1 33181427
chr1 33181435
chr1 33348932
chr1 33348935
chr1 33470561
chr1 33470633
chr1 33470650
chr1 33470655
chr1 33470657
chr1 33470702
chr1 33470746
chr1 33470812
chr1 33472876
chr1 33632851
chr1 33632942
chr1 33632947
chr1 33632998
chr1 33633001
chr1 33633056
chr1 33633166
chr1 33633200
chr1 33633242
chr1 33633260
chr1 33633274
chr1 34567392
chr1 34567982
chr1 34568012
chr1 34924937
chr1 34924951
chr1 34924957
chr1 34924961
chr1 34924980
chr1 34924991
chr1 34925007
chr1 34925010
chr1 34925028
chr1 34925043
chr1 34925052
chr1 34925058
chr1 34925155
chr1 34925168
chr1 34925373
chr1 34984645
chr1 34984673
chr1 34984699
chr1 34985110
chr1 34985118
chr1 34985123
chr1 34985129
chr1 34985136
chr1 35030677
chr1 35030799

chr1 35030804
chr1 35031004
chr1 35031035
chr1 35031087
chr1 35031090
chr1 35031127
chr1 35031183
chr1 35031191
chr1 35031221
chr1 35031227
chr1 35031240
chr1 35031282
chr1 35031320
chr1 35031331
chr1 35031403
chr1 35031408
chr1 35031452
chr1 35031461
chr1 35031472
chr1 35031630
chr1 35031647
chr1 35031657
chr1 35031682
chr1 35031684
chr1 35031695
chr1 35031702
chr1 35031708
chr1 35031717
chr1 35031719
chr1 35031736
chr1 35031742
chr1 35031745
chr1 35031750
chr1 35031755
chr1 35031760
chr1 35031774
chr1 35031779
chr1 35031782
chr1 35031787
chr1 35031802
chr1 35031806
chr1 35031822
chr1 35031836
chr1 35079286
chr1 35079327
chr1 35079335
chr1 35079347
chr1 35079356
chr1 35079359
chr1 35079370
chr1 35079413
chr1 35079420
chr1 35190693
chr1 35190696

chr1 35190724
chr1 35190793
chr1 35190886
chr1 35190919
chr1 35190925
chr1 35190962
chr1 35268293
chr1 35268524
chr1 35268546
chr1 35268842
chr1 35268850
chr1 35268885
chr1 35268887
chr1 35268889
chr1 35268896
chr1 35268916
chr1 35269324
chr1 35269327
chr1 35269463
chr1 35269474
chr1 35269500
chr1 35269524
chr1 35269526
chr1 35269566
chr1 35269584
chr1 35269618
chr1 35269690
chr1 35269714
chr1 35557424
chr1 35637589
chr1 35641770
chr1 35641776
chr1 35641954
chr1 35641958
chr1 35641970
chr1 35642007
chr1 35642092
chr1 35642100
chr1 35642145
chr1 35642162
chr1 35642171
chr1 35642201
chr1 35642209
chr1 35642401
chr1 35646293
chr1 35646324
chr1 35718875
chr1 35718878
chr1 35718884
chr1 35718894
chr1 35718899
chr1 35718903
chr1 35930018
chr1 35930247

chr1 35930256
chr1 35930272
chr1 35930280
chr1 35930305
chr1 35930388
chr1 35930397
chr1 35930440
chr1 35930453
chr1 35930456
chr1 35930460
chr1 35930500
chr1 35930505
chr1 35930538
chr1 35931861
chr1 35931882
chr1 35931902
chr1 35931934
chr1 35932021
chr1 35942793
chr1 35942899
chr1 35942910
chr1 36155594
chr1 36155605
chr1 36157472
chr1 36157539
chr1 36157711
chr1 36157721
chr1 36157780
chr1 36160737
chr1 36160880
chr1 36161022
chr1 36161043
chr1 36161096
chr1 36161103
chr1 36161139
chr1 36161147
chr1 36161169
chr1 36161212
chr1 36161240
chr1 36161246
chr1 36161303
chr1 36161430
chr1 36161518
chr1 36161638
chr1 36223413
chr1 36223440
chr1 36223452
chr1 36223616
chr1 36223655
chr1 36223697
chr1 36374009
chr1 36374044
chr1 36374197
chr1 36374232

chr1 36374655
chr1 36386739
chr1 36386743
chr1 36386862
chr1 36420787
chr1 37255580
chr1 37255591
chr1 37255607
chr1 37255649
chr1 37255657
chr1 37255673
chr1 37255778
chr1 37255800
chr1 37255828
chr1 37255831
chr1 37256070
chr1 37256084
chr1 37256092
chr1 37256162
chr1 37256258
chr1 37311809
chr1 37473474
chr1 37473519
chr1 37473523
chr1 37473577
chr1 37473637
chr1 37473719
chr1 37473753
chr1 37473822
chr1 37473838
chr1 37473922
chr1 37476613
chr1 37476857
chr1 37477162
chr1 37477264
chr1 37477385
chr1 37477396
chr1 37477467
chr1 37477478
chr1 37477505
chr1 37477581
chr1 37477707
chr1 37478539
chr1 37478556
chr1 37478577
chr1 37478600
chr1 37478620
chr1 37478656
chr1 37478670
chr1 37478882
chr1 37479105
chr1 37479137
chr1 37479173
chr1 37479381

chr1 37479391
chr1 37514128
chr1 37553620
chr1 37553631
chr1 37553659
chr1 37553670
chr1 37553756
chr1 37553797
chr1 37553812
chr1 37553822
chr1 37553863
chr1 37553901
chr1 37553911
chr1 37553934
chr1 37553938
chr1 37553951
chr1 37554006
chr1 37554018
chr1 37554024
chr1 37554031
chr1 37554033
chr1 37554041
chr1 37554045
chr1 37554050
chr1 37554055
chr1 37554062
chr1 37554065
chr1 37554071
chr1 37554082
chr1 37554092
chr1 37554096
chr1 37554099
chr1 37554106
chr1 37554115
chr1 37554132
chr1 37554155
chr1 37554158
chr1 37554169
chr1 37554175
chr1 37554180
chr1 37554189
chr1 37595105
chr1 37595222
chr1 37595238
chr1 37595405
chr1 37595418
chr1 37595550
chr1 37595640
chr1 37595644
chr1 37595669
chr1 37595700
chr1 37596252
chr1 37596275
chr1 37596457

chr1 37674037
chr1 37674082
chr1 37674110
chr1 37674122
chr1 37674145
chr1 37674165
chr1 37674182
chr1 37674198
chr1 37674228
chr1 37689916
chr1 37689936
chr1 37689952
chr1 37689962
chr1 37689977
chr1 37690036
chr1 37690041
chr1 37690055
chr1 37692252
chr1 37692275
chr1 37692281
chr1 37692290
chr1 37692296
chr1 37692317
chr1 37692355
chr1 37692357
chr1 37692360
chr1 37692363
chr1 37692366
chr1 37692372
chr1 37692378
chr1 37692398
chr1 37692407
chr1 37692422
chr1 37692457
chr1 37692460
chr1 37692472
chr1 37753570
chr1 37771003
chr1 37859580
chr1 37859611
chr1 37859620
chr1 37859633
chr1 37859658
chr1 37859671
chr1 37859723
chr1 37859727
chr1 37859735
chr1 37859746
chr1 37859767
chr1 37859769
chr1 37859779
chr1 37859783
chr1 37859794
chr1 37859796

chr1 37859803
chr1 37859813
chr1 37859819
chr1 37859821
chr1 37859827
chr1 37859847
chr1 37859867
chr1 37859890
chr1 37859897
chr1 37859903
chr1 37859926
chr1 37859968
chr1 37859985
chr1 37859989
chr1 37859991
chr1 37859994
chr1 37860000
chr1 37860014
chr1 37860022
chr1 37989479
chr1 38012701
chr1 38012704
chr1 38012707
chr1 38012723
chr1 38012745
chr1 38012759
chr1 38012765
chr1 38012768
chr1 38012807
chr1 38012810
chr1 38012818
chr1 38012825
chr1 38012836
chr1 38012842
chr1 38012857
chr1 38012903
chr1 38012933
chr1 38012939
chr1 38012948
chr1 38012978
chr1 38012997
chr1 38013003
chr1 38013014
chr1 38013022
chr1 38013030
chr1 38013048
chr1 38013054
chr1 38013223
chr1 38013240
chr1 38013266
chr1 38013303
chr1 38727934
chr1 38727962
chr1 38728013

chr1 38728191
chr1 38728203
chr1 38728258
chr1 38728280
chr1 38728370
chr1 38728392
chr1 38728394
chr1 38728457
chr1 38728482
chr1 38728530
chr1 38852407
chr1 38852751
chr1 38852822
chr1 38852935
chr1 38853005
chr1 38853087
chr1 38977710
chr1 38977760
chr1 38977794
chr1 38977813
chr1 38991069
chr1 38991801
chr1 38991874
chr1 38991879
chr1 38991893
chr1 38991914
chr1 38991921
chr1 38991932
chr1 38991981
chr1 38991999
chr1 38992016
chr1 38992024
chr1 38992034
chr1 38992036
chr1 38992042
chr1 38992101
chr1 38992104
chr1 38992115
chr1 38992122
chr1 38992157
chr1 38992184
chr1 38992206
chr1 38992239
chr1 38992246
chr1 38996719
chr1 38996750
chr1 39003941
chr1 39026751
chr1 39026756
chr1 39026790
chr1 39026802
chr1 39026849
chr1 39026855
chr1 39026887

chr1 39026898
chr1 39026912
chr1 39026928
chr1 39026948
chr1 39026956
chr1 39098872
chr1 39185777
chr1 39195859
chr1 39195942
chr1 39196265
chr1 39196526
chr1 39196563
chr1 39196677
chr1 39196685
chr1 39196989
chr1 39197565
chr1 39197604
chr1 39204619
chr1 39204727
chr1 39204772
chr1 39204838
chr1 39204909
chr1 39204975
chr1 39205012
chr1 39205076
chr1 39205235
chr1 39205626
chr1 39205630
chr1 39205642
chr1 39206114
chr1 39206167
chr1 39206222
chr1 39206675
chr1 39206688
chr1 39206768
chr1 39215398
chr1 39215414
chr1 39215465
chr1 39215524
chr1 39215777
chr1 39215802
chr1 39216068
chr1 39216770
chr1 39216853
chr1 39216948
chr1 39217014
chr1 39217053
chr1 39231004
chr1 39231122
chr1 39231187
chr1 39231193
chr1 39231209
chr1 39258831
chr1 39258869

chr1 39268138
chr1 39514868
chr1 39514870
chr1 39514878
chr1 39514893
chr1 39514895
chr1 39514897
chr1 39514902
chr1 39514907
chr1 39514911
chr1 39514941
chr1 39514946
chr1 39514951
chr1 39514960
chr1 39514963
chr1 39514968
chr1 39514978
chr1 39514988
chr1 39514992
chr1 39514994
chr1 39515003
chr1 39515006
chr1 39515009
chr1 39515021
chr1 39515023
chr1 39515039
chr1 39515043
chr1 39515064
chr1 39515070
chr1 39515084
chr1 39515086
chr1 39515092
chr1 39515120
chr1 39515125
chr1 39515129
chr1 39515138
chr1 39515147
chr1 39515156
chr1 39515604
chr1 39515615
chr1 39515633
chr1 39515637
chr1 39515648
chr1 39515672
chr1 39515677
chr1 39515680
chr1 39515716
chr1 39515738
chr1 39515740
chr1 39515761
chr1 39515768
chr1 39515777
chr1 39515784
chr1 39515789

chr1 39515809
chr1 39515827
chr1 39515835
chr1 39515837
chr1 39515849
chr1 39515851
chr1 39515872
chr1 39515881
chr1 39515962
chr1 39515982
chr1 39515991
chr1 39516010
chr1 39516073
chr1 39516076
chr1 39516085
chr1 39516095
chr1 39516148
chr1 39516151
chr1 39539433
chr1 39727316
chr1 39727380
chr1 39727473
chr1 39770106
chr1 39770229
chr1 39770310
chr1 39770319
chr1 39770340
chr1 39770342
chr1 39770354
chr1 39770356
chr1 39770364
chr1 39770382
chr1 39770407
chr1 39770414
chr1 39770423
chr1 39770430
chr1 39770451
chr1 39770453
chr1 39770475
chr1 39770511
chr1 39770530
chr1 39770533
chr1 39770538
chr1 39770562
chr1 39770573
chr1 39770577
chr1 39770595
chr1 39770606
chr1 39770982
chr1 39771012
chr1 39771014
chr1 39771028
chr1 39771030
chr1 39771033

chr1 39772133
chr1 39772698
chr1 39772730
chr1 39772768
chr1 39772777
chr1 39772808
chr1 39799937
chr1 39816642
chr1 39816666
chr1 39816727
chr1 39816772
chr1 39817314
chr1 39817316
chr1 39817365
chr1 39817370
chr1 39846281
chr1 39955169
chr1 39955181
chr1 39955183
chr1 39955185
chr1 39955188
chr1 40038956
chr1 40039190
chr1 40039457
chr1 40039488
chr1 40039526
chr1 40039592
chr1 40039614
chr1 40039629
chr1 40039645
chr1 40041371
chr1 40041406
chr1 40041452
chr1 40041521
chr1 40059395
chr1 40059431
chr1 40059497
chr1 40059536
chr1 40060007
chr1 40060100
chr1 40060158
chr1 40060343
chr1 40060381
chr1 40060654
chr1 40060745
chr1 40096664
chr1 40097388
chr1 40097390
chr1 40097402
chr1 40097412
chr1 40097419
chr1 40131165
chr1 40131188
chr1 40144110

chr1 40144233
chr1 40144235
chr1 40144237
chr1 40144291
chr1 40162199
chr1 40162208
chr1 40162216
chr1 40162252
chr1 40162324
chr1 40162409
chr1 40162551
chr1 40162606
chr1 40162609
chr1 40162653
chr1 40162705
chr1 40162760
chr1 40162828
chr1 40163023
chr1 40163231
chr1 40164103
chr1 40164828
chr1 40164871
chr1 40186025
chr1 40186106
chr1 40186120
chr1 40186543
chr1 40186713
chr1 40222330
chr1 40222585
chr1 40222662
chr1 40223258
chr1 40241986
chr1 40242002
chr1 40242024
chr1 40242026
chr1 40242032
chr1 40242049
chr1 40242120
chr1 40242124
chr1 40242198
chr1 40242214
chr1 40256911
chr1 40256926
chr1 40257244
chr1 40373798
chr1 40374848
chr1 40374885
chr1 40374923
chr1 40374947
chr1 40374978
chr1 40375001
chr1 40375251
chr1 40375277
chr1 40375327

chr1 40375334
chr1 40375375
chr1 40381407
chr1 40381526
chr1 40381558
chr1 40381675
chr1 40381793
chr1 40381804
chr1 40381837
chr1 40382260
chr1 40382273
chr1 40382282
chr1 40385814
chr1 40386190
chr1 40386340
chr1 40386353
chr1 40386480
chr1 40386524
chr1 40386572
chr1 40389780
chr1 40389856
chr1 40389924
chr1 40390058
chr1 40390077
chr1 40531920
chr1 40531952
chr1 40600549
chr1 40665592
chr1 40665596
chr1 40665604
chr1 40665616
chr1 40665621
chr1 40665625
chr1 40669033
chr1 40669067
chr1 40669069
chr1 40669245
chr1 40669284
chr1 40669516
chr1 40669557
chr1 40669591
chr1 40692613
chr1 40692622
chr1 40692698
chr1 40692732
chr1 40692767
chr1 40692849
chr1 40693073
chr1 40850073
chr1 40850138
chr1 40850147
chr1 40850191
chr1 40850228
chr1 40850231

chr1 40850240
chr1 40850289
chr1 40850555
chr1 40952735
chr1 40952770
chr1 40952822
chr1 40980935
chr1 41003848
chr1 41241130
chr1 41241135
chr1 41241159
chr1 41241165
chr1 41241176
chr1 41241227
chr1 41241272
chr1 41241286
chr1 41404601
chr1 41673495
chr1 41673526
chr1 41673598
chr1 41673600
chr1 41720974
chr1 41721043
chr1 41739254
chr1 41739264
chr1 41739332
chr1 41739350
chr1 41739360
chr1 41739430
chr1 41739446
chr1 41739458
chr1 41739507
chr1 41739625
chr1 41862840
chr1 41862875
chr1 41891784
chr1 41898294
chr1 41898401
chr1 41898590
chr1 41898594
chr1 41920183
chr1 41920230
chr1 41920253
chr1 41920270
chr1 41920272
chr1 41920392
chr1 41920408
chr1 42333883
chr1 42333899
chr1 42334053
chr1 42334070
chr1 42334146
chr1 42334156
chr1 42334282

chr1 42462737
chr1 42462867
chr1 42462876
chr1 42462890
chr1 42463008
chr1 42463032
chr1 42609283
chr1 42609719
chr1 42612434
chr1 42658788
chr1 42683574
chr1 42683703
chr1 42683734
chr1 42683767
chr1 42683777
chr1 42683826
chr1 42683855
chr1 42683876
chr1 42683924
chr1 42683950
chr1 42815111
chr1 42815134
chr1 42816007
chr1 42845976
chr1 42845982
chr1 42846007
chr1 42846009
chr1 42846016
chr1 42846088
chr1 42846190
chr1 42846197
chr1 42846208
chr1 42847106
chr1 42847115
chr1 42847154
chr1 42847270
chr1 42847283
chr1 42847318
chr1 42847412
chr1 42847698
chr1 42923045
chr1 42923250
chr1 42923286
chr1 42923357
chr1 42923490
chr1 42923613
chr1 42923740
chr1 42923878
chr1 42923905
chr1 42923911
chr1 42923917
chr1 42923923
chr1 42924384
chr1 42924416

chr1 42924430
chr1 42924436
chr1 42924440
chr1 42924443
chr1 42924446
chr1 42924454
chr1 42924467
chr1 42924490
chr1 42924496
chr1 42924502
chr1 42924512
chr1 42924516
chr1 42924543
chr1 42924555
chr1 42924573
chr1 42924584
chr1 42924588
chr1 42924600
chr1 42924603
chr1 42924605
chr1 42924620
chr1 42924634
chr1 42931415
chr1 42931439
chr1 42931563
chr1 42931623
chr1 42931689
chr1 42931697
chr1 42931721
chr1 42931757
chr1 42931989
chr1 42932018
chr1 42932082
chr1 42932130
chr1 42932153
chr1 42932236
chr1 42932240
chr1 42932293
chr1 42932317
chr1 42932383
chr1 42932418
chr1 42932432
chr1 42932456
chr1 42932577
chr1 43295812
chr1 43389451
chr1 43389481
chr1 43389506
chr1 43389592
chr1 43389595
chr1 43389601
chr1 43389616
chr1 43389645
chr1 43389732

chr1 43389740
chr1 43389747
chr1 43389767
chr1 43389770
chr1 43389774
chr1 43389779
chr1 43389800
chr1 43454191
chr1 43454243
chr1 43454278
chr1 43454328
chr1 43454385
chr1 43454419
chr1 43454432
chr1 43454478
chr1 43454490
chr1 43454492
chr1 43454494
chr1 43454503
chr1 43454546
chr1 43454584
chr1 43454590
chr1 43454593
chr1 43454616
chr1 44031075
chr1 44031077
chr1 44031079
chr1 44031090
chr1 44213851
chr1 44214034
chr1 44214187
chr1 44409017
chr1 44409019
chr1 44409024
chr1 44409029
chr1 44409057
chr1 44409066
chr1 44409068
chr1 44409072
chr1 44409078
chr1 44409101
chr1 44409113
chr1 44409150
chr1 44409217
chr1 44409239
chr1 44409284
chr1 44409323
chr1 44409399
chr1 44409436
chr1 44409474
chr1 44409556
chr1 44409594
chr1 44409661
chr1 44409937

chr1 44409943
chr1 44410060
chr1 44429484
chr1 44429505
chr1 44519299
chr1 44674067
chr1 44674099
chr1 44674112
chr1 44674122
chr1 44674129
chr1 44674152
chr1 44674161
chr1 44674168
chr1 44674221
chr1 44674226
chr1 44674236
chr1 44674242
chr1 44674253
chr1 44674267
chr1 44674271
chr1 44674302
chr1 44674304
chr1 44674353
chr1 44674361
chr1 44674383
chr1 44715190
chr1 44715403
chr1 44715486
chr1 44721585
chr1 44721677
chr1 44721679
chr1 44721693
chr1 44721760
chr1 44721846
chr1 44721866
chr1 44721879
chr1 44721958
chr1 44730591
chr1 44730607
chr1 44730645
chr1 44730708
chr1 44730712
chr1 44730755
chr1 44730945
chr1 44731008
chr1 44731047
chr1 44731101
chr1 44731114
chr1 44731134
chr1 44731147
chr1 44731197
chr1 44731204
chr1 44731270
chr1 44731290

chr1 44731349
chr1 44740124
chr1 44740135
chr1 44740331
chr1 44740354
chr1 44786094
chr1 44786100
chr1 44786105
chr1 44786111
chr1 44786115
chr1 44786123
chr1 44786134
chr1 44786154
chr1 44786181
chr1 44800986
chr1 44801014
chr1 44801020
chr1 44801022
chr1 44801031
chr1 44801060
chr1 44801075
chr1 44801083
chr1 44801087
chr1 44801094
chr1 44801110
chr1 44801147
chr1 44801171
chr1 44801366
chr1 44801370
chr1 44807564
chr1 44807568
chr1 44807605
chr1 44807727
chr1 44807774
chr1 44807823
chr1 44807848
chr1 44807854
chr1 44807873
chr1 44807879
chr1 44807882
chr1 44807932
chr1 44807995
chr1 44808008
chr1 44808020
chr1 44819410
chr1 44819442
chr1 44819458
chr1 44819542
chr1 44819606
chr1 44819608
chr1 44819614
chr1 44819631
chr1 44819657
chr1 44819697

chr1 44819699
chr1 44819705
chr1 44985921
chr1 45011468
chr1 45011620
chr1 45011649
chr1 45011953
chr1 45011964
chr1 45012007
chr1 45012585
chr1 45012606
chr1 45116669
chr1 45116794
chr1 45246505
chr1 45246562
chr1 45246735
chr1 45306850
chr1 45306889
chr1 45307097
chr1 45307154
chr1 45307165
chr1 45340515
chr1 45340541
chr1 45499915
chr1 45500174
chr1 45500181
chr1 45500198
chr1 45500200
chr1 45500219
chr1 45500255
chr1 45520882
chr1 45520890
chr1 45520931
chr1 45520933
chr1 45521057
chr1 45521120
chr1 45521171
chr1 45521198
chr1 45521204
chr1 45521230
chr1 45521319
chr1 45521338
chr1 45521354
chr1 45521372
chr1 45521387
chr1 45521395
chr1 45521419
chr1 45543874
chr1 45550975
chr1 45551096
chr1 45551136
chr1 45551190
chr1 45551205
chr1 45551251

chr1 45551270
chr1 45551283
chr1 45551414
chr1 45551421
chr1 45551495
chr1 45551691
chr1 45551800
chr1 45551885
chr1 45583625
chr1 45583629
chr1 45583634
chr1 45583646
chr1 45583685
chr1 45583707
chr1 45583753
chr1 45583761
chr1 45583806
chr1 45583849
chr1 45583872
chr1 45583874
chr1 45585071
chr1 45585119
chr1 45585138
chr1 45585176
chr1 45585280
chr1 45585404
chr1 45585436
chr1 45585546
chr1 45585578
chr1 45585747
chr1 45585918
chr1 45586214
chr1 45586234
chr1 45586239
chr1 45586354
chr1 45586396
chr1 45586410
chr1 45586430
chr1 45586476
chr1 45586512
chr1 45586537
chr1 45586565
chr1 45586568
chr1 45586616
chr1 45684864
chr1 45685062
chr1 45685112
chr1 45687651
chr1 45688717
chr1 45688724
chr1 45803993
chr1 45803996
chr1 45803998
chr1 45804001

chr1 45804004
chr1 45804008
chr1 45804011
chr1 45804015
chr1 45804025
chr1 45804037
chr1 45804041
chr1 45804048
chr1 45804050
chr1 45804057
chr1 45804079
chr1 45804091
chr1 45804116
chr1 45804134
chr1 45804137
chr1 45804147
chr1 45804184
chr1 45804186
chr1 45804214
chr1 45804325
chr1 45804333
chr1 45804366
chr1 45804387
chr1 45804486
chr1 46131817
chr1 46131884
chr1 46131916
chr1 46131922
chr1 46131924
chr1 46131941
chr1 46131953
chr1 46131955
chr1 46131972
chr1 46132052
chr1 46132065
chr1 46132077
chr1 46132092
chr1 46132105
chr1 46132153
chr1 46132209
chr1 46132232
chr1 46132261
chr1 46132268
chr1 46132287
chr1 46132303
chr1 46132305
chr1 46132327
chr1 46132362
chr1 46132376
chr1 46132599
chr1 46132603
chr1 46132629
chr1 46132659
chr1 46132662

chr1 46132664
chr1 46132682
chr1 46132700
chr1 46132708
chr1 46132719
chr1 46132721
chr1 46132724
chr1 46132752
chr1 46132763
chr1 46132766
chr1 46132771
chr1 46132776
chr1 46132784
chr1 46247081
chr1 46247100
chr1 46247108
chr1 46247160
chr1 46247731
chr1 46247768
chr1 46247770
chr1 46247772
chr1 46247815
chr1 46247828
chr1 46247963
chr1 46247976
chr1 46247983
chr1 46248042
chr1 46248047
chr1 46248050
chr1 46248052
chr1 46248075
chr1 46248105
chr1 46248187
chr1 46248192
chr1 46248206
chr1 46248255
chr1 46248283
chr1 46248345
chr1 46248383
chr1 46304059
chr1 46340341
chr1 46340353
chr1 46340357
chr1 46340368
chr1 46340464
chr1 46340483
chr1 46340493
chr1 46340703
chr1 46668008
chr1 46668031
chr1 46668127
chr1 46668133
chr1 46668142
chr1 46668145

chr1 46668154
chr1 46668159
chr1 46668164
chr1 46668169
chr1 46668174
chr1 46668187
chr1 46668189
chr1 46668197
chr1 46668199
chr1 46668208
chr1 46668226
chr1 46668228
chr1 46668234
chr1 46668239
chr1 46668241
chr1 46668252
chr1 46668262
chr1 46668278
chr1 46668281
chr1 46668287
chr1 46668289
chr1 46668296
chr1 46668302
chr1 46668326
chr1 46668332
chr1 46668353
chr1 46888400
chr1 46890063
chr1 47108308
chr1 47108384
chr1 47108462
chr1 47109999
chr1 47171961
chr1 47171982
chr1 47172001
chr1 47172020
chr1 47289478
chr1 47446663
chr1 47446667
chr1 47447081
chr1 47447222
chr1 47447524
chr1 47447877
chr1 48094823
chr1 48094885
chr1 48367231
chr1 48367367
chr1 48481008
chr1 48544993
chr1 48776377
chr1 48776386
chr1 48776994
chr1 48777006
chr1 48777016

chr1 48777024
chr1 48777026
chr1 48777057
chr1 48777085
chr1 48885279
chr1 48885483
chr1 48885523
chr1 48886002
chr1 48899850
chr1 48901052
chr1 50109681
chr1 50109691
chr1 50109702
chr1 50109793
chr1 50109819
chr1 50109886
chr1 50109896
chr1 50110067
chr1 50110086
chr1 50110259
chr1 50110275
chr1 50110284
chr1 50110303
chr1 50110313
chr1 50110414
chr1 50110439
chr1 50110473
chr1 50110541
chr1 50110550
chr1 50110602
chr1 50110842
chr1 50110861
chr1 50110895
chr1 50331538
chr1 50331689
chr1 50331789
chr1 50424164
chr1 50424185
chr1 50429089
chr1 50429286
chr1 50429425
chr1 50429479
chr1 50429558
chr1 50970178
chr1 50970187
chr1 50970194
chr1 50970277
chr1 50970374
chr1 50970389
chr1 50970397
chr1 50970401
chr1 50970458
chr1 50970490
chr1 50970544

chr1 50970551
chr1 50970665
chr1 50970755
chr1 50976125
chr1 50976236
chr1 50976244
chr1 50976267
chr1 50976461
chr1 50976484
chr1 50976511
chr1 50976606
chr1 50976609
chr1 50976646
chr1 50976669
chr1 50976674
chr1 50976686
chr1 50976689
chr1 50976768
chr1 50976787
chr1 50976865
chr1 50976903
chr1 50976937
chr1 50976967
chr1 50976984
chr1 50977000
chr1 50977019
chr1 50977048
chr1 50977058
chr1 50977085
chr1 50977088
chr1 50977120
chr1 50977170
chr1 50977189
chr1 50977243
chr1 50977291
chr1 50977329
chr1 50977340
chr1 50977397
chr1 50977442
chr1 50977444
chr1 50977484
chr1 50977730
chr1 51173590
chr1 51237216
chr1 51237221
chr1 51237272
chr1 51237299
chr1 51237320
chr1 51237368
chr1 51237398
chr1 51237404
chr1 51237521
chr1 51237591
chr1 51237599

chr1 51237688
chr1 51285304
chr1 51430021
chr1 51430098
chr1 51509761
chr1 51516997
chr1 51517162
chr1 51517172
chr1 51517312
chr1 51517499
chr1 51517824
chr1 51517853
chr1 51517956
chr1 51517978
chr1 51517998
chr1 51518001
chr1 51518060
chr1 51518076
chr1 51518085
chr1 51518087
chr1 51518109
chr1 51518121
chr1 51518123
chr1 51518186
chr1 51569623
chr1 51729176
chr1 51729187
chr1 51729207
chr1 51729213
chr1 51729226
chr1 51729267
chr1 51729269
chr1 51729318
chr1 51729357
chr1 51729367
chr1 51729449
chr1 51729474
chr1 51729498
chr1 51729501
chr1 51729504
chr1 51729507
chr1 51729516
chr1 51729518
chr1 51729530
chr1 51729562
chr1 51729599
chr1 51729603
chr1 51729611
chr1 51729619
chr1 51729630
chr1 51729632
chr1 51729726
chr1 51729751
chr1 51729768

chr1 51729773
chr1 51729782
chr1 51729785
chr1 51729796
chr1 51729866
chr1 51729875
chr1 51729882
chr1 51729889
chr1 51729894
chr1 51729919
chr1 51729932
chr1 51729998
chr1 51730014
chr1 51730034
chr1 51730063
chr1 51730067
chr1 51730075
chr1 51730080
chr1 51730082
chr1 51730089
chr1 51730111
chr1 51730114
chr1 51730135
chr1 51730138
chr1 51730148
chr1 51878706
chr1 51878712
chr1 51878714
chr1 51878757
chr1 51878760
chr1 51878770
chr1 51878778
chr1 52055036
chr1 52055075
chr1 52055080
chr1 52055086
chr1 52055088
chr1 52055108
chr1 52055110
chr1 52055118
chr1 52055128
chr1 52055132
chr1 52055140
chr1 52055155
chr1 52055169
chr1 52055172
chr1 52055176
chr1 52055179
chr1 52055374
chr1 52055379
chr1 52055384
chr1 52055413
chr1 52055426
chr1 52055435

chr1 52055445
chr1 52055447
chr1 52055451
chr1 52055483
chr1 52055518
chr1 52055530
chr1 52055546
chr1 52055564
chr1 52055627
chr1 52055663
chr1 52055668
chr1 52055676
chr1 52055681
chr1 52055701
chr1 52055738
chr1 52057509
chr1 52057658
chr1 52139838
chr1 52139886
chr1 52139893
chr1 52140102
chr1 52140322
chr1 52141194
chr1 52141442
chr1 52141618
chr1 52141634
chr1 52141690
chr1 52141729
chr1 52141735
chr1 52141748
chr1 52403408
chr1 52403542
chr1 52403581
chr1 52403663
chr1 52403796
chr1 52403803
chr1 52403815
chr1 52403829
chr1 52403838
chr1 52403842
chr1 52404571
chr1 52404578
chr1 52404581
chr1 52404583
chr1 52404590
chr1 52404608
chr1 52404617
chr1 52404632
chr1 52404723
chr1 52404759
chr1 52404775
chr1 52423774
chr1 52423861
chr1 52423903

chr1 52423931
chr1 52423941
chr1 52423946
chr1 52423972
chr1 52423984
chr1 52423987
chr1 52424204
chr1 52551456
chr1 52551623
chr1 52551673
chr1 52551675
chr1 52551679
chr1 52551681
chr1 52551683
chr1 52551685
chr1 52551719
chr1 52551759
chr1 52551881
chr1 52551939
chr1 52551977
chr1 52552009
chr1 52633752
chr1 52635876
chr1 52636614
chr1 52637043
chr1 52637063
chr1 52637099
chr1 52637289
chr1 52637317
chr1 52637338
chr1 52637378
chr1 52637538
chr1 52637590
chr1 52637622
chr1 52637647
chr1 52702994
chr1 52726352
chr1 52726366
chr1 52726377
chr1 52726389
chr1 52726406
chr1 52726416
chr1 52726418
chr1 52726424
chr1 52726426
chr1 52726428
chr1 52726432
chr1 52726436
chr1 52726444
chr1 52726446
chr1 52726452
chr1 52726461
chr1 52726471
chr1 52726478

chr1 52726484
chr1 52726490
chr1 52726496
chr1 52726511
chr1 52726536
chr1 52726554
chr1 52726560
chr1 52726563
chr1 52726567
chr1 52726570
chr1 52726589
chr1 52726627
chr1 52726630
chr1 52726633
chr1 52726644
chr1 52726659
chr1 52726671
chr1 52726674
chr1 52726709
chr1 52726715
chr1 52726719
chr1 52726734
chr1 52726738
chr1 52726741
chr1 52726743
chr1 52726755
chr1 52726788
chr1 52726816
chr1 52726822
chr1 52726830
chr1 52726865
chr1 52726909
chr1 52726949
chr1 52726963
chr1 52727072
chr1 52727135
chr1 52727153
chr1 52727169
chr1 52727187
chr1 52727197
chr1 52727201
chr1 52727206
chr1 52727209
chr1 52727212
chr1 52727236
chr1 52867344
chr1 52867743
chr1 53014653
chr1 53014683
chr1 53014750
chr1 53014783
chr1 53014791
chr1 53014879
chr1 53014936

chr1 53015031
chr1 53015038
chr1 53015261
chr1 53015305
chr1 53015418
chr1 53015439
chr1 53015527
chr1 53015599
chr1 53028419
chr1 53196644
chr1 53196647
chr1 53196651
chr1 53196684
chr1 53196691
chr1 53196693
chr1 53196713
chr1 53196717
chr1 53196734
chr1 53196751
chr1 53196762
chr1 53196777
chr1 53197310
chr1 53197339
chr1 53197432
chr1 53197435
chr1 53197448
chr1 53413390
chr1 53437034
chr1 53437102
chr1 53501990
chr1 53502209
chr1 53502546
chr1 53837338
chr1 53837343
chr1 53837376
chr1 53837433
chr1 53837443
chr1 53837458
chr1 53837465
chr1 53837501
chr1 53837518
chr1 53837524
chr1 53837526
chr1 53837726
chr1 53837913
chr1 53837971
chr1 53838632
chr1 53838719
chr1 53838858
chr1 53839173
chr1 53839179
chr1 53889914
chr1 53889918
chr1 53889942

chr1 53889962
chr1 53889968
chr1 53889982
chr1 53890022
chr1 53890035
chr1 53946892
chr1 53946980
chr1 54041784
chr1 54042045
chr1 54051734
chr1 54051794
chr1 54051890
chr1 54051912
chr1 54051952
chr1 54051992
chr1 54052072
chr1 54052119
chr1 54052224
chr1 54200604
chr1 54200608
chr1 54200619
chr1 54200798
chr1 54230991
chr1 54231003
chr1 54231158
chr1 54231229
chr1 54231291
chr1 54458972
chr1 54481158
chr1 54488918
chr1 54715753
chr1 54715764
chr1 54716160
chr1 54716166
chr1 54716179
chr1 54716276
chr1 54716298
chr1 54716300
chr1 54716364
chr1 54716388
chr1 54716463
chr1 54764027
chr1 54801310
chr1 54801317
chr1 54801333
chr1 54801357
chr1 54801363
chr1 54801373
chr1 54801436
chr1 54801550
chr1 54801593
chr1 54801612
chr1 54801620
chr1 54818286

chr1 55107101
chr1 55204319
chr1 55211531
chr1 55211692
chr1 55212034
chr1 55212132
chr1 55212235
chr1 55212457
chr1 55212495
chr1 55212663
chr1 55212728
chr1 55212733
chr1 55212785
chr1 55212853
chr1 55212887
chr1 55212917
chr1 55212976
chr1 55213137
chr1 55213182
chr1 55213321
chr1 55213449
chr1 55213552
chr1 55213563
chr1 55213581
chr1 55213662
chr1 55213739
chr1 55213768
chr1 55213783
chr1 55213889
chr1 55213905
chr1 55213907
chr1 55213952
chr1 55213983
chr1 55214162
chr1 55214164
chr1 55217496
chr1 55217664
chr1 55217682
chr1 55217700
chr1 55217849
chr1 55275908
chr1 55276185
chr1 56368638
chr1 56577363
chr1 56577477
chr1 56832177
chr1 56832475
chr1 56832499
chr1 57421234
chr1 57421336
chr1 57421466
chr1 57421503
chr1 57421546
chr1 57421567

chr1 57421580
chr1 57421677
chr1 57421697
chr1 57421902
chr1 58546403
chr1 58546416
chr1 58546425
chr1 58546428
chr1 58546435
chr1 58546456
chr1 58546482
chr1 58546489
chr1 58546494
chr1 58546497
chr1 58546504
chr1 58546519
chr1 58546530
chr1 58546540
chr1 58546550
chr1 58546585
chr1 58546589
chr1 58546595
chr1 58546601
chr1 58546608
chr1 58546613
chr1 58546630
chr1 58546714
chr1 58546760
chr1 58546773
chr1 58546781
chr1 58546785
chr1 58546790
chr1 58546797
chr1 58546802
chr1 58546812
chr1 58546828
chr1 58546838
chr1 58546855
chr1 58546862
chr1 58546864
chr1 58546876
chr1 58546884
chr1 58546890
chr1 58699271
chr1 58699315
chr1 58699456
chr1 58699528
chr1 58699571
chr1 58699576
chr1 58699590
chr1 58699632
chr1 58699636
chr1 58699642
chr1 58699663

chr1 58699688
chr1 58699717
chr1 58699731
chr1 58699737
chr1 58699755
chr1 58699771
chr1 58699801
chr1 58699810
chr1 58699829
chr1 58699832
chr1 58699836
chr1 58740011
chr1 58740249
chr1 58755439
chr1 58755450
chr1 58781611
chr1 58781635
chr1 58781657
chr1 58781753
chr1 58781790
chr1 58781802
chr1 58781807
chr1 58781811
chr1 58781813
chr1 58781815
chr1 58781826
chr1 58781830
chr1 58781837
chr1 58781847
chr1 58781853
chr1 58781857
chr1 58781872
chr1 58781885
chr1 58781887
chr1 58781905
chr1 58781971
chr1 58781992
chr1 58781995
chr1 58815641
chr1 58815706
chr1 58815711
chr1 58815816
chr1 58815822
chr1 58815843
chr1 58815868
chr1 58815897
chr1 58815915
chr1 58815927
chr1 58815932
chr1 58815957
chr1 58816027
chr1 58816058
chr1 58816075
chr1 58816100

chr1 58816124
chr1 58816152
chr1 58816156
chr1 58816185
chr1 58816218
chr1 58816234
chr1 58816247
chr1 58816250
chr1 58816257
chr1 58816271
chr1 58816317
chr1 58816344
chr1 58816346
chr1 58816362
chr1 58903686
chr1 58903692
chr1 58903700
chr1 58903705
chr1 58903709
chr1 58903712
chr1 58903724
chr1 58903737
chr1 58903747
chr1 58903749
chr1 58903758
chr1 59146218
chr1 59146277
chr1 59146353
chr1 59146634
chr1 59146650
chr1 59146663
chr1 59146670
chr1 59146712
chr1 59146736
chr1 59146740
chr1 59146746
chr1 59150414
chr1 59150495
chr1 59759126
chr1 59814650
chr1 59814670
chr1 59814678
chr1 59814701
chr1 59814713
chr1 59814723
chr1 59814729
chr1 59814734
chr1 59814739
chr1 59814816
chr1 59814831
chr1 59814862
chr1 59814883
chr1 59814885
chr1 59814889

chr1 59814912
chr1 59814916
chr1 59814924
chr1 59814929
chr1 59814935
chr1 59814953
chr1 59814956
chr1 59814960
chr1 59814962
chr1 59814965
chr1 59814971
chr1 59814973
chr1 59815013
chr1 59815038
chr1 59815044
chr1 59815047
chr1 59926418
chr1 59926462
chr1 59926468
chr1 59926473
chr1 59926511
chr1 59926555
chr1 59926563
chr1 59926601
chr1 59926612
chr1 59926615
chr1 59926631
chr1 59926634
chr1 59926656
chr1 59926668
chr1 59926691
chr1 60282287
chr1 60637569
chr1 60637628
chr1 60637780
chr1 61057487
chr1 61057498
chr1 61057574
chr1 61057646
chr1 61057654
chr1 61057663
chr1 61057685
chr1 61057687
chr1 61057693
chr1 61057709
chr1 61057716
chr1 61057726
chr1 61057748
chr1 61057768
chr1 61057771
chr1 61057780
chr1 61057803
chr1 61057832
chr1 61057834

chr1 61076657
chr1 61076801
chr1 61076824
chr1 61081459
chr1 61081491
chr1 61081493
chr1 61082927
chr1 61082951
chr1 61082953
chr1 61082971
chr1 61082973
chr1 61082975
chr1 61082983
chr1 61082985
chr1 61083004
chr1 61157386
chr1 61157563
chr1 61157694
chr1 61157789
chr1 61158649
chr1 61452522
chr1 61452691
chr1 61452718
chr1 61452872
chr1 61453188
chr1 61453249
chr1 61588874
chr1 61652540
chr1 62296131
chr1 62296265
chr1 62386539
chr1 62386764
chr1 62438840
chr1 62438891
chr1 62438910
chr1 62438946
chr1 62439076
chr1 62439247
chr1 62439253
chr1 62439270
chr1 62439277
chr1 62439329
chr1 62439333
chr1 62439341
chr1 62439345
chr1 62439404
chr1 62439418
chr1 62439429
chr1 62439434
chr1 62439462
chr1 62439472
chr1 62439478
chr1 62439553
chr1 62440130

chr1 62440145
chr1 62440211
chr1 62440225
chr1 62621166
chr1 62621320
chr1 62672569
chr1 62688978
chr1 62688982
chr1 62688997
chr1 62689062
chr1 62689105
chr1 62689134
chr1 62689250
chr1 62725846
chr1 62783752
chr1 62783766
chr1 62783796
chr1 62783811
chr1 62783853
chr1 62783873
chr1 62783913
chr1 62783935
chr1 62783958
chr1 62783971
chr1 62784022
chr1 62784025
chr1 62784034
chr1 62784047
chr1 62784058
chr1 62784065
chr1 62784493
chr1 62784614
chr1 62784630
chr1 62784790
chr1 62784814
chr1 62784821
chr1 62784924
chr1 62784988
chr1 63305757
chr1 63305767
chr1 63324893
chr1 63324941
chr1 63324974
chr1 63325087
chr1 63325443
chr1 63325960
chr1 63331059
chr1 63331068
chr1 63331197
chr1 63331288
chr1 63331392
chr1 63332009
chr1 63522186
chr1 63522399

chr1 63594591
chr1 63594601
chr1 63594663
chr1 63594679
chr1 63594739
chr1 63594759
chr1 63594770
chr1 63732408
chr1 63757255
chr1 63757952
chr1 64199113
chr1 64199195
chr1 64199363
chr1 64505348
chr1 64505372
chr1 64505449
chr1 64505456
chr1 64505464
chr1 64505505
chr1 64505529
chr1 64505535
chr1 64505540
chr1 64505542
chr1 64505547
chr1 64505552
chr1 64505576
chr1 64505581
chr1 64505584
chr1 64505616
chr1 64505619
chr1 64505639
chr1 64505676
chr1 64505689
chr1 64505693
chr1 64505712
chr1 64505715
chr1 64505736
chr1 64505741
chr1 64505746
chr1 64505753
chr1 64505757
chr1 64505770
chr1 64505798
chr1 64505802
chr1 64527639
chr1 64864058
chr1 64864088
chr1 64864187
chr1 64864332
chr1 64864364
chr1 64864383
chr1 64864390
chr1 64864403
chr1 64864444

chr1 64864478
chr1 64956808
chr1 64956886
chr1 64957241
chr1 64964435
chr1 64965047
chr1 64965142
chr1 64965300
chr1 64965339
chr1 64965365
chr1 64965423
chr1 65068281
chr1 65068341
chr1 65068425
chr1 65068446
chr1 65068519
chr1 65068575
chr1 65068628
chr1 65068698
chr1 65068803
chr1 65068870
chr1 65068886
chr1 65068927
chr1 65068936
chr1 65068944
chr1 65069059
chr1 65069063
chr1 65122848
chr1 65264941
chr1 65264996
chr1 65265409
chr1 65265424
chr1 65265427
chr1 65265564
chr1 65265602
chr1 65265670
chr1 65265693
chr1 65265710
chr1 65265729
chr1 65265740
chr1 65265749
chr1 65265755
chr1 65265786
chr1 65265793
chr1 65265806
chr1 65265808
chr1 65265823
chr1 65265828
chr1 65265834
chr1 65265836
chr1 65265842
chr1 65265846
chr1 65265850
chr1 65265857

chr1 65265860
chr1 65265866
chr1 65265868
chr1 65265870
chr1 65265873
chr1 65265879
chr1 65419664
chr1 65419681
chr1 65419765
chr1 65419811
chr1 65419823
chr1 65419881
chr1 66276222
chr1 66276254
chr1 66330761
chr1 66330793
chr1 66331021
chr1 66331056
chr1 66331345
chr1 66331695
chr1 66331790
chr1 66331798
chr1 66331916
chr1 66331928
chr1 66331994
chr1 66332018
chr1 66332223
chr1 66332232
chr1 66332269
chr1 66332285
chr1 66332422
chr1 66332431
chr1 66332440
chr1 66332469
chr1 66332538
chr1 66332591
chr1 66332822
chr1 66333133
chr1 66333579
chr1 66333720
chr1 66333736
chr1 66334133
chr1 66334468
chr1 66334497
chr1 66334844
chr1 66334872
chr1 66334909
chr1 66335276
chr1 66335320
chr1 66335581
chr1 66335810
chr1 66335833
chr1 66336024
chr1 66336089

chr1 66336144
chr1 66336165
chr1 66336193
chr1 66336242
chr1 66336314
chr1 66336329
chr1 66340322
chr1 66380559
chr1 66533605
chr1 66533635
chr1 66601784
chr1 66929668
chr1 66929694
chr1 66929714
chr1 66929798
chr1 66929838
chr1 66929861
chr1 66929864
chr1 66929884
chr1 66929911
chr1 66931885
chr1 66932081
chr1 66932154
chr1 66932276
chr1 66932445
chr1 66932543
chr1 66933035
chr1 66933147
chr1 66961493
chr1 66981189
chr1 66981389
chr1 66981576
chr1 66981792
chr1 66981803
chr1 66981828
chr1 66981990
chr1 67052754
chr1 67052775
chr1 67053042
chr1 67053062
chr1 67053068
chr1 67053084
chr1 67053102
chr1 67053106
chr1 67053169
chr1 67053183
chr1 67053187
chr1 67053191
chr1 67053209
chr1 67053228
chr1 67053278
chr1 67053370
chr1 67053374
chr1 67053393

chr1 67053417
chr1 67053586
chr1 67053591
chr1 67053595
chr1 67053603
chr1 67110719
chr1 67286481
chr1 67286492
chr1 67333246
chr1 67333293
chr1 67333320
chr1 67339697
chr1 67339723
chr1 67339747
chr1 67339845
chr1 67339878
chr1 67339883
chr1 67427166
chr1 67427175
chr1 67427714
chr1 67427752
chr1 67500441
chr1 67500529
chr1 67500531
chr1 67500560
chr1 67500574
chr1 67500580
chr1 67500585
chr1 67500587
chr1 67500598
chr1 67500714
chr1 67500716
chr1 67674098
chr1 67674352
chr1 67684268
chr1 67684279
chr1 67684306
chr1 67684373
chr1 67684463
chr1 67684477
chr1 67684494
chr1 67684510
chr1 67684717
chr1 67686809
chr1 67686874
chr1 67735950
chr1 67736046
chr1 67766105
chr1 67766293
chr1 67766304
chr1 67766314
chr1 67766347
chr1 67766389
chr1 67766391

chr1 67766422
chr1 67766451
chr1 67766481
chr1 67766484
chr1 68232075
chr1 68232989
chr1 68233007
chr1 68233035
chr1 68233069
chr1 68233072
chr1 68233117
chr1 68233333
chr1 68497322
chr1 68497344
chr1 68497378
chr1 68497515
chr1 69122007
chr1 69131735
chr1 69397016
chr1 69884506
chr1 69884784
chr1 69885010
chr1 69885359
chr1 69886134
chr1 69886188
chr1 69886286
chr1 69886518
chr1 70204760
chr1 70206334
chr1 70206384
chr1 70206435
chr1 70206482
chr1 70222199
chr1 70222341
chr1 70222391
chr1 70222552
chr1 70353391
chr1 70353422
chr1 70353590
chr1 70353721
chr1 70354638
chr1 70354658
chr1 70354661
chr1 70354664
chr1 70354670
chr1 70354673
chr1 70354676
chr1 70354689
chr1 70354703
chr1 70354727
chr1 70354729
chr1 70354765
chr1 70354767
chr1 70354783

chr1 70354795
chr1 70354823
chr1 70354826
chr1 70354829
chr1 70354835
chr1 70410402
chr1 70410735
chr1 70410775
chr1 70411743
chr1 70411759
chr1 70411793
chr1 70509794
chr1 70509956
chr1 70597513
chr1 70598332
chr1 70598427
chr1 70601612
chr1 70601645
chr1 70601781
chr1 70601862
chr1 70602340
chr1 70602410
chr1 70684551
chr1 70684780
chr1 70685385
chr1 70685413
chr1 70704996
chr1 70705069
chr1 70705079
chr1 70705207
chr1 70709089
chr1 71079802
chr1 71079953
chr1 71732908
chr1 71733010
chr1 71733163
chr1 71733304
chr1 72133215
chr1 72133866
chr1 72280982
chr1 72281267
chr1 72281517
chr1 72281671
chr1 72281836
chr1 72281870
chr1 72281934
chr1 72282093
chr1 72282098
chr1 72282119
chr1 72282151
chr1 72282156
chr1 72282164
chr1 72282284
chr1 72282295

chr1 72282304
chr1 72282307
chr1 72282327
chr1 72282330
chr1 72282456
chr1 72282533
chr1 72282559
chr1 72282569
chr1 72282580
chr1 72282583
chr1 72282585
chr1 72282850
chr1 72282858
chr1 72282862
chr1 72282864
chr1 72282867
chr1 72282870
chr1 72282886
chr1 72282888
chr1 72282891
chr1 72282894
chr1 72282911
chr1 72282915
chr1 72282918
chr1 72282921
chr1 72282924
chr1 72282927
chr1 72282981
chr1 72282983
chr1 72282988
chr1 72282993
chr1 72282996
chr1 72283000
chr1 72283003
chr1 72283010
chr1 72283016
chr1 72283018
chr1 72283020
chr1 72283022
chr1 72283032
chr1 72283036
chr1 72283053
chr1 72284482
chr1 72284513
chr1 72284598
chr1 72284607
chr1 72284670
chr1 72284729
chr1 72284741
chr1 72284819
chr1 72284988
chr1 72284995
chr1 72285031
chr1 72285105

chr1 72285273
chr1 72285372
chr1 72285543
chr1 72285702
chr1 72285761
chr1 72305284
chr1 72323247
chr1 72323311
chr1 72324264
chr1 72324288
chr1 72324294
chr1 72324311
chr1 72324328
chr1 72324345
chr1 72324382
chr1 72324515
chr1 72325325
chr1 72326455
chr1 72328002
chr1 72328136
chr1 72328191
chr1 72328625
chr1 72331163
chr1 72331173
chr1 72331393
chr1 72332039
chr1 72332055
chr1 72332335
chr1 72332675
chr1 72332734
chr1 72333142
chr1 72333316
chr1 72333481
chr1 72341396
chr1 72341490
chr1 72341793
chr1 72342069
chr1 72342833
chr1 72343713
chr1 72343729
chr1 72346067
chr1 72590546
chr1 72591751
chr1 72592197
chr1 72721847
chr1 72722865
chr1 72722896
chr1 72754900
chr1 72896144
chr1 72896182
chr1 72896704
chr1 72897146
chr1 72897278
chr1 73103300

chr1 73146005
chr1 73695470
chr1 74197613
chr1 74197660
chr1 74197803
chr1 74197811
chr1 74197830
chr1 74197894
chr1 74197899
chr1 74197938
chr1 74197952
chr1 74197957
chr1 74197996
chr1 74198066
chr1 74198073
chr1 74198093
chr1 74198143
chr1 74198147
chr1 74198159
chr1 74198164
chr1 74198170
chr1 74198178
chr1 74198184
chr1 74198187
chr1 74198191
chr1 74198200
chr1 74198215
chr1 74198219
chr1 74198225
chr1 74198237
chr1 74198243
chr1 74198252
chr1 74198255
chr1 74198257
chr1 74198301
chr1 74198448
chr1 74198476
chr1 74198594
chr1 74198681
chr1 74198735
chr1 74198747
chr1 74198772
chr1 74245424
chr1 74245441
chr1 74462804
chr1 74462836
chr1 74463441
chr1 74463447
chr1 74463527
chr1 74463538
chr1 74463657
chr1 74732586
chr1 74732597
chr1 74732621

chr1 74732645
chr1 74732652
chr1 74732678
chr1 74732719
chr1 74733218
chr1 74733222
chr1 74733237
chr1 74733251
chr1 74733256
chr1 74733258
chr1 74733279
chr1 74733297
chr1 74733308
chr1 74733325
chr1 74733362
chr1 74733391
chr1 74733398
chr1 74733416
chr1 74733433
chr1 74733444
chr1 74733460
chr1 74733493
chr1 74733497
chr1 74733594
chr1 74733636
chr1 74733812
chr1 74733819
chr1 74733933
chr1 74733947
chr1 74733951
chr1 74734047
chr1 75137521
chr1 75137617
chr1 75137656
chr1 75137683
chr1 75137689
chr1 75138329
chr1 75138646
chr1 75138998
chr1 75139208
chr1 75139375
chr1 75617106
chr1 75617170
chr1 75617359
chr1 75617416
chr1 75617478
chr1 75725213
chr1 75787760
chr1 75787851
chr1 75788003
chr1 75788104
chr1 75788220
chr1 75788361
chr1 75788366

chr1 75788471
chr1 75788823
chr1 75845606
chr1 76267654
chr1 76268016
chr1 76268193
chr1 76268212
chr1 76698066
chr1 76867071
chr1 76867128
chr1 76867138
chr1 76867144
chr1 76867197
chr1 77045210
chr1 77219027
chr1 77249482
chr1 77249543
chr1 77282168
chr1 77282188
chr1 77282190
chr1 77282210
chr1 77282217
chr1 77282220
chr1 77282222
chr1 77282250
chr1 77282260
chr1 77282263
chr1 77282270
chr1 77282282
chr1 77282288
chr1 77282300
chr1 77282302
chr1 77282304
chr1 77282325
chr1 77282347
chr1 77282391
chr1 77282394
chr1 77282398
chr1 77282408
chr1 77282430
chr1 77282444
chr1 77282446
chr1 77282451
chr1 77282469
chr1 77282499
chr1 77282505
chr1 77282509
chr1 77282517
chr1 77282527
chr1 77282536
chr1 77282538
chr1 77282640
chr1 77282642
chr1 77282649

chr1 77282680
chr1 77282682
chr1 77282686
chr1 77282691
chr1 77282706
chr1 77282734
chr1 77282744
chr1 77309223
chr1 77309311
chr1 77309328
chr1 77309394
chr1 77309398
chr1 77309552
chr1 77681813
chr1 77681823
chr1 77681847
chr1 77681853
chr1 77681877
chr1 77681886
chr1 77681894
chr1 77682021
chr1 77682042
chr1 77683849
chr1 77683854
chr1 77683881
chr1 77683889
chr1 77683899
chr1 77683916
chr1 77683961
chr1 77683968
chr1 77759229
chr1 77759249
chr1 77759252
chr1 77759254
chr1 77759311
chr1 77759332
chr1 77759339
chr1 77759358
chr1 77759400
chr1 77759404
chr1 77759421
chr1 77759425
chr1 77759436
chr1 77759459
chr1 77759469
chr1 77759491
chr1 77759495
chr1 77759497
chr1 77759503
chr1 77759506
chr1 77759541
chr1 77759545
chr1 77779551
chr1 77779567

chr1 77779579
chr1 77780152
chr1 77780154
chr1 77780173
chr1 77780183
chr1 77780185
chr1 77780220
chr1 77780231
chr1 77780312
chr1 77780503
chr1 77975291
chr1 77975519
chr1 77975580
chr1 77975628
chr1 77975640
chr1 77975957
chr1 77976232
chr1 77977520
chr1 77977567
chr1 77977669
chr1 77977732
chr1 77977744
chr1 77977829
chr1 77977852
chr1 77977899
chr1 77977934
chr1 77980058
chr1 77980073
chr1 77980084
chr1 77980104
chr1 77980110
chr1 77980118
chr1 77980129
chr1 77980134
chr1 77980162
chr1 77980164
chr1 77980174
chr1 77980178
chr1 77980250
chr1 77980449
chr1 77980494
chr1 78003433
chr1 78003509
chr1 78004177
chr1 78004190
chr1 78004365
chr1 78004428
chr1 78004463
chr1 78004502
chr1 78004568
chr1 78004733
chr1 78004746
chr1 78004765
chr1 78004930

chr1 78004956
chr1 78005060
chr1 78005107
chr1 78005208
chr1 78005254
chr1 78005681
chr1 78005870
chr1 78006264
chr1 78040691
chr1 78040899
chr1 78201345
chr1 78650211
chr1 78650228
chr1 78650262
chr1 78650301
chr1 78650643
chr1 78650648
chr1 78650850
chr1 78651065
chr1 78651121
chr1 78651170
chr1 78651262
chr1 78651270
chr1 78651295
chr1 78651399
chr1 78651421
chr1 78651427
chr1 79048052
chr1 79053052
chr1 79053081
chr1 79053203
chr1 79053263
chr1 79193488
chr1 79193593
chr1 79193615
chr1 79193728
chr1 79518077
chr1 79594906
chr1 79595238
chr1 79595383
chr1 79595740
chr1 79595930
chr1 79674714
chr1 79675228
chr1 79861059
chr1 79861268
chr1 79908664
chr1 79908769
chr1 79909125
chr1 79909528
chr1 80129637
chr1 80130710
chr1 80137044
chr1 80137049

chr1 80137059
chr1 80137088
chr1 80137282
chr1 80138896
chr1 80139973
chr1 80389761
chr1 80390027
chr1 80579254
chr1 80739668
chr1 80739802
chr1 80740040
chr1 80740118
chr1 80740746
chr1 80892052
chr1 80892182
chr1 80892585
chr1 80892643
chr1 80892795
chr1 80892884
chr1 80893099
chr1 80893213
chr1 80893415
chr1 81208113
chr1 81305848
chr1 81305868
chr1 81430803
chr1 81431179
chr1 81431360
chr1 81431431
chr1 81431448
chr1 81431496
chr1 81517760
chr1 81517824
chr1 81517861
chr1 81517962
chr1 81518111
chr1 81660967
chr1 81661368
chr1 81760384
chr1 81760685
chr1 81761360
chr1 81761893
chr1 81762187
chr1 81799110
chr1 82161860
chr1 82161883
chr1 82162018
chr1 82162038
chr1 82162043
chr1 82162056
chr1 82162095
chr1 82362278
chr1 82362335
chr1 82362375

chr1 82362669
chr1 82362923
chr1 82548996
chr1 83243675
chr1 83265493
chr1 83265503
chr1 83860330
chr1 83860452
chr1 83860659
chr1 83860662
chr1 83860675
chr1 83860687
chr1 83860701
chr1 84077299
chr1 84077464
chr1 84091013
chr1 84091079
chr1 84144443
chr1 84145284
chr1 84145412
chr1 84146218
chr1 84146443
chr1 84146504
chr1 84146787
chr1 84147012
chr1 84479314
chr1 84479409
chr1 84479415
chr1 84479427
chr1 84479445
chr1 84479465
chr1 84479468
chr1 84479484
chr1 84479486
chr1 84479504
chr1 84479513
chr1 84479515
chr1 84479522
chr1 84479532
chr1 84479536
chr1 84479542
chr1 84479544
chr1 84479556
chr1 84479565
chr1 84479569
chr1 84479571
chr1 84479576
chr1 84479624
chr1 84479638
chr1 84479641
chr1 84479643
chr1 84479650
chr1 84479666
chr1 84479707

chr1 84479748
chr1 84479801
chr1 84479843
chr1 84479854
chr1 84479891
chr1 84479941
chr1 84480002
chr1 84480029
chr1 84480045
chr1 84480059
chr1 84480099
chr1 84480120
chr1 84480183
chr1 84480241
chr1 84480264
chr1 84480598
chr1 84480816
chr1 84501037
chr1 84504772
chr1 84504889
chr1 84504997
chr1 84505278
chr1 84506782
chr1 84506786
chr1 84506799
chr1 84506827
chr1 84506863
chr1 84506926
chr1 84573274
chr1 84573338
chr1 84573575
chr1 84573635
chr1 84573875
chr1 84573884
chr1 84573946
chr1 84944861
chr1 84945178
chr1 84945199
chr1 84945372
chr1 84945441
chr1 84945488
chr1 84995051
chr1 84995480
chr1 84995617
chr1 84995620
chr1 84995724
chr1 84995948
chr1 84995962
chr1 84995986
chr1 84996026
chr1 84996032
chr1 84996135
chr1 84996213
chr1 84996350

chr1 84996382
chr1 84996520
chr1 84996533
chr1 84996542
chr1 84996549
chr1 84996562
chr1 84996579
chr1 84996722
chr1 84996903
chr1 84996961
chr1 84996965
chr1 84997736
chr1 84997812
chr1 84997823
chr1 84997863
chr1 84997878
chr1 84997912
chr1 84997917
chr1 84997985
chr1 84998042
chr1 84998046
chr1 85050885
chr1 85050949
chr1 85092042
chr1 85092050
chr1 85092325
chr1 85109043
chr1 85109065
chr1 85202268
chr1 85202322
chr1 85275515
chr1 85275605
chr1 85275644
chr1 85275649
chr1 85275686
chr1 85275719
chr1 85275722
chr1 85275750
chr1 85275789
chr1 85275912
chr1 85275966
chr1 85275979
chr1 85577365
chr1 85577371
chr1 85577385
chr1 85707314
chr1 85707487
chr1 85707489
chr1 85707493
chr1 85707496
chr1 85707647
chr1 85707661
chr1 85707673
chr1 85707705

chr1 85707895
chr1 85707922
chr1 85707945
chr1 85707951
chr1 85708057
chr1 85708069
chr1 85827534
chr1 85827605
chr1 85827667
chr1 85828494
chr1 85828736
chr1 85828990
chr1 85894010
chr1 85894072
chr1 85894725
chr1 85894849
chr1 85894929
chr1 85894966
chr1 85993511
chr1 86091838
chr1 86152048
chr1 86156412
chr1 86156424
chr1 86156430
chr1 86156582
chr1 86156616
chr1 86156622
chr1 86156626
chr1 86156640
chr1 86156647
chr1 86156656
chr1 86156660
chr1 86156688
chr1 86156693
chr1 86156696
chr1 86156699
chr1 86156705
chr1 86156712
chr1 86156744
chr1 86338530
chr1 86338943
chr1 86396307
chr1 86703758
chr1 86703926
chr1 86703941
chr1 86703983
chr1 86704011
chr1 86704015
chr1 86704069
chr1 86705746
chr1 86705841
chr1 86705847
chr1 86706247
chr1 86706487

chr1 86833414
chr1 86833509
chr1 86833518
chr1 86833579
chr1 86833647
chr1 86913157
chr1 86913227
chr1 86913253
chr1 86913396
chr1 86913416
chr1 86913509
chr1 86913519
chr1 86913529
chr1 86913552
chr1 86965342
chr1 87330244
chr1 87330294
chr1 87330299
chr1 87330308
chr1 87330394
chr1 87330442
chr1 87330544
chr1 87330559
chr1 87330564
chr1 87330575
chr1 87330596
chr1 87330614
chr1 87330627
chr1 87330638
chr1 87330666
chr1 87330683
chr1 87330726
chr1 87330859
chr1 87330883
chr1 87330997
chr1 87331003
chr1 87331039
chr1 87331055
chr1 87331058
chr1 87331062
chr1 87331079
chr1 87331325
chr1 87331339
chr1 87331369
chr1 87331397
chr1 87331404
chr1 87331411
chr1 87331418
chr1 87331430
chr1 87331440
chr1 87331448
chr1 87331486
chr1 87331498
chr1 87331508

chr1 87331516
chr1 87331519
chr1 87331525
chr1 87331528
chr1 87331545
chr1 87331556
chr1 87331563
chr1 87575547
chr1 87827782
chr1 88288874
chr1 88289565
chr1 88655836
chr1 88683434
chr1 88683464
chr1 88683476
chr1 88685900
chr1 88686486
chr1 88686682
chr1 88868165
chr1 88868410
chr1 88868479
chr1 88890191
chr1 88890249
chr1 88890613
chr1 88992103
chr1 88992123
chr1 88992127
chr1 88992135
chr1 88992139
chr1 88992168
chr1 88992179
chr1 88992213
chr1 88992220
chr1 88992224
chr1 88992229
chr1 88992247
chr1 88998574
chr1 89021930
chr1 89021940
chr1 89021970
chr1 89022036
chr1 89022213
chr1 89064681
chr1 89064743
chr1 89064800
chr1 89064981
chr1 89065183
chr1 89124457
chr1 89124619
chr1 89125240
chr1 89125498
chr1 89126750
chr1 89126772
chr1 89126774

chr1 89126776
chr1 89126839
chr1 89127025
chr1 89127042
chr1 89127069
chr1 89127071
chr1 89127081
chr1 89127108
chr1 89127110
chr1 89127210
chr1 89127212
chr1 89127237
chr1 89127239
chr1 89127273
chr1 89127312
chr1 89128883
chr1 89128901
chr1 89128955
chr1 89129081
chr1 89129090
chr1 89197450
chr1 89197577
chr1 89197769
chr1 89197795
chr1 89197876
chr1 89197891
chr1 89197906
chr1 89197930
chr1 89197933
chr1 89197937
chr1 89197944
chr1 89197966
chr1 89197971
chr1 89198151
chr1 89198183
chr1 89198185
chr1 89198209
chr1 89198213
chr1 89198230
chr1 89198319
chr1 89198350
chr1 89199669
chr1 89200811
chr1 89200888
chr1 89201116
chr1 89201197
chr1 89201201
chr1 89258629
chr1 89259397
chr1 89259526
chr1 89259581
chr1 89259662
chr1 89259674
chr1 89259683

chr1 89270408
chr1 89270415
chr1 89270795
chr1 89271435
chr1 89271577
chr1 89271753
chr1 89272120
chr1 89272150
chr1 89272452
chr1 89272458
chr1 89272502
chr1 89272504
chr1 89272514
chr1 89272526
chr1 89272542
chr1 89272571
chr1 89272606
chr1 89272644
chr1 89272646
chr1 89272673
chr1 89272675
chr1 89272707
chr1 89272715
chr1 89272935
chr1 89274305
chr1 89274313
chr1 89274401
chr1 89274727
chr1 89274841
chr1 89363429
chr1 89611029
chr1 89611543
chr1 89611624
chr1 89611668
chr1 89611734
chr1 89634040
chr1 89634066
chr1 89634090
chr1 89634098
chr1 89634136
chr1 89634145
chr1 89634162
chr1 89634175
chr1 89634187
chr1 89634206
chr1 89634260
chr1 89634436
chr1 89634438
chr1 89634453
chr1 89634481
chr1 89634511
chr1 89634525
chr1 89634555
chr1 89634609

chr1 89634680
chr1 89634703
chr1 89635683
chr1 89636063
chr1 89636073
chr1 89719579
chr1 89719790
chr1 89719974
chr1 89720217
chr1 89720237
chr1 89720366
chr1 89720439
chr1 89720849
chr1 89721439
chr1 89843281
chr1 89843297
chr1 89844333
chr1 89855894
chr1 89987426
chr1 89987560
chr1 89995938
chr1 89996039
chr1 89996364
chr1 90061826
chr1 90061933
chr1 90062020
chr1 90062080
chr1 90062107
chr1 90062116
chr1 90062263
chr1 90062288
chr1 90062319
chr1 90062360
chr1 90098344
chr1 90707852
chr1 90708037
chr1 90708044
chr1 90708080
chr1 90708144
chr1 90708259
chr1 90708386
chr1 90708501
chr1 90708540
chr1 90708651
chr1 90708762
chr1 90708844
chr1 90708883
chr1 90711269
chr1 90711367
chr1 90711382
chr1 90711426
chr1 90711624
chr1 90711661
chr1 90711839

chr1 90711850
chr1 90711856
chr1 90711916
chr1 90711921
chr1 90714903
chr1 90715356
chr1 90715408
chr1 90715455
chr1 90715484
chr1 90715571
chr1 90721016
chr1 90721267
chr1 90721291
chr1 90721382
chr1 90721489
chr1 90721496
chr1 90721536
chr1 90721592
chr1 90721609
chr1 90721612
chr1 90721624
chr1 90721628
chr1 90721711
chr1 90721727
chr1 90721749
chr1 90721752
chr1 90722249
chr1 90731976
chr1 90832045
chr1 90833624
chr1 90833636
chr1 90833728
chr1 90833997
chr1 90834002
chr1 90834047
chr1 90840179
chr1 90840239
chr1 90840435
chr1 91020807
chr1 91020834
chr1 91020837
chr1 91020887
chr1 91020911
chr1 91020955
chr1 91021049
chr1 91021128
chr1 91021261
chr1 91021265
chr1 91021302
chr1 91021321
chr1 91021342
chr1 91403750
chr1 91403756
chr1 91404058

chr1 91404068
chr1 91404072
chr1 91404117
chr1 91404133
chr1 91404140
chr1 91404167
chr1 91404225
chr1 91404236
chr1 91404320
chr1 91404337
chr1 91404342
chr1 91404350
chr1 91404352
chr1 91404367
chr1 91404374
chr1 91500837
chr1 91500840
chr1 91500843
chr1 91500855
chr1 91500861
chr1 91500867
chr1 91500871
chr1 91500876
chr1 91500900
chr1 91500903
chr1 91500933
chr1 91500948
chr1 91500958
chr1 91500961
chr1 91500976
chr1 91500978
chr1 91501000
chr1 91501012
chr1 91501020
chr1 91501029
chr1 91501032
chr1 91501059
chr1 91501441
chr1 91501468
chr1 91501553
chr1 91501557
chr1 91501605
chr1 91501615
chr1 91501724
chr1 91501771
chr1 91501777
chr1 91501809
chr1 91501835
chr1 91501973
chr1 91502001
chr1 91502033
chr1 91502287
chr1 91548346
chr1 91548407

chr1 91548617
chr1 91548863
chr1 91684947
chr1 91684992
chr1 91697363
chr1 91697650
chr1 91697692
chr1 91754028
chr1 91754162
chr1 91754455
chr1 91803735
chr1 91803863
chr1 91803960
chr1 91804045
chr1 91804317
chr1 91804375
chr1 91804512
chr1 91804654
chr1 91804676
chr1 91804706
chr1 91804781
chr1 91872771
chr1 91872872
chr1 91872894
chr1 91872937
chr1 91872965
chr1 91873048
chr1 91883537
chr1 91883547
chr1 91883619
chr1 91883713
chr1 91883720
chr1 92080671
chr1 92080674
chr1 92080684
chr1 92080687
chr1 92080693
chr1 92080732
chr1 92117779
chr1 92118054
chr1 92118212
chr1 92298018
chr1 92298060
chr1 92298342
chr1 92298359
chr1 92298370
chr1 92474481
chr1 92474615
chr1 92476814
chr1 92477243
chr1 92483961
chr1 92483982
chr1 92483989
chr1 92483999

chr1 92484007
chr1 92484009
chr1 92484014
chr1 92484026
chr1 92484109
chr1 92484150
chr1 92484153
chr1 92484245
chr1 92484256
chr1 92484272
chr1 92484292
chr1 92484312
chr1 92484340
chr1 92484346
chr1 92484349
chr1 92484402
chr1 92484416
chr1 92484434
chr1 92486881
chr1 92486883
chr1 92486997
chr1 92487005
chr1 92487024
chr1 92487032
chr1 92487036
chr1 92487038
chr1 92536230
chr1 92536305
chr1 92536495
chr1 92536740
chr1 92536884
chr1 92537293
chr1 92537714
chr1 92537956
chr1 92538244
chr1 92538258
chr1 92538752
chr1 92538980
chr1 92584088
chr1 92584156
chr1 92584194
chr1 92584304
chr1 92584374
chr1 92584603
chr1 92585434
chr1 92587646
chr1 92833452
chr1 92833455
chr1 92833568
chr1 92833574
chr1 92858804
chr1 92858877
chr1 92858942
chr1 92858987

chr1 92877813
chr1 92877849
chr1 92877926
chr1 92878018
chr1 92882965
chr1 92975776
chr1 92975833
chr1 92977582
chr1 92977895
chr1 93042649
chr1 93042679
chr1 93042751
chr1 93042765
chr1 93042782
chr1 93081110
chr1 93081234
chr1 93081515
chr1 93081530
chr1 93155482
chr1 93155555
chr1 93155585
chr1 93171295
chr1 93171318
chr1 93171324
chr1 93171352
chr1 93171354
chr1 93171362
chr1 93171364
chr1 93171399
chr1 93171408
chr1 93179354
chr1 93179362
chr1 93179394
chr1 93179457
chr1 93179496
chr1 93179525
chr1 93179548
chr1 93179568
chr1 93179756
chr1 93181383
chr1 93181419
chr1 93181441
chr1 93190942
chr1 93219742
chr1 93345002
chr1 93345269
chr1 93345366
chr1 93348486
chr1 93348730
chr1 93348809
chr1 93349397
chr1 93349472
chr1 93349675
chr1 93349710

chr1 93447587
chr1 93447804
chr1 93447807
chr1 93447857
chr1 93447867
chr1 93447890
chr1 93447913
chr1 93447933
chr1 93447951
chr1 93449700
chr1 93449938
chr1 93615651
chr1 93615665
chr1 93615681
chr1 93615705
chr1 93615732
chr1 93615849
chr1 93846025
chr1 93846050
chr1 93846105
chr1 93846123
chr1 93846136
chr1 93846246
chr1 93848386
chr1 93851195
chr1 93908236
chr1 93908470
chr1 93908648
chr1 93908688
chr1 93908707
chr1 93908712
chr1 93908715
chr1 93908737
chr1 94017261
chr1 94017501
chr1 94017587
chr1 94061686
chr1 94061716
chr1 94061752
chr1 94061959
chr1 94235766
chr1 94235848
chr1 94235879
chr1 94235889
chr1 94235916
chr1 94236116
chr1 94236269
chr1 94236438
chr1 94236441
chr1 94236450
chr1 94236453
chr1 94236557
chr1 94236623
chr1 94236632

chr1 94236651
chr1 94236670
chr1 94236721
chr1 94236771
chr1 94239242
chr1 94239695
chr1 94327692
chr1 94327751
chr1 94328076
chr1 94335846
chr1 94335876
chr1 94335946
chr1 94336010
chr1 94336060
chr1 94336068
chr1 94336070
chr1 94336122
chr1 94419446
chr1 94419452
chr1 94542377
chr1 94542379
chr1 94542391
chr1 94542396
chr1 94542411
chr1 94542423
chr1 94542435
chr1 94542444
chr1 94542449
chr1 94542485
chr1 94542489
chr1 94542494
chr1 94542638
chr1 94542868
chr1 94542923
chr1 94542976
chr1 94543095
chr1 94602909
chr1 94602923
chr1 94962883
chr1 94963004
chr1 94963054
chr1 95116756
chr1 95116847
chr1 95116860
chr1 95116862
chr1 95116910
chr1 95116917
chr1 95116927
chr1 95116936
chr1 95117058
chr1 95117080
chr1 95117085
chr1 95117091
chr1 95117110

chr1 95117144
chr1 95117195
chr1 95117200
chr1 95117206
chr1 95117233
chr1 95756640
chr1 95951361
chr1 95951391
chr1 96193942
chr1 96197169
chr1 96309103
chr1 96417415
chr1 96722525
chr1 96722612
chr1 96722627
chr1 96722731
chr1 96722766
chr1 96722782
chr1 96722829
chr1 96722837
chr1 96722898
chr1 96723016
chr1 96923845
chr1 96924311
chr1 96925235
chr1 96968261
chr1 96968326
chr1 97084904
chr1 97085308
chr1 97911039
chr1 97911557
chr1 97919888
chr1 97919954
chr1 97919969
chr1 97920156
chr1 97920196
chr1 97920247
chr1 97920272
chr1 97921458
chr1 97921493
chr1 97921520
chr1 97921537
chr1 97921558
chr1 97921665
chr1 97921693
chr1 98042639
chr1 98042792
chr1 98043021
chr1 98048089
chr1 98048288
chr1 98048292
chr1 98048313
chr1 98048400
chr1 98048418

chr1 98048423
chr1 98048430
chr1 98048440
chr1 98048454
chr1 98048481
chr1 98048491
chr1 98048508
chr1 98048526
chr1 98048538
chr1 98048576
chr1 98048662
chr1 98048685
chr1 98048712
chr1 98048726
chr1 98048740
chr1 98048762
chr1 98048803
chr1 98048805
chr1 98048832
chr1 98048874
chr1 98048876
chr1 98048887
chr1 98050033
chr1 98050091
chr1 98050220
chr1 98050598
chr1 98050628
chr1 98050705
chr1 98051240
chr1 98051936
chr1 98051984
chr1 98052629
chr1 98052868
chr1 98053001
chr1 98053031
chr1 98053098
chr1 98053212
chr1 98854225
chr1 98854230
chr1 98854291
chr1 98871357
chr1 98871419
chr1 98871526
chr1 98871626
chr1 99647040
chr1 99766346
chr1 99766421
chr1 99766468
chr1 99766482
chr1 99766495
chr1 99766521
chr1 99766536
chr1 99766560
chr1 99766595

chr1 99766616
chr1 99766638
chr1 99766833
chr1 99784753
chr1 99784769
chr1 99784855
chr1 99785306
chr1 99820625
chr1 99820745
chr1 99820750
chr1 99850637
chr1 99850778
chr1 99850788
chr1 99850830
chr1 99850892
chr1 100037363
chr1 100037381
chr1 100037423
chr1 100037542
chr1 100037567
chr1 100039605
chr1 100039934
chr1 100132413
chr1 100133061
chr1 100133063
chr1 100133103
chr1 100133135
chr1 100133149
chr1 100133155
chr1 100133173
chr1 100133180
chr1 100133182
chr1 100133185
chr1 100133188
chr1 100133191
chr1 100133195
chr1 100133197
chr1 100133225
chr1 100133250
chr1 100133273
chr1 100133300
chr1 100133309
chr1 100133311
chr1 100133325
chr1 100133345
chr1 100133369
chr1 100133382
chr1 100133469
chr1 100133474
chr1 100133522
chr1 100133559
chr1 100249262
chr1 100249292
chr1 100249295

chr1 100249312
chr1 100249350
chr1 100249394
chr1 100249425
chr1 100249441
chr1 100249464
chr1 100249495
chr1 100249507
chr1 100249521
chr1 100249542
chr1 100249551
chr1 100249562
chr1 100249607
chr1 100249612
chr1 100249617
chr1 100249663
chr1 100249665
chr1 100249676
chr1 100249686
chr1 100249722
chr1 100250237
chr1 100250270
chr1 100250669
chr1 100265760
chr1 100265774
chr1 100265781
chr1 100265820
chr1 100265831
chr1 100265844
chr1 100265852
chr1 100265893
chr1 100265901
chr1 100265920
chr1 100265978
chr1 100266036
chr1 100266077
chr1 100266079
chr1 100266083
chr1 100266099
chr1 100266111
chr1 100266116
chr1 100266120
chr1 100266134
chr1 100266831
chr1 100266903
chr1 100266920
chr1 100266929
chr1 100266970
chr1 100267283
chr1 100351376
chr1 100351387
chr1 100351395
chr1 100351405
chr1 100351414

chr1 100351456
chr1 100351463
chr1 100351485
chr1 100351495
chr1 100351524
chr1 100351542
chr1 100351744
chr1 100351775
chr1 100353685
chr1 100353764
chr1 100353829
chr1 100353905
chr1 100353960
chr1 100354331
chr1 100894390
chr1 100894521
chr1 100894556
chr1 100896886
chr1 100897026
chr1 100897138
chr1 100897176
chr1 100897212
chr1 101024885
chr1 101025197
chr1 101025295
chr1 101025361
chr1 101025370
chr1 101026183
chr1 101026193
chr1 101026262
chr1 101112161
chr1 101178768
chr1 101179038
chr1 101179096
chr1 101179244
chr1 101179347
chr1 101179432
chr1 101179464
chr1 101179484
chr1 101235091
chr1 101235350
chr1 101235379
chr1 101235476
chr1 101235541
chr1 101235544
chr1 101235550
chr1 101235712
chr1 101235740
chr1 101235751
chr1 101235758
chr1 101235808
chr1 101235829
chr1 101235851
chr1 101235866

chr1 101235939
chr1 101235971
chr1 101236285
chr1 101236302
chr1 101236320
chr1 101236329
chr1 101236361
chr1 101236364
chr1 101236367
chr1 101236374
chr1 101236377
chr1 101236382
chr1 101236384
chr1 101236405
chr1 101236407
chr1 101236409
chr1 101236420
chr1 101238421
chr1 101238559
chr1 101238826
chr1 101238833
chr1 101238885
chr1 101238917
chr1 101238922
chr1 101238932
chr1 101238936
chr1 101238948
chr1 101238956
chr1 101293557
chr1 101293854
chr1 101293862
chr1 101293885
chr1 101309806
chr1 101309808
chr1 101309814
chr1 101309843
chr1 101309852
chr1 101309858
chr1 101309896
chr1 101309902
chr1 101309915
chr1 101309918
chr1 101309957
chr1 101310004
chr1 101310024
chr1 101310036
chr1 101310046
chr1 101310085
chr1 101310216
chr1 101310352
chr1 101310674
chr1 101310685
chr1 101604179
chr1 101606083

chr1 101606160
chr1 101846337
chr1 101981539
chr1 101995959
chr1 101996010
chr1 101996053
chr1 101996204
chr1 101996289
chr1 101996296
chr1 101996302
chr1 101996338
chr1 101996363
chr1 101996431
chr1 102313562
chr1 102314248
chr1 102314288
chr1 102314612
chr1 102314813
chr1 102315636
chr1 102316139
chr1 102316406
chr1 102316631
chr1 102392078
chr1 102881661
chr1 102881671
chr1 102881883
chr1 102882028
chr1 102882338
chr1 102882663
chr1 102948331
chr1 102949390
chr1 102949404
chr1 102949491
chr1 102949726
chr1 102970566
chr1 102970650
chr1 103106471
chr1 103106478
chr1 103106547
chr1 103106748
chr1 103106762
chr1 103106784
chr1 103106815
chr1 103106822
chr1 103106904
chr1 103106946
chr1 103107048
chr1 103107059
chr1 103107077
chr1 103107082
chr1 103107103
chr1 103107113
chr1 103107137
chr1 103107156

chr1 103107167
chr1 103107195
chr1 103107231
chr1 103107307
chr1 103107329
chr1 103107359
chr1 103107380
chr1 103107496
chr1 103107864
chr1 103107882
chr1 103107985
chr1 103108015
chr1 103108032
chr1 103108035
chr1 103108112
chr1 103108124
chr1 103108144
chr1 103108149
chr1 103108170
chr1 103108182
chr1 103108189
chr1 103108216
chr1 103108224
chr1 103108241
chr1 103108258
chr1 103108342
chr1 103108349
chr1 103108390
chr1 103108394
chr1 103108404
chr1 103108408
chr1 103108410
chr1 103108433
chr1 103293656
chr1 103294306
chr1 103294345
chr1 103295321
chr1 103631192
chr1 103631194
chr1 103631208
chr1 103631224
chr1 103634841
chr1 103634882
chr1 103634939
chr1 103635018
chr1 103637776
chr1 103786955
chr1 103788096
chr1 103788247
chr1 104666019
chr1 104666119
chr1 104666327
chr1 104666602
chr1 104667308

chr1 104667661
chr1 104667938
chr1 104667987
chr1 104690342
chr1 104843221
chr1 104845773
chr1 104934631
chr1 105468643
chr1 105468902
chr1 105469057
chr1 105564848
chr1 105564929
chr1 105565132
chr1 105565329
chr1 105565470
chr1 105565555
chr1 105565563
chr1 105565836
chr1 105565925
chr1 105627955
chr1 105628031
chr1 105628052
chr1 105682791
chr1 105683055
chr1 105683747
chr1 106300334
chr1 106300553
chr1 106409685
chr1 106413699
chr1 106745696
chr1 106746070
chr1 106746973
chr1 106746990
chr1 106747110
chr1 106747188
chr1 106747312
chr1 106920222
chr1 107016472
chr1 107141615
chr1 107141620
chr1 107141631
chr1 107141633
chr1 107141635
chr1 107141658
chr1 107141665
chr1 107141691
chr1 107141699
chr1 107141704
chr1 107141709
chr1 107141717
chr1 107141734
chr1 107141745
chr1 107141766
chr1 107141769

chr1 107141785
chr1 107141787
chr1 107141803
chr1 107141824
chr1 107141832
chr1 107141840
chr1 107141859
chr1 107141922
chr1 107141948
chr1 107141977
chr1 107142129
chr1 107142269
chr1 107142301
chr1 107142383
chr1 107142416
chr1 107142467
chr1 107142894
chr1 107142969
chr1 107143088
chr1 107202391
chr1 107202799
chr1 107204266
chr1 107204276
chr1 107963059
chr1 107963130
chr1 107963231
chr1 107963368
chr1 107963432
chr1 107963619
chr1 107963636
chr1 107963669
chr1 107963791
chr1 107963823
chr1 107963944
chr1 107963948
chr1 107963989
chr1 107964301
chr1 107964306
chr1 108199699
chr1 108201204
chr1 108505360
chr1 108505953
chr1 108505968
chr1 108561518
chr1 108561667
chr1 108606097
chr1 108647012
chr1 108647019
chr1 108647177
chr1 108691221
chr1 108691394
chr1 108691399
chr1 108691506
chr1 108691597

chr1 108691670
chr1 108691689
chr1 108691740
chr1 108691836
chr1 108691880
chr1 108691887
chr1 108694422
chr1 108694613
chr1 108694657
chr1 108695150
chr1 108746562
chr1 108746572
chr1 108746590
chr1 108746592
chr1 108746594
chr1 108746599
chr1 108746605
chr1 108746612
chr1 108746614
chr1 108746656
chr1 108746670
chr1 108746673
chr1 108746681
chr1 108746723
chr1 108746731
chr1 108746742
chr1 108746745
chr1 108746748
chr1 108746773
chr1 108746776
chr1 108746794
chr1 108746824
chr1 108746835
chr1 108746840
chr1 108746847
chr1 108746868
chr1 108746884
chr1 108746887
chr1 108746889
chr1 108746974
chr1 108746981
chr1 108746984
chr1 108746989
chr1 108746991
chr1 108747000
chr1 108747006
chr1 108747009
chr1 108747021
chr1 108747026
chr1 108747030
chr1 108747032
chr1 108747041
chr1 108747043
chr1 108747051

chr1 108747056
chr1 108747065
chr1 108747069
chr1 108747074
chr1 108747087
chr1 108747089
chr1 108747096
chr1 108747100
chr1 108747108
chr1 108747112
chr1 108747114
chr1 108828231
chr1 108828264
chr1 108956609
chr1 108956647
chr1 108956655
chr1 108956889
chr1 108962833
chr1 108962865
chr1 108962912
chr1 108962984
chr1 108963060
chr1 108963064
chr1 108963075
chr1 108963086
chr1 108963100
chr1 108963131
chr1 108963136
chr1 108963138
chr1 108963140
chr1 108963146
chr1 108963191
chr1 109041112
chr1 109041123
chr1 109041130
chr1 109041136
chr1 109041157
chr1 109041204
chr1 109041234
chr1 109041244
chr1 109041250
chr1 109041261
chr1 109041280
chr1 109041285
chr1 109041298
chr1 109041300
chr1 109041337
chr1 109041365
chr1 109041374
chr1 109042429
chr1 109075462
chr1 109075479
chr1 109075644
chr1 109075646

chr1 109075685
chr1 109075794
chr1 109075805
chr1 109075816
chr1 109075843
chr1 109075862
chr1 109075874
chr1 109075910
chr1 109075921
chr1 109090588
chr1 109090592
chr1 109090602
chr1 109090624
chr1 109090634
chr1 109090643
chr1 109090651
chr1 109090667
chr1 109090675
chr1 109090685
chr1 109090691
chr1 109090698
chr1 109090718
chr1 109090722
chr1 109090725
chr1 109090729
chr1 109090736
chr1 109090751
chr1 109090758
chr1 109090777
chr1 109090782
chr1 109090787
chr1 109090801
chr1 109090805
chr1 109090819
chr1 109090822
chr1 109090840
chr1 109090869
chr1 109090877
chr1 109090881
chr1 109090903
chr1 109090923
chr1 109090934
chr1 109090939
chr1 109090943
chr1 109090949
chr1 109090958
chr1 109090964
chr1 109090973
chr1 109090988
chr1 109091006
chr1 109091009
chr1 109091042
chr1 109091099
chr1 109091234

chr1 109092004
chr1 109092224
chr1 109214017
chr1 109214671
chr1 109214686
chr1 109214727
chr1 109214741
chr1 109214743
chr1 109214760
chr1 109214808
chr1 109214857
chr1 109214894
chr1 109425386
chr1 109425450
chr1 109425499
chr1 109425614
chr1 109425755
chr1 109425841
chr1 109425851
chr1 109425853
chr1 109425875
chr1 109482792
chr1 109482795
chr1 109482852
chr1 109482858
chr1 109482861
chr1 109482872
chr1 109483020
chr1 109483134
chr1 109483138
chr1 109502377
chr1 109502786
chr1 109502849
chr1 109502879
chr1 109510277
chr1 109547558
chr1 109547566
chr1 109547620
chr1 109547744
chr1 109547793
chr1 109547927
chr1 109547941
chr1 109548001
chr1 109549483
chr1 109549518
chr1 109549815
chr1 109549844
chr1 109621151
chr1 109621157
chr1 109621160
chr1 109621163
chr1 109621233
chr1 109643175
chr1 109655946

chr1 109655949
chr1 109655953
chr1 109687451
chr1 109687457
chr1 109687479
chr1 109687481
chr1 109687515
chr1 109687541
chr1 109687554
chr1 109687585
chr1 109687617
chr1 109687623
chr1 109687630
chr1 109687647
chr1 109687658
chr1 109687664
chr1 109687673
chr1 109687686
chr1 109687718
chr1 109687728
chr1 109687744
chr1 109687751
chr1 109687761
chr1 109687766
chr1 109687773
chr1 109687779
chr1 109687792
chr1 109687807
chr1 109687834
chr1 109687851
chr1 109687858
chr1 109687904
chr1 109687906
chr1 109687915
chr1 109687923
chr1 109687930
chr1 109687938
chr1 109687944
chr1 109687961
chr1 109687972
chr1 109687977
chr1 109687991
chr1 109688011
chr1 109688035
chr1 109688061
chr1 109688077
chr1 109688083
chr1 109688144
chr1 109688146
chr1 109688153
chr1 109688178
chr1 109688185
chr1 109688234
chr1 109688244

chr1 109688259
chr1 109688265
chr1 109688282
chr1 109688377
chr1 109688449
chr1 109688514
chr1 109688601
chr1 109688615
chr1 109985595
chr1 109985613
chr1 109985644
chr1 109985675
chr1 110337503
chr1 110337557
chr1 110337683
chr1 110337744
chr1 110342696
chr1 110343102
chr1 110343113
chr1 110343422
chr1 110343481
chr1 110351630
chr1 110407207
chr1 110407231
chr1 110407271
chr1 110619536
chr1 110619557
chr1 110619561
chr1 110619569
chr1 110619588
chr1 110619592
chr1 110619598
chr1 110619604
chr1 110619620
chr1 110619658
chr1 110619660
chr1 110619674
chr1 110619681
chr1 110619688
chr1 110619740
chr1 110619818
chr1 110619934
chr1 110619942
chr1 110619971
chr1 110620002
chr1 110620047
chr1 110620053
chr1 110620184
chr1 110620309
chr1 110620318
chr1 110620343
chr1 110630904
chr1 110630962
chr1 110630973

chr1 110630978
chr1 110631021
chr1 110631146
chr1 110631197
chr1 110631256
chr1 110631263
chr1 110631411
chr1 110631437
chr1 110668827
chr1 110668851
chr1 110668911
chr1 110669774
chr1 110669958
chr1 110670626
chr1 110671205
chr1 110671296
chr1 110672625
chr1 110672648
chr1 110672668
chr1 110672685
chr1 110672803
chr1 110672826
chr1 110673038
chr1 110673090
chr1 110673136
chr1 110673163
chr1 110673189
chr1 110673213
chr1 110673229
chr1 110673244
chr1 110673263
chr1 110673273
chr1 110673306
chr1 110673344
chr1 110673378
chr1 110673393
chr1 110787325
chr1 110872724
chr1 110873057
chr1 110873102
chr1 110873159
chr1 110873186
chr1 110873218
chr1 110873234
chr1 110873262
chr1 110873299
chr1 110873347
chr1 110873480
chr1 110873559
chr1 110873948
chr1 110874053
chr1 110874079
chr1 110874243
chr1 110874485

chr1 110874867
chr1 110875557
chr1 110876103
chr1 110876174
chr1 110877117
chr1 110879862
chr1 110879872
chr1 110879991
chr1 110880100
chr1 110880194
chr1 110880271
chr1 110880501
chr1 110881123
chr1 110881169
chr1 110881677
chr1 110882259
chr1 110882265
chr1 110882696
chr1 110883226
chr1 110883436
chr1 110883501
chr1 110916373
chr1 110916486
chr1 110917020
chr1 110917225
chr1 110963540
chr1 110963544
chr1 110963550
chr1 110963553
chr1 110963557
chr1 110963576
chr1 110963592
chr1 110963605
chr1 110963611
chr1 110963624
chr1 110963626
chr1 110963631
chr1 110963652
chr1 110963672
chr1 110963733
chr1 110963741
chr1 110963758
chr1 110963770
chr1 110963816
chr1 110963825
chr1 110963832
chr1 110963838
chr1 110963846
chr1 110963869
chr1 110963874
chr1 110963880
chr1 110963882
chr1 110963887
chr1 110963889

chr1 110963898
chr1 110963912
chr1 110963922
chr1 110963938
chr1 110963952
chr1 110963955
chr1 110963965
chr1 110963980
chr1 110963998
chr1 110964001
chr1 110964010
chr1 110964019
chr1 110964071
chr1 110964088
chr1 110964093
chr1 110964106
chr1 110964125
chr1 110964129
chr1 110964133
chr1 110964140
chr1 110964146
chr1 110964151
chr1 110964155
chr1 110964176
chr1 111139320
chr1 111139372
chr1 111139377
chr1 111140871
chr1 111140956
chr1 111141138
chr1 111141179
chr1 111141204
chr1 111141222
chr1 111141302
chr1 111141338
chr1 111199108
chr1 111199528
chr1 111199652
chr1 111199669
chr1 111199869
chr1 111199893
chr1 111200042
chr1 111200110
chr1 111200398
chr1 111200413
chr1 111200416
chr1 111200437
chr1 111200481
chr1 111200544
chr1 111200578
chr1 111200580
chr1 111200649
chr1 111200708
chr1 111203355

chr1 111203449
chr1 111203499
chr1 111203627
chr1 111203655
chr1 111203692
chr1 111203697
chr1 111203716
chr1 111203728
chr1 111203731
chr1 111203736
chr1 111204243
chr1 111204251
chr1 111204260
chr1 111204263
chr1 111204271
chr1 111204273
chr1 111204282
chr1 111204286
chr1 111204305
chr1 111204307
chr1 111204328
chr1 111204334
chr1 111204337
chr1 111204341
chr1 111215649
chr1 111215684
chr1 111215853
chr1 111215894
chr1 111215914
chr1 111216045
chr1 111216170
chr1 111216347
chr1 111216376
chr1 111216578
chr1 111449975
chr1 111450039
chr1 111450084
chr1 111450129
chr1 111450192
chr1 111450257
chr1 111471072
chr1 111471135
chr1 111471219
chr1 111507477
chr1 111507975
chr1 111508150
chr1 111508170
chr1 111508206
chr1 111592717
chr1 111592831
chr1 111592866
chr1 111593041
chr1 111593090
chr1 111593098

chr1 111593179
chr1 111593227
chr1 111593238
chr1 111616373
chr1 111616475
chr1 111621114
chr1 111621204
chr1 111621422
chr1 111621438
chr1 111621502
chr1 111621531
chr1 111621947
chr1 111660005
chr1 111664774
chr1 111665155
chr1 111755596
chr1 111755604
chr1 111755612
chr1 111755618
chr1 111755657
chr1 111755665
chr1 111755667
chr1 111755670
chr1 111755693
chr1 111755784
chr1 111755791
chr1 111755815
chr1 111755819
chr1 112201161
chr1 112201653
chr1 112201755
chr1 112369845
chr1 112396249
chr1 112396256
chr1 112396261
chr1 112396264
chr1 112396349
chr1 112396360
chr1 112396363
chr1 112396366
chr1 112396374
chr1 112396402
chr1 112396408
chr1 112396424
chr1 112396434
chr1 112396442
chr1 112396457
chr1 112396487
chr1 112396490
chr1 112396554
chr1 112396570
chr1 112396590
chr1 112396606
chr1 112396623

chr1 112396631
chr1 112396669
chr1 112396857
chr1 112396884
chr1 112397028
chr1 112466531
chr1 112466787
chr1 112617821
chr1 112617872
chr1 112617992
chr1 112618254
chr1 112618274
chr1 112618294
chr1 112618297
chr1 112618330
chr1 112618394
chr1 112618400
chr1 112620656
chr1 112620871
chr1 112621049
chr1 112621116
chr1 112621205
chr1 112621308
chr1 112621316
chr1 112621320
chr1 112621337
chr1 112621951
chr1 112621982
chr1 112622007
chr1 112622040
chr1 112622050
chr1 112622905
chr1 112636505
chr1 112636542
chr1 112715733
chr1 112715758
chr1 112715797
chr1 112715803
chr1 112715818
chr1 112715830
chr1 112715842
chr1 112715855
chr1 112715864
chr1 112715877
chr1 112715903
chr1 112715913
chr1 112715942
chr1 112716102
chr1 112716174
chr1 112716188
chr1 112716193
chr1 112716211
chr1 112716235
chr1 112716263

chr1 112716315
chr1 112718449
chr1 112718496
chr1 112718610
chr1 112947634
chr1 112954797
chr1 112954860
chr1 112955073
chr1 112955248
chr1 112955271
chr1 112955296
chr1 112955310
chr1 112955340
chr1 112955456
chr1 112957033
chr1 112957037
chr1 112957047
chr1 112957064
chr1 112957083
chr1 112957086
chr1 112957109
chr1 112957179
chr1 112957221
chr1 112957227
chr1 112957276
chr1 112957285
chr1 112957296
chr1 112957361
chr1 112957483
chr1 113042338
chr1 113042383
chr1 113042470
chr1 113073011
chr1 113073087
chr1 113073093
chr1 113073103
chr1 113073120
chr1 113073125
chr1 113073169
chr1 113073177
chr1 113073182
chr1 113073185
chr1 113073197
chr1 113073201
chr1 113073212
chr1 113073220
chr1 113073234
chr1 113073237
chr1 113073243
chr1 113073249
chr1 113073251
chr1 113073256
chr1 113073305
chr1 113073916

chr1 113073942
chr1 113073984
chr1 113074002
chr1 113074074
chr1 113074094
chr1 113074130
chr1 113308517
chr1 113499655
chr1 113499797
chr1 113500442
chr1 113500484
chr1 113501291
chr1 113501748
chr1 113502089
chr1 113502152
chr1 113502768
chr1 113502781
chr1 113503141
chr1 113503293
chr1 113503357
chr1 113503510
chr1 113757903
chr1 113758018
chr1 113758034
chr1 113758076
chr1 113758105
chr1 113758109
chr1 113758121
chr1 113758155
chr1 113758162
chr1 113758179
chr1 113758185
chr1 113758187
chr1 113758193
chr1 113758218
chr1 113758226
chr1 113758420
chr1 113758483
chr1 113758574
chr1 113758674
chr1 113758756
chr1 113810066
chr1 113810266
chr1 113810597
chr1 113810611
chr1 113810657
chr1 113810817
chr1 113811034
chr1 113811210
chr1 113835893
chr1 113835954
chr1 113835962
chr1 113870816
chr1 113870846

chr1 113870898
chr1 113871420
chr1 113871464
chr1 113871718
chr1 113871910
chr1 113872168
chr1 113872180
chr1 113873061
chr1 113873236
chr1 113873454
chr1 113904183
chr1 113904191
chr1 113904246
chr1 113904258
chr1 113904268
chr1 113904290
chr1 113904294
chr1 113928848
chr1 113928948
chr1 113928950
chr1 113929012
chr1 113929032
chr1 113929066
chr1 113929089
chr1 113929092
chr1 113931425
chr1 113931558
chr1 113931592
chr1 113931767
chr1 113931947
chr1 113946669
chr1 113946928
chr1 113947264
chr1 113947373
chr1 114512055
chr1 114512067
chr1 114512095
chr1 114512100
chr1 114512132
chr1 114512163
chr1 114512191
chr1 114512203
chr1 114512259
chr1 114512269
chr1 114581036
chr1 114581136
chr1 114581269
chr1 114581317
chr1 114581354
chr1 114581363
chr1 114581383
chr1 114581453
chr1 114581490
chr1 114581499

chr1 114581506
chr1 114581541
chr1 114581547
chr1 114670195
chr1 114670213
chr1 114670224
chr1 114716441
chr1 114716477
chr1 114716522
chr1 114716547
chr1 114716605
chr1 114716631
chr1 114716703
chr1 114716724
chr1 114716730
chr1 114716756
chr1 114716765
chr1 114716771
chr1 114716775
chr1 114716780
chr1 114716790
chr1 114716793
chr1 114716802
chr1 114716814
chr1 114716819
chr1 114716825
chr1 114716835
chr1 114716842
chr1 114716851
chr1 114716859
chr1 114716871
chr1 114716884
chr1 114716889
chr1 114716906
chr1 114716946
chr1 114716960
chr1 114716997
chr1 114717015
chr1 114717068
chr1 114756031
chr1 114756151
chr1 114756253
chr1 114756326
chr1 114757133
chr1 114757172
chr1 114757175
chr1 114757237
chr1 114757249
chr1 114757350
chr1 114757359
chr1 114757366
chr1 114757403
chr1 114757449
chr1 114757483

chr1 114757485
chr1 114757499
chr1 114757504
chr1 114757508
chr1 114757511
chr1 114757527
chr1 114757544
chr1 114757586
chr1 114757588
chr1 114757592
chr1 114757599
chr1 114757629
chr1 114757644
chr1 114757686
chr1 114757688
chr1 114757694
chr1 114757712
chr1 114757754
chr1 114757761
chr1 114780455
chr1 114780461
chr1 114780470
chr1 114780477
chr1 114780492
chr1 114780495
chr1 114780503
chr1 114780506
chr1 114780509
chr1 114780524
chr1 114780529
chr1 114780532
chr1 114780536
chr1 114780541
chr1 114780549
chr1 114780566
chr1 114780574
chr1 114780592
chr1 114780634
chr1 114780646
chr1 114780648
chr1 114780653
chr1 114803281
chr1 114803308
chr1 115062796
chr1 115062845
chr1 115062872
chr1 115062914
chr1 115062941
chr1 115062956
chr1 115063300
chr1 115063325
chr1 115063469
chr1 115098967
chr1 115098984

chr1 115099127
chr1 115099140
chr1 115099146
chr1 115099187
chr1 115099249
chr1 115099305
chr1 115099357
chr1 115099372
chr1 115099399
chr1 115099497
chr1 115099529
chr1 115099594
chr1 115099810
chr1 115148081
chr1 115148096
chr1 115148105
chr1 115148113
chr1 115148116
chr1 115148129
chr1 115148132
chr1 115148139
chr1 115148151
chr1 115148163
chr1 115148175
chr1 115148181
chr1 115148189
chr1 115148230
chr1 115148236
chr1 115148253
chr1 115148303
chr1 115148483
chr1 115148526
chr1 115148578
chr1 115148582
chr1 115148790
chr1 115148809
chr1 115148865
chr1 115462813
chr1 115462818
chr1 115463004
chr1 115463093
chr1 115642105
chr1 115642109
chr1 115642114
chr1 115642116
chr1 115642119
chr1 115642122
chr1 115642129
chr1 115642139
chr1 115642153
chr1 115642164
chr1 115642171
chr1 115642173
chr1 115642183

chr1 115642194
chr1 115642200
chr1 115642205
chr1 115642208
chr1 115642215
chr1 115642221
chr1 115642229
chr1 115642236
chr1 115642241
chr1 115642246
chr1 115642267
chr1 115642272
chr1 115642277
chr1 115642315
chr1 115642320
chr1 115642338
chr1 115642342
chr1 115642349
chr1 115642352
chr1 115642374
chr1 115642395
chr1 116374275
chr1 116374293
chr1 116374316
chr1 116374332
chr1 116374355
chr1 116374409
chr1 116374438
chr1 116374457
chr1 116374467
chr1 116374470
chr1 116374472
chr1 116374488
chr1 116374504
chr1 116374527
chr1 116374530
chr1 116374532
chr1 116374537
chr1 116374551
chr1 116374559
chr1 116374562
chr1 116374583
chr1 116374612
chr1 116376984
chr1 116377753
chr1 116377803
chr1 116515069
chr1 116515244
chr1 116515282
chr1 116515295
chr1 116515320
chr1 116515333
chr1 116515346
chr1 116515355

chr1 116515610
chr1 116515722
chr1 116515824
chr1 116515852
chr1 116515865
chr1 116568376
chr1 116568465
chr1 116737134
chr1 116737401
chr1 116737497
chr1 116737546
chr1 116737677
chr1 116737695
chr1 116737715
chr1 116737722
chr1 116753872
chr1 116754332
chr1 116754334
chr1 116754398
chr1 116754412
chr1 116754652
chr1 116754734
chr1 116754866
chr1 116755036
chr1 116755115
chr1 116755653
chr1 116755716
chr1 116755829
chr1 116755854
chr1 116755902
chr1 116756207
chr1 116756276
chr1 116756305
chr1 116756307
chr1 116756340
chr1 116756354
chr1 116756357
chr1 116756415
chr1 116756564
chr1 116756724
chr1 116756756
chr1 116757099
chr1 116757153
chr1 116757157
chr1 116757185
chr1 116757193
chr1 116757239
chr1 116757248
chr1 116757285
chr1 116757291
chr1 116757481
chr1 116757502
chr1 116757551
chr1 116757808

chr1 116757914
chr1 116758011
chr1 116758163
chr1 116758243
chr1 116758377
chr1 116758586
chr1 116758655
chr1 116758733
chr1 116758736
chr1 116758794
chr1 116759017
chr1 116759114
chr1 116759125
chr1 116759201
chr1 116769037
chr1 116769090
chr1 116769232
chr1 116769265
chr1 116769282
chr1 116769299
chr1 116769339
chr1 116769374
chr1 116769463
chr1 116769525
chr1 116769830
chr1 116770035
chr1 116770117
chr1 116770131
chr1 116770259
chr1 116770266
chr1 116770273
chr1 116770295
chr1 116799565
chr1 116799658
chr1 116799687
chr1 116799699
chr1 116799787
chr1 116799809
chr1 116799817
chr1 116800400
chr1 116800403
chr1 116800431
chr1 116809211
chr1 116809334
chr1 116809351
chr1 116809384
chr1 116809394
chr1 116809648
chr1 116810097
chr1 116815206
chr1 116815433
chr1 116815527
chr1 116815915
chr1 116816129

chr1 116816273
chr1 116816543
chr1 116816570
chr1 116816604
chr1 116878075
chr1 116878097
chr1 116878099
chr1 116878271
chr1 116878402
chr1 116878421
chr1 116878490
chr1 117051325
chr1 117051378
chr1 117051586
chr1 117051614
chr1 117051653
chr1 117051660
chr1 117051664
chr1 117058693
chr1 117058942
chr1 117059014
chr1 117059045
chr1 117059083
chr1 117059238
chr1 117059289
chr1 117059295
chr1 117059364
chr1 117366097
chr1 117366151
chr1 117368217
chr1 117368245
chr1 117368376
chr1 117368423
chr1 117368444
chr1 117368535
chr1 117368547
chr1 117368589
chr1 117368710
chr1 117368722
chr1 117368747
chr1 117368845
chr1 117368889
chr1 117368933
chr1 117368989
chr1 117369019
chr1 117369488
chr1 117369506
chr1 117377319
chr1 117605573
chr1 117605580
chr1 117605610
chr1 117605612
chr1 117605698
chr1 117605718

chr1 117605753
chr1 117605770
chr1 117605773
chr1 117605790
chr1 117605796
chr1 117605821
chr1 117605831
chr1 117605834
chr1 117607137
chr1 117607143
chr1 117607147
chr1 117607163
chr1 117607182
chr1 117607209
chr1 117607247
chr1 117607278
chr1 117607379
chr1 117607592
chr1 117607612
chr1 117607621
chr1 117607638
chr1 117607686
chr1 117654253
chr1 117654309
chr1 117654334
chr1 117735296
chr1 117758865
chr1 117758881
chr1 117758883
chr1 117758897
chr1 117758908
chr1 117758918
chr1 117758920
chr1 117758968
chr1 117759001
chr1 117759006
chr1 117930415
chr1 117930532
chr1 117930587
chr1 117930601
chr1 117930633
chr1 118166860
chr1 118576634
chr1 118576797
chr1 118576799
chr1 118577289
chr1 118577346
chr1 118577400
chr1 118577654
chr1 118577847
chr1 118577870
chr1 118979007
chr1 118981700
chr1 118982119

chr1 119004863
chr1 119004905
chr1 119005201
chr1 119005528
chr1 119009090
chr1 119009181
chr1 119009183
chr1 119009228
chr1 119009497
chr1 119139612
chr1 119139644
chr1 119139692
chr1 119140895
chr1 119141071
chr1 119141093
chr1 119141095
chr1 119141101
chr1 119675073
chr1 119675202
chr1 119675216
chr1 119675442
chr1 119675484
chr1 119675489
chr1 119675503
chr1 119675512
chr1 119675520
chr1 119675536
chr1 119711996
chr1 119712000
chr1 119712006
chr1 119948817
chr1 120067774
chr1 120067835
chr1 120067841
chr1 120069737
chr1 120142688
chr1 120142696
chr1 120142702
chr1 120142707
chr1 120142948
chr1 120142992
chr1 120142999
chr1 120143019
chr1 120143063
chr1 120143209
chr1 120143256
chr1 120143272
chr1 120175696
chr1 120175699
chr1 120175707
chr1 120175776
chr1 120175779
chr1 120175917
chr1 120184811

chr1 120187011
chr1 120187053
chr1 120187441
chr1 120196705
chr1 120196707
chr1 120196725
chr1 120196976
chr1 120197005
chr1 120197019
chr1 120197048
chr1 120197102
chr1 120197115
chr1 120197118
chr1 120197121
chr1 120197126
chr1 120197145
chr1 120197147
chr1 120197149
chr1 120197154
chr1 120197157
chr1 120197170
chr1 120197178
chr1 120197183
chr1 120197189
chr1 120197191
chr1 120197194
chr1 120197196
chr1 120197214
chr1 120197224
chr1 120197229
chr1 120197238
chr1 120197241
chr1 120197245
chr1 120197255
chr1 120197272
chr1 120197276
chr1 120197296
chr1 120197299
chr1 120233015
chr1 120233019
chr1 120233027
chr1 120233043
chr1 120233054
chr1 120233058
chr1 120233061
chr1 120233063
chr1 120233066
chr1 120233071
chr1 120233110
chr1 120233115
chr1 120233119
chr1 120233127
chr1 120233135
chr1 120233141

chr1 120233235
chr1 120233246
chr1 120233287
chr1 120233298
chr1 120233344
chr1 120233369
chr1 120233372
chr1 120233414
chr1 120233435
chr1 120336683
chr1 120342780
chr1 120342884
chr1 120342907
chr1 120353985
chr1 120354712
chr1 120354718
chr1 120354727
chr1 120354733
chr1 120354753
chr1 120354793
chr1 120354810
chr1 120354838
chr1 120354846
chr1 120354889
chr1 120354900
chr1 120354930
chr1 120354934
chr1 120354950
chr1 120354970
chr1 120354978
chr1 120355011
chr1 120355033
chr1 120355039
chr1 120355132
chr1 120355484
chr1 120355493
chr1 120355500
chr1 120355523
chr1 120355534
chr1 120355545
chr1 120355583
chr1 120355588
chr1 120355612
chr1 120355636
chr1 120355651
chr1 120355668
chr1 120355674
chr1 120355706
chr1 120355714
chr1 120356938
chr1 120356970
chr1 120357276
chr1 120357311
chr1 120357326

chr1 120357373
chr1 120357706
chr1 120358437
chr1 120358454
chr1 120358501
chr1 120358505
chr1 120358759
chr1 120358772
chr1 120358904
chr1 120358954
chr1 120358960
chr1 120359040
chr1 120359043
chr1 120388384
chr1 120388422
chr1 120388875
chr1 120388975
chr1 120388999
chr1 120389014
chr1 120389033
chr1 120389054
chr1 120389056
chr1 120389626
chr1 120411212
chr1 120414626
chr1 120414648
chr1 120414674
chr1 120414680
chr1 120414682
chr1 120414699
chr1 120414718
chr1 120414724
chr1 120414927
chr1 120415168
chr1 120415170
chr1 120415266
chr1 120415272
chr1 120415283
chr1 120415286
chr1 120415288
chr1 120415296
chr1 120415303
chr1 120415312
chr1 120415319
chr1 120415321
chr1 120415338
chr1 120415345
chr1 120415352
chr1 120415366
chr1 120415370
chr1 120415373
chr1 120415380
chr1 120415385
chr1 120415406

chr1 120415415
chr1 120415421
chr1 120415424
chr1 120415442
chr1 120415446
chr1 120415461
chr1 120415467
chr1 120415471
chr1 120415491
chr1 120415507
chr1 120415513
chr1 120415547
chr1 120415557
chr1 120415565
chr1 120415571
chr1 120415598
chr1 120415611
chr1 120415810
chr1 120415821
chr1 120415867
chr1 120415917
chr1 120415928
chr1 120415945
chr1 120415952
chr1 120416067
chr1 120416092
chr1 120416326
chr1 120416330
chr1 120416332
chr1 120416367
chr1 120416379
chr1 120555562
chr1 120555724
chr1 120556022
chr1 120556051
chr1 120556222
chr1 120556634
chr1 120556858
chr1 120556951
chr1 120556959
chr1 120557007
chr1 120557026
chr1 120557304
chr1 120557399
chr1 120557433
chr1 120557486
chr1 120557508
chr1 120557522
chr1 120557535
chr1 120557555
chr1 120557581
chr1 120557744
chr1 120557775
chr1 120557780

chr1 120557789
chr1 120557802
chr1 120557814
chr1 120557828
chr1 120557840
chr1 120557851
chr1 120557881
chr1 120557911
chr1 120557925
chr1 120558911
chr1 120558921
chr1 120558926
chr1 120558928
chr1 120558931
chr1 120558937
chr1 120558944
chr1 120558965
chr1 120558971
chr1 120558976
chr1 120559136
chr1 120559192
chr1 120559274
chr1 120559276
chr1 120561837
chr1 120561971
chr1 120562066
chr1 120664926
chr1 120664953
chr1 120664974
chr1 120665037
chr1 120665040
chr1 120665065
chr1 120665111
chr1 120665163
chr1 120665174
chr1 120665214
chr1 120665217
chr1 120665220
chr1 120665223
chr1 120665268
chr1 120665274
chr1 120665299
chr1 120665303
chr1 120665516
chr1 120665530
chr1 120665548
chr1 120665550
chr1 120665558
chr1 120665570
chr1 120665785
chr1 120665799
chr1 120665810
chr1 120665825
chr1 120667350

chr1 120667770
chr1 120694362
chr1 120694384
chr1 120694404
chr1 120694416
chr1 120694493
chr1 120694502
chr1 120694509
chr1 120694537
chr1 120695671
chr1 120695684
chr1 120695915
chr1 120696810
chr1 120696827
chr1 120697708
chr1 120697725
chr1 120697784
chr1 120698616
chr1 120698688
chr1 120699446
chr1 120699682
chr1 120700339
chr1 120700376
chr1 120700608
chr1 120700612
chr1 120700625
chr1 120700776
chr1 120701118
chr1 120701128
chr1 120701132
chr1 120701135
chr1 120701144
chr1 120701149
chr1 120701159
chr1 120701177
chr1 120701179
chr1 120701182
chr1 120701184
chr1 120701190
chr1 120701195
chr1 120701203
chr1 120701216
chr1 120701219
chr1 120701224
chr1 120701226
chr1 120701228
chr1 120701247
chr1 120701252
chr1 120701255
chr1 120701258
chr1 120701271
chr1 120702527
chr1 120723228
chr1 120723282

chr1 120723351
chr1 120723378
chr1 120723385
chr1 120723425
chr1 120723459
chr1 120723687
chr1 120723692
chr1 120723707
chr1 120724611
chr1 120724614
chr1 120724617
chr1 120724624
chr1 120724637
chr1 120724658
chr1 120724667
chr1 120724679
chr1 120724691
chr1 120724704
chr1 120724716
chr1 120724732
chr1 120724744
chr1 120724773
chr1 120724783
chr1 120724786
chr1 120724788
chr1 120724816
chr1 120724824
chr1 120724833
chr1 120724846
chr1 120724848
chr1 120725366
chr1 120843943
chr1 120844002
chr1 120844135
chr1 120845043
chr1 120845046
chr1 120845054
chr1 120845056
chr1 120845072
chr1 120845079
chr1 120845092
chr1 120845099
chr1 120845127
chr1 120845140
chr1 120845147
chr1 120845159
chr1 120845183
chr1 120845197
chr1 120845207
chr1 120849539
chr1 120849567
chr1 120849619
chr1 120849651
chr1 120850103

chr1 120850135
chr1 120850181
chr1 120850335
chr1 120850364
chr1 120850386
chr1 120850388
chr1 120850408
chr1 120850416
chr1 120851441
chr1 120851490
chr1 120851502
chr1 120851524
chr1 120851953
chr1 120855160
chr1 120855752
chr1 120855926
chr1 120857102
chr1 120857116
chr1 120857132
chr1 120857230
chr1 120857260
chr1 120911970
chr1 120911996
chr1 120912029
chr1 120912107
chr1 120912478
chr1 120912514
chr1 120912570
chr1 120912579
chr1 120913686
chr1 120913688
chr1 120913695
chr1 120913699
chr1 120913711
chr1 120913724
chr1 120913741
chr1 120913743
chr1 120913752
chr1 120913767
chr1 120913787
chr1 120913790
chr1 120913798
chr1 120913814
chr1 120913825
chr1 120913839
chr1 120913842
chr1 120913845
chr1 120913849
chr1 120913867
chr1 120913872
chr1 120913876
chr1 120913885
chr1 120913889
chr1 120913892

chr1 120913895
chr1 120914022
chr1 120914029
chr1 120919230
chr1 120920225
chr1 120920649
chr1 120941720
chr1 120941746
chr1 120941921
chr1 120941964
chr1 120941985
chr1 120942016
chr1 120942024
chr1 120942077
chr1 120942099
chr1 120942144
chr1 120942160
chr1 121008540
chr1 121008546
chr1 121008555
chr1 121008559
chr1 121008593
chr1 121008631
chr1 121008647
chr1 121008660
chr1 121008673
chr1 121008680
chr1 121008685
chr1 121008693
chr1 121008695
chr1 121008709
chr1 121008711
chr1 121008721
chr1 121008724
chr1 121008801
chr1 121008894
chr1 121008902
chr1 121016505
chr1 121016528
chr1 121016634
chr1 121016655
chr1 121016678
chr1 121016687
chr1 121016729
chr1 121016854
chr1 121016867
chr1 121016876
chr1 121016878
chr1 121016887
chr1 121016997
chr1 121017088
chr1 121017151
chr1 121017395
chr1 121020119

chr1 121020121
chr1 121020215
chr1 121020231
chr1 121020238
chr1 121020671
chr1 121020735
chr1 121020739
chr1 121020745
chr1 121020750
chr1 121020761
chr1 121020763
chr1 121020772
chr1 121020776
chr1 121020795
chr1 121020808
chr1 121020819
chr1 121020853
chr1 121097189
chr1 121097208
chr1 121097211
chr1 121097213
chr1 121097218
chr1 121097222
chr1 121097229
chr1 121097231
chr1 121097234
chr1 121097259
chr1 121097281
chr1 121097285
chr1 121097298
chr1 121097304
chr1 121097421
chr1 121118603
chr1 121118626
chr1 121183492
chr1 121183495
chr1 121183584
chr1 121183649
chr1 121183655
chr1 121183937
chr1 121183942
chr1 121183984
chr1 121184033
chr1 121184039
chr1 121184051
chr1 121184056
chr1 121184095
chr1 121184097
chr1 121184120
chr1 121184130
chr1 121184138
chr1 121184150
chr1 121184152
chr1 121185330

chr1 121185340
chr1 121185367
chr1 121185379
chr1 121185384
chr1 121185389
chr1 121185406
chr1 121185423
chr1 121185429
chr1 121185437
chr1 121185439
chr1 121185447
chr1 121185458
chr1 121185461
chr1 121185470
chr1 121185472
chr1 121185474
chr1 121185483
chr1 121185826
chr1 121185833
chr1 121185840
chr1 121185847
chr1 121185851
chr1 121185862
chr1 121185865
chr1 121185894
chr1 121185913
chr1 121185915
chr1 121185929
chr1 121185935
chr1 121185952
chr1 121185955
chr1 121185959
chr1 121186554
chr1 121186557
chr1 121186569
chr1 121186583
chr1 121186592
chr1 121186602
chr1 121186608
chr1 121186623
chr1 121186626
chr1 121186634
chr1 121186643
chr1 121186647
chr1 121186655
chr1 121186657
chr1 121186672
chr1 121395728
chr1 121395872
chr1 121395898
chr1 121395925
chr1 121395935
chr1 121395938
chr1 121395941

chr1 121395946
chr1 121395962
chr1 121395965
chr1 121395968
chr1 121395970
chr1 121395989
chr1 121395994
chr1 121396006
chr1 121396009
chr1 121421587
chr1 121458192
chr1 121458629
chr1 121516563
chr1 121516637
chr1 121516639
chr1 121516692
chr1 121518640
chr1 121612177
chr1 121857792
chr1 121857977
chr1 121858024
chr1 121858144
chr1 121858395
chr1 121858491
chr1 121858519
chr1 121858739
chr1 124798930
chr1 124799493
chr1 124799521
chr1 124799553
chr1 124799583
chr1 124799610
chr1 125077734
chr1 125077992
chr1 125078316
chr1 125079006
chr1 125089459
chr1 125089573
chr1 125089594
chr1 125089674
chr1 125090146
chr1 125090274
chr1 125090282
chr1 125145761
chr1 125146030
chr1 125146288
chr1 125146778
chr1 143277187
chr1 143277756
chr1 143278488
chr1 143278731
chr1 143278776
chr1 143327638
chr1 143327647

chr1 143327649
chr1 143327657
chr1 143327700
chr1 143334474
chr1 143334480
chr1 143334504
chr1 143334515
chr1 143334524
chr1 143334686
chr1 143334723
chr1 143334776
chr1 143334813
chr1 143335796
chr1 143336467
chr1 143336510
chr1 143336556
chr1 143336573
chr1 143336595
chr1 143342147
chr1 143342191
chr1 143342263
chr1 143342361
chr1 143343520
chr1 143343555
chr1 143343609
chr1 143344437
chr1 143424067
chr1 143424773
chr1 143426253
chr1 143426267
chr1 143426350
chr1 143488835
chr1 143489399
chr1 143489455
chr1 143489519
chr1 143489962
chr1 143490727
chr1 143490751
chr1 143493689
chr1 143494374
chr1 143494571
chr1 143494787
chr1 143494834
chr1 143494842
chr1 143494854
chr1 143495190
chr1 143495323
chr1 143495331
chr1 143495796
chr1 143495824
chr1 143495854
chr1 143497036
chr1 143497062
chr1 143497089

chr1 143497096
chr1 143497099
chr1 143497105
chr1 143497108
chr1 143497110
chr1 143497121
chr1 143497129
chr1 143497132
chr1 143497135
chr1 143497137
chr1 143497156
chr1 143497173
chr1 143497176
chr1 143497193
chr1 143497196
chr1 143497213
chr1 143497216
chr1 143497218
chr1 143497234
chr1 143497237
chr1 143497242
chr1 143497252
chr1 143497255
chr1 143497257
chr1 143497272
chr1 143497280
chr1 143497298
chr1 143497313
chr1 143497316
chr1 143497319
chr1 143497344
chr1 143497356
chr1 143497361
chr1 143497379
chr1 143497384
chr1 143497403
chr1 143497408
chr1 143497423
chr1 143497521
chr1 143497524
chr1 143497526
chr1 143497540
chr1 143497543
chr1 143497546
chr1 143497548
chr1 143497560
chr1 143497565
chr1 143497579
chr1 143497582
chr1 143497585
chr1 143497603
chr1 143497606
chr1 143497611
chr1 143497629

chr1 143497632
chr1 143497637
chr1 143497647
chr1 143497653
chr1 143497656
chr1 143497661
chr1 143497681
chr1 143497705
chr1 143497710
chr1 143497720
chr1 143497723
chr1 143497725
chr1 143497737
chr1 143497744
chr1 143497747
chr1 143497752
chr1 143497767
chr1 143497770
chr1 143497785
chr1 143497788
chr1 143497791
chr1 143497793
chr1 143497804
chr1 143497807
chr1 143497830
chr1 143497832
chr1 143497844
chr1 143497847
chr1 143497868
chr1 143497873
chr1 143497885
chr1 143497888
chr1 143497890
chr1 143497905
chr1 143497908
chr1 143497911
chr1 143497913
chr1 143497949
chr1 143498030
chr1 143498335
chr1 143498356
chr1 143498359
chr1 143498375
chr1 143498378
chr1 143498380
chr1 143498396
chr1 143498404
chr1 143498420
chr1 143498426
chr1 143498429
chr1 143498447
chr1 143498450
chr1 143498453
chr1 143498470

chr1 143498472
chr1 143498486
chr1 143498488
chr1 143498500
chr1 143498505
chr1 143498517
chr1 143498520
chr1 143498522
chr1 143498532
chr1 143498537
chr1 143498545
chr1 143498564
chr1 143498569
chr1 143498578
chr1 143498581
chr1 143498584
chr1 143498586
chr1 143498589
chr1 143498600
chr1 143498603
chr1 143498606
chr1 143498622
chr1 143498625
chr1 143498630
chr1 143498642
chr1 143498648
chr1 143498657
chr1 143498676
chr1 143498747
chr1 143498750
chr1 143498753
chr1 143537622
chr1 143538193
chr1 143623926
chr1 143624188
chr1 143624426
chr1 143644031
chr1 143644309
chr1 143644717
chr1 143649450
chr1 143649497
chr1 143720376
chr1 143720387
chr1 143720700
chr1 143728306
chr1 143728314
chr1 143728338
chr1 143728344
chr1 143728368
chr1 143728376
chr1 143728384
chr1 143728929
chr1 143728935
chr1 143728937

chr1 143728983
chr1 143728989
chr1 143729002
chr1 143729030
chr1 143729496
chr1 143729517
chr1 143729537
chr1 143729586
chr1 143729591
chr1 143729606
chr1 143729622
chr1 143729626
chr1 143729639
chr1 143729641
chr1 143729712
chr1 143729719
chr1 143729731
chr1 143729739
chr1 143729744
chr1 143729756
chr1 143729761
chr1 143729793
chr1 143729816
chr1 143729825
chr1 143729836
chr1 143729858
chr1 143729873
chr1 143729890
chr1 143790858
chr1 143790864
chr1 143790866
chr1 143791390
chr1 143791404
chr1 143791444
chr1 143791483
chr1 143791489
chr1 143791495
chr1 143791662
chr1 143791695
chr1 143791720
chr1 143791726
chr1 143791735
chr1 143791739
chr1 143791773
chr1 143791811
chr1 143791827
chr1 143791840
chr1 143803933
chr1 143804118
chr1 143804148
chr1 143804180
chr1 143804243
chr1 143804253
chr1 143804323

chr1 143804331
chr1 143804336
chr1 143804339
chr1 143804345
chr1 143804372
chr1 143804379
chr1 143804381
chr1 143804425
chr1 143804466
chr1 143804507
chr1 143804820
chr1 143804851
chr1 143804862
chr1 143804870
chr1 143804883
chr1 143804892
chr1 143804918
chr1 143804994
chr1 143805029
chr1 143805071
chr1 143806565
chr1 143831417
chr1 143831521
chr1 143838158
chr1 143838185
chr1 143838457
chr1 143838460
chr1 143838470
chr1 143838507
chr1 143838513
chr1 143838520
chr1 143838530
chr1 143838547
chr1 143839059
chr1 143839087
chr1 143839107
chr1 143839137
chr1 143839160
chr1 143839178
chr1 143839186
chr1 143839232
chr1 143839237
chr1 143839499
chr1 143839506
chr1 143839541
chr1 143839544
chr1 143839546
chr1 143839556
chr1 143839612
chr1 143839661
chr1 143839747
chr1 143839756
chr1 143839759
chr1 143839843

chr1 143839891
chr1 143839975
chr1 143884439
chr1 143884634
chr1 143884639
chr1 143884643
chr1 143885102
chr1 143885105
chr1 143885107
chr1 143885110
chr1 143885120
chr1 143885124
chr1 143885146
chr1 143885156
chr1 143885168
chr1 143885184
chr1 143885188
chr1 143885196
chr1 143885198
chr1 143885228
chr1 143885236
chr1 143885239
chr1 143885257
chr1 143885478
chr1 143885485
chr1 143885488
chr1 143885511
chr1 143885526
chr1 143904878
chr1 143904890
chr1 143905301
chr1 143905323
chr1 143905335
chr1 143905339
chr1 143905364
chr1 143905380
chr1 143905391
chr1 143905482
chr1 143905506
chr1 143905536
chr1 143905563
chr1 143905570
chr1 143905572
chr1 143905578
chr1 143905615
chr1 143905617
chr1 143905633
chr1 143905643
chr1 143905651
chr1 143905691
chr1 143905699
chr1 143905703
chr1 143905716
chr1 143905724

chr1 143905845
chr1 143905850
chr1 143905864
chr1 143905868
chr1 143905874
chr1 143905877
chr1 143905879
chr1 143905887
chr1 143905927
chr1 143905930
chr1 143905934
chr1 143905941
chr1 143905959
chr1 143905982
chr1 143906000
chr1 143906096
chr1 143906167
chr1 143906275
chr1 143906414
chr1 143938791
chr1 143938796
chr1 143938956
chr1 143939400
chr1 143939649
chr1 143939694
chr1 143939778
chr1 143941463
chr1 143941503
chr1 143941561
chr1 143942226
chr1 143943885
chr1 143943903
chr1 143943921
chr1 143943928
chr1 143972298
chr1 143972329
chr1 143972352
chr1 143973868
chr1 143973891
chr1 143973902
chr1 143973906
chr1 143973909
chr1 143973916
chr1 143973925
chr1 143973931
chr1 143973949
chr1 143973993
chr1 143973998
chr1 143974094
chr1 143974137
chr1 143974173
chr1 143974177
chr1 143974193
chr1 143974196

chr1 143974208
chr1 143974231
chr1 143974241
chr1 143974514
chr1 143974554
chr1 143974563
chr1 143974570
chr1 143974582
chr1 143974597
chr1 143974634
chr1 143974646
chr1 143974648
chr1 143974655
chr1 143974670
chr1 143974678
chr1 143974699
chr1 143974715
chr1 143974717
chr1 143974935
chr1 143974942
chr1 143974944
chr1 143974951
chr1 143974956
chr1 143974969
chr1 143974991
chr1 143975000
chr1 143975039
chr1 143975062
chr1 143975091
chr1 144076384
chr1 144076421
chr1 144076424
chr1 144076434
chr1 144076441
chr1 144076444
chr1 144076446
chr1 144076472
chr1 144076477
chr1 144076489
chr1 144076506
chr1 144076509
chr1 144076511
chr1 144076526
chr1 144076529
chr1 144076532
chr1 144076553
chr1 144076556
chr1 144076558
chr1 144076577
chr1 144076582
chr1 144077772
chr1 144077785
chr1 144077788
chr1 144077791

chr1 144077793
chr1 144077809
chr1 144077812
chr1 144077815
chr1 144077818
chr1 144077831
chr1 144077834
chr1 144077836
chr1 144077853
chr1 144077856
chr1 144077859
chr1 144077869
chr1 144077878
chr1 144077893
chr1 144077916
chr1 144077919
chr1 144077922
chr1 144077924
chr1 144077948
chr1 144077965
chr1 144077969
chr1 144077971
chr1 144077982
chr1 144078013
chr1 144078016
chr1 144078019
chr1 144078021
chr1 144078031
chr1 144078045
chr1 144078048
chr1 144078051
chr1 144078065
chr1 144078067
chr1 144078099
chr1 144078102
chr1 144078105
chr1 144078107
chr1 144078122
chr1 144078125
chr1 144078128
chr1 144078130
chr1 144078133
chr1 144078170
chr1 144078176
chr1 144078188
chr1 144078190
chr1 144078195
chr1 144078214
chr1 144078217
chr1 144078219
chr1 144078242
chr1 144078254
chr1 144078257
chr1 144078259

chr1 144078276
chr1 144078279
chr1 144078284
chr1 144078776
chr1 144079086
chr1 144081829
chr1 144082178
chr1 144082357
chr1 144082424
chr1 144082441
chr1 144084566
chr1 144129239
chr1 144129736
chr1 144130053
chr1 144130090
chr1 144133296
chr1 144133366
chr1 144133920
chr1 144133934
chr1 144133990
chr1 144134057
chr1 144143151
chr1 144143468
chr1 144144311
chr1 144144322
chr1 144152423
chr1 144152430
chr1 144152433
chr1 144152436
chr1 144152438
chr1 144152441
chr1 144152449
chr1 144152456
chr1 144152459
chr1 144152467
chr1 144152474
chr1 144152477
chr1 144152480
chr1 144152482
chr1 144152485
chr1 144152493
chr1 144152500
chr1 144152503
chr1 144152506
chr1 144152508
chr1 144152511
chr1 144152519
chr1 144152881
chr1 144152888
chr1 144152891
chr1 144152899
chr1 144152906
chr1 144152909
chr1 144152912

chr1 144152914
chr1 144152917
chr1 144153056
chr1 144153059
chr1 144153062
chr1 144153064
chr1 144153067
chr1 144153075
chr1 144153082
chr1 144153085
chr1 144153093
chr1 144153100
chr1 144153103
chr1 144153111
chr1 144153118
chr1 144153121
chr1 144153124
chr1 144153126
chr1 144153129
chr1 144153137
chr1 144153144
chr1 144153147
chr1 144153155
chr1 144153162
chr1 144153165
chr1 144153168
chr1 144153173
chr1 144153175
chr1 144153180
chr1 144153183
chr1 144153186
chr1 144161368
chr1 144162391
chr1 144162449
chr1 144162535
chr1 144167719
chr1 144167763
chr1 144297329
chr1 144297881
chr1 144297899
chr1 144297958
chr1 144297962
chr1 144297974
chr1 144298058
chr1 144298319
chr1 144298343
chr1 144298363
chr1 144298439
chr1 144298455
chr1 144298469
chr1 144298477
chr1 144300262
chr1 144300285
chr1 144300421

chr1 144300719
chr1 144301466
chr1 144301512
chr1 144303082
chr1 144303882
chr1 144304008
chr1 144304012
chr1 144304043
chr1 144304074
chr1 144304089
chr1 144304133
chr1 144304145
chr1 144304149
chr1 144304153
chr1 144306709
chr1 144306734
chr1 144307559
chr1 144307583
chr1 144307591
chr1 144307597
chr1 144307757
chr1 144307821
chr1 144315104
chr1 144315168
chr1 144318043
chr1 144318316
chr1 144320221
chr1 144322686
chr1 144322696
chr1 144322712
chr1 144322746
chr1 144322749
chr1 144322752
chr1 144322755
chr1 144322757
chr1 144322773
chr1 144322776
chr1 144322779
chr1 144322781
chr1 144322800
chr1 144322817
chr1 144322820
chr1 144322831
chr1 144322838
chr1 144322841
chr1 144322843
chr1 144322855
chr1 144322858
chr1 144322860
chr1 144322874
chr1 144322877
chr1 144322918
chr1 144322921
chr1 144322923

chr1 144322947
chr1 144322959
chr1 144322962
chr1 144322964
chr1 144322982
chr1 144322985
chr1 144322990
chr1 144323000
chr1 144323006
chr1 144323009
chr1 144323011
chr1 144323030
chr1 144323033
chr1 144323036
chr1 144323038
chr1 144323050
chr1 144323053
chr1 144323055
chr1 144323071
chr1 144323074
chr1 144323091
chr1 144323096
chr1 144323112
chr1 144323115
chr1 144323120
chr1 144323130
chr1 144323133
chr1 144323135
chr1 144323154
chr1 144323157
chr1 144323162
chr1 144323177
chr1 144323180
chr1 144323194
chr1 144323197
chr1 144323212
chr1 144323215
chr1 144323218
chr1 144323220
chr1 144323231
chr1 144323234
chr1 144323257
chr1 144323259
chr1 144323271
chr1 144323274
chr1 144323276
chr1 144323295
chr1 144323300
chr1 144323312
chr1 144323315
chr1 144323317
chr1 144323335
chr1 144323338
chr1 144323340

chr1 144323358
chr1 144323361
chr1 144323363
chr1 144323382
chr1 144323387
chr1 144323399
chr1 144323402
chr1 144323404
chr1 144323419
chr1 144323422
chr1 144323425
chr1 144323427
chr1 144323446
chr1 144323451
chr1 144323463
chr1 144323468
chr1 144323483
chr1 144323486
chr1 144323489
chr1 144323503
chr1 144323506
chr1 144323508
chr1 144323532
chr1 144323544
chr1 144323547
chr1 144323549
chr1 144323564
chr1 144323567
chr1 144323574
chr1 144323581
chr1 144323584
chr1 144323587
chr1 144323589
chr1 144323598
chr1 144323601
chr1 144323604
chr1 144323626
chr1 144323628
chr1 144323640
chr1 144323643
chr1 144323660
chr1 144323663
chr1 144323666
chr1 144323668
chr1 144323687
chr1 144323692
chr1 144323707
chr1 144323709
chr1 144323722
chr1 144323724
chr1 144323727
chr1 144323730
chr1 144323732
chr1 144323744

chr1 144323747
chr1 144323749
chr1 144323768
chr1 144323771
chr1 144323774
chr1 144323776
chr1 144323795
chr1 144323798
chr1 144323800
chr1 144323816
chr1 144323819
chr1 144323840
chr1 144323859
chr1 144323862
chr1 144323888
chr1 144323904
chr1 144323910
chr1 144323913
chr1 144323931
chr1 144323934
chr1 144323937
chr1 144323951
chr1 144323954
chr1 144323956
chr1 144323970
chr1 144323972
chr1 144323984
chr1 144323989
chr1 144324001
chr1 144324598
chr1 144324825
chr1 144326385
chr1 144329586
chr1 144331783
chr1 144334165
chr1 144334180
chr1 144334221
chr1 144334237
chr1 144334257
chr1 144334259
chr1 144334293
chr1 144334323
chr1 144335712
chr1 144335714
chr1 144335726
chr1 144335746
chr1 144335754
chr1 144335759
chr1 144335770
chr1 144335815
chr1 144335942
chr1 144344633
chr1 144346215
chr1 144351659

chr1 144351751
chr1 144351754
chr1 144351781
chr1 144354318
chr1 144354663
chr1 144408374
chr1 144408469
chr1 144408564
chr1 144413002
chr1 144413206
chr1 144413210
chr1 144413222
chr1 144413287
chr1 144413344
chr1 144413378
chr1 144413424
chr1 144413456
chr1 144413525
chr1 144413553
chr1 144413557
chr1 144413605
chr1 144413620
chr1 144413623
chr1 144413633
chr1 144413653
chr1 144418866
chr1 144418871
chr1 144418883
chr1 144418925
chr1 144418928
chr1 144418930
chr1 144418937
chr1 144419277
chr1 144419283
chr1 144419290
chr1 144419296
chr1 144419307
chr1 144419323
chr1 144419339
chr1 144419466
chr1 144419470
chr1 144419482
chr1 144419584
chr1 144419591
chr1 144419599
chr1 144419658
chr1 144460597
chr1 144460843
chr1 144460854
chr1 144474577
chr1 144474757
chr1 144474812
chr1 144485251
chr1 144485253

chr1 144485302
chr1 144485357
chr1 144485711
chr1 144485753
chr1 144486115
chr1 144486601
chr1 144487087
chr1 144490010
chr1 144490494
chr1 144490519
chr1 144490524
chr1 144490532
chr1 144490565
chr1 144490572
chr1 144490582
chr1 144490598
chr1 144490601
chr1 144490610
chr1 144490755
chr1 144490791
chr1 144490793
chr1 144490807
chr1 144522478
chr1 144522510
chr1 144522535
chr1 144522540
chr1 144522581
chr1 144523138
chr1 144523293
chr1 144523301
chr1 144523333
chr1 144523339
chr1 144523356
chr1 144523371
chr1 144523395
chr1 144523419
chr1 144523424
chr1 144523462
chr1 144523473
chr1 144523484
chr1 144523507
chr1 144523514
chr1 144523523
chr1 144523601
chr1 144523680
chr1 144523699
chr1 144523727
chr1 144523869
chr1 144523871
chr1 144523875
chr1 144523968
chr1 144523974
chr1 144523996
chr1 144524013

chr1 144524029
chr1 144524037
chr1 144524057
chr1 144524073
chr1 144524090
chr1 144524092
chr1 144524107
chr1 144524118
chr1 144524161
chr1 144524169
chr1 144524197
chr1 144524209
chr1 144524254
chr1 144524274
chr1 144524280
chr1 144524289
chr1 144524295
chr1 144524307
chr1 144524326
chr1 144524330
chr1 144524350
chr1 144524354
chr1 144524398
chr1 144546365
chr1 144546390
chr1 144546402
chr1 144546407
chr1 144546422
chr1 144546439
chr1 144546555
chr1 144546560
chr1 144546575
chr1 144546591
chr1 144547271
chr1 144547463
chr1 144547471
chr1 144547479
chr1 144547482
chr1 144547490
chr1 144547507
chr1 144547514
chr1 144547522
chr1 144547546
chr1 144547566
chr1 144547570
chr1 144547575
chr1 144547578
chr1 144547582
chr1 144547611
chr1 144547663
chr1 144547667
chr1 144547700
chr1 144547710
chr1 144547712

chr1 144547716
chr1 144551582
chr1 144551584
chr1 144551743
chr1 144551747
chr1 144551791
chr1 144551868
chr1 144551910
chr1 144552345
chr1 144552358
chr1 144552361
chr1 144552409
chr1 144552415
chr1 144552421
chr1 144552453
chr1 144552480
chr1 144552570
chr1 144552790
chr1 144559498
chr1 144559507
chr1 144559510
chr1 144559588
chr1 144559594
chr1 144559618
chr1 144559626
chr1 144559634
chr1 144559727
chr1 144559729
chr1 144560167
chr1 144560188
chr1 144560194
chr1 144560196
chr1 144560242
chr1 144560261
chr1 144560289
chr1 144560378
chr1 144560676
chr1 144560678
chr1 144560682
chr1 144560713
chr1 144560737
chr1 144560755
chr1 144560776
chr1 144560796
chr1 144560845
chr1 144567196
chr1 144567255
chr1 144567263
chr1 144567270
chr1 144567515
chr1 144567531
chr1 144567547
chr1 144567558
chr1 144567562

chr1 144567564
chr1 144567571
chr1 144567611
chr1 144567625
chr1 144567629
chr1 144567633
chr1 144567645
chr1 144567667
chr1 144567671
chr1 144567679
chr1 144567702
chr1 144567716
chr1 144567724
chr1 144567726
chr1 144567735
chr1 144567748
chr1 144567750
chr1 144567781
chr1 144567792
chr1 144567796
chr1 144567827
chr1 144567833
chr1 144567838
chr1 144567850
chr1 144567855
chr1 144567860
chr1 144567886
chr1 144567890
chr1 144567900
chr1 144567915
chr1 144567924
chr1 144567927
chr1 144567969
chr1 144567981
chr1 144567986
chr1 144567995
chr1 144568007
chr1 144568021
chr1 144568025
chr1 144568049
chr1 144568065
chr1 144568109
chr1 144568127
chr1 144568230
chr1 144568249
chr1 144568251
chr1 144568256
chr1 144568262
chr1 144608696
chr1 144610202
chr1 144610211
chr1 144611855
chr1 144612767
chr1 144612784

chr1 144613085
chr1 144613511
chr1 144614692
chr1 144614736
chr1 144614763
chr1 144614766
chr1 144618750
chr1 144618822
chr1 144621884
chr1 144623767
chr1 144627219
chr1 144627261
chr1 144630701
chr1 144630746
chr1 144630757
chr1 144630762
chr1 144630770
chr1 144630790
chr1 144630802
chr1 144630804
chr1 144630806
chr1 144630810
chr1 144631390
chr1 144632191
chr1 144632221
chr1 144632255
chr1 144632257
chr1 144632277
chr1 144632293
chr1 144632334
chr1 144632349
chr1 144632764
chr1 144639112
chr1 144639197
chr1 144640082
chr1 144640131
chr1 144640140
chr1 144640146
chr1 144640691
chr1 144640899
chr1 144640972
chr1 144641007
chr1 144641387
chr1 144641926
chr1 144641931
chr1 144641949
chr1 144641957
chr1 144641972
chr1 144642001
chr1 144642013
chr1 144642031
chr1 144642055
chr1 144642122
chr1 144642128

chr1 144642131
chr1 144642148
chr1 144642170
chr1 144642185
chr1 144642230
chr1 144642236
chr1 144642353
chr1 144642358
chr1 144642361
chr1 144642377
chr1 144642380
chr1 144642383
chr1 144642394
chr1 144642397
chr1 144642399
chr1 144642402
chr1 144642405
chr1 144642414
chr1 144642438
chr1 144642446
chr1 144642451
chr1 144642461
chr1 144642463
chr1 144642466
chr1 144642478
chr1 144642483
chr1 144642495
chr1 144642497
chr1 144642511
chr1 144642513
chr1 144642516
chr1 144642530
chr1 144642533
chr1 144642536
chr1 144642554
chr1 144642557
chr1 144642563
chr1 144642579
chr1 144642581
chr1 144642587
chr1 144642603
chr1 144642605
chr1 144642608
chr1 144642624
chr1 144642627
chr1 144642648
chr1 144642651
chr1 144642667
chr1 144642669
chr1 144642672
chr1 144642691
chr1 144642693
chr1 144642696
chr1 144642699

chr1 144642718
chr1 144642720
chr1 144642723
chr1 144642735
chr1 144642737
chr1 144642740
chr1 144642775
chr1 144642780
chr1 144642799
chr1 144642801
chr1 144642804
chr1 144642807
chr1 144642824
chr1 144642827
chr1 144642839
chr1 144642841
chr1 144642863
chr1 144642866
chr1 144642869
chr1 144642878
chr1 144642880
chr1 144642883
chr1 144642886
chr1 144642901
chr1 144642904
chr1 144642919
chr1 144642921
chr1 144642924
chr1 144642936
chr1 144642960
chr1 144642962
chr1 144642965
chr1 144642979
chr1 144642982
chr1 144642985
chr1 144643000
chr1 144643005
chr1 144643017
chr1 144643022
chr1 144643041
chr1 144643043
chr1 144643046
chr1 144643064
chr1 144643066
chr1 144643069
chr1 144643081
chr1 144643086
chr1 144643105
chr1 144643107
chr1 144643110
chr1 144643122
chr1 144643124
chr1 144643147
chr1 144643150

chr1 144643161
chr1 144643163
chr1 144643166
chr1 144643169
chr1 144643184
chr1 144643187
chr1 144643201
chr1 144643204
chr1 144643219
chr1 144643224
chr1 144643227
chr1 144643234
chr1 144643246
chr1 144643248
chr1 144643251
chr1 144643261
chr1 144643266
chr1 144643269
chr1 144643285
chr1 144643290
chr1 144643307
chr1 144643310
chr1 144643326
chr1 144643328
chr1 144643331
chr1 144643343
chr1 144643345
chr1 144643348
chr1 144643351
chr1 144643370
chr1 144643372
chr1 144643375
chr1 144643381
chr1 144643391
chr1 144643396
chr1 144643399
chr1 144643417
chr1 144643419
chr1 144643422
chr1 144643434
chr1 144643458
chr1 144643460
chr1 144643463
chr1 144643482
chr1 144643487
chr1 144643499
chr1 144643501
chr1 144643504
chr1 144643507
chr1 144643521
chr1 144643523
chr1 144643526
chr1 144643538
chr1 144643540

chr1 144643543
chr1 144643550
chr1 144643561
chr1 144643564
chr1 144643581
chr1 144643600
chr1 144643602
chr1 144643605
chr1 144643608
chr1 144643624
chr1 144643626
chr1 144643629
chr1 144643632
chr1 144643635
chr1 144643695
chr1 144643839
chr1 144644629
chr1 144646160
chr1 144646223
chr1 144648200
chr1 144648224
chr1 144648237
chr1 144648780
chr1 144649139
chr1 144651213
chr1 144651277
chr1 144651333
chr1 144652060
chr1 144652230
chr1 144652963
chr1 144652968
chr1 144652970
chr1 144656359
chr1 144656364
chr1 144658620
chr1 144658687
chr1 144658691
chr1 144658780
chr1 144658786
chr1 144658794
chr1 144658818
chr1 144659766
chr1 144662224
chr1 144662228
chr1 144662232
chr1 144662244
chr1 144662288
chr1 144662303
chr1 144662334
chr1 144663295
chr1 144663596
chr1 144664825
chr1 144664865
chr1 144664911

chr1 144810401
chr1 144812054
chr1 144812066
chr1 144812076
chr1 144812317
chr1 144812380
chr1 144813663
chr1 144813666
chr1 144813669
chr1 144813673
chr1 144813681
chr1 144813684
chr1 144813686
chr1 144813689
chr1 144813692
chr1 144813699
chr1 144813707
chr1 144813710
chr1 144813713
chr1 144813717
chr1 144813725
chr1 144813728
chr1 144813730
chr1 144813733
chr1 144813736
chr1 144813743
chr1 144813751
chr1 144813754
chr1 144814147
chr1 144814203
chr1 144814211
chr1 144814231
chr1 144814243
chr1 144814249
chr1 144814276
chr1 144814287
chr1 144814321
chr1 144814325
chr1 144814329
chr1 144814334
chr1 144814343
chr1 144814345
chr1 144814351
chr1 144814359
chr1 144815187
chr1 144815207
chr1 144818409
chr1 144819319
chr1 144819375
chr1 144819718
chr1 144819749
chr1 144821952
chr1 144822142
chr1 144822172

chr1 144822197
chr1 144823239
chr1 144823756
chr1 144824268
chr1 144824279
chr1 144831246
chr1 144831406
chr1 144832097
chr1 144877334
chr1 144877338
chr1 144878350
chr1 144878445
chr1 144878817
chr1 144879330
chr1 144880683
chr1 144880688
chr1 144881207
chr1 144881364
chr1 144881621
chr1 144881675
chr1 144881931
chr1 144882424
chr1 144882429
chr1 144882449
chr1 144882451
chr1 144882454
chr1 144882466
chr1 144882489
chr1 144882491
chr1 144882494
chr1 144882513
chr1 144882518
chr1 144882520
chr1 144882532
chr1 144882538
chr1 144883874
chr1 144883876
chr1 144883879
chr1 144883882
chr1 144883889
chr1 144883920
chr1 144883922
chr1 144883925
chr1 144883937
chr1 144883942
chr1 144883996
chr1 144883999
chr1 144884002
chr1 144884017
chr1 144884019
chr1 144884022
chr1 144884034
chr1 144884039
chr1 144884066

chr1 144884068
chr1 144884071
chr1 144884078
chr1 144884088
chr1 144884091
chr1 144884377
chr1 144884398
chr1 144939361
chr1 145010158
chr1 145010164
chr1 145010234
chr1 145010657
chr1 145010752
chr1 145010989
chr1 145092514
chr1 145092567
chr1 145093052
chr1 145093065
chr1 145093071
chr1 145093074
chr1 145093088
chr1 145093095
chr1 145093104
chr1 145093110
chr1 145093113
chr1 145093116
chr1 145093162
chr1 145093176
chr1 145093180
chr1 145093208
chr1 145093217
chr1 145093236
chr1 145093258
chr1 145093270
chr1 145093272
chr1 145093288
chr1 145093396
chr1 145093404
chr1 145093416
chr1 145093423
chr1 145093432
chr1 145093472
chr1 145093556
chr1 145093563
chr1 145093571
chr1 145093575
chr1 145093587
chr1 145093592
chr1 145093995
chr1 145094039
chr1 145094057
chr1 145094063
chr1 145094072
chr1 145094079

chr1 145094082
chr1 145094086
chr1 145094097
chr1 145094100
chr1 145094270
chr1 145094276
chr1 145094280
chr1 145095623
chr1 145095659
chr1 145095690
chr1 145096255
chr1 145096267
chr1 145096281
chr1 145096286
chr1 145096298
chr1 145096304
chr1 145096466
chr1 145096470
chr1 145096502
chr1 145096643
chr1 145096682
chr1 145096688
chr1 145097094
chr1 145097230
chr1 145157042
chr1 145157050
chr1 145157074
chr1 145157104
chr1 145157138
chr1 145157140
chr1 145157149
chr1 145157151
chr1 145157162
chr1 145157185
chr1 145157194
chr1 145157196
chr1 145157207
chr1 145157212
chr1 145157284
chr1 145157355
chr1 145157717
chr1 145157724
chr1 145157740
chr1 145158716
chr1 145158782
chr1 145158839
chr1 145161135
chr1 145161144
chr1 145161146
chr1 145161155
chr1 145161168
chr1 145161293
chr1 145161335
chr1 145161344

chr1 145161367
chr1 145161517
chr1 145161530
chr1 145161556
chr1 145161573
chr1 145161577
chr1 145161591
chr1 145161638
chr1 145161670
chr1 145161873
chr1 145161879
chr1 145161889
chr1 145166534
chr1 145166575
chr1 145191594
chr1 145191694
chr1 145212015
chr1 145212033
chr1 145212361
chr1 145212383
chr1 145212447
chr1 145214437
chr1 145214464
chr1 145215827
chr1 145216518
chr1 145216574
chr1 145216610
chr1 145218932
chr1 145231115
chr1 145231137
chr1 145231178
chr1 145231234
chr1 145231878
chr1 145231919
chr1 145232835
chr1 145232841
chr1 145232843
chr1 145232845
chr1 145232848
chr1 145232853
chr1 145232860
chr1 145232864
chr1 145232874
chr1 145232886
chr1 145232890
chr1 145232893
chr1 145232898
chr1 145232905
chr1 145232920
chr1 145232924
chr1 145232929
chr1 145232931
chr1 145232933
chr1 145232945

chr1 145232947
chr1 145232952
chr1 145232954
chr1 145232960
chr1 145232965
chr1 145232968
chr1 145232972
chr1 145232976
chr1 145232978
chr1 145233026
chr1 145233029
chr1 145233039
chr1 145233087
chr1 145233118
chr1 145233126
chr1 145280297
chr1 145280640
chr1 145281883
chr1 145282092
chr1 145282096
chr1 145282159
chr1 145282162
chr1 145282172
chr1 145282192
chr1 145282214
chr1 145282388
chr1 145282406
chr1 145282423
chr1 145282447
chr1 145282479
chr1 145282497
chr1 145282510
chr1 145282526
chr1 145282531
chr1 145282871
chr1 145282901
chr1 145282909
chr1 145282933
chr1 145282957
chr1 145282989
chr1 145282997
chr1 145283001
chr1 145286716
chr1 145286740
chr1 145286753
chr1 145287025
chr1 145287032
chr1 145287048
chr1 145287050
chr1 145287058
chr1 145287061
chr1 145287088
chr1 145287094
chr1 145287099

chr1 145287101
chr1 145287120
chr1 145287225
chr1 145287243
chr1 145287272
chr1 145287287
chr1 145287303
chr1 145287327
chr1 145287331
chr1 145287345
chr1 145287357
chr1 145287366
chr1 145287371
chr1 145287383
chr1 145287425
chr1 145287428
chr1 145287452
chr1 145287462
chr1 145287466
chr1 145287492
chr1 145287497
chr1 145287502
chr1 145287514
chr1 145287519
chr1 145287525
chr1 145287571
chr1 145287602
chr1 145287604
chr1 145287617
chr1 145287626
chr1 145287628
chr1 145287636
chr1 145287682
chr1 145287686
chr1 145287708
chr1 145287720
chr1 145287724
chr1 145287728
chr1 145287742
chr1 145287776
chr1 145287782
chr1 145287789
chr1 145287791
chr1 145287795
chr1 145287806
chr1 145287822
chr1 145287838
chr1 145288083
chr1 145288090
chr1 145288157
chr1 145377945
chr1 145378005
chr1 145378249
chr1 145385626

chr1 145424680
chr1 145424780
chr1 145424812
chr1 145424880
chr1 145425001
chr1 145425014
chr1 145425027
chr1 145425054
chr1 145425060
chr1 145425068
chr1 145425078
chr1 145425112
chr1 145425118
chr1 145425134
chr1 145425154
chr1 145425158
chr1 145425164
chr1 145425183
chr1 145425201
chr1 145425204
chr1 145425210
chr1 145425219
chr1 145425241
chr1 145425246
chr1 145425253
chr1 145425256
chr1 145425260
chr1 145425274
chr1 145425281
chr1 145425288
chr1 145425305
chr1 145425307
chr1 145425410
chr1 145425412
chr1 145425415
chr1 145425418
chr1 145425420
chr1 145425425
chr1 145425430
chr1 145430934
chr1 145430979
chr1 145430985
chr1 145431018
chr1 145431033
chr1 145431035
chr1 145431037
chr1 145431054
chr1 145431058
chr1 145431081
chr1 145431321
chr1 145431333
chr1 145431385
chr1 145431419
chr1 145431428

chr1 145431448
chr1 145431461
chr1 145431537
chr1 145431552
chr1 145431559
chr1 145431573
chr1 145431577
chr1 145431586
chr1 145431601
chr1 145431613
chr1 145431619
chr1 145459466
chr1 145459492
chr1 145459556
chr1 145460089
chr1 145460102
chr1 145460156
chr1 145460172
chr1 145460215
chr1 145460221
chr1 145464834
chr1 145464922
chr1 145464952
chr1 145464978
chr1 145464994
chr1 145465299
chr1 145465352
chr1 145465401
chr1 145465688
chr1 145465706
chr1 145465727
chr1 145465747
chr1 145465849
chr1 145465851
chr1 145465859
chr1 145466173
chr1 145466214
chr1 145466217
chr1 145466222
chr1 145466230
chr1 145466250
chr1 145466260
chr1 145466277
chr1 145466284
chr1 145466294
chr1 145466340
chr1 145466352
chr1 145466380
chr1 145466411
chr1 145466434
chr1 145466437
chr1 145466443
chr1 145466471
chr1 145466483

chr1 145466518
chr1 145466581
chr1 145475491
chr1 145475591
chr1 145475593
chr1 145475659
chr1 145475663
chr1 145475675
chr1 145475700
chr1 145475705
chr1 145475712
chr1 145475717
chr1 145475723
chr1 145475754
chr1 145475769
chr1 145475784
chr1 145475838
chr1 145475844
chr1 145475849
chr1 145475854
chr1 145475863
chr1 145475875
chr1 145475884
chr1 145475888
chr1 145475894
chr1 145475917
chr1 145475929
chr1 145475933
chr1 145475945
chr1 145475947
chr1 145475976
chr1 145476315
chr1 145476325
chr1 145476381
chr1 145476409
chr1 145476459
chr1 145476470
chr1 145476617
chr1 145476629
chr1 145476633
chr1 145476635
chr1 145476645
chr1 145476673
chr1 145476678
chr1 145476682
chr1 145476689
chr1 145476703
chr1 145476734
chr1 145477640
chr1 145487534
chr1 145487538
chr1 145487704
chr1 145487720
chr1 145487734

chr1 145487738
chr1 145487780
chr1 145487784
chr1 145487791
chr1 145487823
chr1 145487834
chr1 145487852
chr1 145487854
chr1 145487922
chr1 145487930
chr1 145487952
chr1 145487954
chr1 145487960
chr1 145487977
chr1 145487983
chr1 145487992
chr1 145488035
chr1 145488039
chr1 145488051
chr1 145488118
chr1 145488129
chr1 145488149
chr1 145488163
chr1 145488170
chr1 145488179
chr1 145488207
chr1 145488217
chr1 145488219
chr1 145488223
chr1 145488226
chr1 145488247
chr1 145488256
chr1 145488276
chr1 145488290
chr1 145488318
chr1 145488320
chr1 145488331
chr1 145488345
chr1 145607873
chr1 145607889
chr1 145607904
chr1 145607906
chr1 145607909
chr1 145607920
chr1 145607924
chr1 145607938
chr1 145607944
chr1 145607949
chr1 145607967
chr1 145607987
chr1 145608000
chr1 145608003
chr1 145608016
chr1 145608024

chr1 145608052
chr1 145608077
chr1 145608090
chr1 145608436
chr1 145608635
chr1 145608706
chr1 145608725
chr1 145608760
chr1 145817208
chr1 145817216
chr1 145817236
chr1 145817926
chr1 145818090
chr1 145818149
chr1 145818213
chr1 145818314
chr1 145822688
chr1 145823094
chr1 145823110
chr1 145823163
chr1 145823182
chr1 145823353
chr1 145823359
chr1 145823436
chr1 145823529
chr1 145823594
chr1 145823600
chr1 145823606
chr1 145823628
chr1 145823631
chr1 145823657
chr1 145823665
chr1 145823671
chr1 145823675
chr1 145823712
chr1 145823720
chr1 145824675
chr1 145824679
chr1 145824746
chr1 145824813
chr1 145885463
chr1 145885498
chr1 145885581
chr1 145885751
chr1 145885762
chr1 145885768
chr1 145885776
chr1 145927250
chr1 145927258
chr1 145927273
chr1 145927277
chr1 145927317
chr1 145927328
chr1 145927342

chr1 145927360
chr1 145927381
chr1 145927390
chr1 145927402
chr1 145927417
chr1 145927421
chr1 145927429
chr1 145927435
chr1 145927439
chr1 145927447
chr1 145927456
chr1 145927476
chr1 145927487
chr1 145927503
chr1 145927554
chr1 145927565
chr1 145927577
chr1 145927588
chr1 145927599
chr1 145927631
chr1 145932734
chr1 145932808
chr1 145932830
chr1 145932842
chr1 145932849
chr1 145955758
chr1 145955794
chr1 145958181
chr1 145958201
chr1 145958211
chr1 145958214
chr1 145958216
chr1 145958398
chr1 145960883
chr1 145960894
chr1 145960897
chr1 145960906
chr1 145960912
chr1 145960916
chr1 145960940
chr1 145960951
chr1 145960955
chr1 145960962
chr1 145961018
chr1 145962558
chr1 145962563
chr1 145962566
chr1 145962569
chr1 145977539
chr1 145977598
chr1 145978432
chr1 145978466
chr1 145978955
chr1 145979010

chr1 145979081
chr1 145979492
chr1 145979579
chr1 145979615
chr1 145979688
chr1 145979699
chr1 145980085
chr1 145980137
chr1 145994722
chr1 145994755
chr1 145994955
chr1 145994973
chr1 145995087
chr1 145995219
chr1 145995223
chr1 145995320
chr1 145995423
chr1 145995494
chr1 145997772
chr1 145997811
chr1 145997880
chr1 145998247
chr1 146007276
chr1 146007287
chr1 146007331
chr1 146013115
chr1 146013246
chr1 146013326
chr1 146013528
chr1 146035812
chr1 146035890
chr1 146035909
chr1 146035998
chr1 146036006
chr1 146036012
chr1 146036038
chr1 146036040
chr1 146036046
chr1 146036063
chr1 146036070
chr1 146036080
chr1 146036126
chr1 146036134
chr1 146036138
chr1 146036165
chr1 146036189
chr1 146036195
chr1 146036222
chr1 146036244
chr1 146036256
chr1 146036272
chr1 146036348
chr1 146036380
chr1 146036483

chr1 146036550
chr1 146036708
chr1 146038456
chr1 146038473
chr1 146038480
chr1 146038532
chr1 146038536
chr1 146038544
chr1 146038576
chr1 146038607
chr1 146038621
chr1 146038632
chr1 146038637
chr1 146038675
chr1 146038681
chr1 146038711
chr1 146038715
chr1 146038758
chr1 146038768
chr1 146038798
chr1 146038800
chr1 146038822
chr1 146038830
chr1 146038875
chr1 146038880
chr1 146052211
chr1 146052260
chr1 146052265
chr1 146052268
chr1 146052281
chr1 146052297
chr1 146052301
chr1 146052314
chr1 146052316
chr1 146148617
chr1 146219621
chr1 146219870
chr1 146228986
chr1 146228989
chr1 146229214
chr1 146229220
chr1 146229227
chr1 146229230
chr1 146229232
chr1 146229242
chr1 146229257
chr1 146229262
chr1 146229288
chr1 146229300
chr1 146229342
chr1 146229348
chr1 146229961
chr1 146230190
chr1 146230204

chr1 146309223
chr1 146367783
chr1 146368968
chr1 146369051
chr1 146369075
chr1 146369088
chr1 146370418
chr1 146370425
chr1 146370433
chr1 146376321
chr1 146376327
chr1 146376359
chr1 146376384
chr1 146376397
chr1 146376402
chr1 146376425
chr1 146376429
chr1 146376442
chr1 146376447
chr1 146376472
chr1 146376488
chr1 146376494
chr1 146376500
chr1 146376517
chr1 146376532
chr1 146376554
chr1 146376565
chr1 146376574
chr1 146376597
chr1 146376614
chr1 146376619
chr1 146376631
chr1 146376636
chr1 146376644
chr1 146376656
chr1 146376663
chr1 146376750
chr1 146376754
chr1 146376770
chr1 146376785
chr1 146376790
chr1 146377526
chr1 146377533
chr1 146377579
chr1 146377601
chr1 146377759
chr1 146377803
chr1 146377841
chr1 146377905
chr1 146377907
chr1 146377936
chr1 146377995
chr1 146378003
chr1 146378011

chr1 146378035
chr1 146378041
chr1 146378058
chr1 146378065
chr1 146378073
chr1 146378119
chr1 146378131
chr1 146378135
chr1 146378164
chr1 146378175
chr1 146378177
chr1 146378195
chr1 146378209
chr1 146386713
chr1 146387497
chr1 146387577
chr1 146387594
chr1 146387721
chr1 146387735
chr1 146387740
chr1 146387946
chr1 146387948
chr1 146411836
chr1 146411868
chr1 146412199
chr1 146412732
chr1 146412885
chr1 146412938
chr1 146414190
chr1 146414225
chr1 146443300
chr1 146443380
chr1 146443397
chr1 146443436
chr1 146443450
chr1 146443452
chr1 146443480
chr1 146443488
chr1 146443529
chr1 146443539
chr1 146443949
chr1 146443970
chr1 146443972
chr1 146443979
chr1 146443998
chr1 146444004
chr1 146444010
chr1 146444022
chr1 146444025
chr1 146444040
chr1 146444049
chr1 146444080
chr1 146444083
chr1 146444094

chr1 146444098
chr1 146444105
chr1 146444129
chr1 146444156
chr1 146447837
chr1 146448484
chr1 146448526
chr1 146448616
chr1 146448687
chr1 146448880
chr1 146448922
chr1 146472175
chr1 146472197
chr1 146472223
chr1 146472231
chr1 146472248
chr1 146472267
chr1 146472273
chr1 146472305
chr1 146472307
chr1 146472313
chr1 146472318
chr1 146472326
chr1 146472365
chr1 146472379
chr1 146472390
chr1 146472429
chr1 146472451
chr1 146472476
chr1 146472480
chr1 146472501
chr1 146472503
chr1 146472505
chr1 146472511
chr1 146472518
chr1 146472527
chr1 146472549
chr1 146472554
chr1 146472560
chr1 146472578
chr1 146472580
chr1 146472583
chr1 146472587
chr1 146487026
chr1 146487032
chr1 146487037
chr1 146487051
chr1 146487063
chr1 146487077
chr1 146487081
chr1 146487117
chr1 146487121
chr1 146487133
chr1 146487135

chr1 146487164
chr1 146487226
chr1 146487282
chr1 146487303
chr1 146487325
chr1 146487352
chr1 146937599
chr1 146937648
chr1 146937733
chr1 146937898
chr1 146938010
chr1 146938176
chr1 146938245
chr1 146938267
chr1 146938269
chr1 147078507
chr1 147078519
chr1 147078526
chr1 147078555
chr1 147078589
chr1 147089016
chr1 147089056
chr1 147173073
chr1 147173084
chr1 147173129
chr1 147173161
chr1 147173169
chr1 147173171
chr1 147173173
chr1 147173176
chr1 147225301
chr1 147225323
chr1 147225346
chr1 147225377
chr1 147225388
chr1 147225398
chr1 147225444
chr1 147225455
chr1 147225476
chr1 147225490
chr1 147225512
chr1 147225595
chr1 147225605
chr1 147225793
chr1 147225821
chr1 147225825
chr1 147225831
chr1 147225834
chr1 147225843
chr1 147225845
chr1 147225857
chr1 147225866
chr1 147225917
chr1 147225928

chr1 147225955
chr1 147226069
chr1 147226157
chr1 147226232
chr1 147226238
chr1 147226429
chr1 147243653
chr1 147243707
chr1 147243741
chr1 147243864
chr1 147243958
chr1 147244090
chr1 147244222
chr1 147244495
chr1 147244516
chr1 147342572
chr1 147540961
chr1 147540975
chr1 147541227
chr1 147541231
chr1 147541250
chr1 147541353
chr1 147544918
chr1 147544962
chr1 147545087
chr1 147545131
chr1 147545221
chr1 147545268
chr1 147545284
chr1 147545320
chr1 147545356
chr1 147545453
chr1 147545678
chr1 147600373
chr1 147600379
chr1 147600401
chr1 147600403
chr1 147600407
chr1 147600409
chr1 147600421
chr1 147600448
chr1 147600450
chr1 147600457
chr1 147600483
chr1 147600498
chr1 147600512
chr1 147600553
chr1 147600557
chr1 147600680
chr1 147618339
chr1 147669550
chr1 147669568
chr1 147669579
chr1 147669591

chr1 147669607
chr1 147669633
chr1 147928242
chr1 147928248
chr1 147928276
chr1 147928292
chr1 147928307
chr1 147928309
chr1 147928312
chr1 147928817
chr1 147928834
chr1 147928839
chr1 147928842
chr1 147929038
chr1 147929109
chr1 147929128
chr1 147929163
chr1 147929585
chr1 148014621
chr1 148014736
chr1 148014743
chr1 148014757
chr1 148014759
chr1 148014797
chr1 148014827
chr1 148014852
chr1 148014864
chr1 148014880
chr1 148014915
chr1 148014926
chr1 148014932
chr1 148014942
chr1 148014950
chr1 148014958
chr1 148014995
chr1 148015004
chr1 148015022
chr1 148015044
chr1 148015049
chr1 148015056
chr1 148015064
chr1 148015071
chr1 148015077
chr1 148015123
chr1 148015135
chr1 148015253
chr1 148015264
chr1 148015278
chr1 148015335
chr1 148015338
chr1 148015375
chr1 148015383
chr1 148015400
chr1 148015417

chr1 148015439
chr1 148015461
chr1 148015463
chr1 148015471
chr1 148032598
chr1 148032624
chr1 148032688
chr1 148033288
chr1 148033304
chr1 148033347
chr1 148033353
chr1 148033411
chr1 148037620
chr1 148037800
chr1 148038057
chr1 148038487
chr1 148038536
chr1 148038584
chr1 148038610
chr1 148038613
chr1 148038616
chr1 148038630
chr1 148038655
chr1 148038763
chr1 148038765
chr1 148038769
chr1 148038800
chr1 148038824
chr1 148038842
chr1 148038863
chr1 148038883
chr1 148038985
chr1 148038987
chr1 148038995
chr1 148039350
chr1 148039353
chr1 148039358
chr1 148039366
chr1 148039386
chr1 148039396
chr1 148039413
chr1 148039420
chr1 148039430
chr1 148039452
chr1 148039476
chr1 148039488
chr1 148039516
chr1 148039547
chr1 148039607
chr1 148039619
chr1 148044689
chr1 148044693
chr1 148044725
chr1 148044754

chr1 148044764
chr1 148045018
chr1 148045092
chr1 148048794
chr1 148048798
chr1 148048810
chr1 148048829
chr1 148048835
chr1 148048840
chr1 148048847
chr1 148048852
chr1 148048858
chr1 148048889
chr1 148048904
chr1 148048919
chr1 148048973
chr1 148048979
chr1 148048984
chr1 148048998
chr1 148049010
chr1 148049019
chr1 148049023
chr1 148049029
chr1 148049052
chr1 148049064
chr1 148049068
chr1 148049080
chr1 148049082
chr1 148049111
chr1 148049115
chr1 148049139
chr1 148049142
chr1 148049145
chr1 148049224
chr1 148049229
chr1 148049250
chr1 148049258
chr1 148049260
chr1 148049265
chr1 148049272
chr1 148049299
chr1 148049310
chr1 148049312
chr1 148049335
chr1 148049340
chr1 148049383
chr1 148049396
chr1 148049403
chr1 148049434
chr1 148049438
chr1 148049450
chr1 148049460
chr1 148057356
chr1 148057450

chr1 148057458
chr1 148057625
chr1 148057779
chr1 148060329
chr1 148060340
chr1 148060351
chr1 148060355
chr1 148060367
chr1 148060384
chr1 148060392
chr1 148060398
chr1 148060404
chr1 148060409
chr1 148060414
chr1 148060417
chr1 148060422
chr1 148060443
chr1 148060447
chr1 148060471
chr1 148060473
chr1 148060482
chr1 148060485
chr1 148060527
chr1 148060533
chr1 148060539
chr1 148060544
chr1 148060553
chr1 148060565
chr1 148060579
chr1 148060583
chr1 148060619
chr1 148060623
chr1 148060635
chr1 148060637
chr1 148060666
chr1 148060670
chr1 148060694
chr1 148060923
chr1 148060955
chr1 148060966
chr1 148060984
chr1 148060986
chr1 148061033
chr1 148061041
chr1 148061063
chr1 148061065
chr1 148061071
chr1 148061088
chr1 148061094
chr1 148061103
chr1 148061146
chr1 148061150
chr1 148061162
chr1 148061245

chr1 148061256
chr1 148061276
chr1 148061290
chr1 148061297
chr1 148061306
chr1 148061334
chr1 148061344
chr1 148061346
chr1 148061350
chr1 148061353
chr1 148061374
chr1 148061383
chr1 148061403
chr1 148061410
chr1 148061417
chr1 148061445
chr1 148061447
chr1 148061458
chr1 148061472
chr1 148152467
chr1 148152634
chr1 148152911
chr1 148153005
chr1 148153054
chr1 148157138
chr1 148157489
chr1 148157536
chr1 148157564
chr1 148157944
chr1 148157947
chr1 148157961
chr1 148157982
chr1 148158016
chr1 148158018
chr1 148158025
chr1 148158033
chr1 148158036
chr1 148158044
chr1 148158120
chr1 148158136
chr1 148245953
chr1 148245966
chr1 148245988
chr1 148245994
chr1 148255238
chr1 148255240
chr1 148280372
chr1 148280434
chr1 148280448
chr1 148280456
chr1 148280463
chr1 148280465
chr1 148280472
chr1 148280548

chr1 148280595
chr1 148280610
chr1 148280656
chr1 148280701
chr1 148280717
chr1 148280754
chr1 148280770
chr1 148280786
chr1 148280797
chr1 148280814
chr1 148280831
chr1 148280894
chr1 148280900
chr1 148280902
chr1 148280921
chr1 148280923
chr1 148280950
chr1 148280955
chr1 148281046
chr1 148281050
chr1 148281058
chr1 148281089
chr1 148281119
chr1 148281347
chr1 148281364
chr1 148281367
chr1 148281373
chr1 148281394
chr1 148281403
chr1 148281414
chr1 148281419
chr1 148281425
chr1 148281432
chr1 148302497
chr1 148302500
chr1 148302515
chr1 148302523
chr1 148302547
chr1 148302568
chr1 148302737
chr1 148302744
chr1 148302750
chr1 148302755
chr1 148302766
chr1 148302775
chr1 148302796
chr1 148302802
chr1 148302805
chr1 148302822
chr1 148302824
chr1 148302830
chr1 148302854
chr1 148302867
chr1 148302878

chr1 148302884
chr1 148303119
chr1 148303123
chr1 148303129
chr1 148303138
chr1 148303147
chr1 148303167
chr1 148303177
chr1 148303338
chr1 148303355
chr1 148303372
chr1 148303383
chr1 148303399
chr1 148303415
chr1 148303461
chr1 148303464
chr1 148303468
chr1 148303476
chr1 148303502
chr1 148303526
chr1 148303566
chr1 148303634
chr1 148389106
chr1 148389118
chr1 148389140
chr1 148389179
chr1 148389196
chr1 148397454
chr1 148402146
chr1 148402154
chr1 148402274
chr1 148403070
chr1 148456884
chr1 148457677
chr1 148458029
chr1 148459888
chr1 148459912
chr1 148459927
chr1 148459930
chr1 148459962
chr1 148459965
chr1 148459982
chr1 148460005
chr1 148460009
chr1 148460018
chr1 148460021
chr1 148460031
chr1 148460040
chr1 148460050
chr1 148460484
chr1 148460540
chr1 148460576
chr1 148513680
chr1 148513807

chr1 148513864
chr1 148514304
chr1 148515372
chr1 148515646
chr1 148515676
chr1 148515774
chr1 148515790
chr1 148517634
chr1 148517746
chr1 148518472
chr1 148518567
chr1 148521735
chr1 148521782
chr1 148521790
chr1 148522122
chr1 148523196
chr1 148523198
chr1 148523220
chr1 148523550
chr1 148523578
chr1 148523582
chr1 148523630
chr1 148523645
chr1 148523648
chr1 148523658
chr1 148523678
chr1 148523700
chr1 148523747
chr1 148523785
chr1 148523892
chr1 148523909
chr1 148523933
chr1 148523965
chr1 148524098
chr1 148524101
chr1 148524138
chr1 148524357
chr1 148526448
chr1 148526772
chr1 148526822
chr1 148527169
chr1 148528524
chr1 148528540
chr1 148528542
chr1 148528550
chr1 148528553
chr1 148528580
chr1 148528586
chr1 148528591
chr1 148528593
chr1 148528612
chr1 148528944
chr1 148528954
chr1 148528958

chr1 148528984
chr1 148528989
chr1 148528994
chr1 148529006
chr1 148529011
chr1 148529017
chr1 148529048
chr1 148529052
chr1 148529063
chr1 148529094
chr1 148529096
chr1 148529109
chr1 148529118
chr1 148529120
chr1 148529128
chr1 148529142
chr1 148529165
chr1 148529177
chr1 148529199
chr1 148529211
chr1 148529215
chr1 148529219
chr1 148529233
chr1 148529267
chr1 148529273
chr1 148529280
chr1 148529282
chr1 148529286
chr1 148529297
chr1 148529313
chr1 148529329
chr1 148529337
chr1 148529456
chr1 148529460
chr1 148529472
chr1 148529501
chr1 148529574
chr1 148529581
chr1 148529589
chr1 148529648
chr1 148679452
chr1 148679466
chr1 148679468
chr1 148679475
chr1 148679477
chr1 148679493
chr1 148679516
chr1 148679521
chr1 148679528
chr1 148679540
chr1 148679544
chr1 148679553
chr1 148679556
chr1 148679559

chr1 148679562
chr1 148679652
chr1 148679657
chr1 148679662
chr1 148679673
chr1 148679684
chr1 148679692
chr1 148679694
chr1 148679698
chr1 148679719
chr1 148679722
chr1 148679731
chr1 148679735
chr1 148679757
chr1 148679768
chr1 148679779
chr1 148679791
chr1 148679800
chr1 148679813
chr1 148840384
chr1 148840386
chr1 148840392
chr1 148840397
chr1 148840409
chr1 148840424
chr1 148840448
chr1 148840468
chr1 148840472
chr1 148840480
chr1 148840484
chr1 148840513
chr1 148840524
chr1 148840544
chr1 148840558
chr1 148840565
chr1 148840569
chr1 148840574
chr1 148841571
chr1 148843496
chr1 148843600
chr1 148844238
chr1 148844264
chr1 148844286
chr1 148844298
chr1 148844306
chr1 148844308
chr1 148844326
chr1 148844340
chr1 148844366
chr1 148844375
chr1 148844381
chr1 148844385
chr1 148844387
chr1 148844409

chr1 148844418
chr1 148844431
chr1 148844452
chr1 148844454
chr1 148844459
chr1 148844461
chr1 148844501
chr1 148844505
chr1 148844508
chr1 148844510
chr1 148844513
chr1 148844557
chr1 148844562
chr1 148844566
chr1 148844574
chr1 148844582
chr1 148951727
chr1 148951787
chr1 148951920
chr1 148951947
chr1 148951952
chr1 148951958
chr1 148951979
chr1 148951986
chr1 148951992
chr1 148951995
chr1 148951997
chr1 148952002
chr1 148952012
chr1 148952015
chr1 148952023
chr1 148952033
chr1 148952040
chr1 148952052
chr1 148952073
chr1 148952088
chr1 148952090
chr1 148952145
chr1 148952177
chr1 148952192
chr1 148952194
chr1 148952215
chr1 148952221
chr1 148952224
chr1 148952508
chr1 148952513
chr1 148952515
chr1 148952522
chr1 148952550
chr1 148952602
chr1 148952608
chr1 148952734
chr1 148952737
chr1 148952740

chr1 148952743
chr1 148952746
chr1 148952751
chr1 148952767
chr1 148952958
chr1 148952967
chr1 148952972
chr1 148952988
chr1 148952998
chr1 148953012
chr1 148953042
chr1 148953072
chr1 148953083
chr1 148953095
chr1 148953097
chr1 148953109
chr1 148953148
chr1 148953342
chr1 149102130
chr1 149102171
chr1 149102183
chr1 149102458
chr1 149102483
chr1 149102729
chr1 149102740
chr1 149102761
chr1 149102786
chr1 149102796
chr1 149102829
chr1 149103103
chr1 149103107
chr1 149103113
chr1 149103120
chr1 149103128
chr1 149103132
chr1 149103150
chr1 149103153
chr1 149103159
chr1 149103168
chr1 149103189
chr1 149103194
chr1 149103201
chr1 149103204
chr1 149103208
chr1 149103222
chr1 149103229
chr1 149103236
chr1 149124053
chr1 149124078
chr1 149124098
chr1 149124139
chr1 149124166
chr1 149124168
chr1 149124217

chr1 149124272
chr1 149124668
chr1 149125025
chr1 149129049
chr1 149129056
chr1 149129060
chr1 149129071
chr1 149129105
chr1 149129114
chr1 149174792
chr1 149174810
chr1 149174825
chr1 149174903
chr1 149175063
chr1 149175078
chr1 149175274
chr1 149175310
chr1 149175440
chr1 149175451
chr1 149175453
chr1 149175513
chr1 149175516
chr1 149175518
chr1 149175869
chr1 149175886
chr1 149175889
chr1 149175921
chr1 149175924
chr1 149175939
chr1 149175963
chr1 149177699
chr1 149177708
chr1 149178970
chr1 149200653
chr1 149201053
chr1 149201114
chr1 149201873
chr1 149201950
chr1 149201962
chr1 149202004
chr1 149283808
chr1 149283816
chr1 149283855
chr1 149283879
chr1 149283889
chr1 149283910
chr1 149283937
chr1 149283943
chr1 149283947
chr1 149284004
chr1 149284012
chr1 149284027
chr1 149285860
chr1 149285862

chr1 149285866
chr1 149285872
chr1 149285881
chr1 149285907
chr1 149285921
chr1 149285927
chr1 149285938
chr1 149285940
chr1 149285948
chr1 149285960
chr1 149285982
chr1 149286646
chr1 149286748
chr1 149288673
chr1 149289670
chr1 149289675
chr1 149289679
chr1 149289686
chr1 149389432
chr1 149390271
chr1 149390291
chr1 149390297
chr1 149390917
chr1 149390931
chr1 149390943
chr1 149390950
chr1 149390952
chr1 149390955
chr1 149390959
chr1 149390977
chr1 149390993
chr1 149391023
chr1 149391027
chr1 149391030
chr1 149391033
chr1 149391035
chr1 149391040
chr1 149391050
chr1 149391061
chr1 149391076
chr1 149391079
chr1 149391092
chr1 149391102
chr1 149413560
chr1 149413646
chr1 149414459
chr1 149414557
chr1 149539767
chr1 149547909
chr1 149552646
chr1 149558288
chr1 149558292
chr1 149558349
chr1 149558411

chr1 149558419
chr1 149558435
chr1 149558452
chr1 149558463
chr1 149558467
chr1 149558469
chr1 149558482
chr1 149558516
chr1 149558534
chr1 149558538
chr1 149558641
chr1 149558654
chr1 149558656
chr1 149558687
chr1 149558698
chr1 149558702
chr1 149558714
chr1 149558733
chr1 149558739
chr1 149558744
chr1 149558754
chr1 149558756
chr1 149558766
chr1 149558792
chr1 149558796
chr1 149558806
chr1 149558821
chr1 149558830
chr1 149558833
chr1 149559542
chr1 149559625
chr1 149559656
chr1 149559684
chr1 149560576
chr1 149560759
chr1 149560939
chr1 149560989
chr1 149606091
chr1 149606109
chr1 149606124
chr1 149606202
chr1 149606362
chr1 149606466
chr1 149606472
chr1 149606475
chr1 149606499
chr1 149606573
chr1 149606609
chr1 149606665
chr1 149606674
chr1 149607651
chr1 149607655
chr1 149608505
chr1 149610116

chr1 149610271
chr1 149610462
chr1 149610810
chr1 149610832
chr1 149610883
chr1 149612510
chr1 149612587
chr1 149613333
chr1 149613384
chr1 149613409
chr1 149613741
chr1 149614186
chr1 149614208
chr1 149635205
chr1 149635308
chr1 149635403
chr1 149635446
chr1 149635453
chr1 149635470
chr1 149635652
chr1 149635683
chr1 149635886
chr1 149636087
chr1 149636151
chr1 149636182
chr1 149636265
chr1 149636310
chr1 149636326
chr1 149637239
chr1 149637546
chr1 149637548
chr1 149646424
chr1 149646467
chr1 149646483
chr1 149646493
chr1 149646498
chr1 149646500
chr1 149646507
chr1 149646561
chr1 149646581
chr1 149646603
chr1 149646615
chr1 149646653
chr1 149646661
chr1 149646663
chr1 149646672
chr1 149646685
chr1 149646687
chr1 149646732
chr1 149646736
chr1 149646739
chr1 149646749
chr1 149646754
chr1 149646780

chr1 149646784
chr1 149646795
chr1 149646809
chr1 149646880
chr1 149646889
chr1 149646901
chr1 149646919
chr1 149699433
chr1 149699467
chr1 149699473
chr1 149699475
chr1 149699497
chr1 149699999
chr1 149700013
chr1 149700053
chr1 149700092
chr1 149700098
chr1 149700104
chr1 149700304
chr1 149700329
chr1 149700335
chr1 149700344
chr1 149700348
chr1 149700382
chr1 149700420
chr1 149700436
chr1 149700442
chr1 149700449
chr1 149700469
chr1 149700474
chr1 149700482
chr1 149700484
chr1 149700498
chr1 149700500
chr1 149700510
chr1 149700513
chr1 149706911
chr1 149707613
chr1 149707624
chr1 149707630
chr1 149707649
chr1 149707693
chr1 149707707
chr1 149707729
chr1 149707731
chr1 149707754
chr1 149707761
chr1 149707771
chr1 149707817
chr1 149707829
chr1 149707857
chr1 149707906
chr1 149707915
chr1 149707920

chr1 149708449
chr1 149708470
chr1 149708493
chr1 149708502
chr1 149708544
chr1 149708669
chr1 149708682
chr1 149708691
chr1 149708693
chr1 149708702
chr1 149708796
chr1 149708812
chr1 149708908
chr1 149712491
chr1 149712558
chr1 149712562
chr1 149712568
chr1 149712573
chr1 149712584
chr1 149712586
chr1 149712595
chr1 149712599
chr1 149712618
chr1 149739921
chr1 149739957
chr1 149740223
chr1 149740245
chr1 149740248
chr1 149740264
chr1 149740310
chr1 149740447
chr1 149740450
chr1 149740497
chr1 149740502
chr1 149740507
chr1 149740514
chr1 149740535
chr1 149740576
chr1 149740602
chr1 149740751
chr1 149740763
chr1 149747986
chr1 149811460
chr1 149811517
chr1 149811522
chr1 149811556
chr1 149811587
chr1 149811613
chr1 149811636
chr1 149811673
chr1 149811687
chr1 149811694
chr1 149813197
chr1 149813221

chr1 149813251
chr1 149813278
chr1 149813285
chr1 149813287
chr1 149813293
chr1 149813296
chr1 149813330
chr1 149813332
chr1 149813348
chr1 149813358
chr1 149813366
chr1 149813390
chr1 149813406
chr1 149813414
chr1 149813418
chr1 149813439
chr1 149813454
chr1 149813456
chr1 149813462
chr1 149813464
chr1 149813473
chr1 149813491
chr1 149813579
chr1 149813583
chr1 149813602
chr1 149813631
chr1 149813642
chr1 149813674
chr1 149813882
chr1 149813888
chr1 149814128
chr1 149814285
chr1 149814748
chr1 149814753
chr1 149839372
chr1 149840076
chr1 149840081
chr1 149840107
chr1 149840118
chr1 149840126
chr1 149840471
chr1 149840476
chr1 149840486
chr1 149840488
chr1 149840493
chr1 149840496
chr1 149840508
chr1 149840512
chr1 149840519
chr1 149840528
chr1 149840545
chr1 149840551
chr1 149840566
chr1 149840572

chr1 149840587
chr1 149840593
chr1 149840613
chr1 149840628
chr1 149840639
chr1 149840649
chr1 149840660
chr1 149840717
chr1 149840729
chr1 149840967
chr1 149840981
chr1 149840997
chr1 149841000
chr1 149841009
chr1 149841019
chr1 149841025
chr1 149841030
chr1 149841036
chr1 149841121
chr1 149841125
chr1 149841132
chr1 149841150
chr1 149841160
chr1 149841184
chr1 149841189
chr1 149841221
chr1 149841227
chr1 149841252
chr1 149841260
chr1 149841281
chr1 149841283
chr1 149841306
chr1 149841358
chr1 149841367
chr1 149841401
chr1 149841476
chr1 149841483
chr1 149841505
chr1 149841529
chr1 149842071
chr1 149842073
chr1 149842090
chr1 149842095
chr1 149842100
chr1 149842105
chr1 149842110
chr1 149842115
chr1 149842120
chr1 149842125
chr1 149842130
chr1 149842433
chr1 149842454
chr1 149842466
chr1 149842468

chr1 149842484
chr1 149842489
chr1 149842491
chr1 149842498
chr1 149842515
chr1 149842518
chr1 149842530
chr1 149842537
chr1 149843260
chr1 149843262
chr1 149843271
chr1 149843276
chr1 149843283
chr1 149843297
chr1 149843310
chr1 149843315
chr1 149843327
chr1 149843331
chr1 149843335
chr1 149843427
chr1 149843437
chr1 149843441
chr1 149843445
chr1 149843466
chr1 149843468
chr1 149843473
chr1 149843476
chr1 149843520
chr1 149843651
chr1 149843711
chr1 149843721
chr1 149843727
chr1 149843787
chr1 149843830
chr1 149843861
chr1 149843983
chr1 149844078
chr1 149844145
chr1 149844217
chr1 149844297
chr1 149844635
chr1 149844638
chr1 149849233
chr1 149849416
chr1 149849445
chr1 149849858
chr1 149849954
chr1 149850017
chr1 149850056
chr1 149850062
chr1 149850235
chr1 149850335
chr1 149850338
chr1 149850343

chr1 149850345
chr1 149850366
chr1 149850370
chr1 149850374
chr1 149850384
chr1 149850747
chr1 149850750
chr1 149850752
chr1 149850789
chr1 149850795
chr1 149850802
chr1 149850808
chr1 149850819
chr1 149850828
chr1 149850916
chr1 149850925
chr1 149850974
chr1 149851431
chr1 149851434
chr1 149851455
chr1 149851475
chr1 149851513
chr1 149851517
chr1 149851537
chr1 149851544
chr1 149851780
chr1 149851833
chr1 149851842
chr1 149851845
chr1 149851958
chr1 149851986
chr1 149851991
chr1 149852000
chr1 149852012
chr1 149852031
chr1 149852410
chr1 149852418
chr1 149852444
chr1 149852453
chr1 149852484
chr1 149852498
chr1 149852505
chr1 149852781
chr1 149852786
chr1 149852792
chr1 149852802
chr1 149852811
chr1 149852814
chr1 149852830
chr1 149852844
chr1 149854081
chr1 149854125
chr1 149854162
chr1 149869468

chr1 149869496
chr1 149869499
chr1 149869531
chr1 149869829
chr1 149870229
chr1 149870256
chr1 149870285
chr1 149884657
chr1 149884834
chr1 149884970
chr1 149885073
chr1 149885204
chr1 149885210
chr1 149885263
chr1 149885305
chr1 149885323
chr1 149885366
chr1 149885426
chr1 149885428
chr1 149888140
chr1 149888283
chr1 149888332
chr1 149888348
chr1 149888372
chr1 149888406
chr1 149888420
chr1 149888476
chr1 149888493
chr1 149888497
chr1 149888502
chr1 149888505
chr1 149888513
chr1 149888528
chr1 149888541
chr1 149888548
chr1 149888557
chr1 149888602
chr1 149917530
chr1 149917628
chr1 149917654
chr1 149917703
chr1 149917718
chr1 149917754
chr1 149917768
chr1 149917772
chr1 149917778
chr1 149917787
chr1 149917791
chr1 149917793
chr1 149917797
chr1 149917803
chr1 149917809
chr1 149917812
chr1 149917822

chr1 149917836
chr1 149917884
chr1 149917899
chr1 149917901
chr1 149917914
chr1 149917970
chr1 149917992
chr1 149927947
chr1 149927949
chr1 149927962
chr1 149927980
chr1 149927989
chr1 149927994
chr1 149928000
chr1 149928065
chr1 149962599
chr1 149962784
chr1 149962900
chr1 150009705
chr1 150009722
chr1 150009738
chr1 150009741
chr1 150009770
chr1 150009774
chr1 150009785
chr1 150009819
chr1 150009828
chr1 150009862
chr1 150009915
chr1 150009925
chr1 150009979
chr1 150010143
chr1 150010169
chr1 150010205
chr1 150010219
chr1 150010221
chr1 150010232
chr1 150010239
chr1 150010265
chr1 150010270
chr1 150010291
chr1 150010305
chr1 150010321
chr1 150010360
chr1 150010381
chr1 150010395
chr1 150010404
chr1 150010414
chr1 150010421
chr1 150010432
chr1 150010442
chr1 150010451
chr1 150010453
chr1 150010461

chr1 150010479
chr1 150010484
chr1 150010489
chr1 150010491
chr1 150010498
chr1 150010502
chr1 150010519
chr1 150010531
chr1 150010538
chr1 150010542
chr1 150010554
chr1 150010557
chr1 150010560
chr1 150010575
chr1 150010590
chr1 150010594
chr1 150010608
chr1 150010612
chr1 150010645
chr1 150010708
chr1 150011010
chr1 150011125
chr1 150011308
chr1 150066757
chr1 150066784
chr1 150066900
chr1 150066918
chr1 150066924
chr1 150068007
chr1 150068048
chr1 150068061
chr1 150068103
chr1 150068157
chr1 150068200
chr1 150076261
chr1 150076341
chr1 150076755
chr1 150151534
chr1 150151541
chr1 150151563
chr1 150151863
chr1 150233695
chr1 150233792
chr1 150233861
chr1 150233897
chr1 150234030
chr1 150234055
chr1 150234098
chr1 150234114
chr1 150234137
chr1 150234199
chr1 150234208
chr1 150321387
chr1 150321395

chr1 150321398
chr1 150321404
chr1 150321432
chr1 150321438
chr1 150321440
chr1 150321448
chr1 150321453
chr1 150321469
chr1 150321484
chr1 150321487
chr1 150321492
chr1 150321495
chr1 150321498
chr1 150321504
chr1 150321510
chr1 150321513
chr1 150321518
chr1 150321521
chr1 150321528
chr1 150321543
chr1 150321549
chr1 150321557
chr1 150321561
chr1 150321568
chr1 150321607
chr1 150321633
chr1 150321635
chr1 150321647
chr1 150321672
chr1 150321677
chr1 150321690
chr1 150321764
chr1 150321770
chr1 150321782
chr1 150321854
chr1 150321879
chr1 150321931
chr1 150322007
chr1 150322018
chr1 150322035
chr1 150322042
chr1 150322085
chr1 150322124
chr1 150322227
chr1 150322277
chr1 150322493
chr1 150322533
chr1 150322584
chr1 150363728
chr1 150363730
chr1 150363732
chr1 150363743
chr1 150363757
chr1 150366450

chr1 150366491
chr1 150366585
chr1 150366623
chr1 150366639
chr1 150366724
chr1 150366905
chr1 150366930
chr1 150464753
chr1 150487702
chr1 150487759
chr1 150487763
chr1 150487824
chr1 150561196
chr1 150561206
chr1 150561214
chr1 150561228
chr1 150561247
chr1 150561368
chr1 150561397
chr1 150561402
chr1 150561420
chr1 150561437
chr1 150561545
chr1 150561669
chr1 150561673
chr1 150561675
chr1 150561699
chr1 150561703
chr1 150561711
chr1 150561720
chr1 150563459
chr1 150563556
chr1 150563788
chr1 150563958
chr1 150567187
chr1 150567230
chr1 150567232
chr1 150567309
chr1 150567399
chr1 150567576
chr1 150567649
chr1 150567820
chr1 150567881
chr1 150567887
chr1 150567921
chr1 150567993
chr1 150568017
chr1 150568025
chr1 150568298
chr1 150568366
chr1 150568523
chr1 150569155
chr1 150569172
chr1 150569197

chr1 150569252
chr1 150574524
chr1 150574527
chr1 150574768
chr1 150574806
chr1 150574854
chr1 150574861
chr1 150575108
chr1 150575184
chr1 150575201
chr1 150575348
chr1 150575681
chr1 150576511
chr1 150576586
chr1 150576633
chr1 150576645
chr1 150576757
chr1 150611620
chr1 150611686
chr1 150611707
chr1 150622042
chr1 150684093
chr1 150684396
chr1 150695946
chr1 150696531
chr1 150696711
chr1 150696780
chr1 150696782
chr1 150696810
chr1 150696961
chr1 150696982
chr1 150697105
chr1 150697133
chr1 150697449
chr1 150697476
chr1 150697501
chr1 150697649
chr1 150697694
chr1 150697720
chr1 150697783
chr1 150697946
chr1 150765286
chr1 150765393
chr1 150765579
chr1 150765584
chr1 150765697
chr1 150875291
chr1 150875335
chr1 150875441
chr1 150875491
chr1 150875498
chr1 150875828
chr1 150875831
chr1 150875881

chr1 150875972
chr1 150875978
chr1 150875984
chr1 150875990
chr1 150875999
chr1 150876026
chr1 150876029
chr1 150876100
chr1 150876401
chr1 150876412
chr1 150876419
chr1 150876421
chr1 150876428
chr1 150876433
chr1 150876438
chr1 150876441
chr1 150922446
chr1 150922570
chr1 151008726
chr1 151008732
chr1 151008755
chr1 151009023
chr1 151009025
chr1 151009050
chr1 151055757
chr1 151055987
chr1 151056265
chr1 151056453
chr1 151056745
chr1 151056766
chr1 151056851
chr1 151057536
chr1 151057622
chr1 151057879
chr1 151057893
chr1 151057927
chr1 151058221
chr1 151058259
chr1 151060008
chr1 151156586
chr1 151156612
chr1 151156684
chr1 151156732
chr1 151156822
chr1 151156908
chr1 151156962
chr1 151157102
chr1 151157118
chr1 151157197
chr1 151157350
chr1 151157368
chr1 151157438
chr1 151166292
chr1 151166315

chr1 151166345
chr1 151198759
chr1 151198782
chr1 151198787
chr1 151198810
chr1 151198868
chr1 151198877
chr1 151198897
chr1 151198899
chr1 151198951
chr1 151198972
chr1 151199004
chr1 151199014
chr1 151199019
chr1 151199022
chr1 151199028
chr1 151199469
chr1 151199482
chr1 151199554
chr1 151199562
chr1 151199592
chr1 151199636
chr1 151199655
chr1 151199930
chr1 151199950
chr1 151254650
chr1 151254655
chr1 151254664
chr1 151254670
chr1 151254712
chr1 151254715
chr1 151254744
chr1 151254749
chr1 151254757
chr1 151254761
chr1 151254823
chr1 151254850
chr1 151254854
chr1 151254856
chr1 151254863
chr1 151254908
chr1 151254915
chr1 151254927
chr1 151254964
chr1 151254980
chr1 151255159
chr1 151255207
chr1 151281685
chr1 151281697
chr1 151281714
chr1 151281721
chr1 151281738
chr1 151281761
chr1 151281775

chr1 151281794
chr1 151281826
chr1 151281836
chr1 151281884
chr1 151281886
chr1 151326689
chr1 151326927
chr1 151326930
chr1 151326962
chr1 151327006
chr1 151327011
chr1 151327049
chr1 151327090
chr1 151327135
chr1 151327166
chr1 151327172
chr1 151327196
chr1 151340571
chr1 151340594
chr1 151340607
chr1 151340714
chr1 151340961
chr1 151340987
chr1 151341008
chr1 151341028
chr1 151346851
chr1 151346856
chr1 151346866
chr1 151346884
chr1 151346887
chr1 151346929
chr1 151346931
chr1 151346969
chr1 151346988
chr1 151346999
chr1 151347007
chr1 151347018
chr1 151347025
chr1 151347036
chr1 151347062
chr1 151347068
chr1 151347558
chr1 151347573
chr1 151347577
chr1 151347581
chr1 151399857
chr1 151399859
chr1 151399911
chr1 151399923
chr1 151399925
chr1 151399936
chr1 151473199
chr1 151473315
chr1 151473338

chr1 151473396
chr1 151473411
chr1 151473429
chr1 151473436
chr1 151473440
chr1 151473457
chr1 151473477
chr1 151473479
chr1 151473485
chr1 151473487
chr1 151473495
chr1 151473497
chr1 151473500
chr1 151473502
chr1 151473517
chr1 151473519
chr1 151473524
chr1 151473544
chr1 151473583
chr1 151473590
chr1 151473604
chr1 151473608
chr1 151473615
chr1 151473625
chr1 151473628
chr1 151473636
chr1 151473650
chr1 151473665
chr1 151534676
chr1 151594030
chr1 151611449
chr1 151611602
chr1 151611622
chr1 151611629
chr1 151611723
chr1 151611781
chr1 151611795
chr1 151611822
chr1 151612796
chr1 151612848
chr1 151612886
chr1 151612889
chr1 151612894
chr1 151612916
chr1 151612947
chr1 151654833
chr1 151654881
chr1 151762924
chr1 151762950
chr1 151762970
chr1 151791455
chr1 151791465
chr1 151791488
chr1 151791505

chr1 151791507
chr1 151791521
chr1 151791530
chr1 151791546
chr1 151791551
chr1 151791582
chr1 151791586
chr1 151791611
chr1 151791671
chr1 151839878
chr1 151839888
chr1 151839992
chr1 151839999
chr1 151840019
chr1 151840027
chr1 151840037
chr1 151840040
chr1 151840042
chr1 151840044
chr1 151840048
chr1 151840073
chr1 151840152
chr1 151840186
chr1 151840195
chr1 151840234
chr1 151840236
chr1 151840248
chr1 151840370
chr1 151840406
chr1 151905054
chr1 151905132
chr1 151905221
chr1 151905454
chr1 151909115
chr1 151909123
chr1 151909151
chr1 151992896
chr1 151992909
chr1 151992920
chr1 151992958
chr1 151992979
chr1 152034671
chr1 152034739
chr1 152034764
chr1 152034860
chr1 152034914
chr1 152034925
chr1 152035043
chr1 152035656
chr1 152035689
chr1 152035714
chr1 152035717
chr1 152035766
chr1 152035795

chr1 152035833
chr1 152036141
chr1 152036151
chr1 152036153
chr1 152036273
chr1 152036340
chr1 152036363
chr1 152036365
chr1 152036368
chr1 152036373
chr1 152036377
chr1 152036380
chr1 152036383
chr1 152036402
chr1 152036416
chr1 152036423
chr1 152036462
chr1 152036517
chr1 152036556
chr1 152036570
chr1 152036578
chr1 152036580
chr1 152036584
chr1 152036608
chr1 152036611
chr1 152036624
chr1 152036626
chr1 152036628
chr1 152036635
chr1 152036672
chr1 152036698
chr1 152036713
chr1 152036777
chr1 152036808
chr1 152047961
chr1 152048025
chr1 152048062
chr1 152048073
chr1 152048103
chr1 152048185
chr1 152048216
chr1 152048253
chr1 152048318
chr1 152048323
chr1 152048401
chr1 152048456
chr1 152048578
chr1 152048652
chr1 152048699
chr1 152048827
chr1 152143430
chr1 152189115
chr1 152189122
chr1 152189142

chr1 152189156
chr1 152189167
chr1 152189175
chr1 152189185
chr1 152189189
chr1 152189198
chr1 152189207
chr1 152189231
chr1 152189256
chr1 152189259
chr1 152189261
chr1 152189289
chr1 152189293
chr1 152189310
chr1 152189317
chr1 152189319
chr1 152189326
chr1 152189329
chr1 152189335
chr1 152189338
chr1 152189473
chr1 152189502
chr1 152189529
chr1 152189532
chr1 152189640
chr1 152190156
chr1 152190230
chr1 152301851
chr1 152382158
chr1 152382576
chr1 152383044
chr1 152458244
chr1 152461759
chr1 152461767
chr1 152461780
chr1 152461864
chr1 152463927
chr1 152464267
chr1 152464421
chr1 152464444
chr1 152464623
chr1 152464637
chr1 152464745
chr1 152589861
chr1 152589947
chr1 152590143
chr1 152590186
chr1 152590414
chr1 152869350
chr1 152869470
chr1 152869584
chr1 152869600
chr1 152869823
chr1 152869866

chr1 152870019
chr1 152870021
chr1 152870041
chr1 153022176
chr1 153345751
chr1 153346042
chr1 153346065
chr1 153346474
chr1 153346548
chr1 153346564
chr1 153346596
chr1 153346633
chr1 153440405
chr1 153448053
chr1 153511054
chr1 153511068
chr1 153511071
chr1 153511111
chr1 153511136
chr1 153511149
chr1 153511184
chr1 153535739
chr1 153535766
chr1 153535825
chr1 153535833
chr1 153536001
chr1 153536120
chr1 153536122
chr1 153536135
chr1 153536145
chr1 153536156
chr1 153536167
chr1 153536177
chr1 153545317
chr1 153545332
chr1 153545413
chr1 153545419
chr1 153545475
chr1 153545491
chr1 153545571
chr1 153545578
chr1 153545609
chr1 153545772
chr1 153545812
chr1 153545878
chr1 153545908
chr1 153545942
chr1 153566488
chr1 153634152
chr1 153634157
chr1 153634161
chr1 153634186
chr1 153634197
chr1 153634207

chr1 153634213
chr1 153634227
chr1 153634244
chr1 153634257
chr1 153634271
chr1 153634275
chr1 153634282
chr1 153634287
chr1 153634294
chr1 153634300
chr1 153634314
chr1 153634329
chr1 153634342
chr1 153634352
chr1 153634369
chr1 153634380
chr1 153634393
chr1 153634403
chr1 153634406
chr1 153634410
chr1 153634412
chr1 153634445
chr1 153634456
chr1 153634467
chr1 153634478
chr1 153634489
chr1 153634497
chr1 153634527
chr1 153634574
chr1 153634667
chr1 153634702
chr1 153634751
chr1 153634875
chr1 153634883
chr1 153634916
chr1 153634939
chr1 153634946
chr1 153634956
chr1 153634977
chr1 153670482
chr1 153670497
chr1 153670542
chr1 153670546
chr1 153670568
chr1 153671492
chr1 153671504
chr1 153671782
chr1 153671807
chr1 153671828
chr1 153671841
chr1 153671903
chr1 153671908
chr1 153671914
chr1 153671948

chr1 153671953
chr1 153671966
chr1 153671980
chr1 153672011
chr1 153707783
chr1 153708044
chr1 153708162
chr1 153727687
chr1 153727741
chr1 153727754
chr1 153727756
chr1 153727762
chr1 153727770
chr1 153727774
chr1 153727777
chr1 153727780
chr1 153727802
chr1 153727807
chr1 153727809
chr1 153727825
chr1 153727844
chr1 153727854
chr1 153727906
chr1 153727923
chr1 153727926
chr1 153727955
chr1 153727961
chr1 153727963
chr1 153727979
chr1 153727993
chr1 153727995
chr1 153728009
chr1 153728012
chr1 153728014
chr1 153728024
chr1 153728042
chr1 153728049
chr1 153728064
chr1 153728081
chr1 153728104
chr1 153728117
chr1 153728130
chr1 153728162
chr1 153728174
chr1 153728258
chr1 153728309
chr1 153728454
chr1 153728463
chr1 153728469
chr1 153728471
chr1 153849691
chr1 153849874
chr1 153850177
chr1 153921607

chr1 153921619
chr1 153921849
chr1 153921863
chr1 153921867
chr1 153921869
chr1 153921871
chr1 153921896
chr1 153921909
chr1 153921958
chr1 153921987
chr1 153922091
chr1 153922099
chr1 153922103
chr1 153922107
chr1 153922114
chr1 153922117
chr1 153922208
chr1 153922217
chr1 153922221
chr1 153922246
chr1 153922253
chr1 153922308
chr1 153922310
chr1 153922338
chr1 153923560
chr1 153923568
chr1 153923659
chr1 153923725
chr1 153923729
chr1 153945033
chr1 153957451
chr1 153957681
chr1 153957685
chr1 153957700
chr1 153957773
chr1 153957817
chr1 153957822
chr1 153963243
chr1 153963339
chr1 153963354
chr1 153963368
chr1 153963375
chr1 153963381
chr1 153963388
chr1 153963391
chr1 153963393
chr1 153976940
chr1 153976943
chr1 153976948
chr1 153976953
chr1 153978329
chr1 153978367
chr1 153978391
chr1 153991012

chr1 153991015
chr1 153991022
chr1 153991033
chr1 153991059
chr1 153991074
chr1 153991117
chr1 153991175
chr1 153991233
chr1 153991299
chr1 153991335
chr1 153991427
chr1 153991462
chr1 153991523
chr1 153991584
chr1 153991605
chr1 154115343
chr1 154180633
chr1 154180778
chr1 154180781
chr1 154180879
chr1 154181524
chr1 154181615
chr1 154181647
chr1 154181717
chr1 154181740
chr1 154181748
chr1 154181789
chr1 154181845
chr1 154181852
chr1 154181869
chr1 154181899
chr1 154182108
chr1 154182125
chr1 154182149
chr1 154219366
chr1 154219590
chr1 154219686
chr1 154219729
chr1 154219876
chr1 154219894
chr1 154220033
chr1 154220079
chr1 154220121
chr1 154221638
chr1 154221670
chr1 154221680
chr1 154221691
chr1 154221868
chr1 154221879
chr1 154273003
chr1 154273007
chr1 154273017
chr1 154273128
chr1 154325193

chr1 154325239
chr1 154325259
chr1 154325323
chr1 154325360
chr1 154325368
chr1 154325372
chr1 154325398
chr1 154325401
chr1 154325408
chr1 154325436
chr1 154334987
chr1 154353249
chr1 154353294
chr1 154353312
chr1 154353342
chr1 154353352
chr1 154353355
chr1 154353406
chr1 154353421
chr1 154353503
chr1 154403231
chr1 154403235
chr1 154403297
chr1 154403477
chr1 154403630
chr1 154403670
chr1 154403758
chr1 154403762
chr1 154403836
chr1 154463472
chr1 154463709
chr1 154463907
chr1 154463952
chr1 154463959
chr1 154464054
chr1 154464094
chr1 154478978
chr1 154479215
chr1 154479717
chr1 154479742
chr1 154479807
chr1 154479870
chr1 154557751
chr1 154557808
chr1 154557861
chr1 154557902
chr1 154557984
chr1 154557992
chr1 154558005
chr1 154558015
chr1 154606908
chr1 154607032
chr1 154607039
chr1 154607118

chr1 154607141
chr1 154607237
chr1 154607251
chr1 154607259
chr1 154607268
chr1 154607485
chr1 154607499
chr1 154607503
chr1 154607525
chr1 154607568
chr1 154607611
chr1 154607617
chr1 154609113
chr1 154609132
chr1 154609182
chr1 154609318
chr1 154627796
chr1 154627805
chr1 154627807
chr1 154627809
chr1 154628100
chr1 154628206
chr1 154628217
chr1 154628236
chr1 154628312
chr1 154628330
chr1 154628639
chr1 154791183
chr1 154791191
chr1 154791272
chr1 154791366
chr1 154791403
chr1 154791405
chr1 154791433
chr1 154791444
chr1 154791482
chr1 154791537
chr1 154791637
chr1 154791647
chr1 154791657
chr1 154791870
chr1 154791978
chr1 154792032
chr1 154792168
chr1 154942715
chr1 154955007
chr1 154955112
chr1 154955215
chr1 154955233
chr1 154955655
chr1 154955999
chr1 154956034
chr1 154956087
chr1 154956132

chr1 154956191
chr1 154956252
chr1 154956257
chr1 154956289
chr1 154956293
chr1 154956309
chr1 154956391
chr1 154956397
chr1 154956412
chr1 154960996
chr1 154961036
chr1 154961050
chr1 154961119
chr1 154961210
chr1 154961243
chr1 154961400
chr1 154973947
chr1 154973960
chr1 154973968
chr1 154973987
chr1 154973999
chr1 154974016
chr1 154974021
chr1 154975974
chr1 154976018
chr1 154976026
chr1 154976103
chr1 154976272
chr1 154983490
chr1 154983503
chr1 154983515
chr1 154983616
chr1 154983716
chr1 154983746
chr1 154983787
chr1 154983798
chr1 154983800
chr1 154983804
chr1 154983849
chr1 154983912
chr1 154983920
chr1 154999879
chr1 154999882
chr1 154999886
chr1 154999893
chr1 155000170
chr1 155000175
chr1 155000181
chr1 155000407
chr1 155000453
chr1 155000482
chr1 155000489
chr1 155000491
chr1 155000518

chr1 155000523
chr1 155000548
chr1 155000584
chr1 155001342
chr1 155001355
chr1 155002925
chr1 155002931
chr1 155002934
chr1 155002974
chr1 155002990
chr1 155003011
chr1 155003015
chr1 155003098
chr1 155003148
chr1 155003169
chr1 155003222
chr1 155003228
chr1 155003249
chr1 155003252
chr1 155003259
chr1 155003277
chr1 155003303
chr1 155003327
chr1 155003333
chr1 155003352
chr1 155003354
chr1 155003372
chr1 155003374
chr1 155003384
chr1 155003386
chr1 155064041
chr1 155064043
chr1 155064046
chr1 155064087
chr1 155064094
chr1 155064099
chr1 155064101
chr1 155080388
chr1 155080391
chr1 155081371
chr1 155081375
chr1 155081450
chr1 155081466
chr1 155081495
chr1 155081578
chr1 155081663
chr1 155081675
chr1 155081681
chr1 155081684
chr1 155081734
chr1 155081741
chr1 155081762
chr1 155081771
chr1 155081780

chr1 155081818
chr1 155081842
chr1 155081865
chr1 155081884
chr1 155081896
chr1 155136283
chr1 155136514
chr1 155136518
chr1 155136525
chr1 155136559
chr1 155136570
chr1 155136599
chr1 155136603
chr1 155136611
chr1 155136615
chr1 155136635
chr1 155136659
chr1 155136674
chr1 155136691
chr1 155136697
chr1 155136705
chr1 155136721
chr1 155136729
chr1 155136737
chr1 155140114
chr1 155140172
chr1 155172964
chr1 155172969
chr1 155174220
chr1 155174228
chr1 155174231
chr1 155174244
chr1 155174354
chr1 155174413
chr1 155174464
chr1 155174488
chr1 155174496
chr1 155174513
chr1 155174534
chr1 155174544
chr1 155174604
chr1 155174614
chr1 155174625
chr1 155194132
chr1 155194161
chr1 155194165
chr1 155194173
chr1 155194177
chr1 155194218
chr1 155194228
chr1 155194235
chr1 155194252
chr1 155194258
chr1 155194260

chr1 155194282
chr1 155194298
chr1 155240815
chr1 155240948
chr1 155241249
chr1 155241257
chr1 155241288
chr1 155244450
chr1 155244454
chr1 155244485
chr1 155244499
chr1 155244506
chr1 155244509
chr1 155244512
chr1 155244535
chr1 155244546
chr1 155244557
chr1 155244572
chr1 155244580
chr1 155277742
chr1 155277757
chr1 155277777
chr1 155277785
chr1 155277809
chr1 155277819
chr1 155277832
chr1 155277860
chr1 155277867
chr1 155277884
chr1 155277915
chr1 155277917
chr1 155277924
chr1 155277930
chr1 155277937
chr1 155277942
chr1 155277956
chr1 155277976
chr1 155277994
chr1 155309005
chr1 155309011
chr1 155309029
chr1 155309045
chr1 155309079
chr1 155309203
chr1 155309218
chr1 155560878
chr1 155560893
chr1 155560919
chr1 155561232
chr1 155561234
chr1 155563448
chr1 155563452
chr1 155564028
chr1 155564032

chr1 155564116
chr1 155564215
chr1 155564219
chr1 155564221
chr1 155564231
chr1 155564246
chr1 155564268
chr1 155564291
chr1 155610064
chr1 155610076
chr1 155610101
chr1 155610104
chr1 155610109
chr1 155610138
chr1 155610154
chr1 155610167
chr1 155610173
chr1 155610175
chr1 155610191
chr1 155610204
chr1 155610208
chr1 155610211
chr1 155610213
chr1 155610222
chr1 155610224
chr1 155610227
chr1 155610233
chr1 155610243
chr1 155634255
chr1 155634354
chr1 155634431
chr1 155635428
chr1 155635435
chr1 155635469
chr1 155635931
chr1 155687150
chr1 155687152
chr1 155687535
chr1 155687617
chr1 155687641
chr1 155746163
chr1 155746184
chr1 155746186
chr1 155746201
chr1 155746207
chr1 155746235
chr1 155746248
chr1 155746283
chr1 155746294
chr1 155746317
chr1 155859807
chr1 155859861
chr1 155859920
chr1 155860013

chr1 155860089
chr1 155860093
chr1 155860168
chr1 155860205
chr1 155860265
chr1 155860289
chr1 155860292
chr1 155860315
chr1 155860351
chr1 155860382
chr1 155860405
chr1 155860410
chr1 155860429
chr1 155860445
chr1 155860452
chr1 155860473
chr1 155860503
chr1 155860506
chr1 155860509
chr1 155860520
chr1 155860549
chr1 155860577
chr1 155860621
chr1 155860637
chr1 155860647
chr1 155860659
chr1 155860673
chr1 155860686
chr1 155860697
chr1 155910849
chr1 155910852
chr1 155910947
chr1 155911065
chr1 155911070
chr1 155911114
chr1 155911127
chr1 155911186
chr1 155911311
chr1 155911318
chr1 155933792
chr1 155933798
chr1 155933844
chr1 155933847
chr1 155933890
chr1 155933915
chr1 155933961
chr1 155934053
chr1 155934074
chr1 155934098
chr1 155934282
chr1 155934293
chr1 155934365
chr1 155934367
chr1 155934376

chr1 155934380
chr1 155934398
chr1 155934409
chr1 155934423
chr1 155934432
chr1 155934441
chr1 155934449
chr1 155934463
chr1 155934467
chr1 155934475
chr1 155934479
chr1 155934485
chr1 155934497
chr1 155934503
chr1 155934515
chr1 155946614
chr1 155975510
chr1 155975591
chr1 155975702
chr1 155976202
chr1 155978583
chr1 155978597
chr1 155978638
chr1 155978688
chr1 155978698
chr1 155978749
chr1 155978759
chr1 155978780
chr1 155978815
chr1 155978824
chr1 155978827
chr1 155978880
chr1 155978907
chr1 155978930
chr1 155978951
chr1 155978997
chr1 155979040
chr1 155979091
chr1 155979109
chr1 155979138
chr1 155979221
chr1 155979271
chr1 155979321
chr1 155979592
chr1 155979630
chr1 155979846
chr1 155979951
chr1 156005162
chr1 156020701
chr1 156020763
chr1 156020777
chr1 156020779
chr1 156020795
chr1 156053045

chr1 156053084
chr1 156053290
chr1 156053329
chr1 156053362
chr1 156053387
chr1 156053399
chr1 156053401
chr1 156053406
chr1 156053468
chr1 156053480
chr1 156053487
chr1 156053499
chr1 156053509
chr1 156053514
chr1 156053517
chr1 156053522
chr1 156053546
chr1 156053561
chr1 156053563
chr1 156053572
chr1 156053579
chr1 156053603
chr1 156053610
chr1 156053614
chr1 156053653
chr1 156053661
chr1 156053675
chr1 156053680
chr1 156053690
chr1 156053696
chr1 156053704
chr1 156053707
chr1 156053710
chr1 156053717
chr1 156053721
chr1 156053725
chr1 156053733
chr1 156053737
chr1 156053754
chr1 156053758
chr1 156053785
chr1 156081872
chr1 156081890
chr1 156081892
chr1 156081896
chr1 156081913
chr1 156081923
chr1 156081932
chr1 156082001
chr1 156082145
chr1 156082201
chr1 156082211
chr1 156082223
chr1 156082282

chr1 156082291
chr1 156082298
chr1 156082451
chr1 156106129
chr1 156106180
chr1 156106303
chr1 156106317
chr1 156106358
chr1 156106411
chr1 156114397
chr1 156193751
chr1 156193757
chr1 156193882
chr1 156193888
chr1 156193896
chr1 156218028
chr1 156218081
chr1 156225640
chr1 156225643
chr1 156225713
chr1 156226106
chr1 156226253
chr1 156226500
chr1 156257508
chr1 156282958
chr1 156282966
chr1 156282985
chr1 156283017
chr1 156283038
chr1 156283085
chr1 156283138
chr1 156283146
chr1 156283257
chr1 156283282
chr1 156283304
chr1 156283361
chr1 156295639
chr1 156295641
chr1 156295647
chr1 156295651
chr1 156295662
chr1 156295665
chr1 156295694
chr1 156295696
chr1 156295720
chr1 156295726
chr1 156295761
chr1 156338160
chr1 156338171
chr1 156338187
chr1 156338190
chr1 156338192
chr1 156338203
chr1 156338226

chr1 156338920
chr1 156338937
chr1 156338982
chr1 156338985
chr1 156338989
chr1 156339001
chr1 156339029
chr1 156339034
chr1 156430640
chr1 156430695
chr1 156488550
chr1 156488617
chr1 156488875
chr1 156489079
chr1 156489176
chr1 156489214
chr1 156499925
chr1 156501538
chr1 156601175
chr1 156601232
chr1 156601256
chr1 156626230
chr1 156740838
chr1 156740901
chr1 156740932
chr1 156740945
chr1 156740969
chr1 156741007
chr1 156741020
chr1 156741024
chr1 156747456
chr1 156747472
chr1 156747536
chr1 156747540
chr1 156747593
chr1 156747742
chr1 156747861
chr1 156747866
chr1 156747984
chr1 156748468
chr1 156748503
chr1 156748565
chr1 156748645
chr1 156748816
chr1 156748905
chr1 156748917
chr1 156749059
chr1 156749125
chr1 156749232
chr1 156749277
chr1 156766660
chr1 156766719
chr1 156766742
chr1 156766746

chr1 156766796
chr1 156766895
chr1 156766930
chr1 156766979
chr1 156767007
chr1 156767011
chr1 156767069
chr1 156767132
chr1 156767155
chr1 156767161
chr1 156767244
chr1 156767261
chr1 156767276
chr1 156767285
chr1 156767306
chr1 156767319
chr1 156767322
chr1 156767353
chr1 156768334
chr1 156768340
chr1 156768375
chr1 156768512
chr1 156768544
chr1 156768630
chr1 156816353
chr1 156816757
chr1 156816809
chr1 156816825
chr1 156816851
chr1 156816861
chr1 156816881
chr1 156816896
chr1 156816905
chr1 156816920
chr1 156816931
chr1 157045404
chr1 157045408
chr1 157045420
chr1 157045465
chr1 157046239
chr1 157046245
chr1 157046257
chr1 157046265
chr1 157046268
chr1 157046278
chr1 157046311
chr1 157046318
chr1 157046343
chr1 157046354
chr1 157046360
chr1 157046362
chr1 157046367
chr1 157046381
chr1 157046403

chr1 157046410
chr1 157046421
chr1 157046424
chr1 157046428
chr1 157046436
chr1 157046499
chr1 157046530
chr1 157074677
chr1 157074686
chr1 157074740
chr1 157137635
chr1 157137688
chr1 157137699
chr1 157137701
chr1 157137742
chr1 157137762
chr1 157137770
chr1 157137854
chr1 157137864
chr1 157137871
chr1 157137914
chr1 157137958
chr1 157137960
chr1 157137971
chr1 157137997
chr1 157138002
chr1 157138011
chr1 157170574
chr1 157444411
chr1 157444422
chr1 157445006
chr1 157927007
chr1 157927071
chr1 157993214
chr1 157993233
chr1 157993235
chr1 157993237
chr1 157993263
chr1 157993275
chr1 157993278
chr1 157993281
chr1 157993296
chr1 157993303
chr1 157993386
chr1 157993526
chr1 157993528
chr1 157993530
chr1 158118204
chr1 158118218
chr1 158118312
chr1 158200550
chr1 158201685
chr1 158201690
chr1 158201713

chr1 158201909
chr1 158202600
chr1 158202726
chr1 158203683
chr1 158203704
chr1 158203897
chr1 158203924
chr1 158203966
chr1 158225109
chr1 158334565
chr1 158494410
chr1 158494525
chr1 158494640
chr1 158494843
chr1 158494938
chr1 158495200
chr1 158654632
chr1 158877880
chr1 158930854
chr1 158930899
chr1 158931242
chr1 158931302
chr1 158931516
chr1 158931852
chr1 158932148
chr1 158932172
chr1 158932282
chr1 158932289
chr1 158932360
chr1 158932400
chr1 158932554
chr1 158933102
chr1 158933255
chr1 159009022
chr1 159009099
chr1 159009136
chr1 159009167
chr1 159009207
chr1 159009390
chr1 159009627
chr1 159009691
chr1 159009748
chr1 159009857
chr1 159010020
chr1 159010045
chr1 159010051
chr1 159013033
chr1 159013149
chr1 159013191
chr1 159013231
chr1 159013238
chr1 159013254
chr1 159013293
chr1 159013313

chr1 159013358
chr1 159013396
chr1 159013410
chr1 159013419
chr1 159013447
chr1 159013459
chr1 159013779
chr1 159013941
chr1 159013960
chr1 159014205
chr1 159014264
chr1 159014531
chr1 159014773
chr1 159014849
chr1 159014887
chr1 159014901
chr1 159015021
chr1 159015562
chr1 159015570
chr1 159015807
chr1 159015814
chr1 159067862
chr1 159068053
chr1 159068164
chr1 159068297
chr1 159075980
chr1 159076419
chr1 159076588
chr1 159076601
chr1 159076649
chr1 159076661
chr1 159076770
chr1 159076983
chr1 159255748
chr1 159337051
chr1 159337364
chr1 159337974
chr1 159450883
chr1 159451246
chr1 159498081
chr1 159746401
chr1 159746681
chr1 159746829
chr1 159746906
chr1 159800688
chr1 159854223
chr1 159854228
chr1 159854232
chr1 159854251
chr1 159854268
chr1 159854276
chr1 159854289
chr1 159854296
chr1 159854298

chr1 159854311
chr1 159854319
chr1 159923546
chr1 159923639
chr1 159923672
chr1 159923695
chr1 159923716
chr1 159923775
chr1 160031589
chr1 160032229
chr1 160032452
chr1 160098980
chr1 160099011
chr1 160099166
chr1 160099171
chr1 160099190
chr1 160099199
chr1 160099211
chr1 160099482
chr1 160099493
chr1 160101032
chr1 160101140
chr1 160260962
chr1 160260977
chr1 160261235
chr1 160262917
chr1 160262950
chr1 160263049
chr1 160263082
chr1 160263205
chr1 160263249
chr1 160263267
chr1 160263345
chr1 160263349
chr1 160342103
chr1 160342158
chr1 160342293
chr1 160342339
chr1 160342373
chr1 160342392
chr1 160342421
chr1 160342432
chr1 160342448
chr1 160342469
chr1 160342506
chr1 160342630
chr1 160342647
chr1 160342734
chr1 160342740
chr1 160342754
chr1 160342842
chr1 160342934
chr1 160342975
chr1 160343007

chr1 160343017
chr1 160343023
chr1 160343041
chr1 160343072
chr1 160343081
chr1 160343084
chr1 160343308
chr1 160343321
chr1 160343623
chr1 160343669
chr1 160343676
chr1 160343679
chr1 160343684
chr1 160343720
chr1 160343755
chr1 160343791
chr1 160343826
chr1 160343828
chr1 160343846
chr1 160343876
chr1 160343879
chr1 160344089
chr1 160344091
chr1 160381903
chr1 160438050
chr1 160438075
chr1 160438118
chr1 160453640
chr1 160453671
chr1 160453683
chr1 160453687
chr1 160453722
chr1 160453780
chr1 160453795
chr1 160453956
chr1 160454467
chr1 160520070
chr1 160520145
chr1 160520173
chr1 160520583
chr1 160520888
chr1 160521015
chr1 160521032
chr1 160521167
chr1 160521704
chr1 160521840
chr1 160522190
chr1 160522306
chr1 160522454
chr1 160522784
chr1 160522947
chr1 160522968
chr1 160523017
chr1 160523029

chr1 160523071
chr1 160523124
chr1 160523172
chr1 160523199
chr1 160523201
chr1 160523299
chr1 160540208
chr1 160540291
chr1 160540386
chr1 160540564
chr1 160540743
chr1 160540830
chr1 160568595
chr1 160568665
chr1 160568713
chr1 160568777
chr1 160568809
chr1 160577680
chr1 160577853
chr1 160578179
chr1 160578364
chr1 160578507
chr1 160578729
chr1 160578937
chr1 160579010
chr1 160638097
chr1 160638273
chr1 160638394
chr1 160638410
chr1 160638441
chr1 160646325
chr1 160647021
chr1 160647080
chr1 160647236
chr1 160647247
chr1 160647267
chr1 160647302
chr1 160647334
chr1 160647537
chr1 160647566
chr1 160708817
chr1 160708846
chr1 160709131
chr1 160709167
chr1 160709200
chr1 160709315
chr1 160709322
chr1 160709697
chr1 160710091
chr1 160710157
chr1 160710397
chr1 160710707
chr1 160710768
chr1 160711059

chr1 160711066
chr1 160711420
chr1 160711482
chr1 160711740
chr1 160711789
chr1 160711825
chr1 160711837
chr1 160711934
chr1 160711970
chr1 160712107
chr1 160712218
chr1 160712676
chr1 160712851
chr1 160712865
chr1 160712951
chr1 160712976
chr1 160800724
chr1 160800788
chr1 160800962
chr1 160801407
chr1 160837055
chr1 160837200
chr1 160837329
chr1 160837585
chr1 160837605
chr1 160837620
chr1 160837649
chr1 160837675
chr1 160837705
chr1 160837801
chr1 160837893
chr1 160837904
chr1 160971891
chr1 160971947
chr1 160971954
chr1 160971990
chr1 161013540
chr1 161013595
chr1 161013710
chr1 161013723
chr1 161014105
chr1 161014145
chr1 161038752
chr1 161038761
chr1 161038775
chr1 161038792
chr1 161038800
chr1 161038805
chr1 161038825
chr1 161038852
chr1 161038854
chr1 161038871
chr1 161038894
chr1 161038896

chr1 161038905
chr1 161038907
chr1 161038915
chr1 161038922
chr1 161038933
chr1 161038941
chr1 161038944
chr1 161038960
chr1 161038982
chr1 161038988
chr1 161039003
chr1 161039010
chr1 161039012
chr1 161039015
chr1 161039021
chr1 161039034
chr1 161039036
chr1 161039039
chr1 161039059
chr1 161039097
chr1 161039105
chr1 161039130
chr1 161039187
chr1 161039217
chr1 161046744
chr1 161068057
chr1 161068332
chr1 161068444
chr1 161068452
chr1 161068500
chr1 161068716
chr1 161069775
chr1 161069803
chr1 161069811
chr1 161069891
chr1 161069900
chr1 161097666
chr1 161097846
chr1 161097873
chr1 161098062
chr1 161098096
chr1 161098137
chr1 161098154
chr1 161098184
chr1 161098195
chr1 161117960
chr1 161117967
chr1 161153438
chr1 161153443
chr1 161153447
chr1 161153475
chr1 161153494
chr1 161153542
chr1 161153601

chr1 161153722
chr1 161153744
chr1 161166212
chr1 161166216
chr1 161166219
chr1 161166224
chr1 161166245
chr1 161166251
chr1 161166255
chr1 161166270
chr1 161166273
chr1 161166281
chr1 161166308
chr1 161166311
chr1 161166316
chr1 161166348
chr1 161166355
chr1 161166372
chr1 161166405
chr1 161166407
chr1 161166412
chr1 161166414
chr1 161166429
chr1 161185772
chr1 161225825
chr1 161225859
chr1 161225914
chr1 161225953
chr1 161226014
chr1 161226020
chr1 161226026
chr1 161226040
chr1 161226093
chr1 161226103
chr1 161226158
chr1 161226293
chr1 161226361
chr1 161226364
chr1 161305320
chr1 161305458
chr1 161305594
chr1 161305623
chr1 161305681
chr1 161305693
chr1 161305722
chr1 161305763
chr1 161305771
chr1 161305790
chr1 161305803
chr1 161305830
chr1 161305850
chr1 161305858
chr1 161305862
chr1 161305867

chr1 161305874
chr1 161305892
chr1 161305975
chr1 161305990
chr1 161306006
chr1 161306010
chr1 161306053
chr1 161306056
chr1 161306101
chr1 161306112
chr1 161306116
chr1 161306118
chr1 161306123
chr1 161306126
chr1 161306128
chr1 161306192
chr1 161306202
chr1 161306205
chr1 161306220
chr1 161306222
chr1 161382099
chr1 161382443
chr1 161390136
chr1 161390141
chr1 161390152
chr1 161390157
chr1 161390169
chr1 161390178
chr1 161390185
chr1 161390200
chr1 161390205
chr1 161390228
chr1 161390230
chr1 161390246
chr1 161390251
chr1 161390262
chr1 161390267
chr1 161390283
chr1 161390285
chr1 161390294
chr1 161390300
chr1 161390306
chr1 161390311
chr1 161390316
chr1 161390357
chr1 161390381
chr1 161390390
chr1 161390422
chr1 161390424
chr1 161390427
chr1 161390442
chr1 161390463
chr1 161390466
chr1 161390476

chr1 161440498
chr1 161440539
chr1 161440549
chr1 161440553
chr1 161440563
chr1 161440570
chr1 161440586
chr1 161440603
chr1 161440611
chr1 161440618
chr1 161440621
chr1 161440645
chr1 161440652
chr1 161440663
chr1 161440665
chr1 161440669
chr1 161440731
chr1 161440746
chr1 161440761
chr1 161440796
chr1 161440801
chr1 161440804
chr1 161440818
chr1 161440830
chr1 161440833
chr1 161440841
chr1 161440848
chr1 161440856
chr1 161440858
chr1 161440891
chr1 161440897
chr1 161440900
chr1 161440903
chr1 161440906
chr1 161440910
chr1 161440913
chr1 161440918
chr1 161440933
chr1 161440937
chr1 161440939
chr1 161440943
chr1 161440971
chr1 161440974
chr1 161440984
chr1 161440988
chr1 161440990
chr1 161440996
chr1 161441028
chr1 161441035
chr1 161441042
chr1 161441056
chr1 161441063
chr1 161441069
chr1 161441077

chr1 161441087
chr1 161441109
chr1 161441115
chr1 161441125
chr1 161441131
chr1 161441137
chr1 161441144
chr1 161441154
chr1 161441159
chr1 161441162
chr1 161441175
chr1 161441177
chr1 161441179
chr1 161441200
chr1 161441202
chr1 161441209
chr1 161441211
chr1 161441214
chr1 161441229
chr1 161441231
chr1 161441235
chr1 161441249
chr1 161441263
chr1 161441265
chr1 161441283
chr1 161441290
chr1 161441297
chr1 161441303
chr1 161441305
chr1 161441317
chr1 161441321
chr1 161441324
chr1 161441327
chr1 161441330
chr1 161441334
chr1 161441339
chr1 161441342
chr1 161441344
chr1 161441359
chr1 161441370
chr1 161441372
chr1 161441378
chr1 161441384
chr1 161441390
chr1 161441397
chr1 161441403
chr1 161441414
chr1 161441418
chr1 161441427
chr1 161441429
chr1 161441431
chr1 161441436
chr1 161441439
chr1 161441445

chr1 161441450
chr1 161441464
chr1 161441467
chr1 161441477
chr1 161441483
chr1 161441487
chr1 161441489
chr1 161441492
chr1 161441511
chr1 161441514
chr1 161441519
chr1 161441524
chr1 161441527
chr1 161441529
chr1 161441532
chr1 161441544
chr1 161441548
chr1 161441558
chr1 161441563
chr1 161441572
chr1 161441590
chr1 161441598
chr1 161441619
chr1 161441744
chr1 161441747
chr1 161441750
chr1 161441763
chr1 161441767
chr1 161441784
chr1 161441787
chr1 161441817
chr1 161441823
chr1 161441827
chr1 161441829
chr1 161441851
chr1 161441859
chr1 161441880
chr1 161441907
chr1 161441911
chr1 161441915
chr1 161441917
chr1 161441930
chr1 161442040
chr1 161442047
chr1 161442054
chr1 161442064
chr1 161442067
chr1 161442072
chr1 161442085
chr1 161442104
chr1 161442108
chr1 161442132
chr1 161442145
chr1 161442149

chr1 161442154
chr1 161442158
chr1 161442166
chr1 161442174
chr1 161442192
chr1 161445768
chr1 161445785
chr1 161445799
chr1 161445840
chr1 161445923
chr1 161445943
chr1 161445949
chr1 161445997
chr1 161446884
chr1 161446894
chr1 161446902
chr1 161446911
chr1 161446926
chr1 161447470
chr1 161447491
chr1 161447493
chr1 161447495
chr1 161447507
chr1 161447510
chr1 161447520
chr1 161447540
chr1 161447543
chr1 161447545
chr1 161448083
chr1 161448145
chr1 161448153
chr1 161448309
chr1 161448315
chr1 161448318
chr1 161448321
chr1 161448324
chr1 161448328
chr1 161448331
chr1 161448336
chr1 161448351
chr1 161448355
chr1 161448357
chr1 161448361
chr1 161448389
chr1 161448392
chr1 161448402
chr1 161448406
chr1 161448408
chr1 161448414
chr1 161448425
chr1 161448428
chr1 161448432
chr1 161448435
chr1 161448439

chr1 161448453
chr1 161448460
chr1 161448474
chr1 161448481
chr1 161448487
chr1 161448495
chr1 161448505
chr1 161448527
chr1 161448533
chr1 161448543
chr1 161448549
chr1 161448555
chr1 161448562
chr1 161448572
chr1 161448577
chr1 161448580
chr1 161448593
chr1 161448595
chr1 161448597
chr1 161448618
chr1 161448620
chr1 161448627
chr1 161448629
chr1 161448632
chr1 161448647
chr1 161448649
chr1 161448653
chr1 161448667
chr1 161448681
chr1 161448683
chr1 161448690
chr1 161448694
chr1 161448701
chr1 161448708
chr1 161448715
chr1 161448721
chr1 161448723
chr1 161448735
chr1 161448739
chr1 161448742
chr1 161448745
chr1 161448748
chr1 161448752
chr1 161448757
chr1 161448760
chr1 161448762
chr1 161448777
chr1 161448788
chr1 161448790
chr1 161449037
chr1 161449050
chr1 161449054
chr1 161449057
chr1 161449067

chr1 161449097
chr1 161449371
chr1 161449375
chr1 161449390
chr1 161449451
chr1 161449458
chr1 161449465
chr1 161449472
chr1 161449482
chr1 161449485
chr1 161449490
chr1 161449503
chr1 161449522
chr1 161449526
chr1 161449550
chr1 161449584
chr1 161449592
chr1 161449610
chr1 161449637
chr1 161449645
chr1 161449652
chr1 161449656
chr1 161449658
chr1 161449673
chr1 161449690
chr1 161449693
chr1 161449704
chr1 161449724
chr1 161449741
chr1 161449757
chr1 161449761
chr1 161449768
chr1 161449781
chr1 161449800
chr1 161449845
chr1 161449849
chr1 161449866
chr1 161449873
chr1 161449883
chr1 161449893
chr1 161449896
chr1 161449910
chr1 161449918
chr1 161449995
chr1 161450004
chr1 161450009
chr1 161450017
chr1 161450028
chr1 161450030
chr1 161450060
chr1 161450073
chr1 161450078
chr1 161450084
chr1 161450096

chr1 161450161
chr1 161450171
chr1 161450174
chr1 161450180
chr1 161450184
chr1 161450201
chr1 161450244
chr1 161450269
chr1 161450330
chr1 161450376
chr1 161450384
chr1 161450388
chr1 161450390
chr1 161450397
chr1 161450401
chr1 161450422
chr1 161450425
chr1 161450437
chr1 161450447
chr1 161450459
chr1 161450471
chr1 161450491
chr1 161450502
chr1 161450504
chr1 161450509
chr1 161450523
chr1 161450526
chr1 161450531
chr1 161450551
chr1 161450559
chr1 161450572
chr1 161450730
chr1 161450737
chr1 161450751
chr1 161450786
chr1 161450794
chr1 161450798
chr1 161450807
chr1 161450821
chr1 161450965
chr1 161450971
chr1 161450973
chr1 161451031
chr1 161451041
chr1 161451044
chr1 161451058
chr1 161451066
chr1 161451199
chr1 161451258
chr1 161451260
chr1 161451313
chr1 161451315
chr1 161451323
chr1 161451331

chr1 161451333
chr1 161451347
chr1 161451360
chr1 161451367
chr1 161451419
chr1 161451456
chr1 161451483
chr1 161452024
chr1 161452071
chr1 161452080
chr1 161452101
chr1 161452251
chr1 161452253
chr1 161452294
chr1 161452308
chr1 161453039
chr1 161453110
chr1 161453148
chr1 161453165
chr1 161453179
chr1 161453220
chr1 161453276
chr1 161453303
chr1 161453323
chr1 161453329
chr1 161453377
chr1 161455464
chr1 161455526
chr1 161455534
chr1 161455541
chr1 161455548
chr1 161455690
chr1 161455696
chr1 161455699
chr1 161455702
chr1 161455705
chr1 161455709
chr1 161455712
chr1 161455717
chr1 161455732
chr1 161455736
chr1 161455738
chr1 161455742
chr1 161455770
chr1 161455773
chr1 161455834
chr1 161455841
chr1 161455855
chr1 161455862
chr1 161455868
chr1 161455876
chr1 161455886
chr1 161455908
chr1 161455914

chr1 161455924
chr1 161455930
chr1 161455936
chr1 161455943
chr1 161455953
chr1 161455958
chr1 161455961
chr1 161455974
chr1 161455976
chr1 161455978
chr1 161455999
chr1 161456001
chr1 161456008
chr1 161456010
chr1 161456013
chr1 161456028
chr1 161456030
chr1 161456034
chr1 161456048
chr1 161456062
chr1 161456064
chr1 161456071
chr1 161456075
chr1 161456082
chr1 161456089
chr1 161456092
chr1 161456189
chr1 161456196
chr1 161456202
chr1 161456213
chr1 161456217
chr1 161456222
chr1 161456226
chr1 161456230
chr1 161456235
chr1 161456238
chr1 161456244
chr1 161456249
chr1 161456263
chr1 161456418
chr1 161456431
chr1 161456435
chr1 161456438
chr1 161456448
chr1 161456478
chr1 161456502
chr1 161456543
chr1 161456546
chr1 161456549
chr1 161456562
chr1 161456566
chr1 161456583
chr1 161456586
chr1 161456593

chr1 161456650
chr1 161456658
chr1 161456679
chr1 161456706
chr1 161456710
chr1 161456714
chr1 161456716
chr1 161456729
chr1 161456752
chr1 161456756
chr1 161456832
chr1 161456839
chr1 161456846
chr1 161456853
chr1 161456863
chr1 161456866
chr1 161456871
chr1 161456884
chr1 161456903
chr1 161456907
chr1 161456931
chr1 161456944
chr1 161456948
chr1 161456953
chr1 161456957
chr1 161456965
chr1 161456973
chr1 161457085
chr1 161457105
chr1 161457122
chr1 161457138
chr1 161457142
chr1 161457149
chr1 161457162
chr1 161457201
chr1 161457226
chr1 161457230
chr1 161457247
chr1 161457254
chr1 161457264
chr1 161457274
chr1 161457277
chr1 161457291
chr1 161457299
chr1 161457922
chr1 161457933
chr1 161457935
chr1 161457940
chr1 161457954
chr1 161457957
chr1 161457962
chr1 161457990
chr1 161458003
chr1 161458168

chr1 161458182
chr1 161458217
chr1 161458225
chr1 161458229
chr1 161458238
chr1 161458252
chr1 161458257
chr1 161458497
chr1 161458509
chr1 161458525
chr1 161462241
chr1 161462261
chr1 161462282
chr1 161462284
chr1 161462286
chr1 161462298
chr1 161462301
chr1 161462311
chr1 161462823
chr1 161462826
chr1 161462845
chr1 161462849
chr1 161462855
chr1 161462859
chr1 161462868
chr1 161462870
chr1 161462874
chr1 161462936
chr1 161462944
chr1 161462951
chr1 161462958
chr1 161463100
chr1 161463106
chr1 161463109
chr1 161463112
chr1 161463115
chr1 161463119
chr1 161463122
chr1 161463127
chr1 161463142
chr1 161463146
chr1 161463148
chr1 161463152
chr1 161463180
chr1 161463183
chr1 161463384
chr1 161463386
chr1 161463388
chr1 161463409
chr1 161463411
chr1 161463418
chr1 161463420
chr1 161463423
chr1 161463438

chr1 161463440
chr1 161463444
chr1 161463458
chr1 161463472
chr1 161463568
chr1 161463579
chr1 161463581
chr1 161463587
chr1 161463593
chr1 161463599
chr1 161463606
chr1 161463612
chr1 161463623
chr1 161463627
chr1 161463632
chr1 161463636
chr1 161463638
chr1 161463640
chr1 161463645
chr1 161463648
chr1 161463654
chr1 161463659
chr1 161463673
chr1 161464249
chr1 161464256
chr1 161464263
chr1 161464273
chr1 161464276
chr1 161464281
chr1 161464294
chr1 161464313
chr1 161464317
chr1 161464341
chr1 161464358
chr1 161464363
chr1 161464367
chr1 161464375
chr1 161464383
chr1 161464401
chr1 161464428
chr1 161464436
chr1 161464447
chr1 161464449
chr1 161464464
chr1 161464481
chr1 161464484
chr1 161464495
chr1 161464515
chr1 161464532
chr1 161464548
chr1 161464552
chr1 161464559
chr1 161464572
chr1 161464591

chr1 161464611
chr1 161464636
chr1 161464640
chr1 161464657
chr1 161464664
chr1 161464674
chr1 161464684
chr1 161464687
chr1 161464701
chr1 161464709
chr1 161464759
chr1 161464761
chr1 161464786
chr1 161464795
chr1 161464800
chr1 161464808
chr1 161464819
chr1 161464821
chr1 161464851
chr1 161464864
chr1 161464869
chr1 161464875
chr1 161464887
chr1 161464895
chr1 161464975
chr1 161464992
chr1 161465035
chr1 161465060
chr1 161465121
chr1 161465129
chr1 161465131
chr1 161465145
chr1 161465167
chr1 161465175
chr1 161465179
chr1 161465181
chr1 161465188
chr1 161465192
chr1 161465213
chr1 161465216
chr1 161465515
chr1 161465521
chr1 161465528
chr1 161465542
chr1 161465577
chr1 161465585
chr1 161465589
chr1 161465598
chr1 161465612
chr1 161465617
chr1 161465822
chr1 161465832
chr1 161465835
chr1 161465849

chr1 161468067
chr1 161468094
chr1 161468114
chr1 161468120
chr1 161469171
chr1 161469176
chr1 161469388
chr1 161469408
chr1 161469415
chr1 161469421
chr1 161469426
chr1 161470236
chr1 161470240
chr1 161470249
chr1 161470251
chr1 161470255
chr1 161470317
chr1 161470325
chr1 161470332
chr1 161470339
chr1 161470347
chr1 161470481
chr1 161470487
chr1 161470490
chr1 161470493
chr1 161470496
chr1 161470500
chr1 161470503
chr1 161470508
chr1 161470523
chr1 161470527
chr1 161470529
chr1 161470533
chr1 161470561
chr1 161470564
chr1 161470574
chr1 161470578
chr1 161470580
chr1 161470597
chr1 161470600
chr1 161470604
chr1 161470607
chr1 161470611
chr1 161470618
chr1 161470625
chr1 161470632
chr1 161470646
chr1 161470653
chr1 161470659
chr1 161470667
chr1 161470677
chr1 161470699
chr1 161470705
chr1 161470715

chr1 161471980
chr1 161471992
chr1 161472038
chr1 161472045
chr1 161472055
chr1 161472065
chr1 161472068
chr1 161472082
chr1 161529917
chr1 161529923
chr1 161529983
chr1 161530036
chr1 161530218
chr1 161530256
chr1 161530260
chr1 161530328
chr1 161530358
chr1 161530366
chr1 161530384
chr1 161530393
chr1 161530398
chr1 161530408
chr1 161530412
chr1 161530481
chr1 161530550
chr1 161530557
chr1 161530578
chr1 161539610
chr1 161539684
chr1 161539711
chr1 161539733
chr1 161539796
chr1 161539833
chr1 161539865
chr1 161539914
chr1 161539917
chr1 161539940
chr1 161539949
chr1 161539982
chr1 161539993
chr1 161540077
chr1 161540097
chr1 161540114
chr1 161540116
chr1 161540128
chr1 161611822
chr1 161611848
chr1 161611860
chr1 161611864
chr1 161611943
chr1 161611962
chr1 161611970
chr1 161611979
chr1 161611988

chr1 161611997
chr1 161612002
chr1 161612017
chr1 161612033
chr1 161612086
chr1 161612155
chr1 161612162
chr1 161612183
chr1 161621116
chr1 161621144
chr1 161621165
chr1 161621228
chr1 161621265
chr1 161621297
chr1 161621346
chr1 161621349
chr1 161621372
chr1 161621381
chr1 161621414
chr1 161621425
chr1 161621509
chr1 161621511
chr1 161621529
chr1 161621546
chr1 161621548
chr1 161621560
chr1 161621612
chr1 161621664
chr1 161621670
chr1 161621674
chr1 161621685
chr1 161621687
chr1 161621691
chr1 161621698
chr1 161621704
chr1 161621715
chr1 161621731
chr1 161621742
chr1 161621801
chr1 161621803
chr1 161621807
chr1 161621817
chr1 161621820
chr1 161621826
chr1 161621854
chr1 161621856
chr1 161621866
chr1 161621869
chr1 161621892
chr1 161621894
chr1 161621936
chr1 161621964
chr1 161622012
chr1 161622143

chr1 161622189
chr1 161622211
chr1 161622260
chr1 161622327
chr1 161622348
chr1 161622395
chr1 161622447
chr1 161622514
chr1 161622606
chr1 161622837
chr1 161622842
chr1 161622884
chr1 161622996
chr1 161623409
chr1 161623559
chr1 161623586
chr1 161623787
chr1 161744518
chr1 161765300
chr1 161765334
chr1 161765339
chr1 161765344
chr1 161765380
chr1 161765444
chr1 161765462
chr1 161765468
chr1 161765471
chr1 161765500
chr1 161765543
chr1 161765593
chr1 161765626
chr1 161765658
chr1 161765685
chr1 161765832
chr1 161765861
chr1 161765898
chr1 161765900
chr1 161765937
chr1 161765992
chr1 161766090
chr1 161766104
chr1 161766116
chr1 161766135
chr1 161766805
chr1 161766916
chr1 161766955
chr1 161918959
chr1 161919012
chr1 161919062
chr1 161962404
chr1 161962422
chr1 162498107
chr1 162498115
chr1 162498121

chr1 162498129
chr1 162498143
chr1 162498150
chr1 162498153
chr1 162498233
chr1 162498236
chr1 162498277
chr1 162498313
chr1 162498331
chr1 162498341
chr1 162498364
chr1 162498373
chr1 162498436
chr1 162498641
chr1 162561001
chr1 162561014
chr1 162561067
chr1 162561069
chr1 162790566
chr1 162790575
chr1 162790580
chr1 162790588
chr1 162790595
chr1 162790609
chr1 162790612
chr1 162790615
chr1 162790621
chr1 162790794
chr1 162790796
chr1 162790804
chr1 162790824
chr1 162790863
chr1 162790869
chr1 162790882
chr1 162790900
chr1 162790910
chr1 162790914
chr1 162790921
chr1 162790927
chr1 162790931
chr1 162791600
chr1 162791674
chr1 163092185
chr1 163322190
chr1 163322221
chr1 163322227
chr1 163322230
chr1 163322291
chr1 163322317
chr1 163322326
chr1 163322385
chr1 163394671
chr1 163394947
chr1 163395007

chr1 163395459
chr1 163395476
chr1 163496401
chr1 163497093
chr1 164462000
chr1 164462413
chr1 164462506
chr1 164462696
chr1 164462936
chr1 164558318
chr1 164558994
chr1 164559011
chr1 164559445
chr1 164559448
chr1 164559772
chr1 164559777
chr1 164559799
chr1 164559809
chr1 164559828
chr1 164559864
chr1 164559873
chr1 164559882
chr1 164559912
chr1 164559921
chr1 164559933
chr1 164560004
chr1 164560090
chr1 164560103
chr1 164560157
chr1 164560180
chr1 164560189
chr1 164560201
chr1 164560210
chr1 164560294
chr1 164560321
chr1 164560328
chr1 164560428
chr1 164560490
chr1 164560518
chr1 164560755
chr1 164560771
chr1 164561048
chr1 164561069
chr1 164561144
chr1 164561152
chr1 164561187
chr1 164561253
chr1 164561376
chr1 164561712
chr1 164625267
chr1 164651786
chr1 164651816
chr1 164651835
chr1 164651882

chr1 165122712
chr1 165350689
chr1 165351012
chr1 165351014
chr1 165351163
chr1 165351327
chr1 165351337
chr1 165351352
chr1 165351596
chr1 165351688
chr1 165351710
chr1 165377352
chr1 165377463
chr1 165377878
chr1 165393031
chr1 165393041
chr1 165393061
chr1 165393075
chr1 165393097
chr1 165393114
chr1 165393141
chr1 165393183
chr1 165393241
chr1 165393255
chr1 165393390
chr1 165393424
chr1 165393459
chr1 165393470
chr1 165393530
chr1 165593313
chr1 165593464
chr1 165593615
chr1 165593822
chr1 165593886
chr1 165593919
chr1 165597693
chr1 165597711
chr1 165597717
chr1 165597880
chr1 165597950
chr1 165598010
chr1 165598029
chr1 165598037
chr1 165598042
chr1 165598051
chr1 165598072
chr1 165598075
chr1 165598546
chr1 165598557
chr1 165598572
chr1 165598593
chr1 165598605
chr1 165598608
chr1 165598635

chr1 165598660
chr1 165598761
chr1 165630214
chr1 165630306
chr1 165630327
chr1 165630329
chr1 165631606
chr1 165631641
chr1 165631652
chr1 165631673
chr1 165631676
chr1 165631688
chr1 165631693
chr1 165631703
chr1 165768366
chr1 165768507
chr1 165768538
chr1 165768628
chr1 165768643
chr1 165768658
chr1 165768673
chr1 165768688
chr1 165768698
chr1 165768718
chr1 165768734
chr1 165768736
chr1 165768754
chr1 165769096
chr1 165769098
chr1 165769100
chr1 165769111
chr1 165769170
chr1 165769516
chr1 165769599
chr1 165769657
chr1 165828729
chr1 165828773
chr1 165871683
chr1 166489969
chr1 166490002
chr1 166490006
chr1 166490025
chr1 166490045
chr1 166490052
chr1 166490060
chr1 166490088
chr1 166490090
chr1 166490107
chr1 166490134
chr1 166490168
chr1 166490197
chr1 166490242
chr1 166490287
chr1 166490301

chr1 166991798
chr1 166991831
chr1 167043942
chr1 167043982
chr1 167044128
chr1 167044209
chr1 167044214
chr1 167044220
chr1 167044231
chr1 167044233
chr1 167044248
chr1 167044257
chr1 167044296
chr1 167044298
chr1 167044305
chr1 167044321
chr1 167044352
chr1 167044362
chr1 167163367
chr1 167163596
chr1 167163643
chr1 167164215
chr1 167219663
chr1 167219702
chr1 167219765
chr1 167219767
chr1 167219823
chr1 167219834
chr1 167219840
chr1 167219894
chr1 167219905
chr1 167219907
chr1 167219952
chr1 167219955
chr1 167219966
chr1 167222284
chr1 167222364
chr1 167222378
chr1 167222431
chr1 167222434
chr1 167222453
chr1 167222475
chr1 167222504
chr1 167222531
chr1 167222671
chr1 167222887
chr1 167267930
chr1 167267936
chr1 167268019
chr1 167268046
chr1 167438189
chr1 167438271
chr1 167438277
chr1 167455913

chr1 167492245
chr1 167492283
chr1 167492328
chr1 167492350
chr1 167492353
chr1 167492478
chr1 167492587
chr1 167492603
chr1 167492609
chr1 167492614
chr1 167499886
chr1 167516539
chr1 167516583
chr1 167516592
chr1 167516614
chr1 167516616
chr1 167516629
chr1 167516636
chr1 167516799
chr1 167516839
chr1 167516853
chr1 167518249
chr1 167518254
chr1 167518388
chr1 167518391
chr1 167518396
chr1 167518399
chr1 167518415
chr1 167518439
chr1 167518441
chr1 167518451
chr1 167518513
chr1 167518520
chr1 167518525
chr1 167556333
chr1 167556796
chr1 167600211
chr1 167600306
chr1 167600355
chr1 167617135
chr1 167617302
chr1 167617308
chr1 167617527
chr1 167617701
chr1 167617719
chr1 167627766
chr1 167627812
chr1 167628019
chr1 167628047
chr1 167628204
chr1 167628256
chr1 167628280
chr1 167628422
chr1 167628456

chr1 167628506
chr1 167628518
chr1 167628606
chr1 167628830
chr1 167632612
chr1 167632633
chr1 167632682
chr1 167713902
chr1 167713944
chr1 167716016
chr1 167716171
chr1 167716192
chr1 167716237
chr1 167716338
chr1 167716346
chr1 167716351
chr1 167716359
chr1 167716388
chr1 167716405
chr1 167716414
chr1 167716431
chr1 167716439
chr1 167716446
chr1 167716510
chr1 167722667
chr1 167722799
chr1 167722813
chr1 167761545
chr1 167803402
chr1 167803409
chr1 167803412
chr1 167803465
chr1 167803482
chr1 167803541
chr1 167803614
chr1 167803666
chr1 167803694
chr1 167803731
chr1 167803790
chr1 167805213
chr1 167805305
chr1 167805453
chr1 167805478
chr1 167934988
chr1 167935046
chr1 167935048
chr1 167935333
chr1 167937812
chr1 167937883
chr1 167938085
chr1 168146139
chr1 168146159
chr1 168146179
chr1 168178951

chr1 168178957
chr1 168178969
chr1 168178974
chr1 168178995
chr1 168179005
chr1 168179014
chr1 168179021
chr1 168179024
chr1 168179027
chr1 168179030
chr1 168179089
chr1 168179108
chr1 168179122
chr1 168179139
chr1 168179142
chr1 168179166
chr1 168179169
chr1 168179191
chr1 168179198
chr1 168179209
chr1 168179216
chr1 168179233
chr1 168179245
chr1 168179280
chr1 168179288
chr1 168179297
chr1 168179302
chr1 168179323
chr1 168179347
chr1 168225734
chr1 168225747
chr1 168225754
chr1 168341227
chr1 168341294
chr1 168342918
chr1 168342927
chr1 168342937
chr1 168343035
chr1 168343038
chr1 168343114
chr1 168343248
chr1 168348665
chr1 168348678
chr1 168348952
chr1 168348969
chr1 168439624
chr1 168439630
chr1 169044512
chr1 169044612
chr1 169044645
chr1 169309392
chr1 169309474
chr1 169309514
chr1 169309731

chr1 169338501
chr1 169367543
chr1 169367550
chr1 169367572
chr1 169367595
chr1 169367607
chr1 169367616
chr1 169367638
chr1 169367642
chr1 169367680
chr1 169367744
chr1 169367782
chr1 169367805
chr1 169367815
chr1 169367819
chr1 169367824
chr1 169367831
chr1 169367837
chr1 169367850
chr1 169368942
chr1 169369043
chr1 169369107
chr1 169369254
chr1 169369368
chr1 169390160
chr1 169426920
chr1 169426960
chr1 169427003
chr1 169427064
chr1 169427125
chr1 169427135
chr1 169427154
chr1 169427209
chr1 169427248
chr1 169427253
chr1 169427300
chr1 169427303
chr1 169427325
chr1 169427397
chr1 169427399
chr1 169427431
chr1 169427452
chr1 169427516
chr1 169427526
chr1 169427538
chr1 169427547
chr1 169427581
chr1 169427596
chr1 169427607
chr1 169427620
chr1 169427622
chr1 169427630
chr1 169427671
chr1 169486572

chr1 169486610
chr1 169486656
chr1 169486661
chr1 169584833
chr1 169584948
chr1 169584972
chr1 169585114
chr1 169585164
chr1 169586208
chr1 169586250
chr1 169586281
chr1 169586303
chr1 169586367
chr1 169586404
chr1 169586436
chr1 169586443
chr1 169586462
chr1 169586507
chr1 169667443
chr1 169692497
chr1 169692504
chr1 169692525
chr1 169692535
chr1 169692653
chr1 169693272
chr1 169693308
chr1 169693384
chr1 169693386
chr1 169694466
chr1 169694526
chr1 169709865
chr1 169710042
chr1 169710058
chr1 169710412
chr1 169710736
chr1 169710759
chr1 169711094
chr1 169711472
chr1 169712172
chr1 169712177
chr1 169712192
chr1 169712220
chr1 169712286
chr1 169793871
chr1 169794321
chr1 169795318
chr1 169795320
chr1 169795430
chr1 169795432
chr1 169795436
chr1 169795471
chr1 169795508
chr1 169795575
chr1 169795583

chr1 169796419
chr1 169796516
chr1 169809803
chr1 169809806
chr1 169892744
chr1 169892809
chr1 169892863
chr1 169892969
chr1 169892975
chr1 169892983
chr1 169892996
chr1 169893022
chr1 169893165
chr1 169893172
chr1 169893178
chr1 169893213
chr1 169893285
chr1 169893941
chr1 169893947
chr1 169893953
chr1 169893981
chr1 169893984
chr1 169894014
chr1 169894028
chr1 169894048
chr1 169894069
chr1 169894072
chr1 169894074
chr1 169894077
chr1 169894083
chr1 169894121
chr1 169894151
chr1 169894217
chr1 169894272
chr1 169894287
chr1 169894297
chr1 169894318
chr1 169894353
chr1 169894365
chr1 169894375
chr1 169894384
chr1 169894391
chr1 169894402
chr1 169894595
chr1 169894615
chr1 169894626
chr1 169914387
chr1 169914407
chr1 170074305
chr1 170074333
chr1 170074430
chr1 170074452
chr1 170074470
chr1 170074476

chr1 170074482
chr1 170074485
chr1 170074517
chr1 170074519
chr1 170074529
chr1 170074535
chr1 170074565
chr1 170075068
chr1 170214329
chr1 170214349
chr1 170214359
chr1 170214372
chr1 170214398
chr1 170214727
chr1 170214777
chr1 170215265
chr1 170215385
chr1 170494589
chr1 170531475
chr1 170531604
chr1 170531609
chr1 170531616
chr1 170531738
chr1 170531801
chr1 170531807
chr1 170531847
chr1 170531853
chr1 170531859
chr1 170532579
chr1 170532618
chr1 170532659
chr1 170532666
chr1 170532723
chr1 170532745
chr1 170632426
chr1 170632663
chr1 170633755
chr1 170662661
chr1 170662706
chr1 170662752
chr1 170662892
chr1 170662911
chr1 170662951
chr1 170663230
chr1 170663258
chr1 170663282
chr1 170663464
chr1 170663467
chr1 170663632
chr1 170663789
chr1 170663804
chr1 170663844
chr1 170663946
chr1 170663948

chr1 170663951
chr1 170663983
chr1 170663990
chr1 170664004
chr1 170961231
chr1 170961933
chr1 171313468
chr1 171313954
chr1 171313986
chr1 171314014
chr1 171314017
chr1 171314064
chr1 171314096
chr1 171484794
chr1 171484804
chr1 171484896
chr1 171485013
chr1 171486676
chr1 171486748
chr1 171487585
chr1 171487610
chr1 171601468
chr1 171741165
chr1 171741210
chr1 171741227
chr1 171741298
chr1 171741374
chr1 171741408
chr1 171741426
chr1 171741453
chr1 171741465
chr1 171741498
chr1 171741560
chr1 171742701
chr1 171742707
chr1 171742720
chr1 171742758
chr1 171742789
chr1 171742793
chr1 171742836
chr1 171742860
chr1 171742871
chr1 171743070
chr1 171743163
chr1 171842382
chr1 171842389
chr1 171842401
chr1 171842404
chr1 171842434
chr1 171842456
chr1 171842464
chr1 171842489
chr1 171842531
chr1 171842558

chr1 171842603
chr1 171842625
chr1 171842638
chr1 171842663
chr1 171842778
chr1 172180989
chr1 172181008
chr1 172181016
chr1 172181266
chr1 172181428
chr1 172181505
chr1 172181531
chr1 172181570
chr1 172181599
chr1 172182045
chr1 172182071
chr1 172182138
chr1 172338453
chr1 172338592
chr1 172338688
chr1 172358855
chr1 172358926
chr1 172359061
chr1 172359237
chr1 172359254
chr1 172380362
chr1 172380370
chr1 172380465
chr1 172380599
chr1 172380853
chr1 172380884
chr1 172381070
chr1 172381200
chr1 172381249
chr1 172394250
chr1 172394255
chr1 172394279
chr1 172394339
chr1 172443543
chr1 172443607
chr1 172443641
chr1 172443697
chr1 172443728
chr1 172443743
chr1 172443746
chr1 172443749
chr1 172443766
chr1 172443769
chr1 172443784
chr1 172443788
chr1 172443792
chr1 172443815
chr1 172443843
chr1 172443896

chr1 172443899
chr1 172443902
chr1 172443927
chr1 172443954
chr1 172443967
chr1 172443974
chr1 172451134
chr1 172531751
chr1 172531936
chr1 172532009
chr1 172532011
chr1 172532026
chr1 172532062
chr1 172532086
chr1 172534012
chr1 172534017
chr1 172534041
chr1 172534143
chr1 172534171
chr1 172534212
chr1 172534275
chr1 172534292
chr1 172639107
chr1 172639183
chr1 172639292
chr1 172639426
chr1 172639481
chr1 172639493
chr1 172639504
chr1 172639564
chr1 172661911
chr1 172661925
chr1 172661965
chr1 172662050
chr1 172662359
chr1 172662793
chr1 172662849
chr1 172662859
chr1 172662864
chr1 172662890
chr1 172662901
chr1 172675208
chr1 172675383
chr1 172698235
chr1 172698516
chr1 172746471
chr1 172894807
chr1 172895004
chr1 172895030
chr1 172895032
chr1 172895040
chr1 172895042
chr1 172895207
chr1 172895287

chr1 172895366
chr1 172895386
chr1 172895390
chr1 172934520
chr1 172934561
chr1 172934590
chr1 172934597
chr1 173222793
chr1 173331247
chr1 173331253
chr1 173331281
chr1 173331391
chr1 173356466
chr1 173356487
chr1 173420576
chr1 173420727
chr1 173429488
chr1 173429594
chr1 173429666
chr1 173429668
chr1 173429676
chr1 173429900
chr1 173429913
chr1 173715240
chr1 173715242
chr1 173715247
chr1 173715257
chr1 173715263
chr1 173715283
chr1 173715302
chr1 173715391
chr1 173715395
chr1 173715412
chr1 173715422
chr1 173715435
chr1 173715456
chr1 173715536
chr1 173715542
chr1 173823339
chr1 173823548
chr1 173823575
chr1 173823726
chr1 173823784
chr1 173823818
chr1 173823843
chr1 173865447
chr1 173865536
chr1 173865693
chr1 173866073
chr1 173866127
chr1 173866851
chr1 173867082
chr1 173867329
chr1 173867388

chr1 173867498
chr1 173867505
chr1 173869062
chr1 173869107
chr1 173869192
chr1 173869273
chr1 173869306
chr1 173869451
chr1 173869498
chr1 173869513
chr1 174021261
chr1 174021263
chr1 174152355
chr1 174152376
chr1 174152630
chr1 174152835
chr1 174153214
chr1 174153238
chr1 174200731
chr1 174200903
chr1 174206013
chr1 174206197
chr1 174781255
chr1 175000957
chr1 175000976
chr1 175001264
chr1 175022571
chr1 175022725
chr1 175022731
chr1 175023337
chr1 175023373
chr1 175023377
chr1 175023385
chr1 175023403
chr1 175023415
chr1 175023432
chr1 175023455
chr1 175023462
chr1 175023471
chr1 175023499
chr1 175191576
chr1 175191827
chr1 175191834
chr1 175191840
chr1 175191854
chr1 175191881
chr1 175191933
chr1 175192352
chr1 175192361
chr1 175192387
chr1 175192390
chr1 175192397
chr1 175192425
chr1 175192453

chr1 175192455
chr1 175192493
chr1 175192569
chr1 175192590
chr1 175192644
chr1 175192649
chr1 175192651
chr1 175192696
chr1 175192706
chr1 175192711
chr1 175192765
chr1 175514729
chr1 175514735
chr1 175514796
chr1 175652246
chr1 175990768
chr1 175991339
chr1 175991494
chr1 176177686
chr1 176177822
chr1 176177969
chr1 176205348
chr1 176205405
chr1 176205585
chr1 176205784
chr1 176205888
chr1 176205956
chr1 176205968
chr1 176206022
chr1 176552587
chr1 176552680
chr1 176598849
chr1 176947818
chr1 177165260
chr1 177165278
chr1 177165283
chr1 177165318
chr1 177165457
chr1 177165470
chr1 177758091
chr1 177758153
chr1 178094949
chr1 178094962
chr1 178095046
chr1 178095216
chr1 178095258
chr1 178144208
chr1 178230222
chr1 178300240
chr1 178300247
chr1 178371193
chr1 178371392
chr1 178371442
chr1 178371454

chr1 178371527
chr1 178371540
chr1 178371547
chr1 178371645
chr1 178726222
chr1 178726249
chr1 178726305
chr1 178726321
chr1 178726338
chr1 178726373
chr1 178726393
chr1 178726399
chr1 178726418
chr1 178726637
chr1 179142063
chr1 179142942
chr1 179218010
chr1 179228868
chr1 179228871
chr1 179228960
chr1 179228970
chr1 179229029
chr1 179229035
chr1 179229060
chr1 179229062
chr1 179229083
chr1 179229111
chr1 179229118
chr1 179294555
chr1 179294563
chr1 179299128
chr1 179299187
chr1 179299195
chr1 179317659
chr1 179317677
chr1 179317762
chr1 179368353
chr1 179382907
chr1 179444277
chr1 179815408
chr1 179815411
chr1 179867846
chr1 179943646
chr1 179953887
chr1 179953979
chr1 179954021
chr1 179954052
chr1 179954055
chr1 179954081
chr1 179954146
chr1 179954148
chr1 179954162
chr1 179955929
chr1 179955949

chr1 179956081
chr1 179956187
chr1 179956204
chr1 180213523
chr1 180213534
chr1 180213561
chr1 180213576
chr1 180213606
chr1 180213610
chr1 180213620
chr1 180213638
chr1 180213650
chr1 180213683
chr1 180213794
chr1 180227452
chr1 180227631
chr1 180227910
chr1 180228236
chr1 180228272
chr1 180228360
chr1 180237058
chr1 180237060
chr1 180237566
chr1 180237582
chr1 180237936
chr1 180238042
chr1 180238112
chr1 180238161
chr1 180238228
chr1 180238304
chr1 180238438
chr1 180238509
chr1 180482474
chr1 180482583
chr1 180482595
chr1 180482655
chr1 180482665
chr1 180482735
chr1 180482789
chr1 180502707
chr1 180502714
chr1 180503412
chr1 180503415
chr1 180503423
chr1 180503427
chr1 180516228
chr1 180516880
chr1 180632885
chr1 180633553
chr1 180851398
chr1 180852060
chr1 180953895
chr1 180953908
chr1 180953918

chr1 180953920
chr1 180953929
chr1 180953940
chr1 180953964
chr1 180953966
chr1 180953970
chr1 180953975
chr1 180954001
chr1 180954006
chr1 180954012
chr1 180954041
chr1 180954055
chr1 180954071
chr1 180954075
chr1 180954079
chr1 180954094
chr1 180954103
chr1 180954105
chr1 180954112
chr1 180954114
chr1 180954122
chr1 180954128
chr1 180954130
chr1 180954134
chr1 180954138
chr1 180954140
chr1 180954151
chr1 180954154
chr1 180954156
chr1 180954169
chr1 180954204
chr1 180954206
chr1 180954214
chr1 180954216
chr1 180954224
chr1 180954226
chr1 180954228
chr1 180954232
chr1 180954249
chr1 180954254
chr1 180954259
chr1 180954273
chr1 180954285
chr1 180954296
chr1 180954302
chr1 180954304
chr1 180954309
chr1 180954331
chr1 181014761
chr1 181021450
chr1 181021508
chr1 181021714
chr1 181021766
chr1 181022865

chr1 181022872
chr1 181022878
chr1 181022882
chr1 181022885
chr1 181022894
chr1 181022910
chr1 181022920
chr1 181022925
chr1 181022928
chr1 181022949
chr1 181022958
chr1 181022962
chr1 181022964
chr1 181022974
chr1 181022978
chr1 181022981
chr1 181022992
chr1 181022999
chr1 181023006
chr1 181023012
chr1 181033840
chr1 181033992
chr1 181034024
chr1 181034067
chr1 181034074
chr1 181034087
chr1 181034114
chr1 181034186
chr1 181034292
chr1 181044552
chr1 181044561
chr1 181090770
chr1 181090806
chr1 181090817
chr1 181090839
chr1 181090951
chr1 181091001
chr1 181091050
chr1 181091053
chr1 181091174
chr1 181091231
chr1 181099605
chr1 181099613
chr1 181099615
chr1 181099791
chr1 181099798
chr1 181099944
chr1 181142767
chr1 181143012
chr1 181143118
chr1 181143142
chr1 181150399
chr1 181150787
chr1 181936700

chr1 181936975
chr1 181937122
chr1 181937142
chr1 181937294
chr1 181937385
chr1 181970449
chr1 181970877
chr1 182029336
chr1 182029391
chr1 182029405
chr1 182029423
chr1 182029460
chr1 182029464
chr1 182029501
chr1 182029576
chr1 182029580
chr1 182029621
chr1 182029629
chr1 182029666
chr1 182029688
chr1 182248897
chr1 182248915
chr1 182248923
chr1 182249221
chr1 182249340
chr1 182249370
chr1 182259565
chr1 182259705
chr1 182329648
chr1 182329848
chr1 182430811
chr1 182430815
chr1 182430836
chr1 182430917
chr1 182431001
chr1 182588411
chr1 182588477
chr1 182588505
chr1 182588562
chr1 182588715
chr1 182588761
chr1 182588868
chr1 182588876
chr1 182589272
chr1 182589413
chr1 182604075
chr1 182604089
chr1 182604118
chr1 182604129
chr1 182604131
chr1 182604156
chr1 182604241
chr1 182604252
chr1 182604265

chr1 182604269
chr1 182604281
chr1 182604318
chr1 182604320
chr1 182604324
chr1 182604340
chr1 182604342
chr1 182604365
chr1 182604389
chr1 182604402
chr1 182604406
chr1 182604424
chr1 182604432
chr1 182604437
chr1 182604461
chr1 182604478
chr1 182604489
chr1 182604495
chr1 182604502
chr1 182604518
chr1 182604531
chr1 182604533
chr1 182604547
chr1 182604549
chr1 182604564
chr1 182604575
chr1 182604578
chr1 182604580
chr1 182604596
chr1 182604613
chr1 182615443
chr1 182615449
chr1 182615464
chr1 182615518
chr1 182615565
chr1 182615626
chr1 182615675
chr1 182615684
chr1 182615693
chr1 182615812
chr1 182615815
chr1 182615858
chr1 182615868
chr1 182615900
chr1 182616143
chr1 182616236
chr1 182616258
chr1 182616270
chr1 182616281
chr1 182616287
chr1 182616297
chr1 182616299
chr1 182616311
chr1 182616518

chr1 182616545
chr1 182789117
chr1 182841419
chr1 182841469
chr1 182841507
chr1 182841536
chr1 182841576
chr1 182841611
chr1 182841644
chr1 182841651
chr1 182841662
chr1 182841911
chr1 182842089
chr1 182842216
chr1 182842575
chr1 182942163
chr1 182942213
chr1 182942280
chr1 182942335
chr1 182942427
chr1 182942437
chr1 182942442
chr1 182942447
chr1 182942449
chr1 182942452
chr1 182942462
chr1 182942464
chr1 182942483
chr1 182942498
chr1 182942501
chr1 182942505
chr1 183019504
chr1 183019845
chr1 183019964
chr1 183024317
chr1 183024336
chr1 183024340
chr1 183024375
chr1 183024407
chr1 183024447
chr1 183033319
chr1 183033413
chr1 183219162
chr1 183219452
chr1 183219515
chr1 183469878
chr1 183470081
chr1 183632571
chr1 183632866
chr1 183632931
chr1 183633074
chr1 183636426
chr1 183636432
chr1 183636473

chr1 183636478
chr1 183636500
chr1 183636530
chr1 183636536
chr1 183636544
chr1 183685261
chr1 183685337
chr1 183685897
chr1 183685986
chr1 183805379
chr1 183805385
chr1 183805392
chr1 183805409
chr1 183805418
chr1 183805428
chr1 183805463
chr1 183805486
chr1 183805506
chr1 183805606
chr1 183805608
chr1 183805613
chr1 183805621
chr1 183805629
chr1 183805683
chr1 183824907
chr1 183915629
chr1 183915732
chr1 183915815
chr1 183915982
chr1 183916043
chr1 183916076
chr1 183916101
chr1 184051288
chr1 184051310
chr1 184052133
chr1 184052147
chr1 184052164
chr1 184052226
chr1 184052253
chr1 184052303
chr1 184052379
chr1 184052427
chr1 184052454
chr1 184052513
chr1 184052583
chr1 184152451
chr1 184152512
chr1 184152586
chr1 184387676
chr1 184387719
chr1 184387729
chr1 184387773
chr1 184387848
chr1 184387853

chr1 184387890
chr1 184387926
chr1 184387931
chr1 184387953
chr1 184387969
chr1 184387979
chr1 184388012
chr1 184388045
chr1 184388047
chr1 184388055
chr1 184388058
chr1 184388061
chr1 184388063
chr1 184388126
chr1 184388217
chr1 184388258
chr1 184388331
chr1 184388349
chr1 184388394
chr1 184388422
chr1 184388469
chr1 184388556
chr1 184388660
chr1 184662854
chr1 184663183
chr1 184744961
chr1 184753309
chr1 184753355
chr1 184753424
chr1 184753437
chr1 184753626
chr1 184753686
chr1 184753707
chr1 184753772
chr1 184753901
chr1 184754034
chr1 184754270
chr1 184754273
chr1 184755811
chr1 184755846
chr1 184755899
chr1 184755915
chr1 184755949
chr1 184755969
chr1 184755972
chr1 184755987
chr1 184843884
chr1 184843922
chr1 184843937
chr1 184843996
chr1 184844051
chr1 184844110
chr1 184844548
chr1 184844606

chr1 184891093
chr1 184891101
chr1 184891174
chr1 184891265
chr1 184972983
chr1 184973117
chr1 184973243
chr1 184973255
chr1 184973283
chr1 184973417
chr1 184973435
chr1 184973438
chr1 184973443
chr1 184973488
chr1 184973538
chr1 184973561
chr1 184973589
chr1 184973625
chr1 185045934
chr1 185045937
chr1 185045939
chr1 185046531
chr1 185046584
chr1 185046593
chr1 185046661
chr1 185046702
chr1 185046751
chr1 185046768
chr1 185046776
chr1 185046780
chr1 185155896
chr1 185155908
chr1 185155948
chr1 185155965
chr1 185156014
chr1 185156075
chr1 185156091
chr1 185156144
chr1 185156429
chr1 185157995
chr1 185158030
chr1 185158088
chr1 185158090
chr1 185158092
chr1 185158132
chr1 185158177
chr1 185158364
chr1 185158369
chr1 185158529
chr1 185315523
chr1 185315575
chr1 185315625
chr1 185315858
chr1 185315870

chr1 185315898
chr1 185315913
chr1 185315931
chr1 185339120
chr1 185567564
chr1 185567706
chr1 185639696
chr1 185639705
chr1 185639998
chr1 185640158
chr1 185640811
chr1 186288962
chr1 186289072
chr1 186289686
chr1 186289837
chr1 186373653
chr1 186373798
chr1 186376479
chr1 186376502
chr1 186376590
chr1 186376658
chr1 186596130
chr1 186596208
chr1 186596351
chr1 186596596
chr1 186596716
chr1 186615044
chr1 186615384
chr1 186615481
chr1 186618302
chr1 186618317
chr1 186618461
chr1 186618539
chr1 186618552
chr1 186618559
chr1 186618613
chr1 186618653
chr1 186618668
chr1 186618779
chr1 186618880
chr1 186618884
chr1 186626528
chr1 186626556
chr1 186626581
chr1 186627250
chr1 186627325
chr1 186673050
chr1 186680745
chr1 186680749
chr1 186680859
chr1 186680919
chr1 186680930
chr1 186680991
chr1 186680994

chr1 186681151
chr1 186681189
chr1 186681260
chr1 186681306
chr1 186681309
chr1 186681331
chr1 186681347
chr1 186681378
chr1 186681431
chr1 186681563
chr1 186682847
chr1 186693621
chr1 186828686
chr1 186828688
chr1 186828690
chr1 186828694
chr1 186828868
chr1 186828915
chr1 186828975
chr1 186829142
chr1 186829156
chr1 186829235
chr1 186829327
chr1 186829330
chr1 186829341
chr1 186829351
chr1 186829358
chr1 186829429
chr1 186829439
chr1 186829506
chr1 186829542
chr1 186829578
chr1 186838800
chr1 186839017
chr1 186839190
chr1 186960423
chr1 186960441
chr1 186960458
chr1 186960491
chr1 186960580
chr1 186981849
chr1 187051935
chr1 187052157
chr1 187109417
chr1 187109953
chr1 187110790
chr1 187111471
chr1 187297374
chr1 187297903
chr1 187614306
chr1 187614337
chr1 187614642
chr1 187649472
chr1 187833947

chr1 187834239
chr1 187834334
chr1 187849043
chr1 188417041
chr1 188417067
chr1 188525781
chr1 188526020
chr1 188526319
chr1 188653873
chr1 188653932
chr1 188756137
chr1 188756553
chr1 188756969
chr1 188757056
chr1 188757065
chr1 188757265
chr1 188757284
chr1 188759932
chr1 188965680
chr1 188965793
chr1 189003560
chr1 189003971
chr1 189004778
chr1 189006097
chr1 189006303
chr1 189046057
chr1 189046260
chr1 189138274
chr1 189138940
chr1 189532315
chr1 189533076
chr1 189533211
chr1 189534211
chr1 189534361
chr1 189534436
chr1 189630371
chr1 189630825
chr1 189699097
chr1 189699157
chr1 189699416
chr1 189699861
chr1 189940219
chr1 189965760
chr1 189965859
chr1 189995605
chr1 190144890
chr1 190145919
chr1 190146410
chr1 190268173
chr1 190268624
chr1 190476132
chr1 190476206
chr1 190476208
chr1 190476224

chr1 190476278
chr1 190476360
chr1 190476367
chr1 190476408
chr1 190476440
chr1 190476510
chr1 190476829
chr1 190477163
chr1 190477174
chr1 190477626
chr1 190477699
chr1 190477844
chr1 190477957
chr1 190478007
chr1 190478102
chr1 190478156
chr1 190478160
chr1 190478171
chr1 190478189
chr1 190478198
chr1 190478203
chr1 190478222
chr1 190478226
chr1 190478233
chr1 190478249
chr1 190478259
chr1 190478264
chr1 190478271
chr1 190478277
chr1 190478283
chr1 190478291
chr1 190478313
chr1 190478321
chr1 190478339
chr1 190478342
chr1 190478779
chr1 190478875
chr1 190478903
chr1 190478922
chr1 190478952
chr1 190478975
chr1 190478996
chr1 190479003
chr1 190479021
chr1 190479091
chr1 190479161
chr1 190479240
chr1 190479339
chr1 190479582
chr1 190479628
chr1 190479749
chr1 190479799
chr1 190590150
chr1 190590157

chr1 190590785
chr1 191153236
chr1 191153713
chr1 191350894
chr1 191351325
chr1 191351512
chr1 191351940
chr1 191424077
chr1 191597333
chr1 191733349
chr1 191790464
chr1 191790612
chr1 191790728
chr1 191790850
chr1 191892576
chr1 191892676
chr1 191893228
chr1 191893528
chr1 191893620
chr1 191894023
chr1 191894199
chr1 191894601
chr1 191895051
chr1 191895096
chr1 192040870
chr1 192158493
chr1 192159027
chr1 192159052
chr1 192159123
chr1 192159284
chr1 192159500
chr1 192159519
chr1 192159689
chr1 192159822
chr1 192208707
chr1 192478221
chr1 192478341
chr1 192478458
chr1 192516812
chr1 192516856
chr1 192516928
chr1 192516951
chr1 192516995
chr1 192517069
chr1 192517087
chr1 192517168
chr1 192517205
chr1 192517218
chr1 192517241
chr1 192807028
chr1 192807044
chr1 192807048
chr1 192807124
chr1 192807370

chr1 192807463
chr1 192807487
chr1 192807626
chr1 192807729
chr1 192807771
chr1 192807814
chr1 192807853
chr1 192807907
chr1 192808218
chr1 192808325
chr1 192808374
chr1 192808417
chr1 192808421
chr1 192808463
chr1 192808503
chr1 192808505
chr1 192808518
chr1 192808520
chr1 193004363
chr1 193060261
chr1 193060266
chr1 193060298
chr1 193060452
chr1 193060627
chr1 193060688
chr1 193060769
chr1 193105045
chr1 193105047
chr1 193105079
chr1 193105081
chr1 193105109
chr1 193105113
chr1 193105121
chr1 193122566
chr1 193122620
chr1 193122638
chr1 193122678
chr1 193122688
chr1 193122694
chr1 193122753
chr1 193122961
chr1 193122973
chr1 193123061
chr1 193123115
chr1 193123191
chr1 193123266
chr1 193240573
chr1 193240902
chr1 193406889
chr1 193406905
chr1 193406955
chr1 193406989
chr1 193504522
chr1 193537696

chr1 193537744
chr1 193592940
chr1 193593077
chr1 193865711
chr1 194429797
chr1 194429852
chr1 194535683
chr1 194535782
chr1 194536159
chr1 194659070
chr1 194659202
chr1 194672008
chr1 194871099
chr1 194871125
chr1 195195524
chr1 195195568
chr1 195195611
chr1 195195675
chr1 195195703
chr1 195195795
chr1 195195813
chr1 195196159
chr1 195216584
chr1 195216876
chr1 195217529
chr1 195217624
chr1 195217700
chr1 195760516
chr1 196147177
chr1 196147499
chr1 196147568
chr1 196147571
chr1 196569804
chr1 196608118
chr1 196608149
chr1 196608164
chr1 196608219
chr1 196608222
chr1 196608257
chr1 196608291
chr1 196608640
chr1 196608659
chr1 196608662
chr1 196608671
chr1 196608673
chr1 196608675
chr1 196651502
chr1 196651740
chr1 196651833
chr1 196651873
chr1 196651990
chr1 196652069
chr1 196653494
chr1 196653806

chr1 196669280
chr1 196669430
chr1 196784281
chr1 196784899
chr1 196805983
chr1 196806094
chr1 196806185
chr1 196806211
chr1 196878029
chr1 196878042
chr1 196878047
chr1 196878052
chr1 196898116
chr1 196898291
chr1 196898756
chr1 197144698
chr1 197144799
chr1 197145066
chr1 197145193
chr1 197145200
chr1 197145445
chr1 197145527
chr1 197145549
chr1 197200570
chr1 197200611
chr1 197200652
chr1 197200665
chr1 197201070
chr1 197201101
chr1 197201134
chr1 197201143
chr1 197201152
chr1 197201162
chr1 197201165
chr1 197201171
chr1 197201904
chr1 197201933
chr1 197202034
chr1 197202040
chr1 197202052
chr1 197202067
chr1 197202152
chr1 197202192
chr1 197202233
chr1 197202273
chr1 197357434
chr1 197408340
chr1 197408481
chr1 197408887
chr1 197409008
chr1 197409219
chr1 197409460
chr1 197409581
chr1 197448602

chr1 197773480
chr1 197773796
chr1 197773836
chr1 197773929
chr1 197774062
chr1 197774298
chr1 197774375
chr1 197774465
chr1 197774509
chr1 197774559
chr1 197774667
chr1 197774684
chr1 197775895
chr1 197776029
chr1 197776108
chr1 197776136
chr1 197776165
chr1 197776797
chr1 197776868
chr1 197776891
chr1 197777112
chr1 197905087
chr1 197905339
chr1 197907886
chr1 197908112
chr1 197908229
chr1 197908656
chr1 197908704
chr1 197908795
chr1 197908817
chr1 197908866
chr1 197908886
chr1 197908890
chr1 197908903
chr1 197908957
chr1 197908970
chr1 197908973
chr1 197909007
chr1 197909065
chr1 197909074
chr1 197909089
chr1 197909095
chr1 197914678
chr1 197914686
chr1 197914758
chr1 197915004
chr1 197929016
chr1 197929020
chr1 197929047
chr1 197929079
chr1 197929084
chr1 197929087
chr1 197929093
chr1 197929099

chr1 197929115
chr1 197929133
chr1 197929141
chr1 197929190
chr1 197929214
chr1 197929220
chr1 197929584
chr1 197929728
chr1 197929997
chr1 197930052
chr1 197930473
chr1 197930624
chr1 198082340
chr1 198134344
chr1 198134466
chr1 198143324
chr1 198143969
chr1 198156378
chr1 198156429
chr1 198156505
chr1 198157857
chr1 198157872
chr1 198157883
chr1 198157982
chr1 198157990
chr1 198158010
chr1 198158093
chr1 198158126
chr1 198158137
chr1 198158139
chr1 198158155
chr1 198158242
chr1 198158451
chr1 198158554
chr1 198158606
chr1 198158623
chr1 198158690
chr1 198158801
chr1 198158804
chr1 198158842
chr1 198158862
chr1 198158866
chr1 198158923
chr1 198158971
chr1 198158993
chr1 198159018
chr1 198159024
chr1 198159221
chr1 198159231
chr1 198159233
chr1 198159260
chr1 198159269
chr1 198159365
chr1 198159370

chr1 198159428
chr1 198159470
chr1 198159527
chr1 198159534
chr1 198159610
chr1 198159722
chr1 198519431
chr1 198519485
chr1 198520348
chr1 198520361
chr1 198520549
chr1 198520581
chr1 198619880
chr1 198619944
chr1 198619969
chr1 198620419
chr1 198620446
chr1 198620524
chr1 198620816
chr1 198621041
chr1 198621052
chr1 198621070
chr1 198621146
chr1 198621535
chr1 198621596
chr1 198621608
chr1 198621663
chr1 198621699
chr1 198622394
chr1 198622583
chr1 198623107
chr1 198637545
chr1 198637767
chr1 198638418
chr1 198638692
chr1 198639033
chr1 198639114
chr1 198639174
chr1 198639231
chr1 198639253
chr1 198639543
chr1 198639994
chr1 198640171
chr1 198640174
chr1 198640372
chr1 198641355
chr1 198641521
chr1 198641549
chr1 198642115
chr1 198642425
chr1 198642448
chr1 198642478
chr1 198642544
chr1 198643445

chr1 198643599
chr1 198643779
chr1 198643908
chr1 198644809
chr1 198645131
chr1 198646021
chr1 198646461
chr1 198646549
chr1 198646662
chr1 198647371
chr1 198648407
chr1 198648453
chr1 198649576
chr1 198649659
chr1 198649713
chr1 198650099
chr1 198654002
chr1 198654066
chr1 198654451
chr1 198655168
chr1 198655361
chr1 198655999
chr1 198656719
chr1 198656730
chr1 198656744
chr1 198656754
chr1 198656960
chr1 198657311
chr1 198657604
chr1 198657757
chr1 198657947
chr1 198658191
chr1 198658285
chr1 198658480
chr1 198658497
chr1 198658623
chr1 198663731
chr1 198664195
chr1 198664337
chr1 198664347
chr1 198664368
chr1 198664417
chr1 198664637
chr1 198676750
chr1 198677138
chr1 198677835
chr1 198677901
chr1 198678017
chr1 198678347
chr1 198678498
chr1 198678581
chr1 198678592
chr1 198678618
chr1 198678669

chr1 198678688
chr1 198678693
chr1 198679044
chr1 198679091
chr1 198679195
chr1 198679217
chr1 198679223
chr1 198679229
chr1 198679256
chr1 198679272
chr1 198679301
chr1 198680367
chr1 198680369
chr1 198680377
chr1 198680384
chr1 198680414
chr1 198680548
chr1 198680630
chr1 198680796
chr1 198680936
chr1 198681059
chr1 198681198
chr1 198681479
chr1 198681901
chr1 198681931
chr1 198681985
chr1 198682001
chr1 198682517
chr1 198683114
chr1 198683240
chr1 198683345
chr1 198683650
chr1 198683686
chr1 198683941
chr1 198684106
chr1 198684227
chr1 198684602
chr1 198936472
chr1 198936664
chr1 198936943
chr1 198936987
chr1 198936998
chr1 198937205
chr1 198937236
chr1 198937263
chr1 199041592
chr1 199041682
chr1 199041735
chr1 199843440
chr1 199843452
chr1 199843654
chr1 199843676
chr1 199843951
chr1 199844108

chr1 199941419
chr1 199941592
chr1 199941666
chr1 200028764
chr1 200028974
chr1 200029087
chr1 200029098
chr1 200029101
chr1 200029111
chr1 200029114
chr1 200029116
chr1 200029121
chr1 200043289
chr1 200043377
chr1 200043398
chr1 200043518
chr1 200043832
chr1 200146420
chr1 200226285
chr1 200226384
chr1 200226408
chr1 200226469
chr1 200226490
chr1 200226523
chr1 200226590
chr1 200226690
chr1 200226732
chr1 200302332
chr1 200302340
chr1 200302342
chr1 200302344
chr1 200302350
chr1 200302359
chr1 200302364
chr1 200302380
chr1 200302385
chr1 200302391
chr1 200302395
chr1 200302401
chr1 200302409
chr1 200307450
chr1 200352305
chr1 200354145
chr1 200354419
chr1 200354442
chr1 200409505
chr1 200409508
chr1 200409512
chr1 200409529
chr1 200409539
chr1 200409597
chr1 200409606
chr1 200409609
chr1 200409612

chr1 200409615
chr1 200409618
chr1 200409621
chr1 200409633
chr1 200409636
chr1 200409639
chr1 200409645
chr1 200409654
chr1 200409663
chr1 200409672
chr1 200409675
chr1 200409696
chr1 200409710
chr1 200409863
chr1 200409869
chr1 200409875
chr1 200409880
chr1 200409892
chr1 200409910
chr1 200409933
chr1 200409945
chr1 200409952
chr1 200409955
chr1 200409958
chr1 200409961
chr1 200409967
chr1 200409970
chr1 200409973
chr1 200409995
chr1 200409998
chr1 200410029
chr1 200410038
chr1 200410044
chr1 200410046
chr1 200410190
chr1 200410223
chr1 200410228
chr1 200410236
chr1 200410330
chr1 200410348
chr1 200410353
chr1 200410590
chr1 200410631
chr1 200410646
chr1 200410728
chr1 200410732
chr1 200410763
chr1 200410810
chr1 200410844
chr1 200410931
chr1 200437909
chr1 200628217
chr1 200628385
chr1 200628413

chr1 200628446
chr1 200628467
chr1 200670583
chr1 200670656
chr1 200751951
chr1 200862260
chr1 200862834
chr1 200862886
chr1 200862951
chr1 200862957
chr1 200862959
chr1 200863094
chr1 200905719
chr1 200906054
chr1 201019636
chr1 201019822
chr1 201020107
chr1 201119632
chr1 201829689
chr1 201829693
chr1 201829730
chr1 201829810
chr1 201829818
chr1 201829897
chr1 201829926
chr1 201829956
chr1 201830049
chr1 201830101
chr1 201830125
chr1 201830127
chr1 201830141
chr1 201955348
chr1 201955351
chr1 201955353
chr1 201955365
chr1 201955373
chr1 201955410
chr1 201955444
chr1 201955919
chr1 201955924
chr1 201955942
chr1 201956264
chr1 201956347
chr1 202145251
chr1 202145257
chr1 202145303
chr1 202145340
chr1 202145344
chr1 202145359
chr1 202145428
chr1 202159640
chr1 202159653
chr1 202159814
chr1 202159830

chr1 202159925
chr1 202160057
chr1 202160064
chr1 202160076
chr1 202160109
chr1 202160115
chr1 202160127
chr1 202160220
chr1 202160228
chr1 202160259
chr1 202160307
chr1 202160385
chr1 202160438
chr1 202160447
chr1 202160455
chr1 202160489
chr1 202160496
chr1 202160505
chr1 202160597
chr1 202160648
chr1 202160670
chr1 202162405
chr1 202162643
chr1 202162649
chr1 202162693
chr1 202162733
chr1 202162756
chr1 202162833
chr1 202162838
chr1 202162880
chr1 202162943
chr1 202163066
chr1 202312424
chr1 202312436
chr1 202315960
chr1 202316110
chr1 202316117
chr1 202316139
chr1 202316144
chr1 202341253
chr1 202341258
chr1 202341300
chr1 202341304
chr1 202341306
chr1 202341316
chr1 202341344
chr1 202341349
chr1 202341353
chr1 202341360
chr1 202341394
chr1 202341405
chr1 202341434
chr1 202341438
chr1 202341446

chr1 202341450
chr1 202341494
chr1 202341502
chr1 202341509
chr1 202341526
chr1 202341534
chr1 202341556
chr1 202341564
chr1 202341572
chr1 202341626
chr1 202341691
chr1 202341696
chr1 202341713
chr1 202341731
chr1 202341744
chr1 202341786
chr1 202341805
chr1 202341874
chr1 202341916
chr1 202341924
chr1 202341926
chr1 202341928
chr1 202341936
chr1 202341947
chr1 202343774
chr1 202348642
chr1 202348652
chr1 202348658
chr1 202348682
chr1 202349480
chr1 202349482
chr1 202349548
chr1 202349692
chr1 202349702
chr1 202644099
chr1 202644108
chr1 202644116
chr1 202644118
chr1 202644178
chr1 202644205
chr1 202958603
chr1 202958606
chr1 202958670
chr1 202958709
chr1 202958849
chr1 202958891
chr1 202958893
chr1 202967341
chr1 202967351
chr1 202967380
chr1 202967400
chr1 202967403
chr1 202967406
chr1 202967422

chr1 202967429
chr1 202967438
chr1 202967456
chr1 202967458
chr1 202967474
chr1 202967481
chr1 202967498
chr1 202967535
chr1 202967543
chr1 202967551
chr1 202967573
chr1 202967575
chr1 202967581
chr1 202967597
chr1 202967629
chr1 202999417
chr1 202999509
chr1 202999539
chr1 202999557
chr1 203006570
chr1 203006582
chr1 203006630
chr1 203006646
chr1 203006670
chr1 203006693
chr1 203006700
chr1 203006719
chr1 203006732
chr1 203006752
chr1 203006756
chr1 203006764
chr1 203006768
chr1 203006797
chr1 203006808
chr1 203006828
chr1 203006842
chr1 203006849
chr1 203006853
chr1 203006858
chr1 203006886
chr1 203006896
chr1 203006898
chr1 203006902
chr1 203006906
chr1 203007035
chr1 203007124
chr1 203007201
chr1 203007208
chr1 203007211
chr1 203007216
chr1 203007218
chr1 203007259
chr1 203007275
chr1 203007277

chr1 203007284
chr1 203136116
chr1 203136128
chr1 203136192
chr1 203136226
chr1 203136277
chr1 203269247
chr1 203289707
chr1 203289755
chr1 203289775
chr1 203289780
chr1 203289851
chr1 203289910
chr1 203289976
chr1 203289996
chr1 203290077
chr1 203290272
chr1 203290322
chr1 203290400
chr1 203290416
chr1 203306468
chr1 203306473
chr1 203306799
chr1 203307666
chr1 203307748
chr1 203307816
chr1 203321011
chr1 203321158
chr1 203321244
chr1 203328183
chr1 203328227
chr1 203328232
chr1 203627729
chr1 203627812
chr1 203627928
chr1 203627933
chr1 203627957
chr1 203627985
chr1 203627994
chr1 203628053
chr1 203628059
chr1 203628136
chr1 203628141
chr1 203628234
chr1 203628269
chr1 203628274
chr1 203628316
chr1 203628319
chr1 203628362
chr1 203628403
chr1 203628425
chr1 203628448
chr1 203628456
chr1 203628459

chr1 203628470
chr1 203628578
chr1 203628668
chr1 203628670
chr1 203628694
chr1 203628784
chr1 203628931
chr1 203629059
chr1 203629098
chr1 203629121
chr1 203629168
chr1 203629202
chr1 203629259
chr1 203629299
chr1 203629344
chr1 203629353
chr1 203629356
chr1 203629362
chr1 203629365
chr1 203629367
chr1 203629378
chr1 203629411
chr1 203629475
chr1 203629483
chr1 203629493
chr1 203629495
chr1 203629500
chr1 203629505
chr1 203629515
chr1 203629521
chr1 203629523
chr1 203629590
chr1 203629600
chr1 203629616
chr1 203629618
chr1 203629627
chr1 203629633
chr1 203629638
chr1 203629650
chr1 203629653
chr1 203629671
chr1 203629708
chr1 203629713
chr1 203629826
chr1 203650381
chr1 203650424
chr1 203650436
chr1 203650454
chr1 203650474
chr1 203650479
chr1 203650526
chr1 203650549
chr1 203650554
chr1 203650556

chr1 203652815
chr1 203652995
chr1 203653321
chr1 203653342
chr1 203744156
chr1 203744244
chr1 203744464
chr1 203764786
chr1 203764843
chr1 203765039
chr1 203765128
chr1 203765134
chr1 203765209
chr1 203765220
chr1 203765268
chr1 203765283
chr1 203765340
chr1 203765350
chr1 203765377
chr1 203765431
chr1 203765448
chr1 203765467
chr1 203765591
chr1 203765641
chr1 203765767
chr1 203796588
chr1 204031567
chr1 204031571
chr1 204031714
chr1 204031928
chr1 204032217
chr1 204207256
chr1 204207258
chr1 204287187
chr1 204287256
chr1 204287313
chr1 204363586
chr1 204363589
chr1 204363592
chr1 204363599
chr1 204363607
chr1 204363609
chr1 204363630
chr1 204405045
chr1 204405335
chr1 204406210
chr1 204406891
chr1 204407538
chr1 204407666
chr1 204407777
chr1 204407991
chr1 204513369
chr1 204513375
chr1 204513383

chr1 204513393
chr1 204513395
chr1 204516572
chr1 204516576
chr1 204516581
chr1 204516592
chr1 204516599
chr1 204516603
chr1 204516626
chr1 204516630
chr1 204516633
chr1 204516645
chr1 204516647
chr1 204516654
chr1 204516667
chr1 204516700
chr1 204516702
chr1 204516729
chr1 204516768
chr1 204516771
chr1 204516853
chr1 204516871
chr1 204516906
chr1 204516965
chr1 204516967
chr1 204516977
chr1 204517005
chr1 204517038
chr1 204517069
chr1 204517111
chr1 204517135
chr1 204517167
chr1 204517173
chr1 204517190
chr1 204517196
chr1 204517198
chr1 204517236
chr1 204517266
chr1 204517301
chr1 204763671
chr1 204763822
chr1 204763875
chr1 204763922
chr1 204763929
chr1 204763935
chr1 204763944
chr1 204783368
chr1 204783428
chr1 204783472
chr1 205121242
chr1 205121262
chr1 205121291
chr1 205211187
chr1 205288394

chr1 205288686
chr1 205288697
chr1 205319533
chr1 205319562
chr1 205319770
chr1 205396914
chr1 205441187
chr1 205441204
chr1 205630146
chr1 205630537
chr1 205630554
chr1 205630585
chr1 205630707
chr1 205632513
chr1 205632534
chr1 205632546
chr1 205632562
chr1 205747752
chr1 205747954
chr1 205748259
chr1 205748444
chr1 205748490
chr1 205813279
chr1 205813291
chr1 205813296
chr1 205813310
chr1 205813315
chr1 205813328
chr1 205813333
chr1 205813336
chr1 205813344
chr1 206050786
chr1 206121518
chr1 206121725
chr1 206202402
chr1 206202406
chr1 206202467
chr1 206202472
chr1 206205691
chr1 206205695
chr1 206205709
chr1 206205727
chr1 206205733
chr1 206205755
chr1 206205758
chr1 206205761
chr1 206205767
chr1 206205776
chr1 206205783
chr1 206205797
chr1 206205800
chr1 206205806
chr1 206205819
chr1 206205826

chr1 206205828
chr1 206205835
chr1 206205840
chr1 206205853
chr1 206206021
chr1 206471080
chr1 206471083
chr1 206471148
chr1 206471169
chr1 206471200
chr1 206471419
chr1 206471458
chr1 206471482
chr1 206472557
chr1 206545580
chr1 206559387
chr1 206559743
chr1 206559761
chr1 206559811
chr1 206559910
chr1 206559912
chr1 206562853
chr1 206562876
chr1 206562900
chr1 206562908
chr1 206563135
chr1 206563163
chr1 206563188
chr1 206563308
chr1 206563316
chr1 206563470
chr1 206575398
chr1 206575445
chr1 206575452
chr1 206575523
chr1 206575528
chr1 206575700
chr1 206575789
chr1 206575854
chr1 206575861
chr1 206590202
chr1 206590351
chr1 206611946
chr1 206612062
chr1 206612179
chr1 206612190
chr1 206612199
chr1 206612212
chr1 206612229
chr1 206612256
chr1 206612292
chr1 206612307
chr1 206612323
chr1 206612398

chr1 206612404
chr1 206612428
chr1 206612452
chr1 206612456
chr1 206612458
chr1 206612469
chr1 206612486
chr1 206612488
chr1 206612496
chr1 206686413
chr1 206686467
chr1 206686484
chr1 206686555
chr1 206686652
chr1 206757061
chr1 206757586
chr1 206757599
chr1 206757614
chr1 206757714
chr1 206757733
chr1 206757771
chr1 206757888
chr1 206757899
chr1 206757946
chr1 206758026
chr1 206758059
chr1 206765763
chr1 206765924
chr1 206766427
chr1 206766468
chr1 206766511
chr1 206784492
chr1 206784504
chr1 206784529
chr1 206784566
chr1 206784663
chr1 206784669
chr1 206784672
chr1 206784702
chr1 206864738
chr1 206922206
chr1 206922233
chr1 206922323
chr1 206922489
chr1 206922894
chr1 206923013
chr1 207050061
chr1 207050082
chr1 207050241
chr1 207050264
chr1 207050275
chr1 207050334
chr1 207050434
chr1 207050445

chr1 207054781
chr1 207320868
chr1 207320873
chr1 207320878
chr1 207320880
chr1 207454027
chr1 207454116
chr1 207454142
chr1 207454147
chr1 207454187
chr1 207454196
chr1 207454201
chr1 207454206
chr1 207454215
chr1 207454233
chr1 207454236
chr1 207454239
chr1 207454241
chr1 207454256
chr1 207454311
chr1 207454366
chr1 207454370
chr1 207454374
chr1 207454376
chr1 207454383
chr1 207454388
chr1 207454395
chr1 207454398
chr1 207527443
chr1 207527566
chr1 207527621
chr1 207529675
chr1 207530095
chr1 207532602
chr1 207533034
chr1 207538264
chr1 207643566
chr1 207644285
chr1 207752760
chr1 207752762
chr1 207752779
chr1 207825971
chr1 207825976
chr1 207825982
chr1 207826034
chr1 207826051
chr1 207861740
chr1 207861941
chr1 207862336
chr1 207868042
chr1 207868105
chr1 207868229
chr1 207868273
chr1 207868315

chr1 207868322
chr1 207868332
chr1 207868334
chr1 207868344
chr1 207868348
chr1 207868402
chr1 207868477
chr1 207868518
chr1 207868830
chr1 207868832
chr1 207868836
chr1 207868848
chr1 207868850
chr1 207868853
chr1 207868865
chr1 207868876
chr1 207868878
chr1 207868887
chr1 207873151
chr1 207873185
chr1 207873194
chr1 207873363
chr1 207873410
chr1 207873603
chr1 207873634
chr1 207883148
chr1 207883165
chr1 207883309
chr1 207883429
chr1 207883568
chr1 207883865
chr1 207883879
chr1 208086629
chr1 208086638
chr1 208086945
chr1 208087068
chr1 208087094
chr1 208244801
chr1 208245079
chr1 208245103
chr1 208245207
chr1 208245230
chr1 208490422
chr1 208652535
chr1 208652573
chr1 208652807
chr1 208653134
chr1 209127090
chr1 209171345
chr1 209171683
chr1 209171782
chr1 209191453
chr1 209191959
chr1 209192005

chr1 209660144
chr1 209660184
chr1 209660269
chr1 209755550
chr1 209755953
chr1 209755984
chr1 209756673
chr1 209756782
chr1 209757033
chr1 209768286
chr1 209768301
chr1 209768435
chr1 209768468
chr1 209768498
chr1 209768503
chr1 209768623
chr1 209768652
chr1 209768675
chr1 209768721
chr1 209768728
chr1 209768847
chr1 209768868
chr1 209768880
chr1 209768987
chr1 209768997
chr1 209769245
chr1 209769334
chr1 209769381
chr1 209769391
chr1 209769458
chr1 209769781
chr1 209769786
chr1 209769824
chr1 209769872
chr1 209769920
chr1 209770069
chr1 209770094
chr1 209770111
chr1 209784792
chr1 209784814
chr1 209784817
chr1 209784821
chr1 209784840
chr1 209784924
chr1 209784937
chr1 209784998
chr1 209827737
chr1 209827785
chr1 209827792
chr1 209827799
chr1 209827818
chr1 209827847
chr1 209827855
chr1 209827873

chr1 209827889
chr1 209827896
chr1 209827914
chr1 209827918
chr1 209827922
chr1 209827924
chr1 209827927
chr1 209827934
chr1 209827961
chr1 209827964
chr1 209827971
chr1 209827980
chr1 209827988
chr1 209860493
chr1 209860533
chr1 209860540
chr1 209860727
chr1 210051059
chr1 210051181
chr1 210051268
chr1 210051401
chr1 210051799
chr1 210231709
chr1 210231762
chr1 210231867
chr1 210231929
chr1 210232185
chr1 210232189
chr1 210232207
chr1 210232211
chr1 210232217
chr1 210232221
chr1 210232224
chr1 210232273
chr1 210232346
chr1 210232398
chr1 210232426
chr1 210232455
chr1 210232463
chr1 210232469
chr1 210232471
chr1 210232487
chr1 210232504
chr1 210232506
chr1 210232514
chr1 210232521
chr1 210232555
chr1 210251167
chr1 210310627
chr1 210310777
chr1 210310898
chr1 210311140
chr1 210311145
chr1 210311149

chr1 210311151
chr1 210311153
chr1 210311157
chr1 210311205
chr1 210311212
chr1 210311235
chr1 210311396
chr1 210311432
chr1 210311443
chr1 210311449
chr1 210311455
chr1 210311459
chr1 210311475
chr1 210311485
chr1 210311502
chr1 210311514
chr1 210311529
chr1 210311559
chr1 210311575
chr1 210311582
chr1 210311588
chr1 210332944
chr1 210333037
chr1 210333065
chr1 210333104
chr1 210360908
chr1 210361029
chr1 210374213
chr1 210374233
chr1 210374246
chr1 210374305
chr1 210374319
chr1 210374322
chr1 210374339
chr1 210684733
chr1 210994656
chr1 210994714
chr1 211229233
chr1 211229263
chr1 211229273
chr1 211229344
chr1 211229410
chr1 211229469
chr1 211229502
chr1 211229524
chr1 211229596
chr1 211260330
chr1 211323469
chr1 211323584
chr1 211323639
chr1 211323642
chr1 211513708
chr1 211513813
chr1 211513969

chr1 211577341
chr1 211577474
chr1 211577497
chr1 211577562
chr1 211577580
chr1 211577625
chr1 211675170
chr1 211675204
chr1 211675262
chr1 211675275
chr1 211675300
chr1 211675310
chr1 211675316
chr1 211675323
chr1 211675327
chr1 211675332
chr1 211675337
chr1 211675340
chr1 211675361
chr1 211675377
chr1 211675403
chr1 211675743
chr1 211675745
chr1 211675748
chr1 211675751
chr1 211675778
chr1 211675781
chr1 211680931
chr1 211680933
chr1 211680947
chr1 211681076
chr1 211681199
chr1 211681255
chr1 211831066
chr1 211831070
chr1 211831126
chr1 211831161
chr1 211831235
chr1 211831300
chr1 211831306
chr1 211831426
chr1 211831430
chr1 211831440
chr1 211831444
chr1 211831462
chr1 211935702
chr1 212036460
chr1 212036509
chr1 212036561
chr1 212036564
chr1 212036596
chr1 212036606
chr1 212036641
chr1 212036659

chr1 212036684
chr1 212036821
chr1 212036867
chr1 212036897
chr1 212037098
chr1 212037278
chr1 212040953
chr1 212040961
chr1 212186805
chr1 212225509
chr1 212287321
chr1 212287615
chr1 212287745
chr1 212566663
chr1 212566698
chr1 212566753
chr1 212566755
chr1 212566879
chr1 212566883
chr1 212566886
chr1 212566930
chr1 212567022
chr1 212567055
chr1 212567061
chr1 212567118
chr1 212567194
chr1 212567224
chr1 212567342
chr1 212567666
chr1 212596435
chr1 212596531
chr1 212753250
chr1 212753254
chr1 212781268
chr1 212781276
chr1 213014820
chr1 213014854
chr1 213014870
chr1 213014875
chr1 213014955
chr1 213015079
chr1 213051347
chr1 213051350
chr1 213051369
chr1 213051373
chr1 213051380
chr1 213051383
chr1 213051386
chr1 213051417
chr1 213051423
chr1 213051431
chr1 213051441
chr1 213051458
chr1 213051468

chr1 213051475
chr1 213051503
chr1 213051507
chr1 213051518
chr1 213051524
chr1 213051607
chr1 213051638
chr1 213051652
chr1 213051716
chr1 213051791
chr1 213051815
chr1 213052004
chr1 213052050
chr1 213374303
chr1 213783636
chr1 213783790
chr1 213784070
chr1 213784089
chr1 213784717
chr1 213986641
chr1 213986759
chr1 213986761
chr1 213986780
chr1 213986793
chr1 213986805
chr1 213986816
chr1 213986827
chr1 213986961
chr1 213988255
chr1 213990659
chr1 213991094
chr1 213991372
chr1 213991408
chr1 214261038
chr1 214261149
chr1 214261168
chr1 214261214
chr1 214261340
chr1 214261345
chr1 214261644
chr1 214261647
chr1 214261667
chr1 214261683
chr1 214261707
chr1 214261711
chr1 214261740
chr1 214261765
chr1 214261771
chr1 214261839
chr1 214428143
chr1 214428199
chr1 214428371
chr1 214428450
chr1 214439293

chr1 214439463
chr1 214439482
chr1 214439513
chr1 214439542
chr1 214439549
chr1 214439592
chr1 214439625
chr1 214439647
chr1 214439658
chr1 214482547
chr1 214482561
chr1 214482642
chr1 214603290
chr1 214603561
chr1 214603661
chr1 214603675
chr1 214603755
chr1 214603758
chr1 214603781
chr1 214603783
chr1 214603816
chr1 214604113
chr1 214604155
chr1 214604231
chr1 214604253
chr1 214604356
chr1 214632290
chr1 214674347
chr1 215006208
chr1 215006459
chr1 215006747
chr1 215083985
chr1 215567890
chr1 215567906
chr1 215567915
chr1 215567918
chr1 215567922
chr1 215567975
chr1 215567988
chr1 215568188
chr1 215568196
chr1 215570376
chr1 215641245
chr1 216103743
chr1 216104018
chr1 216104114
chr1 216118617
chr1 216118981
chr1 216119602
chr1 216119971
chr1 216600445
chr1 216723251
chr1 216723291
chr1 216723303

chr1 216723307
chr1 216723461
chr1 216723510
chr1 216723519
chr1 216723521
chr1 216723527
chr1 216723561
chr1 216723564
chr1 216723576
chr1 216723581
chr1 216723618
chr1 216723660
chr1 216723672
chr1 216723681
chr1 216723700
chr1 216723919
chr1 216737673
chr1 217174436
chr1 217368764
chr1 217368797
chr1 217369083
chr1 217369448
chr1 217369523
chr1 217369562
chr1 217627215
chr1 217627359
chr1 217627555
chr1 217627604
chr1 217627702
chr1 217630494
chr1 217630590
chr1 217630765
chr1 217925567
chr1 217925733
chr1 217925796
chr1 217925861
chr1 218011132
chr1 218016040
chr1 218016066
chr1 218165492
chr1 218165619
chr1 218165626
chr1 218165634
chr1 218165642
chr1 218165645
chr1 218165659
chr1 218165665
chr1 218165678
chr1 218165690
chr1 218165711
chr1 218165729
chr1 218165754
chr1 218165784
chr1 218165819

chr1 218165852
chr1 218165859
chr1 218165929
chr1 218165978
chr1 218166048
chr1 218166218
chr1 218166279
chr1 218166333
chr1 218166431
chr1 218166488
chr1 218166624
chr1 218166724
chr1 218166806
chr1 218166926
chr1 218166952
chr1 218167149
chr1 218167219
chr1 218285299
chr1 218285406
chr1 218285411
chr1 218285414
chr1 218285427
chr1 218285497
chr1 218285521
chr1 218285527
chr1 218285615
chr1 218285632
chr1 218285732
chr1 218285866
chr1 218285879
chr1 218346722
chr1 218346748
chr1 218346881
chr1 218346895
chr1 218346946
chr1 218346951
chr1 218346958
chr1 218346962
chr1 218346968
chr1 218346972
chr1 218346974
chr1 218346983
chr1 218346986
chr1 218346998
chr1 218347027
chr1 218347043
chr1 218347093
chr1 218347108
chr1 218347120
chr1 218347126
chr1 218347139
chr1 218347141
chr1 218347148
chr1 218347152

chr1 218347163
chr1 218347280
chr1 218347295
chr1 218347332
chr1 218347431
chr1 218347438
chr1 218347445
chr1 218347447
chr1 218347450
chr1 218347499
chr1 218347506
chr1 218347617
chr1 218347703
chr1 218347745
chr1 218347778
chr1 218347874
chr1 218347923
chr1 218347930
chr1 218350388
chr1 218351216
chr1 218351228
chr1 218351343
chr1 218351438
chr1 218351572
chr1 218351587
chr1 218351645
chr1 218351730
chr1 218351759
chr1 218351819
chr1 218560755
chr1 218560829
chr1 218635146
chr1 218994522
chr1 219173889
chr1 219173895
chr1 219173901
chr1 219173904
chr1 219173910
chr1 219173921
chr1 219173929
chr1 219173934
chr1 219173937
chr1 219173953
chr1 219173998
chr1 219174018
chr1 219174036
chr1 219174068
chr1 219174071
chr1 219174104
chr1 219174107
chr1 219316791
chr1 219397844
chr1 219455673
chr1 219960057

chr1 219960117
chr1 219960147
chr1 220089243
chr1 220089294
chr1 220089344
chr1 220089502
chr1 220089509
chr1 220089543
chr1 220089563
chr1 220089612
chr1 220089661
chr1 220089665
chr1 220089675
chr1 220089695
chr1 220089729
chr1 220089731
chr1 220089737
chr1 220089754
chr1 220089769
chr1 220089835
chr1 220089845
chr1 220089847
chr1 220089853
chr1 220089863
chr1 220089895
chr1 220089897
chr1 220089916
chr1 220089936
chr1 220089971
chr1 220090103
chr1 220090153
chr1 220090167
chr1 220090178
chr1 220090183
chr1 220090236
chr1 220090320
chr1 220090340
chr1 220090350
chr1 220090356
chr1 220272913
chr1 220272931
chr1 220273040
chr1 220733192
chr1 220733231
chr1 220733252
chr1 220733260
chr1 220733270
chr1 220733283
chr1 220733295
chr1 220733304
chr1 220733336
chr1 220733355
chr1 220733378
chr1 220733414

chr1 220733492
chr1 220733558
chr1 220733632
chr1 220874924
chr1 221221563
chr1 221221786
chr1 221222353
chr1 221222408
chr1 221222416
chr1 221439291
chr1 221439509
chr1 221439622
chr1 221439695
chr1 221439907
chr1 221439994
chr1 221440147
chr1 221440316
chr1 221712928
chr1 221713297
chr1 221713387
chr1 221713431
chr1 221713504
chr1 221753999
chr1 221754350
chr1 221754358
chr1 221754500
chr1 221754504
chr1 221754506
chr1 221754544
chr1 221754549
chr1 221754553
chr1 221754560
chr1 221754594
chr1 221754605
chr1 221754635
chr1 221754639
chr1 221754651
chr1 221754733
chr1 221754765
chr1 221754773
chr1 221765880
chr1 221778323
chr1 221778495
chr1 221778578
chr1 221778660
chr1 222048544
chr1 222465143
chr1 222465152
chr1 222465219
chr1 222465302
chr1 222465334
chr1 222465365
chr1 222465374
chr1 222465522

chr1 222465532
chr1 222465540
chr1 222465550
chr1 222465556
chr1 222465566
chr1 222465571
chr1 222465574
chr1 222465580
chr1 222465586
chr1 222465589
chr1 222465595
chr1 222465602
chr1 222465613
chr1 222465630
chr1 222465654
chr1 222465682
chr1 222465903
chr1 222465913
chr1 222465915
chr1 222465923
chr1 222466006
chr1 222485228
chr1 222485359
chr1 222485804
chr1 222589560
chr1 222589571
chr1 222589602
chr1 222589864
chr1 222589866
chr1 222617608
chr1 222617610
chr1 222617639
chr1 222617641
chr1 222617652
chr1 222617683
chr1 222617703
chr1 222617709
chr1 222617728
chr1 222617774
chr1 222617800
chr1 222617809
chr1 222617828
chr1 222617830
chr1 222617834
chr1 222617837
chr1 222617847
chr1 222643874
chr1 222643893
chr1 222643897
chr1 222644158
chr1 222644182
chr1 222644197
chr1 222644210
chr1 222644218

chr1 222644231
chr1 222644235
chr1 222644246
chr1 222644597
chr1 222644604
chr1 222644630
chr1 222644702
chr1 222644716
chr1 222644766
chr1 222644800
chr1 222644880
chr1 222645010
chr1 222645042
chr1 222645079
chr1 222645112
chr1 222711073
chr1 222711083
chr1 222711089
chr1 222711110
chr1 222711129
chr1 222711192
chr1 222711389
chr1 222711435
chr1 222711450
chr1 222713716
chr1 222713746
chr1 222713807
chr1 222713856
chr1 222713897
chr1 222815509
chr1 222815524
chr1 222815537
chr1 222815634
chr1 222815639
chr1 222815641
chr1 222815643
chr1 222815654
chr1 222815682
chr1 222815686
chr1 222879970
chr1 222880099
chr1 222880348
chr1 222880413
chr1 222880725
chr1 222880740
chr1 223323807
chr1 223367134
chr1 223716032
chr1 223716145
chr1 223716160
chr1 223716189
chr1 223716523
chr1 223716539
chr1 223908373

chr1 223908650
chr1 223908672
chr1 223908713
chr1 223992248
chr1 223992349
chr1 223992362
chr1 223992389
chr1 223992394
chr1 223992398
chr1 223992400
chr1 223992913
chr1 223992966
chr1 223992970
chr1 223992978
chr1 223992980
chr1 223993003
chr1 223993018
chr1 223993022
chr1 223993030
chr1 223993051
chr1 223993055
chr1 223993065
chr1 223993085
chr1 223993095
chr1 223993101
chr1 223993107
chr1 223993118
chr1 223993121
chr1 223993140
chr1 223993198
chr1 223993238
chr1 223993293
chr1 223993301
chr1 223993313
chr1 223993341
chr1 223993357
chr1 224113999
chr1 224114048
chr1 224114051
chr1 224114939
chr1 224114948
chr1 224114952
chr1 224114965
chr1 224114977
chr1 224115041
chr1 224115051
chr1 224115076
chr1 224115119
chr1 224115346
chr1 224115668
chr1 224115731
chr1 224115746
chr1 224115748
chr1 224116141

chr1 224116287
chr1 224116427
chr1 224116482
chr1 224164358
chr1 224164566
chr1 224182420
chr1 224182447
chr1 224182453
chr1 224182455
chr1 224182540
chr1 224182572
chr1 224182620
chr1 224182763
chr1 224182807
chr1 224182884
chr1 224188598
chr1 224188636
chr1 224245446
chr1 224329599
chr1 224329642
chr1 224329669
chr1 224330460
chr1 224330463
chr1 224330465
chr1 224330472
chr1 224330478
chr1 224330487
chr1 224330490
chr1 224330524
chr1 224330642
chr1 224330668
chr1 224330703
chr1 224330757
chr1 224333537
chr1 224333872
chr1 224334050
chr1 224340963
chr1 224340978
chr1 224340981
chr1 224340991
chr1 224341004
chr1 224341021
chr1 224341024
chr1 224341046
chr1 224341066
chr1 224341079
chr1 224341081
chr1 224341087
chr1 224341115
chr1 224341128
chr1 224341141
chr1 224341152
chr1 224341158
chr1 224341173

chr1 224341175
chr1 224341217
chr1 224341220
chr1 224341241
chr1 224341280
chr1 224341282
chr1 224341297
chr1 224341320
chr1 224341341
chr1 224379986
chr1 224380168
chr1 224380243
chr1 224380288
chr1 224380395
chr1 224432119
chr1 224432175
chr1 224432332
chr1 224432633
chr1 224432712
chr1 224432769
chr1 224432799
chr1 224432880
chr1 224432900
chr1 224432996
chr1 224433077
chr1 224433104
chr1 224433152
chr1 224433220
chr1 224433348
chr1 224433389
chr1 224433441
chr1 224433450
chr1 224435217
chr1 224435278
chr1 224435284
chr1 224435360
chr1 224435371
chr1 224435393
chr1 224435681
chr1 224435944
chr1 224497800
chr1 224498305
chr1 224498349
chr1 224514026
chr1 224514269
chr1 224514286
chr1 224623831
chr1 224623843
chr1 224623979
chr1 224624020
chr1 224624151
chr1 224624237
chr1 224624258
chr1 224949321

chr1 224967473
chr1 224967522
chr1 224968214
chr1 225155602
chr1 225155983
chr1 225156029
chr1 225156336
chr1 225156454
chr1 225156484
chr1 225339861
chr1 225339993
chr1 225425282
chr1 225425506
chr1 225425536
chr1 225425597
chr1 225425868
chr1 225425913
chr1 225425991
chr1 225426131
chr1 225426241
chr1 225426328
chr1 225426559
chr1 225426655
chr1 225426672
chr1 225426676
chr1 225426683
chr1 225426715
chr1 225426751
chr1 225444962
chr1 225445082
chr1 225445187
chr1 225445189
chr1 225467445
chr1 225467448
chr1 225467452
chr1 225467482
chr1 225467572
chr1 225467676
chr1 225467702
chr1 225467704
chr1 225467754
chr1 225467756
chr1 225467790
chr1 225467805
chr1 225467832
chr1 225467893
chr1 225467908
chr1 225467917
chr1 225467962
chr1 225467965
chr1 225467971
chr1 225468036
chr1 225468075
chr1 225468103

chr1 225468197
chr1 225468294
chr1 225563748
chr1 225777804
chr1 225777809
chr1 225777817
chr1 225777852
chr1 225777854
chr1 225777857
chr1 225777864
chr1 225777871
chr1 225777875
chr1 225777894
chr1 225777939
chr1 225777945
chr1 225777974
chr1 225777976
chr1 225777982
chr1 225777997
chr1 225778019
chr1 225778025
chr1 225778031
chr1 225778064
chr1 225778943
chr1 225779003
chr1 225779072
chr1 225779107
chr1 225779115
chr1 225996674
chr1 225996827
chr1 225997081
chr1 225997090
chr1 225997143
chr1 225998610
chr1 225998650
chr1 225998690
chr1 225998711
chr1 225999615
chr1 225999628
chr1 225999634
chr1 225999713
chr1 225999715
chr1 225999730
chr1 225999777
chr1 225999974
chr1 225999991
chr1 226065017
chr1 226065028
chr1 226065126
chr1 226065148
chr1 226100549
chr1 226100591
chr1 226110181
chr1 226110186

chr1 226110188
chr1 226110193
chr1 226110209
chr1 226110216
chr1 226110218
chr1 226110225
chr1 226110227
chr1 226110230
chr1 226186495
chr1 226186501
chr1 226186519
chr1 226186523
chr1 226186528
chr1 226186530
chr1 226186534
chr1 226186547
chr1 226186553
chr1 226333317
chr1 226333426
chr1 226333486
chr1 226333491
chr1 226333506
chr1 226333522
chr1 226333525
chr1 226333555
chr1 226333599
chr1 226333620
chr1 226333638
chr1 226333647
chr1 226333662
chr1 226333682
chr1 226662730
chr1 226662836
chr1 226662909
chr1 226662987
chr1 226663013
chr1 226663040
chr1 226663117
chr1 226663130
chr1 226674892
chr1 226674899
chr1 226674955
chr1 226675001
chr1 226675010
chr1 226675206
chr1 226675214
chr1 226675258
chr1 226675262
chr1 226675307
chr1 226675319
chr1 226675367
chr1 226675390
chr1 226679566
chr1 226679610

chr1 226679721
chr1 226679822
chr1 226679864
chr1 226679914
chr1 226680022
chr1 226680028
chr1 226680034
chr1 226680045
chr1 226680062
chr1 226680105
chr1 226703295
chr1 226703314
chr1 226703332
chr1 226703336
chr1 226731490
chr1 226731735
chr1 226732965
chr1 226732979
chr1 226733075
chr1 226733224
chr1 226739588
chr1 226739626
chr1 226739962
chr1 226739978
chr1 226740053
chr1 226882531
chr1 226882551
chr1 226882587
chr1 226882742
chr1 226882751
chr1 226882832
chr1 226882898
chr1 226882929
chr1 226883056
chr1 226883095
chr1 226883137
chr1 227080082
chr1 227080104
chr1 227080329
chr1 227563709
chr1 227563716
chr1 227563723
chr1 227728615
chr1 227728672
chr1 227735866
chr1 227735911
chr1 227735956
chr1 227735976
chr1 227736041
chr1 228225680
chr1 228225685
chr1 228456735
chr1 228457049
chr1 228457106

chr1 228457113
chr1 228458964
chr1 228459008
chr1 228459018
chr1 228459051
chr1 228459082
chr1 228463285
chr1 228463305
chr1 228463341
chr1 228463358
chr1 228463531
chr1 228463569
chr1 228463652
chr1 228463675
chr1 228463680
chr1 228463747
chr1 228463758
chr1 228463768
chr1 228463774
chr1 228463811
chr1 228463863
chr1 228463869
chr1 228463887
chr1 228500826
chr1 228500857
chr1 228500979
chr1 228501007
chr1 228501051
chr1 228501056
chr1 228501104
chr1 228501108
chr1 228501134
chr1 228501244
chr1 228501251
chr1 228557044
chr1 228557046
chr1 228557078
chr1 228557095
chr1 228557101
chr1 228557123
chr1 228557127
chr1 228557133
chr1 228557160
chr1 228557188
chr1 228557199
chr1 228557210
chr1 228557215
chr1 228557219
chr1 228557231
chr1 228557236
chr1 228557244
chr1 228557248
chr1 228557287
chr1 228557395

chr1 228557401
chr1 228557420
chr1 228557428
chr1 228557440
chr1 228557443
chr1 228557448
chr1 228557453
chr1 228557472
chr1 228608409
chr1 228608423
chr1 228608426
chr1 228608442
chr1 228608754
chr1 228608756
chr1 228608758
chr1 228608760
chr1 228608762
chr1 228608764
chr1 228608802
chr1 228608810
chr1 228608814
chr1 228608816
chr1 228608822
chr1 228608826
chr1 228608838
chr1 228608840
chr1 228608861
chr1 228608885
chr1 228608928
chr1 228609014
chr1 228617708
chr1 228617710
chr1 228617714
chr1 228617718
chr1 228617720
chr1 228617722
chr1 228617724
chr1 228617726
chr1 228617728
chr1 228619063
chr1 228619067
chr1 228619087
chr1 228619101
chr1 228619111
chr1 228619119
chr1 228619126
chr1 228619143
chr1 228619151
chr1 228619173
chr1 228619181
chr1 228619189
chr1 228619945
chr1 228619949
chr1 228619951

chr1 228619955
chr1 228619961
chr1 228619963
chr1 228619965
chr1 228619967
chr1 228619969
chr1 228619997
chr1 228620005
chr1 228620009
chr1 228620011
chr1 228620017
chr1 228626622
chr1 228626626
chr1 228626628
chr1 228626632
chr1 228626636
chr1 228626638
chr1 228626640
chr1 228626642
chr1 228626644
chr1 228626646
chr1 228632330
chr1 228632332
chr1 228632360
chr1 228632365
chr1 228632376
chr1 228632968
chr1 228632979
chr1 228632986
chr1 228632994
chr1 228633002
chr1 228633004
chr1 228633013
chr1 228633027
chr1 228633030
chr1 228633137
chr1 228634690
chr1 228634694
chr1 228634702
chr1 228634706
chr1 228634726
chr1 228634740
chr1 228634750
chr1 228634758
chr1 228634765
chr1 228634782
chr1 228634790
chr1 228634812
chr1 228634820
chr1 228634828
chr1 228635267
chr1 228635270
chr1 228635286
chr1 228635714

chr1 228635734
chr1 228635738
chr1 228635757
chr1 228637641
chr1 228637657
chr1 228637669
chr1 228637674
chr1 228637697
chr1 228637714
chr1 228637717
chr1 228637728
chr1 228637767
chr1 228637809
chr1 228637813
chr1 228637815
chr1 228637819
chr1 228637823
chr1 228637825
chr1 228637827
chr1 228637829
chr1 228637831
chr1 228637833
chr1 228637871
chr1 228637879
chr1 228637883
chr1 228637885
chr1 228637891
chr1 228637895
chr1 228637907
chr1 228637909
chr1 228637930
chr1 228637954
chr1 228637974
chr1 228637978
chr1 228637997
chr1 228639018
chr1 228639022
chr1 228639026
chr1 228639028
chr1 228639036
chr1 228639038
chr1 228639066
chr1 228639071
chr1 228639107
chr1 228639431
chr1 228639674
chr1 228639685
chr1 228639692
chr1 228639700
chr1 228639708
chr1 228639710
chr1 228639719
chr1 228639733
chr1 228639736

chr1 228639752
chr1 228639784
chr1 228639797
chr1 228639803
chr1 228639811
chr1 228639820
chr1 228639822
chr1 228639824
chr1 228642200
chr1 228642239
chr1 228642281
chr1 228642285
chr1 228642287
chr1 228642291
chr1 228642295
chr1 228642297
chr1 228642299
chr1 228642301
chr1 228642303
chr1 228642305
chr1 228642343
chr1 228642351
chr1 228642355
chr1 228642357
chr1 228642363
chr1 228642367
chr1 228642379
chr1 228642381
chr1 228642402
chr1 228642426
chr1 228642446
chr1 228642450
chr1 228642469
chr1 228645595
chr1 228645607
chr1 228645610
chr1 228645626
chr1 228645668
chr1 228645692
chr1 228645721
chr1 228645725
chr1 228645729
chr1 228645731
chr1 228645739
chr1 228645741
chr1 228645769
chr1 228645774
chr1 228645785
chr1 228645810
chr1 228645830
chr1 228645859
chr1 228645863
chr1 228645871
chr1 228645875

chr1 228645895
chr1 228645909
chr1 228645919
chr1 228645927
chr1 228645934
chr1 228645951
chr1 228645959
chr1 228645981
chr1 228645989
chr1 228645997
chr1 228646672
chr1 228772672
chr1 228772737
chr1 228772952
chr1 228773010
chr1 228805169
chr1 228805195
chr1 228805202
chr1 229227695
chr1 229227715
chr1 229227723
chr1 229227803
chr1 229253749
chr1 229253778
chr1 229258250
chr1 229258353
chr1 229258635
chr1 229271163
chr1 229271165
chr1 229271178
chr1 229271187
chr1 229271193
chr1 229271204
chr1 229271207
chr1 229271214
chr1 229271223
chr1 229271227
chr1 229271244
chr1 229271252
chr1 229271272
chr1 229271277
chr1 229271279
chr1 229271282
chr1 229271285
chr1 229271290
chr1 229271292
chr1 229271296
chr1 229271306
chr1 229271314
chr1 229271317
chr1 229271324
chr1 229271327
chr1 229271344
chr1 229271350

chr1 229271360
chr1 229271369
chr1 229271374
chr1 229271381
chr1 229271389
chr1 229271394
chr1 229271396
chr1 229271398
chr1 229271403
chr1 229271408
chr1 229271410
chr1 229271417
chr1 229271420
chr1 229271426
chr1 229271428
chr1 229271434
chr1 229271447
chr1 229271465
chr1 229271471
chr1 229271474
chr1 229271478
chr1 229271484
chr1 229271491
chr1 229271498
chr1 229271520
chr1 229271532
chr1 229271534
chr1 229271539
chr1 229271550
chr1 229271562
chr1 229271568
chr1 229271570
chr1 229271575
chr1 229271577
chr1 229271583
chr1 229271595
chr1 229271603
chr1 229271610
chr1 229271658
chr1 229271718
chr1 229271730
chr1 229271745
chr1 229271990
chr1 229341752
chr1 229341785
chr1 229341823
chr1 229341833
chr1 229341835
chr1 229341861
chr1 229441280
chr1 229441571
chr1 229441649
chr1 229441673
chr1 229507478

chr1 229507567
chr1 229507689
chr1 229507735
chr1 229507761
chr1 229507784
chr1 229627109
chr1 229627126
chr1 229627224
chr1 229627475
chr1 229664251
chr1 229664280
chr1 229664309
chr1 229664328
chr1 229664336
chr1 229664396
chr1 229664505
chr1 229933719
chr1 230395916
chr1 230396038
chr1 230396479
chr1 230428052
chr1 230428065
chr1 230428139
chr1 230428523
chr1 230972798
chr1 230972808
chr1 230972826
chr1 230972991
chr1 230977062
chr1 230977262
chr1 230977293
chr1 230977430
chr1 230979375
chr1 230979441
chr1 230979447
chr1 230979491
chr1 230979494
chr1 230979498
chr1 230979503
chr1 230979508
chr1 230979667
chr1 230979696
chr1 230979712
chr1 230979736
chr1 230979792
chr1 230979842
chr1 230979908
chr1 231053664
chr1 231053713
chr1 231053800
chr1 231053887
chr1 231053991
chr1 231054010
chr1 231106337

chr1 231240661
chr1 231240727
chr1 231240737
chr1 231242106
chr1 231242140
chr1 231242503
chr1 231242631
chr1 231420194
chr1 231420206
chr1 231420215
chr1 231420349
chr1 231420377
chr1 231420467
chr1 231420500
chr1 231420508
chr1 231420532
chr1 231420547
chr1 231420670
chr1 231420733
chr1 231420783
chr1 231420851
chr1 231420862
chr1 231420884
chr1 231493643
chr1 231493687
chr1 231493746
chr1 231529110
chr1 231529126
chr1 231529144
chr1 231604062
chr1 231604131
chr1 231604133
chr1 231604144
chr1 231604158
chr1 231781234
chr1 232172666
chr1 232172765
chr1 232172818
chr1 232458965
chr1 232629163
chr1 232629173
chr1 232950614
chr1 232950626
chr1 232950628
chr1 232950639
chr1 232950657
chr1 232950685
chr1 232950700
chr1 232950717
chr1 232950722
chr1 232950733
chr1 232950778
chr1 232950792
chr1 232950794

chr1 232950796
chr1 232950803
chr1 232950807
chr1 232950821
chr1 232950834
chr1 232950848
chr1 232950876
chr1 232950911
chr1 232950918
chr1 232951003
chr1 232951015
chr1 232951021
chr1 232951045
chr1 232951082
chr1 232951089
chr1 232951091
chr1 232951167
chr1 232951206
chr1 232951278
chr1 232951363
chr1 232951438
chr1 232951486
chr1 233002735
chr1 233002953
chr1 233269443
chr1 233282152
chr1 233295041
chr1 233295079
chr1 233295103
chr1 233295111
chr1 233295207
chr1 233295226
chr1 233295232
chr1 233394916
chr1 233394931
chr1 233395127
chr1 233395262
chr1 233599078
chr1 233599096
chr1 233599121
chr1 233599138
chr1 233599284
chr1 233599296
chr1 233599365
chr1 233599408
chr1 233697472
chr1 233697732
chr1 233698051
chr1 233728222
chr1 233741355
chr1 233951102
chr1 233951243
chr1 233951356
chr1 233952121

chr1 234038533
chr1 234154913
chr1 234154919
chr1 234155157
chr1 234155187
chr1 234170773
chr1 234170807
chr1 234170815
chr1 234170819
chr1 234170861
chr1 234170873
chr1 234171015
chr1 234310439
chr1 234326381
chr1 234326401
chr1 234326425
chr1 234326461
chr1 234326463
chr1 234326473
chr1 234326503
chr1 234326536
chr1 234326545
chr1 234326574
chr1 234326645
chr1 234326647
chr1 234326666
chr1 234326674
chr1 234326679
chr1 234326731
chr1 234326743
chr1 234326748
chr1 234326774
chr1 234326807
chr1 234372882
chr1 234397780
chr1 234397801
chr1 234397947
chr1 234397950
chr1 234398091
chr1 234398455
chr1 234398531
chr1 234599173
chr1 234600507
chr1 234600625
chr1 234600628
chr1 234600678
chr1 234600696
chr1 234600760
chr1 234600781
chr1 234601160
chr1 234601173
chr1 234601385
chr1 234601396
chr1 234601414

chr1 234601427
chr1 234601449
chr1 234601572
chr1 234601591
chr1 234601779
chr1 234601815
chr1 234601855
chr1 234603007
chr1 234603234
chr1 234603869
chr1 234603888
chr1 234603952
chr1 234604035
chr1 234604085
chr1 234604369
chr1 234604809
chr1 234604869
chr1 234604874
chr1 234604908
chr1 234607323
chr1 234607331
chr1 234607335
chr1 234607366
chr1 234607370
chr1 234607392
chr1 234607419
chr1 234607518
chr1 234607527
chr1 234607650
chr1 234607659
chr1 234607669
chr1 234607701
chr1 234607767
chr1 234607781
chr1 234607799
chr1 234607896
chr1 234607930
chr1 234607942
chr1 234607992
chr1 234608029
chr1 234608033
chr1 234608084
chr1 234608126
chr1 234608227
chr1 234608237
chr1 234608289
chr1 234608311
chr1 234608400
chr1 234608438
chr1 234612872
chr1 234612956
chr1 234612992
chr1 234613023
chr1 234613026

chr1 234613029
chr1 234613034
chr1 234676477
chr1 234676584
chr1 234676644
chr1 234723629
chr1 234723641
chr1 234723665
chr1 234723779
chr1 234723785
chr1 234723847
chr1 234724063
chr1 234724260
chr1 234724305
chr1 234724359
chr1 234724406
chr1 234724431
chr1 234724449
chr1 234724453
chr1 234724462
chr1 234724464
chr1 234724489
chr1 234724591
chr1 234724647
chr1 234731166
chr1 234731168
chr1 234731187
chr1 234731233
chr1 234772341
chr1 234772363
chr1 234772438
chr1 234772515
chr1 234772525
chr1 234772528
chr1 234772562
chr1 234772619
chr1 234772629
chr1 234772634
chr1 234772641
chr1 234772656
chr1 234772702
chr1 234772759
chr1 234772767
chr1 234772776
chr1 234772874
chr1 234772907
chr1 234772934
chr1 234772938
chr1 234772953
chr1 234926828
chr1 234926847
chr1 234926899
chr1 234926939
chr1 234926975

chr1 234962818
chr1 234962835
chr1 234962914
chr1 234962921
chr1 234962925
chr1 234962954
chr1 234962962
chr1 234963004
chr1 234963039
chr1 235011156
chr1 235011251
chr1 235011292
chr1 235011302
chr1 235011314
chr1 235011369
chr1 235011394
chr1 235011598
chr1 235011601
chr1 235011656
chr1 235011686
chr1 235011693
chr1 235011712
chr1 235011722
chr1 235011789
chr1 235011880
chr1 235011899
chr1 235011946
chr1 235127344
chr1 235127497
chr1 235127563
chr1 235127628
chr1 235127722
chr1 235127737
chr1 235127760
chr1 235127774
chr1 235127809
chr1 235127835
chr1 235127844
chr1 235127908
chr1 235127950
chr1 235127973
chr1 235127993
chr1 235128004
chr1 235207106
chr1 235207378
chr1 235207382
chr1 235240068
chr1 235240169
chr1 235245555
chr1 235245575
chr1 235246144
chr1 235269400
chr1 235269483
chr1 235298337

chr1 235324872
chr1 235324929
chr1 235325203
chr1 235325382
chr1 235325537
chr1 235325615
chr1 235326216
chr1 235326491
chr1 235329380
chr1 235329400
chr1 235329445
chr1 235329516
chr1 235329614
chr1 235366700
chr1 235366983
chr1 235367108
chr1 235367141
chr1 235367154
chr1 235367172
chr1 235367237
chr1 235367239
chr1 235367280
chr1 235367287
chr1 235367299
chr1 235367309
chr1 235367353
chr1 235367365
chr1 235367383
chr1 235367395
chr1 235367404
chr1 235367425
chr1 235367444
chr1 235367496
chr1 235367498
chr1 235367521
chr1 235367532
chr1 235367711
chr1 235367715
chr1 235492836
chr1 235641986
chr1 235642008
chr1 235642019
chr1 235642028
chr1 235642040
chr1 235642046
chr1 235642066
chr1 235642075
chr1 235642103
chr1 235642107
chr1 235642130
chr1 235642158
chr1 235642163
chr1 235642168
chr1 235642172

chr1 235642189
chr1 235642193
chr1 235642200
chr1 235642209
chr1 235642227
chr1 235642233
chr1 235642244
chr1 235642246
chr1 235642258
chr1 235642260
chr1 235642294
chr1 235642299
chr1 235642336
chr1 235642376
chr1 235642390
chr1 235642394
chr1 235642420
chr1 235642423
chr1 235642443
chr1 235642456
chr1 235642460
chr1 235642528
chr1 235642556
chr1 235642567
chr1 235642592
chr1 235865301
chr1 235865339
chr1 235865474
chr1 235865757
chr1 235865778
chr1 235865905
chr1 235865909
chr1 235865927
chr1 235865983
chr1 235865986
chr1 235866007
chr1 235866011
chr1 235866016
chr1 235866059
chr1 235866070
chr1 235866116
chr1 235866139
chr1 235866150
chr1 235866185
chr1 235866218
chr1 235866228
chr1 235867761
chr1 235867796
chr1 235867800
chr1 235868013
chr1 235868089
chr1 235868127
chr1 235868202
chr1 235868210

chr1 235868233
chr1 235868240
chr1 235873279
chr1 235873457
chr1 235873723
chr1 235888180
chr1 235888233
chr1 235888289
chr1 235888377
chr1 235888395
chr1 235888402
chr1 235888543
chr1 235888591
chr1 235999237
chr1 235999258
chr1 235999427
chr1 235999569
chr1 235999618
chr1 235999639
chr1 235999785
chr1 235999795
chr1 235999818
chr1 235999884
chr1 236370986
chr1 236370999
chr1 236465927
chr1 236465948
chr1 236503185
chr1 236503217
chr1 236503269
chr1 236508890
chr1 236508944
chr1 236508964
chr1 236508969
chr1 236508999
chr1 236509087
chr1 236516924
chr1 236517059
chr1 236517062
chr1 236517082
chr1 236517210
chr1 236517218
chr1 236517250
chr1 236525017
chr1 236525145
chr1 236603916
chr1 236603966
chr1 236603978
chr1 236603988
chr1 236604062
chr1 236604067
chr1 236604102
chr1 236604108
chr1 236604124

chr1 236604137
chr1 236604161
chr1 236604174
chr1 236604179
chr1 236604592
chr1 236604603
chr1 236604610
chr1 236604615
chr1 236604623
chr1 236604635
chr1 236604638
chr1 236609115
chr1 236609317
chr1 236609493
chr1 236796313
chr1 236796565
chr1 236796570
chr1 236796598
chr1 236796630
chr1 237043628
chr1 237043901
chr1 237043917
chr1 237043926
chr1 237043956
chr1 237044090
chr1 237044127
chr1 237044264
chr1 237044292
chr1 237044363
chr1 237044381
chr1 237044583
chr1 237044601
chr1 237044740
chr1 237044878
chr1 237045099
chr1 237045432
chr1 237045752
chr1 237472264
chr1 237472292
chr1 237534542
chr1 237535212
chr1 237535441
chr1 237535518
chr1 237629677
chr1 237652738
chr1 237652985
chr1 238128728
chr1 238591883
chr1 239178720
chr1 239178728
chr1 239262908
chr1 239263080
chr1 239263102
chr1 239263287

chr1 239263398
chr1 239263599
chr1 239263706
chr1 239263726
chr1 239264010
chr1 239385982
chr1 239385984
chr1 239385986
chr1 239385988
chr1 239385990
chr1 239385992
chr1 239385994
chr1 239385996
chr1 239385998
chr1 239386004
chr1 239386079
chr1 239386206
chr1 239386228
chr1 239386277
chr1 239386300
chr1 239386321
chr1 239386324
chr1 239386326
chr1 239386399
chr1 239386441
chr1 239386455
chr1 239386462
chr1 239386464
chr1 239386474
chr1 239386480
chr1 239386482
chr1 239386498
chr1 239386501
chr1 239386530
chr1 239386553
chr1 239386586
chr1 239386611
chr1 239386613
chr1 239386635
chr1 239386651
chr1 239386655
chr1 239718851
chr1 239719146
chr1 239719233
chr1 239719514
chr1 239719540
chr1 239719551
chr1 239719577
chr1 239719675
chr1 239719724
chr1 239719747
chr1 239719762
chr1 239719772
chr1 239720008

chr1 239720113
chr1 239720164
chr1 239720169
chr1 239720195
chr1 239720489
chr1 239720501
chr1 239720701
chr1 239720719
chr1 239898175
chr1 239898216
chr1 239898218
chr1 239898285
chr1 239898304
chr1 239898311
chr1 239898384
chr1 239898474
chr1 239898529
chr1 239898533
chr1 239898604
chr1 239898639
chr1 239898670
chr1 239898708
chr1 239898791
chr1 240090966
chr1 240090987
chr1 240091153
chr1 240091535
chr1 240210626
chr1 240576161
chr1 240576182
chr1 240576364
chr1 240576380
chr1 240576418
chr1 240576503
chr1 240576508
chr1 240613479
chr1 240613578
chr1 240613748
chr1 240614022
chr1 240614064
chr1 240867459
chr1 240902766
chr1 240903440
chr1 240903737
chr1 240946400
chr1 241093424
chr1 241093952
chr1 241093958
chr1 241214532
chr1 241214740
chr1 241214799
chr1 241217618
chr1 241358036
chr1 241358103

chr1 241358155
chr1 241518943
chr1 241518945
chr1 241518952
chr1 241518979
chr1 241520287
chr1 241520293
chr1 241520360
chr1 241520382
chr1 241520405
chr1 241520578
chr1 241520582
chr1 241520808
chr1 241531634
chr1 241531667
chr1 241531708
chr1 241531728
chr1 241531774
chr1 241531864
chr1 241532083
chr1 241532316
chr1 241551907
chr1 241551916
chr1 241551924
chr1 241551964
chr1 241551970
chr1 241636417
chr1 241639254
chr1 241639464
chr1 241639510
chr1 241639537
chr1 241639545
chr1 241848186
chr1 241848188
chr1 241848285
chr1 241848288
chr1 241848293
chr1 241848307
chr1 241848313
chr1 242052455
chr1 242052580
chr1 242052877
chr1 242467528
chr1 242467688
chr1 242467691
chr1 242467770
chr1 242467921
chr1 242522288
chr1 242522448
chr1 242522455
chr1 242522578
chr1 242522586
chr1 242637037
chr1 242637147

chr1 243255889
chr1 243255898
chr1 243256272
chr1 243256295
chr1 243256312
chr1 243256338
chr1 243256344
chr1 243256424
chr1 243256443
chr1 243256493
chr1 243256496
chr1 243256548
chr1 243421852
chr1 243421872
chr1 243421926
chr1 243421928
chr1 243421952
chr1 243421962
chr1 243422226
chr1 243422267
chr1 243422390
chr1 243595972
chr1 243596513
chr1 243596550
chr1 243596790
chr1 243596798
chr1 243596917
chr1 243597039
chr1 243848666
chr1 243849058
chr1 243851289
chr1 243851329
chr1 243851355
chr1 243851374
chr1 243851383
chr1 243851466
chr1 243851503
chr1 243851540
chr1 243851545
chr1 244047090
chr1 244299303
chr1 244299531
chr1 244299576
chr1 244299587
chr1 244299613
chr1 244299620
chr1 244299644
chr1 244299648
chr1 244299664
chr1 244319999
chr1 244320008
chr1 244450783
chr1 244450792
chr1 244450814

chr1 244450821
chr1 244450835
chr1 244600086
chr1 244600091
chr1 244600104
chr1 244600115
chr1 244600239
chr1 244653768
chr1 244653777
chr1 244653795
chr1 244653844
chr1 244833744
chr1 244833850
chr1 244833864
chr1 244833990
chr1 244833998
chr1 244835933
chr1 244836033
chr1 244836105
chr1 244836134
chr1 244836419
chr1 244836463
chr1 244836495
chr1 244836498
chr1 244836659
chr1 244836677
chr1 244836896
chr1 244837218
chr1 244860879
chr1 244861004
chr1 244861087
chr1 244862294
chr1 244862425
chr1 244862488
chr1 244862569
chr1 244862576
chr1 244862580
chr1 244862658
chr1 244862673
chr1 244918707
chr1 244918714
chr1 244918726
chr1 244918734
chr1 244918746
chr1 244918754
chr1 244918766
chr1 244918774
chr1 244918786
chr1 244918794
chr1 244918806
chr1 244918814
chr1 244969480
chr1 244969487
chr1 244969521

chr1 244969557
chr1 244969586
chr1 244969639
chr1 244969642
chr1 244969663
chr1 244969704
chr1 244971818
chr1 244971878
chr1 244972059
chr1 245042913
chr1 245156120
chr1 245156129
chr1 245156145
chr1 245156230
chr1 245310966
chr1 245953451
chr1 245953489
chr1 245953506
chr1 245953564
chr1 245953576
chr1 245953686
chr1 245953708
chr1 245953880
chr1 245954059
chr1 245954121
chr1 245954234
chr1 245954373
chr1 245954444
chr1 246329517
chr1 246329901
chr1 246329914
chr1 246329933
chr1 246567560
chr1 246567634
chr1 246567771
chr1 246567982
chr1 246568090
chr1 246568098
chr1 246569511
chr1 246569644
chr1 246569708
chr1 246569755
chr1 246569817
chr1 246569849
chr1 246569886
chr1 246577294
chr1 246577307
chr1 246577351
chr1 246577552
chr1 246577664
chr1 246817790
chr1 246817960
chr1 247077759
chr1 247077855

chr1 247077865
chr1 247077929
chr1 247077993
chr1 247078076
chr1 247078106
chr1 247078151
chr1 247078172
chr1 247078208
chr1 247078211
chr1 247118290
chr1 247118336
chr1 247118347
chr1 247118612
chr1 247118623
chr1 247118637
chr1 247118658
chr1 247118669
chr1 247126996
chr1 247127019
chr1 247127048
chr1 247127070
chr1 247127555
chr1 247127558
chr1 247127561
chr1 247127563
chr1 247127727
chr1 247171778
chr1 247171789
chr1 247171796
chr1 247171802
chr1 247171832
chr1 247171835
chr1 247171838
chr1 247171862
chr1 247171872
chr1 247171886
chr1 247171922
chr1 247210079
chr1 247210312
chr1 247210390
chr1 247210410
chr1 247210434
chr1 247210437
chr1 247210443
chr1 247210457
chr1 247210465
chr1 247390034
chr1 247390090
chr1 247390135
chr1 247390141
chr1 247390185
chr1 247390199
chr1 247390209
chr1 247390401

chr1 247390418
chr1 247390420
chr1 247390464
chr1 247390482
chr1 247390534
chr1 247390610
chr1 247390641
chr1 247407207
chr1 247407209
chr1 247407233
chr1 247407240
chr1 247407263
chr1 247407276
chr1 247407296
chr1 247407528
chr1 247407532
chr1 247407593
chr1 247407624
chr1 247407835
chr1 247407858
chr1 247408024
chr1 247408127
chr1 247416207
chr1 247416209
chr1 247416223
chr1 247522942
chr1 247523219
chr1 247548197
chr1 247548220
chr1 247548243
chr1 247548276
chr1 247548293
chr1 247548573
chr1 247548609
chr1 247548808
chr1 247548838
chr1 247548935
chr1 247548937
chr1 247548963
chr1 247548979
chr1 247549013
chr1 247549040
chr1 247664899
chr1 247665499
chr1 247859555
chr1 247894391
chr1 247894413
chr1 247894666
chr1 247894802
chr1 247894827
chr1 247895247
chr1 247906082
chr1 247906155
chr1 247906308

chr1 247906361
chr1 247906377
chr1 247906387
chr1 247906409
chr1 247906441
chr1 247906497
chr1 247906533
chr1 247906553
chr1 247906569
chr1 247906609
chr1 247906635
chr1 247906726
chr1 247906906
chr1 247927580
chr1 247927682
chr1 247927697
chr1 247937084
chr1 247937096
chr1 247937103
chr1 247937105
chr1 247937120
chr1 247937125
chr1 247937157
chr1 247937163
chr1 247937170
chr1 247937180
chr1 247937192
chr1 247937199
chr1 247937201
chr1 247937209
chr1 247937219
chr1 247937221
chr1 247937224
chr1 247937243
chr1 247937251
chr1 247937274
chr1 247937276
chr1 247937280
chr1 247937283
chr1 247937289
chr1 247937298
chr1 247937312
chr1 247937351
chr1 247937369
chr1 247937381
chr1 247937393
chr1 247937411
chr1 247937416
chr1 247937423
chr1 247937444
chr1 247937529
chr1 247937620
chr1 247937707
chr1 247937729

chr1 247962590
chr1 247962749
chr1 247963004
chr1 247963024
chr1 247963255
chr1 247963503
chr1 247963595
chr1 247963998
chr1 247993190
chr1 247993338
chr1 248004520
chr1 248004582
chr1 248004610
chr1 248136090
chr1 248232196
chr1 248232428
chr1 248242801
chr1 248242979
chr1 248243050
chr1 248243162
chr1 248243204
chr1 248243251
chr1 248243309
chr1 248250629
chr1 248308165
chr1 248435386
chr1 248435651
chr1 248435667
chr1 248513750
chr1 248513809
chr1 248518160
chr1 248518222
chr1 248518232
chr1 248528717
chr1 248528826
chr1 248528902
chr1 248528974
chr1 248528999
chr1 248529474
chr1 248529600
chr1 248529829
chr1 248529839
chr1 248531048
chr1 248532812
chr1 248533028
chr1 248533199
chr1 248533485
chr1 248533498
chr1 248533874
chr1 248534252
chr1 248534311
chr1 248535116
chr1 248535754
chr1 248539084

chr1 248655263
chr1 248655413
chr1 248655820
chr1 248656102
chr1 248656144
chr1 248837617
chr1 248837717
chr1 248858150
chr1 248858157
chr1 248858179
chr1 248858213
chr1 248858221
chr1 248858227
chr1 248858239
chr1 248858242
chr1 248858244
chr1 248858246
chr1 248858257
chr1 248858260
chr1 248858269
chr1 248858272
chr1 248858285
chr1 248858292
chr1 248858295
chr1 248858339
chr1 248858348
chr1 248858356
chr1 248858362
chr1 248858369
chr1 248858377
chr1 248858390
chr1 248858473
chr1 248858506
chr1 248858523
chr1 248858526
chr1 248858570
chr1 248858647
chr1 248858678
chr1 248858711
chr1 248858721
chr1 248858735
chr1 248858753
chr1 248858760
chr1 248858769
chr1 248858790
chr1 248858806
chr1 248858846
chr1 248862750
chr1 248862883
chr1 248873729
chr1 248873736
chr1 248873747
chr1 248873749
chr1 248873771

chr1 248873775
chr1 248873782
chr1 248873791
chr1 248873793
chr1 248873810
chr1 248873839
chr1 248873850
chr1 248873866
chr1 248873880
chr1 248873909
chr1 248873925
chr1 248873935
chr1 248873943
chr1 248873965
chr1 248873988
chr1 248874011
chr1 248874018
chr1 248874081
chr1 248874098
chr1 248874195
chr1 248925151
chr1 248925264
chr1 248925274
chr1 248925305
chr1 248925322
chr20 237682
chr20 237774
chr20 237822
chr20 237877
chr20 238093
chr20 238314
chr20 238324
chr20 290169
chr20 290171
chr20 290182
chr20 290186
chr20 290221
chr20 290259
chr20 290374
chr20 298762
chr20 298780
chr20 298783
chr20 298789
chr20 298804
chr20 298881
chr20 298941
chr20 298999
chr20 299012
chr20 299052
chr20 299115
chr20 299124
chr20 299200
chr20 299212
chr20 347454

chr20 347478
chr20 347502
chr20 347504
chr20 347511
chr20 347527
chr20 347548
chr20 347575
chr20 347583
chr20 347597
chr20 347601
chr20 347608
chr20 347615
chr20 347811
chr20 347822
chr20 347829
chr20 347840
chr20 347851
chr20 347870
chr20 347875
chr20 347885
chr20 347890
chr20 347897
chr20 347901
chr20 347915
chr20 347930
chr20 347937
chr20 347951
chr20 347955
chr20 347965
chr20 348372
chr20 376296
chr20 381358
chr20 381382
chr20 381396
chr20 381398
chr20 381448
chr20 381522
chr20 381531
chr20 462346
chr20 462348
chr20 462351
chr20 543102
chr20 543114
chr20 543136
chr20 543150
chr20 543171
chr20 543203
chr20 543249
chr20 543251
chr20 543469
chr20 543476
chr20 543505
chr20 543524
chr20 543561

chr20 543564
chr20 543569
chr20 543590
chr20 543599
chr20 543603
chr20 543605
chr20 543610
chr20 543638
chr20 543644
chr20 543650
chr20 543684
chr20 543690
chr20 543693
chr20 543696
chr20 543700
chr20 543732
chr20 543735
chr20 543754
chr20 543759
chr20 543763
chr20 543770
chr20 543784
chr20 543796
chr20 543805
chr20 543811
chr20 543835
chr20 543840
chr20 579255
chr20 579258
chr20 661002
chr20 674756
chr20 674970
chr20 675002
chr20 676394
chr20 676406
chr20 676418
chr20 834268
chr20 834270
chr20 834286
chr20 834304
chr20 834322
chr20 834329
chr20 834341
chr20 834353
chr20 834367
chr20 834395
chr20 834399
chr20 834456
chr20 834561
chr20 834872
chr20 835091
chr20 835478
chr20 1213057
chr20 1213061

chr20 1213063
chr20 1213065
chr20 1213071
chr20 1213080
chr20 1213089
chr20 1213102
chr20 1213134
chr20 1213146
chr20 1213151
chr20 1213188
chr20 1213196
chr20 1213250
chr20 1213270
chr20 1213510
chr20 1266128
chr20 1266135
chr20 1266143
chr20 1391845
chr20 1391910
chr20 1391935
chr20 1391996
chr20 1392105
chr20 1392150
chr20 1392161
chr20 1392178
chr20 1392225
chr20 1392236
chr20 1392321
chr20 1392328
chr20 1392337
chr20 1392363
chr20 1392374
chr20 1434855
chr20 1435071
chr20 1502988
chr20 1503499
chr20 1503626
chr20 1503750
chr20 1503755
chr20 1503778
chr20 1503808
chr20 1503835
chr20 1503967
chr20 1504001
chr20 1588524
chr20 1588665
chr20 1588757
chr20 1588908
chr20 1589044
chr20 1589098
chr20 1589186
chr20 1589311
chr20 1589360
chr20 1593351

chr20 1593729
chr20 1593932
chr20 1594031
chr20 1594072
chr20 1594096
chr20 1594127
chr20 1594980
chr20 1595565
chr20 1595908
chr20 1596508
chr20 1596715
chr20 1596726
chr20 1657747
chr20 1662126
chr20 1662229
chr20 1764251
chr20 2076764
chr20 2076817
chr20 2103672
chr20 2103696
chr20 2103720
chr20 2103737
chr20 2218651
chr20 2379893
chr20 2379928
chr20 2379984
chr20 2647057
chr20 2647093
chr20 2651731
chr20 2751725
chr20 2751764
chr20 2751773
chr20 2751802
chr20 2751807
chr20 2751812
chr20 2751857
chr20 2751872
chr20 2800997
chr20 2801033
chr20 2801039
chr20 2822152
chr20 2822235
chr20 2822278
chr20 2822530
chr20 2874011
chr20 3159650
chr20 3159655
chr20 3159671
chr20 3159675
chr20 3159709
chr20 3159713
chr20 3159719
chr20 3209305
chr20 3209347

chr20 3209366
chr20 3209370
chr20 3209385
chr20 3209391
chr20 3209393
chr20 3209403
chr20 3209407
chr20 3209412
chr20 3209414
chr20 3209426
chr20 3209431
chr20 3209441
chr20 3209454
chr20 3209466
chr20 3209476
chr20 3209486
chr20 3209507
chr20 3209521
chr20 3209541
chr20 3209556
chr20 3209581
chr20 3209592
chr20 3209599
chr20 3209622
chr20 3209651
chr20 3209666
chr20 3209681
chr20 3209697
chr20 3384378
chr20 3408121
chr20 3408126
chr20 3408137
chr20 3408157
chr20 3408193
chr20 3408199
chr20 3408207
chr20 3408217
chr20 3408231
chr20 3408244
chr20 3408253
chr20 3408306
chr20 3408383
chr20 3408406
chr20 3408413
chr20 3408422
chr20 3408425
chr20 3408637
chr20 3408682
chr20 3408688
chr20 3408726
chr20 3408729
chr20 3408761
chr20 3408775
chr20 3408813

chr20 3408841
chr20 3408854
chr20 3408888
chr20 3408900
chr20 3408925
chr20 3787187
chr20 3787299
chr20 3787320
chr20 3787366
chr20 3811263
chr20 3811294
chr20 3811314
chr20 3811395
chr20 3811556
chr20 3811583
chr20 3811600
chr20 3811619
chr20 3811636
chr20 3811652
chr20 3811681
chr20 3811736
chr20 3811750
chr20 3811798
chr20 3811803
chr20 3811828
chr20 3811840
chr20 3811877
chr20 3907250
chr20 3907390
chr20 3907418
chr20 3907575
chr20 3907630
chr20 3907669
chr20 3907676
chr20 3980588
chr20 3980606
chr20 3980892
chr20 3980989
chr20 3981048
chr20 3981142
chr20 3981282
chr20 3989947
chr20 4017911
chr20 4038330
chr20 4038342
chr20 4038371
chr20 4038401
chr20 4038410
chr20 4607229
chr20 4607254
chr20 4665722
chr20 4666082
chr20 4666136
chr20 4666174

chr20 4666190
chr20 4666321
chr20 4666330
chr20 4666346
chr20 4666556
chr20 4727028
chr20 4727125
chr20 4727153
chr20 4727163
chr20 4814480
chr20 4814505
chr20 4814545
chr20 4814571
chr20 4814624
chr20 4814653
chr20 4814658
chr20 4814670
chr20 4814717
chr20 4814729
chr20 4814735
chr20 4814748
chr20 4814798
chr20 4814814
chr20 4814931
chr20 4815037
chr20 4815053
chr20 4815079
chr20 4815085
chr20 4815090
chr20 4815169
chr20 4815218
chr20 4823252
chr20 4823255
chr20 4823264
chr20 4823266
chr20 4823277
chr20 4823291
chr20 4823293
chr20 4823298
chr20 4823300
chr20 4823323
chr20 4962248
chr20 5057349
chr20 5078322
chr20 5078560
chr20 5078648
chr20 5078658
chr20 5078888
chr20 5079255
chr20 5090366
chr20 5090382
chr20 5090413
chr20 5113342
chr20 5113348

chr20 5113390
chr20 5113393
chr20 5113418
chr20 5113424
chr20 5113427
chr20 5113452
chr20 5113464
chr20 5113474
chr20 5113491
chr20 5113493
chr20 5113495
chr20 5113498
chr20 5113647
chr20 5113729
chr20 5113787
chr20 5118355
chr20 5118404
chr20 5118565
chr20 5118741
chr20 5118748
chr20 5118761
chr20 5118788
chr20 5118800
chr20 5118802
chr20 5118811
chr20 5118841
chr20 5118844
chr20 5118900
chr20 5118903
chr20 5118925
chr20 5118929
chr20 5118931
chr20 5593058
chr20 5593254
chr20 5593358
chr20 5593501
chr20 5593666
chr20 5593716
chr20 5593850
chr20 5593886
chr20 5593946
chr20 5609017
chr20 5609022
chr20 5609169
chr20 5609323
chr20 5609410
chr20 5609458
chr20 5609532
chr20 5609587
chr20 5609597
chr20 5609608
chr20 5609621
chr20 5609647
chr20 5609697

chr20 5609700
chr20 5610711
chr20 5610716
chr20 5610721
chr20 5610730
chr20 5610734
chr20 5610736
chr20 5610741
chr20 5610747
chr20 5610750
chr20 5610761
chr20 5610764
chr20 5610766
chr20 5610771
chr20 5610776
chr20 5610794
chr20 5610799
chr20 5610809
chr20 5610813
chr20 5610820
chr20 5610826
chr20 5610831
chr20 5645489
chr20 5645595
chr20 5645606
chr20 5645612
chr20 5645620
chr20 5645637
chr20 5750485
chr20 5750487
chr20 5750522
chr20 5750547
chr20 5750613
chr20 5750622
chr20 5750634
chr20 5750699
chr20 5750835
chr20 5750869
chr20 5750911
chr20 5750922
chr20 5750951
chr20 5750953
chr20 5750991
chr20 5911012
chr20 5911084
chr20 5911856
chr20 5911953
chr20 5911990
chr20 5912019
chr20 5912075
chr20 5912412
chr20 5950023
chr20 5951243
chr20 5951266

chr20 5951336
chr20 5951344
chr20 5951436
chr20 5951494
chr20 5951765
chr20 5951776
chr20 5951826
chr20 5952049
chr20 6006809
chr20 6006843
chr20 6006962
chr20 6007030
chr20 6007092
chr20 6007148
chr20 6007383
chr20 6007397
chr20 6007468
chr20 6007577
chr20 6007695
chr20 6318610
chr20 6319343
chr20 6319645
chr20 6319721
chr20 6426753
chr20 6427106
chr20 6765487
chr20 6765536
chr20 6765562
chr20 6765632
chr20 6765636
chr20 6881986
chr20 7003706
chr20 7051237
chr20 7051352
chr20 7051614
chr20 7051946
chr20 7052563
chr20 7116752
chr20 7116825
chr20 7117323
chr20 7117380
chr20 7793066
chr20 8018020
chr20 8018172
chr20 8018272
chr20 8018561
chr20 8018740
chr20 8018902
chr20 8018912
chr20 8018935
chr20 8018979
chr20 8018994
chr20 8019051
chr20 8019114

chr20 8043243
chr20 8043257
chr20 8043666
chr20 8043820
chr20 8043840
chr20 8043882
chr20 8043892
chr20 8043910
chr20 8131602
chr20 8131634
chr20 8131660
chr20 8131704
chr20 8131715
chr20 8131721
chr20 8131729
chr20 8131745
chr20 8131747
chr20 8131764
chr20 8131766
chr20 8131798
chr20 8131853
chr20 8131908
chr20 8131946
chr20 8131951
chr20 8131958
chr20 8131990
chr20 8132033
chr20 8132049
chr20 8132055
chr20 8132066
chr20 8132085
chr20 8132102
chr20 8132107
chr20 8853360
chr20 8853392
chr20 9053363
chr20 9069762
chr20 9069779
chr20 9512713
chr20 9513068
chr20 9514077
chr20 9514119
chr20 9514179
chr20 9514183
chr20 9514191
chr20 9514314
chr20 9514340
chr20 9514350
chr20 9514363
chr20 9514483
chr20 9913898
chr20 9914253
chr20 9914524
chr20 9914706

chr20 10030427
chr20 10030449
chr20 10035007
chr20 10035012
chr20 10035031
chr20 10035040
chr20 10035052
chr20 10035061
chr20 10035073
chr20 10035075
chr20 10035077
chr20 10035081
chr20 10035088
chr20 10035096
chr20 10035133
chr20 10035135
chr20 10035137
chr20 10035174
chr20 10035182
chr20 10035188
chr20 10035191
chr20 10035198
chr20 10035214
chr20 10172271
chr20 10172279
chr20 10216616
chr20 10216774
chr20 10217087
chr20 10217146
chr20 10217182
chr20 10217196
chr20 10217201
chr20 10217235
chr20 10217249
chr20 10217259
chr20 10217272
chr20 10217358
chr20 10217371
chr20 10217396
chr20 10217399
chr20 10217407
chr20 10219098
chr20 10219106
chr20 10219116
chr20 10219145
chr20 10219173
chr20 10219193
chr20 10219196
chr20 10219209
chr20 10219217
chr20 10219222
chr20 10219279
chr20 10219327
chr20 10219356

chr20 10219418
chr20 10350906
chr20 10351599
chr20 10351639
chr20 10351664
chr20 10351671
chr20 10353260
chr20 10353292
chr20 10353307
chr20 10353344
chr20 10435116
chr20 10435118
chr20 10435161
chr20 10435169
chr20 10435179
chr20 10435220
chr20 10435250
chr20 10435253
chr20 10447273
chr20 10447285
chr20 10453287
chr20 10453307
chr20 10453347
chr20 10453362
chr20 10453385
chr20 10453669
chr20 10453730
chr20 10511606
chr20 10511671
chr20 10511691
chr20 10511746
chr20 10511768
chr20 10512383
chr20 10676665
chr20 10676718
chr20 10676759
chr20 11457112
chr20 11821960
chr20 11822489
chr20 11822550
chr20 11822565
chr20 11823300
chr20 11853158
chr20 11891828
chr20 11891856
chr20 11891864
chr20 11891913
chr20 11891946
chr20 11891997
chr20 11891999
chr20 11892005
chr20 11892131
chr20 11892162
chr20 11892171

chr20 11892262
chr20 11892454
chr20 11892498
chr20 11892603
chr20 11892606
chr20 11892851
chr20 12804591
chr20 12856464
chr20 12856775
chr20 13009015
chr20 13009052
chr20 13009096
chr20 13009196
chr20 13009246
chr20 13009300
chr20 13009426
chr20 13009670
chr20 13009738
chr20 13009847
chr20 13009927
chr20 13010068
chr20 13010104
chr20 13010192
chr20 13228932
chr20 13247364
chr20 13247468
chr20 13247471
chr20 13247681
chr20 13247745
chr20 13247816
chr20 13247999
chr20 13248079
chr20 13248091
chr20 13248146
chr20 13637983
chr20 13637994
chr20 13638215
chr20 13638315
chr20 13638323
chr20 13638332
chr20 13638394
chr20 13638452
chr20 13638457
chr20 13639163
chr20 13639250
chr20 13639293
chr20 13639343
chr20 13639373
chr20 13639384
chr20 13639401
chr20 13684387
chr20 13684435
chr20 13684438
chr20 13684585

chr20 13684696
chr20 13685324
chr20 13685746
chr20 13685795
chr20 13785734
chr20 13788341
chr20 14032008
chr20 14045970
chr20 14046006
chr20 14077477
chr20 14077669
chr20 14337264
chr20 14337299
chr20 14337331
chr20 14337412
chr20 14337440
chr20 14337490
chr20 14337506
chr20 14337528
chr20 14337585
chr20 14337651
chr20 14390252
chr20 14390562
chr20 14598168
chr20 14598241
chr20 15896514
chr20 15896680
chr20 16272479
chr20 16573244
chr20 16573254
chr20 16573267
chr20 16573276
chr20 16573285
chr20 16573295
chr20 16573297
chr20 16573299
chr20 16573323
chr20 16573325
chr20 16573329
chr20 16573332
chr20 16573335
chr20 16624572
chr20 16730356
chr20 16730360
chr20 16730365
chr20 16730381
chr20 16730401
chr20 16730421
chr20 16730449
chr20 16730458
chr20 16730469
chr20 16730544
chr20 16730547
chr20 16730558

chr20 16730583
chr20 16730596
chr20 16730613
chr20 16730618
chr20 16804330
chr20 16804334
chr20 17230235
chr20 17230660
chr20 17477836
chr20 17478213
chr20 17478516
chr20 17505152
chr20 17505166
chr20 17505177
chr20 17505288
chr20 17505308
chr20 17505313
chr20 17505318
chr20 17569300
chr20 17569303
chr20 17569310
chr20 17569660
chr20 17569738
chr20 17569740
chr20 17569742
chr20 17569769
chr20 17569782
chr20 17569785
chr20 17569793
chr20 17965229
chr20 17965231
chr20 17965387
chr20 17965469
chr20 17965603
chr20 17965625
chr20 17965641
chr20 17965776
chr20 17965806
chr20 17965822
chr20 17966013
chr20 17970262
chr20 17970322
chr20 17970428
chr20 17970778
chr20 17970790
chr20 17971175
chr20 17971209
chr20 18138532
chr20 18138575
chr20 18138631
chr20 18138673
chr20 18138731
chr20 18138902
chr20 18466465

chr20 18466522
chr20 18466557
chr20 18466641
chr20 18466643
chr20 18466647
chr20 18467015
chr20 18467018
chr20 18467021
chr20 18467025
chr20 18467039
chr20 18467041
chr20 18467044
chr20 18467049
chr20 18467051
chr20 18467056
chr20 18467113
chr20 18467123
chr20 18467128
chr20 18467146
chr20 18467354
chr20 18467357
chr20 18467659
chr20 18467664
chr20 18467679
chr20 18467681
chr20 18467690
chr20 18467696
chr20 18496566
chr20 18496594
chr20 18496670
chr20 18496679
chr20 18496734
chr20 18496795
chr20 18496802
chr20 18496823
chr20 18496903
chr20 18496935
chr20 18496983
chr20 18497016
chr20 18497019
chr20 18497044
chr20 18497052
chr20 18497055
chr20 18497062
chr20 18497104
chr20 18497122
chr20 18497128
chr20 18497134
chr20 18497349
chr20 18497440
chr20 18497541
chr20 18507607
chr20 18507611
chr20 18507633

chr20 18507648
chr20 18507657
chr20 18507668
chr20 18507673
chr20 18507680
chr20 18507683
chr20 18507704
chr20 18507722
chr20 18507729
chr20 18507740
chr20 18507752
chr20 18507809
chr20 18507823
chr20 18507826
chr20 18507834
chr20 18507978
chr20 18507984
chr20 18507996
chr20 18508001
chr20 18508012
chr20 18508020
chr20 18508028
chr20 18508101
chr20 18508118
chr20 18514877
chr20 18543785
chr20 18567247
chr20 18567252
chr20 18567309
chr20 18567317
chr20 18567341
chr20 18567352
chr20 18567358
chr20 18567362
chr20 18567375
chr20 18567392
chr20 18567395
chr20 18567405
chr20 18567410
chr20 18567413
chr20 18567422
chr20 18567431
chr20 18567440
chr20 18567450
chr20 18567457
chr20 18567466
chr20 18567472
chr20 18567485
chr20 18567498
chr20 18567509
chr20 18567512
chr20 18567519
chr20 18567529
chr20 18567535

chr20 18567575
chr20 18567585
chr20 18567591
chr20 18567608
chr20 18567611
chr20 18567623
chr20 18567632
chr20 18567639
chr20 18567653
chr20 19205937
chr20 19205960
chr20 19441281
chr20 19441293
chr20 19441607
chr20 19441651
chr20 19442035
chr20 19451956
chr20 19452195
chr20 19682840
chr20 19737237
chr20 19737347
chr20 19737372
chr20 19737442
chr20 19937344
chr20 19937351
chr20 19937355
chr20 19937373
chr20 19937380
chr20 19937398
chr20 19937401
chr20 19937422
chr20 19937455
chr20 19937478
chr20 19937587
chr20 19937833
chr20 19937876
chr20 19937972
chr20 20052881
chr20 20052889
chr20 20052901
chr20 20053606
chr20 20083812
chr20 20084077
chr20 20084080
chr20 20084392
chr20 20237851
chr20 20238004
chr20 20277025
chr20 20277028
chr20 20360462
chr20 20360464
chr20 20360508
chr20 20360593
chr20 20360597

chr20 20360623
chr20 20360746
chr20 20361015
chr20 20361023
chr20 20361094
chr20 20361591
chr20 20361715
chr20 20362900
chr20 20362987
chr20 20362990
chr20 20363436
chr20 20452064
chr20 20452225
chr20 20452306
chr20 20452383
chr20 20452418
chr20 20452442
chr20 20452462
chr20 20452471
chr20 20452487
chr20 20452502
chr20 20452521
chr20 20452524
chr20 20452527
chr20 20452559
chr20 20452569
chr20 20452602
chr20 20452608
chr20 20452616
chr20 20452623
chr20 20452644
chr20 20452653
chr20 20452680
chr20 20452684
chr20 20503991
chr20 20504021
chr20 20711281
chr20 20712042
chr20 20712105
chr20 20769719
chr20 20769726
chr20 20769728
chr20 20769776
chr20 20769801
chr20 20769824
chr20 20769853
chr20 20769923
chr20 20769986
chr20 20769994
chr20 21100451
chr20 21100666
chr20 21100675
chr20 21100700
chr20 21236082

chr20 21236106
chr20 21236141
chr20 21236213
chr20 21236231
chr20 21304213
chr20 21304225
chr20 21304231
chr20 21393972
chr20 21394058
chr20 21394246
chr20 21394334
chr20 21394580
chr20 21394667
chr20 21394875
chr20 21395271
chr20 21395506
chr20 21395585
chr20 21395628
chr20 21395650
chr20 21395652
chr20 21395699
chr20 21395721
chr20 21395728
chr20 21395736
chr20 21395746
chr20 21398665
chr20 21398788
chr20 21398913
chr20 21399132
chr20 21399246
chr20 21399287
chr20 21399421
chr20 21399427
chr20 21399597
chr20 21399615
chr20 21548442
chr20 21548469
chr20 21548482
chr20 21548511
chr20 21548542
chr20 21548602
chr20 21548698
chr20 21700334
chr20 21700336
chr20 21700453
chr20 21700635
chr20 21700812
chr20 21700884
chr20 21700943
chr20 21709643
chr20 21709726
chr20 21709728
chr20 21709742
chr20 21709757

chr20 21709807
chr20 21709921
chr20 21709977
chr20 21710092
chr20 21710151
chr20 21710198
chr20 21710443
chr20 21710492
chr20 21710560
chr20 21711014
chr20 21711024
chr20 21711083
chr20 21711089
chr20 21711391
chr20 21711702
chr20 21712106
chr20 21712199
chr20 21712392
chr20 21712469
chr20 21712561
chr20 21712589
chr20 21715241
chr20 21715416
chr20 21715419
chr20 21715426
chr20 21715474
chr20 21715477
chr20 21715613
chr20 21715634
chr20 21715829
chr20 21715860
chr20 21715919
chr20 21715933
chr20 21715970
chr20 21716013
chr20 21716036
chr20 21716257
chr20 21717050
chr20 21768004
chr20 21768027
chr20 21768120
chr20 21768152
chr20 21768478
chr20 21886579
chr20 21886797
chr20 21892543
chr20 21892759
chr20 21893415
chr20 21893578
chr20 21893660
chr20 21894152
chr20 21912288
chr20 21912332
chr20 21912610

chr20 21912789
chr20 21962803
chr20 21964271
chr20 21964768
chr20 21964880
chr20 22559663
chr20 22559681
chr20 22559803
chr20 22559822
chr20 22559873
chr20 22560043
chr20 22560082
chr20 22560120
chr20 22560373
chr20 22560491
chr20 22560645
chr20 22560716
chr20 22561109
chr20 22561220
chr20 22561310
chr20 22561343
chr20 22561901
chr20 22562010
chr20 22562014
chr20 22565820
chr20 22566079
chr20 22566140
chr20 22566275
chr20 22566334
chr20 22566442
chr20 22566860
chr20 22566894
chr20 22566943
chr20 22567141
chr20 22567157
chr20 22567214
chr20 22567239
chr20 22567267
chr20 22567301
chr20 22567504
chr20 22567601
chr20 22569999
chr20 22570220
chr20 22570460
chr20 22570594
chr20 22570662
chr20 22570688
chr20 22570698
chr20 22570864
chr20 22570866
chr20 22570868
chr20 22571050
chr20 22571257
chr20 22571404

chr20 22571429
chr20 22571673
chr20 22571675
chr20 22580142
chr20 22580316
chr20 22580957
chr20 22581190
chr20 22581271
chr20 22581291
chr20 22581297
chr20 22581314
chr20 22581445
chr20 22674912
chr20 22674929
chr20 22674937
chr20 22674959
chr20 22674975
chr20 22675057
chr20 22987300
chr20 22987401
chr20 22987765
chr20 22987860
chr20 23143279
chr20 23143296
chr20 23143315
chr20 23143327
chr20 23349954
chr20 23366531
chr20 23366916
chr20 23420099
chr20 23420181
chr20 23420194
chr20 23420245
chr20 23420283
chr20 23420305
chr20 23420311
chr20 23420350
chr20 23420386
chr20 23420420
chr20 23420423
chr20 23427972
chr20 23428038
chr20 23637856
chr20 23637866
chr20 23637875
chr20 23637877
chr20 23637882
chr20 23637884
chr20 24027424
chr20 24027432
chr20 24027434
chr20 24027452
chr20 24027489
chr20 24027539

chr20 24027686
chr20 24027751
chr20 24212886
chr20 24212955
chr20 24212992
chr20 24213002
chr20 24213053
chr20 24213733
chr20 24219306
chr20 24219464
chr20 24219645
chr20 24219689
chr20 24219691
chr20 24219699
chr20 24219701
chr20 24306378
chr20 24389958
chr20 24948452
chr20 24948667
chr20 24948758
chr20 24948838
chr20 24955065
chr20 24955116
chr20 25090876
chr20 25090913
chr20 25090918
chr20 25090983
chr20 25090987
chr20 25310827
chr20 25391452
chr20 25407953
chr20 25407958
chr20 25407966
chr20 25408065
chr20 25408075
chr20 25425484
chr20 25485188
chr20 25485212
chr20 25485305
chr20 25485448
chr20 25485490
chr20 25485522
chr20 25541863
chr20 25541936
chr20 25697074
chr20 25697102
chr20 25697114
chr20 25697122
chr20 25697144
chr20 25697147
chr20 25697313
chr20 25697623
chr20 25782363
chr20 25982200

chr20 25982634
chr20 25982677
chr20 25983131
chr20 25983199
chr20 25992939
chr20 25992942
chr20 25992949
chr20 25993061
chr20 26010616
chr20 26010658
chr20 26010787
chr20 26010807
chr20 26011257
chr20 26206875
chr20 26207079
chr20 26207370
chr20 26207377
chr20 26207395
chr20 28504769
chr20 28504946
chr20 28600615
chr20 28600893
chr20 28601109
chr20 28602588
chr20 28602614
chr20 28602634
chr20 28602664
chr20 28602678
chr20 28602684
chr20 28602697
chr20 28602699
chr20 28602714
chr20 28602724
chr20 28602737
chr20 28602753
chr20 28602801
chr20 28602816
chr20 28602836
chr20 28602862
chr20 28602883
chr20 28602886
chr20 28602900
chr20 28602910
chr20 28602938
chr20 28602957
chr20 28602967
chr20 28602983
chr20 28602987
chr20 28602992
chr20 28602995
chr20 28603004
chr20 28603010
chr20 28603019
chr20 28603023

chr20	28619015
chr20	28704360
chr20	28704394
chr20	28704798
chr20	28704804
chr20	28705298
chr20	28734606
chr20	28734826
chr20	28735044
chr20	28735133
chr20	28735789
chr20	28757153
chr20	28757185
chr20	28757454
chr20	28983900
chr20	28983917
chr20	28983954
chr20	28995186
chr20	28995250
chr20	28996858
chr20	29000765
chr20	29000802
chr20	29000996
chr20	29004546
chr20	29004740
chr20	29064732
chr20	29064913
chr20	29064983
chr20	29124848
chr20	29125242
chr20	29125576
chr20	29126057
chr20	29126234
chr20	29126332
chr20	29126424
chr20	29126595
chr20	29360878
chr20	29413622
chr20	29413882
chr20	29413884
chr20	29413915
chr20	29629334
chr20	29629626
chr20	29685646
chr20	29685698
chr20	29685779
chr20	29766124
chr20	29766464
chr20	29790259
chr20	29791232
chr20	29797891
chr20	29798483
chr20	29798508
chr20	29873165

chr20 29873198
chr20 29873211
chr20 30252335
chr20 30278469
chr20 30278819
chr20 30279113
chr20 30279299
chr20 30456152
chr20 30456263
chr20 30503516
chr20 30504050
chr20 30504309
chr20 30591932
chr20 30721678
chr20 30817500
chr20 30817584
chr20 30857966
chr20 30858020
chr20 30865482
chr20 30865807
chr20 30895949
chr20 30895952
chr20 30895958
chr20 30895975
chr20 30897167
chr20 30897191
chr20 30897262
chr20 31202374
chr20 31220824
chr20 31220849
chr20 31220869
chr20 31249389
chr20 31249446
chr20 31515153
chr20 31515190
chr20 31515423
chr20 31515769
chr20 31604409
chr20 31604422
chr20 31604427
chr20 31604455
chr20 31604464
chr20 31604466
chr20 31604469
chr20 31604552
chr20 31604608
chr20 31604630
chr20 31604684
chr20 31604703
chr20 31604727
chr20 31604742
chr20 31604798
chr20 31604802
chr20 31604814

chr20 31604841
chr20 31604865
chr20 31604901
chr20 31604909
chr20 31604913
chr20 31605052
chr20 31605059
chr20 31605069
chr20 31605081
chr20 31605095
chr20 31605121
chr20 31605126
chr20 31605141
chr20 31675422
chr20 31676006
chr20 31676015
chr20 31676034
chr20 31676045
chr20 31676071
chr20 31739444
chr20 31739448
chr20 31739450
chr20 31739453
chr20 31739480
chr20 31739487
chr20 31739491
chr20 31739494
chr20 31739502
chr20 31739514
chr20 31739527
chr20 31739534
chr20 31739580
chr20 31739599
chr20 31739610
chr20 31739620
chr20 31739626
chr20 31740619
chr20 31746710
chr20 31967180
chr20 31967439
chr20 31967534
chr20 31967548
chr20 31967602
chr20 31967685
chr20 31967725
chr20 32091954
chr20 32092043
chr20 32109904
chr20 32109954
chr20 32109970
chr20 32109973
chr20 32109978
chr20 32109991
chr20 32110004

chr20 32110007
chr20 32110032
chr20 32277751
chr20 32277756
chr20 32277772
chr20 32277781
chr20 32277785
chr20 32277792
chr20 32277814
chr20 32277832
chr20 32277839
chr20 32277843
chr20 32277845
chr20 32277869
chr20 32277871
chr20 32277876
chr20 32277919
chr20 32277923
chr20 32277953
chr20 32277955
chr20 32277960
chr20 32277973
chr20 32277987
chr20 32278012
chr20 32278043
chr20 32278051
chr20 32278066
chr20 32278073
chr20 32278076
chr20 32278282
chr20 32278458
chr20 32285797
chr20 32285815
chr20 32285958
chr20 32355077
chr20 32355568
chr20 32355602
chr20 32355706
chr20 32355815
chr20 32359502
chr20 32359532
chr20 32359544
chr20 32359563
chr20 32359732
chr20 32359737
chr20 32359745
chr20 32359849
chr20 32359921
chr20 32359934
chr20 32362909
chr20 32362915
chr20 32362939
chr20 32363285
chr20 32485137

chr20 32485143
chr20 32485176
chr20 32485192
chr20 32485218
chr20 32485232
chr20 32485364
chr20 32485366
chr20 32485372
chr20 32485403
chr20 32485411
chr20 32485449
chr20 32485454
chr20 32485468
chr20 32485692
chr20 32534836
chr20 32535239
chr20 32535346
chr20 32535415
chr20 32535478
chr20 32535480
chr20 32535489
chr20 32535492
chr20 32535498
chr20 32535507
chr20 32535512
chr20 32535565
chr20 32583490
chr20 32583493
chr20 32583495
chr20 32583500
chr20 32583505
chr20 32583512
chr20 32583514
chr20 32583519
chr20 32583523
chr20 32583543
chr20 32583554
chr20 32583557
chr20 32583559
chr20 32583564
chr20 32583568
chr20 32583573
chr20 32583579
chr20 32583584
chr20 32583602
chr20 32583605
chr20 32583608
chr20 32583630
chr20 32583635
chr20 32583641
chr20 32583645
chr20 32583688
chr20 32583691
chr20 32583700

chr20 32583702
chr20 32583709
chr20 32583711
chr20 32583714
chr20 32583718
chr20 32583732
chr20 32583737
chr20 32583751
chr20 32583757
chr20 32583761
chr20 32583765
chr20 32583769
chr20 32583786
chr20 32583792
chr20 32583800
chr20 32583814
chr20 32583822
chr20 32583826
chr20 32742606
chr20 32742792
chr20 32742815
chr20 32742921
chr20 33401564
chr20 33401588
chr20 33401606
chr20 33401611
chr20 33401642
chr20 33401652
chr20 33444317
chr20 33444367
chr20 33444369
chr20 33444381
chr20 33444429
chr20 33444438
chr20 33444459
chr20 33444478
chr20 33444488
chr20 33444494
chr20 33444519
chr20 33444536
chr20 33444542
chr20 33444548
chr20 33444637
chr20 33444739
chr20 33444788
chr20 33444805
chr20 33456199
chr20 33456307
chr20 33456522
chr20 33456532
chr20 33456546
chr20 33456572
chr20 33456594
chr20 33456761

chr20 33456882
chr20 33456990
chr20 33457308
chr20 33457331
chr20 33457341
chr20 33457387
chr20 33555071
chr20 33555337
chr20 33667006
chr20 33667062
chr20 33667064
chr20 33667073
chr20 33667079
chr20 33667088
chr20 33667091
chr20 33667097
chr20 33667105
chr20 33667108
chr20 33667110
chr20 33667135
chr20 33667140
chr20 33667142
chr20 33667163
chr20 33667178
chr20 33667185
chr20 33667187
chr20 33667191
chr20 33667202
chr20 33667204
chr20 33667246
chr20 33667250
chr20 33667260
chr20 33667265
chr20 33667268
chr20 33667275
chr20 33667283
chr20 33667287
chr20 33667290
chr20 33667295
chr20 33667297
chr20 33667301
chr20 33667317
chr20 33667320
chr20 33667328
chr20 33667340
chr20 33667360
chr20 33667374
chr20 33667381
chr20 33667404
chr20 33667416
chr20 33667421
chr20 33667424
chr20 33667430
chr20 33667433

chr20 33667437
chr20 33667439
chr20 33667444
chr20 33667446
chr20 33667457
chr20 33667470
chr20 33667488
chr20 33667490
chr20 33667509
chr20 33667511
chr20 33667520
chr20 33667533
chr20 33667536
chr20 33667538
chr20 33667545
chr20 33667554
chr20 33667562
chr20 33667566
chr20 33667568
chr20 33667572
chr20 33667581
chr20 33667586
chr20 33667596
chr20 33667598
chr20 33667607
chr20 33667627
chr20 33667635
chr20 33667655
chr20 33667657
chr20 33667666
chr20 33667682
chr20 33667685
chr20 33667717
chr20 33667744
chr20 33667755
chr20 33667770
chr20 33667776
chr20 33667783
chr20 33667785
chr20 33667791
chr20 33667795
chr20 33667805
chr20 33667818
chr20 33667827
chr20 33667836
chr20 33667855
chr20 33667861
chr20 33667869
chr20 33667886
chr20 33667920
chr20 33667922
chr20 33667933
chr20 33667937
chr20 33667958

chr20 33667963
chr20 33667969
chr20 33667983
chr20 33667995
chr20 33667998
chr20 33668022
chr20 33668025
chr20 33668029
chr20 33668051
chr20 33668059
chr20 33668062
chr20 33690200
chr20 33690227
chr20 33690315
chr20 33690320
chr20 33690506
chr20 33811892
chr20 33812284
chr20 33993359
chr20 33993367
chr20 33993378
chr20 33993396
chr20 33993419
chr20 34110869
chr20 34111073
chr20 34129690
chr20 34129714
chr20 34129721
chr20 34129738
chr20 34129772
chr20 34129793
chr20 34129797
chr20 34129800
chr20 34129916
chr20 34130201
chr20 34130215
chr20 34130408
chr20 34165505
chr20 34216203
chr20 34216207
chr20 34216216
chr20 34216236
chr20 34216238
chr20 34216252
chr20 34216255
chr20 34216258
chr20 34216261
chr20 34216264
chr20 34216267
chr20 34216270
chr20 34216281
chr20 34216283
chr20 34216288
chr20 34216297

chr20 34216308
chr20 34216310
chr20 34216312
chr20 34216316
chr20 34216320
chr20 34216326
chr20 34216349
chr20 34216353
chr20 34216361
chr20 34216363
chr20 34216374
chr20 34216413
chr20 34216510
chr20 34222438
chr20 34222445
chr20 34222484
chr20 34515337
chr20 34515355
chr20 34515393
chr20 34515441
chr20 34515447
chr20 34515455
chr20 34515550
chr20 34515552
chr20 34515557
chr20 34515569
chr20 34515612
chr20 34515622
chr20 34515634
chr20 34515642
chr20 34515678
chr20 34515684
chr20 34515689
chr20 34515728
chr20 34515735
chr20 34515749
chr20 34515779
chr20 34515839
chr20 34515845
chr20 34515871
chr20 34515877
chr20 34515933
chr20 34515955
chr20 34515963
chr20 34515979
chr20 34515995
chr20 34621024
chr20 34676133
chr20 34676336
chr20 34676382
chr20 34676390
chr20 34676399
chr20 34676419
chr20 34676483

chr20 34676496
chr20 34676509
chr20 34676537
chr20 34676582
chr20 34676588
chr20 34676617
chr20 34676619
chr20 34709519
chr20 34709530
chr20 34709535
chr20 34709541
chr20 34709564
chr20 34709620
chr20 34709625
chr20 34709627
chr20 34709631
chr20 34709642
chr20 34709681
chr20 34709692
chr20 34709696
chr20 34824300
chr20 34824472
chr20 34824552
chr20 34875954
chr20 34875993
chr20 34876000
chr20 34876094
chr20 34876099
chr20 34876105
chr20 34876107
chr20 34876154
chr20 34876251
chr20 34876296
chr20 34876306
chr20 34876327
chr20 34876354
chr20 34876360
chr20 34876379
chr20 34876382
chr20 34876385
chr20 34876397
chr20 34876424
chr20 34876461
chr20 34876482
chr20 34876488
chr20 34876503
chr20 34876511
chr20 34876546
chr20 34876556
chr20 34876565
chr20 34876623
chr20 34876625
chr20 34876642
chr20 34876664

chr20 34876670
chr20 34876675
chr20 34876681
chr20 34876688
chr20 35092370
chr20 35092376
chr20 35092552
chr20 35092579
chr20 35092584
chr20 35092609
chr20 35092620
chr20 35096397
chr20 35096491
chr20 35096559
chr20 35096681
chr20 35096711
chr20 35277637
chr20 35277677
chr20 35277680
chr20 35277688
chr20 35277692
chr20 35405108
chr20 35405143
chr20 35411380
chr20 35411383
chr20 35411410
chr20 35411532
chr20 35411739
chr20 35411766
chr20 35411782
chr20 35411805
chr20 35411813
chr20 35411852
chr20 35411872
chr20 35411885
chr20 35411897
chr20 35411915
chr20 35411947
chr20 35411958
chr20 35411983
chr20 35412002
chr20 35412019
chr20 35412033
chr20 35412036
chr20 35412056
chr20 35412062
chr20 35412073
chr20 35412078
chr20 35412080
chr20 35447279
chr20 35447281
chr20 35447340
chr20 35454839
chr20 35454866

chr20 35454901
chr20 35454915
chr20 35454917
chr20 35454936
chr20 35455011
chr20 35455033
chr20 35455045
chr20 35455048
chr20 35455085
chr20 35455103
chr20 35455119
chr20 35455139
chr20 35455151
chr20 35455157
chr20 35455164
chr20 35455180
chr20 35455187
chr20 35455198
chr20 35455200
chr20 35455213
chr20 35455217
chr20 35455223
chr20 35455230
chr20 35455234
chr20 35455273
chr20 35455282
chr20 35455286
chr20 35455293
chr20 35455301
chr20 35455305
chr20 35455314
chr20 35455329
chr20 35455333
chr20 35455340
chr20 35455355
chr20 35455366
chr20 35455389
chr20 35455391
chr20 35455394
chr20 35455404
chr20 35455411
chr20 35455434
chr20 35455442
chr20 35455456
chr20 35455459
chr20 35455462
chr20 35455477
chr20 35455523
chr20 35455526
chr20 35455528
chr20 35490775
chr20 35490833
chr20 35490844
chr20 35491154

chr20 35491267
chr20 35491305
chr20 35491363
chr20 35491400
chr20 35542028
chr20 35542041
chr20 35574358
chr20 35616007
chr20 35616010
chr20 35616019
chr20 35616023
chr20 35616029
chr20 35616035
chr20 35616041
chr20 35616043
chr20 35616053
chr20 35616069
chr20 35616095
chr20 35616115
chr20 35616122
chr20 35616129
chr20 35616144
chr20 35616160
chr20 35616167
chr20 35616174
chr20 35616183
chr20 35616192
chr20 35616194
chr20 35616204
chr20 35616245
chr20 35616250
chr20 35616279
chr20 35616281
chr20 35616288
chr20 35616297
chr20 35616305
chr20 35616311
chr20 35616315
chr20 35616321
chr20 35616327
chr20 35616348
chr20 35616362
chr20 35616372
chr20 35616378
chr20 35616463
chr20 35616471
chr20 35616483
chr20 35616499
chr20 35616535
chr20 35616537
chr20 35616552
chr20 35616603
chr20 35616612
chr20 35616654

chr20 35661334
chr20 35661362
chr20 35661620
chr20 35661686
chr20 35661778
chr20 35661955
chr20 35662128
chr20 35662161
chr20 35662862
chr20 35664470
chr20 35664510
chr20 35664522
chr20 35664533
chr20 35664544
chr20 35664549
chr20 35664567
chr20 35664635
chr20 35664652
chr20 35664662
chr20 35664664
chr20 35664670
chr20 35664680
chr20 35664689
chr20 35664694
chr20 35664714
chr20 35664721
chr20 35664735
chr20 35664748
chr20 35664760
chr20 35664772
chr20 35664774
chr20 35664794
chr20 35664806
chr20 35664808
chr20 35664810
chr20 35664820
chr20 35664825
chr20 35664827
chr20 35664831
chr20 35664845
chr20 35664867
chr20 35664885
chr20 35664888
chr20 35664904
chr20 35664907
chr20 35664912
chr20 35664919
chr20 35664925
chr20 35664927
chr20 35664947
chr20 35664950
chr20 35664959
chr20 35664970
chr20 35664972

chr20 35664978
chr20 35664980
chr20 35738977
chr20 35738990
chr20 35739811
chr20 35740016
chr20 35740495
chr20 35740503
chr20 35740586
chr20 35740632
chr20 35740698
chr20 35740735
chr20 35740768
chr20 35740806
chr20 35740865
chr20 35740885
chr20 35743395
chr20 35743566
chr20 35767931
chr20 35767968
chr20 35768043
chr20 35768151
chr20 35768162
chr20 35768184
chr20 35768188
chr20 35768278
chr20 35768678
chr20 35768691
chr20 35771986
chr20 35771993
chr20 35771995
chr20 35772000
chr20 35772009
chr20 35772039
chr20 35772054
chr20 35772057
chr20 35772063
chr20 35772074
chr20 35772087
chr20 35772089
chr20 35772097
chr20 35772103
chr20 35772107
chr20 35772111
chr20 35772116
chr20 35772126
chr20 35772132
chr20 35772134
chr20 35772139
chr20 35772141
chr20 35772143
chr20 35772145
chr20 35772148
chr20 35772152

chr20 35772156
chr20 35772162
chr20 35772165
chr20 35772168
chr20 35772172
chr20 35772178
chr20 35772186
chr20 35772198
chr20 35772204
chr20 35772211
chr20 35772221
chr20 35772225
chr20 35772265
chr20 35772270
chr20 35772274
chr20 35772278
chr20 35772282
chr20 35772296
chr20 35772306
chr20 35772317
chr20 35772320
chr20 35772327
chr20 35772337
chr20 35772340
chr20 35772344
chr20 35772358
chr20 35772367
chr20 35772373
chr20 35772378
chr20 35772380
chr20 35772385
chr20 35772390
chr20 35772403
chr20 35772421
chr20 35772478
chr20 35772596
chr20 35772675
chr20 35772687
chr20 35772722
chr20 35772749
chr20 35772778
chr20 35773031
chr20 35773064
chr20 35773154
chr20 35773262
chr20 35773420
chr20 35773426
chr20 35773443
chr20 35953055
chr20 35953091
chr20 35953206
chr20 35953395
chr20 35953504
chr20 35953521

chr20 35953535
chr20 35953540
chr20 35953558
chr20 35953597
chr20 35953626
chr20 35953654
chr20 35955251
chr20 35955263
chr20 35992839
chr20 35992882
chr20 35992930
chr20 35992987
chr20 35993102
chr20 35993600
chr20 35995089
chr20 35995186
chr20 35995256
chr20 35995283
chr20 35995373
chr20 36049492
chr20 36050512
chr20 36050523
chr20 36050531
chr20 36050536
chr20 36050542
chr20 36050557
chr20 36050560
chr20 36050562
chr20 36050564
chr20 36050567
chr20 36050571
chr20 36050579
chr20 36050591
chr20 36093652
chr20 36093670
chr20 36093676
chr20 36093680
chr20 36093684
chr20 36093688
chr20 36093714
chr20 36093718
chr20 36093720
chr20 36093722
chr20 36093724
chr20 36093748
chr20 36093794
chr20 36093841
chr20 36093889
chr20 36236817
chr20 36384249
chr20 36384252
chr20 36384271
chr20 36384677
chr20 36644521

chr20 36644714
chr20 36644790
chr20 36644807
chr20 36644832
chr20 36644839
chr20 36644850
chr20 36658522
chr20 36658620
chr20 36658624
chr20 36658748
chr20 36755097
chr20 36835633
chr20 36835697
chr20 36835712
chr20 36835726
chr20 36835857
chr20 36835860
chr20 36835863
chr20 36835866
chr20 36835886
chr20 36919375
chr20 36919871
chr20 36930696
chr20 36930760
chr20 36930804
chr20 36930844
chr20 36931035
chr20 36931277
chr20 36947014
chr20 36947085
chr20 36947233
chr20 36950179
chr20 36950310
chr20 36950594
chr20 36974859
chr20 36974889
chr20 36975028
chr20 36975162
chr20 36975296
chr20 37043426
chr20 37043439
chr20 37043525
chr20 37043592
chr20 37043646
chr20 37043664
chr20 37043694
chr20 37043863
chr20 37044060
chr20 37044145
chr20 37044231
chr20 37178503
chr20 37178510
chr20 37178592
chr20 37178604

chr20 37178629
chr20 37178631
chr20 37178645
chr20 37178654
chr20 37178671
chr20 37178693
chr20 37178725
chr20 37178729
chr20 37567736
chr20 37567743
chr20 37567895
chr20 37568079
chr20 37568121
chr20 37568176
chr20 37568232
chr20 37568262
chr20 37568337
chr20 37693790
chr20 37693798
chr20 37693816
chr20 37693818
chr20 38303403
chr20 38303527
chr20 38303550
chr20 38303632
chr20 38303667
chr20 38303778
chr20 38303921
chr20 38303975
chr20 38303979
chr20 38303983
chr20 38303985
chr20 38303995
chr20 38304015
chr20 38304077
chr20 38321754
chr20 38805002
chr20 38805029
chr20 38805081
chr20 38805102
chr20 38805253
chr20 38805391
chr20 38805417
chr20 38805444
chr20 38805485
chr20 38805502
chr20 38805515
chr20 38805524
chr20 38875010
chr20 38875077
chr20 38875146
chr20 38875280
chr20 38875405
chr20 38875555

chr20 38875589
chr20 38875608
chr20 38875692
chr20 38875748
chr20 38875793
chr20 38875804
chr20 38875870
chr20 38962818
chr20 38962824
chr20 38962832
chr20 38962863
chr20 38963105
chr20 38963191
chr20 39766536
chr20 40193534
chr20 40369804
chr20 40369970
chr20 40621261
chr20 40646776
chr20 40916812
chr20 40968883
chr20 40968945
chr20 40968961
chr20 41003427
chr20 41003561
chr20 41008431
chr20 41028947
chr20 41048221
chr20 41048481
chr20 41048592
chr20 41049040
chr20 41049108
chr20 41138127
chr20 41138225
chr20 41138590
chr20 41138628
chr20 41138696
chr20 41138712
chr20 41138718
chr20 41138744
chr20 41138776
chr20 41138850
chr20 41138852
chr20 41138869
chr20 41138902
chr20 41138968
chr20 41139300
chr20 41139455
chr20 41139537
chr20 41139722
chr20 41232280
chr20 41232499
chr20 41232614
chr20 41329594

chr20 41329609
chr20 41329723
chr20 41329795
chr20 41692962
chr20 41692976
chr20 41692979
chr20 41693017
chr20 41693024
chr20 41693028
chr20 41693063
chr20 41693074
chr20 41693088
chr20 41693095
chr20 41693100
chr20 41693108
chr20 41693115
chr20 41693120
chr20 41693151
chr20 41693156
chr20 41693181
chr20 41693193
chr20 41693200
chr20 41693212
chr20 41693217
chr20 41693247
chr20 41693249
chr20 41693258
chr20 41693266
chr20 41693268
chr20 41693296
chr20 41693311
chr20 41693319
chr20 41693441
chr20 41693505
chr20 41693537
chr20 41693542
chr20 43187508
chr20 43187612
chr20 43187665
chr20 43187790
chr20 43187918
chr20 43187922
chr20 43448509
chr20 43448851
chr20 43590747
chr20 43728501
chr20 43728878
chr20 43924896
chr20 43924974
chr20 43925046
chr20 43950048
chr20 43950058
chr20 43950185
chr20 43950230

chr20 43950267
chr20 43950278
chr20 43950288
chr20 43950315
chr20 43950342
chr20 43950352
chr20 43950385
chr20 44019495
chr20 44019732
chr20 44019751
chr20 44019753
chr20 44069346
chr20 44069350
chr20 44069414
chr20 44069417
chr20 44069420
chr20 44069454
chr20 44069475
chr20 44069497
chr20 44069518
chr20 44069521
chr20 44069547
chr20 44069682
chr20 44069691
chr20 44069697
chr20 44069806
chr20 44069819
chr20 44069822
chr20 44069847
chr20 44069850
chr20 44069886
chr20 44070052
chr20 44070188
chr20 44070204
chr20 44070210
chr20 44070268
chr20 44187179
chr20 44187188
chr20 44187197
chr20 44187200
chr20 44187208
chr20 44209982
chr20 44210017
chr20 44210044
chr20 44210049
chr20 44210915
chr20 44210922
chr20 44210925
chr20 44210929
chr20 44210931
chr20 44210942
chr20 44210947
chr20 44210953
chr20 44210970

chr20 44210974
chr20 44210981
chr20 44211001
chr20 44211022
chr20 44211036
chr20 44211041
chr20 44211052
chr20 44211091
chr20 44211123
chr20 44211128
chr20 44211141
chr20 44211145
chr20 44211155
chr20 44217746
chr20 44217801
chr20 44217884
chr20 44217948
chr20 44217993
chr20 44218027
chr20 44218119
chr20 44218206
chr20 44218486
chr20 44218526
chr20 44218529
chr20 44218644
chr20 44219423
chr20 44301251
chr20 44521841
chr20 44521857
chr20 44521870
chr20 44521877
chr20 44521885
chr20 44521888
chr20 44521896
chr20 44521904
chr20 44521921
chr20 44521957
chr20 44521976
chr20 44522000
chr20 44522041
chr20 44522054
chr20 44522060
chr20 44522076
chr20 44522080
chr20 44522086
chr20 44522091
chr20 44522094
chr20 44522102
chr20 44522109
chr20 44522113
chr20 44522115
chr20 44522119
chr20 44522124
chr20 44522137

chr20 44522327
chr20 44522348
chr20 44522476
chr20 44522510
chr20 44522520
chr20 44522593
chr20 44522617
chr20 44610667
chr20 44646013
chr20 44646072
chr20 44646268
chr20 44646283
chr20 44646541
chr20 44646703
chr20 44647043
chr20 44647048
chr20 44652613
chr20 44652655
chr20 44652692
chr20 44652708
chr20 44652723
chr20 44652725
chr20 44652788
chr20 44652796
chr20 44652804
chr20 44652806
chr20 44745385
chr20 44745405
chr20 44745425
chr20 44745445
chr20 44745586
chr20 44745598
chr20 44745611
chr20 44745635
chr20 44745658
chr20 44745803
chr20 44745807
chr20 44886750
chr20 44886843
chr20 44886960
chr20 44887227
chr20 44959377
chr20 44959480
chr20 44959610
chr20 44959654
chr20 44959667
chr20 44959803
chr20 44959819
chr20 44959832
chr20 44959844
chr20 44959860
chr20 44959953
chr20 44959992
chr20 44966473

chr20 44966480
chr20 44966500
chr20 44966505
chr20 44966520
chr20 44966522
chr20 44966542
chr20 44966552
chr20 44966565
chr20 44966576
chr20 44966594
chr20 44966597
chr20 44966599
chr20 44966602
chr20 44966628
chr20 44966655
chr20 44966694
chr20 44966704
chr20 44966713
chr20 44966744
chr20 44966763
chr20 44966847
chr20 44966882
chr20 44967618
chr20 44967819
chr20 44967873
chr20 44968160
chr20 44968176
chr20 44968198
chr20 44968206
chr20 44968223
chr20 44968240
chr20 44968284
chr20 44968294
chr20 44968327
chr20 44968338
chr20 44968372
chr20 44968379
chr20 44968416
chr20 44968426
chr20 44968432
chr20 44968581
chr20 44968585
chr20 44968607
chr20 44968859
chr20 44968915
chr20 44968989
chr20 44969004
chr20 44969491
chr20 44969606
chr20 44969735
chr20 44969947
chr20 44970033
chr20 44970064
chr20 44970113

chr20 44970118
chr20 44970135
chr20 44970527
chr20 44971021
chr20 44971488
chr20 44971630
chr20 44971723
chr20 44971731
chr20 44971739
chr20 44971771
chr20 44971790
chr20 44971819
chr20 44971827
chr20 44971861
chr20 44971892
chr20 44971906
chr20 44971922
chr20 45415458
chr20 45415688
chr20 45415911
chr20 45415927
chr20 45415946
chr20 45415965
chr20 45416799
chr20 45734739
chr20 45734899
chr20 45735496
chr20 45738770
chr20 45791859
chr20 45791864
chr20 45791866
chr20 45791869
chr20 45791874
chr20 45791892
chr20 45791903
chr20 45791906
chr20 45791908
chr20 45791914
chr20 45791918
chr20 45791922
chr20 45791929
chr20 45791931
chr20 45791942
chr20 45791947
chr20 45791950
chr20 45791960
chr20 45791964
chr20 45791980
chr20 45791995
chr20 45792001
chr20 45792019
chr20 45792022
chr20 45792025
chr20 45792033

chr20 45792035
chr20 45792046
chr20 45792052
chr20 45792061
chr20 45792085
chr20 45792090
chr20 45792262
chr20 45792281
chr20 45833891
chr20 45833904
chr20 45833906
chr20 45833909
chr20 45833911
chr20 45833926
chr20 45857345
chr20 45857350
chr20 45857352
chr20 45857359
chr20 45857361
chr20 45857389
chr20 45857409
chr20 45857429
chr20 45857476
chr20 45857532
chr20 45857558
chr20 45857569
chr20 45857577
chr20 45857650
chr20 45857703
chr20 45857729
chr20 45857785
chr20 45857821
chr20 45857879
chr20 45857924
chr20 45857930
chr20 45857934
chr20 45857967
chr20 45857969
chr20 45857988
chr20 45857992
chr20 45857997
chr20 45858002
chr20 45858035
chr20 45858071
chr20 45858083
chr20 45881362
chr20 45881435
chr20 45881491
chr20 45881594
chr20 45885129
chr20 45933854
chr20 45933877
chr20 45933881
chr20 45933920

chr20 45933965
chr20 45934074
chr20 45934100
chr20 45934150
chr20 45934166
chr20 45934203
chr20 45934254
chr20 45934256
chr20 45934258
chr20 45934285
chr20 45934289
chr20 45971167
chr20 45971169
chr20 45971188
chr20 45971191
chr20 45971199
chr20 45971217
chr20 45971224
chr20 45971226
chr20 45971273
chr20 45971278
chr20 45971282
chr20 45971284
chr20 45971287
chr20 45971290
chr20 45971299
chr20 45971302
chr20 45971308
chr20 46004786
chr20 46005085
chr20 46005104
chr20 46005256
chr20 46022051
chr20 46022057
chr20 46022059
chr20 46022064
chr20 46022103
chr20 46022111
chr20 46022162
chr20 46022183
chr20 46022206
chr20 46022224
chr20 46022235
chr20 46022246
chr20 46022329
chr20 46022332
chr20 46022364
chr20 46022428
chr20 46022434
chr20 46022460
chr20 46022480
chr20 46022488
chr20 46022498
chr20 46022534

chr20 46022541
chr20 46022554
chr20 46022565
chr20 46108491
chr20 46311741
chr20 46311844
chr20 46311846
chr20 46311955
chr20 46311984
chr20 46312150
chr20 46312157
chr20 46312168
chr20 46312256
chr20 46312322
chr20 46312338
chr20 46312415
chr20 46312482
chr20 46363717
chr20 46459590
chr20 46459710
chr20 46459728
chr20 46460020
chr20 46513881
chr20 46513937
chr20 46513989
chr20 46514014
chr20 46545170
chr20 46589427
chr20 46589725
chr20 46690118
chr20 46690131
chr20 46724875
chr20 46994483
chr20 47355400
chr20 47355470
chr20 47356015
chr20 47356103
chr20 47356567
chr20 47356595
chr20 47356606
chr20 47356648
chr20 47356751
chr20 47356767
chr20 47356788
chr20 47356801
chr20 47356809
chr20 47356852
chr20 47356871
chr20 47356992
chr20 47356997
chr20 47357047
chr20 47357088
chr20 47357117
chr20 47357212

chr20 47357285
chr20 47357296
chr20 47415397
chr20 47415481
chr20 47487249
chr20 47487294
chr20 47487418
chr20 47487481
chr20 47502242
chr20 47502260
chr20 47502272
chr20 47502284
chr20 47502286
chr20 47502288
chr20 47502301
chr20 47502312
chr20 47502442
chr20 47502446
chr20 47502460
chr20 47502462
chr20 47502492
chr20 47503390
chr20 47503446
chr20 47503702
chr20 47610678
chr20 47610683
chr20 47620946
chr20 47900229
chr20 48719169
chr20 48719253
chr20 48719336
chr20 48719412
chr20 48719427
chr20 48719432
chr20 48719539
chr20 48760711
chr20 48821958
chr20 48821981
chr20 48822217
chr20 48822371
chr20 48822611
chr20 48822632
chr20 48822858
chr20 48822941
chr20 48825224
chr20 48825250
chr20 48825307
chr20 48825316
chr20 48825356
chr20 48825575
chr20 48825581
chr20 48825655
chr20 48825872
chr20 48921443

chr20 48921462
chr20 48921472
chr20 48921508
chr20 48921514
chr20 48921531
chr20 48921533
chr20 48921549
chr20 48921599
chr20 48921613
chr20 48921627
chr20 48921632
chr20 48921651
chr20 48921656
chr20 48921681
chr20 48921688
chr20 49038120
chr20 49038272
chr20 49045741
chr20 49045774
chr20 49045869
chr20 49045871
chr20 49045881
chr20 49045918
chr20 49045941
chr20 49045956
chr20 49046006
chr20 49046026
chr20 49047288
chr20 49047303
chr20 49047333
chr20 49047414
chr20 49047481
chr20 49165801
chr20 49166422
chr20 49180071
chr20 49219202
chr20 49219213
chr20 49219247
chr20 49219260
chr20 49219267
chr20 49219279
chr20 49219292
chr20 49219523
chr20 49219529
chr20 49276507
chr20 49283833
chr20 49283841
chr20 49534501
chr20 49534803
chr20 49534876
chr20 49534920
chr20 49534954
chr20 49534962
chr20 49535254

chr20 49620740
chr20 49668723
chr20 49668733
chr20 49668749
chr20 49668755
chr20 49668769
chr20 49668841
chr20 49668858
chr20 49668892
chr20 49669588
chr20 49669627
chr20 49669650
chr20 49669652
chr20 49669683
chr20 49669748
chr20 49669871
chr20 49713045
chr20 49713058
chr20 49812995
chr20 49812998
chr20 49813008
chr20 49813012
chr20 49813022
chr20 49813029
chr20 49813035
chr20 49813042
chr20 49813080
chr20 49813089
chr20 49813098
chr20 49813107
chr20 49859709
chr20 49894209
chr20 49928793
chr20 49928805
chr20 49928814
chr20 49928838
chr20 49928873
chr20 50085987
chr20 50086032
chr20 50086052
chr20 50086142
chr20 50086267
chr20 50110157
chr20 50110246
chr20 50110443
chr20 50110626
chr20 50111064
chr20 50111102
chr20 50113560
chr20 50113794
chr20 50338519
chr20 50338801
chr20 50338901
chr20 50339052

chr20 50339113
chr20 50339126
chr20 50339150
chr20 50339154
chr20 50339171
chr20 50339308
chr20 50386244
chr20 50386274
chr20 50386335
chr20 50386338
chr20 50386348
chr20 50386445
chr20 50386452
chr20 50386456
chr20 50386474
chr20 50386483
chr20 50386501
chr20 50386517
chr20 50386556
chr20 50386566
chr20 50386600
chr20 50461213
chr20 50461255
chr20 50461300
chr20 50461345
chr20 50461369
chr20 50483672
chr20 50483679
chr20 50483694
chr20 50483876
chr20 50483910
chr20 50510205
chr20 50510217
chr20 50510219
chr20 50510221
chr20 50511060
chr20 50511063
chr20 50511568
chr20 50511783
chr20 50511914
chr20 50517941
chr20 50518084
chr20 50518091
chr20 50518167
chr20 50519418
chr20 50519527
chr20 50523923
chr20 50546620
chr20 50546821
chr20 50546901
chr20 50725018
chr20 50725074
chr20 50725102
chr20 50794937

chr20 50794942
chr20 50794944
chr20 50794986
chr20 50794988
chr20 50795000
chr20 50795005
chr20 50795019
chr20 50795026
chr20 50795030
chr20 50795038
chr20 50804832
chr20 50804840
chr20 50804912
chr20 50804950
chr20 50804975
chr20 50805235
chr20 50929457
chr20 50929501
chr20 50929528
chr20 50929573
chr20 50929599
chr20 50929609
chr20 50929620
chr20 50929925
chr20 50930009
chr20 50930011
chr20 50930092
chr20 50930205
chr20 50930348
chr20 50931771
chr20 50931774
chr20 50931777
chr20 50931792
chr20 50931797
chr20 51091957
chr20 51091972
chr20 51092053
chr20 51092057
chr20 51092093
chr20 51092099
chr20 51092120
chr20 51092262
chr20 51092304
chr20 51092395
chr20 51092474
chr20 51092592
chr20 51092611
chr20 51220024
chr20 51220152
chr20 51220159
chr20 51220183
chr20 51220245
chr20 51220248
chr20 51400487

chr20 51400504
chr20 51400566
chr20 51400579
chr20 51400675
chr20 51400686
chr20 51400700
chr20 51400717
chr20 51400731
chr20 51400764
chr20 51400825
chr20 51440473
chr20 51440476
chr20 51440720
chr20 51497723
chr20 51497765
chr20 51497813
chr20 51497873
chr20 51497890
chr20 51497953
chr20 51498053
chr20 51498073
chr20 51498101
chr20 51498109
chr20 51498142
chr20 51498209
chr20 51498215
chr20 51498275
chr20 51500589
chr20 51500709
chr20 51500723
chr20 51525709
chr20 51525730
chr20 51525733
chr20 51540560
chr20 51540799
chr20 51540820
chr20 51540870
chr20 51540916
chr20 51540928
chr20 51540933
chr20 51541122
chr20 51541140
chr20 51541149
chr20 51594852
chr20 51595050
chr20 51799497
chr20 51799606
chr20 51799626
chr20 51799636
chr20 51799658
chr20 51799699
chr20 51799719
chr20 51799809
chr20 51799844

chr20 51799851
chr20 51799882
chr20 51800028
chr20 51800043
chr20 51800111
chr20 51800155
chr20 51958865
chr20 51959061
chr20 51959097
chr20 51959211
chr20 51959503
chr20 51959550
chr20 52002227
chr20 52002272
chr20 52002394
chr20 52002455
chr20 52002635
chr20 52047952
chr20 52047956
chr20 52047966
chr20 52048071
chr20 52048073
chr20 52850181
chr20 52971815
chr20 52971855
chr20 52971942
chr20 52972077
chr20 52972109
chr20 52972115
chr20 52972124
chr20 52972131
chr20 52972145
chr20 52972147
chr20 52972151
chr20 52972160
chr20 52972166
chr20 52972171
chr20 52972174
chr20 52972183
chr20 52972202
chr20 52972205
chr20 52972207
chr20 52972210
chr20 52972219
chr20 52972241
chr20 52972246
chr20 52972268
chr20 52972278
chr20 52972285
chr20 52972319
chr20 52972321
chr20 52972369
chr20 52972848
chr20 52972850

chr20 52972852
chr20 52972867
chr20 52972869
chr20 52972875
chr20 52972882
chr20 52972895
chr20 52972901
chr20 52973828
chr20 52974038
chr20 52974189
chr20 52974302
chr20 52974375
chr20 52974445
chr20 52974486
chr20 53070833
chr20 53070851
chr20 53071013
chr20 53071021
chr20 53071101
chr20 53402679
chr20 53402756
chr20 53402862
chr20 53578341
chr20 53578375
chr20 53578576
chr20 53578591
chr20 53578709
chr20 53578755
chr20 53578766
chr20 53578827
chr20 53578880
chr20 53578914
chr20 53579078
chr20 53579164
chr20 53579252
chr20 53579554
chr20 53579569
chr20 53579606
chr20 53579862
chr20 53579886
chr20 53579967
chr20 53580032
chr20 53580195
chr20 53580264
chr20 53580281
chr20 53580306
chr20 53582842
chr20 53582981
chr20 53583055
chr20 53583190
chr20 53583209
chr20 53583239
chr20 53583251
chr20 53583317

chr20 53583828
chr20 53583869
chr20 53584395
chr20 53584604
chr20 53587801
chr20 53588014
chr20 53588411
chr20 53588573
chr20 53588689
chr20 53588762
chr20 53589041
chr20 53589157
chr20 53589569
chr20 53589642
chr20 53589822
chr20 53589843
chr20 53589880
chr20 53589907
chr20 53589931
chr20 53589947
chr20 53589951
chr20 53590292
chr20 53590331
chr20 53593759
chr20 53593765
chr20 53593773
chr20 53593785
chr20 53593787
chr20 53593789
chr20 53593792
chr20 53593795
chr20 53593799
chr20 53593820
chr20 53593825
chr20 53593829
chr20 53593849
chr20 53593853
chr20 53593855
chr20 53593869
chr20 53593878
chr20 53593896
chr20 53593899
chr20 53593901
chr20 53593936
chr20 53594945
chr20 53595028
chr20 53595067
chr20 53595091
chr20 53595109
chr20 53595205
chr20 53595293
chr20 53595330
chr20 53595418
chr20 53595431

chr20 53609145
chr20 53609179
chr20 53609557
chr20 53609631
chr20 53609657
chr20 53609741
chr20 53609813
chr20 53609820
chr20 53609827
chr20 53609921
chr20 53609953
chr20 53622651
chr20 53622736
chr20 53623006
chr20 53623385
chr20 53623403
chr20 53623445
chr20 53623473
chr20 53623483
chr20 53623489
chr20 53623550
chr20 53623557
chr20 53623575
chr20 53623630
chr20 53623691
chr20 53623727
chr20 53623798
chr20 53623909
chr20 53623925
chr20 53642535
chr20 53642551
chr20 53642555
chr20 53642607
chr20 53642814
chr20 53642937
chr20 53642995
chr20 53643039
chr20 53643116
chr20 53643258
chr20 53643262
chr20 53643325
chr20 53643586
chr20 53652168
chr20 53652367
chr20 53652405
chr20 53652468
chr20 53652506
chr20 53652567
chr20 53652612
chr20 53652960
chr20 53653241
chr20 53685956
chr20 53685999
chr20 53686123

chr20 53708429
chr20 53708459
chr20 53708516
chr20 53740536
chr20 53740578
chr20 53740610
chr20 53751354
chr20 53751385
chr20 53751540
chr20 53751601
chr20 53751741
chr20 53751768
chr20 53751812
chr20 53754382
chr20 53814963
chr20 53815026
chr20 53815278
chr20 53828888
chr20 53828962
chr20 53829155
chr20 53829265
chr20 53829272
chr20 53829349
chr20 53829355
chr20 53835719
chr20 53835723
chr20 53835736
chr20 53835882
chr20 53839883
chr20 53869740
chr20 53869873
chr20 53870015
chr20 53872370
chr20 53872486
chr20 53872695
chr20 53872711
chr20 53872752
chr20 53872869
chr20 53872987
chr20 53873024
chr20 53873046
chr20 53873055
chr20 53873063
chr20 53873155
chr20 53873453
chr20 53893019
chr20 53893087
chr20 53893094
chr20 53893315
chr20 53893408
chr20 53893477
chr20 53893521
chr20 53893709
chr20 53899954

chr20 53900014
chr20 53900208
chr20 53915451
chr20 53915595
chr20 53915608
chr20 53915703
chr20 53915710
chr20 53915715
chr20 53916105
chr20 53935076
chr20 53943801
chr20 53943815
chr20 53961071
chr20 54078460
chr20 54078496
chr20 54078533
chr20 54078548
chr20 54078678
chr20 54175132
chr20 54476989
chr20 54477011
chr20 54477052
chr20 54477058
chr20 54477114
chr20 54477126
chr20 54477306
chr20 54477335
chr20 54477387
chr20 54647010
chr20 54649756
chr20 54649817
chr20 54650241
chr20 54650441
chr20 54785746
chr20 54975221
chr20 54975381
chr20 55113622
chr20 55252323
chr20 55502431
chr20 55502510
chr20 55861866
chr20 55862020
chr20 55948458
chr20 56002461
chr20 56002827
chr20 56391585
chr20 56391608
chr20 56391622
chr20 56391627
chr20 56411964
chr20 56412199
chr20 56412214
chr20 56412274
chr20 56455025

chr20 56455160
chr20 56460445
chr20 56468816
chr20 56468829
chr20 56555160
chr20 56927755
chr20 56927790
chr20 56927800
chr20 56927955
chr20 56928169
chr20 56928296
chr20 56928307
chr20 56928310
chr20 56928396
chr20 57345558
chr20 57345825
chr20 57346206
chr20 57346484
chr20 57346575
chr20 57346592
chr20 57389494
chr20 57389496
chr20 57389499
chr20 57389536
chr20 57389563
chr20 57389573
chr20 57389575
chr20 57389579
chr20 57389581
chr20 57389590
chr20 57389599
chr20 57389650
chr20 57389655
chr20 57389666
chr20 57389706
chr20 57391786
chr20 57391793
chr20 57391804
chr20 57391806
chr20 57391867
chr20 57391872
chr20 57391876
chr20 57391879
chr20 57391890
chr20 57391899
chr20 57391912
chr20 57391918
chr20 57391924
chr20 57391933
chr20 57391943
chr20 57391946
chr20 57391950
chr20 57391959
chr20 57391968

chr20 57391972
chr20 57391974
chr20 57391983
chr20 57391989
chr20 57392010
chr20 57392047
chr20 57392065
chr20 57392123
chr20 57392141
chr20 57392169
chr20 57392188
chr20 57392222
chr20 57392253
chr20 57392292
chr20 57392295
chr20 57392322
chr20 57392349
chr20 57392368
chr20 57392392
chr20 57392447
chr20 57392574
chr20 57392585
chr20 57392591
chr20 57392604
chr20 57392622
chr20 57392665
chr20 57392669
chr20 57392673
chr20 57392700
chr20 57392713
chr20 57392716
chr20 57392720
chr20 57392731
chr20 57392752
chr20 57392760
chr20 57392762
chr20 57392775
chr20 57392811
chr20 57392830
chr20 57392840
chr20 57392865
chr20 57392883
chr20 57392906
chr20 57392931
chr20 57392956
chr20 57392977
chr20 57398460
chr20 57398503
chr20 57398508
chr20 57398530
chr20 57398541
chr20 57398544
chr20 57494963
chr20 57495147

chr20 57495213
chr20 57497583
chr20 57497590
chr20 57497894
chr20 57498460
chr20 57498533
chr20 57498540
chr20 57498562
chr20 57498588
chr20 57498636
chr20 57498673
chr20 57498805
chr20 57498897
chr20 57498906
chr20 57628079
chr20 57749155
chr20 58128005
chr20 58128058
chr20 58128071
chr20 58128087
chr20 58128130
chr20 58236435
chr20 58237240
chr20 58237244
chr20 58237247
chr20 58237250
chr20 58237260
chr20 58237280
chr20 58237297
chr20 58237397
chr20 58237524
chr20 58306621
chr20 58306816
chr20 58306893
chr20 58306895
chr20 58306957
chr20 58310650
chr20 58310790
chr20 58310836
chr20 58388785
chr20 58388977
chr20 58388979
chr20 58652805
chr20 58652852
chr20 58652910
chr20 58653016
chr20 58653047
chr20 58653145
chr20 58653157
chr20 58893230
chr20 58893455
chr20 58893733
chr20 58893906
chr20 58893913

chr20 58893918
chr20 58907684
chr20 58907742
chr20 58907777
chr20 58907803
chr20 58907809
chr20 58907814
chr20 58908009
chr20 58908176
chr20 58908184
chr20 58981486
chr20 58981488
chr20 58981490
chr20 58981494
chr20 58981499
chr20 58981501
chr20 58981503
chr20 58981510
chr20 59032892
chr20 59032924
chr20 59032984
chr20 59032988
chr20 59033036
chr20 59078114
chr20 59078414
chr20 59078440
chr20 59078454
chr20 59078611
chr20 59087999
chr20 59088004
chr20 59088027
chr20 59088097
chr20 59120510
chr20 59120513
chr20 59120538
chr20 59120546
chr20 59120561
chr20 59163705
chr20 59163729
chr20 59163771
chr20 59163776
chr20 59163887
chr20 59163971
chr20 59164043
chr20 59164069
chr20 59164305
chr20 59164327
chr20 59164840
chr20 59164856
chr20 59164911
chr20 59164973
chr20 59165014
chr20 59165782
chr20 59165852

chr20 59165948
chr20 59165975
chr20 59166003
chr20 59166031
chr20 59166214
chr20 59166245
chr20 59166257
chr20 59166340
chr20 59166528
chr20 59166538
chr20 59166541
chr20 59166914
chr20 59177936
chr20 59177950
chr20 59178044
chr20 59178047
chr20 59178103
chr20 59178284
chr20 59253463
chr20 59253518
chr20 59253577
chr20 59253588
chr20 59253673
chr20 59253711
chr20 59253755
chr20 59253823
chr20 59254005
chr20 59519657
chr20 59519768
chr20 59533095
chr20 59533098
chr20 59533385
chr20 59533548
chr20 59533850
chr20 59534104
chr20 59534261
chr20 59534563
chr20 59534647
chr20 59534692
chr20 59539388
chr20 59674523
chr20 59940285
chr20 59940287
chr20 59940290
chr20 59940316
chr20 59940333
chr20 59940341
chr20 59940346
chr20 59940358
chr20 59940361
chr20 59940371
chr20 59940379
chr20 59940382
chr20 59940409

chr20 59940417
chr20 59940419
chr20 59940425
chr20 59940443
chr20 59940448
chr20 59940450
chr20 59940453
chr20 59940459
chr20 59940463
chr20 59940469
chr20 59940471
chr20 59940473
chr20 59940477
chr20 59940480
chr20 59940506
chr20 59940511
chr20 59940517
chr20 59940526
chr20 59940528
chr20 59940681
chr20 59941102
chr20 59941115
chr20 59941312
chr20 59941508
chr20 60045643
chr20 60056089
chr20 60056100
chr20 60056109
chr20 60056128
chr20 60056132
chr20 60056161
chr20 60056165
chr20 60056290
chr20 60056366
chr20 60867201
chr20 61059137
chr20 62183032
chr20 62183041
chr20 62183051
chr20 62183056
chr20 62183065
chr20 62183071
chr20 62183078
chr20 62183085
chr20 62183104
chr20 62183106
chr20 62302187
chr20 62302223
chr20 62509144
chr20 62509324
chr20 62509883
chr20 62509908
chr20 62509917
chr20 62510021

chr20 62602759
chr20 62602767
chr20 62602806
chr20 62651745
chr20 62651785
chr20 62651825
chr20 62918549
chr20 62918687
chr20 62918746
chr20 62918942
chr20 62929198
chr20 62929205
chr20 62929212
chr20 62929220
chr20 62929256
chr20 62929275
chr20 62929278
chr20 62929287
chr20 62929294
chr20 62929305
chr20 62929326
chr20 63102720
chr20 63102728
chr20 63102730
chr20 63102732
chr20 63102737
chr20 63102744
chr20 63102746
chr20 63102750
chr20 63102753
chr20 63102766
chr20 63102784
chr20 63102795
chr20 63102797
chr20 63442231
chr20 63442254
chr20 63442259
chr20 63442281
chr20 63442427
chr20 63442429
chr20 63442447
chr20 63442481
chr20 63442507
chr20 63442544
chr20 63537777
chr20 63537796
chr20 63537799
chr20 63627821
chr20 63627830
chr20 63627844
chr20 63627852
chr20 63627869
chr20 63627875
chr20 63627883

chr20 63627895
chr20 63627907
chr20 63627911
chr20 63627932
chr20 63627963
chr20 63627994
chr20 63628014
chr20 63628020
chr20 63628038
chr20 63628045
chr20 63628055
chr20 63628062
chr20 63628068
chr20 63628098
chr20 63628106
chr20 63628116
chr20 63628122
chr20 63628240
chr20 63628276
chr20 63628302
chr20 63628323
chr20 63628330
chr20 63628343
chr20 63628353
chr20 63635549
chr20 63635561
chr20 63635566
chr20 63635622
chr20 63635732
chr20 63635868
chr20 63635886
chr20 63635896
chr20 63635952
chr20 63635957
chr20 63635974
chr20 63635983
chr20 63636048
chr20 63636063
chr20 63636079
chr20 63640939
chr20 63640987
chr20 63641095
chr20 63641099
chr20 63641178
chr20 63641194
chr20 63641204
chr20 63641276
chr20 63641294
chr20 63641298
chr20 63641308
chr20 63641319
chr20 63641322
chr20 63641326
chr20 63641331

chr20 63641375
chr20 63641382
chr20 63641386
chr20 63641388
chr20 63641429
chr20 63641445
chr20 63641451
chr20 63641689
chr20 63736499
chr20 63736510
chr20 63736516
chr20 63736532
chr20 63736536
chr20 63736541
chr20 63736562
chr20 63736602
chr20 63736609
chr20 63736646
chr20 63736703
chr20 63736904
chr20 63737051
chr20 63737106
chr20 63737111
chr20 63737115
chr20 63737192
chr20 63737212
chr20 63737266
chr20 63737270
chr20 63737285
chr20 63737295
chr20 63737305
chr20 63737342
chr20 63737352
chr20 63737427
chr20 63737434
chr20 63737445
chr20 63737451
chr20 63737453
chr20 63737484
chr20 63942196
chr20 63942228
chr20 63942236
chr20 63942251
chr20 63942257
chr20 63981290
chr20 63981296
chr20 63981301
chr20 63981312
chr20 63981330
chr20 63981333
chr20 63981335
chr20 63981337
chr20 63981344
chr20 63981352

chr20 63981364
chr20 63981379
chr20 63981391
chr20 63981405
chr20 63981407
chr20 63981421
chr20 63981433
chr20 63981457
chr20 63981501
chr20 64011841
chr20 64011864
chr20 64012490
chr20 64012513
chr20 64012549
chr20 64012572
chr20 64012605
chr20 64012628
chr20 64012664
chr20 64012782
chr20 64012805
chr20 64012841
chr20 64077213
chr20 64077228
chr20 64077247
chr20 64077277
chr20 64077305
chr20 64077331
chr20 64077335
chr20 64077528
chr20 64077660
chr20 64077713
chr20 64077721
chr20 64077778
chr20 64077804
chr20 64077848
chr20 64077869
chr20 64077898
chr20 64077920
chr20 64077953
chr20 64077963
chr20 64077982
chr20 64078053
chr20 64078393
chr20 64078427
chr20 64078567
chr20 64078587
chr20 64078742
chr20 64078868
chr20 64078873
chr20 64078915
chr20 64078942
chr20 64079107
chr20 64079182
chr20 64079260

chr20 64079297
chr20 64079308
chr20 64079399
chr20 64132276
chr20 64174525
chr20 64175172
chr20 64175525
chr20 64176069
chr20 64326475
chr20 64326508
chr20 64326590
chr20 64327099
chr20 64327122
chr20 64327156
chr20 64327253
chr17 118359
chr17 118404
chr17 118406
chr17 118413
chr17 118432
chr17 118442
chr17 118462
chr17 118476
chr17 118479
chr17 118485
chr17 118487
chr17 118489
chr17 118502
chr17 118521
chr17 118541
chr17 118544
chr17 118552
chr17 118632
chr17 118817
chr17 120302
chr17 120315
chr17 121416
chr17 121502
chr17 121544
chr17 121576
chr17 121804
chr17 122374
chr17 122646
chr17 123102
chr17 123216
chr17 123558
chr17 123598
chr17 123712
chr17 123786
chr17 123826
chr17 123900
chr17 123940
chr17 124356
chr17 124698

chr17 125008
chr17 125040
chr17 125350
chr17 125382
chr17 125952
chr17 125992
chr17 126066
chr17 126106
chr17 126180
chr17 126220
chr17 126294
chr17 126676
chr17 126718
chr17 126750
chr17 126978
chr17 127246
chr17 127662
chr17 129249
chr17 129328
chr17 133209
chr17 168206
chr17 168213
chr17 168254
chr17 168261
chr17 287218
chr17 287274
chr17 287300
chr17 287326
chr17 287352
chr17 287380
chr17 287512
chr17 287538
chr17 287548
chr17 287564
chr17 287574
chr17 287590
chr17 287616
chr17 287642
chr17 287652
chr17 489282
chr17 489299
chr17 489312
chr17 490111
chr17 490119
chr17 490144
chr17 490152
chr17 490184
chr17 490210
chr17 490224
chr17 490238
chr17 490240
chr17 490242
chr17 490248
chr17 490264

chr17 490266
chr17 490274
chr17 490279
chr17 490295
chr17 490304
chr17 490308
chr17 490329
chr17 490334
chr17 490341
chr17 490350
chr17 490356
chr17 490364
chr17 490377
chr17 490380
chr17 490385
chr17 490388
chr17 490394
chr17 491113
chr17 781489
chr17 781503
chr17 781515
chr17 781531
chr17 781533
chr17 781657
chr17 781667
chr17 781734
chr17 781739
chr17 781782
chr17 781814
chr17 1259808
chr17 1259811
chr17 1275952
chr17 1365149
chr17 1365504
chr17 1398234
chr17 1398392
chr17 1398516
chr17 1398523
chr17 1398631
chr17 1398673
chr17 1398691
chr17 1398801
chr17 1485321
chr17 1485324
chr17 1485361
chr17 1485393
chr17 1485398
chr17 1485409
chr17 1485411
chr17 1485414
chr17 1485419
chr17 1485436
chr17 1485438
chr17 1485445

chr17 1485453
chr17 1485459
chr17 1485474
chr17 1485476
chr17 1485486
chr17 1485571
chr17 1485576
chr17 1485588
chr17 1485707
chr17 1485730
chr17 1485734
chr17 1485739
chr17 1485746
chr17 1485748
chr17 1485753
chr17 1485802
chr17 1485809
chr17 1485814
chr17 1485824
chr17 1485837
chr17 1485839
chr17 1485850
chr17 1717481
chr17 1717537
chr17 1717551
chr17 1717577
chr17 1717621
chr17 1717703
chr17 1717774
chr17 1870537
chr17 1870543
chr17 1870593
chr17 1870636
chr17 1870855
chr17 2050146
chr17 2050149
chr17 2050155
chr17 2050168
chr17 2050170
chr17 2050177
chr17 2050180
chr17 2050183
chr17 2050192
chr17 2050197
chr17 2050203
chr17 2050218
chr17 2050273
chr17 2050283
chr17 2050286
chr17 2050293
chr17 2050318
chr17 2050323
chr17 2157877
chr17 2215867

chr17 2215870
chr17 2215905
chr17 2215983
chr17 2216025
chr17 2216027
chr17 2216060
chr17 2216074
chr17 2216100
chr17 2216163
chr17 2216196
chr17 2216394
chr17 2265978
chr17 2266103
chr17 2266185
chr17 2266208
chr17 2266228
chr17 2266271
chr17 2266275
chr17 2266277
chr17 2266299
chr17 2266346
chr17 2266535
chr17 2266558
chr17 2266695
chr17 2266735
chr17 2353484
chr17 2353533
chr17 2353560
chr17 2353637
chr17 2482440
chr17 2482477
chr17 2482502
chr17 2510950
chr17 2510996
chr17 2511008
chr17 2511063
chr17 2511091
chr17 2511155
chr17 2511404
chr17 2511422
chr17 2511425
chr17 2511450
chr17 2511493
chr17 2511530
chr17 2511571
chr17 2511607
chr17 2511609
chr17 2511613
chr17 2511651
chr17 2511657
chr17 2511695
chr17 2511706
chr17 2511736
chr17 2511768

chr17 2511784
chr17 2511787
chr17 2594636
chr17 2594639
chr17 2594654
chr17 2594662
chr17 2594671
chr17 2594687
chr17 2594716
chr17 2594724
chr17 2594728
chr17 2594938
chr17 2595089
chr17 2595122
chr17 2595135
chr17 2595182
chr17 2595337
chr17 2595348
chr17 2595450
chr17 2595464
chr17 2595694
chr17 2595846
chr17 2595921
chr17 2595982
chr17 3049239
chr17 3148844
chr17 3149139
chr17 3412157
chr17 3412254
chr17 3432127
chr17 3432596
chr17 3788414
chr17 3788488
chr17 3845404
chr17 3845406
chr17 3845438
chr17 3845481
chr17 3845504
chr17 3845524
chr17 3845532
chr17 3845588
chr17 3845620
chr17 3845654
chr17 3845697
chr17 3845758
chr17 3845760
chr17 3845777
chr17 3845810
chr17 3845845
chr17 3845848
chr17 3845853
chr17 3845856
chr17 3845868
chr17 3845873

chr17 3845875
chr17 3845891
chr17 3845894
chr17 3845900
chr17 3845911
chr17 3845930
chr17 3845959
chr17 3845966
chr17 3845975
chr17 3845985
chr17 3845988
chr17 3963427
chr17 3963568
chr17 3963593
chr17 3963618
chr17 4044294
chr17 4215419
chr17 4215422
chr17 4215450
chr17 4215469
chr17 4215475
chr17 4215478
chr17 4215575
chr17 4215623
chr17 4215687
chr17 4304564
chr17 4304570
chr17 4304606
chr17 4375369
chr17 4375486
chr17 4375512
chr17 4639635
chr17 4639639
chr17 4639659
chr17 4639672
chr17 4639685
chr17 4639708
chr17 4639714
chr17 4639746
chr17 4639751
chr17 4639758
chr17 4639811
chr17 4639825
chr17 4639831
chr17 4639848
chr17 4639896
chr17 4639908
chr17 4639914
chr17 4639916
chr17 4639925
chr17 4639929
chr17 4639931
chr17 4639943
chr17 4639947

chr17 4639949
chr17 4639965
chr17 4639972
chr17 4639990
chr17 4639994
chr17 4640022
chr17 4640042
chr17 4640107
chr17 4640109
chr17 4640122
chr17 4640125
chr17 4640139
chr17 4640194
chr17 4640204
chr17 4640213
chr17 4640237
chr17 4640247
chr17 4640254
chr17 4640258
chr17 4640301
chr17 4640340
chr17 4640449
chr17 4640482
chr17 4640489
chr17 4640562
chr17 4640564
chr17 4640579
chr17 4640586
chr17 4640605
chr17 4640607
chr17 4640622
chr17 4640626
chr17 4640658
chr17 4640666
chr17 4640674
chr17 4640685
chr17 4640694
chr17 4640709
chr17 4640725
chr17 4640727
chr17 4640731
chr17 4640735
chr17 4640741
chr17 4640746
chr17 4640763
chr17 4640765
chr17 4640795
chr17 4833589
chr17 4833601
chr17 4833605
chr17 4833616
chr17 4833622
chr17 4833631
chr17 4833638

chr17 4833646
chr17 4833648
chr17 4833877
chr17 4833891
chr17 4833919
chr17 4833934
chr17 4833995
chr17 4944617
chr17 4944726
chr17 4944731
chr17 4944756
chr17 4944792
chr17 4944806
chr17 4945060
chr17 4945063
chr17 4945344
chr17 4945585
chr17 4945719
chr17 4945874
chr17 4945895
chr17 4945913
chr17 4945916
chr17 4945962
chr17 4945996
chr17 4946117
chr17 4946197
chr17 4946321
chr17 4946330
chr17 4946385
chr17 4946434
chr17 4946478
chr17 4946576
chr17 4946673
chr17 4946687
chr17 4946729
chr17 4946737
chr17 4946772
chr17 4946786
chr17 4946838
chr17 4946874
chr17 4946920
chr17 4946923
chr17 4946995
chr17 4947184
chr17 4947206
chr17 4947210
chr17 4967866
chr17 4967874
chr17 4967877
chr17 4988297
chr17 4988312
chr17 5112303
chr17 5112315
chr17 5112358

chr17 5112406
chr17 5262721
chr17 5283113
chr17 5330186
chr17 5330451
chr17 5420524
chr17 5420562
chr17 5420575
chr17 5420595
chr17 5420599
chr17 5485814
chr17 5485827
chr17 5485838
chr17 5485841
chr17 5584056
chr17 5584074
chr17 5584115
chr17 5584118
chr17 5584265
chr17 5584286
chr17 5584390
chr17 5584432
chr17 5584498
chr17 5584526
chr17 5584553
chr17 5584638
chr17 5584669
chr17 5584728
chr17 5584778
chr17 5584842
chr17 5770812
chr17 5770914
chr17 5770957
chr17 5770987
chr17 5771099
chr17 5771103
chr17 5771109
chr17 5771112
chr17 5771165
chr17 5771192
chr17 5771207
chr17 5771213
chr17 5771251
chr17 5771253
chr17 5771257
chr17 5771272
chr17 5771274
chr17 5771276
chr17 5771279
chr17 5771285
chr17 5771293
chr17 5771295
chr17 6030132
chr17 6030328

chr17 6374380
chr17 6374568
chr17 6640134
chr17 6640143
chr17 6640147
chr17 6640150
chr17 6640162
chr17 6640168
chr17 6640173
chr17 6641699
chr17 6650692
chr17 6650772
chr17 6650804
chr17 6650810
chr17 6650857
chr17 6650881
chr17 6650915
chr17 6650946
chr17 6650977
chr17 6650983
chr17 6650989
chr17 6651037
chr17 6651052
chr17 6651069
chr17 6651075
chr17 6651077
chr17 6651099
chr17 6651112
chr17 6651172
chr17 6651175
chr17 6651233
chr17 6651271
chr17 6656787
chr17 6656821
chr17 6656849
chr17 6656853
chr17 6656872
chr17 6656879
chr17 6656895
chr17 6656897
chr17 6656901
chr17 6656913
chr17 6656915
chr17 6656919
chr17 6656928
chr17 6656930
chr17 6656936
chr17 6656948
chr17 6657013
chr17 6657019
chr17 6657033
chr17 6657054
chr17 6657057
chr17 6657059

chr17 6755958
chr17 6756007
chr17 6756045
chr17 6756056
chr17 6756095
chr17 6756132
chr17 6756141
chr17 6756149
chr17 6756160
chr17 6756166
chr17 6756243
chr17 6756376
chr17 6756482
chr17 6756526
chr17 6756533
chr17 6756577
chr17 6756589
chr17 6756591
chr17 6756602
chr17 6785246
chr17 6785825
chr17 6831768
chr17 6831770
chr17 6831797
chr17 6831827
chr17 6831833
chr17 6831844
chr17 6831876
chr17 6831904
chr17 6831953
chr17 6831965
chr17 6831988
chr17 6831997
chr17 6832007
chr17 6832009
chr17 6832013
chr17 6832024
chr17 6832029
chr17 6832031
chr17 6832041
chr17 6832057
chr17 6832083
chr17 6947876
chr17 6948090
chr17 7012776
chr17 7012786
chr17 7012807
chr17 7012828
chr17 7012844
chr17 7012855
chr17 7012864
chr17 7012881
chr17 7012922
chr17 7012953

chr17 7012981
chr17 7012985
chr17 7012993
chr17 7012997
chr17 7013039
chr17 7013049
chr17 7013056
chr17 7013073
chr17 7013079
chr17 7013081
chr17 7013111
chr17 7013119
chr17 7013153
chr17 7013224
chr17 7015383
chr17 7015388
chr17 7015402
chr17 7015442
chr17 7015489
chr17 7015521
chr17 7015528
chr17 7015593
chr17 7015600
chr17 7015621
chr17 7015633
chr17 7015654
chr17 7015675
chr17 7015719
chr17 7035089
chr17 7035393
chr17 7035470
chr17 7035493
chr17 7204866
chr17 7204894
chr17 7204909
chr17 7204933
chr17 7204962
chr17 7204986
chr17 7205077
chr17 7205088
chr17 7205109
chr17 7205114
chr17 7251881
chr17 7251887
chr17 7251902
chr17 7251912
chr17 7251954
chr17 7251965
chr17 7252024
chr17 7252033
chr17 7252041
chr17 7252049
chr17 7252068
chr17 7252070

chr17 7252080
chr17 7252115
chr17 7252131
chr17 7252135
chr17 7336502
chr17 7336513
chr17 7336615
chr17 7336617
chr17 7336694
chr17 7336708
chr17 7336739
chr17 7336755
chr17 7336780
chr17 7336786
chr17 7336904
chr17 7337072
chr17 7337161
chr17 7337191
chr17 7337221
chr17 7337230
chr17 7337237
chr17 7337248
chr17 7394231
chr17 7394235
chr17 7394238
chr17 7394241
chr17 7394260
chr17 7435658
chr17 7435776
chr17 7435780
chr17 7435802
chr17 7435808
chr17 7435880
chr17 7435912
chr17 7435915
chr17 7435921
chr17 7435932
chr17 7435936
chr17 7435942
chr17 7435949
chr17 7455825
chr17 7455905
chr17 7455909
chr17 7455935
chr17 7455940
chr17 7455952
chr17 7455979
chr17 7456004
chr17 7456016
chr17 7456022
chr17 7456085
chr17 7456093
chr17 7456117
chr17 7456123

chr17 7456140
chr17 7456146
chr17 7456156
chr17 7456178
chr17 7478956
chr17 7478959
chr17 7478965
chr17 7478978
chr17 7478985
chr17 7478992
chr17 7584315
chr17 7584343
chr17 7584396
chr17 7834204
chr17 7834212
chr17 7834240
chr17 7834250
chr17 7834255
chr17 7834270
chr17 7834291
chr17 7834295
chr17 7836356
chr17 7843722
chr17 7843755
chr17 7843761
chr17 7843768
chr17 7843773
chr17 7843787
chr17 7843793
chr17 7843796
chr17 7843815
chr17 7843817
chr17 7843823
chr17 7843846
chr17 7843894
chr17 7843906
chr17 7843918
chr17 7843929
chr17 7843946
chr17 7843965
chr17 7844029
chr17 7844036
chr17 7844068
chr17 7844088
chr17 7844094
chr17 7844099
chr17 7844111
chr17 7844116
chr17 7844127
chr17 7844151
chr17 7875987
chr17 7876233
chr17 7884300
chr17 7884340

chr17 7884380
chr17 7887673
chr17 7887677
chr17 7887684
chr17 7887692
chr17 7887696
chr17 7887708
chr17 7887731
chr17 7887738
chr17 7887745
chr17 7887752
chr17 7887782
chr17 7887787
chr17 7931992
chr17 7932011
chr17 7932015
chr17 7932019
chr17 7932021
chr17 7932063
chr17 7932094
chr17 7932100
chr17 7932102
chr17 7932106
chr17 7932108
chr17 7932110
chr17 7932340
chr17 8002067
chr17 8002148
chr17 8058981
chr17 8059067
chr17 8059080
chr17 8059197
chr17 8059206
chr17 8059212
chr17 8059245
chr17 8059254
chr17 8059290
chr17 8059356
chr17 8059841
chr17 8059849
chr17 8118205
chr17 8118254
chr17 8118307
chr17 8118349
chr17 8118418
chr17 8119147
chr17 8119158
chr17 8120947
chr17 8121262
chr17 8123721
chr17 8138441
chr17 8138506
chr17 8139037
chr17 8139041

chr17 8139051
chr17 8139053
chr17 8139056
chr17 8139066
chr17 8139072
chr17 8139076
chr17 8139080
chr17 8139090
chr17 8139096
chr17 8139123
chr17 8139143
chr17 8139195
chr17 8139207
chr17 8139274
chr17 8139322
chr17 8139336
chr17 8151444
chr17 8151455
chr17 8151490
chr17 8151497
chr17 8151511
chr17 8151553
chr17 8151563
chr17 8151576
chr17 8151579
chr17 8151604
chr17 8151662
chr17 8151685
chr17 8151691
chr17 8151705
chr17 8151716
chr17 8151827
chr17 8151864
chr17 8151907
chr17 8151916
chr17 8151931
chr17 8151953
chr17 8151956
chr17 8151986
chr17 8151993
chr17 8152021
chr17 8152042
chr17 8152049
chr17 8152062
chr17 8152073
chr17 8152083
chr17 8152095
chr17 8152109
chr17 8152164
chr17 8152177
chr17 8152189
chr17 8152220
chr17 8164215
chr17 8164274

chr17 8164441
chr17 8164458
chr17 8173052
chr17 8173055
chr17 8173073
chr17 8173216
chr17 8173443
chr17 8173493
chr17 8173501
chr17 8173510
chr17 8173551
chr17 8173585
chr17 8173602
chr17 8173631
chr17 8173657
chr17 8173659
chr17 8173687
chr17 8173746
chr17 8173753
chr17 8173788
chr17 8173798
chr17 8173803
chr17 8173807
chr17 8173820
chr17 8173843
chr17 8173871
chr17 8173891
chr17 8173908
chr17 8186009
chr17 8186014
chr17 8199963
chr17 8199997
chr17 8200030
chr17 8200042
chr17 8200061
chr17 8200084
chr17 8200190
chr17 8200231
chr17 8200245
chr17 8200253
chr17 8200263
chr17 8200275
chr17 8200281
chr17 8200295
chr17 8200298
chr17 8200311
chr17 8200317
chr17 8200336
chr17 8200373
chr17 8200381
chr17 8209932
chr17 8209984
chr17 8210026
chr17 8210255

chr17 8210272
chr17 8210315
chr17 8210319
chr17 8210324
chr17 8210330
chr17 8210338
chr17 8210384
chr17 8210396
chr17 8210402
chr17 8210413
chr17 8210437
chr17 8210440
chr17 8210444
chr17 8210446
chr17 8210461
chr17 8210497
chr17 8210500
chr17 8210511
chr17 8210557
chr17 8210561
chr17 8210565
chr17 8210586
chr17 8210589
chr17 8210591
chr17 8210620
chr17 8210625
chr17 8210644
chr17 8210647
chr17 8210672
chr17 8210685
chr17 8210714
chr17 8221052
chr17 8221063
chr17 8221071
chr17 8221119
chr17 8221127
chr17 8221174
chr17 8221492
chr17 8247032
chr17 8247054
chr17 8247056
chr17 8247062
chr17 8247085
chr17 8247124
chr17 8247127
chr17 8247164
chr17 8247283
chr17 8247300
chr17 8247303
chr17 8247340
chr17 8248075
chr17 8248105
chr17 8248109
chr17 8248115

chr17 8248121
chr17 8248128
chr17 8248138
chr17 8248158
chr17 8248173
chr17 8248189
chr17 8248191
chr17 8248196
chr17 8248201
chr17 8248221
chr17 8248263
chr17 8248279
chr17 8248301
chr17 8248303
chr17 8248309
chr17 8248326
chr17 8248351
chr17 8248400
chr17 8248404
chr17 8248580
chr17 8248588
chr17 8248596
chr17 8248615
chr17 8248618
chr17 8248626
chr17 8248642
chr17 8248650
chr17 8249877
chr17 8249901
chr17 8249935
chr17 8249965
chr17 8300393
chr17 8300396
chr17 8300508
chr17 8300655
chr17 8300706
chr17 8300715
chr17 8300802
chr17 8300804
chr17 8300810
chr17 8338230
chr17 8338355
chr17 8383808
chr17 8383824
chr17 8436440
chr17 8436926
chr17 8436945
chr17 8777297
chr17 8777653
chr17 8899157
chr17 8899196
chr17 8899204
chr17 8899337
chr17 8899360

chr17 8998401
chr17 9079714
chr17 9290007
chr17 9290078
chr17 9389420
chr17 9470213
chr17 9470235
chr17 9470325
chr17 9470546
chr17 9559848
chr17 9559855
chr17 9559864
chr17 9575562
chr17 9575583
chr17 9575604
chr17 9575633
chr17 9575671
chr17 9575691
chr17 9575705
chr17 9575713
chr17 9575733
chr17 9575741
chr17 9576450
chr17 10114574
chr17 10114768
chr17 10114808
chr17 10114862
chr17 10114877
chr17 10114944
chr17 10115114
chr17 10115175
chr17 10115215
chr17 10115280
chr17 10115303
chr17 10115314
chr17 10115326
chr17 10115335
chr17 10150101
chr17 10150258
chr17 10150320
chr17 10698445
chr17 10698456
chr17 10698500
chr17 10698533
chr17 10698567
chr17 10698588
chr17 10698684
chr17 10698715
chr17 10698717
chr17 10698782
chr17 10744569
chr17 10744643
chr17 10744676
chr17 10744754

chr17 10875699
chr17 10875775
chr17 10875890
chr17 10903887
chr17 10903924
chr17 10903937
chr17 10904302
chr17 10905115
chr17 11331571
chr17 11996516
chr17 11996575
chr17 12021471
chr17 12021474
chr17 12021486
chr17 12021508
chr17 12021511
chr17 12021527
chr17 12021535
chr17 12021546
chr17 12021553
chr17 12021573
chr17 12021602
chr17 12021627
chr17 12021724
chr17 12021932
chr17 12243975
chr17 12244139
chr17 12244536
chr17 12374954
chr17 12375420
chr17 12375553
chr17 12379243
chr17 12379263
chr17 12394713
chr17 12635188
chr17 12635212
chr17 13018352
chr17 13018358
chr17 13018449
chr17 13018479
chr17 13018513
chr17 13018523
chr17 13018567
chr17 13018575
chr17 13018577
chr17 13018583
chr17 13018592
chr17 13018600
chr17 13018606
chr17 13018608
chr17 13018638
chr17 13018754
chr17 13338204
chr17 13338231

chr17 13338277
chr17 13338358
chr17 13338679
chr17 13421633
chr17 13433838
chr17 13434612
chr17 13594960
chr17 13594981
chr17 13594990
chr17 13594995
chr17 13595036
chr17 13595044
chr17 13595047
chr17 13595239
chr17 14192533
chr17 14192548
chr17 14192561
chr17 14192611
chr17 14192618
chr17 14192704
chr17 14192856
chr17 14193021
chr17 14193095
chr17 14193114
chr17 14193187
chr17 14193206
chr17 14193209
chr17 14193212
chr17 14193232
chr17 14193241
chr17 14193263
chr17 14201342
chr17 14201551
chr17 14201758
chr17 14300197
chr17 14321866
chr17 14322090
chr17 14359319
chr17 14359343
chr17 14359364
chr17 14359449
chr17 14359509
chr17 14359719
chr17 14428349
chr17 14428360
chr17 14428387
chr17 14428511
chr17 14428528
chr17 14428546
chr17 14428568
chr17 14428645
chr17 14428946
chr17 14559715
chr17 14559760

chr17 15171575
chr17 15356908
chr17 15562655
chr17 15562723
chr17 15562771
chr17 15562779
chr17 15562832
chr17 15562875
chr17 15563046
chr17 15563057
chr17 15563063
chr17 15563066
chr17 15563086
chr17 15563118
chr17 15563125
chr17 15589481
chr17 15589496
chr17 15589567
chr17 15589719
chr17 15589884
chr17 15589977
chr17 15590027
chr17 15590050
chr17 15590069
chr17 15590072
chr17 15590075
chr17 15590095
chr17 15590104
chr17 15590126
chr17 15590128
chr17 15590525
chr17 15590592
chr17 15590598
chr17 15684123
chr17 15684129
chr17 15684155
chr17 15684205
chr17 15684219
chr17 15699060
chr17 15699958
chr17 15699962
chr17 15699971
chr17 15699973
chr17 15699994
chr17 15699998
chr17 15700000
chr17 15700033
chr17 15700053
chr17 15700078
chr17 15700124
chr17 15700246
chr17 15700262
chr17 15700433
chr17 15801458

chr17 15801529
chr17 15828384
chr17 15828886
chr17 15828913
chr17 15828934
chr17 15828942
chr17 15868941
chr17 15958960
chr17 15959016
chr17 15959039
chr17 15959075
chr17 15959310
chr17 15959561
chr17 15959563
chr17 15959576
chr17 15959586
chr17 15959625
chr17 15959645
chr17 15959649
chr17 15998823
chr17 15998830
chr17 15998840
chr17 15998847
chr17 15998857
chr17 15999031
chr17 15999047
chr17 16214107
chr17 16214339
chr17 16214374
chr17 16214571
chr17 16214625
chr17 16214773
chr17 16214780
chr17 16214805
chr17 16214824
chr17 16214858
chr17 16214876
chr17 16214911
chr17 16214937
chr17 16214962
chr17 16214975
chr17 16217114
chr17 16217117
chr17 16217134
chr17 16217151
chr17 16217168
chr17 16217201
chr17 16217255
chr17 16217261
chr17 16217305
chr17 16217317
chr17 16217332
chr17 16217343
chr17 16217350

chr17 16217369
chr17 16217379
chr17 16217422
chr17 16217482
chr17 16217492
chr17 16217522
chr17 16217532
chr17 16217543
chr17 16217553
chr17 16217576
chr17 16217619
chr17 16217623
chr17 16217647
chr17 16217650
chr17 16217658
chr17 16217660
chr17 16217729
chr17 16217818
chr17 16217838
chr17 16286463
chr17 16286477
chr17 16287698
chr17 16287714
chr17 16287851
chr17 16287873
chr17 16287905
chr17 16363983
chr17 16364051
chr17 16364094
chr17 16364294
chr17 16380566
chr17 16380574
chr17 16380614
chr17 16380617
chr17 16380638
chr17 16380641
chr17 16380643
chr17 16380651
chr17 16380654
chr17 16380658
chr17 16380660
chr17 16380662
chr17 16380683
chr17 16380686
chr17 16380728
chr17 16380730
chr17 16380747
chr17 16380750
chr17 16380756
chr17 16380762
chr17 16380784
chr17 16380787
chr17 16380813
chr17 16380843

chr17 16380886
chr17 16380929
chr17 16381145
chr17 16381176
chr17 16381194
chr17 16381200
chr17 16381205
chr17 16407345
chr17 16407347
chr17 16407354
chr17 16407364
chr17 16407386
chr17 16407390
chr17 16407399
chr17 16407401
chr17 16407405
chr17 16407410
chr17 16407451
chr17 16407458
chr17 16407466
chr17 16407477
chr17 16407483
chr17 16407499
chr17 16407501
chr17 16407522
chr17 16407524
chr17 16407541
chr17 16407548
chr17 16407560
chr17 16407562
chr17 16407576
chr17 16407597
chr17 16407599
chr17 16407621
chr17 16407631
chr17 16407644
chr17 16407674
chr17 16407755
chr17 16407789
chr17 16415100
chr17 16415354
chr17 16439939
chr17 16439998
chr17 16440195
chr17 16653546
chr17 16653557
chr17 16653559
chr17 16667363
chr17 16667401
chr17 16667405
chr17 16667414
chr17 16667421
chr17 16667423
chr17 16667464

chr17 16667466
chr17 16667501
chr17 16667508
chr17 16667513
chr17 16667523
chr17 16667536
chr17 16667540
chr17 16667545
chr17 16667558
chr17 16667575
chr17 16667611
chr17 16667625
chr17 16667993
chr17 16668030
chr17 16668048
chr17 16668074
chr17 16705139
chr17 16745489
chr17 16745580
chr17 16745597
chr17 16745601
chr17 16745620
chr17 16745667
chr17 16745699
chr17 16745710
chr17 16745712
chr17 16745716
chr17 16745755
chr17 16745773
chr17 16745798
chr17 16745841
chr17 16745849
chr17 16745932
chr17 16745942
chr17 16745966
chr17 16746044
chr17 16746054
chr17 16746075
chr17 16746086
chr17 16746101
chr17 16746110
chr17 16746321
chr17 16746327
chr17 16746579
chr17 16746661
chr17 16746664
chr17 16746667
chr17 16746676
chr17 16746685
chr17 16746698
chr17 16746702
chr17 16746737
chr17 16746747
chr17 16746764

chr17 16746771
chr17 16746773
chr17 16746775
chr17 16746779
chr17 16746788
chr17 16746793
chr17 16746798
chr17 16746823
chr17 16746825
chr17 16746830
chr17 16746844
chr17 16746851
chr17 16906720
chr17 16990944
chr17 16991355
chr17 16991482
chr17 16991488
chr17 16991498
chr17 16991522
chr17 16991527
chr17 16991543
chr17 16991551
chr17 16991573
chr17 16991583
chr17 16991598
chr17 17045029
chr17 17045083
chr17 17045152
chr17 17045183
chr17 17045247
chr17 17045448
chr17 17125957
chr17 17206174
chr17 17206176
chr17 17206180
chr17 17206183
chr17 17206186
chr17 17206195
chr17 17206204
chr17 17206221
chr17 17237908
chr17 17958200
chr17 17958204
chr17 18038156
chr17 18038428
chr17 18071300
chr17 18071419
chr17 18071736
chr17 18410843
chr17 18410853
chr17 18410874
chr17 18410909
chr17 18410942
chr17 18411008

chr17 18411014
chr17 18411017
chr17 18411141
chr17 18411240
chr17 18411262
chr17 18411279
chr17 18411305
chr17 18411336
chr17 18411343
chr17 18411474
chr17 18411484
chr17 18411495
chr17 18411502
chr17 18411519
chr17 18411580
chr17 18411613
chr17 18411769
chr17 18411782
chr17 18411810
chr17 18411812
chr17 18411827
chr17 18411839
chr17 18411858
chr17 18411882
chr17 18411887
chr17 18411895
chr17 18411906
chr17 18411944
chr17 18411972
chr17 18412000
chr17 18412008
chr17 18412253
chr17 18412270
chr17 18412277
chr17 18412280
chr17 18412286
chr17 18412292
chr17 18412295
chr17 18412325
chr17 18412329
chr17 18412346
chr17 18412356
chr17 18412587
chr17 18412591
chr17 18412612
chr17 18412675
chr17 18412683
chr17 18412687
chr17 18412710
chr17 18412728
chr17 18412836
chr17 18412847
chr17 18412881
chr17 18413089

chr17 18433999
chr17 18434358
chr17 18434364
chr17 18434401
chr17 18434408
chr17 18475594
chr17 18475815
chr17 18475845
chr17 18475866
chr17 18475898
chr17 18475909
chr17 18476340
chr17 18476730
chr17 18476845
chr17 18476866
chr17 18476902
chr17 18476908
chr17 18477040
chr17 18493045
chr17 18493104
chr17 18493178
chr17 18493190
chr17 18493194
chr17 18493228
chr17 18493247
chr17 18493343
chr17 18493362
chr17 18493375
chr17 18493446
chr17 18493453
chr17 18564024
chr17 18564049
chr17 18625522
chr17 18625736
chr17 18625784
chr17 18625846
chr17 18634556
chr17 18634558
chr17 18681856
chr17 18681902
chr17 18681927
chr17 18681931
chr17 18681947
chr17 18681980
chr17 18681982
chr17 18681986
chr17 18682007
chr17 18682009
chr17 18682018
chr17 18682022
chr17 18682033
chr17 18682035
chr17 18682042
chr17 18682580

chr17 18682592
chr17 18682835
chr17 18682920
chr17 18697926
chr17 18697939
chr17 18697947
chr17 18697951
chr17 18697956
chr17 18697961
chr17 18697984
chr17 18697987
chr17 18697989
chr17 18697997
chr17 18698027
chr17 18698029
chr17 18698031
chr17 18698036
chr17 18698089
chr17 18698104
chr17 18698154
chr17 18698180
chr17 18698186
chr17 18698238
chr17 18698240
chr17 18712510
chr17 18712581
chr17 18712623
chr17 18712645
chr17 18780455
chr17 18780487
chr17 18780583
chr17 18780698
chr17 18780714
chr17 18780734
chr17 18780764
chr17 18780808
chr17 18780935
chr17 18780949
chr17 18780957
chr17 18780971
chr17 18781010
chr17 18781072
chr17 18781090
chr17 18781096
chr17 18781099
chr17 18781105
chr17 18781110
chr17 18781114
chr17 18781962
chr17 18782018
chr17 18818380
chr17 18818400
chr17 18818433
chr17 18856044

chr17 18856059
chr17 18856077
chr17 18856122
chr17 18856186
chr17 18856189
chr17 18856203
chr17 18856211
chr17 18856220
chr17 18856248
chr17 18856256
chr17 18856260
chr17 18856315
chr17 18856317
chr17 18856328
chr17 18856361
chr17 18856377
chr17 18856388
chr17 18856400
chr17 18856408
chr17 18856519
chr17 18856532
chr17 18856556
chr17 18856581
chr17 18856597
chr17 18858603
chr17 18858619
chr17 18858658
chr17 18858733
chr17 18858798
chr17 18858836
chr17 18858973
chr17 18859009
chr17 18860911
chr17 18921059
chr17 18921093
chr17 18921147
chr17 18921381
chr17 18921706
chr17 18921732
chr17 18921946
chr17 18926719
chr17 19060805
chr17 19060810
chr17 19060828
chr17 19060853
chr17 19060868
chr17 19063868
chr17 19063873
chr17 19063882
chr17 19064472
chr17 19064512
chr17 19064535
chr17 19064543
chr17 19064566

chr17 19064574
chr17 19064596
chr17 19064607
chr17 19064627
chr17 19064640
chr17 19064649
chr17 19065193
chr17 19065208
chr17 19065233
chr17 19065251
chr17 19065256
chr17 19065454
chr17 19065460
chr17 19065470
chr17 19065498
chr17 19065503
chr17 19088191
chr17 19088198
chr17 19088232
chr17 19088243
chr17 19088276
chr17 19088286
chr17 19088288
chr17 19088308
chr17 19088330
chr17 19088408
chr17 19088416
chr17 19088424
chr17 19088474
chr17 19088513
chr17 19088532
chr17 19088998
chr17 19089013
chr17 19089038
chr17 19089043
chr17 19089054
chr17 19089061
chr17 19089079
chr17 19089085
chr17 19089096
chr17 19089101
chr17 19089104
chr17 19089110
chr17 19089116
chr17 19089146
chr17 19089776
chr17 19111060
chr17 19111075
chr17 19111084
chr17 19111096
chr17 19111098
chr17 19112639
chr17 19112648
chr17 19112676

chr17 19112696
chr17 19112707
chr17 19112716
chr17 19113679
chr17 19113694
chr17 19185986
chr17 19185994
chr17 19186017
chr17 19186072
chr17 19186082
chr17 19186175
chr17 19186213
chr17 19186215
chr17 19186456
chr17 19186465
chr17 19186478
chr17 19186510
chr17 19186562
chr17 19186574
chr17 19186578
chr17 19186606
chr17 19186619
chr17 19186662
chr17 19186667
chr17 19186695
chr17 19186705
chr17 19186711
chr17 19186771
chr17 19186778
chr17 19186790
chr17 19186814
chr17 19186818
chr17 19186823
chr17 19186909
chr17 19186914
chr17 19186932
chr17 19186957
chr17 19188073
chr17 19188085
chr17 19188094
chr17 19188110
chr17 19188124
chr17 19188132
chr17 19188143
chr17 19188163
chr17 19188180
chr17 19188443
chr17 19188459
chr17 19188474
chr17 19188477
chr17 19188484
chr17 19188488
chr17 19188507
chr17 19188516

chr17 19188758
chr17 19188763
chr17 19188771
chr17 19188791
chr17 19188807
chr17 19188831
chr17 19188834
chr17 19189426
chr17 19189429
chr17 19189453
chr17 19189469
chr17 19189489
chr17 19189497
chr17 19189503
chr17 19189506
chr17 19189744
chr17 19189753
chr17 19189772
chr17 19189776
chr17 19189783
chr17 19189786
chr17 19189801
chr17 19190136
chr17 19190150
chr17 19190166
chr17 19190175
chr17 19190187
chr17 19191555
chr17 19191565
chr17 19191593
chr17 19191598
chr17 19191641
chr17 19191654
chr17 19191682
chr17 19191686
chr17 19191698
chr17 19191750
chr17 19192178
chr17 19192188
chr17 19192243
chr17 19192266
chr17 19192274
chr17 19192296
chr17 19193051
chr17 19193059
chr17 19193149
chr17 19193851
chr17 19193875
chr17 19193881
chr17 19193883
chr17 19193905
chr17 19193913
chr17 19193996
chr17 19194038

chr17 19194695
chr17 19194748
chr17 19194907
chr17 19194959
chr17 19195246
chr17 19195290
chr17 19196689
chr17 19196693
chr17 19196713
chr17 19196738
chr17 19196743
chr17 19196756
chr17 19196765
chr17 19196779
chr17 19196788
chr17 19196795
chr17 19196818
chr17 19196824
chr17 19196859
chr17 19196938
chr17 19197689
chr17 19197731
chr17 19197739
chr17 19197747
chr17 19197769
chr17 19197771
chr17 19197794
chr17 19197801
chr17 19197809
chr17 19197833
chr17 19197865
chr17 19197895
chr17 19197898
chr17 19197909
chr17 19197943
chr17 19197950
chr17 19197954
chr17 19197959
chr17 19198618
chr17 19198622
chr17 19198634
chr17 19198662
chr17 19198671
chr17 19198678
chr17 19198692
chr17 19198752
chr17 19198756
chr17 19198764
chr17 19198768
chr17 19198788
chr17 19198820
chr17 19198850
chr17 19198852
chr17 19198874

chr17 19198882
chr17 19199040
chr17 19199063
chr17 19199070
chr17 19199197
chr17 19199255
chr17 19200465
chr17 19200480
chr17 19200504
chr17 19200524
chr17 19200528
chr17 19200601
chr17 19200622
chr17 19200626
chr17 19200762
chr17 19201641
chr17 19201665
chr17 19201671
chr17 19201705
chr17 19201747
chr17 19201763
chr17 19201802
chr17 19201849
chr17 19237221
chr17 19237223
chr17 19237238
chr17 19237247
chr17 19237667
chr17 19237672
chr17 19237684
chr17 19237724
chr17 19237731
chr17 19237773
chr17 19237798
chr17 19237810
chr17 19237844
chr17 19237862
chr17 19237877
chr17 19237897
chr17 19237915
chr17 19237930
chr17 19237936
chr17 19237939
chr17 19237971
chr17 19237981
chr17 19237992
chr17 19237997
chr17 19238001
chr17 19238008
chr17 19284744
chr17 19284760
chr17 19284850
chr17 19306445
chr17 19306508

chr17 19306546
chr17 19306576
chr17 19306684
chr17 19306862
chr17 19377258
chr17 19377489
chr17 19377523
chr17 19377559
chr17 19377570
chr17 19377627
chr17 19377634
chr17 19377655
chr17 19440573
chr17 19463186
chr17 19463241
chr17 19463258
chr17 19463277
chr17 19507537
chr17 19507541
chr17 19507564
chr17 19507567
chr17 19507583
chr17 19507585
chr17 19507713
chr17 19507716
chr17 19507725
chr17 19507770
chr17 19507937
chr17 19507939
chr17 19507942
chr17 19507944
chr17 19507953
chr17 19507960
chr17 19507967
chr17 19507971
chr17 19507974
chr17 19507987
chr17 19507992
chr17 19507995
chr17 19507998
chr17 19508023
chr17 19508025
chr17 19508037
chr17 19508042
chr17 19508057
chr17 19508063
chr17 19508098
chr17 19508144
chr17 19508156
chr17 19508161
chr17 19508186
chr17 19508191
chr17 19508202
chr17 19508204

chr17 19508229
chr17 19508242
chr17 19508245
chr17 19508303
chr17 19508361
chr17 19508366
chr17 19508405
chr17 19508408
chr17 19508413
chr17 19508418
chr17 19508443
chr17 19508447
chr17 19508452
chr17 19508454
chr17 19508463
chr17 19508478
chr17 19508483
chr17 19508494
chr17 19508499
chr17 19508503
chr17 19508514
chr17 19508528
chr17 19508569
chr17 19508576
chr17 19513899
chr17 19513924
chr17 19513971
chr17 19514047
chr17 19514077
chr17 19514087
chr17 19514093
chr17 19514117
chr17 19514125
chr17 19514279
chr17 20116733
chr17 20116752
chr17 20271240
chr17 20271255
chr17 20381559
chr17 20381781
chr17 20381786
chr17 20450886
chr17 20450893
chr17 20450924
chr17 20450964
chr17 20450977
chr17 20450996
chr17 20451111
chr17 20451122
chr17 20451145
chr17 20451149
chr17 20451161
chr17 20451235
chr17 20467416

chr17 20467477
chr17 20467492
chr17 20467905
chr17 20508128
chr17 20508183
chr17 20508195
chr17 20508222
chr17 20508510
chr17 20508537
chr17 20508562
chr17 20509250
chr17 20509282
chr17 20509473
chr17 20517592
chr17 20517597
chr17 20517622
chr17 20517646
chr17 20518638
chr17 20518658
chr17 20518667
chr17 20518892
chr17 20518951
chr17 20531357
chr17 20531599
chr17 20531610
chr17 20531736
chr17 20531759
chr17 20531763
chr17 20531771
chr17 20531834
chr17 20531855
chr17 20531859
chr17 20532068
chr17 20532090
chr17 20532100
chr17 20532117
chr17 20532121
chr17 20532151
chr17 20532154
chr17 20532160
chr17 20532166
chr17 20532169
chr17 20532176
chr17 20532193
chr17 20532502
chr17 20532564
chr17 20532588
chr17 20532607
chr17 20532619
chr17 20532740
chr17 20532762
chr17 20532794
chr17 20532797
chr17 20532830

chr17 20532891
chr17 20532908
chr17 20532915
chr17 20532926
chr17 20532936
chr17 20532966
chr17 20532969
chr17 20532977
chr17 20533067
chr17 20533074
chr17 20533105
chr17 20533130
chr17 20533147
chr17 20533169
chr17 20533184
chr17 20533248
chr17 20533268
chr17 20533410
chr17 20533416
chr17 20533550
chr17 20533571
chr17 20533581
chr17 20533666
chr17 20533669
chr17 20533672
chr17 20533711
chr17 20603642
chr17 20603729
chr17 20603804
chr17 20604112
chr17 20824893
chr17 20840553
chr17 20840607
chr17 20840694
chr17 20840697
chr17 20840703
chr17 20840716
chr17 20840728
chr17 20840737
chr17 20840740
chr17 20840744
chr17 20840748
chr17 20840752
chr17 20840756
chr17 20840760
chr17 20840774
chr17 20840861
chr17 20840871
chr17 20840873
chr17 20840900
chr17 20840918
chr17 20840927
chr17 20840933
chr17 20840949

chr17 20840951
chr17 20840956
chr17 20840981
chr17 20840991
chr17 20840995
chr17 20840998
chr17 20841007
chr17 20841016
chr17 20841027
chr17 20841036
chr17 20841039
chr17 20841043
chr17 20841052
chr17 20841060
chr17 20841093
chr17 20841103
chr17 20841107
chr17 20841112
chr17 20841124
chr17 20841135
chr17 20844015
chr17 20844022
chr17 20844031
chr17 20844039
chr17 20844059
chr17 20844062
chr17 20844066
chr17 20844081
chr17 20844086
chr17 20844093
chr17 20844102
chr17 20844110
chr17 20844114
chr17 20844122
chr17 20844129
chr17 20844133
chr17 20844253
chr17 20844255
chr17 20844262
chr17 20844265
chr17 20844275
chr17 20844279
chr17 20844297
chr17 20844304
chr17 20844316
chr17 20844318
chr17 20844321
chr17 20844351
chr17 20844355
chr17 20844360
chr17 20844370
chr17 20844375
chr17 20844393
chr17 20844415

chr17 20844450
chr17 20844458
chr17 20853389
chr17 20853393
chr17 20853501
chr17 20903085
chr17 20903114
chr17 21034397
chr17 21034444
chr17 21034540
chr17 21034543
chr17 21034578
chr17 21034615
chr17 21034637
chr17 21041512
chr17 21041522
chr17 21041529
chr17 21041546
chr17 21041552
chr17 21041554
chr17 21041584
chr17 21041592
chr17 21041650
chr17 21041722
chr17 21041742
chr17 21041744
chr17 21099589
chr17 21099619
chr17 21119882
chr17 21119888
chr17 21119960
chr17 21119969
chr17 21119980
chr17 21120008
chr17 21120145
chr17 21125982
chr17 21126070
chr17 21126085
chr17 21126180
chr17 21126213
chr17 21126307
chr17 21284122
chr17 21284127
chr17 21284133
chr17 21284139
chr17 21285184
chr17 21285317
chr17 21285388
chr17 21285462
chr17 21285485
chr17 21285510
chr17 21285520
chr17 21285666
chr17 21285761

chr17 21285861
chr17 21285882
chr17 21285939
chr17 21285942
chr17 21285967
chr17 21285983
chr17 21285993
chr17 21286102
chr17 21286120
chr17 21286165
chr17 21286176
chr17 21286188
chr17 21286196
chr17 21286231
chr17 21286316
chr17 21286350
chr17 21287730
chr17 21287743
chr17 21287853
chr17 21287934
chr17 21287957
chr17 21287984
chr17 21288022
chr17 21288053
chr17 21288069
chr17 21288077
chr17 21288087
chr17 21288090
chr17 21288101
chr17 21288124
chr17 21288140
chr17 21288163
chr17 21288199
chr17 21288262
chr17 21288465
chr17 21288481
chr17 21288586
chr17 21513111
chr17 21513115
chr17 21513173
chr17 21513275
chr17 21551527
chr17 21551573
chr17 21551602
chr17 21551616
chr17 21551621
chr17 21551629
chr17 21551631
chr17 21551633
chr17 21551656
chr17 21551659
chr17 21551663
chr17 21551667
chr17 21551674

chr17 21551683
chr17 21551689
chr17 21551694
chr17 21551699
chr17 21551708
chr17 21551713
chr17 21551718
chr17 21551727
chr17 21551732
chr17 21551737
chr17 21551746
chr17 21551751
chr17 21551757
chr17 21551761
chr17 21551775
chr17 21551780
chr17 21551790
chr17 21551796
chr17 21551802
chr17 21551809
chr17 21551823
chr17 21551853
chr17 21551871
chr17 21551922
chr17 21551947
chr17 21551953
chr17 21552038
chr17 21552079
chr17 21607391
chr17 21607407
chr17 21607970
chr17 21608521
chr17 21609043
chr17 21609137
chr17 21827771
chr17 21828027
chr17 21828029
chr17 22103351
chr17 22103629
chr17 22103680
chr17 22144054
chr17 22144221
chr17 22151428
chr17 22151727
chr17 22151818
chr17 22154491
chr17 22154493
chr17 22155346
chr17 22155472
chr17 22178071
chr17 22284595
chr17 22284683
chr17 22284891
chr17 22285132

chr17 22285144
chr17 22400523
chr17 22400530
chr17 22400570
chr17 22401748
chr17 22401763
chr17 22401769
chr17 22401793
chr17 22401817
chr17 22401824
chr17 22401830
chr17 22401841
chr17 22401848
chr17 22401850
chr17 22424714
chr17 22424743
chr17 22434627
chr17 22434724
chr17 22541972
chr17 22542096
chr17 22542391
chr17 22542770
chr17 22543051
chr17 22543370
chr17 22543372
chr17 22543774
chr17 22544379
chr17 22554562
chr17 22578345
chr17 22578553
chr17 22578683
chr17 22578893
chr17 22664653
chr17 22664752
chr17 22665011
chr17 22665500
chr17 22666009
chr17 22666018
chr17 22738414
chr17 22738892
chr17 22739018
chr17 23405882
chr17 23406044
chr17 26641000
chr17 26763586
chr17 26763594
chr17 26763722
chr17 26764082
chr17 26764546
chr17 26764605
chr17 26764734
chr17 26765162
chr17 26771825
chr17 26772237

chr17 26961027
chr17 26961094
chr17 26961223
chr17 26961352
chr17 26968571
chr17 26968968
chr17 26968995
chr17 26969064
chr17 26980542
chr17 27091867
chr17 27092012
chr17 27092425
chr17 27092473
chr17 27106086
chr17 27246297
chr17 27295294
chr17 27295298
chr17 27295408
chr17 27295410
chr17 27295672
chr17 27295674
chr17 27295754
chr17 27295821
chr17 27295900
chr17 27295906
chr17 27295923
chr17 27333227
chr17 27333232
chr17 27333240
chr17 27348807
chr17 27466433
chr17 27466448
chr17 27466738
chr17 27466785
chr17 27466798
chr17 27466819
chr17 27466828
chr17 27466866
chr17 27466880
chr17 27466894
chr17 27466898
chr17 27466932
chr17 27473670
chr17 27473715
chr17 27473755
chr17 27473797
chr17 27473983
chr17 27474025
chr17 27474029
chr17 27526623
chr17 27526702
chr17 27616581
chr17 27805672
chr17 27805696

chr17 27805705
chr17 27805737
chr17 27805741
chr17 27805872
chr17 27805919
chr17 27805939
chr17 27805951
chr17 27806126
chr17 27808318
chr17 27808637
chr17 27893448
chr17 27893519
chr17 27893566
chr17 27893569
chr17 27893685
chr17 27893723
chr17 27894063
chr17 27894103
chr17 27894141
chr17 27894174
chr17 27894254
chr17 27894316
chr17 27894405
chr17 27894564
chr17 28042075
chr17 28042080
chr17 28278415
chr17 28576763
chr17 28576783
chr17 28576806
chr17 28576823
chr17 28576842
chr17 28576854
chr17 28576903
chr17 28576910
chr17 28576920
chr17 28576931
chr17 28576937
chr17 28576953
chr17 28576988
chr17 28577001
chr17 28577013
chr17 28577018
chr17 28577021
chr17 28577035
chr17 28577040
chr17 28577051
chr17 28577080
chr17 28577103
chr17 28577132
chr17 28577137
chr17 28599382
chr17 28599393
chr17 28599404

chr17 28599424
chr17 28599429
chr17 28599435
chr17 28599439
chr17 28599463
chr17 28599465
chr17 28599469
chr17 28599474
chr17 28599479
chr17 28599484
chr17 28599493
chr17 28599550
chr17 28599574
chr17 28644136
chr17 28644149
chr17 28644173
chr17 28644278
chr17 28644348
chr17 28644370
chr17 28661517
chr17 28662563
chr17 28662572
chr17 28662600
chr17 28662612
chr17 28662635
chr17 28662691
chr17 28662739
chr17 28662758
chr17 28662848
chr17 28662875
chr17 28662906
chr17 28663082
chr17 28719751
chr17 28719759
chr17 28719853
chr17 28719857
chr17 28719882
chr17 28719895
chr17 28719898
chr17 28719938
chr17 28719940
chr17 28719961
chr17 28719972
chr17 28719980
chr17 28720010
chr17 28720013
chr17 28720019
chr17 28720051
chr17 28720065
chr17 28720075
chr17 28720077
chr17 28720080
chr17 28720087
chr17 28720091

chr17 28720104
chr17 28720175
chr17 28720182
chr17 28720184
chr17 28720198
chr17 28720212
chr17 28720238
chr17 28720240
chr17 28720254
chr17 28720275
chr17 28720322
chr17 28720383
chr17 28720397
chr17 28720409
chr17 28720414
chr17 28720424
chr17 28720445
chr17 28720473
chr17 28720503
chr17 28720531
chr17 28720537
chr17 28720652
chr17 28720662
chr17 28720676
chr17 28720684
chr17 28720737
chr17 28720802
chr17 28720806
chr17 28720820
chr17 28720823
chr17 28720827
chr17 28720838
chr17 28727451
chr17 28727473
chr17 28727497
chr17 28727527
chr17 28727568
chr17 28727658
chr17 28727682
chr17 28727688
chr17 28727705
chr17 28727722
chr17 28727744
chr17 28727764
chr17 28727766
chr17 28727768
chr17 28727774
chr17 28727808
chr17 28727862
chr17 28727866
chr17 28727871
chr17 28727900
chr17 28727910
chr17 28727912

chr17 28727920
chr17 28727946
chr17 28727961
chr17 28727989
chr17 28728056
chr17 28728059
chr17 28728065
chr17 28728067
chr17 28728113
chr17 28744953
chr17 28744997
chr17 28745026
chr17 28745046
chr17 28745075
chr17 28745083
chr17 28745103
chr17 28745116
chr17 28745121
chr17 28745148
chr17 28745157
chr17 28745198
chr17 28745211
chr17 28745214
chr17 28745229
chr17 28745233
chr17 28745241
chr17 28811860
chr17 28811919
chr17 28811985
chr17 28811989
chr17 28812937
chr17 28842471
chr17 28842479
chr17 28842529
chr17 28842575
chr17 28842583
chr17 28854925
chr17 28854962
chr17 28854970
chr17 28854977
chr17 28854990
chr17 28854998
chr17 28855008
chr17 28855015
chr17 28855027
chr17 28855109
chr17 28855111
chr17 28855119
chr17 28855155
chr17 28855163
chr17 28855184
chr17 28855210
chr17 28855218
chr17 28855256

chr17 28855271
chr17 28876501
chr17 28896678
chr17 28896771
chr17 28896808
chr17 28896830
chr17 28896842
chr17 28896887
chr17 28926120
chr17 28926256
chr17 28952318
chr17 28952363
chr17 28952382
chr17 28952386
chr17 28952392
chr17 28952417
chr17 28952419
chr17 28952459
chr17 28952463
chr17 28952489
chr17 28952507
chr17 29139025
chr17 29139108
chr17 29139186
chr17 29139227
chr17 29139641
chr17 29139831
chr17 29139927
chr17 29140021
chr17 29140135
chr17 29140155
chr17 29140189
chr17 29140381
chr17 29140437
chr17 29140448
chr17 29140459
chr17 29140469
chr17 29140474
chr17 29140507
chr17 29140517
chr17 29140682
chr17 29140743
chr17 29140777
chr17 29140797
chr17 29140831
chr17 29140902
chr17 29140927
chr17 29140948
chr17 29140982
chr17 29141025
chr17 29141124
chr17 29141308
chr17 29176538
chr17 29291210

chr17 29291330
chr17 29291498
chr17 29291609
chr17 29291660
chr17 29291826
chr17 29291830
chr17 29291859
chr17 29291869
chr17 29291986
chr17 29292002
chr17 29292012
chr17 29292027
chr17 29292061
chr17 29292091
chr17 29292131
chr17 29292162
chr17 29292175
chr17 29292199
chr17 29292216
chr17 29292299
chr17 29295040
chr17 29324573
chr17 29391188
chr17 29391195
chr17 29391208
chr17 29391243
chr17 29391271
chr17 29391317
chr17 29391328
chr17 29391330
chr17 29391475
chr17 29391484
chr17 29391496
chr17 29391619
chr17 29659289
chr17 29664977
chr17 29665113
chr17 29665573
chr17 29705506
chr17 29705556
chr17 29705740
chr17 29706033
chr17 29706492
chr17 29706544
chr17 29706748
chr17 29706875
chr17 29706879
chr17 29706891
chr17 29706924
chr17 29706928
chr17 29706935
chr17 29706949
chr17 29706969
chr17 29706980

chr17 29707014
chr17 29707022
chr17 29707026
chr17 29707046
chr17 29707069
chr17 29707077
chr17 29722758
chr17 29722791
chr17 29722901
chr17 29722920
chr17 29723006
chr17 29723442
chr17 29723573
chr17 29726955
chr17 29727137
chr17 29727291
chr17 29760530
chr17 29760533
chr17 29760552
chr17 29760574
chr17 29779101
chr17 29779370
chr17 29779460
chr17 29928158
chr17 29928891
chr17 29930606
chr17 29930608
chr17 29930633
chr17 29931629
chr17 30066329
chr17 30066586
chr17 30093313
chr17 30093336
chr17 30093558
chr17 30093592
chr17 30093614
chr17 30093658
chr17 30093723
chr17 30093730
chr17 30104564
chr17 30104590
chr17 30104638
chr17 30104660
chr17 30104698
chr17 30104721
chr17 30104744
chr17 30104753
chr17 30104757
chr17 30104763
chr17 30104802
chr17 30104816
chr17 30104830
chr17 30104878
chr17 30104884

chr17 30104897
chr17 30104902
chr17 30104924
chr17 30104954
chr17 30117359
chr17 30117365
chr17 30117387
chr17 30117433
chr17 30117441
chr17 30117450
chr17 30117469
chr17 30173860
chr17 30173864
chr17 30291808
chr17 30291822
chr17 30291828
chr17 30291835
chr17 30291838
chr17 30291846
chr17 30291869
chr17 30291873
chr17 30291876
chr17 30291884
chr17 30291889
chr17 30291894
chr17 30291910
chr17 30291922
chr17 30291928
chr17 30291933
chr17 30291936
chr17 30291950
chr17 30291955
chr17 30291958
chr17 30291961
chr17 30323406
chr17 30323483
chr17 30323861
chr17 30323868
chr17 30364003
chr17 30364341
chr17 30364401
chr17 30378574
chr17 30378740
chr17 30378763
chr17 30378768
chr17 30477484
chr17 30477490
chr17 30478066
chr17 30627353
chr17 30627408
chr17 30627443
chr17 30667629
chr17 30667638
chr17 30667654

chr17 30667667
chr17 30667718
chr17 30667722
chr17 30667768
chr17 30667770
chr17 30667773
chr17 30667837
chr17 30667839
chr17 30667848
chr17 30667851
chr17 30667871
chr17 30667876
chr17 30667909
chr17 30667915
chr17 30667939
chr17 30667946
chr17 30667957
chr17 30667959
chr17 30667980
chr17 30667996
chr17 30668033
chr17 30668069
chr17 30668071
chr17 30668101
chr17 30668104
chr17 30668140
chr17 30668142
chr17 30668156
chr17 30668182
chr17 30668867
chr17 30668880
chr17 30668900
chr17 30708210
chr17 30709376
chr17 30709382
chr17 30709391
chr17 30709393
chr17 30709401
chr17 30709412
chr17 30709417
chr17 30709423
chr17 30709426
chr17 30709429
chr17 30709434
chr17 30709439
chr17 30709444
chr17 30709449
chr17 30709461
chr17 30709464
chr17 30709481
chr17 30709518
chr17 30709522
chr17 30709534
chr17 30709542

chr17 30731424
chr17 30731442
chr17 30731447
chr17 30731463
chr17 30731465
chr17 30731484
chr17 30731488
chr17 30731494
chr17 30731496
chr17 30731500
chr17 30731506
chr17 30731508
chr17 30731513
chr17 30733171
chr17 30733176
chr17 30733450
chr17 30733463
chr17 30733609
chr17 30809055
chr17 30809149
chr17 30821900
chr17 30821976
chr17 30822034
chr17 30822051
chr17 30822059
chr17 30822135
chr17 30822380
chr17 30822508
chr17 30822688
chr17 30823009
chr17 30823015
chr17 30823082
chr17 30823086
chr17 30823090
chr17 30823092
chr17 30823102
chr17 30823145
chr17 30823276
chr17 30823286
chr17 30823293
chr17 30823313
chr17 30823343
chr17 30823359
chr17 30824154
chr17 30824165
chr17 30824195
chr17 30824210
chr17 30824258
chr17 30824268
chr17 30824280
chr17 30824302
chr17 30824343
chr17 30824368
chr17 30832576

chr17 30832586
chr17 30832627
chr17 30832661
chr17 30832679
chr17 30832695
chr17 30832713
chr17 30832752
chr17 30832759
chr17 30832778
chr17 30832906
chr17 30832917
chr17 30832970
chr17 30833089
chr17 30833132
chr17 30833319
chr17 30939219
chr17 30939271
chr17 31008679
chr17 31008693
chr17 31008696
chr17 31008698
chr17 31008703
chr17 31008706
chr17 31008709
chr17 31008721
chr17 31008730
chr17 31008733
chr17 31008739
chr17 31008742
chr17 31008763
chr17 31008770
chr17 31008778
chr17 31008784
chr17 31008815
chr17 31008818
chr17 31008823
chr17 31008836
chr17 31008839
chr17 31008847
chr17 31008855
chr17 31008890
chr17 31008892
chr17 31008962
chr17 31008967
chr17 31008991
chr17 31009042
chr17 31009047
chr17 31009052
chr17 31009058
chr17 31009071
chr17 31009093
chr17 31009106
chr17 31009113
chr17 31009135

chr17 31009149
chr17 31009171
chr17 31094853
chr17 31094857
chr17 31094874
chr17 31094882
chr17 31094894
chr17 31094918
chr17 31094920
chr17 31094938
chr17 31094942
chr17 31094944
chr17 31094957
chr17 31094962
chr17 31094982
chr17 31094998
chr17 31095001
chr17 31095006
chr17 31095030
chr17 31095033
chr17 31095048
chr17 31095073
chr17 31095117
chr17 31095141
chr17 31095145
chr17 31095148
chr17 31095171
chr17 31095236
chr17 31095243
chr17 31095254
chr17 31095264
chr17 31095267
chr17 31095284
chr17 31095287
chr17 31095294
chr17 31095298
chr17 31095300
chr17 31095345
chr17 31095375
chr17 31095380
chr17 31095385
chr17 31095389
chr17 31095402
chr17 31095444
chr17 31095448
chr17 31095457
chr17 31095459
chr17 31095534
chr17 31095656
chr17 31095687
chr17 31095699
chr17 31095701
chr17 31095758
chr17 31095788

chr17 31095809
chr17 31095813
chr17 31095853
chr17 31095864
chr17 31095957
chr17 31095972
chr17 31096021
chr17 31096024
chr17 31096070
chr17 31096086
chr17 31096157
chr17 31096181
chr17 31308360
chr17 31308432
chr17 31308506
chr17 31308576
chr17 31309126
chr17 31309514
chr17 31309670
chr17 31309845
chr17 31310071
chr17 31310086
chr17 31310289
chr17 31312991
chr17 31313010
chr17 31313431
chr17 31313468
chr17 31313478
chr17 31313566
chr17 31313578
chr17 31313589
chr17 31313660
chr17 31314147
chr17 31314315
chr17 31314455
chr17 31318443
chr17 31318455
chr17 31318470
chr17 31318490
chr17 31318511
chr17 31318633
chr17 31318716
chr17 31318764
chr17 31318913
chr17 31319005
chr17 31319146
chr17 31319314
chr17 31319649
chr17 31319984
chr17 31320295
chr17 31320552
chr17 31320959
chr17 31321279
chr17 31321305

chr17 31321429
chr17 31321448
chr17 31321477
chr17 31321528
chr17 31321729
chr17 31321793
chr17 31322074
chr17 31680185
chr17 31680206
chr17 31680228
chr17 31680300
chr17 31680305
chr17 31680337
chr17 31680351
chr17 31744329
chr17 31744496
chr17 31744577
chr17 31901021
chr17 31901049
chr17 31901309
chr17 31901333
chr17 31901352
chr17 31901356
chr17 31901371
chr17 31901399
chr17 31901405
chr17 31901448
chr17 31901452
chr17 31907576
chr17 31907742
chr17 31907770
chr17 31907891
chr17 31907904
chr17 31908218
chr17 31938158
chr17 31938400
chr17 31938572
chr17 31938611
chr17 31938837
chr17 31938850
chr17 31938904
chr17 32008425
chr17 32008558
chr17 32008688
chr17 32008747
chr17 32008757
chr17 32008845
chr17 32008932
chr17 32008976
chr17 32062586
chr17 32062617
chr17 32062623
chr17 32062626
chr17 32062682

chr17 32062754
chr17 32062827
chr17 32062829
chr17 32062832
chr17 32063006
chr17 32063029
chr17 32063038
chr17 32063051
chr17 32067055
chr17 32067071
chr17 32067073
chr17 32067077
chr17 32067096
chr17 32067109
chr17 32067120
chr17 32067158
chr17 32067171
chr17 32067182
chr17 32067185
chr17 32079751
chr17 32079761
chr17 32079830
chr17 32079865
chr17 32079881
chr17 32079907
chr17 32079915
chr17 32079940
chr17 32079989
chr17 32080040
chr17 32080047
chr17 32083599
chr17 32083634
chr17 32083719
chr17 32083739
chr17 32083765
chr17 32083778
chr17 32083829
chr17 32083833
chr17 32083879
chr17 32083881
chr17 32083884
chr17 32083948
chr17 32083950
chr17 32084026
chr17 32084050
chr17 32084057
chr17 32084068
chr17 32084070
chr17 32084090
chr17 32084106
chr17 32084116
chr17 32084143
chr17 32084179
chr17 32084181

chr17 32084211
chr17 32084214
chr17 32084250
chr17 32084252
chr17 32084266
chr17 32084288
chr17 32084292
chr17 32127753
chr17 32127767
chr17 32127792
chr17 32127805
chr17 32127810
chr17 32127813
chr17 32127818
chr17 32127934
chr17 32128008
chr17 32128234
chr17 32221165
chr17 32221174
chr17 32221185
chr17 32221299
chr17 32221322
chr17 32221361
chr17 32341479
chr17 32341680
chr17 32341714
chr17 32341732
chr17 32341734
chr17 32341808
chr17 32341811
chr17 32341897
chr17 32341901
chr17 32341906
chr17 32351275
chr17 32351308
chr17 32351389
chr17 32351508
chr17 32445509
chr17 32445515
chr17 32445559
chr17 32445572
chr17 32445768
chr17 32445837
chr17 32445915
chr17 32461981
chr17 32822267
chr17 32822290
chr17 32822401
chr17 32822403
chr17 32822410
chr17 32822413
chr17 32822417
chr17 32822421
chr17 32822425

chr17 32822440
chr17 32822450
chr17 32822453
chr17 32822459
chr17 32822476
chr17 32822483
chr17 32822485
chr17 32822487
chr17 32822493
chr17 32822497
chr17 32822500
chr17 32822502
chr17 32822505
chr17 32822513
chr17 32822520
chr17 32822531
chr17 32822541
chr17 32822546
chr17 32822551
chr17 32822556
chr17 32822563
chr17 32822570
chr17 32822583
chr17 32822585
chr17 32822591
chr17 32822595
chr17 32822597
chr17 32822600
chr17 32822605
chr17 32822611
chr17 32822618
chr17 32822621
chr17 32822628
chr17 32822633
chr17 32888128
chr17 32888159
chr17 32888184
chr17 32888205
chr17 32888331
chr17 32888340
chr17 32888367
chr17 32888372
chr17 32888427
chr17 32888451
chr17 32888754
chr17 32889064
chr17 32889093
chr17 32889589
chr17 32889643
chr17 32889855
chr17 32889911
chr17 33751386
chr17 33751484
chr17 34199706

chr17 34199727
chr17 34199738
chr17 34199789
chr17 34378832
chr17 34378871
chr17 34378902
chr17 34378961
chr17 34961024
chr17 34961032
chr17 34961039
chr17 34961047
chr17 34961064
chr17 34961110
chr17 34961156
chr17 34961173
chr17 34961178
chr17 34961180
chr17 34961184
chr17 34961200
chr17 34961214
chr17 34961226
chr17 34961806
chr17 34961818
chr17 34961820
chr17 34961841
chr17 34961845
chr17 34961856
chr17 34961866
chr17 34961880
chr17 34961883
chr17 34961887
chr17 34961893
chr17 34961913
chr17 34981412
chr17 34981453
chr17 35046628
chr17 35046869
chr17 35046886
chr17 35046970
chr17 35047027
chr17 35051672
chr17 35051729
chr17 35051802
chr17 35052115
chr17 35052229
chr17 35063645
chr17 35063691
chr17 35063717
chr17 35063789
chr17 35063835
chr17 35088011
chr17 35088020
chr17 35088050
chr17 35088082

chr17 35088178
chr17 35088196
chr17 35088297
chr17 35088423
chr17 35088431
chr17 35088454
chr17 35089589
chr17 35089596
chr17 35089637
chr17 35089655
chr17 35089677
chr17 35089734
chr17 35089739
chr17 35089750
chr17 35089763
chr17 35089802
chr17 35172031
chr17 35213582
chr17 35213600
chr17 35213602
chr17 35213803
chr17 35213839
chr17 35214096
chr17 35214379
chr17 35214457
chr17 35214473
chr17 35214675
chr17 35214924
chr17 35215023
chr17 35215142
chr17 35218743
chr17 35218818
chr17 35218967
chr17 35242508
chr17 35242519
chr17 35242539
chr17 35242543
chr17 35242554
chr17 35242611
chr17 35242613
chr17 35242618
chr17 35242651
chr17 35242661
chr17 35242672
chr17 35242687
chr17 35242690
chr17 35242705
chr17 35242710
chr17 35242717
chr17 35242728
chr17 35242744
chr17 35242748
chr17 35242880
chr17 35244835

chr17 35245006
chr17 35245319
chr17 35245681
chr17 35280219
chr17 35280743
chr17 35312494
chr17 35312497
chr17 35312522
chr17 35312891
chr17 35312914
chr17 35313116
chr17 35313126
chr17 35313229
chr17 35313281
chr17 35313319
chr17 35313429
chr17 35313478
chr17 35313501
chr17 35313507
chr17 35313520
chr17 35372674
chr17 35372694
chr17 35372827
chr17 35372887
chr17 35372960
chr17 35373031
chr17 35374757
chr17 35374761
chr17 35374846
chr17 35374940
chr17 35375074
chr17 35432834
chr17 35432883
chr17 35432910
chr17 35432912
chr17 35432938
chr17 35432946
chr17 35432967
chr17 35433002
chr17 35460383
chr17 35460400
chr17 35460543
chr17 35460547
chr17 35460550
chr17 35460558
chr17 35460564
chr17 35460566
chr17 35460576
chr17 35460586
chr17 35460588
chr17 35460592
chr17 35460595
chr17 35460606
chr17 35460623

chr17 35460640
chr17 35460663
chr17 35460669
chr17 35460675
chr17 35460681
chr17 35460686
chr17 35460720
chr17 35460727
chr17 35460737
chr17 35460765
chr17 35460767
chr17 35460792
chr17 35460796
chr17 35460798
chr17 35460808
chr17 35460816
chr17 35460818
chr17 35460825
chr17 35460917
chr17 35460945
chr17 35461007
chr17 35521293
chr17 35521893
chr17 35521898
chr17 35521958
chr17 35522025
chr17 35522084
chr17 35536512
chr17 35536700
chr17 35536713
chr17 35536759
chr17 35536891
chr17 35536903
chr17 35536951
chr17 35537018
chr17 35537044
chr17 35537064
chr17 35537112
chr17 35537134
chr17 35537146
chr17 35537158
chr17 35537715
chr17 35537782
chr17 35539048
chr17 35539052
chr17 35539054
chr17 35539094
chr17 35539097
chr17 35539219
chr17 35539221
chr17 35539232
chr17 35539251
chr17 35539258
chr17 35539282

chr17 35539298
chr17 35539304
chr17 35539426
chr17 35539484
chr17 35539488
chr17 35539555
chr17 35539565
chr17 35539568
chr17 35539603
chr17 35539615
chr17 35539627
chr17 35539667
chr17 35540776
chr17 35540805
chr17 35540818
chr17 35541058
chr17 35541179
chr17 35541233
chr17 35541269
chr17 35541401
chr17 35567992
chr17 35568001
chr17 35568008
chr17 35568034
chr17 35568045
chr17 35568051
chr17 35568058
chr17 35568065
chr17 35568068
chr17 35568084
chr17 35568087
chr17 35568094
chr17 35568096
chr17 35568118
chr17 35568122
chr17 35568163
chr17 35568209
chr17 35568238
chr17 35568251
chr17 35568264
chr17 35568330
chr17 35568338
chr17 35578150
chr17 35578202
chr17 35578240
chr17 35578244
chr17 35578266
chr17 35578293
chr17 35578306
chr17 35578339
chr17 35578357
chr17 35578368
chr17 35578386
chr17 35578407

chr17 35578425
chr17 35578441
chr17 35578484
chr17 35587224
chr17 35587239
chr17 35587244
chr17 35587246
chr17 35587249
chr17 35587252
chr17 35587262
chr17 35587311
chr17 35587338
chr17 35587343
chr17 35587384
chr17 35587391
chr17 35587394
chr17 35587403
chr17 35587409
chr17 35587415
chr17 35587421
chr17 35587427
chr17 35587441
chr17 35588722
chr17 35588760
chr17 35588955
chr17 35589094
chr17 35595409
chr17 35803192
chr17 35803509
chr17 35803532
chr17 35810352
chr17 35810532
chr17 35810537
chr17 35810629
chr17 35810726
chr17 35810766
chr17 35810790
chr17 35880251
chr17 35880281
chr17 35880291
chr17 35880293
chr17 35880328
chr17 35880337
chr17 35930431
chr17 35930458
chr17 35930484
chr17 35930530
chr17 35930567
chr17 35930583
chr17 35930624
chr17 35930643
chr17 35930647
chr17 35930655
chr17 35930694

chr17 35930704
chr17 35930722
chr17 35930739
chr17 35930747
chr17 35930749
chr17 35930760
chr17 35930773
chr17 35930779
chr17 35930788
chr17 35930792
chr17 35930794
chr17 35930812
chr17 35930818
chr17 35930825
chr17 35930831
chr17 35930838
chr17 35930843
chr17 35930932
chr17 35946996
chr17 36072510
chr17 36087186
chr17 36087200
chr17 36090133
chr17 36090184
chr17 36090229
chr17 36090672
chr17 36090684
chr17 36090850
chr17 36192599
chr17 36192901
chr17 36193003
chr17 36193259
chr17 36534949
chr17 36534956
chr17 36534960
chr17 36534977
chr17 36534982
chr17 36534990
chr17 36534992
chr17 36534995
chr17 36534997
chr17 36535002
chr17 36535004
chr17 36535008
chr17 36550606
chr17 36551007
chr17 36602394
chr17 36602400
chr17 36602504
chr17 36602530
chr17 36602537
chr17 36602558
chr17 36602586
chr17 36602588

chr17 36602610
chr17 36602634
chr17 36602707
chr17 36602723
chr17 36602751
chr17 36602809
chr17 36602842
chr17 36602931
chr17 36602972
chr17 36602994
chr17 36603089
chr17 36697207
chr17 36697225
chr17 36697261
chr17 36697313
chr17 36722102
chr17 36722123
chr17 36722237
chr17 36722251
chr17 36722299
chr17 36722321
chr17 36722334
chr17 36722339
chr17 36722348
chr17 36722351
chr17 36722355
chr17 36722357
chr17 36722373
chr17 36722380
chr17 36722388
chr17 36722394
chr17 36722403
chr17 36722405
chr17 36722413
chr17 36722417
chr17 36722430
chr17 36722432
chr17 36722438
chr17 36722442
chr17 36722445
chr17 36722452
chr17 36722459
chr17 36722471
chr17 36722473
chr17 36722484
chr17 36722498
chr17 36722505
chr17 36722524
chr17 36722532
chr17 36722539
chr17 36722559
chr17 36722562
chr17 36722579
chr17 36722627

chr17 36722631
chr17 36722641
chr17 36944125
chr17 36944164
chr17 36944832
chr17 36945052
chr17 36945338
chr17 37047700
chr17 37047767
chr17 37047786
chr17 37047816
chr17 37047821
chr17 37047847
chr17 37047901
chr17 37048208
chr17 37373750
chr17 37406154
chr17 37606009
chr17 37606038
chr17 37606203
chr17 37606254
chr17 37606465
chr17 37606778
chr17 37607152
chr17 37607510
chr17 37607514
chr17 37607563
chr17 37607617
chr17 37607649
chr17 37607657
chr17 37607661
chr17 37607703
chr17 37607713
chr17 37607743
chr17 37607792
chr17 37607825
chr17 37607827
chr17 37607850
chr17 37607870
chr17 37607899
chr17 37607904
chr17 37607912
chr17 37607915
chr17 37607918
chr17 37608059
chr17 37608098
chr17 37608207
chr17 37608271
chr17 37608277
chr17 37608318
chr17 37608476
chr17 37608497
chr17 37642421
chr17 37642604

chr17 37642626
chr17 37642750
chr17 37642821
chr17 37642869
chr17 37642922
chr17 37642939
chr17 37643012
chr17 37643621
chr17 37643635
chr17 37643643
chr17 37643649
chr17 37643657
chr17 37643689
chr17 37643717
chr17 37643826
chr17 37644044
chr17 37741099
chr17 37741139
chr17 37741201
chr17 37741258
chr17 37741321
chr17 37741392
chr17 37741472
chr17 37741489
chr17 37741528
chr17 37741595
chr17 37741626
chr17 37741632
chr17 37741646
chr17 37741702
chr17 37741889
chr17 37741917
chr17 37741956
chr17 37761047
chr17 37761104
chr17 37761116
chr17 37761195
chr17 37761200
chr17 37761222
chr17 37761348
chr17 37761390
chr17 37764548
chr17 37764595
chr17 37764751
chr17 37849380
chr17 37849481
chr17 37928210
chr17 37930141
chr17 37930152
chr17 37930186
chr17 37936948
chr17 37936976
chr17 37936994
chr17 37937001

chr17 38256375
chr17 38256383
chr17 38256385
chr17 38256388
chr17 38256429
chr17 38256483
chr17 38256504
chr17 38256534
chr17 38256595
chr17 38256598
chr17 38256608
chr17 38256617
chr17 38256622
chr17 38256625
chr17 38256642
chr17 38256647
chr17 38256649
chr17 38256660
chr17 38256679
chr17 38256685
chr17 38256697
chr17 38256759
chr17 38256762
chr17 38256768
chr17 38256771
chr17 38256778
chr17 38256782
chr17 38256792
chr17 38256797
chr17 38256804
chr17 38256808
chr17 38256816
chr17 38256820
chr17 38256876
chr17 38256883
chr17 38256890
chr17 38256903
chr17 38256906
chr17 38256924
chr17 38256945
chr17 38256984
chr17 38257023
chr17 38257026
chr17 38257046
chr17 38257049
chr17 38257052
chr17 38257061
chr17 38257071
chr17 38257093
chr17 38257101
chr17 38277119
chr17 38277335
chr17 38277441
chr17 38286476

chr17 38286592
chr17 38286733
chr17 38286747
chr17 38286762
chr17 38558551
chr17 38558567
chr17 38558580
chr17 38558588
chr17 38558797
chr17 38558830
chr17 38558838
chr17 38558842
chr17 38558867
chr17 38558878
chr17 38558902
chr17 38558956
chr17 38558985
chr17 38559007
chr17 38559071
chr17 38559095
chr17 38559101
chr17 38559104
chr17 38559268
chr17 38559271
chr17 38564921
chr17 38564932
chr17 38604748
chr17 38604761
chr17 38604776
chr17 38604798
chr17 38604803
chr17 38604838
chr17 38604891
chr17 38604966
chr17 38604982
chr17 38604993
chr17 38605058
chr17 38605065
chr17 38605108
chr17 38605110
chr17 38605125
chr17 38605157
chr17 38605187
chr17 38605286
chr17 38605399
chr17 38605404
chr17 38605438
chr17 38605476
chr17 38605480
chr17 38605499
chr17 38605502
chr17 38605509
chr17 38676382
chr17 38676607

chr17 38676874
chr17 38677046
chr17 38677055
chr17 38677187
chr17 38701434
chr17 38701691
chr17 38701746
chr17 38701788
chr17 38701799
chr17 38701864
chr17 38701889
chr17 38701897
chr17 38701905
chr17 38701932
chr17 38706656
chr17 38706690
chr17 38706739
chr17 38706786
chr17 38706819
chr17 38706875
chr17 38706928
chr17 38706963
chr17 38706977
chr17 38706994
chr17 38751293
chr17 38751423
chr17 38751450
chr17 38751503
chr17 38751551
chr17 38751567
chr17 38851514
chr17 38852244
chr17 38852259
chr17 38852319
chr17 38852348
chr17 38852555
chr17 38852670
chr17 38852687
chr17 38852706
chr17 38852781
chr17 38870941
chr17 38870952
chr17 38870966
chr17 38871048
chr17 38871127
chr17 38871278
chr17 38871317
chr17 38871403
chr17 38871405
chr17 38898519
chr17 38898541
chr17 38898591
chr17 38898713
chr17 38898811

chr17 38898820
chr17 38898869
chr17 38898897
chr17 38898926
chr17 38898999
chr17 38899031
chr17 38943647
chr17 38943754
chr17 39092413
chr17 39092432
chr17 39092454
chr17 39092493
chr17 39092551
chr17 39092626
chr17 39092664
chr17 39092675
chr17 39092854
chr17 39092889
chr17 39096478
chr17 39463162
chr17 39463180
chr17 39463203
chr17 39463316
chr17 39463375
chr17 39463775
chr17 39463838
chr17 39547946
chr17 39548097
chr17 39548103
chr17 39688308
chr17 39688326
chr17 39688395
chr17 39688407
chr17 39688461
chr17 39776235
chr17 39776799
chr17 39777270
chr17 39777680
chr17 39777714
chr17 39777760
chr17 39777854
chr17 39777938
chr17 39794168
chr17 39813863
chr17 39813891
chr17 39813908
chr17 39813910
chr17 39813922
chr17 39813934
chr17 39813943
chr17 39813945
chr17 39813960
chr17 39813996
chr17 39814112

chr17 39862016
chr17 39862954
chr17 39862967
chr17 39863324
chr17 39863422
chr17 39863566
chr17 39863618
chr17 39863647
chr17 39863676
chr17 39863907
chr17 39863933
chr17 39863955
chr17 39863967
chr17 39863970
chr17 39863972
chr17 39864091
chr17 39864104
chr17 39864129
chr17 39864497
chr17 39864505
chr17 39864547
chr17 39864605
chr17 39864819
chr17 39865199
chr17 39865215
chr17 39865243
chr17 39926405
chr17 39926501
chr17 39926525
chr17 39926668
chr17 39926701
chr17 39953095
chr17 39953100
chr17 39981491
chr17 39981506
chr17 39981519
chr17 39981546
chr17 39981669
chr17 39981683
chr17 39981813
chr17 39981872
chr17 39981895
chr17 39981909
chr17 40054220
chr17 40054238
chr17 40054361
chr17 40054390
chr17 40054444
chr17 40054472
chr17 40054477
chr17 40054515
chr17 40054527
chr17 40054530
chr17 40054533

chr17 40054551
chr17 40054559
chr17 40054564
chr17 40111403
chr17 40111495
chr17 40111556
chr17 40111582
chr17 40111584
chr17 40111809
chr17 40111888
chr17 40111914
chr17 40111975
chr17 40113148
chr17 40113306
chr17 40113329
chr17 40113382
chr17 40113416
chr17 40113427
chr17 40113432
chr17 40113434
chr17 40113449
chr17 40113594
chr17 40113607
chr17 40113653
chr17 40113679
chr17 40113705
chr17 40113804
chr17 40123756
chr17 40123769
chr17 40124663
chr17 40139995
chr17 40140042
chr17 40140129
chr17 40140147
chr17 40140160
chr17 40219161
chr17 40219189
chr17 40219247
chr17 40219253
chr17 40219258
chr17 40287728
chr17 40287796
chr17 40287808
chr17 40287840
chr17 40287846
chr17 40287856
chr17 40287869
chr17 40287882
chr17 40287891
chr17 40287893
chr17 40287897
chr17 40287899
chr17 40287919
chr17 40287957

chr17 40287967
chr17 40287978
chr17 40288024
chr17 40288051
chr17 40288070
chr17 40288077
chr17 40288080
chr17 40288118
chr17 40288135
chr17 40288162
chr17 40288184
chr17 40288232
chr17 40288270
chr17 40288335
chr17 40288369
chr17 40288371
chr17 40319065
chr17 40319083
chr17 40319126
chr17 40319184
chr17 40319258
chr17 40319313
chr17 40319525
chr17 40319761
chr17 40319772
chr17 40319823
chr17 40319832
chr17 40320148
chr17 40320151
chr17 40320273
chr17 40322359
chr17 40322402
chr17 40322405
chr17 40322483
chr17 40322498
chr17 40322509
chr17 40322555
chr17 40322567
chr17 40322593
chr17 40322626
chr17 40322657
chr17 40322680
chr17 40322708
chr17 40322948
chr17 40323018
chr17 40416497
chr17 40416780
chr17 40417059
chr17 40417064
chr17 40417209
chr17 40417236
chr17 40417244
chr17 40417252
chr17 40417258

chr17 40417275
chr17 40417293
chr17 40417301
chr17 40417700
chr17 40417705
chr17 40417709
chr17 40417725
chr17 40417730
chr17 40417732
chr17 40417734
chr17 40417746
chr17 40417759
chr17 40417761
chr17 40417766
chr17 40417797
chr17 40417801
chr17 40417828
chr17 40417850
chr17 40418163
chr17 40418167
chr17 40418198
chr17 40480542
chr17 40480593
chr17 40480607
chr17 40480615
chr17 40480623
chr17 40480627
chr17 40480676
chr17 40480701
chr17 40480743
chr17 40480805
chr17 40540647
chr17 40540658
chr17 40540679
chr17 40540712
chr17 40540714
chr17 40540790
chr17 40541094
chr17 40541097
chr17 40541132
chr17 40541148
chr17 40541251
chr17 40541255
chr17 40541279
chr17 40541287
chr17 40541289
chr17 40541311
chr17 40541383
chr17 40541394
chr17 40560814
chr17 40560825
chr17 40560954
chr17 40560973
chr17 40560981

chr17 40560990
chr17 40561016
chr17 40561023
chr17 40561041
chr17 40561054
chr17 40561074
chr17 40561127
chr17 40561189
chr17 40561193
chr17 40561216
chr17 40561247
chr17 40561582
chr17 40561724
chr17 40561735
chr17 40561977
chr17 40562038
chr17 40562176
chr17 40563658
chr17 40563682
chr17 40563692
chr17 40563969
chr17 40564073
chr17 40564191
chr17 40564205
chr17 40564223
chr17 40564342
chr17 40564355
chr17 40564395
chr17 40564453
chr17 40564465
chr17 40564468
chr17 40564565
chr17 40564668
chr17 40564680
chr17 40564776
chr17 40564873
chr17 40564897
chr17 40565105
chr17 40565148
chr17 40565305
chr17 40565314
chr17 40565412
chr17 40565423
chr17 40565451
chr17 40565457
chr17 40565507
chr17 40565636
chr17 40565690
chr17 40565947
chr17 40566222
chr17 40582465
chr17 40582468
chr17 40582471
chr17 40582492

chr17 40582604
chr17 40582651
chr17 40582724
chr17 40645901
chr17 40647387
chr17 40647398
chr17 40647430
chr17 40647448
chr17 40647465
chr17 40647479
chr17 40647527
chr17 40647557
chr17 40647615
chr17 40779447
chr17 40818429
chr17 40818432
chr17 40818436
chr17 41203659
chr17 41203789
chr17 41204123
chr17 41205234
chr17 41205280
chr17 41308761
chr17 41308821
chr17 41308842
chr17 41308911
chr17 41308992
chr17 41309001
chr17 41309078
chr17 41341476
chr17 41470000
chr17 41470072
chr17 41666349
chr17 41666381
chr17 41666398
chr17 41666464
chr17 41666483
chr17 41666487
chr17 41666529
chr17 41666549
chr17 41666600
chr17 41666613
chr17 41666648
chr17 41666653
chr17 41666657
chr17 41666675
chr17 41666748
chr17 41666752
chr17 41666767
chr17 41666782
chr17 41666804
chr17 41666830
chr17 41666856
chr17 41666859

chr17 41666908
chr17 41666934
chr17 41666953
chr17 41679189
chr17 41679221
chr17 41918624
chr17 41918669
chr17 41918671
chr17 41918673
chr17 41918690
chr17 41918693
chr17 41918698
chr17 41918700
chr17 41918707
chr17 41918711
chr17 41918817
chr17 41966108
chr17 42072212
chr17 42072228
chr17 42072230
chr17 42072243
chr17 42112283
chr17 42112302
chr17 42112312
chr17 42112327
chr17 42112346
chr17 42112352
chr17 42112467
chr17 42112608
chr17 42112629
chr17 42112643
chr17 42112660
chr17 42112686
chr17 42154527
chr17 42154530
chr17 42154602
chr17 42154623
chr17 42154682
chr17 42154747
chr17 42154750
chr17 42154779
chr17 42154784
chr17 42154822
chr17 42154854
chr17 42154860
chr17 42154892
chr17 42154895
chr17 42154901
chr17 42154906
chr17 42154909
chr17 42154912
chr17 42154925
chr17 42154932
chr17 42154936

chr17 42154943
chr17 42154955
chr17 42154958
chr17 42154961
chr17 42154976
chr17 42154989
chr17 42155000
chr17 42155005
chr17 42155011
chr17 42183929
chr17 42183940
chr17 42183962
chr17 42183969
chr17 42183994
chr17 42184001
chr17 42184014
chr17 42184027
chr17 42184029
chr17 42184033
chr17 42184058
chr17 42184070
chr17 42184100
chr17 42222329
chr17 42248706
chr17 42248742
chr17 42248754
chr17 42248763
chr17 42248773
chr17 42248824
chr17 42248865
chr17 42248948
chr17 42248960
chr17 42248988
chr17 42248999
chr17 42249285
chr17 42249941
chr17 42250007
chr17 42250199
chr17 42273314
chr17 42273461
chr17 42273634
chr17 42273655
chr17 42273710
chr17 42274024
chr17 42275078
chr17 42275128
chr17 42275207
chr17 42275268
chr17 42275278
chr17 42275337
chr17 42275383
chr17 42275456
chr17 42275468
chr17 42275497

chr17 42286828
chr17 42286843
chr17 42287165
chr17 42287415
chr17 42287474
chr17 42287483
chr17 42287508
chr17 42287589
chr17 42287610
chr17 42287672
chr17 42287678
chr17 42287703
chr17 42287718
chr17 42287747
chr17 42337703
chr17 42337714
chr17 42337724
chr17 42337747
chr17 42337767
chr17 42337781
chr17 42337861
chr17 42378937
chr17 42379034
chr17 42379190
chr17 42379248
chr17 42379254
chr17 42387439
chr17 42387505
chr17 42387573
chr17 42387621
chr17 42387627
chr17 42387662
chr17 42387684
chr17 42387739
chr17 42387752
chr17 42387766
chr17 42389534
chr17 42456735
chr17 42456874
chr17 42457056
chr17 42457201
chr17 42457362
chr17 42457405
chr17 42457502
chr17 42484346
chr17 42484348
chr17 42484352
chr17 42484492
chr17 42484507
chr17 42484627
chr17 42484910
chr17 42484912
chr17 42484918
chr17 42485101

chr17 42678567
chr17 42678571
chr17 42679150
chr17 42679207
chr17 42679278
chr17 42773322
chr17 42773340
chr17 42773356
chr17 42773383
chr17 42773410
chr17 42773414
chr17 42773418
chr17 42773424
chr17 42773431
chr17 42773445
chr17 42773455
chr17 42773474
chr17 42773480
chr17 42773490
chr17 42773498
chr17 42773509
chr17 42773528
chr17 42773554
chr17 42773671
chr17 42773676
chr17 42997968
chr17 42997998
chr17 42998053
chr17 42998137
chr17 42998183
chr17 42998196
chr17 42998213
chr17 42998216
chr17 42998220
chr17 42998276
chr17 42998292
chr17 42998294
chr17 42998301
chr17 42998323
chr17 43006940
chr17 43006968
chr17 43024633
chr17 43024654
chr17 43024657
chr17 43024675
chr17 43024683
chr17 43024691
chr17 43024705
chr17 43024727
chr17 43024731
chr17 43024791
chr17 43024795
chr17 43024797
chr17 43024847

chr17 43024850
chr17 43024887
chr17 43024903
chr17 43024909
chr17 43024911
chr17 43024930
chr17 43024937
chr17 43024939
chr17 43024948
chr17 43024974
chr17 43025002
chr17 43025026
chr17 43025041
chr17 43025065
chr17 43025068
chr17 43025080
chr17 43025109
chr17 43025129
chr17 43025136
chr17 43025142
chr17 43025161
chr17 43025169
chr17 43025171
chr17 43025175
chr17 43025189
chr17 43025206
chr17 43025208
chr17 43025214
chr17 43025219
chr17 43025227
chr17 43025230
chr17 43025232
chr17 43025238
chr17 43025240
chr17 43025248
chr17 43025257
chr17 43025262
chr17 43025266
chr17 43124567
chr17 43124589
chr17 43124610
chr17 43124624
chr17 43170531
chr17 43170586
chr17 43170671
chr17 43170676
chr17 43170744
chr17 43170759
chr17 43170789
chr17 43170791
chr17 43170898
chr17 43170917
chr17 43170924
chr17 43170956

chr17 43170962
chr17 43170971
chr17 43170974
chr17 43176393
chr17 43176527
chr17 43211062
chr17 43211167
chr17 43211325
chr17 43211483
chr17 43232957
chr17 43233004
chr17 43233017
chr17 43233027
chr17 43233051
chr17 43233287
chr17 43233298
chr17 43233412
chr17 43233492
chr17 43233519
chr17 43233530
chr17 43234329
chr17 43234339
chr17 43234345
chr17 43234351
chr17 43234378
chr17 43234381
chr17 43234388
chr17 43234395
chr17 43234413
chr17 43238796
chr17 43238801
chr17 43238841
chr17 43238940
chr17 43238948
chr17 43238998
chr17 43239435
chr17 43239446
chr17 43239570
chr17 43240108
chr17 43240113
chr17 43240117
chr17 43240123
chr17 43240125
chr17 43240129
chr17 43240132
chr17 43240138
chr17 43240164
chr17 43240166
chr17 43240184
chr17 43240211
chr17 43240220
chr17 43240229
chr17 43240243
chr17 43240416

chr17 43240430
chr17 43240440
chr17 43240464
chr17 43240500
chr17 43240503
chr17 43240647
chr17 43240700
chr17 43240711
chr17 43240821
chr17 43240885
chr17 43240912
chr17 43240923
chr17 43241501
chr17 43241521
chr17 43241548
chr17 43241559
chr17 43241562
chr17 43241568
chr17 43241669
chr17 43241677
chr17 43241686
chr17 43241699
chr17 43241704
chr17 43241713
chr17 43241727
chr17 43241737
chr17 43241743
chr17 43241749
chr17 43241776
chr17 43241779
chr17 43241786
chr17 43241793
chr17 43242579
chr17 43242604
chr17 43242643
chr17 43242658
chr17 43242664
chr17 43242701
chr17 43243488
chr17 43243687
chr17 43243694
chr17 43243696
chr17 43243706
chr17 43243770
chr17 43243783
chr17 43244424
chr17 43244437
chr17 43244461
chr17 43244488
chr17 43244500
chr17 43245169
chr17 43245180
chr17 43245301
chr17 43246559

chr17 43246570
chr17 43246572
chr17 43246577
chr17 43246600
chr17 43246615
chr17 43246620
chr17 43246644
chr17 43250733
chr17 43250757
chr17 43251043
chr17 43251057
chr17 43251067
chr17 43251091
chr17 43251327
chr17 43251338
chr17 43251468
chr17 43251617
chr17 43251633
chr17 43251644
chr17 43251653
chr17 43251659
chr17 43251677
chr17 43251684
chr17 43251688
chr17 43251706
chr17 43251736
chr17 43251739
chr17 43251741
chr17 43251754
chr17 43251757
chr17 43251766
chr17 43251771
chr17 43251783
chr17 43252381
chr17 43252387
chr17 43252393
chr17 43252420
chr17 43252423
chr17 43252430
chr17 43252437
chr17 43252455
chr17 43252473
chr17 43252475
chr17 43252616
chr17 43252629
chr17 43252640
chr17 43252663
chr17 43265358
chr17 43265364
chr17 43265378
chr17 43265391
chr17 43265686
chr17 43265728
chr17 43265750

chr17 43265839
chr17 43265853
chr17 43265863
chr17 43265887
chr17 43266123
chr17 43266134
chr17 43266260
chr17 43266437
chr17 43266448
chr17 43266457
chr17 43266463
chr17 43266468
chr17 43266481
chr17 43266488
chr17 43267310
chr17 43267316
chr17 43267325
chr17 43267331
chr17 43267337
chr17 43267345
chr17 43267353
chr17 43267381
chr17 43267401
chr17 43268631
chr17 43268653
chr17 43268668
chr17 43268691
chr17 43268746
chr17 43268774
chr17 43268790
chr17 43268940
chr17 43269334
chr17 43269342
chr17 43269356
chr17 43269358
chr17 43269761
chr17 43269763
chr17 43269786
chr17 43269793
chr17 43269803
chr17 43269825
chr17 43269857
chr17 43269861
chr17 43269902
chr17 43269928
chr17 43269935
chr17 43269954
chr17 43270073
chr17 43270090
chr17 43270094
chr17 43270096
chr17 43270261
chr17 43270285
chr17 43270290

chr17 43270324
chr17 43270771
chr17 43270780
chr17 43270805
chr17 43270858
chr17 43270869
chr17 43270996
chr17 43271173
chr17 43271184
chr17 43271193
chr17 43271199
chr17 43271204
chr17 43271217
chr17 43271224
chr17 43271228
chr17 43271658
chr17 43271667
chr17 43271676
chr17 43271690
chr17 43271710
chr17 43271738
chr17 43271749
chr17 43271752
chr17 43271758
chr17 43277434
chr17 43277487
chr17 43277498
chr17 43277629
chr17 43279078
chr17 43279084
chr17 43280382
chr17 43280411
chr17 43280417
chr17 43280442
chr17 43280625
chr17 43280676
chr17 43280684
chr17 43281127
chr17 43281134
chr17 43281144
chr17 43281166
chr17 43281198
chr17 43281202
chr17 43281242
chr17 43281268
chr17 43281275
chr17 43281386
chr17 43281408
chr17 43281410
chr17 43281416
chr17 43281440
chr17 43281450
chr17 43281492
chr17 43281504

chr17 43281508
chr17 43281537
chr17 43281545
chr17 43281560
chr17 43281578
chr17 43281583
chr17 43281587
chr17 43281592
chr17 43281605
chr17 43281620
chr17 43281630
chr17 43281632
chr17 43281676
chr17 43282439
chr17 43282468
chr17 43282500
chr17 43282829
chr17 43282861
chr17 43282878
chr17 43282884
chr17 43282898
chr17 43282911
chr17 43283148
chr17 43283156
chr17 43283206
chr17 43283643
chr17 43283654
chr17 43283776
chr17 43283844
chr17 43283871
chr17 43283882
chr17 43285126
chr17 43285133
chr17 43285144
chr17 43285159
chr17 43285191
chr17 43285237
chr17 43285239
chr17 43285245
chr17 43286785
chr17 43286836
chr17 43286844
chr17 43286850
chr17 43286858
chr17 43287290
chr17 43287297
chr17 43287307
chr17 43287329
chr17 43287361
chr17 43287365
chr17 43287405
chr17 43287431
chr17 43287548
chr17 43287570

chr17 43287572
chr17 43287578
chr17 43287602
chr17 43287612
chr17 43287654
chr17 43287666
chr17 43287670
chr17 43287838
chr17 43287842
chr17 43287846
chr17 43287865
chr17 43287868
chr17 43287876
chr17 43287881
chr17 43287893
chr17 43287901
chr17 43287911
chr17 43287933
chr17 43287953
chr17 43287965
chr17 43287970
chr17 43288083
chr17 43288096
chr17 43288117
chr17 43288129
chr17 43288155
chr17 43288603
chr17 43288632
chr17 43288762
chr17 43288767
chr17 43288807
chr17 43288809
chr17 43288825
chr17 43288843
chr17 43289807
chr17 43289818
chr17 43289928
chr17 43290245
chr17 43290277
chr17 43290288
chr17 43290302
chr17 43290330
chr17 43290335
chr17 43290346
chr17 43290403
chr17 43290833
chr17 43290843
chr17 43290849
chr17 43290855
chr17 43290882
chr17 43290885
chr17 43290892
chr17 43290899
chr17 43290917

chr17 43290935
chr17 43290937
chr17 43290944
chr17 43290960
chr17 43290963
chr17 43290967
chr17 43290969
chr17 43290973
chr17 43290982
chr17 43290988
chr17 43290994
chr17 43291002
chr17 43291010
chr17 43291058
chr17 43291166
chr17 43291175
chr17 43291188
chr17 43291193
chr17 43291216
chr17 43291231
chr17 43291236
chr17 43291260
chr17 43291264
chr17 43291277
chr17 43291284
chr17 43291295
chr17 43291348
chr17 43291387
chr17 43291389
chr17 43295547
chr17 43295569
chr17 43295604
chr17 43295658
chr17 43295672
chr17 43295682
chr17 43295942
chr17 43295953
chr17 43296062
chr17 43296908
chr17 43296915
chr17 43296924
chr17 43296937
chr17 43296942
chr17 43296950
chr17 43296964
chr17 43296974
chr17 43296980
chr17 43296986
chr17 43297234
chr17 43297247
chr17 43297250
chr17 43297288
chr17 43297297
chr17 43297308

chr17 43297310
chr17 43297315
chr17 43297338
chr17 43299259
chr17 43299269
chr17 43299274
chr17 43299293
chr17 43299310
chr17 43299329
chr17 43299332
chr17 43299342
chr17 43299348
chr17 43299391
chr17 43299397
chr17 43299415
chr17 43299530
chr17 43299541
chr17 43299668
chr17 43300569
chr17 43300583
chr17 43300599
chr17 43300605
chr17 43315698
chr17 43315721
chr17 43315732
chr17 43315767
chr17 43315826
chr17 43315848
chr17 43315858
chr17 43315894
chr17 43315915
chr17 43315985
chr17 43316039
chr17 43316080
chr17 43316100
chr17 43316135
chr17 43316146
chr17 43316171
chr17 43316219
chr17 43316287
chr17 43316311
chr17 43316350
chr17 43360302
chr17 43360339
chr17 43360384
chr17 43360393
chr17 43360429
chr17 43360444
chr17 43360479
chr17 43360509
chr17 43360515
chr17 43360524
chr17 43360554
chr17 43360584

chr17 43360602
chr17 43360614
chr17 43360631
chr17 43360650
chr17 43360657
chr17 43360674
chr17 43360709
chr17 43360721
chr17 43360764
chr17 43360783
chr17 43360787
chr17 43360794
chr17 43360837
chr17 43360844
chr17 43360850
chr17 43360887
chr17 43360892
chr17 43361622
chr17 43361658
chr17 43361693
chr17 43361704
chr17 43361768
chr17 43361784
chr17 43361825
chr17 43361877
chr17 43368102
chr17 43368140
chr17 43368259
chr17 43368291
chr17 43368320
chr17 43368334
chr17 43368341
chr17 43368350
chr17 43368418
chr17 43368426
chr17 43368438
chr17 43368456
chr17 43368464
chr17 43368480
chr17 43368487
chr17 43368495
chr17 43368519
chr17 43368539
chr17 43388739
chr17 43388753
chr17 43388756
chr17 43388764
chr17 43388767
chr17 43388806
chr17 43388815
chr17 43388826
chr17 43388828
chr17 43388833
chr17 43388876

chr17 43388900
chr17 43388917
chr17 43388919
chr17 43388924
chr17 43388935
chr17 43388950
chr17 43388982
chr17 43389028
chr17 43389035
chr17 43389102
chr17 43389106
chr17 43389141
chr17 43389151
chr17 43389184
chr17 43389195
chr17 43389206
chr17 43389214
chr17 43389217
chr17 43389226
chr17 43483909
chr17 43483932
chr17 43483947
chr17 43483949
chr17 43483951
chr17 43483959
chr17 43483979
chr17 43483983
chr17 43483998
chr17 43484001
chr17 43484060
chr17 43484073
chr17 43484078
chr17 43484091
chr17 43484093
chr17 43484112
chr17 43484172
chr17 43484193
chr17 43545914
chr17 43545920
chr17 43545982
chr17 43655872
chr17 43655896
chr17 43655926
chr17 43655970
chr17 43656128
chr17 43656275
chr17 43656382
chr17 43656412
chr17 43713399
chr17 43713407
chr17 43713482
chr17 43713522
chr17 43713555
chr17 43713562

chr17 43713590
chr17 43713633
chr17 43713657
chr17 43713670
chr17 43713700
chr17 43713718
chr17 43779487
chr17 43779490
chr17 43779568
chr17 43779649
chr17 43779687
chr17 43833671
chr17 43833685
chr17 43833687
chr17 43900949
chr17 43907706
chr17 43907717
chr17 43907720
chr17 43907776
chr17 43907789
chr17 43907815
chr17 43907840
chr17 43907854
chr17 43907877
chr17 43907880
chr17 43907895
chr17 43907912
chr17 43907997
chr17 43908040
chr17 43908099
chr17 43928143
chr17 43928209
chr17 43928383
chr17 43928753
chr17 44065884
chr17 44065892
chr17 44065986
chr17 44066000
chr17 44066095
chr17 44066112
chr17 44066180
chr17 44066203
chr17 44066234
chr17 44066252
chr17 44066287
chr17 44066296
chr17 44066298
chr17 44066323
chr17 44066327
chr17 44066333
chr17 44066338
chr17 44066342
chr17 44066345
chr17 44066347

chr17 44066354
chr17 44066358
chr17 44066370
chr17 44066389
chr17 44066392
chr17 44066395
chr17 44066409
chr17 44066506
chr17 44122772
chr17 44122868
chr17 44122916
chr17 44122924
chr17 44122938
chr17 44122977
chr17 44122998
chr17 44123027
chr17 44123031
chr17 44123049
chr17 44123103
chr17 44123126
chr17 44186188
chr17 44186212
chr17 44186234
chr17 44186244
chr17 44186252
chr17 44186269
chr17 44186275
chr17 44186277
chr17 44186308
chr17 44186317
chr17 44186405
chr17 44186408
chr17 44187273
chr17 44217059
chr17 44217154
chr17 44217601
chr17 44345520
chr17 44345525
chr17 44502438
chr17 44502447
chr17 44502449
chr17 44502484
chr17 44502541
chr17 44502617
chr17 44502623
chr17 44502639
chr17 44502657
chr17 44502686
chr17 44502757
chr17 44502759
chr17 44502790
chr17 44502800
chr17 44502901
chr17 44502917

chr17 44502924
chr17 44556242
chr17 44556295
chr17 44556306
chr17 44556330
chr17 44556371
chr17 44556375
chr17 44556393
chr17 44556415
chr17 44556425
chr17 44556445
chr17 44556502
chr17 44556513
chr17 44556527
chr17 44556535
chr17 44556555
chr17 44556573
chr17 44556776
chr17 44556787
chr17 44556810
chr17 44556830
chr17 44556905
chr17 44556940
chr17 44557023
chr17 44557037
chr17 44557043
chr17 44557046
chr17 44557048
chr17 44557057
chr17 44557068
chr17 44688933
chr17 44689009
chr17 44689012
chr17 44689029
chr17 44689203
chr17 44689223
chr17 44689270
chr17 44689285
chr17 44689303
chr17 44689329
chr17 44689693
chr17 44689696
chr17 44689702
chr17 44689708
chr17 44689714
chr17 44689727
chr17 44689729
chr17 44689738
chr17 44689745
chr17 44689748
chr17 44689785
chr17 44689792
chr17 44689804
chr17 44689809

chr17 44689811
chr17 44689814
chr17 44689825
chr17 44689827
chr17 44708010
chr17 44708261
chr17 44708303
chr17 44708457
chr17 44708499
chr17 44708576
chr17 44828625
chr17 44828883
chr17 44828981
chr17 44829005
chr17 44829013
chr17 44829094
chr17 44829105
chr17 44829146
chr17 44829190
chr17 44829199
chr17 44829230
chr17 44829266
chr17 44829295
chr17 44829301
chr17 44829331
chr17 44829337
chr17 45052209
chr17 45052261
chr17 45052274
chr17 45052368
chr17 45052377
chr17 45052432
chr17 45052480
chr17 45150407
chr17 45150432
chr17 45150575
chr17 45150733
chr17 45150739
chr17 45223279
chr17 45223292
chr17 45223348
chr17 45223372
chr17 45223488
chr17 45223550
chr17 45223605
chr17 45223769
chr17 45223790
chr17 45223792
chr17 45223842
chr17 45224157
chr17 45224354
chr17 45224366
chr17 45224385
chr17 45224418

chr17 45224471
chr17 45224535
chr17 45224803
chr17 45224832
chr17 45224911
chr17 45225124
chr17 45225366
chr17 45225409
chr17 45225451
chr17 45227287
chr17 45227493
chr17 45248448
chr17 45248592
chr17 45312316
chr17 45312349
chr17 45312374
chr17 45312423
chr17 45312604
chr17 45312653
chr17 45312692
chr17 45312804
chr17 45314233
chr17 45314286
chr17 45314450
chr17 45314580
chr17 45314668
chr17 45314681
chr17 45314799
chr17 45314921
chr17 45314966
chr17 45315045
chr17 45315067
chr17 45315180
chr17 45315247
chr17 45315351
chr17 45315424
chr17 45315489
chr17 45315523
chr17 45315792
chr17 45315848
chr17 45316074
chr17 45316171
chr17 45316191
chr17 45316243
chr17 45409866
chr17 45409887
chr17 45409915
chr17 45410097
chr17 45410202
chr17 45410238
chr17 45410248
chr17 45410273
chr17 45410308
chr17 45410397

chr17 45410405
chr17 45491130
chr17 45491153
chr17 45491172
chr17 45491179
chr17 45491186
chr17 45491206
chr17 45491213
chr17 45491263
chr17 45491291
chr17 45491302
chr17 45491315
chr17 45491358
chr17 45491417
chr17 45491441
chr17 45491447
chr17 45491471
chr17 45583611
chr17 45583618
chr17 45583660
chr17 45583685
chr17 45583773
chr17 45583802
chr17 45583820
chr17 45583822
chr17 45583832
chr17 45583892
chr17 45583955
chr17 45583961
chr17 45583971
chr17 45584030
chr17 45584143
chr17 45584273
chr17 45584326
chr17 45584333
chr17 45584346
chr17 45584357
chr17 45584395
chr17 45585392
chr17 45585408
chr17 45585410
chr17 45585435
chr17 45585439
chr17 45585454
chr17 45585466
chr17 45585468
chr17 45585473
chr17 45585512
chr17 45585546
chr17 45585564
chr17 45585574
chr17 45585579
chr17 45585583
chr17 45585595

chr17 45585609
chr17 45585613
chr17 45585627
chr17 45585715
chr17 45585761
chr17 45585771
chr17 45585778
chr17 45585787
chr17 45585842
chr17 45585881
chr17 45585883
chr17 45585929
chr17 45585932
chr17 45585941
chr17 45585963
chr17 45585980
chr17 45585998
chr17 45586027
chr17 45586030
chr17 45586033
chr17 45586168
chr17 45586178
chr17 45586188
chr17 45586190
chr17 45586193
chr17 45586195
chr17 45586198
chr17 45586200
chr17 45586207
chr17 45586213
chr17 45586217
chr17 45586232
chr17 45586237
chr17 45586241
chr17 45586250
chr17 45586264
chr17 45592532
chr17 45592546
chr17 45592651
chr17 45592891
chr17 45592902
chr17 45599857
chr17 45619946
chr17 45619965
chr17 45619968
chr17 45619990
chr17 45620492
chr17 45620508
chr17 45620516
chr17 45620518
chr17 45620525
chr17 45620532
chr17 45620536
chr17 45620539

chr17 45620542
chr17 45620557
chr17 45620560
chr17 46122011
chr17 46122050
chr17 46179258
chr17 46190407
chr17 46190433
chr17 46190446
chr17 46190519
chr17 46190764
chr17 46190772
chr17 46191025
chr17 46191122
chr17 46191242
chr17 46191339
chr17 46194829
chr17 46195160
chr17 46195241
chr17 46224453
chr17 46224476
chr17 46224520
chr17 46224657
chr17 46224664
chr17 46224695
chr17 46224891
chr17 46224895
chr17 46224897
chr17 46224901
chr17 46225766
chr17 46243614
chr17 46243617
chr17 46243620
chr17 46243623
chr17 46243625
chr17 46243633
chr17 46260181
chr17 46260184
chr17 46260198
chr17 46260216
chr17 46260224
chr17 46260231
chr17 46260238
chr17 46260247
chr17 46260251
chr17 46260256
chr17 46260271
chr17 46260275
chr17 46260281
chr17 46260288
chr17 46260290
chr17 46260293
chr17 46260295
chr17 46260298

chr17 46260300
chr17 46260310
chr17 46260320
chr17 46260326
chr17 46260328
chr17 46260380
chr17 46260433
chr17 46260455
chr17 46260458
chr17 46260461
chr17 46260490
chr17 46264970
chr17 46265011
chr17 46359315
chr17 46359351
chr17 46359389
chr17 46359455
chr17 46359540
chr17 46359600
chr17 46359875
chr17 46359906
chr17 46361800
chr17 46361830
chr17 46361847
chr17 46361897
chr17 46361900
chr17 46361906
chr17 46361909
chr17 46362006
chr17 46362043
chr17 46362064
chr17 46362067
chr17 46362085
chr17 46362090
chr17 46362103
chr17 46362122
chr17 46362158
chr17 46362160
chr17 46362173
chr17 46362571
chr17 46537388
chr17 46537402
chr17 46537409
chr17 46577276
chr17 46577314
chr17 46577380
chr17 46577394
chr17 46577402
chr17 46577465
chr17 46577525
chr17 46579725
chr17 46579755
chr17 46579772
chr17 46579788

chr17 46579822
chr17 46579825
chr17 46579831
chr17 46579834
chr17 46579931
chr17 46579968
chr17 46579989
chr17 46579992
chr17 46580010
chr17 46580015
chr17 46580028
chr17 46580047
chr17 46580083
chr17 46580085
chr17 46580098
chr17 46580267
chr17 46580282
chr17 46580288
chr17 46580351
chr17 46580649
chr17 46580657
chr17 46580687
chr17 46580709
chr17 46580964
chr17 46580968
chr17 46580980
chr17 46581062
chr17 46581250
chr17 46581274
chr17 46581280
chr17 46581285
chr17 46922388
chr17 46922447
chr17 46922607
chr17 46922697
chr17 46922719
chr17 46923105
chr17 46923109
chr17 46923116
chr17 46923123
chr17 46923143
chr17 46923150
chr17 46923165
chr17 46923171
chr17 46923180
chr17 46923186
chr17 46923189
chr17 46923215
chr17 46923230
chr17 46923234
chr17 46923241
chr17 46923256
chr17 46923261
chr17 46923298

chr17 46923302
chr17 46923321
chr17 46923331
chr17 46923357
chr17 46923376
chr17 46923540
chr17 46923563
chr17 46923589
chr17 47067297
chr17 47067304
chr17 47067318
chr17 47067320
chr17 47067323
chr17 47067377
chr17 47067425
chr17 47067453
chr17 47067463
chr17 47067475
chr17 47067491
chr17 47067540
chr17 47067605
chr17 47067684
chr17 47067917
chr17 47068069
chr17 47188326
chr17 47188380
chr17 47188555
chr17 47188602
chr17 47188617
chr17 47188629
chr17 47188678
chr17 47188686
chr17 47205409
chr17 47205469
chr17 47205480
chr17 47205509
chr17 47205514
chr17 47205656
chr17 47205674
chr17 47205746
chr17 47220714
chr17 47323532
chr17 47324068
chr17 47324073
chr17 47324114
chr17 47324121
chr17 47324147
chr17 47324178
chr17 47324330
chr17 47324336
chr17 47324367
chr17 47324374
chr17 47324409
chr17 47324414

chr17 47324422
chr17 47324458
chr17 47324467
chr17 47324500
chr17 47324527
chr17 47324535
chr17 47324538
chr17 47324569
chr17 47324581
chr17 47324636
chr17 47324654
chr17 47324665
chr17 47324669
chr17 47324674
chr17 47324678
chr17 47324763
chr17 47324837
chr17 47324889
chr17 47324913
chr17 47423415
chr17 47423423
chr17 47423426
chr17 47423432
chr17 47423436
chr17 47423438
chr17 47423443
chr17 47423448
chr17 47423461
chr17 47423463
chr17 47423473
chr17 47423477
chr17 47423481
chr17 47423485
chr17 47423489
chr17 47423549
chr17 47423555
chr17 47492084
chr17 47492141
chr17 47492164
chr17 47492181
chr17 47492193
chr17 47492226
chr17 47492240
chr17 47492274
chr17 47492290
chr17 47492338
chr17 47502464
chr17 47502478
chr17 47502542
chr17 47502551
chr17 47502619
chr17 47502735
chr17 47511911
chr17 47531285

chr17 47531289
chr17 47531291
chr17 47531295
chr17 47531330
chr17 47531334
chr17 47531337
chr17 47531351
chr17 47531359
chr17 47531381
chr17 47531391
chr17 47531400
chr17 47531403
chr17 47531406
chr17 47531426
chr17 47531429
chr17 47531431
chr17 47531437
chr17 47531453
chr17 47531457
chr17 47531468
chr17 47531507
chr17 47649050
chr17 47649243
chr17 47649258
chr17 47651178
chr17 47651208
chr17 47651229
chr17 47651791
chr17 47660142
chr17 47660184
chr17 47660196
chr17 47660224
chr17 47660256
chr17 47734112
chr17 47734120
chr17 47734278
chr17 47734283
chr17 47734369
chr17 47734380
chr17 47734393
chr17 47789484
chr17 47789508
chr17 47789520
chr17 47790536
chr17 47790822
chr17 47821187
chr17 47821226
chr17 47821247
chr17 47821347
chr17 47821445
chr17 47821475
chr17 47821531
chr17 47821652
chr17 47821681

chr17 47821703
chr17 47896059
chr17 47896100
chr17 47896103
chr17 47896112
chr17 47941618
chr17 47941640
chr17 47941651
chr17 47941658
chr17 47941664
chr17 47941667
chr17 47941680
chr17 47941691
chr17 47941696
chr17 47941701
chr17 47941704
chr17 47941707
chr17 47941711
chr17 47941715
chr17 47941723
chr17 47941757
chr17 47941787
chr17 47941799
chr17 47941801
chr17 47941808
chr17 47941818
chr17 47941821
chr17 47941847
chr17 47941865
chr17 47941880
chr17 47941906
chr17 47941925
chr17 47941957
chr17 47942003
chr17 47942011
chr17 47942044
chr17 47942061
chr17 47942146
chr17 47942197
chr17 47942253
chr17 47942303
chr17 47942321
chr17 47970771
chr17 47970783
chr17 47970815
chr17 47970835
chr17 48011885
chr17 48011895
chr17 48011898
chr17 48011903
chr17 48011923
chr17 48011943
chr17 48011965
chr17 48011978

chr17 48012067
chr17 48047555
chr17 48047569
chr17 48047609
chr17 48047634
chr17 48047640
chr17 48047645
chr17 48047651
chr17 48047656
chr17 48047662
chr17 48047685
chr17 48047689
chr17 48107586
chr17 48107589
chr17 48107616
chr17 48107627
chr17 48107642
chr17 48107648
chr17 48107650
chr17 48107657
chr17 48107663
chr17 48107683
chr17 48107697
chr17 48107702
chr17 48107707
chr17 48107715
chr17 48107726
chr17 48107733
chr17 48107736
chr17 48107743
chr17 48107751
chr17 48107754
chr17 48107763
chr17 48107776
chr17 48107801
chr17 48107805
chr17 48107811
chr17 48107848
chr17 48107854
chr17 48108101
chr17 48108136
chr17 48108191
chr17 48108222
chr17 48108236
chr17 48108267
chr17 48108271
chr17 48108383
chr17 48108447
chr17 48108581
chr17 48108612
chr17 48295165
chr17 48295198
chr17 48295245
chr17 48295532

chr17 48295642
chr17 48295736
chr17 48295786
chr17 48295820
chr17 48335037
chr17 48335922
chr17 48335928
chr17 48335932
chr17 48335973
chr17 48426580
chr17 48426846
chr17 48426932
chr17 48427429
chr17 48427494
chr17 48428026
chr17 48428224
chr17 48428325
chr17 48428354
chr17 48428882
chr17 48428893
chr17 48429027
chr17 48429123
chr17 48429205
chr17 48429279
chr17 48429303
chr17 48429312
chr17 48429364
chr17 48429374
chr17 48429392
chr17 48429532
chr17 48429567
chr17 48429585
chr17 48429629
chr17 48429649
chr17 48429701
chr17 48429723
chr17 48429771
chr17 48429829
chr17 48429848
chr17 48429983
chr17 48429989
chr17 48430006
chr17 48430030
chr17 48492338
chr17 48492340
chr17 48492496
chr17 48492513
chr17 48492562
chr17 48510927
chr17 48511263
chr17 48511278
chr17 48511498
chr17 48511512
chr17 48548626

chr17 48586120
chr17 48597000
chr17 48597317
chr17 48612532
chr17 48612534
chr17 48612640
chr17 48612690
chr17 48612695
chr17 48612705
chr17 48612711
chr17 48612717
chr17 48612721
chr17 48612725
chr17 48612761
chr17 48612764
chr17 48612808
chr17 48613497
chr17 48613501
chr17 48613504
chr17 48613529
chr17 48613588
chr17 48613594
chr17 48613639
chr17 48613643
chr17 48613649
chr17 48613658
chr17 48613669
chr17 48613671
chr17 48613675
chr17 48613678
chr17 48613692
chr17 48613749
chr17 48718110
chr17 48732021
chr17 48732260
chr17 48735494
chr17 48742978
chr17 48745990
chr17 48746092
chr17 48746210
chr17 48746225
chr17 48746268
chr17 48746435
chr17 48746480
chr17 48751408
chr17 48751430
chr17 48751458
chr17 48751751
chr17 48751801
chr17 48816742
chr17 48816753
chr17 48816786
chr17 48816792
chr17 48816800

chr17 48816819
chr17 48816829
chr17 48816847
chr17 48816872
chr17 48816890
chr17 48816932
chr17 48816949
chr17 48816961
chr17 48816971
chr17 48841506
chr17 48841537
chr17 48852434
chr17 48852679
chr17 48893426
chr17 48893429
chr17 48893466
chr17 48893468
chr17 48893471
chr17 48893506
chr17 48893545
chr17 48893736
chr17 48909263
chr17 48909278
chr17 48909310
chr17 48909335
chr17 48909381
chr17 48909390
chr17 48909445
chr17 48909563
chr17 48909568
chr17 48995186
chr17 48995330
chr17 48995339
chr17 48995359
chr17 49002209
chr17 49002514
chr17 49002597
chr17 49003569
chr17 49003611
chr17 49003747
chr17 49003758
chr17 49003814
chr17 49003833
chr17 49003854
chr17 49003880
chr17 49003892
chr17 49003898
chr17 49191499
chr17 49191638
chr17 49191697
chr17 49191723
chr17 49191765
chr17 49191808
chr17 49191820

chr17 49192019
chr17 49193204
chr17 49193232
chr17 49193237
chr17 49193241
chr17 49193264
chr17 49193295
chr17 49193323
chr17 49193327
chr17 49193335
chr17 49193337
chr17 49193391
chr17 49193415
chr17 49193421
chr17 49315605
chr17 49315729
chr17 49315774
chr17 49315811
chr17 49395175
chr17 49414608
chr17 49414693
chr17 49414698
chr17 49414731
chr17 49414739
chr17 49414743
chr17 49414754
chr17 49414806
chr17 49414817
chr17 49414882
chr17 49566042
chr17 49566048
chr17 49569565
chr17 49569583
chr17 49569598
chr17 49569630
chr17 49569663
chr17 49569690
chr17 49569699
chr17 49676478
chr17 49676602
chr17 49676649
chr17 49676796
chr17 49676851
chr17 49676883
chr17 49676958
chr17 49677068
chr17 49677099
chr17 49677261
chr17 49677343
chr17 49677354
chr17 49677359
chr17 49696075
chr17 49707322
chr17 49707359

chr17 49707535
chr17 49707620
chr17 49707627
chr17 49707652
chr17 49707673
chr17 49707706
chr17 49707711
chr17 49707717
chr17 49707723
chr17 49741738
chr17 49741815
chr17 49741893
chr17 49742143
chr17 49742146
chr17 49742261
chr17 49746686
chr17 49746768
chr17 49746817
chr17 49748411
chr17 49748453
chr17 49748463
chr17 49748903
chr17 49748914
chr17 49749083
chr17 49749105
chr17 49749122
chr17 49762684
chr17 49762692
chr17 49762731
chr17 49763120
chr17 49763163
chr17 49763167
chr17 49763177
chr17 49763226
chr17 49763281
chr17 49763291
chr17 49763308
chr17 49763352
chr17 49763356
chr17 49763531
chr17 49763542
chr17 49763544
chr17 49764215
chr17 49764282
chr17 49764294
chr17 49764323
chr17 49764332
chr17 49764393
chr17 49764410
chr17 49764425
chr17 49764465
chr17 49764470
chr17 49764494
chr17 49764496

chr17 49764499
chr17 49764547
chr17 49764605
chr17 49764772
chr17 49764783
chr17 49764812
chr17 49779962
chr17 49788377
chr17 50151625
chr17 50151639
chr17 50151679
chr17 50151713
chr17 50151737
chr17 50151756
chr17 50151796
chr17 50151824
chr17 50151847
chr17 50151904
chr17 50151996
chr17 50152019
chr17 50152047
chr17 50152192
chr17 50152211
chr17 50152345
chr17 50152347
chr17 50152379
chr17 50200189
chr17 50200191
chr17 50200224
chr17 50200270
chr17 50200296
chr17 50200310
chr17 50200326
chr17 50200348
chr17 50200383
chr17 50200412
chr17 50200418
chr17 50200438
chr17 50372735
chr17 50373379
chr17 50373384
chr17 50373399
chr17 50373411
chr17 50373423
chr17 50421787
chr17 50425978
chr17 50425980
chr17 50425996
chr17 50426016
chr17 50426045
chr17 50426066
chr17 50426068
chr17 50426074
chr17 50426092

chr17 50426094
chr17 50426096
chr17 50426102
chr17 50426105
chr17 50426108
chr17 50426111
chr17 50426113
chr17 50426118
chr17 50426123
chr17 50426128
chr17 50426131
chr17 50426134
chr17 50426141
chr17 50426147
chr17 50426150
chr17 50426179
chr17 50426187
chr17 50426191
chr17 50426196
chr17 50426198
chr17 50426202
chr17 50426207
chr17 50426212
chr17 50426234
chr17 50426239
chr17 50426244
chr17 50426256
chr17 50426273
chr17 50426276
chr17 50426286
chr17 50426303
chr17 50426306
chr17 50426314
chr17 50426331
chr17 50426336
chr17 50426346
chr17 50426379
chr17 50426402
chr17 50426404
chr17 50479120
chr17 50479127
chr17 50479135
chr17 50479143
chr17 50479147
chr17 50479222
chr17 50479227
chr17 50479311
chr17 50479394
chr17 50479417
chr17 50479476
chr17 50479532
chr17 50479567
chr17 50479603
chr17 50479605

chr17 50479609
chr17 50508594
chr17 50508604
chr17 50508610
chr17 50508613
chr17 50508621
chr17 50508635
chr17 50508638
chr17 50508651
chr17 50508664
chr17 50508668
chr17 50508699
chr17 50508715
chr17 50508756
chr17 50508772
chr17 50508776
chr17 50508786
chr17 50508798
chr17 50508805
chr17 50508823
chr17 50508869
chr17 50508934
chr17 50532386
chr17 50532394
chr17 50532456
chr17 50708631
chr17 50708737
chr17 50708870
chr17 50719610
chr17 50719635
chr17 50719646
chr17 50719651
chr17 50719663
chr17 50719666
chr17 50719672
chr17 50719676
chr17 50719684
chr17 50719690
chr17 50719716
chr17 50719720
chr17 50719740
chr17 50719744
chr17 50719746
chr17 50719772
chr17 50719778
chr17 50719791
chr17 50719795
chr17 50719802
chr17 50719810
chr17 50719814
chr17 50719825
chr17 50719831
chr17 50719840
chr17 50719842

chr17 50719860
chr17 50719867
chr17 50719882
chr17 50719886
chr17 50719888
chr17 50719908
chr17 50719910
chr17 50719913
chr17 50719915
chr17 50719930
chr17 50719936
chr17 50719942
chr17 50719946
chr17 50719955
chr17 50719989
chr17 50721449
chr17 50721699
chr17 50721705
chr17 50721979
chr17 50852346
chr17 50864673
chr17 50864788
chr17 50864887
chr17 50864987
chr17 50865018
chr17 50865028
chr17 50865051
chr17 50865062
chr17 50867734
chr17 50867737
chr17 50867753
chr17 50867823
chr17 50867868
chr17 50867871
chr17 50867908
chr17 50867927
chr17 50867950
chr17 50867973
chr17 50868007
chr17 50868278
chr17 50871517
chr17 50871662
chr17 50871760
chr17 50871800
chr17 50871818
chr17 50872004
chr17 50931274
chr17 50931278
chr17 50931291
chr17 50931300
chr17 50931303
chr17 50931309
chr17 50931315
chr17 50931319

chr17 51029753
chr17 51030004
chr17 51120012
chr17 51120019
chr17 51121446
chr17 51121532
chr17 51121620
chr17 51121643
chr17 51121719
chr17 51121775
chr17 51121829
chr17 51121919
chr17 51121936
chr17 51121955
chr17 51121961
chr17 51121970
chr17 51121989
chr17 51122005
chr17 51122009
chr17 51154400
chr17 51154412
chr17 51154457
chr17 51154487
chr17 51154528
chr17 51259170
chr17 51259202
chr17 51259242
chr17 51259264
chr17 51259330
chr17 51259334
chr17 51259345
chr17 51259372
chr17 51259381
chr17 51259386
chr17 51259388
chr17 51259433
chr17 51259454
chr17 51259514
chr17 51259561
chr17 51259669
chr17 51259676
chr17 51259700
chr17 51259715
chr17 51259742
chr17 51259758
chr17 52160704
chr17 52160754
chr17 52160956
chr17 52160965
chr17 52161835
chr17 52162197
chr17 52162265
chr17 52162482
chr17 53104729

chr17 53717473
chr17 53718085
chr17 53747078
chr17 53755522
chr17 53755634
chr17 53755959
chr17 53805776
chr17 53805963
chr17 53806206
chr17 53970814
chr17 53971086
chr17 53971277
chr17 54697778
chr17 54697821
chr17 54967590
chr17 54967608
chr17 54967633
chr17 54967685
chr17 54967712
chr17 54967777
chr17 54967815
chr17 54967853
chr17 54967939
chr17 54967947
chr17 54967978
chr17 55238743
chr17 55238750
chr17 55238797
chr17 55238799
chr17 55238804
chr17 55238824
chr17 55238828
chr17 55238836
chr17 55238947
chr17 55239009
chr17 55239046
chr17 55239109
chr17 55420212
chr17 55420240
chr17 55420304
chr17 55420346
chr17 55420372
chr17 55420727
chr17 55420808
chr17 55420854
chr17 55420883
chr17 55420886
chr17 55420898
chr17 55420959
chr17 55420973
chr17 55421014
chr17 55421034
chr17 55421055
chr17 55421065

chr17 55504902
chr17 55505310
chr17 55559311
chr17 55559474
chr17 55577094
chr17 55577178
chr17 55577203
chr17 55577258
chr17 55577269
chr17 55577389
chr17 55577487
chr17 55577514
chr17 55577535
chr17 55577655
chr17 55577663
chr17 55577686
chr17 55577694
chr17 55577696
chr17 55577719
chr17 55577725
chr17 55577772
chr17 55577790
chr17 55735234
chr17 55735359
chr17 55735375
chr17 55735407
chr17 55735672
chr17 55735831
chr17 55735988
chr17 55735998
chr17 55736290
chr17 55750519
chr17 55750902
chr17 55750906
chr17 55750920
chr17 55750947
chr17 55750967
chr17 55750995
chr17 55751007
chr17 55751016
chr17 55751026
chr17 55751035
chr17 55751062
chr17 55751064
chr17 55751213
chr17 55751230
chr17 55751258
chr17 55751269
chr17 55751271
chr17 55751285
chr17 55751288
chr17 55751294
chr17 55751303
chr17 55751314

chr17 55751319
chr17 55751326
chr17 55751328
chr17 55751343
chr17 55751345
chr17 55751354
chr17 55751372
chr17 55751376
chr17 55751404
chr17 55751412
chr17 55751432
chr17 55751486
chr17 55751489
chr17 55751524
chr17 55751548
chr17 55751556
chr17 55766592
chr17 55766710
chr17 56206236
chr17 56206503
chr17 56206533
chr17 56297337
chr17 56297405
chr17 56512259
chr17 56592992
chr17 56593007
chr17 56593009
chr17 56593013
chr17 56593022
chr17 56593053
chr17 56593067
chr17 56593074
chr17 56593093
chr17 56593158
chr17 56593178
chr17 56593198
chr17 56593207
chr17 56593210
chr17 56593213
chr17 56593215
chr17 56593241
chr17 56593246
chr17 56593290
chr17 56593348
chr17 56593350
chr17 56593365
chr17 56593371
chr17 56593374
chr17 56593377
chr17 56593383
chr17 56593386
chr17 56593392
chr17 56593395
chr17 56593398

chr17 56593401
chr17 56593404
chr17 56593407
chr17 56595014
chr17 56595179
chr17 56595192
chr17 56595313
chr17 56595320
chr17 56595365
chr17 56595412
chr17 56595441
chr17 56595662
chr17 56595674
chr17 56595705
chr17 56595729
chr17 56595777
chr17 56595792
chr17 56595930
chr17 56595937
chr17 56595952
chr17 56595956
chr17 56595995
chr17 56596021
chr17 56596029
chr17 56596046
chr17 56596075
chr17 56596077
chr17 56596092
chr17 56596104
chr17 56596109
chr17 56596156
chr17 56596165
chr17 56596216
chr17 56596232
chr17 56596239
chr17 56596255
chr17 56596259
chr17 56596309
chr17 56596342
chr17 56596352
chr17 56596414
chr17 56596456
chr17 56596462
chr17 56596473
chr17 56596503
chr17 56596521
chr17 56596573
chr17 56596576
chr17 56596587
chr17 56596593
chr17 56596601
chr17 56596619
chr17 56596623
chr17 56596639

chr17 56596667
chr17 56596698
chr17 56596738
chr17 56596740
chr17 56596751
chr17 56596754
chr17 56596798
chr17 56596806
chr17 56596809
chr17 56596837
chr17 56596851
chr17 56596868
chr17 56596874
chr17 56596878
chr17 56596896
chr17 56596899
chr17 56596914
chr17 56596917
chr17 56596925
chr17 56596955
chr17 56596961
chr17 56596974
chr17 56596977
chr17 56596986
chr17 56596993
chr17 56597004
chr17 56597030
chr17 56597040
chr17 56597075
chr17 56597077
chr17 56597156
chr17 56597173
chr17 56597207
chr17 56597241
chr17 56597296
chr17 56597340
chr17 56597393
chr17 56597427
chr17 56597434
chr17 56597473
chr17 56597482
chr17 56597513
chr17 56597515
chr17 56597524
chr17 56597548
chr17 56597563
chr17 56597570
chr17 56597572
chr17 56597575
chr17 56679520
chr17 56679535
chr17 56679739
chr17 56716048
chr17 56716098

chr17 56716102
chr17 56716139
chr17 56819802
chr17 56819824
chr17 56914589
chr17 56914595
chr17 56914603
chr17 56914617
chr17 56914632
chr17 56914787
chr17 56914791
chr17 56914795
chr17 56914813
chr17 56914815
chr17 56914817
chr17 56914819
chr17 56914821
chr17 56977646
chr17 56977712
chr17 56977725
chr17 56978338
chr17 56978456
chr17 56978468
chr17 56978534
chr17 56978577
chr17 56978601
chr17 57054726
chr17 57086576
chr17 57086626
chr17 57086628
chr17 57086636
chr17 57086717
chr17 57131279
chr17 57131365
chr17 57131414
chr17 57131434
chr17 57131467
chr17 57131482
chr17 57131530
chr17 57131673
chr17 57131707
chr17 57131722
chr17 57131770
chr17 57131793
chr17 57131803
chr17 57131818
chr17 57131841
chr17 57131889
chr17 57131913
chr17 57131923
chr17 57131938
chr17 57131971
chr17 57131986
chr17 57132009

chr17 57257619
chr17 57257633
chr17 57257783
chr17 57257786
chr17 57257832
chr17 57257944
chr17 57257955
chr17 57257970
chr17 57258065
chr17 57258079
chr17 57258103
chr17 57802137
chr17 57802728
chr17 57802937
chr17 57955638
chr17 57955654
chr17 57955658
chr17 57955678
chr17 57955690
chr17 57955696
chr17 57955706
chr17 57955714
chr17 57955716
chr17 57955723
chr17 57955730
chr17 57955755
chr17 57955768
chr17 57955788
chr17 57955796
chr17 57955798
chr17 57955822
chr17 57955862
chr17 57955923
chr17 57955996
chr17 57956005
chr17 57956103
chr17 57956155
chr17 57956395
chr17 57956428
chr17 57956433
chr17 57986797
chr17 57986826
chr17 57986837
chr17 57986839
chr17 57987086
chr17 57987121
chr17 57987167
chr17 57987204
chr17 57987208
chr17 57987230
chr17 57987235
chr17 57987246
chr17 58005242
chr17 58005413

chr17 58005419
chr17 58005434
chr17 58005446
chr17 58005482
chr17 58005506
chr17 58005518
chr17 58005524
chr17 58005530
chr17 58005536
chr17 58005581
chr17 58005787
chr17 58005835
chr17 58005862
chr17 58005875
chr17 58005892
chr17 58005928
chr17 58006022
chr17 58006146
chr17 58006153
chr17 58006165
chr17 58006203
chr17 58006278
chr17 58006305
chr17 58006369
chr17 58006396
chr17 58084576
chr17 58141245
chr17 58171026
chr17 58333538
chr17 58333603
chr17 58334056
chr17 58334102
chr17 58334152
chr17 58334381
chr17 58334440
chr17 58334494
chr17 58334504
chr17 58334645
chr17 58334679
chr17 58334689
chr17 58334826
chr17 58334832
chr17 58334835
chr17 58334845
chr17 58334900
chr17 58335130
chr17 58335141
chr17 58335145
chr17 58335215
chr17 58335266
chr17 58335272
chr17 58335378
chr17 58335439
chr17 58335500

chr17 58335646
chr17 58335907
chr17 58336097
chr17 58336394
chr17 58336429
chr17 58336529
chr17 58336616
chr17 58336728
chr17 58336793
chr17 58336955
chr17 58337252
chr17 58337329
chr17 58338101
chr17 58338165
chr17 58338187
chr17 58339431
chr17 58339582
chr17 58339634
chr17 58339703
chr17 58339753
chr17 58339781
chr17 58339911
chr17 58339998
chr17 58340003
chr17 58340161
chr17 58341821
chr17 58344067
chr17 58344079
chr17 58344179
chr17 58344344
chr17 58344564
chr17 58344568
chr17 58344669
chr17 58344749
chr17 58344965
chr17 58351394
chr17 58351441
chr17 58351589
chr17 58351699
chr17 58351711
chr17 58351760
chr17 58351778
chr17 58351790
chr17 58351813
chr17 58417603
chr17 58417710
chr17 58417718
chr17 58417783
chr17 58417789
chr17 58417922
chr17 58418512
chr17 58513659
chr17 58513716
chr17 58513729

chr17 58513794
chr17 58513811
chr17 58513830
chr17 58513971
chr17 58513980
chr17 58514046
chr17 58514069
chr17 58514098
chr17 58514134
chr17 58514174
chr17 58514184
chr17 58514250
chr17 58514254
chr17 58514260
chr17 58514274
chr17 58514297
chr17 58514314
chr17 58514322
chr17 58514362
chr17 58514389
chr17 58514391
chr17 58514395
chr17 58514411
chr17 58514413
chr17 58514417
chr17 58514430
chr17 58514442
chr17 58514448
chr17 58514453
chr17 58514460
chr17 58514469
chr17 58514473
chr17 58514477
chr17 58556991
chr17 58557040
chr17 58631671
chr17 58631703
chr17 58631730
chr17 58631757
chr17 58631759
chr17 58631769
chr17 58631778
chr17 58631795
chr17 58631864
chr17 58631904
chr17 58631954
chr17 58659337
chr17 58659394
chr17 58659399
chr17 58659408
chr17 58659416
chr17 58659453
chr17 58659490
chr17 58659502

chr17 58659537
chr17 58659578
chr17 58659583
chr17 58692857
chr17 58692862
chr17 58692881
chr17 58692903
chr17 58692918
chr17 58692933
chr17 58753513
chr17 58875786
chr17 59001503
chr17 59001548
chr17 59001554
chr17 59001605
chr17 59001634
chr17 59001643
chr17 59001703
chr17 59001783
chr17 59001875
chr17 59001886
chr17 59004523
chr17 59004742
chr17 59004903
chr17 59005015
chr17 59105505
chr17 59105716
chr17 59155917
chr17 59155971
chr17 59156015
chr17 59156024
chr17 59156102
chr17 59156109
chr17 59156145
chr17 59156267
chr17 59156308
chr17 59156340
chr17 59209570
chr17 59209577
chr17 59209827
chr17 59209837
chr17 59221190
chr17 59221195
chr17 59221198
chr17 59221230
chr17 59221251
chr17 59221265
chr17 59221278
chr17 59221283
chr17 59221314
chr17 59221323
chr17 59221387
chr17 59287598
chr17 59311213

chr17 59362184
chr17 59435465
chr17 59435595
chr17 59435652
chr17 59435723
chr17 59440928
chr17 59440949
chr17 59440961
chr17 59441035
chr17 59484086
chr17 59484125
chr17 59484166
chr17 59565773
chr17 59565776
chr17 59565787
chr17 59565792
chr17 59565794
chr17 59565799
chr17 59565804
chr17 59565814
chr17 59565823
chr17 59565826
chr17 59565856
chr17 59565891
chr17 59565896
chr17 59565918
chr17 59565923
chr17 59565932
chr17 59565963
chr17 59565984
chr17 59566003
chr17 59566021
chr17 59566025
chr17 59566095
chr17 59566098
chr17 59566103
chr17 59566196
chr17 59618412
chr17 59618492
chr17 59618495
chr17 59618500
chr17 59620562
chr17 59620601
chr17 59620670
chr17 59620729
chr17 59620786
chr17 59620791
chr17 59620810
chr17 59620847
chr17 59620870
chr17 59620980
chr17 59621019
chr17 59621178
chr17 59621346

chr17 59642070
chr17 59642321
chr17 59642747
chr17 59708057
chr17 59708151
chr17 59708259
chr17 59708264
chr17 59708348
chr17 59708390
chr17 59708399
chr17 59762518
chr17 59783174
chr17 59783182
chr17 59783272
chr17 59783427
chr17 59783640
chr17 59783762
chr17 59784004
chr17 59784280
chr17 59784296
chr17 59785120
chr17 59785172
chr17 59785389
chr17 59785597
chr17 59785604
chr17 59785614
chr17 59785775
chr17 59785790
chr17 59786194
chr17 59786319
chr17 59786384
chr17 59786731
chr17 59786787
chr17 59786917
chr17 59787002
chr17 59787117
chr17 59787132
chr17 59787245
chr17 59787312
chr17 59787478
chr17 59787559
chr17 59787592
chr17 59787769
chr17 59787775
chr17 59826114
chr17 59826183
chr17 59828486
chr17 59828595
chr17 59833113
chr17 59833272
chr17 59837413
chr17 59837425
chr17 59837475
chr17 59837521

chr17 59837733
chr17 59837773
chr17 59837811
chr17 59838010
chr17 59838232
chr17 59838304
chr17 59838356
chr17 59838379
chr17 59838412
chr17 59838439
chr17 59838527
chr17 59838880
chr17 59838945
chr17 59838986
chr17 59839024
chr17 59839030
chr17 59839055
chr17 59839057
chr17 59839080
chr17 59839087
chr17 59839097
chr17 59839141
chr17 59839193
chr17 59839233
chr17 59839237
chr17 59839280
chr17 59839282
chr17 59839286
chr17 59839461
chr17 59839806
chr17 59839870
chr17 59839882
chr17 59839981
chr17 59840105
chr17 59840228
chr17 59840601
chr17 59840613
chr17 59840901
chr17 59840979
chr17 59841139
chr17 59841239
chr17 59841269
chr17 59841321
chr17 59841702
chr17 59841736
chr17 59841805
chr17 59841920
chr17 59842058
chr17 59842087
chr17 59842242
chr17 59842355
chr17 59842394
chr17 59842398
chr17 59842402

chr17 59842404
chr17 59842497
chr17 59842534
chr17 59842538
chr17 59842550
chr17 59842626
chr17 59842634
chr17 59843221
chr17 59846328
chr17 59893295
chr17 59893297
chr17 59893334
chr17 59893343
chr17 59893351
chr17 59893365
chr17 59893370
chr17 59893373
chr17 59893376
chr17 59893378
chr17 59893391
chr17 59893393
chr17 59893408
chr17 59893445
chr17 59893447
chr17 59893449
chr17 59893467
chr17 59893471
chr17 59893476
chr17 59964573
chr17 59964589
chr17 59964699
chr17 59964708
chr17 60020433
chr17 60020469
chr17 60025095
chr17 60025283
chr17 60025582
chr17 60025595
chr17 60025598
chr17 60025604
chr17 60025607
chr17 60025618
chr17 60025636
chr17 60025648
chr17 60025653
chr17 60025655
chr17 60025671
chr17 60025686
chr17 60026025
chr17 60026028
chr17 60026032
chr17 60026035
chr17 60026038
chr17 60026049

chr17 60026052
chr17 60026056
chr17 60026058
chr17 60026064
chr17 60026076
chr17 60026088
chr17 60026206
chr17 60026212
chr17 60026231
chr17 60026239
chr17 60026246
chr17 60026252
chr17 60026264
chr17 60026277
chr17 60026279
chr17 60026282
chr17 60026285
chr17 60026287
chr17 60026310
chr17 60026313
chr17 60026674
chr17 60026679
chr17 60026687
chr17 60026694
chr17 60026696
chr17 60026698
chr17 60026703
chr17 60026720
chr17 60026723
chr17 60026725
chr17 60026728
chr17 60026753
chr17 60026772
chr17 60026781
chr17 60026784
chr17 60026786
chr17 60026789
chr17 60026802
chr17 60026804
chr17 60026819
chr17 60026825
chr17 60026829
chr17 60027127
chr17 60027176
chr17 60027180
chr17 60027204
chr17 60027206
chr17 60027491
chr17 60027511
chr17 60027520
chr17 60078268
chr17 60078309
chr17 60078329
chr17 60078349

chr17 60078421
chr17 60078425
chr17 60078479
chr17 60078503
chr17 60078507
chr17 60078571
chr17 60078597
chr17 60078599
chr17 60078650
chr17 60078664
chr17 60078670
chr17 60078684
chr17 60078686
chr17 60078689
chr17 60078699
chr17 60078707
chr17 60078719
chr17 60102508
chr17 60102541
chr17 60102551
chr17 60102594
chr17 60102622
chr17 60102637
chr17 60102665
chr17 60102669
chr17 60102671
chr17 60102676
chr17 60102678
chr17 60102682
chr17 60102684
chr17 60102687
chr17 60102698
chr17 60102704
chr17 60102707
chr17 60102718
chr17 60102721
chr17 60102724
chr17 60102727
chr17 60102748
chr17 60102751
chr17 60102770
chr17 60103057
chr17 60111369
chr17 60111442
chr17 60111468
chr17 60111518
chr17 60111729
chr17 60111752
chr17 60111867
chr17 60112386
chr17 60112408
chr17 60112525
chr17 60112600
chr17 60112604

chr17 60112689
chr17 60112942
chr17 60114231
chr17 60114235
chr17 60114295
chr17 60114306
chr17 60119716
chr17 60119720
chr17 60119726
chr17 60119741
chr17 60119743
chr17 60119756
chr17 60119759
chr17 60119761
chr17 60119764
chr17 60119773
chr17 60119792
chr17 60119817
chr17 60119820
chr17 60119822
chr17 60119825
chr17 60119842
chr17 60119847
chr17 60119849
chr17 60119851
chr17 60119858
chr17 60119866
chr17 60119871
chr17 60120403
chr17 60120411
chr17 60120415
chr17 60120421
chr17 60120446
chr17 60120457
chr17 60120469
chr17 60120481
chr17 60120487
chr17 60120489
chr17 60120493
chr17 60120496
chr17 60120507
chr17 60120510
chr17 60120513
chr17 60120517
chr17 60135031
chr17 60135038
chr17 60135060
chr17 60135137
chr17 60135159
chr17 60135180
chr17 60135202
chr17 60135242
chr17 60135248
chr17 60135276

chr17 60135292
chr17 60135297
chr17 60135314
chr17 60135318
chr17 60135343
chr17 60135402
chr17 60135442
chr17 60135458
chr17 60135482
chr17 60135494
chr17 60135503
chr17 60135520
chr17 60135541
chr17 60135567
chr17 60135578
chr17 60135581
chr17 60135597
chr17 60135601
chr17 60135608
chr17 60135612
chr17 60135620
chr17 60142590
chr17 60142624
chr17 60391653
chr17 60391656
chr17 60391691
chr17 60392431
chr17 60392437
chr17 60392443
chr17 60392446
chr17 60392560
chr17 60392580
chr17 60392614
chr17 60392672
chr17 60392690
chr17 60392751
chr17 60392784
chr17 60392803
chr17 60392813
chr17 60392821
chr17 60392872
chr17 60392913
chr17 60392941
chr17 60392949
chr17 60392973
chr17 60393019
chr17 60524850
chr17 60524853
chr17 60524855
chr17 60525315
chr17 60525325
chr17 60525348
chr17 60599279
chr17 60599371

chr17 60599376
chr17 60599552
chr17 60599573
chr17 60601750
chr17 60601951
chr17 60602056
chr17 60602144
chr17 60602204
chr17 60602266
chr17 60602274
chr17 60864306
chr17 60864599
chr17 60864744
chr17 60864971
chr17 61139178
chr17 61139319
chr17 61168719
chr17 61168813
chr17 61435781
chr17 61536755
chr17 61550685
chr17 61551187
chr17 61551282
chr17 61862749
chr17 61862870
chr17 61862942
chr17 61863086
chr17 61863091
chr17 61863095
chr17 61863114
chr17 61863195
chr17 61863230
chr17 61863262
chr17 61863276
chr17 61863933
chr17 61863945
chr17 61863997
chr17 61864036
chr17 61864113
chr17 62008465
chr17 62008467
chr17 62008683
chr17 62008686
chr17 62008699
chr17 62009082
chr17 62063718
chr17 62063788
chr17 62063976
chr17 62064013
chr17 62064025
chr17 62064470
chr17 62065484
chr17 62065489
chr17 62065496

chr17 62065503
chr17 62065509
chr17 62065513
chr17 62065530
chr17 62165245
chr17 62388963
chr17 62424289
chr17 62424314
chr17 62424501
chr17 62424576
chr17 62424612
chr17 62424614
chr17 62470950
chr17 62471334
chr17 62478602
chr17 62478605
chr17 62478608
chr17 62478610
chr17 62478635
chr17 62478641
chr17 62478643
chr17 62478647
chr17 62478663
chr17 62478668
chr17 62478671
chr17 62478679
chr17 62478702
chr17 62478729
chr17 62478732
chr17 62478761
chr17 62478772
chr17 62478783
chr17 62478785
chr17 62478795
chr17 62478797
chr17 62478820
chr17 62478835
chr17 62478840
chr17 62478845
chr17 62478850
chr17 62478863
chr17 62478869
chr17 62478886
chr17 62478892
chr17 62478901
chr17 62478908
chr17 62478910
chr17 62478913
chr17 62478919
chr17 62478934
chr17 62478938
chr17 62478958
chr17 62478975
chr17 62478995

chr17 62478998
chr17 62479009
chr17 62479015
chr17 62479017
chr17 62479024
chr17 62479026
chr17 62479030
chr17 62479034
chr17 62479040
chr17 62479046
chr17 62479048
chr17 62479051
chr17 62479055
chr17 62479069
chr17 62479071
chr17 62479077
chr17 62479087
chr17 62479094
chr17 62479110
chr17 62479114
chr17 62479122
chr17 62479131
chr17 62479134
chr17 62479137
chr17 62479140
chr17 62479143
chr17 62479151
chr17 62479155
chr17 62479165
chr17 62479168
chr17 62479184
chr17 62479190
chr17 62479195
chr17 62479216
chr17 62479221
chr17 62479226
chr17 62479232
chr17 62479237
chr17 62479882
chr17 62479921
chr17 62480175
chr17 62480237
chr17 62480302
chr17 62480544
chr17 62553131
chr17 62553298
chr17 62553380
chr17 62643553
chr17 62643619
chr17 62643701
chr17 62643742
chr17 62643809
chr17 62653043
chr17 62653074

chr17 62653105
chr17 62653109
chr17 62653116
chr17 62653134
chr17 62653140
chr17 62653159
chr17 62653161
chr17 62653165
chr17 62653181
chr17 62653210
chr17 62653279
chr17 62653295
chr17 62653297
chr17 62653333
chr17 62653355
chr17 62653372
chr17 62653384
chr17 62653389
chr17 62653413
chr17 62653415
chr17 62653431
chr17 62653446
chr17 62965670
chr17 62965686
chr17 62965711
chr17 62965741
chr17 62965776
chr17 62965805
chr17 62965841
chr17 62965845
chr17 62965865
chr17 62968110
chr17 62968249
chr17 63425123
chr17 63425134
chr17 63425178
chr17 63425226
chr17 63425277
chr17 63425523
chr17 63598959
chr17 63611940
chr17 63612026
chr17 63612058
chr17 63698315
chr17 63698370
chr17 63698376
chr17 63698430
chr17 63698469
chr17 63698482
chr17 63698508
chr17 63698523
chr17 63698558
chr17 63698569
chr17 63698613

chr17 63698626
chr17 63698629
chr17 63698686
chr17 63698693
chr17 63698695
chr17 63698698
chr17 63698729
chr17 63698732
chr17 63702226
chr17 63742300
chr17 63742334
chr17 63742362
chr17 63742425
chr17 63742478
chr17 63742480
chr17 63742484
chr17 63742505
chr17 63742507
chr17 63742536
chr17 63742574
chr17 63742651
chr17 63742655
chr17 63742657
chr17 63742667
chr17 63742695
chr17 63742698
chr17 63742700
chr17 63742725
chr17 63742745
chr17 63742748
chr17 63742756
chr17 63742786
chr17 63742790
chr17 63742802
chr17 63742806
chr17 63742854
chr17 63742861
chr17 63742884
chr17 63742886
chr17 63742909
chr17 63751907
chr17 63751950
chr17 63751983
chr17 63752013
chr17 63752016
chr17 63752076
chr17 63752082
chr17 63752169
chr17 63752333
chr17 63752646
chr17 63775093
chr17 63775120
chr17 63775141
chr17 63826769

chr17 63826782
chr17 64016896
chr17 64017160
chr17 64017173
chr17 64017189
chr17 64019962
chr17 64020014
chr17 64020073
chr17 64020078
chr17 64020125
chr17 64020132
chr17 64020195
chr17 64020558
chr17 64020591
chr17 64020593
chr17 64020615
chr17 64020661
chr17 64020677
chr17 64020721
chr17 64020750
chr17 64020838
chr17 64020862
chr17 64020870
chr17 64020891
chr17 64020902
chr17 64020926
chr17 64020989
chr17 64021021
chr17 64021061
chr17 64021148
chr17 64120744
chr17 64133806
chr17 64497437
chr17 64497481
chr17 64497511
chr17 64497522
chr17 64497534
chr17 64497546
chr17 64497556
chr17 64504191
chr17 64504259
chr17 64504375
chr17 64504635
chr17 64504639
chr17 64504684
chr17 64504828
chr17 64504859
chr17 64507679
chr17 64507723
chr17 64507765
chr17 64507776
chr17 64507781
chr17 64507819
chr17 64507900

chr17 64507960
chr17 64508020
chr17 64508114
chr17 64508182
chr17 64508217
chr17 64580443
chr17 64580512
chr17 64580577
chr17 64599104
chr17 64599175
chr17 64599317
chr17 64599366
chr17 64599483
chr17 64599508
chr17 64599519
chr17 64599535
chr17 64599558
chr17 64608662
chr17 64661306
chr17 64661309
chr17 64661312
chr17 64661334
chr17 64661367
chr17 64661379
chr17 64661391
chr17 64661398
chr17 64661425
chr17 64661427
chr17 64661440
chr17 64661456
chr17 64661461
chr17 64661483
chr17 64661497
chr17 64661519
chr17 64661526
chr17 64661536
chr17 64661562
chr17 64661574
chr17 64661580
chr17 64661585
chr17 64661590
chr17 64661624
chr17 64661629
chr17 64661640
chr17 64708997
chr17 64709016
chr17 64709107
chr17 64709156
chr17 64709162
chr17 64709168
chr17 64709232
chr17 64709276
chr17 64736742
chr17 64737070

chr17 64737106
chr17 64737159
chr17 64737172
chr17 64780807
chr17 64780867
chr17 64780889
chr17 64780919
chr17 64780929
chr17 64780950
chr17 64780966
chr17 64780974
chr17 64780981
chr17 64780986
chr17 64780996
chr17 64781018
chr17 64781033
chr17 64781043
chr17 64781078
chr17 64781095
chr17 64781097
chr17 64781129
chr17 64781144
chr17 64781147
chr17 64781159
chr17 64781162
chr17 64781168
chr17 64781177
chr17 64781180
chr17 64793031
chr17 64793053
chr17 64798651
chr17 64808792
chr17 64836966
chr17 64836968
chr17 64836977
chr17 64836989
chr17 64836993
chr17 64836997
chr17 64836999
chr17 64837004
chr17 64837021
chr17 64837024
chr17 64837033
chr17 64837409
chr17 64837535
chr17 64837558
chr17 64837577
chr17 64837584
chr17 64837591
chr17 64837611
chr17 64837613
chr17 64837618
chr17 64837668
chr17 64837696

chr17 64837707
chr17 64837720
chr17 64837763
chr17 64837831
chr17 64837855
chr17 64837861
chr17 64837885
chr17 64837894
chr17 64837917
chr17 64837935
chr17 64837947
chr17 64918772
chr17 64918805
chr17 64918809
chr17 64918814
chr17 64918832
chr17 64961654
chr17 64961932
chr17 64961936
chr17 64961941
chr17 64961958
chr17 64961961
chr17 64962017
chr17 64965860
chr17 64965862
chr17 64966256
chr17 64966259
chr17 64966270
chr17 64966358
chr17 64966373
chr17 64969167
chr17 64974399
chr17 64974496
chr17 64974580
chr17 64974637
chr17 64975058
chr17 64975121
chr17 64975142
chr17 64975181
chr17 64975208
chr17 64984990
chr17 64984994
chr17 64985026
chr17 64985091
chr17 64985246
chr17 64985702
chr17 64985736
chr17 64985901
chr17 64985927
chr17 64985978
chr17 64985992
chr17 64986095
chr17 64986105
chr17 64986111

chr17 64986136
chr17 64986151
chr17 64986405
chr17 64986425
chr17 64986454
chr17 64986474
chr17 64986598
chr17 64986607
chr17 64986616
chr17 64986675
chr17 64986800
chr17 64986802
chr17 65041642
chr17 65041656
chr17 65042109
chr17 65054698
chr17 65054734
chr17 65055206
chr17 65055257
chr17 65055265
chr17 65055310
chr17 65099912
chr17 65100006
chr17 65100062
chr17 65100065
chr17 65100200
chr17 65100283
chr17 65100317
chr17 65100338
chr17 65100407
chr17 65100465
chr17 65100505
chr17 65100517
chr17 65100551
chr17 65100671
chr17 65100685
chr17 65120798
chr17 65121214
chr17 65121420
chr17 65121447
chr17 65121503
chr17 65121676
chr17 65121694
chr17 65121756
chr17 65121883
chr17 65228953
chr17 65228986
chr17 65228993
chr17 65229006
chr17 65229008
chr17 65229274
chr17 65229491
chr17 65229654
chr17 65494897

chr17 65495033
chr17 65495067
chr17 65495138
chr17 65495145
chr17 65495227
chr17 65495428
chr17 65495433
chr17 65495438
chr17 65495457
chr17 65495468
chr17 65495496
chr17 65495531
chr17 65495540
chr17 65495546
chr17 65495570
chr17 65657056
chr17 65657096
chr17 65935720
chr17 65935964
chr17 65936072
chr17 65936107
chr17 65936178
chr17 66255276
chr17 66255463
chr17 66285324
chr17 66285340
chr17 66404013
chr17 66404033
chr17 66542035
chr17 66542042
chr17 66542128
chr17 66542256
chr17 66542414
chr17 66798435
chr17 66798453
chr17 66798489
chr17 66798510
chr17 66798519
chr17 66798540
chr17 66799113
chr17 66799146
chr17 67244214
chr17 67374636
chr17 67374708
chr17 67374723
chr17 67390976
chr17 67390980
chr17 67390993
chr17 67391060
chr17 67421847
chr17 67421850
chr17 67421985
chr17 67422010
chr17 67422148

chr17 67422350
chr17 67431572
chr17 67431646
chr17 67440684
chr17 67440827
chr17 67440839
chr17 67440881
chr17 67440987
chr17 67441119
chr17 67441239
chr17 67441257
chr17 67441286
chr17 67441325
chr17 67441358
chr17 67689683
chr17 67826959
chr17 67826975
chr17 67827014
chr17 67827057
chr17 67827110
chr17 67827112
chr17 67827114
chr17 67827132
chr17 67827161
chr17 67827211
chr17 67827246
chr17 67827274
chr17 67827316
chr17 67827323
chr17 67827389
chr17 67827414
chr17 67827422
chr17 67827491
chr17 67827614
chr17 67829368
chr17 67829386
chr17 67829411
chr17 67829440
chr17 67829444
chr17 67829481
chr17 67829673
chr17 67829811
chr17 67856106
chr17 67856229
chr17 67963442
chr17 68035152
chr17 68035155
chr17 68035320
chr17 68035332
chr17 68035349
chr17 68035370
chr17 68035374
chr17 68035387
chr17 68035395

chr17 68035397
chr17 68035407
chr17 68035425
chr17 68035430
chr17 68035435
chr17 68035442
chr17 68035453
chr17 68035457
chr17 68035461
chr17 68035472
chr17 68035476
chr17 68035480
chr17 68035533
chr17 68035537
chr17 68035550
chr17 68036265
chr17 68036328
chr17 68036355
chr17 68036395
chr17 68036402
chr17 68036463
chr17 68036616
chr17 68036635
chr17 68050294
chr17 68101950
chr17 68101970
chr17 68101972
chr17 68101975
chr17 68101980
chr17 68101993
chr17 68101995
chr17 68102004
chr17 68104410
chr17 68183103
chr17 68183106
chr17 68183208
chr17 68183279
chr17 68183343
chr17 68183454
chr17 68183462
chr17 68183527
chr17 68183529
chr17 68183531
chr17 68185226
chr17 68185290
chr17 68206371
chr17 68206419
chr17 68206468
chr17 68206472
chr17 68206540
chr17 68206958
chr17 68207004
chr17 68207012
chr17 68207014

chr17 68207034
chr17 68207063
chr17 68207176
chr17 68207219
chr17 68207226
chr17 68237383
chr17 68237388
chr17 68237442
chr17 68237460
chr17 68237463
chr17 68237535
chr17 68237633
chr17 68237653
chr17 68237777
chr17 68237865
chr17 68237907
chr17 68237947
chr17 68237953
chr17 68237963
chr17 68238048
chr17 68238080
chr17 68238376
chr17 68238719
chr17 68238796
chr17 68238944
chr17 68238967
chr17 68239078
chr17 68248398
chr17 68248440
chr17 68248448
chr17 68248505
chr17 68248514
chr17 68248535
chr17 68248657
chr17 68248917
chr17 68249014
chr17 68249027
chr17 68249070
chr17 68258743
chr17 68258907
chr17 68258968
chr17 68258979
chr17 68258982
chr17 68259052
chr17 68259067
chr17 68259069
chr17 68259103
chr17 68259303
chr17 68259309
chr17 68259337
chr17 68259342
chr17 68259369
chr17 68259376
chr17 68259378

chr17 68259525
chr17 68259580
chr17 68259721
chr17 68412480
chr17 68412740
chr17 68412766
chr17 68413193
chr17 68413259
chr17 68413432
chr17 68413468
chr17 68413488
chr17 68413648
chr17 68413657
chr17 68413698
chr17 68413715
chr17 68413721
chr17 68413762
chr17 68413770
chr17 68413776
chr17 68413788
chr17 68413819
chr17 68413822
chr17 68413838
chr17 68413846
chr17 68413855
chr17 68413870
chr17 68413885
chr17 68413942
chr17 68413959
chr17 68413969
chr17 68413974
chr17 68413976
chr17 68413980
chr17 68413985
chr17 68413987
chr17 68414003
chr17 68414038
chr17 68414069
chr17 68414078
chr17 68414081
chr17 68414088
chr17 68414105
chr17 68414121
chr17 68414200
chr17 68414227
chr17 68437928
chr17 68457908
chr17 68457938
chr17 68457947
chr17 68457950
chr17 68457956
chr17 68457959
chr17 68457978
chr17 68457980

chr17 68457995
chr17 68458084
chr17 68458166
chr17 68458170
chr17 68458198
chr17 68458200
chr17 68458203
chr17 68463036
chr17 68463127
chr17 68463142
chr17 68463450
chr17 68463480
chr17 68487970
chr17 68488026
chr17 68488034
chr17 68488202
chr17 68488320
chr17 68488475
chr17 68488493
chr17 68512005
chr17 68512018
chr17 68512046
chr17 68512054
chr17 68512065
chr17 68512069
chr17 68512078
chr17 68512080
chr17 68512091
chr17 68512102
chr17 68512116
chr17 68512180
chr17 68512196
chr17 68512212
chr17 68512216
chr17 68512243
chr17 68513650
chr17 68513659
chr17 69194156
chr17 69194806
chr17 69414462
chr17 69414477
chr17 69414482
chr17 69414496
chr17 69414547
chr17 69414559
chr17 69414574
chr17 69414581
chr17 69414585
chr17 69414590
chr17 69414596
chr17 69414617
chr17 69414626
chr17 69414629
chr17 69414649

chr17 69414656
chr17 69414658
chr17 69414665
chr17 69414676
chr17 69414682
chr17 69414766
chr17 69414854
chr17 69414889
chr17 69414995
chr17 69415076
chr17 69415083
chr17 69415206
chr17 69415286
chr17 69415469
chr17 69415474
chr17 69415508
chr17 69415589
chr17 69415640
chr17 69415818
chr17 69415854
chr17 69416129
chr17 69569136
chr17 69569139
chr17 69569250
chr17 69569263
chr17 69569275
chr17 69569285
chr17 69569373
chr17 69569467
chr17 69581413
chr17 69581458
chr17 69581490
chr17 69581559
chr17 69581579
chr17 69581595
chr17 69581700
chr17 69581703
chr17 69581735
chr17 69581750
chr17 69581793
chr17 69581801
chr17 69581817
chr17 69581854
chr17 69581883
chr17 69582079
chr17 69582215
chr17 69582338
chr17 69582570
chr17 69582573
chr17 69582591
chr17 69582594
chr17 69613872
chr17 69614339
chr17 69614687

chr17 69614729
chr17 69615118
chr17 69615137
chr17 69615515
chr17 70086645
chr17 70086727
chr17 70390404
chr17 70390426
chr17 70390486
chr17 70548121
chr17 70559134
chr17 70592442
chr17 70592537
chr17 70592823
chr17 70592911
chr17 70594133
chr17 70598290
chr17 70758734
chr17 70758997
chr17 71286667
chr17 71473416
chr17 71473534
chr17 71473897
chr17 71534205
chr17 72030282
chr17 72030292
chr17 72030350
chr17 72030418
chr17 72030443
chr17 72030481
chr17 72030487
chr17 72030497
chr17 72030512
chr17 72030538
chr17 72030560
chr17 72030570
chr17 72030580
chr17 72030627
chr17 72525655
chr17 72719793
chr17 72719846
chr17 72719884
chr17 72719894
chr17 72719897
chr17 73191927
chr17 73191977
chr17 73191980
chr17 73191993
chr17 73192049
chr17 73192053
chr17 73192062
chr17 73192127
chr17 73192132
chr17 73192152

chr17 73232989
chr17 74012490
chr17 74012497
chr17 74012515
chr17 74012588
chr17 74012676
chr17 74012764
chr17 74121944
chr17 74737591
chr17 74737640
chr17 74737671
chr17 74737673
chr17 74737682
chr17 74737718
chr17 74737722
chr17 74737730
chr17 74737739
chr17 74737764
chr17 74737780
chr17 74737858
chr17 74737895
chr17 74737902
chr17 74737929
chr17 74737956
chr17 74737989
chr17 74737996
chr17 74738094
chr17 74738122
chr17 74738216
chr17 74738257
chr17 74738269
chr17 74749947
chr17 74749980
chr17 74750006
chr17 74750095
chr17 74750107
chr17 74750113
chr17 74750122
chr17 74750130
chr17 74750187
chr17 74750327
chr17 74750398
chr17 74750618
chr17 74750652
chr17 74750706
chr17 74750710
chr17 74750798
chr17 74750858
chr17 74750862
chr17 74750884
chr17 74750888
chr17 74751091
chr17 74751129
chr17 74751183

chr17 74751331
chr17 74751350
chr17 74776424
chr17 74776430
chr17 74776436
chr17 74776438
chr17 74776443
chr17 74776454
chr17 74776456
chr17 74776458
chr17 74776460
chr17 74776587
chr17 74776592
chr17 74776597
chr17 75012950
chr17 75012964
chr17 75012967
chr17 75013012
chr17 75013024
chr17 75013039
chr17 75013047
chr17 75013049
chr17 75013066
chr17 75013074
chr17 75013113
chr17 75013117
chr17 75013233
chr17 75034447
chr17 75034503
chr17 75034505
chr17 75034513
chr17 75034524
chr17 75034563
chr17 75034582
chr17 75034584
chr17 75034611
chr17 75034624
chr17 75034635
chr17 75034637
chr17 75034640
chr17 75034649
chr17 75034671
chr17 75034677
chr17 75034680
chr17 75034682
chr17 75034684
chr17 75034699
chr17 75034701
chr17 75034717
chr17 75034731
chr17 75034734
chr17 75034738
chr17 75034747
chr17 75034780

chr17 75034786
chr17 75046980
chr17 75114393
chr17 75114415
chr17 75114418
chr17 75114459
chr17 75114462
chr17 75114478
chr17 75114492
chr17 75114494
chr17 75114503
chr17 75114541
chr17 75114552
chr17 75114556
chr17 75114571
chr17 75114582
chr17 75114585
chr17 75153387
chr17 75153401
chr17 75153409
chr17 75180895
chr17 75180919
chr17 75180931
chr17 75181061
chr17 75181141
chr17 75181164
chr17 75181233
chr17 75181239
chr17 75181308
chr17 75181349
chr17 75181391
chr17 75260785
chr17 75260889
chr17 75260938
chr17 75260944
chr17 75261006
chr17 75261051
chr17 75261057
chr17 75295601
chr17 75295713
chr17 75338568
chr17 75338591
chr17 75338607
chr17 75338822
chr17 75338941
chr17 75338944
chr17 75338987
chr17 75338995
chr17 75339042
chr17 75339256
chr17 75339262
chr17 75339279
chr17 75339294
chr17 75339338

chr17 75339342
chr17 75339350
chr17 75339354
chr17 75339414
chr17 75393912
chr17 75393915
chr17 75393938
chr17 75393949
chr17 75393965
chr17 75394019
chr17 75394035
chr17 75394046
chr17 75394069
chr17 75394116
chr17 75394166
chr17 75394200
chr17 75394386
chr17 75394389
chr17 75394420
chr17 75412638
chr17 75412659
chr17 75412730
chr17 75412733
chr17 75412768
chr17 75412816
chr17 75412835
chr17 75777695
chr17 75777819
chr17 75777924
chr17 75778455
chr17 75778617
chr17 75778620
chr17 75778663
chr17 75778690
chr17 75778709
chr17 75778714
chr17 75778734
chr17 75778741
chr17 75779959
chr17 75780017
chr17 75780039
chr17 75780178
chr17 75780232
chr17 75780295
chr17 75783083
chr17 75783271
chr17 75783450
chr17 75783537
chr17 75783644
chr17 75843549
chr17 75843935
chr17 75843980
chr17 75844109
chr17 75844132

chr17 75844161
chr17 75844167
chr17 75844224
chr17 75844239
chr17 75844267
chr17 75844270
chr17 75844291
chr17 75844306
chr17 75844314
chr17 75844332
chr17 75844364
chr17 76071453
chr17 76071482
chr17 76071522
chr17 76071528
chr17 76071575
chr17 76071632
chr17 76071638
chr17 76071727
chr17 76071780
chr17 76071803
chr17 76071815
chr17 76071884
chr17 76071915
chr17 76072045
chr17 76145775
chr17 76145784
chr17 76145815
chr17 76182413
chr17 76182441
chr17 76182546
chr17 76191595
chr17 76191677
chr17 76191855
chr17 76191906
chr17 76191935
chr17 76192072
chr17 76192153
chr17 76192250
chr17 76192284
chr17 76515498
chr17 76515501
chr17 76515505
chr17 76515510
chr17 76557242
chr17 76557252
chr17 76557256
chr17 76557258
chr17 76557263
chr17 76557272
chr17 76557278
chr17 76557315
chr17 76557324
chr17 76557351

chr17 76557392
chr17 76557416
chr17 76557427
chr17 76557454
chr17 76557479
chr17 76557540
chr17 76557554
chr17 76557585
chr17 76557609
chr17 76557639
chr17 76557641
chr17 76557650
chr17 76557652
chr17 76557667
chr17 76557670
chr17 76557712
chr17 76557714
chr17 76557723
chr17 76557735
chr17 76734362
chr17 76734514
chr17 76734560
chr17 76734807
chr17 76734939
chr17 76736173
chr17 76738297
chr17 76738302
chr17 76738335
chr17 76738360
chr17 76738447
chr17 76738516
chr17 76738535
chr17 76738537
chr17 76791303
chr17 76791479
chr17 76840332
chr17 76840948
chr17 76969140
chr17 76969145
chr17 76969151
chr17 76969155
chr17 76969163
chr17 76969170
chr17 76969181
chr17 76969212
chr17 76969215
chr17 76969220
chr17 76969252
chr17 76969275
chr17 76969299
chr17 76969312
chr17 76969351
chr17 76969363
chr17 76969392

chr17 76969513
chr17 76969532
chr17 76969543
chr17 76969597
chr17 76969599
chr17 76969617
chr17 76969621
chr17 76969626
chr17 76969646
chr17 76969653
chr17 76969657
chr17 76969674
chr17 77088875
chr17 77088911
chr17 77088914
chr17 77088918
chr17 77088926
chr17 77088932
chr17 77088945
chr17 77089100
chr17 77089103
chr17 77089181
chr17 77089184
chr17 77089230
chr17 77089246
chr17 77331677
chr17 77331699
chr17 77331703
chr17 77331732
chr17 77331754
chr17 77331764
chr17 77331782
chr17 77331792
chr17 77331801
chr17 77331831
chr17 77331959
chr17 77332084
chr17 77332169
chr17 77332255
chr17 77332416
chr17 77332505
chr17 77332579
chr17 77332584
chr17 77332613
chr17 77342255
chr17 77342337
chr17 77342347
chr17 77342389
chr17 77342536
chr17 77342575
chr17 77342695
chr17 77342747
chr17 77342852
chr17 77389556

chr17 77389661
chr17 77389693
chr17 77389704
chr17 77389727
chr17 77389785
chr17 77389792
chr17 77389848
chr17 77389863
chr17 77440751
chr17 77440837
chr17 77441065
chr17 77441248
chr17 77441347
chr17 77441359
chr17 77441430
chr17 77441451
chr17 77441617
chr17 77441651
chr17 77441703
chr17 77441758
chr17 77441771
chr17 77441784
chr17 77441824
chr17 77441873
chr17 77441888
chr17 77441907
chr17 77441942
chr17 77442051
chr17 77442073
chr17 77442106
chr17 77442117
chr17 77445100
chr17 77445111
chr17 77445218
chr17 77445223
chr17 77445286
chr17 77445304
chr17 77445306
chr17 77445310
chr17 77445460
chr17 77445521
chr17 77445525
chr17 77445541
chr17 77445545
chr17 77445625
chr17 77445810
chr17 77445883
chr17 77445988
chr17 77445998
chr17 77446016
chr17 77446054
chr17 77446072
chr17 77446098
chr17 77446111

chr17 77446202
chr17 77450770
chr17 77450795
chr17 77450965
chr17 77451082
chr17 77451089
chr17 77451122
chr17 77451216
chr17 77451234
chr17 77451243
chr17 77451264
chr17 77451267
chr17 77451276
chr17 77451283
chr17 77451321
chr17 77451350
chr17 77451372
chr17 77451396
chr17 77451398
chr17 77451420
chr17 77451422
chr17 77452561
chr17 77460538
chr17 77460558
chr17 77460565
chr17 77460625
chr17 77460627
chr17 77460754
chr17 77460780
chr17 77645389
chr17 77645482
chr17 77645518
chr17 77645528
chr17 77645545
chr17 77645584
chr17 77645601
chr17 77645611
chr17 77645614
chr17 77645616
chr17 77645621
chr17 77645633
chr17 77645672
chr17 77666652
chr17 77666702
chr17 77667055
chr17 77667070
chr17 77667085
chr17 77667091
chr17 77667273
chr17 77667280
chr17 77667394
chr17 77667580
chr17 77963304
chr17 77963341

chr17 77963459
chr17 77963674
chr17 78127613
chr17 78127620
chr17 78127666
chr17 78127676
chr17 78127701
chr17 78127732
chr17 78127736
chr17 78127748
chr17 78127757
chr17 78127772
chr17 78127778
chr17 78127783
chr17 78128836
chr17 78128841
chr17 78128846
chr17 78128848
chr17 78128851
chr17 78128857
chr17 78128867
chr17 78128871
chr17 78128876
chr17 78128878
chr17 78128881
chr17 78128910
chr17 78129208
chr17 78129289
chr17 78129549
chr17 78129644
chr17 78129951
chr17 78130081
chr17 78130182
chr17 78130220
chr17 78130334
chr17 78130368
chr17 78130409
chr17 78130496
chr17 78130505
chr17 78130576
chr17 78130954
chr17 78130991
chr17 78130993
chr17 78131049
chr17 78131078
chr17 78131118
chr17 78131136
chr17 78131198
chr17 78214608
chr17 78214630
chr17 78214638
chr17 78214648
chr17 78214658
chr17 78214685

chr17 78356416
chr17 78356440
chr17 78356579
chr17 78356619
chr17 78356628
chr17 78356636
chr17 78631569
chr17 78631619
chr17 78736100
chr17 78736175
chr17 78736321
chr17 78736332
chr17 78736404
chr17 78736469
chr17 78736654
chr17 78736665
chr17 78736669
chr17 78736678
chr17 78736692
chr17 78736704
chr17 78736735
chr17 78736742
chr17 78736751
chr17 78736772
chr17 78736823
chr17 78736902
chr17 78736937
chr17 78736976
chr17 78736993
chr17 78737179
chr17 78737198
chr17 78747953
chr17 78748132
chr17 78748152
chr17 78748227
chr17 78748271
chr17 78748312
chr17 78748724
chr17 78748732
chr17 78748947
chr17 78748949
chr17 78748972
chr17 78749000
chr17 78772232
chr17 78772242
chr17 78772247
chr17 78772272
chr17 78774123
chr17 78774418
chr17 78774663
chr17 78774816
chr17 78775241
chr17 78775307
chr17 78775397

chr17 78775428
chr17 78775437
chr17 78775501
chr17 78775692
chr17 78775882
chr17 78775983
chr17 78776011
chr17 78776111
chr17 78779797
chr17 78779921
chr17 78780023
chr17 78780141
chr17 78986510
chr17 79796709
chr17 79796748
chr17 79796755
chr17 79796775
chr17 79796794
chr17 79796797
chr17 79796800
chr17 79796803
chr17 79796806
chr17 79796811
chr17 79796815
chr17 79796817
chr17 79796821
chr17 79796874
chr17 79796879
chr17 79796894
chr17 79796898
chr17 79796902
chr17 79796917
chr17 79796934
chr17 79796940
chr17 79796943
chr17 79796959
chr17 79796963
chr17 79796965
chr17 79796973
chr17 79796984
chr17 79797006
chr17 79797009
chr17 79797025
chr17 79797055
chr17 79797059
chr17 79804945
chr17 79804965
chr17 79804995
chr17 79805018
chr17 79805028
chr17 80220434
chr17 80260578
chr17 80260581
chr17 80260584

chr17 80260587
chr17 80260596
chr17 80260752
chr17 80260755
chr17 80260758
chr17 80260761
chr17 80260764
chr17 80260767
chr17 80260776
chr17 80260782
chr17 80260785
chr17 80260788
chr17 80260791
chr17 80260794
chr17 80260797
chr17 80260806
chr17 80260812
chr17 80260815
chr17 80260818
chr17 80260821
chr17 80260824
chr17 80260827
chr17 80260836
chr17 80260851
chr17 80261740
chr17 80261744
chr17 80263487
chr17 80263569
chr17 80263590
chr17 80263598
chr17 80263633
chr17 80263695
chr17 80263744
chr17 80263888
chr17 80264021
chr17 80264028
chr17 80264038
chr17 80264057
chr17 80264114
chr17 80264384
chr17 80264399
chr17 80264478
chr17 80264594
chr17 80264608
chr17 80264662
chr17 80264670
chr17 80264741
chr17 80264753
chr17 80264784
chr17 80264793
chr17 80264809
chr17 80264901
chr17 80264934
chr17 80264980

chr17 80264996
chr17 80408991
chr17 80409012
chr17 80488438
chr17 80488456
chr17 80488458
chr17 80488474
chr17 80488510
chr17 80488528
chr17 80488530
chr17 80488546
chr17 80488799
chr17 80488835
chr17 80488855
chr17 80488907
chr17 80488979
chr17 80488999
chr17 80489052
chr17 80489072
chr17 80505151
chr17 80764623
chr17 80764843
chr17 80764996
chr17 80866464
chr17 81018425
chr17 81510010
chr17 81510227
chr17 81510256
chr17 81510320
chr17 81510395
chr17 81510412
chr17 81510430
chr17 81510577
chr17 81510596
chr17 81510672
chr17 81510687
chr17 81510703
chr17 81510710
chr17 81510723
chr17 81510728
chr17 81510731
chr17 81510794
chr17 81510804
chr17 81510814
chr17 81510827
chr17 81510853
chr17 81510871
chr17 81510891
chr17 81510919
chr17 81510948
chr17 81553395
chr17 81553439
chr17 81553474
chr17 82297348

chr17 82297367
chr17 82297388
chr17 82297440
chr17 82297998
chr17 82298002
chr17 82298006
chr17 82298015
chr17 82298034
chr17 82298047
chr17 82298051
chr17 82298060
chr17 82298066
chr17 82298091
chr17 82298095
chr17 82298106
chr17 82298121
chr17 82298144
chr17 82298152
chr17 82298170
chr17 82298230
chr17 82298254
chr17 82298260
chr17 82298268
chr17 82298362
chr17 82298430
chr17 82419152
chr17 82419169
chr17 82419192
chr17 82419389
chr17 82419423
chr17 82448807
chr17 82448832
chr17 82449475
chr17 82449714
chr17 82449718
chr17 82449730
chr17 82449748
chr17 82449818
chr17 82449828
chr17 82449831
chr17 82449861
chr17 82449869
chr17 82449873
chr17 82449877
chr17 82449899
chr17 82449903
chr17 82449930
chr17 82450007
chr17 82450009
chr17 82450047
chr17 82450052
chr17 82450056
chr17 82450072
chr17 82450097

chr17 82450102
chr17 82450110
chr17 82450139
chr17 82450154
chr17 82450206
chr17 82450236
chr17 82450274
chr17 82716498
chr17 82716524
chr17 82716526
chr17 82716541
chr17 82716557
chr17 82716573
chr17 82716585
chr17 82716591
chr17 82716595
chr17 82716605
chr17 82716621
chr17 82716633
chr17 82716637
chr17 82716644
chr17 82716654
chr17 82716661
chr17 82716672
chr17 82716674
chr17 82716676
chr17 82716692
chr17 82716696
chr17 82716700
chr17 82716703
chr17 82716710
chr17 82716718
chr17 82716727
chr17 82716732
chr17 82716738
chr17 82716740
chr17 82716744
chr17 82716746
chr17 82716749
chr17 82716774
chr17 82716776
chr17 82716805
chr17 82716814
chr17 82716821
chr17 82716840
chr17 82716848
chr17 82716853
chr17 82716864
chr17 82716872
chr17 82716892
chr17 82716906
chr17 82716911
chr17 82716915
chr17 82716920

chr17 82716928
chr17 82716939
chr17 83120264
chr17 83244220
chr17 83244322
chr17 83244777
chr17 83244927
chr17 83245328
chr17 83245349
chr7 31067
chr7 31077
chr7 31107
chr7 31181
chr7 31184
chr7 31191
chr7 31248
chr7 31251
chr7 31260
chr7 31277
chr7 31282
chr7 31288
chr7 31301
chr7 31308
chr7 31403
chr7 31405
chr7 31409
chr7 351514
chr7 351517
chr7 351529
chr7 351578
chr7 351711
chr7 351723
chr7 351749
chr7 351759
chr7 351766
chr7 351782
chr7 351788
chr7 351797
chr7 351814
chr7 351836
chr7 351859
chr7 351877
chr7 351895
chr7 351918
chr7 786765
chr7 786817
chr7 786965
chr7 812969
chr7 813026
chr7 813039
chr7 813077
chr7 813088
chr7 813173
chr7 817719

chr7 876938
chr7 876942
chr7 876945
chr7 876959
chr7 876989
chr7 876992
chr7 876998
chr7 877006
chr7 877020
chr7 877025
chr7 877148
chr7 877155
chr7 877172
chr7 877176
chr7 877185
chr7 877201
chr7 877244
chr7 877256
chr7 877266
chr7 877274
chr7 877288
chr7 877306
chr7 877311
chr7 877338
chr7 877349
chr7 877352
chr7 877370
chr7 877403
chr7 877417
chr7 877440
chr7 877470
chr7 877504
chr7 877535
chr7 877564
chr7 877573
chr7 877623
chr7 877640
chr7 877650
chr7 877653
chr7 877706
chr7 877728
chr7 877737
chr7 877792
chr7 877823
chr7 877831
chr7 877858
chr7 877894
chr7 923033
chr7 923078
chr7 923089
chr7 923107
chr7 923118
chr7 923297
chr7 923328

chr7 923414
chr7 923446
chr7 923517
chr7 923524
chr7 923568
chr7 923590
chr7 923660
chr7 923667
chr7 941010
chr7 1940160
chr7 1940165
chr7 1940173
chr7 1940248
chr7 1940260
chr7 1940268
chr7 1940285
chr7 1940287
chr7 1940413
chr7 1940455
chr7 1940477
chr7 1940481
chr7 1940495
chr7 1940542
chr7 1940550
chr7 1940586
chr7 1940595
chr7 1940612
chr7 1940637
chr7 1940648
chr7 1940653
chr7 1961863
chr7 1961908
chr7 1961929
chr7 1961947
chr7 1961995
chr7 1962040
chr7 1962055
chr7 1962063
chr7 1962106
chr7 1962147
chr7 1962194
chr7 1962200
chr7 2314313
chr7 2314320
chr7 2314335
chr7 2405742
chr7 2405758
chr7 2405768
chr7 2405773
chr7 2405808
chr7 2405834
chr7 2405948
chr7 2405969
chr7 2405987

chr7 2406064
chr7 2406092
chr7 2406117
chr7 2406133
chr7 2406157
chr7 2406208
chr7 2406410
chr7 2406456
chr7 2406479
chr7 2406502
chr7 2406513
chr7 2406515
chr7 2406536
chr7 2406568
chr7 2406734
chr7 2406741
chr7 2508775
chr7 2521836
chr7 2521878
chr7 2521881
chr7 2521895
chr7 2522083
chr7 2522137
chr7 2522151
chr7 2522234
chr7 2522287
chr7 2522363
chr7 2522441
chr7 2529862
chr7 2529892
chr7 2529930
chr7 2529933
chr7 2529942
chr7 2529958
chr7 2529985
chr7 2530016
chr7 2530101
chr7 2530130
chr7 2530172
chr7 2530182
chr7 2530190
chr7 2530235
chr7 2530280
chr7 2530318
chr7 2530396
chr7 2530427
chr7 2555296
chr7 2555298
chr7 2555302
chr7 2555311
chr7 2555328
chr7 2555340
chr7 2555350
chr7 2555362

chr7 2555373
chr7 2555375
chr7 2555379
chr7 2555384
chr7 2555406
chr7 2555410
chr7 2555419
chr7 2555423
chr7 2555439
chr7 2555441
chr7 2555454
chr7 2555460
chr7 2555467
chr7 2555477
chr7 2555479
chr7 2555487
chr7 2555491
chr7 2555497
chr7 2555504
chr7 2555507
chr7 2555520
chr7 2555532
chr7 2555538
chr7 2555561
chr7 2555570
chr7 2555577
chr7 3065655
chr7 3065797
chr7 3092778
chr7 3092784
chr7 3092810
chr7 3092914
chr7 3094923
chr7 3094958
chr7 3095011
chr7 3095123
chr7 3095129
chr7 3095167
chr7 3095186
chr7 3095216
chr7 3095226
chr7 3095267
chr7 3095308
chr7 3095329
chr7 3095440
chr7 3117219
chr7 3117247
chr7 3117735
chr7 3117748
chr7 3117865
chr7 3117869
chr7 3118009
chr7 3118044
chr7 4129208

chr7 4129298
chr7 4599473
chr7 4599485
chr7 4599546
chr7 4599557
chr7 4683220
chr7 4683314
chr7 4683373
chr7 4683394
chr7 4683406
chr7 4683408
chr7 4683432
chr7 4683448
chr7 4683471
chr7 4683644
chr7 4683693
chr7 4683820
chr7 4683829
chr7 4683875
chr7 4683885
chr7 4683946
chr7 4683975
chr7 4683981
chr7 4684019
chr7 4684036
chr7 4684056
chr7 4684069
chr7 4684078
chr7 4684109
chr7 4684128
chr7 4684296
chr7 4684303
chr7 4684401
chr7 4684454
chr7 4684484
chr7 4684595
chr7 4745138
chr7 4745190
chr7 4745195
chr7 4745201
chr7 4745203
chr7 4745272
chr7 4745282
chr7 4745288
chr7 4745315
chr7 4745332
chr7 4745377
chr7 4745384
chr7 4745386
chr7 4745396
chr7 4745409
chr7 4745416
chr7 4745426
chr7 4745627

chr7 4745631
chr7 4745678
chr7 4745701
chr7 4745863
chr7 4745871
chr7 4746120
chr7 4746146
chr7 4746155
chr7 4746158
chr7 4959318
chr7 4959337
chr7 4959382
chr7 4959397
chr7 4959402
chr7 4959420
chr7 4959422
chr7 4959424
chr7 4959426
chr7 4959438
chr7 4959453
chr7 4959465
chr7 4959478
chr7 4959527
chr7 4959567
chr7 4959573
chr7 4959705
chr7 5046536
chr7 5046545
chr7 5046553
chr7 5046577
chr7 5046619
chr7 5046623
chr7 5046626
chr7 5046639
chr7 5046672
chr7 5046686
chr7 5046700
chr7 5396809
chr7 5422144
chr7 5422152
chr7 5422157
chr7 5422168
chr7 5422221
chr7 5425789
chr7 5527290
chr7 5527336
chr7 5527372
chr7 5527481
chr7 5527537
chr7 5527566
chr7 5527575
chr7 5527594
chr7 5527695
chr7 5527727

chr7 5527745
chr7 5527761
chr7 5527768
chr7 5527780
chr7 5527786
chr7 5527832
chr7 5527849
chr7 5527852
chr7 5527861
chr7 5527872
chr7 5527965
chr7 5528057
chr7 5528084
chr7 5528129
chr7 5528162
chr7 5528231
chr7 5528316
chr7 5528322
chr7 5528350
chr7 5528362
chr7 5528396
chr7 5528413
chr7 5528425
chr7 5528428
chr7 5528454
chr7 5528458
chr7 5528466
chr7 5528470
chr7 5528474
chr7 5528494
chr7 5528496
chr7 5528500
chr7 5528535
chr7 5528553
chr7 5528584
chr7 5528611
chr7 5528617
chr7 5528629
chr7 5528643
chr7 5528653
chr7 5528683
chr7 5528780
chr7 5528809
chr7 5528887
chr7 5528918
chr7 5529063
chr7 5529070
chr7 5529099
chr7 5529107
chr7 5531333
chr7 5531361
chr7 5531468
chr7 5531492
chr7 5531497

chr7 5531536
chr7 5531540
chr7 5531558
chr7 5531599
chr7 5531650
chr7 5531655
chr7 5531745
chr7 5531760
chr7 5531770
chr7 5531812
chr7 5531822
chr7 5531844
chr7 5531962
chr7 5531977
chr7 5531979
chr7 5532006
chr7 5532080
chr7 5532236
chr7 5532325
chr7 5532355
chr7 5532363
chr7 5532370
chr7 5532380
chr7 5532387
chr7 5532435
chr7 5532442
chr7 5532494
chr7 5532532
chr7 5532644
chr7 5532649
chr7 5532704
chr7 5532772
chr7 5532792
chr7 5570012
chr7 5570026
chr7 5695204
chr7 5695272
chr7 5695322
chr7 5695341
chr7 5695347
chr7 5695352
chr7 5695393
chr7 5695402
chr7 5695410
chr7 5695450
chr7 5695457
chr7 5695481
chr7 5695492
chr7 5695498
chr7 5695527
chr7 5695563
chr7 5704785
chr7 5898438
chr7 5898442

chr7 5898467
chr7 5898469
chr7 5898543
chr7 5898547
chr7 5898562
chr7 5898564
chr7 5898573
chr7 5898596
chr7 5898598
chr7 5898619
chr7 5898638
chr7 5898657
chr7 5898662
chr7 5898667
chr7 5898680
chr7 5898688
chr7 5898691
chr7 5898695
chr7 5898701
chr7 5898706
chr7 5898712
chr7 5898715
chr7 5898721
chr7 5898729
chr7 5898741
chr7 5898761
chr7 5898766
chr7 5899298
chr7 5899325
chr7 5899402
chr7 5899405
chr7 5899425
chr7 5899483
chr7 5899495
chr7 5899523
chr7 5969919
chr7 5969975
chr7 5970043
chr7 5970125
chr7 5970129
chr7 5970134
chr7 5970163
chr7 5970173
chr7 5970175
chr7 5970179
chr7 5970193
chr7 5970222
chr7 5970228
chr7 5970370
chr7 5970382
chr7 5970445
chr7 5970453
chr7 5970463
chr7 5970485

chr7 5970501
chr7 5970651
chr7 5970667
chr7 5970675
chr7 5970678
chr7 5970689
chr7 5970695
chr7 5971290
chr7 5971353
chr7 5971406
chr7 5971424
chr7 5971431
chr7 5971441
chr7 5971447
chr7 5971450
chr7 5971555
chr7 6080924
chr7 6080941
chr7 6106207
chr7 6106558
chr7 6106625
chr7 6221499
chr7 6221518
chr7 6221822
chr7 6221884
chr7 6257064
chr7 6257072
chr7 6257101
chr7 6257149
chr7 6257218
chr7 6257496
chr7 6375278
chr7 6375286
chr7 6375368
chr7 6375380
chr7 6375445
chr7 6375455
chr7 6375492
chr7 6375506
chr7 6375508
chr7 6375512
chr7 6375531
chr7 6375586
chr7 6375632
chr7 6504087
chr7 6504140
chr7 6504165
chr7 6504172
chr7 6504187
chr7 6504205
chr7 6504228
chr7 6504251
chr7 6504270
chr7 6504274

chr7 6504287
chr7 6504308
chr7 6504328
chr7 6504346
chr7 6504372
chr7 6504381
chr7 6504391
chr7 6504401
chr7 6504412
chr7 6536735
chr7 6536746
chr7 6536748
chr7 6536757
chr7 6577300
chr7 6577310
chr7 6577316
chr7 6577337
chr7 6730457
chr7 6730466
chr7 6730472
chr7 6753501
chr7 6753993
chr7 6754015
chr7 6754031
chr7 6754037
chr7 6754039
chr7 6754041
chr7 6754409
chr7 6754421
chr7 6754542
chr7 6754548
chr7 6754563
chr7 6754569
chr7 6754598
chr7 6754612
chr7 6754616
chr7 6754618
chr7 6754628
chr7 6754657
chr7 6754662
chr7 6754666
chr7 6825501
chr7 6825529
chr7 6825541
chr7 6825599
chr7 6825619
chr7 6825622
chr7 6825671
chr7 6825698
chr7 6825745
chr7 6826217
chr7 6826230
chr7 6826235
chr7 6826255

chr7 6826267
chr7 6826275
chr7 6826281
chr7 6826284
chr7 6826290
chr7 6826295
chr7 6826305
chr7 6826308
chr7 6826316
chr7 6826329
chr7 6826334
chr7 6826339
chr7 6826358
chr7 6826386
chr7 6826407
chr7 6826409
chr7 6826432
chr7 6826441
chr7 6826443
chr7 6826458
chr7 6826462
chr7 6846559
chr7 6846573
chr7 6846594
chr7 6855126
chr7 6855134
chr7 6855136
chr7 6855141
chr7 6855147
chr7 6855149
chr7 6855153
chr7 6855175
chr7 6855236
chr7 6855238
chr7 6855258
chr7 6855276
chr7 6855387
chr7 6855396
chr7 6855420
chr7 7026196
chr7 7026288
chr7 7026590
chr7 7181801
chr7 7183435
chr7 7183453
chr7 7183471
chr7 7183601
chr7 7183632
chr7 7183686
chr7 7183804
chr7 7183907
chr7 7183981
chr7 7184032
chr7 7258228

chr7 7258259
chr7 7258491
chr7 7258560
chr7 7258830
chr7 7258996
chr7 7259107
chr7 7259135
chr7 7259195
chr7 7259198
chr7 7424733
chr7 7424769
chr7 7424818
chr7 7567425
chr7 7567438
chr7 7567471
chr7 7567483
chr7 7567637
chr7 7567669
chr7 7567675
chr7 7567687
chr7 7567824
chr7 7567853
chr7 7568160
chr7 7639489
chr7 7639585
chr7 7639598
chr7 7639625
chr7 7719364
chr7 7719590
chr7 7719655
chr7 7944420
chr7 7944662
chr7 7944721
chr7 7944754
chr7 7944881
chr7 7945050
chr7 7967709
chr7 7967750
chr7 7967816
chr7 7967906
chr7 7967978
chr7 7968073
chr7 7968088
chr7 7968309
chr7 7968367
chr7 7970534
chr7 7970540
chr7 7970811
chr7 7971044
chr7 7971064
chr7 8015161
chr7 8125854
chr7 8125932
chr7 8125972

chr7 8126065
chr7 8126223
chr7 8126291
chr7 8126380
chr7 8129058
chr7 8133710
chr7 8134151
chr7 8134331
chr7 8134447
chr7 8134453
chr7 8134505
chr7 8134546
chr7 8134574
chr7 8134587
chr7 8134636
chr7 8134679
chr7 8165157
chr7 8165181
chr7 8165404
chr7 8165471
chr7 8176442
chr7 8176461
chr7 8176520
chr7 8176571
chr7 8176612
chr7 8176742
chr7 8176879
chr7 8176979
chr7 8176986
chr7 8177028
chr7 8177154
chr7 8177193
chr7 8177235
chr7 8198499
chr7 8437804
chr7 8437889
chr7 8437930
chr7 8437967
chr7 8438072
chr7 8438151
chr7 8438213
chr7 8438798
chr7 8439110
chr7 8459849
chr7 8459859
chr7 8459909
chr7 8460366
chr7 8460746
chr7 8463256
chr7 8463699
chr7 8729082
chr7 8729857
chr7 8891435
chr7 8959224

chr7 8959451
chr7 9193477
chr7 9405787
chr7 9405807
chr7 9406393
chr7 9406541
chr7 9407073
chr7 9452472
chr7 9497549
chr7 9498372
chr7 9498541
chr7 9498599
chr7 9617512
chr7 9617617
chr7 9617828
chr7 9619014
chr7 9619028
chr7 9619109
chr7 9619236
chr7 9619240
chr7 9619245
chr7 9730795
chr7 9731018
chr7 9823991
chr7 9978044
chr7 9978399
chr7 9978472
chr7 9978626
chr7 9978680
chr7 10318490
chr7 10319384
chr7 10454351
chr7 10454718
chr7 10467807
chr7 10467839
chr7 10889058
chr7 10889127
chr7 10889429
chr7 10889453
chr7 10939188
chr7 10939338
chr7 10939381
chr7 10939404
chr7 10939418
chr7 10974560
chr7 10974587
chr7 10974610
chr7 10974650
chr7 11830465
chr7 11830686
chr7 11830798
chr7 11830980
chr7 11830986
chr7 11831016

chr7 11831038
chr7 11831045
chr7 11831200
chr7 11831205
chr7 11831280
chr7 11831506
chr7 11831508
chr7 11831530
chr7 11831596
chr7 11831675
chr7 11831782
chr7 11831788
chr7 11831793
chr7 11831800
chr7 11831803
chr7 11831814
chr7 11831817
chr7 11831821
chr7 11831825
chr7 11831848
chr7 12210235
chr7 12210305
chr7 12210374
chr7 12210535
chr7 12210547
chr7 12210584
chr7 12210608
chr7 12210619
chr7 12210653
chr7 12210682
chr7 12210705
chr7 12210710
chr7 12211574
chr7 12211587
chr7 12211618
chr7 12211624
chr7 12211672
chr7 12211716
chr7 12211724
chr7 12211793
chr7 12211943
chr7 12402927
chr7 12403096
chr7 12403173
chr7 12403246
chr7 12403263
chr7 12403310
chr7 12569834
chr7 12569837
chr7 12570034
chr7 12570052
chr7 12570192
chr7 12570273
chr7 12570281

chr7 12570306
chr7 12570308
chr7 12635994
chr7 12636023
chr7 12636064
chr7 12636086
chr7 12636109
chr7 12687622
chr7 12687675
chr7 12687683
chr7 12687723
chr7 12687736
chr7 12687808
chr7 12687862
chr7 12715771
chr7 12715828
chr7 12715835
chr7 12742437
chr7 12896608
chr7 13104403
chr7 13104822
chr7 13153167
chr7 13153409
chr7 13153451
chr7 13593986
chr7 13594073
chr7 13594540
chr7 13988398
chr7 13988873
chr7 13988921
chr7 13988961
chr7 13988964
chr7 13989065
chr7 13989220
chr7 13989256
chr7 13989312
chr7 13989329
chr7 13989340
chr7 13989361
chr7 13989374
chr7 13989376
chr7 13989380
chr7 13989390
chr7 13989405
chr7 13989411
chr7 13989432
chr7 13989439
chr7 13989460
chr7 13989474
chr7 13989506
chr7 13989573
chr7 13989580
chr7 13989654
chr7 13989660

chr7 13989681
chr7 13989735
chr7 13989754
chr7 13989756
chr7 13989780
chr7 13989796
chr7 13989814
chr7 13989816
chr7 13989827
chr7 13989851
chr7 13989855
chr7 13989862
chr7 13989884
chr7 13989889
chr7 13989899
chr7 13989916
chr7 13989934
chr7 13989953
chr7 13989962
chr7 13989967
chr7 13990032
chr7 13990055
chr7 13990057
chr7 13990071
chr7 13990076
chr7 13990146
chr7 13990194
chr7 13990230
chr7 13990276
chr7 13990280
chr7 13990410
chr7 13990482
chr7 13990498
chr7 13990502
chr7 13990530
chr7 13990536
chr7 13990562
chr7 13990613
chr7 13990617
chr7 13990630
chr7 13990655
chr7 13991182
chr7 13991193
chr7 13991296
chr7 13991341
chr7 13991385
chr7 13991397
chr7 14394592
chr7 14394985
chr7 14395069
chr7 14646200
chr7 14647061
chr7 14647415
chr7 14688462

chr7 14688640
chr7 14691303
chr7 14691758
chr7 14691821
chr7 14691825
chr7 14697499
chr7 14705985
chr7 14742543
chr7 14855514
chr7 14856228
chr7 15098763
chr7 15098852
chr7 15098921
chr7 15139476
chr7 15139539
chr7 15140556
chr7 15688317
chr7 15688361
chr7 15688451
chr7 15688469
chr7 15688503
chr7 15688529
chr7 15688552
chr7 15688562
chr7 15688608
chr7 15688663
chr7 15688666
chr7 15688962
chr7 15689086
chr7 15689433
chr7 15798605
chr7 16147002
chr7 16147313
chr7 16332178
chr7 16563849
chr7 16644835
chr7 16644837
chr7 16644872
chr7 16644879
chr7 16644901
chr7 16644939
chr7 16645041
chr7 16645058
chr7 16645064
chr7 16645071
chr7 16645092
chr7 16645099
chr7 16645127
chr7 16645158
chr7 16645160
chr7 16645174
chr7 16645213
chr7 16645222
chr7 16645237

chr7 16645242
chr7 16645252
chr7 16645256
chr7 16645268
chr7 16645281
chr7 16645337
chr7 16645372
chr7 16645382
chr7 16645410
chr7 16645452
chr7 16645457
chr7 16646121
chr7 16646856
chr7 16646918
chr7 16646989
chr7 16647081
chr7 16647146
chr7 16647287
chr7 16647372
chr7 16647379
chr7 16647386
chr7 16647424
chr7 16647746
chr7 16647937
chr7 16648037
chr7 16711728
chr7 16711738
chr7 16711834
chr7 16711890
chr7 16753337
chr7 16852214
chr7 17031619
chr7 17233823
chr7 17233936
chr7 17234243
chr7 17234378
chr7 17234432
chr7 17234458
chr7 17234517
chr7 17234560
chr7 17234586
chr7 17234589
chr7 17234641
chr7 17234664
chr7 17234713
chr7 17234796
chr7 17301685
chr7 17301848
chr7 17302757
chr7 17302866
chr7 17302912
chr7 17302916
chr7 17302940
chr7 17302958

chr7 17402185
chr7 17402393
chr7 17934139
chr7 17934174
chr7 17939580
chr7 17939654
chr7 17939699
chr7 17939708
chr7 17939822
chr7 17939826
chr7 17939831
chr7 18025361
chr7 18025652
chr7 18607259
chr7 18939631
chr7 18939742
chr7 18939876
chr7 18940778
chr7 18940968
chr7 18970645
chr7 18970765
chr7 19098585
chr7 19099188
chr7 19099417
chr7 19099434
chr7 19099568
chr7 19099637
chr7 19099781
chr7 19099859
chr7 19104911
chr7 19105090
chr7 19105742
chr7 19105847
chr7 19105878
chr7 19105907
chr7 19106062
chr7 19106130
chr7 19106201
chr7 19106250
chr7 19106266
chr7 19106268
chr7 19106272
chr7 19106274
chr7 19106330
chr7 19106332
chr7 19109582
chr7 19109614
chr7 19109632
chr7 19110234
chr7 19110247
chr7 19110872
chr7 19110895
chr7 19110910
chr7 19111025

chr7 19111094
chr7 19111151
chr7 19111177
chr7 19111259
chr7 19111302
chr7 19113384
chr7 19113530
chr7 19113619
chr7 19114130
chr7 19114370
chr7 19114473
chr7 19114589
chr7 19114687
chr7 19114861
chr7 19114872
chr7 19114912
chr7 19115557
chr7 19115807
chr7 19115857
chr7 19115933
chr7 19115940
chr7 19115960
chr7 19115971
chr7 19115991
chr7 19115998
chr7 19116162
chr7 19116183
chr7 19119911
chr7 19119946
chr7 19120036
chr7 19120091
chr7 19120104
chr7 19120107
chr7 19120239
chr7 19120258
chr7 19120374
chr7 19120386
chr7 19120787
chr7 19120998
chr7 19121141
chr7 19121194
chr7 19121198
chr7 19121569
chr7 19121601
chr7 19121611
chr7 19121974
chr7 19122087
chr7 19142010
chr7 19142026
chr7 19142212
chr7 19142388
chr7 19142394
chr7 19142417
chr7 19142711

chr7 19142781
chr7 19142839
chr7 19143221
chr7 19143307
chr7 19143319
chr7 19143403
chr7 19146133
chr7 19146269
chr7 19146354
chr7 19146411
chr7 19146426
chr7 19146431
chr7 19146445
chr7 19146479
chr7 19146573
chr7 19146581
chr7 19146656
chr7 19146843
chr7 19146868
chr7 19146947
chr7 19147068
chr7 19152636
chr7 19395642
chr7 19395877
chr7 19396357
chr7 19397619
chr7 19397942
chr7 19397996
chr7 19398018
chr7 19398138
chr7 19405061
chr7 19405158
chr7 19405169
chr7 19405498
chr7 19405754
chr7 19405781
chr7 19708524
chr7 19708599
chr7 19708618
chr7 19708623
chr7 19708625
chr7 19708666
chr7 19708695
chr7 19708698
chr7 19708726
chr7 19708757
chr7 19708775
chr7 19708786
chr7 19708802
chr7 19708809
chr7 19708814
chr7 19708832
chr7 19708837
chr7 19708843

chr7 19708845
chr7 19708861
chr7 19708866
chr7 19708886
chr7 19708900
chr7 19708911
chr7 19708921
chr7 19708944
chr7 19708988
chr7 19708990
chr7 19708993
chr7 19771492
chr7 19771879
chr7 19771929
chr7 19772030
chr7 19772222
chr7 19772375
chr7 19772474
chr7 19772476
chr7 20065463
chr7 20065766
chr7 20239018
chr7 20301629
chr7 20301710
chr7 20301727
chr7 20329401
chr7 20329413
chr7 20329415
chr7 20329445
chr7 20329497
chr7 20329504
chr7 20329530
chr7 20329595
chr7 20329597
chr7 20329645
chr7 20329678
chr7 20329727
chr7 20329761
chr7 20329772
chr7 20329807
chr7 20329842
chr7 20329864
chr7 20329872
chr7 20329890
chr7 20329898
chr7 20329900
chr7 20329909
chr7 20329933
chr7 20330014
chr7 20330044
chr7 20330062
chr7 20330117
chr7 20330314
chr7 20330363

chr7 20330381
chr7 20333398
chr7 20333520
chr7 20382920
chr7 20438721
chr7 20438817
chr7 20438925
chr7 20438934
chr7 20438955
chr7 20438963
chr7 20438966
chr7 20585524
chr7 20598554
chr7 20598767
chr7 20598914
chr7 20598963
chr7 20598979
chr7 20599016
chr7 20774585
chr7 20774949
chr7 20775298
chr7 20775399
chr7 20775606
chr7 20775984
chr7 20775990
chr7 20783206
chr7 20783210
chr7 20783259
chr7 20788728
chr7 20788846
chr7 20788862
chr7 20788881
chr7 20788899
chr7 20788912
chr7 20788962
chr7 20789230
chr7 20789276
chr7 20789373
chr7 20789883
chr7 20789897
chr7 20790226
chr7 20791583
chr7 20791631
chr7 20791647
chr7 20791699
chr7 20791754
chr7 20791991
chr7 20792196
chr7 20792387
chr7 20792613
chr7 20792629
chr7 20792646
chr7 20792906
chr7 20792912

chr7 20795715
chr7 20795945
chr7 20796008
chr7 20796112
chr7 20796135
chr7 20796614
chr7 20796716
chr7 20796736
chr7 20796789
chr7 20796804
chr7 20796877
chr7 20796962
chr7 20797053
chr7 20797176
chr7 20807804
chr7 20808232
chr7 20808635
chr7 20822683
chr7 20822994
chr7 20834078
chr7 20834230
chr7 20836558
chr7 20836696
chr7 20836869
chr7 20837125
chr7 20837179
chr7 20837242
chr7 20945802
chr7 20954113
chr7 20996976
chr7 20997608
chr7 21428479
chr7 21428493
chr7 21428824
chr7 21428968
chr7 21944721
chr7 21944732
chr7 21944736
chr7 21944739
chr7 21944750
chr7 21944760
chr7 21944766
chr7 21944772
chr7 21944898
chr7 21945081
chr7 21945108
chr7 21945153
chr7 21945183
chr7 21945225
chr7 21945276
chr7 22159530
chr7 22452532
chr7 22500037
chr7 22500041

chr7 22500061
chr7 22500067
chr7 22500073
chr7 22500084
chr7 22501741
chr7 22502113
chr7 22524189
chr7 22524205
chr7 22524211
chr7 22524260
chr7 22524280
chr7 22562928
chr7 22563096
chr7 22563215
chr7 22563276
chr7 22563304
chr7 22563339
chr7 22563352
chr7 22563382
chr7 22563392
chr7 22563397
chr7 22640490
chr7 22640500
chr7 22640567
chr7 22640577
chr7 22640613
chr7 22640621
chr7 22662874
chr7 22662886
chr7 22663307
chr7 22822114
chr7 22822269
chr7 22822279
chr7 22822322
chr7 22822340
chr7 22822416
chr7 22822500
chr7 22822508
chr7 22822516
chr7 22822532
chr7 22822563
chr7 22822569
chr7 22822601
chr7 22822604
chr7 22822629
chr7 22822658
chr7 22822709
chr7 22854210
chr7 22854215
chr7 22854243
chr7 22885944
chr7 22926444
chr7 22926690
chr7 22926774

chr7 23014355
chr7 23014369
chr7 23014382
chr7 23014405
chr7 23014446
chr7 23014578
chr7 23014597
chr7 23086216
chr7 23086379
chr7 23086404
chr7 23105305
chr7 23105323
chr7 23105337
chr7 23106598
chr7 23106617
chr7 23106650
chr7 23106678
chr7 23106713
chr7 23106766
chr7 23106786
chr7 23106789
chr7 23106812
chr7 23106822
chr7 23106828
chr7 23106880
chr7 23107132
chr7 23128407
chr7 23128465
chr7 23128648
chr7 23182461
chr7 23182474
chr7 23182498
chr7 23262040
chr7 23262058
chr7 23262061
chr7 23262074
chr7 23262210
chr7 23262311
chr7 23273236
chr7 23273242
chr7 23273283
chr7 23273299
chr7 23273301
chr7 23273430
chr7 23299966
chr7 23299976
chr7 23299985
chr7 23300032
chr7 23300132
chr7 23300149
chr7 23300211
chr7 23357295
chr7 23357334
chr7 23357616

chr7 23404750
chr7 23471074
chr7 23471082
chr7 23471103
chr7 23471114
chr7 23471167
chr7 23471171
chr7 23471174
chr7 23471176
chr7 23472140
chr7 23472281
chr7 23474739
chr7 23474743
chr7 23474764
chr7 23474771
chr7 23474775
chr7 23474792
chr7 23474822
chr7 23474859
chr7 23474935
chr7 23474940
chr7 23474949
chr7 23475026
chr7 23475036
chr7 23475063
chr7 23475069
chr7 23475104
chr7 23475121
chr7 23475156
chr7 23475169
chr7 23475218
chr7 23475361
chr7 23475537
chr7 23477320
chr7 23530463
chr7 23531667
chr7 23531669
chr7 23531692
chr7 23531694
chr7 23531704
chr7 23531712
chr7 23531716
chr7 23531720
chr7 23531722
chr7 23531732
chr7 23531742
chr7 23531754
chr7 23531757
chr7 23531765
chr7 23531772
chr7 23531774
chr7 23531797
chr7 23539567
chr7 23539700

chr7 23539813
chr7 23539945
chr7 23545277
chr7 23680349
chr7 23680351
chr7 23680363
chr7 23680366
chr7 23680370
chr7 23680374
chr7 23680376
chr7 23680386
chr7 23680466
chr7 23680468
chr7 23680600
chr7 23680608
chr7 23680659
chr7 23680673
chr7 23680675
chr7 23680687
chr7 23680715
chr7 23680749
chr7 23680761
chr7 23680767
chr7 23680786
chr7 23680788
chr7 23895068
chr7 23895094
chr7 23895113
chr7 23895420
chr7 24286141
chr7 24286218
chr7 24286805
chr7 24287001
chr7 24357085
chr7 24572238
chr7 24572263
chr7 24572356
chr7 24572401
chr7 24572526
chr7 24572556
chr7 24820212
chr7 24820224
chr7 24820331
chr7 24820396
chr7 24820460
chr7 24820480
chr7 24820490
chr7 24820501
chr7 24820520
chr7 24820548
chr7 24820703
chr7 24821061
chr7 24821216
chr7 24821285

chr7 24821381
chr7 24821553
chr7 24821577
chr7 24836015
chr7 24836144
chr7 24836241
chr7 24878131
chr7 24878166
chr7 24878267
chr7 24878352
chr7 24878378
chr7 24907730
chr7 24907764
chr7 24907786
chr7 24907850
chr7 24966492
chr7 24966494
chr7 24966784
chr7 24966908
chr7 24982052
chr7 25094318
chr7 25094410
chr7 25094494
chr7 25094504
chr7 25094516
chr7 25094520
chr7 25094659
chr7 25098741
chr7 25124331
chr7 25124349
chr7 25124359
chr7 25124425
chr7 25124452
chr7 25124481
chr7 25124549
chr7 25124575
chr7 25124624
chr7 25124659
chr7 25124667
chr7 25124706
chr7 25180173
chr7 25180175
chr7 25180190
chr7 25180273
chr7 25180316
chr7 25180323
chr7 25180328
chr7 25180331
chr7 25180336
chr7 25180342
chr7 25180357
chr7 25180364
chr7 25180377
chr7 25566108

chr7 25566398
chr7 25566466
chr7 25566571
chr7 25602923
chr7 25603041
chr7 25603057
chr7 25603080
chr7 25603083
chr7 25603092
chr7 25603123
chr7 25603158
chr7 25603174
chr7 25603176
chr7 25603185
chr7 25603187
chr7 25603192
chr7 25603226
chr7 25603305
chr7 25603314
chr7 25603326
chr7 25603338
chr7 25603341
chr7 25603348
chr7 25603350
chr7 25603372
chr7 25603384
chr7 25603390
chr7 25603398
chr7 25603512
chr7 25854796
chr7 25854909
chr7 25855059
chr7 25855080
chr7 25855107
chr7 25855115
chr7 25861762
chr7 25861780
chr7 25861785
chr7 25861818
chr7 25861904
chr7 25862025
chr7 25862079
chr7 25862100
chr7 25862107
chr7 25862192
chr7 25862198
chr7 25862220
chr7 25862243
chr7 25862312
chr7 25862331
chr7 25862345
chr7 25862358
chr7 25862376
chr7 25862387

chr7 25862393
chr7 25862411
chr7 25863015
chr7 25863070
chr7 25863305
chr7 25863409
chr7 25863424
chr7 25863436
chr7 25863492
chr7 25863530
chr7 25863532
chr7 25863663
chr7 25863670
chr7 25863791
chr7 25863838
chr7 25863957
chr7 25864408
chr7 25864754
chr7 25865319
chr7 25865577
chr7 25865624
chr7 25865854
chr7 26078765
chr7 26079030
chr7 26079059
chr7 26079080
chr7 26079082
chr7 26079084
chr7 26079118
chr7 26079121
chr7 26079123
chr7 26079132
chr7 26079136
chr7 26079138
chr7 26079153
chr7 26079155
chr7 26079170
chr7 26079188
chr7 26091221
chr7 26153383
chr7 26153412
chr7 26153426
chr7 26153432
chr7 26153441
chr7 26153456
chr7 26153489
chr7 26153498
chr7 26153506
chr7 26156543
chr7 26156574
chr7 26196382
chr7 26196437
chr7 26196449
chr7 26196457

chr7 26196810
chr7 26196966
chr7 26197086
chr7 26197101
chr7 26197273
chr7 26197331
chr7 26197651
chr7 26197713
chr7 26203568
chr7 26203574
chr7 26203735
chr7 26203827
chr7 26203940
chr7 26279241
chr7 26279255
chr7 26279415
chr7 26279492
chr7 26376610
chr7 26376617
chr7 26376619
chr7 26376626
chr7 26376641
chr7 26376656
chr7 26376658
chr7 26376660
chr7 26376677
chr7 26473001
chr7 26855686
chr7 26855960
chr7 26856227
chr7 26856616
chr7 26856721
chr7 26857021
chr7 26857110
chr7 26857261
chr7 26857357
chr7 26857452
chr7 26857467
chr7 26857508
chr7 26857513
chr7 26857519
chr7 26857523
chr7 26857527
chr7 26862910
chr7 26863066
chr7 26863421
chr7 26863558
chr7 26863613
chr7 26864480
chr7 26864483
chr7 26864496
chr7 26864502
chr7 26864521
chr7 26864526

chr7 26864540
chr7 26864548
chr7 26864553
chr7 26864564
chr7 26864569
chr7 26864604
chr7 26864623
chr7 26864634
chr7 26864675
chr7 26864699
chr7 26864714
chr7 26864723
chr7 26864756
chr7 26864763
chr7 26864768
chr7 26864771
chr7 26864808
chr7 26864821
chr7 26864836
chr7 26864851
chr7 26864855
chr7 26864860
chr7 26864877
chr7 26864905
chr7 26864964
chr7 26864970
chr7 26864979
chr7 26864997
chr7 27096383
chr7 27134550
chr7 27134572
chr7 27135104
chr7 27135199
chr7 27196370
chr7 27196372
chr7 27196456
chr7 27196482
chr7 27196559
chr7 27196912
chr7 27197055
chr7 27197313
chr7 27197359
chr7 27197444
chr7 27197530
chr7 27203903
chr7 27206925
chr7 27207149
chr7 27207180
chr7 27207280
chr7 27207299
chr7 27223606
chr7 27223844
chr7 27223937
chr7 27224824

chr7 27224968
chr7 27225044
chr7 27225098
chr7 27225122
chr7 27225140
chr7 27225147
chr7 27490302
chr7 27490561
chr7 27739399
chr7 27739430
chr7 27739503
chr7 27739505
chr7 27739523
chr7 27739535
chr7 27739736
chr7 27900919
chr7 27900998
chr7 27901061
chr7 27901145
chr7 28096883
chr7 28239659
chr7 28239890
chr7 28239913
chr7 28239943
chr7 28239952
chr7 28239955
chr7 28293625
chr7 28330218
chr7 28330236
chr7 28330439
chr7 28330584
chr7 28330725
chr7 28330871
chr7 28410601
chr7 28410607
chr7 28410634
chr7 28410646
chr7 28410664
chr7 28410754
chr7 28410787
chr7 28410874
chr7 28410882
chr7 28410885
chr7 28410945
chr7 28410954
chr7 28410971
chr7 28410977
chr7 28411002
chr7 28411008
chr7 28411068
chr7 28411070
chr7 28411077
chr7 28411128
chr7 28411178

chr7 28411200
chr7 28411256
chr7 28411327
chr7 28411358
chr7 28411392
chr7 28411396
chr7 28411445
chr7 28411451
chr7 28411480
chr7 28560923
chr7 28560925
chr7 28560929
chr7 28560937
chr7 28560941
chr7 28560951
chr7 28560961
chr7 28560973
chr7 28560981
chr7 28560985
chr7 28560999
chr7 28561005
chr7 28561017
chr7 28561025
chr7 28561462
chr7 28661779
chr7 28662095
chr7 28662281
chr7 28662304
chr7 28662366
chr7 28662413
chr7 28684874
chr7 28684936
chr7 28685118
chr7 28685148
chr7 28685505
chr7 28685741
chr7 28685789
chr7 28685815
chr7 28737538
chr7 28853318
chr7 28853351
chr7 28853370
chr7 28853491
chr7 28853707
chr7 28853714
chr7 28853779
chr7 28853837
chr7 28853844
chr7 28853932
chr7 28853938
chr7 28853943
chr7 28853951
chr7 28853970
chr7 28853982

chr7 28853989
chr7 28853996
chr7 28854002
chr7 28854022
chr7 28854031
chr7 28854039
chr7 28854088
chr7 28854111
chr7 28854121
chr7 28854137
chr7 28854147
chr7 28854166
chr7 28854203
chr7 28875244
chr7 28875699
chr7 28922311
chr7 28922314
chr7 28922454
chr7 28922560
chr7 28926945
chr7 28926953
chr7 28926956
chr7 28926988
chr7 28927004
chr7 28927040
chr7 28927042
chr7 28927047
chr7 28927059
chr7 28927074
chr7 28927076
chr7 28927093
chr7 28927098
chr7 28927108
chr7 28927156
chr7 28927180
chr7 28927203
chr7 28927240
chr7 29091210
chr7 29178535
chr7 29178542
chr7 29179292
chr7 29195481
chr7 29195564
chr7 29195595
chr7 29195791
chr7 29196086
chr7 29196088
chr7 29196305
chr7 29196331
chr7 29196425
chr7 29196428
chr7 29196627
chr7 29196761
chr7 29243916

chr7	29244177
chr7	29244191
chr7	29408777
chr7	29409162
chr7	29409227
chr7	29487589
chr7	29580887
chr7	29580909
chr7	29580959
chr7	29580984
chr7	29684230
chr7	29684380
chr7	29684410
chr7	29684461
chr7	29684573
chr7	29684581
chr7	29684583
chr7	29684589
chr7	29684595
chr7	29684606
chr7	29685955
chr7	29685971
chr7	29686165
chr7	30020450
chr7	30020725
chr7	30025898
chr7	30026139
chr7	30026143
chr7	30026201
chr7	30026294
chr7	30026311
chr7	30026331
chr7	30026390
chr7	30026469
chr7	30026485
chr7	30026487
chr7	30026546
chr7	30026558
chr7	30026578
chr7	30027388
chr7	30027403
chr7	30027434
chr7	30027452
chr7	30027576
chr7	30027605
chr7	30027665
chr7	30027668
chr7	30027684
chr7	30027738
chr7	30027748
chr7	30027836
chr7	30027959
chr7	30028064
chr7	30029297

chr7 30029367
chr7 30029407
chr7 30029442
chr7 30061962
chr7 30061973
chr7 30062000
chr7 30135157
chr7 30135160
chr7 30135165
chr7 30135186
chr7 30135193
chr7 30322111
chr7 30322311
chr7 30322426
chr7 30322539
chr7 30322563
chr7 30323042
chr7 30323165
chr7 30330832
chr7 30330856
chr7 30330891
chr7 30331203
chr7 30479356
chr7 30479372
chr7 30594023
chr7 30595598
chr7 30595609
chr7 30595618
chr7 30595625
chr7 30595704
chr7 30595755
chr7 30595826
chr7 30595852
chr7 30595877
chr7 30595929
chr7 30596096
chr7 30596255
chr7 30727292
chr7 30727326
chr7 30727480
chr7 30938990
chr7 30939017
chr7 30939019
chr7 30939069
chr7 30939089
chr7 31249354
chr7 31260211
chr7 31260398
chr7 31260464
chr7 31261366
chr7 31261413
chr7 31629755
chr7 31813264
chr7 32087186

chr7 32087443
chr7 32210445
chr7 32210521
chr7 32210545
chr7 32210894
chr7 32210906
chr7 32310335
chr7 32311000
chr7 32311046
chr7 32353434
chr7 32354035
chr7 32490610
chr7 32490651
chr7 32490670
chr7 32490724
chr7 32490759
chr7 32490899
chr7 32494794
chr7 32494820
chr7 32494876
chr7 32494906
chr7 32494947
chr7 32496271
chr7 32496440
chr7 32688045
chr7 32729033
chr7 32729039
chr7 32729048
chr7 32729050
chr7 32729058
chr7 32729065
chr7 32729071
chr7 32729074
chr7 32729087
chr7 32729106
chr7 32729109
chr7 32729114
chr7 32729117
chr7 32729131
chr7 32729141
chr7 32729158
chr7 32729168
chr7 32729170
chr7 32729173
chr7 32729197
chr7 32729202
chr7 32729213
chr7 32729219
chr7 32729225
chr7 32729227
chr7 32729347
chr7 32729398
chr7 32729429
chr7 32729501

chr7 32729579
chr7 32762462
chr7 32762766
chr7 32762775
chr7 32762804
chr7 32762855
chr7 32762865
chr7 32762878
chr7 32762887
chr7 32762890
chr7 32762896
chr7 32762900
chr7 32762952
chr7 32762957
chr7 32762975
chr7 32846728
chr7 32846738
chr7 32892898
chr7 32893085
chr7 32905087
chr7 32905121
chr7 32905250
chr7 32905278
chr7 32905281
chr7 32905292
chr7 32905423
chr7 32905556
chr7 33039374
chr7 33039482
chr7 33039592
chr7 33039701
chr7 33039724
chr7 33040140
chr7 33040859
chr7 33040884
chr7 33040888
chr7 33061418
chr7 33061594
chr7 33061603
chr7 33061735
chr7 33061743
chr7 33061781
chr7 33061837
chr7 33108001
chr7 33108145
chr7 33108204
chr7 33108274
chr7 33108314
chr7 33108340
chr7 33108525
chr7 33108530
chr7 33108625
chr7 33108633
chr7 33108641

chr7 33108708
chr7 33108724
chr7 33108778
chr7 33109991
chr7 33110022
chr7 33128996
chr7 33129013
chr7 33129018
chr7 33129063
chr7 33129074
chr7 33129080
chr7 33129086
chr7 33129090
chr7 33129096
chr7 33129098
chr7 33129108
chr7 33129113
chr7 33129117
chr7 33129119
chr7 33129121
chr7 33129132
chr7 33129883
chr7 33130350
chr7 33130453
chr7 33130559
chr7 33130726
chr7 33130854
chr7 33905501
chr7 33905505
chr7 33905521
chr7 33905538
chr7 33905551
chr7 33905568
chr7 33905571
chr7 33905574
chr7 33905576
chr7 34173632
chr7 34174211
chr7 34174298
chr7 34174358
chr7 34174385
chr7 34174541
chr7 34174566
chr7 34174597
chr7 34174616
chr7 34174824
chr7 34174876
chr7 34174919
chr7 34854915
chr7 34855031
chr7 34992080
chr7 34992144
chr7 34992264
chr7 35187257

chr7 35187382
chr7 35190238
chr7 35250184
chr7 35250381
chr7 35250563
chr7 35250624
chr7 35251313
chr7 35251349
chr7 35251478
chr7 35251551
chr7 35251879
chr7 35251888
chr7 35252033
chr7 35252141
chr7 35252231
chr7 35252656
chr7 35252693
chr7 35688546
chr7 35691701
chr7 35691830
chr7 35691882
chr7 35691946
chr7 35692196
chr7 35692298
chr7 35692392
chr7 35692398
chr7 35709599
chr7 35709621
chr7 35709696
chr7 35800087
chr7 35800114
chr7 35800153
chr7 35800167
chr7 35800177
chr7 35800389
chr7 35800391
chr7 35802386
chr7 35802824
chr7 35802828
chr7 35802833
chr7 36153322
chr7 36153330
chr7 36153334
chr7 36153343
chr7 36389917
chr7 36389992
chr7 36390001
chr7 36390006
chr7 36390013
chr7 36390016
chr7 36390025
chr7 36390034
chr7 36390040
chr7 36390058

chr7 36390082
chr7 36390123
chr7 36390239
chr7 36390346
chr7 36390362
chr7 36390372
chr7 36390377
chr7 36390414
chr7 36390421
chr7 36390427
chr7 36390562
chr7 36390613
chr7 36390629
chr7 36390667
chr7 36390687
chr7 36390715
chr7 36390799
chr7 36390834
chr7 36391016
chr7 36391269
chr7 36391276
chr7 36391460
chr7 36391540
chr7 36391808
chr7 36391814
chr7 36391924
chr7 36391961
chr7 36391969
chr7 36391977
chr7 36392014
chr7 36392179
chr7 36671131
chr7 36678602
chr7 36678604
chr7 36678606
chr7 36678608
chr7 36678610
chr7 36678612
chr7 36678614
chr7 36678695
chr7 36724516
chr7 36724525
chr7 36724535
chr7 36724600
chr7 36725008
chr7 36725100
chr7 36784757
chr7 36785025
chr7 36785050
chr7 36785062
chr7 36785076
chr7 36785102
chr7 37341221
chr7 37341245

chr7 37341332
chr7 37341429
chr7 37341616
chr7 37341648
chr7 37341669
chr7 37342348
chr7 37342958
chr7 37342966
chr7 37342983
chr7 37342993
chr7 37343010
chr7 37343017
chr7 37343019
chr7 37343127
chr7 37352939
chr7 37352961
chr7 37353078
chr7 37353161
chr7 37353241
chr7 37353247
chr7 37353276
chr7 37353284
chr7 37353286
chr7 37360386
chr7 37360450
chr7 37360587
chr7 37408243
chr7 37408375
chr7 37408401
chr7 37408438
chr7 37408474
chr7 37408542
chr7 37449333
chr7 37449339
chr7 37449359
chr7 37449375
chr7 37449388
chr7 37449402
chr7 37449463
chr7 37449466
chr7 37449571
chr7 37449699
chr7 37449747
chr7 37449767
chr7 37449801
chr7 37664208
chr7 37732204
chr7 37732212
chr7 37732263
chr7 37732295
chr7 37732367
chr7 37833546
chr7 37833575
chr7 37833578

chr7 37833586
chr7 37833668
chr7 37833674
chr7 37833676
chr7 37833703
chr7 37833717
chr7 37833725
chr7 37833752
chr7 37833775
chr7 37833902
chr7 37834008
chr7 37834054
chr7 37834457
chr7 37834903
chr7 37835101
chr7 37847132
chr7 37847227
chr7 37847236
chr7 37847245
chr7 37847254
chr7 37847335
chr7 37847344
chr7 37847353
chr7 37847362
chr7 37847578
chr7 37847677
chr7 37847686
chr7 37915564
chr7 37915647
chr7 37915690
chr7 37915702
chr7 37915756
chr7 37917046
chr7 37917080
chr7 37917086
chr7 37917102
chr7 37917248
chr7 37917267
chr7 37917304
chr7 37917338
chr7 37917361
chr7 37917419
chr7 38177614
chr7 38177670
chr7 38177711
chr7 38177741
chr7 38177813
chr7 38178890
chr7 38232766
chr7 38233028
chr7 38233083
chr7 38233132
chr7 38233169
chr7 38233180

chr7 38233249
chr7 38233266
chr7 38233282
chr7 38233324
chr7 38233661
chr7 38309761
chr7 38309769
chr7 38309772
chr7 38309820
chr7 38309830
chr7 38309999
chr7 38310267
chr7 38310364
chr7 38310496
chr7 38310541
chr7 38310797
chr7 38310821
chr7 38310863
chr7 38310902
chr7 38311020
chr7 38311074
chr7 38311103
chr7 38311153
chr7 38311240
chr7 38311320
chr7 38311322
chr7 38311331
chr7 38311337
chr7 38311343
chr7 38311359
chr7 38311363
chr7 38311373
chr7 38311376
chr7 38311398
chr7 38311403
chr7 38311405
chr7 38311408
chr7 38311413
chr7 38311432
chr7 38311435
chr7 38311437
chr7 38311469
chr7 38311475
chr7 38311482
chr7 38311493
chr7 38311502
chr7 38311510
chr7 38311531
chr7 38311541
chr7 38311553
chr7 38311561
chr7 38311574
chr7 38311586
chr7 38311618

chr7 38311625
chr7 38311656
chr7 38311658
chr7 38311691
chr7 38311697
chr7 38311704
chr7 38311723
chr7 38311729
chr7 38311763
chr7 38311867
chr7 38311937
chr7 38311957
chr7 38312372
chr7 38312395
chr7 38312547
chr7 38312634
chr7 38312661
chr7 38353639
chr7 38353697
chr7 38353767
chr7 38353801
chr7 38353846
chr7 38353878
chr7 38353924
chr7 38353991
chr7 38358154
chr7 38358436
chr7 38358649
chr7 38358658
chr7 38358675
chr7 38358742
chr7 38358788
chr7 38358928
chr7 38358975
chr7 38358990
chr7 38359009
chr7 38359235
chr7 38359238
chr7 38359471
chr7 38359489
chr7 38359544
chr7 38359818
chr7 38362866
chr7 38398796
chr7 38398824
chr7 38398910
chr7 38398939
chr7 38398948
chr7 38399029
chr7 38463524
chr7 38463763
chr7 38463796
chr7 38463804
chr7 38463808

chr7 38908557
chr7 38908670
chr7 38908695
chr7 38908750
chr7 38908815
chr7 38908860
chr7 38908914
chr7 38908929
chr7 38908933
chr7 38908935
chr7 38908951
chr7 38908972
chr7 38908997
chr7 38909010
chr7 38909023
chr7 38909031
chr7 38909033
chr7 38909046
chr7 38909063
chr7 38909169
chr7 38909177
chr7 38909182
chr7 38909204
chr7 38909295
chr7 38909540
chr7 38909735
chr7 38914325
chr7 39413899
chr7 39413917
chr7 39413922
chr7 39413933
chr7 39413958
chr7 39413961
chr7 39413974
chr7 39413982
chr7 39465021
chr7 39465102
chr7 39465133
chr7 39465237
chr7 39465239
chr7 39465243
chr7 39465247
chr7 39465253
chr7 39465261
chr7 39465265
chr7 39465280
chr7 39565556
chr7 39565576
chr7 39565851
chr7 39565930
chr7 39565937
chr7 39565982
chr7 39566046
chr7 39566053

chr7 39566084
chr7 39566086
chr7 39566088
chr7 39566119
chr7 39566679
chr7 39566699
chr7 39566716
chr7 39566733
chr7 39566746
chr7 39566752
chr7 39566759
chr7 39566776
chr7 39566886
chr7 39566892
chr7 39567213
chr7 39567366
chr7 39567382
chr7 39589829
chr7 39589876
chr7 39589917
chr7 39589934
chr7 39590007
chr7 39590213
chr7 39609503
chr7 39609536
chr7 39609625
chr7 39733612
chr7 39733623
chr7 39733628
chr7 39733637
chr7 39733652
chr7 39733692
chr7 39733712
chr7 39755018
chr7 39755022
chr7 39755041
chr7 39755493
chr7 39832970
chr7 39833216
chr7 39833219
chr7 39833222
chr7 39833225
chr7 39833228
chr7 39833231
chr7 39833234
chr7 39833243
chr7 39833252
chr7 39833255
chr7 39833261
chr7 39833267
chr7 39833271
chr7 39833275
chr7 39833277
chr7 39833279

chr7 39833283
chr7 39833287
chr7 39833294
chr7 39833298
chr7 39854983
chr7 39855013
chr7 39855044
chr7 39855057
chr7 39855059
chr7 39855062
chr7 39855066
chr7 39855068
chr7 39855073
chr7 39855084
chr7 39855088
chr7 39855091
chr7 39855095
chr7 39855103
chr7 39855114
chr7 39855193
chr7 39855202
chr7 39855204
chr7 39855206
chr7 39855211
chr7 39855218
chr7 39855224
chr7 39855232
chr7 39855235
chr7 39855255
chr7 39855389
chr7 39952423
chr7 39952709
chr7 39952913
chr7 39953083
chr7 39953167
chr7 40133451
chr7 40133579
chr7 40133727
chr7 40644723
chr7 40644730
chr7 40734064
chr7 41386450
chr7 41688708
chr7 41688923
chr7 41689054
chr7 41689339
chr7 41699712
chr7 41699734
chr7 41699778
chr7 41699782
chr7 41699831
chr7 41699834
chr7 41699843
chr7 41699993

chr7 41700000
chr7 41700284
chr7 41700296
chr7 41700436
chr7 41700438
chr7 41700628
chr7 41732104
chr7 41732145
chr7 41799200
chr7 41799280
chr7 41845314
chr7 41881571
chr7 41881709
chr7 41881733
chr7 41881745
chr7 41881761
chr7 41881782
chr7 41881879
chr7 41881923
chr7 41881926
chr7 41881963
chr7 41943260
chr7 41943262
chr7 41943274
chr7 41943278
chr7 41943578
chr7 41943612
chr7 41943762
chr7 42405496
chr7 42405578
chr7 42452534
chr7 42452579
chr7 42452628
chr7 42453655
chr7 42453657
chr7 42453714
chr7 42453716
chr7 42453895
chr7 42453908
chr7 42671911
chr7 42857931
chr7 42869401
chr7 42869535
chr7 42869560
chr7 42869650
chr7 42869901
chr7 42887776
chr7 42887874
chr7 42887880
chr7 42887986
chr7 42888094
chr7 42888096
chr7 42888098
chr7 42888100

chr7 42888102
chr7 42888104
chr7 42888108
chr7 42888110
chr7 42888207
chr7 42888210
chr7 42888292
chr7 42888350
chr7 42888352
chr7 42888369
chr7 42888383
chr7 42888434
chr7 42888436
chr7 42888438
chr7 42888451
chr7 42931024
chr7 42931096
chr7 42931134
chr7 42931244
chr7 42931351
chr7 42931354
chr7 42931368
chr7 42931385
chr7 42931483
chr7 42931486
chr7 42932883
chr7 42932903
chr7 42932908
chr7 42932976
chr7 42932994
chr7 42933025
chr7 42933073
chr7 42933111
chr7 42933131
chr7 42933178
chr7 42933191
chr7 42933233
chr7 42933271
chr7 43075923
chr7 43075960
chr7 43414769
chr7 43414850
chr7 43414939
chr7 43454820
chr7 43455018
chr7 43455026
chr7 43584319
chr7 43584382
chr7 43584390
chr7 43584448
chr7 43584474
chr7 43584489
chr7 43584863
chr7 43584918

chr7 43584922
chr7 43584934
chr7 43584967
chr7 43585025
chr7 43585106
chr7 43585121
chr7 43585156
chr7 43585218
chr7 43585264
chr7 43585283
chr7 43585427
chr7 43585612
chr7 43585720
chr7 43586116
chr7 43586142
chr7 43586432
chr7 43586553
chr7 43586602
chr7 43586748
chr7 43587185
chr7 43587193
chr7 43587215
chr7 43587217
chr7 43587231
chr7 43587240
chr7 43587255
chr7 43587279
chr7 43587299
chr7 43587633
chr7 43647955
chr7 43648046
chr7 43648162
chr7 43648279
chr7 43648398
chr7 43648413
chr7 43648545
chr7 43648754
chr7 43648856
chr7 43648907
chr7 43649934
chr7 43650058
chr7 43650139
chr7 43650153
chr7 43650286
chr7 43650536
chr7 43650595
chr7 43650616
chr7 43650648
chr7 43650667
chr7 43650689
chr7 43650698
chr7 43650856
chr7 43650923
chr7 43659241

chr7 43834638
chr7 43834678
chr7 43834800
chr7 43838756
chr7 43838871
chr7 43868923
chr7 43869023
chr7 43869033
chr7 43869053
chr7 43869055
chr7 43869061
chr7 43869066
chr7 43869070
chr7 43869092
chr7 43869102
chr7 43869111
chr7 44039904
chr7 44039929
chr7 44039935
chr7 44039985
chr7 44039987
chr7 44039998
chr7 44040010
chr7 44040026
chr7 44040034
chr7 44040068
chr7 44040119
chr7 44040125
chr7 44040139
chr7 44040142
chr7 44040148
chr7 44040389
chr7 44040391
chr7 44040545
chr7 44040601
chr7 44040605
chr7 44040607
chr7 44040611
chr7 44040613
chr7 44040616
chr7 44040628
chr7 44040638
chr7 44040640
chr7 44040647
chr7 44040650
chr7 44040657
chr7 44040659
chr7 44040666
chr7 44040670
chr7 44040678
chr7 44040680
chr7 44040704
chr7 44040706
chr7 44040711

chr7 44040717
chr7 44040719
chr7 44040737
chr7 44040743
chr7 44040750
chr7 44040754
chr7 44044572
chr7 44044581
chr7 44044591
chr7 44044593
chr7 44044599
chr7 44044619
chr7 44044683
chr7 44044688
chr7 44044693
chr7 44044698
chr7 44044713
chr7 44044716
chr7 44044718
chr7 44083452
chr7 44200481
chr7 44200539
chr7 44200553
chr7 44200621
chr7 44200680
chr7 44200781
chr7 44200820
chr7 44226937
chr7 44227086
chr7 44227271
chr7 44227378
chr7 44489762
chr7 44489779
chr7 44489872
chr7 44489908
chr7 44490957
chr7 44491065
chr7 44491075
chr7 44491131
chr7 44491158
chr7 44491183
chr7 44491224
chr7 44491234
chr7 44491250
chr7 44491287
chr7 44609553
chr7 44631247
chr7 44631264
chr7 44631346
chr7 44631456
chr7 44638997
chr7 44639025
chr7 44639084
chr7 44797713

chr7 44797715
chr7 44797742
chr7 44797748
chr7 44797783
chr7 44797861
chr7 44797890
chr7 44797903
chr7 44798025
chr7 44798151
chr7 44798185
chr7 44798190
chr7 44798274
chr7 44798285
chr7 44798289
chr7 44798296
chr7 44798316
chr7 44798373
chr7 44798381
chr7 44798427
chr7 44798438
chr7 44798444
chr7 44798461
chr7 44798467
chr7 44798549
chr7 44798674
chr7 44798867
chr7 44845877
chr7 44845986
chr7 44846104
chr7 44846210
chr7 44846295
chr7 44846339
chr7 44846343
chr7 44846345
chr7 44846355
chr7 44846383
chr7 44846392
chr7 44846413
chr7 44846477
chr7 44846485
chr7 44846489
chr7 44846503
chr7 44846525
chr7 44846535
chr7 44846542
chr7 44846565
chr7 44846603
chr7 44846791
chr7 44846798
chr7 44846871
chr7 44846878
chr7 44846955
chr7 44847095
chr7 44876468

chr7 44876475
chr7 44876600
chr7 44876621
chr7 44886043
chr7 44886118
chr7 44886168
chr7 44886205
chr7 44891515
chr7 44922162
chr7 44977585
chr7 44977602
chr7 44977818
chr7 44977940
chr7 44978046
chr7 44978079
chr7 44978095
chr7 44978143
chr7 44978155
chr7 44978242
chr7 44978448
chr7 44978560
chr7 44978563
chr7 44978637
chr7 44978701
chr7 44978777
chr7 44978806
chr7 44978812
chr7 44978952
chr7 44978967
chr7 44979053
chr7 44979059
chr7 44979135
chr7 44979158
chr7 44985746
chr7 44985881
chr7 44985969
chr7 44986011
chr7 44986021
chr7 44986074
chr7 44986088
chr7 44986129
chr7 44986185
chr7 44986213
chr7 44986226
chr7 44986243
chr7 44986266
chr7 44986319
chr7 44986336
chr7 44986360
chr7 44986371
chr7 44986385
chr7 44986389
chr7 44986391
chr7 44986399

chr7 44999210
chr7 44999280
chr7 44999347
chr7 44999364
chr7 44999396
chr7 44999417
chr7 44999522
chr7 44999592
chr7 44999595
chr7 44999600
chr7 44999605
chr7 44999622
chr7 44999630
chr7 44999632
chr7 44999643
chr7 44999653
chr7 44999657
chr7 44999659
chr7 44999669
chr7 44999672
chr7 44999679
chr7 44999686
chr7 44999693
chr7 44999700
chr7 44999726
chr7 44999738
chr7 44999740
chr7 44999873
chr7 44999954
chr7 44999956
chr7 44999962
chr7 45000714
chr7 45000722
chr7 45000743
chr7 45000799
chr7 45000844
chr7 45000951
chr7 45000981
chr7 45001064
chr7 45001100
chr7 45001132
chr7 45001361
chr7 45027130
chr7 45027139
chr7 45027242
chr7 45027244
chr7 45027259
chr7 45027296
chr7 45027456
chr7 45027458
chr7 45027720
chr7 45027823
chr7 45027999
chr7 45028016

chr7 45111122
chr7 45111124
chr7 45111196
chr7 45111201
chr7 45111226
chr7 45111239
chr7 45111241
chr7 45111245
chr7 45111249
chr7 45165643
chr7 45166017
chr7 45166063
chr7 45166101
chr7 45166259
chr7 45166326
chr7 45922963
chr7 45922974
chr7 45922977
chr7 45923022
chr7 45923078
chr7 45923099
chr7 45923114
chr7 45923149
chr7 45923355
chr7 45923386
chr7 45923401
chr7 45923416
chr7 45923425
chr7 45923440
chr7 45923449
chr7 45950971
chr7 45951301
chr7 46823718
chr7 46823748
chr7 46824516
chr7 46824579
chr7 46824636
chr7 47128253
chr7 47647187
chr7 47979343
chr7 47979357
chr7 47979359
chr7 47991674
chr7 47991702
chr7 47991711
chr7 47991725
chr7 47991738
chr7 47991758
chr7 47991783
chr7 47991797
chr7 47991834
chr7 47991871
chr7 47991879
chr7 47991893

chr7 47991945
chr7 47991948
chr7 47991951
chr7 47991962
chr7 47992001
chr7 47992003
chr7 47992113
chr7 47992116
chr7 47992126
chr7 47992141
chr7 47992154
chr7 47992157
chr7 47992171
chr7 47992175
chr7 47992177
chr7 47992192
chr7 47992203
chr7 47992270
chr7 47992277
chr7 47992313
chr7 47992319
chr7 47992481
chr7 48110406
chr7 48110410
chr7 48110452
chr7 48110464
chr7 48110525
chr7 50068991
chr7 50069060
chr7 50069075
chr7 50069340
chr7 50069407
chr7 50069534
chr7 50069558
chr7 50092184
chr7 50092190
chr7 50092240
chr7 50092543
chr7 50092632
chr7 50092673
chr7 50092686
chr7 50092844
chr7 50092913
chr7 50092945
chr7 50092977
chr7 50092985
chr7 50093007
chr7 50093026
chr7 50093038
chr7 50093044
chr7 50093052
chr7 50093055
chr7 50093057
chr7 50093082

chr7 50093095
chr7 50093116
chr7 50093121
chr7 50093137
chr7 50093140
chr7 50093145
chr7 50093151
chr7 50093154
chr7 50093163
chr7 50093166
chr7 50093168
chr7 50093182
chr7 50093200
chr7 50093203
chr7 50093206
chr7 50093209
chr7 50093220
chr7 50093224
chr7 50093226
chr7 50093228
chr7 50093236
chr7 50266282
chr7 50266293
chr7 50266452
chr7 50266615
chr7 50266617
chr7 50266688
chr7 50266714
chr7 50266881
chr7 50266937
chr7 50266939
chr7 50266951
chr7 50267067
chr7 50267335
chr7 50267440
chr7 50267575
chr7 50267605
chr7 50268273
chr7 50268366
chr7 50268378
chr7 50268406
chr7 50308216
chr7 50308223
chr7 50308330
chr7 50308578
chr7 50308599
chr7 50308659
chr7 50308825
chr7 50308889
chr7 50308897
chr7 50308919
chr7 50308967
chr7 50309047
chr7 50309074

chr7 50309082
chr7 50309093
chr7 50309125
chr7 50309130
chr7 50309132
chr7 50309140
chr7 50309207
chr7 50309274
chr7 50309389
chr7 50309967
chr7 50310009
chr7 50310070
chr7 50310199
chr7 50310396
chr7 50310602
chr7 50310607
chr7 50310620
chr7 50310670
chr7 50310689
chr7 50310902
chr7 50311089
chr7 50311506
chr7 50311680
chr7 50311761
chr7 50311894
chr7 50312106
chr7 50312173
chr7 50312216
chr7 50312339
chr7 50312357
chr7 50312366
chr7 50312411
chr7 50312472
chr7 50312811
chr7 50312845
chr7 50313012
chr7 50313055
chr7 50313550
chr7 50313625
chr7 50315599
chr7 50315657
chr7 50315662
chr7 50315844
chr7 50315910
chr7 50315922
chr7 50316083
chr7 50316150
chr7 50316177
chr7 50316215
chr7 50316786
chr7 50316804
chr7 50317191
chr7 50317265
chr7 50317280

chr7 50317282
chr7 50317351
chr7 50317425
chr7 50317471
chr7 50317537
chr7 50318135
chr7 50318139
chr7 50318160
chr7 50318229
chr7 50318264
chr7 50318322
chr7 50318364
chr7 50318382
chr7 50318598
chr7 50318620
chr7 50318622
chr7 50318650
chr7 50337787
chr7 50337950
chr7 50337960
chr7 50337989
chr7 50338288
chr7 50338377
chr7 50338468
chr7 50338679
chr7 50338699
chr7 50338869
chr7 50338877
chr7 50338955
chr7 50340098
chr7 50340121
chr7 50340233
chr7 50340305
chr7 50340362
chr7 50340413
chr7 50340444
chr7 50342268
chr7 50342623
chr7 50350544
chr7 50350582
chr7 50350587
chr7 50350608
chr7 50350611
chr7 50350622
chr7 50350653
chr7 50350784
chr7 50350833
chr7 50350894
chr7 50350961
chr7 50351039
chr7 50351450
chr7 50351509
chr7 50351701
chr7 50351867

chr7 50351906
chr7 50352403
chr7 50368855
chr7 50369163
chr7 50396061
chr7 50396408
chr7 50396523
chr7 50446746
chr7 50446757
chr7 50446773
chr7 50446779
chr7 50449544
chr7 50449609
chr7 50449714
chr7 50634260
chr7 50740139
chr7 50740210
chr7 50740261
chr7 50740603
chr7 50761651
chr7 51121456
chr7 51121634
chr7 51121903
chr7 51121940
chr7 51122047
chr7 51122062
chr7 51807720
chr7 51890903
chr7 51890934
chr7 51890959
chr7 51890980
chr7 51891107
chr7 51891143
chr7 51891148
chr7 52936886
chr7 52937032
chr7 52937297
chr7 52937443
chr7 52971937
chr7 53059898
chr7 53060329
chr7 53168880
chr7 53169931
chr7 53635896
chr7 53636239
chr7 54050461
chr7 54050716
chr7 54051459
chr7 54051486
chr7 54261429
chr7 54261462
chr7 54261718
chr7 54416296
chr7 54416329

chr7 54416414
chr7 54416909
chr7 54416969
chr7 54417010
chr7 54442328
chr7 54543337
chr7 54543353
chr7 54543369
chr7 54543478
chr7 54543789
chr7 54544113
chr7 54544139
chr7 54545090
chr7 54545099
chr7 54545118
chr7 54545139
chr7 54545146
chr7 54545182
chr7 54545207
chr7 54545242
chr7 54545287
chr7 54545359
chr7 54545388
chr7 54545420
chr7 54545605
chr7 54545645
chr7 54545814
chr7 54546083
chr7 54546202
chr7 54546244
chr7 54546332
chr7 54547186
chr7 54547213
chr7 54547654
chr7 54549154
chr7 54567203
chr7 54567827
chr7 54758310
chr7 54758484
chr7 54758525
chr7 54758628
chr7 54758661
chr7 54758665
chr7 54758680
chr7 54758727
chr7 54758765
chr7 54758771
chr7 54758774
chr7 54773900
chr7 54773929
chr7 54773961
chr7 54774003
chr7 54933184
chr7 54933186

chr7 54933196
chr7 54933204
chr7 54933222
chr7 54933227
chr7 54933234
chr7 54933245
chr7 54933255
chr7 54933261
chr7 54933305
chr7 54933312
chr7 54933330
chr7 54933334
chr7 54933343
chr7 54933352
chr7 54933374
chr7 54933376
chr7 54933379
chr7 54933383
chr7 54933390
chr7 54933392
chr7 54933395
chr7 54933406
chr7 54933429
chr7 54933432
chr7 54933435
chr7 54933441
chr7 55105406
chr7 55105527
chr7 55255375
chr7 55255412
chr7 55255415
chr7 55255456
chr7 55255463
chr7 55255487
chr7 55280277
chr7 55280848
chr7 55280933
chr7 55365154
chr7 55365198
chr7 55533799
chr7 55533868
chr7 55569843
chr7 55569954
chr7 55570026
chr7 55571170
chr7 55571182
chr7 55571425
chr7 55641266
chr7 55641301
chr7 55887273
chr7 55887287
chr7 55887307
chr7 55887313
chr7 55887315

chr7 55887322
chr7 55887329
chr7 55887339
chr7 55887398
chr7 55887416
chr7 55887421
chr7 55887424
chr7 55887430
chr7 55887436
chr7 55887458
chr7 55887465
chr7 55887468
chr7 55887476
chr7 55887505
chr7 55887507
chr7 55887510
chr7 55887522
chr7 55887543
chr7 55887547
chr7 55887565
chr7 55887574
chr7 55887591
chr7 55887600
chr7 55887602
chr7 55887605
chr7 55887608
chr7 55887611
chr7 55887614
chr7 55887617
chr7 55887620
chr7 55887623
chr7 55887626
chr7 55887629
chr7 55887632
chr7 55887635
chr7 55887638
chr7 55887648
chr7 55887658
chr7 55887663
chr7 55887676
chr7 55887688
chr7 55887690
chr7 55887693
chr7 55999840
chr7 56033989
chr7 56034003
chr7 56034020
chr7 56034023
chr7 56034029
chr7 56034032
chr7 56034036
chr7 56034040
chr7 56034086
chr7 56034090

chr7 56034115
chr7 56034188
chr7 56034201
chr7 56034212
chr7 56034254
chr7 56034287
chr7 56034305
chr7 56034329
chr7 56034349
chr7 56034351
chr7 56034357
chr7 56034434
chr7 56034437
chr7 56034460
chr7 56034474
chr7 56034538
chr7 56034546
chr7 56034554
chr7 56034578
chr7 56034584
chr7 56034601
chr7 56034616
chr7 56064517
chr7 56064519
chr7 56064524
chr7 56064532
chr7 56064595
chr7 56064599
chr7 56064601
chr7 56064609
chr7 56064639
chr7 56064644
chr7 56064689
chr7 56064739
chr7 56064743
chr7 56064748
chr7 56064751
chr7 56064775
chr7 56064797
chr7 56064814
chr7 56064831
chr7 56064837
chr7 56064861
chr7 56064869
chr7 56064914
chr7 56064958
chr7 56064963
chr7 56064967
chr7 56064974
chr7 56064988
chr7 56065019
chr7 56065048
chr7 56106014
chr7 56106041

chr7 56106060
chr7 56106089
chr7 56106138
chr7 56106152
chr7 56106177
chr7 56106220
chr7 56106235
chr7 56106248
chr7 56106256
chr7 56106259
chr7 56106265
chr7 56528901
chr7 56529099
chr7 56529539
chr7 56576019
chr7 56577036
chr7 56579242
chr7 56579369
chr7 56580262
chr7 56907205
chr7 56907375
chr7 56907665
chr7 56907673
chr7 56908007
chr7 56908025
chr7 56908084
chr7 56908143
chr7 56908150
chr7 56908167
chr7 56960216
chr7 56960220
chr7 56960228
chr7 56960455
chr7 56960760
chr7 56960777
chr7 56961618
chr7 56961783
chr7 56961977
chr7 56997475
chr7 56997532
chr7 56997654
chr7 56997828
chr7 56998067
chr7 56998083
chr7 56998194
chr7 56998398
chr7 56998565
chr7 57079498
chr7 57079798
chr7 57081096
chr7 57081216
chr7 57081682
chr7 57082306
chr7 57084036

chr7 57153052
chr7 57536484
chr7 57614345
chr7 57614364
chr7 57614895
chr7 57615472
chr7 57615915
chr7 57616650
chr7 57616653
chr7 57619099
chr7 57619456
chr7 57619980
chr7 57620670
chr7 57621022
chr7 57637526
chr7 57816800
chr7 57816819
chr7 57817171
chr7 57871172
chr7 57871529
chr7 57871548
chr7 57871731
chr7 57872144
chr7 57872353
chr7 57872515
chr7 58085000
chr7 58103363
chr7 58104086
chr7 58104210
chr7 58169745
chr7 58811999
chr7 58812034
chr7 58812274
chr7 58812278
chr7 58812313
chr7 60878760
chr7 60878785
chr7 60879250
chr7 60879273
chr7 60879500
chr7 60879597
chr7 60880188
chr7 60880360
chr7 60880702
chr7 60881494
chr7 60881651
chr7 60881712
chr7 60882063
chr7 60882066
chr7 60882077
chr7 60882152
chr7 60882340
chr7 60882542
chr7 60882735

chr7 60882744
chr7 60882998
chr7 60883248
chr7 60884340
chr7 60884667
chr7 60884756
chr7 60885449
chr7 60885877
chr7 60887234
chr7 60887521
chr7 60888008
chr7 60888069
chr7 60888373
chr7 60889257
chr7 60889365
chr7 60889494
chr7 60890048
chr7 60890568
chr7 60890641
chr7 60890649
chr7 60904832
chr7 60904862
chr7 60905004
chr7 60906503
chr7 60907248
chr7 60907831
chr7 60907951
chr7 60907955
chr7 60908643
chr7 60910199
chr7 60910210
chr7 60910223
chr7 60910285
chr7 60910311
chr7 60910528
chr7 60910567
chr7 60910577
chr7 60910616
chr7 60910642
chr7 60910694
chr7 60910756
chr7 60910779
chr7 60939971
chr7 60940235
chr7 60940700
chr7 60990530
chr7 60990569
chr7 60990659
chr7 60991098
chr7 60996954
chr7 60998546
chr7 60999061
chr7 60999162
chr7 60999172

chr7 61071053
chr7 61071079
chr7 61071089
chr7 61075213
chr7 61075221
chr7 61118409
chr7 61118411
chr7 61118482
chr7 61118620
chr7 61119052
chr7 61119433
chr7 61119442
chr7 61120095
chr7 61120130
chr7 61120376
chr7 61120506
chr7 61120780
chr7 61120951
chr7 61121014
chr7 61121035
chr7 61121046
chr7 61121450
chr7 61121651
chr7 61122864
chr7 61123240
chr7 61123505
chr7 61123514
chr7 61123748
chr7 61123769
chr7 61124040
chr7 61125460
chr7 61126239
chr7 61126248
chr7 61126419
chr7 61126424
chr7 61579070
chr7 61579220
chr7 61579272
chr7 61579298
chr7 61581891
chr7 61584006
chr7 61584660
chr7 61584674
chr7 61584839
chr7 61584891
chr7 61585033
chr7 61585063
chr7 61585304
chr7 61585455
chr7 61586405
chr7 61586687
chr7 61586785
chr7 61588234
chr7 61588825

chr7 61588994
chr7 61589829
chr7 61589976
chr7 61590876
chr7 61590917
chr7 61701985
chr7 61702058
chr7 61703443
chr7 61703702
chr7 61703726
chr7 61704148
chr7 61705340
chr7 61707109
chr7 61707807
chr7 61707844
chr7 61708192
chr7 61708262
chr7 61708670
chr7 61709342
chr7 61710239
chr7 61710384
chr7 61710438
chr7 61712739
chr7 61712985
chr7 61713722
chr7 61714127
chr7 61715794
chr7 61716213
chr7 61716385
chr7 61717174
chr7 61717188
chr7 61717195
chr7 61717211
chr7 62026925
chr7 62027473
chr7 62027860
chr7 62028077
chr7 62028085
chr7 62028216
chr7 62028586
chr7 62028678
chr7 62029361
chr7 62029469
chr7 62029493
chr7 62030353
chr7 62030718
chr7 62031205
chr7 62223299
chr7 62224307
chr7 62224559
chr7 62224659
chr7 62224932
chr7 62224997
chr7 62225399

chr7 62226168
chr7 62226344
chr7 62226780
chr7 62280615
chr7 62284138
chr7 62284230
chr7 62284256
chr7 62285281
chr7 62285398
chr7 62286361
chr7 62286377
chr7 62286476
chr7 62286914
chr7 62288377
chr7 62289459
chr7 62289467
chr7 62289559
chr7 62291609
chr7 62291779
chr7 62291911
chr7 62291933
chr7 62292832
chr7 62292984
chr7 62293782
chr7 62294642
chr7 62294940
chr7 62316018
chr7 62316283
chr7 62316340
chr7 62316971
chr7 62316975
chr7 62317095
chr7 62317127
chr7 62334436
chr7 62334614
chr7 62334707
chr7 62359848
chr7 62360130
chr7 62360313
chr7 62360386
chr7 62361518
chr7 62361688
chr7 62369065
chr7 62369542
chr7 62372405
chr7 62372460
chr7 62372680
chr7 62373295
chr7 62373616
chr7 62373732
chr7 62400303
chr7 62400748
chr7 62409183
chr7 62409307

chr7 62409372
chr7 62409459
chr7 62409641
chr7 62409766
chr7 62436396
chr7 62456663
chr7 62456706
chr7 62456712
chr7 62456722
chr7 62983355
chr7 62983554
chr7 62983574
chr7 62984142
chr7 63017728
chr7 63018385
chr7 63018469
chr7 63018517
chr7 63019367
chr7 63019379
chr7 63150740
chr7 63252589
chr7 63252967
chr7 63335686
chr7 63336021
chr7 63336056
chr7 63336440
chr7 63336809
chr7 63336821
chr7 63336880
chr7 63337362
chr7 63337828
chr7 63338196
chr7 63338282
chr7 63338596
chr7 63339497
chr7 63339601
chr7 63485455
chr7 63485702
chr7 63485935
chr7 63486170
chr7 63488177
chr7 63489266
chr7 63506462
chr7 63554324
chr7 63554839
chr7 63554977
chr7 63555158
chr7 63734913
chr7 63735032
chr7 63735086
chr7 63794614
chr7 63877239
chr7 63877462
chr7 64306972

chr7 64306994
chr7 64306996
chr7 64307002
chr7 64307019
chr7 64307025
chr7 64307051
chr7 64307071
chr7 64307075
chr7 64307083
chr7 64307085
chr7 64307087
chr7 64307116
chr7 64307127
chr7 64307147
chr7 64307161
chr7 64307168
chr7 64307177
chr7 64307205
chr7 64307215
chr7 64307217
chr7 64307225
chr7 64307233
chr7 64307241
chr7 64307277
chr7 64307283
chr7 64307293
chr7 64307331
chr7 64314002
chr7 64314020
chr7 64314022
chr7 64314045
chr7 64314061
chr7 64314126
chr7 64314129
chr7 64314161
chr7 64314177
chr7 64314205
chr7 64314209
chr7 64314215
chr7 64314221
chr7 64314241
chr7 64314243
chr7 64314268
chr7 64314276
chr7 64314278
chr7 64314309
chr7 64314340
chr7 64314345
chr7 64314367
chr7 64314411
chr7 64314419
chr7 64314468
chr7 64314500
chr7 64427665

chr7 64427843
chr7 64486458
chr7 64563446
chr7 64570361
chr7 64570371
chr7 64570380
chr7 64570389
chr7 64570392
chr7 64570395
chr7 64570398
chr7 64570406
chr7 64570412
chr7 64570419
chr7 64570423
chr7 64570428
chr7 64570438
chr7 64570440
chr7 64570449
chr7 64570454
chr7 64570479
chr7 64570484
chr7 64570489
chr7 64570491
chr7 64570493
chr7 64570545
chr7 64570551
chr7 64570560
chr7 64570565
chr7 64570571
chr7 64570591
chr7 64570593
chr7 64666046
chr7 64666257
chr7 64666260
chr7 64666652
chr7 64666892
chr7 64666935
chr7 64667138
chr7 64795291
chr7 64806195
chr7 64810867
chr7 64895465
chr7 64895569
chr7 64895687
chr7 64903491
chr7 64903499
chr7 64903527
chr7 64903529
chr7 64903549
chr7 64903556
chr7 64903603
chr7 64903611
chr7 64903615
chr7 64903633

chr7 64903642
chr7 64903682
chr7 64903687
chr7 64903690
chr7 64903702
chr7 64903710
chr7 64903798
chr7 64903800
chr7 65005945
chr7 65006124
chr7 65006173
chr7 65006233
chr7 65006237
chr7 65006259
chr7 65006271
chr7 65006326
chr7 65006348
chr7 65006355
chr7 65006368
chr7 65006372
chr7 65006403
chr7 65006410
chr7 65006424
chr7 65006430
chr7 65006432
chr7 65006451
chr7 65006468
chr7 65006503
chr7 65006523
chr7 65006529
chr7 65006568
chr7 65006604
chr7 65006819
chr7 65006908
chr7 65006931
chr7 65007146
chr7 65038299
chr7 65038303
chr7 65038306
chr7 65038309
chr7 65038324
chr7 65038334
chr7 65038343
chr7 65038353
chr7 65038359
chr7 65038362
chr7 65038365
chr7 65038376
chr7 65038379
chr7 65038383
chr7 65038395
chr7 65038400
chr7 65038406
chr7 65038408

chr7 65038417
chr7 65038426
chr7 65038440
chr7 65038446
chr7 65038467
chr7 65038495
chr7 65038503
chr7 65038510
chr7 65038521
chr7 65038548
chr7 65038551
chr7 65038571
chr7 65038589
chr7 65038595
chr7 65038599
chr7 65038603
chr7 65038614
chr7 65038622
chr7 65038882
chr7 65038884
chr7 65039012
chr7 65039241
chr7 65039335
chr7 65081252
chr7 65081258
chr7 65081290
chr7 65081302
chr7 65081311
chr7 65081321
chr7 65120530
chr7 65122394
chr7 65122971
chr7 65123081
chr7 65123083
chr7 65123094
chr7 65123099
chr7 65123118
chr7 65123120
chr7 65123143
chr7 65123146
chr7 65123149
chr7 65123151
chr7 65123154
chr7 65123207
chr7 65123210
chr7 65123212
chr7 65123224
chr7 65123227
chr7 65123229
chr7 65123244
chr7 65123250
chr7 65123252
chr7 65123260
chr7 65123264

chr7 65123269
chr7 65123664
chr7 65139742
chr7 65140732
chr7 65140734
chr7 65140757
chr7 65140763
chr7 65140765
chr7 65140768
chr7 65140780
chr7 65140783
chr7 65140786
chr7 65140788
chr7 65140838
chr7 65140841
chr7 65140843
chr7 65140857
chr7 65140862
chr7 65140865
chr7 65140876
chr7 65140881
chr7 65140894
chr7 65140897
chr7 65140900
chr7 65140902
chr7 65140932
chr7 65140935
chr7 65140954
chr7 65140957
chr7 65140960
chr7 65140962
chr7 65140977
chr7 65140980
chr7 65140983
chr7 65140985
chr7 65140996
chr7 65140999
chr7 65141001
chr7 65141015
chr7 65141018
chr7 65141023
chr7 65141035
chr7 65141041
chr7 65141051
chr7 65141069
chr7 65141086
chr7 65141089
chr7 65141096
chr7 65141102
chr7 65141116
chr7 65141121
chr7 65141140
chr7 65141151
chr7 65141165

chr7 65141169
chr7 65141188
chr7 65141196
chr7 65141198
chr7 65141200
chr7 65141206
chr7 65141228
chr7 65141234
chr7 65141248
chr7 65141252
chr7 65141264
chr7 65141275
chr7 65141284
chr7 65141321
chr7 65141343
chr7 65141345
chr7 65141363
chr7 65141375
chr7 65141384
chr7 65269935
chr7 65269963
chr7 65269965
chr7 65269971
chr7 65270147
chr7 65270153
chr7 65270227
chr7 65270232
chr7 65270375
chr7 65373664
chr7 65373728
chr7 65373742
chr7 65373750
chr7 65373762
chr7 65373779
chr7 65373808
chr7 65373819
chr7 65373835
chr7 65373861
chr7 65374053
chr7 65374072
chr7 65374079
chr7 65374089
chr7 65374296
chr7 65374299
chr7 65374367
chr7 65374449
chr7 65475441
chr7 65475797
chr7 65475895
chr7 65508436
chr7 65508473
chr7 65508490
chr7 65508508
chr7 65508510

chr7 65508512
chr7 65508534
chr7 65508541
chr7 65508552
chr7 65508581
chr7 65508633
chr7 65508641
chr7 65508673
chr7 65508675
chr7 65508678
chr7 65508680
chr7 65508699
chr7 65508735
chr7 65508740
chr7 65509382
chr7 65509384
chr7 65509396
chr7 65510072
chr7 65510077
chr7 65510080
chr7 65510337
chr7 65554820
chr7 65554834
chr7 65554841
chr7 65554845
chr7 65554864
chr7 65554869
chr7 65554883
chr7 65554889
chr7 65554896
chr7 65554899
chr7 65554916
chr7 65554934
chr7 65554944
chr7 65554950
chr7 65554962
chr7 65554964
chr7 65554967
chr7 65554970
chr7 65554984
chr7 65554986
chr7 65554989
chr7 65555000
chr7 65555002
chr7 65555005
chr7 65555008
chr7 65555023
chr7 65555025
chr7 65555028
chr7 65555031
chr7 65571284
chr7 65572113
chr7 65572322
chr7 65572456

chr7 65572712
chr7 65572717
chr7 65572721
chr7 65572729
chr7 65572731
chr7 65572737
chr7 65572752
chr7 65572756
chr7 65572768
chr7 65572776
chr7 65572779
chr7 65572807
chr7 65572809
chr7 65572812
chr7 65572815
chr7 65572829
chr7 65572831
chr7 65572834
chr7 65572837
chr7 65572849
chr7 65572852
chr7 65572854
chr7 65572857
chr7 65572860
chr7 65572883
chr7 65572885
chr7 65572904
chr7 65572920
chr7 65572922
chr7 65573032
chr7 65573125
chr7 65574656
chr7 65647616
chr7 65647619
chr7 65647622
chr7 65647624
chr7 65647635
chr7 65647638
chr7 65647640
chr7 65647654
chr7 65647657
chr7 65647660
chr7 65647662
chr7 65651321
chr7 65651339
chr7 65835522
chr7 65835552
chr7 65981125
chr7 65981141
chr7 65981170
chr7 65981252
chr7 65981268
chr7 65981324
chr7 65981474

chr7 65981479
chr7 65981483
chr7 65981526
chr7 65981563
chr7 65981596
chr7 65981605
chr7 65981630
chr7 65981638
chr7 66030633
chr7 66099077
chr7 66099175
chr7 66099214
chr7 66099238
chr7 66099309
chr7 66099635
chr7 66099638
chr7 66099825
chr7 66099837
chr7 66099931
chr7 66100040
chr7 66100087
chr7 66100099
chr7 66100112
chr7 66100154
chr7 66100192
chr7 66100204
chr7 66100254
chr7 66100366
chr7 66100382
chr7 66202328
chr7 66207444
chr7 66207454
chr7 66207708
chr7 66348475
chr7 66628655
chr7 66628658
chr7 66628719
chr7 66628728
chr7 66628735
chr7 66628774
chr7 66628777
chr7 66631523
chr7 66631563
chr7 66631742
chr7 66631819
chr7 66921438
chr7 66921440
chr7 66921450
chr7 66921453
chr7 66921458
chr7 66921468
chr7 66921512
chr7 66921514
chr7 66921520

chr7 66921526
chr7 66921533
chr7 66921542
chr7 66921548
chr7 66921557
chr7 66921560
chr7 66921573
chr7 66921594
chr7 66921609
chr7 66921616
chr7 66921626
chr7 66921630
chr7 66921636
chr7 66921670
chr7 66921672
chr7 66921679
chr7 66921697
chr7 66921712
chr7 66921724
chr7 66921734
chr7 66921750
chr7 66921761
chr7 66921777
chr7 66921791
chr7 66921810
chr7 66921821
chr7 66921868
chr7 66921872
chr7 66921928
chr7 66921978
chr7 66922064
chr7 66922072
chr7 66922075
chr7 66922084
chr7 66994973
chr7 66995024
chr7 67163363
chr7 67163405
chr7 67163434
chr7 67303364
chr7 67303417
chr7 67303454
chr7 67303459
chr7 67303472
chr7 67303487
chr7 67303497
chr7 67303499
chr7 67303503
chr7 68230210
chr7 68230249
chr7 68230652
chr7 68789680
chr7 68789772
chr7 69599853

chr7 69599858
chr7 69599871
chr7 69599873
chr7 69599882
chr7 69599898
chr7 69599955
chr7 69599985
chr7 69600002
chr7 69600012
chr7 69600018
chr7 69600022
chr7 69600040
chr7 69600043
chr7 69600045
chr7 69600050
chr7 69600125
chr7 69600127
chr7 69600153
chr7 69600161
chr7 69600173
chr7 69600181
chr7 69600185
chr7 69600198
chr7 69600249
chr7 69600266
chr7 69600365
chr7 69600375
chr7 69600408
chr7 69600464
chr7 69600474
chr7 69600511
chr7 69600578
chr7 69696115
chr7 69696179
chr7 69696189
chr7 69696321
chr7 69824520
chr7 69824533
chr7 69824550
chr7 69824553
chr7 69824646
chr7 69824880
chr7 69824889
chr7 69824971
chr7 70006982
chr7 70006998
chr7 70007143
chr7 70362106
chr7 70362240
chr7 71448350
chr7 71680184
chr7 71680204
chr7 71680374
chr7 71680376

chr7 71680396
chr7 71733156
chr7 71733183
chr7 71733188
chr7 71733194
chr7 71733221
chr7 71733284
chr7 71733302
chr7 71733332
chr7 71733349
chr7 71733360
chr7 71733372
chr7 71733400
chr7 71733405
chr7 71733407
chr7 71733409
chr7 71742217
chr7 72058890
chr7 72058952
chr7 72058957
chr7 72059126
chr7 72286757
chr7 72286981
chr7 72411315
chr7 72827460
chr7 72827530
chr7 72827677
chr7 72827716
chr7 72827764
chr7 72827792
chr7 72827837
chr7 72827915
chr7 72827934
chr7 72827937
chr7 72827945
chr7 72827970
chr7 72827984
chr7 72827990
chr7 72827998
chr7 72829934
chr7 72829941
chr7 72829950
chr7 72829985
chr7 72830100
chr7 72830143
chr7 72830182
chr7 72830222
chr7 72830302
chr7 72830307
chr7 72830334
chr7 72830389
chr7 72830401
chr7 72830457
chr7 72830464

chr7 72830468
chr7 72830481
chr7 72830487
chr7 72830489
chr7 72830538
chr7 72879651
chr7 72879654
chr7 72879662
chr7 72879665
chr7 72879669
chr7 72879672
chr7 72879679
chr7 72879697
chr7 72879710
chr7 72937340
chr7 72937680
chr7 72953073
chr7 72953121
chr7 72953131
chr7 72953163
chr7 72953179
chr7 72953564
chr7 72953579
chr7 72953597
chr7 72953607
chr7 72953866
chr7 72953871
chr7 72953952
chr7 72954006
chr7 72954052
chr7 72954092
chr7 72954520
chr7 72954530
chr7 72954533
chr7 72954547
chr7 72954557
chr7 72954563
chr7 72954578
chr7 72954609
chr7 72954611
chr7 72954613
chr7 72954619
chr7 72954668
chr7 72954678
chr7 72954681
chr7 72954690
chr7 72954700
chr7 72955005
chr7 72955016
chr7 72955147
chr7 72955149
chr7 72955157
chr7 72955159
chr7 73005255

chr7 73005262
chr7 73005266
chr7 73005294
chr7 73005325
chr7 73005565
chr7 73005567
chr7 73005605
chr7 73005608
chr7 73005617
chr7 73154468
chr7 73154504
chr7 73155060
chr7 73155080
chr7 73155085
chr7 73155090
chr7 73155094
chr7 73155097
chr7 73155520
chr7 73155529
chr7 73155546
chr7 73155551
chr7 73155570
chr7 73155600
chr7 73155614
chr7 73155634
chr7 73155646
chr7 73155652
chr7 73155670
chr7 73155674
chr7 73155705
chr7 73155711
chr7 73155726
chr7 73156266
chr7 73234140
chr7 73234222
chr7 73234891
chr7 73234897
chr7 73234908
chr7 73234911
chr7 73234918
chr7 73234955
chr7 73234967
chr7 73234975
chr7 73234992
chr7 73235002
chr7 73235259
chr7 73235286
chr7 73235289
chr7 73235292
chr7 73235321
chr7 73235329
chr7 73235333
chr7 73235345
chr7 73235377

chr7 73235395
chr7 73235421
chr7 73235426
chr7 73235439
chr7 73235457
chr7 73235470
chr7 73235491
chr7 73235520
chr7 73235544
chr7 73235551
chr7 73235562
chr7 73235564
chr7 73235583
chr7 73235585
chr7 73235588
chr7 73235593
chr7 73235606
chr7 73247185
chr7 73247227
chr7 73519459
chr7 73520242
chr7 73520463
chr7 73520544
chr7 73520552
chr7 73520721
chr7 73520814
chr7 73520895
chr7 73520901
chr7 73520935
chr7 73588092
chr7 73588150
chr7 73588166
chr7 73588173
chr7 73588180
chr7 73588242
chr7 73588271
chr7 73648033
chr7 73648041
chr7 73648071
chr7 73648084
chr7 73648088
chr7 73648102
chr7 73648109
chr7 73648112
chr7 73648117
chr7 73648123
chr7 73648152
chr7 73648160
chr7 73648167
chr7 73648178
chr7 73648180
chr7 73648191
chr7 73648206
chr7 73648243

chr7 73648252
chr7 73648257
chr7 73648281
chr7 73648364
chr7 73648707
chr7 73649530
chr7 74083696
chr7 74083715
chr7 74083721
chr7 74083723
chr7 74083731
chr7 74083737
chr7 74083740
chr7 74083743
chr7 74083747
chr7 74083751
chr7 74083753
chr7 74083758
chr7 74083764
chr7 74083769
chr7 74083785
chr7 74083790
chr7 74083795
chr7 74083803
chr7 74083807
chr7 74083819
chr7 74083822
chr7 74083824
chr7 74083827
chr7 74083830
chr7 74083835
chr7 74083844
chr7 74083847
chr7 74083854
chr7 74083861
chr7 74083867
chr7 74083872
chr7 74083877
chr7 74175561
chr7 74175574
chr7 74175659
chr7 74175707
chr7 74175754
chr7 74175792
chr7 74649886
chr7 74650063
chr7 74650147
chr7 74650149
chr7 74650157
chr7 74650182
chr7 74650209
chr7 74650216
chr7 74650227
chr7 74650245

chr7 74650376
chr7 74650557
chr7 74650568
chr7 74650634
chr7 74650661
chr7 74650734
chr7 74656456
chr7 74656468
chr7 74656532
chr7 74656550
chr7 74656567
chr7 74656625
chr7 74656638
chr7 74656644
chr7 74788243
chr7 74788245
chr7 74788261
chr7 74788267
chr7 74788278
chr7 74788281
chr7 74788288
chr7 74788325
chr7 74788337
chr7 74788345
chr7 74788362
chr7 74788372
chr7 74788406
chr7 74788715
chr7 74788747
chr7 74788756
chr7 74788765
chr7 74788791
chr7 74788796
chr7 74788809
chr7 74788827
chr7 74788840
chr7 74788861
chr7 74788871
chr7 74788876
chr7 74788890
chr7 74788914
chr7 74788921
chr7 74789494
chr7 74789509
chr7 74789542
chr7 74849598
chr7 74849642
chr7 74849646
chr7 74849658
chr7 74849691
chr7 74849700
chr7 74849708
chr7 74849724
chr7 74849929

chr7 74849962
chr7 74850532
chr7 74850570
chr7 74850582
chr7 74850592
chr7 74850622
chr7 74850635
chr7 74850696
chr7 74850720
chr7 74850779
chr7 74850802
chr7 74850819
chr7 74850830
chr7 74850837
chr7 74850871
chr7 74851030
chr7 74851032
chr7 74851040
chr7 74851045
chr7 74851047
chr7 74851051
chr7 74851058
chr7 74851062
chr7 74851065
chr7 74851069
chr7 74851074
chr7 74851084
chr7 74851094
chr7 74851098
chr7 74851125
chr7 74851130
chr7 74851134
chr7 74851138
chr7 74851262
chr7 74851267
chr7 74851271
chr7 74851275
chr7 74851308
chr7 74851313
chr7 74851325
chr7 74851337
chr7 74851340
chr7 74851343
chr7 74851346
chr7 74851349
chr7 74851355
chr7 74851362
chr7 74851374
chr7 74851382
chr7 74851384
chr7 74851387
chr7 74851392
chr7 74851396
chr7 74851398

chr7 74851405
chr7 74851420
chr7 74851431
chr7 74851438
chr7 74851444
chr7 74851447
chr7 74851451
chr7 74851460
chr7 74851462
chr7 74851752
chr7 74851769
chr7 74851776
chr7 74851786
chr7 74851831
chr7 74851851
chr7 74851870
chr7 74851872
chr7 74851912
chr7 74851933
chr7 74851954
chr7 74851958
chr7 74889926
chr7 74889936
chr7 74889946
chr7 74889950
chr7 74889957
chr7 74889961
chr7 74889989
chr7 74890020
chr7 74890043
chr7 74890047
chr7 74890300
chr7 74890303
chr7 74890312
chr7 74890363
chr7 74890405
chr7 74890494
chr7 74890505
chr7 74891128
chr7 74891142
chr7 74891147
chr7 74891259
chr7 74891268
chr7 74891336
chr7 74891351
chr7 74891433
chr7 74891742
chr7 74891803
chr7 74891815
chr7 74891857
chr7 74964322
chr7 74964341
chr7 74964353
chr7 74964370

chr7 74964376
chr7 74964511
chr7 74964513
chr7 74964528
chr7 74964535
chr7 74964543
chr7 74964552
chr7 74964556
chr7 74964567
chr7 74964569
chr7 74964593
chr7 74964613
chr7 74964627
chr7 74964635
chr7 74964637
chr7 74964639
chr7 74964641
chr7 74964646
chr7 74964648
chr7 74964654
chr7 74964659
chr7 74964666
chr7 74964669
chr7 74965960
chr7 74966707
chr7 74966743
chr7 74966773
chr7 75092301
chr7 75092345
chr7 75092355
chr7 75092362
chr7 75092379
chr7 75092466
chr7 75092468
chr7 75092483
chr7 75092492
chr7 75092498
chr7 75092501
chr7 75092517
chr7 75092522
chr7 75092530
chr7 75092609
chr7 75092616
chr7 75092629
chr7 75092633
chr7 75092644
chr7 75092649
chr7 75092654
chr7 75092660
chr7 75092667
chr7 75092669
chr7 75092671
chr7 75092680
chr7 75092684

chr7 75092687
chr7 75092693
chr7 75092700
chr7 75092711
chr7 75092726
chr7 75092733
chr7 75092735
chr7 75092739
chr7 75092744
chr7 75092747
chr7 75092749
chr7 75092757
chr7 75092769
chr7 75092776
chr7 75092782
chr7 75092785
chr7 75092788
chr7 75092791
chr7 75092794
chr7 75092797
chr7 75092800
chr7 75092803
chr7 75092815
chr7 75092827
chr7 75092832
chr7 75092865
chr7 75092869
chr7 75092873
chr7 75092878
chr7 75093071
chr7 75093075
chr7 75093078
chr7 75093082
chr7 75093089
chr7 75093093
chr7 75093095
chr7 75093100
chr7 75093108
chr7 75093110
chr7 75093134
chr7 75093141
chr7 75093144
chr7 75093163
chr7 75093168
chr7 75093174
chr7 75093184
chr7 75093189
chr7 75093191
chr7 75093420
chr7 75093444
chr7 75093505
chr7 75093518
chr7 75093548
chr7 75093558

chr7 75093570
chr7 75093608
chr7 75093634
chr7 75093815
chr7 75093825
chr7 75094178
chr7 75094211
chr7 75094285
chr7 75094542
chr7 75094606
chr7 75094644
chr7 75095163
chr7 75095356
chr7 75095380
chr7 75095432
chr7 75095444
chr7 75095448
chr7 75095461
chr7 75095476
chr7 75095507
chr7 75095518
chr7 75095579
chr7 75095583
chr7 75095633
chr7 75095819
chr7 75095836
chr7 75096254
chr7 75096324
chr7 75096341
chr7 75096402
chr7 75096452
chr7 75096508
chr7 75096515
chr7 75096535
chr7 75096548
chr7 75096580
chr7 75156533
chr7 75156919
chr7 75157039
chr7 75157063
chr7 75157077
chr7 75157082
chr7 75157092
chr7 75157113
chr7 75157126
chr7 75157144
chr7 75157157
chr7 75157162
chr7 75157188
chr7 75157197
chr7 75157206
chr7 75157238
chr7 75157250
chr7 75157254

chr7 75157262
chr7 75157272
chr7 75157278
chr7 75157282
chr7 75157284
chr7 75157291
chr7 75157294
chr7 75157297
chr7 75157312
chr7 75157316
chr7 75157324
chr7 75157327
chr7 75157334
chr7 75157348
chr7 75157354
chr7 75157356
chr7 75157360
chr7 75157377
chr7 75157379
chr7 75157384
chr7 75157392
chr7 75157405
chr7 75157410
chr7 75157415
chr7 75157418
chr7 75157428
chr7 75157434
chr7 75157581
chr7 75157591
chr7 75157608
chr7 75157616
chr7 75157628
chr7 75157665
chr7 75157672
chr7 75157675
chr7 75157686
chr7 75157692
chr7 75157708
chr7 75157710
chr7 75157718
chr7 75157720
chr7 75157738
chr7 75157776
chr7 75157781
chr7 75157811
chr7 75157817
chr7 75157824
chr7 75157829
chr7 75157869
chr7 75157874
chr7 75157877
chr7 75157884
chr7 75157896
chr7 75157912

chr7 75157922
chr7 75157943
chr7 75157960
chr7 75157963
chr7 75158298
chr7 75158361
chr7 75158443
chr7 75235135
chr7 75235139
chr7 75236416
chr7 75236869
chr7 75236872
chr7 75236878
chr7 75236887
chr7 75236908
chr7 75236923
chr7 75236929
chr7 75236960
chr7 75236964
chr7 75236982
chr7 75237724
chr7 75237731
chr7 75237736
chr7 75237742
chr7 75237744
chr7 75237749
chr7 75237751
chr7 75237753
chr7 75237755
chr7 75237763
chr7 75237777
chr7 75237795
chr7 75237828
chr7 75237830
chr7 75237841
chr7 75237845
chr7 75237854
chr7 75237862
chr7 75237869
chr7 75237884
chr7 75237886
chr7 75237914
chr7 75237956
chr7 75237958
chr7 75237982
chr7 75238008
chr7 75238021
chr7 75238027
chr7 75238044
chr7 75238206
chr7 75238225
chr7 75238227
chr7 75238229
chr7 75238267

chr7 75238287
chr7 75356745
chr7 75356759
chr7 75356766
chr7 75356770
chr7 75357142
chr7 75357320
chr7 75357933
chr7 75357975
chr7 75357987
chr7 75358048
chr7 75358118
chr7 75358357
chr7 75358379
chr7 75358439
chr7 75358454
chr7 75358522
chr7 75358531
chr7 75358793
chr7 75358798
chr7 75358806
chr7 75358818
chr7 75358823
chr7 75358834
chr7 75358839
chr7 75358849
chr7 75358851
chr7 75358856
chr7 75358893
chr7 75358911
chr7 75358913
chr7 75358915
chr7 75358924
chr7 75358928
chr7 75358930
chr7 75358946
chr7 75358978
chr7 75358984
chr7 75358989
chr7 75359059
chr7 75359062
chr7 75359081
chr7 75359090
chr7 75359106
chr7 75359119
chr7 75359597
chr7 75359608
chr7 75359617
chr7 75359622
chr7 75359624
chr7 75359635
chr7 75359647
chr7 75359651
chr7 75359700

chr7 75359704
chr7 75359707
chr7 75359716
chr7 75359728
chr7 75359744
chr7 75359749
chr7 75359753
chr7 75359776
chr7 75359807
chr7 75359835
chr7 75359839
chr7 75359846
chr7 75359850
chr7 75359860
chr7 75359870
chr7 75359909
chr7 75359932
chr7 75359946
chr7 75360133
chr7 75360137
chr7 75360139
chr7 75360149
chr7 75360164
chr7 75360177
chr7 75360182
chr7 75409907
chr7 75409930
chr7 75409932
chr7 75409940
chr7 75409942
chr7 75410068
chr7 75410079
chr7 75410152
chr7 75410357
chr7 75410359
chr7 75410361
chr7 75410379
chr7 75410384
chr7 75410394
chr7 75410403
chr7 75410406
chr7 75410416
chr7 75410465
chr7 75410471
chr7 75410475
chr7 75410506
chr7 75410521
chr7 75410527
chr7 75410537
chr7 75410551
chr7 75410554
chr7 75410564
chr7 75410680
chr7 75410683

chr7 75410697
chr7 75410712
chr7 75410734
chr7 75410739
chr7 75410754
chr7 75410757
chr7 75410992
chr7 75411032
chr7 75411078
chr7 75411132
chr7 75427295
chr7 75427303
chr7 75427327
chr7 75427351
chr7 75427366
chr7 75427389
chr7 75427702
chr7 75427899
chr7 75427931
chr7 75427952
chr7 75428068
chr7 75428105
chr7 75428119
chr7 75428122
chr7 75441995
chr7 75442048
chr7 75442070
chr7 75442091
chr7 75442106
chr7 75442135
chr7 75442145
chr7 75442161
chr7 75442169
chr7 75442231
chr7 75442241
chr7 75442243
chr7 75442246
chr7 75442249
chr7 75442253
chr7 75442257
chr7 75442558
chr7 75442564
chr7 75442575
chr7 75442593
chr7 75442600
chr7 75442608
chr7 75442615
chr7 75442618
chr7 75442623
chr7 75442627
chr7 75442632
chr7 75442635
chr7 75442643
chr7 75442666

chr7 75442672
chr7 75442676
chr7 75442679
chr7 75442682
chr7 75442686
chr7 75442699
chr7 75442702
chr7 75442708
chr7 75442711
chr7 75443108
chr7 75443156
chr7 75443163
chr7 75443200
chr7 75443225
chr7 75443250
chr7 75443344
chr7 75443384
chr7 75443703
chr7 75443709
chr7 75443713
chr7 75443742
chr7 75443767
chr7 75443807
chr7 75443809
chr7 75485487
chr7 75485555
chr7 75485559
chr7 75485569
chr7 75485573
chr7 75485587
chr7 75485620
chr7 75485633
chr7 75485636
chr7 75485640
chr7 75485900
chr7 75485908
chr7 75485913
chr7 75485924
chr7 75485930
chr7 75485932
chr7 75485934
chr7 75485936
chr7 75486002
chr7 75486010
chr7 75618069
chr7 75618132
chr7 75618216
chr7 76048330
chr7 76048561
chr7 76048576
chr7 76153286
chr7 76281221
chr7 76281242
chr7 76281894

chr7 76281896
chr7 76281934
chr7 76281978
chr7 76282013
chr7 76282030
chr7 76282051
chr7 76282100
chr7 76282142
chr7 76282145
chr7 76282233
chr7 76282236
chr7 76282330
chr7 76282388
chr7 76282415
chr7 76282450
chr7 76282460
chr7 76282501
chr7 76282503
chr7 76302727
chr7 76302738
chr7 76302746
chr7 76302770
chr7 76302772
chr7 76302783
chr7 76302791
chr7 76303240
chr7 76303245
chr7 76303853
chr7 76303855
chr7 76303865
chr7 76303877
chr7 76303941
chr7 76303956
chr7 76357442
chr7 76357596
chr7 76357606
chr7 76357610
chr7 76357644
chr7 76357806
chr7 76357873
chr7 76357875
chr7 76357898
chr7 76357912
chr7 76357917
chr7 76379921
chr7 76379963
chr7 76380013
chr7 76380016
chr7 76380018
chr7 76380025
chr7 76380056
chr7 76380062
chr7 76380068
chr7 76380153

chr7 76380155
chr7 76380161
chr7 76380170
chr7 76380172
chr7 76380177
chr7 76380191
chr7 76445718
chr7 76445725
chr7 76445745
chr7 76446219
chr7 76461812
chr7 76461835
chr7 76461846
chr7 76461861
chr7 76461863
chr7 76461866
chr7 76461877
chr7 76461889
chr7 76461891
chr7 76461899
chr7 76461901
chr7 76461909
chr7 76461926
chr7 76461936
chr7 76461940
chr7 76461958
chr7 76461961
chr7 76461965
chr7 76461970
chr7 76461973
chr7 76461983
chr7 76462793
chr7 76462831
chr7 76535469
chr7 76535517
chr7 76535523
chr7 76535766
chr7 76605609
chr7 76605611
chr7 76605649
chr7 76605712
chr7 76605820
chr7 76606161
chr7 76606294
chr7 76606314
chr7 76606321
chr7 76606357
chr7 76626654
chr7 76626669
chr7 76626698
chr7 76626724
chr7 76626732
chr7 76626772
chr7 76626774

chr7 76627909
chr7 76627917
chr7 76628236
chr7 76628242
chr7 76628246
chr7 76628275
chr7 76628420
chr7 76628452
chr7 76628460
chr7 77122003
chr7 77122034
chr7 77122041
chr7 77122055
chr7 77122076
chr7 77122119
chr7 77122121
chr7 77122130
chr7 77122175
chr7 77122200
chr7 77122249
chr7 77122254
chr7 77122271
chr7 77122289
chr7 77122398
chr7 77122409
chr7 77122419
chr7 77122454
chr7 77122466
chr7 77122491
chr7 77122502
chr7 77122506
chr7 77122542
chr7 77122576
chr7 77122589
chr7 77122744
chr7 77193090
chr7 77193146
chr7 77194172
chr7 77194325
chr7 77194365
chr7 77194789
chr7 77194829
chr7 77199324
chr7 77199428
chr7 77199457
chr7 77199475
chr7 77199529
chr7 77199568
chr7 77199587
chr7 77199646
chr7 77199742
chr7 77351712
chr7 77352562
chr7 77536692

chr7 77536714
chr7 77536891
chr7 77536895
chr7 77536903
chr7 77536908
chr7 77536933
chr7 77536937
chr7 77696094
chr7 77696100
chr7 77696108
chr7 77696112
chr7 77696120
chr7 77696143
chr7 77696148
chr7 77696172
chr7 77696178
chr7 77696180
chr7 77696188
chr7 77696194
chr7 77696216
chr7 77696221
chr7 77696223
chr7 77696228
chr7 77696238
chr7 77696245
chr7 77696248
chr7 77696262
chr7 77696280
chr7 77696286
chr7 77696294
chr7 77696340
chr7 77697381
chr7 77697584
chr7 77697595
chr7 77697602
chr7 77697610
chr7 77697640
chr7 77697645
chr7 77697755
chr7 77697763
chr7 77697775
chr7 77697807
chr7 77697865
chr7 77697901
chr7 77697935
chr7 77697967
chr7 77697980
chr7 77799589
chr7 77799667
chr7 77799752
chr7 77799757
chr7 77799887
chr7 77799991
chr7 77800248

chr7 77800280
chr7 77800307
chr7 77800449
chr7 77975952
chr7 77983106
chr7 77983116
chr7 77983135
chr7 77983255
chr7 78020656
chr7 78128596
chr7 78331797
chr7 78332043
chr7 78741821
chr7 78741936
chr7 78742075
chr7 78769607
chr7 78769612
chr7 78769719
chr7 78769812
chr7 78769853
chr7 78769990
chr7 78770087
chr7 78770309
chr7 78770411
chr7 78770956
chr7 78771208
chr7 78771241
chr7 78771245
chr7 78771252
chr7 78771303
chr7 78771305
chr7 78771350
chr7 78771453
chr7 79099929
chr7 79451981
chr7 79452092
chr7 79452127
chr7 79452139
chr7 79452332
chr7 79452350
chr7 79452354
chr7 79452359
chr7 79452361
chr7 79452363
chr7 79452379
chr7 79452383
chr7 79452389
chr7 79452404
chr7 79452437
chr7 79452445
chr7 79452475
chr7 79452481
chr7 79452491
chr7 79452522

chr7 79452545
chr7 79452552
chr7 79452562
chr7 79452593
chr7 79452605
chr7 79452701
chr7 79452706
chr7 79452722
chr7 79452768
chr7 79452815
chr7 79453249
chr7 79453331
chr7 79453336
chr7 79453438
chr7 79453452
chr7 79741533
chr7 79741650
chr7 79904848
chr7 79905618
chr7 79905724
chr7 79911097
chr7 79912322
chr7 79912404
chr7 79912580
chr7 79973900
chr7 80078540
chr7 80078917
chr7 80135693
chr7 80135699
chr7 80135799
chr7 80135824
chr7 80135847
chr7 80135867
chr7 80135883
chr7 80135976
chr7 80136003
chr7 80136014
chr7 80136038
chr7 80136047
chr7 80136078
chr7 80136114
chr7 80136121
chr7 80136126
chr7 80136152
chr7 80136226
chr7 80136571
chr7 80136752
chr7 80136950
chr7 80569026
chr7 80590263
chr7 80590610
chr7 80590763
chr7 80590880
chr7 80591076

chr7 80591792
chr7 80592067
chr7 80592117
chr7 80714400
chr7 80714678
chr7 80714727
chr7 80714994
chr7 80715037
chr7 80715348
chr7 80715562
chr7 80715828
chr7 80715866
chr7 80715884
chr7 80716202
chr7 80716219
chr7 80716391
chr7 80918012
chr7 80918373
chr7 80918461
chr7 80918511
chr7 80918542
chr7 80918636
chr7 80918648
chr7 80918665
chr7 80918706
chr7 80918708
chr7 80918804
chr7 80933544
chr7 80933710
chr7 80934069
chr7 81110180
chr7 81121586
chr7 81391062
chr7 81391110
chr7 81391426
chr7 81439118
chr7 81439332
chr7 81439949
chr7 81440120
chr7 81607751
chr7 81607860
chr7 81608651
chr7 81769070
chr7 81769159
chr7 81769212
chr7 81769277
chr7 81769307
chr7 81769417
chr7 81769504
chr7 82135994
chr7 82219923
chr7 82220120
chr7 82220161
chr7 82572497

chr7 82572503
chr7 82595933
chr7 82595956
chr7 82596034
chr7 82596412
chr7 82957931
chr7 83061201
chr7 83061866
chr7 83063021
chr7 83161369
chr7 83161522
chr7 83162053
chr7 83306901
chr7 83647288
chr7 83647509
chr7 83647936
chr7 83648143
chr7 83648145
chr7 83648357
chr7 83648442
chr7 83648448
chr7 83648531
chr7 83648533
chr7 83648548
chr7 83648557
chr7 83648569
chr7 83648625
chr7 83648629
chr7 83648649
chr7 83648653
chr7 83648655
chr7 83648730
chr7 83648750
chr7 83648806
chr7 83648822
chr7 83648825
chr7 83648917
chr7 83648925
chr7 83648940
chr7 83648957
chr7 83649074
chr7 83649146
chr7 83649181
chr7 83649188
chr7 83649224
chr7 83649278
chr7 83650111
chr7 83670315
chr7 83748112
chr7 83748265
chr7 83936355
chr7 83936584
chr7 83936703
chr7 83936709

chr7 84053874
chr7 84054118
chr7 84060782
chr7 84060967
chr7 84061084
chr7 84149576
chr7 84149673
chr7 84194171
chr7 84194595
chr7 84194640
chr7 84194817
chr7 84194829
chr7 84194939
chr7 84194974
chr7 84194980
chr7 84195057
chr7 84195066
chr7 84195068
chr7 84195083
chr7 84195165
chr7 84195220
chr7 84195235
chr7 84195254
chr7 84195301
chr7 84764992
chr7 84879649
chr7 84997747
chr7 84997826
chr7 85082196
chr7 85252630
chr7 85252747
chr7 85420954
chr7 85424910
chr7 85425610
chr7 85953671
chr7 85954386
chr7 85955002
chr7 86016378
chr7 86124774
chr7 86125072
chr7 86125343
chr7 86193986
chr7 86194195
chr7 86279704
chr7 86280111
chr7 86280147
chr7 86280652
chr7 86443131
chr7 86443527
chr7 86643488
chr7 86643490
chr7 86643515
chr7 86643521
chr7 86645485

chr7 86645533
chr7 86645544
chr7 86784986
chr7 86785077
chr7 86785235
chr7 86785645
chr7 86785799
chr7 87152309
chr7 87152311
chr7 87152313
chr7 87152316
chr7 87152325
chr7 87152334
chr7 87152336
chr7 87152349
chr7 87152357
chr7 87152363
chr7 87152372
chr7 87152376
chr7 87152388
chr7 87152392
chr7 87152406
chr7 87152411
chr7 87152413
chr7 87152417
chr7 87152425
chr7 87152431
chr7 87152439
chr7 87152442
chr7 87153517
chr7 87153604
chr7 87153678
chr7 87153696
chr7 87153761
chr7 87153802
chr7 87153841
chr7 87154036
chr7 87154161
chr7 87154331
chr7 87154448
chr7 87154573
chr7 87154667
chr7 87216744
chr7 87216915
chr7 87217135
chr7 87217219
chr7 87217281
chr7 87218248
chr7 87218323
chr7 87218334
chr7 87218377
chr7 87218510
chr7 87218530
chr7 87218666

chr7 87218786
chr7 87218879
chr7 87219051
chr7 87344649
chr7 87344943
chr7 87345015
chr7 87345035
chr7 87345066
chr7 87345128
chr7 87345174
chr7 87345193
chr7 87345217
chr7 87345237
chr7 87345240
chr7 87345272
chr7 87345308
chr7 87345314
chr7 87345319
chr7 87345321
chr7 87345358
chr7 87345396
chr7 87345425
chr7 87345428
chr7 87475081
chr7 87598418
chr7 87598444
chr7 87598738
chr7 87598812
chr7 87599409
chr7 87599411
chr7 87599433
chr7 87599484
chr7 87599492
chr7 87599548
chr7 87599606
chr7 87599656
chr7 87599687
chr7 87599697
chr7 87599811
chr7 87599833
chr7 87599848
chr7 87599999
chr7 87600077
chr7 87600165
chr7 87600187
chr7 87600203
chr7 87600236
chr7 87600238
chr7 87600268
chr7 87600271
chr7 87600297
chr7 87600301
chr7 87600304
chr7 87600328

chr7 87600330
chr7 87600358
chr7 87600360
chr7 87600380
chr7 87600406
chr7 87600408
chr7 87600424
chr7 87600427
chr7 87600435
chr7 87600448
chr7 87600458
chr7 87600461
chr7 87600463
chr7 87600467
chr7 87600487
chr7 87600491
chr7 87600493
chr7 87600514
chr7 87600519
chr7 87600521
chr7 87600523
chr7 87600532
chr7 87600534
chr7 87600539
chr7 87600564
chr7 87600573
chr7 87600652
chr7 87600673
chr7 87600808
chr7 87600836
chr7 87600843
chr7 87600873
chr7 87600880
chr7 87600913
chr7 87600918
chr7 87600989
chr7 87600994
chr7 87601018
chr7 87601028
chr7 87601067
chr7 87601101
chr7 87601103
chr7 87601146
chr7 87601193
chr7 87601214
chr7 87601221
chr7 87601250
chr7 87601487
chr7 87601502
chr7 87874551
chr7 87874874
chr7 87874877
chr7 87875233
chr7 87875282

chr7 87875352
chr7 87875417
chr7 87875422
chr7 87877803
chr7 87877824
chr7 87877864
chr7 87878238
chr7 88218131
chr7 88567302
chr7 88568757
chr7 88568764
chr7 88568786
chr7 88569474
chr7 88569943
chr7 88757874
chr7 88758374
chr7 88758381
chr7 88758431
chr7 88758439
chr7 88758459
chr7 88758518
chr7 88758547
chr7 88758560
chr7 88758562
chr7 88760326
chr7 88760399
chr7 88760440
chr7 88760687
chr7 88761782
chr7 88763670
chr7 88764156
chr7 88764168
chr7 88764190
chr7 89230027
chr7 89770586
chr7 90119864
chr7 90119900
chr7 90119907
chr7 90119986
chr7 90120075
chr7 90120285
chr7 90120412
chr7 90120491
chr7 90120796
chr7 90152111
chr7 90152253
chr7 90152628
chr7 90152826
chr7 90152915
chr7 90152929
chr7 90152992
chr7 90153000
chr7 90153102
chr7 90245525

chr7 90245532
chr7 90245540
chr7 90245597
chr7 90245602
chr7 90346934
chr7 90346959
chr7 90346970
chr7 90347024
chr7 90347129
chr7 90347168
chr7 90347174
chr7 90347211
chr7 90347228
chr7 90347241
chr7 90347247
chr7 90347316
chr7 90347325
chr7 90347355
chr7 90403153
chr7 90403221
chr7 90403247
chr7 90403265
chr7 90403290
chr7 90403331
chr7 90403347
chr7 90403354
chr7 90403642
chr7 90403652
chr7 90403739
chr7 90403752
chr7 90403758
chr7 90403783
chr7 90403785
chr7 90403841
chr7 90403860
chr7 90403906
chr7 90403956
chr7 90404277
chr7 90404313
chr7 90404350
chr7 90404371
chr7 90404396
chr7 90736970
chr7 90737105
chr7 90737491
chr7 90737759
chr7 90746295
chr7 90755951
chr7 91263515
chr7 91263521
chr7 91263579
chr7 91263582
chr7 91263585
chr7 91263596

chr7 91263606
chr7 91263609
chr7 91263615
chr7 91263629
chr7 91263643
chr7 91263647
chr7 91263650
chr7 91263727
chr7 91263774
chr7 91263832
chr7 91263838
chr7 91263844
chr7 91263864
chr7 91263866
chr7 91263877
chr7 91263893
chr7 91263935
chr7 91263960
chr7 91263964
chr7 91264004
chr7 91264025
chr7 91264075
chr7 91264083
chr7 91264085
chr7 91456878
chr7 91457357
chr7 91458606
chr7 91830120
chr7 91830158
chr7 91830231
chr7 91830673
chr7 91880183
chr7 91880196
chr7 91880202
chr7 91880218
chr7 91880273
chr7 91880310
chr7 91880318
chr7 91880324
chr7 91880363
chr7 91880428
chr7 91880431
chr7 91880455
chr7 91880477
chr7 91880525
chr7 91940238
chr7 91940273
chr7 91940280
chr7 91940321
chr7 91940327
chr7 91940349
chr7 91940473
chr7 91940477
chr7 91940498

chr7 91940509
chr7 91940529
chr7 91941252
chr7 91941359
chr7 91941361
chr7 91941389
chr7 91941460
chr7 91941559
chr7 91941592
chr7 92133731
chr7 92133755
chr7 92133782
chr7 92133789
chr7 92133794
chr7 92133810
chr7 92133848
chr7 92133862
chr7 92133880
chr7 92133888
chr7 92133894
chr7 92133916
chr7 92133931
chr7 92133954
chr7 92244534
chr7 92244771
chr7 92245027
chr7 92247035
chr7 92247060
chr7 92247075
chr7 92247155
chr7 92247168
chr7 92448574
chr7 92448782
chr7 92448813
chr7 92448915
chr7 92527299
chr7 92527421
chr7 92527424
chr7 92527605
chr7 92527608
chr7 92529123
chr7 92529204
chr7 92610339
chr7 92610445
chr7 92610888
chr7 92611004
chr7 92632195
chr7 92632819
chr7 92633410
chr7 92633656
chr7 92634293
chr7 92635028
chr7 92809370
chr7 92809833

chr7 92809888
chr7 92809909
chr7 92809943
chr7 92809987
chr7 92810130
chr7 92810448
chr7 92813226
chr7 92813458
chr7 92813590
chr7 92832416
chr7 92837124
chr7 92837147
chr7 92837153
chr7 92837193
chr7 92837374
chr7 92837460
chr7 92837481
chr7 92837528
chr7 92904003
chr7 92904479
chr7 93147366
chr7 93147505
chr7 93147662
chr7 93147814
chr7 93147855
chr7 93147902
chr7 93148085
chr7 93148146
chr7 93148217
chr7 93231432
chr7 93231446
chr7 93231535
chr7 93231542
chr7 93231717
chr7 93231726
chr7 93231735
chr7 93231756
chr7 93231774
chr7 93231778
chr7 93231854
chr7 93231901
chr7 93231903
chr7 93231907
chr7 93231918
chr7 93231971
chr7 93231973
chr7 93231984
chr7 93231997
chr7 93232126
chr7 93232131
chr7 93232134
chr7 93232175
chr7 93232178
chr7 93232182

chr7 93232191
chr7 93232794
chr7 93232816
chr7 93232840
chr7 93232905
chr7 93232971
chr7 93233036
chr7 93233198
chr7 93537436
chr7 93537458
chr7 93537871
chr7 93538271
chr7 93539068
chr7 93591935
chr7 93593075
chr7 93784684
chr7 93784707
chr7 93921948
chr7 93921954
chr7 93921963
chr7 93922093
chr7 93922099
chr7 93922116
chr7 93922122
chr7 93922133
chr7 93922155
chr7 93922204
chr7 93922249
chr7 93922283
chr7 93922553
chr7 93922625
chr7 93922706
chr7 93922794
chr7 93922962
chr7 93922967
chr7 94003447
chr7 94003471
chr7 94003507
chr7 94003523
chr7 94003619
chr7 94003635
chr7 94003652
chr7 94003679
chr7 94003766
chr7 94003779
chr7 94003814
chr7 94004797
chr7 94004890
chr7 94013085
chr7 94510743
chr7 94510753
chr7 94510780
chr7 94510847
chr7 94510860

chr7 94510930
chr7 94654618
chr7 94654707
chr7 94654719
chr7 94654773
chr7 94654823
chr7 94654946
chr7 94654962
chr7 94654964
chr7 94655006
chr7 94655345
chr7 94655366
chr7 94655398
chr7 94655411
chr7 94655419
chr7 94655429
chr7 94655438
chr7 94655444
chr7 94662773
chr7 94846905
chr7 94847051
chr7 95095406
chr7 95095465
chr7 95435168
chr7 95435297
chr7 95477143
chr7 95477156
chr7 95477184
chr7 95477220
chr7 95477252
chr7 95477276
chr7 95477351
chr7 95477578
chr7 95477631
chr7 95477638
chr7 95553588
chr7 95719129
chr7 95754747
chr7 95754823
chr7 95833287
chr7 95834365
chr7 95834454
chr7 95834807
chr7 95835146
chr7 95835182
chr7 95836039
chr7 95916309
chr7 95916321
chr7 95916484
chr7 95916566
chr7 96075647
chr7 96075651
chr7 96075818
chr7 96075832

chr7 96075873
chr7 96075919
chr7 96075932
chr7 96075947
chr7 96076008
chr7 96076022
chr7 96076038
chr7 96076067
chr7 96076104
chr7 96076122
chr7 96076133
chr7 96076206
chr7 96076404
chr7 96076417
chr7 96076428
chr7 96076453
chr7 96076460
chr7 96076509
chr7 96076561
chr7 96076564
chr7 96076620
chr7 96076646
chr7 96077111
chr7 96320632
chr7 96320769
chr7 96321022
chr7 96321276
chr7 96321306
chr7 96321311
chr7 96321371
chr7 96321411
chr7 96321443
chr7 96321468
chr7 96321470
chr7 96321478
chr7 96321492
chr7 96495678
chr7 96495680
chr7 96495775
chr7 96495992
chr7 96496041
chr7 96496142
chr7 96496175
chr7 96496195
chr7 96709424
chr7 96709426
chr7 96709448
chr7 96709473
chr7 96709505
chr7 96709521
chr7 96709532
chr7 96709549
chr7 96709555
chr7 96709560

chr7 96709567
chr7 96709590
chr7 96709593
chr7 96709612
chr7 96709635
chr7 96709929
chr7 96709937
chr7 96709946
chr7 96709955
chr7 96709964
chr7 96710019
chr7 96710049
chr7 96710122
chr7 96988299
chr7 96988480
chr7 96988490
chr7 96988602
chr7 96990264
chr7 96990277
chr7 96990327
chr7 96990393
chr7 96990407
chr7 96990412
chr7 96990474
chr7 96990530
chr7 96990577
chr7 96991441
chr7 96991608
chr7 96991715
chr7 96991737
chr7 96991788
chr7 96991825
chr7 96991837
chr7 96991851
chr7 96991883
chr7 96995160
chr7 96995470
chr7 96995726
chr7 96995922
chr7 96995924
chr7 96995945
chr7 96995952
chr7 96996185
chr7 96996207
chr7 96996212
chr7 96996218
chr7 96996241
chr7 96996254
chr7 96996274
chr7 96996277
chr7 96996310
chr7 96996318
chr7 96996322
chr7 96996325

chr7 96999699
chr7 96999847
chr7 96999863
chr7 96999865
chr7 96999867
chr7 96999869
chr7 96999871
chr7 96999873
chr7 96999875
chr7 96999877
chr7 97000194
chr7 97000348
chr7 97000799
chr7 97000843
chr7 97001434
chr7 97001438
chr7 97004668
chr7 97004728
chr7 97004746
chr7 97004804
chr7 97004889
chr7 97004894
chr7 97004907
chr7 97004915
chr7 97004937
chr7 97004980
chr7 97004990
chr7 97005000
chr7 97005073
chr7 97005102
chr7 97005118
chr7 97010328
chr7 97010550
chr7 97010641
chr7 97010849
chr7 97010932
chr7 97011035
chr7 97011284
chr7 97011580
chr7 97011625
chr7 97011653
chr7 97011764
chr7 97011775
chr7 97011834
chr7 97011862
chr7 97011893
chr7 97011920
chr7 97013848
chr7 97014142
chr7 97014196
chr7 97014227
chr7 97014374
chr7 97014451
chr7 97014509

chr7 97014770
chr7 97014939
chr7 97014968
chr7 97015438
chr7 97015470
chr7 97015613
chr7 97015707
chr7 97015837
chr7 97015915
chr7 97015934
chr7 97016009
chr7 97016141
chr7 97016152
chr7 97016183
chr7 97019417
chr7 97019689
chr7 97019738
chr7 97019741
chr7 97027046
chr7 97027053
chr7 97027121
chr7 97027226
chr7 97027238
chr7 97027378
chr7 97093915
chr7 97094281
chr7 97094333
chr7 97117628
chr7 97117652
chr7 97117766
chr7 97117784
chr7 97117797
chr7 97117816
chr7 97117825
chr7 97117834
chr7 97117855
chr7 97117867
chr7 97117877
chr7 97117906
chr7 97117943
chr7 97117976
chr7 97118016
chr7 97118056
chr7 97118091
chr7 97118177
chr7 97118180
chr7 97118242
chr7 97118299
chr7 97118322
chr7 97118451
chr7 97118467
chr7 97118489
chr7 97118504
chr7 97118542

chr7 97118598
chr7 97118607
chr7 97118734
chr7 97118742
chr7 97730812
chr7 97731303
chr7 97731352
chr7 97731500
chr7 97731502
chr7 97731537
chr7 97731604
chr7 97731648
chr7 97731651
chr7 97734183
chr7 97734200
chr7 97734204
chr7 97734235
chr7 97734417
chr7 97734429
chr7 97759175
chr7 97759204
chr7 97759216
chr7 97778227
chr7 97778290
chr7 97778304
chr7 97778329
chr7 97778345
chr7 97778360
chr7 97778379
chr7 97778546
chr7 97778641
chr7 97778649
chr7 97778658
chr7 97778676
chr7 97778696
chr7 97778767
chr7 97779970
chr7 97780827
chr7 97781253
chr7 97781519
chr7 97781571
chr7 97781644
chr7 97784659
chr7 97871961
chr7 97871964
chr7 97871974
chr7 97871979
chr7 97871981
chr7 97872000
chr7 97872003
chr7 97872056
chr7 97872070
chr7 97872075
chr7 97872080

chr7 97872088
chr7 97872092
chr7 97872094
chr7 97872105
chr7 97872140
chr7 97872156
chr7 97872165
chr7 97872172
chr7 97872176
chr7 97872181
chr7 97872193
chr7 97872202
chr7 97872217
chr7 97872221
chr7 97872223
chr7 97872228
chr7 97872230
chr7 97872237
chr7 97872256
chr7 97872278
chr7 97872296
chr7 97872298
chr7 97872356
chr7 97872380
chr7 97879395
chr7 98133368
chr7 98133371
chr7 98133750
chr7 98168746
chr7 98168788
chr7 98168823
chr7 98202408
chr7 98250917
chr7 98250944
chr7 98251180
chr7 98251236
chr7 98251251
chr7 98251333
chr7 98251376
chr7 98251381
chr7 98251465
chr7 98251480
chr7 98251503
chr7 98251536
chr7 98252082
chr7 98252092
chr7 98252095
chr7 98252109
chr7 98252133
chr7 98252135
chr7 98252138
chr7 98252142
chr7 98252147
chr7 98252153

chr7 98252155
chr7 98252169
chr7 98252172
chr7 98252176
chr7 98252185
chr7 98252190
chr7 98252199
chr7 98252206
chr7 98252223
chr7 98252229
chr7 98252242
chr7 98252261
chr7 98281592
chr7 98281595
chr7 98281601
chr7 98281609
chr7 98281627
chr7 98281635
chr7 98281646
chr7 98281648
chr7 98281653
chr7 98281655
chr7 98281659
chr7 98281664
chr7 98281669
chr7 98281674
chr7 98281676
chr7 98281695
chr7 98281704
chr7 98281709
chr7 98281712
chr7 98281714
chr7 98281720
chr7 98281723
chr7 98281726
chr7 98281729
chr7 98281754
chr7 98281759
chr7 98281764
chr7 98281769
chr7 98281774
chr7 98281777
chr7 98281781
chr7 98281783
chr7 98281787
chr7 98281792
chr7 98281809
chr7 98281823
chr7 98281827
chr7 98281831
chr7 98281849
chr7 98281858
chr7 98281864
chr7 98281866

chr7 98281870
chr7 98281875
chr7 98281879
chr7 98281885
chr7 98281888
chr7 98281897
chr7 98281900
chr7 98281902
chr7 98281908
chr7 98281911
chr7 98281914
chr7 98281930
chr7 98281945
chr7 98619162
chr7 98619227
chr7 98619285
chr7 98619356
chr7 98619396
chr7 98619540
chr7 98641829
chr7 98642074
chr7 98642081
chr7 98642102
chr7 98642117
chr7 98642130
chr7 98642157
chr7 98642211
chr7 98642227
chr7 98642239
chr7 98642242
chr7 98879683
chr7 98879714
chr7 98879815
chr7 98879864
chr7 98879909
chr7 98879990
chr7 98880105
chr7 98880111
chr7 98880116
chr7 99142399
chr7 99142542
chr7 99142555
chr7 99142563
chr7 99142585
chr7 99142605
chr7 99142632
chr7 99142656
chr7 99142886
chr7 99142902
chr7 99142918
chr7 99142945
chr7 99142948
chr7 99142980
chr7 99143003

chr7 99143028
chr7 99143076
chr7 99143092
chr7 99143105
chr7 99143117
chr7 99143125
chr7 99143146
chr7 99143162
chr7 99143188
chr7 99143259
chr7 99143278
chr7 99143311
chr7 99143375
chr7 99143395
chr7 99143402
chr7 99143429
chr7 99143456
chr7 99143467
chr7 99143475
chr7 99143489
chr7 99143497
chr7 99143507
chr7 99143509
chr7 99143515
chr7 99143523
chr7 99143525
chr7 99143529
chr7 99143540
chr7 99143546
chr7 99143553
chr7 99143556
chr7 99143564
chr7 99143583
chr7 99143590
chr7 99143594
chr7 99143597
chr7 99144201
chr7 99144206
chr7 99144213
chr7 99144221
chr7 99144224
chr7 99144228
chr7 99144232
chr7 99144241
chr7 99144245
chr7 99144247
chr7 99144249
chr7 99144256
chr7 99144263
chr7 99144265
chr7 99144294
chr7 99144317
chr7 99144323
chr7 99144348

chr7 99144358
chr7 99144360
chr7 99144362
chr7 99144366
chr7 99375788
chr7 99379753
chr7 99379756
chr7 99379896
chr7 99379924
chr7 99379940
chr7 99380096
chr7 99380139
chr7 99380153
chr7 99380163
chr7 99380217
chr7 99380334
chr7 99380342
chr7 99380405
chr7 99380629
chr7 99380653
chr7 99380689
chr7 99380813
chr7 99380975
chr7 99380993
chr7 99381082
chr7 99381125
chr7 99381142
chr7 99381212
chr7 99409575
chr7 99409607
chr7 99409624
chr7 99409769
chr7 99472188
chr7 99472194
chr7 99472202
chr7 99472210
chr7 99472226
chr7 99472236
chr7 99499628
chr7 99499655
chr7 99499659
chr7 99499663
chr7 99499675
chr7 99499682
chr7 99499689
chr7 99499808
chr7 99499822
chr7 99499825
chr7 99499837
chr7 99505112
chr7 99505119
chr7 99505121
chr7 99505134
chr7 99505137

chr7 99505146
chr7 99505153
chr7 99505157
chr7 99505172
chr7 99505190
chr7 99505192
chr7 99505204
chr7 99505224
chr7 99505235
chr7 99505269
chr7 99505272
chr7 99505293
chr7 99505299
chr7 99505310
chr7 99505322
chr7 99505338
chr7 99505344
chr7 99552256
chr7 99552273
chr7 99552304
chr7 99552410
chr7 99552454
chr7 99552475
chr7 99552488
chr7 99552543
chr7 99558908
chr7 99558916
chr7 99637506
chr7 99810175
chr7 99810225
chr7 99810250
chr7 99912986
chr7 99929588
chr7 99929598
chr7 99929601
chr7 100015891
chr7 100015894
chr7 100015910
chr7 100015916
chr7 100015937
chr7 100015948
chr7 100015974
chr7 100015982
chr7 100015993
chr7 100016012
chr7 100016020
chr7 100016026
chr7 100016036
chr7 100016048
chr7 100016054
chr7 100016056
chr7 100016060
chr7 100016065
chr7 100016071

chr7 100016100
chr7 100016118
chr7 100016142
chr7 100016151
chr7 100016159
chr7 100016173
chr7 100016207
chr7 100016213
chr7 100016384
chr7 100016389
chr7 100016518
chr7 100016543
chr7 100041069
chr7 100041468
chr7 100049888
chr7 100049939
chr7 100049957
chr7 100049989
chr7 100049995
chr7 100089205
chr7 100089212
chr7 100089215
chr7 100089244
chr7 100089253
chr7 100089300
chr7 100089312
chr7 100089333
chr7 100089336
chr7 100119267
chr7 100119273
chr7 100119330
chr7 100119333
chr7 100119337
chr7 100119340
chr7 100119354
chr7 100119362
chr7 100119412
chr7 100119437
chr7 100120217
chr7 100148714
chr7 100148732
chr7 100148771
chr7 100148775
chr7 100148781
chr7 100148791
chr7 100148796
chr7 100148818
chr7 100148821
chr7 100148852
chr7 100148871
chr7 100148889
chr7 100148894
chr7 100148906
chr7 100148912

chr7 100148925
chr7 100148962
chr7 100148971
chr7 100148987
chr7 100148993
chr7 100148995
chr7 100149005
chr7 100149008
chr7 100149019
chr7 100149063
chr7 100149085
chr7 100149089
chr7 100149124
chr7 100149127
chr7 100149171
chr7 100149187
chr7 100149191
chr7 100149197
chr7 100149300
chr7 100149324
chr7 100149365
chr7 100149407
chr7 100149445
chr7 100149450
chr7 100149476
chr7 100149506
chr7 100149526
chr7 100149543
chr7 100149578
chr7 100149583
chr7 100149623
chr7 100149657
chr7 100217921
chr7 100217933
chr7 100218102
chr7 100218163
chr7 100218414
chr7 100220140
chr7 100220154
chr7 100220162
chr7 100220596
chr7 100221487
chr7 100221493
chr7 100334827
chr7 100334869
chr7 100334881
chr7 100334942
chr7 100345178
chr7 100345234
chr7 100345246
chr7 100345260
chr7 100396450
chr7 100396468
chr7 100427008

chr7 100476261
chr7 100476313
chr7 100476623
chr7 100476655
chr7 100476855
chr7 100477311
chr7 100477317
chr7 100477338
chr7 100477379
chr7 100477406
chr7 100477416
chr7 100477427
chr7 100477438
chr7 100477478
chr7 100477548
chr7 100477581
chr7 100477616
chr7 100477630
chr7 100477639
chr7 100477681
chr7 100477684
chr7 100477701
chr7 100477720
chr7 100477723
chr7 100477746
chr7 100477766
chr7 100477773
chr7 100477777
chr7 100477779
chr7 100539784
chr7 100539798
chr7 100539812
chr7 100539816
chr7 100539827
chr7 100539839
chr7 100539848
chr7 100539881
chr7 100539900
chr7 100539918
chr7 100540005
chr7 100540020
chr7 100540038
chr7 100540137
chr7 100540148
chr7 100540258
chr7 100544004
chr7 100544086
chr7 100544162
chr7 100544243
chr7 100546152
chr7 100546159
chr7 100546168
chr7 100546172
chr7 100583639

chr7 100583673
chr7 100583742
chr7 100583786
chr7 100583800
chr7 100583839
chr7 100583855
chr7 100583883
chr7 100583889
chr7 100583908
chr7 100584005
chr7 100584078
chr7 100584083
chr7 100584088
chr7 100584115
chr7 100584140
chr7 100584165
chr7 100584180
chr7 100584250
chr7 100584362
chr7 100584389
chr7 100584405
chr7 100584429
chr7 100584435
chr7 100584452
chr7 100584462
chr7 100584704
chr7 100584844
chr7 100584888
chr7 100585136
chr7 100585155
chr7 100585262
chr7 100585279
chr7 100585349
chr7 100585383
chr7 100585391
chr7 100585412
chr7 100585447
chr7 100585457
chr7 100585464
chr7 100585472
chr7 100585526
chr7 100585547
chr7 100585599
chr7 100604685
chr7 100604709
chr7 100605104
chr7 100605111
chr7 100605142
chr7 100605155
chr7 100605161
chr7 100605172
chr7 100605200
chr7 100605202
chr7 100605208

chr7 100605220
chr7 100605233
chr7 100605256
chr7 100605259
chr7 100605309
chr7 100605318
chr7 100605324
chr7 100605364
chr7 100605411
chr7 100605436
chr7 100605438
chr7 100605459
chr7 100605484
chr7 100605487
chr7 100605491
chr7 100673587
chr7 100673594
chr7 100673605
chr7 100673627
chr7 100853132
chr7 100853182
chr7 100853225
chr7 100853256
chr7 100853335
chr7 100853365
chr7 101017213
chr7 101017231
chr7 101017264
chr7 101017302
chr7 101017306
chr7 101017314
chr7 101017323
chr7 101017334
chr7 101017358
chr7 101017373
chr7 101017383
chr7 101017387
chr7 101017416
chr7 101017437
chr7 101017449
chr7 101017465
chr7 101017479
chr7 101017559
chr7 101017600
chr7 101017612
chr7 101017628
chr7 101017676
chr7 101017732
chr7 101018228
chr7 101018248
chr7 101085246
chr7 101085310
chr7 101085354
chr7 101085371

chr7 101085381
chr7 101085387
chr7 101085403
chr7 101085420
chr7 101085425
chr7 101085430
chr7 101085450
chr7 101085511
chr7 101085514
chr7 101085587
chr7 101085608
chr7 101085612
chr7 101085646
chr7 101085648
chr7 101085680
chr7 101085694
chr7 101085732
chr7 101085743
chr7 101085748
chr7 101085764
chr7 101085778
chr7 101085873
chr7 101085984
chr7 101086011
chr7 101086024
chr7 101086036
chr7 101138829
chr7 101144291
chr7 101166509
chr7 101166550
chr7 101166571
chr7 101166573
chr7 101166594
chr7 101166629
chr7 101166633
chr7 101166685
chr7 101166698
chr7 101166704
chr7 101166716
chr7 101166746
chr7 101166764
chr7 101166770
chr7 101166788
chr7 101166885
chr7 101166901
chr7 101166906
chr7 101166912
chr7 101166946
chr7 101166959
chr7 101166966
chr7 101166982
chr7 101166992
chr7 101167003
chr7 101167070

chr7 101167077
chr7 101167146
chr7 101167179
chr7 101167196
chr7 101167228
chr7 101167413
chr7 101167418
chr7 101167436
chr7 101167450
chr7 101167473
chr7 101167538
chr7 101167701
chr7 101167756
chr7 101167822
chr7 101167827
chr7 101167984
chr7 101167986
chr7 101167988
chr7 101167990
chr7 101168813
chr7 101168823
chr7 101168855
chr7 101168884
chr7 101169078
chr7 101169148
chr7 101169199
chr7 101169212
chr7 101169215
chr7 101169272
chr7 101169465
chr7 101169605
chr7 101169626
chr7 101169645
chr7 101169647
chr7 101169670
chr7 101169683
chr7 101169700
chr7 101169712
chr7 101169720
chr7 101169744
chr7 101171086
chr7 101171134
chr7 101171204
chr7 101171208
chr7 101171213
chr7 101171229
chr7 101171241
chr7 101171440
chr7 101171450
chr7 101171453
chr7 101171466
chr7 101171616
chr7 101171618
chr7 101171627

chr7 101171633
chr7 101171637
chr7 101171643
chr7 101171684
chr7 101245074
chr7 101245088
chr7 101245091
chr7 101245095
chr7 101363563
chr7 101363577
chr7 101363581
chr7 101363607
chr7 101363609
chr7 101363617
chr7 101363625
chr7 101363633
chr7 101363648
chr7 101363654
chr7 101363660
chr7 101363668
chr7 101363682
chr7 101363684
chr7 101363709
chr7 101363724
chr7 101363732
chr7 101363737
chr7 101363744
chr7 101363760
chr7 101363764
chr7 101363767
chr7 101363791
chr7 101363796
chr7 101363800
chr7 101363804
chr7 101363810
chr7 101363821
chr7 101363831
chr7 101363833
chr7 101363839
chr7 101363881
chr7 101363883
chr7 101363888
chr7 101754332
chr7 101754502
chr7 101754578
chr7 101754616
chr7 101754694
chr7 101754750
chr7 101754797
chr7 101813756
chr7 101813834
chr7 101813895
chr7 101814022
chr7 102287316

chr7 102287320
chr7 102287325
chr7 102287333
chr7 102287345
chr7 102287350
chr7 102287367
chr7 102287373
chr7 102287391
chr7 102287395
chr7 102287407
chr7 102287417
chr7 102287475
chr7 102287484
chr7 102287497
chr7 102287525
chr7 102287536
chr7 102287565
chr7 102287596
chr7 102287609
chr7 102287621
chr7 102287630
chr7 102287637
chr7 102287646
chr7 102287667
chr7 102287670
chr7 102363837
chr7 102363841
chr7 102363847
chr7 102363849
chr7 102363851
chr7 102363855
chr7 102363869
chr7 102363871
chr7 102363880
chr7 102364705
chr7 102426715
chr7 102426819
chr7 102426958
chr7 102478053
chr7 102478140
chr7 102478144
chr7 102478210
chr7 102478225
chr7 102478229
chr7 102478234
chr7 102478272
chr7 102478278
chr7 102478292
chr7 102478411
chr7 102478420
chr7 102478433
chr7 102478442
chr7 102478449
chr7 102478460

chr7 102479154
chr7 102479157
chr7 102479204
chr7 102479263
chr7 102480017
chr7 102480104
chr7 102480122
chr7 102480155
chr7 102480161
chr7 102480198
chr7 102480214
chr7 102480237
chr7 102480408
chr7 102480567
chr7 102480594
chr7 102480807
chr7 102480816
chr7 102480821
chr7 102480839
chr7 102480857
chr7 102481462
chr7 102481493
chr7 102481537
chr7 102481557
chr7 102481894
chr7 102481905
chr7 102481960
chr7 102482003
chr7 102483235
chr7 102483283
chr7 102484185
chr7 102484208
chr7 102493333
chr7 102493348
chr7 102493534
chr7 102493574
chr7 102493620
chr7 102493626
chr7 102493669
chr7 102493712
chr7 102493727
chr7 102493743
chr7 102493846
chr7 102493874
chr7 102493879
chr7 102493891
chr7 102493904
chr7 102493937
chr7 102493940
chr7 102493957
chr7 102494210
chr7 102494221
chr7 102494235
chr7 102494241

chr7 102494276
chr7 102494291
chr7 102494293
chr7 102494304
chr7 102494687
chr7 102494695
chr7 102494697
chr7 102494750
chr7 102494763
chr7 102494795
chr7 102494813
chr7 102494827
chr7 102517065
chr7 102517633
chr7 102517637
chr7 102517639
chr7 102517656
chr7 102517677
chr7 102517682
chr7 102517693
chr7 102517700
chr7 102517711
chr7 102517713
chr7 102517721
chr7 102517743
chr7 102517750
chr7 102517758
chr7 102517762
chr7 102517765
chr7 102517769
chr7 102517781
chr7 102517784
chr7 102517795
chr7 102517800
chr7 102517811
chr7 102517817
chr7 102517823
chr7 102517828
chr7 102517831
chr7 102517837
chr7 102517845
chr7 102518092
chr7 102518146
chr7 102518148
chr7 102518156
chr7 102518179
chr7 102518188
chr7 102518251
chr7 102518289
chr7 102518553
chr7 102571873
chr7 102571888
chr7 102571892
chr7 102571897

chr7 102572112
chr7 102572123
chr7 102572148
chr7 102572152
chr7 102572222
chr7 102572232
chr7 102572235
chr7 102572782
chr7 102572788
chr7 102572795
chr7 102572798
chr7 102572811
chr7 102572814
chr7 102592838
chr7 102614862
chr7 102614897
chr7 102616136
chr7 102616612
chr7 102616616
chr7 102616618
chr7 102616635
chr7 102616656
chr7 102616661
chr7 102616672
chr7 102616679
chr7 102616690
chr7 102616692
chr7 102616700
chr7 102616705
chr7 102616722
chr7 102616729
chr7 102616737
chr7 102616741
chr7 102616744
chr7 102616748
chr7 102616760
chr7 102616763
chr7 102616774
chr7 102616779
chr7 102616790
chr7 102616796
chr7 102616802
chr7 102617071
chr7 102617125
chr7 102617127
chr7 102617135
chr7 102617158
chr7 102617167
chr7 102617230
chr7 102617268
chr7 102617528
chr7 102670615
chr7 102670690
chr7 102670702

chr7 102670712
chr7 102670748
chr7 102670756
chr7 102670787
chr7 102670796
chr7 102670883
chr7 102670953
chr7 102670968
chr7 102670972
chr7 102670977
chr7 102671015
chr7 102671021
chr7 102671035
chr7 102671192
chr7 102671228
chr7 102671232
chr7 102671440
chr7 102671466
chr7 102671501
chr7 102671508
chr7 102671516
chr7 102671518
chr7 102671533
chr7 102671539
chr7 102671542
chr7 102671556
chr7 102671562
chr7 102671575
chr7 102671580
chr7 102671595
chr7 102671607
chr7 102671610
chr7 102671646
chr7 102671677
chr7 102671687
chr7 102671690
chr7 102671701
chr7 102671712
chr7 102671720
chr7 102671727
chr7 102671747
chr7 102671753
chr7 102671755
chr7 102671766
chr7 102671787
chr7 102671793
chr7 102671802
chr7 102671806
chr7 102671811
chr7 102671826
chr7 102671833
chr7 102671840
chr7 102671855
chr7 102671861

chr7 102671868
chr7 102671871
chr7 102671884
chr7 102671934
chr7 102672347
chr7 102672403
chr7 102672589
chr7 102672602
chr7 102672610
chr7 102672745
chr7 102673372
chr7 102673407
chr7 102673475
chr7 102673483
chr7 102673510
chr7 102687904
chr7 102687933
chr7 102687991
chr7 102688024
chr7 102688034
chr7 102688040
chr7 102688664
chr7 102688699
chr7 102688705
chr7 102689568
chr7 102689593
chr7 102689597
chr7 102689603
chr7 102689612
chr7 102689639
chr7 102689648
chr7 102689652
chr7 102689656
chr7 102689658
chr7 102689663
chr7 102689669
chr7 102689689
chr7 102689700
chr7 102689766
chr7 102689774
chr7 102689776
chr7 102689842
chr7 102689847
chr7 102689874
chr7 102689892
chr7 102689906
chr7 102689926
chr7 102689944
chr7 102689955
chr7 102689962
chr7 102689971
chr7 102690311
chr7 102690317
chr7 102690327

chr7 102748381
chr7 102748591
chr7 102748843
chr7 102748868
chr7 102748880
chr7 102748925
chr7 102748945
chr7 102748948
chr7 102748957
chr7 102749034
chr7 102749036
chr7 102749052
chr7 102749069
chr7 102749074
chr7 102749102
chr7 102749123
chr7 102749140
chr7 102749148
chr7 102749193
chr7 102749202
chr7 102749204
chr7 102749247
chr7 102749259
chr7 102749268
chr7 102749282
chr7 102749289
chr7 102749320
chr7 102749339
chr7 102749343
chr7 102749351
chr7 102749356
chr7 102749374
chr7 102749383
chr7 102749388
chr7 102749391
chr7 102749425
chr7 102749433
chr7 102749439
chr7 102749443
chr7 102749445
chr7 102749452
chr7 102749465
chr7 102972571
chr7 102972657
chr7 103010062
chr7 103010130
chr7 103065856
chr7 103075244
chr7 103075247
chr7 103075290
chr7 103075292
chr7 103075304
chr7 103148389
chr7 103148412

chr7 103148441
chr7 103148517
chr7 103148756
chr7 103148769
chr7 103148949
chr7 103148956
chr7 103148958
chr7 103148965
chr7 103148988
chr7 103149000
chr7 103149032
chr7 103149052
chr7 103149062
chr7 103149065
chr7 103279354
chr7 103279622
chr7 103279711
chr7 103279790
chr7 103279792
chr7 103279797
chr7 103280373
chr7 103280431
chr7 103280448
chr7 103280515
chr7 103280531
chr7 103280533
chr7 103280543
chr7 103280554
chr7 103280577
chr7 103280592
chr7 103280605
chr7 103280607
chr7 103280635
chr7 103280639
chr7 103280651
chr7 103280676
chr7 103280689
chr7 103280696
chr7 103280706
chr7 103280721
chr7 103280723
chr7 103280725
chr7 103280728
chr7 103280739
chr7 103280744
chr7 103280763
chr7 103280785
chr7 103280805
chr7 103280807
chr7 103280847
chr7 103280857
chr7 103297088
chr7 103297212
chr7 103298058

chr7 103298073
chr7 103298103
chr7 103298183
chr7 103298371
chr7 103343843
chr7 103347489
chr7 103348013
chr7 103348031
chr7 103348074
chr7 103348081
chr7 103348129
chr7 103348252
chr7 103348496
chr7 103348527
chr7 103348581
chr7 103348632
chr7 103348634
chr7 103348642
chr7 103348655
chr7 103348664
chr7 103348666
chr7 103348679
chr7 103348684
chr7 103348694
chr7 103348718
chr7 103454630
chr7 104069583
chr7 104069831
chr7 104070086
chr7 104070853
chr7 104207218
chr7 104207250
chr7 104207589
chr7 104207602
chr7 104207614
chr7 104207631
chr7 104207635
chr7 104207717
chr7 104207725
chr7 104337130
chr7 104560465
chr7 104669826
chr7 104669907
chr7 104857709
chr7 104857821
chr7 104857868
chr7 104857902
chr7 104858268
chr7 104943963
chr7 104944084
chr7 104944120
chr7 104944276
chr7 104944288
chr7 104944452

chr7 104944557
chr7 104944590
chr7 104944593
chr7 104944682
chr7 104944719
chr7 104944736
chr7 104944768
chr7 104944833
chr7 104944882
chr7 104944887
chr7 104944891
chr7 104944896
chr7 104944899
chr7 104944936
chr7 104945035
chr7 104945068
chr7 104945103
chr7 104945140
chr7 104984493
chr7 104984516
chr7 105009848
chr7 105009857
chr7 105009860
chr7 105010297
chr7 105010367
chr7 105010486
chr7 105010874
chr7 105010908
chr7 105016709
chr7 105016861
chr7 105016997
chr7 105017230
chr7 105017530
chr7 105017808
chr7 105017880
chr7 105017922
chr7 105018124
chr7 105267119
chr7 105267399
chr7 105268424
chr7 105268563
chr7 105268708
chr7 105268820
chr7 105268983
chr7 105269248
chr7 105269368
chr7 105269545
chr7 105352116
chr7 105352279
chr7 105352333
chr7 105352637
chr7 105388838
chr7 105388840
chr7 105388843

chr7 105388846
chr7 105388849
chr7 105388851
chr7 105388855
chr7 105388858
chr7 105531675
chr7 105531721
chr7 105531788
chr7 105531845
chr7 105531865
chr7 105531956
chr7 105532022
chr7 105532024
chr7 105532036
chr7 105532073
chr7 105532088
chr7 105532093
chr7 105532100
chr7 105532126
chr7 105532130
chr7 105532133
chr7 105532186
chr7 105691236
chr7 105691243
chr7 105691282
chr7 105691292
chr7 105691325
chr7 105691331
chr7 105691397
chr7 105692110
chr7 105692195
chr7 105692227
chr7 105708038
chr7 105708071
chr7 105708073
chr7 105708106
chr7 105708160
chr7 105708195
chr7 105708205
chr7 105708254
chr7 105809434
chr7 105875751
chr7 105875980
chr7 106022251
chr7 106022269
chr7 106022325
chr7 106022363
chr7 106022438
chr7 106022645
chr7 106071429
chr7 106071520
chr7 106071529
chr7 106071534
chr7 106071547

chr7 106071669
chr7 106071671
chr7 106071873
chr7 106110909
chr7 106111014
chr7 106111111
chr7 106111113
chr7 106111174
chr7 106111195
chr7 106111266
chr7 106111334
chr7 106111336
chr7 106111354
chr7 106111408
chr7 106111420
chr7 106283616
chr7 106283657
chr7 106283716
chr7 106283817
chr7 106283825
chr7 106283957
chr7 106284017
chr7 106284022
chr7 106285680
chr7 106285686
chr7 106285696
chr7 106285699
chr7 106285754
chr7 106285769
chr7 106285779
chr7 106285832
chr7 106285842
chr7 106285859
chr7 106285882
chr7 106285901
chr7 106285927
chr7 106285962
chr7 106286008
chr7 106286021
chr7 106286029
chr7 106286061
chr7 106286100
chr7 106286138
chr7 106425373
chr7 106425470
chr7 106425540
chr7 106542533
chr7 106542559
chr7 106542674
chr7 106866222
chr7 106866265
chr7 106866800
chr7 106866924
chr7 106867029

chr7 106867142
chr7 107045162
chr7 107045168
chr7 107045275
chr7 107045286
chr7 107045296
chr7 107045303
chr7 107045351
chr7 107045406
chr7 107126337
chr7 107126345
chr7 107126352
chr7 107126375
chr7 107126446
chr7 107126457
chr7 107126561
chr7 107126574
chr7 107126648
chr7 107167130
chr7 107167363
chr7 107167392
chr7 107167428
chr7 107167473
chr7 107167475
chr7 107167755
chr7 107170190
chr7 107170230
chr7 107170252
chr7 107170295
chr7 107170473
chr7 107170615
chr7 107333160
chr7 107333297
chr7 107563482
chr7 107563495
chr7 107563497
chr7 107563519
chr7 107563560
chr7 107580686
chr7 107580691
chr7 107580696
chr7 107580710
chr7 107580814
chr7 107580882
chr7 107580904
chr7 107580951
chr7 107580980
chr7 107581038
chr7 107659842
chr7 107659921
chr7 107659963
chr7 107660035
chr7 107660273
chr7 107660316

chr7 107660347
chr7 107743029
chr7 107743064
chr7 107743213
chr7 107743225
chr7 107743227
chr7 107743245
chr7 107743256
chr7 107743262
chr7 107743276
chr7 107744519
chr7 107744530
chr7 107744537
chr7 107744542
chr7 107744544
chr7 107744558
chr7 107744571
chr7 107744575
chr7 107744619
chr7 107744645
chr7 107744653
chr7 107744715
chr7 107815640
chr7 107815829
chr7 107815874
chr7 107857258
chr7 107857311
chr7 107857327
chr7 107891774
chr7 107891838
chr7 107891940
chr7 107891984
chr7 107892068
chr7 107892071
chr7 108456442
chr7 108456444
chr7 108456447
chr7 108456450
chr7 108456452
chr7 108456454
chr7 108456460
chr7 108456463
chr7 108456469
chr7 108456478
chr7 108456491
chr7 108456493
chr7 108456496
chr7 108456513
chr7 108456526
chr7 108456534
chr7 108525439
chr7 108526692
chr7 108526732
chr7 108526753

chr7 108526788
chr7 108526834
chr7 108526903
chr7 108527017
chr7 108569720
chr7 108570787
chr7 108570799
chr7 108570847
chr7 108570875
chr7 108899289
chr7 109243177
chr7 109298009
chr7 109298661
chr7 109428211
chr7 109428721
chr7 109716987
chr7 109717192
chr7 110564706
chr7 110564822
chr7 110583692
chr7 111138379
chr7 111138531
chr7 111139047
chr7 111139192
chr7 111580224
chr7 111580284
chr7 112203866
chr7 112204013
chr7 112204104
chr7 112204146
chr7 112204196
chr7 112204228
chr7 112204334
chr7 112204396
chr7 112204407
chr7 112204416
chr7 112204503
chr7 112204662
chr7 112204765
chr7 112204772
chr7 112204922
chr7 112205015
chr7 112205030
chr7 112205085
chr7 112205113
chr7 112205127
chr7 112205218
chr7 112205244
chr7 112205248
chr7 112449489
chr7 112449639
chr7 112449743
chr7 112449751
chr7 112449787

chr7 112449808
chr7 112449866
chr7 112449965
chr7 112449981
chr7 112450022
chr7 112451590
chr7 112451595
chr7 112451733
chr7 112451803
chr7 112451806
chr7 112451866
chr7 112550541
chr7 112789652
chr7 112790095
chr7 112790097
chr7 112790128
chr7 112790131
chr7 112790139
chr7 112938842
chr7 112938863
chr7 112938879
chr7 112939082
chr7 112939093
chr7 112939155
chr7 112939167
chr7 112939197
chr7 112939236
chr7 112939239
chr7 112939255
chr7 112939277
chr7 112939702
chr7 112939712
chr7 112939716
chr7 112939728
chr7 112939732
chr7 112939735
chr7 112939753
chr7 112939759
chr7 112939762
chr7 112939765
chr7 112939768
chr7 112939771
chr7 112939774
chr7 112939789
chr7 112939795
chr7 112939937
chr7 112939943
chr7 112940281
chr7 112940321
chr7 112940334
chr7 112940420
chr7 112940473
chr7 113086341
chr7 113086480

chr7 113086483
chr7 113086486
chr7 113086492
chr7 113086494
chr7 113086519
chr7 113086521
chr7 113086534
chr7 113086539
chr7 113086545
chr7 113086560
chr7 113086569
chr7 113086571
chr7 113086574
chr7 113086577
chr7 113086580
chr7 113086586
chr7 113086592
chr7 113086598
chr7 113086601
chr7 113086604
chr7 113087859
chr7 113087889
chr7 113087961
chr7 113087998
chr7 113088187
chr7 113118265
chr7 113118282
chr7 113118435
chr7 113118447
chr7 113118512
chr7 113118518
chr7 113118601
chr7 113118604
chr7 113118607
chr7 113118624
chr7 113118627
chr7 113118629
chr7 113118654
chr7 113118673
chr7 113118720
chr7 113118822
chr7 113118848
chr7 113118865
chr7 113118899
chr7 113118927
chr7 113119011
chr7 113119013
chr7 113119030
chr7 113214605
chr7 113214846
chr7 113216847
chr7 113216885
chr7 113217130
chr7 113217310

chr7 113217366
chr7 113360353
chr7 113741667
chr7 113967376
chr7 113967423
chr7 113968296
chr7 114073882
chr7 114074007
chr7 114074139
chr7 114074436
chr7 114074448
chr7 114074450
chr7 114082087
chr7 114082169
chr7 114082229
chr7 114082657
chr7 114082659
chr7 114082663
chr7 114082666
chr7 114082703
chr7 114082716
chr7 114082718
chr7 114082727
chr7 114082749
chr7 114241720
chr7 114241743
chr7 114241764
chr7 114298841
chr7 114298909
chr7 114298954
chr7 114326433
chr7 114387572
chr7 114835515
chr7 114835955
chr7 114839438
chr7 114909089
chr7 114924564
chr7 114924609
chr7 114924933
chr7 114925037
chr7 114926106
chr7 114931289
chr7 114931333
chr7 114931491
chr7 114931512
chr7 114931735
chr7 114931794
chr7 114931825
chr7 114931859
chr7 115009519
chr7 115009720
chr7 115009739
chr7 115476887
chr7 115476952

chr7 115476970
chr7 115477002
chr7 115477041
chr7 115477093
chr7 115477234
chr7 115477268
chr7 115477273
chr7 115477280
chr7 115477311
chr7 115477323
chr7 115477579
chr7 115477596
chr7 115477616
chr7 115477659
chr7 115477675
chr7 115477687
chr7 115477720
chr7 115477785
chr7 115477842
chr7 115477851
chr7 115477863
chr7 115477870
chr7 115477959
chr7 115478090
chr7 115760235
chr7 115760533
chr7 115760960
chr7 115761320
chr7 115761641
chr7 115839362
chr7 115845690
chr7 115846032
chr7 115933282
chr7 116146068
chr7 116146224
chr7 116146295
chr7 116210757
chr7 116210760
chr7 116210775
chr7 116210777
chr7 116210782
chr7 116210784
chr7 116210786
chr7 116210789
chr7 116210791
chr7 116210793
chr7 116210796
chr7 116210800
chr7 116210911
chr7 116210917
chr7 116210934
chr7 116210940
chr7 116210955
chr7 116210957

chr7 116210961
chr7 116210967
chr7 116210971
chr7 116210973
chr7 116210996
chr7 116211850
chr7 116211880
chr7 116211912
chr7 116211978
chr7 116212077
chr7 116212095
chr7 116212252
chr7 116212460
chr7 116212476
chr7 116212525
chr7 116212698
chr7 116212780
chr7 116212938
chr7 116212999
chr7 116213012
chr7 116213166
chr7 116225102
chr7 116393695
chr7 116393737
chr7 116393762
chr7 116524246
chr7 116524291
chr7 116524296
chr7 116524318
chr7 116524368
chr7 116524382
chr7 116524479
chr7 116524546
chr7 116524553
chr7 116524579
chr7 116524689
chr7 116525249
chr7 116525709
chr7 116525724
chr7 116525736
chr7 116525757
chr7 116525761
chr7 116525790
chr7 116525801
chr7 116525851
chr7 116525870
chr7 116525887
chr7 116525890
chr7 116525909
chr7 116588449
chr7 116588474
chr7 116588656
chr7 116588688
chr7 116588852

chr7 116589494
chr7 116591670
chr7 116591755
chr7 116591784
chr7 116591791
chr7 116591876
chr7 116591893
chr7 116591997
chr7 116592366
chr7 116592483
chr7 116592526
chr7 116592538
chr7 116592566
chr7 116592588
chr7 116842914
chr7 116843475
chr7 116843627
chr7 117014915
chr7 117014920
chr7 117014936
chr7 117672596
chr7 117674724
chr7 117674871
chr7 117874599
chr7 117971635
chr7 118184650
chr7 118184654
chr7 118184675
chr7 118184826
chr7 118184855
chr7 118184896
chr7 118184914
chr7 118185010
chr7 118185020
chr7 118214711
chr7 118214725
chr7 118214764
chr7 118214769
chr7 118214778
chr7 118214798
chr7 118214927
chr7 118225952
chr7 118226265
chr7 118629168
chr7 118629174
chr7 118629981
chr7 118630435
chr7 118636250
chr7 118668833
chr7 118673801
chr7 118693461
chr7 118693540
chr7 118704605
chr7 118704633

chr7 118704777
chr7 118705341
chr7 118705391
chr7 118705658
chr7 118947860
chr7 118948198
chr7 118948330
chr7 118948415
chr7 119044236
chr7 119044302
chr7 119044762
chr7 119045471
chr7 119049555
chr7 119049968
chr7 119050571
chr7 119955009
chr7 119955280
chr7 119955342
chr7 119955421
chr7 120271884
chr7 120271954
chr7 120272244
chr7 120272351
chr7 120272360
chr7 120272386
chr7 120272417
chr7 120277324
chr7 120277435
chr7 120277927
chr7 120278071
chr7 120278515
chr7 120552630
chr7 120553806
chr7 120588121
chr7 120588599
chr7 120792037
chr7 120792052
chr7 120792062
chr7 120949474
chr7 120949510
chr7 120949585
chr7 120949598
chr7 120949627
chr7 120951455
chr7 120951504
chr7 120951537
chr7 120951563
chr7 120951578
chr7 120951583
chr7 120951754
chr7 120987465
chr7 120987618
chr7 120987653
chr7 120987785

chr7 120987790
chr7 120987821
chr7 120987861
chr7 120987948
chr7 120988002
chr7 120988059
chr7 120988067
chr7 120988165
chr7 120988292
chr7 120988429
chr7 120988518
chr7 120988598
chr7 120988604
chr7 120988622
chr7 120988656
chr7 120988668
chr7 120988673
chr7 120988682
chr7 120988688
chr7 120988698
chr7 120988704
chr7 120988720
chr7 120988723
chr7 120988727
chr7 120988734
chr7 120988738
chr7 120988740
chr7 120988742
chr7 120988744
chr7 120988746
chr7 120988768
chr7 120988820
chr7 120988941
chr7 120988997
chr7 120991669
chr7 120991786
chr7 121045590
chr7 121055309
chr7 121330570
chr7 121330589
chr7 121330600
chr7 121330619
chr7 121330630
chr7 121330632
chr7 121330640
chr7 121330643
chr7 121330648
chr7 121330650
chr7 121330661
chr7 121330663
chr7 121330666
chr7 121330675
chr7 121330680
chr7 121330688

chr7 121330706
chr7 121330815
chr7 121330871
chr7 121395373
chr7 121396593
chr7 121396596
chr7 121396607
chr7 121396615
chr7 121396675
chr7 121396749
chr7 121396801
chr7 121438434
chr7 121438523
chr7 121438534
chr7 121438593
chr7 121438612
chr7 121438662
chr7 121438679
chr7 121438685
chr7 121438726
chr7 121438734
chr7 121443203
chr7 121443424
chr7 121443472
chr7 121443487
chr7 121443501
chr7 121836558
chr7 121837330
chr7 121837752
chr7 121843037
chr7 121868781
chr7 121871900
chr7 121872128
chr7 121873942
chr7 121874019
chr7 121874150
chr7 121874337
chr7 121874367
chr7 121874392
chr7 121874435
chr7 121874437
chr7 121874458
chr7 121874492
chr7 121874577
chr7 121874678
chr7 121874708
chr7 121874861
chr7 121875032
chr7 121875119
chr7 121991228
chr7 121991530
chr7 121992030
chr7 121995155
chr7 122143182

chr7 122143206
chr7 122241941
chr7 122241952
chr7 122242049
chr7 122307332
chr7 122307426
chr7 122307428
chr7 122307474
chr7 122307481
chr7 122307941
chr7 122308040
chr7 122308252
chr7 122308547
chr7 122308752
chr7 122308833
chr7 122308959
chr7 122308977
chr7 122309051
chr7 122309130
chr7 122309150
chr7 122309515
chr7 122309703
chr7 122309770
chr7 122309852
chr7 122309871
chr7 122309971
chr7 122309976
chr7 122310000
chr7 122312079
chr7 122312086
chr7 122312095
chr7 122312101
chr7 122312253
chr7 122312278
chr7 122312319
chr7 122312856
chr7 122312972
chr7 122312977
chr7 122313519
chr7 122313521
chr7 122314257
chr7 122314268
chr7 122314310
chr7 122314435
chr7 122314579
chr7 122314582
chr7 122314800
chr7 122314806
chr7 122314832
chr7 122314905
chr7 122314911
chr7 122398608
chr7 122398766
chr7 122398769

chr7 122882646
chr7 122883252
chr7 122887468
chr7 122887471
chr7 122887484
chr7 122887505
chr7 122887636
chr7 122907255
chr7 122907271
chr7 122907647
chr7 122907823
chr7 122908470
chr7 123532438
chr7 123532513
chr7 123532586
chr7 123532625
chr7 123532823
chr7 123532830
chr7 123532835
chr7 123532840
chr7 123532847
chr7 123532854
chr7 123532871
chr7 123532892
chr7 123532894
chr7 123532901
chr7 123532904
chr7 123532914
chr7 123532920
chr7 123532930
chr7 123532937
chr7 123532948
chr7 123532956
chr7 123532959
chr7 123532974
chr7 123533020
chr7 123533024
chr7 123533035
chr7 123533044
chr7 123533076
chr7 123533079
chr7 123533082
chr7 123533098
chr7 123533112
chr7 123533115
chr7 123533141
chr7 123533157
chr7 123533168
chr7 123533176
chr7 123534117
chr7 123534142
chr7 123534203
chr7 123534299
chr7 123534318

chr7 123534322
chr7 123534340
chr7 123534355
chr7 123534373
chr7 123534419
chr7 123534421
chr7 123534431
chr7 123534452
chr7 123534455
chr7 123534468
chr7 123534499
chr7 123534516
chr7 123534519
chr7 123534545
chr7 123534560
chr7 123534563
chr7 123534565
chr7 123534582
chr7 123534595
chr7 123534620
chr7 123534627
chr7 123534658
chr7 123534689
chr7 123534700
chr7 123534713
chr7 123534715
chr7 123534749
chr7 123534755
chr7 123534770
chr7 123534799
chr7 123534813
chr7 123534828
chr7 123534857
chr7 123534865
chr7 123534871
chr7 123534884
chr7 123534886
chr7 123534915
chr7 123534927
chr7 123534946
chr7 123534948
chr7 123535001
chr7 123535003
chr7 123535032
chr7 123535040
chr7 123535083
chr7 123535103
chr7 123535105
chr7 123535133
chr7 123535137
chr7 123535141
chr7 123535156
chr7 123535172
chr7 123535184

chr7 123535192
chr7 123535205
chr7 123535207
chr7 123535235
chr7 123535317
chr7 123556987
chr7 123557065
chr7 123557081
chr7 123557109
chr7 123557131
chr7 123557240
chr7 123557248
chr7 123557264
chr7 123557268
chr7 123557285
chr7 123557287
chr7 123557322
chr7 123557380
chr7 123557406
chr7 123557506
chr7 123557527
chr7 123557536
chr7 123558251
chr7 123558283
chr7 123558321
chr7 123558330
chr7 123747317
chr7 123747386
chr7 123747419
chr7 123747435
chr7 123747604
chr7 123747707
chr7 123747720
chr7 123747793
chr7 123747938
chr7 123747948
chr7 123748063
chr7 123748099
chr7 123748124
chr7 123748179
chr7 123749879
chr7 124031042
chr7 124031121
chr7 124031258
chr7 124031301
chr7 124031490
chr7 124177576
chr7 124178347
chr7 124178620
chr7 124479530
chr7 124480192
chr7 124565801
chr7 124929321
chr7 124929342

chr7 124929424
chr7 124929476
chr7 124929509
chr7 124929528
chr7 124929550
chr7 124929590
chr7 124929659
chr7 124929672
chr7 124929682
chr7 124929692
chr7 124929732
chr7 124930172
chr7 124930305
chr7 125290570
chr7 125383355
chr7 125383598
chr7 125384036
chr7 125384149
chr7 125561192
chr7 125561225
chr7 125561287
chr7 125561445
chr7 125561453
chr7 125561456
chr7 125593843
chr7 125594071
chr7 125774285
chr7 125774418
chr7 125774640
chr7 125774939
chr7 125775111
chr7 125775113
chr7 125776378
chr7 125776409
chr7 125776558
chr7 125776655
chr7 125776836
chr7 125950109
chr7 125951057
chr7 125951659
chr7 126037629
chr7 126037835
chr7 126037848
chr7 126038627
chr7 126038665
chr7 126038737
chr7 126039031
chr7 126039094
chr7 126039210
chr7 126145798
chr7 126700284
chr7 126701219
chr7 126701285
chr7 126959323

chr7 126989250
chr7 127348166
chr7 127348178
chr7 127348208
chr7 127391004
chr7 127391147
chr7 127391260
chr7 127391478
chr7 127391482
chr7 127391512
chr7 127391570
chr7 127391608
chr7 127391611
chr7 127391637
chr7 127391644
chr7 127391687
chr7 127391707
chr7 127537030
chr7 127537048
chr7 127537090
chr7 127652722
chr7 127652751
chr7 127652776
chr7 127857231
chr7 127859506
chr7 128294026
chr7 128343234
chr7 128343256
chr7 128343292
chr7 128343331
chr7 128343451
chr7 128343453
chr7 128343471
chr7 128343482
chr7 128343524
chr7 128343580
chr7 128343601
chr7 128343630
chr7 128343634
chr7 128343658
chr7 128343662
chr7 128343667
chr7 128343691
chr7 128343966
chr7 128343985
chr7 128459013
chr7 128459054
chr7 128459216
chr7 128523883
chr7 128532399
chr7 128532444
chr7 128532494
chr7 128532759
chr7 128533279

chr7 128533479
chr7 128608959
chr7 128609126
chr7 128609169
chr7 128609206
chr7 128609296
chr7 128609418
chr7 128739782
chr7 128739790
chr7 128739830
chr7 128739867
chr7 128739885
chr7 128739911
chr7 128739955
chr7 128739962
chr7 128740027
chr7 128753895
chr7 128777729
chr7 128777859
chr7 128777982
chr7 128778025
chr7 129298653
chr7 129612820
chr7 129615359
chr7 129844231
chr7 129844484
chr7 129844498
chr7 129844510
chr7 129949222
chr7 129949245
chr7 129949377
chr7 129949386
chr7 129950767
chr7 129950912
chr7 129950955
chr7 129951337
chr7 129951504
chr7 129951524
chr7 129951526
chr7 129951677
chr7 129951828
chr7 129951901
chr7 129952024
chr7 129952086
chr7 129952105
chr7 129989626
chr7 130050914
chr7 130050975
chr7 130051004
chr7 130051018
chr7 130051024
chr7 130051046
chr7 130071212
chr7 130071245

chr7 130071250
chr7 130071275
chr7 130071670
chr7 130071744
chr7 130071939
chr7 130071999
chr7 130072048
chr7 130072088
chr7 130072228
chr7 130072251
chr7 130072331
chr7 130072429
chr7 130072445
chr7 130072473
chr7 130204962
chr7 130204985
chr7 130205011
chr7 130205083
chr7 130205102
chr7 130205113
chr7 130205144
chr7 130205146
chr7 130205160
chr7 130205162
chr7 130205195
chr7 130205208
chr7 130458469
chr7 130458495
chr7 130641765
chr7 130641847
chr7 130641863
chr7 130641967
chr7 130641972
chr7 130734609
chr7 130734613
chr7 130734616
chr7 130734694
chr7 130734700
chr7 130734724
chr7 130734729
chr7 130734753
chr7 130734799
chr7 130734833
chr7 130734853
chr7 130734914
chr7 130734939
chr7 130734942
chr7 130734993
chr7 130735031
chr7 130735171
chr7 130735331
chr7 130735353
chr7 130735556
chr7 130735766

chr7 130735810
chr7 130735822
chr7 130913872
chr7 130913877
chr7 130913879
chr7 130913910
chr7 130913993
chr7 130914000
chr7 130914149
chr7 130914185
chr7 130914367
chr7 130943087
chr7 130943259
chr7 130943305
chr7 130943484
chr7 130943512
chr7 130943527
chr7 130943698
chr7 130943755
chr7 130943796
chr7 130956972
chr7 130956986
chr7 130957240
chr7 130957406
chr7 130957789
chr7 130957811
chr7 130957907
chr7 130958030
chr7 130959578
chr7 130959663
chr7 130959699
chr7 130959905
chr7 130960048
chr7 130960111
chr7 130960115
chr7 130960118
chr7 130960470
chr7 130960594
chr7 130960596
chr7 130960669
chr7 130960739
chr7 130960891
chr7 130960990
chr7 130961124
chr7 130961226
chr7 130961241
chr7 130961272
chr7 130961292
chr7 130961319
chr7 130961354
chr7 130961430
chr7 130961460
chr7 130961586
chr7 130961603

chr7 131007546
chr7 131011144
chr7 131031707
chr7 131031760
chr7 131031919
chr7 131031975
chr7 131040443
chr7 131040476
chr7 131040522
chr7 131040559
chr7 131040655
chr7 131040687
chr7 131040766
chr7 131057289
chr7 131057348
chr7 131057384
chr7 131057415
chr7 131057859
chr7 131057911
chr7 131058152
chr7 131058160
chr7 131108549
chr7 131108564
chr7 131108640
chr7 131108664
chr7 131108727
chr7 131108792
chr7 131108884
chr7 131108901
chr7 131108973
chr7 131109316
chr7 131109334
chr7 131109369
chr7 131109575
chr7 131109631
chr7 131109645
chr7 131109825
chr7 131109833
chr7 131109838
chr7 131109868
chr7 131109870
chr7 131109931
chr7 131109933
chr7 131109945
chr7 131109963
chr7 131109970
chr7 131109976
chr7 131109979
chr7 131231582
chr7 131231609
chr7 131231613
chr7 131231625
chr7 131231888
chr7 131231906

chr7 131231998
chr7 131232162
chr7 131232248
chr7 131328343
chr7 131328355
chr7 131328357
chr7 131328371
chr7 131328385
chr7 131328389
chr7 131328398
chr7 131328401
chr7 131328404
chr7 131328420
chr7 131328430
chr7 131328448
chr7 131328514
chr7 131328557
chr7 131328583
chr7 131328637
chr7 131328643
chr7 131328661
chr7 131328698
chr7 131328710
chr7 131328762
chr7 131557778
chr7 131557842
chr7 131557859
chr7 131747828
chr7 131747952
chr7 131753331
chr7 131753691
chr7 132318652
chr7 132318733
chr7 132318795
chr7 132318838
chr7 132318840
chr7 132318939
chr7 132318946
chr7 132318967
chr7 132318977
chr7 132318982
chr7 132319004
chr7 132319028
chr7 132319067
chr7 132319172
chr7 132319273
chr7 132319302
chr7 132438327
chr7 132460821
chr7 132461046
chr7 132461361
chr7 132561781
chr7 132562726
chr7 132563933

chr7 132655022
chr7 132655219
chr7 132655259
chr7 132655285
chr7 132655356
chr7 132655454
chr7 132655482
chr7 132655520
chr7 132655548
chr7 132655561
chr7 132655605
chr7 132655620
chr7 133081537
chr7 133081557
chr7 133081587
chr7 133081635
chr7 133081639
chr7 133081874
chr7 133081892
chr7 133081896
chr7 133081901
chr7 133081912
chr7 133081915
chr7 133081943
chr7 133081958
chr7 133081969
chr7 133081979
chr7 133081988
chr7 133081997
chr7 133082007
chr7 133082009
chr7 133082011
chr7 133082036
chr7 133082043
chr7 133082057
chr7 133253092
chr7 133253094
chr7 133253163
chr7 133253228
chr7 133253233
chr7 133253240
chr7 133253303
chr7 133253398
chr7 133253442
chr7 133253457
chr7 133253600
chr7 133253615
chr7 133253665
chr7 133253734
chr7 133253760
chr7 133253869
chr7 133253903
chr7 134231503
chr7 134231547

chr7 134231576
chr7 134459366
chr7 134459482
chr7 134459536
chr7 134547386
chr7 134547443
chr7 134547504
chr7 134547510
chr7 134547523
chr7 134547669
chr7 134547711
chr7 134547751
chr7 134547802
chr7 134547848
chr7 134614149
chr7 134614185
chr7 134646033
chr7 134646122
chr7 134646262
chr7 134646283
chr7 134646330
chr7 134646350
chr7 134646467
chr7 134646487
chr7 134646507
chr7 134646514
chr7 134646549
chr7 134646555
chr7 134646571
chr7 134646574
chr7 134646618
chr7 134646624
chr7 134646627
chr7 134647157
chr7 134647313
chr7 134647319
chr7 134647384
chr7 134647390
chr7 134647512
chr7 134669764
chr7 134669931
chr7 134669946
chr7 134711503
chr7 134711564
chr7 134927168
chr7 134986273
chr7 134986306
chr7 134987125
chr7 134987180
chr7 134987192
chr7 134987211
chr7 134987236
chr7 134987259
chr7 134987283

chr7 135147290
chr7 135147379
chr7 135147469
chr7 135147530
chr7 135147680
chr7 135147792
chr7 135147832
chr7 135147919
chr7 135148018
chr7 135148092
chr7 135148098
chr7 135148187
chr7 135148279
chr7 135148309
chr7 135148547
chr7 135148583
chr7 135148660
chr7 135148698
chr7 135148938
chr7 135330654
chr7 135508774
chr7 135508802
chr7 135508898
chr7 135508919
chr7 135508929
chr7 135508969
chr7 135508977
chr7 135509003
chr7 135509009
chr7 135509019
chr7 135509053
chr7 135509119
chr7 135509181
chr7 135509294
chr7 135510521
chr7 135510551
chr7 135510558
chr7 135510560
chr7 135510599
chr7 135510609
chr7 135510628
chr7 135510657
chr7 135510751
chr7 135510760
chr7 135510776
chr7 135557875
chr7 135557900
chr7 135557913
chr7 135557935
chr7 135557949
chr7 135557952
chr7 135557961
chr7 135557970
chr7 135557996

chr7 135558009
chr7 135558034
chr7 135558040
chr7 135558052
chr7 135558077
chr7 135558136
chr7 135558138
chr7 135558140
chr7 135558143
chr7 135558151
chr7 135558164
chr7 135558203
chr7 135975317
chr7 135975353
chr7 135975776
chr7 135975923
chr7 135976041
chr7 135980104
chr7 135980464
chr7 135980735
chr7 135980859
chr7 135980934
chr7 136505014
chr7 136505228
chr7 136867142
chr7 136868212
chr7 136868341
chr7 137343232
chr7 137347900
chr7 137545005
chr7 137935163
chr7 137935189
chr7 137935225
chr7 137935337
chr7 137935601
chr7 137935651
chr7 137935716
chr7 137935872
chr7 137935889
chr7 138162507
chr7 138624992
chr7 138625010
chr7 138625258
chr7 138625294
chr7 138625375
chr7 138752708
chr7 138752789
chr7 138752806
chr7 138752812
chr7 138815153
chr7 139044279
chr7 139044327
chr7 139044389
chr7 139044405

chr7 139044623
chr7 139044633
chr7 139044647
chr7 139044743
chr7 139044788
chr7 139044822
chr7 139044874
chr7 139045223
chr7 139045317
chr7 139045378
chr7 139075005
chr7 139075146
chr7 139075173
chr7 139075195
chr7 139094895
chr7 139094918
chr7 139094975
chr7 139095012
chr7 139095088
chr7 139095225
chr7 139095227
chr7 139095246
chr7 139095297
chr7 139095374
chr7 139100138
chr7 139100257
chr7 139100259
chr7 139100269
chr7 139100313
chr7 139100327
chr7 139100341
chr7 139103451
chr7 139103568
chr7 139103678
chr7 139103696
chr7 139104154
chr7 139104180
chr7 139104239
chr7 139104290
chr7 139104300
chr7 139104435
chr7 139104439
chr7 139104441
chr7 139104451
chr7 139104488
chr7 139104542
chr7 139104583
chr7 139104595
chr7 139104613
chr7 139104615
chr7 139104637
chr7 139104646
chr7 139104654
chr7 139104666

chr7 139104679
chr7 139104708
chr7 139104759
chr7 139104765
chr7 139104925
chr7 139104947
chr7 139104956
chr7 139104964
chr7 139104976
chr7 139104989
chr7 139105070
chr7 139105082
chr7 139105316
chr7 139105524
chr7 139105695
chr7 139105846
chr7 139105908
chr7 139108548
chr7 139108551
chr7 139108593
chr7 139232442
chr7 139232445
chr7 139232467
chr7 139232491
chr7 139232637
chr7 139232691
chr7 139340609
chr7 139340626
chr7 139340640
chr7 139340646
chr7 139340755
chr7 139340813
chr7 139361386
chr7 139361493
chr7 139361708
chr7 139361790
chr7 139361855
chr7 139494936
chr7 139495175
chr7 139776075
chr7 139776092
chr7 139776096
chr7 139776111
chr7 139776130
chr7 139776147
chr7 139776170
chr7 139776183
chr7 139776329
chr7 139776361
chr7 139776499
chr7 139776529
chr7 139776573
chr7 139776604
chr7 139776615

chr7 139776626
chr7 139776648
chr7 139776682
chr7 139776690
chr7 139776754
chr7 139916812
chr7 139916822
chr7 139916839
chr7 140229465
chr7 140229489
chr7 140229511
[truncated: 97,730 more chars]
